# Loss of cadherin 17 downregulates LGR5 expression, stem cell properties and drug resistance in metastatic colorectal cancer cells

**DOI:** 10.1038/s41419-025-07811-w

**Published:** 2025-07-01

**Authors:** Ruben A. Bartolomé, Laura Pintado-Berninches, Javier Robles, Tania Calvo-López, Issam Boukich, Pablo Otero-Núñez, Jose Manuel Gonzalez-Sancho, J. Ignacio Casal

**Affiliations:** 1 Department of Molecular Biomedicine. Centro de Investigaciones Biológicas (CIB-CSIC), Madrid, Spain; 2 Instituto de Investigaciones Biomédicas Sols-Morreale, Consejo Superior de Investigaciones Científicas–Universidad Autónoma de Madrid, Madrid, Spain; 3 Departamento de Bioquímica, Facultad de Medicina, Universidad Autónoma de Madrid, 28029 Madrid, Spain; 4 Protein Alternatives SL. Tres Cantos, Madrid, Spain; 5 Centro de Investigación Biomédica en Red de Cáncer (CIBERONC), Madrid, Spain; 6 Instituto de Investigación Sanitaria del Hospital Universitario La Paz-IdiPAZ, Madrid, Spain

**Keywords:** Colon cancer, Mechanisms of disease, Targeted therapies

## Abstract

Cadherin 17 (CDH17) plays a crucial role in the metastatic progression of colorectal cancer (CRC) through its interaction with α2β1 integrin and desmocollin 1. To further elucidate the molecular mechanisms involving CDH17 functions in CRC, we examined global expression alterations following CDH17 silencing in various metastatic cell lines. Loss of CDH17 resulted in a marked down-regulation of the intestinal cancer stem cell (CSC) marker LGR5, leading to the inhibition of Wnt/β-catenin signaling, suppression of pluripotency genes such as MYC, and a subsequent reduction in stemness properties. Treatment with CDH17/integrin blocking antibodies produced similar effects, decreasing both, LGR5 expression and Wnt signaling. CDH17 silencing also down-regulated various transporters associated with drug-resistance, including the glutamine-transporter SLC38A5. Consequently, the loss of CDH17 increased sensitivity to 5-FU, irinotecan, oxidative stress and anoikis in CRC cells. Notably, SLC38A5 silencing was necessary for CDH17-driven effects on drug resistance and survival. Pharmacological inhibition of SLC38A5 with amiloride, significantly increased cell sensitivity to 5-FU and irinotecan, and improved mouse survival in metastasis models. In conclusion, CDH17 plays a crucial role in maintaining colorectal cancer cell stemness and chemoresistance via LGR5/Wnt/MYC signaling and SLC38A5 expression. These findings underscore the therapeutic potential of CDH17 targeting in metastatic CRC, and support the use of amiloride for inhibiting liver metastasis.

## Introduction

Cancer stem cells (CSCs) constitute a distinct subset of tumor cells characterized by self-renewal capacity, drive tumor growth and significantly contribute to disease progression and relapse, including metastasis [1, 2]. Additionally, CSCs are also associated with drug resistance [3], which is mediated by the increased expression of various protein families, notably the ATP-binding cassette transporters (ABCs) and the solute carrier (SLC) proteins [4]. Actually, a major problem of current therapies is the failure to eradicate CSC subpopulations in tumors, with the subsequent recurrence and metastatic progression [5, 6]. The Wnt/β-catenin pathway is essential to regulate the balance between stemness, proliferation and differentiation in embryonic and adult stem cells, by regulating MYC expression among other genes [7]. Any misbalance may lead to developmental alterations or malignancies [8]. Wnt signaling and cadherin-mediated cell-cell adhesion are crucial regulators in the organization and maintenance of CSCs, including colorectal cancer (CRC) stem cells (SCs). These inter-related signaling pathways not only govern the self-renewal and differentiation processes of CSCs, but also play pivotal roles in promoting their invasive and metastatic potential [7, 9, 10].

The regulation of pluripotency is intimately linked to cell-cell adhesion and colony integrity of human embryonic stem cells (hESCs) during undifferentiated growth. Disruption of cellular association and compact structures of hESCs causes the down-regulation of pluripotency transcription factors [11]. For example, E-cadherin (CDH1) is recognized as a key regulator of pluripotency, stem cell maintenance and differentiation in ESCs [11, 12]. The presence of catenin-binding sites in E-cadherin contributes to the regulation of the stemness capacity through both, the cadherin-mediated adhesion processes, and the Wnt/β-catenin signaling pathway [12]. However, the role of “non-canonical”, tissue-specific cadherins, such as cadherin 17 (CDH17) in the context of cancer stem cell biology remains vastly unexplored. CDH17 is almost exclusively expressed in the intestinal cells of both embryonic and adult small intestine and colon [13], and is considered a selective marker of colon cancer cell lines, being expressed in 35 of the 54 epithelial-like colon cell lines described in the cancer cell line encyclopedia [14]. In colorectal cancer, CDH17 expression declines at early stages and is re-expressed at late stages. CDH17 is also overexpressed in some other tumors of the gastrointestinal tract and neuroendocrine tumors [15, 16].

Structurally, CDH17 differs from classical cadherins, like E-cadherin, by containing a very short cytoplasmic domain lacking catenin-binding sites [17]. However, immunoprecipitation (IP) studies showed the association of CDH17 with β-catenin and various proteins of the actin cytoskeleton in CRC metastatic cells [18]. The short cytoplasmic domain, together with the CDH17 localization in lipid rafts of the plasma membrane, facilitates lateral mobility and positioning in different cell-cell junctions [19]. CDH17 promotes CRC liver metastasis formation by binding and regulating the activation of the α2β1 integrin pathway through the RGD motif present in CDH17 ectodomain [18, 20]. Moreover, CDH17 associates with desmocollin 1 (DSC1) and p120-catenin to modulate migration and invasion in CRC cells exhibiting a mesenchymal phenotype [19]. Thus, CDH17 constitutes a promising therapeutic candidate for preventing metastatic progression in CRC by using either specific monoclonal antibodies against the RGD motif of CDH17 [21], CAR-T cells [22], or an anti-NLV peptide blocking CDH17/DSC1 binding [19].

To further explain the underlying molecular mechanisms regulated by CDH17 in CRC, we conducted a global expression analysis in two stably CDH17 knocked-down CRC metastatic cell lines, namely KM12SM and SW620. KM12SM is a highly-metastatic cell line, classified as consensus molecular subtype 1 (CMS1), and characterized by displaying epithelial colon-like [23] and stem cell markers [24], including LGR5, CD133, ALCAM, ALDH1, EPHB2 and SOX2 [25]. SW620, derived from the lymph nodes of a patient [26], has been classified within the mesenchymal CMS4 subtype. SW620 cells express lower levels of CDH17 and E-cadherin compared to KM12SM, indicating a different expression pattern associated with a lower metastatic capacity. For validation studies, we included the metastatic HT29 cell line, representative of the CMS3 subtype [23], which is characterized by an epithelial phenotype and metabolic deregulation. The inclusion of three different subtypes helps to capture, at some extent, the heterogeneity of the disease.

In this report, the depletion of CDH17 led to the downregulation of key intestinal stem cell markers, such as LGR5, in both metastatic cell lines, thereby impairing Wnt signaling and stemness maintenance. Additionally, among the most down-regulated genes after CDH17 silencing, we identified SLC38A5, a glutamine (Gln)-transporter, involved in the regulation of drug resistance. Treatment with the SLC38A5 inhibitor, amiloride, increased survival in mouse models of metastasis.

## Results

### Gene expression profile of CDH17 knocked-down colorectal cancer metastatic cells

**Fig. 1:**
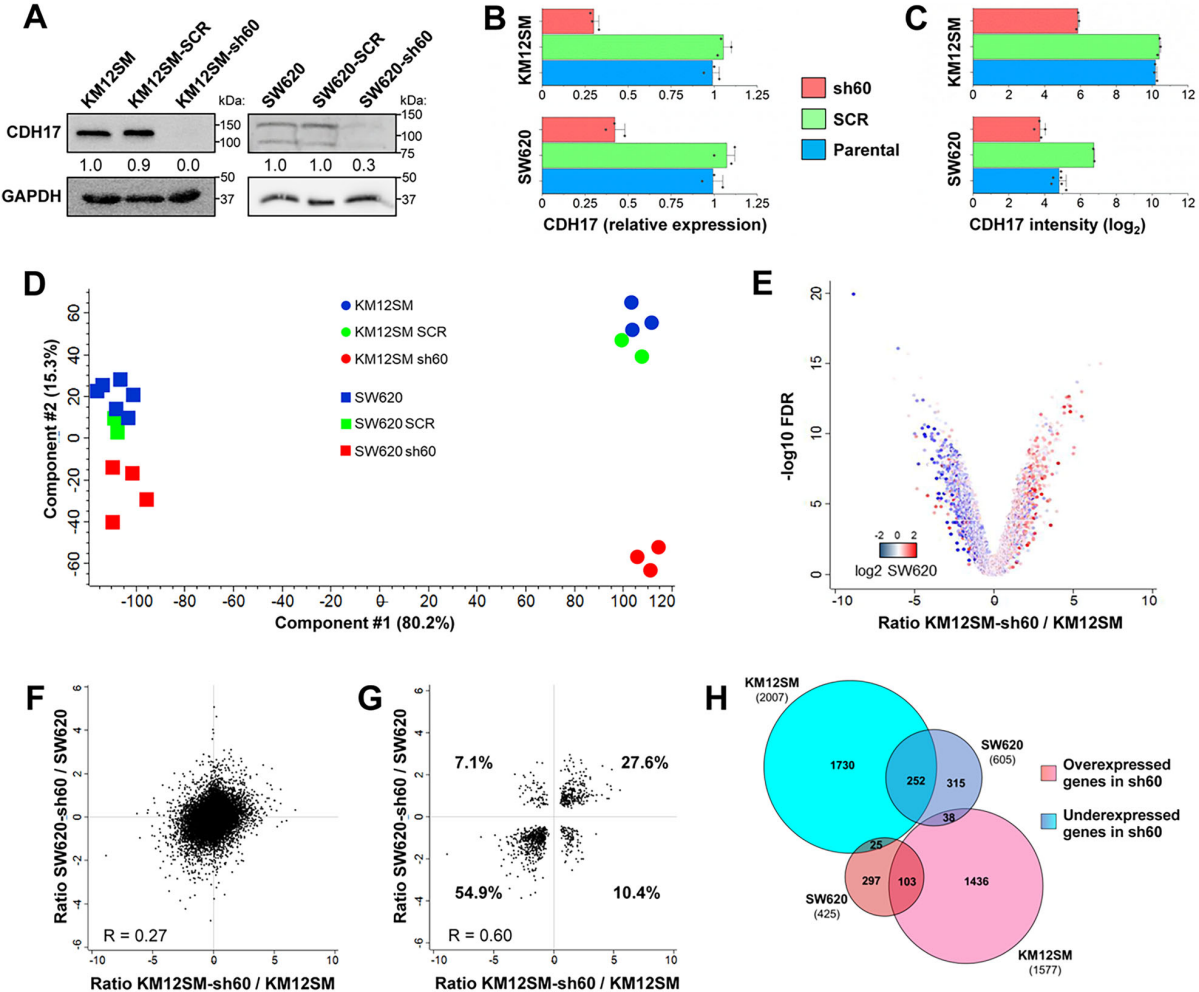
Transcriptomic analysis of the KM12SM and SW620 cell lines silenced for CDH17. Confirmation of CDH17 silencing by (**A**) western blot, (**B**) qPCR and (**C**) expression microarrays. **D** PCA of KM12SM and SW620 samples. **E** Volcano plot showing the distribution of the KM12SM-sh60/KM12SM ratio. Dots are colored according to the SW620-sh60/SW620 ratio. Correlation between the ratios (sh60/parental) of KM12SM and SW620 analyzing (**F**) all quantified genes and (**G**) only significantly-altered genes. Pearson correlation coefficients are shown. **H** Venn diagram of the genes over- (red) and under-expressed (blue) in the CDH17-silenced lines compared to the control lines KM12SM and SW620 based on an FDR < 0.05 and a ratio > |2 | .

Parental KM12SM and SW620 cells were compared with CDH17-knocked down (KD) cells, referred to as sh60 (shRNA against CDH17), and scrambled control (SCR) cells using global transcriptomic analysis (Supplementary Table S1). Confirmation of CDH17 silencing was done by western blot (Fig. 1A), qPCR (Fig. 1B) and examination of the expression microarray values (Fig. 1C). Principal component analysis (PCA) showed an effective separation of the CDH17-silenced lines from controls (parental and SCR) (Fig. 1D). Volcano (Fig. 1E) and scatter plot analyses (Fig. 1F) indicated a moderate correlation (*R* = 0.27) in gene alterations between the two cell lines. However, this correlation improved substantially (*R* = 0.60) when the analysis was focused on significantly-altered genes (Fig. 1G). Thus, we selected only those differentially-expressed genes with a fold-change > 2 or < 0.5, based on a FDR < 0.05, for analysis (Fig. 1H, Supplementary Table S1). KM12SM KD cells exhibited 2007 down-regulated and 1577 up-regulated genes relative to control cells (*p*-value ≤ 0.05), where SW620 KD cells exhibited 425 up-regulated and 605 down-regulated genes (*p*-value ≤ 0.05). The lower number of differentially-expressed genes in SW620 KD cells can be explained by the lower basal expression of CDH17 and stem cell markers.

**Fig. 2:**
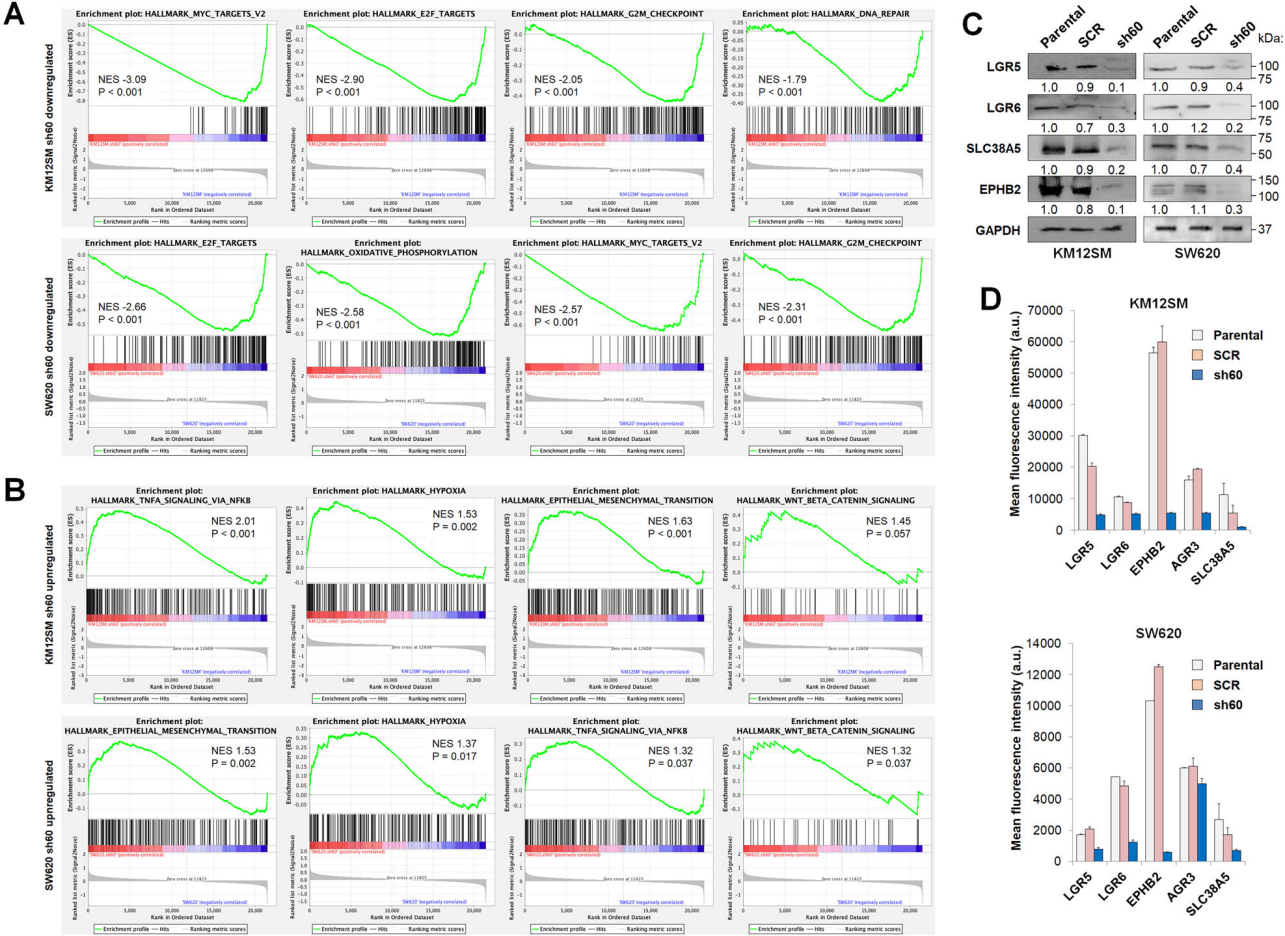
Gene set enrichment analysis and validation of CSC markers in CDH17-silenced cells. Gene set enrichment analysis (GSEA) using hallmark gene sets from the molecular signature database for the downregulated (**A**) or upregulated (**B**) genes in CDH17-silenced (sh60) colon cancer cells. Normalized Enrichment Scores (NES) and normalized *p-*values are shown inside each panel. Validation of selected targets affected by CDH17 silencing using (**C**) Western blot and (**D**) flow cytometry analyses. Below each lane, band quantification relative to control cells is shown. Data are representative of three independent experiments.

KEGG analysis indicated the upregulation of MAPK signaling pathway, autophagy, ECM-receptor interactions and circadian rhythm genes in both cell lines, and the down-regulation of cell cycle, DNA replication and mismatch repair (Supplementary Fig. S1A). Using gene set enrichment analysis (GSEA), we observed a significant overlap in the altered signaling pathways within both cell lines after CDH17 silencing (Supplementary Fig. S1B). Many of them were related with inflammation and metabolism. Heatmaps representative of the most altered genes within each category are shown in Supplementary Fig. S1C. Specifically, among the significantly down-regulated categories in CDH17-silenced cells we found “MYC targets”, “E2F targets” and “G2M checkpoint”, both in KM12SM and SW620 (Fig. 2A) cells. These enriched pathways support the decline in cell proliferation associated with MYC inhibition [18]. Among the GSEA up-regulated enriched categories in CDH17 KD cells for both cell lines, we found pathways associated with “TNFα signaling” “hypoxia”, “EMT” or “Wnt-β-catenin signaling” (Fig. 2B). However, the up-regulation of Wnt/β-catenin signaling was associated with the enrichment of some Wnt repressors and genes from the β-catenin destruction complex. Regarding EMT alterations, an increased expression of EMT mediators, such as *SNAI1* (ratio 3.0; FDR 0.01), *ZEB1* (ratio 3.51; FDR 1.5E-03) and *TGFB1* (ratio 4.66; FDR 4.2E-05) [27] was observed in KM12SM KD cells. These data were confirmed by the increased expression of genes associated with EMT according to the GSEA analysis in both cell lines (Supplementary Fig. S1B). Although EMT slightly increased the migratory and invasive capacities in KM12SM CDH17 KD cells (Supplementary Fig. S2), overall they were less metastatic [18].

### Loss of CDH17 decreases the expression of LGR5 and other stem cell markers

Given the established link between E-cadherin-mediated cell-cell adhesion and the modulation of stemness and pluripotency in embryonic cells [28], as well as the observed Wnt/β-catenin signaling alterations, we searched for modifications in stem cell marker genes. KM12SM KD CDH17 cells showed a significant decrease in the expression ratios of various stem cell marker genes when compared to SCR and parental cells (Supplementary Table S1**)**: *LGR5* (ratio 0.07; FDR 6.4E-07), *LGR6* (ratio 0.20; FDR 5.0E-06); *EPHB2* (ratio 0.11; FDR 4.4E-05), *CD133* (ratio 0.14; FDR 6.4E-07), *SOX2* (ratio 0.29; FDR 5.1E-03), and *MYC* (ratio 0.15; FDR 8.9E-07). In SW620 KD cells, a similar reduction was observed in *LGR5* (ratio 0.14; FDR 9.9E-06), *LGR6* (ratio 0.31; FDR 5.0E-04), *EPHB2* (ratio 0.26; FDR 8.5E-03), *SOX2* (ratio 0.33; FDR 9.0E-04), as well as *CD24* (ratio 0.36; FDR 3.0E-04) and *CD44* (ratio 0.41; FDR 3.1E-03). Interestingly, two factors of the OCT4, SOX2, KLF4 and MYC (OSKM) cocktail for pluripotency [29], SOX2 and MYC, along with KLF4 at a lower extent, displayed reduced expression in KM12SM CDH17 KD cells, suggesting a potential inhibition of the cellular reprogramming capacity after CDH17 inhibition. SOX2 expression was also reduced in SW620 KD cells. Next, selected gene alterations were validated by western blot and flow cytometry analyses. Protein expression of LGR5, LGR6, EPHB2, AGR3, and SLC38A5 confirmed a severe repression following CDH17 silencing, as demonstrated by Western blot (Fig. 2C) and flow cytometry (Fig. 2D).

### Loss of CDH17 downregulates the Wnt signaling pathway in colorectal cancer by inhibiting LGR5 expression

**Fig. 3:**
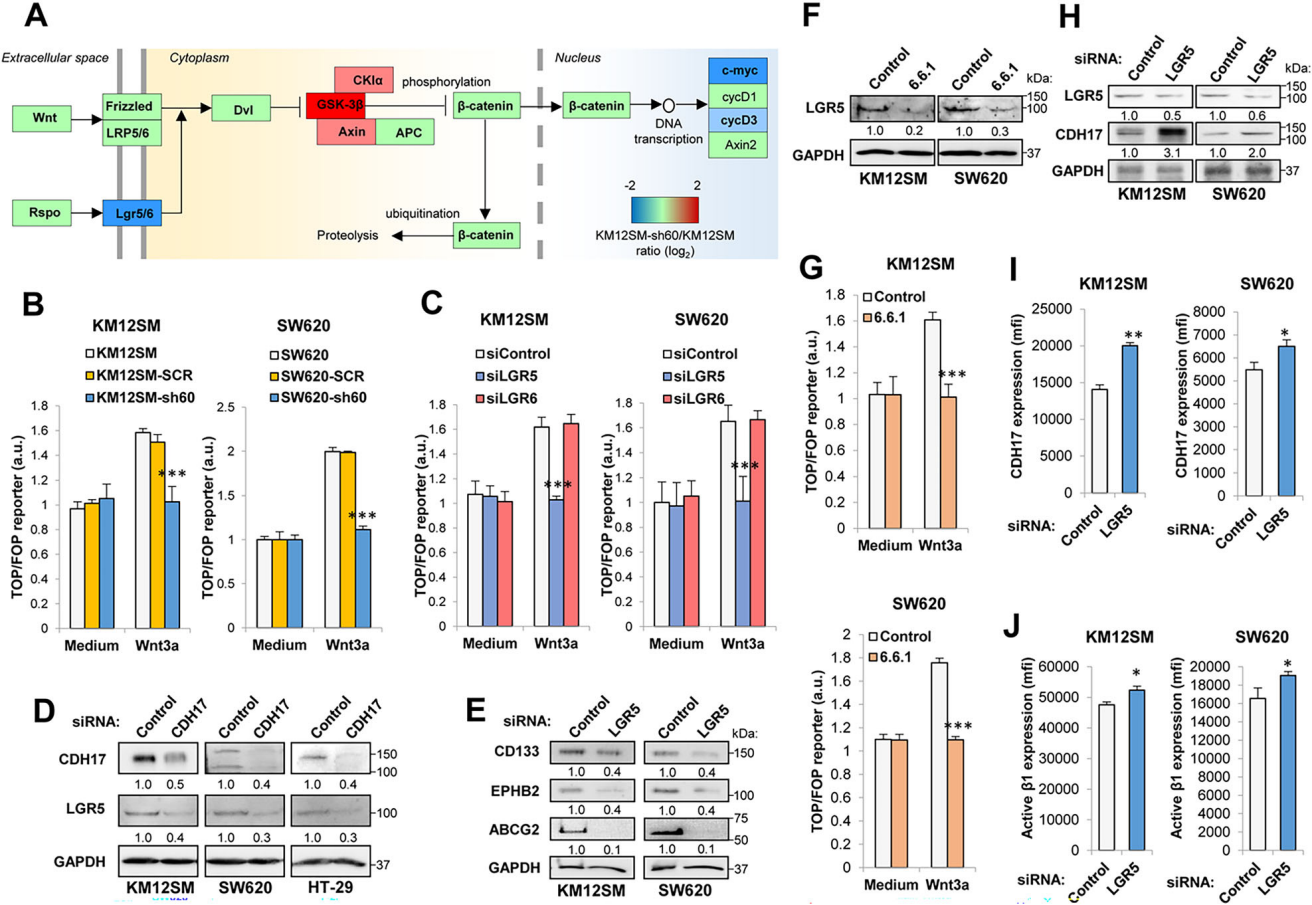
CDH17 regulates Wnt/β-catenin signaling through LGR5 expression. **A** Wnt signaling pathway representation according to KEGG (hsa04310) showing significantly-altered genes after CDH17 silencing (FDR < 0.05) in KM12SM cells. **B** TOP/FOP assays in the indicated cell lines after treatment with Wnt3a (500 ng/mL). **C** TOP/FOP assays in cells transfected with control, LGR5- or LGR6-targeting siRNAs in the presence of Wnt3a. **D** Western blot analysis of CDH17 and LGR5 using cell extracts from KM12SM and SW620 cells transfected with control, or CDH17-specific siRNAs. GAPDH was used as a loading control. Band quantification relative to control cells is shown below each lane. **E** Western blot analysis in control or LGR5-silenced cell extracts. GAPDH was used as loading control. **F** Western blot analysis of LGR5 expression in the indicated cell lines treated with control or anti-CDH17 antibody 6.6.1. **G** TOP/FOP assays in the indicated cell lines after treatment with 6.6.1 antibody and/or Wnt3a. The ratio TOP/FOP was significantly decreased in treated cells after CDH17 or LGR5 silencing or 6.6.1 treatment (***p < 0.001). **H** Western blot analysis of LGR5 and CDH17 following LGR5-silencing. GAPDH was used as loading control. Flow cytometry assays of LGR5-silenced cells to detect (**I**) CDH17 expression or (**J**) β1 integrin in high affinity conformation. LGR5 silencing increased CDH17 and active β1 integrin expression (*p < 0.05; **p < 0.01). Histograms show the average of six replicates. Error bars indicate standard deviation. Data are representative of three independent experiments.

Both cell lines showed a reduced expression of LGR5 and LGR6, which have been reported to play a role in Wnt signaling activation [30–33]. Although expression of Wnt receptors was mostly not affected after CDH17 silencing (Supplementary Table S1), KM12SM CDH17 KD cells exhibited an increased expression of genes related to the β-catenin destruction complex, including *GSK3B* (ratio 3.05; FDR 2.9E-05), *AXIN* (ratio 2.30; FDR 3.7E-03) and *CSNK1A* (ratio 1.64; FDR 0.02) (Fig. 3A, Supplementary Table S1). In addition, SW620 KD cells showed increased expression of the Wnt inhibitors *DKK4* (ratio 4.08) and *DKK1* (ratio 2.31) (Supplementary Table S1), suggesting cell-specific Wnt inhibition mechanisms. We assessed the inhibition of the Wnt pathway after CDH17 silencing using TOP/FOP assays to determine the β-catenin promoter activity in both cell lines. CDH17-silenced cells showed similar levels of basal Wnt activation to SCR and parental cells, but they failed to respond to Wnt3a activation (Fig. 3B). To further clarify the individual role of LGR5 and LGR6 down-regulation in the CDH17-mediated inhibition of Wnt activation, we carried out siRNA-silencing of LGR5 and LGR6 expression (Supplementary Fig. S3A). TOP/FOP assays revealed that LGR5 silencing, but not LGR6, inhibited Wnt pathway activation in both CRC cell lines (Fig. 3C). Consistent with these findings, we observed a significant correlation between LGR5 expression levels and the expression of β-catenin and related Wnt signaling target genes (*MYC, AXIN2, CCND1*) in CRC datasets (Supplementary Fig. S3B), whereas LGR6 expression exhibited only a slight correlation with Cyclin D1. We identified a direct regulation of LGR5 expression following siRNA-mediated silencing of CDH17 in three different cell lines (Fig. 3D). In turn, siRNA LGR5 silencing led to the reduced expression of stem cell markers CD133, EPHB2 and ABCG2, replicating CDH17 downregulation (Fig. 3E). Notably, cell treatment with the Wnt inhibitor ICG-001 did not cause LGR5 inhibition, but reduced the expression of EPHB2, CD133 or ABCG2 (Supplementary Fig. S4A, B), confirming the role of CDH17 and LGR5 as upstream positive regulators of the Wnt signaling pathway.

In addition, given the capacity of CDH17-specific antibodies to block α2β1 integrin activation and liver metastasis progression [21], we investigated whether the treatment with the anti-CDH17 antibody 6.6.1 might affect LGR5 and Wnt signaling. Metastatic cells treated with the 6.6.1 antibody revealed a significant down-regulation of LGR5 (Fig. 3F), together with a clear decline of Wnt activation using a TOP/FOP assay (Fig. 3G). These findings support that integrin signaling activation induced by CDH17 participates in the regulation of LGR5 expression and the subsequent Wnt signaling activation. Interestingly, specific siRNA-silencing of LGR5 caused the up-regulation of CDH17 expression (Fig. 3H, I), which was accompanied by an increase in β1 integrin activation (Fig. 3J), suggesting a negative feedback loop. Collectively, these results indicate that the loss of CDH17 causes the reduction of LGR5 expression, which drives the inhibition of Wnt/β-catenin signaling and, in turn, a reduction of MYC expression and stemness characteristics. This mechanism is mirrored by the treatment with anti-CDH17 RGD 6.6.1 antibody, which inhibits liver metastasis [21].

### CDH17 silencing attenuates drug resistance in colorectal cancer cells

A significant downregulation in the expression of various ABC and SLC transporters involved in drug efflux [34] and drug uptake [4], respectively (Supplementary Table S1), including *ABCC2* (ratio 0.26; FDR 0.01), *ABCE1* (ratio 0.20; FDR 2.2E-05) and *ABCG2* (ratio 0.38; FDR 2.0E-04) was observed in KM12SM CDH17 KD cells. Likewise, other transporters associated with drug-resistance, such as *SLC6A20* (ratio 0.08; FDR 9.3E-10), *SLC27A2* (ratio 0.02; FDR 4.9E-07), and *SLC38A5* (ratio 0.01; FDR 2.6E-16), exhibited reduced expression in KM12SM KD cells. Although the alterations in SW620 KD cells were less pronounced, there were still significant reductions in *ABCC2* (ratio 0.08, FDR 5.3E-05), *SLC22A3* (ratio 0.07; FDR 2.6E-07), and *SLC38A5* (ratio 0.30; FDR 1.3E-06). Many of these transporters are MYC targets showing a good agreement with GSEA analysis and the Wnt/β-catenin signaling inhibition after CDH17 silencing.

**Fig. 4:**
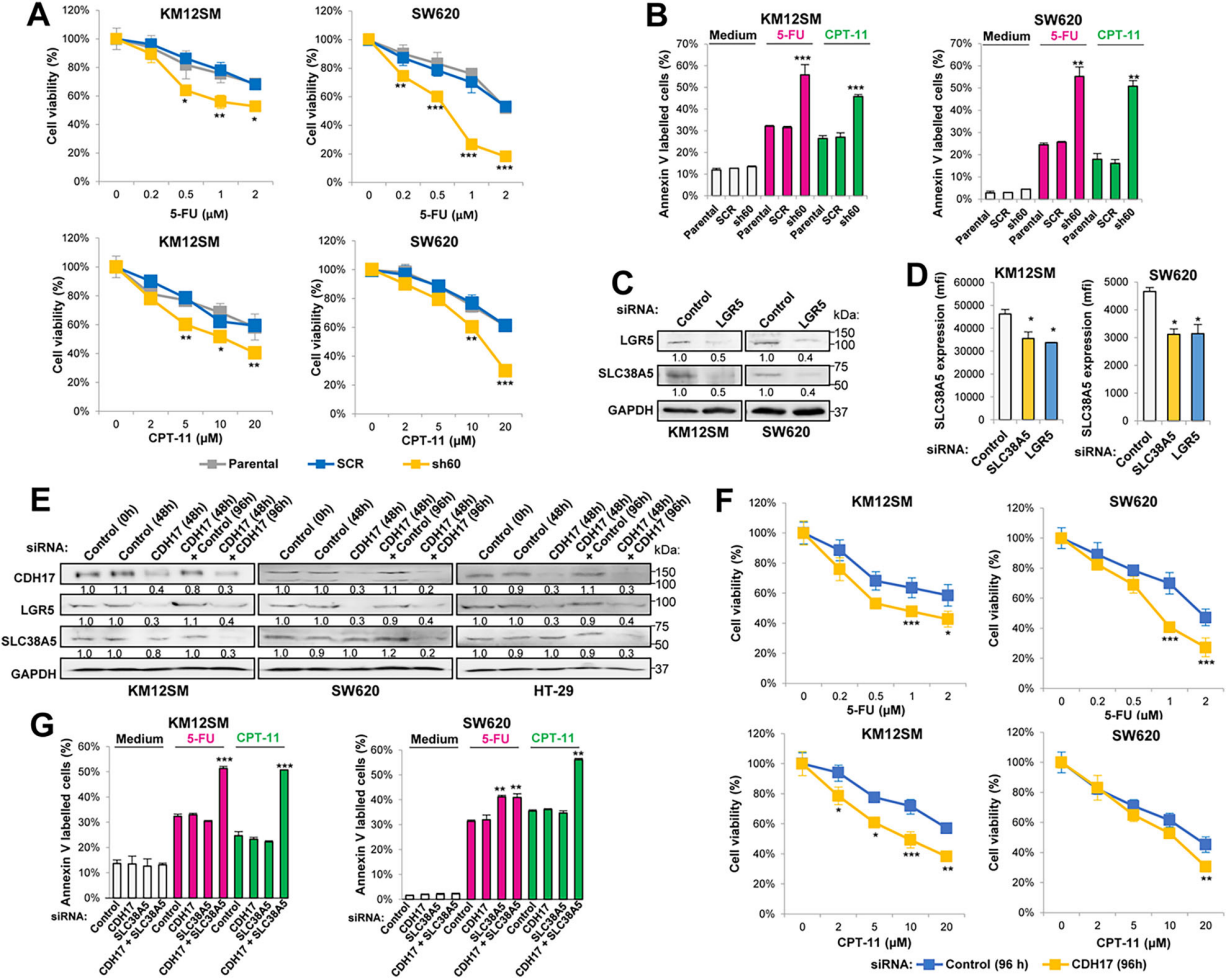
SLC38A5 and CDH17 participate in drug resistance in colon cancer. **A** Cell viability assays were performed on the indicated cell lines treated with different concentrations of 5-FU or CPT-11 for 48 h. **B** Apoptosis assays were conducted on the same cell lines treated with 5-FU (2 μM) or CPT-11 (20 μM) for 48 h. Analysis of SLC38A5 expression in LGR5-silenced cells by Western blot (**C**) and flow cytometry (**D**). SLC38A5 expression in the cell surface was significantly inhibited (*p < 0.05) following SLC38A5 or LGR5 silencing. **E** Western blot analysis of LGR5 and SLC38A5 in cell lines transfected with control or CDH17-targeting siRNAs and re-transfected after 48 h. Cell lysates were taken at 0, 48 and 96 h from the first transfection. Band quantification relative to control cells is shown. Cell viability (**F**) and apoptosis detection (**G**) assays were performed on KM12SM and SW620 cells previously transfected twice in 48 h with control or CDH17-targeting siRNAs and incubated for 48 h more in the presence of the indicated drugs. Stable or extended CDH17 silencing resulted in an increased percentage of apoptotic cells or a reduction in the percentage of viable cells following siRNA treatment (*p < 0.05;**p < 0.01;***p < 0.001). Data are representative of three independent experiments.

To explore the involvement of CDH17 in drug resistance, we first investigated the sensitivity of stably-silenced CDH17-sh60 KM12SM and SW620 KD cells, compared to SCR and parental cells, to treatment with 5-fluorouracil (5-FU) and irinotecan (CPT-11). Whereas parental and SCR cells exhibited similar sensitivity to these drugs after 48 h, CDH17-sh60 KD cells were significantly more sensitive (Fig. 4A). Treatment with 5-FU and irinotecan significantly increased the number of apoptotic cells in CDH17-sh60 KD cells compared to parental and SCR cells, as determined by Annexin V labeling (Fig. 4B), without affecting the necrosis rate (data not shown). Next, we examined whether LGR5 mediates the effects of CDH17 on drug resistance. Silencing LGR5 in both cell lines caused a similar decrease in cell viability after treatment with irinotecan, as well as increased sensitivity to 5-FU in SW620 cells (Supplementary Fig. S5A). This heightened irinotecan sensitivity might be explained by the downregulation of ABCG2 (Fig. 3E), a well-known mediator of irinotecan resistance [35], and the reduced expression (Fig. 4C**)** and membrane accessibility of SLC38A5 **(**Fig. 4D) after siRNA silencing of LRG5 and SLC38A5. In contrast, SLC38A5 silencing did not cause any alteration in CDH17 expression (Supplementary Fig. S5B). Therefore, CDH17 KD cells exhibited increased sensitivity to drug treatments, a process driven by CDH17-mediated LGR5 downregulation.

### SLC38A5 mediates drug resistance associated with CDH17

To further explore the mechanisms underlying drug resistance, we focused on SLC38A5, a MYC-regulated [36] Gln-transporter [4], which was the most down-regulated gene following CDH17 depletion. First, we assessed the individual and combined contributions of CDH17 and SLC38A5 to 5-FU and CPT-11 resistance in CRC using single and combined silencing with two different siRNAs (Supplementary Fig. S6A, B). SLC38A5 silencing significantly reduced SW620 cell viability following 5-FU treatment compared to non-treated cells, while KM12SM required double silencing for a comparable effect (Supplementary Fig. S6C, D). Both cell lines showed increased sensitivity to CPT-11 only after double silencing. These results appear to contradict those obtained with stably CDH17-silenced cells, which exhibited increased sensibility to these drugs, However, since SLC38A5 is not a direct target of CDH17, but a downstream target of Wnt signaling and MYC, we hypothesized that effective inhibition of SLC38A5 expression might require a CDH17-silencing time longer than the 48 h siRNA transfection used in this experiment. Therefore, to determine whether extended siRNA inhibition (96 h) would replicate the effects obtained with stable CDH17-sh60 KD and double siRNA silencing, we performed two consecutive 48 h siRNA CDH17 transfections in three different cell lines (Fig. 4E). We found that effective SLC38A5 silencing was only achieved after 96 h. These cells exhibited reduced viability (Fig. 4F), mirroring the effects observed in stable CDH17-sh60 KD cells (Fig. 4A) and doubly-silenced cells with two different siRNAs (Supplementary Fig. S6C, D). This reduction in cell viability was associated with enhanced apoptosis (Fig. 4G), without affecting the number of necrotic cells (data not shown). Collectively, these findings support that CDH17 requires SLC38A5 for mediating drug resistance to 5-FU and irinotecan.

### SLC38A5 modulates apoptosis and anoikis in colorectal cancer cells

**Fig. 5:**
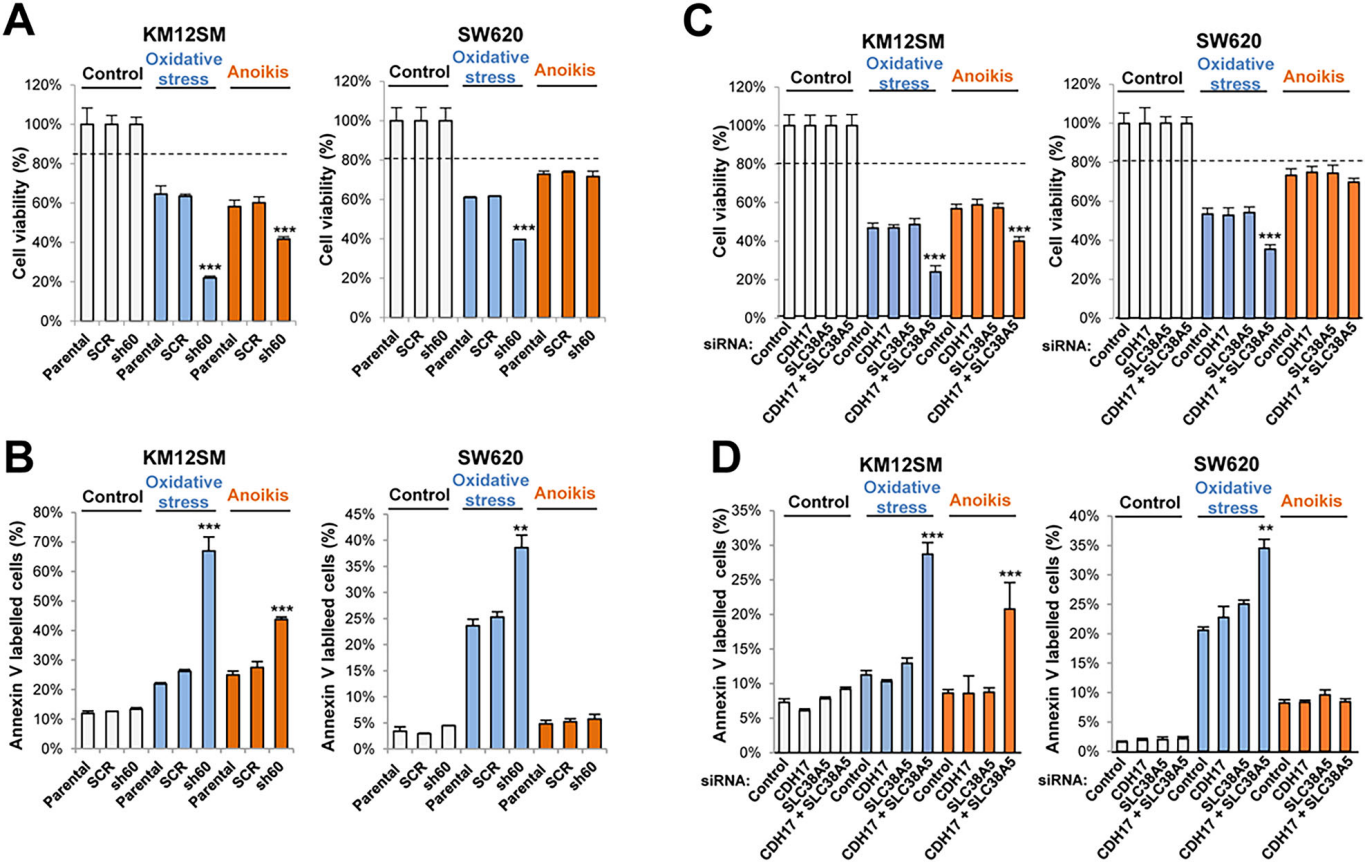
SLC38A5 and CDH17 collaborate to promote cell survival. **A** Cell viability and **B** apoptosis detection assays were performed on the indicated cell lines in the presence or absence of 1 mM H_2_O_2_ or under detachment conditions (anoikis) for 16 h. KM12SM and SW620 cells transfected with control, CDH17 and/or SLC38A5-targeting siRNAs were incubated with 1 mM H_2_O_2_ or in detachment conditions for 16 h and analyzed for cell viability (**C**) or apoptosis detection (**D**). Stable CDH17 silencing (sh60) or transient silencing of SLC38A5 combined with CDH17 led to a significant increment in the percentage of apoptotic cells or a reduction in the percentage of viable cells (**p < 0.01; ***p < 0.001). Data are representative of three independent experiments.

KM12SM-sh60 CDH17 KD cells exhibited lower viability (Fig. 5A) and increased apoptosis (Fig. 5B) after oxidative stress induced by hydrogen peroxide. To note that SW620-sh60 CDH17 KD cells were more resistant to anoikis than KM12SM-sh60 cells, in both analyses. As above, we assessed the individual contributions of CDH17 and SLC38A5 to these effects using specific siRNAs. A lower cell viability following oxidative stress and anoikis was exclusively observed in doubly-silenced cells for CDH17 and SLC38A5, a combination that replicates CDH17-sh60 KD cells gene profile (Fig. 5C, Supplementary Fig. S7). Again, SW620 cells were more resistant to anoikis. Consistent results were observed after quantifying apoptosis in both cell lines (Fig. 5D). Together, these results support the relevance of SLC38A5 in mechanisms of survival to stress and anoikis promoted by CDH17.

### SLC38A5 inhibition increases cell sensitivity to chemotherapy

**Fig. 6:**
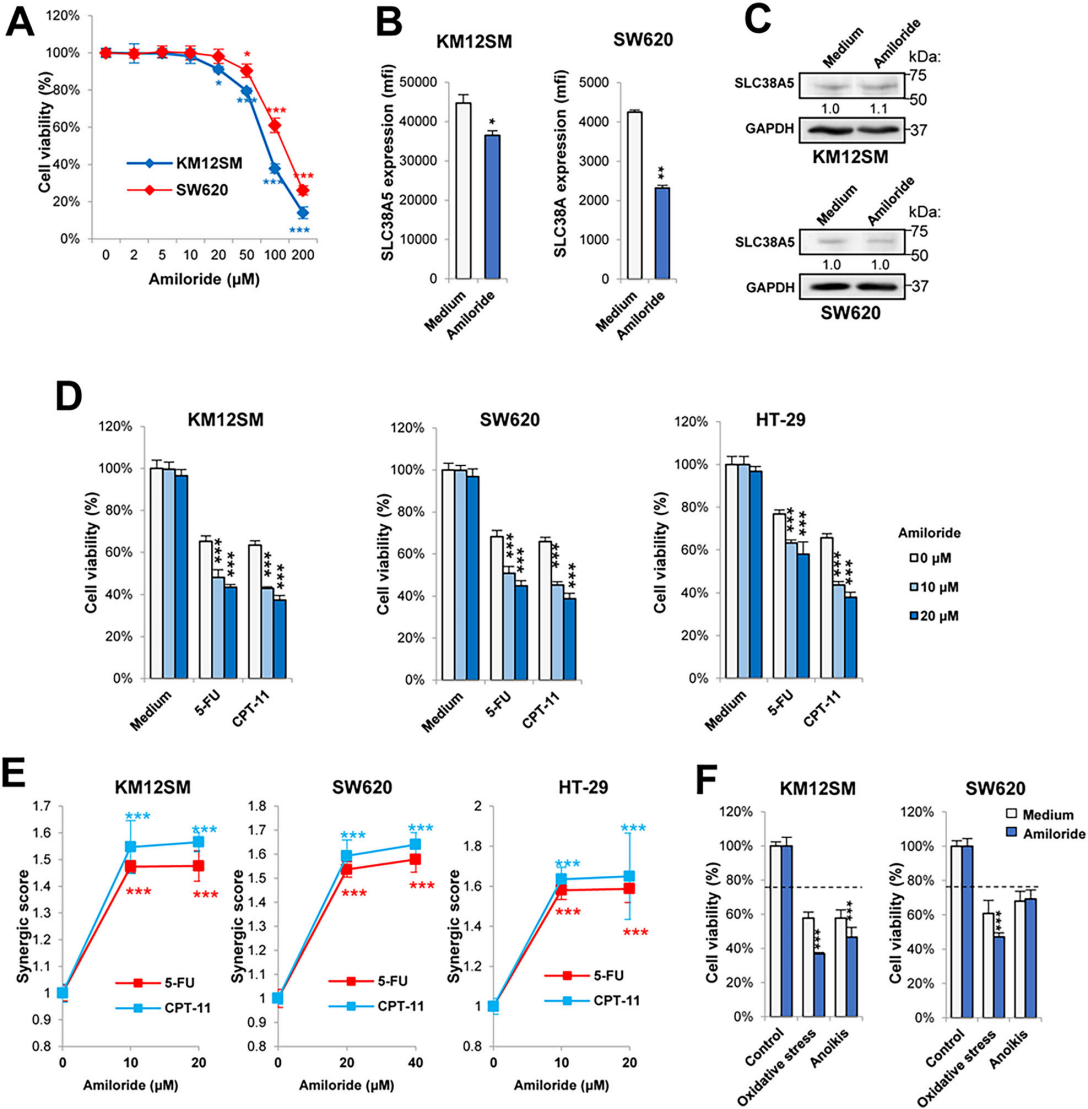
Amiloride reduces drug resistance in colon cancer cells. **A** Cell viability of KM12SM and SW620 cells incubated with increasing concentrations of amiloride. **B** Flow cytometry and (**C**) Western blot analysis of SLC38A5 expression in the indicated cells treated with amiloride (10–20 μM). Amiloride caused a significant reduction of SLC38A5 expression in cell membranes (*p < 0.05; **p < 0.01). **D** The indicated cell lines were subjected to cell viability assays following treatment with amiloride (10–20 μM), 5-FU (2 μM), and/or CPT-11 (20 μM) for 48 h. **E** Synergic scores of amiloride plus 5-FU or CPT-11 were significantly greater than 1 (***, p < 0.001), indicating synergy between both agents in cell death promotion. **F** Cell viability of KM12SM and SW620 cells exposed to amiloride (10–20 μM), 1 mM H_2_O_2_ or in detachment conditions for 16 h. Addition of amiloride reduced the percentage of viable cells (*p < 0.05; **p < 0.01; ***p < 0.001). Data are representative of three independent experiments.

SLC38A5 plays two different functions in cancer cells: as amino acid transporter and as amino acid-dependent Na^+^/H^+^ exchanger. The Na^+^/H^+^ exchange activity facilitates the uptake of extracellular proteins into the cells by macropinocytosis [37]. The upregulation of this process renders cells sensitive to amiloride, an inhibitor of SLC38A5 [37]. Therefore, we investigated the impact of amiloride, a drug used in hypertension [37], on cell viability and liver metastasis. A dose-response curve indicated that concentrations of amiloride exceeding 10-20 µM reduced cell viability after 16 h (Fig. 6A) and surface expression of SLC38A5 (Fig. 6B), suggesting a reduced functional activity for SLC38A5, while total SLC38A5 remained unchanged (Fig. 6C). Treatment with 10–20 µM amiloride further reduced cell viability in a similar way when combined with 5-FU and irinotecan in three cell lines (Fig. 6D). Furthermore, we have calculated the synergy score for each cell line and chemotherapeutic agent combination. Our results showed synergistic effects for both, 5-FU and CPT-11, in the three cell lines tested, as the synergy scores were between 1.47 and 1.65 (Fig. 6E), supporting an enhanced effect of chemotherapy in the presence of amiloride. Additionally, amiloride enhanced the sensitivity to oxidative stress in both cell lines, and to anoikis specifically in KM12SM, as SW620 exhibited resistance to anoikis (Fig. 6F).

### SLC38A5 inhibition displays anti-metastatic activity in mouse models

**Fig. 7:**
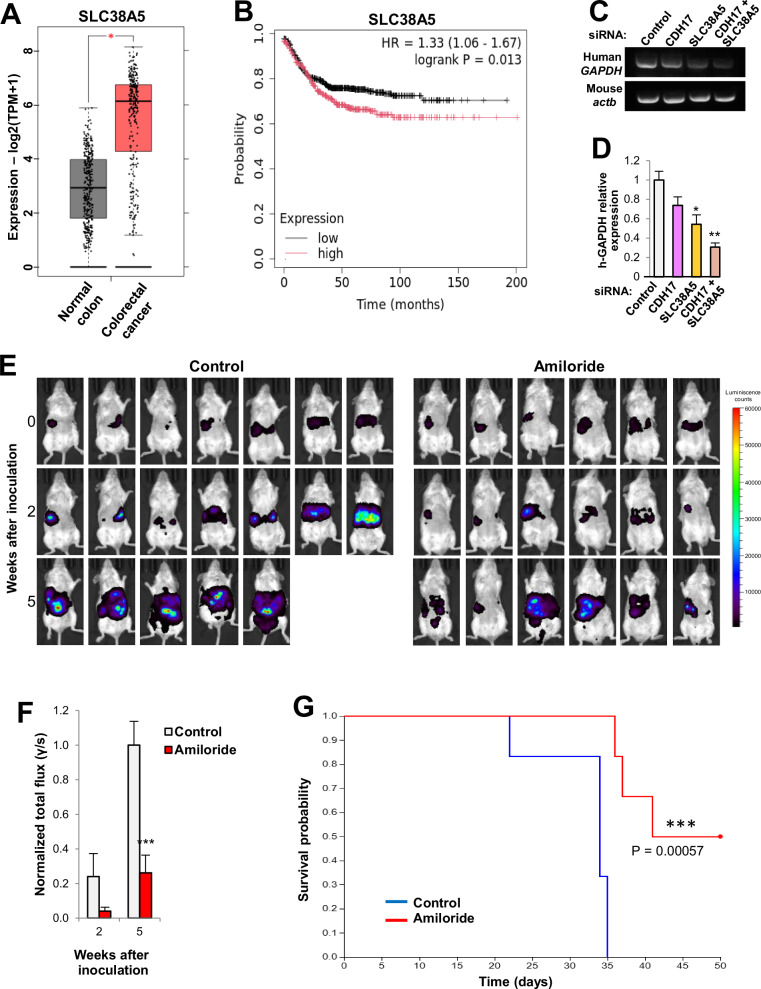
Inhibition of SLC38A5 reduces liver metastasis of colorectal cancer cells. **A** SLC38A5 mRNA expression in colorectal cancer or normal tissue was analyzed using TCGA colorectal cancer database according to the GEPIA2 website. SLC38A5 expression was significantly higher in tumors than normal tissues (*p < 0.05). **B** High SLC38A5 expression associates with poor colorectal cancer patient survival using the Kaplan-Meier Plotter website, which includes 1336 colorectal cancer patients. Log-rank test and Hazard Ratio values are shown inside the panel. KM12SM cells transfected with the indicated siRNAs were inoculated into the spleen of mice. After 72 h, mRNA from livers was isolated and subjected to (**C**) RT-PCR or (**D**) qPCR to detect human GAPDH, as a surrogate for liver colonization. Murine β-actin was used as loading control. Transient silencing of SLC38A5 combined or not with CDH17 silencing caused a significant reduction in liver colonization (*p < 0.05; **p < 0.01). Data are representative of three independent experiments. **E** Luminescence images of liver metastasis taken after inoculation of KM12SM GFP-Luc cells in the spleen of SCID mice (*n* = 7) treated intraperitoneally with amiloride (5 mg/kg) or vehicle for 4 weeks. Images were taken at the indicated times after inoculation. **F** Photon flux determinations were normalized and represented as mean ± SEM. Amiloride treatment significantly reduces the photon flux (***p < 0.001), indicating a reduction in tumor volume, compared to control mice. **G** Amiloride treatment significantly increased the survival of mice according to the log-rank test (***p < 0.001).

To assess the clinical relevance of our findings in CRC, we analyzed SLC38A5 expression in a dataset containing 624 samples of CRC patients. An increased expression of SLC38A5 in cancer tissues compared to normal mucosa was detected (Fig. 7A). Moreover, high SLC38A5 expression correlated significantly with poor survival outcomes in CRC patients **(**Fig. 7B). This led us to explore the potential therapeutic benefits of inhibiting SLC38A5. We first evaluated the liver homing capacity of metastatic KM12SM cells transfected with siRNAs targeting CDH17, SLC38A5 and both CDH17/SLC38A5, compared to cells transfected with scrambled/control siRNA. Notably, SLC38A5 inhibition alone and, more effectively, combined CDH17/SLC38A5 silencing significantly reduced liver homing, which was associated with decreased adhesion capacity as confirmed by semi-quantitative PCR (Fig. 7C) and quantitative PCR (Fig. 7D). Finally, we tested the efficacy of amiloride in preventing liver metastasis in mice inoculated with KM12SM GFP-Luc cells. Amiloride-treated mice showed a marked reduction of bioluminescence after 2 and 5 weeks, following intraperitoneal treatment (Fig. 7E, F). Kaplan-Meier analysis revealed a significant increase in survival among amiloride-treated mice compared to vehicle-treated controls, with 50% of treated mice remaining alive at the experimental endpoint (Fig. 7G). In summary, inhibiting SLC38A5 activity impaired liver homing and significantly improved survival in CRC metastasis models.

## Discussion

**Fig. 8:**
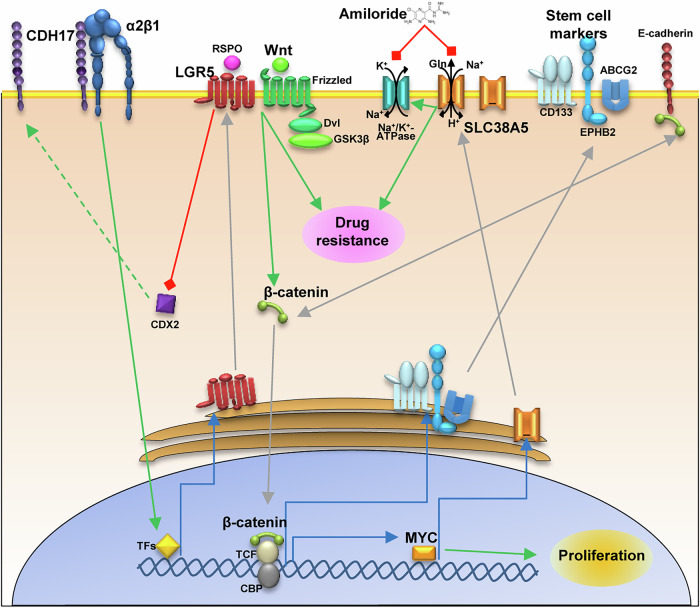
CDH17-mediated regulation of LGR5 expression, Wnt signaling and drug resistance. The interaction of CDH17 with α2β1 integrin regulates the expression of LGR5, which in turn enhances the Wnt/β-catenin signaling. This pathway drives cell proliferation and upregulates stem cell markers, as well as proteins involved in drug resistance, such as SLC38A5, through MYC activation. Any disruption of CDH17 expression or its interaction with integrins would destabilize this regulatory cascade. Moreover, amiloride, an inhibitor of SLC38A5, increases tumor cell susceptibility to chemotherapy agents and, likely, intracellular Ca^2+^ alterations that impact metastatic progression.

In this study, we found that CDH17 silencing resulted in the downregulation of the intestinal stem cell marker LGR5, inhibition of Wnt signaling and subsequent repression of MYC, as evidenced by the decreased expression of MYC target genes revealed by transcriptomic analysis. Furthermore, disruption of the CDH17/α2β1 integrin interaction similarly reduced LGR5 expression and inhibited Wnt activity. As a downstream effect of MYC suppression, CDH17 silencing also repressed several ABC and SLC transporter genes, such as SLC38A5, which are implicated in drug resistance. This led to enhanced sensitivity of CDH17-silenced cancer cells to 5-FU and irinotecan treatments. The effects of CDH17 required SLC38A5 silencing for increased cellular apoptosis, following treatment with 5-FU, irinotecan, oxidative stress and anoikis. Amiloride, an SLC38A5 inhibitor, increased cell sensitivity to chemotherapeutic drugs and liver metastasis survival in a mouse model. In summary, tissue-specific CDH17 works as a regulator of CRC SC biology by modulating LGR5 expression, Wnt signaling, MYC expression, and, therefore, stemness, and drug resistance capacities through SLC38A5 in metastatic cells (Fig. 8).

Development, organization and homeostasis of tissues and organs depend on the formation of specific cell-cell junctions [12]. Similar to embryonic development, CSCs require specific cell-cell junctions for a proper homeostasis, self-renewal and pluripotency maintenance. In the case of intestinal CSCs, we hypothesized that CDH17 is an essential cadherin for the formation of homotypic junctions between disseminated CSCs. Consequently, the loss of CDH17 expression disrupts the stem cell program and impairs metastatic potential by downregulating LGR5, which in turn inhibits Wnt signaling. Wnt/β-catenin, Hedgehog, and Notch pathways signaling are interconnected and play crucial roles in regulating CSCs functions [38]. The three pathways were altered to varying extents after silencing CDH17 in both cell lines according to the GSEA analysis. In hepatocellular carcinoma, CDH17 loss was also associated with Wnt inhibition, though using distinct mechanisms [39]. In cancer cells, the interaction of CDH17 with the integrin α2β1 promotes the activation of several pathways, ultimately leading to increased cell adhesion and proliferation. Here, treatment with a CDH17-specific antibody led to reduced LGR5 expression, thereby inhibiting Wnt signaling. This inhibition likely explains the effectiveness of CDH17-integrin blocking antibodies in suppressing liver metastasis, highlighting their potential as a therapeutic strategy [21].

To identify transcription factors (TFs) involved in LGR5 regulation, we analyzed the LGR5 promotor using Catrin (Catalogue of Transcriptional Regulatory Interactors, https://ophid.utoronto.ca/Catrin). Among the highest scoring TFs, we identified three --FOXA2 (Pearson R = 0.22, *p* = 2.6E-04). HNF4A (Pearson R = 0.27, *p* = 2.6E-04) and SRF (Pearson R = 0.14, *p* = 0.022) -- whose expression significantly correlate with LGR5 levels, according to GEPIA2 web tool. The expression and/or activation of these TFs has been previously associated with integrin signaling [40, 41], supporting a potential link between CDH17-integrin interaction and LGR5 expression. This interaction induces β1 integrins into high affinity conformation for activation of the integrin signaling upon CDH17 binding. Interestingly, although many of the CDH17 KD effects were mediated by ablation of LGR5 expression, siRNA silencing of LGR5 led to an increase in CDH17 expression, in a self-regulatory loop for maintaining cell plasticity. Indeed, LGR5 KO mice have shown strong induction of Paneth cell differentiation in embryonic intestinal cells [42]. The increased expression of CDH17 after LGR5 inhibition was confirmed by the activation of its receptor, α2β1 integrin.

MYC is a transcriptional target of β-catenin that regulates the expression of various Gln transporters crucial for the well-known “glutamine addiction” in cancer cells [43, 44], including SLC38A5 [36]. MYC downregulation in CDH17 KD cells can be explained by the inhibition of Wnt/β-catenin signaling and the observed upregulation of circadian transcription factors. We hypothesized that MYC depletion in CDH17 KD cells leads to the downregulation of SLC38A5 and other transporters, as well as G2/M checkpoint regulators. Consistent with this, our transcriptomic data revealed the concurrent down-regulation of various SLC genes in CDH17 KD cells, underscoring the complexity of this regulatory network. Notably, SLC38A5 downregulation is associated with defective mitosis and an increased proportion of cells in the S and G2/M phases [37], mirroring the effects observed after knocking down CDH17 in CRC cells.

Beyond some promising approaches based on natural products [45–47], chemotherapy remains the most commonly-used strategy for advanced CRC treatment. Therefore, understanding the mechanisms of drug resistance is critical for an improved outcome of the patients. ABC transporters and SLC38A5 have been associated with drug resistance to cisplatin and gemcitabine in breast and pancreatic cancer, respectively [48, 49]. The pronounced down-regulation of ABC transporters (ABCC2, ABCG2), and SLC38A5 observed after CDH17 silencing increases drug sensitivity in CRC SCs. Our findings demonstrate that both *CDH17* and *SLC38A5* silencing are required for enhanced cancer cell sensitivity to 5-FU, irinotecan, oxidative stress, and anoikis. These results suggest that targeting CDH17 could improve chemotherapy resistance in CSCs.

As previously mentioned, *SLC38A5* primarily functions as an amino acid transporter, which also possesses a Na^+^/H^+^ exchange activity that can be inhibited by amiloride [37]. Moreover, elevated *SLC38A5* expression has been linked to hypertrophy and hyperplasia of intestinal crypts [43], where SLC38A5 may act indirectly on Na^+^/K^+^-ATPases [50], suggesting a broader role in ion homeostasis and epithelial cell function. In our experiments, pharmacological inhibition of SLC38A5 using amiloride was highly effective in reducing *SLC38A5* expression at the membrane level, significantly enhancing drug sensitivity. Notably, amiloride and its derivatives have been proposed as potential anti-tumor and anti-metastasis agents, highlighting their therapeutic relevance in cancer treatment strategies [51]. Moreover, some studies have reported an increase of intracellular Ca^2+^ activity following amiloride treatment [52], which impairs calcium efflux via the sodium-calcium exchanger, leading to intracellular calcium accumulation and subsequent cell death [53]. Similar alterations of intracellular Ca^2+^ levels, commonly observed with Na^+^/K^+^-ATPase inhibitors, such as digoxin, influence metastasis formation by disrupting the clustering of circulating tumor cells [54]. Although still hypothetical, these effects could explain the extended survival observed in metastatic mice treated with amiloride.

Collectively, our results highlight a crucial role for CDH17 in maintaining CRC stem cells, resembling the function of E-cadherin in embryonic development [12]. Loss of CDH17 inhibited LGR5 expression and Wnt/β-catenin signaling, leading to MYC down-regulation and a subsequent reduction of pluripotency and CSC properties (e.g. SOX2, CD133, EPHB2). Furthermore, the decline in stemness capacity increased drug sensitivity by downregulating key transporters such as ABCG2 and SLC38A5. SLC38A5 inhibition, either by siRNA silencing or amiloride treatment, demonstrated a metastatic suppressor effect. In summary, these findings provide deeper insights into the molecular mechanisms underlying CDH17 functions in CRC and its potential as a therapeutic target to hinder CRC metastatic progression [19, 21]. Our results underscore the critical role of tissue-specific cadherins, such as CDH17, in regulating pluripotency and stem cell characteristics, highlighting their broader significance in cancer biology.

## Materials and methods

### Cell cultures, transfection vectors and antibodies

KM12SM cells were obtained from the MD Anderson Cancer Center (Houston, TX, USA), SW620 cells were obtained from the ECACC (Germany) and HT29 cells were obtained from the ATCC (USA). Cell lines were cultured in DMEM containing 10% FBS (Invitrogen, Carlsbad, CA, USA) and antibiotics at 37 °C in a 5% CO_2_ humidified atmosphere. KM12SM, SW620 cells and HT29 were authenticated by short tandem repeat analysis and periodically tested for mycoplasma. CDH17-knocked down (KD) KM12SM-sh60 and SW620-sh60 cells, which express the CDH17-specific sh60 shRNA, and control cells, which express a scrambled shRNA, were obtained as previously described [18]. KM12SM GFP-Luc cells were obtained after lentiviral transduction, using the pFUGW-FerH-ffLuc2-eGFP plasmid (Addgene) for lentivirus production with the Lenti-X Packaging Single Shots (Takara Bio, Japan). GFP-positive KM12SM cells were selected using the FACSAria Fusion Sorter (Becton-Dickinson, USA). For transient silencing, siRNAs targeting CDH17 (#1 SASI_Hs01_00166354 and #2 5’-GCAGUUGUGUUUAUCCGCAUdTdT-3’), SLC38A5 (#1 SASI_HS01_0024688, and #2, SASI_HS01_0024689), LGR5 (SASI-HS01_0019980), LGR6 (SASI_Hs01_00189108) or scrambled siRNA (5’-AUUGUAUGCGAUCGCA-GACCdTdT-3’) were purchased from Merck (Darmstadt, Germany) and transfected in cancer cells using JetPrime (PolyPlus, Illkirch, France).

Antibodies against CDH17 (14339-1-AP) for Western blot, LGR6 (17658-1-AP), and SLC38A5 (28102-1-AP) were purchased from Proteintech (Rosemont, IL, USA). Antibodies against CDH17 (H-1) for flow cytometry, EPHB2 (2D12C6), RhoGDI (G-2), AGR3 (G-10) and ABCG2 (B-1) were from Santa Cruz Biotechnologies (Dallas, TX, USA). Blocking monoclonal antibody 6.6.1 against CDH17 [21] was a kind gift of Protein Alternatives SL (Tres Cantos, Madrid, Spain). Anti-GAPDH (ab8245) and anti-LGR5 (144-10545-20) were acquired from Abcam (Cambridge, UK) and RayBiotech (Norcross, GA, USA), respectively. Anti-β1 integrin in high affinity conformation (556078, HUTS21) and anti-α2 integrin (MA5-32306) were from BD Biosciences (Franklin Lakes, NJ, USA) and Thermo-Fisher Scientific (Waltham, MA, USA), respectively. Anti-β-actin (OTI1) was purchased from OriGene (Rockville, MD, USA). The Wnt inhibitor, ICG-001, was purchased from Selleckchem (Frankfurt am Main, Germany).

### Gene expression analysis

For global gene expression analysis, total RNA was isolated from each cell line using NucleoSpin RNA kit (Macherey-Nagel, Germany). Quality assessment of the RNA was assessed with an Agilent 2100 bio-analyzer. Samples were processed with “GeneChip® WT PLUS Reagent Kit” (Applied Biosystems, Foster City, CA, USA), hybridized with “Clariom™ S Array, human” (Applied Biosystems) and scanned with a “GeneChip® Scanner 3000 7 G” (Applied Biosystems) at the UCM genomics facility (Madrid, Spain). Raw data were processed with the RMA algorithm included in Transcriptome Analysis Console (Applied Biosystems) for normalization and gene level analysis. For each experimental condition, three independent RNA replicates were processed and analyzed. Fold-changes between experimental conditions were calculated as a ratio between the mean of the gene expression signals. Statistical analysis was performed with e-bayes limma included in Transcriptome Analysis Console (Applied Biosystems).

### In silico analysis of gene alterations

The altered genes and proteins were analyzed by systems biology in order to obtain the prediction of the enriched functions. Then, genes were ordered based on the ratio, from most overexpressed to most repressed, and the distribution of genes belonging to each category were analyzed. GSEA and KEGG analyses of transcriptomic data were carried out by EGO Genomics (Salamanca. Spain). Venn diagrams were illustrated using BioVenn. Molecular pathways were represented using KEGG templates. The expression of SLC38A5 in normal colon mucosa and CRC samples was evaluated using GEPIA2 web tool (http://gepia.cancer-pku.cn) with the TCGA CRC dataset (*n* = 624). Additionally, Kaplan-Meier analysis was performed using the KM plotter tool (http://kmplot.com) with a dataset of 1167 CRC patients. The best cut-off method was used to split the samples into high and low SLC38A5 expression groups.

### Western blot

For whole lysates, cells were treated with lysis buffer (1% Igepal, 50 mM Tris-HCl, 100 mM NaCl, 2 mM MgCl_2_, 10% glycerol, containing protease (complete mini, EDTA free (Roche Diagnostics, Mannheim, Germany)) and phosphatase (Phosphatase Inhibitor Cocktail 2 (Merck)) inhibitors. After SDS/PAGE, proteins were transferred to nitrocellulose membranes and incubated with 5% skimmed milk in PBS for blocking. Incubation with primary antibodies and, finally, with HRP-coupled secondary antibodies (Thermo-Fisher Scientific) was done. Bands were visualized using West Pico Chemiluminescent Substrate (Thermo Fisher Scientific) and quantified using Quantity One software (Bio-Rad, CA, USA). Band quantification was carried out with MultiGauge software (Fujifilm Life Sciences, MA, USA).

### Quantitative PCR

Quantitative PCR was performed as previously described [55], using CDH17 specific primers (5’-AGGGATCTCAGGAATTGAATC-3’, 5’-GAGGATCAGTTGAGTTTTCC-3’), and GAPDH amplification as a loading control.

### Flow cytometry

Colon cancer cells were incubated with primary antibodies (10 μg/mL) in incubation buffer (human gamma-globulin (Merck, 20 μg/mL) in PBS), followed by incubation with Alexa-468-conjugated secondary antibodies. After washing, mean fluorescence intensity (mfi) was evaluated in a Cytoflex-S (Beckman-Coulter, CA, USA) cytofluorometer.

### Invasion and migration assays

Invasion and migration (wound healing) were carried out as previously described [24].

### TOP/FOP assays

TOP/FOP assays were performed as previously described [55]. Briefly, cells were transfected with 8xTOPFlash or 8xFOPFlash plasmids and treated, or not, with Wnt3a (500 ng/mL, Peprotech, UK), lysed, and the luciferase activity of Firefly and Renilla were measured using the Dual-Luciferase Reporter Assay System (Promega, Madison, WI, USA). Luminescence of Firefly luciferase (encoded under a β-catenin controlled promoter) was normalized using luminescence of Renilla luciferase (under a constitutive promoter). The normalized values are represented as TOP/FOP ratios, since 8xFOPFlash vector contains a mutated promoter that represents the basal luminescence.

### Cell viability and apoptosis detection assays

For cell viability quantification, 5 × 10^3^ cells were incubated with 2% FBS in DMEM in 96-well plates in the presence or not of 5-Fluorouracil (5-FU) (Selleckhem) or irinotecan (CPT-11) (Selleckhem) for 48 h, or in detachment conditions (anoikis), 1 mM H_2_O_2_ (oxidative stress) or 10–20 µM amiloride (Selleckhem) for 16 h. Then, methyl thiazolyl blue tretazolium (MTT) bromide at 1 mg/mL (Merck) was added to the medium for 1.5 h. Optical density at 560 nm was measured for cell viability. The percentage of apoptotic cells was determined using Annexin V-FITC Kit (Miltenyi Biomedicine, Bergisch Gladbach, Germany) after 48 h exposition to the chemotherapy agents, or incubation for 16 h in detachment conditions, 1 mM H_2_O_2_ or amiloride (10–20 μM). Apoptotic cells were quantified in a Cytoflex-S cytometer. Synergy scores for amiloride combinations with chemotherapy agents were calculated by dividing the effect on cell viability of the combination by the sum of the effects of each agent when used separately.

### Metastasis experiments

The Ethics Committees of the CSIC and Community of Madrid approved all protocols used in animal experimentation (PROEX 140/18 and 190.1/20). For liver homing assays, Swiss nude mice were inoculated in the spleen with 1 × 10^6^ siRNA-transfected KM12SM cells. Then, 72 h after inoculation, mice were euthanized, and the mRNA from the liver was isolated using TriReagent (Ambion, Carlsbad, CA, USA), and retrotranscribed with M-MLV RT (Promega, Madison, WI, USA). The cDNA was subjected to 30-cycle PCR with NZY Taq II 2x Green Master Mix kit (NZYTech, Lisbon, Portugal) or to real-time qPCR using FastStart Master Mix (Roche) to amplify human GAPDH and, as a loading control, murine ACTB using specific primers. Human GAPDH amplification was used as a surrogate for mouse liver colonization.

To investigate amiloride metastasis inhibition, 5 × 10^5^ KM12SM GFP-Luc cells per mice were inoculated in the spleen of SCID mice. Spleens were removed 24 h after inoculation. Then, mice were treated with amiloride (5 mg/kg) (*n* = 6) or vehicle (*n* = 7), intraperitoneally, three times a week for 4 weeks. Metastatic progression was examined at time zero, two and five weeks after inoculation using IVIS Lumina Series III (PerkinElmer, USA) and the normalized total photon flux was analyzed with the Living Image 4.7.4 software (PerkinElmer). XenoLight D-Luciferin (3 mg) (PerkinElmer) was used as luciferase substrate. Mice were euthanized when bioluminescence reached saturation levels.

### Statistical analysis

Gene expression correlations were calculated by using the Pearson correlation coefficient and p-value. Kaplan-Meier survival analyses were assessed by the log-rank test. All other data were tested with F- test and analyzed by the Student *t*-test (for two conditions) or one-way ANOVA followed by Tukey-Kramer multiple comparison test (for more than two conditions) post-hoc analysis. Experiments were carried out in hexaplicates. In all statistical analyses, the minimum acceptable level of significance was *p* < 0.05, except for GSEA hallmarks, where enrichments were considered statistically significant with FDR *p* < 0.25 following standard practices.

## Supplementary information

Supplementary Figures

Table S1

Original WB data

## Data Availability

The datasets generated during the current study are available from the corresponding author on reasonable request.
