# Supplementary material for: Loss of cadherin 17 downregulates LGR5 expression, stem cell properties and drug resistance in metastatic colorectal cancer cells
Source: Cell Death Dis. 2025 Jul 1;16(1):475. doi: 10.1038/s41419-025-07811-w (PMC12217925; doi:10.1038/s41419-025-07811-w)
Supplement: Supplementary file 1 — Supplementary Figures [file 41419_2025_7811_MOESM1_ESM.pdf]

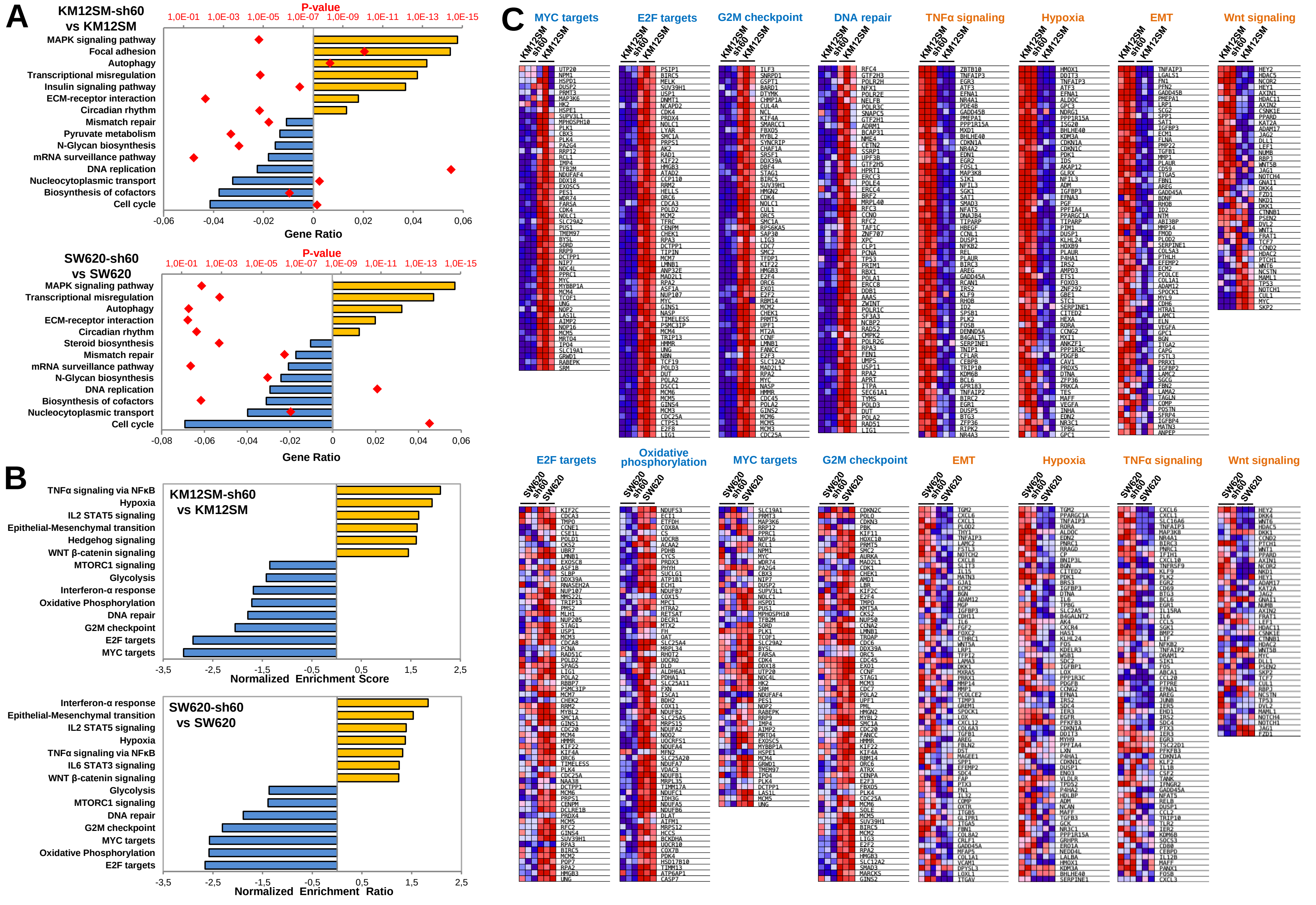

Figure S1. Transcriptomic analysis of CDH17-silenced colon cancer cells. KEGG (A) and GSEA (B) Analysis of significantly altered genes between CDH17-silenced (sh60) and control KM12SM and SW620 cells. (C) Heatmaps of selected GSEA hallmarks significantly altered in CDH17-silenced cells.

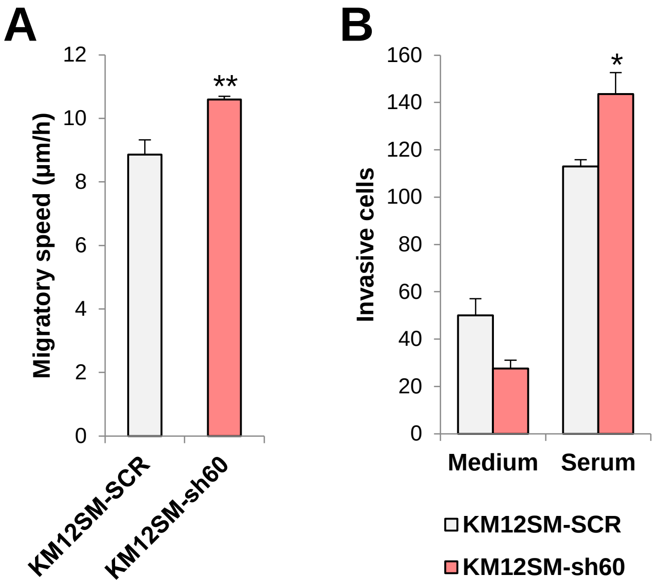

Figure S2. CDH17 silencing promotes cell migration and invasion. CDH17-silenced and control KM12SM cells were subjected to wound-healing (A) and cell invasion (B) assays. Cell migration and invasion were significantly increased after CDH17 silencing (*, $p < 0.05$; **, $p < 0.01$).

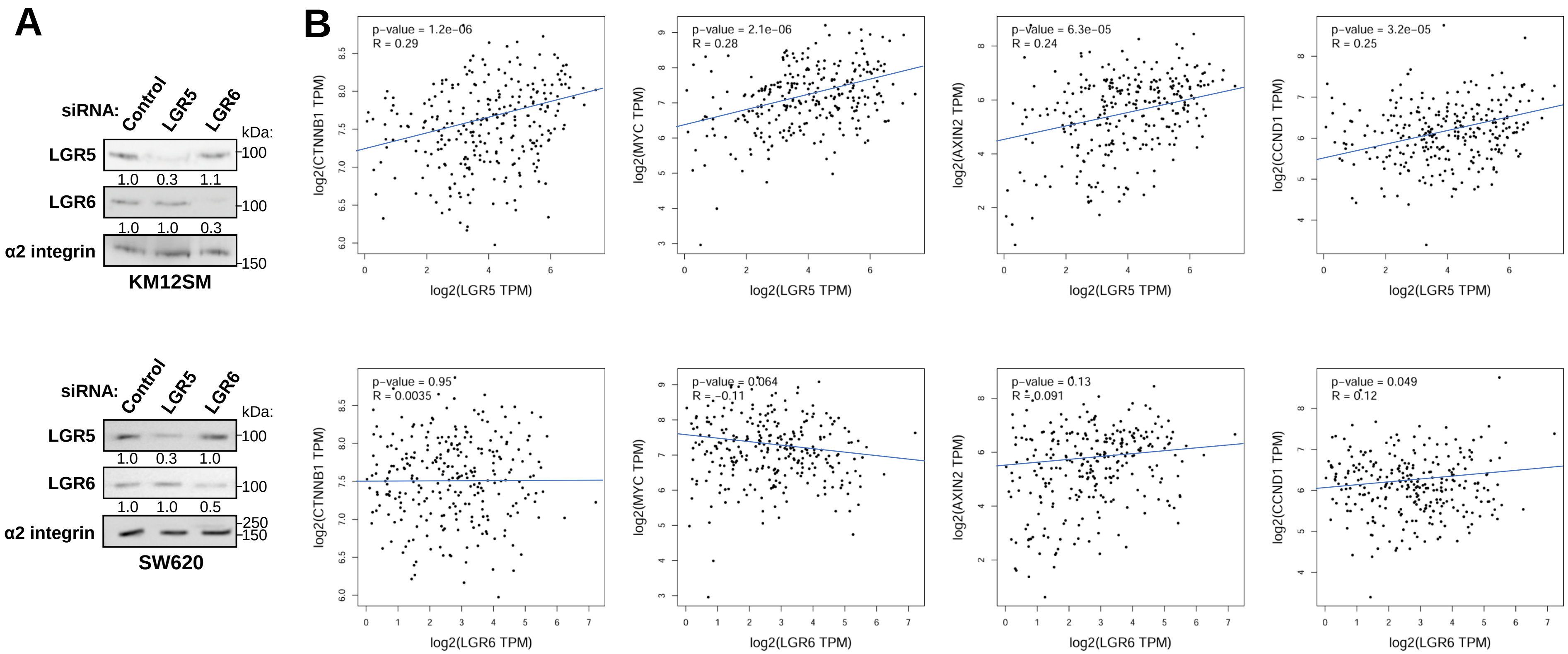

Figure S3. LGR5 correlates with Wnt signaling pathway activation. (A) KM12SM and SW620 cells were transfected with LRG5, LGR6 or control-targeting siRNAs. After 48 h, cell lysates were subjected to Western blot analysis to confirm the decrement in expression levels of these proteins. α2 integrin was used as loading control. (B) Correlations between the expression levels of LGR5 or LGR6 and β-catenin (*CTNNB1*) or β-catenin-controlled genes: c-myc (*MYC*), axin-2 (*AXIN2*) and cyclin-D1 (*CCND1*) according to the TCGA colon adenocarcinoma dataset.

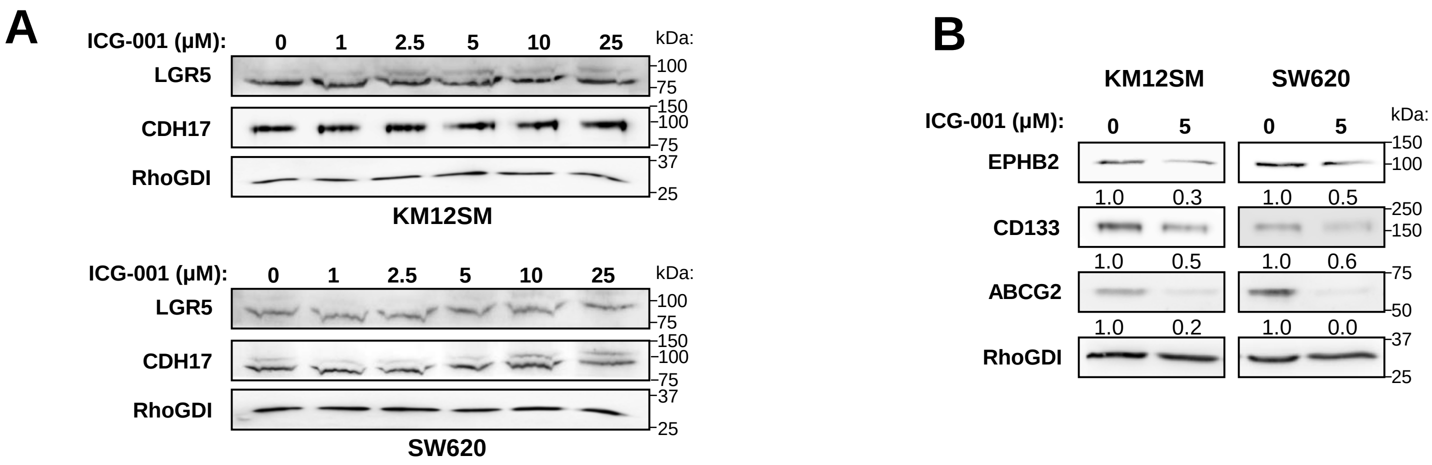

Figure S4. Inhibition of Wnt signaling pathway reduces the expression of cancer stem cell markers. (B) The same cells were treated with the indicated concentrations of the inhibitor ICG-001 for 48 h. Western blot analyses showed no alterations on LGR5 or CDH17 expression. RhoGDI was used as loading control. (B) The same cells were treated as before with or without ICG-001 (5 μM). Cell lysates were analyzed by Western blot to test the expression of the indicated cancer stem cell markers. Relative expression is shown below each band. RhoGDI was used as loading control.

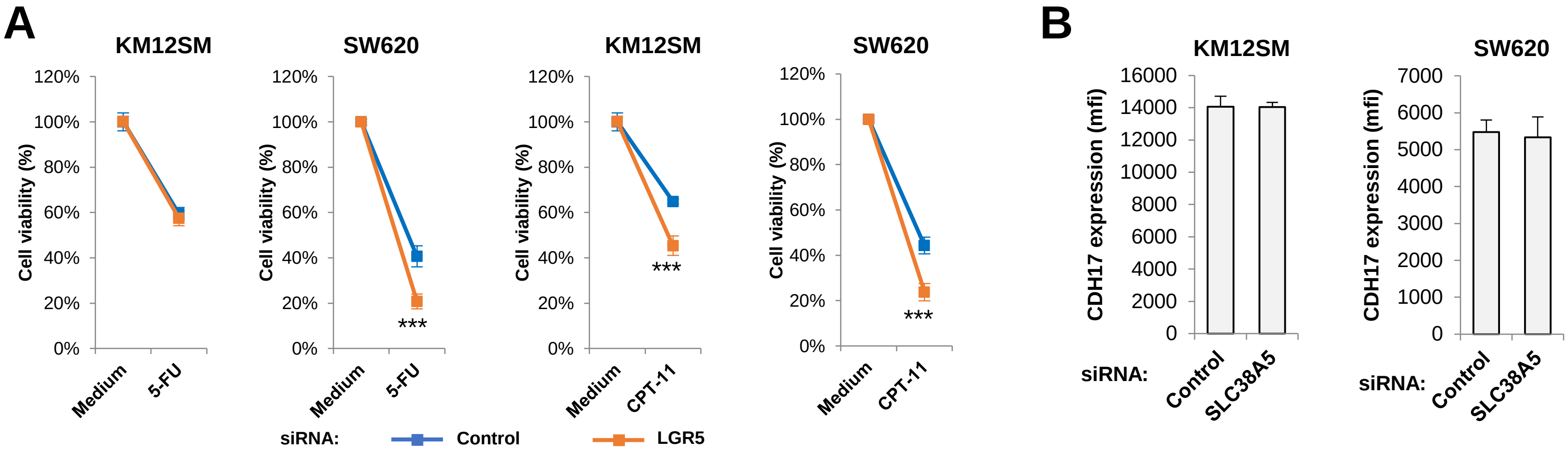

Figure S5. LGR5 knock down reduce drug resistance. (A) Cell viability assays were carried out in the indicated cell lines previously transfected with control or LGR5-targeting siRNAs and treated with 5-FU (5 μM) or CPT-11 (20 μM) for 48 h. (B) The indicated cell lines were transfected with control or SLC38A-targeting siRNAs and subjected to flow cytometry assays to detect the expression of CDH17 in the cell surface.

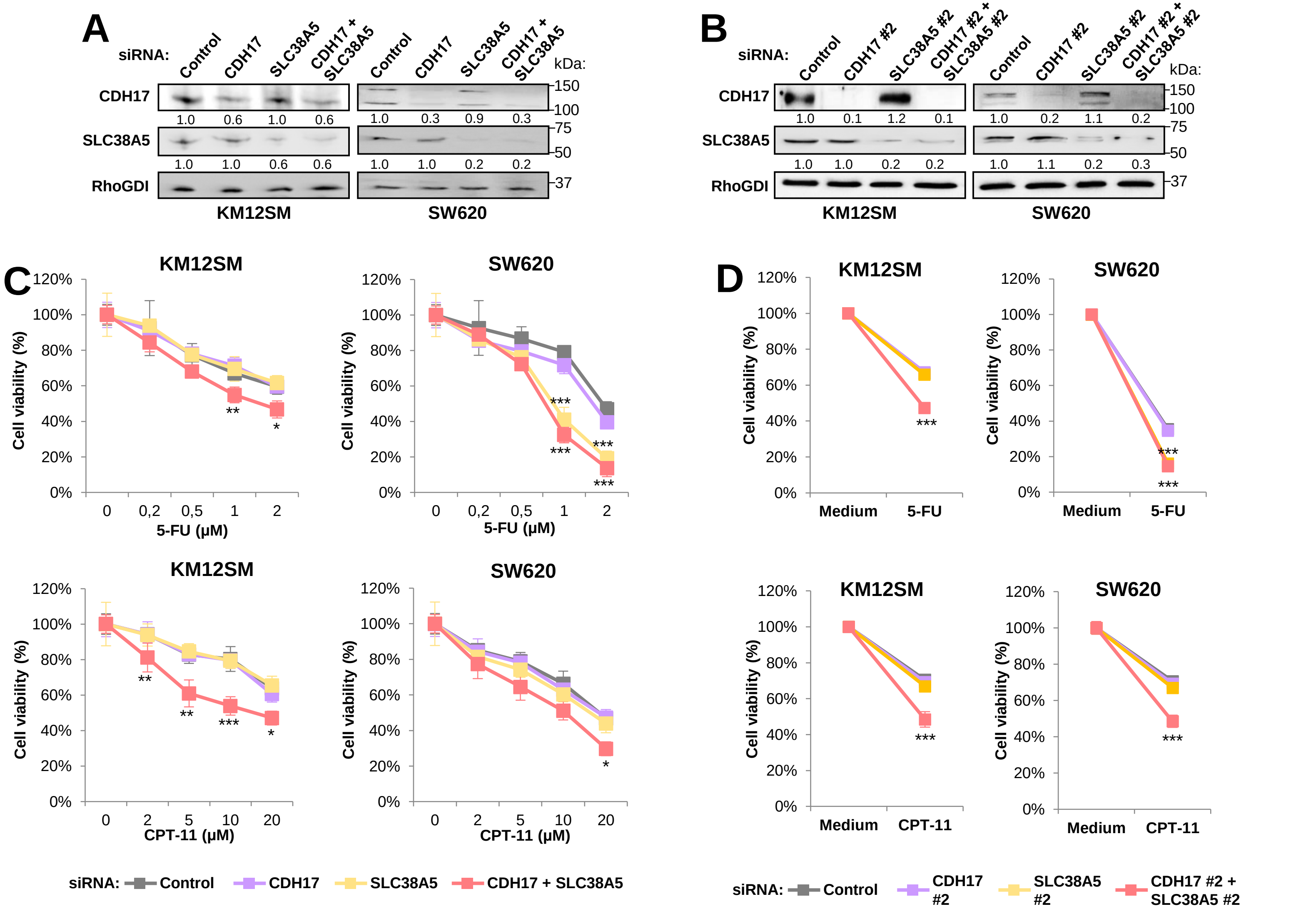

Figure S6. CDH17 and SLC38A collaborate to promote chemoresistance. (A, B) Western blot analysis of KM12SM and SW620 cells previously transfected with the indicated siRNAs. As loading control, RhoGDI was used. Band quantification is shown below each band. (C,D) The indicated transfectants were incubated with the chemotherapy drugs 5-FU (5 µM or the indicated concentrations) or CPT-11 (20 µM or the indicated concentrations) for 48 h and subjected to cell viability assays. The silencing of SLC38A alone or combined with CDH17 silencing caused a significant reduction in the percentage of viable cells (*, $p < 0.05$; **, $p < 0.001$; ***, $p < 0.001$) after exposition to the chemotherapy agents.

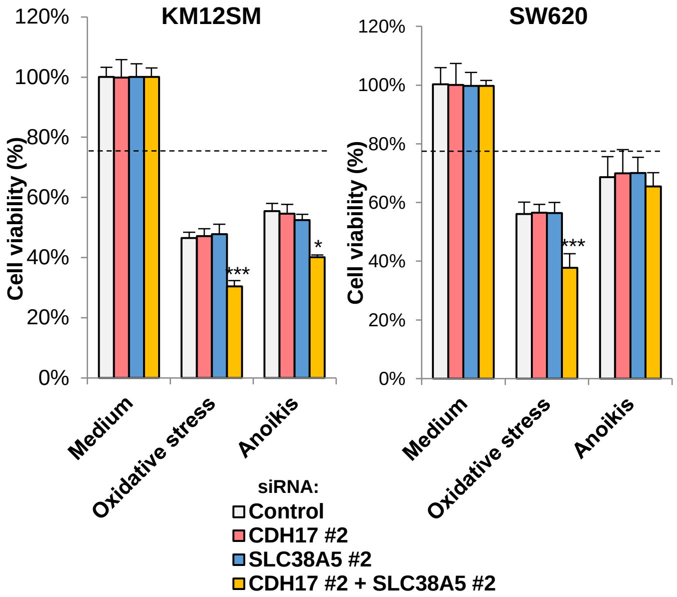

Figure S7. CDH17 and SLC38A collaborate to promote cell survival to oxidative stress and anoikis. The indicated transfectants were incubated in presence or absence of H_2O_2 (0.5 mM) or in detachment conditions for 16 h, and subjected to cell viability assays. Dashed lines indicate the input, i.e. the value of cell viability at the beginning of the assay. The simultaneous silencing of SLC38A and CDH17 caused a significant reduction in the percentage of viable cells (*, $p < 0.05$; ***, $p < 0.001$) after exposition to the different treatments.
