# Supplementary material for: Loss of cadherin 17 downregulates LGR5 expression, stem cell properties and drug resistance in metastatic colorectal cancer cells
Source: Cell Death Dis. 2025 Jul 1;16(1):475. doi: 10.1038/s41419-025-07811-w (PMC12217925; doi:10.1038/s41419-025-07811-w)
Supplement: Supplementary file 2 — Table S1 [file 41419_2025_7811_MOESM2_ESM.pdf]

**Supplementary Table S1: Transcriptomic analysis of KM12SM and SW620 cells knocked down for CDH17**

| ID | Gene Symbol | Description | Group | KM12SM-sh60 | KM12SM-SCR | KM12SM | FC KM12SM | p KM12SM | FDR KM12SM | SW620-sh60 | SW620-SCR | SW620 | FC SW620 | p SW620 | FDR SW620 |
|---|---|---|---|---|---|---|---|---|---|---|---|---|---|---|---|
| TC0800008062.hg.1 | ZBTB10 | zinc finger and BTB domain containing 10 | Coding | 11.14 | 5.86 | 4.41 | 106.15 | 1.06E-15 | 5.19E-12 | 8.85 | 8.44 | 8.53 | 1.25 | 0.1513 | 0.3893 |
| TC0X00010515.hg.1 | CAPN6 | calpain 6 | Coding | 8.82 | 2.8 | 2.79 | 65.34 | 1.21E-15 | 5.19E-12 | 3.82 | 3.47 | 3.99 | 0.89 | 0.2032 | 0.4587 |
| TC0700007034.hg.1 | CREB5 | cAMP responsive element binding protein 5 | Multiple_C | 11.82 | 5.43 | 6.21 | 48.84 | 1.22E-11 | 6.61E-09 | 7.29 | 6.85 | 7.5 | 0.86 | 0.2618 | 0.5261 |
| TC1600008301.hg.1 | CALB2 | calbindin 2 | Multiple_C | 9.99 | 5.08 | 4.44 | 46.85 | 9.94E-14 | 2.13E-10 | 4.06 | 3.48 | 3.43 | 1.55 | 0.0215 | 0.1256 |
| TC1200010968.hg.1 | DDIT3 | DNA-damage-inducible transcript 3 | Coding | 12.14 | 7.48 | 6.61 | 46.21 | 6.13E-12 | 3.99E-09 | 8.05 | 6.08 | 6.78 | 2.41 | 0.0008 | 0.0142 |
| TC0200011138.hg.1 | UGT1A1; UGT | UDP glucuronosyltransferase 1 family, polypeptide A1; UDP gluc | Multiple_C | 10.34 | 4.29 | 5.09 | 38.05 | 2.10E-14 | 5.62E-11 | 3.68 | 3.46 | 4.11 | 0.74 | 0.0792 | 0.2712 |
| TC1500007814.hg.1 | NEO1 | neogenin 1 | Multiple_C | 10.75 | 6.09 | 5.56 | 36.50 | 2.74E-12 | 2.11E-09 | 9.62 | 9.27 | 9.32 | 1.23 | 0.9245 | 0.969 |
| TC0300012048.hg.1 | ZBTB20; MIR5 | zinc finger and BTB domain containing 20; microRNA 568 | Multiple_C | 9 | 5.13 | 3.96 | 32.90 | 8.86E-10 | 1.66E-07 | 12.01 | 9.95 | 11.24 | 1.71 | 0.3289 | 0.5927 |
| TC0100017116.hg.1 | C1orf116 | chromosome 1 open reading frame 116 | Multiple_C | 10.81 | 5.44 | 5.8 | 32.22 | 2.72E-09 | 3.53E-07 | 10.87 | 10.15 | 10.11 | 1.69 | 0.0104 | 0.0804 |
| TC1200006787.hg.1 | GABARAPL1 | GABA(A) receptor-associated protein like 1 | Multiple_C | 11.02 | 6.89 | 6.19 | 28.44 | 2.76E-12 | 2.11E-09 | 9.58 | 8.04 | 7.34 | 4.72 | 1.81E-08 | 7.60E-06 |
| TC0200007132.hg.1 | YPEL5 | yippee like 5 | Multiple_C | 13.57 | 10.54 | 8.76 | 28.05 | 1.10E-12 | 1.09E-09 | 9.63 | 8.87 | 8.35 | 2.43 | 4.71E-06 | 0.0004 |
| TC1900011734.hg.1 | MIA; RAB4B; I | melanoma inhibitory activity; RAB4B, member RAS oncogene fa | Multiple_C | 9.88 | 5.28 | 5.11 | 27.28 | 3.48E-13 | 4.97E-10 | 4.77 | 4.75 | 4.42 | 1.27 | 0.2398 | 0.5033 |
| TC1200007653.hg.1 | NR4A1 | nuclear receptor subfamily 4, group A, member 1 | Multiple_C | 13.11 | 7.61 | 8.38 | 26.54 | 2.28E-13 | 3.97E-10 | 8.38 | 6.90 | 6.92 | 2.75 | 4.35E-06 | 0.0004 |
| TC1200008678.hg.1 | EID3 | EP300 interacting inhibitor of differentiation 3 | Coding | 11 | 6.2 | 6.35 | 25.11 | 8.42E-13 | 9.51E-10 | 4.5 | 3.82 | 4.02 | 1.39 | 0.1469 | 0.3831 |
| TC0500009488.hg.1 | CREBRF | CREB3 regulatory factor | Multiple_C | 10.51 | 7.2 | 5.93 | 23.92 | 2.45E-13 | 3.97E-10 | 6.4 | 5.33 | 5.53 | 1.83 | 0.0005 | 0.0106 |
| TC1100012019.hg.1 | SESN3 | sestrin 3 | Multiple_C | 7.83 | 3.64 | 3.38 | 21.86 | 3.32E-12 | 2.38E-09 | 10.48 | 10.63 | 9.62 | 1.82 | 0.0001 | 0.0038 |
| TC1700010200.hg.1 | ALDOC | aldolase C, fructose-bisphosphate | Multiple_C | 12.96 | 9.13 | 8.51 | 21.86 | 1.17E-12 | 1.09E-09 | 8.66 | 7.39 | 7.11 | 2.93 | 1.47E-06 | 0.0002 |
| TC0100011533.hg.1 | ATF3 | activating transcription factor 3 | Multiple_C | 11.71 | 6.75 | 7.35 | 20.53 | 3.19E-11 | 1.34E-08 | 7.03 | 5.71 | 6.96 | 1.05 | 0.8027 | 0.9119 |
| TC0100017984.hg.1 | AKT3 | v-akt murine thymoma viral oncogene homolog 3 | Multiple_C | 8.09 | 3.68 | 3.81 | 19.43 | 3.00E-11 | 1.30E-08 | 9.2 | 8.04 | 8.49 | 1.64 | 0.084 | 0.2789 |
| TC0400007775.hg.1 | ODAM | odontogenic, ameloblast asssociated | Multiple_C | 7.79 | 3.42 | 3.56 | 18.77 | 1.37E-12 | 1.22E-09 | 4.29 | 4.55 | 4.06 | 1.17 | 0.0908 | 0.2915 |
| TC0600009597.hg.1 | TNFAIP3 | tumor necrosis factor, alpha-induced protein 3 | Multiple_C | 10.02 | 6 | 5.95 | 16.80 | 1.22E-08 | 1.05E-06 | 10.76 | 9.07 | 8.35 | 5.31 | 8.45E-07 | 0.0001 |
| TC1900011766.hg.1 | MARK4 | MAP/microtubule affinity-regulating kinase 4 | Multiple_C | 10.07 | 6.26 | 6.05 | 16.22 | 1.58E-11 | 8.27E-09 | 8.92 | 8.56 | 8.53 | 1.31 | 0.3684 | 0.6305 |
| TC2200007312.hg.1 | LGALS1 | lectin, galactoside-binding, soluble, 1 | Multiple_C | 10.28 | 6.16 | 6.27 | 16.11 | 9.76E-11 | 3.12E-08 | 13.5 | 12.77 | 12.46 | 2.06 | 0.0017 | 0.0237 |
| TC2200009356.hg.1 | ARFGAP3 | ADP-ribosylation factor GTPase activating protein 3 | Multiple_C | 12.49 | 10.28 | 8.55 | 15.35 | 5.99E-08 | 3.32E-06 | 8.04 | 7.71 | 8.3 | 0.84 | 0.2697 | 0.5347 |
| TC0200009905.hg.1 | DHRS9 | dehydrogenase/reductase (SDR family) member 9 | Coding | 8.76 | 5.48 | 4.85 | 15.03 | 8.46E-14 | 2.02E-10 | 3.28 | 3.22 | 3.34 | 0.96 | 0.9992 | 0.9997 |
| TC1200011812.hg.1 | CRY1 | cryptochrome circadian clock 1 | Multiple_C | 9.29 | 6.04 | 5.4 | 14.83 | 2.49E-10 | 6.60E-08 | 6.98 | 5.73 | 6.59 | 1.31 | 0.1008 | 0.3105 |
| TC0200006671.hg.1 | GRHL1 | grainyhead-like transcription factor 1 | Multiple_C | 8.29 | 4.17 | 4.49 | 13.93 | 3.04E-10 | 7.08E-08 | 4.36 | 3.89 | 4.52 | 0.90 | 0.8424 | 0.9313 |
| TC0200016679.hg.1 | SERTAD2 | SERTA domain containing 2 | Multiple_C | 10.64 | 6.06 | 6.87 | 13.64 | 5.02E-11 | 1.89E-08 | 7.13 | 6.70 | 7.12 | 1.01 | 0.3191 | 0.5832 |
| TC0200016471.hg.1 | MXD1 | MAX dimerization protein 1 | Multiple_C | 12.44 | 8.14 | 8.75 | 12.91 | 2.45E-11 | 1.14E-08 | 10.46 | 9.28 | 10.31 | 1.11 | 0.9865 | 0.9943 |
| TC0100010111.hg.1 | EFNA1 | ephrin-A1 | Multiple_C | 10.2 | 7.14 | 6.53 | 12.73 | 1.14E-10 | 3.54E-08 | 5.49 | 5.21 | 4.82 | 1.59 | 0.0113 | 0.0843 |

| | | | | | | | | | | | | | | | |
|---|---|---|---|---|---|---|---|---|---|---|---|---|---|---|---|
| TC2200007204.hg.1 | HMOX1 | heme oxygenase 1 | Multiple_C | 7.88 | 4.18 | 4.21 | 12.73 | 2.59E-13 | 3.97E-10 | 3.94 | 3.58 | 3.65 | 1.22 | 0.0553 | 0.2211 |
| TC2100008297.hg.1 | SIK1 | salt-inducible kinase 1 | Multiple_C | 9.23 | 5.27 | 5.57 | 12.64 | 1.59E-09 | 2.49E-07 | 7.98 | 6.50 | 7.33 | 1.57 | 0.0117 | 0.0862 |
| TC1900010807.hg.1 | CEACAM1 | carcinoembryonic antigen-related cell adhesion molecule 1 (bili | Multiple_C | 13.3 | 8.37 | 9.67 | 12.38 | 6.52E-11 | 2.22E-08 | 7.96 | 8.20 | 8.38 | 0.75 | 0.0509 | 0.2104 |
| TC0100010360.hg.1 | HSPA6 | heat shock 70kDa protein 6 (HSP70B) | Multiple_C | 9.72 | 5.61 | 6.14 | 11.96 | 0.0003 | 0.0027 | 8.92 | 6.35 | 6.95 | 3.92 | 0.1289 | 0.3552 |
| TC0800008126.hg.1 | CA2 | carbonic anhydrase II | Multiple_C | 10.67 | 6.26 | 7.1 | 11.88 | 4.63E-11 | 1.77E-08 | 5.65 | 5.88 | 6.03 | 0.77 | 0.1511 | 0.3892 |
| TC0200011062.hg.1 | B3GNT7 | UDP-GlcNAc:betaGal beta-1,3-N-acetylglucosaminyltransferase | Multiple_C | 9.47 | 6.31 | 5.91 | 11.79 | 1.78E-11 | 8.87E-09 | 4.07 | 3.84 | 3.83 | 1.18 | 0.1422 | 0.3749 |
| TC1500010788.hg.1 | CHD2; MIR31 | chromodomain helicase DNA binding protein 2; microRNA 3175 | Multiple_C | 12.28 | 9.69 | 8.72 | 11.79 | 3.33E-11 | 1.37E-08 | 10.63 | 9.40 | 9.6 | 2.04 | 0.0003 | 0.0079 |
| TC1100008044.hg.1 | DRAP1 | DR1-associated protein 1 (negative cofactor 2 alpha) | Multiple_C | 14.58 | 11.31 | 11.03 | 11.71 | 4.09E-10 | 9.04E-08 | 10.95 | 10.66 | 10.19 | 1.69 | 0.0038 | 0.0416 |
| TC1000012536.hg.1 | MPP7 | membrane protein, palmitoylated 7 | Multiple_C | 10.19 | 7.02 | 6.65 | 11.63 | 5.81E-11 | 2.08E-08 | 8.82 | 9.65 | 9.64 | 0.57 | 0.0016 | 0.0228 |
| TC1900006588.hg.1 | GADD45B | growth arrest and DNA-damage-inducible, beta | Multiple_C | 11.22 | 7.98 | 7.69 | 11.55 | 7.94E-09 | 7.37E-07 | 9.16 | 8.27 | 8.92 | 1.18 | 0.1393 | 0.371 |
| TC0X00006799.hg.1 | SAT1 | spermidine/spermine N1-acetyltransferase 1 | Multiple_C | 16.78 | 15.28 | 13.27 | 11.39 | 4.95E-06 | 9.99E-05 | 12.18 | 11.33 | 11.41 | 1.71 | 0.0619 | 0.2363 |
| TC0700010005.hg.1 | ZFAND2A | zinc finger, AN1-type domain 2A | Multiple_C | 11.93 | 8.97 | 8.43 | 11.31 | 4.51E-07 | 1.50E-05 | 9.36 | 8.21 | 9.16 | 1.15 | 0.7221 | 0.8705 |
| TC2000009180.hg.1 | OSER1 | oxidative stress responsive serine-rich 1 | Coding | 12.85 | 10.59 | 9.35 | 11.31 | 1.53E-08 | 1.21E-06 | 9.35 | 8.89 | 9.3 | 1.04 | 0.8781 | 0.9479 |
| TC0800011064.hg.1 | CALB1 | calbindin 1 | Multiple_C | 15.03 | 12.87 | 11.55 | 11.16 | 5.05E-10 | 1.09E-07 | 11.37 | 9.98 | 11 | 1.29 | 0.9755 | 0.9902 |
| TC0200009078.hg.1 | EPB41L5 | erythrocyte membrane protein band 4.1 like 5 | Multiple_C | 11.3 | 7.89 | 7.84 | 11.00 | 5.49E-09 | 5.69E-07 | 9.14 | 8.74 | 9.25 | 0.93 | 0.3678 | 0.6301 |
| TC0200006674.hg.1 | KLF11 | Kruppel-like factor 11 | Multiple_C | 11.08 | 7.48 | 7.64 | 10.85 | 2.32E-09 | 3.19E-07 | 8.03 | 7.68 | 7.56 | 1.39 | 0.9991 | 0.9997 |
| TC0600010235.hg.1 | MLLT4 | myeloid/lymphoid or mixed-lineage leukemia; translocated to, | Multiple_C | 12.21 | 9.5 | 8.78 | 10.78 | 1.97E-08 | 1.42E-06 | 8.71 | 9.04 | 9.01 | 0.81 | 0.7862 | 0.9045 |
| TC2000007202.hg.1 | ACSS2 | acyl-CoA synthetase short-chain family member 2 | Multiple_C | 11.52 | 10.16 | 8.13 | 10.48 | 5.79E-06 | 0.0001 | 8.41 | 7.54 | 7.24 | 2.25 | 0.288 | 0.5527 |
| TC0800010506.hg.1 | PLAG1 | pleiomorphic adenoma gene 1 | Multiple_C | 7.1 | 3.67 | 3.73 | 10.34 | 2.16E-10 | 5.80E-08 | 7.27 | 6.60 | 7.19 | 1.06 | 0.7019 | 0.8589 |
| TC0100017167.hg.1 | LAMB3; MIR4 | laminin, beta 3; microRNA 4260 | Multiple_C | 12.9 | 9.97 | 9.54 | 10.27 | 2.92E-05 | 0.0004 | 7.1 | 6.33 | 7.48 | 0.77 | 0.8826 | 0.9496 |
| TC1100010207.hg.1 | SOX6; MIR60 | SRY box 6; microRNA 6073 | Multiple_C | 7.58 | 4.13 | 4.24 | 10.13 | 3.77E-08 | 2.33E-06 | 4.11 | 3.98 | 3.77 | 1.27 | 0.1224 | 0.3452 |
| TC1200009457.hg.1 | ULK1 | unc-51 like autophagy activating kinase 1 | Multiple_C | 9.63 | 6.57 | 6.31 | 9.99 | 4.55E-08 | 2.69E-06 | 7.66 | 7.51 | 7.03 | 1.55 | 0.0097 | 0.0762 |
| TC0200015402.hg.1 | FAM126B | family with sequence similarity 126, member B | Multiple_C | 12.36 | 9.17 | 9.08 | 9.71 | 7.96E-12 | 4.88E-09 | 8.36 | 7.55 | 8.58 | 0.86 | 0.2841 | 0.5495 |
| TC1200007592.hg.1 | AQP5 | aquaporin 5 | Multiple_C | 11.26 | 8.64 | 7.98 | 9.71 | 1.29E-09 | 2.19E-07 | 5.2 | 5.22 | 5.31 | 0.93 | 0.7067 | 0.8619 |
| TC1400010071.hg.1 | CLMN | calmin (calponin-like, transmembrane) | Multiple_C | 10.87 | 9.42 | 7.59 | 9.71 | 2.08E-08 | 1.46E-06 | 5.08 | 4.05 | 4.06 | 2.03 | 0.0013 | 0.0202 |
| TC1900011305.hg.1 | ZNF83 | zinc finger protein 83 | Multiple_C | 10.96 | 9.29 | 7.68 | 9.71 | 3.11E-09 | 3.84E-07 | 5.41 | 5.20 | 5.62 | 0.86 | 0.54 | 0.7594 |
| TC1200012637.hg.1 | HOXC10 | homeobox C10 | Multiple_C | 10.19 | 7.35 | 6.92 | 9.65 | 2.10E-11 | 1.02E-08 | 9.44 | 9.02 | 9.97 | 0.69 | 0.0129 | 0.0916 |
| TC0100015234.hg.1 | SLC16A4 | solute carrier family 16, member 4 | Multiple_C | 9.26 | 9.15 | 6.02 | 9.45 | 5.18E-07 | 1.66E-05 | 5.81 | 4.90 | 5.19 | 1.54 | 0.1769 | 0.4256 |
| TC1700010604.hg.1 | NR1D1 | nuclear receptor subfamily 1, group D, member 1 | Multiple_C | 10.09 | 6.16 | 6.89 | 9.19 | 6.41E-12 | 4.04E-09 | 8.52 | 6.71 | 8.35 | 1.13 | 0.2567 | 0.5215 |
| TC1100010992.hg.1 | TCN1 | transcobalamin I (vitamin B12 binding protein, R binder family) | Multiple_C | 9.07 | 4.58 | 5.88 | 9.13 | 8.38E-10 | 1.61E-07 | 4.05 | 4.58 | 3.83 | 1.16 | 0.4001 | 0.657 |
| TC1500010800.hg.1 | ALDH1A3 | aldehyde dehydrogenase 1 family, member A3 | Multiple_C | 14 | 10.74 | 10.81 | 9.13 | 2.81E-10 | 6.92E-08 | 7.25 | 6.88 | 6.68 | 1.48 | 0.0414 | 0.1872 |
| TC0200014819.hg.1 | SCN9A | sodium channel, voltage gated, type IX alpha subunit | Multiple_C | 7.15 | 3.53 | 3.97 | 9.06 | 1.39E-07 | 6.20E-06 | 4.75 | 4.29 | 3.89 | 1.82 | 0.0449 | 0.1962 |
| TC2000007246.hg.1 | DLGAP4 | discs, large (Drosophila) homolog-associated protein 4 | Multiple_C | 8.64 | 5.02 | 5.46 | 9.06 | 1.18E-09 | 2.07E-07 | 7.32 | 7.06 | 7.48 | 0.90 | 0.8488 | 0.9347 |

| | | | | | | | | | | | | | | | | |
|---|---|---|---|---|---|---|---|---|---|---|---|---|---|---|---|---|
| TC0200016464.hg.1 | APLF | aprataxin and PNKP like factor | Multiple_C | 7.9 | 4.8 | 4.73 | 9.00 | 6.70E-09 | 6.48E-07 | 7.31 | 6.53 | 6.42 | 1.85 | 0.0405 | 0.1845 |
| TC0100017118.hg.1 | YOD1 | YOD1 deubiquitinase | Multiple_C | 11.06 | 8.33 | 7.9 | 8.94 | 5.09E-09 | 5.35E-07 | 9.03 | 7.58 | 8.16 | 1.83 | 0.0523 | 0.2136 |
| TC1200007844.hg.1 | RBMS2 | RNA binding motif, single stranded interacting protein 2 | Multiple_C | 10.25 | 7.52 | 7.1 | 8.88 | 1.06E-07 | 4.99E-06 | 11.08 | 9.81 | 10.04 | 2.06 | 0.0022 | 0.0285 |
| TC1400010776.hg.1 | CATSPERB | catsper channel auxiliary subunit beta | Multiple_C | 8.45 | 5.61 | 5.31 | 8.82 | 2.37E-09 | 3.22E-07 | 4.38 | 4.63 | 4.67 | 0.82 | 0.9121 | 0.9641 |
| TC1200010292.hg.1 | AMN1 | antagonist of mitotic exit network 1 homolog | Multiple_C | 10.19 | 9.93 | 7.06 | 8.75 | 0.0002 | 0.0018 | 6 | 4.95 | 5.75 | 1.19 | 0.459 | 0.7024 |
| TC1500010744.hg.1 | GCOM1; MYZ/ | GRINL1A complex locus 1; myocardial zonula adherens protein; | Multiple_C | 13.55 | 11.86 | 10.42 | 8.75 | 3.72E-06 | 7.94E-05 | 6.44 | 6.40 | 7.18 | 0.60 | 0.1829 | 0.4337 |
| TC0100017836.hg.1 | LYST | lysosomal trafficking regulator | Multiple_C | 9.93 | 7.58 | 6.81 | 8.69 | 4.73E-07 | 1.56E-05 | 6.77 | 5.68 | 6.4 | 1.29 | 0.6156 | 0.8091 |
| TC0300014064.hg.1 | PFN2 | profilin 2 | Multiple_C | 8.73 | 5.94 | 5.61 | 8.69 | 6.15E-09 | 6.13E-07 | 10.05 | 9.04 | 9.51 | 1.45 | 0.0266 | 0.1423 |
| TC0300009282.hg.1 | TIPARP | TCDD-inducible poly(ADP-ribose) polymerase | Multiple_C | 14.62 | 12.37 | 11.51 | 8.63 | 3.30E-10 | 7.52E-08 | 12.18 | 10.77 | 12.08 | 1.07 | 0.4628 | 0.7054 |
| TC1900008496.hg.1 | PPP1R15A | protein phosphatase 1, regulatory subunit 15A | Multiple_C | 11.96 | 8.38 | 8.85 | 8.63 | 2.95E-11 | 1.30E-08 | 8.23 | 7.22 | 7.79 | 1.36 | 0.0293 | 0.1516 |
| TC1000012561.hg.1 | JMJD1C | jumonji domain containing 1C | Multiple_C | 12.41 | 9.67 | 9.32 | 8.51 | 2.99E-08 | 1.97E-06 | 12.09 | 11.27 | 11.67 | 1.34 | 0.2625 | 0.5268 |
| TC1100011259.hg.1 | FOSL1 | FOS-like antigen 1 | Multiple_C | 12.51 | 7.94 | 9.42 | 8.51 | 5.13E-08 | 2.93E-06 | 10.84 | 8.98 | 11 | 0.90 | 0.8307 | 0.9247 |
| TC0200015721.hg.1 | TMBIM1; MIR | transmembrane BAX inhibitor motif containing 1; microRNA 65: | Multiple_C | 12.29 | 9.54 | 9.22 | 8.40 | 3.36E-09 | 4.09E-07 | 7.45 | 7.47 | 7.33 | 1.09 | 0.8274 | 0.9237 |
| TC0100016018.hg.1 | CRABP2 | cellular retinoic acid binding protein 2 | Coding | 6.98 | 4.23 | 3.94 | 8.22 | 1.51E-07 | 6.57E-06 | 10.01 | 8.71 | 8.22 | 3.46 | 2.27E-05 | 0.0013 |
| TC0300006483.hg.1 | BHLHE40 | basic helix-loop-helix family, member e40 | Multiple_C | 11.43 | 7.93 | 8.39 | 8.22 | 2.05E-08 | 1.46E-06 | 13.52 | 12.28 | 12.79 | 1.66 | 0.1204 | 0.3425 |
| TC0200015397.hg.1 | CLK1 | CDC like kinase 1 | Multiple_C | 9.35 | 10.31 | 6.32 | 8.17 | 3.72E-08 | 2.31E-06 | 6.12 | 5.13 | 5.93 | 1.14 | 0.2487 | 0.5134 |
| TC1500007851.hg.1 | ARID3B | AT rich interactive domain 3B (BRIGHT-like) | Multiple_C | 8.06 | 4.62 | 5.04 | 8.11 | 1.31E-06 | 3.42E-05 | 7.31 | 6.64 | 7.24 | 1.05 | 0.7942 | 0.9085 |
| TC1600009952.hg.1 | YPEL3 | yippee like 3 | Multiple_C | 11.45 | 9.27 | 8.43 | 8.11 | 1.93E-06 | 4.61E-05 | 8.84 | 8.16 | 6.96 | 3.68 | 9.50E-05 | 0.0035 |
| TC1700010811.hg.1 | HDAC5 | histone deacetylase 5 | Multiple_C | 10.39 | 8.26 | 7.38 | 8.06 | 1.10E-07 | 5.12E-06 | 6.09 | 5.64 | 5.31 | 1.72 | 0.0124 | 0.0894 |
| TC2000009604.hg.1 | PMEPA1 | prostate transmembrane protein, androgen induced 1 | Multiple_C | 10.29 | 7.74 | 7.28 | 8.06 | 8.58E-08 | 4.31E-06 | 8.61 | 7.26 | 9.02 | 0.75 | 0.1688 | 0.4145 |
| TC0700010965.hg.1 | IGFBP3 | insulin like growth factor binding protein 3 | Multiple_C | 14.72 | 10.73 | 11.72 | 8.00 | 5.20E-08 | 2.95E-06 | 4.79 | 4.37 | 4.27 | 1.43 | 0.006 | 0.0551 |
| TC1200012308.hg.1 | NCOR2 | nuclear receptor corepressor 2 | Multiple_C | 10.69 | 7.74 | 7.69 | 8.00 | 1.35E-09 | 2.23E-07 | 9.33 | 9.35 | 9.11 | 1.16 | 0.7967 | 0.9092 |
| TC1600010006.hg.1 | PRSS8 | protease, serine, 8 | Multiple_C | 11.77 | 8.58 | 8.77 | 8.00 | 1.23E-11 | 6.61E-09 | 4.94 | 4.66 | 4.79 | 1.11 | 0.7727 | 0.8979 |
| TC0500011497.hg.1 | GLRX | glutaredoxin | Multiple_C | 13.56 | 12.38 | 10.58 | 7.89 | 1.27E-08 | 1.08E-06 | 8.39 | 8.47 | 7.95 | 1.36 | 0.0683 | 0.2489 |
| TC0700008747.hg.1 | HBP1 | HMG-box transcription factor 1 | Multiple_C | 13.98 | 13.1 | 11.03 | 7.73 | 4.41E-12 | 3.05E-09 | 10.1 | 9.12 | 9.02 | 2.11 | 7.33E-06 | 0.0006 |
| TC1100009453.hg.1 | ST3GAL4 | ST3 beta-galactoside alpha-2,3-sialyltransferase 4 | Multiple_C | 14.99 | 13.19 | 12.04 | 7.73 | 1.33E-08 | 1.11E-06 | 7.86 | 7.30 | 7.69 | 1.13 | 0.9641 | 0.9847 |
| TC0X00007310.hg.1 | TSPYL2 | TSPY-like 2 | Multiple_C | 12.96 | 10.32 | 10.03 | 7.62 | 7.97E-08 | 4.04E-06 | 12.39 | 10.84 | 11.91 | 1.39 | 0.0206 | 0.1226 |
| TC1800008285.hg.1 | NPC1 | Niemann-Pick disease, type C1 | Multiple_C | 13.75 | 12.15 | 10.82 | 7.62 | 2.71E-10 | 6.91E-08 | 9.62 | 9.30 | 9.68 | 0.96 | 0.6925 | 0.8535 |
| TC1300009810.hg.1 | ANKRD10 | ankyrin repeat domain 10 | Multiple_C | 14.4 | 12.47 | 11.48 | 7.57 | 7.93E-07 | 2.32E-05 | 11.96 | 11.56 | 10.98 | 1.97 | 0.0014 | 0.0213 |
| TC0900009860.hg.1 | AQP3 | aquaporin 3 (Gill blood group) | Multiple_C | 10.05 | 7.1 | 7.14 | 7.52 | 5.11E-08 | 2.93E-06 | 5.46 | 5.53 | 6.07 | 0.66 | 0.0186 | 0.115 |
| TC1000006911.hg.1 | TMEM236 | transmembrane protein 236 | Coding | 6.4 | 3.58 | 3.49 | 7.52 | 1.64E-08 | 1.26E-06 | 4.21 | 3.84 | 3.6 | 1.53 | 0.0631 | 0.2391 |
| TC0300009224.hg.1 | P2RY1 | purinergic receptor P2Y, G-protein coupled, 1 | Multiple_C | 7.06 | 3.37 | 4.17 | 7.41 | 6.25E-11 | 2.16E-08 | 3.57 | 3.43 | 3.43 | 1.10 | 0.4261 | 0.6785 |
| TC0900008441.hg.1 | UGCG | UDP-glucose ceramide glucosyltransferase | Multiple_C | 11.8 | 9.01 | 8.91 | 7.41 | 1.63E-08 | 1.26E-06 | 7.89 | 7.40 | 7.8 | 1.06 | 0.4482 | 0.695 |

| | | | | | | | | | | | | | | | |
|---|---|---|---|---|---|---|---|---|---|---|---|---|---|---|---|
| TC1500010783.hg.1 | SEMA4B | sema domain, immunoglobulin domain (Ig), transmembrane do | Multiple_C | 13.48 | 10.35 | 10.6 | 7.36 | 3.24E-09 | 3.98E-07 | 7.77 | 7.65 | 7.35 | 1.34 | 0.0822 | 0.2762 |
| TC1900011153.hg.1 | RRAS | related RAS viral (r-ras) oncogene homolog | Multiple_C | 8.65 | 5.78 | 5.77 | 7.36 | 4.53E-07 | 1.51E-05 | 7.51 | 7.25 | 6.7 | 1.75 | 0.2997 | 0.564 |
| TC2000008815.hg.1 | BCL2L1 | BCL2-like 1 | Multiple_C | 12.7 | 10.24 | 9.83 | 7.31 | 2.06E-09 | 2.91E-07 | 11.36 | 11.47 | 11.52 | 0.90 | 0.3078 | 0.5717 |
| TC0200015476.hg.1 | RAPH1 | Ras association (RalGDS/AF-6) and pleckstrin homology domain | Multiple_C | 10.66 | 7.52 | 7.87 | 6.92 | 1.55E-07 | 6.69E-06 | 9.03 | 8.16 | 8.89 | 1.10 | 0.2344 | 0.4969 |
| TC0900010769.hg.1 | NFIL3 | nuclear factor, interleukin 3 regulated | Coding | 12.41 | 9.41 | 9.62 | 6.92 | 5.90E-09 | 5.97E-07 | 9.02 | 7.72 | 9.05 | 0.98 | 0.9326 | 0.9717 |
| TC1600009943.hg.1 | SEZ6L2 | seizure related 6 homolog (mouse)-like 2 | Multiple_C | 7.45 | 4.78 | 4.66 | 6.92 | 3.28E-08 | 2.12E-06 | 4.17 | 4.27 | 3.91 | 1.20 | 0.1592 | 0.401 |
| TC0100013123.hg.1 | UBR4 | ubiquitin protein ligase E3 component n-recognin 4 | Multiple_C | 15.62 | 13 | 12.84 | 6.87 | 6.01E-10 | 1.28E-07 | 11.24 | 11.13 | 10.97 | 1.21 | 0.1992 | 0.4545 |
| TC0600009862.hg.1 | AKAP12 | A kinase (PRKA) anchor protein 12 | Multiple_C | 10.92 | 7.5 | 8.14 | 6.87 | 4.45E-08 | 2.64E-06 | 11.53 | 12.05 | 11.92 | 0.76 | 0.0307 | 0.1561 |
| TC1500010369.hg.1 | MFGE8 | milk fat globule-EGF factor 8 protein | Multiple_C | 11.15 | 7.9 | 8.37 | 6.87 | 8.64E-10 | 1.64E-07 | 9.97 | 9.45 | 9.2 | 1.71 | 0.0007 | 0.0133 |
| TC0200009978.hg.1 | PDK1 | pyruvate dehydrogenase kinase, isozyme 1 | Multiple_C | 10.91 | 8.51 | 8.14 | 6.82 | 1.90E-09 | 2.76E-07 | 11.44 | 11.13 | 10.3 | 2.20 | 1.35E-05 | 0.0009 |
| TC0200014719.hg.1 | BAZ2B | bromodomain adjacent to zinc finger domain 2B | Multiple_C | 10.89 | 8.49 | 8.12 | 6.82 | 6.40E-09 | 6.29E-07 | 8.71 | 8.01 | 7.69 | 2.03 | 0.0005 | 0.0107 |
| TC1200010615.hg.1 | LMBR1L | limb development membrane protein 1-like | Multiple_C | 7.67 | 5.28 | 4.9 | 6.82 | 5.75E-05 | 0.0007 | 6.01 | 5.98 | 5.97 | 1.03 | 0.4764 | 0.7154 |
| TC1400008583.hg.1 | CCNB1IP1; SN | cyclin B1 interacting protein 1, E3 ubiquitin protein ligase; small | Multiple_C | 15.07 | 14.56 | 12.31 | 6.77 | 2.17E-07 | 8.67E-06 | 12.67 | 11.70 | 12.01 | 1.58 | 0.1063 | 0.3202 |
| TC0100008625.hg.1 | PDE4B | phosphodiesterase 4B, cAMP-specific | Multiple_C | 8.94 | 5.87 | 6.2 | 6.68 | 2.72E-09 | 3.53E-07 | 3.71 | 3.76 | 3.86 | 0.90 | 0.8387 | 0.9292 |
| TC0100015023.hg.1 | DPYD | dihydropyrimidine dehydrogenase | Multiple_C | 7.37 | 4.55 | 4.63 | 6.68 | 1.91E-10 | 5.27E-08 | 5.67 | 6.60 | 4.7 | 1.96 | 0.001 | 0.0167 |
| TC1100009744.hg.1 | DUSP8 | dual specificity phosphatase 8 | Multiple_C | 5.94 | 2.8 | 3.2 | 6.68 | 4.97E-09 | 5.31E-07 | 5.26 | 4.49 | 4.58 | 1.60 | 0.0187 | 0.1153 |
| TC1600007147.hg.1 | TMEM159 | transmembrane protein 159 | Multiple_C | 11.97 | 10.14 | 9.24 | 6.63 | 2.32E-09 | 3.19E-07 | 5.44 | 4.60 | 4.68 | 1.69 | 0.013 | 0.092 |
| TC2100008562.hg.1 | RUNX1 | runt-related transcription factor 1 | Multiple_C | 12.91 | 9.05 | 10.18 | 6.63 | 1.04E-08 | 9.37E-07 | 14.83 | 13.50 | 14.06 | 1.71 | 0.0012 | 0.0192 |
| TC0300012918.hg.1 | CCNL1 | cyclin L1 | Multiple_C | 13.78 | 12.31 | 11.06 | 6.59 | 3.85E-09 | 4.40E-07 | 11.64 | 10.43 | 11.26 | 1.30 | 0.176 | 0.4244 |
| TC0600009362.hg.1 | HEY2 | hes-related family bHLH transcription factor with YRPW motif 2 | Multiple_C | 6.42 | 3.5 | 3.71 | 6.54 | 1.31E-06 | 3.43E-05 | 7.85 | 7.01 | 5.56 | 4.89 | 1.34E-07 | 3.23E-05 |
| TC0100015754.hg.1 | CTSK | cathepsin K | Multiple_C | 8.06 | 5.91 | 5.36 | 6.50 | 4.77E-07 | 1.57E-05 | 5.18 | 5.24 | 4.94 | 1.18 | 0.2394 | 0.5028 |
| TC0200015559.hg.1 | PLEKHM3 | pleckstrin homology domain containing, family M, member 3 | Multiple_C | 8.68 | 5.82 | 5.99 | 6.45 | 5.12E-09 | 5.35E-07 | 6.83 | 6.93 | 6.67 | 1.12 | 0.1726 | 0.4197 |
| TC0X00011279.hg.1 | TSPAN7 | tetraspanin 7 | Multiple_C | 7.23 | 4.27 | 4.55 | 6.41 | 5.39E-05 | 0.0006 | 6.45 | 5.04 | 4.5 | 3.86 | 0.0036 | 0.0401 |
| TC0600007862.hg.1 | PIM1 | Pim-1 proto-oncogene, serine/threonine kinase | Multiple_C | 13.82 | 12.09 | 11.15 | 6.36 | 3.27E-08 | 2.12E-06 | 4.2 | 3.98 | 4.46 | 0.84 | 0.1793 | 0.4283 |
| TC1700007787.hg.1 | WIPF2 | WAS/WASL interacting protein family, member 2 | Multiple_C | 8.2 | 5.38 | 5.54 | 6.32 | 7.10E-08 | 3.73E-06 | 6.11 | 6.14 | 6.15 | 0.97 | 0.6073 | 0.8037 |
| TC1700008342.hg.1 | PCTP | phosphatidylcholine transfer protein | Multiple_C | 11.16 | 9.28 | 8.5 | 6.32 | 9.03E-05 | 0.001 | 6.51 | 6.71 | 7.16 | 0.64 | 0.9264 | 0.9696 |
| TC1800006889.hg.1 | RIOK3 | RIO kinase 3 | Multiple_C | 13.68 | 12.13 | 11.02 | 6.32 | 3.34E-07 | 1.18E-05 | 10.44 | 9.46 | 9.6 | 1.79 | 0.0112 | 0.0835 |
| TC1800007013.hg.1 | DSG3 | desmoglein 3 | Multiple_C | 10.89 | 8.4 | 8.23 | 6.32 | 2.08E-09 | 2.92E-07 | 4.15 | 4.25 | 3.85 | 1.23 | 0.3871 | 0.646 |
| TC0800009856.hg.1 | EGR3 | early growth response 3 | Multiple_C | 6.38 | 3.5 | 3.73 | 6.28 | 6.57E-08 | 3.53E-06 | 5.56 | 5.49 | 5.32 | 1.18 | 0.2216 | 0.4819 |
| TC1600008231.hg.1 | NFAT5 | nuclear factor of activated T-cells 5, tonicity-responsive | Multiple_C | 11.87 | 8.66 | 9.22 | 6.28 | 1.23E-05 | 0.0002 | 12.82 | 11.26 | 11.92 | 1.87 | 0.0189 | 0.1163 |
| TC1200011574.hg.1 | FGD6 | FYVE, RhoGEF and PH domain containing 6 | Multiple_C | 10.99 | 8.17 | 8.37 | 6.15 | 2.88E-08 | 1.92E-06 | 7.22 | 6.58 | 7.34 | 0.92 | 0.3416 | 0.6053 |
| TC1500008187.hg.1 | AKAP13; MIR7 | A kinase (PRKA) anchor protein 13; microRNA 7706 | Multiple_C | 10.97 | 8.56 | 8.35 | 6.15 | 6.22E-10 | 1.30E-07 | 8.67 | 8.38 | 8.57 | 1.07 | 0.4824 | 0.7202 |
| TC0800009511.hg.1 | SGK223 | homolog of rat pragma of Rnd2; Tyrosine-protein kinase SgK223 | Coding | 8.49 | 5.88 | 5.88 | 6.11 | 4.80E-09 | 5.15E-07 | 6.52 | 6.72 | 6.85 | 0.80 | 0.0654 | 0.2435 |

| | | | | | | | | | | | | | | | |
|---|---|---|---|---|---|---|---|---|---|---|---|---|---|---|---|
| TC1500008521.hg.1 | MEF2A | myocyte enhancer factor 2A | Multiple_C | 13.05 | 10.4 | 10.44 | 6.11 | 2.94E-10 | 6.93E-08 | 11.53 | 10.29 | 10.61 | 1.89 | 1.09E-05 | 0.0008 |
| TC0300011853.hg.1 | SENP7 | SUMO1/sentrin specific peptidase 7 | Multiple_C | 8.2 | 7.46 | 5.6 | 6.06 | 6.50E-09 | 6.35E-07 | 6.54 | 5.62 | 6.23 | 1.24 | 0.1553 | 0.3955 |
| TC0700007345.hg.1 | STK17A | serine/threonine kinase 17a | Multiple_C | 12.22 | 10.55 | 9.62 | 6.06 | 7.68E-10 | 1.54E-07 | 10.05 | 8.56 | 9.54 | 1.42 | 0.0138 | 0.0952 |
| TC1700009850.hg.1 | PMP22 | peripheral myelin protein 22 | Multiple_C | 13.73 | 11.75 | 11.13 | 6.06 | 3.49E-06 | 7.54E-05 | 7.24 | 5.68 | 6.7 | 1.45 | 0.5412 | 0.7602 |
| TC1700010676.hg.1 | KRT15; MIR65 | keratin 15, type I; microRNA 6510 | Multiple_C | 8.04 | 5.61 | 5.44 | 6.06 | 4.03E-08 | 2.45E-06 | 5.74 | 4.79 | 5.03 | 1.64 | 0.0196 | 0.1186 |
| TC1300008272.hg.1 | LATS2 | large tumor suppressor kinase 2 | Multiple_C | 9.69 | 7.29 | 7.1 | 6.02 | 7.09E-08 | 3.73E-06 | 6.48 | 6.27 | 6.03 | 1.37 | 0.721 | 0.8698 |
| TC1500007695.hg.1 | PAQR5 | progestin and adipoQ receptor family member V | Multiple_C | 7.45 | 4.36 | 4.86 | 6.02 | 9.57E-07 | 2.68E-05 | 7.82 | 5.01 | 5.89 | 3.81 | 1.25E-07 | 3.14E-05 |
| TC0100011406.hg.1 | CD55 | CD55 molecule, decay accelerating factor for complement (Cror | Multiple_C | 16.61 | 13.12 | 14.03 | 5.98 | 3.90E-07 | 1.34E-05 | 6.81 | 4.99 | 7.25 | 0.74 | 0.4064 | 0.6621 |
| TC1700006729.hg.1 | ACADVL | acyl-CoA dehydrogenase, very long chain | Multiple_C | 12.11 | 11.28 | 9.53 | 5.98 | 6.91E-07 | 2.10E-05 | 10.83 | 10.29 | 10.22 | 1.53 | 0.0632 | 0.2391 |
| TC1100013161.hg.1 | CD59 | CD59 molecule, complement regulatory protein | Multiple_C | 13.99 | 11.83 | 11.42 | 5.94 | 5.13E-08 | 2.93E-06 | 9.61 | 9.42 | 9.69 | 0.95 | 0.8647 | 0.9423 |
| TC0100007449.hg.1 | SH3BGRL3 | SH3 domain binding glutamate-rich protein like 3 | Multiple_C | 14.67 | 10.09 | 12.11 | 5.90 | 5.95E-05 | 0.0007 | 13.02 | 12.55 | 12.8 | 1.16 | 0.4824 | 0.7202 |
| TC0600008166.hg.1 | SLC25A27 | solute carrier family 25, member 27 | Multiple_C | 8.64 | 8.94 | 6.08 | 5.90 | 6.88E-07 | 2.10E-05 | 6.45 | 5.47 | 4.89 | 2.95 | 7.46E-05 | 0.0029 |
| TC0900009339.hg.1 | WASH1 | WAS protein family homolog 1 | Multiple_C | 10.48 | 8.29 | 7.92 | 5.90 | 4.74E-08 | 2.79E-06 | 8.97 | 9.14 | 8.84 | 1.09 | 0.931 | 0.9709 |
| TC1700011561.hg.1 | WIPI1 | WD repeat domain, phosphoinositide interacting 1 | Multiple_C | 8.38 | 6.14 | 5.82 | 5.90 | 0.0012 | 0.0077 | 8.25 | 6.29 | 6.68 | 2.97 | 0.0002 | 0.0055 |
| TC1900010847.hg.1 | LYPD3 | LY6/PLAUR domain containing 3 | Multiple_C | 7.29 | 4.35 | 4.73 | 5.90 | 3.54E-08 | 2.24E-06 | 3.62 | 3.47 | 3.69 | 0.95 | 0.2507 | 0.5149 |
| TC0300013970.hg.1 | PFKFB4; MIR6 | 6-phosphofructo-2-kinase/fructose-2,6-biphosphatase 4; microl | Multiple_C | 7.81 | 4.84 | 5.27 | 5.82 | 1.57E-06 | 3.96E-05 | 8.22 | 8.72 | 7.11 | 2.16 | 0.0003 | 0.0079 |
| TC0600008972.hg.1 | PRDM1 | PR domain containing 1, with ZNF domain | Multiple_C | 9.23 | 5.38 | 6.69 | 5.82 | 3.07E-09 | 3.81E-07 | 9.37 | 8.01 | 7.91 | 2.75 | 3.19E-07 | 6.71E-05 |
| TC0700012798.hg.1 | KDM7A | lysine (K)-specific demethylase 7A | Multiple_C | 9.37 | 6.99 | 6.83 | 5.82 | 5.86E-08 | 3.28E-06 | 9.4 | 8.89 | 9.13 | 1.21 | 0.0399 | 0.183 |
| TC0X00009942.hg.1 | PJA1 | praja ring finger 1, E3 ubiquitin protein ligase | Multiple_C | 7.27 | 4.7 | 4.73 | 5.82 | 2.33E-07 | 9.17E-06 | 7.52 | 8.45 | 8.43 | 0.53 | 0.0295 | 0.1522 |
| TC2100007263.hg.1 | PDE9A | phosphodiesterase 9A | Multiple_C | 8.11 | 6.17 | 5.57 | 5.82 | 2.75E-09 | 3.53E-07 | 6.92 | 6.02 | 5.68 | 2.36 | 4.39E-05 | 0.002 |
| TC2200007037.hg.1 | MTMR3 | myotubularin related protein 3 | Multiple_C | 9.82 | 7.8 | 7.28 | 5.82 | 7.26E-05 | 0.0008 | 7.71 | 7.36 | 7.42 | 1.22 | 0.6091 | 0.8043 |
| TC0400008318.hg.1 | TET2 | tet methylcytosine dioxygenase 2 | Multiple_C | 8.63 | 6.01 | 6.1 | 5.78 | 0.0001 | 0.0012 | 8.04 | 6.40 | 5.89 | 4.44 | 3.26E-05 | 0.0016 |
| TC1700008455.hg.1 | YPEL2 | yippee like 2 | Multiple_C | 5.38 | 3.38 | 2.87 | 5.70 | 3.66E-09 | 4.31E-07 | 3.87 | 3.28 | 3.24 | 1.55 | 0.0025 | 0.0311 |
| TC1700012353.hg.1 | PER1; MIR688 | period circadian clock 1; microRNA 6883 | Multiple_C | 7.43 | 4.83 | 4.92 | 5.70 | 1.62E-07 | 6.91E-06 | 6.66 | 5.85 | 5.87 | 1.73 | 0.007 | 0.0611 |
| TC0100016000.hg.1 | MEF2D | myocyte enhancer factor 2D | Multiple_C | 8.62 | 5.84 | 6.12 | 5.66 | 1.09E-07 | 5.09E-06 | 7.43 | 6.62 | 6.87 | 1.47 | 0.1676 | 0.413 |
| TC0100016944.hg.1 | ARL8A | ADP-ribosylation factor like GTPase 8A | Multiple_C | 9.42 | 6.98 | 6.92 | 5.66 | 1.76E-05 | 0.0003 | 8.91 | 8.88 | 9.15 | 0.85 | 0.6365 | 0.8215 |
| TC0200010511.hg.1 | NBEAL1 | neurobeachin like 1 | Multiple_C | 8.68 | 7.96 | 6.18 | 5.66 | 1.86E-05 | 0.0003 | 7.19 | 6.16 | 6.02 | 2.25 | 0.0926 | 0.295 |
| TC0200015650.hg.1 | FN1 | fibronectin 1 | Multiple_C | 6.41 | 4.38 | 3.91 | 5.66 | 5.83E-06 | 0.0001 | 4.68 | 3.91 | 4.23 | 1.37 | 0.1058 | 0.3194 |
| TC0300009412.hg.1 | SERPINI1 | serpin peptidase inhibitor, clade I (neuroserpin), member 1 | Multiple_C | 6.79 | 4.68 | 4.29 | 5.66 | 7.97E-05 | 0.0009 | 8.25 | 9.82 | 7.19 | 2.08 | 0.0094 | 0.0748 |
| TC0600013231.hg.1 | SGK1 | serum/glucocorticoid regulated kinase 1 | Multiple_C | 10.76 | 7.16 | 8.26 | 5.66 | 0.0027 | 0.0149 | 5.58 | 4.67 | 4.72 | 1.82 | 0.0822 | 0.2762 |
| TC1700012460.hg.1 | ABCA5 | ATP binding cassette subfamily A member 5 | Multiple_C | 7.79 | 6.14 | 5.29 | 5.66 | 4.21E-08 | 2.53E-06 | 7.57 | 7.24 | 6.83 | 1.67 | 0.0015 | 0.0223 |
| TC0100017849.hg.1 | ERO1B | endoplasmic reticulum oxidoreductase beta | Multiple_C | 7.72 | 8.19 | 5.23 | 5.62 | 0.0035 | 0.0181 | 5.26 | 5.08 | 4.46 | 1.74 | 0.311 | 0.5752 |
| TC0200009331.hg.1 | POTEE | POTE ankyrin domain family, member E | Coding | 9.12 | 7.38 | 6.63 | 5.62 | 3.69E-06 | 7.89E-05 | 7.2 | 6.19 | 6.57 | 1.55 | 0.0949 | 0.2994 |

| | | | | | | | | | | | | | | | |
|---|---|---|---|---|---|---|---|---|---|---|---|---|---|---|---|
| TC1000009863.hg.1 | FAM107B | family with sequence similarity 107, member B | Multiple_( | 11.58 | 9.35 | 9.09 | 5.62 | 3.37E-07 | 1.19E-05 | 5.72 | 4.93 | 5.64 | 1.06 | 0.4149 | 0.6694 |
| TC0600007701.hg.1 | PHF1 | PHD finger protein 1 | Multiple_( | 8.21 | 5.93 | 5.73 | 5.58 | 2.17E-08 | 1.50E-06 | 6.47 | 6.24 | 6.25 | 1.16 | 0.5741 | 0.7814 |
| TC1200010926.hg.1 | BAZ2A | bromodomain adjacent to zinc finger domain 2A | Multiple_( | 13.05 | 11.02 | 10.57 | 5.58 | 1.35E-08 | 1.12E-06 | 11.72 | 10.96 | 11.09 | 1.55 | 0.0109 | 0.0821 |
| TC0500007881.hg.1 | JMY | junction mediating and regulatory protein, p53 cofactor | Multiple_( | 10.96 | 9.02 | 8.49 | 5.54 | 1.03E-07 | 4.88E-06 | 9.14 | 8.31 | 8.14 | 2.00 | 6.32E-05 | 0.0026 |
| TC0X00006715.hg.1 | SCML1 | sex comb on midleg-like 1 (Drosophila) | Multiple_( | 12.62 | 11.24 | 10.15 | 5.54 | 4.71E-06 | 9.57E-05 | 9.17 | 10.10 | 11.18 | 0.25 | 7.95E-05 | 0.0031 |
| TC1900011449.hg.1 | TNNT1 | troponin T type 1 (skeletal, slow) | Multiple_( | 8.2 | 6.64 | 5.73 | 5.54 | 3.00E-08 | 1.97E-06 | 6.1 | 5.32 | 5.02 | 2.11 | 0.0002 | 0.0053 |
| TC1600007448.hg.1 | CORO1A | coronin, actin binding protein, 1A | Multiple_( | 9.03 | 6.47 | 6.57 | 5.50 | 1.02E-05 | 0.0002 | 7.92 | 7.43 | 6.92 | 2.00 | 0.0118 | 0.0864 |
| TC1600009954.hg.1 | MAPK3 | mitogen-activated protein kinase 3 | Multiple_( | 12 | 9 | 9.55 | 5.46 | 5.14E-09 | 5.35E-07 | 11.09 | 11.08 | 10.69 | 1.32 | 0.2635 | 0.5282 |
| TC0300013831.hg.1 | SEMA3B; MIR | sema domain, immunoglobulin domain (Ig), short basic domain, | Multiple_( | 6.37 | 4.35 | 3.93 | 5.43 | 8.43E-07 | 2.44E-05 | 8 | 6.70 | 6.28 | 3.29 | 1.94E-05 | 0.0012 |
| TC1100010688.hg.1 | PHF21A | PHD finger protein 21A | Multiple_( | 10.96 | 8.77 | 8.52 | 5.43 | 1.36E-07 | 6.06E-06 | 10.75 | 9.64 | 9.71 | 2.06 | 0.0004 | 0.0085 |
| TC1300006481.hg.1 | ZMYM2 | zinc finger, MYM-type 2 | Multiple_( | 11.09 | 9.84 | 8.65 | 5.43 | 3.35E-06 | 7.30E-05 | 8.11 | 8.16 | 7.93 | 1.13 | 0.4723 | 0.7127 |
| TC0300009890.hg.1 | HRASLS | HRAS-like suppressor | Coding | 6.39 | 4.35 | 3.96 | 5.39 | 6.80E-10 | 1.39E-07 | 3.86 | 3.85 | 3.57 | 1.22 | 0.0818 | 0.2755 |
| TC0500013195.hg.1 | ATP6AP1L | ATPase, H+ transporting, lysosomal accessory protein 1-like | Multiple_( | 7.25 | 5.49 | 4.82 | 5.39 | 2.35E-07 | 9.21E-06 | 5.55 | 5.79 | 5.35 | 1.15 | 0.0627 | 0.2382 |
| TC0200007717.hg.1 | AHSA2 | AHA1, activator of heat shock 90kDa protein ATPase homolog 2 | Multiple_( | 9.49 | 8.41 | 7.07 | 5.35 | 4.58E-06 | 9.33E-05 | 9.01 | 8.85 | 8.54 | 1.39 | 0.0539 | 0.2173 |
| TC0300012924.hg.1 | VEPH1 | ventricular zone expressed PH domain containing 1 | Multiple_( | 6.32 | 3.63 | 3.9 | 5.35 | 2.91E-06 | 6.50E-05 | 8.9 | 6.70 | 7.29 | 3.05 | 1.30E-05 | 0.0009 |
| TC2000009768.hg.1 | SLCO4A1-AS1 | SLCO4A1 antisense RNA 1 | Multiple_( | 7.32 | 5.02 | 4.9 | 5.35 | 1.10E-07 | 5.12E-06 | 4.39 | 4.20 | 3.89 | 1.41 | 0.2223 | 0.4826 |
| TC0100015976.hg.1 | KIAA0907; SC/ | KIAA0907; small Cajal body-specific RNA 4 | Multiple_( | 10.36 | 7.75 | 7.95 | 5.31 | 8.72E-08 | 4.36E-06 | 10.31 | 10.23 | 10.01 | 1.23 | 0.4764 | 0.7154 |
| TC0200007805.hg.1 | AFTPH | aftiphilin | Multiple_( | 14.93 | 13.07 | 12.53 | 5.28 | 1.92E-10 | 5.27E-08 | 10.93 | 10.74 | 10.83 | 1.07 | 0.3121 | 0.5763 |
| TC0800011881.hg.1 | NDRG1 | N-myc downstream regulated 1 | Multiple_( | 6.39 | 3.53 | 3.99 | 5.28 | 5.43E-05 | 0.0007 | 5 | 5.52 | 5 | 1.00 | 0.6357 | 0.8212 |
| TC1000007176.hg.1 | MAP3K8 | mitogen-activated protein kinase kinase kinase 8 | Multiple_( | 10.31 | 9.2 | 7.91 | 5.28 | 1.88E-08 | 1.38E-06 | 7.62 | 6.56 | 6.12 | 2.83 | 3.37E-07 | 7.01E-05 |
| TC2100007850.hg.1 | LTN1 | listerin E3 ubiquitin protein ligase 1 | Multiple_( | 11.54 | 10.39 | 9.14 | 5.28 | 1.13E-08 | 1.01E-06 | 11.5 | 10.83 | 11.5 | 1.00 | 0.7882 | 0.9056 |
| TC0200008291.hg.1 | KDM3A | lysine (K)-specific demethylase 3A | Multiple_( | 9.31 | 7.55 | 6.92 | 5.24 | 3.73E-10 | 8.33E-08 | 7.34 | 7.71 | 7.32 | 1.01 | 0.4896 | 0.7253 |
| TC0200010489.hg.1 | BMPR2 | bone morphogenetic protein receptor type II | Multiple_( | 8.35 | 6.16 | 5.96 | 5.24 | 4.69E-07 | 1.55E-05 | 7.8 | 7.87 | 8.02 | 0.86 | 0.5154 | 0.7446 |
| TC0400010704.hg.1 | LNX1 | ligand of numb-protein X 1, E3 ubiquitin protein ligase | Multiple_( | 9.42 | 7.78 | 7.03 | 5.24 | 6.60E-08 | 3.53E-06 | 3.42 | 3.57 | 3.25 | 1.13 | 0.7136 | 0.866 |
| TC2000008242.hg.1 | RNF24 | ring finger protein 24 | Multiple_( | 9.04 | 6.38 | 6.65 | 5.24 | 6.50E-07 | 2.01E-05 | 7.91 | 7.36 | 8 | 0.94 | 0.9538 | 0.9811 |
| TC2100008249.hg.1 | TFF3 | trefoil factor 3 | Multiple_( | 13.95 | 11.76 | 11.56 | 5.24 | 4.41E-07 | 1.48E-05 | 5.54 | 6.59 | 5.75 | 0.86 | 0.5406 | 0.7599 |
| TC0800012280.hg.1 | PDLIM2 | PDZ and LIM domain 2 (mystique) | Multiple_( | 9.78 | 7 | 7.4 | 5.21 | 7.15E-08 | 3.75E-06 | 5.41 | 5.51 | 5.87 | 0.73 | 0.0184 | 0.1144 |
| TC1100006831.hg.1 | ADM | adrenomedullin | Coding | 10.98 | 8.23 | 8.6 | 5.21 | 1.86E-09 | 2.73E-07 | 6.53 | 6.02 | 6.27 | 1.20 | 0.1587 | 0.4002 |
| TC1200007861.hg.1 | LRP1 | LDL receptor related protein 1 | Multiple_( | 7.89 | 5.59 | 5.51 | 5.21 | 0.0002 | 0.0019 | 7.65 | 7.78 | 6.94 | 1.64 | 0.0096 | 0.0757 |
| TC0300013812.hg.1 | TRAK1 | trafficking protein, kinesin binding 1 | Multiple_( | 11.43 | 8.85 | 9.06 | 5.17 | 1.04E-09 | 1.86E-07 | 8.71 | 8.84 | 8.83 | 0.92 | 0.3003 | 0.5646 |
| TC1000011005.hg.1 | P4HA1 | prolyl 4-hydroxylase, alpha polypeptide I | Multiple_( | 13.13 | 11.96 | 10.76 | 5.17 | 3.41E-08 | 2.19E-06 | 11.75 | 11.30 | 10.82 | 1.91 | 0.0035 | 0.0391 |
| TC2000006721.hg.1 | BTBD3 | BTB (POZ) domain containing 3 | Multiple_( | 11.84 | 9.37 | 9.47 | 5.17 | 1.28E-09 | 2.19E-07 | 11.82 | 11.48 | 11.38 | 1.36 | 0.011 | 0.083 |
| TC0400010904.hg.1 | YTHDC1 | YTH domain containing 1 | Multiple_( | 12.37 | 10.63 | 10.01 | 5.13 | 3.45E-09 | 4.19E-07 | 12.44 | 11.80 | 12.17 | 1.21 | 0.1736 | 0.421 |

| | | | | | | | | | | | | | | | |
|---|---|---|---|---|---|---|---|---|---|---|---|---|---|---|---|
| TC0500011502.hg.1 | ELL2 | elongation factor, RNA polymerase II, 2 | Multiple_C | 14.03 | 11.12 | 11.67 | 5.13 | 1.28E-06 | 3.38E-05 | 10.75 | 11.54 | 12.45 | 0.31 | 2.48E-06 | 0.0003 |
| TC1600011442.hg.1 | MAP1LC3B | microtubule-associated protein 1 light chain 3 beta | Multiple_C | 14.49 | 12.36 | 12.13 | 5.13 | 1.93E-09 | 2.78E-07 | 11.06 | 10.73 | 10.47 | 1.51 | 0.0012 | 0.0184 |
| TC1700010682.hg.1 | KRT17 | keratin 17, type I | Multiple_C | 9.52 | 6.94 | 7.16 | 5.13 | 1.50E-06 | 3.83E-05 | 7.4 | 6.74 | 6.43 | 1.96 | 0.0633 | 0.2393 |
| TC0100013349.hg.1 | RSRP1 | arginine/serine-rich protein 1 | Multiple_C | 10.47 | 10.17 | 8.12 | 5.10 | 7.42E-07 | 2.22E-05 | 9.55 | 9.59 | 9.25 | 1.23 | 0.2361 | 0.4989 |
| TC0200014004.hg.1 | CCDC93 | coiled-coil domain containing 93 | Multiple_C | 11.15 | 9.14 | 8.8 | 5.10 | 0.0005 | 0.0041 | 9.92 | 8.72 | 9.69 | 1.17 | 0.7045 | 0.8606 |
| TC1100009813.hg.1 | CDKN1C | cyclin-dependent kinase inhibitor 1C (p57, Kip2) | Multiple_C | 9.59 | 7.4 | 7.24 | 5.10 | 0.0001 | 0.0011 | 6.91 | 6.58 | 6.3 | 1.53 | 0.0885 | 0.2877 |
| TC1600006561.hg.1 | MAPK8IP3 | mitogen-activated protein kinase 8 interacting protein 3 | Multiple_C | 9.22 | 7.57 | 6.88 | 5.06 | 3.55E-07 | 1.24E-05 | 9.77 | 9.16 | 8.79 | 1.97 | 0.0029 | 0.0351 |
| TC0400010884.hg.1 | CENPC | centromere protein C | Multiple_C | 9.28 | 7.66 | 6.95 | 5.03 | 2.81E-10 | 6.92E-08 | 8.36 | 8.46 | 7.97 | 1.31 | 0.015 | 0.1002 |
| TC1200006771.hg.1 | CLEC2D | C-type lectin domain family 2, member D | Multiple_C | 6.38 | 4.09 | 4.05 | 5.03 | 6.88E-06 | 0.0001 | 6.58 | 6.15 | 6.02 | 1.47 | 0.1369 | 0.3676 |
| TC0100012434.hg.1 | HES4 | hes family bHLH transcription factor 4 | Multiple_C | 9.44 | 6.98 | 7.13 | 4.96 | 7.37E-08 | 3.84E-06 | 7.43 | 7.87 | 7.68 | 0.84 | 0.3518 | 0.6153 |
| TC0500011775.hg.1 | SEMA6A | sema domain, transmembrane domain (TM), and cytoplasmic d | Multiple_C | 7.63 | 5.22 | 5.32 | 4.96 | 4.84E-07 | 1.58E-05 | 8.34 | 6.24 | 5.77 | 5.94 | 6.95E-08 | 2.07E-05 |
| TC0X00011404.hg.1 | IDS | iduronate 2-sulfatase | Multiple_C | 10.26 | 7.65 | 7.95 | 4.96 | 6.17E-08 | 3.39E-06 | 7.75 | 7.14 | 9.74 | 0.25 | 2.02E-08 | 8.33E-06 |
| TC1100013032.hg.1 | DNAJC4 | DnaJ (Hsp40) homolog, subfamily C, member 4 | Multiple_C | 10.96 | 8.78 | 8.65 | 4.96 | 5.74E-09 | 5.91E-07 | 8.03 | 8.42 | 7.8 | 1.17 | 0.933 | 0.972 |
| TC1700007204.hg.1 | SPECC1 | sperm antigen with calponin homology and coiled-coil domains | Multiple_C | 7.12 | 4.68 | 4.81 | 4.96 | 2.28E-07 | 8.98E-06 | 8.99 | 8.41 | 8.85 | 1.10 | 0.6087 | 0.8042 |
| TC1700011919.hg.1 | CEP295NL; TIM | CEP295 N-terminal like; TIMP metallopeptidase inhibitor 2 | Multiple_C | 7.84 | 5.77 | 5.53 | 4.96 | 2.84E-09 | 3.59E-07 | 12.1 | 11.15 | 11.27 | 1.78 | 0.0006 | 0.0111 |
| TC2200008409.hg.1 | ZMAT5 | zinc finger, matrin-type 5 | Multiple_C | 7.88 | 6.13 | 5.57 | 4.96 | 0.0002 | 0.0017 | 6.12 | 6.57 | 5.8 | 1.25 | 0.0793 | 0.2712 |
| TC0100008664.hg.1 | GADD45A | growth arrest and DNA-damage-inducible, alpha | Multiple_C | 14.29 | 11.88 | 11.99 | 4.92 | 1.32E-09 | 2.19E-07 | 12.58 | 11.03 | 11.04 | 2.91 | 2.32E-09 | 1.88E-06 |
| TC0100017420.hg.1 | DUSP10 | dual specificity phosphatase 10 | Multiple_C | 12.34 | 9.63 | 10.04 | 4.92 | 1.20E-07 | 5.50E-06 | 3.58 | 3.35 | 3.58 | 1.00 | 0.9618 | 0.984 |
| TC0500010835.hg.1 | PLK2 | polo-like kinase 2 | Multiple_C | 12.16 | 9.09 | 9.86 | 4.92 | 0.0002 | 0.0022 | 10.83 | 9.18 | 8.85 | 3.94 | 0.0004 | 0.0094 |
| TC0500012975.hg.1 | DBN1 | drebrin 1 | Multiple_C | 7.58 | 5 | 5.28 | 4.92 | 2.19E-08 | 1.51E-06 | 10.89 | 11.02 | 11.4 | 0.70 | 0.0413 | 0.1869 |
| TC0600007887.hg.1 | ZFAND3 | zinc finger, AN1-type domain 3 | Multiple_C | 12.7 | 11.11 | 10.4 | 4.92 | 6.21E-08 | 3.40E-06 | 11.56 | 10.57 | 11.28 | 1.21 | 0.2577 | 0.5226 |
| TC0700009493.hg.1 | ZYX | zyxin | Multiple_C | 10.26 | 7.47 | 7.96 | 4.92 | 3.79E-06 | 8.03E-05 | 8.48 | 8.75 | 9.4 | 0.53 | 0.0016 | 0.0229 |
| TC1900006819.hg.1 | TRIP10 | thyroid hormone receptor interactor 10 | Multiple_C | 15.2 | 13.58 | 12.9 | 4.92 | 1.87E-08 | 1.38E-06 | 10.86 | 9.88 | 10.1 | 1.69 | 0.002 | 0.0267 |
| TC0500013235.hg.1 | FAM53C | family with sequence similarity 53, member C | Multiple_C | 12.04 | 9.2 | 9.75 | 4.89 | 8.07E-05 | 0.0009 | 11.17 | 11.53 | 11.32 | 0.90 | 0.5588 | 0.7714 |
| TC0600014318.hg.1 | FAM46A | family with sequence similarity 46, member A | Coding | 11.2 | 8.4 | 8.91 | 4.89 | 1.66E-07 | 7.01E-06 | 5.27 | 4.86 | 5.48 | 0.86 | 0.3176 | 0.5818 |
| TC0800009231.hg.1 | GRINA | glutamate receptor, ionotropic, N-methyl D-aspartate-associate | Multiple_C | 8.52 | 6.53 | 6.23 | 4.89 | 6.41E-08 | 3.48E-06 | 7.32 | 7.19 | 7.42 | 0.93 | 0.2686 | 0.5334 |
| TC1700010198.hg.1 | UNC119 | unc-119 lipid binding chaperone | Multiple_C | 11.26 | 8.9 | 8.97 | 4.89 | 1.88E-08 | 1.38E-06 | 8.85 | 8.87 | 9.02 | 0.89 | 0.2692 | 0.5342 |
| TC0200016224.hg.1 | PER2 | period circadian clock 2 | Multiple_C | 5.93 | 3.77 | 3.65 | 4.86 | 3.47E-06 | 7.51E-05 | 5.35 | 5.45 | 5.54 | 0.88 | 0.052 | 0.2129 |
| TC0300007165.hg.1 | NKTR | natural killer cell triggering receptor | Multiple_C | 13.31 | 10.84 | 11.03 | 4.86 | 2.93E-07 | 1.07E-05 | 12.1 | 11.23 | 11.67 | 1.35 | 0.043 | 0.1914 |
| TC0800009919.hg.1 | GNRH1 | gonadotropin releasing hormone 1 | Coding | 8.7 | 7.3 | 6.42 | 4.86 | 1.28E-05 | 0.0002 | 5.94 | 5.65 | 5.76 | 1.13 | 0.7038 | 0.86 |
| TC0X00008836.hg.1 | PLXNA3 | plexin A3 | Multiple_C | 7.65 | 4.9 | 5.37 | 4.86 | 1.14E-05 | 0.0002 | 6.65 | 7.11 | 7.52 | 0.55 | 0.099 | 0.3075 |
| TC1000007404.hg.1 | CSGALNACT2 | chondroitin sulfate N-acetylgalactosaminyltransferase 2 | Multiple_C | 9.77 | 7.83 | 7.49 | 4.86 | 6.63E-07 | 2.05E-05 | 9.73 | 8.15 | 9.08 | 1.57 | 0.0947 | 0.299 |
| TC1100011980.hg.1 | TAF1D; SNORA | TATA box binding protein associated factor 1D; small nucleolar | Multiple_C | 14.87 | 14.99 | 12.59 | 4.86 | 2.49E-08 | 1.69E-06 | 12.81 | 10.74 | 11.63 | 2.27 | 9.15E-06 | 0.0007 |

| | | | | | | | | | | | | | | | |
|---|---|---|---|---|---|---|---|---|---|---|---|---|---|---|---|
| TC1200012153.hg.1 | PXN | paxillin | Multiple_C | 12.69 | 9.94 | 10.42 | 4.82 | 1.10E-07 | 5.10E-06 | 10.29 | 10.75 | 10.8 | 0.70 | 0.0242 | 0.1343 |
| TC1300006979.hg.1 | RGCC | regulator of cell cycle | Multiple_C | 10.64 | 9.52 | 8.37 | 4.82 | 2.10E-06 | 4.97E-05 | 5.47 | 5.66 | 4.96 | 1.42 | 0.0629 | 0.2386 |
| TC1300007774.hg.1 | MBNL2 | muscleblind-like splicing regulator 2 | Multiple_C | 14.01 | 12.61 | 11.74 | 4.82 | 1.16E-07 | 5.32E-06 | 13.08 | 11.38 | 11.51 | 2.97 | 2.83E-06 | 0.0003 |
| TC1500010429.hg.1 | CIB1 | calcium and integrin binding 1 (calmyrin) | Multiple_C | 12.17 | 10.55 | 9.9 | 4.82 | 9.40E-08 | 4.62E-06 | 7.13 | 7.33 | 7.02 | 1.08 | 0.6951 | 0.855 |
| TC0200008262.hg.1 | RNF181 | ring finger protein 181 | Multiple_C | 10.76 | 9.37 | 8.5 | 4.79 | 7.48E-08 | 3.88E-06 | 7.25 | 7.57 | 7.19 | 1.04 | 0.8952 | 0.9556 |
| TC0200010840.hg.1 | DNAJB2 | DnaJ (Hsp40) homolog, subfamily B, member 2 | Multiple_C | 9.05 | 6.88 | 6.79 | 4.79 | 2.18E-05 | 0.0003 | 6.11 | 5.43 | 6.03 | 1.06 | 0.8367 | 0.9281 |
| TC0200013298.hg.1 | ST3GAL5 | ST3 beta-galactoside alpha-2,3-sialyltransferase 5 | Multiple_C | 6.84 | 4.84 | 4.58 | 4.79 | 0.0001 | 0.0014 | 4.4 | 3.79 | 4.06 | 1.27 | 0.1836 | 0.4345 |
| TC0500011448.hg.1 | ARRDC3 | arrestin domain containing 3 | Multiple_C | 9.04 | 6.63 | 6.78 | 4.79 | 2.66E-05 | 0.0004 | 9.35 | 8.90 | 10.24 | 0.54 | 0.0064 | 0.0577 |
| TC0500011483.hg.1 | MCTP1 | multiple C2 domains, transmembrane 1 | Multiple_C | 11.33 | 10.12 | 9.07 | 4.79 | 2.41E-07 | 9.37E-06 | 10.47 | 9.74 | 7.78 | 6.45 | 1.26E-09 | 1.22E-06 |
| TC0700007157.hg.1 | NPSR1 | neuropeptide S receptor 1 | Multiple_C | 6.79 | 4.11 | 4.53 | 4.79 | 3.49E-06 | 7.54E-05 | 4.39 | 4.71 | 4.72 | 0.80 | 0.7129 | 0.8655 |
| TC0200012713.hg.1 | BCL11A | B-cell CLL/lymphoma 11A (zinc finger protein) | Multiple_C | 9.77 | 7.69 | 7.52 | 4.76 | 9.44E-07 | 2.66E-05 | 9.46 | 9.81 | 9.9 | 0.74 | 0.1004 | 0.3098 |
| TC0400012826.hg.1 | HERC3 | HECT and RLD domain containing E3 ubiquitin protein ligase 3 | Multiple_C | 7.94 | 5.17 | 5.69 | 4.76 | 3.92E-08 | 2.39E-06 | 7.29 | 7.44 | 7.09 | 1.15 | 0.7268 | 0.8731 |
| TC2000009504.hg.1 | ZNF217 | zinc finger protein 217 | Multiple_C | 13.4 | 11.36 | 11.15 | 4.76 | 1.09E-07 | 5.09E-06 | 10.65 | 10.29 | 10.54 | 1.08 | 0.423 | 0.6756 |
| TC0100018238.hg.1 | DNAJB4 | DnaJ (Hsp40) homolog, subfamily B, member 4 | Multiple_C | 11.17 | 10.49 | 8.93 | 4.72 | 1.72E-05 | 0.0003 | 10.07 | 8.27 | 9.01 | 2.08 | 0.0236 | 0.1326 |
| TC0400010242.hg.1 | PPARGC1A | peroxisome proliferator-activated receptor gamma, coactivator | Multiple_C | 10.96 | 8.37 | 8.72 | 4.72 | 1.66E-06 | 4.13E-05 | 7.92 | 5.21 | 5.77 | 4.44 | 4.24E-08 | 1.49E-05 |
| TC0600013409.hg.1 | HIVEP2 | human immunodeficiency virus type I enhancer binding protein | Multiple_C | 7.91 | 5.57 | 5.67 | 4.72 | 0.0005 | 0.0037 | 7.97 | 7.36 | 7.27 | 1.62 | 0.3966 | 0.6544 |
| TC0800011713.hg.1 | MTSS1 | metastasis suppressor 1 | Multiple_C | 7.07 | 4.72 | 4.83 | 4.72 | 3.36E-07 | 1.19E-05 | 7.89 | 7.65 | 6.6 | 2.45 | 0.0005 | 0.0101 |
| TC0300007669.hg.1 | SYNPR | synaptoporin | Multiple_C | 8.28 | 6.72 | 6.05 | 4.69 | 3.49E-08 | 2.22E-06 | 4.65 | 4.89 | 4.93 | 0.82 | 0.198 | 0.4531 |
| TC0400007857.hg.1 | AREG | amphiregulin | Multiple_C | 14.46 | 13.63 | 12.23 | 4.69 | 2.39E-08 | 1.64E-06 | 7.71 | 5.63 | 6.16 | 2.93 | 1.16E-06 | 0.0002 |
| TC1400010390.hg.1 | AHNAK2 | AHNAK nucleoprotein 2 | Multiple_C | 8.88 | 6.81 | 6.65 | 4.69 | 2.85E-07 | 1.05E-05 | 4.78 | 4.42 | 4.61 | 1.13 | 0.3448 | 0.6086 |
| TC1500008232.hg.1 | ISG20 | interferon stimulated exonuclease gene 20kDa | Multiple_C | 8.51 | 6.4 | 6.28 | 4.69 | 8.81E-08 | 4.40E-06 | 5.84 | 5.74 | 5.61 | 1.17 | 0.5228 | 0.7489 |
| TC2000007150.hg.1 | BPIFB1 | BPI fold containing family B, member 1 | Multiple_C | 5.49 | 3.34 | 3.26 | 4.69 | 1.89E-07 | 7.79E-06 | 3.46 | 3.12 | 3.34 | 1.09 | 0.631 | 0.8184 |
| TC0500012032.hg.1 | AFF4 | AF4/FMR2 family, member 4 | Multiple_C | 12.18 | 9.59 | 9.96 | 4.66 | 1.84E-08 | 1.38E-06 | 10.98 | 10.00 | 10.83 | 1.11 | 0.5572 | 0.771 |
| TC0X00011194.hg.1 | FLNA | filamin A, alpha | Multiple_C | 12.51 | 11.57 | 10.29 | 4.66 | 1.03E-05 | 0.0002 | 12.02 | 12.59 | 13.27 | 0.42 | 0.0007 | 0.0124 |
| TC1700007478.hg.1 | NF1 | neurofibromin 1 | Multiple_C | 11.28 | 9.72 | 9.06 | 4.66 | 1.51E-07 | 6.58E-06 | 11.9 | 10.77 | 11.3 | 1.52 | 0.0487 | 0.2051 |
| TC1900010743.hg.1 | TGFB1 | transforming growth factor beta 1 | Multiple_C | 9.88 | 7.31 | 7.66 | 4.66 | 1.70E-06 | 4.20E-05 | 10.42 | 10.25 | 9.77 | 1.57 | 0.0038 | 0.0414 |
| TC1200012590.hg.1 | LRRC23 | leucine rich repeat containing 23 | Multiple_C | 7.69 | 6.42 | 5.48 | 4.63 | 1.55E-06 | 3.92E-05 | 7.34 | 6.51 | 6.46 | 1.84 | 0.0866 | 0.2837 |
| TC1400010737.hg.1 | KLHL28 | kelch-like family member 28 | Multiple_C | 9.52 | 7.69 | 7.31 | 4.63 | 5.53E-06 | 0.0001 | 7.26 | 6.52 | 7.44 | 0.88 | 0.944 | 0.9771 |
| TC1900011635.hg.1 | CHMP2A | charged multivesicular body protein 2A | Multiple_C | 13.52 | 11.5 | 11.31 | 4.63 | 6.89E-07 | 2.10E-05 | 9.47 | 9.35 | 8.95 | 1.43 | 0.0207 | 0.1229 |
| TC0400011299.hg.1 | FAM13A | family with sequence similarity 13, member A | Multiple_C | 9.08 | 6.98 | 6.88 | 4.59 | 2.11E-08 | 1.48E-06 | 4.37 | 4.62 | 4.54 | 0.89 | 0.9214 | 0.9676 |
| TC0600007847.hg.1 | CDKN1A | cyclin-dependent kinase inhibitor 1A (p21, Cip1) | Multiple_C | 9.06 | 5.9 | 6.86 | 4.59 | 5.87E-05 | 0.0007 | 4.45 | 4.06 | 4.11 | 1.27 | 0.0627 | 0.2382 |
| TC0600009821.hg.1 | ULBP1 | UL16 binding protein 1 | Coding | 9.05 | 6.83 | 6.85 | 4.59 | 0.0003 | 0.0023 | 7.08 | 6.16 | 6.8 | 1.21 | 0.2173 | 0.4766 |
| TC1700009650.hg.1 | SAT2 | spermidine/spermine N1-acetyltransferase family member 2 | Multiple_C | 9.96 | 9.04 | 7.76 | 4.59 | 1.20E-05 | 0.0002 | 6.34 | 6.48 | 6.52 | 0.88 | 0.5257 | 0.7504 |

| | | | | | | | | | | | | | | | |
|---|---|---|---|---|---|---|---|---|---|---|---|---|---|---|---|
| TC0300013933.hg.1 | THUMPD3-AS | THUMPD3 antisense RNA 1 | Multiple_C | 8.35 | 6.78 | 6.16 | 4.56 | 2.53E-06 | 5.83E-05 | 9.28 | 9.21 | 8.14 | 2.20 | 0.008 | 0.0671 |
| TC2000009744.hg.1 | LAMA5; MIR4 | laminin, alpha 5; microRNA 4758 | Multiple_C | 9.44 | 7.44 | 7.25 | 4.56 | 0.0058 | 0.0272 | 8.54 | 7.77 | 7.3 | 2.36 | 0.0591 | 0.2292 |
| TC0100018234.hg.1 | TNNI3K | TNNI3 interacting kinase | Multiple_C | 6.09 | 4.2 | 3.91 | 4.53 | 5.32E-07 | 1.70E-05 | 4.03 | 3.99 | 4.18 | 0.90 | 0.6272 | 0.8158 |
| TC0200013567.hg.1 | ANKRD36B | ankyrin repeat domain 36B | Multiple_C | 10.82 | 8.8 | 8.64 | 4.53 | 7.45E-07 | 2.23E-05 | 10.65 | 9.78 | 9.53 | 2.17 | 0.0002 | 0.0055 |
| TC0900008811.hg.1 | STXBP1 | syntaxin binding protein 1 | Multiple_C | 11.99 | 9.75 | 9.81 | 4.53 | 1.64E-09 | 2.52E-07 | 12.95 | 11.84 | 12.04 | 1.88 | 0.0002 | 0.0058 |
| TC1100010998.hg.1 | MS4A4E | membrane-spanning 4-domains, subfamily A, member 4E | Multiple_C | 6.03 | 3.28 | 3.85 | 4.53 | 9.01E-05 | 0.001 | 5.08 | 4.86 | 4.18 | 1.87 | 0.0078 | 0.066 |
| TC1600009162.hg.1 | HCFC1R1 | host cell factor C1 regulator 1 (XPO1 dependent) | Multiple_C | 8.21 | 5.49 | 6.03 | 4.53 | 1.40E-07 | 6.20E-06 | 4.65 | 4.22 | 3.95 | 1.62 | 0.005 | 0.0489 |
| TC1000006816.hg.1 | OPTN | optineurin | Multiple_C | 10.36 | 9.67 | 8.19 | 4.50 | 2.28E-06 | 5.34E-05 | 9.72 | 8.71 | 7.89 | 3.56 | 8.37E-07 | 0.0001 |
| TC1500009358.hg.1 | SECISBP2L | SECIS binding protein 2-like | Multiple_C | 11.19 | 8.77 | 9.02 | 4.50 | 5.64E-06 | 0.0001 | 11.45 | 10.55 | 11.13 | 1.25 | 0.2022 | 0.4579 |
| TC0100018463.hg.1 | RHOC | ras homolog family member C | Multiple_C | 14.02 | 11.43 | 11.86 | 4.47 | 1.35E-07 | 6.02E-06 | 10.4 | 10.20 | 10.95 | 0.68 | 0.0295 | 0.1522 |
| TC0400007938.hg.1 | BMP2K | BMP2 inducible kinase | Multiple_C | 12.22 | 9.06 | 10.06 | 4.47 | 1.96E-06 | 4.68E-05 | 12.83 | 11.95 | 12.33 | 1.41 | 0.0527 | 0.2146 |
| TC1500010780.hg.1 | GOLGA6L9 | golgin A6 family-like 9 | Multiple_C | 8.87 | 7.22 | 6.71 | 4.47 | 5.18E-05 | 0.0006 | 6.28 | 5.71 | 5.71 | 1.48 | 0.0202 | 0.1213 |
| TC0700010716.hg.1 | DPY19L1 | dpy-19-like 1 (C. elegans) | Multiple_C | 12.63 | 10.82 | 10.48 | 4.44 | 0.0001 | 0.0014 | 10.42 | 10.52 | 10.86 | 0.74 | 0.3693 | 0.6314 |
| TC1700012321.hg.1 | TBC1D3B; TBC | TBC1 domain family, member 3B; TBC1 domain family, member | Multiple_C | 9.89 | 8.59 | 7.74 | 4.44 | 3.36E-06 | 7.30E-05 | 8.31 | 8.26 | 7.88 | 1.35 | 0.2271 | 0.4877 |
| TC0100010149.hg.1 | RAB25 | RAB25, member RAS oncogene family | Multiple_C | 14.12 | 11.8 | 11.98 | 4.41 | 1.42E-07 | 6.26E-06 | 4.57 | 4.52 | 4.33 | 1.18 | 0.1481 | 0.3847 |
| TC1700012241.hg.1 | TBC1D3L; TBC | TBC1 domain family, member 3L; TBC1 domain family, member | Coding | 9.24 | 7.77 | 7.1 | 4.41 | 6.61E-06 | 0.0001 | 8.09 | 7.51 | 7.08 | 2.01 | 0.002 | 0.0266 |
| TC2000008985.hg.1 | RBM39 | RNA binding motif protein 39 | Multiple_C | 13.4 | 12.33 | 11.26 | 4.41 | 2.06E-08 | 1.46E-06 | 11.86 | 11.50 | 11.9 | 0.97 | 0.853 | 0.9365 |
| TC0100006725.hg.1 | PER3 | period circadian clock 3 | Multiple_C | 6.97 | 4.89 | 4.84 | 4.38 | 1.40E-07 | 6.20E-06 | 5.8 | 4.92 | 4.54 | 2.39 | 9.93E-05 | 0.0036 |
| TC0X00010169.hg.1 | BRWD3 | bromodomain and WD repeat domain containing 3 | Multiple_C | 8.77 | 7.11 | 6.64 | 4.38 | 0.0002 | 0.0015 | 9.78 | 8.90 | 8.94 | 1.79 | 0.0992 | 0.3077 |
| TC1500007379.hg.1 | FAM63B | family with sequence similarity 63, member B | Multiple_C | 8.69 | 7.31 | 6.56 | 4.38 | 3.26E-07 | 1.16E-05 | 6.76 | 4.95 | 5.8 | 1.95 | 0.0001 | 0.004 |
| TC1000012567.hg.1 | AGAP5 | ArfGAP with GTPase domain, ankyrin repeat and PH domain 5 | Multiple_C | 11.37 | 9.33 | 9.25 | 4.35 | 2.74E-09 | 3.53E-07 | 11.86 | 11.60 | 11.59 | 1.21 | 0.1676 | 0.413 |
| TC0200009933.hg.1 | ERICH2 | glutamate rich 2 | Multiple_C | 6.75 | 4.79 | 4.64 | 4.32 | 5.23E-05 | 0.0006 | 7.31 | 7.06 | 6.19 | 2.17 | 0.1216 | 0.3442 |
| TC0400012763.hg.1 | S100P | S100 calcium binding protein P | Multiple_C | 13.14 | 13.25 | 11.03 | 4.32 | 8.25E-06 | 0.0001 | 5.59 | 4.98 | 5.36 | 1.17 | 0.6655 | 0.8363 |
| TC0300013025.hg.1 | BCHE | butyrylcholinesterase | Coding | 5.68 | 3.87 | 3.58 | 4.29 | 2.76E-05 | 0.0004 | 5.58 | 5.54 | 5.35 | 1.17 | 0.1566 | 0.3975 |
| TC0400010626.hg.1 | FRYL | FRY like transcription coactivator | Multiple_C | 9.72 | 7.8 | 7.62 | 4.29 | 2.93E-07 | 1.07E-05 | 9.22 | 8.57 | 8.57 | 1.57 | 0.0044 | 0.0455 |
| TC0600010707.hg.1 | NRN1 | neuritin 1 | Multiple_C | 10.93 | 9.51 | 8.83 | 4.29 | 0.0004 | 0.0031 | 5.63 | 5.39 | 4.66 | 1.96 | 0.0043 | 0.0451 |
| TC1100010551.hg.1 | TRAF6 | TNF receptor-associated factor 6, E3 ubiquitin protein ligase | Multiple_C | 9.33 | 7.66 | 7.23 | 4.29 | 1.32E-06 | 3.44E-05 | 9.17 | 8.27 | 8.95 | 1.16 | 0.2526 | 0.5168 |
| TC1700007702.hg.1 | SOCS7 | suppressor of cytokine signaling 7 | Multiple_C | 9.38 | 7.36 | 7.28 | 4.29 | 2.09E-07 | 8.44E-06 | 7.48 | 7.16 | 7.87 | 0.76 | 0.0131 | 0.0924 |
| TC0100017289.hg.1 | PTPN14 | protein tyrosine phosphatase, non-receptor type 14 | Multiple_C | 9.24 | 7.38 | 7.15 | 4.26 | 7.21E-06 | 0.0001 | 8.62 | 9.49 | 9 | 0.77 | 0.0187 | 0.1155 |
| TC0200016717.hg.1 | TBC1D8 | TBC1 domain family, member 8 (with GRAM domain) | Multiple_C | 11.06 | 9.84 | 8.97 | 4.26 | 5.79E-07 | 1.83E-05 | 8.69 | 8.49 | 8.5 | 1.14 | 0.4328 | 0.684 |
| TC0300007189.hg.1 | SNRK | SNF related kinase | Multiple_C | 9.1 | 6.87 | 7.01 | 4.26 | 1.32E-08 | 1.11E-06 | 6.87 | 6.78 | 7.15 | 0.82 | 0.1916 | 0.4445 |
| TC0600012372.hg.1 | LCA5 | Leber congenital amaurosis 5 | Multiple_C | 9.61 | 9.26 | 7.52 | 4.26 | 6.53E-06 | 0.0001 | 6.91 | 5.43 | 5.89 | 2.03 | 0.0005 | 0.0105 |
| TC0800008032.hg.1 | ZC2HC1A | zinc finger, C2HC-type containing 1A | Multiple_C | 7.85 | 5.72 | 5.76 | 4.26 | 7.17E-07 | 2.16E-05 | 8.54 | 8.13 | 7.99 | 1.46 | 0.0924 | 0.2945 |

| | | | | | | | | | | | | | | | |
|---|---|---|---|---|---|---|---|---|---|---|---|---|---|---|---|
| TC0900011192.hg.1 | LPAR1 | lysophosphatidic acid receptor 1 | Multiple_C | 6.35 | 3.94 | 4.26 | 4.26 | 0.0079 | 0.0347 | 10.02 | 7.86 | 7.62 | 5.28 | 0.0438 | 0.1934 |
| TC0X00008078.hg.1 | MID2 | midline 2 | Multiple_C | 5.82 | 3.67 | 3.73 | 4.26 | 7.06E-05 | 0.0008 | 3.64 | 3.36 | 3.44 | 1.15 | 0.0687 | 0.2496 |
| TC1400006851.hg.1 | ARHGAP5 | Rho GTPase activating protein 5 | Multiple_C | 12.4 | 10.36 | 10.31 | 4.26 | 2.26E-05 | 0.0003 | 10.29 | 10.01 | 10.97 | 0.62 | 0.0632 | 0.2391 |
| TC1500007791.hg.1 | ARIH1; MIR63 | ariadne RBR E3 ubiquitin protein ligase 1; microRNA 630 | Multiple_C | 12.46 | 10.38 | 10.37 | 4.26 | 3.58E-05 | 0.0005 | 11.73 | 10.52 | 11.38 | 1.27 | 0.2281 | 0.4889 |
| TC1700008734.hg.1 | AMZ2 | archaelysin family metallopeptidase 2 | Multiple_C | 11.21 | 9.82 | 9.12 | 4.26 | 1.33E-07 | 5.99E-06 | 11.77 | 11.32 | 11.4 | 1.29 | 0.1015 | 0.3116 |
| TC2000006446.hg.1 | RBCK1 | RanBP-type and C3HC4-type zinc finger containing 1 | Multiple_C | 12.44 | 11.79 | 10.35 | 4.26 | 2.59E-05 | 0.0004 | 10.39 | 10.33 | 9.99 | 1.32 | 0.5789 | 0.7844 |
| TC0200008233.hg.1 | KCMF1 | potassium channel modulatory factor 1 | Multiple_C | 15.08 | 13.08 | 13 | 4.23 | 5.97E-09 | 5.99E-07 | 13.18 | 12.34 | 13.18 | 1.00 | 0.8219 | 0.9213 |
| TC0200012863.hg.1 | SPRED2 | sprouty-related, EVH1 domain containing 2 | Multiple_C | 10.96 | 8.8 | 8.88 | 4.23 | 1.07E-07 | 5.00E-06 | 11.48 | 11.27 | 11.31 | 1.13 | 0.6766 | 0.8443 |
| TC0400012759.hg.1 | FAM193A | family with sequence similarity 193, member A | Multiple_C | 9.48 | 7.06 | 7.4 | 4.23 | 0.0001 | 0.0011 | 9.31 | 9.10 | 9.18 | 1.09 | 0.986 | 0.9943 |
| TC1200012602.hg.1 | SLCO1B7; SLC | solute carrier organic anion transporter family, member 1B7 (nc | Multiple_C | 8.29 | 6.94 | 6.21 | 4.23 | 4.72E-07 | 1.56E-05 | 3.65 | 3.76 | 3.41 | 1.18 | 0.0256 | 0.1395 |
| TC1700010675.hg.1 | KRT13 | keratin 13, type I | Multiple_C | 6.51 | 4.45 | 4.43 | 4.23 | 7.11E-06 | 0.0001 | 4.14 | 4.19 | 4.17 | 0.98 | 0.9336 | 0.972 |
| TC2100008490.hg.1 | BACH1 | BTB and CNC homology 1, basic leucine zipper transcription fact | Multiple_C | 11.58 | 9.85 | 9.5 | 4.23 | 7.42E-05 | 0.0008 | 8.57 | 8.22 | 9.07 | 0.71 | 0.0826 | 0.2767 |
| TC2200007495.hg.1 | SREBF2 | sterol regulatory element binding transcription factor 2 | Multiple_C | 12.36 | 10.79 | 10.28 | 4.23 | 9.90E-05 | 0.001 | 10.56 | 10.10 | 10 | 1.47 | 0.0307 | 0.1561 |
| TC0200016452.hg.1 | REL | v-rel avian reticuloendotheliosis viral oncogene homolog | Multiple_C | 10.92 | 9.01 | 8.85 | 4.20 | 4.77E-08 | 2.80E-06 | 9.46 | 8.32 | 9.19 | 1.21 | 0.1331 | 0.362 |
| TC0700010334.hg.1 | AGMO | alkylglycerol monooxygenase | Multiple_C | 7.64 | 5.64 | 5.57 | 4.20 | 2.01E-05 | 0.0003 | 6.03 | 7.67 | 7.64 | 0.33 | 8.79E-05 | 0.0033 |
| TC1500007874.hg.1 | C15orf39 | chromosome 15 open reading frame 39 | Coding | 9.39 | 6.65 | 7.32 | 4.20 | 6.94E-07 | 2.11E-05 | 8.45 | 8.40 | 9.06 | 0.66 | 0.0602 | 0.2322 |
| TC2000006551.hg.1 | ATRN | attractin | Multiple_C | 9.59 | 7.74 | 7.52 | 4.20 | 1.54E-06 | 3.91E-05 | 11.12 | 10.65 | 10.13 | 1.99 | 0.001 | 0.0168 |
| TC2000009392.hg.1 | B4GALT5 | UDP-Gal:betaGlcNAc beta 1,4- galactosyltransferase, polypeptic | Multiple_C | 12.78 | 9.97 | 10.71 | 4.20 | 3.07E-09 | 3.81E-07 | 11.87 | 10.63 | 11.96 | 0.94 | 0.599 | 0.7986 |
| TC0200013606.hg.1 | LYG1 | lysozyme G-like 1 | Multiple_C | 8.54 | 7 | 6.48 | 4.17 | 1.72E-07 | 7.15E-06 | 6.59 | 6.40 | 6.04 | 1.46 | 0.0563 | 0.2231 |
| TC1400008963.hg.1 | MBIP | MAP3K12 binding inhibitory protein 1 | Multiple_C | 10.33 | 9.17 | 8.27 | 4.17 | 5.25E-08 | 2.96E-06 | 9.93 | 9.00 | 9.42 | 1.42 | 0.0163 | 0.1061 |
| TC1900008166.hg.1 | CD79A | CD79a molecule, immunoglobulin-associated alpha | Multiple_C | 8.28 | 5.99 | 6.22 | 4.17 | 2.49E-05 | 0.0003 | 7.04 | 6.69 | 6.65 | 1.31 | 0.4635 | 0.7057 |
| TC1900010531.hg.1 | ZNF461 | zinc finger protein 461 | Multiple_C | 8.9 | 7.33 | 6.84 | 4.17 | 6.20E-07 | 1.94E-05 | 8.63 | 8.28 | 8.45 | 1.13 | 0.3915 | 0.6501 |
| TC2000006571.hg.1 | SMOX | spermine oxidase | Multiple_C | 9.52 | 7.25 | 7.46 | 4.17 | 2.71E-07 | 1.01E-05 | 9.12 | 9.32 | 9.1 | 1.01 | 0.7917 | 0.9074 |
| TC2000007572.hg.1 | NCOA3 | nuclear receptor coactivator 3 | Multiple_C | 12.98 | 10.92 | 10.92 | 4.17 | 1.68E-07 | 7.04E-06 | 11.44 | 10.05 | 10.66 | 1.72 | 0.0027 | 0.0331 |
| TC0100011855.hg.1 | GUK1 | guanylate kinase 1 | Multiple_C | 14.43 | 12.22 | 12.38 | 4.14 | 2.81E-07 | 1.04E-05 | 11.39 | 11.58 | 11.46 | 0.95 | 0.8786 | 0.948 |
| TC0500009274.hg.1 | FABP6 | fatty acid binding protein 6, ileal | Multiple_C | 6.83 | 4.43 | 4.78 | 4.14 | 6.62E-06 | 0.0001 | 4.21 | 3.94 | 3.92 | 1.22 | 0.378 | 0.638 |
| TC0700009411.hg.1 | MGAM2 | maltase-glucoamylase 2 (putative) | Multiple_C | 14.03 | 12.19 | 11.98 | 4.14 | 4.18E-07 | 1.42E-05 | 3.32 | 3.84 | 3.54 | 0.86 | 0.4288 | 0.6806 |
| TC1000011807.hg.1 | SMNDC1 | survival motor neuron domain containing 1 | Multiple_C | 13.16 | 11.71 | 11.11 | 4.14 | 7.80E-07 | 2.30E-05 | 11.63 | 10.81 | 11.22 | 1.33 | 0.1486 | 0.3856 |
| TC1700011436.hg.1 | ERN1 | endoplasmic reticulum to nucleus signaling 1 | Multiple_C | 9.48 | 7.59 | 7.43 | 4.14 | 2.75E-05 | 0.0004 | 7.25 | 7.05 | 8.18 | 0.52 | 0.0552 | 0.2207 |
| TC2000009268.hg.1 | ELMO2 | engulfment and cell motility 2 | Multiple_C | 10.41 | 7.96 | 8.36 | 4.14 | 1.14E-06 | 3.08E-05 | 8.83 | 8.62 | 8.79 | 1.03 | 0.9257 | 0.9694 |
| TC1200012664.hg.1 | MYRFL | myelin regulatory factor-like | Multiple_C | 5.73 | 3.61 | 3.69 | 4.11 | 9.22E-07 | 2.61E-05 | 4.55 | 4.10 | 3.92 | 1.55 | 0.0693 | 0.251 |
| TC1900010998.hg.1 | PRKD2 | protein kinase D2 | Multiple_C | 9.22 | 7.11 | 7.18 | 4.11 | 4.30E-06 | 8.86E-05 | 8.04 | 8.16 | 7.95 | 1.06 | 0.7884 | 0.9057 |
| TC0100013182.hg.1 | CAMK2N1 | calcium/calmodulin-dependent protein kinase II inhibitor 1 | Multiple_C | 15.27 | 13.1 | 13.24 | 4.08 | 2.66E-05 | 0.0004 | 10.6 | 9.97 | 9.08 | 2.87 | 1.63E-05 | 0.001 |

| | | | | | | | | | | | | | | | |
|---|---|---|---|---|---|---|---|---|---|---|---|---|---|---|---|
| TC0300012073.hg.1 | LSAMP | limbic system-associated membrane protein | Multiple_C | 8.27 | 6.64 | 6.24 | 4.08 | 1.66E-07 | 7.01E-06 | 4.23 | 4.85 | 5.44 | 0.43 | 2.08E-05 | 0.0012 |
| TC0X00010851.hg.1 | GPC3 | glypican 3 | Multiple_C | 7.48 | 5.44 | 5.45 | 4.08 | 3.89E-08 | 2.39E-06 | 3.78 | 3.81 | 3.82 | 0.97 | 0.9535 | 0.981 |
| TC1000012428.hg.1 | AKR1C3 | aldo-keto reductase family 1, member C3 | Multiple_C | 11.28 | 11.34 | 9.25 | 4.08 | 3.42E-07 | 1.20E-05 | 7.91 | 9.85 | 10.06 | 0.23 | 7.93E-09 | 4.23E-06 |
| TC1500010160.hg.1 | CTSH | cathepsin H | Multiple_C | 6.71 | 4.25 | 4.68 | 4.08 | 0.0002 | 0.0015 | 4.64 | 4.77 | 3.83 | 1.75 | 0.0012 | 0.0194 |
| TC2000007704.hg.1 | PARD6B | par-6 family cell polarity regulator beta | Multiple_C | 11.65 | 9.47 | 9.62 | 4.08 | 7.64E-08 | 3.94E-06 | 9.61 | 8.43 | 9.26 | 1.27 | 0.037 | 0.1752 |
| TC0200012956.hg.1 | GFPT1 | glutamine--fructose-6-phosphate transaminase 1 | Multiple_C | 14.85 | 14.09 | 12.83 | 4.06 | 1.05E-05 | 0.0002 | 12.71 | 11.52 | 12.39 | 1.25 | 0.0661 | 0.2449 |
| TC0400006930.hg.1 | CPEB2 | cytoplasmic polyadenylation element binding protein 2 | Multiple_C | 6.44 | 5.13 | 4.42 | 4.06 | 7.58E-07 | 2.25E-05 | 3.94 | 3.36 | 3.21 | 1.66 | 0.0019 | 0.026 |
| TC0400012947.hg.1 | GPRIN3 | GPRIN family member 3 | Coding | 8.41 | 6.41 | 6.39 | 4.06 | 4.30E-08 | 2.57E-06 | 3.91 | 3.76 | 3.75 | 1.12 | 0.0671 | 0.247 |
| TC0500012238.hg.1 | HBEGF | heparin-binding EGF-like growth factor | Multiple_C | 10.5 | 7.93 | 8.48 | 4.06 | 2.45E-07 | 9.44E-06 | 6.19 | 5.87 | 6.15 | 1.03 | 0.8966 | 0.9564 |
| TC1300009765.hg.1 | IRS2 | insulin receptor substrate 2 | Multiple_C | 10.96 | 9.55 | 8.94 | 4.06 | 1.99E-06 | 4.74E-05 | 8.82 | 8.68 | 7.96 | 1.82 | 0.0012 | 0.0189 |
| TC2100008030.hg.1 | RCAN1 | regulator of calcineurin 1 | Multiple_C | 12.21 | 10.83 | 10.19 | 4.06 | 3.12E-08 | 2.03E-06 | 7.98 | 8.56 | 9.08 | 0.47 | 1.33E-05 | 0.0009 |
| TC0100007645.hg.1 | TINAGL1 | tubulointerstitial nephritis antigen-like 1 | Multiple_C | 13.77 | 12.47 | 11.76 | 4.03 | 0.0003 | 0.0024 | 9.78 | 8.42 | 8.28 | 2.83 | 0.0007 | 0.0129 |
| TC0100011382.hg.1 | DYRK3 | dual specificity tyrosine-(Y)-phosphorylation regulated kinase 3 | Multiple_C | 8.59 | 7.44 | 6.58 | 4.03 | 2.68E-05 | 0.0004 | 8.24 | 8.10 | 8.14 | 1.07 | 0.5056 | 0.7371 |
| TC0100014344.hg.1 | MYSM1 | Myb-like, SWIRM and MPN domains 1 | Multiple_C | 12.37 | 10.63 | 10.36 | 4.03 | 1.22E-05 | 0.0002 | 11.8 | 10.47 | 11.22 | 1.49 | 0.0524 | 0.2138 |
| TC0100017172.hg.1 | IRF6 | interferon regulatory factor 6 | Multiple_C | 10.86 | 8.72 | 8.85 | 4.03 | 9.25E-06 | 0.0002 | 4.84 | 4.74 | 4.7 | 1.10 | 0.1151 | 0.3349 |
| TC0600009327.hg.1 | PKIB | protein kinase (cAMP-dependent, catalytic) inhibitor beta | Multiple_C | 6.24 | 4.63 | 4.23 | 4.03 | 1.72E-05 | 0.0003 | 4.08 | 4.09 | 3.93 | 1.11 | 0.1222 | 0.345 |
| TC0800009872.hg.1 | TNFRSF10D | tumor necrosis factor receptor superfamily, member 10d, decoy | Coding | 11.76 | 10.61 | 9.75 | 4.03 | 9.52E-08 | 4.65E-06 | 10.62 | 9.46 | 9.7 | 1.89 | 0.0003 | 0.008 |
| TC1000008431.hg.1 | PCGF5 | polycomb group ring finger 5 | Multiple_C | 10.03 | 7.7 | 8.02 | 4.03 | 2.33E-05 | 0.0003 | 7.73 | 8.20 | 8.33 | 0.66 | 0.3548 | 0.6181 |
| TC1100013043.hg.1 | FAM89B | family with sequence similarity 89, member B | Coding | 7.84 | 5.29 | 5.83 | 4.03 | 0.0024 | 0.0133 | 6.21 | 6.44 | 6.61 | 0.76 | 0.7456 | 0.8834 |
| TC1300008229.hg.1 | ZMYM5 | zinc finger, MYM-type 5 | Multiple_C | 11.41 | 10.5 | 9.4 | 4.03 | 0.0012 | 0.0077 | 7.46 | 6.72 | 6.83 | 1.55 | 0.8165 | 0.9187 |
| TC1600007874.hg.1 | CHD9 | chromodomain helicase DNA binding protein 9 | Multiple_C | 9.81 | 8.33 | 7.8 | 4.03 | 1.76E-05 | 0.0003 | 9.05 | 8.84 | 9.89 | 0.56 | 0.0048 | 0.0477 |
| TC0100015945.hg.1 | THBS3 | thrombospondin 3 | Multiple_C | 7.5 | 6.61 | 5.5 | 4.00 | 3.70E-06 | 7.91E-05 | 9.08 | 9.03 | 7.96 | 2.17 | 4.73E-05 | 0.0021 |
| TC0100013417.hg.1 | SLC9A1 | solute carrier family 9, subfamily A (NHE1, cation proton antipo | Multiple_C | 9.74 | 7.4 | 7.75 | 3.97 | 7.74E-05 | 0.0009 | 6.92 | 7.50 | 8.28 | 0.39 | 2.95E-05 | 0.0015 |
| TC0300006544.hg.1 | SETD5 | SET domain containing 5 | Multiple_C | 10.19 | 8.01 | 8.2 | 3.97 | 3.62E-07 | 1.26E-05 | 9.1 | 8.55 | 9.14 | 0.97 | 0.7325 | 0.8763 |
| TC1700009256.hg.1 | NARF | nuclear prelamin A recognition factor | Multiple_C | 11.69 | 9.47 | 9.7 | 3.97 | 6.38E-08 | 3.47E-06 | 12.31 | 12.75 | 11.94 | 1.29 | 0.0175 | 0.1107 |
| TC1700010738.hg.1 | EZH1 | enhancer of zeste 1 polycomb repressive complex 2 subunit | Multiple_C | 8.69 | 6.92 | 6.7 | 3.97 | 1.03E-06 | 2.86E-05 | 7.31 | 6.50 | 6.76 | 1.46 | 0.0287 | 0.1493 |
| TC0100008912.hg.1 | CYR61 | cysteine-rich, angiogenic inducer, 61 | Multiple_C | 7.69 | 5.71 | 5.71 | 3.94 | 3.13E-06 | 6.93E-05 | 5.32 | 5.28 | 5.07 | 1.19 | 0.1095 | 0.3258 |
| TC0200016704.hg.1 | RNF103 | ring finger protein 103 | Multiple_C | 10.52 | 9.49 | 8.54 | 3.94 | 9.50E-07 | 2.67E-05 | 8.74 | 8.70 | 8.26 | 1.39 | 0.3027 | 0.567 |
| TC0400010085.hg.1 | BOD1L1 | biorientation of chromosomes in cell division 1-like 1 | Multiple_C | 8.67 | 7.37 | 6.69 | 3.94 | 3.01E-07 | 1.09E-05 | 9.03 | 9.20 | 8.64 | 1.31 | 0.3007 | 0.5651 |
| TC0800012285.hg.1 | HMBOX1 | homeobox containing 1 | Multiple_C | 9.51 | 7.83 | 7.53 | 3.94 | 2.40E-07 | 9.32E-06 | 7.36 | 6.79 | 7.68 | 0.80 | 0.1279 | 0.354 |
| TC1200012240.hg.1 | RSRC2 | arginine/serine-rich coiled-coil 2 | Multiple_C | 14.87 | 13.97 | 12.89 | 3.94 | 1.53E-06 | 3.89E-05 | 13.87 | 12.66 | 13.49 | 1.30 | 0.1181 | 0.3396 |
| TC1500008335.hg.1 | FURIN | furin (paired basic amino acid cleaving enzyme) | Multiple_C | 7.34 | 5.11 | 5.36 | 3.94 | 4.55E-08 | 2.69E-06 | 5.6 | 5.74 | 5.8 | 0.87 | 0.3347 | 0.599 |
| TC1700008794.hg.1 | SOX9 | SRY box 9 | Multiple_C | 12.51 | 9.92 | 10.53 | 3.94 | 2.31E-05 | 0.0003 | 12.87 | 12.69 | 12.21 | 1.58 | 0.0112 | 0.0835 |

| | | | | | | | | | | | | | | | |
|---|---|---|---|---|---|---|---|---|---|---|---|---|---|---|---|
| TC1700011465.hg.1 | LRRC37A3 | leucine rich repeat containing 37, member A3 | Multiple_C | 6.81 | 4.96 | 4.83 | 3.94 | 3.25E-07 | 1.16E-05 | 6.91 | 7.10 | 7.19 | 0.82 | 0.1117 | 0.3293 |
| TC2200008898.hg.1 | TTLL1 | tubulin tyrosine ligase-like family member 1 | Multiple_C | 6.71 | 5.38 | 4.73 | 3.94 | 0.0009 | 0.0061 | 5.69 | 5.47 | 4.83 | 1.82 | 0.0139 | 0.0957 |
| TC0300011151.hg.1 | RPL29 | ribosomal protein L29 | Multiple_C | 15.16 | 13.87 | 13.19 | 3.92 | 8.12E-06 | 0.0001 | 11.97 | 12.56 | 12.07 | 0.93 | 0.6216 | 0.8128 |
| TC0300011525.hg.1 | RYBP | RING1 and YY1 binding protein | Multiple_C | 10.99 | 8.69 | 9.02 | 3.92 | 1.10E-05 | 0.0002 | 8.26 | 7.41 | 8.38 | 0.92 | 0.7228 | 0.8707 |
| TC0900011713.hg.1 | FNBP1 | formin binding protein 1 | Multiple_C | 8.69 | 7.02 | 6.72 | 3.92 | 1.51E-05 | 0.0002 | 5.73 | 6.02 | 6.53 | 0.57 | 0.0058 | 0.0544 |
| TC1700008254.hg.1 | ACSF2 | acyl-CoA synthetase family member 2 | Multiple_C | 8.03 | 7.04 | 6.06 | 3.92 | 5.08E-07 | 1.64E-05 | 5.85 | 5.77 | 5.36 | 1.40 | 0.0372 | 0.1754 |
| TC1800009290.hg.1 | MYO5B | myosin VB | Multiple_C | 13.05 | 11.65 | 11.08 | 3.92 | 6.14E-06 | 0.0001 | 6.31 | 7.22 | 7.71 | 0.38 | 1.36E-05 | 0.0009 |
| TC1900008860.hg.1 | LENG8 | leukocyte receptor cluster (LRC) member 8 | Multiple_C | 12.94 | 11.06 | 10.97 | 3.92 | 0.0002 | 0.0016 | 12.74 | 12.79 | 12.43 | 1.24 | 0.5651 | 0.7747 |
| TC2200008888.hg.1 | A4GALT | alpha 1,4-galactosyltransferase | Multiple_C | 7.21 | 4.87 | 5.25 | 3.89 | 9.87E-08 | 4.76E-06 | 4.12 | 3.66 | 3.58 | 1.45 | 0.0077 | 0.0653 |
| TC0600009059.hg.1 | FOXO3 | forkhead box O3 | Multiple_C | 11.46 | 9.35 | 9.51 | 3.86 | 8.36E-06 | 0.0001 | 9.54 | 9.35 | 9.49 | 1.04 | 0.7854 | 0.9041 |
| TC0700013361.hg.1 | ZNF713 | zinc finger protein 713 | Multiple_C | 6.91 | 4.73 | 4.96 | 3.86 | 0.0002 | 0.0018 | 6.91 | 7.38 | 7 | 0.94 | 0.6615 | 0.8347 |
| TC0900007085.hg.1 | TESK1; MIR46 | testis-specific kinase 1; microRNA 4667 | Multiple_C | 10.89 | 8.84 | 8.94 | 3.86 | 2.67E-07 | 1.00E-05 | 10.85 | 10.95 | 11.56 | 0.61 | 0.0011 | 0.0173 |
| TC0X00008001.hg.1 | WBP5 | WW domain binding protein 5 | Coding | 7.57 | 6.03 | 5.62 | 3.86 | 4.41E-06 | 9.05E-05 | 7.8 | 8.01 | 7.85 | 0.97 | 0.6456 | 0.826 |
| TC1700011369.hg.1 | MED13 | mediator complex subunit 13 | Multiple_C | 13.26 | 10.85 | 11.31 | 3.86 | 7.29E-07 | 2.19E-05 | 13.81 | 13.54 | 14.03 | 0.86 | 0.7465 | 0.8837 |
| TC0200012307.hg.1 | SOS1 | SOS Ras/Rac guanine nucleotide exchange factor 1 | Multiple_C | 11.89 | 10.01 | 9.95 | 3.84 | 3.91E-05 | 0.0005 | 9.78 | 9.07 | 9.67 | 1.08 | 0.7692 | 0.8964 |
| TC0800010945.hg.1 | SLC10A5 | solute carrier family 10, member 5 | Coding | 7.77 | 5.14 | 5.83 | 3.84 | 0.0014 | 0.0089 | 5.34 | 6.34 | 6.34 | 0.50 | 0.2817 | 0.5474 |
| TC1300009218.hg.1 | DACH1 | dachshund family transcription factor 1 | Multiple_C | 5.1 | 2.98 | 3.16 | 3.84 | 4.18E-06 | 8.69E-05 | 9.28 | 11.67 | 9.23 | 1.04 | 0.5221 | 0.7487 |
| TC0500013245.hg.1 | PCDHB9 | protocadherin beta 9 | Multiple_C | 9.65 | 7.3 | 7.72 | 3.81 | 8.25E-08 | 4.16E-06 | 4.8 | 4.92 | 5.1 | 0.81 | 0.1665 | 0.4116 |
| TC1200007137.hg.1 | FGFR1OP2 | FGFR1 oncogene partner 2 | Multiple_C | 11.59 | 10.57 | 9.66 | 3.81 | 1.51E-07 | 6.58E-06 | 12.53 | 11.82 | 11.59 | 1.92 | 0.0002 | 0.0064 |
| TC1200007897.hg.1 | TSPAN31 | tetraspanin 31 | Multiple_C | 9.24 | 7.58 | 7.31 | 3.81 | 8.02E-08 | 4.05E-06 | 9.44 | 8.87 | 8.58 | 1.82 | 0.0003 | 0.0067 |
| TC1200008474.hg.1 | SOCS2 | suppressor of cytokine signaling 2 | Coding | 7.08 | 6.2 | 5.15 | 3.81 | 3.07E-06 | 6.82E-05 | 5.43 | 5.09 | 6.25 | 0.57 | 0.0014 | 0.0214 |
| TC1200012763.hg.1 | TAS2R30; TAS | taste receptor, type 2, member 30; taste receptor, type 2, mem | Multiple_C | 8.08 | 6.1 | 6.15 | 3.81 | 5.56E-05 | 0.0007 | 5.59 | 5.79 | 5.99 | 0.76 | 0.9817 | 0.9925 |
| TC2200007083.hg.1 | INPP5J | inositol polyphosphate-5-phosphatase J | Multiple_C | 6.47 | 4.73 | 4.54 | 3.81 | 4.80E-06 | 9.71E-05 | 5.31 | 5.15 | 5.21 | 1.07 | 0.7762 | 0.8996 |
| TC0100007207.hg.1 | PINK1; MIR60 | PTEN induced putative kinase 1; microRNA 6084 | Multiple_C | 8.21 | 6.46 | 6.29 | 3.78 | 3.75E-05 | 0.0005 | 6.06 | 6.12 | 5.81 | 1.19 | 0.8602 | 0.9402 |
| TC0500007465.hg.1 | GPBP1 | GC-rich promoter binding protein 1 | Multiple_C | 12.77 | 11.92 | 10.85 | 3.78 | 8.24E-06 | 0.0001 | 11.38 | 10.65 | 10.87 | 1.42 | 0.3718 | 0.6332 |
| TC0500008882.hg.1 | PCDHB11 | protocadherin beta 11 | Coding | 9.66 | 7.7 | 7.74 | 3.78 | 9.08E-06 | 0.0002 | 9.56 | 10.17 | 10.05 | 0.71 | 0.079 | 0.2709 |
| TC0600010121.hg.1 | QKI | QKI, KH domain containing, RNA binding | Multiple_C | 10.83 | 8.93 | 8.91 | 3.78 | 0.0009 | 0.006 | 7.34 | 5.25 | 8.41 | 0.48 | 0.1226 | 0.3456 |
| TC1100013129.hg.1 | CTSD | cathepsin D | Multiple_C | 14.27 | 11.06 | 12.35 | 3.78 | 6.83E-07 | 2.09E-05 | 12.55 | 12.36 | 12.23 | 1.25 | 0.0642 | 0.2411 |
| TC1500009200.hg.1 | TTBK2 | tau tubulin kinase 2 | Multiple_C | 8.84 | 7.69 | 6.92 | 3.78 | 2.81E-06 | 6.33E-05 | 8.62 | 7.69 | 8.16 | 1.38 | 0.085 | 0.2806 |
| TC0200007999.hg.1 | ZNF638 | Transcript Identified by AceView, Entrez Gene ID(s) 27332 | Coding | 7.4 | 5.82 | 5.49 | 3.76 | 2.87E-05 | 0.0004 | 5.99 | 5.54 | 5.63 | 1.28 | 0.0519 | 0.2128 |
| TC0200013610.hg.1 | REV1 | REV1, DNA directed polymerase | Multiple_C | 9.87 | 8.41 | 7.96 | 3.76 | 4.12E-07 | 1.40E-05 | 8.52 | 8.04 | 8.41 | 1.08 | 0.9232 | 0.9685 |
| TC0200015815.hg.1 | EPHA4 | EPH receptor A4 | Multiple_C | 8.12 | 7.2 | 6.21 | 3.76 | 0.0001 | 0.0011 | 7.91 | 4.99 | 7.1 | 1.75 | 0.0089 | 0.0719 |
| TC0300014082.hg.1 | ETV5 | ets variant 5 | Multiple_C | 10.68 | 8.15 | 8.77 | 3.76 | 8.57E-06 | 0.0002 | 12.25 | 12.42 | 11.81 | 1.36 | 0.0444 | 0.1949 |

| | | | | | | | | | | | | | | | |
|---|---|---|---|---|---|---|---|---|---|---|---|---|---|---|---|
| TC0400011685.hg.1 | PRSS12 | protease, serine, 12 (neurotrypsin, motopsin) | Multiple_C | 11.45 | 10.4 | 9.54 | 3.76 | 7.77E-06 | 0.0001 | 4.73 | 4.29 | 4.51 | 1.16 | 0.1254 | 0.3499 |
| TC1100010107.hg.1 | CSNK2A3 | casein kinase 2, alpha 3 polypeptide | Coding | 11.99 | 9.98 | 10.08 | 3.76 | 6.84E-06 | 0.0001 | 11.34 | 10.83 | 11.3 | 1.03 | 0.9979 | 0.999 |
| TC1100011260.hg.1 | C11orf68 | chromosome 11 open reading frame 68 | Multiple_C | 8.45 | 6.22 | 6.54 | 3.76 | 7.57E-07 | 2.25E-05 | 5.54 | 5.60 | 5.45 | 1.06 | 0.8441 | 0.9318 |
| TC1100013140.hg.1 | TAF10 | TAF10 RNA polymerase II, TATA box binding protein (TBP)-assoc | Multiple_C | 13.24 | 10.67 | 11.33 | 3.76 | 3.43E-06 | 7.42E-05 | 10.95 | 11.33 | 11.08 | 0.91 | 0.9275 | 0.9698 |
| TC1200012808.hg.1 | R3HDM2 | R3H domain containing 2 | Multiple_C | 8.51 | 6.81 | 6.6 | 3.76 | 1.84E-06 | 4.43E-05 | 8.17 | 8.61 | 8.02 | 1.11 | 0.7964 | 0.9092 |
| TC1500010909.hg.1 | STARD5 | StAR-related lipid transfer domain containing 5 | Multiple_C | 7.91 | 5.74 | 6 | 3.76 | 0.0039 | 0.0197 | 6.11 | 5.97 | 5.83 | 1.21 | 0.3908 | 0.6493 |
| TC1700010540.hg.1 | PCGF2 | polycomb group ring finger 2 | Coding | 10.4 | 8.29 | 8.49 | 3.76 | 2.67E-05 | 0.0004 | 9.23 | 9.70 | 8.7 | 1.44 | 0.0087 | 0.0712 |
| TC1700012093.hg.1 | FASN | fatty acid synthase | Multiple_C | 13.03 | 11.1 | 11.12 | 3.76 | 1.47E-08 | 1.20E-06 | 12.35 | 12.09 | 12.39 | 0.97 | 0.2451 | 0.5091 |
| TC1900008042.hg.1 | PAK4 | p21 protein (Cdc42/Rac)-activated kinase 4 | Multiple_C | 11.41 | 9.26 | 9.5 | 3.76 | 4.22E-06 | 8.75E-05 | 9.99 | 10.28 | 10.15 | 0.90 | 0.6075 | 0.8037 |
| TC2000006736.hg.1 | SPTLC3 | serine palmitoyltransferase, long chain base subunit 3 | Multiple_C | 6.42 | 4.08 | 4.51 | 3.76 | 3.72E-06 | 7.93E-05 | 3.9 | 3.59 | 3.52 | 1.30 | 0.1685 | 0.4142 |
| TC0200010966.hg.1 | RHBDD1 | rhomboid domain containing 1 | Multiple_C | 8.56 | 7.24 | 6.66 | 3.73 | 2.02E-06 | 4.81E-05 | 9.14 | 8.56 | 9.56 | 0.75 | 0.1346 | 0.3643 |
| TC0300013888.hg.1 | TSC22D2 | TSC22 domain family, member 2 | Multiple_C | 13.19 | 11.35 | 11.29 | 3.73 | 6.68E-07 | 2.05E-05 | 9.33 | 9.02 | 9.89 | 0.68 | 0.2387 | 0.5021 |
| TC0600007617.hg.1 | C6orf48; SNOI | chromosome 6 open reading frame 48; small nucleolar RNA, C/I | Multiple_C | 13.01 | 13.57 | 11.11 | 3.73 | 5.57E-05 | 0.0007 | 10.03 | 9.56 | 9.64 | 1.31 | 0.0316 | 0.159 |
| TC1000008182.hg.1 | ZMIZ1 | zinc finger, MIZ-type containing 1 | Multiple_C | 8.73 | 6.65 | 6.83 | 3.73 | 2.66E-06 | 6.05E-05 | 7.06 | 6.94 | 7.04 | 1.01 | 0.5704 | 0.7788 |
| TC1000008727.hg.1 | NFKB2 | nuclear factor of kappa light polypeptide gene enhancer in B-ce | Multiple_C | 10.18 | 8.85 | 8.28 | 3.73 | 0.001 | 0.0066 | 6.75 | 5.48 | 6.06 | 1.61 | 0.0421 | 0.1893 |
| TC1200006450.hg.1 | WNK1 | WNK lysine deficient protein kinase 1 | Multiple_C | 9.59 | 7.41 | 7.69 | 3.73 | 3.63E-07 | 1.26E-05 | 11.28 | 11.88 | 11.92 | 0.64 | 0.0033 | 0.038 |
| TC1200012254.hg.1 | VPS37B | vacuolar protein sorting 37 homolog B (S. cerevisiae) | Multiple_C | 9.84 | 7.61 | 7.94 | 3.73 | 7.02E-07 | 2.12E-05 | 8.3 | 8.18 | 8.68 | 0.77 | 0.1091 | 0.3252 |
| TC1400009421.hg.1 | GPX2 | glutathione peroxidase 2 | Multiple_C | 15.84 | 13.71 | 13.94 | 3.73 | 2.84E-07 | 1.05E-05 | 13 | 12.69 | 13.12 | 0.92 | 0.6327 | 0.8192 |
| TC0500008700.hg.1 | SEC24A | SEC24 homolog A, COPII coat complex component | Multiple_C | 10.85 | 8.73 | 8.96 | 3.71 | 4.35E-05 | 0.0005 | 9.34 | 8.93 | 10.13 | 0.58 | 0.032 | 0.16 |
| TC0100017113.hg.1 | PIGR | polymeric immunoglobulin receptor | Multiple_C | 6.58 | 4.38 | 4.7 | 3.68 | 6.67E-07 | 2.05E-05 | 4.85 | 5.15 | 4.67 | 1.13 | 0.0521 | 0.2131 |
| TC0800011249.hg.1 | RNF19A | ring finger protein 19A, RBR E3 ubiquitin protein ligase | Multiple_C | 9.07 | 8.03 | 7.19 | 3.68 | 1.28E-07 | 5.79E-06 | 6.85 | 5.92 | 6.32 | 1.44 | 0.0081 | 0.0678 |
| TC1200007251.hg.1 | KIAA1551 | KIAA1551 | Multiple_C | 10.87 | 9.2 | 8.99 | 3.68 | 1.59E-06 | 4.00E-05 | 7.3 | 7.13 | 7.41 | 0.93 | 0.5429 | 0.7614 |
| TC1600011427.hg.1 | IST1 | increased sodium tolerance 1 homolog (yeast) | Multiple_C | 13.34 | 11.75 | 11.46 | 3.68 | 9.98E-08 | 4.80E-06 | 13.36 | 12.77 | 13.32 | 1.03 | 0.5064 | 0.7377 |
| TC1700012316.hg.1 | ANAPC11 | anaphase promoting complex subunit 11 | Multiple_C | 11.02 | 9.4 | 9.14 | 3.68 | 0.0001 | 0.0014 | 8.13 | 8.34 | 8.28 | 0.90 | 0.9269 | 0.9697 |
| TC2200008091.hg.1 | PI4KAP2 | phosphatidylinositol 4-kinase, catalytic, alpha pseudogene 2 | Multiple_C | 10.28 | 8.47 | 8.4 | 3.68 | 5.75E-06 | 0.0001 | 8.26 | 8.85 | 8.51 | 0.84 | 0.8954 | 0.9558 |
| TC0600014333.hg.1 | MICAL1 | microtubule associated monooxygenase, calponin and LIM dom | Multiple_C | 8.48 | 6.85 | 6.61 | 3.66 | 2.38E-05 | 0.0003 | 6.35 | 6.45 | 5.77 | 1.49 | 0.4303 | 0.6821 |
| TC0700011500.hg.1 | STX1A | syntaxin 1A (brain) | Multiple_C | 8.57 | 6.25 | 6.7 | 3.66 | 0.0004 | 0.003 | 7.86 | 7.79 | 7.88 | 0.99 | 0.9903 | 0.9961 |
| TC0900009333.hg.1 | FAM157B | family with sequence similarity 157, member B | Multiple_C | 10.75 | 8.59 | 8.88 | 3.66 | 2.25E-07 | 8.90E-06 | 8.49 | 8.34 | 8.33 | 1.12 | 0.2296 | 0.4906 |
| TC1100013087.hg.1 | BIRC3 | baculoviral IAP repeat containing 3 | Multiple_C | 8.07 | 7.1 | 6.2 | 3.66 | 0.0001 | 0.0012 | 6.89 | 4.29 | 4.81 | 4.23 | 5.62E-05 | 0.0025 |
| TC1500007633.hg.1 | SMAD3 | SMAD family member 3 | Multiple_C | 8.34 | 6.13 | 6.47 | 3.66 | 3.22E-06 | 7.07E-05 | 6.92 | 8.09 | 8.93 | 0.25 | 3.81E-08 | 1.36E-05 |
| TC1500010712.hg.1 | GOLGA8N | golgin A8 family, member N | Multiple_C | 7.85 | 6.92 | 5.98 | 3.66 | 4.24E-05 | 0.0005 | 7.64 | 6.86 | 6.79 | 1.80 | 0.0054 | 0.0514 |
| TC1700007757.hg.1 | GRB7 | growth factor receptor bound protein 7 | Multiple_C | 9.76 | 8.26 | 7.89 | 3.66 | 1.75E-05 | 0.0003 | 8.44 | 8.36 | 8.22 | 1.16 | 0.6686 | 0.838 |
| TC1700012279.hg.1 | HOXB-AS3 | HOXB cluster antisense RNA 3 | Multiple_C | 5.68 | 3.61 | 3.81 | 3.66 | 0.0004 | 0.0032 | 7.02 | 6.98 | 7.25 | 0.85 | 0.5982 | 0.7979 |

| | | | | | | | | | | | | | | | |
|---|---|---|---|---|---|---|---|---|---|---|---|---|---|---|---|
| TC1900007847.hg.1 | USF2 | upstream transcription factor 2, c-fos interacting | Multiple_C | 10.61 | 9.04 | 8.74 | 3.66 | 8.68E-07 | 2.50E-05 | 9.8 | 10.02 | 10.06 | 0.84 | 0.4045 | 0.66 |
| TC1000008467.hg.1 | MARCHF5 | membrane associated ring finger 5 | Multiple_C | 12.67 | 11.61 | 10.81 | 3.63 | 3.76E-05 | 0.0005 | 10.7 | 9.55 | 10.62 | 1.06 | 0.8798 | 0.9484 |
| TC1300007531.hg.1 | LMO7DN | LMO7 downstream neighbor | Multiple_C | 9.29 | 7.17 | 7.43 | 3.63 | 1.16E-05 | 0.0002 | 5.39 | 5.58 | 5.55 | 0.90 | 0.4529 | 0.6979 |
| TC1300008533.hg.1 | HSPH1 | heat shock 105kDa/110kDa protein 1 | Multiple_C | 13.12 | 11.69 | 11.26 | 3.63 | 1.70E-05 | 0.0003 | 15.9 | 14.23 | 14.9 | 2.00 | 0.0052 | 0.0506 |
| TC1500010613.hg.1 | TTC23 | tetratricopeptide repeat domain 23 | Multiple_C | 9.6 | 8.15 | 7.74 | 3.63 | 3.62E-05 | 0.0005 | 8.33 | 7.44 | 7.91 | 1.34 | 0.3649 | 0.6273 |
| TC1500010915.hg.1 | GOLGA6L5P; ( | golgin A6 family-like 5, pseudogene; golgin A6 family-like 17, ps | Multiple_C | 8.73 | 7.76 | 6.87 | 3.63 | 9.30E-07 | 2.63E-05 | 7.54 | 6.81 | 6.78 | 1.69 | 0.002 | 0.0269 |
| TC1600008698.hg.1 | GSE1 | Gse1 coiled-coil protein | Multiple_C | 9.21 | 7.59 | 7.35 | 3.63 | 4.27E-05 | 0.0005 | 11.47 | 11.45 | 10.58 | 1.85 | 0.0354 | 0.1705 |
| TC2100008487.hg.1 | TPTE | transmembrane phosphatase with tensin homology | Multiple_C | 6.45 | 4.35 | 4.59 | 3.63 | 9.74E-05 | 0.001 | 6.1 | 5.23 | 5.2 | 1.87 | 0.018 | 0.1127 |
| TC0200013902.hg.1 | RGPD5; RGPD | RANBP2-like and GRIP domain containing 5; RANBP2-like and G | Multiple_C | 13.74 | 12.08 | 11.89 | 3.61 | 1.29E-06 | 3.39E-05 | 12.63 | 11.11 | 11.79 | 1.79 | 0.0097 | 0.0762 |
| TC0400011087.hg.1 | CCNI | cyclin I | Multiple_C | 16.65 | 15.04 | 14.8 | 3.61 | 7.49E-08 | 3.88E-06 | 12.32 | 12.71 | 12.63 | 0.81 | 0.1387 | 0.3698 |
| TC1300008840.hg.1 | LCP1 | lymphocyte cytosolic protein 1 (L-plastin) | Multiple_C | 5.51 | 3.65 | 3.66 | 3.61 | 4.40E-06 | 9.03E-05 | 14.13 | 13.77 | 13.48 | 1.57 | 0.0404 | 0.1842 |
| TC1600008799.hg.1 | BANP | BTG3 associated nuclear protein | Multiple_C | 11.59 | 9.72 | 9.74 | 3.61 | 3.98E-06 | 8.37E-05 | 11.39 | 11.03 | 11.44 | 0.97 | 0.6788 | 0.8453 |
| TC0200014269.hg.1 | POTEI | POTE ankyrin domain family, member I | Multiple_C | 7.8 | 7.06 | 5.96 | 3.58 | 5.24E-05 | 0.0006 | 6.12 | 5.52 | 5.45 | 1.59 | 0.0996 | 0.3085 |
| TC0200015506.hg.1 | INO80D | INO80 complex subunit D | Multiple_C | 10.2 | 7.45 | 8.36 | 3.58 | 3.35E-06 | 7.29E-05 | 8.98 | 8.39 | 9 | 0.99 | 0.9784 | 0.9909 |
| TC0400006591.hg.1 | HTT | huntingtin | Multiple_C | 9.29 | 7.28 | 7.45 | 3.58 | 1.47E-06 | 3.78E-05 | 9.66 | 9.43 | 9.49 | 1.13 | 0.9677 | 0.9866 |
| TC0700008003.hg.1 | CLIP2 | CAP-GLY domain containing linker protein 2 | Multiple_C | 9.25 | 8.12 | 7.41 | 3.58 | 7.07E-06 | 0.0001 | 8.78 | 8.50 | 8.81 | 0.98 | 0.608 | 0.804 |
| TC0900008793.hg.1 | ZBTB34 | zinc finger and BTB domain containing 34 | Multiple_C | 6.45 | 4.96 | 4.61 | 3.58 | 0.0051 | 0.0243 | 4.95 | 4.86 | 5.13 | 0.88 | 0.5203 | 0.7477 |
| TC1000011740.hg.1 | COL17A1; MIF | collagen, type XVII, alpha 1; microRNA 936 | Multiple_C | 13.95 | 11.99 | 12.11 | 3.58 | 6.63E-06 | 0.0001 | 4.24 | 3.97 | 4.01 | 1.17 | 0.2694 | 0.5343 |
| TC1000012471.hg.1 | ADIRF; AGAP1 | adipogenesis regulatory factor; ankyrin repeat and GTPase dom | Multiple_C | 7.29 | 5.46 | 5.45 | 3.58 | 1.85E-05 | 0.0003 | 6.18 | 6.20 | 6.42 | 0.85 | 0.3049 | 0.5689 |
| TC1500008167.hg.1 | ZNF592 | zinc finger protein 592 | Multiple_C | 10.32 | 7.49 | 8.48 | 3.58 | 2.84E-07 | 1.05E-05 | 9.23 | 9.65 | 10.06 | 0.56 | 0.0094 | 0.0748 |
| TC1900008727.hg.1 | ERVV-2 | endogenous retrovirus group V, member 2 | Coding | 5.59 | 4.89 | 3.75 | 3.58 | 2.38E-07 | 9.29E-06 | 3.84 | 3.56 | 3.39 | 1.37 | 0.1893 | 0.4419 |
| TC0100017028.hg.1 | PPP1R15B | protein phosphatase 1, regulatory subunit 15B | Coding | 13.34 | 11.86 | 11.51 | 3.56 | 3.83E-07 | 1.32E-05 | 11.29 | 10.33 | 11.47 | 0.88 | 0.8205 | 0.9208 |
| TC0200006627.hg.1 | ID2 | inhibitor of DNA binding 2, dominant negative helix-loop-helix p | Multiple_C | 11.74 | 10.19 | 9.91 | 3.56 | 2.88E-05 | 0.0004 | 9.41 | 9.43 | 9.28 | 1.09 | 0.1494 | 0.3869 |
| TC0200009774.hg.1 | TANC1 | tetratricopeptide repeat, ankyrin repeat and coiled-coil containi | Multiple_C | 10.25 | 8.3 | 8.42 | 3.56 | 2.28E-05 | 0.0003 | 8.16 | 7.23 | 7.38 | 1.72 | 0.0111 | 0.0832 |
| TC0400010125.hg.1 | FGFBP1 | fibroblast growth factor binding protein 1 | Multiple_C | 9.03 | 8.5 | 7.2 | 3.56 | 5.13E-06 | 0.0001 | 4.05 | 3.96 | 4.31 | 0.84 | 0.7523 | 0.8874 |
| TC0500013246.hg.1 | PCDHB10 | protocadherin beta 10 | Coding | 6.81 | 4.45 | 4.98 | 3.56 | 0.0001 | 0.0014 | 4.46 | 4.09 | 4.14 | 1.25 | 0.3157 | 0.5797 |
| TC1000007199.hg.1 | ZEB1 | zinc finger E-box binding homeobox 1 | Multiple_C | 5.49 | 3.58 | 3.66 | 3.56 | 0.0002 | 0.0015 | 11.1 | 10.30 | 10.71 | 1.31 | 0.6249 | 0.8147 |
| TC1000012427.hg.1 | AKR1C1 | aldo-keto reductase family 1, member C1 | Multiple_C | 8.3 | 7.62 | 6.47 | 3.56 | 1.46E-06 | 3.75E-05 | 6.81 | 6.87 | 7.68 | 0.55 | 0.0228 | 0.13 |
| TC1700011953.hg.1 | CBX4 | chromobox homolog 4 | Multiple_C | 10.78 | 8.72 | 8.95 | 3.56 | 8.22E-05 | 0.0009 | 10.44 | 9.76 | 10.24 | 1.15 | 0.4186 | 0.6724 |
| TC1700012191.hg.1 | CD68 | CD68 molecule | Coding | 11.13 | 9.94 | 9.3 | 3.56 | 0.0002 | 0.0017 | 6.37 | 5.95 | 6.81 | 0.74 | 0.2315 | 0.4928 |
| TC0700011876.hg.1 | ASNS | asparagine synthetase (glutamine-hydrolyzing) | Multiple_C | 14.86 | 15.65 | 13.04 | 3.53 | 0.0001 | 0.0011 | 10.99 | 9.42 | 11.23 | 0.85 | 0.4391 | 0.6895 |
| TC0700013442.hg.1 | LSMEM1 | leucine-rich single-pass membrane protein 1 | Multiple_C | 6.86 | 5.41 | 5.04 | 3.53 | 0.0003 | 0.0027 | 5.42 | 5.40 | 5.87 | 0.73 | 0.1197 | 0.3417 |
| TC1000006466.hg.1 | WDR37 | WD repeat domain 37 | Multiple_C | 9.73 | 7.69 | 7.91 | 3.53 | 7.90E-06 | 0.0001 | 8.2 | 8.28 | 8.5 | 0.81 | 0.0793 | 0.2712 |

| | | | | | | | | | | | | | | | |
|---|---|---|---|---|---|---|---|---|---|---|---|---|---|---|---|
| TC1200012647.hg.1 | MYL6B | myosin light chain 6B | Multiple_C | 10.29 | 9.14 | 8.47 | 3.53 | 5.39E-05 | 0.0006 | 8.69 | 9.29 | 8.96 | 0.83 | 0.4566 | 0.7005 |
| TC1600006593.hg.1 | RAB26 | RAB26, member RAS oncogene family | Multiple_C | 7.17 | 5.96 | 5.35 | 3.53 | 0.0048 | 0.0233 | 7.89 | 6.57 | 6.24 | 3.14 | 0.0003 | 0.0074 |
| TC1700009528.hg.1 | CXCL16 | chemokine (C-X-C motif) ligand 16 | Multiple_C | 12.47 | 10.16 | 10.65 | 3.53 | 0.0063 | 0.0288 | 5.46 | 5.03 | 5.14 | 1.25 | 0.0356 | 0.171 |
| TC1900008329.hg.1 | VASP | vasodilator-stimulated phosphoprotein | Multiple_C | 12.64 | 11.11 | 10.82 | 3.53 | 0.0142 | 0.0548 | 10.97 | 11.61 | 11.28 | 0.81 | 0.4901 | 0.7257 |
| TC1900009824.hg.1 | DNAJB1 | DnaJ (Hsp40) homolog, subfamily B, member 1 | Multiple_C | 14.8 | 13.14 | 12.98 | 3.53 | 0.0061 | 0.0284 | 15.19 | 12.77 | 14.74 | 1.37 | 0.6658 | 0.8364 |
| TC0100018256.hg.1 | NBPF6; NBPF5 | neuroblastoma breakpoint family, member 6; neuroblastoma b | Multiple_C | 7.11 | 5.93 | 5.3 | 3.51 | 5.08E-07 | 1.64E-05 | 7.68 | 8.29 | 7.68 | 1.00 | 0.9737 | 0.9896 |
| TC0200010443.hg.1 | CFLAR | CASP8 and FADD like apoptosis regulator | Multiple_C | 12.86 | 11.99 | 11.05 | 3.51 | 3.10E-06 | 6.88E-05 | 9.37 | 9.34 | 9.71 | 0.79 | 0.0343 | 0.1668 |
| TC0900007010.hg.1 | UBE2R2 | ubiquitin-conjugating enzyme E2R 2 | Multiple_C | 10.76 | 9.57 | 8.95 | 3.51 | 2.04E-06 | 4.85E-05 | 10.15 | 9.94 | 10.44 | 0.82 | 0.0186 | 0.1153 |
| TC1200007820.hg.1 | RAB5B | RAB5B, member RAS oncogene family | Multiple_C | 11.64 | 9.89 | 9.83 | 3.51 | 4.18E-05 | 0.0005 | 12.67 | 12.91 | 12.13 | 1.45 | 0.0946 | 0.2988 |
| TC1500010842.hg.1 | GOLGA8R | golgin A8 family, member R | Multiple_C | 5.15 | 3.68 | 3.34 | 3.51 | 7.16E-06 | 0.0001 | 4.97 | 5.05 | 5.14 | 0.89 | 0.5608 | 0.7721 |
| TC1900009859.hg.1 | BRD4 | bromodomain containing 4 | Multiple_C | 13.63 | 10.86 | 11.82 | 3.51 | 7.76E-07 | 2.29E-05 | 13.09 | 12.64 | 12.89 | 1.15 | 0.2502 | 0.5146 |
| TC0100015975.hg.1 | RIT1 | Ras-like without CAAX 1 | Multiple_C | 9.87 | 8.97 | 8.07 | 3.48 | 7.32E-07 | 2.20E-05 | 9.13 | 8.37 | 8.94 | 1.14 | 0.0487 | 0.2051 |
| TC0200012522.hg.1 | FBXO11 | F-box protein 11 | Multiple_C | 10.66 | 9.58 | 8.86 | 3.48 | 2.43E-06 | 5.63E-05 | 9.34 | 9.06 | 9.51 | 0.89 | 0.1926 | 0.4459 |
| TC0300009247.hg.1 | ARHGEF26 | Rho guanine nucleotide exchange factor 26 | Multiple_C | 6.22 | 4.36 | 4.42 | 3.48 | 2.23E-05 | 0.0003 | 5.62 | 4.05 | 4.63 | 1.99 | 0.0023 | 0.029 |
| TC0500010924.hg.1 | ADAMTS6 | ADAM metallopeptidase with thrombospondin type 1 motif 6 | Multiple_C | 5.86 | 4.86 | 4.06 | 3.48 | 3.10E-05 | 0.0004 | 5.54 | 5.15 | 4.71 | 1.78 | 0.001 | 0.0168 |
| TC0500012842.hg.1 | DUSP1 | dual specificity phosphatase 1 | Multiple_C | 11.57 | 7.99 | 9.77 | 3.48 | 9.56E-06 | 0.0002 | 7.93 | 6.63 | 6.72 | 2.31 | 0.0005 | 0.0106 |
| TC1100012473.hg.1 | MPZL3 | myelin protein zero-like 3 | Multiple_C | 9.7 | 7.85 | 7.9 | 3.48 | 2.79E-05 | 0.0004 | 7.93 | 7.11 | 7.46 | 1.39 | 0.3736 | 0.6342 |
| TC1400008693.hg.1 | CDH24 | cadherin 24, type 2 | Multiple_C | 9.79 | 7.59 | 7.99 | 3.48 | 1.74E-06 | 4.26E-05 | 7.68 | 7.39 | 7.31 | 1.29 | 0.2215 | 0.4819 |
| TC1500010723.hg.1 | CHAC1 | ChaC glutathione-specific gamma-glutamylcyclotransferase 1 | Multiple_C | 11.45 | 8.24 | 9.65 | 3.48 | 0.0023 | 0.0131 | 9.23 | 8.53 | 8.99 | 1.18 | 0.4236 | 0.6763 |
| TC1900010856.hg.1 | PLAUR | plasminogen activator, urokinase receptor | Multiple_C | 10.54 | 8.5 | 8.74 | 3.48 | 6.59E-07 | 2.04E-05 | 6.31 | 5.71 | 6.28 | 1.02 | 0.9263 | 0.9696 |
| TC0500012980.hg.1 | FAM193B | family with sequence similarity 193, member B | Multiple_C | 7.91 | 6.3 | 6.12 | 3.46 | 3.89E-08 | 2.39E-06 | 6.81 | 6.92 | 6.33 | 1.39 | 0.0246 | 0.1362 |
| TC1000010844.hg.1 | RUFY2 | RUN and FYVE domain containing 2 | Multiple_C | 11.61 | 10.94 | 9.82 | 3.46 | 0.002 | 0.0116 | 11.5 | 9.85 | 10.79 | 1.64 | 0.0265 | 0.1423 |
| TC1400010584.hg.1 | IRF9 | interferon regulatory factor 9 | Multiple_C | 8.74 | 5.76 | 6.95 | 3.46 | 6.29E-07 | 1.96E-05 | 6.7 | 6.05 | 6.38 | 1.25 | 0.1652 | 0.4099 |
| TC1500007619.hg.1 | SMAD6 | SMAD family member 6 | Multiple_C | 13.16 | 11.45 | 11.37 | 3.46 | 0.0003 | 0.0024 | 11.02 | 9.81 | 11.22 | 0.87 | 0.2881 | 0.5527 |
| TC1700012141.hg.1 | WDR45B | WD repeat domain 45B | Multiple_C | 13.51 | 11.85 | 11.72 | 3.46 | 1.55E-08 | 1.22E-06 | 12.55 | 12.33 | 12.6 | 0.97 | 0.7248 | 0.872 |
| TC1900008435.hg.1 | SEPW1 | selenoprotein W, 1 | Multiple_C | 11.44 | 8.99 | 9.65 | 3.46 | 2.05E-06 | 4.86E-05 | 8.39 | 8.38 | 8.04 | 1.27 | 0.0919 | 0.2937 |
| TC0100014774.hg.1 | C1orf52 | chromosome 1 open reading frame 52 | Multiple_C | 11.23 | 10.27 | 9.45 | 3.43 | 0.0009 | 0.0061 | 9.58 | 9.04 | 9.29 | 1.22 | 0.0895 | 0.2894 |
| TC0700011905.hg.1 | BAIAP2L1 | BAI1-associated protein 2-like 1 | Multiple_C | 12.8 | 11 | 11.02 | 3.43 | 4.04E-06 | 8.47E-05 | 9.75 | 8.47 | 10.05 | 0.81 | 0.6006 | 0.7997 |
| TC1000008881.hg.1 | MXI1 | MAX interactor 1, dimerization protein | Multiple_C | 12.96 | 11.98 | 11.18 | 3.43 | 0.0011 | 0.007 | 9.34 | 8.85 | 9.33 | 1.01 | 0.5143 | 0.7435 |
| TC1200008081.hg.1 | DYRK2 | dual specificity tyrosine-(Y)-phosphorylation regulated kinase 2 | Multiple_C | 9.48 | 8.19 | 7.7 | 3.43 | 3.05E-05 | 0.0004 | 6.38 | 6.37 | 6.67 | 0.82 | 0.1227 | 0.3456 |
| TC1200012617.hg.1 | FGD4 | FYVE, RhoGEF and PH domain containing 4 | Multiple_C | 10.56 | 8.65 | 8.78 | 3.43 | 0.0003 | 0.0025 | 10.02 | 8.75 | 8.83 | 2.28 | 0.0123 | 0.0886 |
| TC1300006690.hg.1 | POLR1D | polymerase (RNA) I polypeptide D | Multiple_C | 12.73 | 11.75 | 10.95 | 3.43 | 0.0003 | 0.0022 | 9.47 | 9.94 | 9.94 | 0.72 | 0.9051 | 0.9607 |
| TC1700012285.hg.1 | PRR11 | proline rich 11 | Multiple_C | 9.93 | 8.82 | 8.15 | 3.43 | 7.37E-05 | 0.0008 | 11.56 | 12.07 | 11.84 | 0.82 | 0.0156 | 0.1029 |

| | | | | | | | | | | | | | | | |
|---|---|---|---|---|---|---|---|---|---|---|---|---|---|---|---|
| TC2000009957.hg.1 | LINC00266-1 | long intergenic non-protein coding RNA 266-1 | Multiple_C | 9.94 | 8.33 | 8.16 | 3.43 | 3.26E-06 | 7.15E-05 | 8.75 | 7.93 | 8.3 | 1.37 | 0.0656 | 0.2437 |
| TC1200007899.hg.1 | MARCHF9 | membrane associated ring finger 9 | Multiple_C | 8.04 | 6.35 | 6.27 | 3.41 | 0.0002 | 0.0018 | 5.68 | 5.68 | 5.18 | 1.41 | 0.083 | 0.2774 |
| TC1600007353.hg.1 | NPIPB8 | nuclear pore complex interacting protein family, member B8 | Coding | 12.43 | 10.38 | 10.66 | 3.41 | 1.23E-06 | 3.28E-05 | 12.92 | 12.24 | 12.63 | 1.22 | 0.1599 | 0.4022 |
| TC2000008030.hg.1 | OGFR | opioid growth factor receptor | Multiple_C | 12.73 | 10.45 | 10.96 | 3.41 | 5.45E-05 | 0.0007 | 10.77 | 11.53 | 11.45 | 0.62 | 0.0017 | 0.0241 |
| TC0100008271.hg.1 | RNF11 | ring finger protein 11 | Multiple_C | 11.73 | 11.38 | 9.97 | 3.39 | 6.38E-06 | 0.0001 | 8.77 | 7.84 | 8.24 | 1.44 | 0.045 | 0.1962 |
| TC0100015786.hg.1 | POGZ | pogo transposable element with ZNF domain | Multiple_C | 11.88 | 9.39 | 10.12 | 3.39 | 4.33E-08 | 2.59E-06 | 11.22 | 10.73 | 11.13 | 1.06 | 0.6032 | 0.8015 |
| TC0200006891.hg.1 | RHOB | ras homolog family member B | Coding | 10.76 | 8.36 | 9 | 3.39 | 0.0003 | 0.0023 | 10.54 | 10.28 | 9.89 | 1.57 | 0.0437 | 0.1932 |
| TC0600008109.hg.1 | VEGFA | vascular endothelial growth factor A | Multiple_C | 15.62 | 14.71 | 13.86 | 3.39 | 5.54E-05 | 0.0007 | 11.25 | 10.47 | 11.62 | 0.77 | 0.9729 | 0.989 |
| TC1100009016.hg.1 | COLCA2 | colorectal cancer associated 2 | Coding | 7.08 | 6.53 | 5.32 | 3.39 | 0.0002 | 0.0018 | 4.31 | 4.94 | 4.62 | 0.81 | 0.1917 | 0.4446 |
| TC1300009638.hg.1 | RPS26 | Homo sapiens ribosomal protein S26, mRNA (cDNA clone MGC: | Multiple_C | 15.3 | 13.46 | 13.54 | 3.39 | 0.0073 | 0.0325 | 9.69 | 10.50 | 10.86 | 0.44 | 0.0289 | 0.1504 |
| TC1600009916.hg.1 | NPIPB11 | nuclear pore complex interacting protein family, member B11 | Coding | 11.62 | 9.48 | 9.86 | 3.39 | 3.07E-06 | 6.83E-05 | 12.34 | 11.59 | 11.96 | 1.30 | 0.0623 | 0.2371 |
| TC1700007042.hg.1 | MPRIP | myosin phosphatase Rho interacting protein | Multiple_C | 14.81 | 13.25 | 13.05 | 3.39 | 3.65E-05 | 0.0005 | 13.21 | 12.77 | 13.02 | 1.14 | 0.4392 | 0.6895 |
| TC1900011726.hg.1 | C19orf33 | chromosome 19 open reading frame 33 | Multiple_C | 13.5 | 12.83 | 11.74 | 3.39 | 7.94E-07 | 2.32E-05 | 8.17 | 8.44 | 8.27 | 0.93 | 0.8915 | 0.9539 |
| TC0100015162.hg.1 | NBPF4 | neuroblastoma breakpoint family, member 4 | Multiple_C | 6.71 | 5.27 | 4.96 | 3.36 | 4.27E-06 | 8.82E-05 | 8.63 | 9.64 | 8.22 | 1.33 | 0.239 | 0.5026 |
| TC0500009211.hg.1 | CYFIP2 | cytoplasmic FMR1 interacting protein 2 | Multiple_C | 7.42 | 5.3 | 5.67 | 3.36 | 0.0001 | 0.0011 | 8.48 | 6.93 | 7.2 | 2.43 | 0.0039 | 0.042 |
| TC1400009524.hg.1 | ZFP36L1 | ZFP36 ring finger protein-like 1 | Coding | 10.64 | 7.78 | 8.89 | 3.36 | 9.18E-06 | 0.0002 | 11.43 | 10.28 | 11.95 | 0.70 | 0.0143 | 0.0973 |
| TC0200007188.hg.1 | BIRC6 | Transcript Identified by AceView, Entrez Gene ID(s) 57448 | Coding | 6.95 | 5.09 | 5.21 | 3.34 | 3.21E-05 | 0.0004 | 5.53 | 5.44 | 5.66 | 0.91 | 0.7124 | 0.8651 |
| TC0200014990.hg.1 | CIR1 | corepressor interacting with RBPJ, 1 | Multiple_C | 11.35 | 10.96 | 9.61 | 3.34 | 1.81E-05 | 0.0003 | 9.16 | 8.56 | 8.67 | 1.40 | 0.0966 | 0.3028 |
| TC0300009673.hg.1 | KLHL24 | kelch-like family member 24 | Multiple_C | 10.06 | 9.49 | 8.32 | 3.34 | 1.06E-06 | 2.92E-05 | 8.2 | 7.58 | 7.24 | 1.95 | 0.0003 | 0.0084 |
| TC0700012909.hg.1 | OR2A7; ARHG | olfactory receptor, family 2, subfamily A, member 7; Rho guanir | Multiple_C | 10.8 | 8.62 | 9.06 | 3.34 | 3.78E-06 | 8.02E-05 | 10.33 | 10.54 | 10.03 | 1.23 | 0.1935 | 0.447 |
| TC0X00010029.hg.1 | RPS4X | ribosomal protein S4, X-linked | Multiple_C | 17.44 | 15.41 | 15.7 | 3.34 | 0.0019 | 0.0111 | 13.74 | 14.07 | 14.43 | 0.62 | 0.0479 | 0.2032 |
| TC1000008942.hg.1 | TCF7L2 | transcription factor 7-like 2 (T-cell specific, HMG-box) | Multiple_C | 8.95 | 6.71 | 7.21 | 3.34 | 0.004 | 0.0201 | 8.38 | 8.43 | 8.33 | 1.04 | 0.9068 | 0.9614 |
| TC1800009215.hg.1 | ANKRD12 | ankyrin repeat domain 12 | Multiple_C | 8.72 | 8.11 | 6.98 | 3.34 | 1.80E-06 | 4.36E-05 | 8.89 | 8.42 | 8.77 | 1.09 | 0.478 | 0.7167 |
| TC2000007169.hg.1 | CHMP4B | charged multivesicular body protein 4B | Multiple_C | 12.97 | 11 | 11.23 | 3.34 | 1.85E-05 | 0.0003 | 11.27 | 11.76 | 11.38 | 0.93 | 0.0378 | 0.1771 |
| TC0100009064.hg.1 | FNBP1L | formin binding protein 1-like | Multiple_C | 12.54 | 11.41 | 10.81 | 3.32 | 9.81E-05 | 0.001 | 11.92 | 11.49 | 12.09 | 0.89 | 0.0563 | 0.2232 |
| TC0200012608.hg.1 | PSME4 | proteasome activator subunit 4 | Multiple_C | 11.06 | 9.41 | 9.33 | 3.32 | 0.0002 | 0.0017 | 10.05 | 9.18 | 10.13 | 0.95 | 0.4654 | 0.7072 |
| TC0200012809.hg.1 | PELI1 | pellino E3 ubiquitin protein ligase 1 | Multiple_C | 12.81 | 10.7 | 11.08 | 3.32 | 1.04E-05 | 0.0002 | 10.01 | 9.46 | 9.58 | 1.35 | 0.3248 | 0.5885 |
| TC0400008450.hg.1 | ANK2 | ankyrin 2, neuronal | Multiple_C | 6.15 | 4.69 | 4.42 | 3.32 | 2.76E-05 | 0.0004 | 6.49 | 5.55 | 6.41 | 1.06 | 0.719 | 0.8689 |
| TC0900007977.hg.1 | CARD19 | caspase recruitment domain family, member 19 | Multiple_C | 10.71 | 9 | 8.98 | 3.32 | 0.0002 | 0.0016 | 7.15 | 7.55 | 7.59 | 0.74 | 0.1478 | 0.3842 |
| TC1700006627.hg.1 | SPNS2 | spinster homolog 2 (Drosophila) | Multiple_C | 8.14 | 6.48 | 6.41 | 3.32 | 0.0033 | 0.0173 | 9.4 | 8.49 | 9.88 | 0.72 | 0.131 | 0.3587 |
| TC1700012395.hg.1 | TBC1D3L | TBC1 domain family, member 3L | Coding | 10.29 | 9.17 | 8.56 | 3.32 | 1.75E-06 | 4.28E-05 | 11.15 | 10.77 | 10.42 | 1.66 | 0.0028 | 0.0341 |
| TC1900009346.hg.1 | UBXN6 | UBX domain protein 6 | Multiple_C | 9.28 | 8.45 | 7.55 | 3.32 | 0.0003 | 0.0022 | 6.11 | 6.54 | 6.3 | 0.88 | 0.0537 | 0.2169 |
| TC0200008534.hg.1 | ANKRD36 | ankyrin repeat domain 36 | Multiple_C | 11.09 | 9.28 | 9.37 | 3.29 | 6.71E-06 | 0.0001 | 10.6 | 9.96 | 9.8 | 1.74 | 0.0008 | 0.0145 |

| | | | | | | | | | | | | | | | |
|---|---|---|---|---|---|---|---|---|---|---|---|---|---|---|---|
| TC0200014790.hg.1 | GRB14 | growth factor receptor bound protein 14 | Multiple_C | 6.92 | 5.48 | 5.2 | 3.29 | 6.28E-05 | 0.0007 | 3.42 | 3.17 | 3.63 | 0.86 | 0.9758 | 0.9902 |
| TC1100013045.hg.1 | SIPA1 | signal-induced proliferation-associated 1 | Multiple_C | 6.45 | 4.72 | 4.73 | 3.29 | 0.0101 | 0.0421 | 5.34 | 5.11 | 4.83 | 1.42 | 0.2807 | 0.5466 |
| TC1200009132.hg.1 | RNF10 | ring finger protein 10 | Multiple_C | 12.86 | 11.79 | 11.14 | 3.29 | 1.07E-06 | 2.94E-05 | 10.21 | 10.40 | 10.44 | 0.85 | 0.2429 | 0.5066 |
| TC1500010042.hg.1 | CYP1A1 | cytochrome P450, family 1, subfamily A, polypeptide 1 | Multiple_C | 6.16 | 4.11 | 4.44 | 3.29 | 7.97E-05 | 0.0009 | 4.68 | 3.97 | 4.17 | 1.42 | 0.0633 | 0.2393 |
| TC1500010097.hg.1 | SCAPER | S-phase cyclin A-associated protein in the ER | Multiple_C | 7 | 5.29 | 5.28 | 3.29 | 0.0013 | 0.0083 | 6.33 | 6.06 | 5.98 | 1.27 | 0.2667 | 0.5312 |
| TC2200007196.hg.1 | TOM1 | target of myb1 membrane trafficking protein | Multiple_C | 9.24 | 7.85 | 7.52 | 3.29 | 7.02E-05 | 0.0008 | 8.98 | 8.83 | 8.47 | 1.42 | 0.1014 | 0.3115 |
| TC0100011243.hg.1 | PPFIA4 | protein tyrosine phosphatase, receptor type, f polypeptide (PTP | Multiple_C | 7.06 | 5.29 | 5.35 | 3.27 | 0.0004 | 0.0034 | 6.05 | 6.01 | 5.64 | 1.33 | 0.3295 | 0.5936 |
| TC0700009536.hg.1 | ARHGEF5 | Rho guanine nucleotide exchange factor 5 | Multiple_C | 12.1 | 9.91 | 10.39 | 3.27 | 4.23E-06 | 8.75E-05 | 11.83 | 12.12 | 12.04 | 0.86 | 0.2873 | 0.5523 |
| TC1000006924.hg.1 | ARL5B | ADP-ribosylation factor like GTPase 5B | Multiple_C | 12.98 | 11.28 | 11.27 | 3.27 | 1.80E-06 | 4.36E-05 | 11.93 | 11.20 | 11.87 | 1.04 | 0.3588 | 0.6216 |
| TC1400006529.hg.1 | ANG; RNASE4 | angiogenin, ribonuclease, RNase A family, 5; ribonuclease, RNas | Multiple_C | 10.82 | 9.76 | 9.11 | 3.27 | 9.71E-06 | 0.0002 | 4.06 | 3.78 | 3.96 | 1.07 | 0.7923 | 0.9077 |
| TC1400008606.hg.1 | RNASE1 | ribonuclease, RNase A family, 1 (pancreatic) | Multiple_C | 6.02 | 3.95 | 4.31 | 3.27 | 0.0014 | 0.0088 | 4.27 | 4.28 | 4.18 | 1.06 | 0.5646 | 0.7745 |
| TC1500008029.hg.1 | FAH | fumarylacetoacetate hydrolase (fumarylacetoacetase) | Multiple_C | 8.88 | 7.93 | 7.17 | 3.27 | 0.0007 | 0.0051 | 6.42 | 6.56 | 6.32 | 1.07 | 0.851 | 0.9358 |
| TC1600007531.hg.1 | ITGAX | integrin alpha X | Multiple_C | 6.48 | 4.74 | 4.77 | 3.27 | 1.40E-06 | 3.61E-05 | 6.76 | 6.89 | 6.42 | 1.27 | 0.2722 | 0.5378 |
| TC1900008824.hg.1 | MYADM | myeloid-associated differentiation marker | Multiple_C | 10.26 | 7.36 | 8.55 | 3.27 | 0.0006 | 0.0047 | 10.36 | 10.57 | 11.33 | 0.51 | 0.0202 | 0.1212 |
| TC2100007446.hg.1 | COL6A1 | collagen, type VI, alpha 1 | Multiple_C | 10.22 | 9.39 | 8.51 | 3.27 | 1.40E-05 | 0.0002 | 8.02 | 7.08 | 8.02 | 1.00 | 0.9008 | 0.9589 |
| TC0100013028.hg.1 | EPHA2 | EPH receptor A2 | Multiple_C | 12.31 | 10.73 | 10.61 | 3.25 | 5.61E-05 | 0.0007 | 10.83 | 9.65 | 10.06 | 1.71 | 0.0004 | 0.0097 |
| TC0200008065.hg.1 | TET3 | tet methylcytosine dioxygenase 3 | Multiple_C | 6.19 | 4.34 | 4.49 | 3.25 | 0.0006 | 0.0047 | 6.35 | 6.59 | 6.71 | 0.78 | 0.2185 | 0.4782 |
| TC0200013109.hg.1 | PCGF1 | polycomb group ring finger 1 | Multiple_C | 10.68 | 9.87 | 8.98 | 3.25 | 4.12E-06 | 8.59E-05 | 7.03 | 6.70 | 7.01 | 1.01 | 0.1774 | 0.4261 |
| TC0300007110.hg.1 | MYRIP | myosin VIIA and Rab interacting protein | Multiple_C | 6.98 | 5.33 | 5.28 | 3.25 | 0.0005 | 0.004 | 7.39 | 7.90 | 7.38 | 1.01 | 0.2625 | 0.5268 |
| TC0300007842.hg.1 | EBLN2 | endogenous Bornavirus-like nucleoprotein 2 | Coding | 7.7 | 7.05 | 6 | 3.25 | 1.52E-06 | 3.88E-05 | 5.77 | 5.37 | 5.38 | 1.31 | 0.048 | 0.2033 |
| TC0400008376.hg.1 | SEC24B | SEC24 homolog B, COPII coat complex component | Multiple_C | 11.18 | 10.31 | 9.48 | 3.25 | 0.001 | 0.0069 | 8.82 | 8.49 | 8.98 | 0.90 | 0.6619 | 0.835 |
| TC0600006855.hg.1 | RREB1 | ras responsive element binding protein 1 | Multiple_C | 12.61 | 10.31 | 10.91 | 3.25 | 2.24E-07 | 8.90E-06 | 11.78 | 11.88 | 12.04 | 0.84 | 0.379 | 0.6387 |
| TC0600008183.hg.1 | CD2AP | CD2-associated protein | Multiple_C | 11.22 | 10.06 | 9.52 | 3.25 | 1.70E-06 | 4.19E-05 | 10.83 | 10.85 | 10.88 | 0.97 | 0.5174 | 0.7458 |
| TC0900012225.hg.1 | RNF38 | ring finger protein 38 | Multiple_C | 9.98 | 7.49 | 8.28 | 3.25 | 0.0009 | 0.0062 | 10.49 | 10.07 | 10.88 | 0.76 | 0.9026 | 0.9596 |
| TC1400008355.hg.1 | RCOR1 | REST corepressor 1 | Multiple_C | 9.78 | 7.82 | 8.08 | 3.25 | 0.0002 | 0.0016 | 8.83 | 8.39 | 9.3 | 0.72 | 0.2863 | 0.5515 |
| TC1600007037.hg.1 | NPIPA7 | nuclear pore complex interacting protein family, member A7 | Coding | 12.07 | 10.28 | 10.37 | 3.25 | 3.60E-08 | 2.26E-06 | 12.33 | 12.09 | 12.11 | 1.16 | 0.2974 | 0.562 |
| TC1700012296.hg.1 | PRKAR1A; ARS | protein kinase, cAMP-dependent, regulatory, type I, alpha; aryls | Multiple_C | 9.68 | 7.84 | 7.98 | 3.25 | 8.16E-05 | 0.0009 | 11.47 | 10.84 | 10.14 | 2.51 | 0.0002 | 0.0048 |
| TC0300010770.hg.1 | CSRNP1 | cysteine-serine-rich nuclear protein 1 | Multiple_C | 6.94 | 4.94 | 5.25 | 3.23 | 1.30E-06 | 3.41E-05 | 6.67 | 6.25 | 6.51 | 1.12 | 0.6241 | 0.8145 |
| TC0300011032.hg.1 | NCKIPSD | NCK interacting protein with SH3 domain | Multiple_C | 9.31 | 7.1 | 7.62 | 3.23 | 2.38E-05 | 0.0003 | 8.8 | 9.16 | 9.27 | 0.72 | 0.2482 | 0.5127 |
| TC0300013471.hg.1 | BCL6 | B-cell CLL/lymphoma 6 | Multiple_C | 12.38 | 9.71 | 10.69 | 3.23 | 0.0002 | 0.0019 | 10.51 | 8.75 | 8.84 | 3.18 | 0.0002 | 0.005 |
| TC0800006864.hg.1 | ZDHHC2 | zinc finger, DHHC-type containing 2 | Multiple_C | 8.06 | 7.13 | 6.37 | 3.23 | 4.28E-06 | 8.84E-05 | 7.2 | 7.36 | 6.67 | 1.44 | 0.0183 | 0.1141 |
| TC1100010761.hg.1 | FNBP4 | formin binding protein 4 | Multiple_C | 12.83 | 11.44 | 11.14 | 3.23 | 6.06E-08 | 3.35E-06 | 12.61 | 12.12 | 12.45 | 1.12 | 0.2062 | 0.4628 |
| TC1400008397.hg.1 | MARK3 | MAP/microtubule affinity-regulating kinase 3 | Multiple_C | 11.42 | 9.94 | 9.73 | 3.23 | 1.35E-05 | 0.0002 | 10.98 | 9.84 | 11.07 | 0.94 | 0.871 | 0.9445 |

| | | | | | | | | | | | | | | | |
|---|---|---|---|---|---|---|---|---|---|---|---|---|---|---|---|
| TC1700010762.hg.1 | VAT1 | vesicle amine transport 1 | Multiple_C | 12.81 | 12.17 | 11.12 | 3.23 | 1.85E-05 | 0.0003 | 10.58 | 9.35 | 9.34 | 2.36 | 9.72E-05 | 0.0035 |
| TC0200009049.hg.1 | INSIG2 | insulin induced gene 2 | Multiple_C | 10.92 | 10.1 | 9.24 | 3.20 | 0.0002 | 0.0017 | 9.54 | 8.81 | 8.54 | 2.00 | 0.0507 | 0.2097 |
| TC0200009601.hg.1 | MBD5 | methyl-CpG binding domain protein 5 | Multiple_C | 7.69 | 6.56 | 6.01 | 3.20 | 9.65E-06 | 0.0002 | 6.77 | 6.73 | 6.28 | 1.40 | 0.0448 | 0.1958 |
| TC0300007115.hg.1 | EIF1B | eukaryotic translation initiation factor 1B | Multiple_C | 14.51 | 14.18 | 12.83 | 3.20 | 6.94E-07 | 2.11E-05 | 11.43 | 10.83 | 11.13 | 1.23 | 0.0162 | 0.1057 |
| TC0500008014.hg.1 | RASA1 | RAS p21 protein activator (GTPase activating protein) 1 | Multiple_C | 11.07 | 10.15 | 9.39 | 3.20 | 1.53E-06 | 3.89E-05 | 9.78 | 8.99 | 8.91 | 1.83 | 6.56E-05 | 0.0027 |
| TC0600009622.hg.1 | CCDC28A | coiled-coil domain containing 28A | Multiple_C | 10.63 | 10.62 | 8.95 | 3.20 | 5.66E-06 | 0.0001 | 7.49 | 7.38 | 6.71 | 1.72 | 0.002 | 0.0266 |
| TC0800012176.hg.1 | PLEC | plectin | Multiple_C | 12.21 | 10.38 | 10.53 | 3.20 | 0.0019 | 0.0113 | 11.12 | 9.95 | 9.76 | 2.57 | 0.0037 | 0.041 |
| TC1700011476.hg.1 | GNA13 | guanine nucleotide binding protein (G protein), alpha 13 | Multiple_C | 12.56 | 10.86 | 10.88 | 3.20 | 2.12E-07 | 8.53E-06 | 11.73 | 11.15 | 12.38 | 0.64 | 0.0051 | 0.0498 |
| TC1800009275.hg.1 | ST8SIA5 | ST8 alpha-N-acetyl-neuraminide alpha-2,8-sialyltransferase 5 | Multiple_C | 6 | 4.72 | 4.32 | 3.20 | 2.57E-05 | 0.0004 | 4.69 | 4.69 | 4.92 | 0.85 | 0.4187 | 0.6725 |
| TC1900012050.hg.1 | PPP6R1 | protein phosphatase 6, regulatory subunit 1 | Multiple_C | 12.39 | 10.5 | 10.71 | 3.20 | 2.98E-07 | 1.08E-05 | 10.76 | 11.17 | 11.45 | 0.62 | 0.0117 | 0.0862 |
| TC2200008885.hg.1 | POLDIP3 | polymerase (DNA-directed), delta interacting protein 3 | Multiple_C | 12.21 | 9.56 | 10.53 | 3.20 | 0.0001 | 0.0014 | 11.37 | 11.44 | 12.13 | 0.59 | 0.0437 | 0.1932 |
| TC0100015891.hg.1 | CRTC2 | CREB regulated transcription coactivator 2 | Multiple_C | 10.09 | 7.56 | 8.42 | 3.18 | 0.0061 | 0.0281 | 8.44 | 8.05 | 8.86 | 0.75 | 0.288 | 0.5527 |
| TC0100018210.hg.1 | SH3D21 | SH3 domain containing 21 | Multiple_C | 7.17 | 5.14 | 5.5 | 3.18 | 0.001 | 0.007 | 5.64 | 6.13 | 5.66 | 0.99 | 0.3615 | 0.6242 |
| TC0200015330.hg.1 | C2orf66 | chromosome 2 open reading frame 66 | Coding | 5.74 | 4.14 | 4.07 | 3.18 | 0.0018 | 0.0107 | 5 | 4.72 | 4.17 | 1.78 | 0.1196 | 0.3417 |
| TC0800012324.hg.1 | LRRC69; MIR4 | leucine rich repeat containing 69; microRNA 4661 | Multiple_C | 5.52 | 4.91 | 3.85 | 3.18 | 3.88E-05 | 0.0005 | 5.09 | 4.85 | 5.01 | 1.06 | 0.5784 | 0.7843 |
| TC0X00008832.hg.1 | GDI1 | GDP dissociation inhibitor 1 | Multiple_C | 13.26 | 11.56 | 11.59 | 3.18 | 8.99E-07 | 2.57E-05 | 10.48 | 9.40 | 10.83 | 0.78 | 0.1194 | 0.3415 |
| TC1200011608.hg.1 | CDK17 | cyclin-dependent kinase 17 | Multiple_C | 12.91 | 11.49 | 11.24 | 3.18 | 2.69E-06 | 6.11E-05 | 12.15 | 11.05 | 11.5 | 1.57 | 0.0088 | 0.0715 |
| TC1700008920.hg.1 | ITGB4 | integrin beta 4 | Multiple_C | 11.97 | 11.85 | 10.3 | 3.18 | 0.0023 | 0.0129 | 10.66 | 10.99 | 9.98 | 1.60 | 0.2058 | 0.4622 |
| TC0100009646.hg.1 | NBPF26 | neuroblastoma breakpoint family, member 26 | Coding | 11.05 | 9.79 | 9.39 | 3.16 | 1.69E-05 | 0.0003 | 9.92 | 9.55 | 9.77 | 1.11 | 0.3302 | 0.5944 |
| TC0100013432.hg.1 | WASF2 | WAS protein family, member 2 | Multiple_C | 12.11 | 9.79 | 10.45 | 3.16 | 1.58E-07 | 6.74E-06 | 10.76 | 10.60 | 10.84 | 0.95 | 0.5914 | 0.7934 |
| TC0200009789.hg.1 | MARCHF7 | membrane associated ring finger 7 | Multiple_C | 13.73 | 12.69 | 12.07 | 3.16 | 3.81E-05 | 0.0005 | 11.83 | 10.96 | 11.28 | 1.46 | 0.0318 | 0.1597 |
| TC0200011419.hg.1 | ATG4B | autophagy related 4B, cysteine peptidase | Multiple_C | 10.15 | 8.77 | 8.49 | 3.16 | 0.0008 | 0.0054 | 9.38 | 8.94 | 8.85 | 1.44 | 0.8919 | 0.9539 |
| TC0400008609.hg.1 | KIAA1109 | KIAA1109 | Multiple_C | 9.25 | 8.28 | 7.59 | 3.16 | 7.98E-06 | 0.0001 | 6.63 | 5.83 | 6.24 | 1.31 | 0.0659 | 0.2444 |
| TC0700007398.hg.1 | ZMIZ2 | zinc finger, MIZ-type containing 2 | Multiple_C | 7.45 | 5.39 | 5.79 | 3.16 | 0.002 | 0.0115 | 7.94 | 8.58 | 8.35 | 0.75 | 0.1498 | 0.3876 |
| TC0800010630.hg.1 | GGH | gamma-glutamyl hydrolase (conjugase, folylpolygammaglutamy | Multiple_C | 11.67 | 10.44 | 10.01 | 3.16 | 7.91E-05 | 0.0009 | 11.75 | 10.81 | 9.37 | 5.21 | 1.55E-07 | 3.66E-05 |
| TC1200010983.hg.1 | AVIL | advillin | Multiple_C | 5.82 | 4.02 | 4.16 | 3.16 | 9.72E-05 | 0.001 | 4.8 | 4.79 | 4.56 | 1.18 | 0.7077 | 0.8624 |
| TC1500007659.hg.1 | PIAS1 | protein inhibitor of activated STAT 1 | Multiple_C | 13.07 | 11.05 | 11.41 | 3.16 | 0.0002 | 0.0017 | 12.08 | 10.95 | 12.26 | 0.88 | 0.6441 | 0.8255 |
| TC1500010090.hg.1 | NRG4 | neuregulin 4 | Multiple_C | 8.27 | 8.14 | 6.61 | 3.16 | 0.0017 | 0.01 | 6.16 | 5.91 | 5.87 | 1.22 | 0.1637 | 0.4076 |
| TC1900008103.hg.1 | PLD3 | phospholipase D family, member 3 | Multiple_C | 11.25 | 9.82 | 9.59 | 3.16 | 0.0005 | 0.0037 | 10.63 | 10.34 | 9.63 | 2.00 | 0.1202 | 0.3423 |
| TC2000008121.hg.1 | C20orf96 | chromosome 20 open reading frame 96 | Multiple_C | 5.52 | 3.6 | 3.86 | 3.16 | 1.29E-05 | 0.0002 | 7.45 | 6.23 | 6.17 | 2.43 | 8.92E-05 | 0.0033 |
| TC2000008445.hg.1 | FLRT3 | fibronectin leucine rich transmembrane protein 3 | Multiple_C | 6.68 | 4.74 | 5.02 | 3.16 | 7.03E-06 | 0.0001 | 4.41 | 4.23 | 4.08 | 1.26 | 0.1964 | 0.4511 |
| TC0200008823.hg.1 | RGPD6; RGPD | RANBP2-like and GRIP domain containing 6; RANBP2-like and G | Multiple_C | 10.71 | 9.58 | 9.06 | 3.14 | 0.0001 | 0.0012 | 9.88 | 8.43 | 9.47 | 1.33 | 0.0734 | 0.2596 |
| TC0200012009.hg.1 | DTNB | dystrobrevin beta | Multiple_C | 7.5 | 6.4 | 5.85 | 3.14 | 0.0001 | 0.0014 | 8.02 | 7.24 | 7.68 | 1.27 | 0.4725 | 0.7127 |

| | | | | | | | | | | | | | | | |
|---|---|---|---|---|---|---|---|---|---|---|---|---|---|---|---|
| TC0200014085.hg.1 | TFCP2L1 | transcription factor CP2-like 1 | Multiple_C | 5.93 | 4.41 | 4.28 | 3.14 | 0.0039 | 0.0197 | 5.63 | 6.11 | 6.65 | 0.49 | 0.001 | 0.0168 |
| TC0200015870.hg.1 | WDFY1 | WD repeat and FYVE domain containing 1 | Multiple_C | 11.13 | 10.28 | 9.48 | 3.14 | 2.32E-05 | 0.0003 | 8.85 | 8.49 | 8.38 | 1.39 | 0.1157 | 0.3358 |
| TC0400011643.hg.1 | CAMK2D | calcium/calmodulin-dependent protein kinase II delta | Multiple_C | 12.46 | 11.65 | 10.81 | 3.14 | 2.22E-07 | 8.81E-06 | 10.7 | 10.59 | 10.91 | 0.86 | 0.2417 | 0.5056 |
| TC0700008870.hg.1 | CAV2 | caveolin 2 | Multiple_C | 10.73 | 10.29 | 9.08 | 3.14 | 3.72E-05 | 0.0005 | 8.01 | 6.87 | 7.89 | 1.09 | 0.3537 | 0.6171 |
| TC1200007693.hg.1 | IGFBP6 | insulin like growth factor binding protein 6 | Multiple_C | 5.31 | 3.67 | 3.66 | 3.14 | 0.0003 | 0.0025 | 10.01 | 8.76 | 7.42 | 6.02 | 1.16E-08 | 5.55E-06 |
| TC2000009953.hg.1 | OSBPL2 | oxysterol binding protein-like 2 | Multiple_C | 12.22 | 10.31 | 10.57 | 3.14 | 2.84E-06 | 6.37E-05 | 10.34 | 9.78 | 10.43 | 0.94 | 0.8448 | 0.9321 |
| TC0100006486.hg.1 | AGRN | agrin | Multiple_C | 8.22 | 6.42 | 6.58 | 3.12 | 7.32E-05 | 0.0008 | 7.76 | 8.05 | 7.8 | 0.97 | 0.5835 | 0.7878 |
| TC0100015895.hg.1 | RAB13 | RAB13, member RAS oncogene family | Multiple_C | 13.6 | 12.06 | 11.96 | 3.12 | 4.61E-07 | 1.53E-05 | 11.23 | 11.55 | 10.83 | 1.32 | 0.0401 | 0.1837 |
| TC0200007477.hg.1 | SOCS5 | suppressor of cytokine signaling 5 | Coding | 10.47 | 9.73 | 8.83 | 3.12 | 3.73E-06 | 7.94E-05 | 8.26 | 7.40 | 7.9 | 1.28 | 0.0732 | 0.2593 |
| TC0200014703.hg.1 | CCDC148 | coiled-coil domain containing 148 | Multiple_C | 5.89 | 4.04 | 4.25 | 3.12 | 9.98E-05 | 0.0011 | 4.5 | 4.95 | 4.76 | 0.84 | 0.4319 | 0.6835 |
| TC0600008473.hg.1 | SMAP1 | small ArfGAP 1 | Multiple_C | 7.67 | 7.19 | 6.03 | 3.12 | 2.72E-06 | 6.17E-05 | 9.68 | 9.90 | 10.09 | 0.75 | 0.0902 | 0.2906 |
| TC0800008179.hg.1 | RIPK2 | receptor-interacting serine-threonine kinase 2 | Multiple_C | 14.1 | 12.75 | 12.46 | 3.12 | 1.83E-05 | 0.0003 | 10.65 | 10.14 | 10.61 | 1.03 | 0.8235 | 0.922 |
| TC0900010404.hg.1 | ZFAND5 | zinc finger, AN1-type domain 5 | Multiple_C | 14.22 | 13.45 | 12.58 | 3.12 | 8.63E-06 | 0.0002 | 11.24 | 10.60 | 11.36 | 0.92 | 0.8259 | 0.9233 |
| TC1200007959.hg.1 | MON2 | MON2 homolog, regulator of endosome-to-Golgi trafficking | Multiple_C | 9.31 | 8.44 | 7.67 | 3.12 | 0.0006 | 0.0048 | 8.9 | 8.48 | 8.64 | 1.20 | 0.9286 | 0.9701 |
| TC1400010632.hg.1 | GPATCH2L | G-patch domain containing 2 like | Multiple_C | 11.04 | 10.02 | 9.4 | 3.12 | 9.13E-07 | 2.60E-05 | 10.07 | 9.76 | 9.99 | 1.06 | 0.6681 | 0.8377 |
| TC1900011714.hg.1 | LIN37 | lin-37 DREAM MuvB core complex component | Multiple_C | 9.57 | 7.42 | 7.93 | 3.12 | 7.00E-05 | 0.0008 | 7.45 | 7.13 | 6.59 | 1.82 | 0.0263 | 0.1418 |
| TC0100006773.hg.1 | SPSB1 | splA/ryanodine receptor domain and SOCS box containing 1 | Multiple_C | 10.36 | 8.45 | 8.73 | 3.10 | 1.72E-06 | 4.23E-05 | 8.38 | 7.91 | 8.79 | 0.75 | 0.0339 | 0.1658 |
| TC0100018555.hg.1 | LEMD1 | LEM domain containing 1 | Multiple_C | 6.21 | 6.84 | 4.58 | 3.10 | 0.0123 | 0.0493 | 6.51 | 5.22 | 4.67 | 3.58 | 0.0002 | 0.0051 |
| TC0200012639.hg.1 | CLHC1 | clathrin heavy chain linker domain containing 1 | Multiple_C | 5.14 | 3.58 | 3.51 | 3.10 | 4.46E-05 | 0.0006 | 3.65 | 3.85 | 3.57 | 1.06 | 0.7367 | 0.8786 |
| TC0200015601.hg.1 | KANSL1L | KAT8 regulatory NSL complex subunit 1 like | Multiple_C | 7.66 | 6.39 | 6.03 | 3.10 | 0.0001 | 0.0011 | 7.72 | 7.73 | 7.38 | 1.27 | 0.4229 | 0.6756 |
| TC0400012917.hg.1 | ARAP2 | ArfGAP with RhoGAP domain, ankyrin repeat and PH domain 2 | Multiple_C | 7.14 | 5.77 | 5.51 | 3.10 | 0.0023 | 0.0127 | 6.63 | 5.83 | 6.67 | 0.97 | 0.9405 | 0.9753 |
| TC0600007060.hg.1 | MYLIP; MIR46 | myosin regulatory light chain interacting protein; microRNA 463 | Multiple_C | 9.86 | 8.24 | 8.23 | 3.10 | 0.001 | 0.0067 | 6.98 | 6.27 | 6.46 | 1.43 | 0.0663 | 0.2456 |
| TC0600007675.hg.1 | BRD2 | bromodomain containing 2 | Multiple_C | 11.63 | 9.9 | 10 | 3.10 | 1.91E-06 | 4.58E-05 | 11.07 | 10.82 | 11.5 | 0.74 | 0.0319 | 0.1598 |
| TC0600013361.hg.1 | CITED2 | Cbp/p300-interacting transactivator, with Glu/Asp rich carboxy- | Coding | 11.3 | 9.64 | 9.67 | 3.10 | 3.64E-05 | 0.0005 | 8.53 | 8.61 | 7.28 | 2.38 | 1.60E-05 | 0.001 |
| TC0600014277.hg.1 | HLA-DMA | major histocompatibility complex, class II, DM alpha | Multiple_C | 7.39 | 5.34 | 5.76 | 3.10 | 0.0008 | 0.0055 | 5.13 | 5.50 | 5.21 | 0.95 | 0.1552 | 0.3954 |
| TC0900010366.hg.1 | KLF9 | Kruppel-like factor 9 | Multiple_C | 8.69 | 6.93 | 7.06 | 3.10 | 0.0004 | 0.0033 | 5.63 | 4.81 | 4.73 | 1.87 | 0.0091 | 0.0732 |
| TC1000012581.hg.1 | ARHGAP19-SL | ARHGAP19-SLIT1 readthrough (NMD candidate) | Multiple_C | 10.25 | 9.47 | 8.62 | 3.10 | 1.08E-06 | 2.95E-05 | 8.88 | 8.56 | 9.37 | 0.71 | 0.0713 | 0.2556 |
| TC1200007709.hg.1 | PFDN5 | prefoldin subunit 5 | Multiple_C | 15.37 | 15.67 | 13.74 | 3.10 | 1.36E-05 | 0.0002 | 12.86 | 12.94 | 12.24 | 1.54 | 0.0008 | 0.0139 |
| TC1500008183.hg.1 | GOLGA6L3 | golgin A6 family-like 3 | Multiple_C | 9.3 | 8.19 | 7.67 | 3.10 | 0.0041 | 0.0204 | 6.99 | 6.08 | 5.78 | 2.31 | 0.0065 | 0.058 |
| TC2000007157.hg.1 | CBFA2T2 | core-binding factor, runt domain, alpha subunit 2; translocated | Multiple_C | 8.99 | 6.88 | 7.36 | 3.10 | 0.0007 | 0.005 | 7.94 | 7.95 | 8.01 | 0.95 | 0.3121 | 0.5763 |
| TC2000008035.hg.1 | GID8 | GID complex subunit 8 | Multiple_C | 11.53 | 9.94 | 9.9 | 3.10 | 6.93E-06 | 0.0001 | 10.03 | 10.09 | 10.15 | 0.92 | 0.791 | 0.9067 |
| TC2100006442.hg.1 | SIK1 | salt-inducible kinase 1 | Multiple_C | 7.09 | 5.25 | 5.46 | 3.10 | 4.09E-06 | 8.54E-05 | 6.51 | 5.90 | 6.09 | 1.34 | 0.1329 | 0.3618 |
| TC0100010863.hg.1 | LAMC1 | laminin, gamma 1 (formerly LAMB2) | Multiple_C | 12.16 | 10.79 | 10.54 | 3.07 | 1.60E-05 | 0.0002 | 9.88 | 9.17 | 9.37 | 1.42 | 0.0686 | 0.2494 |

| | | | | | | | | | | | | | | | |
|---|---|---|---|---|---|---|---|---|---|---|---|---|---|---|---|
| TC0100018507.hg.1 | ARHGEF2 | Rho/Rac guanine nucleotide exchange factor 2 | Multiple_C | 10.42 | 9.32 | 8.8 | 3.07 | 0.0002 | 0.0019 | 10.91 | 10.48 | 10.37 | 1.45 | 0.1996 | 0.455 |
| TC0300009731.hg.1 | MAP3K13 | mitogen-activated protein kinase kinase kinase 13 | Multiple_C | 6.77 | 4.94 | 5.15 | 3.07 | 2.92E-05 | 0.0004 | 4.79 | 4.65 | 4.6 | 1.14 | 0.2818 | 0.5474 |
| TC0700008063.hg.1 | SPDYE5 | speedy/RINGO cell cycle regulator family member E5 | Coding | 6.15 | 4.23 | 4.53 | 3.07 | 2.75E-05 | 0.0004 | 6.51 | 6.62 | 6.58 | 0.95 | 0.1723 | 0.4193 |
| TC0X00011278.hg.1 | PRRG1 | proline rich Gla (G-carboxyglutamic acid) 1 | Multiple_C | 9.85 | 8.7 | 8.23 | 3.07 | 1.40E-06 | 3.61E-05 | 9.7 | 9.91 | 10.66 | 0.51 | 0.0012 | 0.0194 |
| TC1200011968.hg.1 | HECTD4; MIR6 | HECT domain containing E3 ubiquitin protein ligase 4; microRNA | Multiple_C | 10.87 | 8.91 | 9.25 | 3.07 | 6.71E-07 | 2.06E-05 | 9.31 | 9.61 | 9.29 | 1.01 | 0.888 | 0.9523 |
| TC1600009855.hg.1 | NPIPB6 | nuclear pore complex interacting protein family, member B6 | Coding | 11.5 | 9.63 | 9.88 | 3.07 | 8.92E-06 | 0.0002 | 11.93 | 11.17 | 11.42 | 1.42 | 0.0187 | 0.1154 |
| TC1700010254.hg.1 | SSH2 | slingshot protein phosphatase 2 | Multiple_C | 10.42 | 8.67 | 8.8 | 3.07 | 1.75E-05 | 0.0003 | 9.82 | 9.99 | 10.01 | 0.88 | 0.3515 | 0.6152 |
| TC1700010515.hg.1 | TBC1D3 | TBC1 domain family, member 3 | Coding | 9.45 | 8.59 | 7.83 | 3.07 | 2.76E-06 | 6.24E-05 | 8.51 | 8.22 | 7.96 | 1.46 | 0.0483 | 0.204 |
| TC2200007457.hg.1 | EP300; MIR12 | E1A binding protein p300; microRNA 1281 | Multiple_C | 9.53 | 7.69 | 7.91 | 3.07 | 1.02E-07 | 4.88E-06 | 9.08 | 9.13 | 9.17 | 0.94 | 0.7793 | 0.9011 |
| TSUnmapped00000050.h | MLXIP | MLX interacting protein | Coding | 9.58 | 8.35 | 7.96 | 3.07 | 7.95E-05 | 0.0009 | 6.78 | 7.32 | 7.86 | 0.47 | 0.0001 | 0.0038 |
| TC0100015971.hg.1 | GON4L | gon-4-like (C. elegans) | Multiple_C | 14.02 | 11.8 | 12.41 | 3.05 | 9.59E-07 | 2.68E-05 | 13.42 | 12.54 | 13.38 | 1.03 | 0.7721 | 0.8976 |
| TC0100018303.hg.1 | EFNA3 | ephrin-A3 | Multiple_C | 7.94 | 6.12 | 6.33 | 3.05 | 3.44E-05 | 0.0005 | 4.31 | 4.27 | 4.48 | 0.89 | 0.3758 | 0.6364 |
| TC0300012132.hg.1 | GSK3B | glycogen synthase kinase 3 beta | Multiple_C | 12.96 | 11.6 | 11.35 | 3.05 | 1.05E-06 | 2.90E-05 | 11.26 | 11.25 | 11.48 | 0.86 | 0.0623 | 0.237 |
| TC0700008690.hg.1 | KMT2E | lysine (K)-specific methyltransferase 2E | Multiple_C | 11.97 | 10.56 | 10.36 | 3.05 | 7.23E-06 | 0.0001 | 10.45 | 9.87 | 10.54 | 0.94 | 0.7204 | 0.8698 |
| TC1000010982.hg.1 | ASCC1 | Transcript Identified by AceView, Entrez Gene ID(s) 51008 | Coding | 6.5 | 4.8 | 4.89 | 3.05 | 0.0009 | 0.0062 | 4.18 | 4.05 | 4.95 | 0.59 | 0.0376 | 0.1766 |
| TC1200010265.hg.1 | CAPRIN2 | caprin family member 2 | Multiple_C | 13.03 | 12.12 | 11.42 | 3.05 | 3.20E-05 | 0.0004 | 10.83 | 8.81 | 9.29 | 2.91 | 8.71E-05 | 0.0033 |
| TC1300009249.hg.1 | KLF12 | Kruppel-like factor 12 | Multiple_C | 7.31 | 5.57 | 5.7 | 3.05 | 0.0003 | 0.0023 | 7.57 | 7.78 | 6.69 | 1.84 | 0.028 | 0.1474 |
| TC2000007666.hg.1 | RNF114 | ring finger protein 114 | Multiple_C | 13.07 | 11.55 | 11.46 | 3.05 | 1.58E-05 | 0.0002 | 11.19 | 11.05 | 11.71 | 0.70 | 0.0145 | 0.0981 |
| TC0100011384.hg.1 | MAPKAPK2 | mitogen-activated protein kinase-activated protein kinase 2 | Multiple_C | 11.32 | 9.72 | 9.72 | 3.03 | 8.74E-07 | 2.50E-05 | 9.81 | 9.70 | 10.02 | 0.86 | 0.1166 | 0.3375 |
| TC0100018521.hg.1 | TSTD1 | thiosulfate sulfurtransferase (rhodanese)-like domain containin | Multiple_C | 11.71 | 11.39 | 10.11 | 3.03 | 1.03E-05 | 0.0002 | 7.1 | 7.10 | 6.6 | 1.41 | 0.0098 | 0.0768 |
| TC0200011974.hg.1 | SF3B6 | splicing factor 3b subunit 6 | Multiple_C | 17.39 | 18.07 | 15.79 | 3.03 | 2.93E-05 | 0.0004 | 12.69 | 12.11 | 11.44 | 2.38 | 6.43E-06 | 0.0005 |
| TC0200014246.hg.1 | POTEF | POTE ankyrin domain family, member F | Multiple_C | 8.83 | 8.2 | 7.23 | 3.03 | 0.0001 | 0.0013 | 8.59 | 7.70 | 7.24 | 2.55 | 0.0039 | 0.0422 |
| TC0300011859.hg.1 | ZBTB11 | zinc finger and BTB domain containing 11 | Multiple_C | 9.78 | 8.63 | 8.18 | 3.03 | 0.0012 | 0.0077 | 7.92 | 6.55 | 7.94 | 0.99 | 0.7811 | 0.9017 |
| TC0300012781.hg.1 | WWTR1 | WW domain containing transcription regulator 1 | Multiple_C | 9.11 | 6.71 | 7.51 | 3.03 | 0.0008 | 0.0059 | 5.29 | 5.24 | 5.57 | 0.82 | 0.6564 | 0.8321 |
| TC0400010470.hg.1 | SMIM14 | small integral membrane protein 14 | Multiple_C | 9.33 | 9.03 | 7.73 | 3.03 | 4.39E-06 | 9.02E-05 | 5.51 | 5.56 | 5.27 | 1.18 | 0.4219 | 0.6749 |
| TC0400012818.hg.1 | CCNG2 | cyclin G2 | Multiple_C | 12.73 | 12.27 | 11.13 | 3.03 | 0.0011 | 0.0072 | 8.33 | 8.76 | 7.42 | 1.88 | 0.1794 | 0.4284 |
| TC0500007163.hg.1 | NIPBL | Nipped-B homolog (Drosophila) | Multiple_C | 11.03 | 9.66 | 9.43 | 3.03 | 0.0001 | 0.0015 | 11.82 | 11.44 | 11.51 | 1.24 | 0.274 | 0.5395 |
| TC0600011817.hg.1 | C6orf132 | chromosome 6 open reading frame 132 | Multiple_C | 10.67 | 8.43 | 9.07 | 3.03 | 2.09E-05 | 0.0003 | 5.61 | 5.13 | 5 | 1.53 | 0.0505 | 0.2092 |
| TC0600013001.hg.1 | CEP85L | centrosomal protein 85kDa-like | Multiple_C | 6.47 | 4.54 | 4.87 | 3.03 | 0.0007 | 0.0052 | 4.65 | 4.49 | 4.3 | 1.27 | 0.1991 | 0.4544 |
| TC0X00008394.hg.1 | BCORL1 | BCL6 corepressor-like 1 | Multiple_C | 8.08 | 6.19 | 6.48 | 3.03 | 5.24E-06 | 0.0001 | 8.06 | 8.53 | 8.45 | 0.76 | 0.2724 | 0.5378 |
| TC1100006484.hg.1 | EPS8L2 | EPS8-like 2 | Multiple_C | 10.23 | 8.68 | 8.63 | 3.03 | 0.0002 | 0.0019 | 10.61 | 10.62 | 9.81 | 1.74 | 0.0336 | 0.165 |
| TC1500006728.hg.1 | GOLGA8N; GC | golgin A8 family, member N; golgin A8 family, member H | Multiple_C | 5.89 | 4.56 | 4.29 | 3.03 | 0.0001 | 0.0013 | 5.99 | 5.13 | 5.3 | 1.61 | 0.0202 | 0.1214 |
| TC1600010476.hg.1 | CSNK2A2 | casein kinase 2, alpha prime polypeptide | Multiple_C | 13.31 | 12.29 | 11.71 | 3.03 | 3.79E-05 | 0.0005 | 14.44 | 14.12 | 14.34 | 1.07 | 0.9041 | 0.9605 |

| | | | | | | | | | | | | | | | |
|---|---|---|---|---|---|---|---|---|---|---|---|---|---|---|---|
| TC1700007949.hg.1 | ARL4D | ADP-ribosylation factor like GTPase 4D | Coding | 7.22 | 5.92 | 5.62 | 3.03 | 6.72E-05 | 0.0008 | 5.84 | 5.46 | 5.98 | 0.91 | 0.4235 | 0.6763 |
| TC1700010565.hg.1 | FBXL20 | F-box and leucine-rich repeat protein 20 | Multiple_C | 9.48 | 8.2 | 7.88 | 3.03 | 7.76E-06 | 0.0001 | 7.3 | 7.12 | 7.34 | 0.97 | 0.9064 | 0.9614 |
| TC1900007512.hg.1 | ZNF431; VN1R | zinc finger protein 431; vomeronasal 1 receptor 82 pseudogene | Multiple_C | 9.64 | 8.64 | 8.04 | 3.03 | 4.04E-06 | 8.47E-05 | 8.13 | 7.77 | 8.4 | 0.83 | 0.5481 | 0.765 |
| TC2000007015.hg.1 | PYGB | phosphorylase, glycogen; brain | Multiple_C | 14.48 | 13.98 | 12.88 | 3.03 | 0.0047 | 0.0229 | 10.07 | 10.37 | 10.1 | 0.98 | 0.5769 | 0.7832 |
| TC0200014792.hg.1 | COBLL1 | cordon-bleu WH2 repeat protein like 1 | Multiple_C | 9.01 | 7.05 | 7.42 | 3.01 | 9.66E-06 | 0.0002 | 5.72 | 5.23 | 7.12 | 0.38 | 8.58E-06 | 0.0006 |
| TC0300008371.hg.1 | GRAMD1C | GRAM domain containing 1C | Multiple_C | 7.83 | 7.75 | 6.24 | 3.01 | 0.0002 | 0.0016 | 4.73 | 4.52 | 3.97 | 1.69 | 0.0019 | 0.0258 |
| TC0300013787.hg.1 | TTLL3 | tubulin tyrosine ligase-like family member 3 | Multiple_C | 8.76 | 7.65 | 7.17 | 3.01 | 0.0001 | 0.0012 | 8.56 | 8.18 | 7.6 | 1.95 | 0.0002 | 0.0061 |
| TC0300014084.hg.1 | CRYGS | crystallin gamma S | Multiple_C | 6 | 4.82 | 4.41 | 3.01 | 4.78E-05 | 0.0006 | 4.89 | 4.93 | 4.3 | 1.51 | 0.0337 | 0.1653 |
| TC0400012857.hg.1 | RAPGEF2 | Rap guanine nucleotide exchange factor 2 | Multiple_C | 9.07 | 8.09 | 7.48 | 3.01 | 0.0004 | 0.0032 | 8.99 | 8.20 | 8.23 | 1.69 | 0.0071 | 0.0615 |
| TC0600009819.hg.1 | ULBP2 | UL16 binding protein 2 | Coding | 11.48 | 9.33 | 9.89 | 3.01 | 0.0001 | 0.0012 | 6.87 | 6.73 | 7.38 | 0.70 | 0.1002 | 0.3094 |
| TC1100013012.hg.1 | STX3 | syntaxin 3 | Multiple_C | 12.41 | 9.96 | 10.82 | 3.01 | 2.64E-06 | 6.00E-05 | 11.74 | 10.07 | 11.15 | 1.51 | 0.0143 | 0.0973 |
| TC1700012438.hg.1 | SUPT4H1 | SPT4 homolog, DSIF elongation factor subunit | Multiple_C | 12.65 | 11.1 | 11.06 | 3.01 | 2.38E-07 | 9.29E-06 | 11.38 | 11.72 | 11.65 | 0.83 | 0.0365 | 0.1737 |
| TC2000007670.hg.1 | SNAI1 | snail family zinc finger 1 | Coding | 7.14 | 5.01 | 5.55 | 3.01 | 0.0023 | 0.0127 | 7.26 | 6.58 | 7.07 | 1.14 | 0.2092 | 0.4664 |
| TC0100010872.hg.1 | SMG7 | SMG7 nonsense mediated mRNA decay factor | Multiple_C | 12.01 | 9.6 | 10.43 | 2.99 | 2.06E-05 | 0.0003 | 11.61 | 11.10 | 11.74 | 0.91 | 0.9211 | 0.9675 |
| TC0100014743.hg.1 | TTLL7 | tubulin tyrosine ligase-like family member 7 | Multiple_C | 4.97 | 3.01 | 3.39 | 2.99 | 9.97E-05 | 0.0011 | 10.55 | 9.92 | 9.41 | 2.20 | 0.0102 | 0.0788 |
| TC0200008870.hg.1 | BCL2L11 | BCL2-like 11 (apoptosis facilitator) | Multiple_C | 9.65 | 6.71 | 8.07 | 2.99 | 2.64E-07 | 9.93E-06 | 9.27 | 9.21 | 9.1 | 1.13 | 0.3097 | 0.5735 |
| TC0200010788.hg.1 | ARPC2 | actin related protein 2/3 complex subunit 2 | Multiple_C | 14.5 | 13.2 | 12.92 | 2.99 | 8.18E-05 | 0.0009 | 12.99 | 12.25 | 12.69 | 1.23 | 0.1946 | 0.4485 |
| TC0500010585.hg.1 | RPL37 | ribosomal protein L37 | Multiple_C | 16.98 | 15.55 | 15.4 | 2.99 | 0.0005 | 0.0041 | 14.79 | 14.39 | 14.48 | 1.24 | 0.4461 | 0.6935 |
| TC0600006967.hg.1 | EDN1 | endothelin 1 | Multiple_C | 5.87 | 4.32 | 4.29 | 2.99 | 5.11E-07 | 1.64E-05 | 4.48 | 4.61 | 5.03 | 0.68 | 0.0021 | 0.0273 |
| TC0700007137.hg.1 | BBS9 | Bardet-Biedl syndrome 9 | Multiple_C | 8.01 | 7.55 | 6.43 | 2.99 | 0.0099 | 0.0413 | 6.34 | 6.10 | 6.21 | 1.09 | 0.411 | 0.6656 |
| TC0X00010648.hg.1 | RPL39; SNORA | ribosomal protein L39; small nucleolar RNA, H/ACA box 69 | Multiple_C | 16.08 | 15.76 | 14.5 | 2.99 | 0.0003 | 0.0024 | 12.38 | 12.66 | 12.46 | 0.95 | 0.6562 | 0.8321 |
| TC1000008363.hg.1 | PTEN | phosphatase and tensin homolog | Multiple_C | 12.47 | 11.89 | 10.89 | 2.99 | 0.0002 | 0.0015 | 13.01 | 12.76 | 13.55 | 0.69 | 0.0193 | 0.1175 |
| TC1100012227.hg.1 | C11orf65 | chromosome 11 open reading frame 65 | Multiple_C | 6.85 | 5.75 | 5.27 | 2.99 | 4.13E-06 | 8.60E-05 | 6.36 | 5.85 | 5.6 | 1.69 | 0.0005 | 0.0103 |
| TC1200008026.hg.1 | LEMD3 | LEM domain containing 3 | Multiple_C | 10.78 | 10.12 | 9.2 | 2.99 | 3.20E-05 | 0.0004 | 10.24 | 9.39 | 9.67 | 1.48 | 0.0385 | 0.1787 |
| TC1400010730.hg.1 | EGLN3 | egl-9 family hypoxia-inducible factor 3 | Multiple_C | 8.66 | 6.89 | 7.08 | 2.99 | 6.57E-05 | 0.0008 | 8.35 | 9.34 | 8.03 | 1.25 | 0.7444 | 0.8831 |
| TC1500010764.hg.1 | CLK3 | CDC like kinase 3 | Multiple_C | 11.86 | 10.21 | 10.28 | 2.99 | 1.31E-06 | 3.43E-05 | 10.13 | 9.27 | 9.8 | 1.26 | 0.1628 | 0.4062 |
| TC1600009958.hg.1 | NPIPB4 | nuclear pore complex interacting protein family, member B4 | Multiple_C | 13.15 | 11.27 | 11.57 | 2.99 | 4.65E-07 | 1.54E-05 | 13.1 | 12.39 | 12.7 | 1.32 | 0.0099 | 0.0774 |
| TC1600010175.hg.1 | C16orf87 | chromosome 16 open reading frame 87 | Multiple_C | 10.17 | 9.61 | 8.59 | 2.99 | 3.34E-05 | 0.0004 | 8.83 | 7.92 | 8.92 | 0.94 | 0.3852 | 0.6441 |
| TC1700011756.hg.1 | CASKIN2 | CASK interacting protein 2 | Multiple_C | 7.62 | 5.25 | 6.04 | 2.99 | 0.0002 | 0.0016 | 8.5 | 8.83 | 8.89 | 0.76 | 0.0939 | 0.2976 |
| TC1800008677.hg.1 | CFAP53 | cilia and flagella associated protein 53 | Coding | 6.78 | 6.05 | 5.2 | 2.99 | 2.37E-05 | 0.0003 | 4.84 | 5.05 | 4.76 | 1.06 | 0.6166 | 0.8098 |
| TC2200006681.hg.1 | CRKL | v-crk avian sarcoma virus CT10 oncogene homolog-like | Multiple_C | 13.76 | 11.76 | 12.18 | 2.99 | 1.16E-07 | 5.32E-06 | 13.06 | 12.94 | 13.46 | 0.76 | 0.0169 | 0.1089 |
| TC0700013604.hg.1 | UPK3BL; POLR | uroplakin 3B-like; polymerase (RNA) II (DNA directed) polypepti | Multiple_C | 11.89 | 9.93 | 10.32 | 2.97 | 0.0003 | 0.0027 | 10.14 | 10.70 | 11.16 | 0.49 | 0.0004 | 0.0085 |
| TC0900011631.hg.1 | GOLGA2 | golgin A2 | Multiple_C | 10.99 | 10.02 | 9.42 | 2.97 | 4.88E-05 | 0.0006 | 9.77 | 9.22 | 9.36 | 1.33 | 0.669 | 0.8382 |

| | | | | | | | | | | | | | | | |
|---|---|---|---|---|---|---|---|---|---|---|---|---|---|---|---|
| TC1000011454.hg.1 | FRA10AC1 | fragile site, folic acid type, rare, fra(10)(q23.3) or fra(10)(q24.2) | Multiple_C | 9.84 | 10.2 | 8.27 | 2.97 | 5.24E-05 | 0.0006 | 8.14 | 7.67 | 7.44 | 1.62 | 0.0009 | 0.0151 |
| TC1400009705.hg.1 | PGF | placental growth factor | Multiple_C | 9.31 | 7.76 | 7.74 | 2.97 | 9.67E-06 | 0.0002 | 7.04 | 6.61 | 6.98 | 1.04 | 0.6988 | 0.8572 |
| TC1600011365.hg.1 | NPIPB9 | nuclear pore complex interacting protein family, member B9 | Coding | 11.18 | 9.36 | 9.61 | 2.97 | 4.43E-06 | 9.08E-05 | 11.08 | 10.49 | 10.58 | 1.41 | 0.0622 | 0.237 |
| TC1900011470.hg.1 | UBE2S | ubiquitin-conjugating enzyme E2S | Multiple_C | 12.56 | 10.49 | 10.99 | 2.97 | 0.0012 | 0.0077 | 10.59 | 11.49 | 11.74 | 0.45 | 0.0086 | 0.0708 |
| TC2000006823.hg.1 | ZNF133 | zinc finger protein 133 | Multiple_C | 6.04 | 5.53 | 4.47 | 2.97 | 0.0002 | 0.0018 | 5.23 | 5.42 | 5.45 | 0.86 | 0.3201 | 0.584 |
| TC2100008222.hg.1 | RIPK4 | receptor-interacting serine-threonine kinase 4 | Coding | 9.67 | 8.42 | 8.1 | 2.97 | 3.04E-05 | 0.0004 | 6.5 | 6.73 | 7.47 | 0.51 | 0.0016 | 0.0233 |
| TC2200008055.hg.1 | PI4KA | phosphatidylinositol 4-kinase, catalytic, alpha | Multiple_C | 10.49 | 8.83 | 8.92 | 2.97 | 0.0005 | 0.0036 | 9.71 | 9.71 | 8.98 | 1.66 | 0.0248 | 0.1366 |
| TC0200009487.hg.1 | SPOPL | speckle-type POZ protein-like | Multiple_C | 11.1 | 9.6 | 9.54 | 2.95 | 0.0001 | 0.0013 | 9.02 | 7.84 | 8.88 | 1.10 | 0.3495 | 0.6137 |
| TC0200010518.hg.1 | ABI2 | abl-interactor 2 | Multiple_C | 9.35 | 7.52 | 7.79 | 2.95 | 0.0001 | 0.0011 | 9.8 | 8.94 | 9.82 | 0.99 | 0.8843 | 0.9504 |
| TC0300013904.hg.1 | NAALADL2 | N-acetylated alpha-linked acidic dipeptidase-like 2 | Multiple_C | 5.31 | 3.66 | 3.75 | 2.95 | 0.0022 | 0.0127 | 3.58 | 3.47 | 3.49 | 1.06 | 0.2242 | 0.4851 |
| TC0400009001.hg.1 | FAM160A1 | family with sequence similarity 160, member A1 | Multiple_C | 6.3 | 4.38 | 4.74 | 2.95 | 0.0303 | 0.0992 | 5.45 | 4.14 | 4.63 | 1.77 | 0.0834 | 0.278 |
| TC0X00007075.hg.1 | KDM6A | lysine (K)-specific demethylase 6A | Multiple_C | 10.08 | 9.55 | 8.52 | 2.95 | 1.12E-06 | 3.04E-05 | 7.34 | 7.71 | 8.07 | 0.60 | 0.0042 | 0.0442 |
| TC1200006520.hg.1 | TSPAN9 | tetraspanin 9 | Multiple_C | 7.14 | 5.76 | 5.58 | 2.95 | 0.0002 | 0.0017 | 8.87 | 8.36 | 8.69 | 1.13 | 0.5251 | 0.7498 |
| TC1200009461.hg.1 | EP400; SNORA | E1A binding protein p400; small nucleolar RNA, H/ACA box 49 | Multiple_C | 9.29 | 7.28 | 7.73 | 2.95 | 0.0004 | 0.0033 | 9.57 | 9.72 | 9.53 | 1.03 | 0.7066 | 0.8619 |
| TC1500008511.hg.1 | LRRC28 | leucine rich repeat containing 28 | Multiple_C | 10.24 | 9.3 | 8.68 | 2.95 | 0.0022 | 0.0126 | 8.08 | 8.19 | 8.5 | 0.75 | 0.5675 | 0.7765 |
| TC1600011501.hg.1 | NPIPB3 | nuclear pore complex interacting protein family, member B3 | Multiple_C | 13.22 | 11.39 | 11.66 | 2.95 | 1.08E-06 | 2.95E-05 | 13.12 | 12.28 | 12.66 | 1.38 | 0.0084 | 0.0698 |
| TC0100011153.hg.1 | C1orf106 | chromosome 1 open reading frame 106 | Multiple_C | 9.75 | 7.82 | 8.2 | 2.93 | 5.81E-06 | 0.0001 | 6.93 | 7.44 | 7.4 | 0.72 | 0.0617 | 0.2358 |
| TC0200013376.hg.1 | EIF2AK3 | eukaryotic translation initiation factor 2-alpha kinase 3 | Multiple_C | 9.27 | 8.25 | 7.72 | 2.93 | 6.74E-05 | 0.0008 | 8.23 | 7.63 | 7.88 | 1.27 | 0.3397 | 0.6039 |
| TC0300009661.hg.1 | B3GNT5 | UDP-GlcNAc:betaGal beta-1,3-N-acetylglucosaminyltransferase | Multiple_C | 12.76 | 11.13 | 11.21 | 2.93 | 4.12E-07 | 1.40E-05 | 8.62 | 9.99 | 11.16 | 0.17 | 1.64E-11 | 4.41E-08 |
| TC0700010561.hg.1 | HOXA5 | homeobox A5 | Multiple_C | 11.31 | 10.57 | 9.76 | 2.93 | 0.0018 | 0.0105 | 7.29 | 6.56 | 6.65 | 1.56 | 0.7878 | 0.9054 |
| TC0900011613.hg.1 | FAM102A | family with sequence similarity 102, member A | Multiple_C | 7.37 | 5.53 | 5.82 | 2.93 | 0.0002 | 0.0017 | 7.21 | 6.42 | 7.5 | 0.82 | 0.5381 | 0.759 |
| TC1200009734.hg.1 | VAMP1 | vesicle associated membrane protein 1 | Multiple_C | 8.64 | 6.69 | 7.09 | 2.93 | 3.80E-05 | 0.0005 | 8.43 | 8.19 | 8.38 | 1.04 | 0.4857 | 0.7228 |
| TC1200010598.hg.1 | RND1 | Rho family GTPase 1 | Multiple_C | 5.89 | 3.88 | 4.34 | 2.93 | 2.56E-05 | 0.0004 | 6.06 | 5.67 | 5.44 | 1.54 | 0.0101 | 0.0785 |
| TC1200011311.hg.1 | NAP1L1 | nucleosome assembly protein 1-like 1 | Multiple_C | 15.58 | 14.76 | 14.03 | 2.93 | 0.0004 | 0.0034 | 14.96 | 14.23 | 14.08 | 1.84 | 0.0011 | 0.0181 |
| TC1500010784.hg.1 | NGRN; TTLL13 | neugrin, neurite outgrowth associated; tubulin tyrosine ligase-li | Multiple_C | 12.18 | 10.46 | 10.63 | 2.93 | 1.15E-05 | 0.0002 | 10.09 | 9.73 | 10.59 | 0.71 | 0.1777 | 0.4263 |
| TC1500010792.hg.1 | MCTP2 | multiple C2 domains, transmembrane 2 | Multiple_C | 9.5 | 8.48 | 7.95 | 2.93 | 6.10E-05 | 0.0007 | 5 | 3.95 | 4.83 | 1.13 | 0.3437 | 0.6073 |
| TC1700012228.hg.1 | LRRC37B | leucine rich repeat containing 37B | Multiple_C | 8.72 | 7.15 | 7.17 | 2.93 | 4.29E-06 | 8.85E-05 | 6.86 | 6.82 | 7.12 | 0.84 | 0.7547 | 0.8885 |
| TC1700012239.hg.1 | TBC1D3K | TBC1 domain family, member 3K | Coding | 7.77 | 6.47 | 6.22 | 2.93 | 0.0045 | 0.0221 | 7.43 | 6.91 | 6.38 | 2.07 | 0.1002 | 0.3095 |
| TC1900011084.hg.1 | DBP | D site of albumin promoter (albumin D-box) binding protein | Multiple_C | 8.58 | 7.07 | 7.03 | 2.93 | 0.0001 | 0.0012 | 6.96 | 7.23 | 6.9 | 1.04 | 0.8946 | 0.9554 |
| TC1900011750.hg.1 | ZNF155 | zinc finger protein 155 | Multiple_C | 6.76 | 5.78 | 5.21 | 2.93 | 1.05E-05 | 0.0002 | 6.43 | 6.16 | 6.07 | 1.28 | 0.0597 | 0.2308 |
| TC2000009959.hg.1 | CSNK2A1 | casein kinase 2, alpha 1 polypeptide | Multiple_C | 15.24 | 14.15 | 13.69 | 2.93 | 5.74E-05 | 0.0007 | 15.59 | 14.74 | 15.35 | 1.18 | 0.7714 | 0.8972 |
| TC2200007932.hg.1 | PI4KAP1 | phosphatidylinositol 4-kinase, catalytic, alpha pseudogene 1 | Multiple_C | 10.74 | 9.14 | 9.19 | 2.93 | 0.0002 | 0.0017 | 9.63 | 9.96 | 9.65 | 0.99 | 0.3639 | 0.6265 |
| TC0100007747.hg.1 | GJB3 | gap junction protein beta 3 | Multiple_C | 9.68 | 7.99 | 8.14 | 2.91 | 6.71E-06 | 0.0001 | 5.4 | 4.53 | 5.32 | 1.06 | 0.8664 | 0.9427 |

| | | | | | | | | | | | | | | | |
|---|---|---|---|---|---|---|---|---|---|---|---|---|---|---|---|
| TC0100018248.hg.1 | RNPC3 | RNA binding region (RNP1, RRM) containing 3 | Multiple_C | 8.87 | 8.79 | 7.33 | 2.91 | 9.30E-07 | 2.63E-05 | 8.58 | 7.87 | 7.52 | 2.08 | 9.38E-05 | 0.0035 |
| TC0200012734.hg.1 | USP34 | ubiquitin specific peptidase 34 | Multiple_C | 11.03 | 9.55 | 9.49 | 2.91 | 1.18E-05 | 0.0002 | 10.48 | 9.67 | 10.15 | 1.26 | 0.8088 | 0.9155 |
| TC0200014550.hg.1 | ORC4 | origin recognition complex subunit 4 | Multiple_C | 12.65 | 12.1 | 11.11 | 2.91 | 0.0003 | 0.0024 | 11 | 9.96 | 10.69 | 1.24 | 0.7413 | 0.881 |
| TC0300009651.hg.1 | ATP11B | ATPase, class VI, type 11B | Multiple_C | 10.24 | 9.52 | 8.7 | 2.91 | 3.96E-05 | 0.0005 | 6.83 | 6.95 | 7.5 | 0.63 | 0.0051 | 0.0499 |
| TC0600007864.hg.1 | TBC1D22B | TBC1 domain family, member 22B | Multiple_C | 9.78 | 8.19 | 8.24 | 2.91 | 0.004 | 0.02 | 7.69 | 7.22 | 7.99 | 0.81 | 0.0454 | 0.197 |
| TC0900007029.hg.1 | UBAP1 | ubiquitin associated protein 1 | Multiple_C | 11.84 | 10.4 | 10.3 | 2.91 | 2.61E-06 | 5.96E-05 | 9.42 | 9.22 | 9.55 | 0.91 | 0.088 | 0.2866 |
| TC1000007020.hg.1 | OTUD1 | OTU deubiquitinase 1 | Coding | 7.86 | 6.91 | 6.32 | 2.91 | 0.0037 | 0.0192 | 5.06 | 4.26 | 4.13 | 1.91 | 0.0231 | 0.1308 |
| TC1000008088.hg.1 | SAMD8 | sterile alpha motif domain containing 8 | Multiple_C | 10.97 | 9.84 | 9.43 | 2.91 | 5.82E-06 | 0.0001 | 9.33 | 7.89 | 8.58 | 1.68 | 0.0011 | 0.0175 |
| TC1300008688.hg.1 | FOXO1 | forkhead box O1 | Multiple_C | 9.16 | 7.59 | 7.62 | 2.91 | 7.68E-05 | 0.0009 | 5.13 | 4.94 | 4.74 | 1.31 | 0.1341 | 0.3637 |
| TC1500007833.hg.1 | PML | promyelocytic leukemia | Multiple_C | 9.45 | 7.1 | 7.91 | 2.91 | 0.0004 | 0.003 | 7.19 | 7.02 | 8.01 | 0.57 | 0.0028 | 0.0341 |
| TC1500008933.hg.1 | GOLGA8O | golgin A8 family, member O | Multiple_C | 6.56 | 5.58 | 5.02 | 2.91 | 8.54E-05 | 0.0009 | 6.14 | 4.97 | 5.02 | 2.17 | 0.0028 | 0.0335 |
| TC1600009202.hg.1 | CREBBP | CREB binding protein | Multiple_C | 11.89 | 9.74 | 10.35 | 2.91 | 0.0119 | 0.0481 | 11.22 | 11.48 | 11.89 | 0.63 | 0.1217 | 0.3444 |
| TC1600011560.hg.1 | MTSS1L | metastasis suppressor 1-like | Multiple_C | 9.67 | 7.39 | 8.13 | 2.91 | 2.00E-05 | 0.0003 | 10.5 | 10.74 | 11.59 | 0.47 | 8.36E-06 | 0.0006 |
| TC1700012243.hg.1 | TBC1D3E | TBC1 domain family, member 3E | Coding | 11.03 | 10.09 | 9.49 | 2.91 | 2.00E-05 | 0.0003 | 10.56 | 10.07 | 9.52 | 2.06 | 0.0013 | 0.0199 |
| TC0100009442.hg.1 | WNT2B | wingless-type MMTV integration site family, member 2B | Multiple_C | 4.62 | 3.06 | 3.09 | 2.89 | 0.0009 | 0.0062 | 3.55 | 3.98 | 3.49 | 1.04 | 0.181 | 0.431 |
| TC0100010008.hg.1 | LCE1E | late cornified envelope 1E | Coding | 13.82 | 12.06 | 12.29 | 2.89 | 0.0002 | 0.0016 | 14.13 | 14.20 | 13.89 | 1.18 | 0.3375 | 0.6018 |
| TC0200007418.hg.1 | SLC3A1 | solute carrier family 3 (amino acid transporter heavy chain), me | Coding | 5.82 | 4.85 | 4.29 | 2.89 | 0.0001 | 0.0011 | 4.46 | 4.37 | 4.49 | 0.98 | 0.3804 | 0.64 |
| TC0300008336.hg.1 | C3orf52 | chromosome 3 open reading frame 52 | Multiple_C | 7.65 | 5.96 | 6.12 | 2.89 | 2.01E-05 | 0.0003 | 5.78 | 5.26 | 5.72 | 1.04 | 0.1971 | 0.4519 |
| TC0500009281.hg.1 | PTTG1 | pituitary tumor-transforming 1 | Multiple_C | 13.16 | 12.15 | 11.63 | 2.89 | 0.0001 | 0.0011 | 10.14 | 10.19 | 10.02 | 1.09 | 0.9838 | 0.9934 |
| TC0700008450.hg.1 | LMTK2 | lemur tyrosine kinase 2 | Multiple_C | 11.63 | 9.32 | 10.1 | 2.89 | 0.0001 | 0.0011 | 9.88 | 9.64 | 10.4 | 0.70 | 0.0115 | 0.0848 |
| TC0700008868.hg.1 | TES | testin LIM domain protein | Multiple_C | 14.03 | 13.39 | 12.5 | 2.89 | 4.01E-06 | 8.43E-05 | 12.23 | 11.45 | 12.21 | 1.01 | 0.9865 | 0.9943 |
| TC0X00011223.hg.1 | MPP1 | membrane protein, palmitoylated 1 | Multiple_C | 8.28 | 6.22 | 6.75 | 2.89 | 0.0007 | 0.005 | 7.85 | 8.20 | 7.67 | 1.13 | 0.7542 | 0.8883 |
| TC1000012580.hg.1 | ARHGAP19 | Rho GTPase activating protein 19 | Multiple_C | 10.89 | 10.35 | 9.36 | 2.89 | 1.87E-05 | 0.0003 | 9.08 | 8.86 | 9.84 | 0.59 | 0.0106 | 0.0811 |
| TC1100008330.hg.1 | IL18BP | interleukin 18 binding protein | Multiple_C | 6.87 | 5.83 | 5.34 | 2.89 | 7.67E-05 | 0.0009 | 7.11 | 6.89 | 6.83 | 1.21 | 0.3037 | 0.5678 |
| TC1200007887.hg.1 | MBD6 | methyl-CpG binding domain protein 6 | Multiple_C | 9.34 | 7.32 | 7.81 | 2.89 | 1.08E-05 | 0.0002 | 7.78 | 7.75 | 7.87 | 0.94 | 0.3182 | 0.5822 |
| TC1500010890.hg.1 | HEXA | hexosaminidase A (alpha polypeptide) | Multiple_C | 12.14 | 11.34 | 10.61 | 2.89 | 3.18E-05 | 0.0004 | 10.46 | 11.10 | 10.4 | 1.04 | 0.6784 | 0.8452 |
| TC1700011205.hg.1 | C17orf67 | chromosome 17 open reading frame 67 | Multiple_C | 7.37 | 6.27 | 5.84 | 2.89 | 8.91E-05 | 0.001 | 5.59 | 5.10 | 5.18 | 1.33 | 0.0356 | 0.171 |
| TC1900008399.hg.1 | ARHGAP35 | Rho GTPase activating protein 35 | Multiple_C | 11.03 | 8.71 | 9.5 | 2.89 | 0.0005 | 0.004 | 9.34 | 10.03 | 10.2 | 0.55 | 0.0001 | 0.0046 |
| TC0100014772.hg.1 | SYDE2 | synapse defective 1, Rho GTPase, homolog 2 (C. elegans) | Multiple_C | 9.34 | 8.49 | 7.82 | 2.87 | 6.36E-06 | 0.0001 | 7.33 | 5.88 | 5.3 | 4.08 | 4.05E-09 | 2.63E-06 |
| TC0200008930.hg.1 | SLC20A1 | solute carrier family 20 (phosphate transporter), member 1 | Multiple_C | 12.84 | 10.62 | 11.32 | 2.87 | 9.59E-06 | 0.0002 | 11.31 | 9.98 | 11.62 | 0.81 | 0.0131 | 0.0921 |
| TC0300010913.hg.1 | CDCP1 | CUB domain containing protein 1 | Multiple_C | 13.4 | 10.91 | 11.88 | 2.87 | 9.48E-08 | 4.64E-06 | 12.56 | 12.66 | 12.9 | 0.79 | 0.0208 | 0.1233 |
| TC0400006703.hg.1 | KIAA0232 | KIAA0232 | Multiple_C | 8.49 | 6.58 | 6.97 | 2.87 | 1.19E-05 | 0.0002 | 7.6 | 7.68 | 7.62 | 0.99 | 0.6542 | 0.8316 |
| TC0600009710.hg.1 | STX11 | syntaxin 11 | Coding | 4.55 | 3.29 | 3.03 | 2.87 | 0.0009 | 0.0061 | 3.31 | 3.20 | 3.4 | 0.94 | 0.6544 | 0.8317 |

| | | | | | | | | | | | | | | | |
|---|---|---|---|---|---|---|---|---|---|---|---|---|---|---|---|
| TC0600014258.hg.1 | HLA-B | major histocompatibility complex, class I, B | Multiple_C | 10.4 | 9.08 | 8.88 | 2.87 | 8.34E-05 | 0.0009 | 11.05 | 10.23 | 10.26 | 1.73 | 0.118 | 0.3395 |
| TC1100009245.hg.1 | CBL | Cbl proto-oncogene, E3 ubiquitin protein ligase | Multiple_C | 9.51 | 7.2 | 7.99 | 2.87 | 1.22E-05 | 0.0002 | 9.18 | 8.64 | 9.35 | 0.89 | 0.5672 | 0.7763 |
| TC1200006688.hg.1 | NANOG | Nanog homeobox | Multiple_C | 5.89 | 3.77 | 4.37 | 2.87 | 1.48E-05 | 0.0002 | 3.75 | 3.78 | 4.01 | 0.84 | 0.4019 | 0.6582 |
| TC1200010024.hg.1 | ARHGDIB | Rho GDP dissociation inhibitor (GDI) beta | Multiple_C | 5.9 | 4.26 | 4.38 | 2.87 | 1.50E-05 | 0.0002 | 4.63 | 4.41 | 4.7 | 0.95 | 0.8287 | 0.9242 |
| TC1300006675.hg.1 | SNORD102; S | small nucleolar RNA, C/D box 102; small nucleolar RNA, H/ACA | Multiple_C | 17.29 | 16.36 | 15.77 | 2.87 | 0.0021 | 0.0119 | 12.58 | 13.16 | 12.64 | 0.96 | 0.6649 | 0.8362 |
| TC1600009512.hg.1 | NTAN1 | N-terminal asparagine amidase | Multiple_C | 11.53 | 10.44 | 10.01 | 2.87 | 0.0002 | 0.0015 | 8.69 | 7.86 | 8.21 | 1.39 | 0.3625 | 0.6254 |
| TC1900009315.hg.1 | DAPK3; MIR63 | death-associated protein kinase 3; microRNA 637 | Multiple_C | 11.17 | 9.34 | 9.65 | 2.87 | 0.0013 | 0.0081 | 8.47 | 8.44 | 8.82 | 0.78 | 0.0151 | 0.1004 |
| TC2000009673.hg.1 | SYCP2 | synaptonemal complex protein 2 | Multiple_C | 6.77 | 6.71 | 5.25 | 2.87 | 8.16E-06 | 0.0001 | 5.39 | 4.12 | 4.22 | 2.25 | 7.24E-05 | 0.0029 |
| TC2100007663.hg.1 | BTG3 | BTG family, member 3 | Multiple_C | 11.9 | 10.52 | 10.38 | 2.87 | 0.001 | 0.0067 | 7.81 | 6.42 | 6.52 | 2.45 | 0.0001 | 0.0046 |
| TC0100014065.hg.1 | EFCAB14 | EF-hand calcium binding domain 14 | Multiple_C | 10.95 | 9.41 | 9.44 | 2.85 | 7.20E-06 | 0.0001 | 10.17 | 9.62 | 10.18 | 0.99 | 0.9712 | 0.9882 |
| TC0400012644.hg.1 | SORBS2 | sorbin and SH3 domain containing 2 | Multiple_C | 7.09 | 5.84 | 5.58 | 2.85 | 0.0014 | 0.0087 | 8.97 | 9.59 | 9.03 | 0.96 | 0.6847 | 0.8488 |
| TC0600007512.hg.1 | TRIM15 | tripartite motif containing 15 | Multiple_C | 10.36 | 9.01 | 8.85 | 2.85 | 0.0245 | 0.0841 | 7.63 | 7.45 | 7.46 | 1.13 | 0.9623 | 0.9842 |
| TC0800007080.hg.1 | BNIP3L | BCL2/adenovirus E1B 19kDa interacting protein 3-like | Multiple_C | 15.23 | 14.28 | 13.72 | 2.85 | 7.18E-06 | 0.0001 | 12.02 | 11.72 | 10.28 | 3.34 | 1.38E-07 | 3.29E-05 |
| TC0800011074.hg.1 | C8orf88 | chromosome 8 open reading frame 88 | Multiple_C | 4.53 | 2.78 | 3.02 | 2.85 | 0.0009 | 0.0059 | 4.04 | 3.82 | 3.65 | 1.31 | 0.0621 | 0.2366 |
| TC1100006899.hg.1 | ARNTL | aryl hydrocarbon receptor nuclear translocator-like | Multiple_C | 8.32 | 6.54 | 6.81 | 2.85 | 5.15E-06 | 0.0001 | 7.22 | 7.42 | 7.49 | 0.83 | 0.8722 | 0.945 |
| TC1400007584.hg.1 | TTC9 | tetratricopeptide repeat domain 9 | Coding | 6.63 | 5.28 | 5.12 | 2.85 | 0.0038 | 0.0193 | 6.15 | 5.61 | 6.44 | 0.82 | 0.9525 | 0.9806 |
| TC1600011440.hg.1 | OSGIN1 | oxidative stress induced growth inhibitor 1 | Multiple_C | 7.61 | 5.67 | 6.1 | 2.85 | 0.0051 | 0.0245 | 6.21 | 5.99 | 5.87 | 1.27 | 0.7737 | 0.8983 |
| TC1700009377.hg.1 | INPP5K | inositol polyphosphate-5-phosphatase K | Multiple_C | 9.67 | 7.73 | 8.16 | 2.85 | 0.0021 | 0.0122 | 8.16 | 8.27 | 8.05 | 1.08 | 0.9883 | 0.995 |
| TC1700012240.hg.1 | TBC1D3H; TBC | Homo sapiens TBC1 domain family, member 3H (TBC1D3H), mR | Coding | 9.94 | 8.82 | 8.43 | 2.85 | 1.56E-05 | 0.0002 | 9.29 | 8.86 | 8.35 | 1.92 | 8.56E-05 | 0.0032 |
| TC2000008852.hg.1 | NOL4L | nucleolar protein 4-like | Multiple_C | 9.81 | 8.01 | 8.3 | 2.85 | 8.29E-05 | 0.0009 | 10.36 | 9.90 | 9.43 | 1.91 | 0.0006 | 0.0114 |
| TC0100011205.hg.1 | ELF3 | E74-like factor 3 (ets domain transcription factor, epithelial-spe | Multiple_C | 11.72 | 9.97 | 10.22 | 2.83 | 0.0003 | 0.0025 | 5.2 | 5.18 | 5.26 | 0.96 | 0.7147 | 0.8667 |
| TC0300009500.hg.1 | FNDC3B | fibronectin type III domain containing 3B | Multiple_C | 13.45 | 11.43 | 11.95 | 2.83 | 4.34E-05 | 0.0005 | 12.04 | 10.72 | 11.68 | 1.28 | 0.7799 | 0.9013 |
| TC0400007245.hg.1 | TBC1D1 | TBC1 (tre-2/USP6, BUB2, cdc16) domain family, member 1 | Multiple_C | 9.24 | 8.14 | 7.74 | 2.83 | 2.86E-05 | 0.0004 | 8.1 | 7.74 | 8.3 | 0.87 | 0.3715 | 0.6332 |
| TC0600013431.hg.1 | PLAGL1; HYM | pleiomorphic adenoma gene-like 1; hydatidiform mole associat | Multiple_C | 7.32 | 4.96 | 5.82 | 2.83 | 0.0016 | 0.0095 | 5.19 | 4.97 | 5.48 | 0.82 | 0.6409 | 0.8239 |
| TC0700008786.hg.1 | DNAJB9 | DnaJ (Hsp40) homolog, subfamily B, member 9 | Multiple_C | 9.67 | 9.57 | 8.17 | 2.83 | 8.24E-06 | 0.0001 | 8.19 | 7.47 | 7.84 | 1.27 | 0.1043 | 0.3166 |
| TC0700013441.hg.1 | IFRD1 | interferon-related developmental regulator 1 | Multiple_C | 14.65 | 15.19 | 13.15 | 2.83 | 1.17E-05 | 0.0002 | 13.49 | 11.80 | 12.99 | 1.41 | 0.0674 | 0.2474 |
| TC0700013629.hg.1 | BRAF | B-Raf proto-oncogene, serine/threonine kinase | Multiple_C | 9.62 | 8.18 | 8.12 | 2.83 | 0.0021 | 0.0122 | 9.52 | 8.76 | 9.46 | 1.04 | 0.9505 | 0.9797 |
| TC0900008109.hg.1 | HABP4 | hyaluronan binding protein 4 | Multiple_C | 7.11 | 6.28 | 5.61 | 2.83 | 0.0006 | 0.0047 | 5.51 | 5.40 | 5.5 | 1.01 | 0.8373 | 0.9283 |
| TC1000006796.hg.1 | SEC61A2 | Sec61 translocon alpha 2 subunit | Multiple_C | 8.28 | 6.86 | 6.78 | 2.83 | 0.0005 | 0.0037 | 8.11 | 7.04 | 7.79 | 1.25 | 0.0913 | 0.2924 |
| TC1200009881.hg.1 | CLEC2B | C-type lectin domain family 2, member B | Multiple_C | 5.09 | 4.21 | 3.59 | 2.83 | 4.04E-05 | 0.0005 | 4.87 | 4.44 | 3.95 | 1.89 | 0.0022 | 0.0285 |
| TC1300009714.hg.1 | ARGLU1 | arginine and glutamate rich 1 | Multiple_C | 11.89 | 11.59 | 10.39 | 2.83 | 9.33E-05 | 0.001 | 13.01 | 12.37 | 11.75 | 2.39 | 0.0001 | 0.0041 |
| TC1400009669.hg.1 | ELMSAN1 | ELM2 and Myb/SANT-like domain containing 1 | Multiple_C | 7.74 | 6.14 | 6.24 | 2.83 | 3.58E-05 | 0.0005 | 7.83 | 7.39 | 7.45 | 1.30 | 0.223 | 0.4835 |
| TC1600011505.hg.1 | NPIPB4 | nuclear pore complex interacting protein family, member B4 | Multiple_C | 13.67 | 11.71 | 12.17 | 2.83 | 1.63E-07 | 6.94E-06 | 14.44 | 13.71 | 13.85 | 1.51 | 0.0054 | 0.0517 |

| | | | | | | | | | | | | | | | |
|---|---|---|---|---|---|---|---|---|---|---|---|---|---|---|---|
| TC1700007779.hg.1 | MSL1 | male-specific lethal 1 homolog (Drosophila) | Multiple_C | 10.09 | 9.49 | 8.59 | 2.83 | 0.0648 | 0.1755 | 9.94 | 8.72 | 10.02 | 0.95 | 0.3312 | 0.5953 |
| TC1700010654.hg.1 | KRTAP2-4 | keratin associated protein 2-4 | Coding | 8.29 | 6.44 | 6.79 | 2.83 | 0.0009 | 0.0063 | 9.05 | 8.78 | 8.53 | 1.43 | 0.0564 | 0.2234 |
| TSUnmapped00000491.h | ZDHHC3 | zinc finger, DHHC-type containing 3 | Coding | 7.76 | 6.16 | 6.26 | 2.83 | 2.68E-05 | 0.0004 | 5.09 | 4.93 | 5.17 | 0.95 | 0.9556 | 0.9815 |
| TC0100009958.hg.1 | CGN | cingulin | Multiple_C | 7.55 | 6.21 | 6.06 | 2.81 | 5.34E-06 | 0.0001 | 5.12 | 5.54 | 5.47 | 0.78 | 0.0753 | 0.2637 |
| TC0100016035.hg.1 | ETV3 | ets variant 3 | Multiple_C | 11.59 | 9.74 | 10.1 | 2.81 | 0.0036 | 0.0187 | 10.42 | 10.20 | 10.49 | 0.95 | 0.1811 | 0.4311 |
| TC0100018478.hg.1 | NBPF10 | neuroblastoma breakpoint family, member 10 | Multiple_C | 11.36 | 10.17 | 9.87 | 2.81 | 4.84E-05 | 0.0006 | 10.14 | 9.71 | 9.89 | 1.19 | 0.3402 | 0.604 |
| TC0200007533.hg.1 | FOXN2 | forkhead box N2 | Multiple_C | 12.22 | 11.13 | 10.73 | 2.81 | 7.39E-05 | 0.0008 | 10.93 | 10.06 | 11.47 | 0.69 | 0.045 | 0.1963 |
| TC0200012961.hg.1 | AAK1 | AP2 associated kinase 1 | Multiple_C | 9.63 | 7.24 | 8.14 | 2.81 | 0.0002 | 0.0016 | 8.99 | 8.12 | 8.81 | 1.13 | 0.1468 | 0.3828 |
| TC0200014672.hg.1 | NR4A2 | nuclear receptor subfamily 4, group A, member 2 | Multiple_C | 6.25 | 4.09 | 4.76 | 2.81 | 9.77E-05 | 0.001 | 4.81 | 4.70 | 4.84 | 0.98 | 0.5203 | 0.7477 |
| TC0200016673.hg.1 | RTN4 | reticulon 4 | Multiple_C | 8.74 | 6.86 | 7.25 | 2.81 | 2.73E-05 | 0.0004 | 7.54 | 7.12 | 6.85 | 1.61 | 0.1736 | 0.421 |
| TC0500012285.hg.1 | FCHSD1 | FCH and double SH3 domains 1 | Multiple_C | 7.56 | 6.29 | 6.07 | 2.81 | 7.71E-05 | 0.0009 | 8.07 | 7.52 | 7.24 | 1.78 | 0.0021 | 0.0277 |
| TC0500012497.hg.1 | TNIP1 | TNFAIP3 interacting protein 1 | Multiple_C | 9.28 | 7.52 | 7.79 | 2.81 | 0.0004 | 0.003 | 8.14 | 8.02 | 7.93 | 1.16 | 0.9593 | 0.9834 |
| TC0700008873.hg.1 | CAV1 | caveolin 1 | Multiple_C | 12.3 | 11.37 | 10.81 | 2.81 | 0.0002 | 0.0015 | 10.09 | 9.56 | 10.69 | 0.66 | 0.0041 | 0.0435 |
| TC0900008684.hg.1 | GPR21; RABG/ | G protein-coupled receptor 21; RAB GTPase activating protein 1 | Multiple_C | 12.44 | 12.37 | 10.95 | 2.81 | 0.0015 | 0.0095 | 10.95 | 10.18 | 10.39 | 1.47 | 0.0942 | 0.2981 |
| TC1200012080.hg.1 | TESC | tescalcin | Multiple_C | 13.81 | 12.21 | 12.32 | 2.81 | 4.16E-05 | 0.0005 | 11.36 | 11.17 | 11.03 | 1.26 | 0.0901 | 0.2906 |
| TC1400009579.hg.1 | ADAM20 | ADAM metallopeptidase domain 20 | Coding | 5.18 | 3.57 | 3.69 | 2.81 | 8.53E-05 | 0.0009 | 3.46 | 3.27 | 3.44 | 1.01 | 0.1347 | 0.3644 |
| TC1500010291.hg.1 | NMB | neuromedin B | Coding | 8.65 | 7.27 | 7.16 | 2.81 | 0.0002 | 0.0016 | 5.83 | 5.85 | 5.5 | 1.26 | 0.2032 | 0.4587 |
| TC1600011426.hg.1 | ATXN1L | ataxin 1-like | Multiple_C | 10.14 | 7.9 | 8.65 | 2.81 | 0.0066 | 0.03 | 10.38 | 10.69 | 11.37 | 0.50 | 0.1124 | 0.3304 |
| TSUnmapped00000236.h | TCF20 | transcription factor 20 (AR1) | Coding | 10.96 | 9.17 | 9.47 | 2.81 | 1.15E-05 | 0.0002 | 11.57 | 11.42 | 11.53 | 1.03 | 0.8115 | 0.9166 |
| TSUnmapped00000511.h | TCF20 | transcription factor 20 (AR1) | Coding | 10.96 | 9.17 | 9.47 | 2.81 | 1.15E-05 | 0.0002 | 11.57 | 11.42 | 11.53 | 1.03 | 0.8115 | 0.9166 |
| TC0200015631.hg.1 | IKZF2 | IKAROS family zinc finger 2 | Multiple_C | 5.62 | 4.13 | 4.14 | 2.79 | 2.07E-06 | 4.89E-05 | 3.47 | 3.68 | 3.49 | 0.99 | 0.9566 | 0.982 |
| TC0300010632.hg.1 | OSBPL10 | oxysterol binding protein-like 10 | Multiple_C | 13.31 | 11.7 | 11.83 | 2.79 | 0.0001 | 0.0013 | 9.92 | 10.10 | 11.24 | 0.40 | 3.62E-05 | 0.0018 |
| TC0500011751.hg.1 | FEM1C | fem-1 homolog c (C. elegans) | Multiple_C | 11.45 | 10.18 | 9.97 | 2.79 | 2.05E-05 | 0.0003 | 10.71 | 8.59 | 10.2 | 1.42 | 0.0148 | 0.0996 |
| TC0600008722.hg.1 | ZNF292 | zinc finger protein 292 | Multiple_C | 9.35 | 7.91 | 7.87 | 2.79 | 0.0002 | 0.0015 | 7.87 | 7.51 | 7.57 | 1.23 | 0.2937 | 0.5586 |
| TC0600013179.hg.1 | OR2A4 | olfactory receptor, family 2, subfamily A, member 4 | Coding | 6.86 | 5.11 | 5.38 | 2.79 | 0.0042 | 0.0211 | 5.32 | 4.61 | 5.03 | 1.22 | 0.1075 | 0.3224 |
| TC0700009827.hg.1 | RBM33 | RNA binding motif protein 33 | Multiple_C | 9.95 | 8.21 | 8.47 | 2.79 | 2.38E-05 | 0.0003 | 11.09 | 10.10 | 11.03 | 1.04 | 0.7844 | 0.9033 |
| TC1000007598.hg.1 | AGAP6 | ArfGAP with GTPase domain, ankyrin repeat and PH domain 6 | Multiple_C | 11.42 | 10.05 | 9.94 | 2.79 | 1.40E-06 | 3.61E-05 | 12.85 | 12.07 | 12.44 | 1.33 | 0.004 | 0.0433 |
| TC1100008626.hg.1 | PCF11 | PCF11 cleavage and polyadenylation factor subunit | Multiple_C | 11.66 | 10.66 | 10.18 | 2.79 | 2.30E-05 | 0.0003 | 11.68 | 12.06 | 11.99 | 0.81 | 0.6818 | 0.8474 |
| TC1500008658.hg.1 | POTEB3 | POTE ankyrin domain family, member B3 | Multiple_C | 4.6 | 3.23 | 3.12 | 2.79 | 0.0014 | 0.0089 | 5.39 | 5.34 | 5.08 | 1.24 | 0.2118 | 0.4695 |
| TC1500010941.hg.1 | LYSMD4 | LysM, putative peptidoglycan-binding, domain containing 4 | Coding | 5.12 | 3.81 | 3.64 | 2.79 | 0.0001 | 0.0011 | 4.18 | 4.16 | 3.89 | 1.22 | 0.6526 | 0.8305 |
| TC1900006522.hg.1 | MIDN | midnolin | Multiple_C | 11.82 | 9.48 | 10.34 | 2.79 | 3.14E-06 | 6.95E-05 | 11.34 | 11.27 | 11.63 | 0.82 | 0.0779 | 0.269 |
| TC2000007474.hg.1 | PABPC1L | poly(A) binding protein, cytoplasmic 1-like | Multiple_C | 8.84 | 7.75 | 7.36 | 2.79 | 1.03E-05 | 0.0002 | 9 | 8.87 | 7.99 | 2.01 | 6.51E-05 | 0.0027 |
| TC0100007943.hg.1 | RLF | rearranged L-myc fusion | Multiple_C | 11.06 | 9.98 | 9.59 | 2.77 | 8.70E-07 | 2.50E-05 | 10.81 | 9.63 | 10.57 | 1.18 | 0.1096 | 0.3259 |

| | | | | | | | | | | | | | | | |
|---|---|---|---|---|---|---|---|---|---|---|---|---|---|---|---|
| TC0700006836.hg.1 | RPL21 | Homo sapiens ribosomal protein L21, mRNA (cDNA clone MGC: | Multiple_C | 16.49 | 16.18 | 15.02 | 2.77 | 0.0006 | 0.0048 | 10.24 | 10.89 | 10.58 | 0.79 | 0.7747 | 0.8988 |
| TC0900010737.hg.1 | DIRAS2 | DIRAS family, GTP-binding RAS-like 2 | Coding | 5.24 | 3.93 | 3.77 | 2.77 | 0.0048 | 0.0233 | 4.67 | 4.56 | 4.36 | 1.24 | 0.2427 | 0.5062 |
| TC1300008424.hg.1 | USP12 | ubiquitin specific peptidase 12 | Multiple_C | 10.46 | 9.44 | 8.99 | 2.77 | 0.0032 | 0.0171 | 7.88 | 7.64 | 8.17 | 0.82 | 0.4763 | 0.7154 |
| TC1400009967.hg.1 | TTC7B | tetratricopeptide repeat domain 7B | Multiple_C | 5.96 | 4.73 | 4.49 | 2.77 | 0.0002 | 0.0016 | 5.82 | 6.59 | 7.46 | 0.32 | 2.13E-06 | 0.0002 |
| TC1500008105.hg.1 | FAM103A1 | family with sequence similarity 103, member A1 | Coding | 12.27 | 11.16 | 10.8 | 2.77 | 7.78E-06 | 0.0001 | 9.9 | 9.86 | 10.16 | 0.84 | 0.4756 | 0.7147 |
| TC0100007898.hg.1 | MACF1; KIAA( | microtubule-actin crosslinking factor 1; KIAA0754 | Multiple_C | 11.99 | 11.07 | 10.53 | 2.75 | 0.0009 | 0.0064 | 11.4 | 11.74 | 11.31 | 1.06 | 0.703 | 0.8595 |
| TC0100011901.hg.1 | RAB4A; SPHAI | RAB4A, member RAS oncogene family; S-phase response (cyclir | Multiple_C | 11.98 | 12.34 | 10.52 | 2.75 | 0.0004 | 0.0035 | 8.9 | 8.67 | 8.34 | 1.47 | 0.0204 | 0.1221 |
| TC0100015572.hg.1 | SRGAP2B | SLIT-ROBO Rho GTPase activating protein 2B | Multiple_C | 11.22 | 10.1 | 9.76 | 2.75 | 0.0003 | 0.0026 | 10.03 | 9.81 | 9.56 | 1.39 | 0.2824 | 0.5479 |
| TC0300011450.hg.1 | TMF1 | TATA element modulatory factor 1 | Multiple_C | 11.45 | 10.71 | 9.99 | 2.75 | 8.96E-07 | 2.56E-05 | 9.61 | 8.91 | 9.19 | 1.34 | 0.0129 | 0.0916 |
| TC0300014020.hg.1 | CLDND1 | claudin domain containing 1 | Multiple_C | 13.24 | 12.34 | 11.78 | 2.75 | 1.23E-05 | 0.0002 | 12.7 | 11.56 | 12.39 | 1.24 | 0.1382 | 0.369 |
| TC0500007673.hg.1 | RAD17 | RAD17 checkpoint clamp loader component | Multiple_C | 11.43 | 10.7 | 9.97 | 2.75 | 0.0005 | 0.0039 | 11.1 | 11.18 | 10.58 | 1.43 | 0.0116 | 0.0857 |
| TC0700011780.hg.1 | PEX1 | peroxisomal biogenesis factor 1 | Multiple_C | 8.84 | 9.3 | 7.38 | 2.75 | 0.0008 | 0.0054 | 8.05 | 7.98 | 7.08 | 1.96 | 0.0177 | 0.1117 |
| TC0X00008084.hg.1 | COL4A5 | collagen, type IV, alpha 5 | Multiple_C | 4.57 | 2.68 | 3.11 | 2.75 | 0.0014 | 0.0087 | 3.35 | 3.21 | 3.45 | 0.93 | 0.7447 | 0.8832 |
| TC0X00010473.hg.1 | TSC22D3 | TSC22 domain family, member 3 | Coding | 7.43 | 6.21 | 5.97 | 2.75 | 5.30E-06 | 0.0001 | 6.31 | 5.73 | 5.76 | 1.46 | 0.0327 | 0.1623 |
| TC1000010509.hg.1 | AGAP4 | ArfGAP with GTPase domain, ankyrin repeat and PH domain 4 | Multiple_C | 11.38 | 10.03 | 9.92 | 2.75 | 5.70E-06 | 0.0001 | 12.48 | 12.03 | 12.1 | 1.30 | 0.0695 | 0.2515 |
| TC1300006543.hg.1 | FGF9 | fibroblast growth factor 9 | Multiple_C | 10.01 | 8.73 | 8.55 | 2.75 | 0.0034 | 0.0177 | 9.95 | 8.12 | 8.61 | 2.53 | 0.0004 | 0.0085 |
| TC1300008668.hg.1 | LHFP | lipoma HMGIC fusion partner | Multiple_C | 4.99 | 3.48 | 3.53 | 2.75 | 0.0036 | 0.0187 | 8.58 | 5.78 | 5.8 | 6.87 | 4.02E-09 | 2.63E-06 |
| TC1400007328.hg.1 | DAAM1 | dishevelled associated activator of morphogenesis 1 | Multiple_C | 10.58 | 10.42 | 9.12 | 2.75 | 0.0001 | 0.0014 | 9.77 | 8.81 | 8.79 | 1.97 | 0.0043 | 0.0449 |
| TC1500009514.hg.1 | RFX7 | regulatory factor X, 7 | Multiple_C | 10.9 | 9.3 | 9.44 | 2.75 | 0.0002 | 0.0021 | 11.07 | 10.53 | 11.01 | 1.04 | 0.7445 | 0.8831 |
| TC1500010236.hg.1 | GOLGA6L10 | golgin A6 family-like 10 | Multiple_C | 7.26 | 6.32 | 5.8 | 2.75 | 7.14E-05 | 0.0008 | 6.23 | 5.45 | 5.43 | 1.74 | 0.0101 | 0.0784 |
| TC1700009969.hg.1 | FLII | flightless I actin binding protein | Multiple_C | 11.26 | 10.58 | 9.8 | 2.75 | 0.0002 | 0.0021 | 10.78 | 11.10 | 10.46 | 1.25 | 0.2773 | 0.5432 |
| TC1700011237.hg.1 | VEZF1 | vascular endothelial zinc finger 1 | Multiple_C | 11.65 | 9.69 | 10.19 | 2.75 | 0.0004 | 0.0035 | 10.9 | 10.24 | 11.23 | 0.80 | 0.45 | 0.6962 |
| TC2000007117.hg.1 | ASXL1 | additional sex combs like transcriptional regulator 1 | Multiple_C | 11.18 | 10.66 | 9.72 | 2.75 | 0.0012 | 0.0076 | 11.25 | 11.68 | 12.27 | 0.49 | 0.0013 | 0.0203 |
| TC2000008023.hg.1 | SLCO4A1 | solute carrier organic anion transporter family, member 4A1 | Multiple_C | 11.52 | 10.07 | 10.06 | 2.75 | 0.0003 | 0.0029 | 10.17 | 8.94 | 9.63 | 1.45 | 0.0486 | 0.2049 |
| TC2100008408.hg.1 | POFUT2 | protein O-fucosyltransferase 2 | Multiple_C | 7.47 | 6.13 | 6.01 | 2.75 | 0.0021 | 0.0121 | 6.13 | 6.32 | 6.13 | 1.00 | 0.6864 | 0.8497 |
| TC2200009194.hg.1 | MED15 | mediator complex subunit 15 | Multiple_C | 12.95 | 11.23 | 11.49 | 2.75 | 0.0036 | 0.0186 | 12.89 | 12.62 | 12.84 | 1.04 | 0.9382 | 0.9743 |
| TC0100009658.hg.1 | SRGAP2C | SLIT-ROBO Rho GTPase activating protein 2C | Multiple_C | 11.96 | 10.65 | 10.51 | 2.73 | 4.08E-05 | 0.0005 | 10.9 | 10.78 | 10.33 | 1.48 | 0.0455 | 0.1973 |
| TC0100018418.hg.1 | IFFO2 | intermediate filament family orphan 2 | Multiple_C | 8.67 | 7.18 | 7.22 | 2.73 | 1.81E-06 | 4.38E-05 | 7.11 | 6.98 | 7.19 | 0.95 | 0.5425 | 0.7614 |
| TC0200011965.hg.1 | ATAD2B | ATPase family, AAA domain containing 2B | Multiple_C | 7 | 5.79 | 5.55 | 2.73 | 0.0002 | 0.0015 | 5.6 | 6.15 | 6.95 | 0.39 | 2.06E-05 | 0.0012 |
| TC0400011175.hg.1 | TMEM150C | transmembrane protein 150C | Multiple_C | 5.49 | 4.25 | 4.04 | 2.73 | 0.0027 | 0.0147 | 4.61 | 6.19 | 5.33 | 0.61 | 0.0191 | 0.117 |
| TC0600012240.hg.1 | LMBRD1 | LMBR1 domain containing 1 | Multiple_C | 13.12 | 13.43 | 11.67 | 2.73 | 0.0013 | 0.0082 | 10.84 | 9.66 | 10.23 | 1.53 | 0.2479 | 0.5124 |
| TC1200011573.hg.1 | NR2C1 | nuclear receptor subfamily 2, group C, member 1 | Multiple_C | 13 | 12.25 | 11.55 | 2.73 | 4.51E-06 | 9.23E-05 | 10.84 | 9.91 | 10.3 | 1.45 | 0.0026 | 0.0325 |
| TC1200012636.hg.1 | PCBP2; PCBP2 | poly(rC) binding protein 2; PCBP2 overlapping transcript 1 | Multiple_C | 16.42 | 15.32 | 14.97 | 2.73 | 3.20E-06 | 7.05E-05 | 17.51 | 17.75 | 17.65 | 0.91 | 0.5251 | 0.7498 |

| | | | | | | | | | | | | | | | |
|---|---|---|---|---|---|---|---|---|---|---|---|---|---|---|---|
| TC1300008819.hg.1 | TPT1; SNORA3 | tumor protein, translationally-controlled 1; small nucleolar RNA | Multiple_C | 17.98 | 17.37 | 16.53 | 2.73 | 3.40E-05 | 0.0004 | 13.87 | 13.38 | 12.8 | 2.10 | 5.45E-05 | 0.0024 |
| TC1400007710.hg.1 | JDP2 | Jun dimerization protein 2 | Multiple_C | 8.37 | 7.47 | 6.92 | 2.73 | 0.0006 | 0.0042 | 7.59 | 6.96 | 6.96 | 1.55 | 0.341 | 0.6048 |
| TC1500008545.hg.1 | ASB7 | ankyrin repeat and SOCS box containing 7 | Multiple_C | 10.82 | 9.19 | 9.37 | 2.73 | 6.18E-06 | 0.0001 | 8.46 | 8.04 | 8.5 | 0.97 | 0.6534 | 0.8312 |
| TC1700006877.hg.1 | ADPRM | ADP-ribose/CDP-alcohol diphosphatase, manganese-dependent | Multiple_C | 9.21 | 10.47 | 7.76 | 2.73 | 0.0261 | 0.0882 | 6.73 | 5.86 | 6.13 | 1.52 | 0.1366 | 0.3674 |
| TC1700007791.hg.1 | RARA | retinoic acid receptor, alpha | Multiple_C | 7.62 | 5.79 | 6.17 | 2.73 | 3.39E-06 | 7.36E-05 | 8.16 | 7.99 | 8.11 | 1.04 | 0.8721 | 0.9449 |
| TC1900009201.hg.1 | MKNK2 | MAP kinase interacting serine/threonine kinase 2 | Multiple_C | 9.02 | 7.85 | 7.57 | 2.73 | 0.008 | 0.0349 | 8.21 | 8.47 | 8.8 | 0.66 | 0.1419 | 0.3746 |
| TC1900009670.hg.1 | DOCK6 | dedicator of cytokinesis 6 | Multiple_C | 9.8 | 8.18 | 8.35 | 2.73 | 3.49E-05 | 0.0005 | 9.03 | 9.32 | 8.71 | 1.25 | 0.1632 | 0.4068 |
| TC2000009964.hg.1 | SDCBP2 | syndecan binding protein (syntenin) 2 | Multiple_C | 6.17 | 5.09 | 4.72 | 2.73 | 2.10E-05 | 0.0003 | 5.34 | 4.66 | 4.34 | 2.00 | 0.0031 | 0.0366 |
| TC0100018506.hg.1 | YY1AP1 | YY1 associated protein 1 | Multiple_C | 12.99 | 11.31 | 11.55 | 2.71 | 3.67E-06 | 7.86E-05 | 12.07 | 11.64 | 12.15 | 0.95 | 0.9992 | 0.9997 |
| TC0200007998.hg.1 | ZNF638 | zinc finger protein 638 | Multiple_C | 10.47 | 10.61 | 9.03 | 2.71 | 0.003 | 0.0161 | 8.85 | 8.77 | 8.39 | 1.38 | 0.9768 | 0.9904 |
| TC0200016470.hg.1 | SNRNP27 | small nuclear ribonucleoprotein, U4/U6.U5 27kDa subunit | Multiple_C | 9.83 | 8.84 | 8.39 | 2.71 | 1.77E-05 | 0.0003 | 8.31 | 7.93 | 8.78 | 0.72 | 0.1706 | 0.4168 |
| TC0300009677.hg.1 | YEATS2 | YEATS domain containing 2 | Multiple_C | 9.78 | 8.11 | 8.34 | 2.71 | 1.29E-05 | 0.0002 | 9.61 | 9.64 | 9.33 | 1.21 | 0.0546 | 0.2194 |
| TC1100011155.hg.1 | TRPT1 | tRNA phosphotransferase 1 | Multiple_C | 10.1 | 9.03 | 8.66 | 2.71 | 0.004 | 0.0201 | 8.23 | 8.22 | 7.59 | 1.56 | 0.7618 | 0.8923 |
| TC1600007982.hg.1 | HERPUD1 | homocysteine-inducible, endoplasmic reticulum stress-inducible | Multiple_C | 13.6 | 12.95 | 12.16 | 2.71 | 0.0195 | 0.07 | 11.13 | 10.43 | 10.91 | 1.16 | 0.3814 | 0.6408 |
| TC1700012396.hg.1 | TBC1D3C | TBC1 domain family, member 3C | Coding | 9.25 | 8.21 | 7.81 | 2.71 | 7.16E-06 | 0.0001 | 8.33 | 8.19 | 7.85 | 1.39 | 0.0222 | 0.1282 |
| TC1800008978.hg.1 | RTTN | rotatin | Multiple_C | 9.09 | 7.94 | 7.65 | 2.71 | 0.0016 | 0.0098 | 6.27 | 6.78 | 6.67 | 0.76 | 0.3496 | 0.6137 |
| TC2000010022.hg.1 | ZNFX1 | zinc finger, NFX1-type containing 1 | Multiple_C | 9.15 | 7.36 | 7.71 | 2.71 | 0.0023 | 0.013 | 7.23 | 6.99 | 7.21 | 1.01 | 0.9173 | 0.9663 |
| TSUnmapped00000169.h | TCF20 | transcription factor 20 (AR1) | Coding | 10.91 | 9.22 | 9.47 | 2.71 | 2.64E-05 | 0.0004 | 11.64 | 11.61 | 11.51 | 1.09 | 0.9894 | 0.9956 |
| TSUnmapped00000402.h | TCF20 | transcription factor 20 (AR1) | Coding | 10.91 | 9.22 | 9.47 | 2.71 | 2.64E-05 | 0.0004 | 11.64 | 11.61 | 11.51 | 1.09 | 0.9894 | 0.9956 |
| TC0100009770.hg.1 | NBPF12 | neuroblastoma breakpoint family, member 12 | Multiple_C | 11.87 | 10.81 | 10.44 | 2.69 | 1.87E-05 | 0.0003 | 10.98 | 10.73 | 10.88 | 1.07 | 0.1543 | 0.3939 |
| TC0100013437.hg.1 | AHDC1 | AT hook, DNA binding motif, containing 1 | Multiple_C | 6.76 | 4.96 | 5.33 | 2.69 | 0.0012 | 0.0079 | 5.5 | 5.57 | 5.81 | 0.81 | 0.7543 | 0.8884 |
| TC0100018519.hg.1 | F11R | F11 receptor | Multiple_C | 13.7 | 12.1 | 12.27 | 2.69 | 0.0009 | 0.0064 | 10.58 | 10.80 | 11.53 | 0.52 | 0.0014 | 0.0215 |
| TC0200011040.hg.1 | ITM2C | integral membrane protein 2C | Multiple_C | 16.05 | 14.69 | 14.62 | 2.69 | 2.08E-05 | 0.0003 | 14.9 | 14.52 | 14.76 | 1.10 | 0.4115 | 0.6662 |
| TC0200015773.hg.1 | PTPRN | protein tyrosine phosphatase, receptor type, N | Multiple_C | 5.92 | 4.28 | 4.49 | 2.69 | 8.71E-05 | 0.0009 | 5.25 | 5.88 | 5.61 | 0.78 | 0.5194 | 0.7469 |
| TC0500012642.hg.1 | RNF145 | ring finger protein 145 | Multiple_C | 10.9 | 9.27 | 9.47 | 2.69 | 0.0062 | 0.0287 | 9.03 | 7.41 | 8.46 | 1.48 | 0.0776 | 0.2683 |
| TC0600011945.hg.1 | RCAN2 | regulator of calcineurin 2 | Coding | 5.47 | 4.21 | 4.04 | 2.69 | 0.0003 | 0.0026 | 3.7 | 3.49 | 3.53 | 1.13 | 0.074 | 0.261 |
| TC0700008548.hg.1 | NYAP1 | neuronal tyrosine-phosphorylated phosphoinositide-3-kinase ad | Multiple_C | 7.21 | 5.52 | 5.78 | 2.69 | 0.0013 | 0.0085 | 7.14 | 7.10 | 6.99 | 1.11 | 0.9274 | 0.9698 |
| TC1500008485.hg.1 | IGF1R | insulin-like growth factor 1 receptor | Multiple_C | 11.59 | 9.23 | 10.16 | 2.69 | 2.30E-05 | 0.0003 | 11.44 | 11.01 | 11.07 | 1.29 | 0.1407 | 0.3728 |
| TC1600011364.hg.1 | NPIPB5 | nuclear pore complex interacting protein family, member B5 | Multiple_C | 12.91 | 11.66 | 11.48 | 2.69 | 4.58E-06 | 9.34E-05 | 12.59 | 12.04 | 12.17 | 1.34 | 0.0202 | 0.1212 |
| TC1700008085.hg.1 | STH | saitohin | Coding | 7.47 | 5.89 | 6.04 | 2.69 | 0.0002 | 0.0018 | 6.41 | 6.38 | 6.15 | 1.20 | 0.2356 | 0.4983 |
| TC0100009913.hg.1 | ADAMTSL4 | ADAMTS like 4 | Multiple_C | 6.34 | 5.17 | 4.92 | 2.68 | 3.89E-05 | 0.0005 | 5.1 | 4.93 | 5.03 | 1.05 | 0.0743 | 0.2616 |
| TC0100015871.hg.1 | S100A16 | S100 calcium binding protein A16 | Multiple_C | 14.29 | 12.35 | 12.87 | 2.68 | 0.0002 | 0.0021 | 12.43 | 12.62 | 12.43 | 1.00 | 0.9218 | 0.9677 |
| TC0200009871.hg.1 | CSRNP3 | cysteine-serine-rich nuclear protein 3 | Multiple_C | 5.41 | 3.79 | 3.99 | 2.68 | 2.33E-05 | 0.0003 | 6.34 | 5.93 | 5.78 | 1.47 | 0.0087 | 0.0712 |

| | | | | | | | | | | | | | | | |
|---|---|---|---|---|---|---|---|---|---|---|---|---|---|---|---|
| TC0200012252.hg.1 | STRN | striatin, calmodulin binding protein | Multiple_C | 11.81 | 10.17 | 10.39 | 2.68 | 3.84E-05 | 0.0005 | 10.6 | 10.15 | 10.29 | 1.24 | 0.1873 | 0.4391 |
| TC0200015009.hg.1 | ATF2 | activating transcription factor 2 | Multiple_C | 12.72 | 11.65 | 11.3 | 2.68 | 1.89E-05 | 0.0003 | 11.95 | 11.45 | 12.29 | 0.79 | 0.1262 | 0.3514 |
| TC0500012498.hg.1 | ANXA6 | annexin A6 | Multiple_C | 4.95 | 3.53 | 3.53 | 2.68 | 0.1366 | 0.3014 | 8.86 | 6.70 | 5.76 | 8.57 | 0.0003 | 0.0081 |
| TC0700006928.hg.1 | CCDC126 | coiled-coil domain containing 126 | Multiple_C | 10.31 | 10.8 | 8.89 | 2.68 | 0.0085 | 0.0366 | 8.34 | 7.62 | 7.97 | 1.29 | 0.2511 | 0.5151 |
| TC0900008891.hg.1 | LRRC8A | leucine rich repeat containing 8 family, member A | Multiple_C | 9.94 | 8.47 | 8.52 | 2.68 | 0.0168 | 0.0624 | 8.8 | 8.33 | 8.94 | 0.91 | 0.8752 | 0.9465 |
| TC0900012276.hg.1 | SCAI; GOLGA1 | suppressor of cancer cell invasion; golgin A1 | Multiple_C | 8.68 | 7.59 | 7.26 | 2.68 | 0.0003 | 0.0026 | 7.59 | 6.55 | 7.91 | 0.80 | 0.2956 | 0.5605 |
| TC1000007510.hg.1 | AGAP9; CTGLF | Salzman2013 ANNOTATED, CDS, coding, INTERNAL, OVCODE, O | Multiple_C | 11.01 | 9.72 | 9.59 | 2.68 | 1.77E-05 | 0.0003 | 11.26 | 10.83 | 10.94 | 1.25 | 0.5883 | 0.7917 |
| TC1100006629.hg.1 | ART1 | ADP-ribosyltransferase 1 | Multiple_C | 6.94 | 4.97 | 5.52 | 2.68 | 0.0058 | 0.027 | 5.95 | 6.15 | 5.82 | 1.09 | 0.7123 | 0.8651 |
| TC1100013186.hg.1 | SYVN1 | synovial apoptosis inhibitor 1, synoviolin | Multiple_C | 12.18 | 10.19 | 10.76 | 2.68 | 0.0003 | 0.0024 | 12.12 | 11.66 | 12.32 | 0.87 | 0.5436 | 0.762 |
| TC1200012761.hg.1 | TAS2R31 | taste receptor, type 2, member 31 | Coding | 8.62 | 7.34 | 7.2 | 2.68 | 0.0001 | 0.0014 | 6.59 | 6.53 | 7.01 | 0.75 | 0.1854 | 0.437 |
| TC1500009508.hg.1 | NEDD4 | neural precursor cell expressed, developmentally down-regulat | Multiple_C | 6.97 | 5.68 | 5.55 | 2.68 | 0.0004 | 0.0032 | 7 | 6.36 | 7.25 | 0.84 | 0.0608 | 0.2341 |
| TC0100009959.hg.1 | TUFT1 | tuftelin 1 | Multiple_C | 9.08 | 7.77 | 7.67 | 2.66 | 5.98E-05 | 0.0007 | 6.45 | 6.98 | 7.8 | 0.39 | 2.01E-05 | 0.0012 |
| TC0200015887.hg.1 | CUL3 | cullin 3 | Multiple_C | 13.68 | 13.59 | 12.27 | 2.66 | 2.17E-05 | 0.0003 | 11.8 | 11.18 | 11.5 | 1.23 | 0.1058 | 0.3193 |
| TC0300014050.hg.1 | NPHP3 | nephronophthisis 3 (adolescent) | Multiple_C | 7.4 | 7.5 | 5.99 | 2.66 | 0.0016 | 0.0095 | 7.02 | 6.81 | 6.76 | 1.20 | 0.7035 | 0.8598 |
| TC0400008053.hg.1 | AFF1 | AF4/FMR2 family, member 1 | Multiple_C | 11.3 | 8.68 | 9.89 | 2.66 | 0.0001 | 0.0011 | 10.48 | 10.44 | 11.08 | 0.66 | 0.0298 | 0.1531 |
| TC0400011159.hg.1 | PRKG2 | protein kinase, cGMP-dependent, type II | Multiple_C | 5.58 | 4.33 | 4.17 | 2.66 | 0.0002 | 0.002 | 6.49 | 6.38 | 6.54 | 0.97 | 0.8949 | 0.9555 |
| TC0900011582.hg.1 | FAM129B | family with sequence similarity 129, member B | Multiple_C | 11.09 | 9.43 | 9.68 | 2.66 | 0.0186 | 0.0676 | 7.95 | 8.25 | 8.87 | 0.53 | 0.0035 | 0.0395 |
| TC1200008142.hg.1 | FRS2 | fibroblast growth factor receptor substrate 2 | Multiple_C | 9.85 | 8.73 | 8.44 | 2.66 | 0.0002 | 0.0015 | 8.49 | 7.51 | 8.08 | 1.33 | 0.1314 | 0.3593 |
| TSUnmapped00000154.h | LRP6 | LDL receptor related protein 6 | Coding | 11.46 | 9.58 | 10.05 | 2.66 | 0.0002 | 0.0019 | 11.44 | 11.45 | 11.43 | 1.01 | 0.6212 | 0.8128 |
| TC0100010526.hg.1 | TBX19 | T-box 19 | Multiple_C | 4.83 | 3.37 | 3.43 | 2.64 | 0.0056 | 0.0263 | 3.65 | 3.90 | 4.29 | 0.64 | 0.1729 | 0.4202 |
| TC0200016476.hg.1 | WBP1 | WW domain binding protein 1 | Multiple_C | 13.21 | 11.71 | 11.81 | 2.64 | 0.0001 | 0.0013 | 12.14 | 12.20 | 11.76 | 1.30 | 0.0636 | 0.24 |
| TC0300008989.hg.1 | SLC25A36 | solute carrier family 25 (pyrimidine nucleotide carrier), member | Multiple_C | 11.91 | 11.83 | 10.51 | 2.64 | 0.0015 | 0.0094 | 10.46 | 9.34 | 9.63 | 1.78 | 0.0154 | 0.1021 |
| TC0700009174.hg.1 | MKLN1 | muskelin 1, intracellular mediator containing kelch motifs | Multiple_C | 11.86 | 11.02 | 10.46 | 2.64 | 8.77E-05 | 0.001 | 10.3 | 9.92 | 11.24 | 0.52 | 0.001 | 0.0163 |
| TC0700013102.hg.1 | KMT2C | lysine (K)-specific methyltransferase 2C | Multiple_C | 10.53 | 8.33 | 9.13 | 2.64 | 4.24E-05 | 0.0005 | 11.8 | 11.01 | 11.51 | 1.22 | 0.2049 | 0.4612 |
| TC0900008497.hg.1 | ZNF618 | zinc finger protein 618 | Multiple_C | 7.25 | 5.49 | 5.85 | 2.64 | 0.0001 | 0.0012 | 7.53 | 7.40 | 6.92 | 1.53 | 0.0415 | 0.1876 |
| TC1200008671.hg.1 | HCFC2 | host cell factor C2 | Multiple_C | 8.19 | 7.08 | 6.79 | 2.64 | 0.001 | 0.0064 | 7.83 | 7.53 | 7.67 | 1.12 | 0.8629 | 0.9412 |
| TC1200010838.hg.1 | ZNF385A | zinc finger protein 385A | Multiple_C | 6.27 | 4.78 | 4.87 | 2.64 | 0.0002 | 0.0015 | 7.48 | 6.97 | 6.11 | 2.58 | 6.64E-05 | 0.0027 |
| TC1400006664.hg.1 | REM2 | RAS (RAD and GEM)-like GTP binding 2 | Coding | 5.29 | 3.91 | 3.89 | 2.64 | 0.0003 | 0.0028 | 4.5 | 4.53 | 4.43 | 1.05 | 0.6469 | 0.8269 |
| TC0100008066.hg.1 | PTPRF | protein tyrosine phosphatase, receptor type, F | Multiple_C | 10.93 | 10.38 | 9.54 | 2.62 | 1.31E-05 | 0.0002 | 8.78 | 8.52 | 8.43 | 1.27 | 0.2374 | 0.5006 |
| TC0100008621.hg.1 | LEPR; LEPROT | leptin receptor; leptin receptor overlapping transcript | Multiple_C | 6.86 | 7.49 | 5.47 | 2.62 | 0.0001 | 0.0014 | 6.42 | 6.46 | 6.57 | 0.90 | 0.625 | 0.8148 |
| TC0100009936.hg.1 | BNIPL | BCL2/adenovirus E1B 19kD interacting protein like | Multiple_C | 5.42 | 4.49 | 4.03 | 2.62 | 0.0027 | 0.0146 | 4.83 | 4.32 | 4.47 | 1.28 | 0.8523 | 0.9361 |
| TC0100017072.hg.1 | ELK4 | ELK4, ETS-domain protein (SRF accessory protein 1) | Multiple_C | 13.68 | 11.88 | 12.29 | 2.62 | 5.35E-05 | 0.0006 | 13.11 | 11.92 | 13.19 | 0.95 | 0.7125 | 0.8651 |
| TC0200007746.hg.1 | B3GNT2 | UDP-GlcNAc:betaGal beta-1,3-N-acetylglucosaminyltransferase | Coding | 11.88 | 11.47 | 10.49 | 2.62 | 0.0001 | 0.0015 | 9.46 | 7.98 | 8.12 | 2.53 | 4.20E-05 | 0.002 |

| | | | | | | | | | | | | | | | |
|---|---|---|---|---|---|---|---|---|---|---|---|---|---|---|---|
| TC0200012256.hg.1 | HEATR5B | HEAT repeat containing 5B | Multiple_C | 8.57 | 7.78 | 7.18 | 2.62 | 0.0024 | 0.0136 | 8.37 | 7.94 | 7.74 | 1.55 | 0.3023 | 0.5667 |
| TC0500010549.hg.1 | RICTOR | RPTOR independent companion of MTOR, complex 2 | Multiple_C | 10.77 | 10.22 | 9.38 | 2.62 | 2.82E-05 | 0.0004 | 11.15 | 9.96 | 10.07 | 2.11 | 9.99E-05 | 0.0036 |
| TC0500012977.hg.1 | PDLIM7 | PDZ and LIM domain 7 (enigma) | Multiple_C | 10.53 | 8.83 | 9.14 | 2.62 | 0.0002 | 0.0017 | 8.39 | 8.77 | 8.5 | 0.93 | 0.8428 | 0.9316 |
| TC0600011846.hg.1 | C6orf226 | chromosome 6 open reading frame 226 | Coding | 7.63 | 6.35 | 6.24 | 2.62 | 4.51E-05 | 0.0006 | 4.94 | 5.12 | 4.99 | 0.97 | 0.7249 | 0.872 |
| TC0600012495.hg.1 | CGA | glycoprotein hormones, alpha polypeptide | Multiple_C | 5.12 | 3.73 | 3.73 | 2.62 | 0.0095 | 0.04 | 4.54 | 4.28 | 4.5 | 1.03 | 0.5842 | 0.7882 |
| TC0700012812.hg.1 | MKRN1 | makorin ring finger protein 1 | Multiple_C | 13.77 | 13.01 | 12.38 | 2.62 | 4.01E-06 | 8.43E-05 | 12.15 | 11.64 | 12.3 | 0.90 | 0.8493 | 0.9347 |
| TC0900010580.hg.1 | GKAP1 | G kinase anchoring protein 1 | Multiple_C | 6.82 | 6.72 | 5.43 | 2.62 | 0.0002 | 0.002 | 5.94 | 5.12 | 5.85 | 1.06 | 0.7365 | 0.8786 |
| TC1100013148.hg.1 | PIK3C2A | phosphatidylinositol-4-phosphate 3-kinase, catalytic subunit typ | Multiple_C | 12.39 | 11.44 | 11 | 2.62 | 0.0041 | 0.0204 | 11.67 | 10.56 | 11.47 | 1.15 | 0.1283 | 0.3545 |
| TC1200009796.hg.1 | SLC2A14 | solute carrier family 2 (facilitated glucose transporter), member | Multiple_C | 5.49 | 3.41 | 4.1 | 2.62 | 7.83E-05 | 0.0009 | 14.17 | 14.05 | 14.05 | 1.09 | 0.6981 | 0.857 |
| TC1300009980.hg.1 | LMO7 | LIM domain 7 | Multiple_C | 12.79 | 11.61 | 11.4 | 2.62 | 0.0001 | 0.0012 | 13.88 | 13.41 | 13.09 | 1.73 | 0.0169 | 0.1089 |
| TC1300010030.hg.1 | N4BP2L2 | NEDD4 binding protein 2-like 2 | Multiple_C | 13.26 | 13.7 | 11.87 | 2.62 | 0.0002 | 0.0015 | 12.82 | 12.15 | 11.11 | 3.27 | 5.21E-06 | 0.0005 |
| TC1700008549.hg.1 | TANC2 | tetratricopeptide repeat, ankyrin repeat and coiled-coil containi | Multiple_C | 9.21 | 7.66 | 7.82 | 2.62 | 0.0003 | 0.0025 | 9.73 | 8.88 | 9.21 | 1.43 | 0.0261 | 0.1412 |
| TC1800007089.hg.1 | C18orf21 | chromosome 18 open reading frame 21 | Multiple_C | 9.67 | 9.05 | 8.28 | 2.62 | 0.0002 | 0.002 | 6.8 | 7.85 | 7.57 | 0.59 | 0.0009 | 0.0152 |
| TC1800007101.hg.1 | KIAA1328 | KIAA1328 | Multiple_C | 5.26 | 3.9 | 3.87 | 2.62 | 0.0005 | 0.0037 | 4.06 | 4.47 | 4.17 | 0.93 | 0.2626 | 0.527 |
| TC1900009576.hg.1 | ZNF699 | zinc finger protein 699 | Multiple_C | 7.88 | 7.05 | 6.49 | 2.62 | 1.21E-05 | 0.0002 | 7.33 | 7.23 | 7.11 | 1.16 | 0.219 | 0.4789 |
| TC1900010078.hg.1 | ZNF14 | zinc finger protein 14 | Multiple_C | 7.22 | 7.04 | 5.83 | 2.62 | 7.17E-05 | 0.0008 | 3.97 | 3.81 | 3.6 | 1.29 | 0.016 | 0.1051 |
| TC0200013772.hg.1 | RGPD3 | RANBP2-like and GRIP domain containing 3 | Coding | 9.06 | 7.53 | 7.68 | 2.60 | 0.001 | 0.0068 | 8.77 | 8.18 | 8.69 | 1.06 | 0.9751 | 0.9902 |
| TC0400007247.hg.1 | PTTG2 | pituitary tumor-transforming 2 | Coding | 6.91 | 6.16 | 5.53 | 2.60 | 0.001 | 0.007 | 5.52 | 6.19 | 5.7 | 0.88 | 0.4851 | 0.7222 |
| TC0400007933.hg.1 | ANXA3 | annexin A3 | Multiple_C | 13.21 | 14.11 | 11.83 | 2.60 | 6.11E-06 | 0.0001 | 13.35 | 11.67 | 12.05 | 2.46 | 4.40E-07 | 8.59E-05 |
| TC0500010635.hg.1 | HMGCS1 | 3-hydroxy-3-methylglutaryl-CoA synthase 1 (soluble) | Multiple_C | 12.61 | 13.13 | 11.23 | 2.60 | 0.0032 | 0.0172 | 8.99 | 8.33 | 10.67 | 0.31 | 0.0006 | 0.0112 |
| TC0700010510.hg.1 | C7orf31 | chromosome 7 open reading frame 31 | Multiple_C | 7.28 | 5.91 | 5.9 | 2.60 | 0.0072 | 0.0322 | 7.18 | 7.56 | 6.58 | 1.52 | 0.1081 | 0.3234 |
| TC0700011562.hg.1 | TMEM120A | transmembrane protein 120A | Multiple_C | 9.01 | 7.55 | 7.63 | 2.60 | 1.76E-05 | 0.0003 | 6.56 | 6.54 | 7.01 | 0.73 | 0.0464 | 0.1993 |
| TC0700013324.hg.1 | GET4 | golgi to ER traffic protein 4 | Multiple_C | 11.26 | 9.43 | 9.88 | 2.60 | 0.0007 | 0.0049 | 8.83 | 9.29 | 9.4 | 0.67 | 0.0452 | 0.1966 |
| TC0700013605.hg.1 | PNPLA8 | patatin-like phospholipase domain containing 8 | Multiple_C | 11.84 | 12.23 | 10.46 | 2.60 | 0.0002 | 0.0018 | 8.33 | 7.76 | 8.31 | 1.01 | 0.6975 | 0.8566 |
| TC1000011040.hg.1 | CAMK2G | calcium/calmodulin-dependent protein kinase II gamma | Multiple_C | 9.52 | 8.55 | 8.14 | 2.60 | 4.11E-05 | 0.0005 | 8.62 | 8.64 | 8.56 | 1.04 | 0.4039 | 0.6596 |
| TC1000011420.hg.1 | CPEB3 | cytoplasmic polyadenylation element binding protein 3 | Multiple_C | 6.6 | 5.89 | 5.22 | 2.60 | 5.02E-05 | 0.0006 | 4.55 | 3.83 | 4.01 | 1.45 | 0.049 | 0.2058 |
| TC1200010866.hg.1 | CD63 | CD63 molecule | Multiple_C | 12.66 | 11.12 | 11.28 | 2.60 | 2.89E-05 | 0.0004 | 12.38 | 12.03 | 11.92 | 1.38 | 0.0357 | 0.1711 |
| TC1400009629.hg.1 | ZFYVE1 | zinc finger, FYVE domain containing 1 | Multiple_C | 7.35 | 6.69 | 5.97 | 2.60 | 0.0002 | 0.0019 | 5.13 | 5.06 | 4.92 | 1.16 | 0.4488 | 0.6956 |
| TC1900008507.hg.1 | FTL | ferritin, light polypeptide | Multiple_C | 16.33 | 15.14 | 14.95 | 2.60 | 5.62E-06 | 0.0001 | 12.31 | 12.38 | 12.85 | 0.69 | 0.0192 | 0.117 |
| TC1900011390.hg.1 | TARM1 | T cell-interacting, activating receptor on myeloid cells 1 | Multiple_C | 8.23 | 6.86 | 6.85 | 2.60 | 0.0026 | 0.0142 | 7.81 | 7.53 | 7.51 | 1.23 | 0.3445 | 0.6081 |
| TC2100008240.hg.1 | UMODL1-AS1 | UMODL1 antisense RNA 1 | Multiple_C | 5.43 | 3.91 | 4.05 | 2.60 | 0.0154 | 0.0584 | 5.49 | 5.19 | 4.75 | 1.67 | 0.0122 | 0.0884 |
| TC0200011188.hg.1 | AGAP1 | ArfGAP with GTPase domain, ankyrin repeat and PH domain 1 | Multiple_C | 8.57 | 7 | 7.2 | 2.58 | 2.68E-05 | 0.0004 | 8.38 | 7.53 | 7.72 | 1.58 | 0.0051 | 0.0495 |
| TC0500011554.hg.1 | CHD1 | chromodomain helicase DNA binding protein 1 | Multiple_C | 13.1 | 12.12 | 11.73 | 2.58 | 1.43E-06 | 3.68E-05 | 12.88 | 12.63 | 12.92 | 0.97 | 0.7824 | 0.9022 |

| | | | | | | | | | | | | | | | |
|---|---|---|---|---|---|---|---|---|---|---|---|---|---|---|---|
| TC0600013539.hg.1 | RAET1L | retinoic acid early transcript 1L | Coding | 9.39 | 7.94 | 8.02 | 2.58 | 0.0212 | 0.075 | 6.11 | 5.76 | 6.37 | 0.84 | 0.3201 | 0.584 |
| TC0700010015.hg.1 | INTS1 | integrator complex subunit 1 | Multiple_C | 9.25 | 8 | 7.88 | 2.58 | 7.32E-05 | 0.0008 | 8.37 | 9.17 | 8.22 | 1.11 | 0.5303 | 0.7534 |
| TC1000006785.hg.1 | PROSER2 | proline and serine rich 2 | Multiple_C | 8.18 | 6.47 | 6.81 | 2.58 | 0.0001 | 0.0013 | 6.33 | 6.36 | 6.45 | 0.92 | 0.8394 | 0.9294 |
| TC1400009258.hg.1 | TBPL2 | TATA box binding protein like 2 | Multiple_C | 4.51 | 3.05 | 3.14 | 2.58 | 0.0153 | 0.0579 | 3.38 | 3.59 | 3.32 | 1.04 | 0.7967 | 0.9092 |
| TC1700011743.hg.1 | GGA3 | golgi-associated, gamma adaptin ear containing, ARF binding pr | Multiple_C | 8.64 | 7.05 | 7.27 | 2.58 | 9.76E-06 | 0.0002 | 9.17 | 9.01 | 9 | 1.13 | 0.3562 | 0.6194 |
| TC1900007511.hg.1 | ZNF714; VN1R | zinc finger protein 714; vomeronasal 1 receptor 81 pseudogene | Multiple_C | 6.3 | 4.99 | 4.93 | 2.58 | 0.0082 | 0.0358 | 7.03 | 7.30 | 8.13 | 0.47 | 0.0027 | 0.0333 |
| TSUnmapped00000437.h | DGKD | diacylglycerol kinase, delta 130kDa | Coding | 8.2 | 7.49 | 6.83 | 2.58 | 0.0064 | 0.0292 | 7.03 | 7.28 | 7.29 | 0.84 | 0.6152 | 0.8089 |
| TC0100011272.hg.1 | ZBED6; ZC3H1 | zinc finger, BED-type containing 6; zinc finger CCCH-type contai | Multiple_C | 13.73 | 12.97 | 12.37 | 2.57 | 0.0001 | 0.0011 | 12.9 | 12.15 | 12.56 | 1.27 | 0.3034 | 0.5677 |
| TC0100011333.hg.1 | CDK18 | cyclin-dependent kinase 18 | Multiple_C | 6.65 | 5.31 | 5.29 | 2.57 | 0.0002 | 0.0016 | 5.85 | 5.77 | 5.09 | 1.69 | 0.0044 | 0.0456 |
| TC0200009973.hg.1 | ITGA6 | integrin alpha 6 | Multiple_C | 14.5 | 13.1 | 13.14 | 2.57 | 0.0002 | 0.0017 | 11.82 | 11.60 | 12.6 | 0.58 | 0.0014 | 0.0211 |
| TC0200010012.hg.1 | AC010894.3; S | novel transcript; Transcript Identified by AceView, Entrez Gene | Multiple_C | 5.18 | 3.64 | 3.82 | 2.57 | 0.0011 | 0.007 | 4.41 | 4.80 | 4.86 | 0.73 | 0.8694 | 0.9437 |
| TC0200010624.hg.1 | PIKFYVE | phosphoinositide kinase, FYVE finger containing | Multiple_C | 9.69 | 8.1 | 8.33 | 2.57 | 7.00E-05 | 0.0008 | 9.25 | 9.28 | 9.81 | 0.68 | 0.0041 | 0.0436 |
| TC0200012058.hg.1 | OST4 | oligosaccharyltransferase complex subunit 4 (non-catalytic) | Multiple_C | 15.93 | 14.03 | 14.57 | 2.57 | 0.0027 | 0.0146 | 12.64 | 12.53 | 12.61 | 1.02 | 0.9271 | 0.9697 |
| TC0200016477.hg.1 | INO80B-WBP1 | INO80B-WBP1 readthrough (NMD candidate) | Multiple_C | 9.49 | 8.25 | 8.13 | 2.57 | 0.0003 | 0.0028 | 8.21 | 8.62 | 8.32 | 0.93 | 0.1417 | 0.3744 |
| TC0600011697.hg.1 | CCDC167 | coiled-coil domain containing 167 | Coding | 7.38 | 6.45 | 6.02 | 2.57 | 0.0091 | 0.0386 | 6.03 | 6.29 | 6.16 | 0.91 | 0.9084 | 0.9623 |
| TC1400007204.hg.1 | CGRRF1 | cell growth regulator with ring finger domain 1 | Multiple_C | 8.63 | 8.6 | 7.27 | 2.57 | 0.0002 | 0.0021 | 6.93 | 6.34 | 6.47 | 1.38 | 0.0446 | 0.1954 |
| TC1600006594.hg.1 | TRAF7 | TNF receptor-associated factor 7, E3 ubiquitin protein ligase | Multiple_C | 10.67 | 8.97 | 9.31 | 2.57 | 0.038 | 0.1179 | 9.44 | 9.63 | 9.62 | 0.88 | 0.9691 | 0.9872 |
| TC1700012432.hg.1 | HOXB3; HOXB | homeobox B3; homeobox B4; microRNA 10a | Multiple_C | 8.27 | 6.75 | 6.91 | 2.57 | 3.16E-06 | 6.99E-05 | 9.52 | 9.46 | 9.08 | 1.36 | 0.0891 | 0.2889 |
| TC1800007504.hg.1 | KIAA1468 | KIAA1468 | Multiple_C | 8.81 | 7.98 | 7.45 | 2.57 | 0.0081 | 0.0354 | 6.15 | 6.85 | 6.6 | 0.73 | 0.0641 | 0.241 |
| TC1900009137.hg.1 | CBARP | calcium channel, voltage-dependent, beta subunit associated re | Multiple_C | 5.89 | 4.32 | 4.53 | 2.57 | 0.0001 | 0.0012 | 5.79 | 5.66 | 5.48 | 1.24 | 0.3518 | 0.6153 |
| TC1900010926.hg.1 | TRAPPC6A | trafficking protein particle complex 6A | Multiple_C | 8.71 | 7.57 | 7.35 | 2.57 | 1.90E-05 | 0.0003 | 8.75 | 8.96 | 8.6 | 1.11 | 0.3817 | 0.6409 |
| TC2000007191.hg.1 | DYNLRB1 | dynein, light chain, roadblock-type 1 | Multiple_C | 13.56 | 11.49 | 12.2 | 2.57 | 0.0001 | 0.0011 | 10.64 | 10.50 | 10.47 | 1.13 | 0.4222 | 0.675 |
| TC0100006707.hg.1 | CAMTA1 | calmodulin binding transcription activator 1 | Multiple_C | 14.94 | 14.31 | 13.59 | 2.55 | 3.88E-05 | 0.0005 | 10.34 | 10.30 | 10.35 | 0.99 | 0.2238 | 0.4846 |
| TC0100013018.hg.1 | ZBTB17 | zinc finger and BTB domain containing 17 | Multiple_C | 6.12 | 5.04 | 4.77 | 2.55 | 5.26E-05 | 0.0006 | 5.62 | 5.98 | 5.66 | 0.97 | 0.4424 | 0.6912 |
| TC0100013882.hg.1 | FOXJ3 | forkhead box J3 | Multiple_C | 12.47 | 10.25 | 11.12 | 2.55 | 0.0034 | 0.0178 | 11.5 | 10.74 | 11.53 | 0.98 | 0.6124 | 0.8064 |
| TC0100015645.hg.1 | NBPF11 | neuroblastoma breakpoint family, member 11 | Multiple_C | 9.03 | 7.82 | 7.68 | 2.55 | 0.0012 | 0.0075 | 7.93 | 7.76 | 7.73 | 1.15 | 0.8575 | 0.9395 |
| TC0900007680.hg.1 | TLE4 | transducin-like enhancer of split 4 | Multiple_C | 7.91 | 6.11 | 6.56 | 2.55 | 5.10E-06 | 0.0001 | 3.47 | 3.10 | 3.59 | 0.92 | 0.3368 | 0.6012 |
| TC0900011754.hg.1 | QRFP | pyroglutamylated RFamide peptide | Coding | 10.12 | 8.58 | 8.77 | 2.55 | 0.0005 | 0.0041 | 9.59 | 9.78 | 10.03 | 0.74 | 0.1325 | 0.3613 |
| TC1100007948.hg.1 | PRDX5 | peroxiredoxin 5 | Coding | 11.9 | 11.11 | 10.55 | 2.55 | 3.35E-05 | 0.0004 | 9.49 | 9.02 | 9.18 | 1.24 | 0.035 | 0.1689 |
| TC1100011410.hg.1 | MRGPRF | MAS-related GPR, member F | Coding | 6.02 | 4.48 | 4.67 | 2.55 | 0.0001 | 0.0012 | 5.66 | 5.60 | 5.47 | 1.14 | 0.2117 | 0.4695 |
| TC1500008286.hg.1 | ZNF710 | zinc finger protein 710 | Multiple_C | 9.32 | 7.89 | 7.97 | 2.55 | 0.0009 | 0.0064 | 7.94 | 8.19 | 7.99 | 0.97 | 0.9401 | 0.9753 |
| TC1600008407.hg.1 | NPIPB15 | nuclear pore complex interacting protein family, member B15 | Multiple_C | 10.82 | 9.2 | 9.47 | 2.55 | 1.07E-05 | 0.0002 | 10.82 | 10.47 | 10.64 | 1.13 | 0.2244 | 0.4851 |
| TC1600011354.hg.1 | NPIPA2 | nuclear pore complex interacting protein family, member A2 | Coding | 10.35 | 9.05 | 9 | 2.55 | 8.69E-05 | 0.0009 | 10.92 | 10.56 | 10.49 | 1.35 | 0.064 | 0.2407 |

| | | | | | | | | | | | | | | | |
|---|---|---|---|---|---|---|---|---|---|---|---|---|---|---|---|
| TC1900007397.hg.1 | UBA52 | ubiquitin A-52 residue ribosomal protein fusion product 1 | Multiple_C | 17.37 | 15.48 | 16.02 | 2.55 | 3.27E-05 | 0.0004 | 13.88 | 14.01 | 14.17 | 0.82 | 0.0391 | 0.1802 |
| TC2200007069.hg.1 | OSBP2 | oxysterol binding protein 2 | Multiple_C | 5.35 | 3.83 | 4 | 2.55 | 0.005 | 0.0241 | 5.88 | 6.50 | 6.99 | 0.46 | 0.0008 | 0.0142 |
| TC0200008911.hg.1 | ZC3H6 | zinc finger CCCH-type containing 6 | Multiple_C | 7.05 | 7.08 | 5.71 | 2.53 | 0.0011 | 0.0071 | 5.53 | 5.54 | 5.13 | 1.32 | 0.3638 | 0.6265 |
| TC0200009428.hg.1 | CCNT2 | cyclin T2 | Multiple_C | 11.96 | 10.76 | 10.62 | 2.53 | 0.0002 | 0.0018 | 11.67 | 10.72 | 11.51 | 1.12 | 0.2897 | 0.5543 |
| TC0200009936.hg.1 | GAD1 | glutamate decarboxylase 1 | Multiple_C | 9.53 | 8.11 | 8.19 | 2.53 | 8.49E-06 | 0.0002 | 10.01 | 10.47 | 10 | 1.01 | 0.8565 | 0.9389 |
| TC0200010275.hg.1 | GLS | glutaminase | Multiple_C | 12.41 | 11.56 | 11.07 | 2.53 | 1.97E-05 | 0.0003 | 11.33 | 10.00 | 10.91 | 1.34 | 0.1574 | 0.3985 |
| TC0200014848.hg.1 | ABCB11 | ATP binding cassette subfamily B member 11 | Multiple_C | 4.43 | 3.45 | 3.09 | 2.53 | 6.84E-05 | 0.0008 | 3.67 | 3.79 | 3.39 | 1.21 | 0.0968 | 0.3032 |
| TC0200016423.hg.1 | SPDYA | speedy/RINGO cell cycle regulator family member A | Multiple_C | 5.47 | 3.85 | 4.13 | 2.53 | 1.80E-05 | 0.0003 | 3.92 | 4.43 | 4.64 | 0.61 | 0.027 | 0.1435 |
| TC0800009793.hg.1 | SLC18A1 | solute carrier family 18 (vesicular monoamine transporter), mer | Multiple_C | 6.57 | 5.18 | 5.23 | 2.53 | 0.001 | 0.0066 | 4.4 | 4.25 | 3.99 | 1.33 | 0.1108 | 0.328 |
| TC0900006583.hg.1 | UHRF2 | ubiquitin-like with PHD and ring finger domains 2, E3 ubiquitin p | Multiple_C | 11.32 | 10.46 | 9.98 | 2.53 | 7.17E-05 | 0.0008 | 10.84 | 10.29 | 10.62 | 1.16 | 0.3099 | 0.5737 |
| TC1200010984.hg.1 | CTDSP2 | CTD small phosphatase 2 | Multiple_C | 9.52 | 8.08 | 8.18 | 2.53 | 0.0017 | 0.0102 | 8.89 | 9.66 | 9.51 | 0.65 | 0.1116 | 0.3291 |
| TC1700012344.hg.1 | GABARAP | GABA(A) receptor-associated protein | Multiple_C | 14.26 | 12.43 | 12.92 | 2.53 | 1.48E-05 | 0.0002 | 13.28 | 13.39 | 13.09 | 1.14 | 0.1384 | 0.3693 |
| TC1900011675.hg.1 | C19orf53 | chromosome 19 open reading frame 53 | Coding | 14.09 | 11.84 | 12.75 | 2.53 | 0.0013 | 0.0082 | 12.23 | 11.66 | 11.55 | 1.60 | 0.0234 | 0.1319 |
| TC2000008666.hg.1 | NAPB | N-ethylmaleimide-sensitive factor attachment protein, beta | Multiple_C | 8.69 | 8.26 | 7.35 | 2.53 | 6.02E-05 | 0.0007 | 10.09 | 8.94 | 9.01 | 2.11 | 0.0002 | 0.0066 |
| TC0200007067.hg.1 | NRBP1 | nuclear receptor binding protein 1 | Multiple_C | 10.25 | 9.25 | 8.92 | 2.51 | 4.79E-06 | 9.69E-05 | 10.52 | 10.59 | 10.71 | 0.88 | 0.4017 | 0.6582 |
| TC0200012980.hg.1 | ASPRV1; PCBF | aspartic peptidase, retroviral-like 1; PCBP1 antisense RNA 1 | Multiple_C | 8.63 | 7.46 | 7.3 | 2.51 | 8.80E-06 | 0.0002 | 8.89 | 9.25 | 9.3 | 0.75 | 0.04 | 0.1833 |
| TC0300011941.hg.1 | MYH15 | myosin, heavy chain 15 | Multiple_C | 5.24 | 3.99 | 3.91 | 2.51 | 0.0001 | 0.0012 | 3.66 | 3.83 | 3.68 | 0.99 | 0.7299 | 0.8746 |
| TC0400011043.hg.1 | CDKL2 | cyclin-dependent kinase-like 2 (CDC2-related kinase) | Multiple_C | 6.88 | 5.83 | 5.55 | 2.51 | 0.0036 | 0.0186 | 7.17 | 5.34 | 5.62 | 2.93 | 0.005 | 0.0489 |
| TC0400011207.hg.1 | HELQ | helicase, POLQ-like | Multiple_C | 8.57 | 7.61 | 7.24 | 2.51 | 1.18E-05 | 0.0002 | 7.58 | 7.47 | 7.5 | 1.06 | 0.4609 | 0.7039 |
| TC0600011948.hg.1 | CYP39A1 | cytochrome P450, family 39, subfamily A, polypeptide 1 | Multiple_C | 5.29 | 4.44 | 3.96 | 2.51 | 0.0189 | 0.0686 | 4.44 | 3.93 | 3.8 | 1.56 | 0.0588 | 0.2287 |
| TC0X00009117.hg.1 | FANCB | Fanconi anemia complementation group B | Multiple_C | 8.34 | 6.53 | 7.01 | 2.51 | 0.0083 | 0.0359 | 6.66 | 7.00 | 7.88 | 0.43 | 0.0006 | 0.0115 |
| TC1000011736.hg.1 | OBFC1 | oligonucleotide/oligosaccharide-binding fold containing 1 | Multiple_C | 12.13 | 12.78 | 10.8 | 2.51 | 0.0101 | 0.0421 | 7.84 | 7.50 | 7.48 | 1.28 | 0.5909 | 0.7931 |
| TC1100007262.hg.1 | EHF | ets homologous factor | Multiple_C | 16.57 | 14.94 | 15.24 | 2.51 | 0.0062 | 0.0286 | 11.86 | 11.34 | 11.33 | 1.44 | 0.0083 | 0.069 |
| TC1100008139.hg.1 | AIP | aryl hydrocarbon receptor interacting protein | Multiple_C | 11.03 | 10.45 | 9.7 | 2.51 | 0.006 | 0.0278 | 10.04 | 9.89 | 8.99 | 2.07 | 0.0013 | 0.0203 |
| TC1100012131.hg.1 | MMP1 | matrix metallopeptidase 1 | Multiple_C | 6.44 | 4.99 | 5.11 | 2.51 | 0.0054 | 0.0256 | 5.48 | 5.29 | 4.95 | 1.44 | 0.0952 | 0.2999 |
| TC1200007051.hg.1 | PYROXD1 | pyridine nucleotide-disulphide oxidoreductase domain 1 | Multiple_C | 9.29 | 9.49 | 7.96 | 2.51 | 3.02E-05 | 0.0004 | 8.27 | 7.04 | 7.37 | 1.87 | 0.0018 | 0.0249 |
| TC1200009220.hg.1 | CCDC62 | coiled-coil domain containing 62 | Multiple_C | 4.82 | 3.4 | 3.49 | 2.51 | 1.45E-05 | 0.0002 | 4.62 | 4.65 | 4.43 | 1.14 | 0.659 | 0.8338 |
| TC1300008497.hg.1 | UBL3 | ubiquitin-like 3 | Multiple_C | 12.21 | 12.62 | 10.88 | 2.51 | 0.0342 | 0.1089 | 9.24 | 9.36 | 9.08 | 1.12 | 0.2369 | 0.5 |
| TC0100008503.hg.1 | HOOK1 | hook microtubule-tethering protein 1 | Multiple_C | 10.39 | 9.78 | 9.07 | 2.50 | 0.0065 | 0.0297 | 11.09 | 10.34 | 10.41 | 1.60 | 0.0254 | 0.1387 |
| TC0100011400.hg.1 | PFKFB2 | 6-phosphofructo-2-kinase/fructose-2,6-biphosphatase 2 | Multiple_C | 8.66 | 7.57 | 7.34 | 2.50 | 3.38E-05 | 0.0004 | 6.02 | 5.61 | 6.49 | 0.72 | 0.0452 | 0.1966 |
| TC0200008507.hg.1 | ARID5A | AT rich interactive domain 5A (MRF1-like) | Multiple_C | 8.02 | 6.55 | 6.7 | 2.50 | 0.0002 | 0.0015 | 6.59 | 7.23 | 7.12 | 0.69 | 0.0222 | 0.1283 |
| TC0200008790.hg.1 | RGPD4 | RANBP2-like and GRIP domain containing 4 | Multiple_C | 5.55 | 3.84 | 4.23 | 2.50 | 7.69E-05 | 0.0009 | 5.35 | 5.04 | 5.42 | 0.95 | 0.9116 | 0.9638 |
| TC0200010219.hg.1 | FAM171B | family with sequence similarity 171, member B | Multiple_C | 8.07 | 6.58 | 6.75 | 2.50 | 0.0003 | 0.0026 | 8.44 | 6.71 | 5.49 | 7.73 | 8.80E-10 | 9.43E-07 |

| | | | | | | | | | | | | | | | |
|---|---|---|---|---|---|---|---|---|---|---|---|---|---|---|---|
| TC0300011047.hg.1 | IMPDH2 | IMP (inosine 5-monophosphate) dehydrogenase 2 | Multiple_C | 14.27 | 13.01 | 12.95 | 2.50 | 0.0013 | 0.0085 | 10.27 | 10.29 | 10.34 | 0.95 | 0.291 | 0.5557 |
| TC0300013855.hg.1 | NFKBIZ | nuclear factor of kappa light polypeptide gene enhancer in B-ce | Multiple_C | 10.85 | 9.24 | 9.53 | 2.50 | 2.30E-05 | 0.0003 | 9.5 | 9.07 | 8.82 | 1.60 | 0.0041 | 0.0436 |
| TC0500013253.hg.1 | RBM27 | RNA binding motif protein 27 | Multiple_C | 12.92 | 12.08 | 11.6 | 2.50 | 1.05E-05 | 0.0002 | 14.17 | 13.73 | 14.11 | 1.04 | 0.4508 | 0.6963 |
| TC0600011482.hg.1 | AGER | advanced glycosylation end product-specific receptor | Multiple_C | 5.52 | 4.17 | 4.2 | 2.50 | 0.0009 | 0.0059 | 4.38 | 4.41 | 4.51 | 0.91 | 0.9101 | 0.9629 |
| TC0600011943.hg.1 | ENPP5 | ectonucleotide pyrophosphatase/phosphodiesterase 5 (putativ | Multiple_C | 10.47 | 9.46 | 9.15 | 2.50 | 0.001 | 0.0065 | 7.97 | 6.47 | 5.29 | 6.41 | 7.40E-09 | 4.15E-06 |
| TC0700009333.hg.1 | UBN2 | ubinuclein 2 | Multiple_C | 9.62 | 8.33 | 8.3 | 2.50 | 0.0002 | 0.0017 | 9.6 | 8.90 | 9.26 | 1.27 | 0.1811 | 0.4311 |
| TC0700012584.hg.1 | UBE2H | ubiquitin conjugating enzyme E2H | Multiple_C | 14.37 | 13.5 | 13.05 | 2.50 | 0.0001 | 0.0011 | 9.77 | 9.42 | 10.04 | 0.83 | 0.4913 | 0.7263 |
| TC1100007217.hg.1 | QSER1 | glutamine and serine rich 1 | Multiple_C | 10.87 | 9.15 | 9.55 | 2.50 | 0.0001 | 0.0014 | 12.16 | 12.25 | 12.64 | 0.72 | 0.0241 | 0.1343 |
| TC1200009931.hg.1 | TAS2R46 | taste receptor, type 2, member 46 | Coding | 4.67 | 3.78 | 3.35 | 2.50 | 0.0005 | 0.004 | 3.81 | 3.53 | 3.41 | 1.32 | 0.0547 | 0.2195 |
| TC1200012713.hg.1 | MAP1LC3B2 | microtubule-associated protein 1 light chain 3 beta 2 | Coding | 6.94 | 6 | 5.62 | 2.50 | 0.0012 | 0.0077 | 5.31 | 4.99 | 5.19 | 1.09 | 0.9617 | 0.984 |
| TC1500007602.hg.1 | SLC24A1 | solute carrier family 24 (sodium/potassium/calcium exchanger) | Multiple_C | 6.03 | 4.81 | 4.71 | 2.50 | 0.0037 | 0.0191 | 6.15 | 6.18 | 5.68 | 1.39 | 0.0177 | 0.1117 |
| TC1700008888.hg.1 | ARMC7 | armadillo repeat containing 7 | Multiple_C | 9.47 | 8.21 | 8.15 | 2.50 | 4.39E-06 | 9.02E-05 | 9.32 | 9.34 | 9.26 | 1.04 | 0.9499 | 0.9795 |
| TC1900011926.hg.1 | ZNF91 | zinc finger protein 91 | Multiple_C | 11.32 | 10.14 | 10 | 2.50 | 0.0014 | 0.0088 | 10.55 | 9.69 | 10.56 | 0.99 | 0.8629 | 0.9412 |
| TC2000008755.hg.1 | ZNF337 | zinc finger protein 337 | Multiple_C | 7.94 | 6.79 | 6.62 | 2.50 | 1.63E-06 | 4.07E-05 | 7.48 | 6.92 | 6.75 | 1.66 | 0.0017 | 0.0243 |
| TC0100011364.hg.1 | SRGAP2 | SLIT-ROBO Rho GTPase activating protein 2 | Multiple_C | 10.28 | 9.11 | 8.97 | 2.48 | 8.47E-05 | 0.0009 | 9.2 | 9.12 | 8.83 | 1.29 | 0.1164 | 0.337 |
| TC0100012211.hg.1 | ZBTB18 | zinc finger and BTB domain containing 18 | Multiple_C | 7.07 | 6.1 | 5.76 | 2.48 | 0.0011 | 0.0072 | 8.97 | 8.66 | 8.93 | 1.03 | 0.9606 | 0.9837 |
| TC0100014775.hg.1 | BCL10 | B-cell CLL/lymphoma 10 | Coding | 10.46 | 9.12 | 9.15 | 2.48 | 0.0054 | 0.0256 | 8.86 | 8.71 | 8.99 | 0.91 | 0.8444 | 0.9318 |
| TC0100016899.hg.1 | KIF21B | kinesin family member 21B | Multiple_C | 5.65 | 4.2 | 4.34 | 2.48 | 0.0019 | 0.0113 | 5.73 | 5.52 | 5.46 | 1.21 | 0.5028 | 0.7346 |
| TC0100016963.hg.1 | KDM5B | lysine (K)-specific demethylase 5B | Multiple_C | 11.84 | 10.8 | 10.53 | 2.48 | 0.0001 | 0.0014 | 8.79 | 7.72 | 7.2 | 3.01 | 6.91E-07 | 0.0001 |
| TC0100017471.hg.1 | WDR26; MIR4 | WD repeat domain 26; microRNA 4742 | Multiple_C | 11.13 | 9.99 | 9.82 | 2.48 | 1.96E-05 | 0.0003 | 10.79 | 10.43 | 10.29 | 1.41 | 0.0572 | 0.2252 |
| TC0200006679.hg.1 | C2orf48 | chromosome 2 open reading frame 48 | Multiple_C | 6.98 | 5.39 | 5.67 | 2.48 | 0.0003 | 0.0026 | 6.98 | 7.19 | 6.99 | 0.99 | 0.7354 | 0.8779 |
| TC0200009431.hg.1 | RAB3GAP1 | RAB3 GTPase activating protein subunit 1 (catalytic) | Multiple_C | 10.58 | 10.64 | 9.27 | 2.48 | 0.0053 | 0.0252 | 9.27 | 8.43 | 9.14 | 1.09 | 0.534 | 0.7563 |
| TC0200010745.hg.1 | IGFBP2 | insulin like growth factor binding protein 2 | Multiple_C | 12.76 | 11.59 | 11.45 | 2.48 | 7.09E-05 | 0.0008 | 5.68 | 7.26 | 5.77 | 0.94 | 0.4602 | 0.7034 |
| TC0200015136.hg.1 | FRZB | frizzled-related protein | Coding | 5.23 | 4.17 | 3.92 | 2.48 | 0.0004 | 0.0034 | 4.64 | 4.26 | 3.98 | 1.58 | 0.0458 | 0.198 |
| TC0300010082.hg.1 | FAM157A | family with sequence similarity 157, member A | Multiple_C | 7.21 | 5.78 | 5.9 | 2.48 | 0.0061 | 0.0284 | 5.3 | 5.56 | 5.51 | 0.86 | 0.5664 | 0.7759 |
| TC0300011052.hg.1 | LAMB2 | laminin, beta 2 (laminin S) | Multiple_C | 7.12 | 6.09 | 5.81 | 2.48 | 0.0019 | 0.0112 | 6.73 | 6.40 | 6.17 | 1.47 | 0.0061 | 0.0559 |
| TC0600006554.hg.1 | FOXC1 | forkhead box C1 | Multiple_C | 6.77 | 5.32 | 5.46 | 2.48 | 4.24E-05 | 0.0005 | 5.26 | 5.03 | 5.46 | 0.87 | 0.615 | 0.8087 |
| TC0700009462.hg.1 | MTRNR2L6 | MT-RNR2-like 6 | Coding | 9.3 | 7.58 | 7.99 | 2.48 | 0.004 | 0.0202 | 7.93 | 8.20 | 8.5 | 0.67 | 0.0376 | 0.1767 |
| TC1100006750.hg.1 | NLRP14 | NLR family, pyrin domain containing 14 | Multiple_C | 5.04 | 3.28 | 3.73 | 2.48 | 6.49E-06 | 0.0001 | 4.17 | 4.39 | 4.3 | 0.91 | 0.9449 | 0.9777 |
| TC1100007307.hg.1 | C11orf74 | chromosome 11 open reading frame 74 | Multiple_C | 8.9 | 9.29 | 7.59 | 2.48 | 0.0027 | 0.0149 | 8.79 | 8.30 | 7.69 | 2.14 | 0.0061 | 0.0558 |
| TC1200012859.hg.1 | RHOF | ras homolog family member F (in filopodia) | Multiple_C | 12.61 | 10.96 | 11.3 | 2.48 | 0.0003 | 0.0023 | 10.34 | 9.30 | 9.41 | 1.91 | 7.22E-05 | 0.0029 |
| TC1400008945.hg.1 | RALGAPA1 | Ral GTPase activating protein, alpha subunit 1 (catalytic) | Multiple_C | 9.52 | 8.55 | 8.21 | 2.48 | 0.0004 | 0.0033 | 8.57 | 7.58 | 8.16 | 1.33 | 0.6311 | 0.8184 |
| TC1700008769.hg.1 | KCNJ2 | potassium channel, inwardly rectifying subfamily J, member 2 | Coding | 5.53 | 4 | 4.22 | 2.48 | 0.0008 | 0.0056 | 3.16 | 4.10 | 3.8 | 0.64 | 0.1413 | 0.3739 |

| | | | | | | | | | | | | | | | |
|---|---|---|---|---|---|---|---|---|---|---|---|---|---|---|---|
| TC2000007089.hg.1 | TPX2 | TPX2, microtubule-associated | Multiple_C | 13.21 | 11.43 | 11.9 | 2.48 | 0.0211 | 0.0747 | 13.08 | 13.04 | 13.12 | 0.97 | 0.3244 | 0.5884 |
| TC2000007320.hg.1 | RALGAPB | Ral GTPase activating protein, beta subunit (non-catalytic) | Multiple_C | 12.14 | 10.96 | 10.83 | 2.48 | 1.64E-05 | 0.0003 | 11.94 | 11.14 | 11.27 | 1.59 | 0.0033 | 0.0377 |
| TC2000007682.hg.1 | CEBPB | CCAAT/enhancer binding protein (C/EBP), beta | Multiple_C | 9.48 | 8 | 8.17 | 2.48 | 0.0002 | 0.0017 | 7.87 | 8.20 | 8.08 | 0.86 | 0.2119 | 0.4695 |
| TC2100008506.hg.1 | MORC3 | MORC family CW-type zinc finger 3 | Multiple_C | 10.68 | 10.19 | 9.37 | 2.48 | 6.90E-06 | 0.0001 | 10.17 | 8.38 | 9.24 | 1.91 | 0.0001 | 0.004 |
| TC0100009142.hg.1 | PTBP2 | polypyrimidine tract binding protein 2 | Multiple_C | 9.26 | 8.3 | 7.96 | 2.46 | 0.0006 | 0.0047 | 8.74 | 8.24 | 8.6 | 1.10 | 0.7208 | 0.8698 |
| TC0100010867.hg.1 | LAMC2 | laminin, gamma 2 | Multiple_C | 12.71 | 11.52 | 11.41 | 2.46 | 0.003 | 0.016 | 9.7 | 6.67 | 7.54 | 4.47 | 1.51E-06 | 0.0002 |
| TC0100013411.hg.1 | KDF1 | keratinocyte differentiation factor 1 | Coding | 7.85 | 6.65 | 6.55 | 2.46 | 0.0015 | 0.0091 | 6.51 | 6.79 | 6.84 | 0.80 | 0.0727 | 0.2584 |
| TC0500012294.hg.1 | PCDH1 | protocadherin 1 | Multiple_C | 7.81 | 6.3 | 6.51 | 2.46 | 0.0026 | 0.0144 | 5.44 | 5.49 | 5.3 | 1.10 | 0.3922 | 0.6506 |
| TC0X00008862.hg.1 | FUNDC2 | FUN14 domain containing 2 | Multiple_C | 10.1 | 9.13 | 8.8 | 2.46 | 0.0005 | 0.0036 | 9.6 | 9.32 | 10.3 | 0.62 | 0.0307 | 0.1561 |
| TC1100008012.hg.1 | DPF2 | D4, zinc and double PHD fingers family 2 | Multiple_C | 13.18 | 11.6 | 11.88 | 2.46 | 6.03E-05 | 0.0007 | 12.77 | 12.73 | 13.41 | 0.64 | 0.053 | 0.2151 |
| TC1300010000.hg.1 | TNFSF13B | tumor necrosis factor (ligand) superfamily, member 13b | Multiple_C | 5.77 | 4.4 | 4.47 | 2.46 | 0.0008 | 0.0056 | 4.57 | 4.45 | 4.11 | 1.38 | 0.0112 | 0.0838 |
| TC1500007518.hg.1 | RAB8B | RAB8B, member RAS oncogene family | Multiple_C | 11.3 | 10.35 | 10 | 2.46 | 0.0004 | 0.0033 | 9.2 | 7.93 | 8.28 | 1.89 | 0.001 | 0.0161 |
| TC1600006870.hg.1 | ATF7IP2 | activating transcription factor 7 interacting protein 2 | Multiple_C | 8.01 | 6.92 | 6.71 | 2.46 | 0.0026 | 0.0144 | 5.96 | 5.49 | 5.78 | 1.13 | 0.055 | 0.2201 |
| TC1700007918.hg.1 | AOC2 | amine oxidase, copper containing 2 (retina-specific) | Multiple_C | 5.34 | 4.04 | 4.04 | 2.46 | 8.42E-05 | 0.0009 | 4.37 | 3.89 | 3.97 | 1.32 | 0.0874 | 0.2854 |
| TC1700010910.hg.1 | KANSL1 | KAT8 regulatory NSL complex subunit 1 | Multiple_C | 9.67 | 7.93 | 8.37 | 2.46 | 0.0007 | 0.005 | 10.69 | 10.45 | 10.52 | 1.13 | 0.4714 | 0.7122 |
| TC1900009487.hg.1 | XAB2 | XPA binding protein 2 | Multiple_C | 8.85 | 7.85 | 7.55 | 2.46 | 0.0052 | 0.0247 | 6.56 | 6.60 | 6.51 | 1.04 | 0.9617 | 0.984 |
| TC1900011398.hg.1 | TMC4 | transmembrane channel like 4 | Multiple_C | 7.49 | 6.53 | 6.19 | 2.46 | 0.0023 | 0.0129 | 5.98 | 5.20 | 4.87 | 2.16 | 0.0065 | 0.0583 |
| TC2200009349.hg.1 | CSNK1E | casein kinase 1, epsilon | Multiple_C | 11.44 | 10.16 | 10.14 | 2.46 | 3.45E-05 | 0.0005 | 8.82 | 8.63 | 8.81 | 1.01 | 0.2304 | 0.4919 |
| TC0100007505.hg.1 | WDTC1 | WD and tetratricopeptide repeats 1 | Multiple_C | 10.21 | 9.01 | 8.92 | 2.45 | 0.0034 | 0.0179 | 8.86 | 9.40 | 8.72 | 1.10 | 0.3778 | 0.6378 |
| TC0100010416.hg.1 | PBX1 | pre-B-cell leukemia homeobox 1 | Multiple_C | 6.05 | 4.89 | 4.76 | 2.45 | 4.26E-05 | 0.0005 | 4.66 | 4.81 | 4.6 | 1.04 | 0.1325 | 0.3613 |
| TC0100018480.hg.1 | NBPF14 | neuroblastoma breakpoint family, member 14 | Coding | 12.08 | 10.93 | 10.79 | 2.45 | 8.59E-05 | 0.0009 | 10.07 | 9.49 | 9.53 | 1.45 | 0.053 | 0.215 |
| TC0200010785.hg.1 | CXCR2 | chemokine (C-X-C motif) receptor 2 | Multiple_C | 5.32 | 4.22 | 4.03 | 2.45 | 0.026 | 0.0878 | 4.27 | 4.41 | 4.01 | 1.20 | 0.4239 | 0.6766 |
| TC0200015865.hg.1 | SCG2 | secretogranin II | Coding | 6.18 | 4.75 | 4.89 | 2.45 | 0.0587 | 0.1632 | 5.29 | 7.20 | 6.53 | 0.42 | 0.0017 | 0.0234 |
| TC0400010681.hg.1 | LRRC66 | leucine rich repeat containing 66 | Coding | 5.53 | 3.76 | 4.24 | 2.45 | 0.0006 | 0.0043 | 3.4 | 3.30 | 3.37 | 1.02 | 0.8296 | 0.9244 |
| TC0400011108.hg.1 | CNOT6L | CCR4-NOT transcription complex subunit 6-like | Multiple_C | 10.13 | 9.47 | 8.84 | 2.45 | 0.0018 | 0.0104 | 8.89 | 9.08 | 9.18 | 0.82 | 0.1365 | 0.3673 |
| TC0600008757.hg.1 | PNRC1 | proline-rich nuclear receptor coactivator 1 | Multiple_C | 11.15 | 9.76 | 9.86 | 2.45 | 0.0011 | 0.0073 | 10.61 | 9.73 | 8.89 | 3.29 | 2.45E-06 | 0.0003 |
| TC0600014256.hg.1 | GABBR1 | gamma-aminobutyric acid (GABA) B receptor, 1 | Multiple_C | 7.58 | 5.83 | 6.29 | 2.45 | 3.55E-05 | 0.0005 | 8.11 | 7.13 | 7.29 | 1.77 | 0.0218 | 0.1268 |
| TC0700009072.hg.1 | IRF5 | interferon regulatory factor 5 | Multiple_C | 5.47 | 4.02 | 4.18 | 2.45 | 0.0179 | 0.0656 | 5.2 | 5.42 | 5.23 | 0.98 | 0.5399 | 0.7594 |
| TC0800009764.hg.1 | PSD3 | pleckstrin and Sec7 domain containing 3 | Multiple_C | 11.29 | 10.77 | 10 | 2.45 | 0.001 | 0.0068 | 9.54 | 8.51 | 9.76 | 0.86 | 0.346 | 0.6098 |
| TC0900012029.hg.1 | ENTPD2 | ectonucleoside triphosphate diphosphohydrolase 2 | Multiple_C | 6.23 | 4.69 | 4.94 | 2.45 | 0.0005 | 0.0037 | 3.87 | 3.43 | 3.49 | 1.30 | 0.0143 | 0.0972 |
| TC1100008025.hg.1 | SCYL1 | SCY1-like, kinase-like 1 | Multiple_C | 8.52 | 7.19 | 7.23 | 2.45 | 0.0019 | 0.0109 | 7.25 | 7.48 | 7.67 | 0.75 | 0.118 | 0.3395 |
| TC1100013149.hg.1 | SERGEF | secretion regulating guanine nucleotide exchange factor | Multiple_C | 7.83 | 6.71 | 6.54 | 2.45 | 0.0015 | 0.0093 | 7.86 | 7.08 | 7.43 | 1.35 | 0.7785 | 0.901 |
| TC1400007890.hg.1 | GPR65 | G protein-coupled receptor 65 | Coding | 4.81 | 3.68 | 3.52 | 2.45 | 0.0016 | 0.0099 | 4.97 | 4.47 | 4.96 | 1.01 | 0.9132 | 0.9645 |

| | | | | | | | | | | | | | | | |
|---|---|---|---|---|---|---|---|---|---|---|---|---|---|---|---|
| TC1600007096.hg.1 | TMC7 | transmembrane channel like 7 | Multiple_C | 10.39 | 9.05 | 9.1 | 2.45 | 0.0002 | 0.0019 | 8.37 | 7.17 | 8.67 | 0.81 | 0.1033 | 0.315 |
| TC1700006471.hg.1 | WNT9B | wingless-type MMTV integration site family, member 9B | Coding | 16.57 | 14.86 | 15.28 | 2.45 | 3.68E-05 | 0.0005 | 16.71 | 16.52 | 16.51 | 1.15 | 0.4904 | 0.7257 |
| TC1700006751.hg.1 | POLR2A | polymerase (RNA) II (DNA directed) polypeptide A, 220kDa | Multiple_C | 13.72 | 11.72 | 12.43 | 2.45 | 1.36E-06 | 3.52E-05 | 14.42 | 14.77 | 14.5 | 0.95 | 0.6593 | 0.8339 |
| TC1700008603.hg.1 | CEP95 | centrosomal protein 95kDa | Multiple_C | 10.57 | 10.32 | 9.28 | 2.45 | 0.0009 | 0.0059 | 11 | 10.37 | 10.57 | 1.35 | 0.18 | 0.4293 |
| TC1800006508.hg.1 | MYL12B | myosin light chain 12B | Coding | 16.7 | 16.04 | 15.41 | 2.45 | 8.12E-06 | 0.0001 | 14 | 14.07 | 13.39 | 1.53 | 0.0016 | 0.0229 |
| TC1800008707.hg.1 | MEX3C | mex-3 RNA binding family member C | Multiple_C | 8.11 | 7.13 | 6.82 | 2.45 | 0.0098 | 0.041 | 7.81 | 8.38 | 8.21 | 0.76 | 0.0134 | 0.0936 |
| TC1900008164.hg.1 | RPS19 | ribosomal protein S19 | Multiple_C | 17.81 | 16.36 | 16.52 | 2.45 | 0.0001 | 0.0011 | 14.77 | 14.98 | 14.53 | 1.18 | 0.2659 | 0.5305 |
| TC1900012021.hg.1 | ZNF841 | zinc finger protein 841 | Multiple_C | 10.38 | 9.13 | 9.09 | 2.45 | 7.82E-06 | 0.0001 | 8.09 | 7.96 | 8 | 1.06 | 0.876 | 0.9469 |
| TC2000008130.hg.1 | TBC1D20 | TBC1 domain family, member 20 | Multiple_C | 10.39 | 8.54 | 9.1 | 2.45 | 0.0025 | 0.0139 | 10.12 | 9.44 | 10.06 | 1.04 | 0.8544 | 0.9376 |
| TC2200009350.hg.1 | CSNK1E | casein kinase 1, epsilon | Multiple_C | 14.17 | 12.72 | 12.88 | 2.45 | 0.0003 | 0.0022 | 12.75 | 12.48 | 12.91 | 0.90 | 0.4732 | 0.7131 |
| TC0100015885.hg.1 | GATAD2B | GATA zinc finger domain containing 2B | Multiple_C | 11.36 | 9.97 | 10.08 | 2.43 | 1.44E-05 | 0.0002 | 11.1 | 10.68 | 11.17 | 0.95 | 0.9049 | 0.9607 |
| TC0200016648.hg.1 | CDC42EP3 | CDC42 effector protein (Rho GTPase binding) 3 | Coding | 8.07 | 6.09 | 6.79 | 2.43 | 0.0002 | 0.0016 | 6.15 | 6.43 | 7.45 | 0.41 | 2.81E-05 | 0.0015 |
| TC0300007410.hg.1 | GNAI2 | guanine nucleotide binding protein (G protein), alpha inhibiting | Multiple_C | 11.36 | 9.71 | 10.08 | 2.43 | 1.32E-05 | 0.0002 | 13.3 | 14.56 | 13.43 | 0.91 | 0.5648 | 0.7746 |
| TC0300013701.hg.1 | UBXN7 | UBX domain protein 7 | Multiple_C | 9.45 | 8.95 | 8.17 | 2.43 | 0.0001 | 0.0014 | 9.2 | 9.08 | 9.23 | 0.98 | 0.6988 | 0.8572 |
| TC0600007480.hg.1 | HLA-F | major histocompatibility complex, class I, F | Multiple_C | 8.01 | 6.94 | 6.73 | 2.43 | 0.0007 | 0.0049 | 9.51 | 8.82 | 8.87 | 1.56 | 0.0219 | 0.127 |
| TC0600009169.hg.1 | FAM229B | family with sequence similarity 229, member B | Coding | 7.55 | 7.44 | 6.27 | 2.43 | 0.005 | 0.0242 | 6.16 | 6.00 | 5.74 | 1.34 | 0.0072 | 0.0623 |
| TC0600012702.hg.1 | HACE1 | HECT domain and ankyrin repeat containing E3 ubiquitin protein | Multiple_C | 7.58 | 7.49 | 6.3 | 2.43 | 0.0113 | 0.0461 | 5.99 | 5.42 | 5.36 | 1.55 | 0.0047 | 0.0476 |
| TC0X00011277.hg.1 | CA5B | carbonic anhydrase VB, mitochondrial | Multiple_C | 9.14 | 8.01 | 7.86 | 2.43 | 0.0037 | 0.0189 | 8.42 | 8.29 | 9.03 | 0.66 | 0.3815 | 0.6408 |
| TC1000011659.hg.1 | MGEA5 | meningioma expressed antigen 5 (hyaluronidase) | Multiple_C | 9.97 | 10.04 | 8.69 | 2.43 | 0.0031 | 0.0166 | 7.6 | 7.54 | 7.91 | 0.81 | 0.3237 | 0.588 |
| TC1000011718.hg.1 | CALHM2 | calcium homeostasis modulator 2 | Multiple_C | 6.64 | 5.32 | 5.36 | 2.43 | 0.0014 | 0.0088 | 7.05 | 7.25 | 7.7 | 0.64 | 0.2521 | 0.5163 |
| TC1100013088.hg.1 | BIRC2 | baculoviral IAP repeat containing 2 | Multiple_C | 10.35 | 10.72 | 9.07 | 2.43 | 0.0061 | 0.0284 | 9.12 | 8.56 | 8.58 | 1.45 | 0.931 | 0.9709 |
| TC1200006905.hg.1 | FAM234B | family with sequence similarity 234, member B | Multiple_C | 8.22 | 6.77 | 6.94 | 2.43 | 0.0002 | 0.0016 | 7.99 | 7.31 | 7.89 | 1.07 | 0.6995 | 0.8575 |
| TC1200008165.hg.1 | KCNMB4 | potassium channel subfamily M regulatory beta subunit 4 | Multiple_C | 10.47 | 10.4 | 9.19 | 2.43 | 0.0001 | 0.0011 | 7.57 | 8.76 | 6.29 | 2.43 | 3.14E-06 | 0.0003 |
| TC1600006633.hg.1 | SRRM2 | serine/arginine repetitive matrix 2 | Multiple_C | 12.53 | 10.12 | 11.25 | 2.43 | 0.0019 | 0.0111 | 12 | 11.89 | 12.63 | 0.65 | 0.0178 | 0.1121 |
| TC1900007878.hg.1 | KMT2B | lysine (K)-specific methyltransferase 2B | Multiple_C | 6.98 | 5.35 | 5.7 | 2.43 | 0.0079 | 0.0346 | 6.02 | 6.30 | 6.65 | 0.65 | 0.0262 | 0.1415 |
| TC1900011231.hg.1 | KLK7 | kallikrein related peptidase 7 | Multiple_C | 6.38 | 4.97 | 5.1 | 2.43 | 0.0061 | 0.0283 | 5.53 | 5.05 | 4.99 | 1.45 | 0.0745 | 0.2621 |
| TC2000009955.hg.1 | SLC2A4RG | SLC2A4 regulator | Multiple_C | 5.65 | 4.59 | 4.37 | 2.43 | 0.003 | 0.0158 | 5.27 | 5.62 | 5.63 | 0.78 | 0.5204 | 0.7477 |
| TC2200007309.hg.1 | SH3BP1; PDXF | SH3-domain binding protein 1; pyridoxal (pyridoxine, vitamin B6 | Multiple_C | 9.06 | 6.92 | 7.78 | 2.43 | 0.0219 | 0.077 | 10.11 | 10.26 | 9.59 | 1.43 | 0.36 | 0.6228 |
| TSUnmapped00000606.h | FMN1 | formin 1 | Coding | 5.37 | 4.31 | 4.09 | 2.43 | 0.0035 | 0.0181 | 4.7 | 4.55 | 4.83 | 0.91 | 0.5738 | 0.7813 |
| TC0100009441.hg.1 | CTTNBP2NL | CTTNBP2 N-terminal like | Multiple_C | 10.23 | 8.69 | 8.96 | 2.41 | 0.0017 | 0.0101 | 8.77 | 7.84 | 8.19 | 1.49 | 0.0526 | 0.2141 |
| TC0100015805.hg.1 | CELF3 | CUGBP, Elav-like family member 3 | Multiple_C | 5.87 | 4.38 | 4.6 | 2.41 | 0.0059 | 0.0276 | 5.67 | 5.93 | 5.54 | 1.09 | 0.8217 | 0.9212 |
| TC0300013829.hg.1 | RBM5 | RNA binding motif protein 5 | Multiple_C | 12.02 | 11.54 | 10.75 | 2.41 | 0.0002 | 0.0016 | 11.65 | 11.04 | 11.34 | 1.24 | 0.0794 | 0.2713 |
| TC0600007307.hg.1 | BTN2A1 | butyrophilin, subfamily 2, member A1 | Multiple_C | 7.35 | 6.32 | 6.08 | 2.41 | 0.0004 | 0.003 | 8.05 | 7.86 | 8.23 | 0.88 | 0.0812 | 0.2748 |

| | | | | | | | | | | | | | | |
|---|---|---|---|---|---|---|---|---|---|---|---|---|---|---|
| TC0600012971.hg.1 | ZUFSP | zinc finger with UFM1-specific peptidase domain | Multiple_C | 9.54 | 9.63 | 8.27 | 2.41 | 0.0048 | 0.0231 | 8.53 | 8.49 | 8.81 | 0.82 | 0.624 | 0.8143 |
| TC0Y00006642.hg.1 | TMSB4Y | thymosin beta 4, Y-linked | Coding | 14.32 | 14.03 | 13.05 | 2.41 | 5.21E-05 | 0.0006 | 11.48 | 11.70 | 12.13 | 0.64 | 0.021 | 0.1239 |
| TC1000009869.hg.1 | CDNF | cerebral dopamine neurotrophic factor | Multiple_C | 6.43 | 6.15 | 5.16 | 2.41 | 0.0002 | 0.0017 | 4.07 | 4.19 | 3.94 | 1.09 | 0.4331 | 0.6841 |
| TC1000012516.hg.1 | AKR1C2 | aldo-keto reductase family 1, member C2 | Multiple_C | 6.64 | 5.51 | 5.37 | 2.41 | 0.006 | 0.0278 | 7.18 | 6.80 | 7.27 | 0.94 | 0.2451 | 0.5091 |
| TC1000012542.hg.1 | ZNF438 | zinc finger protein 438 | Multiple_C | 8.36 | 7.11 | 7.09 | 2.41 | 5.57E-05 | 0.0007 | 6.9 | 6.53 | 6.49 | 1.33 | 0.0366 | 0.1739 |
| TC1400010619.hg.1 | HIF1A | hypoxia inducible factor 1, alpha subunit (basic helix-loop-helix | Multiple_C | 14.52 | 12.72 | 13.25 | 2.41 | 0.0052 | 0.025 | 12.82 | 11.53 | 13.4 | 0.67 | 0.0432 | 0.1918 |
| TC1600007944.hg.1 | GNAO1 | guanine nucleotide binding protein (G protein), alpha activating | Multiple_C | 7.15 | 5.92 | 5.88 | 2.41 | 0.0178 | 0.0652 | 6.62 | 6.47 | 6.34 | 1.21 | 0.7774 | 0.9003 |
| TC1600009677.hg.1 | CRYM | crystallin mu | Multiple_C | 8.03 | 7.57 | 6.76 | 2.41 | 0.0002 | 0.0019 | 5.61 | 5.01 | 5.27 | 1.27 | 0.1659 | 0.4107 |
| TC1700008563.hg.1 | DCAF7 | DDB1 and CUL4 associated factor 7 | Multiple_C | 12.4 | 10.76 | 11.13 | 2.41 | 0.0017 | 0.0103 | 12.86 | 13.12 | 13.07 | 0.86 | 0.0823 | 0.2763 |
| TC1700011448.hg.1 | POLG2 | polymerase (DNA directed), gamma 2, accessory subunit | Multiple_C | 8.56 | 8.4 | 7.29 | 2.41 | 0.0038 | 0.0193 | 8.56 | 7.54 | 7.96 | 1.52 | 0.059 | 0.2289 |
| TC1700011773.hg.1 | WBP2 | WW domain binding protein 2 | Multiple_C | 11.06 | 9.08 | 9.79 | 2.41 | 0.0012 | 0.0079 | 11.77 | 11.09 | 11.67 | 1.07 | 0.3925 | 0.6507 |
| TC1800008578.hg.1 | EPG5 | ectopic P-granules autophagy protein 5 homolog (C. elegans) | Multiple_C | 7.21 | 5.87 | 5.94 | 2.41 | 0.0002 | 0.0018 | 5.95 | 6.71 | 6.37 | 0.75 | 0.0797 | 0.272 |
| TC1900006730.hg.1 | KDM4B | lysine (K)-specific demethylase 4B | Multiple_C | 6.5 | 5.04 | 5.23 | 2.41 | 0.0035 | 0.0181 | 5.78 | 5.86 | 6.03 | 0.84 | 0.4494 | 0.6958 |
| TC1900011683.hg.1 | HSH2D | hematopoietic SH2 domain containing | Multiple_C | 7.7 | 6.24 | 6.43 | 2.41 | 0.0017 | 0.0104 | 3.7 | 3.64 | 3.59 | 1.08 | 0.3397 | 0.6039 |
| TC0100013902.hg.1 | SVBP | small vasohibin binding protein | Multiple_C | 6.94 | 6.29 | 5.68 | 2.39 | 0.0002 | 0.0015 | 4.55 | 5.37 | 5.5 | 0.52 | 0.0007 | 0.0128 |
| TC0200015447.hg.1 | SUMO1 | small ubiquitin-like modifier 1 | Multiple_C | 15.64 | 15.02 | 14.38 | 2.39 | 0.0007 | 0.0051 | 14.2 | 14.15 | 13.86 | 1.27 | 0.2391 | 0.5027 |
| TC0400006578.hg.1 | SH3BP2 | SH3-domain binding protein 2 | Multiple_C | 9.57 | 8.1 | 8.31 | 2.39 | 0.0019 | 0.0109 | 10.59 | 10.73 | 10.58 | 1.01 | 0.1894 | 0.4419 |
| TC0600009322.hg.1 | HSF2 | heat shock transcription factor 2 | Multiple_C | 11.72 | 11.51 | 10.46 | 2.39 | 0.0016 | 0.0098 | 10.5 | 10.12 | 10.33 | 1.13 | 0.3929 | 0.6511 |
| TC0700007870.hg.1 | STAG3L4 | stromal antigen 3-like 4 (pseudogene) | Multiple_C | 10.97 | 10.73 | 9.71 | 2.39 | 0.0002 | 0.0015 | 9.28 | 8.87 | 9.24 | 1.03 | 0.4974 | 0.7307 |
| TC0700009056.hg.1 | FAM71F2 | family with sequence similarity 71, member F2 | Multiple_C | 5.7 | 4.4 | 4.44 | 2.39 | 0.0001 | 0.0014 | 4.99 | 5.29 | 5.25 | 0.84 | 0.268 | 0.5326 |
| TC0700012461.hg.1 | WASL | Wiskott-Aldrich syndrome-like | Multiple_C | 12.26 | 10.54 | 11 | 2.39 | 0.003 | 0.016 | 9.83 | 8.59 | 9.89 | 0.96 | 0.3726 | 0.6337 |
| TC0800010949.hg.1 | SNX16 | sorting nexin 16 | Multiple_C | 9.07 | 9.5 | 7.81 | 2.39 | 1.48E-05 | 0.0002 | 7.22 | 6.67 | 6.96 | 1.20 | 0.1396 | 0.3712 |
| TC0900011673.hg.1 | CRAT | carnitine O-acetyltransferase | Multiple_C | 9.97 | 8.83 | 8.71 | 2.39 | 0.0002 | 0.0019 | 7.66 | 7.99 | 7.65 | 1.01 | 0.7686 | 0.8963 |
| TC1000008020.hg.1 | FAM149B1 | family with sequence similarity 149, member B1 | Multiple_C | 7.87 | 7.58 | 6.61 | 2.39 | 0.0369 | 0.1157 | 6.94 | 7.19 | 6.92 | 1.01 | 0.4325 | 0.6839 |
| TC1000008758.hg.1 | CNNM2 | cyclin and CBS domain divalent metal cation transport mediator | Multiple_C | 8.57 | 7.31 | 7.31 | 2.39 | 0.0012 | 0.0076 | 7.06 | 6.76 | 7.43 | 0.77 | 0.0565 | 0.2237 |
| TC1300008253.hg.1 | CRYL1 | crystallin lambda 1 | Multiple_C | 10.26 | 10.63 | 9 | 2.39 | 0.005 | 0.024 | 5.57 | 5.63 | 5.15 | 1.34 | 0.0123 | 0.0888 |
| TC1400010748.hg.1 | LINC01588 | long intergenic non-protein coding RNA 1588 | Multiple_C | 7.45 | 7.05 | 6.19 | 2.39 | 0.0001 | 0.0014 | 5.33 | 4.95 | 4.53 | 1.74 | 0.003 | 0.0354 |
| TC1500007700.hg.1 | RPLP1 | ribosomal protein, large, P1 | Multiple_C | 17.69 | 16.91 | 16.43 | 2.39 | 0.0006 | 0.0044 | 14.07 | 14.27 | 13.57 | 1.41 | 0.1011 | 0.3108 |
| TC1800006710.hg.1 | CHMP1B | charged multivesicular body protein 1B | Multiple_C | 11.73 | 11.55 | 10.47 | 2.39 | 1.82E-05 | 0.0003 | 12.28 | 12.14 | 11.25 | 2.04 | 3.94E-06 | 0.0004 |
| TC1900006576.hg.1 | DOT1L | DOT1-like histone H3K79 methyltransferase | Multiple_C | 9.53 | 7.92 | 8.27 | 2.39 | 0.0005 | 0.004 | 8.34 | 8.43 | 8.91 | 0.67 | 0.4109 | 0.6655 |
| TC1900008326.hg.1 | FOSB | FBJ murine osteosarcoma viral oncogene homolog B | Multiple_C | 8.11 | 6.22 | 6.85 | 2.39 | 8.25E-05 | 0.0009 | 5.76 | 5.67 | 5.34 | 1.34 | 0.1073 | 0.3223 |
| TC1900010632.hg.1 | SIRT2 | sirtuin 2 | Multiple_C | 10.53 | 8.6 | 9.27 | 2.39 | 0.0002 | 0.0017 | 7.47 | 7.89 | 8.23 | 0.59 | 0.0082 | 0.0685 |
| TC2100007056.hg.1 | SIM2 | single-minded family bHLH transcription factor 2 | Multiple_C | 10.19 | 8.68 | 8.93 | 2.39 | 0.0048 | 0.0232 | 4.17 | 4.12 | 4.23 | 0.96 | 0.794 | 0.9084 |

| | | | | | | | | | | | | | | | |
|---|---|---|---|---|---|---|---|---|---|---|---|---|---|---|---|
| TC0100014392.hg.1 | TM2D1 | TM2 domain containing 1 | Multiple_C | 11.49 | 11.79 | 10.24 | 2.38 | 0.0008 | 0.0057 | 10.49 | 9.37 | 9.19 | 2.46 | 4.49E-06 | 0.0004 |
| TC0100014995.hg.1 | CNN3 | calponin 3, acidic | Multiple_C | 14.01 | 13.02 | 12.76 | 2.38 | 1.71E-05 | 0.0003 | 12.5 | 12.63 | 13.4 | 0.54 | 0.0002 | 0.0052 |
| TC0500007368.hg.1 | ITGA2 | integrin, alpha 2 (CD49B, alpha 2 subunit of VLA-2 receptor) | Multiple_C | 10.61 | 9.65 | 9.36 | 2.38 | 0.0037 | 0.0189 | 7.51 | 6.87 | 7.2 | 1.24 | 0.2506 | 0.5149 |
| TC0700010081.hg.1 | CARD11 | caspase recruitment domain family, member 11 | Multiple_C | 7.31 | 6.09 | 6.06 | 2.38 | 0.0005 | 0.0035 | 5.32 | 5.46 | 5.42 | 0.93 | 0.6129 | 0.8066 |
| TC0700013388.hg.1 | STAG3L1 | stromal antigen 3-like 1 (pseudogene) | Multiple_C | 11.21 | 10.14 | 9.96 | 2.38 | 0.0005 | 0.0037 | 11.12 | 11.74 | 11.23 | 0.93 | 0.2337 | 0.4961 |
| TC0800010417.hg.1 | PCMTD1 | protein-L-isoaspartate (D-aspartate) O-methyltransferase doma | Multiple_C | 11.16 | 11.45 | 9.91 | 2.38 | 0.001 | 0.0067 | 9.89 | 9.07 | 8.63 | 2.39 | 0.0003 | 0.0075 |
| TC1100011485.hg.1 | SHANK2 | SH3 and multiple ankyrin repeat domains 2 | Multiple_C | 6.24 | 4.92 | 4.99 | 2.38 | 0.0042 | 0.0208 | 5.91 | 5.85 | 6.21 | 0.81 | 0.4325 | 0.6839 |
| TC1200008353.hg.1 | NTS | neurotensin | Multiple_C | 7.72 | 6.88 | 6.47 | 2.38 | 0.0003 | 0.0023 | 3.85 | 4.00 | 3.67 | 1.13 | 0.4885 | 0.7247 |
| TC2000008268.hg.1 | SLC23A2 | solute carrier family 23 (ascorbic acid transporter), member 2 | Multiple_C | 7.01 | 5.55 | 5.76 | 2.38 | 0.0013 | 0.0081 | 8.87 | 8.26 | 8.5 | 1.29 | 0.0668 | 0.2465 |
| TC2200007419.hg.1 | TNRC6B | trinucleotide repeat containing 6B | Multiple_C | 9.72 | 8.27 | 8.47 | 2.38 | 0.0016 | 0.0096 | 9.23 | 8.73 | 8.43 | 1.74 | 0.0375 | 0.1763 |
| TC2200009365.hg.1 | CHKB | choline kinase beta | Multiple_C | 8.74 | 7.9 | 7.49 | 2.38 | 2.62E-05 | 0.0004 | 7.28 | 7.87 | 7.12 | 1.12 | 0.4959 | 0.7296 |
| TC0100013072.hg.1 | ATP13A2 | ATPase type 13A2 | Multiple_C | 11.13 | 9.91 | 9.89 | 2.36 | 0.003 | 0.016 | 9.13 | 9.17 | 8.95 | 1.13 | 0.9926 | 0.9968 |
| TC0200008248.hg.1 | ELMOD3 | ELMO/CED-12 domain containing 3 | Multiple_C | 7.22 | 6.16 | 5.98 | 2.36 | 0.0002 | 0.0018 | 6.17 | 6.59 | 6.99 | 0.57 | 0.003 | 0.0354 |
| TC0200011120.hg.1 | NEU2 | sialidase 2 (cytosolic sialidase) | Coding | 5.66 | 4.07 | 4.42 | 2.36 | 0.0226 | 0.0788 | 4.44 | 4.69 | 4.57 | 0.91 | 0.3615 | 0.6242 |
| TC0200014697.hg.1 | ACVR1 | activin A receptor type I | Multiple_C | 8.43 | 8.14 | 7.19 | 2.36 | 0.0026 | 0.0144 | 5.18 | 5.06 | 5.62 | 0.74 | 0.1825 | 0.4332 |
| TC0200016475.hg.1 | INO80B | INO80 complex subunit B | Multiple_C | 8.73 | 7.82 | 7.49 | 2.36 | 4.46E-05 | 0.0006 | 7.32 | 7.53 | 7.64 | 0.80 | 0.189 | 0.4415 |
| TC0500008785.hg.1 | EGR1 | early growth response 1 | Coding | 12.02 | 8.14 | 10.78 | 2.36 | 0.0009 | 0.0063 | 12.64 | 10.96 | 10.41 | 4.69 | 3.14E-06 | 0.0003 |
| TC0500009319.hg.1 | CCNG1 | cyclin G1 | Multiple_C | 12.99 | 13.06 | 11.75 | 2.36 | 0.0084 | 0.0362 | 11.13 | 11.02 | 10.92 | 1.16 | 0.7111 | 0.8644 |
| TC0600007616.hg.1 | HSPA1B; HSPA | heat shock 70kDa protein 1B; heat shock 70kDa protein 1A | Coding | 14.07 | 12.8 | 12.83 | 2.36 | 0.0512 | 0.1471 | 13.5 | 10.52 | 13.28 | 1.16 | 0.8558 | 0.9386 |
| TC1000012032.hg.1 | RPL21 | ribosomal protein L21 | Multiple_C | 16.27 | 15.73 | 15.03 | 2.36 | 0.0017 | 0.0103 | 11.56 | 12.17 | 11.75 | 0.88 | 0.3491 | 0.6132 |
| TC1100012994.hg.1 | CRY2 | cryptochrome circadian clock 2 | Multiple_C | 4.82 | 3.38 | 3.58 | 2.36 | 0.0002 | 0.0018 | 4.21 | 4.31 | 4.49 | 0.82 | 0.1336 | 0.363 |
| TC1100013036.hg.1 | PPP2R5B | protein phosphatase 2, regulatory subunit B, beta | Multiple_C | 6.74 | 5.66 | 5.5 | 2.36 | 0.0013 | 0.0084 | 5.45 | 5.21 | 5.17 | 1.21 | 0.2397 | 0.5033 |
| TC1200006737.hg.1 | PHC1 | polyhomeotic homolog 1 (Drosophila) | Multiple_C | 8.74 | 7.32 | 7.5 | 2.36 | 2.66E-05 | 0.0004 | 9.44 | 8.71 | 8.35 | 2.13 | 5.41E-06 | 0.0005 |
| TC1200012864.hg.1 | CCDC92 | coiled-coil domain containing 92 | Multiple_C | 7.94 | 6.71 | 6.7 | 2.36 | 0.0034 | 0.0178 | 5.95 | 6.31 | 5.56 | 1.31 | 0.1326 | 0.3614 |
| TC1400006663.hg.1 | LRP10 | LDL receptor related protein 10 | Multiple_C | 11.47 | 9.76 | 10.23 | 2.36 | 0.002 | 0.0114 | 9.08 | 8.95 | 8.2 | 1.84 | 0.1543 | 0.3939 |
| TC1600011487.hg.1 | NPIPA8 | nuclear pore complex interacting protein family, member A8 | Coding | 10.43 | 9.3 | 9.19 | 2.36 | 1.24E-05 | 0.0002 | 10.88 | 10.72 | 10.71 | 1.13 | 0.2175 | 0.4769 |
| TC1700006772.hg.1 | KDM6B | lysine (K)-specific demethylase 6B | Multiple_C | 9.33 | 7.29 | 8.09 | 2.36 | 6.70E-05 | 0.0008 | 9.6 | 8.86 | 8.86 | 1.67 | 0.0059 | 0.055 |
| TC1700011234.hg.1 | CUEDC1 | CUE domain containing 1 | Multiple_C | 10.36 | 9.56 | 9.12 | 2.36 | 0.0008 | 0.0053 | 8.74 | 9.10 | 8.88 | 0.91 | 0.0865 | 0.2836 |
| TC1900007957.hg.1 | ZNF568 | zinc finger protein 568 | Multiple_C | 6.96 | 5.69 | 5.72 | 2.36 | 0.0021 | 0.012 | 4.68 | 4.48 | 4.76 | 0.95 | 0.8403 | 0.9302 |
| TC0100015586.hg.1 | PDZK1 | PDZ domain containing 1 | Multiple_C | 6.34 | 5.37 | 5.11 | 2.35 | 0.0074 | 0.033 | 5.35 | 5.91 | 6.1 | 0.59 | 0.052 | 0.213 |
| TC0200007628.hg.1 | RPS27A | ribosomal protein S27a | Multiple_C | 16.39 | 15.79 | 15.16 | 2.35 | 0.002 | 0.0115 | 11.21 | 11.54 | 11.58 | 0.77 | 0.1092 | 0.3252 |
| TC0200011688.hg.1 | ADAM17 | ADAM metallopeptidase domain 17 | Multiple_C | 10.55 | 10.02 | 9.32 | 2.35 | 0.0053 | 0.0253 | 10.4 | 9.42 | 10.05 | 1.27 | 0.3441 | 0.6077 |
| TC0200014991.hg.1 | GPR155 | G protein-coupled receptor 155 | Multiple_C | 5.59 | 4.53 | 4.36 | 2.35 | 0.0055 | 0.0261 | 11.01 | 10.59 | 9.4 | 3.05 | 0.0008 | 0.0139 |

| | | | | | | | | | | | | | | | |
|---|---|---|---|---|---|---|---|---|---|---|---|---|---|---|---|
| TC0300012216.hg.1 | CCDC14 | coiled-coil domain containing 14 | Multiple_C | 12.49 | 11.84 | 11.26 | 2.35 | 3.37E-06 | 7.32E-05 | 11.43 | 11.49 | 11.41 | 1.01 | 0.9475 | 0.9789 |
| TC0500008342.hg.1 | DCP2 | decapping mRNA 2 | Multiple_C | 9.64 | 8.77 | 8.41 | 2.35 | 0.0028 | 0.0154 | 9.31 | 8.76 | 8.75 | 1.47 | 0.1222 | 0.345 |
| TC0500011702.hg.1 | STARD4 | StAR-related lipid transfer domain containing 4 | Multiple_C | 11.09 | 10.81 | 9.86 | 2.35 | 0.0003 | 0.0027 | 9.74 | 9.11 | 9.44 | 1.23 | 0.2887 | 0.5533 |
| TC0600008569.hg.1 | MYO6 | myosin VI | Multiple_C | 10.26 | 9.41 | 9.03 | 2.35 | 0.0006 | 0.0042 | 6.69 | 6.57 | 6.61 | 1.06 | 0.9654 | 0.9854 |
| TC0700008454.hg.1 | BHLHA15 | basic helix-loop-helix family, member a15 | Multiple_C | 10.98 | 9.32 | 9.75 | 2.35 | 0.0004 | 0.0031 | 11.57 | 11.63 | 11.26 | 1.24 | 0.0541 | 0.2177 |
| TC0900007488.hg.1 | PIP5K1B | phosphatidylinositol-4-phosphate 5-kinase, type I, beta | Multiple_C | 11.76 | 11.28 | 10.53 | 2.35 | 0.0017 | 0.01 | 4.44 | 4.62 | 4.53 | 0.94 | 0.8998 | 0.9583 |
| TC1000010101.hg.1 | ABI1 | abl-interactor 1 | Multiple_C | 13.52 | 12.89 | 12.29 | 2.35 | 9.76E-07 | 2.72E-05 | 11.67 | 11.42 | 11.26 | 1.33 | 0.0531 | 0.2154 |
| TC1200012593.hg.1 | CLEC4A | C-type lectin domain family 4, member A | Coding | 5.39 | 4.59 | 4.16 | 2.35 | 0.0019 | 0.0111 | 5.24 | 4.61 | 4.9 | 1.27 | 0.1206 | 0.3428 |
| TC1400007588.hg.1 | PCNX | pecanex homolog (Drosophila) | Multiple_C | 11.36 | 10.15 | 10.13 | 2.35 | 4.94E-05 | 0.0006 | 11.9 | 11.46 | 11.48 | 1.34 | 0.048 | 0.2033 |
| TC1400008557.hg.1 | POTEG; POTEI | POTE ankyrin domain family, member G; POTE ankyrin domain f | Multiple_C | 5.08 | 4.11 | 3.85 | 2.35 | 0.0014 | 0.009 | 4.47 | 4.14 | 4.38 | 1.06 | 0.6398 | 0.8234 |
| TC1700010681.hg.1 | KRT16 | keratin 16, type I | Multiple_C | 8.4 | 6.99 | 7.17 | 2.35 | 0.0023 | 0.0131 | 6.63 | 6.57 | 6.6 | 1.02 | 0.5576 | 0.7711 |
| TC1900008862.hg.1 | LAIR2 | leukocyte-associated immunoglobulin-like receptor 2 | Coding | 8.07 | 6.66 | 6.84 | 2.35 | 0.001 | 0.0066 | 6.22 | 6.29 | 6.4 | 0.88 | 0.441 | 0.6904 |
| TC2100008165.hg.1 | BRWD1 | bromodomain and WD repeat domain containing 1 | Multiple_C | 10.39 | 9.73 | 9.16 | 2.35 | 3.05E-05 | 0.0004 | 10.16 | 8.94 | 9.37 | 1.73 | 0.0092 | 0.0739 |
| TC0100009274.hg.1 | AMY1C; AMY1 | amylase, alpha 1C (salivary); amylase, alpha 1A (salivary) | Multiple_C | 5.29 | 5.1 | 4.07 | 2.33 | 2.18E-05 | 0.0003 | 3.5 | 3.69 | 3.63 | 0.91 | 0.9528 | 0.9808 |
| TC0200010362.hg.1 | COQ10B | coenzyme Q10B | Multiple_C | 11.51 | 11.55 | 10.29 | 2.33 | 1.85E-05 | 0.0003 | 9.98 | 8.70 | 9.25 | 1.66 | 0.0022 | 0.0286 |
| TC0300007068.hg.1 | OXSR1 | oxidative stress responsive 1 | Multiple_C | 14.47 | 13.45 | 13.25 | 2.33 | 0.0001 | 0.0013 | 14.72 | 13.78 | 14.44 | 1.21 | 0.3521 | 0.6154 |
| TC0300013763.hg.1 | RUBCN; MIR9 | RUN domain and cysteine-rich domain containing, Beclin 1-inte | Multiple_C | 8.81 | 6.71 | 7.59 | 2.33 | 0.0112 | 0.0455 | 8.28 | 8.38 | 8.53 | 0.84 | 0.7917 | 0.9074 |
| TC0400008359.hg.1 | RPL34 | ribosomal protein L34 | Multiple_C | 18.45 | 18.43 | 17.23 | 2.33 | 3.19E-05 | 0.0004 | 13 | 12.96 | 12.56 | 1.36 | 0.0165 | 0.1071 |
| TC0600008655.hg.1 | TPBG | trophoblast glycoprotein | Multiple_C | 9.69 | 9.22 | 8.47 | 2.33 | 0.0338 | 0.1078 | 5.61 | 4.50 | 4.25 | 2.57 | 0.009 | 0.0727 |
| TC0600013537.hg.1 | RAET1G | retinoic acid early transcript 1G | Multiple_C | 6.52 | 5.05 | 5.3 | 2.33 | 0.0006 | 0.0046 | 4.21 | 3.98 | 4.31 | 0.93 | 0.4224 | 0.6752 |
| TC0600014098.hg.1 | ATAT1 | alpha tubulin acetyltransferase 1 | Multiple_C | 5.88 | 4.69 | 4.66 | 2.33 | 2.69E-05 | 0.0004 | 7.15 | 6.64 | 6.9 | 1.19 | 0.2389 | 0.5024 |
| TC0700008494.hg.1 | BUD31 | BUD31 homolog | Multiple_C | 12.05 | 11.96 | 10.83 | 2.33 | 0.0037 | 0.0191 | 8.6 | 8.12 | 8.94 | 0.79 | 0.0558 | 0.222 |
| TC0900011819.hg.1 | TSC1 | tuberous sclerosis 1 | Multiple_C | 10.84 | 9.62 | 9.62 | 2.33 | 0.001 | 0.0067 | 11.21 | 10.30 | 10.45 | 1.69 | 0.0399 | 0.183 |
| TC0X00007529.hg.1 | IGBP1 | immunoglobulin (CD79A) binding protein 1 | Multiple_C | 12.77 | 13.21 | 11.55 | 2.33 | 0.006 | 0.0278 | 9.84 | 9.93 | 9.94 | 0.93 | 0.5206 | 0.7478 |
| TC1200011911.hg.1 | PPTC7 | PTC7 protein phosphatase homolog | Multiple_C | 13.25 | 12.23 | 12.03 | 2.33 | 0.001 | 0.0068 | 10.08 | 9.48 | 10.25 | 0.89 | 0.0859 | 0.2824 |
| TC1300006520.hg.1 | SAP18 | Sin3A associated protein 18kDa | Multiple_C | 15.11 | 14.11 | 13.89 | 2.33 | 0.0096 | 0.0404 | 10.89 | 10.96 | 10.57 | 1.25 | 0.7393 | 0.8802 |
| TC1300007794.hg.1 | FARP1; MIR31 | FERM, ARH/RhoGEF and pleckstrin domain protein 1; microRNA | Multiple_C | 11.32 | 10.39 | 10.1 | 2.33 | 0.0005 | 0.0036 | 11.55 | 11.97 | 11.37 | 1.13 | 0.4089 | 0.6642 |
| TC1400010741.hg.1 | RPS29; RPL32I | ribosomal protein S29; ribosomal protein L32 pseudogene 29 | Multiple_C | 16.71 | 16.87 | 15.49 | 2.33 | 0.0016 | 0.0097 | 13.85 | 13.65 | 13.07 | 1.72 | 0.0042 | 0.044 |
| TC1600010955.hg.1 | MAFTRR | MAF transcriptional regulator RNA | Multiple_C | 5.21 | 4.3 | 3.99 | 2.33 | 0.0035 | 0.0183 | 4.3 | 4.38 | 4.48 | 0.88 | 0.8763 | 0.947 |
| TC1700006646.hg.1 | MINK1 | misshapen-like kinase 1 | Multiple_C | 10.25 | 9.75 | 9.03 | 2.33 | 0.0009 | 0.0061 | 8.5 | 8.50 | 8.57 | 0.95 | 0.7265 | 0.8729 |
| TC1700009541.hg.1 | CAMTA2 | calmodulin binding transcription activator 2 | Multiple_C | 10.42 | 8.45 | 9.2 | 2.33 | 0.0003 | 0.0027 | 9.31 | 9.40 | 9.35 | 0.97 | 0.9438 | 0.9771 |
| TC1700012246.hg.1 | STARD3 | StAR-related lipid transfer domain containing 3 | Multiple_C | 8.98 | 7.38 | 7.76 | 2.33 | 0.019 | 0.0688 | 7.93 | 8.31 | 7.67 | 1.20 | 0.4224 | 0.6752 |
| TSUnmapped00000374.I | DUSP16 | dual specificity phosphatase 16 | Coding | 9.33 | 8.38 | 8.11 | 2.33 | 0.0022 | 0.0125 | 8.9 | 8.23 | 8.49 | 1.33 | 0.0556 | 0.2215 |

| | | | | | | | | | | | | | | | |
|---|---|---|---|---|---|---|---|---|---|---|---|---|---|---|---|
| TSUnmapped00000398.I | KAT6B | K(lysine) acetyltransferase 6B | Coding | 7.75 | 6.12 | 6.53 | 2.33 | 0.0458 | 0.1354 | 9.3 | 8.68 | 9.14 | 1.12 | 0.6691 | 0.8382 |
| TC0200007261.hg.1 | QPCT | glutaminyl-peptide cyclotransferase | Multiple_C | 6.16 | 6.36 | 4.95 | 2.31 | 0.0045 | 0.022 | 7.19 | 7.87 | 8.19 | 0.50 | 0.002 | 0.0264 |
| TC0300007278.hg.1 | NBEAL2 | neurobeachin like 2 | Multiple_C | 10.7 | 9.88 | 9.49 | 2.31 | 0.0064 | 0.0292 | 6.77 | 6.27 | 6.43 | 1.27 | 0.0672 | 0.247 |
| TC0700008556.hg.1 | PCOLCE | procollagen C-endopeptidase enhancer | Multiple_C | 8.41 | 6.92 | 7.2 | 2.31 | 0.0004 | 0.003 | 7.49 | 7.69 | 7.41 | 1.06 | 0.8972 | 0.9566 |
| TC0700008875.hg.1 | MET | MET proto-oncogene, receptor tyrosine kinase | Multiple_C | 13.13 | 11.84 | 11.92 | 2.31 | 0.0002 | 0.0019 | 11.18 | 11.24 | 12.73 | 0.34 | 9.00E-07 | 0.0001 |
| TC0700010728.hg.1 | HERPUD2 | HERPUD family member 2 | Multiple_C | 11.58 | 10.06 | 10.37 | 2.31 | 5.65E-05 | 0.0007 | 9.3 | 8.75 | 8.95 | 1.27 | 0.1119 | 0.3294 |
| TC0700012182.hg.1 | NAMPT | nicotinamide phosphoribosyltransferase | Multiple_C | 11.94 | 11.06 | 10.73 | 2.31 | 0.0046 | 0.0227 | 9.46 | 8.57 | 9.64 | 0.88 | 0.344 | 0.6076 |
| TC0X00007040.hg.1 | GPR82 | G protein-coupled receptor 82 | Multiple_C | 4.62 | 3.24 | 3.41 | 2.31 | 0.0013 | 0.0081 | 4.79 | 4.60 | 4.41 | 1.30 | 0.1984 | 0.4533 |
| TC0X00007325.hg.1 | RIBC1 | RIB43A domain with coiled-coils 1 | Multiple_C | 7.5 | 5.99 | 6.29 | 2.31 | 0.0018 | 0.0108 | 5.63 | 5.95 | 5.96 | 0.80 | 0.4802 | 0.7187 |
| TC0X00011198.hg.1 | DNASE1L1 | deoxyribonuclease I-like 1 | Multiple_C | 9.14 | 8.41 | 7.93 | 2.31 | 0.0001 | 0.0014 | 7.25 | 6.78 | 7.06 | 1.14 | 0.5186 | 0.7464 |
| TC1000007761.hg.1 | ARID5B | AT rich interactive domain 5B (MRF1-like) | Multiple_C | 10.74 | 9.17 | 9.53 | 2.31 | 0.0029 | 0.0157 | 9.91 | 9.65 | 9.78 | 1.09 | 0.6347 | 0.8208 |
| TC1100012722.hg.1 | CDON | cell adhesion associated, oncogene regulated | Multiple_C | 7.56 | 6.93 | 6.35 | 2.31 | 0.0014 | 0.0087 | 6.95 | 5.84 | 6.58 | 1.29 | 0.1331 | 0.3621 |
| TC1200008726.hg.1 | TCP11L2 | t-complex 11, testis-specific-like 2 | Multiple_C | 10.07 | 10.5 | 8.86 | 2.31 | 0.0072 | 0.0322 | 7.66 | 6.77 | 6.43 | 2.35 | 0.0054 | 0.0514 |
| TC1200012760.hg.1 | PRR4 | proline rich 4 (lacrimal) | Multiple_C | 6.65 | 5.97 | 5.44 | 2.31 | 0.0016 | 0.0096 | 5.82 | 5.79 | 5.71 | 1.08 | 0.124 | 0.3478 |
| TC1500007645.hg.1 | MAP2K5 | mitogen-activated protein kinase kinase 5 | Multiple_C | 6.97 | 6.45 | 5.76 | 2.31 | 0.0035 | 0.0181 | 5.2 | 5.01 | 4.99 | 1.16 | 0.4674 | 0.7088 |
| TC1600011353.hg.1 | NPIPA3 | nuclear pore complex interacting protein family, member A3 | Multiple_C | 10.2 | 9.05 | 8.99 | 2.31 | 0.0001 | 0.0014 | 10.95 | 10.61 | 10.52 | 1.35 | 0.0426 | 0.1904 |
| TC1700006775.hg.1 | CHD3 | chromodomain helicase DNA binding protein 3 | Multiple_C | 9.16 | 8.34 | 7.95 | 2.31 | 0.0248 | 0.0848 | 7.08 | 7.52 | 7.31 | 0.85 | 0.0567 | 0.224 |
| TC1700007419.hg.1 | TAOK1; MIR45 | TAO kinase 1; microRNA 4523 | Multiple_C | 11.57 | 10.67 | 10.36 | 2.31 | 8.12E-05 | 0.0009 | 11.6 | 10.90 | 11.17 | 1.35 | 0.1902 | 0.4429 |
| TC1900006499.hg.1 | ARID3A | AT rich interactive domain 3A (BRIGHT-like) | Multiple_C | 10.44 | 9.29 | 9.23 | 2.31 | 0.0008 | 0.0057 | 11.47 | 11.85 | 11.57 | 0.93 | 0.7352 | 0.8777 |
| TC1900006848.hg.1 | CAMSAP3 | calmodulin regulated spectrin-associated protein family, memb | Multiple_C | 7.23 | 5.7 | 6.02 | 2.31 | 0.0063 | 0.029 | 5.65 | 6.61 | 6.97 | 0.40 | 3.53E-05 | 0.0017 |
| TC1900011328.hg.1 | ZNF160 | Transcript Identified by AceView, Entrez Gene ID(s) 90338 | Coding | 15.33 | 13.21 | 14.12 | 2.31 | 0.0003 | 0.0022 | 14.79 | 14.57 | 14.84 | 0.97 | 0.7507 | 0.8866 |
| TC2000007251.hg.1 | MYL9 | myosin light chain 9 | Multiple_C | 9.52 | 7.61 | 8.31 | 2.31 | 9.46E-05 | 0.001 | 9.8 | 9.30 | 9.33 | 1.39 | 0.0151 | 0.1008 |
| TC2000009440.hg.1 | ADNP | activity-dependent neuroprotector homeobox | Multiple_C | 12.96 | 11.55 | 11.75 | 2.31 | 8.61E-05 | 0.0009 | 13.74 | 13.58 | 13.9 | 0.90 | 0.5734 | 0.7811 |
| TC0200008062.hg.1 | ACTG2 | actin, gamma 2, smooth muscle, enteric | Multiple_C | 7.49 | 6.2 | 6.29 | 2.30 | 2.74E-05 | 0.0004 | 6.63 | 6.62 | 6.26 | 1.29 | 0.0117 | 0.086 |
| TC0200008632.hg.1 | RPL31 | ribosomal protein L31 | Multiple_C | 17.5 | 17.92 | 16.3 | 2.30 | 3.19E-05 | 0.0004 | 14.19 | 13.79 | 13.56 | 1.55 | 0.0019 | 0.0258 |
| TC0200010907.hg.1 | ACSL3 | acyl-CoA synthetase long-chain family member 3 | Multiple_C | 12.95 | 12.55 | 11.75 | 2.30 | 0.0248 | 0.0847 | 10.24 | 9.72 | 10.4 | 0.90 | 0.6358 | 0.8212 |
| TC0200014029.hg.1 | C2orf76 | chromosome 2 open reading frame 76 | Multiple_C | 8.28 | 7.38 | 7.08 | 2.30 | 0.0016 | 0.0097 | 6.14 | 5.67 | 5.66 | 1.39 | 0.0792 | 0.2712 |
| TC0200016595.hg.1 | RPL37A | ribosomal protein L37a | Multiple_C | 17.75 | 16.78 | 16.55 | 2.30 | 6.10E-05 | 0.0007 | 14.81 | 14.54 | 14.36 | 1.37 | 0.0402 | 0.1838 |
| TC0400007848.hg.1 | MTHFD2L | methylenetetrahydrofolate dehydrogenase (NADP+ dependent) | Multiple_C | 6.75 | 6.07 | 5.55 | 2.30 | 0.0003 | 0.0029 | 6.29 | 5.25 | 5.6 | 1.61 | 0.088 | 0.2864 |
| TC0400008331.hg.1 | NPNT | nephronectin | Multiple_C | 10.88 | 9.17 | 9.68 | 2.30 | 0.0004 | 0.0031 | 3.75 | 3.31 | 3.27 | 1.39 | 0.0268 | 0.143 |
| TC0500013387.hg.1 | RPS14 | ribosomal protein S14 | Multiple_C | 15.27 | 14.63 | 14.07 | 2.30 | 0.0017 | 0.0101 | 13.42 | 13.70 | 12.77 | 1.57 | 0.0053 | 0.0508 |
| TC0700011550.hg.1 | POM121C | POM121 transmembrane nucleoporin C | Multiple_C | 12.62 | 10.33 | 11.42 | 2.30 | 0.0001 | 0.0013 | 14.42 | 14.88 | 15.1 | 0.62 | 0.0219 | 0.1271 |
| TC0700013382.hg.1 | KCTD7 | potassium channel tetramerization domain containing 7 | Multiple_C | 6.28 | 4.83 | 5.08 | 2.30 | 0.0002 | 0.0017 | 6.36 | 6.77 | 6.73 | 0.77 | 0.1419 | 0.3746 |

| | | | | | | | | | | | | | | | |
|---|---|---|---|---|---|---|---|---|---|---|---|---|---|---|---|
| TC0800008279.hg.1 | PLEKHF2 | pleckstrin homology domain containing, family F (with FYVE dor | Multiple_C | 10.21 | 9.6 | 9.01 | 2.30 | 0.003 | 0.016 | 7.27 | 6.74 | 7.8 | 0.69 | 0.0046 | 0.0467 |
| TC0900012167.hg.1 | GSN | gelsolin | Multiple_C | 11.21 | 10.19 | 10.01 | 2.30 | 0.0082 | 0.0357 | 8.55 | 7.42 | 6.26 | 4.89 | 2.54E-06 | 0.0003 |
| TC1100010274.hg.1 | SPTY2D1 | SPT2 chromatin protein domain containing 1 | Multiple_C | 9.87 | 8.89 | 8.67 | 2.30 | 6.92E-05 | 0.0008 | 9.25 | 8.77 | 9.43 | 0.88 | 0.9849 | 0.9937 |
| TC1300006974.hg.1 | NAA16 | N(alpha)-acetyltransferase 16, NatA auxiliary subunit | Multiple_C | 10.47 | 9.01 | 9.27 | 2.30 | 0.0006 | 0.0045 | 9.1 | 8.61 | 8.87 | 1.17 | 0.2293 | 0.4905 |
| TC1400010041.hg.1 | IFI27L2 | interferon, alpha-inducible protein 27-like 2 | Multiple_C | 10.69 | 9.27 | 9.49 | 2.30 | 0.0001 | 0.0014 | 8.02 | 8.31 | 8.11 | 0.94 | 0.639 | 0.823 |
| TC1500009623.hg.1 | BNIP2 | BCL2/adenovirus E1B 19kDa interacting protein 2 | Multiple_C | 9.42 | 9.36 | 8.22 | 2.30 | 0.0001 | 0.0013 | 9.37 | 8.48 | 8.79 | 1.49 | 0.0202 | 0.1214 |
| TC1600008938.hg.1 | AXIN1 | axin 1 | Multiple_C | 8.55 | 7.24 | 7.35 | 2.30 | 0.0005 | 0.0037 | 8.37 | 8.57 | 8.55 | 0.88 | 0.1074 | 0.3223 |
| TC1700008082.hg.1 | MAPT | microtubule associated protein tau | Multiple_C | 8.07 | 6.97 | 6.87 | 2.30 | 0.0001 | 0.0012 | 5.63 | 5.26 | 5.15 | 1.39 | 0.0334 | 0.1643 |
| TSUnmapped00000353.I | DUSP16 | dual specificity phosphatase 16 | Coding | 8.85 | 8.2 | 7.65 | 2.30 | 0.0001 | 0.0011 | 8.09 | 7.21 | 7.54 | 1.46 | 0.0218 | 0.1268 |
| TSUnmapped00000422.I | VPS11 | VPS11, CORVET/HOPS core subunit [Source:HGNC Symbol;Acc:I | Coding | 5.65 | 4.52 | 4.45 | 2.30 | 0.01 | 0.0418 | 5.84 | 5.76 | 5.66 | 1.13 | 0.6856 | 0.8495 |
| TC0100016833.hg.1 | ZBTB41 | zinc finger and BTB domain containing 41 | Multiple_C | 8.6 | 8.11 | 7.41 | 2.28 | 0.0002 | 0.002 | 8.03 | 6.63 | 7.42 | 1.53 | 0.002 | 0.0268 |
| TC0200015424.hg.1 | ALS2CR12 | amyotrophic lateral sclerosis 2 chromosome region candidate 1 | Multiple_C | 4.78 | 3.33 | 3.59 | 2.28 | 0.0012 | 0.0076 | 4.46 | 4.71 | 4.84 | 0.77 | 0.2128 | 0.4706 |
| TC0500007804.hg.1 | HMGCR | 3-hydroxy-3-methylglutaryl-CoA reductase | Multiple_C | 13.04 | 13.43 | 11.85 | 2.28 | 0.0006 | 0.0042 | 11.25 | 10.87 | 11.45 | 0.87 | 0.0617 | 0.2361 |
| TC0500007890.hg.1 | PAPD4 | PAP associated domain containing 4 | Multiple_C | 10.81 | 10.43 | 9.62 | 2.28 | 0.0003 | 0.0024 | 10.12 | 9.53 | 9.65 | 1.39 | 0.0021 | 0.0278 |
| TC0500008540.hg.1 | PHAX | phosphorylated adaptor for RNA export | Multiple_C | 12.56 | 12.02 | 11.37 | 2.28 | 0.0082 | 0.0355 | 12.43 | 12.03 | 12.5 | 0.95 | 0.7924 | 0.9077 |
| TC0500008854.hg.1 | ANKHD1; EIF4 | ankyrin repeat and KH domain containing 1; eukaryotic translati | Multiple_C | 13.41 | 12.73 | 12.22 | 2.28 | 8.11E-05 | 0.0009 | 11 | 10.35 | 11 | 1.00 | 0.5754 | 0.7822 |
| TC0600009789.hg.1 | TAB2 | TGF-beta activated kinase 1/MAP3K7 binding protein 2 | Multiple_C | 11.87 | 10.66 | 10.68 | 2.28 | 0.0004 | 0.0033 | 10.82 | 10.95 | 10.79 | 1.02 | 0.9763 | 0.9903 |
| TC0700013623.hg.1 | KCP | kielin/chordin-like protein | Multiple_C | 5.86 | 4.52 | 4.67 | 2.28 | 0.0051 | 0.0243 | 4.98 | 4.81 | 4.77 | 1.16 | 0.4507 | 0.6963 |
| TC0900012157.hg.1 | AKAP2 | A kinase (PRKA) anchor protein 2 | Multiple_C | 6.03 | 4.48 | 4.84 | 2.28 | 0.0009 | 0.0064 | 8.81 | 8.31 | 9.26 | 0.73 | 0.0283 | 0.1482 |
| TC1100008541.hg.1 | AAMDC | adipogenesis associated, Mth938 domain containing | Multiple_C | 9.78 | 8.99 | 8.59 | 2.28 | 0.0017 | 0.01 | 6.17 | 6.40 | 6.03 | 1.10 | 0.0575 | 0.2261 |
| TC1100011054.hg.1 | RAB3IL1 | RAB3A interacting protein (rabin3)-like 1 | Multiple_C | 9.17 | 7.78 | 7.98 | 2.28 | 0.0128 | 0.0506 | 8.25 | 8.04 | 8.27 | 0.99 | 0.7694 | 0.8966 |
| TC1700008102.hg.1 | LRRC37A2 | leucine rich repeat containing 37, member A2 | Multiple_C | 8.18 | 6.77 | 6.99 | 2.28 | 0.0142 | 0.0548 | 8.4 | 8.34 | 8.42 | 0.99 | 0.1929 | 0.4461 |
| TC1700008938.hg.1 | UBALD2 | UBA-like domain containing 2 | Multiple_C | 9.9 | 8.19 | 8.71 | 2.28 | 0.0016 | 0.0095 | 10.21 | 10.69 | 10.08 | 1.09 | 0.6078 | 0.8039 |
| TC2200007468.hg.1 | TEF | thyrotrophic embryonic factor | Multiple_C | 4.77 | 4.18 | 3.58 | 2.28 | 0.0002 | 0.0016 | 5.3 | 5.95 | 5.62 | 0.80 | 0.1433 | 0.3767 |
| TC2200008475.hg.1 | PIK3IP1 | phosphoinositide-3-kinase interacting protein 1 | Multiple_C | 5.38 | 4.1 | 4.19 | 2.28 | 0.0045 | 0.022 | 4.67 | 4.38 | 4.31 | 1.28 | 0.3071 | 0.5713 |
| TC2200008734.hg.1 | CBX7 | chromobox homolog 7 | Multiple_C | 7.32 | 6.22 | 6.13 | 2.28 | 0.0042 | 0.0209 | 6.02 | 5.86 | 5.84 | 1.13 | 0.4683 | 0.7093 |
| TC0100010903.hg.1 | RNF2 | ring finger protein 2 | Multiple_C | 11.05 | 10.15 | 9.87 | 2.27 | 5.88E-05 | 0.0007 | 8.85 | 8.44 | 8.53 | 1.25 | 0.1548 | 0.3946 |
| TC0100011219.hg.1 | PPP1R12B | protein phosphatase 1, regulatory subunit 12B | Multiple_C | 6.8 | 5.57 | 5.62 | 2.27 | 0.0003 | 0.0029 | 5.49 | 5.29 | 5.66 | 0.89 | 0.3267 | 0.5903 |
| TC0100013636.hg.1 | RNF19B | ring finger protein 19B | Multiple_C | 11.31 | 10.28 | 10.13 | 2.27 | 0.0001 | 0.0012 | 6.03 | 6.25 | 7.07 | 0.49 | 3.76E-05 | 0.0018 |
| TC0100016709.hg.1 | TPR | translocated promoter region, nuclear basket protein | Multiple_C | 11.58 | 10.72 | 10.4 | 2.27 | 0.0068 | 0.0308 | 10.59 | 10.32 | 10.58 | 1.01 | 0.9431 | 0.9767 |
| TC0200012318.hg.1 | CDKL4 | cyclin-dependent kinase-like 4 | Multiple_C | 5.58 | 5.27 | 4.4 | 2.27 | 0.0013 | 0.0081 | 4.98 | 4.68 | 4.37 | 1.53 | 0.0558 | 0.222 |
| TC0200016484.hg.1 | RGPD2; RGPD | RANBP2-like and GRIP domain containing 2; RANBP2-like and G | Multiple_C | 5.13 | 3.95 | 3.95 | 2.27 | 0.0458 | 0.1357 | 6.76 | 5.68 | 6.03 | 1.66 | 0.0473 | 0.2017 |
| TC0300008017.hg.1 | ZNF654 | zinc finger protein 654 | Coding | 8.75 | 7.55 | 7.57 | 2.27 | 0.0008 | 0.0057 | 7.79 | 7.33 | 7.45 | 1.27 | 0.7405 | 0.8807 |

| | | | | | | | | | | | | | | | |
|---|---|---|---|---|---|---|---|---|---|---|---|---|---|---|---|
| TC0300009035.hg.1 | TRPC1 | transient receptor potential cation channel, subfamily C, memb | Multiple_C | 6.87 | 6.5 | 5.69 | 2.27 | 0.0083 | 0.036 | 5.64 | 5.90 | 6.24 | 0.66 | 0.3137 | 0.5778 |
| TC0300010968.hg.1 | SETD2 | SET domain containing 2 | Multiple_C | 8.93 | 7.63 | 7.75 | 2.27 | 7.72E-06 | 0.0001 | 8.58 | 8.44 | 8.87 | 0.82 | 0.0311 | 0.1571 |
| TC0300011899.hg.1 | CBLB | Cbl proto-oncogene B, E3 ubiquitin protein ligase | Multiple_C | 10.57 | 9.48 | 9.39 | 2.27 | 0.0003 | 0.0024 | 7.37 | 6.50 | 6.61 | 1.69 | 0.0023 | 0.0296 |
| TC0300012166.hg.1 | HCLS1 | hematopoietic cell-specific Lyn substrate 1 | Multiple_C | 5.13 | 3.8 | 3.95 | 2.27 | 0.0008 | 0.0058 | 3.94 | 3.87 | 3.92 | 1.01 | 0.8389 | 0.9292 |
| TC0400009694.hg.1 | ATP5I | ATP synthase, H+ transporting, mitochondrial Fo complex subur | Multiple_C | 12.45 | 11.91 | 11.27 | 2.27 | 0.0001 | 0.0011 | 9.76 | 10.07 | 9.38 | 1.30 | 0.2569 | 0.5218 |
| TC0600011714.hg.1 | BTBD9 | BTB (POZ) domain containing 9 | Multiple_C | 8.94 | 8.21 | 7.76 | 2.27 | 0.0052 | 0.0249 | 7.42 | 6.47 | 6.41 | 2.01 | 0.0796 | 0.2719 |
| TC0700010899.hg.1 | POLR2J4 | polymerase (RNA) II (DNA directed) polypeptide J4, pseudogene | Multiple_C | 6 | 5.71 | 4.82 | 2.27 | 0.0049 | 0.0237 | 6.36 | 6.16 | 6.18 | 1.13 | 0.2753 | 0.5414 |
| TC0800011216.hg.1 | STK3 | serine/threonine kinase 3 | Multiple_C | 11.47 | 11.21 | 10.29 | 2.27 | 8.30E-05 | 0.0009 | 10.41 | 9.19 | 10.12 | 1.22 | 0.2194 | 0.4793 |
| TC0X00007574.hg.1 | ACRC | acidic repeat containing | Multiple_C | 5.75 | 4.51 | 4.57 | 2.27 | 0.0008 | 0.0055 | 6.18 | 6.29 | 6.27 | 0.94 | 0.5505 | 0.7665 |
| TC0X00008798.hg.1 | SSR4 | signal sequence receptor, delta | Multiple_C | 12.8 | 11.28 | 11.62 | 2.27 | 0.0033 | 0.0172 | 8.74 | 8.99 | 9.3 | 0.68 | 0.027 | 0.1436 |
| TC1400006457.hg.1 | POTEM; POTE | POTE ankyrin domain family, member M; POTE ankyrin domain | Coding | 5.7 | 5.18 | 4.52 | 2.27 | 0.041 | 0.1248 | 4.54 | 4.33 | 4.32 | 1.16 | 0.9662 | 0.9858 |
| TC1500008023.hg.1 | ZFAND6 | zinc finger, AN1-type domain 6 | Multiple_C | 14.24 | 14.23 | 13.06 | 2.27 | 0.0007 | 0.0049 | 10.9 | 10.77 | 11.15 | 0.84 | 0.4958 | 0.7296 |
| TC1700010997.hg.1 | HOXB9 | homeobox B9 | Multiple_C | 6.06 | 5.02 | 4.88 | 2.27 | 0.0045 | 0.0222 | 8.49 | 7.99 | 8.62 | 0.91 | 0.1816 | 0.4318 |
| TC1900006602.hg.1 | ZNF555 | zinc finger protein 555 | Coding | 10.7 | 9.98 | 9.52 | 2.27 | 0.0003 | 0.0026 | 8.19 | 7.43 | 7.84 | 1.27 | 0.031 | 0.1567 |
| TC0100015580.hg.1 | NBPF20 | neuroblastoma breakpoint family, member 20 | Coding | 10.86 | 10 | 9.69 | 2.25 | 0.0032 | 0.0168 | 10.11 | 9.75 | 9.84 | 1.21 | 0.7365 | 0.8786 |
| TC0100018273.hg.1 | ATP1A1 | ATPase, Na+/K+ transporting, alpha 1 polypeptide | Multiple_C | 15.89 | 14.62 | 14.72 | 2.25 | 0.0002 | 0.002 | 14.57 | 14.19 | 14.82 | 0.84 | 0.0209 | 0.1234 |
| TC0200010837.hg.1 | ANKZF1 | ankyrin repeat and zinc finger domain containing 1 | Multiple_C | 10.73 | 9.65 | 9.56 | 2.25 | 0.0074 | 0.0331 | 9.17 | 8.57 | 8.33 | 1.79 | 0.3734 | 0.6341 |
| TC0200015388.hg.1 | KCTD18 | potassium channel tetramerization domain containing 18 | Multiple_C | 8.61 | 8.3 | 7.44 | 2.25 | 0.0126 | 0.0502 | 7.52 | 8.01 | 7.14 | 1.30 | 0.0586 | 0.2283 |
| TC0200016662.hg.1 | CALM2 | calmodulin 2 (phosphorylase kinase, delta) | Multiple_C | 16 | 16.51 | 14.83 | 2.25 | 0.0004 | 0.0029 | 14.29 | 13.78 | 14.08 | 1.16 | 0.0425 | 0.1902 |
| TC0400006637.hg.1 | ZBTB49 | zinc finger and BTB domain containing 49 | Multiple_C | 5.4 | 4.36 | 4.23 | 2.25 | 0.0039 | 0.0198 | 5.74 | 5.49 | 5.23 | 1.42 | 0.0183 | 0.1141 |
| TC0400008175.hg.1 | PDLIM5 | PDZ and LIM domain 5 | Multiple_C | 13.23 | 11.24 | 12.06 | 2.25 | 0.0001 | 0.0011 | 12.81 | 12.33 | 12.67 | 1.10 | 0.6076 | 0.8038 |
| TC1200011599.hg.1 | LTA4H | leukotriene A4 hydrolase | Multiple_C | 15.46 | 15.74 | 14.29 | 2.25 | 0.0134 | 0.0524 | 13.59 | 12.79 | 12.55 | 2.06 | 0.0409 | 0.1856 |
| TC1400007466.hg.1 | FUT8 | fucosyltransferase 8 (alpha (1,6) fucosyltransferase) | Multiple_C | 12.58 | 12.42 | 11.41 | 2.25 | 0.0001 | 0.0013 | 10.25 | 9.56 | 10.99 | 0.60 | 0.0002 | 0.005 |
| TC1500007866.hg.1 | PPCDC | phosphopantothenoylcysteine decarboxylase | Multiple_C | 10.24 | 9.14 | 9.07 | 2.25 | 0.0002 | 0.002 | 7.19 | 7.14 | 6.94 | 1.19 | 0.7637 | 0.8932 |
| TC1600006842.hg.1 | C16orf72 | chromosome 16 open reading frame 72 | Multiple_C | 10.85 | 9.48 | 9.68 | 2.25 | 0.0003 | 0.0022 | 10.12 | 10.12 | 10.32 | 0.87 | 0.0523 | 0.2136 |
| TC1700007016.hg.1 | TRPV2 | transient receptor potential cation channel, subfamily V, memb | Multiple_C | 6.21 | 4.89 | 5.04 | 2.25 | 0.0006 | 0.0043 | 6.98 | 6.95 | 7.06 | 0.95 | 0.5625 | 0.7731 |
| TC1700011208.hg.1 | TRIM25; MIR3 | tripartite motif containing 25; microRNA 3614 | Multiple_C | 11.28 | 8.79 | 10.11 | 2.25 | 0.0011 | 0.0071 | 10.7 | 10.35 | 11.55 | 0.55 | 0.0015 | 0.0222 |
| TC1900007150.hg.1 | CC2D1A | coiled-coil and C2 domain containing 1A | Multiple_C | 9.66 | 8.27 | 8.49 | 2.25 | 0.0122 | 0.0488 | 8.67 | 8.91 | 8.74 | 0.95 | 0.7294 | 0.8744 |
| TC1900008113.hg.1 | LTBP4 | latent transforming growth factor beta binding protein 4 | Multiple_C | 7.27 | 6.41 | 6.1 | 2.25 | 0.3095 | 0.5227 | 5.54 | 4.58 | 4.33 | 2.31 | 0.0004 | 0.0086 |
| TC2200009351.hg.1 | CSNK1E | casein kinase 1, epsilon | Coding | 13.29 | 12.27 | 12.12 | 2.25 | 0.0002 | 0.0021 | 12.06 | 11.62 | 11.99 | 1.05 | 0.4948 | 0.7288 |
| TC0100007552.hg.1 | SESN2 | sestrin 2 | Multiple_C | 9.73 | 7.77 | 8.57 | 2.23 | 0.0047 | 0.0231 | 6.41 | 6.18 | 6.85 | 0.74 | 0.1126 | 0.3306 |
| TC0100009908.hg.1 | ECM1 | extracellular matrix protein 1 | Multiple_C | 5.56 | 4 | 4.4 | 2.23 | 0.0006 | 0.0045 | 4.27 | 4.31 | 4.17 | 1.07 | 0.5652 | 0.7747 |
| TC0200010009.hg.1 | SCRN3 | secernin 3 | Multiple_C | 9.67 | 8.98 | 8.51 | 2.23 | 0.0078 | 0.0343 | 8.74 | 9.04 | 9.03 | 0.82 | 0.8871 | 0.9518 |

| | | | | | | | | | | | | | | | |
|---|---|---|---|---|---|---|---|---|---|---|---|---|---|---|---|
| TC0200012095.hg.1 | SUPT7L | SPT7-like STAGA complex gamma subunit | Multiple_C | 12.43 | 11.73 | 11.27 | 2.23 | 0.0006 | 0.0047 | 10.54 | 10.38 | 10.29 | 1.19 | 0.8113 | 0.9166 |
| TC0200014834.hg.1 | STK39 | serine threonine kinase 39 | Multiple_C | 13.08 | 12.25 | 11.92 | 2.23 | 0.0177 | 0.065 | 9.81 | 8.81 | 9.23 | 1.49 | 0.5719 | 0.7798 |
| TC0200015194.hg.1 | TFPI | tissue factor pathway inhibitor (lipoprotein-associated coagulat | Multiple_C | 11.13 | 10.46 | 9.97 | 2.23 | 0.0235 | 0.0814 | 5.98 | 4.78 | 5.81 | 1.13 | 0.2897 | 0.5543 |
| TC0600009628.hg.1 | HECA | hdc homolog, cell cycle regulator | Multiple_C | 10.57 | 9.75 | 9.41 | 2.23 | 8.20E-05 | 0.0009 | 8.22 | 8.04 | 7.86 | 1.28 | 0.0582 | 0.2275 |
| TC0600013898.hg.1 | FAM103A1 | Homo sapiens family with sequence similarity 103, member A1, | Multiple_C | 10.72 | 10.17 | 9.56 | 2.23 | 0.0199 | 0.0712 | 7.68 | 7.68 | 8.1 | 0.75 | 0.4221 | 0.6749 |
| TC0700013602.hg.1 | POLR2J3 | polymerase (RNA) II (DNA directed) polypeptide J3 | Multiple_C | 12.95 | 11.52 | 11.79 | 2.23 | 0.0011 | 0.0072 | 11.36 | 12.04 | 12.4 | 0.49 | 0.0021 | 0.0279 |
| TC0900008790.hg.1 | ZBTB43 | zinc finger and BTB domain containing 43 | Multiple_C | 8.03 | 6.9 | 6.87 | 2.23 | 0.0114 | 0.0463 | 6.16 | 5.59 | 6.6 | 0.74 | 0.1325 | 0.3613 |
| TC1000008798.hg.1 | SFR1 | SWI5-dependent homologous recombination repair protein 1 | Multiple_C | 12.09 | 11.22 | 10.93 | 2.23 | 0.0006 | 0.0045 | 7.58 | 7.65 | 7.73 | 0.90 | 0.58 | 0.7853 |
| TC1000011323.hg.1 | GLUD1 | glutamate dehydrogenase 1 | Multiple_C | 13.99 | 14.42 | 12.83 | 2.23 | 0.0008 | 0.0059 | 12.58 | 12.42 | 12.89 | 0.81 | 0.2102 | 0.4673 |
| TC1100008342.hg.1 | INPPL1 | inositol polyphosphate phosphatase-like 1 | Multiple_C | 9.18 | 8.14 | 8.02 | 2.23 | 0.0003 | 0.0024 | 9.97 | 9.94 | 9.46 | 1.42 | 0.0057 | 0.0534 |
| TC1100008985.hg.1 | ATM | ATM serine/threonine kinase | Multiple_C | 10.43 | 10.6 | 9.27 | 2.23 | 0.0017 | 0.0101 | 11.67 | 11.13 | 11.31 | 1.28 | 0.0164 | 0.1068 |
| TC1200007890.hg.1 | PIP4K2C | phosphatidylinositol-5-phosphate 4-kinase, type II, gamma | Multiple_C | 9.11 | 7.54 | 7.95 | 2.23 | 0.0014 | 0.0088 | 8.81 | 9.00 | 9.29 | 0.72 | 0.0581 | 0.2274 |
| TC1300008439.hg.1 | MTIF3 | mitochondrial translational initiation factor 3 | Multiple_C | 11.63 | 12.15 | 10.47 | 2.23 | 0.0276 | 0.0922 | 5.69 | 5.69 | 5.45 | 1.18 | 0.5873 | 0.7911 |
| TC1700008271.hg.1 | WFIKKN2 | WAP, follistatin/kazal, immunoglobulin, kunitz and netrin domai | Coding | 4.53 | 3.31 | 3.37 | 2.23 | 0.0015 | 0.0095 | 4.61 | 4.34 | 4.2 | 1.33 | 0.2168 | 0.4761 |
| TC1700008690.hg.1 | PITPNC1 | phosphatidylinositol transfer protein, cytoplasmic 1 | Multiple_C | 10.72 | 9.8 | 9.56 | 2.23 | 0.0039 | 0.0198 | 7.97 | 7.89 | 7.61 | 1.28 | 0.1412 | 0.3738 |
| TC1900011957.hg.1 | GSK3A | glycogen synthase kinase 3 alpha | Multiple_C | 10.1 | 8.1 | 8.94 | 2.23 | 0.0038 | 0.0194 | 9.4 | 10.14 | 10.63 | 0.43 | 1.57E-05 | 0.001 |
| TC2200008866.hg.1 | TCF20 | transcription factor 20 (AR1) | Multiple_C | 9.84 | 9.04 | 8.68 | 2.23 | 0.0005 | 0.0036 | 10.51 | 10.90 | 10.75 | 0.85 | 0.186 | 0.4378 |
| TC2200009337.hg.1 | GATSL3 | GATS protein-like 3 | Multiple_C | 6.3 | 5.05 | 5.14 | 2.23 | 0.0074 | 0.0329 | 4.92 | 4.65 | 4.42 | 1.41 | 0.09 | 0.2904 |
| TC0100015756.hg.1 | ARNT | aryl hydrocarbon receptor nuclear translocator | Multiple_C | 12.32 | 11.31 | 11.17 | 2.22 | 0.0048 | 0.0232 | 10.43 | 10.55 | 10.67 | 0.85 | 0.701 | 0.8583 |
| TC0100018568.hg.1 | ARID4B | AT rich interactive domain 4B (RBP1-like) | Multiple_C | 9.67 | 8.92 | 8.52 | 2.22 | 0.0456 | 0.135 | 8.88 | 8.12 | 8.53 | 1.27 | 0.2514 | 0.5155 |
| TC0200007184.hg.1 | BIRC6 | baculoviral IAP repeat containing 6 | Multiple_C | 10.64 | 10.2 | 9.49 | 2.22 | 0.0002 | 0.0021 | 9.69 | 9.42 | 9.74 | 0.97 | 0.7059 | 0.8617 |
| TC0200011195.hg.1 | AGAP1; noygl | Jeck2013 ALT_ACCEPTOR, ALT_DONOR, coding, INTERNAL, intro | Multiple_C | 5.45 | 4.19 | 4.3 | 2.22 | 0.0008 | 0.0057 | 4.07 | 3.78 | 3.88 | 1.14 | 0.0744 | 0.2619 |
| TC0200013875.hg.1 | RGPD8; RGPD | RANBP2-like and GRIP domain containing 8; RANBP2-like and G | Multiple_C | 10.47 | 9.75 | 9.32 | 2.22 | 0.0007 | 0.0051 | 9.85 | 8.63 | 9.61 | 1.18 | 0.2874 | 0.5523 |
| TC0300009451.hg.1 | MYNN | myoneurin | Multiple_C | 10.51 | 10.55 | 9.36 | 2.22 | 0.0007 | 0.0049 | 9.15 | 7.97 | 8.73 | 1.34 | 0.1115 | 0.3291 |
| TC0300009724.hg.1 | VPS8 | vacuolar protein sorting 8 homolog (S. cerevisiae) | Multiple_C | 8.17 | 8.54 | 7.02 | 2.22 | 0.0076 | 0.0336 | 9.39 | 8.58 | 9.25 | 1.10 | 0.4479 | 0.6949 |
| TC0400011130.hg.1 | PAQR3 | progestin and adipoQ receptor family member III | Multiple_C | 12.34 | 11.21 | 11.19 | 2.22 | 0.0009 | 0.0062 | 11.3 | 9.52 | 10.79 | 1.42 | 0.1428 | 0.3759 |
| TC0400012810.hg.1 | MOB1B | MOB kinase activator 1B | Multiple_C | 9.08 | 8.3 | 7.93 | 2.22 | 0.0004 | 0.0031 | 8.31 | 7.71 | 7.98 | 1.26 | 0.124 | 0.3477 |
| TC1000006904.hg.1 | STAM | signal transducing adaptor molecule (SH3 domain and ITAM mo | Multiple_C | 12.22 | 10.86 | 11.07 | 2.22 | 0.0012 | 0.0078 | 9.9 | 8.69 | 10.22 | 0.80 | 0.2871 | 0.5523 |
| TC1000008984.hg.1 | FAM160B1 | family with sequence similarity 160, member B1 | Multiple_C | 8.43 | 7.25 | 7.28 | 2.22 | 0.0002 | 0.0017 | 7.48 | 6.98 | 7.34 | 1.10 | 0.3416 | 0.6053 |
| TC1500007986.hg.1 | MORF4L1 | mortality factor 4 like 1 | Multiple_C | 14.75 | 14.29 | 13.6 | 2.22 | 0.0002 | 0.0016 | 13.33 | 13.09 | 12.87 | 1.38 | 0.0299 | 0.1533 |
| TC1600007262.hg.1 | TNRC6A | trinucleotide repeat containing 6A | Multiple_C | 10.39 | 8.73 | 9.24 | 2.22 | 0.0003 | 0.0023 | 11.41 | 10.75 | 11.41 | 1.00 | 0.9738 | 0.9896 |
| TC1600009134.hg.1 | PRSS27 | protease, serine 27 | Multiple_C | 8.03 | 6.44 | 6.88 | 2.22 | 0.0004 | 0.0029 | 7.79 | 7.58 | 7.46 | 1.26 | 0.431 | 0.6826 |
| TC1700012481.hg.1 | TMC6 | transmembrane channel like 6 | Multiple_C | 9.63 | 8.2 | 8.48 | 2.22 | 0.0014 | 0.0086 | 8.61 | 8.78 | 8.24 | 1.29 | 0.5823 | 0.7869 |

| | | | | | | | | | | | | | | | |
|---|---|---|---|---|---|---|---|---|---|---|---|---|---|---|---|
| TC1900009368.hg.1 | TICAM1 | toll-like receptor adaptor molecule 1 | Coding | 5.67 | 4.32 | 4.52 | 2.22 | 0.0079 | 0.0347 | 4.92 | 4.98 | 5.03 | 0.93 | 0.5947 | 0.7953 |
| TC2000009882.hg.1 | PTPRA; VPS16 | protein tyrosine phosphatase, receptor type, A; vacuolar protei | Multiple_C | 12.13 | 10.82 | 10.98 | 2.22 | 0.0009 | 0.0064 | 13.54 | 13.15 | 13.21 | 1.26 | 0.2141 | 0.4725 |
| TC2000009912.hg.1 | CNBD2 | cyclic nucleotide binding domain containing 2 | Coding | 6.84 | 6.15 | 5.69 | 2.22 | 0.0008 | 0.0055 | 4.97 | 5.24 | 4.46 | 1.42 | 0.0816 | 0.2753 |
| TC2100007999.hg.1 | DNAJC28 | DnaJ (Hsp40) homolog, subfamily C, member 28 | Coding | 7.18 | 7.04 | 6.03 | 2.22 | 0.0105 | 0.0433 | 5.73 | 6.01 | 5.28 | 1.37 | 0.1105 | 0.3274 |
| TC2200007324.hg.1 | MICALL1 | MICAL-like 1 | Multiple_C | 7.76 | 6.65 | 6.61 | 2.22 | 0.0006 | 0.0046 | 7.69 | 7.95 | 7.79 | 0.93 | 0.5079 | 0.7384 |
| TC2200009353.hg.1 | SUN2 | Sad1 and UNC84 domain containing 2 | Multiple_C | 6.8 | 5.59 | 5.65 | 2.22 | 0.0125 | 0.0497 | 6.62 | 6.63 | 6.34 | 1.21 | 0.4114 | 0.666 |
| TSUnmapped00000054.h | ZNF780B | zinc finger protein 780B | Coding | 7.46 | 6.31 | 6.31 | 2.22 | 0.0172 | 0.0636 | 6.75 | 6.90 | 6.95 | 0.87 | 0.8215 | 0.9211 |
| TSUnmapped00000088.h | DUSP16 | dual specificity phosphatase 16 | Coding | 8.01 | 7.52 | 6.86 | 2.22 | 0.0005 | 0.0036 | 6.63 | 6.11 | 6.44 | 1.14 | 0.1335 | 0.3628 |
| TSUnmapped00000177.h | ZNF780B | zinc finger protein 780B | Coding | 7.46 | 6.31 | 6.31 | 2.22 | 0.0172 | 0.0636 | 6.75 | 6.90 | 6.95 | 0.87 | 0.8215 | 0.9211 |
| TC0100010060.hg.1 | RPS27 | ribosomal protein S27 | Multiple_C | 18.41 | 17.58 | 17.27 | 2.20 | 0.0019 | 0.0113 | 13.78 | 13.61 | 13.56 | 1.16 | 0.4615 | 0.7043 |
| TC0100018243.hg.1 | LINC01140 | long intergenic non-protein coding RNA 1140 | Multiple_C | 4.31 | 3.07 | 3.17 | 2.20 | 0.0064 | 0.0292 | 3.6 | 3.66 | 3.35 | 1.19 | 0.0749 | 0.2631 |
| TC0200009091.hg.1 | RALB | v-ral simian leukemia viral oncogene homolog B | Multiple_C | 12.38 | 11.5 | 11.24 | 2.20 | 0.0017 | 0.0102 | 9.65 | 9.89 | 10.36 | 0.61 | 0.1632 | 0.4067 |
| TC0200013531.hg.1 | KANSL3 | KAT8 regulatory NSL complex subunit 3 | Multiple_C | 9.31 | 7.55 | 8.17 | 2.20 | 0.0018 | 0.0104 | 9.01 | 8.92 | 9.28 | 0.83 | 0.2936 | 0.5585 |
| TC0200013750.hg.1 | FHL2 | four and a half LIM domains 2 | Multiple_C | 13.26 | 13.29 | 12.12 | 2.20 | 0.0048 | 0.0235 | 9.21 | 8.45 | 8.67 | 1.45 | 0.4269 | 0.6791 |
| TC0200014597.hg.1 | RND3 | Rho family GTPase 3 | Multiple_C | 10.18 | 9.97 | 9.04 | 2.20 | 0.0161 | 0.0604 | 9.51 | 8.76 | 8.96 | 1.46 | 0.5977 | 0.7976 |
| TC0200016654.hg.1 | COX7A2L | cytochrome c oxidase subunit VIIa polypeptide 2 like | Multiple_C | 14.92 | 15.43 | 13.78 | 2.20 | 0.0011 | 0.007 | 12.53 | 12.18 | 12.12 | 1.33 | 0.0412 | 0.1868 |
| TC0300006781.hg.1 | KCNH8 | potassium channel, voltage gated eag related subfamily H, mem | Multiple_C | 8.1 | 8.09 | 6.96 | 2.20 | 0.0096 | 0.0402 | 6.76 | 6.48 | 5.7 | 2.08 | 0.0337 | 0.1653 |
| TC0500012271.hg.1 | TAF7 | TAF7 RNA polymerase II, TATA box binding protein (TBP)-associ | Multiple_C | 13.89 | 14.6 | 12.75 | 2.20 | 0.0041 | 0.0206 | 11.21 | 11.08 | 11.15 | 1.04 | 0.3361 | 0.6005 |
| TC0600007792.hg.1 | PPARD | peroxisome proliferator-activated receptor delta | Multiple_C | 9.64 | 7.56 | 8.5 | 2.20 | 0.0018 | 0.0107 | 9.24 | 8.00 | 8.37 | 1.83 | 0.0273 | 0.1447 |
| TC0800007370.hg.1 | ADAM32 | ADAM metallopeptidase domain 32 | Multiple_C | 5.02 | 4.42 | 3.88 | 2.20 | 0.0002 | 0.0017 | 4.39 | 4.62 | 4.65 | 0.84 | 0.0479 | 0.2032 |
| TC0800009868.hg.1 | TNFRSF10B | tumor necrosis factor receptor superfamily, member 10b | Multiple_C | 11.84 | 11.19 | 10.7 | 2.20 | 0.0254 | 0.0864 | 9.94 | 9.55 | 9.91 | 1.02 | 0.7528 | 0.8876 |
| TC0800010427.hg.1 | RB1CC1 | RB1-inducible coiled-coil 1 | Multiple_C | 10.38 | 11.59 | 9.24 | 2.20 | 0.0003 | 0.0024 | 9.94 | 9.61 | 8.9 | 2.06 | 0.0001 | 0.0044 |
| TC1000009983.hg.1 | NEBL | nebulette | Multiple_C | 6 | 5.52 | 4.86 | 2.20 | 0.0033 | 0.0174 | 4.57 | 5.00 | 4.91 | 0.79 | 0.035 | 0.1691 |
| TC1000010065.hg.1 | ARHGAP21 | Rho GTPase activating protein 21 | Multiple_C | 11.59 | 10.74 | 10.45 | 2.20 | 0.0363 | 0.114 | 9.14 | 8.26 | 8.9 | 1.18 | 0.6081 | 0.8041 |
| TC1000011487.hg.1 | SORBS1 | sorbin and SH3 domain containing 1 | Multiple_C | 6.4 | 5.49 | 5.26 | 2.20 | 0.0017 | 0.0102 | 4.7 | 4.77 | 4.84 | 0.91 | 0.8885 | 0.9524 |
| TC1100007490.hg.1 | FAM180B | family with sequence similarity 180, member B | Coding | 5.34 | 4.02 | 4.2 | 2.20 | 0.001 | 0.0066 | 5.13 | 5.21 | 4.93 | 1.15 | 0.5195 | 0.7469 |
| TC1100012422.hg.1 | APOA4 | apolipoprotein A-IV | Coding | 8.89 | 7.5 | 7.75 | 2.20 | 0.0146 | 0.0559 | 7.75 | 7.75 | 7.82 | 0.95 | 0.1933 | 0.4467 |
| TC1200008508.hg.1 | VEZT | vezatin, adherens junctions transmembrane protein | Multiple_C | 11.23 | 10.22 | 10.09 | 2.20 | 0.0031 | 0.0163 | 9.39 | 9.24 | 9.54 | 0.90 | 0.0937 | 0.2972 |
| TC1200012334.hg.1 | UBC | ubiquitin C | Multiple_C | 18.11 | 17.42 | 16.97 | 2.20 | 0.0018 | 0.0109 | 16.35 | 16.03 | 16.62 | 0.83 | 0.7408 | 0.8808 |
| TC1200012790.hg.1 | KMT2D | lysine (K)-specific methyltransferase 2D | Multiple_C | 8.64 | 7.3 | 7.5 | 2.20 | 0.0006 | 0.0044 | 10.58 | 11.12 | 10.9 | 0.80 | 0.1911 | 0.4441 |
| TC1300006930.hg.1 | COG6 | component of oligomeric golgi complex 6 | Multiple_C | 9.78 | 10.16 | 8.64 | 2.20 | 0.0078 | 0.0344 | 7.87 | 7.70 | 7.35 | 1.43 | 0.0076 | 0.0647 |
| TC1700007851.hg.1 | EIF1 | eukaryotic translation initiation factor 1 | Multiple_C | 17.38 | 17.05 | 16.24 | 2.20 | 4.01E-05 | 0.0005 | 15.52 | 14.79 | 15.18 | 1.27 | 0.215 | 0.4736 |
| TC2000006510.hg.1 | TGM6 | transglutaminase 6 | Multiple_C | 8.54 | 7.03 | 7.4 | 2.20 | 0.0265 | 0.0892 | 6.94 | 7.07 | 7.13 | 0.88 | 0.4467 | 0.6939 |

| | | | | | | | | | | | | | | |
|---|---|---|---|---|---|---|---|---|---|---|---|---|---|---|
| TC2000009376.hg.1 | STAU1 | staufen double-stranded RNA binding protein 1 | Multiple_C | 14.36 | 12.81 | 13.22 | 2.20 | 7.67E-05 | 0.0009 | 14.15 | 14.05 | 14.37 | 0.86 | 0.2911 | 0.5557 |
| TC2100007082.hg.1 | DYRK1A | dual specificity tyrosine-(Y)-phosphorylation regulated kinase 1/ | Multiple_C | 11.57 | 10.32 | 10.43 | 2.20 | 0.0005 | 0.0041 | 11.67 | 11.23 | 11.39 | 1.21 | 0.2693 | 0.5343 |
| TC0200016607.hg.1 | UBE2F | ubiquitin-conjugating enzyme E2F (putative) | Multiple_C | 9.62 | 8.84 | 8.49 | 2.19 | 0.0008 | 0.0056 | 7.93 | 7.79 | 8.19 | 0.84 | 0.2093 | 0.4664 |
| TC0400011620.hg.1 | ZGRF1 | zinc finger, GRF-type containing 1 | Multiple_C | 8.98 | 8.28 | 7.85 | 2.19 | 0.0036 | 0.0187 | 7.53 | 7.75 | 8.01 | 0.72 | 0.0408 | 0.1855 |
| TC0500011672.hg.1 | PJA2 | praja ring finger 2, E3 ubiquitin protein ligase | Multiple_C | 11.87 | 11.46 | 10.74 | 2.19 | 0.0041 | 0.0205 | 10.51 | 9.60 | 9.5 | 2.01 | 0.0347 | 0.1684 |
| TC0600012885.hg.1 | LAMA4 | laminin, alpha 4 | Multiple_C | 5.99 | 4.74 | 4.86 | 2.19 | 0.0007 | 0.0051 | 6.3 | 6.79 | 6.75 | 0.73 | 0.0561 | 0.2227 |
| TC0700011692.hg.1 | KIAA1324L | KIAA1324-like | Multiple_C | 8.79 | 7.95 | 7.66 | 2.19 | 0.0009 | 0.0064 | 9.54 | 8.97 | 9.47 | 1.05 | 0.6011 | 0.8001 |
| TC0700011779.hg.1 | ERVW-1 | endogenous retrovirus group W, member 1 | Multiple_C | 5.83 | 4.78 | 4.7 | 2.19 | 0.004 | 0.0202 | 4.54 | 4.51 | 4.46 | 1.06 | 0.8188 | 0.9199 |
| TC0800010575.hg.1 | CA8 | carbonic anhydrase VIII | Multiple_C | 4.82 | 3.56 | 3.69 | 2.19 | 0.0009 | 0.0064 | 5.05 | 4.31 | 4.52 | 1.44 | 0.0184 | 0.1143 |
| TC0800012410.hg.1 | WHSC1L1 | Wolf-Hirschhorn syndrome candidate 1-like 1 | Multiple_C | 13.83 | 12.32 | 12.7 | 2.19 | 2.30E-05 | 0.0003 | 13.69 | 13.10 | 13.51 | 1.13 | 0.4086 | 0.664 |
| TC0X00006656.hg.1 | RAB9A | RAB9A, member RAS oncogene family | Multiple_C | 8.14 | 7.53 | 7.01 | 2.19 | 0.0438 | 0.1313 | 5.98 | 5.78 | 5.57 | 1.33 | 0.219 | 0.4789 |
| TC0X00009750.hg.1 | KDM5C | lysine (K)-specific demethylase 5C | Multiple_C | 12.1 | 10.97 | 10.97 | 2.19 | 0.0018 | 0.0108 | 10.85 | 11.01 | 11.72 | 0.55 | 0.0032 | 0.0376 |
| TC1000006617.hg.1 | NET1 | neuroepithelial cell transforming 1 | Multiple_C | 15.08 | 13.85 | 13.95 | 2.19 | 5.16E-05 | 0.0006 | 15.01 | 14.09 | 14.53 | 1.39 | 0.0109 | 0.0823 |
| TC1100012052.hg.1 | CCDC82 | coiled-coil domain containing 82 | Multiple_C | 11.39 | 11.17 | 10.26 | 2.19 | 0.0078 | 0.0344 | 12.18 | 11.64 | 11.23 | 1.93 | 0.005 | 0.0489 |
| TC1100012775.hg.1 | ETS1 | v-ets avian erythroblastosis virus E26 oncogene homolog 1 | Multiple_C | 4.79 | 3.56 | 3.66 | 2.19 | 0.0094 | 0.0395 | 10.25 | 10.69 | 10.62 | 0.77 | 0.564 | 0.7743 |
| TC1200012638.hg.1 | HOXC9 | homeobox C9 | Multiple_C | 6.6 | 5.35 | 5.47 | 2.19 | 0.0123 | 0.0492 | 6.12 | 6.09 | 6.57 | 0.73 | 0.2077 | 0.4642 |
| TC1700011519.hg.1 | HELZ | helicase with zinc finger | Multiple_C | 11.1 | 10.08 | 9.97 | 2.19 | 0.0016 | 0.0099 | 10.5 | 10.26 | 10.86 | 0.78 | 0.5104 | 0.7403 |
| TC1700011910.hg.1 | CYTH1 | cytohesin 1 | Multiple_C | 10.2 | 9.18 | 9.07 | 2.19 | 0.0005 | 0.0037 | 10.26 | 9.42 | 9.22 | 2.06 | 1.40E-05 | 0.0009 |
| TC1800007905.hg.1 | LPIN2 | lipin 2 | Multiple_C | 8.63 | 7.32 | 7.5 | 2.19 | 0.0002 | 0.0016 | 9.92 | 9.41 | 9.35 | 1.48 | 0.0044 | 0.0457 |
| TC1900006652.hg.1 | TJP3 | tight junction protein 3 | Multiple_C | 6.65 | 5.8 | 5.52 | 2.19 | 0.002 | 0.0116 | 4.37 | 3.60 | 3.67 | 1.62 | 0.0162 | 0.1059 |
| TC2000007380.hg.1 | TOP1 | topoisomerase (DNA) I | Multiple_C | 12.52 | 10.67 | 11.39 | 2.19 | 0.0044 | 0.0218 | 10.68 | 10.39 | 11.22 | 0.69 | 0.0517 | 0.2124 |
| TC2000007488.hg.1 | PIGT | phosphatidylinositol glycan anchor biosynthesis class T | Multiple_C | 10.88 | 9.13 | 9.75 | 2.19 | 0.004 | 0.0201 | 10.85 | 11.25 | 12.08 | 0.43 | 3.12E-05 | 0.0016 |
| TC0100007784.hg.1 | AGO4 | argonaute RISC catalytic component 4 | Multiple_C | 6.19 | 5.56 | 5.07 | 2.17 | 0.0069 | 0.0311 | 5.93 | 5.86 | 5.16 | 1.71 | 0.1077 | 0.3228 |
| TC0200014809.hg.1 | GALNT3 | polypeptide N-acetylgalactosaminyltransferase 3 | Multiple_C | 13.44 | 14.07 | 12.32 | 2.17 | 0.0409 | 0.1247 | 4.84 | 3.26 | 4.1 | 1.67 | 0.0287 | 0.1494 |
| TC0300007596.hg.1 | FLNB | filamin B, beta | Multiple_C | 12.27 | 12.47 | 11.15 | 2.17 | 0.0006 | 0.0047 | 8.29 | 7.86 | 7.82 | 1.39 | 0.0178 | 0.1119 |
| TC0300011834.hg.1 | FILIP1L | filamin A interacting protein 1-like | Multiple_C | 10.74 | 7.85 | 9.62 | 2.17 | 0.1213 | 0.2768 | 10.98 | 12.06 | 10.33 | 1.57 | 0.2003 | 0.4557 |
| TC0500013360.hg.1 | CDKL3 | cyclin-dependent kinase-like 3 | Multiple_C | 6.78 | 7.38 | 5.66 | 2.17 | 0.0177 | 0.0651 | 6.43 | 6.44 | 5.71 | 1.65 | 0.0015 | 0.0225 |
| TC0600008156.hg.1 | ENPP4 | ectonucleotide pyrophosphatase/phosphodiesterase 4 (putativ | Coding | 10.43 | 9.78 | 9.31 | 2.17 | 0.0003 | 0.0024 | 8.92 | 7.87 | 8.54 | 1.30 | 0.5827 | 0.7873 |
| TC0600009353.hg.1 | TPD52L1 | tumor protein D52-like 1 | Multiple_C | 13.51 | 12.79 | 12.39 | 2.17 | 0.0004 | 0.0033 | 11.58 | 11.33 | 10.85 | 1.66 | 0.0003 | 0.0081 |
| TC0600014257.hg.1 | HLA-C | major histocompatibility complex, class I, C | Multiple_C | 7.52 | 6.66 | 6.4 | 2.17 | 0.0468 | 0.1379 | 11.92 | 11.55 | 11.37 | 1.46 | 0.0634 | 0.2395 |
| TC0700011982.hg.1 | GATS | GATS, stromal antigen 3 opposite strand | Multiple_C | 7.29 | 5.72 | 6.17 | 2.17 | 0.0048 | 0.0232 | 8.03 | 8.80 | 8.56 | 0.69 | 0.0232 | 0.1314 |
| TC0800010002.hg.1 | DUSP4 | dual specificity phosphatase 4 | Multiple_C | 15.18 | 14.12 | 14.06 | 2.17 | 0.0008 | 0.0058 | 10.63 | 9.92 | 10.45 | 1.13 | 0.0818 | 0.2755 |
| TC1000012329.hg.1 | BNIP3 | BCL2/adenovirus E1B 19kDa interacting protein 3 | Multiple_C | 5.58 | 4.41 | 4.46 | 2.17 | 0.0755 | 0.1957 | 6.01 | 5.89 | 5.45 | 1.47 | 0.315 | 0.5788 |

| | | | | | | | | | | | | | | | |
|---|---|---|---|---|---|---|---|---|---|---|---|---|---|---|---|
| TC1100007462.hg.1 | ZNF408 | zinc finger protein 408 | Multiple_C | 9.19 | 8.28 | 8.07 | 2.17 | 0.0624 | 0.1703 | 9.33 | 9.57 | 9.35 | 0.99 | 0.8132 | 0.9171 |
| TC1100008999.hg.1 | ZC3H12C | zinc finger CCCH-type containing 12C | Multiple_C | 4.38 | 2.82 | 3.26 | 2.17 | 0.0027 | 0.0148 | 6.47 | 6.29 | 6.3 | 1.13 | 0.7053 | 0.8612 |
| TC1100009101.hg.1 | ZBTB16 | zinc finger and BTB domain containing 16 | Multiple_C | 4.42 | 2.83 | 3.3 | 2.17 | 0.0011 | 0.0073 | 6.05 | 6.01 | 6.02 | 1.02 | 0.7983 | 0.9096 |
| TC1300007070.hg.1 | COG3 | component of oligomeric golgi complex 3 | Multiple_C | 10.36 | 10.05 | 9.24 | 2.17 | 0.0044 | 0.0218 | 9.04 | 8.72 | 8.82 | 1.16 | 0.0959 | 0.3014 |
| TC1300008837.hg.1 | ZC3H13 | zinc finger CCCH-type containing 13 | Multiple_C | 11.94 | 10.68 | 10.82 | 2.17 | 0.0001 | 0.0012 | 11.42 | 11.25 | 10.66 | 1.69 | 0.0004 | 0.0096 |
| TC1500006703.hg.1 | GOLGA8T | golgin A8 family, member T | Multiple_C | 6.88 | 6.06 | 5.76 | 2.17 | 0.0005 | 0.0039 | 6.58 | 6.12 | 6.02 | 1.47 | 0.0193 | 0.1176 |
| TC1500009946.hg.1 | MYO9A | myosin IXA | Multiple_C | 8.57 | 8.02 | 7.45 | 2.17 | 0.0053 | 0.0252 | 7.76 | 7.60 | 7.83 | 0.95 | 0.7006 | 0.858 |
| TC1600007275.hg.1 | LCMT1 | leucine carboxyl methyltransferase 1 | Multiple_C | 11.37 | 10.75 | 10.25 | 2.17 | 0.0008 | 0.0057 | 10.81 | 10.30 | 10.2 | 1.53 | 0.2803 | 0.5462 |
| TC1600007362.hg.1 | ATXN2L | ataxin 2-like | Multiple_C | 13.43 | 11.15 | 12.31 | 2.17 | 0.0003 | 0.0028 | 13.87 | 13.27 | 14.27 | 0.76 | 0.0359 | 0.1718 |
| TC1700007930.hg.1 | RPL27 | ribosomal protein L27 | Multiple_C | 14.45 | 14.69 | 13.33 | 2.17 | 0.0011 | 0.0074 | 12.05 | 11.39 | 11.32 | 1.66 | 0.0105 | 0.0807 |
| TC1700008128.hg.1 | NPEPPS | aminopeptidase puromycin sensitive | Multiple_C | 10.1 | 9.63 | 8.98 | 2.17 | 0.0065 | 0.0298 | 10.72 | 10.20 | 10.36 | 1.28 | 0.9482 | 0.979 |
| TC1700009436.hg.1 | MNT | MAX network transcriptional repressor | Multiple_C | 6.85 | 5.58 | 5.73 | 2.17 | 0.011 | 0.0452 | 7.16 | 7.47 | 7.46 | 0.81 | 0.7677 | 0.8958 |
| TC1800006702.hg.1 | SLC35G4 | solute carrier family 35, member G4 | Multiple_C | 6.61 | 5.44 | 5.49 | 2.17 | 0.0011 | 0.0071 | 6.08 | 5.74 | 5.82 | 1.20 | 0.0828 | 0.2771 |
| TC1800007396.hg.1 | ONECUT2 | one cut homeobox 2 | Multiple_C | 6.52 | 4.93 | 5.4 | 2.17 | 0.0287 | 0.0948 | 5.05 | 4.92 | 5.26 | 0.86 | 0.1259 | 0.351 |
| TC1900008057.hg.1 | ZFP36 | ZFP36 ring finger protein | Coding | 10.11 | 7.59 | 8.99 | 2.17 | 0.0024 | 0.0134 | 7.06 | 5.82 | 6.96 | 1.07 | 0.8564 | 0.9389 |
| TC1900009963.hg.1 | ANO8 | anoctamin 8 | Multiple_C | 9.87 | 8.88 | 8.75 | 2.17 | 0.0007 | 0.005 | 7.03 | 6.96 | 6.82 | 1.16 | 0.3377 | 0.6018 |
| TC1900010375.hg.1 | RHPN2 | rhophilin, Rho GTPase binding protein 2 | Multiple_C | 12.79 | 11.64 | 11.67 | 2.17 | 0.012 | 0.0484 | 8.45 | 8.56 | 9.26 | 0.57 | 0.0057 | 0.0534 |
| TC1900011819.hg.1 | ZNF211 | zinc finger protein 211 | Multiple_C | 8.78 | 9.05 | 7.66 | 2.17 | 0.0006 | 0.0044 | 6.55 | 6.59 | 6.59 | 0.97 | 0.6316 | 0.8186 |
| TC2000007325.hg.1 | ADIG | adipogenin | Multiple_C | 4.91 | 3.98 | 3.79 | 2.17 | 0.0006 | 0.0042 | 4.63 | 4.62 | 4.56 | 1.05 | 0.5623 | 0.7729 |
| TSUnmapped00000274.h | ZNF197 | zinc finger protein 197 | Coding | 6.73 | 6.46 | 5.61 | 2.17 | 0.0005 | 0.0038 | 5.27 | 5.08 | 4.38 | 1.85 | 0.0007 | 0.0128 |
| TC0100015627.hg.1 | PRKAB2 | protein kinase, AMP-activated, beta 2 non-catalytic subunit | Multiple_C | 9.32 | 8.3 | 8.21 | 2.16 | 0.0002 | 0.0016 | 8.81 | 7.82 | 8.35 | 1.38 | 0.1395 | 0.3711 |
| TC0100018282.hg.1 | POLR3GL | polymerase (RNA) III (DNA directed) polypeptide G (32kD)-like | Multiple_C | 8.67 | 9.13 | 7.56 | 2.16 | 0.0019 | 0.011 | 6.71 | 6.89 | 6.43 | 1.21 | 0.2795 | 0.5455 |
| TC0200016300.hg.1 | OR6B2 | olfactory receptor, family 6, subfamily B, member 2 | Coding | 5.57 | 4.25 | 4.46 | 2.16 | 0.0019 | 0.0109 | 4.24 | 3.99 | 3.77 | 1.39 | 0.0431 | 0.1916 |
| TC0300013941.hg.1 | MKRN2OS | MKRN2 opposite strand | Coding | 5.02 | 4.11 | 3.91 | 2.16 | 0.0039 | 0.0199 | 4.24 | 4.34 | 4.23 | 1.01 | 0.3254 | 0.5891 |
| TC0500012957.hg.1 | UIMC1 | ubiquitin interaction motif containing 1 | Multiple_C | 9.65 | 9.33 | 8.54 | 2.16 | 0.0087 | 0.0374 | 7.99 | 7.59 | 8.15 | 0.90 | 0.4622 | 0.7049 |
| TC0700013390.hg.1 | TRIM73 | tripartite motif containing 73 | Multiple_C | 4.72 | 3.75 | 3.61 | 2.16 | 0.009 | 0.0385 | 4.65 | 4.55 | 4.12 | 1.44 | 0.1055 | 0.3189 |
| TC0800006680.hg.1 | TNKS | tankyrase, TRF1-interacting ankyrin-related ADP-ribose polyme | Multiple_C | 8.14 | 7.6 | 7.03 | 2.16 | 0.0212 | 0.0751 | 7.39 | 7.23 | 7.18 | 1.16 | 0.5562 | 0.7706 |
| TC0800007466.hg.1 | POTEA | POTE ankyrin domain family, member A | Multiple_C | 6.19 | 4.89 | 5.08 | 2.16 | 0.0003 | 0.0026 | 5.72 | 5.36 | 5.19 | 1.44 | 0.0183 | 0.1141 |
| TC0800008352.hg.1 | VPS13B | vacuolar protein sorting 13 homolog B (yeast) | Multiple_C | 11.36 | 10.68 | 10.25 | 2.16 | 0.0004 | 0.0029 | 10.8 | 10.01 | 10.34 | 1.38 | 0.0356 | 0.171 |
| TC0800010894.hg.1 | HEY1 | hes-related family bHLH transcription factor with YRPW motif 1 | Multiple_C | 5.65 | 4.57 | 4.54 | 2.16 | 0.0011 | 0.007 | 5.75 | 5.40 | 5.54 | 1.16 | 0.4715 | 0.7122 |
| TC0X00011317.hg.1 | TCEAL1 | transcription elongation factor A (SII)-like 1 | Multiple_C | 11.43 | 11.27 | 10.32 | 2.16 | 4.94E-05 | 0.0006 | 10.71 | 11.14 | 10.73 | 0.99 | 0.9997 | 1 |
| TC1100008054.hg.1 | PACS1 | phosphofurin acidic cluster sorting protein 1 | Multiple_C | 8.58 | 7.21 | 7.47 | 2.16 | 0.0005 | 0.0039 | 9.92 | 9.54 | 9.15 | 1.71 | 0.0085 | 0.07 |
| TC1200007359.hg.1 | CNTN1 | contactin 1 | Multiple_C | 5.44 | 4.56 | 4.33 | 2.16 | 0.0045 | 0.022 | 4.96 | 4.11 | 4.54 | 1.34 | 0.2581 | 0.5228 |

| | | | | | | | | | | | | | | | |
|---|---|---|---|---|---|---|---|---|---|---|---|---|---|---|---|
| TC1200007590.hg.1 | AQP2 | aquaporin 2 (collecting duct) | Coding | 5.8 | 4.78 | 4.69 | 2.16 | 0.0017 | 0.0103 | 4.5 | 4.58 | 4.42 | 1.06 | 0.6347 | 0.8208 |
| TC1200008002.hg.1 | XPOT | exportin, tRNA | Multiple_C | 13.83 | 14.02 | 12.72 | 2.16 | 0.0009 | 0.006 | 13.5 | 12.47 | 13.67 | 0.89 | 0.4081 | 0.6638 |
| TC1300009979.hg.1 | UCHL3 | ubiquitin C-terminal hydrolase L3 | Multiple_C | 14.17 | 14.36 | 13.06 | 2.16 | 6.07E-05 | 0.0007 | 12.32 | 11.22 | 11.45 | 1.83 | 2.86E-05 | 0.0015 |
| TC1500010237.hg.1 | RPL9 | ribosomal protein L9 | Multiple_C | 14.85 | 15.59 | 13.74 | 2.16 | 0.0022 | 0.0127 | 11.55 | 11.17 | 11.05 | 1.41 | 0.0069 | 0.0606 |
| TC1700012242.hg.1 | LOC10106038 | TBC1 domain family member-like; TBC1 domain family, member | Coding | 10.2 | 9.61 | 9.09 | 2.16 | 0.0014 | 0.0087 | 9.44 | 9.12 | 8.55 | 1.85 | 0.0101 | 0.0783 |
| TC1800007734.hg.1 | ZNF236 | zinc finger protein 236 | Multiple_C | 11.29 | 10.14 | 10.18 | 2.16 | 5.02E-05 | 0.0006 | 7.72 | 8.15 | 8.36 | 0.64 | 0.0033 | 0.0376 |
| TC2200009317.hg.1 | KLHL22 | kelch-like family member 22 | Multiple_C | 6.81 | 5.27 | 5.7 | 2.16 | 0.0126 | 0.05 | 6.73 | 6.86 | 6.31 | 1.34 | 0.1354 | 0.3655 |
| TC0100009054.hg.1 | MTF2 | metal response element binding transcription factor 2 | Multiple_C | 11.82 | 10.48 | 10.72 | 2.14 | 0.0008 | 0.0054 | 10.57 | 9.81 | 9.93 | 1.56 | 0.0029 | 0.0346 |
| TC0100014214.hg.1 | ECHDC2 | enoyl-CoA hydratase domain containing 2 | Multiple_C | 9.61 | 8.94 | 8.51 | 2.14 | 0.0001 | 0.0014 | 8.88 | 8.57 | 8.43 | 1.37 | 0.0844 | 0.2797 |
| TC0200011097.hg.1 | ALPPL2 | alkaline phosphatase, placental like 2 | Coding | 6.89 | 5.48 | 5.79 | 2.14 | 0.0009 | 0.0061 | 7.38 | 6.90 | 6.45 | 1.91 | 0.0041 | 0.0434 |
| TC0200011980.hg.1 | ITSN2 | intersectin 2 | Multiple_C | 10.94 | 10.06 | 9.84 | 2.14 | 8.97E-05 | 0.001 | 10.92 | 10.36 | 10.59 | 1.26 | 0.0404 | 0.1842 |
| TC0300009782.hg.1 | EIF4A2; SNOR | eukaryotic translation initiation factor 4A2; small nucleolar RNA | Multiple_C | 15.42 | 15.79 | 14.32 | 2.14 | 0.0024 | 0.0134 | 13.22 | 11.92 | 12.24 | 1.97 | 0.0023 | 0.0291 |
| TC0300010310.hg.1 | RPL32; SNORA | ribosomal protein L32; small nucleolar RNA, H/ACA box 7A | Multiple_C | 18.2 | 16.94 | 17.1 | 2.14 | 0.0005 | 0.0041 | 15.11 | 14.94 | 14.76 | 1.27 | 0.1739 | 0.4213 |
| TC0400012769.hg.1 | BST1 | bone marrow stromal cell antigen 1 | Multiple_C | 4.5 | 3.55 | 3.4 | 2.14 | 0.1181 | 0.2717 | 4.42 | 4.57 | 4.5 | 0.95 | 0.7606 | 0.8915 |
| TC0500012153.hg.1 | FAM13B | family with sequence similarity 13, member B | Multiple_C | 8.38 | 7.26 | 7.28 | 2.14 | 0.0076 | 0.0336 | 8.26 | 7.61 | 8.25 | 1.01 | 0.9649 | 0.985 |
| TC0700006645.hg.1 | USP42 | ubiquitin specific peptidase 42 | Multiple_C | 9.97 | 8.28 | 8.87 | 2.14 | 0.001 | 0.007 | 9.72 | 9.10 | 9.78 | 0.96 | 0.0669 | 0.2467 |
| TC0700007374.hg.1 | YKT6 | YKT6 v-SNARE homolog (S. cerevisiae) | Multiple_C | 10.87 | 9.68 | 9.77 | 2.14 | 0.0091 | 0.0387 | 10.79 | 10.98 | 11.26 | 0.72 | 0.0525 | 0.214 |
| TC0700008321.hg.1 | ANKIB1 | ankyrin repeat and IBR domain containing 1 | Multiple_C | 11.1 | 10.59 | 10 | 2.14 | 0.0017 | 0.0101 | 9.81 | 8.88 | 9.83 | 0.99 | 0.7343 | 0.8775 |
| TC0700010181.hg.1 | RSPH10B2; RS | radial spoke head 10 homolog B2 (Chlamydomonas); radial spok | Multiple_C | 5.11 | 4.11 | 4.01 | 2.14 | 0.0012 | 0.0079 | 5.21 | 5.84 | 5.36 | 0.90 | 0.2847 | 0.5497 |
| TC0Y00007179.hg.1 | PRORY | proline rich, Y-linked | Coding | 5.86 | 4.6 | 4.76 | 2.14 | 0.0236 | 0.0815 | 4.92 | 4.38 | 4.49 | 1.35 | 0.2213 | 0.4814 |
| TC1000008452.hg.1 | BTAF1 | BTAF1 RNA polymerase II, B-TFIID transcription factor-associate | Multiple_C | 11.38 | 11.18 | 10.28 | 2.14 | 0.0087 | 0.0374 | 10.94 | 9.71 | 10.54 | 1.32 | 0.0634 | 0.2396 |
| TC1100009004.hg.1 | FDX1 | ferredoxin 1 | Coding | 9.81 | 10 | 8.71 | 2.14 | 0.0088 | 0.0378 | 8.89 | 8.87 | 8.6 | 1.22 | 0.351 | 0.6149 |
| TC1100011797.hg.1 | RAB30 | RAB30, member RAS oncogene family | Multiple_C | 7.78 | 6.93 | 6.68 | 2.14 | 0.0109 | 0.0446 | 8.79 | 8.87 | 10.41 | 0.33 | 1.60E-05 | 0.001 |
| TC1200012829.hg.1 | NDUFA12 | NADH dehydrogenase (ubiquinone) 1 alpha subcomplex, 12 | Multiple_C | 14.02 | 13.12 | 12.92 | 2.14 | 0.01 | 0.0416 | 7.64 | 7.76 | 8.54 | 0.54 | 0.0337 | 0.1654 |
| TC1300007845.hg.1 | CLYBL | citrate lyase beta like | Multiple_C | 6.98 | 7.09 | 5.88 | 2.14 | 0.0326 | 0.105 | 5.51 | 5.29 | 5.22 | 1.22 | 0.2592 | 0.5233 |
| TC1500007822.hg.1 | CD276 | CD276 molecule | Multiple_C | 5.39 | 4.12 | 4.29 | 2.14 | 0.0455 | 0.1348 | 6.35 | 6.12 | 5.94 | 1.33 | 0.2391 | 0.5027 |
| TC1500010923.hg.1 | DET1 | de-etiolated homolog 1 (Arabidopsis) | Multiple_C | 5.79 | 4.53 | 4.69 | 2.14 | 0.0113 | 0.046 | 7.12 | 6.85 | 6.88 | 1.18 | 0.6523 | 0.8305 |
| TC1600006822.hg.1 | METTL22 | methyltransferase like 22 | Multiple_C | 8.9 | 8.86 | 7.8 | 2.14 | 0.025 | 0.0853 | 7.93 | 8.07 | 7.73 | 1.15 | 0.2917 | 0.5563 |
| TC1600009524.hg.1 | NPIPA5 | nuclear pore complex interacting protein family, member A5 | Coding | 10.5 | 9.58 | 9.4 | 2.14 | 0.0003 | 0.0023 | 11.14 | 10.75 | 10.74 | 1.32 | 0.0732 | 0.2594 |
| TC1700008984.hg.1 | SEC14L1; SCAI | SEC14-like lipid binding 1; small Cajal body-specific RNA 16; sma | Multiple_C | 10.06 | 9.62 | 8.96 | 2.14 | 0.0001 | 0.0011 | 11.26 | 11.02 | 10.87 | 1.31 | 0.015 | 0.1002 |
| TC1900009192.hg.1 | ABHD17A | abhydrolase domain containing 17A | Multiple_C | 7.45 | 6.32 | 6.35 | 2.14 | 0.1196 | 0.2741 | 5.51 | 5.79 | 5.46 | 1.04 | 0.9175 | 0.9663 |
| TC2000008587.hg.1 | RALGAPA2 | Ral GTPase activating protein, alpha subunit 2 (catalytic) | Multiple_C | 10.1 | 8.89 | 9 | 2.14 | 0.024 | 0.0827 | 8.04 | 7.76 | 7.8 | 1.18 | 0.0462 | 0.1987 |
| TC2100008494.hg.1 | IFNAR2 | interferon (alpha, beta and omega) receptor 2 | Multiple_C | 11.27 | 10.46 | 10.17 | 2.14 | 0.0001 | 0.0012 | 10.65 | 10.19 | 9.86 | 1.73 | 0.0003 | 0.0075 |

| | | | | | | | | | | | | | | | |
|---|---|---|---|---|---|---|---|---|---|---|---|---|---|---|---|
| TC2200008181.hg.1 | ZNF70 | zinc finger protein 70 | Multiple_C | 6.45 | 5.48 | 5.35 | 2.14 | 0.0036 | 0.0184 | 6.41 | 6.59 | 6.34 | 1.05 | 0.3892 | 0.6479 |
| TC0100006437.hg.1 | OR4F5 | olfactory receptor, family 4, subfamily F, member 5 | Coding | 4.24 | 3.15 | 3.15 | 2.13 | 0.0032 | 0.0167 | 3.52 | 3.59 | 3.55 | 0.98 | 0.8187 | 0.9199 |
| TC0100018246.hg.1 | LRRC8C | leucine rich repeat containing 8 family, member C | Multiple_C | 4.82 | 3.68 | 3.73 | 2.13 | 0.0147 | 0.0561 | 8.24 | 8.00 | 8.01 | 1.17 | 0.412 | 0.6665 |
| TC0200010572.hg.1 | ZDBF2 | zinc finger, DBF-type containing 2 | Multiple_C | 3.44 | 2.23 | 2.35 | 2.13 | 0.004 | 0.0203 | 7.26 | 7.21 | 6.54 | 1.65 | 0.0614 | 0.2353 |
| TC0200012022.hg.1 | KIF3C | kinesin family member 3C | Multiple_C | 6.47 | 5.21 | 5.38 | 2.13 | 0.0017 | 0.0102 | 8.93 | 8.62 | 8.66 | 1.21 | 0.3426 | 0.6064 |
| TC0400007120.hg.1 | RBPJ | recombination signal binding protein for immunoglobulin kappa | Multiple_C | 10.92 | 9.85 | 9.83 | 2.13 | 0.0038 | 0.0195 | 9.79 | 9.66 | 9.95 | 0.90 | 0.3058 | 0.5698 |
| TC0600011463.hg.1 | NEU1 | sialidase 1 (lysosomal sialidase) | Multiple_C | 10.18 | 9.23 | 9.09 | 2.13 | 0.0008 | 0.0058 | 9.01 | 9.58 | 9.71 | 0.62 | 0.0255 | 0.1391 |
| TC0600011927.hg.1 | SUPT3H | SPT3 homolog, SAGA and STAGA complex component | Multiple_C | 7.87 | 7.7 | 6.78 | 2.13 | 0.023 | 0.0799 | 6.69 | 6.85 | 7.46 | 0.59 | 0.0633 | 0.2393 |
| TC0600014293.hg.1 | CRIP3 | cysteine-rich protein 3 | Multiple_C | 4.82 | 3.75 | 3.73 | 2.13 | 0.0008 | 0.0059 | 4.12 | 4.17 | 3.81 | 1.24 | 0.5263 | 0.7508 |
| TC0800011452.hg.1 | RSPO2 | R-spondin 2 | Coding | 4.16 | 2.9 | 3.07 | 2.13 | 0.0057 | 0.0266 | 3.8 | 3.59 | 3.48 | 1.25 | 0.0549 | 0.22 |
| TC0X00008828.hg.1 | EMD | emerin | Multiple_C | 13 | 11.55 | 11.91 | 2.13 | 0.0134 | 0.0523 | 11.32 | 11.52 | 12.84 | 0.35 | 5.80E-06 | 0.0005 |
| TC1000007272.hg.1 | CREM | cAMP responsive element modulator | Multiple_C | 9.84 | 9.69 | 8.75 | 2.13 | 0.0001 | 0.0011 | 8.21 | 7.37 | 7.18 | 2.04 | 1.49E-05 | 0.0009 |
| TC1000007785.hg.1 | NRBF2 | nuclear receptor binding factor 2 | Multiple_C | 9.42 | 9.01 | 8.33 | 2.13 | 0.0044 | 0.0217 | 8.42 | 8.11 | 7.83 | 1.51 | 0.1078 | 0.3229 |
| TC1100010187.hg.1 | CALCA | calcitonin-related polypeptide alpha | Multiple_C | 5.77 | 4.44 | 4.68 | 2.13 | 0.0266 | 0.0892 | 5.41 | 5.36 | 5.41 | 1.00 | 0.2222 | 0.4825 |
| TC1200012798.hg.1 | ATP5G2 | ATP synthase, H+ transporting, mitochondrial Fo complex subur | Multiple_C | 14.74 | 13.41 | 13.65 | 2.13 | 0.0001 | 0.0011 | 13.65 | 13.55 | 14.01 | 0.78 | 0.4461 | 0.6935 |
| TC1300009273.hg.1 | COMMD6 | COMM domain containing 6 | Multiple_C | 15.4 | 16.7 | 14.31 | 2.13 | 4.75E-05 | 0.0006 | 10.18 | 9.51 | 9.52 | 1.58 | 2.45E-05 | 0.0013 |
| TC1400007354.hg.1 | PPM1A | protein phosphatase, Mg2+/Mn2+ dependent, 1A | Multiple_C | 12.88 | 12.55 | 11.79 | 2.13 | 0.0004 | 0.003 | 11.27 | 10.54 | 11.08 | 1.14 | 0.047 | 0.2006 |
| TC1400010797.hg.1 | NUDT14 | nudix hydrolase 14 | Multiple_C | 9.15 | 7.77 | 8.06 | 2.13 | 0.0156 | 0.0589 | 9.04 | 9.11 | 8.54 | 1.41 | 0.2551 | 0.5196 |
| TC1600006585.hg.1 | SLC9A3R2 | solute carrier family 9, subfamily A (NHE3, cation proton antipo | Multiple_C | 8.26 | 7.29 | 7.17 | 2.13 | 0.0408 | 0.1244 | 9.11 | 8.19 | 8 | 2.16 | 0.0032 | 0.0376 |
| TC1700008709.hg.1 | BPTF | bromodomain PHD finger transcription factor | Multiple_C | 10.4 | 8.77 | 9.31 | 2.13 | 0.0034 | 0.0177 | 12.23 | 12.01 | 12.12 | 1.08 | 0.9484 | 0.979 |
| TC1700012282.hg.1 | SPATA20 | spermatogenesis associated 20 | Multiple_C | 6.94 | 6.47 | 5.85 | 2.13 | 0.0013 | 0.0081 | 5.65 | 6.48 | 6.18 | 0.69 | 0.0479 | 0.2032 |
| TC1800007198.hg.1 | SLC14A1 | solute carrier family 14 (urea transporter), member 1 (Kidd bloc | Multiple_C | 5.43 | 4.26 | 4.34 | 2.13 | 0.0058 | 0.027 | 3.9 | 3.79 | 3.78 | 1.09 | 0.9847 | 0.9937 |
| TC1800008679.hg.1 | MBD1 | methyl-CpG binding domain protein 1 | Multiple_C | 9.31 | 7.84 | 8.22 | 2.13 | 0.0022 | 0.0126 | 7.76 | 7.94 | 7.99 | 0.85 | 0.2875 | 0.5523 |
| TC1900006462.hg.1 | MADCAM1 | mucosal vascular addressin cell adhesion molecule 1 | Multiple_C | 7.6 | 6.45 | 6.51 | 2.13 | 0.0102 | 0.0424 | 6.85 | 6.88 | 6.42 | 1.35 | 0.0338 | 0.1655 |
| TC1900006640.hg.1 | FZR1 | fizzy/cell division cycle 20 related 1 | Multiple_C | 8.93 | 7.77 | 7.84 | 2.13 | 0.0078 | 0.0344 | 9.2 | 9.41 | 8.9 | 1.23 | 0.5537 | 0.7686 |
| TC1900007802.hg.1 | KIAA0355 | KIAA0355 | Multiple_C | 5.65 | 4.61 | 4.56 | 2.13 | 0.0003 | 0.0023 | 7.03 | 6.70 | 6.29 | 1.67 | 0.0015 | 0.0217 |
| TC1900012047.hg.1 | TNNI3 | troponin I type 3 (cardiac) | Multiple_C | 7.57 | 6.16 | 6.48 | 2.13 | 0.0019 | 0.0113 | 6.41 | 6.50 | 5.91 | 1.41 | 0.0943 | 0.2982 |
| TC2000009966.hg.1 | FKBP1A-SDCB | FKBP1A-SDCBP2 readthrough (NMD candidate) | Multiple_C | 6.79 | 5.49 | 5.7 | 2.13 | 0.0017 | 0.0103 | 6.22 | 6.37 | 6.03 | 1.14 | 0.2064 | 0.4631 |
| TC2200009314.hg.1 | RTN4R | reticulon 4 receptor | Multiple_C | 6.74 | 5.33 | 5.65 | 2.13 | 0.0064 | 0.0293 | 6.86 | 6.52 | 6.74 | 1.09 | 0.5286 | 0.7524 |
| TC0200010836.hg.1 | ZFAND2B | zinc finger, AN1-type domain 2B | Multiple_C | 8.82 | 8.33 | 7.74 | 2.11 | 0.0019 | 0.0109 | 7.37 | 7.71 | 7.59 | 0.86 | 0.3961 | 0.654 |
| TC0200016446.hg.1 | ACYP2 | acylphosphatase 2, muscle type | Multiple_C | 5.69 | 4.33 | 4.61 | 2.11 | 0.0002 | 0.0021 | 5.7 | 5.80 | 5.54 | 1.12 | 0.3563 | 0.6194 |
| TC0300006994.hg.1 | FBXL2 | F-box and leucine-rich repeat protein 2 | Multiple_C | 6.94 | 5.69 | 5.86 | 2.11 | 0.0024 | 0.0133 | 8.04 | 6.88 | 6.44 | 3.03 | 3.84E-05 | 0.0018 |
| TC0300011516.hg.1 | EIF4E3 | eukaryotic translation initiation factor 4E family member 3 | Multiple_C | 6.64 | 6.01 | 5.56 | 2.11 | 0.0027 | 0.0149 | 4.85 | 4.77 | 5.06 | 0.86 | 0.3189 | 0.583 |

| | | | | | | | | | | | | | | | |
|---|---|---|---|---|---|---|---|---|---|---|---|---|---|---|---|
| TC0600008348.hg.1 | ZNF451 | zinc finger protein 451 | Multiple_C | 11.96 | 11.42 | 10.88 | 2.11 | 0.0003 | 0.0024 | 12.15 | 11.40 | 11.98 | 1.13 | 0.4096 | 0.6647 |
| TC0700012684.hg.1 | AKR1B1 | aldo-keto reductase family 1, member B1 (aldose reductase) | Multiple_C | 4.77 | 3.88 | 3.69 | 2.11 | 0.0035 | 0.0181 | 10.4 | 6.25 | 9.51 | 1.85 | 0.0041 | 0.0436 |
| TC0700013383.hg.1 | RABGEF1 | RAB guanine nucleotide exchange factor (GEF) 1 | Multiple_C | 11.91 | 10.87 | 10.83 | 2.11 | 0.007 | 0.0314 | 11.25 | 11.00 | 11.55 | 0.81 | 0.0991 | 0.3076 |
| TC0800009113.hg.1 | PTP4A3 | protein tyrosine phosphatase type IVA, member 3 | Multiple_C | 8.2 | 7.26 | 7.12 | 2.11 | 0.0003 | 0.0029 | 11.32 | 9.67 | 8.91 | 5.31 | 5.77E-10 | 7.62E-07 |
| TC0X00009000.hg.1 | VCX3A; VCX | variable charge, X-linked 3A; variable charge, X-linked | Coding | 6.08 | 4.8 | 5 | 2.11 | 0.0361 | 0.1137 | 5.69 | 5.51 | 5.39 | 1.23 | 0.1831 | 0.434 |
| TC1000008159.hg.1 | RPS24 | ribosomal protein S24 | Multiple_C | 18.19 | 17.21 | 17.11 | 2.11 | 0.0001 | 0.0014 | 16.71 | 16.61 | 16.35 | 1.28 | 0.0641 | 0.2411 |
| TC1000012365.hg.1 | CFAP46 | cilia and flagella associated protein 46 | Multiple_C | 6.38 | 6.18 | 5.3 | 2.11 | 0.0012 | 0.0078 | 3.84 | 3.46 | 3.79 | 1.04 | 0.3387 | 0.6028 |
| TC1100011144.hg.1 | MACROD1 | MACRO domain containing 1 | Multiple_C | 9.11 | 8.79 | 8.03 | 2.11 | 0.004 | 0.0202 | 10.05 | 9.80 | 9.75 | 1.23 | 0.2315 | 0.4928 |
| TC1200008790.hg.1 | USP30 | ubiquitin specific peptidase 30 | Multiple_C | 10.19 | 9.59 | 9.11 | 2.11 | 1.74E-05 | 0.0003 | 9.02 | 9.01 | 9.05 | 0.98 | 0.9172 | 0.9663 |
| TC1500007856.hg.1 | CSK | c-src tyrosine kinase | Multiple_C | 8.77 | 7.62 | 7.69 | 2.11 | 0.0011 | 0.0073 | 9.22 | 9.78 | 9.71 | 0.71 | 0.0071 | 0.0615 |
| TC1500007919.hg.1 | ISL2 | ISL LIM homeobox 2 | Multiple_C | 7.6 | 6.68 | 6.52 | 2.11 | 0.0004 | 0.0032 | 7.23 | 7.08 | 6.93 | 1.23 | 0.2013 | 0.4567 |
| TC1500009036.hg.1 | MEIS2 | Meis homeobox 2 | Multiple_C | 10.68 | 10.03 | 9.6 | 2.11 | 0.0128 | 0.0507 | 7.8 | 7.90 | 7.5 | 1.23 | 0.6977 | 0.8567 |
| TC1600009944.hg.1 | KCTD13 | potassium channel tetramerization domain containing 13 | Multiple_C | 9.98 | 8.58 | 8.9 | 2.11 | 0.0007 | 0.0049 | 7.96 | 7.93 | 8.15 | 0.88 | 0.5298 | 0.7532 |
| TC1600011527.hg.1 | VKORC1 | vitamin K epoxide reductase complex subunit 1 | Multiple_C | 14.76 | 13.53 | 13.68 | 2.11 | 0.0003 | 0.0023 | 12.19 | 12.01 | 12.21 | 0.99 | 0.9497 | 0.9794 |
| TC1700008882.hg.1 | KCTD2 | potassium channel tetramerization domain containing 2 | Multiple_C | 9.37 | 8.5 | 8.29 | 2.11 | 0.0341 | 0.1087 | 8.8 | 9.39 | 9.31 | 0.70 | 0.228 | 0.4887 |
| TC1700011210.hg.1 | COIL | coilin | Multiple_C | 10.8 | 10.68 | 9.72 | 2.11 | 9.78E-05 | 0.001 | 8.15 | 7.89 | 8.48 | 0.80 | 0.341 | 0.6049 |
| TC1800007518.hg.1 | ZCCHC2 | zinc finger, CCHC domain containing 2 | Multiple_C | 8.08 | 6.53 | 7 | 2.11 | 0.0078 | 0.0343 | 6.84 | 5.99 | 6.88 | 0.97 | 0.3042 | 0.5679 |
| TC2200008887.hg.1 | ATP5L2 | ATP synthase, H+ transporting, mitochondrial Fo complex subur | Coding | 6.95 | 6.44 | 5.87 | 2.11 | 0.0139 | 0.0539 | 7 | 6.59 | 6.39 | 1.53 | 0.0191 | 0.117 |
| TC2200009366.hg.1 | CHKB-CPT1B | CHKB-CPT1B readthrough (NMD candidate) | Multiple_C | 7.43 | 6.71 | 6.35 | 2.11 | 0.0106 | 0.0435 | 7.62 | 7.51 | 6.96 | 1.58 | 0.2014 | 0.4568 |
| TC0100018443.hg.1 | PLPP3 | phospholipid phosphatase 3 | Multiple_C | 8.9 | 7.24 | 7.83 | 2.10 | 0.0088 | 0.0376 | 9.35 | 11.40 | 11.11 | 0.30 | 4.23E-07 | 8.32E-05 |
| TC0200011080.hg.1 | COPS7B | COP9 signalosome subunit 7B | Multiple_C | 10.99 | 9.66 | 9.92 | 2.10 | 5.47E-05 | 0.0007 | 12.37 | 12.46 | 12.76 | 0.76 | 0.034 | 0.1659 |
| TC0200014509.hg.1 | GTDC1 | glycosyltransferase like domain containing 1 | Multiple_C | 9.32 | 8.13 | 8.25 | 2.10 | 0.0057 | 0.0266 | 8.71 | 7.41 | 8.25 | 1.38 | 0.0146 | 0.0986 |
| TC0300007073.hg.1 | SLC22A13 | solute carrier family 22 (organic anion/urate transporter), mem | Multiple_C | 4.34 | 3.35 | 3.27 | 2.10 | 0.0009 | 0.0064 | 4.29 | 4.31 | 4.02 | 1.21 | 0.1067 | 0.321 |
| TC0300012813.hg.1 | P2RY14 | purinergic receptor P2Y, G-protein coupled, 14 | Coding | 4.04 | 3.02 | 2.97 | 2.10 | 0.008 | 0.035 | 4.72 | 4.64 | 4.38 | 1.27 | 0.1064 | 0.3204 |
| TC0400012670.hg.1 | FAT1 | FAT atypical cadherin 1 | Multiple_C | 13.23 | 11.53 | 12.16 | 2.10 | 5.92E-05 | 0.0007 | 11.4 | 11.97 | 12.04 | 0.64 | 0.0047 | 0.0472 |
| TC0500011655.hg.1 | FBXL17 | F-box and leucine-rich repeat protein 17 | Multiple_C | 6.6 | 5.82 | 5.53 | 2.10 | 0.0017 | 0.0101 | 6.59 | 7.22 | 6.97 | 0.77 | 0.0905 | 0.2911 |
| TC0600009611.hg.1 | HEBP2 | heme binding protein 2 | Multiple_C | 11.98 | 11.54 | 10.91 | 2.10 | 0.0054 | 0.0256 | 8.21 | 8.93 | 8.34 | 0.91 | 0.0931 | 0.2962 |
| TC0600012801.hg.1 | SESN1 | sestrin 1 | Multiple_C | 7.31 | 7.35 | 6.24 | 2.10 | 0.0033 | 0.0175 | 6.65 | 6.21 | 6.04 | 1.53 | 0.0397 | 0.1823 |
| TC0600012871.hg.1 | TRAF3IP2 | TRAF3 interacting protein 2 | Multiple_C | 9.47 | 9.27 | 8.4 | 2.10 | 0.0003 | 0.0026 | 6.85 | 6.79 | 6.52 | 1.26 | 0.2969 | 0.5615 |
| TC0700009647.hg.1 | ZNF862 | zinc finger protein 862 | Multiple_C | 5.9 | 5.41 | 4.83 | 2.10 | 0.0238 | 0.082 | 5.79 | 5.51 | 5.37 | 1.34 | 0.1643 | 0.4086 |
| TC0700013332.hg.1 | RBAK; RBAK-R | RB-associated KRAB zinc finger; RBAK-RBAKDN readthrough; RE | Multiple_C | 12.81 | 11.51 | 11.74 | 2.10 | 0.0001 | 0.0012 | 13.4 | 13.50 | 13.74 | 0.79 | 0.0327 | 0.1622 |
| TC0700013575.hg.1 | STAG3L3; STA | stromal antigen 3-like 3 (pseudogene); stromal antigen 3-like 2 ( | Multiple_C | 10.96 | 10.28 | 9.89 | 2.10 | 0.0047 | 0.0228 | 11.21 | 11.80 | 11.36 | 0.90 | 0.0819 | 0.2757 |
| TC0800010043.hg.1 | PPP2CB | protein phosphatase 2, catalytic subunit, beta isozyme | Multiple_C | 12.39 | 12.62 | 11.32 | 2.10 | 0.0008 | 0.0057 | 9.04 | 8.40 | 8.97 | 1.05 | 0.6863 | 0.8497 |

| | | | | | | | | | | | | | | | |
|---|---|---|---|---|---|---|---|---|---|---|---|---|---|---|---|
| TC0800010502.hg.1 | RPS20; SNORI | ribosomal protein S20; small nucleolar RNA, C/D box 54 | Multiple_C | 17.03 | 16.62 | 15.96 | 2.10 | 0.0017 | 0.0101 | 15.15 | 15.13 | 14.63 | 1.43 | 0.0825 | 0.2766 |
| TC1000009742.hg.1 | KIN | Kin17 DNA and RNA binding protein | Multiple_C | 8.62 | 8.67 | 7.55 | 2.10 | 0.0071 | 0.032 | 7.3 | 7.44 | 7.61 | 0.81 | 0.5703 | 0.7787 |
| TC1000011524.hg.1 | PIK3AP1 | phosphoinositide-3-kinase adaptor protein 1 | Multiple_C | 12.56 | 10.89 | 11.49 | 2.10 | 6.06E-05 | 0.0007 | 6.36 | 6.43 | 6.68 | 0.80 | 0.0196 | 0.1186 |
| TC1100009746.hg.1 | KRTAP5-2 | keratin associated protein 5-2 | Coding | 5.65 | 4.47 | 4.58 | 2.10 | 0.0051 | 0.0246 | 4.85 | 4.55 | 4.31 | 1.45 | 0.0161 | 0.1054 |
| TC1100013190.hg.1 | CFL1 | cofilin 1 (non-muscle) | Multiple_C | 15.2 | 12.34 | 14.13 | 2.10 | 0.0016 | 0.0095 | 15.25 | 15.89 | 15.43 | 0.88 | 0.7011 | 0.8583 |
| TC1200010806.hg.1 | MAP3K12 | mitogen-activated protein kinase kinase kinase 12 | Multiple_C | 5.31 | 4.42 | 4.24 | 2.10 | 0.045 | 0.134 | 5.87 | 5.56 | 4.76 | 2.16 | 0.003 | 0.0353 |
| TC1200012755.hg.1 | PRR4 | proline rich 4 (lacrimal) | Multiple_C | 5.25 | 4.25 | 4.18 | 2.10 | 0.0746 | 0.1941 | 5.26 | 4.72 | 4.62 | 1.56 | 0.12 | 0.3422 |
| TC1400008333.hg.1 | PPP2R5C | protein phosphatase 2, regulatory subunit B, gamma | Multiple_C | 14.2 | 14.3 | 13.13 | 2.10 | 0.0034 | 0.0177 | 13.49 | 13.21 | 13.47 | 1.01 | 0.772 | 0.8975 |
| TC1700012477.hg.1 | CYGB; RP11-6 | Transcript Identified by AceView, Entrez Gene ID(s) 114757; nov | Multiple_C | 4.54 | 3.29 | 3.47 | 2.10 | 0.0608 | 0.1671 | 4.96 | 4.86 | 4.41 | 1.46 | 0.2076 | 0.4642 |
| TC1800008231.hg.1 | ESCO1 | establishment of sister chromatid cohesion N-acetyltransferase | Multiple_C | 12.6 | 12.53 | 11.53 | 2.10 | 0.0079 | 0.0345 | 9.97 | 9.36 | 8.95 | 2.03 | 0.0065 | 0.0581 |
| TC1900006510.hg.1 | GPX4 | glutathione peroxidase 4 | Multiple_C | 14.56 | 13.64 | 13.49 | 2.10 | 0.0004 | 0.003 | 11.98 | 12.21 | 11.94 | 1.03 | 0.9608 | 0.9837 |
| TC1900010941.hg.1 | ERCC1 | excision repair cross-complementation group 1 | Multiple_C | 10.64 | 9.68 | 9.57 | 2.10 | 0.0012 | 0.0076 | 9.31 | 9.27 | 8.62 | 1.61 | 0.0114 | 0.0844 |
| TC0100007512.hg.1 | GPR3 | G protein-coupled receptor 3 | Coding | 8.37 | 5.81 | 7.31 | 2.08 | 0.0038 | 0.0192 | 6.63 | 6.51 | 7.48 | 0.55 | 0.0508 | 0.2102 |
| TC0100010184.hg.1 | TTC24 | tetratricopeptide repeat domain 24 | Multiple_C | 10.64 | 9.54 | 9.58 | 2.08 | 0.0011 | 0.0071 | 9.23 | 9.57 | 9.38 | 0.90 | 0.3022 | 0.5667 |
| TC0200006969.hg.1 | NCOA1 | nuclear receptor coactivator 1 | Multiple_C | 8.83 | 7.1 | 7.77 | 2.08 | 0.0136 | 0.0531 | 8.14 | 8.22 | 8.05 | 1.06 | 0.2715 | 0.5369 |
| TC0200013265.hg.1 | CAPG | capping protein (actin filament), gelsolin-like | Multiple_C | 10.31 | 10.18 | 9.25 | 2.08 | 0.0103 | 0.0428 | 6.23 | 6.02 | 6.28 | 0.97 | 0.4661 | 0.7078 |
| TC0200015729.hg.1 | USP37 | ubiquitin specific peptidase 37 | Multiple_C | 9.51 | 7.4 | 8.45 | 2.08 | 0.0023 | 0.0132 | 8.43 | 8.21 | 9.12 | 0.62 | 0.0151 | 0.1007 |
| TC0400008913.hg.1 | C4orf51 | chromosome 4 open reading frame 51 | Multiple_C | 4.22 | 3.1 | 3.16 | 2.08 | 0.0286 | 0.0947 | 4.2 | 4.38 | 4.1 | 1.07 | 0.8799 | 0.9484 |
| TC0400012768.hg.1 | FAM200B | family with sequence similarity 200, member B | Multiple_C | 10.51 | 10.34 | 9.45 | 2.08 | 0.0006 | 0.0046 | 9.52 | 9.55 | 9.29 | 1.17 | 0.1958 | 0.4501 |
| TC0500007552.hg.1 | LOC10042156 | family with sequence similarity 133, member A pseudogene | Multiple_C | 8.38 | 7.58 | 7.32 | 2.08 | 0.0348 | 0.1104 | 7.03 | 6.39 | 6.46 | 1.48 | 0.0732 | 0.2593 |
| TC0700009526.hg.1 | CTAGE8; CTAC | CTAGE family, member 8; CTAGE family, member 4 | Coding | 8.02 | 6.9 | 6.96 | 2.08 | 0.0327 | 0.1053 | 7.81 | 8.21 | 7.83 | 0.99 | 0.8315 | 0.9249 |
| TC0700009581.hg.1 | C7orf33 | chromosome 7 open reading frame 33 | Coding | 5.26 | 4.03 | 4.2 | 2.08 | 0.0047 | 0.0228 | 4.76 | 4.56 | 4.57 | 1.14 | 0.7408 | 0.8808 |
| TC0700009977.hg.1 | PDGFA | platelet-derived growth factor alpha polypeptide | Multiple_C | 4.76 | 4.22 | 3.7 | 2.08 | 0.0535 | 0.1518 | 12.25 | 12.66 | 12.01 | 1.18 | 0.1097 | 0.3261 |
| TC0700012701.hg.1 | CNOT4 | CCR4-NOT transcription complex subunit 4 | Multiple_C | 10.04 | 8.61 | 8.98 | 2.08 | 0.0465 | 0.1372 | 8.66 | 7.88 | 9.22 | 0.68 | 0.6845 | 0.8487 |
| TC0900010910.hg.1 | SLC35D2 | solute carrier family 35 (UDP-GlcNAc/UDP-glucose transporter), | Multiple_C | 11.11 | 10.94 | 10.05 | 2.08 | 0.0023 | 0.0132 | 8.73 | 9.25 | 10.17 | 0.37 | 7.78E-05 | 0.003 |
| TC0M00006443.hg.1 | CYTB | cytochrome b | Multiple_C | 18.16 | 17.08 | 17.1 | 2.08 | 0.0051 | 0.0246 | 17.91 | 18.27 | 18.29 | 0.77 | 0.2051 | 0.4614 |
| TC1000009472.hg.1 | CYP2E1 | cytochrome P450, family 2, subfamily E, polypeptide 1 | Multiple_C | 5.13 | 4.6 | 4.07 | 2.08 | 0.006 | 0.0278 | 4.42 | 4.40 | 4.26 | 1.12 | 0.6737 | 0.8419 |
| TC1000011704.hg.1 | NT5C2 | 5-nucleotidase, cytosolic II | Multiple_C | 12.07 | 10.44 | 11.01 | 2.08 | 0.0874 | 0.2182 | 10.68 | 9.89 | 11.16 | 0.72 | 0.4972 | 0.7306 |
| TC1100010087.hg.1 | MTRNR2L8; N | MT-RNR2-like 8; microRNA 4485 | Multiple_C | 17.56 | 16.08 | 16.5 | 2.08 | 0.0013 | 0.0083 | 18.19 | 18.54 | 18.74 | 0.68 | 0.0225 | 0.129 |
| TC1200007640.hg.1 | SLC4A8 | solute carrier family 4, sodium bicarbonate cotransporter, mem | Multiple_C | 8.31 | 7.05 | 7.25 | 2.08 | 0.0098 | 0.0408 | 7.98 | 8.24 | 7.53 | 1.37 | 0.2773 | 0.5432 |
| TC1200012716.hg.1 | COX6A1 | cytochrome c oxidase subunit VIa polypeptide 1 | Multiple_C | 17 | 16.18 | 15.94 | 2.08 | 0.0006 | 0.0045 | 12.8 | 12.80 | 12.51 | 1.22 | 0.0224 | 0.1288 |
| TC1500007691.hg.1 | GLCE | glucuronic acid epimerase | Multiple_C | 9.36 | 8.57 | 8.3 | 2.08 | 0.0396 | 0.1219 | 7.23 | 6.67 | 7.19 | 1.03 | 0.7923 | 0.9077 |
| TC1600009620.hg.1 | SMG1 | SMG1 phosphatidylinositol 3-kinase-related kinase | Multiple_C | 14.42 | 13.71 | 13.36 | 2.08 | 0.0011 | 0.007 | 13.57 | 12.50 | 13.42 | 1.11 | 0.9135 | 0.9646 |

| | | | | | | | | | | | | | | | |
|---|---|---|---|---|---|---|---|---|---|---|---|---|---|---|---|
| TC1700006645.hg.1 | PLD2 | phospholipase D2 | Multiple_C | 8.07 | 7.84 | 7.01 | 2.08 | 0.0262 | 0.0884 | 4.9 | 4.99 | 4.91 | 0.99 | 0.7309 | 0.8753 |
| TC1700011815.hg.1 | MXRA7 | matrix-remodelling associated 7 | Multiple_C | 8.98 | 7.89 | 7.92 | 2.08 | 8.81E-05 | 0.001 | 11.13 | 11.20 | 10.97 | 1.12 | 0.2818 | 0.5474 |
| TC1800007233.hg.1 | KATNAL2 | katanin p60 subunit A-like 2 | Multiple_C | 5.04 | 3.74 | 3.98 | 2.08 | 0.0022 | 0.0126 | 4.57 | 4.41 | 4.57 | 1.00 | 0.3579 | 0.6207 |
| TC1900006642.hg.1 | C19orf71 | chromosome 19 open reading frame 71 | Coding | 5.24 | 3.99 | 4.18 | 2.08 | 0.0004 | 0.0034 | 4.78 | 4.97 | 4.73 | 1.04 | 0.9674 | 0.9865 |
| TC1900008085.hg.1 | ZNF546 | zinc finger protein 546 | Multiple_C | 5.68 | 3.84 | 4.62 | 2.08 | 0.001 | 0.0067 | 5.94 | 6.17 | 5.96 | 0.99 | 0.8156 | 0.9182 |
| TC1900008433.hg.1 | GLTSCR2; SNC | glioma tumor suppressor candidate region gene 2; small nucleo | Multiple_C | 9.2 | 8.84 | 8.14 | 2.08 | 0.2168 | 0.4128 | 6.45 | 6.51 | 6.63 | 0.88 | 0.4856 | 0.7228 |
| TC1900009186.hg.1 | ATP8B3 | ATPase, aminophospholipid transporter, class I, type 8B, memb | Multiple_C | 5.23 | 4.1 | 4.17 | 2.08 | 0.0028 | 0.0154 | 5.64 | 5.58 | 5.43 | 1.16 | 0.1033 | 0.315 |
| TC2000007219.hg.1 | ERGIC3 | ERGIC and golgi 3 | Multiple_C | 14.16 | 13.3 | 13.1 | 2.08 | 0.002 | 0.0115 | 12.2 | 12.22 | 12.27 | 0.95 | 0.591 | 0.7931 |
| TSUnmapped00000215.h | TCF20 | transcription factor 20 (AR1) | Coding | 9.33 | 8.61 | 8.27 | 2.08 | 9.65E-05 | 0.001 | 9.97 | 10.26 | 9.75 | 1.16 | 0.918 | 0.9665 |
| TSUnmapped00000554.h | TCF20 | transcription factor 20 (AR1) | Coding | 9.33 | 8.61 | 8.27 | 2.08 | 9.65E-05 | 0.001 | 9.97 | 10.26 | 9.75 | 1.16 | 0.918 | 0.9665 |
| TSUnmapped00000753.h | RPS25 | ribosomal protein S25 | Coding | 16.88 | 16.91 | 15.82 | 2.08 | 0.0002 | 0.002 | 14.7 | 14.23 | 13.94 | 1.69 | 0.0008 | 0.0147 |
| TC0100014191.hg.1 | RAB3B | RAB3B, member RAS oncogene family | Multiple_C | 7.06 | 5.35 | 6.01 | 2.07 | 0.0027 | 0.0149 | 4.37 | 3.63 | 3.87 | 1.41 | 0.0306 | 0.1556 |
| TC0100015258.hg.1 | DRAM2 | DNA-damage regulated autophagy modulator 2 | Multiple_C | 13.13 | 14.42 | 12.08 | 2.07 | 0.0237 | 0.0819 | 10.36 | 8.83 | 9.25 | 2.16 | 0.05 | 0.2083 |
| TC0100016406.hg.1 | VAMP4 | vesicle associated membrane protein 4 | Multiple_C | 7.55 | 7.36 | 6.5 | 2.07 | 0.0129 | 0.0511 | 6.41 | 5.98 | 5.48 | 1.91 | 0.0037 | 0.0408 |
| TC0200015202.hg.1 | DIRC1 | disrupted in renal carcinoma 1 | Coding | 6.38 | 4.89 | 5.33 | 2.07 | 0.0253 | 0.0862 | 6.27 | 6.23 | 6.41 | 0.91 | 0.9484 | 0.979 |
| TC0300008316.hg.1 | PVRL3 | poliovirus receptor-related 3 | Multiple_C | 10.33 | 9.12 | 9.28 | 2.07 | 0.0039 | 0.0196 | 3.12 | 2.86 | 3.08 | 1.03 | 0.8072 | 0.915 |
| TC0300008324.hg.1 | PHLDB2; PLCX | pleckstrin homology-like domain, family B, member 2; phosphat | Multiple_C | 4.59 | 3.49 | 3.54 | 2.07 | 0.0007 | 0.005 | 3.82 | 3.99 | 3.71 | 1.08 | 0.6627 | 0.8353 |
| TC0500009544.hg.1 | MSX2 | msh homeobox 2 | Coding | 8.38 | 6.93 | 7.33 | 2.07 | 0.0042 | 0.0209 | 8.04 | 7.79 | 7.93 | 1.08 | 0.7952 | 0.9087 |
| TC0500012166.hg.1 | CDC25C | cell division cycle 25C | Multiple_C | 9.56 | 10.14 | 8.51 | 2.07 | 0.021 | 0.0744 | 7.91 | 9.50 | 8.27 | 0.78 | 0.2959 | 0.5607 |
| TC0500012371.hg.1 | PLAC8L1 | PLAC8-like 1 | Coding | 4.5 | 3.05 | 3.45 | 2.07 | 0.005 | 0.024 | 4.37 | 4.30 | 4.31 | 1.04 | 0.5373 | 0.7588 |
| TC0600008379.hg.1 | MTRNR2L9 | MT-RNR2-like 9 | Multiple_C | 13.65 | 12.16 | 12.6 | 2.07 | 0.005 | 0.0241 | 12.93 | 13.59 | 13.51 | 0.67 | 0.0111 | 0.0833 |
| TC0900010352.hg.1 | PTAR1 | protein prenyltransferase alpha subunit repeat containing 1 | Multiple_C | 10.52 | 9.43 | 9.47 | 2.07 | 0.0007 | 0.005 | 10.78 | 9.08 | 10.39 | 1.31 | 0.0875 | 0.2854 |
| TC1200008771.hg.1 | ISCU | iron-sulfur cluster assembly enzyme | Multiple_C | 13.46 | 13.73 | 12.41 | 2.07 | 0.0005 | 0.0036 | 9.92 | 9.35 | 9.67 | 1.19 | 0.2074 | 0.4641 |
| TC1200010740.hg.1 | KRT80 | keratin 80, type II | Multiple_C | 5.97 | 4.95 | 4.92 | 2.07 | 0.0329 | 0.1056 | 10.73 | 9.50 | 10.49 | 1.18 | 0.462 | 0.7047 |
| TC1400008940.hg.1 | NFKBIA | nuclear factor of kappa light polypeptide gene enhancer in B-ce | Multiple_C | 11.86 | 10.14 | 10.81 | 2.07 | 0.0004 | 0.003 | 8.78 | 8.09 | 8.73 | 1.04 | 0.8744 | 0.9459 |
| TC1400009123.hg.1 | SOS2 | SOS Ras/Rho guanine nucleotide exchange factor 2 | Multiple_C | 7.89 | 7.33 | 6.84 | 2.07 | 0.0011 | 0.007 | 5.49 | 5.85 | 6.46 | 0.51 | 0.0064 | 0.0576 |
| TC1600011355.hg.1 | NPIPA1 | nuclear pore complex interacting protein family, member A1 | Multiple_C | 10.51 | 9.7 | 9.46 | 2.07 | 0.0035 | 0.0183 | 10.76 | 10.48 | 10.64 | 1.09 | 0.8733 | 0.9453 |
| TC1900011755.hg.1 | ZNF226 | zinc finger protein 226 | Multiple_C | 8.48 | 8.36 | 7.43 | 2.07 | 0.0026 | 0.0144 | 6.64 | 6.39 | 6.29 | 1.27 | 0.1759 | 0.4243 |
| TC2100008233.hg.1 | ZBTB21 | zinc finger and BTB domain containing 21 | Multiple_C | 11.15 | 9.39 | 10.1 | 2.07 | 0.0013 | 0.008 | 10.1 | 9.03 | 10.58 | 0.72 | 0.231 | 0.4923 |
| TC2100008251.hg.1 | TFF1 | trefoil factor 1 | Multiple_C | 15.22 | 14.79 | 14.17 | 2.07 | 0.0004 | 0.0029 | 3.64 | 3.59 | 3.3 | 1.27 | 0.1585 | 0.4 |
| TC0100017446.hg.1 | SUSD4 | sushi domain containing 4 | Multiple_C | 6.8 | 5.74 | 5.76 | 2.06 | 0.1058 | 0.251 | 3.53 | 3.50 | 3.91 | 0.77 | 0.7239 | 0.8714 |
| TC0300006687.hg.1 | CCDC174 | coiled-coil domain containing 174 | Multiple_C | 8.57 | 7.93 | 7.53 | 2.06 | 0.0339 | 0.1083 | 7.34 | 7.05 | 7.33 | 1.01 | 0.4863 | 0.7234 |
| TC0400012952.hg.1 | BDH2 | 3-hydroxybutyrate dehydrogenase, type 2 | Multiple_C | 10.72 | 11.57 | 9.68 | 2.06 | 0.0073 | 0.0328 | 5.41 | 5.98 | 5.27 | 1.10 | 0.4725 | 0.7127 |

| | | | | | | | | | | | | | | | |
|---|---|---|---|---|---|---|---|---|---|---|---|---|---|---|---|
| TC0600014241.hg.1 | TBC1D7 | TBC1 domain family, member 7 | Multiple_C | 9.65 | 8.48 | 8.61 | 2.06 | 0.0576 | 0.1607 | 6.93 | 6.66 | 6.84 | 1.06 | 0.3425 | 0.6061 |
| TC0700012761.hg.1 | ZC3HAV1 | zinc finger CCCH-type, antiviral 1 | Multiple_C | 10.41 | 8.93 | 9.37 | 2.06 | 0.0008 | 0.0055 | 10.83 | 10.48 | 10.44 | 1.31 | 0.459 | 0.7024 |
| TC0700013493.hg.1 | WDR60 | WD repeat domain 60 | Multiple_C | 7.27 | 6.61 | 6.23 | 2.06 | 0.3117 | 0.5251 | 7.76 | 7.66 | 7.32 | 1.36 | 0.045 | 0.1963 |
| TC0800009854.hg.1 | BIN3-IT1 | BIN3 intronic transcript 1 | Multiple_C | 4.6 | 3.67 | 3.56 | 2.06 | 0.003 | 0.0159 | 4.39 | 4.36 | 4.14 | 1.19 | 0.1758 | 0.4242 |
| TC0900008467.hg.1 | KIAA1958 | KIAA1958 | Multiple_C | 7.9 | 6.71 | 6.86 | 2.06 | 0.0009 | 0.0059 | 8.08 | 7.98 | 7.54 | 1.45 | 0.1815 | 0.4316 |
| TC0X00007238.hg.1 | PAGE4 | P antigen family, member 4 (prostate associated) | Multiple_C | 4.18 | 3.2 | 3.14 | 2.06 | 0.0044 | 0.0218 | 4 | 4.20 | 4.1 | 0.93 | 0.7772 | 0.9002 |
| TC1000010768.hg.1 | EGR2 | early growth response 2 | Multiple_C | 4.87 | 3.56 | 3.83 | 2.06 | 0.006 | 0.0278 | 6.77 | 6.22 | 5.87 | 1.87 | 0.0002 | 0.006 |
| TC1200007594.hg.1 | ASIC1 | acid sensing ion channel 1 | Multiple_C | 7.77 | 7.17 | 6.73 | 2.06 | 0.2383 | 0.44 | 6.58 | 6.34 | 6.34 | 1.18 | 0.9838 | 0.9934 |
| TC1200009913.hg.1 | YBX3 | Y box binding protein 3 | Multiple_C | 14.61 | 13.02 | 13.57 | 2.06 | 0.0083 | 0.0359 | 14.25 | 13.64 | 13.89 | 1.28 | 0.666 | 0.8365 |
| TC1200011327.hg.1 | CSRP2 | cysteine and glycine-rich protein 2 | Multiple_C | 11.17 | 9.72 | 10.13 | 2.06 | 0.003 | 0.0162 | 9.41 | 9.24 | 9.23 | 1.13 | 0.2264 | 0.487 |
| TC1400009256.hg.1 | ATG14 | autophagy related 14 | Multiple_C | 9.78 | 9.66 | 8.74 | 2.06 | 0.0011 | 0.0071 | 9.7 | 8.94 | 8.89 | 1.75 | 0.0004 | 0.0092 |
| TC1400009649.hg.1 | NUMB | numb homolog (Drosophila) | Multiple_C | 11.49 | 10.62 | 10.45 | 2.06 | 0.0053 | 0.0252 | 9.91 | 10.20 | 10.33 | 0.75 | 0.6581 | 0.833 |
| TC1400009701.hg.1 | AREL1 | apoptosis resistant E3 ubiquitin protein ligase 1 | Multiple_C | 10.95 | 9.33 | 9.91 | 2.06 | 0.0012 | 0.0079 | 10.14 | 9.80 | 10.12 | 1.01 | 0.7837 | 0.9026 |
| TC1700010239.hg.1 | NUFIP2 | nuclear fragile X mental retardation protein interacting protein | Coding | 11.88 | 11.03 | 10.84 | 2.06 | 7.99E-05 | 0.0009 | 12.22 | 11.49 | 12.35 | 0.91 | 0.4881 | 0.7244 |
| TC1700010358.hg.1 | MYO1D | myosin ID | Multiple_C | 12.39 | 11.51 | 11.35 | 2.06 | 0.0039 | 0.0196 | 9.64 | 9.59 | 10.03 | 0.76 | 0.0459 | 0.1982 |
| TC1900006796.hg.1 | ALKBH7 | alkB homolog 7 | Multiple_C | 6.36 | 5.96 | 5.32 | 2.06 | 0.0407 | 0.1242 | 4.4 | 4.63 | 4.46 | 0.96 | 0.8839 | 0.9503 |
| TC1900007508.hg.1 | ZNF430 | zinc finger protein 430 | Multiple_C | 11.93 | 10.97 | 10.89 | 2.06 | 0.0034 | 0.0178 | 9.11 | 8.94 | 9.31 | 0.87 | 0.6554 | 0.8321 |
| TC1900008053.hg.1 | SAMD4B | sterile alpha motif domain containing 4B | Multiple_C | 13 | 11.35 | 11.96 | 2.06 | 0.0058 | 0.027 | 12.16 | 11.94 | 11.9 | 1.20 | 0.9269 | 0.9697 |
| TC1900009828.hg.1 | NDUFB7 | NADH dehydrogenase (ubiquinone) 1 beta subcomplex, 7, 18kD | Multiple_C | 9.25 | 8.35 | 8.21 | 2.06 | 0.0023 | 0.0128 | 6.69 | 7.36 | 7.09 | 0.76 | 0.372 | 0.6333 |
| TC1900011049.hg.1 | TPRX1 | tetra-peptide repeat homeobox 1 | Coding | 4.79 | 3.46 | 3.75 | 2.06 | 0.0008 | 0.0055 | 4.46 | 4.41 | 4.16 | 1.23 | 0.2417 | 0.5056 |
| TC2000007184.hg.1 | ITCH | itchy E3 ubiquitin protein ligase | Multiple_C | 11.17 | 10.67 | 10.13 | 2.06 | 0.0001 | 0.0014 | 10.28 | 9.73 | 10.52 | 0.85 | 0.1654 | 0.4102 |
| TC2000008094.hg.1 | ABHD16B | abhydrolase domain containing 16B | Coding | 6.82 | 5.65 | 5.78 | 2.06 | 0.002 | 0.0114 | 6.52 | 6.62 | 6.23 | 1.22 | 0.1703 | 0.4168 |
| TC2000010002.hg.1 | NCOA6 | nuclear receptor coactivator 6 | Multiple_C | 9.34 | 7.65 | 8.3 | 2.06 | 0.0136 | 0.0531 | 9.34 | 9.78 | 9.69 | 0.78 | 0.4282 | 0.6802 |
| TC2100007917.hg.1 | KRTAP11-1 | keratin associated protein 11-1 | Coding | 5.48 | 4.62 | 4.44 | 2.06 | 0.0001 | 0.0011 | 5.3 | 5.22 | 4.94 | 1.28 | 0.0835 | 0.2782 |
| TC2100008495.hg.1 | IL10RB | interleukin 10 receptor, beta | Multiple_C | 8.93 | 8.29 | 7.89 | 2.06 | 0.0273 | 0.0913 | 7.91 | 7.72 | 7.5 | 1.33 | 0.8735 | 0.9454 |
| TC0100007789.hg.1 | AGO3 | argonaute RISC catalytic component 3 | Multiple_C | 9.59 | 8.59 | 8.56 | 2.04 | 0.0133 | 0.0521 | 10.08 | 9.25 | 10.19 | 0.93 | 0.306 | 0.5702 |
| TC0100015182.hg.1 | TAF13 | TAF13 RNA polymerase II, TATA box binding protein (TBP)-assoc | Multiple_C | 11.81 | 11.88 | 10.78 | 2.04 | 0.003 | 0.016 | 12.89 | 11.89 | 12.44 | 1.37 | 0.2336 | 0.4959 |
| TC0200007702.hg.1 | PAPOLG | poly(A) polymerase gamma | Multiple_C | 10.82 | 9.67 | 9.79 | 2.04 | 0.0199 | 0.0713 | 11 | 9.79 | 10.79 | 1.16 | 0.7879 | 0.9054 |
| TC0200016727.hg.1 | IWS1 | IWS1 homolog (S. cerevisiae) | Multiple_C | 11.15 | 9.5 | 10.12 | 2.04 | 0.0027 | 0.0148 | 10.92 | 10.47 | 10.67 | 1.19 | 0.0237 | 0.1329 |
| TC0300007748.hg.1 | MITF | microphthalmia-associated transcription factor | Multiple_C | 4.78 | 3.5 | 3.75 | 2.04 | 9.06E-05 | 0.001 | 3.68 | 3.44 | 3.22 | 1.38 | 0.0177 | 0.1117 |
| TC0300013962.hg.1 | ANO10 | anoctamin 10 | Multiple_C | 8.39 | 8.08 | 7.36 | 2.04 | 0.0227 | 0.0791 | 7.92 | 6.87 | 7.47 | 1.37 | 0.6654 | 0.8363 |
| TC0400011695.hg.1 | SEC24D | SEC24 homolog D, COPII coat complex component | Multiple_C | 10.96 | 10.74 | 9.93 | 2.04 | 0.006 | 0.0278 | 7.88 | 7.47 | 8.56 | 0.62 | 0.4922 | 0.727 |
| TC0500007811.hg.1 | ANKDD1B | ankyrin repeat and death domain containing 1B | Multiple_C | 5.5 | 4.26 | 4.47 | 2.04 | 0.0422 | 0.128 | 5.77 | 6.27 | 5.61 | 1.12 | 0.1599 | 0.4022 |

| | | | | | | | | | | | | | | | |
|---|---|---|---|---|---|---|---|---|---|---|---|---|---|---|---|
| TC0700012296.hg.1 | C7orf60 | chromosome 7 open reading frame 60 | Multiple_C | 6.15 | 5.34 | 5.12 | 2.04 | 0.0014 | 0.0086 | 7.01 | 6.97 | 7.03 | 0.99 | 0.4047 | 0.6602 |
| TC0700013599.hg.1 | ACHE | acetylcholinesterase (Yt blood group) | Coding | 7.52 | 6.31 | 6.49 | 2.04 | 0.0119 | 0.0478 | 5.45 | 5.58 | 5.57 | 0.92 | 0.6857 | 0.8495 |
| TC0900010069.hg.1 | ZNF658B | zinc finger protein 658B, pseudogene | Multiple_C | 5.71 | 5.53 | 4.68 | 2.04 | 0.0126 | 0.0502 | 4.02 | 4.69 | 4.68 | 0.63 | 0.065 | 0.2427 |
| TC0X00007937.hg.1 | DRP2 | dystrophin related protein 2 | Multiple_C | 4.77 | 3.66 | 3.74 | 2.04 | 0.0098 | 0.041 | 4.62 | 4.61 | 4.39 | 1.17 | 0.5305 | 0.7535 |
| TC1000011023.hg.1 | MSS51 | MSS51 mitochondrial translational activator | Coding | 6.41 | 5.09 | 5.38 | 2.04 | 0.0159 | 0.0597 | 6.28 | 5.84 | 5.48 | 1.74 | 0.0146 | 0.0986 |
| TC1100010713.hg.1 | CKAP5; SNORI | cytoskeleton associated protein 5; small nucleolar RNA, C/D box | Multiple_C | 12.32 | 11.38 | 11.29 | 2.04 | 0.0023 | 0.0131 | 13.18 | 13.24 | 13.13 | 1.04 | 0.6404 | 0.8236 |
| TC1100012037.hg.1 | MAML2 | mastermind-like transcriptional coactivator 2 | Multiple_C | 8.04 | 6.86 | 7.01 | 2.04 | 0.0265 | 0.0891 | 10.42 | 10.32 | 9.88 | 1.45 | 0.2383 | 0.5017 |
| TC1100013230.hg.1 | BCL9L | B-cell CLL/lymphoma 9-like | Multiple_C | 9.39 | 7.75 | 8.36 | 2.04 | 0.0019 | 0.011 | 11.64 | 11.63 | 11.66 | 0.99 | 0.963 | 0.9844 |
| TC1200010857.hg.1 | PHC1 | Homo sapiens polyhomeotic homolog 1 (Drosophila), mRNA (cD | Multiple_C | 9.6 | 8.26 | 8.57 | 2.04 | 0.0005 | 0.0037 | 10.36 | 9.98 | 9.74 | 1.54 | 0.0031 | 0.0368 |
| TC1200011891.hg.1 | GIT2 | G protein-coupled receptor kinase interacting ArfGAP 2 | Multiple_C | 10.8 | 9.8 | 9.77 | 2.04 | 0.009 | 0.0382 | 9.15 | 8.69 | 9.17 | 0.99 | 0.8024 | 0.9117 |
| TC1300007969.hg.1 | ABHD13 | abhydrolase domain containing 13 | Coding | 9.4 | 9.21 | 8.37 | 2.04 | 0.0005 | 0.0038 | 10.72 | 9.84 | 9.88 | 1.79 | 0.0008 | 0.0139 |
| TC1400007298.hg.1 | ACTR10 | actin-related protein 10 homolog (S. cerevisiae) | Multiple_C | 11.9 | 12.49 | 10.87 | 2.04 | 0.0027 | 0.0146 | 9.2 | 9.42 | 8.97 | 1.17 | 0.1357 | 0.3662 |
| TC1500010725.hg.1 | CAPN3 | calpain 3 | Multiple_C | 5.56 | 4.16 | 4.53 | 2.04 | 0.0033 | 0.0173 | 3.84 | 3.97 | 3.67 | 1.13 | 0.9596 | 0.9834 |
| TC1600009951.hg.1 | TBX6 | T-box 6 | Multiple_C | 5.22 | 3.95 | 4.19 | 2.04 | 0.0056 | 0.0265 | 4.5 | 4.41 | 4.1 | 1.32 | 0.1278 | 0.3537 |
| TC1700010068.hg.1 | CCDC144NL | coiled-coil domain containing 144 family, N-terminal like | Multiple_C | 7.08 | 6.01 | 6.05 | 2.04 | 0.0041 | 0.0206 | 5.83 | 6.12 | 6.03 | 0.87 | 0.89 | 0.9532 |
| TC1800007620.hg.1 | SOCS6 | suppressor of cytokine signaling 6 | Multiple_C | 9.72 | 8.82 | 8.69 | 2.04 | 0.0037 | 0.019 | 5.33 | 6.00 | 6.24 | 0.53 | 0.0007 | 0.0131 |
| TC1900010574.hg.1 | ZNF571 | zinc finger protein 571 | Multiple_C | 9.18 | 9.57 | 8.15 | 2.04 | 0.0083 | 0.036 | 7.68 | 6.90 | 6.55 | 2.19 | 0.0466 | 0.1997 |
| TC2000008213.hg.1 | DDRGK1 | DDRGK domain containing 1 | Multiple_C | 9.19 | 8.84 | 8.16 | 2.04 | 0.0201 | 0.0717 | 9.04 | 8.87 | 8.79 | 1.19 | 0.7428 | 0.8822 |
| TC2200008186.hg.1 | CHCHD10 | coiled-coil-helix-coiled-coil-helix domain containing 10 | Multiple_C | 14.5 | 13.18 | 13.47 | 2.04 | 0.0024 | 0.0136 | 6.99 | 7.18 | 7.2 | 0.86 | 0.4686 | 0.7096 |
| TC0100010115.hg.1 | TRIM46 | tripartite motif containing 46 | Multiple_C | 4.58 | 3.63 | 3.56 | 2.03 | 0.0015 | 0.0093 | 4.25 | 4.11 | 3.85 | 1.32 | 0.2163 | 0.4754 |
| TC0100013747.hg.1 | SNIP1 | Smad nuclear interacting protein 1 | Multiple_C | 9.11 | 8.06 | 8.09 | 2.03 | 0.0024 | 0.0132 | 8.58 | 8.85 | 8.74 | 0.90 | 0.5123 | 0.7418 |
| TC0100018286.hg.1 | NBPF19 | neuroblastoma breakpoint family, member 19 | Coding | 10.36 | 9.87 | 9.34 | 2.03 | 0.0006 | 0.0043 | 9.16 | 8.69 | 8.57 | 1.51 | 0.038 | 0.1775 |
| TC0100018433.hg.1 | RRAGC | Ras-related GTP binding C | Multiple_C | 12.16 | 12.06 | 11.14 | 2.03 | 0.0054 | 0.0254 | 9.1 | 8.43 | 9.41 | 0.81 | 0.218 | 0.4775 |
| TC0200008573.hg.1 | UNC50 | unc-50 homolog (C. elegans) | Multiple_C | 8.57 | 8.62 | 7.55 | 2.03 | 0.002 | 0.0117 | 6.77 | 6.34 | 6.39 | 1.30 | 0.0516 | 0.2121 |
| TC0200010049.hg.1 | HOXD8 | homeobox D8 | Coding | 8.07 | 8.36 | 7.05 | 2.03 | 0.0281 | 0.0933 | 4.97 | 5.01 | 4.56 | 1.33 | 0.1703 | 0.4168 |
| TC0200014244.hg.1 | FAR2P1 | fatty acyl-CoA reductase 2 pseudogene 1 | Multiple_C | 7.26 | 7.28 | 6.24 | 2.03 | 0.0012 | 0.0077 | 6.75 | 6.73 | 5.93 | 1.77 | 0.0261 | 0.1411 |
| TC0200015332.hg.1 | PGAP1 | post-GPI attachment to proteins 1 | Multiple_C | 10.89 | 10.25 | 9.87 | 2.03 | 0.0044 | 0.0219 | 10.12 | 9.18 | 9.61 | 1.42 | 0.1693 | 0.4151 |
| TC0300009855.hg.1 | IL1RAP | interleukin 1 receptor accessory protein | Multiple_C | 8.13 | 7.59 | 7.11 | 2.03 | 0.0112 | 0.0455 | 6.82 | 4.88 | 5.25 | 2.97 | 0.0001 | 0.0042 |
| TC0300012482.hg.1 | RAB6B | RAB6B, member RAS oncogene family | Multiple_C | 6.96 | 5.83 | 5.94 | 2.03 | 0.0008 | 0.0059 | 6.94 | 6.38 | 5.95 | 1.99 | 0.0011 | 0.0181 |
| TC0400007928.hg.1 | FRAS1 | Fraser extracellular matrix complex subunit 1 | Multiple_C | 8.52 | 8.01 | 7.5 | 2.03 | 0.0021 | 0.0121 | 7.9 | 7.75 | 7.6 | 1.23 | 0.0264 | 0.1419 |
| TC0400009894.hg.1 | C4orf50 | chromosome 4 open reading frame 50 | Coding | 5.43 | 4.57 | 4.41 | 2.03 | 0.0168 | 0.0624 | 4.52 | 4.85 | 4.78 | 0.84 | 0.2491 | 0.5137 |
| TC0500008883.hg.1 | PCDHB12 | protocadherin beta 12 | Coding | 9.09 | 8.27 | 8.07 | 2.03 | 0.0007 | 0.0052 | 8.1 | 8.09 | 7.81 | 1.22 | 0.6363 | 0.8215 |
| TC0500013064.hg.1 | MGAT4B | mannosyl (alpha-1,3-)-glycoprotein beta-1,4-N-acetylglucosamii | Multiple_C | 11.09 | 10.56 | 10.07 | 2.03 | 0.0119 | 0.048 | 8.96 | 9.35 | 9.55 | 0.66 | 0.0513 | 0.2115 |

| | | | | | | | | | | | | | | | |
|---|---|---|---|---|---|---|---|---|---|---|---|---|---|---|---|
| TC0700011574.hg.1 | SPDYE16 | speedy/RINGO cell cycle regulator family member E16 [Source:I | Multiple_C | 7.89 | 6.62 | 6.87 | 2.03 | 0.0008 | 0.0059 | 6.82 | 7.04 | 7.01 | 0.88 | 0.3385 | 0.6028 |
| TC0700011948.hg.1 | FAM200A | family with sequence similarity 200, member A | Coding | 4.59 | 3.48 | 3.57 | 2.03 | 0.0169 | 0.0628 | 5.03 | 5.34 | 5.42 | 0.76 | 0.6275 | 0.816 |
| TC0700012086.hg.1 | POLR2J | polymerase (RNA) II (DNA directed) polypeptide J, 13.3kDa | Multiple_C | 14.86 | 13.48 | 13.84 | 2.03 | 0.0009 | 0.0061 | 12.92 | 13.58 | 13.95 | 0.49 | 0.0017 | 0.0239 |
| TC0700013456.hg.1 | ARF5 | ADP-ribosylation factor 5 | Multiple_C | 13.33 | 12 | 12.31 | 2.03 | 0.0015 | 0.009 | 12.57 | 13.24 | 12.82 | 0.84 | 0.2036 | 0.4592 |
| TC0800007382.hg.1 | IDO1 | indoleamine 2,3-dioxygenase 1 | Multiple_C | 4.72 | 3.74 | 3.7 | 2.03 | 0.0366 | 0.1147 | 4.47 | 4.19 | 4.08 | 1.31 | 0.2711 | 0.5366 |
| TC0900009275.hg.1 | C9orf173 | chromosome 9 open reading frame 173 | Multiple_C | 6.85 | 6.1 | 5.83 | 2.03 | 0.0262 | 0.0883 | 6.34 | 6.41 | 5.97 | 1.29 | 0.4832 | 0.7207 |
| TC0X00007523.hg.1 | EDA | ectodysplasin A | Multiple_C | 4.14 | 2.96 | 3.12 | 2.03 | 0.0245 | 0.0841 | 4.49 | 4.32 | 4.28 | 1.16 | 0.8181 | 0.9197 |
| TC1000012117.hg.1 | CHST15 | carbohydrate (N-acetylgalactosamine 4-sulfate 6-O) sulfotransfe | Multiple_C | 7.77 | 6.39 | 6.75 | 2.03 | 0.0139 | 0.0538 | 11.62 | 10.32 | 10.49 | 2.19 | 0.0003 | 0.0083 |
| TC1100007227.hg.1 | HIPK3 | homeodomain interacting protein kinase 3 | Multiple_C | 10.24 | 8.81 | 9.22 | 2.03 | 0.1362 | 0.3009 | 10.06 | 9.56 | 10.3 | 0.85 | 0.0978 | 0.3051 |
| TC1100007868.hg.1 | GNG3 | guanine nucleotide binding protein (G protein), gamma 3 | Multiple_C | 4.45 | 3.26 | 3.43 | 2.03 | 0.0028 | 0.0152 | 4.38 | 4.42 | 4.11 | 1.21 | 0.1748 | 0.4228 |
| TC1300007344.hg.1 | TDRD3 | tudor domain containing 3 | Multiple_C | 8.3 | 8.03 | 7.28 | 2.03 | 0.0063 | 0.0291 | 8.13 | 9.00 | 8.6 | 0.72 | 0.1404 | 0.3725 |
| TC1300008552.hg.1 | ZAR1L | zygote arrest 1-like | Coding | 4.54 | 3.48 | 3.52 | 2.03 | 0.0017 | 0.0103 | 3.73 | 3.64 | 3.6 | 1.09 | 0.5785 | 0.7843 |
| TC1400010692.hg.1 | APOPT1 | apoptogenic 1, mitochondrial | Multiple_C | 10.05 | 10.3 | 9.03 | 2.03 | 0.0118 | 0.0477 | 10.32 | 9.84 | 10.22 | 1.07 | 0.9279 | 0.9698 |
| TC1600010854.hg.1 | CLEC18B | C-type lectin domain family 18, member B | Multiple_C | 4.36 | 3.5 | 3.34 | 2.03 | 0.0007 | 0.0049 | 4.25 | 4.24 | 4.05 | 1.15 | 0.0887 | 0.2882 |
| TC1700008912.hg.1 | MYO15B | myosin XVB | Multiple_C | 6.17 | 4.95 | 5.15 | 2.03 | 0.071 | 0.1874 | 6.71 | 7.16 | 6.65 | 1.04 | 0.6609 | 0.8345 |
| TC1700010686.hg.1 | JUP | junction plakoglobin | Multiple_C | 11.31 | 9.37 | 10.29 | 2.03 | 0.01 | 0.0416 | 9.37 | 9.37 | 8.86 | 1.42 | 0.1777 | 0.4263 |
| TC1900008432.hg.1 | EHD2 | EH domain containing 2 | Multiple_C | 11.57 | 10.29 | 10.55 | 2.03 | 0.0073 | 0.0325 | 5.63 | 4.77 | 4.85 | 1.72 | 0.0407 | 0.185 |
| TC1900011312.hg.1 | ZNF611 | zinc finger protein 611 | Multiple_C | 13.43 | 12.81 | 12.41 | 2.03 | 0.0091 | 0.0386 | 11.27 | 11.33 | 11.27 | 1.00 | 0.7981 | 0.9095 |
| TC1900011746.hg.1 | ARHGEF1 | Rho guanine nucleotide exchange factor 1 | Multiple_C | 6.26 | 5.52 | 5.24 | 2.03 | 0.0228 | 0.0793 | 5.22 | 5.19 | 5.2 | 1.01 | 0.1013 | 0.3114 |
| TC2000008007.hg.1 | RPS21 | ribosomal protein S21 | Multiple_C | 19 | 18.78 | 17.98 | 2.03 | 0.0008 | 0.0059 | 15.15 | 15.13 | 14.36 | 1.73 | 0.0009 | 0.0155 |
| TC2000008193.hg.1 | IDH3B | isocitrate dehydrogenase 3 (NAD+) beta | Multiple_C | 11.53 | 10.28 | 10.51 | 2.03 | 0.0021 | 0.0119 | 12.33 | 12.33 | 11.99 | 1.27 | 0.264 | 0.5288 |
| TC2000010031.hg.1 | TAF4 | TAF4 RNA polymerase II, TATA box binding protein (TBP)-associ | Multiple_C | 9.97 | 8.85 | 8.95 | 2.03 | 0.0009 | 0.0062 | 10.2 | 10.11 | 10.58 | 0.77 | 0.2492 | 0.5137 |
| TC2200009152.hg.1 | PLXNB2 | plexin B2 | Multiple_C | 9.29 | 8.28 | 8.27 | 2.03 | 0.0309 | 0.1006 | 8.57 | 9.07 | 8.43 | 1.10 | 0.3115 | 0.5756 |
| TC0100006982.hg.1 | EFHD2 | EF-hand domain family member D2 | Coding | 10.73 | 9.19 | 9.72 | 2.01 | 0.074 | 0.193 | 9.83 | 10.35 | 10.42 | 0.66 | 0.003 | 0.0354 |
| TC0100009417.hg.1 | C1orf162 | chromosome 1 open reading frame 162 | Multiple_C | 4.71 | 3.77 | 3.7 | 2.01 | 0.0017 | 0.0102 | 4.22 | 4.35 | 3.94 | 1.21 | 0.2383 | 0.5017 |
| TC0100009950.hg.1 | ZNF687 | zinc finger protein 687 | Multiple_C | 7.99 | 6.84 | 6.98 | 2.01 | 0.0009 | 0.0062 | 7.02 | 7.81 | 7.51 | 0.71 | 0.0411 | 0.1864 |
| TC0100012468.hg.1 | DVL1; MIR680 | dishevelled segment polarity protein 1; microRNA 6808 | Multiple_C | 10.07 | 9.43 | 9.06 | 2.01 | 0.0545 | 0.1539 | 9.72 | 10.03 | 9.7 | 1.01 | 0.2056 | 0.4621 |
| TC0100015957.hg.1 | ASH1L; MIR55 | ash1 (absent, small, or homeotic)-like (Drosophila); microRNA 5 | Multiple_C | 8.76 | 7.75 | 7.75 | 2.01 | 0.004 | 0.02 | 7.43 | 7.33 | 7.35 | 1.06 | 0.7578 | 0.8901 |
| TC0100016447.hg.1 | RC3H1 | ring finger and CCCH-type domains 1 | Multiple_C | 9.45 | 7.64 | 8.44 | 2.01 | 0.0021 | 0.0122 | 9.61 | 9.05 | 9.71 | 0.93 | 0.0548 | 0.2195 |
| TC0100016839.hg.1 | DENND1B | DENN/MADD domain containing 1B | Multiple_C | 8.53 | 7.67 | 7.52 | 2.01 | 0.0534 | 0.1516 | 6.32 | 6.33 | 6.44 | 0.92 | 0.1423 | 0.375 |
| TC0100016851.hg.1 | ATP6V1G3 | ATPase, H+ transporting, lysosomal 13kDa, V1 subunit G3 | Coding | 7.19 | 5.76 | 6.18 | 2.01 | 0.0016 | 0.0095 | 6.16 | 5.85 | 5.9 | 1.20 | 0.2093 | 0.4664 |
| TC0100016988.hg.1 | FMOD | fibromodulin | Multiple_C | 4.9 | 4.13 | 3.89 | 2.01 | 0.0553 | 0.1557 | 4.52 | 4.56 | 4.42 | 1.07 | 0.3205 | 0.5845 |
| TC0200008059.hg.1 | STAMBP | STAM binding protein | Multiple_C | 11.28 | 9.94 | 10.27 | 2.01 | 0.0034 | 0.0178 | 10.94 | 10.45 | 10.76 | 1.13 | 0.3589 | 0.6217 |

| | | | | | | | | | | | | | | | |
|---|---|---|---|---|---|---|---|---|---|---|---|---|---|---|---|
| TC0200008260.hg.1 | VAMP8 | vesicle associated membrane protein 8 | Multiple_C | 10.47 | 9.64 | 9.46 | 2.01 | 0.0011 | 0.0071 | 9.22 | 9.18 | 8.63 | 1.51 | 0.0265 | 0.1421 |
| TC0200008924.hg.1 | CHCHD5 | coiled-coil-helix-coiled-coil-helix domain containing 5 | Multiple_C | 6.94 | 5.91 | 5.93 | 2.01 | 0.0013 | 0.008 | 6.77 | 7.31 | 7.13 | 0.78 | 0.4413 | 0.6905 |
| TC0200010473.hg.1 | FZD7 | frizzled class receptor 7 | Coding | 7.15 | 7.18 | 6.14 | 2.01 | 0.0352 | 0.1114 | 4.03 | 3.76 | 3.86 | 1.13 | 0.1564 | 0.397 |
| TC0200014694.hg.1 | ACVR1C | activin A receptor type IC | Multiple_C | 7.1 | 6.09 | 6.09 | 2.01 | 0.0037 | 0.0191 | 5.12 | 4.69 | 4.71 | 1.33 | 0.0654 | 0.2435 |
| TC0300013821.hg.1 | ZNF197 | zinc finger protein 197 | Multiple_C | 7.68 | 7.49 | 6.67 | 2.01 | 0.0028 | 0.0152 | 5.89 | 6.08 | 6.01 | 0.92 | 0.7958 | 0.9088 |
| TC0300013828.hg.1 | RBM6 | RNA binding motif protein 6 | Multiple_C | 11.75 | 10.38 | 10.74 | 2.01 | 0.0002 | 0.0019 | 12.06 | 11.85 | 12.17 | 0.93 | 0.622 | 0.8128 |
| TC0400011791.hg.1 | ANKRD50 | ankyrin repeat domain 50 | Multiple_C | 7.36 | 6.71 | 6.35 | 2.01 | 0.0437 | 0.1311 | 6.37 | 6.75 | 6.91 | 0.69 | 0.0429 | 0.191 |
| TC0500007549.hg.1 | ZSWIM6 | zinc finger, SWIM-type containing 6 | Multiple_C | 10.75 | 9.61 | 9.74 | 2.01 | 0.0284 | 0.0941 | 10.84 | 9.82 | 9.82 | 2.03 | 0.0218 | 0.1267 |
| TC0500010788.hg.1 | IL6ST | interleukin 6 signal transducer | Multiple_C | 10 | 8.69 | 8.99 | 2.01 | 0.0005 | 0.004 | 9.74 | 8.62 | 9.19 | 1.46 | 0.0162 | 0.106 |
| TC0500012236.hg.1 | PFDN1 | prefoldin subunit 1 | Multiple_C | 12.28 | 11.54 | 11.27 | 2.01 | 0.0161 | 0.0604 | 10.16 | 10.48 | 10.14 | 1.01 | 0.3973 | 0.6551 |
| TC0600008146.hg.1 | RUNX2 | runt-related transcription factor 2 | Multiple_C | 9.3 | 7.99 | 8.29 | 2.01 | 0.0002 | 0.0016 | 5.24 | 5.35 | 5.23 | 1.01 | 0.8871 | 0.9518 |
| TC0600009488.hg.1 | RPS12 | ribosomal protein S12 | Multiple_C | 17.27 | 17.03 | 16.26 | 2.01 | 0.0049 | 0.0236 | 14.53 | 14.06 | 13.32 | 2.31 | 0.0004 | 0.0085 |
| TC0600014276.hg.1 | HLA-DMB | major histocompatibility complex, class II, DM beta | Multiple_C | 5.98 | 4.4 | 4.97 | 2.01 | 0.0011 | 0.0073 | 5.44 | 5.34 | 5.31 | 1.09 | 0.6863 | 0.8497 |
| TC0700008517.hg.1 | ZKSCAN1 | zinc finger with KRAB and SCAN domains 1 | Multiple_C | 12.15 | 11.56 | 11.14 | 2.01 | 0.0011 | 0.0073 | 12.09 | 12.17 | 11.74 | 1.27 | 0.0824 | 0.2765 |
| TC0900007505.hg.1 | FAM189A2 | family with sequence similarity 189, member A2 | Multiple_C | 5.49 | 4.46 | 4.48 | 2.01 | 0.0119 | 0.0478 | 4.66 | 4.67 | 4.24 | 1.34 | 0.1368 | 0.3676 |
| TC0X00006789.hg.1 | FAM3C2 | FAM3C pseudogene | Multiple_C | 13.24 | 12.94 | 12.23 | 2.01 | 0.0062 | 0.0286 | 11.36 | 10.67 | 10.61 | 1.68 | 0.0006 | 0.0122 |
| TC0X00008481.hg.1 | FAM122C | family with sequence similarity 122C | Multiple_C | 7.52 | 6.75 | 6.51 | 2.01 | 0.0035 | 0.0181 | 8.19 | 8.39 | 8.38 | 0.88 | 0.064 | 0.2409 |
| TC1000009985.hg.1 | C10orf113 | chromosome 10 open reading frame 113 | Coding | 9.13 | 8.06 | 8.12 | 2.01 | 0.0007 | 0.0048 | 6.65 | 6.83 | 6.58 | 1.05 | 0.3179 | 0.582 |
| TC1000010993.hg.1 | MICU1 | mitochondrial calcium uptake 1 | Multiple_C | 10.23 | 9.68 | 9.22 | 2.01 | 0.0006 | 0.0044 | 10.82 | 10.19 | 10.22 | 1.52 | 0.0426 | 0.1905 |
| TC1100008786.hg.1 | CEP295 | centrosomal protein 295kDa | Multiple_C | 8.04 | 7.19 | 7.03 | 2.01 | 0.0196 | 0.0704 | 8.01 | 8.27 | 8.08 | 0.95 | 0.9317 | 0.9711 |
| TC1100010505.hg.1 | ABTB2 | ankyrin repeat and BTB (POZ) domain containing 2 | Multiple_C | 8.32 | 6.83 | 7.31 | 2.01 | 0.0078 | 0.0342 | 4.72 | 4.56 | 5.26 | 0.69 | 0.0025 | 0.0311 |
| TC1100011094.hg.1 | UBXN1 | UBX domain protein 1 | Multiple_C | 12.63 | 11.89 | 11.62 | 2.01 | 0.0029 | 0.0158 | 12.88 | 12.58 | 12.35 | 1.44 | 0.0165 | 0.1069 |
| TC1200012663.hg.1 | RAB3IP | RAB3A interacting protein | Multiple_C | 11.1 | 10.51 | 10.09 | 2.01 | 0.0003 | 0.0024 | 10.32 | 9.31 | 8.98 | 2.53 | 1.39E-06 | 0.0002 |
| TC1300006767.hg.1 | USPL1 | ubiquitin specific peptidase like 1 | Multiple_C | 11.38 | 11.16 | 10.37 | 2.01 | 0.0255 | 0.0865 | 10.67 | 8.74 | 9.76 | 1.88 | 0.004 | 0.0432 |
| TC1400008452.hg.1 | ADSSL1 | adenylosuccinate synthase like 1 | Multiple_C | 7.14 | 7.35 | 6.13 | 2.01 | 0.0096 | 0.0403 | 5.84 | 5.33 | 5.26 | 1.49 | 0.0016 | 0.0228 |
| TC1500009224.hg.1 | STRC | stereocilin | Multiple_C | 4.72 | 4.3 | 3.71 | 2.01 | 0.0003 | 0.0028 | 3.37 | 3.17 | 3.33 | 1.03 | 0.8861 | 0.9512 |
| TC1600010966.hg.1 | CDYL2 | chromodomain protein, Y-like 2 | Multiple_C | 10.21 | 9.61 | 9.2 | 2.01 | 0.0001 | 0.0013 | 7.48 | 9.31 | 9.58 | 0.23 | 8.42E-10 | 9.43E-07 |
| TC1700011652.hg.1 | FAM104A | family with sequence similarity 104, member A | Multiple_C | 8.64 | 7.64 | 7.63 | 2.01 | 0.0021 | 0.0121 | 7.57 | 7.33 | 7.26 | 1.24 | 0.1431 | 0.3764 |
| TC1900009692.hg.1 | ZNF823 | zinc finger protein 823 | Multiple_C | 10.43 | 10.1 | 9.42 | 2.01 | 0.0007 | 0.0053 | 8.84 | 8.68 | 8.88 | 0.97 | 0.9369 | 0.9738 |
| TC2000009134.hg.1 | CHD6 | chromodomain helicase DNA binding protein 6 | Multiple_C | 9.73 | 9.18 | 8.72 | 2.01 | 0.002 | 0.0115 | 8.92 | 8.88 | 8.8 | 1.09 | 0.279 | 0.5452 |
| TC2200009352.hg.1 | LOC400927; C | TPTE and PTEN homologous inositol lipid phosphatase pseudog | Multiple_C | 7.14 | 6.57 | 6.13 | 2.01 | 0.0004 | 0.0029 | 7.32 | 6.80 | 6.29 | 2.04 | 0.0044 | 0.0456 |
| TC0100013441.hg.1 | FGR | FGR proto-oncogene, Src family tyrosine kinase | Multiple_C | 7.92 | 6.53 | 6.92 | 2.00 | 0.0026 | 0.0141 | 6.89 | 6.95 | 6.66 | 1.17 | 0.2846 | 0.5496 |
| TC0100015401.hg.1 | IGSF3 | immunoglobulin superfamily, member 3 | Multiple_C | 8.46 | 8.08 | 7.46 | 2.00 | 0.0131 | 0.0516 | 4.86 | 4.83 | 4.55 | 1.24 | 0.2827 | 0.548 |

| | | | | | | | | | | | | | | | |
|---|---|---|---|---|---|---|---|---|---|---|---|---|---|---|---|
| TC0100018285.hg.1 | NBPF19 | neuroblastoma breakpoint family, member 19 | Coding | 10.6 | 9.86 | 9.6 | 2.00 | 0.0013 | 0.0081 | 8.1 | 7.13 | 6.7 | 2.64 | 6.07E-07 | 0.0001 |
| TC0200010975.hg.1 | AGFG1; MIR5 | ArfGAP with FG repeats 1; microRNA 5703 | Multiple_C | 11.65 | 10.31 | 10.65 | 2.00 | 0.0067 | 0.0306 | 10.97 | 10.83 | 11.53 | 0.68 | 0.0416 | 0.1879 |
| TC0300009815.hg.1 | LPP | LIM domain containing preferred translocation partner in lipom | Multiple_C | 9.3 | 7.31 | 8.3 | 2.00 | 0.0088 | 0.0375 | 8.28 | 7.99 | 8.63 | 0.78 | 0.1284 | 0.3547 |
| TC0300012155.hg.1 | HGD | homogentisate 1,2-dioxygenase | Multiple_C | 7.27 | 6.35 | 6.27 | 2.00 | 0.0292 | 0.0963 | 5.88 | 8.01 | 7.62 | 0.30 | 0.0005 | 0.0106 |
| TC0400010168.hg.1 | LCORL | ligand dependent nuclear receptor corepressor like | Multiple_C | 12.08 | 11.23 | 11.08 | 2.00 | 0.0017 | 0.0101 | 11.67 | 10.09 | 10.76 | 1.88 | 0.0078 | 0.0661 |
| TC0500007875.hg.1 | BHMT2 | betaine--homocysteine S-methyltransferase 2 | Multiple_C | 8.56 | 7.68 | 7.56 | 2.00 | 0.0045 | 0.0222 | 6.92 | 7.03 | 6.81 | 1.08 | 0.572 | 0.7798 |
| TC0600011583.hg.1 | C6orf1 | chromosome 6 open reading frame 1 | Multiple_C | 7.41 | 6.22 | 6.41 | 2.00 | 0.0112 | 0.0458 | 6.24 | 5.80 | 6.03 | 1.16 | 0.6634 | 0.8355 |
| TC0600013249.hg.1 | ALDH8A1 | aldehyde dehydrogenase 8 family, member A1 | Multiple_C | 5.11 | 4.3 | 4.11 | 2.00 | 0.0024 | 0.0134 | 4.32 | 4.30 | 3.85 | 1.39 | 0.067 | 0.247 |
| TC0600014089.hg.1 | PGBD1 | piggyBac transposable element derived 1 | Multiple_C | 7.13 | 5.95 | 6.13 | 2.00 | 0.0043 | 0.0212 | 6.95 | 6.95 | 6.86 | 1.06 | 0.8357 | 0.9277 |
| TC0900009859.hg.1 | AQP7 | aquaporin 7 | Multiple_C | 4.98 | 3.96 | 3.98 | 2.00 | 0.028 | 0.0932 | 4.35 | 4.69 | 4.37 | 0.99 | 0.3534 | 0.6169 |
| TC0X00008735.hg.1 | PRRG3 | proline rich Gla (G-carboxyglutamic acid) 3 (transmembrane) | Multiple_C | 5.47 | 4.43 | 4.47 | 2.00 | 0.0285 | 0.0945 | 4.28 | 4.44 | 4.76 | 0.72 | 0.2762 | 0.5424 |
| TC1100009040.hg.1 | C11orf1 | chromosome 11 open reading frame 1 | Coding | 13.1 | 13.07 | 12.1 | 2.00 | 0.0978 | 0.2367 | 9.09 | 9.09 | 8.89 | 1.15 | 0.8611 | 0.9406 |
| TC1100011535.hg.1 | LOC10013331 | transient receptor potential cation channel, subfamily C, memb | Multiple_C | 6.82 | 5.48 | 5.82 | 2.00 | 0.0068 | 0.0306 | 5.57 | 5.74 | 5.5 | 1.05 | 0.9465 | 0.9782 |
| TC1100013160.hg.1 | C11orf91 | chromosome 11 open reading frame 91 | Multiple_C | 4.98 | 3.93 | 3.98 | 2.00 | 0.0002 | 0.0018 | 3.95 | 3.69 | 3.53 | 1.34 | 0.0247 | 0.1364 |
| TC1200009967.hg.1 | DUSP16 | dual specificity phosphatase 16 | Multiple_C | 9.09 | 8.81 | 8.09 | 2.00 | 0.003 | 0.0162 | 8.77 | 7.93 | 8.02 | 1.68 | 0.005 | 0.0491 |
| TC1200011657.hg.1 | ANKS1B | ankyrin repeat and sterile alpha motif domain containing 1B | Multiple_C | 6.19 | 5.14 | 5.19 | 2.00 | 0.0199 | 0.0711 | 5.62 | 6.09 | 5.85 | 0.85 | 0.2341 | 0.4967 |
| TC1200012803.hg.1 | NACA | nascent polypeptide-associated complex alpha subunit | Multiple_C | 16.37 | 16.11 | 15.37 | 2.00 | 7.24E-05 | 0.0008 | 15.33 | 14.95 | 14.84 | 1.40 | 0.0111 | 0.0835 |
| TC1400006659.hg.1 | MMP14 | matrix metallopeptidase 14 (membrane-inserted) | Multiple_C | 6.31 | 5.41 | 5.31 | 2.00 | 0.0017 | 0.0102 | 4.16 | 4.14 | 3.73 | 1.35 | 0.1373 | 0.368 |
| TC1500010845.hg.1 | OTUD7A | OTU deubiquitinase 7A | Multiple_C | 5.9 | 4.7 | 4.9 | 2.00 | 0.0002 | 0.0015 | 5.13 | 5.28 | 5.12 | 1.01 | 0.2483 | 0.5129 |
| TC1500010942.hg.1 | LYSMD4 | LysM, putative peptidoglycan-binding, domain containing 4 | Multiple_C | 5.59 | 4.48 | 4.59 | 2.00 | 0.0186 | 0.0676 | 4.05 | 4.19 | 4.01 | 1.03 | 0.6005 | 0.7997 |
| TC1700009275.hg.1 | FN3K | fructosamine 3 kinase | Multiple_C | 8.48 | 7.66 | 7.48 | 2.00 | 0.015 | 0.0572 | 7.84 | 7.44 | 7.02 | 1.77 | 0.2141 | 0.4725 |
| TC1700010865.hg.1 | PLCD3 | phospholipase C, delta 3 | Multiple_C | 7.47 | 6.59 | 6.47 | 2.00 | 0.0186 | 0.0677 | 6.64 | 7.06 | 7.01 | 0.77 | 0.0743 | 0.2616 |
| TC1800009232.hg.1 | LOC10050554 | uncharacterized LOC100505549; Salzman2013 ANNOTATED, CD | Multiple_C | 5.68 | 4.51 | 4.68 | 2.00 | 0.0005 | 0.004 | 6.26 | 6.36 | 6.56 | 0.81 | 0.1916 | 0.4445 |
| TC1900010781.hg.1 | RABAC1 | Rab acceptor 1 (prenylated) | Multiple_C | 12.61 | 12.08 | 11.61 | 2.00 | 0.0008 | 0.0058 | 10.07 | 10.14 | 9.7 | 1.29 | 0.075 | 0.2632 |
| TC1900010805.hg.1 | CXCL17 | chemokine (C-X-C motif) ligand 17 | Coding | 5.5 | 4.99 | 4.5 | 2.00 | 0.0034 | 0.0178 | 4.87 | 5.02 | 4.85 | 1.01 | 0.7346 | 0.8776 |
| TC2000007497.hg.1 | WFDC13 | WAP four-disulfide core domain 13 | Coding | 3.85 | 2.74 | 2.85 | 2.00 | 0.0244 | 0.0838 | 3.56 | 3.65 | 3.72 | 0.90 | 0.8212 | 0.921 |
| TC2000009946.hg.1 | FAM209B | family with sequence similarity 209, member B | Coding | 4.87 | 4.13 | 3.87 | 2.00 | 0.0031 | 0.0163 | 3.64 | 3.43 | 3.43 | 1.16 | 0.3219 | 0.5858 |
| TC0100008648.hg.1 | IL23R | interleukin 23 receptor | Multiple_C | 5 | 3.83 | 4.01 | 1.99 | 0.0009 | 0.0062 | 4.34 | 4.46 | 3.98 | 1.28 | 0.0841 | 0.279 |
| TC0100011397.hg.1 | C4BPB | complement component 4 binding protein, beta | Multiple_C | 5.5 | 4.63 | 4.51 | 1.99 | 0.0018 | 0.0105 | 3.57 | 3.18 | 3.41 | 1.12 | 0.0678 | 0.248 |
| TC0100013611.hg.1 | BSDC1 | BSD domain containing 1 | Multiple_C | 9.13 | 8.21 | 8.14 | 1.99 | 0.0036 | 0.0185 | 8.08 | 7.99 | 8.18 | 0.93 | 0.8879 | 0.9523 |
| TC0100015254.hg.1 | LRIF1 | ligand dependent nuclear receptor interacting factor 1 | Multiple_C | 8.49 | 8.03 | 7.5 | 1.99 | 0.112 | 0.2617 | 6.31 | 5.91 | 6.58 | 0.83 | 0.487 | 0.7237 |
| TC0100015680.hg.1 | NBPF9 | neuroblastoma breakpoint family, member 9 | Multiple_C | 9.38 | 8.75 | 8.39 | 1.99 | 0.0128 | 0.0506 | 7.98 | 8.00 | 8.67 | 0.62 | 0.0171 | 0.1095 |
| TC0200007486.hg.1 | TTC7A | tetratricopeptide repeat domain 7A | Multiple_C | 7.04 | 5.68 | 6.05 | 1.99 | 0.052 | 0.1486 | 6.3 | 6.78 | 6.69 | 0.76 | 0.0451 | 0.1963 |

| | | | | | | | | | | | | | | | |
|---|---|---|---|---|---|---|---|---|---|---|---|---|---|---|---|
| TC0200009596.hg.1 | ACVR2A | activin A receptor type IIA | Multiple_C | 9.49 | 9.22 | 8.5 | 1.99 | 0.0023 | 0.0132 | 7.5 | 6.74 | 6.97 | 1.44 | 0.2035 | 0.4591 |
| TC0400009526.hg.1 | C4orf47 | chromosome 4 open reading frame 47 | Multiple_C | 6.12 | 5.29 | 5.13 | 1.99 | 0.0108 | 0.0443 | 6.1 | 6.03 | 5.42 | 1.60 | 0.0076 | 0.0651 |
| TC0500013369.hg.1 | SIL1 | SIL1 nucleotide exchange factor | Multiple_C | 9.28 | 8.58 | 8.29 | 1.99 | 0.0771 | 0.199 | 8.05 | 8.08 | 8.01 | 1.03 | 0.4568 | 0.7007 |
| TC0600010777.hg.1 | TFAP2A | transcription factor AP-2 alpha (activating enhancer binding pro | Multiple_C | 8.15 | 6.91 | 7.16 | 1.99 | 0.0037 | 0.0192 | 5.5 | 5.74 | 5.68 | 0.88 | 0.9484 | 0.979 |
| TC0600011802.hg.1 | FRS3 | fibroblast growth factor receptor substrate 3 | Multiple_C | 7.96 | 6.41 | 6.97 | 1.99 | 0.0014 | 0.0086 | 7.03 | 6.99 | 6.74 | 1.22 | 0.4006 | 0.6573 |
| TC0700012971.hg.1 | ZNF425 | zinc finger protein 425 | Multiple_C | 7.1 | 6.84 | 6.11 | 1.99 | 0.001 | 0.0067 | 5.51 | 6.47 | 5.79 | 0.82 | 0.525 | 0.7498 |
| TC0900009933.hg.1 | FAM214B | family with sequence similarity 214, member B | Multiple_C | 6.97 | 6.09 | 5.98 | 1.99 | 0.0015 | 0.0091 | 5.31 | 5.28 | 5.28 | 1.02 | 0.3976 | 0.6552 |
| TC0900010814.hg.1 | ZNF484 | zinc finger protein 484 | Multiple_C | 8.36 | 8.39 | 7.37 | 1.99 | 0.0291 | 0.096 | 7.3 | 6.67 | 6.46 | 1.79 | 0.0414 | 0.1872 |
| TC0900012278.hg.1 | ST6GALNAC6 | ST6 (alpha-N-acetyl-neuraminyl-2,3-beta-galactosyl-1,3)-N-acet | Multiple_C | 8.33 | 7.3 | 7.34 | 1.99 | 0.0003 | 0.0027 | 6.78 | 6.66 | 6.59 | 1.14 | 0.3816 | 0.6409 |
| TC0X00007573.hg.1 | OGT | O-linked N-acetylglucosamine (GlcNAc) transferase | Multiple_C | 11.57 | 11.34 | 10.58 | 1.99 | 0.0192 | 0.0692 | 11.88 | 11.81 | 12.3 | 0.75 | 0.8614 | 0.9407 |
| TC1000008400.hg.1 | IFIT1 | interferon-induced protein with tetratricopeptide repeats 1 | Coding | 5.38 | 3.81 | 4.39 | 1.99 | 0.0082 | 0.0358 | 5.62 | 6.44 | 4.83 | 1.73 | 0.0054 | 0.0514 |
| TC1000008716.hg.1 | GBF1 | golgi brefeldin A resistant guanine nucleotide exchange factor 1 | Multiple_C | 12.35 | 10.33 | 11.36 | 1.99 | 0.0002 | 0.0015 | 11.2 | 11.41 | 11.58 | 0.77 | 0.0435 | 0.1927 |
| TC1100009168.hg.1 | SIDT2 | SID1 transmembrane family, member 2 | Multiple_C | 8.17 | 7.71 | 7.18 | 1.99 | 0.0043 | 0.0214 | 8.87 | 8.53 | 8.53 | 1.27 | 0.1117 | 0.3293 |
| TC1100011234.hg.1 | LTBP3 | latent transforming growth factor beta binding protein 3 | Multiple_C | 8.77 | 7.42 | 7.78 | 1.99 | 0.0148 | 0.0565 | 8.6 | 7.33 | 6.45 | 4.44 | 1.72E-05 | 0.0011 |
| TC1200011255.hg.1 | ZFC3H1 | zinc finger, C3H1-type containing | Multiple_C | 10.97 | 10.06 | 9.98 | 1.99 | 0.1082 | 0.2553 | 12.53 | 11.55 | 11.78 | 1.68 | 0.0702 | 0.2533 |
| TC1400008155.hg.1 | C14orf177 | chromosome 14 open reading frame 177 | Multiple_C | 5.88 | 4.75 | 4.89 | 1.99 | 0.002 | 0.0117 | 5.75 | 6.03 | 5.72 | 1.02 | 0.5266 | 0.7511 |
| TC1600009326.hg.1 | USP7 | ubiquitin specific peptidase 7 (herpes virus-associated) | Multiple_C | 10.28 | 9.21 | 9.29 | 1.99 | 0.0152 | 0.0578 | 11.67 | 12.37 | 12.16 | 0.71 | 0.0089 | 0.0719 |
| TC1600009396.hg.1 | TNP2 | transition protein 2 (during histone to protamine replacement) | Multiple_C | 4.6 | 3.3 | 3.61 | 1.99 | 0.0001 | 0.0013 | 3.6 | 3.60 | 3.66 | 0.96 | 0.9204 | 0.9672 |
| TC1700010230.hg.1 | TIAF1; MYO18 | TGFB1-induced anti-apoptotic factor 1; myosin XVIIIA | Multiple_C | 9.1 | 8.81 | 8.11 | 1.99 | 0.0301 | 0.0986 | 8.48 | 8.62 | 8.19 | 1.22 | 0.2391 | 0.5027 |
| TC1700010584.hg.1 | MIEN1 | migration and invasion enhancer 1 | Multiple_C | 10.32 | 9.23 | 9.33 | 1.99 | 0.0005 | 0.0041 | 7.05 | 7.22 | 7.24 | 0.88 | 0.3743 | 0.635 |
| TC1700012304.hg.1 | COG1 | component of oligomeric golgi complex 1 | Multiple_C | 9.73 | 8.89 | 8.74 | 1.99 | 0.0003 | 0.0025 | 8.56 | 8.39 | 8.36 | 1.15 | 0.1856 | 0.4372 |
| TC1900007061.hg.1 | ZNF844 | zinc finger protein 844 | Coding | 9.22 | 9.02 | 8.23 | 1.99 | 0.0002 | 0.0019 | 5.06 | 5.02 | 4.89 | 1.13 | 0.5569 | 0.7707 |
| TC0100009383.hg.1 | RBM15 | RNA binding motif protein 15 | Coding | 11.65 | 10.47 | 10.67 | 1.97 | 0.0006 | 0.0044 | 10.93 | 11.35 | 11.32 | 0.76 | 0.1787 | 0.4276 |
| TC0100018465.hg.1 | BCAS2 | breast carcinoma amplified sequence 2 | Multiple_C | 13.21 | 12.37 | 12.23 | 1.97 | 0.0096 | 0.0404 | 10.71 | 10.52 | 10.95 | 0.85 | 0.6532 | 0.8309 |
| TC0200007249.hg.1 | GPATCH11 | G-patch domain containing 11 | Multiple_C | 10.34 | 10.05 | 9.36 | 1.97 | 0.0165 | 0.0616 | 10.51 | 9.52 | 9.96 | 1.46 | 0.0479 | 0.2032 |
| TC0200008094.hg.1 | SEMA4F | sema domain, immunoglobulin domain (Ig), transmembrane do | Multiple_C | 5.36 | 4.25 | 4.38 | 1.97 | 0.0002 | 0.0019 | 5.28 | 5.17 | 5.12 | 1.12 | 0.7766 | 0.8998 |
| TC0200011034.hg.1 | CAB39 | calcium binding protein 39 | Multiple_C | 10.77 | 10.04 | 9.79 | 1.97 | 5.92E-05 | 0.0007 | 10.66 | 10.53 | 11.31 | 0.64 | 0.0012 | 0.019 |
| TC0200016360.hg.1 | STK25 | serine/threonine kinase 25 | Multiple_C | 10.86 | 9.92 | 9.88 | 1.97 | 0.0011 | 0.0072 | 12.46 | 13.11 | 12.13 | 1.26 | 0.1063 | 0.3201 |
| TC0200016667.hg.1 | GPR75-ASB3 | GPR75-ASB3 readthrough | Multiple_C | 10.26 | 9.5 | 9.28 | 1.97 | 0.0007 | 0.0048 | 8.71 | 8.46 | 8.64 | 1.05 | 0.8479 | 0.934 |
| TC0500009346.hg.1 | WWC1 | WW and C2 domain containing 1 | Multiple_C | 10.14 | 8.92 | 9.16 | 1.97 | 0.0014 | 0.009 | 8.63 | 8.08 | 8.58 | 1.04 | 0.8987 | 0.9576 |
| TC0700006737.hg.1 | ARL4A | ADP-ribosylation factor like GTPase 4A | Coding | 9.67 | 9.87 | 8.69 | 1.97 | 0.0391 | 0.1205 | 9.86 | 9.53 | 9.28 | 1.49 | 0.8625 | 0.9412 |
| TC0700013363.hg.1 | GBAS | glioblastoma amplified sequence | Multiple_C | 12.92 | 13.05 | 11.94 | 1.97 | 0.014 | 0.0541 | 12.3 | 13.04 | 12.55 | 0.84 | 0.2845 | 0.5496 |
| TC0900008854.hg.1 | SWI5 | SWI5 homologous recombination repair protein | Multiple_C | 6.1 | 4.77 | 5.12 | 1.97 | 0.0041 | 0.0205 | 4.54 | 4.86 | 5.08 | 0.69 | 0.013 | 0.0921 |

| | | | | | | | | | | | | | | | |
|---|---|---|---|---|---|---|---|---|---|---|---|---|---|---|---|
| TC1000008744.hg.1 | TRIM8 | tripartite motif containing 8 | Multiple_C | 8.28 | 6.85 | 7.3 | 1.97 | 0.0111 | 0.0452 | 7.57 | 8.24 | 8.22 | 0.64 | 0.0279 | 0.1466 |
| TC1000010239.hg.1 | ARHGAP12 | Rho GTPase activating protein 12 | Multiple_C | 12.84 | 12.33 | 11.86 | 1.97 | 0.0013 | 0.0082 | 11.72 | 10.86 | 10.8 | 1.89 | 0.0017 | 0.0243 |
| TC1000012522.hg.1 | BEND7 | BEN domain containing 7 | Multiple_C | 5.13 | 3.92 | 4.15 | 1.97 | 0.0017 | 0.0104 | 5.09 | 4.94 | 5.04 | 1.04 | 0.7312 | 0.8754 |
| TC1000012552.hg.1 | FAM25G; FAN | family with sequence similarity 25, member G; family with sequ | Multiple_C | 6.74 | 5.61 | 5.76 | 1.97 | 0.0012 | 0.0079 | 5.28 | 5.44 | 5.76 | 0.72 | 0.1168 | 0.3377 |
| TC1100006812.hg.1 | ZNF143 | zinc finger protein 143 | Multiple_C | 12.23 | 12.03 | 11.25 | 1.97 | 0.0138 | 0.0537 | 11.7 | 10.41 | 10.75 | 1.93 | 0.0106 | 0.0809 |
| TC1100013128.hg.1 | IFITM10 | interferon induced transmembrane protein 10 | Multiple_C | 6.75 | 5.59 | 5.77 | 1.97 | 0.0052 | 0.0248 | 5.04 | 4.79 | 4.69 | 1.27 | 0.1523 | 0.3912 |
| TC1200006730.hg.1 | RIMKLB | ribosomal modification protein rimK-like family member B | Multiple_C | 10.62 | 9.78 | 9.64 | 1.97 | 0.0026 | 0.0144 | 5.18 | 4.63 | 4.71 | 1.39 | 0.1083 | 0.3237 |
| TC1200008677.hg.1 | RPL18A | ribosomal protein L18a | Multiple_C | 17.04 | 15.78 | 16.06 | 1.97 | 0.0012 | 0.0079 | 13.04 | 13.02 | 13.01 | 1.02 | 0.0425 | 0.1903 |
| TC1200009928.hg.1 | TAS2R19 | taste receptor, type 2, member 19 | Coding | 4.7 | 4.2 | 3.72 | 1.97 | 0.004 | 0.0201 | 3.97 | 3.61 | 3.69 | 1.21 | 0.4193 | 0.6729 |
| TC1300007491.hg.1 | KLF5 | Kruppel-like factor 5 (intestinal) | Multiple_C | 15.97 | 14.24 | 14.99 | 1.97 | 0.0028 | 0.0154 | 14.82 | 14.06 | 14.45 | 1.29 | 0.119 | 0.341 |
| TC1300008720.hg.1 | MTRF1 | mitochondrial translational release factor 1 | Multiple_C | 8.77 | 8.63 | 7.79 | 1.97 | 0.0005 | 0.0036 | 8.67 | 8.86 | 7.83 | 1.79 | 0.0004 | 0.0084 |
| TC1400009585.hg.1 | MAP3K9 | mitogen-activated protein kinase kinase kinase 9 | Multiple_C | 7.09 | 5.89 | 6.11 | 1.97 | 0.0127 | 0.0504 | 6.55 | 6.37 | 6.69 | 0.91 | 0.4684 | 0.7094 |
| TC1500006666.hg.1 | GOLGA8F; GO | golgin A8 family, member F; golgin A8 family, member G | Multiple_C | 7.33 | 7.06 | 6.35 | 1.97 | 0.0037 | 0.019 | 7.27 | 6.83 | 6.79 | 1.39 | 0.0517 | 0.2124 |
| TC1500008983.hg.1 | SLC12A6 | solute carrier family 12 (potassium/chloride transporter), meml | Multiple_C | 8.88 | 8.54 | 7.9 | 1.97 | 0.0006 | 0.0045 | 8.98 | 8.62 | 8.29 | 1.61 | 0.0146 | 0.0988 |
| TC1600010798.hg.1 | ZNF821 | zinc finger protein 821 | Multiple_C | 5.37 | 4.73 | 4.39 | 1.97 | 0.0087 | 0.0374 | 4.29 | 4.66 | 4.74 | 0.73 | 0.3003 | 0.5646 |
| TC1800008129.hg.1 | PTPN2 | Transcript Identified by AceView, Entrez Gene ID(s) 5771 | Coding | 4.68 | 3.9 | 3.7 | 1.97 | 0.1043 | 0.2485 | 5.01 | 4.44 | 4.41 | 1.52 | 0.1122 | 0.33 |
| TC1900009637.hg.1 | CDKN2D | cyclin-dependent kinase inhibitor 2D (p19, inhibits CDK4) | Coding | 7.56 | 6.38 | 6.58 | 1.97 | 0.0025 | 0.0141 | 5.78 | 5.86 | 5.71 | 1.05 | 0.5544 | 0.7691 |
| TC1900010067.hg.1 | PBX4 | pre-B-cell leukemia homeobox 4 | Multiple_C | 7.18 | 5.99 | 6.2 | 1.97 | 0.0021 | 0.0122 | 6.28 | 5.87 | 5.46 | 1.77 | 0.0032 | 0.0373 |
| TC1900011452.hg.1 | PTPRH | protein tyrosine phosphatase, receptor type, H | Multiple_C | 7.48 | 6.65 | 6.5 | 1.97 | 0.0005 | 0.0038 | 6.22 | 6.04 | 5.68 | 1.45 | 0.0643 | 0.2413 |
| TC1900012024.hg.1 | ZNF600 | zinc finger protein 600 | Multiple_C | 7.16 | 6.35 | 6.18 | 1.97 | 0.0145 | 0.0556 | 5.7 | 5.98 | 6.12 | 0.75 | 0.103 | 0.3145 |
| TC2000007492.hg.1 | WFDC2 | WAP four-disulfide core domain 2 | Multiple_C | 4.5 | 3.59 | 3.52 | 1.97 | 0.1585 | 0.3345 | 3.62 | 3.45 | 3.71 | 0.94 | 0.2858 | 0.5508 |
| TC2000007792.hg.1 | PFDN4 | prefoldin subunit 4 | Multiple_C | 13.15 | 16.14 | 12.17 | 1.97 | 0.0138 | 0.0536 | 10.16 | 9.74 | 10.06 | 1.07 | 0.7858 | 0.9043 |
| TC2100007996.hg.1 | TMEM50B | transmembrane protein 50B | Multiple_C | 10.86 | 11.42 | 9.88 | 1.97 | 0.0596 | 0.1648 | 9.17 | 7.84 | 8.3 | 1.83 | 0.0942 | 0.2981 |
| TC2200008489.hg.1 | PRR14L | proline rich 14-like | Multiple_C | 10.03 | 8.77 | 9.05 | 1.97 | 0.0058 | 0.027 | 7.84 | 7.81 | 7.9 | 0.96 | 0.4507 | 0.6963 |
| TC2200009338.hg.1 | TBC1D10A | TBC1 domain family, member 10A | Multiple_C | 7.44 | 6.19 | 6.46 | 1.97 | 0.0066 | 0.0301 | 5.96 | 5.58 | 5.84 | 1.09 | 0.4043 | 0.6599 |
| TSUnmapped00000267.I | LRP6 | LDL receptor related protein 6 | Coding | 11.92 | 11.04 | 10.94 | 1.97 | 0.0088 | 0.0378 | 12.58 | 12.45 | 12.02 | 1.47 | 0.0407 | 0.185 |
| TSUnmapped00000405.I | DGKD | diacylglycerol kinase, delta 130kDa | Coding | 8.07 | 7.83 | 7.09 | 1.97 | 0.0149 | 0.0568 | 6.75 | 6.90 | 6.73 | 1.01 | 0.6296 | 0.8175 |
| TC0100007184.hg.1 | OTUD3 | OTU deubiquitinase 3 | Multiple_C | 9.36 | 8.9 | 8.39 | 1.96 | 0.0029 | 0.0157 | 8.89 | 7.48 | 8.71 | 1.13 | 0.8455 | 0.9325 |
| TC0100008641.hg.1 | MIER1 | mesoderm induction early response 1, transcriptional regulator | Multiple_C | 10.65 | 10.04 | 9.68 | 1.96 | 0.0037 | 0.0189 | 11.1 | 10.25 | 11.06 | 1.03 | 0.9149 | 0.9651 |
| TC0100010925.hg.1 | OCLM | oculomedin | Coding | 6.05 | 5.28 | 5.08 | 1.96 | 0.0034 | 0.0178 | 6.37 | 6.33 | 6.22 | 1.11 | 0.7551 | 0.8887 |
| TC0100013908.hg.1 | SLC2A1 | solute carrier family 2 (facilitated glucose transporter), member | Multiple_C | 13.78 | 11.86 | 12.81 | 1.96 | 0.0021 | 0.0122 | 16.74 | 16.80 | 16.16 | 1.49 | 0.1404 | 0.3725 |
| TC0200007050.hg.1 | AGBL5 | ATP/GTP binding protein-like 5 | Multiple_C | 11.42 | 10.45 | 10.45 | 1.96 | 0.0008 | 0.0059 | 9.65 | 10.39 | 10.09 | 0.74 | 0.069 | 0.2501 |
| TC0200007712.hg.1 | PEX13 | peroxisomal biogenesis factor 13 | Multiple_C | 10.42 | 9.66 | 9.45 | 1.96 | 0.0104 | 0.043 | 10.11 | 10.49 | 10.19 | 0.95 | 0.5269 | 0.7512 |

| | | | | | | | | | | | | | | | |
|---|---|---|---|---|---|---|---|---|---|---|---|---|---|---|---|
| TC0200008464.hg.1 | KCNIP3 | Kv channel interacting protein 3, calsenilin | Multiple_C | 4.87 | 3.87 | 3.9 | 1.96 | 0.4197 | 0.6309 | 4.44 | 4.30 | 4.41 | 1.02 | 0.8034 | 0.9123 |
| TC0200013257.hg.1 | TGOLN2 | trans-golgi network protein 2 | Multiple_C | 10.98 | 9.59 | 10.01 | 1.96 | 0.0068 | 0.031 | 9.28 | 9.53 | 9.64 | 0.78 | 0.128 | 0.354 |
| TC0200013602.hg.1 | TSGA10 | testis specific 10 | Multiple_C | 7.24 | 7.19 | 6.27 | 1.96 | 0.0113 | 0.046 | 7.24 | 7.62 | 6.67 | 1.48 | 0.032 | 0.1599 |
| TC0200014190.hg.1 | AMMECR1L | AMMECR1 like | Multiple_C | 9.58 | 8.09 | 8.61 | 1.96 | 0.0009 | 0.0063 | 10.4 | 9.83 | 10.63 | 0.85 | 0.2378 | 0.5013 |
| TC0200016610.hg.1 | CAPN10 | calpain 10 | Multiple_C | 6.67 | 6.05 | 5.7 | 1.96 | 0.0406 | 0.1242 | 6.13 | 5.86 | 5.63 | 1.41 | 0.5102 | 0.7402 |
| TC0300006622.hg.1 | PPARG | peroxisome proliferator-activated receptor gamma | Multiple_C | 13.39 | 12.88 | 12.42 | 1.96 | 0.0068 | 0.0309 | 8.23 | 7.91 | 9.59 | 0.39 | 0.0002 | 0.0065 |
| TC0300007050.hg.1 | C3orf35 | chromosome 3 open reading frame 35 | Multiple_C | 5.6 | 4.98 | 4.63 | 1.96 | 0.0104 | 0.0431 | 3.44 | 3.77 | 3.85 | 0.75 | 0.3746 | 0.6353 |
| TC0400008534.hg.1 | NDST3 | N-deacetylase/N-sulfotransferase (heparan glucosaminyl) 3 | Multiple_C | 6.05 | 5.18 | 5.08 | 1.96 | 0.0063 | 0.0289 | 4.69 | 4.86 | 4.75 | 0.96 | 0.9536 | 0.981 |
| TC0400012381.hg.1 | NEK1 | NIMA-related kinase 1 | Multiple_C | 8.99 | 8.11 | 8.02 | 1.96 | 0.0302 | 0.0989 | 9.94 | 9.37 | 9.74 | 1.15 | 0.8376 | 0.9285 |
| TC0500009095.hg.1 | NDST1 | N-deacetylase/N-sulfotransferase (heparan glucosaminyl) 1 | Multiple_C | 4.24 | 3.26 | 3.27 | 1.96 | 0.007 | 0.0315 | 3.66 | 3.67 | 3.74 | 0.95 | 0.7611 | 0.8918 |
| TC0500011398.hg.1 | CCNH | cyclin H | Multiple_C | 12.09 | 13.25 | 11.12 | 1.96 | 0.0011 | 0.007 | 10.46 | 9.80 | 10.34 | 1.09 | 0.2137 | 0.4719 |
| TC0600009843.hg.1 | PLEKHG1 | pleckstrin homology domain containing, family G (with RhoGef ( | Multiple_C | 9.47 | 8.26 | 8.5 | 1.96 | 0.0076 | 0.0335 | 3.8 | 4.02 | 3.53 | 1.21 | 0.2791 | 0.5452 |
| TC0700006618.hg.1 | WIPI2 | WD repeat domain, phosphoinositide interacting 2 | Multiple_C | 11.38 | 10.24 | 10.41 | 1.96 | 0.0005 | 0.0037 | 10.04 | 10.69 | 10.49 | 0.73 | 0.0466 | 0.1995 |
| TC0700010189.hg.1 | CYTH3 | cytohesin 3 | Multiple_C | 9.92 | 9.4 | 8.95 | 1.96 | 0.0011 | 0.0071 | 7.95 | 8.14 | 8.03 | 0.95 | 0.6822 | 0.8475 |
| TC0X00007149.hg.1 | TIMP1 | TIMP metallopeptidase inhibitor 1 | Multiple_C | 15.21 | 11.29 | 14.24 | 1.96 | 0.0116 | 0.0469 | 8.15 | 9.02 | 10.32 | 0.22 | 9.17E-08 | 2.49E-05 |
| TC0X00011134.hg.1 | CSAG1 | chondrosarcoma associated gene 1 | Coding | 4.07 | 2.89 | 3.1 | 1.96 | 0.004 | 0.02 | 3.97 | 3.83 | 3.67 | 1.23 | 0.469 | 0.7101 |
| TC1100009306.hg.1 | SC5D | sterol-C5-desaturase | Multiple_C | 10.32 | 10.35 | 9.35 | 1.96 | 0.0018 | 0.0108 | 7.39 | 7.10 | 8.13 | 0.60 | 0.0089 | 0.0719 |
| TC1100009412.hg.1 | PKNOX2 | PBX/knotted 1 homeobox 2 | Multiple_C | 7.7 | 6.71 | 6.73 | 1.96 | 0.0041 | 0.0206 | 7.25 | 7.32 | 7.06 | 1.14 | 0.6507 | 0.8295 |
| TC1100011048.hg.1 | TMEM258; MI | transmembrane protein 258; microRNA 611 | Multiple_C | 13.51 | 13.05 | 12.54 | 1.96 | 0.0098 | 0.0409 | 9.73 | 9.70 | 9.48 | 1.19 | 0.8592 | 0.9398 |
| TC1200007262.hg.1 | BICD1 | bicaudal D homolog 1 (Drosophila) | Multiple_C | 8.39 | 7.11 | 7.42 | 1.96 | 0.0031 | 0.0165 | 8.13 | 8.46 | 8.55 | 0.75 | 0.0132 | 0.0926 |
| TC1500008988.hg.1 | GOLGA8A; GC | golgin A8 family, member A; golgin A8 family, member B | Multiple_C | 9.22 | 9.43 | 8.25 | 1.96 | 0.0028 | 0.0152 | 12.19 | 10.80 | 9.94 | 4.76 | 2.45E-09 | 1.88E-06 |
| TC1500010906.hg.1 | RP11-351M8.: | Transcript Identified by AceView, Entrez Gene ID(s) 23184; nove | Multiple_C | 6.48 | 5.9 | 5.51 | 1.96 | 0.0021 | 0.0122 | 5.94 | 6.00 | 6.03 | 0.94 | 0.5552 | 0.7697 |
| TC1700009417.hg.1 | SMG6 | SMG6 nonsense mediated mRNA decay factor | Multiple_C | 9.31 | 8.27 | 8.34 | 1.96 | 0.0014 | 0.0088 | 8.51 | 9.17 | 8.95 | 0.74 | 0.0923 | 0.2945 |
| TC1800006749.hg.1 | CEP192 | centrosomal protein 192kDa | Multiple_C | 7.48 | 6.83 | 6.51 | 1.96 | 0.0023 | 0.0129 | 8.06 | 9.02 | 8.01 | 1.04 | 0.729 | 0.8742 |
| TC1900008856.hg.1 | TTYH1 | tweety family member 1 | Multiple_C | 9.02 | 8.09 | 8.05 | 1.96 | 0.0033 | 0.0174 | 8.72 | 9.01 | 8.68 | 1.03 | 0.8728 | 0.9452 |
| TC1900009459.hg.1 | INSR | insulin receptor | Multiple_C | 6.53 | 5.74 | 5.56 | 1.96 | 0.0106 | 0.0436 | 5.38 | 5.65 | 4.99 | 1.31 | 0.4161 | 0.6701 |
| TC2000008072.hg.1 | PPDPF | pancreatic progenitor cell differentiation and proliferation facto | Multiple_C | 11.18 | 9.54 | 10.21 | 1.96 | 0.0246 | 0.0844 | 7 | 7.95 | 7.99 | 0.50 | 0.0396 | 0.1819 |
| TC2200006501.hg.1 | IL17RA | interleukin 17 receptor A | Multiple_C | 9.21 | 7.64 | 8.24 | 1.96 | 0.0003 | 0.0022 | 7.34 | 7.42 | 7.75 | 0.75 | 0.0621 | 0.2366 |
| TC2200007406.hg.1 | ATF4 | activating transcription factor 4 | Multiple_C | 14.84 | 15.06 | 13.87 | 1.96 | 0.0022 | 0.0126 | 12.59 | 12.33 | 12.68 | 0.94 | 0.7211 | 0.87 |
| TC2200008465.hg.1 | SELM | selenoprotein M; selenoprotein M [Source:EntrezGene;Acc:140 | Multiple_C | 5.37 | 4.3 | 4.4 | 1.96 | 0.0016 | 0.0098 | 3.45 | 3.44 | 3.07 | 1.30 | 0.2579 | 0.5227 |
| TC2200008799.hg.1 | ST13 | suppression of tumorigenicity 13 (colon carcinoma) (Hsp70 inte | Multiple_C | 13.49 | 13.26 | 12.52 | 1.96 | 0.0002 | 0.0018 | 13.94 | 13.95 | 13.86 | 1.06 | 0.5957 | 0.796 |
| TSUnmapped00000411.h | DGKD | diacylglycerol kinase, delta 130kDa | Coding | 7.33 | 6.93 | 6.36 | 1.96 | 0.0169 | 0.0626 | 6.94 | 7.16 | 6.79 | 1.11 | 0.8442 | 0.9318 |
| TC0200011013.hg.1 | FBXO36 | F-box protein 36 | Multiple_C | 6.99 | 6.61 | 6.03 | 1.95 | 0.0193 | 0.0695 | 6.24 | 6.16 | 6.09 | 1.11 | 0.5078 | 0.7384 |

| | | | | | | | | | | | | | | | |
|---|---|---|---|---|---|---|---|---|---|---|---|---|---|---|---|
| TC0200013330.hg.1 | PLGLB1; PLGLI | plasminogen-like B1; plasminogen-like B2 | Multiple_C | 4.74 | 3.66 | 3.78 | 1.95 | 0.033 | 0.1061 | 5.02 | 4.73 | 4.2 | 1.77 | 0.0045 | 0.046 |
| TC0300011651.hg.1 | GBE1 | glucan (1,4-alpha-), branching enzyme 1 | Multiple_C | 7.49 | 6.15 | 6.53 | 1.95 | 0.0002 | 0.0015 | 8.25 | 8.66 | 8.56 | 0.81 | 0.1088 | 0.3246 |
| TC0400011548.hg.1 | LEF1 | lymphoid enhancer-binding factor 1 | Multiple_C | 9.3 | 8.08 | 8.34 | 1.95 | 0.0524 | 0.1494 | 8.26 | 8.16 | 8.35 | 0.94 | 0.6464 | 0.8265 |
| TC0600008071.hg.1 | PTK7 | protein tyrosine kinase 7 (inactive) | Multiple_C | 5.43 | 4.22 | 4.47 | 1.95 | 0.0096 | 0.0404 | 10.03 | 10.66 | 9.57 | 1.38 | 0.0854 | 0.2813 |
| TC0600014166.hg.1 | CCDC162P | coiled-coil domain containing 162, pseudogene | Multiple_C | 5.92 | 5.56 | 4.96 | 1.95 | 0.0063 | 0.0288 | 4.91 | 5.81 | 4.79 | 1.09 | 0.6514 | 0.83 |
| TC0700008147.hg.1 | RSBN1L | round spermatid basic protein 1-like | Multiple_C | 10.45 | 10.6 | 9.49 | 1.95 | 0.0024 | 0.0134 | 10.72 | 9.95 | 11 | 0.82 | 0.0444 | 0.1951 |
| TC0700010585.hg.1 | JAZF1 | JAZF zinc finger 1 | Multiple_C | 7.46 | 6.15 | 6.5 | 1.95 | 0.0011 | 0.0073 | 7.15 | 7.37 | 7.27 | 0.92 | 0.6951 | 0.855 |
| TC0700012477.hg.1 | GPR37 | G protein-coupled receptor 37 (endothelin receptor type B-like) | Coding | 4.28 | 3.43 | 3.32 | 1.95 | 0.0002 | 0.0015 | 3.77 | 3.87 | 3.69 | 1.06 | 0.5923 | 0.7938 |
| TC0900006559.hg.1 | CD274 | CD274 molecule | Multiple_C | 6.91 | 5.53 | 5.95 | 1.95 | 0.0025 | 0.0138 | 5.01 | 4.45 | 4.72 | 1.22 | 0.3732 | 0.6341 |
| TC0900009073.hg.1 | RPL7A; SNORI | ribosomal protein L7a; small nucleolar RNA, C/D box 36C; small | Multiple_C | 16.05 | 15.86 | 15.09 | 1.95 | 0.0133 | 0.0523 | 14.26 | 14.38 | 13.92 | 1.27 | 0.229 | 0.4901 |
| TC0X00009255.hg.1 | RPL9P7 | ribosomal protein L9 pseudogene 7 | Multiple_C | 14.19 | 14.77 | 13.23 | 1.95 | 0.0054 | 0.0254 | 11.12 | 10.46 | 10.46 | 1.58 | 0.0205 | 0.1222 |
| TC1000007700.hg.1 | UBE2D1 | ubiquitin conjugating enzyme E2D 1 | Multiple_C | 13.13 | 12.61 | 12.17 | 1.95 | 0.0031 | 0.0166 | 12 | 11.48 | 11.93 | 1.05 | 0.2426 | 0.5061 |
| TC1000008478.hg.1 | CYP26A1 | cytochrome P450, family 26, subfamily A, polypeptide 1 | Multiple_C | 4.01 | 2.87 | 3.05 | 1.95 | 0.0467 | 0.1375 | 4.46 | 4.35 | 4.06 | 1.32 | 0.0069 | 0.0604 |
| TC1000010367.hg.1 | ZNF25 | zinc finger protein 25 | Multiple_C | 4.84 | 3.73 | 3.88 | 1.95 | 0.014 | 0.0542 | 5.95 | 5.65 | 5.18 | 1.71 | 0.0223 | 0.1283 |
| TC1100011202.hg.1 | TMEM262 | transmembrane protein 262 | Multiple_C | 5.71 | 4.65 | 4.75 | 1.95 | 0.003 | 0.0162 | 5.08 | 5.52 | 4.98 | 1.07 | 0.8153 | 0.9182 |
| TC1200007838.hg.1 | IL23A | interleukin 23, alpha subunit p19 | Multiple_C | 6.73 | 5.93 | 5.77 | 1.95 | 0.1361 | 0.3008 | 6.2 | 6.15 | 6.48 | 0.82 | 0.5835 | 0.7878 |
| TC1400009074.hg.1 | RPL10L | ribosomal protein L10-like | Coding | 4.42 | 3.5 | 3.46 | 1.95 | 0.0085 | 0.0367 | 4.31 | 4.26 | 4.09 | 1.16 | 0.2649 | 0.5295 |
| TC1400009842.hg.1 | SEL1L | sel-1 suppressor of lin-12-like (C. elegans) | Multiple_C | 11.48 | 11.6 | 10.52 | 1.95 | 0.0217 | 0.0763 | 12.06 | 12.21 | 12.06 | 1.00 | 0.7747 | 0.8988 |
| TC1500006956.hg.1 | BAHD1 | bromo adjacent homology domain containing 1 | Multiple_C | 7.85 | 6.6 | 6.89 | 1.95 | 0.0019 | 0.0111 | 8.09 | 8.11 | 8.27 | 0.88 | 0.4188 | 0.6725 |
| TC1500007967.hg.1 | DNAJA4 | DnaJ (Hsp40) homolog, subfamily A, member 4 | Multiple_C | 11.49 | 10.71 | 10.53 | 1.95 | 0.008 | 0.0348 | 10.39 | 7.73 | 10.26 | 1.09 | 0.725 | 0.8721 |
| TC1600009399.hg.1 | PRM2 | protamine 2 | Multiple_C | 5.88 | 5.04 | 4.92 | 1.95 | 0.0061 | 0.0281 | 5.82 | 6.09 | 5.72 | 1.07 | 0.8532 | 0.9367 |
| TC1700012207.hg.1 | EPN2; EPN2-IT | epsin 2; EPN2 intronic transcript 1 | Multiple_C | 8.66 | 7.31 | 7.7 | 1.95 | 0.0119 | 0.0478 | 7.22 | 6.86 | 7.33 | 0.93 | 0.1208 | 0.3429 |
| TC1900008031.hg.1 | LGALS7B | lectin, galactoside-binding, soluble, 7B | Multiple_C | 6.88 | 5.75 | 5.92 | 1.95 | 0.003 | 0.0159 | 6.51 | 6.60 | 6.29 | 1.16 | 0.6198 | 0.8119 |
| TC1900011656.hg.1 | STXBP2 | syntaxin binding protein 2 | Multiple_C | 7.65 | 6.61 | 6.69 | 1.95 | 0.0985 | 0.238 | 4.36 | 4.53 | 4.57 | 0.86 | 0.4284 | 0.6803 |
| TC1900011794.hg.1 | ZNF701; ZNF1 | zinc finger protein 701; zinc finger protein 137, pseudogene | Multiple_C | 12 | 11.4 | 11.04 | 1.95 | 0.0008 | 0.0053 | 8.67 | 8.75 | 8.84 | 0.89 | 0.3787 | 0.6384 |
| TC2000008845.hg.1 | PLAGL2 | pleiomorphic adenoma gene-like 2 | Coding | 8.2 | 6.19 | 7.24 | 1.95 | 0.0168 | 0.0626 | 8.81 | 9.64 | 9.95 | 0.45 | 0.0003 | 0.0079 |
| TC2200009033.hg.1 | TBC1D22A-AS | TBC1D22A antisense RNA 1 | Multiple_C | 3.91 | 2.84 | 2.95 | 1.95 | 0.0052 | 0.0249 | 4.84 | 4.49 | 4.36 | 1.39 | 0.2202 | 0.4803 |
| TC2200009262.hg.1 | EIF3L | eukaryotic translation initiation factor 3, subunit L | Multiple_C | 13.95 | 13.16 | 12.99 | 1.95 | 0.0065 | 0.0297 | 12.99 | 12.69 | 12.97 | 1.01 | 0.3366 | 0.601 |
| TC2200009340.hg.1 | RNF215 | ring finger protein 215 | Multiple_C | 7.26 | 6.65 | 6.3 | 1.95 | 0.0015 | 0.009 | 4.8 | 5.14 | 4.8 | 1.00 | 0.9973 | 0.9987 |
| TC0100010100.hg.1 | CKS1B | CDC28 protein kinase regulatory subunit 1B | Multiple_C | 13.74 | 13.26 | 12.79 | 1.93 | 0.0089 | 0.0381 | 11.76 | 11.95 | 11.58 | 1.13 | 0.5724 | 0.7801 |
| TC0100011419.hg.1 | CD46 | CD46 molecule, complement regulatory protein | Multiple_C | 12.77 | 12.93 | 11.82 | 1.93 | 0.0093 | 0.0392 | 13.46 | 12.99 | 13.26 | 1.15 | 0.0557 | 0.2218 |
| TC0100013676.hg.1 | ZMYM6NB; ZI | ZMYM6 neighbor; zinc finger, MYM-type 6 | Multiple_C | 8.3 | 7.54 | 7.35 | 1.93 | 0.0074 | 0.033 | 8.1 | 7.52 | 8.02 | 1.06 | 0.316 | 0.58 |
| TC0100014502.hg.1 | WDR78 | WD repeat domain 78 | Multiple_C | 4.69 | 4.22 | 3.74 | 1.93 | 0.028 | 0.0931 | 4.84 | 4.75 | 3.95 | 1.85 | 0.0007 | 0.0129 |

| | | | | | | | | | | | | | | | | | |
|---|---|---|---|---|---|---|---|---|---|---|---|---|---|---|---|---|---|
| TC0100017100.hg.1 | EIF2D | eukaryotic translation initiation factor 2D | Multiple_C | 11.29 | 11.58 | 10.34 | 1.93 | 0.0015 | 0.0095 | 9.77 | 10.38 | 9.8 | 0.98 | 0.6192 | 0.8113 |
| TC0200008904.hg.1 | FBLN7 | fibulin 7 | Multiple_C | 4.34 | 3.21 | 3.39 | 1.93 | 0.0021 | 0.0122 | 4.18 | 4.16 | 4.12 | 1.04 | 0.6911 | 0.8526 |
| TC0200011130.hg.1 | DGKD | diacylglycerol kinase, delta 130kDa | Multiple_C | 9.73 | 9.07 | 8.78 | 1.93 | 0.0021 | 0.012 | 7.95 | 8.08 | 8.37 | 0.75 | 0.0516 | 0.2123 |
| TC0200011449.hg.1 | SH3YL1 | SH3 and SYLF domain containing 1 | Multiple_C | 9.43 | 10.04 | 8.48 | 1.93 | 0.0532 | 0.1512 | 5.24 | 4.90 | 4.72 | 1.43 | 0.0326 | 0.1617 |
| TC0400011421.hg.1 | LAMTOR3 | late endosomal/lysosomal adaptor, MAPK and MTOR activator | Multiple_C | 9.6 | 10.42 | 8.65 | 1.93 | 0.0497 | 0.144 | 6.66 | 6.64 | 7.15 | 0.71 | 0.0168 | 0.1081 |
| TC0500008880.hg.1 | PCDHB7 | protocadherin beta 7 | Coding | 5.01 | 4.13 | 4.06 | 1.93 | 0.0003 | 0.0023 | 5.99 | 5.42 | 5.08 | 1.88 | 0.0004 | 0.0085 |
| TC0600013945.hg.1 | KIF25-AS1 | KIF25 antisense RNA 1 | Multiple_C | 5.41 | 4.06 | 4.46 | 1.93 | 0.0055 | 0.0259 | 5.91 | 5.94 | 5.9 | 1.01 | 0.8372 | 0.9283 |
| TC0700010604.hg.1 | CPVL | carboxypeptidase, vitellogenic-like | Multiple_C | 11.4 | 11.63 | 10.45 | 1.93 | 0.0419 | 0.1272 | 6.02 | 7.30 | 6.77 | 0.59 | 0.0031 | 0.0366 |
| TC0800008263.hg.1 | ESRP1 | epithelial splicing regulatory protein 1 | Multiple_C | 13.27 | 12.85 | 12.32 | 1.93 | 0.2247 | 0.4234 | 11.95 | 9.12 | 11.89 | 1.04 | 0.1539 | 0.3932 |
| TC0900011610.hg.1 | ST6GALNAC4 | ST6 (alpha-N-acetyl-neuraminyl-2,3-beta-galactosyl-1,3)-N-acet | Multiple_C | 5.21 | 4.12 | 4.26 | 1.93 | 0.0023 | 0.0127 | 6.11 | 6.20 | 5.96 | 1.11 | 0.268 | 0.5326 |
| TC0M00006432.hg.1 | ND1 | NADH dehydrogenase, subunit 1 (complex I) | Multiple_C | 17.98 | 16.65 | 17.03 | 1.93 | 0.0007 | 0.0053 | 18.7 | 18.92 | 19.1 | 0.76 | 0.0858 | 0.2822 |
| TC0X00009384.hg.1 | DYNLT3 | dynein, light chain, Tctex-type 3 | Coding | 12.68 | 12.95 | 11.73 | 1.93 | 0.0058 | 0.0273 | 7.82 | 7.94 | 8.21 | 0.76 | 0.6353 | 0.821 |
| TC1000008048.hg.1 | ZSWIM8 | zinc finger, SWIM-type containing 8 | Multiple_C | 8.06 | 7.08 | 7.11 | 1.93 | 0.0028 | 0.0151 | 8.23 | 8.80 | 8.23 | 1.00 | 0.942 | 0.9761 |
| TC1100006731.hg.1 | ILK | integrin linked kinase | Multiple_C | 13.2 | 12 | 12.25 | 1.93 | 0.0015 | 0.0093 | 12.84 | 13.56 | 13.05 | 0.86 | 0.4324 | 0.6839 |
| TC1200008592.hg.1 | SCYL2 | SCY1-like, kinase-like 2 | Multiple_C | 12.44 | 11.39 | 11.49 | 1.93 | 0.0574 | 0.1601 | 11.82 | 10.58 | 11.6 | 1.16 | 0.6537 | 0.8314 |
| TC1200009547.hg.1 | SLC6A13 | solute carrier family 6 (neurotransmitter transporter), member | Multiple_C | 4.75 | 3.55 | 3.8 | 1.93 | 0.0155 | 0.0585 | 4.49 | 4.27 | 4.1 | 1.31 | 0.0607 | 0.2337 |
| TC1200011367.hg.1 | PPP1R12A | protein phosphatase 1, regulatory subunit 12A | Multiple_C | 10.7 | 10.64 | 9.75 | 1.93 | 0.0052 | 0.0248 | 8.67 | 8.60 | 8.82 | 0.90 | 0.8124 | 0.9166 |
| TC1500008554.hg.1 | LRRK1 | leucine-rich repeat kinase 1 | Multiple_C | 9.05 | 8.05 | 8.1 | 1.93 | 0.0052 | 0.0249 | 6.92 | 6.98 | 7.13 | 0.86 | 0.6223 | 0.8128 |
| TC1500010869.hg.1 | CCPG1; MIR62 | cell cycle progression 1; microRNA 628 | Multiple_C | 9.56 | 10.15 | 8.61 | 1.93 | 0.0216 | 0.0761 | 7.1 | 6.21 | 6.73 | 1.29 | 0.174 | 0.4216 |
| TC1500010910.hg.1 | RPS17 | ribosomal protein S17 | Multiple_C | 19.37 | 18.81 | 18.42 | 1.93 | 0.0015 | 0.0093 | 15.13 | 14.86 | 14.64 | 1.40 | 0.0423 | 0.1899 |
| TC1600008822.hg.1 | ZFPM1 | zinc finger protein, FOG family member 1 | Multiple_C | 6.18 | 5.02 | 5.23 | 1.93 | 0.0047 | 0.0228 | 6.08 | 6.51 | 6.53 | 0.73 | 0.0375 | 0.1764 |
| TC1700007319.hg.1 | WSB1 | WD repeat and SOCS box containing 1 | Multiple_C | 11.26 | 11.74 | 10.31 | 1.93 | 0.0954 | 0.2329 | 8.41 | 7.96 | 8.17 | 1.18 | 0.0437 | 0.1933 |
| TC1700009701.hg.1 | CCDC42 | coiled-coil domain containing 42 | Coding | 6.21 | 5.29 | 5.26 | 1.93 | 0.0286 | 0.0947 | 5.18 | 5.29 | 5.28 | 0.93 | 0.4031 | 0.6593 |
| TC1800008219.hg.1 | ROCK1 | Rho-associated, coiled-coil containing protein kinase 1 | Multiple_C | 9.51 | 8.84 | 8.56 | 1.93 | 0.0062 | 0.0287 | 8.62 | 8.36 | 8.65 | 0.98 | 0.5632 | 0.7737 |
| TC2000006957.hg.1 | GZF1 | GDNF-inducible zinc finger protein 1 | Multiple_C | 7.42 | 6.22 | 6.47 | 1.93 | 0.0063 | 0.0288 | 7.78 | 7.60 | 7.8 | 0.99 | 0.7089 | 0.8633 |
| TC2000007336.hg.1 | PPP1R16B | protein phosphatase 1, regulatory subunit 16B | Multiple_C | 7.63 | 6.5 | 6.68 | 1.93 | 0.0419 | 0.1272 | 7.03 | 7.10 | 7.39 | 0.78 | 0.4727 | 0.7128 |
| TC2000008228.hg.1 | SIGLEC1 | sialic acid binding Ig-like lectin 1, sialoadhesin | Coding | 8.37 | 7.17 | 7.42 | 1.93 | 0.0021 | 0.0118 | 7.43 | 7.37 | 7.31 | 1.09 | 0.6141 | 0.8078 |
| TSUnmapped00000496.h | KIF15 | kinesin family member 15 | Coding | 5.55 | 3.95 | 4.6 | 1.93 | 0.0011 | 0.0071 | 4.72 | 5.00 | 5.04 | 0.80 | 0.3284 | 0.5921 |
| TC0100007292.hg.1 | C1QB | complement component 1, q subcomponent, B chain | Coding | 4.68 | 3.59 | 3.74 | 1.92 | 0.0619 | 0.1694 | 4.25 | 4.60 | 4.56 | 0.81 | 0.7892 | 0.9059 |
| TC0100007794.hg.1 | MAP7D1 | MAP7 domain containing 1 | Multiple_C | 11.69 | 10.45 | 10.75 | 1.92 | 0.0094 | 0.0396 | 11.21 | 11.46 | 11.51 | 0.81 | 0.0298 | 0.1533 |
| TC0100009641.hg.1 | NBPF8 | neuroblastoma breakpoint family, member 8 | Multiple_C | 7.58 | 7.02 | 6.64 | 1.92 | 0.0864 | 0.2163 | 6.82 | 6.94 | 7.1 | 0.82 | 0.6898 | 0.852 |
| TC0100010499.hg.1 | POU2F1 | POU class 2 homeobox 1 | Multiple_C | 12.45 | 9.94 | 11.51 | 1.92 | 0.0023 | 0.013 | 11.99 | 11.93 | 12.38 | 0.76 | 0.1775 | 0.4261 |
| TC0200016502.hg.1 | IL18R1 | interleukin 18 receptor 1 | Multiple_C | 4.78 | 4.03 | 3.84 | 1.92 | 0.0042 | 0.021 | 3.83 | 4.11 | 3.77 | 1.04 | 0.5452 | 0.7629 |

| | | | | | | | | | | | | | | | |
|---|---|---|---|---|---|---|---|---|---|---|---|---|---|---|---|
| TC0300013792.hg.1 | TPRXL | tetra-peptide repeat homeobox-like | Multiple_C | 7.78 | 6.43 | 6.84 | 1.92 | 0.0012 | 0.0077 | 6.21 | 5.85 | 5.96 | 1.19 | 0.5589 | 0.7714 |
| TC0400006793.hg.1 | USP17L15 | ubiquitin specific peptidase 17-like family member 15 | Coding | 4.1 | 3.1 | 3.16 | 1.92 | 0.0068 | 0.031 | 4 | 4.38 | 4.44 | 0.74 | 0.6044 | 0.8022 |
| TC0400008429.hg.1 | ALPK1 | alpha kinase 1 | Multiple_C | 6.67 | 5.71 | 5.73 | 1.92 | 0.0025 | 0.0139 | 4.72 | 4.58 | 4.67 | 1.04 | 0.8073 | 0.915 |
| TC0400010463.hg.1 | RPL9 | ribosomal protein L9 | Multiple_C | 16.51 | 17.05 | 15.57 | 1.92 | 0.0034 | 0.0176 | 13.41 | 12.99 | 12.94 | 1.39 | 0.0138 | 0.0952 |
| TC0600007540.hg.1 | C6orf136 | chromosome 6 open reading frame 136 | Multiple_C | 8.67 | 7.74 | 7.73 | 1.92 | 0.0311 | 0.1012 | 7.91 | 7.49 | 8.39 | 0.72 | 0.0203 | 0.1216 |
| TC0600007800.hg.1 | RPL10A | ribosomal protein L10a | Multiple_C | 15.62 | 15.61 | 14.68 | 1.92 | 0.001 | 0.0069 | 12.11 | 12.26 | 11.68 | 1.35 | 0.017 | 0.1091 |
| TC0800009831.hg.1 | LGI3 | leucine-rich repeat LGI family, member 3 | Multiple_C | 4.5 | 3.44 | 3.56 | 1.92 | 0.0256 | 0.0868 | 4.21 | 4.14 | 4.28 | 0.95 | 0.5683 | 0.7771 |
| TC0800012076.hg.1 | ARC | activity-regulated cytoskeleton-associated protein | Multiple_C | 5.5 | 4.4 | 4.56 | 1.92 | 0.0362 | 0.1139 | 5.5 | 5.17 | 5.06 | 1.36 | 0.1531 | 0.3921 |
| TC0X00009339.hg.1 | TAB3 | TGF-beta activated kinase 1/MAP3K7 binding protein 3 | Multiple_C | 10.79 | 9.64 | 9.85 | 1.92 | 0.0013 | 0.0081 | 9.76 | 10.13 | 10.7 | 0.52 | 0.0008 | 0.0138 |
| TC1000007596.hg.1 | TIMM23B | translocase of inner mitochondrial membrane 23 homolog B (ye | Multiple_C | 11.24 | 10.43 | 10.3 | 1.92 | 0.0001 | 0.0013 | 11.5 | 10.92 | 11.37 | 1.09 | 0.4895 | 0.7251 |
| TC1000008750.hg.1 | WBP1L | WW domain binding protein 1-like | Multiple_C | 8.84 | 8.04 | 7.9 | 1.92 | 0.0073 | 0.0325 | 9.69 | 10.24 | 9.52 | 1.13 | 0.7206 | 0.8698 |
| TC1000009221.hg.1 | ZRANB1 | zinc finger, RAN-binding domain containing 1 | Multiple_C | 10.39 | 9.77 | 9.45 | 1.92 | 0.0048 | 0.0233 | 9.65 | 8.25 | 9.33 | 1.25 | 0.1189 | 0.341 |
| TC1100009679.hg.1 | LMNTD2 | lamin tail domain containing 2 | Multiple_C | 6.09 | 4.79 | 5.15 | 1.92 | 0.0022 | 0.0124 | 5.9 | 5.95 | 5.61 | 1.22 | 0.2074 | 0.4641 |
| TC1100012516.hg.1 | CCDC153 | coiled-coil domain containing 153 | Multiple_C | 7.57 | 6.59 | 6.63 | 1.92 | 0.0425 | 0.1285 | 7.11 | 6.58 | 6.38 | 1.66 | 0.4094 | 0.6646 |
| TC1100013031.hg.1 | NUDT22 | nudix hydrolase 22 | Multiple_C | 7.52 | 6.36 | 6.58 | 1.92 | 0.0101 | 0.0419 | 6.82 | 7.03 | 6.4 | 1.34 | 0.4892 | 0.725 |
| TC1200007992.hg.1 | SRGAP1 | SLIT-ROBO Rho GTPase activating protein 1 | Multiple_C | 8.95 | 7.77 | 8.01 | 1.92 | 0.0036 | 0.0185 | 6.16 | 5.68 | 6.3 | 0.91 | 0.0424 | 0.1899 |
| TC1200009724.hg.1 | SCNN1A | sodium channel, non voltage gated 1 alpha subunit | Multiple_C | 11.67 | 10.89 | 10.73 | 1.92 | 0.0148 | 0.0566 | 9.36 | 9.59 | 9.94 | 0.67 | 0.2248 | 0.4856 |
| TC1200011435.hg.1 | RASSF9 | Ras association (RalGDS/AF-6) domain family (N-terminal) mem | Coding | 5.62 | 5.89 | 4.68 | 1.92 | 0.0042 | 0.0207 | 3.34 | 3.44 | 3.46 | 0.92 | 0.5645 | 0.7745 |
| TC1200011454.hg.1 | CEP290 | centrosomal protein 290kDa | Multiple_C | 9.68 | 9.66 | 8.74 | 1.92 | 0.0235 | 0.0812 | 9.56 | 9.00 | 9.29 | 1.21 | 0.5678 | 0.7767 |
| TC1500010109.hg.1 | PEAK1 | pseudopodium-enriched atypical kinase 1 | Multiple_C | 10.09 | 8.94 | 9.15 | 1.92 | 0.0014 | 0.0088 | 8.6 | 7.69 | 8.22 | 1.30 | 0.2342 | 0.4968 |
| TC1600011513.hg.1 | BOLA2; BOLA2 | bolA family member 2; bolA family member 2B | Multiple_C | 13.45 | 13.21 | 12.51 | 1.92 | 0.0978 | 0.2367 | 7.7 | 8.24 | 8.26 | 0.68 | 0.0021 | 0.0278 |
| TC1700006911.hg.1 | ARHGAP44 | Rho GTPase activating protein 44 | Multiple_C | 7.71 | 6.5 | 6.77 | 1.92 | 0.0068 | 0.0309 | 6.08 | 4.84 | 4.67 | 2.66 | 0.0002 | 0.0053 |
| TC1800007523.hg.1 | PHLPP1 | PH domain and leucine rich repeat protein phosphatase 1 | Multiple_C | 10.47 | 9.7 | 9.53 | 1.92 | 0.0073 | 0.0325 | 8.19 | 8.63 | 7.99 | 1.15 | 0.8588 | 0.9397 |
| TC1900006481.hg.1 | MISP | mitotic spindle positioning | Multiple_C | 10.8 | 10.02 | 9.86 | 1.92 | 0.0371 | 0.116 | 7.78 | 8.51 | 8.76 | 0.51 | 0.0058 | 0.0543 |
| TC1900007328.hg.1 | SLC27A1 | solute carrier family 27 (fatty acid transporter), member 1 | Multiple_C | 7.27 | 6.3 | 6.33 | 1.92 | 0.0007 | 0.0052 | 6.68 | 6.29 | 5.88 | 1.74 | 0.0002 | 0.0048 |
| TC1900010659.hg.1 | RPS16 | ribosomal protein S16 | Multiple_C | 17.75 | 16.78 | 16.81 | 1.92 | 0.0049 | 0.0238 | 14.57 | 14.14 | 13.9 | 1.59 | 0.0384 | 0.1783 |
| TC1900011224.hg.1 | KLK15 | kallikrein related peptidase 15 | Multiple_C | 9.73 | 8.38 | 8.79 | 1.92 | 0.0545 | 0.1541 | 8.38 | 8.86 | 8.51 | 0.91 | 0.485 | 0.7222 |
| TC2000009821.hg.1 | GMEB2 | glucocorticoid modulatory element binding protein 2 | Multiple_C | 6.98 | 5.32 | 6.04 | 1.92 | 0.0327 | 0.1053 | 6.54 | 6.52 | 6.73 | 0.88 | 0.2221 | 0.4824 |
| TC2200007138.hg.1 | FBXO7 | F-box protein 7 | Multiple_C | 13.09 | 11.42 | 12.15 | 1.92 | 0.0004 | 0.0033 | 11.83 | 11.58 | 12.45 | 0.65 | 0.0054 | 0.0515 |
| TSUnmapped00000129.h | ADAMTS13 | ADAM metallopeptidase with thrombospondin type 1 motif 13 | Coding | 6.85 | 5.73 | 5.91 | 1.92 | 0.001 | 0.0066 | 5.76 | 5.92 | 5.97 | 0.86 | 0.2494 | 0.5139 |
| TC0100009996.hg.1 | LCE3B | late cornified envelope 3B | Coding | 7.34 | 6.23 | 6.41 | 1.91 | 0.001 | 0.0067 | 7.92 | 8.04 | 7.6 | 1.25 | 0.0319 | 0.1598 |
| TC0100018497.hg.1 | SHC1 | SHC (Src homology 2 domain containing) transforming protein 1 | Multiple_C | 10.39 | 9.81 | 9.46 | 1.91 | 0.0049 | 0.0237 | 10.2 | 10.53 | 10.35 | 0.90 | 0.5752 | 0.7822 |
| TC0200011889.hg.1 | LAPTM4A | lysosomal protein transmembrane 4 alpha | Multiple_C | 15.47 | 14.7 | 14.54 | 1.91 | 0.0034 | 0.0176 | 13.27 | 12.91 | 12.73 | 1.45 | 0.0108 | 0.0821 |

| | | | | | | | | | | | | | | | |
|---|---|---|---|---|---|---|---|---|---|---|---|---|---|---|---|
| TC0200016414.hg.1 | FAM228B | family with sequence similarity 228, member B | Multiple_C | 4.02 | 3.05 | 3.09 | 1.91 | 0.0214 | 0.0756 | 4.36 | 4.89 | 4.75 | 0.76 | 0.0765 | 0.2662 |
| TC0300013555.hg.1 | MB21D2 | Mab-21 domain containing 2 | Coding | 8.56 | 6.93 | 7.63 | 1.91 | 0.0184 | 0.067 | 5.16 | 5.64 | 5.56 | 0.76 | 0.1594 | 0.4014 |
| TC0400010519.hg.1 | APBB2 | amyloid beta (A4) precursor protein-binding, family B, member | Multiple_C | 9.95 | 9.22 | 9.02 | 1.91 | 0.0053 | 0.0251 | 9.46 | 9.88 | 9.89 | 0.74 | 0.4398 | 0.6898 |
| TC0600007748.hg.1 | HMGA1 | high mobility group AT-hook 1 | Multiple_C | 13.99 | 12.62 | 13.06 | 1.91 | 0.0039 | 0.0199 | 16.44 | 15.72 | 15.89 | 1.46 | 0.1072 | 0.3221 |
| TC0600008729.hg.1 | C6orf163 | chromosome 6 open reading frame 163 | Multiple_C | 5.22 | 4.25 | 4.29 | 1.91 | 0.0042 | 0.0211 | 4.49 | 4.38 | 4.17 | 1.25 | 0.2276 | 0.4882 |
| TC0600011857.hg.1 | CUL7 | cullin 7 | Multiple_C | 6.64 | 5.8 | 5.71 | 1.91 | 0.0391 | 0.1205 | 6.61 | 7.21 | 7 | 0.76 | 0.1218 | 0.3445 |
| TC0700013323.hg.1 | SUN1 | Sad1 and UNC84 domain containing 1 | Multiple_C | 13.16 | 12.99 | 12.23 | 1.91 | 0.1652 | 0.3437 | 13.38 | 13.18 | 12.88 | 1.41 | 0.9329 | 0.972 |
| TC0900011501.hg.1 | NR6A1 | nuclear receptor subfamily 6, group A, member 1 | Multiple_C | 7.33 | 6.63 | 6.4 | 1.91 | 0.0021 | 0.0121 | 7.22 | 7.10 | 6.29 | 1.91 | 0.0007 | 0.0129 |
| TC0X00007599.hg.1 | DMRTC1; FAM | DMRT-like family C1; family with sequence similarity 226, meml | Multiple_C | 4.4 | 3.39 | 3.47 | 1.91 | 0.0067 | 0.0304 | 5.03 | 4.90 | 4.81 | 1.16 | 0.3114 | 0.5756 |
| TC1100007819.hg.1 | MYRF | myelin regulatory factor | Multiple_C | 5.92 | 5.01 | 4.99 | 1.91 | 0.1155 | 0.2676 | 7.91 | 9.23 | 10.04 | 0.23 | 0.0044 | 0.0456 |
| TC1100010058.hg.1 | DENND5A | DENN/MADD domain containing 5A | Multiple_C | 7.11 | 6.59 | 6.18 | 1.91 | 0.0032 | 0.017 | 8.83 | 8.59 | 8.93 | 0.93 | 0.8275 | 0.9237 |
| TC1300010048.hg.1 | FBXL3 | F-box and leucine-rich repeat protein 3 | Multiple_C | 9.35 | 8.66 | 8.42 | 1.91 | 0.009 | 0.0383 | 9.55 | 9.29 | 9.3 | 1.19 | 0.363 | 0.6258 |
| TC1400007504.hg.1 | RDH12 | retinol dehydrogenase 12 (all-trans/9-cis/11-cis) | Multiple_C | 4.9 | 4.01 | 3.97 | 1.91 | 0.0779 | 0.2006 | 4.21 | 3.92 | 3.57 | 1.56 | 0.5589 | 0.7714 |
| TC1400009351.hg.1 | SIX1 | SIX homeobox 1 | Multiple_C | 6.54 | 5.17 | 5.61 | 1.91 | 0.0009 | 0.0064 | 9.78 | 9.34 | 9.11 | 1.59 | 0.0023 | 0.029 |
| TC1500009813.hg.1 | MEGF11 | multiple EGF-like-domains 11 | Multiple_C | 4.18 | 3.08 | 3.25 | 1.91 | 0.004 | 0.02 | 4.03 | 4.68 | 4.27 | 0.85 | 0.266 | 0.5305 |
| TC1500010093.hg.1 | ETFA | electron-transfer-flavoprotein, alpha polypeptide | Multiple_C | 14.49 | 15.41 | 13.56 | 1.91 | 5.44E-05 | 0.0007 | 11.92 | 11.91 | 12.07 | 0.90 | 0.7286 | 0.8741 |
| TC1600007285.hg.1 | HS3ST4 | heparan sulfate (glucosamine) 3-O-sulfotransferase 4 | Multiple_C | 5.84 | 4.58 | 4.91 | 1.91 | 0.0006 | 0.0044 | 5.44 | 5.55 | 5.3 | 1.10 | 0.5091 | 0.7392 |
| TC1700010996.hg.1 | HOXB8 | homeobox B8 | Coding | 3.92 | 2.86 | 2.99 | 1.91 | 0.0409 | 0.1247 | 6.54 | 6.57 | 6.37 | 1.13 | 0.4479 | 0.6949 |
| TC1700012473.hg.1 | FBF1 | Fas (TNFRSF6) binding factor 1 | Multiple_C | 6.03 | 4.99 | 5.1 | 1.91 | 0.009 | 0.0383 | 5.84 | 6.25 | 5.97 | 0.91 | 0.644 | 0.8254 |
| TC2000007393.hg.1 | LPIN3 | lipin 3 | Multiple_C | 6.23 | 5.12 | 5.3 | 1.91 | 0.0353 | 0.1117 | 5.71 | 5.60 | 5.05 | 1.58 | 0.4101 | 0.665 |
| TC2000007915.hg.1 | GNAS | GNAS complex locus | Multiple_C | 14.65 | 13.4 | 13.72 | 1.91 | 0.0034 | 0.0178 | 12.56 | 12.97 | 12.89 | 0.80 | 0.0657 | 0.2441 |
| TC2000009292.hg.1 | ZMYND8 | zinc finger, MYND-type containing 8 | Multiple_C | 12.11 | 10.65 | 11.18 | 1.91 | 0.0018 | 0.0107 | 14.47 | 13.83 | 14.02 | 1.37 | 0.025 | 0.1373 |
| TC2200009345.hg.1 | C1QTNF6 | C1q and tumor necrosis factor related protein 6 | Multiple_C | 4.66 | 3.73 | 3.73 | 1.91 | 0.0061 | 0.0281 | 6.09 | 6.06 | 5.72 | 1.29 | 0.3482 | 0.6121 |
| TC0100008803.hg.1 | FAM73A | family with sequence similarity 73, member A | Multiple_C | 8.52 | 8.64 | 7.6 | 1.89 | 0.0089 | 0.038 | 8.13 | 7.45 | 7.58 | 1.46 | 0.0107 | 0.0814 |
| TC0100009788.hg.1 | BCL9 | B-cell CLL/lymphoma 9 | Multiple_C | 9.17 | 7.61 | 8.25 | 1.89 | 0.0039 | 0.0199 | 9.41 | 9.19 | 9.68 | 0.83 | 0.0464 | 0.1993 |
| TC0100013805.hg.1 | OXCT2 | 3-oxoacid CoA-transferase 2 | Coding | 5.07 | 4.08 | 4.15 | 1.89 | 0.0064 | 0.0292 | 4.63 | 4.43 | 4.31 | 1.25 | 0.3824 | 0.6414 |
| TC0100015115.hg.1 | AMY1B; AMY1 | amylase, alpha 1B (salivary); amylase, alpha 1A (salivary) | Multiple_C | 5.28 | 4.76 | 4.36 | 1.89 | 0.004 | 0.02 | 4.06 | 3.86 | 4.28 | 0.86 | 0.7006 | 0.858 |
| TC0200007471.hg.1 | RHOQ | ras homolog family member Q | Multiple_C | 9.12 | 8.4 | 8.2 | 1.89 | 0.0024 | 0.0134 | 9.11 | 8.23 | 8.16 | 1.93 | 0.0002 | 0.0061 |
| TC0600008078.hg.1 | SLC22A7 | solute carrier family 22 (organic anion transporter), member 7 | Multiple_C | 4.42 | 3.08 | 3.5 | 1.89 | 0.1746 | 0.3565 | 4.95 | 4.56 | 4.03 | 1.89 | 0.0077 | 0.0653 |
| TC0600012655.hg.1 | PNISR | PNN-interacting serine/arginine-rich protein | Multiple_C | 11.02 | 10.66 | 10.1 | 1.89 | 0.0196 | 0.0703 | 9.49 | 8.82 | 9.19 | 1.23 | 0.119 | 0.341 |
| TC0600012814.hg.1 | CD164 | CD164 molecule, sialomucin | Multiple_C | 15.12 | 14.95 | 14.2 | 1.89 | 0.0187 | 0.0679 | 12.55 | 12.27 | 12.5 | 1.04 | 0.4583 | 0.7021 |
| TC0700008246.hg.1 | DMTF1 | cyclin D binding myb-like transcription factor 1 | Multiple_C | 13.01 | 12.23 | 12.09 | 1.89 | 0.0348 | 0.1104 | 13.39 | 11.99 | 12.91 | 1.39 | 0.0704 | 0.2536 |
| TC0700013400.hg.1 | GATAD1 | GATA zinc finger domain containing 1 | Multiple_C | 10.91 | 10.32 | 9.99 | 1.89 | 0.0025 | 0.0138 | 10.75 | 10.09 | 10.67 | 1.06 | 0.5574 | 0.7711 |

| | | | | | | | | | | | | | | | |
|---|---|---|---|---|---|---|---|---|---|---|---|---|---|---|---|
| TC0900006564.hg.1 | RIC1 | RIC1 homolog, RAB6A GEF complex partner 1 | Multiple_C | 8.08 | 7.24 | 7.16 | 1.89 | 0.0058 | 0.027 | 7.53 | 6.95 | 7.41 | 1.09 | 0.5114 | 0.7412 |
| TC0900007006.hg.1 | PRSS3 | protease, serine, 3 | Multiple_C | 15.05 | 14.03 | 14.13 | 1.89 | 0.0195 | 0.0701 | 9.03 | 9.84 | 9.09 | 0.96 | 0.6995 | 0.8575 |
| TC0900007640.hg.1 | FOXB2 | forkhead box B2 | Coding | 4.63 | 3.55 | 3.71 | 1.89 | 0.0355 | 0.1121 | 4.25 | 4.25 | 3.91 | 1.27 | 0.1267 | 0.3522 |
| TC0900009915.hg.1 | CCL19 | chemokine (C-C motif) ligand 19 | Multiple_C | 7.58 | 6.66 | 6.66 | 1.89 | 0.0208 | 0.0739 | 6.19 | 6.04 | 5.69 | 1.41 | 0.0822 | 0.2762 |
| TC0900012135.hg.1 | ANKRD20A4 | ankyrin repeat domain 20 family, member A4 | Multiple_C | 4.32 | 3.13 | 3.4 | 1.89 | 0.0292 | 0.0963 | 5.92 | 5.52 | 5.39 | 1.44 | 0.0325 | 0.1616 |
| TC0X00008699.hg.1 | MAGEA8 | MAGE family member A8 | Multiple_C | 7.17 | 6.21 | 6.25 | 1.89 | 0.0012 | 0.0078 | 6.97 | 6.99 | 6.88 | 1.06 | 0.6498 | 0.829 |
| TC0X00011308.hg.1 | RPL36A | ribosomal protein L36a | Multiple_C | 16.6 | 16.3 | 15.68 | 1.89 | 0.0252 | 0.0857 | 12.21 | 11.76 | 12.15 | 1.04 | 0.8423 | 0.9313 |
| TC1000007132.hg.1 | BAMBI | BMP and activin membrane-bound inhibitor | Multiple_C | 10.75 | 10.65 | 9.83 | 1.89 | 0.0209 | 0.0742 | 11.75 | 11.72 | 11.16 | 1.51 | 0.0932 | 0.2965 |
| TC1100010077.hg.1 | SBF2 | SET binding factor 2 | Multiple_C | 10.54 | 10.31 | 9.62 | 1.89 | 0.0003 | 0.0026 | 9.77 | 8.98 | 9.6 | 1.13 | 0.2137 | 0.4719 |
| TC1100010222.hg.1 | RPL36A | ribosomal protein L36a | Multiple_C | 10.31 | 9.77 | 9.39 | 1.89 | 0.0688 | 0.183 | 8.13 | 7.69 | 7.83 | 1.23 | 0.2011 | 0.4565 |
| TC1100011257.hg.1 | EFEMP2 | EGF containing fibulin-like extracellular matrix protein 2 | Multiple_C | 5.74 | 4.8 | 4.82 | 1.89 | 0.0083 | 0.0358 | 5.33 | 5.23 | 4.81 | 1.43 | 0.0417 | 0.1881 |
| TC1200006896.hg.1 | GPRC5A; MIR( | G protein-coupled receptor, class C, group 5, member A; microF | Multiple_C | 14.79 | 12.89 | 13.87 | 1.89 | 0.0619 | 0.1694 | 9.38 | 7.96 | 9 | 1.30 | 0.163 | 0.4066 |
| TC1200011400.hg.1 | CCDC59 | coiled-coil domain containing 59 | Multiple_C | 14.22 | 14.01 | 13.3 | 1.89 | 0.0072 | 0.0323 | 12.35 | 11.45 | 12.54 | 0.88 | 0.8731 | 0.9452 |
| TC1200011727.hg.1 | IGF1 | insulin-like growth factor 1 (somatomedin C) | Multiple_C | 6.31 | 5.39 | 5.39 | 1.89 | 0.0707 | 0.1868 | 5.54 | 5.42 | 5.43 | 1.08 | 0.7756 | 0.8993 |
| TC1300006715.hg.1 | PAN3; RNU6-8 | PAN3 poly(A) specific ribonuclease subunit; RNA, U6 small nucle | Multiple_C | 9.4 | 9.02 | 8.48 | 1.89 | 0.0815 | 0.2074 | 8.16 | 8.83 | 8.49 | 0.80 | 0.2276 | 0.4882 |
| TC1400008173.hg.1 | CCNK | cyclin K | Multiple_C | 12.52 | 11.02 | 11.6 | 1.89 | 0.0303 | 0.0991 | 12.62 | 11.97 | 12.45 | 1.13 | 0.4839 | 0.7215 |
| TC1700008661.hg.1 | PRKCA | protein kinase C, alpha | Multiple_C | 7.42 | 6.44 | 6.5 | 1.89 | 0.003 | 0.0161 | 6.21 | 6.87 | 6.99 | 0.58 | 0.0009 | 0.0155 |
| TC1900009823.hg.1 | GIPC1 | GIPC PDZ domain containing family, member 1 | Multiple_C | 10.91 | 9.61 | 9.99 | 1.89 | 0.0067 | 0.0305 | 8.13 | 8.57 | 8.33 | 0.87 | 0.77 | 0.8968 |
| TC1900011751.hg.1 | ZNF222 | zinc finger protein 222 | Multiple_C | 11.28 | 10.59 | 10.36 | 1.89 | 0.0046 | 0.0224 | 9.22 | 9.40 | 9.21 | 1.01 | 0.9961 | 0.9982 |
| TC2200008781.hg.1 | MKL1 | megakaryoblastic leukemia (translocation) 1 | Multiple_C | 7.47 | 6.01 | 6.55 | 1.89 | 0.0056 | 0.0263 | 9.17 | 9.17 | 9.01 | 1.12 | 0.9624 | 0.9842 |
| TSUnmapped00000315.h | OBP2B | odorant binding protein 2B | Coding | 5.74 | 4.79 | 4.82 | 1.89 | 0.0112 | 0.0457 | 5.13 | 5.25 | 5.34 | 0.86 | 0.7703 | 0.8969 |
| TC0100007638.hg.1 | SERINC2 | serine incorporator 2 | Multiple_C | 10.34 | 8.4 | 9.43 | 1.88 | 0.092 | 0.2267 | 7.42 | 6.98 | 7.82 | 0.76 | 0.4193 | 0.6729 |
| TC0100012760.hg.1 | RERE | arginine-glutamic acid dipeptide (RE) repeats | Multiple_C | 8.5 | 7.07 | 7.59 | 1.88 | 0.0591 | 0.1638 | 9.08 | 9.91 | 9.07 | 1.01 | 0.9572 | 0.9823 |
| TC0100018440.hg.1 | STIL | SCL/TAL1 interrupting locus | Multiple_C | 9.99 | 8.18 | 9.08 | 1.88 | 0.1513 | 0.3242 | 9.42 | 9.28 | 9.74 | 0.80 | 0.2125 | 0.4702 |
| TC0200008894.hg.1 | MERTK | MER proto-oncogene, tyrosine kinase | Multiple_C | 6.3 | 6.09 | 5.39 | 1.88 | 0.048 | 0.1403 | 6.72 | 5.73 | 6.25 | 1.39 | 0.1115 | 0.3291 |
| TC0200014717.hg.1 | WDSUB1 | WD repeat, sterile alpha motif and U-box domain containing 1 | Multiple_C | 8.05 | 8.61 | 7.14 | 1.88 | 0.016 | 0.06 | 6.07 | 5.71 | 5.43 | 1.56 | 0.0369 | 0.1748 |
| TC0300010916.hg.1 | TMEM158 | transmembrane protein 158 (gene/pseudogene) | Coding | 6.17 | 5 | 5.26 | 1.88 | 0.011 | 0.045 | 5.22 | 5.51 | 5.38 | 0.90 | 0.7797 | 0.9012 |
| TC0400009221.hg.1 | MSMO1 | methylsterol monooxygenase 1 | Multiple_C | 14.92 | 14.93 | 14.01 | 1.88 | 0.0244 | 0.0838 | 13.4 | 10.84 | 13.2 | 1.15 | 0.9933 | 0.997 |
| TC0400010886.hg.1 | UBA6 | ubiquitin-like modifier activating enzyme 6 | Multiple_C | 10.28 | 9.51 | 9.37 | 1.88 | 0.0301 | 0.0986 | 9.46 | 9.21 | 9.6 | 0.91 | 0.1812 | 0.4313 |
| TC0400012875.hg.1 | KLKB1 | kallikrein B1 | Multiple_C | 5.09 | 4.86 | 4.18 | 1.88 | 0.0057 | 0.0267 | 3.89 | 3.97 | 3.82 | 1.05 | 0.9835 | 0.9933 |
| TC0500008846.hg.1 | IGIP | IgA-inducing protein | Coding | 5.89 | 5.88 | 4.98 | 1.88 | 0.0089 | 0.0379 | 4.28 | 4.28 | 4.04 | 1.18 | 0.2949 | 0.5598 |
| TC0500010043.hg.1 | SEMA5A | sema domain, seven thrombospondin repeats (type 1 and type | Multiple_C | 4.18 | 3.13 | 3.27 | 1.88 | 0.037 | 0.1158 | 3.29 | 3.38 | 3.63 | 0.79 | 0.1844 | 0.4357 |
| TC0600007610.hg.1 | MSH5; MSH5- | mutS homolog 5; MSH5-SAPCD1 readthrough (NMD candidate) | Multiple_C | 6.33 | 5.69 | 5.42 | 1.88 | 0.0026 | 0.0144 | 6.27 | 6.70 | 6.04 | 1.17 | 0.2794 | 0.5454 |

| | | | | | | | | | | | | | | | |
|---|---|---|---|---|---|---|---|---|---|---|---|---|---|---|---|
| TC0600011535.hg.1 | RGL2 | ral guanine nucleotide dissociation stimulator-like 2 | Multiple_C | 7.61 | 6.79 | 6.7 | 1.88 | 0.0139 | 0.0537 | 7.33 | 7.70 | 7.25 | 1.06 | 0.9636 | 0.9846 |
| TC0700011066.hg.1 | COBL | cordon-bleu WH2 repeat protein | Multiple_C | 11.19 | 9.92 | 10.28 | 1.88 | 9.57E-05 | 0.001 | 11.89 | 11.68 | 11.66 | 1.17 | 0.5894 | 0.7923 |
| TC0800012211.hg.1 | CPSF1; MIR93 | cleavage and polyadenylation specific factor 1; microRNA 939; r | Multiple_C | 11.45 | 10.44 | 10.54 | 1.88 | 0.0127 | 0.0505 | 9.64 | 10.25 | 9.83 | 0.88 | 0.6418 | 0.8245 |
| TC0X00007205.hg.1 | HDAC6 | histone deacetylase 6 | Multiple_C | 9 | 8.81 | 8.09 | 1.88 | 0.0084 | 0.0363 | 8.31 | 9.10 | 9.04 | 0.60 | 0.1944 | 0.4484 |
| TC0X00010532.hg.1 | AMOT; MIR43 | angiomotin; microRNA 4329 | Multiple_C | 4.39 | 3.29 | 3.48 | 1.88 | 0.0229 | 0.0796 | 5.73 | 6.45 | 5.47 | 1.20 | 0.6111 | 0.8057 |
| TC1100011157.hg.1 | PPP1R14B | protein phosphatase 1, regulatory (inhibitor) subunit 14B | Multiple_C | 14.01 | 12.97 | 13.1 | 1.88 | 0.0041 | 0.0205 | 13.32 | 14.06 | 13.81 | 0.71 | 0.5915 | 0.7934 |
| TC1200008526.hg.1 | AMDHD1 | amidohydrolase domain containing 1 | Multiple_C | 6.53 | 5.91 | 5.62 | 1.88 | 0.0214 | 0.0756 | 5.76 | 5.58 | 5.43 | 1.26 | 0.1001 | 0.3094 |
| TC1200010809.hg.1 | ATF7; NPFF | activating transcription factor 7; neuropeptide FF-amide peptid | Multiple_C | 9.42 | 8.58 | 8.51 | 1.88 | 0.0037 | 0.019 | 9.35 | 9.41 | 9.51 | 0.90 | 0.4236 | 0.6763 |
| TC1400006513.hg.1 | PARP2 | poly(ADP-ribose) polymerase 2 | Multiple_C | 10.71 | 9.39 | 9.8 | 1.88 | 0.0208 | 0.0738 | 9.08 | 8.00 | 8.94 | 1.10 | 0.3154 | 0.5794 |
| TC1400008588.hg.1 | TEP1 | telomerase-associated protein 1 | Multiple_C | 7.2 | 6.6 | 6.29 | 1.88 | 0.0006 | 0.0047 | 5.69 | 5.48 | 5.4 | 1.22 | 0.1147 | 0.3341 |
| TC1400010796.hg.1 | JAG2 | jagged 2 | Multiple_C | 7.94 | 6.92 | 7.03 | 1.88 | 0.0041 | 0.0205 | 8.94 | 8.64 | 8.72 | 1.16 | 0.6068 | 0.8035 |
| TC1500007939.hg.1 | HMG20A | high mobility group 20A | Multiple_C | 10.49 | 10.34 | 9.58 | 1.88 | 0.0033 | 0.0176 | 10.23 | 9.86 | 9.16 | 2.10 | 0.0003 | 0.0083 |
| TC1500008893.hg.1 | MTMR10 | myotubularin related protein 10 | Multiple_C | 8.94 | 8.06 | 8.03 | 1.88 | 0.0261 | 0.0882 | 7.46 | 7.29 | 8.11 | 0.64 | 0.0382 | 0.178 |
| TC1600007557.hg.1 | ZNF267 | zinc finger protein 267 | Multiple_C | 11.49 | 11.12 | 10.58 | 1.88 | 0.0119 | 0.0479 | 10.13 | 8.95 | 9.75 | 1.30 | 0.4037 | 0.6596 |
| TC1600009147.hg.1 | PRSS22 | protease, serine, 22 | Multiple_C | 9.7 | 9.58 | 8.79 | 1.88 | 0.0009 | 0.0063 | 4.33 | 3.31 | 3.74 | 1.51 | 0.014 | 0.0959 |
| TC1600010316.hg.1 | TOX3 | TOX high mobility group box family member 3 | Multiple_C | 10.58 | 8.92 | 9.67 | 1.88 | 0.0501 | 0.1447 | 7.56 | 9.06 | 11.13 | 0.08 | 8.09E-09 | 4.23E-06 |
| TC1700010704.hg.1 | ZNF385C | zinc finger protein 385C | Multiple_C | 6.1 | 5.07 | 5.19 | 1.88 | 0.0121 | 0.0485 | 6.86 | 6.10 | 5.79 | 2.10 | 0.0139 | 0.0957 |
| TC1700010716.hg.1 | GHDC | GH3 domain containing | Multiple_C | 7 | 6.39 | 6.09 | 1.88 | 0.0512 | 0.1471 | 6.66 | 6.70 | 6.52 | 1.10 | 0.5329 | 0.7557 |
| TC1700011128.hg.1 | SPAG9 | sperm associated antigen 9 | Multiple_C | 12.1 | 11.78 | 11.19 | 1.88 | 0.0008 | 0.0055 | 10.95 | 10.91 | 11.32 | 0.77 | 0.0568 | 0.2242 |
| TC1800006506.hg.1 | MYL12A | myosin light chain 12A | Multiple_C | 14.39 | 14.29 | 13.48 | 1.88 | 0.0019 | 0.0111 | 12.49 | 12.44 | 11.66 | 1.78 | 0.0059 | 0.0546 |
| TC1900008235.hg.1 | IRGC | immunity-related GTPase family, cinema | Coding | 4.03 | 3.14 | 3.12 | 1.88 | 0.0113 | 0.0459 | 3.36 | 3.47 | 3.12 | 1.18 | 0.9801 | 0.9915 |
| TC1900008533.hg.1 | CD37 | CD37 molecule | Multiple_C | 5.51 | 4.56 | 4.6 | 1.88 | 0.0184 | 0.0671 | 4.73 | 4.64 | 4.95 | 0.86 | 0.3583 | 0.6212 |
| TC1900008604.hg.1 | MGC45922; C | uncharacterized LOC284365; uncharacterized LOC284365 [Sour | Multiple_C | 4.81 | 3.78 | 3.9 | 1.88 | 0.0186 | 0.0675 | 4.43 | 4.33 | 4.32 | 1.08 | 0.3727 | 0.6337 |
| TC1900008904.hg.1 | KMT5C | lysine (K)-specific methyltransferase 5C | Multiple_C | 6.37 | 5.69 | 5.46 | 1.88 | 0.0036 | 0.0187 | 6.43 | 6.63 | 6.03 | 1.32 | 0.8048 | 0.9134 |
| TC2000009218.hg.1 | SDC4 | syndecan 4 | Multiple_C | 13.46 | 12.94 | 12.55 | 1.88 | 0.017 | 0.063 | 10.39 | 8.50 | 9.45 | 1.92 | 0.0006 | 0.0116 |
| TC2200007909.hg.1 | MICAL3 | microtubule associated monooxygenase, calponin and LIM dom | Multiple_C | 8.01 | 6.68 | 7.1 | 1.88 | 0.0262 | 0.0883 | 9.08 | 8.73 | 8.77 | 1.24 | 0.6464 | 0.8265 |
| TC2200009279.hg.1 | SMDT1 | single-pass membrane protein with aspartate-rich tail 1 | Multiple_C | 11.64 | 11.4 | 10.73 | 1.88 | 0.0006 | 0.0042 | 7.86 | 8.23 | 7.92 | 0.96 | 0.9754 | 0.9902 |
| TSUnmapped00000167.h | ZDHHC3 | zinc finger, DHHC-type containing 3 | Coding | 6.85 | 6.03 | 5.94 | 1.88 | 0.0104 | 0.0431 | 4.74 | 4.83 | 4.59 | 1.11 | 0.5978 | 0.7976 |
| TC0100014958.hg.1 | BCAR3 | breast cancer anti-estrogen resistance 3 | Multiple_C | 7.83 | 7.62 | 6.93 | 1.87 | 0.0069 | 0.0314 | 5.06 | 5.10 | 5.69 | 0.65 | 0.1104 | 0.3273 |
| TC0100015850.hg.1 | SPRR2B | small proline-rich protein 2B | Coding | 4.43 | 3.08 | 3.53 | 1.87 | 0.0061 | 0.0284 | 3.56 | 3.75 | 3.79 | 0.85 | 0.3164 | 0.5802 |
| TC0100017079.hg.1 | SLC41A1 | solute carrier family 41 (magnesium transporter), member 1 | Multiple_C | 10.42 | 9.07 | 9.52 | 1.87 | 0.0123 | 0.049 | 9.14 | 8.79 | 9.43 | 0.82 | 0.1882 | 0.4403 |
| TC0100018271.hg.1 | OLFML3 | olfactomedin like 3 | Multiple_C | 4.48 | 3.51 | 3.58 | 1.87 | 0.0103 | 0.0427 | 4.6 | 5.16 | 4.73 | 0.91 | 0.7505 | 0.8865 |
| TC0200011106.hg.1 | EIF4E2 | eukaryotic translation initiation factor 4E family member 2 | Multiple_C | 12.2 | 10.97 | 11.3 | 1.87 | 0.0011 | 0.0073 | 10.64 | 10.90 | 10.72 | 0.95 | 0.8628 | 0.9412 |

| | | | | | | | | | | | | | | | |
|---|---|---|---|---|---|---|---|---|---|---|---|---|---|---|---|
| TC0200015525.hg.1 | DYTN | dystrotelin | Multiple_C | 5.49 | 4.18 | 4.59 | 1.87 | 0.0093 | 0.0392 | 4.87 | 4.85 | 4.83 | 1.03 | 0.6885 | 0.8511 |
| TC0200016668.hg.1 | GPR75 | G protein-coupled receptor 75 | Coding | 6.14 | 4.5 | 5.24 | 1.87 | 0.0286 | 0.0946 | 5.7 | 6.23 | 6.46 | 0.59 | 0.0231 | 0.1308 |
| TC0300010208.hg.1 | CAMK1 | calcium/calmodulin-dependent protein kinase I | Multiple_C | 8.43 | 7.52 | 7.53 | 1.87 | 0.044 | 0.1318 | 7.5 | 7.61 | 7.34 | 1.12 | 0.1368 | 0.3676 |
| TC0300011119.hg.1 | C3orf18 | chromosome 3 open reading frame 18 | Multiple_C | 5.84 | 5.07 | 4.94 | 1.87 | 0.0815 | 0.2074 | 5.29 | 5.65 | 4.93 | 1.28 | 0.0129 | 0.0914 |
| TC0300013948.hg.1 | TBC1D5 | TBC1 domain family, member 5 | Multiple_C | 7.97 | 7.71 | 7.07 | 1.87 | 0.0239 | 0.0825 | 8.61 | 8.46 | 8.35 | 1.20 | 0.9681 | 0.9867 |
| TC0400007104.hg.1 | SLC34A2 | solute carrier family 34 (type II sodium/phosphate cotransporte | Multiple_C | 3.98 | 2.78 | 3.08 | 1.87 | 0.0257 | 0.087 | 3.56 | 3.71 | 3.38 | 1.13 | 0.1676 | 0.413 |
| TC0400010484.hg.1 | PDS5A | PDS5 cohesin associated factor A | Multiple_C | 12.67 | 11.58 | 11.77 | 1.87 | 0.0374 | 0.1166 | 12.34 | 11.97 | 12.82 | 0.72 | 0.0764 | 0.266 |
| TC0400010618.hg.1 | TEC | tec protein tyrosine kinase | Multiple_C | 7.52 | 7.05 | 6.62 | 1.87 | 0.011 | 0.0451 | 6.13 | 5.66 | 5.85 | 1.21 | 0.2508 | 0.515 |
| TC0600007508.hg.1 | PPP1R11 | protein phosphatase 1, regulatory (inhibitor) subunit 11 | Coding | 9.46 | 8.22 | 8.56 | 1.87 | 0.0009 | 0.0063 | 10.17 | 10.48 | 10.46 | 0.82 | 0.1149 | 0.3348 |
| TC0600008014.hg.1 | MDFI | MyoD family inhibitor | Multiple_C | 6.34 | 5.45 | 5.44 | 1.87 | 0.0063 | 0.0288 | 6.61 | 6.89 | 6.64 | 0.98 | 0.5212 | 0.7482 |
| TC0600011173.hg.1 | GUSBP2 | glucuronidase, beta pseudogene 2 | Multiple_C | 11.81 | 11.52 | 10.91 | 1.87 | 0.0009 | 0.0059 | 15.23 | 15.13 | 14.85 | 1.30 | 0.0803 | 0.2731 |
| TC0600013054.hg.1 | SERINC1 | serine incorporator 1 | Multiple_C | 13.05 | 13.56 | 12.15 | 1.87 | 0.0949 | 0.2319 | 11.75 | 11.25 | 11.25 | 1.41 | 0.0685 | 0.2492 |
| TC0700007807.hg.1 | ZNF92 | zinc finger protein 92 | Multiple_C | 10.65 | 9.63 | 9.75 | 1.87 | 0.0513 | 0.1472 | 10.34 | 9.74 | 10.8 | 0.73 | 0.3812 | 0.6408 |
| TC0700012755.hg.1 | KIAA1549 | KIAA1549 | Multiple_C | 10.3 | 8.92 | 9.4 | 1.87 | 0.0236 | 0.0815 | 10.51 | 10.77 | 10.77 | 0.84 | 0.0675 | 0.2474 |
| TC0800007210.hg.1 | WRN | Transcript Identified by AceView, Entrez Gene ID(s) 7486 | Coding | 4.61 | 4.01 | 3.71 | 1.87 | 0.0715 | 0.1884 | 4.1 | 3.59 | 3.58 | 1.43 | 0.0124 | 0.0892 |
| TC1000012551.hg.1 | ANXA8 | annexin A8 | Multiple_C | 5.27 | 4.37 | 4.37 | 1.87 | 0.0326 | 0.1051 | 4.4 | 4.62 | 4.57 | 0.89 | 0.7837 | 0.9026 |
| TC1100008120.hg.1 | KDM2A | lysine (K)-specific demethylase 2A | Multiple_C | 10.13 | 9.01 | 9.23 | 1.87 | 0.007 | 0.0315 | 10.68 | 10.56 | 10.81 | 0.91 | 0.7352 | 0.8777 |
| TC1100008523.hg.1 | CAPN5 | calpain 5 | Multiple_C | 6.91 | 6.18 | 6.01 | 1.87 | 0.0169 | 0.0627 | 5.08 | 5.25 | 5.02 | 1.04 | 0.9347 | 0.9725 |
| TC1100013126.hg.1 | KRTAP5-3 | keratin associated protein 5-3 | Coding | 6.59 | 5.69 | 5.69 | 1.87 | 0.0118 | 0.0475 | 6.03 | 5.83 | 5.73 | 1.23 | 0.2778 | 0.5438 |
| TC1200011140.hg.1 | GRIP1 | glutamate receptor interacting protein 1 | Multiple_C | 4.67 | 3.77 | 3.77 | 1.87 | 0.0069 | 0.0314 | 4.26 | 4.04 | 3.8 | 1.38 | 0.1508 | 0.3888 |
| TC1400007029.hg.1 | C14orf28 | chromosome 14 open reading frame 28 | Multiple_C | 6.84 | 7.58 | 5.94 | 1.87 | 0.0075 | 0.0331 | 5.19 | 5.70 | 5.14 | 1.04 | 0.7688 | 0.8964 |
| TC1400008347.hg.1 | ZNF839 | zinc finger protein 839 | Multiple_C | 8.15 | 7.56 | 7.25 | 1.87 | 0.0061 | 0.0284 | 5.97 | 6.19 | 6.07 | 0.93 | 0.3414 | 0.6052 |
| TC1400009563.hg.1 | SLC10A1 | solute carrier family 10 (sodium/bile acid cotransporter), memb | Coding | 4.38 | 3.51 | 3.48 | 1.87 | 0.0009 | 0.0061 | 4.52 | 4.41 | 4.26 | 1.20 | 0.2896 | 0.5543 |
| TC1400010764.hg.1 | VTI1B | vesicle transport through interaction with t-SNAREs 1B | Multiple_C | 10.95 | 10.08 | 10.05 | 1.87 | 0.0038 | 0.0196 | 11.21 | 11.38 | 11.44 | 0.85 | 0.281 | 0.5467 |
| TC1500008995.hg.1 | GOLGA8B | golgin A8 family, member B | Multiple_C | 9.3 | 9.5 | 8.4 | 1.87 | 0.0025 | 0.0137 | 12.51 | 11.08 | 9.83 | 6.41 | 1.50E-10 | 2.68E-07 |
| TC1500010465.hg.1 | VPS33B | vacuolar protein sorting 33 homolog B (yeast) | Multiple_C | 8.48 | 7.94 | 7.58 | 1.87 | 0.0058 | 0.027 | 8.32 | 8.29 | 7.89 | 1.35 | 0.0872 | 0.2851 |
| TC1700008533.hg.1 | TLK2 | tousled-like kinase 2 | Multiple_C | 11.5 | 11.16 | 10.6 | 1.87 | 0.0003 | 0.0026 | 12.45 | 12.44 | 12.7 | 0.84 | 0.2816 | 0.5474 |
| TC1700008842.hg.1 | RPL38 | ribosomal protein L38 | Multiple_C | 18.81 | 17.86 | 17.91 | 1.87 | 0.001 | 0.0065 | 16.83 | 16.59 | 16.27 | 1.47 | 0.0192 | 0.117 |
| TC1900009568.hg.1 | OR7D4 | olfactory receptor, family 7, subfamily D, member 4 | Coding | 5.9 | 4.84 | 5 | 1.87 | 0.0319 | 0.1031 | 5.43 | 5.20 | 5.48 | 0.97 | 0.9868 | 0.9944 |
| TC1900010939.hg.1 | PPP1R13L | protein phosphatase 1, regulatory subunit 13 like | Multiple_C | 7.32 | 6.29 | 6.42 | 1.87 | 0.0373 | 0.1165 | 5.69 | 5.69 | 5.59 | 1.07 | 0.7176 | 0.8682 |
| TC1900011915.hg.1 | ZNF737 | zinc finger protein 737 | Multiple_C | 10.61 | 9.84 | 9.71 | 1.87 | 0.0046 | 0.0226 | 6.52 | 6.83 | 7.32 | 0.57 | 0.0034 | 0.0383 |
| TC1900012028.hg.1 | ZNF320 | zinc finger protein 320 | Multiple_C | 11.21 | 11.07 | 10.31 | 1.87 | 0.0144 | 0.0552 | 9.26 | 9.19 | 9.45 | 0.88 | 0.3213 | 0.5853 |
| TC2000009819.hg.1 | HELZ2 | helicase with zinc finger 2, transcriptional coactivator | Multiple_C | 5.52 | 4.51 | 4.62 | 1.87 | 0.0052 | 0.0249 | 4.09 | 4.01 | 4.08 | 1.01 | 0.4725 | 0.7127 |

| | | | | | | | | | | | | | | | |
|---|---|---|---|---|---|---|---|---|---|---|---|---|---|---|---|
| TC2000009895.hg.1 | LINC00493 | long intergenic non-protein coding RNA 493 | Multiple_C | 9.98 | 10.64 | 9.08 | 1.87 | 0.0596 | 0.1648 | 7.59 | 7.20 | 6.71 | 1.84 | 0.0144 | 0.0979 |
| TC2200009267.hg.1 | GTPBP1 | GTP binding protein 1 | Coding | 5.54 | 4.47 | 4.64 | 1.87 | 0.1353 | 0.2997 | 4.85 | 5.07 | 5.27 | 0.75 | 0.4167 | 0.6705 |
| TC0100010887.hg.1 | C1orf21 | chromosome 1 open reading frame 21 | Multiple_C | 13.04 | 13.2 | 12.15 | 1.85 | 0.0407 | 0.1242 | 4.62 | 3.65 | 3.7 | 1.89 | 0.0204 | 0.1221 |
| TC0100014752.hg.1 | GNG5 | guanine nucleotide binding protein (G protein), gamma 5 | Multiple_C | 14.33 | 14.24 | 13.44 | 1.85 | 0.0122 | 0.049 | 10.28 | 10.05 | 10.08 | 1.15 | 0.2572 | 0.5221 |
| TC0200013517.hg.1 | SNRNP200 | small nuclear ribonucleoprotein, U5 200kDa subunit | Multiple_C | 12.84 | 11.55 | 11.95 | 1.85 | 0.0028 | 0.0154 | 13.39 | 14.20 | 13.74 | 0.78 | 0.0605 | 0.2333 |
| TC0200016511.hg.1 | IL1RN | interleukin 1 receptor antagonist | Multiple_C | 4.34 | 3.09 | 3.45 | 1.85 | 0.0051 | 0.0243 | 3.91 | 3.61 | 3.33 | 1.49 | 0.0101 | 0.0784 |
| TC0300007324.hg.1 | CAMP | cathelicidin antimicrobial peptide | Coding | 3.87 | 2.88 | 2.98 | 1.85 | 0.0158 | 0.0594 | 3.54 | 3.47 | 3.24 | 1.23 | 0.0387 | 0.1792 |
| TC0300007617.hg.1 | KCTD6 | potassium channel tetramerization domain containing 6 | Multiple_C | 8.59 | 7.79 | 7.7 | 1.85 | 0.0126 | 0.0502 | 6.95 | 6.84 | 6.96 | 0.99 | 0.82 | 0.9205 |
| TC0300012970.hg.1 | KPNA4 | karyopherin alpha 4 (importin alpha 3) | Multiple_C | 12.63 | 12.08 | 11.74 | 1.85 | 0.0004 | 0.003 | 11.69 | 10.84 | 11.76 | 0.95 | 0.6846 | 0.8488 |
| TC0400011518.hg.1 | INTS12 | integrator complex subunit 12 | Multiple_C | 12.98 | 12.22 | 12.09 | 1.85 | 0.0012 | 0.0079 | 9.74 | 9.44 | 10.26 | 0.70 | 0.1741 | 0.4217 |
| TC0500008691.hg.1 | UBE2B | ubiquitin conjugating enzyme E2B | Multiple_C | 14.05 | 14.57 | 13.16 | 1.85 | 0.0047 | 0.0231 | 11.47 | 11.24 | 11.17 | 1.23 | 0.0883 | 0.2871 |
| TC0500009837.hg.1 | SLC12A7 | solute carrier family 12 (potassium/chloride transporter), meml | Multiple_C | 8.51 | 7.65 | 7.62 | 1.85 | 0.0208 | 0.0738 | 12.42 | 12.43 | 12.52 | 0.93 | 0.8923 | 0.9539 |
| TC0600012747.hg.1 | CD24 | CD24 molecule | Multiple_C | 15.92 | 14.1 | 15.03 | 1.85 | 0.0743 | 0.1934 | 10.81 | 13.01 | 12.28 | 0.36 | 2.82E-06 | 0.0003 |
| TC0700013419.hg.1 | ARPC1A | actin related protein 2/3 complex subunit 1A | Multiple_C | 14.27 | 13.92 | 13.38 | 1.85 | 0.0094 | 0.0397 | 9.7 | 9.67 | 9.55 | 1.11 | 0.3546 | 0.618 |
| TC0800012474.hg.1 | AGO2 | argonaute RISC catalytic component 2 | Multiple_C | 12.2 | 11.06 | 11.31 | 1.85 | 0.0006 | 0.0044 | 11.13 | 10.72 | 11.12 | 1.01 | 0.9318 | 0.9711 |
| TC0X00006681.hg.1 | ZRSR2 | zinc finger (CCCH type), RNA binding motif and serine/arginine r | Multiple_C | 7.04 | 6.51 | 6.15 | 1.85 | 0.0832 | 0.2104 | 6.35 | 6.32 | 7.1 | 0.59 | 0.0852 | 0.2811 |
| TC0X00008790.hg.1 | DUSP9 | dual specificity phosphatase 9 | Multiple_C | 5.83 | 4.87 | 4.94 | 1.85 | 0.0877 | 0.2188 | 5.19 | 5.27 | 5.22 | 0.98 | 0.7587 | 0.8905 |
| TC1000007678.hg.1 | MTRNR2L5 | MT-RNR2-like 5 | Coding | 6.98 | 5.92 | 6.09 | 1.85 | 0.022 | 0.0771 | 5.93 | 6.36 | 6.25 | 0.80 | 0.5005 | 0.7332 |
| TC1000009090.hg.1 | BAG3 | BCL2-associated athanogene 3 | Coding | 12.03 | 10.03 | 11.14 | 1.85 | 0.0703 | 0.186 | 11.93 | 9.99 | 12.48 | 0.68 | 0.0163 | 0.1061 |
| TC1100012812.hg.1 | PRDM10 | PR domain containing 10 | Multiple_C | 7.24 | 6.23 | 6.35 | 1.85 | 0.0138 | 0.0535 | 6.82 | 7.04 | 7.39 | 0.67 | 0.0425 | 0.1903 |
| TC1100013205.hg.1 | ARAP1 | ArfGAP with RhoGAP domain, ankyrin repeat and PH domain 1 | Multiple_C | 7.41 | 6.75 | 6.52 | 1.85 | 0.0345 | 0.1098 | 7.74 | 7.87 | 7.35 | 1.31 | 0.9466 | 0.9782 |
| TC1200009980.hg.1 | GPRC5D | G protein-coupled receptor, class C, group 5, member D | Coding | 4.39 | 3.84 | 3.5 | 1.85 | 0.0022 | 0.0126 | 3.53 | 3.06 | 3.02 | 1.42 | 0.068 | 0.2485 |
| TC1200010130.hg.1 | SOX5 | SRY box 5 | Multiple_C | 4.6 | 3.56 | 3.71 | 1.85 | 0.0829 | 0.2099 | 8.85 | 8.93 | 8.3 | 1.46 | 0.0456 | 0.1977 |
| TC1200010901.hg.1 | RNF41 | ring finger protein 41, E3 ubiquitin protein ligase | Multiple_C | 10.71 | 9.65 | 9.82 | 1.85 | 0.0046 | 0.0224 | 10.62 | 10.70 | 10.66 | 0.97 | 0.9728 | 0.989 |
| TC1200011330.hg.1 | E2F7 | E2F transcription factor 7 | Multiple_C | 9.44 | 6.78 | 8.55 | 1.85 | 0.0084 | 0.0364 | 8.65 | 8.01 | 9.04 | 0.76 | 0.0996 | 0.3085 |
| TC1300009348.hg.1 | SPRY2 | sprouty RTK signaling antagonist 2 | Coding | 10.24 | 10.18 | 9.35 | 1.85 | 0.0073 | 0.0325 | 10.08 | 9.49 | 8.87 | 2.31 | 7.78E-05 | 0.003 |
| TC1500010729.hg.1 | SERF2 | small EDRK-rich factor 2 | Multiple_C | 13.83 | 13.32 | 12.94 | 1.85 | 0.0238 | 0.082 | 10.71 | 10.95 | 10.81 | 0.93 | 0.9738 | 0.9896 |
| TC1500010881.hg.1 | TIPIN; RPL9P2 | TIMELESS interacting protein; ribosomal protein L9 pseudogene | Multiple_C | 8.79 | 8.65 | 7.9 | 1.85 | 0.0066 | 0.0301 | 5.75 | 5.69 | 5.68 | 1.05 | 0.1507 | 0.3887 |
| TC1700008095.hg.1 | LRRC37A | leucine rich repeat containing 37A | Multiple_C | 10.03 | 8.44 | 9.14 | 1.85 | 0.0027 | 0.0149 | 10.2 | 10.01 | 10.53 | 0.80 | 0.1046 | 0.3171 |
| TC1700009398.hg.1 | MIR22HG | MIR22 host gene | Multiple_C | 5.15 | 3.79 | 4.26 | 1.85 | 0.0589 | 0.1634 | 4.36 | 4.11 | 4.06 | 1.23 | 0.1685 | 0.4142 |
| TC1800009086.hg.1 | ZNF516 | zinc finger protein 516 | Multiple_C | 6.58 | 5.57 | 5.69 | 1.85 | 0.0006 | 0.0047 | 5.54 | 5.34 | 4.9 | 1.56 | 0.0138 | 0.0952 |
| TC1900011673.hg.1 | CCDC130 | coiled-coil domain containing 130 | Multiple_C | 10.16 | 8.99 | 9.27 | 1.85 | 0.0245 | 0.0839 | 7.51 | 7.45 | 7.53 | 0.99 | 0.1672 | 0.4126 |
| TC1900011885.hg.1 | ZNF709 | zinc finger protein 709 | Multiple_C | 6.59 | 5.76 | 5.7 | 1.85 | 0.0019 | 0.0111 | 5.02 | 4.52 | 4.69 | 1.26 | 0.1528 | 0.3919 |

| | | | | | | | | | | | | | | | |
|---|---|---|---|---|---|---|---|---|---|---|---|---|---|---|---|
| TC2000007658.hg.1 | SLC9A8 | solute carrier family 9, subfamily A (NHE8, cation proton antipo | Multiple_C | 8.39 | 7.02 | 7.5 | 1.85 | 0.0097 | 0.0407 | 8.63 | 9.24 | 8.93 | 0.81 | 0.3467 | 0.6103 |
| TC2000007945.hg.1 | C20orf197 | chromosome 20 open reading frame 197 | Multiple_C | 3.81 | 2.93 | 2.92 | 1.85 | 0.0245 | 0.0841 | 3.94 | 3.92 | 3.77 | 1.13 | 0.9035 | 0.9602 |
| TC2000009885.hg.1 | PANK2 | pantothenate kinase 2 | Multiple_C | 10.9 | 10.37 | 10.01 | 1.85 | 0.0094 | 0.0396 | 10.67 | 10.89 | 10.81 | 0.91 | 0.6907 | 0.8526 |
| TC2200006833.hg.1 | BCR | breakpoint cluster region | Multiple_C | 8.52 | 6.98 | 7.63 | 1.85 | 0.0115 | 0.0465 | 8.27 | 8.47 | 7.97 | 1.23 | 0.3739 | 0.6345 |
| TC2200007001.hg.1 | HSCB | HscB mitochondrial iron-sulfur cluster co-chaperone | Multiple_C | 9.04 | 9.54 | 8.15 | 1.85 | 0.158 | 0.3339 | 7.01 | 7.11 | 6.73 | 1.21 | 0.4935 | 0.728 |
| TC0100006550.hg.1 | PRKCZ | protein kinase C, zeta | Multiple_C | 11.21 | 10.21 | 10.33 | 1.84 | 7.09E-05 | 0.0008 | 10.34 | 10.27 | 10.27 | 1.05 | 0.3717 | 0.6332 |
| TC0100008536.hg.1 | INADL | InaD-like (Drosophila) | Multiple_C | 10.36 | 9.72 | 9.48 | 1.84 | 0.0139 | 0.0539 | 8.92 | 7.61 | 8.11 | 1.75 | 0.024 | 0.1338 |
| TC0100009760.hg.1 | NUDT4P1; NU | nudix hydrolase 4 pseudogene 1; nudix hydrolase 4 pseudogene | Multiple_C | 15.33 | 14.37 | 14.45 | 1.84 | 0.0017 | 0.0103 | 16.84 | 16.17 | 16.23 | 1.53 | 0.0049 | 0.0487 |
| TC0100009833.hg.1 | NUDT4; NUDT | nudix hydrolase 4; nudix hydrolase 4 pseudogene 1 | Multiple_C | 15.33 | 14.37 | 14.45 | 1.84 | 0.0017 | 0.0103 | 16.84 | 16.17 | 16.23 | 1.53 | 0.0049 | 0.0487 |
| TC0100011566.hg.1 | PROX1 | prospero homeobox 1 | Multiple_C | 5.67 | 4.84 | 4.79 | 1.84 | 0.0798 | 0.2047 | 14.27 | 14.65 | 14.47 | 0.87 | 0.1683 | 0.4141 |
| TC0100016252.hg.1 | LMX1A | LIM homeobox transcription factor 1, alpha | Multiple_C | 6.05 | 5.1 | 5.17 | 1.84 | 0.1107 | 0.2595 | 5.87 | 5.74 | 5.47 | 1.32 | 0.0496 | 0.2074 |
| TC0100017058.hg.1 | TMEM81 | transmembrane protein 81 | Coding | 6.64 | 5.96 | 5.76 | 1.84 | 0.0233 | 0.0807 | 5.36 | 5.35 | 5.56 | 0.87 | 0.4312 | 0.6826 |
| TC0100018526.hg.1 | ILDR2 | immunoglobulin-like domain containing receptor 2 | Multiple_C | 4.3 | 3.12 | 3.42 | 1.84 | 0.0179 | 0.0657 | 4.72 | 5.62 | 5.68 | 0.51 | 0.0002 | 0.0065 |
| TC0200006687.hg.1 | HPCAL1 | hippocalcin-like 1 | Multiple_C | 7.65 | 6.58 | 6.77 | 1.84 | 0.0211 | 0.0748 | 8.75 | 8.62 | 9.46 | 0.61 | 0.0126 | 0.0901 |
| TC0200016719.hg.1 | SNORD89; RN | small nucleolar RNA, C/D box 89; ring finger protein 149 | Multiple_C | 7.39 | 6.99 | 6.51 | 1.84 | 0.0213 | 0.0752 | 7.41 | 6.74 | 7.49 | 0.95 | 0.683 | 0.8479 |
| TC0300010076.hg.1 | RPL35A | ribosomal protein L35a | Multiple_C | 16.74 | 16.7 | 15.86 | 1.84 | 0.0033 | 0.0175 | 14.37 | 14.20 | 13.43 | 1.92 | 9.61E-05 | 0.0035 |
| TC0500011208.hg.1 | WDR41 | WD repeat domain 41 | Multiple_C | 11.73 | 11.35 | 10.85 | 1.84 | 0.0053 | 0.0252 | 11.66 | 10.44 | 10.49 | 2.25 | 1.14E-05 | 0.0008 |
| TC0500012491.hg.1 | ZNF300 | zinc finger protein 300 | Coding | 4.79 | 4.15 | 3.91 | 1.84 | 0.009 | 0.0384 | 4.02 | 4.15 | 4.2 | 0.88 | 0.4417 | 0.6907 |
| TC0500013248.hg.1 | PCDHB15 | protocadherin beta 15 | Coding | 6.87 | 6.38 | 5.99 | 1.84 | 0.0158 | 0.0596 | 3.58 | 3.81 | 3.51 | 1.05 | 0.4926 | 0.7272 |
| TC0600010066.hg.1 | IGF2R | insulin-like growth factor 2 receptor | Multiple_C | 6.86 | 5.97 | 5.98 | 1.84 | 0.0004 | 0.003 | 10.86 | 10.95 | 9.86 | 2.00 | 1.91E-05 | 0.0011 |
| TC0700007198.hg.1 | ANLN | anillin actin binding protein | Multiple_C | 14.83 | 12.85 | 13.95 | 1.84 | 0.0372 | 0.1162 | 15.32 | 14.60 | 15.24 | 1.06 | 0.2609 | 0.525 |
| TC0700010182.hg.1 | PMS2 | PMS1 homolog 2, mismatch repair system component | Multiple_C | 10 | 9.15 | 9.12 | 1.84 | 0.0034 | 0.0179 | 9.8 | 9.89 | 10.46 | 0.63 | 0.0006 | 0.0115 |
| TC0700011054.hg.1 | GRB10 | growth factor receptor bound protein 10 | Multiple_C | 9.93 | 10.09 | 9.05 | 1.84 | 0.0993 | 0.2397 | 4.28 | 4.86 | 5.46 | 0.44 | 0.0515 | 0.2119 |
| TC0900006969.hg.1 | CHMP5 | charged multivesicular body protein 5 | Multiple_C | 13.24 | 14.75 | 12.36 | 1.84 | 0.006 | 0.0279 | 10.66 | 10.38 | 10.15 | 1.42 | 0.002 | 0.0267 |
| TC0900007520.hg.1 | SMC5 | structural maintenance of chromosomes 5 | Multiple_C | 9.74 | 8.76 | 8.86 | 1.84 | 0.0033 | 0.0175 | 9.45 | 8.61 | 9.39 | 1.04 | 0.8156 | 0.9182 |
| TC0900009225.hg.1 | EGFL7 | EGF-like-domain, multiple 7 | Multiple_C | 9.81 | 8.79 | 8.93 | 1.84 | 0.0014 | 0.0088 | 8.76 | 8.70 | 8.54 | 1.16 | 0.2497 | 0.5142 |
| TC0M00006434.hg.1 | ND2 | MTND2 | Multiple_C | 12.84 | 11.67 | 11.96 | 1.84 | 0.0134 | 0.0523 | 11.56 | 11.40 | 12.11 | 0.68 | 0.0147 | 0.099 |
| TC1000007564.hg.1 | MAPK8 | mitogen-activated protein kinase 8 | Multiple_C | 11.14 | 10.98 | 10.26 | 1.84 | 0.0126 | 0.0502 | 10.81 | 9.76 | 10.65 | 1.12 | 0.5984 | 0.798 |
| TC1000010961.hg.1 | PSAP | prosaposin | Multiple_C | 14.42 | 14.13 | 13.54 | 1.84 | 0.0204 | 0.0726 | 10.74 | 10.50 | 10.84 | 0.93 | 0.645 | 0.8258 |
| TC1000011585.hg.1 | GOT1 | glutamic-oxaloacetic transaminase 1, soluble | Multiple_C | 14.2 | 13.54 | 13.32 | 1.84 | 0.0129 | 0.0511 | 12.14 | 11.14 | 12.08 | 1.04 | 0.9028 | 0.9597 |
| TC1000012011.hg.1 | RGS10 | regulator of G-protein signaling 10 | Multiple_C | 11.69 | 12.63 | 10.81 | 1.84 | 0.0085 | 0.0366 | 5.45 | 4.66 | 5.02 | 1.35 | 0.1006 | 0.3101 |
| TC1100007840.hg.1 | SCGB1D2 | secretoglobin, family 1D, member 2 | Coding | 5.96 | 4.95 | 5.08 | 1.84 | 0.0921 | 0.2268 | 6.06 | 6.07 | 5.83 | 1.17 | 0.4195 | 0.673 |
| TC1100011838.hg.1 | PICALM | phosphatidylinositol binding clathrin assembly protein | Multiple_C | 13.88 | 13.2 | 13 | 1.84 | 0.0021 | 0.0121 | 13.15 | 12.57 | 12.86 | 1.22 | 0.1108 | 0.3281 |

| | | | | | | | | | | | | | | | |
|---|---|---|---|---|---|---|---|---|---|---|---|---|---|---|---|
| TC1200006472.hg.1 | ADIPOR2 | adiponectin receptor 2 | Multiple_C | 11.61 | 9.78 | 10.73 | 1.84 | 0.0117 | 0.0472 | 12.57 | 12.52 | 12.35 | 1.16 | 0.7409 | 0.8808 |
| TC1200009550.hg.1 | KDM5A | lysine (K)-specific demethylase 5A | Multiple_C | 8.71 | 7.76 | 7.83 | 1.84 | 0.0173 | 0.0637 | 8.28 | 8.60 | 8.77 | 0.71 | 0.0889 | 0.2885 |
| TC1200011767.hg.1 | SLC41A2 | solute carrier family 41 (magnesium transporter), member 2 | Multiple_C | 10.03 | 9.49 | 9.15 | 1.84 | 0.0064 | 0.0294 | 7.49 | 7.28 | 8.01 | 0.70 | 0.0028 | 0.0335 |
| TC1400008631.hg.1 | CHD8 | chromodomain helicase DNA binding protein 8 | Multiple_C | 10.48 | 9.37 | 9.6 | 1.84 | 0.0187 | 0.0679 | 11.15 | 10.90 | 10.79 | 1.28 | 0.9259 | 0.9694 |
| TC1500008202.hg.1 | AGBL1 | ATP/GTP binding protein-like 1 | Multiple_C | 3.94 | 3.01 | 3.06 | 1.84 | 0.1959 | 0.386 | 3.53 | 3.29 | 3.18 | 1.27 | 0.3729 | 0.6339 |
| TC1600007030.hg.1 | NPIPA7; NPIP/ | nuclear pore complex interacting protein family, member A7; nu | Multiple_C | 10.7 | 10.19 | 9.82 | 1.84 | 0.0157 | 0.0591 | 11.09 | 10.94 | 11.01 | 1.06 | 0.8212 | 0.921 |
| TC1600009196.hg.1 | C16orf90 | chromosome 16 open reading frame 90 | Coding | 4.69 | 3.69 | 3.81 | 1.84 | 0.0011 | 0.0073 | 4.39 | 4.41 | 4.31 | 1.06 | 0.3615 | 0.6242 |
| TC1600009199.hg.1 | SLX4 | SLX4 structure-specific endonuclease subunit | Multiple_C | 8.36 | 7.16 | 7.48 | 1.84 | 0.0053 | 0.0254 | 8.54 | 8.76 | 8.22 | 1.25 | 0.5547 | 0.7692 |
| TC1700007296.hg.1 | MTRNR2L1 | MT-RNR2-like 1 | Coding | 13.47 | 12.19 | 12.59 | 1.84 | 0.0081 | 0.0353 | 12.89 | 13.14 | 13.32 | 0.74 | 0.061 | 0.2345 |
| TC1900008303.hg.1 | CLASRP | CLK4-associating serine/arginine rich protein | Multiple_C | 6.78 | 6.14 | 5.9 | 1.84 | 0.0147 | 0.0564 | 6.61 | 6.76 | 6.46 | 1.11 | 0.3702 | 0.6325 |
| TC2000007486.hg.1 | RBPJL | recombination signal binding protein for immunoglobulin kappa | Multiple_C | 7.28 | 6.35 | 6.4 | 1.84 | 0.0086 | 0.0369 | 6.46 | 6.77 | 6.62 | 0.90 | 0.3938 | 0.652 |
| TC2200008886.hg.1 | CYB5R3 | cytochrome b5 reductase 3 | Multiple_C | 12.17 | 9.96 | 11.29 | 1.84 | 0.0009 | 0.0061 | 10.66 | 11.31 | 11.16 | 0.71 | 0.0511 | 0.211 |
| TC2200009252.hg.1 | SEC14L2 | SEC14-like lipid binding 2 | Multiple_C | 7.64 | 6.47 | 6.76 | 1.84 | 0.0033 | 0.0173 | 6.76 | 6.76 | 6.49 | 1.21 | 0.1177 | 0.3391 |
| TSUnmapped00000147.h | ATG16L1 | autophagy related 16-like 1 | Coding | 7.13 | 6.3 | 6.25 | 1.84 | 0.0564 | 0.1581 | 5.75 | 5.21 | 5.63 | 1.09 | 0.44 | 0.6898 |
| TSUnmapped00000262.h | MLXIP | MLX interacting protein | Coding | 6.26 | 5.31 | 5.38 | 1.84 | 0.0545 | 0.1539 | 5.21 | 4.72 | 4.97 | 1.18 | 0.5591 | 0.7714 |
| TSUnmapped00000449.h | DGKD | diacylglycerol kinase, delta 130kDa | Coding | 7.03 | 6.09 | 6.15 | 1.84 | 0.0264 | 0.089 | 6.8 | 6.81 | 7.01 | 0.86 | 0.5176 | 0.7459 |
| TSUnmapped00000638.h | DYRK1B | dual specificity tyrosine-(Y)-phosphorylation regulated kinase 1I | Coding | 5.06 | 4.14 | 4.18 | 1.84 | 0.032 | 0.1035 | 4.31 | 4.37 | 4.23 | 1.06 | 0.2229 | 0.4834 |
| TC0100010518.hg.1 | DCAF6 | DDB1 and CUL4 associated factor 6 | Multiple_C | 11.2 | 10.45 | 10.33 | 1.83 | 0.0012 | 0.0077 | 11.1 | 10.41 | 10.3 | 1.74 | 0.0015 | 0.022 |
| TC0100012898.hg.1 | NPPB | natriuretic peptide B | Coding | 5.6 | 4.6 | 4.73 | 1.83 | 0.0178 | 0.0653 | 5.85 | 5.60 | 5.2 | 1.57 | 0.0251 | 0.1377 |
| TC0100015060.hg.1 | LRRC39 | leucine rich repeat containing 39 | Coding | 5.91 | 5.38 | 5.04 | 1.83 | 0.0669 | 0.1795 | 5.77 | 5.84 | 5.52 | 1.19 | 0.425 | 0.6777 |
| TC0100015346.hg.1 | TRIM33 | tripartite motif containing 33 | Multiple_C | 12.84 | 12.2 | 11.97 | 1.83 | 0.0029 | 0.0157 | 11.09 | 11.31 | 11.29 | 0.87 | 0.2918 | 0.5564 |
| TC0100018249.hg.1 | AMY2B; ACTG | amylase, alpha 2B (pancreatic); actin gamma 1 pseudogene 4 | Multiple_C | 6.31 | 6.28 | 5.44 | 1.83 | 0.0134 | 0.0524 | 3.93 | 4.02 | 3.8 | 1.09 | 0.9459 | 0.9781 |
| TC0200008323.hg.1 | PLGLB2 | plasminogen-like B2 | Multiple_C | 4.35 | 3.51 | 3.48 | 1.83 | 0.004 | 0.02 | 4.65 | 4.39 | 4.09 | 1.47 | 0.0105 | 0.0807 |
| TC0200008734.hg.1 | C2orf49 | chromosome 2 open reading frame 49 | Coding | 9.56 | 9.18 | 8.69 | 1.83 | 0.0002 | 0.0018 | 9.54 | 9.28 | 9.67 | 0.91 | 0.5375 | 0.7589 |
| TC0200011803.hg.1 | NBAS | neuroblastoma amplified sequence | Multiple_C | 7.3 | 7.61 | 6.43 | 1.83 | 0.0102 | 0.0424 | 6.67 | 6.38 | 6.2 | 1.39 | 0.0764 | 0.2659 |
| TC0200016707.hg.1 | ZNF514 | zinc finger protein 514 | Multiple_C | 6.17 | 5.64 | 5.3 | 1.83 | 0.0174 | 0.0642 | 5.35 | 5.22 | 4.75 | 1.52 | 0.0397 | 0.1822 |
| TC0300013639.hg.1 | PPP1R2 | protein phosphatase 1, regulatory (inhibitor) subunit 2 | Multiple_C | 11.36 | 10.68 | 10.49 | 1.83 | 0.0146 | 0.056 | 11.66 | 11.87 | 11.5 | 1.12 | 0.1075 | 0.3224 |
| TC0400007880.hg.1 | USO1 | Transcript Identified by AceView, Entrez Gene ID(s) 8615 | Coding | 8.03 | 6.74 | 7.16 | 1.83 | 0.0076 | 0.0335 | 7.76 | 7.85 | 7.87 | 0.93 | 0.9277 | 0.9698 |
| TC0400012881.hg.1 | DUX4 | double homeobox 4 | Multiple_C | 4.44 | 3.49 | 3.57 | 1.83 | 0.6483 | 0.805 | 3.72 | 3.66 | 3.44 | 1.21 | 0.2511 | 0.5151 |
| TC0500009566.hg.1 | CPLX2 | complexin 2 | Multiple_C | 6.62 | 5.47 | 5.75 | 1.83 | 0.0167 | 0.0622 | 6.38 | 7.11 | 7.44 | 0.48 | 0.0024 | 0.0303 |
| TC0500012317.hg.1 | FGF1 | fibroblast growth factor 1 (acidic) | Multiple_C | 4.48 | 3.37 | 3.61 | 1.83 | 0.0076 | 0.0338 | 4.53 | 4.70 | 4.52 | 1.01 | 0.5189 | 0.7466 |
| TC0600013936.hg.1 | LOC441178; R | uncharacterized LOC441178; novel tanscript | Multiple_C | 5.41 | 4.18 | 4.54 | 1.83 | 0.0122 | 0.049 | 4.2 | 4.13 | 4.06 | 1.10 | 0.4516 | 0.6969 |
| TC0700008318.hg.1 | AKAP9 | A kinase (PRKA) anchor protein 9 | Multiple_C | 9.2 | 8.49 | 8.33 | 1.83 | 0.0241 | 0.0828 | 7.48 | 7.09 | 7.7 | 0.86 | 0.8444 | 0.9318 |

| | | | | | | | | | | | | | | | |
|---|---|---|---|---|---|---|---|---|---|---|---|---|---|---|---|
| TC0800007688.hg.1 | LYN | LYN proto-oncogene, Src family tyrosine kinase | Multiple_C | 12.19 | 11.23 | 11.32 | 1.83 | 0.034 | 0.1085 | 9.09 | 8.98 | 9.81 | 0.61 | 0.0142 | 0.0967 |
| TC0800011139.hg.1 | KIAA1429 | KIAA1429 | Multiple_C | 10.71 | 10.73 | 9.84 | 1.83 | 0.034 | 0.1085 | 9.87 | 9.87 | 9.78 | 1.06 | 0.4104 | 0.6652 |
| TC0800012299.hg.1 | HOOK3 | hook microtubule-tethering protein 3 | Multiple_C | 8.26 | 7.85 | 7.39 | 1.83 | 0.0315 | 0.1022 | 9.34 | 7.92 | 8.85 | 1.40 | 0.6113 | 0.8057 |
| TC0900010056.hg.1 | ANKRD18A; F/ | ankyrin repeat domain 18A; family with sequence similarity 95, | Multiple_C | 8.21 | 7.41 | 7.34 | 1.83 | 0.0014 | 0.0088 | 7.24 | 7.32 | 7.28 | 0.97 | 0.7933 | 0.9078 |
| TC0900012025.hg.1 | ABCA2 | ATP binding cassette subfamily A member 2 | Multiple_C | 7.86 | 6.92 | 6.99 | 1.83 | 0.0112 | 0.0457 | 6.35 | 5.80 | 5.51 | 1.79 | 0.0011 | 0.0181 |
| TC0X00011082.hg.1 | HSFX2; HSFX1 | heat shock transcription factor family, X-linked 2; heat shock tra | Coding | 5.79 | 4.76 | 4.92 | 1.83 | 0.0011 | 0.0072 | 5.06 | 5.17 | 5.09 | 0.98 | 0.9534 | 0.981 |
| TC0X00011389.hg.1 | NKAP | NFKB activating protein | Multiple_C | 8.23 | 9.83 | 7.36 | 1.83 | 0.0471 | 0.1386 | 7.13 | 7.36 | 7.64 | 0.70 | 0.0631 | 0.239 |
| TC1000011892.hg.1 | CCDC186; MIF | coiled-coil domain containing 186; microRNA 2110 | Multiple_C | 11.67 | 11.47 | 10.8 | 1.83 | 0.0742 | 0.1933 | 11.21 | 9.51 | 9.55 | 3.16 | 0.001 | 0.0167 |
| TC1500007977.hg.1 | HYKK | hydroxylysine kinase | Multiple_C | 9.98 | 9.35 | 9.11 | 1.83 | 0.1527 | 0.3261 | 6.24 | 5.50 | 5.74 | 1.41 | 0.0191 | 0.117 |
| TC1600011493.hg.1 | RPS15A | ribosomal protein S15a | Multiple_C | 18.22 | 17.97 | 17.35 | 1.83 | 0.0004 | 0.0031 | 14.75 | 14.28 | 14.02 | 1.66 | 0.0049 | 0.0486 |
| TC1700012247.hg.1 | TCAP | titin-cap | Coding | 8.24 | 7.14 | 7.37 | 1.83 | 0.1216 | 0.2773 | 8.6 | 8.50 | 8.14 | 1.38 | 0.0755 | 0.2642 |
| TC1800006852.hg.1 | GATA6 | GATA binding protein 6 | Coding | 12.47 | 11.53 | 11.6 | 1.83 | 0.007 | 0.0315 | 5 | 5.75 | 5.49 | 0.71 | 0.4167 | 0.6705 |
| TC1900008546.hg.1 | PRRG2 | proline rich Gla (G-carboxyglutamic acid) 2 | Multiple_C | 6.2 | 5.19 | 5.33 | 1.83 | 0.003 | 0.016 | 5.6 | 5.41 | 5.27 | 1.26 | 0.057 | 0.2248 |
| TC1900011071.hg.1 | KDELR1 | KDEL (Lys-Asp-Glu-Leu) endoplasmic reticulum protein retentio | Multiple_C | 13.69 | 12.97 | 12.82 | 1.83 | 0.0033 | 0.0173 | 12.34 | 12.70 | 12.86 | 0.70 | 0.0048 | 0.0482 |
| TC1900011752.hg.1 | ZNF223 | zinc finger protein 223 | Multiple_C | 8.39 | 7.85 | 7.52 | 1.83 | 0.0835 | 0.2109 | 6.21 | 6.62 | 6.16 | 1.04 | 0.7787 | 0.901 |
| TC2000007476.hg.1 | STK4 | serine/threonine kinase 4 | Multiple_C | 11.12 | 10.73 | 10.25 | 1.83 | 0.0861 | 0.2158 | 11.74 | 10.50 | 10.93 | 1.75 | 0.0223 | 0.1284 |
| TC2200008513.hg.1 | RFPL3S | RFPL3 antisense | Multiple_C | 6.38 | 6.25 | 5.51 | 1.83 | 0.0018 | 0.0108 | 4.97 | 4.18 | 4.51 | 1.38 | 0.4215 | 0.6745 |
| TC2200009157.hg.1 | SBF1 | SET binding factor 1 | Multiple_C | 7.58 | 6.61 | 6.71 | 1.83 | 0.0089 | 0.0382 | 6.9 | 7.25 | 7.45 | 0.68 | 0.0168 | 0.1082 |
| TSUnmapped00000504.h | ZNF197 | zinc finger protein 197 | Coding | 5.13 | 4.32 | 4.26 | 1.83 | 0.003 | 0.016 | 4.24 | 4.46 | 4.64 | 0.76 | 0.7893 | 0.9059 |
| TSUnmapped00000639.h | SERTAD4 | SERTA domain containing 4 | Coding | 6.76 | 6.14 | 5.89 | 1.83 | 0.0013 | 0.0084 | 3.25 | 3.50 | 3.24 | 1.01 | 0.5933 | 0.7945 |
| TC0100008927.hg.1 | SH3GLB1 | SH3-domain GRB2-like endophilin B1 | Multiple_C | 11.26 | 11.35 | 10.4 | 1.82 | 0.0218 | 0.0767 | 9.29 | 9.39 | 9.88 | 0.66 | 0.1608 | 0.4034 |
| TC0100008955.hg.1 | PKN2 | protein kinase N2 | Multiple_C | 12.93 | 12.11 | 12.07 | 1.82 | 0.0159 | 0.0597 | 11.95 | 10.76 | 11.43 | 1.43 | 0.0486 | 0.2049 |
| TC0100010387.hg.1 | DDR2 | discoidin domain receptor tyrosine kinase 2 | Multiple_C | 4.75 | 4.63 | 3.89 | 1.82 | 0.0044 | 0.0217 | 4.69 | 4.19 | 4.57 | 1.09 | 0.5086 | 0.7389 |
| TC0200010165.hg.1 | PPP1R1C | protein phosphatase 1, regulatory (inhibitor) subunit 1C | Multiple_C | 6.82 | 6.11 | 5.96 | 1.82 | 0.0014 | 0.0086 | 6.98 | 6.79 | 7.06 | 0.95 | 0.9284 | 0.97 |
| TC0200015571.hg.1 | CRYGC | crystallin gamma C | Coding | 6.04 | 5.05 | 5.18 | 1.82 | 0.0058 | 0.027 | 4.76 | 4.86 | 4.84 | 0.95 | 0.8252 | 0.9229 |
| TC0300012718.hg.1 | PLOD2 | procollagen-lysine, 2-oxoglutarate 5-dioxygenase 2 | Multiple_C | 4.7 | 3.76 | 3.84 | 1.82 | 0.0551 | 0.1554 | 12.79 | 10.02 | 9.72 | 8.40 | 1.31E-09 | 1.22E-06 |
| TC0300012770.hg.1 | TM4SF18 | transmembrane 4 L six family member 18 | Multiple_C | 4.26 | 3.58 | 3.4 | 1.82 | 0.0351 | 0.1112 | 3.89 | 4.07 | 3.74 | 1.11 | 0.5034 | 0.7353 |
| TC0400006773.hg.1 | LOC650293 | seven transmembrane helix receptor; Transcript Identified by A | Multiple_C | 4.88 | 4.02 | 4.02 | 1.82 | 0.0447 | 0.1334 | 3.9 | 3.86 | 3.72 | 1.13 | 0.5026 | 0.7346 |
| TC0500009987.hg.1 | MED10 | mediator complex subunit 10 | Multiple_C | 12.08 | 11.95 | 11.22 | 1.82 | 0.0053 | 0.0254 | 12.92 | 12.76 | 13.03 | 0.93 | 0.9186 | 0.9666 |
| TC0500011282.hg.1 | MTRNR2L2 | MT-RNR2-like 2 | Multiple_C | 13.69 | 12.49 | 12.83 | 1.82 | 0.002 | 0.0117 | 12.59 | 12.73 | 13.26 | 0.63 | 0.0023 | 0.0297 |
| TC0600006976.hg.1 | PHACTR1 | phosphatase and actin regulator 1 | Multiple_C | 4.35 | 3.45 | 3.49 | 1.82 | 0.0664 | 0.1786 | 4.38 | 4.10 | 3.93 | 1.37 | 0.0427 | 0.1905 |
| TC0600009808.hg.1 | PCMT1 | protein-L-isoaspartate (D-aspartate) O-methyltransferase | Multiple_C | 13.25 | 13.02 | 12.39 | 1.82 | 0.055 | 0.1552 | 11.16 | 11.58 | 11.2 | 0.97 | 0.9041 | 0.9605 |
| TC0600011197.hg.1 | ZNF184 | zinc finger protein 184 | Multiple_C | 9.48 | 9.58 | 8.62 | 1.82 | 0.0108 | 0.0445 | 8.85 | 7.59 | 8.43 | 1.34 | 0.0412 | 0.1865 |

| | | | | | | | | | | | | | | | |
|---|---|---|---|---|---|---|---|---|---|---|---|---|---|---|---|
| TC0600013523.hg.1 | PPIL4 | peptidylprolyl isomerase (cyclophilin)-like 4 | Multiple_C | 10.02 | 9.94 | 9.16 | 1.82 | 0.2419 | 0.4442 | 9.92 | 9.44 | 9.85 | 1.05 | 0.5736 | 0.7812 |
| TC0700009398.hg.1 | TAS2R3 | taste receptor, type 2, member 3 | Coding | 5.2 | 4.63 | 4.34 | 1.82 | 0.0247 | 0.0844 | 4.24 | 3.90 | 4.17 | 1.05 | 0.0888 | 0.2882 |
| TC0800011206.hg.1 | RPL30 | ribosomal protein L30 | Multiple_C | 17.71 | 17.81 | 16.85 | 1.82 | 0.0004 | 0.003 | 14.01 | 13.46 | 13.19 | 1.77 | 0.0004 | 0.0085 |
| TC0900007064.hg.1 | DNAJB5 | DnaJ (Hsp40) homolog, subfamily B, member 5 | Coding | 6.13 | 5.12 | 5.27 | 1.82 | 0.0048 | 0.0233 | 5.9 | 5.11 | 5.67 | 1.17 | 0.1437 | 0.3774 |
| TC0900007105.hg.1 | HRCT1 | histidine rich carboxyl terminus 1 | Coding | 6.6 | 5.43 | 5.74 | 1.82 | 0.0067 | 0.0303 | 5.16 | 5.14 | 5.05 | 1.08 | 0.9235 | 0.9685 |
| TC0900007863.hg.1 | CKS2 | CDC28 protein kinase regulatory subunit 2 | Multiple_C | 13.49 | 13.31 | 12.63 | 1.82 | 0.098 | 0.237 | 11.15 | 10.95 | 11.59 | 0.74 | 0.4086 | 0.664 |
| TC0900011038.hg.1 | GRIN3A | glutamate receptor, ionotropic, N-methyl-D-aspartate 3A | Multiple_C | 3.66 | 2.53 | 2.8 | 1.82 | 0.0186 | 0.0676 | 3.04 | 2.78 | 2.84 | 1.15 | 0.1789 | 0.4278 |
| TC1000007885.hg.1 | SRGN | Transcript Identified by AceView, Entrez Gene ID(s) 5552 | Coding | 6.26 | 5.14 | 5.4 | 1.82 | 0.0091 | 0.0387 | 5.18 | 5.08 | 5.55 | 0.77 | 0.1637 | 0.4076 |
| TC1000008668.hg.1 | SFXN3 | sideroflexin 3 | Multiple_C | 9.09 | 8.59 | 8.23 | 1.82 | 0.1911 | 0.3793 | 10.24 | 10.16 | 10.11 | 1.09 | 0.6328 | 0.8192 |
| TC1100006791.hg.1 | RPL27A; SNOF | ribosomal protein L27a; small nucleolar RNA, H/ACA box 3A; sm | Multiple_C | 15.87 | 16.08 | 15.01 | 1.82 | 0.0082 | 0.0358 | 13.22 | 12.88 | 12.08 | 2.20 | 0.0002 | 0.0063 |
| TC1100010531.hg.1 | SLC1A2 | solute carrier family 1 (glial high affinity glutamate transporter), | Multiple_C | 4.78 | 4.34 | 3.92 | 1.82 | 0.0189 | 0.0686 | 4.63 | 4.55 | 4.06 | 1.48 | 0.0192 | 0.117 |
| TC1200007891.hg.1 | DTX3 | deltex 3, E3 ubiquitin ligase | Multiple_C | 5.11 | 4.38 | 4.25 | 1.82 | 0.0332 | 0.1065 | 6.81 | 7.11 | 6.79 | 1.01 | 0.5494 | 0.766 |
| TC1200008466.hg.1 | NUDT4 | nudix hydrolase 4 | Multiple_C | 15.11 | 14.3 | 14.25 | 1.82 | 8.56E-05 | 0.0009 | 16.56 | 15.99 | 16 | 1.47 | 0.0008 | 0.0146 |
| TC1300008044.hg.1 | ARHGEF7 | Rho guanine nucleotide exchange factor 7 | Multiple_C | 10.42 | 9.88 | 9.56 | 1.82 | 0.0184 | 0.0671 | 11.71 | 11.46 | 11.5 | 1.16 | 0.153 | 0.3921 |
| TC1300008449.hg.1 | CDX2 | caudal type homeobox 2 | Multiple_C | 8.41 | 6.61 | 7.55 | 1.82 | 0.0942 | 0.2307 | 7.95 | 8.99 | 8.45 | 0.71 | 0.1929 | 0.4461 |
| TC1400009538.hg.1 | DCAF5 | DDB1 and CUL4 associated factor 5 | Multiple_C | 10.18 | 8.91 | 9.32 | 1.82 | 0.0074 | 0.0329 | 9.09 | 9.21 | 9.29 | 0.87 | 0.493 | 0.7276 |
| TC1500008309.hg.1 | ZNF774 | zinc finger protein 774 | Multiple_C | 8.86 | 7.88 | 8 | 1.82 | 0.0679 | 0.1814 | 6.54 | 6.33 | 6.27 | 1.21 | 0.5863 | 0.7902 |
| TC1500009370.hg.1 | FAM227B | family with sequence similarity 227, member B | Multiple_C | 4.51 | 4.22 | 3.65 | 1.82 | 0.0009 | 0.0062 | 4.69 | 5.13 | 4.59 | 1.07 | 0.1396 | 0.3712 |
| TC1500009395.hg.1 | TRPM7 | transient receptor potential cation channel, subfamily M, meml | Multiple_C | 8.38 | 8.38 | 7.52 | 1.82 | 0.0105 | 0.0435 | 8.55 | 8.68 | 8.81 | 0.84 | 0.2471 | 0.5117 |
| TC1600009683.hg.1 | IGSF6 | immunoglobulin superfamily, member 6 | Multiple_C | 5 | 4.23 | 4.14 | 1.82 | 0.0303 | 0.0992 | 4.21 | 4.32 | 4.6 | 0.76 | 0.8856 | 0.9509 |
| TC1700006667.hg.1 | USP6 | ubiquitin specific peptidase 6 | Multiple_C | 6.86 | 6.02 | 6 | 1.82 | 0.0004 | 0.0031 | 6.4 | 6.43 | 6.34 | 1.04 | 0.7665 | 0.8952 |
| TC1700012366.hg.1 | TBC1D27 | TBC1 domain family, member 27 | Multiple_C | 5.7 | 4.84 | 4.84 | 1.82 | 0.0314 | 0.1021 | 5.59 | 5.33 | 4.92 | 1.59 | 0.0493 | 0.2066 |
| TC1800008605.hg.1 | PIAS2 | protein inhibitor of activated STAT 2 | Multiple_C | 5.75 | 5.12 | 4.89 | 1.82 | 0.0568 | 0.1588 | 4.66 | 4.78 | 4.96 | 0.81 | 0.3303 | 0.5944 |
| TC2200008571.hg.1 | MB | myoglobin | Multiple_C | 6.04 | 4.79 | 5.18 | 1.82 | 0.0722 | 0.1897 | 4.55 | 4.38 | 4.2 | 1.27 | 0.533 | 0.7557 |
| TC2200009364.hg.1 | CPT1B | carnitine palmitoyltransferase 1B (muscle) | Multiple_C | 8.05 | 7.58 | 7.19 | 1.82 | 0.0136 | 0.053 | 7.84 | 7.54 | 7.43 | 1.33 | 0.3428 | 0.6065 |
| TSUnmapped00000359.I | SLC2A6 | solute carrier family 2 (facilitated glucose transporter), member | Coding | 8.14 | 7.02 | 7.28 | 1.82 | 0.0025 | 0.014 | 7.93 | 8.30 | 8.23 | 0.81 | 0.0653 | 0.2433 |
| TSUnmapped00000465.I | EIF3F | Eukaryotic translation initiation factor 3 subunit F [Source:UniP | Coding | 6.31 | 5.43 | 5.45 | 1.82 | 0.0091 | 0.0386 | 6.29 | 6.25 | 6.13 | 1.12 | 0.1263 | 0.3515 |
| TC0100013415.hg.1 | FAM46B | family with sequence similarity 46, member B | Multiple_C | 5.48 | 4.63 | 4.63 | 1.80 | 0.0017 | 0.01 | 4.95 | 4.93 | 4.8 | 1.11 | 0.1927 | 0.446 |
| TC0200008063.hg.1 | DGUOK | deoxyguanosine kinase | Multiple_C | 11.83 | 11.28 | 10.98 | 1.80 | 0.0266 | 0.0894 | 9.39 | 9.95 | 9.73 | 0.79 | 0.8826 | 0.9496 |
| TC0200010164.hg.1 | SSFA2 | sperm specific antigen 2 | Multiple_C | 10.16 | 10.87 | 9.31 | 1.80 | 0.0045 | 0.0223 | 9.52 | 8.96 | 9.83 | 0.81 | 0.1326 | 0.3614 |
| TC0200012418.hg.1 | LRPPRC | leucine-rich pentatricopeptide repeat containing | Multiple_C | 13.27 | 13.56 | 12.42 | 1.80 | 0.083 | 0.2101 | 12.29 | 12.19 | 12.52 | 0.85 | 0.6858 | 0.8495 |
| TC0200016669.hg.1 | ASB3 | ankyrin repeat and SOCS box containing 3 | Multiple_C | 9.72 | 9.84 | 8.87 | 1.80 | 0.0321 | 0.1038 | 8.64 | 8.42 | 8.6 | 1.03 | 0.7743 | 0.8986 |
| TC0400010230.hg.1 | GBA3 | Jeck2013 ANTISENSE, coding, INTERNAL, intronic best transcript | Multiple_C | 14.52 | 13.82 | 13.67 | 1.80 | 0.002 | 0.0117 | 13.92 | 14.24 | 14.54 | 0.65 | 0.0026 | 0.0326 |

| | | | | | | | | | | | | | | | |
|---|---|---|---|---|---|---|---|---|---|---|---|---|---|---|---|
| TC0400012564.hg.1 | CLDN22 | claudin 22 | Coding | 4.56 | 4.57 | 3.71 | 1.80 | 0.0018 | 0.0108 | 3.5 | 3.32 | 3.5 | 1.00 | 0.975 | 0.9902 |
| TC0600007423.hg.1 | GPX5 | glutathione peroxidase 5 | Multiple_C | 3.98 | 3.23 | 3.13 | 1.80 | 0.0029 | 0.0157 | 3.54 | 3.39 | 3.47 | 1.05 | 0.3623 | 0.6252 |
| TC0700010581.hg.1 | HIBADH | 3-hydroxyisobutyrate dehydrogenase | Multiple_C | 12.48 | 13.3 | 11.63 | 1.80 | 0.0445 | 0.1328 | 8.6 | 8.75 | 8.92 | 0.80 | 0.9458 | 0.9781 |
| TC0700013080.hg.1 | RHEB | Ras homolog enriched in brain | Multiple_C | 13.52 | 13.33 | 12.67 | 1.80 | 0.0721 | 0.1894 | 10.96 | 10.58 | 11.48 | 0.70 | 0.1646 | 0.409 |
| TC0700013338.hg.1 | GLCCI1 | glucocorticoid induced 1 | Multiple_C | 8.08 | 7.46 | 7.23 | 1.80 | 0.0156 | 0.0588 | 5.63 | 6.11 | 6.88 | 0.42 | 0.0003 | 0.0075 |
| TC0900007143.hg.1 | ZCCHC7 | zinc finger, CCHC domain containing 7 | Multiple_C | 11.99 | 12.44 | 11.14 | 1.80 | 0.0273 | 0.0913 | 10.21 | 9.20 | 9.67 | 1.45 | 0.6732 | 0.8415 |
| TC0900008740.hg.1 | OLFML2A | olfactomedin like 2A | Coding | 5.36 | 4.31 | 4.51 | 1.80 | 0.0028 | 0.0153 | 5.18 | 4.93 | 4.88 | 1.23 | 0.2024 | 0.4581 |
| TC0900008849.hg.1 | C9orf16 | chromosome 9 open reading frame 16 | Multiple_C | 9.76 | 8.71 | 8.91 | 1.80 | 0.008 | 0.0349 | 8.54 | 8.36 | 8.18 | 1.28 | 0.6507 | 0.8296 |
| TC1000010327.hg.1 | FZD8; MIR468 | frizzled class receptor 8; microRNA 4683 | Multiple_C | 4.67 | 3.61 | 3.82 | 1.80 | 0.0036 | 0.0184 | 3.92 | 3.95 | 3.89 | 1.02 | 0.6415 | 0.8242 |
| TC1100012443.hg.1 | LOC10065276 | uncharacterized LOC100652768; Salzman2013 ANNOTATED, ncl | Multiple_C | 5.9 | 5.2 | 5.05 | 1.80 | 0.0014 | 0.0089 | 5.65 | 5.86 | 5.6 | 1.04 | 0.8706 | 0.9444 |
| TC1100013196.hg.1 | TMEM134 | transmembrane protein 134 | Multiple_C | 9 | 8.04 | 8.15 | 1.80 | 0.0004 | 0.0033 | 7.64 | 8.10 | 7.59 | 1.04 | 0.8013 | 0.9112 |
| TC1200006653.hg.1 | ENO2 | enolase 2 (gamma, neuronal) | Multiple_C | 6.1 | 5.14 | 5.25 | 1.80 | 0.131 | 0.2929 | 5.64 | 5.66 | 5.57 | 1.05 | 0.7637 | 0.8932 |
| TC1200006654.hg.1 | ATN1 | atrophin 1 | Multiple_C | 7.02 | 5.81 | 6.17 | 1.80 | 0.0012 | 0.0077 | 8.14 | 8.58 | 8.14 | 1.00 | 0.8023 | 0.9117 |
| TC1200010397.hg.1 | CPNE8 | copine VIII | Multiple_C | 4.05 | 3.2 | 3.2 | 1.80 | 0.0688 | 0.183 | 4.9 | 4.90 | 4.71 | 1.14 | 0.8252 | 0.9229 |
| TC1200011710.hg.1 | SYCP3 | synaptonemal complex protein 3 | Multiple_C | 5.1 | 4.54 | 4.25 | 1.80 | 0.004 | 0.0203 | 4.15 | 3.75 | 4.09 | 1.04 | 0.8228 | 0.9219 |
| TC1400010693.hg.1 | KLC1 | kinesin light chain 1 | Multiple_C | 11.11 | 10.71 | 10.26 | 1.80 | 0.0029 | 0.0157 | 10.88 | 9.82 | 10.07 | 1.75 | 0.0022 | 0.0282 |
| TC1500008616.hg.1 | HERC2P3 | hect domain and RLD 2 pseudogene 3 | Multiple_C | 9.16 | 8.47 | 8.31 | 1.80 | 0.2212 | 0.4185 | 9.18 | 9.35 | 8.77 | 1.33 | 0.258 | 0.5228 |
| TC1600008925.hg.1 | RHBDF1 | rhomboid 5 homolog 1 (Drosophila) | Multiple_C | 9.81 | 8.7 | 8.96 | 1.80 | 0.0157 | 0.0591 | 8.2 | 8.36 | 8.14 | 1.04 | 0.539 | 0.7594 |
| TC1600011535.hg.1 | SIAH1 | siah E3 ubiquitin protein ligase 1 | Multiple_C | 11.34 | 11.41 | 10.49 | 1.80 | 0.0178 | 0.0653 | 9.72 | 9.24 | 9.48 | 1.18 | 0.9669 | 0.9862 |
| TC1700007982.hg.1 | C17orf53 | chromosome 17 open reading frame 53 | Multiple_C | 8.29 | 6.79 | 7.44 | 1.80 | 0.1606 | 0.3373 | 6.55 | 7.19 | 7.15 | 0.66 | 0.0348 | 0.1684 |
| TC1700010038.hg.1 | ULK2 | unc-51 like autophagy activating kinase 2 | Multiple_C | 4.82 | 4.03 | 3.97 | 1.80 | 0.0333 | 0.1067 | 4.08 | 4.38 | 4.28 | 0.87 | 0.6439 | 0.8254 |
| TC1700010802.hg.1 | MPP2 | membrane protein, palmitoylated 2 | Multiple_C | 4.59 | 3.72 | 3.74 | 1.80 | 0.0532 | 0.1513 | 4.69 | 4.76 | 4.92 | 0.85 | 0.3537 | 0.6171 |
| TC1700011731.hg.1 | ATP5H | ATP synthase, H+ transporting, mitochondrial Fo complex subur | Multiple_C | 14.97 | 14.82 | 14.12 | 1.80 | 0.0122 | 0.0489 | 10.56 | 10.39 | 10.1 | 1.38 | 0.1704 | 0.4168 |
| TC1800007298.hg.1 | LIPG | lipase, endothelial | Multiple_C | 10.55 | 9.57 | 9.7 | 1.80 | 0.0024 | 0.0136 | 3.21 | 3.05 | 3.16 | 1.04 | 0.5081 | 0.7386 |
| TC1900009443.hg.1 | C3 | complement component 3 | Multiple_C | 5.49 | 5.22 | 4.64 | 1.80 | 0.0214 | 0.0756 | 3.59 | 3.33 | 3.34 | 1.19 | 0.0374 | 0.1761 |
| TC1900010620.hg.1 | CAPN12 | calpain 12 | Multiple_C | 5.19 | 5.28 | 4.34 | 1.80 | 0.0034 | 0.0178 | 4.74 | 4.52 | 4.24 | 1.41 | 0.0356 | 0.1709 |
| TC1900010951.hg.1 | SNRPD2 | small nuclear ribonucleoprotein D2 polypeptide | Multiple_C | 12.91 | 12.38 | 12.06 | 1.80 | 0.0015 | 0.0094 | 10.65 | 10.98 | 10.24 | 1.33 | 0.0235 | 0.1326 |
| TC2000007288.hg.1 | NNAT | neuronatin | Multiple_C | 7.14 | 6.09 | 6.29 | 1.80 | 0.0068 | 0.031 | 5.54 | 5.81 | 5.84 | 0.81 | 0.2293 | 0.4905 |
| TC2000008097.hg.1 | DNAJC5; MIR9 | DnaJ (Hsp40) homolog, subfamily C, member 5; microRNA 941-: | Multiple_C | 9.46 | 8.36 | 8.61 | 1.80 | 0.0283 | 0.0938 | 8.64 | 9.07 | 9.2 | 0.68 | 0.0151 | 0.1005 |
| TC2000009555.hg.1 | GCNT7 | glucosaminyl (N-acetyl) transferase family member 7 | Coding | 3.69 | 2.7 | 2.84 | 1.80 | 0.0142 | 0.0547 | 3.45 | 3.42 | 3.26 | 1.14 | 0.2639 | 0.5287 |
| TC2000009648.hg.1 | PRELID3B; ATI | PRELI domain containing 3B; ATP synthase, H+ transporting, mit | Multiple_C | 14.3 | 14.34 | 13.45 | 1.80 | 0.0005 | 0.0041 | 13.67 | 13.08 | 13.69 | 0.99 | 0.9095 | 0.9628 |
| TC2100007964.hg.1 | SYNJ1 | synaptojanin 1 | Multiple_C | 7.53 | 6.8 | 6.68 | 1.80 | 0.0403 | 0.1234 | 6.63 | 5.95 | 6.56 | 1.05 | 0.6014 | 0.8003 |
| TC2200006908.hg.1 | KIAA1671 | KIAA1671 | Multiple_C | 6.58 | 5.16 | 5.73 | 1.80 | 0.0301 | 0.0987 | 6.99 | 7.27 | 7.54 | 0.68 | 0.1953 | 0.4496 |

| | | | | | | | | | | | | | | | |
|---|---|---|---|---|---|---|---|---|---|---|---|---|---|---|---|
| TC0100010674.hg.1 | GPR52 | G protein-coupled receptor 52 | Coding | 6.4 | 5.38 | 5.56 | 1.79 | 0.0377 | 0.1174 | 5.18 | 4.88 | 5.01 | 1.13 | 0.9289 | 0.9702 |
| TC0100011698.hg.1 | DISP1 | dispatched homolog 1 (Drosophila) | Multiple_C | 8.07 | 7.7 | 7.23 | 1.79 | 0.0081 | 0.0353 | 6.55 | 5.88 | 6.4 | 1.11 | 0.9805 | 0.9917 |
| TC0100013000.hg.1 | CASP9 | caspase 9 | Multiple_C | 6.31 | 5.7 | 5.47 | 1.79 | 0.0514 | 0.1475 | 5.41 | 5.39 | 5.35 | 1.04 | 0.8143 | 0.9176 |
| TC0100017519.hg.1 | ACBD3 | acyl-CoA binding domain containing 3 | Multiple_C | 11.42 | 10.66 | 10.58 | 1.79 | 0.0448 | 0.1336 | 9.52 | 9.18 | 9.66 | 0.91 | 0.109 | 0.3249 |
| TC0100018565.hg.1 | AGT | angiotensinogen (serpin peptidase inhibitor, clade A, member 8 | Multiple_C | 4.59 | 4.1 | 3.75 | 1.79 | 0.0017 | 0.0102 | 4.23 | 4.95 | 4.57 | 0.79 | 0.3201 | 0.584 |
| TC0200010507.hg.1 | CARF | calcium responsive transcription factor | Multiple_C | 9.31 | 8.74 | 8.47 | 1.79 | 0.0184 | 0.0671 | 9.44 | 9.85 | 9.73 | 0.82 | 0.46 | 0.7031 |
| TC0300010474.hg.1 | PP2D1 | protein phosphatase 2C-like domain containing 1 | Multiple_C | 4.44 | 3.8 | 3.6 | 1.79 | 0.0049 | 0.0235 | 4.75 | 4.37 | 4.42 | 1.26 | 0.1129 | 0.3312 |
| TC0400007572.hg.1 | EXOC1 | exocyst complex component 1 | Multiple_C | 11.79 | 12.55 | 10.95 | 1.79 | 0.0434 | 0.1306 | 9.39 | 8.80 | 8.99 | 1.32 | 0.1518 | 0.3903 |
| TC0500007396.hg.1 | SNX18 | sorting nexin 18 | Coding | 6.96 | 6.17 | 6.12 | 1.79 | 0.003 | 0.0159 | 5.86 | 6.23 | 5.91 | 0.97 | 0.9343 | 0.9724 |
| TC0500008830.hg.1 | UBE2D2 | ubiquitin conjugating enzyme E2D 2 | Multiple_C | 13.55 | 13.26 | 12.71 | 1.79 | 0.0019 | 0.0113 | 12.44 | 12.34 | 12.48 | 0.97 | 0.646 | 0.8263 |
| TC0500011725.hg.1 | MCC | mutated in colorectal cancers | Multiple_C | 5.5 | 4.44 | 4.66 | 1.79 | 0.013 | 0.0513 | 9.13 | 7.96 | 7.97 | 2.23 | 7.36E-05 | 0.0029 |
| TC0600007108.hg.1 | RNF144B | ring finger protein 144B | Multiple_C | 5.84 | 4.74 | 5 | 1.79 | 0.0191 | 0.069 | 5.49 | 5.36 | 5.43 | 1.04 | 0.9852 | 0.9938 |
| TC0600014096.hg.1 | TRIM39-RPP2 | TRIM39-RPP21 readthrough | Coding | 8.1 | 7.36 | 7.26 | 1.79 | 0.0295 | 0.097 | 7.5 | 7.68 | 7.34 | 1.12 | 0.4201 | 0.6734 |
| TC0700011119.hg.1 | SEC61G | Sec61 translocon gamma subunit | Multiple_C | 13.65 | 13.25 | 12.81 | 1.79 | 0.0351 | 0.1112 | 12.76 | 13.01 | 12.06 | 1.62 | 0.0168 | 0.1081 |
| TC0700011519.hg.1 | STAG3L2 | stromal antigen 3-like 2 (pseudogene) | Multiple_C | 9.58 | 8.74 | 8.74 | 1.79 | 0.0013 | 0.0085 | 10.97 | 11.24 | 11.21 | 0.85 | 0.2124 | 0.4702 |
| TC0800010258.hg.1 | KAT6A | K(lysine) acetyltransferase 6A | Multiple_C | 10.07 | 9.18 | 9.23 | 1.79 | 0.0406 | 0.124 | 9.86 | 9.36 | 9.7 | 1.12 | 0.7049 | 0.8608 |
| TC0900011575.hg.1 | RPL12 | ribosomal protein L12 | Multiple_C | 17.78 | 17.6 | 16.94 | 1.79 | 0.001 | 0.0069 | 15.86 | 15.46 | 15.3 | 1.47 | 0.0042 | 0.0439 |
| TC0900012159.hg.1 | DNAJC25 | DnaJ (Hsp40) homolog, subfamily C , member 25 | Multiple_C | 9.58 | 9.48 | 8.74 | 1.79 | 0.0485 | 0.1414 | 8.79 | 8.32 | 8.5 | 1.22 | 0.9315 | 0.9711 |
| TC0X00008298.hg.1 | GLUD2 | glutamate dehydrogenase 2 | Multiple_C | 8.81 | 8.65 | 7.97 | 1.79 | 0.0012 | 0.008 | 7.95 | 7.70 | 8.16 | 0.86 | 0.1503 | 0.3882 |
| TC1000006624.hg.1 | CALML3 | calmodulin-like 3 | Coding | 6.36 | 5.51 | 5.52 | 1.79 | 0.004 | 0.0202 | 5.92 | 6.13 | 5.87 | 1.04 | 0.7711 | 0.8972 |
| TC1000010113.hg.1 | ANKRD26 | ankyrin repeat domain 26 | Multiple_C | 7.16 | 7.37 | 6.32 | 1.79 | 0.0039 | 0.0198 | 7.76 | 7.62 | 7.65 | 1.08 | 0.5278 | 0.7516 |
| TC1000011012.hg.1 | ECD | ecdysoneless homolog (Drosophila) | Multiple_C | 12.37 | 11.83 | 11.53 | 1.79 | 0.0008 | 0.0055 | 13.27 | 12.83 | 13.53 | 0.84 | 0.2011 | 0.4565 |
| TC1100009971.hg.1 | TRIM3 | tripartite motif containing 3 | Multiple_C | 4.58 | 3.61 | 3.74 | 1.79 | 0.0013 | 0.0082 | 4.07 | 3.47 | 3.76 | 1.24 | 0.3389 | 0.6029 |
| TC1100011183.hg.1 | SF1 | splicing factor 1 | Multiple_C | 6.59 | 4.95 | 5.75 | 1.79 | 0.4373 | 0.6467 | 6.71 | 7.02 | 7.37 | 0.63 | 0.0977 | 0.3049 |
| TC1200009183.hg.1 | SETD1B | SET domain containing 1B | Multiple_C | 9 | 7.47 | 8.16 | 1.79 | 0.0284 | 0.0942 | 7.63 | 8.60 | 8.72 | 0.47 | 0.0007 | 0.0129 |
| TC1200012043.hg.1 | MED13L | mediator complex subunit 13-like | Multiple_C | 12.09 | 11.08 | 11.25 | 1.79 | 0.0278 | 0.0926 | 10.32 | 9.93 | 9.94 | 1.30 | 0.0911 | 0.292 |
| TC1200012744.hg.1 | C1R | complement component 1, r subcomponent | Multiple_C | 3.99 | 3.17 | 3.15 | 1.79 | 0.0147 | 0.0562 | 3.48 | 3.37 | 3.45 | 1.02 | 0.8736 | 0.9454 |
| TC1400006945.hg.1 | MIPOL1 | mirror-image polydactyly 1 | Multiple_C | 3.82 | 3.18 | 2.98 | 1.79 | 0.0138 | 0.0536 | 3.81 | 3.28 | 3.17 | 1.56 | 0.0126 | 0.09 |
| TC1400007495.hg.1 | PLEKHH1 | pleckstrin homology domain containing, family H (with MyTH4 c | Multiple_C | 9.83 | 8.76 | 8.99 | 1.79 | 0.0031 | 0.0165 | 6.51 | 6.42 | 6.38 | 1.09 | 0.9547 | 0.9814 |
| TC1500010672.hg.1 | TARSL2 | threonyl-tRNA synthetase-like 2 | Multiple_C | 7.52 | 8.91 | 6.68 | 1.79 | 0.1674 | 0.3469 | 6.91 | 5.93 | 5.9 | 2.01 | 0.115 | 0.3348 |
| TC1500010728.hg.1 | SERF2 | small EDRK-rich factor 2 | Multiple_C | 8.33 | 7.76 | 7.49 | 1.79 | 0.0026 | 0.0145 | 5.65 | 5.71 | 5.59 | 1.04 | 0.1913 | 0.4443 |
| TC1700010241.hg.1 | NUFIP2; rerdy | Memczak2013 ALT_ACCEPTOR, ALT_DONOR, coding, INTERNAL | Multiple_C | 6.09 | 5.18 | 5.25 | 1.79 | 0.0267 | 0.0895 | 5.33 | 4.92 | 4.63 | 1.62 | 0.1956 | 0.45 |
| TC1700011038.hg.1 | ZNF652 | zinc finger protein 652 | Multiple_C | 8.03 | 6.84 | 7.19 | 1.79 | 0.0256 | 0.0869 | 8.2 | 8.74 | 9.29 | 0.47 | 0.0003 | 0.0069 |

| | | | | | | | | | | | | | | | |
|---|---|---|---|---|---|---|---|---|---|---|---|---|---|---|---|
| TC1900007025.hg.1 | PLPPR2 | phospholipid phosphatase related 2 | Multiple_C | 7.75 | 6.95 | 6.91 | 1.79 | 0.0088 | 0.0378 | 6.11 | 5.66 | 6.05 | 1.04 | 0.7094 | 0.8635 |
| TC1900010706.hg.1 | SERTAD1 | SERTA domain containing 1 | Multiple_C | 7.85 | 6.69 | 7.01 | 1.79 | 0.039 | 0.1202 | 5.94 | 5.61 | 6.11 | 0.89 | 0.6092 | 0.8044 |
| TC1900010824.hg.1 | PSG11 | pregnancy specific beta-1-glycoprotein 11 | Multiple_C | 4.84 | 3.46 | 4 | 1.79 | 0.0267 | 0.0895 | 4.84 | 4.64 | 4.66 | 1.13 | 0.0849 | 0.2804 |
| TC1900011650.hg.1 | ARHGEF18 | Rho/Rac guanine nucleotide exchange factor 18 | Multiple_C | 11.49 | 10.56 | 10.65 | 1.79 | 0.0394 | 0.1211 | 11.2 | 10.42 | 10.84 | 1.28 | 0.489 | 0.725 |
| TC2000009462.hg.1 | SALL4 | spalt-like transcription factor 4 | Multiple_C | 7.88 | 7.18 | 7.04 | 1.79 | 0.0409 | 0.1246 | 6.65 | 6.90 | 7.02 | 0.77 | 0.1057 | 0.3191 |
| TC2000009588.hg.1 | MTRNR2L3 | MT-RNR2-like 3 | Coding | 7.38 | 6.27 | 6.54 | 1.79 | 0.0286 | 0.0946 | 6.78 | 7.29 | 7.79 | 0.50 | 0.0016 | 0.0232 |
| TC2000010027.hg.1 | TMEM189-UB | TMEM189-UBE2V1 readthrough | Multiple_C | 13.26 | 11.74 | 12.42 | 1.79 | 0.0012 | 0.0076 | 12.78 | 12.52 | 13.52 | 0.60 | 0.0004 | 0.0089 |
| TC2200008643.hg.1 | ELFN2 | extracellular leucine-rich repeat and fibronectin type III domain | Multiple_C | 6.34 | 5.5 | 5.5 | 1.79 | 0.0472 | 0.1386 | 5.46 | 5.80 | 5.9 | 0.74 | 0.0369 | 0.175 |
| TC2200008864.hg.1 | CYP2D6 | cytochrome P450, family 2, subfamily D, polypeptide 6 | Multiple_C | 6.32 | 5.56 | 5.48 | 1.79 | 0.0271 | 0.0906 | 5.49 | 6.13 | 6 | 0.70 | 0.1667 | 0.4117 |
| TSUnmapped00000537.h | PRAMEF6 | PRAME family member 6 [Source:HGNC Symbol;Acc:HGNC:305 | Coding | 7.27 | 6.51 | 6.43 | 1.79 | 0.0601 | 0.1658 | 5.83 | 6.15 | 6.14 | 0.81 | 0.3385 | 0.6028 |
| TSUnmapped00000781.h | PRAMEF6 | PRAME family member 6 [Source:HGNC Symbol;Acc:HGNC:305 | Coding | 7.27 | 6.51 | 6.43 | 1.79 | 0.0601 | 0.1658 | 5.83 | 6.15 | 6.14 | 0.81 | 0.3385 | 0.6028 |
| TC0100007669.hg.1 | CCDC28B | coiled-coil domain containing 28B | Multiple_C | 6.34 | 5.71 | 5.51 | 1.78 | 0.0052 | 0.025 | 4.41 | 4.41 | 4.61 | 0.87 | 0.3558 | 0.6189 |
| TC0100008103.hg.1 | RPS8; SNORD | ribosomal protein S8; small nucleolar RNA, C/D box 55; small nu | Multiple_C | 16.56 | 16.41 | 15.73 | 1.78 | 0.0038 | 0.0195 | 14.1 | 13.76 | 13.32 | 1.72 | 0.003 | 0.0357 |
| TC0100010068.hg.1 | HAX1 | HCLS1 associated protein X-1 | Multiple_C | 10.27 | 10.73 | 9.44 | 1.78 | 0.0078 | 0.0342 | 7.58 | 7.50 | 7.62 | 0.97 | 0.9251 | 0.9693 |
| TC0100012670.hg.1 | RPL22 | ribosomal protein L22 | Multiple_C | 18.09 | 17.78 | 17.26 | 1.78 | 0.0015 | 0.0094 | 16.61 | 16.52 | 16.51 | 1.07 | 0.9789 | 0.9911 |
| TC0100013348.hg.1 | SYF2 | SYF2 pre-mRNA-splicing factor | Multiple_C | 12.42 | 12.99 | 11.59 | 1.78 | 0.0497 | 0.1441 | 9.46 | 9.33 | 8.63 | 1.78 | 0.0009 | 0.0154 |
| TC0200011743.hg.1 | FLJ33534; ACC | uncharacterized LOC285150; uncharacterized LOC285150 [Sour | Multiple_C | 6.16 | 5.1 | 5.33 | 1.78 | 0.0012 | 0.0078 | 5.42 | 5.35 | 5.46 | 0.97 | 0.8979 | 0.9571 |
| TC0200013605.hg.1 | LYG2 | lysozyme G-like 2 | Coding | 4.55 | 3.47 | 3.72 | 1.78 | 0.019 | 0.0687 | 4.59 | 4.75 | 4.72 | 0.91 | 0.833 | 0.9257 |
| TC0200015741.hg.1 | RNF25 | ring finger protein 25 | Multiple_C | 9.48 | 8.52 | 8.65 | 1.78 | 0.0311 | 0.1013 | 9.16 | 9.36 | 9.5 | 0.79 | 0.5568 | 0.7706 |
| TC0300006702.hg.1 | CAPN7 | calpain 7 | Multiple_C | 10.75 | 11.06 | 9.92 | 1.78 | 0.1039 | 0.2479 | 9.26 | 9.33 | 9.58 | 0.80 | 0.0492 | 0.2063 |
| TC0300010033.hg.1 | PAK2 | p21 protein (Cdc42/Rac)-activated kinase 2 | Multiple_C | 10.15 | 9.64 | 9.32 | 1.78 | 0.0145 | 0.0557 | 11.74 | 11.88 | 11.93 | 0.88 | 0.3463 | 0.61 |
| TC0300010722.hg.1 | LRRFIP2 | leucine rich repeat (in FLII) interacting protein 2 | Multiple_C | 11.34 | 10.48 | 10.51 | 1.78 | 0.069 | 0.1834 | 10.21 | 9.85 | 10.02 | 1.14 | 0.6139 | 0.8076 |
| TC0400007879.hg.1 | USO1 | USO1 vesicle transport factor | Multiple_C | 14.05 | 13.82 | 13.22 | 1.78 | 0.0301 | 0.0987 | 14.46 | 13.06 | 13.1 | 2.57 | 0.0007 | 0.0126 |
| TC0400009462.hg.1 | ING2 | inhibitor of growth family member 2 | Coding | 8.75 | 8.82 | 7.92 | 1.78 | 0.0093 | 0.0393 | 7.56 | 7.07 | 7.48 | 1.06 | 0.8691 | 0.9436 |
| TC0500008849.hg.1 | CYSTM1 | cysteine-rich transmembrane module containing 1 | Multiple_C | 15.16 | 15.77 | 14.33 | 1.78 | 0.0025 | 0.014 | 9.6 | 9.51 | 9.53 | 1.05 | 0.7602 | 0.8913 |
| TC0500010932.hg.1 | SGTB | small glutamine-rich tetratricopeptide repeat (TPR)-containing, | Multiple_C | 9.33 | 9.52 | 8.5 | 1.78 | 0.0034 | 0.0178 | 10.24 | 9.11 | 9.42 | 1.77 | 0.0112 | 0.0835 |
| TC0500013142.hg.1 | EXOC3 | exocyst complex component 3 | Multiple_C | 7.08 | 6.65 | 6.25 | 1.78 | 0.0306 | 0.0998 | 8.78 | 8.80 | 8.64 | 1.10 | 0.569 | 0.7777 |
| TC0500013239.hg.1 | PCDHB3 | protocadherin beta 3 | Coding | 4.27 | 3.42 | 3.44 | 1.78 | 0.0735 | 0.1919 | 3.62 | 3.79 | 3.38 | 1.18 | 0.1201 | 0.3422 |
| TC0600010802.hg.1 | ELOVL2 | ELOVL fatty acid elongase 2 | Multiple_C | 5.15 | 4.2 | 4.32 | 1.78 | 0.0106 | 0.0437 | 4.79 | 4.74 | 4.35 | 1.36 | 0.0949 | 0.2995 |
| TC0600012064.hg.1 | GCM1 | glial cells missing homolog 1 (Drosophila) | Coding | 4.19 | 3.17 | 3.36 | 1.78 | 0.0231 | 0.0801 | 3.37 | 3.40 | 3.5 | 0.91 | 0.8254 | 0.923 |
| TC0600012540.hg.1 | GABRR2 | gamma-aminobutyric acid (GABA) A receptor, rho 2 | Multiple_C | 4.03 | 3.3 | 3.2 | 1.78 | 0.0231 | 0.0802 | 3.81 | 3.62 | 3.3 | 1.42 | 0.0056 | 0.0532 |
| TC0700008564.hg.1 | ZAN | zonadhesin (gene/pseudogene) | Multiple_C | 4.35 | 3.37 | 3.52 | 1.78 | 0.047 | 0.1382 | 3.97 | 3.54 | 3.55 | 1.34 | 0.0088 | 0.0715 |
| TC0800009927.hg.1 | EBF2 | early B-cell factor 2 | Multiple_C | 4.42 | 3.39 | 3.59 | 1.78 | 0.0035 | 0.0183 | 3.63 | 3.63 | 3.6 | 1.02 | 0.8069 | 0.9148 |

| | | | | | | | | | | | | | | | |
|---|---|---|---|---|---|---|---|---|---|---|---|---|---|---|---|
| TC0900009414.hg.1 | RFX3 | regulatory factor X, 3 (influences HLA class II expression) | Multiple_C | 9.17 | 8.91 | 8.34 | 1.78 | 0.0072 | 0.0323 | 8.72 | 9.17 | 8.19 | 1.44 | 0.0121 | 0.0881 |
| TC0X00007582.hg.1 | CXorf49B | chromosome X open reading frame 49B | Multiple_C | 4.59 | 3.69 | 3.76 | 1.78 | 0.0375 | 0.1169 | 3.66 | 3.86 | 4.02 | 0.78 | 0.0786 | 0.2701 |
| TC0X00007781.hg.1 | ZNF711 | zinc finger protein 711 | Multiple_C | 5.15 | 4.2 | 4.32 | 1.78 | 0.0542 | 0.1535 | 10.47 | 9.80 | 9.85 | 1.54 | 0.1344 | 0.3642 |
| TC0X00011395.hg.1 | USP26 | ubiquitin specific peptidase 26 | Coding | 4.15 | 3.46 | 3.32 | 1.78 | 0.0369 | 0.1156 | 4.05 | 4.26 | 4.08 | 0.98 | 0.8819 | 0.9494 |
| TC0Y00006648.hg.1 | VCY1B | variable charge, Y-linked 1B | Coding | 4.01 | 3.09 | 3.18 | 1.78 | 0.0095 | 0.0401 | 3.76 | 3.80 | 3.65 | 1.08 | 0.5814 | 0.786 |
| TC0Y00007319.hg.1 | AKAP17A; SFR | Homo sapiens A kinase (PRKA) anchor protein 17A (AKAP17A), t | Multiple_C | 8.3 | 7.38 | 7.47 | 1.78 | 0.028 | 0.0931 | 6.83 | 7.34 | 7.67 | 0.56 | 0.0038 | 0.0415 |
| TC1000012519.hg.1 | IL15RA | interleukin 15 receptor, alpha | Multiple_C | 10.19 | 9.37 | 9.36 | 1.78 | 0.0221 | 0.0773 | 8.81 | 8.09 | 7.86 | 1.93 | 0.0005 | 0.0102 |
| TC1100007220.hg.1 | DEPDC7 | DEP domain containing 7 | Multiple_C | 9.6 | 9.38 | 8.77 | 1.78 | 0.0022 | 0.0127 | 10.28 | 10.32 | 10.9 | 0.65 | 0.0012 | 0.0191 |
| TC1200008804.hg.1 | FAM222A | family with sequence similarity 222, member A | Multiple_C | 5.29 | 4.21 | 4.46 | 1.78 | 0.003 | 0.016 | 5.96 | 6.58 | 6.25 | 0.82 | 0.268 | 0.5326 |
| TC1200010774.hg.1 | KRT76 | keratin 76, type II | Coding | 6.44 | 5.54 | 5.61 | 1.78 | 0.138 | 0.3036 | 5.53 | 5.83 | 5.95 | 0.75 | 0.1498 | 0.3876 |
| TC1400007145.hg.1 | FRMD6 | FERM domain containing 6 | Multiple_C | 6.35 | 5.24 | 5.52 | 1.78 | 0.0033 | 0.0174 | 6.43 | 5.82 | 6.08 | 1.27 | 0.0929 | 0.2957 |
| TC1400010780.hg.1 | ATXN3 | ataxin 3 | Multiple_C | 8.75 | 8.66 | 7.92 | 1.78 | 0.0161 | 0.0602 | 7.77 | 7.06 | 7.47 | 1.23 | 0.3316 | 0.5957 |
| TC1500006699.hg.1 | GOLGA8J | golgin A8 family, member J | Multiple_C | 6.19 | 5.76 | 5.36 | 1.78 | 0.0211 | 0.0748 | 6.59 | 5.98 | 5.83 | 1.69 | 0.0047 | 0.0472 |
| TC1500008312.hg.1 | IQGAP1 | IQ motif containing GTPase activating protein 1 | Multiple_C | 14.35 | 13.68 | 13.52 | 1.78 | 0.0011 | 0.0071 | 12.46 | 12.36 | 12.47 | 0.99 | 0.8785 | 0.9479 |
| TC1500010018.hg.1 | SEMA7A | semaphorin 7A, GPI membrane anchor (John Milton Hagen bloc | Multiple_C | 4.85 | 3.83 | 4.02 | 1.78 | 0.0043 | 0.0213 | 5.03 | 4.72 | 4.87 | 1.12 | 0.379 | 0.6386 |
| TC1600008888.hg.1 | RPL13; SNORD | ribosomal protein L13; small nucleolar RNA, C/D box 68 | Multiple_C | 16.6 | 16.25 | 15.77 | 1.78 | 0.0037 | 0.0189 | 13.49 | 13.86 | 12.7 | 1.73 | 0.0007 | 0.0138 |
| TC1600009120.hg.1 | CEMP1 | cementum protein 1 | Coding | 4.55 | 3.8 | 3.72 | 1.78 | 0.0132 | 0.052 | 3.66 | 3.50 | 3.33 | 1.26 | 0.1674 | 0.4129 |
| TC1600009659.hg.1 | ACSM2B | acyl-CoA synthetase medium-chain family member 2B | Multiple_C | 5.06 | 4.23 | 4.23 | 1.78 | 0.0348 | 0.1103 | 4.93 | 5.09 | 5.18 | 0.84 | 0.4229 | 0.6756 |
| TC1900009627.hg.1 | TYK2 | tyrosine kinase 2 | Multiple_C | 10.47 | 9.29 | 9.64 | 1.78 | 0.0227 | 0.0792 | 8.14 | 8.81 | 8.7 | 0.68 | 0.0599 | 0.2314 |
| TC1900011795.hg.1 | NDUFA3 | NADH dehydrogenase (ubiquinone) 1 alpha subcomplex, 3, 9kDa | Multiple_C | 14.53 | 11.94 | 13.7 | 1.78 | 0.0603 | 0.1663 | 10.24 | 10.30 | 10.28 | 0.97 | 0.976 | 0.9902 |
| TC1900011972.hg.1 | ZNF404 | zinc finger protein 404 | Coding | 4.19 | 3.83 | 3.36 | 1.78 | 0.0158 | 0.0594 | 3.36 | 3.34 | 3.36 | 1.00 | 0.8787 | 0.948 |
| TC2200009271.hg.1 | APOBEC3C | apolipoprotein B mRNA editing enzyme, catalytic polypeptide-li | Multiple_C | 4.5 | 3.79 | 3.67 | 1.78 | 0.0171 | 0.0634 | 12.7 | 11.03 | 11.6 | 2.14 | 0.0003 | 0.0071 |
| TC0100010282.hg.1 | ATP1A4 | ATPase, Na+/K+ transporting, alpha 4 polypeptide | Multiple_C | 5.86 | 4.95 | 5.04 | 1.77 | 0.0186 | 0.0677 | 5.81 | 6.06 | 5.73 | 1.06 | 0.7822 | 0.9022 |
| TC0100012889.hg.1 | MAD2L2 | MAD2 mitotic arrest deficient-like 2 (yeast) | Multiple_C | 10.89 | 10.39 | 10.07 | 1.77 | 0.0125 | 0.0499 | 10.72 | 10.52 | 9.88 | 1.79 | 0.0001 | 0.0042 |
| TC0100015865.hg.1 | S100A5 | S100 calcium binding protein A5 | Coding | 3.92 | 3.34 | 3.1 | 1.77 | 0.0086 | 0.037 | 3.86 | 3.67 | 3.81 | 1.04 | 0.7609 | 0.8917 |
| TC0100018339.hg.1 | MDM4 | MDM4, p53 regulator | Multiple_C | 10.18 | 9.68 | 9.36 | 1.77 | 0.0007 | 0.0049 | 10.11 | 9.53 | 9.71 | 1.32 | 0.0583 | 0.2278 |
| TC0200006536.hg.1 | RPS7 | ribosomal protein S7 | Multiple_C | 17.73 | 17.74 | 16.91 | 1.77 | 0.0005 | 0.0041 | 15.62 | 15.17 | 14.66 | 1.95 | 6.32E-05 | 0.0026 |
| TC0200007768.hg.1 | OTX1 | orthodenticle homeobox 1 | Multiple_C | 8.35 | 6.87 | 7.53 | 1.77 | 0.0468 | 0.1379 | 8.12 | 7.84 | 7.57 | 1.46 | 0.0161 | 0.1057 |
| TC0200008089.hg.1 | TLX2 | T-cell leukemia homeobox 2 | Multiple_C | 5.09 | 4.1 | 4.27 | 1.77 | 0.0015 | 0.0092 | 4.17 | 4.30 | 4.24 | 0.95 | 0.5585 | 0.7714 |
| TC0200008230.hg.1 | TMSB10 | thymosin beta 10 | Multiple_C | 17.76 | 17.1 | 16.94 | 1.77 | 0.0013 | 0.0081 | 14.02 | 13.76 | 12.93 | 2.13 | 0.001 | 0.0164 |
| TC0300012527.hg.1 | MSL2 | male-specific lethal 2 homolog (Drosophila) | Coding | 10.88 | 9.81 | 10.06 | 1.77 | 0.0075 | 0.0334 | 11.05 | 9.94 | 11.22 | 0.89 | 0.0836 | 0.2782 |
| TC0300013975.hg.1 | IP6K2 | inositol hexakisphosphate kinase 2 | Multiple_C | 10.24 | 9.74 | 9.42 | 1.77 | 0.0161 | 0.0605 | 8.73 | 8.77 | 8.51 | 1.16 | 0.6993 | 0.8574 |
| TC0400010609.hg.1 | CORIN | corin, serine peptidase | Multiple_C | 5.61 | 4.53 | 4.79 | 1.77 | 0.0057 | 0.0268 | 5.01 | 5.28 | 5.02 | 0.99 | 0.7208 | 0.8698 |

| | | | | | | | | | | | | | | | |
|---|---|---|---|---|---|---|---|---|---|---|---|---|---|---|---|
| TC0500008782.hg.1 | KDM3B | lysine (K)-specific demethylase 3B | Multiple_C | 10.79 | 9.87 | 9.97 | 1.77 | 0.0062 | 0.0285 | 10.83 | 11.06 | 10.57 | 1.20 | 0.303 | 0.5673 |
| TC0500013100.hg.1 | FLT4 | fms-related tyrosine kinase 4 | Multiple_C | 4.56 | 3.93 | 3.74 | 1.77 | 0.0161 | 0.0603 | 4.62 | 4.79 | 4.7 | 0.95 | 0.9229 | 0.9684 |
| TC0600007402.hg.1 | ZKSCAN8 | zinc finger with KRAB and SCAN domains 8 | Multiple_C | 9.1 | 8.67 | 8.28 | 1.77 | 0.0367 | 0.1151 | 9.7 | 9.52 | 9.74 | 0.97 | 0.7886 | 0.9058 |
| TC0600010921.hg.1 | ATXN1 | ataxin 1 | Multiple_C | 7.15 | 6.12 | 6.33 | 1.77 | 0.1839 | 0.3695 | 9.98 | 9.38 | 9.42 | 1.47 | 0.0029 | 0.0346 |
| TC0600011631.hg.1 | TEAD3 | TEA domain family member 3 | Multiple_C | 7.2 | 6.23 | 6.38 | 1.77 | 0.0116 | 0.0469 | 5.84 | 5.91 | 6 | 0.90 | 0.4734 | 0.7133 |
| TC0600011822.hg.1 | MRPS10 | mitochondrial ribosomal protein S10 | Multiple_C | 12.32 | 12.3 | 11.5 | 1.77 | 0.0009 | 0.0064 | 10.1 | 9.55 | 9.99 | 1.08 | 0.4212 | 0.6744 |
| TC0600013528.hg.1 | LATS1 | large tumor suppressor kinase 1 | Multiple_C | 9.78 | 7.99 | 8.96 | 1.77 | 0.0015 | 0.0091 | 10.96 | 10.07 | 10.8 | 1.12 | 0.5001 | 0.7328 |
| TC0700009067.hg.1 | ATP6V1F | ATPase, H+ transporting, lysosomal 14kDa, V1 subunit F | Multiple_C | 13.68 | 13.08 | 12.86 | 1.77 | 0.0007 | 0.0051 | 12.21 | 12.30 | 12.79 | 0.67 | 0.0049 | 0.0487 |
| TC0800010807.hg.1 | RPL7 | ribosomal protein L7 | Multiple_C | 17.5 | 17.45 | 16.68 | 1.77 | 0.0055 | 0.026 | 16.35 | 15.82 | 15.73 | 1.54 | 0.0069 | 0.0604 |
| TC0900010624.hg.1 | ZCCHC6 | zinc finger, CCHC domain containing 6 | Multiple_C | 10.99 | 11.26 | 10.17 | 1.77 | 0.011 | 0.0451 | 10.37 | 9.42 | 9.7 | 1.59 | 0.1155 | 0.3355 |
| TC0900011841.hg.1 | SURF1 | surfeit 1 | Multiple_C | 7.09 | 6.61 | 6.27 | 1.77 | 0.1368 | 0.3016 | 6.58 | 7.12 | 6.47 | 1.08 | 0.7832 | 0.9026 |
| TC0900012291.hg.1 | NPDC1 | neural proliferation, differentiation and control, 1 | Multiple_C | 9.44 | 8.27 | 8.62 | 1.77 | 0.0022 | 0.0126 | 6.12 | 6.80 | 6.84 | 0.61 | 0.0106 | 0.0812 |
| TC0X00010097.hg.1 | RLIM | ring finger protein, LIM domain interacting | Multiple_C | 10.29 | 9.68 | 9.47 | 1.77 | 0.0661 | 0.1781 | 8.96 | 9.47 | 10.35 | 0.38 | 0.0008 | 0.0144 |
| TC0Y00006433.hg.1 | PLCXD1 | phosphatidylinositol-specific phospholipase C, X domain contair | Multiple_C | 5.34 | 4.77 | 4.52 | 1.77 | 0.0283 | 0.0939 | 4.93 | 5.76 | 6.07 | 0.45 | 0.0003 | 0.0067 |
| TC0Y00006882.hg.1 | SLC25A6 | solute carrier family 25 (mitochondrial carrier; adenine nucleoti | Multiple_C | 15.64 | 15.11 | 14.82 | 1.77 | 0.007 | 0.0314 | 14.62 | 15.17 | 15.39 | 0.59 | 0.011 | 0.083 |
| TC1000008595.hg.1 | R3HCC1L | R3H domain and coiled-coil containing 1-like | Multiple_C | 7.41 | 6.83 | 6.59 | 1.77 | 0.0317 | 0.1026 | 5.12 | 5.17 | 6.32 | 0.44 | 0.1169 | 0.3379 |
| TC1000008795.hg.1 | SLK | STE20-like kinase | Multiple_C | 14.6 | 14.01 | 13.78 | 1.77 | 0.0793 | 0.2038 | 12.64 | 11.69 | 12.37 | 1.21 | 0.5196 | 0.7469 |
| TC1000010515.hg.1 | NCOA4 | nuclear receptor coactivator 4 | Multiple_C | 12.37 | 11.11 | 11.55 | 1.77 | 0.0196 | 0.0703 | 12.12 | 11.82 | 11.93 | 1.14 | 0.3163 | 0.5802 |
| TC1100007786.hg.1 | TMEM132A | transmembrane protein 132A | Multiple_C | 8.1 | 7.01 | 7.28 | 1.77 | 0.0057 | 0.0269 | 8.19 | 8.57 | 8.57 | 0.77 | 0.4183 | 0.6722 |
| TC1100010514.hg.1 | ELF5 | E74-like factor 5 (ets domain transcription factor) | Multiple_C | 11.16 | 9.5 | 10.34 | 1.77 | 0.0042 | 0.0208 | 3.96 | 3.40 | 3.53 | 1.35 | 0.0719 | 0.2565 |
| TC1100011374.hg.1 | KMT5B | lysine (K)-specific methyltransferase 5B | Multiple_C | 12.15 | 12.5 | 11.33 | 1.77 | 0.0153 | 0.0579 | 13.44 | 12.01 | 12.53 | 1.88 | 0.0031 | 0.0369 |
| TC1200011037.hg.1 | FAM19A2 | family with sequence similarity 19 (chemokine (C-C motif)-like), | Multiple_C | 4.57 | 3.84 | 3.75 | 1.77 | 0.191 | 0.3793 | 4.3 | 4.50 | 4.52 | 0.86 | 0.6034 | 0.8016 |
| TC1200012580.hg.1 | NTF3 | neurotrophin 3 | Multiple_C | 9.97 | 9.02 | 9.15 | 1.77 | 0.0053 | 0.0251 | 8.82 | 8.98 | 8.92 | 0.93 | 0.7928 | 0.9077 |
| TC1400009780.hg.1 | NOXRED1 | NADP-dependent oxidoreductase domain containing 1 | Multiple_C | 5.17 | 4.71 | 4.35 | 1.77 | 0.0151 | 0.0573 | 4.53 | 4.45 | 4.56 | 0.98 | 0.3058 | 0.5698 |
| TC1400009784.hg.1 | VIPAS39 | VPS33B interacting protein, apical-basolateral polarity regulator | Multiple_C | 10.3 | 9.75 | 9.48 | 1.77 | 0.0038 | 0.0192 | 9.3 | 9.55 | 9.56 | 0.84 | 0.2167 | 0.4759 |
| TC1400009922.hg.1 | PTPN21 | protein tyrosine phosphatase, non-receptor type 21 | Multiple_C | 6.58 | 5.61 | 5.76 | 1.77 | 0.059 | 0.1638 | 7.74 | 8.10 | 8.05 | 0.81 | 0.2993 | 0.5639 |
| TC1500006994.hg.1 | CHP1 | calcineurin-like EF-hand protein 1 | Multiple_C | 14.52 | 13.71 | 13.7 | 1.77 | 0.0016 | 0.0095 | 13.49 | 12.89 | 13.69 | 0.87 | 0.0825 | 0.2767 |
| TC1500010630.hg.1 | ADAMTS17 | ADAM metallopeptidase with thrombospondin type 1 motif 17 | Multiple_C | 6.21 | 4.76 | 5.39 | 1.77 | 0.0121 | 0.0485 | 6.11 | 5.95 | 5.88 | 1.17 | 0.5409 | 0.7601 |
| TC1600006747.hg.1 | MGRN1 | mahogunin ring finger 1, E3 ubiquitin protein ligase | Multiple_C | 10.92 | 9.02 | 10.1 | 1.77 | 0.0025 | 0.0138 | 11.05 | 11.01 | 10.96 | 1.06 | 0.6444 | 0.8255 |
| TC1700011430.hg.1 | CD79B | CD79b molecule, immunoglobulin-associated beta | Multiple_C | 6.56 | 5.69 | 5.74 | 1.77 | 0.0643 | 0.1743 | 6.08 | 5.91 | 5.77 | 1.24 | 0.7161 | 0.8674 |
| TC1800007201.hg.1 | SIGLEC15 | sialic acid binding Ig-like lectin 15 | Multiple_C | 5.12 | 4.07 | 4.3 | 1.77 | 0.0996 | 0.2402 | 5.29 | 5.21 | 4.83 | 1.38 | 0.0549 | 0.22 |
| TC1800007316.hg.1 | SKA1 | spindle and kinetochore associated complex subunit 1 | Multiple_C | 8.01 | 6.42 | 7.19 | 1.77 | 0.0035 | 0.018 | 7.28 | 7.39 | 7.73 | 0.73 | 0.0726 | 0.2583 |
| TC1800008487.hg.1 | CELF4 | CUGBP, Elav-like family member 4 | Multiple_C | 3.98 | 2.97 | 3.16 | 1.77 | 0.0047 | 0.023 | 3.41 | 3.69 | 3.62 | 0.86 | 0.7809 | 0.9017 |

| | | | | | | | | | | | | | | | |
|---|---|---|---|---|---|---|---|---|---|---|---|---|---|---|---|
| TC1900006955.hg.1 | UBL5 | ubiquitin-like 5 | Multiple_C | 14.42 | 14.12 | 13.6 | 1.77 | 0.0279 | 0.0931 | 10.64 | 11.26 | 10.15 | 1.40 | 0.0534 | 0.2159 |
| TC1900008547.hg.1 | PRR12 | proline rich 12 | Multiple_C | 9.25 | 8.15 | 8.43 | 1.77 | 0.0381 | 0.1181 | 9.12 | 9.95 | 9.87 | 0.59 | 0.0011 | 0.0175 |
| TC1900008931.hg.1 | RFPL4A | ret finger protein-like 4A | Coding | 4.09 | 3.35 | 3.27 | 1.77 | 0.0805 | 0.2058 | 4.54 | 3.21 | 4.55 | 0.99 | 0.4009 | 0.6575 |
| TC1900009083.hg.1 | C2CD4C | C2 calcium-dependent domain containing 4C | Coding | 4.28 | 3.44 | 3.46 | 1.77 | 0.0045 | 0.0222 | 4.31 | 4.39 | 4.2 | 1.08 | 0.15 | 0.3879 |
| TC1900009198.hg.1 | BTBD2 | BTB (POZ) domain containing 2 | Multiple_C | 11.76 | 11.1 | 10.94 | 1.77 | 0.0075 | 0.0333 | 9.67 | 10.14 | 10.32 | 0.64 | 0.032 | 0.1599 |
| TC1900011001.hg.1 | STRN4 | striatin, calmodulin binding protein 4 | Multiple_C | 9.52 | 8.55 | 8.7 | 1.77 | 0.0474 | 0.1391 | 9.06 | 9.06 | 9.19 | 0.91 | 0.1772 | 0.426 |
| TC1900011748.hg.1 | SRRM5 | serine/arginine repetitive matrix 5 | Coding | 4.45 | 3.56 | 3.63 | 1.77 | 0.0123 | 0.0492 | 4.28 | 4.11 | 3.94 | 1.27 | 0.0274 | 0.1451 |
| TC2000007227.hg.1 | ROMO1 | reactive oxygen species modulator 1 | Multiple_C | 12.9 | 12.11 | 12.08 | 1.77 | 0.0007 | 0.0049 | 10.47 | 11.31 | 9.92 | 1.46 | 0.0211 | 0.1242 |
| TC2000010021.hg.1 | ZNFX1 | zinc finger, NFX1-type containing 1 | Multiple_C | 4.96 | 4.21 | 4.14 | 1.77 | 0.012 | 0.0481 | 4.38 | 4.04 | 4.25 | 1.09 | 0.6141 | 0.8078 |
| TC2100008314.hg.1 | CSTB | cystatin B (stefin B) | Multiple_C | 16.11 | 14.85 | 15.29 | 1.77 | 0.0005 | 0.0039 | 11.01 | 10.93 | 10.85 | 1.12 | 0.347 | 0.6105 |
| TC2100008519.hg.1 | WRB | tryptophan rich basic protein | Multiple_C | 4.62 | 3.66 | 3.8 | 1.77 | 0.0096 | 0.0403 | 4.21 | 3.77 | 3.95 | 1.20 | 0.1711 | 0.4176 |
| TC0100009116.hg.1 | RWDD3; TMEI | RWD domain containing 3; transmembrane protein 56; TMEM5 | Multiple_C | 7.98 | 8.92 | 7.17 | 1.75 | 0.0012 | 0.0079 | 6.04 | 6.53 | 6.36 | 0.80 | 0.1561 | 0.3967 |
| TC0100010815.hg.1 | MR1 | major histocompatibility complex, class I-related | Multiple_C | 7.25 | 6.99 | 6.44 | 1.75 | 0.0027 | 0.0149 | 6.77 | 6.90 | 6.31 | 1.38 | 0.0382 | 0.178 |
| TC0100015049.hg.1 | FRRS1 | ferric-chelate reductase 1 | Multiple_C | 8.11 | 7.2 | 7.3 | 1.75 | 0.1135 | 0.2642 | 6.74 | 6.62 | 7.16 | 0.75 | 0.0348 | 0.1684 |
| TC0100015397.hg.1 | CD58 | CD58 molecule | Multiple_C | 10.26 | 10.47 | 9.45 | 1.75 | 0.0176 | 0.0646 | 8.51 | 8.03 | 9.02 | 0.70 | 0.1156 | 0.3355 |
| TC0100015840.hg.1 | LCE1C | late cornified envelope 1C | Coding | 10.35 | 9.41 | 9.54 | 1.75 | 0.0066 | 0.0302 | 10.59 | 10.81 | 10.61 | 0.99 | 0.7165 | 0.8676 |
| TC0200009470.hg.1 | HNMT | histamine N-methyltransferase | Multiple_C | 9.47 | 9.54 | 8.66 | 1.75 | 0.0117 | 0.0472 | 5.43 | 6.57 | 5.55 | 0.92 | 0.9594 | 0.9834 |
| TC0200012335.hg.1 | THUMPD2 | THUMP domain containing 2 | Multiple_C | 9.07 | 9.31 | 8.26 | 1.75 | 0.0119 | 0.0478 | 9.1 | 8.30 | 8.5 | 1.52 | 0.1645 | 0.409 |
| TC0300008945.hg.1 | MRAS | muscle RAS oncogene homolog | Multiple_C | 3.72 | 3.06 | 2.91 | 1.75 | 0.068 | 0.1815 | 4.34 | 3.91 | 3.19 | 2.22 | 0.0004 | 0.0095 |
| TC0400009953.hg.1 | AFAP1 | actin filament associated protein 1 | Multiple_C | 5.56 | 4.73 | 4.75 | 1.75 | 0.0669 | 0.1796 | 4.49 | 4.20 | 3.84 | 1.57 | 0.0248 | 0.1366 |
| TC0500007725.hg.1 | BDP1 | B double prime 1, subunit of RNA polymerase III transcription in | Multiple_C | 8.74 | 7.77 | 7.93 | 1.75 | 0.0073 | 0.0325 | 10.56 | 10.08 | 10.3 | 1.20 | 0.4006 | 0.6574 |
| TC0500009743.hg.1 | BTNL8 | butyrophilin-like 8 | Multiple_C | 5.76 | 4.58 | 4.95 | 1.75 | 0.0045 | 0.0221 | 5.01 | 5.10 | 5.05 | 0.97 | 0.8886 | 0.9524 |
| TC0600007556.hg.1 | MUC21 | mucin 21, cell surface associated | Multiple_C | 4.54 | 3.96 | 3.73 | 1.75 | 0.0678 | 0.1812 | 4.2 | 3.78 | 3.75 | 1.37 | 0.1393 | 0.371 |
| TC0600007613.hg.1 | HSPA1A; HSPA | heat shock 70kDa protein 1A; heat shock 70kDa protein 1B | Coding | 12.76 | 11.9 | 11.95 | 1.75 | 0.2915 | 0.5026 | 14 | 11.02 | 12.93 | 2.10 | 0.427 | 0.6791 |
| TC0600012652.hg.1 | FAXC | failed axon connections homolog | Multiple_C | 6.16 | 5.51 | 5.35 | 1.75 | 0.0113 | 0.0459 | 8.74 | 7.95 | 8.38 | 1.28 | 0.0825 | 0.2767 |
| TC0700010558.hg.1 | HOXA2 | homeobox A2 | Multiple_C | 7.4 | 6.43 | 6.59 | 1.75 | 0.0241 | 0.083 | 4.73 | 4.05 | 4.42 | 1.24 | 0.0774 | 0.2682 |
| TC0700013342.hg.1 | CHN2 | chimerin 2 | Multiple_C | 6.95 | 7.67 | 6.14 | 1.75 | 0.0137 | 0.0534 | 5.46 | 6.59 | 6.94 | 0.36 | 0.0002 | 0.0064 |
| TC0700013458.hg.1 | HILPDA | hypoxia inducible lipid droplet-associated | Multiple_C | 8.54 | 7.76 | 7.73 | 1.75 | 0.0203 | 0.0724 | 6.72 | 6.56 | 6.7 | 1.01 | 0.8447 | 0.932 |
| TC0700013593.hg.1 | OR2AE1 | olfactory receptor, family 2, subfamily AE, member 1 | Coding | 3.92 | 2.76 | 3.11 | 1.75 | 0.0628 | 0.1711 | 3.53 | 3.30 | 3.64 | 0.93 | 0.4266 | 0.6788 |
| TC0800011239.hg.1 | COX6C | cytochrome c oxidase subunit VIc | Multiple_C | 15.71 | 16.17 | 14.9 | 1.75 | 0.0461 | 0.1363 | 8.82 | 8.85 | 8.05 | 1.71 | 0.0057 | 0.0533 |
| TC0800012288.hg.1 | DCTN6 | dynactin 6 | Multiple_C | 13.74 | 14.56 | 12.93 | 1.75 | 0.0019 | 0.011 | 9.59 | 9.56 | 10 | 0.75 | 0.1394 | 0.371 |
| TC0X00007562.hg.1 | ITGB1BP2 | integrin beta 1 binding protein (melusin) 2 | Multiple_C | 3.87 | 3.37 | 3.06 | 1.75 | 0.1132 | 0.2636 | 4.14 | 4.36 | 4.22 | 0.95 | 0.755 | 0.8886 |
| TC0X00008908.hg.1 | SLC25A6 | solute carrier family 25 (mitochondrial carrier; adenine nucleoti | Multiple_C | 15.63 | 15.09 | 14.82 | 1.75 | 0.0082 | 0.0358 | 14.72 | 15.17 | 15.38 | 0.63 | 0.0077 | 0.0656 |

| | | | | | | | | | | | | | | | |
|---|---|---|---|---|---|---|---|---|---|---|---|---|---|---|---|
| TC0X00010833.hg.1 | FRMD7 | FERM domain containing 7 | Multiple_C | 3.64 | 2.62 | 2.83 | 1.75 | 0.0215 | 0.0758 | 3.78 | 4.02 | 3.5 | 1.21 | 0.287 | 0.5522 |
| TC1100007472.hg.1 | NR1H3 | nuclear receptor subfamily 1, group H, member 3 | Multiple_C | 6.71 | 5.62 | 5.9 | 1.75 | 0.0007 | 0.0051 | 6.45 | 6.36 | 5.93 | 1.43 | 0.0267 | 0.1428 |
| TC1100007780.hg.1 | MS4A10 | membrane-spanning 4-domains, subfamily A, member 10 | Coding | 4.4 | 3.71 | 3.59 | 1.75 | 0.0277 | 0.0926 | 4.9 | 4.89 | 5.1 | 0.87 | 0.6849 | 0.8489 |
| TC1100012961.hg.1 | AMPD3 | adenosine monophosphate deaminase 3 | Multiple_C | 4.45 | 3.18 | 3.64 | 1.75 | 0.0077 | 0.0341 | 4.24 | 3.98 | 4.06 | 1.13 | 0.7046 | 0.8606 |
| TC1500008360.hg.1 | SLCO3A1 | solute carrier organic anion transporter family, member 3A1 | Multiple_C | 4.88 | 3.91 | 4.07 | 1.75 | 0.0131 | 0.0514 | 6.36 | 6.77 | 7.14 | 0.58 | 0.0006 | 0.012 |
| TC1500008809.hg.1 | GOLGA8G | golgin A8 family, member G | Multiple_C | 5.91 | 5.61 | 5.1 | 1.75 | 0.0262 | 0.0884 | 6.35 | 5.60 | 5.91 | 1.36 | 0.1749 | 0.4229 |
| TC1500010659.hg.1 | SNRPA1 | small nuclear ribonucleoprotein polypeptide A | Multiple_C | 14.98 | 14.34 | 14.17 | 1.75 | 0.0049 | 0.0238 | 12.49 | 12.10 | 12.85 | 0.78 | 0.1589 | 0.4004 |
| TC1700009619.hg.1 | DLG4 | discs, large homolog 4 (Drosophila) | Multiple_C | 5.04 | 4.69 | 4.23 | 1.75 | 0.1319 | 0.2943 | 9.79 | 8.70 | 8.52 | 2.41 | 0.0017 | 0.0234 |
| TC1700012275.hg.1 | EFCAB13 | EF-hand calcium binding domain 13 | Multiple_C | 4.5 | 3.83 | 3.69 | 1.75 | 0.0221 | 0.0776 | 4.04 | 3.85 | 4.25 | 0.86 | 0.3673 | 0.6295 |
| TC1800008764.hg.1 | TXNL1 | thioredoxin-like 1 | Multiple_C | 13.2 | 13.11 | 12.39 | 1.75 | 0.0051 | 0.0244 | 10.54 | 10.74 | 10.71 | 0.89 | 0.6674 | 0.8374 |
| TC1900009546.hg.1 | PRAM1 | PML-RARA regulated adaptor molecule 1 | Multiple_C | 4.18 | 3.36 | 3.37 | 1.75 | 0.0549 | 0.1548 | 4.53 | 4.32 | 4.46 | 1.05 | 0.9486 | 0.9791 |
| TC1900010833.hg.1 | PSG5 | pregnancy specific beta-1-glycoprotein 5 | Multiple_C | 5.37 | 4.67 | 4.56 | 1.75 | 0.0014 | 0.0087 | 5.08 | 5.07 | 5.19 | 0.93 | 0.3909 | 0.6495 |
| TC2200007904.hg.1 | ATP6V1E1 | ATPase, H+ transporting, lysosomal 31kDa, V1 subunit E1 | Multiple_C | 11.51 | 11.43 | 10.7 | 1.75 | 0.0172 | 0.0636 | 10.43 | 10.84 | 11.02 | 0.66 | 0.1249 | 0.3493 |
| TSUnmapped00000213.h | DGKD | diacylglycerol kinase, delta 130kDa | Coding | 4.35 | 3.38 | 3.54 | 1.75 | 0.0519 | 0.1484 | 3.94 | 3.91 | 3.89 | 1.04 | 0.7594 | 0.8908 |
| TC0100006483.hg.1 | ISG15 | ISG15 ubiquitin-like modifier | Multiple_C | 7.58 | 6.47 | 6.78 | 1.74 | 0.0186 | 0.0676 | 5.58 | 5.54 | 5.32 | 1.20 | 0.2679 | 0.5326 |
| TC0100009029.hg.1 | BRDT | bromodomain, testis-specific | Multiple_C | 9.9 | 8.91 | 9.1 | 1.74 | 0.0651 | 0.1761 | 9.02 | 9.03 | 8.75 | 1.21 | 0.4441 | 0.6923 |
| TC0100011378.hg.1 | RASSF5 | Ras association (RalGDS/AF-6) domain family member 5 | Multiple_C | 6.4 | 5.37 | 5.6 | 1.74 | 0.0049 | 0.0236 | 7.79 | 7.81 | 7.93 | 0.91 | 0.8001 | 0.9105 |
| TC0100013713.hg.1 | STK40 | serine/threonine kinase 40 | Multiple_C | 8.92 | 7.6 | 8.12 | 1.74 | 0.1281 | 0.2881 | 8.69 | 8.89 | 9.33 | 0.64 | 0.0681 | 0.2486 |
| TC0100017613.hg.1 | TRIM17 | tripartite motif containing 17 | Coding | 7.03 | 5.82 | 6.23 | 1.74 | 0.0125 | 0.0498 | 5.87 | 6.04 | 5.49 | 1.30 | 0.2463 | 0.5109 |
| TC0200011898.hg.1 | SDC1 | syndecan 1 | Multiple_C | 10.83 | 9.52 | 10.03 | 1.74 | 0.042 | 0.1274 | 8.18 | 9.11 | 9.49 | 0.40 | 4.29E-05 | 0.002 |
| TC0200015072.hg.1 | TTC30A | tetratricopeptide repeat domain 30A | Coding | 4.25 | 3.66 | 3.45 | 1.74 | 0.0387 | 0.1195 | 4.13 | 4.28 | 4.26 | 0.91 | 0.6512 | 0.8299 |
| TC0200016645.hg.1 | ASXL2 | additional sex combs like transcriptional regulator 2 | Multiple_C | 10.29 | 8.77 | 9.49 | 1.74 | 0.035 | 0.1109 | 10.92 | 10.09 | 10.61 | 1.24 | 0.2055 | 0.4618 |
| TC0300010228.hg.1 | FANCD2OS | FANCD2 opposite strand | Multiple_C | 5.51 | 4.39 | 4.71 | 1.74 | 0.0015 | 0.0093 | 4.79 | 5.38 | 5.2 | 0.75 | 0.0187 | 0.1154 |
| TC0300011028.hg.1 | COL7A1; MIR7 | collagen, type VII, alpha 1; microRNA 711 | Multiple_C | 8.42 | 7.74 | 7.62 | 1.74 | 0.0181 | 0.0662 | 7.58 | 7.96 | 7.5 | 1.06 | 0.8464 | 0.933 |
| TC0300014002.hg.1 | HESX1 | HESX homeobox 1 | Coding | 5.31 | 5.58 | 4.51 | 1.74 | 0.0102 | 0.0424 | 5.03 | 5.45 | 4.93 | 1.07 | 0.5736 | 0.7812 |
| TC0400006487.hg.1 | TMEM175 | transmembrane protein 175 | Multiple_C | 8.36 | 7.32 | 7.56 | 1.74 | 0.0789 | 0.2029 | 7.02 | 8.19 | 7.94 | 0.53 | 0.0072 | 0.0625 |
| TC0400012967.hg.1 | MGARP | mitochondria localized glutamic acid rich protein | Coding | 4.76 | 3.96 | 3.96 | 1.74 | 0.018 | 0.0659 | 4.35 | 4.10 | 4.51 | 0.90 | 0.594 | 0.795 |
| TC0600008120.hg.1 | TMEM63B | transmembrane protein 63B | Multiple_C | 9.56 | 8.81 | 8.76 | 1.74 | 0.0058 | 0.0273 | 8.33 | 7.05 | 7.43 | 1.87 | 0.0011 | 0.0181 |
| TC0600011594.hg.1 | C6orf106 | chromosome 6 open reading frame 106 | Multiple_C | 10.82 | 10.35 | 10.02 | 1.74 | 0.0183 | 0.0667 | 10.32 | 10.50 | 10.31 | 1.01 | 0.2724 | 0.5378 |
| TC0600013180.hg.1 | CTAGE9 | CTAGE family, member 9 | Coding | 7.51 | 7.25 | 6.71 | 1.74 | 0.0451 | 0.1341 | 6.3 | 6.67 | 6.32 | 0.99 | 0.3313 | 0.5954 |
| TC0700008762.hg.1 | CBLL1 | Cbl proto-oncogene-like 1, E3 ubiquitin protein ligase | Multiple_C | 11.24 | 9.65 | 10.44 | 1.74 | 0.0741 | 0.1931 | 12.74 | 11.84 | 12.96 | 0.86 | 0.9638 | 0.9846 |
| TC0700010621.hg.1 | FKBP14 | FK506 binding protein 14 | Multiple_C | 10.72 | 11.28 | 9.92 | 1.74 | 0.0227 | 0.0791 | 10.13 | 8.74 | 9.95 | 1.13 | 0.3272 | 0.5907 |
| TC0X00006658.hg.1 | OFD1 | oral-facial-digital syndrome 1 | Multiple_C | 8.35 | 8.87 | 7.55 | 1.74 | 0.0555 | 0.1563 | 7.75 | 7.73 | 7.33 | 1.34 | 0.2817 | 0.5474 |

| | | | | | | | | | | | | | | |
|---|---|---|---|---|---|---|---|---|---|---|---|---|---|---|
| TC1000008224.hg.1 | PLAC9 | placenta specific 9 | Multiple_C | 4.15 | 3.18 | 3.35 | 1.74 | 0.0088 | 0.0379 | 4.14 | 4.29 | 3.94 | 1.15 | 0.6839 | 0.8485 |
| TC1000012554.hg.1 | FAM25C | family with sequence similarity 25, member C | Coding | 6.96 | 5.92 | 6.16 | 1.74 | 0.3044 | 0.5173 | 5.94 | 6.23 | 6.12 | 0.88 | 0.8992 | 0.9579 |
| TC1000012579.hg.1 | SLIT1; ARHGA | slit guidance ligand 1; ARHGAP19-SLIT1 readthrough (NMD canc | Multiple_C | 5.19 | 3.91 | 4.39 | 1.74 | 0.0258 | 0.0873 | 6.4 | 6.41 | 5.86 | 1.45 | 0.032 | 0.1601 |
| TC1100007826.hg.1 | BEST1 | bestrophin 1 | Multiple_C | 5.69 | 5.04 | 4.89 | 1.74 | 0.0977 | 0.2367 | 4.74 | 4.14 | 4.93 | 0.88 | 0.5019 | 0.7343 |
| TC1100008067.hg.1 | RAB1B | RAB1B, member RAS oncogene family | Multiple_C | 13.67 | 12.04 | 12.87 | 1.74 | 0.0961 | 0.234 | 13.49 | 13.63 | 14.05 | 0.68 | 0.0635 | 0.2398 |
| TC1200006935.hg.1 | ATF7IP | activating transcription factor 7 interacting protein | Multiple_C | 11.65 | 11 | 10.85 | 1.74 | 0.009 | 0.0384 | 11.91 | 10.47 | 11.6 | 1.24 | 0.6357 | 0.8212 |
| TC1200010971.hg.1 | DCTN2 | dynactin 2 (p50) | Multiple_C | 11.85 | 11.62 | 11.05 | 1.74 | 0.0145 | 0.0556 | 11.39 | 11.33 | 11.28 | 1.08 | 0.653 | 0.8308 |
| TC1200012841.hg.1 | SSH1 | slingshot protein phosphatase 1 | Multiple_C | 9.33 | 7.78 | 8.53 | 1.74 | 0.0112 | 0.0457 | 8.71 | 8.67 | 8.29 | 1.34 | 0.0321 | 0.1605 |
| TC1400009655.hg.1 | HEATR4 | HEAT repeat containing 4 | Multiple_C | 5.49 | 4.69 | 4.69 | 1.74 | 0.1892 | 0.377 | 5.05 | 4.99 | 4.68 | 1.29 | 0.0756 | 0.2643 |
| TC1500009492.hg.1 | RSL24D1 | ribosomal L24 domain containing 1 | Multiple_C | 13.89 | 15.95 | 13.09 | 1.74 | 0.0031 | 0.0165 | 11.83 | 11.25 | 11.29 | 1.45 | 0.0006 | 0.0113 |
| TC1600007368.hg.1 | ATP2A1 | ATPase, Ca++ transporting, cardiac muscle, fast twitch 1 | Multiple_C | 5.39 | 4.17 | 4.59 | 1.74 | 0.0048 | 0.0232 | 4.67 | 4.76 | 4.85 | 0.88 | 0.7466 | 0.8837 |
| TC1600009977.hg.1 | ZNF688 | zinc finger protein 688 | Multiple_C | 6.37 | 5.52 | 5.57 | 1.74 | 0.0445 | 0.133 | 5.84 | 5.90 | 5.49 | 1.27 | 0.0733 | 0.2595 |
| TC1600010598.hg.1 | CCDC79 | coiled-coil domain containing 79 | Multiple_C | 4.7 | 3.94 | 3.9 | 1.74 | 0.2905 | 0.5013 | 4.67 | 4.87 | 4.68 | 0.99 | 0.5043 | 0.7364 |
| TC1700006897.hg.1 | DNAH9 | dynein, axonemal, heavy chain 9 | Multiple_C | 5.61 | 4.58 | 4.81 | 1.74 | 0.1718 | 0.3531 | 5.69 | 5.77 | 5.32 | 1.29 | 0.8186 | 0.9198 |
| TC1700007102.hg.1 | ALKBH5 | alkB homolog 5, RNA demethylase | Multiple_C | 11.54 | 10.44 | 10.74 | 1.74 | 0.0036 | 0.0184 | 10.53 | 10.67 | 11.01 | 0.72 | 0.1923 | 0.4456 |
| TC1700008186.hg.1 | IGF2BP1 | insulin-like growth factor 2 mRNA binding protein 1 | Multiple_C | 3.73 | 2.67 | 2.93 | 1.74 | 0.0259 | 0.0876 | 11.96 | 12.25 | 12.69 | 0.60 | 0.0219 | 0.1271 |
| TC1700011749.hg.1 | GRB2 | growth factor receptor bound protein 2 | Multiple_C | 13.59 | 12.45 | 12.79 | 1.74 | 0.008 | 0.0351 | 13.94 | 13.95 | 14.11 | 0.89 | 0.3126 | 0.5767 |
| TC1900009583.hg.1 | ZNF266 | zinc finger protein 266 | Multiple_C | 10.49 | 10.43 | 9.69 | 1.74 | 0.0285 | 0.0944 | 7.27 | 7.44 | 7.27 | 1.00 | 0.9771 | 0.9906 |
| TC1900009588.hg.1 | ZNF121 | zinc finger protein 121 | Multiple_C | 12.38 | 11.95 | 11.58 | 1.74 | 0.0324 | 0.1045 | 11.43 | 10.50 | 12.12 | 0.62 | 0.0084 | 0.0693 |
| TC1900011658.hg.1 | TRAPPC5 | trafficking protein particle complex 5 | Multiple_C | 11.86 | 11.57 | 11.06 | 1.74 | 0.014 | 0.054 | 5.76 | 6.51 | 6.14 | 0.77 | 0.5327 | 0.7555 |
| TC2000007161.hg.1 | C20orf144 | chromosome 20 open reading frame 144 | Multiple_C | 6.54 | 5.6 | 5.74 | 1.74 | 0.0045 | 0.0221 | 6.38 | 6.18 | 5.82 | 1.47 | 0.0136 | 0.0944 |
| TC2000008381.hg.1 | JAG1 | jagged 1 | Multiple_C | 9.9 | 9.33 | 9.1 | 1.74 | 0.0172 | 0.0636 | 9.06 | 8.90 | 10.09 | 0.49 | 3.37E-05 | 0.0017 |
| TC2200007783.hg.1 | PIM3 | Pim-3 proto-oncogene, serine/threonine kinase | Multiple_C | 9.79 | 8.58 | 8.99 | 1.74 | 0.0094 | 0.0397 | 7.4 | 7.16 | 7.39 | 1.01 | 0.7227 | 0.8707 |
| TC2200008611.hg.1 | EIF3D | eukaryotic translation initiation factor 3, subunit D | Multiple_C | 14.68 | 14.8 | 13.88 | 1.74 | 0.0503 | 0.1453 | 14 | 14.43 | 14.29 | 0.82 | 0.2414 | 0.5055 |
| TSUnmapped00000406.h | ZNF660 | zinc finger protein 660 | Coding | 4.6 | 3.84 | 3.8 | 1.74 | 0.0022 | 0.0126 | 4.83 | 4.83 | 5.27 | 0.74 | 0.0982 | 0.3058 |
| TSUnmapped00000421.h | ATG16L1 | autophagy related 16-like 1 | Coding | 9.21 | 8.19 | 8.41 | 1.74 | 0.0183 | 0.0667 | 7.64 | 7.32 | 7.72 | 0.95 | 0.984 | 0.9935 |
| TC0100007910.hg.1 | OXCT2P1 | 3-oxoacid CoA-transferase 2 pseudogene 1 | Multiple_C | 5.69 | 4.8 | 4.9 | 1.73 | 0.0074 | 0.0329 | 5.76 | 6.00 | 6.03 | 0.83 | 0.315 | 0.5788 |
| TC0100009035.hg.1 | KIAA1107 | KIAA1107 | Coding | 6.07 | 7.09 | 5.28 | 1.73 | 0.0012 | 0.0078 | 6.03 | 5.40 | 4.58 | 2.73 | 2.63E-08 | 1.01E-05 |
| TC0100012221.hg.1 | C1orf101 | chromosome 1 open reading frame 101 | Multiple_C | 4.95 | 4.35 | 4.16 | 1.73 | 0.0139 | 0.0538 | 4.75 | 4.55 | 4.74 | 1.01 | 0.2618 | 0.5261 |
| TC0100013229.hg.1 | HSPG2 | heparan sulfate proteoglycan 2 | Multiple_C | 9.06 | 8.14 | 8.27 | 1.73 | 0.1379 | 0.3035 | 10.01 | 9.87 | 8.83 | 2.27 | 0.0004 | 0.0085 |
| TC0100018134.hg.1 | OR2T35 | olfactory receptor, family 2, subfamily T, member 35 | Coding | 4.08 | 3.18 | 3.29 | 1.73 | 0.0279 | 0.0929 | 4.42 | 4.07 | 3.73 | 1.61 | 0.0329 | 0.1629 |
| TC0200009440.hg.1 | R3HDM1 | R3H domain containing 1 | Multiple_C | 10.29 | 8.02 | 9.5 | 1.73 | 0.0429 | 0.1295 | 12.08 | 11.15 | 12.46 | 0.77 | 0.2845 | 0.5496 |
| TC0200009829.hg.1 | GCA | grancalcin, EF-hand calcium binding protein | Multiple_C | 9.61 | 10.33 | 8.82 | 1.73 | 0.0709 | 0.1873 | 6.35 | 5.65 | 5.88 | 1.39 | 0.8804 | 0.9486 |

| | | | | | | | | | | | | | | | |
|---|---|---|---|---|---|---|---|---|---|---|---|---|---|---|---|
| TC0200010897.hg.1 | SGPP2 | sphingosine-1-phosphate phosphatase 2 | Multiple_C | 11.47 | 10.07 | 10.68 | 1.73 | 0.0006 | 0.0047 | 4.79 | 5.14 | 5.3 | 0.70 | 0.0034 | 0.0387 |
| TC0200012936.hg.1 | FBXO48 | F-box protein 48 | Coding | 6.27 | 6.61 | 5.48 | 1.73 | 0.0126 | 0.05 | 5.04 | 5.06 | 5.4 | 0.78 | 0.2127 | 0.4706 |
| TC0300009147.hg.1 | RNF13 | ring finger protein 13 | Multiple_C | 13.39 | 13.46 | 12.6 | 1.73 | 0.0087 | 0.0373 | 12.39 | 12.05 | 12.27 | 1.09 | 0.7762 | 0.8996 |
| TC0300013923.hg.1 | IL5RA | interleukin 5 receptor, alpha | Multiple_C | 4.36 | 3.56 | 3.57 | 1.73 | 0.0076 | 0.0335 | 5.04 | 5.17 | 5.08 | 0.97 | 0.7317 | 0.8758 |
| TC0400010784.hg.1 | NOA1 | nitric oxide associated 1 | Multiple_C | 13.13 | 13.78 | 12.34 | 1.73 | 0.034 | 0.1084 | 9.54 | 9.78 | 9.52 | 1.01 | 0.3133 | 0.5775 |
| TC0500013288.hg.1 | TAS2R1 | taste receptor, type 2, member 1 | Coding | 4.04 | 2.89 | 3.25 | 1.73 | 0.0118 | 0.0477 | 4.44 | 4.61 | 4.32 | 1.09 | 0.4159 | 0.67 |
| TC0600012113.hg.1 | BMP5 | bone morphogenetic protein 5 | Multiple_C | 4.07 | 3.36 | 3.28 | 1.73 | 0.0932 | 0.2289 | 8.27 | 7.77 | 7.37 | 1.87 | 0.0015 | 0.022 |
| TC0600012247.hg.1 | COL9A1 | collagen, type IX, alpha 1 | Multiple_C | 5.15 | 4.12 | 4.36 | 1.73 | 0.0742 | 0.1933 | 4.52 | 4.43 | 4.16 | 1.28 | 0.5497 | 0.7662 |
| TC0600014143.hg.1 | PHF3 | PHD finger protein 3 | Multiple_C | 11.94 | 11.5 | 11.15 | 1.73 | 0.1573 | 0.333 | 12.23 | 10.49 | 11.13 | 2.14 | 0.1705 | 0.4168 |
| TC0700008597.hg.1 | CUX1 | cut-like homeobox 1 | Multiple_C | 10.32 | 10.18 | 9.53 | 1.73 | 0.0315 | 0.1023 | 11.12 | 10.11 | 11.09 | 1.02 | 0.8215 | 0.9211 |
| TC0800010631.hg.1 | TTPA | tocopherol (alpha) transfer protein | Multiple_C | 5.91 | 5.09 | 5.12 | 1.73 | 0.0096 | 0.0404 | 9.87 | 9.17 | 8.51 | 2.57 | 0.0004 | 0.0093 |
| TC0800012310.hg.1 | C8orf44 | chromosome 8 open reading frame 44 | Multiple_C | 6.2 | 5.7 | 5.41 | 1.73 | 0.0429 | 0.1294 | 5.7 | 5.78 | 5.31 | 1.31 | 0.0141 | 0.0964 |
| TC0X00008040.hg.1 | MUM1L1 | melanoma associated antigen (mutated) 1-like 1 | Coding | 5.53 | 4.92 | 4.74 | 1.73 | 0.0493 | 0.1432 | 5.31 | 6.07 | 5.81 | 0.71 | 0.0269 | 0.1433 |
| TC0X00010643.hg.1 | SEPT6 | septin 6 | Multiple_C | 9.29 | 8.43 | 8.5 | 1.73 | 0.0306 | 0.0998 | 11.37 | 11.62 | 11.71 | 0.79 | 0.9395 | 0.975 |
| TC0X00010877.hg.1 | FAM122B | family with sequence similarity 122B | Multiple_C | 8.48 | 7.86 | 7.69 | 1.73 | 0.0176 | 0.0646 | 10.9 | 12.01 | 11.88 | 0.51 | 5.79E-05 | 0.0025 |
| TC0Y00007255.hg.1 | BPY2C | basic charge, Y-linked, 2C | Multiple_C | 3.08 | 2.3 | 2.29 | 1.73 | 0.0965 | 0.2347 | 3.7 | 3.74 | 3.21 | 1.40 | 0.0729 | 0.2587 |
| TC1000010265.hg.1 | ITGB1 | integrin beta 1 | Multiple_C | 15.01 | 14.98 | 14.22 | 1.73 | 0.0029 | 0.0156 | 15.21 | 14.19 | 14.64 | 1.48 | 0.0479 | 0.2032 |
| TC1000010593.hg.1 | C10orf128 | chromosome 10 open reading frame 128 | Multiple_C | 5.63 | 4.87 | 4.84 | 1.73 | 0.0687 | 0.1829 | 5.58 | 5.41 | 5.21 | 1.29 | 0.2238 | 0.4846 |
| TC1100007899.hg.1 | RARRES3 | retinoic acid receptor responder (tazarotene induced) 3 | Multiple_C | 9.05 | 8.77 | 8.26 | 1.73 | 0.003 | 0.0161 | 5.42 | 5.41 | 5.09 | 1.26 | 0.3039 | 0.5679 |
| TC1100009740.hg.1 | MOB2 | MOB kinase activator 2 | Multiple_C | 7.28 | 6.9 | 6.49 | 1.73 | 0.0153 | 0.058 | 7.4 | 7.29 | 6.75 | 1.57 | 0.0094 | 0.075 |
| TC1100011176.hg.1 | NRXN2 | neurexin 2 | Multiple_C | 4.91 | 3.87 | 4.12 | 1.73 | 0.0066 | 0.0302 | 4.6 | 4.71 | 4.46 | 1.10 | 0.3465 | 0.6102 |
| TC1100013191.hg.1 | POLD4 | polymerase (DNA-directed), delta 4, accessory subunit | Multiple_C | 9.35 | 9.11 | 8.56 | 1.73 | 0.019 | 0.0686 | 6.65 | 6.81 | 6.26 | 1.31 | 0.2294 | 0.4906 |
| TC1200009926.hg.1 | TAS2R50 | taste receptor, type 2, member 50 | Coding | 4.43 | 4.77 | 3.64 | 1.73 | 0.0042 | 0.0211 | 3.7 | 3.85 | 3.65 | 1.04 | 0.9391 | 0.9749 |
| TC1200010221.hg.1 | MANSC4 | MANSC domain containing 4 | Coding | 4.03 | 3.25 | 3.24 | 1.73 | 0.036 | 0.1135 | 3.49 | 3.54 | 4.06 | 0.67 | 0.1898 | 0.4424 |
| TC1200011971.hg.1 | RPL6 | ribosomal protein L6 | Multiple_C | 16.43 | 16.3 | 15.64 | 1.73 | 0.0167 | 0.0621 | 14.11 | 13.99 | 13.84 | 1.21 | 0.1189 | 0.341 |
| TC1200012725.hg.1 | FAM101A | family with sequence similarity 101, member A | Multiple_C | 5.93 | 5 | 5.14 | 1.73 | 0.0013 | 0.0084 | 4.61 | 4.71 | 4.75 | 0.91 | 0.6541 | 0.8315 |
| TC1300008371.hg.1 | CENPJ | centromere protein J | Multiple_C | 8.57 | 8.16 | 7.78 | 1.73 | 0.2706 | 0.4791 | 8.03 | 8.05 | 7.6 | 1.35 | 0.6562 | 0.8321 |
| TC1400009122.hg.1 | VCPKMT | valosin containing protein lysine (K) methyltransferase | Multiple_C | 10.15 | 10 | 9.36 | 1.73 | 0.0034 | 0.0177 | 8.23 | 6.87 | 7.7 | 1.44 | 0.1248 | 0.3491 |
| TC1500010655.hg.1 | CHSY1 | chondroitin sulfate synthase 1 | Multiple_C | 10.52 | 9.39 | 9.73 | 1.73 | 0.0042 | 0.0209 | 7.32 | 7.29 | 8.22 | 0.54 | 0.0003 | 0.0081 |
| TC1600010467.hg.1 | CNGB1 | cyclic nucleotide gated channel beta 1 | Multiple_C | 6.17 | 5.5 | 5.38 | 1.73 | 0.0211 | 0.0746 | 5.61 | 5.52 | 5.34 | 1.21 | 0.4909 | 0.726 |
| TC1700009260.hg.1 | FOXK2 | forkhead box K2 | Multiple_C | 12.62 | 11.64 | 11.83 | 1.73 | 0.0026 | 0.0145 | 13 | 12.40 | 13.2 | 0.87 | 0.4493 | 0.6958 |
| TC1700011066.hg.1 | TAC4 | tachykinin 4 (hemokinin) | Multiple_C | 5.78 | 4.86 | 4.99 | 1.73 | 0.0029 | 0.0156 | 5.25 | 5.32 | 5.21 | 1.03 | 0.7872 | 0.9049 |
| TC1700011363.hg.1 | NACA2 | nascent polypeptide-associated complex alpha subunit 2 | Coding | 12.25 | 13.35 | 11.46 | 1.73 | 0.0375 | 0.1168 | 9.86 | 9.01 | 8.82 | 2.06 | 0.0393 | 0.1808 |

| | | | | | | | | | | | | | | | |
|---|---|---|---|---|---|---|---|---|---|---|---|---|---|---|---|
| TC1900010433.hg.1 | SCGB2B2 | secretoglobin, family 2B, member 2 | Multiple_C | 4.32 | 3.61 | 3.53 | 1.73 | 0.1458 | 0.3159 | 4.34 | 4.38 | 4.3 | 1.03 | 0.8025 | 0.9118 |
| TC1900012025.hg.1 | ZNF28 | zinc finger protein 28 | Multiple_C | 11.12 | 10.24 | 10.33 | 1.73 | 0.0248 | 0.0847 | 9.63 | 8.96 | 9.42 | 1.16 | 0.6383 | 0.8228 |
| TC2000007012.hg.1 | ENTPD6 | ectonucleoside triphosphate diphosphohydrolase 6 (putative) | Multiple_C | 9.72 | 9.2 | 8.93 | 1.73 | 0.0085 | 0.0366 | 8.26 | 8.28 | 8.59 | 0.80 | 0.0355 | 0.1708 |
| TC2000007231.hg.1 | PHF20 | PHD finger protein 20 | Multiple_C | 10.22 | 10.13 | 9.43 | 1.73 | 0.0234 | 0.0811 | 10.72 | 9.59 | 10.24 | 1.39 | 0.1478 | 0.3841 |
| TC2000008052.hg.1 | BIRC7 | baculoviral IAP repeat containing 7 | Coding | 3.94 | 2.98 | 3.15 | 1.73 | 0.0089 | 0.038 | 3.25 | 3.68 | 3.76 | 0.70 | 0.0936 | 0.297 |
| TC2000009204.hg.1 | TOMM34 | translocase of outer mitochondrial membrane 34 | Multiple_C | 13.25 | 11.35 | 12.46 | 1.73 | 0.0027 | 0.0148 | 14.06 | 13.65 | 13.83 | 1.17 | 0.697 | 0.8563 |
| TC2200009233.hg.1 | ADORA2A | adenosine A2a receptor | Multiple_C | 8.32 | 7.27 | 7.53 | 1.73 | 0.0209 | 0.0743 | 6.92 | 7.21 | 7.35 | 0.74 | 0.0689 | 0.2499 |
| TC0100007315.hg.1 | ZNF436-AS1 | ZNF436 antisense RNA 1 | Multiple_C | 5.63 | 5.04 | 4.85 | 1.72 | 0.0371 | 0.1161 | 4.52 | 4.49 | 4.57 | 0.97 | 0.7449 | 0.8832 |
| TC0100010798.hg.1 | QSOX1 | quiescin Q6 sulfhydryl oxidase 1 | Multiple_C | 11.07 | 10.03 | 10.29 | 1.72 | 0.0205 | 0.0729 | 9.34 | 9.56 | 9.74 | 0.76 | 0.1972 | 0.452 |
| TC0100015017.hg.1 | RPL7 | ribosomal protein L7 | Multiple_C | 14.45 | 15.94 | 13.67 | 1.72 | 0.0472 | 0.1387 | 11.11 | 10.44 | 10.64 | 1.39 | 0.0524 | 0.2137 |
| TC0200010273.hg.1 | NAB1 | NGFI-A binding protein 1 | Multiple_C | 12.68 | 12.94 | 11.9 | 1.72 | 0.0043 | 0.0214 | 10.23 | 9.92 | 9.6 | 1.55 | 0.0098 | 0.0767 |
| TC0200014091.hg.1 | CLASP1 | cytoplasmic linker associated protein 1 | Multiple_C | 10.06 | 8.54 | 9.28 | 1.72 | 0.0134 | 0.0525 | 9.99 | 9.27 | 10.18 | 0.88 | 0.4678 | 0.7089 |
| TC0200014893.hg.1 | TLK1 | tousled-like kinase 1 | Multiple_C | 9.22 | 8.32 | 8.44 | 1.72 | 0.0117 | 0.0471 | 8.54 | 8.67 | 9.37 | 0.56 | 0.0078 | 0.0659 |
| TC0300007485.hg.1 | SMIM4 | small integral membrane protein 4 | Multiple_C | 7.14 | 7.51 | 6.36 | 1.72 | 0.502 | 0.7002 | 5.55 | 6.22 | 6.48 | 0.52 | 0.0019 | 0.0258 |
| TC0300009696.hg.1 | VWA5B2 | von Willebrand factor A domain containing 5B2 | Multiple_C | 5.03 | 4.34 | 4.25 | 1.72 | 0.0282 | 0.0937 | 5.29 | 4.96 | 5 | 1.22 | 0.3639 | 0.6265 |
| TC0300012170.hg.1 | GOLGB1 | golgin B1 | Multiple_C | 9.3 | 9.52 | 8.52 | 1.72 | 0.0497 | 0.1438 | 8.48 | 7.92 | 7.81 | 1.59 | 0.1531 | 0.3922 |
| TC0400006731.hg.1 | LOC389199; A | uncharacterized LOC389199; Transcript Identified by AceView, E | Multiple_C | 5.33 | 4.38 | 4.55 | 1.72 | 0.0241 | 0.0829 | 4.61 | 4.95 | 4.55 | 1.04 | 0.7505 | 0.8865 |
| TC0400006798.hg.1 | USP17L17 | ubiquitin specific peptidase 17-like family member 17 | Coding | 4.29 | 3.45 | 3.51 | 1.72 | 0.0358 | 0.1131 | 4.25 | 4.36 | 4.41 | 0.90 | 0.6754 | 0.8433 |
| TC0400009352.hg.1 | CEP44 | centrosomal protein 44kDa | Multiple_C | 7.01 | 6.85 | 6.23 | 1.72 | 0.2583 | 0.4648 | 7.49 | 7.11 | 7.59 | 0.93 | 0.4936 | 0.728 |
| TC0500007808.hg.1 | POLK | polymerase (DNA directed) kappa | Multiple_C | 11.44 | 11.42 | 10.66 | 1.72 | 0.0074 | 0.033 | 12.13 | 10.75 | 11.05 | 2.11 | 9.65E-05 | 0.0035 |
| TC0500008702.hg.1 | CAMLG | calcium modulating ligand | Multiple_C | 12.9 | 14.41 | 12.12 | 1.72 | 0.026 | 0.0879 | 9.02 | 8.74 | 8.62 | 1.32 | 0.1029 | 0.3143 |
| TC0600008255.hg.1 | EFHC1 | EF-hand domain (C-terminal) containing 1 | Multiple_C | 7.31 | 7.3 | 6.53 | 1.72 | 0.0182 | 0.0665 | 8.12 | 7.42 | 6.92 | 2.30 | 6.02E-05 | 0.0025 |
| TC0600009076.hg.1 | ARMC2 | armadillo repeat containing 2 | Multiple_C | 5.16 | 4.03 | 4.38 | 1.72 | 0.18 | 0.364 | 6.09 | 6.42 | 6.25 | 0.90 | 0.5143 | 0.7435 |
| TC0600012199.hg.1 | EYS | eyes shut homolog (Drosophila) | Multiple_C | 5.02 | 3.98 | 4.24 | 1.72 | 0.0323 | 0.1043 | 4.3 | 4.12 | 4.3 | 1.00 | 0.5749 | 0.782 |
| TC0700009065.hg.1 | CCDC136 | coiled-coil domain containing 136 | Multiple_C | 4.8 | 4.23 | 4.02 | 1.72 | 0.1501 | 0.3221 | 6.94 | 6.30 | 5.97 | 1.96 | 0.0073 | 0.0631 |
| TC0700009099.hg.1 | NRF1 | nuclear respiratory factor 1 | Multiple_C | 9.05 | 8.18 | 8.27 | 1.72 | 0.0261 | 0.0882 | 8.11 | 8.76 | 9.13 | 0.49 | 0.0002 | 0.0054 |
| TC0700011476.hg.1 | NSUN5P2; NSI | NOP2/Sun domain family, member 5 pseudogene 2; NOP2/Sun | Multiple_C | 8.75 | 8.05 | 7.97 | 1.72 | 0.0125 | 0.0499 | 9.08 | 9.23 | 9.01 | 1.05 | 0.6874 | 0.8502 |
| TC0700011929.hg.1 | SMURF1 | SMAD specific E3 ubiquitin protein ligase 1 | Multiple_C | 11.75 | 11.16 | 10.97 | 1.72 | 0.0074 | 0.0331 | 10.04 | 10.16 | 10.75 | 0.61 | 0.0017 | 0.0239 |
| TC0800007316.hg.1 | EIF4EBP1 | eukaryotic translation initiation factor 4E binding protein 1 | Multiple_C | 13.17 | 12.77 | 12.39 | 1.72 | 0.0776 | 0.2001 | 10.21 | 10.09 | 10.41 | 0.87 | 0.1659 | 0.4107 |
| TC0800010103.hg.1 | LSM12 | Homo sapiens LSM12 homolog (S. cerevisiae), mRNA (cDNA clo | Multiple_C | 13.73 | 12.39 | 12.95 | 1.72 | 0.0081 | 0.0352 | 13.21 | 12.47 | 13.43 | 0.86 | 0.3339 | 0.5981 |
| TC0800012316.hg.1 | XKR9 | X-linked Kx blood group related 9 | Coding | 5.2 | 5.9 | 4.42 | 1.72 | 0.0121 | 0.0487 | 3.93 | 3.42 | 3.58 | 1.27 | 0.1414 | 0.3739 |
| TC0900012002.hg.1 | LCN8 | lipocalin 8 | Multiple_C | 4.96 | 4.2 | 4.18 | 1.72 | 0.0233 | 0.0807 | 4.74 | 4.31 | 4 | 1.67 | 0.0019 | 0.026 |
| TC0X00007194.hg.1 | TBC1D25 | TBC1 domain family, member 25 | Multiple_C | 8.98 | 8.55 | 8.2 | 1.72 | 0.1025 | 0.2452 | 8.88 | 9.04 | 9.19 | 0.81 | 0.1841 | 0.4351 |

| | | | | | | | | | | | | | | | |
|---|---|---|---|---|---|---|---|---|---|---|---|---|---|---|---|
| TC0X00008405.hg.1 | RBMX2 | RNA binding motif protein, X-linked 2 | Multiple_C | 12.07 | 12.14 | 11.29 | 1.72 | 0.3769 | 0.5899 | 9.89 | 10.84 | 11.48 | 0.33 | 0.0042 | 0.0439 |
| TC0X00009025.hg.1 | ANOS1 | anosmin 1 | Multiple_C | 4.49 | 3.81 | 3.71 | 1.72 | 0.0093 | 0.0392 | 3.84 | 4.01 | 4 | 0.90 | 0.8419 | 0.9311 |
| TC0X00009101.hg.1 | GPM6B | glycoprotein M6B | Multiple_C | 5.57 | 5.24 | 4.79 | 1.72 | 0.0629 | 0.1713 | 6.07 | 5.52 | 5.84 | 1.17 | 0.3128 | 0.577 |
| TC0X00009733.hg.1 | XAGE3 | X antigen family, member 3 | Coding | 5.89 | 5.08 | 5.11 | 1.72 | 0.0661 | 0.1781 | 5.22 | 5.14 | 4.91 | 1.24 | 0.119 | 0.341 |
| TC1100006491.hg.1 | RPLP2; SNORA | ribosomal protein, large, P2; small nucleolar RNA, H/ACA box 52 | Multiple_C | 17.03 | 15.47 | 16.25 | 1.72 | 0.015 | 0.0571 | 11.98 | 11.59 | 11.73 | 1.19 | 0.5874 | 0.7912 |
| TC1100006539.hg.1 | TNNI2 | troponin I type 2 (skeletal, fast) | Multiple_C | 4.94 | 4.09 | 4.16 | 1.72 | 0.0436 | 0.1309 | 4.71 | 4.84 | 4.52 | 1.14 | 0.4329 | 0.6841 |
| TC1100007482.hg.1 | SLC39A13 | solute carrier family 39 (zinc transporter), member 13 | Multiple_C | 8.36 | 7.52 | 7.58 | 1.72 | 0.0195 | 0.0702 | 6.67 | 6.55 | 6.25 | 1.34 | 0.5591 | 0.7714 |
| TC1100012452.hg.1 | DSCAML1 | Down syndrome cell adhesion molecule like 1 | Coding | 4.69 | 3.76 | 3.91 | 1.72 | 0.033 | 0.106 | 3.62 | 3.76 | 3.62 | 1.00 | 0.2251 | 0.486 |
| TC1200007823.hg.1 | RPS26 | ribosomal protein S26 | Multiple_C | 17.58 | 16.06 | 16.8 | 1.72 | 0.0865 | 0.2163 | 14.46 | 14.43 | 14.25 | 1.16 | 0.9043 | 0.9607 |
| TC1200012629.hg.1 | DAZAP2 | DAZ associated protein 2 | Multiple_C | 14.94 | 13.79 | 14.16 | 1.72 | 0.0077 | 0.0341 | 15.68 | 15.46 | 15.86 | 0.88 | 0.1251 | 0.3497 |
| TC1200012706.hg.1 | ACAD10 | acyl-CoA dehydrogenase family, member 10 | Multiple_C | 8.22 | 7.91 | 7.44 | 1.72 | 0.0832 | 0.2104 | 6.2 | 6.83 | 6.12 | 1.06 | 0.9127 | 0.9642 |
| TC1400009697.hg.1 | NPC2; MIR470 | Niemann-Pick disease, type C2; microRNA 4709 | Multiple_C | 12.02 | 10.89 | 11.24 | 1.72 | 0.0754 | 0.1957 | 6.08 | 6.25 | 5.99 | 1.06 | 0.8766 | 0.9471 |
| TC1500007365.hg.1 | LIPC | lipase, hepatic | Multiple_C | 4.1 | 3.03 | 3.32 | 1.72 | 0.1365 | 0.3014 | 7.38 | 9.01 | 6.91 | 1.39 | 0.5156 | 0.7446 |
| TC1600010227.hg.1 | N4BP1 | NEDD4 binding protein 1 | Multiple_C | 11.62 | 9.83 | 10.84 | 1.72 | 0.0069 | 0.0311 | 11.77 | 11.81 | 11.73 | 1.03 | 0.7569 | 0.8897 |
| TC1700008418.hg.1 | DYNLL2 | dynein, light chain, LC8-type 2 | Multiple_C | 11.41 | 10.85 | 10.63 | 1.72 | 0.2137 | 0.4089 | 11.2 | 11.52 | 11.85 | 0.64 | 0.0012 | 0.0189 |
| TC1700010562.hg.1 | ARL5C | ADP-ribosylation factor like GTPase 5C | Multiple_C | 4.4 | 3.28 | 3.62 | 1.72 | 0.1047 | 0.2493 | 4.15 | 4.06 | 3.96 | 1.14 | 0.3913 | 0.6499 |
| TC1700012476.hg.1 | CYGB | cytoglobin | Multiple_C | 4.97 | 3.99 | 4.19 | 1.72 | 0.0213 | 0.0752 | 4.53 | 4.31 | 4.18 | 1.27 | 0.0208 | 0.1233 |
| TC1900008287.hg.1 | PVRL2 | poliovirus receptor-related 2 (herpesvirus entry mediator B) | Multiple_C | 11.47 | 9.73 | 10.69 | 1.72 | 0.0488 | 0.1421 | 8.99 | 9.34 | 9.59 | 0.66 | 0.0448 | 0.1958 |
| TC1900010480.hg.1 | SBSN | suprabasin | Coding | 4.41 | 3.43 | 3.63 | 1.72 | 0.0027 | 0.0149 | 3.67 | 3.74 | 3.43 | 1.18 | 0.3671 | 0.6295 |
| TC1900010715.hg.1 | NUMBL | numb homolog (Drosophila)-like | Multiple_C | 7.98 | 7.35 | 7.2 | 1.72 | 0.0928 | 0.2282 | 7.1 | 7.15 | 7.06 | 1.03 | 0.4305 | 0.6823 |
| TC2000008678.hg.1 | CST3 | cystatin C | Multiple_C | 11.05 | 8.93 | 10.27 | 1.72 | 0.0255 | 0.0867 | 8.03 | 9.44 | 9.05 | 0.49 | 0.1906 | 0.4437 |
| TC2000008684.hg.1 | CST2 | cystatin SA | Coding | 4.85 | 4 | 4.07 | 1.72 | 0.0742 | 0.1933 | 4.79 | 5.01 | 4.84 | 0.97 | 0.9505 | 0.9797 |
| TC2100006967.hg.1 | IFNGR2 | interferon gamma receptor 2 (interferon gamma transducer 1) | Multiple_C | 13.27 | 13.36 | 12.49 | 1.72 | 0.003 | 0.016 | 12.76 | 12.24 | 12.7 | 1.04 | 0.4365 | 0.6871 |
| TSUnmapped00000291.h | VPS11 | VPS11, CORVET/HOPS core subunit [Source:HGNC Symbol;Acc:H | Coding | 8.44 | 7.14 | 7.66 | 1.72 | 0.003 | 0.0159 | 7.5 | 7.57 | 7.73 | 0.85 | 0.5765 | 0.783 |
| TC0100006937.hg.1 | PRDM2 | PR domain containing 2, with ZNF domain | Multiple_C | 9.62 | 8.8 | 8.85 | 1.71 | 0.025 | 0.0853 | 10.35 | 9.77 | 9.99 | 1.28 | 0.0694 | 0.2513 |
| TC0100010155.hg.1 | SEMA4A | sema domain, immunoglobulin domain (Ig), transmembrane do | Multiple_C | 5.32 | 4.8 | 4.55 | 1.71 | 0.0432 | 0.1301 | 4.94 | 4.83 | 4.96 | 0.99 | 0.3898 | 0.6485 |
| TC0100010586.hg.1 | FMO4 | flavin containing monooxygenase 4 | Multiple_C | 4.25 | 3.34 | 3.48 | 1.71 | 0.0423 | 0.1282 | 4.51 | 4.16 | 3.97 | 1.45 | 0.0629 | 0.2387 |
| TC0100013445.hg.1 | IFI6 | interferon, alpha-inducible protein 6 | Multiple_C | 11.98 | 8.41 | 11.21 | 1.71 | 0.0458 | 0.1356 | 7.59 | 7.64 | 6.95 | 1.56 | 0.0123 | 0.0885 |
| TC0100014988.hg.1 | F3 | coagulation factor III (thromboplastin, tissue factor) | Multiple_C | 12.19 | 10.91 | 11.42 | 1.71 | 0.0571 | 0.1594 | 5.32 | 5.13 | 5.47 | 0.90 | 0.3174 | 0.5815 |
| TC0100016205.hg.1 | C1orf111 | chromosome 1 open reading frame 111 | Multiple_C | 5.26 | 4.49 | 4.49 | 1.71 | 0.0236 | 0.0816 | 4.39 | 4.57 | 4.25 | 1.10 | 0.3762 | 0.6365 |
| TC0100017491.hg.1 | ENAH | enabled homolog (Drosophila) | Multiple_C | 13.8 | 12.8 | 13.03 | 1.71 | 0.0182 | 0.0665 | 12.64 | 12.59 | 12.87 | 0.85 | 0.5586 | 0.7714 |
| TC0300007466.hg.1 | ALAS1 | 5-aminolevulinate synthase 1 | Multiple_C | 11.2 | 10.08 | 10.43 | 1.71 | 0.0046 | 0.0227 | 9.96 | 9.31 | 9.33 | 1.55 | 0.0039 | 0.0422 |
| TC0300014052.hg.1 | RYK | receptor-like tyrosine kinase | Multiple_C | 13.14 | 13.4 | 12.37 | 1.71 | 0.0009 | 0.0063 | 11.98 | 11.39 | 11.59 | 1.31 | 0.0088 | 0.0719 |

| | | | | | | | | | | | | | | | |
|---|---|---|---|---|---|---|---|---|---|---|---|---|---|---|---|
| TC0400008237.hg.1 | MTTP | microsomal triglyceride transfer protein | Coding | 6.61 | 6.08 | 5.84 | 1.71 | 0.0008 | 0.0054 | 5.01 | 4.86 | 4.91 | 1.07 | 0.6008 | 0.7998 |
| TC0400011466.hg.1 | UBE2D3 | ubiquitin conjugating enzyme E2D 3 | Multiple_C | 15.88 | 15.47 | 15.11 | 1.71 | 0.0004 | 0.0034 | 14.15 | 14.11 | 14.51 | 0.78 | 0.1173 | 0.3387 |
| TC0500007911.hg.1 | SPZ1 | spermatogenic leucine zipper 1 | Coding | 4.56 | 3.61 | 3.79 | 1.71 | 0.0672 | 0.18 | 4.82 | 5.11 | 4.4 | 1.34 | 0.604 | 0.8019 |
| TC0500009598.hg.1 | EIF4E1B | eukaryotic translation initiation factor 4E family member 1B | Multiple_C | 6.77 | 5.62 | 6 | 1.71 | 0.0117 | 0.0474 | 5.71 | 6.01 | 5.77 | 0.96 | 0.817 | 0.9189 |
| TC0600013464.hg.1 | SHPRH | SNF2 histone linker PHD RING helicase, E3 ubiquitin protein liga | Multiple_C | 8.22 | 7.86 | 7.45 | 1.71 | 0.2634 | 0.4706 | 8.66 | 8.39 | 8.77 | 0.93 | 0.3078 | 0.5717 |
| TC0700006649.hg.1 | ZDHHC4 | zinc finger, DHHC-type containing 4 | Multiple_C | 11.87 | 11.38 | 11.1 | 1.71 | 0.0115 | 0.0465 | 10.32 | 10.79 | 10.95 | 0.65 | 0.022 | 0.1273 |
| TC0700011976.hg.1 | C7orf43; MIR4 | chromosome 7 open reading frame 43; microRNA 4658 | Multiple_C | 6.54 | 5.47 | 5.77 | 1.71 | 0.0512 | 0.147 | 5.64 | 5.71 | 5.55 | 1.06 | 0.086 | 0.2827 |
| TC0700013584.hg.1 | CYP51A1 | cytochrome P450, family 51, subfamily A, polypeptide 1 | Multiple_C | 13.97 | 14.2 | 13.2 | 1.71 | 0.0321 | 0.1038 | 10.77 | 10.06 | 11.83 | 0.48 | 7.46E-05 | 0.0029 |
| TC0900011013.hg.1 | ERP44 | endoplasmic reticulum protein 44 | Multiple_C | 12.43 | 12.6 | 11.66 | 1.71 | 0.0015 | 0.0092 | 10.57 | 10.07 | 10.33 | 1.18 | 0.1016 | 0.3118 |
| TC0X00007709.hg.1 | PGK1 | phosphoglycerate kinase 1 | Multiple_C | 13.91 | 12.86 | 13.14 | 1.71 | 0.1197 | 0.2743 | 13.3 | 14.93 | 13.85 | 0.68 | 0.0064 | 0.0574 |
| TC1000011287.hg.1 | GRID1 | glutamate receptor, ionotropic, delta 1 | Multiple_C | 4.42 | 3.65 | 3.65 | 1.71 | 0.0406 | 0.1241 | 4.68 | 4.83 | 4.6 | 1.06 | 0.824 | 0.9222 |
| TC1100009611.hg.1 | JAM3 | junctional adhesion molecule 3 | Multiple_C | 4.61 | 3.66 | 3.84 | 1.71 | 0.0294 | 0.0967 | 4.02 | 4.14 | 4.45 | 0.74 | 0.1791 | 0.4281 |
| TC1100010410.hg.1 | BDNF | brain-derived neurotrophic factor | Multiple_C | 4.2 | 3.29 | 3.43 | 1.71 | 0.0565 | 0.1581 | 3.65 | 3.67 | 3.78 | 0.91 | 0.7237 | 0.8712 |
| TC1300006633.hg.1 | PABPC3 | poly(A) binding protein, cytoplasmic 3 | Coding | 11.98 | 11.59 | 11.21 | 1.71 | 0.0133 | 0.0522 | 8.66 | 8.63 | 8.9 | 0.85 | 0.2416 | 0.5056 |
| TC1300009376.hg.1 | SLITRK1 | SLIT and NTRK-like family, member 1 | Coding | 4.56 | 3.74 | 3.79 | 1.71 | 0.0148 | 0.0564 | 3.86 | 4.04 | 3.95 | 0.94 | 0.8264 | 0.9234 |
| TC1500007901.hg.1 | SNX33 | sorting nexin 33 | Multiple_C | 6.35 | 5.85 | 5.58 | 1.71 | 0.0793 | 0.2037 | 6.59 | 7.00 | 6.24 | 1.27 | 0.8202 | 0.9206 |
| TC1500010049.hg.1 | FAM219B | family with sequence similarity 219, member B | Multiple_C | 6.48 | 5.9 | 5.71 | 1.71 | 0.2456 | 0.4492 | 4.29 | 4.66 | 4.86 | 0.67 | 0.2316 | 0.4928 |
| TC1500010838.hg.1 | GOLGA8M | golgin A8 family, member M | Multiple_C | 4.44 | 3.95 | 3.67 | 1.71 | 0.0035 | 0.0181 | 5.6 | 5.37 | 5.07 | 1.44 | 0.0529 | 0.215 |
| TC1600009412.hg.1 | TXNDC11 | thioredoxin domain containing 11 | Multiple_C | 9.71 | 8.43 | 8.94 | 1.71 | 0.0667 | 0.1793 | 9.95 | 9.74 | 10.27 | 0.80 | 0.0629 | 0.2387 |
| TC1600011430.hg.1 | CNTNAP4 | contactin associated protein-like 4 | Multiple_C | 3.63 | 2.77 | 2.86 | 1.71 | 0.0284 | 0.0941 | 4 | 3.62 | 3.52 | 1.39 | 0.0041 | 0.0436 |
| TC1700012215.hg.1 | KSR1 | kinase suppressor of ras 1 | Multiple_C | 6.9 | 6.02 | 6.13 | 1.71 | 0.0166 | 0.062 | 6.1 | 5.94 | 6.11 | 0.99 | 0.6118 | 0.806 |
| TC1900006578.hg.1 | SF3A2 | splicing factor 3a subunit 2 | Multiple_C | 14.05 | 12.61 | 13.28 | 1.71 | 0.0664 | 0.1787 | 13.49 | 14.32 | 14.21 | 0.61 | 0.1733 | 0.4206 |
| TC1900007871.hg.1 | COX6B1 | cytochrome c oxidase subunit VIb polypeptide 1 (ubiquitous) | Multiple_C | 14.62 | 13.77 | 13.85 | 1.71 | 0.0669 | 0.1795 | 10.26 | 10.98 | 10.21 | 1.04 | 0.4186 | 0.6724 |
| TC1900011767.hg.1 | BLOC1S3 | biogenesis of lysosomal organelles complex-1, subunit 3 | Multiple_C | 6.94 | 5.91 | 6.17 | 1.71 | 0.0254 | 0.0864 | 7.07 | 7.12 | 7.72 | 0.64 | 0.0018 | 0.0247 |
| TC1900011873.hg.1 | ZNF433 | zinc finger protein 433 | Coding | 5.93 | 5.44 | 5.16 | 1.71 | 0.1747 | 0.3566 | 3.82 | 4.18 | 3.96 | 0.91 | 0.7412 | 0.881 |
| TC1900011890.hg.1 | WDR83OS | WD repeat domain 83 opposite strand | Multiple_C | 10.13 | 9.05 | 9.36 | 1.71 | 0.0337 | 0.1078 | 8.22 | 8.72 | 8.43 | 0.86 | 0.8884 | 0.9524 |
| TC1900011936.hg.1 | ZNF781 | zinc finger protein 781 | Multiple_C | 4.91 | 4.59 | 4.14 | 1.71 | 0.005 | 0.0241 | 4.52 | 4.75 | 4.78 | 0.84 | 0.5267 | 0.7511 |
| TC2200006883.hg.1 | SUSD2 | sushi domain containing 2 | Multiple_C | 4.89 | 4.21 | 4.12 | 1.71 | 0.1272 | 0.2865 | 4.51 | 4.19 | 3.94 | 1.48 | 0.1628 | 0.4062 |
| TC2200009259.hg.1 | SMTN | smoothelin | Multiple_C | 11.04 | 9.77 | 10.27 | 1.71 | 0.0064 | 0.0292 | 10.86 | 11.16 | 11.3 | 0.74 | 0.0225 | 0.1292 |
| TC0100010244.hg.1 | IFI16 | interferon, gamma-inducible protein 16 | Multiple_C | 4.75 | 3.9 | 3.99 | 1.69 | 0.0111 | 0.0455 | 3.83 | 3.65 | 3.56 | 1.21 | 0.4195 | 0.673 |
| TC0100010924.hg.1 | C1orf27 | chromosome 1 open reading frame 27 | Multiple_C | 10.19 | 10.52 | 9.43 | 1.69 | 0.0088 | 0.0377 | 8.68 | 8.08 | 8.33 | 1.27 | 0.0284 | 0.1485 |
| TC0100013993.hg.1 | PTCH2 | patched 2 | Multiple_C | 6.55 | 5.49 | 5.79 | 1.69 | 0.0436 | 0.131 | 5.5 | 5.34 | 5.32 | 1.13 | 0.291 | 0.5557 |
| TC0100015866.hg.1 | S100A4 | S100 calcium binding protein A4 | Multiple_C | 9.12 | 9.33 | 8.36 | 1.69 | 0.0628 | 0.1711 | 12.14 | 12.08 | 11.28 | 1.82 | 0.0049 | 0.0485 |

| | | | | | | | | | | | | | | | |
|---|---|---|---|---|---|---|---|---|---|---|---|---|---|---|---|
| TC0100016065.hg.1 | CD1B | CD1b molecule | Coding | 3.66 | 3.04 | 2.9 | 1.69 | 0.0237 | 0.0817 | 3.56 | 3.40 | 3.5 | 1.04 | 0.1603 | 0.4027 |
| TC0200010545.hg.1 | NRP2 | neuropilin 2 | Multiple_C | 8.66 | 7.81 | 7.9 | 1.69 | 0.0435 | 0.1308 | 10.68 | 7.61 | 9.21 | 2.77 | 0.0054 | 0.0514 |
| TC0200016403.hg.1 | LPIN1; MIR54 | lipin 1; microRNA 548s | Multiple_C | 11.45 | 11.05 | 10.69 | 1.69 | 0.0171 | 0.0633 | 6.63 | 5.73 | 5.98 | 1.57 | 0.119 | 0.341 |
| TC0300012189.hg.1 | KPNA1 | karyopherin alpha 1 | Multiple_C | 10.16 | 9.56 | 9.4 | 1.69 | 0.0158 | 0.0596 | 9.01 | 8.68 | 9.36 | 0.78 | 0.3476 | 0.6113 |
| TC0300014077.hg.1 | WDR49 | WD repeat domain 49 | Multiple_C | 4.88 | 4.17 | 4.12 | 1.69 | 0.0062 | 0.0284 | 4.36 | 4.40 | 4.28 | 1.06 | 0.0576 | 0.2261 |
| TC0400006789.hg.1 | USP17L13 | ubiquitin specific peptidase 17-like family member 13 | Coding | 3.94 | 3.26 | 3.18 | 1.69 | 0.0272 | 0.091 | 4.17 | 3.92 | 4.18 | 0.99 | 0.5196 | 0.7469 |
| TC0400007789.hg.1 | RUFY3 | RUN and FYVE domain containing 3 | Multiple_C | 7.4 | 8.84 | 6.64 | 1.69 | 0.0512 | 0.1471 | 4.12 | 4.97 | 5.79 | 0.31 | 0.0001 | 0.0047 |
| TC0400007853.hg.1 | EREG | epiregulin | Multiple_C | 13.04 | 12.2 | 12.28 | 1.69 | 0.1534 | 0.3271 | 8.68 | 5.60 | 6.09 | 6.02 | 0.0002 | 0.0062 |
| TC0500008483.hg.1 | SNX24 | sorting nexin 24 | Multiple_C | 10.01 | 9.8 | 9.25 | 1.69 | 0.0605 | 0.1667 | 7.48 | 6.79 | 6.48 | 2.00 | 0.0021 | 0.028 |
| TC0500008975.hg.1 | HMHB1 | histocompatibility (minor) HB-1 | Multiple_C | 7.37 | 6.51 | 6.61 | 1.69 | 0.0034 | 0.0179 | 6.18 | 6.42 | 6.27 | 0.94 | 0.9909 | 0.9963 |
| TC0500013215.hg.1 | LVRN | laeverin | Multiple_C | 5.61 | 4.68 | 4.85 | 1.69 | 0.0155 | 0.0584 | 4.77 | 4.89 | 4.78 | 0.99 | 0.7834 | 0.9026 |
| TC0600007203.hg.1 | ACOT13 | acyl-CoA thioesterase 13 | Multiple_C | 11.32 | 11.96 | 10.56 | 1.69 | 0.0867 | 0.2168 | 8.44 | 7.77 | 7.68 | 1.69 | 0.0409 | 0.1856 |
| TC0600009238.hg.1 | RSPH4A | radial spoke head 4 homolog A (Chlamydomonas) | Coding | 4.15 | 3.22 | 3.39 | 1.69 | 0.005 | 0.0242 | 3.54 | 3.74 | 3.62 | 0.95 | 0.5184 | 0.7463 |
| TC0600010085.hg.1 | PLG | plasminogen | Multiple_C | 4.52 | 3.65 | 3.76 | 1.69 | 0.0084 | 0.0362 | 4.95 | 4.57 | 4.63 | 1.25 | 0.2405 | 0.504 |
| TC0700007285.hg.1 | CDK13 | cyclin-dependent kinase 13 | Multiple_C | 11.42 | 10.08 | 10.66 | 1.69 | 0.0664 | 0.1786 | 11.65 | 11.24 | 11.67 | 0.99 | 0.5267 | 0.7511 |
| TC0700008011.hg.1 | NCF1 | neutrophil cytosolic factor 1 | Multiple_C | 4.55 | 4.14 | 3.79 | 1.69 | 0.0105 | 0.0432 | 4.53 | 4.49 | 4.36 | 1.13 | 0.4464 | 0.6936 |
| TC0700013530.hg.1 | HOXA4 | homeobox A4 | Coding | 5.34 | 4.76 | 4.58 | 1.69 | 0.1049 | 0.2495 | 5.7 | 5.15 | 5.08 | 1.54 | 0.0329 | 0.1627 |
| TC0800011150.hg.1 | TP53INP1 | tumor protein p53 inducible nuclear protein 1 | Multiple_C | 5.73 | 5.73 | 4.97 | 1.69 | 0.0021 | 0.012 | 5.82 | 5.05 | 4.62 | 2.30 | 0.0003 | 0.0066 |
| TC0900009914.hg.1 | CCL27 | chemokine (C-C motif) ligand 27 | Multiple_C | 6.81 | 6.34 | 6.05 | 1.69 | 0.0126 | 0.0502 | 5.84 | 6.04 | 5.92 | 0.95 | 0.9492 | 0.9793 |
| TC0900012202.hg.1 | KIAA2026 | KIAA2026 | Multiple_C | 8.27 | 7.51 | 7.51 | 1.69 | 0.069 | 0.1833 | 8.58 | 7.89 | 7.75 | 1.78 | 0.0417 | 0.188 |
| TC0M00006437.hg.1 | COX2 | cytochrome c oxidase subunit II | Multiple_C | 17.7 | 16.52 | 16.94 | 1.69 | 0.0803 | 0.2055 | 16.93 | 17.03 | 17.1 | 0.89 | 0.5136 | 0.7431 |
| TC0X00006631.hg.1 | TMSB4X | thymosin beta 4, X-linked | Multiple_C | 16.87 | 17.15 | 16.11 | 1.69 | 0.003 | 0.0159 | 15.98 | 15.95 | 16.32 | 0.79 | 0.1138 | 0.3328 |
| TC0X00008608.hg.1 | SPANXA2 | SPANX family, member A2 | Coding | 6.79 | 5.91 | 6.03 | 1.69 | 0.0048 | 0.0233 | 5.61 | 5.82 | 6.01 | 0.76 | 0.3906 | 0.6493 |
| TC1000010482.hg.1 | C10orf10 | chromosome 10 open reading frame 10 | Multiple_C | 5.68 | 4.72 | 4.92 | 1.69 | 0.0082 | 0.0358 | 6.38 | 5.89 | 6.04 | 1.27 | 0.1785 | 0.4272 |
| TC1000012015.hg.1 | TIAL1 | TIA1 cytotoxic granule-associated RNA binding protein-like 1 | Multiple_C | 11.42 | 11.62 | 10.66 | 1.69 | 0.0371 | 0.1161 | 11.16 | 10.72 | 11.14 | 1.01 | 0.6918 | 0.8529 |
| TC1100009207.hg.1 | KMT2A | lysine (K)-specific methyltransferase 2A | Multiple_C | 9.92 | 8.58 | 9.16 | 1.69 | 0.0668 | 0.1793 | 10.01 | 9.60 | 9.83 | 1.13 | 0.4508 | 0.6963 |
| TC1200012655.hg.1 | HELB | helicase (DNA) B | Multiple_C | 7.4 | 7.05 | 6.64 | 1.69 | 0.0636 | 0.1729 | 7.77 | 7.62 | 7.66 | 1.08 | 0.7926 | 0.9077 |
| TC1300007148.hg.1 | CYSLTR2 | cysteinyl leukotriene receptor 2 | Coding | 3.84 | 2.69 | 3.08 | 1.69 | 0.039 | 0.1204 | 4.27 | 4.21 | 4.47 | 0.87 | 0.4701 | 0.7109 |
| TC1300007218.hg.1 | FAM124A | family with sequence similarity 124 member A | Multiple_C | 6.16 | 5.38 | 5.4 | 1.69 | 0.0123 | 0.049 | 6.1 | 6.06 | 5.89 | 1.16 | 0.1714 | 0.4179 |
| TC1300008696.hg.1 | MRPS31 | mitochondrial ribosomal protein S31 | Multiple_C | 7.95 | 8.33 | 7.19 | 1.69 | 0.0675 | 0.1807 | 5.76 | 5.71 | 5.34 | 1.34 | 0.5211 | 0.7481 |
| TC1300009570.hg.1 | STK24 | serine/threonine kinase 24 | Multiple_C | 9.04 | 8.37 | 8.28 | 1.69 | 0.0082 | 0.0358 | 10.22 | 9.97 | 9.62 | 1.52 | 0.0779 | 0.269 |
| TC1400006943.hg.1 | MIPOL1 | mirror-image polydactyly 1 | Multiple_C | 6.45 | 5.99 | 5.69 | 1.69 | 0.0293 | 0.0963 | 4.09 | 3.69 | 3.95 | 1.10 | 0.1134 | 0.3321 |
| TC1400010085.hg.1 | TCL1A | T-cell leukemia/lymphoma 1A | Multiple_C | 6.48 | 5.57 | 5.72 | 1.69 | 0.0988 | 0.2387 | 5.43 | 5.88 | 5.95 | 0.70 | 0.1087 | 0.3245 |

| | | | | | | | | | | | | | | | |
|---|---|---|---|---|---|---|---|---|---|---|---|---|---|---|---|
| TC1500008139.hg.1 | GOLGA6L4 | Homo sapiens golgin A6 family-like 4 (GOLGA6L4), mRNA.; golgi | Multiple_C | 7.97 | 7.18 | 7.21 | 1.69 | 0.0106 | 0.0437 | 6.71 | 6.44 | 6.32 | 1.31 | 0.1886 | 0.441 |
| TC1500010463.hg.1 | PRC1 | protein regulator of cytokinesis 1 | Multiple_C | 13.38 | 12.74 | 12.62 | 1.69 | 0.1209 | 0.2762 | 12.5 | 12.20 | 13.05 | 0.68 | 0.0289 | 0.1504 |
| TC1500010947.hg.1 | TM2D3 | TM2 domain containing 3 | Multiple_C | 8.69 | 9.29 | 7.93 | 1.69 | 0.0158 | 0.0595 | 7.17 | 7.09 | 7.23 | 0.96 | 0.9175 | 0.9663 |
| TC1600006733.hg.1 | GLIS2 | GLIS family zinc finger 2 | Multiple_C | 3.84 | 2.97 | 3.08 | 1.69 | 0.013 | 0.0514 | 4.17 | 3.94 | 3.92 | 1.19 | 0.3512 | 0.615 |
| TC1600009076.hg.1 | RPL3L | ribosomal protein L3-like | Multiple_C | 8.53 | 7.52 | 7.77 | 1.69 | 0.0207 | 0.0738 | 7.51 | 7.67 | 7.17 | 1.27 | 0.5499 | 0.7662 |
| TC1700007915.hg.1 | WNK4 | WNK lysine deficient protein kinase 4 | Multiple_C | 4.96 | 4.21 | 4.2 | 1.69 | 0.002 | 0.0114 | 3.83 | 4.49 | 4.19 | 0.78 | 0.2669 | 0.5314 |
| TC1700008635.hg.1 | RGS9 | regulator of G-protein signaling 9 | Multiple_C | 4.06 | 3.5 | 3.3 | 1.69 | 0.0254 | 0.0863 | 3.93 | 3.13 | 3.42 | 1.42 | 0.0044 | 0.0456 |
| TC1700009830.hg.1 | CDRT15 | CMT1A duplicated region transcript 15 | Coding | 5.69 | 4.88 | 4.93 | 1.69 | 0.0048 | 0.0231 | 5.73 | 6.00 | 5.7 | 1.02 | 0.9258 | 0.9694 |
| TC1700010891.hg.1 | PLEKHM1; MII | pleckstrin homology domain containing, family M (with RUN do | Multiple_C | 9.1 | 8.23 | 8.34 | 1.69 | 0.0037 | 0.0189 | 7.55 | 7.57 | 7.62 | 0.95 | 0.7948 | 0.9087 |
| TC1900007305.hg.1 | MYO9B | myosin IXB | Multiple_C | 9.5 | 8.34 | 8.74 | 1.69 | 0.1186 | 0.2725 | 10.02 | 10.08 | 10.01 | 1.01 | 0.5703 | 0.7787 |
| TC1900008992.hg.1 | ZNF543 | zinc finger protein 543 | Multiple_C | 8.35 | 7.49 | 7.59 | 1.69 | 0.0657 | 0.1773 | 6.8 | 6.93 | 7.03 | 0.85 | 0.3008 | 0.5652 |
| TC1900011235.hg.1 | KLK12 | kallikrein related peptidase 12 | Coding | 5.78 | 5.43 | 5.02 | 1.69 | 0.0492 | 0.1428 | 4.92 | 4.70 | 4.72 | 1.15 | 0.1278 | 0.3538 |
| TC2000009253.hg.1 | ZNF335 | zinc finger protein 335 | Multiple_C | 6.22 | 5.04 | 5.46 | 1.69 | 0.0826 | 0.2093 | 6.13 | 6.36 | 6.54 | 0.75 | 0.9374 | 0.9741 |
| TC2100007166.hg.1 | SH3BGR | SH3 domain binding glutamate-rich protein | Coding | 5.97 | 5.68 | 5.21 | 1.69 | 0.0105 | 0.0434 | 6.03 | 6.42 | 6.23 | 0.87 | 0.3894 | 0.6481 |
| TC2200008687.hg.1 | TMEM184B | transmembrane protein 184B | Multiple_C | 10 | 8.31 | 9.24 | 1.69 | 0.0619 | 0.1694 | 8.46 | 8.75 | 9.64 | 0.44 | 0.0002 | 0.0061 |
| TC2200009231.hg.1 | MIF | macrophage migration inhibitory factor (glycosylation-inhibiting | Multiple_C | 14.42 | 13.82 | 13.66 | 1.69 | 0.0085 | 0.0367 | 11.5 | 12.05 | 11.09 | 1.33 | 0.0669 | 0.2469 |
| TSUnmapped00000228.h | NDUFA10 | NADH dehydrogenase (ubiquinone) 1 alpha subcomplex, 10, 42l | Coding | 5.9 | 5.16 | 5.14 | 1.69 | 0.0049 | 0.0236 | 5.37 | 5.13 | 4.93 | 1.36 | 0.0649 | 0.2427 |
| TC0100006994.hg.1 | PLEKHM2 | pleckstrin homology domain containing, family M (with RUN do | Multiple_C | 9.03 | 8.1 | 8.28 | 1.68 | 0.0025 | 0.0139 | 8.28 | 8.59 | 8.7 | 0.75 | 0.0507 | 0.2098 |
| TC0100009273.hg.1 | AMY2A | amylase, alpha 2A (pancreatic) | Multiple_C | 5.51 | 5.34 | 4.76 | 1.68 | 0.0056 | 0.0263 | 4.98 | 4.68 | 4.66 | 1.25 | 0.1263 | 0.3515 |
| TC0100010252.hg.1 | FCER1A | Fc fragment of IgE, high affinity I, receptor for; alpha polypeptid | Coding | 3.38 | 2.39 | 2.63 | 1.68 | 0.0564 | 0.1579 | 3.7 | 3.56 | 3.71 | 0.99 | 0.8872 | 0.9518 |
| TC0100014895.hg.1 | ZNF644 | zinc finger protein 644 | Multiple_C | 11.95 | 12.12 | 11.2 | 1.68 | 0.0222 | 0.0777 | 10.37 | 9.81 | 10.17 | 1.15 | 0.2586 | 0.523 |
| TC0100016711.hg.1 | PDC | phosducin | Coding | 3.92 | 3.16 | 3.17 | 1.68 | 0.0696 | 0.1846 | 4.01 | 3.74 | 3.64 | 1.29 | 0.0948 | 0.2992 |
| TC0100016916.hg.1 | PHLDA3 | pleckstrin homology-like domain, family A, member 3 | Multiple_C | 6.06 | 5.23 | 5.31 | 1.68 | 0.059 | 0.1636 | 5.36 | 5.64 | 5.49 | 0.91 | 0.4725 | 0.7127 |
| TC0100018485.hg.1 | ADAMTSL4-AS | ADAMTSL4 antisense RNA 1 | Multiple_C | 5 | 4.14 | 4.25 | 1.68 | 0.0237 | 0.082 | 4.71 | 4.46 | 4.27 | 1.36 | 0.6694 | 0.8384 |
| TC0200008481.hg.1 | TRIM43 | tripartite motif containing 43 | Coding | 3.91 | 2.85 | 3.16 | 1.68 | 0.0142 | 0.0547 | 4.44 | 4.26 | 4.31 | 1.09 | 0.8633 | 0.9414 |
| TC0200008554.hg.1 | COX5B | cytochrome c oxidase subunit Vb | Multiple_C | 11.43 | 11.12 | 10.68 | 1.68 | 0.0685 | 0.1825 | 7.85 | 7.91 | 7.56 | 1.22 | 0.1569 | 0.3978 |
| TC0200010053.hg.1 | HOXD1; MIR7 | homeobox D1; microRNA 7704 | Multiple_C | 7.67 | 6.82 | 6.92 | 1.68 | 0.0219 | 0.077 | 4.35 | 4.49 | 4.61 | 0.84 | 0.2886 | 0.5532 |
| TC0200010216.hg.1 | ITGAV | integrin alpha V | Multiple_C | 10.09 | 9.73 | 9.34 | 1.68 | 0.0071 | 0.0319 | 8.27 | 7.47 | 7.68 | 1.51 | 0.0117 | 0.0862 |
| TC0200011992.hg.1 | DNAJC27 | DnaJ (Hsp40) homolog, subfamily C, member 27 | Multiple_C | 7.04 | 6.11 | 6.29 | 1.68 | 0.0372 | 0.1161 | 7.13 | 6.68 | 6.97 | 1.12 | 0.4953 | 0.7291 |
| TC0200016006.hg.1 | HTR2B | 5-hydroxytryptamine (serotonin) receptor 2B, G protein-couple | Multiple_C | 4.33 | 3.37 | 3.58 | 1.68 | 0.001 | 0.0069 | 4.09 | 3.87 | 3.72 | 1.29 | 0.0786 | 0.2701 |
| TC0200016318.hg.1 | PP14571; AC1 | uncharacterized LOC100130449; novel transcript, antisense to ( | Multiple_C | 5.09 | 4.04 | 4.34 | 1.68 | 0.0337 | 0.1077 | 4.23 | 4.38 | 4.46 | 0.85 | 0.4728 | 0.7128 |
| TC0300006714.hg.1 | EAF1 | ELL associated factor 1 | Multiple_C | 10.69 | 9.76 | 9.94 | 1.68 | 0.0316 | 0.1024 | 9.94 | 9.52 | 10.07 | 0.91 | 0.8504 | 0.9355 |
| TC0300011223.hg.1 | SELK | selenoprotein K; selenoprotein K [Source:EntrezGene;Acc:5851 | Multiple_C | 11.02 | 10.85 | 10.27 | 1.68 | 0.0152 | 0.0577 | 8.98 | 9.15 | 8.76 | 1.16 | 0.3792 | 0.6388 |

| | | | | | | | | | | | | | | | |
|---|---|---|---|---|---|---|---|---|---|---|---|---|---|---|---|
| TC0300013417.hg.1 | LIPH | lipase, member H | Multiple_C | 12.08 | 11.68 | 11.33 | 1.68 | 0.0602 | 0.166 | 6.75 | 6.08 | 5.98 | 1.71 | 0.0267 | 0.1429 |
| TC0300013528.hg.1 | GMNC | geminin coiled-coil domain containing | Multiple_C | 3.68 | 2.71 | 2.93 | 1.68 | 0.0184 | 0.0671 | 3.07 | 3.00 | 3.14 | 0.95 | 0.8384 | 0.929 |
| TC0400011524.hg.1 | TBCK | TBC1 domain containing kinase | Multiple_C | 7.85 | 7.26 | 7.1 | 1.68 | 0.0381 | 0.1181 | 6.73 | 6.53 | 7.02 | 0.82 | 0.0901 | 0.2906 |
| TC0500007378.hg.1 | NDUFS4 | NADH dehydrogenase (ubiquinone) Fe-S protein 4, 18kDa (NAD | Multiple_C | 11.62 | 12.45 | 10.87 | 1.68 | 0.0052 | 0.0249 | 9.21 | 9.21 | 8.56 | 1.57 | 0.0131 | 0.0921 |
| TC0500009009.hg.1 | TCERG1 | transcription elongation regulator 1 | Multiple_C | 12.38 | 12.38 | 11.63 | 1.68 | 0.1241 | 0.2812 | 12.86 | 12.45 | 12.64 | 1.16 | 0.205 | 0.4613 |
| TC0500013332.hg.1 | DMGDH | dimethylglycine dehydrogenase | Multiple_C | 4.13 | 4.46 | 3.38 | 1.68 | 0.0312 | 0.1015 | 3.89 | 3.74 | 3.75 | 1.10 | 0.9808 | 0.9918 |
| TC0600006869.hg.1 | DSP | desmoplakin | Multiple_C | 10.79 | 10.41 | 10.04 | 1.68 | 0.0287 | 0.0949 | 12.83 | 14.03 | 12.86 | 0.98 | 0.8593 | 0.9398 |
| TC0600009851.hg.1 | MTHFD1L | methylenetetrahydrofolate dehydrogenase (NADP+ dependent) | Multiple_C | 12.08 | 12.35 | 11.33 | 1.68 | 0.0284 | 0.0942 | 11.54 | 11.93 | 12.28 | 0.60 | 0.1422 | 0.3749 |
| TC0700009395.hg.1 | WEE2 | WEE1 homolog 2 (S. pombe) | Coding | 3.54 | 2.64 | 2.79 | 1.68 | 0.0284 | 0.094 | 3.86 | 3.49 | 3.5 | 1.28 | 0.1414 | 0.3739 |
| TC0700010472.hg.1 | TRA2A | transformer 2 alpha homolog (Drosophila) | Multiple_C | 12.72 | 12.06 | 11.97 | 1.68 | 0.0064 | 0.0294 | 11.59 | 11.66 | 11.84 | 0.84 | 0.2976 | 0.5623 |
| TC0X00009440.hg.1 | CXorf38 | chromosome X open reading frame 38 | Coding | 9.71 | 9.95 | 8.96 | 1.68 | 0.0803 | 0.2055 | 8.13 | 8.14 | 8.88 | 0.59 | 0.0458 | 0.198 |
| TC1000006611.hg.1 | AKR1C4 | aldo-keto reductase family 1, member C4 | Multiple_C | 4.52 | 3.81 | 3.77 | 1.68 | 0.0553 | 0.1558 | 4.65 | 5.90 | 5.1 | 0.73 | 0.1103 | 0.3272 |
| TC1300006802.hg.1 | BRCA2 | breast cancer 2, early onset | Multiple_C | 8.5 | 6.54 | 7.75 | 1.68 | 0.2102 | 0.4042 | 8.44 | 9.39 | 8.56 | 0.92 | 0.792 | 0.9076 |
| TC1300010008.hg.1 | GAS6-AS1 | GAS6 antisense RNA 1 | Multiple_C | 6.31 | 5.33 | 5.56 | 1.68 | 0.1142 | 0.2653 | 5.59 | 5.22 | 5.35 | 1.18 | 0.2697 | 0.5347 |
| TC1500007777.hg.1 | SENP8 | SUMO/sentrin peptidase family member, NEDD8 specific | Coding | 7.96 | 8.15 | 7.21 | 1.68 | 0.0333 | 0.1066 | 6 | 6.67 | 6.58 | 0.67 | 0.1527 | 0.3918 |
| TC1500010390.hg.1 | RHCG | Rh family, C glycoprotein | Multiple_C | 3.8 | 3.15 | 3.05 | 1.68 | 0.2899 | 0.5008 | 3.49 | 3.38 | 3.6 | 0.93 | 0.6521 | 0.8304 |
| TC1500010733.hg.1 | C15orf48; MIF | chromosome 15 open reading frame 48; microRNA 147b | Multiple_C | 5.3 | 4.88 | 4.55 | 1.68 | 0.0015 | 0.0093 | 3.61 | 3.46 | 3.42 | 1.14 | 0.3677 | 0.6301 |
| TC1500010770.hg.1 | GOLGA6D | golgin A6 family, member D | Coding | 6.11 | 5.1 | 5.36 | 1.68 | 0.0162 | 0.0607 | 6.65 | 6.27 | 6.18 | 1.39 | 0.0817 | 0.2753 |
| TC1600009777.hg.1 | ARHGAP17 | Rho GTPase activating protein 17 | Multiple_C | 9.93 | 9.12 | 9.18 | 1.68 | 0.0347 | 0.1101 | 9.01 | 9.43 | 9.13 | 0.92 | 0.1753 | 0.4233 |
| TC1600011362.hg.1 | ACSM3 | acyl-CoA synthetase medium-chain family member 3 | Multiple_C | 8.03 | 10.28 | 7.28 | 1.68 | 0.008 | 0.0348 | 4.43 | 4.08 | 3.78 | 1.57 | 0.0184 | 0.1144 |
| TC1700011418.hg.1 | LIMD2 | LIM domain containing 2 | Multiple_C | 5.48 | 4.65 | 4.73 | 1.68 | 0.0436 | 0.131 | 6.07 | 6.26 | 5.94 | 1.09 | 0.41 | 0.665 |
| TC1900008544.hg.1 | RCN3 | reticulocalbin 3, EF-hand calcium binding domain | Multiple_C | 10.8 | 10.29 | 10.05 | 1.68 | 0.0554 | 0.156 | 7.54 | 6.71 | 6.51 | 2.04 | 0.1275 | 0.3534 |
| TC1900011952.hg.1 | ZNF780A | zinc finger protein 780A | Multiple_C | 9.27 | 9.06 | 8.52 | 1.68 | 0.0857 | 0.2149 | 7.44 | 7.76 | 7.85 | 0.75 | 0.1084 | 0.3238 |
| TC2100008227.hg.1 | PRDM15 | PR domain containing 15 | Multiple_C | 7.02 | 6.37 | 6.27 | 1.68 | 0.0188 | 0.0681 | 6.64 | 6.71 | 7.06 | 0.75 | 0.0768 | 0.2669 |
| TSUnmapped00000104.h | CCDC84 | coiled-coil domain containing 84 | Coding | 10.16 | 9.47 | 9.41 | 1.68 | 0.1583 | 0.3343 | 8.71 | 8.73 | 8.96 | 0.84 | 0.7253 | 0.8722 |
| TC0100006864.hg.1 | DRAXIN | dorsal inhibitory axon guidance protein | Multiple_C | 4.53 | 3.56 | 3.79 | 1.67 | 0.0221 | 0.0774 | 4.75 | 4.97 | 5.06 | 0.81 | 0.1424 | 0.3753 |
| TC0100009468.hg.1 | LRIG2 | leucine-rich repeats and immunoglobulin-like domains 2 | Multiple_C | 7.96 | 7.95 | 7.22 | 1.67 | 0.0077 | 0.0338 | 5.96 | 5.95 | 6.43 | 0.72 | 0.0082 | 0.068 |
| TC0100010755.hg.1 | RALGPS2 | Ral GEF with PH domain and SH3 binding motif 2 | Multiple_C | 9.94 | 9.5 | 9.2 | 1.67 | 0.0102 | 0.0423 | 6.55 | 7.28 | 6.38 | 1.13 | 0.3799 | 0.6396 |
| TC0100012329.hg.1 | OR2W3 | olfactory receptor, family 2, subfamily W, member 3 | Coding | 5.6 | 4.59 | 4.86 | 1.67 | 0.0264 | 0.089 | 5.73 | 5.47 | 5.26 | 1.39 | 0.1693 | 0.4151 |
| TC0100013038.hg.1 | FBXO42 | F-box protein 42 | Multiple_C | 7.85 | 6.65 | 7.11 | 1.67 | 0.0079 | 0.0346 | 6.07 | 6.38 | 6.6 | 0.69 | 0.0044 | 0.0456 |
| TC0100015271.hg.1 | OVGP1 | oviductal glycoprotein 1 | Multiple_C | 5.3 | 6.01 | 4.56 | 1.67 | 0.0332 | 0.1065 | 4.57 | 3.85 | 4.24 | 1.26 | 0.0341 | 0.1662 |
| TC0100018549.hg.1 | TNNT2 | troponin T type 2 (cardiac) | Multiple_C | 8.93 | 8.36 | 8.19 | 1.67 | 0.0095 | 0.0399 | 8.09 | 7.78 | 7.35 | 1.67 | 0.0035 | 0.0392 |
| TC0200013603.hg.1 | MITD1 | microtubule interacting and trafficking domain containing 1 | Multiple_C | 9.71 | 10.4 | 8.97 | 1.67 | 0.0392 | 0.1207 | 8.01 | 7.28 | 7.68 | 1.26 | 0.2542 | 0.5184 |

| | | | | | | | | | | | | | | | |
|---|---|---|---|---|---|---|---|---|---|---|---|---|---|---|---|
| TC0200014617.hg.1 | NEB | nebulin | Multiple_C | 5.11 | 4.27 | 4.37 | 1.67 | 0.0284 | 0.094 | 4.54 | 5.11 | 4.92 | 0.77 | 0.839 | 0.9292 |
| TC0200016626.hg.1 | MBOAT2 | membrane bound O-acyltransferase domain containing 2 | Multiple_C | 10.89 | 10.52 | 10.15 | 1.67 | 0.0081 | 0.0355 | 13 | 11.47 | 11.89 | 2.16 | 5.94E-05 | 0.0025 |
| TC0300007470.hg.1 | PPM1M | protein phosphatase, Mg2+/Mn2+ dependent, 1M | Multiple_C | 4.3 | 4.51 | 3.56 | 1.67 | 0.0215 | 0.0758 | 4.68 | 4.63 | 4.46 | 1.16 | 0.8908 | 0.9535 |
| TC0300013259.hg.1 | KCNMB3 | potassium channel subfamily M regulatory beta subunit 3 | Multiple_C | 5.48 | 4.37 | 4.74 | 1.67 | 0.0735 | 0.192 | 5.72 | 6.65 | 5.98 | 0.84 | 0.8657 | 0.9425 |
| TC0300014053.hg.1 | PIK3CB | phosphatidylinositol-4,5-bisphosphate 3-kinase, catalytic subun | Multiple_C | 10.87 | 10.3 | 10.13 | 1.67 | 0.0378 | 0.1176 | 9.26 | 8.98 | 9.61 | 0.78 | 0.0425 | 0.1902 |
| TC0400006476.hg.1 | PCGF3 | polycomb group ring finger 3 | Multiple_C | 9.25 | 8.43 | 8.51 | 1.67 | 0.2776 | 0.4868 | 10.07 | 9.89 | 9.53 | 1.45 | 0.8444 | 0.9318 |
| TC0400008091.hg.1 | SPP1 | secreted phosphoprotein 1 | Multiple_C | 4.5 | 3.94 | 3.76 | 1.67 | 0.0047 | 0.023 | 4.34 | 4.40 | 4.12 | 1.16 | 0.0802 | 0.273 |
| TC0400010612.hg.1 | NFXL1 | nuclear transcription factor, X-box binding-like 1 | Multiple_C | 7.96 | 8.4 | 7.22 | 1.67 | 0.3112 | 0.5245 | 7.71 | 6.85 | 7.77 | 0.96 | 0.6132 | 0.807 |
| TC0400011048.hg.1 | PPEF2 | protein phosphatase, EF-hand calcium binding domain 2 | Multiple_C | 4.71 | 3.82 | 3.97 | 1.67 | 0.104 | 0.2481 | 4.99 | 4.75 | 4.75 | 1.18 | 0.3389 | 0.6029 |
| TC0500006479.hg.1 | NKD2 | naked cuticle homolog 2 (Drosophila) | Multiple_C | 5.69 | 4.95 | 4.95 | 1.67 | 0.0006 | 0.0046 | 8.75 | 9.27 | 8.45 | 1.23 | 0.323 | 0.5871 |
| TC0500009342.hg.1 | TENM2 | teneurin transmembrane protein 2 | Multiple_C | 5.02 | 4.39 | 4.28 | 1.67 | 0.0013 | 0.0083 | 4.87 | 4.94 | 4.87 | 1.00 | 0.6637 | 0.8355 |
| TC0500009814.hg.1 | TPPP | tubulin polymerization promoting protein | Multiple_C | 6.05 | 5.11 | 5.31 | 1.67 | 0.3177 | 0.5312 | 5.53 | 5.04 | 5.19 | 1.27 | 0.4024 | 0.6586 |
| TC0500011984.hg.1 | HINT1 | histidine triad nucleotide binding protein 1 | Multiple_C | 14.57 | 14.31 | 13.83 | 1.67 | 0.1649 | 0.3434 | 10.61 | 11.01 | 10.86 | 0.84 | 0.2844 | 0.5496 |
| TC0600009240.hg.1 | KPNA5 | karyopherin alpha 5 (importin alpha 6) | Multiple_C | 9.43 | 10.81 | 8.69 | 1.67 | 0.0462 | 0.1365 | 9.14 | 8.63 | 8.78 | 1.28 | 0.3563 | 0.6195 |
| TC0600011823.hg.1 | TRERF1 | transcriptional regulating factor 1 | Multiple_C | 9.98 | 8.8 | 9.24 | 1.67 | 0.0606 | 0.1669 | 8.05 | 8.09 | 8.47 | 0.75 | 0.3124 | 0.5765 |
| TC0600013780.hg.1 | LPAL2 | lipoprotein, Lp(a)-like 2, pseudogene | Multiple_C | 6.58 | 5.6 | 5.84 | 1.67 | 0.0234 | 0.0809 | 5.11 | 5.24 | 5.62 | 0.70 | 0.798 | 0.9095 |
| TC0700012080.hg.1 | SPDYE6; SPDY | speedy/RINGO cell cycle regulator family member E6; speedy/R | Coding | 6.8 | 5.93 | 6.06 | 1.67 | 0.002 | 0.0113 | 5.57 | 5.67 | 6.07 | 0.71 | 0.0355 | 0.1708 |
| TC0700012427.hg.1 | FAM3C | family with sequence similarity 3, member C | Multiple_C | 14.08 | 14.07 | 13.34 | 1.67 | 0.0143 | 0.0551 | 12.54 | 11.77 | 11.78 | 1.69 | 0.0022 | 0.0288 |
| TC0800007185.hg.1 | RBPMS | RNA binding protein with multiple splicing | Multiple_C | 12.16 | 11.72 | 11.42 | 1.67 | 0.0416 | 0.1263 | 6.93 | 8.86 | 8.74 | 0.29 | 1.06E-06 | 0.0002 |
| TC0800010987.hg.1 | C8orf59 | chromosome 8 open reading frame 59 | Multiple_C | 10.41 | 11.24 | 9.67 | 1.67 | 0.0997 | 0.2404 | 5.98 | 6.29 | 6.36 | 0.77 | 0.1474 | 0.3836 |
| TC0800012467.hg.1 | OC90 | otoconin 90 | Coding | 5.11 | 4.59 | 4.37 | 1.67 | 0.0365 | 0.1147 | 4.29 | 4.14 | 4.03 | 1.20 | 0.1685 | 0.4142 |
| TC0M00006440.hg.1 | ND3 | NADH dehydrogenase, subunit 3 (complex I) | Multiple_C | 15.19 | 14.23 | 14.45 | 1.67 | 0.0571 | 0.1595 | 12.63 | 13.21 | 13.48 | 0.55 | 0.0045 | 0.046 |
| TC0X00009638.hg.1 | OTUD5 | OTU deubiquitinase 5 | Multiple_C | 10.97 | 10.69 | 10.23 | 1.67 | 0.0475 | 0.1393 | 10.93 | 11.43 | 11.59 | 0.63 | 0.0059 | 0.0544 |
| TC0X00010046.hg.1 | DMRTC1B; DN | DMRT-like family C1B; DMRT-like family C1 | Multiple_C | 5.23 | 4.2 | 4.49 | 1.67 | 0.0077 | 0.034 | 5.46 | 5.16 | 5.12 | 1.27 | 0.1011 | 0.3109 |
| TC1000011556.hg.1 | AVPI1 | arginine vasopressin-induced 1 | Multiple_C | 6.3 | 5.15 | 5.56 | 1.67 | 0.0044 | 0.0216 | 4.74 | 4.70 | 4.7 | 1.03 | 0.7754 | 0.8992 |
| TC1100006576.hg.1 | CD81 | CD81 molecule | Multiple_C | 12.08 | 11.22 | 11.34 | 1.67 | 0.0089 | 0.038 | 12.09 | 12.63 | 12.22 | 0.91 | 0.4628 | 0.7054 |
| TC1100008042.hg.1 | CCDC85B | coiled-coil domain containing 85B | Coding | 5.92 | 4.9 | 5.18 | 1.67 | 0.0254 | 0.0863 | 4.55 | 4.90 | 5 | 0.73 | 0.2302 | 0.4914 |
| TC1200007622.hg.1 | ATF1 | activating transcription factor 1 | Multiple_C | 13.18 | 12.91 | 12.44 | 1.67 | 0.0049 | 0.0236 | 13.2 | 13.30 | 13.44 | 0.85 | 0.4185 | 0.6723 |
| TC1200009524.hg.1 | ZNF84 | zinc finger protein 84 | Multiple_C | 11.63 | 11.56 | 10.89 | 1.67 | 0.0035 | 0.0181 | 8.74 | 8.79 | 8.9 | 0.90 | 0.795 | 0.9087 |
| TC1200011741.hg.1 | C12orf42 | chromosome 12 open reading frame 42 | Multiple_C | 5.04 | 4.49 | 4.3 | 1.67 | 0.019 | 0.0687 | 4.28 | 4.78 | 4.68 | 0.76 | 0.9861 | 0.9943 |
| TC1200011961.hg.1 | TMEM116 | transmembrane protein 116 | Multiple_C | 8.64 | 8.08 | 7.9 | 1.67 | 0.0358 | 0.1131 | 6.02 | 6.23 | 6.66 | 0.64 | 0.0334 | 0.1642 |
| TC1200012742.hg.1 | SPSB2 | splA/ryanodine receptor domain and SOCS box containing 2 | Multiple_C | 4.69 | 4.05 | 3.95 | 1.67 | 0.1962 | 0.3864 | 4.44 | 4.66 | 4.63 | 0.88 | 0.2059 | 0.4623 |
| TC1300009012.hg.1 | THSD1 | thrombospondin type 1 domain containing 1 | Multiple_C | 5.76 | 5.75 | 5.02 | 1.67 | 0.0361 | 0.1137 | 6.11 | 5.47 | 4.97 | 2.20 | 2.51E-05 | 0.0014 |

| | | | | | | | | | | | | | | | |
|---|---|---|---|---|---|---|---|---|---|---|---|---|---|---|---|
| TC1400007120.hg.1 | ATL1 | atlastin GTPase 1 | Multiple_C | 5.33 | 4.53 | 4.59 | 1.67 | 0.0974 | 0.2361 | 7.49 | 8.63 | 7.45 | 1.03 | 0.7928 | 0.9077 |
| TC1500009130.hg.1 | RHOV | ras homolog family member V | Multiple_C | 5.48 | 4.56 | 4.74 | 1.67 | 0.0174 | 0.0643 | 4.26 | 4.52 | 4.32 | 0.96 | 0.3817 | 0.6409 |
| TC1600011080.hg.1 | FAM92B | family with sequence similarity 92, member B | Coding | 7.29 | 6.08 | 6.55 | 1.67 | 0.0009 | 0.0064 | 6.87 | 6.94 | 6.89 | 0.99 | 0.8759 | 0.9469 |
| TC1700007536.hg.1 | SPACA3 | sperm acrosome associated 3 | Multiple_C | 4.81 | 4.02 | 4.07 | 1.67 | 0.0426 | 0.1288 | 4.08 | 3.88 | 3.57 | 1.42 | 0.007 | 0.0611 |
| TC1700008251.hg.1 | EME1 | essential meiotic structure-specific endonuclease 1 | Multiple_C | 9.96 | 9.4 | 9.22 | 1.67 | 0.0891 | 0.2214 | 9.48 | 9.84 | 9.57 | 0.94 | 0.7749 | 0.8989 |
| TC1700009870.hg.1 | UBE2S; UBE2S | ubiquitin-conjugating enzyme E2S; ubiquitin conjugating enzym | Multiple_C | 7.64 | 6.8 | 6.9 | 1.67 | 0.0074 | 0.033 | 6.37 | 6.66 | 6.55 | 0.88 | 0.2371 | 0.5003 |
| TC1700012356.hg.1 | RPL26 | ribosomal protein L26 | Multiple_C | 17.78 | 17.67 | 17.04 | 1.67 | 0.006 | 0.028 | 15.77 | 15.74 | 15.44 | 1.26 | 0.2776 | 0.5436 |
| TC1800008734.hg.1 | STARD6 | StAR-related lipid transfer domain containing 6 | Multiple_C | 3.97 | 3.19 | 3.23 | 1.67 | 0.0354 | 0.1118 | 3.79 | 3.89 | 3.81 | 0.99 | 0.8804 | 0.9486 |
| TC1800008804.hg.1 | ALPK2 | alpha kinase 2 | Multiple_C | 4.15 | 3.41 | 3.41 | 1.67 | 0.0721 | 0.1894 | 4.04 | 3.96 | 3.74 | 1.23 | 0.095 | 0.2995 |
| TC1900008249.hg.1 | ZNF230 | zinc finger protein 230 | Multiple_C | 7.18 | 6.88 | 6.44 | 1.67 | 0.0561 | 0.1574 | 5.54 | 5.74 | 5.94 | 0.76 | 0.7619 | 0.8923 |
| TC1900009586.hg.1 | ZNF426 | zinc finger protein 426 | Multiple_C | 3.9 | 3.35 | 3.16 | 1.67 | 0.1763 | 0.3588 | 3.28 | 3.59 | 3.46 | 0.88 | 0.5324 | 0.7552 |
| TSUnmapped00000089.h | ZNF546 | zinc finger protein 546 | Coding | 3.88 | 2.91 | 3.14 | 1.67 | 0.0483 | 0.141 | 3.6 | 3.70 | 3.46 | 1.10 | 0.1608 | 0.4033 |
| TC0100013497.hg.1 | SRSF4 | serine/arginine-rich splicing factor 4 | Multiple_C | 13.28 | 12.67 | 12.55 | 1.66 | 0.0051 | 0.0244 | 13.22 | 12.96 | 13.81 | 0.66 | 0.0027 | 0.0328 |
| TC0100014023.hg.1 | GPBP1L1 | GC-rich promoter binding protein 1-like 1 | Multiple_C | 10.55 | 10.54 | 9.82 | 1.66 | 0.0048 | 0.0231 | 9.23 | 9.03 | 8.89 | 1.27 | 0.0293 | 0.1517 |
| TC0100018393.hg.1 | FAAP20 | Fanconi anemia core complex associated protein 20 | Multiple_C | 7.68 | 6.81 | 6.95 | 1.66 | 0.012 | 0.0482 | 6.87 | 7.27 | 6.96 | 0.94 | 0.6644 | 0.8358 |
| TC0200007609.hg.1 | SPTBN1 | spectrin, beta, non-erythrocytic 1 | Multiple_C | 14.31 | 13.09 | 13.58 | 1.66 | 0.0286 | 0.0946 | 13.43 | 13.48 | 13.46 | 0.98 | 0.2603 | 0.5245 |
| TC0200008300.hg.1 | RMND5A | required for meiotic nuclear division 5 homolog A | Multiple_C | 11.61 | 11 | 10.88 | 1.66 | 0.0597 | 0.1651 | 11.35 | 10.73 | 11.19 | 1.12 | 0.761 | 0.8917 |
| TC0200014800.hg.1 | SLC38A11 | solute carrier family 38, member 11 | Multiple_C | 4.19 | 3.23 | 3.46 | 1.66 | 0.0079 | 0.0348 | 3.37 | 3.56 | 3.51 | 0.91 | 0.7986 | 0.9098 |
| TC0300013051.hg.1 | PDCD10 | programmed cell death 10 | Multiple_C | 15.09 | 15.46 | 14.36 | 1.66 | 0.0501 | 0.1449 | 14.57 | 14.08 | 13.66 | 1.88 | 0.0002 | 0.0064 |
| TC0300013932.hg.1 | SRGAP3 | SLIT-ROBO Rho GTPase activating protein 3 | Multiple_C | 5.93 | 4.99 | 5.2 | 1.66 | 0.0157 | 0.0591 | 5.52 | 4.92 | 4.61 | 1.88 | 0.0012 | 0.0191 |
| TC0600008092.hg.1 | TJAP1 | tight junction associated protein 1 (peripheral) | Multiple_C | 9.62 | 7.91 | 8.89 | 1.66 | 0.0932 | 0.2289 | 9.2 | 9.74 | 9.1 | 1.07 | 0.7491 | 0.8855 |
| TC0700010687.hg.1 | KBTBD2 | kelch repeat and BTB (POZ) domain containing 2 | Multiple_C | 10.33 | 10.05 | 9.6 | 1.66 | 0.1682 | 0.3479 | 10.02 | 9.85 | 9.87 | 1.11 | 0.3241 | 0.5883 |
| TC0700010697.hg.1 | RP9 | retinitis pigmentosa 9 (autosomal dominant) | Multiple_C | 11.03 | 10.3 | 10.3 | 1.66 | 0.0886 | 0.2207 | 8.78 | 9.04 | 9.45 | 0.63 | 0.0338 | 0.1656 |
| TC0700013472.hg.1 | MGAM | maltase-glucoamylase | Multiple_C | 4.71 | 3.79 | 3.98 | 1.66 | 0.0169 | 0.0628 | 4.12 | 3.84 | 3.73 | 1.31 | 0.5238 | 0.7491 |
| TC0800006968.hg.1 | FGF17 | fibroblast growth factor 17 | Multiple_C | 5.41 | 4.7 | 4.68 | 1.66 | 0.0201 | 0.0717 | 4.74 | 4.48 | 4.83 | 0.94 | 0.7209 | 0.8698 |
| TC0800007735.hg.1 | UBXN2B | UBX domain protein 2B | Multiple_C | 10.01 | 9.55 | 9.28 | 1.66 | 0.0064 | 0.0292 | 9.23 | 8.94 | 9.97 | 0.60 | 0.0015 | 0.0225 |
| TC0800010081.hg.1 | RNF122 | ring finger protein 122 | Multiple_C | 7.08 | 7 | 6.35 | 1.66 | 0.0315 | 0.1023 | 5.72 | 5.58 | 5.65 | 1.05 | 0.5694 | 0.7779 |
| TC0900008337.hg.1 | ZNF462 | zinc finger protein 462 | Multiple_C | 10.18 | 9.61 | 9.45 | 1.66 | 0.0021 | 0.012 | 4.2 | 3.95 | 3.97 | 1.17 | 0.6437 | 0.8253 |
| TC0900008750.hg.1 | GAPVD1 | GTPase activating protein and VPS9 domains 1 | Multiple_C | 9.73 | 8.54 | 9 | 1.66 | 0.0268 | 0.0899 | 8.73 | 8.47 | 8.87 | 0.91 | 0.5087 | 0.7389 |
| TC0900008887.hg.1 | TBC1D13 | TBC1 domain family, member 13 | Multiple_C | 9.76 | 8.28 | 9.03 | 1.66 | 0.0307 | 0.1002 | 8.94 | 10.00 | 9.52 | 0.67 | 0.0205 | 0.1225 |
| TC0900009489.hg.1 | GLDC | glycine dehydrogenase (decarboxylating) | Multiple_C | 4.98 | 4.2 | 4.25 | 1.66 | 0.109 | 0.257 | 4.37 | 4.42 | 4.44 | 0.95 | 0.4699 | 0.7107 |
| TC0900011803.hg.1 | SETX | senataxin | Multiple_C | 9.49 | 8.89 | 8.76 | 1.66 | 0.0449 | 0.1338 | 9.65 | 10.03 | 9.45 | 1.15 | 0.3831 | 0.6421 |
| TC0X00009755.hg.1 | IQSEC2 | IQ motif and Sec7 domain 2 | Multiple_C | 4.4 | 3.79 | 3.67 | 1.66 | 0.0256 | 0.0868 | 3.48 | 3.73 | 3.77 | 0.82 | 0.2212 | 0.4813 |

| | | | | | | | | | | | | | | | |
|---|---|---|---|---|---|---|---|---|---|---|---|---|---|---|---|
| TC1000007905.hg.1 | C10orf35 | chromosome 10 open reading frame 35 | Multiple_C | 8.05 | 7.48 | 7.32 | 1.66 | 0.0011 | 0.0071 | 7.91 | 7.79 | 7.51 | 1.32 | 0.2002 | 0.4557 |
| TC1100007390.hg.1 | EXT2 | exostosin glycosyltransferase 2 | Multiple_C | 9.53 | 9.37 | 8.8 | 1.66 | 0.0086 | 0.0371 | 8.66 | 8.20 | 9.05 | 0.76 | 0.1066 | 0.3208 |
| TC1100007400.hg.1 | TSPAN18 | tetraspanin 18 | Multiple_C | 5.55 | 4.81 | 4.82 | 1.66 | 0.0402 | 0.1232 | 4.69 | 4.71 | 4.79 | 0.93 | 0.4589 | 0.7024 |
| TC1100009169.hg.1 | TAGLN | transgelin | Multiple_C | 5.83 | 5.19 | 5.1 | 1.66 | 0.1297 | 0.2906 | 5.37 | 5.69 | 6.93 | 0.34 | 4.76E-06 | 0.0004 |
| TC1100009666.hg.1 | SIGIRR | single immunoglobulin and toll-interleukin 1 receptor (TIR) dom | Multiple_C | 9.23 | 8.09 | 8.5 | 1.66 | 0.0796 | 0.2042 | 6.62 | 6.39 | 6.31 | 1.24 | 0.2959 | 0.5607 |
| TC1100011029.hg.1 | VWCE | von Willebrand factor C and EGF domains | Multiple_C | 4.66 | 3.72 | 3.93 | 1.66 | 0.0486 | 0.1417 | 5.43 | 5.43 | 5.16 | 1.21 | 0.4378 | 0.6883 |
| TC1100012424.hg.1 | SIK3 | SIK family kinase 3 | Multiple_C | 7.96 | 6.67 | 7.23 | 1.66 | 0.1156 | 0.2679 | 8.13 | 8.09 | 8.56 | 0.74 | 0.071 | 0.2549 |
| TC1100013016.hg.1 | PGA4 | pepsinogen 4, group I (pepsinogen A) | Multiple_C | 5.5 | 4.6 | 4.77 | 1.66 | 0.057 | 0.1593 | 5.04 | 5.02 | 4.83 | 1.16 | 0.2895 | 0.5543 |
| TC1100013164.hg.1 | PRG2 | proteoglycan 2, bone marrow (natural killer cell activator, eosin | Multiple_C | 4.02 | 3.26 | 3.29 | 1.66 | 0.0035 | 0.018 | 3.84 | 3.80 | 3.9 | 0.96 | 0.6323 | 0.819 |
| TC1200007769.hg.1 | PDE1B | phosphodiesterase 1B, calmodulin-dependent | Multiple_C | 4.93 | 3.84 | 4.2 | 1.66 | 0.0905 | 0.2239 | 3.4 | 3.62 | 3.88 | 0.72 | 0.0694 | 0.2513 |
| TC1200008162.hg.1 | CNOT2 | CCR4-NOT transcription complex subunit 2 | Multiple_C | 13.04 | 12.37 | 12.31 | 1.66 | 0.0065 | 0.0297 | 11.89 | 11.98 | 12.05 | 0.90 | 0.2685 | 0.5332 |
| TC1300006652.hg.1 | CDK8 | cyclin-dependent kinase 8 | Multiple_C | 10.67 | 10.88 | 9.94 | 1.66 | 0.2273 | 0.4266 | 10.33 | 10.97 | 10.28 | 1.04 | 0.9757 | 0.9902 |
| TC1400006537.hg.1 | RNASE3 | ribonuclease, RNase A family, 3 | Coding | 4.49 | 3.89 | 3.76 | 1.66 | 0.0332 | 0.1065 | 5.62 | 5.38 | 5.22 | 1.32 | 0.0283 | 0.1482 |
| TC1400008635.hg.1 | RAB2B | RAB2B, member RAS oncogene family | Multiple_C | 9.22 | 9.38 | 8.49 | 1.66 | 0.0521 | 0.149 | 9.11 | 8.72 | 8.8 | 1.24 | 0.2373 | 0.5006 |
| TC1500006683.hg.1 | APBA2 | amyloid beta (A4) precursor protein-binding, family A, member | Multiple_C | 6.89 | 6.16 | 6.16 | 1.66 | 0.1003 | 0.2414 | 5.68 | 5.79 | 5.68 | 1.00 | 0.5883 | 0.7917 |
| TC1500007671.hg.1 | FEM1B | fem-1 homolog b (C. elegans) | Coding | 11.21 | 10.57 | 10.48 | 1.66 | 0.0016 | 0.0095 | 10.82 | 10.30 | 11.28 | 0.73 | 0.0238 | 0.1333 |
| TC1500009348.hg.1 | FBN1 | fibrillin 1 | Multiple_C | 5.47 | 4.71 | 4.74 | 1.66 | 0.0071 | 0.0317 | 4.94 | 4.85 | 4.54 | 1.32 | 0.1115 | 0.3291 |
| TC1600006616.hg.1 | NTN3 | netrin 3 | Coding | 4.19 | 3.2 | 3.46 | 1.66 | 0.066 | 0.178 | 3.85 | 3.62 | 3.58 | 1.21 | 0.265 | 0.5296 |
| TC1700010816.hg.1 | ATXN7L3 | ataxin 7-like 3 | Multiple_C | 7.07 | 6.35 | 6.34 | 1.66 | 0.0435 | 0.1308 | 6.74 | 7.22 | 7.34 | 0.66 | 0.024 | 0.1338 |
| TC1800007066.hg.1 | MAPRE2 | microtubule-associated protein, RP/EB family, member 2 | Multiple_C | 10.76 | 9.43 | 10.03 | 1.66 | 0.0442 | 0.1323 | 9.81 | 10.05 | 10.3 | 0.71 | 0.018 | 0.1127 |
| TC1800007186.hg.1 | SETBP1 | SET binding protein 1 | Multiple_C | 5.74 | 4.78 | 5.01 | 1.66 | 0.0331 | 0.1061 | 4.85 | 4.62 | 4.99 | 0.91 | 0.6112 | 0.8057 |
| TC1900007839.hg.1 | FXYD5 | FXYD domain containing ion transport regulator 5 | Multiple_C | 13.48 | 12.11 | 12.75 | 1.66 | 0.0247 | 0.0846 | 11.23 | 8.69 | 9.45 | 3.43 | 5.99E-08 | 1.89E-05 |
| TC1900008726.hg.1 | ERVV-1 | endogenous retrovirus group V, member 1 | Coding | 4.27 | 4.06 | 3.54 | 1.66 | 0.0057 | 0.0267 | 3.47 | 3.63 | 3.36 | 1.08 | 0.9703 | 0.9878 |
| TC1900009196.hg.1 | CSNK1G2-AS1 | CSNK1G2 antisense RNA 1 | Multiple_C | 5.55 | 4.66 | 4.82 | 1.66 | 0.2492 | 0.4532 | 5.46 | 5.43 | 5.45 | 1.01 | 0.9953 | 0.9978 |
| TC1900010855.hg.1 | CADM4 | cell adhesion molecule 4 | Multiple_C | 5.86 | 5.04 | 5.13 | 1.66 | 0.0197 | 0.0705 | 6.1 | 5.80 | 5.62 | 1.39 | 0.079 | 0.2709 |
| TC1900011733.hg.1 | RAB4B; MIA-R | RAB4B, member RAS oncogene family; MIA-RAB4B readthrough | Multiple_C | 6.29 | 5.68 | 5.56 | 1.66 | 0.0035 | 0.0181 | 5 | 4.99 | 5.19 | 0.88 | 0.818 | 0.9196 |
| TC1900011780.hg.1 | RPL13A; SNOR | ribosomal protein L13a; small nucleolar RNA, C/D box 35A; sma | Multiple_C | 18 | 17.35 | 17.27 | 1.66 | 0.0258 | 0.0873 | 16.24 | 16.19 | 15.78 | 1.38 | 0.0812 | 0.2748 |
| TC1900011910.hg.1 | HOMER3 | homer scaffolding protein 3 | Multiple_C | 5.09 | 4.44 | 4.36 | 1.66 | 0.0064 | 0.0293 | 6.31 | 6.22 | 5.93 | 1.30 | 0.0715 | 0.2559 |
| TC2200008078.hg.1 | GGT2 | gamma-glutamyltransferase 2 | Multiple_C | 9.04 | 8.04 | 8.31 | 1.66 | 0.1166 | 0.2693 | 9.51 | 9.59 | 9.51 | 1.00 | 0.86 | 0.9402 |
| TC2200009229.hg.1 | MMP11 | matrix metallopeptidase 11 | Multiple_C | 4.49 | 4.15 | 3.76 | 1.66 | 0.0141 | 0.0543 | 3.59 | 3.57 | 3.54 | 1.04 | 0.9612 | 0.9838 |
| TC0100007532.hg.1 | STX12 | syntaxin 12 | Multiple_C | 11.17 | 11.54 | 10.45 | 1.65 | 0.0129 | 0.0509 | 9.26 | 8.72 | 8.73 | 1.44 | 0.0225 | 0.1292 |
| TC0100009329.hg.1 | FNDC7 | fibronectin type III domain containing 7 | Multiple_C | 4.81 | 3.77 | 4.09 | 1.65 | 0.081 | 0.2069 | 3.35 | 3.57 | 3.42 | 0.95 | 0.4941 | 0.7282 |
| TC0100015861.hg.1 | S100A7L2 | S100 calcium binding protein A7 like 2 | Coding | 3.85 | 3 | 3.13 | 1.65 | 0.5222 | 0.7153 | 4.12 | 3.80 | 3.53 | 1.51 | 0.0182 | 0.1135 |

| | | | | | | | | | | | | | | | |
|---|---|---|---|---|---|---|---|---|---|---|---|---|---|---|---|
| TC0200014728.hg.1 | LY75-CD302; ( | LY75-CD302 readthrough; CD302 molecule; lymphocyte antigen | Multiple_( | 9.44 | 9.31 | 8.72 | 1.65 | 0.1005 | 0.2418 | 9.66 | 7.55 | 7.95 | 3.27 | 5.99E-05 | 0.0025 |
| TC0200016750.hg.1 | NFE2L2 | nuclear factor, erythroid 2-like 2 | Multiple_( | 11.86 | 11.05 | 11.14 | 1.65 | 0.0109 | 0.0448 | 9.53 | 8.71 | 9.76 | 0.85 | 0.4218 | 0.6748 |
| TC0300013330.hg.1 | DCUN1D1 | DCN1, defective in cullin neddylation 1, domain containing 1 | Multiple_( | 10.49 | 10.32 | 9.77 | 1.65 | 0.0531 | 0.1512 | 10.13 | 9.95 | 10.03 | 1.07 | 0.7006 | 0.8581 |
| TC0300013973.hg.1 | CELSR3; MIR4 | cadherin, EGF LAG seven-pass G-type receptor 3; microRNA 479 | Multiple_( | 4.69 | 3.88 | 3.97 | 1.65 | 0.1273 | 0.2865 | 5.61 | 6.28 | 6.16 | 0.68 | 0.3243 | 0.5884 |
| TC0400007447.hg.1 | SLAIN2 | SLAIN motif family member 2 | Multiple_( | 12.86 | 11.53 | 12.14 | 1.65 | 0.1113 | 0.2604 | 12.31 | 11.20 | 12.43 | 0.92 | 0.8899 | 0.9532 |
| TC0500008431.hg.1 | DMXL1 | Dmx-like 1 | Multiple_( | 9.57 | 9.64 | 8.85 | 1.65 | 0.039 | 0.1202 | 8.88 | 8.46 | 8.64 | 1.18 | 0.6947 | 0.8548 |
| TC0500008633.hg.1 | C5orf56 | chromosome 5 open reading frame 56 | Multiple_( | 6.46 | 5.57 | 5.74 | 1.65 | 0.0226 | 0.079 | 6.08 | 5.69 | 5.58 | 1.41 | 0.0277 | 0.146 |
| TC0500009562.hg.1 | HRH2 | histamine receptor H2 | Multiple_( | 3.8 | 2.99 | 3.08 | 1.65 | 0.0187 | 0.0678 | 4.44 | 4.29 | 4.05 | 1.31 | 0.084 | 0.2789 |
| TC0500013015.hg.1 | PHYKPL | 5-phosphohydroxy-L-lysine phospho-lyase | Multiple_( | 9.16 | 10.16 | 8.44 | 1.65 | 0.1412 | 0.3087 | 7.06 | 6.93 | 6.29 | 1.71 | 0.0118 | 0.0866 |
| TC0500013016.hg.1 | COL23A1 | collagen, type XXIII, alpha 1 | Multiple_( | 9.58 | 8.92 | 8.86 | 1.65 | 0.011 | 0.045 | 8.65 | 8.55 | 8.37 | 1.21 | 0.1378 | 0.3688 |
| TC0600007485.hg.1 | HLA-V | major histocompatibility complex, class I, V (pseudogene) | Multiple_( | 8.38 | 7.34 | 7.66 | 1.65 | 0.0016 | 0.0098 | 9.02 | 8.66 | 8.51 | 1.42 | 0.0987 | 0.3067 |
| TC0600009206.hg.1 | MARCKS | myristoylated alanine-rich protein kinase C substrate | Multiple_( | 10.01 | 8.94 | 9.29 | 1.65 | 0.0065 | 0.0295 | 7.02 | 8.33 | 8.65 | 0.32 | 1.19E-06 | 0.0002 |
| TC0600011546.hg.1 | CUTA | cutA divalent cation tolerance homolog (E. coli) | Multiple_( | 10.99 | 10.65 | 10.27 | 1.65 | 0.1362 | 0.3009 | 9.09 | 9.19 | 9.75 | 0.63 | 0.0209 | 0.1233 |
| TC0600013177.hg.1 | MED23 | mediator complex subunit 23 | Multiple_( | 10.07 | 9.87 | 9.35 | 1.65 | 0.0447 | 0.1333 | 9.17 | 8.83 | 9.21 | 0.97 | 0.7366 | 0.8786 |
| TC0700010912.hg.1 | MYL7 | myosin light chain 7 | Multiple_( | 5.93 | 5.06 | 5.21 | 1.65 | 0.1061 | 0.2517 | 5.3 | 5.27 | 5.34 | 0.97 | 0.5613 | 0.7723 |
| TC0700013615.hg.1 | ZNF800 | zinc finger protein 800 | Multiple_( | 11.03 | 10.08 | 10.31 | 1.65 | 0.0182 | 0.0665 | 11.02 | 10.33 | 11.49 | 0.72 | 0.0368 | 0.1746 |
| TC0800011241.hg.1 | RGS22 | regulator of G-protein signaling 22 | Multiple_( | 4.33 | 3.58 | 3.61 | 1.65 | 0.0513 | 0.1472 | 4.33 | 3.85 | 3.48 | 1.80 | 0.012 | 0.0871 |
| TC0800011334.hg.1 | KLF10 | Kruppel-like factor 10 | Multiple_( | 11.23 | 10.18 | 10.51 | 1.65 | 0.0326 | 0.1051 | 11.71 | 10.56 | 11.37 | 1.27 | 0.0311 | 0.157 |
| TC0900007167.hg.1 | DCAF10 | DDB1 and CUL4 associated factor 10 | Multiple_( | 8.54 | 8.89 | 7.82 | 1.65 | 0.0121 | 0.0487 | 8.22 | 8.22 | 8.04 | 1.13 | 0.4121 | 0.6666 |
| TC0900007828.hg.1 | SPATA31C1 | SPATA31 subfamily C, member 1 | Multiple_( | 5.29 | 4.45 | 4.57 | 1.65 | 0.0867 | 0.2169 | 4.68 | 4.20 | 4.49 | 1.14 | 0.4338 | 0.6847 |
| TC0900008784.hg.1 | LMX1B | LIM homeobox transcription factor 1, beta | Multiple_( | 6.16 | 5.3 | 5.44 | 1.65 | 0.0043 | 0.0215 | 6.12 | 5.88 | 5.22 | 1.87 | 0.0004 | 0.0095 |
| TC0900011523.hg.1 | HSPA5 | heat shock 70kDa protein 5 (glucose-regulated protein, 78kDa) | Multiple_( | 15.81 | 16.09 | 15.09 | 1.65 | 0.016 | 0.0601 | 14.39 | 13.73 | 14.38 | 1.01 | 0.9044 | 0.9607 |
| TC0900011660.hg.1 | CCBL1 | cysteine conjugate-beta lyase, cytoplasmic | Multiple_( | 8.57 | 9 | 7.85 | 1.65 | 0.2077 | 0.4011 | 7.98 | 7.63 | 8.19 | 0.86 | 0.1162 | 0.3366 |
| TC0X00006558.hg.1 | VCX; VCX3A | variable charge, X-linked; variable charge, X-linked 3A | Coding | 5.71 | 4.84 | 4.99 | 1.65 | 0.0604 | 0.1665 | 6.08 | 6.07 | 5.99 | 1.06 | 0.7147 | 0.8667 |
| TC1000008054.hg.1 | PLAU | plasminogen activator, urokinase | Multiple_( | 6.49 | 5.85 | 5.77 | 1.65 | 0.0409 | 0.1246 | 7.06 | 6.75 | 8.09 | 0.49 | 0.0003 | 0.007 |
| TC1000008579.hg.1 | UBTD1 | ubiquitin domain containing 1 | Multiple_( | 9.55 | 8.83 | 8.83 | 1.65 | 0.007 | 0.0315 | 9.96 | 9.96 | 9.7 | 1.20 | 0.2356 | 0.4983 |
| TC1100010268.hg.1 | TSG101 | tumor susceptibility 101 | Multiple_( | 12.85 | 13.16 | 12.13 | 1.65 | 0.0066 | 0.0301 | 11.59 | 11.49 | 11.02 | 1.48 | 0.0173 | 0.1103 |
| TC1100010705.hg.1 | AMBRA1 | autophagy/beclin-1 regulator 1 | Multiple_( | 12.08 | 10.96 | 11.36 | 1.65 | 0.007 | 0.0315 | 11.28 | 11.46 | 11.9 | 0.65 | 0.0098 | 0.0771 |
| TC1100013042.hg.1 | SSSCA1 | Sjogren syndrome/scleroderma autoantigen 1 | Multiple_( | 11.59 | 9.34 | 10.87 | 1.65 | 0.1475 | 0.3184 | 7.5 | 8.80 | 8.89 | 0.38 | 0.0031 | 0.0367 |
| TC1200009122.hg.1 | SIRT4 | sirtuin 4 | Multiple_( | 5.04 | 4.48 | 4.32 | 1.65 | 0.0079 | 0.0347 | 4.89 | 4.44 | 4.03 | 1.82 | 0.001 | 0.0163 |
| TC1200010252.hg.1 | TMTC1 | transmembrane and tetratricopeptide repeat containing 1 | Multiple_( | 5.62 | 4.95 | 4.9 | 1.65 | 0.0536 | 0.1522 | 4.71 | 4.78 | 5.26 | 0.68 | 0.1106 | 0.3276 |
| TC1200012238.hg.1 | ZCCHC8 | zinc finger, CCHC domain containing 8 | Multiple_( | 13.57 | 12.76 | 12.85 | 1.65 | 0.0584 | 0.1624 | 12.07 | 11.06 | 11.86 | 1.16 | 0.3982 | 0.6556 |
| TC1400008913.hg.1 | SNX6 | sorting nexin 6 | Multiple_( | 13.43 | 13.66 | 12.71 | 1.65 | 0.0057 | 0.0269 | 11.56 | 11.05 | 11.07 | 1.40 | 0.0057 | 0.0534 |

| | | | | | | | | | | | | | | | |
|---|---|---|---|---|---|---|---|---|---|---|---|---|---|---|---|
| TC1400010624.hg.1 | PTGR2 | prostaglandin reductase 2 | Multiple_C | 6.8 | 6.88 | 6.08 | 1.65 | 0.1023 | 0.245 | 10.16 | 9.39 | 9.54 | 1.54 | 0.6826 | 0.8476 |
| TC1500007554.hg.1 | ZNF609 | zinc finger protein 609 | Multiple_C | 9.02 | 8.19 | 8.3 | 1.65 | 0.0225 | 0.0787 | 10.91 | 10.10 | 10.27 | 1.56 | 0.1513 | 0.3894 |
| TC1500007975.hg.1 | IREB2 | iron responsive element binding protein 2 | Multiple_C | 9.8 | 8.54 | 9.08 | 1.65 | 0.0263 | 0.0887 | 11 | 11.22 | 11.06 | 0.96 | 0.6429 | 0.8248 |
| TC1500009429.hg.1 | DMXL2 | Dmx-like 2 | Multiple_C | 9.8 | 10.7 | 9.08 | 1.65 | 0.0047 | 0.0228 | 8.61 | 7.89 | 7.67 | 1.92 | 0.0016 | 0.0228 |
| TC1500009998.hg.1 | GOLGA6A | golgin A6 family, member A | Multiple_C | 6.88 | 6.14 | 6.16 | 1.65 | 0.0854 | 0.2145 | 7.74 | 6.68 | 6.51 | 2.35 | 0.0016 | 0.0229 |
| TC1600007183.hg.1 | UQCRC2 | ubiquinol-cytochrome c reductase core protein II | Multiple_C | 16.1 | 15.79 | 15.38 | 1.65 | 0.0098 | 0.0411 | 13.13 | 13.00 | 13.19 | 0.96 | 0.5277 | 0.7515 |
| TC1600007485.hg.1 | FBRS | fibrosin | Multiple_C | 6.02 | 4.86 | 5.3 | 1.65 | 0.1933 | 0.3825 | 6.13 | 6.34 | 6.82 | 0.62 | 0.0013 | 0.0198 |
| TC1600010825.hg.1 | ZFHX3 | zinc finger homeobox 3 | Multiple_C | 5.47 | 4.18 | 4.75 | 1.65 | 0.0572 | 0.1599 | 10.03 | 8.98 | 7.64 | 5.24 | 6.58E-08 | 2.02E-05 |
| TC1700009903.hg.1 | ZNF624 | zinc finger protein 624 | Multiple_C | 5.56 | 5.37 | 4.84 | 1.65 | 0.0657 | 0.1773 | 4.73 | 4.46 | 4.7 | 1.02 | 0.8515 | 0.9358 |
| TC1700011189.hg.1 | TMEM100 | transmembrane protein 100 | Multiple_C | 4.78 | 4.01 | 4.06 | 1.65 | 0.0098 | 0.0408 | 5.05 | 5.05 | 5.06 | 0.99 | 0.8935 | 0.9547 |
| TC1700011783.hg.1 | SRP68 | signal recognition particle 68kDa | Multiple_C | 11.12 | 10.38 | 10.4 | 1.65 | 0.0256 | 0.0867 | 10.45 | 10.86 | 11.09 | 0.64 | 0.0027 | 0.0328 |
| TC1800009286.hg.1 | RPL17-C18orf: | RPL17-C18orf32 readthrough | Coding | 17.93 | 18.76 | 17.21 | 1.65 | 0.0112 | 0.0456 | 13.1 | 13.04 | 12.15 | 1.93 | 3.14E-05 | 0.0016 |
| TC1900006532.hg.1 | RPS15 | ribosomal protein S15 | Multiple_C | 18.79 | 17.64 | 18.07 | 1.65 | 0.006 | 0.0278 | 13.57 | 13.89 | 13.48 | 1.06 | 0.2931 | 0.5579 |
| TC1900007004.hg.1 | C19orf52 | chromosome 19 open reading frame 52 | Multiple_C | 7.23 | 6.34 | 6.51 | 1.65 | 0.0501 | 0.1448 | 5.74 | 6.29 | 6.15 | 0.75 | 0.5539 | 0.7687 |
| TC1900008112.hg.1 | SHKBP1 | SH3KBP1 binding protein 1 | Multiple_C | 7.81 | 6.7 | 7.09 | 1.65 | 0.0131 | 0.0515 | 6.82 | 6.78 | 6.78 | 1.03 | 0.9553 | 0.9814 |
| TC2000007495.hg.1 | WFDC10A | WAP four-disulfide core domain 10A | Coding | 5.54 | 4.87 | 4.82 | 1.65 | 0.0611 | 0.1679 | 5.72 | 5.49 | 5.46 | 1.20 | 0.1159 | 0.3362 |
| TC2100007886.hg.1 | KRTAP27-1 | keratin associated protein 27-1 | Coding | 4.54 | 3.92 | 3.82 | 1.65 | 0.0789 | 0.2028 | 4.76 | 4.53 | 4.9 | 0.91 | 0.9301 | 0.9704 |
| TC2200006912.hg.1 | CRYBB3 | crystallin beta B3 | Multiple_C | 4.55 | 3.77 | 3.83 | 1.65 | 0.0136 | 0.0531 | 3.77 | 4.06 | 4.03 | 0.84 | 0.185 | 0.4364 |
| TC2200007337.hg.1 | MAFF | v-maf avian musculoaponeurotic fibrosarcoma oncogene homo | Multiple_C | 7.83 | 6.97 | 7.11 | 1.65 | 0.0034 | 0.0178 | 7 | 6.83 | 6.75 | 1.19 | 0.1114 | 0.329 |
| TC2200009121.hg.1 | BRD1 | bromodomain containing 1 | Multiple_C | 8.98 | 8.15 | 8.26 | 1.65 | 0.0719 | 0.1891 | 7.69 | 7.88 | 7.95 | 0.84 | 0.3957 | 0.6536 |
| TC2200009339.hg.1 | RNF215 | ring finger protein 215 | Multiple_C | 3.71 | 2.81 | 2.99 | 1.65 | 0.0454 | 0.1348 | 3.62 | 3.69 | 3.18 | 1.36 | 0.0148 | 0.0994 |
| TC0100010017.hg.1 | SPRR1A | small proline-rich protein 1A | Multiple_C | 4.1 | 3.2 | 3.39 | 1.64 | 0.0067 | 0.0305 | 3.65 | 3.62 | 3.72 | 0.95 | 0.3669 | 0.6293 |
| TC0100011770.hg.1 | EPHX1 | epoxide hydrolase 1, microsomal (xenobiotic) | Multiple_C | 11.67 | 9.98 | 10.96 | 1.64 | 0.0576 | 0.1607 | 9.98 | 9.96 | 10.27 | 0.82 | 0.972 | 0.9885 |
| TC0100012452.hg.1 | TNFRSF18 | tumor necrosis factor receptor superfamily, member 18 | Coding | 6.47 | 5.75 | 5.76 | 1.64 | 0.0553 | 0.1558 | 7.58 | 7.54 | 7.33 | 1.19 | 0.281 | 0.5467 |
| TC0100012816.hg.1 | CLSTN1 | calsyntenin 1 | Multiple_C | 12.52 | 11.48 | 11.81 | 1.64 | 0.0028 | 0.0153 | 10.21 | 10.24 | 10.26 | 0.97 | 0.3565 | 0.6197 |
| TC0100014349.hg.1 | JUN | jun proto-oncogene | Multiple_C | 12.55 | 10.53 | 11.84 | 1.64 | 0.0355 | 0.1121 | 12.17 | 11.35 | 11.63 | 1.45 | 0.77 | 0.8968 |
| TC0100016003.hg.1 | IQGAP3 | IQ motif containing GTPase activating protein 3 | Multiple_C | 8.49 | 7.54 | 7.78 | 1.64 | 0.0062 | 0.0288 | 6.91 | 7.59 | 7.02 | 0.93 | 0.7908 | 0.9066 |
| TC0100016887.hg.1 | ZNF281 | zinc finger protein 281 | Multiple_C | 9.43 | 8.85 | 8.72 | 1.64 | 0.0225 | 0.0785 | 9.01 | 9.11 | 9.18 | 0.89 | 0.3749 | 0.6356 |
| TC0200007411.hg.1 | PPM1B | protein phosphatase, Mg2+/Mn2+ dependent, 1B | Multiple_C | 11.11 | 11.25 | 10.4 | 1.64 | 0.1449 | 0.3148 | 10.27 | 9.66 | 10.03 | 1.18 | 0.791 | 0.9067 |
| TC0200007664.hg.1 | VRK2 | vaccinia related kinase 2 | Multiple_C | 10.91 | 11.22 | 10.2 | 1.64 | 0.005 | 0.0239 | 10.94 | 10.60 | 10.91 | 1.02 | 0.8861 | 0.9512 |
| TC0200009680.hg.1 | FMNL2 | formin like 2 | Multiple_C | 9.15 | 9.61 | 8.44 | 1.64 | 0.0348 | 0.1104 | 10.28 | 9.94 | 9.71 | 1.48 | 0.0008 | 0.0145 |
| TC0200010927.hg.1 | MRPL44 | mitochondrial ribosomal protein L44 | Multiple_C | 11.12 | 10.97 | 10.41 | 1.64 | 0.0941 | 0.2305 | 9.11 | 9.36 | 9.27 | 0.90 | 0.3206 | 0.5845 |
| TC0200016768.hg.1 | PECR | peroxisomal trans-2-enoyl-CoA reductase | Multiple_C | 8.8 | 8.58 | 8.09 | 1.64 | 0.0347 | 0.1103 | 8.23 | 7.86 | 7.56 | 1.59 | 0.0314 | 0.1583 |

| | | | | | | | | | | | | | | | |
|---|---|---|---|---|---|---|---|---|---|---|---|---|---|---|---|
| TC0300014055.hg.1 | PIK3CB | phosphatidylinositol-4,5-bisphosphate 3-kinase, catalytic subun | Coding | 10.82 | 9.79 | 10.11 | 1.64 | 0.0148 | 0.0565 | 10.6 | 10.57 | 10.92 | 0.80 | 0.0948 | 0.2993 |
| TC0300014076.hg.1 | SERPINI2 | serpin peptidase inhibitor, clade I (pancpin), member 2 | Multiple_C | 4.87 | 4.43 | 4.16 | 1.64 | 0.028 | 0.0932 | 4.35 | 4.25 | 4.03 | 1.25 | 0.2315 | 0.4928 |
| TC0400007360.hg.1 | DCAF4L1 | DDB1 and CUL4 associated factor 4-like 1 | Coding | 4.53 | 3.62 | 3.82 | 1.64 | 0.116 | 0.2684 | 4.01 | 3.84 | 4.12 | 0.93 | 0.3759 | 0.6364 |
| TC0400011313.hg.1 | SNCA | synuclein alpha | Multiple_C | 6 | 5.3 | 5.29 | 1.64 | 0.0473 | 0.1389 | 4.87 | 4.66 | 4.59 | 1.21 | 0.127 | 0.3528 |
| TC0500007610.hg.1 | ERBB2IP | erbb2 interacting protein | Multiple_C | 11.61 | 11.96 | 10.9 | 1.64 | 0.0114 | 0.0461 | 12.72 | 12.91 | 12.14 | 1.49 | 0.0014 | 0.021 |
| TC0500010427.hg.1 | ZFR | zinc finger RNA binding protein | Multiple_C | 12.24 | 12.12 | 11.53 | 1.64 | 0.0232 | 0.0805 | 11.46 | 11.27 | 11.31 | 1.11 | 0.8121 | 0.9166 |
| TC0500011758.hg.1 | CDO1 | cysteine dioxygenase type 1 | Multiple_C | 3.87 | 3.44 | 3.16 | 1.64 | 0.0049 | 0.0239 | 4.33 | 4.18 | 4.22 | 1.08 | 0.759 | 0.8906 |
| TC0500012425.hg.1 | CSNK1A1 | casein kinase 1, alpha 1 | Multiple_C | 14.1 | 14.15 | 13.39 | 1.64 | 0.0046 | 0.0224 | 12.96 | 12.45 | 12.7 | 1.20 | 0.1446 | 0.3792 |
| TC0600011661.hg.1 | STK38 | serine/threonine kinase 38 | Multiple_C | 10.78 | 10.24 | 10.07 | 1.64 | 0.0016 | 0.0095 | 9.25 | 9.02 | 9.36 | 0.93 | 0.7723 | 0.8976 |
| TC0600014123.hg.1 | NFYA | nuclear transcription factor Y subunit alpha | Multiple_C | 9.31 | 8.33 | 8.6 | 1.64 | 0.0112 | 0.0456 | 9.98 | 9.28 | 9.79 | 1.14 | 0.1192 | 0.3412 |
| TC0600014134.hg.1 | SPATS1 | spermatogenesis associated, serine-rich 1 | Coding | 4.96 | 3.74 | 4.25 | 1.64 | 0.0256 | 0.0869 | 3.92 | 3.98 | 4.06 | 0.91 | 0.7038 | 0.86 |
| TC0700008375.hg.1 | PPP1R9A | protein phosphatase 1, regulatory subunit 9A | Multiple_C | 8.48 | 8.35 | 7.77 | 1.64 | 0.0209 | 0.0743 | 6.5 | 7.54 | 6.68 | 0.88 | 0.1295 | 0.3563 |
| TC0700008465.hg.1 | NPTX2 | neuronal pentraxin II | Multiple_C | 5.07 | 4.18 | 4.36 | 1.64 | 0.0187 | 0.0679 | 5.31 | 5.82 | 5.89 | 0.67 | 0.1467 | 0.3827 |
| TC0700008743.hg.1 | PIK3CG | phosphatidylinositol-4,5-bisphosphate 3-kinase, catalytic subun | Multiple_C | 3.73 | 3.26 | 3.02 | 1.64 | 0.1253 | 0.2833 | 4.09 | 4.01 | 4.01 | 1.06 | 0.6982 | 0.857 |
| TC0700009472.hg.1 | EPHB6 | EPH receptor B6 | Multiple_C | 5.8 | 5.24 | 5.09 | 1.64 | 0.0434 | 0.1306 | 8.92 | 9.51 | 8.33 | 1.51 | 0.4859 | 0.723 |
| TC0700013603.hg.1 | RASA4 | RAS p21 protein activator 4 | Multiple_C | 4.47 | 4.55 | 3.76 | 1.64 | 0.0181 | 0.0663 | 5.37 | 4.61 | 4.93 | 1.36 | 0.2527 | 0.517 |
| TC0800007414.hg.1 | GOLGA7 | golgin A7 | Multiple_C | 11.94 | 11.81 | 11.23 | 1.64 | 0.023 | 0.08 | 11.27 | 11.62 | 11.69 | 0.75 | 0.0309 | 0.1566 |
| TC0800009212.hg.1 | MAPK15 | mitogen-activated protein kinase 15 | Multiple_C | 5.49 | 4.81 | 4.78 | 1.64 | 0.0538 | 0.1526 | 6.04 | 5.40 | 4.88 | 2.23 | 0.0006 | 0.0124 |
| TC0800011954.hg.1 | COL22A1 | collagen, type XXII, alpha 1 | Multiple_C | 3.91 | 3.24 | 3.2 | 1.64 | 0.0558 | 0.1567 | 3.32 | 3.37 | 3.2 | 1.09 | 0.8812 | 0.9491 |
| TC0900011181.hg.1 | TXNDC8 | thioredoxin domain containing 8 (spermatozoa) | Coding | 4.88 | 3.82 | 4.17 | 1.64 | 0.2135 | 0.4086 | 4.72 | 4.59 | 4.1 | 1.54 | 0.1682 | 0.414 |
| TC0900011217.hg.1 | PTBP3 | polypyrimidine tract binding protein 3 | Multiple_C | 14.39 | 13.25 | 13.68 | 1.64 | 0.0029 | 0.0158 | 13.64 | 13.35 | 14.26 | 0.65 | 0.0132 | 0.0929 |
| TC0900011918.hg.1 | FCN1 | ficolin (collagen/fibrinogen domain containing) 1 | Coding | 5.33 | 4.58 | 4.62 | 1.64 | 0.1071 | 0.2534 | 5.34 | 5.29 | 5.17 | 1.13 | 0.5738 | 0.7813 |
| TC0900012197.hg.1 | PTGDS | prostaglandin D2 synthase 21kDa (brain) | Multiple_C | 4.03 | 3.22 | 3.32 | 1.64 | 0.0795 | 0.2041 | 3.88 | 3.72 | 3.96 | 0.95 | 0.5022 | 0.7344 |
| TC0X00008688.hg.1 | MAGEA9B; M. | MAGE family member A9B; MAGE family member A9 | Coding | 5.11 | 4.1 | 4.4 | 1.64 | 0.0562 | 0.1576 | 4.72 | 4.85 | 4.55 | 1.13 | 0.4611 | 0.7039 |
| TC1000012108.hg.1 | CPXM2 | carboxypeptidase X (M14 family), member 2 | Multiple_C | 4.1 | 3.18 | 3.39 | 1.64 | 0.0988 | 0.2387 | 3.54 | 3.73 | 3.68 | 0.91 | 0.8101 | 0.916 |
| TC1000012472.hg.1 | FAM25A | family with sequence similarity 25, member A | Coding | 9.34 | 8.47 | 8.63 | 1.64 | 0.0703 | 0.1861 | 7.78 | 7.65 | 7.41 | 1.29 | 0.8958 | 0.956 |
| TC1100008127.hg.1 | SSH3 | slingshot protein phosphatase 3 | Multiple_C | 9.65 | 8.94 | 8.94 | 1.64 | 0.0591 | 0.1638 | 10.16 | 9.87 | 9.26 | 1.87 | 0.0015 | 0.0215 |
| TC1200007740.hg.1 | HOXC8 | homeobox C8 | Multiple_C | 4.85 | 4.23 | 4.14 | 1.64 | 0.0671 | 0.1798 | 6.71 | 6.51 | 6.31 | 1.32 | 0.1243 | 0.3484 |
| TC1200012594.hg.1 | ZNF705A; FAI | zinc finger protein 705A; family with sequence similarity 66, me | Multiple_C | 4.49 | 3.75 | 3.78 | 1.64 | 0.0437 | 0.1311 | 4.53 | 4.38 | 4.55 | 0.99 | 0.2429 | 0.5066 |
| TC1200012789.hg.1 | PRKAG1 | protein kinase, AMP-activated, gamma 1 non-catalytic subunit | Multiple_C | 12.86 | 12.83 | 12.15 | 1.64 | 0.0068 | 0.0308 | 13.71 | 13.48 | 13.98 | 0.83 | 0.1393 | 0.371 |
| TC1300008702.hg.1 | ELF1 | E74-like factor 1 (ets domain transcription factor) | Multiple_C | 12.46 | 11.61 | 11.75 | 1.64 | 0.0072 | 0.0323 | 12.9 | 12.90 | 12.06 | 1.79 | 0.0001 | 0.0045 |
| TC1300009905.hg.1 | LOC10192884 | collagen alpha-1(II) chain-like; novel transcript | Multiple_C | 7.56 | 6.56 | 6.85 | 1.64 | 0.0248 | 0.0848 | 6.61 | 6.69 | 6.28 | 1.26 | 0.1125 | 0.3304 |
| TC1400007441.hg.1 | AKAP5 | A kinase (PRKA) anchor protein 5 | Coding | 5.44 | 4.46 | 4.73 | 1.64 | 0.0196 | 0.0703 | 4.76 | 4.39 | 4.75 | 1.01 | 0.9617 | 0.984 |

| | | | | | | | | | | | | | | | |
|---|---|---|---|---|---|---|---|---|---|---|---|---|---|---|---|
| TC1400008748.hg.1 | TGM1 | transglutaminase 1 | Multiple_C | 5.44 | 4.63 | 4.73 | 1.64 | 0.3012 | 0.5135 | 4.56 | 4.58 | 4.57 | 0.99 | 0.7723 | 0.8976 |
| TC1500006969.hg.1 | ZFYVE19 | zinc finger, FYVE domain containing 19 | Multiple_C | 10.07 | 9.77 | 9.36 | 1.64 | 0.0039 | 0.0196 | 9.13 | 9.19 | 8.82 | 1.24 | 0.1418 | 0.3745 |
| TC1600010837.hg.1 | C16orf47 | chromosome 16 open reading frame 47 | Multiple_C | 4.04 | 3.1 | 3.33 | 1.64 | 0.1531 | 0.3267 | 4.36 | 4.35 | 4.09 | 1.21 | 0.1871 | 0.4389 |
| TC1600011315.hg.1 | FAM234A; AR | family with sequence similarity 234, member A; Rho GDP dissoc | Multiple_C | 11.75 | 11.23 | 11.04 | 1.64 | 0.0055 | 0.0261 | 11.39 | 11.51 | 11.62 | 0.85 | 0.1363 | 0.3669 |
| TC1700006744.hg.1 | NLGN2 | neuroligin 2 | Multiple_C | 5.37 | 4.65 | 4.66 | 1.64 | 0.0011 | 0.0072 | 6.12 | 6.22 | 5.65 | 1.39 | 0.0087 | 0.0709 |
| TC1700011131.hg.1 | MBTD1 | mbt domain containing 1 | Multiple_C | 9.07 | 9.49 | 8.36 | 1.64 | 0.0041 | 0.0206 | 10.04 | 9.66 | 9.03 | 2.01 | 0.0002 | 0.0064 |
| TC1800007059.hg.1 | DTNA | dystrobrevin, alpha | Multiple_C | 3.61 | 2.9 | 2.9 | 1.64 | 0.1008 | 0.2423 | 3.12 | 3.09 | 3.35 | 0.85 | 0.6608 | 0.8345 |
| TC1900006827.hg.1 | ADGRE1 | adhesion G protein-coupled receptor E1 | Multiple_C | 4.12 | 3.31 | 3.41 | 1.64 | 0.0597 | 0.165 | 3.72 | 3.76 | 3.32 | 1.32 | 0.0415 | 0.1876 |
| TC1900009854.hg.1 | NOTCH3 | notch 3 | Multiple_C | 4.56 | 3.79 | 3.85 | 1.64 | 0.0073 | 0.0325 | 4.37 | 4.19 | 4.02 | 1.27 | 0.0547 | 0.2195 |
| TC1900011085.hg.1 | CA11 | carbonic anhydrase XI | Multiple_C | 5.7 | 4.99 | 4.99 | 1.64 | 0.0116 | 0.0469 | 5.1 | 5.23 | 5.01 | 1.06 | 0.9436 | 0.977 |
| TC1900011103.hg.1 | TULP2 | tubby like protein 2 | Multiple_C | 6.21 | 5.36 | 5.5 | 1.64 | 0.0408 | 0.1244 | 5.76 | 5.82 | 5.68 | 1.06 | 0.6376 | 0.8222 |
| TC2000006559.hg.1 | CDC25B | cell division cycle 25B | Multiple_C | 11.87 | 10.98 | 11.16 | 1.64 | 0.0033 | 0.0174 | 14.26 | 15.06 | 13.99 | 1.21 | 0.2183 | 0.4781 |
| TC2000009646.hg.1 | CTSZ | cathepsin Z | Multiple_C | 10.93 | 10.09 | 10.22 | 1.64 | 0.0515 | 0.1477 | 12.57 | 13.21 | 12.68 | 0.93 | 0.0501 | 0.2086 |
| TC2100008486.hg.1 | BAGE; BAGE4; | B melanoma antigen; B melanoma antigen family, member 4; B | Multiple_C | 8.27 | 7.51 | 7.56 | 1.64 | 0.0051 | 0.0244 | 6.88 | 6.85 | 6.57 | 1.24 | 0.8191 | 0.9201 |
| TC2100008560.hg.1 | DONSON; CRY | downstream neighbor of SON; crystallin zeta like 1 | Multiple_C | 10.88 | 11.8 | 10.17 | 1.64 | 0.0147 | 0.0562 | 9.68 | 8.93 | 8.78 | 1.87 | 0.001 | 0.0162 |
| TC2200008007.hg.1 | ARVCF | armadillo repeat gene deleted in velocardiofacial syndrome | Multiple_C | 4.58 | 3.77 | 3.87 | 1.64 | 0.1326 | 0.2956 | 4.53 | 4.69 | 4.47 | 1.04 | 0.8358 | 0.9277 |
| TC2200008859.hg.1 | NDUFA6 | NADH dehydrogenase (ubiquinone) 1 alpha subcomplex, 6, 14kI | Multiple_C | 15.13 | 15 | 14.42 | 1.64 | 0.0128 | 0.0508 | 10.48 | 10.81 | 10.41 | 1.05 | 0.5799 | 0.7852 |
| TC0100009480.hg.1 | HIPK1 | homeodomain interacting protein kinase 1 | Multiple_C | 12.73 | 11.17 | 12.03 | 1.62 | 0.0138 | 0.0535 | 11.3 | 10.92 | 11.49 | 0.88 | 0.2763 | 0.5424 |
| TC0100015943.hg.1 | DPM3 | dolichyl-phosphate mannosyltransferase polypeptide 3 | Multiple_C | 7.85 | 7.04 | 7.15 | 1.62 | 0.0344 | 0.1095 | 5.91 | 6.11 | 6 | 0.94 | 0.8145 | 0.9177 |
| TC0200007759.hg.1 | EHBP1 | EH domain binding protein 1 | Multiple_C | 9.74 | 9.27 | 9.04 | 1.62 | 0.0339 | 0.1083 | 9.11 | 8.23 | 9.1 | 1.01 | 0.751 | 0.8866 |
| TC0200007854.hg.1 | MEIS1 | Meis homeobox 1 | Multiple_C | 4.86 | 4.25 | 4.16 | 1.62 | 0.1425 | 0.3105 | 6.33 | 5.98 | 6.12 | 1.16 | 0.1023 | 0.3132 |
| TC0200010607.hg.1 | CREB1 | cAMP responsive element binding protein 1 | Multiple_C | 10.53 | 9.86 | 9.83 | 1.62 | 0.0094 | 0.0395 | 10.44 | 10.39 | 10.55 | 0.93 | 0.3773 | 0.6376 |
| TC0200014385.hg.1 | CCNT2-AS1 | CCNT2 antisense RNA 1 | Multiple_C | 7.61 | 6.88 | 6.91 | 1.62 | 0.0014 | 0.0089 | 7.76 | 8.00 | 7.79 | 0.98 | 0.8233 | 0.9219 |
| TC0200016604.hg.1 | RBM44 | RNA binding motif protein 44 | Multiple_C | 4.31 | 3.69 | 3.61 | 1.62 | 0.215 | 0.4105 | 4.34 | 3.93 | 3.82 | 1.43 | 0.0245 | 0.1358 |
| TC0300006694.hg.1 | NR2C2 | nuclear receptor subfamily 2, group C, member 2 | Multiple_C | 10.83 | 9.95 | 10.13 | 1.62 | 0.0189 | 0.0685 | 11.22 | 11.29 | 11.5 | 0.82 | 0.2486 | 0.5134 |
| TC0300006847.hg.1 | NR1D2 | nuclear receptor subfamily 1, group D, member 2 | Multiple_C | 10.97 | 11.64 | 10.27 | 1.62 | 0.0455 | 0.1349 | 8.71 | 7.22 | 7.84 | 1.83 | 0.0009 | 0.0155 |
| TC0300011860.hg.1 | RPL24 | ribosomal protein L24 | Multiple_C | 16.33 | 16.22 | 15.63 | 1.62 | 0.0733 | 0.1916 | 13.59 | 13.04 | 12.73 | 1.82 | 0.0013 | 0.0201 |
| TC0300012021.hg.1 | CFAP44 | cilia and flagella associated protein 44 | Multiple_C | 6.72 | 6.14 | 6.02 | 1.62 | 0.1707 | 0.3515 | 5.93 | 5.51 | 5.46 | 1.39 | 0.631 | 0.8184 |
| TC0400006785.hg.1 | USP17L11; US | ubiquitin specific peptidase 17-like family member 11; ubiquitin | Coding | 4.35 | 3.77 | 3.65 | 1.62 | 0.08 | 0.2051 | 4.05 | 4.20 | 4.28 | 0.85 | 0.8764 | 0.9471 |
| TC0400007459.hg.1 | OCIAD1 | OCIA domain containing 1 | Multiple_C | 12.35 | 12.66 | 11.65 | 1.62 | 0.0382 | 0.1184 | 11.01 | 10.80 | 10.47 | 1.45 | 0.0275 | 0.1451 |
| TC0400010867.hg.1 | EPHA5 | EPH receptor A5 | Coding | 4.45 | 3.52 | 3.75 | 1.62 | 0.01 | 0.0418 | 4.19 | 3.87 | 4.1 | 1.06 | 0.2439 | 0.5078 |
| TC0500007077.hg.1 | NPR3 | natriuretic peptide receptor 3 | Multiple_C | 4.37 | 4.04 | 3.67 | 1.62 | 0.0133 | 0.0522 | 4.25 | 3.85 | 3.88 | 1.29 | 0.3112 | 0.5754 |
| TC0500009521.hg.1 | CPEB4 | cytoplasmic polyadenylation element binding protein 4 | Multiple_C | 6.96 | 6.38 | 6.26 | 1.62 | 0.0283 | 0.0939 | 9.33 | 7.88 | 8.6 | 1.66 | 0.0044 | 0.0456 |

| | | | | | | | | | | | | | | | |
|---|---|---|---|---|---|---|---|---|---|---|---|---|---|---|---|
| TC0500009594.hg.1 | CDHR2 | cadherin-related family member 2 | Multiple_C | 5.44 | 4.51 | 4.74 | 1.62 | 0.0732 | 0.1915 | 6.32 | 7.63 | 6.84 | 0.70 | 0.0028 | 0.0341 |
| TC0600007629.hg.1 | CYP21A2 | cytochrome P450, family 21, subfamily A, polypeptide 2 | Multiple_C | 4.09 | 3.58 | 3.39 | 1.62 | 0.156 | 0.3309 | 4.28 | 4.36 | 4.26 | 1.01 | 0.3764 | 0.6367 |
| TC0600008102.hg.1 | MAD2L1BP | MAD2L1 binding protein | Multiple_C | 11.64 | 12.19 | 10.94 | 1.62 | 0.0164 | 0.0613 | 10.14 | 10.56 | 10.68 | 0.69 | 0.138 | 0.3689 |
| TC0600013186.hg.1 | CTGF | connective tissue growth factor | Multiple_C | 8.14 | 7.71 | 7.44 | 1.62 | 0.0806 | 0.206 | 5.66 | 6.00 | 5.65 | 1.01 | 0.1294 | 0.3562 |
| TC0700006795.hg.1 | AHR | aryl hydrocarbon receptor | Multiple_C | 16.56 | 15.63 | 15.86 | 1.62 | 0.1111 | 0.26 | 13.92 | 11.34 | 12.11 | 3.51 | 3.93E-05 | 0.0019 |
| TC0700008818.hg.1 | ZNF277 | zinc finger protein 277 | Multiple_C | 10.62 | 11.42 | 9.92 | 1.62 | 0.0862 | 0.216 | 8.77 | 8.23 | 8.24 | 1.44 | 0.4591 | 0.7024 |
| TC0700011554.hg.1 | PMS2P3 | PMS1 homolog 2, mismatch repair system component pseudog | Multiple_C | 11.11 | 10.44 | 10.41 | 1.62 | 0.0084 | 0.0362 | 11.03 | 10.81 | 11.28 | 0.84 | 0.138 | 0.3689 |
| TC0700013597.hg.1 | SAP25 | Sin3A associated protein 25kDa | Multiple_C | 4.3 | 3.79 | 3.6 | 1.62 | 0.044 | 0.1317 | 5.17 | 5.14 | 4.71 | 1.38 | 0.1725 | 0.4197 |
| TC0900012234.hg.1 | CBWD6; CBW | COBW domain containing 6; COBW domain containing 5; COBW | Multiple_C | 11.45 | 12.32 | 10.75 | 1.62 | 0.2076 | 0.4011 | 8.78 | 8.58 | 8.57 | 1.16 | 0.1275 | 0.3534 |
| TC0X00009069.hg.1 | ARHGAP6 | Rho GTPase activating protein 6 | Multiple_C | 9.15 | 9.02 | 8.45 | 1.62 | 0.0962 | 0.2341 | 3.82 | 3.82 | 3.44 | 1.30 | 0.1609 | 0.4036 |
| TC0X00010954.hg.1 | FGF13; LINC0 | fibroblast growth factor 13; long intergenic non-protein coding | Multiple_C | 6.11 | 5.56 | 5.41 | 1.62 | 0.0428 | 0.1292 | 6.5 | 6.68 | 6.5 | 1.00 | 0.9545 | 0.9814 |
| TC1100006516.hg.1 | BRSK2 | BR serine/threonine kinase 2 | Multiple_C | 5.51 | 4.7 | 4.81 | 1.62 | 0.2768 | 0.486 | 5.54 | 5.13 | 5.01 | 1.44 | 0.1134 | 0.3321 |
| TC1100013020.hg.1 | PPP1R32 | protein phosphatase 1, regulatory subunit 32 | Multiple_C | 4.07 | 3.44 | 3.37 | 1.62 | 0.0115 | 0.0464 | 4.6 | 4.27 | 3.91 | 1.61 | 0.0073 | 0.0629 |
| TC1200012181.hg.1 | SPPL3 | signal peptide peptidase like 3 | Multiple_C | 10.28 | 8.74 | 9.58 | 1.62 | 0.022 | 0.0773 | 8.36 | 7.99 | 9.25 | 0.54 | 0.0004 | 0.0096 |
| TC1300007052.hg.1 | GTF2F2 | general transcription factor IIF subunit 2 | Multiple_C | 11.83 | 12.6 | 11.13 | 1.62 | 0.149 | 0.3205 | 11.11 | 9.98 | 9.36 | 3.36 | 6.84E-06 | 0.0005 |
| TC1400006571.hg.1 | OR4E2 | olfactory receptor, family 4, subfamily E, member 2 | Coding | 5.3 | 4.22 | 4.6 | 1.62 | 0.0212 | 0.075 | 4.43 | 4.32 | 4.53 | 0.93 | 0.6284 | 0.8166 |
| TC1400010773.hg.1 | DIO2 | deiodinase, iodothyronine, type II | Multiple_C | 4.49 | 3.64 | 3.79 | 1.62 | 0.014 | 0.0541 | 4.73 | 4.51 | 4.37 | 1.28 | 0.1306 | 0.358 |
| TC1500007346.hg.1 | CGNL1 | cingulin-like 1 | Multiple_C | 4.02 | 3.3 | 3.32 | 1.62 | 0.1331 | 0.2963 | 8.5 | 8.30 | 7.56 | 1.92 | 0.0029 | 0.0345 |
| TC1500009353.hg.1 | CEP152 | centrosomal protein 152kDa | Multiple_C | 9.46 | 9.04 | 8.76 | 1.62 | 0.0656 | 0.1771 | 9.57 | 8.71 | 8.99 | 1.49 | 0.0319 | 0.1598 |
| TC1500010945.hg.1 | LINS1 | lines homolog 1 | Multiple_C | 9.43 | 9.36 | 8.73 | 1.62 | 0.0897 | 0.2224 | 8.15 | 7.29 | 7.78 | 1.29 | 0.0148 | 0.0996 |
| TC1600011058.hg.1 | TLDC1 | TBC/LysM-associated domain containing 1 | Multiple_C | 8.56 | 7.86 | 7.86 | 1.62 | 0.015 | 0.0571 | 6.7 | 6.71 | 6.53 | 1.13 | 0.6427 | 0.8248 |
| TC1700007630.hg.1 | TBC1D3G | TBC1 domain family, member 3G | Coding | 8.34 | 7.9 | 7.64 | 1.62 | 0.0047 | 0.0228 | 8.38 | 8.07 | 7.64 | 1.67 | 0.0018 | 0.0251 |
| TC1700007777.hg.1 | THRA | thyroid hormone receptor, alpha | Multiple_C | 9.74 | 9.14 | 9.04 | 1.62 | 0.0129 | 0.0511 | 10.26 | 9.67 | 9.78 | 1.39 | 0.4756 | 0.7147 |
| TC1700012458.hg.1 | CEP112 | centrosomal protein 112kDa | Multiple_C | 3.95 | 3.01 | 3.25 | 1.62 | 0.0533 | 0.1516 | 6.32 | 6.82 | 6.29 | 1.02 | 0.3819 | 0.641 |
| TC1800006487.hg.1 | SMCHD1 | structural maintenance of chromosomes flexible hinge domain | Multiple_C | 9.54 | 9.13 | 8.84 | 1.62 | 0.0143 | 0.0549 | 10.47 | 10.53 | 10.69 | 0.86 | 0.9751 | 0.9902 |
| TC1800009285.hg.1 | RPL17; SNORD | ribosomal protein L17; small nucleolar RNA, C/D box 58A; small | Multiple_C | 18.26 | 18.52 | 17.56 | 1.62 | 0.0181 | 0.0662 | 14.98 | 14.99 | 14.31 | 1.59 | 0.0085 | 0.07 |
| TC1900007963.hg.1 | ZNF383 | zinc finger protein 383 | Multiple_C | 9.62 | 10.04 | 8.92 | 1.62 | 0.0326 | 0.1051 | 8.31 | 7.75 | 8.13 | 1.13 | 0.3592 | 0.622 |
| TC1900008020.hg.1 | ACTN4 | actinin, alpha 4 | Multiple_C | 15.13 | 13.38 | 14.43 | 1.62 | 0.0147 | 0.0562 | 16.05 | 15.64 | 15.17 | 1.84 | 0.0009 | 0.0159 |
| TC1900008696.hg.1 | ZNF880 | zinc finger protein 880 | Multiple_C | 7.66 | 7.1 | 6.96 | 1.62 | 0.1178 | 0.2713 | 6.29 | 6.28 | 6.1 | 1.14 | 0.5342 | 0.7563 |
| TC2200008827.hg.1 | TOB2 | transducer of ERBB2, 2 | Coding | 10.66 | 9.3 | 9.96 | 1.62 | 0.0794 | 0.2039 | 8.76 | 9.25 | 9.52 | 0.59 | 0.03 | 0.1536 |
| TC2200009248.hg.1 | KREMEN1 | kringle containing transmembrane protein 1 | Multiple_C | 7.66 | 7 | 6.96 | 1.62 | 0.0996 | 0.2404 | 11.35 | 11.14 | 11.43 | 0.95 | 0.9344 | 0.9724 |
| TC0100006977.hg.1 | TMEM51 | transmembrane protein 51 | Coding | 8.93 | 7.78 | 8.24 | 1.61 | 0.409 | 0.6213 | 6.11 | 6.22 | 6.19 | 0.95 | 0.9197 | 0.9671 |
| TC0100009944.hg.1 | PIP5K1A | phosphatidylinositol-4-phosphate 5-kinase, type I, alpha | Multiple_C | 12.94 | 11.34 | 12.25 | 1.61 | 0.0121 | 0.0485 | 12.18 | 11.05 | 12.21 | 0.98 | 0.7413 | 0.881 |

| | | | | | | | | | | | | | | | |
|---|---|---|---|---|---|---|---|---|---|---|---|---|---|---|---|
| TC0100018298.hg.1 | INTS3 | integrator complex subunit 3 | Multiple_C | 10.82 | 9.51 | 10.13 | 1.61 | 0.0335 | 0.1071 | 10.6 | 10.95 | 10.33 | 1.21 | 0.8943 | 0.9552 |
| TC0200007363.hg.1 | MTA3 | metastasis associated 1 family member 3 | Multiple_C | 9.34 | 9.29 | 8.65 | 1.61 | 0.0098 | 0.0409 | 7.82 | 7.64 | 8.1 | 0.82 | 0.2155 | 0.4742 |
| TC0200015211.hg.1 | SLC40A1 | solute carrier family 40 (iron-regulated transporter), member 1 | Multiple_C | 9.3 | 8.68 | 8.61 | 1.61 | 0.0554 | 0.156 | 8.05 | 7.66 | 6.91 | 2.20 | 9.27E-05 | 0.0034 |
| TC0200016454.hg.1 | KIAA1841 | KIAA1841 | Multiple_C | 7.08 | 6.74 | 6.39 | 1.61 | 0.0447 | 0.1334 | 7.93 | 8.08 | 7.66 | 1.21 | 0.8602 | 0.9402 |
| TC0200016603.hg.1 | LRRFIP1 | leucine rich repeat (in FLII) interacting protein 1 | Multiple_C | 10.92 | 9.77 | 10.23 | 1.61 | 0.128 | 0.2879 | 8.13 | 8.16 | 8.39 | 0.84 | 0.0639 | 0.2404 |
| TC0300011517.hg.1 | PROK2 | prokineticin 2 | Coding | 5.15 | 4.66 | 4.46 | 1.61 | 0.0646 | 0.1751 | 4.42 | 4.41 | 4.72 | 0.81 | 0.1982 | 0.4532 |
| TC0300012245.hg.1 | ZNF148 | zinc finger protein 148 | Multiple_C | 12.57 | 12.59 | 11.88 | 1.61 | 0.0038 | 0.0194 | 11.37 | 11.02 | 11.79 | 0.75 | 0.0069 | 0.0605 |
| TC0300012591.hg.1 | PRR23C | proline rich 23C | Coding | 5.64 | 5.04 | 4.95 | 1.61 | 0.0072 | 0.0323 | 5.4 | 5.34 | 4.99 | 1.33 | 0.0339 | 0.1658 |
| TC0400009458.hg.1 | CDKN2AIP | CDKN2A interacting protein | Multiple_C | 10.51 | 10.17 | 9.82 | 1.61 | 0.0261 | 0.0881 | 8.72 | 8.70 | 9.05 | 0.80 | 0.1834 | 0.4342 |
| TC0400012756.hg.1 | CFAP99 | cilia and flagella associated protein 99 | Multiple_C | 5.92 | 4.9 | 5.23 | 1.61 | 0.0228 | 0.0795 | 5.65 | 5.31 | 5.04 | 1.53 | 0.2502 | 0.5146 |
| TC0500008008.hg.1 | COX7C; MIR36 | cytochrome c oxidase subunit VIIc; microRNA 3607 | Multiple_C | 16.53 | 17.48 | 15.84 | 1.61 | 0.0107 | 0.0442 | 11.84 | 11.11 | 11.18 | 1.58 | 0.015 | 0.1001 |
| TC0500008494.hg.1 | CSNK1G3 | casein kinase 1, gamma 3 | Multiple_C | 10.76 | 10.86 | 10.07 | 1.61 | 0.0839 | 0.2116 | 10.32 | 8.96 | 9.47 | 1.80 | 0.0127 | 0.0908 |
| TC0500008558.hg.1 | PRRC1 | proline-rich coiled-coil 1 | Multiple_C | 10.77 | 10.85 | 10.08 | 1.61 | 0.0691 | 0.1834 | 11.35 | 10.47 | 11.33 | 1.01 | 0.6528 | 0.8307 |
| TC0500008730.hg.1 | SLC25A48 | solute carrier family 25, member 48 | Multiple_C | 5.09 | 4.4 | 4.4 | 1.61 | 0.0848 | 0.2134 | 4.46 | 4.10 | 4.09 | 1.29 | 0.5483 | 0.7651 |
| TC0500011049.hg.1 | NAIP | NLR family, apoptosis inhibitory protein | Multiple_C | 5.92 | 5.68 | 5.23 | 1.61 | 0.2271 | 0.4263 | 8.16 | 8.41 | 7.88 | 1.21 | 0.4754 | 0.7146 |
| TC0600008319.hg.1 | FAM83B | family with sequence similarity 83, member B | Multiple_C | 10.82 | 9.49 | 10.13 | 1.61 | 0.0147 | 0.0561 | 3.31 | 2.72 | 2.75 | 1.47 | 0.019 | 0.1166 |
| TC0700008090.hg.1 | HSPB1 | heat shock 27kDa protein 1 | Multiple_C | 16.18 | 13.71 | 15.49 | 1.61 | 0.0383 | 0.1187 | 11.99 | 12.52 | 13.51 | 0.35 | 0.0005 | 0.011 |
| TC0700008181.hg.1 | GNAI1 | guanine nucleotide binding protein (G protein), alpha inhibiting | Multiple_C | 12.98 | 12.46 | 12.29 | 1.61 | 0.0615 | 0.1688 | 13.93 | 13.05 | 13.23 | 1.62 | 0.2317 | 0.4929 |
| TC0700010245.hg.1 | COL28A1 | collagen, type XXVIII, alpha 1 | Multiple_C | 6.38 | 5.63 | 5.69 | 1.61 | 0.0507 | 0.146 | 5.67 | 5.72 | 5.27 | 1.32 | 0.1546 | 0.3942 |
| TC0700011842.hg.1 | PDK4 | pyruvate dehydrogenase kinase, isozyme 4 | Multiple_C | 8.18 | 8.7 | 7.49 | 1.61 | 0.0589 | 0.1634 | 4.83 | 4.72 | 5.28 | 0.73 | 0.0506 | 0.2096 |
| TC0700013394.hg.1 | CCDC146 | coiled-coil domain containing 146 | Multiple_C | 4.9 | 5.32 | 4.21 | 1.61 | 0.09 | 0.223 | 4.86 | 4.12 | 3.7 | 2.23 | 0.0167 | 0.1081 |
| TC0800007077.hg.1 | PPP2R2A | protein phosphatase 2, regulatory subunit B, alpha | Multiple_C | 13.86 | 13.41 | 13.17 | 1.61 | 0.0077 | 0.0338 | 12.88 | 11.77 | 12.56 | 1.25 | 0.0276 | 0.1456 |
| TC0800009816.hg.1 | GFRA2 | GDNF family receptor alpha 2 | Multiple_C | 3.28 | 2.48 | 2.59 | 1.61 | 0.0092 | 0.0388 | 3.25 | 3.61 | 3.4 | 0.90 | 0.3054 | 0.5695 |
| TC0900010925.hg.1 | ZNF782 | zinc finger protein 782 | Multiple_C | 5.9 | 5.34 | 5.21 | 1.61 | 0.0043 | 0.0214 | 5.37 | 5.07 | 5.06 | 1.24 | 0.2417 | 0.5056 |
| TC0X00009227.hg.1 | SMPX | small muscle protein, X-linked | Multiple_C | 3.42 | 2.7 | 2.73 | 1.61 | 0.1542 | 0.3282 | 3.69 | 3.60 | 3.25 | 1.36 | 0.1407 | 0.3728 |
| TC1000009881.hg.1 | PPIAP30 | peptidylprolyl isomerase A (cyclophilin A) pseudogene 30 | Multiple_C | 5.61 | 4.59 | 4.92 | 1.61 | 0.1771 | 0.3597 | 4.52 | 4.35 | 4.5 | 1.01 | 0.801 | 0.9111 |
| TC1100006995.hg.1 | SAA1 | serum amyloid A1 | Multiple_C | 4.91 | 4.25 | 4.22 | 1.61 | 0.1128 | 0.263 | 4 | 4.09 | 3.91 | 1.06 | 0.6464 | 0.8265 |
| TC1100008126.hg.1 | ANKRD13D | ankyrin repeat domain 13 family, member D | Multiple_C | 6.9 | 5.94 | 6.21 | 1.61 | 0.025 | 0.0853 | 5.95 | 5.89 | 5.9 | 1.04 | 0.9451 | 0.9777 |
| TC1100009068.hg.1 | NCAM1 | neural cell adhesion molecule 1 | Multiple_C | 4.04 | 3.18 | 3.35 | 1.61 | 0.0719 | 0.1892 | 4.02 | 4.02 | 3.73 | 1.22 | 0.249 | 0.5137 |
| TC1100011337.hg.1 | PITPNM1 | phosphatidylinositol transfer protein, membrane-associated 1 | Multiple_C | 5.51 | 4.65 | 4.82 | 1.61 | 0.0614 | 0.1684 | 4.77 | 4.77 | 4.58 | 1.14 | 0.0573 | 0.2255 |
| TC1200006438.hg.1 | IQSEC3 | IQ motif and Sec7 domain 3 | Multiple_C | 7.23 | 6.48 | 6.54 | 1.61 | 0.0102 | 0.0424 | 7.01 | 7.09 | 7.06 | 0.97 | 0.7706 | 0.8971 |
| TC1200010588.hg.1 | KANSL2; SNOI | KAT8 regulatory NSL complex subunit 2; small nucleolar RNA, H, | Multiple_C | 11.69 | 11.21 | 11 | 1.61 | 0.003 | 0.0159 | 11.42 | 11.27 | 11.64 | 0.86 | 0.3361 | 0.6005 |
| TC1200011527.hg.1 | RPL41P5 | ribosomal protein L41 pseudogene 5 | Multiple_C | 16.67 | 16.78 | 15.98 | 1.61 | 0.0436 | 0.1309 | 14.81 | 14.70 | 14.41 | 1.32 | 0.1202 | 0.3423 |

| | | | | | | | | | | | | | | | |
|---|---|---|---|---|---|---|---|---|---|---|---|---|---|---|---|
| TC1300008025.hg.1 | ING1 | inhibitor of growth family member 1 | Coding | 9.91 | 9.2 | 9.22 | 1.61 | 0.0514 | 0.1475 | 7.54 | 7.70 | 7.59 | 0.97 | 0.692 | 0.853 |
| TC1300009924.hg.1 | GAS6 | growth arrest-specific 6 | Multiple_C | 7.36 | 6.54 | 6.67 | 1.61 | 0.1522 | 0.3256 | 9.14 | 8.75 | 7.97 | 2.25 | 0.0002 | 0.0051 |
| TC1400007320.hg.1 | DACT1 | dishevelled-binding antagonist of beta-catenin 1 | Multiple_C | 5.09 | 4.44 | 4.4 | 1.61 | 0.0064 | 0.0295 | 4.34 | 4.56 | 4.26 | 1.06 | 0.7677 | 0.8958 |
| TC1400008019.hg.1 | GOLGA5 | golgin A5 | Multiple_C | 11.9 | 12.78 | 11.21 | 1.61 | 0.1748 | 0.3566 | 8.84 | 8.92 | 8.61 | 1.17 | 0.2134 | 0.4716 |
| TC1500009082.hg.1 | BMF | Bcl2 modifying factor | Multiple_C | 5.94 | 5.33 | 5.25 | 1.61 | 0.0092 | 0.0388 | 5.87 | 5.34 | 5.35 | 1.43 | 0.0502 | 0.2087 |
| TC1500009263.hg.1 | PATL2 | protein associated with topoisomerase II homolog 2 (yeast) | Multiple_C | 4.19 | 3.82 | 3.5 | 1.61 | 0.1108 | 0.2596 | 3.99 | 4.05 | 4.18 | 0.88 | 0.5136 | 0.7431 |
| TC1500010119.hg.1 | LINGO1 | leucine rich repeat and Ig domain containing 1 | Multiple_C | 7.84 | 7.05 | 7.15 | 1.61 | 0.0378 | 0.1175 | 8.03 | 7.75 | 7.58 | 1.37 | 0.0081 | 0.0675 |
| TC1600006893.hg.1 | CLEC16A | C-type lectin domain family 16, member A | Multiple_C | 8.9 | 7.72 | 8.21 | 1.61 | 0.0097 | 0.0407 | 9.09 | 8.84 | 8.97 | 1.09 | 0.7856 | 0.9042 |
| TC1600008735.hg.1 | FOXF1 | forkhead box F1 | Multiple_C | 8.04 | 7.11 | 7.35 | 1.61 | 0.0036 | 0.0185 | 7.38 | 7.35 | 7.29 | 1.06 | 0.7189 | 0.8689 |
| TC1700006556.hg.1 | PAFAH1B1 | platelet-activating factor acetylhydrolase 1b, regulatory subunit | Multiple_C | 12.93 | 12.81 | 12.24 | 1.61 | 0.0135 | 0.0528 | 13.14 | 12.71 | 12.97 | 1.13 | 0.3431 | 0.6068 |
| TC1700010734.hg.1 | PLEKHH3 | pleckstrin homology domain containing, family H (with MyTH4 c | Multiple_C | 5.56 | 4.79 | 4.87 | 1.61 | 0.0017 | 0.0101 | 4.32 | 4.35 | 4.38 | 0.96 | 0.5952 | 0.7959 |
| TC1700011795.hg.1 | PRPSAP1 | phosphoribosyl pyrophosphate synthetase-associated protein 1 | Multiple_C | 10.23 | 10.39 | 9.54 | 1.61 | 0.0054 | 0.0254 | 8.68 | 8.70 | 8.78 | 0.93 | 0.3774 | 0.6376 |
| TC1700012439.hg.1 | RNF43 | ring finger protein 43 | Multiple_C | 9.91 | 8.69 | 9.22 | 1.61 | 0.0017 | 0.0101 | 10.14 | 10.85 | 10.75 | 0.66 | 0.0057 | 0.0533 |
| TC1700012469.hg.1 | SUMO2 | small ubiquitin-like modifier 2 | Multiple_C | 16.43 | 16.98 | 15.74 | 1.61 | 0.0041 | 0.0204 | 15.2 | 15.01 | 14.98 | 1.16 | 0.2768 | 0.5428 |
| TC1900010597.hg.1 | YIF1B | Yip1 interacting factor homolog B (S. cerevisiae) | Multiple_C | 10.64 | 8.89 | 9.95 | 1.61 | 0.03 | 0.0984 | 8.64 | 8.37 | 8.74 | 0.93 | 0.7894 | 0.9059 |
| TC1900011924.hg.1 | ZNF91 | zinc finger protein 91 | Multiple_C | 4.24 | 3.67 | 3.55 | 1.61 | 0.0406 | 0.1242 | 4.05 | 3.76 | 3.55 | 1.41 | 0.1878 | 0.4397 |
| TC2000006520.hg.1 | EBF4 | early B-cell factor 4 | Multiple_C | 4.08 | 3.08 | 3.39 | 1.61 | 0.3487 | 0.5618 | 4.36 | 4.04 | 4.02 | 1.27 | 0.0226 | 0.1296 |
| TC2000007760.hg.1 | TSHZ2 | teashirt zinc finger homeobox 2 | Coding | 3.48 | 2.6 | 2.79 | 1.61 | 0.0192 | 0.0692 | 3.61 | 4.62 | 3.76 | 0.90 | 0.7451 | 0.8832 |
| TC2000009447.hg.1 | DPM1 | dolichyl-phosphate mannosyltransferase polypeptide 1, catalyti | Multiple_C | 13.63 | 14.79 | 12.94 | 1.61 | 0.0061 | 0.0282 | 11.33 | 11.14 | 10.57 | 1.69 | 0.0001 | 0.0042 |
| TC2000009848.hg.1 | UCKL1; MIR19 | uridine-cytidine kinase 1-like 1; microRNA 1914 | Multiple_C | 9.89 | 9.26 | 9.2 | 1.61 | 0.0731 | 0.1912 | 8.46 | 9.55 | 9.05 | 0.66 | 0.3633 | 0.626 |
| TC2200009234.hg.1 | SPECC1L-ADO | SPECC1L-ADORA2A readthrough (NMD candidate) | Multiple_C | 4.24 | 3.61 | 3.55 | 1.61 | 0.0807 | 0.2061 | 5.21 | 5.33 | 5.85 | 0.64 | 0.8044 | 0.913 |
| TSUnmapped00000432.h | NDUFA6 | NADH dehydrogenase (ubiquinone) 1 alpha subcomplex, 6, 14kI | Coding | 6.13 | 5.98 | 5.44 | 1.61 | 0.0061 | 0.0284 | 5.44 | 5.52 | 5.15 | 1.22 | 0.3803 | 0.64 |
| TC0100009339.hg.1 | TMEM167B | transmembrane protein 167B | Multiple_C | 9.71 | 10.26 | 9.03 | 1.60 | 0.0245 | 0.084 | 8.12 | 7.25 | 8.23 | 0.93 | 0.7605 | 0.8915 |
| TC0100015815.hg.1 | THEM5 | thioesterase superfamily member 5 | Coding | 6.08 | 5.17 | 5.4 | 1.60 | 0.0761 | 0.1969 | 5.47 | 5.50 | 5.72 | 0.84 | 0.5788 | 0.7844 |
| TC0100018572.hg.1 | LYPD8 | LY6/PLAUR domain containing 8 | Coding | 4.58 | 4.06 | 3.9 | 1.60 | 0.0533 | 0.1516 | 4.23 | 4.21 | 4.27 | 0.97 | 0.8692 | 0.9436 |
| TC0200009883.hg.1 | XIRP2 | xin actin binding repeat containing 2 | Coding | 4.8 | 4.05 | 4.12 | 1.60 | 0.0226 | 0.0789 | 4.42 | 3.98 | 3.85 | 1.48 | 0.0838 | 0.2785 |
| TC0200014870.hg.1 | METTL5 | methyltransferase like 5 | Multiple_C | 14.06 | 15.07 | 13.38 | 1.60 | 0.0605 | 0.1667 | 10.5 | 10.18 | 10.22 | 1.21 | 0.0932 | 0.2965 |
| TC0200016494.hg.1 | CNNM4 | cyclin and CBS domain divalent metal cation transport mediator | Multiple_C | 10.51 | 8.47 | 9.83 | 1.60 | 0.0223 | 0.078 | 8.48 | 8.08 | 8.14 | 1.27 | 0.038 | 0.1776 |
| TC0300006520.hg.1 | LMCD1; LINC0 | LIM and cysteine-rich domains 1; long intergenic non-protein cc | Multiple_C | 4.6 | 3.8 | 3.92 | 1.60 | 0.0517 | 0.148 | 6.92 | 6.72 | 7.17 | 0.84 | 0.7661 | 0.895 |
| TC0300009289.hg.1 | LEKR1 | leucine, glutamate and lysine rich 1 | Multiple_C | 5.51 | 5.46 | 4.83 | 1.60 | 0.2356 | 0.4367 | 5.43 | 5.25 | 4.9 | 1.44 | 0.0186 | 0.115 |
| TC0300013859.hg.1 | CD200 | CD200 molecule | Multiple_C | 3.57 | 2.63 | 2.89 | 1.60 | 0.0298 | 0.0977 | 3.78 | 3.62 | 3.39 | 1.31 | 0.4415 | 0.6906 |
| TC0400007495.hg.1 | DCUN1D4 | DCN1, defective in cullin neddylation 1, domain containing 4 | Multiple_C | 10.01 | 10.64 | 9.33 | 1.60 | 0.089 | 0.2212 | 8.45 | 8.12 | 8.75 | 0.81 | 0.5567 | 0.7706 |
| TC0400007851.hg.1 | EPGN | epithelial mitogen | Multiple_C | 4.01 | 3.35 | 3.33 | 1.60 | 0.0054 | 0.0254 | 3.34 | 3.24 | 3.45 | 0.93 | 0.9748 | 0.9901 |

| | | | | | | | | | | | | | | | |
|---|---|---|---|---|---|---|---|---|---|---|---|---|---|---|---|
| TC0400010939.hg.1 | UGT2A2; UGT | UDP glucuronosyltransferase 2 family, polypeptide A2; UDP gluc | Multiple_C | 5.33 | 4.84 | 4.65 | 1.60 | 0.0786 | 0.2021 | 4.06 | 4.54 | 4.16 | 0.93 | 0.7351 | 0.8777 |
| TC0400011637.hg.1 | RPS26 | Homo sapiens ribosomal protein S26, mRNA (cDNA clone MGC: | Multiple_C | 17.17 | 15.69 | 16.49 | 1.60 | 0.1585 | 0.3346 | 14.08 | 13.96 | 13.69 | 1.31 | 0.9867 | 0.9943 |
| TC0500012935.hg.1 | KIAA1191 | KIAA1191 | Multiple_C | 12.29 | 11.88 | 11.61 | 1.60 | 0.0114 | 0.0463 | 9.69 | 9.75 | 9.95 | 0.84 | 0.7288 | 0.8742 |
| TC0500012979.hg.1 | DDX41 | DEAD (Asp-Glu-Ala-Asp) box polypeptide 41 | Multiple_C | 11.6 | 10.58 | 10.92 | 1.60 | 0.0628 | 0.1711 | 11.29 | 11.42 | 11.82 | 0.69 | 0.0667 | 0.2464 |
| TC0500013174.hg.1 | KIF2A | kinesin heavy chain member 2A | Multiple_C | 11.25 | 10.32 | 10.57 | 1.60 | 0.0176 | 0.0646 | 12.3 | 11.84 | 12.32 | 0.99 | 0.8201 | 0.9205 |
| TC0500013231.hg.1 | CATSPER3 | cation channel, sperm associated 3 | Multiple_C | 5.88 | 4.52 | 5.2 | 1.60 | 0.0083 | 0.0359 | 4.83 | 5.16 | 5.51 | 0.62 | 0.0005 | 0.0101 |
| TC0600009102.hg.1 | FIG4 | FIG4 phosphoinositide 5-phosphatase | Multiple_C | 9.6 | 10.76 | 8.92 | 1.60 | 0.1224 | 0.2788 | 9.11 | 8.32 | 8.47 | 1.56 | 0.0295 | 0.1522 |
| TC0600011402.hg.1 | CDSN | corneodesmosin | Coding | 6.1 | 5.22 | 5.42 | 1.60 | 0.0888 | 0.2208 | 6.27 | 6.20 | 6.13 | 1.10 | 0.3199 | 0.5839 |
| TC0600014260.hg.1 | ATP6V1G2 | ATPase, H+ transporting, lysosomal 13kDa, V1 subunit G2 | Multiple_C | 4.68 | 4 | 4 | 1.60 | 0.0073 | 0.0325 | 4.6 | 4.89 | 4.53 | 1.05 | 0.3851 | 0.644 |
| TC0700011383.hg.1 | SBDS | Shwachman-Bodian-Diamond syndrome | Multiple_C | 13.4 | 13.85 | 12.72 | 1.60 | 0.0735 | 0.192 | 11.49 | 10.94 | 11.57 | 0.95 | 0.954 | 0.9812 |
| TC0700013587.hg.1 | SHFM1 | split hand/foot malformation (ectrodactyly) type 1 | Multiple_C | 4.54 | 4 | 3.86 | 1.60 | 0.0232 | 0.0803 | 3.64 | 3.83 | 4.24 | 0.66 | 0.05 | 0.2083 |
| TC0800009194.hg.1 | RHPN1 | rhophilin, Rho GTPase binding protein 1 | Multiple_C | 4.37 | 3.39 | 3.69 | 1.60 | 0.0322 | 0.1039 | 5.41 | 5.43 | 5.16 | 1.19 | 0.6458 | 0.8261 |
| TC0800011260.hg.1 | SNX31 | sorting nexin 31 | Multiple_C | 4.58 | 4.09 | 3.9 | 1.60 | 0.0455 | 0.1349 | 4.08 | 3.91 | 3.97 | 1.08 | 0.6991 | 0.8573 |
| TC0900008043.hg.1 | C9orf3; MIR24 | chromosome 9 open reading frame 3; microRNA 24-1; microRN | Multiple_C | 9.91 | 9.34 | 9.23 | 1.60 | 0.121 | 0.2764 | 9.8 | 9.83 | 9.68 | 1.09 | 0.4903 | 0.7257 |
| TC0X00009667.hg.1 | FOXP3 | forkhead box P3 | Coding | 4.97 | 4.16 | 4.29 | 1.60 | 0.0478 | 0.1399 | 5.37 | 5.60 | 5.56 | 0.88 | 0.6686 | 0.838 |
| TC0X00010012.hg.1 | CXorf49B; CXo | chromosome X open reading frame 49B; chromosome X open r | Multiple_C | 4.61 | 3.97 | 3.93 | 1.60 | 0.0091 | 0.0386 | 4.43 | 4.57 | 4.54 | 0.93 | 0.4712 | 0.7121 |
| TC1000009893.hg.1 | ITGA8 | integrin alpha 8 | Multiple_C | 3.94 | 3.48 | 3.26 | 1.60 | 0.1003 | 0.2414 | 4.91 | 4.84 | 4.31 | 1.52 | 0.1026 | 0.3136 |
| TC1000010507.hg.1 | buskee; FAM2 | Transcript Identified by AceView; family with sequence similarit | Multiple_C | 7.46 | 6.66 | 6.78 | 1.60 | 0.0146 | 0.0561 | 5.65 | 5.66 | 5.69 | 0.97 | 0.7163 | 0.8675 |
| TC1000011719.hg.1 | CALHM1 | calcium homeostasis modulator 1 | Coding | 3.68 | 2.77 | 3 | 1.60 | 0.0204 | 0.0727 | 4.03 | 3.98 | 3.93 | 1.07 | 0.077 | 0.2673 |
| TC1100008761.hg.1 | FAT3 | FAT atypical cadherin 3 | Multiple_C | 4.87 | 4.15 | 4.19 | 1.60 | 0.0195 | 0.07 | 4.23 | 4.09 | 4.08 | 1.11 | 0.6936 | 0.8542 |
| TC1100009540.hg.1 | C11orf44 | chromosome 11 open reading frame 44 | Multiple_C | 5.31 | 4.68 | 4.63 | 1.60 | 0.113 | 0.2635 | 5.53 | 5.44 | 5.46 | 1.05 | 0.6057 | 0.8027 |
| TC1100012308.hg.1 | CRYAB | crystallin alpha B | Coding | 3.74 | 3.28 | 3.06 | 1.60 | 0.0192 | 0.0691 | 3.98 | 3.56 | 3.59 | 1.31 | 0.0518 | 0.2125 |
| TC1200007615.hg.1 | DIP2B | disco-interacting protein 2 homolog B | Multiple_C | 14.04 | 12.89 | 13.36 | 1.60 | 0.0138 | 0.0535 | 13.17 | 12.82 | 13.38 | 0.86 | 0.1449 | 0.3797 |
| TC1300007197.hg.1 | DLEU1 | deleted in lymphocytic leukemia 1 (non-protein coding) | Multiple_C | 6.91 | 6.7 | 6.23 | 1.60 | 0.0401 | 0.1229 | 6.79 | 7.39 | 7.16 | 0.77 | 0.0512 | 0.2112 |
| TC1400008055.hg.1 | IFI27L1 | interferon, alpha-inducible protein 27-like 1 | Multiple_C | 11.07 | 10.52 | 10.39 | 1.60 | 0.0199 | 0.0713 | 8.35 | 8.44 | 7.77 | 1.49 | 0.0128 | 0.0914 |
| TC1500006968.hg.1 | C15orf62 | chromosome 15 open reading frame 62 | Coding | 5.2 | 4.45 | 4.52 | 1.60 | 0.1676 | 0.3471 | 4.71 | 4.93 | 4.85 | 0.91 | 0.2872 | 0.5523 |
| TC1500009221.hg.1 | TP53BP1 | tumor protein p53 binding protein 1 | Multiple_C | 7.63 | 7.59 | 6.95 | 1.60 | 0.2811 | 0.4911 | 8.4 | 8.27 | 7.89 | 1.42 | 0.0209 | 0.1235 |
| TC1500009458.hg.1 | ARPP19 | cAMP-regulated phosphoprotein 19kDa | Multiple_C | 12.12 | 11.64 | 11.44 | 1.60 | 0.2014 | 0.3935 | 12.2 | 11.86 | 12.21 | 0.99 | 0.1871 | 0.4389 |
| TC1600007803.hg.1 | CNEP1R1 | CTD nuclear envelope phosphatase 1 regulatory subunit 1 | Multiple_C | 10.38 | 11.24 | 9.7 | 1.60 | 0.0811 | 0.2069 | 10.34 | 9.32 | 10.13 | 1.16 | 0.9772 | 0.9906 |
| TC1700006580.hg.1 | OR3A4P; OR3A | olfactory receptor, family 3, subfamily A, member 4 pseudogene | Multiple_C | 4 | 3.45 | 3.32 | 1.60 | 0.3223 | 0.5361 | 4.09 | 4.20 | 4.17 | 0.95 | 0.3308 | 0.5949 |
| TC1700007771.hg.1 | GSDMA | gasdermin A | Coding | 6.07 | 5.36 | 5.39 | 1.60 | 0.0431 | 0.13 | 5.27 | 5.62 | 5.46 | 0.88 | 0.0811 | 0.2746 |
| TC1700011261.hg.1 | SEPT4 | septin 4 | Multiple_C | 5.12 | 4.16 | 4.44 | 1.60 | 0.1774 | 0.3601 | 5.75 | 6.01 | 5.23 | 1.43 | 0.107 | 0.3219 |
| TC1700012445.hg.1 | STRADA | STE20-related kinase adaptor alpha | Multiple_C | 7.71 | 6.36 | 7.03 | 1.60 | 0.0024 | 0.0134 | 10.82 | 10.67 | 10.64 | 1.13 | 0.3872 | 0.6461 |

| | | | | | | | | | | | | | | | |
|---|---|---|---|---|---|---|---|---|---|---|---|---|---|---|---|
| TC1800006757.hg.1 | LDLRAD4 | low density lipoprotein receptor class A domain containing 4 | Multiple_C | 4.39 | 3.81 | 3.71 | 1.60 | 0.0307 | 0.1002 | 4.72 | 4.44 | 4.57 | 1.11 | 0.4638 | 0.7061 |
| TC1800008842.hg.1 | MC4R | melanocortin 4 receptor | Coding | 3.88 | 3.38 | 3.2 | 1.60 | 0.1717 | 0.3529 | 3.94 | 3.96 | 3.83 | 1.08 | 0.4404 | 0.6901 |
| TC1800009258.hg.1 | ZNF519 | zinc finger protein 519 | Multiple_C | 7 | 6.22 | 6.32 | 1.60 | 0.0155 | 0.0586 | 8.53 | 8.65 | 8.87 | 0.79 | 0.2417 | 0.5056 |
| TC1800009278.hg.1 | TCEB3CL | transcription elongation factor B polypeptide 3C-like | Coding | 5.38 | 4.59 | 4.7 | 1.60 | 0.0099 | 0.0413 | 4.51 | 4.67 | 4.41 | 1.07 | 0.7287 | 0.8742 |
| TC1900008012.hg.1 | CATSPERG | catsper channel auxiliary subunit gamma | Multiple_C | 4.18 | 3.57 | 3.5 | 1.60 | 0.0207 | 0.0737 | 4.41 | 4.27 | 3.83 | 1.49 | 0.0149 | 0.0997 |
| TC1900009330.hg.1 | TMIGD2 | transmembrane and immunoglobulin domain containing 2 | Coding | 6.57 | 5.6 | 5.89 | 1.60 | 0.0021 | 0.012 | 4.93 | 5.15 | 4.92 | 1.01 | 0.9565 | 0.9819 |
| TC1900009352.hg.1 | SEMA6B | sema domain, transmembrane domain (TM), and cytoplasmic d | Multiple_C | 4.18 | 3.4 | 3.5 | 1.60 | 0.0776 | 0.2 | 6.65 | 5.78 | 5.61 | 2.06 | 0.0009 | 0.0158 |
| TC1900009528.hg.1 | KANK3 | KN motif and ankyrin repeat domains 3 | Multiple_C | 5.64 | 4.9 | 4.96 | 1.60 | 0.026 | 0.088 | 5.63 | 5.77 | 5.66 | 0.98 | 0.3413 | 0.6051 |
| TC1900010670.hg.1 | CLC | Charcot-Leyden crystal galectin | Coding | 4.8 | 4.29 | 4.12 | 1.60 | 0.1079 | 0.2548 | 5.09 | 5.39 | 4.96 | 1.09 | 0.648 | 0.8279 |
| TC1900011709.hg.1 | FXYD1 | FXYD domain containing ion transport regulator 1 | Multiple_C | 4.51 | 3.61 | 3.83 | 1.60 | 0.096 | 0.2337 | 4.26 | 4.42 | 4.33 | 0.95 | 0.6966 | 0.8561 |
| TC2000006826.hg.1 | LINC00851 | long intergenic non-protein coding RNA 851 | Multiple_C | 5.32 | 4.69 | 4.64 | 1.60 | 0.0044 | 0.0218 | 5.59 | 5.67 | 5.27 | 1.25 | 0.0551 | 0.2203 |
| TC2000009220.hg.1 | TP53TG5 | TP53 target 5 | Multiple_C | 5.22 | 4.28 | 4.54 | 1.60 | 0.0855 | 0.2147 | 5.19 | 4.95 | 4.75 | 1.36 | 0.0519 | 0.2128 |
| TSUnmapped00000253.h | HYOU1 | hypoxia up-regulated 1 | Coding | 14.16 | 13.6 | 13.48 | 1.60 | 0.0515 | 0.1477 | 13.46 | 14.01 | 13.59 | 0.91 | 0.1277 | 0.3536 |
| TC0100010074.hg.1 | AQP10 | aquaporin 10 | Multiple_C | 4.45 | 3.76 | 3.78 | 1.59 | 0.1716 | 0.3529 | 4.02 | 4.35 | 4.19 | 0.89 | 0.1706 | 0.4168 |
| TC0100015591.hg.1 | PIAS3 | protein inhibitor of activated STAT 3 | Multiple_C | 8.89 | 7.71 | 8.22 | 1.59 | 0.2394 | 0.4414 | 7.47 | 7.52 | 7.94 | 0.72 | 0.067 | 0.2469 |
| TC0100018237.hg.1 | RABGGTB; SN | Rab geranylgeranyltransferase, beta subunit; small nucleolar RN | Multiple_C | 13.05 | 13.38 | 12.38 | 1.59 | 0.1251 | 0.2829 | 13.43 | 12.21 | 13.49 | 0.96 | 0.3982 | 0.6556 |
| TC0200008675.hg.1 | IL18RAP | interleukin 18 receptor accessory protein | Multiple_C | 5 | 4.28 | 4.33 | 1.59 | 0.01 | 0.0417 | 4.37 | 4.40 | 4.28 | 1.06 | 0.1926 | 0.4459 |
| TC0200008803.hg.1 | GCC2 | GRIP and coiled-coil domain containing 2 | Multiple_C | 9.71 | 9.46 | 9.04 | 1.59 | 0.1101 | 0.2585 | 10.32 | 9.66 | 9.21 | 2.16 | 0.041 | 0.1862 |
| TC0200009806.hg.1 | TANK | TRAF family member-associated NFKB activator | Multiple_C | 10.73 | 11.1 | 10.06 | 1.59 | 0.0342 | 0.1091 | 9.56 | 8.91 | 8.88 | 1.60 | 0.0051 | 0.0496 |
| TC0200011901.hg.1 | PUM2 | pumilio RNA binding family member 2 | Multiple_C | 12.86 | 12.57 | 12.19 | 1.59 | 0.0191 | 0.069 | 12.88 | 12.33 | 12.84 | 1.03 | 0.8804 | 0.9486 |
| TC0200015348.hg.1 | SF3B1 | splicing factor 3b, subunit 1, 155kDa | Multiple_C | 11.44 | 11.31 | 10.77 | 1.59 | 0.1222 | 0.2784 | 11.46 | 10.89 | 11.24 | 1.16 | 0.2963 | 0.561 |
| TC0300007247.hg.1 | CCR9 | chemokine (C-C motif) receptor 9 | Multiple_C | 4.62 | 4.34 | 3.95 | 1.59 | 0.0917 | 0.226 | 4.66 | 4.67 | 4.7 | 0.97 | 0.8518 | 0.9359 |
| TC0300008532.hg.1 | FBXO40 | F-box protein 40 | Coding | 3.41 | 2.46 | 2.74 | 1.59 | 0.0227 | 0.0791 | 3.66 | 3.46 | 3.36 | 1.23 | 0.224 | 0.4849 |
| TC0300009799.hg.1 | RTP4 | receptor (chemosensory) transporter protein 4 | Coding | 3.77 | 3.14 | 3.1 | 1.59 | 0.1179 | 0.2714 | 3.81 | 3.88 | 3.8 | 1.01 | 0.2712 | 0.5367 |
| TC0300013421.hg.1 | dorsnarby; IGI | Transcript Identified by AceView; Zhang2013 ALT_ACCEPTOR, A | Multiple_C | 6.15 | 5.43 | 5.48 | 1.59 | 0.0111 | 0.0454 | 5.69 | 5.45 | 5.54 | 1.11 | 0.8675 | 0.9431 |
| TC0300013784.hg.1 | CAV3 | caveolin 3 | Coding | 4.62 | 3.86 | 3.95 | 1.59 | 0.0691 | 0.1835 | 5.66 | 5.40 | 5.6 | 1.04 | 0.8848 | 0.9505 |
| TC0400006471.hg.1 | PDE6B | phosphodiesterase 6B, cGMP-specific, rod, beta | Multiple_C | 5.89 | 5.02 | 5.22 | 1.59 | 0.0413 | 0.1257 | 4.51 | 4.74 | 4.98 | 0.72 | 0.0663 | 0.2456 |
| TC0400006616.hg.1 | ADRA2C | adrenoceptor alpha 2C | Coding | 5.33 | 4.75 | 4.66 | 1.59 | 0.2259 | 0.4247 | 7.27 | 7.18 | 7.35 | 0.95 | 0.6807 | 0.8464 |
| TC0400007830.hg.1 | ALB | albumin | Multiple_C | 4.67 | 4.07 | 4 | 1.59 | 0.0176 | 0.0646 | 5.69 | 4.90 | 4.86 | 1.78 | 0.0021 | 0.0273 |
| TC0400009376.hg.1 | WDR17 | WD repeat domain 17 | Multiple_C | 3.81 | 2.69 | 3.14 | 1.59 | 0.2985 | 0.5102 | 6.26 | 4.70 | 5.37 | 1.85 | 0.0139 | 0.0957 |
| TC0500008736.hg.1 | TGFBI | transforming growth factor, beta-induced, 68kDa | Multiple_C | 13.8 | 13.24 | 13.13 | 1.59 | 0.0289 | 0.0953 | 10.44 | 12.13 | 11.93 | 0.36 | 1.32E-06 | 0.0002 |
| TC0600009368.hg.1 | HINT3 | histidine triad nucleotide binding protein 3 | Multiple_C | 10.69 | 11.33 | 10.02 | 1.59 | 0.2471 | 0.4507 | 10.28 | 10.09 | 9.85 | 1.35 | 0.268 | 0.5326 |
| TC0600011470.hg.1 | NELFE; MIR12 | negative elongation factor complex member E; microRNA 1236 | Multiple_C | 7.49 | 6.87 | 6.82 | 1.59 | 0.0214 | 0.0754 | 6.81 | 7.16 | 7.36 | 0.68 | 0.0014 | 0.021 |

| | | | | | | | | | | | | | | | |
|---|---|---|---|---|---|---|---|---|---|---|---|---|---|---|---|
| TC0600012827.hg.1 | WASF1 | WAS protein family, member 1 | Coding | 9.45 | 8.25 | 8.78 | 1.59 | 0.1547 | 0.3288 | 9.76 | 10.10 | 10.04 | 0.82 | 0.8903 | 0.9532 |
| TC0700012734.hg.1 | CREB3L2 | cAMP responsive element binding protein 3-like 2 | Multiple_C | 7.13 | 6.19 | 6.46 | 1.59 | 0.0175 | 0.0645 | 6.52 | 6.26 | 6.82 | 0.81 | 0.0994 | 0.3082 |
| TC0800006866.hg.1 | VPS37A | vacuolar protein sorting 37 homolog A (S. cerevisiae) | Multiple_C | 9.64 | 9.3 | 8.97 | 1.59 | 0.0676 | 0.1809 | 8.3 | 7.43 | 8.88 | 0.67 | 0.0167 | 0.1079 |
| TC0800009600.hg.1 | FAM167A | family with sequence similarity 167, member A | Multiple_C | 3.95 | 3.31 | 3.28 | 1.59 | 0.1046 | 0.249 | 4.83 | 4.67 | 4.42 | 1.33 | 0.0114 | 0.0848 |
| TC0800010282.hg.1 | DKK4 | dickkopf WNT signaling pathway inhibitor 4 | Coding | 5.92 | 5.79 | 5.25 | 1.59 | 0.5276 | 0.7191 | 7.15 | 6.07 | 5.12 | 4.08 | 2.08E-06 | 0.0002 |
| TC0900007094.hg.1 | CA9 | carbonic anhydrase IX | Multiple_C | 5.64 | 4.91 | 4.97 | 1.59 | 0.0773 | 0.1994 | 7.09 | 8.74 | 8.02 | 0.52 | 0.0013 | 0.0195 |
| TC0900008243.hg.1 | PLPPR1 | phospholipid phosphatase related 1 | Multiple_C | 4.25 | 4 | 3.58 | 1.59 | 0.0128 | 0.0507 | 4.42 | 4.05 | 3.85 | 1.48 | 0.0057 | 0.0534 |
| TC0X00009537.hg.1 | ZNF674 | zinc finger protein 674 | Multiple_C | 7.15 | 7 | 6.48 | 1.59 | 0.1465 | 0.3168 | 8.24 | 8.39 | 8.66 | 0.75 | 0.099 | 0.3074 |
| TC0X00009841.hg.1 | SPIN2B | spindlin family, member 2B | Multiple_C | 7.1 | 6.62 | 6.43 | 1.59 | 0.2153 | 0.4109 | 7.16 | 7.71 | 7.6 | 0.74 | 0.3107 | 0.5747 |
| TC1000007760.hg.1 | C10orf107 | chromosome 10 open reading frame 107 | Coding | 3.7 | 2.89 | 3.03 | 1.59 | 0.3283 | 0.5422 | 4.3 | 4.31 | 4.02 | 1.21 | 0.1133 | 0.332 |
| TC1000010912.hg.1 | SAR1A | secretion associated, Ras related GTPase 1A | Multiple_C | 13.53 | 12.56 | 12.86 | 1.59 | 0.0074 | 0.033 | 13.22 | 12.72 | 13.24 | 0.99 | 0.905 | 0.9607 |
| TC1000011574.hg.1 | LOXL4 | lysyl oxidase-like 4 | Multiple_C | 4.49 | 3.66 | 3.82 | 1.59 | 0.0959 | 0.2336 | 4.97 | 4.87 | 4.47 | 1.41 | 0.0113 | 0.084 |
| TC1100008037.hg.1 | OVOL1 | ovo-like zinc finger 1 | Multiple_C | 4.91 | 3.89 | 4.24 | 1.59 | 0.0089 | 0.038 | 3.53 | 3.40 | 3.35 | 1.13 | 0.5104 | 0.7403 |
| TC1100013028.hg.1 | OTUB1 | OTU deubiquitinase, ubiquitin aldehyde binding 1 | Multiple_C | 12.07 | 11.11 | 11.4 | 1.59 | 0.004 | 0.0203 | 12.05 | 12.61 | 12.2 | 0.90 | 0.2875 | 0.5523 |
| TC1100013052.hg.1 | RBM4 | RNA binding motif protein 4 | Multiple_C | 12.05 | 11.35 | 11.38 | 1.59 | 0.0663 | 0.1783 | 11.66 | 11.32 | 11.63 | 1.02 | 0.5616 | 0.7725 |
| TC1200007120.hg.1 | RASSF8 | Ras association (RalGDS/AF-6) domain family (N-terminal) mem | Multiple_C | 4.98 | 4.38 | 4.31 | 1.59 | 0.0067 | 0.0305 | 7.86 | 6.92 | 6.74 | 2.17 | 0.0022 | 0.0282 |
| TC1200007826.hg.1 | RPL41 | ribosomal protein L41 | Multiple_C | 16.83 | 16.55 | 16.16 | 1.59 | 0.0347 | 0.1101 | 15.18 | 15.11 | 14.93 | 1.19 | 0.6866 | 0.8498 |
| TC1200009997.hg.1 | GRIN2B | glutamate receptor, ionotropic, N-methyl D-aspartate 2B | Multiple_C | 7.05 | 5.78 | 6.38 | 1.59 | 0.1454 | 0.3154 | 7.03 | 7.11 | 7.87 | 0.56 | 0.0038 | 0.0416 |
| TC1200012100.hg.1 | KSR2 | kinase suppressor of ras 2 | Multiple_C | 6.45 | 5.02 | 5.78 | 1.59 | 0.4036 | 0.6165 | 5.54 | 5.61 | 5.6 | 0.96 | 0.1222 | 0.345 |
| TC1200012267.hg.1 | SBNO1; MIR8( | strawberry notch homolog 1 (Drosophila); microRNA 8072 | Multiple_C | 12.88 | 12.65 | 12.21 | 1.59 | 0.0274 | 0.0915 | 11.01 | 10.41 | 10.54 | 1.39 | 0.18 | 0.4292 |
| TC1200012273.hg.1 | RILPL1 | Rab interacting lysosomal protein-like 1 | Multiple_C | 6.06 | 5.79 | 5.39 | 1.59 | 0.0815 | 0.2074 | 5.02 | 4.76 | 4.36 | 1.58 | 0.0082 | 0.0681 |
| TC1300006987.hg.1 | DGKH | diacylglycerol kinase, eta | Multiple_C | 8.07 | 7.34 | 7.4 | 1.59 | 0.0419 | 0.1272 | 7.34 | 6.74 | 6.73 | 1.53 | 0.0032 | 0.0369 |
| TC1400006551.hg.1 | ARHGEF40 | Rho guanine nucleotide exchange factor (GEF) 40 | Multiple_C | 4.57 | 3.94 | 3.9 | 1.59 | 0.0116 | 0.0468 | 4.77 | 4.83 | 4.73 | 1.03 | 0.4647 | 0.7067 |
| TC1400010777.hg.1 | TC2N | tandem C2 domains, nuclear | Multiple_C | 13.98 | 13.86 | 13.31 | 1.59 | 0.372 | 0.5846 | 7.26 | 4.87 | 7.46 | 0.87 | 0.1841 | 0.4351 |
| TC1500008100.hg.1 | WHAMM | WAS protein homolog associated with actin, golgi membranes a | Multiple_C | 7.76 | 7.14 | 7.09 | 1.59 | 0.0088 | 0.0378 | 7.22 | 6.73 | 7.24 | 0.99 | 0.8459 | 0.9327 |
| TC1500009189.hg.1 | ZNF106 | zinc finger protein 106 | Multiple_C | 10.48 | 10.04 | 9.81 | 1.59 | 0.0201 | 0.0719 | 9.21 | 9.10 | 9.05 | 1.12 | 0.9439 | 0.9771 |
| TC1500009204.hg.1 | UBR1 | ubiquitin protein ligase E3 component n-recognin 1 | Multiple_C | 11.29 | 10.51 | 10.62 | 1.59 | 0.0409 | 0.1246 | 11.75 | 11.12 | 11.76 | 0.99 | 0.6524 | 0.8305 |
| TC1500010925.hg.1 | ARPIN | actin-related protein 2/3 complex inhibitor | Multiple_C | 9.43 | 8.76 | 8.76 | 1.59 | 0.1452 | 0.3152 | 8.55 | 8.93 | 8.85 | 0.81 | 0.5532 | 0.7684 |
| TC1600008675.hg.1 | KIAA0513 | KIAA0513 | Multiple_C | 7.67 | 6.75 | 7 | 1.59 | 0.0108 | 0.0442 | 5 | 5.15 | 5.43 | 0.74 | 0.0697 | 0.2518 |
| TC1700007916.hg.1 | CNTD1 | cyclin N-terminal domain containing 1 | Multiple_C | 3.42 | 2.93 | 2.75 | 1.59 | 0.0605 | 0.1667 | 3.54 | 3.87 | 3.78 | 0.85 | 0.5916 | 0.7935 |
| TC1700009119.hg.1 | CARD14 | caspase recruitment domain family, member 14 | Multiple_C | 5.36 | 4.54 | 4.69 | 1.59 | 0.0099 | 0.0414 | 4.99 | 5.01 | 4.95 | 1.03 | 0.9675 | 0.9865 |
| TC1700009694.hg.1 | MYH10 | myosin, heavy chain 10, non-muscle | Multiple_C | 3.59 | 2.68 | 2.92 | 1.59 | 0.1064 | 0.2522 | 14.39 | 14.42 | 13.93 | 1.38 | 0.0581 | 0.2273 |
| TC1700010529.hg.1 | SRCIN1 | SRC kinase signaling inhibitor 1 | Multiple_C | 4.76 | 4.06 | 4.09 | 1.59 | 0.1215 | 0.2771 | 5.38 | 5.53 | 5.24 | 1.10 | 0.3707 | 0.6327 |

| | | | | | | | | | | | | | | | |
|---|---|---|---|---|---|---|---|---|---|---|---|---|---|---|---|
| TC1700011313.hg.1 | USP32 | ubiquitin specific peptidase 32 | Multiple_C | 8.42 | 7.98 | 7.75 | 1.59 | 0.0693 | 0.1839 | 8.45 | 7.87 | 8.51 | 0.96 | 0.8921 | 0.9539 |
| TC1900007189.hg.1 | ZNF333 | zinc finger protein 333 | Multiple_C | 4.45 | 3.57 | 3.78 | 1.59 | 0.0718 | 0.1889 | 4.19 | 4.15 | 4 | 1.14 | 0.3036 | 0.5678 |
| TC1900007349.hg.1 | RPL18A; SNOR | ribosomal protein L18a; small nucleolar RNA, H/ACA box 68 | Multiple_C | 18.25 | 17.26 | 17.58 | 1.59 | 0.0228 | 0.0795 | 17.31 | 16.89 | 16.65 | 1.58 | 0.0144 | 0.0978 |
| TC1900008607.hg.1 | KLK3 | kallikrein related peptidase 3 | Multiple_C | 7.16 | 6.29 | 6.49 | 1.59 | 0.0347 | 0.1103 | 7.64 | 7.41 | 7.39 | 1.19 | 0.4608 | 0.7039 |
| TC1900009078.hg.1 | MIER2 | mesoderm induction early response 1, family member 2 | Multiple_C | 7.98 | 7.09 | 7.31 | 1.59 | 0.1861 | 0.3726 | 6.11 | 6.49 | 6.87 | 0.59 | 0.0503 | 0.209 |
| TC1900009382.hg.1 | PTPRS | protein tyrosine phosphatase, receptor type, S | Multiple_C | 8.22 | 7.35 | 7.55 | 1.59 | 0.173 | 0.3546 | 10.01 | 8.54 | 8.24 | 3.41 | 2.81E-06 | 0.0003 |
| TC1900010494.hg.1 | HSPB6 | heat shock protein, alpha-crystallin-related, B6 | Multiple_C | 3.96 | 3.18 | 3.29 | 1.59 | 0.0256 | 0.0867 | 4.96 | 5.21 | 4.94 | 1.01 | 0.974 | 0.9897 |
| TC1900011724.hg.1 | ZNF540; ZNF5 | zinc finger protein 540; ZNF571 antisense RNA 1 | Multiple_C | 5.66 | 5.79 | 4.99 | 1.59 | 0.0241 | 0.0828 | 5.09 | 4.60 | 4.46 | 1.55 | 0.0061 | 0.0557 |
| TC1900011857.hg.1 | NDUFA7 | NADH dehydrogenase (ubiquinone) 1 alpha subcomplex, 7, 14.5 | Multiple_C | 13.19 | 12.97 | 12.52 | 1.59 | 0.0083 | 0.0361 | 7.77 | 8.36 | 7.99 | 0.86 | 0.1031 | 0.3145 |
| TC2000007132.hg.1 | DNMT3B | DNA (cytosine-5-)-methyltransferase 3 beta | Multiple_C | 7.11 | 6.06 | 6.44 | 1.59 | 0.0302 | 0.099 | 6.27 | 6.49 | 7.25 | 0.51 | 0.0004 | 0.0084 |
| TC2200008966.hg.1 | KIAA0930 | KIAA0930 | Multiple_C | 9.97 | 8.88 | 9.3 | 1.59 | 0.3135 | 0.527 | 9.56 | 9.91 | 10.49 | 0.52 | 0.0035 | 0.0392 |
| TSUnmapped00000495.h | ZDHHC3 | zinc finger, DHHC-type containing 3 | Coding | 5.75 | 5.11 | 5.08 | 1.59 | 0.0787 | 0.2024 | 4.6 | 4.33 | 4.3 | 1.23 | 0.4032 | 0.6593 |
| TC0100006787.hg.1 | PIK3CD | phosphatidylinositol-4,5-bisphosphate 3-kinase, catalytic subun | Multiple_C | 5.93 | 5.26 | 5.27 | 1.58 | 0.1056 | 0.2508 | 6.5 | 6.47 | 6.3 | 1.15 | 0.1847 | 0.436 |
| TC0100008692.hg.1 | SRSF11 | serine/arginine-rich splicing factor 11 | Multiple_C | 12.98 | 12.79 | 12.32 | 1.58 | 0.0147 | 0.0563 | 12.01 | 11.60 | 12.22 | 0.86 | 0.2259 | 0.4863 |
| TC0100009341.hg.1 | KIAA1324 | KIAA1324 | Multiple_C | 5.13 | 3.94 | 4.47 | 1.58 | 0.0148 | 0.0566 | 4.07 | 4.15 | 3.86 | 1.16 | 0.3344 | 0.5985 |
| TC0100010000.hg.1 | LCE2B | late cornified envelope 2B | Coding | 4.64 | 4.04 | 3.98 | 1.58 | 0.1219 | 0.2778 | 4.74 | 4.56 | 4.78 | 0.97 | 0.8462 | 0.9329 |
| TC0100010571.hg.1 | PRRX1 | paired related homeobox 1 | Multiple_C | 4.2 | 3.31 | 3.54 | 1.58 | 0.0936 | 0.2295 | 4.72 | 4.26 | 4.33 | 1.31 | 0.0359 | 0.1716 |
| TC0100011446.hg.1 | MIR205HG; M | MIR205 host gene; microRNA 205 | Multiple_C | 6.73 | 6.02 | 6.07 | 1.58 | 0.1645 | 0.3428 | 5.64 | 5.51 | 5.51 | 1.09 | 0.4286 | 0.6805 |
| TC0100014457.hg.1 | JAK1 | Janus kinase 1 | Multiple_C | 11.6 | 11.59 | 10.94 | 1.58 | 0.3062 | 0.5193 | 9.82 | 9.97 | 10.65 | 0.56 | 0.1447 | 0.3793 |
| TC0100014849.hg.1 | GBP3 | guanylate binding protein 3 | Multiple_C | 10.21 | 9.1 | 9.55 | 1.58 | 0.0394 | 0.1213 | 4.91 | 4.48 | 4.6 | 1.24 | 0.2093 | 0.4664 |
| TC0100015774.hg.1 | LYSMD1 | LysM, putative peptidoglycan-binding, domain containing 1 | Multiple_C | 4.55 | 4.1 | 3.89 | 1.58 | 0.1342 | 0.2979 | 5.01 | 5.08 | 4.88 | 1.09 | 0.4806 | 0.719 |
| TC0100015832.hg.1 | CRNN | cornulin | Coding | 5.21 | 4.45 | 4.55 | 1.58 | 0.0392 | 0.1207 | 4.35 | 4.54 | 4.51 | 0.90 | 0.3177 | 0.5819 |
| TC0100015952.hg.1 | CLK2 | CDC like kinase 2 | Multiple_C | 11.11 | 10.93 | 10.45 | 1.58 | 0.0016 | 0.0098 | 11.38 | 11.43 | 11.04 | 1.27 | 0.1882 | 0.4404 |
| TC0100015956.hg.1 | RUSC1-AS1 | RUSC1 antisense RNA 1 | Multiple_C | 5.26 | 4.47 | 4.6 | 1.58 | 0.0107 | 0.044 | 4.76 | 5.02 | 4.76 | 1.00 | 0.9137 | 0.9647 |
| TC0100018200.hg.1 | SEPN1 | selenoprotein N, 1 | Multiple_C | 5.52 | 4.66 | 4.86 | 1.58 | 0.3404 | 0.5544 | 5.96 | 6.74 | 6.7 | 0.60 | 0.4381 | 0.6884 |
| TC0200008223.hg.1 | DNAH6 | dynein, axonemal, heavy chain 6 | Multiple_C | 5.64 | 5.19 | 4.98 | 1.58 | 0.0727 | 0.1904 | 5.26 | 5.01 | 5.03 | 1.17 | 0.0273 | 0.1448 |
| TC0200010445.hg.1 | CASP10 | caspase 10 | Multiple_C | 5.83 | 5.01 | 5.17 | 1.58 | 0.0571 | 0.1596 | 3.54 | 3.50 | 3.39 | 1.11 | 0.3865 | 0.6455 |
| TC0200010852.hg.1 | GMPPA | GDP-mannose pyrophosphorylase A | Multiple_C | 9.64 | 8.91 | 8.98 | 1.58 | 0.0122 | 0.049 | 9.27 | 9.80 | 9.38 | 0.93 | 0.1603 | 0.4027 |
| TC0200011391.hg.1 | ANO7 | anoctamin 7 | Multiple_C | 6.08 | 4.98 | 5.42 | 1.58 | 0.0898 | 0.2224 | 7.99 | 7.76 | 7.66 | 1.26 | 0.2003 | 0.4557 |
| TC0200012925.hg.1 | C1D | C1D nuclear receptor corepressor | Multiple_C | 10.17 | 11.15 | 9.51 | 1.58 | 0.18 | 0.3639 | 8.01 | 8.17 | 8.01 | 1.00 | 0.4038 | 0.6596 |
| TC0200015266.hg.1 | TMEFF2 | transmembrane protein with EGF-like and two follistatin-like do | Multiple_C | 4.01 | 3.28 | 3.35 | 1.58 | 0.1229 | 0.2796 | 3.86 | 3.77 | 3.64 | 1.16 | 0.2545 | 0.5186 |
| TC0200015377.hg.1 | TYW5 | tRNA-yW synthesizing protein 5 | Multiple_C | 9.15 | 8.97 | 8.49 | 1.58 | 0.0085 | 0.0368 | 7.89 | 7.17 | 7.6 | 1.22 | 0.037 | 0.1751 |
| TC0200015869.hg.1 | AP1S3 | adaptor-related protein complex 1 sigma 3 subunit | Multiple_C | 11.81 | 11.51 | 11.15 | 1.58 | 0.0893 | 0.2217 | 10.37 | 8.86 | 10.12 | 1.19 | 0.5183 | 0.7463 |

| | | | | | | | | | | | | | | | |
|---|---|---|---|---|---|---|---|---|---|---|---|---|---|---|---|
| TC0300007044.hg.1 | GOLGA4 | golgin A4 | Multiple_C | 10.75 | 10.19 | 10.09 | 1.58 | 0.0484 | 0.1413 | 9.28 | 8.45 | 8.79 | 1.40 | 0.1833 | 0.4342 |
| TC0300007482.hg.1 | NISCH | nischarin | Multiple_C | 8.91 | 8.26 | 8.25 | 1.58 | 0.0336 | 0.1075 | 7.73 | 7.87 | 7.84 | 0.93 | 0.8249 | 0.9228 |
| TC0300010714.hg.1 | TRANK1 | tetratricopeptide repeat and ankyrin repeat containing 1 | Multiple_C | 4.19 | 3.31 | 3.53 | 1.58 | 0.4734 | 0.6766 | 3.7 | 3.67 | 3.69 | 1.01 | 0.9378 | 0.9742 |
| TC0400010614.hg.1 | CNGA1 | cyclic nucleotide gated channel alpha 1 | Multiple_C | 4.04 | 3.33 | 3.38 | 1.58 | 0.2129 | 0.4078 | 3.68 | 3.81 | 3.79 | 0.93 | 0.4219 | 0.6748 |
| TC0400010682.hg.1 | SGCB | sarcoglycan beta | Coding | 11.36 | 10.36 | 10.7 | 1.58 | 0.052 | 0.1486 | 8.99 | 9.07 | 8.83 | 1.12 | 0.3987 | 0.6559 |
| TC0500009097.hg.1 | SYNPO | synaptopodin | Multiple_C | 4.47 | 3.76 | 3.81 | 1.58 | 0.0065 | 0.0297 | 4.12 | 3.83 | 3.85 | 1.21 | 0.5479 | 0.7648 |
| TC0500012039.hg.1 | ZCCHC10 | zinc finger, CCHC domain containing 10 | Multiple_C | 9.38 | 9.37 | 8.72 | 1.58 | 0.1092 | 0.2571 | 8.35 | 8.39 | 8.68 | 0.80 | 0.5634 | 0.7738 |
| TC0500013226.hg.1 | CDC42SE2 | CDC42 small effector 2 | Multiple_C | 12.63 | 12.43 | 11.97 | 1.58 | 0.0682 | 0.182 | 12.39 | 11.91 | 12.39 | 1.00 | 0.4154 | 0.6697 |
| TC0600007599.hg.1 | PRRC2A | proline-rich coiled-coil 2A | Multiple_C | 9.62 | 8.34 | 8.96 | 1.58 | 0.3606 | 0.574 | 10.72 | 11.02 | 11.2 | 0.72 | 0.0803 | 0.2731 |
| TC0600012358.hg.1 | PHIP | pleckstrin homology domain interacting protein | Multiple_C | 12 | 10.39 | 11.34 | 1.58 | 0.0352 | 0.1114 | 10.78 | 10.00 | 10.54 | 1.18 | 0.2978 | 0.5625 |
| TC0600012670.hg.1 | SIM1 | single-minded family bHLH transcription factor 1 | Multiple_C | 4.84 | 4.03 | 4.18 | 1.58 | 0.0248 | 0.0847 | 4.51 | 4.70 | 4.75 | 0.85 | 0.4236 | 0.6763 |
| TC0600014198.hg.1 | TULP4 | tubby like protein 4 | Multiple_C | 9.12 | 7.99 | 8.46 | 1.58 | 0.0056 | 0.0265 | 10.32 | 10.12 | 9.76 | 1.47 | 0.0256 | 0.1394 |
| TC0800010937.hg.1 | FABP9 | fatty acid binding protein 9, testis | Coding | 3.6 | 2.98 | 2.94 | 1.58 | 0.0766 | 0.198 | 3.76 | 3.68 | 3.67 | 1.06 | 0.3465 | 0.6101 |
| TC0800012374.hg.1 | GLI4 | GLI family zinc finger 4 | Multiple_C | 8.2 | 7.26 | 7.54 | 1.58 | 0.0035 | 0.0183 | 7.17 | 7.52 | 7.49 | 0.80 | 0.1243 | 0.3484 |
| TC0900007710.hg.1 | SPATA31D4 | SPATA31 subfamily D, member 4 | Coding | 4.8 | 3.79 | 4.14 | 1.58 | 0.0257 | 0.0871 | 3.99 | 3.68 | 3.87 | 1.09 | 0.5732 | 0.7809 |
| TC0900009775.hg.1 | EQTN | equatorin, sperm acrosome associated | Multiple_C | 3.98 | 3.25 | 3.32 | 1.58 | 0.4763 | 0.679 | 3.86 | 3.97 | 4.11 | 0.84 | 0.5239 | 0.7491 |
| TC0900011393.hg.1 | TRAF1 | TNF receptor-associated factor 1 | Multiple_C | 5.13 | 4.94 | 4.47 | 1.58 | 0.0873 | 0.2181 | 4.14 | 4.27 | 4.17 | 0.98 | 0.5249 | 0.7497 |
| TC0900012146.hg.1 | SPATA31D1 | SPATA31 subfamily D, member 1 | Multiple_C | 6.01 | 5.35 | 5.35 | 1.58 | 0.0443 | 0.1323 | 6.23 | 5.89 | 5.71 | 1.43 | 0.0615 | 0.2355 |
| TC0X00009644.hg.1 | KCND1 | potassium channel, voltage gated Shal related subfamily D, mer | Coding | 4.49 | 3.92 | 3.83 | 1.58 | 0.0175 | 0.0643 | 4.72 | 4.46 | 4.51 | 1.16 | 0.1274 | 0.3534 |
| TC0X00010525.hg.1 | TRPC5 | transient receptor potential cation channel, subfamily C, memb | Coding | 4.4 | 3.75 | 3.74 | 1.58 | 0.0203 | 0.0724 | 4.7 | 4.25 | 4.43 | 1.21 | 0.2632 | 0.5277 |
| TC0Y00006487.hg.1 | ZFY | zinc finger protein, Y-linked | Multiple_C | 5.38 | 4.78 | 4.72 | 1.58 | 0.0233 | 0.0807 | 4.99 | 5.74 | 5.88 | 0.54 | 0.0136 | 0.0946 |
| TC1100008038.hg.1 | SNX32 | sorting nexin 32 | Multiple_C | 7.03 | 6.59 | 6.37 | 1.58 | 0.0132 | 0.0519 | 5.79 | 5.71 | 5.43 | 1.28 | 0.1681 | 0.4137 |
| TC1100008480.hg.1 | UVRAG | UV radiation resistance associated | Multiple_C | 7.95 | 7.8 | 7.29 | 1.58 | 0.0143 | 0.0549 | 10.43 | 9.78 | 10.3 | 1.09 | 0.9013 | 0.959 |
| TC1100009385.hg.1 | OR8G5 | olfactory receptor, family 8, subfamily G, member 5 | Coding | 3.6 | 2.36 | 2.94 | 1.58 | 0.1297 | 0.2905 | 3.77 | 3.71 | 3.7 | 1.05 | 0.6768 | 0.8443 |
| TC1100010850.hg.1 | OR5T2 | olfactory receptor, family 5, subfamily T, member 2 | Coding | 3.58 | 2.87 | 2.92 | 1.58 | 0.0407 | 0.1243 | 3.73 | 3.74 | 3.37 | 1.28 | 0.0409 | 0.1856 |
| TC1100011331.hg.1 | PPP1CA | protein phosphatase 1, catalytic subunit, alpha isozyme | Multiple_C | 14.47 | 12.33 | 13.81 | 1.58 | 0.0077 | 0.0338 | 15.18 | 15.60 | 15.62 | 0.74 | 0.0369 | 0.1751 |
| TC1200008574.hg.1 | APAF1 | apoptotic peptidase activating factor 1 | Multiple_C | 9.91 | 8.12 | 9.25 | 1.58 | 0.0716 | 0.1885 | 8.59 | 8.89 | 8.48 | 1.08 | 0.7294 | 0.8743 |
| TC1200010229.hg.1 | PTHLH | parathyroid hormone-like hormone | Multiple_C | 5.36 | 4.83 | 4.7 | 1.58 | 0.1573 | 0.333 | 5.7 | 5.88 | 6.2 | 0.71 | 0.0135 | 0.094 |
| TC1200012143.hg.1 | CIT; MIR1178 | citron rho-interacting serine/threonine kinase; microRNA 1178 | Multiple_C | 9.41 | 8.47 | 8.75 | 1.58 | 0.1702 | 0.3509 | 9.7 | 9.80 | 9.62 | 1.06 | 0.9318 | 0.9711 |
| TC1200012736.hg.1 | ZNF10 | zinc finger protein 10 | Multiple_C | 7.31 | 6.24 | 6.65 | 1.58 | 0.0266 | 0.0895 | 4.65 | 4.26 | 4.65 | 1.00 | 0.8661 | 0.9425 |
| TC1400006486.hg.1 | OR4M1 | olfactory receptor, family 4, subfamily M, member 1 | Coding | 3.24 | 2.25 | 2.58 | 1.58 | 0.0558 | 0.1568 | 3.47 | 3.51 | 3.47 | 1.00 | 0.6557 | 0.8321 |
| TC1400007595.hg.1 | SIPA1L1 | signal-induced proliferation-associated 1 like 1 | Multiple_C | 7.34 | 6.18 | 6.68 | 1.58 | 0.0693 | 0.184 | 5.8 | 5.47 | 5.21 | 1.51 | 0.0291 | 0.1511 |
| TC1400008718.hg.1 | MYH7 | myosin, heavy chain 7, cardiac muscle, beta | Multiple_C | 5.86 | 4.96 | 5.2 | 1.58 | 0.0671 | 0.1798 | 4.48 | 4.73 | 4.6 | 0.92 | 0.6935 | 0.8541 |

| | | | | | | | | | | | | | | | |
|---|---|---|---|---|---|---|---|---|---|---|---|---|---|---|---|
| TC1400009839.hg.1 | STON2 | stonin 2 | Multiple_C | 9.3 | 8.23 | 8.64 | 1.58 | 0.0201 | 0.0716 | 8.42 | 9.29 | 9.57 | 0.45 | 0.0001 | 0.0037 |
| TC1500007614.hg.1 | MAP2K1 | mitogen-activated protein kinase kinase 1 | Multiple_C | 11.3 | 10.72 | 10.64 | 1.58 | 0.0361 | 0.1137 | 10.78 | 10.90 | 10.46 | 1.25 | 0.7578 | 0.8901 |
| TC1500007804.hg.1 | BBS4 | Bardet-Biedl syndrome 4 | Multiple_C | 8.25 | 8.71 | 7.59 | 1.58 | 0.0672 | 0.18 | 8.17 | 8.20 | 7.8 | 1.29 | 0.8122 | 0.9166 |
| TC1500009089.hg.1 | PLCB2 | phospholipase C, beta 2 | Multiple_C | 9.22 | 8.62 | 8.56 | 1.58 | 0.0059 | 0.0274 | 7.56 | 7.45 | 7.34 | 1.16 | 0.2087 | 0.4658 |
| TC1500010755.hg.1 | ANKDD1A | ankyrin repeat and death domain containing 1A | Multiple_C | 4.34 | 3.43 | 3.68 | 1.58 | 0.097 | 0.2355 | 5.01 | 4.76 | 4.64 | 1.29 | 0.1538 | 0.3931 |
| TC1600007538.hg.1 | SLC5A2 | solute carrier family 5 (sodium/glucose cotransporter), member | Multiple_C | 4.25 | 3.63 | 3.59 | 1.58 | 0.011 | 0.0451 | 4.84 | 4.70 | 4.72 | 1.09 | 0.8659 | 0.9425 |
| TC1700008848.hg.1 | GPR142 | G protein-coupled receptor 142 | Coding | 4.03 | 3.33 | 3.37 | 1.58 | 0.0564 | 0.158 | 4.01 | 3.76 | 3.58 | 1.35 | 0.007 | 0.0611 |
| TC1700010563.hg.1 | CACNB1 | calcium channel, voltage-dependent, beta 1 subunit | Multiple_C | 7.61 | 7.07 | 6.95 | 1.58 | 0.0095 | 0.0398 | 8.76 | 8.53 | 8.13 | 1.55 | 0.0052 | 0.0503 |
| TC1700012039.hg.1 | LOC10013037 | uncharacterized LOC100130370; novel transcript | Multiple_C | 6.15 | 5.29 | 5.49 | 1.58 | 0.0217 | 0.0763 | 5.84 | 5.91 | 5.74 | 1.07 | 0.3239 | 0.5882 |
| TC1700012472.hg.1 | MRPL38 | mitochondrial ribosomal protein L38 | Multiple_C | 10.14 | 9.67 | 9.48 | 1.58 | 0.1433 | 0.3119 | 10.53 | 11.00 | 10.88 | 0.78 | 0.6447 | 0.8257 |
| TC1900006605.hg.1 | ZNF57 | zinc finger protein 57 | Coding | 11.46 | 11.12 | 10.8 | 1.58 | 0.0135 | 0.0527 | 8.73 | 8.15 | 8.67 | 1.04 | 0.9549 | 0.9814 |
| TC1900007260.hg.1 | TPM4 | tropomyosin 4 | Multiple_C | 14.55 | 14.35 | 13.89 | 1.58 | 0.0634 | 0.1723 | 14.14 | 13.62 | 13.59 | 1.46 | 0.0942 | 0.2981 |
| TC1900007622.hg.1 | ZNF254 | zinc finger protein 254 | Multiple_C | 11.61 | 11.42 | 10.95 | 1.58 | 0.1076 | 0.2542 | 10.91 | 10.38 | 10.64 | 1.21 | 0.2401 | 0.5039 |
| TC1900008188.hg.1 | MEGF8 | multiple EGF-like-domains 8 | Multiple_C | 6.37 | 5.52 | 5.71 | 1.58 | 0.0569 | 0.1592 | 5.67 | 5.78 | 5.76 | 0.94 | 0.3095 | 0.5734 |
| TC1900009493.hg.1 | CD209 | CD209 molecule | Multiple_C | 4.23 | 3.79 | 3.57 | 1.58 | 0.0228 | 0.0794 | 4.12 | 4.01 | 3.82 | 1.23 | 0.0978 | 0.3051 |
| TC1900010005.hg.1 | RAB3A | RAB3A, member RAS oncogene family | Multiple_C | 8.04 | 6.92 | 7.38 | 1.58 | 0.0143 | 0.0551 | 6.41 | 5.41 | 5.84 | 1.48 | 0.0219 | 0.1271 |
| TC1900010761.hg.1 | CEACAM4 | carcinoembryonic antigen-related cell adhesion molecule 4 | Multiple_C | 4.48 | 3.73 | 3.82 | 1.58 | 0.0388 | 0.1198 | 4.3 | 4.64 | 4.92 | 0.65 | 0.0741 | 0.2612 |
| TC1900011190.hg.1 | IZUMO2 | IZUMO family member 2 | Coding | 4.27 | 3.53 | 3.61 | 1.58 | 0.0131 | 0.0516 | 4.5 | 4.38 | 4.4 | 1.07 | 0.5671 | 0.7763 |
| TC1900011858.hg.1 | ZNF846 | zinc finger protein 846 | Multiple_C | 5.4 | 5.65 | 4.74 | 1.58 | 0.0423 | 0.1282 | 4.56 | 4.36 | 4.1 | 1.38 | 0.0495 | 0.207 |
| TC1900012009.hg.1 | SYT3 | synaptotagmin III | Multiple_C | 6.1 | 5.23 | 5.44 | 1.58 | 0.5527 | 0.7367 | 5.27 | 5.47 | 5.28 | 0.99 | 0.7085 | 0.863 |
| TC2000007193.hg.1 | MAP1LC3A | microtubule-associated protein 1 light chain 3 alpha | Multiple_C | 4.81 | 4.3 | 4.15 | 1.58 | 0.6951 | 0.8362 | 3.49 | 3.60 | 4 | 0.70 | 0.7556 | 0.8889 |
| TC2000007503.hg.1 | UBE2C | ubiquitin-conjugating enzyme E2C | Multiple_C | 11.73 | 10.88 | 11.07 | 1.58 | 0.0942 | 0.2307 | 10.42 | 10.33 | 9.84 | 1.49 | 0.2921 | 0.5568 |
| TC2000008892.hg.1 | NECAB3 | N-terminal EF-hand calcium binding protein 3 | Multiple_C | 10.35 | 9.88 | 9.69 | 1.58 | 0.0123 | 0.0491 | 8.96 | 9.15 | 8.72 | 1.18 | 0.0879 | 0.2864 |
| TC2000009852.hg.1 | RGS19 | regulator of G-protein signaling 19 | Multiple_C | 6.3 | 5.47 | 5.64 | 1.58 | 0.0353 | 0.1117 | 6.99 | 7.13 | 6.95 | 1.03 | 0.3617 | 0.6245 |
| TC2000010008.hg.1 | NFS1 | NFS1 cysteine desulfurase | Multiple_C | 9.75 | 8.74 | 9.09 | 1.58 | 0.0087 | 0.0373 | 8.16 | 8.93 | 9.18 | 0.49 | 4.54E-05 | 0.0021 |
| TC2200009149.hg.1 | TUBGCP6 | tubulin, gamma complex associated protein 6 | Multiple_C | 5.52 | 5.26 | 4.86 | 1.58 | 0.0491 | 0.1428 | 5.5 | 5.89 | 5.52 | 0.99 | 0.4632 | 0.7055 |
| TC2200009200.hg.1 | TMEM191C | transmembrane protein 191C | Multiple_C | 4.4 | 3.88 | 3.74 | 1.58 | 0.0043 | 0.0212 | 3.75 | 3.63 | 3.43 | 1.25 | 0.5206 | 0.7478 |
| TSUnmapped00000225.h | ATG16L1 | autophagy related 16-like 1 | Coding | 5.44 | 4.74 | 4.78 | 1.58 | 0.0799 | 0.2048 | 4.36 | 4.46 | 4.81 | 0.73 | 0.0622 | 0.237 |
| TSUnmapped00000711.h | ATG16L1 | autophagy related 16-like 1 | Coding | 9.94 | 9.17 | 9.28 | 1.58 | 0.036 | 0.1134 | 8.68 | 8.05 | 8.61 | 1.05 | 0.3036 | 0.5678 |
| TC0100008697.hg.1 | CTH | cystathionine gamma-lyase | Multiple_C | 11.75 | 12.12 | 11.1 | 1.57 | 0.0891 | 0.2214 | 5.61 | 5.18 | 5.05 | 1.47 | 0.0386 | 0.1788 |
| TC0100011148.hg.1 | CAMSAP2 | calmodulin regulated spectrin-associated protein family, memb | Multiple_C | 9.77 | 9.13 | 9.12 | 1.57 | 0.0163 | 0.0609 | 10.92 | 10.12 | 10.56 | 1.28 | 0.1246 | 0.3488 |
| TC0100013928.hg.1 | HYI | hydroxypyruvate isomerase (putative) | Multiple_C | 6.32 | 5.46 | 5.67 | 1.57 | 0.0522 | 0.1492 | 6.48 | 6.36 | 6.13 | 1.27 | 0.366 | 0.6283 |
| TC0200007015.hg.1 | RAB10 | RAB10, member RAS oncogene family | Multiple_C | 13.93 | 13.65 | 13.28 | 1.57 | 0.0092 | 0.0389 | 13.18 | 13.16 | 13.3 | 0.92 | 0.2651 | 0.5297 |

| | | | | | | | | | | | | | | | |
|---|---|---|---|---|---|---|---|---|---|---|---|---|---|---|---|
| TC0200008724.hg.1 | MRPS9 | mitochondrial ribosomal protein S9 | Multiple_C | 10.73 | 11.95 | 10.08 | 1.57 | 0.1188 | 0.2729 | 10.12 | 9.52 | 9.16 | 1.95 | 0.0002 | 0.0066 |
| TC0200010116.hg.1 | DFNB59 | deafness, autosomal recessive 59 | Multiple_C | 4.37 | 4.18 | 3.72 | 1.57 | 0.0337 | 0.1077 | 3.93 | 3.91 | 3.67 | 1.20 | 0.7307 | 0.8751 |
| TC0200016661.hg.1 | C2orf61 | chromosome 2 open reading frame 61 | Multiple_C | 3.53 | 2.57 | 2.88 | 1.57 | 0.1102 | 0.2587 | 3.54 | 3.56 | 3.39 | 1.11 | 0.6491 | 0.8288 |
| TC0300006982.hg.1 | TRIM71 | tripartite motif containing 71, E3 ubiquitin protein ligase | Multiple_C | 5.93 | 5.37 | 5.28 | 1.57 | 0.0154 | 0.0583 | 6.01 | 6.37 | 6.39 | 0.77 | 0.067 | 0.247 |
| TC0300008551.hg.1 | FAM162A | family with sequence similarity 162, member A | Multiple_C | 13.17 | 13.26 | 12.52 | 1.57 | 0.0354 | 0.1118 | 9.16 | 9.05 | 8.7 | 1.38 | 0.02 | 0.1207 |
| TC0300008919.hg.1 | SLC35G2 | solute carrier family 35, member G2 | Multiple_C | 6.78 | 5.98 | 6.13 | 1.57 | 0.0515 | 0.1477 | 8.1 | 8.48 | 8.51 | 0.75 | 0.0829 | 0.2772 |
| TC0300011065.hg.1 | AMT; NICN1 | aminomethyltransferase; nicolin 1 | Multiple_C | 5.93 | 5.52 | 5.28 | 1.57 | 0.0184 | 0.0669 | 8.27 | 8.42 | 8.18 | 1.06 | 0.423 | 0.6756 |
| TC0300013849.hg.1 | ROBO2 | roundabout guidance receptor 2 | Multiple_C | 3.38 | 2.6 | 2.73 | 1.57 | 0.1583 | 0.3343 | 5.84 | 5.34 | 5.17 | 1.59 | 0.0041 | 0.0435 |
| TC0400009681.hg.1 | ZNF721; ABCA | zinc finger protein 721; ATP binding cassette subfamily A memb | Multiple_C | 10.81 | 10.59 | 10.16 | 1.57 | 0.0922 | 0.227 | 9.31 | 8.98 | 9.14 | 1.13 | 0.4093 | 0.6646 |
| TC0400010418.hg.1 | RELL1 | RELT-like 1 | Multiple_C | 10.33 | 9.35 | 9.68 | 1.57 | 0.0786 | 0.2021 | 6.45 | 6.79 | 7.43 | 0.51 | 0.0056 | 0.0529 |
| TC0400011188.hg.1 | LIN54 | lin-54 DREAM MuvB core complex component | Multiple_C | 8.38 | 7.23 | 7.73 | 1.57 | 0.0093 | 0.0392 | 7.79 | 7.88 | 8.45 | 0.63 | 0.0093 | 0.0742 |
| TC0400011263.hg.1 | SPARCL1 | SPARC like 1 | Multiple_C | 4.38 | 4.04 | 3.73 | 1.57 | 0.246 | 0.4496 | 4.66 | 4.71 | 4.43 | 1.17 | 0.4543 | 0.699 |
| TC0500013205.hg.1 | CAST | calpastatin | Multiple_C | 12.5 | 12.25 | 11.85 | 1.57 | 0.0542 | 0.1535 | 12.28 | 11.08 | 11.55 | 1.66 | 0.055 | 0.22 |
| TC0500013211.hg.1 | APC | adenomatous polyposis coli | Multiple_C | 10.15 | 9.12 | 9.5 | 1.57 | 0.0322 | 0.104 | 10.01 | 9.65 | 10 | 1.01 | 0.6793 | 0.8456 |
| TC0600007487.hg.1 | HLA-G | major histocompatibility complex, class I, G | Multiple_C | 9.03 | 8.34 | 8.38 | 1.57 | 0.0651 | 0.176 | 8.56 | 8.46 | 8.59 | 0.98 | 0.6544 | 0.8316 |
| TC0700009700.hg.1 | NUB1 | negative regulator of ubiquitin-like proteins 1 | Multiple_C | 10.26 | 11.01 | 9.61 | 1.57 | 0.0429 | 0.1295 | 11.33 | 10.31 | 11.2 | 1.09 | 0.4915 | 0.7265 |
| TC0700011675.hg.1 | SEMA3D | sema domain, immunoglobulin domain (Ig), short basic domain, | Multiple_C | 3.88 | 3.06 | 3.23 | 1.57 | 0.2375 | 0.4391 | 6.64 | 6.51 | 7.16 | 0.70 | 0.0944 | 0.2985 |
| TC0800006496.hg.1 | MYOM2 | myomesin 2 | Multiple_C | 5.41 | 4.55 | 4.76 | 1.57 | 0.0244 | 0.0838 | 4.54 | 3.89 | 4.08 | 1.38 | 0.0179 | 0.1126 |
| TC0800010823.hg.1 | TCEB1 | transcription elongation factor B (SIII), polypeptide 1 (15kDa, el | Multiple_C | 14.42 | 14.46 | 13.77 | 1.57 | 0.0292 | 0.0962 | 10.48 | 10.14 | 10.5 | 0.99 | 0.6077 | 0.8038 |
| TC0800011579.hg.1 | EXT1 | exostosin glycosyltransferase 1 | Multiple_C | 11.65 | 11.01 | 11 | 1.57 | 0.389 | 0.6028 | 7.93 | 7.10 | 7.96 | 0.98 | 0.8431 | 0.9317 |
| TC0900011276.hg.1 | AMBP | alpha-1-microglobulin/bikunin precursor | Multiple_C | 5.08 | 4.28 | 4.43 | 1.57 | 0.0592 | 0.1641 | 4.93 | 4.85 | 4.68 | 1.19 | 0.0436 | 0.1931 |
| TC0900012288.hg.1 | LCN10 | lipocalin 10 | Multiple_C | 3.86 | 3.14 | 3.21 | 1.57 | 0.0141 | 0.0544 | 3.54 | 3.66 | 3.38 | 1.12 | 0.4882 | 0.7245 |
| TC0X00008747.hg.1 | GABRQ | gamma-aminobutyric acid (GABA) A receptor, theta | Coding | 3.8 | 3.06 | 3.15 | 1.57 | 0.0037 | 0.0189 | 3.21 | 3.29 | 3.43 | 0.86 | 0.2513 | 0.5154 |
| TC0X00009262.hg.1 | SUPT20HL2 | SPT20 homolog, SAGA complex component-like 2 | Multiple_C | 7.04 | 6.03 | 6.39 | 1.57 | 0.2228 | 0.4206 | 7.18 | 7.13 | 6.93 | 1.19 | 0.2646 | 0.5292 |
| TC0Y00007250.hg.1 | DAZ3 | deleted in azoospermia 3 | Coding | 3.84 | 3.31 | 3.19 | 1.57 | 0.0125 | 0.0499 | 3.61 | 3.35 | 3.35 | 1.20 | 0.0638 | 0.2404 |
| TC1000008736.hg.1 | SUFU | suppressor of fused homolog (Drosophila) | Multiple_C | 7.46 | 6.7 | 6.81 | 1.57 | 0.0028 | 0.015 | 6.33 | 6.71 | 6.8 | 0.72 | 0.0063 | 0.0572 |
| TC1100006656.hg.1 | OR52K2 | olfactory receptor, family 52, subfamily K, member 2 | Coding | 6.29 | 5.69 | 5.64 | 1.57 | 0.0616 | 0.1688 | 5.47 | 5.48 | 5.52 | 0.97 | 0.8443 | 0.9318 |
| TC1100006698.hg.1 | OR52B6 | olfactory receptor, family 52, subfamily B, member 6 | Coding | 3.14 | 2.43 | 2.49 | 1.57 | 0.0303 | 0.099 | 3.57 | 3.64 | 3.66 | 0.94 | 0.2561 | 0.521 |
| TC1100009233.hg.1 | CCDC84 | coiled-coil domain containing 84 | Multiple_C | 8.08 | 7.29 | 7.43 | 1.57 | 0.017 | 0.0631 | 6.24 | 6.27 | 6.33 | 0.94 | 0.9488 | 0.9793 |
| TC1100009667.hg.1 | ANO9 | anoctamin 9 | Multiple_C | 5.43 | 4.76 | 4.78 | 1.57 | 0.06 | 0.1656 | 5.06 | 4.93 | 4.58 | 1.39 | 0.003 | 0.0357 |
| TC1100010787.hg.1 | TRIM64C | tripartite motif containing 64C | Coding | 4.48 | 3.97 | 3.83 | 1.57 | 0.1886 | 0.3763 | 5.91 | 5.89 | 5.77 | 1.10 | 0.5954 | 0.796 |
| TC1100011125.hg.1 | HRASLS2 | HRAS-like suppressor 2 | Coding | 7.58 | 7.52 | 6.93 | 1.57 | 0.0171 | 0.0633 | 4.23 | 3.84 | 4.18 | 1.04 | 0.6643 | 0.8357 |
| TC1100013192.hg.1 | CLCF1 | cardiotrophin-like cytokine factor 1 | Coding | 6.29 | 5.31 | 5.64 | 1.57 | 0.0392 | 0.1208 | 5.79 | 5.94 | 6.64 | 0.55 | 0.0022 | 0.0283 |

| | | | | | | | | | | | | | | | |
|---|---|---|---|---|---|---|---|---|---|---|---|---|---|---|---|
| TC1200007146.hg.1 | STK38L | serine/threonine kinase 38 like | Multiple_C | 9.59 | 9.13 | 8.94 | 1.57 | 0.0286 | 0.0947 | 13.31 | 13.26 | 13.28 | 1.02 | 0.9546 | 0.9814 |
| TC1200012167.hg.1 | COQ5 | coenzyme Q5, methyltransferase | Multiple_C | 10.22 | 11.2 | 9.57 | 1.57 | 0.0176 | 0.0649 | 8.22 | 8.81 | 7.84 | 1.30 | 0.0494 | 0.2069 |
| TC1300006698.hg.1 | PDX1 | pancreatic and duodenal homeobox 1 | Coding | 3.42 | 2.72 | 2.77 | 1.57 | 0.014 | 0.0542 | 3.21 | 3.18 | 3.05 | 1.12 | 0.2075 | 0.4641 |
| TC1300008443.hg.1 | LNX2 | ligand of numb-protein X 2 | Multiple_C | 8.55 | 7.51 | 7.9 | 1.57 | 0.0306 | 0.0999 | 6.86 | 6.42 | 6.74 | 1.09 | 0.5757 | 0.7824 |
| TC1300008873.hg.1 | SUCLA2 | succinate-CoA ligase, ADP-forming, beta subunit | Multiple_C | 13.28 | 13.11 | 12.63 | 1.57 | 0.0528 | 0.1504 | 11.75 | 11.57 | 10.55 | 2.30 | 2.21E-05 | 0.0012 |
| TC1400010754.hg.1 | C14orf37 | chromosome 14 open reading frame 37 | Multiple_C | 4.09 | 3.22 | 3.44 | 1.57 | 0.0831 | 0.2101 | 3.59 | 3.52 | 3.57 | 1.01 | 0.7548 | 0.8886 |
| TC1500009179.hg.1 | PLA2G4D | phospholipase A2, group IVD (cytosolic) | Multiple_C | 5.13 | 4.19 | 4.48 | 1.57 | 0.1024 | 0.2452 | 5.66 | 5.52 | 5.06 | 1.52 | 0.0343 | 0.1668 |
| TC1500010233.hg.1 | MEX3B | mex-3 RNA binding family member B | Coding | 4.85 | 4.53 | 4.2 | 1.57 | 0.043 | 0.1298 | 4.98 | 5.05 | 5.26 | 0.82 | 0.2947 | 0.5597 |
| TC1500010841.hg.1 | CHRFAM7A | CHRNA7 (cholinergic receptor, nicotinic, alpha 7, exons 5-10) ar | Multiple_C | 7.94 | 7.75 | 7.29 | 1.57 | 0.172 | 0.3533 | 7.78 | 6.83 | 7.44 | 1.27 | 0.1042 | 0.3165 |
| TC1700008914.hg.1 | SMIM5 | small integral membrane protein 5 | Multiple_C | 7.61 | 6.83 | 6.96 | 1.57 | 0.0887 | 0.2207 | 7.45 | 7.49 | 7.23 | 1.16 | 0.9763 | 0.9903 |
| TC1700010497.hg.1 | SYNRG | synergin, gamma | Multiple_C | 9.85 | 9.14 | 9.2 | 1.57 | 0.0399 | 0.1225 | 10.4 | 9.81 | 10.32 | 1.06 | 0.2575 | 0.5225 |
| TC1700012269.hg.1 | MGC57346-CI | Homo sapiens MGC57346-CRHR1 readthrough (MGC57346-CRI | Multiple_C | 10.07 | 9.33 | 9.42 | 1.57 | 0.0148 | 0.0565 | 9.9 | 9.27 | 9.71 | 1.14 | 0.8197 | 0.9205 |
| TC1700012369.hg.1 | FLCN | folliculin | Multiple_C | 7.72 | 6.95 | 7.07 | 1.57 | 0.1826 | 0.3676 | 6.4 | 6.74 | 6.84 | 0.74 | 0.4614 | 0.7042 |
| TC1900007810.hg.1 | WTIP | Wilms tumor 1 interacting protein | Multiple_C | 5.02 | 4.33 | 4.37 | 1.57 | 0.0099 | 0.0414 | 5.05 | 5.15 | 5.15 | 0.93 | 0.4629 | 0.7054 |
| TC1900008151.hg.1 | ERICH4 | glutamate rich 4 | Coding | 4.77 | 3.99 | 4.12 | 1.57 | 0.0194 | 0.0697 | 4.36 | 4.79 | 4.47 | 0.93 | 0.8625 | 0.9412 |
| TC1900008542.hg.1 | RPS11; SNORI | ribosomal protein S11; small nucleolar RNA, C/D box 35B | Multiple_C | 17.15 | 16.84 | 16.5 | 1.57 | 0.0078 | 0.0344 | 15.16 | 14.79 | 14.73 | 1.35 | 0.452 | 0.6973 |
| TC1900009080.hg.1 | THEG | theg spermatid protein | Multiple_C | 3.73 | 2.84 | 3.08 | 1.57 | 0.0541 | 0.1533 | 3.96 | 3.99 | 4 | 0.97 | 0.9697 | 0.9875 |
| TC1900011124.hg.1 | C19orf73 | chromosome 19 open reading frame 73 | Coding | 5.49 | 4.87 | 4.84 | 1.57 | 0.0167 | 0.0622 | 4.75 | 5.11 | 5.05 | 0.81 | 0.5205 | 0.7478 |
| TC2000008759.hg.1 | FAM182B | family with sequence similarity 182, member B | Multiple_C | 4.65 | 4.07 | 4 | 1.57 | 0.0842 | 0.2122 | 5.04 | 5.05 | 4.73 | 1.24 | 0.5204 | 0.7477 |
| TC2200008300.hg.1 | TFIP11 | tuftelin interacting protein 11 | Multiple_C | 6.92 | 6.07 | 6.27 | 1.57 | 0.3969 | 0.6106 | 6.06 | 6.33 | 6.58 | 0.70 | 0.0411 | 0.1862 |
| TC2200009281.hg.1 | PNPLA3 | patatin-like phospholipase domain containing 3 | Multiple_C | 6.62 | 7.3 | 5.97 | 1.57 | 0.3995 | 0.6132 | 5.91 | 5.34 | 6 | 0.94 | 0.3933 | 0.6515 |
| TSUnmapped00000040.h | ZNF660 | zinc finger protein 660 | Coding | 3.76 | 3.13 | 3.11 | 1.57 | 0.0856 | 0.2148 | 3.73 | 3.63 | 3.53 | 1.15 | 0.4553 | 0.6998 |
| TSUnmapped00000052.h | HHAT | hedgehog acyltransferase | Coding | 4.14 | 3.29 | 3.49 | 1.57 | 0.0281 | 0.0933 | 4.13 | 4.18 | 4.16 | 0.98 | 0.8489 | 0.9347 |
| TSUnmapped00000057.h | SURF4 | surfeit 4 | Coding | 6.44 | 6.02 | 5.79 | 1.57 | 0.003 | 0.016 | 5.59 | 5.69 | 5.97 | 0.77 | 0.0231 | 0.1308 |
| TC0100006699.hg.1 | THAP3 | THAP domain containing, apoptosis associated protein 3 | Multiple_C | 5.98 | 6.16 | 5.34 | 1.56 | 0.058 | 0.1615 | 5.8 | 5.68 | 5.33 | 1.39 | 0.1285 | 0.3548 |
| TC0100007268.hg.1 | CDC42 | cell division cycle 42 | Multiple_C | 15.61 | 14.94 | 14.97 | 1.56 | 0.0055 | 0.026 | 15.25 | 15.47 | 15.67 | 0.75 | 0.0447 | 0.1956 |
| TC0100008193.hg.1 | FOXE3 | forkhead box E3 | Coding | 4.8 | 3.97 | 4.16 | 1.56 | 0.006 | 0.0281 | 5.17 | 5.43 | 5.12 | 1.04 | 0.8394 | 0.9294 |
| TC0100009199.hg.1 | MFSD14A | major facilitator superfamily domain containing 14A | Multiple_C | 12.1 | 11.18 | 11.46 | 1.56 | 0.008 | 0.0351 | 10.69 | 10.27 | 11.15 | 0.73 | 0.002 | 0.0268 |
| TC0100011064.hg.1 | CFH | complement factor H | Multiple_C | 4.18 | 3.41 | 3.54 | 1.56 | 0.0104 | 0.0429 | 3.19 | 3.10 | 3.24 | 0.97 | 0.8286 | 0.9242 |
| TC0100011162.hg.1 | IGFN1 | immunoglobulin-like and fibronectin type III domain containing | Multiple_C | 4.22 | 3.43 | 3.58 | 1.56 | 0.0442 | 0.1322 | 3.96 | 4.12 | 3.94 | 1.01 | 0.9555 | 0.9815 |
| TC0100011692.hg.1 | MIA3 | melanoma inhibitory activity family, member 3 | Multiple_C | 10.34 | 9.94 | 9.7 | 1.56 | 0.2676 | 0.4757 | 8.27 | 8.36 | 7.92 | 1.27 | 0.304 | 0.5679 |
| TC0100013561.hg.1 | FABP3 | fatty acid binding protein 3, muscle and heart | Multiple_C | 4.58 | 4.07 | 3.94 | 1.56 | 0.0398 | 0.1221 | 4.44 | 3.71 | 3.78 | 1.58 | 0.0035 | 0.0394 |
| TC0100014058.hg.1 | KNCN | kinocilin | Multiple_C | 6.73 | 5.97 | 6.09 | 1.56 | 0.0127 | 0.0502 | 5.98 | 6.14 | 6.03 | 0.97 | 0.9875 | 0.9947 |

| | | | | | | | | | | | | | | | |
|---|---|---|---|---|---|---|---|---|---|---|---|---|---|---|---|
| TC0100017451.hg.1 | TP53BP2 | tumor protein p53 binding protein 2 | Multiple_C | 10.38 | 9 | 9.74 | 1.56 | 0.0616 | 0.1689 | 9.91 | 9.32 | 10.14 | 0.85 | 0.6817 | 0.8473 |
| TC0200016097.hg.1 | DNAJB3 | DnaJ (Hsp40) homolog, subfamily B, member 3 | Multiple_C | 4.66 | 3.77 | 4.02 | 1.56 | 0.0108 | 0.0442 | 3.47 | 3.45 | 3.4 | 1.05 | 0.6803 | 0.8462 |
| TC0200016672.hg.1 | RTN4 | reticulon 4 | Multiple_C | 14.44 | 14.21 | 13.8 | 1.56 | 0.132 | 0.2945 | 12.36 | 11.90 | 12.45 | 0.94 | 0.7884 | 0.9057 |
| TC0300011029.hg.1 | UQCRC1 | ubiquinol-cytochrome c reductase core protein I | Multiple_C | 13.82 | 12.93 | 13.18 | 1.56 | 0.0532 | 0.1513 | 12.72 | 12.75 | 12.13 | 1.51 | 0.0668 | 0.2465 |
| TC0300011792.hg.1 | MTRNR2L2; M | MT-RNR2-like 2; MT-RNR2-like 12 | Multiple_C | 17.49 | 16.42 | 16.85 | 1.56 | 0.0044 | 0.0216 | 18.14 | 18.44 | 18.49 | 0.78 | 0.1723 | 0.4192 |
| TC0300013090.hg.1 | PHC3 | polyhomeotic homolog 3 (Drosophila) | Multiple_C | 12.08 | 11.02 | 11.44 | 1.56 | 0.036 | 0.1135 | 12.22 | 10.86 | 11.89 | 1.26 | 0.2257 | 0.4863 |
| TC0400008137.hg.1 | CCSER1 | coiled-coil serine rich protein 1 | Multiple_C | 6.61 | 7.02 | 5.97 | 1.56 | 0.0509 | 0.1465 | 3.44 | 3.54 | 3.56 | 0.92 | 0.576 | 0.7827 |
| TC0400012902.hg.1 | FGFBP2 | fibroblast growth factor binding protein 2 | Coding | 3.85 | 3.33 | 3.21 | 1.56 | 0.0274 | 0.0917 | 3.4 | 3.22 | 3.21 | 1.14 | 0.1973 | 0.4522 |
| TC0500010651.hg.1 | FGF10 | fibroblast growth factor 10 | Coding | 4.97 | 4.27 | 4.33 | 1.56 | 0.088 | 0.2193 | 4.46 | 4.74 | 4.79 | 0.80 | 0.8819 | 0.9494 |
| TC0500011742.hg.1 | TRIM36 | tripartite motif containing 36 | Multiple_C | 5.44 | 5.14 | 4.8 | 1.56 | 0.3111 | 0.5245 | 6.22 | 5.56 | 5.9 | 1.25 | 0.1341 | 0.3636 |
| TC0600006805.hg.1 | PPP1R3G | protein phosphatase 1, regulatory subunit 3G | Coding | 3.96 | 3.23 | 3.32 | 1.56 | 0.0062 | 0.0286 | 4.23 | 4.38 | 4.27 | 0.97 | 0.4631 | 0.7054 |
| TC0600012428.hg.1 | UBE3D | ubiquitin protein ligase E3D | Multiple_C | 8.61 | 8.14 | 7.97 | 1.56 | 0.0177 | 0.0651 | 7.62 | 7.77 | 8.09 | 0.72 | 0.0331 | 0.1634 |
| TC0600014268.hg.1 | PRRT1 | proline-rich transmembrane protein 1 | Multiple_C | 4.12 | 3.38 | 3.48 | 1.56 | 0.0556 | 0.1563 | 5.06 | 5.21 | 5.25 | 0.88 | 0.9533 | 0.981 |
| TC0700008004.hg.1 | GTF2IRD1 | GTF2I repeat domain containing 1 | Multiple_C | 8.54 | 7.88 | 7.9 | 1.56 | 0.17 | 0.3506 | 10.83 | 10.67 | 10.35 | 1.39 | 0.4167 | 0.6705 |
| TC0700011977.hg.1 | GAL3ST4 | galactose-3-O-sulfotransferase 4 | Multiple_C | 5.41 | 4.77 | 4.77 | 1.56 | 0.2251 | 0.4238 | 3.7 | 3.77 | 3.8 | 0.93 | 0.4716 | 0.7122 |
| TC0800007328.hg.1 | BAG4 | BCL2-associated athanogene 4 | Multiple_C | 10.9 | 10.1 | 10.26 | 1.56 | 0.1127 | 0.263 | 8.93 | 9.18 | 9.72 | 0.58 | 0.0486 | 0.2049 |
| TC0800009619.hg.1 | CTSB | cathepsin B | Multiple_C | 15.03 | 14.35 | 14.39 | 1.56 | 0.2032 | 0.3954 | 11.57 | 11.20 | 11.27 | 1.23 | 0.5244 | 0.7494 |
| TC0800011683.hg.1 | FBXO32 | F-box protein 32 | Multiple_C | 8.68 | 9.07 | 8.04 | 1.56 | 0.0283 | 0.0939 | 3.75 | 3.84 | 3.97 | 0.86 | 0.4433 | 0.6916 |
| TC0900006815.hg.1 | DMRTA1 | DMRT-like family A1 | Coding | 3.38 | 2.7 | 2.74 | 1.56 | 0.054 | 0.1531 | 5.85 | 5.15 | 5.09 | 1.69 | 0.0222 | 0.128 |
| TC0900008219.hg.1 | NR4A3 | nuclear receptor subfamily 4, group A, member 3 | Multiple_C | 4.73 | 4.02 | 4.09 | 1.56 | 0.1413 | 0.3088 | 3.45 | 3.26 | 3.5 | 0.97 | 0.4461 | 0.6935 |
| TC0900010920.hg.1 | AAED1 | AhpC/TSA antioxidant enzyme domain containing 1 | Multiple_C | 8.67 | 8.66 | 8.03 | 1.56 | 0.2617 | 0.4686 | 7 | 7.65 | 7.95 | 0.52 | 0.001 | 0.0163 |
| TC0900011829.hg.1 | RALGDS | ral guanine nucleotide dissociation stimulator | Multiple_C | 8.44 | 7.75 | 7.8 | 1.56 | 0.1253 | 0.2832 | 8.04 | 7.77 | 7.36 | 1.60 | 0.0041 | 0.0433 |
| TC0900012042.hg.1 | RNF208 | ring finger protein 208 | Coding | 5 | 3.8 | 4.36 | 1.56 | 0.0814 | 0.2074 | 5 | 4.67 | 4.59 | 1.33 | 0.2407 | 0.5042 |
| TC0X00006803.hg.1 | EIF2S3 | eukaryotic translation initiation factor 2, subunit 3 gamma, 52kD | Multiple_C | 14.99 | 14.77 | 14.35 | 1.56 | 0.315 | 0.5284 | 16.06 | 16.16 | 16.15 | 0.94 | 0.3811 | 0.6406 |
| TC0X00009205.hg.1 | SH3KBP1 | SH3-domain kinase binding protein 1 | Multiple_C | 12.57 | 11.86 | 11.93 | 1.56 | 0.0378 | 0.1175 | 11.79 | 11.48 | 12.35 | 0.68 | 0.0702 | 0.2534 |
| TC0X00011402.hg.1 | SPANXA1 | sperm protein associated with the nucleus, X-linked, family mer | Coding | 7.47 | 6.75 | 6.83 | 1.56 | 0.0448 | 0.1334 | 6.76 | 7.21 | 7.09 | 0.80 | 0.1321 | 0.3606 |
| TC0X00011403.hg.1 | SPANXD | SPANX family, member D | Coding | 6.05 | 5.41 | 5.41 | 1.56 | 0.0326 | 0.1051 | 7.03 | 6.95 | 7.06 | 0.98 | 0.894 | 0.9551 |
| TC1000010758.hg.1 | RTKN2 | rhotekin 2 | Coding | 10.21 | 9 | 9.57 | 1.56 | 0.0901 | 0.2231 | 11.27 | 11.91 | 11.14 | 1.09 | 0.4775 | 0.7164 |
| TC1000012480.hg.1 | CYP2C18 | cytochrome P450, family 2, subfamily C, polypeptide 18 | Coding | 3.95 | 3.23 | 3.31 | 1.56 | 0.0512 | 0.147 | 3.63 | 3.70 | 3.44 | 1.14 | 0.4326 | 0.6839 |
| TC1100008136.hg.1 | RPS6KB2 | ribosomal protein S6 kinase, 70kDa, polypeptide 2 | Multiple_C | 10.67 | 9.78 | 10.03 | 1.56 | 0.081 | 0.2068 | 10.41 | 11.09 | 10.6 | 0.88 | 0.8419 | 0.9311 |
| TC1100008612.hg.1 | RPS28 | ribosomal protein S28 | Multiple_C | 17.87 | 16.35 | 17.23 | 1.56 | 0.0239 | 0.0823 | 13.29 | 13.28 | 13.37 | 0.95 | 0.9298 | 0.9703 |
| TC1100010838.hg.1 | OR5F1 | olfactory receptor, family 5, subfamily F, member 1 | Coding | 3.45 | 2.57 | 2.81 | 1.56 | 0.3395 | 0.5535 | 3.47 | 3.64 | 3.58 | 0.93 | 0.7073 | 0.8621 |
| TC1100012969.hg.1 | CALCB | calcitonin-related polypeptide beta | Multiple_C | 3.63 | 2.88 | 2.99 | 1.56 | 0.0213 | 0.0753 | 3.51 | 3.57 | 3.29 | 1.16 | 0.3533 | 0.6167 |

| | | | | | | | | | | | | | | | |
|---|---|---|---|---|---|---|---|---|---|---|---|---|---|---|---|
| TC1200012749.hg.1 | CD163 | CD163 molecule | Multiple_C | 6.15 | 5.63 | 5.51 | 1.56 | 0.1193 | 0.2736 | 6.31 | 6.61 | 6.27 | 1.03 | 0.5379 | 0.759 |
| TC1300009598.hg.1 | GPR183 | G protein-coupled receptor 183 | Coding | 5.05 | 4.16 | 4.41 | 1.56 | 0.1193 | 0.2736 | 7.59 | 8.79 | 7.73 | 0.91 | 0.2155 | 0.4742 |
| TC1400010039.hg.1 | DDX24 | DEAD (Asp-Glu-Ala-Asp) box helicase 24 | Multiple_C | 10.41 | 9.39 | 9.77 | 1.56 | 0.0011 | 0.007 | 9.77 | 9.75 | 9.69 | 1.06 | 0.7518 | 0.8869 |
| TC1400010625.hg.1 | ZNF410 | zinc finger protein 410 | Multiple_C | 7.28 | 6.72 | 6.64 | 1.56 | 0.4186 | 0.6301 | 7.04 | 7.09 | 7.73 | 0.62 | 0.0584 | 0.2278 |
| TC1400010644.hg.1 | SERPINA3 | serpin peptidase inhibitor, clade A (alpha-1 antiproteinase, antit | Multiple_C | 5.09 | 4.35 | 4.45 | 1.56 | 0.0175 | 0.0646 | 4.93 | 4.83 | 4.77 | 1.12 | 0.5533 | 0.7684 |
| TC1500006972.hg.1 | SPINT1 | serine peptidase inhibitor, Kunitz type 1 | Multiple_C | 11.78 | 10.54 | 11.14 | 1.56 | 0.1643 | 0.3425 | 9.04 | 9.03 | 9.1 | 0.96 | 0.8608 | 0.9405 |
| TC1500007699.hg.1 | KIF23 | kinesin family member 23 | Multiple_C | 12.46 | 11.44 | 11.82 | 1.56 | 0.2113 | 0.4057 | 10.98 | 11.18 | 11.09 | 0.93 | 0.3416 | 0.6053 |
| TC1500009238.hg.1 | FRMD5 | FERM domain containing 5 | Multiple_C | 10.33 | 10.54 | 9.69 | 1.56 | 0.321 | 0.5348 | 9.5 | 9.29 | 10 | 0.71 | 0.0386 | 0.1788 |
| TC1600008943.hg.1 | TMEM8A | transmembrane protein 8A | Multiple_C | 9.6 | 8.32 | 8.96 | 1.56 | 0.0444 | 0.1327 | 7.97 | 8.33 | 8.55 | 0.67 | 0.0516 | 0.2123 |
| TC1600010174.hg.1 | MYLK3 | myosin light chain kinase 3 | Multiple_C | 5.08 | 4.32 | 4.44 | 1.56 | 0.2449 | 0.4485 | 4.86 | 4.72 | 4.58 | 1.21 | 0.262 | 0.5263 |
| TC1600011345.hg.1 | DNASE1 | deoxyribonuclease I | Multiple_C | 5 | 4.18 | 4.36 | 1.56 | 0.002 | 0.0114 | 4.54 | 5.10 | 4.59 | 0.97 | 0.6757 | 0.8435 |
| TC1700007740.hg.1 | RPL19 | ribosomal protein L19 | Multiple_C | 16.55 | 16.04 | 15.91 | 1.56 | 0.0241 | 0.0828 | 13.22 | 13.40 | 12.85 | 1.29 | 0.0919 | 0.2937 |
| TC1700009393.hg.1 | RILP | Rab interacting lysosomal protein | Multiple_C | 8.38 | 8.05 | 7.74 | 1.56 | 0.0169 | 0.0628 | 5.93 | 5.80 | 5.67 | 1.20 | 0.3514 | 0.6151 |
| TC1700009671.hg.1 | HES7 | hes family bHLH transcription factor 7 | Coding | 6.16 | 5.38 | 5.52 | 1.56 | 0.0204 | 0.0727 | 4.87 | 4.35 | 4.54 | 1.26 | 0.3449 | 0.6087 |
| TC1800006589.hg.1 | PTPRM | protein tyrosine phosphatase, receptor type, M | Multiple_C | 4.44 | 3.77 | 3.8 | 1.56 | 0.0675 | 0.1807 | 4.35 | 4.17 | 6.24 | 0.27 | 5.74E-06 | 0.0005 |
| TC1800008650.hg.1 | SMAD7 | SMAD family member 7 | Multiple_C | 5.98 | 5.41 | 5.34 | 1.56 | 0.2029 | 0.3952 | 6.24 | 6.58 | 6.57 | 0.80 | 0.0621 | 0.2366 |
| TC1800008977.hg.1 | CD226 | CD226 molecule | Multiple_C | 5.25 | 4.58 | 4.61 | 1.56 | 0.1366 | 0.3014 | 4.5 | 4.85 | 4.51 | 0.99 | 0.1391 | 0.3705 |
| TC1900006711.hg.1 | TNFAIP8L1 | tumor necrosis factor, alpha-induced protein 8-like 1 | Multiple_C | 7.98 | 7.77 | 7.34 | 1.56 | 0.0701 | 0.1857 | 7.36 | 8.27 | 7.55 | 0.88 | 0.8238 | 0.9222 |
| TC1900010707.hg.1 | SERTAD3 | SERTA domain containing 3 | Multiple_C | 6.34 | 6.08 | 5.7 | 1.56 | 0.5862 | 0.7626 | 4.48 | 4.87 | 5.23 | 0.59 | 0.0226 | 0.1296 |
| TC1900011852.hg.1 | RFX2 | regulatory factor X, 2 (influences HLA class II expression) | Multiple_C | 6.28 | 5.25 | 5.64 | 1.56 | 0.0482 | 0.1408 | 5.46 | 5.45 | 5.43 | 1.02 | 0.9075 | 0.9617 |
| TC1900011975.hg.1 | ZNF112 | zinc finger protein 112 | Coding | 6.46 | 5.89 | 5.82 | 1.56 | 0.033 | 0.1059 | 4.19 | 3.83 | 3.99 | 1.15 | 0.1856 | 0.4372 |
| TC2000007083.hg.1 | ID1 | inhibitor of DNA binding 1, dominant negative helix-loop-helix p | Multiple_C | 11.61 | 11.49 | 10.97 | 1.56 | 0.034 | 0.1085 | 8.15 | 7.87 | 7.3 | 1.80 | 0.0039 | 0.0424 |
| TC2200008578.hg.1 | RPL41 | Synthetic construct Homo sapiens clone IMAGE:100063377, MC | Multiple_C | 15.02 | 15.02 | 14.38 | 1.56 | 0.0166 | 0.0619 | 12.18 | 12.22 | 12.07 | 1.08 | 0.4411 | 0.6904 |
| TC2200009328.hg.1 | HPS4 | Hermansky-Pudlak syndrome 4 | Multiple_C | 9.12 | 8.44 | 8.48 | 1.56 | 0.1366 | 0.3014 | 10.64 | 10.13 | 10.26 | 1.30 | 0.1279 | 0.354 |
| TC0100013634.hg.1 | TMEM54 | transmembrane protein 54 | Multiple_C | 11.19 | 9.72 | 10.56 | 1.55 | 0.0991 | 0.2394 | 5.61 | 5.52 | 5.72 | 0.93 | 0.7267 | 0.873 |
| TC0100014022.hg.1 | CCDC17 | coiled-coil domain containing 17 | Multiple_C | 6.72 | 5.74 | 6.09 | 1.55 | 0.0126 | 0.05 | 5.81 | 6.01 | 5.83 | 0.99 | 0.6048 | 0.8024 |
| TC0100014660.hg.1 | FUBP1 | far upstream element (FUSE) binding protein 1 | Multiple_C | 12.17 | 11.67 | 11.54 | 1.55 | 0.0075 | 0.0333 | 11.76 | 11.97 | 12.39 | 0.65 | 0.001 | 0.0168 |
| TC0100015236.hg.1 | LAMTOR5 | late endosomal/lysosomal adaptor, MAPK and MTOR activator | Multiple_C | 14.33 | 14.72 | 13.7 | 1.55 | 0.1592 | 0.3354 | 10.17 | 10.05 | 9.53 | 1.56 | 0.011 | 0.083 |
| TC0100018518.hg.1 | PEX19 | peroxisomal biogenesis factor 19 | Multiple_C | 9.85 | 9.59 | 9.22 | 1.55 | 0.0213 | 0.0753 | 9.34 | 9.63 | 9.54 | 0.87 | 0.2287 | 0.4896 |
| TC0200007591.hg.1 | ERLEC1 | endoplasmic reticulum lectin 1 | Multiple_C | 10.05 | 10.06 | 9.42 | 1.55 | 0.1053 | 0.2504 | 9.28 | 8.97 | 8.94 | 1.27 | 0.2128 | 0.4706 |
| TC0200008945.hg.1 | IL36G | interleukin 36, gamma | Coding | 4.82 | 4.22 | 4.19 | 1.55 | 0.0812 | 0.207 | 4.25 | 4.53 | 4.21 | 1.03 | 0.2742 | 0.5397 |
| TC0200009055.hg.1 | MARCO | macrophage receptor with collagenous structure | Multiple_C | 5.69 | 5 | 5.06 | 1.55 | 0.0485 | 0.1414 | 5.28 | 5.36 | 4.93 | 1.27 | 0.227 | 0.4875 |
| TC0200010056.hg.1 | MTX2 | metaxin 2 | Multiple_C | 10.85 | 11.58 | 10.22 | 1.55 | 0.0351 | 0.1111 | 8.47 | 8.62 | 8.71 | 0.85 | 0.5602 | 0.772 |

| | | | | | | | | | | | | | | | |
|---|---|---|---|---|---|---|---|---|---|---|---|---|---|---|---|
| TC0200010502.hg.1 | FAM117B | family with sequence similarity 117, member B | Multiple_C | 10.18 | 9.27 | 9.55 | 1.55 | 0.1709 | 0.3518 | 9.65 | 9.93 | 10.15 | 0.71 | 0.2905 | 0.5554 |
| TC0200010839.hg.1 | TUBA4B | tubulin, alpha 4b | Multiple_C | 5.41 | 4.34 | 4.78 | 1.55 | 0.0838 | 0.2116 | 5.02 | 5.22 | 5.18 | 0.90 | 0.5259 | 0.7505 |
| TC0200011121.hg.1 | INPP5D | inositol polyphosphate-5-phosphatase D | Multiple_C | 6.41 | 5.79 | 5.78 | 1.55 | 0.0041 | 0.0204 | 9.84 | 9.55 | 9.9 | 0.96 | 0.8915 | 0.9539 |
| TC0200012382.hg.1 | OXER1 | oxoeicosanoid (OXE) receptor 1 | Coding | 6.09 | 5.04 | 5.46 | 1.55 | 0.1771 | 0.3598 | 5.86 | 5.53 | 5.49 | 1.29 | 0.4549 | 0.6996 |
| TC0200016382.hg.1 | PDCD1 | programmed cell death 1 | Coding | 7.35 | 6.7 | 6.72 | 1.55 | 0.146 | 0.3162 | 6.64 | 6.66 | 6.58 | 1.04 | 0.9556 | 0.9815 |
| TC0200016420.hg.1 | MRPL33 | mitochondrial ribosomal protein L33 | Multiple_C | 7.01 | 6.7 | 6.38 | 1.55 | 0.042 | 0.1274 | 6.16 | 6.05 | 5.73 | 1.35 | 0.0347 | 0.1682 |
| TC0200016501.hg.1 | IL1RL1 | interleukin 1 receptor-like 1 | Multiple_C | 4.09 | 3.35 | 3.46 | 1.55 | 0.0761 | 0.197 | 4.12 | 3.95 | 4.05 | 1.05 | 0.7743 | 0.8986 |
| TC0200016624.hg.1 | KIDINS220 | kinase D-interacting substrate 220kDa | Multiple_C | 10.73 | 9.95 | 10.1 | 1.55 | 0.0436 | 0.131 | 10.23 | 10.25 | 10.46 | 0.85 | 0.2004 | 0.4558 |
| TC0400008389.hg.1 | RRH | retinal pigment epithelium-derived rhodopsin homolog | Coding | 4.47 | 3.79 | 3.84 | 1.55 | 0.2195 | 0.4164 | 3.95 | 4.10 | 4.03 | 0.95 | 0.6622 | 0.835 |
| TC0400010636.hg.1 | OCIAD2 | OCIA domain containing 2 | Multiple_C | 12.17 | 11.76 | 11.54 | 1.55 | 0.0378 | 0.1175 | 8.37 | 8.26 | 8.62 | 0.84 | 0.2884 | 0.5529 |
| TC0400011144.hg.1 | ANTXR2 | anthrax toxin receptor 2 | Multiple_C | 9.71 | 9.02 | 9.08 | 1.55 | 0.0478 | 0.1398 | 5.37 | 5.34 | 4.78 | 1.51 | 0.039 | 0.18 |
| TC0400012765.hg.1 | CPZ; GPR78 | carboxypeptidase Z; G protein-coupled receptor 78 | Multiple_C | 6.11 | 5.16 | 5.48 | 1.55 | 0.0997 | 0.2404 | 5.96 | 5.76 | 5.78 | 1.13 | 0.7065 | 0.8618 |
| TC0500007623.hg.1 | MAST4 | microtubule associated serine/threonine kinase family member | Multiple_C | 5.97 | 5.09 | 5.34 | 1.55 | 0.0152 | 0.0576 | 7.99 | 7.39 | 7.93 | 1.04 | 0.6389 | 0.823 |
| TC0500009470.hg.1 | ERGIC1 | endoplasmic reticulum-golgi intermediate compartment 1 | Multiple_C | 13.33 | 12.29 | 12.7 | 1.55 | 0.0014 | 0.0088 | 13.44 | 13.12 | 13.03 | 1.33 | 0.0133 | 0.0931 |
| TC0500009648.hg.1 | N4BP3 | NEDD4 binding protein 3 | Coding | 6.02 | 4.99 | 5.39 | 1.55 | 0.1192 | 0.2734 | 5.9 | 5.83 | 5.86 | 1.03 | 0.296 | 0.5609 |
| TC0500011490.hg.1 | TTC37 | tetratricopeptide repeat domain 37 | Multiple_C | 8.26 | 8.88 | 7.63 | 1.55 | 0.0184 | 0.0671 | 10.97 | 10.21 | 10.31 | 1.58 | 0.0239 | 0.1337 |
| TC0500012390.hg.1 | JAKMIP2 | janus kinase and microtubule interacting protein 2 | Multiple_C | 3.88 | 3.58 | 3.25 | 1.55 | 0.1568 | 0.332 | 6.61 | 5.65 | 4.82 | 3.46 | 0.0002 | 0.0059 |
| TC0600007500.hg.1 | HCG9 | HLA complex group 9 (non-protein coding) | Multiple_C | 4.47 | 3.84 | 3.84 | 1.55 | 0.2016 | 0.3938 | 4.8 | 4.67 | 4.3 | 1.41 | 0.2705 | 0.5357 |
| TC0600007711.hg.1 | ITPR3 | inositol 1,4,5-trisphosphate receptor, type 3 | Multiple_C | 7.21 | 6 | 6.58 | 1.55 | 0.5 | 0.699 | 10.25 | 9.45 | 9.5 | 1.68 | 0.0048 | 0.0478 |
| TC0600008663.hg.1 | DOPEY1 | dopey family member 1 | Multiple_C | 8.22 | 8.55 | 7.59 | 1.55 | 0.1257 | 0.2838 | 6.69 | 6.61 | 7.1 | 0.75 | 0.0839 | 0.2787 |
| TC0600009019.hg.1 | SOBP | sine oculis binding protein homolog | Multiple_C | 8.34 | 7.6 | 7.71 | 1.55 | 0.2824 | 0.4926 | 7.59 | 7.73 | 7.59 | 1.00 | 0.2077 | 0.4643 |
| TC0600012709.hg.1 | POPDC3 | popeye domain containing 3 | Multiple_C | 4.73 | 4.21 | 4.1 | 1.55 | 0.0569 | 0.159 | 8.3 | 7.23 | 6.77 | 2.89 | 0.0001 | 0.0044 |
| TC0700007178.hg.1 | SEPT7 | septin 7 | Multiple_C | 13.44 | 13.44 | 12.81 | 1.55 | 0.0482 | 0.1409 | 11.58 | 11.19 | 11.1 | 1.39 | 0.0757 | 0.2646 |
| TC0700010257.hg.1 | ICA1 | islet cell autoantigen 1 | Multiple_C | 11.22 | 11.41 | 10.59 | 1.55 | 0.2523 | 0.4571 | 9.85 | 8.82 | 8.14 | 3.27 | 0.0006 | 0.0122 |
| TC0700013354.hg.1 | GPR141 | G protein-coupled receptor 141 | Multiple_C | 3.99 | 3.59 | 3.36 | 1.55 | 0.1283 | 0.2884 | 4.15 | 4.85 | 4.57 | 0.75 | 0.1037 | 0.3157 |
| TC0900007475.hg.1 | CBWD3 | COBW domain containing 3 | Multiple_C | 13.63 | 14.11 | 13 | 1.55 | 0.2624 | 0.4695 | 12.48 | 11.96 | 12.37 | 1.08 | 0.2094 | 0.4664 |
| TC0900012290.hg.1 | FUT7 | fucosyltransferase 7 (alpha (1,3) fucosyltransferase) | Coding | 3.96 | 3.46 | 3.33 | 1.55 | 0.0627 | 0.1708 | 3.75 | 3.88 | 3.66 | 1.06 | 0.2152 | 0.4738 |
| TC0M00006442.hg.1 | ND5 | NADH dehydrogenase, subunit 5 (complex I) | Multiple_C | 17.68 | 16.59 | 17.05 | 1.55 | 0.0386 | 0.1194 | 18.33 | 17.98 | 18.53 | 0.87 | 0.2259 | 0.4863 |
| TC0X00010409.hg.1 | BEX2 | brain expressed X-linked 2 | Coding | 4.36 | 3.87 | 3.73 | 1.55 | 0.1363 | 0.301 | 6.05 | 5.71 | 5.35 | 1.62 | 0.0411 | 0.1862 |
| TC1000008320.hg.1 | SNCG | synuclein gamma | Multiple_C | 7.91 | 7.32 | 7.28 | 1.55 | 0.0077 | 0.0341 | 6.32 | 6.24 | 6.31 | 1.01 | 0.9212 | 0.9675 |
| TC1000011017.hg.1 | CFAP70 | cilia and flagella associated protein 70 | Multiple_C | 4.51 | 4.36 | 3.88 | 1.55 | 0.0432 | 0.1301 | 4.21 | 4.42 | 4.22 | 0.99 | 0.7967 | 0.9092 |
| TC1100006659.hg.1 | OR52M1 | olfactory receptor, family 52, subfamily M, member 1 | Coding | 4.01 | 3.43 | 3.38 | 1.55 | 0.0542 | 0.1533 | 3.41 | 3.63 | 3.37 | 1.03 | 0.8331 | 0.9258 |
| TC1100008505.hg.1 | EMSY | EMSY BRCA2-interacting transcriptional repressor | Multiple_C | 11.03 | 9.46 | 10.4 | 1.55 | 0.0739 | 0.1926 | 11.47 | 10.92 | 11.3 | 1.13 | 0.4711 | 0.7119 |

| | | | | | | | | | | | | | | | |
|---|---|---|---|---|---|---|---|---|---|---|---|---|---|---|---|
| TC1100008557.hg.1 | USP35 | ubiquitin specific peptidase 35 | Multiple_C | 8.93 | 8.52 | 8.3 | 1.55 | 0.0147 | 0.0563 | 8.29 | 8.12 | 7.93 | 1.28 | 0.2562 | 0.521 |
| TC1100013033.hg.1 | KCNK4 | potassium channel, two pore domain subfamily K, member 4 | Multiple_C | 5.24 | 4.45 | 4.61 | 1.55 | 0.0052 | 0.025 | 5.13 | 5.44 | 5.34 | 0.86 | 0.4429 | 0.6914 |
| TC1200006670.hg.1 | CLSTN3 | calsyntenin 3 | Multiple_C | 6.55 | 5.93 | 5.92 | 1.55 | 0.009 | 0.0384 | 6.96 | 6.82 | 6.41 | 1.46 | 0.0063 | 0.057 |
| TC1200008373.hg.1 | TMTC3 | transmembrane and tetratricopeptide repeat containing 3 | Multiple_C | 11.99 | 11.57 | 11.36 | 1.55 | 0.0123 | 0.0493 | 9.35 | 7.91 | 9.27 | 1.06 | 0.8423 | 0.9313 |
| TC1200009722.hg.1 | TNFRSF1A | tumor necrosis factor receptor superfamily, member 1A | Multiple_C | 10.03 | 8.8 | 9.4 | 1.55 | 0.1724 | 0.3537 | 7.49 | 7.99 | 8.88 | 0.38 | 0.0002 | 0.0065 |
| TC1200011894.hg.1 | C12orf76 | chromosome 12 open reading frame 76 | Multiple_C | 6.99 | 8.21 | 6.36 | 1.55 | 0.1482 | 0.3192 | 6.06 | 5.80 | 5.55 | 1.42 | 0.2955 | 0.5604 |
| TC1200011993.hg.1 | SLC8B1 | solute carrier family 8 (sodium/lithium/calcium exchanger), mei | Multiple_C | 9.89 | 8.98 | 9.26 | 1.55 | 0.0448 | 0.1336 | 7.38 | 7.24 | 7.14 | 1.18 | 0.2959 | 0.5607 |
| TC1300007221.hg.1 | SERPINE3 | serpin peptidase inhibitor, clade E (nexin, plasminogen activatoı | Coding | 5.12 | 4.34 | 4.49 | 1.55 | 0.0024 | 0.0134 | 4.85 | 4.69 | 4.46 | 1.31 | 0.2661 | 0.5306 |
| TC1400010339.hg.1 | C14orf2 | chromosome 14 open reading frame 2 | Multiple_C | 15.53 | 16.71 | 14.9 | 1.55 | 0.0069 | 0.0312 | 11.02 | 10.84 | 10.85 | 1.13 | 0.7625 | 0.8927 |
| TC1500007240.hg.1 | TMOD3 | tropomodulin 3 (ubiquitous) | Multiple_C | 11.57 | 11.71 | 10.94 | 1.55 | 0.0648 | 0.1754 | 11.72 | 10.93 | 11.2 | 1.43 | 0.0429 | 0.191 |
| TC1500007392.hg.1 | CCNB2 | cyclin B2 | Multiple_C | 12.6 | 13.33 | 11.97 | 1.55 | 0.0627 | 0.1708 | 11.01 | 11.70 | 11.25 | 0.85 | 0.0999 | 0.309 |
| TC1500009457.hg.1 | MYO5A | myosin VA | Multiple_C | 5.71 | 4.93 | 5.08 | 1.55 | 0.0229 | 0.0796 | 9.41 | 9.20 | 9 | 1.33 | 0.1882 | 0.4403 |
| TC1500010128.hg.1 | TBC1D2B | TBC1 domain family, member 2B | Multiple_C | 8.34 | 7.29 | 7.71 | 1.55 | 0.1483 | 0.3193 | 9.13 | 8.53 | 8.87 | 1.20 | 0.304 | 0.5679 |
| TC1600007255.hg.1 | RBBP6 | retinoblastoma binding protein 6 | Multiple_C | 12.16 | 11.07 | 11.53 | 1.55 | 0.1834 | 0.3688 | 11.82 | 11.78 | 11.97 | 0.90 | 0.3935 | 0.6518 |
| TC1600009021.hg.1 | TPSG1 | tryptase gamma 1 | Multiple_C | 4.9 | 4.3 | 4.27 | 1.55 | 0.0097 | 0.0406 | 4.8 | 4.90 | 4.57 | 1.17 | 0.2468 | 0.5114 |
| TC1600009395.hg.1 | SOCS1 | suppressor of cytokine signaling 1 | Coding | 3.62 | 3.01 | 2.99 | 1.55 | 0.0176 | 0.0648 | 3.66 | 3.54 | 3.54 | 1.09 | 0.4611 | 0.7039 |
| TC1600009533.hg.1 | MYH11 | myosin, heavy chain 11, smooth muscle | Multiple_C | 5.36 | 4.89 | 4.73 | 1.55 | 0.0496 | 0.1438 | 4.24 | 4.48 | 4.04 | 1.15 | 0.3653 | 0.6278 |
| TC1700008917.hg.1 | SAP30BP | SAP30 binding protein | Multiple_C | 11.4 | 10.94 | 10.77 | 1.55 | 0.0138 | 0.0537 | 11.21 | 11.05 | 11.32 | 0.93 | 0.45 | 0.6962 |
| TC1700009861.hg.1 | TVP23C; CDRT | trans-golgi network vesicle protein 23 homolog C (S. cerevisiae) | Multiple_C | 13.56 | 14.46 | 12.93 | 1.55 | 0.0071 | 0.0318 | 11.2 | 9.99 | 10.9 | 1.23 | 0.0262 | 0.1415 |
| TC1700011802.hg.1 | RHBDF2 | rhomboid 5 homolog 2 (Drosophila) | Multiple_C | 10.18 | 9.2 | 9.55 | 1.55 | 0.0089 | 0.0382 | 12.28 | 12.42 | 11.97 | 1.24 | 0.4857 | 0.7229 |
| TC1800006841.hg.1 | MIB1 | mindbomb E3 ubiquitin protein ligase 1 | Multiple_C | 10.54 | 11.09 | 9.91 | 1.55 | 0.2223 | 0.4201 | 9.76 | 9.28 | 9.17 | 1.51 | 0.4574 | 0.7014 |
| TC1900007426.hg.1 | NCAN | neurocan | Multiple_C | 7.38 | 6.46 | 6.75 | 1.55 | 0.073 | 0.1912 | 6.88 | 6.89 | 6.3 | 1.49 | 0.137 | 0.3677 |
| TC1900008456.hg.1 | ELSPBP1 | epididymal sperm binding protein 1 | Coding | 5.35 | 4.58 | 4.72 | 1.55 | 0.4483 | 0.6564 | 4.57 | 4.57 | 4.57 | 1.00 | 0.2804 | 0.5462 |
| TC1900009841.hg.1 | OR7A5 | olfactory receptor, family 7, subfamily A, member 5 | Multiple_C | 3.66 | 3.02 | 3.03 | 1.55 | 0.0728 | 0.1907 | 3.59 | 3.58 | 3.35 | 1.18 | 0.2953 | 0.5604 |
| TC1900010016.hg.1 | ISYNA1 | inositol-3-phosphate synthase 1 | Multiple_C | 8.83 | 8.42 | 8.2 | 1.55 | 0.0082 | 0.0357 | 9.11 | 7.73 | 6.93 | 4.53 | 3.69E-08 | 1.34E-05 |
| TC1900011651.hg.1 | C19orf45 | chromosome 19 open reading frame 45 | Multiple_C | 6.84 | 6.09 | 6.21 | 1.55 | 0.0477 | 0.1398 | 6.38 | 6.18 | 5.97 | 1.33 | 0.0602 | 0.2322 |
| TC2100008380.hg.1 | KRTAP12-2 | keratin associated protein 12-2 | Coding | 6.64 | 5.6 | 6.01 | 1.55 | 0.1711 | 0.352 | 6.56 | 6.88 | 6.68 | 0.92 | 0.3424 | 0.6061 |
| TC2200009195.hg.1 | AIFM3 | apoptosis-inducing factor, mitochondrion-associated, 3 | Multiple_C | 5.52 | 4.67 | 4.89 | 1.55 | 0.0916 | 0.2259 | 5.96 | 5.56 | 5.22 | 1.67 | 0.0022 | 0.0282 |
| TC0100010281.hg.1 | ATP1A2 | ATPase, Na+/K+ transporting, alpha 2 polypeptide | Multiple_C | 5.16 | 4.87 | 4.54 | 1.54 | 0.0666 | 0.179 | 4.07 | 4.02 | 4.02 | 1.04 | 0.7351 | 0.8777 |
| TC0100010369.hg.1 | ATF6 | activating transcription factor 6 | Multiple_C | 11.04 | 10.93 | 10.42 | 1.54 | 0.038 | 0.118 | 10.13 | 9.83 | 10.12 | 1.01 | 0.9956 | 0.998 |
| TC0100011663.hg.1 | C1orf115 | chromosome 1 open reading frame 115 | Multiple_C | 5.37 | 4.75 | 4.75 | 1.54 | 0.0515 | 0.1476 | 5.16 | 5.21 | 4.75 | 1.33 | 0.1553 | 0.3955 |
| TC0100015332.hg.1 | RSBN1 | round spermatid basic protein 1 | Multiple_C | 9.49 | 9.36 | 8.87 | 1.54 | 0.0606 | 0.1668 | 8.02 | 8.11 | 8.4 | 0.77 | 0.1609 | 0.4036 |
| TC0100017014.hg.1 | LINC00303 | long intergenic non-protein coding RNA 303 | Multiple_C | 3.55 | 3.35 | 2.93 | 1.54 | 0.0133 | 0.0521 | 3.55 | 3.79 | 3.45 | 1.07 | 0.518 | 0.7463 |

| | | | | | | | | | | | | | | | |
|---|---|---|---|---|---|---|---|---|---|---|---|---|---|---|---|
| TC0200012499.hg.1 | MCFD2 | multiple coagulation factor deficiency 2 | Multiple_C | 10.49 | 10.58 | 9.87 | 1.54 | 0.0444 | 0.1327 | 10.91 | 11.06 | 11.24 | 0.80 | 0.1614 | 0.4043 |
| TC0200013095.hg.1 | BOLA3 | bolA family member 3 | Multiple_C | 12.97 | 12.42 | 12.35 | 1.54 | 0.0141 | 0.0544 | 8.18 | 8.42 | 8.81 | 0.65 | 0.2777 | 0.5437 |
| TC0200015246.hg.1 | STAT4 | signal transducer and activator of transcription 4 | Multiple_C | 4.34 | 3.69 | 3.72 | 1.54 | 0.0462 | 0.1365 | 4.15 | 4.09 | 4.1 | 1.04 | 0.9022 | 0.9594 |
| TC0200016710.hg.1 | ANKRD36C | ankyrin repeat domain 36C | Multiple_C | 11.77 | 10.59 | 11.15 | 1.54 | 0.1166 | 0.2693 | 11.97 | 10.56 | 11.08 | 1.85 | 0.0044 | 0.0453 |
| TC0300006605.hg.1 | ATG7 | autophagy related 7 | Multiple_C | 9.36 | 9.25 | 8.74 | 1.54 | 0.05 | 0.1445 | 8.55 | 8.62 | 8.63 | 0.95 | 0.8076 | 0.9152 |
| TC0300008130.hg.1 | COL8A1 | collagen, type VIII, alpha 1 | Multiple_C | 5.53 | 4.95 | 4.91 | 1.54 | 0.0831 | 0.2102 | 5.04 | 4.98 | 4.97 | 1.05 | 0.995 | 0.9976 |
| TC0300011731.hg.1 | CGGBP1 | CGG triplet repeat binding protein 1 | Multiple_C | 11.69 | 11.03 | 11.07 | 1.54 | 0.0131 | 0.0515 | 11.77 | 12.06 | 12.19 | 0.75 | 0.0343 | 0.1668 |
| TC0300012112.hg.1 | B4GALT4 | UDP-Gal:betaGlcNAc beta 1,4- galactosyltransferase, polypeptic | Multiple_C | 7.91 | 8.26 | 7.29 | 1.54 | 0.0511 | 0.147 | 6.29 | 6.28 | 6.07 | 1.16 | 0.9814 | 0.9923 |
| TC0300013873.hg.1 | TF | transferrin | Multiple_C | 4.64 | 4.11 | 4.02 | 1.54 | 0.023 | 0.08 | 3.61 | 3.51 | 3.82 | 0.86 | 0.7154 | 0.8671 |
| TC0400006756.hg.1 | TRMT44 | tRNA methyltransferase 44 homolog (S. cerevisiae) | Multiple_C | 5.72 | 5.46 | 5.1 | 1.54 | 0.0401 | 0.123 | 5.87 | 5.85 | 6.1 | 0.85 | 0.2891 | 0.5538 |
| TC0400007783.hg.1 | AMTN | amelotin | Coding | 3.71 | 3 | 3.09 | 1.54 | 0.0187 | 0.0678 | 3.87 | 3.61 | 3.46 | 1.33 | 0.0909 | 0.2916 |
| TC0400008004.hg.1 | MRPS18C | mitochondrial ribosomal protein S18C | Multiple_C | 10.58 | 10.71 | 9.96 | 1.54 | 0.1402 | 0.3075 | 7.62 | 7.56 | 7.77 | 0.90 | 0.8668 | 0.9429 |
| TC0400008785.hg.1 | NOCT | nocturnin | Multiple_C | 9.22 | 7.67 | 8.6 | 1.54 | 0.0432 | 0.1301 | 8.2 | 7.75 | 8.68 | 0.72 | 0.2951 | 0.56 |
| TC0400009767.hg.1 | ZFYVE28 | zinc finger, FYVE domain containing 28 | Multiple_C | 5.2 | 4.62 | 4.58 | 1.54 | 0.5292 | 0.72 | 5.51 | 5.17 | 4.69 | 1.77 | 0.0174 | 0.1107 |
| TC0400012820.hg.1 | FGF5 | fibroblast growth factor 5 | Multiple_C | 4.15 | 3.41 | 3.53 | 1.54 | 0.007 | 0.0315 | 3.4 | 3.27 | 3.24 | 1.12 | 0.64 | 0.8234 |
| TC0500008114.hg.1 | FAM81B | family with sequence similarity 81, member B | Multiple_C | 4.55 | 4.35 | 3.93 | 1.54 | 0.4905 | 0.6905 | 4.3 | 4.25 | 4.13 | 1.13 | 0.9945 | 0.9975 |
| TC0500009038.hg.1 | SPINK9 | serine peptidase inhibitor, Kazal type 9 | Coding | 4.2 | 3.23 | 3.58 | 1.54 | 0.0887 | 0.2207 | 4.14 | 3.72 | 3.65 | 1.40 | 0.1119 | 0.3294 |
| TC0500012207.hg.1 | SPATA24 | spermatogenesis associated 24 | Multiple_C | 5.24 | 4.72 | 4.62 | 1.54 | 0.0162 | 0.0608 | 4.41 | 4.49 | 4.55 | 0.91 | 0.3732 | 0.6341 |
| TC0500013240.hg.1 | PCDHB4 | protocadherin beta 4 | Multiple_C | 4.26 | 3.73 | 3.64 | 1.54 | 0.0537 | 0.1523 | 4.7 | 4.61 | 4.53 | 1.13 | 0.2069 | 0.4637 |
| TC0500013416.hg.1 | MXD3 | MAX dimerization protein 3 | Multiple_C | 8.1 | 7.46 | 7.48 | 1.54 | 0.3137 | 0.5271 | 6.34 | 6.85 | 6.52 | 0.88 | 0.5035 | 0.7354 |
| TC0600010211.hg.1 | TTLL2 | tubulin tyrosine ligase-like family member 2 | Coding | 4.74 | 4.09 | 4.12 | 1.54 | 0.0502 | 0.145 | 4.81 | 4.69 | 4.46 | 1.27 | 0.0783 | 0.2694 |
| TC0600011821.hg.1 | GUCA1B | guanylate cyclase activator 1B (retina) | Coding | 4.08 | 3.56 | 3.46 | 1.54 | 0.0419 | 0.1272 | 3.94 | 3.59 | 3.5 | 1.36 | 0.0786 | 0.2701 |
| TC0600014006.hg.1 | WDR27 | WD repeat domain 27 | Multiple_C | 7.67 | 7.68 | 7.05 | 1.54 | 0.0742 | 0.1932 | 7.43 | 7.44 | 7.25 | 1.13 | 0.534 | 0.7563 |
| TC0700006656.hg.1 | RSPH10B2 | radial spoke head 10 homolog B2 (Chlamydomonas) | Multiple_C | 4.98 | 4.31 | 4.36 | 1.54 | 0.2261 | 0.4251 | 4.27 | 4.46 | 4.41 | 0.91 | 0.4162 | 0.6701 |
| TC0700013051.hg.1 | ATG9B | autophagy related 9B | Multiple_C | 4.13 | 3.29 | 3.51 | 1.54 | 0.096 | 0.2338 | 4.71 | 4.19 | 4.14 | 1.48 | 0.0601 | 0.2321 |
| TC0700013578.hg.1 | SEMA3A | sema domain, immunoglobulin domain (Ig), short basic domain, | Multiple_C | 10.99 | 10.88 | 10.37 | 1.54 | 0.3038 | 0.5166 | 14.79 | 13.39 | 14.4 | 1.31 | 0.1311 | 0.3587 |
| TC0700013596.hg.1 | TSC22D4 | TSC22 domain family, member 4 | Multiple_C | 4.68 | 4.14 | 4.06 | 1.54 | 0.0327 | 0.1053 | 4.91 | 5.35 | 5.01 | 0.93 | 0.9921 | 0.9968 |
| TC0800007007.hg.1 | TNFRSF10C | tumor necrosis factor receptor superfamily, member 10c, decoy | Multiple_C | 7.49 | 7.07 | 6.87 | 1.54 | 0.0246 | 0.0842 | 6.75 | 6.62 | 6.46 | 1.22 | 0.3115 | 0.5756 |
| TC0900008532.hg.1 | DEC1 | deleted in esophageal cancer 1 | Multiple_C | 4.58 | 3.84 | 3.96 | 1.54 | 0.0217 | 0.0763 | 4.08 | 4.05 | 3.91 | 1.13 | 0.5512 | 0.767 |
| TC0900008795.hg.1 | RALGPS1 | Ral GEF with PH domain and SH3 binding motif 1 | Multiple_C | 7.68 | 7.17 | 7.06 | 1.54 | 0.0249 | 0.085 | 6.39 | 6.03 | 6.26 | 1.09 | 0.7202 | 0.8697 |
| TC0900010762.hg.1 | AUH | AU RNA binding protein/enoyl-CoA hydratase | Multiple_C | 10.27 | 10.19 | 9.65 | 1.54 | 0.0135 | 0.0528 | 8.21 | 8.29 | 8.11 | 1.07 | 0.3442 | 0.6077 |
| TC0900010800.hg.1 | ECM2 | extracellular matrix protein 2, female organ and adipocyte spec | Coding | 3.76 | 3.08 | 3.14 | 1.54 | 0.0661 | 0.1781 | 4.64 | 3.88 | 3.84 | 1.74 | 0.005 | 0.0489 |
| TC0X00007204.hg.1 | GATA1 | GATA binding protein 1 (globin transcription factor 1) | Multiple_C | 6.53 | 5.43 | 5.91 | 1.54 | 0.1175 | 0.2707 | 6.69 | 6.46 | 6.43 | 1.20 | 0.3123 | 0.5765 |

| | | | | | | | | | | | | | | | |
|---|---|---|---|---|---|---|---|---|---|---|---|---|---|---|---|
| TC0Y00006722.hg.1 | TXLNGY | taxilin gamma pseudogene, Y-linked | Multiple_C | 3.91 | 2.89 | 3.29 | 1.54 | 0.0965 | 0.2346 | 3.91 | 3.99 | 3.7 | 1.16 | 0.4036 | 0.6596 |
| TC1000007226.hg.1 | CCDC7 | coiled-coil domain containing 7 | Multiple_C | 7.09 | 6.9 | 6.47 | 1.54 | 0.0729 | 0.191 | 5.66 | 4.83 | 5.7 | 0.97 | 0.8622 | 0.9411 |
| TC1000009002.hg.1 | PNLIPRP3 | pancreatic lipase-related protein 3 | Multiple_C | 3.18 | 2.2 | 2.56 | 1.54 | 0.0328 | 0.1055 | 3.46 | 3.71 | 3.41 | 1.04 | 0.9273 | 0.9698 |
| TC1100008948.hg.1 | GRIA4 | glutamate receptor, ionotropic, AMPA 4 | Multiple_C | 3.81 | 3.05 | 3.19 | 1.54 | 0.142 | 0.3098 | 3.97 | 4.06 | 4.11 | 0.91 | 0.5759 | 0.7826 |
| TC1100009237.hg.1 | VPS11 | vacuolar protein sorting 11 homolog (S. cerevisiae) | Multiple_C | 8.47 | 7.86 | 7.85 | 1.54 | 0.0059 | 0.0276 | 10.27 | 10.69 | 10.3 | 0.98 | 0.6959 | 0.8556 |
| TC1100009561.hg.1 | NTM | neurotrimin | Multiple_C | 3.71 | 3.13 | 3.09 | 1.54 | 0.008 | 0.0349 | 3.37 | 3.42 | 3.72 | 0.78 | 0.119 | 0.341 |
| TC1100009729.hg.1 | TOLLIP | toll interacting protein | Multiple_C | 8.4 | 7.5 | 7.78 | 1.54 | 0.0809 | 0.2066 | 8.01 | 8.06 | 7.88 | 1.09 | 0.8603 | 0.9402 |
| TC1100013157.hg.1 | DCDC1 | doublecortin domain containing 1 | Coding | 5.65 | 5 | 5.03 | 1.54 | 0.0168 | 0.0624 | 5.16 | 4.43 | 4.53 | 1.55 | 0.0024 | 0.0299 |
| TC1200006656.hg.1 | C12orf57; RNU | chromosome 12 open reading frame 57; RNA, U7 small nuclear | Multiple_C | 14.31 | 14.11 | 13.69 | 1.54 | 0.0237 | 0.0817 | 9.06 | 8.89 | 8.04 | 2.03 | 0.0048 | 0.0479 |
| TC1200006773.hg.1 | KLRF1 | killer cell lectin-like receptor subfamily F, member 1 | Multiple_C | 3.79 | 3.05 | 3.17 | 1.54 | 0.2292 | 0.4289 | 4.49 | 4.09 | 3.93 | 1.47 | 0.0285 | 0.1489 |
| TC1200007647.hg.1 | ACVR1B | activin A receptor type IB | Multiple_C | 8.61 | 8.1 | 7.99 | 1.54 | 0.1658 | 0.3446 | 8.28 | 8.35 | 8.58 | 0.81 | 0.8787 | 0.948 |
| TC1200008255.hg.1 | ZDHHC17 | zinc finger, DHHC-type containing 17 | Multiple_C | 7.68 | 8.1 | 7.06 | 1.54 | 0.2088 | 0.4025 | 7.67 | 6.92 | 6.58 | 2.13 | 0.0934 | 0.2968 |
| TC1200010518.hg.1 | SLC38A2 | solute carrier family 38, member 2 | Multiple_C | 14.53 | 13.85 | 13.91 | 1.54 | 0.2316 | 0.4321 | 16.17 | 15.01 | 16.18 | 0.99 | 0.9755 | 0.9902 |
| TC1200011982.hg.1 | RASAL1 | RAS protein activator like 1 (GAP1 like) | Multiple_C | 7.48 | 6.81 | 6.86 | 1.54 | 0.0115 | 0.0465 | 6.72 | 6.29 | 6.6 | 1.09 | 0.3505 | 0.6145 |
| TC1300007161.hg.1 | CDADC1 | cytidine and dCMP deaminase domain containing 1 | Multiple_C | 10.26 | 10.47 | 9.64 | 1.54 | 0.027 | 0.0905 | 9.05 | 8.81 | 8.96 | 1.06 | 0.6152 | 0.8089 |
| TC1300007824.hg.1 | UBAC2 | UBA domain containing 2 | Multiple_C | 10.68 | 10.77 | 10.06 | 1.54 | 0.0064 | 0.0292 | 10.72 | 10.79 | 10.45 | 1.21 | 0.0422 | 0.1894 |
| TC1400006691.hg.1 | THTPA | thiamine triphosphatase | Multiple_C | 7.28 | 6.42 | 6.66 | 1.54 | 0.0944 | 0.231 | 7.44 | 7.64 | 7.13 | 1.24 | 0.9946 | 0.9976 |
| TC1400007036.hg.1 | PRPF39 | pre-mRNA processing factor 39 | Multiple_C | 8.35 | 9.55 | 7.73 | 1.54 | 0.0363 | 0.1142 | 8.45 | 7.68 | 8.51 | 0.96 | 0.4167 | 0.6705 |
| TC1400008205.hg.1 | YY1; MIR6764 | YY1 transcription factor; microRNA 6764 | Multiple_C | 13.74 | 13.99 | 13.12 | 1.54 | 0.0013 | 0.0084 | 11.97 | 12.20 | 12.22 | 0.84 | 0.3305 | 0.5945 |
| TC1500008690.hg.1 | GOLGA8EP; G | golgin A8 family, member E, pseudogene; golgin A8 family, mem | Multiple_C | 6.14 | 5.28 | 5.52 | 1.54 | 0.0379 | 0.1177 | 6.18 | 5.72 | 5.57 | 1.53 | 0.0063 | 0.0572 |
| TC1500009460.hg.1 | FAM214A | family with sequence similarity 214, member A | Multiple_C | 6.72 | 6.71 | 6.1 | 1.54 | 0.0402 | 0.1231 | 5.95 | 6.29 | 5.91 | 1.03 | 0.309 | 0.5731 |
| TC1500009996.hg.1 | STOML1 | stomatin (EPB72)-like 1 | Multiple_C | 6.86 | 6.09 | 6.24 | 1.54 | 0.5215 | 0.7149 | 6.1 | 5.61 | 5.42 | 1.60 | 0.2556 | 0.5202 |
| TC1500010757.hg.1 | RAB11A | RAB11A, member RAS oncogene family | Multiple_C | 14.78 | 14.71 | 14.16 | 1.54 | 0.0159 | 0.0596 | 11.56 | 11.26 | 10.95 | 1.53 | 0.0012 | 0.0189 |
| TC1600007440.hg.1 | ALDOA | aldolase A, fructose-bisphosphate | Multiple_C | 16.25 | 14.89 | 15.63 | 1.54 | 0.0478 | 0.1399 | 15.44 | 16.21 | 15.63 | 0.88 | 0.4702 | 0.711 |
| TC1600010463.hg.1 | KIFC3 | kinesin family member C3 | Multiple_C | 5.59 | 5.14 | 4.97 | 1.54 | 0.0704 | 0.1863 | 6.09 | 5.71 | 5.42 | 1.59 | 0.019 | 0.1166 |
| TC1600011422.hg.1 | CLEC18A | C-type lectin domain family 18, member A | Multiple_C | 6.08 | 5.8 | 5.46 | 1.54 | 0.1248 | 0.2823 | 5.34 | 5.60 | 5.44 | 0.93 | 0.9228 | 0.9683 |
| TC1600011462.hg.1 | BRICD5 | BRICHOS domain containing 5 | Multiple_C | 5.24 | 4.5 | 4.62 | 1.54 | 0.031 | 0.1009 | 5.2 | 5.23 | 5.08 | 1.09 | 0.7084 | 0.8629 |
| TC1700006740.hg.1 | ACAP1 | ArfGAP with coiled-coil, ankyrin repeat and PH domains 1 | Multiple_C | 5.1 | 4.32 | 4.48 | 1.54 | 0.0426 | 0.1288 | 5.48 | 5.08 | 5.02 | 1.38 | 0.1196 | 0.3417 |
| TC1900006916.hg.1 | MBD3L1 | methyl-CpG binding domain protein 3-like 1 | Coding | 4.6 | 4.26 | 3.98 | 1.54 | 0.03 | 0.0984 | 4.77 | 4.88 | 5.02 | 0.84 | 0.1566 | 0.3974 |
| TC1900007151.hg.1 | DCAF15 | DDB1 and CUL4 associated factor 15 | Multiple_C | 9.8 | 8.73 | 9.18 | 1.54 | 0.1881 | 0.3755 | 9.22 | 9.52 | 9.51 | 0.82 | 0.0271 | 0.144 |
| TC1900008061.hg.1 | DLL3 | delta-like 3 (Drosophila) | Multiple_C | 4.43 | 3.71 | 3.81 | 1.54 | 0.0346 | 0.11 | 4.76 | 4.58 | 4.39 | 1.29 | 0.3516 | 0.6152 |
| TC1900009871.hg.1 | WIZ | widely interspaced zinc finger motifs | Multiple_C | 8.92 | 8.1 | 8.3 | 1.54 | 0.0144 | 0.0553 | 8.85 | 9.05 | 8.93 | 0.95 | 0.3656 | 0.628 |
| TC2000006435.hg.1 | DEFB127 | defensin, beta 127 | Coding | 3.6 | 3.28 | 2.98 | 1.54 | 0.047 | 0.1382 | 3.14 | 3.40 | 3.23 | 0.94 | 0.6264 | 0.8154 |

| | | | | | | | | | | | | | | | |
|---|---|---|---|---|---|---|---|---|---|---|---|---|---|---|---|
| TC2000006916.hg.1 | RPL41P1 | ribosomal protein L41 pseudogene 1 | Multiple_C | 15.87 | 15.86 | 15.25 | 1.54 | 0.0233 | 0.0808 | 13.69 | 13.81 | 13.69 | 1.00 | 0.9289 | 0.9702 |
| TC2000007094.hg.1 | TTLL9 | tubulin tyrosine ligase-like family member 9 | Multiple_C | 3.72 | 3.35 | 3.1 | 1.54 | 0.1236 | 0.2806 | 4.33 | 3.79 | 3.74 | 1.51 | 0.0109 | 0.0823 |
| TC2000009887.hg.1 | PLCB1 | phospholipase C, beta 1 (phosphoinositide-specific) | Multiple_C | 7 | 6.75 | 6.38 | 1.54 | 0.0632 | 0.172 | 8.9 | 8.50 | 8.1 | 1.74 | 0.0033 | 0.0382 |
| TC2000009905.hg.1 | EFCAB8 | EF-hand calcium binding domain 8 | Coding | 5.82 | 4.7 | 5.2 | 1.54 | 0.0398 | 0.1222 | 5.06 | 4.72 | 5.08 | 0.99 | 0.8499 | 0.9351 |
| TC2000009997.hg.1 | DUSP15 | dual specificity phosphatase 15 | Multiple_C | 5.2 | 4.27 | 4.58 | 1.54 | 0.1491 | 0.3205 | 5.21 | 5.03 | 4.75 | 1.38 | 0.0421 | 0.1893 |
| TC2200008036.hg.1 | USP41 | ubiquitin specific peptidase 41 | Multiple_C | 8.35 | 7.58 | 7.73 | 1.54 | 0.5044 | 0.7019 | 7.75 | 7.74 | 8.69 | 0.52 | 0.0463 | 0.1989 |
| TC2200008229.hg.1 | LRRC75B | leucine rich repeat containing 75B | Multiple_C | 5.08 | 4.36 | 4.46 | 1.54 | 0.0097 | 0.0405 | 5.22 | 5.85 | 5.41 | 0.88 | 0.7593 | 0.8908 |
| TC2200008507.hg.1 | SLC5A4 | solute carrier family 5 (glucose activated ion channel), member | Coding | 4.45 | 3.88 | 3.83 | 1.54 | 0.0411 | 0.1251 | 3.42 | 3.28 | 3.49 | 0.95 | 0.5366 | 0.7584 |
| TC2200008594.hg.1 | MYH9 | myosin, heavy chain 9, non-muscle | Multiple_C | 13.02 | 12.13 | 12.4 | 1.54 | 0.0101 | 0.0419 | 13.41 | 13.30 | 13.17 | 1.18 | 0.6556 | 0.8321 |
| TSUnmapped00000122.h | CCDC84 | coiled-coil domain containing 84 | Coding | 7.64 | 6.88 | 7.02 | 1.54 | 0.0175 | 0.0644 | 7.62 | 6.69 | 7.18 | 1.36 | 0.1175 | 0.3388 |
| TSUnmapped00000483.h | RPS6KA1 | ribosomal protein S6 kinase, 90kDa, polypeptide 1 | Coding | 7.35 | 6.34 | 6.73 | 1.54 | 0.0794 | 0.2039 | 5.98 | 6.14 | 6.12 | 0.91 | 0.604 | 0.8019 |
| TSUnmapped00000675.h | ADAMTS13 | ADAM metallopeptidase with thrombospondin type 1 motif 13 | Coding | 6.03 | 5.45 | 5.41 | 1.54 | 0.2836 | 0.4937 | 4.95 | 4.86 | 4.72 | 1.17 | 0.4127 | 0.6674 |
| TC0100010085.hg.1 | CHRNB2 | cholinergic receptor, nicotinic beta 2 | Coding | 4.81 | 4.1 | 4.2 | 1.53 | 0.0355 | 0.1121 | 5.33 | 5.29 | 5.26 | 1.05 | 0.8228 | 0.9219 |
| TC0100010623.hg.1 | SUCO | SUN domain containing ossification factor | Multiple_C | 7.59 | 7.29 | 6.98 | 1.53 | 0.2333 | 0.434 | 6.55 | 6.37 | 7.21 | 0.63 | 0.0193 | 0.1174 |
| TC0100012401.hg.1 | OR4F29; OR4F | olfactory receptor, family 4, subfamily F, member 29; olfactory | Coding | 4.79 | 3.99 | 4.18 | 1.53 | 0.3212 | 0.5349 | 4.04 | 4.79 | 4.37 | 0.80 | 0.8468 | 0.9333 |
| TC0100012410.hg.1 | OR4F16; OR4F | olfactory receptor, family 4, subfamily F, member 16; olfactory | Coding | 4.79 | 3.99 | 4.18 | 1.53 | 0.3212 | 0.5349 | 4.04 | 4.79 | 4.37 | 0.80 | 0.8468 | 0.9333 |
| TC0100013873.hg.1 | HIVEP3 | human immunodeficiency virus type I enhancer binding protein | Multiple_C | 6.06 | 5.28 | 5.45 | 1.53 | 0.1311 | 0.293 | 5.49 | 5.34 | 5.02 | 1.39 | 0.4298 | 0.6817 |
| TC0100015561.hg.1 | NBPF15 | neuroblastoma breakpoint family, member 15 | Multiple_C | 10.28 | 9.99 | 9.67 | 1.53 | 0.0936 | 0.2295 | 9.82 | 9.63 | 10.47 | 0.64 | 0.1524 | 0.3913 |
| TC0100015781.hg.1 | PI4KB | phosphatidylinositol 4-kinase, catalytic, beta | Multiple_C | 8.15 | 7.88 | 7.54 | 1.53 | 0.1744 | 0.3562 | 7.25 | 7.46 | 7.71 | 0.73 | 0.1647 | 0.4092 |
| TC0100015825.hg.1 | TCHHL1 | trichohyalin like 1 | Coding | 4.04 | 3.36 | 3.43 | 1.53 | 0.236 | 0.4373 | 3.31 | 3.56 | 3.79 | 0.72 | 0.1995 | 0.4548 |
| TC0100016150.hg.1 | ITLN2 | intelectin 2 | Multiple_C | 3.64 | 2.78 | 3.03 | 1.53 | 0.0397 | 0.1219 | 3.69 | 3.62 | 3.59 | 1.07 | 0.4478 | 0.6948 |
| TC0100017241.hg.1 | TMEM206 | transmembrane protein 206 | Multiple_C | 9.22 | 8.73 | 8.61 | 1.53 | 0.097 | 0.2355 | 7.37 | 7.13 | 7.27 | 1.07 | 0.8118 | 0.9166 |
| TC0100018307.hg.1 | ACKR1 | atypical chemokine receptor 1 (Duffy blood group) | Coding | 3.64 | 3.12 | 3.03 | 1.53 | 0.0514 | 0.1474 | 3.63 | 3.71 | 3.35 | 1.21 | 0.1255 | 0.3501 |
| TC0100018515.hg.1 | CFAP45 | cilia and flagella associated protein 45 | Multiple_C | 5.26 | 4.71 | 4.65 | 1.53 | 0.0017 | 0.0102 | 3.53 | 3.45 | 3.52 | 1.01 | 0.9005 | 0.9587 |
| TC0200006951.hg.1 | UBXN2A | UBX domain protein 2A | Multiple_C | 8.66 | 8.55 | 8.05 | 1.53 | 0.0287 | 0.0948 | 9.8 | 9.70 | 9.82 | 0.99 | 0.6878 | 0.8506 |
| TC0200012085.hg.1 | FNDC4 | fibronectin type III domain containing 4 | Multiple_C | 4.97 | 4.22 | 4.36 | 1.53 | 0.0181 | 0.0663 | 4.86 | 4.84 | 4.53 | 1.26 | 0.2312 | 0.4925 |
| TC0200013111.hg.1 | DQX1 | DEAQ box RNA-dependent ATPase 1 | Multiple_C | 5 | 4.31 | 4.39 | 1.53 | 0.0717 | 0.1888 | 4.24 | 4.42 | 4.51 | 0.83 | 0.2979 | 0.5626 |
| TC0200013836.hg.1 | SEPT10 | septin 10 | Multiple_C | 11.77 | 12.69 | 11.16 | 1.53 | 0.0892 | 0.2216 | 9.64 | 9.09 | 9.07 | 1.48 | 0.2704 | 0.5357 |
| TC0300008355.hg.1 | BOC | BOC cell adhesion associated, oncogene regulated | Multiple_C | 4.88 | 4.19 | 4.27 | 1.53 | 0.0311 | 0.1013 | 4.61 | 4.70 | 4.45 | 1.12 | 0.7833 | 0.9026 |
| TC0300011103.hg.1 | HYAL1 | hyaluronoglucosaminidase 1 | Multiple_C | 8.21 | 7.81 | 7.6 | 1.53 | 0.0159 | 0.0597 | 7.88 | 7.85 | 7.55 | 1.26 | 0.0269 | 0.1433 |
| TC0400007264.hg.1 | KLF3 | Kruppel-like factor 3 (basic) | Multiple_C | 12.13 | 10.84 | 11.52 | 1.53 | 0.1085 | 0.2558 | 10.58 | 10.44 | 10.87 | 0.82 | 0.2495 | 0.514 |
| TC0400008661.hg.1 | INTU | inturned planar cell polarity protein | Multiple_C | 5.61 | 5.21 | 5 | 1.53 | 0.0847 | 0.2132 | 5.18 | 4.84 | 5.07 | 1.08 | 0.3699 | 0.6322 |
| TC0400010132.hg.1 | TAPT1 | transmembrane anterior posterior transformation 1 | Multiple_C | 10.3 | 9.96 | 9.69 | 1.53 | 0.1057 | 0.2509 | 10.91 | 9.79 | 10.24 | 1.59 | 0.2825 | 0.548 |

| | | | | | | | | | | | | | | | |
|---|---|---|---|---|---|---|---|---|---|---|---|---|---|---|---|
| TC0500007494.hg.1 | RAB3C | RAB3C, member RAS oncogene family | Multiple_C | 4.22 | 3.28 | 3.61 | 1.53 | 0.1317 | 0.294 | 3.77 | 3.61 | 3.81 | 0.97 | 0.8133 | 0.9171 |
| TC0500007780.hg.1 | ARHGEF28 | Rho guanine nucleotide exchange factor 28 | Multiple_C | 8.18 | 7.68 | 7.57 | 1.53 | 0.1596 | 0.336 | 6.82 | 6.67 | 6.57 | 1.19 | 0.6086 | 0.8042 |
| TC0500009621.hg.1 | SLC34A1 | solute carrier family 34 (type II sodium/phosphate cotransporte | Multiple_C | 5.68 | 4.71 | 5.07 | 1.53 | 0.0172 | 0.0636 | 4.95 | 5.24 | 5.64 | 0.62 | 0.0137 | 0.0949 |
| TC0500011125.hg.1 | ANKRA2 | ankyrin repeat, family A (RFXANK-like), 2 | Multiple_C | 9.16 | 10.41 | 8.55 | 1.53 | 0.1219 | 0.2778 | 7.98 | 7.69 | 6.41 | 2.97 | 0.0004 | 0.0089 |
| TC0600010056.hg.1 | WTAP | Wilms tumor 1 associated protein | Multiple_C | 12.8 | 12.52 | 12.19 | 1.53 | 0.0647 | 0.1752 | 11.65 | 12.41 | 12.24 | 0.66 | 0.0088 | 0.0718 |
| TC0700012478.hg.1 | C7orf77 | chromosome 7 open reading frame 77 | Multiple_C | 3.5 | 2.92 | 2.89 | 1.53 | 0.4454 | 0.6539 | 4.27 | 3.78 | 3.56 | 1.64 | 0.0753 | 0.2637 |
| TC0700013490.hg.1 | ZNF775 | zinc finger protein 775 | Multiple_C | 7.19 | 6.46 | 6.58 | 1.53 | 0.0732 | 0.1915 | 6.03 | 6.14 | 6.05 | 0.99 | 0.7573 | 0.89 |
| TC0700013491.hg.1 | GIMAP1-GIM/ | GIMAP1-GIMAP5 readthrough; GTPase, IMAP family member 5 | Multiple_C | 5.61 | 5.19 | 5 | 1.53 | 0.0625 | 0.1706 | 4.94 | 4.65 | 4.4 | 1.45 | 0.0995 | 0.3084 |
| TC0800009166.hg.1 | GML | glycosylphosphatidylinositol anchored molecule like | Coding | 3.88 | 3.36 | 3.27 | 1.53 | 0.2054 | 0.3983 | 3.57 | 3.61 | 3.6 | 0.98 | 0.8262 | 0.9234 |
| TC0800010153.hg.1 | STAR | steroidogenic acute regulatory protein | Multiple_C | 6.7 | 6.07 | 6.09 | 1.53 | 0.0437 | 0.1311 | 6.21 | 5.97 | 6.02 | 1.14 | 0.3272 | 0.5907 |
| TC0800010607.hg.1 | ASPH | aspartate beta-hydroxylase | Multiple_C | 17.95 | 17.7 | 17.34 | 1.53 | 0.0496 | 0.1438 | 17.81 | 16.06 | 17.63 | 1.13 | 0.2276 | 0.4882 |
| TC0900009996.hg.1 | PAX5 | paired box 5 | Multiple_C | 6.17 | 5.07 | 5.56 | 1.53 | 0.0597 | 0.1651 | 5.24 | 5.42 | 5.47 | 0.85 | 0.4125 | 0.6672 |
| TC0X00007655.hg.1 | SLC16A2 | solute carrier family 16, member 2 (thyroid hormone transporte | Multiple_C | 3.93 | 3.36 | 3.32 | 1.53 | 0.1125 | 0.2625 | 4.25 | 4.12 | 4.04 | 1.16 | 0.9593 | 0.9834 |
| TC0X00009124.hg.1 | PIGA | phosphatidylinositol glycan anchor biosynthesis class A | Multiple_C | 11.38 | 10.7 | 10.77 | 1.53 | 0.1037 | 0.2475 | 10.1 | 10.08 | 10.52 | 0.75 | 0.0256 | 0.1392 |
| TC0X00011406.hg.1 | CXorf40B | chromosome X open reading frame 40B | Multiple_C | 9.94 | 9.66 | 9.33 | 1.53 | 0.0983 | 0.2378 | 8.08 | 8.41 | 9.05 | 0.51 | 0.0003 | 0.0073 |
| TC0X00011413.hg.1 | L1CAM | L1 cell adhesion molecule | Multiple_C | 4.37 | 3.82 | 3.76 | 1.53 | 0.0894 | 0.2218 | 6.41 | 7.31 | 8.13 | 0.30 | 1.63E-07 | 3.76E-05 |
| TC1000009017.hg.1 | SLC18A2 | solute carrier family 18 (vesicular monoamine transporter), mer | Multiple_C | 4.74 | 4.1 | 4.13 | 1.53 | 0.1337 | 0.2971 | 4.84 | 4.71 | 4.89 | 0.97 | 0.7671 | 0.8956 |
| TC1000009916.hg.1 | CUBN | cubilin (intrinsic factor-cobalamin receptor) | Multiple_C | 4.02 | 3.5 | 3.41 | 1.53 | 0.0723 | 0.1897 | 3.75 | 3.53 | 3.64 | 1.08 | 0.6054 | 0.8027 |
| TC1000010358.hg.1 | MTRNR2L7 | MT-RNR2-like 7 | Coding | 6.15 | 5.41 | 5.54 | 1.53 | 0.2435 | 0.4464 | 5.13 | 5.10 | 5.43 | 0.81 | 0.4488 | 0.6956 |
| TC1000010362.hg.1 | ZNF248 | zinc finger protein 248 | Multiple_C | 7.22 | 7.22 | 6.61 | 1.53 | 0.0241 | 0.0828 | 7.09 | 6.82 | 7.05 | 1.03 | 0.4422 | 0.6911 |
| TC1100007095.hg.1 | LUZP2 | leucine zipper protein 2 | Multiple_C | 3.69 | 2.92 | 3.08 | 1.53 | 0.1921 | 0.3809 | 3.48 | 3.58 | 3.23 | 1.19 | 0.1851 | 0.4365 |
| TC1100009394.hg.1 | PANX3 | pannexin 3 | Coding | 4.13 | 3.57 | 3.52 | 1.53 | 0.5 | 0.699 | 4.1 | 3.70 | 3.91 | 1.14 | 0.3623 | 0.6252 |
| TC1200006890.hg.1 | APOLD1; DDX | apolipoprotein L domain containing 1; DEAD (Asp-Glu-Ala-Asp) | Multiple_C | 14.1 | 14.28 | 13.49 | 1.53 | 0.0194 | 0.0698 | 12.89 | 12.23 | 13.19 | 0.81 | 0.2283 | 0.4892 |
| TC1200010642.hg.1 | FAIM2 | Fas apoptotic inhibitory molecule 2 | Coding | 6.84 | 6.5 | 6.23 | 1.53 | 0.02 | 0.0714 | 3.42 | 3.10 | 3.47 | 0.97 | 0.3602 | 0.6229 |
| TC1300007483.hg.1 | BORA | bora, aurora kinase A activator | Multiple_C | 8.42 | 8.57 | 7.81 | 1.53 | 0.0204 | 0.0727 | 9.4 | 9.50 | 9.11 | 1.22 | 0.1021 | 0.3127 |
| TC1400009353.hg.1 | SIX4 | SIX homeobox 4 | Multiple_C | 8.95 | 8.35 | 8.34 | 1.53 | 0.0726 | 0.1904 | 8.83 | 7.37 | 8.22 | 1.53 | 0.0041 | 0.0434 |
| TC1500009984.hg.1 | NPTN | neuroplastin | Multiple_C | 11.36 | 10.75 | 10.75 | 1.53 | 0.0639 | 0.1734 | 10.8 | 11.24 | 11.43 | 0.65 | 0.05 | 0.2084 |
| TC1500010754.hg.1 | PLEKHO2 | pleckstrin homology domain containing, family O member 2 | Multiple_C | 4.91 | 4.24 | 4.3 | 1.53 | 0.0272 | 0.0911 | 4.83 | 5.08 | 5.14 | 0.81 | 0.1564 | 0.397 |
| TC1500010863.hg.1 | SERINC4 | serine incorporator 4 | Multiple_C | 5.45 | 4.82 | 4.84 | 1.53 | 0.0254 | 0.0864 | 5.18 | 4.85 | 4.59 | 1.51 | 0.0054 | 0.0514 |
| TC1600006533.hg.1 | UBE2I | ubiquitin conjugating enzyme E2I | Multiple_C | 13.82 | 12.75 | 13.21 | 1.53 | 0.0189 | 0.0683 | 12.97 | 13.05 | 13.63 | 0.63 | 0.0008 | 0.0148 |
| TC1600010406.hg.1 | AMFR | autocrine motility factor receptor, E3 ubiquitin protein ligase | Multiple_C | 9.91 | 9.95 | 9.3 | 1.53 | 0.0619 | 0.1694 | 8.96 | 8.79 | 8.69 | 1.21 | 0.0671 | 0.247 |
| TC1600011412.hg.1 | SLC9A5 | solute carrier family 9, subfamily A (NHE5, cation proton antipo | Multiple_C | 6.3 | 5.74 | 5.69 | 1.53 | 0.0623 | 0.1702 | 7.12 | 6.53 | 6.33 | 1.73 | 0.0278 | 0.1462 |
| TC1600011521.hg.1 | ZNF768 | zinc finger protein 768 | Coding | 9.63 | 9.15 | 9.02 | 1.53 | 0.1027 | 0.2458 | 8.29 | 8.52 | 8.59 | 0.81 | 0.1931 | 0.4464 |

| | | | | | | | | | | | | | | |
|---|---|---|---|---|---|---|---|---|---|---|---|---|---|---|
| TC1700006654.hg.1 | RNF167 | ring finger protein 167 | Multiple_C | 10.1 | 8.89 | 9.49 | 1.53 | 0.0325 | 0.1049 | 10.19 | 10.18 | 10.51 | 0.80 | 0.0964 | 0.3024 |
| TC1700009730.hg.1 | RCVRN | recoverin | Multiple_C | 5.01 | 4.92 | 4.4 | 1.53 | 0.3935 | 0.6074 | 5.55 | 5.38 | 5.12 | 1.35 | 0.1546 | 0.3942 |
| TC1700011818.hg.1 | JMJD6 | jumonji domain containing 6 | Multiple_C | 8.69 | 7.94 | 8.08 | 1.53 | 0.1463 | 0.3165 | 7.25 | 7.06 | 7.35 | 0.93 | 0.9833 | 0.9931 |
| TC1700012286.hg.1 | SMG8 | SMG8 nonsense mediated mRNA decay factor | Multiple_C | 7.3 | 7.25 | 6.69 | 1.53 | 0.0621 | 0.1696 | 7.45 | 7.45 | 7.64 | 0.88 | 0.3343 | 0.5983 |
| TC1700012433.hg.1 | HOXB6 | homeobox B6 | Multiple_C | 5.63 | 4.89 | 5.02 | 1.53 | 0.0622 | 0.1699 | 7.14 | 7.24 | 7.33 | 0.88 | 0.5092 | 0.7394 |
| TC1800009306.hg.1 | RNF152 | ring finger protein 152 | Multiple_C | 4.61 | 3.83 | 4 | 1.53 | 0.0768 | 0.1985 | 4.58 | 4.70 | 4.47 | 1.08 | 0.7882 | 0.9056 |
| TC1900006856.hg.1 | CLEC4M | C-type lectin domain family 4, member M | Multiple_C | 6.37 | 5.66 | 5.76 | 1.53 | 0.0492 | 0.1428 | 5.17 | 5.23 | 5.14 | 1.02 | 0.7419 | 0.8815 |
| TC1900007365.hg.1 | PIK3R2; IFI30 | phosphoinositide-3-kinase, regulatory subunit 2 (beta); interfer | Multiple_C | 7.95 | 7.2 | 7.34 | 1.53 | 0.0849 | 0.2135 | 8.42 | 9.10 | 8.89 | 0.72 | 0.0233 | 0.1318 |
| TC1900008118.hg.1 | ITPKC | inositol-trisphosphate 3-kinase C | Multiple_C | 5.95 | 5.24 | 5.34 | 1.53 | 0.0019 | 0.011 | 5.06 | 4.96 | 5.01 | 1.04 | 0.5781 | 0.7843 |
| TC1900008623.hg.1 | SIGLEC9 | sialic acid binding Ig-like lectin 9 | Coding | 4.63 | 3.86 | 4.02 | 1.53 | 0.1144 | 0.2656 | 4.58 | 4.21 | 3.94 | 1.56 | 0.0776 | 0.2683 |
| TC1900008989.hg.1 | ZNF460 | zinc finger protein 460 | Multiple_C | 10.62 | 10.47 | 10.01 | 1.53 | 0.0379 | 0.1177 | 10.01 | 9.64 | 10 | 1.01 | 0.6448 | 0.8257 |
| TC1900009003.hg.1 | ZNF134 | zinc finger protein 134 | Multiple_C | 9.76 | 9.1 | 9.15 | 1.53 | 0.0289 | 0.0954 | 8.22 | 8.78 | 9.15 | 0.52 | 7.36E-05 | 0.0029 |
| TC1900009272.hg.1 | AES | amino-terminal enhancer of split | Multiple_C | 12.77 | 11.82 | 12.16 | 1.53 | 0.0414 | 0.1259 | 12.61 | 12.78 | 12.49 | 1.09 | 0.6258 | 0.815 |
| TC1900009806.hg.1 | PRKACA | protein kinase, cAMP-dependent, catalytic, alpha | Multiple_C | 9.51 | 8.15 | 8.9 | 1.53 | 0.0658 | 0.1775 | 10.21 | 10.97 | 11.1 | 0.54 | 0.0032 | 0.0375 |
| TC1900010513.hg.1 | COX7A1 | cytochrome c oxidase subunit VIIa polypeptide 1 (muscle) | Multiple_C | 5.31 | 4.22 | 4.7 | 1.53 | 0.0235 | 0.0812 | 5.5 | 5.52 | 5.27 | 1.17 | 0.642 | 0.8245 |
| TC1900010886.hg.1 | ZNF235 | zinc finger protein 235 | Multiple_C | 6.76 | 6.67 | 6.15 | 1.53 | 0.0069 | 0.0312 | 6.66 | 6.53 | 6.82 | 0.90 | 0.4325 | 0.6839 |
| TC1900011018.hg.1 | BBC3; MIR319 | BCL2 binding component 3; microRNA 3191 | Multiple_C | 6.5 | 6.21 | 5.89 | 1.53 | 0.112 | 0.2618 | 4.67 | 4.72 | 4.4 | 1.21 | 0.5101 | 0.7401 |
| TC1900011339.hg.1 | BIRC8 | baculoviral IAP repeat containing 8 | Coding | 3.68 | 3.16 | 3.07 | 1.53 | 0.0713 | 0.188 | 4.16 | 3.73 | 3.91 | 1.19 | 0.3084 | 0.5724 |
| TC1900011690.hg.1 | ZNF493 | zinc finger protein 493 | Multiple_C | 6.56 | 5.92 | 5.95 | 1.53 | 0.0955 | 0.233 | 4.61 | 4.74 | 4.76 | 0.90 | 0.8366 | 0.9281 |
| TC1900011799.hg.1 | LILRB1 | leukocyte immunoglobulin-like receptor, subfamily B (with TM a | Multiple_C | 4.74 | 3.82 | 4.13 | 1.53 | 0.0371 | 0.116 | 4.38 | 4.17 | 4.02 | 1.28 | 0.2534 | 0.5176 |
| TC2000007620.hg.1 | ARFGEF2 | ADP-ribosylation factor guanine nucleotide-exchange factor 2 (I | Multiple_C | 10.6 | 9.81 | 9.99 | 1.53 | 0.2947 | 0.5061 | 11.35 | 10.51 | 11.45 | 0.93 | 0.4843 | 0.7219 |
| TC2000007942.hg.1 | FAM217B | family with sequence similarity 217, member B | Multiple_C | 7.94 | 7.29 | 7.33 | 1.53 | 0.07 | 0.1856 | 8.56 | 8.53 | 8.12 | 1.36 | 0.1524 | 0.3913 |
| TC2100007297.hg.1 | CRYAA | crystallin alpha A | Multiple_C | 4.48 | 3.68 | 3.87 | 1.53 | 0.0314 | 0.1021 | 3.87 | 4.01 | 3.96 | 0.94 | 0.572 | 0.7798 |
| TC2200008862.hg.1 | CYP2D6 | cytochrome P450, family 2, subfamily D, polypeptide 6 | Multiple_C | 5.87 | 5.3 | 5.26 | 1.53 | 0.0536 | 0.1522 | 5.82 | 6.31 | 6.06 | 0.85 | 0.3944 | 0.6525 |
| TC0100007006.hg.1 | SPEN | spen family transcriptional repressor | Multiple_C | 11.72 | 10.82 | 11.12 | 1.52 | 0.0608 | 0.1673 | 10.68 | 10.11 | 10.74 | 0.96 | 0.3288 | 0.5926 |
| TC0100007254.hg.1 | LDLRAD2 | low density lipoprotein receptor class A domain containing 2 | Multiple_C | 5.96 | 5.21 | 5.36 | 1.52 | 0.1522 | 0.3256 | 6.33 | 6.32 | 6.52 | 0.88 | 0.9684 | 0.9868 |
| TC0100008773.hg.1 | MSH4 | mutS homolog 4 | Multiple_C | 3.96 | 2.7 | 3.36 | 1.52 | 0.003 | 0.016 | 3.47 | 3.75 | 3.14 | 1.26 | 0.0817 | 0.2753 |
| TC0100009386.hg.1 | PROK1 | prokineticin 1 | Coding | 5.7 | 5.07 | 5.1 | 1.52 | 0.0063 | 0.029 | 5.31 | 5.61 | 5.48 | 0.89 | 0.9532 | 0.981 |
| TC0100010810.hg.1 | KIAA1614 | KIAA1614 | Multiple_C | 5.73 | 5 | 5.13 | 1.52 | 0.1086 | 0.2562 | 5.24 | 5.12 | 4.92 | 1.25 | 0.5813 | 0.786 |
| TC0100015853.hg.1 | SPRR2G | small proline-rich protein 2G | Multiple_C | 4.2 | 3.18 | 3.6 | 1.52 | 0.0728 | 0.1908 | 4 | 3.98 | 3.96 | 1.03 | 0.8309 | 0.9247 |
| TC0100016086.hg.1 | AIM2 | absent in melanoma 2 | Multiple_C | 4.11 | 3.75 | 3.51 | 1.52 | 0.0133 | 0.0521 | 3.94 | 3.60 | 3.99 | 0.97 | 0.7576 | 0.8901 |
| TC0100016155.hg.1 | USF1 | upstream transcription factor 1 | Multiple_C | 7.79 | 7.02 | 7.19 | 1.52 | 0.1075 | 0.254 | 6.55 | 7.34 | 7.29 | 0.60 | 0.0896 | 0.2896 |
| TC0100018464.hg.1 | PPM1J | protein phosphatase, Mg2+/Mn2+ dependent, 1J | Multiple_C | 5.11 | 4.37 | 4.51 | 1.52 | 0.0041 | 0.0204 | 4.08 | 4.04 | 4.13 | 0.97 | 0.6258 | 0.815 |

| | | | | | | | | | | | | | | | |
|---|---|---|---|---|---|---|---|---|---|---|---|---|---|---|---|
| TC0200014170.hg.1 | MAP3K2 | mitogen-activated protein kinase kinase kinase 2 | Multiple_C | 9.2 | 8.12 | 8.6 | 1.52 | 0.0656 | 0.1772 | 9.05 | 8.21 | 9.09 | 0.97 | 0.1538 | 0.3931 |
| TC0200016643.hg.1 | DNMT3A | DNA (cytosine-5-)-methyltransferase 3 alpha | Multiple_C | 6.8 | 5.95 | 6.2 | 1.52 | 0.0415 | 0.1262 | 9.16 | 9.20 | 8.74 | 1.34 | 0.0863 | 0.2833 |
| TC0300006688.hg.1 | C3orf20 | chromosome 3 open reading frame 20 | Multiple_C | 3.89 | 3.2 | 3.29 | 1.52 | 0.0597 | 0.1651 | 3.62 | 3.57 | 3.57 | 1.04 | 0.9917 | 0.9964 |
| TC0300009471.hg.1 | SKIL | SKI-like proto-oncogene | Multiple_C | 8.34 | 7.94 | 7.74 | 1.52 | 0.0537 | 0.1525 | 10.1 | 9.58 | 10.16 | 0.96 | 0.7727 | 0.8979 |
| TC0300013879.hg.1 | IL20RB | interleukin 20 receptor beta | Multiple_C | 5.41 | 4.86 | 4.81 | 1.52 | 0.1719 | 0.3531 | 4.69 | 4.53 | 4.95 | 0.84 | 0.3749 | 0.6356 |
| TC0300013989.hg.1 | ABHD14B | abhydrolase domain containing 14B | Multiple_C | 8.81 | 8.4 | 8.21 | 1.52 | 0.0699 | 0.1851 | 8.21 | 8.46 | 8.15 | 1.04 | 0.5584 | 0.7714 |
| TC0400007777.hg.1 | CSN3 | casein kappa | Coding | 3.58 | 2.9 | 2.98 | 1.52 | 0.5368 | 0.7253 | 4.68 | 4.38 | 4.38 | 1.23 | 0.4021 | 0.6584 |
| TC0400008088.hg.1 | MEPE | matrix extracellular phosphoglycoprotein | Multiple_C | 3.58 | 2.88 | 2.98 | 1.52 | 0.1349 | 0.2989 | 3.62 | 3.67 | 3.71 | 0.94 | 0.2003 | 0.4557 |
| TC0400009210.hg.1 | TRIM60 | tripartite motif containing 60 | Coding | 4.01 | 3.29 | 3.41 | 1.52 | 0.0199 | 0.0713 | 3.59 | 3.60 | 3.44 | 1.11 | 0.8622 | 0.9411 |
| TC0400009978.hg.1 | ACOX3 | acyl-CoA oxidase 3, pristanoyl | Multiple_C | 6.15 | 6.23 | 5.55 | 1.52 | 0.0973 | 0.236 | 6.97 | 7.61 | 7.05 | 0.95 | 0.2197 | 0.4798 |
| TC0500009984.hg.1 | FLJ33360; CTD | FLJ33360 protein; FLJ33360 protein [Source:EntrezGene;Acc:40 | Multiple_C | 5.11 | 4.46 | 4.51 | 1.52 | 0.1168 | 0.2694 | 5.83 | 5.44 | 5.2 | 1.55 | 0.0539 | 0.2173 |
| TC0600007305.hg.1 | BTN2A3P | butyrophilin, subfamily 2, member A3, pseudogene | Multiple_C | 6.09 | 6.28 | 5.49 | 1.52 | 0.0634 | 0.1724 | 6.42 | 6.09 | 6.32 | 1.07 | 0.6803 | 0.8462 |
| TC0600007693.hg.1 | RPS18 | ribosomal protein S18 | Multiple_C | 17.24 | 17.56 | 16.64 | 1.52 | 0.0014 | 0.0089 | 16.67 | 16.48 | 16 | 1.59 | 0.0051 | 0.0498 |
| TC0600008211.hg.1 | GLYATL3 | glycine-N-acyltransferase-like 3 | Coding | 3.5 | 3.09 | 2.9 | 1.52 | 0.0459 | 0.1358 | 3.93 | 4.03 | 4.22 | 0.82 | 0.3655 | 0.6279 |
| TC0600008665.hg.1 | RWDD2A | RWD domain containing 2A | Coding | 7.61 | 8.28 | 7.01 | 1.52 | 0.1498 | 0.3216 | 6.87 | 6.67 | 6.37 | 1.41 | 0.1448 | 0.3796 |
| TC0600011401.hg.1 | C6orf15 | chromosome 6 open reading frame 15 | Coding | 5.21 | 4.43 | 4.61 | 1.52 | 0.0596 | 0.1648 | 5.76 | 5.80 | 5.39 | 1.29 | 0.4226 | 0.6753 |
| TC0600013926.hg.1 | TCP10 | t-complex 10 | Multiple_C | 8.43 | 7.71 | 7.83 | 1.52 | 0.1819 | 0.3666 | 7.72 | 8.34 | 8.17 | 0.73 | 0.2449 | 0.509 |
| TC0600014348.hg.1 | NHSL1 | NHS-like 1 | Multiple_C | 10.34 | 9.69 | 9.74 | 1.52 | 0.4292 | 0.6394 | 6.82 | 8.10 | 9.1 | 0.21 | 0.0022 | 0.0285 |
| TC0700006629.hg.1 | FSCN1 | fascin actin-bundling protein 1 | Multiple_C | 11.26 | 10.31 | 10.66 | 1.52 | 0.3505 | 0.5633 | 10.93 | 12.59 | 12.07 | 0.45 | 0.006 | 0.0552 |
| TC0700009502.hg.1 | CTAGE15 | CTAGE family, member 15 | Coding | 5.38 | 4.86 | 4.78 | 1.52 | 0.0108 | 0.0442 | 5.09 | 4.94 | 4.94 | 1.11 | 0.6117 | 0.806 |
| TC0700011563.hg.1 | STYXL1 | serine/threonine/tyrosine interacting-like 1 | Multiple_C | 7.55 | 7.33 | 6.95 | 1.52 | 0.0979 | 0.2369 | 6.41 | 7.29 | 7.46 | 0.48 | 0.0001 | 0.0037 |
| TC0700011932.hg.1 | KPNA7 | karyopherin alpha 7 (importin alpha 8) | Coding | 5.31 | 4.78 | 4.71 | 1.52 | 0.0278 | 0.0926 | 4.59 | 5.18 | 5.16 | 0.67 | 0.0281 | 0.1476 |
| TC0700013178.hg.1 | BLACE | B-cell acute lymphoblastic leukemia expressed | Multiple_C | 3.99 | 3.34 | 3.39 | 1.52 | 0.025 | 0.0854 | 4.8 | 4.86 | 4.5 | 1.23 | 0.0327 | 0.1621 |
| TC0800009529.hg.1 | PPP1R3B | protein phosphatase 1, regulatory subunit 3B | Coding | 7.29 | 7.04 | 6.69 | 1.52 | 0.0105 | 0.0435 | 4.45 | 4.27 | 4.66 | 0.86 | 0.1586 | 0.4002 |
| TC0800009735.hg.1 | MTUS1 | microtubule associated tumor suppressor 1 | Multiple_C | 12.11 | 11.57 | 11.51 | 1.52 | 0.3386 | 0.5527 | 6.71 | 6.94 | 6.52 | 1.14 | 0.7739 | 0.8984 |
| TC0800012216.hg.1 | CYHR1 | cysteine/histidine-rich 1 | Multiple_C | 13.97 | 13.2 | 13.37 | 1.52 | 0.0135 | 0.0526 | 13.93 | 14.05 | 13.91 | 1.01 | 0.9778 | 0.9908 |
| TC0900006458.hg.1 | DMRT2 | doublesex and mab-3 related transcription factor 2 | Multiple_C | 7.85 | 6.9 | 7.25 | 1.52 | 0.0255 | 0.0865 | 4.15 | 4.20 | 4.13 | 1.01 | 0.3162 | 0.5801 |
| TC0900007069.hg.1 | C9orf131 | chromosome 9 open reading frame 131 | Coding | 3.58 | 2.68 | 2.98 | 1.52 | 0.049 | 0.1425 | 3.81 | 3.88 | 3.6 | 1.16 | 0.2057 | 0.4621 |
| TC0900009779.hg.1 | C9orf72 | chromosome 9 open reading frame 72 | Multiple_C | 7.13 | 8.6 | 6.53 | 1.52 | 0.3458 | 0.5596 | 5.31 | 4.47 | 4.33 | 1.97 | 0.0073 | 0.0631 |
| TC0900012067.hg.1 | PNPLA7 | patatin-like phospholipase domain containing 7 | Multiple_C | 6.15 | 5.21 | 5.55 | 1.52 | 0.0316 | 0.1025 | 6.42 | 6.27 | 6.29 | 1.09 | 0.9133 | 0.9645 |
| TC0900012120.hg.1 | IL11RA | interleukin 11 receptor, alpha | Multiple_C | 6.07 | 5.52 | 5.47 | 1.52 | 0.1359 | 0.3007 | 6.91 | 6.64 | 6.44 | 1.39 | 0.2578 | 0.5227 |
| TC0900012192.hg.1 | PHPT1 | phosphohistidine phosphatase 1 | Multiple_C | 11.7 | 10.99 | 11.1 | 1.52 | 0.164 | 0.3423 | 10.33 | 11.19 | 11.03 | 0.62 | 0.0034 | 0.0389 |
| TC0X00011010.hg.1 | SPANXN3 | SPANX family, member N3 | Coding | 6.37 | 5.95 | 5.77 | 1.52 | 0.0128 | 0.0506 | 6.36 | 6.57 | 6.11 | 1.19 | 0.584 | 0.788 |

| | | | | | | | | | | | | | | | |
|---|---|---|---|---|---|---|---|---|---|---|---|---|---|---|---|
| TC0X00011310.hg.1 | RPL36A-HNRN | RPL36A-HNRNPH2 readthrough | Coding | 15.4 | 15.51 | 14.8 | 1.52 | 0.0511 | 0.1469 | 12.19 | 11.88 | 12.36 | 0.89 | 0.7378 | 0.8792 |
| TC1000006707.hg.1 | GATA3 | GATA binding protein 3 | Multiple_C | 4.84 | 4.42 | 4.24 | 1.52 | 0.0672 | 0.1801 | 5.04 | 5.00 | 5.06 | 0.99 | 0.7743 | 0.8986 |
| TC1000007544.hg.1 | GDF2 | growth differentiation factor 2 | Coding | 4.08 | 3.32 | 3.48 | 1.52 | 0.0139 | 0.0539 | 4.53 | 4.72 | 4.33 | 1.15 | 0.3198 | 0.5839 |
| TC1000009880.hg.1 | NMT2 | N-myristoyltransferase 2 | Multiple_C | 9.08 | 9.22 | 8.48 | 1.52 | 0.3518 | 0.5647 | 8.98 | 9.05 | 9.27 | 0.82 | 0.7854 | 0.9041 |
| TC1000009927.hg.1 | ST8SIA6 | ST8 alpha-N-acetyl-neuraminide alpha-2,8-sialyltransferase 6 | Multiple_C | 4.6 | 3.82 | 4 | 1.52 | 0.1527 | 0.3262 | 4.46 | 4.26 | 3.94 | 1.43 | 0.0655 | 0.2437 |
| TC1100010491.hg.1 | FBXO3 | F-box protein 3 | Multiple_C | 9.94 | 10.53 | 9.34 | 1.52 | 0.0333 | 0.1067 | 7.76 | 7.60 | 7.65 | 1.08 | 0.3255 | 0.5891 |
| TC1100012508.hg.1 | RPS25 | ribosomal protein S25 | Multiple_C | 18 | 18.05 | 17.4 | 1.52 | 0.0018 | 0.0108 | 16.04 | 15.58 | 15.32 | 1.65 | 0.0007 | 0.013 |
| TC1200007050.hg.1 | IAPP | islet amyloid polypeptide | Coding | 4.29 | 3.27 | 3.69 | 1.52 | 0.0604 | 0.1665 | 4.82 | 4.04 | 3.83 | 1.99 | 0.0004 | 0.0092 |
| TC1200012648.hg.1 | MYL6 | myosin light chain 6 | Multiple_C | 13.72 | 13.75 | 13.12 | 1.52 | 0.1024 | 0.2452 | 11.07 | 11.48 | 11.28 | 0.86 | 0.6654 | 0.8363 |
| TC1200012807.hg.1 | STAC3 | SH3 and cysteine rich domain 3 | Multiple_C | 6.07 | 5.64 | 5.47 | 1.52 | 0.1248 | 0.2824 | 4.71 | 5.28 | 5.31 | 0.66 | 0.2019 | 0.4575 |
| TC1400006723.hg.1 | REC8 | REC8 meiotic recombination protein | Multiple_C | 6.67 | 5.45 | 6.07 | 1.52 | 0.0059 | 0.0273 | 5.86 | 5.87 | 6.02 | 0.90 | 0.527 | 0.7512 |
| TC1400006933.hg.1 | PAX9 | paired box 9 | Multiple_C | 7.87 | 6.56 | 7.27 | 1.52 | 0.0621 | 0.1696 | 6.89 | 5.98 | 6.16 | 1.66 | 0.0243 | 0.1348 |
| TC1400007548.hg.1 | GALNT16 | polypeptide N-acetylgalactosaminyltransferase 16 | Multiple_C | 6.05 | 5.32 | 5.45 | 1.52 | 0.0946 | 0.2312 | 8.08 | 6.86 | 6.74 | 2.53 | 1.33E-05 | 0.0009 |
| TC1400009386.hg.1 | GPHB5 | glycoprotein hormone beta 5 | Multiple_C | 4.7 | 3.89 | 4.1 | 1.52 | 0.0152 | 0.0576 | 4.4 | 4.28 | 4.32 | 1.06 | 0.988 | 0.9949 |
| TC1500010892.hg.1 | MAN2C1 | mannosidase, alpha, class 2C, member 1 | Multiple_C | 6.12 | 5.89 | 5.52 | 1.52 | 0.1413 | 0.3088 | 5.8 | 6.25 | 6.05 | 0.84 | 0.5885 | 0.7918 |
| TC1600009367.hg.1 | TEKT5 | tektin 5 | Multiple_C | 3.76 | 3.1 | 3.16 | 1.52 | 0.1516 | 0.3246 | 3.55 | 3.82 | 3.3 | 1.19 | 0.3813 | 0.6408 |
| TC1700008357.hg.1 | DGKE | diacylglycerol kinase, epsilon 64kDa | Multiple_C | 7.61 | 6.96 | 7.01 | 1.52 | 0.0851 | 0.2139 | 7.26 | 6.53 | 6.88 | 1.30 | 0.1118 | 0.3294 |
| TC1700012143.hg.1 | RAB40B | RAB40B, member RAS oncogene family | Multiple_C | 7.46 | 6.65 | 6.86 | 1.52 | 0.1336 | 0.297 | 6.43 | 6.34 | 6.11 | 1.25 | 0.3889 | 0.6475 |
| TC1700012354.hg.1 | VAMP2 | vesicle associated membrane protein 2 | Multiple_C | 6.98 | 5.77 | 6.38 | 1.52 | 0.0943 | 0.2308 | 5.99 | 5.89 | 6.07 | 0.95 | 0.6323 | 0.819 |
| TC1700012380.hg.1 | SPAG5 | sperm associated antigen 5 | Multiple_C | 8.06 | 7.99 | 7.46 | 1.52 | 0.492 | 0.6921 | 6.02 | 7.22 | 6.55 | 0.69 | 0.0154 | 0.1023 |
| TC1800007673.hg.1 | C18orf63 | chromosome 18 open reading frame 63 | Coding | 3.69 | 2.56 | 3.09 | 1.52 | 0.0814 | 0.2073 | 3.93 | 3.80 | 4.21 | 0.82 | 0.5468 | 0.7641 |
| TC1900007787.hg.1 | KCTD15 | potassium channel tetramerization domain containing 15 | Multiple_C | 3.94 | 3.38 | 3.34 | 1.52 | 0.4797 | 0.6818 | 4.45 | 5.20 | 5.37 | 0.53 | 0.0458 | 0.1981 |
| TC1900009676.hg.1 | CCDC151 | coiled-coil domain containing 151 | Multiple_C | 4.19 | 3.42 | 3.59 | 1.52 | 0.1475 | 0.3184 | 5.04 | 5.21 | 4.73 | 1.24 | 0.5595 | 0.7716 |
| TC1900010925.hg.1 | NKPD1 | NTPase, KAP family P-loop domain containing 1 | Multiple_C | 3.53 | 2.9 | 2.93 | 1.52 | 0.0186 | 0.0676 | 3.51 | 3.48 | 3.52 | 0.99 | 0.485 | 0.7222 |
| TC1900011234.hg.1 | KLK11 | kallikrein related peptidase 11 | Multiple_C | 4.49 | 3.64 | 3.89 | 1.52 | 0.0902 | 0.2234 | 4.01 | 3.94 | 4.05 | 0.97 | 0.9761 | 0.9902 |
| TC1900011486.hg.1 | ZNF784 | zinc finger protein 784 | Multiple_C | 4.38 | 3.61 | 3.78 | 1.52 | 0.0097 | 0.0405 | 3.86 | 4.07 | 3.88 | 0.99 | 0.9666 | 0.986 |
| TC1900011657.hg.1 | MCEMP1 | mast cell-expressed membrane protein 1 | Multiple_C | 6.03 | 5.49 | 5.43 | 1.52 | 0.0517 | 0.148 | 5.37 | 5.37 | 5.35 | 1.01 | 0.8833 | 0.95 |
| TC1900011693.hg.1 | ZNF429 | zinc finger protein 429 | Coding | 6.15 | 5.11 | 5.55 | 1.52 | 0.027 | 0.0905 | 5 | 5.43 | 5.38 | 0.77 | 0.6765 | 0.8442 |
| TC2000006561.hg.1 | MAVS | mitochondrial antiviral signaling protein | Multiple_C | 8.08 | 7.1 | 7.48 | 1.52 | 0.0711 | 0.1877 | 8.85 | 9.98 | 9.64 | 0.58 | 0.0285 | 0.1487 |
| TC2000007085.hg.1 | COX4I2 | cytochrome c oxidase subunit IV isoform 2 (lung) | Multiple_C | 4.77 | 4.26 | 4.17 | 1.52 | 0.0939 | 0.23 | 4.36 | 4.73 | 4.55 | 0.88 | 0.5174 | 0.7458 |
| TC2000007167.hg.1 | ZNF341 | zinc finger protein 341 | Multiple_C | 5.71 | 5.43 | 5.11 | 1.52 | 0.1076 | 0.2542 | 4.95 | 4.68 | 4.68 | 1.21 | 0.545 | 0.7628 |
| TC2100006787.hg.1 | GABPA | GA binding protein transcription factor alpha subunit | Multiple_C | 10.24 | 9.87 | 9.64 | 1.52 | 0.0294 | 0.0967 | 9.68 | 9.36 | 9.07 | 1.53 | 0.0045 | 0.0457 |
| TC2200007013.hg.1 | EWSR1 | EWS RNA binding protein 1 | Multiple_C | 11.9 | 10.15 | 11.3 | 1.52 | 0.3772 | 0.5901 | 12.24 | 12.34 | 12.71 | 0.72 | 0.1774 | 0.4261 |

| | | | | | | | | | | | | | | | |
|---|---|---|---|---|---|---|---|---|---|---|---|---|---|---|---|
| TSUnmapped00000524.l | INPP5D | inositol polyphosphate-5-phosphatase D | Coding | 5.62 | 4.94 | 5.02 | 1.52 | 0.3209 | 0.5347 | 8.39 | 8.32 | 8.55 | 0.90 | 0.438 | 0.6884 |
| TC0100006900.hg.1 | AADACL4 | arylacetamide deacetylase-like 4 | Coding | 3.36 | 2.64 | 2.77 | 1.51 | 0.2461 | 0.4497 | 3.05 | 3.20 | 3.07 | 0.99 | 0.6933 | 0.8541 |
| TC0100007240.hg.1 | NBPF3 | neuroblastoma breakpoint family, member 3 | Multiple_C | 8.1 | 7.75 | 7.51 | 1.51 | 0.242 | 0.4445 | 7.53 | 7.03 | 7.21 | 1.25 | 0.5177 | 0.7461 |
| TC0100011847.hg.1 | ARF1; MIR362 | ADP-ribosylation factor 1; microRNA 3620 | Multiple_C | 15.47 | 14.47 | 14.88 | 1.51 | 0.037 | 0.1159 | 14.88 | 14.76 | 15.1 | 0.86 | 0.2491 | 0.5137 |
| TC0100014403.hg.1 | KANK4 | KN motif and ankyrin repeat domains 4 | Multiple_C | 4.6 | 3.86 | 4.01 | 1.51 | 0.0648 | 0.1753 | 4.1 | 4.16 | 3.83 | 1.21 | 0.4788 | 0.7172 |
| TC0100015716.hg.1 | MTMR11 | myotubularin related protein 11 | Multiple_C | 8.09 | 8.11 | 7.5 | 1.51 | 0.0789 | 0.2029 | 4.65 | 5.40 | 5.25 | 0.66 | 0.0483 | 0.204 |
| TC0100016969.hg.1 | KLHL12 | kelch-like family member 12 | Multiple_C | 10.06 | 10.16 | 9.47 | 1.51 | 0.1331 | 0.2963 | 7.66 | 7.54 | 7.99 | 0.80 | 0.2686 | 0.5334 |
| TC0100018081.hg.1 | ZNF670; ZNF6 | zinc finger protein 670; zinc finger protein 695; ZNF670-ZNF695 | Multiple_C | 9.89 | 8.84 | 9.3 | 1.51 | 0.1134 | 0.264 | 7.44 | 7.45 | 8.21 | 0.59 | 0.0001 | 0.0046 |
| TC0100018490.hg.1 | LINGO4 | leucine rich repeat and Ig domain containing 4 | Coding | 4.74 | 4.5 | 4.15 | 1.51 | 0.0585 | 0.1626 | 4 | 3.85 | 3.68 | 1.25 | 0.2096 | 0.4666 |
| TC0200007353.hg.1 | EML4 | echinoderm microtubule associated protein like 4 | Multiple_C | 12.93 | 12.27 | 12.34 | 1.51 | 0.0369 | 0.1157 | 13.02 | 12.61 | 12.73 | 1.22 | 0.2004 | 0.4558 |
| TC0200008955.hg.1 | PSD4 | pleckstrin and Sec7 domain containing 4 | Multiple_C | 3.34 | 2.87 | 2.75 | 1.51 | 0.1561 | 0.3311 | 5.72 | 5.34 | 5.33 | 1.31 | 0.4536 | 0.6986 |
| TC0200013595.hg.1 | MGAT4A | mannosyl (alpha-1,3-)-glycoprotein beta-1,4-N-acetylglucosami | Multiple_C | 8.33 | 7.99 | 7.74 | 1.51 | 0.0426 | 0.1288 | 4.16 | 3.65 | 4.28 | 0.92 | 0.2374 | 0.5007 |
| TC0200016413.hg.1 | FKBP1B | FK506 binding protein 1B | Multiple_C | 5.65 | 5.24 | 5.06 | 1.51 | 0.0436 | 0.131 | 5.3 | 5.07 | 4.92 | 1.30 | 0.0119 | 0.0871 |
| TC0200016640.hg.1 | HS1BP3 | HCLS1 binding protein 3 | Coding | 7.5 | 6.84 | 6.91 | 1.51 | 0.0608 | 0.1673 | 6.76 | 6.53 | 6.34 | 1.34 | 0.1438 | 0.3778 |
| TC0300008284.hg.1 | HHLA2 | HERV-H LTR-associating 2 | Coding | 2.92 | 1.79 | 2.33 | 1.51 | 0.0936 | 0.2296 | 3.23 | 3.19 | 3.1 | 1.09 | 0.31 | 0.5739 |
| TC0300010304.hg.1 | TMEM40 | transmembrane protein 40 | Multiple_C | 4.62 | 4.14 | 4.03 | 1.51 | 0.0517 | 0.148 | 4.6 | 4.42 | 4.18 | 1.34 | 0.0876 | 0.2855 |
| TC0300011019.hg.1 | PLXNB1 | plexin B1 | Multiple_C | 5.98 | 5.72 | 5.39 | 1.51 | 0.0233 | 0.0806 | 7.5 | 7.64 | 6.6 | 1.87 | 0.0014 | 0.021 |
| TC0300012665.hg.1 | XRN1 | 5-3 exoribonuclease 1 | Multiple_C | 9.09 | 9.51 | 8.5 | 1.51 | 0.1077 | 0.2544 | 8.32 | 7.70 | 8.09 | 1.17 | 0.2095 | 0.4665 |
| TC0300012834.hg.1 | TMEM14EP | transmembrane protein 14E, pseudogene | Coding | 4.06 | 3.22 | 3.47 | 1.51 | 0.0513 | 0.1473 | 3.55 | 2.89 | 3.03 | 1.43 | 0.0396 | 0.1818 |
| TC0300013955.hg.1 | ZNF385D | zinc finger protein 385D | Multiple_C | 3.84 | 3.06 | 3.25 | 1.51 | 0.1972 | 0.388 | 3.42 | 3.49 | 3.56 | 0.91 | 0.8248 | 0.9227 |
| TC0400006800.hg.1 | USP17L18; US | ubiquitin specific peptidase 17-like family member 18; ubiquitin | Coding | 4.68 | 4.25 | 4.09 | 1.51 | 0.0823 | 0.2087 | 4.25 | 4.50 | 4.37 | 0.92 | 0.7184 | 0.8686 |
| TC0400007169.hg.1 | PCDH7 | protocadherin 7 | Multiple_C | 3.49 | 2.62 | 2.9 | 1.51 | 0.1003 | 0.2414 | 3.55 | 3.89 | 3.52 | 1.02 | 0.8644 | 0.9422 |
| TC0400007282.hg.1 | KLB | klotho beta | Coding | 4.26 | 3.91 | 3.67 | 1.51 | 0.0303 | 0.099 | 3.93 | 3.89 | 4.22 | 0.82 | 0.3557 | 0.6189 |
| TC0400011395.hg.1 | EIF4E | eukaryotic translation initiation factor 4E | Multiple_C | 13.47 | 13.5 | 12.88 | 1.51 | 0.0428 | 0.1294 | 11.13 | 10.78 | 11.26 | 0.91 | 0.5975 | 0.7975 |
| TC0500008491.hg.1 | PRDM6 | PR domain containing 6 | Multiple_C | 5.05 | 4.27 | 4.46 | 1.51 | 0.2339 | 0.4348 | 4.76 | 4.98 | 5.02 | 0.84 | 0.2622 | 0.5265 |
| TC0500011163.hg.1 | COL4A3BP | collagen, type IV, alpha 3 (Goodpasture antigen) binding proteir | Multiple_C | 11.31 | 10.92 | 10.72 | 1.51 | 0.0019 | 0.0111 | 10.9 | 10.53 | 10.08 | 1.77 | 0.0001 | 0.0037 |
| TC0500012942.hg.1 | RNF44 | ring finger protein 44 | Multiple_C | 6.56 | 6.43 | 5.97 | 1.51 | 0.0096 | 0.0404 | 7.09 | 7.22 | 7.11 | 0.99 | 0.2305 | 0.4919 |
| TC0600007832.hg.1 | BRPF3 | bromodomain and PHD finger containing 3 | Multiple_C | 6.77 | 5.79 | 6.18 | 1.51 | 0.0508 | 0.1463 | 6.97 | 7.42 | 7.55 | 0.67 | 0.0083 | 0.0688 |
| TC0600013727.hg.1 | EZR | ezrin | Multiple_C | 16.47 | 15.72 | 15.88 | 1.51 | 0.0266 | 0.0893 | 16.38 | 15.81 | 16.15 | 1.17 | 0.2734 | 0.5389 |
| TC0700009400.hg.1 | TAS2R5 | taste receptor, type 2, member 5 | Coding | 5.39 | 4.88 | 4.8 | 1.51 | 0.1079 | 0.2548 | 4.72 | 4.87 | 4.69 | 1.02 | 0.3318 | 0.5959 |
| TC0700012009.hg.1 | PCOLCE-AS1 | PCOLCE antisense RNA 1 | Multiple_C | 3.72 | 3.06 | 3.13 | 1.51 | 0.0618 | 0.1694 | 3.28 | 3.33 | 3.38 | 0.93 | 0.783 | 0.9025 |
| TC0800011697.hg.1 | FER1L6-AS1 | FER1L6 antisense RNA 1 | Multiple_C | 4.36 | 3.54 | 3.77 | 1.51 | 0.0824 | 0.2089 | 4.97 | 5.20 | 4.9 | 1.05 | 0.9229 | 0.9684 |
| TC0M00006435.hg.1 | COX1 | cytochrome c oxidase subunit I | Coding | 18.06 | 17.16 | 17.47 | 1.51 | 0.0108 | 0.0444 | 18.57 | 18.37 | 18.73 | 0.90 | 0.6565 | 0.8321 |

| | | | | | | | | | | | | | | | |
|---|---|---|---|---|---|---|---|---|---|---|---|---|---|---|---|
| TC0X00009123.hg.1 | ASB11 | ankyrin repeat and SOCS box containing 11, E3 ubiquitin protein | Coding | 4.3 | 3.62 | 3.71 | 1.51 | 0.0199 | 0.0712 | 4.1 | 3.69 | 3.5 | 1.52 | 0.0032 | 0.0375 |
| TC1100010864.hg.1 | OR5M10 | olfactory receptor, family 5, subfamily M, member 10 | Coding | 4.11 | 3.74 | 3.52 | 1.51 | 0.3678 | 0.5806 | 3.66 | 3.53 | 3.49 | 1.13 | 0.6172 | 0.8099 |
| TC1100011180.hg.1 | RASGRP2 | RAS guanyl releasing protein 2 (calcium and DAG-regulated) | Multiple_C | 5.93 | 5.3 | 5.34 | 1.51 | 0.0186 | 0.0677 | 5.85 | 6.08 | 5.83 | 1.01 | 0.6372 | 0.822 |
| TC1200010205.hg.1 | C12orf71 | chromosome 12 open reading frame 71 | Coding | 5.1 | 4.67 | 4.51 | 1.51 | 0.1916 | 0.3802 | 4.59 | 4.01 | 3.94 | 1.57 | 0.0203 | 0.1216 |
| TC1200010569.hg.1 | ASB8 | ankyrin repeat and SOCS box containing 8 | Multiple_C | 10.8 | 10.11 | 10.21 | 1.51 | 0.0364 | 0.1144 | 11.76 | 11.94 | 11.94 | 0.88 | 0.3082 | 0.5724 |
| TC1200012799.hg.1 | SARNP | SAP domain containing ribonucleoprotein | Multiple_C | 11.51 | 11.78 | 10.92 | 1.51 | 0.01 | 0.0416 | 10.02 | 9.98 | 10.05 | 0.98 | 0.8627 | 0.9412 |
| TC1300010005.hg.1 | MCF2L | MCF.2 cell line derived transforming sequence like | Coding | 4.3 | 3.42 | 3.71 | 1.51 | 0.1383 | 0.304 | 4.17 | 3.66 | 3.51 | 1.58 | 0.1081 | 0.3234 |
| TC1400008591.hg.1 | TMEM55B | transmembrane protein 55B | Multiple_C | 9.16 | 9.53 | 8.57 | 1.51 | 0.0843 | 0.2124 | 6.56 | 6.52 | 6.88 | 0.80 | 0.1895 | 0.4421 |
| TC1500006663.hg.1 | RPL41P2 | ribosomal protein L41 pseudogene 2 | Multiple_C | 15.87 | 16.01 | 15.28 | 1.51 | 0.0221 | 0.0775 | 12.94 | 12.84 | 12.78 | 1.12 | 0.0824 | 0.2765 |
| TC1500007202.hg.1 | USP8 | ubiquitin specific peptidase 8 | Multiple_C | 9.49 | 9.7 | 8.9 | 1.51 | 0.1677 | 0.3472 | 9.45 | 9.16 | 9.62 | 0.89 | 0.612 | 0.8063 |
| TC1600006826.hg.1 | ABAT | 4-aminobutyrate aminotransferase | Multiple_C | 4.38 | 3.88 | 3.79 | 1.51 | 0.049 | 0.1425 | 3.85 | 3.97 | 3.86 | 0.99 | 0.3066 | 0.5709 |
| TC1600007465.hg.1 | ZNF48 | zinc finger protein 48 | Multiple_C | 6.5 | 5.67 | 5.91 | 1.51 | 0.0877 | 0.2188 | 6.01 | 6.00 | 6.08 | 0.95 | 0.6133 | 0.807 |
| TC1600011383.hg.1 | TMEM265; SR | transmembrane protein 265; Snf2-related CREBBP activator pro | Multiple_C | 10.05 | 8.54 | 9.46 | 1.51 | 0.003 | 0.0159 | 11.1 | 11.51 | 11.26 | 0.90 | 0.3249 | 0.5885 |
| TC1600011433.hg.1 | SYCE1L | synaptonemal complex central element protein 1-like | Multiple_C | 4.86 | 4.33 | 4.27 | 1.51 | 0.0048 | 0.0234 | 4.58 | 5.00 | 4.46 | 1.09 | 0.7729 | 0.8979 |
| TC1700007262.hg.1 | MAP2K3 | mitogen-activated protein kinase kinase 3 | Multiple_C | 9.32 | 7.92 | 8.73 | 1.51 | 0.2629 | 0.47 | 7.75 | 7.32 | 7.39 | 1.28 | 0.4524 | 0.6977 |
| TC1700007769.hg.1 | LRRC3C | leucine rich repeat containing 3C | Coding | 3.72 | 3.12 | 3.13 | 1.51 | 0.3494 | 0.562 | 3.52 | 3.94 | 3.3 | 1.16 | 0.5099 | 0.7401 |
| TC1700007796.hg.1 | IGFBP4 | insulin like growth factor binding protein 4 | Multiple_C | 7.22 | 6.75 | 6.63 | 1.51 | 0.0131 | 0.0516 | 7.48 | 8.04 | 8.3 | 0.57 | 8.37E-05 | 0.0032 |
| TC1700007966.hg.1 | C17orf105 | chromosome 17 open reading frame 105 | Coding | 3.74 | 2.97 | 3.15 | 1.51 | 0.0395 | 0.1214 | 4 | 3.85 | 3.68 | 1.25 | 0.0502 | 0.2087 |
| TC1700010990.hg.1 | HOXB1 | homeobox B1 | Coding | 4.46 | 3.64 | 3.87 | 1.51 | 0.1109 | 0.2598 | 4.6 | 4.57 | 4.23 | 1.29 | 0.0236 | 0.1328 |
| TC1700011793.hg.1 | QRICH2 | glutamine rich 2 | Multiple_C | 5.21 | 4.45 | 4.62 | 1.51 | 0.2463 | 0.4498 | 4.91 | 4.82 | 4.45 | 1.38 | 0.0517 | 0.2124 |
| TC1800007448.hg.1 | GRP | gastrin-releasing peptide | Multiple_C | 5.72 | 5.18 | 5.13 | 1.51 | 0.3411 | 0.5549 | 5.57 | 5.50 | 5.61 | 0.97 | 0.8744 | 0.9459 |
| TC1900006833.hg.1 | MBD3L5 | methyl-CpG binding domain protein 3-like 5 | Coding | 4.93 | 4.31 | 4.34 | 1.51 | 0.485 | 0.6863 | 4.25 | 4.17 | 4.23 | 1.01 | 0.4527 | 0.6978 |
| TC1900008551.hg.1 | BCL2L12 | BCL2-like 12 (proline rich) | Multiple_C | 10.55 | 9.33 | 9.96 | 1.51 | 0.0163 | 0.0609 | 7.02 | 6.96 | 6.82 | 1.15 | 0.2608 | 0.525 |
| TC1900008827.hg.1 | CACNG8; MIR | calcium channel, voltage-dependent, gamma subunit 8; microRI | Multiple_C | 5.91 | 5.29 | 5.32 | 1.51 | 0.297 | 0.5085 | 5.16 | 5.37 | 5.43 | 0.83 | 0.9151 | 0.9653 |
| TC1900011619.hg.1 | ZNF497; A1BG | zinc finger protein 497; alpha-1-B glycoprotein | Multiple_C | 3.82 | 3.16 | 3.23 | 1.51 | 0.1245 | 0.2819 | 3.92 | 3.87 | 3.49 | 1.35 | 0.0379 | 0.1773 |
| TC1900011679.hg.1 | CYP4F3 | cytochrome P450, family 4, subfamily F, polypeptide 3 | Multiple_C | 5.02 | 4.34 | 4.43 | 1.51 | 0.0801 | 0.2053 | 5.14 | 5.17 | 5.12 | 1.01 | 0.966 | 0.9858 |
| TC2000009060.hg.1 | KIAA1755 | KIAA1755 | Multiple_C | 4.03 | 3.15 | 3.44 | 1.51 | 0.1065 | 0.2523 | 3.55 | 3.53 | 3.57 | 0.99 | 0.9243 | 0.9688 |
| TC2200006614.hg.1 | GP1BB; SEPT5 | glycoprotein Ib (platelet), beta polypeptide; septin 5 | Multiple_C | 4.71 | 4.22 | 4.12 | 1.51 | 0.014 | 0.0541 | 5.19 | 5.09 | 4.62 | 1.48 | 0.094 | 0.2979 |
| TC2200007802.hg.1 | PPP6R2 | protein phosphatase 6, regulatory subunit 2 | Multiple_C | 6.84 | 6.52 | 6.25 | 1.51 | 0.2218 | 0.4194 | 7.2 | 7.22 | 6.86 | 1.27 | 0.1082 | 0.3236 |
| TC2200008425.hg.1 | LIF | leukemia inhibitory factor | Coding | 8.02 | 7.75 | 7.43 | 1.51 | 0.028 | 0.0931 | 6.18 | 5.32 | 5.65 | 1.44 | 0.002 | 0.0265 |
| TC2200008703.hg.1 | DDX17 | DEAD (Asp-Glu-Ala-Asp) box helicase 17 | Multiple_C | 12.95 | 12.32 | 12.36 | 1.51 | 0.0098 | 0.0409 | 14.28 | 13.88 | 14.32 | 0.97 | 0.94 | 0.9753 |
| TSUnmapped00000572.h | RPS6KA1 | ribosomal protein S6 kinase, 90kDa, polypeptide 1 | Coding | 10.23 | 9.64 | 9.64 | 1.51 | 0.0214 | 0.0755 | 6.74 | 6.60 | 6.36 | 1.30 | 0.5296 | 0.7532 |
| TC0100006862.hg.1 | FBXO6 | F-box protein 6 | Multiple_C | 5.95 | 5.43 | 5.37 | 1.49 | 0.1054 | 0.2504 | 5.63 | 5.45 | 5.26 | 1.29 | 0.0444 | 0.195 |

| | | | | | | | | | | | | | | | |
|---|---|---|---|---|---|---|---|---|---|---|---|---|---|---|---|
| TC0100007691.hg.1 | KIAA1522 | KIAA1522 | Multiple_C | 7.6 | 6.56 | 7.02 | 1.49 | 0.1976 | 0.3884 | 4.98 | 5.12 | 4.92 | 1.04 | 0.9971 | 0.9987 |
| TC0100009903.hg.1 | RPRD2 | regulation of nuclear pre-mRNA domain containing 2 | Multiple_C | 10.2 | 8.87 | 9.62 | 1.49 | 0.0802 | 0.2055 | 10.46 | 9.85 | 10.58 | 0.92 | 0.5783 | 0.7843 |
| TC0100016157.hg.1 | PVRL4 | poliovirus receptor-related 4 | Multiple_C | 6.62 | 5.93 | 6.04 | 1.49 | 0.0654 | 0.1768 | 5.54 | 5.78 | 5.41 | 1.09 | 0.5095 | 0.7397 |
| TC0100017815.hg.1 | TOMM20 | translocase of outer mitochondrial membrane 20 homolog (yea | Multiple_C | 12.42 | 13.53 | 11.84 | 1.49 | 0.0496 | 0.1438 | 11.91 | 12.08 | 11.68 | 1.17 | 0.4969 | 0.7304 |
| TC0200007473.hg.1 | CRIPT | cysteine-rich PDZ-binding protein | Multiple_C | 11.31 | 10.49 | 10.73 | 1.49 | 0.1817 | 0.3664 | 10.38 | 9.71 | 9.56 | 1.77 | 0.0053 | 0.0513 |
| TC0200008168.hg.1 | REG1A | regenerating islet-derived 1 alpha | Multiple_C | 4.19 | 3.46 | 3.61 | 1.49 | 0.3098 | 0.5229 | 4.19 | 3.90 | 4.02 | 1.13 | 0.1926 | 0.4459 |
| TC0200009539.hg.1 | ARHGAP15 | Rho GTPase activating protein 15 | Multiple_C | 3.72 | 3.28 | 3.14 | 1.49 | 0.077 | 0.199 | 3.63 | 3.98 | 3.9 | 0.83 | 0.7702 | 0.8969 |
| TC0200010255.hg.1 | PMS1 | PMS1 homolog 1, mismatch repair system component | Multiple_C | 8.76 | 8.74 | 8.18 | 1.49 | 0.4834 | 0.685 | 7.78 | 7.38 | 7.76 | 1.01 | 0.8767 | 0.9471 |
| TC0200016742.hg.1 | PLA2R1 | phospholipase A2 receptor 1 | Multiple_C | 3.99 | 3.91 | 3.41 | 1.49 | 0.073 | 0.1911 | 4.08 | 3.77 | 3.5 | 1.49 | 0.0052 | 0.0502 |
| TC0300008660.hg.1 | ALDH1L1-AS1 | ALDH1L1 antisense RNA 1 | Multiple_C | 5.46 | 4.51 | 4.88 | 1.49 | 0.0843 | 0.2124 | 4.19 | 4.21 | 4.26 | 0.95 | 0.7305 | 0.875 |
| TC0300009004.hg.1 | RASA2 | RAS p21 protein activator 2 | Multiple_C | 7.49 | 7.3 | 6.91 | 1.49 | 0.0403 | 0.1233 | 6.35 | 6.16 | 6.57 | 0.86 | 0.3889 | 0.6475 |
| TC0300009686.hg.1 | HTR3C | 5-hydroxytryptamine (serotonin) receptor 3C, ionotropic | Coding | 3.95 | 3.29 | 3.37 | 1.49 | 0.2328 | 0.4335 | 3.96 | 3.80 | 3.76 | 1.15 | 0.5689 | 0.7776 |
| TC0300013107.hg.1 | SLC2A2 | solute carrier family 2 (facilitated glucose transporter), member | Multiple_C | 3.63 | 3.24 | 3.05 | 1.49 | 0.5023 | 0.7004 | 4.22 | 4.32 | 4.22 | 1.00 | 0.4046 | 0.6601 |
| TC0300013563.hg.1 | ATP13A5 | ATPase type 13A5 | Multiple_C | 5.15 | 4.47 | 4.57 | 1.49 | 0.3055 | 0.5186 | 4.36 | 4.46 | 4.48 | 0.92 | 0.6911 | 0.8526 |
| TC0400007770.hg.1 | STATH | statherin | Multiple_C | 4.22 | 3.34 | 3.64 | 1.49 | 0.0169 | 0.0626 | 3.56 | 3.51 | 3.93 | 0.77 | 0.0418 | 0.1883 |
| TC0400009525.hg.1 | ANKRD37 | ankyrin repeat domain 37 | Multiple_C | 7.99 | 7.43 | 7.41 | 1.49 | 0.3942 | 0.6079 | 6.77 | 7.09 | 6.31 | 1.38 | 0.4368 | 0.6874 |
| TC0400009855.hg.1 | TMEM128 | transmembrane protein 128 | Multiple_C | 9.5 | 10.46 | 8.92 | 1.49 | 0.1068 | 0.2529 | 8.39 | 8.63 | 8.24 | 1.11 | 0.5788 | 0.7844 |
| TC0400010459.hg.1 | RFC1 | replication factor C subunit 1 | Multiple_C | 11.37 | 10.86 | 10.79 | 1.49 | 0.1171 | 0.2701 | 10.98 | 10.46 | 11.18 | 0.87 | 0.2824 | 0.5479 |
| TC0500007452.hg.1 | MAP3K1 | mitogen-activated protein kinase kinase kinase 1, E3 ubiquitin p | Multiple_C | 8.03 | 7.99 | 7.45 | 1.49 | 0.0594 | 0.1644 | 9.31 | 9.06 | 8.82 | 1.40 | 0.1705 | 0.4168 |
| TC0500008794.hg.1 | CTNNA1 | catenin (cadherin-associated protein), alpha 1 | Multiple_C | 14.23 | 14.21 | 13.65 | 1.49 | 0.4354 | 0.645 | 12.74 | 12.70 | 13.08 | 0.79 | 0.7403 | 0.8807 |
| TC0500009603.hg.1 | UNC5A | unc-5 netrin receptor A | Coding | 4.35 | 3.68 | 3.77 | 1.49 | 0.3169 | 0.5303 | 4.19 | 4.16 | 4 | 1.14 | 0.0872 | 0.285 |
| TC0500012312.hg.1 | SPRY4 | sprouty RTK signaling antagonist 4 | Multiple_C | 9.38 | 8.29 | 8.8 | 1.49 | 0.0531 | 0.151 | 8.5 | 7.58 | 8.41 | 1.06 | 0.554 | 0.7688 |
| TC0600011381.hg.1 | FLOT1 | flotillin 1 | Multiple_C | 10.03 | 9.69 | 9.45 | 1.49 | 0.031 | 0.1009 | 10.49 | 10.14 | 9.73 | 1.69 | 0.0016 | 0.0225 |
| TC0600014206.hg.1 | TCP10L2 | t-complex 10-like 2 | Multiple_C | 6.7 | 5.91 | 6.12 | 1.49 | 0.1284 | 0.2884 | 6.84 | 6.97 | 6.5 | 1.27 | 0.8831 | 0.9499 |
| TC0700007370.hg.1 | AEBP1; MIR46 | AE binding protein 1; microRNA 4649 | Multiple_C | 7.25 | 6.48 | 6.67 | 1.49 | 0.0731 | 0.1914 | 6.5 | 6.72 | 6.85 | 0.78 | 0.1366 | 0.3673 |
| TC0700013457.hg.1 | FSCN3 | fascin actin-bundling protein 3, testicular | Multiple_C | 3.86 | 3.43 | 3.28 | 1.49 | 0.0805 | 0.2058 | 3.48 | 3.59 | 3.62 | 0.91 | 0.7636 | 0.8932 |
| TC0800011170.hg.1 | GDF6 | growth differentiation factor 6 | Coding | 6.48 | 5.77 | 5.9 | 1.49 | 0.4443 | 0.6527 | 5.98 | 6.01 | 5.9 | 1.06 | 0.9944 | 0.9975 |
| TC0800011814.hg.1 | GSDMC | gasdermin C | Multiple_C | 4.32 | 3.71 | 3.74 | 1.49 | 0.14 | 0.3072 | 4.37 | 4.29 | 4.26 | 1.08 | 0.5569 | 0.7707 |
| TC0800011826.hg.1 | ASAP1; ASAP1 | ArfGAP with SH3 domain, ankyrin repeat and PH domain 1; ASA | Multiple_C | 10.22 | 9.56 | 9.64 | 1.49 | 0.1468 | 0.3173 | 8.99 | 8.90 | 9.46 | 0.72 | 0.1183 | 0.34 |
| TC0900007082.hg.1 | RUSC2 | RUN and SH3 domain containing 2 | Multiple_C | 4.4 | 3.52 | 3.82 | 1.49 | 0.2192 | 0.4161 | 4.81 | 4.62 | 4.85 | 0.97 | 0.7061 | 0.8618 |
| TC0900007435.hg.1 | CBWD5 | COBW domain containing 5 | Multiple_C | 14.38 | 14.68 | 13.8 | 1.49 | 0.1808 | 0.365 | 13.59 | 13.08 | 13.51 | 1.06 | 0.1773 | 0.426 |
| TC0900007646.hg.1 | VPS13A | vacuolar protein sorting 13 homolog A (S. cerevisiae) | Multiple_C | 6.94 | 6.73 | 6.36 | 1.49 | 0.2149 | 0.4105 | 7.55 | 6.53 | 6.64 | 1.88 | 0.0062 | 0.0564 |
| TC0900008238.hg.1 | MURC | muscle-related coiled-coil protein | Coding | 3.96 | 3.02 | 3.38 | 1.49 | 0.1649 | 0.3433 | 4.59 | 4.31 | 4.39 | 1.15 | 0.7205 | 0.8698 |

| | | | | | | | | | | | | | | | |
|---|---|---|---|---|---|---|---|---|---|---|---|---|---|---|---|
| TC0900008379.hg.1 | ACTL7A | actin-like 7A | Coding | 4.23 | 3.83 | 3.65 | 1.49 | 0.0109 | 0.0447 | 4.4 | 4.43 | 4.39 | 1.01 | 0.5966 | 0.7968 |
| TC0900008653.hg.1 | MORN5 | MORN repeat containing 5 | Multiple_C | 4.57 | 3.59 | 3.99 | 1.49 | 0.017 | 0.0629 | 4.77 | 4.96 | 4.52 | 1.19 | 0.5976 | 0.7976 |
| TC0900010971.hg.1 | TBC1D2 | TBC1 domain family, member 2 | Multiple_C | 6.28 | 5.65 | 5.7 | 1.49 | 0.6974 | 0.8377 | 5.1 | 5.18 | 4.84 | 1.20 | 0.3929 | 0.6511 |
| TC0900011029.hg.1 | BAAT | bile acid-CoA:amino acid N-acyltransferase | Coding | 3.84 | 3.33 | 3.26 | 1.49 | 0.1664 | 0.3455 | 3.78 | 3.67 | 3.57 | 1.16 | 0.3917 | 0.6502 |
| TC0X00007363.hg.1 | MAGED2 | MAGE family member D2 | Multiple_C | 9.7 | 10.55 | 9.12 | 1.49 | 0.2543 | 0.4597 | 9.01 | 9.43 | 8.71 | 1.23 | 0.6558 | 0.8321 |
| TC0X00008388.hg.1 | SASH3 | SAM and SH3 domain containing 3 | Multiple_C | 3.77 | 3.03 | 3.19 | 1.49 | 0.0771 | 0.199 | 3.85 | 4.03 | 3.98 | 0.91 | 0.4525 | 0.6977 |
| TC0Y00007240.hg.1 | CSPG4P1Y; DN | chondroitin sulfate proteoglycan 4 pseudogene 1, Y-linked; dyna | Multiple_C | 3.73 | 3.43 | 3.15 | 1.49 | 0.2171 | 0.413 | 3.97 | 4.06 | 3.93 | 1.03 | 0.8668 | 0.943 |
| TC1000007846.hg.1 | SIRT1 | sirtuin 1 | Multiple_C | 10.61 | 10.44 | 10.03 | 1.49 | 0.1214 | 0.277 | 9.8 | 9.27 | 10.57 | 0.59 | 0.0066 | 0.0585 |
| TC1000008005.hg.1 | MCU | mitochondrial calcium uniporter | Multiple_C | 14.23 | 13.9 | 13.65 | 1.49 | 0.0209 | 0.0742 | 12.3 | 11.92 | 12.68 | 0.77 | 0.0315 | 0.1587 |
| TC1000008558.hg.1 | C10orf12 | chromosome 10 open reading frame 12 | Coding | 9.12 | 7.74 | 8.54 | 1.49 | 0.2362 | 0.4375 | 8.51 | 9.10 | 9.39 | 0.54 | 0.0038 | 0.0416 |
| TC1000010597.hg.1 | DRGX | dorsal root ganglia homeobox | Coding | 5.11 | 4.45 | 4.53 | 1.49 | 0.1157 | 0.2679 | 5.51 | 5.32 | 4.77 | 1.67 | 0.0092 | 0.074 |
| TC1100007763.hg.1 | MS4A3 | membrane-spanning 4-domains, subfamily A, member 3 (hemat | Multiple_C | 3.46 | 2.93 | 2.88 | 1.49 | 0.1073 | 0.2537 | 4.02 | 4.13 | 4.05 | 0.98 | 0.7758 | 0.8993 |
| TC1100008147.hg.1 | NDUFV1 | NADH dehydrogenase (ubiquinone) flavoprotein 1, 51kDa | Multiple_C | 12.25 | 12.07 | 11.67 | 1.49 | 0.2168 | 0.4128 | 12.93 | 12.97 | 12.47 | 1.38 | 0.9999 | 1 |
| TC1100009171.hg.1 | RNF214 | ring finger protein 214 | Multiple_C | 7.85 | 7.6 | 7.27 | 1.49 | 0.1561 | 0.331 | 7.73 | 7.70 | 7.49 | 1.18 | 0.5115 | 0.7413 |
| TC1100011199.hg.1 | NAALADL1 | N-acetylated alpha-linked acidic dipeptidase-like 1 | Multiple_C | 5.37 | 4.66 | 4.79 | 1.49 | 0.0403 | 0.1234 | 5.06 | 5.59 | 4.68 | 1.30 | 0.0303 | 0.1545 |
| TC1100011658.hg.1 | MAP6 | microtubule associated protein 6 | Multiple_C | 5.61 | 4.93 | 5.03 | 1.49 | 0.1316 | 0.2938 | 4.69 | 4.91 | 4.83 | 0.91 | 0.2238 | 0.4846 |
| TC1100012470.hg.1 | SCN2B | sodium channel, voltage gated, type II beta subunit | Coding | 4.69 | 3.95 | 4.11 | 1.49 | 0.2243 | 0.4227 | 5.07 | 4.99 | 4.76 | 1.24 | 0.376 | 0.6364 |
| TC1200010944.hg.1 | ZBTB39 | zinc finger and BTB domain containing 39 | Coding | 6.63 | 5.65 | 6.05 | 1.49 | 0.0685 | 0.1826 | 7.28 | 7.26 | 7.71 | 0.74 | 0.5156 | 0.7446 |
| TC1200011002.hg.1 | LRIG3 | leucine-rich repeats and immunoglobulin-like domains 3 | Multiple_C | 10.46 | 10.21 | 9.88 | 1.49 | 0.0526 | 0.1499 | 3.6 | 3.44 | 3.4 | 1.15 | 0.1277 | 0.3536 |
| TC1200012626.hg.1 | PRPF40B | PRP40 homolog, pre-mRNA processing factor B | Multiple_C | 5.37 | 4.82 | 4.79 | 1.49 | 0.1204 | 0.2754 | 6.11 | 6.19 | 6.08 | 1.02 | 0.9404 | 0.9753 |
| TC1300009293.hg.1 | MYCBP2 | MYC binding protein 2, E3 ubiquitin protein ligase | Multiple_C | 10.25 | 9.17 | 9.67 | 1.49 | 0.0141 | 0.0543 | 11.42 | 11.29 | 10.97 | 1.37 | 0.2865 | 0.5517 |
| TC14_GL000194v1_rand | MAFIP; TEKT4 | MAFF interacting protein (pseudogene); tektin 4 pseudogene 2 | Multiple_C | 9.16 | 8.8 | 8.58 | 1.49 | 0.0505 | 0.1456 | 11.54 | 11.79 | 11.45 | 1.06 | 0.3289 | 0.5927 |
| TC1400008382.hg.1 | TNFAIP2 | tumor necrosis factor, alpha-induced protein 2 | Multiple_C | 6.84 | 5.91 | 6.26 | 1.49 | 0.0928 | 0.2281 | 6.58 | 5.27 | 5.51 | 2.10 | 0.0259 | 0.1402 |
| TC1400008615.hg.1 | NDRG2; MIR6 | NDRG family member 2; microRNA 6717 | Multiple_C | 4.02 | 3.46 | 3.44 | 1.49 | 0.015 | 0.0571 | 3.65 | 3.91 | 3.72 | 0.95 | 0.8437 | 0.9318 |
| TC1500010727.hg.1 | CCNDBP1 | cyclin D-type binding-protein 1 | Multiple_C | 11.14 | 12.76 | 10.56 | 1.49 | 0.0213 | 0.0753 | 10.38 | 10.71 | 9.73 | 1.57 | 0.0043 | 0.0452 |
| TC1600010622.hg.1 | LRRC29 | leucine rich repeat containing 29 | Multiple_C | 6.03 | 5.36 | 5.45 | 1.49 | 0.108 | 0.2549 | 5.71 | 5.69 | 5.64 | 1.05 | 0.7832 | 0.9026 |
| TC1600010752.hg.1 | AARS | alanyl-tRNA synthetase | Multiple_C | 14.44 | 15.07 | 13.86 | 1.49 | 0.1806 | 0.3648 | 14 | 13.17 | 14.14 | 0.91 | 0.5943 | 0.7952 |
| TC1700007415.hg.1 | CRYBA1 | crystallin beta A1 | Coding | 3.59 | 3.29 | 3.01 | 1.49 | 0.026 | 0.0879 | 3.76 | 4.12 | 4.04 | 0.82 | 0.0978 | 0.305 |
| TC1700008326.hg.1 | TOM1L1 | target of myb1 like 1 membrane trafficking protein | Multiple_C | 11.24 | 12.08 | 10.66 | 1.49 | 0.2049 | 0.3976 | 9.76 | 9.25 | 9.4 | 1.28 | 0.2033 | 0.4589 |
| TC1700010856.hg.1 | C1QL1 | complement component 1, q subcomponent-like 1 | Coding | 5.45 | 4.71 | 4.87 | 1.49 | 0.064 | 0.1738 | 5.05 | 4.89 | 5.17 | 0.92 | 0.9054 | 0.9607 |
| TC1700012358.hg.1 | GSG1L2 | GSG1-like 2 | Multiple_C | 4.58 | 4.09 | 4 | 1.49 | 0.0387 | 0.1197 | 4.59 | 4.46 | 4.61 | 0.99 | 0.9927 | 0.9969 |
| TC1900008603.hg.1 | ACPT | acid phosphatase, testicular | Coding | 5.84 | 4.91 | 5.26 | 1.49 | 0.1904 | 0.3783 | 5.86 | 6.29 | 5.93 | 0.95 | 0.9523 | 0.9804 |
| TC1900011847.hg.1 | LRG1 | leucine-rich alpha-2-glycoprotein 1 | Multiple_C | 5.58 | 4.68 | 5 | 1.49 | 0.3771 | 0.5901 | 5.67 | 5.53 | 5.18 | 1.40 | 0.0894 | 0.2892 |

| | | | | | | | | | | | | | | | |
|---|---|---|---|---|---|---|---|---|---|---|---|---|---|---|---|
| TC2000007058.hg.1 | FRG1BP | FSHD region gene 1 family member B, pseudogene | Multiple_C | 7.71 | 7.48 | 7.13 | 1.49 | 0.1526 | 0.326 | 8.67 | 8.67 | 8.7 | 0.98 | 0.6842 | 0.8486 |
| TC2000010025.hg.1 | UBE2V1 | ubiquitin conjugating enzyme E2 variant 1 | Multiple_C | 14.56 | 14.42 | 13.98 | 1.49 | 0.0267 | 0.0896 | 15.03 | 14.76 | 15.26 | 0.85 | 0.357 | 0.6202 |
| TC2200007792.hg.1 | SELO | selenoprotein O; selenoprotein O [Source:EntrezGene;Acc:8364 | Multiple_C | 5.98 | 5.5 | 5.4 | 1.49 | 0.0535 | 0.1519 | 5.73 | 5.99 | 5.7 | 1.02 | 0.7896 | 0.9059 |
| TC2200008676.hg.1 | SOX10 | SRY box 10 | Multiple_C | 6.4 | 5.35 | 5.82 | 1.49 | 0.4288 | 0.6391 | 6 | 5.95 | 5.66 | 1.27 | 0.2177 | 0.4772 |
| TC2200009128.hg.1 | ALG12 | ALG12, alpha-1,6-mannosyltransferase | Multiple_C | 6.3 | 5.51 | 5.72 | 1.49 | 0.0226 | 0.079 | 6.66 | 6.64 | 6.72 | 0.96 | 0.8851 | 0.9507 |
| TSUnmapped00000075.h | ZNF780A | zinc finger protein 780A | Coding | 6.73 | 6.61 | 6.15 | 1.49 | 0.3167 | 0.5301 | 6.14 | 6.42 | 6.19 | 0.97 | 0.2817 | 0.5474 |
| TSUnmapped00000186.h | ZNF197 | zinc finger protein 197 | Coding | 5.77 | 5.62 | 5.19 | 1.49 | 0.0997 | 0.2405 | 4.08 | 4.08 | 4.05 | 1.02 | 0.6564 | 0.8321 |
| TSUnmapped00000387.h | ADAMTS13 | ADAM metallopeptidase with thrombospondin type 1 motif 13 | Coding | 5.35 | 4.68 | 4.77 | 1.49 | 0.0719 | 0.1891 | 4.84 | 4.77 | 4.66 | 1.13 | 0.511 | 0.7409 |
| TSUnmapped00000412.h | BCL2L14 | BCL2-like 14 (apoptosis facilitator) | Coding | 9.05 | 9.11 | 8.47 | 1.49 | 0.1074 | 0.2538 | 6.82 | 6.57 | 6.35 | 1.39 | 0.1155 | 0.3355 |
| TC0100006801.hg.1 | NMNAT1 | nicotinamide nucleotide adenylyltransferase 1 | Multiple_C | 8.23 | 8.2 | 7.66 | 1.48 | 0.118 | 0.2714 | 7.03 | 8.39 | 7.67 | 0.64 | 0.0051 | 0.0495 |
| TC0100006999.hg.1 | FBLIM1 | filamin binding LIM protein 1 | Multiple_C | 5.97 | 5.39 | 5.4 | 1.48 | 0.0329 | 0.1058 | 4.97 | 5.01 | 4.56 | 1.33 | 0.0735 | 0.2598 |
| TC0100009276.hg.1 | AMY1A; AMY1 | amylase, alpha 1A (salivary); amylase, alpha 1C (salivary) | Coding | 5.29 | 5.71 | 4.72 | 1.48 | 0.0436 | 0.131 | 3.55 | 3.20 | 3.07 | 1.39 | 0.0129 | 0.0918 |
| TC0100011388.hg.1 | IL19 | interleukin 19 | Multiple_C | 4.63 | 4.06 | 4.06 | 1.48 | 0.0231 | 0.0801 | 5.02 | 4.75 | 4.61 | 1.33 | 0.1056 | 0.3189 |
| TC0100011554.hg.1 | RPS6KC1 | ribosomal protein S6 kinase, 52kDa, polypeptide 1 | Multiple_C | 5.66 | 5.44 | 5.09 | 1.48 | 0.285 | 0.4953 | 6.27 | 5.75 | 6.08 | 1.14 | 0.3723 | 0.6335 |
| TC0100013382.hg.1 | AIM1L | absent in melanoma 1-like | Multiple_C | 5.62 | 5.13 | 5.05 | 1.48 | 0.0366 | 0.1148 | 5.46 | 5.67 | 6.13 | 0.63 | 0.2221 | 0.4823 |
| TC0100013483.hg.1 | TAF12 | TAF12 RNA polymerase II, TATA box binding protein (TBP)-assoc | Multiple_C | 10.11 | 10.27 | 9.54 | 1.48 | 0.1204 | 0.2753 | 9.68 | 9.47 | 9.09 | 1.51 | 0.0586 | 0.2284 |
| TC0100015434.hg.1 | GDAP2 | ganglioside induced differentiation associated protein 2 | Multiple_C | 10.2 | 9.57 | 9.63 | 1.48 | 0.0358 | 0.1131 | 10.32 | 8.95 | 9.78 | 1.45 | 0.0283 | 0.1482 |
| TC0100015864.hg.1 | S100A6 | S100 calcium binding protein A6 | Multiple_C | 15.87 | 16.35 | 15.3 | 1.48 | 0.046 | 0.1361 | 9.79 | 10.23 | 9.43 | 1.28 | 0.0237 | 0.1328 |
| TC0100016914.hg.1 | TNNI1 | troponin I type 1 (skeletal, slow) | Coding | 4.55 | 3.96 | 3.98 | 1.48 | 0.2048 | 0.3976 | 4 | 4.11 | 4.36 | 0.78 | 0.1514 | 0.3895 |
| TC0100017730.hg.1 | EGLN1 | egl-9 family hypoxia-inducible factor 1 | Multiple_C | 10.1 | 9.53 | 9.53 | 1.48 | 0.019 | 0.0686 | 9.17 | 9.78 | 9.22 | 0.97 | 0.6503 | 0.8292 |
| TC0100017761.hg.1 | PCNXL2 | pecanex-like 2 (Drosophila) | Multiple_C | 8.57 | 8.14 | 8 | 1.48 | 0.0451 | 0.134 | 7.7 | 7.40 | 7.87 | 0.89 | 0.8754 | 0.9465 |
| TC0200008627.hg.1 | NPAS2 | neuronal PAS domain protein 2 | Multiple_C | 12.64 | 11.83 | 12.07 | 1.48 | 0.0373 | 0.1165 | 12.13 | 11.35 | 11.47 | 1.58 | 0.0041 | 0.0435 |
| TC0200009350.hg.1 | C2orf27A | chromosome 2 open reading frame 27A | Multiple_C | 4.31 | 4.11 | 3.74 | 1.48 | 0.0333 | 0.1066 | 5.55 | 5.50 | 5.21 | 1.27 | 0.3112 | 0.5754 |
| TC0200016252.hg.1 | HDAC4 | histone deacetylase 4 | Multiple_C | 8.29 | 7.38 | 7.72 | 1.48 | 0.0863 | 0.216 | 12.59 | 12.16 | 12.12 | 1.39 | 0.28 | 0.546 |
| TC0200016747.hg.1 | TTC21B | tetratricopeptide repeat domain 21B | Multiple_C | 6.68 | 6.71 | 6.11 | 1.48 | 0.1975 | 0.3883 | 7.16 | 6.40 | 6.92 | 1.18 | 0.9439 | 0.9771 |
| TC0300008152.hg.1 | TFG | TRK-fused gene | Multiple_C | 12.15 | 10.58 | 11.58 | 1.48 | 0.0854 | 0.2145 | 10.97 | 10.22 | 11.14 | 0.89 | 0.4889 | 0.725 |
| TC0300011039.hg.1 | ARIH2OS | ariadne homolog 2 opposite strand | Coding | 4.6 | 4.11 | 4.03 | 1.48 | 0.0245 | 0.0839 | 3.76 | 3.33 | 3.23 | 1.44 | 0.079 | 0.2709 |
| TC0300011849.hg.1 | ABI3BP | ABI family, member 3 (NESH) binding protein | Multiple_C | 3.77 | 3.44 | 3.2 | 1.48 | 0.0633 | 0.1721 | 4.37 | 4.34 | 4.46 | 0.94 | 0.6933 | 0.8541 |
| TC0300013865.hg.1 | SEC22A | SEC22 homolog A, vesicle trafficking protein | Multiple_C | 10.27 | 10 | 9.7 | 1.48 | 0.0856 | 0.2148 | 9.15 | 8.51 | 8.88 | 1.21 | 0.3808 | 0.6405 |
| TC0400006472.hg.1 | MYL5 | myosin light chain 5 | Multiple_C | 8.44 | 8.29 | 7.87 | 1.48 | 0.2085 | 0.4021 | 7.1 | 6.96 | 6.61 | 1.40 | 0.4824 | 0.7202 |
| TC0500007395.hg.1 | HSPB3 | heat shock 27kDa protein 3 | Coding | 3.86 | 3.4 | 3.29 | 1.48 | 0.0757 | 0.1963 | 3.84 | 3.93 | 4.18 | 0.79 | 0.0581 | 0.2273 |
| TC0500008229.hg.1 | PAM | peptidylglycine alpha-amidating monooxygenase | Multiple_C | 9.49 | 9.71 | 8.92 | 1.48 | 0.2696 | 0.4779 | 11.37 | 11.88 | 11.25 | 1.09 | 0.8152 | 0.9182 |
| TC0500008875.hg.1 | PCDHB2 | protocadherin beta 2 | Coding | 4.26 | 3.83 | 3.69 | 1.48 | 0.0117 | 0.0473 | 3.61 | 3.55 | 3.33 | 1.21 | 0.163 | 0.4066 |

| | | | | | | | | | | | | | | | |
|---|---|---|---|---|---|---|---|---|---|---|---|---|---|---|---|
| TC0500010592.hg.1 | C6 | complement component 6 | Multiple_C | 3.97 | 3.12 | 3.4 | 1.48 | 0.3382 | 0.5521 | 3.43 | 3.45 | 3.57 | 0.91 | 0.2566 | 0.5214 |
| TC0500013191.hg.1 | NSA2 | NSA2 ribosome biogenesis homolog | Multiple_C | 14.49 | 15.04 | 13.92 | 1.48 | 0.3356 | 0.5496 | 11.78 | 11.66 | 11.58 | 1.15 | 0.2566 | 0.5214 |
| TC0600007518.hg.1 | HLA-L | major histocompatibility complex, class I, L (pseudogene) | Multiple_C | 8.29 | 8.05 | 7.72 | 1.48 | 0.0818 | 0.2079 | 10.13 | 9.68 | 9.47 | 1.58 | 0.0114 | 0.0845 |
| TC0600007625.hg.1 | CYP21A1P; CY | cytochrome P450, family 21, subfamily A, polypeptide 1 pseudo | Multiple_C | 4.22 | 3.65 | 3.65 | 1.48 | 0.0409 | 0.1246 | 4.43 | 4.59 | 4.41 | 1.01 | 0.8558 | 0.9386 |
| TC0600009426.hg.1 | LAMA2 | laminin, alpha 2 | Multiple_C | 4.17 | 3.42 | 3.6 | 1.48 | 0.2138 | 0.4089 | 4.59 | 5.76 | 5.38 | 0.58 | 0.0407 | 0.1852 |
| TC0600011525.hg.1 | RXRB | retinoid X receptor beta | Multiple_C | 10.02 | 10.33 | 9.45 | 1.48 | 0.1285 | 0.2887 | 9.41 | 9.84 | 9.35 | 1.04 | 0.4083 | 0.664 |
| TC0600014101.hg.1 | MICA | MHC class I polypeptide-related sequence A | Coding | 9.38 | 8.92 | 8.81 | 1.48 | 0.0464 | 0.137 | 7.93 | 6.57 | 7.88 | 1.04 | 0.5133 | 0.7428 |
| TC0700010719.hg.1 | TBX20 | T-box 20 | Multiple_C | 3.56 | 3.01 | 2.99 | 1.48 | 0.4408 | 0.6494 | 6.15 | 6.28 | 7.02 | 0.55 | 0.1099 | 0.3264 |
| TC0700012522.hg.1 | PRRT4 | proline-rich transmembrane protein 4 | Coding | 5.44 | 4.53 | 4.87 | 1.48 | 0.0107 | 0.0438 | 5.21 | 4.96 | 4.57 | 1.56 | 0.0042 | 0.0443 |
| TC0800007065.hg.1 | CDCA2 | cell division cycle associated 2 | Multiple_C | 10.18 | 9.49 | 9.61 | 1.48 | 0.0848 | 0.2134 | 8.97 | 9.83 | 9.9 | 0.52 | 0.0017 | 0.0243 |
| TC0800010298.hg.1 | THAP1 | THAP domain containing, apoptosis associated protein 1 | Multiple_C | 10.82 | 10.76 | 10.25 | 1.48 | 0.0209 | 0.0742 | 10.15 | 9.83 | 10.29 | 0.91 | 0.999 | 0.9997 |
| TC0800011314.hg.1 | UBR5 | ubiquitin protein ligase E3 component n-recognin 5 | Multiple_C | 9.86 | 10.51 | 9.29 | 1.48 | 0.1838 | 0.3694 | 8.44 | 7.88 | 8.26 | 1.13 | 0.9883 | 0.995 |
| TC0800012168.hg.1 | NRBP2; MIR68 | nuclear receptor binding protein 2; microRNA 6845 | Multiple_C | 8.39 | 7.51 | 7.82 | 1.48 | 0.1679 | 0.3475 | 6.72 | 7.01 | 6.9 | 0.88 | 0.2358 | 0.4984 |
| TC0800012173.hg.1 | EPPK1 | epiplakin 1 | Coding | 8.51 | 8.23 | 7.94 | 1.48 | 0.1031 | 0.2464 | 6.69 | 5.10 | 4.33 | 5.13 | 4.62E-07 | 8.66E-05 |
| TC0800012371.hg.1 | LY6E | lymphocyte antigen 6 complex, locus E | Multiple_C | 10.99 | 9.7 | 10.42 | 1.48 | 0.1306 | 0.2922 | 8.7 | 8.69 | 8.88 | 0.88 | 0.3164 | 0.5802 |
| TC0900006538.hg.1 | JAK2 | Janus kinase 2 | Multiple_C | 7.71 | 8.12 | 7.14 | 1.48 | 0.0328 | 0.1055 | 5.04 | 4.84 | 4.49 | 1.46 | 0.0133 | 0.0932 |
| TC0900008148.hg.1 | TDRD7 | tudor domain containing 7 | Multiple_C | 9.07 | 8.03 | 8.5 | 1.48 | 0.0878 | 0.219 | 7.29 | 7.31 | 6.69 | 1.52 | 0.0247 | 0.1364 |
| TC0900010773.hg.1 | ROR2 | receptor tyrosine kinase-like orphan receptor 2 | Multiple_C | 3.78 | 2.96 | 3.21 | 1.48 | 0.4291 | 0.6394 | 4.69 | 4.52 | 4.55 | 1.10 | 0.63 | 0.8175 |
| TC0900011284.hg.1 | AKNA | AT-hook transcription factor | Multiple_C | 6.53 | 5.96 | 5.96 | 1.48 | 0.16 | 0.3366 | 5.66 | 5.68 | 5.61 | 1.04 | 0.5424 | 0.7614 |
| TC0X00007163.hg.1 | SPACA5 | sperm acrosome associated 5 | Coding | 5.09 | 4.99 | 4.52 | 1.48 | 0.31 | 0.5232 | 4.5 | 4.58 | 4.66 | 0.90 | 0.4584 | 0.7022 |
| TC0X00009582.hg.1 | UXT | ubiquitously expressed prefoldin like chaperone | Multiple_C | 12.41 | 12.46 | 11.84 | 1.48 | 0.065 | 0.1758 | 9.26 | 10.29 | 10.04 | 0.58 | 0.0482 | 0.204 |
| TC0X00009981.hg.1 | IL2RG | interleukin 2 receptor, gamma | Multiple_C | 5.3 | 4.37 | 4.73 | 1.48 | 0.1184 | 0.2721 | 5.5 | 4.55 | 4.38 | 2.17 | 0.0009 | 0.0154 |
| TC0X00011306.hg.1 | NHSL2 | NHS-like 2 | Multiple_C | 3.54 | 2.98 | 2.97 | 1.48 | 0.0186 | 0.0676 | 3.65 | 3.43 | 3.43 | 1.16 | 0.0902 | 0.2906 |
| TC0Y00006807.hg.1 | BPY2B | basic charge, Y-linked, 2B | Multiple_C | 3.3 | 2.48 | 2.73 | 1.48 | 0.1071 | 0.2535 | 3.63 | 3.37 | 3.55 | 1.06 | 0.9108 | 0.9633 |
| TC0Y00007331.hg.1 | CSPG4P1Y | chondroitin sulfate proteoglycan 4 pseudogene 1, Y-linked | Multiple_C | 3.36 | 2.99 | 2.79 | 1.48 | 0.025 | 0.0854 | 4.12 | 4.42 | 4.01 | 1.08 | 0.6495 | 0.8289 |
| TC1000008206.hg.1 | SFTPA1 | surfactant protein A1 | Multiple_C | 3.82 | 3.37 | 3.25 | 1.48 | 0.0213 | 0.0753 | 3.9 | 3.67 | 3.84 | 1.04 | 0.2993 | 0.5639 |
| TC1000008276.hg.1 | CCSER2 | coiled-coil serine rich protein 2 | Multiple_C | 11.76 | 10.58 | 11.19 | 1.48 | 0.089 | 0.2212 | 12.5 | 11.22 | 12.35 | 1.11 | 0.5979 | 0.7976 |
| TC1000008488.hg.1 | PDE6C | phosphodiesterase 6C, cGMP-specific, cone, alpha prime | Multiple_C | 4.94 | 4.41 | 4.37 | 1.48 | 0.1489 | 0.3203 | 5.42 | 5.43 | 5.06 | 1.28 | 0.052 | 0.213 |
| TC1000009447.hg.1 | KNDC1 | kinase non-catalytic C-lobe domain (KIND) containing 1 | Multiple_C | 5.11 | 4.18 | 4.54 | 1.48 | 0.0562 | 0.1577 | 5.64 | 5.71 | 5.32 | 1.25 | 0.0729 | 0.2587 |
| TC1100008111.hg.1 | SYT12 | synaptotagmin XII | Multiple_C | 4.39 | 3.72 | 3.82 | 1.48 | 0.0931 | 0.2286 | 4.25 | 4.17 | 3.94 | 1.24 | 0.1528 | 0.3919 |
| TC1100008264.hg.1 | PPFIA1 | protein tyrosine phosphatase, receptor type, f polypeptide (PTP | Multiple_C | 8.73 | 8.49 | 8.16 | 1.48 | 0.025 | 0.0853 | 8.24 | 8.00 | 8.51 | 0.83 | 0.3536 | 0.6171 |
| TC1100009745.hg.1 | KRTAP5-1 | keratin associated protein 5-1 | Coding | 9.45 | 8.51 | 8.88 | 1.48 | 0.1423 | 0.3103 | 8.86 | 8.88 | 8.66 | 1.15 | 0.6483 | 0.8281 |
| TC1100010739.hg.1 | RAPSN | receptor-associated protein of the synapse | Multiple_C | 4.93 | 4.16 | 4.36 | 1.48 | 0.0672 | 0.1801 | 4.97 | 4.91 | 4.5 | 1.39 | 0.0222 | 0.128 |

| | | | | | | | | | | | | | | | |
|---|---|---|---|---|---|---|---|---|---|---|---|---|---|---|---|
| TC1100010885.hg.1 | TNKS1BP1 | tankyrase 1 binding protein 1 | Multiple_C | 6.28 | 5.88 | 5.71 | 1.48 | 0.0568 | 0.1588 | 6.16 | 5.94 | 5.72 | 1.36 | 0.1874 | 0.4392 |
| TC1200012793.hg.1 | KRT83 | keratin 83, type II | Multiple_C | 3.93 | 3.16 | 3.36 | 1.48 | 0.0383 | 0.1187 | 4.83 | 4.26 | 3.92 | 1.88 | 0.0002 | 0.006 |
| TC1500007914.hg.1 | TMEM266 | transmembrane protein 266 | Multiple_C | 4.46 | 3.7 | 3.89 | 1.48 | 0.0896 | 0.2222 | 3.83 | 3.70 | 3.71 | 1.09 | 0.7509 | 0.8866 |
| TC1500010251.hg.1 | HOMER2 | homer scaffolding protein 2 | Multiple_C | 5.13 | 4.37 | 4.56 | 1.48 | 0.4604 | 0.6662 | 10.69 | 10.80 | 10.71 | 0.99 | 0.8847 | 0.9505 |
| TC1600006666.hg.1 | CASP16P | caspase 16, pseudogene | Multiple_C | 8.83 | 8.13 | 8.26 | 1.48 | 0.0162 | 0.0606 | 8.57 | 8.63 | 8.07 | 1.41 | 0.0117 | 0.086 |
| TC1600011562.hg.1 | ZNF19 | zinc finger protein 19 | Multiple_C | 12.02 | 11.31 | 11.45 | 1.48 | 0.1424 | 0.3103 | 11.33 | 11.12 | 11.21 | 1.09 | 0.6412 | 0.8242 |
| TC1700010810.hg.1 | LSM12 | LSM12 homolog | Multiple_C | 14.27 | 13.18 | 13.7 | 1.48 | 0.0243 | 0.0836 | 14.83 | 14.07 | 14.61 | 1.16 | 0.5228 | 0.7489 |
| TC1700012110.hg.1 | SECTM1 | secreted and transmembrane 1 | Multiple_C | 7.15 | 5.42 | 6.58 | 1.48 | 0.0696 | 0.1846 | 5.21 | 4.09 | 4.35 | 1.82 | 0.0001 | 0.0038 |
| TC1700012457.hg.1 | AXIN2 | axin 2 | Multiple_C | 4.7 | 3.81 | 4.13 | 1.48 | 0.0853 | 0.2143 | 12.96 | 13.76 | 12.61 | 1.27 | 0.7527 | 0.8876 |
| TC1800007793.hg.1 | SALL3 | spalt-like transcription factor 3 | Multiple_C | 6.51 | 5.94 | 5.94 | 1.48 | 0.047 | 0.1382 | 5.77 | 5.93 | 6.01 | 0.85 | 0.1564 | 0.397 |
| TC1900006537.hg.1 | REEP6 | receptor accessory protein 6 | Multiple_C | 8.69 | 7.93 | 8.12 | 1.48 | 0.0142 | 0.0548 | 9.22 | 8.41 | 8.01 | 2.31 | 5.67E-05 | 0.0025 |
| TC1900006670.hg.1 | PIAS4 | protein inhibitor of activated STAT 4 | Multiple_C | 10.19 | 9.26 | 9.62 | 1.48 | 0.0397 | 0.122 | 8.35 | 8.58 | 8.81 | 0.73 | 0.0254 | 0.1388 |
| TC1900007005.hg.1 | SMARCA4 | SWI/SNF related, matrix associated, actin dependent regulator c | Multiple_C | 11.36 | 10.77 | 10.79 | 1.48 | 0.0389 | 0.12 | 11.29 | 11.82 | 11.34 | 0.97 | 0.7027 | 0.8594 |
| TC1900007399.hg.1 | TMEM59L | transmembrane protein 59-like | Multiple_C | 5.06 | 4.54 | 4.49 | 1.48 | 0.0626 | 0.1706 | 5.27 | 4.95 | 4.77 | 1.41 | 0.2028 | 0.4584 |
| TC1900007462.hg.1 | ZNF90 | zinc finger protein 90 | Multiple_C | 7.25 | 6.4 | 6.68 | 1.48 | 0.0547 | 0.1544 | 7.15 | 7.53 | 7.5 | 0.78 | 0.2447 | 0.5089 |
| TC1900008240.hg.1 | ZNF283 | zinc finger protein 283 | Multiple_C | 12.26 | 12.36 | 11.69 | 1.48 | 0.1487 | 0.32 | 11.47 | 11.16 | 11.12 | 1.27 | 0.5291 | 0.7526 |
| TC1900008694.hg.1 | ZNF610 | zinc finger protein 610 | Multiple_C | 5.49 | 5.12 | 4.92 | 1.48 | 0.0755 | 0.1958 | 4.89 | 5.06 | 5.26 | 0.77 | 0.0601 | 0.232 |
| TC1900008882.hg.1 | NCR1 | natural cytotoxicity triggering receptor 1 | Multiple_C | 4.74 | 4.01 | 4.17 | 1.48 | 0.0177 | 0.065 | 4.56 | 4.56 | 4.33 | 1.17 | 0.1799 | 0.4292 |
| TC1900009190.hg.1 | KLF16 | Kruppel-like factor 16 | Coding | 8.83 | 7.56 | 8.26 | 1.48 | 0.2066 | 0.4 | 8.05 | 7.79 | 8.24 | 0.88 | 0.1096 | 0.326 |
| TC2000008802.hg.1 | DEFB116 | defensin, beta 116 | Coding | 4.37 | 3.36 | 3.8 | 1.48 | 0.0466 | 0.1374 | 4.13 | 4.15 | 4.22 | 0.94 | 0.7426 | 0.8821 |
| TC2000008975.hg.1 | C20orf173 | chromosome 20 open reading frame 173 | Multiple_C | 4.68 | 3.69 | 4.11 | 1.48 | 0.187 | 0.374 | 4.23 | 4.33 | 4.49 | 0.84 | 0.7537 | 0.888 |
| TC2200007088.hg.1 | LIMK2 | LIM domain kinase 2 | Multiple_C | 9.97 | 9.25 | 9.4 | 1.48 | 0.0301 | 0.0985 | 7.36 | 7.44 | 7.47 | 0.93 | 0.5633 | 0.7738 |
| TC2200008136.hg.1 | PRAME | preferentially expressed antigen in melanoma | Multiple_C | 4.29 | 3.71 | 3.72 | 1.48 | 0.1415 | 0.3091 | 4.71 | 4.11 | 4.39 | 1.25 | 0.2279 | 0.4886 |
| TC2200009348.hg.1 | MIR659; ANKI | microRNA 659; ankyrin repeat domain 54 | Multiple_C | 5.66 | 5.05 | 5.09 | 1.48 | 0.0125 | 0.0499 | 5.13 | 5.45 | 5.25 | 0.92 | 0.8318 | 0.925 |
| TSUnmapped00000012.h | LRP6 | LDL receptor related protein 6 | Coding | 9.43 | 10.39 | 8.86 | 1.48 | 0.0451 | 0.134 | 9.32 | 9.46 | 9.31 | 1.01 | 0.7971 | 0.9093 |
| TSUnmapped00000045.h | CCDC84 | coiled-coil domain containing 84 | Coding | 10 | 9.46 | 9.43 | 1.48 | 0.5169 | 0.7112 | 8.34 | 7.88 | 8.04 | 1.23 | 0.0954 | 0.3003 |
| TSUnmapped00000059.h | HYOU1 | hypoxia up-regulated 1 | Coding | 4.6 | 4.09 | 4.03 | 1.48 | 0.0282 | 0.0937 | 5.3 | 5.39 | 5.12 | 1.13 | 0.82 | 0.9205 |
| TC0100010231.hg.1 | CD1E | CD1e molecule | Multiple_C | 5.21 | 4.59 | 4.65 | 1.47 | 0.1111 | 0.26 | 4.69 | 4.59 | 4.34 | 1.27 | 0.0771 | 0.2674 |
| TC0100011861.hg.1 | OBSCN | obscurin, cytoskeletal calmodulin and titin-interacting RhoGEF | Multiple_C | 5.04 | 4.45 | 4.48 | 1.47 | 0.0579 | 0.1613 | 4.49 | 4.57 | 4.18 | 1.24 | 0.0763 | 0.2659 |
| TC0100013588.hg.1 | PTP4A2 | protein tyrosine phosphatase type IVA, member 2 | Multiple_C | 14.87 | 14.37 | 14.31 | 1.47 | 0.1268 | 0.2856 | 13.71 | 13.03 | 13.92 | 0.86 | 0.9211 | 0.9675 |
| TC0100013749.hg.1 | RSPO1 | R-spondin 1 | Coding | 5.87 | 5.26 | 5.31 | 1.47 | 0.2303 | 0.4304 | 7.29 | 7.51 | 7.26 | 1.02 | 0.7281 | 0.8739 |
| TC0100013992.hg.1 | TCTEX1D4 | Tctex1 domain containing 4 | Coding | 4.02 | 3.39 | 3.46 | 1.47 | 0.3141 | 0.5275 | 4.47 | 4.49 | 4.63 | 0.90 | 0.8599 | 0.9402 |
| TC0100014218.hg.1 | SCP2 | Jeck2013 ANTISENSE, coding, INTERNAL, OVEXON, UTR3 best tra | Multiple_C | 11.73 | 12.14 | 11.17 | 1.47 | 0.0219 | 0.077 | 10.31 | 9.90 | 10.57 | 0.84 | 0.536 | 0.7579 |

| | | | | | | | | | | | | | | | |
|---|---|---|---|---|---|---|---|---|---|---|---|---|---|---|---|
| TC0100018297.hg.1 | LCE1B | late cornified envelope 1B | Coding | 9.06 | 7.96 | 8.5 | 1.47 | 0.069 | 0.1834 | 7.88 | 8.15 | 8.25 | 0.77 | 0.0902 | 0.2906 |
| TC0200007096.hg.1 | FOSL2 | FOS-like antigen 2 | Multiple_C | 10.3 | 9.46 | 9.74 | 1.47 | 0.016 | 0.06 | 8.17 | 7.90 | 8.1 | 1.05 | 0.8156 | 0.9182 |
| TC0200007803.hg.1 | LGALSL | lectin, galactoside-binding-like | Multiple_C | 9.68 | 10.05 | 9.12 | 1.47 | 0.0913 | 0.2254 | 8.98 | 8.24 | 8.9 | 1.06 | 0.1951 | 0.4492 |
| TC0200007907.hg.1 | GKN1 | gastrokine 1 | Multiple_C | 3.34 | 2.48 | 2.78 | 1.47 | 0.249 | 0.453 | 3.41 | 2.84 | 3.32 | 1.06 | 0.4282 | 0.6802 |
| TC0200008253.hg.1 | SH2D6 | SH2 domain containing 6 | Multiple_C | 4.49 | 3.9 | 3.93 | 1.47 | 0.0919 | 0.2264 | 4.36 | 4.70 | 4.58 | 0.86 | 0.8113 | 0.9166 |
| TC0200009662.hg.1 | TNFAIP6 | tumor necrosis factor, alpha-induced protein 6 | Multiple_C | 4.12 | 3.72 | 3.56 | 1.47 | 0.2333 | 0.434 | 4.24 | 4.28 | 4.21 | 1.02 | 0.4011 | 0.6577 |
| TC0200009911.hg.1 | PPIG | peptidylprolyl isomerase G (cyclophilin G) | Multiple_C | 12.39 | 12.76 | 11.83 | 1.47 | 0.1115 | 0.2609 | 9.6 | 9.23 | 10.14 | 0.69 | 0.0584 | 0.2278 |
| TC0200010536.hg.1 | PARD3B | par-3 family cell polarity regulator beta | Multiple_C | 3.99 | 3.89 | 3.43 | 1.47 | 0.013 | 0.0514 | 4.28 | 3.31 | 3.41 | 1.83 | 0.0002 | 0.0061 |
| TC0200011023.hg.1 | SP100 | SP100 nuclear antigen | Multiple_C | 8.12 | 7.16 | 7.56 | 1.47 | 0.1679 | 0.3476 | 6.53 | 5.82 | 7.07 | 0.69 | 0.4746 | 0.7142 |
| TC0200013311.hg.1 | REEP1 | receptor accessory protein 1 | Multiple_C | 4.44 | 3.76 | 3.88 | 1.47 | 0.3942 | 0.6079 | 4.25 | 4.15 | 4.31 | 0.96 | 0.9291 | 0.9702 |
| TC0200014260.hg.1 | CCDC115 | coiled-coil domain containing 115 | Multiple_C | 8.72 | 8.66 | 8.16 | 1.47 | 0.008 | 0.0351 | 7.09 | 7.16 | 6.75 | 1.27 | 0.0494 | 0.2069 |
| TC0200014814.hg.1 | SCN1A | sodium channel, voltage gated, type I alpha subunit | Multiple_C | 3.76 | 3.44 | 3.2 | 1.47 | 0.0643 | 0.1742 | 3.92 | 3.85 | 3.3 | 1.54 | 0.0112 | 0.0835 |
| TC0300012720.hg.1 | PLSCR4 | phospholipid scramblase 4 | Multiple_C | 4.09 | 3.5 | 3.53 | 1.47 | 0.0053 | 0.0253 | 3.34 | 3.21 | 3.18 | 1.12 | 0.3602 | 0.6229 |
| TC0400008834.hg.1 | SCOC | short coiled-coil protein | Coding | 9.77 | 10.73 | 9.21 | 1.47 | 0.0172 | 0.0635 | 8.08 | 7.49 | 7.56 | 1.43 | 0.004 | 0.0432 |
| TC0400009790.hg.1 | MFSD10 | major facilitator superfamily domain containing 10 | Multiple_C | 10.69 | 10.08 | 10.13 | 1.47 | 0.21 | 0.4039 | 9.39 | 9.28 | 9.64 | 0.84 | 0.946 | 0.9781 |
| TC0500007267.hg.1 | CCDC152 | coiled-coil domain containing 152 | Multiple_C | 4.26 | 3.7 | 3.7 | 1.47 | 0.007 | 0.0317 | 3.87 | 4.01 | 3.94 | 0.95 | 0.5522 | 0.7677 |
| TC0500007691.hg.1 | SMN1; SMN2 | survival of motor neuron 1, telomeric; survival of motor neuron | Multiple_C | 13.42 | 13.51 | 12.86 | 1.47 | 0.0362 | 0.1138 | 14.65 | 14.16 | 14.07 | 1.49 | 0.0008 | 0.0139 |
| TC0500008627.hg.1 | SLC22A4 | solute carrier family 22 (organic cation/zwitterion transporter), | Multiple_C | 4.78 | 3.88 | 4.22 | 1.47 | 0.1952 | 0.3852 | 4.59 | 4.77 | 4.29 | 1.23 | 0.1711 | 0.4176 |
| TC0500009455.hg.1 | NEURL1B | neuralized E3 ubiquitin protein ligase 1B | Multiple_C | 3.29 | 2.73 | 2.73 | 1.47 | 0.18 | 0.364 | 3.36 | 3.70 | 3.77 | 0.75 | 0.0363 | 0.173 |
| TC0500012120.hg.1 | IL9 | interleukin 9 | Coding | 3.9 | 2.95 | 3.34 | 1.47 | 0.0789 | 0.2029 | 3.54 | 3.77 | 4.14 | 0.66 | 0.0685 | 0.2492 |
| TC0500013104.hg.1 | OR2Y1 | olfactory receptor, family 2, subfamily Y, member 1 | Coding | 3.21 | 2.73 | 2.65 | 1.47 | 0.215 | 0.4105 | 3.12 | 3.44 | 3.3 | 0.88 | 0.5469 | 0.7641 |
| TC0600010061.hg.1 | PNLDC1 | poly(A)-specific ribonuclease (PARN)-like domain containing 1 | Multiple_C | 3.42 | 2.76 | 2.86 | 1.47 | 0.3466 | 0.5599 | 3.42 | 3.29 | 3 | 1.34 | 0.1387 | 0.3699 |
| TC0600013199.hg.1 | TAAR3 | trace amine associated receptor 3 (gene/pseudogene) | Multiple_C | 3.57 | 2.69 | 3.01 | 1.47 | 0.3406 | 0.5546 | 3.76 | 3.74 | 3.77 | 0.99 | 0.9627 | 0.9842 |
| TC0700008809.hg.1 | LRRN3 | leucine rich repeat neuronal 3 | Multiple_C | 5.58 | 4.76 | 5.02 | 1.47 | 0.0143 | 0.0552 | 5.65 | 5.22 | 5.01 | 1.56 | 0.2382 | 0.5016 |
| TC0700010453.hg.1 | TOMM7 | translocase of outer mitochondrial membrane 7 homolog (yeas | Multiple_C | 13.66 | 15.18 | 13.1 | 1.47 | 0.0733 | 0.1916 | 9.12 | 8.76 | 8.46 | 1.58 | 0.0122 | 0.0882 |
| TC0800009636.hg.1 | USP17L7 | ubiquitin specific peptidase 17-like family member 7 | Coding | 3.54 | 2.83 | 2.98 | 1.47 | 0.0229 | 0.0796 | 3.39 | 3.21 | 3.68 | 0.82 | 0.2132 | 0.4713 |
| TC0800011437.hg.1 | ABRA | actin binding Rho activating protein | Coding | 5.28 | 4.35 | 4.72 | 1.47 | 0.0106 | 0.0438 | 4.21 | 4.25 | 4.15 | 1.04 | 0.9364 | 0.9735 |
| TC0900009272.hg.1 | CYSRT1 | cysteine-rich tail protein 1 | Multiple_C | 4.81 | 4.12 | 4.25 | 1.47 | 0.398 | 0.6115 | 4.06 | 4.11 | 4.04 | 1.01 | 0.9776 | 0.9908 |
| TC0900012222.hg.1 | APTX | aprataxin | Multiple_C | 4.68 | 4.06 | 4.12 | 1.47 | 0.0524 | 0.1495 | 3.99 | 3.81 | 3.71 | 1.21 | 0.8103 | 0.9161 |
| TC0M00006439.hg.1 | ATP8; ATP6; C | ATP synthase F0 subunit 8; ATP synthase F0 subunit 6; cytochro | Multiple_C | 18.15 | 17.23 | 17.59 | 1.47 | 0.0651 | 0.1761 | 18.63 | 18.58 | 18.77 | 0.91 | 0.5411 | 0.7602 |
| TC0X00006704.hg.1 | REPS2 | RALBP1 associated Eps domain containing 2 | Multiple_C | 7.41 | 7.39 | 6.85 | 1.47 | 0.3055 | 0.5186 | 3.5 | 3.61 | 3.45 | 1.04 | 0.1992 | 0.4545 |
| TC0X00008689.hg.1 | MAGEA11 | MAGE family member A11 | Multiple_C | 5.55 | 4.78 | 4.99 | 1.47 | 0.0684 | 0.1823 | 4.47 | 4.22 | 4.41 | 1.04 | 0.7221 | 0.8705 |
| TC1000011726.hg.1 | SH3PXD2A | SH3 and PX domains 2A | Multiple_C | 7.78 | 7.01 | 7.22 | 1.47 | 0.0727 | 0.1904 | 6.47 | 6.63 | 6.55 | 0.95 | 0.2822 | 0.5478 |

| | | | | | | | | | | | | | | | |
|---|---|---|---|---|---|---|---|---|---|---|---|---|---|---|---|
| TC1000011743.hg.1 | CFAP43 | cilia and flagella associated protein 43 | Multiple_C | 6.09 | 6.31 | 5.53 | 1.47 | 0.3177 | 0.5312 | 5.6 | 5.31 | 4.85 | 1.68 | 0.0469 | 0.2004 |
| TC1100007216.hg.1 | PRRG4 | proline rich Gla (G-carboxyglutamic acid) 4 (transmembrane) | Multiple_C | 10.36 | 8.97 | 9.8 | 1.47 | 0.048 | 0.1403 | 6.66 | 4.33 | 5.69 | 1.96 | 0.0001 | 0.0039 |
| TC1100007940.hg.1 | VEGFB | vascular endothelial growth factor B | Multiple_C | 8.17 | 7.37 | 7.61 | 1.47 | 0.0094 | 0.0397 | 7.65 | 7.95 | 7.62 | 1.02 | 0.6325 | 0.8191 |
| TC1100008082.hg.1 | NPAS4 | neuronal PAS domain protein 4 | Multiple_C | 4.18 | 3.68 | 3.62 | 1.47 | 0.0855 | 0.2147 | 3.42 | 3.59 | 3.49 | 0.95 | 0.8744 | 0.9459 |
| TC1100011158.hg.1 | BAD | BCL2-associated agonist of cell death | Multiple_C | 5.98 | 5.55 | 5.42 | 1.47 | 0.2159 | 0.4116 | 5.42 | 5.79 | 5.4 | 1.01 | 0.1535 | 0.3928 |
| TC1100013040.hg.1 | TM7SF2 | transmembrane 7 superfamily member 2 | Multiple_C | 9.92 | 9.25 | 9.36 | 1.47 | 0.1272 | 0.2865 | 9.09 | 9.00 | 8.54 | 1.46 | 0.0113 | 0.084 |
| TC1200006574.hg.1 | KCNA5 | potassium channel, voltage gated shaker related subfamily A, m | Coding | 5.95 | 5.43 | 5.39 | 1.47 | 0.0733 | 0.1915 | 6.29 | 6.48 | 6.22 | 1.05 | 0.7951 | 0.9087 |
| TC1200009587.hg.1 | CACNA2D4 | calcium channel, voltage-dependent, alpha 2/delta subunit 4 | Multiple_C | 3.95 | 3.24 | 3.39 | 1.47 | 0.4105 | 0.6227 | 8.4 | 9.63 | 10.2 | 0.29 | 0.0113 | 0.0841 |
| TC1200009846.hg.1 | LINC00612 | long intergenic non-protein coding RNA 612 | Multiple_C | 3.75 | 3.25 | 3.19 | 1.47 | 0.0993 | 0.2397 | 3.32 | 3.09 | 3.48 | 0.90 | 0.7429 | 0.8822 |
| TC1200010777.hg.1 | KRT79 | keratin 79, type II | Multiple_C | 4.83 | 4.43 | 4.27 | 1.47 | 0.0172 | 0.0635 | 5.38 | 5.23 | 5.36 | 1.01 | 0.9797 | 0.9914 |
| TC1200012579.hg.1 | NTF3 | neurotrophin 3 | Multiple_C | 3.85 | 3.23 | 3.29 | 1.47 | 0.049 | 0.1425 | 3.7 | 3.78 | 3.45 | 1.19 | 0.2029 | 0.4585 |
| TC1200012635.hg.1 | PRR13 | proline rich 13 | Multiple_C | 13.46 | 12.93 | 12.9 | 1.47 | 0.0821 | 0.2082 | 12.85 | 13.21 | 13.21 | 0.78 | 0.2031 | 0.4587 |
| TC1400007632.hg.1 | PAPLN | papilin, proteoglycan-like sulfated glycoprotein | Multiple_C | 3.74 | 3.24 | 3.18 | 1.47 | 0.0113 | 0.0459 | 3.49 | 3.31 | 3.57 | 0.95 | 0.8424 | 0.9313 |
| TC1400008036.hg.1 | COX8C | cytochrome c oxidase subunit VIIIC | Coding | 4.88 | 4.33 | 4.32 | 1.47 | 0.1128 | 0.2631 | 4.48 | 4.51 | 4.3 | 1.13 | 0.5784 | 0.7843 |
| TC1400008838.hg.1 | STRN3 | striatin, calmodulin binding protein 3 | Multiple_C | 10.8 | 10.71 | 10.24 | 1.47 | 0.1239 | 0.2808 | 10.5 | 9.66 | 10.44 | 1.04 | 0.8876 | 0.9521 |
| TC1500007409.hg.1 | GCNT3 | glucosaminyl (N-acetyl) transferase 3, mucin type | Multiple_C | 13.77 | 14.25 | 13.21 | 1.47 | 0.1779 | 0.3609 | 4.68 | 4.88 | 5.08 | 0.76 | 0.2668 | 0.5312 |
| TC1500010399.hg.1 | PLIN1 | perilipin 1 | Multiple_C | 3.8 | 3.34 | 3.24 | 1.47 | 0.0593 | 0.1643 | 3.66 | 3.62 | 3.53 | 1.09 | 0.5015 | 0.734 |
| TC1500010680.hg.1 | OR4F4; OR4F1 | olfactory receptor, family 4, subfamily F, member 4; olfactory re | Coding | 3.83 | 3.14 | 3.27 | 1.47 | 0.3078 | 0.5209 | 4.32 | 3.91 | 4.18 | 1.10 | 0.5754 | 0.7822 |
| TC1600008656.hg.1 | KLHL36 | kelch-like family member 36 | Multiple_C | 7.58 | 6.77 | 7.02 | 1.47 | 0.1624 | 0.34 | 6.05 | 6.02 | 5.83 | 1.16 | 0.7638 | 0.8932 |
| TC1600009252.hg.1 | PPL | periplakin | Multiple_C | 4.68 | 4.65 | 4.12 | 1.47 | 0.0125 | 0.0498 | 8.83 | 8.99 | 7.48 | 2.55 | 0.0002 | 0.0064 |
| TC1600010215.hg.1 | ABCC12 | ATP binding cassette subfamily C member 12 | Multiple_C | 5.9 | 5.25 | 5.34 | 1.47 | 0.0342 | 0.1089 | 4.33 | 4.54 | 4.27 | 1.04 | 0.7568 | 0.8896 |
| TC1600011349.hg.1 | RBFOX1 | RNA binding protein, fox-1 homolog (C. elegans) 1 | Multiple_C | 4.82 | 4.12 | 4.26 | 1.47 | 0.204 | 0.3966 | 4.34 | 4.61 | 4.94 | 0.66 | 0.0068 | 0.06 |
| TC1700007942.hg.1 | NBR1 | neighbor of BRCA1 gene 1 | Multiple_C | 10.86 | 11.11 | 10.3 | 1.47 | 0.0587 | 0.163 | 8.69 | 8.96 | 8.46 | 1.17 | 0.6296 | 0.8175 |
| TC1700007944.hg.1 | TMEM106A | transmembrane protein 106A | Multiple_C | 6.41 | 5.71 | 5.85 | 1.47 | 0.1817 | 0.3663 | 5.54 | 5.88 | 6.2 | 0.63 | 0.001 | 0.0163 |
| TC1700008105.hg.1 | WNT9B | wingless-type MMTV integration site family, member 9B | Coding | 4.73 | 4.05 | 4.17 | 1.47 | 0.3678 | 0.5806 | 4.64 | 4.74 | 4.74 | 0.93 | 0.569 | 0.7777 |
| TC1700009949.hg.1 | SREBF1 | sterol regulatory element binding transcription factor 1 | Multiple_C | 12.21 | 12.67 | 11.65 | 1.47 | 0.482 | 0.6836 | 12.1 | 12.00 | 11.66 | 1.36 | 0.2728 | 0.5382 |
| TC1700010707.hg.1 | DHX58 | DEXH (Asp-Glu-X-His) box polypeptide 58 | Multiple_C | 6.21 | 5.73 | 5.65 | 1.47 | 0.0134 | 0.0524 | 5.16 | 5.00 | 5.05 | 1.08 | 0.8117 | 0.9166 |
| TC1800008952.hg.1 | DSEL | dermatan sulfate epimerase-like | Coding | 3.49 | 3.1 | 2.93 | 1.47 | 0.1241 | 0.2813 | 5.22 | 5.48 | 5.47 | 0.84 | 0.4502 | 0.6962 |
| TC1900006601.hg.1 | ZNF554 | zinc finger protein 554 | Multiple_C | 7.08 | 6.76 | 6.52 | 1.47 | 0.2662 | 0.4739 | 6.11 | 5.74 | 5.73 | 1.30 | 0.2385 | 0.5019 |
| TC1900007340.hg.1 | MAP1S | microtubule-associated protein 1S | Multiple_C | 11.06 | 9.48 | 10.5 | 1.47 | 0.4328 | 0.6428 | 8.47 | 9.23 | 10 | 0.35 | 0.0045 | 0.0462 |
| TC1900008549.hg.1 | SCAF1 | SR-related CTD-associated factor 1 | Multiple_C | 6.41 | 5.72 | 5.85 | 1.47 | 0.0254 | 0.0864 | 5.06 | 5.44 | 5.68 | 0.65 | 0.0188 | 0.1156 |
| TC1900010783.hg.1 | GRIK5 | glutamate receptor, ionotropic, kainate 5 | Multiple_C | 6.72 | 5.83 | 6.16 | 1.47 | 0.0499 | 0.1444 | 5.65 | 5.71 | 5.67 | 0.99 | 0.1429 | 0.3761 |
| TC1900011668.hg.1 | ZNF763 | zinc finger protein 763 | Coding | 6.79 | 6.63 | 6.23 | 1.47 | 0.0802 | 0.2055 | 5.76 | 5.62 | 5.6 | 1.12 | 0.1727 | 0.42 |

| | | | | | | | | | | | | | | | |
|---|---|---|---|---|---|---|---|---|---|---|---|---|---|---|---|
| TC2000006799.hg.1 | DSTN | destrin (actin depolymerizing factor) | Multiple_C | 14.54 | 14.39 | 13.98 | 1.47 | 0.0435 | 0.1308 | 13.15 | 12.16 | 13.12 | 1.02 | 0.4355 | 0.6863 |
| TC2000009016.hg.1 | DSN1 | DSN1 homolog, MIS12 kinetochore complex component | Multiple_C | 11.22 | 10.2 | 10.66 | 1.47 | 0.0962 | 0.234 | 10.18 | 11.13 | 11.1 | 0.53 | 0.0016 | 0.0233 |
| TC2000009198.hg.1 | ADA | adenosine deaminase | Multiple_C | 3.78 | 3.18 | 3.22 | 1.47 | 0.1742 | 0.3561 | 5.66 | 5.97 | 5.57 | 1.06 | 0.9308 | 0.9708 |
| TC2000009243.hg.1 | TNNC2 | troponin C type 2 (fast) | Coding | 5.14 | 4.5 | 4.58 | 1.47 | 0.1061 | 0.2517 | 5.11 | 4.99 | 4.93 | 1.13 | 0.3982 | 0.6556 |
| TC2000009945.hg.1 | FAM209A | family with sequence similarity 209, member A | Coding | 4.05 | 3.67 | 3.49 | 1.47 | 0.0027 | 0.0146 | 3.47 | 3.62 | 3.24 | 1.17 | 0.2134 | 0.4716 |
| TC2100006881.hg.1 | KRTAP20-4 | keratin associated protein 20-4 | Multiple_C | 4.91 | 4.53 | 4.35 | 1.47 | 0.0251 | 0.0855 | 4.26 | 4.45 | 4.13 | 1.09 | 0.6341 | 0.8203 |
| TC2100007114.hg.1 | DSCR10 | Down syndrome critical region 10 (non-protein coding) | Multiple_C | 6.53 | 5.9 | 5.97 | 1.47 | 0.0654 | 0.1766 | 5.8 | 6.04 | 5.64 | 1.12 | 0.9626 | 0.9842 |
| TC2200007615.hg.1 | RIBC2 | RIB43A domain with coiled-coils 2 | Multiple_C | 4.63 | 4.05 | 4.07 | 1.47 | 0.0538 | 0.1526 | 8.09 | 8.91 | 8.6 | 0.70 | 0.0872 | 0.2851 |
| TC2200009284.hg.1 | ARHGAP8 | Rho GTPase activating protein 8 | Multiple_C | 8.83 | 7.51 | 8.27 | 1.47 | 0.1788 | 0.3623 | 7.71 | 7.03 | 7.59 | 1.09 | 0.1285 | 0.3548 |
| TSUnmapped00000282.h | KAT6B | K(lysine) acetyltransferase 6B | Coding | 7.5 | 7.01 | 6.94 | 1.47 | 0.1142 | 0.2653 | 8.35 | 8.09 | 7.86 | 1.40 | 0.1546 | 0.3942 |
| TC0100007326.hg.1 | RPL11 | ribosomal protein L11 | Multiple_C | 17.2 | 17.08 | 16.65 | 1.46 | 0.0058 | 0.027 | 14.63 | 14.16 | 14.36 | 1.21 | 0.1312 | 0.359 |
| TC0100007477.hg.1 | ARID1A | AT rich interactive domain 1A (SWI-like) | Multiple_C | 10.97 | 10 | 10.42 | 1.46 | 0.0255 | 0.0865 | 10.73 | 11.61 | 11.32 | 0.66 | 0.0177 | 0.1117 |
| TC0100008599.hg.1 | UBE2U | ubiquitin-conjugating enzyme E2U (putative) | Multiple_C | 3.49 | 2.62 | 2.94 | 1.46 | 0.5901 | 0.7654 | 3.38 | 3.57 | 3.47 | 0.94 | 0.7456 | 0.8834 |
| TC0100009929.hg.1 | SETDB1 | SET domain, bifurcated 1 | Multiple_C | 9.93 | 9.64 | 9.38 | 1.46 | 0.0365 | 0.1147 | 10.13 | 9.74 | 9.88 | 1.19 | 0.3168 | 0.5809 |
| TC0100013610.hg.1 | FAM229A | family with sequence similarity 229, member A | Multiple_C | 4.51 | 3.73 | 3.96 | 1.46 | 0.0077 | 0.0339 | 3.83 | 4.07 | 3.86 | 0.98 | 0.6586 | 0.8334 |
| TC0100015819.hg.1 | S100A10 | S100 calcium binding protein A10 | Multiple_C | 17.49 | 16.63 | 16.94 | 1.46 | 0.0812 | 0.2071 | 14.26 | 13.94 | 13.29 | 1.96 | 0.0072 | 0.0624 |
| TC0100018162.hg.1 | TNFRSF14 | tumor necrosis factor receptor superfamily, member 14 | Multiple_C | 5.51 | 5.35 | 4.96 | 1.46 | 0.0352 | 0.1115 | 5.49 | 5.37 | 5.39 | 1.07 | 0.5143 | 0.7435 |
| TC0200008071.hg.1 | MTHFD2 | methylenetetrahydrofolate dehydrogenase (NADP+ dependent) | Multiple_C | 15.39 | 15.99 | 14.84 | 1.46 | 0.038 | 0.118 | 14.66 | 14.13 | 14.4 | 1.20 | 0.0814 | 0.275 |
| TC0200011976.hg.1 | PFN4 | profilin family, member 4 | Multiple_C | 4.18 | 3.79 | 3.63 | 1.46 | 0.0187 | 0.0678 | 4.39 | 4.14 | 3.96 | 1.35 | 0.1971 | 0.4519 |
| TC0200012189.hg.1 | NLRC4 | NLR family, CARD domain containing 4 | Coding | 4.55 | 3.79 | 4 | 1.46 | 0.4658 | 0.6704 | 3.58 | 3.58 | 3.71 | 0.91 | 0.4346 | 0.6853 |
| TC0200016099.hg.1 | HJURP | Holliday junction recognition protein | Multiple_C | 13.08 | 11.97 | 12.53 | 1.46 | 0.078 | 0.2008 | 12.24 | 12.22 | 12.46 | 0.86 | 0.2211 | 0.4812 |
| TC0200016598.hg.1 | SPEG | SPEG complex locus | Multiple_C | 9.62 | 9.12 | 9.07 | 1.46 | 0.1474 | 0.3183 | 8.35 | 8.80 | 8.36 | 0.99 | 0.2206 | 0.4807 |
| TC0200016772.hg.1 | NHEJ1 | nonhomologous end-joining factor 1 | Multiple_C | 9.31 | 8.69 | 8.76 | 1.46 | 0.0106 | 0.0436 | 8.49 | 8.27 | 9.05 | 0.68 | 0.0064 | 0.0579 |
| TC0300007512.hg.1 | PRKCD | protein kinase C, delta | Multiple_C | 8.45 | 7.83 | 7.9 | 1.46 | 0.0488 | 0.1421 | 8.29 | 8.64 | 8.49 | 0.87 | 0.5506 | 0.7665 |
| TC0300008332.hg.1 | TAGLN3 | transgelin 3 | Coding | 4.54 | 4.23 | 3.99 | 1.46 | 0.0498 | 0.1443 | 4.34 | 4.28 | 4.23 | 1.08 | 0.6956 | 0.8555 |
| TC0300008514.hg.1 | NDUFB4 | NADH dehydrogenase (ubiquinone) 1 beta subcomplex, 4, 15kD | Multiple_C | 14.78 | 15.21 | 14.23 | 1.46 | 0.0261 | 0.0882 | 10.13 | 10.07 | 9.89 | 1.18 | 0.2045 | 0.4606 |
| TC0300012572.hg.1 | CEP70 | centrosomal protein 70kDa | Multiple_C | 10.36 | 11.4 | 9.81 | 1.46 | 0.0769 | 0.1988 | 9.45 | 8.90 | 8.82 | 1.55 | 0.0065 | 0.0579 |
| TC0300013814.hg.1 | ZNF662 | zinc finger protein 662 | Multiple_C | 5.33 | 4.87 | 4.78 | 1.46 | 0.0453 | 0.1345 | 4.81 | 4.65 | 4.65 | 1.12 | 0.1897 | 0.4423 |
| TC0300013978.hg.1 | USP4 | ubiquitin specific peptidase 4 (proto-oncogene) | Multiple_C | 9.42 | 9.21 | 8.87 | 1.46 | 0.1591 | 0.3353 | 9.19 | 8.88 | 9.58 | 0.76 | 0.0859 | 0.2825 |
| TC0300014048.hg.1 | NPHP3-ACAD1 | NPHP3-ACAD11 readthrough (NMD candidate) | Multiple_C | 7.09 | 8.17 | 6.54 | 1.46 | 0.19 | 0.3778 | 6.4 | 6.35 | 6.38 | 1.01 | 0.4173 | 0.6711 |
| TC0400006718.hg.1 | SORCS2 | sortilin-related VPS10 domain containing receptor 2 | Multiple_C | 4.87 | 4.21 | 4.32 | 1.46 | 0.0744 | 0.1935 | 7.08 | 6.71 | 6.64 | 1.36 | 0.5349 | 0.7569 |
| TC0400008086.hg.1 | IBSP | integrin-binding sialoprotein | Coding | 3.97 | 3.37 | 3.42 | 1.46 | 0.1929 | 0.3818 | 4.57 | 4.55 | 4.03 | 1.45 | 0.1199 | 0.3419 |
| TC0400008093.hg.1 | PKD2 | polycystic kidney disease 2 (autosomal dominant) | Multiple_C | 6.46 | 6.54 | 5.91 | 1.46 | 0.2041 | 0.3966 | 6.25 | 6.69 | 5.66 | 1.51 | 0.8087 | 0.9155 |

| | | | | | | | | | | | | | | | |
|---|---|---|---|---|---|---|---|---|---|---|---|---|---|---|---|
| TC0400011405.hg.1 | ADH6 | alcohol dehydrogenase 6 (class V) | Multiple_C | 3.88 | 3.2 | 3.33 | 1.46 | 0.0227 | 0.0793 | 4.1 | 4.11 | 3.98 | 1.09 | 0.9584 | 0.9831 |
| TC0400012945.hg.1 | HSD17B11 | hydroxysteroid (17-beta) dehydrogenase 11 | Multiple_C | 14.52 | 15.64 | 13.97 | 1.46 | 0.2891 | 0.4998 | 11.52 | 11.39 | 11.62 | 0.93 | 0.8297 | 0.9244 |
| TC0500009055.hg.1 | ABLIM3 | actin binding LIM protein family, member 3 | Multiple_C | 4.87 | 3.95 | 4.32 | 1.46 | 0.0424 | 0.1284 | 5.4 | 5.84 | 5.83 | 0.74 | 0.0589 | 0.2289 |
| TC0500012282.hg.1 | HDAC3 | histone deacetylase 3 | Multiple_C | 11.31 | 10.87 | 10.76 | 1.46 | 0.1222 | 0.2785 | 10.06 | 10.96 | 11.09 | 0.49 | 0.007 | 0.061 |
| TC0600009886.hg.1 | VIP | vasoactive intestinal peptide | Coding | 3.24 | 2.82 | 2.69 | 1.46 | 0.5249 | 0.7172 | 3.92 | 3.59 | 3.51 | 1.33 | 0.115 | 0.3348 |
| TC0600012032.hg.1 | PKHD1 | polycystic kidney and hepatic disease 1 (autosomal recessive) | Multiple_C | 4.2 | 3.42 | 3.65 | 1.46 | 0.0977 | 0.2367 | 3.99 | 3.80 | 3.86 | 1.09 | 0.5797 | 0.785 |
| TC0700008625.hg.1 | PRKRIP1 | PRKR interacting protein 1 (IL11 inducible) | Multiple_C | 9.31 | 9.29 | 8.76 | 1.46 | 0.0138 | 0.0537 | 8.87 | 8.74 | 8.9 | 0.98 | 0.8843 | 0.9504 |
| TC0700009606.hg.1 | ZNF282 | zinc finger protein 282 | Multiple_C | 9.59 | 8.87 | 9.04 | 1.46 | 0.1389 | 0.3052 | 9.54 | 9.62 | 10.03 | 0.71 | 0.0786 | 0.2702 |
| TC0700013501.hg.1 | COX19 | COX19 cytochrome c oxidase assembly factor | Multiple_C | 7.77 | 7.56 | 7.22 | 1.46 | 0.0145 | 0.0555 | 6.27 | 6.60 | 5.99 | 1.21 | 0.5734 | 0.781 |
| TC0800007579.hg.1 | C8orf22 | chromosome 8 open reading frame 22 | Coding | 3.45 | 2.69 | 2.9 | 1.46 | 0.349 | 0.5618 | 4.36 | 4.41 | 4.09 | 1.21 | 0.1173 | 0.3386 |
| TC0800008493.hg.1 | DCSTAMP | dendrocyte expressed seven transmembrane protein | Multiple_C | 4.99 | 4.48 | 4.44 | 1.46 | 0.0257 | 0.0872 | 5.59 | 5.30 | 5.07 | 1.43 | 0.0477 | 0.2026 |
| TC0800009465.hg.1 | SPAG11B | sperm associated antigen 11B | Coding | 4.42 | 3.61 | 3.87 | 1.46 | 0.2177 | 0.4138 | 4.2 | 4.50 | 4.28 | 0.95 | 0.6239 | 0.8143 |
| TC0900009643.hg.1 | SAXO1 | stabilizer of axonemal microtubules 1 | Multiple_C | 4.36 | 3.56 | 3.81 | 1.46 | 0.3273 | 0.5412 | 3.89 | 3.58 | 3.64 | 1.19 | 0.1672 | 0.4126 |
| TC1000009444.hg.1 | ADGRA1 | adhesion G protein-coupled receptor A1 | Multiple_C | 5.52 | 4.84 | 4.97 | 1.46 | 0.0474 | 0.1391 | 7.02 | 7.13 | 6.94 | 1.06 | 0.282 | 0.5475 |
| TC1000010249.hg.1 | KIF5B | kinesin family member 5B | Multiple_C | 14.69 | 14.26 | 14.14 | 1.46 | 0.0338 | 0.1078 | 14.2 | 13.31 | 13.59 | 1.53 | 0.0193 | 0.1174 |
| TC1000011696.hg.1 | CYP17A1 | cytochrome P450, family 17, subfamily A, polypeptide 1 | Multiple_C | 5.66 | 4.95 | 5.11 | 1.46 | 0.0477 | 0.1396 | 5.14 | 5.28 | 5.14 | 1.00 | 0.6434 | 0.8252 |
| TC1100008208.hg.1 | IGHMBP2 | immunoglobulin mu binding protein 2 | Multiple_C | 9.6 | 8.96 | 9.05 | 1.46 | 0.2084 | 0.4021 | 8.82 | 8.88 | 8.83 | 0.99 | 0.5225 | 0.7487 |
| TC1100012990.hg.1 | MIR670HG; H: | MIR670 host gene; hydroxysteroid (17-beta) dehydrogenase 12 | Multiple_C | 3.69 | 3.07 | 3.14 | 1.46 | 0.1377 | 0.3032 | 3.3 | 3.51 | 3.67 | 0.77 | 0.9507 | 0.9797 |
| TC1100013127.hg.1 | KRTAP5-4 | keratin associated protein 5-4 | Coding | 6.1 | 5.37 | 5.55 | 1.46 | 0.0402 | 0.1232 | 6.08 | 5.47 | 5.48 | 1.52 | 0.0011 | 0.0175 |
| TC1200008477.hg.1 | CRADD | CASP2 and RIPK1 domain containing adaptor with death domair | Multiple_C | 7.48 | 7.44 | 6.93 | 1.46 | 0.5336 | 0.7232 | 5.54 | 6.14 | 6.27 | 0.60 | 0.0098 | 0.0766 |
| TC1200009892.hg.1 | OLR1 | oxidized low density lipoprotein (lectin-like) receptor 1 | Multiple_C | 4.13 | 3.55 | 3.58 | 1.46 | 0.1145 | 0.2657 | 3.68 | 3.55 | 3.43 | 1.19 | 0.2477 | 0.5122 |
| TC1200012876.hg.1 | CHFR | checkpoint with forkhead and ring finger domains, E3 ubiquitin | Multiple_C | 4.87 | 5.2 | 4.32 | 1.46 | 0.1138 | 0.2646 | 7.71 | 7.92 | 8.2 | 0.71 | 0.156 | 0.3966 |
| TC1500008986.hg.1 | LPCAT4 | lysophosphatidylcholine acyltransferase 4 | Multiple_C | 9.9 | 9.55 | 9.35 | 1.46 | 0.2444 | 0.4478 | 8.47 | 7.66 | 7.97 | 1.41 | 0.0788 | 0.2704 |
| TC1500009641.hg.1 | RORA | RAR-related orphan receptor A | Multiple_C | 3.42 | 2.79 | 2.87 | 1.46 | 0.3412 | 0.5549 | 7.87 | 7.03 | 6.27 | 3.03 | 0.0003 | 0.008 |
| TC1500010396.hg.1 | KIF7 | kinesin family member 7 | Multiple_C | 7.07 | 6.48 | 6.52 | 1.46 | 0.0378 | 0.1177 | 6.09 | 6.11 | 6.06 | 1.02 | 0.9025 | 0.9596 |
| TC1600009022.hg.1 | TPSB2; TPSAB | tryptase beta 2 (gene/pseudogene); tryptase alpha/beta 1 | Coding | 4.53 | 3.81 | 3.98 | 1.46 | 0.0432 | 0.1301 | 5.02 | 4.89 | 4.83 | 1.14 | 0.0679 | 0.2481 |
| TC1600009137.hg.1 | TCEB2 | transcription elongation factor B (SIII), polypeptide 2 (18kDa, ele | Multiple_C | 13.19 | 13.16 | 12.64 | 1.46 | 0.0604 | 0.1666 | 8.01 | 8.67 | 7.93 | 1.06 | 0.3704 | 0.6327 |
| TC1600011223.hg.1 | SNAI3 | snail family zinc finger 3 | Coding | 4.97 | 4.24 | 4.42 | 1.46 | 0.0216 | 0.076 | 4.56 | 4.59 | 4.41 | 1.11 | 0.4434 | 0.6916 |
| TC1700006703.hg.1 | C17orf100 | chromosome 17 open reading frame 100 | Multiple_C | 3.55 | 2.93 | 3 | 1.46 | 0.0365 | 0.1146 | 4.38 | 4.22 | 3.7 | 1.60 | 0.0225 | 0.1292 |
| TC1700007068.hg.1 | MED9 | mediator complex subunit 9 | Multiple_C | 6.54 | 5.65 | 5.99 | 1.46 | 0.0483 | 0.141 | 6.37 | 6.27 | 6.33 | 1.03 | 0.4813 | 0.7197 |
| TC1700008834.hg.1 | LOC10013439 | uncharacterized LOC100134391; novel transcript | Multiple_C | 4.26 | 3.59 | 3.71 | 1.46 | 0.0083 | 0.0361 | 3.54 | 3.72 | 3.81 | 0.83 | 0.0809 | 0.2742 |
| TC1700008933.hg.1 | GALR2 | galanin receptor 2 | Coding | 5.8 | 5.18 | 5.25 | 1.46 | 0.1894 | 0.3771 | 4.19 | 4.29 | 4.07 | 1.09 | 0.9976 | 0.9988 |
| TC1700009544.hg.1 | INCA1 | inhibitor of CDK, cyclin A1 interacting protein 1 | Coding | 6.38 | 5.45 | 5.83 | 1.46 | 0.1732 | 0.3549 | 6.27 | 6.65 | 6.76 | 0.71 | 0.5439 | 0.7622 |

| | | | | | | | | | | | | | | |
|---|---|---|---|---|---|---|---|---|---|---|---|---|---|---|
| TC1700011117.hg.1 | LINC00483 | long intergenic non-protein coding RNA 483 | Multiple_C | 4.64 | 3.84 | 4.09 | 1.46 | 0.5597 | 0.7423 | 4.06 | 4.15 | 3.8 | 1.20 | 0.5573 | 0.771 |
| TC1700011697.hg.1 | CD300LB | CD300 molecule-like family member b | Multiple_C | 5.93 | 5.53 | 5.38 | 1.46 | 0.1238 | 0.2808 | 6.19 | 5.84 | 5.96 | 1.17 | 0.1579 | 0.3992 |
| TC1700012226.hg.1 | ADAP2 | ArfGAP with dual PH domains 2 | Multiple_C | 4.47 | 3.97 | 3.92 | 1.46 | 0.1323 | 0.295 | 3.76 | 3.55 | 3.6 | 1.12 | 0.7741 | 0.8985 |
| TC1900007988.hg.1 | SIPA1L3 | signal-induced proliferation-associated 1 like 3 | Multiple_C | 8.92 | 7.81 | 8.37 | 1.46 | 0.0063 | 0.0289 | 10.69 | 10.89 | 10.89 | 0.87 | 0.2105 | 0.4678 |
| TC1900008946.hg.1 | GALP | galanin-like peptide | Coding | 5.87 | 5.3 | 5.32 | 1.46 | 0.0661 | 0.1781 | 5.66 | 5.62 | 5.61 | 1.04 | 0.8523 | 0.9361 |
| TC1900009442.hg.1 | TNFSF14 | tumor necrosis factor (ligand) superfamily, member 14 | Multiple_C | 3.7 | 3.04 | 3.15 | 1.46 | 0.0716 | 0.1886 | 4.37 | 4.35 | 4.45 | 0.95 | 0.9014 | 0.959 |
| TC1900010818.hg.1 | PSG1 | pregnancy specific beta-1-glycoprotein 1 | Multiple_C | 3.75 | 2.87 | 3.2 | 1.46 | 0.088 | 0.2193 | 3.97 | 4.15 | 4.1 | 0.91 | 0.7632 | 0.8931 |
| TC1900011758.hg.1 | APOE | apolipoprotein E | Multiple_C | 5.74 | 5.4 | 5.19 | 1.46 | 0.0656 | 0.1772 | 7.32 | 6.63 | 6.41 | 1.88 | 0.0014 | 0.0205 |
| TC2000008669.hg.1 | CST11 | cystatin 11 | Coding | 3.77 | 3.3 | 3.22 | 1.46 | 0.0373 | 0.1165 | 4.13 | 4.05 | 3.72 | 1.33 | 0.0609 | 0.2344 |
| TC2100006882.hg.1 | KRTAP20-2 | keratin associated protein 20-2 | Coding | 3.93 | 3.29 | 3.38 | 1.46 | 0.4094 | 0.6217 | 4.67 | 4.65 | 4.51 | 1.12 | 0.2428 | 0.5064 |
| TSUnmapped00000801.h | VPS11 | VPS11, CORVET/HOPS core subunit [Source:HGNC Symbol;Acc:H | Coding | 9.63 | 8.83 | 9.08 | 1.46 | 0.1421 | 0.31 | 10.46 | 11.05 | 10.79 | 0.80 | 0.2306 | 0.4919 |
| TC0100007188.hg.1 | PLA2G5 | phospholipase A2, group V | Multiple_C | 5.74 | 5.25 | 5.2 | 1.45 | 0.0198 | 0.0711 | 4.98 | 5.06 | 5.02 | 0.97 | 0.3263 | 0.5903 |
| TC0100009474.hg.1 | MAGI3 | membrane associated guanylate kinase, WW and PDZ domain c | Multiple_C | 8.22 | 7.49 | 7.68 | 1.45 | 0.0724 | 0.1899 | 6.75 | 6.87 | 7.71 | 0.51 | 0.0014 | 0.0215 |
| TC0100009560.hg.1 | CD101 | CD101 molecule | Multiple_C | 5.79 | 4.78 | 5.25 | 1.45 | 0.0535 | 0.1519 | 4.6 | 4.71 | 4.35 | 1.19 | 0.2276 | 0.4882 |
| TC0100013192.hg.1 | SH2D5 | SH2 domain containing 5 | Multiple_C | 5.45 | 4.97 | 4.91 | 1.45 | 0.0123 | 0.049 | 5.65 | 5.46 | 5.3 | 1.27 | 0.2456 | 0.51 |
| TC0100013757.hg.1 | MTF1 | metal-regulatory transcription factor 1 | Multiple_C | 10.12 | 8.68 | 9.58 | 1.45 | 0.1645 | 0.3428 | 9.87 | 8.69 | 9.71 | 1.12 | 0.8078 | 0.9153 |
| TC0100015824.hg.1 | LOC10013110 | putative UPF0607 protein ENSP00000383783 | Coding | 3.35 | 2.52 | 2.81 | 1.45 | 0.0456 | 0.135 | 4.29 | 3.89 | 3.72 | 1.48 | 0.0101 | 0.0782 |
| TC0100016053.hg.1 | CD5L | CD5 molecule-like | Multiple_C | 4.57 | 4 | 4.03 | 1.45 | 0.0383 | 0.1187 | 4.04 | 4.10 | 4.21 | 0.89 | 0.4223 | 0.6752 |
| TC0200007205.hg.1 | RASGRP3 | RAS guanyl releasing protein 3 (calcium and DAG-regulated) | Multiple_C | 4.92 | 4.35 | 4.38 | 1.45 | 0.1535 | 0.3271 | 4.56 | 4.30 | 4.23 | 1.26 | 0.93 | 0.9704 |
| TC0200007458.hg.1 | EPAS1 | endothelial PAS domain protein 1 | Multiple_C | 8.68 | 7.94 | 8.14 | 1.45 | 0.0423 | 0.128 | 5.25 | 5.06 | 4.84 | 1.33 | 0.0553 | 0.2209 |
| TC0200010253.hg.1 | ANKAR | ankyrin and armadillo repeat containing | Multiple_C | 5.82 | 5.65 | 5.28 | 1.45 | 0.0083 | 0.0361 | 5.42 | 5.64 | 5.75 | 0.80 | 0.1754 | 0.4234 |
| TC0300009363.hg.1 | OTOL1 | otolin 1 | Coding | 3.94 | 3.06 | 3.4 | 1.45 | 0.0433 | 0.1304 | 3.14 | 3.18 | 3.2 | 0.96 | 0.925 | 0.9693 |
| TC0300010739.hg.1 | PLCD1 | phospholipase C, delta 1 | Multiple_C | 5.83 | 6.02 | 5.29 | 1.45 | 0.1491 | 0.3206 | 4.08 | 3.99 | 3.87 | 1.16 | 0.3027 | 0.5669 |
| TC0300010772.hg.1 | XIRP1 | xin actin binding repeat containing 1 | Coding | 4.43 | 4.06 | 3.89 | 1.45 | 0.0369 | 0.1156 | 4.19 | 4.25 | 4.13 | 1.04 | 0.3352 | 0.5994 |
| TC0300010955.hg.1 | ALS2CL | ALS2 C-terminal like | Multiple_C | 4.2 | 4.46 | 3.66 | 1.45 | 0.0337 | 0.1076 | 3.97 | 3.62 | 3.6 | 1.29 | 0.0286 | 0.149 |
| TC0300013908.hg.1 | FXR1 | fragile X mental retardation, autosomal homolog 1 | Multiple_C | 14.5 | 16.03 | 13.96 | 1.45 | 0.0862 | 0.216 | 14.27 | 12.82 | 13.14 | 2.19 | 0.0018 | 0.025 |
| TC0400012212.hg.1 | ASIC5 | acid sensing ion channel family member 5 | Coding | 3.68 | 3.33 | 3.14 | 1.45 | 0.1003 | 0.2414 | 3.52 | 3.69 | 3.61 | 0.94 | 0.7491 | 0.8855 |
| TC0400012922.hg.1 | TLR6 | toll-like receptor 6 | Multiple_C | 8.23 | 8.3 | 7.69 | 1.45 | 0.2899 | 0.5008 | 4.37 | 4.54 | 5.15 | 0.58 | 0.0072 | 0.0624 |
| TC0500009031.hg.1 | SPINK5 | serine peptidase inhibitor, Kazal type 5 | Multiple_C | 4.88 | 4.3 | 4.34 | 1.45 | 0.2027 | 0.3949 | 4.12 | 4.67 | 4.76 | 0.64 | 0.2367 | 0.4998 |
| TC0500009464.hg.1 | LOC10192809 | uncharacterized LOC101928093; novel transcript | Multiple_C | 5.09 | 4.66 | 4.55 | 1.45 | 0.1032 | 0.2466 | 4.92 | 5.40 | 5.05 | 0.91 | 0.8001 | 0.9105 |
| TC0500010540.hg.1 | LIFR | leukemia inhibitory factor receptor alpha | Multiple_C | 3.42 | 2.89 | 2.88 | 1.45 | 0.038 | 0.118 | 3.56 | 3.56 | 3.44 | 1.09 | 0.529 | 0.7526 |
| TC0600007412.hg.1 | NKAPL | NFKB activating protein-like | Coding | 3.88 | 3.41 | 3.34 | 1.45 | 0.0104 | 0.0431 | 3.49 | 3.74 | 3.9 | 0.75 | 0.0358 | 0.1712 |
| TC0600007848.hg.1 | RAB44 | RAB44, member RAS oncogene family | Multiple_C | 5.32 | 4.91 | 4.78 | 1.45 | 0.1228 | 0.2795 | 4.79 | 4.59 | 4.64 | 1.11 | 0.9343 | 0.9724 |

| | | | | | | | | | | | | | | | |
|---|---|---|---|---|---|---|---|---|---|---|---|---|---|---|---|
| TC0600008050.hg.1 | UBR2 | ubiquitin protein ligase E3 component n-recognin 2 | Multiple_C | 10.72 | 10.44 | 10.18 | 1.45 | 0.1096 | 0.2577 | 10.26 | 9.47 | 10.37 | 0.93 | 0.3094 | 0.5733 |
| TC0600008447.hg.1 | ADGRB3 | adhesion G protein-coupled receptor B3 | Multiple_C | 3.05 | 2.4 | 2.51 | 1.45 | 0.0909 | 0.2246 | 3.63 | 3.91 | 3.68 | 0.97 | 0.6123 | 0.8064 |
| TC0600010572.hg.1 | TUBB2B | tubulin, beta 2B class IIb | Multiple_C | 5.43 | 4.9 | 4.89 | 1.45 | 0.1365 | 0.3014 | 6.26 | 5.17 | 5.12 | 2.20 | 0.0002 | 0.0058 |
| TC0700010463.hg.1 | IGF2BP3 | insulin-like growth factor 2 mRNA binding protein 3 | Multiple_C | 8.41 | 8.12 | 7.87 | 1.45 | 0.1168 | 0.2694 | 10.85 | 10.69 | 11.13 | 0.82 | 0.1048 | 0.3174 |
| TC0700012813.hg.1 | DENND2A | DENN/MADD domain containing 2A | Multiple_C | 6.1 | 5.63 | 5.56 | 1.45 | 0.0209 | 0.0742 | 5.17 | 4.79 | 4.64 | 1.44 | 0.0233 | 0.1319 |
| TC0800008243.hg.1 | PDP1 | pyruvate dehyrogenase phosphatase catalytic subunit 1 | Multiple_C | 11.79 | 11.17 | 11.25 | 1.45 | 0.0557 | 0.1566 | 10.11 | 8.12 | 9.07 | 2.06 | 0.0005 | 0.0104 |
| TC0800009462.hg.1 | ZNF705G | zinc finger protein 705G | Coding | 4.89 | 4.52 | 4.35 | 1.45 | 0.3579 | 0.5714 | 4.26 | 3.80 | 3.88 | 1.30 | 0.5492 | 0.7657 |
| TC0800012206.hg.1 | DGAT1; MIR68 | diacylglycerol O-acyltransferase 1; microRNA 6848 | Multiple_C | 12.06 | 11.08 | 11.52 | 1.45 | 0.2595 | 0.4659 | 8.91 | 9.16 | 9.53 | 0.65 | 0.0102 | 0.0789 |
| TC0900007050.hg.1 | DNAI1 | Transcript Identified by AceView, Entrez Gene ID(s) 27019 | Coding | 7.39 | 6.51 | 6.85 | 1.45 | 0.147 | 0.3176 | 6.18 | 6.15 | 6.07 | 1.08 | 0.2965 | 0.5611 |
| TC0900007593.hg.1 | RORB | RAR-related orphan receptor B | Coding | 4.73 | 4.03 | 4.19 | 1.45 | 0.0066 | 0.0299 | 4.6 | 4.41 | 4.43 | 1.13 | 0.2853 | 0.5503 |
| TC0900008839.hg.1 | SLC25A25 | solute carrier family 25 (mitochondrial carrier; phosphate carrie | Multiple_C | 6.95 | 6.05 | 6.41 | 1.45 | 0.0811 | 0.2069 | 6.4 | 5.93 | 6.35 | 1.04 | 0.9448 | 0.9776 |
| TC0900008951.hg.1 | HMCN2 | hemicentin 2 | Multiple_C | 4.27 | 3.55 | 3.73 | 1.45 | 0.2849 | 0.4953 | 3.84 | 3.71 | 3.85 | 0.99 | 0.7079 | 0.8625 |
| TC0900010147.hg.1 | CNTNAP3B | contactin associated protein-like 3B | Multiple_C | 4.77 | 4.17 | 4.23 | 1.45 | 0.0794 | 0.2039 | 5.63 | 5.43 | 4.66 | 1.96 | 0.0069 | 0.0606 |
| TC0900010933.hg.1 | CTSV | cathepsin V | Multiple_C | 10.08 | 10.4 | 9.54 | 1.45 | 0.2224 | 0.4202 | 6.39 | 6.05 | 5.86 | 1.44 | 0.1002 | 0.3094 |
| TC0X00007213.hg.1 | CCDC120 | coiled-coil domain containing 120 | Multiple_C | 6.48 | 5.87 | 5.94 | 1.45 | 0.2341 | 0.435 | 6.52 | 6.63 | 6.78 | 0.84 | 0.2603 | 0.5245 |
| TC0X00008786.hg.1 | ATP2B3 | ATPase, Ca++ transporting, plasma membrane 3 | Multiple_C | 7.16 | 6.46 | 6.62 | 1.45 | 0.037 | 0.1159 | 5.79 | 5.92 | 6.09 | 0.81 | 0.3948 | 0.653 |
| TC0X00010375.hg.1 | ZMAT1 | zinc finger, matrin-type 1 | Multiple_C | 4.73 | 4.52 | 4.19 | 1.45 | 0.4102 | 0.6225 | 5.16 | 4.77 | 4.29 | 1.83 | 0.0026 | 0.0319 |
| TC1000008891.hg.1 | DUSP5 | dual specificity phosphatase 5 | Multiple_C | 4.83 | 4.66 | 4.29 | 1.45 | 0.0805 | 0.2059 | 9.16 | 7.07 | 8.78 | 1.30 | 0.0238 | 0.1333 |
| TC1100006529.hg.1 | KRTAP5-6 | keratin associated protein 5-6 | Coding | 5.25 | 5.07 | 4.71 | 1.45 | 0.1236 | 0.2806 | 4.91 | 5.00 | 4.64 | 1.21 | 0.3672 | 0.6295 |
| TC1100008904.hg.1 | YAP1 | Yes-associated protein 1 | Multiple_C | 9.34 | 9.16 | 8.8 | 1.45 | 0.3242 | 0.5382 | 12.59 | 13.04 | 12.49 | 1.07 | 0.8114 | 0.9166 |
| TC1100009407.hg.1 | CCDC15 | coiled-coil domain containing 15 | Multiple_C | 5.23 | 4.51 | 4.69 | 1.45 | 0.0058 | 0.027 | 4.41 | 4.73 | 4.83 | 0.75 | 0.2134 | 0.4716 |
| TC1100010236.hg.1 | KCNJ11 | potassium channel, inwardly rectifying subfamily J, member 11 | Multiple_C | 5.61 | 4.83 | 5.07 | 1.45 | 0.0704 | 0.1862 | 5.19 | 5.00 | 5.15 | 1.03 | 0.9964 | 0.9983 |
| TC1100013133.hg.1 | HBG2; HBG1 | hemoglobin, gamma G; hemoglobin, gamma A | Multiple_C | 4.24 | 3.49 | 3.7 | 1.45 | 0.0751 | 0.195 | 3.47 | 3.48 | 3.62 | 0.90 | 0.7512 | 0.8867 |
| TC1200006629.hg.1 | GAPDH | glyceraldehyde-3-phosphate dehydrogenase | Multiple_C | 17.66 | 16.5 | 17.12 | 1.45 | 0.0591 | 0.1638 | 18.31 | 18.75 | 18.48 | 0.89 | 0.1826 | 0.4332 |
| TC1200007535.hg.1 | CACNB3 | calcium channel, voltage-dependent, beta 3 subunit | Multiple_C | 8.64 | 7.89 | 8.1 | 1.45 | 0.0741 | 0.193 | 6.37 | 6.24 | 6.02 | 1.27 | 0.0698 | 0.2523 |
| TC1200008222.hg.1 | GLIPR1L1 | GLI pathogenesis-related 1 like 1 | Multiple_C | 4.76 | 4.12 | 4.22 | 1.45 | 0.0736 | 0.1922 | 5.08 | 5.68 | 5.1 | 0.99 | 0.5432 | 0.7617 |
| TC1200008873.hg.1 | SH2B3 | SH2B adaptor protein 3 | Multiple_C | 6.97 | 6.38 | 6.43 | 1.45 | 0.0687 | 0.1829 | 6.58 | 6.74 | 6.93 | 0.78 | 0.2028 | 0.4584 |
| TC1300006810.hg.1 | PDS5B | PDS5 cohesin associated factor B | Multiple_C | 11.32 | 11.33 | 10.78 | 1.45 | 0.1671 | 0.3466 | 11.23 | 10.93 | 10.32 | 1.88 | 0.1094 | 0.3256 |
| TC1300008212.hg.1 | TUBA3C | tubulin, alpha 3c | Coding | 7.88 | 7.16 | 7.34 | 1.45 | 0.0464 | 0.1368 | 6.8 | 7.20 | 7.23 | 0.74 | 0.2349 | 0.4975 |
| TC1300008793.hg.1 | TSC22D1 | TSC22 domain family, member 1 | Multiple_C | 12.96 | 12.58 | 12.42 | 1.45 | 0.0507 | 0.146 | 12.4 | 12.84 | 11.63 | 1.71 | 0.0027 | 0.0329 |
| TC1300008813.hg.1 | KCTD4 | potassium channel tetramerization domain containing 4 | Coding | 4.08 | 3.44 | 3.54 | 1.45 | 0.0223 | 0.078 | 3.96 | 4.08 | 4.06 | 0.93 | 0.2628 | 0.5273 |
| TC1300009725.hg.1 | FAM155A | family with sequence similarity 155, member A | Coding | 5.38 | 5.08 | 4.84 | 1.45 | 0.1447 | 0.3144 | 4.65 | 4.97 | 4.75 | 0.93 | 0.4871 | 0.7237 |
| TC1400009066.hg.1 | MIS18BP1 | MIS18 binding protein 1 | Multiple_C | 9 | 8.41 | 8.46 | 1.45 | 0.2611 | 0.4679 | 9.64 | 10.04 | 9.76 | 0.92 | 0.6682 | 0.8377 |

| | | | | | | | | | | | | | | | |
|---|---|---|---|---|---|---|---|---|---|---|---|---|---|---|---|
| TC1400010019.hg.1 | MOAP1 | modulator of apoptosis 1 | Coding | 7.54 | 7.56 | 7 | 1.45 | 0.088 | 0.2193 | 6.55 | 6.48 | 6.06 | 1.40 | 0.0427 | 0.1906 |
| TC1500007304.hg.1 | C15orf65 | chromosome 15 open reading frame 65 | Multiple_C | 4.89 | 5.9 | 4.35 | 1.45 | 0.0578 | 0.1611 | 4.96 | 4.98 | 5.01 | 0.97 | 0.9683 | 0.9868 |
| TC1600007189.hg.1 | VWA3A | von Willebrand factor A domain containing 3A | Multiple_C | 3.36 | 2.63 | 2.82 | 1.45 | 0.0328 | 0.1055 | 3.63 | 3.76 | 3.74 | 0.93 | 0.5694 | 0.778 |
| TC1600010007.hg.1 | PRSS36 | protease, serine 36 | Multiple_C | 3.69 | 3.01 | 3.15 | 1.45 | 0.085 | 0.2138 | 3.82 | 4.12 | 3.67 | 1.11 | 0.6655 | 0.8363 |
| TC1600010664.hg.1 | DPEP3 | dipeptidase 3 | Multiple_C | 3.7 | 3 | 3.16 | 1.45 | 0.0213 | 0.0753 | 4.12 | 4.08 | 3.92 | 1.15 | 0.1868 | 0.4387 |
| TC1700007288.hg.1 | FAM27L | family with sequence similarity 27-like | Multiple_C | 3.9 | 3.51 | 3.36 | 1.45 | 0.0174 | 0.0641 | 4.17 | 3.97 | 3.95 | 1.16 | 0.6469 | 0.8269 |
| TC1700008539.hg.1 | MRC2 | mannose receptor, C type 2 | Multiple_C | 4.92 | 4.09 | 4.38 | 1.45 | 0.1161 | 0.2685 | 4.66 | 4.65 | 4.62 | 1.03 | 0.7389 | 0.88 |
| TC1700009221.hg.1 | GPS1 | G protein pathway suppressor 1 | Multiple_C | 12.11 | 10.61 | 11.57 | 1.45 | 0.0857 | 0.2148 | 12.46 | 13.16 | 13.27 | 0.57 | 0.0123 | 0.0886 |
| TC1700009982.hg.1 | USP32P2; FAN | ubiquitin specific peptidase 32 pseudogene 2; family with seque | Multiple_C | 10.06 | 10.03 | 9.52 | 1.45 | 0.0281 | 0.0933 | 9.25 | 8.56 | 8.85 | 1.32 | 0.3249 | 0.5885 |
| TC1700012442.hg.1 | TBC1D3P1-DH | TBC1D3P1-DHX40P1 readthrough transcribed pseudogene | Multiple_C | 9.13 | 9.18 | 8.59 | 1.45 | 0.5445 | 0.7308 | 8.19 | 8.17 | 7.97 | 1.16 | 0.7969 | 0.9093 |
| TC1900006688.hg.1 | MPND | MPN domain containing | Multiple_C | 6.81 | 6.35 | 6.27 | 1.45 | 0.0853 | 0.2144 | 5.71 | 5.98 | 5.73 | 0.99 | 0.4785 | 0.7171 |
| TC1900007001.hg.1 | C19orf38 | chromosome 19 open reading frame 38 | Multiple_C | 4.59 | 4.45 | 4.05 | 1.45 | 0.0139 | 0.0539 | 3.56 | 3.54 | 3.41 | 1.11 | 0.2753 | 0.5414 |
| TC1900008746.hg.1 | ZNF845 | zinc finger protein 845 | Coding | 10.94 | 11.21 | 10.4 | 1.45 | 0.6932 | 0.8348 | 8.65 | 8.50 | 8.75 | 0.93 | 0.1504 | 0.3883 |
| TC1900011606.hg.1 | ZNF329 | zinc finger protein 329 | Multiple_C | 7.09 | 7.25 | 6.55 | 1.45 | 0.0871 | 0.2176 | 5.47 | 5.76 | 6.17 | 0.62 | 0.0677 | 0.2478 |
| TC1900012055.hg.1 | VN1R1 | vomeronasal 1 receptor 1 | Multiple_C | 5.77 | 5.44 | 5.23 | 1.45 | 0.0185 | 0.0673 | 4.61 | 4.78 | 4.78 | 0.89 | 0.9791 | 0.9912 |
| TC2000007073.hg.1 | REM1 | RAS (RAD and GEM)-like GTP-binding 1 | Coding | 5.36 | 4.59 | 4.82 | 1.45 | 0.0291 | 0.0958 | 4.6 | 4.71 | 4.97 | 0.77 | 0.056 | 0.2224 |
| TC2000008499.hg.1 | RRBP1 | ribosome binding protein 1 | Multiple_C | 12.1 | 12.16 | 11.56 | 1.45 | 0.1807 | 0.365 | 9.37 | 10.13 | 10.33 | 0.51 | 0.0169 | 0.1084 |
| TC2100006918.hg.1 | MRAP | melanocortin 2 receptor accessory protein | Multiple_C | 5.44 | 4.77 | 4.9 | 1.45 | 0.0591 | 0.1639 | 4.57 | 4.93 | 4.64 | 0.95 | 0.5255 | 0.7501 |
| TC2200008706.hg.1 | FAM227A | family with sequence similarity 227, member A | Multiple_C | 5.16 | 4.59 | 4.62 | 1.45 | 0.158 | 0.3339 | 7.01 | 6.72 | 5.97 | 2.06 | 0.039 | 0.18 |
| TSUnmapped00000031.h | SAG | S-antigen; retina and pineal gland (arrestin) | Coding | 4.85 | 4.31 | 4.31 | 1.45 | 0.1634 | 0.3416 | 4.01 | 4.05 | 3.86 | 1.11 | 0.5575 | 0.7711 |
| TC0100007457.hg.1 | LIN28A | lin-28 homolog A (C. elegans) | Multiple_C | 4.87 | 4.46 | 4.34 | 1.44 | 0.2774 | 0.4867 | 4.09 | 4.26 | 4.45 | 0.78 | 0.053 | 0.215 |
| TC0100008084.hg.1 | CCDC24 | coiled-coil domain containing 24 | Multiple_C | 5.21 | 4.77 | 4.68 | 1.44 | 0.0538 | 0.1525 | 5.56 | 5.42 | 5.02 | 1.45 | 0.0211 | 0.124 |
| TC0100009344.hg.1 | SARS | seryl-tRNA synthetase | Multiple_C | 13.09 | 14.01 | 12.56 | 1.44 | 0.543 | 0.7298 | 9.83 | 9.43 | 10.33 | 0.71 | 0.2769 | 0.5428 |
| TC0100011242.hg.1 | TMEM183A | transmembrane protein 183A | Multiple_C | 11.8 | 11.07 | 11.27 | 1.44 | 0.0409 | 0.1247 | 9.88 | 9.72 | 10.36 | 0.72 | 0.0618 | 0.2362 |
| TC0100012473.hg.1 | CCNL2 | cyclin L2 | Multiple_C | 11.73 | 12.54 | 11.2 | 1.44 | 0.0751 | 0.1951 | 11.56 | 11.29 | 10.91 | 1.57 | 0.0035 | 0.0392 |
| TC0100014109.hg.1 | TRABD2B | TraB domain containing 2B | Multiple_C | 4.21 | 3.74 | 3.68 | 1.44 | 0.0274 | 0.0916 | 4.57 | 4.53 | 4.19 | 1.30 | 0.1528 | 0.3919 |
| TC0100016171.hg.1 | MPZ | myelin protein zero | Multiple_C | 4.71 | 4.15 | 4.18 | 1.44 | 0.041 | 0.1248 | 4.38 | 4.17 | 4.18 | 1.15 | 0.6985 | 0.8571 |
| TC0100016633.hg.1 | RGS8 | regulator of G-protein signaling 8 | Multiple_C | 3.82 | 3.1 | 3.29 | 1.44 | 0.0677 | 0.1811 | 3.76 | 3.57 | 3.31 | 1.37 | 0.0962 | 0.3022 |
| TC0100018403.hg.1 | ERRFI1 | ERBB receptor feedback inhibitor 1 | Multiple_C | 16.26 | 13.89 | 15.73 | 1.44 | 0.0519 | 0.1483 | 13.56 | 12.54 | 14.73 | 0.44 | 1.32E-05 | 0.0009 |
| TC0100018466.hg.1 | DENND2C | DENN/MADD domain containing 2C | Multiple_C | 4.1 | 3.13 | 3.57 | 1.44 | 0.0841 | 0.2122 | 4.63 | 4.76 | 4.42 | 1.16 | 0.3548 | 0.6181 |
| TC0200008263.hg.1 | USP39 | ubiquitin specific peptidase 39 | Multiple_C | 13.42 | 12.35 | 12.89 | 1.44 | 0.0662 | 0.1783 | 12.88 | 13.13 | 13.5 | 0.65 | 0.0101 | 0.0784 |
| TC0200008996.hg.1 | ACTR3 | ARP3 actin-related protein 3 homolog (yeast) | Multiple_C | 14.37 | 13.91 | 13.84 | 1.44 | 0.3514 | 0.5642 | 14.05 | 13.49 | 14.02 | 1.02 | 0.6257 | 0.815 |
| TC0200014912.hg.1 | DLX2 | distal-less homeobox 2 | Coding | 6.96 | 5.85 | 6.43 | 1.44 | 0.0617 | 0.169 | 6.89 | 7.15 | 6.8 | 1.06 | 0.7259 | 0.8725 |

| | | | | | | | | | | | | | | | |
|---|---|---|---|---|---|---|---|---|---|---|---|---|---|---|---|
| TC0200016419.hg.1 | GPN1 | GPN-loop GTPase 1 | Multiple_C | 10.73 | 10.97 | 10.2 | 1.44 | 0.1487 | 0.32 | 10.15 | 10.11 | 10.51 | 0.78 | 0.0476 | 0.2025 |
| TC0300008933.hg.1 | CLDN18 | claudin 18 | Coding | 4.19 | 3.57 | 3.66 | 1.44 | 0.1256 | 0.2836 | 4.55 | 4.76 | 4.71 | 0.90 | 0.3401 | 0.604 |
| TC0300010068.hg.1 | LRCH3 | leucine-rich repeats and calponin homology (CH) domain contai | Multiple_C | 9.91 | 10.18 | 9.38 | 1.44 | 0.0634 | 0.1725 | 10.68 | 10.72 | 10.69 | 0.99 | 0.3143 | 0.5782 |
| TC0300011950.hg.1 | GUCA1C | guanylate cyclase activator 1C | Coding | 3.49 | 3.04 | 2.96 | 1.44 | 0.4569 | 0.6633 | 4.59 | 4.59 | 4.57 | 1.01 | 0.314 | 0.578 |
| TC0300013547.hg.1 | FGF12 | fibroblast growth factor 12 | Multiple_C | 3.8 | 3.1 | 3.27 | 1.44 | 0.0748 | 0.1944 | 3.45 | 3.20 | 3.37 | 1.06 | 0.2507 | 0.5149 |
| TC0300013837.hg.1 | IQCF3 | IQ motif containing F3 | Multiple_C | 6.83 | 6.16 | 6.3 | 1.44 | 0.186 | 0.3726 | 6.22 | 6.31 | 6.31 | 0.94 | 0.4737 | 0.7135 |
| TC0300013906.hg.1 | KCNMB2 | potassium channel subfamily M regulatory beta subunit 2 | Multiple_C | 3.9 | 3.26 | 3.37 | 1.44 | 0.0311 | 0.1011 | 3.79 | 3.88 | 3.74 | 1.04 | 0.9798 | 0.9914 |
| TC0300013935.hg.1 | LOC401052; A | uncharacterized LOC401052; Salzman2013 ANNOTATED, coding | Multiple_C | 5.11 | 4.58 | 4.58 | 1.44 | 0.0485 | 0.1414 | 4.33 | 4.39 | 4.22 | 1.08 | 0.2306 | 0.492 |
| TC0400007128.hg.1 | TBC1D19 | TBC1 domain family, member 19 | Multiple_C | 7.38 | 7.6 | 6.85 | 1.44 | 0.0516 | 0.1479 | 8.01 | 7.52 | 7.8 | 1.16 | 0.0711 | 0.2552 |
| TC0400007962.hg.1 | BMP3 | bone morphogenetic protein 3 | Coding | 4.53 | 3.96 | 4 | 1.44 | 0.0683 | 0.1822 | 3.85 | 3.90 | 3.76 | 1.06 | 0.4087 | 0.664 |
| TC0400010097.hg.1 | LINC00504 | long intergenic non-protein coding RNA 504 | Multiple_C | 4.08 | 3.64 | 3.55 | 1.44 | 0.0151 | 0.0575 | 3.59 | 3.35 | 3.15 | 1.36 | 0.067 | 0.2469 |
| TC0400011005.hg.1 | RASSF6 | Ras association (RalGDS/AF-6) domain family member 6 | Multiple_C | 6.9 | 6.9 | 6.37 | 1.44 | 0.2382 | 0.4399 | 9.71 | 9.39 | 10.01 | 0.81 | 0.237 | 0.5003 |
| TC0400011920.hg.1 | SLC7A11 | solute carrier family 7 (anionic amino acid transporter light chai | Multiple_C | 15.31 | 15.11 | 14.78 | 1.44 | 0.3617 | 0.5753 | 8.43 | 7.99 | 9.53 | 0.47 | 0.0453 | 0.1967 |
| TC0400012992.hg.1 | MFAP3L | microfibrillar associated protein 3 like | Coding | 4.48 | 3.77 | 3.95 | 1.44 | 0.1864 | 0.3731 | 3.94 | 3.78 | 3.63 | 1.24 | 0.2052 | 0.4615 |
| TC0500008324.hg.1 | EPB41L4A-AS2 | EPB41L4A antisense RNA 2 (head to head) | Multiple_C | 3.5 | 2.86 | 2.97 | 1.44 | 0.0115 | 0.0465 | 3.75 | 3.89 | 3.54 | 1.16 | 0.4563 | 0.7004 |
| TC0500010795.hg.1 | ANKRD55 | ankyrin repeat domain 55 | Multiple_C | 3.49 | 3.07 | 2.96 | 1.44 | 0.1015 | 0.2435 | 4.64 | 4.43 | 4.12 | 1.43 | 0.0057 | 0.0533 |
| TC0500011255.hg.1 | MTX3 | metaxin 3 | Multiple_C | 5.93 | 5.39 | 5.4 | 1.44 | 0.5181 | 0.7123 | 6.57 | 6.41 | 6.63 | 0.96 | 0.3042 | 0.5679 |
| TC0500013316.hg.1 | CD180 | CD180 molecule | Multiple_C | 3.23 | 2.25 | 2.7 | 1.44 | 0.1906 | 0.3787 | 3.83 | 3.67 | 3.76 | 1.05 | 0.81 | 0.9158 |
| TC0600007819.hg.1 | CLPSL2 | colipase-like 2 | Multiple_C | 4.35 | 3.81 | 3.82 | 1.44 | 0.7914 | 0.8932 | 3.82 | 4.01 | 4.01 | 0.88 | 0.3811 | 0.6406 |
| TC0600011439.hg.1 | NCR3 | natural cytotoxicity triggering receptor 3 | Multiple_C | 5.21 | 4.7 | 4.68 | 1.44 | 0.0704 | 0.1862 | 4.71 | 4.93 | 4.82 | 0.93 | 0.7377 | 0.8792 |
| TC0600013126.hg.1 | PTPRK | protein tyrosine phosphatase, receptor type, K | Multiple_C | 11.07 | 10.73 | 10.54 | 1.44 | 0.1479 | 0.3187 | 8.9 | 10.27 | 9.51 | 0.66 | 0.0023 | 0.0292 |
| TC0600014265.hg.1 | CLIC1 | chloride intracellular channel 1 | Coding | 14.54 | 14.36 | 14.01 | 1.44 | 0.0443 | 0.1325 | 12.94 | 13.54 | 13.25 | 0.81 | 0.1139 | 0.3328 |
| TC0600014282.hg.1 | RPS10 | ribosomal protein S10 | Multiple_C | 17.53 | 17.78 | 17 | 1.44 | 0.0243 | 0.0836 | 15.53 | 15.02 | 14.46 | 2.10 | 9.03E-05 | 0.0034 |
| TC0700010162.hg.1 | FBXL18 | F-box and leucine-rich repeat protein 18 | Multiple_C | 7.99 | 7 | 7.46 | 1.44 | 0.0288 | 0.0951 | 7.21 | 7.49 | 7.53 | 0.80 | 0.0503 | 0.209 |
| TC0700011156.hg.1 | SEPT14 | septin 14 | Multiple_C | 4.22 | 3.78 | 3.69 | 1.44 | 0.0317 | 0.1026 | 3.82 | 3.59 | 3.6 | 1.16 | 0.2499 | 0.5144 |
| TC0700012754.hg.1 | ATP6V0A4 | ATPase, H+ transporting, lysosomal V0 subunit a4 | Multiple_C | 3.76 | 3.33 | 3.23 | 1.44 | 0.0758 | 0.1964 | 9.73 | 7.30 | 7.57 | 4.47 | 3.95E-07 | 7.92E-05 |
| TC0700013347.hg.1 | AQP1 | aquaporin 1 (Colton blood group) | Multiple_C | 5.61 | 4.87 | 5.08 | 1.44 | 0.0618 | 0.1693 | 5.27 | 5.36 | 5.03 | 1.18 | 0.2735 | 0.5389 |
| TC0700013483.hg.1 | PRSS2 | protease, serine, 2 (trypsin 2) | Multiple_C | 9.87 | 9.56 | 9.34 | 1.44 | 0.7128 | 0.8481 | 7.13 | 7.10 | 7.79 | 0.63 | 0.2629 | 0.5273 |
| TC0800006718.hg.1 | C8orf74 | chromosome 8 open reading frame 74 | Multiple_C | 4.96 | 4.38 | 4.43 | 1.44 | 0.4776 | 0.68 | 4.75 | 4.60 | 4.86 | 0.93 | 0.728 | 0.8739 |
| TC0800007384.hg.1 | IDO2 | indoleamine 2,3-dioxygenase 2 | Multiple_C | 5.48 | 4.78 | 4.95 | 1.44 | 0.0138 | 0.0537 | 5.1 | 5.32 | 5.34 | 0.85 | 0.1776 | 0.4261 |
| TC0800008510.hg.1 | OXR1 | oxidation resistance 1 | Multiple_C | 10.04 | 11.01 | 9.51 | 1.44 | 0.0588 | 0.1633 | 11.31 | 10.22 | 10.08 | 2.35 | 4.56E-05 | 0.0021 |
| TC0800009437.hg.1 | DEFA1B; DEFA | defensin, alpha 1B; defensin, alpha 1; defensin, alpha 3, neutrop | Coding | 4.57 | 3.8 | 4.04 | 1.44 | 0.0812 | 0.2071 | 4.13 | 4.29 | 4.07 | 1.04 | 0.972 | 0.9885 |
| TC0800009466.hg.1 | DEFB104A; DE | defensin, beta 104A; defensin, beta 104B | Coding | 3.77 | 3.08 | 3.24 | 1.44 | 0.043 | 0.1297 | 3.43 | 3.66 | 3.87 | 0.74 | 0.0904 | 0.291 |

| | | | | | | | | | | | | | | | |
|---|---|---|---|---|---|---|---|---|---|---|---|---|---|---|---|
| TC0800010426.hg.1 | FAM150A | family with sequence similarity 150, member A | Coding | 3.82 | 3.51 | 3.29 | 1.44 | 0.2316 | 0.4321 | 3.58 | 3.64 | 3.82 | 0.85 | 0.428 | 0.6801 |
| TC0800012281.hg.1 | C8orf58 | chromosome 8 open reading frame 58 | Multiple_C | 4.15 | 3.39 | 3.62 | 1.44 | 0.1032 | 0.2466 | 3.58 | 3.90 | 3.72 | 0.91 | 0.9311 | 0.9709 |
| TC0800012381.hg.1 | DEFA1; DEFA1 | defensin, alpha 1; defensin, alpha 1B | Coding | 4.57 | 3.8 | 4.04 | 1.44 | 0.0812 | 0.2071 | 4.13 | 4.29 | 4.07 | 1.04 | 0.972 | 0.9885 |
| TC0900008277.hg.1 | CYLC2 | cylicin, basic protein of sperm head cytoskeleton 2 | Multiple_C | 4.88 | 4.23 | 4.35 | 1.44 | 0.1008 | 0.2423 | 4.85 | 4.77 | 4.74 | 1.08 | 0.782 | 0.9022 |
| TC0900011955.hg.1 | CAMSAP1 | calmodulin regulated spectrin-associated protein 1 | Multiple_C | 7.84 | 7.36 | 7.31 | 1.44 | 0.3418 | 0.5556 | 7.7 | 7.68 | 7.7 | 1.00 | 0.0897 | 0.2896 |
| TC0X00006607.hg.1 | AMELX | amelogenin, X-linked | Coding | 4.84 | 3.97 | 4.31 | 1.44 | 0.0852 | 0.2142 | 3.84 | 3.99 | 4.17 | 0.80 | 0.7952 | 0.9087 |
| TC0X00008330.hg.1 | XIAP | X-linked inhibitor of apoptosis, E3 ubiquitin protein ligase | Multiple_C | 9.7 | 10.17 | 9.17 | 1.44 | 0.0649 | 0.1756 | 8.83 | 8.81 | 10.05 | 0.43 | 0.0007 | 0.0125 |
| TC0X00010462.hg.1 | RBM41 | RNA binding motif protein 41 | Multiple_C | 8.74 | 9 | 8.21 | 1.44 | 0.2656 | 0.4732 | 8.89 | 8.58 | 9.11 | 0.86 | 0.1261 | 0.3513 |
| TC0X00011314.hg.1 | BHLHB9 | basic helix-loop-helix domain containing, class B, 9 | Multiple_C | 6.03 | 5.47 | 5.5 | 1.44 | 0.1406 | 0.3079 | 7.31 | 7.62 | 7.52 | 0.86 | 0.7103 | 0.8642 |
| TC1100006748.hg.1 | OR2D3 | olfactory receptor, family 2, subfamily D, member 3 | Coding | 4.4 | 3.87 | 3.87 | 1.44 | 0.1352 | 0.2995 | 3.64 | 3.93 | 3.77 | 0.91 | 0.5444 | 0.7625 |
| TC1100008378.hg.1 | P2RY2 | purinergic receptor P2Y, G-protein coupled, 2 | Multiple_C | 3.66 | 3.24 | 3.13 | 1.44 | 0.0151 | 0.0574 | 4.37 | 4.43 | 4.29 | 1.06 | 0.6308 | 0.8183 |
| TC1100008800.hg.1 | IZUMO1R | IZUMO1 receptor, JUNO | Coding | 5.24 | 4.48 | 4.71 | 1.44 | 0.0209 | 0.0742 | 4.38 | 4.57 | 4.47 | 0.94 | 0.8128 | 0.9168 |
| TC1100009685.hg.1 | IRF7 | interferon regulatory factor 7 | Multiple_C | 4.5 | 3.6 | 3.97 | 1.44 | 0.0181 | 0.0663 | 3.7 | 3.41 | 3.47 | 1.17 | 0.2143 | 0.4727 |
| TC1100009817.hg.1 | PHLDA2 | pleckstrin homology-like domain, family A, member 2 | Multiple_C | 9.82 | 8.07 | 9.29 | 1.44 | 0.1858 | 0.3723 | 6.59 | 6.40 | 7.07 | 0.72 | 0.0639 | 0.2407 |
| TC1100009970.hg.1 | HPX | hemopexin | Multiple_C | 7.2 | 6.37 | 6.67 | 1.44 | 0.0386 | 0.1193 | 5.52 | 5.74 | 5.84 | 0.80 | 0.1863 | 0.4382 |
| TC1100011074.hg.1 | SCGB1D4 | secretoglobin, family 1D, member 4 | Coding | 4.15 | 3.56 | 3.62 | 1.44 | 0.1336 | 0.297 | 4.04 | 4.03 | 3.6 | 1.36 | 0.122 | 0.3447 |
| TC1100011652.hg.1 | GDPD5 | glycerophosphodiester phosphodiesterase domain containing 5 | Multiple_C | 6.2 | 5.33 | 5.67 | 1.44 | 0.0593 | 0.1641 | 5.29 | 5.47 | 5.48 | 0.88 | 0.8922 | 0.9539 |
| TC1100011811.hg.1 | CCDC90B | coiled-coil domain containing 90B | Multiple_C | 11.46 | 12.48 | 10.93 | 1.44 | 0.092 | 0.2266 | 11.75 | 11.63 | 10.88 | 1.83 | 0.018 | 0.1127 |
| TC1100012660.hg.1 | OR8D2 | olfactory receptor, family 8, subfamily D, member 2 (gene/pseu | Multiple_C | 3.84 | 3.38 | 3.31 | 1.44 | 0.0252 | 0.086 | 3.2 | 3.38 | 3.29 | 0.94 | 0.5072 | 0.7382 |
| TC1100013064.hg.1 | KRTAP5-8 | keratin associated protein 5-8 | Coding | 5.51 | 4.79 | 4.98 | 1.44 | 0.4078 | 0.62 | 5.21 | 4.63 | 4.58 | 1.55 | 0.0332 | 0.1639 |
| TC1100013155.hg.1 | DCDC5; DCDC | doublecortin domain containing 5; doublecortin domain contair | Multiple_C | 4.06 | 3.53 | 3.53 | 1.44 | 0.0511 | 0.147 | 4.83 | 4.35 | 4.07 | 1.69 | 0.0038 | 0.0418 |
| TC1200010778.hg.1 | KRT78 | keratin 78, type II | Multiple_C | 6.48 | 5.58 | 5.95 | 1.44 | 0.1071 | 0.2535 | 5.79 | 5.84 | 5.44 | 1.27 | 0.0674 | 0.2474 |
| TC1200010839.hg.1 | ITGA5 | integrin alpha 5 | Multiple_C | 3.12 | 2.34 | 2.59 | 1.44 | 0.0265 | 0.0892 | 8.89 | 8.72 | 8.35 | 1.45 | 0.0163 | 0.1063 |
| TC1200012234.hg.1 | CLIP1 | CAP-GLY domain containing linker protein 1 | Multiple_C | 9.45 | 9.63 | 8.92 | 1.44 | 0.4887 | 0.6891 | 7.13 | 6.89 | 7.3 | 0.89 | 0.2817 | 0.5474 |
| TC1400007168.hg.1 | GPR137C | G protein-coupled receptor 137C | Multiple_C | 5.97 | 5.09 | 5.44 | 1.44 | 0.0619 | 0.1694 | 5.96 | 6.03 | 5.62 | 1.27 | 0.0642 | 0.2411 |
| TC1400009186.hg.1 | ERO1A | endoplasmic reticulum oxidoreductase alpha | Multiple_C | 15.09 | 14.77 | 14.56 | 1.44 | 0.0728 | 0.1908 | 14.96 | 14.06 | 14.42 | 1.45 | 0.0138 | 0.0952 |
| TC1400010022.hg.1 | BTBD7 | BTB (POZ) domain containing 7 | Multiple_C | 10.43 | 9.7 | 9.9 | 1.44 | 0.1245 | 0.2819 | 9.32 | 9.24 | 9.8 | 0.72 | 0.1571 | 0.398 |
| TC1500008635.hg.1 | POTEB2; POTE | POTE ankyrin domain family, member B2; POTE ankyrin domain | Multiple_C | 3.78 | 3.06 | 3.25 | 1.44 | 0.3893 | 0.6032 | 4.5 | 3.83 | 3.67 | 1.78 | 0.1227 | 0.3457 |
| TC1500009386.hg.1 | GABPB1 | GA binding protein transcription factor, beta subunit 1 | Multiple_C | 10.12 | 9.54 | 9.59 | 1.44 | 0.0139 | 0.0538 | 9.55 | 9.32 | 10.13 | 0.67 | 0.0045 | 0.0462 |
| TC1500010319.hg.1 | KLHL25 | kelch-like family member 25 | Multiple_C | 5.13 | 4.41 | 4.6 | 1.44 | 0.0207 | 0.0735 | 5.36 | 5.79 | 6 | 0.64 | 0.0129 | 0.0918 |
| TC1600008633.hg.1 | NECAB2 | N-terminal EF-hand calcium binding protein 2 | Multiple_C | 8 | 7.25 | 7.47 | 1.44 | 0.1185 | 0.2723 | 7.65 | 8.06 | 7.96 | 0.81 | 0.1875 | 0.4394 |
| TC1600011460.hg.1 | SPSB3 | splA/ryanodine receptor domain and SOCS box containing 3 | Multiple_C | 8.52 | 7.92 | 7.99 | 1.44 | 0.0163 | 0.061 | 8.34 | 8.28 | 7.72 | 1.54 | 0.1824 | 0.433 |
| TC1600011461.hg.1 | IGFALS | insulin-like growth factor binding protein, acid labile subunit | Multiple_C | 4.5 | 3.6 | 3.97 | 1.44 | 0.3089 | 0.5221 | 4.43 | 4.33 | 4.32 | 1.08 | 0.6678 | 0.8375 |

| | | | | | | | | | | | | | | | |
|---|---|---|---|---|---|---|---|---|---|---|---|---|---|---|---|
| TC1700006577.hg.1 | OR1A2 | olfactory receptor, family 1, subfamily A, member 2 | Coding | 3.4 | 2.38 | 2.87 | 1.44 | 0.0158 | 0.0594 | 3.51 | 3.71 | 3.45 | 1.04 | 0.301 | 0.5655 |
| TC1700006583.hg.1 | ASPA | aspartoacylase | Coding | 3.39 | 2.9 | 2.86 | 1.44 | 0.0715 | 0.1884 | 3.83 | 3.52 | 3.36 | 1.39 | 0.0492 | 0.2064 |
| TC1700007057.hg.1 | NT5M | 5,3-nucleotidase, mitochondrial | Multiple_C | 7.31 | 6.97 | 6.78 | 1.44 | 0.0275 | 0.092 | 7.09 | 6.90 | 6.43 | 1.58 | 0.0036 | 0.0402 |
| TC1700008511.hg.1 | TBX2 | T-box 2 | Multiple_C | 4.72 | 3.82 | 4.19 | 1.44 | 0.1056 | 0.2508 | 8.6 | 10.24 | 10.13 | 0.35 | 2.82E-06 | 0.0003 |
| TC1700009318.hg.1 | FAM101B | family with sequence similarity 101, member B | Multiple_C | 5.07 | 3.98 | 4.54 | 1.44 | 0.0321 | 0.1038 | 8.98 | 7.67 | 7.36 | 3.07 | 4.50E-08 | 1.53E-05 |
| TC1700010973.hg.1 | COPZ2 | coatomer protein complex subunit zeta 2 | Multiple_C | 4.17 | 3.53 | 3.64 | 1.44 | 0.5496 | 0.7346 | 4.81 | 4.86 | 4.38 | 1.35 | 0.1054 | 0.3186 |
| TC1700012089.hg.1 | DCXR | dicarbonyl/L-xylulose reductase | Multiple_C | 12.18 | 10.81 | 11.65 | 1.44 | 0.0452 | 0.1343 | 10.97 | 10.96 | 10.84 | 1.09 | 0.4853 | 0.7224 |
| TC1700012186.hg.1 | TNFSF13 | tumor necrosis factor (ligand) superfamily, member 13 | Multiple_C | 6.1 | 5.79 | 5.57 | 1.44 | 0.055 | 0.1552 | 5.59 | 5.76 | 5.35 | 1.18 | 0.3433 | 0.607 |
| TC1800006679.hg.1 | NAPG | N-ethylmaleimide-sensitive factor attachment protein, gamma | Multiple_C | 9.26 | 9.39 | 8.73 | 1.44 | 0.0265 | 0.0892 | 11.99 | 10.94 | 10.74 | 2.38 | 2.90E-06 | 0.0003 |
| TC1800006891.hg.1 | C18orf8 | chromosome 18 open reading frame 8 | Multiple_C | 9.91 | 9.36 | 9.38 | 1.44 | 0.1832 | 0.3685 | 7.14 | 6.86 | 7.26 | 0.92 | 0.6029 | 0.8013 |
| TC1900006477.hg.1 | FSTL3 | follistatin-like 3 (secreted glycoprotein) | Multiple_C | 6.27 | 5.91 | 5.74 | 1.44 | 0.0261 | 0.0882 | 7.37 | 6.46 | 5.73 | 3.12 | 2.80E-06 | 0.0003 |
| TC1900006645.hg.1 | HMG20B | high mobility group 20B | Multiple_C | 10.78 | 10.66 | 10.25 | 1.44 | 0.1442 | 0.3136 | 8.83 | 9.19 | 8.68 | 1.11 | 0.5718 | 0.7797 |
| TC1900007777.hg.1 | CEBPG | CCAAT/enhancer binding protein (C/EBP), gamma | Coding | 12.33 | 12.74 | 11.8 | 1.44 | 0.3373 | 0.5512 | 10.53 | 9.82 | 11.03 | 0.71 | 0.8506 | 0.9355 |
| TC1900011014.hg.1 | ZC3H4 | zinc finger CCCH-type containing 4 | Multiple_C | 9.57 | 8.72 | 9.04 | 1.44 | 0.0548 | 0.1547 | 9.8 | 10.87 | 10.25 | 0.73 | 0.0982 | 0.3058 |
| TC1900011725.hg.1 | SPINT2 | serine peptidase inhibitor, Kunitz type, 2 | Multiple_C | 12.54 | 12.59 | 12.01 | 1.44 | 0.1895 | 0.3773 | 8.78 | 8.43 | 8.52 | 1.20 | 0.6287 | 0.8168 |
| TC1900011826.hg.1 | ZNF544 | zinc finger protein 544 | Multiple_C | 5.93 | 5.38 | 5.4 | 1.44 | 0.0584 | 0.1625 | 5.77 | 5.92 | 6 | 0.85 | 0.4329 | 0.6841 |
| TC2000006464.hg.1 | FAM110A | family with sequence similarity 110, member A | Multiple_C | 6.96 | 6.01 | 6.43 | 1.44 | 0.0439 | 0.1316 | 7.26 | 7.50 | 7.24 | 1.01 | 0.7538 | 0.8881 |
| TC2000007102.hg.1 | HCK | HCK proto-oncogene, Src family tyrosine kinase | Multiple_C | 5.11 | 4.27 | 4.58 | 1.44 | 0.1619 | 0.3392 | 3.93 | 3.98 | 4.49 | 0.68 | 0.0599 | 0.2316 |
| TC2000007327.hg.1 | ARHGAP40 | Rho GTPase activating protein 40 | Multiple_C | 5.52 | 4.87 | 4.99 | 1.44 | 0.0563 | 0.1578 | 6.29 | 5.45 | 5.29 | 2.00 | 0.0015 | 0.0219 |
| TC2100007597.hg.1 | HSPA13 | heat shock protein 70kDa family, member 13 | Multiple_C | 11.34 | 12.26 | 10.81 | 1.44 | 0.4878 | 0.6889 | 10.39 | 9.82 | 10.2 | 1.14 | 0.4153 | 0.6696 |
| TC2100008530.hg.1 | KRTAP10-11; I | keratin associated protein 10-11; keratin associated protein 12- | Multiple_C | 8.12 | 7.23 | 7.59 | 1.44 | 0.1171 | 0.27 | 9.53 | 9.12 | 8.86 | 1.59 | 0.152 | 0.3905 |
| TC2200007287.hg.1 | CYTH4 | cytohesin 4 | Multiple_C | 5.74 | 5.4 | 5.21 | 1.44 | 0.063 | 0.1716 | 5.61 | 5.27 | 5.33 | 1.21 | 0.9637 | 0.9846 |
| TC2200008655.hg.1 | CARD10 | caspase recruitment domain family, member 10 | Multiple_C | 7.56 | 6.83 | 7.03 | 1.44 | 0.0689 | 0.1832 | 7.46 | 7.54 | 7.35 | 1.08 | 0.6999 | 0.8578 |
| TC2200009310.hg.1 | GAB4 | GRB2-associated binding protein family, member 4 | Multiple_C | 7.71 | 6.94 | 7.18 | 1.44 | 0.0164 | 0.0612 | 8.06 | 8.18 | 8.01 | 1.04 | 0.8006 | 0.9108 |
| TSUnmapped00000091.h | HHAT | hedgehog acyltransferase | Coding | 5.19 | 4.61 | 4.66 | 1.44 | 0.036 | 0.1135 | 4.67 | 4.61 | 4.38 | 1.22 | 0.8096 | 0.9156 |
| TC0100007444.hg.1 | CNKSR1 | connector enhancer of kinase suppressor of Ras 1 | Multiple_C | 5.86 | 4.77 | 5.34 | 1.43 | 0.929 | 0.9663 | 4.74 | 4.78 | 4.57 | 1.13 | 0.1668 | 0.412 |
| TC0100007838.hg.1 | DNALI1 | dynein, axonemal, light intermediate chain 1 | Multiple_C | 6.81 | 6.29 | 6.29 | 1.43 | 0.1929 | 0.3818 | 6.22 | 6.13 | 6.06 | 1.12 | 0.2802 | 0.546 |
| TC0100010793.hg.1 | CEP350 | centrosomal protein 350kDa | Multiple_C | 8.47 | 7.99 | 7.95 | 1.43 | 0.1415 | 0.3091 | 8.37 | 7.70 | 8.22 | 1.11 | 0.5299 | 0.7532 |
| TC0100010874.hg.1 | RGL1 | ral guanine nucleotide dissociation stimulator-like 1 | Multiple_C | 6.8 | 5.63 | 6.28 | 1.43 | 0.2194 | 0.4164 | 9.88 | 10.15 | 9.63 | 1.19 | 0.5048 | 0.7368 |
| TC0100013266.hg.1 | C1orf234 | chromosome 1 open reading frame 234 | Multiple_C | 4.94 | 4.61 | 4.42 | 1.43 | 0.2086 | 0.4023 | 5.03 | 4.78 | 4.91 | 1.09 | 0.619 | 0.8113 |
| TC0100018513.hg.1 | C1orf204 | chromosome 1 open reading frame 204 | Multiple_C | 5.65 | 5.2 | 5.13 | 1.43 | 0.039 | 0.1204 | 5.17 | 5.14 | 4.85 | 1.25 | 0.2493 | 0.5138 |
| TC0200010340.hg.1 | CCDC150 | coiled-coil domain containing 150 | Multiple_C | 6.32 | 5.88 | 5.8 | 1.43 | 0.1246 | 0.282 | 6.07 | 6.22 | 6.11 | 0.97 | 0.498 | 0.7313 |
| TC0200010857.hg.1 | INHA | inhibin alpha | Multiple_C | 4.86 | 4.24 | 4.34 | 1.43 | 0.045 | 0.1339 | 4.97 | 5.40 | 4.84 | 1.09 | 0.5344 | 0.7565 |

| | | | | | | | | | | | | | | | |
|---|---|---|---|---|---|---|---|---|---|---|---|---|---|---|---|
| TC0200011878.hg.1 | OSR1 | odd-skipped related transciption factor 1 | Multiple_C | 5.62 | 4.84 | 5.1 | 1.43 | 0.2035 | 0.3958 | 3.46 | 3.37 | 3.26 | 1.15 | 0.9863 | 0.9943 |
| TC0200015556.hg.1 | FZD5 | frizzled class receptor 5 | Coding | 8.06 | 7.23 | 7.54 | 1.43 | 0.2731 | 0.4819 | 6.35 | 7.03 | 7.69 | 0.40 | 2.85E-05 | 0.0015 |
| TC0200016366.hg.1 | THAP4 | THAP domain containing 4 | Multiple_C | 10.98 | 10.35 | 10.46 | 1.43 | 0.1337 | 0.2971 | 9.9 | 10.37 | 10.73 | 0.56 | 0.0044 | 0.0457 |
| TC0300006669.hg.1 | LSM3 | LSM3 homolog, U6 small nuclear RNA and mRNA degradation a | Multiple_C | 14.9 | 14.08 | 14.38 | 1.43 | 0.2835 | 0.4936 | 10.69 | 11.13 | 11.15 | 0.73 | 0.0549 | 0.2199 |
| TC0300007255.hg.1 | CCR3 | chemokine (C-C motif) receptor 3 | Multiple_C | 3.01 | 2.46 | 2.49 | 1.43 | 0.1157 | 0.268 | 3.2 | 3.21 | 3.16 | 1.03 | 0.356 | 0.6192 |
| TC0300011172.hg.1 | TNNC1 | troponin C type 1 (slow) | Multiple_C | 7.4 | 7.2 | 6.88 | 1.43 | 0.4177 | 0.6295 | 9.05 | 7.94 | 6.91 | 4.41 | 6.14E-05 | 0.0026 |
| TC0300013736.hg.1 | DLG1 | discs, large homolog 1 (Drosophila) | Multiple_C | 11.69 | 11.53 | 11.17 | 1.43 | 0.0553 | 0.1559 | 13.06 | 12.60 | 12.7 | 1.28 | 0.0656 | 0.2437 |
| TC0400007918.hg.1 | CXCL13 | chemokine (C-X-C motif) ligand 13 | Multiple_C | 3.49 | 3.03 | 2.97 | 1.43 | 0.7164 | 0.8501 | 3.65 | 3.84 | 3.79 | 0.91 | 0.4967 | 0.7303 |
| TC0400008228.hg.1 | METAP1 | methionyl aminopeptidase 1 | Multiple_C | 11.36 | 10.97 | 10.84 | 1.43 | 0.0368 | 0.1154 | 8.67 | 8.10 | 8.88 | 0.86 | 0.0505 | 0.2094 |
| TC0400008329.hg.1 | GSTCD | glutathione S-transferase, C-terminal domain containing | Multiple_C | 11.43 | 10.36 | 10.91 | 1.43 | 0.1182 | 0.2718 | 10.15 | 10.59 | 10.38 | 0.85 | 0.0464 | 0.1992 |
| TC0500010453.hg.1 | ADAMTS12 | ADAM metallopeptidase with thrombospondin type 1 motif 12 | Multiple_C | 5.54 | 5.1 | 5.02 | 1.43 | 0.1887 | 0.3764 | 4.27 | 4.30 | 4.47 | 0.87 | 0.8295 | 0.9243 |
| TC0500013364.hg.1 | TIFAB | TRAF-interacting protein with forkhead-associated domain, fam | Coding | 3.94 | 3.29 | 3.42 | 1.43 | 0.0483 | 0.141 | 3.67 | 3.97 | 3.91 | 0.85 | 0.2929 | 0.5578 |
| TC0600009505.hg.1 | TCF21 | transcription factor 21 | Multiple_C | 5.04 | 4.44 | 4.52 | 1.43 | 0.2041 | 0.3966 | 5.39 | 5.45 | 5.24 | 1.11 | 0.2255 | 0.4862 |
| TC0600013258.hg.1 | AHI1 | Abelson helper integration site 1 | Multiple_C | 8.55 | 8.79 | 8.03 | 1.43 | 0.2085 | 0.4021 | 8.81 | 8.01 | 7.69 | 2.17 | 0.0401 | 0.1837 |
| TC0700008583.hg.1 | AP1S1 | adaptor-related protein complex 1 sigma 1 subunit | Coding | 9.36 | 9.21 | 8.84 | 1.43 | 0.4391 | 0.6483 | 6.7 | 7.22 | 7.11 | 0.75 | 0.0379 | 0.1774 |
| TC0700013485.hg.1 | LOC155060; Z | AI894139 pseudogene; Transcript Identified by AceView, Entrez | Multiple_C | 8.47 | 7.85 | 7.95 | 1.43 | 0.1293 | 0.2898 | 7.43 | 7.21 | 7.01 | 1.34 | 0.3916 | 0.6501 |
| TC0800008266.hg.1 | DPY19L4 | dpy-19-like 4 (C. elegans) | Multiple_C | 11.41 | 11.98 | 10.89 | 1.43 | 0.0474 | 0.1392 | 11.67 | 11.01 | 11.38 | 1.22 | 0.2613 | 0.5255 |
| TC0900006597.hg.1 | KDM4C | lysine (K)-specific demethylase 4C | Multiple_C | 9.13 | 9 | 8.61 | 1.43 | 0.6196 | 0.7872 | 8.98 | 9.21 | 9.3 | 0.80 | 0.4756 | 0.7147 |
| TC0900007098.hg.1 | RGP1 | RGP1 homolog, RAB6A GEF complex partner 1 | Multiple_C | 8.4 | 7.54 | 7.88 | 1.43 | 0.1017 | 0.2441 | 7.84 | 7.78 | 8.08 | 0.85 | 0.4565 | 0.7005 |
| TC0900008953.hg.1 | ASS1 | argininosuccinate synthase 1 | Multiple_C | 11.49 | 12.66 | 10.97 | 1.43 | 0.2655 | 0.4732 | 10.94 | 10.83 | 10.74 | 1.15 | 0.2187 | 0.4787 |
| TC0900012215.hg.1 | IFNA16 | interferon, alpha 16 | Coding | 3.64 | 3.28 | 3.12 | 1.43 | 0.3463 | 0.5597 | 3.79 | 3.49 | 3.52 | 1.21 | 0.3462 | 0.61 |
| TC0X00010990.hg.1 | SPANXC; SPAN | SPANX family, member C; SPANX family, member D | Coding | 6.77 | 6.28 | 6.25 | 1.43 | 0.0138 | 0.0537 | 7.53 | 7.56 | 7.52 | 1.01 | 0.7194 | 0.8691 |
| TC0X00011238.hg.1 | CLIC2 | chloride intracellular channel 2 | Multiple_C | 3.56 | 3 | 3.04 | 1.43 | 0.2137 | 0.4089 | 5.64 | 5.74 | 5.94 | 0.81 | 0.046 | 0.1983 |
| TC0Y00006812.hg.1 | DAZ2; DAZ4 | deleted in azoospermia 2; deleted in azoospermia 4 | Multiple_C | 3.54 | 3.08 | 3.02 | 1.43 | 0.0283 | 0.0939 | 3.53 | 3.37 | 3.37 | 1.12 | 0.1743 | 0.422 |
| TC0Y00007078.hg.1 | VCY; VCY1B | variable charge, Y-linked; variable charge, Y-linked 1B | Coding | 4.11 | 3.46 | 3.59 | 1.43 | 0.0487 | 0.1418 | 4.07 | 4.06 | 3.85 | 1.16 | 0.2634 | 0.5281 |
| TC0Y00007160.hg.1 | KDM5D | lysine (K)-specific demethylase 5D | Multiple_C | 4.58 | 3.92 | 4.06 | 1.43 | 0.1414 | 0.3089 | 4.57 | 4.56 | 4.41 | 1.12 | 0.3965 | 0.6543 |
| TC0Y00007324.hg.1 | TSPY8 | testis specific protein, Y-linked 8 | Multiple_C | 3.62 | 2.91 | 3.1 | 1.43 | 0.0665 | 0.1788 | 3.89 | 3.64 | 3.71 | 1.13 | 0.2808 | 0.5467 |
| TC1000006917.hg.1 | CACNB2 | calcium channel, voltage-dependent, beta 2 subunit | Multiple_C | 3.43 | 2.81 | 2.91 | 1.43 | 0.1642 | 0.3424 | 3.65 | 3.54 | 3.27 | 1.30 | 0.7032 | 0.8595 |
| TC1000008099.hg.1 | C10orf11 | chromosome 10 open reading frame 11 | Multiple_C | 4.43 | 3.81 | 3.91 | 1.43 | 0.0083 | 0.0358 | 4.67 | 4.30 | 4.5 | 1.13 | 0.3539 | 0.6173 |
| TC1000008272.hg.1 | RGR | retinal G protein coupled receptor | Multiple_C | 6.55 | 5.84 | 6.03 | 1.43 | 0.66 | 0.8137 | 5.47 | 5.46 | 5.42 | 1.04 | 0.8724 | 0.9451 |
| TC1000011081.hg.1 | ZNF503 | zinc finger protein 503 | Multiple_C | 5.73 | 5.03 | 5.21 | 1.43 | 0.1786 | 0.3618 | 10.47 | 10.46 | 9.88 | 1.51 | 0.0762 | 0.2656 |
| TC1100009911.hg.1 | OR52A5 | olfactory receptor, family 52, subfamily A, member 5 | Coding | 3.39 | 2.85 | 2.87 | 1.43 | 0.2024 | 0.3947 | 3.44 | 3.31 | 3.15 | 1.22 | 0.0576 | 0.2261 |
| TC1100011131.hg.1 | C11orf95 | chromosome 11 open reading frame 95 | Multiple_C | 13.65 | 12.95 | 13.13 | 1.43 | 0.048 | 0.1403 | 13.56 | 13.85 | 13.49 | 1.05 | 0.9617 | 0.984 |

| | | | | | | | | | | | | | | | |
|---|---|---|---|---|---|---|---|---|---|---|---|---|---|---|---|
| TC1100011282.hg.1 | CD248 | CD248 molecule, endosialin | Coding | 4.62 | 3.83 | 4.1 | 1.43 | 0.0245 | 0.084 | 5.65 | 5.29 | 5.12 | 1.44 | 0.0144 | 0.098 |
| TC1100012750.hg.1 | KIRREL3 | kin of IRRE like 3 (Drosophila) | Multiple_C | 5.79 | 5.17 | 5.27 | 1.43 | 0.0519 | 0.1485 | 5.36 | 5.55 | 5.46 | 0.93 | 0.5613 | 0.7723 |
| TC1200008706.hg.1 | KIAA1033 | KIAA1033 | Multiple_C | 9.85 | 9.68 | 9.33 | 1.43 | 0.0239 | 0.0824 | 9.6 | 8.97 | 9.69 | 0.94 | 0.6173 | 0.8099 |
| TC1300007171.hg.1 | ARL11 | ADP-ribosylation factor like GTPase 11 | Multiple_C | 3.59 | 3.06 | 3.07 | 1.43 | 0.0171 | 0.0634 | 4.22 | 4.38 | 3.84 | 1.30 | 0.0234 | 0.1319 |
| TC1400008589.hg.1 | KLHL33 | kelch-like family member 33 | Coding | 4.87 | 4.53 | 4.35 | 1.43 | 0.0129 | 0.051 | 4.57 | 4.75 | 4.51 | 1.04 | 0.4952 | 0.7291 |
| TC1400010630.hg.1 | TTLL5 | tubulin tyrosine ligase-like family member 5 | Multiple_C | 10.75 | 10 | 10.23 | 1.43 | 0.1356 | 0.3 | 11.31 | 11.03 | 10.82 | 1.40 | 0.0465 | 0.1994 |
| TC1500007091.hg.1 | CTDSPL2 | CTD small phosphatase like 2 | Multiple_C | 11.36 | 11.15 | 10.84 | 1.43 | 0.5518 | 0.7363 | 11 | 10.46 | 11.19 | 0.88 | 0.6255 | 0.8149 |
| TC1500010010.hg.1 | CYP11A1 | cytochrome P450, family 11, subfamily A, polypeptide 1 | Multiple_C | 5.2 | 4.74 | 4.68 | 1.43 | 0.0677 | 0.1811 | 4.07 | 4.36 | 4.16 | 0.94 | 0.6564 | 0.8321 |
| TC1500010743.hg.1 | MAPK6 | mitogen-activated protein kinase 6 | Multiple_C | 13.07 | 12.92 | 12.55 | 1.43 | 0.0974 | 0.2361 | 12.1 | 11.37 | 12.3 | 0.87 | 0.1932 | 0.4466 |
| TC1600009192.hg.1 | MTRNR2L4 | MT-RNR2-like 4 | Coding | 5.12 | 4.42 | 4.6 | 1.43 | 0.1459 | 0.3161 | 5.16 | 5.10 | 5.36 | 0.87 | 0.6192 | 0.8113 |
| TC1700008465.hg.1 | CLTC | clathrin, heavy chain (Hc) | Multiple_C | 13.71 | 13.62 | 13.19 | 1.43 | 0.0389 | 0.1202 | 13.14 | 13.22 | 13.45 | 0.81 | 0.0687 | 0.2497 |
| TC1700009857.hg.1 | TEKT3 | tektin 3 | Multiple_C | 4.86 | 4.07 | 4.34 | 1.43 | 0.2469 | 0.4504 | 4.24 | 4.28 | 3.93 | 1.24 | 0.7056 | 0.8614 |
| TC1700012100.hg.1 | CSNK1D | casein kinase 1, delta | Multiple_C | 12.33 | 11.98 | 11.81 | 1.43 | 0.0939 | 0.23 | 11.64 | 11.69 | 12.03 | 0.76 | 0.2558 | 0.5204 |
| TC1800007411.hg.1 | NEDD4L | neural precursor cell expressed, developmentally down-regulat | Multiple_C | 11.38 | 9.78 | 10.86 | 1.43 | 0.0814 | 0.2074 | 12.09 | 11.58 | 11.67 | 1.34 | 0.0572 | 0.2252 |
| TC1800008806.hg.1 | LOC10192732 | uncharacterized LOC101927322; novel transcript, antisense to N | Multiple_C | 4.48 | 3.87 | 3.96 | 1.43 | 0.036 | 0.1134 | 4.23 | 4.79 | 4.3 | 0.95 | 0.9811 | 0.9921 |
| TC1900007043.hg.1 | ZNF833P | zinc finger protein 833, pseudogene | Multiple_C | 4.54 | 4.03 | 4.02 | 1.43 | 0.0898 | 0.2225 | 4.14 | 4.34 | 4.31 | 0.89 | 0.1942 | 0.4481 |
| TC1900007048.hg.1 | ZNF491 | zinc finger protein 491 | Multiple_C | 4.12 | 3.44 | 3.6 | 1.43 | 0.0682 | 0.1819 | 4.23 | 3.85 | 3.89 | 1.27 | 0.1261 | 0.3513 |
| TC1900007356.hg.1 | KCNN1 | potassium channel, calcium activated intermediate/small condu | Multiple_C | 5.8 | 5.12 | 5.28 | 1.43 | 0.7 | 0.8395 | 5 | 4.80 | 4.86 | 1.10 | 0.6915 | 0.8528 |
| TC1900007891.hg.1 | HCST | hematopoietic cell signal transducer | Multiple_C | 4.59 | 4.07 | 4.07 | 1.43 | 0.2281 | 0.4278 | 5.18 | 5.10 | 4.87 | 1.24 | 0.6518 | 0.8303 |
| TC1900008321.hg.1 | KLC3 | kinesin light chain 3 | Multiple_C | 6.64 | 5.88 | 6.12 | 1.43 | 0.1427 | 0.3109 | 5.5 | 5.39 | 5.14 | 1.28 | 0.1297 | 0.3566 |
| TC1900009491.hg.1 | FCER2 | Fc fragment of IgE, low affinity II, receptor for (CD23) | Multiple_C | 5.07 | 4.76 | 4.55 | 1.43 | 0.2523 | 0.4571 | 5.21 | 4.97 | 5.15 | 1.04 | 0.5236 | 0.7491 |
| TC1900010602.hg.1 | GGN | gametogenetin | Multiple_C | 5.64 | 5.13 | 5.12 | 1.43 | 0.0686 | 0.1827 | 5.05 | 4.85 | 4.95 | 1.07 | 0.8471 | 0.9335 |
| TC1900011233.hg.1 | KLK10 | kallikrein related peptidase 10 | Multiple_C | 5.76 | 4.97 | 5.24 | 1.43 | 0.1529 | 0.3263 | 4.44 | 3.86 | 3.88 | 1.47 | 0.0213 | 0.1248 |
| TC1900011261.hg.1 | SIGLEC6 | sialic acid binding Ig-like lectin 6 | Multiple_C | 4.87 | 4.18 | 4.35 | 1.43 | 0.1334 | 0.2968 | 3.62 | 3.65 | 3.71 | 0.94 | 0.371 | 0.633 |
| TC1900011676.hg.1 | RLN3 | relaxin 3 | Coding | 6.52 | 5.96 | 6 | 1.43 | 0.0231 | 0.0803 | 6.67 | 6.86 | 6.78 | 0.93 | 0.625 | 0.8148 |
| TC1900011762.hg.1 | APOC2 | apolipoprotein C-II | Multiple_C | 6.09 | 5.26 | 5.57 | 1.43 | 0.0021 | 0.0122 | 7.38 | 8.59 | 7.39 | 0.99 | 0.5978 | 0.7976 |
| TC1900011846.hg.1 | PLIN5 | perilipin 5 | Multiple_C | 5.09 | 4.45 | 4.57 | 1.43 | 0.0213 | 0.0752 | 4.59 | 4.44 | 4.4 | 1.14 | 0.3148 | 0.5787 |
| TC1900012017.hg.1 | CLDND2 | claudin domain containing 2 | Coding | 7.52 | 6.87 | 7 | 1.43 | 0.0427 | 0.1291 | 7.01 | 7.34 | 7.02 | 0.99 | 0.8828 | 0.9497 |
| TC2000007213.hg.1 | MMP24 | matrix metallopeptidase 24 (membrane-inserted) | Multiple_C | 3.97 | 3.58 | 3.45 | 1.43 | 0.5421 | 0.7291 | 4.36 | 4.46 | 4.54 | 0.88 | 0.9833 | 0.9931 |
| TC2000008029.hg.1 | MRGBP | MRG/MORF4L binding protein | Multiple_C | 8.22 | 8.15 | 7.7 | 1.43 | 0.0903 | 0.2234 | 7.5 | 7.68 | 7.77 | 0.83 | 0.0353 | 0.1703 |
| TC2000008993.hg.1 | SCAND1 | SCAN domain containing 1 | Coding | 9.82 | 9.12 | 9.3 | 1.43 | 0.1338 | 0.2973 | 6.82 | 7.67 | 7.7 | 0.54 | 0.0024 | 0.0305 |
| TC2000009800.hg.1 | YTHDF1 | YTH N(6)-methyladenosine RNA binding protein 1 | Multiple_C | 12.65 | 11.01 | 12.13 | 1.43 | 0.0506 | 0.1459 | 11.87 | 12.10 | 12.35 | 0.72 | 0.0043 | 0.0447 |
| TC2000009909.hg.1 | RALY | RALY heterogeneous nuclear ribonucleoprotein | Multiple_C | 13.01 | 12.1 | 12.49 | 1.43 | 0.1065 | 0.2523 | 12.62 | 13.19 | 13.25 | 0.65 | 0.0033 | 0.0377 |

| | | | | | | | | | | | | | | |
|---|---|---|---|---|---|---|---|---|---|---|---|---|---|---|
| TC2100007072.hg.1 | TTC3 | tetratricopeptide repeat domain 3 | Multiple_C | 12.42 | 12.56 | 11.9 | 1.43 | 0.1483 | 0.3194 | 12.34 | 11.44 | 12.31 | 1.02 | 0.7716 | 0.8972 |
| TC2100007935.hg.1 | SCAF4 | SR-related CTD-associated factor 4 | Multiple_C | 12.12 | 10.45 | 11.6 | 1.43 | 0.1822 | 0.3669 | 12.92 | 12.45 | 13.3 | 0.77 | 0.254 | 0.5181 |
| TC2200009232.hg.1 | SPECC1L | sperm antigen with calponin homology and coiled-coil domains | Multiple_C | 9.88 | 8.98 | 9.36 | 1.43 | 0.0777 | 0.2004 | 10.63 | 10.72 | 10.99 | 0.78 | 0.5387 | 0.7592 |
| TSUnmapped00000530.h | ATG16L1 | autophagy related 16-like 1 | Coding | 8.09 | 7.55 | 7.57 | 1.43 | 0.0269 | 0.0901 | 6.28 | 5.58 | 5.85 | 1.35 | 0.0224 | 0.1287 |
| TC0100011105.hg.1 | PTPRC | protein tyrosine phosphatase, receptor type, C | Multiple_C | 4.65 | 4.12 | 4.14 | 1.42 | 0.1779 | 0.3609 | 3.97 | 4.05 | 4.03 | 0.96 | 0.3264 | 0.5903 |
| TC0100011797.hg.1 | C1orf95 | chromosome 1 open reading frame 95 | Multiple_C | 6.92 | 6.24 | 6.41 | 1.42 | 0.4028 | 0.6157 | 7.48 | 7.68 | 7.33 | 1.11 | 0.5223 | 0.7487 |
| TC0100015851.hg.1 | SPRR2E | small proline-rich protein 2E | Coding | 4.98 | 4.45 | 4.47 | 1.42 | 0.1178 | 0.2712 | 4.35 | 4.28 | 4.54 | 0.88 | 0.798 | 0.9095 |
| TC0100017538.hg.1 | ITPKB | Transcript Identified by AceView, Entrez Gene ID(s) 3707 | Coding | 3.86 | 3.29 | 3.35 | 1.42 | 0.0913 | 0.2254 | 3.7 | 3.82 | 3.95 | 0.84 | 0.8485 | 0.9346 |
| TC0100018207.hg.1 | TSSK3 | testis-specific serine kinase 3 | Coding | 5.03 | 4.27 | 4.52 | 1.42 | 0.0841 | 0.2121 | 4.07 | 4.19 | 4.32 | 0.84 | 0.5721 | 0.7798 |
| TC0200008971.hg.1 | CBWD2 | COBW domain containing 2 | Multiple_C | 13.62 | 14.16 | 13.11 | 1.42 | 0.2163 | 0.4121 | 12.47 | 12.02 | 12.3 | 1.13 | 0.1512 | 0.3892 |
| TC0300010908.hg.1 | ZDHHC3 | zinc finger, DHHC-type containing 3 | Multiple_C | 13.92 | 12.89 | 13.41 | 1.42 | 0.013 | 0.0514 | 11.76 | 11.73 | 12.3 | 0.69 | 0.0047 | 0.0472 |
| TC0300010966.hg.1 | CCDC12 | coiled-coil domain containing 12 | Multiple_C | 11.03 | 10.52 | 10.52 | 1.42 | 0.1674 | 0.3469 | 9.2 | 9.10 | 8.97 | 1.17 | 0.7512 | 0.8867 |
| TC0300011314.hg.1 | C3orf67 | chromosome 3 open reading frame 67 | Multiple_C | 4.18 | 3.8 | 3.67 | 1.42 | 0.0891 | 0.2213 | 6.11 | 7.00 | 7.08 | 0.51 | 0.0001 | 0.0044 |
| TC0300012218.hg.1 | ROPN1 | rhophilin associated tail protein 1 | Multiple_C | 5.57 | 5.03 | 5.06 | 1.42 | 0.0895 | 0.222 | 6.1 | 6.50 | 6.44 | 0.79 | 0.0342 | 0.1664 |
| TC0400007617.hg.1 | POLR2B | polymerase (RNA) II (DNA directed) polypeptide B, 140kDa | Multiple_C | 13.42 | 13 | 12.91 | 1.42 | 0.2381 | 0.4398 | 12.19 | 11.97 | 12.32 | 0.91 | 0.3454 | 0.6092 |
| TC0400008030.hg.1 | ARHGAP24 | Rho GTPase activating protein 24 | Multiple_C | 3.96 | 3.28 | 3.45 | 1.42 | 0.077 | 0.1989 | 3.57 | 3.52 | 3.12 | 1.37 | 0.0586 | 0.2284 |
| TC0400008557.hg.1 | USP53 | ubiquitin specific peptidase 53 | Multiple_C | 10.1 | 9.69 | 9.59 | 1.42 | 0.1982 | 0.3891 | 9.51 | 8.40 | 8.98 | 1.44 | 0.1179 | 0.3395 |
| TC0400009258.hg.1 | PALLD | palladin, cytoskeletal associated protein | Multiple_C | 10.81 | 10.04 | 10.3 | 1.42 | 0.1052 | 0.2501 | 8.48 | 7.58 | 7.71 | 1.71 | 0.3566 | 0.6198 |
| TC0400012565.hg.1 | CLDN24 | claudin 24 | Coding | 5.23 | 4.85 | 4.72 | 1.42 | 0.0545 | 0.1539 | 3.95 | 3.87 | 3.81 | 1.10 | 0.4189 | 0.6725 |
| TC0400012891.hg.1 | NELFA; MIR94 | negative elongation factor complex member A; microRNA 943 | Multiple_C | 10.8 | 9.66 | 10.29 | 1.42 | 0.0819 | 0.2079 | 10.18 | 10.34 | 10.71 | 0.69 | 0.062 | 0.2365 |
| TC0500008356.hg.1 | KCNN2 | potassium channel, calcium activated intermediate/small condu | Multiple_C | 3.78 | 3.3 | 3.27 | 1.42 | 0.1856 | 0.372 | 3.29 | 3.69 | 3.51 | 0.86 | 0.3265 | 0.5903 |
| TC0600008418.hg.1 | LOC441155 | zinc finger CCCH-type domain-containing-like | Multiple_C | 6.19 | 6.2 | 5.68 | 1.42 | 0.0371 | 0.1161 | 4.87 | 4.84 | 4.91 | 0.97 | 0.656 | 0.8321 |
| TC0600012012.hg.1 | DEFB112 | defensin, beta 112 | Multiple_C | 4.05 | 3.91 | 3.54 | 1.42 | 0.2325 | 0.4333 | 4.02 | 4.09 | 4.1 | 0.95 | 0.4254 | 0.678 |
| TC0600013010.hg.1 | MAN1A1 | mannosidase, alpha, class 1A, member 1 | Multiple_C | 8.23 | 8.45 | 7.72 | 1.42 | 0.1029 | 0.2461 | 11.92 | 12.38 | 11.53 | 1.31 | 0.0413 | 0.1869 |
| TC0700008023.hg.1 | GATSL2 | GATS protein-like 2 | Multiple_C | 7.17 | 5.96 | 6.66 | 1.42 | 0.2593 | 0.4657 | 7.22 | 8.06 | 8 | 0.58 | 0.0109 | 0.0822 |
| TC0700008677.hg.1 | LHFPL3 | lipoma HMGIC fusion partner-like 3 | Coding | 4.28 | 4.57 | 3.77 | 1.42 | 0.0297 | 0.0975 | 3.61 | 3.80 | 4 | 0.76 | 0.1164 | 0.3372 |
| TC0700009678.hg.1 | GIMAP2 | GTPase, IMAP family member 2 | Multiple_C | 3.96 | 3.07 | 3.45 | 1.42 | 0.0303 | 0.0992 | 3.99 | 4.27 | 4.21 | 0.86 | 0.8028 | 0.9119 |
| TC0700011705.hg.1 | ABCB4 | ATP binding cassette subfamily B member 4 | Multiple_C | 4.29 | 3.58 | 3.78 | 1.42 | 0.1233 | 0.2802 | 6.66 | 6.02 | 5.3 | 2.57 | 2.03E-05 | 0.0012 |
| TC0700011782.hg.1 | FAM133B | family with sequence similarity 133, member B | Multiple_C | 9.37 | 9.02 | 8.86 | 1.42 | 0.0737 | 0.1925 | 8.77 | 8.39 | 9.24 | 0.72 | 0.1337 | 0.3631 |
| TC0800008770.hg.1 | RNF139 | ring finger protein 139 | Multiple_C | 11.78 | 12.04 | 11.27 | 1.42 | 0.062 | 0.1696 | 8.69 | 8.88 | 9.35 | 0.63 | 0.0044 | 0.0454 |
| TC0800009891.hg.1 | STC1 | stanniocalcin 1 | Multiple_C | 4.32 | 3.65 | 3.81 | 1.42 | 0.0223 | 0.078 | 3.79 | 3.95 | 4.02 | 0.85 | 0.9134 | 0.9645 |
| TC0800010783.hg.1 | MSC | musculin | Multiple_C | 4.5 | 3.82 | 3.99 | 1.42 | 0.2501 | 0.4544 | 4.1 | 4.27 | 4.21 | 0.93 | 0.4906 | 0.7259 |
| TC0900007101.hg.1 | TMEM8B | transmembrane protein 8B | Multiple_C | 4.51 | 4.38 | 4 | 1.42 | 0.0559 | 0.1569 | 3.35 | 3.52 | 3.46 | 0.93 | 0.5341 | 0.7563 |

| | | | | | | | | | | | | | | | |
|---|---|---|---|---|---|---|---|---|---|---|---|---|---|---|---|
| TC0900007877.hg.1 | GADD45G | growth arrest and DNA-damage-inducible, gamma | Multiple_C | 4.91 | 4.4 | 4.4 | 1.42 | 0.0566 | 0.1585 | 4.17 | 4.18 | 4.04 | 1.09 | 0.8805 | 0.9487 |
| TC0900009279.hg.1 | TOR4A | torsin family 4, member A | Multiple_C | 7.26 | 6.6 | 6.75 | 1.42 | 0.1553 | 0.3299 | 6.29 | 6.75 | 6.67 | 0.77 | 0.2077 | 0.4642 |
| TC0900009673.hg.1 | MLLT3 | myeloid/lymphoid or mixed-lineage leukemia; translocated to, 3 | Multiple_C | 11.17 | 10.93 | 10.66 | 1.42 | 0.054 | 0.1531 | 10.34 | 9.79 | 9.61 | 1.66 | 0.0013 | 0.0196 |
| TC0900009944.hg.1 | CD72 | CD72 molecule | Multiple_C | 6.75 | 6.08 | 6.24 | 1.42 | 0.4188 | 0.6301 | 5.42 | 5.92 | 5.92 | 0.71 | 0.1488 | 0.3858 |
| TC0900011597.hg.1 | SH2D3C | SH2 domain containing 3C | Multiple_C | 4.59 | 3.99 | 4.08 | 1.42 | 0.0678 | 0.1812 | 4.65 | 4.71 | 4.38 | 1.21 | 0.3075 | 0.5716 |
| TC0900012155.hg.1 | MSANTD3-TM | MSANTD3-TMEFF1 readthrough | Coding | 4.58 | 4.32 | 4.07 | 1.42 | 0.2483 | 0.4522 | 8.22 | 6.76 | 6.46 | 3.39 | 3.86E-05 | 0.0018 |
| TC0900012162.hg.1 | ORM1 | orosomucoid 1 | Multiple_C | 3.05 | 2.77 | 2.54 | 1.42 | 0.0566 | 0.1585 | 3.37 | 3.62 | 3.16 | 1.16 | 0.1926 | 0.4459 |
| TC0900012256.hg.1 | CDC14B | cell division cycle 14B | Multiple_C | 9.76 | 9.82 | 9.25 | 1.42 | 0.3538 | 0.5666 | 7.14 | 7.17 | 7.79 | 0.64 | 0.0047 | 0.0475 |
| TC0X00006433.hg.1 | PLCXD1 | phosphatidylinositol-specific phospholipase C, X domain contair | Multiple_C | 4.54 | 4.13 | 4.03 | 1.42 | 0.1225 | 0.279 | 4.9 | 5.60 | 6.01 | 0.46 | 0.0008 | 0.0138 |
| TC0X00007235.hg.1 | GAGE4; GAGE | G antigen 4; G antigen 7; G antigen 12G | Coding | 3.93 | 3.51 | 3.42 | 1.42 | 0.2351 | 0.4361 | 3.6 | 3.52 | 3.58 | 1.01 | 0.9814 | 0.9923 |
| TC0X00008125.hg.1 | PAK3 | p21 protein (Cdc42/Rac)-activated kinase 3 | Multiple_C | 3.77 | 3.3 | 3.26 | 1.42 | 0.7859 | 0.8902 | 4.13 | 4.01 | 3.86 | 1.21 | 0.9088 | 0.9625 |
| TC0X00008495.hg.1 | FAM127A | family with sequence similarity 127, member A | Multiple_C | 3.44 | 2.93 | 2.93 | 1.42 | 0.2166 | 0.4125 | 3.17 | 3.57 | 3.58 | 0.75 | 0.098 | 0.3053 |
| TC0X00009213.hg.1 | MAP7D2 | MAP7 domain containing 2 | Multiple_C | 4.97 | 4.58 | 4.46 | 1.42 | 0.3263 | 0.5403 | 8.7 | 7.99 | 7.77 | 1.91 | 0.0046 | 0.0468 |
| TC0X00009256.hg.1 | KLHL15 | kelch-like family member 15 | Coding | 11.31 | 10.73 | 10.8 | 1.42 | 0.1162 | 0.2687 | 9.63 | 10.20 | 10.95 | 0.40 | 0.0002 | 0.0051 |
| TC0X00009603.hg.1 | SSX9 | synovial sarcoma, X breakpoint 9 | Multiple_C | 4.39 | 3.5 | 3.88 | 1.42 | 0.0841 | 0.2122 | 4.92 | 4.88 | 4.75 | 1.13 | 0.5318 | 0.7546 |
| TC1000008875.hg.1 | ADD3 | adducin 3 (gamma) | Multiple_C | 11.06 | 11.47 | 10.55 | 1.42 | 0.2353 | 0.4364 | 9.66 | 9.99 | 9.12 | 1.45 | 0.0801 | 0.2728 |
| TC1000012604.hg.1 | METTL10 | methyltransferase like 10 | Multiple_C | 9.17 | 9.11 | 8.66 | 1.42 | 0.1742 | 0.3561 | 8.11 | 7.82 | 8.75 | 0.64 | 0.0374 | 0.1763 |
| TC1100006455.hg.1 | B4GALNT4 | beta-1,4-N-acetyl-galactosaminyl transferase 4 | Multiple_C | 6.24 | 5.48 | 5.73 | 1.42 | 0.089 | 0.2213 | 6.5 | 6.57 | 6.31 | 1.14 | 0.3736 | 0.6342 |
| TC1100006938.hg.1 | LOC10272495 | uncharacterized LOC102724957; novel transcript | Multiple_C | 3.69 | 2.78 | 3.18 | 1.42 | 0.013 | 0.0513 | 4.27 | 3.66 | 3.82 | 1.37 | 0.0244 | 0.1352 |
| TC1100009824.hg.1 | CARS | cysteinyl-tRNA synthetase | Multiple_C | 12.22 | 12.83 | 11.71 | 1.42 | 0.328 | 0.5418 | 11.21 | 10.43 | 10.6 | 1.53 | 0.9952 | 0.9977 |
| TC1100012012.hg.1 | CWC15 | CWC15 spliceosome-associated protein | Multiple_C | 9.2 | 9.64 | 8.69 | 1.42 | 0.5349 | 0.7241 | 7.75 | 7.54 | 7.44 | 1.24 | 0.1928 | 0.4461 |
| TC1100012121.hg.1 | TMEM123 | transmembrane protein 123 | Multiple_C | 15.69 | 15.27 | 15.18 | 1.42 | 0.0358 | 0.1131 | 17 | 16.86 | 17.21 | 0.86 | 0.3123 | 0.5765 |
| TC1100012535.hg.1 | PVRL1 | poliovirus receptor-related 1 (herpesvirus entry mediator C) | Multiple_C | 8.17 | 6.66 | 7.66 | 1.42 | 0.0985 | 0.2382 | 10.88 | 12.91 | 12.97 | 0.23 | 1.73E-05 | 0.0011 |
| TC1200006996.hg.1 | PIK3C2G | phosphatidylinositol-4-phosphate 3-kinase, catalytic subunit typ | Multiple_C | 3.05 | 2.48 | 2.54 | 1.42 | 0.2629 | 0.47 | 3.37 | 3.06 | 3.34 | 1.02 | 0.3341 | 0.5982 |
| TC1200007406.hg.1 | IRAK4 | interleukin 1 receptor associated kinase 4 | Multiple_C | 7.32 | 7.87 | 6.81 | 1.42 | 0.0798 | 0.2048 | 8.21 | 8.04 | 8.28 | 0.95 | 0.9692 | 0.9873 |
| TC1200007641.hg.1 | SCN8A | sodium channel, voltage gated, type VIII alpha subunit | Multiple_C | 3.8 | 3.54 | 3.29 | 1.42 | 0.1136 | 0.2643 | 4.57 | 5.51 | 4.85 | 0.82 | 0.0875 | 0.2855 |
| TC1200007864.hg.1 | NXPH4 | neurexophilin 4 | Multiple_C | 4.4 | 3.95 | 3.89 | 1.42 | 0.1718 | 0.3531 | 4.49 | 4.31 | 4.44 | 1.04 | 0.5888 | 0.7918 |
| TC1200009445.hg.1 | SFSWAP | splicing factor, suppressor of white-apricot family | Multiple_C | 11.96 | 10.99 | 11.45 | 1.42 | 0.1938 | 0.3832 | 10.93 | 10.47 | 11.09 | 0.90 | 0.0762 | 0.2656 |
| TC1200012575.hg.1 | DYRK4 | dual specificity tyrosine-(Y)-phosphorylation regulated kinase 4 | Multiple_C | 8.99 | 9.49 | 8.48 | 1.42 | 0.1236 | 0.2806 | 7.32 | 7.59 | 7.41 | 0.94 | 0.683 | 0.8479 |
| TC1400008056.hg.1 | IFI27 | interferon, alpha-inducible protein 27 | Multiple_C | 5.92 | 4.06 | 5.41 | 1.42 | 0.0815 | 0.2074 | 4.71 | 4.83 | 4.78 | 0.95 | 0.818 | 0.9196 |
| TC1400009104.hg.1 | RPL36AL | ribosomal protein L36a-like | Coding | 16.09 | 16.2 | 15.58 | 1.42 | 0.2397 | 0.4417 | 11.51 | 11.48 | 11.41 | 1.07 | 0.3849 | 0.6438 |
| TC1400010582.hg.1 | FITM1 | fat storage-inducing transmembrane protein 1 | Coding | 4.27 | 3.62 | 3.76 | 1.42 | 0.1234 | 0.2803 | 4.22 | 4.38 | 4.13 | 1.06 | 0.5587 | 0.7714 |
| TC1400010703.hg.1 | MTA1 | metastasis associated 1 | Multiple_C | 10.82 | 10.1 | 10.31 | 1.42 | 0.4101 | 0.6224 | 10.94 | 10.73 | 10.66 | 1.21 | 0.7121 | 0.865 |

| | | | | | | | | | | | | | | | |
|---|---|---|---|---|---|---|---|---|---|---|---|---|---|---|---|
| TC1400010793.hg.1 | MIR1247 | microRNA 1247 | Multiple_C | 5.07 | 4.41 | 4.56 | 1.42 | 0.0684 | 0.1823 | 6.48 | 6.77 | 6.56 | 0.95 | 0.2503 | 0.5146 |
| TC1500006981.hg.1 | DLL4 | delta-like 4 (Drosophila) | Multiple_C | 4.69 | 4.15 | 4.18 | 1.42 | 0.3526 | 0.5653 | 5.83 | 6.96 | 7.62 | 0.29 | 0.0001 | 0.0044 |
| TC1500009079.hg.1 | SRP14 | signal recognition particle 14kDa | Multiple_C | 15.22 | 14.68 | 14.71 | 1.42 | 0.4113 | 0.6236 | 14.6 | 15.01 | 15.04 | 0.74 | 0.252 | 0.5163 |
| TC1500010893.hg.1 | PTPN9 | protein tyrosine phosphatase, non-receptor type 9 | Multiple_C | 8.86 | 8.26 | 8.35 | 1.42 | 0.1316 | 0.2939 | 7.63 | 8.10 | 7.93 | 0.81 | 0.0956 | 0.3008 |
| TC1600007449.hg.1 | SLX1A; SLX1B; | SLX1 homolog A, structure-specific endonuclease subunit; SLX1 | Multiple_C | 9.52 | 9.5 | 9.01 | 1.42 | 0.5349 | 0.7241 | 8.01 | 8.20 | 8.26 | 0.84 | 0.1318 | 0.3601 |
| TC1800006565.hg.1 | LINC01387 | long intergenic non-protein coding RNA 1387 | Multiple_C | 4.09 | 3.73 | 3.58 | 1.42 | 0.0066 | 0.03 | 4.2 | 3.79 | 3.92 | 1.21 | 0.0794 | 0.2713 |
| TC1800007471.hg.1 | PMAIP1 | phorbol-12-myristate-13-acetate-induced protein 1 | Multiple_C | 8.03 | 7.92 | 7.52 | 1.42 | 0.297 | 0.5085 | 9.2 | 7.86 | 7.57 | 3.10 | 0.0033 | 0.0378 |
| TC1900007203.hg.1 | CCDC105 | coiled-coil domain containing 105 | Coding | 3.32 | 2.62 | 2.81 | 1.42 | 0.0262 | 0.0884 | 3.54 | 3.62 | 3.32 | 1.16 | 0.1507 | 0.3887 |
| TC1900008257.hg.1 | ZNF227 | zinc finger protein 227 | Multiple_C | 10.27 | 9.87 | 9.76 | 1.42 | 0.3919 | 0.6057 | 7.45 | 7.72 | 8.75 | 0.41 | 0.0093 | 0.0744 |
| TC1900010031.hg.1 | COMP | cartilage oligomeric matrix protein | Multiple_C | 5.27 | 4.5 | 4.76 | 1.42 | 0.0971 | 0.2357 | 6.97 | 6.85 | 6.42 | 1.46 | 0.0206 | 0.1225 |
| TC1900011535.hg.1 | ZNF835 | zinc finger protein 835 | Coding | 4.52 | 3.72 | 4.01 | 1.42 | 0.1453 | 0.3153 | 4.63 | 4.66 | 4.59 | 1.03 | 0.2986 | 0.5633 |
| TC2000006527.hg.1 | TMEM239; C2 | transmembrane protein 239; chromosome 20 open reading frar | Coding | 5.94 | 5.19 | 5.43 | 1.42 | 0.0093 | 0.0395 | 5.49 | 5.64 | 5.62 | 0.91 | 0.5934 | 0.7945 |
| TC2000007498.hg.1 | SPINT4 | serine peptidase inhibitor, Kunitz type 4 | Coding | 3.74 | 3.11 | 3.23 | 1.42 | 0.0887 | 0.2207 | 4.07 | 3.90 | 3.74 | 1.26 | 0.1653 | 0.4101 |
| TC2000009759.hg.1 | C20orf166-AS | C20orf166 antisense RNA 1 | Multiple_C | 4.87 | 4.1 | 4.36 | 1.42 | 0.0309 | 0.1008 | 5.6 | 4.99 | 4.82 | 1.72 | 0.0057 | 0.0534 |
| TC2000009949.hg.1 | STX16 | syntaxin 16 | Multiple_C | 11.4 | 11.99 | 10.89 | 1.42 | 0.0599 | 0.1655 | 12.39 | 11.66 | 11.82 | 1.48 | 0.0069 | 0.0605 |
| TC2000009965.hg.1 | FKBP1A; MIR6 | FK506 binding protein 1A; microRNA 6869 | Multiple_C | 11.29 | 10.11 | 10.78 | 1.42 | 0.0557 | 0.1566 | 12.27 | 12.38 | 12.33 | 0.96 | 0.6357 | 0.8212 |
| TC2100008027.hg.1 | KCNE1 | potassium channel, voltage gated subfamily E regulatory beta su | Multiple_C | 3.08 | 2.58 | 2.57 | 1.42 | 0.0239 | 0.0824 | 3.06 | 2.88 | 2.9 | 1.12 | 0.4299 | 0.6817 |
| TC2200008442.hg.1 | SEC14L3 | SEC14-like lipid binding 3 | Coding | 3.33 | 3.16 | 2.82 | 1.42 | 0.1076 | 0.2542 | 3.62 | 3.59 | 3.28 | 1.27 | 0.4407 | 0.6902 |
| TSUnmapped00000107.h | DUSP16 | dual specificity phosphatase 16 | Coding | 8.09 | 9.31 | 7.58 | 1.42 | 0.2631 | 0.4704 | 7.08 | 7.02 | 6.84 | 1.18 | 0.9466 | 0.9782 |
| TSUnmapped00000151.h | ADAMTS13 | ADAM metallopeptidase with thrombospondin type 1 motif 13 | Coding | 6.02 | 5.51 | 5.51 | 1.42 | 0.1044 | 0.2488 | 5.07 | 5.16 | 4.8 | 1.21 | 0.3671 | 0.6294 |
| TC0100007786.hg.1 | AGO1 | argonaute RISC catalytic component 1 | Multiple_C | 8.99 | 8.45 | 8.49 | 1.41 | 0.0637 | 0.173 | 10.35 | 10.08 | 10.35 | 1.00 | 0.9551 | 0.9814 |
| TC0100009366.hg.1 | STRIP1 | striatin interacting protein 1 | Multiple_C | 10.5 | 10.37 | 10 | 1.41 | 0.2007 | 0.3927 | 11.23 | 11.86 | 11.14 | 1.06 | 0.2881 | 0.5527 |
| TC0100012544.hg.1 | MMEL1 | membrane metallo-endopeptidase-like 1 | Multiple_C | 4.11 | 3.54 | 3.61 | 1.41 | 0.0977 | 0.2367 | 4.17 | 4.03 | 3.86 | 1.24 | 0.1763 | 0.4249 |
| TC0100015872.hg.1 | S100A14 | S100 calcium binding protein A14 | Multiple_C | 12.58 | 13.59 | 12.08 | 1.41 | 0.3073 | 0.5205 | 5.98 | 5.03 | 5.02 | 1.95 | 0.0014 | 0.0206 |
| TC0100015932.hg.1 | PBXIP1 | pre-B-cell leukemia homeobox interacting protein 1 | Multiple_C | 5.57 | 5.17 | 5.07 | 1.41 | 0.3091 | 0.5223 | 5.56 | 5.43 | 4.83 | 1.66 | 0.0466 | 0.1995 |
| TC0100017445.hg.1 | TLR5 | toll-like receptor 5 | Multiple_C | 7.15 | 6.54 | 6.65 | 1.41 | 0.2326 | 0.4333 | 5.61 | 5.62 | 5.78 | 0.89 | 0.197 | 0.4519 |
| TC0100018389.hg.1 | CDK11A | cyclin-dependent kinase 11A | Multiple_C | 11.87 | 12.12 | 11.37 | 1.41 | 0.1742 | 0.3561 | 11.49 | 12.14 | 11.61 | 0.92 | 0.6609 | 0.8345 |
| TC0100018451.hg.1 | GBP2 | guanylate binding protein 2, interferon-inducible | Multiple_C | 5.53 | 4.77 | 5.03 | 1.41 | 0.038 | 0.1179 | 4.59 | 4.35 | 4.91 | 0.80 | 0.3759 | 0.6364 |
| TC0200008452.hg.1 | MAL | mal, T-cell differentiation protein | Multiple_C | 4.66 | 4.26 | 4.16 | 1.41 | 0.0334 | 0.1068 | 4.03 | 4.15 | 4.17 | 0.91 | 0.4642 | 0.7065 |
| TC0200010152.hg.1 | UBE2E3 | ubiquitin-conjugating enzyme E2E 3 | Multiple_C | 10.35 | 10.4 | 9.85 | 1.41 | 0.0623 | 0.17 | 10.36 | 10.64 | 10.43 | 0.95 | 0.7444 | 0.8831 |
| TC0200016757.hg.1 | NCKAP1 | NCK-associated protein 1 | Multiple_C | 13.42 | 14.27 | 12.92 | 1.41 | 0.217 | 0.4129 | 10.81 | 10.00 | 10.43 | 1.30 | 0.9837 | 0.9934 |
| TC0300007086.hg.1 | TTC21A; MIR6 | tetratricopeptide repeat domain 21A; microRNA 6822 | Multiple_C | 5.36 | 5.16 | 4.86 | 1.41 | 0.6304 | 0.794 | 5.88 | 5.63 | 5.02 | 1.82 | 0.033 | 0.1629 |
| TC0300007679.hg.1 | ATXN7 | ataxin 7 | Multiple_C | 10.08 | 8.07 | 9.58 | 1.41 | 0.155 | 0.3293 | 9.78 | 9.05 | 10.05 | 0.83 | 0.4541 | 0.6989 |

| | | | | | | | | | | | | | | | |
|---|---|---|---|---|---|---|---|---|---|---|---|---|---|---|---|
| TC0300008530.hg.1 | ARGFX | arginine-fifty homeobox | Coding | 3.76 | 3.01 | 3.26 | 1.41 | 0.0458 | 0.1354 | 3.4 | 3.63 | 3.51 | 0.93 | 0.4106 | 0.6652 |
| TC0300009279.hg.1 | KCNAB1 | potassium channel, voltage gated subfamily A regulatory beta s | Multiple_C | 5 | 4.32 | 4.5 | 1.41 | 0.0843 | 0.2124 | 4.1 | 4.03 | 3.85 | 1.19 | 0.0925 | 0.2949 |
| TC0300010123.hg.1 | CRBN | cereblon | Multiple_C | 10.34 | 11.46 | 9.84 | 1.41 | 0.0971 | 0.2357 | 9.51 | 8.76 | 9.32 | 1.14 | 0.1227 | 0.3456 |
| TC0300013983.hg.1 | NAT6 | N-acetyltransferase 6 (GCN5-related) | Coding | 7 | 6.07 | 6.5 | 1.41 | 0.1421 | 0.3099 | 6.57 | 6.80 | 6.53 | 1.03 | 0.7451 | 0.8832 |
| TC0400007225.hg.1 | DTHD1 | death domain containing 1 | Multiple_C | 3.61 | 2.94 | 3.11 | 1.41 | 0.1048 | 0.2495 | 3.55 | 3.71 | 3.96 | 0.75 | 0.1573 | 0.3982 |
| TC0400008892.hg.1 | HHIP | hedgehog interacting protein | Multiple_C | 3.72 | 3.19 | 3.22 | 1.41 | 0.1723 | 0.3536 | 4.56 | 4.43 | 4.13 | 1.35 | 0.0195 | 0.118 |
| TC0400009904.hg.1 | PPP2R2C | protein phosphatase 2, regulatory subunit B, gamma | Multiple_C | 3.45 | 2.78 | 2.95 | 1.41 | 0.0197 | 0.0707 | 3.17 | 2.96 | 3.05 | 1.09 | 0.788 | 0.9054 |
| TC0400011208.hg.1 | FAM175A | family with sequence similarity 175, member A | Multiple_C | 9.98 | 11.71 | 9.48 | 1.41 | 0.3104 | 0.5238 | 8.56 | 7.40 | 7.99 | 1.48 | 0.1517 | 0.3902 |
| TC0500009098.hg.1 | MYOZ3 | myozenin 3 | Multiple_C | 3.62 | 3.22 | 3.12 | 1.41 | 0.1555 | 0.3302 | 3.61 | 3.60 | 3.62 | 0.99 | 0.605 | 0.8024 |
| TC0500009478.hg.1 | RPL26L1 | ribosomal protein L26-like 1 | Coding | 11.02 | 11.6 | 10.52 | 1.41 | 0.161 | 0.3379 | 9.77 | 10.41 | 9.74 | 1.02 | 0.7346 | 0.8776 |
| TC0500011316.hg.1 | RPS23 | ribosomal protein S23 | Multiple_C | 18.24 | 18.43 | 17.74 | 1.41 | 0.0189 | 0.0686 | 17.37 | 16.97 | 16.76 | 1.53 | 0.0214 | 0.1252 |
| TC0500012189.hg.1 | LRRTM2 | leucine rich repeat transmembrane neuronal 2 | Multiple_C | 4.51 | 3.8 | 4.01 | 1.41 | 0.3041 | 0.517 | 4.48 | 4.04 | 3.74 | 1.67 | 0.0649 | 0.2427 |
| TC0500012205.hg.1 | MZB1 | marginal zone B and B1 cell-specific protein | Multiple_C | 4.77 | 4.48 | 4.27 | 1.41 | 0.0902 | 0.2233 | 4.82 | 4.93 | 5.06 | 0.85 | 0.3471 | 0.6107 |
| TC0600008562.hg.1 | SENP6 | SUMO1/sentrin specific peptidase 6 | Multiple_C | 10.06 | 10.22 | 9.56 | 1.41 | 0.1193 | 0.2736 | 10.42 | 10.28 | 10.88 | 0.73 | 0.0914 | 0.2927 |
| TC0600009931.hg.1 | CLDN20 | claudin 20 | Coding | 3.92 | 3.28 | 3.42 | 1.41 | 0.1224 | 0.2788 | 4.03 | 4.01 | 3.83 | 1.15 | 0.2914 | 0.556 |
| TC0600011308.hg.1 | MAS1L | MAS1 proto-oncogene like, G protein-coupled receptor | Coding | 5.25 | 5.11 | 4.75 | 1.41 | 0.1063 | 0.252 | 4.46 | 4.67 | 4.52 | 0.96 | 0.8121 | 0.9166 |
| TC0600012010.hg.1 | DEFB113 | defensin, beta 113 | Coding | 4.45 | 3.94 | 3.95 | 1.41 | 0.122 | 0.2781 | 4.49 | 4.69 | 4.55 | 0.96 | 0.7121 | 0.865 |
| TC0600012123.hg.1 | DST | dystonin | Multiple_C | 10.93 | 10.07 | 10.43 | 1.41 | 0.03 | 0.0984 | 13.38 | 11.74 | 12.31 | 2.10 | 1.85E-05 | 0.0011 |
| TC0600014177.hg.1 | DSE | dermatan sulfate epimerase | Multiple_C | 5.03 | 4.93 | 4.53 | 1.41 | 0.0413 | 0.1257 | 5.56 | 5.32 | 5.14 | 1.34 | 0.0634 | 0.2395 |
| TC0700007111.hg.1 | CCDC129 | coiled-coil domain containing 129 | Multiple_C | 4.37 | 4.02 | 3.87 | 1.41 | 0.0776 | 0.2 | 3.66 | 4.09 | 3.76 | 0.93 | 0.446 | 0.6935 |
| TC0700007996.hg.1 | ELN | elastin | Multiple_C | 5.79 | 5.2 | 5.29 | 1.41 | 0.0544 | 0.1538 | 5.18 | 5.26 | 5.05 | 1.09 | 0.178 | 0.4266 |
| TC0700009886.hg.1 | DNAJB6 | DnaJ (Hsp40) homolog, subfamily B, member 6 | Multiple_C | 12.03 | 12.3 | 11.53 | 1.41 | 0.1927 | 0.3817 | 12.89 | 12.35 | 12.61 | 1.21 | 0.0491 | 0.2061 |
| TC0700011898.hg.1 | TECPR1 | tectonin beta-propeller repeat containing 1 | Multiple_C | 5.82 | 5.22 | 5.32 | 1.41 | 0.3152 | 0.5285 | 5.31 | 5.79 | 5.79 | 0.72 | 0.1179 | 0.3395 |
| TC0700013481.hg.1 | PRSS1 | protease, serine, 1 (trypsin 1) | Multiple_C | 4 | 4.01 | 3.5 | 1.41 | 0.4729 | 0.6761 | 3.67 | 3.27 | 3.27 | 1.32 | 0.0197 | 0.119 |
| TC0900009694.hg.1 | IFNA4 | interferon, alpha 4 | Coding | 3.89 | 3.36 | 3.39 | 1.41 | 0.0272 | 0.091 | 3.95 | 3.56 | 3.45 | 1.41 | 0.015 | 0.1004 |
| TC0900010854.hg.1 | NUTM2F | NUT family member 2F | Coding | 4.96 | 4.45 | 4.46 | 1.41 | 0.249 | 0.453 | 4.89 | 5.14 | 4.97 | 0.95 | 0.4358 | 0.6864 |
| TC0900012055.hg.1 | EXD3 | exonuclease 3-5 domain containing 3 | Multiple_C | 6.49 | 5.95 | 5.99 | 1.41 | 0.061 | 0.1677 | 7.23 | 7.33 | 7.08 | 1.11 | 0.2657 | 0.5303 |
| TC0X00006758.hg.1 | CNKSR2 | connector enhancer of kinase suppressor of Ras 2 | Multiple_C | 4.9 | 4.29 | 4.4 | 1.41 | 0.0958 | 0.2335 | 4.72 | 4.62 | 4.51 | 1.16 | 0.1081 | 0.3234 |
| TC0X00007231.hg.1 | GAGE12H; GA | G antigen 12H; G antigen 12B; G antigen 12C | Coding | 3.73 | 3.33 | 3.23 | 1.41 | 0.2057 | 0.3987 | 3.47 | 3.56 | 3.54 | 0.95 | 0.8434 | 0.9317 |
| TC0X00007232.hg.1 | GAGE12D | G antigen 12D | Coding | 3.73 | 3.33 | 3.23 | 1.41 | 0.2057 | 0.3987 | 3.47 | 3.56 | 3.54 | 0.95 | 0.8434 | 0.9317 |
| TC0X00007233.hg.1 | GAGE12G; GA | G antigen 12G; G antigen 12E | Coding | 3.73 | 3.33 | 3.23 | 1.41 | 0.2057 | 0.3987 | 3.47 | 3.56 | 3.54 | 0.95 | 0.8434 | 0.9317 |
| TC0X00010642.hg.1 | NKRF | NFKB repressing factor | Multiple_C | 9.48 | 9.62 | 8.98 | 1.41 | 0.3429 | 0.5568 | 9.47 | 10.11 | 10.94 | 0.36 | 0.0012 | 0.0189 |
| TC0Y00006444.hg.1 | CSF2RA | colony stimulating factor 2 receptor, alpha, low-affinity (granulc | Multiple_C | 6.42 | 6.23 | 5.92 | 1.41 | 0.8005 | 0.8977 | 3.57 | 3.63 | 3.54 | 1.02 | 0.7717 | 0.8973 |

| | | | | | | | | | | | | | | | |
|---|---|---|---|---|---|---|---|---|---|---|---|---|---|---|---|
| TC1_KI270713v1_randor | FAM231A | family with sequence similarity 231, member A | Coding | 6.1 | 5.35 | 5.6 | 1.41 | 0.0239 | 0.0824 | 5.49 | 5.80 | 5.5 | 0.99 | 0.6041 | 0.802 |
| TC1100006437.hg.1 | ODF3 | outer dense fiber of sperm tails 3 | Multiple_C | 4.57 | 3.96 | 4.07 | 1.41 | 0.9994 | 0.9997 | 4.15 | 4.46 | 4.45 | 0.81 | 0.5062 | 0.7376 |
| TC1100007385.hg.1 | C11orf96 | chromosome 11 open reading frame 96 | Multiple_C | 3.61 | 2.88 | 3.11 | 1.41 | 0.3218 | 0.5356 | 4.51 | 4.53 | 4.51 | 1.00 | 0.2792 | 0.5452 |
| TC1100010929.hg.1 | OR10Q1 | olfactory receptor, family 10, subfamily Q, member 1 | Coding | 4.99 | 4.5 | 4.49 | 1.41 | 0.0503 | 0.1452 | 4.67 | 4.95 | 4.67 | 1.00 | 0.78 | 0.9013 |
| TC1100010962.hg.1 | MPEG1 | macrophage expressed 1 | Coding | 4.43 | 3.69 | 3.93 | 1.41 | 0.2731 | 0.4819 | 4 | 4.02 | 3.88 | 1.09 | 0.7691 | 0.8964 |
| TC1100011914.hg.1 | TRIM64B | tripartite motif containing 64B | Multiple_C | 4.46 | 4.06 | 3.96 | 1.41 | 0.0647 | 0.1753 | 4.51 | 4.53 | 4.43 | 1.06 | 0.5382 | 0.759 |
| TC1100012959.hg.1 | TIMM10B | translocase of inner mitochondrial membrane 10 homolog B (ye | Multiple_C | 11.15 | 10.29 | 10.65 | 1.41 | 0.1881 | 0.3755 | 10.97 | 10.56 | 11.09 | 0.92 | 0.7104 | 0.8642 |
| TC1100013209.hg.1 | PAK1 | p21 protein (Cdc42/Rac)-activated kinase 1 | Multiple_C | 10.58 | 10.94 | 10.08 | 1.41 | 0.143 | 0.3114 | 8.06 | 7.60 | 8.04 | 1.01 | 0.6124 | 0.8064 |
| TC1100013224.hg.1 | POU2AF1 | POU class 2 associating factor 1 | Multiple_C | 3.59 | 2.96 | 3.09 | 1.41 | 0.0464 | 0.1368 | 4.05 | 4.14 | 3.99 | 1.04 | 0.6299 | 0.8175 |
| TC1200009910.hg.1 | KLRAP1 | killer cell lectin-like receptor subfamily A pseudogene 1 | Multiple_C | 5.23 | 4.4 | 4.73 | 1.41 | 0.1159 | 0.2682 | 4.24 | 4.60 | 4.5 | 0.84 | 0.8696 | 0.9438 |
| TC1200011965.hg.1 | NAA25 | N(alpha)-acetyltransferase 25, NatB auxiliary subunit | Multiple_C | 7.6 | 7.46 | 7.1 | 1.41 | 0.0387 | 0.1195 | 5.51 | 5.26 | 5.56 | 0.97 | 0.7173 | 0.8682 |
| TC1200012642.hg.1 | BLOC1S1-RDH | BLOC1S1-RDH5 readthrough; Uncharacterized protein [Source: | Multiple_C | 8.41 | 7.6 | 7.91 | 1.41 | 0.455 | 0.6623 | 7.15 | 7.83 | 7.32 | 0.89 | 0.3914 | 0.65 |
| TC1500010707.hg.1 | ARHGAP11B | Rho GTPase activating protein 11B | Multiple_C | 10.26 | 8.98 | 9.76 | 1.41 | 0.0179 | 0.0655 | 10.13 | 9.96 | 10.25 | 0.92 | 0.265 | 0.5295 |
| TC1600007001.hg.1 | NOMO1 | NODAL modulator 1 | Multiple_C | 14.5 | 13.56 | 14 | 1.41 | 0.0219 | 0.0768 | 14.74 | 14.69 | 14.95 | 0.86 | 0.299 | 0.5637 |
| TC1600008438.hg.1 | CTRB1 | chymotrypsinogen B1 | Coding | 3.58 | 3.05 | 3.08 | 1.41 | 0.0773 | 0.1995 | 3.48 | 3.67 | 3.65 | 0.89 | 0.8521 | 0.936 |
| TC1600010000.hg.1 | STX1B | syntaxin 1B | Multiple_C | 4.13 | 3.66 | 3.63 | 1.41 | 0.5312 | 0.7212 | 4.59 | 4.64 | 4.47 | 1.09 | 0.2462 | 0.5108 |
| TC1600010665.hg.1 | DPEP2 | dipeptidase 2 | Multiple_C | 6.05 | 5.57 | 5.55 | 1.41 | 0.5131 | 0.7086 | 5.57 | 5.77 | 5.44 | 1.09 | 0.838 | 0.9288 |
| TC1700009938.hg.1 | RASD1 | RAS, dexamethasone-induced 1 | Coding | 4.21 | 3.42 | 3.71 | 1.41 | 0.197 | 0.3878 | 3.99 | 4.08 | 4.36 | 0.77 | 0.3247 | 0.5885 |
| TC1700010041.hg.1 | AKAP10 | A kinase (PRKA) anchor protein 10 | Multiple_C | 8.49 | 8.77 | 7.99 | 1.41 | 0.2068 | 0.4 | 8.19 | 8.43 | 8.13 | 1.04 | 0.8872 | 0.9518 |
| TC1700010222.hg.1 | PHF12 | PHD finger protein 12 | Multiple_C | 8.76 | 7.93 | 8.26 | 1.41 | 0.0281 | 0.0933 | 8.46 | 8.74 | 8.51 | 0.97 | 0.7398 | 0.8805 |
| TC1700010927.hg.1 | CDC27 | cell division cycle 27 | Multiple_C | 11.01 | 10.11 | 10.51 | 1.41 | 0.6106 | 0.7808 | 11.5 | 11.13 | 11.67 | 0.89 | 0.5503 | 0.7664 |
| TC1700011250.hg.1 | BZRAP1 | benzodiazepine receptor (peripheral) associated protein 1 | Multiple_C | 5.55 | 4.95 | 5.05 | 1.41 | 0.0457 | 0.1352 | 5.28 | 5.31 | 5.16 | 1.09 | 0.9285 | 0.9701 |
| TC1700011294.hg.1 | PTRH2 | peptidyl-tRNA hydrolase 2 | Multiple_C | 11.58 | 12.07 | 11.08 | 1.41 | 0.0428 | 0.1294 | 9.99 | 10.18 | 9.94 | 1.04 | 0.8362 | 0.9278 |
| TC1900006565.hg.1 | CSNK1G2 | casein kinase 1, gamma 2 | Multiple_C | 8.74 | 7.83 | 8.24 | 1.41 | 0.7937 | 0.8942 | 8.24 | 9.14 | 8.97 | 0.60 | 0.0684 | 0.2491 |
| TC1900008646.hg.1 | LOC10012908 | uncharacterized LOC100129083; Transcript Identified by AceVie | Multiple_C | 5.52 | 4.94 | 5.02 | 1.41 | 0.0847 | 0.2134 | 5.85 | 6.08 | 5.95 | 0.93 | 0.4327 | 0.6839 |
| TC1900009100.hg.1 | POLRMT | polymerase (RNA) mitochondrial (DNA directed) | Multiple_C | 9.41 | 8.81 | 8.91 | 1.41 | 0.4283 | 0.6386 | 7.3 | 7.64 | 7.11 | 1.14 | 0.6964 | 0.856 |
| TC1900011208.hg.1 | ASPDH | aspartate dehydrogenase domain containing | Multiple_C | 5.36 | 4.65 | 4.86 | 1.41 | 0.0288 | 0.0951 | 5.01 | 5.03 | 4.98 | 1.02 | 0.6399 | 0.8234 |
| TC2000006507.hg.1 | LOC388780; R | uncharacterized LOC388780; putative novel transcript | Multiple_C | 4.87 | 4.4 | 4.37 | 1.41 | 0.2011 | 0.393 | 4.4 | 4.22 | 4.18 | 1.16 | 0.0999 | 0.309 |
| TC2000007133.hg.1 | MAPRE1 | microtubule-associated protein, RP/EB family, member 1 | Multiple_C | 14.06 | 13.62 | 13.56 | 1.41 | 0.0772 | 0.1991 | 14.62 | 14.48 | 14.89 | 0.83 | 0.3987 | 0.6559 |
| TC2000007145.hg.1 | BPIFA2 | BPI fold containing family A, member 2 | Coding | 2.94 | 2.33 | 2.44 | 1.41 | 0.0616 | 0.1689 | 3.49 | 3.52 | 3.43 | 1.04 | 0.8576 | 0.9396 |
| TC2000007386.hg.1 | PLCG1; RPL23. | phospholipase C, gamma 1; ribosomal protein L23a pseudogene | Multiple_C | 8.34 | 7.86 | 7.84 | 1.41 | 0.2447 | 0.4481 | 8.64 | 9.27 | 9.13 | 0.71 | 0.0063 | 0.0572 |
| TC2000007545.hg.1 | EYA2 | EYA transcriptional coactivator and phosphatase 2 | Multiple_C | 5.18 | 4.52 | 4.68 | 1.41 | 0.0388 | 0.1199 | 4.91 | 5.05 | 5.02 | 0.93 | 0.3773 | 0.6376 |
| TC2100007967.hg.1 | PAXBP1 | PAX3 and PAX7 binding protein 1 | Multiple_C | 11.15 | 11.61 | 10.65 | 1.41 | 0.0453 | 0.1345 | 10.08 | 9.49 | 9.99 | 1.06 | 0.4432 | 0.6916 |

| | | | | | | | | | | | | | | | |
|---|---|---|---|---|---|---|---|---|---|---|---|---|---|---|---|
| TC2200007808.hg.1 | ADM2 | adrenomedullin 2 | Multiple_C | 4.39 | 3.78 | 3.89 | 1.41 | 0.0661 | 0.1781 | 4.36 | 4.32 | 4.1 | 1.20 | 0.2597 | 0.5239 |
| TC2200008920.hg.1 | SULT4A1 | sulfotransferase family 4A member 1 | Multiple_C | 5.42 | 4.88 | 4.92 | 1.41 | 0.136 | 0.3007 | 5.47 | 5.50 | 5.05 | 1.34 | 0.0536 | 0.2168 |
| TC2200009151.hg.1 | MAPK11 | mitogen-activated protein kinase 11 | Multiple_C | 3.88 | 3.08 | 3.38 | 1.41 | 0.1035 | 0.2472 | 4.27 | 4.34 | 4.25 | 1.01 | 0.3375 | 0.6018 |
| TSUnmapped00000273.h | NDUFA10 | NADH dehydrogenase (ubiquinone) 1 alpha subcomplex, 10, 42l | Coding | 6.03 | 5.3 | 5.53 | 1.41 | 0.2841 | 0.4944 | 4.84 | 4.77 | 4.76 | 1.06 | 0.8807 | 0.9487 |
| TSUnmapped00000488.h | ZDHHC3 | zinc finger, DHHC-type containing 3 | Coding | 14.15 | 12.95 | 13.65 | 1.41 | 0.164 | 0.3423 | 11.93 | 12.07 | 12.68 | 0.59 | 0.001 | 0.0166 |
| TC0100007206.hg.1 | CDA | cytidine deaminase | Multiple_C | 4.2 | 3.58 | 3.71 | 1.40 | 0.3769 | 0.5899 | 5.31 | 4.51 | 4.16 | 2.22 | 0.0005 | 0.011 |
| TC0100008061.hg.1 | SZT2 | seizure threshold 2 homolog (mouse) | Multiple_C | 8.82 | 7.84 | 8.33 | 1.40 | 0.3697 | 0.5825 | 9.08 | 9.64 | 8.97 | 1.08 | 0.7922 | 0.9077 |
| TC0100009479.hg.1 | DCLRE1B | DNA cross-link repair 1B | Multiple_C | 9.21 | 7.7 | 8.72 | 1.40 | 0.1384 | 0.3043 | 7.27 | 8.15 | 8.64 | 0.39 | 2.00E-06 | 0.0002 |
| TC0100011282.hg.1 | SOX13 | SRY box 13 | Multiple_C | 11.18 | 10.13 | 10.69 | 1.40 | 0.2268 | 0.4259 | 10.96 | 11.22 | 10.95 | 1.01 | 0.3066 | 0.5709 |
| TC0100011534.hg.1 | FAM71A | family with sequence similarity 71, member A | Coding | 3.72 | 3.08 | 3.23 | 1.40 | 0.0314 | 0.1019 | 3.86 | 4.13 | 4.09 | 0.85 | 0.8853 | 0.9508 |
| TC0100011669.hg.1 | HLX | H2.0-like homeobox | Multiple_C | 3.83 | 3.24 | 3.34 | 1.40 | 0.0326 | 0.1051 | 4.29 | 4.12 | 4.15 | 1.10 | 0.8492 | 0.9347 |
| TC0100013323.hg.1 | STPG1 | sperm-tail PG-rich repeat containing 1 | Multiple_C | 9.85 | 9.3 | 9.36 | 1.40 | 0.1972 | 0.388 | 8.81 | 7.65 | 8.5 | 1.24 | 0.1709 | 0.4172 |
| TC0100015109.hg.1 | LOC10192843 | uncharacterized LOC101928436; novel transcript; Transcript Ide | Multiple_C | 3.44 | 2.71 | 2.95 | 1.40 | 0.0524 | 0.1494 | 3.54 | 3.77 | 3.53 | 1.01 | 0.5788 | 0.7844 |
| TC0100015155.hg.1 | VAV3 | vav 3 guanine nucleotide exchange factor | Multiple_C | 5.26 | 4.81 | 4.77 | 1.40 | 0.1112 | 0.2602 | 4.89 | 4.86 | 4.87 | 1.01 | 0.2236 | 0.4845 |
| TC0100016977.hg.1 | MYOG | myogenin (myogenic factor 4) | Coding | 6.21 | 5.82 | 5.72 | 1.40 | 0.274 | 0.4827 | 4.64 | 4.52 | 4.39 | 1.19 | 0.9764 | 0.9903 |
| TC0100017021.hg.1 | KISS1 | KiSS-1 metastasis-suppressor | Coding | 5.25 | 4.57 | 4.76 | 1.40 | 0.0132 | 0.052 | 4.77 | 4.80 | 4.81 | 0.97 | 0.9491 | 0.9793 |
| TC0100017115.hg.1 | FCAMR | Fc receptor, IgA, IgM, high affinity | Multiple_C | 6.39 | 5.76 | 5.9 | 1.40 | 0.0983 | 0.2377 | 5.44 | 5.50 | 5.31 | 1.09 | 0.8798 | 0.9484 |
| TC0200007446.hg.1 | PRKCE | protein kinase C, epsilon | Multiple_C | 9.93 | 9.11 | 9.44 | 1.40 | 0.1605 | 0.3371 | 7.72 | 7.96 | 7.71 | 1.01 | 0.4457 | 0.6935 |
| TC0200011237.hg.1 | COPS8 | COP9 signalosome subunit 8 | Multiple_C | 12.45 | 12.66 | 11.96 | 1.40 | 0.1001 | 0.2411 | 12.68 | 12.49 | 12.7 | 0.99 | 0.6753 | 0.8432 |
| TC0200011356.hg.1 | GPC1 | glypican 1 | Multiple_C | 4.35 | 3.79 | 3.86 | 1.40 | 0.0812 | 0.207 | 4.9 | 4.72 | 4.74 | 1.12 | 0.4414 | 0.6905 |
| TC0200011445.hg.1 | FAM110C | family with sequence similarity 110, member C | Multiple_C | 4.3 | 3.68 | 3.81 | 1.40 | 0.1414 | 0.3089 | 5.42 | 4.77 | 4.69 | 1.66 | 0.0069 | 0.0607 |
| TC0200012320.hg.1 | MAP4K3 | mitogen-activated protein kinase kinase kinase kinase 3 | Multiple_C | 9.78 | 8.95 | 9.29 | 1.40 | 0.1967 | 0.3873 | 9.04 | 8.15 | 8.86 | 1.13 | 0.5766 | 0.783 |
| TC0200012742.hg.1 | XPO1 | exportin 1 | Multiple_C | 12.73 | 12.99 | 12.24 | 1.40 | 0.1231 | 0.2799 | 13.65 | 13.66 | 13.75 | 0.93 | 0.1611 | 0.404 |
| TC0200012854.hg.1 | RAB1A | RAB1A, member RAS oncogene family | Multiple_C | 15.9 | 16.01 | 15.41 | 1.40 | 0.0665 | 0.1789 | 15.02 | 14.16 | 14.49 | 1.44 | 0.5068 | 0.738 |
| TC0200015744.hg.1 | PRKAG3 | protein kinase, AMP-activated, gamma 3 non-catalytic subunit | Multiple_C | 5.53 | 4.86 | 5.04 | 1.40 | 0.0397 | 0.122 | 4.67 | 4.64 | 4.59 | 1.06 | 0.5261 | 0.7507 |
| TC0200016775.hg.1 | ATG9A | autophagy related 9A | Multiple_C | 11.39 | 9.98 | 10.9 | 1.40 | 0.0058 | 0.0273 | 9.7 | 9.30 | 9.88 | 0.88 | 0.1363 | 0.3669 |
| TC0300007166.hg.1 | ZBTB47 | zinc finger and BTB domain containing 47 | Coding | 4.94 | 4.54 | 4.45 | 1.40 | 0.1276 | 0.2871 | 5.46 | 5.84 | 5.39 | 1.05 | 0.5667 | 0.7761 |
| TC0300010744.hg.1 | ACAA1 | acetyl-CoA acyltransferase 1 | Multiple_C | 13.41 | 13.2 | 12.92 | 1.40 | 0.0397 | 0.1221 | 10.93 | 11.82 | 11.45 | 0.70 | 0.0104 | 0.0803 |
| TC0400009198.hg.1 | APELA | apelin receptor early endogenous ligand | Multiple_C | 3.8 | 3.03 | 3.31 | 1.40 | 0.0614 | 0.1684 | 3.56 | 3.61 | 3.57 | 0.99 | 0.8191 | 0.9201 |
| TC0400011464.hg.1 | MANBA | mannosidase, beta A, lysosomal | Multiple_C | 10.67 | 10.7 | 10.18 | 1.40 | 0.0922 | 0.2271 | 7.28 | 7.04 | 7.8 | 0.70 | 0.0065 | 0.0583 |
| TC0500008202.hg.1 | FAM174A | family with sequence similarity 174, member A | Multiple_C | 5.6 | 4.79 | 5.11 | 1.40 | 0.57 | 0.7502 | 7.15 | 6.99 | 6.86 | 1.22 | 0.7681 | 0.896 |
| TC0500013039.hg.1 | ADAMTS2 | ADAM metallopeptidase with thrombospondin type 1 motif 2 | Multiple_C | 4.06 | 3.52 | 3.57 | 1.40 | 0.1605 | 0.3371 | 3.97 | 3.76 | 4.07 | 0.93 | 0.7343 | 0.8775 |
| TC0500013124.hg.1 | TRIM7 | tripartite motif containing 7 | Multiple_C | 6.52 | 5.86 | 6.03 | 1.40 | 0.27 | 0.4783 | 5.57 | 5.96 | 5.96 | 0.76 | 0.1015 | 0.3116 |

| | | | | | | | | | | | | | | | |
|---|---|---|---|---|---|---|---|---|---|---|---|---|---|---|---|
| TC0600009262.hg.1 | SLC35F1 | solute carrier family 35, member F1 | Coding | 4.52 | 3.72 | 4.03 | 1.40 | 0.1346 | 0.2985 | 3.42 | 3.33 | 3.36 | 1.04 | 0.5537 | 0.7686 |
| TC0600009381.hg.1 | CENPW | centromere protein W | Multiple_C | 11.21 | 11.91 | 10.72 | 1.40 | 0.32 | 0.5335 | 7.14 | 7.89 | 6.95 | 1.14 | 0.9288 | 0.9702 |
| TC0600014266.hg.1 | TNXB | tenascin XB | Multiple_C | 4.02 | 3.49 | 3.53 | 1.40 | 0.1031 | 0.2465 | 3.95 | 3.57 | 3.86 | 1.06 | 0.2065 | 0.4631 |
| TC0700007024.hg.1 | TAX1BP1 | Tax1 (human T-cell leukemia virus type I) binding protein 1 | Multiple_C | 14.79 | 16.21 | 14.3 | 1.40 | 0.2399 | 0.4419 | 10.52 | 10.10 | 9.95 | 1.48 | 0.067 | 0.2469 |
| TC0700008639.hg.1 | SPDYE2; SPDY | speedy/RINGO cell cycle regulator family member E2; speedy/R | Coding | 3.56 | 2.79 | 3.07 | 1.40 | 0.0121 | 0.0487 | 4.24 | 4.04 | 4.1 | 1.10 | 0.3194 | 0.5834 |
| TC0700008647.hg.1 | SPDYE2B | speedy/RINGO cell cycle regulator family member E2B | Coding | 3.56 | 2.79 | 3.07 | 1.40 | 0.0121 | 0.0487 | 4.24 | 4.04 | 4.1 | 1.10 | 0.3194 | 0.5834 |
| TC0700009796.hg.1 | HTR5A | 5-hydroxytryptamine (serotonin) receptor 5A, G protein-couple | Multiple_C | 3.79 | 3.25 | 3.3 | 1.40 | 0.0152 | 0.0575 | 3.82 | 3.89 | 3.79 | 1.02 | 0.7472 | 0.8841 |
| TC0700011575.hg.1 | POMZP3 | POM121 and ZP3 fusion | Multiple_C | 10.71 | 9.66 | 10.22 | 1.40 | 0.0466 | 0.1375 | 11.54 | 11.71 | 12.08 | 0.69 | 0.0256 | 0.1394 |
| TC0700013609.hg.1 | TFEC | transcription factor EC | Multiple_C | 3.61 | 2.9 | 3.12 | 1.40 | 0.1507 | 0.3232 | 3.86 | 3.79 | 3.28 | 1.49 | 0.0585 | 0.2281 |
| TC0800006603.hg.1 | DEFB105B; DE | defensin, beta 105B; defensin, beta 105A | Coding | 4.7 | 4.65 | 4.21 | 1.40 | 0.0947 | 0.2315 | 3.56 | 3.61 | 3.55 | 1.01 | 0.6517 | 0.8303 |
| TC0800008011.hg.1 | ZFHX4 | zinc finger homeobox 4 | Multiple_C | 4.11 | 3.61 | 3.62 | 1.40 | 0.2032 | 0.3954 | 5.1 | 4.99 | 4.24 | 1.82 | 0.0054 | 0.0516 |
| TC0900008992.hg.1 | PLPP7 | phospholipid phosphatase 7 (inactive) | Coding | 6.24 | 5.67 | 5.75 | 1.40 | 0.0904 | 0.2236 | 5.91 | 5.69 | 5.44 | 1.39 | 0.0479 | 0.2032 |
| TC0900009173.hg.1 | LCN9 | lipocalin 9 | Multiple_C | 4.22 | 3.43 | 3.73 | 1.40 | 0.1703 | 0.351 | 3.85 | 4.01 | 4.07 | 0.86 | 0.4793 | 0.7179 |
| TC0X00006446.hg.1 | CSF2RA | colony stimulating factor 2 receptor, alpha, low-affinity (granulo | Multiple_C | 6.38 | 6.48 | 5.89 | 1.40 | 0.6806 | 0.8263 | 3.48 | 3.59 | 3.35 | 1.09 | 0.9925 | 0.9968 |
| TC0X00008807.hg.1 | AVPR2 | arginine vasopressin receptor 2 | Multiple_C | 5.03 | 4.12 | 4.54 | 1.40 | 0.0847 | 0.2133 | 5.5 | 5.39 | 5.45 | 1.04 | 0.6123 | 0.8064 |
| TC0X00011214.hg.1 | CTAG1B | cancer/testis antigen 1B | Multiple_C | 5.99 | 5.58 | 5.5 | 1.40 | 0.075 | 0.1949 | 5.27 | 5.49 | 5.32 | 0.97 | 0.5452 | 0.7628 |
| TC1000009025.hg.1 | EMX2 | empty spiracles homeobox 2 | Multiple_C | 4.03 | 3.58 | 3.54 | 1.40 | 0.4795 | 0.6816 | 4.32 | 4.09 | 3.76 | 1.47 | 0.1249 | 0.3493 |
| TC1100007637.hg.1 | OR8U1; OR8U | olfactory receptor, family 8, subfamily U, member 1; olfactory r | Coding | 5.52 | 5.22 | 5.03 | 1.40 | 0.0504 | 0.1455 | 4.75 | 4.50 | 4.58 | 1.13 | 0.156 | 0.3966 |
| TC1100007943.hg.1 | PLCB3 | phospholipase C, beta 3 (phosphatidylinositol-specific) | Multiple_C | 10.91 | 9.54 | 10.42 | 1.40 | 0.0129 | 0.0511 | 9.27 | 9.22 | 8.44 | 1.78 | 0.0019 | 0.0254 |
| TC1100008847.hg.1 | CEP57 | centrosomal protein 57kDa | Multiple_C | 11.49 | 13.2 | 11 | 1.40 | 0.0702 | 0.1859 | 11.88 | 11.79 | 11.34 | 1.45 | 0.0031 | 0.0362 |
| TC1100009159.hg.1 | APOC3 | apolipoprotein C-III | Multiple_C | 4.81 | 4.17 | 4.32 | 1.40 | 0.2198 | 0.4169 | 5.14 | 5.37 | 5.2 | 0.96 | 0.4282 | 0.6802 |
| TC1100010047.hg.1 | C11orf16 | chromosome 11 open reading frame 16 | Multiple_C | 4.58 | 3.93 | 4.09 | 1.40 | 0.0331 | 0.1062 | 4.45 | 4.24 | 4.12 | 1.26 | 0.2355 | 0.4983 |
| TC1100010981.hg.1 | PATL1 | protein associated with topoisomerase II homolog 1 (yeast) | Multiple_C | 10.6 | 8.58 | 10.11 | 1.40 | 0.1056 | 0.2508 | 10.62 | 10.12 | 10.84 | 0.86 | 0.9459 | 0.9781 |
| TC1100011605.hg.1 | UCP3 | uncoupling protein 3 (mitochondrial, proton carrier) | Multiple_C | 5.73 | 4.8 | 5.24 | 1.40 | 0.2268 | 0.4259 | 5.64 | 5.75 | 5.5 | 1.10 | 0.0754 | 0.2638 |
| TC1100013096.hg.1 | RBM7 | RNA binding motif protein 7 | Multiple_C | 10.9 | 11.29 | 10.41 | 1.40 | 0.0704 | 0.1862 | 9.71 | 9.30 | 9.87 | 0.90 | 0.7631 | 0.8931 |
| TC1200007833.hg.1 | SLC39A5 | solute carrier family 39 (zinc transporter), member 5 | Multiple_C | 7.1 | 6.58 | 6.61 | 1.40 | 0.0126 | 0.0502 | 5.66 | 5.61 | 5.53 | 1.09 | 0.5429 | 0.7614 |
| TC1200009829.hg.1 | CLEC4E | C-type lectin domain family 4, member E | Multiple_C | 4.3 | 3.21 | 3.81 | 1.40 | 0.0857 | 0.2148 | 4.78 | 4.72 | 4.61 | 1.13 | 0.1167 | 0.3376 |
| TC1200009919.hg.1 | TAS2R10 | taste receptor, type 2, member 10 | Coding | 4.91 | 4.41 | 4.42 | 1.40 | 0.1655 | 0.3441 | 4.37 | 4.32 | 4.4 | 0.98 | 0.314 | 0.5781 |
| TC1200010490.hg.1 | NELL2 | neural EGFL like 2 | Multiple_C | 3.47 | 2.82 | 2.98 | 1.40 | 0.0195 | 0.0701 | 3.94 | 3.80 | 3.94 | 1.00 | 0.7787 | 0.901 |
| TC1200012861.hg.1 | DIABLO | diablo, IAP-binding mitochondrial protein | Multiple_C | 11.68 | 11.15 | 11.19 | 1.40 | 0.088 | 0.2193 | 9.54 | 9.46 | 10.27 | 0.60 | 0.0014 | 0.0206 |
| TC1300006734.hg.1 | MTUS2 | microtubule associated tumor suppressor candidate 2 | Multiple_C | 4.01 | 3.27 | 3.52 | 1.40 | 0.0419 | 0.1272 | 4.13 | 4.26 | 4.41 | 0.82 | 0.2432 | 0.5068 |
| TC1300007104.hg.1 | LRCH1 | leucine-rich repeats and calponin homology (CH) domain contai | Multiple_C | 9.32 | 9.27 | 8.83 | 1.40 | 0.0348 | 0.1104 | 10.05 | 9.72 | 8.97 | 2.11 | 0.0003 | 0.0077 |
| TC1300007158.hg.1 | MLNR | motilin receptor | Coding | 3.19 | 2.29 | 2.7 | 1.40 | 0.0344 | 0.1095 | 3.61 | 3.66 | 3.57 | 1.03 | 0.4665 | 0.708 |

| | | | | | | | | | | | | | | | |
|---|---|---|---|---|---|---|---|---|---|---|---|---|---|---|---|
| TC1300008108.hg.1 | F7 | coagulation factor VII (serum prothrombin conversion accelerat | Multiple_C | 5.92 | 5.23 | 5.43 | 1.40 | 0.0874 | 0.2182 | 4.9 | 5.01 | 4.96 | 0.96 | 0.6818 | 0.8474 |
| TC1300009673.hg.1 | TEX30 | testis expressed 30 | Multiple_C | 4.69 | 5.21 | 4.2 | 1.40 | 0.1234 | 0.2804 | 10.92 | 11.42 | 10.45 | 1.39 | 0.0356 | 0.171 |
| TC1400007093.hg.1 | KLHDC1 | kelch domain containing 1 | Multiple_C | 5.5 | 6.69 | 5.01 | 1.40 | 0.0493 | 0.1431 | 5.21 | 5.04 | 4.79 | 1.34 | 0.1279 | 0.354 |
| TC1500007060.hg.1 | MAP1A | microtubule associated protein 1A | Multiple_C | 4.17 | 2.94 | 3.68 | 1.40 | 0.3313 | 0.5454 | 4.34 | 4.42 | 4.06 | 1.21 | 0.0891 | 0.2889 |
| TC1600006587.hg.1 | TSC2 | tuberous sclerosis 2 | Multiple_C | 8.53 | 7.73 | 8.04 | 1.40 | 0.1535 | 0.3271 | 7.51 | 7.75 | 7.21 | 1.23 | 0.7383 | 0.8794 |
| TC1600006685.hg.1 | ZNF75A | zinc finger protein 75a | Multiple_C | 5.36 | 5.01 | 4.87 | 1.40 | 0.0426 | 0.1289 | 6.46 | 6.76 | 7.25 | 0.58 | 0.0054 | 0.0514 |
| TC1600009244.hg.1 | SEPT12 | septin 12 | Multiple_C | 5.28 | 4.9 | 4.79 | 1.40 | 0.0141 | 0.0543 | 5.05 | 5.11 | 4.82 | 1.17 | 0.2526 | 0.5168 |
| TC1600010618.hg.1 | EXOC3L1 | exocyst complex component 3-like 1 | Multiple_C | 6.7 | 6.04 | 6.21 | 1.40 | 0.0868 | 0.217 | 5.84 | 5.87 | 5.77 | 1.05 | 0.3217 | 0.5856 |
| TC1600011374.hg.1 | SPN | sialophorin | Multiple_C | 6.24 | 5.52 | 5.75 | 1.40 | 0.0421 | 0.1275 | 8.04 | 8.07 | 7.92 | 1.09 | 0.5796 | 0.785 |
| TC1700007383.hg.1 | RPL23A; SNOF | ribosomal protein L23a; small nucleolar RNA, C/D box 4B; small | Multiple_C | 17.49 | 17.12 | 17 | 1.40 | 0.0052 | 0.0248 | 15.04 | 14.86 | 15 | 1.03 | 0.9348 | 0.9725 |
| TC1700007387.hg.1 | TRAF4 | TNF receptor-associated factor 4 | Multiple_C | 7.74 | 6.83 | 7.25 | 1.40 | 0.3664 | 0.5796 | 5.75 | 5.85 | 6.07 | 0.80 | 0.47 | 0.7108 |
| TC1700008218.hg.1 | FLJ45513; RP1 | uncharacterized LOC729220; novel transcript antisense to TAC4 | Multiple_C | 4.49 | 3.84 | 4 | 1.40 | 0.0934 | 0.2292 | 4.44 | 4.47 | 3.93 | 1.42 | 0.7511 | 0.8866 |
| TC1700010649.hg.1 | KRTAP1-4 | keratin associated protein 1-4 | Coding | 5.65 | 4.83 | 5.16 | 1.40 | 0.1286 | 0.2889 | 5.34 | 5.31 | 5.23 | 1.08 | 0.3985 | 0.6559 |
| TC1700011699.hg.1 | CD300C | CD300c molecule | Coding | 4.49 | 3.89 | 4 | 1.40 | 0.0825 | 0.2092 | 5.08 | 4.91 | 4.82 | 1.20 | 0.1152 | 0.3349 |
| TC1700012287.hg.1 | RPS6KB1 | ribosomal protein S6 kinase, 70kDa, polypeptide 1 | Multiple_C | 12.4 | 12.06 | 11.91 | 1.40 | 0.0388 | 0.1199 | 12.6 | 12.57 | 12.84 | 0.85 | 0.2268 | 0.4874 |
| TC1700012307.hg.1 | TSEN54 | TSEN54 tRNA splicing endonuclease subunit | Multiple_C | 7.17 | 6.59 | 6.68 | 1.40 | 0.2048 | 0.3976 | 7.24 | 7.98 | 7.93 | 0.62 | 0.1215 | 0.3442 |
| TC1800006639.hg.1 | RALBP1 | ralA binding protein 1 | Multiple_C | 10.24 | 10.02 | 9.75 | 1.40 | 0.2752 | 0.4842 | 8.94 | 9.34 | 9.17 | 0.85 | 0.4502 | 0.6962 |
| TC1900007045.hg.1 | ZNF441 | zinc finger protein 441 | Multiple_C | 4.79 | 4.12 | 4.3 | 1.40 | 0.3014 | 0.5138 | 5.38 | 5.37 | 5.35 | 1.02 | 0.1684 | 0.4141 |
| TC1900009808.hg.1 | ADGRL1 | adhesion G protein-coupled receptor L1 | Multiple_C | 8.38 | 7.75 | 7.89 | 1.40 | 0.2112 | 0.4056 | 7.49 | 7.04 | 7.21 | 1.21 | 0.3638 | 0.6265 |
| TC1900011397.hg.1 | LENG1 | leukocyte receptor cluster (LRC) member 1 | Coding | 6.91 | 6.46 | 6.42 | 1.40 | 0.029 | 0.0956 | 6.41 | 6.44 | 6.6 | 0.88 | 0.2042 | 0.4602 |
| TC1900011757.hg.1 | CEACAM19 | carcinoembryonic antigen-related cell adhesion molecule 19 | Multiple_C | 5.88 | 5.26 | 5.39 | 1.40 | 0.4901 | 0.6901 | 5.97 | 5.85 | 5.97 | 1.00 | 0.8643 | 0.9422 |
| TC2000007297.hg.1 | VSTM2L | V-set and transmembrane domain containing 2 like | Coding | 5.78 | 5.36 | 5.29 | 1.40 | 0.2209 | 0.4183 | 4.66 | 4.74 | 4.61 | 1.04 | 0.9171 | 0.9663 |
| TC2000008149.hg.1 | TMEM74B | transmembrane protein 74B | Multiple_C | 3.72 | 3.44 | 3.23 | 1.40 | 0.2204 | 0.4178 | 3.78 | 4.00 | 3.5 | 1.21 | 0.1481 | 0.3847 |
| TC2100006854.hg.1 | MAP3K7CL | MAP3K7 C-terminal like | Multiple_C | 4.49 | 4.22 | 4 | 1.40 | 0.07 | 0.1855 | 3.69 | 3.81 | 3.85 | 0.90 | 0.6742 | 0.8422 |
| TC2100008376.hg.1 | KRTAP10-2 | keratin associated protein 10-2 | Multiple_C | 5.29 | 4.46 | 4.8 | 1.40 | 0.0618 | 0.1693 | 5.28 | 5.27 | 5.28 | 1.00 | 0.9761 | 0.9902 |
| TC2200007068.hg.1 | SLC35E4 | solute carrier family 35, member E4 | Multiple_C | 3.84 | 3.44 | 3.35 | 1.40 | 0.0589 | 0.1634 | 5.14 | 5.39 | 5.8 | 0.63 | 0.0591 | 0.2291 |
| TC2200007307.hg.1 | GGA1 | golgi-associated, gamma adaptin ear containing, ARF binding pr | Multiple_C | 9 | 8.2 | 8.51 | 1.40 | 0.2557 | 0.4615 | 8.77 | 9.13 | 8.82 | 0.97 | 0.6369 | 0.8219 |
| TSUnmapped00000285.h | MLXIP | MLX interacting protein | Coding | 5.88 | 4.97 | 5.39 | 1.40 | 0.0253 | 0.0861 | 4.2 | 4.60 | 4.63 | 0.74 | 0.2748 | 0.5406 |
| TSUnmapped00000804.h | PRAMEF5 | PRAME family member 5 | Coding | 6.03 | 5.28 | 5.54 | 1.40 | 0.188 | 0.3754 | 5.72 | 5.76 | 5.8 | 0.95 | 0.7745 | 0.8987 |
| TC0100010366.hg.1 | FCRLB | Fc receptor-like B | Multiple_C | 5.53 | 5 | 5.05 | 1.39 | 0.0162 | 0.0605 | 5.87 | 6.11 | 6.12 | 0.84 | 0.2188 | 0.4787 |
| TC0100010579.hg.1 | FMO3 | flavin containing monooxygenase 3 | Multiple_C | 4.52 | 3.8 | 4.04 | 1.39 | 0.4827 | 0.6842 | 3.88 | 3.57 | 3.96 | 0.95 | 0.2791 | 0.5452 |
| TC0100011857.hg.1 | GJC2 | gap junction protein gamma 2 | Multiple_C | 4.5 | 3.9 | 4.02 | 1.39 | 0.0721 | 0.1894 | 4.27 | 4.48 | 4.71 | 0.74 | 0.073 | 0.259 |
| TC0100013744.hg.1 | MEAF6 | MYST/Esa1-associated factor 6 | Multiple_C | 11.46 | 11.41 | 10.98 | 1.39 | 0.0502 | 0.145 | 10.88 | 10.46 | 10.91 | 0.98 | 0.4612 | 0.7041 |

| | | | | | | | | | | | | | | | |
|---|---|---|---|---|---|---|---|---|---|---|---|---|---|---|---|
| TC0100013925.hg.1 | MED8 | mediator complex subunit 8 | Multiple_C | 9.64 | 9.14 | 9.16 | 1.39 | 0.1072 | 0.2535 | 8.68 | 8.51 | 9.13 | 0.73 | 0.2702 | 0.5354 |
| TC0100014323.hg.1 | C8B | complement component 8, beta polypeptide | Multiple_C | 3.44 | 2.82 | 2.96 | 1.39 | 0.0662 | 0.1783 | 3.63 | 3.58 | 3.48 | 1.11 | 0.6768 | 0.8443 |
| TC0100014368.hg.1 | C1orf87 | chromosome 1 open reading frame 87 | Multiple_C | 3.58 | 3.12 | 3.1 | 1.39 | 0.1617 | 0.3388 | 3.9 | 3.99 | 3.74 | 1.12 | 0.112 | 0.3296 |
| TC0100016755.hg.1 | BRINP3 | bone morphogenetic protein/retinoic acid inducible neural-spec | Multiple_C | 3.31 | 3.15 | 2.83 | 1.39 | 0.0838 | 0.2116 | 3.55 | 3.38 | 3.28 | 1.21 | 0.1805 | 0.43 |
| TC0100018177.hg.1 | CELA2A | chymotrypsin-like elastase family, member 2A | Multiple_C | 6.33 | 5.61 | 5.85 | 1.39 | 0.1135 | 0.2642 | 6.33 | 6.33 | 6.2 | 1.09 | 0.366 | 0.6283 |
| TC0100018180.hg.1 | CLCNKA | chloride channel, voltage-sensitive Ka | Multiple_C | 5.29 | 4.57 | 4.81 | 1.39 | 0.0554 | 0.156 | 5.02 | 4.93 | 4.91 | 1.08 | 0.5586 | 0.7714 |
| TC0100018199.hg.1 | SRRM1 | serine/arginine repetitive matrix 1 | Multiple_C | 13.6 | 12.61 | 13.12 | 1.39 | 0.1002 | 0.2414 | 14.38 | 14.04 | 14.77 | 0.76 | 0.0774 | 0.2682 |
| TC0100018441.hg.1 | CDCP2 | CUB domain containing protein 2 | Multiple_C | 7.8 | 7.17 | 7.32 | 1.39 | 0.044 | 0.1317 | 7.5 | 7.38 | 7.33 | 1.13 | 0.5144 | 0.7435 |
| TC0200010045.hg.1 | HOXD13 | homeobox D13 | Coding | 3.52 | 2.93 | 3.04 | 1.39 | 0.0376 | 0.1171 | 4.13 | 4.05 | 4.07 | 1.04 | 0.6785 | 0.8452 |
| TC0200010615.hg.1 | CCNYL1; MIR4 | cyclin Y like 1; microRNA 4775 | Multiple_C | 8.3 | 8 | 7.82 | 1.39 | 0.3229 | 0.5367 | 7.61 | 8.05 | 8.48 | 0.55 | 0.0011 | 0.0183 |
| TC0200010859.hg.1 | SLC4A3 | solute carrier family 4 (anion exchanger), member 3 | Multiple_C | 6.66 | 6.29 | 6.18 | 1.39 | 0.0591 | 0.1638 | 5.87 | 5.96 | 5.57 | 1.23 | 0.1871 | 0.4388 |
| TC0200016159.hg.1 | GBX2 | gastrulation brain homeobox 2 | Multiple_C | 3.67 | 2.82 | 3.19 | 1.39 | 0.1594 | 0.3357 | 4.28 | 4.10 | 4.02 | 1.20 | 0.179 | 0.4279 |
| TC0300006446.hg.1 | CNTN6 | contactin 6 | Multiple_C | 4 | 3.44 | 3.52 | 1.39 | 0.06 | 0.1656 | 3.9 | 3.89 | 3.73 | 1.13 | 0.0996 | 0.3085 |
| TC0300006784.hg.1 | RAB5A | RAB5A, member RAS oncogene family | Multiple_C | 14.59 | 14.46 | 14.11 | 1.39 | 0.1023 | 0.245 | 13.94 | 13.31 | 13.54 | 1.32 | 0.1795 | 0.4285 |
| TC0300006846.hg.1 | RPL15 | ribosomal protein L15 | Multiple_C | 17.61 | 17.42 | 17.13 | 1.39 | 0.03 | 0.0984 | 15.89 | 15.63 | 15.46 | 1.35 | 0.1803 | 0.4297 |
| TC0300009713.hg.1 | EPHB3 | EPH receptor B3 | Multiple_C | 4.68 | 4.03 | 4.2 | 1.39 | 0.1119 | 0.2614 | 7.04 | 7.29 | 7.06 | 0.99 | 0.6276 | 0.816 |
| TC0300012654.hg.1 | TFDP2 | transcription factor Dp-2 (E2F dimerization partner 2) | Multiple_C | 11.15 | 11.74 | 10.67 | 1.39 | 0.2192 | 0.4161 | 9.82 | 9.72 | 9.29 | 1.44 | 0.1239 | 0.3475 |
| TC0300013386.hg.1 | THPO | thrombopoietin | Multiple_C | 4.37 | 3.95 | 3.89 | 1.39 | 0.2386 | 0.4403 | 3.76 | 3.74 | 3.64 | 1.09 | 0.6621 | 0.835 |
| TC0400008084.hg.1 | DMP1 | dentin matrix acidic phosphoprotein 1 | Coding | 3.4 | 2.93 | 2.92 | 1.39 | 0.3942 | 0.6079 | 4.03 | 3.85 | 4.14 | 0.93 | 0.6444 | 0.8255 |
| TC0400011013.hg.1 | PPBP | pro-platelet basic protein | Coding | 4.45 | 3.71 | 3.97 | 1.39 | 0.0168 | 0.0624 | 4.21 | 3.91 | 4.35 | 0.91 | 0.458 | 0.702 |
| TC0500006442.hg.1 | SDHA | succinate dehydrogenase complex subunit A, flavoprotein (Fp) | Multiple_C | 11.15 | 11.13 | 10.67 | 1.39 | 0.1095 | 0.2576 | 10.92 | 10.99 | 10.49 | 1.35 | 0.3042 | 0.5679 |
| TC0500012501.hg.1 | CCDC69 | coiled-coil domain containing 69 | Multiple_C | 4.86 | 4.21 | 4.38 | 1.39 | 0.0999 | 0.2407 | 5.53 | 5.39 | 4.71 | 1.77 | 0.0005 | 0.0104 |
| TC0500012662.hg.1 | ZBED8 | zinc finger, BED-type containing 8 | Coding | 5.53 | 6.02 | 5.05 | 1.39 | 0.661 | 0.814 | 5.42 | 6.03 | 5.21 | 1.16 | 0.2624 | 0.5267 |
| TC0600007416.hg.1 | ZKSCAN3 | zinc finger with KRAB and SCAN domains 3 | Multiple_C | 4.43 | 3.6 | 3.95 | 1.39 | 0.0437 | 0.1311 | 4.71 | 4.85 | 4.84 | 0.91 | 0.6979 | 0.8569 |
| TC0600014360.hg.1 | IPCEF1 | interaction protein for cytohesin exchange factors 1 | Multiple_C | 4.71 | 4.48 | 4.23 | 1.39 | 0.1402 | 0.3074 | 5.12 | 5.20 | 5.04 | 1.06 | 0.6916 | 0.8528 |
| TC0700006520.hg.1 | MAFK | v-maf avian musculoaponeurotic fibrosarcoma oncogene homo | Multiple_C | 7.99 | 7.14 | 7.51 | 1.39 | 0.3661 | 0.5794 | 7.15 | 7.25 | 8 | 0.55 | 0.0119 | 0.0867 |
| TC0700008928.hg.1 | CPED1 | cadherin-like and PC-esterase domain containing 1 | Multiple_C | 4.52 | 3.93 | 4.04 | 1.39 | 0.3629 | 0.5765 | 4.54 | 3.88 | 3.85 | 1.61 | 0.0028 | 0.0339 |
| TC0700008963.hg.1 | LMOD2 | leiomodin 2 (cardiac) | Coding | 3.68 | 2.94 | 3.2 | 1.39 | 0.3897 | 0.6034 | 3.46 | 3.31 | 3.54 | 0.95 | 0.9395 | 0.975 |
| TC0700013391.hg.1 | NSUN5P1 | NOP2/Sun domain family, member 5 pseudogene 1 | Multiple_C | 9.08 | 8.76 | 8.6 | 1.39 | 0.0758 | 0.1964 | 7.48 | 7.81 | 7.67 | 0.88 | 0.9672 | 0.9863 |
| TC0800006978.hg.1 | POLR3D | polymerase (RNA) III (DNA directed) polypeptide D, 44kDa | Multiple_C | 10.19 | 10.12 | 9.71 | 1.39 | 0.2482 | 0.452 | 9.68 | 9.16 | 9.6 | 1.06 | 0.5674 | 0.7764 |
| TC0800011171.hg.1 | UQCRB | ubiquinol-cytochrome c reductase binding protein | Multiple_C | 14.79 | 16.09 | 14.31 | 1.39 | 0.1454 | 0.3154 | 10.83 | 10.55 | 10.39 | 1.36 | 0.7618 | 0.8923 |
| TC0900006569.hg.1 | MLANA | melan-A | Multiple_C | 4.57 | 3.85 | 4.09 | 1.39 | 0.0309 | 0.1007 | 4.24 | 4.24 | 4.25 | 0.99 | 0.923 | 0.9684 |
| TC0900008727.hg.1 | GPR144; ADGI | Transcript Identified by AceView, Entrez Gene ID(s) 347088; adh | Multiple_C | 4.22 | 3.8 | 3.74 | 1.39 | 0.6684 | 0.8187 | 3.78 | 3.62 | 3.46 | 1.25 | 0.1286 | 0.3548 |

| | | | | | | | | | | | | | | |
|---|---|---|---|---|---|---|---|---|---|---|---|---|---|---|
| TC0900008910.hg.1 | C9orf106 | chromosome 9 open reading frame 106 | Multiple_C | 5.81 | 5.36 | 5.33 | 1.39 | 0.0806 | 0.2061 | 5.51 | 5.48 | 5.5 | 1.01 | 0.3236 | 0.588 |
| TC0900012289.hg.1 | LCN6 | lipocalin 6 | Multiple_C | 4.87 | 4.21 | 4.39 | 1.39 | 0.0319 | 0.1033 | 3.99 | 4.08 | 4.11 | 0.92 | 0.5096 | 0.7397 |
| TC0900012293.hg.1 | TMEM210 | transmembrane protein 210 | Multiple_C | 4.67 | 3.78 | 4.19 | 1.39 | 0.0737 | 0.1924 | 4.61 | 4.72 | 4.65 | 0.97 | 0.8841 | 0.9504 |
| TC0X00008027.hg.1 | ZCCHC18 | zinc finger, CCHC domain containing 18 | Multiple_C | 3.81 | 3.23 | 3.33 | 1.39 | 0.1032 | 0.2466 | 3.61 | 3.94 | 3.74 | 0.91 | 0.94 | 0.9753 |
| TC0X00008344.hg.1 | SH2D1A | SH2 domain containing 1A | Multiple_C | 4.42 | 3.55 | 3.94 | 1.39 | 0.0164 | 0.0612 | 4.34 | 4.42 | 4.37 | 0.98 | 0.738 | 0.8792 |
| TC0X00008737.hg.1 | CNGA2 | cyclic nucleotide gated channel alpha 2 | Coding | 7.14 | 6.71 | 6.66 | 1.39 | 0.1509 | 0.3234 | 7.41 | 7.24 | 7.27 | 1.10 | 0.4105 | 0.6652 |
| TC0X00009659.hg.1 | PRICKLE3 | prickle homolog 3 | Multiple_C | 6.3 | 5.5 | 5.82 | 1.39 | 0.0476 | 0.1395 | 6.23 | 6.46 | 6.46 | 0.85 | 0.3924 | 0.6507 |
| TC1000006794.hg.1 | DHTKD1 | dehydrogenase E1 and transketolase domain containing 1 | Multiple_C | 9.66 | 9.4 | 9.18 | 1.39 | 0.168 | 0.3477 | 8.56 | 8.99 | 8.46 | 1.07 | 0.7329 | 0.8766 |
| TC1000007583.hg.1 | C10orf71 | chromosome 10 open reading frame 71 | Multiple_C | 4.7 | 3.96 | 4.22 | 1.39 | 0.0382 | 0.1185 | 3.66 | 3.81 | 3.76 | 0.93 | 0.7687 | 0.8963 |
| TC1000008253.hg.1 | NRG3 | neuregulin 3 | Coding | 4.46 | 3.84 | 3.98 | 1.39 | 0.0356 | 0.1125 | 4.93 | 4.15 | 4.21 | 1.65 | 0.0048 | 0.0482 |
| TC1100007774.hg.1 | MS4A13 | membrane-spanning 4-domains, subfamily A, member 13 | Coding | 3.54 | 3.31 | 3.06 | 1.39 | 0.289 | 0.4998 | 3.53 | 3.42 | 3.47 | 1.04 | 0.9187 | 0.9666 |
| TC1100008045.hg.1 | TSGA10IP | testis specific 10 interacting protein | Multiple_C | 6.12 | 5.7 | 5.64 | 1.39 | 0.0181 | 0.0663 | 5.67 | 5.89 | 5.97 | 0.81 | 0.3163 | 0.5802 |
| TC1100009901.hg.1 | OR51G1 | olfactory receptor, family 51, subfamily G, member 1 (gene/pse | Multiple_C | 3.76 | 2.93 | 3.28 | 1.39 | 0.282 | 0.492 | 4.04 | 4.02 | 4 | 1.03 | 0.8096 | 0.9156 |
| TC1100011122.hg.1 | HRASLS5 | HRAS-like suppressor family, member 5 | Multiple_C | 7.21 | 6.56 | 6.73 | 1.39 | 0.0562 | 0.1577 | 3.96 | 3.75 | 4.01 | 0.97 | 0.5238 | 0.7491 |
| TC1100011910.hg.1 | TRIM49 | tripartite motif containing 49 | Coding | 5.02 | 4.04 | 4.54 | 1.39 | 0.0348 | 0.1103 | 6.52 | 6.36 | 5.92 | 1.52 | 0.1582 | 0.3998 |
| TC1100012420.hg.1 | APOA5 | apolipoprotein A-V | Coding | 6.68 | 5.99 | 6.2 | 1.39 | 0.3093 | 0.5224 | 5.96 | 6.03 | 5.91 | 1.04 | 0.9241 | 0.9688 |
| TC1200007595.hg.1 | SMARCD1 | SWI/SNF related, matrix associated, actin dependent regulator c | Multiple_C | 11.19 | 10.2 | 10.71 | 1.39 | 0.4237 | 0.6348 | 12.27 | 13.44 | 13.07 | 0.57 | 0.0077 | 0.0653 |
| TC1200010961.hg.1 | NDUFA4L2 | NADH dehydrogenase (ubiquinone) 1 alpha subcomplex, 4-like 2 | Multiple_C | 4.56 | 4.3 | 4.08 | 1.39 | 0.2498 | 0.4541 | 4.81 | 5.33 | 4.71 | 1.07 | 0.6869 | 0.85 |
| TC1200011224.hg.1 | LRRC10 | leucine rich repeat containing 10 | Coding | 4.95 | 4.52 | 4.47 | 1.39 | 0.2813 | 0.4913 | 5.59 | 4.73 | 4.95 | 1.56 | 0.1663 | 0.4114 |
| TC1200012764.hg.1 | PRH1; TAS2R1 | proline-rich protein HaeIII subfamily 1; taste receptor, type 2, m | Multiple_C | 6.88 | 6.71 | 6.4 | 1.39 | 0.1734 | 0.3552 | 7.05 | 6.99 | 6.86 | 1.14 | 0.3068 | 0.5711 |
| TC1300006676.hg.1 | RASL11A | RAS-like, family 11, member A | Multiple_C | 7.21 | 6.9 | 6.73 | 1.39 | 0.0876 | 0.2187 | 5.08 | 5.67 | 5.1 | 0.99 | 0.3076 | 0.5716 |
| TC1300007248.hg.1 | CKAP2 | cytoskeleton associated protein 2 | Multiple_C | 11.26 | 10.7 | 10.78 | 1.39 | 0.5733 | 0.7525 | 11.78 | 12.00 | 12.06 | 0.82 | 0.3839 | 0.6429 |
| TC1300008342.hg.1 | C1QTNF9B | C1q and tumor necrosis factor related protein 9B | Multiple_C | 8.8 | 8.08 | 8.32 | 1.39 | 0.0198 | 0.071 | 7.09 | 7.24 | 7.32 | 0.85 | 0.3429 | 0.6066 |
| TC1300009088.hg.1 | DIAPH3 | diaphanous-related formin 3 | Multiple_C | 10.06 | 8.73 | 9.58 | 1.39 | 0.1864 | 0.3731 | 10.98 | 11.45 | 10.78 | 1.15 | 0.8489 | 0.9347 |
| TC1300009672.hg.1 | CCDC168 | coiled-coil domain containing 168 | Coding | 3.75 | 3.32 | 3.27 | 1.39 | 0.1449 | 0.3148 | 3.44 | 3.93 | 3.79 | 0.78 | 0.7064 | 0.8618 |
| TC1400008844.hg.1 | HECTD1 | HECT domain containing E3 ubiquitin protein ligase 1 | Multiple_C | 11.56 | 11.44 | 11.08 | 1.39 | 0.3128 | 0.5263 | 9.35 | 9.50 | 9.57 | 0.86 | 0.3278 | 0.5912 |
| TC1500006786.hg.1 | SCG5 | secretogranin V | Multiple_C | 4.85 | 4.93 | 4.37 | 1.39 | 0.2551 | 0.4608 | 4.35 | 4.41 | 4.31 | 1.03 | 0.2104 | 0.4677 |
| TC1500008135.hg.1 | UBE2Q2L | ubiquitin conjugating enzyme E2Q family member 2-like | Coding | 5.25 | 4.8 | 4.77 | 1.39 | 0.1242 | 0.2813 | 6.3 | 6.32 | 6.22 | 1.06 | 0.2735 | 0.5389 |
| TC1500009926.hg.1 | LARP6 | La ribonucleoprotein domain family, member 6 | Multiple_C | 6.44 | 5.81 | 5.96 | 1.39 | 0.177 | 0.3596 | 6.93 | 7.16 | 7.27 | 0.79 | 0.0501 | 0.2086 |
| TC1600006486.hg.1 | RHBDL1 | rhomboid, veinlet-like 1 (Drosophila) | Multiple_C | 5.85 | 5.64 | 5.37 | 1.39 | 0.0906 | 0.2241 | 5.24 | 5.05 | 5.28 | 0.97 | 0.2379 | 0.5014 |
| TC1600007499.hg.1 | CTF1 | cardiotrophin 1 | Coding | 4.93 | 4.23 | 4.45 | 1.39 | 0.3453 | 0.559 | 5.01 | 4.91 | 4.97 | 1.03 | 0.5933 | 0.7945 |
| TC1600011190.hg.1 | CA5A | carbonic anhydrase VA, mitochondrial | Multiple_C | 4.01 | 3.5 | 3.53 | 1.39 | 0.5947 | 0.7689 | 4.02 | 4.03 | 3.81 | 1.16 | 0.5606 | 0.7721 |
| TC1600011377.hg.1 | PRRT2 | proline-rich transmembrane protein 2 | Multiple_C | 5.2 | 4.43 | 4.72 | 1.39 | 0.0074 | 0.033 | 4.24 | 4.39 | 4.41 | 0.89 | 0.6437 | 0.8253 |

| | | | | | | | | | | | | | | | |
|---|---|---|---|---|---|---|---|---|---|---|---|---|---|---|---|
| TC1700008238.hg.1 | SGCA | sarcoglycan alpha | Multiple_C | 3.41 | 2.73 | 2.93 | 1.39 | 0.0933 | 0.2291 | 3.72 | 3.74 | 3.81 | 0.94 | 0.3341 | 0.5982 |
| TC1700008423.hg.1 | OR4D2 | olfactory receptor, family 4, subfamily D, member 2 | Coding | 5.14 | 4.66 | 4.66 | 1.39 | 0.1082 | 0.2553 | 4.84 | 5.01 | 4.88 | 0.97 | 0.9212 | 0.9675 |
| TC1700008846.hg.1 | KIF19 | kinesin family member 19 | Multiple_C | 5.79 | 5.01 | 5.31 | 1.39 | 0.2454 | 0.4489 | 5.72 | 5.38 | 5.18 | 1.45 | 0.0356 | 0.171 |
| TC1700011708.hg.1 | CD300LF | CD300 molecule-like family member f | Multiple_C | 5.46 | 5.16 | 4.98 | 1.39 | 0.129 | 0.2893 | 4.97 | 5.20 | 5.37 | 0.76 | 0.1601 | 0.4027 |
| TC1900007240.hg.1 | OR10H5 | olfactory receptor, family 10, subfamily H, member 5 | Coding | 3.82 | 3.32 | 3.34 | 1.39 | 0.1334 | 0.2968 | 3.58 | 3.60 | 3.25 | 1.26 | 0.1222 | 0.345 |
| TC1900008575.hg.1 | MYH14 | myosin, heavy chain 14, non-muscle | Multiple_C | 6.13 | 6.11 | 5.65 | 1.39 | 0.0522 | 0.1491 | 3.73 | 3.56 | 3.4 | 1.26 | 0.0347 | 0.1682 |
| TC1900008902.hg.1 | BRSK1 | BR serine/threonine kinase 1 | Multiple_C | 4.17 | 3.57 | 3.69 | 1.39 | 0.1731 | 0.3548 | 4.38 | 4.26 | 3.94 | 1.36 | 0.0392 | 0.1804 |
| TC1900008987.hg.1 | AURKC | aurora kinase C | Coding | 3.61 | 2.88 | 3.13 | 1.39 | 0.0461 | 0.1362 | 4.06 | 3.44 | 3.76 | 1.23 | 0.1518 | 0.3903 |
| TC1900009307.hg.1 | APBA3 | amyloid beta (A4) precursor protein-binding, family A, member | Multiple_C | 6.81 | 6.17 | 6.33 | 1.39 | 0.1165 | 0.2692 | 5.87 | 6.04 | 5.61 | 1.20 | 0.9267 | 0.9697 |
| TC1900009604.hg.1 | COL5A3 | collagen, type V, alpha 3 | Multiple_C | 3.96 | 3.7 | 3.48 | 1.39 | 0.0211 | 0.0747 | 4.01 | 4.35 | 4.43 | 0.75 | 0.0973 | 0.3041 |
| TC1900011099.hg.1 | HSD17B14 | hydroxysteroid (17-beta) dehydrogenase 14 | Multiple_C | 3.41 | 3.11 | 2.93 | 1.39 | 0.1378 | 0.3033 | 3.04 | 3.13 | 3.08 | 0.97 | 0.7326 | 0.8763 |
| TC1900011581.hg.1 | ZNF154 | zinc finger protein 154 | Multiple_C | 3.69 | 3.37 | 3.21 | 1.39 | 0.245 | 0.4485 | 3.44 | 3.46 | 3.41 | 1.02 | 0.2646 | 0.5292 |
| TC1900011688.hg.1 | NDUFA13 | NADH dehydrogenase (ubiquinone) 1 alpha subcomplex, 13 | Multiple_C | 10.21 | 10.25 | 9.73 | 1.39 | 0.1806 | 0.3648 | 8.93 | 8.99 | 8.33 | 1.52 | 0.014 | 0.0959 |
| TC1900011753.hg.1 | ZNF284 | zinc finger protein 284 | Multiple_C | 4.72 | 4.64 | 4.24 | 1.39 | 0.085 | 0.2139 | 6.19 | 6.16 | 6.17 | 1.01 | 0.4872 | 0.7238 |
| TC1900011928.hg.1 | ZNF675 | zinc finger protein 675 | Multiple_C | 12.28 | 12.05 | 11.8 | 1.39 | 0.1235 | 0.2804 | 10.04 | 10.10 | 10.26 | 0.86 | 0.4142 | 0.6687 |
| TC1900011984.hg.1 | DMPK | dystrophia myotonica-protein kinase | Multiple_C | 5.81 | 5.23 | 5.33 | 1.39 | 0.123 | 0.2797 | 6.06 | 6.03 | 5.91 | 1.11 | 0.6084 | 0.8042 |
| TC2000008134.hg.1 | TCF15 | transcription factor 15 (basic helix-loop-helix) | Coding | 5.74 | 5.37 | 5.26 | 1.39 | 0.176 | 0.3583 | 5.67 | 5.67 | 5.61 | 1.04 | 0.7442 | 0.883 |
| TC2000009239.hg.1 | WFDC10B | WAP four-disulfide core domain 10B | Coding | 3.96 | 3.31 | 3.48 | 1.39 | 0.4652 | 0.67 | 4.3 | 3.92 | 3.99 | 1.24 | 0.2581 | 0.5228 |
| TC2100006627.hg.1 | MIR99AHG | mir-99a-let-7c cluster host gene | Multiple_C | 5.5 | 5.07 | 5.02 | 1.39 | 0.2662 | 0.474 | 5.94 | 5.29 | 4.83 | 2.16 | 0.0041 | 0.0436 |
| TC2200006457.hg.1 | POTEH | POTE ankyrin domain family, member H | Multiple_C | 4.96 | 4.41 | 4.48 | 1.39 | 0.0274 | 0.0915 | 4.21 | 4.23 | 4.21 | 1.00 | 0.7729 | 0.8979 |
| TC2200006617.hg.1 | TBX1 | T-box 1 | Multiple_C | 4.5 | 3.85 | 4.02 | 1.39 | 0.0809 | 0.2066 | 4.5 | 4.60 | 4.7 | 0.87 | 0.4576 | 0.7015 |
| TSUnmapped00000342.h | TCF20 | transcription factor 20 (AR1) | Coding | 9.45 | 8.53 | 8.97 | 1.39 | 0.1343 | 0.298 | 9.97 | 10.01 | 10.18 | 0.86 | 0.4311 | 0.6826 |
| TSUnmapped00000362.h | TCF20 | transcription factor 20 (AR1) | Coding | 9.45 | 8.53 | 8.97 | 1.39 | 0.1343 | 0.298 | 9.97 | 10.01 | 10.18 | 0.86 | 0.4311 | 0.6826 |
| TC0100006739.hg.1 | SLC45A1 | solute carrier family 45, member 1 | Multiple_C | 3.68 | 2.93 | 3.21 | 1.39 | 0.0189 | 0.0683 | 4.24 | 4.38 | 4.16 | 1.06 | 0.4586 | 0.7022 |
| TC0100007451.hg.1 | CD52 | CD52 molecule | Multiple_C | 5.9 | 5.02 | 5.43 | 1.39 | 0.2192 | 0.4161 | 4.85 | 4.77 | 5.08 | 0.85 | 0.8583 | 0.9397 |
| TC0100007671.hg.1 | DCDC2B | doublecortin domain containing 2B | Multiple_C | 4.09 | 3.67 | 3.62 | 1.39 | 0.2736 | 0.4822 | 4.74 | 4.46 | 4.45 | 1.22 | 0.7069 | 0.862 |
| TC0100008559.hg.1 | ANGPTL3 | angiopoietin like 3 | Multiple_C | 5.26 | 4.35 | 4.79 | 1.39 | 0.0955 | 0.233 | 5.07 | 5.22 | 5.14 | 0.95 | 0.2597 | 0.524 |
| TC0100008757.hg.1 | LHX8 | LIM homeobox 8 | Multiple_C | 4.25 | 3.66 | 3.78 | 1.39 | 0.2851 | 0.4954 | 4.28 | 4.26 | 4.27 | 1.01 | 0.8386 | 0.9291 |
| TC0100010381.hg.1 | UAP1 | UDP-N-acetylglucosamine pyrophosphorylase 1 | Multiple_C | 11.86 | 13.29 | 11.39 | 1.39 | 0.0376 | 0.117 | 9.71 | 9.15 | 9.34 | 1.29 | 0.0865 | 0.2836 |
| TC0100012485.hg.1 | TMEM240 | transmembrane protein 240 | Coding | 4.6 | 4.02 | 4.13 | 1.39 | 0.1351 | 0.2992 | 4.52 | 5.05 | 4.98 | 0.73 | 0.1568 | 0.3977 |
| TC0100014321.hg.1 | C1orf168 | chromosome 1 open reading frame 168 | Multiple_C | 4.1 | 4.06 | 3.63 | 1.39 | 0.0544 | 0.1538 | 4.99 | 5.58 | 5.25 | 0.84 | 0.3988 | 0.656 |
| TC0100016199.hg.1 | OLFML2B | olfactomedin like 2B | Coding | 4.82 | 4.41 | 4.35 | 1.39 | 0.2092 | 0.4029 | 4.72 | 5.34 | 5.03 | 0.81 | 0.3971 | 0.655 |
| TC0200007785.hg.1 | UGP2 | UDP-glucose pyrophosphorylase 2 | Multiple_C | 9.34 | 10.02 | 8.87 | 1.39 | 0.2197 | 0.4168 | 7.44 | 7.69 | 7.77 | 0.80 | 0.0128 | 0.0912 |

| | | | | | | | | | | | | | | | |
|---|---|---|---|---|---|---|---|---|---|---|---|---|---|---|---|
| TC0200008682.hg.1 | TMEM182 | transmembrane protein 182 | Multiple_C | 9.81 | 9.85 | 9.34 | 1.39 | 0.0528 | 0.1504 | 8.5 | 7.52 | 7.9 | 1.52 | 0.0053 | 0.0513 |
| TC0200009305.hg.1 | CFC1B; CFC1 | cripto, FRL-1, cryptic family 1B; cripto, FRL-1, cryptic family 1 | Coding | 4.61 | 4.27 | 4.14 | 1.39 | 0.3106 | 0.524 | 3.71 | 3.98 | 3.8 | 0.94 | 0.8059 | 0.9142 |
| TC0200010157.hg.1 | ITGA4 | integrin alpha 4 | Multiple_C | 4.95 | 4.34 | 4.48 | 1.39 | 0.2776 | 0.4868 | 4.32 | 4.49 | 4.46 | 0.91 | 0.3782 | 0.638 |
| TC0200015019.hg.1 | KIAA1715 | KIAA1715 | Multiple_C | 9.14 | 8.76 | 8.67 | 1.39 | 0.06 | 0.1656 | 8.89 | 8.67 | 8.83 | 1.04 | 0.4468 | 0.6939 |
| TC0200015951.hg.1 | PID1 | phosphotyrosine interaction domain containing 1 | Multiple_C | 4.57 | 3.47 | 4.1 | 1.39 | 0.3274 | 0.5413 | 3.98 | 3.82 | 3.76 | 1.16 | 0.0615 | 0.2356 |
| TC0300008271.hg.1 | BBX | bobby sox homolog (Drosophila) | Multiple_C | 12.01 | 12.15 | 11.54 | 1.39 | 0.045 | 0.134 | 10.35 | 9.30 | 9.56 | 1.73 | 0.0002 | 0.0061 |
| TC0300012145.hg.1 | FSTL1; MIR198 | follistatin like 1; microRNA 198 | Multiple_C | 4.14 | 3.47 | 3.67 | 1.39 | 0.1955 | 0.3856 | 4.22 | 4.43 | 3.98 | 1.18 | 0.078 | 0.2692 |
| TC0300013903.hg.1 | NAALADL2 | N-acetylated alpha-linked acidic dipeptidase-like 2 | Multiple_C | 7.04 | 6.49 | 6.57 | 1.39 | 0.2625 | 0.4697 | 7.75 | 8.06 | 7.45 | 1.23 | 0.0925 | 0.2949 |
| TC0300014062.hg.1 | ANKUB1 | ankyrin repeat and ubiquitin domain containing 1 | Multiple_C | 3.77 | 3.53 | 3.3 | 1.39 | 0.3427 | 0.5567 | 3.95 | 4.27 | 4.19 | 0.85 | 0.627 | 0.8157 |
| TC0400006802.hg.1 | USP17L19 | ubiquitin specific peptidase 17-like family member 19 | Coding | 3.33 | 2.93 | 2.86 | 1.39 | 0.3643 | 0.5778 | 3.88 | 4.16 | 4.16 | 0.82 | 0.0841 | 0.2789 |
| TC0400007596.hg.1 | ARL9 | ADP-ribosylation factor like GTPase 9 | Coding | 3.47 | 2.95 | 3 | 1.39 | 0.0748 | 0.1944 | 3.33 | 3.34 | 3.55 | 0.86 | 0.2456 | 0.51 |
| TC0400009450.hg.1 | WWC2 | WW and C2 domain containing 2 | Multiple_C | 7.85 | 7.39 | 7.38 | 1.39 | 0.5966 | 0.7701 | 8.3 | 7.99 | 8.11 | 1.14 | 0.3002 | 0.5646 |
| TC0500007314.hg.1 | MRPS30 | mitochondrial ribosomal protein S30 | Multiple_C | 14.26 | 14.52 | 13.79 | 1.39 | 0.1464 | 0.3167 | 14.55 | 13.58 | 14.44 | 1.08 | 0.5235 | 0.7491 |
| TC0500008919.hg.1 | RNF14 | ring finger protein 14 | Multiple_C | 10.63 | 10.62 | 10.16 | 1.39 | 0.0818 | 0.2078 | 9.78 | 9.26 | 10.18 | 0.76 | 0.0934 | 0.2968 |
| TC0500008941.hg.1 | ARHGAP26 | Rho GTPase activating protein 26 | Multiple_C | 11.29 | 10.44 | 10.82 | 1.39 | 0.0387 | 0.1195 | 10.14 | 9.48 | 10.58 | 0.74 | 0.065 | 0.2427 |
| TC0500012030.hg.1 | GDF9 | growth differentiation factor 9 | Multiple_C | 3.62 | 3.19 | 3.15 | 1.39 | 0.4199 | 0.6313 | 3.04 | 3.33 | 3.11 | 0.95 | 0.324 | 0.5883 |
| TC0500012272.hg.1 | SLC25A2 | solute carrier family 25 (mitochondrial carrier; ornithine transpo | Coding | 4.04 | 3.59 | 3.57 | 1.39 | 0.2925 | 0.5037 | 3.18 | 3.33 | 3.21 | 0.98 | 0.5706 | 0.7789 |
| TC0500012553.hg.1 | HAND1 | heart and neural crest derivatives expressed 1 | Multiple_C | 4.19 | 3.44 | 3.72 | 1.39 | 0.0701 | 0.1856 | 3.58 | 3.61 | 3.58 | 1.00 | 0.8563 | 0.9389 |
| TC0500013247.hg.1 | PCDHB14 | protocadherin beta 14 | Coding | 8.06 | 6.92 | 7.59 | 1.39 | 0.0619 | 0.1695 | 5.22 | 5.40 | 5.39 | 0.89 | 0.0874 | 0.2854 |
| TC0600007346.hg.1 | VN1R10P | vomeronasal 1 receptor 10 pseudogene | Multiple_C | 4.01 | 4.45 | 3.54 | 1.39 | 0.2023 | 0.3946 | 4.02 | 4.15 | 4.17 | 0.90 | 0.4363 | 0.6869 |
| TC0600008525.hg.1 | KHDC3L | KH domain containing 3-like, subcortical maternal complex men | Coding | 6.12 | 5.6 | 5.65 | 1.39 | 0.0189 | 0.0683 | 5.94 | 6.34 | 6.4 | 0.73 | 0.0633 | 0.2394 |
| TC0600009456.hg.1 | ENPP3 | ectonucleotide pyrophosphatase/phosphodiesterase 3 | Multiple_C | 5.66 | 4.73 | 5.19 | 1.39 | 0.3822 | 0.5953 | 6.07 | 6.31 | 5.85 | 1.16 | 0.3588 | 0.6216 |
| TC0600010064.hg.1 | MAS1 | MAS1 proto-oncogene, G protein-coupled receptor | Coding | 4.63 | 4.32 | 4.16 | 1.39 | 0.1383 | 0.304 | 5.07 | 4.72 | 4.55 | 1.43 | 0.0584 | 0.2278 |
| TC0600010867.hg.1 | MCUR1 | mitochondrial calcium uniporter regulator 1 | Multiple_C | 9.16 | 9.09 | 8.69 | 1.39 | 0.198 | 0.3889 | 10.34 | 9.44 | 10.25 | 1.06 | 0.7092 | 0.8634 |
| TC0600011733.hg.1 | KCNK5 | potassium channel, two pore domain subfamily K, member 5 | Multiple_C | 8.3 | 7.5 | 7.83 | 1.39 | 0.268 | 0.4761 | 5.95 | 5.91 | 6.56 | 0.66 | 0.0713 | 0.2555 |
| TC0600011960.hg.1 | TNFRSF21 | tumor necrosis factor receptor superfamily, member 21 | Multiple_C | 12.26 | 11.72 | 11.79 | 1.39 | 0.0548 | 0.1547 | 7.32 | 7.19 | 8.53 | 0.43 | 8.66E-06 | 0.0006 |
| TC0600012597.hg.1 | EPHA7 | EPH receptor A7 | Multiple_C | 4.84 | 4.68 | 4.37 | 1.39 | 0.416 | 0.6279 | 4.89 | 4.37 | 4.31 | 1.49 | 0.1032 | 0.3149 |
| TC0600013305.hg.1 | OLIG3 | oligodendrocyte transcription factor 3 | Coding | 4.52 | 3.87 | 4.05 | 1.39 | 0.199 | 0.39 | 3.73 | 3.55 | 3.47 | 1.20 | 0.2465 | 0.511 |
| TC0600014328.hg.1 | OSTM1 | osteopetrosis associated transmembrane protein 1 | Multiple_C | 8.61 | 9.01 | 8.14 | 1.39 | 0.165 | 0.3435 | 10.32 | 8.93 | 9.49 | 1.78 | 0.0012 | 0.019 |
| TC0700007121.hg.1 | AVL9 | AVL9 homolog (S. cerevisiase) | Multiple_C | 9.14 | 9.18 | 8.67 | 1.39 | 0.5226 | 0.7156 | 8.18 | 8.68 | 9.06 | 0.54 | 0.0186 | 0.1151 |
| TC0700008358.hg.1 | COL1A2 | collagen, type I, alpha 2 | Multiple_C | 8.28 | 7.78 | 7.81 | 1.39 | 0.1196 | 0.2743 | 8.6 | 8.35 | 8.13 | 1.39 | 0.1978 | 0.4529 |
| TC0700009243.hg.1 | TMEM140 | transmembrane protein 140 | Multiple_C | 4.74 | 4.12 | 4.27 | 1.39 | 0.2141 | 0.4093 | 5.53 | 5.29 | 5.29 | 1.18 | 0.8163 | 0.9185 |
| TC0700012696.hg.1 | WDR91 | WD repeat domain 91 | Multiple_C | 6.22 | 5.91 | 5.75 | 1.39 | 0.2552 | 0.4609 | 6.87 | 6.71 | 6.61 | 1.20 | 0.7372 | 0.8788 |

| | | | | | | | | | | | | | | | |
|---|---|---|---|---|---|---|---|---|---|---|---|---|---|---|---|
| TC0700012998.hg.1 | ZNF767P | zinc finger family member 767, pseudogene | Multiple_C | 6.66 | 6.46 | 6.19 | 1.39 | 0.0316 | 0.1025 | 8.16 | 7.38 | 7.89 | 1.21 | 0.5358 | 0.7578 |
| TC0800010940.hg.1 | FABP12 | fatty acid binding protein 12 | Coding | 4.16 | 3.63 | 3.69 | 1.39 | 0.0108 | 0.0445 | 3.41 | 3.32 | 3.53 | 0.92 | 0.6022 | 0.8007 |
| TC0800011764.hg.1 | FAM84B | family with sequence similarity 84, member B | Multiple_C | 11.59 | 11.94 | 11.12 | 1.39 | 0.2028 | 0.3951 | 6.39 | 5.64 | 5.41 | 1.97 | 0.0029 | 0.0345 |
| TC0800012110.hg.1 | LY6H | lymphocyte antigen 6 complex, locus H | Multiple_C | 4.51 | 4.23 | 4.04 | 1.39 | 0.2743 | 0.4831 | 4.25 | 4.43 | 4.19 | 1.04 | 0.5862 | 0.7902 |
| TC0900009139.hg.1 | FCN2 | ficolin (collagen/fibrinogen domain containing lectin) 2 | Coding | 5.08 | 4.44 | 4.61 | 1.39 | 0.0428 | 0.1293 | 5.13 | 5.20 | 5.16 | 0.98 | 0.8155 | 0.9182 |
| TC0900011603.hg.1 | ENG | endoglin | Multiple_C | 6.45 | 5.8 | 5.98 | 1.39 | 0.3124 | 0.5258 | 7.4 | 7.70 | 7.35 | 1.04 | 0.8294 | 0.9243 |
| TC0900011950.hg.1 | SOHLH1 | spermatogenesis and oogenesis specific basic helix-loop-helix 1 | Coding | 4.93 | 4.38 | 4.46 | 1.39 | 0.0515 | 0.1476 | 4.67 | 4.79 | 4.63 | 1.03 | 0.6985 | 0.8571 |
| TC0X00006839.hg.1 | MAGEB6 | MAGE family member B6 | Coding | 3.28 | 2.6 | 2.81 | 1.39 | 0.1109 | 0.2598 | 3.36 | 3.14 | 2.91 | 1.37 | 0.0545 | 0.2191 |
| TC0X00007203.hg.1 | GLOD5 | glyoxalase domain containing 5 | Multiple_C | 6.79 | 6.32 | 6.32 | 1.39 | 0.0278 | 0.0926 | 6.43 | 6.56 | 6.41 | 1.01 | 0.9766 | 0.9904 |
| TC0Y00006775.hg.1 | BPY2C; BPY2 | basic charge, Y-linked, 2C; basic charge, Y-linked, 2 | Multiple_C | 3.13 | 2.59 | 2.66 | 1.39 | 0.0659 | 0.1778 | 3.47 | 3.22 | 3.57 | 0.93 | 0.4505 | 0.6963 |
| TC1000010006.hg.1 | DNAJC1 | DnaJ (Hsp40) homolog, subfamily C, member 1 | Multiple_C | 11.97 | 12.7 | 11.5 | 1.39 | 0.0672 | 0.18 | 8.86 | 8.55 | 9.64 | 0.58 | 0.0007 | 0.0131 |
| TC1000012511.hg.1 | PRAP1 | proline-rich acidic protein 1 | Multiple_C | 4.74 | 4.12 | 4.27 | 1.39 | 0.4486 | 0.6565 | 7.79 | 7.57 | 6.01 | 3.43 | 6.09E-06 | 0.0005 |
| TC1100007787.hg.1 | CD6 | CD6 molecule | Multiple_C | 4.15 | 3.67 | 3.68 | 1.39 | 0.0677 | 0.1811 | 3.97 | 3.96 | 4.02 | 0.97 | 0.8368 | 0.9282 |
| TC1100009791.hg.1 | ASCL2 | achaete-scute family bHLH transcription factor 2 | Coding | 4.09 | 3.54 | 3.62 | 1.39 | 0.1476 | 0.3184 | 4.88 | 5.00 | 4.63 | 1.19 | 0.3974 | 0.6551 |
| TC1100009918.hg.1 | HBD | hemoglobin, delta | Coding | 3.7 | 3.18 | 3.23 | 1.39 | 0.175 | 0.357 | 3.97 | 3.89 | 3.81 | 1.12 | 0.3005 | 0.5648 |
| TC1100010928.hg.1 | OR1S2 | olfactory receptor, family 1, subfamily S, member 2 | Coding | 3.47 | 3.11 | 3 | 1.39 | 0.0964 | 0.2345 | 3.67 | 3.90 | 3.71 | 0.97 | 0.779 | 0.9011 |
| TC1100013147.hg.1 | RPS13; SNORD | ribosomal protein S13; small nucleolar RNA, C/D box 14B | Multiple_C | 17.49 | 17.99 | 17.02 | 1.39 | 0.1319 | 0.2943 | 13.74 | 13.57 | 13.41 | 1.26 | 0.1128 | 0.3308 |
| TC1200008434.hg.1 | C12orf74 | chromosome 12 open reading frame 74 | Coding | 4.62 | 3.99 | 4.15 | 1.39 | 0.0395 | 0.1215 | 4.06 | 4.90 | 4.42 | 0.78 | 0.1345 | 0.3643 |
| TC1200008488.hg.1 | PLXNC1 | plexin C1 | Multiple_C | 4.66 | 4.04 | 4.19 | 1.39 | 0.1424 | 0.3104 | 3.84 | 3.96 | 3.78 | 1.04 | 0.7505 | 0.8865 |
| TC1200009670.hg.1 | FGF23 | fibroblast growth factor 23 | Coding | 4.61 | 4.17 | 4.14 | 1.39 | 0.4328 | 0.6428 | 4.61 | 4.39 | 4.07 | 1.45 | 0.0574 | 0.2259 |
| TC1200009875.hg.1 | CLECL1 | C-type lectin-like 1 | Coding | 4.73 | 4.15 | 4.26 | 1.39 | 0.2384 | 0.4401 | 4.37 | 4.02 | 4.07 | 1.23 | 0.4585 | 0.7022 |
| TC1200012592.hg.1 | C1S | complement component 1, s subcomponent | Multiple_C | 3.85 | 3.47 | 3.38 | 1.39 | 0.2904 | 0.5013 | 3.92 | 3.38 | 3.32 | 1.52 | 0.0289 | 0.1502 |
| TC1300006643.hg.1 | ATP8A2 | ATPase, aminophospholipid transporter, class I, type 8A, membe | Multiple_C | 3.41 | 3.28 | 2.94 | 1.39 | 0.0433 | 0.1304 | 4.01 | 3.68 | 4.01 | 1.00 | 0.7767 | 0.8999 |
| TC1400009787.hg.1 | SPTLC2 | serine palmitoyltransferase, long chain base subunit 2 | Multiple_C | 12.59 | 11.38 | 12.12 | 1.39 | 0.164 | 0.3423 | 11.08 | 10.41 | 10.37 | 1.64 | 0.005 | 0.049 |
| TC1500007513.hg.1 | LACTB | lactamase, beta | Multiple_C | 9.7 | 11.01 | 9.23 | 1.39 | 0.1789 | 0.3623 | 6.42 | 5.97 | 6.67 | 0.84 | 0.7733 | 0.8981 |
| TC1500008695.hg.1 | GOLGA8J; GOI | golgin A8 family, member J; golgin A8 family, member I, pseudo | Multiple_C | 5.7 | 5.15 | 5.23 | 1.39 | 0.0293 | 0.0965 | 5.34 | 5.04 | 5.07 | 1.21 | 0.2254 | 0.4862 |
| TC1600007723.hg.1 | GPT2 | glutamic pyruvate transaminase (alanine aminotransferase) 2 | Multiple_C | 9.95 | 10.11 | 9.48 | 1.39 | 0.4555 | 0.6625 | 9.11 | 9.20 | 9.15 | 0.97 | 0.0728 | 0.2586 |
| TC1600009163.hg.1 | CCDC64B | coiled-coil domain containing 64B | Multiple_C | 4.8 | 4.31 | 4.33 | 1.39 | 0.3125 | 0.526 | 4.34 | 4.28 | 4.21 | 1.09 | 0.4304 | 0.6821 |
| TC1600011328.hg.1 | NPW | neuropeptide W | Coding | 3.53 | 2.9 | 3.06 | 1.39 | 0.1527 | 0.3261 | 4.49 | 4.51 | 4.37 | 1.09 | 0.6155 | 0.8091 |
| TC1700006641.hg.1 | ZMYND15 | zinc finger, MYND-type containing 15 | Multiple_C | 6.98 | 6.36 | 6.51 | 1.39 | 0.1934 | 0.3826 | 7.14 | 6.93 | 6.56 | 1.49 | 0.0678 | 0.248 |
| TC1700007827.hg.1 | KRTAP9-6 | keratin associated protein 9-6 | Coding | 4.18 | 3.7 | 3.71 | 1.39 | 0.1907 | 0.3789 | 3.85 | 3.93 | 3.93 | 0.95 | 0.9574 | 0.9823 |
| TC1700009708.hg.1 | PIK3R5 | phosphoinositide-3-kinase, regulatory subunit 5 | Multiple_C | 4.45 | 3.87 | 3.98 | 1.39 | 0.0384 | 0.1189 | 4.12 | 4.04 | 4.55 | 0.74 | 0.0703 | 0.2534 |
| TC1700010666.hg.1 | KRT33A | keratin 33A, type I | Coding | 3.69 | 3.02 | 3.22 | 1.39 | 0.0547 | 0.1544 | 3.14 | 3.39 | 3.34 | 0.87 | 0.6882 | 0.8508 |

| | | | | | | | | | | | | | | | |
|---|---|---|---|---|---|---|---|---|---|---|---|---|---|---|---|
| TC1700012414.hg.1 | KRT31 | keratin 31, type I | Multiple_C | 4.73 | 4.27 | 4.26 | 1.39 | 0.1934 | 0.3826 | 4.36 | 4.20 | 4.34 | 1.01 | 0.4422 | 0.691 |
| TC1800007010.hg.1 | DSG1 | desmoglein 1 | Coding | 4.26 | 4.13 | 3.79 | 1.39 | 0.2306 | 0.4309 | 4.5 | 4.46 | 4.53 | 0.98 | 0.713 | 0.8656 |
| TC1800007826.hg.1 | KCNG2 | potassium channel, voltage gated modifier subfamily G, membe | Multiple_C | 5.91 | 5.39 | 5.44 | 1.39 | 0.3649 | 0.5785 | 6.08 | 5.80 | 5.43 | 1.57 | 0.1274 | 0.3534 |
| TC1900007619.hg.1 | ZNF726; ZNF9 | zinc finger protein 726; zinc finger protein 92 pseudogene 3 | Multiple_C | 5.05 | 4.62 | 4.58 | 1.39 | 0.1713 | 0.3524 | 5.46 | 5.30 | 5.13 | 1.26 | 0.1238 | 0.3474 |
| TC1900010595.hg.1 | PPP1R14A | protein phosphatase 1, regulatory (inhibitor) subunit 14A | Coding | 5.79 | 5.44 | 5.32 | 1.39 | 0.0689 | 0.1833 | 6.57 | 7.09 | 6.85 | 0.82 | 0.1317 | 0.3597 |
| TC1900011639.hg.1 | STK11 | serine/threonine kinase 11 | Multiple_C | 10.03 | 9.45 | 9.56 | 1.39 | 0.4678 | 0.6721 | 9.66 | 9.82 | 9.61 | 1.04 | 0.8923 | 0.9539 |
| TC1900011689.hg.1 | YJEFN3 | YjeF N-terminal domain containing 3 | Coding | 7.52 | 6.85 | 7.05 | 1.39 | 0.1009 | 0.2424 | 6.57 | 6.83 | 6.8 | 0.85 | 0.3483 | 0.6121 |
| TC1900011777.hg.1 | CYTH2 | cytohesin 2 | Multiple_C | 10.48 | 10.45 | 10.01 | 1.39 | 0.1893 | 0.377 | 9.89 | 10.36 | 9.93 | 0.97 | 0.2504 | 0.5148 |
| TC1900011990.hg.1 | RPL18 | ribosomal protein L18 | Multiple_C | 17.86 | 16.7 | 17.39 | 1.39 | 0.0242 | 0.0832 | 13.93 | 13.71 | 13.77 | 1.12 | 0.4154 | 0.6697 |
| TC2000006533.hg.1 | GNRH2 | gonadotropin releasing hormone 2 | Multiple_C | 3.35 | 2.67 | 2.88 | 1.39 | 0.1254 | 0.2834 | 3.59 | 3.51 | 3.53 | 1.04 | 0.3454 | 0.6092 |
| TC2000007925.hg.1 | TUBB1 | tubulin, beta 1 class VI | Coding | 4.1 | 3.21 | 3.63 | 1.39 | 0.1554 | 0.33 | 4.16 | 4.02 | 3.95 | 1.16 | 0.3089 | 0.5731 |
| TC2000008211.hg.1 | UBOX5; FASTK | U-box domain containing 5; FAST kinase domains 5 | Multiple_C | 6.55 | 5.87 | 6.08 | 1.39 | 0.1429 | 0.3112 | 6.8 | 7.70 | 7.66 | 0.55 | 0.0108 | 0.0821 |
| TC2000008316.hg.1 | LRRN4 | leucine rich repeat neuronal 4 | Coding | 3.94 | 3.3 | 3.47 | 1.39 | 0.2591 | 0.4656 | 4.23 | 4.49 | 4.84 | 0.66 | 0.0108 | 0.0821 |
| TC2100007241.hg.1 | ABCG1 | ATP binding cassette subfamily G member 1 | Multiple_C | 4.01 | 3.5 | 3.54 | 1.39 | 0.3433 | 0.557 | 4.64 | 3.98 | 4.14 | 1.41 | 0.0324 | 0.1611 |
| TC2200007871.hg.1 | CCT8L2 | chaperonin containing TCP1, subunit 8 (theta)-like 2 | Coding | 5.16 | 4.36 | 4.69 | 1.39 | 0.0735 | 0.1919 | 5.1 | 5.65 | 5.28 | 0.88 | 0.6006 | 0.7997 |
| TC2200008356.hg.1 | TTC28 | tetratricopeptide repeat domain 28 | Multiple_C | 5.59 | 4.85 | 5.12 | 1.39 | 0.0693 | 0.1839 | 5.34 | 5.37 | 5.09 | 1.19 | 0.2994 | 0.5639 |
| TC2200008831.hg.1 | PMM1 | phosphomannomutase 1 | Multiple_C | 4.23 | 4.12 | 3.76 | 1.39 | 0.0829 | 0.2099 | 3.55 | 3.38 | 3.69 | 0.91 | 0.632 | 0.8189 |
| TC0100006695.hg.1 | TAS1R1 | taste receptor, type 1, member 1 | Coding | 4.79 | 4 | 4.33 | 1.38 | 0.0375 | 0.117 | 3.65 | 3.70 | 3.46 | 1.14 | 0.134 | 0.3636 |
| TC0100008369.hg.1 | LRRC42 | leucine rich repeat containing 42 | Multiple_C | 11.16 | 11.43 | 10.7 | 1.38 | 0.1139 | 0.2648 | 10.3 | 10.40 | 10.27 | 1.02 | 0.4979 | 0.7312 |
| TC0100008620.hg.1 | DNAJC6 | DnaJ (Hsp40) homolog, subfamily C, member 6 | Multiple_C | 5.6 | 5.06 | 5.14 | 1.38 | 0.1604 | 0.3371 | 7.71 | 7.50 | 8.14 | 0.74 | 0.0086 | 0.0703 |
| TC0100009433.hg.1 | LOC643355; R | uncharacterized LOC643355; putative novel transcript; Transcri | Multiple_C | 7.3 | 7.18 | 6.84 | 1.38 | 0.0824 | 0.2089 | 7.67 | 8.19 | 7.68 | 0.99 | 0.9736 | 0.9895 |
| TC0100012934.hg.1 | PRAMEF4 | PRAME family member 4 | Coding | 3.41 | 3.01 | 2.95 | 1.38 | 0.0541 | 0.1532 | 3.32 | 3.51 | 3.34 | 0.99 | 0.4119 | 0.6664 |
| TC0100015243.hg.1 | KCNA10 | potassium channel, voltage gated shaker related subfamily A, m | Coding | 5.85 | 5.01 | 5.39 | 1.38 | 0.334 | 0.5477 | 4.83 | 5.44 | 5.9 | 0.48 | 0.0033 | 0.0381 |
| TC0100016158.hg.1 | PFDN2 | prefoldin subunit 2 | Multiple_C | 11.62 | 11.79 | 11.16 | 1.38 | 0.0664 | 0.1786 | 9.47 | 8.70 | 8.63 | 1.79 | 0.0004 | 0.0092 |
| TC0100016350.hg.1 | CCDC181 | coiled-coil domain containing 181 | Multiple_C | 3.91 | 3.38 | 3.45 | 1.38 | 0.1549 | 0.3291 | 3.82 | 3.72 | 3.74 | 1.06 | 0.9336 | 0.972 |
| TC0100017066.hg.1 | KLHDC8A | kelch domain containing 8A | Multiple_C | 4.46 | 4.06 | 4 | 1.38 | 0.0531 | 0.151 | 4.25 | 4.47 | 4.2 | 1.04 | 0.9399 | 0.9753 |
| TC0100017193.hg.1 | KCNH1 | potassium channel, voltage gated eag related subfamily H, mem | Multiple_C | 3.3 | 2.69 | 2.84 | 1.38 | 0.0568 | 0.1589 | 3.79 | 3.84 | 3.99 | 0.87 | 0.2143 | 0.4727 |
| TC0100017587.hg.1 | MRPL55 | mitochondrial ribosomal protein L55 | Multiple_C | 8.2 | 7.69 | 7.74 | 1.38 | 0.0696 | 0.1846 | 6.36 | 6.61 | 6.49 | 0.91 | 0.5688 | 0.7776 |
| TC0100018479.hg.1 | NOTCH2NL | notch 2 N-terminal like | Multiple_C | 11.74 | 11.28 | 11.28 | 1.38 | 0.1494 | 0.3211 | 9.64 | 8.65 | 8.07 | 2.97 | 4.76E-07 | 8.66E-05 |
| TC0200012261.hg.1 | PRKD3 | protein kinase D3 | Multiple_C | 9.82 | 8.93 | 9.36 | 1.38 | 0.0639 | 0.1735 | 9.86 | 9.67 | 10.12 | 0.84 | 0.1367 | 0.3675 |
| TC0200013638.hg.1 | LONRF2 | LON peptidase N-terminal domain and ring finger 2 | Multiple_C | 4.87 | 4.47 | 4.41 | 1.38 | 0.0086 | 0.0369 | 5.04 | 5.29 | 5.2 | 0.90 | 0.519 | 0.7466 |
| TC0200016093.hg.1 | USP40 | ubiquitin specific peptidase 40 | Multiple_C | 8.36 | 7.59 | 7.9 | 1.38 | 0.1042 | 0.2485 | 7.12 | 8.18 | 7.21 | 0.94 | 0.929 | 0.9702 |
| TC0300006655.hg.1 | HDAC11 | histone deacetylase 11 | Multiple_C | 4.59 | 4.47 | 4.13 | 1.38 | 0.085 | 0.2139 | 4.56 | 4.28 | 4.44 | 1.09 | 0.7277 | 0.8736 |

| | | | | | | | | | | | | | | | |
|---|---|---|---|---|---|---|---|---|---|---|---|---|---|---|---|
| TC0300007484.hg.1 | STAB1 | stabilin 1 | Multiple_C | 4.79 | 4.37 | 4.33 | 1.38 | 0.6769 | 0.8244 | 3.84 | 4.01 | 4.4 | 0.68 | 0.0472 | 0.2015 |
| TC0300008320.hg.1 | CD96 | CD96 molecule | Multiple_C | 3.62 | 3.29 | 3.16 | 1.38 | 0.2007 | 0.3926 | 3.29 | 3.29 | 3.5 | 0.86 | 0.5681 | 0.777 |
| TC0300008856.hg.1 | BFSP2 | beaded filament structural protein 2, phakinin | Multiple_C | 5.43 | 4.7 | 4.97 | 1.38 | 0.0355 | 0.1122 | 4.69 | 4.82 | 4.51 | 1.13 | 0.1834 | 0.4342 |
| TC0300012200.hg.1 | SEMA5B | sema domain, seven thrombospondin repeats (type 1 and type | Multiple_C | 3.94 | 3.5 | 3.48 | 1.38 | 0.0293 | 0.0965 | 3.71 | 3.78 | 3.92 | 0.86 | 0.1115 | 0.3291 |
| TC0300012238.hg.1 | HEG1 | heart development protein with EGF-like domains 1 | Multiple_C | 5.27 | 4.53 | 4.81 | 1.38 | 0.1283 | 0.2884 | 5.84 | 5.73 | 5.46 | 1.30 | 0.1774 | 0.4261 |
| TC0400006538.hg.1 | TACC3 | transforming, acidic coiled-coil containing protein 3 | Multiple_C | 11.03 | 9.54 | 10.57 | 1.38 | 0.443 | 0.6519 | 11.04 | 13.11 | 12.09 | 0.48 | 0.048 | 0.2032 |
| TC0500008541.hg.1 | TEX43 | testis expressed 43 | Multiple_C | 3.98 | 3.59 | 3.52 | 1.38 | 0.0878 | 0.2191 | 4.32 | 3.97 | 4.07 | 1.19 | 0.4181 | 0.672 |
| TC0500009077.hg.1 | HMGXB3 | HMG box domain containing 3 | Multiple_C | 9.4 | 8.83 | 8.94 | 1.38 | 0.3691 | 0.582 | 9.23 | 9.17 | 9.28 | 0.97 | 0.9314 | 0.9711 |
| TC0500011470.hg.1 | FAM172A; PO | family with sequence similarity 172, member A; POU domain cla | Multiple_C | 7.28 | 7.8 | 6.82 | 1.38 | 0.9847 | 0.9928 | 5.07 | 4.88 | 4.73 | 1.27 | 0.3621 | 0.6249 |
| TC0500012775.hg.1 | FAM196B | family with sequence similarity 196, member B | Multiple_C | 4.34 | 3.43 | 3.88 | 1.38 | 0.0587 | 0.1631 | 4.13 | 4.14 | 4.49 | 0.78 | 0.1359 | 0.3665 |
| TC0600007067.hg.1 | GMPR | guanosine monophosphate reductase | Multiple_C | 6.51 | 5.87 | 6.05 | 1.38 | 0.0819 | 0.208 | 4.93 | 5.15 | 5.13 | 0.87 | 0.7955 | 0.9087 |
| TC0600011474.hg.1 | TNXA | tenascin XA (pseudogene) | Multiple_C | 7.14 | 6.8 | 6.68 | 1.38 | 0.017 | 0.0631 | 6.17 | 6.15 | 6 | 1.13 | 0.3244 | 0.5884 |
| TC0600014106.hg.1 | CFB | complement factor B | Multiple_C | 3.68 | 3.4 | 3.22 | 1.38 | 0.0853 | 0.2144 | 3.99 | 3.95 | 3.62 | 1.29 | 0.5352 | 0.7572 |
| TC0700008536.hg.1 | SPDYE3 | speedy/RINGO cell cycle regulator family member E3 | Multiple_C | 3.73 | 3.18 | 3.27 | 1.38 | 0.0184 | 0.0671 | 4.14 | 4.70 | 4.18 | 0.97 | 0.5483 | 0.7651 |
| TC0700009066.hg.1 | FLNC | filamin C, gamma | Multiple_C | 5.49 | 4.93 | 5.03 | 1.38 | 0.1783 | 0.3615 | 6.84 | 6.02 | 6.14 | 1.62 | 0.113 | 0.3313 |
| TC0700009068.hg.1 | LOC10013070 | uncharacterized LOC100130705; putative novel transcript | Multiple_C | 3.9 | 3.45 | 3.44 | 1.38 | 0.0435 | 0.1309 | 3.86 | 4.07 | 4.08 | 0.86 | 0.4401 | 0.6898 |
| TC0700009346.hg.1 | CLEC2L | C-type lectin domain family 2, member L | Multiple_C | 3.67 | 3.22 | 3.21 | 1.38 | 0.0591 | 0.1638 | 3.87 | 3.84 | 3.85 | 1.01 | 0.2145 | 0.4731 |
| TC0700013595.hg.1 | C7orf61 | chromosome 7 open reading frame 61 | Coding | 3.54 | 2.88 | 3.08 | 1.38 | 0.1111 | 0.26 | 3.86 | 3.73 | 3.69 | 1.13 | 0.3413 | 0.6051 |
| TC0800008632.hg.1 | SLC30A8 | solute carrier family 30 (zinc transporter), member 8 | Multiple_C | 3.95 | 3.68 | 3.49 | 1.38 | 0.4618 | 0.667 | 3.81 | 3.74 | 3.62 | 1.14 | 0.2509 | 0.5151 |
| TC0800010193.hg.1 | TM2D2 | TM2 domain containing 2 | Multiple_C | 10.67 | 9.67 | 10.21 | 1.38 | 0.341 | 0.5549 | 7.12 | 7.35 | 8.18 | 0.48 | 5.76E-05 | 0.0025 |
| TC0800010251.hg.1 | NKX6-3 | NK6 homeobox 3 | Coding | 6.56 | 6 | 6.1 | 1.38 | 0.1013 | 0.2433 | 6.77 | 7.01 | 6.75 | 1.01 | 0.6185 | 0.8109 |
| TC0900007472.hg.1 | ANKRD20A1 | ankyrin repeat domain 20 family, member A1 | Multiple_C | 4.33 | 3.98 | 3.87 | 1.38 | 0.2789 | 0.4885 | 5.68 | 5.47 | 5.66 | 1.01 | 0.5666 | 0.7761 |
| TC0900008804.hg.1 | ZNF79 | zinc finger protein 79 | Multiple_C | 5.7 | 4.86 | 5.24 | 1.38 | 0.4283 | 0.6386 | 5.6 | 5.68 | 5.51 | 1.06 | 0.4527 | 0.6978 |
| TC0900011378.hg.1 | CDK5RAP2 | CDK5 regulatory subunit associated protein 2 | Multiple_C | 7.06 | 6.82 | 6.6 | 1.38 | 0.4432 | 0.652 | 6.71 | 6.72 | 6.37 | 1.27 | 0.7346 | 0.8776 |
| TC0900011947.hg.1 | GLT6D1 | glycosyltransferase 6 domain containing 1 | Coding | 4.23 | 3.96 | 3.77 | 1.38 | 0.3494 | 0.562 | 4.13 | 3.86 | 3.87 | 1.20 | 0.6476 | 0.8276 |
| TC0X00007507.hg.1 | EFNB1 | ephrin-B1 | Multiple_C | 8.83 | 8.42 | 8.37 | 1.38 | 0.246 | 0.4496 | 5.9 | 6.63 | 7.07 | 0.44 | 0.0021 | 0.0278 |
| TC0X00007558.hg.1 | NLGN3 | neuroligin 3 | Multiple_C | 5.2 | 5 | 4.74 | 1.38 | 0.0816 | 0.2075 | 3.78 | 4.04 | 4.21 | 0.74 | 0.1304 | 0.3577 |
| TC0X00007609.hg.1 | CDX4 | caudal type homeobox 4 | Coding | 4.84 | 4.16 | 4.38 | 1.38 | 0.1247 | 0.2823 | 4.23 | 4.11 | 3.9 | 1.26 | 0.1868 | 0.4387 |
| TC0X00008081.hg.1 | ATG4A | autophagy related 4A, cysteine peptidase | Multiple_C | 9.49 | 8.83 | 9.03 | 1.38 | 0.2075 | 0.4011 | 8.19 | 7.89 | 9.12 | 0.52 | 0.002 | 0.0265 |
| TC0X00009804.hg.1 | PAGE3 | P antigen family, member 3 (prostate associated) | Coding | 3.29 | 3.04 | 2.83 | 1.38 | 0.0661 | 0.1781 | 3.56 | 3.47 | 3.31 | 1.19 | 0.2003 | 0.4557 |
| TC0X00011364.hg.1 | ZNF630 | zinc finger protein 630 | Multiple_C | 4.1 | 3.77 | 3.64 | 1.38 | 0.1501 | 0.3222 | 4.78 | 4.24 | 4.42 | 1.28 | 0.2297 | 0.4908 |
| TC1000006704.hg.1 | TAF3 | TATA box binding protein associated factor 3 | Multiple_C | 8.09 | 7.25 | 7.63 | 1.38 | 0.12 | 0.2747 | 7.39 | 7.30 | 7.5 | 0.93 | 0.4999 | 0.7326 |
| TC1000007461.hg.1 | RASSF4 | Ras association (RalGDS/AF-6) domain family member 4 | Multiple_C | 5.63 | 5.25 | 5.17 | 1.38 | 0.2094 | 0.403 | 7.01 | 6.57 | 6.28 | 1.66 | 0.0027 | 0.0335 |

| | | | | | | | | | | | | | | | |
|---|---|---|---|---|---|---|---|---|---|---|---|---|---|---|---|
| TC1000007776.hg.1 | ZNF365 | zinc finger protein 365 | Multiple_C | 5.12 | 4.58 | 4.66 | 1.38 | 0.0685 | 0.1825 | 4.94 | 4.60 | 4.62 | 1.25 | 0.0893 | 0.2892 |
| TC1000007883.hg.1 | SRGN | serglycin | Multiple_C | 4.4 | 3.82 | 3.94 | 1.38 | 0.2707 | 0.4791 | 4.96 | 4.40 | 5.24 | 0.82 | 0.4421 | 0.691 |
| TC1000008508.hg.1 | CYP2C9 | cytochrome P450, family 2, subfamily C, polypeptide 9 | Multiple_C | 3.43 | 3.46 | 2.97 | 1.38 | 0.3642 | 0.5778 | 3.63 | 3.32 | 3.33 | 1.23 | 0.0818 | 0.2755 |
| TC1100011723.hg.1 | RSF1 | remodeling and spacing factor 1 | Multiple_C | 10.82 | 11.18 | 10.36 | 1.38 | 0.1417 | 0.3095 | 10.26 | 10.00 | 10.07 | 1.14 | 0.1833 | 0.4342 |
| TC1200006909.hg.1 | EMP1 | epithelial membrane protein 1 | Multiple_C | 10.7 | 10.29 | 10.24 | 1.38 | 0.6579 | 0.8122 | 7.85 | 6.01 | 7.36 | 1.40 | 0.0972 | 0.3039 |
| TC1200009375.hg.1 | GLT1D1 | glycosyltransferase 1 domain containing 1 | Multiple_C | 5.21 | 4.78 | 4.75 | 1.38 | 0.3271 | 0.541 | 4.97 | 4.91 | 4.91 | 1.04 | 0.2881 | 0.5527 |
| TC1200010158.hg.1 | KRAS | Kirsten rat sarcoma viral oncogene homolog | Coding | 11.82 | 11.16 | 11.36 | 1.38 | 0.4943 | 0.6943 | 10.95 | 10.65 | 10.61 | 1.27 | 0.0877 | 0.2858 |
| TC1200011298.hg.1 | CAPS2 | calcyphosine 2 | Multiple_C | 4.41 | 4.37 | 3.95 | 1.38 | 0.0702 | 0.1859 | 3.89 | 4.05 | 3.92 | 0.98 | 0.4093 | 0.6646 |
| TC1200012293.hg.1 | DNAH10OS | dynein, axonemal, heavy chain 10 opposite strand | Coding | 3.8 | 3.19 | 3.34 | 1.38 | 0.2617 | 0.4686 | 3.37 | 3.52 | 3.55 | 0.88 | 0.4197 | 0.6731 |
| TC1300008010.hg.1 | COL4A2 | collagen, type IV, alpha 2 | Multiple_C | 7.71 | 7.45 | 7.25 | 1.38 | 0.1357 | 0.3003 | 6.43 | 6.84 | 6.58 | 0.90 | 0.4015 | 0.6582 |
| TC1400009052.hg.1 | FSCB | fibrous sheath CABYR binding protein | Coding | 4.09 | 3.59 | 3.63 | 1.38 | 0.0549 | 0.1549 | 4.39 | 4.32 | 4.29 | 1.07 | 0.6619 | 0.835 |
| TC1400009709.hg.1 | MLH3 | mutL homolog 3 | Multiple_C | 7.9 | 8.08 | 7.44 | 1.38 | 0.2472 | 0.4507 | 7.17 | 7.61 | 7.72 | 0.68 | 0.1845 | 0.4357 |
| TC1400010767.hg.1 | ZFYVE26 | zinc finger, FYVE domain containing 26 | Multiple_C | 7.95 | 7.5 | 7.49 | 1.38 | 0.1412 | 0.3087 | 8.03 | 8.55 | 8.55 | 0.70 | 0.2316 | 0.4928 |
| TC1500008317.hg.1 | CRTC3 | CREB regulated transcription coactivator 3 | Multiple_C | 8.91 | 7.96 | 8.45 | 1.38 | 0.0132 | 0.0518 | 6.76 | 6.55 | 7.21 | 0.73 | 0.1614 | 0.4043 |
| TC1600007464.hg.1 | MYLPF | myosin light chain, phosphorylatable, fast skeletal muscle | Multiple_C | 3.4 | 3.04 | 2.94 | 1.38 | 0.1287 | 0.2889 | 3.26 | 3.38 | 3.51 | 0.84 | 0.0884 | 0.2876 |
| TC1600009187.hg.1 | TIGD7 | tigger transposable element derived 7 | Multiple_C | 4.84 | 4.1 | 4.38 | 1.38 | 0.8454 | 0.9241 | 4.84 | 4.91 | 5.4 | 0.68 | 0.2715 | 0.5369 |
| TC1600009530.hg.1 | KIAA0430; MI | KIAA0430; microRNA 6506 | Multiple_C | 7.2 | 6.22 | 6.74 | 1.38 | 0.8676 | 0.9355 | 7.68 | 7.34 | 7.45 | 1.17 | 0.388 | 0.6467 |
| TC1600011423.hg.1 | CLEC18C | C-type lectin domain family 18, member C | Multiple_C | 5.44 | 5.17 | 4.98 | 1.38 | 0.15 | 0.322 | 4.68 | 4.79 | 4.68 | 1.00 | 0.8807 | 0.9487 |
| TC1700007559.hg.1 | CCL11 | chemokine (C-C motif) ligand 11 | Multiple_C | 3.72 | 3.01 | 3.26 | 1.38 | 0.5063 | 0.7033 | 3.61 | 4.05 | 3.83 | 0.86 | 0.4532 | 0.6983 |
| TC1700009113.hg.1 | GAA | glucosidase, alpha; acid | Multiple_C | 9.7 | 9.48 | 9.24 | 1.38 | 0.0969 | 0.2353 | 9.46 | 9.71 | 9.42 | 1.03 | 0.7665 | 0.8952 |
| TC1700009219.hg.1 | RAC3 | ras-related C3 botulinum toxin substrate 3 (rho family, small GT | Multiple_C | 6.54 | 5.97 | 6.08 | 1.38 | 0.499 | 0.6985 | 6.84 | 6.95 | 7.08 | 0.85 | 0.1929 | 0.4461 |
| TC1700010592.hg.1 | ORMDL3 | ORMDL sphingolipid biosynthesis regulator 3 | Multiple_C | 9.48 | 9.29 | 9.02 | 1.38 | 0.2864 | 0.4969 | 9.66 | 9.77 | 9.62 | 1.03 | 0.5074 | 0.7384 |
| TC1700012070.hg.1 | FAM195B | family with sequence similarity 195, member B | Multiple_C | 6.27 | 5.27 | 5.81 | 1.38 | 0.1274 | 0.2868 | 7.31 | 7.64 | 7.83 | 0.70 | 0.187 | 0.4388 |
| TC1700012255.hg.1 | KRTAP9-2 | keratin associated protein 9-2 | Coding | 5.14 | 4.8 | 4.68 | 1.38 | 0.1043 | 0.2485 | 5.46 | 5.23 | 5.2 | 1.20 | 0.2967 | 0.5614 |
| TC1800009243.hg.1 | SERPINB10 | serpin peptidase inhibitor, clade B (ovalbumin), member 10 | Multiple_C | 3.95 | 3.89 | 3.49 | 1.38 | 0.0656 | 0.1771 | 3.93 | 3.50 | 3.68 | 1.19 | 0.1129 | 0.3311 |
| TC1900006507.hg.1 | CNN2 | calponin 2 | Multiple_C | 11.92 | 11.04 | 11.46 | 1.38 | 0.0674 | 0.1804 | 11.05 | 11.56 | 11.93 | 0.54 | 0.006 | 0.0553 |
| TC1900008880.hg.1 | KIR2DL4 | killer cell immunoglobulin-like receptor, two domains, long cyto | Multiple_C | 6.16 | 6.2 | 5.7 | 1.38 | 0.0448 | 0.1335 | 4.99 | 4.90 | 4.93 | 1.04 | 0.1614 | 0.4043 |
| TC1900009369.hg.1 | PLIN3 | perilipin 3 | Multiple_C | 8.82 | 8.03 | 8.36 | 1.38 | 0.3879 | 0.6015 | 8.21 | 8.22 | 7.93 | 1.21 | 0.3879 | 0.6466 |
| TC1900010017.hg.1 | ELL | elongation factor RNA polymerase II | Multiple_C | 8.13 | 7.24 | 7.67 | 1.38 | 0.0385 | 0.1191 | 7.66 | 7.75 | 7.58 | 1.06 | 0.9395 | 0.975 |
| TC1900011030.hg.1 | SLC8A2 | solute carrier family 8 (sodium/calcium exchanger), member 2 | Multiple_C | 5.68 | 5.39 | 5.22 | 1.38 | 0.174 | 0.356 | 5.19 | 5.11 | 5.15 | 1.03 | 0.7862 | 0.9045 |
| TC1900011507.hg.1 | ZSCAN5B | zinc finger and SCAN domain containing 5B | Coding | 3.87 | 3.43 | 3.41 | 1.38 | 0.0448 | 0.1336 | 3.52 | 3.55 | 3.63 | 0.93 | 0.6558 | 0.8321 |
| TC1900011736.hg.1 | EGLN2 | egl-9 family hypoxia-inducible factor 2 | Multiple_C | 10.2 | 9.67 | 9.74 | 1.38 | 0.035 | 0.1109 | 8.02 | 8.29 | 8.03 | 0.99 | 0.8144 | 0.9177 |
| TC2000006642.hg.1 | BMP2 | bone morphogenetic protein 2 | Coding | 7.34 | 6.82 | 6.88 | 1.38 | 0.0239 | 0.0824 | 4.44 | 4.40 | 3.94 | 1.41 | 0.0211 | 0.1241 |

| | | | | | | | | | | | | | | | |
|---|---|---|---|---|---|---|---|---|---|---|---|---|---|---|---|
| TC2000008746.hg.1 | NINL | ninein-like | Multiple_C | 6.21 | 5.68 | 5.75 | 1.38 | 0.3437 | 0.5574 | 7.11 | 6.66 | 6.5 | 1.53 | 0.0504 | 0.2091 |
| TC2000009817.hg.1 | SRMS | src-related kinase lacking C-terminal regulatory tyrosine and N-t | Coding | 7 | 6.45 | 6.54 | 1.38 | 0.0558 | 0.1568 | 6.57 | 6.68 | 6.53 | 1.03 | 0.3265 | 0.5903 |
| TC2100008577.hg.1 | KRTAP10-6 | keratin associated protein 10-6 | Coding | 6.1 | 5.26 | 5.64 | 1.38 | 0.1575 | 0.3332 | 4.99 | 5.10 | 5.1 | 0.93 | 0.9376 | 0.9741 |
| TC2200006577.hg.1 | DGCR5; DGCR | DiGeorge syndrome critical region gene 5 (non-protein coding); | Multiple_C | 4.81 | 4.31 | 4.35 | 1.38 | 0.2674 | 0.4755 | 5.32 | 5.97 | 6.18 | 0.55 | 0.003 | 0.0353 |
| TC2200008211.hg.1 | GGT5 | gamma-glutamyltransferase 5 | Multiple_C | 4.88 | 4.56 | 4.42 | 1.38 | 0.0494 | 0.1434 | 4.55 | 4.34 | 4.08 | 1.39 | 0.0349 | 0.1686 |
| TSUnmapped00000323.h | RPS6KA1 | ribosomal protein S6 kinase, 90kDa, polypeptide 1 | Coding | 7.49 | 7.03 | 7.03 | 1.38 | 0.1297 | 0.2906 | 7.28 | 7.18 | 6.85 | 1.35 | 0.2181 | 0.4778 |
| TSUnmapped00000519.h | CACFD1 | calcium channel flower domain containing 1 | Coding | 5.4 | 4.83 | 4.94 | 1.38 | 0.1489 | 0.3204 | 5.25 | 5.19 | 5.18 | 1.05 | 0.7015 | 0.8585 |
| TC0100007716.hg.1 | ZNF362 | zinc finger protein 362 | Multiple_C | 6.87 | 6.06 | 6.42 | 1.37 | 0.1109 | 0.2598 | 7.44 | 7.61 | 6.81 | 1.55 | 0.005 | 0.0489 |
| TC0100010018.hg.1 | SPRR3 | small proline-rich protein 3 | Multiple_C | 3.86 | 3.26 | 3.41 | 1.37 | 0.0752 | 0.1954 | 3.7 | 3.54 | 3.59 | 1.08 | 0.4687 | 0.7098 |
| TC0100010345.hg.1 | PCP4L1 | Purkinje cell protein 4 like 1 | Coding | 6.46 | 5.49 | 6.01 | 1.37 | 0.0534 | 0.1516 | 6.26 | 5.94 | 6.17 | 1.06 | 0.6072 | 0.8036 |
| TC0100012176.hg.1 | BECN2 | beclin 2 | Coding | 3.38 | 2.79 | 2.93 | 1.37 | 0.109 | 0.257 | 4.06 | 4.21 | 3.97 | 1.06 | 0.1174 | 0.3388 |
| TC0100015594.hg.1 | ITGA10 | integrin alpha 10 | Multiple_C | 4.41 | 3.99 | 3.96 | 1.37 | 0.1286 | 0.2888 | 4.37 | 4.60 | 4.38 | 0.99 | 0.962 | 0.9841 |
| TC0100016794.hg.1 | B3GALT2 | UDP-Gal:betaGlcNAc beta 1,3-galactosyltransferase 2 | Coding | 5.41 | 4.46 | 4.96 | 1.37 | 0.0526 | 0.1499 | 4.83 | 5.04 | 5.01 | 0.88 | 0.9483 | 0.979 |
| TC0100018349.hg.1 | CNIH3 | cornichon family AMPA receptor auxiliary protein 3 | Multiple_C | 5.35 | 5.05 | 4.9 | 1.37 | 0.5066 | 0.7035 | 5.83 | 5.63 | 6.12 | 0.82 | 0.3534 | 0.6169 |
| TC0200007432.hg.1 | SIX3 | SIX homeobox 3 | Coding | 4.32 | 3.74 | 3.87 | 1.37 | 0.4263 | 0.6369 | 4.64 | 4.31 | 3.97 | 1.59 | 0.0224 | 0.1287 |
| TC0200008468.hg.1 | FAHD2A | fumarylacetoacetate hydrolase domain containing 2A | Multiple_C | 8.24 | 9.01 | 7.79 | 1.37 | 0.1252 | 0.283 | 7.42 | 7.33 | 7.13 | 1.22 | 0.3874 | 0.6462 |
| TC0200009167.hg.1 | CNTNAP5 | contactin associated protein-like 5 | Multiple_C | 3.86 | 3.63 | 3.41 | 1.37 | 0.6674 | 0.8181 | 4.15 | 4.26 | 4.25 | 0.93 | 0.8758 | 0.9468 |
| TC0200011104.hg.1 | CHRND | cholinergic receptor, nicotinic delta | Multiple_C | 6 | 5.48 | 5.55 | 1.37 | 0.326 | 0.54 | 7.27 | 7.52 | 7.24 | 1.02 | 0.2259 | 0.4863 |
| TC0200013863.hg.1 | NPHP1 | nephronophthisis 1 (juvenile) | Multiple_C | 4.26 | 3.9 | 3.81 | 1.37 | 0.0724 | 0.1899 | 5.37 | 5.03 | 4.89 | 1.39 | 0.0069 | 0.0607 |
| TC0200014641.hg.1 | RPRM | reprimo, TP53 dependent G2 arrest mediator candidate | Coding | 3.36 | 2.76 | 2.91 | 1.37 | 0.0262 | 0.0883 | 3.72 | 3.70 | 3.69 | 1.02 | 0.9531 | 0.981 |
| TC0200014735.hg.1 | RBMS1; MIR4 | RNA binding motif, single stranded interacting protein 1; microR | Multiple_C | 8.74 | 8.72 | 8.29 | 1.37 | 0.2618 | 0.4687 | 7.92 | 6.97 | 7.42 | 1.41 | 0.2622 | 0.5265 |
| TC0200014971.hg.1 | SP3 | Sp3 transcription factor | Multiple_C | 13.46 | 13.13 | 13.01 | 1.37 | 0.0179 | 0.0656 | 12.75 | 12.98 | 13.21 | 0.73 | 0.0086 | 0.0708 |
| TC0300006555.hg.1 | BRPF1 | bromodomain and PHD finger containing 1 | Multiple_C | 5.63 | 4.88 | 5.18 | 1.37 | 0.2472 | 0.4508 | 6.08 | 6.31 | 6.43 | 0.78 | 0.4401 | 0.6898 |
| TC0300006968.hg.1 | CMTM7 | CKLF-like MARVEL transmembrane domain containing 7 | Multiple_C | 11.44 | 10.04 | 10.99 | 1.37 | 0.4402 | 0.649 | 9.35 | 8.43 | 8.44 | 1.88 | 0.0109 | 0.0821 |
| TC0300007455.hg.1 | GPR62 | G protein-coupled receptor 62 | Coding | 4.67 | 4.21 | 4.22 | 1.37 | 0.1266 | 0.2853 | 4.21 | 4.64 | 4.53 | 0.80 | 0.7418 | 0.8815 |
| TC0300008147.hg.1 | LNP1 | leukemia NUP98 fusion partner 1 | Coding | 4.83 | 4.52 | 4.38 | 1.37 | 0.2067 | 0.4 | 6.16 | 6.77 | 6.61 | 0.73 | 0.1397 | 0.3714 |
| TC0300008524.hg.1 | STXBP5L | syntaxin binding protein 5-like | Multiple_C | 4.71 | 4.13 | 4.26 | 1.37 | 0.0953 | 0.2326 | 4.91 | 5.94 | 5.25 | 0.79 | 0.1368 | 0.3676 |
| TC0300009600.hg.1 | ZNF639 | zinc finger protein 639 | Multiple_C | 10.65 | 10.69 | 10.2 | 1.37 | 0.4079 | 0.6202 | 9.99 | 9.55 | 10.33 | 0.79 | 0.4918 | 0.7268 |
| TC0300012178.hg.1 | ILDR1 | immunoglobulin-like domain containing receptor 1 | Multiple_C | 5.71 | 5.33 | 5.26 | 1.37 | 0.0599 | 0.1655 | 4.45 | 4.25 | 4.7 | 0.84 | 0.2269 | 0.4874 |
| TC0400007713.hg.1 | STAP1 | signal transducing adaptor family member 1 | Coding | 3.9 | 4.07 | 3.45 | 1.37 | 0.1124 | 0.2624 | 4.31 | 4.06 | 3.92 | 1.31 | 0.034 | 0.1659 |
| TC0400007988.hg.1 | THAP9 | THAP domain containing 9 | Multiple_C | 5.29 | 5.58 | 4.84 | 1.37 | 0.1283 | 0.2884 | 4.52 | 4.69 | 4.66 | 0.91 | 0.2995 | 0.5639 |
| TC0400008007.hg.1 | GPAT3 | glycerol-3-phosphate acyltransferase 3 | Multiple_C | 10.04 | 9.45 | 9.59 | 1.37 | 0.1955 | 0.3856 | 7.28 | 5.97 | 8.1 | 0.57 | 0.0099 | 0.0773 |
| TC0400008984.hg.1 | RPS3A; SNORI | ribosomal protein S3A; small nucleolar RNA, C/D box 73A | Multiple_C | 17.16 | 18.01 | 16.71 | 1.37 | 0.027 | 0.0904 | 16.34 | 16.17 | 15.63 | 1.64 | 0.0005 | 0.0104 |

| | | | | | | | | | | | | | | | |
|---|---|---|---|---|---|---|---|---|---|---|---|---|---|---|---|
| TC0400011014.hg.1 | CXCL5 | chemokine (C-X-C motif) ligand 5 | Multiple_C | 4.06 | 4.03 | 3.61 | 1.37 | 0.2784 | 0.4878 | 5.06 | 4.71 | 4.73 | 1.26 | 0.0142 | 0.0969 |
| TC0400012931.hg.1 | G3BP2 | GTPase activating protein (SH3 domain) binding protein 2 | Multiple_C | 14.83 | 14.44 | 14.38 | 1.37 | 0.0439 | 0.1315 | 15.68 | 15.26 | 15.52 | 1.12 | 0.3304 | 0.5945 |
| TC0500007044.hg.1 | CDH6 | cadherin 6, type 2, K-cadherin (fetal kidney) | Multiple_C | 4.23 | 3.5 | 3.78 | 1.37 | 0.0803 | 0.2055 | 3.77 | 3.81 | 3.93 | 0.90 | 0.7214 | 0.8701 |
| TC0500008784.hg.1 | REEP2 | receptor accessory protein 2 | Multiple_C | 3.86 | 3.42 | 3.41 | 1.37 | 0.1318 | 0.2941 | 7.49 | 7.25 | 6.65 | 1.79 | 0.0017 | 0.0239 |
| TC0500008921.hg.1 | NDFIP1 | Nedd4 family interacting protein 1 | Multiple_C | 12.2 | 11.38 | 11.75 | 1.37 | 0.1102 | 0.2587 | 9.11 | 8.82 | 9.24 | 0.91 | 0.6179 | 0.8103 |
| TC0500012138.hg.1 | SPOCK1 | sparc/osteonectin, cwcv and kazal-like domains proteoglycan (t | Multiple_C | 3.58 | 2.98 | 3.13 | 1.37 | 0.3064 | 0.5196 | 4.25 | 4.33 | 4.26 | 0.99 | 0.2418 | 0.5056 |
| TC0500012587.hg.1 | TIMD4 | T-cell immunoglobulin and mucin domain containing 4 | Coding | 3.19 | 2.61 | 2.74 | 1.37 | 0.331 | 0.545 | 3.52 | 3.26 | 3.52 | 1.00 | 0.1768 | 0.4255 |
| TC0500012663.hg.1 | SLU7 | SLU7 homolog, splicing factor | Multiple_C | 10.12 | 10.07 | 9.67 | 1.37 | 0.1372 | 0.3024 | 10.19 | 9.59 | 9.11 | 2.11 | 7.37E-06 | 0.0006 |
| TC0500013172.hg.1 | C5orf64 | chromosome 5 open reading frame 64 | Multiple_C | 4.83 | 4.5 | 4.38 | 1.37 | 0.1191 | 0.2734 | 5.38 | 5.18 | 5.35 | 1.02 | 0.7338 | 0.8772 |
| TC0600007038.hg.1 | JARID2 | jumonji, AT rich interactive domain 2 | Multiple_C | 7.25 | 6.5 | 6.8 | 1.37 | 0.1224 | 0.2788 | 7.71 | 7.36 | 7.51 | 1.15 | 0.3009 | 0.5652 |
| TC0600011739.hg.1 | KCNK16 | potassium channel, two pore domain subfamily K, member 16 | Coding | 4.46 | 3.87 | 4.01 | 1.37 | 0.0831 | 0.2102 | 4.56 | 4.54 | 4.32 | 1.18 | 0.4414 | 0.6905 |
| TC0600011913.hg.1 | NFKBIE | nuclear factor of kappa light polypeptide gene enhancer in B-ce | Multiple_C | 6.06 | 5.68 | 5.61 | 1.37 | 0.1858 | 0.3723 | 5.13 | 5.15 | 5.12 | 1.01 | 0.9777 | 0.9908 |
| TC0600013527.hg.1 | KATNA1 | katanin p60 (ATPase containing) subunit A 1 | Multiple_C | 8.03 | 8.36 | 7.58 | 1.37 | 0.2107 | 0.405 | 5.77 | 5.79 | 5.69 | 1.06 | 0.6339 | 0.8202 |
| TC0600013870.hg.1 | T | T brachyury transcription factor | Multiple_C | 4.82 | 4.4 | 4.37 | 1.37 | 0.0039 | 0.0197 | 7.92 | 8.52 | 7.54 | 1.30 | 0.3278 | 0.5912 |
| TC0600014148.hg.1 | CYB5R4 | cytochrome b5 reductase 4 | Multiple_C | 8.46 | 8.4 | 8.01 | 1.37 | 0.0341 | 0.1086 | 7.67 | 7.34 | 7.62 | 1.04 | 0.8158 | 0.9183 |
| TC0600014238.hg.1 | BLOC1S5 | biogenesis of lysosomal organelles complex-1, subunit 5, muted | Multiple_C | 6.98 | 6.7 | 6.53 | 1.37 | 0.2077 | 0.4011 | 9.59 | 9.70 | 9.75 | 0.90 | 0.1253 | 0.3498 |
| TC0700007949.hg.1 | POM121 | POM121 transmembrane nucleoporin | Multiple_C | 11.14 | 9.48 | 10.69 | 1.37 | 0.1223 | 0.2786 | 13.63 | 14.34 | 14.55 | 0.53 | 0.0014 | 0.021 |
| TC0700008240.hg.1 | GRM3 | glutamate receptor, metabotropic 3 | Coding | 4.33 | 3.89 | 3.88 | 1.37 | 0.067 | 0.1797 | 5.26 | 5.06 | 5.01 | 1.19 | 0.2173 | 0.4765 |
| TC0700009410.hg.1 | OR9A4 | olfactory receptor, family 9, subfamily A, member 4 | Coding | 3.73 | 3.33 | 3.28 | 1.37 | 0.4642 | 0.6693 | 3.94 | 3.74 | 3.55 | 1.31 | 0.0528 | 0.2148 |
| TC0800006760.hg.1 | FDFT1 | farnesyl-diphosphate farnesyltransferase 1 | Multiple_C | 14.59 | 14.89 | 14.14 | 1.37 | 0.2001 | 0.3918 | 11.84 | 11.67 | 12.65 | 0.57 | 0.0109 | 0.0823 |
| TC0800008782.hg.1 | ZNF572 | zinc finger protein 572 | Coding | 6.84 | 6.76 | 6.39 | 1.37 | 0.1132 | 0.2636 | 5.99 | 5.99 | 5.33 | 1.58 | 0.0351 | 0.1693 |
| TC0800009886.hg.1 | NKX2-6 | NK2 homeobox 6 | Coding | 5.01 | 4.4 | 4.56 | 1.37 | 0.2886 | 0.4993 | 5.29 | 5.12 | 4.65 | 1.56 | 0.126 | 0.3511 |
| TC0800012088.hg.1 | LY6D | lymphocyte antigen 6 complex, locus D | Multiple_C | 5.94 | 5.21 | 5.49 | 1.37 | 0.1442 | 0.3136 | 5.95 | 5.95 | 6.06 | 0.93 | 0.9803 | 0.9916 |
| TC0900009076.hg.1 | STKLD1 | serine/threonine kinase-like domain containing 1 | Multiple_C | 3.25 | 2.76 | 2.8 | 1.37 | 0.2205 | 0.4179 | 4.15 | 4.34 | 4.02 | 1.09 | 0.4457 | 0.6935 |
| TC0900009910.hg.1 | ARID3C | AT rich interactive domain 3C (BRIGHT-like) | Coding | 3.23 | 2.88 | 2.78 | 1.37 | 0.0611 | 0.1679 | 3.43 | 3.16 | 3 | 1.35 | 0.0089 | 0.0719 |
| TC0900011394.hg.1 | C5 | complement component 5 | Multiple_C | 4.65 | 4.94 | 4.2 | 1.37 | 0.0362 | 0.1139 | 4.62 | 4.86 | 5.35 | 0.60 | 0.0211 | 0.1242 |
| TC0X00006567.hg.1 | VCX3B; VCX | variable charge, X-linked 3B; variable charge, X-linked | Coding | 5.02 | 4.45 | 4.57 | 1.37 | 0.1094 | 0.2576 | 4.34 | 4.31 | 4.4 | 0.96 | 0.939 | 0.9748 |
| TC0X00006857.hg.1 | MAGEB10 | MAGE family member B10 | Coding | 3.98 | 3.06 | 3.53 | 1.37 | 0.1034 | 0.247 | 3.84 | 4.26 | 3.96 | 0.92 | 0.9863 | 0.9943 |
| TC0X00007026.hg.1 | USP9X | ubiquitin specific peptidase 9, X-linked | Multiple_C | 13 | 12.34 | 12.55 | 1.37 | 0.1695 | 0.3498 | 13.17 | 13.11 | 13.13 | 1.03 | 0.7689 | 0.8964 |
| TC0X00009341.hg.1 | DMD | dystrophin | Multiple_C | 8.41 | 7.72 | 7.96 | 1.37 | 0.0688 | 0.183 | 4.21 | 4.00 | 3.96 | 1.19 | 0.31 | 0.5739 |
| TC0X00009650.hg.1 | WDR45; PRAF | WD repeat domain 45; PRA1 domain family, member 2 | Multiple_C | 8.7 | 8.28 | 8.25 | 1.37 | 0.5826 | 0.7599 | 6.93 | 8.47 | 8.07 | 0.45 | 0.0006 | 0.012 |
| TC0X00011332.hg.1 | PLXNB3 | plexin B3 | Multiple_C | 3.86 | 3.22 | 3.41 | 1.37 | 0.0386 | 0.1193 | 4.91 | 5.40 | 5.37 | 0.73 | 0.0319 | 0.1597 |
| TC1000009851.hg.1 | FRMD4A | FERM domain containing 4A | Multiple_C | 4.08 | 3.41 | 3.63 | 1.37 | 0.0619 | 0.1694 | 7.68 | 7.97 | 8.02 | 0.79 | 0.2397 | 0.5033 |

| | | | | | | | | | | | | | | | |
|---|---|---|---|---|---|---|---|---|---|---|---|---|---|---|---|
| TC1000011215.hg.1 | DYDC1 | DPY30 domain containing 1 | Multiple_C | 3.82 | 3.23 | 3.37 | 1.37 | 0.107 | 0.2533 | 3.44 | 3.25 | 3.44 | 1.00 | 0.9517 | 0.9799 |
| TC1100008462.hg.1 | RPS3; SNORD1 | ribosomal protein S3; small nucleolar RNA, C/D box 15A | Multiple_C | 16.42 | 16.33 | 15.97 | 1.37 | 0.0928 | 0.2281 | 16.48 | 16.19 | 16.45 | 1.02 | 0.5801 | 0.7853 |
| TC1100011338.hg.1 | CDK2AP2 | cyclin-dependent kinase 2 associated protein 2 | Multiple_C | 9.15 | 7.73 | 8.7 | 1.37 | 0.0619 | 0.1695 | 7.99 | 8.17 | 7.85 | 1.10 | 0.6088 | 0.8043 |
| TC1100011744.hg.1 | GAB2 | GRB2-associated binding protein 2 | Multiple_C | 5.36 | 4.7 | 4.91 | 1.37 | 0.1923 | 0.3811 | 5.04 | 4.78 | 4.9 | 1.10 | 0.0466 | 0.1995 |
| TC1100012445.hg.1 | BACE1 | beta-site APP-cleaving enzyme 1 | Multiple_C | 5.64 | 5.11 | 5.19 | 1.37 | 0.2317 | 0.4322 | 6.05 | 6.51 | 6.5 | 0.73 | 0.6313 | 0.8184 |
| TC1100012495.hg.1 | DDX6 | DEAD (Asp-Glu-Ala-Asp) box helicase 6 | Multiple_C | 11.82 | 11.35 | 11.37 | 1.37 | 0.4354 | 0.645 | 12.13 | 12.68 | 13.22 | 0.47 | 0.0008 | 0.0145 |
| TC1100013134.hg.1 | HBE1 | hemoglobin, epsilon 1 | Multiple_C | 5.17 | 4.5 | 4.72 | 1.37 | 0.3875 | 0.6011 | 6.93 | 5.64 | 6.45 | 1.39 | 0.0525 | 0.214 |
| TC1200006700.hg.1 | FOXJ2 | forkhead box J2 | Multiple_C | 6.78 | 5.67 | 6.33 | 1.37 | 0.1525 | 0.326 | 7.67 | 8.03 | 8.13 | 0.73 | 0.1118 | 0.3294 |
| TC1200007694.hg.1 | SOAT2 | sterol O-acyltransferase 2 | Multiple_C | 3.91 | 3.47 | 3.46 | 1.37 | 0.1035 | 0.2472 | 5.71 | 5.64 | 5.08 | 1.55 | 0.0088 | 0.0719 |
| TC1200008798.hg.1 | MYO1H | myosin IH | Multiple_C | 3.27 | 2.7 | 2.82 | 1.37 | 0.0859 | 0.2154 | 3.68 | 3.65 | 3.6 | 1.06 | 0.2012 | 0.4566 |
| TC1200009103.hg.1 | PRKAB1 | protein kinase, AMP-activated, beta 1 non-catalytic subunit | Multiple_C | 10.68 | 10.35 | 10.23 | 1.37 | 0.082 | 0.2081 | 9.83 | 9.93 | 10.27 | 0.74 | 0.0893 | 0.2891 |
| TC1200009927.hg.1 | TAS2R20 | taste receptor, type 2, member 20 | Coding | 5.67 | 5.97 | 5.22 | 1.37 | 0.013 | 0.0512 | 4.8 | 4.99 | 4.87 | 0.95 | 0.2821 | 0.5476 |
| TC1200009985.hg.1 | GSG1 | germ cell associated 1 | Coding | 4.92 | 4.36 | 4.47 | 1.37 | 0.4668 | 0.6713 | 4.41 | 4.33 | 4.2 | 1.16 | 0.3022 | 0.5667 |
| TC1200010065.hg.1 | PLCZ1 | phospholipase C, zeta 1 | Multiple_C | 3.53 | 3.15 | 3.08 | 1.37 | 0.2862 | 0.4965 | 4.39 | 4.40 | 4.29 | 1.07 | 0.6494 | 0.8289 |
| TC1200012210.hg.1 | MORN3 | MORN repeat containing 3 | Multiple_C | 6.27 | 5.87 | 5.82 | 1.37 | 0.1521 | 0.3255 | 5.46 | 5.58 | 5.7 | 0.85 | 0.192 | 0.4451 |
| TC1200012634.hg.1 | KRT7 | keratin 7, type II | Multiple_C | 12.47 | 13.07 | 12.02 | 1.37 | 0.0267 | 0.0897 | 8.84 | 8.30 | 8.36 | 1.39 | 0.0119 | 0.0867 |
| TC1400010289.hg.1 | CDC42BPB | CDC42 binding protein kinase beta (DMPK-like) | Multiple_C | 10.45 | 9.68 | 10 | 1.37 | 0.0693 | 0.184 | 9.52 | 9.16 | 9.1 | 1.34 | 0.1686 | 0.4143 |
| TC1500007529.hg.1 | USP3 | ubiquitin specific peptidase 3 | Multiple_C | 12.6 | 12.33 | 12.15 | 1.37 | 0.5324 | 0.7221 | 12.27 | 11.71 | 11.98 | 1.22 | 0.7924 | 0.9077 |
| TC1500008086.hg.1 | SAXO2 | stabilizer of axonemal microtubules 2 | Multiple_C | 10.53 | 10.09 | 10.08 | 1.37 | 0.0449 | 0.1338 | 10.33 | 10.55 | 10.57 | 0.85 | 0.4203 | 0.6735 |
| TC1500009096.hg.1 | C15orf52 | chromosome 15 open reading frame 52 | Multiple_C | 6.88 | 6.36 | 6.43 | 1.37 | 0.097 | 0.2354 | 6.57 | 6.93 | 7.07 | 0.71 | 0.1425 | 0.3753 |
| TC1500010350.hg.1 | NTRK3 | neurotrophic tyrosine kinase, receptor, type 3 | Multiple_C | 3.83 | 3.45 | 3.38 | 1.37 | 0.0819 | 0.208 | 4.39 | 3.80 | 3.82 | 1.48 | 0.0076 | 0.0648 |
| TC1500010858.hg.1 | CATSPER2 | cation channel, sperm associated 2 | Multiple_C | 5.52 | 4.78 | 5.07 | 1.37 | 0.0516 | 0.1479 | 4.93 | 4.66 | 4.99 | 0.96 | 0.4694 | 0.7105 |
| TC1500010884.hg.1 | RPL4; SNORD1 | ribosomal protein L4; small nucleolar RNA, C/D box 16; small nu | Multiple_C | 16.74 | 16.77 | 16.29 | 1.37 | 0.0712 | 0.1879 | 15.97 | 15.84 | 15.75 | 1.16 | 0.2903 | 0.5552 |
| TC1600007439.hg.1 | C16orf92 | chromosome 16 open reading frame 92 | Coding | 3.72 | 3.25 | 3.27 | 1.37 | 0.1882 | 0.3755 | 3.76 | 3.65 | 3.29 | 1.39 | 0.0025 | 0.0314 |
| TC1600008580.hg.1 | GAN; MIR472( | gigaxonin; microRNA 4720 | Multiple_C | 10.13 | 9.83 | 9.68 | 1.37 | 0.1136 | 0.2643 | 10.25 | 9.43 | 10.04 | 1.16 | 0.7447 | 0.8831 |
| TC1600008617.hg.1 | CDH13 | cadherin 13 | Multiple_C | 3.91 | 3.1 | 3.46 | 1.37 | 0.2943 | 0.5057 | 10.36 | 9.64 | 9.02 | 2.53 | 0.0006 | 0.0115 |
| TC1600008936.hg.1 | RGS11 | regulator of G-protein signaling 11 | Multiple_C | 5.5 | 5.26 | 5.05 | 1.37 | 0.351 | 0.5639 | 4.73 | 4.46 | 4.7 | 1.02 | 0.2112 | 0.4687 |
| TC1600008977.hg.1 | CCDC78 | coiled-coil domain containing 78 | Multiple_C | 4.79 | 4.18 | 4.34 | 1.37 | 0.2399 | 0.4419 | 5.07 | 5.39 | 5.07 | 1.00 | 0.2024 | 0.4581 |
| TC1600009956.hg.1 | BOLA2B; BOL/ | bolA family member 2B; bolA family member 2 | Multiple_C | 15.46 | 16.12 | 15.01 | 1.37 | 0.0127 | 0.0504 | 10.27 | 10.87 | 11.04 | 0.59 | 0.0062 | 0.0564 |
| TC1600011043.hg.1 | SLC38A8 | solute carrier family 38, member 8 | Multiple_C | 4.05 | 3.29 | 3.6 | 1.37 | 0.0453 | 0.1345 | 4.09 | 4.28 | 4.25 | 0.90 | 0.4049 | 0.6603 |
| TC1700008995.hg.1 | SEPT9 | septin 9 | Multiple_C | 9.68 | 8.86 | 9.23 | 1.37 | 0.1723 | 0.3536 | 13.05 | 12.97 | 12.44 | 1.53 | 0.1208 | 0.343 |
| TC1700009037.hg.1 | C17orf99 | chromosome 17 open reading frame 99 | Multiple_C | 3.75 | 3.23 | 3.3 | 1.37 | 0.147 | 0.3176 | 3.92 | 3.83 | 4.12 | 0.87 | 0.4089 | 0.6643 |
| TC1700009954.hg.1 | TOM1L2 | target of myb1 like 2 membrane trafficking protein | Multiple_C | 8.47 | 7.98 | 8.02 | 1.37 | 0.2179 | 0.4143 | 8.16 | 8.18 | 8.17 | 0.99 | 0.9725 | 0.9888 |

| | | | | | | | | | | | | | | | |
|---|---|---|---|---|---|---|---|---|---|---|---|---|---|---|---|
| TC1700010670.hg.1 | KRT38 | keratin 38, type I | Coding | 4.77 | 4.36 | 4.32 | 1.37 | 0.0363 | 0.1142 | 4.3 | 4.24 | 4.36 | 0.96 | 0.6062 | 0.8029 |
| TC1700010680.hg.1 | KRT14 | keratin 14, type I | Multiple_C | 3.41 | 2.69 | 2.96 | 1.37 | 0.0835 | 0.2109 | 3.34 | 3.47 | 3.21 | 1.09 | 0.3875 | 0.6463 |
| TC1700012334.hg.1 | TRPV1 | transient receptor potential cation channel, subfamily V, memb | Multiple_C | 5.79 | 5.15 | 5.34 | 1.37 | 0.1185 | 0.2724 | 6.5 | 6.68 | 6.39 | 1.08 | 0.4275 | 0.6796 |
| TC1800007835.hg.1 | ADNP2 | ADNP homeobox 2 | Multiple_C | 9.39 | 8.67 | 8.94 | 1.37 | 0.1366 | 0.3014 | 9.69 | 9.85 | 10.05 | 0.78 | 0.1784 | 0.4272 |
| TC1900007297.hg.1 | SIN3B | SIN3 transcription regulator family member B | Multiple_C | 4.59 | 4.47 | 4.14 | 1.37 | 0.0738 | 0.1925 | 4.91 | 4.93 | 4.71 | 1.15 | 0.2005 | 0.4558 |
| TC1900008692.hg.1 | ZNF480 | zinc finger protein 480 | Multiple_C | 4.7 | 3.71 | 4.25 | 1.37 | 0.2657 | 0.4732 | 9.91 | 10.07 | 10.41 | 0.71 | 0.457 | 0.701 |
| TC1900009224.hg.1 | C19orf35 | chromosome 19 open reading frame 35 | Coding | 4 | 3.56 | 3.55 | 1.37 | 0.5843 | 0.7611 | 4.21 | 4.07 | 4.23 | 0.99 | 0.6096 | 0.8047 |
| TC1900009625.hg.1 | ICAM3 | intercellular adhesion molecule 3 | Multiple_C | 3.92 | 3.52 | 3.47 | 1.37 | 0.01 | 0.0418 | 3.92 | 3.90 | 3.84 | 1.06 | 0.3681 | 0.6304 |
| TC1900011226.hg.1 | KLK4 | kallikrein related peptidase 4 | Multiple_C | 5.12 | 4.58 | 4.67 | 1.37 | 0.036 | 0.1135 | 4.98 | 4.84 | 4.56 | 1.34 | 0.1201 | 0.3422 |
| TC1900011236.hg.1 | KLK13 | kallikrein related peptidase 13 | Coding | 4.39 | 3.83 | 3.94 | 1.37 | 0.1872 | 0.3743 | 4.51 | 4.22 | 4.16 | 1.27 | 0.1499 | 0.3879 |
| TC2000007457.hg.1 | PKIG | protein kinase (cAMP-dependent, catalytic) inhibitor gamma | Multiple_C | 3.69 | 3.19 | 3.24 | 1.37 | 0.2693 | 0.4775 | 6.96 | 6.78 | 5.32 | 3.12 | 4.77E-07 | 8.66E-05 |
| TC2000008967.hg.1 | FAM83C | family with sequence similarity 83, member C | Coding | 4.22 | 3.52 | 3.77 | 1.37 | 0.0947 | 0.2314 | 3.79 | 3.85 | 3.74 | 1.04 | 0.2204 | 0.4804 |
| TC2100008390.hg.1 | PTTG1IP | pituitary tumor-transforming 1 interacting protein | Multiple_C | 14.13 | 13.65 | 13.68 | 1.37 | 0.4441 | 0.6525 | 13.84 | 13.78 | 14.14 | 0.81 | 0.173 | 0.4202 |
| TC2100008568.hg.1 | TMPRSS2 | transmembrane protease, serine 2 | Multiple_C | 10.07 | 10.3 | 9.62 | 1.37 | 0.4752 | 0.678 | 8.54 | 8.69 | 8.41 | 1.09 | 0.8782 | 0.9479 |
| TC22_KI270734v1_randc | DGCR6; LOC1( | Homo sapiens DiGeorge syndrome critical region gene 6 (DGCR | Coding | 5.44 | 4.69 | 4.99 | 1.37 | 0.2525 | 0.4573 | 7.05 | 6.92 | 5.88 | 2.25 | 9.68E-05 | 0.0035 |
| TC2200007894.hg.1 | CECR1 | cat eye syndrome chromosome region, candidate 1 | Multiple_C | 3.11 | 2.66 | 2.66 | 1.37 | 0.0643 | 0.1742 | 3.32 | 3.15 | 3.09 | 1.17 | 0.21 | 0.467 |
| TC2200008201.hg.1 | GSTT2; GSTT2 | glutathione S-transferase theta 2 (gene/pseudogene); glutathio | Multiple_C | 6.8 | 6.15 | 6.35 | 1.37 | 0.1297 | 0.2905 | 5.04 | 5.17 | 4.8 | 1.18 | 0.0369 | 0.1751 |
| TC2200008837.hg.1 | SNU13 | SNU13 homolog, small nuclear ribonucleoprotein (U4/U6.U5) | Multiple_C | 13.93 | 13.87 | 13.48 | 1.37 | 0.2099 | 0.4038 | 12.13 | 12.05 | 12.42 | 0.82 | 0.8338 | 0.9262 |
| TC2200009270.hg.1 | APOBEC3B | apolipoprotein B mRNA editing enzyme, catalytic polypeptide-li | Coding | 10.07 | 9.18 | 9.62 | 1.37 | 0.698 | 0.8381 | 8.13 | 7.10 | 7.42 | 1.64 | 0.0027 | 0.0331 |
| TSUnmapped00000370.h | SLC16A1 | solute carrier family 16 (monocarboxylate transporter), membe | Coding | 9.43 | 9.2 | 8.98 | 1.37 | 0.1235 | 0.2805 | 7.49 | 7.16 | 7.26 | 1.17 | 0.5656 | 0.7751 |
| TSUnmapped00000474.h | SLC16A1 | solute carrier family 16 (monocarboxylate transporter), membe | Coding | 8.13 | 8.51 | 7.68 | 1.37 | 0.0837 | 0.2113 | 5.81 | 5.70 | 5.64 | 1.13 | 0.9145 | 0.9649 |
| TC0100007790.hg.1 | TEKT2 | tektin 2 (testicular) | Coding | 3.31 | 2.85 | 2.87 | 1.36 | 0.1585 | 0.3345 | 3.17 | 3.19 | 3.14 | 1.02 | 0.6902 | 0.8524 |
| TC0100009534.hg.1 | SLC22A15 | solute carrier family 22, member 15 | Multiple_C | 4.86 | 4.55 | 4.42 | 1.36 | 0.1532 | 0.3268 | 6.28 | 6.48 | 7.49 | 0.43 | 0.0664 | 0.2457 |
| TC0100009742.hg.1 | CD160 | CD160 molecule | Multiple_C | 3.95 | 3.41 | 3.51 | 1.36 | 0.109 | 0.2569 | 4.41 | 4.32 | 3.82 | 1.51 | 0.1893 | 0.4419 |
| TC0100010299.hg.1 | NHLH1 | nescient helix-loop-helix 1 | Coding | 5.43 | 4.99 | 4.99 | 1.36 | 0.135 | 0.2991 | 5.12 | 5.22 | 5.31 | 0.88 | 0.4892 | 0.725 |
| TC0100011696.hg.1 | FAM177B | family with sequence similarity 177, member B | Multiple_C | 4.59 | 3.62 | 4.15 | 1.36 | 0.0827 | 0.2095 | 4.12 | 4.27 | 3.89 | 1.17 | 0.154 | 0.3933 |
| TC0100012010.hg.1 | SLC35F3 | solute carrier family 35, member F3 | Multiple_C | 3.94 | 3.36 | 3.5 | 1.36 | 0.3113 | 0.5247 | 3.96 | 3.81 | 3.91 | 1.04 | 0.5359 | 0.7579 |
| TC0100014257.hg.1 | SSBP3 | single stranded DNA binding protein 3 | Multiple_C | 10.47 | 9.67 | 10.03 | 1.36 | 0.2435 | 0.4464 | 11.16 | 11.21 | 10.89 | 1.21 | 0.7961 | 0.909 |
| TC0100014522.hg.1 | GNG12 | guanine nucleotide binding protein (G protein), gamma 12 | Multiple_C | 13.09 | 13.23 | 12.65 | 1.36 | 0.2597 | 0.4661 | 10.87 | 10.69 | 10.2 | 1.59 | 0.1618 | 0.4048 |
| TC0100014857.hg.1 | GBP5 | guanylate binding protein 5 | Multiple_C | 3.94 | 3.63 | 3.5 | 1.36 | 0.1623 | 0.3397 | 3.64 | 3.74 | 3.78 | 0.91 | 0.6105 | 0.8053 |
| TC0100015918.hg.1 | UBE2Q1 | ubiquitin-conjugating enzyme E2Q family member 1 | Multiple_C | 10.46 | 9.67 | 10.02 | 1.36 | 0.3084 | 0.5214 | 10.32 | 9.89 | 10.14 | 1.13 | 0.4017 | 0.6582 |
| TC0200009074.hg.1 | PTPN4 | protein tyrosine phosphatase, non-receptor type 4 (megakaryo | Multiple_C | 10.77 | 10.86 | 10.33 | 1.36 | 0.0918 | 0.2262 | 9.53 | 8.72 | 8.95 | 1.49 | 0.1148 | 0.3344 |
| TC0200011362.hg.1 | RNPEPL1 | arginyl aminopeptidase (aminopeptidase B)-like 1 | Multiple_C | 8.77 | 8.69 | 8.33 | 1.36 | 0.3303 | 0.5442 | 7.96 | 8.20 | 7.76 | 1.15 | 0.2983 | 0.563 |

| | | | | | | | | | | | | | | | |
|---|---|---|---|---|---|---|---|---|---|---|---|---|---|---|---|
| TC0200015073.hg.1 | PDE11A | phosphodiesterase 11A | Multiple_C | 3.38 | 2.94 | 2.94 | 1.36 | 0.1904 | 0.3783 | 4.3 | 4.62 | 3.88 | 1.34 | 0.0188 | 0.1156 |
| TC0200015929.hg.1 | C2orf83 | chromosome 2 open reading frame 83 | Multiple_C | 4.9 | 4.57 | 4.46 | 1.36 | 0.2722 | 0.4808 | 4.01 | 4.16 | 4.1 | 0.94 | 0.631 | 0.8184 |
| TC0200016319.hg.1 | ANKMY1 | ankyrin repeat and MYND domain containing 1 | Multiple_C | 8.42 | 7.72 | 7.98 | 1.36 | 0.39 | 0.6037 | 7.99 | 7.53 | 7.45 | 1.45 | 0.7689 | 0.8964 |
| TC0200016732.hg.1 | MZT2A; MIR4 | mitotic spindle organizing protein 2A; microRNA 4784 | Multiple_C | 12.06 | 11.78 | 11.62 | 1.36 | 0.1073 | 0.2536 | 9.92 | 10.66 | 10.08 | 0.90 | 0.6373 | 0.822 |
| TC0300006489.hg.1 | ARL8B | ADP-ribosylation factor like GTPase 8B | Multiple_C | 10.65 | 10.46 | 10.21 | 1.36 | 0.256 | 0.462 | 8.67 | 8.60 | 9.99 | 0.40 | 8.92E-05 | 0.0033 |
| TC0300007269.hg.1 | TMIE | transmembrane inner ear | Coding | 4.68 | 4.24 | 4.24 | 1.36 | 0.0144 | 0.0552 | 4.28 | 4.56 | 4.23 | 1.04 | 0.7238 | 0.8713 |
| TC0300012397.hg.1 | PLXND1 | plexin D1 | Multiple_C | 4.06 | 3.7 | 3.62 | 1.36 | 0.4868 | 0.6881 | 7 | 8.20 | 8.16 | 0.45 | 0.0267 | 0.1428 |
| TC0300012495.hg.1 | AMOTL2; MIR | angiomotin like 2; microRNA 6827; ribosomal protein L39 pseuc | Multiple_C | 6.43 | 5.91 | 5.99 | 1.36 | 0.073 | 0.1912 | 5.23 | 5.31 | 4.9 | 1.26 | 0.0584 | 0.2278 |
| TC0300013125.hg.1 | PLD1 | phospholipase D1, phosphatidylcholine-specific | Coding | 4.17 | 3.48 | 3.73 | 1.36 | 0.1099 | 0.2583 | 3.48 | 3.54 | 3.43 | 1.04 | 0.9481 | 0.979 |
| TC0400006603.hg.1 | DOK7 | docking protein 7 | Multiple_C | 6.48 | 5.77 | 6.04 | 1.36 | 0.0327 | 0.1053 | 7.24 | 7.43 | 6.98 | 1.20 | 0.2027 | 0.4583 |
| TC0400007371.hg.1 | SHISA3 | shisa family member 3 | Coding | 4.74 | 4.14 | 4.3 | 1.36 | 0.4662 | 0.6708 | 5.57 | 5.69 | 5.69 | 0.92 | 0.9796 | 0.9914 |
| TC0400008641.hg.1 | FAT4 | FAT atypical cadherin 4 | Multiple_C | 5.49 | 4.8 | 5.05 | 1.36 | 0.0476 | 0.1396 | 4.77 | 4.65 | 4.9 | 0.91 | 0.9906 | 0.9962 |
| TC0400012310.hg.1 | MARCHF1 | membrane associated ring finger 1 | Multiple_C | 4.2 | 3.54 | 3.76 | 1.36 | 0.0974 | 0.2361 | 4.76 | 4.75 | 4.83 | 0.95 | 0.4254 | 0.678 |
| TC0400012478.hg.1 | ASB5 | ankyrin repeat and SOCS box containing 5 | Multiple_C | 3.4 | 3.1 | 2.96 | 1.36 | 0.0971 | 0.2357 | 4.65 | 4.43 | 4.17 | 1.39 | 0.0187 | 0.1154 |
| TC0500013221.hg.1 | ZNF474 | zinc finger protein 474 | Multiple_C | 4.05 | 3.4 | 3.61 | 1.36 | 0.3883 | 0.602 | 4.89 | 4.89 | 4.42 | 1.39 | 0.1714 | 0.4179 |
| TC0600007127.hg.1 | ID4 | inhibitor of DNA binding 4, dominant negative helix-loop-helix p | Coding | 5.31 | 4.93 | 4.87 | 1.36 | 0.0416 | 0.1263 | 4.58 | 4.78 | 4.57 | 1.01 | 0.8825 | 0.9496 |
| TC0600007836.hg.1 | KCTD20 | potassium channel tetramerization domain containing 20 | Multiple_C | 8.46 | 7.78 | 8.02 | 1.36 | 0.1879 | 0.3753 | 9.04 | 8.89 | 9.04 | 1.00 | 0.8115 | 0.9166 |
| TC0600009623.hg.1 | ECT2L | epithelial cell transforming 2 like | Multiple_C | 3.12 | 2.85 | 2.68 | 1.36 | 0.1406 | 0.3079 | 3.91 | 3.66 | 3.54 | 1.29 | 0.1105 | 0.3274 |
| TC0600011287.hg.1 | ZNF311 | zinc finger protein 311 | Multiple_C | 4.42 | 4.18 | 3.98 | 1.36 | 0.174 | 0.356 | 4.69 | 4.63 | 4.81 | 0.92 | 0.7158 | 0.8673 |
| TC0600011459.hg.1 | HSPA1L | heat shock 70kDa protein 1-like | Coding | 4.32 | 3.93 | 3.88 | 1.36 | 0.1547 | 0.329 | 4.8 | 4.19 | 4.59 | 1.16 | 0.6001 | 0.7995 |
| TC0600014031.hg.1 | DLL1 | delta-like 1 (Drosophila) | Multiple_C | 4.43 | 4.09 | 3.99 | 1.36 | 0.0353 | 0.1116 | 4.2 | 4.28 | 4.27 | 0.95 | 0.5123 | 0.7418 |
| TC0600014313.hg.1 | KHDC1 | KH homology domain containing 1 | Coding | 3.5 | 3.05 | 3.06 | 1.36 | 0.1371 | 0.3024 | 4.28 | 4.20 | 3.94 | 1.27 | 0.1158 | 0.336 |
| TC0700010170.hg.1 | RNF216 | ring finger protein 216 | Multiple_C | 9.27 | 8.71 | 8.83 | 1.36 | 0.0713 | 0.188 | 9.56 | 9.47 | 10.11 | 0.68 | 0.0208 | 0.1233 |
| TC0700010665.hg.1 | NEUROD6 | neuronal differentiation 6 | Coding | 4.45 | 3.87 | 4.01 | 1.36 | 0.0093 | 0.0392 | 4.37 | 4.44 | 4.24 | 1.09 | 0.6515 | 0.8301 |
| TC0700012276.hg.1 | DOCK4 | dedicator of cytokinesis 4 | Multiple_C | 8.86 | 8.27 | 8.42 | 1.36 | 0.0492 | 0.1429 | 9.44 | 8.97 | 9.24 | 1.15 | 0.8304 | 0.9247 |
| TC0700012870.hg.1 | KEL | Kell blood group, metallo-endopeptidase | Multiple_C | 4.01 | 3.88 | 3.57 | 1.36 | 0.2134 | 0.4085 | 4.2 | 4.35 | 4.11 | 1.06 | 0.1141 | 0.3334 |
| TC0700013526.hg.1 | FAM126A | family with sequence similarity 126, member A | Multiple_C | 4.69 | 4.21 | 4.25 | 1.36 | 0.6501 | 0.8063 | 8.32 | 7.45 | 7.63 | 1.61 | 0.1775 | 0.4261 |
| TC0700013601.hg.1 | RASA4B; RAS/ | RAS p21 protein activator 4B; RAS p21 protein activator 4 | Multiple_C | 5.05 | 4.54 | 4.61 | 1.36 | 0.2114 | 0.4057 | 5.66 | 4.89 | 4.41 | 2.38 | 0.0021 | 0.0273 |
| TC0800006966.hg.1 | NPM2 | nucleophosmin/nucleoplasmin 2 | Multiple_C | 6.4 | 5.6 | 5.96 | 1.36 | 0.1459 | 0.3161 | 6.44 | 6.89 | 6.6 | 0.90 | 0.0992 | 0.3079 |
| TC0900006986.hg.1 | ANKRD18B | ankyrin repeat domain 18B | Multiple_C | 7.02 | 6.9 | 6.58 | 1.36 | 0.1834 | 0.3688 | 6.04 | 6.05 | 5.77 | 1.21 | 0.1151 | 0.3349 |
| TC0900010284.hg.1 | ANKRD20A1; / | ankyrin repeat domain 20 family, member A1; ankyrin repeat d | Multiple_C | 4.13 | 3.81 | 3.69 | 1.36 | 0.2273 | 0.4265 | 7.26 | 6.66 | 6.84 | 1.34 | 0.0145 | 0.0982 |
| TC0900011256.hg.1 | RNF183 | ring finger protein 183 | Multiple_C | 5.41 | 4.84 | 4.97 | 1.36 | 0.2458 | 0.4494 | 6.26 | 6.13 | 5.83 | 1.35 | 0.2263 | 0.4869 |
| TC0900011708.hg.1 | PTGES | prostaglandin E synthase | Multiple_C | 6.19 | 5.59 | 5.75 | 1.36 | 0.4348 | 0.6447 | 5.9 | 5.67 | 5.69 | 1.16 | 0.0512 | 0.2111 |

| | | | | | | | | | | | | | | | |
|---|---|---|---|---|---|---|---|---|---|---|---|---|---|---|---|
| TC0X00010005.hg.1 | CXCR3 | chemokine (C-X-C motif) receptor 3 | Coding | 4.63 | 4 | 4.19 | 1.36 | 0.1256 | 0.2837 | 4.46 | 4.44 | 4.17 | 1.22 | 0.3378 | 0.602 |
| TC1000006652.hg.1 | PFKFB3 | 6-phosphofructo-2-kinase/fructose-2,6-biphosphatase 3 | Multiple_C | 7.46 | 6.34 | 7.02 | 1.36 | 0.102 | 0.2445 | 7.5 | 7.35 | 7.41 | 1.06 | 0.622 | 0.8128 |
| TC1000008310.hg.1 | LDB3 | LIM domain binding 3 | Coding | 3.88 | 3.31 | 3.44 | 1.36 | 0.4109 | 0.6232 | 4.17 | 3.91 | 3.59 | 1.49 | 0.0694 | 0.2513 |
| TC1000008927.hg.1 | VTI1A | vesicle transport through interaction with t-SNAREs 1A | Multiple_C | 7.17 | 6.42 | 6.73 | 1.36 | 0.1349 | 0.299 | 8.1 | 8.16 | 8.56 | 0.73 | 0.0527 | 0.2145 |
| TC1000012575.hg.1 | ATAD1 | ATPase family, AAA domain containing 1 | Multiple_C | 12.39 | 13.33 | 11.95 | 1.36 | 0.177 | 0.3596 | 13.17 | 12.13 | 12.99 | 1.13 | 0.9208 | 0.9673 |
| TC1100007951.hg.1 | CCDC88B | coiled-coil domain containing 88B | Multiple_C | 5.01 | 4.45 | 4.57 | 1.36 | 0.292 | 0.5032 | 5.25 | 5.18 | 5.03 | 1.16 | 0.4638 | 0.7061 |
| TC1100010535.hg.1 | PAMR1 | peptidase domain containing associated with muscle regenerati | Multiple_C | 3.86 | 3.14 | 3.42 | 1.36 | 0.062 | 0.1695 | 3.73 | 3.57 | 3.52 | 1.16 | 0.1895 | 0.442 |
| TC1100011694.hg.1 | LRRC32 | leucine rich repeat containing 32 | Multiple_C | 5.38 | 4.98 | 4.94 | 1.36 | 0.2811 | 0.4911 | 5.09 | 4.94 | 5.09 | 1.00 | 0.9206 | 0.9672 |
| TC1100012976.hg.1 | NUCB2 | nucleobindin 2 | Multiple_C | 12.4 | 13.45 | 11.96 | 1.36 | 0.1488 | 0.3202 | 11.26 | 10.37 | 10.75 | 1.42 | 0.2107 | 0.4681 |
| TC1100013030.hg.1 | FLRT1 | fibronectin leucine rich transmembrane protein 1 | Coding | 4.25 | 3.78 | 3.81 | 1.36 | 0.1018 | 0.2441 | 4.11 | 3.83 | 4.06 | 1.04 | 0.7312 | 0.8754 |
| TC1100013226.hg.1 | ALG9 | ALG9, alpha-1,2-mannosyltransferase | Multiple_C | 8.22 | 7.12 | 7.78 | 1.36 | 0.4707 | 0.6747 | 6.69 | 7.13 | 7.3 | 0.66 | 0.0102 | 0.0789 |
| TC1200007954.hg.1 | USP15; MIR61 | ubiquitin specific peptidase 15; microRNA 6125 | Multiple_C | 11.6 | 11.7 | 11.16 | 1.36 | 0.3805 | 0.5933 | 11.16 | 10.33 | 11 | 1.12 | 0.4993 | 0.7319 |
| TC1300007484.hg.1 | PIBF1 | progesterone immunomodulatory binding factor 1 | Multiple_C | 8.04 | 9.07 | 7.6 | 1.36 | 0.3073 | 0.5205 | 8.91 | 8.47 | 7.15 | 3.39 | 0.0005 | 0.0107 |
| TC1300008511.hg.1 | KATNAL1 | katanin p60 subunit A-like 1 | Multiple_C | 7.98 | 7.68 | 7.54 | 1.36 | 0.0356 | 0.1124 | 7.65 | 7.68 | 7.24 | 1.33 | 0.0781 | 0.2692 |
| TC1400007308.hg.1 | TOMM20L | translocase of outer mitochondrial membrane 20 homolog (yea | Coding | 4.33 | 3.68 | 3.89 | 1.36 | 0.1162 | 0.2687 | 3.96 | 4.69 | 4.14 | 0.88 | 0.3825 | 0.6415 |
| TC1400008341.hg.1 | DYNC1H1 | dynein, cytoplasmic 1, heavy chain 1 | Multiple_C | 12.52 | 12.19 | 12.08 | 1.36 | 0.0818 | 0.2079 | 12.63 | 12.60 | 12.29 | 1.27 | 0.4508 | 0.6963 |
| TC1500007926.hg.1 | RCN2 | reticulocalbin 2, EF-hand calcium binding domain | Multiple_C | 12.87 | 14.35 | 12.43 | 1.36 | 0.2653 | 0.473 | 11.22 | 10.93 | 11.01 | 1.16 | 0.2543 | 0.5184 |
| TC1500009363.hg.1 | COPS2 | COP9 signalosome subunit 2 | Multiple_C | 13.71 | 15.24 | 13.27 | 1.36 | 0.1377 | 0.3032 | 13.36 | 12.66 | 13.17 | 1.14 | 0.0441 | 0.1945 |
| TC1600006965.hg.1 | SHISA9 | shisa family member 9 | Multiple_C | 4.85 | 3.92 | 4.41 | 1.36 | 0.5199 | 0.7134 | 5.16 | 6.48 | 6.66 | 0.35 | 8.99E-06 | 0.0007 |
| TC1600007026.hg.1 | NOMO3; NON | NODAL modulator 3; NODAL modulator 2 | Multiple_C | 13.47 | 13.11 | 13.03 | 1.36 | 0.0956 | 0.2332 | 13.67 | 13.41 | 13.66 | 1.01 | 0.7795 | 0.9011 |
| TC1600009978.hg.1 | ZNF785 | zinc finger protein 785 | Coding | 3.95 | 3.37 | 3.51 | 1.36 | 0.2634 | 0.4706 | 5.59 | 5.89 | 5.73 | 0.91 | 0.7546 | 0.8885 |
| TC1600011561.hg.1 | ZNF23 | zinc finger protein 23 | Multiple_C | 8.27 | 8.14 | 7.83 | 1.36 | 0.5023 | 0.7004 | 8.56 | 8.42 | 7.89 | 1.59 | 0.1604 | 0.4028 |
| TC1700007171.hg.1 | RNF112 | ring finger protein 112 | Multiple_C | 4.82 | 4.38 | 4.38 | 1.36 | 0.4544 | 0.662 | 4.02 | 3.94 | 3.76 | 1.20 | 0.8691 | 0.9436 |
| TC1700007438.hg.1 | EFCAB5 | EF-hand calcium binding domain 5 | Multiple_C | 5.78 | 5.35 | 5.34 | 1.36 | 0.7675 | 0.8803 | 3.58 | 3.86 | 3.7 | 0.92 | 0.3801 | 0.6398 |
| TC1700007725.hg.1 | LASP1 | LIM and SH3 protein 1 | Multiple_C | 12.9 | 12.37 | 12.46 | 1.36 | 0.0428 | 0.1292 | 13.15 | 13.77 | 13.06 | 1.06 | 0.7677 | 0.8958 |
| TC1700009556.hg.1 | SCIMP | SLP adaptor and CSK interacting membrane protein | Multiple_C | 3.66 | 3.29 | 3.22 | 1.36 | 0.2979 | 0.5096 | 4.59 | 4.20 | 4.01 | 1.49 | 0.0206 | 0.1225 |
| TC1700010445.hg.1 | HEATR9 | HEAT repeat containing 9 | Multiple_C | 3.39 | 3.16 | 2.95 | 1.36 | 0.4828 | 0.6844 | 4 | 3.67 | 3.73 | 1.21 | 0.0721 | 0.2573 |
| TC1700011088.hg.1 | COL1A1 | collagen, type I, alpha 1 | Multiple_C | 5.24 | 4.84 | 4.8 | 1.36 | 0.0451 | 0.134 | 5.19 | 5.11 | 5.12 | 1.05 | 0.5885 | 0.7918 |
| TC1700011578.hg.1 | ABCA6 | ATP binding cassette subfamily A member 6 | Multiple_C | 4.48 | 4.17 | 4.04 | 1.36 | 0.2157 | 0.4113 | 4.61 | 4.15 | 4.11 | 1.41 | 0.2605 | 0.5248 |
| TC1700011704.hg.1 | CD300E | CD300e molecule | Coding | 4.64 | 4.1 | 4.2 | 1.36 | 0.24 | 0.442 | 4.13 | 4.07 | 4.27 | 0.91 | 0.5235 | 0.7491 |
| TC1700012109.hg.1 | CD7 | CD7 molecule | Multiple_C | 6.69 | 6.18 | 6.25 | 1.36 | 0.1887 | 0.3764 | 6.26 | 6.21 | 6.29 | 0.98 | 0.298 | 0.5626 |
| TC1700012339.hg.1 | NUP88 | nucleoporin 88kDa | Multiple_C | 4.84 | 4.92 | 4.4 | 1.36 | 0.178 | 0.3609 | 5.27 | 4.76 | 5.11 | 1.12 | 0.4849 | 0.7222 |
| TC1800009284.hg.1 | C18orf32 | chromosome 18 open reading frame 32 | Multiple_C | 10.44 | 12.33 | 10 | 1.36 | 0.1556 | 0.3303 | 7.38 | 8.01 | 7.22 | 1.12 | 0.3099 | 0.5737 |

| | | | | | | | | | | | | | | | |
|---|---|---|---|---|---|---|---|---|---|---|---|---|---|---|---|
| TC1900006859.hg.1 | EVI5L | ecotropic viral integration site 5-like | Multiple_C | 6.73 | 6.29 | 6.29 | 1.36 | 0.5257 | 0.7178 | 5.75 | 5.39 | 4.89 | 1.82 | 0.0518 | 0.2125 |
| TC1900007237.hg.1 | OR10H2 | olfactory receptor, family 10, subfamily H, member 2 | Coding | 4.51 | 3.95 | 4.07 | 1.36 | 0.2511 | 0.4556 | 3.78 | 3.94 | 3.91 | 0.91 | 0.5362 | 0.758 |
| TC1900008366.hg.1 | IGFL1 | IGF like family member 1 | Coding | 5.74 | 5.21 | 5.3 | 1.36 | 0.0814 | 0.2073 | 5.41 | 5.83 | 5.61 | 0.87 | 0.5937 | 0.7948 |
| TC1900010851.hg.1 | ETHE1 | ethylmalonic encephalopathy 1 | Multiple_C | 14.05 | 14.91 | 13.61 | 1.36 | 0.2337 | 0.4345 | 5.75 | 5.76 | 5.85 | 0.93 | 0.8486 | 0.9346 |
| TC1900011834.hg.1 | C19orf25 | chromosome 19 open reading frame 25 | Multiple_C | 9.82 | 9.21 | 9.38 | 1.36 | 0.0924 | 0.2274 | 9.42 | 9.84 | 9.5 | 0.95 | 0.7068 | 0.8619 |
| TC1900012018.hg.1 | ZNF577 | zinc finger protein 577 | Multiple_C | 9.73 | 10.4 | 9.29 | 1.36 | 0.0863 | 0.216 | 6.28 | 6.01 | 6.57 | 0.82 | 0.3103 | 0.5744 |
| TC2000006781.hg.1 | SNRPB2 | small nuclear ribonucleoprotein polypeptide B | Multiple_C | 12.95 | 12.38 | 12.51 | 1.36 | 0.0482 | 0.1409 | 13.62 | 14.44 | 14.02 | 0.76 | 0.116 | 0.3363 |
| TC2000007895.hg.1 | RAB22A | RAB22A, member RAS oncogene family | Multiple_C | 12.68 | 12.27 | 12.24 | 1.36 | 0.0445 | 0.133 | 11.2 | 10.52 | 10.91 | 1.22 | 0.1083 | 0.3237 |
| TC2000007900.hg.1 | VAPB | VAMP (vesicle-associated membrane protein)-associated prote | Multiple_C | 13.99 | 13.85 | 13.55 | 1.36 | 0.0664 | 0.1787 | 15.21 | 14.99 | 14.87 | 1.27 | 0.0474 | 0.202 |
| TC2000008675.hg.1 | CST9L | cystatin 9-like | Coding | 3.35 | 2.95 | 2.91 | 1.36 | 0.0238 | 0.0822 | 3.62 | 3.57 | 3.62 | 1.00 | 0.9051 | 0.9607 |
| TC2000009149.hg.1 | PTPRT | protein tyrosine phosphatase, receptor type, T | Multiple_C | 3.5 | 2.88 | 3.06 | 1.36 | 0.0595 | 0.1646 | 3.28 | 3.45 | 3.21 | 1.05 | 0.9514 | 0.9798 |
| TC2000010015.hg.1 | WFDC6 | WAP four-disulfide core domain 6 | Coding | 5.04 | 4.74 | 4.6 | 1.36 | 0.189 | 0.3767 | 4.6 | 4.23 | 4.8 | 0.87 | 0.8089 | 0.9155 |
| TC2100007376.hg.1 | KRTAP10-9 | keratin associated protein 10-9 | Multiple_C | 4.02 | 3.35 | 3.58 | 1.36 | 0.2991 | 0.5107 | 4.39 | 4.09 | 4.14 | 1.19 | 0.1623 | 0.4056 |
| TC2200008146.hg.1 | RSPH14 | radial spoke head 14 homolog (Chlamydomonas) | Coding | 3.88 | 3.17 | 3.44 | 1.36 | 0.0498 | 0.1442 | 3.74 | 3.90 | 3.5 | 1.18 | 0.1652 | 0.4099 |
| TC2200009230.hg.1 | SLC2A11 | solute carrier family 2 (facilitated glucose transporter), member | Multiple_C | 8.31 | 7.5 | 7.87 | 1.36 | 0.0731 | 0.1914 | 7.63 | 7.85 | 7.49 | 1.10 | 0.3972 | 0.655 |
| TC0100008549.hg.1 | L1TD1 | LINE-1 type transposase domain containing 1 | Multiple_C | 2.97 | 2.55 | 2.54 | 1.35 | 0.4085 | 0.6207 | 4.22 | 4.08 | 3.56 | 1.58 | 0.0403 | 0.184 |
| TC0100009902.hg.1 | PRPF3 | pre-mRNA processing factor 3 | Multiple_C | 11.28 | 10.39 | 10.85 | 1.35 | 0.182 | 0.3667 | 11.47 | 11.49 | 11.8 | 0.80 | 0.141 | 0.3734 |
| TC0100012604.hg.1 | C1orf174 | chromosome 1 open reading frame 174 | Multiple_C | 9.48 | 9.02 | 9.05 | 1.35 | 0.0836 | 0.2111 | 7.81 | 7.92 | 8.44 | 0.65 | 0.0215 | 0.1257 |
| TC0100013275.hg.1 | ZNF436 | zinc finger protein 436 | Coding | 7.26 | 6.88 | 6.83 | 1.35 | 0.2599 | 0.4663 | 4.92 | 5.43 | 5.93 | 0.50 | 0.0025 | 0.0314 |
| TC0100013632.hg.1 | FNDC5 | fibronectin type III domain containing 5 | Multiple_C | 6.07 | 5.51 | 5.64 | 1.35 | 0.3227 | 0.5364 | 5.65 | 5.83 | 5.82 | 0.89 | 0.8949 | 0.9555 |
| TC0100014250.hg.1 | TMEM59 | transmembrane protein 59 | Multiple_C | 14.53 | 15.51 | 14.1 | 1.35 | 0.046 | 0.1359 | 10.49 | 9.72 | 9.76 | 1.66 | 0.004 | 0.0431 |
| TC0100018182.hg.1 | SZRD1 | SUZ RNA binding domain containing 1 | Multiple_C | 10.41 | 9.2 | 9.98 | 1.35 | 0.2878 | 0.4986 | 9.37 | 9.92 | 10.52 | 0.45 | 0.0015 | 0.0225 |
| TC0100018267.hg.1 | KCNC4 | potassium channel, voltage gated Shaw related subfamily C, me | Multiple_C | 5.39 | 4.99 | 4.96 | 1.35 | 0.2396 | 0.4416 | 4.43 | 4.65 | 4.48 | 0.97 | 0.997 | 0.9987 |
| TC0200007061.hg.1 | TRIM54 | tripartite motif containing 54 | Multiple_C | 4.96 | 4.91 | 4.53 | 1.35 | 0.2163 | 0.4121 | 4.54 | 4.53 | 4.34 | 1.15 | 0.8364 | 0.9279 |
| TC0200007908.hg.1 | ANTXR1 | anthrax toxin receptor 1 | Multiple_C | 2.97 | 2.55 | 2.54 | 1.35 | 0.4257 | 0.6364 | 12.47 | 10.81 | 10.27 | 4.59 | 3.14E-08 | 1.16E-05 |
| TC0200013878.hg.1 | BUB1 | BUB1 mitotic checkpoint serine/threonine kinase | Multiple_C | 11.07 | 10.81 | 10.64 | 1.35 | 0.2526 | 0.4574 | 10.82 | 11.13 | 11.66 | 0.56 | 0.002 | 0.0265 |
| TC0300007474.hg.1 | DNAH1 | dynein, axonemal, heavy chain 1 | Multiple_C | 5.97 | 5.53 | 5.54 | 1.35 | 0.06 | 0.1656 | 6.98 | 7.03 | 6.89 | 1.06 | 0.3717 | 0.6332 |
| TC0300011236.hg.1 | LRTM1 | leucine-rich repeats and transmembrane domains 1 | Coding | 3.53 | 3.02 | 3.1 | 1.35 | 0.271 | 0.4795 | 3.4 | 3.24 | 3.3 | 1.07 | 0.9308 | 0.9708 |
| TC0300012266.hg.1 | SLC41A3 | solute carrier family 41, member 3 | Multiple_C | 9.33 | 8.61 | 8.9 | 1.35 | 0.2855 | 0.4959 | 8.7 | 8.87 | 8.75 | 0.97 | 0.9093 | 0.9626 |
| TC0300013882.hg.1 | CHST2 | carbohydrate (N-acetylglucosamine-6-O) sulfotransferase 2 | Coding | 7.83 | 7.18 | 7.4 | 1.35 | 0.1329 | 0.296 | 8.27 | 8.49 | 8.47 | 0.87 | 0.3466 | 0.6103 |
| TC0300013988.hg.1 | PCBP4 | poly(rC) binding protein 4 | Multiple_C | 7.79 | 7.18 | 7.36 | 1.35 | 0.2085 | 0.4021 | 10.34 | 10.79 | 10.02 | 1.25 | 0.1112 | 0.3287 |
| TC0300014078.hg.1 | CCDC39 | coiled-coil domain containing 39 | Coding | 3.43 | 3.15 | 3 | 1.35 | 0.1124 | 0.2625 | 3.79 | 3.97 | 3.62 | 1.13 | 0.3644 | 0.627 |
| TC0400007306.hg.1 | N4BP2 | NEDD4 binding protein 2 | Multiple_C | 6.59 | 7.28 | 6.16 | 1.35 | 0.1155 | 0.2677 | 7.88 | 8.24 | 8.08 | 0.87 | 0.5195 | 0.7469 |

| | | | | | | | | | | | | | | | |
|---|---|---|---|---|---|---|---|---|---|---|---|---|---|---|---|
| TC0400007455.hg.1 | SLC10A4 | solute carrier family 10, member 4 | Coding | 3.16 | 2.66 | 2.73 | 1.35 | 0.5687 | 0.7491 | 3.21 | 3.31 | 3.16 | 1.04 | 0.8065 | 0.9146 |
| TC0400010601.hg.1 | COX7B2 | cytochrome c oxidase subunit VIIb2 | Coding | 3.67 | 3.41 | 3.24 | 1.35 | 0.0859 | 0.2154 | 3.02 | 2.83 | 3.11 | 0.94 | 0.5372 | 0.7587 |
| TC0400013001.hg.1 | LRP2BP | LRP2 binding protein | Coding | 3.77 | 3.48 | 3.34 | 1.35 | 0.1719 | 0.3531 | 4.08 | 4.20 | 3.89 | 1.14 | 0.0681 | 0.2486 |
| TC0500009673.hg.1 | ZNF454 | zinc finger protein 454 | Multiple_C | 3.74 | 3.38 | 3.31 | 1.35 | 0.0528 | 0.1504 | 3.65 | 3.57 | 3.76 | 0.93 | 0.7739 | 0.8984 |
| TC0600007595.hg.1 | LTA | lymphotoxin alpha | Multiple_C | 3.91 | 3.44 | 3.48 | 1.35 | 0.1062 | 0.2518 | 3.77 | 3.54 | 3.73 | 1.03 | 0.9564 | 0.9819 |
| TC0600011118.hg.1 | SLC17A2 | solute carrier family 17, member 2 | Multiple_C | 3.23 | 2.59 | 2.8 | 1.35 | 0.2752 | 0.4842 | 3.26 | 3.12 | 3.2 | 1.04 | 0.2906 | 0.5555 |
| TC0600012316.hg.1 | TMEM30A | transmembrane protein 30A | Multiple_C | 12.28 | 12.43 | 11.85 | 1.35 | 0.1013 | 0.2432 | 11.64 | 11.74 | 11.81 | 0.89 | 0.5155 | 0.7446 |
| TC0600012664.hg.1 | MCHR2 | melanin-concentrating hormone receptor 2 | Coding | 3.51 | 3.18 | 3.08 | 1.35 | 0.088 | 0.2193 | 4.05 | 3.85 | 3.94 | 1.08 | 0.3441 | 0.6077 |
| TC0700006484.hg.1 | GPR146 | G protein-coupled receptor 146 | Multiple_C | 3.98 | 3.38 | 3.55 | 1.35 | 0.1843 | 0.37 | 5.12 | 5.06 | 4.73 | 1.31 | 0.0561 | 0.2226 |
| TC0700007364.hg.1 | SPDYE1 | speedy/RINGO cell cycle regulator family member E1 | Coding | 4.46 | 3.9 | 4.03 | 1.35 | 0.3665 | 0.5796 | 5.29 | 5.48 | 5.57 | 0.82 | 0.1401 | 0.3718 |
| TC0700009478.hg.1 | C7orf34 | chromosome 7 open reading frame 34 | Coding | 4.88 | 4.19 | 4.45 | 1.35 | 0.1946 | 0.3844 | 5.09 | 5.11 | 4.81 | 1.21 | 0.0146 | 0.0988 |
| TC0700013013.hg.1 | ZNF467 | zinc finger protein 467 | Coding | 3.71 | 3.52 | 3.28 | 1.35 | 0.2987 | 0.5103 | 4.02 | 3.87 | 3.84 | 1.13 | 0.6445 | 0.8256 |
| TC0700013529.hg.1 | HOXA3 | homeobox A3 | Multiple_C | 8.7 | 7.83 | 8.27 | 1.35 | 0.146 | 0.3161 | 8.42 | 8.12 | 7.95 | 1.39 | 0.0269 | 0.1434 |
| TC0800009418.hg.1 | ANGPT2 | angiopoietin 2 | Multiple_C | 4.71 | 4.22 | 4.28 | 1.35 | 0.5443 | 0.7306 | 5.02 | 6.07 | 5.24 | 0.86 | 0.7276 | 0.8736 |
| TC0800009589.hg.1 | AF131215.3; X | Memczak2013 ALT_ACCEPTOR, ALT_DONOR, coding, INTERNAL | Multiple_C | 3.75 | 3.52 | 3.32 | 1.35 | 0.222 | 0.4196 | 3.92 | 3.86 | 3.28 | 1.56 | 0.0074 | 0.0632 |
| TC0800009971.hg.1 | SCARA5 | scavenger receptor class A, member 5 | Coding | 4.58 | 3.97 | 4.15 | 1.35 | 0.1039 | 0.2479 | 4.09 | 4.11 | 4.03 | 1.04 | 0.5871 | 0.7909 |
| TC0800010749.hg.1 | NCOA2 | nuclear receptor coactivator 2 | Multiple_C | 9.52 | 9.13 | 9.09 | 1.35 | 0.0867 | 0.2169 | 8.11 | 9.06 | 8.77 | 0.63 | 0.0002 | 0.0062 |
| TC0800012130.hg.1 | MAFA | v-maf avian musculoaponeurotic fibrosarcoma oncogene homo | Multiple_C | 6.01 | 5.5 | 5.58 | 1.35 | 0.2427 | 0.4452 | 4.79 | 4.95 | 5.29 | 0.71 | 0.4754 | 0.7146 |
| TC0800012301.hg.1 | FAM110B | family with sequence similarity 110, member B | Multiple_C | 4.24 | 3.81 | 3.81 | 1.35 | 0.383 | 0.5962 | 4.28 | 4.21 | 4.33 | 0.97 | 0.4301 | 0.6819 |
| TC0900007733.hg.1 | IDNK | idnK, gluconokinase homolog (E. coli) | Multiple_C | 5.71 | 6.4 | 5.28 | 1.35 | 0.0432 | 0.1301 | 4.69 | 5.70 | 4.99 | 0.81 | 0.565 | 0.7747 |
| TC0900009434.hg.1 | SPATA6L | spermatogenesis associated 6-like | Multiple_C | 9.84 | 9.38 | 9.41 | 1.35 | 0.1364 | 0.3011 | 9.72 | 9.55 | 9.2 | 1.43 | 0.027 | 0.1437 |
| TC0900009654.hg.1 | RPS6 | ribosomal protein S6 | Multiple_C | 17.03 | 17.21 | 16.6 | 1.35 | 0.1024 | 0.2451 | 15.67 | 15.44 | 14.98 | 1.61 | 0.0302 | 0.1545 |
| TC0900010959.hg.1 | HEMGN | hemogen | Multiple_C | 3.12 | 2.62 | 2.69 | 1.35 | 0.2731 | 0.4819 | 3.63 | 3.87 | 4.18 | 0.68 | 0.9253 | 0.9693 |
| TC0900012048.hg.1 | FAM166A | family with sequence similarity 166, member A | Multiple_C | 3.85 | 3.37 | 3.42 | 1.35 | 0.0943 | 0.2309 | 3.43 | 3.80 | 3.87 | 0.74 | 0.0668 | 0.2465 |
| TC0900012154.hg.1 | TMEFF1 | transmembrane protein with EGF-like and two follistatin-like do | Multiple_C | 5.9 | 5.61 | 5.47 | 1.35 | 0.028 | 0.0932 | 9.32 | 7.79 | 7.39 | 3.81 | 2.96E-07 | 6.34E-05 |
| TC0X00006585.hg.1 | SHROOM2 | shroom family member 2 | Multiple_C | 4.62 | 4.09 | 4.19 | 1.35 | 0.0926 | 0.2277 | 3.9 | 3.98 | 3.95 | 0.97 | 0.9795 | 0.9914 |
| TC0X00007990.hg.1 | RAB40AL | RAB40A, member RAS oncogene family-like | Coding | 3.44 | 3.13 | 3.01 | 1.35 | 0.0513 | 0.1472 | 3.48 | 3.87 | 3.84 | 0.78 | 0.05 | 0.2085 |
| TC0X00008881.hg.1 | SPRY3 | sprouty RTK signaling antagonist 3 | Coding | 4.09 | 3.49 | 3.66 | 1.35 | 0.1755 | 0.3579 | 4.14 | 3.93 | 3.29 | 1.80 | 0.0044 | 0.0456 |
| TC0X00010818.hg.1 | IGSF1 | immunoglobulin superfamily, member 1 | Multiple_C | 3.68 | 3.39 | 3.25 | 1.35 | 0.0496 | 0.1438 | 3.34 | 3.56 | 3.43 | 0.94 | 0.536 | 0.7579 |
| TC0X00011121.hg.1 | GABRE; MIR2: | gamma-aminobutyric acid (GABA) A receptor, epsilon; microRN | Multiple_C | 4.49 | 3.92 | 4.06 | 1.35 | 0.2861 | 0.4965 | 4.87 | 6.25 | 7.89 | 0.12 | 9.58E-08 | 2.57E-05 |
| TC0Y00006684.hg.1 | CDY2A | chromodomain protein, Y-linked, 2A | Coding | 3.69 | 3.61 | 3.26 | 1.35 | 0.1459 | 0.316 | 3.56 | 3.70 | 3.41 | 1.11 | 0.6038 | 0.8019 |
| TC0Y00006855.hg.1 | SPRY3 | Homo sapiens sprouty RTK signaling antagonist 3 (SPRY3), trans | Coding | 4.09 | 3.49 | 3.66 | 1.35 | 0.1755 | 0.3579 | 4.14 | 3.93 | 3.29 | 1.80 | 0.0044 | 0.0456 |
| TC1000007910.hg.1 | COL13A1 | collagen, type XIII, alpha 1 | Multiple_C | 3.31 | 2.71 | 2.88 | 1.35 | 0.1167 | 0.2693 | 3.78 | 3.85 | 3.51 | 1.21 | 0.137 | 0.3678 |

| | | | | | | | | | | | | | | | |
|---|---|---|---|---|---|---|---|---|---|---|---|---|---|---|---|
| TC1000008670.hg.1 | TLX1 | T-cell leukemia homeobox 1 | Multiple_C | 4.97 | 4.65 | 4.54 | 1.35 | 0.1876 | 0.3749 | 5.9 | 5.40 | 5.34 | 1.47 | 0.207 | 0.4637 |
| TC1000008968.hg.1 | ADRB1 | adrenoceptor beta 1 | Multiple_C | 6.35 | 5.9 | 5.92 | 1.35 | 0.172 | 0.3533 | 6.25 | 6.23 | 5.74 | 1.42 | 0.0237 | 0.1331 |
| TC1000010976.hg.1 | SPOCK2 | sparc/osteonectin, cwcv and kazal-like domains proteoglycan (t | Multiple_C | 6.22 | 5.78 | 5.79 | 1.35 | 0.0383 | 0.1187 | 5.71 | 5.28 | 5.32 | 1.31 | 0.0109 | 0.0821 |
| TC1000012496.hg.1 | AS3MT | arsenite methyltransferase | Multiple_C | 4.59 | 4.91 | 4.16 | 1.35 | 0.0888 | 0.2209 | 6.45 | 6.74 | 6.42 | 1.02 | 0.8687 | 0.9434 |
| TC1100008669.hg.1 | PRSS23 | protease, serine, 23 | Multiple_C | 5.93 | 5.44 | 5.5 | 1.35 | 0.8163 | 0.9074 | 4.96 | 5.61 | 5.84 | 0.54 | 0.0195 | 0.1182 |
| TC1100008927.hg.1 | DYNC2H1 | dynein, cytoplasmic 2, heavy chain 1 | Multiple_C | 4.04 | 3.66 | 3.61 | 1.35 | 0.4338 | 0.6438 | 5.4 | 4.70 | 4.4 | 2.00 | 7.97E-06 | 0.0006 |
| TC1100009301.hg.1 | TBCEL | tubulin folding cofactor E-like | Multiple_C | 8.33 | 8.14 | 7.9 | 1.35 | 0.2517 | 0.4563 | 9.8 | 9.62 | 9.61 | 1.14 | 0.7622 | 0.8925 |
| TC1100012133.hg.1 | MMP3 | matrix metallopeptidase 3 | Multiple_C | 5.27 | 4.81 | 4.84 | 1.35 | 0.1913 | 0.3797 | 4.43 | 4.26 | 4.36 | 1.05 | 0.9993 | 0.9997 |
| TC1100013063.hg.1 | KRTAP5-7 | keratin associated protein 5-7 | Coding | 4.87 | 4.18 | 4.44 | 1.35 | 0.2395 | 0.4416 | 5.52 | 5.29 | 5.31 | 1.16 | 0.571 | 0.779 |
| TC1100013100.hg.1 | ATP5L | ATP synthase, H+ transporting, mitochondrial Fo complex subur | Multiple_C | 15.92 | 16.65 | 15.49 | 1.35 | 0.013 | 0.0513 | 11.8 | 11.88 | 11.9 | 0.93 | 0.5886 | 0.7918 |
| TC1200006610.hg.1 | PLEKHG6 | pleckstrin homology domain containing, family G (with RhoGef ( | Multiple_C | 6.85 | 6.23 | 6.42 | 1.35 | 0.508 | 0.7045 | 4.16 | 4.00 | 3.62 | 1.45 | 0.0752 | 0.2635 |
| TC1200008107.hg.1 | RAP1B | RAP1B, member of RAS oncogene family | Multiple_C | 13.9 | 13.99 | 13.47 | 1.35 | 0.0897 | 0.2224 | 12.05 | 12.07 | 12.35 | 0.81 | 0.3552 | 0.6185 |
| TC1200008295.hg.1 | OTOGL | otogelin-like | Multiple_C | 3.52 | 3.2 | 3.09 | 1.35 | 0.0775 | 0.1998 | 3.96 | 4.04 | 4.07 | 0.93 | 0.3939 | 0.6521 |
| TC1200008855.hg.1 | CCDC63 | coiled-coil domain containing 63 | Multiple_C | 4.28 | 3.85 | 3.85 | 1.35 | 0.5444 | 0.7308 | 4.32 | 4.23 | 4.11 | 1.16 | 0.0265 | 0.1423 |
| TC1200011845.hg.1 | SELPLG | selectin P ligand | Coding | 5.38 | 4.84 | 4.95 | 1.35 | 0.03 | 0.0984 | 4.67 | 4.36 | 4.24 | 1.35 | 0.0639 | 0.2406 |
| TC1300006619.hg.1 | ATP12A | ATPase, H+/K+ transporting, nongastric, alpha polypeptide | Multiple_C | 3.47 | 2.9 | 3.04 | 1.35 | 0.1868 | 0.3738 | 3.8 | 3.91 | 3.77 | 1.02 | 0.8529 | 0.9365 |
| TC1300008179.hg.1 | UPF3A | UPF3 regulator of nonsense transcripts homolog A (yeast) | Multiple_C | 10.23 | 10.47 | 9.8 | 1.35 | 0.0796 | 0.2042 | 12.03 | 12.57 | 12.07 | 0.97 | 0.5967 | 0.7968 |
| TC1300008983.hg.1 | INTS6 | integrator complex subunit 6 | Multiple_C | 9.84 | 9.02 | 9.41 | 1.35 | 0.1541 | 0.3279 | 10.34 | 9.84 | 10.02 | 1.25 | 0.0418 | 0.1882 |
| TC1400006823.hg.1 | SCFD1 | sec1 family domain containing 1 | Multiple_C | 11.13 | 11.47 | 10.7 | 1.35 | 0.1379 | 0.3035 | 9.81 | 8.73 | 9.11 | 1.62 | 0.0267 | 0.1428 |
| TC1400007725.hg.1 | ESRRB | estrogen-related receptor beta | Multiple_C | 5.68 | 5.06 | 5.25 | 1.35 | 0.1521 | 0.3255 | 5.66 | 5.68 | 5.56 | 1.07 | 0.7358 | 0.8781 |
| TC1400008704.hg.1 | CEBPE | CCAAT/enhancer binding protein (C/EBP), epsilon | Coding | 3.32 | 2.98 | 2.89 | 1.35 | 0.7413 | 0.8663 | 3.31 | 3.39 | 3.44 | 0.91 | 0.6234 | 0.8139 |
| TC1400009490.hg.1 | PIGH | phosphatidylinositol glycan anchor biosynthesis class H | Multiple_C | 7.89 | 8.07 | 7.46 | 1.35 | 0.4531 | 0.6606 | 5.44 | 5.33 | 5.52 | 0.95 | 0.347 | 0.6105 |
| TC1500006726.hg.1 | ULK4P1; ULK4 | ULK4 pseudogene 1; ULK4 pseudogene 2 | Multiple_C | 7.35 | 6.96 | 6.92 | 1.35 | 0.2011 | 0.393 | 7.29 | 7.22 | 7.32 | 0.98 | 0.7252 | 0.8722 |
| TC1500007640.hg.1 | IQCH | IQ motif containing H | Multiple_C | 6.74 | 7.17 | 6.31 | 1.35 | 0.6791 | 0.8257 | 8.4 | 9.61 | 9.78 | 0.38 | 0.0897 | 0.2896 |
| TC1500007652.hg.1 | SKOR1 | SKI family transcriptional corepressor 1 | Multiple_C | 6.16 | 5.63 | 5.73 | 1.35 | 0.1416 | 0.3092 | 5.52 | 5.77 | 6 | 0.72 | 0.4622 | 0.7049 |
| TC1500009904.hg.1 | TLE3 | transducin-like enhancer of split 3 | Multiple_C | 9.69 | 8.69 | 9.26 | 1.35 | 0.4551 | 0.6624 | 12.16 | 11.71 | 11.61 | 1.46 | 0.0461 | 0.1987 |
| TC1500010041.hg.1 | EDC3 | enhancer of mRNA decapping 3 | Multiple_C | 10.8 | 10.18 | 10.37 | 1.35 | 0.1144 | 0.2657 | 10.58 | 10.30 | 10.79 | 0.86 | 0.2816 | 0.5474 |
| TC1500010501.hg.1 | RGMA | repulsive guidance molecule family member a | Multiple_C | 4.9 | 4.31 | 4.47 | 1.35 | 0.3473 | 0.5604 | 9.33 | 9.34 | 8.51 | 1.77 | 0.0206 | 0.1226 |
| TC1500010747.hg.1 | RNF111 | ring finger protein 111 | Multiple_C | 9.73 | 9.71 | 9.3 | 1.35 | 0.1123 | 0.2623 | 8.43 | 8.47 | 8.67 | 0.85 | 0.2116 | 0.4694 |
| TC1600007955.hg.1 | MT3 | metallothionein 3 | Multiple_C | 6.12 | 5.34 | 5.69 | 1.35 | 0.2265 | 0.4255 | 5.78 | 5.36 | 5.29 | 1.40 | 0.0453 | 0.1967 |
| TC1600008008.hg.1 | DRC7 | dynein regulatory complex subunit 7 | Multiple_C | 4.75 | 4.51 | 4.32 | 1.35 | 0.1462 | 0.3165 | 4.08 | 4.47 | 4.03 | 1.04 | 0.77 | 0.8968 |
| TC1600008779.hg.1 | JPH3 | junctophilin 3 | Multiple_C | 3.85 | 3.14 | 3.42 | 1.35 | 0.8461 | 0.9244 | 4.88 | 4.64 | 4.12 | 1.69 | 0.0008 | 0.0138 |
| TC1600010216.hg.1 | ABCC11 | ATP binding cassette subfamily C member 11 | Multiple_C | 4.58 | 4.15 | 4.15 | 1.35 | 0.0168 | 0.0624 | 4.1 | 4.37 | 4.46 | 0.78 | 0.1345 | 0.3643 |

| | | | | | | | | | | | | | | | |
|---|---|---|---|---|---|---|---|---|---|---|---|---|---|---|---|
| TC1600011494.hg.1 | ARL6IP1 | ADP-ribosylation factor like GTPase 6 interacting protein 1 | Multiple_C | 14.86 | 15.12 | 14.43 | 1.35 | 0.0593 | 0.1641 | 12.48 | 13.36 | 13.2 | 0.61 | 0.0019 | 0.0258 |
| TC1700006855.hg.1 | CFAP52 | cilia and flagella associated protein 52 | Multiple_C | 3.85 | 3.52 | 3.42 | 1.35 | 0.274 | 0.4827 | 4.83 | 4.51 | 4.45 | 1.30 | 0.4019 | 0.6582 |
| TC1700007919.hg.1 | AOC3 | amine oxidase, copper containing 3 | Multiple_C | 5.64 | 5.06 | 5.21 | 1.35 | 0.4506 | 0.6583 | 5.25 | 4.86 | 4.96 | 1.22 | 0.3712 | 0.633 |
| TC1700007971.hg.1 | FAM215A | family with sequence similarity 215, member A (non-protein co | Multiple_C | 4.51 | 4.08 | 4.08 | 1.35 | 0.219 | 0.4159 | 4.18 | 4.50 | 4.48 | 0.81 | 0.0211 | 0.1241 |
| TC1700009681.hg.1 | CTC1 | CTS telomere maintenance complex component 1 | Multiple_C | 7.86 | 6.68 | 7.43 | 1.35 | 0.2141 | 0.4093 | 8.52 | 8.87 | 8.62 | 0.93 | 0.7646 | 0.8937 |
| TC1800007155.hg.1 | PIK3C3 | phosphatidylinositol 3-kinase, catalytic subunit type 3 | Multiple_C | 8.25 | 9.49 | 7.82 | 1.35 | 0.2717 | 0.4802 | 8.1 | 8.37 | 7.06 | 2.06 | 0.1578 | 0.3992 |
| TC1900006882.hg.1 | CERS4 | ceramide synthase 4 | Multiple_C | 5.8 | 5.22 | 5.37 | 1.35 | 0.1772 | 0.3599 | 5.97 | 5.85 | 5.82 | 1.11 | 0.8278 | 0.924 |
| TC1900007442.hg.1 | CILP2 | cartilage intermediate layer protein 2 | Multiple_C | 5.95 | 5.36 | 5.52 | 1.35 | 0.4854 | 0.6868 | 5.5 | 5.47 | 5.28 | 1.16 | 0.728 | 0.8738 |
| TC1900008536.hg.1 | CCDC155 | coiled-coil domain containing 155 | Multiple_C | 4.17 | 3.79 | 3.74 | 1.35 | 0.5914 | 0.7662 | 3.75 | 4.04 | 3.76 | 0.99 | 0.6499 | 0.8291 |
| TC1900009555.hg.1 | ACTL9 | actin-like 9 | Coding | 3.76 | 3.16 | 3.33 | 1.35 | 0.0797 | 0.2044 | 3.6 | 3.81 | 3.86 | 0.84 | 0.1363 | 0.3669 |
| TC1900011801.hg.1 | LILRB4 | leukocyte immunoglobulin-like receptor, subfamily B (with TM a | Multiple_C | 3.95 | 3.64 | 3.52 | 1.35 | 0.0905 | 0.2239 | 4.12 | 4.20 | 4.31 | 0.88 | 0.3323 | 0.5965 |
| TC1900011963.hg.1 | PSG6 | pregnancy specific beta-1-glycoprotein 6 | Multiple_C | 5.46 | 4.98 | 5.03 | 1.35 | 0.2266 | 0.4255 | 4.31 | 4.18 | 4.01 | 1.23 | 0.0857 | 0.2821 |
| TC2000008264.hg.1 | PRNT | prion protein (testis specific) | Multiple_C | 4.49 | 3.89 | 4.06 | 1.35 | 0.0436 | 0.131 | 4.12 | 4.00 | 4.1 | 1.01 | 0.5117 | 0.7415 |
| TC2000008473.hg.1 | KIF16B | kinesin family member 16B | Multiple_C | 8.57 | 8.74 | 8.14 | 1.35 | 0.0598 | 0.1654 | 9.27 | 9.20 | 9.31 | 0.97 | 0.468 | 0.7092 |
| TC2000008955.hg.1 | TRPC4AP | transient receptor potential cation channel, subfamily C, memb | Multiple_C | 12.79 | 12.93 | 12.36 | 1.35 | 0.0664 | 0.1787 | 11.55 | 11.75 | 11.62 | 0.95 | 0.9269 | 0.9697 |
| TC2000009858.hg.1 | NPBWR2 | neuropeptides B/W receptor 2 | Coding | 3.18 | 2.35 | 2.75 | 1.35 | 0.2119 | 0.4064 | 3.11 | 3.34 | 3.15 | 0.97 | 0.5883 | 0.7917 |
| TC2100007908.hg.1 | KRTAP21-3 | keratin associated protein 21-3 | Coding | 4.06 | 3.87 | 3.63 | 1.35 | 0.5456 | 0.7313 | 3.96 | 3.74 | 3.56 | 1.32 | 0.1585 | 0.4001 |
| TC2200007876.hg.1 | XKR3 | X-linked Kx blood group related 3 | Multiple_C | 3.65 | 3.24 | 3.22 | 1.35 | 0.1747 | 0.3566 | 3.6 | 3.55 | 3.37 | 1.17 | 0.3737 | 0.6343 |
| TSUnmapped00000097.h | CYP2D6 | cytochrome P450, family 2, subfamily D, polypeptide 6 | Coding | 4.2 | 3.57 | 3.77 | 1.35 | 0.0358 | 0.1129 | 4.16 | 4.35 | 4.28 | 0.92 | 0.5644 | 0.7745 |
| TSUnmapped00000219.h | MLXIP | MLX interacting protein | Coding | 3.84 | 3.63 | 3.41 | 1.35 | 0.1889 | 0.3766 | 3.35 | 3.01 | 3.21 | 1.10 | 0.0743 | 0.2616 |
| TSUnmapped00000339.h | SURF1 | surfeit 1 | Coding | 7.3 | 7.35 | 6.87 | 1.35 | 0.4815 | 0.6832 | 6.76 | 7.09 | 6.14 | 1.54 | 0.0322 | 0.1608 |
| TC0100007283.hg.1 | ZBTB40 | zinc finger and BTB domain containing 40 | Multiple_C | 8.33 | 7.66 | 7.91 | 1.34 | 0.1983 | 0.3891 | 8.65 | 7.59 | 8.1 | 1.46 | 0.0116 | 0.0857 |
| TC0100008078.hg.1 | ARTN | artemin | Multiple_C | 5.8 | 5.11 | 5.38 | 1.34 | 0.5217 | 0.715 | 5.52 | 5.42 | 5.17 | 1.27 | 0.5629 | 0.7735 |
| TC0100008139.hg.1 | MAST2 | microtubule associated serine/threonine kinase 2 | Multiple_C | 9.14 | 8.26 | 8.72 | 1.34 | 0.3192 | 0.5329 | 9.19 | 8.92 | 9.35 | 0.90 | 0.1336 | 0.3629 |
| TC0100008332.hg.1 | ZYG11B | zyg-11 family member B, cell cycle regulator | Multiple_C | 9.38 | 9.22 | 8.96 | 1.34 | 0.1042 | 0.2484 | 8.76 | 8.96 | 9.3 | 0.69 | 0.0883 | 0.2871 |
| TC0100009352.hg.1 | SYPL2 | synaptophysin-like 2 | Multiple_C | 5.05 | 4.55 | 4.63 | 1.34 | 0.2413 | 0.4436 | 4.92 | 4.60 | 4.83 | 1.06 | 0.6776 | 0.8448 |
| TC0100009998.hg.1 | LCE2D | late cornified envelope 2D | Coding | 3.97 | 3.46 | 3.55 | 1.34 | 0.1825 | 0.3675 | 4.44 | 4.23 | 3.96 | 1.39 | 0.0286 | 0.1491 |
| TC0100010009.hg.1 | LCE1D | late cornified envelope 1D | Coding | 3.85 | 3.35 | 3.43 | 1.34 | 0.3467 | 0.5599 | 3.92 | 3.87 | 4.1 | 0.88 | 0.4367 | 0.6873 |
| TC0100011316.hg.1 | NFASC | neurofascin | Multiple_C | 3.42 | 2.96 | 3 | 1.34 | 0.4387 | 0.648 | 3.33 | 3.55 | 3.65 | 0.80 | 0.3213 | 0.5853 |
| TC0100011335.hg.1 | MFSD4 | major facilitator superfamily domain containing 4 | Multiple_C | 4.28 | 3.69 | 3.86 | 1.34 | 0.0639 | 0.1734 | 3.89 | 3.58 | 3.62 | 1.21 | 0.1226 | 0.3456 |
| TC0100012335.hg.1 | OR2AK2 | olfactory receptor, family 2, subfamily AK, member 2 | Coding | 3.69 | 3.32 | 3.27 | 1.34 | 0.311 | 0.5245 | 3.96 | 3.84 | 4.04 | 0.95 | 0.8801 | 0.9485 |
| TC0100013854.hg.1 | SCMH1 | sex comb on midleg homolog 1 (Drosophila) | Multiple_C | 7.74 | 7.21 | 7.32 | 1.34 | 0.431 | 0.6412 | 8.74 | 8.98 | 9.74 | 0.50 | 0.001 | 0.0166 |
| TC0100018312.hg.1 | C1orf226 | chromosome 1 open reading frame 226 | Coding | 6.12 | 5.44 | 5.7 | 1.34 | 0.1047 | 0.2493 | 5.63 | 5.62 | 5.34 | 1.22 | 0.4459 | 0.6935 |

| | | | | | | | | | | | | | | | |
|---|---|---|---|---|---|---|---|---|---|---|---|---|---|---|---|
| TC0100018486.hg.1 | CDC42SE1 | CDC42 small effector 1 | Multiple_C | 10.57 | 9.62 | 10.15 | 1.34 | 0.3225 | 0.5363 | 10.59 | 10.43 | 11.03 | 0.74 | 0.1397 | 0.3712 |
| TC0200009986.hg.1 | ZAK; pk | sterile alpha motif and leucine zipper containing kinase AZK; Mi | Multiple_C | 10.7 | 10.28 | 10.28 | 1.34 | 0.1578 | 0.3337 | 10.34 | 10.11 | 10.8 | 0.73 | 0.019 | 0.1166 |
| TC0200010803.hg.1 | PLCD4 | phospholipase C, delta 4 | Multiple_C | 4.58 | 4.33 | 4.16 | 1.34 | 0.1865 | 0.3733 | 5.06 | 4.90 | 4.46 | 1.52 | 0.0246 | 0.1359 |
| TC0200011372.hg.1 | AQP12A | aquaporin 12A | Multiple_C | 5.59 | 5.12 | 5.17 | 1.34 | 0.2528 | 0.4577 | 5.77 | 5.47 | 5.44 | 1.26 | 0.1135 | 0.3322 |
| TC0200012248.hg.1 | FEZ2 | fasciculation and elongation protein zeta 2 (zygin II) | Multiple_C | 7.53 | 8.14 | 7.11 | 1.34 | 0.3504 | 0.5632 | 5.29 | 5.41 | 5.75 | 0.73 | 0.459 | 0.7024 |
| TC0200012684.hg.1 | FANCL | Fanconi anemia complementation group L | Multiple_C | 7.92 | 8.41 | 7.5 | 1.34 | 0.327 | 0.541 | 8.01 | 8.15 | 7.98 | 1.02 | 0.7755 | 0.8992 |
| TC0200013899.hg.1 | ZC3H8 | zinc finger CCCH-type containing 8 | Multiple_C | 9.33 | 9.98 | 8.91 | 1.34 | 0.2819 | 0.4919 | 10.24 | 9.60 | 9.91 | 1.26 | 0.4717 | 0.7122 |
| TC0200015225.hg.1 | HIBCH | 3-hydroxyisobutyryl-CoA hydrolase | Multiple_C | 10.32 | 10.44 | 9.9 | 1.34 | 0.2332 | 0.434 | 7.4 | 7.64 | 8.15 | 0.59 | 0.037 | 0.1751 |
| TC0200016039.hg.1 | PDE6D | phosphodiesterase 6D, cGMP-specific, rod, delta | Multiple_C | 11.12 | 10.66 | 10.7 | 1.34 | 0.147 | 0.3176 | 9.42 | 9.65 | 9.12 | 1.23 | 0.4932 | 0.7276 |
| TC0200016330.hg.1 | AQP12B | aquaporin 12B | Multiple_C | 5.47 | 4.82 | 5.05 | 1.34 | 0.071 | 0.1875 | 5.26 | 5.09 | 4.81 | 1.37 | 0.1077 | 0.3228 |
| TC0200016770.hg.1 | MIR6809; TNS | microRNA 6809; tensin 1 | Multiple_C | 3.84 | 3.14 | 3.42 | 1.34 | 0.2198 | 0.4169 | 3.81 | 4.00 | 3.3 | 1.42 | 0.1921 | 0.4452 |
| TC0300011815.hg.1 | DCBLD2 | discoidin, CUB and LCCL domain containing 2 | Multiple_C | 12.84 | 11.61 | 12.42 | 1.34 | 0.1288 | 0.2891 | 16.19 | 15.64 | 15.92 | 1.21 | 0.2987 | 0.5633 |
| TC0300013809.hg.1 | RPL14 | ribosomal protein L14 | Multiple_C | 16.91 | 16.5 | 16.49 | 1.34 | 0.0342 | 0.109 | 16.18 | 15.72 | 15.85 | 1.26 | 0.0918 | 0.2936 |
| TC0300013852.hg.1 | ST3GAL6 | ST3 beta-galactoside alpha-2,3-sialyltransferase 6 | Multiple_C | 3.89 | 3.59 | 3.47 | 1.34 | 0.4628 | 0.668 | 4.64 | 3.98 | 3.96 | 1.60 | 0.0033 | 0.038 |
| TC0400009764.hg.1 | HAUS3; POLN | HAUS augmin like complex subunit 3; polymerase (DNA directe | Multiple_C | 5.27 | 4.97 | 4.85 | 1.34 | 0.1737 | 0.3556 | 4.79 | 4.83 | 4.7 | 1.06 | 0.4494 | 0.6958 |
| TC0500006664.hg.1 | ADCY2 | Transcript Identified by AceView, Entrez Gene ID(s) 108 | Coding | 9.07 | 8.7 | 8.65 | 1.34 | 0.0862 | 0.216 | 7.81 | 8.22 | 8.18 | 0.77 | 0.0635 | 0.2397 |
| TC0500007758.hg.1 | FCHO2 | FCH domain only 2 | Multiple_C | 9.11 | 8.55 | 8.69 | 1.34 | 0.4171 | 0.6289 | 8.44 | 8.08 | 8.19 | 1.19 | 0.7232 | 0.8709 |
| TC0500007915.hg.1 | FAM151B | family with sequence similarity 151, member B | Multiple_C | 4.88 | 4.3 | 4.46 | 1.34 | 0.1649 | 0.3433 | 4.94 | 5.26 | 5.08 | 0.91 | 0.9538 | 0.9811 |
| TC0500008619.hg.1 | IL3 | interleukin 3 | Coding | 4.66 | 4.52 | 4.24 | 1.34 | 0.1417 | 0.3095 | 4.18 | 4.13 | 4.13 | 1.04 | 0.1728 | 0.42 |
| TC0500008626.hg.1 | PDLIM4 | PDZ and LIM domain 4 | Multiple_C | 6.13 | 5.67 | 5.71 | 1.34 | 0.0907 | 0.2241 | 6.51 | 6.72 | 6.35 | 1.12 | 0.4336 | 0.6846 |
| TC0500008863.hg.1 | IK; MIR3655 | IK cytokine, down-regulator of HLA II; microRNA 3655 | Multiple_C | 12.78 | 12.41 | 12.36 | 1.34 | 0.5561 | 0.7392 | 12.14 | 12.01 | 12.32 | 0.88 | 0.7678 | 0.8959 |
| TC0500013372.hg.1 | ECSCR | endothelial cell surface expressed chemotaxis and apoptosis re | Multiple_C | 4.09 | 3.79 | 3.67 | 1.34 | 0.0888 | 0.2208 | 4.56 | 4.69 | 4.4 | 1.12 | 0.1662 | 0.4113 |
| TC0500013381.hg.1 | PRELID2 | PRELI domain containing 2 | Multiple_C | 10.33 | 10.72 | 9.91 | 1.34 | 0.106 | 0.2514 | 6.4 | 5.93 | 6.02 | 1.30 | 0.2287 | 0.4897 |
| TC0600007244.hg.1 | SCGN | secretagogin, EF-hand calcium binding protein | Coding | 3.92 | 3.5 | 3.5 | 1.34 | 0.1947 | 0.3845 | 3.13 | 3.37 | 3.31 | 0.88 | 0.5703 | 0.7787 |
| TC0600007791.hg.1 | DEF6 | DEF6 guanine nucleotide exchange factor | Multiple_C | 6.52 | 6.01 | 6.1 | 1.34 | 0.06 | 0.1657 | 5.94 | 6.15 | 6.1 | 0.90 | 0.4594 | 0.7026 |
| TC0600007946.hg.1 | GLP1R | glucagon-like peptide 1 receptor | Coding | 5.41 | 4.96 | 4.99 | 1.34 | 0.5234 | 0.7161 | 5.24 | 5.45 | 5.17 | 1.05 | 0.7304 | 0.875 |
| TC0600008468.hg.1 | FAM135A | family with sequence similarity 135, member A | Multiple_C | 10.27 | 9.98 | 9.85 | 1.34 | 0.3143 | 0.5277 | 8.2 | 7.09 | 8.44 | 0.85 | 0.2802 | 0.546 |
| TC0600011503.hg.1 | HLA-DQB2 | major histocompatibility complex, class II, DQ beta 2 | Coding | 6.3 | 5.49 | 5.88 | 1.34 | 0.2389 | 0.4409 | 6.49 | 6.22 | 6.09 | 1.32 | 0.1718 | 0.4185 |
| TC0600011884.hg.1 | MRPS18A | mitochondrial ribosomal protein S18A | Multiple_C | 12.66 | 12 | 12.24 | 1.34 | 0.4789 | 0.6811 | 10.99 | 11.94 | 11.74 | 0.59 | 0.0537 | 0.2169 |
| TC0600012116.hg.1 | COL21A1 | collagen, type XXI, alpha 1 | Multiple_C | 3.38 | 3.34 | 2.96 | 1.34 | 0.3921 | 0.6059 | 3.83 | 3.40 | 3.38 | 1.37 | 0.0694 | 0.2513 |
| TC0600014111.hg.1 | SYNGAP1; MII | synaptic Ras GTPase activating protein 1; microRNA 5004 | Multiple_C | 5.58 | 5.04 | 5.16 | 1.34 | 0.1497 | 0.3215 | 8.8 | 7.77 | 7.46 | 2.53 | 1.39E-05 | 0.0009 |
| TC0600014316.hg.1 | FILIP1 | filamin A interacting protein 1 | Multiple_C | 3.84 | 3.3 | 3.42 | 1.34 | 0.0978 | 0.2367 | 3.38 | 3.67 | 3.48 | 0.93 | 0.7717 | 0.8973 |
| TC0600014320.hg.1 | TBX18 | T-box 18 | Multiple_C | 3.38 | 2.96 | 2.96 | 1.34 | 0.0462 | 0.1365 | 3.69 | 3.70 | 3.53 | 1.12 | 0.3718 | 0.6332 |

| | | | | | | | | | | | | | | | |
|---|---|---|---|---|---|---|---|---|---|---|---|---|---|---|---|
| TC0700006636.hg.1 | ANKRD61 | ankyrin repeat domain 61 | Coding | 3.9 | 3.3 | 3.48 | 1.34 | 0.1988 | 0.3897 | 4.53 | 4.10 | 4.66 | 0.91 | 0.6968 | 0.8563 |
| TC0700006727.hg.1 | TMEM106B | transmembrane protein 106B | Multiple_C | 8.03 | 8.38 | 7.61 | 1.34 | 0.0299 | 0.098 | 8.27 | 7.86 | 7.66 | 1.53 | 0.0211 | 0.1242 |
| TC0700007262.hg.1 | POU6F2 | POU class 6 homeobox 2 | Multiple_C | 5.52 | 4.74 | 5.1 | 1.34 | 0.1745 | 0.3563 | 5.16 | 5.50 | 5.52 | 0.78 | 0.0673 | 0.2473 |
| TC0700010926.hg.1 | TMED4 | transmembrane p24 trafficking protein 4 | Multiple_C | 11.69 | 11.2 | 11.27 | 1.34 | 0.7818 | 0.8876 | 9.57 | 10.13 | 10.45 | 0.54 | 0.006 | 0.0552 |
| TC0700012855.hg.1 | PRSS58 | protease, serine, 58 | Coding | 4.68 | 3.98 | 4.26 | 1.34 | 0.1183 | 0.2721 | 4.79 | 5.07 | 4.15 | 1.56 | 0.0246 | 0.1358 |
| TC0800007366.hg.1 | HTRA4 | HtrA serine peptidase 4 | Coding | 4.34 | 3.61 | 3.92 | 1.34 | 0.194 | 0.3834 | 4.03 | 4.05 | 4.03 | 1.00 | 0.6211 | 0.8127 |
| TC0800007641.hg.1 | RGS20 | regulator of G-protein signaling 20 | Multiple_C | 3.48 | 3.01 | 3.06 | 1.34 | 0.302 | 0.5145 | 3.83 | 3.87 | 3.91 | 0.95 | 0.9441 | 0.9771 |
| TC0800008547.hg.1 | PKHD1L1 | polycystic kidney and hepatic disease 1 (autosomal recessive)-li | Multiple_C | 4.07 | 3.97 | 3.65 | 1.34 | 0.1715 | 0.3527 | 4.15 | 4.38 | 4.12 | 1.02 | 0.3182 | 0.5822 |
| TC0800009637.hg.1 | USP17L2 | ubiquitin specific peptidase 17-like family member 2 | Coding | 4.3 | 3.6 | 3.88 | 1.34 | 0.209 | 0.4028 | 3.78 | 3.56 | 3.48 | 1.23 | 0.2101 | 0.4671 |
| TC0800009921.hg.1 | KCTD9 | potassium channel tetramerization domain containing 9 | Multiple_C | 10.4 | 10.69 | 9.98 | 1.34 | 0.1749 | 0.3568 | 10.04 | 9.78 | 9.74 | 1.23 | 0.1733 | 0.4206 |
| TC0800010936.hg.1 | PMP2 | peripheral myelin protein 2 | Coding | 3.15 | 2.98 | 2.73 | 1.34 | 0.2051 | 0.3979 | 3.84 | 3.53 | 3.49 | 1.27 | 0.0256 | 0.1392 |
| TC0900009324.hg.1 | CACNA1B | calcium channel, voltage-dependent, N type, alpha 1B subunit | Multiple_C | 4.39 | 4.28 | 3.97 | 1.34 | 0.2622 | 0.4693 | 3.8 | 3.88 | 3.66 | 1.10 | 0.5513 | 0.767 |
| TC0900010439.hg.1 | TRPM6 | transient receptor potential cation channel, subfamily M, meml | Multiple_C | 4.19 | 3.79 | 3.77 | 1.34 | 0.238 | 0.4396 | 4.09 | 4.14 | 3.84 | 1.19 | 0.2826 | 0.548 |
| TC0900011039.hg.1 | PPP3R2 | protein phosphatase 3, regulatory subunit B, beta | Coding | 4.19 | 3.45 | 3.77 | 1.34 | 0.9076 | 0.9557 | 4.31 | 4.40 | 4.18 | 1.09 | 0.9458 | 0.9781 |
| TC0900011444.hg.1 | OR1B1 | olfactory receptor, family 1, subfamily B, member 1 (gene/pseu | Multiple_C | 3.4 | 3.52 | 2.98 | 1.34 | 0.2253 | 0.4241 | 3.82 | 3.85 | 3.39 | 1.35 | 0.0768 | 0.2669 |
| TC0900012113.hg.1 | IFNA8 | interferon, alpha 8 | Coding | 4.24 | 3.45 | 3.82 | 1.34 | 0.02 | 0.0715 | 4.32 | 4.14 | 4.16 | 1.12 | 0.4104 | 0.6652 |
| TC0900012180.hg.1 | PHYHD1 | phytanoyl-CoA dioxygenase domain containing 1 | Multiple_C | 5.01 | 4.45 | 4.59 | 1.34 | 0.2976 | 0.509 | 4.9 | 5.12 | 4.68 | 1.16 | 0.2227 | 0.4833 |
| TC0M00006441.hg.1 | ND4L; ND4 | NADH dehydrogenase, subunit 4L (complex I); NADH dehydroge | Multiple_C | 18.34 | 17.73 | 17.92 | 1.34 | 0.0626 | 0.1706 | 18.73 | 18.64 | 19.08 | 0.78 | 0.3615 | 0.6242 |
| TC0X00007532.hg.1 | AWAT1 | acyl-CoA wax alcohol acyltransferase 1 | Multiple_C | 4.62 | 4.22 | 4.2 | 1.34 | 0.628 | 0.7924 | 4.01 | 3.81 | 3.87 | 1.10 | 0.553 | 0.7684 |
| TC0X00008253.hg.1 | UBE2A | ubiquitin conjugating enzyme E2A | Multiple_C | 12.72 | 12.13 | 12.3 | 1.34 | 0.1236 | 0.2806 | 10.58 | 11.41 | 11.95 | 0.39 | 1.95E-05 | 0.0012 |
| TC0X00009185.hg.1 | SCML2 | sex comb on midleg-like 2 (Drosophila) | Multiple_C | 3.89 | 3.26 | 3.47 | 1.34 | 0.0499 | 0.1443 | 5.66 | 5.53 | 5.88 | 0.86 | 0.6171 | 0.8099 |
| TC0X00009486.hg.1 | MAOB | monoamine oxidase B | Multiple_C | 4.15 | 3.69 | 3.73 | 1.34 | 0.1472 | 0.318 | 4.24 | 4.66 | 4.19 | 1.04 | 0.4118 | 0.6664 |
| TC0X00010431.hg.1 | RAB9B | RAB9B, member RAS oncogene family | Multiple_C | 4.81 | 4.39 | 4.39 | 1.34 | 0.1993 | 0.3905 | 4.93 | 5.11 | 4.44 | 1.40 | 0.2942 | 0.5591 |
| TC0X00010596.hg.1 | CT83 | cancer/testis antigen 83 | Coding | 3.62 | 2.96 | 3.2 | 1.34 | 0.2859 | 0.4962 | 4.19 | 4.27 | 4.43 | 0.85 | 0.9887 | 0.9952 |
| TC1000006934.hg.1 | MALRD1 | MAM and LDL receptor class A domain containing 1 | Multiple_C | 3.4 | 3.2 | 2.98 | 1.34 | 0.1114 | 0.2606 | 4.02 | 3.82 | 4.08 | 0.96 | 0.6102 | 0.805 |
| TC1000007833.hg.1 | LRRTM3 | leucine rich repeat transmembrane neuronal 3 | Multiple_C | 3.81 | 3.24 | 3.39 | 1.34 | 0.213 | 0.408 | 4.06 | 3.89 | 3.63 | 1.35 | 0.0113 | 0.084 |
| TC1000010926.hg.1 | TBATA | thymus, brain and testes associated | Multiple_C | 4.44 | 3.7 | 4.02 | 1.34 | 0.1153 | 0.2674 | 4.12 | 4.12 | 3.71 | 1.33 | 0.0377 | 0.177 |
| TC1000012213.hg.1 | FAM196A | family with sequence similarity 196, member A | Multiple_C | 4.3 | 3.79 | 3.88 | 1.34 | 0.7669 | 0.8801 | 4.22 | 3.81 | 4.3 | 0.95 | 0.6401 | 0.8234 |
| TC1100007507.hg.1 | PTPRJ | protein tyrosine phosphatase, receptor type, J | Multiple_C | 10.21 | 9.64 | 9.79 | 1.34 | 0.0894 | 0.2218 | 10.72 | 9.74 | 9.94 | 1.72 | 0.0004 | 0.0085 |
| TC1100010276.hg.1 | IGSF22 | immunoglobulin superfamily, member 22 | Multiple_C | 3.83 | 3.38 | 3.41 | 1.34 | 0.5533 | 0.7371 | 3.8 | 3.66 | 3.58 | 1.16 | 0.491 | 0.726 |
| TC1100012520.hg.1 | MCAM; MIR6 | melanoma cell adhesion molecule; microRNA 6756 | Multiple_C | 4.4 | 3.94 | 3.98 | 1.34 | 0.4994 | 0.6987 | 6.99 | 5.80 | 5.23 | 3.39 | 0.0002 | 0.0051 |
| TC1100012526.hg.1 | USP2 | ubiquitin specific peptidase 2 | Multiple_C | 4.23 | 3.52 | 3.81 | 1.34 | 0.1879 | 0.3753 | 3.53 | 3.18 | 3.46 | 1.05 | 0.6928 | 0.8538 |
| TC1200007514.hg.1 | H1FNT | H1 histone family, member N, testis-specific | Coding | 4.48 | 3.95 | 4.06 | 1.34 | 0.2096 | 0.4033 | 4.51 | 4.55 | 4.53 | 0.99 | 0.8092 | 0.9156 |

| | | | | | | | | | | | | | | | |
|---|---|---|---|---|---|---|---|---|---|---|---|---|---|---|---|
| TC1200007596.hg.1 | GPD1 | glycerol-3-phosphate dehydrogenase 1 | Multiple_C | 3.58 | 3.15 | 3.16 | 1.34 | 0.2462 | 0.4498 | 4.03 | 3.81 | 3.75 | 1.21 | 0.5584 | 0.7714 |
| TC1200007654.hg.1 | ATG101 | autophagy related 101 | Multiple_C | 10.49 | 10.24 | 10.07 | 1.34 | 0.0316 | 0.1024 | 9.2 | 8.88 | 9.18 | 1.01 | 0.3912 | 0.6499 |
| TC1200007713.hg.1 | SP1 | Sp1 transcription factor | Multiple_C | 12.49 | 11.6 | 12.07 | 1.34 | 0.0609 | 0.1676 | 12.87 | 12.87 | 13.01 | 0.91 | 0.4824 | 0.7202 |
| TC1200008814.hg.1 | ANKRD13A | ankyrin repeat domain 13A | Multiple_C | 10.19 | 9.58 | 9.77 | 1.34 | 0.1601 | 0.3367 | 8.44 | 8.01 | 8.31 | 1.09 | 0.8407 | 0.9305 |
| TC1200009921.hg.1 | TAS2R13 | taste receptor, type 2, member 13 | Coding | 4.03 | 3.88 | 3.61 | 1.34 | 0.024 | 0.0827 | 3.37 | 4.10 | 3.4 | 0.98 | 0.8424 | 0.9313 |
| TC1200012776.hg.1 | ST8SIA1 | ST8 alpha-N-acetyl-neuraminide alpha-2,8-sialyltransferase 1 | Multiple_C | 4.22 | 3.76 | 3.8 | 1.34 | 0.5057 | 0.7029 | 4.06 | 4.04 | 3.98 | 1.06 | 0.4676 | 0.7088 |
| TC1300006883.hg.1 | SERTM1 | serine-rich and transmembrane domain containing 1 | Coding | 4.64 | 4.32 | 4.22 | 1.34 | 0.4941 | 0.694 | 5.38 | 5.03 | 4.95 | 1.35 | 0.0517 | 0.2124 |
| TC1300007152.hg.1 | FNDC3A | fibronectin type III domain containing 3A | Multiple_C | 8.55 | 8.08 | 8.13 | 1.34 | 0.1202 | 0.2751 | 10.88 | 10.18 | 10.33 | 1.46 | 0.1623 | 0.4055 |
| TC1300008766.hg.1 | ENOX1 | ecto-NOX disulfide-thiol exchanger 1 | Multiple_C | 4.98 | 4.34 | 4.56 | 1.34 | 0.1463 | 0.3165 | 5.08 | 4.98 | 5.15 | 0.95 | 0.6019 | 0.8005 |
| TC1400008070.hg.1 | SERPINA13P | serpin peptidase inhibitor, clade A (alpha-1 antiproteinase, antit | Multiple_C | 3.57 | 3.17 | 3.15 | 1.34 | 0.1741 | 0.356 | 3.6 | 3.53 | 3.77 | 0.89 | 0.7756 | 0.8993 |
| TC1400010635.hg.1 | TMEM63C | transmembrane protein 63C | Multiple_C | 6.28 | 5.41 | 5.86 | 1.34 | 0.1571 | 0.3326 | 6.76 | 6.38 | 6.23 | 1.44 | 0.6679 | 0.8376 |
| TC1500007097.hg.1 | B2M | beta-2-microglobulin | Multiple_C | 16.7 | 17.4 | 16.28 | 1.34 | 0.1816 | 0.3662 | 13.55 | 13.24 | 13.08 | 1.39 | 0.0067 | 0.0595 |
| TC1500008470.hg.1 | ARRDC4 | arrestin domain containing 4 | Coding | 7.89 | 7.29 | 7.47 | 1.34 | 0.6326 | 0.795 | 6.12 | 5.85 | 6.24 | 0.92 | 0.1367 | 0.3674 |
| TC1500008984.hg.1 | NOP10 | NOP10 ribonucleoprotein | Multiple_C | 15.88 | 15.26 | 15.46 | 1.34 | 0.1233 | 0.2802 | 12.63 | 12.58 | 13.26 | 0.65 | 0.0282 | 0.1477 |
| TC1500009504.hg.1 | PYGO1 | pygopus family PHD finger 1 | Multiple_C | 3.85 | 3.5 | 3.43 | 1.34 | 0.15 | 0.3219 | 3.96 | 3.71 | 3.78 | 1.13 | 0.2125 | 0.4703 |
| TC1500009951.hg.1 | GRAMD2 | GRAM domain containing 2 | Multiple_C | 4.66 | 3.97 | 4.24 | 1.34 | 0.077 | 0.1988 | 4.48 | 4.27 | 4.14 | 1.27 | 0.1948 | 0.449 |
| TC1500010157.hg.1 | ADAMTS7 | ADAM metallopeptidase with thrombospondin type 1 motif 7 | Multiple_C | 3.6 | 2.92 | 3.18 | 1.34 | 0.2987 | 0.5103 | 3.79 | 4.08 | 3.78 | 1.01 | 0.9659 | 0.9857 |
| TC1500010162.hg.1 | RASGRF1 | Ras protein-specific guanine nucleotide-releasing factor 1 | Multiple_C | 4.9 | 4.19 | 4.48 | 1.34 | 0.1247 | 0.2823 | 4.76 | 4.80 | 4.57 | 1.14 | 0.6858 | 0.8495 |
| TC1600006459.hg.1 | RPL23AP5 | ribosomal protein L23a pseudogene 5 | Multiple_C | 3.48 | 2.99 | 3.06 | 1.34 | 0.0211 | 0.0747 | 3.57 | 3.74 | 3.65 | 0.95 | 0.6576 | 0.8327 |
| TC1600008640.hg.1 | ADAD2 | adenosine deaminase domain containing 2 | Multiple_C | 5.49 | 5 | 5.07 | 1.34 | 0.1508 | 0.3233 | 5.09 | 5.16 | 5 | 1.06 | 0.1045 | 0.3169 |
| TC1600009041.hg.1 | CCDC154 | coiled-coil domain containing 154 | Multiple_C | 4.57 | 4.18 | 4.15 | 1.34 | 0.0813 | 0.2072 | 4.33 | 4.27 | 4.18 | 1.11 | 0.3075 | 0.5716 |
| TC1600010951.hg.1 | MAF | v-maf avian musculoaponeurotic fibrosarcoma oncogene homo | Coding | 3.97 | 3.48 | 3.55 | 1.34 | 0.1937 | 0.3831 | 4.91 | 4.73 | 4.62 | 1.22 | 0.3338 | 0.598 |
| TC1600011290.hg.1 | VPS9D1 | VPS9 domain containing 1 | Multiple_C | 5.24 | 4.56 | 4.82 | 1.34 | 0.2675 | 0.4756 | 4.67 | 4.67 | 4.56 | 1.08 | 0.9912 | 0.9963 |
| TC1600011380.hg.1 | TMEM219 | transmembrane protein 219 | Multiple_C | 5.16 | 5.13 | 4.74 | 1.34 | 0.2413 | 0.4436 | 5.05 | 5.15 | 4.8 | 1.19 | 0.2058 | 0.4622 |
| TC1600011557.hg.1 | TERF2 | telomeric repeat binding factor 2 | Multiple_C | 11.16 | 10.41 | 10.74 | 1.34 | 0.334 | 0.5477 | 12.83 | 12.71 | 13.13 | 0.81 | 0.042 | 0.1887 |
| TC1700007230.hg.1 | CDRT15L2 | CMT1A duplicated region transcript 15-like 2 | Coding | 4.96 | 4.36 | 4.54 | 1.34 | 0.1313 | 0.2934 | 3.24 | 3.26 | 3.55 | 0.81 | 0.1941 | 0.448 |
| TC1700011558.hg.1 | SLC16A6 | solute carrier family 16, member 6 | Multiple_C | 3.23 | 2.99 | 2.81 | 1.34 | 0.1973 | 0.388 | 11.22 | 7.94 | 8.73 | 5.62 | 9.87E-10 | 1.01E-06 |
| TC1900006586.hg.1 | TMPRSS9 | transmembrane protease, serine 9 | Multiple_C | 3.99 | 3.39 | 3.57 | 1.34 | 0.0615 | 0.1687 | 3.59 | 3.74 | 3.51 | 1.06 | 0.7582 | 0.8903 |
| TC1900008750.hg.1 | ZNF761; TPM3 | zinc finger protein 761; tropomyosin 3 pseudogene 9 | Multiple_C | 11.33 | 11.57 | 10.91 | 1.34 | 0.0545 | 0.1539 | 9.39 | 9.19 | 9.26 | 1.09 | 0.9607 | 0.9837 |
| TC1900008986.hg.1 | ZNF264 | zinc finger protein 264 | Coding | 10.4 | 10.19 | 9.98 | 1.34 | 0.2547 | 0.4603 | 7.72 | 7.62 | 7.48 | 1.18 | 0.3947 | 0.6528 |
| TC1900009165.hg.1 | ADAMTSL5 | ADAMTS like 5 | Multiple_C | 6.41 | 5.84 | 5.99 | 1.34 | 0.1083 | 0.2554 | 6.5 | 6.70 | 6.21 | 1.22 | 0.3792 | 0.6388 |
| TC1900011483.hg.1 | ZNF579 | zinc finger protein 579 | Multiple_C | 7.74 | 6.94 | 7.32 | 1.34 | 0.0438 | 0.1313 | 7.84 | 8.05 | 7.84 | 1.00 | 0.2249 | 0.4856 |
| TC2000006444.hg.1 | TRIB3 | tribbles pseudokinase 3 | Multiple_C | 10.82 | 10.72 | 10.4 | 1.34 | 0.2689 | 0.4771 | 9.77 | 8.67 | 10.12 | 0.78 | 0.5288 | 0.7524 |

| | | | | | | | | | | | | | | | |
|---|---|---|---|---|---|---|---|---|---|---|---|---|---|---|---|
| TC2000007448.hg.1 | GDAP1L1 | ganglioside induced differentiation associated protein 1-like 1 | Multiple_C | 5.83 | 5.18 | 5.41 | 1.34 | 0.1688 | 0.3487 | 4.91 | 4.74 | 4.9 | 1.01 | 0.3135 | 0.5777 |
| TC2000008144.hg.1 | ANGPT4 | angiopoietin 4 | Coding | 3.38 | 2.98 | 2.96 | 1.34 | 0.3692 | 0.582 | 3.56 | 3.32 | 3.41 | 1.11 | 0.7526 | 0.8876 |
| TC2000009911.hg.1 | ASIP | agouti signaling protein | Coding | 4.65 | 4.14 | 4.23 | 1.34 | 0.1355 | 0.3 | 5.01 | 4.90 | 4.66 | 1.27 | 0.1973 | 0.4522 |
| TC2100006878.hg.1 | KRTAP6-3 | keratin associated protein 6-3 | Multiple_C | 6.85 | 6.07 | 6.43 | 1.34 | 0.1059 | 0.2514 | 6.19 | 5.82 | 5.72 | 1.39 | 0.0863 | 0.2833 |
| TC2200006643.hg.1 | ZDHHC8 | zinc finger, DHHC-type containing 8 | Multiple_C | 6.69 | 5.92 | 6.27 | 1.34 | 0.4414 | 0.6501 | 6.41 | 6.29 | 6.08 | 1.26 | 0.9776 | 0.9908 |
| TC2200007303.hg.1 | CDC42EP1 | CDC42 effector protein (Rho GTPase binding) 1 | Multiple_C | 9.82 | 9.07 | 9.4 | 1.34 | 0.3843 | 0.5976 | 7.79 | 8.36 | 9.1 | 0.40 | 0.0011 | 0.0181 |
| TC2200009261.hg.1 | SFI1 | SFI1 centrin binding protein | Multiple_C | 7.12 | 6.25 | 6.7 | 1.34 | 0.3179 | 0.5313 | 6.25 | 6.13 | 5.75 | 1.41 | 0.1681 | 0.4137 |
| TSUnmapped00000146.h | ZNF780A | zinc finger protein 780A | Coding | 5.97 | 5.67 | 5.55 | 1.34 | 0.0951 | 0.2322 | 5.83 | 6.32 | 5.93 | 0.93 | 0.4311 | 0.6826 |
| TSUnmapped00000242.h | KIF15 | kinesin family member 15 | Coding | 5.75 | 5.62 | 5.33 | 1.34 | 0.2207 | 0.4181 | 4.8 | 5.05 | 4.59 | 1.16 | 0.8241 | 0.9222 |
| TSUnmapped00000372.h | SLC16A1 | solute carrier family 16 (monocarboxylate transporter), membe | Coding | 5.45 | 5.15 | 5.03 | 1.34 | 0.2458 | 0.4494 | 5.47 | 5.12 | 5.58 | 0.93 | 0.6782 | 0.8451 |
| TC0100006540.hg.1 | CALML6 | calmodulin-like 6 | Multiple_C | 5.38 | 4.89 | 4.97 | 1.33 | 0.2049 | 0.3976 | 4.87 | 5.04 | 4.88 | 0.99 | 0.2896 | 0.5543 |
| TC0100006998.hg.1 | TMEM82 | transmembrane protein 82 | Multiple_C | 3.76 | 3.33 | 3.35 | 1.33 | 0.298 | 0.5096 | 3.7 | 3.54 | 3.58 | 1.09 | 0.0829 | 0.2772 |
| TC0100007372.hg.1 | NCMAP | noncompact myelin associated protein | Multiple_C | 7.36 | 6.91 | 6.95 | 1.33 | 0.1424 | 0.3103 | 4.85 | 4.98 | 4.85 | 1.00 | 0.822 | 0.9213 |
| TC0100007832.hg.1 | ZC3H12A; MIF | zinc finger CCCH-type containing 12A; microRNA 6732 | Multiple_C | 7.57 | 6.83 | 7.16 | 1.33 | 0.3971 | 0.6109 | 7.67 | 7.19 | 7.57 | 1.07 | 0.4072 | 0.6631 |
| TC0100009651.hg.1 | PPIAL4A | peptidylprolyl isomerase A (cyclophilin A)-like 4A | Coding | 5.24 | 4.7 | 4.83 | 1.33 | 0.3154 | 0.5286 | 4.26 | 4.37 | 4.62 | 0.78 | 0.1227 | 0.3456 |
| TC0100009746.hg.1 | NUDT17 | nudix hydrolase 17 | Multiple_C | 6.2 | 5.73 | 5.79 | 1.33 | 0.176 | 0.3583 | 6.08 | 6.11 | 6.21 | 0.91 | 0.5243 | 0.7494 |
| TC0100010016.hg.1 | SPRR4 | small proline-rich protein 4 | Coding | 4.39 | 3.75 | 3.98 | 1.33 | 0.3419 | 0.5556 | 4.21 | 4.45 | 4.45 | 0.85 | 0.7283 | 0.8739 |
| TC0100010234.hg.1 | OR10R2 | olfactory receptor, family 10, subfamily R, member 2 | Coding | 4.95 | 4.81 | 4.54 | 1.33 | 0.1417 | 0.3094 | 5.2 | 5.36 | 5.32 | 0.92 | 0.373 | 0.634 |
| TC0100010806.hg.1 | XPR1 | xenotropic and polytropic retrovirus receptor 1 | Multiple_C | 11.62 | 10.37 | 11.21 | 1.33 | 0.5504 | 0.7353 | 11.56 | 10.44 | 11.81 | 0.84 | 0.1467 | 0.3826 |
| TC0100014653.hg.1 | USP33 | ubiquitin specific peptidase 33 | Multiple_C | 11.86 | 12.42 | 11.45 | 1.33 | 0.2796 | 0.4893 | 10.41 | 9.57 | 9.7 | 1.64 | 0.0312 | 0.1577 |
| TC0100014769.hg.1 | MCOLN3 | mucolipin 3 | Multiple_C | 7.4 | 7.37 | 6.99 | 1.33 | 0.2447 | 0.4481 | 3.8 | 3.22 | 3.54 | 1.20 | 0.7638 | 0.8932 |
| TC0100014926.hg.1 | GFI1 | growth factor independent 1 transcription repressor | Multiple_C | 9.18 | 8.41 | 8.77 | 1.33 | 0.0677 | 0.181 | 9.79 | 10.08 | 9.74 | 1.04 | 0.9194 | 0.9668 |
| TC0100014977.hg.1 | ABCA4 | ATP binding cassette subfamily A member 4 | Multiple_C | 4.66 | 3.77 | 4.25 | 1.33 | 0.4008 | 0.614 | 4.47 | 4.40 | 4.37 | 1.07 | 0.7305 | 0.875 |
| TC0100015593.hg.1 | ANKRD35 | ankyrin repeat domain 35 | Coding | 3.73 | 3.2 | 3.32 | 1.33 | 0.022 | 0.0773 | 3.87 | 3.99 | 3.89 | 0.99 | 0.8829 | 0.9497 |
| TC0100018045.hg.1 | SMYD3 | SET and MYND domain containing 3 | Multiple_C | 9.85 | 9.98 | 9.44 | 1.33 | 0.2137 | 0.4089 | 9.01 | 8.72 | 8.75 | 1.20 | 0.3774 | 0.6376 |
| TC0200011075.hg.1 | PTMA | prothymosin, alpha | Multiple_C | 16.61 | 15.53 | 16.2 | 1.33 | 0.184 | 0.3695 | 16.01 | 16.24 | 16.26 | 0.84 | 0.7855 | 0.9042 |
| TC0200011171.hg.1 | SH3BP4 | SH3-domain binding protein 4 | Multiple_C | 11.89 | 10.81 | 11.48 | 1.33 | 0.08 | 0.2051 | 12.09 | 12.31 | 12.53 | 0.74 | 0.6185 | 0.8109 |
| TC0200011276.hg.1 | FAM132B | family with sequence similarity 132, member B | Multiple_C | 6.86 | 6.48 | 6.45 | 1.33 | 0.0663 | 0.1783 | 6.13 | 6.42 | 6.37 | 0.85 | 0.5228 | 0.7489 |
| TC0200012433.hg.1 | PREPL | prolyl endopeptidase-like | Multiple_C | 10.84 | 10.47 | 10.43 | 1.33 | 0.202 | 0.3942 | 11.83 | 10.43 | 10.98 | 1.80 | 0.022 | 0.1274 |
| TC0200016597.hg.1 | SPEG | SPEG complex locus | Multiple_C | 4.75 | 4.15 | 4.34 | 1.33 | 0.2255 | 0.4243 | 4.23 | 4.24 | 4.2 | 1.02 | 0.9073 | 0.9617 |
| TC0300006454.hg.1 | CNTN4 | contactin 4 | Multiple_C | 3.04 | 2.5 | 2.63 | 1.33 | 0.0998 | 0.2406 | 3.01 | 3.10 | 2.78 | 1.17 | 0.118 | 0.3395 |
| TC0300007406.hg.1 | SEMA3F | sema domain, immunoglobulin domain (Ig), short basic domain, | Multiple_C | 3.51 | 3.07 | 3.1 | 1.33 | 0.0901 | 0.2232 | 8.07 | 6.90 | 6.61 | 2.75 | 0.0001 | 0.0038 |
| TC0300007529.hg.1 | CACNA2D3 | calcium channel, voltage-dependent, alpha 2/delta subunit 3 | Multiple_C | 3.8 | 3.31 | 3.39 | 1.33 | 0.9287 | 0.9663 | 4.2 | 4.21 | 3.87 | 1.26 | 0.2615 | 0.5256 |

| | | | | | | | | | | | | | | | |
|---|---|---|---|---|---|---|---|---|---|---|---|---|---|---|---|
| TC0300008107.hg.1 | OR5K4 | olfactory receptor, family 5, subfamily K, member 4 | Coding | 4.08 | 3.53 | 3.67 | 1.33 | 0.4298 | 0.6401 | 3.99 | 3.72 | 3.43 | 1.47 | 0.3158 | 0.5798 |
| TC0300009597.hg.1 | PIK3CA | phosphatidylinositol-4,5-bisphosphate 3-kinase, catalytic subun | Multiple_C | 10.51 | 10.95 | 10.1 | 1.33 | 0.1619 | 0.3391 | 10.11 | 9.63 | 9.44 | 1.59 | 0.0252 | 0.1379 |
| TC0300010952.hg.1 | LRRC2 | leucine rich repeat containing 2 | Multiple_C | 3.85 | 3.36 | 3.44 | 1.33 | 0.1258 | 0.2839 | 5.15 | 5.29 | 5.28 | 0.91 | 0.377 | 0.6374 |
| TC0300013991.hg.1 | TWF2 | twinfilin actin binding protein 2 | Multiple_C | 10.98 | 10.9 | 10.57 | 1.33 | 0.1685 | 0.3483 | 10.83 | 10.96 | 10 | 1.78 | 0.0046 | 0.0464 |
| TC0400007500.hg.1 | SPATA18 | spermatogenesis associated 18 | Multiple_C | 4.07 | 3.5 | 3.66 | 1.33 | 0.2464 | 0.4499 | 4.03 | 3.96 | 4.01 | 1.01 | 0.9991 | 0.9997 |
| TC0400010935.hg.1 | UGT2B4 | UDP glucuronosyltransferase 2 family, polypeptide B4 | Multiple_C | 4.92 | 4.24 | 4.51 | 1.33 | 0.3697 | 0.5825 | 4.73 | 4.77 | 4.8 | 0.95 | 0.5581 | 0.7714 |
| TC0400011040.hg.1 | RCHY1 | ring finger and CHY zinc finger domain containing 1, E3 ubiquitir | Multiple_C | 9.23 | 10.04 | 8.82 | 1.33 | 0.2394 | 0.4414 | 8.27 | 7.61 | 8.06 | 1.16 | 0.4731 | 0.7131 |
| TC0400012798.hg.1 | ADGRL3 | adhesion G protein-coupled receptor L3 | Multiple_C | 3.57 | 3.58 | 3.16 | 1.33 | 0.2481 | 0.4519 | 3.88 | 4.27 | 3.88 | 1.00 | 0.1478 | 0.3841 |
| TC0500011730.hg.1 | TSSK1B | testis-specific serine kinase 1B | Coding | 3.29 | 2.86 | 2.88 | 1.33 | 0.451 | 0.6586 | 3.59 | 3.65 | 3.53 | 1.04 | 0.2447 | 0.5089 |
| TC0500012286.hg.1 | ARAP3 | ArfGAP with RhoGAP domain, ankyrin repeat and PH domain 3 | Multiple_C | 5.15 | 4.94 | 4.74 | 1.33 | 0.5259 | 0.7179 | 5.72 | 5.90 | 5.97 | 0.84 | 0.929 | 0.9702 |
| TC0600007602.hg.1 | APOM | apolipoprotein M | Multiple_C | 3.37 | 2.96 | 2.96 | 1.33 | 0.105 | 0.2497 | 3.82 | 4.32 | 4.13 | 0.81 | 0.206 | 0.4624 |
| TC0600009545.hg.1 | PDE7B | phosphodiesterase 7B | Multiple_C | 3.59 | 3.14 | 3.18 | 1.33 | 0.3132 | 0.5266 | 4.09 | 3.85 | 3.93 | 1.12 | 0.3201 | 0.584 |
| TC0600012740.hg.1 | RTN4IP1 | reticulon 4 interacting protein 1 | Multiple_C | 7.11 | 6.34 | 6.7 | 1.33 | 0.1324 | 0.2952 | 5.72 | 5.87 | 5.6 | 1.09 | 0.5185 | 0.7464 |
| TC0600013341.hg.1 | GVQW2 | GVQW motif containing 2 | Multiple_C | 7.31 | 6.22 | 6.9 | 1.33 | 0.0855 | 0.2147 | 6.58 | 6.62 | 6.82 | 0.85 | 0.6072 | 0.8036 |
| TC0600013800.hg.1 | PARK2 | parkin RBR E3 ubiquitin protein ligase | Coding | 4.61 | 4.23 | 4.2 | 1.33 | 0.1419 | 0.3098 | 4.82 | 4.40 | 4.16 | 1.58 | 0.0344 | 0.1673 |
| TC0700008096.hg.1 | UPK3B | uroplakin 3B | Multiple_C | 4.83 | 4.45 | 4.42 | 1.33 | 0.082 | 0.2082 | 4.64 | 4.85 | 4.54 | 1.07 | 0.3005 | 0.5649 |
| TC0700009292.hg.1 | RPL41 | Synthetic construct Homo sapiens clone IMAGE:100063377, MC | Coding | 16.82 | 16.91 | 16.41 | 1.33 | 0.0405 | 0.1238 | 15.23 | 15.17 | 15.04 | 1.14 | 0.4305 | 0.6823 |
| TC0700013027.hg.1 | RARRES2 | retinoic acid receptor responder (tazarotene induced) 2 | Multiple_C | 4.87 | 4.35 | 4.46 | 1.33 | 0.3106 | 0.524 | 6.64 | 7.04 | 6.67 | 0.98 | 0.4397 | 0.6898 |
| TC0700013598.hg.1 | LRCH4 | leucine-rich repeats and calponin homology (CH) domain contai | Multiple_C | 4.44 | 3.94 | 4.03 | 1.33 | 0.1894 | 0.3771 | 5.34 | 5.00 | 5.01 | 1.26 | 0.2656 | 0.5302 |
| TC0800012428.hg.1 | UBE2W | ubiquitin-conjugating enzyme E2W (putative) | Multiple_C | 11.51 | 12.43 | 11.1 | 1.33 | 0.2454 | 0.449 | 9.24 | 8.25 | 9.06 | 1.13 | 0.1252 | 0.3497 |
| TC0900006886.hg.1 | TEK | TEK tyrosine kinase, endothelial | Multiple_C | 3.14 | 3.04 | 2.73 | 1.33 | 0.1116 | 0.261 | 3.9 | 3.95 | 3.68 | 1.16 | 0.5542 | 0.769 |
| TC0900006945.hg.1 | TOPORS-AS1; | TOPORS antisense RNA 1; GVQW motif containing 1 | Multiple_C | 6.62 | 6.08 | 6.21 | 1.33 | 0.1052 | 0.2502 | 5.63 | 5.62 | 5.68 | 0.97 | 0.9128 | 0.9642 |
| TC0900009202.hg.1 | GPSM1 | G-protein signaling modulator 1 | Multiple_C | 3.28 | 2.8 | 2.87 | 1.33 | 0.1062 | 0.2518 | 4.19 | 4.09 | 3.76 | 1.35 | 0.1201 | 0.3423 |
| TC0900010618.hg.1 | C9orf153 | chromosome 9 open reading frame 153 | Multiple_C | 4.99 | 4.71 | 4.58 | 1.33 | 0.0554 | 0.156 | 4.63 | 4.55 | 4.34 | 1.22 | 0.2874 | 0.5523 |
| TC0900011033.hg.1 | ALDOB | aldolase B, fructose-bisphosphate | Multiple_C | 3.71 | 3.38 | 3.3 | 1.33 | 0.2417 | 0.444 | 3.44 | 3.49 | 3.9 | 0.73 | 0.0241 | 0.1343 |
| TC0X00006977.hg.1 | MID1IP1 | MID1 interacting protein 1 | Coding | 9.27 | 9.22 | 8.86 | 1.33 | 0.3051 | 0.5182 | 8.1 | 8.47 | 8.94 | 0.56 | 0.0253 | 0.1386 |
| TC0X00007651.hg.1 | ZCCHC13 | zinc finger, CCHC domain containing 13 | Coding | 4.15 | 3.87 | 3.74 | 1.33 | 0.4102 | 0.6225 | 4.14 | 4.08 | 4.21 | 0.95 | 0.7965 | 0.9092 |
| TC0X00007851.hg.1 | FAM133A | family with sequence similarity 133, member A | Coding | 3.64 | 3.31 | 3.23 | 1.33 | 0.7892 | 0.8922 | 3.43 | 3.39 | 3.42 | 1.01 | 0.408 | 0.6637 |
| TC0X00008192.hg.1 | RBMXL3 | RNA binding motif protein, X-linked-like 3 | Coding | 3.87 | 3.49 | 3.46 | 1.33 | 0.3645 | 0.578 | 4.61 | 4.40 | 3.83 | 1.72 | 0.0035 | 0.0397 |
| TC0X00008727.hg.1 | GPR50 | G protein-coupled receptor 50 | Coding | 2.95 | 2.45 | 2.54 | 1.33 | 0.5215 | 0.7149 | 3.73 | 3.51 | 3.23 | 1.41 | 0.0912 | 0.2922 |
| TC0X00010675.hg.1 | CUL4B | cullin 4B | Multiple_C | 11.69 | 12.48 | 11.28 | 1.33 | 0.1641 | 0.3424 | 11.67 | 12.40 | 12.28 | 0.66 | 0.134 | 0.3636 |
| TC0X00011185.hg.1 | MECP2 | methyl-CpG binding protein 2 | Multiple_C | 6.77 | 6.34 | 6.36 | 1.33 | 0.093 | 0.2284 | 6.35 | 6.23 | 7.12 | 0.59 | 0.0058 | 0.054 |
| TC0Y00006780.hg.1 | DAZ2; DAZ3 | deleted in azoospermia 2; deleted in azoospermia 3 | Coding | 3.39 | 2.81 | 2.98 | 1.33 | 0.0075 | 0.0333 | 3.53 | 3.48 | 3.41 | 1.09 | 0.2412 | 0.5051 |

| | | | | | | | | | | | | | | | |
|---|---|---|---|---|---|---|---|---|---|---|---|---|---|---|---|
| TC1000006754.hg.1 | CELF2 | CUGBP, Elav-like family member 2 | Multiple_C | 7.54 | 6.36 | 7.13 | 1.33 | 0.3067 | 0.5198 | 4.33 | 4.57 | 4.61 | 0.82 | 0.2219 | 0.4822 |
| TC1000008377.hg.1 | LIPJ | lipase, family member J | Multiple_C | 3.3 | 2.79 | 2.89 | 1.33 | 0.5679 | 0.7484 | 4.1 | 4.02 | 4.12 | 0.99 | 0.7183 | 0.8686 |
| TC1000009408.hg.1 | C10orf91 | chromosome 10 open reading frame 91 | Multiple_C | 4.45 | 3.92 | 4.04 | 1.33 | 0.5621 | 0.7441 | 5.45 | 5.16 | 5.23 | 1.16 | 0.4657 | 0.7074 |
| TC1000010956.hg.1 | C10orf54 | chromosome 10 open reading frame 54 | Multiple_C | 5.91 | 5.76 | 5.5 | 1.33 | 0.057 | 0.1594 | 5.34 | 4.81 | 4.99 | 1.27 | 0.0554 | 0.2211 |
| TC1000012600.hg.1 | FAM24B | family with sequence similarity 24, member B | Multiple_C | 7.54 | 7.5 | 7.13 | 1.33 | 0.1538 | 0.3276 | 7.59 | 6.59 | 7.04 | 1.46 | 0.0055 | 0.0524 |
| TC1100007729.hg.1 | DTX4 | deltex 4, E3 ubiquitin ligase | Multiple_C | 8.66 | 8.03 | 8.25 | 1.33 | 0.2404 | 0.4424 | 4.22 | 4.22 | 3.91 | 1.24 | 0.1405 | 0.3726 |
| TC1100007912.hg.1 | C11orf84 | chromosome 11 open reading frame 84 | Multiple_C | 7.77 | 6.92 | 7.36 | 1.33 | 0.3122 | 0.5256 | 8.64 | 9.29 | 10.12 | 0.36 | 0.0003 | 0.0068 |
| TC1100009075.hg.1 | NCAM1 | Transcript Identified by AceView, Entrez Gene ID(s) 4684 | Coding | 3.53 | 3.12 | 3.12 | 1.33 | 0.509 | 0.7051 | 3.71 | 3.55 | 3.52 | 1.14 | 0.3635 | 0.6263 |
| TC1100011052.hg.1 | FADS3 | fatty acid desaturase 3 | Multiple_C | 9.01 | 8.35 | 8.6 | 1.33 | 0.2205 | 0.4179 | 8.98 | 8.67 | 9.2 | 0.86 | 0.5218 | 0.7485 |
| TC1100011343.hg.1 | C11orf72 | chromosome 11 open reading frame 72 | Multiple_C | 3.83 | 3.18 | 3.42 | 1.33 | 0.1528 | 0.3262 | 3.55 | 3.60 | 3.53 | 1.01 | 0.4027 | 0.6588 |
| TC1100012610.hg.1 | BSX | brain-specific homeobox | Multiple_C | 4.92 | 4.35 | 4.51 | 1.33 | 0.487 | 0.6882 | 4.39 | 4.87 | 4.82 | 0.74 | 0.047 | 0.2007 |
| TC1200007809.hg.1 | GDF11 | growth differentiation factor 11 | Coding | 4.78 | 4.36 | 4.37 | 1.33 | 0.1567 | 0.332 | 6.13 | 6.94 | 6.62 | 0.71 | 0.0115 | 0.0849 |
| TC1200008675.hg.1 | TXNRD1 | thioredoxin reductase 1 | Multiple_C | 14.57 | 14.13 | 14.16 | 1.33 | 0.1168 | 0.2695 | 11.85 | 10.73 | 12.4 | 0.68 | 0.0332 | 0.1639 |
| TC1200008803.hg.1 | MVK | mevalonate kinase | Multiple_C | 9.66 | 9.96 | 9.25 | 1.33 | 0.2574 | 0.4636 | 6.7 | 6.83 | 7.28 | 0.67 | 0.0861 | 0.2829 |
| TC1200008918.hg.1 | RPH3A | rabphilin 3A | Multiple_C | 3.98 | 3.38 | 3.57 | 1.33 | 0.0312 | 0.1013 | 4.04 | 4.14 | 4.04 | 1.00 | 0.6734 | 0.8416 |
| TC1200009748.hg.1 | ZNF384 | zinc finger protein 384 | Multiple_C | 8 | 6.39 | 7.59 | 1.33 | 0.6124 | 0.7825 | 9.69 | 10.36 | 10.67 | 0.51 | 0.0137 | 0.0948 |
| TC1200011207.hg.1 | CPM | carboxypeptidase M | Multiple_C | 3.94 | 3.27 | 3.53 | 1.33 | 0.6237 | 0.7895 | 3.69 | 3.44 | 3.88 | 0.88 | 0.3596 | 0.6224 |
| TC1200012158.hg.1 | PLA2G1B | phospholipase A2, group IB (pancreas) | Coding | 3.87 | 3.25 | 3.46 | 1.33 | 0.8442 | 0.9232 | 4.17 | 4.34 | 4.22 | 0.97 | 0.3339 | 0.5981 |
| TC1200012805.hg.1 | TAC3 | tachykinin 3 | Multiple_C | 3.67 | 3.15 | 3.26 | 1.33 | 0.3963 | 0.6099 | 3.59 | 3.70 | 3.52 | 1.05 | 0.7185 | 0.8687 |
| TC1300009678.hg.1 | SLC10A2 | solute carrier family 10 (sodium/bile acid cotransporter), memb | Coding | 5.32 | 4.97 | 4.91 | 1.33 | 0.4376 | 0.647 | 4.84 | 4.92 | 4.91 | 0.95 | 0.2034 | 0.459 |
| TC1400006970.hg.1 | PNN | pinin, desmosome associated protein | Multiple_C | 13.15 | 13.59 | 12.74 | 1.33 | 0.1391 | 0.3054 | 13.45 | 12.90 | 13.21 | 1.18 | 0.2594 | 0.5236 |
| TC1400007232.hg.1 | FBXO34 | F-box protein 34 | Multiple_C | 10.17 | 10.13 | 9.76 | 1.33 | 0.2224 | 0.4202 | 8.51 | 8.34 | 8.34 | 1.13 | 0.2862 | 0.5512 |
| TC1400007404.hg.1 | RHOJ | ras homolog family member J | Multiple_C | 4.47 | 3.87 | 4.06 | 1.33 | 0.1165 | 0.2691 | 5.68 | 5.09 | 4.77 | 1.88 | 0.0078 | 0.0661 |
| TC1400008343.hg.1 | WDR20 | WD repeat domain 20 | Multiple_C | 9.11 | 8.53 | 8.7 | 1.33 | 0.1198 | 0.2745 | 7.12 | 6.76 | 7.62 | 0.71 | 0.025 | 0.1375 |
| TC1400010045.hg.1 | SERPINA1 | serpin peptidase inhibitor, clade A (alpha-1 antiproteinase, antit | Multiple_C | 4.58 | 3.85 | 4.17 | 1.33 | 0.4436 | 0.6521 | 8.07 | 8.39 | 7.02 | 2.07 | 0.237 | 0.5003 |
| TC1400010715.hg.1 | AJUBA | ajuba LIM protein | Multiple_C | 8.26 | 8.3 | 7.85 | 1.33 | 0.2503 | 0.4547 | 7.93 | 8.21 | 8.24 | 0.81 | 0.0686 | 0.2494 |
| TC1600006658.hg.1 | IL32 | interleukin 32 | Multiple_C | 6.6 | 6.22 | 6.19 | 1.33 | 0.0898 | 0.2224 | 5.96 | 5.37 | 5.33 | 1.55 | 0.0028 | 0.0338 |
| TC1600007537.hg.1 | TGFB1I1 | transforming growth factor beta 1 induced transcript 1 | Multiple_C | 8.41 | 7.98 | 8 | 1.33 | 0.2621 | 0.4692 | 6.42 | 7.00 | 6.73 | 0.81 | 0.2491 | 0.5137 |
| TC1600007887.hg.1 | RBL2 | retinoblastoma-like 2 | Multiple_C | 9.92 | 10.23 | 9.51 | 1.33 | 0.1528 | 0.3262 | 9.8 | 9.86 | 9.32 | 1.39 | 0.0233 | 0.1319 |
| TC1600007936.hg.1 | SLC6A2 | solute carrier family 6 (neurotransmitter transporter), member | Multiple_C | 5.3 | 4.95 | 4.89 | 1.33 | 0.0926 | 0.2277 | 4.44 | 4.59 | 4.37 | 1.05 | 0.6352 | 0.8209 |
| TC1600010636.hg.1 | AGRP | agouti related neuropeptide | Coding | 4.23 | 3.64 | 3.82 | 1.33 | 0.0405 | 0.1238 | 4.45 | 4.43 | 4.29 | 1.12 | 0.8784 | 0.9479 |
| TC1600010734.hg.1 | NOB1 | NIN1/RPN12 binding protein 1 homolog | Multiple_C | 13.7 | 14.44 | 13.29 | 1.33 | 0.4605 | 0.6663 | 14.66 | 14.67 | 14.72 | 0.96 | 0.8308 | 0.9247 |
| TC1700009498.hg.1 | ATP2A3 | ATPase, Ca++ transporting, ubiquitous | Multiple_C | 6.6 | 5.88 | 6.19 | 1.33 | 0.4688 | 0.6728 | 6.24 | 6.02 | 5.8 | 1.36 | 0.1283 | 0.3545 |

| | | | | | | | | | | | | | | | |
|---|---|---|---|---|---|---|---|---|---|---|---|---|---|---|---|
| TC1700010960.hg.1 | MRPL10 | mitochondrial ribosomal protein L10 | Multiple_C | 10.28 | 10.69 | 9.87 | 1.33 | 0.1144 | 0.2656 | 9.5 | 9.80 | 9.36 | 1.10 | 0.4736 | 0.7135 |
| TC1700011917.hg.1 | USP36 | ubiquitin specific peptidase 36 | Multiple_C | 10.01 | 9.02 | 9.6 | 1.33 | 0.1189 | 0.2731 | 11.58 | 11.34 | 11.84 | 0.84 | 0.199 | 0.4542 |
| TC1700012015.hg.1 | AATK | apoptosis-associated tyrosine kinase | Multiple_C | 6.69 | 6.08 | 6.28 | 1.33 | 0.2516 | 0.4561 | 5.26 | 5.42 | 5.52 | 0.84 | 0.0916 | 0.2931 |
| TC1700012235.hg.1 | UNC45B | unc-45 myosin chaperone B | Coding | 3.21 | 2.76 | 2.8 | 1.33 | 0.1404 | 0.3077 | 3.48 | 3.65 | 3.31 | 1.13 | 0.807 | 0.9148 |
| TC1900007743.hg.1 | ZNF507 | zinc finger protein 507 | Multiple_C | 10.36 | 10.11 | 9.95 | 1.33 | 0.0539 | 0.1528 | 10.8 | 10.70 | 10.93 | 0.91 | 0.219 | 0.4789 |
| TC1900008011.hg.1 | KCNK6 | potassium channel, two pore domain subfamily K, member 6 | Multiple_C | 8.72 | 8.14 | 8.31 | 1.33 | 0.3618 | 0.5754 | 6.86 | 6.94 | 7.37 | 0.70 | 0.1137 | 0.3325 |
| TC1900008932.hg.1 | RFPL4AL1 | ret finger protein-like 4A-like 1 | Coding | 3.82 | 3.25 | 3.41 | 1.33 | 0.1535 | 0.3271 | 4.27 | 3.21 | 4.56 | 0.82 | 0.3195 | 0.5836 |
| TC1900008937.hg.1 | NLRP5 | NLR family, pyrin domain containing 5 | Coding | 3.37 | 2.86 | 2.96 | 1.33 | 0.0148 | 0.0564 | 3.76 | 3.72 | 3.93 | 0.89 | 0.6174 | 0.8101 |
| TC1900008969.hg.1 | SMIM17 | small integral membrane protein 17 | Multiple_C | 5.44 | 4.97 | 5.03 | 1.33 | 0.0943 | 0.2308 | 5.85 | 5.59 | 5.39 | 1.38 | 0.0892 | 0.2891 |
| TC1900010172.hg.1 | ZNF676 | zinc finger protein 676 | Coding | 5.72 | 5.34 | 5.31 | 1.33 | 0.5901 | 0.7654 | 5.82 | 6.05 | 5.94 | 0.92 | 0.7965 | 0.9092 |
| TC1900010745.hg.1 | B9D2 | B9 protein domain 2 | Multiple_C | 6.44 | 5.96 | 6.03 | 1.33 | 0.5211 | 0.7145 | 5.84 | 6.23 | 6.28 | 0.74 | 0.1232 | 0.3466 |
| TC2000006436.hg.1 | DEFB129 | defensin, beta 129 | Multiple_C | 4.05 | 3.65 | 3.64 | 1.33 | 0.1324 | 0.2952 | 4.24 | 4.18 | 4.07 | 1.13 | 0.8149 | 0.9181 |
| TC2000007992.hg.1 | SS18L1 | synovial sarcoma translocation gene on chromosome 18-like 1 | Multiple_C | 8.24 | 7.78 | 7.83 | 1.33 | 0.4016 | 0.6147 | 8.69 | 8.98 | 8.58 | 1.08 | 0.8233 | 0.9219 |
| TC2000008200.hg.1 | PCED1A | PC-esterase domain containing 1A | Multiple_C | 7.58 | 7.66 | 7.17 | 1.33 | 0.2167 | 0.4126 | 11.91 | 12.04 | 10.9 | 2.01 | 0.0003 | 0.0066 |
| TC2100006991.hg.1 | KCNE2 | potassium channel, voltage gated subfamily E regulatory beta su | Coding | 3.09 | 2.6 | 2.68 | 1.33 | 0.145 | 0.3148 | 3.31 | 3.43 | 3.41 | 0.93 | 0.892 | 0.9539 |
| TC2100008285.hg.1 | CBS | cystathionine-beta-synthase | Multiple_C | 5.14 | 4.33 | 4.73 | 1.33 | 0.2366 | 0.4379 | 9.98 | 9.32 | 8.65 | 2.51 | 3.64E-06 | 0.0003 |
| TC2200006878.hg.1 | GSTT2 | glutathione S-transferase theta 2 (gene/pseudogene) | Multiple_C | 6.23 | 5.36 | 5.82 | 1.33 | 0.1025 | 0.2452 | 4.09 | 4.08 | 3.91 | 1.13 | 0.253 | 0.5172 |
| TC2200007439.hg.1 | MCHR1 | melanin-concentrating hormone receptor 1 | Multiple_C | 4.37 | 3.91 | 3.96 | 1.33 | 0.4162 | 0.628 | 4.31 | 4.32 | 4.36 | 0.97 | 0.3274 | 0.591 |
| TC2200008203.hg.1 | DDT | D-dopachrome tautomerase | Multiple_C | 13.04 | 12.13 | 12.63 | 1.33 | 0.2246 | 0.4232 | 8.26 | 8.98 | 8.63 | 0.77 | 0.4408 | 0.6903 |
| TC2200008682.hg.1 | PLA2G6 | phospholipase A2, group VI (cytosolic, calcium-independent) | Multiple_C | 6 | 5.56 | 5.59 | 1.33 | 0.2973 | 0.5087 | 5.55 | 5.72 | 5.5 | 1.04 | 0.4385 | 0.6889 |
| TC2200008995.hg.1 | WNT7B | wingless-type MMTV integration site family, member 7B | Multiple_C | 4.31 | 3.69 | 3.9 | 1.33 | 0.1021 | 0.2448 | 4.68 | 4.55 | 4.56 | 1.09 | 0.1012 | 0.311 |
| TSUnmapped00000394.h | ATG16L1 | autophagy related 16-like 1 | Coding | 8.16 | 7.72 | 7.75 | 1.33 | 0.2474 | 0.451 | 6.41 | 5.58 | 5.89 | 1.43 | 0.0552 | 0.2207 |
| TSUnmapped00000455.h | NDUFA10 | NADH dehydrogenase (ubiquinone) 1 alpha subcomplex, 10, 42k | Coding | 3.31 | 3.08 | 2.9 | 1.33 | 0.5248 | 0.7171 | 3.97 | 3.78 | 3.72 | 1.19 | 0.1485 | 0.3854 |
| TSUnmapped00000726.h | ZDHHC3 | zinc finger, DHHC-type containing 3 | Coding | 12.72 | 11.95 | 12.31 | 1.33 | 0.4138 | 0.6258 | 9.56 | 9.79 | 10.58 | 0.49 | 0.0007 | 0.0131 |
| TSUnmapped00000741.h | ZNF546 | zinc finger protein 546 | Coding | 4.08 | 3.86 | 3.67 | 1.33 | 0.0868 | 0.2169 | 5.21 | 5.09 | 4.82 | 1.31 | 0.4942 | 0.7282 |
| TC0100010335.hg.1 | USP21 | ubiquitin specific peptidase 21 | Multiple_C | 10.45 | 9.9 | 10.05 | 1.32 | 0.0689 | 0.1832 | 8.22 | 8.17 | 8.28 | 0.96 | 0.7991 | 0.9102 |
| TC0100011587.hg.1 | KCNK2 | potassium channel, two pore domain subfamily K, member 2 | Multiple_C | 5.18 | 4.71 | 4.78 | 1.32 | 0.0739 | 0.1927 | 5.55 | 5.86 | 5.6 | 0.97 | 0.9922 | 0.9968 |
| TC0100012991.hg.1 | C1orf195 | chromosome 1 open reading frame 195 | Multiple_C | 3.79 | 3.78 | 3.39 | 1.32 | 0.0854 | 0.2146 | 3.84 | 3.63 | 3.64 | 1.15 | 0.2348 | 0.4973 |
| TC0100015990.hg.1 | PAQR6 | progestin and adipoQ receptor family member VI | Multiple_C | 4.73 | 4.1 | 4.33 | 1.32 | 0.1421 | 0.3099 | 5.56 | 5.61 | 5.32 | 1.18 | 0.4843 | 0.7219 |
| TC0100018309.hg.1 | FCGR2C | Fc fragment of IgG, low affinity IIc, receptor for (CD32) (gene/ps | Multiple_C | 3.53 | 3.14 | 3.13 | 1.32 | 0.1083 | 0.2554 | 3.52 | 3.59 | 3.52 | 1.00 | 0.7038 | 0.86 |
| TC0100018481.hg.1 | NOTCH2NL | notch 2 N-terminal like | Coding | 8.48 | 8.3 | 8.08 | 1.32 | 0.1153 | 0.2674 | 6.94 | 6.00 | 5.69 | 2.38 | 5.02E-06 | 0.0004 |
| TC0100018567.hg.1 | RBM34 | RNA binding motif protein 34 | Multiple_C | 11.17 | 12.06 | 10.77 | 1.32 | 0.4649 | 0.6697 | 10.25 | 10.09 | 10.33 | 0.95 | 0.8198 | 0.9205 |
| TC0200006648.hg.1 | IAH1 | isoamyl acetate-hydrolyzing esterase 1 homolog | Multiple_C | 10.43 | 10.86 | 10.03 | 1.32 | 0.1132 | 0.2636 | 9.23 | 9.53 | 8.91 | 1.25 | 0.0628 | 0.2386 |

| | | | | | | | | | | | | | | | |
|---|---|---|---|---|---|---|---|---|---|---|---|---|---|---|---|
| TC0200007535.hg.1 | PPP1R21 | protein phosphatase 1, regulatory subunit 21 | Multiple_C | 6.86 | 7.56 | 6.46 | 1.32 | 0.0926 | 0.2278 | 6.28 | 6.53 | 6.48 | 0.87 | 0.2716 | 0.5369 |
| TC0200008742.hg.1 | NCK2 | NCK adaptor protein 2 | Multiple_C | 9.8 | 8.86 | 9.4 | 1.32 | 0.0517 | 0.1479 | 7.42 | 7.81 | 8.32 | 0.54 | 0.0118 | 0.0862 |
| TC0200012098.hg.1 | RBKS; BRE-AS: | ribokinase; BRE antisense RNA 1 | Multiple_C | 5.49 | 5.61 | 5.09 | 1.32 | 0.273 | 0.4817 | 4.47 | 4.66 | 3.96 | 1.42 | 0.0976 | 0.3047 |
| TC0200014775.hg.1 | KCNH7 | potassium channel, voltage gated eag related subfamily H, mem | Multiple_C | 4.12 | 3.74 | 3.72 | 1.32 | 0.513 | 0.7086 | 3.6 | 4.06 | 3.81 | 0.86 | 0.2909 | 0.5557 |
| TC0200014978.hg.1 | OLA1 | Obg-like ATPase 1 | Multiple_C | 14.48 | 14.83 | 14.08 | 1.32 | 0.355 | 0.5681 | 13.74 | 13.02 | 13.6 | 1.10 | 0.6075 | 0.8037 |
| TC0200015427.hg.1 | TRAK2 | Jeck2013 ALT_ACCEPTOR, ALT_DONOR, coding, INTERNAL, intrc | Multiple_C | 6.6 | 6.39 | 6.2 | 1.32 | 0.0833 | 0.2107 | 5.84 | 5.28 | 5.87 | 0.98 | 0.9175 | 0.9663 |
| TC0300007074.hg.1 | SLC22A14 | solute carrier family 22, member 14 | Multiple_C | 4.28 | 3.76 | 3.88 | 1.32 | 0.0388 | 0.1199 | 3.56 | 3.53 | 3.48 | 1.06 | 0.5836 | 0.7879 |
| TC0300010066.hg.1 | FYTTD1 | forty-two-three domain containing 1 | Multiple_C | 11.96 | 12.53 | 11.56 | 1.32 | 0.1739 | 0.3559 | 12.64 | 12.01 | 12.14 | 1.41 | 0.0044 | 0.0454 |
| TC0300010239.hg.1 | GHRL | ghrelin/obestatin prepropeptide | Multiple_C | 4.55 | 3.84 | 4.15 | 1.32 | 0.0621 | 0.1696 | 4.19 | 4.17 | 3.93 | 1.20 | 0.2475 | 0.512 |
| TC0300012897.hg.1 | SSR3 | signal sequence receptor, gamma (translocon-associated protei | Multiple_C | 15.06 | 15.12 | 14.66 | 1.32 | 0.0361 | 0.1137 | 14.27 | 13.78 | 14.63 | 0.78 | 0.0639 | 0.2404 |
| TC0300013977.hg.1 | C3orf62 | chromosome 3 open reading frame 62 | Multiple_C | 6.86 | 6.83 | 6.46 | 1.32 | 0.0626 | 0.1708 | 5.99 | 6.32 | 5.8 | 1.14 | 0.2813 | 0.5472 |
| TC0400008183.hg.1 | BMPR1B | bone morphogenetic protein receptor type IB | Multiple_C | 4.95 | 4.7 | 4.55 | 1.32 | 0.0743 | 0.1934 | 6.01 | 5.76 | 4.48 | 2.89 | 6.85E-06 | 0.0005 |
| TC0400011220.hg.1 | WDFY3 | WD repeat and FYVE domain containing 3 | Multiple_C | 7.51 | 7.24 | 7.11 | 1.32 | 0.1569 | 0.3323 | 4.68 | 5.05 | 4.89 | 0.86 | 0.3975 | 0.6552 |
| TC0400011486.hg.1 | CXXC4 | CXXC finger protein 4 | Multiple_C | 3.69 | 3.64 | 3.29 | 1.32 | 0.0716 | 0.1885 | 3.26 | 3.43 | 3.28 | 0.99 | 0.5384 | 0.759 |
| TC0400011538.hg.1 | PAPSS1 | 3-phosphoadenosine 5-phosphosulfate synthase 1 | Multiple_C | 11.4 | 11.79 | 11 | 1.32 | 0.329 | 0.5428 | 9.22 | 8.58 | 8.65 | 1.48 | 0.1366 | 0.3674 |
| TC0400012143.hg.1 | FBXW7 | F-box and WD repeat domain containing 7, E3 ubiquitin protein | Multiple_C | 7.56 | 7.59 | 7.16 | 1.32 | 0.1164 | 0.269 | 7.3 | 6.88 | 7.21 | 1.06 | 0.6839 | 0.8485 |
| TC0500009020.hg.1 | STK32A | serine/threonine kinase 32A | Multiple_C | 3.81 | 3.28 | 3.41 | 1.32 | 0.1534 | 0.3271 | 3.7 | 3.53 | 3.5 | 1.15 | 0.5023 | 0.7345 |
| TC0500010456.hg.1 | SLC45A2 | solute carrier family 45, member 2 | Multiple_C | 3.45 | 2.79 | 3.05 | 1.32 | 0.1253 | 0.2832 | 3.7 | 3.88 | 3.81 | 0.93 | 0.367 | 0.6294 |
| TC0500010615.hg.1 | SEPP1 | selenoprotein P, plasma, 1 | Multiple_C | 3.94 | 3.39 | 3.54 | 1.32 | 0.4608 | 0.6664 | 6.49 | 5.85 | 4.88 | 3.05 | 0.0004 | 0.0089 |
| TC0500011240.hg.1 | HOMER1 | homer scaffolding protein 1 | Multiple_C | 10.66 | 10.39 | 10.26 | 1.32 | 0.6493 | 0.8057 | 10.55 | 9.87 | 9.88 | 1.59 | 0.0349 | 0.1688 |
| TC0500013349.hg.1 | FBN2 | fibrillin 2 | Multiple_C | 4.5 | 4.21 | 4.1 | 1.32 | 0.0763 | 0.1975 | 4.94 | 4.85 | 4.88 | 1.04 | 0.5546 | 0.7692 |
| TC0600013196.hg.1 | TAAR5 | trace amine associated receptor 5 | Coding | 3.64 | 3.27 | 3.24 | 1.32 | 0.1818 | 0.3664 | 3.59 | 3.53 | 3.52 | 1.05 | 0.1888 | 0.4413 |
| TC0600013300.hg.1 | IFNGR1 | interferon gamma receptor 1 | Multiple_C | 10.45 | 10.41 | 10.05 | 1.32 | 0.137 | 0.3021 | 8.15 | 8.37 | 8.37 | 0.86 | 0.8958 | 0.956 |
| TC0700010504.hg.1 | OSBPL3 | oxysterol binding protein-like 3 | Multiple_C | 9.52 | 9.51 | 9.12 | 1.32 | 0.6165 | 0.7851 | 10.56 | 10.39 | 10.63 | 0.95 | 0.7215 | 0.8701 |
| TC0700012013.hg.1 | GIGYF1 | GRB10 interacting GYF protein 1 | Multiple_C | 4.29 | 4 | 3.89 | 1.32 | 0.288 | 0.4987 | 4.69 | 4.97 | 5.07 | 0.77 | 0.0826 | 0.2768 |
| TC0700013061.hg.1 | GBX1 | gastrulation brain homeobox 1 | Multiple_C | 3.48 | 3.1 | 3.08 | 1.32 | 0.3761 | 0.5889 | 3.85 | 4.09 | 3.95 | 0.93 | 0.2002 | 0.4557 |
| TC0800006887.hg.1 | NAT1 | N-acetyltransferase 1 (arylamine N-acetyltransferase) | Multiple_C | 8.46 | 8.82 | 8.06 | 1.32 | 0.4511 | 0.6586 | 7.14 | 6.90 | 6.91 | 1.17 | 0.2548 | 0.5192 |
| TC0900008173.hg.1 | ANP32B | acidic nuclear phosphoprotein 32 family member B | Multiple_C | 14.69 | 14.74 | 14.29 | 1.32 | 0.574 | 0.7531 | 13.58 | 13.76 | 13.88 | 0.81 | 0.4053 | 0.6606 |
| TC0900008741.hg.1 | WDR38 | WD repeat domain 38 | Coding | 4.71 | 4.25 | 4.31 | 1.32 | 0.1651 | 0.3435 | 4.9 | 4.96 | 4.95 | 0.97 | 0.6282 | 0.8165 |
| TC0900010907.hg.1 | HSD17B3 | hydroxysteroid (17-beta) dehydrogenase 3 | Multiple_C | 4.06 | 3.44 | 3.66 | 1.32 | 0.0763 | 0.1975 | 4.11 | 4.15 | 4.25 | 0.91 | 0.8204 | 0.9207 |
| TC0900011396.hg.1 | RAB14 | RAB14, member RAS oncogene family | Coding | 12.21 | 12.19 | 11.81 | 1.32 | 0.0641 | 0.1739 | 11.05 | 10.60 | 11.19 | 0.91 | 0.0682 | 0.2488 |
| TC0900012184.hg.1 | PRRC2B | proline-rich coiled-coil 2B | Multiple_C | 8.5 | 7.95 | 8.1 | 1.32 | 0.7554 | 0.8745 | 9.18 | 9.42 | 9.57 | 0.76 | 0.1001 | 0.3094 |
| TC0X00008144.hg.1 | TRPC5OS | TRPC5 opposite strand | Multiple_C | 4.11 | 3.61 | 3.71 | 1.32 | 0.2743 | 0.483 | 3.41 | 3.24 | 3.46 | 0.97 | 0.4226 | 0.6753 |

| | | | | | | | | | | | | | | | |
|---|---|---|---|---|---|---|---|---|---|---|---|---|---|---|---|
| TC0X00008785.hg.1 | BGN | biglycan | Multiple_C | 3.64 | 3.01 | 3.24 | 1.32 | 0.3808 | 0.5937 | 5.26 | 4.62 | 4.08 | 2.27 | 0.0009 | 0.0155 |
| TC0X00010406.hg.1 | TCEAL8 | transcription elongation factor A (SII)-like 8 | Multiple_C | 4.16 | 3.47 | 3.76 | 1.32 | 0.5793 | 0.757 | 6.29 | 6.81 | 6.5 | 0.86 | 0.7056 | 0.8614 |
| TC0X00011181.hg.1 | HCFC1 | host cell factor C1 | Multiple_C | 9.86 | 8.62 | 9.46 | 1.32 | 0.4026 | 0.6154 | 10.65 | 11.76 | 12.4 | 0.30 | 1.06E-06 | 0.0002 |
| TC0X00011315.hg.1 | TCEAL3 | transcription elongation factor A (SII)-like 3 | Coding | 6.8 | 7.3 | 6.4 | 1.32 | 0.4992 | 0.6986 | 4.56 | 4.51 | 4.35 | 1.16 | 0.1934 | 0.4469 |
| TC1000007518.hg.1 | SYT15 | synaptotagmin XV | Multiple_C | 4.32 | 3.8 | 3.92 | 1.32 | 0.1316 | 0.2939 | 3.96 | 4.10 | 4.05 | 0.94 | 0.8311 | 0.9247 |
| TC1000008489.hg.1 | LGI1 | leucine-rich, glioma inactivated 1 | Multiple_C | 4.04 | 3.53 | 3.64 | 1.32 | 0.2247 | 0.4234 | 4.33 | 4.43 | 4.4 | 0.95 | 0.2179 | 0.4775 |
| TC1000008556.hg.1 | LCOR | ligand dependent nuclear receptor corepressor | Multiple_C | 11.87 | 10.72 | 11.47 | 1.32 | 0.528 | 0.7195 | 10.86 | 10.15 | 11.35 | 0.71 | 0.0457 | 0.1978 |
| TC1000009666.hg.1 | CALML5 | calmodulin-like 5 | Coding | 7.35 | 6.86 | 6.95 | 1.32 | 0.2001 | 0.3918 | 6.44 | 6.64 | 6.54 | 0.93 | 0.3841 | 0.643 |
| TC1000010073.hg.1 | GPR158-AS1 | GPR158 antisense RNA 1 | Multiple_C | 3.62 | 3.05 | 3.22 | 1.32 | 0.1008 | 0.2423 | 3.54 | 3.51 | 3.16 | 1.30 | 0.266 | 0.5305 |
| TC1100006977.hg.1 | KCNC1 | potassium channel, voltage gated Shaw related subfamily C, me | Multiple_C | 3.77 | 2.83 | 3.37 | 1.32 | 0.0681 | 0.1819 | 3.49 | 3.82 | 3.55 | 0.96 | 0.7561 | 0.889 |
| TC1100007632.hg.1 | OR8K3 | olfactory receptor, family 8, subfamily K, member 3 (gene/pseu | Multiple_C | 3.1 | 2.57 | 2.7 | 1.32 | 0.2253 | 0.4241 | 3.16 | 3.13 | 3.04 | 1.09 | 0.1233 | 0.3467 |
| TC1100008370.hg.1 | ATG16L2 | autophagy related 16-like 2 | Multiple_C | 5.95 | 5.42 | 5.55 | 1.32 | 0.2067 | 0.4 | 4.8 | 4.69 | 4.68 | 1.09 | 0.4219 | 0.6748 |
| TC1100008713.hg.1 | TRIM77 | tripartite motif containing 77 | Coding | 4.29 | 3.6 | 3.89 | 1.32 | 0.4018 | 0.6147 | 4.64 | 4.29 | 4.29 | 1.27 | 0.1221 | 0.345 |
| TC1100009954.hg.1 | OR56A1 | olfactory receptor, family 56, subfamily A, member 1 | Coding | 4.49 | 3.47 | 4.09 | 1.32 | 0.1184 | 0.2721 | 3.54 | 3.54 | 4.04 | 0.71 | 0.6478 | 0.8278 |
| TC1100011833.hg.1 | SYTL2 | synaptotagmin-like 2 | Multiple_C | 8.89 | 9.31 | 8.49 | 1.32 | 0.4348 | 0.6447 | 6.78 | 6.48 | 6.44 | 1.27 | 0.1587 | 0.4002 |
| TC1100012648.hg.1 | OR10S1 | olfactory receptor, family 10, subfamily S, member 1 | Coding | 3.68 | 3.12 | 3.28 | 1.32 | 0.2994 | 0.5111 | 4.05 | 3.89 | 4.47 | 0.75 | 0.696 | 0.8557 |
| TC1100012793.hg.1 | TP53AIP1 | tumor protein p53 regulated apoptosis inducing protein 1 | Multiple_C | 5.01 | 4.39 | 4.61 | 1.32 | 0.112 | 0.2618 | 3.9 | 4.08 | 3.91 | 0.99 | 0.9184 | 0.9665 |
| TC1100013017.hg.1 | PGA5 | pepsinogen 5, group I (pepsinogen A) | Multiple_C | 4.95 | 4.64 | 4.55 | 1.32 | 0.5536 | 0.7374 | 5.14 | 4.61 | 4.61 | 1.44 | 0.4526 | 0.6977 |
| TC1100013038.hg.1 | SNX15 | sorting nexin 15 | Multiple_C | 7.69 | 7.22 | 7.29 | 1.32 | 0.1443 | 0.3138 | 6.87 | 7.20 | 7.09 | 0.86 | 0.1184 | 0.3401 |
| TC1200008650.hg.1 | ASCL1 | achaete-scute family bHLH transcription factor 1 | Multiple_C | 3.22 | 2.54 | 2.82 | 1.32 | 0.1333 | 0.2967 | 3.19 | 3.09 | 3.2 | 0.99 | 0.7565 | 0.8894 |
| TC1200009814.hg.1 | FAM90A1 | family with sequence similarity 90, member A1 | Coding | 3.52 | 3.18 | 3.12 | 1.32 | 0.111 | 0.2599 | 3.49 | 3.37 | 3.43 | 1.04 | 0.559 | 0.7714 |
| TC1200011420.hg.1 | SLC6A15 | solute carrier family 6 (neutral amino acid transporter), membe | Multiple_C | 5.39 | 4.86 | 4.99 | 1.32 | 0.3149 | 0.5283 | 4.6 | 4.19 | 4.23 | 1.29 | 0.1277 | 0.3536 |
| TC1200012719.hg.1 | WDR66 | WD repeat domain 66 | Multiple_C | 4.72 | 4.97 | 4.32 | 1.32 | 0.3645 | 0.578 | 5.91 | 5.56 | 5.34 | 1.48 | 0.0547 | 0.2195 |
| TC1300009580.hg.1 | SLC15A1 | solute carrier family 15 (oligopeptide transporter), member 1 | Multiple_C | 3.9 | 3.61 | 3.5 | 1.32 | 0.6561 | 0.811 | 4.26 | 4.23 | 4.36 | 0.93 | 0.8301 | 0.9246 |
| TC1300009907.hg.1 | DCUN1D2 | DCN1, defective in cullin neddylation 1, domain containing 2 | Multiple_C | 7.7 | 8.08 | 7.3 | 1.32 | 0.264 | 0.4714 | 9.12 | 8.40 | 8.98 | 1.10 | 0.8392 | 0.9294 |
| TC1500007818.hg.1 | REC114 | REC114 meiotic recombination protein | Coding | 3.34 | 2.8 | 2.94 | 1.32 | 0.0462 | 0.1365 | 3.59 | 3.54 | 3.4 | 1.14 | 0.4538 | 0.6987 |
| TC1600007437.hg.1 | INO80E | INO80 complex subunit E | Multiple_C | 11.58 | 10.73 | 11.18 | 1.32 | 0.3587 | 0.5723 | 10.3 | 11.11 | 11.27 | 0.51 | 0.1205 | 0.3425 |
| TC1600008156.hg.1 | LRRC36 | leucine rich repeat containing 36 | Multiple_C | 4.07 | 4.02 | 3.67 | 1.32 | 0.3476 | 0.5607 | 3.9 | 3.82 | 3.2 | 1.62 | 0.0008 | 0.0147 |
| TC1600008498.hg.1 | VAT1L | vesicle amine transport 1-like | Multiple_C | 3.81 | 3.3 | 3.41 | 1.32 | 0.1581 | 0.334 | 4.22 | 3.94 | 3.63 | 1.51 | 0.013 | 0.0921 |
| TC1600008585.hg.1 | CMIP | c-Maf inducing protein | Multiple_C | 10.14 | 9.86 | 9.74 | 1.32 | 0.1996 | 0.391 | 14.2 | 13.95 | 13.9 | 1.23 | 0.0776 | 0.2683 |
| TC1600009400.hg.1 | PRM1 | protamine 1 | Coding | 3.89 | 3.45 | 3.49 | 1.32 | 0.1601 | 0.3367 | 3.77 | 3.74 | 3.74 | 1.02 | 0.815 | 0.9181 |
| TC1700007014.hg.1 | UBB | ubiquitin B | Multiple_C | 16.42 | 15.88 | 16.02 | 1.32 | 0.1293 | 0.2899 | 15.49 | 15.45 | 15.45 | 1.03 | 0.8411 | 0.9306 |
| TC1700007826.hg.1 | KRTAP9-4 | keratin associated protein 9-4 | Coding | 3.41 | 2.87 | 3.01 | 1.32 | 0.4206 | 0.6319 | 3.62 | 3.17 | 3.18 | 1.36 | 0.2236 | 0.4845 |

| | | | | | | | | | | | | | | | |
|---|---|---|---|---|---|---|---|---|---|---|---|---|---|---|---|
| TC1700009254.hg.1 | HEXDC | hexosaminidase (glycosyl hydrolase family 20, catalytic domain) | Multiple_C | 5.92 | 5.51 | 5.52 | 1.32 | 0.2672 | 0.4753 | 5.63 | 5.82 | 5.59 | 1.03 | 0.6945 | 0.8547 |
| TC1700009877.hg.1 | ZSWIM7 | zinc finger, SWIM-type containing 7 | Multiple_C | 8.48 | 9.16 | 8.08 | 1.32 | 0.1947 | 0.3845 | 7 | 7.02 | 6.47 | 1.44 | 0.307 | 0.5713 |
| TC1700010639.hg.1 | KRT10 | keratin 10, type I | Multiple_C | 3.43 | 2.61 | 3.03 | 1.32 | 0.2067 | 0.4 | 4.28 | 4.15 | 3.58 | 1.62 | 0.0165 | 0.107 |
| TC1700011768.hg.1 | GALK1 | galactokinase 1 | Multiple_C | 8.33 | 8.09 | 7.93 | 1.32 | 0.2665 | 0.4743 | 6.92 | 7.24 | 7.22 | 0.81 | 0.262 | 0.5263 |
| TC1800008121.hg.1 | SPIRE1 | spire-type actin nucleation factor 1 | Multiple_C | 4.82 | 4.38 | 4.42 | 1.32 | 0.1721 | 0.3534 | 7.63 | 7.57 | 7.33 | 1.23 | 0.6142 | 0.8079 |
| TC1800008348.hg.1 | AQP4 | aquaporin 4 | Multiple_C | 4.3 | 3.78 | 3.9 | 1.32 | 0.0217 | 0.0763 | 4.63 | 4.69 | 4.58 | 1.04 | 0.9053 | 0.9607 |
| TC1800008358.hg.1 | CDH2 | cadherin 2, type 1, N-cadherin (neuronal) | Multiple_C | 3.81 | 3.29 | 3.41 | 1.32 | 0.3453 | 0.559 | 4.1 | 4.09 | 4.14 | 0.97 | 0.4908 | 0.726 |
| TC1800008680.hg.1 | CXXC1 | CXXC finger protein 1 | Multiple_C | 9.47 | 8.89 | 9.07 | 1.32 | 0.1212 | 0.2767 | 8.46 | 9.02 | 8.44 | 1.01 | 0.692 | 0.853 |
| TC1900006700.hg.1 | HDGFRP2 | hepatoma-derived growth factor-related protein 2; Hepatoma-( | Multiple_C | 9.56 | 9.03 | 9.16 | 1.32 | 0.0639 | 0.1735 | 8.38 | 8.76 | 8.67 | 0.82 | 0.2069 | 0.4637 |
| TC1900008013.hg.1 | PSMD8 | proteasome 26S subunit, non-ATPase 8 | Multiple_C | 14.22 | 13.57 | 13.82 | 1.32 | 0.0601 | 0.1659 | 13.25 | 13.74 | 13.71 | 0.73 | 0.572 | 0.7798 |
| TC1900010050.hg.1 | SUGP1 | SURP and G-patch domain containing 1 | Multiple_C | 8.55 | 7.93 | 8.15 | 1.32 | 0.1132 | 0.2636 | 8.06 | 8.63 | 8.58 | 0.70 | 0.3233 | 0.5876 |
| TC1900010646.hg.1 | IFNL3 | interferon, lambda 3 | Coding | 4.34 | 3.98 | 3.94 | 1.32 | 0.4057 | 0.6185 | 5.27 | 5.76 | 5.63 | 0.78 | 0.0258 | 0.14 |
| TC1900011237.hg.1 | KLK14 | kallikrein related peptidase 14 | Coding | 4.82 | 4.19 | 4.42 | 1.32 | 0.4028 | 0.6157 | 4.68 | 4.40 | 4.31 | 1.29 | 0.3367 | 0.601 |
| TC1900011868.hg.1 | EPOR | erythropoietin receptor | Multiple_C | 6.68 | 6.07 | 6.28 | 1.32 | 0.1099 | 0.2583 | 5.64 | 5.45 | 5.28 | 1.28 | 0.1264 | 0.3516 |
| TC1900011889.hg.1 | MAN2B1 | mannosidase, alpha, class 2B, member 1 | Multiple_C | 9.52 | 8.86 | 9.12 | 1.32 | 0.0971 | 0.2357 | 8.28 | 8.61 | 8.18 | 1.07 | 0.9577 | 0.9825 |
| TC1900012030.hg.1 | ZNF321P | zinc finger protein 321, pseudogene | Multiple_C | 10 | 9.87 | 9.6 | 1.32 | 0.1145 | 0.2657 | 7.77 | 8.16 | 7.91 | 0.91 | 0.4581 | 0.702 |
| TC2000007197.hg.1 | TP53INP2 | tumor protein p53 inducible nuclear protein 2 | Multiple_C | 5.31 | 4.43 | 4.91 | 1.32 | 0.1686 | 0.3486 | 4.86 | 4.66 | 4.33 | 1.44 | 0.2476 | 0.512 |
| TC2000008690.hg.1 | GGTLC1 | gamma-glutamyltransferase light chain 1 | Coding | 5.06 | 4.54 | 4.66 | 1.32 | 0.2733 | 0.4819 | 4.81 | 4.55 | 4.36 | 1.37 | 0.0225 | 0.1292 |
| TC2000009247.hg.1 | SPATA25 | spermatogenesis associated 25 | Coding | 4.5 | 4.58 | 4.1 | 1.32 | 0.2272 | 0.4264 | 6.16 | 6.73 | 6.36 | 0.87 | 0.4579 | 0.7019 |
| TC2000009755.hg.1 | GATA5 | GATA binding protein 5 | Coding | 4.1 | 3.29 | 3.7 | 1.32 | 0.1105 | 0.2593 | 4.17 | 4.10 | 4.22 | 0.97 | 0.7295 | 0.8744 |
| TC2000009947.hg.1 | STX16-NPEPL1 | STX16-NPEPL1 readthrough (NMD candidate) | Multiple_C | 6.47 | 6.69 | 6.07 | 1.32 | 0.6041 | 0.7758 | 5.73 | 5.69 | 5.49 | 1.18 | 0.868 | 0.9433 |
| TC2000009996.hg.1 | DUSP15 | dual specificity phosphatase 15 | Coding | 3.72 | 3.18 | 3.32 | 1.32 | 0.0566 | 0.1585 | 4.08 | 4.11 | 4.13 | 0.97 | 0.7024 | 0.8591 |
| TC2100008200.hg.1 | PLAC4 | placenta specific 4 | Multiple_C | 3.7 | 3.28 | 3.3 | 1.32 | 0.1165 | 0.2692 | 3.86 | 3.91 | 3.72 | 1.10 | 0.4008 | 0.6575 |
| TC2200008022.hg.1 | CCDC188 | coiled-coil domain containing 188 | Multiple_C | 5.62 | 5.03 | 5.22 | 1.32 | 0.0105 | 0.0435 | 7.48 | 7.54 | 7.48 | 1.00 | 0.9146 | 0.965 |
| TC2200008169.hg.1 | LOC388882; A | uncharacterized LOC388882; novel transcript | Multiple_C | 3.37 | 2.95 | 2.97 | 1.32 | 0.2716 | 0.4802 | 3.19 | 3.20 | 3.26 | 0.95 | 0.231 | 0.4923 |
| TC2200008661.hg.1 | LGALS2 | lectin, galactoside-binding, soluble, 2 | Coding | 6.44 | 5.98 | 6.04 | 1.32 | 0.2918 | 0.5029 | 6.15 | 6.37 | 6.03 | 1.09 | 0.497 | 0.7305 |
| TSUnmapped00000582.h | ZNF780A | zinc finger protein 780A | Coding | 6.21 | 6.09 | 5.81 | 1.32 | 0.2905 | 0.5013 | 6.57 | 6.22 | 5.91 | 1.58 | 0.0305 | 0.1555 |
| TSUnmapped00000591.h | HHAT | hedgehog acyltransferase | Coding | 5.06 | 4.45 | 4.66 | 1.32 | 0.2355 | 0.4366 | 5 | 5.43 | 5.41 | 0.75 | 0.0671 | 0.247 |
| TSUnmapped00000615.h | LRIG1 | | Coding | 10.21 | 9.58 | 9.81 | 1.32 | 0.2258 | 0.4246 | 8.09 | 8.83 | 8.93 | 0.56 | 0.0013 | 0.0204 |
| TC0100007948.hg.1 | TMCO2 | transmembrane and coiled-coil domains 2 | Multiple_C | 3.37 | 2.79 | 2.98 | 1.31 | 0.2366 | 0.438 | 3.8 | 3.95 | 3.8 | 1.00 | 0.9784 | 0.9909 |
| TC0100008815.hg.1 | IFI44L | interferon-induced protein 44-like | Multiple_C | 3.92 | 2.68 | 3.53 | 1.31 | 0.0563 | 0.1578 | 3.7 | 3.66 | 3.65 | 1.04 | 0.5427 | 0.7614 |
| TC0100009418.hg.1 | RAP1A | RAP1A, member of RAS oncogene family | Multiple_C | 12.64 | 13.36 | 12.25 | 1.31 | 0.3992 | 0.6129 | 10.97 | 10.48 | 10.4 | 1.48 | 0.0026 | 0.032 |
| TC0100009656.hg.1 | FCGR1B | Fc fragment of IgG, high affinity Ib, receptor (CD64) | Multiple_C | 3.07 | 2.67 | 2.68 | 1.31 | 0.1142 | 0.2652 | 3.48 | 3.38 | 3.37 | 1.08 | 0.7129 | 0.8655 |

| | | | | | | | | | | | | | | | |
|---|---|---|---|---|---|---|---|---|---|---|---|---|---|---|---|
| TC0100010543.hg.1 | ATP1B1 | ATPase, Na+/K+ transporting, beta 1 polypeptide | Multiple_C | 15.94 | 15.16 | 15.55 | 1.31 | 0.0841 | 0.2122 | 10.03 | 10.07 | 10.77 | 0.60 | 0.0425 | 0.1902 |
| TC0100010559.hg.1 | METTL11B | methyltransferase like 11B | Multiple_C | 4.17 | 3.59 | 3.78 | 1.31 | 0.1782 | 0.3612 | 3.63 | 3.53 | 3.58 | 1.04 | 0.729 | 0.8742 |
| TC0100012941.hg.1 | PRAMEF13 | PRAME family member 13 | Multiple_C | 5.01 | 4.31 | 4.62 | 1.31 | 0.3354 | 0.5494 | 4.81 | 4.90 | 4.6 | 1.16 | 0.1307 | 0.358 |
| TC0100014848.hg.1 | CCBL2; RBMXI | cysteine conjugate-beta lyase 2; RNA binding motif protein, X-lii | Multiple_C | 9.76 | 11.02 | 9.37 | 1.31 | 0.1444 | 0.314 | 6.26 | 6.50 | 5.92 | 1.27 | 0.135 | 0.3648 |
| TC0100015771.hg.1 | SEMA6C | sema domain, transmembrane domain (TM), and cytoplasmic d | Multiple_C | 5.34 | 4.82 | 4.95 | 1.31 | 0.0305 | 0.0997 | 4.5 | 4.33 | 4.46 | 1.03 | 0.9537 | 0.9811 |
| TC0100015985.hg.1 | MEX3A | mex-3 RNA binding family member A | Multiple_C | 3.43 | 3.08 | 3.04 | 1.31 | 0.0657 | 0.1773 | 6.6 | 6.71 | 5.87 | 1.66 | 0.0065 | 0.0583 |
| TC0100016034.hg.1 | ETV3L | ets variant 3-like | Coding | 3.34 | 2.71 | 2.95 | 1.31 | 0.1829 | 0.3681 | 4.4 | 4.28 | 4.07 | 1.26 | 0.1711 | 0.4176 |
| TC0100017597.hg.1 | C1orf145 | chromosome 1 open reading frame 145 | Multiple_C | 4.19 | 4 | 3.8 | 1.31 | 0.0855 | 0.2147 | 4 | 3.79 | 3.69 | 1.24 | 0.0774 | 0.2682 |
| TC0100018224.hg.1 | FAM183A | family with sequence similarity 183, member A | Multiple_C | 3.17 | 2.54 | 2.78 | 1.31 | 0.7189 | 0.8519 | 4.27 | 4.23 | 3.87 | 1.32 | 0.0919 | 0.2937 |
| TC0100018484.hg.1 | MCL1 | myeloid cell leukemia 1 | Multiple_C | 13.41 | 12.69 | 13.02 | 1.31 | 0.1493 | 0.321 | 11.93 | 11.80 | 12.17 | 0.85 | 0.134 | 0.3636 |
| TC0200006524.hg.1 | TRAPPC12 | trafficking protein particle complex 12 | Multiple_C | 8.29 | 8.3 | 7.9 | 1.31 | 0.8814 | 0.9424 | 8.58 | 8.30 | 7.84 | 1.67 | 0.0299 | 0.1534 |
| TC0200008335.hg.1 | THNSL2 | threonine synthase-like 2 | Multiple_C | 5.72 | 4.96 | 5.33 | 1.31 | 0.2931 | 0.5044 | 5.19 | 5.53 | 5.42 | 0.85 | 0.5691 | 0.7777 |
| TC0200011129.hg.1 | SAG | S-antigen; retina and pineal gland (arrestin) | Multiple_C | 4.23 | 3.91 | 3.84 | 1.31 | 0.4376 | 0.647 | 4.26 | 4.07 | 4.03 | 1.17 | 0.3738 | 0.6344 |
| TC0200014402.hg.1 | LCT | lactase | Coding | 4.31 | 4.02 | 3.92 | 1.31 | 0.128 | 0.2878 | 5.73 | 5.46 | 4.94 | 1.73 | 0.0271 | 0.1441 |
| TC0200015876.hg.1 | SERPINE2 | serpin peptidase inhibitor, clade E (nexin, plasminogen activatoı | Multiple_C | 5.53 | 5.2 | 5.14 | 1.31 | 0.5128 | 0.7085 | 8.04 | 7.75 | 8.52 | 0.72 | 0.0516 | 0.2122 |
| TC0200016529.hg.1 | ARHGEF4 | Rho guanine nucleotide exchange factor 4 | Multiple_C | 4.49 | 4.19 | 4.1 | 1.31 | 0.1296 | 0.2904 | 4.1 | 3.95 | 4.21 | 0.93 | 0.4553 | 0.6998 |
| TC0200016601.hg.1 | GIGYF2 | GRB10 interacting GYF protein 2 | Multiple_C | 9.27 | 9.63 | 8.88 | 1.31 | 0.176 | 0.3584 | 10.58 | 10.24 | 10.22 | 1.28 | 0.6837 | 0.8484 |
| TC0300007101.hg.1 | RPSA; SNORA( | ribosomal protein SA; small nucleolar RNA, H/ACA box 62; smal | Multiple_C | 17.22 | 17.23 | 16.83 | 1.31 | 0.0218 | 0.0765 | 16.33 | 16.28 | 16.54 | 0.86 | 0.4259 | 0.6784 |
| TC0300009531.hg.1 | NLGN1 | neuroligin 1 | Multiple_C | 3.93 | 3.83 | 3.54 | 1.31 | 0.1 | 0.2411 | 4.06 | 3.80 | 3.78 | 1.21 | 0.2072 | 0.464 |
| TC0300011075.hg.1 | MST1 | macrophage stimulating 1 | Multiple_C | 4.07 | 3.98 | 3.68 | 1.31 | 0.1904 | 0.3783 | 3.72 | 4.10 | 3.85 | 0.91 | 0.4232 | 0.6759 |
| TC0300013804.hg.1 | OXSM | 3-oxoacyl-ACP synthase, mitochondrial | Multiple_C | 9.51 | 10.4 | 9.12 | 1.31 | 0.1816 | 0.3662 | 8.16 | 8.02 | 7.83 | 1.26 | 0.197 | 0.4519 |
| TC0400006546.hg.1 | WHSC1 | Wolf-Hirschhorn syndrome candidate 1 | Multiple_C | 10.98 | 10.19 | 10.59 | 1.31 | 0.1781 | 0.361 | 11.36 | 11.49 | 11.79 | 0.74 | 0.0163 | 0.1061 |
| TC0400006804.hg.1 | USP17L22; US | ubiquitin specific peptidase 17-like family member 22; ubiquitin | Coding | 5.03 | 4.9 | 4.64 | 1.31 | 0.1241 | 0.2812 | 4.49 | 4.47 | 4.42 | 1.05 | 0.1738 | 0.4212 |
| TC0400011359.hg.1 | UNC5C | unc-5 netrin receptor C | Multiple_C | 3.84 | 3.77 | 3.45 | 1.31 | 0.3831 | 0.5962 | 3.83 | 3.95 | 3.51 | 1.25 | 0.3774 | 0.6376 |
| TC0500006803.hg.1 | TRIO | trio Rho guanine nucleotide exchange factor | Multiple_C | 6.98 | 6.87 | 6.59 | 1.31 | 0.2791 | 0.4887 | 10.19 | 10.70 | 9.78 | 1.33 | 0.1075 | 0.3224 |
| TC0500008732.hg.1 | FBXL21 | F-box and leucine-rich repeat protein 21 (gene/pseudogene) | Multiple_C | 3.39 | 3.16 | 3 | 1.31 | 0.1794 | 0.3631 | 4.14 | 4.00 | 3.74 | 1.32 | 0.0844 | 0.2797 |
| TC0600007495.hg.1 | HLA-A | major histocompatibility complex, class I, A | Multiple_C | 7.06 | 6.9 | 6.67 | 1.31 | 0.2593 | 0.4657 | 13.9 | 13.58 | 13.62 | 1.21 | 0.5025 | 0.7346 |
| TC0600007694.hg.1 | B3GALT4 | UDP-Gal:betaGlcNAc beta 1,3-galactosyltransferase 4 | Multiple_C | 3.59 | 3.37 | 3.2 | 1.31 | 0.0862 | 0.2159 | 3.93 | 4.01 | 3.85 | 1.06 | 0.2219 | 0.4822 |
| TC0600007855.hg.1 | FGD2 | FYVE, RhoGEF and PH domain containing 2 | Multiple_C | 3.89 | 3.41 | 3.5 | 1.31 | 0.1583 | 0.3343 | 4.07 | 3.98 | 3.85 | 1.16 | 0.1038 | 0.3157 |
| TC0600008038.hg.1 | GUCA1A | guanylate cyclase activator 1A (retina) | Coding | 4.61 | 4.39 | 4.22 | 1.31 | 0.1574 | 0.3331 | 4.46 | 4.91 | 4.38 | 1.06 | 0.3888 | 0.6475 |
| TC0600008073.hg.1 | SRF | serum response factor | Multiple_C | 8.28 | 7.11 | 7.89 | 1.31 | 0.3664 | 0.5796 | 6.65 | 7.52 | 8.53 | 0.27 | 7.07E-06 | 0.0006 |
| TC0600008630.hg.1 | TTK | TTK protein kinase | Multiple_C | 12.57 | 13.13 | 12.18 | 1.31 | 0.2336 | 0.4343 | 11.72 | 11.30 | 11.81 | 0.94 | 0.835 | 0.9272 |
| TC0600009082.hg.1 | C6orf183 | chromosome 6 open reading frame 183 | Multiple_C | 4.07 | 3.63 | 3.68 | 1.31 | 0.1201 | 0.2749 | 3.91 | 3.73 | 3.76 | 1.11 | 0.5811 | 0.786 |

| | | | | | | | | | | | | | | | |
|---|---|---|---|---|---|---|---|---|---|---|---|---|---|---|---|
| TC0600010520.hg.1 | MYLK4 | myosin light chain kinase family member 4 | Multiple_C | 4.19 | 3.88 | 3.8 | 1.31 | 0.2237 | 0.422 | 4.26 | 4.10 | 4.24 | 1.01 | 0.8777 | 0.9476 |
| TC0600010864.hg.1 | RANBP9 | RAN binding protein 9 | Multiple_C | 10.48 | 10.9 | 10.09 | 1.31 | 0.0749 | 0.1946 | 11.29 | 10.89 | 10.9 | 1.31 | 0.1423 | 0.375 |
| TC0600011255.hg.1 | ZSCAN23 | zinc finger and SCAN domain containing 23 | Multiple_C | 3.61 | 2.93 | 3.22 | 1.31 | 0.3529 | 0.5657 | 4.53 | 4.37 | 3.89 | 1.56 | 0.0157 | 0.1034 |
| TC0600011880.hg.1 | GTPBP2 | GTP binding protein 2 | Multiple_C | 9.72 | 9.66 | 9.33 | 1.31 | 0.2605 | 0.4673 | 8.97 | 8.32 | 8.59 | 1.30 | 0.2026 | 0.4583 |
| TC0600014074.hg.1 | HIVEP1 | human immunodeficiency virus type I enhancer binding protein | Multiple_C | 7.79 | 7.38 | 7.4 | 1.31 | 0.3789 | 0.5919 | 7.52 | 7.02 | 7.08 | 1.36 | 0.902 | 0.9592 |
| TC0700006481.hg.1 | CYP2W1 | cytochrome P450, family 2, subfamily W, polypeptide 1 | Multiple_C | 6.55 | 6.17 | 6.16 | 1.31 | 0.1331 | 0.2963 | 6.99 | 7.21 | 6.63 | 1.28 | 0.0338 | 0.1655 |
| TC0700012165.hg.1 | ATXN7L1 | ataxin 7-like 1 | Multiple_C | 5.44 | 4.51 | 5.05 | 1.31 | 0.6264 | 0.7915 | 5.75 | 4.79 | 5.88 | 0.91 | 0.6101 | 0.805 |
| TC0700012203.hg.1 | COG5 | component of oligomeric golgi complex 5 | Multiple_C | 12.83 | 13.13 | 12.44 | 1.31 | 0.2144 | 0.4098 | 12.49 | 11.31 | 12.33 | 1.12 | 0.1142 | 0.3335 |
| TC0700012506.hg.1 | PAX4 | paired box 4 | Multiple_C | 4.56 | 3.64 | 4.17 | 1.31 | 0.1793 | 0.3631 | 4.08 | 4.22 | 4.36 | 0.82 | 0.5226 | 0.7489 |
| TC0800007284.hg.1 | KCNU1 | potassium channel, subfamily U, member 1 | Coding | 3.08 | 2.63 | 2.69 | 1.31 | 0.1265 | 0.2851 | 3.31 | 3.40 | 2.99 | 1.25 | 0.1504 | 0.3883 |
| TC0800009157.hg.1 | LY6K | lymphocyte antigen 6 complex, locus K | Multiple_C | 3.4 | 2.69 | 3.01 | 1.31 | 0.1769 | 0.3596 | 4.08 | 3.98 | 3.76 | 1.25 | 0.221 | 0.4812 |
| TC0800009625.hg.1 | DEFB136 | defensin, beta 136 | Coding | 3.98 | 3.48 | 3.59 | 1.31 | 0.2169 | 0.4128 | 4.2 | 4.28 | 4.19 | 1.01 | 0.916 | 0.9656 |
| TC0800011285.hg.1 | ZNF706 | zinc finger protein 706 | Multiple_C | 12.86 | 13.44 | 12.47 | 1.31 | 0.1769 | 0.3596 | 11.38 | 10.85 | 10.69 | 1.61 | 0.0064 | 0.0573 |
| TC0900008670.hg.1 | OR1Q1 | olfactory receptor, family 1, subfamily Q, member 1 | Coding | 3.09 | 2.77 | 2.7 | 1.31 | 0.1405 | 0.3078 | 3.96 | 3.17 | 3 | 1.95 | 0.0006 | 0.0113 |
| TC0900009707.hg.1 | IFNA2 | interferon, alpha 2 | Coding | 4.75 | 4.15 | 4.36 | 1.31 | 0.2964 | 0.5079 | 4.96 | 4.53 | 4.66 | 1.23 | 0.6024 | 0.8008 |
| TC0900009950.hg.1 | TLN1; MIR685 | talin 1; microRNA 6852 | Multiple_C | 7.94 | 7.55 | 7.55 | 1.31 | 0.8275 | 0.9133 | 8.24 | 8.62 | 8.15 | 1.06 | 0.8948 | 0.9555 |
| TC0900011805.hg.1 | TTF1 | transcription termination factor, RNA polymerase I | Multiple_C | 7.16 | 7.45 | 6.77 | 1.31 | 0.299 | 0.5106 | 7.29 | 7.13 | 7.03 | 1.20 | 0.3105 | 0.5746 |
| TC0900012200.hg.1 | SLC34A3 | solute carrier family 34 (type II sodium/phosphate cotransporte | Coding | 3.82 | 3.38 | 3.43 | 1.31 | 0.1372 | 0.3025 | 4.07 | 4.00 | 4.16 | 0.94 | 0.4964 | 0.7301 |
| TC0X00006625.hg.1 | TLR7 | toll-like receptor 7 | Multiple_C | 3.67 | 3.05 | 3.28 | 1.31 | 0.312 | 0.5254 | 3.78 | 3.57 | 3.41 | 1.29 | 0.0328 | 0.1625 |
| TC0X00007522.hg.1 | FAM155B | family with sequence similarity 155, member B | Coding | 5.69 | 5.14 | 5.3 | 1.31 | 0.3162 | 0.5294 | 5.94 | 6.15 | 5.85 | 1.06 | 0.946 | 0.9781 |
| TC0X00008900.hg.1 | CRLF2 | cytokine receptor-like factor 2 | Multiple_C | 7.29 | 6.66 | 6.9 | 1.31 | 0.3525 | 0.5652 | 5.67 | 5.60 | 5.73 | 0.96 | 0.3572 | 0.6202 |
| TC0X00010687.hg.1 | CT47A7; CT47 | cancer/testis antigen family 47, member A7; cancer/testis antig | Coding | 3.96 | 3.57 | 3.57 | 1.31 | 0.5879 | 0.7641 | 3.8 | 4.07 | 3.99 | 0.88 | 0.6524 | 0.8305 |
| TC1000007950.hg.1 | UNC5B | unc-5 netrin receptor B | Multiple_C | 5.16 | 4.47 | 4.77 | 1.31 | 0.0314 | 0.1019 | 4.75 | 4.75 | 4.89 | 0.91 | 0.6353 | 0.821 |
| TC1000008643.hg.1 | SCD | stearoyl-CoA desaturase (delta-9-desaturase) | Multiple_C | 14.19 | 13.98 | 13.8 | 1.31 | 0.2744 | 0.4831 | 13.53 | 12.48 | 14.06 | 0.69 | 0.248 | 0.5126 |
| TC1000010533.hg.1 | GPRIN2 | G protein regulated inducer of neurite outgrowth 2 | Multiple_C | 3.41 | 3.05 | 3.02 | 1.31 | 0.1256 | 0.2836 | 3.61 | 3.44 | 3.45 | 1.12 | 0.157 | 0.3979 |
| TC1000010831.hg.1 | HERC4 | HECT and RLD domain containing E3 ubiquitin protein ligase 4 | Multiple_C | 10.76 | 11.63 | 10.37 | 1.31 | 0.1454 | 0.3154 | 10.42 | 9.20 | 10.1 | 1.25 | 0.3264 | 0.5903 |
| TC1000011520.hg.1 | OPALIN | oligodendrocytic myelin paranodal and inner loop protein | Multiple_C | 5.62 | 5.04 | 5.23 | 1.31 | 0.2155 | 0.4112 | 5.76 | 5.61 | 5.18 | 1.49 | 0.0588 | 0.2287 |
| TC1000011521.hg.1 | TLL2 | tolloid like 2 | Multiple_C | 6.12 | 5.69 | 5.73 | 1.31 | 0.2067 | 0.4 | 5.37 | 5.52 | 5.58 | 0.86 | 0.95 | 0.9795 |
| TC1100006442.hg.1 | NLRP6 | NLR family, pyrin domain containing 6 | Multiple_C | 4.93 | 4.39 | 4.54 | 1.31 | 0.3259 | 0.5399 | 4.63 | 4.48 | 4.44 | 1.14 | 0.1813 | 0.4314 |
| TC1100006709.hg.1 | OR52E4 | olfactory receptor, family 52, subfamily E, member 4 | Coding | 3.42 | 3.13 | 3.03 | 1.31 | 0.3613 | 0.5748 | 3.93 | 4.26 | 4.28 | 0.78 | 0.9961 | 0.9982 |
| TC1100007453.hg.1 | MDK | midkine (neurite growth-promoting factor 2) | Multiple_C | 10.42 | 9.53 | 10.03 | 1.31 | 0.0697 | 0.1847 | 8.95 | 9.66 | 9.22 | 0.83 | 0.3775 | 0.6376 |
| TC1100007913.hg.1 | MARK2 | MAP/microtubule affinity-regulating kinase 2 | Multiple_C | 10.33 | 8.77 | 9.94 | 1.31 | 0.4849 | 0.6862 | 10.45 | 10.57 | 10.69 | 0.85 | 0.0775 | 0.2683 |
| TC1100008341.hg.1 | FOLR2 | folate receptor 2 (fetal) | Coding | 3.81 | 3.28 | 3.42 | 1.31 | 0.0211 | 0.0748 | 3.37 | 3.35 | 3.25 | 1.09 | 0.4018 | 0.6582 |

| | | | | | | | | | | | | | | | |
|---|---|---|---|---|---|---|---|---|---|---|---|---|---|---|---|
| TC1100009926.hg.1 | OR51B2 | olfactory receptor, family 51, subfamily B, member 2 (gene/pse | Multiple_C | 3.71 | 3.09 | 3.32 | 1.31 | 0.2691 | 0.4773 | 5.54 | 4.09 | 5.12 | 1.34 | 0.184 | 0.4351 |
| TC1100010156.hg.1 | BTBD10 | BTB (POZ) domain containing 10 | Multiple_C | 9.98 | 9.99 | 9.59 | 1.31 | 0.1856 | 0.372 | 9.06 | 8.50 | 9.2 | 0.91 | 0.0606 | 0.2336 |
| TC1100011295.hg.1 | CCDC87 | coiled-coil domain containing 87 | Coding | 4.27 | 3.41 | 3.88 | 1.31 | 0.1876 | 0.3749 | 4.33 | 4.21 | 4.28 | 1.04 | 0.2829 | 0.5483 |
| TC1100011917.hg.1 | TRIM49D2; TR | tripartite motif containing 49D2; tripartite motif containing 49D | Coding | 4.24 | 3.41 | 3.85 | 1.31 | 0.2221 | 0.4198 | 3.75 | 3.63 | 3.48 | 1.21 | 0.3355 | 0.5998 |
| TC1100012659.hg.1 | OR8D1 | olfactory receptor, family 8, subfamily D, member 1 | Coding | 4.05 | 3.83 | 3.66 | 1.31 | 0.2466 | 0.4502 | 4.28 | 3.64 | 3.68 | 1.52 | 0.1173 | 0.3387 |
| TC1100013204.hg.1 | ZNF705E | zinc finger protein 705E | Multiple_C | 5.47 | 5.25 | 5.08 | 1.31 | 0.1814 | 0.3659 | 4.53 | 4.33 | 4.31 | 1.16 | 0.4676 | 0.7088 |
| TC1200009793.hg.1 | CLEC4C | C-type lectin domain family 4, member C | Coding | 3.92 | 3.59 | 3.53 | 1.31 | 0.1292 | 0.2897 | 4.06 | 3.81 | 3.52 | 1.45 | 0.0111 | 0.0833 |
| TC1200010413.hg.1 | ABCD2 | ATP binding cassette subfamily D member 2 | Multiple_C | 4.26 | 4.28 | 3.87 | 1.31 | 0.647 | 0.8043 | 3.12 | 3.33 | 3.64 | 0.70 | 0.0833 | 0.278 |
| TC1200010794.hg.1 | ITGB7 | integrin beta 7 | Multiple_C | 4.19 | 3.6 | 3.8 | 1.31 | 0.1558 | 0.3305 | 4.57 | 4.34 | 4.62 | 0.97 | 0.1989 | 0.4542 |
| TC1200011786.hg.1 | NUAK1 | NUAK family, SNF1-like kinase, 1 | Multiple_C | 6.25 | 6.17 | 5.86 | 1.31 | 0.1554 | 0.33 | 4.01 | 4.16 | 3.81 | 1.15 | 0.1911 | 0.4441 |
| TC1200012105.hg.1 | WSB2 | WD repeat and SOCS box containing 2 | Multiple_C | 13.01 | 12.49 | 12.62 | 1.31 | 0.1494 | 0.321 | 10.32 | 10.37 | 10.85 | 0.69 | 0.0308 | 0.1562 |
| TC1200012248.hg.1 | HCAR3 | hydroxycarboxylic acid receptor 3 | Coding | 3.87 | 3.08 | 3.48 | 1.31 | 0.5089 | 0.7051 | 4.31 | 4.48 | 4.4 | 0.94 | 0.6577 | 0.8328 |
| TC1300008111.hg.1 | PROZ | protein Z, vitamin K-dependent plasma glycoprotein | Multiple_C | 4.1 | 3.82 | 3.71 | 1.31 | 0.4986 | 0.6981 | 3.82 | 3.75 | 3.65 | 1.13 | 0.3293 | 0.5934 |
| TC1300008644.hg.1 | POSTN | periostin, osteoblast specific factor | Multiple_C | 3.61 | 3.11 | 3.22 | 1.31 | 0.227 | 0.4261 | 4.16 | 4.08 | 3.92 | 1.18 | 0.3124 | 0.5765 |
| TC1400007628.hg.1 | PSEN1 | presenilin 1 | Multiple_C | 11.38 | 10.78 | 10.99 | 1.31 | 0.1118 | 0.2614 | 9.96 | 10.33 | 10.07 | 0.93 | 0.8208 | 0.9209 |
| TC1500007237.hg.1 | SCG3 | secretogranin III | Multiple_C | 3.59 | 3.01 | 3.2 | 1.31 | 0.2843 | 0.4946 | 3.6 | 3.47 | 4.06 | 0.73 | 0.1666 | 0.4117 |
| TC1500007546.hg.1 | SNX22 | sorting nexin 22 | Multiple_C | 4.29 | 3.79 | 3.9 | 1.31 | 0.5105 | 0.7064 | 4.25 | 4.44 | 4.45 | 0.87 | 0.8182 | 0.9197 |
| TC1500007802.hg.1 | GOLGA6B | golgin A6 family, member B | Multiple_C | 5.99 | 5.84 | 5.6 | 1.31 | 0.0328 | 0.1055 | 7.21 | 6.07 | 6.01 | 2.30 | 8.07E-06 | 0.0006 |
| TC1500008015.hg.1 | ST20-AS1 | ST20 antisense RNA 1 | Multiple_C | 3.43 | 2.72 | 3.04 | 1.31 | 0.5453 | 0.7312 | 3.71 | 3.93 | 3.59 | 1.09 | 0.3084 | 0.5724 |
| TC1600009231.hg.1 | CDIP1 | cell death-inducing p53 target 1 | Multiple_C | 3.18 | 2.76 | 2.79 | 1.31 | 0.1564 | 0.3314 | 3.94 | 3.95 | 3.82 | 1.09 | 0.4403 | 0.69 |
| TC1600010394.hg.1 | CES1 | carboxylesterase 1 | Multiple_C | 5.53 | 5.16 | 5.14 | 1.31 | 0.3389 | 0.553 | 5.27 | 5.14 | 4.49 | 1.72 | 0.007 | 0.0611 |
| TC1600011237.hg.1 | PABPN1L | poly(A) binding protein, nuclear 1-like (cytoplasmic) | Coding | 3.89 | 3.47 | 3.5 | 1.31 | 0.0968 | 0.2353 | 3.87 | 4.04 | 3.94 | 0.95 | 0.9628 | 0.9842 |
| TC1700006499.hg.1 | BHLHA9 | basic helix-loop-helix family, member a9 | Coding | 4.34 | 3.79 | 3.95 | 1.31 | 0.2172 | 0.413 | 4.02 | 3.86 | 3.85 | 1.13 | 0.4903 | 0.7257 |
| TC1700008080.hg.1 | SPPL2C | signal peptide peptidase like 2C | Coding | 4.44 | 4.07 | 4.05 | 1.31 | 0.335 | 0.5491 | 3.74 | 3.83 | 3.82 | 0.95 | 0.9647 | 0.985 |
| TC1700010548.hg.1 | CWC25 | CWC25 spliceosome-associated protein homolog | Multiple_C | 8.05 | 7.71 | 7.66 | 1.31 | 0.1515 | 0.3245 | 6.89 | 6.96 | 7.28 | 0.76 | 0.3804 | 0.64 |
| TC1700010807.hg.1 | PYY | peptide YY | Coding | 5.44 | 4.88 | 5.05 | 1.31 | 0.0892 | 0.2215 | 5.17 | 5.32 | 5.2 | 0.98 | 0.7929 | 0.9077 |
| TC1700012229.hg.1 | RHBDL3 | rhomboid, veinlet-like 3 (Drosophila) | Multiple_C | 5.44 | 5.13 | 5.05 | 1.31 | 0.477 | 0.6795 | 4.94 | 5.12 | 5.3 | 0.78 | 0.6497 | 0.829 |
| TC1800007037.hg.1 | MEP1B | meprin A, beta | Multiple_C | 3.92 | 3.58 | 3.53 | 1.31 | 0.65 | 0.8063 | 4.78 | 3.98 | 4.4 | 1.30 | 0.2509 | 0.5151 |
| TC1800007722.hg.1 | C18orf65 | chromosome 18 open reading frame 65 | Multiple_C | 3.87 | 3.59 | 3.48 | 1.31 | 0.0562 | 0.1577 | 4.47 | 5.00 | 4.3 | 1.13 | 0.8359 | 0.9277 |
| TC1900006626.hg.1 | CELF5 | CUGBP, Elav-like family member 5 | Multiple_C | 5.36 | 4.8 | 4.97 | 1.31 | 0.2036 | 0.3959 | 4.53 | 4.56 | 4.79 | 0.84 | 0.1522 | 0.391 |
| TC1900006772.hg.1 | NRTN | neurturin | Coding | 5.46 | 4.9 | 5.07 | 1.31 | 0.3816 | 0.5947 | 7.15 | 7.16 | 7.14 | 1.01 | 0.9781 | 0.9908 |
| TC1900007329.hg.1 | PGLS | 6-phosphogluconolactonase | Multiple_C | 10.8 | 10.85 | 10.41 | 1.31 | 0.7849 | 0.8894 | 7.57 | 8.60 | 7.87 | 0.81 | 0.2643 | 0.529 |
| TC1900007502.hg.1 | ZNF85 | zinc finger protein 85 | Multiple_C | 9.92 | 10.06 | 9.53 | 1.31 | 0.1082 | 0.2554 | 5.57 | 5.54 | 5.81 | 0.85 | 0.2962 | 0.561 |

| | | | | | | | | | | | | | | | |
|---|---|---|---|---|---|---|---|---|---|---|---|---|---|---|---|
| TC1900008187.hg.1 | TMEM145 | transmembrane protein 145 | Multiple_C | 4.6 | 3.93 | 4.21 | 1.31 | 0.2081 | 0.4017 | 4.46 | 4.61 | 4.82 | 0.78 | 0.1254 | 0.3501 |
| TC1900008252.hg.1 | ZNF224 | zinc finger protein 224 | Multiple_C | 9.6 | 9.84 | 9.21 | 1.31 | 0.1474 | 0.3183 | 7.97 | 8.33 | 7.72 | 1.19 | 0.0071 | 0.0615 |
| TC1900008566.hg.1 | SIGLEC16 | sialic acid binding Ig-like lectin 16 (gene/pseudogene) | Multiple_C | 6.27 | 5.86 | 5.88 | 1.31 | 0.2264 | 0.4253 | 6.56 | 6.57 | 6.67 | 0.93 | 0.9127 | 0.9642 |
| TC1900008982.hg.1 | USP29 | ubiquitin specific peptidase 29 | Coding | 3.72 | 3.39 | 3.33 | 1.31 | 0.3434 | 0.5571 | 3.98 | 3.84 | 3.81 | 1.13 | 0.353 | 0.6166 |
| TC1900009302.hg.1 | PIP5K1C | phosphatidylinositol-4-phosphate 5-kinase, type I, gamma | Multiple_C | 8.98 | 7.64 | 8.59 | 1.31 | 0.2686 | 0.4768 | 7.74 | 8.57 | 8.59 | 0.55 | 0.0673 | 0.2473 |
| TC1900011467.hg.1 | IL11 | interleukin 11 | Coding | 6.62 | 5.99 | 6.23 | 1.31 | 0.2398 | 0.4419 | 6.34 | 6.24 | 6.42 | 0.95 | 0.8258 | 0.9233 |
| TC1900011934.hg.1 | ZNF585B | zinc finger protein 585B | Multiple_C | 12.03 | 11.89 | 11.64 | 1.31 | 0.1611 | 0.338 | 9.99 | 9.91 | 9.84 | 1.11 | 0.431 | 0.6826 |
| TC1900011996.hg.1 | CGB8 | chorionic gonadotropin, beta polypeptide 8 | Coding | 4.74 | 4.24 | 4.35 | 1.31 | 0.4298 | 0.6401 | 4.38 | 4.21 | 4.28 | 1.07 | 0.1789 | 0.4278 |
| TC2000009231.hg.1 | WFDC8 | WAP four-disulfide core domain 8 | Coding | 3.39 | 3.41 | 3 | 1.31 | 0.0894 | 0.2218 | 4.59 | 4.71 | 4.38 | 1.16 | 0.221 | 0.4812 |
| TC2000009874.hg.1 | PSMF1 | proteasome inhibitor subunit 1 | Multiple_C | 12.02 | 10.17 | 11.63 | 1.31 | 0.2464 | 0.4499 | 14.3 | 14.51 | 14.64 | 0.79 | 0.0654 | 0.2435 |
| TC2100008526.hg.1 | SLC37A1 | solute carrier family 37 (glucose-6-phosphate transporter), men | Multiple_C | 5.11 | 5.08 | 4.72 | 1.31 | 0.4685 | 0.6724 | 4.01 | 4.17 | 4.26 | 0.84 | 0.2047 | 0.4609 |
| TC2200007273.hg.1 | CSF2RB | colony stimulating factor 2 receptor, beta, low-affinity (granuloc | Multiple_C | 4.89 | 4.4 | 4.5 | 1.31 | 0.0783 | 0.2016 | 4.07 | 4.27 | 4.39 | 0.80 | 0.1714 | 0.4179 |
| TC2200008370.hg.1 | XBP1 | X-box binding protein 1 | Multiple_C | 12.97 | 12.59 | 12.58 | 1.31 | 0.2111 | 0.4056 | 10.78 | 10.17 | 10.44 | 1.27 | 0.1842 | 0.4353 |
| TC2200009303.hg.1 | MAPK8IP2 | mitogen-activated protein kinase 8 interacting protein 2 | Coding | 3.75 | 3.11 | 3.36 | 1.31 | 0.0459 | 0.1358 | 3.48 | 3.46 | 3.63 | 0.90 | 0.3733 | 0.6341 |
| TC0100006492.hg.1 | TTLL10 | tubulin tyrosine ligase-like family member 10 | Multiple_C | 5.7 | 4.95 | 5.32 | 1.30 | 0.0725 | 0.1901 | 5.58 | 5.97 | 5.82 | 0.85 | 0.3795 | 0.639 |
| TC0100007199.hg.1 | VWA5B1 | von Willebrand factor A domain containing 5B1 | Multiple_C | 4.47 | 3.83 | 4.09 | 1.30 | 0.1167 | 0.2694 | 4.6 | 4.69 | 4.77 | 0.89 | 0.634 | 0.8203 |
| TC0100007641.hg.1 | LINC01225 | long intergenic non-protein coding RNA 1225 | Multiple_C | 5.61 | 4.97 | 5.23 | 1.30 | 0.3471 | 0.5604 | 4.63 | 4.09 | 4.38 | 1.19 | 0.8069 | 0.9148 |
| TC0100007964.hg.1 | ZNF684 | zinc finger protein 684 | Multiple_C | 8.71 | 8.41 | 8.33 | 1.30 | 0.288 | 0.4987 | 7.14 | 7.11 | 7.15 | 0.99 | 0.4664 | 0.708 |
| TC0100008416.hg.1 | BSND | barttin CLCNK-type chloride channel accessory beta subunit | Coding | 4.57 | 3.92 | 4.19 | 1.30 | 0.0953 | 0.2326 | 3.73 | 3.77 | 3.56 | 1.13 | 0.5346 | 0.7567 |
| TC0100009324.hg.1 | FAM102B | family with sequence similarity 102, member B | Multiple_C | 12.33 | 11.53 | 11.95 | 1.30 | 0.2381 | 0.4398 | 7.47 | 7.82 | 7.62 | 0.90 | 0.7104 | 0.8642 |
| TC0100010001.hg.1 | LCE2A | late cornified envelope 2A | Coding | 4.12 | 3.56 | 3.74 | 1.30 | 0.5774 | 0.7556 | 5.01 | 4.90 | 4.78 | 1.17 | 0.185 | 0.4364 |
| TC0100010241.hg.1 | MNDA | myeloid cell nuclear differentiation antigen | Multiple_C | 3.24 | 2.57 | 2.86 | 1.30 | 0.6685 | 0.8188 | 3.82 | 3.59 | 3.67 | 1.11 | 0.6597 | 0.834 |
| TC0100010268.hg.1 | FCRL6 | Fc receptor-like 6 | Multiple_C | 5.7 | 5.29 | 5.32 | 1.30 | 0.2182 | 0.4147 | 5.33 | 5.36 | 5.51 | 0.88 | 0.8186 | 0.9198 |
| TC0100010284.hg.1 | PEA15 | phosphoprotein enriched in astrocytes 15 | Multiple_C | 11.7 | 10.7 | 11.32 | 1.30 | 0.1157 | 0.268 | 10.23 | 9.60 | 10.96 | 0.60 | 0.0003 | 0.008 |
| TC0100010843.hg.1 | LINC00272 | long intergenic non-protein coding RNA 272 | Multiple_C | 4.26 | 3.75 | 3.88 | 1.30 | 0.487 | 0.6881 | 4.07 | 3.82 | 3.52 | 1.46 | 0.2418 | 0.5056 |
| TC0100011012.hg.1 | RGS1 | regulator of G-protein signaling 1 | Multiple_C | 3.4 | 3.22 | 3.02 | 1.30 | 0.3769 | 0.5899 | 3.22 | 3.31 | 3.31 | 0.94 | 0.6599 | 0.8341 |
| TC0100011073.hg.1 | CRB1 | crumbs family member 1, photoreceptor morphogenesis associ | Multiple_C | 4.19 | 3.51 | 3.81 | 1.30 | 0.2447 | 0.4482 | 4.38 | 4.29 | 4.41 | 0.98 | 0.4561 | 0.7004 |
| TC0100012222.hg.1 | DESI2 | desumoylating isopeptidase 2 | Multiple_C | 12.14 | 11.88 | 11.76 | 1.30 | 0.0653 | 0.1765 | 11.38 | 11.13 | 11.69 | 0.81 | 0.1709 | 0.4172 |
| TC0100012328.hg.1 | TRIM58 | tripartite motif containing 58 | Multiple_C | 6.75 | 6.37 | 6.37 | 1.30 | 0.1475 | 0.3184 | 6.82 | 6.69 | 6.34 | 1.39 | 0.0306 | 0.1556 |
| TC0100013920.hg.1 | C1orf210 | chromosome 1 open reading frame 210 | Coding | 7.09 | 6.33 | 6.71 | 1.30 | 0.7125 | 0.8481 | 5.54 | 5.76 | 5.82 | 0.82 | 0.5591 | 0.7714 |
| TC0100014013.hg.1 | CCDC163P | coiled-coil domain containing 163, pseudogene | Multiple_C | 6.05 | 5.79 | 5.67 | 1.30 | 0.8378 | 0.9193 | 5.53 | 5.78 | 5.8 | 0.83 | 0.1449 | 0.3797 |
| TC0100014790.hg.1 | COL24A1 | collagen, type XXIV, alpha 1 | Multiple_C | 3.32 | 2.75 | 2.94 | 1.30 | 0.4384 | 0.6477 | 4.05 | 3.61 | 3.69 | 1.28 | 0.167 | 0.4123 |
| TC0100015803.hg.1 | TDRKH | tudor and KH domain containing | Multiple_C | 8.56 | 9.59 | 8.18 | 1.30 | 0.1258 | 0.2839 | 7.52 | 7.05 | 7.49 | 1.02 | 0.405 | 0.6604 |

| | | | | | | | | | | | | | | | |
|---|---|---|---|---|---|---|---|---|---|---|---|---|---|---|---|
| TC0100015901.hg.1 | C1orf189 | chromosome 1 open reading frame 189 | Coding | 4.61 | 4.23 | 4.23 | 1.30 | 0.3283 | 0.5422 | 4.3 | 4.23 | 4.48 | 0.88 | 0.539 | 0.7594 |
| TC0100016077.hg.1 | OR6K3 | olfactory receptor, family 6, subfamily K, member 3 | Coding | 3.31 | 2.77 | 2.93 | 1.30 | 0.2445 | 0.4479 | 2.84 | 3.05 | 3.19 | 0.78 | 0.9298 | 0.9703 |
| TC0100016548.hg.1 | ABL2 | ABL proto-oncogene 2, non-receptor tyrosine kinase | Multiple_C | 8.61 | 8.24 | 8.23 | 1.30 | 0.1018 | 0.2441 | 8.12 | 7.25 | 8.13 | 0.99 | 0.8657 | 0.9425 |
| TC0100016903.hg.1 | TMEM9 | transmembrane protein 9 | Multiple_C | 11.12 | 11.73 | 10.74 | 1.30 | 0.3853 | 0.5986 | 8.72 | 8.27 | 7.96 | 1.69 | 0.122 | 0.3447 |
| TC0100017548.hg.1 | CDC42BPA | CDC42 binding protein kinase alpha (DMPK-like) | Multiple_C | 9.31 | 8.74 | 8.93 | 1.30 | 0.6073 | 0.7782 | 9.39 | 9.37 | 9.39 | 1.00 | 0.9182 | 0.9665 |
| TC0200007068.hg.1 | KRTCAP3 | keratinocyte associated protein 3 | Multiple_C | 7.22 | 6.96 | 6.84 | 1.30 | 0.4947 | 0.6946 | 4.34 | 4.17 | 3.91 | 1.35 | 0.1359 | 0.3665 |
| TC0200009214.hg.1 | PROC | protein C (inactivator of coagulation factors Va and VIIIa) | Multiple_C | 4.81 | 4.31 | 4.43 | 1.30 | 0.1598 | 0.3362 | 4.75 | 4.69 | 4.28 | 1.39 | 0.0155 | 0.1024 |
| TC0200010214.hg.1 | ZC3H15 | zinc finger CCCH-type containing 15 | Multiple_C | 13.31 | 14.04 | 12.93 | 1.30 | 0.2684 | 0.4765 | 12.8 | 11.34 | 12.31 | 1.40 | 0.0317 | 0.1591 |
| TC0200010627.hg.1 | PTH2R | parathyroid hormone 2 receptor | Multiple_C | 4.55 | 4.19 | 4.17 | 1.30 | 0.2395 | 0.4416 | 4.02 | 4.12 | 3.6 | 1.34 | 0.0771 | 0.2674 |
| TC0200014276.hg.1 | CFC1 | cripto, FRL-1, cryptic family 1 | Coding | 4.19 | 3.8 | 3.81 | 1.30 | 0.0955 | 0.233 | 3.73 | 3.83 | 3.68 | 1.04 | 0.6703 | 0.839 |
| TC0200014857.hg.1 | FASTKD1 | FAST kinase domains 1 | Multiple_C | 9.73 | 10.18 | 9.35 | 1.30 | 0.6791 | 0.8257 | 9.67 | 8.39 | 8.99 | 1.60 | 0.8097 | 0.9157 |
| TC0200015608.hg.1 | LANCL1 | LanC lantibiotic synthetase component C-like 1 (bacterial) | Multiple_C | 8.22 | 8.51 | 7.84 | 1.30 | 0.4714 | 0.6752 | 8.57 | 8.99 | 8.97 | 0.76 | 0.0831 | 0.2776 |
| TC0200016743.hg.1 | ITGB6 | integrin beta 6 | Multiple_C | 7.11 | 8.16 | 6.73 | 1.30 | 0.3279 | 0.5417 | 4.62 | 4.09 | 3.88 | 1.67 | 0.0638 | 0.2404 |
| TC0300006566.hg.1 | CRELD1 | cysteine rich with EGF-like domains 1 | Multiple_C | 6.18 | 6.82 | 5.8 | 1.30 | 0.0197 | 0.0708 | 5.48 | 4.71 | 4.95 | 1.44 | 0.3746 | 0.6353 |
| TC0300009698.hg.1 | ECE2 | endothelin converting enzyme 2 | Multiple_C | 5.4 | 4.81 | 5.02 | 1.30 | 0.2027 | 0.3949 | 5.09 | 5.34 | 5.17 | 0.95 | 0.5155 | 0.7446 |
| TC0300009843.hg.1 | TP63 | tumor protein p63 | Multiple_C | 5.8 | 5.49 | 5.42 | 1.30 | 0.2944 | 0.5059 | 4.9 | 5.39 | 5.53 | 0.65 | 0.4776 | 0.7164 |
| TC0300011276.hg.1 | DENND6A | DENN/MADD domain containing 6A | Multiple_C | 10.18 | 10.14 | 9.8 | 1.30 | 0.2342 | 0.435 | 10.41 | 9.92 | 10.3 | 1.08 | 0.9462 | 0.9782 |
| TC0300013599.hg.1 | GP5 | glycoprotein V (platelet) | Coding | 3.21 | 2.77 | 2.83 | 1.30 | 0.1531 | 0.3266 | 3.45 | 3.20 | 3.37 | 1.06 | 0.814 | 0.9174 |
| TC0300013994.hg.1 | ITIH4 | inter-alpha-trypsin inhibitor heavy chain family, member 4 | Multiple_C | 3.95 | 3.22 | 3.57 | 1.30 | 0.154 | 0.3278 | 3.85 | 3.73 | 3.77 | 1.06 | 0.3811 | 0.6406 |
| TC0400007520.hg.1 | RASL11B | RAS-like, family 11, member B | Multiple_C | 3.84 | 3.62 | 3.46 | 1.30 | 0.3511 | 0.5639 | 6.64 | 7.17 | 5.62 | 2.03 | 0.0034 | 0.0383 |
| TC0400008213.hg.1 | RAP1GDS1 | RAP1, GTP-GDP dissociation stimulator 1 | Multiple_C | 6.82 | 7.32 | 6.44 | 1.30 | 0.2525 | 0.4573 | 5.48 | 5.31 | 5.97 | 0.71 | 0.0901 | 0.2906 |
| TC0400008994.hg.1 | PRSS48 | protease, serine, 48 | Coding | 3.49 | 2.82 | 3.11 | 1.30 | 0.113 | 0.2635 | 3.9 | 3.89 | 3.89 | 1.01 | 0.9425 | 0.9764 |
| TC0400011057.hg.1 | SCARB2 | scavenger receptor class B, member 2 | Multiple_C | 10.53 | 10.45 | 10.15 | 1.30 | 0.0754 | 0.1956 | 8.34 | 8.27 | 8.35 | 0.99 | 0.9948 | 0.9976 |
| TC0400011750.hg.1 | TRPC3 | transient receptor potential cation channel, subfamily C, membe | Multiple_C | 3.78 | 3.4 | 3.4 | 1.30 | 0.0638 | 0.1732 | 3.57 | 4.19 | 3.62 | 0.97 | 0.6055 | 0.8027 |
| TC0500007699.hg.1 | SMN1 | survival of motor neuron 1, telomeric | Multiple_C | 13.14 | 13.34 | 12.76 | 1.30 | 0.4335 | 0.6435 | 14.65 | 14.21 | 14.25 | 1.32 | 0.0648 | 0.2424 |
| TC0500008842.hg.1 | PSD2 | pleckstrin and Sec7 domain containing 2 | Multiple_C | 3.58 | 3 | 3.2 | 1.30 | 0.4253 | 0.6363 | 6.21 | 6.11 | 4.82 | 2.62 | 5.62E-05 | 0.0025 |
| TC0500010115.hg.1 | CTNND2 | catenin (cadherin-associated protein), delta 2 | Multiple_C | 3.2 | 2.58 | 2.82 | 1.30 | 0.0183 | 0.0667 | 3.87 | 3.95 | 3.88 | 0.99 | 0.6573 | 0.8327 |
| TC0500012280.hg.1 | DIAPH1 | diaphanous-related formin 1 | Multiple_C | 11.21 | 10.64 | 10.83 | 1.30 | 0.0682 | 0.1819 | 10.94 | 10.82 | 10.94 | 1.00 | 0.6371 | 0.822 |
| TC0500013029.hg.1 | AACSP1 | acetoacetyl-CoA synthetase pseudogene 1 | Multiple_C | 5.15 | 4.47 | 4.77 | 1.30 | 0.1978 | 0.3887 | 4.52 | 4.39 | 4.27 | 1.19 | 0.3308 | 0.5949 |
| TC0500013204.hg.1 | CAST | calpastatin | Multiple_C | 12.9 | 12.59 | 12.52 | 1.30 | 0.2141 | 0.4093 | 13.25 | 11.63 | 12.72 | 1.44 | 0.0803 | 0.2731 |
| TC0500013351.hg.1 | FNIP1 | folliculin interacting protein 1 | Multiple_C | 10.36 | 10.54 | 9.98 | 1.30 | 0.1284 | 0.2884 | 10.21 | 9.76 | 10.06 | 1.11 | 0.7066 | 0.8619 |
| TC0500013429.hg.1 | RNF130 | ring finger protein 130 | Multiple_C | 12.47 | 12.73 | 12.09 | 1.30 | 0.0559 | 0.1571 | 10.58 | 10.53 | 10.31 | 1.21 | 0.0401 | 0.1834 |
| TC0600009740.hg.1 | GRM1 | glutamate receptor, metabotropic 1 | Multiple_C | 3.16 | 2.94 | 2.78 | 1.30 | 0.4633 | 0.6684 | 3.11 | 3.01 | 2.99 | 1.09 | 0.6324 | 0.819 |

| | | | | | | | | | | | | | | | |
|---|---|---|---|---|---|---|---|---|---|---|---|---|---|---|---|
| TC0600010263.hg.1 | SMOC2 | SPARC related modular calcium binding 2 | Multiple_C | 6.74 | 6.04 | 6.36 | 1.30 | 0.502 | 0.7002 | 7.17 | 9.05 | 7.96 | 0.58 | 0.8701 | 0.944 |
| TC0600011294.hg.1 | OR2B3 | olfactory receptor, family 2, subfamily B, member 3 | Coding | 3.92 | 3.6 | 3.54 | 1.30 | 0.1776 | 0.3603 | 4.18 | 3.80 | 3.64 | 1.45 | 0.0358 | 0.1712 |
| TC0600011372.hg.1 | PPP1R10 | protein phosphatase 1, regulatory subunit 10 | Multiple_C | 8.89 | 7.89 | 8.51 | 1.30 | 0.2371 | 0.4386 | 11.04 | 11.90 | 11.07 | 0.98 | 0.5221 | 0.7487 |
| TC0600013433.hg.1 | SF3B5 | splicing factor 3b subunit 5 | Coding | 10.57 | 10.12 | 10.19 | 1.30 | 0.4166 | 0.6284 | 8.73 | 9.52 | 9.22 | 0.71 | 0.0308 | 0.1563 |
| TC0600014194.hg.1 | TIAM2 | T-cell lymphoma invasion and metastasis 2 | Multiple_C | 4.46 | 4.29 | 4.08 | 1.30 | 0.1063 | 0.252 | 4.55 | 4.78 | 4.76 | 0.86 | 0.9184 | 0.9665 |
| TC0600014270.hg.1 | GPSM3 | G-protein signaling modulator 3 | Coding | 5.12 | 4.79 | 4.74 | 1.30 | 0.1687 | 0.3486 | 5.77 | 5.43 | 5.21 | 1.47 | 0.0296 | 0.1525 |
| TC0700008306.hg.1 | FZD1 | frizzled class receptor 1 | Coding | 8.92 | 8.23 | 8.54 | 1.30 | 0.2104 | 0.4044 | 4.95 | 5.52 | 5.46 | 0.70 | 0.0252 | 0.1379 |
| TC0700008961.hg.1 | ASB15 | ankyrin repeat and SOCS box containing 15 | Coding | 4.21 | 3.85 | 3.83 | 1.30 | 0.3772 | 0.5901 | 3.78 | 3.80 | 3.71 | 1.05 | 0.9003 | 0.9586 |
| TC0700012451.hg.1 | TAS2R16 | taste receptor, type 2, member 16 | Coding | 3.73 | 4.07 | 3.35 | 1.30 | 0.203 | 0.3953 | 3.99 | 3.89 | 3.78 | 1.16 | 0.5243 | 0.7494 |
| TC0700012456.hg.1 | IQUB | IQ motif and ubiquitin domain containing | Multiple_C | 3.93 | 3.5 | 3.55 | 1.30 | 0.595 | 0.7691 | 3.79 | 3.58 | 3.73 | 1.04 | 0.8005 | 0.9108 |
| TC0700013428.hg.1 | PILRB; STAG3I | paired immunoglobin-like type 2 receptor beta; stromal antigen | Multiple_C | 9.92 | 9.59 | 9.54 | 1.30 | 0.3917 | 0.6054 | 10.27 | 10.83 | 10.51 | 0.85 | 0.3809 | 0.6405 |
| TC0800006739.hg.1 | SLC35G5 | solute carrier family 35, member G5 | Coding | 4.92 | 4.6 | 4.54 | 1.30 | 0.2189 | 0.4159 | 4.87 | 4.59 | 4.33 | 1.45 | 0.0105 | 0.0806 |
| TC0800007774.hg.1 | CHD7 | chromodomain helicase DNA binding protein 7 | Multiple_C | 7.86 | 7.1 | 7.48 | 1.30 | 0.3435 | 0.5572 | 7.7 | 7.74 | 7.94 | 0.85 | 0.322 | 0.5858 |
| TC0800007787.hg.1 | CLVS1 | clavesin 1 | Multiple_C | 4.16 | 3.3 | 3.78 | 1.30 | 0.1232 | 0.28 | 4.09 | 3.66 | 3.64 | 1.37 | 0.0492 | 0.2064 |
| TC0800009464.hg.1 | DEFB103A; DE | defensin, beta 103A; defensin, beta 103B | Coding | 5.21 | 4.82 | 4.83 | 1.30 | 0.6885 | 0.8321 | 6.1 | 5.77 | 5.62 | 1.39 | 0.0418 | 0.1883 |
| TC0900006708.hg.1 | SNAPC3 | small nuclear RNA activating complex polypeptide 3 | Multiple_C | 9.63 | 9.39 | 9.25 | 1.30 | 0.2458 | 0.4494 | 8.32 | 8.51 | 9.14 | 0.57 | 0.0033 | 0.038 |
| TC0900011440.hg.1 | OR1J1 | olfactory receptor, family 1, subfamily J, member 1 | Coding | 4.11 | 3.9 | 3.73 | 1.30 | 0.5406 | 0.7282 | 4.18 | 3.93 | 3.75 | 1.35 | 0.0356 | 0.171 |
| TC0900011998.hg.1 | SNORA17A; SI | small nucleolar RNA, H/ACA box 17A; small nucleolar RNA, H/AC | Multiple_C | 11.18 | 11.71 | 10.8 | 1.30 | 0.0817 | 0.2077 | 8.65 | 8.83 | 8.4 | 1.19 | 0.332 | 0.5961 |
| TC0900012191.hg.1 | C9orf172 | chromosome 9 open reading frame 172 | Coding | 5 | 4.42 | 4.62 | 1.30 | 0.8194 | 0.9093 | 4.96 | 4.76 | 4.63 | 1.26 | 0.5468 | 0.7641 |
| TC0X00006841.hg.1 | MAGEB5 | MAGE family member B5 | Coding | 4.51 | 4.13 | 4.13 | 1.30 | 0.1498 | 0.3216 | 4.44 | 4.22 | 4.24 | 1.15 | 0.7156 | 0.8673 |
| TC0X00006924.hg.1 | FAM47B | family with sequence similarity 47, member B | Coding | 5.85 | 5.14 | 5.47 | 1.30 | 0.2171 | 0.413 | 5.15 | 5.02 | 5.17 | 0.99 | 0.9448 | 0.9776 |
| TC0X00007227.hg.1 | GAGE10 | G antigen 10 | Coding | 5.42 | 5.26 | 5.04 | 1.30 | 0.1622 | 0.3396 | 4.66 | 4.60 | 4.45 | 1.16 | 0.6831 | 0.848 |
| TC0X00008052.hg.1 | CXorf57 | chromosome X open reading frame 57 | Multiple_C | 9.55 | 8.9 | 9.17 | 1.30 | 0.2429 | 0.4456 | 8.09 | 8.24 | 8.56 | 0.72 | 0.1316 | 0.3597 |
| TC0X00009356.hg.1 | TMEM47 | transmembrane protein 47 | Coding | 6.66 | 6.29 | 6.28 | 1.30 | 0.3275 | 0.5413 | 14.61 | 13.97 | 14.18 | 1.35 | 0.0461 | 0.1987 |
| TC0X00009793.hg.1 | PFKFB1 | 6-phosphofructo-2-kinase/fructose-2,6-biphosphatase 1 | Multiple_C | 4.1 | 3.64 | 3.72 | 1.30 | 0.2126 | 0.4074 | 3.79 | 3.79 | 4.02 | 0.85 | 0.427 | 0.6791 |
| TC0X00009869.hg.1 | ARHGEF9; ARI | Cdc42 guanine nucleotide exchange factor 9; ARHGEF9 intronic | Multiple_C | 5.41 | 5.19 | 5.03 | 1.30 | 0.0428 | 0.1293 | 9.52 | 9.71 | 9.46 | 1.04 | 0.448 | 0.6949 |
| TC0X00010401.hg.1 | NXF3 | nuclear RNA export factor 3 | Multiple_C | 3.88 | 3.74 | 3.5 | 1.30 | 0.1538 | 0.3277 | 4.18 | 4.38 | 4.12 | 1.04 | 0.6639 | 0.8355 |
| TC0X00010559.hg.1 | LRCH2 | leucine-rich repeats and calponin homology (CH) domain contai | Multiple_C | 3.02 | 2.69 | 2.64 | 1.30 | 0.167 | 0.3464 | 3.87 | 3.56 | 3.29 | 1.49 | 0.0095 | 0.0754 |
| TC0X00011245.hg.1 | H2AFB3 | H2A histone family, member B3 | Coding | 4.08 | 3.62 | 3.7 | 1.30 | 0.1262 | 0.2845 | 3.44 | 3.51 | 3.65 | 0.86 | 0.8905 | 0.9533 |
| TC1000007403.hg.1 | RET | ret proto-oncogene | Multiple_C | 4.44 | 3.89 | 4.06 | 1.30 | 0.2827 | 0.4929 | 4.85 | 5.08 | 5.14 | 0.82 | 0.9892 | 0.9955 |
| TC1000009387.hg.1 | PPP2R2D | protein phosphatase 2, regulatory subunit B, delta | Multiple_C | 9.79 | 9.76 | 9.41 | 1.30 | 0.0621 | 0.1696 | 9.26 | 8.34 | 9.13 | 1.09 | 0.9459 | 0.9781 |
| TC1000009612.hg.1 | KLF6 | Kruppel-like factor 6 | Multiple_C | 14.03 | 12.72 | 13.65 | 1.30 | 0.0638 | 0.1733 | 11.41 | 10.72 | 11.52 | 0.93 | 0.7335 | 0.8771 |
| TC1000010039.hg.1 | C10orf67 | chromosome 10 open reading frame 67 | Coding | 3.28 | 2.86 | 2.9 | 1.30 | 0.2862 | 0.4965 | 3.48 | 4.12 | 3.65 | 0.89 | 0.5889 | 0.7918 |

| | | | | | | | | | | | | | | | |
|---|---|---|---|---|---|---|---|---|---|---|---|---|---|---|---|
| TC1100006720.hg.1 | C11orf42 | chromosome 11 open reading frame 42 | Coding | 3.68 | 3.39 | 3.3 | 1.30 | 0.101 | 0.2426 | 4.11 | 4.38 | 4.32 | 0.86 | 0.3242 | 0.5884 |
| TC1100008174.hg.1 | ALDH3B1 | aldehyde dehydrogenase 3 family, member B1 | Multiple_C | 7.95 | 8.18 | 7.57 | 1.30 | 0.8099 | 0.904 | 6.64 | 6.59 | 6.04 | 1.52 | 0.055 | 0.2201 |
| TC1100009431.hg.1 | PATE4 | prostate and testis expressed 4 | Multiple_C | 5.77 | 5.27 | 5.39 | 1.30 | 0.2721 | 0.4807 | 6.4 | 6.56 | 6.33 | 1.05 | 0.6639 | 0.8355 |
| TC1100010703.hg.1 | CHRM4 | cholinergic receptor, muscarinic 4 | Coding | 3.35 | 2.91 | 2.97 | 1.30 | 0.1986 | 0.3896 | 3.39 | 3.51 | 3.66 | 0.83 | 0.7381 | 0.8793 |
| TC1100011103.hg.1 | STX5 | syntaxin 5 | Multiple_C | 7.44 | 7.39 | 7.06 | 1.30 | 0.1201 | 0.275 | 7.95 | 7.91 | 7.71 | 1.18 | 0.5886 | 0.7918 |
| TC1200006888.hg.1 | CDKN1B | cyclin-dependent kinase inhibitor 1B (p27, Kip1) | Multiple_C | 11.1 | 11.61 | 10.72 | 1.30 | 0.1602 | 0.3368 | 9.93 | 9.55 | 9.75 | 1.13 | 0.246 | 0.5105 |
| TC1200007687.hg.1 | EIF4B | eukaryotic translation initiation factor 4B | Multiple_C | 15.33 | 15.69 | 14.95 | 1.30 | 0.1526 | 0.326 | 15.77 | 15.45 | 15.99 | 0.86 | 0.4437 | 0.692 |
| TC1200007707.hg.1 | ESPL1 | extra spindle pole bodies like 1, separase | Multiple_C | 9.26 | 8.31 | 8.88 | 1.30 | 0.2854 | 0.4958 | 10.06 | 11.23 | 10.42 | 0.78 | 0.1342 | 0.364 |
| TC1200007895.hg.1 | OS9 | osteosarcoma amplified 9, endoplasmic reticulum lectin | Multiple_C | 11.7 | 10.89 | 11.32 | 1.30 | 0.3858 | 0.5992 | 11.38 | 11.88 | 11.81 | 0.74 | 0.2035 | 0.4591 |
| TC1200011755.hg.1 | NFYB | nuclear transcription factor Y subunit beta | Multiple_C | 10.97 | 11.92 | 10.59 | 1.30 | 0.2283 | 0.428 | 8.39 | 8.44 | 8.7 | 0.81 | 0.1441 | 0.3781 |
| TC1200011855.hg.1 | SVOP | SV2 related protein | Multiple_C | 5.44 | 5.08 | 5.06 | 1.30 | 0.2133 | 0.4084 | 5.02 | 4.76 | 4.43 | 1.51 | 0.0281 | 0.1475 |
| TC1200012151.hg.1 | RPLP0 | ribosomal protein, large, P0 | Multiple_C | 16.96 | 17.18 | 16.58 | 1.30 | 0.0616 | 0.1689 | 15.13 | 15.42 | 14.87 | 1.20 | 0.9138 | 0.9647 |
| TC1200012570.hg.1 | CACNA1C | calcium channel, voltage-dependent, L type, alpha 1C subunit | Multiple_C | 4.04 | 3.24 | 3.66 | 1.30 | 0.1432 | 0.3117 | 4.42 | 4.17 | 4.24 | 1.13 | 0.3033 | 0.5677 |
| TC1400006484.hg.1 | OR4Q3 | olfactory receptor, family 4, subfamily Q, member 3 | Coding | 3.75 | 3.28 | 3.37 | 1.30 | 0.0545 | 0.154 | 3.86 | 3.81 | 3.72 | 1.10 | 0.1595 | 0.4016 |
| TC1400007491.hg.1 | MPP5 | membrane protein, palmitoylated 5 | Multiple_C | 10.65 | 10.74 | 10.27 | 1.30 | 0.1307 | 0.2923 | 9.53 | 8.96 | 9.4 | 1.09 | 0.997 | 0.9987 |
| TC1400008053.hg.1 | LINC00521 | long intergenic non-protein coding RNA 521 | Multiple_C | 4.59 | 4.24 | 4.21 | 1.30 | 0.2536 | 0.4588 | 4.74 | 4.72 | 4.33 | 1.33 | 0.0754 | 0.2638 |
| TC1400008395.hg.1 | EIF5 | eukaryotic translation initiation factor 5 | Multiple_C | 15.67 | 14.61 | 15.29 | 1.30 | 0.0503 | 0.1451 | 15.49 | 14.75 | 15.52 | 0.98 | 0.2965 | 0.5611 |
| TC1400010756.hg.1 | GPR135 | G protein-coupled receptor 135 | Multiple_C | 5.49 | 5.11 | 5.11 | 1.30 | 0.1382 | 0.3039 | 4.45 | 4.64 | 4.46 | 0.99 | 0.9233 | 0.9685 |
| TC1500006788.hg.1 | GREM1 | gremlin 1, DAN family BMP antagonist | Multiple_C | 4.56 | 4.07 | 4.18 | 1.30 | 0.5554 | 0.7389 | 4.62 | 4.52 | 4.58 | 1.03 | 0.9268 | 0.9697 |
| TC1500009728.hg.1 | DAPK2 | death-associated protein kinase 2 | Multiple_C | 5.06 | 4.54 | 4.68 | 1.30 | 0.2376 | 0.4391 | 4.17 | 4.36 | 4.09 | 1.06 | 0.2802 | 0.546 |
| TC1600008034.hg.1 | NDRG4 | NDRG family member 4 | Multiple_C | 4.41 | 3.8 | 4.03 | 1.30 | 0.3694 | 0.5823 | 6.57 | 5.47 | 5.1 | 2.77 | 0.0064 | 0.0573 |
| TC1600009217.hg.1 | SRL | sarcalumenin | Coding | 3.14 | 2.85 | 2.76 | 1.30 | 0.0636 | 0.1729 | 3.09 | 3.21 | 3.22 | 0.91 | 0.9856 | 0.994 |
| TC1600010307.hg.1 | C16orf97 | chromosome 16 open reading frame 97 | Multiple_C | 4.15 | 3.79 | 3.77 | 1.30 | 0.2317 | 0.4321 | 4.23 | 4.65 | 4.17 | 1.04 | 0.8898 | 0.9531 |
| TC1700007871.hg.1 | HSPB9 | heat shock protein, alpha-crystallin-related, B9 | Coding | 2.78 | 2.21 | 2.4 | 1.30 | 0.0827 | 0.2096 | 2.94 | 3.27 | 3.19 | 0.84 | 0.5896 | 0.7924 |
| TC1700008673.hg.1 | CACNG5 | calcium channel, voltage-dependent, gamma subunit 5 | Coding | 3.62 | 3.26 | 3.24 | 1.30 | 0.1593 | 0.3355 | 4.81 | 4.60 | 4.26 | 1.46 | 0.0376 | 0.1767 |
| TC1700008757.hg.1 | MAP2K6 | mitogen-activated protein kinase kinase 6 | Multiple_C | 8.71 | 9.09 | 8.33 | 1.30 | 0.527 | 0.7187 | 7.2 | 8.96 | 8.1 | 0.54 | 0.0028 | 0.0335 |
| TC1700009469.hg.1 | OR1D5 | olfactory receptor, family 1, subfamily D, member 5 | Coding | 3.83 | 3.44 | 3.45 | 1.30 | 0.0607 | 0.1671 | 4.22 | 4.37 | 4.25 | 0.98 | 0.6069 | 0.8036 |
| TC1700010792.hg.1 | MEOX1 | mesenchyme homeobox 1 | Coding | 5.68 | 5.12 | 5.3 | 1.30 | 0.4447 | 0.6532 | 5.67 | 5.61 | 5.56 | 1.08 | 0.1986 | 0.4536 |
| TC1700011002.hg.1 | PRAC1 | prostate cancer susceptibility candidate 1 | Coding | 3.36 | 2.55 | 2.98 | 1.30 | 0.1444 | 0.314 | 3.64 | 3.73 | 3.7 | 0.96 | 0.5436 | 0.762 |
| TC1700012403.hg.1 | KRTAP1-3 | keratin associated protein 1-3 | Coding | 4.37 | 3.88 | 3.99 | 1.30 | 0.2696 | 0.4779 | 3.92 | 3.94 | 4.13 | 0.86 | 0.2531 | 0.5174 |
| TC1700012482.hg.1 | SIRT7 | sirtuin 7 | Multiple_C | 7.03 | 6.58 | 6.65 | 1.30 | 0.971 | 0.9861 | 8.62 | 8.21 | 8.24 | 1.30 | 0.1332 | 0.3621 |
| TC1900007764.hg.1 | WDR88 | WD repeat domain 88 | Coding | 3.59 | 3.14 | 3.21 | 1.30 | 0.0877 | 0.2188 | 3.54 | 3.89 | 3.86 | 0.80 | 0.1248 | 0.3491 |
| TC1900008526.hg.1 | LIN7B | lin-7 homolog B (C. elegans) | Multiple_C | 5.97 | 5.53 | 5.59 | 1.30 | 0.0683 | 0.1822 | 5.18 | 4.99 | 4.76 | 1.34 | 0.0604 | 0.2331 |

| | | | | | | | | | | | | | | | |
|---|---|---|---|---|---|---|---|---|---|---|---|---|---|---|---|
| TC1900008608.hg.1 | KLK2 | kallikrein related peptidase 2 | Multiple_C | 4.6 | 4.24 | 4.22 | 1.30 | 0.221 | 0.4184 | 4.74 | 5.14 | 5.16 | 0.75 | 0.2059 | 0.4624 |
| TC1900009417.hg.1 | MLLT1 | myeloid/lymphoid or mixed-lineage leukemia; translocated to, 1 | Multiple_C | 6.54 | 5.66 | 6.16 | 1.30 | 0.2772 | 0.4864 | 7.57 | 8.48 | 8.23 | 0.63 | 0.0101 | 0.0786 |
| TC1900009682.hg.1 | ELOF1 | ELF1 homolog, elongation factor 1 | Multiple_C | 10.47 | 9.59 | 10.09 | 1.30 | 0.2981 | 0.5096 | 7.42 | 7.55 | 8.06 | 0.64 | 0.0314 | 0.1581 |
| TC1900010849.hg.1 | PHLDB3 | pleckstrin homology-like domain, family B, member 3 | Multiple_C | 4.33 | 3.7 | 3.95 | 1.30 | 0.0714 | 0.1883 | 4.9 | 4.94 | 4.8 | 1.07 | 0.9547 | 0.9814 |
| TC1900011209.hg.1 | LRRC4B | leucine rich repeat containing 4B | Coding | 3.71 | 3.16 | 3.33 | 1.30 | 0.1426 | 0.3107 | 3.92 | 3.75 | 3.97 | 0.97 | 0.8274 | 0.9237 |
| TC1900012011.hg.1 | KLK8 | kallikrein related peptidase 8 | Multiple_C | 7.33 | 7.04 | 6.95 | 1.30 | 0.1481 | 0.3191 | 7.06 | 7.03 | 7.12 | 0.96 | 0.8177 | 0.9193 |
| TC2000009031.hg.1 | GHRH | growth hormone releasing hormone | Coding | 5.39 | 4.9 | 5.01 | 1.30 | 0.2091 | 0.4028 | 4.21 | 4.53 | 4.53 | 0.80 | 0.5303 | 0.7534 |
| TC2000010024.hg.1 | KCNB1 | potassium channel, voltage gated Shab related subfamily B, mem | Coding | 4.3 | 3.91 | 3.92 | 1.30 | 0.4044 | 0.6173 | 4.35 | 4.08 | 3.88 | 1.39 | 0.5698 | 0.7783 |
| TC2200006716.hg.1 | CCDC116 | coiled-coil domain containing 116 | Coding | 4.79 | 4.6 | 4.41 | 1.30 | 0.1398 | 0.3067 | 4.7 | 4.78 | 4.71 | 0.99 | 0.9027 | 0.9596 |
| TC2200007976.hg.1 | SLC25A1 | solute carrier family 25 (mitochondrial carrier; citrate transporte | Multiple_C | 10.89 | 10.19 | 10.51 | 1.30 | 0.1174 | 0.2705 | 6.78 | 7.87 | 8.16 | 0.38 | 9.60E-05 | 0.0035 |
| TC2200008741.hg.1 | PDGFB | platelet-derived growth factor beta polypeptide | Coding | 5.23 | 4.71 | 4.85 | 1.30 | 0.061 | 0.1677 | 6.45 | 5.94 | 5.87 | 1.49 | 0.0431 | 0.1915 |
| TSUnmapped00000111.h | RPS6KA1 | ribosomal protein S6 kinase, 90kDa, polypeptide 1 | Coding | 4.88 | 4.51 | 4.5 | 1.30 | 0.2243 | 0.4227 | 4.41 | 4.36 | 4.03 | 1.30 | 0.0357 | 0.1711 |
| TSUnmapped00000762.h | SLC25A26 | solute carrier family 25 (S-adenosylmethionine carrier), membe | Coding | 4.15 | 4.17 | 3.77 | 1.30 | 0.2296 | 0.4295 | 3.51 | 3.91 | 3.85 | 0.79 | 0.102 | 0.3125 |
| TSUnmapped00000808.h | PRAMEF8 | PRAME family member 8 | Coding | 3.39 | 2.7 | 3.01 | 1.30 | 0.1435 | 0.3122 | 3.44 | 3.60 | 3.31 | 1.09 | 0.5195 | 0.7469 |
| TSUnmapped00000823.h | DUX1 | double homeobox 1 | Coding | 4.84 | 4.45 | 4.46 | 1.30 | 0.0401 | 0.123 | 4.18 | 4.56 | 4.61 | 0.74 | 0.0373 | 0.1759 |
| TC0100006624.hg.1 | SMIM1 | small integral membrane protein 1 (Vel blood group) | Multiple_C | 4.86 | 4.25 | 4.49 | 1.29 | 0.1331 | 0.2963 | 4.61 | 4.73 | 4.58 | 1.02 | 0.6232 | 0.8138 |
| TC0100006861.hg.1 | FBXO44 | F-box protein 44 | Multiple_C | 6.14 | 5.93 | 5.77 | 1.29 | 0.1631 | 0.3411 | 8.54 | 7.65 | 7.08 | 2.75 | 1.70E-06 | 0.0002 |
| TC0100007290.hg.1 | C1QA | complement component 1, q subcomponent, A chain | Coding | 4.64 | 4.24 | 4.27 | 1.29 | 0.2422 | 0.4447 | 4.56 | 4.75 | 4.36 | 1.15 | 0.199 | 0.4542 |
| TC0100008937.hg.1 | LOC10192784 | uncharacterized LOC101927844; Transcript Identified by AceVie | Coding | 4.01 | 3.57 | 3.64 | 1.29 | 0.6499 | 0.8063 | 3.98 | 3.70 | 3.77 | 1.16 | 0.5672 | 0.7763 |
| TC0100009061.hg.1 | DR1 | down-regulator of transcription 1 | Multiple_C | 12.65 | 12.75 | 12.28 | 1.29 | 0.1239 | 0.2808 | 11.97 | 11.44 | 11.8 | 1.13 | 0.3543 | 0.6178 |
| TC0100010024.hg.1 | LELP1 | late cornified envelope-like proline-rich 1 | Coding | 4.23 | 4 | 3.86 | 1.29 | 0.1086 | 0.2562 | 4.55 | 4.36 | 4.83 | 0.82 | 0.2335 | 0.4958 |
| TC0100011001.hg.1 | RGS18 | regulator of G-protein signaling 18 | Multiple_C | 3.55 | 3.04 | 3.18 | 1.29 | 0.1358 | 0.3004 | 3.97 | 3.71 | 3.87 | 1.07 | 0.7051 | 0.861 |
| TC0100011026.hg.1 | TROVE2 | TROVE domain family, member 2 | Multiple_C | 10.64 | 10.29 | 10.27 | 1.29 | 0.1264 | 0.2849 | 11.28 | 9.96 | 10.76 | 1.43 | 0.0035 | 0.0391 |
| TC0100011842.hg.1 | WNT3A | wingless-type MMTV integration site family, member 3A | Coding | 3.18 | 2.54 | 2.81 | 1.29 | 0.0815 | 0.2074 | 2.91 | 3.11 | 3.08 | 0.89 | 0.7628 | 0.8929 |
| TC0100012849.hg.1 | CASZ1 | castor zinc finger 1 | Multiple_C | 8.07 | 6.95 | 7.7 | 1.29 | 0.2302 | 0.4304 | 10.08 | 10.18 | 10.33 | 0.84 | 0.3998 | 0.6568 |
| TC0100014127.hg.1 | SPATA6 | spermatogenesis associated 6 | Multiple_C | 7.38 | 7.78 | 7.01 | 1.29 | 0.3975 | 0.6111 | 7.94 | 6.95 | 6.38 | 2.95 | 7.04E-07 | 0.0001 |
| TC0100015353.hg.1 | CSDE1 | cold shock domain containing E1, RNA binding | Multiple_C | 14.17 | 13.95 | 13.8 | 1.29 | 0.5985 | 0.7712 | 12.92 | 12.46 | 12.7 | 1.16 | 0.8293 | 0.9243 |
| TC0100015847.hg.1 | SPRR2D | small proline-rich protein 2D | Coding | 4.16 | 3.81 | 3.79 | 1.29 | 0.035 | 0.1109 | 4.08 | 4.21 | 4.31 | 0.85 | 0.3884 | 0.6471 |
| TC0100016122.hg.1 | IGSF8 | immunoglobulin superfamily, member 8 | Multiple_C | 7.74 | 7.4 | 7.37 | 1.29 | 0.0401 | 0.123 | 4.69 | 4.72 | 4.75 | 0.96 | 0.7725 | 0.8978 |
| TC0100017310.hg.1 | ESRRG | estrogen-related receptor gamma | Multiple_C | 3.68 | 3.2 | 3.31 | 1.29 | 0.1742 | 0.3561 | 4.06 | 3.78 | 3.59 | 1.39 | 0.0146 | 0.0988 |
| TC0100018220.hg.1 | CCDC30 | coiled-coil domain containing 30 | Multiple_C | 3.84 | 3.48 | 3.47 | 1.29 | 0.2549 | 0.4605 | 4.28 | 3.71 | 3.71 | 1.48 | 0.0734 | 0.2596 |
| TC0100018221.hg.1 | C1orf50 | chromosome 1 open reading frame 50 | Multiple_C | 7.74 | 7.99 | 7.37 | 1.29 | 0.1422 | 0.3101 | 6.17 | 6.13 | 6.3 | 0.91 | 0.836 | 0.9278 |
| TC0200006835.hg.1 | MSGN1 | mesogenin 1 | Coding | 7.96 | 7.32 | 7.59 | 1.29 | 0.2363 | 0.4375 | 7.69 | 7.86 | 7.75 | 0.96 | 0.7371 | 0.8788 |

| | | | | | | | | | | | | | | | |
|---|---|---|---|---|---|---|---|---|---|---|---|---|---|---|---|
| TC0200013114.hg.1 | M1AP | meiosis 1 associated protein | Multiple_C | 3.97 | 3.67 | 3.6 | 1.29 | 0.4687 | 0.6728 | 3.91 | 3.91 | 3.85 | 1.04 | 0.9204 | 0.9672 |
| TC0200013351.hg.1 | RGPD1; RGPD | RANBP2-like and GRIP domain containing 1; RANBP2-like and G | Multiple_C | 6.26 | 6.08 | 5.89 | 1.29 | 0.6024 | 0.7745 | 7.93 | 7.04 | 7.33 | 1.52 | 0.0539 | 0.2173 |
| TC0200013524.hg.1 | NEURL3 | neuralized E3 ubiquitin protein ligase 3 | Multiple_C | 3.83 | 3.32 | 3.46 | 1.29 | 0.3058 | 0.5189 | 3.89 | 3.69 | 3.66 | 1.17 | 0.2915 | 0.5561 |
| TC0200013552.hg.1 | FAHD2B | fumarylacetoacetate hydrolase domain containing 2B | Multiple_C | 7.14 | 7.2 | 6.77 | 1.29 | 0.253 | 0.4579 | 6.61 | 6.65 | 6.31 | 1.23 | 0.0652 | 0.2431 |
| TC0200013865.hg.1 | LINC00116 | long intergenic non-protein coding RNA 116 | Multiple_C | 4.8 | 4.15 | 4.43 | 1.29 | 0.1858 | 0.3723 | 4.92 | 5.26 | 5.04 | 0.92 | 0.7508 | 0.8866 |
| TC0200016605.hg.1 | UBE2F-SCLY | UBE2F-SCLY readthrough (NMD candidate) | Multiple_C | 8.71 | 8.16 | 8.34 | 1.29 | 0.2044 | 0.3969 | 7.98 | 7.76 | 8.19 | 0.86 | 0.6069 | 0.8036 |
| TC0300007096.hg.1 | CCR8 | chemokine (C-C motif) receptor 8 | Multiple_C | 3.04 | 2.51 | 2.67 | 1.29 | 0.2582 | 0.4647 | 4.05 | 3.72 | 3.6 | 1.37 | 0.0631 | 0.2391 |
| TC0300007430.hg.1 | HEMK1 | HemK methyltransferase family member 1 | Multiple_C | 7.07 | 7.03 | 6.7 | 1.29 | 0.2493 | 0.4532 | 6.39 | 6.86 | 6.48 | 0.94 | 0.7636 | 0.8932 |
| TC0300007797.hg.1 | GPR27 | G protein-coupled receptor 27 | Coding | 5.01 | 4.43 | 4.64 | 1.29 | 0.2002 | 0.3919 | 5.86 | 5.77 | 5.63 | 1.17 | 0.1777 | 0.4263 |
| TC0300009194.hg.1 | SUCNR1 | succinate receptor 1 | Coding | 3.2 | 2.9 | 2.83 | 1.29 | 0.448 | 0.6561 | 3.6 | 3.42 | 3.47 | 1.09 | 0.1306 | 0.3579 |
| TC0300010185.hg.1 | OXTR | oxytocin receptor | Multiple_C | 5.65 | 5.21 | 5.28 | 1.29 | 0.0812 | 0.2071 | 5.55 | 5.39 | 5.1 | 1.37 | 0.047 | 0.2006 |
| TC0300010949.hg.1 | LTF | lactotransferrin | Multiple_C | 3.75 | 3.44 | 3.38 | 1.29 | 0.1387 | 0.3047 | 3.65 | 3.69 | 3.66 | 0.99 | 0.8833 | 0.95 |
| TC0300011455.hg.1 | FRMD4B | FERM domain containing 4B | Multiple_C | 6.36 | 5.73 | 5.99 | 1.29 | 0.4155 | 0.6276 | 5.21 | 5.27 | 5.07 | 1.10 | 0.5443 | 0.7625 |
| TC0300011988.hg.1 | GCSAM | germinal center-associated, signaling and motility | Coding | 3.67 | 3.18 | 3.3 | 1.29 | 0.1896 | 0.3774 | 3.33 | 3.22 | 3.36 | 0.98 | 0.6663 | 0.8365 |
| TC0300012006.hg.1 | CD200R1L | CD200 receptor 1 like | Coding | 3.45 | 3.19 | 3.08 | 1.29 | 0.349 | 0.5618 | 4.12 | 3.78 | 3.51 | 1.53 | 0.0076 | 0.0651 |
| TC0300012807.hg.1 | SIAH2 | siah E3 ubiquitin protein ligase 2 | Multiple_C | 11.06 | 10.09 | 10.69 | 1.29 | 0.111 | 0.2599 | 9.11 | 8.41 | 9.65 | 0.69 | 0.0488 | 0.2053 |
| TC0300013337.hg.1 | MCF2L2 | MCF.2 cell line derived transforming sequence-like 2 | Multiple_C | 4.75 | 4.47 | 4.38 | 1.29 | 0.0274 | 0.0915 | 4.5 | 4.57 | 4.59 | 0.94 | 0.6632 | 0.8355 |
| TC0400010694.hg.1 | ERVMER34-1 | endogenous retrovirus group MER34, member 1 | Multiple_C | 3.04 | 2.6 | 2.67 | 1.29 | 0.3244 | 0.5384 | 8.75 | 9.56 | 9.08 | 0.80 | 0.1887 | 0.4411 |
| TC0400012829.hg.1 | ARHGEF38 | Rho guanine nucleotide exchange factor 38 | Multiple_C | 10.29 | 10.9 | 9.92 | 1.29 | 0.6378 | 0.7991 | 7.73 | 5.23 | 6.35 | 2.60 | 0.0002 | 0.0063 |
| TC0500007292.hg.1 | NIM1K | NIM1 serine/threonine protein kinase | Multiple_C | 3.39 | 2.89 | 3.02 | 1.29 | 0.1938 | 0.3831 | 3.45 | 3.37 | 3.34 | 1.08 | 0.5336 | 0.7561 |
| TC0500007919.hg.1 | MSH3 | mutS homolog 3 | Multiple_C | 8.36 | 8.5 | 7.99 | 1.29 | 0.0706 | 0.1865 | 9.02 | 8.95 | 9.22 | 0.87 | 0.1788 | 0.4276 |
| TC0500009108.hg.1 | GPX3 | glutathione peroxidase 3 | Multiple_C | 3.89 | 3.36 | 3.52 | 1.29 | 0.1223 | 0.2785 | 5.13 | 4.45 | 4.62 | 1.42 | 0.0171 | 0.1094 |
| TC0500010766.hg.1 | PLPP1; RNF13 | phospholipid phosphatase 1; ring finger protein 138, E3 ubiquiti | Multiple_C | 9.44 | 8.82 | 9.07 | 1.29 | 0.1762 | 0.3587 | 8.72 | 8.51 | 8.28 | 1.36 | 0.0498 | 0.208 |
| TC0500011478.hg.1 | KIAA0825 | KIAA0825 | Multiple_C | 3.6 | 3.47 | 3.23 | 1.29 | 0.437 | 0.6464 | 4.2 | 4.27 | 3.84 | 1.28 | 0.1004 | 0.3098 |
| TC0500013222.hg.1 | LOC10050584 | zinc finger protein 474-like | Multiple_C | 5.06 | 4.29 | 4.69 | 1.29 | 0.5132 | 0.7086 | 7.82 | 6.96 | 6.7 | 2.17 | 0.3206 | 0.5845 |
| TC0600014350.hg.1 | TXLNB | taxilin beta | Coding | 3.58 | 3.07 | 3.21 | 1.29 | 0.3152 | 0.5285 | 4.02 | 4.13 | 3.91 | 1.08 | 0.3135 | 0.5777 |
| TC0700006449.hg.1 | WI2-2373I1.2; | forkhead box L1-like; novel transcript; Transcript Identified by A | Multiple_C | 5.05 | 4.69 | 4.68 | 1.29 | 0.1523 | 0.3257 | 4.42 | 4.66 | 4.32 | 1.07 | 0.3519 | 0.6153 |
| TC0700009232.hg.1 | BPGM | 2,3-bisphosphoglycerate mutase | Coding | 10.06 | 10.16 | 9.69 | 1.29 | 0.4389 | 0.6482 | 9.12 | 8.40 | 8.9 | 1.16 | 0.7352 | 0.8777 |
| TC0700012652.hg.1 | PLXNA4 | plexin A4 | Multiple_C | 3.49 | 3.33 | 3.12 | 1.29 | 0.0655 | 0.177 | 3.85 | 3.63 | 3.69 | 1.12 | 0.6736 | 0.8419 |
| TC0800009885.hg.1 | NKX3-1 | NK3 homeobox 1 | Multiple_C | 4.38 | 3.88 | 4.01 | 1.29 | 0.2115 | 0.4058 | 5.34 | 5.39 | 5.14 | 1.15 | 0.377 | 0.6374 |
| TC0800010765.hg.1 | TRAM1 | translocation associated membrane protein 1 | Multiple_C | 13.85 | 14.1 | 13.48 | 1.29 | 0.0846 | 0.2131 | 12.7 | 12.58 | 13.15 | 0.73 | 0.021 | 0.124 |
| TC0800012033.hg.1 | GPR20 | G protein-coupled receptor 20 | Coding | 3.77 | 2.97 | 3.4 | 1.29 | 0.2516 | 0.4561 | 3.73 | 3.72 | 3.72 | 1.01 | 0.666 | 0.8365 |
| TC0900006560.hg.1 | PDCD1LG2 | programmed cell death 1 ligand 2 | Multiple_C | 3.51 | 2.96 | 3.14 | 1.29 | 0.1737 | 0.3557 | 3.55 | 3.44 | 3.5 | 1.04 | 0.7467 | 0.8838 |

| | | | | | | | | | | | | | | | |
|---|---|---|---|---|---|---|---|---|---|---|---|---|---|---|---|
| TC0900009145.hg.1 | OLFM1 | olfactomedin 1 | Multiple_C | 3.31 | 2.96 | 2.94 | 1.29 | 0.3362 | 0.55 | 3.76 | 3.23 | 3.96 | 0.87 | 0.4947 | 0.7288 |
| TC0900009255.hg.1 | C9orf139 | chromosome 9 open reading frame 139 | Multiple_C | 3.73 | 3.69 | 3.36 | 1.29 | 0.2551 | 0.4608 | 3.32 | 3.32 | 3.32 | 1.00 | 0.4442 | 0.6923 |
| TC0900009520.hg.1 | PTPRD | protein tyrosine phosphatase, receptor type, D | Multiple_C | 4.54 | 4.19 | 4.17 | 1.29 | 0.1758 | 0.3582 | 4.86 | 4.56 | 4.78 | 1.06 | 0.1285 | 0.3548 |
| TC0900012163.hg.1 | ORM2 | orosomucoid 2 | Multiple_C | 2.76 | 2.22 | 2.39 | 1.29 | 0.164 | 0.3423 | 3.19 | 3.17 | 3.14 | 1.04 | 0.7822 | 0.9022 |
| TC0X00007298.hg.1 | SSX2B | synovial sarcoma, X breakpoint 2B | Multiple_C | 3.83 | 3.5 | 3.46 | 1.29 | 0.2595 | 0.4659 | 3.39 | 3.68 | 3.51 | 0.92 | 0.9805 | 0.9917 |
| TC0X00008136.hg.1 | ALG13 | ALG13, UDP-N-acetylglucosaminyltransferase subunit | Multiple_C | 9.61 | 9.61 | 9.24 | 1.29 | 0.5511 | 0.7359 | 8.48 | 8.70 | 9.42 | 0.52 | 0.0005 | 0.01 |
| TC0X00008210.hg.1 | SLC6A14 | solute carrier family 6 (amino acid transporter), member 14 | Multiple_C | 3.29 | 3.21 | 2.92 | 1.29 | 0.3524 | 0.5651 | 3.28 | 3.01 | 3.06 | 1.16 | 0.3089 | 0.5731 |
| TC1000007557.hg.1 | NPY4R; CH17- | Homo sapiens neuropeptide Y receptor Y4 (NPY4R), transcript v | Multiple_C | 3.51 | 2.96 | 3.14 | 1.29 | 0.0811 | 0.2069 | 3.6 | 3.58 | 3.23 | 1.29 | 0.277 | 0.5429 |
| TC1000008271.hg.1 | CDHR1 | cadherin-related family member 1 | Multiple_C | 4.14 | 3.94 | 3.77 | 1.29 | 0.3007 | 0.513 | 3.99 | 3.83 | 4.18 | 0.88 | 0.5919 | 0.7937 |
| TC1000008908.hg.1 | SHOC2 | SHOC2 leucine-rich repeat scaffold protein | Multiple_C | 11.83 | 11.76 | 11.46 | 1.29 | 0.0511 | 0.1469 | 10.09 | 9.87 | 10.5 | 0.75 | 0.0168 | 0.1084 |
| TC1000008951.hg.1 | HABP2 | hyaluronan binding protein 2 | Multiple_C | 3.01 | 2.57 | 2.64 | 1.29 | 0.2547 | 0.4602 | 3.5 | 3.45 | 3.39 | 1.08 | 0.1951 | 0.4492 |
| TC1000010840.hg.1 | ATOH7 | atonal bHLH transcription factor 7 | Coding | 4.19 | 3.61 | 3.82 | 1.29 | 0.3569 | 0.5703 | 3.64 | 3.52 | 3.54 | 1.07 | 0.4163 | 0.6702 |
| TC1000012414.hg.1 | SYCE1; SPRNP | synaptonemal complex central element protein 1; shadow of pr | Multiple_C | 4.43 | 3.69 | 4.06 | 1.29 | 0.0579 | 0.1613 | 4.32 | 3.99 | 3.74 | 1.49 | 0.0371 | 0.1752 |
| TC1100006546.hg.1 | TNNT3 | troponin T type 3 (skeletal, fast) | Multiple_C | 4.31 | 3.74 | 3.94 | 1.29 | 0.1115 | 0.2608 | 4.4 | 4.33 | 4.1 | 1.23 | 0.3098 | 0.5737 |
| TC1100007139.hg.1 | METTL15 | methyltransferase like 15 | Multiple_C | 9.66 | 10.05 | 9.29 | 1.29 | 0.0582 | 0.162 | 10.68 | 10.36 | 10.47 | 1.16 | 0.4592 | 0.7026 |
| TC1100007790.hg.1 | CD5 | CD5 molecule | Multiple_C | 4.93 | 4.58 | 4.56 | 1.29 | 0.2885 | 0.4993 | 5.42 | 5.06 | 4.72 | 1.62 | 0.0016 | 0.0228 |
| TC1100010003.hg.1 | CYB5R2 | cytochrome b5 reductase 2 | Multiple_C | 11.3 | 10.61 | 10.93 | 1.29 | 0.4741 | 0.6771 | 5.71 | 6.10 | 6.02 | 0.81 | 0.2056 | 0.4621 |
| TC1100011520.hg.1 | KRTAP5-11 | keratin associated protein 5-11 | Multiple_C | 6.61 | 6.14 | 6.24 | 1.29 | 0.0373 | 0.1164 | 6.47 | 6.17 | 5.89 | 1.49 | 0.0071 | 0.0617 |
| TC1100012471.hg.1 | AMICA1 | adhesion molecule, interacts with CXADR antigen 1 | Multiple_C | 3.54 | 3.29 | 3.17 | 1.29 | 0.3786 | 0.5916 | 3.13 | 3.39 | 3.5 | 0.77 | 0.1692 | 0.4151 |
| TC1100012646.hg.1 | TMEM225 | transmembrane protein 225 | Coding | 3.48 | 3.29 | 3.11 | 1.29 | 0.7462 | 0.8695 | 3.4 | 3.73 | 3.64 | 0.85 | 0.343 | 0.6067 |
| TC1200007151.hg.1 | SMCO2 | single-pass membrane protein with coiled-coil domains 2 | Multiple_C | 4.92 | 4.58 | 4.55 | 1.29 | 0.0876 | 0.2186 | 5.1 | 4.49 | 5.01 | 1.06 | 0.87 | 0.944 |
| TC1200008223.hg.1 | GLIPR1L2 | GLI pathogenesis-related 1 like 2 | Multiple_C | 4.58 | 4.83 | 4.21 | 1.29 | 0.2885 | 0.4993 | 4.89 | 4.93 | 4.81 | 1.06 | 0.0955 | 0.3006 |
| TC1200008906.hg.1 | TRAFD1 | TRAF-type zinc finger domain containing 1 | Multiple_C | 9.28 | 8.95 | 8.91 | 1.29 | 0.4741 | 0.6771 | 8.08 | 8.40 | 8.59 | 0.70 | 0.2532 | 0.5175 |
| TC1200010910.hg.1 | APOF | apolipoprotein F | Coding | 4.44 | 3.54 | 4.07 | 1.29 | 0.6526 | 0.8079 | 4.45 | 4.35 | 4.34 | 1.08 | 0.9009 | 0.9589 |
| TC1300008772.hg.1 | CCDC122 | coiled-coil domain containing 122 | Multiple_C | 5.67 | 5.64 | 5.3 | 1.29 | 0.6922 | 0.8342 | 7.74 | 8.17 | 6.61 | 2.19 | 0.0003 | 0.0069 |
| TC1500006528.hg.1 | GOLGA8S | golgin A8 family, member S | Multiple_C | 5.35 | 4.93 | 4.98 | 1.29 | 0.2233 | 0.4215 | 6 | 5.86 | 5.86 | 1.10 | 0.1298 | 0.3569 |
| TC1500007757.hg.1 | NR2E3 | nuclear receptor subfamily 2, group E, member 3 | Multiple_C | 4.29 | 3.74 | 3.92 | 1.29 | 0.4556 | 0.6625 | 4.64 | 4.36 | 4.3 | 1.27 | 0.1036 | 0.3157 |
| TC1500007860.hg.1 | MPI | mannose phosphate isomerase | Multiple_C | 10.16 | 9.39 | 9.79 | 1.29 | 0.34 | 0.554 | 10.42 | 10.95 | 10.59 | 0.89 | 0.5902 | 0.7927 |
| TC1500009981.hg.1 | HCN4 | hyperpolarization activated cyclic nucleotide gated potassium c | Multiple_C | 3.9 | 3.73 | 3.53 | 1.29 | 0.2287 | 0.4284 | 3.71 | 3.74 | 3.65 | 1.04 | 0.9508 | 0.9797 |
| TC1600006439.hg.1 | MPG | N-methylpurine DNA glycosylase | Multiple_C | 6.58 | 6.16 | 6.21 | 1.29 | 0.2226 | 0.4203 | 5.69 | 6.38 | 5.76 | 0.95 | 0.7204 | 0.8698 |
| TC1600006641.hg.1 | FLYWCH2 | FLYWCH family member 2 | Multiple_C | 9.18 | 8.57 | 8.81 | 1.29 | 0.4494 | 0.6573 | 7.35 | 7.36 | 6.92 | 1.35 | 0.7035 | 0.8598 |
| TC1600008335.hg.1 | DHX38 | DEAH (Asp-Glu-Ala-His) box polypeptide 38 | Multiple_C | 8.12 | 7.99 | 7.75 | 1.29 | 0.4156 | 0.6276 | 9.12 | 9.74 | 9.25 | 0.91 | 0.9325 | 0.9717 |
| TC1600009210.hg.1 | ADCY9 | adenylate cyclase 9 | Multiple_C | 6.94 | 6.47 | 6.57 | 1.29 | 0.1745 | 0.3563 | 5.7 | 5.99 | 6.4 | 0.62 | 0.001 | 0.0167 |

| | | | | | | | | | | | | | | | |
|---|---|---|---|---|---|---|---|---|---|---|---|---|---|---|---|
| TC1600011323.hg.1 | HAGHL | hydroxyacylglutathione hydrolase-like | Multiple_C | 6.61 | 6.26 | 6.24 | 1.29 | 0.0668 | 0.1793 | 6.71 | 6.95 | 6.55 | 1.12 | 0.3663 | 0.6283 |
| TC1700006524.hg.1 | SERPINF1 | serpin peptidase inhibitor, clade F (alpha-2 antiplasmin, pigmen | Multiple_C | 5.33 | 4.98 | 4.96 | 1.29 | 0.143 | 0.3114 | 11.6 | 11.93 | 11.61 | 0.99 | 1 | 1 |
| TC1700006784.hg.1 | GUCY2D | guanylate cyclase 2D, membrane (retina-specific) | Multiple_C | 7.97 | 7.72 | 7.6 | 1.29 | 0.1372 | 0.3024 | 7.4 | 7.58 | 7.49 | 0.94 | 0.897 | 0.9566 |
| TC1700007558.hg.1 | CCL7 | chemokine (C-C motif) ligand 7 | Multiple_C | 4.6 | 3.96 | 4.23 | 1.29 | 0.6795 | 0.8259 | 4.56 | 4.82 | 4.56 | 1.00 | 0.919 | 0.9668 |
| TC1700008216.hg.1 | KAT7 | K(lysine) acetyltransferase 7 | Multiple_C | 8.24 | 7.41 | 7.87 | 1.29 | 0.309 | 0.5221 | 8.42 | 7.62 | 8.31 | 1.08 | 0.2132 | 0.4713 |
| TC1700009249.hg.1 | UTS2R | urotensin 2 receptor | Coding | 4.44 | 3.99 | 4.07 | 1.29 | 0.483 | 0.6845 | 4.7 | 4.93 | 4.56 | 1.10 | 0.2597 | 0.524 |
| TC1700010425.hg.1 | SLFN12 | schlafen family member 12 | Multiple_C | 4.36 | 4.02 | 3.99 | 1.29 | 0.7038 | 0.842 | 4.08 | 3.95 | 3.93 | 1.11 | 0.769 | 0.8964 |
| TC1700011726.hg.1 | HID1 | HID1 domain containing | Multiple_C | 5.85 | 5.32 | 5.48 | 1.29 | 0.5855 | 0.7619 | 5.64 | 5.13 | 4.59 | 2.07 | 0.0023 | 0.029 |
| TC1700012413.hg.1 | KRT34 | keratin 34, type I | Multiple_C | 5.26 | 4.78 | 4.89 | 1.29 | 0.2821 | 0.4921 | 5.07 | 5.33 | 5.44 | 0.77 | 0.1087 | 0.3246 |
| TC1700012485.hg.1 | MYADML2 | myeloid-associated differentiation marker-like 2 | Coding | 5.64 | 5.03 | 5.27 | 1.29 | 0.0781 | 0.201 | 4.77 | 4.69 | 4.93 | 0.90 | 0.6304 | 0.818 |
| TC1800006675.hg.1 | APCDD1 | adenomatosis polyposis coli down-regulated 1 | Multiple_C | 4.43 | 3.82 | 4.06 | 1.29 | 0.4561 | 0.6629 | 11.15 | 13.15 | 10.29 | 1.82 | 0.001 | 0.0165 |
| TC1800006728.hg.1 | CIDEA | cell death-inducing DFFA-like effector a | Multiple_C | 5.6 | 5.03 | 5.23 | 1.29 | 0.3912 | 0.6049 | 4.27 | 4.43 | 4.5 | 0.85 | 0.9185 | 0.9666 |
| TC1900007831.hg.1 | HPN | hepsin | Multiple_C | 6.01 | 5.49 | 5.64 | 1.29 | 0.1163 | 0.2689 | 5.88 | 5.71 | 5.36 | 1.43 | 0.0395 | 0.1817 |
| TC1900008840.hg.1 | RPS9 | ribosomal protein S9 | Multiple_C | 15.94 | 17.14 | 15.57 | 1.29 | 0.0692 | 0.1838 | 13.35 | 13.52 | 12.93 | 1.34 | 0.0555 | 0.2213 |
| TC1900009805.hg.1 | SAMD1 | sterile alpha motif domain containing 1 | Multiple_C | 6.48 | 5.69 | 6.11 | 1.29 | 0.2802 | 0.4899 | 8.11 | 8.53 | 8.3 | 0.88 | 0.1922 | 0.4454 |
| TC1900011605.hg.1 | ZSCAN18 | zinc finger and SCAN domain containing 18 | Multiple_C | 5.78 | 5.16 | 5.41 | 1.29 | 0.1823 | 0.3672 | 3.87 | 3.99 | 4.12 | 0.84 | 0.0539 | 0.2175 |
| TC1900011870.hg.1 | ELAVL3 | ELAV like neuron-specific RNA binding protein 3 | Multiple_C | 2.94 | 2.41 | 2.57 | 1.29 | 0.2714 | 0.4799 | 3.03 | 3.11 | 3.01 | 1.01 | 0.383 | 0.642 |
| TC1900011997.hg.1 | CGB7 | chorionic gonadotropin, beta polypeptide 7 | Multiple_C | 4.22 | 3.84 | 3.85 | 1.29 | 0.0232 | 0.0804 | 4.27 | 4.21 | 4.14 | 1.09 | 0.3172 | 0.5812 |
| TC2000006508.hg.1 | TGM3 | transglutaminase 3 | Multiple_C | 5.27 | 4.71 | 4.9 | 1.29 | 0.0299 | 0.098 | 5.21 | 5.10 | 4.84 | 1.29 | 0.1179 | 0.3395 |
| TC2000007855.hg.1 | SPO11 | SPO11 meiotic protein covalently bound to DSB | Coding | 4.62 | 4.75 | 4.25 | 1.29 | 0.3361 | 0.55 | 3.91 | 4.05 | 3.79 | 1.09 | 0.1984 | 0.4533 |
| TC2000009544.hg.1 | CBLN4 | cerebellin 4 precursor | Coding | 4.54 | 4.23 | 4.17 | 1.29 | 0.4028 | 0.6157 | 4.87 | 4.89 | 4.66 | 1.16 | 0.7971 | 0.9093 |
| TC2000009802.hg.1 | NKAIN4 | Na+/K+ transporting ATPase interacting 4 | Multiple_C | 6.27 | 5.67 | 5.9 | 1.29 | 0.5487 | 0.734 | 4.75 | 4.75 | 4.86 | 0.93 | 0.7133 | 0.8657 |
| TC2100008576.hg.1 | KRTAP10-5 | keratin associated protein 10-5 | Multiple_C | 4.92 | 4.48 | 4.55 | 1.29 | 0.1813 | 0.3657 | 3.92 | 4.02 | 4.3 | 0.77 | 0.9908 | 0.9962 |
| TC2200008874.hg.1 | NFAM1 | NFAT activating protein with ITAM motif 1 | Multiple_C | 4.06 | 3.6 | 3.69 | 1.29 | 0.1655 | 0.3441 | 4.05 | 4.07 | 4.1 | 0.97 | 0.8476 | 0.9338 |
| TC2200009000.hg.1 | PRR34 | proline rich 34 | Coding | 5.64 | 5.31 | 5.27 | 1.29 | 0.2709 | 0.4794 | 5.53 | 5.38 | 5.19 | 1.27 | 0.9716 | 0.9882 |
| TSUnmapped00000159.h | ADAMTS13 | ADAM metallopeptidase with thrombospondin type 1 motif 13 | Coding | 3.86 | 3.44 | 3.49 | 1.29 | 0.1366 | 0.3014 | 3.64 | 3.91 | 3.55 | 1.06 | 0.5697 | 0.7783 |
| TSUnmapped00000161.h | SURF1 | surfeit 1 | Coding | 5.43 | 5.91 | 5.06 | 1.29 | 0.7049 | 0.8425 | 5.43 | 5.84 | 5.1 | 1.26 | 0.2094 | 0.4664 |
| TSUnmapped00000233.h | HYOU1 | hypoxia up-regulated 1 | Coding | 6.8 | 6.78 | 6.43 | 1.29 | 0.4608 | 0.6664 | 6.67 | 6.43 | 6.43 | 1.18 | 0.1115 | 0.3291 |
| TSUnmapped00000390.h | RPS6KA1 | ribosomal protein S6 kinase, 90kDa, polypeptide 1 | Coding | 8.46 | 8.42 | 8.09 | 1.29 | 0.1299 | 0.2909 | 6.54 | 6.31 | 6.01 | 1.44 | 0.1765 | 0.4251 |
| TSUnmapped00000485.h | RPS6KA1 | ribosomal protein S6 kinase, 90kDa, polypeptide 1 | Coding | 10.54 | 9.8 | 10.17 | 1.29 | 0.3354 | 0.5494 | 7.89 | 8.39 | 8.04 | 0.90 | 0.2158 | 0.4748 |
| TSUnmapped00000660.h | PRAMEF18 | PRAME family member 18 | Coding | 3.33 | 3.06 | 2.96 | 1.29 | 0.5212 | 0.7146 | 4.21 | 4.37 | 3.73 | 1.39 | 0.0436 | 0.193 |
| TSUnmapped00000677.h | RPL7A | ribosomal protein L7a | Coding | 15.75 | 16.01 | 15.38 | 1.29 | 0.1609 | 0.3378 | 13.82 | 13.91 | 13.38 | 1.36 | 0.0334 | 0.1641 |
| TC0100006997.hg.1 | SLC25A34 | solute carrier family 25, member 34 | Multiple_C | 7.91 | 7.04 | 7.55 | 1.28 | 0.0514 | 0.1474 | 6.39 | 6.67 | 6.88 | 0.71 | 0.0361 | 0.1724 |

| | | | | | | | | | | | | | | | |
|---|---|---|---|---|---|---|---|---|---|---|---|---|---|---|---|
| TC0100007725.hg.1 | ZSCAN20 | zinc finger and SCAN domain containing 20 | Multiple_C | 5.37 | 4.75 | 5.01 | 1.28 | 0.1712 | 0.3522 | 4.73 | 4.89 | 4.82 | 0.94 | 0.5828 | 0.7874 |
| TC0100009943.hg.1 | SCNM1; TNFA | sodium channel modifier 1; tumor necrosis factor, alpha-induce | Multiple_C | 11.53 | 11.05 | 11.17 | 1.28 | 0.3906 | 0.6042 | 9.82 | 10.29 | 10.2 | 0.77 | 0.0886 | 0.288 |
| TC0100010846.hg.1 | RGSL1 | regulator of G-protein signaling like 1 | Multiple_C | 3.79 | 3.53 | 3.43 | 1.28 | 0.264 | 0.4713 | 3.7 | 3.78 | 3.55 | 1.11 | 0.7514 | 0.8867 |
| TC0100013061.hg.1 | MST1L | macrophage stimulating 1-like | Multiple_C | 4.21 | 5.02 | 3.85 | 1.28 | 0.0635 | 0.1727 | 3.76 | 3.94 | 3.89 | 0.91 | 0.8136 | 0.9172 |
| TC0100014081.hg.1 | PDZK1IP1 | PDZK1 interacting protein 1 | Multiple_C | 4.53 | 4.1 | 4.17 | 1.28 | 0.2353 | 0.4364 | 4.62 | 4.52 | 4.41 | 1.16 | 0.297 | 0.5616 |
| TC0100016095.hg.1 | OR10J5 | olfactory receptor, family 10, subfamily J, member 5 | Coding | 3.47 | 2.76 | 3.11 | 1.28 | 0.2633 | 0.4706 | 3.47 | 3.20 | 3.07 | 1.32 | 0.074 | 0.261 |
| TC0100016567.hg.1 | TOR1AIP2 | torsin A interacting protein 2 | Multiple_C | 10.18 | 9.6 | 9.82 | 1.28 | 0.1826 | 0.3676 | 9.59 | 9.07 | 9.56 | 1.02 | 0.7004 | 0.858 |
| TC0100018322.hg.1 | C1orf220 | chromosome 1 open reading frame 220 | Multiple_C | 7.23 | 6.66 | 6.87 | 1.28 | 0.0594 | 0.1644 | 5.12 | 5.22 | 5.62 | 0.71 | 0.1161 | 0.3365 |
| TC0100018406.hg.1 | HNRNPCL1 | heterogeneous nuclear ribonucleoprotein C-like 1 | Coding | 7 | 7.07 | 6.64 | 1.28 | 0.5441 | 0.7305 | 6.42 | 6.93 | 6.52 | 0.93 | 0.6055 | 0.8027 |
| TC0200007976.hg.1 | ATP6V1B1 | ATPase, H+ transporting, lysosomal 56/58kDa, V1 subunit B1 | Multiple_C | 4.92 | 4.28 | 4.56 | 1.28 | 0.2828 | 0.4929 | 5.65 | 5.99 | 5.77 | 0.92 | 0.7263 | 0.8728 |
| TC0200014802.hg.1 | SCN3A | sodium channel, voltage gated, type III alpha subunit | Multiple_C | 3.34 | 2.93 | 2.98 | 1.28 | 0.3319 | 0.5458 | 6.13 | 6.11 | 4.52 | 3.05 | 7.83E-05 | 0.003 |
| TC0300006993.hg.1 | CRTAP | cartilage associated protein | Multiple_C | 12.97 | 12.46 | 12.61 | 1.28 | 0.1532 | 0.3269 | 9.75 | 9.96 | 10.36 | 0.66 | 0.009 | 0.0729 |
| TC0300008148.hg.1 | TMEM45A | transmembrane protein 45A | Multiple_C | 4.57 | 4.11 | 4.21 | 1.28 | 0.4254 | 0.6363 | 5.43 | 5.14 | 5.23 | 1.15 | 0.9007 | 0.9589 |
| TC0300008904.hg.1 | PPP2R3A | protein phosphatase 2, regulatory subunit B, alpha | Multiple_C | 3.92 | 3.27 | 3.56 | 1.28 | 0.1183 | 0.272 | 3.46 | 3.34 | 3.47 | 0.99 | 0.6918 | 0.8529 |
| TC0300011403.hg.1 | MAGI1 | membrane associated guanylate kinase, WW and PDZ domain c | Multiple_C | 8.46 | 8.52 | 8.1 | 1.28 | 0.1808 | 0.365 | 9.22 | 9.96 | 9.58 | 0.78 | 0.0996 | 0.3085 |
| TC0300013813.hg.1 | ACKR2 | atypical chemokine receptor 2 | Multiple_C | 6.41 | 5.65 | 6.05 | 1.28 | 0.2615 | 0.4684 | 5.93 | 6.12 | 5.76 | 1.13 | 0.2888 | 0.5534 |
| TC0400006712.hg.1 | TADA2B | transcriptional adaptor 2B | Multiple_C | 8.61 | 8.04 | 8.25 | 1.28 | 0.0333 | 0.1066 | 8.81 | 8.99 | 9.07 | 0.84 | 0.1195 | 0.3416 |
| TC0400007345.hg.1 | LIMCH1 | LIM and calponin homology domains 1 | Multiple_C | 4.12 | 3.63 | 3.76 | 1.28 | 0.009 | 0.0384 | 3.24 | 3.35 | 3.14 | 1.07 | 0.5462 | 0.7637 |
| TC0400007901.hg.1 | SHROOM3 | shroom family member 3 | Multiple_C | 11.24 | 10.26 | 10.88 | 1.28 | 0.2198 | 0.4169 | 8.68 | 9.12 | 8.37 | 1.24 | 0.135 | 0.3648 |
| TC0400007958.hg.1 | PRDM8 | PR domain containing 8 | Multiple_C | 4.59 | 4.03 | 4.23 | 1.28 | 0.139 | 0.3052 | 4.11 | 4.17 | 3.91 | 1.15 | 0.4879 | 0.7244 |
| TC0400009765.hg.1 | MXD4; MIR48 | MAX dimerization protein 4; microRNA 4800 | Multiple_C | 9.58 | 9.28 | 9.22 | 1.28 | 0.294 | 0.5053 | 8.5 | 8.80 | 8.27 | 1.17 | 0.0595 | 0.2304 |
| TC0400010580.hg.1 | GNPDA2 | glucosamine-6-phosphate deaminase 2 | Multiple_C | 8.86 | 9.49 | 8.5 | 1.28 | 0.4557 | 0.6625 | 7.37 | 6.86 | 7.58 | 0.86 | 0.9573 | 0.9823 |
| TC0400010925.hg.1 | UGT2B11 | UDP glucuronosyltransferase 2 family, polypeptide B11 | Multiple_C | 4.11 | 3.88 | 3.75 | 1.28 | 0.4391 | 0.6483 | 4.32 | 4.28 | 4.18 | 1.10 | 0.9216 | 0.9676 |
| TC0400011217.hg.1 | NKX6-1 | NK6 homeobox 1 | Coding | 4.51 | 4.24 | 4.15 | 1.28 | 0.3128 | 0.5263 | 4.99 | 4.98 | 4.6 | 1.31 | 0.2175 | 0.4769 |
| TC0400012257.hg.1 | C4orf45 | chromosome 4 open reading frame 45 | Multiple_C | 4.44 | 4.17 | 4.08 | 1.28 | 0.0909 | 0.2245 | 4.87 | 4.69 | 4.51 | 1.28 | 0.214 | 0.4723 |
| TC0400012868.hg.1 | STOX2 | storkhead box 2 | Multiple_C | 4.63 | 4.32 | 4.27 | 1.28 | 0.0265 | 0.0891 | 4.29 | 4.14 | 4.35 | 0.96 | 0.519 | 0.7466 |
| TC0400012900.hg.1 | FBXL5 | F-box and leucine-rich repeat protein 5 | Multiple_C | 9 | 9.45 | 8.64 | 1.28 | 0.1976 | 0.3884 | 7.27 | 6.50 | 7.21 | 1.04 | 0.8084 | 0.9155 |
| TC0500010920.hg.1 | SREK1IP1 | SREK1-interacting protein 1 | Multiple_C | 12.47 | 13.23 | 12.11 | 1.28 | 0.1637 | 0.342 | 10.08 | 10.40 | 10.44 | 0.78 | 0.8065 | 0.9146 |
| TC0500012145.hg.1 | KLHL3 | kelch-like family member 3 | Multiple_C | 3.18 | 3.04 | 2.82 | 1.28 | 0.2058 | 0.3988 | 3.3 | 3.47 | 3.5 | 0.87 | 0.5558 | 0.77 |
| TC0500012340.hg.1 | NR3C1 | nuclear receptor subfamily 3, group C, member 1 (glucocorticoi | Multiple_C | 3.65 | 3.29 | 3.29 | 1.28 | 0.1852 | 0.3714 | 4.68 | 3.99 | 4.62 | 1.04 | 0.1883 | 0.4404 |
| TC0500012392.hg.1 | SPINK1 | serine peptidase inhibitor, Kazal type 1 | Multiple_C | 4.2 | 3.87 | 3.84 | 1.28 | 0.5067 | 0.7035 | 4.46 | 4.14 | 3.89 | 1.48 | 0.1532 | 0.3922 |
| TC0500013020.hg.1 | CLK4 | CDC like kinase 4 | Multiple_C | 9.98 | 11.42 | 9.62 | 1.28 | 0.1003 | 0.2414 | 8.73 | 7.59 | 8.2 | 1.44 | 0.0114 | 0.0844 |
| TC0500013391.hg.1 | PWWP2A | PWWP domain containing 2A | Multiple_C | 9.84 | 9.3 | 9.48 | 1.28 | 0.1153 | 0.2673 | 9.67 | 9.80 | 9.78 | 0.93 | 0.4923 | 0.727 |

| | | | | | | | | | | | | | | | |
|---|---|---|---|---|---|---|---|---|---|---|---|---|---|---|---|
| TC0600006442.hg.1 | IRF4 | interferon regulatory factor 4 | Multiple_C | 5.45 | 4.93 | 5.09 | 1.28 | 0.2152 | 0.4107 | 5.1 | 5.29 | 4.92 | 1.13 | 0.5231 | 0.7491 |
| TC0600008022.hg.1 | TOMM6; PRIC | translocase of outer mitochondrial membrane 6 homolog (yeas | Multiple_C | 4.83 | 4.51 | 4.47 | 1.28 | 0.2405 | 0.4425 | 4.15 | 3.95 | 3.8 | 1.27 | 0.0392 | 0.1805 |
| TC0600008035.hg.1 | TAF8 | TATA box binding protein associated factor 8 | Multiple_C | 10.72 | 9.95 | 10.36 | 1.28 | 0.2114 | 0.4058 | 10.68 | 10.84 | 10.81 | 0.91 | 0.2244 | 0.4851 |
| TC0600010960.hg.1 | TPMT | thiopurine S-methyltransferase | Multiple_C | 13.78 | 13.66 | 13.42 | 1.28 | 0.2489 | 0.4529 | 12.05 | 11.34 | 11.82 | 1.17 | 0.4269 | 0.6791 |
| TC0600012296.hg.1 | EEF1A1 | eukaryotic translation elongation factor 1 alpha 1 | Multiple_C | 18.31 | 18.11 | 17.95 | 1.28 | 0.0199 | 0.0712 | 18.59 | 18.45 | 18.6 | 0.99 | 0.642 | 0.8245 |
| TC0600013062.hg.1 | TRDN | triadin | Coding | 3.62 | 3.18 | 3.26 | 1.28 | 0.8866 | 0.9453 | 4.57 | 4.12 | 3.77 | 1.74 | 0.0213 | 0.1249 |
| TC0600013604.hg.1 | RGS17 | regulator of G-protein signaling 17 | Multiple_C | 4.11 | 3.7 | 3.75 | 1.28 | 0.2106 | 0.4048 | 4.41 | 3.87 | 4.23 | 1.13 | 0.4215 | 0.6745 |
| TC0700006913.hg.1 | GPNMB | glycoprotein (transmembrane) nmb | Multiple_C | 5.14 | 4.59 | 4.78 | 1.28 | 0.455 | 0.6623 | 5.03 | 5.24 | 5.08 | 0.97 | 0.5504 | 0.7664 |
| TC0700008582.hg.1 | SERPINE1 | serpin peptidase inhibitor, clade E (nexin, plasminogen activato | Multiple_C | 5.12 | 4.5 | 4.76 | 1.28 | 0.0835 | 0.2109 | 9.82 | 7.68 | 8.37 | 2.73 | 0.0013 | 0.0196 |
| TC0700011831.hg.1 | PON1 | paraoxonase 1 | Multiple_C | 4.25 | 3.8 | 3.89 | 1.28 | 0.2452 | 0.4487 | 3.47 | 3.71 | 3.68 | 0.86 | 0.6727 | 0.8412 |
| TC0700012712.hg.1 | LUZP6; MTPN | leucine zipper protein 6; myotrophin | Multiple_C | 13.04 | 12.73 | 12.68 | 1.28 | 0.3816 | 0.5947 | 13.68 | 13.02 | 13.63 | 1.04 | 0.6956 | 0.8555 |
| TC0700012751.hg.1 | SVOPL | SVOP-like | Multiple_C | 3.66 | 3.27 | 3.3 | 1.28 | 0.373 | 0.5855 | 4.22 | 3.71 | 3.45 | 1.71 | 0.0006 | 0.0119 |
| TC0800007439.hg.1 | POLB | polymerase (DNA directed), beta | Multiple_C | 9.4 | 10.9 | 9.04 | 1.28 | 0.0688 | 0.183 | 7.59 | 6.60 | 7.08 | 1.42 | 0.0685 | 0.2492 |
| TC0800007738.hg.1 | SDCBP | syndecan binding protein | Multiple_C | 15.61 | 16 | 15.25 | 1.28 | 0.1324 | 0.2952 | 11.3 | 10.65 | 10.55 | 1.68 | 0.0012 | 0.0192 |
| TC0800008641.hg.1 | MED30 | mediator complex subunit 30 | Multiple_C | 10.92 | 11.88 | 10.56 | 1.28 | 0.1274 | 0.2867 | 7.09 | 7.41 | 7.06 | 1.02 | 0.3057 | 0.5697 |
| TC0800008946.hg.1 | TG | thyroglobulin | Multiple_C | 3.03 | 2.44 | 2.67 | 1.28 | 0.1949 | 0.3848 | 3.23 | 3.41 | 3.42 | 0.88 | 0.333 | 0.5972 |
| TC0900006872.hg.1 | LOC10050642 | putative deoxyuridine 5-triphosphate nucleotidohydrolase-like | Multiple_C | 4.22 | 3.84 | 3.86 | 1.28 | 0.2744 | 0.4831 | 4.49 | 4.69 | 4.47 | 1.01 | 0.867 | 0.9431 |
| TC0900007437.hg.1 | FOXD4L4 | forkhead box D4-like 4 | Coding | 6.18 | 5.68 | 5.82 | 1.28 | 0.2261 | 0.4251 | 6.18 | 5.91 | 6.11 | 1.05 | 0.4193 | 0.6729 |
| TC0900011441.hg.1 | OR1N1 | olfactory receptor, family 1, subfamily N, member 1 | Coding | 3.68 | 3.23 | 3.32 | 1.28 | 0.3326 | 0.5464 | 3.37 | 3.07 | 3.21 | 1.12 | 0.0495 | 0.2073 |
| TC0900012175.hg.1 | CDK9 | cyclin-dependent kinase 9 | Multiple_C | 11.67 | 11.12 | 11.31 | 1.28 | 0.233 | 0.4338 | 11.56 | 12.19 | 11.97 | 0.75 | 0.0544 | 0.2187 |
| TC0X00007292.hg.1 | SSX8 | synovial sarcoma, X breakpoint 8 | Multiple_C | 4.07 | 3.6 | 3.71 | 1.28 | 0.3537 | 0.5666 | 3.86 | 3.84 | 3.97 | 0.93 | 0.806 | 0.9142 |
| TC0X00007504.hg.1 | STARD8 | StAR-related lipid transfer domain containing 8 | Multiple_C | 4.43 | 3.96 | 4.07 | 1.28 | 0.2891 | 0.4998 | 4.12 | 4.15 | 3.92 | 1.15 | 0.2586 | 0.523 |
| TC0X00008034.hg.1 | NRK | Nik related kinase | Multiple_C | 4.49 | 4.17 | 4.13 | 1.28 | 0.3789 | 0.5919 | 5.65 | 5.26 | 4.89 | 1.69 | 0.0081 | 0.0677 |
| TC0X00009036.hg.1 | FAM9B | family with sequence similarity 9, member B | Multiple_C | 4.1 | 3.67 | 3.74 | 1.28 | 0.1055 | 0.2506 | 4.4 | 4.29 | 4.19 | 1.16 | 0.257 | 0.5218 |
| TC1100009903.hg.1 | OR51A4 | olfactory receptor, family 51, subfamily A, member 4 | Coding | 3.93 | 3.61 | 3.57 | 1.28 | 0.9469 | 0.9756 | 3.75 | 3.29 | 3.58 | 1.13 | 0.6422 | 0.8245 |
| TC1100009990.hg.1 | OR6A2 | olfactory receptor, family 6, subfamily A, member 2 | Coding | 3.84 | 3.36 | 3.48 | 1.28 | 0.3372 | 0.551 | 3.63 | 3.56 | 3.33 | 1.23 | 0.1457 | 0.3808 |
| TC1100011095.hg.1 | LRRN4CL | LRRN4 C-terminal like | Coding | 3.39 | 3.09 | 3.03 | 1.28 | 0.041 | 0.125 | 3.57 | 3.72 | 3.69 | 0.92 | 0.7248 | 0.872 |
| TC1100012677.hg.1 | OR8B12 | olfactory receptor, family 8, subfamily B, member 12 | Coding | 4.69 | 4.63 | 4.33 | 1.28 | 0.1664 | 0.3455 | 4.64 | 4.61 | 4.69 | 0.97 | 0.8785 | 0.9479 |
| TC1100013066.hg.1 | KRTAP5-10 | keratin associated protein 5-10 | Coding | 4.16 | 4.02 | 3.8 | 1.28 | 0.4613 | 0.6669 | 4.1 | 3.91 | 3.61 | 1.40 | 0.1944 | 0.4485 |
| TC1100013165.hg.1 | SLC43A3 | solute carrier family 43, member 3 | Multiple_C | 3.81 | 3.58 | 3.45 | 1.28 | 0.0491 | 0.1427 | 14.04 | 13.76 | 14.21 | 0.89 | 0.2488 | 0.5135 |
| TC1200006951.hg.1 | PDE6H | phosphodiesterase 6H, cGMP-specific, cone, gamma | Coding | 3.85 | 3.17 | 3.49 | 1.28 | 0.0966 | 0.2348 | 3.52 | 3.48 | 3.36 | 1.12 | 0.0326 | 0.1618 |
| TC1200009852.hg.1 | PZP | pregnancy-zone protein | Multiple_C | 3.64 | 3.01 | 3.28 | 1.28 | 0.7254 | 0.8555 | 6.32 | 4.93 | 4.45 | 3.66 | 3.87E-05 | 0.0018 |
| TC1200012639.hg.1 | HOXC6; HOXC | homeobox C6; homeobox C4; homeobox C5 | Multiple_C | 5.1 | 4.39 | 4.74 | 1.28 | 0.2373 | 0.4388 | 7.77 | 8.04 | 7.94 | 0.89 | 0.5187 | 0.7464 |

| | | | | | | | | | | | | | | | |
|---|---|---|---|---|---|---|---|---|---|---|---|---|---|---|---|
| TC1200012758.hg.1 | TAS2R14 | taste receptor, type 2, member 14 | Coding | 5.73 | 5.59 | 5.37 | 1.28 | 0.1852 | 0.3714 | 5.34 | 5.09 | 4.8 | 1.45 | 0.0174 | 0.1104 |
| TC1200012765.hg.1 | PRB4 | proline-rich protein BstNI subfamily 4 | Multiple_C | 5.06 | 4.61 | 4.7 | 1.28 | 0.1817 | 0.3664 | 4.84 | 4.49 | 4.62 | 1.16 | 0.4987 | 0.7316 |
| TC1200012827.hg.1 | CCER1 | coiled-coil glutamate rich protein 1 | Multiple_C | 3.58 | 3.18 | 3.22 | 1.28 | 0.126 | 0.2842 | 3.39 | 3.35 | 3.36 | 1.02 | 0.4931 | 0.7276 |
| TC1300006583.hg.1 | SGCG | sarcoglycan gamma | Coding | 3.38 | 2.75 | 3.02 | 1.28 | 0.1919 | 0.3805 | 3.48 | 3.37 | 3.21 | 1.21 | 0.7075 | 0.8624 |
| TC1300008659.hg.1 | STOML3 | stomatin (EPB72)-like 3 | Coding | 4.09 | 3.46 | 3.73 | 1.28 | 0.3822 | 0.5953 | 3.91 | 3.82 | 3.84 | 1.05 | 0.1352 | 0.3652 |
| TC1300009026.hg.1 | PCDH8 | protocadherin 8 | Multiple_C | 2.76 | 2.34 | 2.4 | 1.28 | 0.1529 | 0.3264 | 3.11 | 3.02 | 2.9 | 1.16 | 0.4605 | 0.7039 |
| TC1400007343.hg.1 | LRRC9 | leucine rich repeat containing 9 | Multiple_C | 3.37 | 3.03 | 3.01 | 1.28 | 0.527 | 0.7187 | 3.84 | 3.48 | 3.09 | 1.68 | 0.0217 | 0.1264 |
| TC1400007443.hg.1 | HSPA2 | heat shock 70kDa protein 2 | Multiple_C | 7.25 | 6.67 | 6.89 | 1.28 | 0.2488 | 0.4529 | 9.97 | 8.86 | 9.43 | 1.45 | 0.0026 | 0.032 |
| TC1400008443.hg.1 | C14orf180 | chromosome 14 open reading frame 180 | Coding | 5.86 | 5.01 | 5.5 | 1.28 | 0.1358 | 0.3004 | 5.08 | 4.94 | 4.9 | 1.13 | 0.4198 | 0.6731 |
| TC1400008714.hg.1 | SLC22A17 | solute carrier family 22, member 17 | Multiple_C | 7.46 | 6.47 | 7.1 | 1.28 | 0.0613 | 0.1682 | 8.35 | 8.47 | 8.34 | 1.01 | 0.5608 | 0.7721 |
| TC1400010774.hg.1 | FOXN3 | forkhead box N3 | Multiple_C | 7.6 | 6.55 | 7.24 | 1.28 | 0.3967 | 0.6104 | 4.74 | 4.76 | 4.62 | 1.09 | 0.9259 | 0.9694 |
| TC1500009403.hg.1 | TNFAIP8L3 | tumor necrosis factor, alpha-induced protein 8-like 3 | Multiple_C | 5.27 | 4.7 | 4.91 | 1.28 | 0.0335 | 0.1072 | 4.41 | 4.38 | 4.12 | 1.22 | 0.3277 | 0.5912 |
| TC1600006502.hg.1 | PRR25 | proline rich 25 | Coding | 3.89 | 3.84 | 3.53 | 1.28 | 0.1452 | 0.3152 | 3.69 | 3.90 | 3.94 | 0.84 | 0.2069 | 0.4637 |
| TC1600007317.hg.1 | KIAA0556 | KIAA0556 | Multiple_C | 8.74 | 8.34 | 8.38 | 1.28 | 0.1259 | 0.284 | 9.67 | 9.13 | 8.98 | 1.61 | 0.001 | 0.0161 |
| TC1600008427.hg.1 | ZNRF1 | zinc and ring finger 1, E3 ubiquitin protein ligase | Multiple_C | 8.12 | 8.24 | 7.76 | 1.28 | 0.1348 | 0.2989 | 7.07 | 7.34 | 7.36 | 0.82 | 0.0247 | 0.1364 |
| TC1600009068.hg.1 | MEIOB; LINC0 | meiosis specific with OB domains; long intergenic non-protein c | Multiple_C | 4.37 | 3.72 | 4.01 | 1.28 | 0.4644 | 0.6694 | 5.33 | 4.91 | 4.3 | 2.04 | 0.0032 | 0.0372 |
| TC1600009397.hg.1 | PRM3 | protamine 3 | Coding | 5.95 | 5.33 | 5.59 | 1.28 | 0.2044 | 0.3969 | 4.82 | 5.11 | 4.93 | 0.93 | 0.7446 | 0.8831 |
| TC1600011523.hg.1 | ZNF764 | zinc finger protein 764 | Multiple_C | 6.62 | 6.3 | 6.26 | 1.28 | 0.3475 | 0.5605 | 6.23 | 6.49 | 6.34 | 0.93 | 0.5015 | 0.734 |
| TC1700006857.hg.1 | USP43 | ubiquitin specific peptidase 43 | Multiple_C | 6.7 | 6.71 | 6.34 | 1.28 | 0.5647 | 0.7463 | 5.27 | 5.28 | 5.17 | 1.07 | 0.789 | 0.9058 |
| TC1700008859.hg.1 | C17orf77 | chromosome 17 open reading frame 77 | Multiple_C | 5.29 | 4.83 | 4.93 | 1.28 | 0.2146 | 0.41 | 4.96 | 4.85 | 4.86 | 1.07 | 0.4362 | 0.6868 |
| TC1700008907.hg.1 | TMEM94; MIF | transmembrane protein 94; microRNA 6785 | Multiple_C | 6.95 | 6.52 | 6.59 | 1.28 | 0.3565 | 0.5699 | 7.48 | 7.77 | 7.38 | 1.07 | 0.4585 | 0.7022 |
| TC1700009184.hg.1 | TSPAN10 | tetraspanin 10 | Multiple_C | 4.28 | 3.84 | 3.92 | 1.28 | 0.0968 | 0.2351 | 4.13 | 4.32 | 4.36 | 0.85 | 0.2119 | 0.4696 |
| TC1700009520.hg.1 | ALOX15 | arachidonate 15-lipoxygenase | Multiple_C | 6.82 | 6.51 | 6.46 | 1.28 | 0.1356 | 0.3001 | 5.43 | 5.47 | 5.49 | 0.96 | 0.6266 | 0.8154 |
| TC1700009713.hg.1 | STX8 | syntaxin 8 | Multiple_C | 9.31 | 10.1 | 8.95 | 1.28 | 0.3178 | 0.5313 | 7.4 | 7.31 | 7.35 | 1.04 | 0.7674 | 0.8958 |
| TC1700010645.hg.1 | KRTAP3-2 | keratin associated protein 3-2 | Coding | 4.11 | 3.72 | 3.75 | 1.28 | 0.3286 | 0.5425 | 9.27 | 8.79 | 6.88 | 5.24 | 4.92E-07 | 8.86E-05 |
| TC1700011260.hg.1 | MTMR4 | myotubularin related protein 4 | Multiple_C | 10.25 | 9.4 | 9.89 | 1.28 | 0.2592 | 0.4657 | 8.99 | 10.17 | 9.89 | 0.54 | 0.0523 | 0.2136 |
| TC1800006656.hg.1 | VAPA | VAMP associated protein A | Multiple_C | 12.96 | 13.42 | 12.6 | 1.28 | 0.5271 | 0.7187 | 12.09 | 12.11 | 12.11 | 0.99 | 0.6502 | 0.8292 |
| TC1800009218.hg.1 | ANKRD20A5P; | ankyrin repeat domain 20 family, member A5, pseudogene; ras | Multiple_C | 4.88 | 4.58 | 4.52 | 1.28 | 0.1687 | 0.3487 | 5.69 | 5.79 | 5.35 | 1.27 | 0.2924 | 0.5572 |
| TC1900006712.hg.1 | DPP9-AS1 | DPP9 antisense RNA 1 | Multiple_C | 3.3 | 2.57 | 2.94 | 1.28 | 0.0955 | 0.233 | 3.4 | 3.40 | 3.53 | 0.91 | 0.5316 | 0.7544 |
| TC1900007363.hg.1 | MAST3 | microtubule associated serine/threonine kinase 3 | Multiple_C | 4.93 | 4.51 | 4.57 | 1.28 | 0.2542 | 0.4596 | 6.2 | 6.26 | 6.21 | 0.99 | 0.8974 | 0.9568 |
| TC1900008059.hg.1 | SUPT5H | SPT5 homolog, DSIF elongation factor subunit | Multiple_C | 12.67 | 12.09 | 12.31 | 1.28 | 0.2094 | 0.403 | 12.63 | 12.30 | 12.18 | 1.37 | 0.541 | 0.7601 |
| TC1900008090.hg.1 | MAP3K10 | mitogen-activated protein kinase kinase kinase 10 | Multiple_C | 5.47 | 5.14 | 5.11 | 1.28 | 0.1669 | 0.3463 | 4.97 | 5.15 | 5.17 | 0.87 | 0.3315 | 0.5955 |
| TC1900008428.hg.1 | GLTSCR1 | glioma tumor suppressor candidate region gene 1 | Multiple_C | 5.38 | 4.87 | 5.02 | 1.28 | 0.3058 | 0.5189 | 5.76 | 6.02 | 5.93 | 0.89 | 0.6485 | 0.8283 |

| | | | | | | | | | | | | | | | |
|---|---|---|---|---|---|---|---|---|---|---|---|---|---|---|---|
| TC1900008745.hg.1 | ZNF525 | zinc finger protein 525 | Multiple_C | 8.56 | 8.47 | 8.2 | 1.28 | 0.213 | 0.4079 | 6.8 | 6.89 | 7.04 | 0.85 | 0.9067 | 0.9614 |
| TC1900008747.hg.1 | ZNF765 | zinc finger protein 765 | Multiple_C | 7.53 | 7.36 | 7.17 | 1.28 | 0.3193 | 0.533 | 6.39 | 6.31 | 6.48 | 0.94 | 0.7556 | 0.8889 |
| TC1900009328.hg.1 | SIRT6 | sirtuin 6 | Multiple_C | 6.3 | 5.63 | 5.94 | 1.28 | 0.3859 | 0.5993 | 5.23 | 5.18 | 5.05 | 1.13 | 0.5393 | 0.7594 |
| TC1900010499.hg.1 | PRODH2 | proline dehydrogenase (oxidase) 2 | Multiple_C | 3.97 | 3.49 | 3.61 | 1.28 | 0.3078 | 0.5208 | 3.86 | 4.08 | 3.8 | 1.04 | 0.7326 | 0.8763 |
| TC1900011916.hg.1 | ZNF626 | zinc finger protein 626 | Multiple_C | 10.47 | 10.25 | 10.11 | 1.28 | 0.2975 | 0.5089 | 7.77 | 7.66 | 8.29 | 0.70 | 0.0276 | 0.1456 |
| TC1900011998.hg.1 | NTF4 | neurotrophin 4 | Multiple_C | 4.71 | 4.38 | 4.35 | 1.28 | 0.1539 | 0.3277 | 4.1 | 4.40 | 4.38 | 0.82 | 0.0952 | 0.3 |
| TC2000007139.hg.1 | BPIFB6 | BPI fold containing family B, member 6 | Coding | 3.88 | 3.55 | 3.52 | 1.28 | 0.2512 | 0.4556 | 4.45 | 4.24 | 3.95 | 1.41 | 0.0333 | 0.164 |
| TC2000007300.hg.1 | RPRD1B | regulation of nuclear pre-mRNA domain containing 1B | Multiple_C | 10.69 | 10.26 | 10.33 | 1.28 | 0.4712 | 0.6751 | 10.07 | 10.67 | 11.21 | 0.45 | 4.93E-06 | 0.0004 |
| TC2000007936.hg.1 | PHACTR3 | phosphatase and actin regulator 3 | Multiple_C | 4.37 | 3.72 | 4.01 | 1.28 | 0.1847 | 0.3706 | 6.74 | 6.10 | 6.21 | 1.44 | 0.0118 | 0.0864 |
| TC2000009975.hg.1 | TMEM230 | transmembrane protein 230 | Multiple_C | 13.43 | 13.35 | 13.07 | 1.28 | 0.1274 | 0.2867 | 13.16 | 13.24 | 12.69 | 1.39 | 0.036 | 0.1723 |
| TC2000010035.hg.1 | SOX18 | SRY box 18 | Multiple_C | 3.5 | 2.88 | 3.14 | 1.28 | 0.3131 | 0.5266 | 4.07 | 4.12 | 3.53 | 1.45 | 0.0063 | 0.0569 |
| TC2100007915.hg.1 | KRTAP8-1 | keratin associated protein 8-1 | Coding | 3.41 | 2.9 | 3.05 | 1.28 | 0.8267 | 0.9129 | 3.27 | 3.25 | 3.2 | 1.05 | 0.7024 | 0.8591 |
| TC2100008379.hg.1 | KRTAP12-4 | keratin associated protein 12-4 | Multiple_C | 3.08 | 2.49 | 2.72 | 1.28 | 0.3659 | 0.5794 | 3.6 | 3.69 | 3.3 | 1.23 | 0.1005 | 0.31 |
| TC2200006917.hg.1 | CRYBB2 | crystallin beta B2 | Multiple_C | 4.48 | 4.1 | 4.12 | 1.28 | 0.2041 | 0.3966 | 5.24 | 5.42 | 5.34 | 0.93 | 0.6617 | 0.8349 |
| TC2200008515.hg.1 | BPIFC | BPI fold containing family C | Coding | 3.67 | 3.18 | 3.31 | 1.28 | 0.3163 | 0.5296 | 4.58 | 4.33 | 4.41 | 1.13 | 0.4358 | 0.6864 |
| TSUnmapped00000046.I | ZNF501 | zinc finger protein 501 [Source:HGNC Symbol;Acc:HGNC:23717] | Coding | 4.55 | 4.25 | 4.19 | 1.28 | 0.3447 | 0.5584 | 4.02 | 3.77 | 4.25 | 0.85 | 0.8737 | 0.9454 |
| TC0100007791.hg.1 | ADPRHL2 | ADP-ribosylhydrolase like 2 | Multiple_C | 6.91 | 6.87 | 6.56 | 1.27 | 0.59 | 0.7653 | 5.31 | 5.55 | 5.64 | 0.80 | 0.3082 | 0.5724 |
| TC0100010075.hg.1 | ATP8B2 | ATPase, aminophospholipid transporter, class I, type 8B, memb | Multiple_C | 5.28 | 5.4 | 4.93 | 1.27 | 0.4627 | 0.6679 | 5.57 | 5.04 | 5.27 | 1.23 | 0.0303 | 0.1545 |
| TC0100013021.hg.1 | HSPB7 | heat shock 27kDa protein family, member 7 (cardiovascular) | Multiple_C | 5.31 | 4.69 | 4.96 | 1.27 | 0.5705 | 0.7504 | 4.93 | 5.00 | 5.06 | 0.91 | 0.4712 | 0.712 |
| TC0100013651.hg.1 | PHC2; MIR360 | polyhomeotic homolog 2 (Drosophila); microRNA 3605 | Multiple_C | 10.42 | 10.03 | 10.07 | 1.27 | 0.3998 | 0.6133 | 8.25 | 8.79 | 9.25 | 0.50 | 7.11E-05 | 0.0028 |
| TC0100013801.hg.1 | NT5C1A | 5-nucleotidase, cytosolic IA | Multiple_C | 4.57 | 3.76 | 4.22 | 1.27 | 0.428 | 0.6384 | 3.89 | 3.76 | 3.87 | 1.01 | 0.58 | 0.7853 |
| TC0100014203.hg.1 | ZCCHC11 | zinc finger, CCHC domain containing 11 | Multiple_C | 6.38 | 6.89 | 6.03 | 1.27 | 0.7805 | 0.887 | 6.58 | 6.33 | 6.62 | 0.97 | 0.5607 | 0.7721 |
| TC0100014846.hg.1 | GTF2B | general transcription factor IIB | Multiple_C | 12.09 | 12.83 | 11.74 | 1.27 | 0.0382 | 0.1185 | 9.72 | 9.42 | 9.55 | 1.13 | 0.4931 | 0.7276 |
| TC0100014889.hg.1 | BARHL2 | BarH-like homeobox 2 | Coding | 4.55 | 3.84 | 4.2 | 1.27 | 0.3722 | 0.5848 | 4.02 | 4.18 | 4.11 | 0.94 | 0.8291 | 0.9243 |
| TC0100016162.hg.1 | B4GALT3 | UDP-Gal:betaGlcNAc beta 1,4- galactosyltransferase, polypeptic | Multiple_C | 8.97 | 8.01 | 8.62 | 1.27 | 0.1166 | 0.2693 | 6.85 | 6.57 | 7.6 | 0.59 | 0.001 | 0.0169 |
| TC0100016427.hg.1 | TNFSF18 | tumor necrosis factor (ligand) superfamily, member 18 | Coding | 3.05 | 3.19 | 2.7 | 1.27 | 0.1289 | 0.2893 | 2.97 | 3.23 | 2.9 | 1.05 | 0.6898 | 0.852 |
| TC0100018124.hg.1 | OR2M7 | olfactory receptor, family 2, subfamily M, member 7 | Coding | 4.19 | 3.96 | 3.84 | 1.27 | 0.0147 | 0.0562 | 4.21 | 4.51 | 4.26 | 0.97 | 0.9665 | 0.986 |
| TC0100018129.hg.1 | OR2T29 | olfactory receptor, family 2, subfamily T, member 29 | Coding | 4.29 | 3.88 | 3.94 | 1.27 | 0.4381 | 0.6475 | 3.58 | 3.57 | 3.44 | 1.10 | 0.5188 | 0.7465 |
| TC0200007055.hg.1 | TCF23 | transcription factor 23 | Multiple_C | 4.22 | 3.93 | 3.87 | 1.27 | 0.2457 | 0.4494 | 4 | 3.90 | 4.02 | 0.99 | 0.8819 | 0.9494 |
| TC0200007058.hg.1 | ATRAID | all-trans retinoic acid-induced differentiation factor | Multiple_C | 12.52 | 12.45 | 12.17 | 1.27 | 0.233 | 0.4338 | 11.39 | 11.56 | 11.54 | 0.90 | 0.6424 | 0.8246 |
| TC0200008942.hg.1 | IL37 | interleukin 37 | Coding | 4.65 | 4.2 | 4.3 | 1.27 | 0.2502 | 0.4547 | 3.53 | 3.61 | 3.49 | 1.03 | 0.5227 | 0.7489 |
| TC0200011020.hg.1 | SP140 | SP140 nuclear body protein | Multiple_C | 4.07 | 3.54 | 3.72 | 1.27 | 0.3402 | 0.5543 | 4.13 | 3.68 | 3.79 | 1.27 | 0.1459 | 0.3812 |
| TC0200015087.hg.1 | SESTD1 | SEC14 and spectrin domains 1 | Multiple_C | 8.6 | 8.43 | 8.25 | 1.27 | 0.1617 | 0.3388 | 6.44 | 6.02 | 6.14 | 1.23 | 0.5569 | 0.7707 |

| | | | | | | | | | | | | | | | |
|---|---|---|---|---|---|---|---|---|---|---|---|---|---|---|---|
| TC0200016068.hg.1 | TIGD1; MIR50 | tigger transposable element derived 1; microRNA 5001 | Multiple_C | 6.11 | 5.68 | 5.76 | 1.27 | 0.3975 | 0.6111 | 5.26 | 5.68 | 5.33 | 0.95 | 0.3579 | 0.6207 |
| TC0200016676.hg.1 | WDPCP | WD repeat containing planar cell polarity effector | Multiple_C | 5.98 | 5.63 | 5.63 | 1.27 | 0.3341 | 0.5479 | 6.87 | 7.08 | 6.78 | 1.06 | 0.7275 | 0.8736 |
| TC0300006791.hg.1 | KAT2B | K(lysine) acetyltransferase 2B | Multiple_C | 8.29 | 7.7 | 7.94 | 1.27 | 0.3632 | 0.5766 | 5.51 | 5.83 | 5.23 | 1.21 | 0.6383 | 0.8228 |
| TC0300007450.hg.1 | IQCF2 | IQ motif containing F2 | Multiple_C | 5.2 | 4.57 | 4.85 | 1.27 | 0.3118 | 0.5251 | 4.95 | 4.93 | 4.73 | 1.16 | 0.0403 | 0.184 |
| TC0300007610.hg.1 | PXK | PX domain containing serine/threonine kinase | Multiple_C | 9.78 | 8.73 | 9.43 | 1.27 | 0.6698 | 0.8198 | 10.19 | 8.79 | 9.03 | 2.23 | 0.5532 | 0.7684 |
| TC0300010002.hg.1 | LINC00885 | long intergenic non-protein coding RNA 885 | Multiple_C | 4.17 | 3.99 | 3.82 | 1.27 | 0.2004 | 0.3922 | 4.56 | 4.52 | 4.65 | 0.94 | 0.8674 | 0.9431 |
| TC0300011144.hg.1 | IQCF5 | IQ motif containing F5 | Coding | 4.91 | 4.53 | 4.56 | 1.27 | 0.3443 | 0.558 | 5.4 | 5.23 | 5.16 | 1.18 | 0.0922 | 0.2942 |
| TC0300012281.hg.1 | ZXDC | ZXD family zinc finger C | Multiple_C | 9.28 | 8.46 | 8.93 | 1.27 | 0.769 | 0.881 | 10.61 | 10.26 | 10.2 | 1.33 | 0.0844 | 0.2797 |
| TC0300013965.hg.1 | PRSS46 | protease, serine 46 | Coding | 3.03 | 2.68 | 2.68 | 1.27 | 0.1147 | 0.2662 | 4 | 4.26 | 4.23 | 0.85 | 0.8825 | 0.9496 |
| TC0400006579.hg.1 | ADD1 | adducin 1 (alpha) | Multiple_C | 11.39 | 10.64 | 11.04 | 1.27 | 0.0578 | 0.1611 | 11.2 | 11.62 | 11.28 | 0.95 | 0.6425 | 0.8246 |
| TC0400011077.hg.1 | SOWAHB | sosondowah ankyrin repeat domain family member B | Coding | 5.39 | 6.08 | 5.04 | 1.27 | 0.171 | 0.3519 | 3.61 | 3.34 | 3.43 | 1.13 | 0.0428 | 0.191 |
| TC0400011754.hg.1 | IL2 | interleukin 2 | Multiple_C | 3.42 | 3.12 | 3.07 | 1.27 | 0.7826 | 0.8881 | 3.14 | 3.24 | 3.17 | 0.98 | 0.5011 | 0.7336 |
| TC0400012641.hg.1 | PDLIM3 | PDZ and LIM domain 3 | Multiple_C | 4.28 | 4.05 | 3.93 | 1.27 | 0.2027 | 0.395 | 7.6 | 6.43 | 6.01 | 3.01 | 0.0006 | 0.0114 |
| TC0400012795.hg.1 | FIP1L1 | factor interacting with PAPOLA and CPSF1 | Multiple_C | 11.6 | 11.27 | 11.25 | 1.27 | 0.5527 | 0.7367 | 12.28 | 11.67 | 12.31 | 0.98 | 0.5131 | 0.7428 |
| TC0500006493.hg.1 | SLC6A19 | solute carrier family 6 (neutral amino acid transporter), membe | Coding | 3.57 | 3.06 | 3.22 | 1.27 | 0.1363 | 0.301 | 3.46 | 3.55 | 3.55 | 0.94 | 0.997 | 0.9987 |
| TC0500007434.hg.1 | RPL41 | Synthetic construct Homo sapiens clone IMAGE:100063377, MC | Multiple_C | 17.08 | 17.38 | 16.73 | 1.27 | 0.0679 | 0.1814 | 15.37 | 15.30 | 15.07 | 1.23 | 0.184 | 0.4351 |
| TC0500011352.hg.1 | EDIL3 | EGF-like repeats and discoidin I-like domains 3 | Multiple_C | 4.66 | 4.44 | 4.31 | 1.27 | 0.7026 | 0.8413 | 3.92 | 3.96 | 4.2 | 0.82 | 0.2935 | 0.5583 |
| TC0500011528.hg.1 | LIX1 | limb and CNS expressed 1 | Coding | 3.18 | 2.81 | 2.83 | 1.27 | 0.9862 | 0.9932 | 7.62 | 7.62 | 6.41 | 2.31 | 0.019 | 0.1166 |
| TC0500013300.hg.1 | C9 | complement component 9 | Multiple_C | 3.45 | 3.26 | 3.1 | 1.27 | 0.2318 | 0.4323 | 3.56 | 3.64 | 3.31 | 1.19 | 0.0874 | 0.2853 |
| TC0600007644.hg.1 | XXbac-BPG154 | putative novel transcript; Transcript Identified by AceView, Entr | Multiple_C | 4.17 | 3.94 | 3.82 | 1.27 | 0.1899 | 0.3776 | 3.97 | 3.75 | 3.91 | 1.04 | 0.7776 | 0.9003 |
| TC0600008064.hg.1 | PPP2R5D | protein phosphatase 2, regulatory subunit B, delta | Multiple_C | 10.41 | 10.17 | 10.06 | 1.27 | 0.2282 | 0.4279 | 10.08 | 10.27 | 10.48 | 0.76 | 0.0251 | 0.1376 |
| TC0600010770.hg.1 | OFCC1 | orofacial cleft 1 candidate 1 | Multiple_C | 4.62 | 4.16 | 4.27 | 1.27 | 0.2445 | 0.4479 | 4.68 | 4.71 | 4.56 | 1.09 | 0.582 | 0.7867 |
| TC0600014299.hg.1 | GSTA3 | glutathione S-transferase alpha 3 | Coding | 4.21 | 3.7 | 3.86 | 1.27 | 0.1756 | 0.358 | 4.23 | 4.14 | 3.86 | 1.29 | 0.0891 | 0.2889 |
| TC0700012856.hg.1 | TRY2P | trypsinogen-like pseudogene | Multiple_C | 4.05 | 3.57 | 3.7 | 1.27 | 0.258 | 0.4644 | 4.25 | 4.06 | 3.94 | 1.24 | 0.0985 | 0.3065 |
| TC0800009432.hg.1 | DEFA6 | defensin, alpha 6, Paneth cell-specific | Coding | 3.83 | 3.29 | 3.48 | 1.27 | 0.1876 | 0.3749 | 3.59 | 3.67 | 3.6 | 0.99 | 0.3385 | 0.6028 |
| TC0800012382.hg.1 | DEFA3 | defensin, alpha 3, neutrophil-specific | Coding | 4.36 | 3.7 | 4.01 | 1.27 | 0.1042 | 0.2484 | 4.07 | 4.10 | 4.02 | 1.04 | 0.8012 | 0.9112 |
| TC0900008865.hg.1 | CERCAM | cerebral endothelial cell adhesion molecule | Multiple_C | 4.56 | 4.15 | 4.21 | 1.27 | 0.4082 | 0.6204 | 8.12 | 8.16 | 7.75 | 1.29 | 0.7559 | 0.8889 |
| TC0900009706.hg.1 | IFNA13 | interferon, alpha 13 | Coding | 4.76 | 4.26 | 4.41 | 1.27 | 0.3914 | 0.6052 | 5.12 | 5.02 | 4.75 | 1.29 | 0.0967 | 0.3028 |
| TC0900010578.hg.1 | UBQLN1 | ubiquilin 1 | Multiple_C | 13.81 | 13.8 | 13.46 | 1.27 | 0.2397 | 0.4417 | 13.76 | 14.02 | 14.19 | 0.74 | 0.0321 | 0.1603 |
| TC0900010903.hg.1 | RP11-569G13. | uncharacterized protein LOC158434 [Source:RefSeq peptide;Ac | Multiple_C | 4.99 | 4.51 | 4.64 | 1.27 | 0.3341 | 0.5479 | 5.02 | 5.12 | 4.92 | 1.07 | 0.8021 | 0.9117 |
| TC0X00006809.hg.1 | ZFX | zinc finger protein, X-linked | Multiple_C | 9.5 | 9 | 9.15 | 1.27 | 0.2749 | 0.4838 | 8.93 | 9.34 | 10.17 | 0.42 | 0.0005 | 0.0105 |
| TC0X00007261.hg.1 | NUDT10 | nudix hydrolase 10 | Coding | 10.8 | 10.4 | 10.45 | 1.27 | 0.1364 | 0.3011 | 10.68 | 10.70 | 10.26 | 1.34 | 0.0284 | 0.1485 |
| TC0X00007378.hg.1 | MAGEH1 | MAGE family member H1 | Coding | 4.58 | 3.98 | 4.23 | 1.27 | 0.4787 | 0.6809 | 4.07 | 4.26 | 4.26 | 0.88 | 0.307 | 0.5713 |

| | | | | | | | | | | | | | | | |
|---|---|---|---|---|---|---|---|---|---|---|---|---|---|---|---|
| TC0X00008765.hg.1 | PNMA6A | paraneoplastic Ma antigen family member 6A | Coding | 3.39 | 3.32 | 3.04 | 1.27 | 0.7501 | 0.8717 | 3.77 | 4.02 | 3.52 | 1.19 | 0.1827 | 0.4333 |
| TC0X00008821.hg.1 | OPN1MW | opsin 1 (cone pigments), medium-wave-sensitive | Multiple_C | 4.98 | 4.8 | 4.63 | 1.27 | 0.5752 | 0.7542 | 4.72 | 4.54 | 4.6 | 1.09 | 0.7346 | 0.8776 |
| TC0X00010374.hg.1 | NXF5 | nuclear RNA export factor 5 | Multiple_C | 3.76 | 3.11 | 3.41 | 1.27 | 0.2586 | 0.465 | 3.99 | 3.53 | 3.29 | 1.62 | 0.0251 | 0.1376 |
| TC0X00010875.hg.1 | PLAC1 | placenta specific 1 | Multiple_C | 5.33 | 5.04 | 4.98 | 1.27 | 0.1154 | 0.2674 | 5.2 | 5.11 | 5.2 | 1.00 | 0.9963 | 0.9983 |
| TC0X00010982.hg.1 | CDR1 | cerebellar degeneration related protein 1 | Coding | 3.72 | 3.53 | 3.37 | 1.27 | 0.425 | 0.6362 | 3.82 | 4.15 | 3.94 | 0.92 | 0.91 | 0.9628 |
| TC0X00011408.hg.1 | MAGEA5 | MAGE family member A5 | Multiple_C | 4.12 | 3.67 | 3.77 | 1.27 | 0.1608 | 0.3377 | 3.36 | 3.59 | 3.63 | 0.83 | 0.1276 | 0.3536 |
| TC1000007934.hg.1 | ADAMTS14 | ADAM metallopeptidase with thrombospondin type 1 motif 14 | Multiple_C | 3.25 | 2.77 | 2.9 | 1.27 | 0.3823 | 0.5953 | 6.07 | 6.32 | 6.81 | 0.60 | 0.0017 | 0.0238 |
| TC1000009296.hg.1 | PTPRE | protein tyrosine phosphatase, receptor type, E | Multiple_C | 6.29 | 5.82 | 5.94 | 1.27 | 0.7285 | 0.8573 | 7.84 | 7.53 | 7.05 | 1.73 | 0.0087 | 0.0709 |
| TC1000009894.hg.1 | FAM188A | family with sequence similarity 188, member A | Multiple_C | 8.29 | 7.94 | 7.94 | 1.27 | 0.3948 | 0.6083 | 6.7 | 6.22 | 6.83 | 0.91 | 0.5412 | 0.7602 |
| TC1100008113.hg.1 | RHOD | ras homolog family member D | Multiple_C | 5.93 | 5.37 | 5.58 | 1.27 | 0.4773 | 0.6799 | 9.16 | 8.44 | 9.28 | 0.92 | 0.8315 | 0.9249 |
| TC1100008382.hg.1 | ARHGEF17 | Rho guanine nucleotide exchange factor 17 | Multiple_C | 5.92 | 5.83 | 5.57 | 1.27 | 0.4683 | 0.6723 | 5.31 | 5.00 | 4.65 | 1.58 | 0.0774 | 0.2682 |
| TC1100009368.hg.1 | OR10G4 | olfactory receptor, family 10, subfamily G, member 4 | Coding | 4.45 | 4.16 | 4.1 | 1.27 | 0.0856 | 0.2148 | 3.72 | 3.76 | 3.78 | 0.96 | 0.7625 | 0.8927 |
| TC1100010405.hg.1 | LIN7C | lin-7 homolog C (C. elegans) | Multiple_C | 12.47 | 12.43 | 12.12 | 1.27 | 0.3391 | 0.5531 | 12.82 | 12.43 | 12.87 | 0.97 | 0.8493 | 0.9347 |
| TC1100010897.hg.1 | UBE2L6 | ubiquitin-conjugating enzyme E2L 6 | Multiple_C | 11.44 | 10.76 | 11.09 | 1.27 | 0.4164 | 0.6283 | 6.82 | 4.67 | 4.48 | 5.06 | 4.04E-07 | 8.01E-05 |
| TC1100012643.hg.1 | OR6M1 | olfactory receptor, family 6, subfamily M, member 1 | Coding | 3.04 | 2.76 | 2.69 | 1.27 | 0.9998 | 0.9998 | 3.51 | 3.54 | 3.62 | 0.93 | 0.3203 | 0.5842 |
| TC1100012957.hg.1 | TRIM22 | tripartite motif containing 22 | Multiple_C | 3.29 | 2.95 | 2.94 | 1.27 | 0.072 | 0.1892 | 4.01 | 4.36 | 4.21 | 0.87 | 0.3869 | 0.6459 |
| TC1200006686.hg.1 | DPPA3 | developmental pluripotency associated 3 | Coding | 4.4 | 3.98 | 4.05 | 1.27 | 0.2945 | 0.506 | 4.44 | 4.16 | 4.27 | 1.13 | 0.9778 | 0.9908 |
| TC1200007053.hg.1 | SPX | spexin hormone | Multiple_C | 4.59 | 3.78 | 4.24 | 1.27 | 0.3279 | 0.5417 | 5.68 | 3.85 | 3.98 | 3.25 | 1.39E-05 | 0.0009 |
| TC1200007567.hg.1 | KCNH3 | potassium channel, voltage gated eag related subfamily H, mem | Multiple_C | 5.3 | 4.82 | 4.95 | 1.27 | 0.012 | 0.0483 | 5.43 | 5.52 | 5.38 | 1.04 | 0.3483 | 0.6121 |
| TC1200008028.hg.1 | MSRB3 | methionine sulfoxide reductase B3 | Multiple_C | 3.12 | 2.77 | 2.77 | 1.27 | 0.1697 | 0.3501 | 9.12 | 8.14 | 8.62 | 1.41 | 0.0293 | 0.1517 |
| TC1200010481.hg.1 | TWF1 | twinfilin actin binding protein 1 | Multiple_C | 12.96 | 13.58 | 12.61 | 1.27 | 0.1548 | 0.3291 | 12.16 | 10.53 | 11.4 | 1.69 | 0.0004 | 0.0085 |
| TC1200010761.hg.1 | KRT71 | keratin 71, type II | Coding | 5.3 | 4.94 | 4.95 | 1.27 | 0.4807 | 0.6826 | 4.64 | 4.93 | 4.65 | 0.99 | 0.6773 | 0.8446 |
| TC1200011385.hg.1 | LIN7A | lin-7 homolog A (C. elegans) | Multiple_C | 3.19 | 2.92 | 2.84 | 1.27 | 0.4045 | 0.6175 | 3.27 | 3.04 | 3.21 | 1.04 | 0.7236 | 0.8712 |
| TC1200012653.hg.1 | METTL21B | methyltransferase like 21B | Multiple_C | 8.27 | 8.15 | 7.92 | 1.27 | 0.2929 | 0.5042 | 7.16 | 7.37 | 7.5 | 0.79 | 0.1732 | 0.4205 |
| TC1200012867.hg.1 | RIMBP2 | RIMS binding protein 2 | Multiple_C | 5.1 | 4.85 | 4.75 | 1.27 | 0.113 | 0.2635 | 9.87 | 9.41 | 9.19 | 1.60 | 0.0256 | 0.1394 |
| TC1300007135.hg.1 | ITM2B | integral membrane protein 2B | Multiple_C | 15.3 | 15.45 | 14.95 | 1.27 | 0.0816 | 0.2075 | 11.65 | 11.74 | 10.42 | 2.35 | 2.68E-05 | 0.0014 |
| TC1300008109.hg.1 | F10 | coagulation factor X | Multiple_C | 5.62 | 5.06 | 5.27 | 1.27 | 0.5989 | 0.7714 | 6.17 | 6.98 | 5.87 | 1.23 | 0.1244 | 0.3485 |
| TC1400006546.hg.1 | TPPP2 | tubulin polymerization-promoting protein family member 2 | Multiple_C | 4.23 | 4.06 | 3.88 | 1.27 | 0.5518 | 0.7363 | 4.74 | 4.45 | 4.57 | 1.13 | 0.5904 | 0.7928 |
| TC1400008758.hg.1 | CBLN3 | cerebellin 3 precursor | Coding | 3.93 | 3.7 | 3.58 | 1.27 | 0.2534 | 0.4583 | 4.03 | 3.73 | 3.51 | 1.43 | 0.013 | 0.0919 |
| TC1500007015.hg.1 | JMJD7; PLA2G | jumonji domain containing 7; phospholipase A2, group IVB (cyto | Multiple_C | 5.22 | 5.22 | 4.87 | 1.27 | 0.1171 | 0.27 | 4.19 | 4.58 | 4.39 | 0.87 | 0.1923 | 0.4456 |
| TC1500007584.hg.1 | KBTBD13 | kelch repeat and BTB (POZ) domain containing 13 | Coding | 3.56 | 3.49 | 3.21 | 1.27 | 0.952 | 0.9779 | 3.69 | 3.49 | 3.6 | 1.06 | 0.9886 | 0.9952 |
| TC1500007839.hg.1 | ISLR | immunoglobulin superfamily containing leucine-rich repeat | Multiple_C | 5.54 | 4.97 | 5.19 | 1.27 | 0.0739 | 0.1927 | 4.8 | 4.94 | 5.05 | 0.84 | 0.2173 | 0.4765 |
| TC1500007841.hg.1 | CCDC33 | coiled-coil domain containing 33 | Multiple_C | 6.45 | 5.76 | 6.1 | 1.27 | 0.0872 | 0.2178 | 7.8 | 8.05 | 7.92 | 0.92 | 0.3036 | 0.5678 |

| | | | | | | | | | | | | | | | |
|---|---|---|---|---|---|---|---|---|---|---|---|---|---|---|---|
| TC1500010020.hg.1 | UBL7 | ubiquitin-like 7 | Multiple_C | 11.4 | 9.73 | 11.05 | 1.27 | 0.221 | 0.4184 | 10.25 | 10.55 | 10.21 | 1.03 | 0.489 | 0.725 |
| TC1600007131.hg.1 | ACSM2A | acyl-CoA synthetase medium-chain family member 2A | Multiple_C | 7.54 | 6.99 | 7.19 | 1.27 | 0.5312 | 0.7212 | 7.68 | 7.57 | 7.62 | 1.04 | 0.1151 | 0.3349 |
| TC1600008015.hg.1 | LOC388282; C | uncharacterized LOC388282; novel transcript | Multiple_C | 4.1 | 3.63 | 3.75 | 1.27 | 0.269 | 0.4771 | 5.32 | 5.22 | 5.24 | 1.06 | 0.8434 | 0.9317 |
| TC1600009486.hg.1 | PLA2G10 | phospholipase A2, group X | Multiple_C | 6.47 | 6.23 | 6.12 | 1.27 | 0.1674 | 0.3469 | 4.63 | 4.46 | 4.35 | 1.21 | 0.2111 | 0.4687 |
| TC1600009856.hg.1 | EIF3C; EIF3CL | eukaryotic translation initiation factor 3, subunit C; eukaryotic t | Multiple_C | 14.44 | 14.33 | 14.09 | 1.27 | 0.0952 | 0.2325 | 14.96 | 15.05 | 15.1 | 0.91 | 0.339 | 0.6031 |
| TC1600010184.hg.1 | ITFG1 | integrin alpha FG-GAP repeat containing 1 | Multiple_C | 9.01 | 9.52 | 8.66 | 1.27 | 0.2413 | 0.4436 | 7.67 | 7.36 | 7.79 | 0.92 | 0.5513 | 0.767 |
| TC1600010291.hg.1 | SALL1 | spalt-like transcription factor 1 | Multiple_C | 4.32 | 3.67 | 3.97 | 1.27 | 0.4069 | 0.6194 | 4.45 | 4.34 | 4.08 | 1.29 | 0.4794 | 0.7179 |
| TC1600011306.hg.1 | PRDM7 | PR domain containing 7 | Multiple_C | 4.25 | 4 | 3.9 | 1.27 | 0.0736 | 0.1922 | 4.63 | 4.57 | 4.29 | 1.27 | 0.3655 | 0.6279 |
| TC1600011325.hg.1 | CRAMP1 | cramped chromatin regulator homolog 1 | Multiple_C | 7 | 6.14 | 6.65 | 1.27 | 0.2876 | 0.4984 | 7.52 | 7.50 | 7.83 | 0.81 | 0.0697 | 0.2518 |
| TC1700007913.hg.1 | RAMP2 | receptor (G protein-coupled) activity modifying protein 2 | Multiple_C | 4.47 | 4.04 | 4.12 | 1.27 | 0.1061 | 0.2516 | 3.6 | 3.90 | 3.61 | 0.99 | 0.8983 | 0.9574 |
| TC1700008852.hg.1 | CD300A | CD300a molecule | Multiple_C | 4.49 | 4.12 | 4.14 | 1.27 | 0.0942 | 0.2307 | 4.73 | 4.55 | 4.58 | 1.11 | 0.4005 | 0.6573 |
| TC1700008874.hg.1 | OTOP2 | otopetrin 2 | Multiple_C | 4.61 | 4.41 | 4.26 | 1.27 | 0.0916 | 0.226 | 4.58 | 4.67 | 4.42 | 1.12 | 0.0991 | 0.3077 |
| TC1700009612.hg.1 | CLEC10A | C-type lectin domain family 10, member A | Multiple_C | 3.4 | 3 | 3.05 | 1.27 | 0.4439 | 0.6523 | 3.77 | 3.72 | 3.33 | 1.36 | 0.1127 | 0.3307 |
| TC1700009744.hg.1 | MYH1 | myosin, heavy chain 1, skeletal muscle, adult | Coding | 3.82 | 3.72 | 3.47 | 1.27 | 0.1774 | 0.3601 | 3.69 | 3.57 | 3.78 | 0.94 | 0.8001 | 0.9105 |
| TC1700010651.hg.1 | KRTAP2-1 | keratin associated protein 2-1 | Coding | 5.97 | 5.56 | 5.62 | 1.27 | 0.1461 | 0.3162 | 7.03 | 6.88 | 7.08 | 0.97 | 0.4107 | 0.6652 |
| TC1700011263.hg.1 | TEX14 | testis expressed 14 | Multiple_C | 3.9 | 3.68 | 3.55 | 1.27 | 0.5224 | 0.7154 | 3.64 | 3.34 | 3.58 | 1.04 | 0.7298 | 0.8746 |
| TC1700012244.hg.1 | CDK12 | cyclin-dependent kinase 12 | Multiple_C | 10.45 | 9.98 | 10.1 | 1.27 | 0.2765 | 0.4858 | 10.83 | 11.74 | 11.35 | 0.70 | 0.0174 | 0.1105 |
| TC1700012333.hg.1 | TRPV3 | transient receptor potential cation channel, subfamily V, memb | Multiple_C | 3.82 | 3.54 | 3.47 | 1.27 | 0.1799 | 0.3639 | 3.32 | 3.28 | 3.35 | 0.98 | 0.4674 | 0.7088 |
| TC1900006801.hg.1 | CRB3 | crumbs family member 3 | Coding | 11.42 | 10.99 | 11.07 | 1.27 | 0.4799 | 0.682 | 9.8 | 9.78 | 9.67 | 1.09 | 0.6678 | 0.8375 |
| TC1900007238.hg.1 | OR10H3 | olfactory receptor, family 10, subfamily H, member 3 | Coding | 4.49 | 3.87 | 4.14 | 1.27 | 0.3031 | 0.5158 | 4.26 | 4.49 | 4.47 | 0.86 | 0.1097 | 0.3261 |
| TC1900008392.hg.1 | FKRP | fukutin related protein | Multiple_C | 4.07 | 3.38 | 3.72 | 1.27 | 0.1682 | 0.3479 | 4.95 | 5.38 | 5.32 | 0.77 | 0.0374 | 0.1763 |
| TC1900008445.hg.1 | ZNF114 | zinc finger protein 114 | Multiple_C | 5 | 4.6 | 4.65 | 1.27 | 0.1612 | 0.3382 | 5.53 | 5.48 | 5.68 | 0.90 | 0.6497 | 0.829 |
| TC1900008701.hg.1 | ZNF534 | zinc finger protein 534 | Coding | 4.93 | 4.72 | 4.58 | 1.27 | 0.142 | 0.3099 | 4.5 | 4.42 | 4.74 | 0.85 | 0.9358 | 0.9731 |
| TC1900009046.hg.1 | RNF225 | ring finger protein 225 | Coding | 5.01 | 4.5 | 4.66 | 1.27 | 0.5334 | 0.7231 | 5.04 | 5.08 | 5.2 | 0.90 | 0.9895 | 0.9956 |
| TC1900010508.hg.1 | CLIP3 | CAP-GLY domain containing linker protein 3 | Multiple_C | 3.78 | 3.23 | 3.43 | 1.27 | 0.153 | 0.3265 | 4.26 | 4.08 | 3.82 | 1.36 | 0.0393 | 0.181 |
| TC1900010943.hg.1 | RTN2 | reticulon 2 | Multiple_C | 6.48 | 5.95 | 6.13 | 1.27 | 0.2499 | 0.4542 | 5.18 | 5.01 | 5.03 | 1.11 | 0.2424 | 0.5061 |
| TC1900010967.hg.1 | PGLYRP1 | peptidoglycan recognition protein 1 | Coding | 5.14 | 4.83 | 4.79 | 1.27 | 0.9388 | 0.9716 | 4.64 | 4.95 | 4.83 | 0.88 | 0.4379 | 0.6884 |
| TC1900011066.hg.1 | CCDC114 | coiled-coil domain containing 114 | Multiple_C | 5.43 | 5.02 | 5.08 | 1.27 | 0.5995 | 0.772 | 4.4 | 4.41 | 4.34 | 1.04 | 0.2593 | 0.5234 |
| TC1900012008.hg.1 | SIGLEC11 | sialic acid binding Ig-like lectin 11 | Multiple_C | 5.26 | 4.88 | 4.91 | 1.27 | 0.2879 | 0.4987 | 5.53 | 5.54 | 5.55 | 0.99 | 0.6256 | 0.8149 |
| TC2000006558.hg.1 | HSPA12B | heat shock 70kD protein 12B | Multiple_C | 5.28 | 4.77 | 4.93 | 1.27 | 0.0402 | 0.1232 | 4.74 | 5.05 | 4.9 | 0.90 | 0.245 | 0.5091 |
| TC2000007069.hg.1 | DEFB118 | defensin, beta 118 | Coding | 4.24 | 3.54 | 3.89 | 1.27 | 0.2243 | 0.4227 | 3.89 | 3.52 | 3.73 | 1.12 | 0.9874 | 0.9947 |
| TC2100006976.hg.1 | ITSN1 | intersectin 1 | Multiple_C | 9.68 | 9.31 | 9.33 | 1.27 | 0.6228 | 0.789 | 7.95 | 8.20 | 8.48 | 0.69 | 0.0312 | 0.1576 |
| TC2100007906.hg.1 | KRTAP6-1 | keratin associated protein 6-1 | Coding | 4.4 | 4.2 | 4.05 | 1.27 | 0.2175 | 0.4135 | 4 | 4.03 | 4.56 | 0.68 | 0.029 | 0.1507 |

| | | | | | | | | | | | | | | | |
|---|---|---|---|---|---|---|---|---|---|---|---|---|---|---|---|
| TC2200007413.hg.1 | GRAP2 | GRB2-related adaptor protein 2 | Multiple_C | 3.36 | 3.26 | 3.01 | 1.27 | 0.093 | 0.2284 | 3.65 | 3.86 | 3.51 | 1.10 | 0.1075 | 0.3224 |
| TSUnmapped00000098.h | DGKD | diacylglycerol kinase, delta 130kDa | Coding | 5.9 | 5.37 | 5.55 | 1.27 | 0.2552 | 0.4609 | 5.54 | 5.36 | 5.19 | 1.27 | 0.3105 | 0.5746 |
| TSUnmapped00000358.h | ZNF197 | zinc finger protein 197 | Coding | 6.76 | 6.85 | 6.41 | 1.27 | 0.354 | 0.567 | 5.54 | 5.26 | 5.58 | 0.97 | 0.6636 | 0.8355 |
| TSUnmapped00000492.h | KAT6B | K(lysine) acetyltransferase 6B | Coding | 5.47 | 4.78 | 5.12 | 1.27 | 0.4551 | 0.6624 | 7.02 | 6.46 | 6.48 | 1.45 | 0.016 | 0.1051 |
| TC0100007102.hg.1 | PADI1 | peptidyl arginine deiminase, type I | Multiple_C | 5.15 | 4.61 | 4.81 | 1.27 | 0.371 | 0.5837 | 5.77 | 4.62 | 4.91 | 1.82 | 0.0014 | 0.0211 |
| TC0100011527.hg.1 | NENF | neudesin neurotrophic factor | Multiple_C | 7.99 | 8 | 7.65 | 1.27 | 0.2708 | 0.4793 | 5.27 | 6.25 | 5.97 | 0.62 | 0.0066 | 0.0585 |
| TC0100013042.hg.1 | SPATA21 | spermatogenesis associated 21 | Multiple_C | 3.76 | 3.13 | 3.42 | 1.27 | 0.3929 | 0.6068 | 3.82 | 3.94 | 3.8 | 1.01 | 0.5536 | 0.7686 |
| TC0100013246.hg.1 | WNT4 | wingless-type MMTV integration site family, member 4 | Multiple_C | 4.45 | 3.82 | 4.11 | 1.27 | 0.1707 | 0.3515 | 4.49 | 4.32 | 4.17 | 1.25 | 0.0563 | 0.2231 |
| TC0100015186.hg.1 | C1orf194 | chromosome 1 open reading frame 194 | Multiple_C | 3.77 | 3.22 | 3.43 | 1.27 | 0.3117 | 0.5251 | 4.49 | 4.77 | 4.72 | 0.85 | 0.4929 | 0.7275 |
| TC0100015854.hg.1 | PGLYRP3 | peptidoglycan recognition protein 3 | Multiple_C | 2.7 | 2.57 | 2.36 | 1.27 | 0.4547 | 0.6621 | 2.92 | 2.99 | 2.87 | 1.04 | 0.9926 | 0.9968 |
| TC0100016137.hg.1 | CD84 | CD84 molecule | Multiple_C | 3.18 | 2.5 | 2.84 | 1.27 | 0.3117 | 0.5251 | 3.19 | 3.27 | 3.09 | 1.07 | 0.7801 | 0.9013 |
| TC0100016139.hg.1 | CD48 | CD48 molecule | Multiple_C | 3.58 | 2.99 | 3.24 | 1.27 | 0.7021 | 0.8409 | 3.41 | 3.31 | 3.27 | 1.10 | 0.1592 | 0.4011 |
| TC0100017793.hg.1 | IRF2BP2 | interferon regulatory factor 2 binding protein 2 | Multiple_C | 9.6 | 9.03 | 9.26 | 1.27 | 0.8666 | 0.9349 | 9.44 | 10.14 | 9.84 | 0.76 | 0.0115 | 0.0852 |
| TC0100018301.hg.1 | DCST1 | DC-STAMP domain containing 1 | Multiple_C | 4.56 | 4.13 | 4.22 | 1.27 | 0.4581 | 0.6642 | 4.38 | 4.48 | 4.43 | 0.97 | 0.7351 | 0.8777 |
| TC0200006912.hg.1 | TDRD15 | tudor domain containing 15 | Multiple_C | 3 | 2.47 | 2.66 | 1.27 | 0.3021 | 0.5145 | 3.38 | 3.26 | 3.47 | 0.94 | 0.3433 | 0.607 |
| TC0200009281.hg.1 | MZT2B | mitotic spindle organizing protein 2B | Multiple_C | 12.72 | 12.91 | 12.38 | 1.27 | 0.1613 | 0.3382 | 10.1 | 10.75 | 10.03 | 1.05 | 0.9863 | 0.9943 |
| TC0200011107.hg.1 | EFHD1 | EF-hand domain family member D1 | Multiple_C | 7.08 | 6.57 | 6.74 | 1.27 | 0.1428 | 0.311 | 5.86 | 6.23 | 6.01 | 0.90 | 0.2034 | 0.4591 |
| TC0200011888.hg.1 | MATN3 | matrilin 3 | Multiple_C | 3.66 | 3.32 | 3.32 | 1.27 | 0.3078 | 0.5209 | 4.27 | 3.79 | 3.5 | 1.71 | 0.0039 | 0.0426 |
| TC0200013062.hg.1 | FBXO41 | F-box protein 41 | Multiple_C | 6.74 | 6.09 | 6.4 | 1.27 | 0.2278 | 0.4274 | 6.51 | 7.09 | 6.97 | 0.73 | 0.0677 | 0.2478 |
| TC0200014024.hg.1 | C1QL2 | complement component 1, q subcomponent-like 2 | Coding | 4.14 | 3.71 | 3.8 | 1.27 | 0.1653 | 0.3438 | 4.04 | 4.03 | 3.8 | 1.18 | 0.2289 | 0.4901 |
| TC0200015399.hg.1 | PPIL3 | peptidylprolyl isomerase (cyclophilin)-like 3 | Multiple_C | 12.66 | 14.08 | 12.32 | 1.27 | 0.3242 | 0.5382 | 10.14 | 10.24 | 9.78 | 1.28 | 0.0574 | 0.2259 |
| TC0200016332.hg.1 | KIF1A | kinesin family member 1A | Multiple_C | 6.97 | 6.67 | 6.63 | 1.27 | 0.2054 | 0.3984 | 6.74 | 6.79 | 6.38 | 1.28 | 0.1817 | 0.4318 |
| TC0200016498.hg.1 | LIPT1 | lipoyltransferase 1 | Multiple_C | 7.05 | 7.97 | 6.71 | 1.27 | 0.3174 | 0.5308 | 5.21 | 5.18 | 5.1 | 1.08 | 0.0943 | 0.2982 |
| TC0200016602.hg.1 | C2orf82 | chromosome 2 open reading frame 82 | Multiple_C | 7.39 | 6.88 | 7.05 | 1.27 | 0.0987 | 0.2384 | 7.09 | 7.31 | 7.04 | 1.04 | 0.7679 | 0.8959 |
| TC0300007336.hg.1 | ATRIP; TREX1 | ATR interacting protein; three prime repair exonuclease 1 | Multiple_C | 7.25 | 6.53 | 6.91 | 1.27 | 0.9585 | 0.9803 | 7.95 | 8.14 | 8.18 | 0.85 | 0.5459 | 0.7634 |
| TC0300008818.hg.1 | COL6A5 | collagen, type VI, alpha 5 | Multiple_C | 3.45 | 3.25 | 3.11 | 1.27 | 0.1331 | 0.2963 | 3.17 | 3.00 | 3.02 | 1.11 | 0.5466 | 0.764 |
| TC0300010209.hg.1 | TADA3 | transcriptional adaptor 3 | Multiple_C | 10.54 | 9.64 | 10.2 | 1.27 | 0.1373 | 0.3026 | 9.6 | 10.38 | 9.53 | 1.05 | 0.3032 | 0.5676 |
| TC0300010242.hg.1 | ATP2B2 | ATPase, Ca++ transporting, plasma membrane 2 | Multiple_C | 5.49 | 5.18 | 5.15 | 1.27 | 0.4394 | 0.6485 | 4.89 | 5.11 | 5.16 | 0.83 | 0.6547 | 0.8317 |
| TC0300012790.hg.1 | TMEM183B | transmembrane protein 183B | Multiple_C | 11.96 | 12.15 | 11.62 | 1.27 | 0.1421 | 0.3099 | 9.86 | 9.79 | 10.01 | 0.90 | 0.3297 | 0.5939 |
| TC0300013842.hg.1 | SLMAP | sarcolemma associated protein | Multiple_C | 9.29 | 9.05 | 8.95 | 1.27 | 0.455 | 0.6623 | 8.86 | 7.89 | 8.86 | 1.00 | 0.6722 | 0.8408 |
| TC0400006437.hg.1 | ZNF718 | zinc finger protein 718 | Multiple_C | 6.01 | 5.37 | 5.67 | 1.27 | 0.1318 | 0.2941 | 6.4 | 6.82 | 7.54 | 0.45 | 0.0002 | 0.0065 |
| TC0400006491.hg.1 | IDUA | iduronidase, alpha-L- | Multiple_C | 5.79 | 5.56 | 5.45 | 1.27 | 0.1238 | 0.2808 | 5.44 | 5.05 | 4.86 | 1.49 | 0.0064 | 0.0579 |
| TC0400007542.hg.1 | KIT | v-kit Hardy-Zuckerman 4 feline sarcoma viral oncogene homolo | Multiple_C | 2.97 | 2.67 | 2.63 | 1.27 | 0.2432 | 0.446 | 3.06 | 2.96 | 2.99 | 1.05 | 0.4079 | 0.6637 |

| | | | | | | | | | | | | | | | |
|---|---|---|---|---|---|---|---|---|---|---|---|---|---|---|---|
| TC0400010282.hg.1 | SEL1L3 | sel-1 suppressor of lin-12-like 3 (C. elegans) | Multiple_C | 12.98 | 12.64 | 12.64 | 1.27 | 0.5785 | 0.7565 | 10.53 | 10.75 | 11.03 | 0.71 | 0.3396 | 0.6039 |
| TC0400010539.hg.1 | PHOX2B | paired-like homeobox 2b | Multiple_C | 6.21 | 5.54 | 5.87 | 1.27 | 0.2333 | 0.434 | 6.05 | 6.26 | 6.17 | 0.92 | 0.87 | 0.944 |
| TC0400012222.hg.1 | PDGFC | platelet derived growth factor C | Multiple_C | 3.77 | 3.27 | 3.43 | 1.27 | 0.3019 | 0.5144 | 4.3 | 3.98 | 3.67 | 1.55 | 0.0065 | 0.0582 |
| TC0500007123.hg.1 | DNAJC21 | DnaJ (Hsp40) homolog, subfamily C, member 21 | Multiple_C | 9.55 | 10.79 | 9.21 | 1.27 | 0.4006 | 0.6139 | 9.52 | 8.90 | 9.4 | 1.09 | 0.2099 | 0.467 |
| TC0500007772.hg.1 | BTF3 | basic transcription factor 3 | Multiple_C | 16.23 | 16.22 | 15.89 | 1.27 | 0.1447 | 0.3144 | 14.82 | 14.87 | 14.74 | 1.06 | 0.6843 | 0.8486 |
| TC0500009417.hg.1 | TLX3 | T-cell leukemia homeobox 3 | Coding | 3.22 | 2.96 | 2.88 | 1.27 | 0.0887 | 0.2207 | 3.44 | 3.27 | 3.44 | 1.00 | 0.6907 | 0.8526 |
| TC0500009587.hg.1 | HIGD2A | HIG1 hypoxia inducible domain family, member 2A | Multiple_C | 12.87 | 12.39 | 12.53 | 1.27 | 0.3789 | 0.5919 | 7.19 | 7.49 | 7.71 | 0.70 | 0.0171 | 0.1096 |
| TC0500009669.hg.1 | ZNF354B | zinc finger protein 354B | Multiple_C | 6.59 | 7.66 | 6.25 | 1.27 | 0.3394 | 0.5535 | 5.96 | 5.39 | 6.16 | 0.87 | 0.9203 | 0.9672 |
| TC0500010018.hg.1 | C5orf49 | chromosome 5 open reading frame 49 | Coding | 3.59 | 3.39 | 3.25 | 1.27 | 0.2163 | 0.4121 | 4.09 | 4.53 | 4.45 | 0.78 | 0.2484 | 0.513 |
| TC0500012631.hg.1 | EBF1 | early B-cell factor 1 | Multiple_C | 3.81 | 3.51 | 3.47 | 1.27 | 0.1725 | 0.3539 | 4.56 | 4.54 | 4.51 | 1.04 | 0.5336 | 0.7561 |
| TC0500013193.hg.1 | CRHBP | corticotropin releasing hormone binding protein | Multiple_C | 3.42 | 3.12 | 3.08 | 1.27 | 0.0693 | 0.1839 | 3.7 | 3.87 | 3.97 | 0.83 | 0.4299 | 0.6817 |
| TC0600007677.hg.1 | HLA-DPB1 | major histocompatibility complex, class II, DP beta 1 | Multiple_C | 3.69 | 3.32 | 3.35 | 1.27 | 0.4406 | 0.6494 | 3.84 | 3.61 | 3.75 | 1.06 | 0.637 | 0.822 |
| TC0600009127.hg.1 | AMD1 | adenosylmethionine decarboxylase 1 | Multiple_C | 13.77 | 12.84 | 13.43 | 1.27 | 0.3182 | 0.5317 | 12.19 | 11.56 | 12.92 | 0.60 | 0.0077 | 0.0656 |
| TC0600014105.hg.1 | C2 | complement component 2 | Multiple_C | 4.97 | 4.62 | 4.63 | 1.27 | 0.1404 | 0.3077 | 4.92 | 5.19 | 4.9 | 1.01 | 0.5992 | 0.7987 |
| TC0600014290.hg.1 | UNC5CL | unc-5 family C-terminal like | Multiple_C | 4.21 | 3.61 | 3.87 | 1.27 | 0.1202 | 0.275 | 4.06 | 4.30 | 4.19 | 0.91 | 0.7226 | 0.8707 |
| TC0700007149.hg.1 | BMPER | BMP binding endothelial regulator | Multiple_C | 5.26 | 4.77 | 4.92 | 1.27 | 0.1584 | 0.3344 | 5.56 | 5.23 | 5.02 | 1.45 | 0.0609 | 0.2344 |
| TC0700013413.hg.1 | TRRAP | transformation/transcription domain-associated protein | Multiple_C | 7.76 | 7.11 | 7.42 | 1.27 | 0.3241 | 0.5381 | 7.32 | 7.64 | 8.1 | 0.58 | 0.0004 | 0.0097 |
| TC0800006557.hg.1 | MCPH1 | microcephalin 1 | Multiple_C | 9.87 | 10.07 | 9.53 | 1.27 | 0.3586 | 0.5723 | 10.7 | 9.50 | 10.17 | 1.44 | 0.0231 | 0.1308 |
| TC0800008088.hg.1 | CHMP4C | charged multivesicular body protein 4C | Coding | 8.66 | 9.25 | 8.32 | 1.27 | 0.5785 | 0.7565 | 8.77 | 8.34 | 8.65 | 1.09 | 0.5579 | 0.7714 |
| TC0800010171.hg.1 | C8orf86 | chromosome 8 open reading frame 86 | Coding | 3.92 | 3.54 | 3.58 | 1.27 | 0.1056 | 0.2508 | 3.74 | 3.55 | 3.53 | 1.16 | 0.3498 | 0.6139 |
| TC0900006741.hg.1 | SH3GL2 | SH3-domain GRB2-like 2 | Multiple_C | 3.83 | 3.47 | 3.49 | 1.27 | 0.2588 | 0.4653 | 3.77 | 4.00 | 3.82 | 0.97 | 0.4117 | 0.6662 |
| TC0900009077.hg.1 | ADAMTS13 | ADAM metallopeptidase with thrombospondin type 1 motif 13 | Multiple_C | 3.1 | 2.97 | 2.76 | 1.27 | 0.2029 | 0.3952 | 4.16 | 4.11 | 3.59 | 1.48 | 0.0113 | 0.0841 |
| TC0900009580.hg.1 | CER1 | cerberus 1, DAN family BMP antagonist | Coding | 3.76 | 3.43 | 3.42 | 1.27 | 0.3796 | 0.5927 | 4.53 | 4.25 | 4.45 | 1.06 | 0.0752 | 0.2634 |
| TC0900010390.hg.1 | ABHD17B | abhydrolase domain containing 17B | Multiple_C | 11.26 | 11.23 | 10.92 | 1.27 | 0.3836 | 0.5967 | 9.46 | 9.30 | 9.89 | 0.74 | 0.0984 | 0.3062 |
| TC0900012011.hg.1 | EDF1 | endothelial differentiation-related factor 1 | Multiple_C | 12.82 | 12.33 | 12.48 | 1.27 | 0.0376 | 0.117 | 10.81 | 10.98 | 10.94 | 0.91 | 0.3496 | 0.6137 |
| TC0X00007540.hg.1 | DLG3 | discs, large homolog 3 (Drosophila) | Multiple_C | 11.12 | 11.54 | 10.78 | 1.27 | 0.4007 | 0.614 | 8.86 | 9.08 | 9.67 | 0.57 | 0.0431 | 0.1915 |
| TC0X00007690.hg.1 | MAGEE1 | MAGE family member E1 | Multiple_C | 3.46 | 3.03 | 3.12 | 1.27 | 0.494 | 0.694 | 3.43 | 3.99 | 3.75 | 0.80 | 0.4207 | 0.6739 |
| TC0X00007946.hg.1 | ARMCX4 | armadillo repeat containing, X-linked 4 | Multiple_C | 3.08 | 3.01 | 2.74 | 1.27 | 0.6981 | 0.8381 | 4.81 | 4.96 | 4.97 | 0.90 | 0.2873 | 0.5523 |
| TC0X00009780.hg.1 | FGD1 | FYVE, RhoGEF and PH domain containing 1 | Multiple_C | 3.98 | 3.71 | 3.64 | 1.27 | 0.4603 | 0.6662 | 5.3 | 5.17 | 4.89 | 1.33 | 0.1847 | 0.436 |
| TC0X00010493.hg.1 | GUCY2F | guanylate cyclase 2F, retinal | Coding | 3.19 | 2.81 | 2.85 | 1.27 | 0.2267 | 0.4257 | 3.16 | 3.35 | 3.08 | 1.06 | 0.361 | 0.6241 |
| TC1000007450.hg.1 | C10orf142 | chromosome 10 open reading frame 142 | Multiple_C | 7.16 | 6.68 | 6.82 | 1.27 | 0.4499 | 0.6577 | 6.48 | 6.74 | 6.4 | 1.06 | 0.9098 | 0.9628 |
| TC1000009152.hg.1 | HTRA1 | HtrA serine peptidase 1 | Multiple_C | 3.67 | 3.27 | 3.33 | 1.27 | 0.4076 | 0.6199 | 7.14 | 7.48 | 7.14 | 1.00 | 0.2255 | 0.4862 |
| TC1000010256.hg.1 | EPC1 | enhancer of polycomb homolog 1 (Drosophila) | Multiple_C | 9.39 | 8.62 | 9.05 | 1.27 | 0.2021 | 0.3944 | 8.55 | 8.03 | 8.77 | 0.86 | 0.1982 | 0.4532 |

| | | | | | | | | | | | | | | | |
|---|---|---|---|---|---|---|---|---|---|---|---|---|---|---|---|
| TC1000010955.hg.1 | C10orf105 | chromosome 10 open reading frame 105 | Coding | 3.62 | 3.36 | 3.28 | 1.27 | 0.1722 | 0.3535 | 3.87 | 3.93 | 3.85 | 1.01 | 0.7314 | 0.8755 |
| TC1000011661.hg.1 | KCNIP2 | Kv channel interacting protein 2 | Multiple_C | 4.71 | 4.41 | 4.37 | 1.27 | 0.1023 | 0.245 | 4.57 | 4.45 | 4.01 | 1.47 | 0.0215 | 0.1256 |
| TC1000012612.hg.1 | STK32C | serine/threonine kinase 32C | Coding | 7.78 | 7.21 | 7.44 | 1.27 | 0.0713 | 0.188 | 6.74 | 6.85 | 6.49 | 1.19 | 0.1308 | 0.3583 |
| TC1100006436.hg.1 | SCGB1C1 | secretoglobin, family 1C, member 1 | Coding | 8.16 | 7.69 | 7.82 | 1.27 | 0.0759 | 0.1965 | 7.6 | 7.69 | 7.8 | 0.87 | 0.4426 | 0.6913 |
| TC1100007518.hg.1 | OR4S1 | olfactory receptor, family 4, subfamily S, member 1 | Coding | 4.6 | 4.08 | 4.26 | 1.27 | 0.1331 | 0.2963 | 4.1 | 4.06 | 4.07 | 1.02 | 0.9048 | 0.9607 |
| TC1100007555.hg.1 | OR4C45 | olfactory receptor, family 4, subfamily C, member 45 | Multiple_C | 3.25 | 2.57 | 2.91 | 1.27 | 0.2827 | 0.4929 | 3.43 | 3.45 | 3.24 | 1.14 | 0.2968 | 0.5614 |
| TC1100007619.hg.1 | TRIM51 | tripartite motif-containing 51 | Coding | 4.84 | 4.59 | 4.5 | 1.27 | 0.5167 | 0.7109 | 4.39 | 4.42 | 4.15 | 1.18 | 0.3048 | 0.5688 |
| TC1100008502.hg.1 | LOC10050612 | putative uncharacterized protein FLJ37770-like; Transcript Ident | Multiple_C | 5.23 | 4.79 | 4.89 | 1.27 | 0.7572 | 0.8753 | 5.79 | 5.90 | 5.66 | 1.09 | 0.5234 | 0.7491 |
| TC1100009924.hg.1 | OR51B4 | olfactory receptor, family 51, subfamily B, member 4 | Coding | 5.14 | 4.93 | 4.8 | 1.27 | 0.8788 | 0.9411 | 5.73 | 5.15 | 6.28 | 0.68 | 0.0759 | 0.2653 |
| TC1100011611.hg.1 | PGM2L1 | phosphoglucomutase 2-like 1 | Multiple_C | 6.94 | 6.63 | 6.6 | 1.27 | 0.9188 | 0.9623 | 6.52 | 5.79 | 6.55 | 0.98 | 0.9261 | 0.9695 |
| TC1100012230.hg.1 | EXPH5 | exophilin 5 | Multiple_C | 11.69 | 11.02 | 11.35 | 1.27 | 0.5814 | 0.7589 | 10.98 | 9.42 | 10.21 | 1.71 | 0.0704 | 0.2536 |
| TC1200006687.hg.1 | NANOGNB | NANOG neighbor homeobox | Coding | 4.08 | 3.52 | 3.74 | 1.27 | 0.3221 | 0.5359 | 4.52 | 4.74 | 4.52 | 1.00 | 0.8239 | 0.9222 |
| TC1200008739.hg.1 | TMEM263 | transmembrane protein 263 | Multiple_C | 9.03 | 9.17 | 8.69 | 1.27 | 0.5569 | 0.7397 | 8.39 | 8.34 | 8.23 | 1.12 | 0.8969 | 0.9566 |
| TC1300006919.hg.1 | FREM2 | FRAS1 related extracellular matrix protein 2 | Multiple_C | 3.96 | 3.8 | 3.62 | 1.27 | 0.1611 | 0.338 | 3.24 | 3.51 | 3.54 | 0.81 | 0.9651 | 0.9852 |
| TC1300007032.hg.1 | SERP2 | stress-associated endoplasmic reticulum protein family membe | Coding | 4.34 | 4.13 | 4 | 1.27 | 0.0911 | 0.2249 | 7.13 | 7.54 | 6.21 | 1.89 | 9.84E-05 | 0.0036 |
| TC1300007228.hg.1 | WDFY2 | WD repeat and FYVE domain containing 2 | Multiple_C | 8.1 | 7.72 | 7.76 | 1.27 | 0.2888 | 0.4995 | 11.43 | 11.08 | 10.17 | 2.39 | 6.67E-05 | 0.0027 |
| TC1400007395.hg.1 | SYT16 | synaptotagmin XVI | Multiple_C | 3.98 | 3.88 | 3.64 | 1.27 | 0.3134 | 0.5269 | 3.7 | 3.65 | 3.74 | 0.97 | 0.9016 | 0.9591 |
| TC1400007671.hg.1 | VSX2 | visual system homeobox 2 | Coding | 7.07 | 6.44 | 6.73 | 1.27 | 0.3698 | 0.5825 | 8.15 | 8.23 | 8.12 | 1.02 | 0.8683 | 0.9434 |
| TC1400010340.hg.1 | RD3L | retinal degeneration 3-like | Coding | 3.52 | 3.71 | 3.18 | 1.27 | 0.2856 | 0.496 | 3.62 | 3.49 | 3.61 | 1.01 | 0.2255 | 0.4862 |
| TC1500007905.hg.1 | ODF3L1 | outer dense fiber of sperm tails 3-like 1 | Coding | 3.76 | 3.57 | 3.42 | 1.27 | 0.295 | 0.5064 | 4.67 | 4.39 | 4.58 | 1.06 | 0.4948 | 0.7288 |
| TC1500009056.hg.1 | RASGRP1 | RAS guanyl releasing protein 1 (calcium and DAG-regulated) | Multiple_C | 3.83 | 3.99 | 3.49 | 1.27 | 0.1808 | 0.365 | 3.82 | 3.29 | 3.49 | 1.26 | 0.3458 | 0.6096 |
| TC1600007354.hg.1 | EIF3C; EIF3CL | eukaryotic translation initiation factor 3, subunit C; eukaryotic t | Multiple_C | 14.86 | 14.5 | 14.52 | 1.27 | 0.1193 | 0.2736 | 15.02 | 15.16 | 15.05 | 0.98 | 0.5834 | 0.7878 |
| TC1600008829.hg.1 | ZC3H18 | zinc finger CCCH-type containing 18 | Multiple_C | 7.95 | 7.49 | 7.61 | 1.27 | 0.7875 | 0.8913 | 7.67 | 8.11 | 7.92 | 0.84 | 0.0929 | 0.2956 |
| TC1600009168.hg.1 | ZSCAN10 | zinc finger and SCAN domain containing 10 | Multiple_C | 3.62 | 3.42 | 3.28 | 1.27 | 0.3369 | 0.5507 | 3.96 | 3.99 | 3.94 | 1.01 | 0.259 | 0.5233 |
| TC1600010604.hg.1 | FAM96B | family with sequence similarity 96, member B | Multiple_C | 12.77 | 13.03 | 12.43 | 1.27 | 0.3136 | 0.527 | 12.26 | 11.70 | 11.44 | 1.77 | 0.0676 | 0.2477 |
| TC1600011002.hg.1 | SDR42E1 | short chain dehydrogenase/reductase family 42E, member 1 | Multiple_C | 4.25 | 3.82 | 3.91 | 1.27 | 0.9882 | 0.9937 | 4.15 | 4.28 | 4.1 | 1.04 | 0.8769 | 0.9471 |
| TC1600011342.hg.1 | NAA60 | N(alpha)-acetyltransferase 60, NatF catalytic subunit | Multiple_C | 10.68 | 9.29 | 10.34 | 1.27 | 0.3043 | 0.5172 | 11.51 | 12.02 | 11.88 | 0.77 | 0.0527 | 0.2146 |
| TC1700006652.hg.1 | C17orf107 | chromosome 17 open reading frame 107 | Multiple_C | 4.62 | 4.39 | 4.28 | 1.27 | 0.3753 | 0.588 | 4.05 | 4.24 | 4.15 | 0.93 | 0.5242 | 0.7494 |
| TC1700006743.hg.1 | TNK1 | tyrosine kinase, non-receptor, 1 | Multiple_C | 6.08 | 5.2 | 5.74 | 1.27 | 0.6457 | 0.8036 | 5.5 | 5.91 | 5.67 | 0.89 | 0.4404 | 0.6901 |
| TC1700007080.hg.1 | RAI1 | retinoic acid induced 1 | Multiple_C | 6.34 | 5.5 | 6 | 1.27 | 0.1287 | 0.2889 | 7.25 | 7.34 | 7.45 | 0.87 | 0.1081 | 0.3234 |
| TC1700007859.hg.1 | KLHL10 | kelch-like family member 10 | Multiple_C | 3.22 | 3 | 2.88 | 1.27 | 0.7682 | 0.8807 | 3.82 | 3.74 | 3.8 | 1.01 | 0.1868 | 0.4387 |
| TC1700007922.hg.1 | G6PC | glucose-6-phosphatase, catalytic subunit | Multiple_C | 3.3 | 2.73 | 2.96 | 1.27 | 0.1236 | 0.2806 | 3.58 | 3.66 | 3.65 | 0.95 | 0.7799 | 0.9013 |
| TC1700007967.hg.1 | CD300LG | CD300 molecule-like family member g | Multiple_C | 4.81 | 4.26 | 4.47 | 1.27 | 0.3297 | 0.5435 | 4.53 | 4.78 | 4.83 | 0.81 | 0.3735 | 0.6341 |

| | | | | | | | | | | | | | | | |
|---|---|---|---|---|---|---|---|---|---|---|---|---|---|---|---|
| TC1700008014.hg.1 | ADAM11 | ADAM metallopeptidase domain 11 | Multiple_C | 3.75 | 3.42 | 3.41 | 1.27 | 0.4273 | 0.6379 | 3.44 | 3.31 | 3.34 | 1.07 | 0.1328 | 0.3616 |
| TC1700008032.hg.1 | ACBD4 | acyl-CoA binding domain containing 4 | Multiple_C | 6.37 | 6.88 | 6.03 | 1.27 | 0.4035 | 0.6165 | 5.81 | 5.80 | 5.01 | 1.74 | 0.0263 | 0.1418 |
| TC1700008598.hg.1 | MILR1 | mast cell immunoglobulin-like receptor 1 | Multiple_C | 5.56 | 5.03 | 5.22 | 1.27 | 0.4674 | 0.6717 | 7.14 | 7.33 | 6.38 | 1.69 | 0.0027 | 0.0328 |
| TC1700009740.hg.1 | MYH13 | myosin, heavy chain 13, skeletal muscle | Coding | 4.13 | 3.9 | 3.79 | 1.27 | 0.2342 | 0.435 | 4.19 | 4.39 | 3.92 | 1.21 | 0.4044 | 0.66 |
| TC1700010214.hg.1 | FAM222B | family with sequence similarity 222, member B | Multiple_C | 5.31 | 4.42 | 4.97 | 1.27 | 0.9535 | 0.9784 | 5.84 | 5.79 | 6.13 | 0.82 | 0.9462 | 0.9782 |
| TC1700010461.hg.1 | CCL3L3; CCL3L | chemokine (C-C motif) ligand 3-like 3; chemokine (C-C motif) lig | Multiple_C | 3.04 | 2.75 | 2.7 | 1.27 | 0.4556 | 0.6625 | 3.39 | 3.67 | 3.69 | 0.81 | 0.3917 | 0.6502 |
| TC1700010672.hg.1 | KRT32 | keratin 32, type I | Coding | 3.93 | 3.69 | 3.59 | 1.27 | 0.1668 | 0.3462 | 3.85 | 3.62 | 3.79 | 1.04 | 0.9491 | 0.9793 |
| TC1700010679.hg.1 | KRT9 | keratin 9, type I | Coding | 4.72 | 4.36 | 4.38 | 1.27 | 0.144 | 0.3132 | 4.41 | 4.57 | 4.34 | 1.05 | 0.3354 | 0.5997 |
| TC1700012295.hg.1 | ARSG | arylsulfatase G | Multiple_C | 3.54 | 2.94 | 3.2 | 1.27 | 0.4319 | 0.642 | 6.22 | 5.31 | 5.54 | 1.60 | 0.0243 | 0.1346 |
| TC1800007015.hg.1 | TTR | transthyretin | Multiple_C | 5 | 4.84 | 4.66 | 1.27 | 0.1048 | 0.2494 | 4.25 | 4.26 | 4.17 | 1.06 | 0.5483 | 0.7651 |
| TC1800008906.hg.1 | VPS4B | vacuolar protein sorting 4 homolog B (S. cerevisiae) | Multiple_C | 9.58 | 9.82 | 9.24 | 1.27 | 0.3846 | 0.5979 | 8.8 | 9.00 | 8.56 | 1.18 | 0.0834 | 0.278 |
| TC1800009307.hg.1 | SERPINB4 | serpin peptidase inhibitor, clade B (ovalbumin), member 4 | Multiple_C | 4.3 | 3.73 | 3.96 | 1.27 | 0.7803 | 0.887 | 4.42 | 4.35 | 4.12 | 1.23 | 0.1936 | 0.4472 |
| TC1800009309.hg.1 | CBLN2 | cerebellin 2 precursor | Multiple_C | 3.93 | 3.41 | 3.59 | 1.27 | 0.4274 | 0.638 | 3.79 | 3.70 | 3.69 | 1.07 | 0.1105 | 0.3275 |
| TC1900006660.hg.1 | ATCAY | ataxia, cerebellar, Cayman type | Multiple_C | 4.27 | 3.6 | 3.93 | 1.27 | 0.2863 | 0.4968 | 4.95 | 4.80 | 4 | 1.93 | 0.013 | 0.092 |
| TC1900008363.hg.1 | IGFL2 | IGF like family member 2 | Multiple_C | 3.77 | 3.49 | 3.43 | 1.27 | 0.0942 | 0.2307 | 4.07 | 4.06 | 4.08 | 0.99 | 0.8497 | 0.935 |
| TC1900008513.hg.1 | CGB2 | chorionic gonadotropin, beta polypeptide 2 | Coding | 4.45 | 4.07 | 4.11 | 1.27 | 0.2775 | 0.4868 | 4.7 | 4.59 | 4.34 | 1.28 | 0.0384 | 0.1784 |
| TC1900008888.hg.1 | EPS8L1 | EPS8-like 1 | Multiple_C | 5.4 | 4.78 | 5.06 | 1.27 | 0.3613 | 0.5748 | 4.39 | 4.38 | 4.1 | 1.22 | 0.1088 | 0.3247 |
| TC1900009548.hg.1 | MYO1F | myosin IF | Multiple_C | 3.13 | 2.66 | 2.79 | 1.27 | 0.2837 | 0.4938 | 3.68 | 3.79 | 3.37 | 1.24 | 0.1666 | 0.4117 |
| TC1900010704.hg.1 | PRX | periaxin | Multiple_C | 6.43 | 5.95 | 6.09 | 1.27 | 0.1629 | 0.3407 | 6.14 | 6.37 | 6.26 | 0.92 | 0.5538 | 0.7687 |
| TC1900010771.hg.1 | LYPD4 | LY6/PLAUR domain containing 4 | Coding | 4.55 | 4.14 | 4.21 | 1.27 | 0.4879 | 0.6889 | 3.78 | 3.57 | 3.51 | 1.21 | 0.277 | 0.543 |
| TC1900012029.hg.1 | ZNF816-ZNF32 | ZNF816-ZNF321P readthrough | Coding | 9.91 | 10.32 | 9.57 | 1.27 | 0.3723 | 0.5849 | 7.93 | 8.08 | 7.89 | 1.03 | 0.4941 | 0.7282 |
| TC2000007506.hg.1 | ZSWIM3 | zinc finger, SWIM-type containing 3 | Multiple_C | 5.34 | 5.14 | 5 | 1.27 | 0.293 | 0.5042 | 5.16 | 5.05 | 4.91 | 1.19 | 0.3181 | 0.5822 |
| TC2000009209.hg.1 | KCNS1 | potassium voltage-gated channel, modifier subfamily S, membe | Coding | 4.23 | 3.85 | 3.89 | 1.27 | 0.1641 | 0.3424 | 4.06 | 4.05 | 4.03 | 1.02 | 0.6531 | 0.8309 |
| TC2100006447.hg.1 | CRYAA | crystallin alpha A | Multiple_C | 3.62 | 3.2 | 3.28 | 1.27 | 0.2122 | 0.407 | 3.38 | 3.52 | 3.28 | 1.07 | 0.6032 | 0.8015 |
| TC2200007607.hg.1 | NUP50 | nucleoporin 50kDa | Multiple_C | 12.59 | 11.47 | 12.25 | 1.27 | 0.285 | 0.4953 | 11.74 | 12.05 | 12.71 | 0.51 | 0.0007 | 0.0126 |
| TSUnmapped00000341.h | ZNF780A | zinc finger protein 780A | Coding | 6.45 | 5.82 | 6.11 | 1.27 | 0.0859 | 0.2154 | 5.67 | 6.40 | 6.11 | 0.74 | 0.0747 | 0.2628 |
| TSUnmapped00000366.h | BCL2L14 | BCL2-like 14 (apoptosis facilitator) | Coding | 4.08 | 3.39 | 3.74 | 1.27 | 0.2172 | 0.413 | 3.48 | 3.66 | 3.69 | 0.86 | 0.635 | 0.8208 |
| TSUnmapped00000529.h | FMN1 | formin 1 | Coding | 4.72 | 4.24 | 4.38 | 1.27 | 0.2686 | 0.4768 | 5.42 | 5.38 | 5.18 | 1.18 | 0.2425 | 0.5061 |
| TC0100007136.hg.1 | IGSF21 | immunoglobin superfamily, member 21 | Multiple_C | 4.67 | 3.86 | 4.34 | 1.26 | 0.1091 | 0.2571 | 4.44 | 4.43 | 4.41 | 1.02 | 0.6156 | 0.8091 |
| TC0100010778.hg.1 | AXDND1 | axonemal dynein light chain domain containing 1 | Multiple_C | 3.39 | 3.04 | 3.06 | 1.26 | 0.1735 | 0.3553 | 3.57 | 3.92 | 3.63 | 0.96 | 0.9876 | 0.9947 |
| TC0100011873.hg.1 | RNF187 | ring finger protein 187 | Multiple_C | 10.3 | 11.14 | 9.97 | 1.26 | 0.3993 | 0.6129 | 8.82 | 9.53 | 9.67 | 0.55 | 0.1612 | 0.4041 |
| TC0100012950.hg.1 | LRRC38 | leucine rich repeat containing 38 | Coding | 4.54 | 3.9 | 4.21 | 1.26 | 0.4824 | 0.6838 | 4.29 | 4.08 | 4.13 | 1.12 | 0.3142 | 0.5781 |
| TC0100013699.hg.1 | C1orf216 | chromosome 1 open reading frame 216 | Multiple_C | 6.08 | 5.71 | 5.75 | 1.26 | 0.0251 | 0.0855 | 6.02 | 6.23 | 5.77 | 1.19 | 0.7798 | 0.9012 |

| | | | | | | | | | | | | | | | |
|---|---|---|---|---|---|---|---|---|---|---|---|---|---|---|---|
| TC0100016971.hg.1 | ADIPOR1 | adiponectin receptor 1 | Multiple_C | 13.56 | 12.05 | 13.23 | 1.26 | 0.133 | 0.2962 | 13.04 | 12.18 | 12.51 | 1.44 | 0.018 | 0.1127 |
| TC0100017018.hg.1 | ETNK2 | ethanolamine kinase 2 | Multiple_C | 3.25 | 2.83 | 2.92 | 1.26 | 0.2361 | 0.4374 | 5.31 | 4.97 | 4.39 | 1.89 | 0.0117 | 0.0862 |
| TC0100017080.hg.1 | PM20D1 | peptidase M20 domain containing 1 | Multiple_C | 4.13 | 3.67 | 3.8 | 1.26 | 0.0657 | 0.1773 | 4.33 | 4.05 | 4.07 | 1.20 | 0.3379 | 0.6022 |
| TC0200009955.hg.1 | CYBRD1 | cytochrome b reductase 1 | Multiple_C | 2.96 | 2.68 | 2.63 | 1.26 | 0.8255 | 0.9123 | 8.53 | 7.26 | 7.47 | 2.08 | 0.001 | 0.0168 |
| TC0200009982.hg.1 | RAPGEF4 | Rap guanine nucleotide exchange factor 4 | Multiple_C | 3.87 | 3.54 | 3.54 | 1.26 | 0.2424 | 0.4448 | 6.69 | 6.94 | 6.87 | 0.88 | 0.3139 | 0.578 |
| TC0200010818.hg.1 | WNT10A | wingless-type MMTV integration site family, member 10A | Multiple_C | 4.7 | 4.04 | 4.37 | 1.26 | 0.2723 | 0.4808 | 6.9 | 7.09 | 6.63 | 1.21 | 0.3148 | 0.5787 |
| TC0200011093.hg.1 | ALPP | alkaline phosphatase, placental | Multiple_C | 4.85 | 4.42 | 4.52 | 1.26 | 0.3025 | 0.5151 | 5.41 | 5.37 | 4.99 | 1.34 | 0.0827 | 0.2769 |
| TC0200011423.hg.1 | D2HGDH | D-2-hydroxyglutarate dehydrogenase | Multiple_C | 5.78 | 5.61 | 5.45 | 1.26 | 0.3648 | 0.5783 | 5.1 | 5.44 | 5.12 | 0.99 | 0.1681 | 0.4137 |
| TC0200015963.hg.1 | TRIP12 | thyroid hormone receptor interactor 12 | Multiple_C | 11.14 | 10.67 | 10.81 | 1.26 | 0.4126 | 0.6246 | 11.46 | 11.42 | 11.35 | 1.08 | 0.9472 | 0.9786 |
| TC0200016408.hg.1 | KCNS3 | potassium voltage-gated channel, modifier subfamily S, membe | Multiple_C | 3.62 | 3.39 | 3.29 | 1.26 | 0.3149 | 0.5283 | 6.69 | 5.46 | 5.86 | 1.78 | 0.0009 | 0.0156 |
| TC0200016418.hg.1 | ZNF512 | zinc finger protein 512 | Multiple_C | 3.01 | 2.7 | 2.68 | 1.26 | 0.2077 | 0.4011 | 9.44 | 9.63 | 9.82 | 0.77 | 0.082 | 0.2761 |
| TC0300007263.hg.1 | RTP3 | receptor (chemosensory) transporter protein 3 | Coding | 4.49 | 4.07 | 4.16 | 1.26 | 0.4397 | 0.6488 | 4.41 | 4.27 | 4.18 | 1.17 | 0.3631 | 0.6258 |
| TC0300007385.hg.1 | BSN | bassoon presynaptic cytomatrix protein | Multiple_C | 4.21 | 3.75 | 3.88 | 1.26 | 0.1672 | 0.3467 | 5.06 | 5.03 | 5.1 | 0.97 | 0.5809 | 0.7859 |
| TC0300008654.hg.1 | ROPN1B | rhophilin associated tail protein 1B | Multiple_C | 4.65 | 4.06 | 4.32 | 1.26 | 0.058 | 0.1616 | 5.07 | 5.29 | 5.08 | 0.99 | 0.9334 | 0.972 |
| TC0300009179.hg.1 | MED12L | mediator complex subunit 12 like | Multiple_C | 4.05 | 3.1 | 3.72 | 1.26 | 0.6481 | 0.8049 | 7.17 | 5.88 | 5.47 | 3.25 | 0.0058 | 0.0541 |
| TC0400007232.hg.1 | C4orf19 | chromosome 4 open reading frame 19 | Coding | 8.33 | 8.29 | 8 | 1.26 | 0.3697 | 0.5825 | 4.02 | 4.35 | 4.44 | 0.75 | 0.4242 | 0.677 |
| TC0500007820.hg.1 | SV2C | synaptic vesicle glycoprotein 2C | Multiple_C | 4.41 | 4.36 | 4.08 | 1.26 | 0.4042 | 0.6172 | 4.04 | 3.69 | 3.4 | 1.56 | 0.0032 | 0.0371 |
| TC0500009037.hg.1 | SPINK7 | serine peptidase inhibitor, Kazal type 7 (putative) | Multiple_C | 3.26 | 3.23 | 2.93 | 1.26 | 0.3194 | 0.5331 | 3.41 | 3.59 | 3.4 | 1.01 | 0.7695 | 0.8967 |
| TC0500012450.hg.1 | TIGD6 | tigger transposable element derived 6 | Multiple_C | 5.99 | 5.59 | 5.66 | 1.26 | 0.2067 | 0.4 | 4.52 | 4.62 | 4.44 | 1.06 | 0.7836 | 0.9026 |
| TC0600006950.hg.1 | TMEM170B | transmembrane protein 170B | Coding | 5.22 | 4.68 | 4.89 | 1.26 | 0.1239 | 0.281 | 4.14 | 3.99 | 4.38 | 0.85 | 0.2726 | 0.538 |
| TC0600007557.hg.1 | MUC22 | mucin 22 | Multiple_C | 4.85 | 4.41 | 4.52 | 1.26 | 0.53 | 0.7204 | 4.76 | 4.47 | 4.49 | 1.21 | 0.0722 | 0.2574 |
| TC0600010948.hg.1 | NUP153 | nucleoporin 153kDa | Multiple_C | 10.68 | 9.6 | 10.35 | 1.26 | 0.1332 | 0.2965 | 12.51 | 11.82 | 13.42 | 0.53 | 0.0025 | 0.0315 |
| TC0600011406.hg.1 | POU5F1 | POU class 5 homeobox 1 | Multiple_C | 5.69 | 5.57 | 5.36 | 1.26 | 0.2551 | 0.4608 | 5.18 | 5.28 | 4.93 | 1.19 | 0.465 | 0.7068 |
| TC0600011853.hg.1 | MEA1 | male-enhanced antigen 1 | Multiple_C | 8.12 | 7.06 | 7.79 | 1.26 | 0.482 | 0.6836 | 6.91 | 7.72 | 7.14 | 0.85 | 0.1601 | 0.4027 |
| TC0600012036.hg.1 | IL17F | interleukin 17F | Multiple_C | 5.75 | 5.33 | 5.42 | 1.26 | 0.2834 | 0.4935 | 4.98 | 4.95 | 4.94 | 1.03 | 0.733 | 0.8767 |
| TC0600012815.hg.1 | PPIL6 | peptidylprolyl isomerase (cyclophilin)-like 6 | Multiple_C | 6.13 | 7.67 | 5.8 | 1.26 | 0.4178 | 0.6295 | 6.51 | 5.29 | 5.13 | 2.60 | 8.07E-05 | 0.0031 |
| TC0600013155.hg.1 | TMEM244 | transmembrane protein 244 | Coding | 3.79 | 3.63 | 3.46 | 1.26 | 0.853 | 0.9281 | 4.25 | 4.22 | 4.09 | 1.12 | 0.2417 | 0.5056 |
| TC0700006651.hg.1 | ZNF853 | zinc finger protein 853 | Multiple_C | 5.92 | 5.23 | 5.59 | 1.26 | 0.0511 | 0.1469 | 6.51 | 6.27 | 5.7 | 1.75 | 0.0041 | 0.0435 |
| TC0700007831.hg.1 | TPST1 | tyrosylprotein sulfotransferase 1 | Multiple_C | 3.98 | 3.75 | 3.65 | 1.26 | 0.9224 | 0.9639 | 5.16 | 4.59 | 4.94 | 1.16 | 0.7479 | 0.8847 |
| TC0700011584.hg.1 | FGL2 | fibrinogen-like 2 | Multiple_C | 3.38 | 3.03 | 3.05 | 1.26 | 0.5787 | 0.7565 | 3.72 | 3.93 | 3.94 | 0.86 | 0.505 | 0.7368 |
| TC0700011642.hg.1 | HGF | hepatocyte growth factor (hepapoietin A; scatter factor) | Multiple_C | 3.99 | 3.63 | 3.66 | 1.26 | 0.6617 | 0.8145 | 4.11 | 3.66 | 3.46 | 1.57 | 0.0037 | 0.0411 |
| TC0700013425.hg.1 | STAG3 | stromal antigen 3 | Multiple_C | 6.44 | 5.96 | 6.11 | 1.26 | 0.0666 | 0.179 | 5.1 | 5.29 | 5.56 | 0.73 | 0.2601 | 0.5244 |
| TC0800009569.hg.1 | RP1L1 | retinitis pigmentosa 1-like 1 | Multiple_C | 4.13 | 3.89 | 3.8 | 1.26 | 0.1476 | 0.3184 | 3.73 | 3.78 | 3.67 | 1.04 | 0.5922 | 0.7938 |

| | | | | | | | | | | | | | | | |
|---|---|---|---|---|---|---|---|---|---|---|---|---|---|---|---|
| TC0800011041.hg.1 | MMP16 | matrix metallopeptidase 16 (membrane-inserted) | Multiple_C | 3.55 | 3.15 | 3.22 | 1.26 | 0.7259 | 0.8558 | 3.66 | 3.78 | 3.73 | 0.95 | 0.7539 | 0.8881 |
| TC0800012422.hg.1 | VCPIP1 | valosin containing protein (p97)/p47 complex interacting protei | Multiple_C | 9.08 | 9.15 | 8.75 | 1.26 | 0.5836 | 0.7607 | 9.14 | 8.56 | 8.74 | 1.32 | 0.2911 | 0.5557 |
| TC0900010470.hg.1 | PRUNE2 | prune homolog 2 (Drosophila) | Multiple_C | 6.6 | 6.3 | 6.27 | 1.26 | 0.3846 | 0.5979 | 5.85 | 6.08 | 5.92 | 0.95 | 0.5807 | 0.7859 |
| TC0900010667.hg.1 | SPATA31C2 | SPATA31 subfamily C, member 2 | Multiple_C | 3.43 | 3.01 | 3.1 | 1.26 | 0.1579 | 0.3338 | 3.39 | 3.69 | 3.62 | 0.85 | 0.3828 | 0.6418 |
| TC0900011776.hg.1 | UCK1 | uridine-cytidine kinase 1 | Multiple_C | 8.97 | 8.22 | 8.64 | 1.26 | 0.8593 | 0.9313 | 7.83 | 8.05 | 8.62 | 0.58 | 0.0037 | 0.0405 |
| TC0900012126.hg.1 | ANKRD20A3; A | ankyrin repeat domain 20 family, member A3; ankyrin repeat do | Multiple_C | 3.85 | 3.63 | 3.52 | 1.26 | 0.4214 | 0.6325 | 5.81 | 4.62 | 5.54 | 1.21 | 0.1461 | 0.3816 |
| TC0X00007169.hg.1 | SSX6 | synovial sarcoma, X breakpoint 6 (pseudogene) | Multiple_C | 3.56 | 3.03 | 3.23 | 1.26 | 0.298 | 0.5096 | 3.56 | 3.70 | 3.68 | 0.92 | 0.9346 | 0.9725 |
| TC0X00008230.hg.1 | IL13RA1 | interleukin 13 receptor, alpha 1 | Multiple_C | 9.73 | 9.66 | 9.4 | 1.26 | 0.5403 | 0.728 | 7.74 | 8.87 | 10.12 | 0.19 | 6.24E-07 | 0.0001 |
| TC0X00008750.hg.1 | MAGEA3 | MAGE family member A3 | Coding | 6.32 | 5.95 | 5.99 | 1.26 | 0.0753 | 0.1955 | 5.96 | 5.93 | 5.57 | 1.31 | 0.0178 | 0.1121 |
| TC0X00008767.hg.1 | MAGEA1 | MAGE family member A1 | Multiple_C | 4.9 | 4.61 | 4.57 | 1.26 | 0.1606 | 0.3373 | 4.27 | 4.31 | 4.29 | 0.99 | 0.4028 | 0.659 |
| TC0X00008859.hg.1 | H2AFB1; H2AF | H2A histone family, member B1; H2A histone family, member B | Coding | 4.55 | 3.9 | 4.22 | 1.26 | 0.1929 | 0.3818 | 5.08 | 5.42 | 5.35 | 0.83 | 0.2969 | 0.5615 |
| TC0X00009581.hg.1 | ELK1 | ELK1, member of ETS oncogene family | Multiple_C | 9.45 | 7.89 | 9.12 | 1.26 | 0.5361 | 0.725 | 9.74 | 10.55 | 10.65 | 0.53 | 0.0006 | 0.0117 |
| TC0X00009963.hg.1 | P2RY4 | pyrimidinergic receptor P2Y, G-protein coupled, 4 | Coding | 6.38 | 5.84 | 6.05 | 1.26 | 0.3241 | 0.5381 | 4.6 | 4.75 | 4.51 | 1.06 | 0.8503 | 0.9354 |
| TC0X00010155.hg.1 | ITM2A | integral membrane protein 2A | Multiple_C | 4.77 | 4.22 | 4.44 | 1.26 | 0.2586 | 0.465 | 4.73 | 4.90 | 4.64 | 1.06 | 0.6982 | 0.857 |
| TC0X00010341.hg.1 | XKRX | X-linked Kx blood group related, X-linked | Multiple_C | 3.06 | 2.62 | 2.73 | 1.26 | 0.2642 | 0.4716 | 4.13 | 4.11 | 3.68 | 1.37 | 0.2011 | 0.4565 |
| TC0X00011305.hg.1 | NHSL2 | NHS-like 2 | Multiple_C | 4.57 | 4.21 | 4.24 | 1.26 | 0.599 | 0.7715 | 3.77 | 4.15 | 4.19 | 0.75 | 0.0645 | 0.2418 |
| TC0Y00006954.hg.1 | AMELY | amelogenin, Y-linked | Coding | 3.83 | 3.43 | 3.5 | 1.26 | 0.2607 | 0.4675 | 3.97 | 3.76 | 3.83 | 1.10 | 0.4701 | 0.7109 |
| TC0Y00007322.hg.1 | RPS4Y1 | ribosomal protein S4, Y-linked 1 | Multiple_C | 5 | 4.72 | 4.67 | 1.26 | 0.4468 | 0.6551 | 4.13 | 4.36 | 4.24 | 0.93 | 0.8137 | 0.9172 |
| TC1000007072.hg.1 | APBB1IP | amyloid beta (A4) precursor protein-binding, family B, member | Multiple_C | 3.19 | 2.57 | 2.86 | 1.26 | 0.2891 | 0.4998 | 3.73 | 3.64 | 3.58 | 1.11 | 0.6061 | 0.8029 |
| TC1000007589.hg.1 | CHAT | choline O-acetyltransferase | Multiple_C | 5.49 | 5.16 | 5.16 | 1.26 | 0.1191 | 0.2734 | 4.75 | 4.53 | 4.77 | 0.99 | 0.6421 | 0.8245 |
| TC1000008382.hg.1 | LIPN | lipase, family member N | Coding | 4.09 | 3.59 | 3.76 | 1.26 | 0.1523 | 0.3257 | 4.35 | 4.13 | 3.59 | 1.69 | 0.0008 | 0.0147 |
| TC1000008539.hg.1 | DNTT | DNA nucleotidylexotransferase | Multiple_C | 3.71 | 3.32 | 3.38 | 1.26 | 0.2264 | 0.4254 | 3.21 | 3.27 | 3.44 | 0.85 | 0.6707 | 0.8394 |
| TC1000010716.hg.1 | FAM13C | family with sequence similarity 13, member C | Multiple_C | 3.38 | 2.94 | 3.05 | 1.26 | 0.5447 | 0.7309 | 8.77 | 9.08 | 8.45 | 1.25 | 0.1755 | 0.4236 |
| TC1000011649.hg.1 | POLL | polymerase (DNA directed), lambda | Multiple_C | 7.39 | 7.39 | 7.06 | 1.26 | 0.1198 | 0.2745 | 6.76 | 6.94 | 6.58 | 1.13 | 0.7132 | 0.8657 |
| TC1000012363.hg.1 | NKX6-2 | NK6 homeobox 2 | Multiple_C | 5 | 4.58 | 4.67 | 1.26 | 0.58 | 0.7576 | 4.74 | 4.89 | 4.96 | 0.86 | 0.1344 | 0.3642 |
| TC1100006444.hg.1 | ATHL1 | ATH1, acid trehalase-like 1 (yeast) | Multiple_C | 5.65 | 5.55 | 5.32 | 1.26 | 0.2904 | 0.5013 | 5.26 | 4.94 | 4.93 | 1.26 | 0.7535 | 0.8879 |
| TC1100008873.hg.1 | CNTN5 | contactin 5 | Multiple_C | 3.06 | 2.91 | 2.73 | 1.26 | 0.6221 | 0.7886 | 3 | 2.92 | 2.97 | 1.02 | 0.9916 | 0.9964 |
| TC1100009485.hg.1 | FLI1 | Fli-1 proto-oncogene, ETS transcription factor | Multiple_C | 3.75 | 3.72 | 3.42 | 1.26 | 0.1158 | 0.268 | 4.25 | 4.11 | 3.98 | 1.21 | 0.0726 | 0.2582 |
| TC1100009717.hg.1 | MUC6 | mucin 6, oligomeric mucus/gel-forming | Multiple_C | 3.57 | 3.11 | 3.24 | 1.26 | 0.2157 | 0.4113 | 3.85 | 3.84 | 3.68 | 1.13 | 0.1613 | 0.4041 |
| TC1100009789.hg.1 | TH | tyrosine hydroxylase | Multiple_C | 5.92 | 5.34 | 5.59 | 1.26 | 0.6369 | 0.7983 | 6.54 | 6.21 | 5.84 | 1.62 | 0.024 | 0.1338 |
| TC1100009917.hg.1 | HBB | hemoglobin, beta | Multiple_C | 3.98 | 3.45 | 3.65 | 1.26 | 0.7296 | 0.8583 | 5.38 | 4.68 | 5.06 | 1.25 | 0.2083 | 0.4652 |
| TC1100013006.hg.1 | CNTF | ciliary neurotrophic factor | Multiple_C | 4.65 | 4.01 | 4.32 | 1.26 | 0.4621 | 0.6672 | 4.52 | 3.95 | 4 | 1.43 | 0.1769 | 0.4257 |
| TC1200007652.hg.1 | GRASP | GRP1 (general receptor for phosphoinositides 1)-associated sca | Multiple_C | 4.33 | 3.91 | 4 | 1.26 | 0.2083 | 0.4019 | 4.37 | 4.27 | 4.29 | 1.06 | 0.4016 | 0.6582 |

| | | | | | | | | | | | | | | | |
|---|---|---|---|---|---|---|---|---|---|---|---|---|---|---|---|
| TC1200010110.hg.1 | KCNJ8 | potassium channel, inwardly rectifying subfamily J, member 8 | Coding | 5.86 | 5.49 | 5.53 | 1.26 | 0.3192 | 0.5329 | 5.65 | 5.75 | 5.67 | 0.99 | 0.6017 | 0.8005 |
| TC1200010556.hg.1 | HDAC7 | histone deacetylase 7 | Multiple_C | 5.56 | 4.9 | 5.23 | 1.26 | 0.5382 | 0.7264 | 6.4 | 6.91 | 7.08 | 0.62 | 0.0213 | 0.1251 |
| TC1200010859.hg.1 | OR6C70 | olfactory receptor, family 6, subfamily C, member 70 | Coding | 3.36 | 3.01 | 3.03 | 1.26 | 0.5137 | 0.7089 | 3.97 | 3.73 | 3.61 | 1.28 | 0.1299 | 0.3569 |
| TC1200011121.hg.1 | LOC10012994 | uncharacterized LOC100129940; Transcript Identified by AceVie | Multiple_C | 5.71 | 5.45 | 5.38 | 1.26 | 0.042 | 0.1274 | 5.85 | 5.90 | 5.43 | 1.34 | 0.0452 | 0.1966 |
| TC1300006641.hg.1 | NUP58 | nucleoporin 58kDa | Multiple_C | 11.76 | 11.14 | 11.43 | 1.26 | 0.5203 | 0.7137 | 10.21 | 10.19 | 10.99 | 0.58 | 0.0423 | 0.1896 |
| TC1300008827.hg.1 | ERICH6B | glutamate rich 6B | Multiple_C | 3.72 | 3.04 | 3.39 | 1.26 | 0.3176 | 0.5311 | 3.81 | 3.96 | 3.9 | 0.94 | 0.4982 | 0.7314 |
| TC1400009481.hg.1 | PLEK2 | pleckstrin 2 | Multiple_C | 14.61 | 13.9 | 14.28 | 1.26 | 0.1203 | 0.2752 | 10.83 | 11.21 | 10.66 | 1.13 | 0.2574 | 0.5224 |
| TC1400009732.hg.1 | C14orf1 | chromosome 14 open reading frame 1 | Multiple_C | 14.07 | 14.05 | 13.74 | 1.26 | 0.2077 | 0.4011 | 12.41 | 12.18 | 13.39 | 0.51 | 0.0381 | 0.1776 |
| TC1500006562.hg.1 | NPAP1 | nuclear pore associated protein 1 | Coding | 3.66 | 3.17 | 3.33 | 1.26 | 0.3729 | 0.5854 | 3.39 | 3.61 | 3.7 | 0.81 | 0.2197 | 0.4798 |
| TC1500006822.hg.1 | NUTM1 | NUT midline carcinoma, family member 1 | Coding | 4.92 | 4.39 | 4.59 | 1.26 | 0.1402 | 0.3074 | 4.92 | 5.05 | 5.59 | 0.63 | 0.0428 | 0.191 |
| TC1500007825.hg.1 | TBC1D21 | TBC1 domain family, member 21 | Coding | 4.6 | 4.09 | 4.27 | 1.26 | 0.1102 | 0.2587 | 4.54 | 4.62 | 4.39 | 1.11 | 0.4552 | 0.6998 |
| TC1500008043.hg.1 | ABHD17C | abhydrolase domain containing 17C | Multiple_C | 12.21 | 12.14 | 11.88 | 1.26 | 0.2721 | 0.4807 | 8.79 | 8.61 | 9.2 | 0.75 | 0.0834 | 0.278 |
| TC1500008757.hg.1 | ATP10A | ATPase, class V, type 10A | Multiple_C | 4.98 | 4.46 | 4.65 | 1.26 | 0.2763 | 0.4856 | 3.95 | 3.68 | 3.66 | 1.22 | 0.481 | 0.7193 |
| TC1500008896.hg.1 | TRPM1 | transient receptor potential cation channel, subfamily M, memb | Multiple_C | 4.85 | 4.36 | 4.52 | 1.26 | 0.1486 | 0.3199 | 4.57 | 5.02 | 5.01 | 0.74 | 0.2151 | 0.4738 |
| TC1500010407.hg.1 | ANPEP | alanyl (membrane) aminopeptidase | Multiple_C | 4.01 | 3.73 | 3.68 | 1.26 | 0.2468 | 0.4504 | 5.02 | 5.52 | 5.12 | 0.93 | 0.4525 | 0.6977 |
| TC1600006646.hg.1 | KREMEN2 | kringle containing transmembrane protein 2 | Coding | 3.15 | 2.74 | 2.82 | 1.26 | 0.1206 | 0.2756 | 3.35 | 3.50 | 3.36 | 0.99 | 0.9335 | 0.972 |
| TC1600006651.hg.1 | CLDN9 | claudin 9 | Coding | 3.96 | 3.52 | 3.63 | 1.26 | 0.335 | 0.5491 | 4.03 | 4.34 | 4.27 | 0.85 | 0.2092 | 0.4664 |
| TC1600007103.hg.1 | CLEC19A | C-type lectin domain family 19, member A | Multiple_C | 3.17 | 3.04 | 2.84 | 1.26 | 0.4316 | 0.6419 | 3 | 3.22 | 3.09 | 0.94 | 0.5534 | 0.7684 |
| TC1600009008.hg.1 | C1QTNF8 | C1q and tumor necrosis factor related protein 8 | Coding | 4.57 | 3.87 | 4.24 | 1.26 | 0.2813 | 0.4913 | 5.54 | 5.33 | 5.27 | 1.21 | 0.0915 | 0.2928 |
| TC1600009580.hg.1 | XYLT1 | xylosyltransferase I | Multiple_C | 8.17 | 7.86 | 7.84 | 1.26 | 0.0215 | 0.0758 | 3.56 | 3.48 | 3.91 | 0.78 | 0.1626 | 0.4059 |
| TC1600011550.hg.1 | BEAN1-AS1 | BEAN1 antisense RNA 1 | Multiple_C | 4.42 | 4.12 | 4.09 | 1.26 | 0.2771 | 0.4862 | 4.67 | 4.59 | 4.63 | 1.03 | 0.8007 | 0.9108 |
| TC1700006860.hg.1 | GLP2R | glucagon-like peptide 2 receptor | Multiple_C | 3.85 | 3.43 | 3.52 | 1.26 | 0.2335 | 0.4343 | 4.11 | 3.93 | 3.99 | 1.09 | 0.5831 | 0.7875 |
| TC1700007138.hg.1 | TVP23B | trans-golgi network vesicle protein 23 homolog B (S. cerevisiae) | Multiple_C | 14.1 | 15.38 | 13.77 | 1.26 | 0.249 | 0.453 | 11.83 | 10.66 | 11.14 | 1.61 | 0.0132 | 0.0927 |
| TC1700007761.hg.1 | ZPBP2 | zona pellucida binding protein 2 | Coding | 3.33 | 2.98 | 3 | 1.26 | 0.1797 | 0.3636 | 3.8 | 3.77 | 3.68 | 1.09 | 0.6428 | 0.8248 |
| TC1700008005.hg.1 | FZD2 | frizzled class receptor 2 | Coding | 5.74 | 5.17 | 5.41 | 1.26 | 0.5909 | 0.7659 | 4.61 | 4.83 | 4.84 | 0.85 | 0.4459 | 0.6935 |
| TC1700008573.hg.1 | DDX42 | DEAD (Asp-Glu-Ala-Asp) box helicase 42 | Multiple_C | 10.45 | 10.31 | 10.12 | 1.26 | 0.208 | 0.4015 | 12.48 | 12.21 | 12.42 | 1.04 | 0.745 | 0.8832 |
| TC1700010507.hg.1 | YWHAEP7 | tyrosine 3-monooxygenase/tryptophan 5-monooxygenase activ | Multiple_C | 8.89 | 8.23 | 8.56 | 1.26 | 0.5979 | 0.7711 | 11.54 | 11.40 | 11.43 | 1.08 | 0.9629 | 0.9844 |
| TC1700010518.hg.1 | GPR179 | G protein-coupled receptor 179 | Multiple_C | 4.36 | 4.16 | 4.03 | 1.26 | 0.5985 | 0.7712 | 4.48 | 4.27 | 4.32 | 1.12 | 0.7153 | 0.8671 |
| TC1700010652.hg.1 | KRTAP2-2 | keratin associated protein 2-2 | Coding | 5.92 | 5.34 | 5.59 | 1.26 | 0.1722 | 0.3535 | 6.05 | 5.99 | 6.14 | 0.94 | 0.3026 | 0.5669 |
| TC1700011428.hg.1 | CSH1 | chorionic somatomammotropin hormone 1 (placental lactogen) | Multiple_C | 3.21 | 2.53 | 2.88 | 1.26 | 0.1736 | 0.3556 | 3.49 | 3.20 | 3.09 | 1.32 | 0.063 | 0.2389 |
| TC1700011451.hg.1 | SMURF2 | SMAD specific E3 ubiquitin protein ligase 2 | Multiple_C | 10.05 | 10.47 | 9.72 | 1.26 | 0.1956 | 0.3856 | 11.48 | 10.01 | 10.19 | 2.45 | 6.77E-05 | 0.0027 |
| TC1700011719.hg.1 | GRIN2C | glutamate receptor, ionotropic, N-methyl D-aspartate 2C | Multiple_C | 3.72 | 3.5 | 3.39 | 1.26 | 0.3526 | 0.5653 | 3.55 | 3.66 | 3.54 | 1.01 | 0.8695 | 0.9438 |
| TC1700011720.hg.1 | FDXR | ferredoxin reductase | Multiple_C | 7.74 | 7.3 | 7.41 | 1.26 | 0.2137 | 0.4089 | 8.12 | 8.03 | 7.48 | 1.56 | 0.2122 | 0.4699 |

| | | | | | | | | | | | | | | | |
|---|---|---|---|---|---|---|---|---|---|---|---|---|---|---|---|
| TC1700011736.hg.1 | NT5C | 5, 3-nucleotidase, cytosolic | Multiple_C | 9.93 | 10.17 | 9.6 | 1.26 | 0.6289 | 0.7929 | 8.9 | 9.61 | 9.4 | 0.71 | 0.1618 | 0.4048 |
| TC1700012184.hg.1 | CHRNB1 | cholinergic receptor, nicotinic beta 1 | Multiple_C | 4.18 | 3.92 | 3.85 | 1.26 | 0.2327 | 0.4335 | 8.25 | 7.76 | 8.04 | 1.16 | 0.2088 | 0.4659 |
| TC1700012447.hg.1 | CSH2 | chorionic somatomammotropin hormone 2 | Multiple_C | 4.59 | 4.13 | 4.26 | 1.26 | 0.2361 | 0.4374 | 4.54 | 4.31 | 4.29 | 1.19 | 0.487 | 0.7237 |
| TC1900008907.hg.1 | RPL28; MIR68 | ribosomal protein L28; microRNA 6805 | Multiple_C | 17.83 | 16.86 | 17.5 | 1.26 | 0.077 | 0.1989 | 17.03 | 17.29 | 17.26 | 0.85 | 0.8904 | 0.9533 |
| TC1900008927.hg.1 | EPN1 | epsin 1 | Multiple_C | 8.47 | 7.82 | 8.14 | 1.26 | 0.311 | 0.5245 | 8.71 | 9.40 | 9.73 | 0.49 | 0.0163 | 0.1063 |
| TC1900009045.hg.1 | RPS5 | ribosomal protein S5 | Multiple_C | 16.1 | 16.57 | 15.77 | 1.26 | 0.058 | 0.1615 | 13.92 | 14.05 | 13.29 | 1.55 | 0.0107 | 0.0817 |
| TC1900009456.hg.1 | MBD3L3 | methyl-CpG binding domain protein 3-like 3 | Coding | 4.36 | 4.09 | 4.03 | 1.26 | 0.2335 | 0.4342 | 3.73 | 3.84 | 3.89 | 0.90 | 0.8664 | 0.9427 |
| TC1900011160.hg.1 | TSKS | testis-specific serine kinase substrate | Multiple_C | 3.36 | 3.13 | 3.03 | 1.26 | 0.3901 | 0.6038 | 4 | 4.14 | 4.11 | 0.93 | 0.2338 | 0.4962 |
| TC1900011207.hg.1 | JOSD2 | Josephin domain containing 2 | Multiple_C | 7.6 | 7.06 | 7.27 | 1.26 | 0.504 | 0.7015 | 6.71 | 7.00 | 6.82 | 0.93 | 0.699 | 0.8573 |
| TC1900011329.hg.1 | ZNF415 | zinc finger protein 415 | Multiple_C | 5.89 | 5.54 | 5.56 | 1.26 | 0.67 | 0.82 | 3.91 | 4.41 | 4.48 | 0.67 | 0.3861 | 0.6451 |
| TC1900011761.hg.1 | APOC4 | apolipoprotein C-IV | Multiple_C | 9.17 | 8.51 | 8.84 | 1.26 | 0.4256 | 0.6363 | 7.41 | 7.48 | 7.45 | 0.97 | 0.4843 | 0.7219 |
| TC1900011779.hg.1 | FLT3LG | fms-related tyrosine kinase 3 ligand | Multiple_C | 6.43 | 5.99 | 6.1 | 1.26 | 0.5003 | 0.6992 | 4.96 | 4.91 | 4.91 | 1.04 | 0.7741 | 0.8985 |
| TC1900011805.hg.1 | CCDC106 | coiled-coil domain containing 106 | Multiple_C | 8.17 | 7.67 | 7.84 | 1.26 | 0.3381 | 0.5521 | 9.13 | 9.30 | 8.97 | 1.12 | 0.5509 | 0.7668 |
| TC1900011886.hg.1 | ZNF564 | zinc finger protein 564 | Multiple_C | 8.55 | 8.54 | 8.22 | 1.26 | 0.3714 | 0.5841 | 7.15 | 7.37 | 7.38 | 0.85 | 0.1463 | 0.3818 |
| TC1900012020.hg.1 | ZNF432 | zinc finger protein 432 | Multiple_C | 10.11 | 10.41 | 9.78 | 1.26 | 0.1839 | 0.3695 | 7.38 | 7.46 | 7.26 | 1.09 | 0.4513 | 0.6966 |
| TC2000007125.hg.1 | LOC149950; R | uncharacterized LOC149950; novel transcript | Multiple_C | 4.59 | 4.22 | 4.26 | 1.26 | 0.3751 | 0.5879 | 3.91 | 4.17 | 4 | 0.94 | 0.5408 | 0.7601 |
| TC2100006655.hg.1 | LINC01549 | long intergenic non-protein coding RNA 1549 | Multiple_C | 3.19 | 2.77 | 2.86 | 1.26 | 0.509 | 0.7051 | 4.11 | 4.29 | 3.89 | 1.16 | 0.2009 | 0.4563 |
| TC2100006974.hg.1 | SON; MIR650: | SON DNA binding protein; microRNA 6501 | Multiple_C | 10.85 | 10.68 | 10.52 | 1.26 | 0.1292 | 0.2897 | 10.61 | 11.15 | 10.3 | 1.24 | 0.643 | 0.8248 |
| TC2100007039.hg.1 | DOPEY2 | dopey family member 2 | Multiple_C | 5.99 | 5.52 | 5.66 | 1.26 | 0.3657 | 0.5794 | 6.1 | 6.56 | 6.63 | 0.69 | 0.1203 | 0.3425 |
| TC2100007822.hg.1 | ADAMTS5 | ADAM metallopeptidase with thrombospondin type 1 motif 5 | Multiple_C | 3.47 | 3.12 | 3.14 | 1.26 | 0.5357 | 0.7247 | 3.71 | 3.69 | 3.65 | 1.04 | 0.5708 | 0.779 |
| TC2200006881.hg.1 | CABIN1 | calcineurin binding protein 1 | Multiple_C | 7.27 | 6.57 | 6.94 | 1.26 | 0.2847 | 0.495 | 6.93 | 7.43 | 7.3 | 0.77 | 0.037 | 0.1752 |
| TC2200007114.hg.1 | YWHAH | tyrosine 3-monooxygenase/tryptophan 5-monooxygenase activ | Multiple_C | 13.79 | 12.46 | 13.46 | 1.26 | 0.5454 | 0.7312 | 13.3 | 13.02 | 13.47 | 0.89 | 0.524 | 0.7492 |
| TC2200007525.hg.1 | SERHL2 | serine hydrolase-like 2 | Multiple_C | 5.22 | 5.2 | 4.89 | 1.26 | 0.1112 | 0.2602 | 5.64 | 5.29 | 5.29 | 1.27 | 0.2754 | 0.5415 |
| TC2200008701.hg.1 | KCNJ4 | potassium channel, inwardly rectifying subfamily J, member 4 | Coding | 4.55 | 4.01 | 4.22 | 1.26 | 0.363 | 0.5765 | 4.01 | 4.11 | 4.04 | 0.98 | 0.9852 | 0.9938 |
| TC2200009139.hg.1 | IL17REL | interleukin 17 receptor E-like | Coding | 5.97 | 5.43 | 5.64 | 1.26 | 0.182 | 0.3667 | 6.1 | 5.50 | 5.55 | 1.46 | 0.0203 | 0.1217 |
| TC2200009360.hg.1 | HDAC10 | histone deacetylase 10 | Multiple_C | 3.73 | 3.38 | 3.4 | 1.26 | 0.139 | 0.3052 | 3.84 | 3.93 | 3.61 | 1.17 | 0.5002 | 0.7328 |
| TSUnmapped00000196.h | ZNF546 | zinc finger protein 546 | Coding | 4.45 | 4.36 | 4.12 | 1.26 | 0.4127 | 0.6248 | 5.44 | 5.67 | 5.5 | 0.96 | 0.4197 | 0.6731 |
| TSUnmapped00000480.h | ZNF780B | zinc finger protein 780B | Coding | 7.56 | 7.15 | 7.23 | 1.26 | 0.1248 | 0.2824 | 6.17 | 6.48 | 6.52 | 0.78 | 0.7913 | 0.907 |
| TSUnmapped00000751.h | PRAMEF9 | PRAME family member 9 | Coding | 3.98 | 3.73 | 3.65 | 1.26 | 0.564 | 0.7457 | 4.67 | 4.99 | 4.62 | 1.04 | 0.1688 | 0.4145 |
| TC0100008414.hg.1 | TMEM61 | transmembrane protein 61 | Coding | 6.49 | 6.22 | 6.17 | 1.25 | 0.0512 | 0.147 | 6.46 | 6.74 | 6.95 | 0.71 | 0.2471 | 0.5117 |
| TC0100008845.hg.1 | ADGRL2 | adhesion G protein-coupled receptor L2 | Multiple_C | 3.24 | 3.11 | 2.92 | 1.25 | 0.5788 | 0.7565 | 4.88 | 8.13 | 5.34 | 0.73 | 0.0161 | 0.1056 |
| TC0100009373.hg.1 | SLC6A17 | solute carrier family 6 (neutral amino acid transporter), membe | Multiple_C | 4.06 | 3.48 | 3.74 | 1.25 | 0.0953 | 0.2326 | 4.15 | 4.20 | 4.17 | 0.99 | 0.9124 | 0.9642 |
| TC0100010025.hg.1 | PRR9 | proline rich 9 | Coding | 3.82 | 3.5 | 3.5 | 1.25 | 0.3503 | 0.5631 | 3.59 | 3.52 | 3.49 | 1.07 | 0.5603 | 0.772 |

| | | | | | | | | | | | | | | | |
|---|---|---|---|---|---|---|---|---|---|---|---|---|---|---|---|
| TC0100010140.hg.1 | SYT11 | synaptotagmin XI | Coding | 4.37 | 3.77 | 4.05 | 1.25 | 0.5441 | 0.7305 | 11.57 | 10.76 | 10.43 | 2.20 | 2.37E-05 | 0.0013 |
| TC0100011068.hg.1 | CFHR4 | complement factor H-related 4 | Coding | 3.93 | 3.56 | 3.61 | 1.25 | 0.1412 | 0.3087 | 4.14 | 4.59 | 4.09 | 1.04 | 0.2501 | 0.5145 |
| TC0100012432.hg.1 | PERM1 | PPARGC1 and ESRR induced regulator, muscle 1 | Multiple_C | 4.31 | 3.59 | 3.99 | 1.25 | 0.2204 | 0.4178 | 4.34 | 4.05 | 3.92 | 1.34 | 0.1284 | 0.3547 |
| TC0100013659.hg.1 | CSMD2 | CUB and Sushi multiple domains 2 | Multiple_C | 3.11 | 2.75 | 2.79 | 1.25 | 0.1918 | 0.3804 | 3.45 | 3.29 | 3.15 | 1.23 | 0.11 | 0.3266 |
| TC0100016431.hg.1 | TNFSF4 | tumor necrosis factor (ligand) superfamily, member 4 | Multiple_C | 3.09 | 2.61 | 2.77 | 1.25 | 0.3576 | 0.5711 | 3.59 | 3.36 | 3.56 | 1.02 | 0.9689 | 0.9871 |
| TC0100017212.hg.1 | SLC30A1 | solute carrier family 30 (zinc transporter), member 1 | Multiple_C | 8.58 | 7.63 | 8.26 | 1.25 | 0.416 | 0.6279 | 8.86 | 8.53 | 8.96 | 0.93 | 0.3552 | 0.6185 |
| TC0100018508.hg.1 | ARHGEF2 | Rho/Rac guanine nucleotide exchange factor 2 | Multiple_C | 4.3 | 4.03 | 3.98 | 1.25 | 0.2736 | 0.4822 | 5.15 | 5.04 | 5.13 | 1.01 | 0.4411 | 0.6904 |
| TC0200008512.hg.1 | FER1L5 | fer-1-like family member 5 | Multiple_C | 5.61 | 5.47 | 5.29 | 1.25 | 0.3433 | 0.557 | 6.11 | 6.21 | 5.96 | 1.11 | 0.5813 | 0.786 |
| TC0200008801.hg.1 | SULT1C4 | sulfotransferase family 1C member 4 | Multiple_C | 3.83 | 3.61 | 3.51 | 1.25 | 0.1644 | 0.3426 | 4.82 | 4.41 | 4.11 | 1.64 | 0.0022 | 0.0282 |
| TC0200009889.hg.1 | B3GALT1 | UDP-Gal:betaGlcNAc beta 1,3-galactosyltransferase 1 | Coding | 3.59 | 3.12 | 3.27 | 1.25 | 0.7949 | 0.8948 | 3.76 | 3.63 | 3.52 | 1.18 | 0.1716 | 0.4182 |
| TC0200010580.hg.1 | LOC200726; A | hCG1657980; Transcript Identified by AceView, Entrez Gene ID( | Multiple_C | 3.64 | 3.28 | 3.32 | 1.25 | 0.1442 | 0.3136 | 3.81 | 3.59 | 3.73 | 1.06 | 0.529 | 0.7526 |
| TC0200010696.hg.1 | SPAG16 | sperm associated antigen 16 | Multiple_C | 3.86 | 3.68 | 3.54 | 1.25 | 0.617 | 0.7853 | 7.03 | 6.75 | 6.4 | 1.55 | 0.0434 | 0.1926 |
| TC0200010854.hg.1 | TMEM198 | transmembrane protein 198 | Coding | 4.3 | 3.82 | 3.98 | 1.25 | 0.5732 | 0.7525 | 5.23 | 5.36 | 5.37 | 0.91 | 0.5759 | 0.7826 |
| TC0200010952.hg.1 | NYAP2 | neuronal tyrosine-phosphorylated phosphoinositide-3-kinase ac | Coding | 3.45 | 3.07 | 3.13 | 1.25 | 0.092 | 0.2266 | 3.31 | 3.53 | 3.23 | 1.06 | 0.5804 | 0.7855 |
| TC0200013870.hg.1 | LIMS3L | LIM and senescent cell antigen-like domains 3-like | Multiple_C | 3.65 | 3.06 | 3.33 | 1.25 | 0.1106 | 0.2594 | 4.36 | 4.54 | 4.36 | 1.00 | 0.4796 | 0.7181 |
| TC0200014650.hg.1 | LOC10014459 | uncharacterized LOC100144595; novel transcript; Transcript Ide | Multiple_C | 5.55 | 5.38 | 5.23 | 1.25 | 0.2817 | 0.4916 | 5.73 | 5.66 | 5.44 | 1.22 | 0.272 | 0.5375 |
| TC0200015432.hg.1 | MPP4 | membrane protein, palmitoylated 4 | Multiple_C | 5.18 | 4.73 | 4.86 | 1.25 | 0.2046 | 0.3972 | 5.83 | 5.73 | 5.64 | 1.14 | 0.3026 | 0.5669 |
| TC0200015434.hg.1 | ALS2 | ALS2, alsin Rho guanine nucleotide exchange factor | Multiple_C | 7.7 | 7.33 | 7.38 | 1.25 | 0.4369 | 0.6463 | 7.56 | 7.26 | 7.6 | 0.97 | 0.1442 | 0.3784 |
| TC0200016302.hg.1 | PRR21 | proline rich 21 | Coding | 4.1 | 3.81 | 3.78 | 1.25 | 0.4271 | 0.6377 | 4.57 | 4.64 | 4.42 | 1.11 | 0.6317 | 0.8187 |
| TC0200016567.hg.1 | HOXD10 | homeobox D10 | Coding | 3.38 | 3.12 | 3.06 | 1.25 | 0.114 | 0.2651 | 3.97 | 4.04 | 3.8 | 1.13 | 0.3331 | 0.5973 |
| TC0300007117.hg.1 | ENTPD3 | ectonucleoside triphosphate diphosphohydrolase 3 | Coding | 3.99 | 3.76 | 3.67 | 1.25 | 0.2914 | 0.5025 | 4.3 | 4.11 | 4.23 | 1.05 | 0.7668 | 0.8954 |
| TC0300009238.hg.1 | C3orf79 | chromosome 3 open reading frame 79 | Multiple_C | 3.4 | 3.42 | 3.08 | 1.25 | 0.1663 | 0.3455 | 3.38 | 3.67 | 3.52 | 0.91 | 0.3842 | 0.6432 |
| TC0300009258.hg.1 | MME | membrane metallo-endopeptidase | Multiple_C | 3.25 | 2.83 | 2.93 | 1.25 | 0.2075 | 0.4011 | 9.7 | 11.17 | 9.21 | 1.40 | 0.1657 | 0.4105 |
| TC0300009974.hg.1 | MUC20; SDHA | mucin 20, cell surface associated; succinate dehydrogenase con | Multiple_C | 3.61 | 3.17 | 3.29 | 1.25 | 0.1895 | 0.3772 | 4.17 | 3.94 | 3.71 | 1.38 | 0.0784 | 0.2696 |
| TC0300012123.hg.1 | CD80 | CD80 molecule | Multiple_C | 3.74 | 3.29 | 3.42 | 1.25 | 0.2201 | 0.4173 | 4 | 4.08 | 3.91 | 1.06 | 0.1968 | 0.4516 |
| TC0300013950.hg.1 | EFHB | EF-hand domain family, member B | Multiple_C | 4.68 | 5.31 | 4.36 | 1.25 | 0.4698 | 0.6738 | 3.82 | 3.52 | 3.88 | 0.96 | 0.8284 | 0.9241 |
| TC0400008083.hg.1 | DSPP | dentin sialophosphoprotein | Coding | 5.4 | 5.34 | 5.08 | 1.25 | 0.3361 | 0.55 | 4.82 | 4.84 | 4.83 | 0.99 | 0.6349 | 0.8208 |
| TC0400008348.hg.1 | HADH | hydroxyacyl-CoA dehydrogenase | Multiple_C | 12.02 | 12.18 | 11.7 | 1.25 | 0.0783 | 0.2015 | 8.7 | 9.11 | 8.63 | 1.05 | 0.744 | 0.8829 |
| TC0400010688.hg.1 | USP46 | ubiquitin specific peptidase 46 | Multiple_C | 8.26 | 7.79 | 7.94 | 1.25 | 0.2854 | 0.4958 | 8.17 | 7.81 | 8.76 | 0.66 | 0.107 | 0.3219 |
| TC0400012760.hg.1 | EVC | Ellis van Creveld protein | Multiple_C | 5.98 | 5.51 | 5.66 | 1.25 | 0.1757 | 0.3581 | 6.91 | 6.90 | 6.89 | 1.01 | 0.6393 | 0.8231 |
| TC0400012880.hg.1 | DUX4 | double homeobox 4 | Multiple_C | 5.64 | 5.61 | 5.32 | 1.25 | 0.1534 | 0.3271 | 4.47 | 4.73 | 4.91 | 0.74 | 0.276 | 0.5422 |
| TC0400012938.hg.1 | RASGEF1B | RasGEF domain family member 1B | Multiple_C | 6.08 | 5.32 | 5.76 | 1.25 | 0.0836 | 0.2112 | 4.56 | 4.53 | 4.33 | 1.17 | 0.6504 | 0.8293 |
| TC0500006579.hg.1 | IRX1 | iroquois homeobox 1 | Coding | 3.76 | 3.37 | 3.44 | 1.25 | 0.3772 | 0.5901 | 3.91 | 4.00 | 3.93 | 0.99 | 0.856 | 0.9387 |

| | | | | | | | | | | | | | | |
|---|---|---|---|---|---|---|---|---|---|---|---|---|---|---|
| TC0500008766.hg.1 | MYOT | myotilin | Multiple_C | 3.08 | 2.79 | 2.76 | 1.25 | 0.2793 | 0.4889 | 3.72 | 3.80 | 3.55 | 1.13 | 0.1246 | 0.3488 |
| TC0500009422.hg.1 | NPM1 | nucleophosmin (nucleolar phosphoprotein B23, numatrin) | Multiple_C | 15.63 | 15.91 | 15.31 | 1.25 | 0.9978 | 0.9989 | 14.89 | 15.06 | 15.39 | 0.71 | 0.0778 | 0.2686 |
| TC0500009424.hg.1 | FGF18 | fibroblast growth factor 18 | Coding | 4.62 | 4.28 | 4.3 | 1.25 | 0.4138 | 0.6258 | 5.52 | 5.41 | 5.12 | 1.32 | 0.1692 | 0.4151 |
| TC0600007257.hg.1 | TRIM38 | tripartite motif containing 38 | Multiple_C | 5.72 | 4.44 | 5.4 | 1.25 | 0.3432 | 0.557 | 5.51 | 5.48 | 6.01 | 0.71 | 0.2342 | 0.4967 |
| TC0600008883.hg.1 | KLHL32 | kelch-like family member 32 | Multiple_C | 3.5 | 3.02 | 3.18 | 1.25 | 0.493 | 0.6928 | 3.69 | 3.82 | 3.67 | 1.01 | 0.8762 | 0.947 |
| TC0600010383.hg.1 | HUS1B | HUS1 checkpoint clamp component B | Coding | 3.46 | 2.83 | 3.14 | 1.25 | 0.4072 | 0.6196 | 4.94 | 4.57 | 4.08 | 1.82 | 0.0046 | 0.0464 |
| TC0600011800.hg.1 | PGC | progastricsin (pepsinogen C) | Multiple_C | 4.96 | 4.86 | 4.64 | 1.25 | 0.1548 | 0.329 | 5.36 | 5.47 | 5.18 | 1.13 | 0.8012 | 0.9112 |
| TC0600011805.hg.1 | USP49 | ubiquitin specific peptidase 49 | Multiple_C | 6.06 | 5.62 | 5.74 | 1.25 | 0.3506 | 0.5634 | 5.9 | 6.10 | 6.01 | 0.93 | 0.0916 | 0.2931 |
| TC0600014240.hg.1 | LOC10013035 | uncharacterized LOC100130357; novel transcript, antisense to F | Multiple_C | 5.09 | 4.8 | 4.77 | 1.25 | 0.08 | 0.2051 | 3.8 | 3.64 | 3.61 | 1.14 | 0.2906 | 0.5555 |
| TC0700008849.hg.1 | FOXP2 | forkhead box P2 | Multiple_C | 5.02 | 4.13 | 4.7 | 1.25 | 0.4058 | 0.6186 | 7.31 | 7.08 | 7.72 | 0.75 | 0.1345 | 0.3643 |
| TC0700009323.hg.1 | TMEM213 | transmembrane protein 213 | Multiple_C | 4.42 | 3.95 | 4.1 | 1.25 | 0.1549 | 0.3291 | 4.87 | 4.80 | 4.76 | 1.08 | 0.8299 | 0.9245 |
| TC0700011961.hg.1 | GJC3 | gap junction protein gamma 3 | Coding | 5 | 4.79 | 4.68 | 1.25 | 0.3406 | 0.5546 | 4.85 | 4.93 | 4.7 | 1.11 | 0.827 | 0.9237 |
| TC0700013072.hg.1 | WDR86 | WD repeat domain 86 | Multiple_C | 4.68 | 4.24 | 4.36 | 1.25 | 0.0479 | 0.1402 | 4.75 | 4.84 | 4.72 | 1.02 | 0.6609 | 0.8345 |
| TC0800006873.hg.1 | PDGFRL | platelet-derived growth factor receptor-like | Multiple_C | 7.54 | 7.38 | 7.22 | 1.25 | 0.5671 | 0.7479 | 3.73 | 3.59 | 3.44 | 1.22 | 0.2849 | 0.55 |
| TC0800006989.hg.1 | CCAR2 | cell cycle and apoptosis regulator 2 | Multiple_C | 10.46 | 9.98 | 10.14 | 1.25 | 0.4114 | 0.6237 | 10.07 | 10.88 | 10.67 | 0.66 | 0.0275 | 0.1451 |
| TC0800009941.hg.1 | PNMA2 | paraneoplastic Ma antigen 2 | Multiple_C | 4.75 | 4.16 | 4.43 | 1.25 | 0.1781 | 0.361 | 4.37 | 4.11 | 4.15 | 1.16 | 0.0487 | 0.2051 |
| TC0800012446.hg.1 | YWHAZ | tyrosine 3-monooxygenase/tryptophan 5-monooxygenase activ | Multiple_C | 16.29 | 15.64 | 15.97 | 1.25 | 0.0774 | 0.1996 | 16.48 | 15.90 | 16.56 | 0.95 | 0.7403 | 0.8807 |
| TC0800012475.hg.1 | JRK | Jrk helix-turn-helix protein | Multiple_C | 8.84 | 8.44 | 8.52 | 1.25 | 0.11 | 0.2584 | 9.29 | 9.37 | 9.15 | 1.10 | 0.4742 | 0.7139 |
| TC0900007116.hg.1 | CCIN | calicin | Coding | 3.71 | 3.13 | 3.39 | 1.25 | 0.2828 | 0.4929 | 4.55 | 4.51 | 4.31 | 1.18 | 0.4903 | 0.7257 |
| TC0900007489.hg.1 | FAM122A | family with sequence similarity 122A | Coding | 8.8 | 7.84 | 8.48 | 1.25 | 0.2567 | 0.4629 | 7.69 | 7.74 | 7.62 | 1.05 | 0.6773 | 0.8446 |
| TC0900007807.hg.1 | DAPK1 | death-associated protein kinase 1 | Multiple_C | 10.64 | 10.05 | 10.32 | 1.25 | 0.1733 | 0.3551 | 9.1 | 9.13 | 8.94 | 1.12 | 0.105 | 0.3179 |
| TC0900011066.hg.1 | OR13C5 | olfactory receptor, family 13, subfamily C, member 5 | Coding | 4.07 | 3.64 | 3.75 | 1.25 | 0.3954 | 0.6089 | 3.74 | 3.63 | 3.8 | 0.96 | 0.8173 | 0.9191 |
| TC0900011860.hg.1 | SARDH | sarcosine dehydrogenase | Multiple_C | 5.93 | 5.97 | 5.61 | 1.25 | 0.59 | 0.7654 | 6.48 | 6.09 | 5.6 | 1.84 | 0.0024 | 0.0302 |
| TC0900012024.hg.1 | CLIC3 | chloride intracellular channel 3 | Multiple_C | 3.27 | 2.82 | 2.95 | 1.25 | 0.0619 | 0.1694 | 4.54 | 4.34 | 4.04 | 1.41 | 0.0806 | 0.2736 |
| TC0900012179.hg.1 | SPTAN1 | spectrin, alpha, non-erythrocytic 1 | Multiple_C | 11.78 | 11.35 | 11.46 | 1.25 | 0.1264 | 0.285 | 11.28 | 11.77 | 10.72 | 1.47 | 0.052 | 0.2129 |
| TC0X00007668.hg.1 | UPRT | uracil phosphoribosyltransferase (FUR1) homolog (S. cerevisiae | Multiple_C | 4.19 | 4.21 | 3.87 | 1.25 | 0.4538 | 0.6613 | 8.5 | 9.56 | 8.78 | 0.82 | 0.4606 | 0.7039 |
| TC0X00008873.hg.1 | H2AFB2 | H2A histone family, member B2 | Coding | 3.51 | 2.9 | 3.19 | 1.25 | 0.1659 | 0.3446 | 4.18 | 4.44 | 4.39 | 0.86 | 0.6114 | 0.8057 |
| TC0X00009022.hg.1 | VCX2 | variable charge, X-linked 2 | Coding | 5.17 | 4.55 | 4.85 | 1.25 | 0.1463 | 0.3166 | 4.52 | 4.47 | 4.23 | 1.22 | 0.1216 | 0.3442 |
| TC0X00011380.hg.1 | IRS4 | insulin receptor substrate 4 | Coding | 2.94 | 2.68 | 2.62 | 1.25 | 0.3204 | 0.534 | 2.8 | 2.76 | 2.68 | 1.09 | 0.1375 | 0.3685 |
| TC0Y00007326.hg.1 | TSPY1 | testis specific protein, Y-linked 1 | Coding | 4.9 | 4.31 | 4.58 | 1.25 | 0.3674 | 0.5805 | 4.06 | 3.97 | 3.94 | 1.09 | 0.3056 | 0.5697 |
| TC1000007509.hg.1 | ANTXRL | anthrax toxin receptor-like | Multiple_C | 4.1 | 3.37 | 3.78 | 1.25 | 0.2768 | 0.486 | 4.58 | 4.37 | 4.12 | 1.38 | 0.0718 | 0.2564 |
| TC1000008399.hg.1 | IFIT1B | interferon-induced protein with tetratricopeptide repeats 1B | Coding | 3.88 | 3.35 | 3.56 | 1.25 | 0.3164 | 0.5297 | 3.75 | 3.86 | 3.63 | 1.09 | 0.1852 | 0.4367 |
| TC1000011411.hg.1 | PPP1R3C | protein phosphatase 1, regulatory subunit 3C | Multiple_C | 2.96 | 2.51 | 2.64 | 1.25 | 0.1097 | 0.2579 | 3.13 | 3.22 | 2.99 | 1.10 | 0.1627 | 0.4061 |

| | | | | | | | | | | | | | | | |
|---|---|---|---|---|---|---|---|---|---|---|---|---|---|---|---|
| TC1000011478.hg.1 | CYP2C8 | cytochrome P450, family 2, subfamily C, polypeptide 8 | Multiple_C | 3.05 | 2.86 | 2.73 | 1.25 | 0.2968 | 0.5084 | 3.1 | 3.10 | 2.98 | 1.09 | 0.5943 | 0.7952 |
| TC1100006820.hg.1 | SWAP70 | SWAP switching B-cell complex 70kDa subunit | Multiple_C | 10.76 | 10.19 | 10.44 | 1.25 | 0.5983 | 0.7712 | 10.64 | 9.73 | 9.9 | 1.67 | 0.0157 | 0.1032 |
| TC1100007069.hg.1 | GAS2 | growth arrest-specific 2 | Multiple_C | 3.5 | 3.46 | 3.18 | 1.25 | 0.3139 | 0.5274 | 4.72 | 5.87 | 5.09 | 0.77 | 0.061 | 0.2345 |
| TC1100007463.hg.1 | F2 | coagulation factor II (thrombin) | Multiple_C | 3.52 | 3.25 | 3.2 | 1.25 | 0.2078 | 0.4013 | 3.87 | 3.77 | 3.69 | 1.13 | 0.5039 | 0.736 |
| TC1100007613.hg.1 | OR5L2 | olfactory receptor, family 5, subfamily L, member 2 | Coding | 3.95 | 3.54 | 3.63 | 1.25 | 0.4736 | 0.6767 | 4.18 | 4.05 | 3.92 | 1.20 | 0.1301 | 0.3573 |
| TC1100008979.hg.1 | CUL5 | cullin 5 | Multiple_C | 11.49 | 12.26 | 11.17 | 1.25 | 0.3248 | 0.5389 | 13.19 | 12.10 | 12.43 | 1.69 | 0.0891 | 0.2889 |
| TC1100009302.hg.1 | TECTA | tectorin alpha | Multiple_C | 3.71 | 3.34 | 3.39 | 1.25 | 0.0344 | 0.1095 | 3.98 | 4.37 | 4.04 | 0.96 | 0.7622 | 0.8925 |
| TC1100009771.hg.1 | PRR33 | proline rich 33 | Coding | 3.4 | 2.79 | 3.08 | 1.25 | 0.4152 | 0.6273 | 3.62 | 3.53 | 3.32 | 1.23 | 0.3823 | 0.6414 |
| TC1100009912.hg.1 | OR52A1 | olfactory receptor, family 52, subfamily A, member 1 | Coding | 3.89 | 3.6 | 3.57 | 1.25 | 0.7031 | 0.8416 | 3.66 | 3.35 | 3.27 | 1.31 | 0.1916 | 0.4445 |
| TC1100010015.hg.1 | NLRP10 | NLR family, pyrin domain containing 10 | Coding | 4.29 | 4.04 | 3.97 | 1.25 | 0.2335 | 0.4342 | 3.62 | 3.75 | 3.79 | 0.89 | 0.8844 | 0.9504 |
| TC1100013057.hg.1 | TCIRG1 | T-cell, immune regulator 1, ATPase, H+ transporting, lysosomal | Multiple_C | 7.95 | 7.45 | 7.63 | 1.25 | 0.3365 | 0.5503 | 10.71 | 11.12 | 10.2 | 1.42 | 0.0112 | 0.0837 |
| TC1200007153.hg.1 | PPFIBP1 | PTPRF interacting protein, binding protein 1 (liprin beta 1) | Multiple_C | 10.56 | 10.76 | 10.24 | 1.25 | 0.2212 | 0.4185 | 13.92 | 12.82 | 12.77 | 2.22 | 6.04E-06 | 0.0005 |
| TC1200009326.hg.1 | LOC10012855 | uncharacterized LOC100128554; Transcript Identified by AceVie | Multiple_C | 4.36 | 4.12 | 4.04 | 1.25 | 0.1824 | 0.3673 | 4.6 | 5.17 | 4.54 | 1.04 | 0.2601 | 0.5243 |
| TC1200009800.hg.1 | SLC2A3 | solute carrier family 2 (facilitated glucose transporter), member | Multiple_C | 3.42 | 2.86 | 3.1 | 1.25 | 0.4805 | 0.6825 | 12.81 | 12.55 | 12.97 | 0.90 | 0.9158 | 0.9655 |
| TC1200011946.hg.1 | BRAP | BRCA1 associated protein | Multiple_C | 10.34 | 10.25 | 10.02 | 1.25 | 0.4154 | 0.6276 | 8.96 | 8.69 | 9.06 | 0.93 | 0.6311 | 0.8184 |
| TC1300008083.hg.1 | ATP11AUN | ATP11A upstream neighbor | Multiple_C | 3.24 | 3 | 2.92 | 1.25 | 0.9379 | 0.9711 | 3.44 | 3.35 | 3.26 | 1.13 | 0.7921 | 0.9076 |
| TC1300009984.hg.1 | SLITRK5 | SLIT and NTRK-like family, member 5 | Coding | 3.95 | 4.18 | 3.63 | 1.25 | 0.2041 | 0.3966 | 4.38 | 4.81 | 4.37 | 1.01 | 0.802 | 0.9117 |
| TC1400006553.hg.1 | TMEM253 | transmembrane protein 253 | Multiple_C | 3.5 | 3.25 | 3.18 | 1.25 | 0.5177 | 0.7119 | 4.43 | 4.19 | 3.97 | 1.38 | 0.2145 | 0.4731 |
| TC1400010317.hg.1 | CKB | creatine kinase, brain | Multiple_C | 6.47 | 6.07 | 6.15 | 1.25 | 0.1973 | 0.388 | 9.63 | 8.56 | 7.91 | 3.29 | 1.05E-05 | 0.0007 |
| TC1400010726.hg.1 | ADCY4 | adenylate cyclase 4 | Multiple_C | 4.06 | 4.12 | 3.74 | 1.25 | 0.1908 | 0.3789 | 4.08 | 3.72 | 3.68 | 1.32 | 0.0926 | 0.295 |
| TC15_KI270727v1_randc | POTEB3; POTE | Homo sapiens POTE ankyrin domain family, member B3 (POTEB | Coding | 3.45 | 3.32 | 3.13 | 1.25 | 0.1739 | 0.3559 | 4.68 | 4.67 | 4.63 | 1.04 | 0.7423 | 0.8818 |
| TC1500007885.hg.1 | NEIL1 | nei-like DNA glycosylase 1 | Multiple_C | 7.25 | 7.05 | 6.93 | 1.25 | 0.7944 | 0.8945 | 6.31 | 6.38 | 5.94 | 1.29 | 0.5512 | 0.767 |
| TC1500008719.hg.1 | GOLGA6L2 | golgin A6 family-like 2 | Coding | 5.19 | 4.63 | 4.87 | 1.25 | 0.1639 | 0.3422 | 5.17 | 5.10 | 5.15 | 1.01 | 0.8099 | 0.9158 |
| TC1600007217.hg.1 | SCNN1B | sodium channel, non voltage gated 1 beta subunit | Multiple_C | 5.05 | 4.85 | 4.73 | 1.25 | 0.4481 | 0.6563 | 5.04 | 4.93 | 4.94 | 1.07 | 0.8555 | 0.9385 |
| TC1600009138.hg.1 | PRSS33 | protease, serine, 33 | Coding | 5.08 | 4.61 | 4.76 | 1.25 | 0.302 | 0.5144 | 5.27 | 5.45 | 5.38 | 0.93 | 0.4105 | 0.6652 |
| TC1600010978.hg.1 | PKD1L2 | polycystic kidney disease 1-like 2 (gene/pseudogene) | Multiple_C | 3.53 | 3.16 | 3.21 | 1.25 | 0.5009 | 0.6995 | 5.11 | 4.23 | 4.38 | 1.66 | 0.0057 | 0.0536 |
| TC1700007560.hg.1 | CCL8 | chemokine (C-C motif) ligand 8 | Coding | 3.68 | 3.06 | 3.36 | 1.25 | 0.9384 | 0.9713 | 3.29 | 3.78 | 3.6 | 0.81 | 0.1494 | 0.3869 |
| TC1700008033.hg.1 | HEXIM1 | hexamethylene bis-acetamide inducible 1 | Coding | 10.35 | 10.75 | 10.03 | 1.25 | 0.3953 | 0.6088 | 9.51 | 10.17 | 9.51 | 1.00 | 0.9392 | 0.9749 |
| TC1700008250.hg.1 | XYLT2 | xylosyltransferase II | Multiple_C | 5.53 | 5.21 | 5.21 | 1.25 | 0.1541 | 0.3279 | 4.92 | 4.89 | 4.96 | 0.97 | 0.8857 | 0.9509 |
| TC1700010293.hg.1 | CRLF3 | cytokine receptor-like factor 3 | Multiple_C | 8.2 | 9.26 | 7.88 | 1.25 | 0.7912 | 0.8931 | 7.94 | 6.62 | 7.63 | 1.24 | 0.4501 | 0.6962 |
| TC1700012076.hg.1 | ARHGDIA | Rho GDP dissociation inhibitor (GDI) alpha | Multiple_C | 12.96 | 11.42 | 12.64 | 1.25 | 0.2082 | 0.4017 | 14.53 | 15.20 | 15.16 | 0.65 | 0.0267 | 0.1429 |
| TC1700012126.hg.1 | C17orf62 | chromosome 17 open reading frame 62 | Multiple_C | 10.16 | 8.4 | 9.84 | 1.25 | 0.7799 | 0.8868 | 9.33 | 10.98 | 9.88 | 0.68 | 0.3633 | 0.626 |
| TC1700012200.hg.1 | CCDC144A | coiled-coil domain containing 144A | Multiple_C | 3.72 | 3.03 | 3.4 | 1.25 | 0.3794 | 0.5925 | 4.36 | 3.95 | 4.27 | 1.06 | 0.8121 | 0.9166 |

| | | | | | | | | | | | | | | | |
|---|---|---|---|---|---|---|---|---|---|---|---|---|---|---|---|
| TC1700012274.hg.1 | ITGB3 | integrin beta 3 | Multiple_C | 4.05 | 3.66 | 3.73 | 1.25 | 0.5624 | 0.7443 | 4.09 | 3.96 | 4.03 | 1.04 | 0.6795 | 0.8459 |
| TC1700012409.hg.1 | KRTAP4-4 | keratin associated protein 4-4 | Coding | 3.94 | 3.28 | 3.62 | 1.25 | 0.2288 | 0.4285 | 3.36 | 3.71 | 3.4 | 0.97 | 0.9783 | 0.9909 |
| TC1800006905.hg.1 | CABYR | calcium binding tyrosine-(Y)-phosphorylation regulated | Multiple_C | 5.12 | 4.65 | 4.8 | 1.25 | 0.3047 | 0.5177 | 5.63 | 4.68 | 5.74 | 0.93 | 0.8198 | 0.9205 |
| TC1800007506.hg.1 | TNFRSF11A | tumor necrosis factor receptor superfamily, member 11a, NFKB | Multiple_C | 9.75 | 9.48 | 9.43 | 1.25 | 0.0967 | 0.235 | 3.87 | 3.61 | 3.58 | 1.22 | 0.148 | 0.3846 |
| TC1900006490.hg.1 | AZU1 | azurocidin 1 | Coding | 3.49 | 2.94 | 3.17 | 1.25 | 0.3416 | 0.5554 | 4.53 | 4.29 | 4.32 | 1.16 | 0.3688 | 0.6308 |
| TC1900006926.hg.1 | OR7D2 | olfactory receptor, family 7, subfamily D, member 2 | Coding | 4.09 | 3.34 | 3.77 | 1.25 | 0.1539 | 0.3277 | 4.04 | 4.07 | 4.21 | 0.89 | 0.3574 | 0.6203 |
| TC1900007270.hg.1 | KLF2 | Kruppel-like factor 2 | Coding | 6.68 | 6 | 6.36 | 1.25 | 0.4466 | 0.655 | 6.66 | 6.50 | 6.2 | 1.38 | 0.2268 | 0.4874 |
| TC1900007885.hg.1 | ARHGAP33 | Rho GTPase activating protein 33 | Multiple_C | 6.23 | 5.76 | 5.91 | 1.25 | 0.3736 | 0.5862 | 6.68 | 6.87 | 6.95 | 0.83 | 0.6274 | 0.8158 |
| TC1900008279.hg.1 | BCL3; MIR808 | B-cell CLL/lymphoma 3; microRNA 8085 | Multiple_C | 5.43 | 4.48 | 5.11 | 1.25 | 0.1365 | 0.3013 | 3.84 | 3.65 | 3.72 | 1.09 | 0.5513 | 0.767 |
| TC1900008634.hg.1 | SIGLECL1 | SIGLEC family like 1 | Multiple_C | 4.03 | 3.43 | 3.71 | 1.25 | 0.1162 | 0.2687 | 4.71 | 4.40 | 4.21 | 1.41 | 0.0296 | 0.1525 |
| TC1900009333.hg.1 | STAP2 | signal transducing adaptor family member 2 | Multiple_C | 9.32 | 9.62 | 9 | 1.25 | 0.1422 | 0.3101 | 6.16 | 5.76 | 5.91 | 1.19 | 0.942 | 0.9761 |
| TC1900009607.hg.1 | ANGPTL6 | angiopoietin like 6 | Multiple_C | 3.93 | 3.43 | 3.61 | 1.25 | 0.0946 | 0.2313 | 4.23 | 4.13 | 3.94 | 1.22 | 0.2449 | 0.509 |
| TC1900009842.hg.1 | OR7C1 | olfactory receptor, family 7, subfamily C, member 1 | Multiple_C | 4.29 | 4.12 | 3.97 | 1.25 | 0.4262 | 0.6369 | 3.85 | 4.30 | 4.34 | 0.71 | 0.4623 | 0.7049 |
| TC1900009906.hg.1 | CIB3 | calcium and integrin binding family member 3 | Multiple_C | 5.04 | 4.3 | 4.72 | 1.25 | 0.0432 | 0.1301 | 5.16 | 5.17 | 4.79 | 1.29 | 0.3182 | 0.5822 |
| TC1900010163.hg.1 | ZNF43 | zinc finger protein 43 | Multiple_C | 7.59 | 7.28 | 7.27 | 1.25 | 0.3152 | 0.5285 | 6.2 | 6.58 | 6.97 | 0.59 | 0.0008 | 0.0139 |
| TC1900011132.hg.1 | SLC6A16 | solute carrier family 6, member 16 | Multiple_C | 3.93 | 3.78 | 3.61 | 1.25 | 0.2539 | 0.4591 | 4.4 | 4.43 | 4.34 | 1.04 | 0.4372 | 0.6879 |
| TC1900011759.hg.1 | APOC1 | apolipoprotein C-I | Multiple_C | 5.41 | 5.81 | 5.09 | 1.25 | 0.5497 | 0.7347 | 5.72 | 5.95 | 5.59 | 1.09 | 0.4341 | 0.685 |
| TC1900011798.hg.1 | LILRA1 | leukocyte immunoglobulin-like receptor, subfamily A (with TM c | Multiple_C | 3.48 | 3.3 | 3.16 | 1.25 | 0.2792 | 0.4888 | 3.41 | 3.42 | 3.5 | 0.94 | 0.2839 | 0.5494 |
| TC1900011899.hg.1 | CALR3 | calreticulin 3 | Multiple_C | 5.1 | 5.3 | 4.78 | 1.25 | 0.2785 | 0.488 | 4.18 | 4.17 | 4.32 | 0.91 | 0.865 | 0.9424 |
| TC1900011906.hg.1 | NXNL1 | nucleoredoxin-like 1 | Multiple_C | 3.76 | 3.4 | 3.44 | 1.25 | 0.245 | 0.4485 | 5.27 | 5.17 | 4.84 | 1.35 | 0.0577 | 0.2263 |
| TC1900012052.hg.1 | FAM71E2 | family with sequence similarity 71, member E2 | Coding | 4.81 | 4.27 | 4.49 | 1.25 | 0.1987 | 0.3896 | 5.11 | 5.15 | 4.89 | 1.16 | 0.2563 | 0.5211 |
| TC2000006437.hg.1 | DEFB132 | defensin, beta 132 | Coding | 3.43 | 3.06 | 3.11 | 1.25 | 0.6615 | 0.8144 | 3.64 | 3.58 | 3.42 | 1.16 | 0.2504 | 0.5148 |
| TC2000006501.hg.1 | SIRPA | signal-regulatory protein alpha | Coding | 4.18 | 3.92 | 3.86 | 1.25 | 0.3536 | 0.5664 | 6.7 | 8.02 | 8.2 | 0.35 | 0.0001 | 0.0046 |
| TC2000007100.hg.1 | CCM2L | cerebral cavernous malformation 2-like | Coding | 4 | 3.6 | 3.68 | 1.25 | 0.0494 | 0.1434 | 4.7 | 4.77 | 4.91 | 0.86 | 0.1647 | 0.4092 |
| TC2100006997.hg.1 | CLIC6 | chloride intracellular channel 6 | Coding | 4.32 | 4.05 | 4 | 1.25 | 0.2143 | 0.4097 | 4.5 | 4.23 | 4.15 | 1.27 | 0.1927 | 0.4461 |
| TC2200006520.hg.1 | SLC25A18 | solute carrier family 25 (glutamate carrier), member 18 | Multiple_C | 5.78 | 5.23 | 5.46 | 1.25 | 0.2368 | 0.4381 | 5.61 | 5.60 | 5.39 | 1.16 | 0.3754 | 0.636 |
| TC2200007032.hg.1 | CABP7 | calcium binding protein 7 | Coding | 3.75 | 3.43 | 3.43 | 1.25 | 0.2002 | 0.392 | 4.09 | 4.20 | 3.97 | 1.09 | 0.7637 | 0.8932 |
| TC2200008495.hg.1 | C22orf24 | chromosome 22 open reading frame 24 | Multiple_C | 4.58 | 3.72 | 4.26 | 1.25 | 0.1755 | 0.3579 | 4.57 | 4.01 | 4.18 | 1.31 | 0.1189 | 0.341 |
| TC2200008800.hg.1 | DNAJB7 | DnaJ (Hsp40) homolog, subfamily B, member 7 | Coding | 3.47 | 3.51 | 3.15 | 1.25 | 0.1803 | 0.3644 | 3.74 | 4.18 | 4.08 | 0.79 | 0.8334 | 0.9259 |
| TSUnmapped00000363.h | EIF3F | Eukaryotic translation initiation factor 3 subunit F [Source:UniP | Coding | 11.25 | 11.33 | 10.93 | 1.25 | 0.1352 | 0.2995 | 9.95 | 10.36 | 9.95 | 1.00 | 0.9844 | 0.9935 |
| TSUnmapped00000461.h | ZNF35 | zinc finger protein 35 | Coding | 8.8 | 7.83 | 8.48 | 1.25 | 0.2506 | 0.455 | 7.7 | 7.45 | 7.81 | 0.93 | 0.3993 | 0.6563 |
| TSUnmapped00000817.h | F10 | coagulation factor X | Coding | 3.27 | 2.89 | 2.95 | 1.25 | 0.5545 | 0.7381 | 5.15 | 5.89 | 4.36 | 1.73 | 0.0016 | 0.0234 |
| TC0100006604.hg.1 | PRDM16 | PR domain containing 16 | Multiple_C | 4.6 | 4.26 | 4.29 | 1.24 | 0.2809 | 0.4909 | 7.44 | 7.01 | 6.97 | 1.39 | 0.0366 | 0.174 |

| | | | | | | | | | | | | | | | |
|---|---|---|---|---|---|---|---|---|---|---|---|---|---|---|---|
| TC0100007971.hg.1 | KCNQ4 | potassium channel, voltage gated KQT-like subfamily Q, membe | Multiple_C | 4.05 | 3.6 | 3.74 | 1.24 | 0.4377 | 0.647 | 4.21 | 4.27 | 4.66 | 0.73 | 0.0434 | 0.1927 |
| TC0100008366.hg.1 | DIO1 | deiodinase, iodothyronine, type I | Multiple_C | 3.4 | 2.7 | 3.09 | 1.24 | 0.0632 | 0.172 | 3.91 | 3.80 | 3.87 | 1.03 | 0.7368 | 0.8787 |
| TC0100010197.hg.1 | PEAR1 | platelet endothelial aggregation receptor 1 | Multiple_C | 5.28 | 4.6 | 4.97 | 1.24 | 0.2607 | 0.4675 | 5.47 | 5.38 | 5.22 | 1.19 | 0.3708 | 0.6328 |
| TC0100012456.hg.1 | FAM132A | family with sequence similarity 132, member A | Multiple_C | 5.44 | 4.75 | 5.13 | 1.24 | 0.1022 | 0.2449 | 5.19 | 5.10 | 5.11 | 1.06 | 0.6177 | 0.8101 |
| TC0100013134.hg.1 | CAPZB | capping protein (actin filament) muscle Z-line, beta | Multiple_C | 14.04 | 13.05 | 13.73 | 1.24 | 0.38 | 0.5929 | 14.49 | 14.17 | 14.54 | 0.97 | 0.6452 | 0.8258 |
| TC0100013799.hg.1 | HEYL | Transcript Identified by AceView, Entrez Gene ID(s) 26508 | Multiple_C | 4.9 | 4.32 | 4.59 | 1.24 | 0.3633 | 0.5768 | 4.72 | 4.38 | 4.75 | 0.98 | 0.3783 | 0.6381 |
| TC0100014069.hg.1 | CYP4A11 | cytochrome P450, family 4, subfamily A, polypeptide 11 | Multiple_C | 3.7 | 3.35 | 3.39 | 1.24 | 0.5823 | 0.7597 | 3.64 | 3.71 | 3.85 | 0.86 | 0.9696 | 0.9875 |
| TC0100015577.hg.1 | PPIAL4F; PPIA | peptidylprolyl isomerase A (cyclophilin A)-like 4F; peptidylprolyl | Coding | 5.37 | 4.96 | 5.06 | 1.24 | 0.1404 | 0.3077 | 4.82 | 4.69 | 4.86 | 0.97 | 0.3901 | 0.6488 |
| TC0100018499.hg.1 | MUC1 | mucin 1, cell surface associated | Multiple_C | 4.67 | 4.11 | 4.36 | 1.24 | 0.6058 | 0.7771 | 5.78 | 6.16 | 6 | 0.86 | 0.0605 | 0.2332 |
| TC0200007113.hg.1 | FAM179A | family with sequence similarity 179, member A | Multiple_C | 4.63 | 4.17 | 4.32 | 1.24 | 0.5345 | 0.7239 | 5.22 | 5.11 | 4.93 | 1.22 | 0.6046 | 0.8023 |
| TC0200008261.hg.1 | VAMP5 | vesicle associated membrane protein 5 | Multiple_C | 5.13 | 5.17 | 4.82 | 1.24 | 0.2872 | 0.4979 | 7.2 | 7.39 | 6.95 | 1.19 | 0.2938 | 0.5587 |
| TC0200010263.hg.1 | INPP1 | inositol polyphosphate-1-phosphatase | Multiple_C | 11.49 | 11.59 | 11.18 | 1.24 | 0.3075 | 0.5207 | 7.54 | 7.43 | 7.53 | 1.01 | 0.5934 | 0.7945 |
| TC0200011413.hg.1 | BOK | BCL2-related ovarian killer | Multiple_C | 5.72 | 5.38 | 5.41 | 1.24 | 0.3356 | 0.5496 | 4.9 | 4.95 | 4.59 | 1.24 | 0.8673 | 0.9431 |
| TC0200011837.hg.1 | FAM49A | family with sequence similarity 49, member A | Multiple_C | 4.02 | 3.53 | 3.71 | 1.24 | 0.1066 | 0.2524 | 4.47 | 4.10 | 4.36 | 1.08 | 0.2002 | 0.4557 |
| TC0200012035.hg.1 | ADGRF3 | adhesion G protein-coupled receptor F3 | Multiple_C | 4.24 | 4.08 | 3.93 | 1.24 | 0.1757 | 0.3581 | 5.09 | 4.82 | 4.54 | 1.46 | 0.0755 | 0.2641 |
| TC0200012068.hg.1 | SLC30A3 | solute carrier family 30 (zinc transporter), member 3 | Multiple_C | 4.2 | 3.88 | 3.89 | 1.24 | 0.2285 | 0.4282 | 6.65 | 6.25 | 5.94 | 1.64 | 0.0173 | 0.1103 |
| TC0200015616.hg.1 | ERBB4 | erb-b2 receptor tyrosine kinase 4 | Multiple_C | 6.04 | 5.5 | 5.73 | 1.24 | 0.2714 | 0.4799 | 4.35 | 3.96 | 4.28 | 1.05 | 0.4078 | 0.6637 |
| TC0200016682.hg.1 | PPP3R1 | protein phosphatase 3, regulatory subunit B, alpha | Coding | 11.84 | 11.93 | 11.53 | 1.24 | 0.1405 | 0.3078 | 12.5 | 12.03 | 12.73 | 0.85 | 0.4645 | 0.7066 |
| TC0300006577.hg.1 | BRK1 | BRICK1, SCAR/WAVE actin-nucleating complex subunit | Multiple_C | 11.9 | 11.82 | 11.59 | 1.24 | 0.3501 | 0.563 | 9.37 | 9.81 | 9.21 | 1.12 | 0.8948 | 0.9555 |
| TC0300006773.hg.1 | LOC339862; R | uncharacterized LOC339862; novel transcript; Transcript Identif | Multiple_C | 3.48 | 3.03 | 3.17 | 1.24 | 0.3121 | 0.5255 | 3.42 | 4.20 | 4.57 | 0.45 | 0.0111 | 0.0833 |
| TC0300006948.hg.1 | ZNF860 | zinc finger protein 860 | Multiple_C | 8.75 | 7.9 | 8.44 | 1.24 | 0.3196 | 0.5333 | 6.65 | 6.86 | 7.28 | 0.65 | 0.0013 | 0.0194 |
| TC0300008536.hg.1 | EAF2 | ELL associated factor 2 | Multiple_C | 8.73 | 8.93 | 8.42 | 1.24 | 0.6854 | 0.83 | 6.17 | 5.94 | 6.14 | 1.02 | 0.4678 | 0.7089 |
| TC0300012812.hg.1 | GPR171 | G protein-coupled receptor 171 | Coding | 4.03 | 3.47 | 3.72 | 1.24 | 0.6039 | 0.7757 | 4.56 | 4.49 | 4.74 | 0.88 | 0.4135 | 0.668 |
| TC0300013095.hg.1 | SLC7A14 | solute carrier family 7, member 14 | Multiple_C | 3.22 | 2.85 | 2.91 | 1.24 | 0.1745 | 0.3563 | 3.68 | 3.56 | 3.66 | 1.01 | 0.478 | 0.7166 |
| TC0400007978.hg.1 | ENOPH1 | enolase-phosphatase 1 | Multiple_C | 12.96 | 13.19 | 12.65 | 1.24 | 0.455 | 0.6623 | 11.35 | 10.96 | 11.13 | 1.16 | 0.3573 | 0.6202 |
| TC0400010733.hg.1 | KDR | kinase insert domain receptor | Multiple_C | 3.18 | 2.77 | 2.87 | 1.24 | 0.3212 | 0.5349 | 3.56 | 3.42 | 3.23 | 1.26 | 0.2402 | 0.5039 |
| TC0400011348.hg.1 | HPGDS | hematopoietic prostaglandin D synthase | Multiple_C | 4.16 | 4.01 | 3.85 | 1.24 | 0.295 | 0.5064 | 3.8 | 3.73 | 3.56 | 1.18 | 0.4556 | 0.6999 |
| TC0500008884.hg.1 | PCDHB13 | protocadherin beta 13 | Coding | 9.55 | 8.6 | 9.24 | 1.24 | 0.5766 | 0.755 | 4.07 | 4.08 | 4.3 | 0.85 | 0.9023 | 0.9595 |
| TC0600007194.hg.1 | KAAG1 | kidney associated antigen 1 | Coding | 3.84 | 3.59 | 3.53 | 1.24 | 0.3072 | 0.5205 | 3.7 | 3.76 | 3.87 | 0.89 | 0.3084 | 0.5724 |
| TC0600011704.hg.1 | MDGA1 | MAM domain containing glycosylphosphatidylinositol anchor 1 | Multiple_C | 3.82 | 3.51 | 3.51 | 1.24 | 0.657 | 0.8117 | 3.47 | 3.66 | 3.56 | 0.94 | 0.5502 | 0.7664 |
| TC0600011798.hg.1 | TFEB | transcription factor EB | Multiple_C | 2.76 | 2.5 | 2.45 | 1.24 | 0.0968 | 0.2352 | 2.76 | 2.82 | 2.69 | 1.05 | 0.361 | 0.6241 |
| TC0600012002.hg.1 | CRISP2 | cysteine-rich secretory protein 2 | Coding | 3.65 | 3.4 | 3.34 | 1.24 | 0.2218 | 0.4194 | 3.94 | 3.86 | 3.44 | 1.41 | 0.0238 | 0.1331 |
| TC0600012288.hg.1 | DPPA5 | developmental pluripotency associated 5 | Coding | 4.06 | 4.06 | 3.75 | 1.24 | 0.5881 | 0.7642 | 4.23 | 4.42 | 4.21 | 1.01 | 0.319 | 0.583 |

| | | | | | | | | | | | | | | | |
|---|---|---|---|---|---|---|---|---|---|---|---|---|---|---|---|
| TC0600013395.hg.1 | NMBR | neuromedin B receptor | Multiple_C | 4.67 | 4.27 | 4.36 | 1.24 | 0.8806 | 0.9419 | 5.14 | 5.21 | 4.98 | 1.12 | 0.518 | 0.7463 |
| TC0600013757.hg.1 | SOD2 | superoxide dismutase 2, mitochondrial | Multiple_C | 13.12 | 13.17 | 12.81 | 1.24 | 0.3248 | 0.5389 | 10.49 | 11.11 | 11.1 | 0.66 | 0.1018 | 0.3121 |
| TC0700006864.hg.1 | SP4 | Sp4 transcription factor | Multiple_C | 6.27 | 5.89 | 5.96 | 1.24 | 0.2376 | 0.4391 | 7.19 | 7.37 | 7.45 | 0.84 | 0.052 | 0.2129 |
| TC0700009689.hg.1 | NOS3 | Transcript Identified by AceView, Entrez Gene ID(s) 4846 | Coding | 3.42 | 2.88 | 3.11 | 1.24 | 0.4071 | 0.6195 | 4.61 | 4.54 | 4.13 | 1.39 | 0.3393 | 0.6035 |
| TC0700010770.hg.1 | SFRP4 | secreted frizzled-related protein 4 | Multiple_C | 3.45 | 3.18 | 3.14 | 1.24 | 0.2775 | 0.4867 | 3.21 | 3.35 | 3.47 | 0.84 | 0.8251 | 0.9228 |
| TC0700012224.hg.1 | LAMB1 | laminin, beta 1 | Multiple_C | 12.06 | 13.32 | 11.75 | 1.24 | 0.5239 | 0.7165 | 8.31 | 8.04 | 9.29 | 0.51 | 0.0498 | 0.2079 |
| TC0700012377.hg.1 | CTTNBP2 | cortactin binding protein 2 | Multiple_C | 3.9 | 3.43 | 3.59 | 1.24 | 0.4356 | 0.6452 | 3.29 | 3.36 | 3.75 | 0.73 | 0.3734 | 0.6341 |
| TC0700013618.hg.1 | MGC27345 | uncharacterized protein MGC27345 | Multiple_C | 6.77 | 5.66 | 6.46 | 1.24 | 0.6792 | 0.8258 | 6 | 6.41 | 7.59 | 0.33 | 0.0002 | 0.0059 |
| TC0800006738.hg.1 | MTMR9 | myotubularin related protein 9 | Multiple_C | 7.44 | 7.4 | 7.13 | 1.24 | 0.1228 | 0.2794 | 7.38 | 7.23 | 7.74 | 0.78 | 0.0388 | 0.1793 |
| TC0800007040.hg.1 | ADAM28 | ADAM metallopeptidase domain 28 | Multiple_C | 3.31 | 3 | 3 | 1.24 | 0.2488 | 0.4529 | 3.53 | 3.55 | 3.61 | 0.95 | 0.605 | 0.8024 |
| TC0800007377.hg.1 | ADAM18 | ADAM metallopeptidase domain 18 | Multiple_C | 3.74 | 3.93 | 3.43 | 1.24 | 0.218 | 0.4143 | 3.72 | 3.56 | 3.63 | 1.06 | 0.6061 | 0.8029 |
| TC0800008658.hg.1 | COLEC10 | collectin sub-family member 10 (C-type lectin) | Multiple_C | 5.15 | 4.84 | 4.84 | 1.24 | 0.2371 | 0.4385 | 3.83 | 3.90 | 3.48 | 1.27 | 0.5498 | 0.7662 |
| TC0800009326.hg.1 | TDRP | testis development related protein | Multiple_C | 4.33 | 4.07 | 4.02 | 1.24 | 0.2087 | 0.4025 | 4.22 | 4.39 | 4.35 | 0.91 | 0.6253 | 0.8149 |
| TC0800009997.hg.1 | KIF13B | kinesin family member 13B | Multiple_C | 10.61 | 10.96 | 10.3 | 1.24 | 0.406 | 0.6186 | 9.29 | 8.87 | 9.07 | 1.16 | 0.3995 | 0.6564 |
| TC0800010142.hg.1 | GOT1L1 | glutamic-oxaloacetic transaminase 1-like 1 | Coding | 4.56 | 4.4 | 4.25 | 1.24 | 0.5203 | 0.7137 | 4.14 | 4.13 | 4.31 | 0.89 | 0.9773 | 0.9906 |
| TC0800011023.hg.1 | CNGB3 | cyclic nucleotide gated channel beta 3 | Multiple_C | 4.82 | 4.11 | 4.51 | 1.24 | 0.3918 | 0.6055 | 4.03 | 4.21 | 4 | 1.02 | 0.9277 | 0.9698 |
| TC0800012279.hg.1 | SORBS3 | sorbin and SH3 domain containing 3 | Multiple_C | 5.19 | 4.31 | 4.88 | 1.24 | 0.2005 | 0.3923 | 4.27 | 4.63 | 4.41 | 0.91 | 0.7583 | 0.8903 |
| TC0800012325.hg.1 | SLC26A7 | solute carrier family 26 (anion exchanger), member 7 | Multiple_C | 3.27 | 2.95 | 2.96 | 1.24 | 0.3459 | 0.5596 | 3.26 | 3.05 | 3.29 | 0.98 | 0.3175 | 0.5815 |
| TC0900008935.hg.1 | USP20 | ubiquitin specific peptidase 20 | Multiple_C | 5.9 | 5.49 | 5.59 | 1.24 | 0.5088 | 0.705 | 5.72 | 6.04 | 5.72 | 1.00 | 0.5156 | 0.7446 |
| TC0900009165.hg.1 | LCN1 | lipocalin 1 | Coding | 4.21 | 3.88 | 3.9 | 1.24 | 0.2153 | 0.4109 | 3.84 | 4.03 | 3.95 | 0.93 | 0.6621 | 0.835 |
| TC0900012158.hg.1 | PALM2-AKAP2 | PALM2-AKAP2 readthrough | Coding | 4.19 | 3.72 | 3.88 | 1.24 | 0.3686 | 0.5815 | 7.39 | 6.79 | 7.06 | 1.26 | 0.2123 | 0.4702 |
| TC0X00010661.hg.1 | RHOXF2B; RH | Rhox homeobox family, member 2B; Rhox homeobox family, m | Multiple_C | 3.29 | 3.01 | 2.98 | 1.24 | 0.3928 | 0.6066 | 3.8 | 3.80 | 3.58 | 1.16 | 0.0547 | 0.2195 |
| TC0X00010787.hg.1 | APLN | apelin | Multiple_C | 4.88 | 4.17 | 4.57 | 1.24 | 0.1583 | 0.3343 | 5.82 | 6.13 | 5.9 | 0.95 | 0.5214 | 0.7482 |
| TC0Y00006873.hg.1 | CRLF2 | cytokine receptor-like factor 2 | Multiple_C | 7.22 | 6.69 | 6.91 | 1.24 | 0.6896 | 0.8329 | 5.52 | 5.52 | 5.75 | 0.85 | 0.2307 | 0.4921 |
| TC1000007007.hg.1 | ARMC3 | armadillo repeat containing 3 | Multiple_C | 4.39 | 3.85 | 4.08 | 1.24 | 0.1099 | 0.2583 | 5.43 | 5.64 | 5.45 | 0.99 | 0.5086 | 0.7389 |
| TC1000008447.hg.1 | TNKS2 | tankyrase, TRF1-interacting ankyrin-related ADP-ribose polymer | Multiple_C | 10.88 | 10.82 | 10.57 | 1.24 | 0.1234 | 0.2803 | 10.9 | 10.07 | 10.35 | 1.46 | 0.0032 | 0.0369 |
| TC1000009737.hg.1 | ITIH5 | inter-alpha-trypsin inhibitor heavy chain family, member 5 | Multiple_C | 4.54 | 4.04 | 4.23 | 1.24 | 0.4626 | 0.6679 | 5.11 | 5.15 | 5.06 | 1.04 | 0.4255 | 0.678 |
| TC1000011973.hg.1 | PRLHR | prolactin releasing hormone receptor | Coding | 3.05 | 2.9 | 2.74 | 1.24 | 0.2255 | 0.4243 | 3.03 | 3.16 | 3.13 | 0.93 | 0.6609 | 0.8345 |
| TC1100006492.hg.1 | PNPLA2 | patatin-like phospholipase domain containing 2 | Multiple_C | 6.66 | 6.16 | 6.35 | 1.24 | 0.8257 | 0.9124 | 6.56 | 6.57 | 6.04 | 1.43 | 0.2442 | 0.5084 |
| TC1100007184.hg.1 | DNAJC24 | DnaJ (Hsp40) homolog, subfamily C, member 24 | Multiple_C | 8.77 | 9.7 | 8.46 | 1.24 | 0.5411 | 0.7285 | 10.47 | 9.21 | 9.86 | 1.53 | 0.0643 | 0.2413 |
| TC1100007266.hg.1 | PDHX | pyruvate dehydrogenase complex, component X | Multiple_C | 11.24 | 11.61 | 10.93 | 1.24 | 0.1879 | 0.3753 | 12.03 | 12.30 | 11.97 | 1.04 | 0.8969 | 0.9566 |
| TC1100007611.hg.1 | OR5L1 | olfactory receptor, family 5, subfamily L, member 1 (gene/pseu | Multiple_C | 4.34 | 4.27 | 4.03 | 1.24 | 0.2269 | 0.4259 | 3.86 | 3.77 | 3.64 | 1.16 | 0.0775 | 0.2682 |
| TC1100007732.hg.1 | OR5AN1 | olfactory receptor, family 5, subfamily AN, member 1 | Coding | 2.83 | 2.64 | 2.52 | 1.24 | 0.0441 | 0.1321 | 3.22 | 3.76 | 3.71 | 0.71 | 0.0808 | 0.274 |

| | | | | | | | | | | | | | | | |
|---|---|---|---|---|---|---|---|---|---|---|---|---|---|---|---|
| TC1100010760.hg.1 | AGBL2 | ATP/GTP binding protein-like 2 | Multiple_C | 5 | 5.07 | 4.69 | 1.24 | 0.3118 | 0.5251 | 8.05 | 6.98 | 6.31 | 3.34 | 8.30E-05 | 0.0032 |
| TC1100011196.hg.1 | C11orf85 | chromosome 11 open reading frame 85 | Multiple_C | 3.58 | 3.24 | 3.27 | 1.24 | 0.4588 | 0.6649 | 4.29 | 4.19 | 4.04 | 1.19 | 0.2997 | 0.564 |
| TC1100011241.hg.1 | MAP3K11 | mitogen-activated protein kinase kinase kinase 11 | Multiple_C | 11.25 | 10.78 | 10.94 | 1.24 | 0.0799 | 0.2048 | 10.83 | 11.32 | 11.28 | 0.73 | 0.119 | 0.341 |
| TC1100011310.hg.1 | PC | pyruvate carboxylase | Multiple_C | 4.45 | 4.08 | 4.14 | 1.24 | 0.4408 | 0.6494 | 6.38 | 6.47 | 6.25 | 1.09 | 0.5558 | 0.77 |
| TC1200006850.hg.1 | ETV6 | ets variant 6 | Multiple_C | 9.63 | 8.53 | 9.32 | 1.24 | 0.3419 | 0.5556 | 9.86 | 9.41 | 9.48 | 1.30 | 0.3952 | 0.6531 |
| TC1200007644.hg.1 | ANKRD33 | ankyrin repeat domain 33 | Multiple_C | 2.98 | 2.46 | 2.67 | 1.24 | 0.1915 | 0.38 | 2.89 | 3.05 | 2.79 | 1.07 | 0.6168 | 0.8098 |
| TC1200007822.hg.1 | IKZF4 | IKAROS family zinc finger 4 | Multiple_C | 4.59 | 3.98 | 4.28 | 1.24 | 0.5378 | 0.726 | 5.47 | 5.91 | 5.47 | 1.00 | 0.3136 | 0.5777 |
| TC1300007546.hg.1 | IRG1 | immunoresponsive 1 homolog (mouse) | Coding | 3.19 | 2.91 | 2.88 | 1.24 | 0.6031 | 0.7751 | 3.77 | 3.47 | 3.61 | 1.12 | 0.0672 | 0.2471 |
| TC1300008219.hg.1 | TPTE2 | transmembrane phosphoinositide 3-phosphatase and tensin ho | Multiple_C | 3.55 | 3.19 | 3.24 | 1.24 | 0.4288 | 0.6391 | 3.81 | 3.71 | 3.67 | 1.10 | 0.5379 | 0.759 |
| TC1300008858.hg.1 | HTR2A | 5-hydroxytryptamine (serotonin) receptor 2A, G protein-couple | Multiple_C | 3.55 | 3.12 | 3.24 | 1.24 | 0.5325 | 0.7222 | 3.96 | 4.00 | 3.96 | 1.00 | 0.9073 | 0.9617 |
| TC1400006729.hg.1 | TSSK4 | testis-specific serine kinase 4 | Multiple_C | 4.48 | 4.03 | 4.17 | 1.24 | 0.4956 | 0.6954 | 4.48 | 4.55 | 4.38 | 1.07 | 0.9113 | 0.9636 |
| TC1400008478.hg.1 | PACS2 | phosphofurin acidic cluster sorting protein 2 | Multiple_C | 8.1 | 7.48 | 7.79 | 1.24 | 0.1557 | 0.3304 | 8.89 | 8.92 | 8.63 | 1.20 | 0.1882 | 0.4403 |
| TC1400008486.hg.1 | CRIP2 | cysteine-rich protein 2 | Multiple_C | 4.1 | 3.85 | 3.79 | 1.24 | 0.5848 | 0.7614 | 5.73 | 5.11 | 4.75 | 1.97 | 0.0005 | 0.01 |
| TC1400008919.hg.1 | CFL2 | cofilin 2 (muscle) | Multiple_C | 8.44 | 7.96 | 8.13 | 1.24 | 0.5501 | 0.7351 | 8.6 | 7.56 | 8.38 | 1.16 | 0.4665 | 0.708 |
| TC1400010714.hg.1 | HAUS4; MIR47 | HAUS augmin like complex subunit 4; microRNA 4707 | Multiple_C | 8.42 | 9.2 | 8.11 | 1.24 | 0.2732 | 0.4819 | 5.21 | 5.91 | 5.62 | 0.75 | 0.3497 | 0.6137 |
| TC1500010368.hg.1 | HAPLN3 | hyaluronan and proteoglycan link protein 3 | Multiple_C | 5.04 | 4.59 | 4.73 | 1.24 | 0.3001 | 0.512 | 3.81 | 3.58 | 3.69 | 1.09 | 0.3012 | 0.5655 |
| TC1600006525.hg.1 | CACNA1H | calcium channel, voltage-dependent, T type, alpha 1H subunit | Multiple_C | 3.1 | 2.78 | 2.79 | 1.24 | 0.5882 | 0.7642 | 5.04 | 4.82 | 5.03 | 1.01 | 0.6994 | 0.8574 |
| TC1600006995.hg.1 | BFAR | bifunctional apoptosis regulator | Multiple_C | 13.23 | 13.38 | 12.92 | 1.24 | 0.3947 | 0.6083 | 11.64 | 11.91 | 11.97 | 0.80 | 0.1801 | 0.4293 |
| TC1600007186.hg.1 | C16orf52 | chromosome 16 open reading frame 52 | Multiple_C | 10.48 | 10.45 | 10.17 | 1.24 | 0.4592 | 0.6653 | 7.27 | 7.22 | 7.39 | 0.92 | 0.8618 | 0.9408 |
| TC1600007541.hg.1 | AHSP | alpha hemoglobin stabilizing protein | Multiple_C | 2.98 | 2.46 | 2.67 | 1.24 | 0.2415 | 0.4438 | 3 | 3.13 | 2.78 | 1.16 | 0.0377 | 0.1769 |
| TC1600007619.hg.1 | TP53TG3B | TP53 target 3B | Multiple_C | 4 | 3.65 | 3.69 | 1.24 | 0.8132 | 0.9059 | 3.8 | 3.67 | 3.93 | 0.91 | 0.4717 | 0.7122 |
| TC1600008005.hg.1 | ADGRG1 | adhesion G protein-coupled receptor G1 | Multiple_C | 11.08 | 9.77 | 10.77 | 1.24 | 0.7216 | 0.8535 | 12.96 | 12.96 | 13.35 | 0.76 | 0.0914 | 0.2927 |
| TC1600008170.hg.1 | C16orf86 | chromosome 16 open reading frame 86 | Multiple_C | 5.15 | 4.9 | 4.84 | 1.24 | 0.3579 | 0.5714 | 4.82 | 5.13 | 4.95 | 0.91 | 0.9398 | 0.9752 |
| TC1600010623.hg.1 | FHOD1 | formin homology 2 domain containing 1 | Multiple_C | 6.13 | 5.65 | 5.82 | 1.24 | 0.1987 | 0.3896 | 7.89 | 8.08 | 7.87 | 1.01 | 0.9773 | 0.9906 |
| TC1700007017.hg.1 | SNORD49A; SI | small nucleolar RNA, C/D box 49A; small nucleolar RNA, C/D bo: | Multiple_C | 16.34 | 17.17 | 16.03 | 1.24 | 0.2128 | 0.4078 | 14.47 | 14.62 | 14.81 | 0.79 | 0.7303 | 0.875 |
| TC1700007671.hg.1 | C17orf78 | chromosome 17 open reading frame 78 | Coding | 3.56 | 3.16 | 3.25 | 1.24 | 0.2462 | 0.4497 | 3.73 | 3.81 | 3.8 | 0.95 | 0.696 | 0.8557 |
| TC1700009516.hg.1 | GGT6 | gamma-glutamyltransferase 6 | Multiple_C | 4.3 | 3.98 | 3.99 | 1.24 | 0.3132 | 0.5267 | 3.93 | 3.84 | 3.6 | 1.26 | 0.1294 | 0.3562 |
| TC1700012423.hg.1 | LRRC37A4P | leucine rich repeat containing 37, member A4, pseudogene | Multiple_C | 6.17 | 5.3 | 5.86 | 1.24 | 0.5955 | 0.7695 | 6.39 | 6.59 | 6.59 | 0.87 | 0.6087 | 0.8042 |
| TC1700012448.hg.1 | GH2 | growth hormone 2 | Coding | 3.2 | 2.77 | 2.89 | 1.24 | 0.3829 | 0.5961 | 3.14 | 3.12 | 2.91 | 1.17 | 0.0663 | 0.2456 |
| TC1800006788.hg.1 | MC5R | melanocortin 5 receptor | Coding | 3.63 | 3.11 | 3.32 | 1.24 | 0.6494 | 0.8058 | 4.57 | 4.47 | 4 | 1.48 | 0.0318 | 0.1597 |
| TC1800007276.hg.1 | CTIF; MIR474 | CBP80/20-dependent translation initiation factor; microRNA 47 | Multiple_C | 4.17 | 3.83 | 3.86 | 1.24 | 0.2129 | 0.4078 | 3.84 | 3.98 | 3.77 | 1.05 | 0.7824 | 0.9022 |
| TC1800007797.hg.1 | ATP9B | ATPase, class II, type 9B | Multiple_C | 7.64 | 8.22 | 7.33 | 1.24 | 0.5046 | 0.702 | 6.24 | 7.16 | 7.21 | 0.51 | 0.0015 | 0.0219 |
| TC1800008614.hg.1 | SKOR2 | SKI family transcriptional corepressor 2 | Coding | 3.75 | 3.65 | 3.44 | 1.24 | 0.3809 | 0.5938 | 4 | 3.98 | 3.7 | 1.23 | 0.0749 | 0.2631 |

| | | | | | | | | | | | | | | | |
|---|---|---|---|---|---|---|---|---|---|---|---|---|---|---|---|
| TC1900007207.hg.1 | OR1I1 | olfactory receptor, family 1, subfamily I, member 1 | Coding | 3.36 | 2.95 | 3.05 | 1.24 | 0.3676 | 0.5806 | 3.5 | 3.50 | 3.41 | 1.06 | 0.5463 | 0.7637 |
| TC1900007906.hg.1 | TBCB | tubulin folding cofactor B | Multiple_C | 12.03 | 11.98 | 11.72 | 1.24 | 0.4555 | 0.6625 | 10.25 | 10.33 | 10.02 | 1.17 | 0.2571 | 0.5221 |
| TC1900008404.hg.1 | NPAS1 | neuronal PAS domain protein 1 | Multiple_C | 4.21 | 3.66 | 3.9 | 1.24 | 0.2969 | 0.5085 | 4.3 | 4.04 | 3.68 | 1.54 | 0.0866 | 0.2837 |
| TC1900008702.hg.1 | ZNF578 | zinc finger protein 578 | Multiple_C | 6.78 | 6.64 | 6.47 | 1.24 | 0.8168 | 0.9075 | 3.99 | 3.89 | 4.09 | 0.93 | 0.531 | 0.7539 |
| TC1900009432.hg.1 | DENND1C | DENN/MADD domain containing 1C | Multiple_C | 5.09 | 4.71 | 4.78 | 1.24 | 0.1129 | 0.2632 | 4.85 | 5.39 | 5.24 | 0.76 | 0.7158 | 0.8673 |
| TC1900010801.hg.1 | CNFN | cornifelin | Coding | 6.14 | 5.41 | 5.83 | 1.24 | 0.2076 | 0.4011 | 5.47 | 5.44 | 5.23 | 1.18 | 0.5809 | 0.7859 |
| TC1900011842.hg.1 | DIRAS1 | DIRAS family, GTP-binding RAS-like 1 | Coding | 5.49 | 5.14 | 5.18 | 1.24 | 0.5302 | 0.7206 | 4.72 | 4.80 | 4.8 | 0.95 | 0.8085 | 0.9155 |
| TC1900011922.hg.1 | ZNF728 | zinc finger protein 728 | Multiple_C | 7.05 | 6.71 | 6.74 | 1.24 | 0.4464 | 0.6548 | 6.05 | 5.99 | 6.28 | 0.85 | 0.1646 | 0.4092 |
| TC1900011930.hg.1 | U2AF1L4 | U2 small nuclear RNA auxiliary factor 1-like 4 | Multiple_C | 8.67 | 8.21 | 8.36 | 1.24 | 0.1981 | 0.3889 | 8.7 | 9.22 | 8.89 | 0.88 | 0.7129 | 0.8655 |
| TC1900011994.hg.1 | CGB1 | chorionic gonadotropin, beta polypeptide 1 | Coding | 4.32 | 3.84 | 4.01 | 1.24 | 0.1105 | 0.2593 | 4.11 | 4.10 | 4.07 | 1.03 | 0.7439 | 0.8829 |
| TC2000006782.hg.1 | OTOR | otoraplin | Multiple_C | 4.44 | 4.38 | 4.13 | 1.24 | 0.126 | 0.2842 | 5.04 | 4.71 | 4.14 | 1.87 | 0.0102 | 0.0787 |
| TC2000007482.hg.1 | SEMG2 | semenogelin II | Coding | 4.61 | 4.18 | 4.3 | 1.24 | 0.1846 | 0.3705 | 4.69 | 4.61 | 4.73 | 0.97 | 0.6765 | 0.8442 |
| TC2000008056.hg.1 | COL20A1 | collagen, type XX, alpha 1 | Multiple_C | 6.12 | 5.59 | 5.81 | 1.24 | 0.1798 | 0.3638 | 6.19 | 6.34 | 5.95 | 1.18 | 0.9949 | 0.9976 |
| TC2000008367.hg.1 | PAK7 | p21 protein (Cdc42/Rac)-activated kinase 7 | Coding | 3.38 | 2.45 | 3.07 | 1.24 | 0.6554 | 0.8104 | 3.57 | 3.15 | 3.36 | 1.16 | 0.4635 | 0.7057 |
| TC2000008885.hg.1 | SNTA1 | syntrophin, alpha 1 | Multiple_C | 4.69 | 4.18 | 4.38 | 1.24 | 0.1361 | 0.3009 | 4.74 | 5.22 | 5.26 | 0.70 | 0.0022 | 0.0285 |
| TC2000008895.hg.1 | PXMP4 | peroxisomal membrane protein 4 | Multiple_C | 8.78 | 7.97 | 8.47 | 1.24 | 0.0959 | 0.2337 | 7.39 | 7.75 | 7.96 | 0.67 | 0.0167 | 0.108 |
| TC2000009986.hg.1 | SNX5 | sorting nexin 5 | Multiple_C | 13.91 | 14.52 | 13.6 | 1.24 | 0.5614 | 0.7437 | 13.29 | 13.15 | 13.51 | 0.86 | 0.1781 | 0.4268 |
| TC2000009994.hg.1 | DEFB124 | defensin, beta 124 | Multiple_C | 5.13 | 4.93 | 4.82 | 1.24 | 0.8926 | 0.9483 | 4.99 | 5.04 | 5.41 | 0.75 | 0.1025 | 0.3136 |
| TC2200006713.hg.1 | UBE2L3 | ubiquitin conjugating enzyme E2L 3 | Multiple_C | 13.74 | 12.65 | 13.43 | 1.24 | 0.3012 | 0.5136 | 13.16 | 13.21 | 13.71 | 0.68 | 0.0111 | 0.0833 |
| TC2200007790.hg.1 | TRABD | TraB domain containing | Multiple_C | 10.16 | 9.76 | 9.85 | 1.24 | 0.3249 | 0.5389 | 9.94 | 10.35 | 10.25 | 0.81 | 0.2286 | 0.4896 |
| TC2200007952.hg.1 | CLTCL1 | clathrin, heavy chain-like 1 | Multiple_C | 6.34 | 6.49 | 6.03 | 1.24 | 0.1985 | 0.3895 | 5.58 | 5.92 | 5.79 | 0.86 | 0.7431 | 0.8822 |
| TC2200008444.hg.1 | SEC14L4 | SEC14-like lipid binding 4 | Multiple_C | 3.7 | 3.41 | 3.39 | 1.24 | 0.3156 | 0.5288 | 6.68 | 7.33 | 6.42 | 1.20 | 0.6543 | 0.8316 |
| TC2200009140.hg.1 | TTLL8 | tubulin tyrosine ligase-like family member 8 | Multiple_C | 3.96 | 3.15 | 3.65 | 1.24 | 0.8232 | 0.9112 | 5.03 | 4.97 | 4.61 | 1.34 | 0.3761 | 0.6364 |
| TSUnmapped00000220.h | ADAMTS13 | ADAM metallopeptidase with thrombospondin type 1 motif 13 | Coding | 3.84 | 3.29 | 3.53 | 1.24 | 0.2025 | 0.3947 | 4.47 | 4.58 | 4.43 | 1.03 | 0.7637 | 0.8932 |
| TSUnmapped00000345.h | SAG | S-antigen; retina and pineal gland (arrestin) | Coding | 3.08 | 2.56 | 2.77 | 1.24 | 0.2584 | 0.465 | 3.5 | 3.44 | 3.36 | 1.10 | 0.0685 | 0.2492 |
| TC0100008358.hg.1 | DMRTB1 | DMRT-like family B with proline-rich C-terminal, 1 | Multiple_C | 3.72 | 3.19 | 3.42 | 1.23 | 0.4227 | 0.6339 | 3.81 | 3.92 | 3.87 | 0.96 | 0.6351 | 0.8208 |
| TC0100009899.hg.1 | C1orf54 | chromosome 1 open reading frame 54 | Multiple_C | 5.18 | 4.97 | 4.88 | 1.23 | 0.6314 | 0.7943 | 3.63 | 3.73 | 3.83 | 0.87 | 0.874 | 0.9457 |
| TC0100009994.hg.1 | CRCT1 | cysteine rich C-terminal 1 | Coding | 3.33 | 2.87 | 3.03 | 1.23 | 0.1987 | 0.3896 | 4.12 | 4.20 | 4 | 1.09 | 0.2644 | 0.529 |
| TC0100010103.hg.1 | LENEP | lens epithelial protein | Coding | 3.6 | 3.19 | 3.3 | 1.23 | 0.225 | 0.4238 | 4.22 | 4.39 | 4.47 | 0.84 | 0.3801 | 0.6398 |
| TC0100010621.hg.1 | C1orf105 | chromosome 1 open reading frame 105 | Multiple_C | 3.89 | 3.26 | 3.59 | 1.23 | 0.4587 | 0.6648 | 3.63 | 3.79 | 4.03 | 0.76 | 0.363 | 0.6257 |
| TC0100011129.hg.1 | NR5A2 | nuclear receptor subfamily 5, group A, member 2 | Multiple_C | 4.6 | 4.73 | 4.3 | 1.23 | 0.1362 | 0.3009 | 3.47 | 3.62 | 3.89 | 0.75 | 0.0274 | 0.145 |
| TC0100011466.hg.1 | SERTAD4 | SERTA domain containing 4 | Multiple_C | 6.97 | 5.97 | 6.67 | 1.23 | 0.331 | 0.545 | 3.62 | 3.66 | 3.46 | 1.12 | 0.1466 | 0.3825 |
| TC0100012113.hg.1 | RYR2 | ryanodine receptor 2 (cardiac) | Multiple_C | 2.97 | 2.89 | 2.67 | 1.23 | 0.3776 | 0.5905 | 3.36 | 3.32 | 3.15 | 1.16 | 0.2127 | 0.4706 |

| | | | | | | | | | | | | | | | |
|---|---|---|---|---|---|---|---|---|---|---|---|---|---|---|---|
| TC0100012330.hg.1 | OR2T8 | olfactory receptor, family 2, subfamily T, member 8 | Coding | 5.29 | 4.86 | 4.99 | 1.23 | 0.5206 | 0.714 | 5.93 | 5.43 | 4.97 | 1.95 | 0.1482 | 0.3847 |
| TC0100013073.hg.1 | SDHB | succinate dehydrogenase complex subunit B, iron sulfur (Ip) | Multiple_C | 14.1 | 14.21 | 13.8 | 1.23 | 0.2609 | 0.4676 | 12.77 | 12.64 | 12.5 | 1.21 | 0.2791 | 0.5452 |
| TC0100013802.hg.1 | HPCAL4 | hippocalcin like 4 | Coding | 4.79 | 4.51 | 4.49 | 1.23 | 0.416 | 0.6279 | 5.65 | 5.25 | 5.31 | 1.27 | 0.0814 | 0.275 |
| TC0100015747.hg.1 | GOLPH3L | golgi phosphoprotein 3-like | Multiple_C | 11.76 | 12.28 | 11.46 | 1.23 | 0.363 | 0.5765 | 9.26 | 9.27 | 9.65 | 0.76 | 0.4225 | 0.6753 |
| TC0100016163.hg.1 | ADAMTS4 | ADAM metallopeptidase with thrombospondin type 1 motif 4 | Multiple_C | 3.32 | 2.71 | 3.02 | 1.23 | 0.6113 | 0.7814 | 4.14 | 4.07 | 3.59 | 1.46 | 0.2745 | 0.5402 |
| TC0100017145.hg.1 | PLXNA2 | plexin A2 | Multiple_C | 3.09 | 2.49 | 2.79 | 1.23 | 0.2906 | 0.5014 | 4.29 | 3.84 | 3.98 | 1.24 | 0.1397 | 0.3712 |
| TC0200007038.hg.1 | DRC1 | dynein regulatory complex subunit 1 | Multiple_C | 4.85 | 4.61 | 4.55 | 1.23 | 0.3259 | 0.54 | 3.91 | 3.92 | 3.98 | 0.95 | 0.9788 | 0.9911 |
| TC0200008458.hg.1 | ZNF2 | zinc finger protein 2 | Coding | 4.85 | 4.94 | 4.55 | 1.23 | 0.9487 | 0.9763 | 4.82 | 4.72 | 4.76 | 1.04 | 0.4745 | 0.7141 |
| TC0200011450.hg.1 | FAM150B | family with sequence similarity 150, member B | Multiple_C | 3.26 | 2.91 | 2.96 | 1.23 | 0.457 | 0.6633 | 3.53 | 3.68 | 3.62 | 0.94 | 0.4668 | 0.7084 |
| TC0200015128.hg.1 | PDE1A | phosphodiesterase 1A, calmodulin-dependent | Multiple_C | 3.51 | 3 | 3.21 | 1.23 | 0.4228 | 0.634 | 3.59 | 3.20 | 3.36 | 1.17 | 0.0646 | 0.2421 |
| TC0200016421.hg.1 | BRE | brain and reproductive organ-expressed (TNFRSF1A modulator) | Multiple_C | 11.46 | 10.44 | 11.16 | 1.23 | 0.2605 | 0.4673 | 11.48 | 10.80 | 11.31 | 1.13 | 0.0864 | 0.2834 |
| TC0300010840.hg.1 | CCK | cholecystokinin | Multiple_C | 6.27 | 5.8 | 5.97 | 1.23 | 0.1107 | 0.2595 | 5.2 | 5.29 | 5.47 | 0.83 | 0.1175 | 0.3388 |
| TC0400006639.hg.1 | NSG1; D4S234 | neuron specific gene family member 1; Neuron-specific protein | Multiple_C | 4.82 | 4.66 | 4.52 | 1.23 | 0.281 | 0.491 | 4.62 | 4.68 | 4.85 | 0.85 | 0.4346 | 0.6853 |
| TC0400006742.hg.1 | SH3TC1 | SH3 domain and tetratricopeptide repeats 1 | Multiple_C | 6.02 | 5.49 | 5.72 | 1.23 | 0.2697 | 0.478 | 6.69 | 6.64 | 6.55 | 1.10 | 0.9251 | 0.9693 |
| TC0400007231.hg.1 | NWD2 | NACHT and WD repeat domain containing 2 | Coding | 3.18 | 2.85 | 2.88 | 1.23 | 0.7879 | 0.8914 | 3.64 | 4.13 | 3.43 | 1.16 | 0.3429 | 0.6066 |
| TC0400009338.hg.1 | HAND2-AS1 | HAND2 antisense RNA 1 (head to head) | Multiple_C | 3.58 | 3.08 | 3.28 | 1.23 | 0.322 | 0.5359 | 3.9 | 3.74 | 3.57 | 1.26 | 0.0293 | 0.1516 |
| TC0400009431.hg.1 | TENM3 | teneurin transmembrane protein 3 | Multiple_C | 8.48 | 8.58 | 8.18 | 1.23 | 0.3332 | 0.5469 | 4.78 | 5.14 | 4.96 | 0.88 | 0.7498 | 0.886 |
| TC0400010166.hg.1 | FAM184B | family with sequence similarity 184, member B | Multiple_C | 4.33 | 4.01 | 4.03 | 1.23 | 0.1761 | 0.3584 | 4.84 | 4.79 | 4.66 | 1.13 | 0.6263 | 0.8154 |
| TC0400011061.hg.1 | CCDC158 | coiled-coil domain containing 158 | Multiple_C | 4.03 | 4.01 | 3.73 | 1.23 | 0.1688 | 0.3487 | 4.12 | 4.33 | 3.93 | 1.14 | 0.7475 | 0.8843 |
| TC0400012015.hg.1 | GYPE | glycophorin E (MNS blood group) | Multiple_C | 4.02 | 3.6 | 3.72 | 1.23 | 0.614 | 0.7836 | 4.62 | 4.44 | 4.19 | 1.35 | 0.0386 | 0.1789 |
| TC0500007668.hg.1 | CDK7 | cyclin-dependent kinase 7 | Multiple_C | 12.71 | 14.02 | 12.41 | 1.23 | 0.5014 | 0.6999 | 11.67 | 10.83 | 11.07 | 1.52 | 0.0207 | 0.1229 |
| TC0500008654.hg.1 | LEAP2 | liver expressed antimicrobial peptide 2 | Multiple_C | 5.09 | 5.53 | 4.79 | 1.23 | 0.5304 | 0.7207 | 4.38 | 4.38 | 4.09 | 1.22 | 0.7505 | 0.8865 |
| TC0500009751.hg.1 | BTNL9 | butyrophilin-like 9 | Multiple_C | 11.12 | 10.7 | 10.82 | 1.23 | 0.0147 | 0.0563 | 10.21 | 10.15 | 10.03 | 1.13 | 0.3056 | 0.5697 |
| TC0500011277.hg.1 | ANKRD34B | ankyrin repeat domain 34B | Coding | 3.12 | 2.87 | 2.82 | 1.23 | 0.855 | 0.9291 | 3.48 | 3.59 | 3.11 | 1.29 | 0.1123 | 0.3301 |
| TC0500011529.hg.1 | RIOK2 | RIO kinase 2 | Multiple_C | 8.44 | 9.79 | 8.14 | 1.23 | 0.541 | 0.7285 | 8.56 | 7.97 | 8.46 | 1.07 | 0.5059 | 0.7373 |
| TC0500012470.hg.1 | CD74 | CD74 molecule, major histocompatibility complex, class II invari | Multiple_C | 5.35 | 5.17 | 5.05 | 1.23 | 0.9239 | 0.9644 | 5.07 | 4.58 | 4.53 | 1.45 | 0.0509 | 0.2104 |
| TC0600009332.hg.1 | FABP7 | fatty acid binding protein 7, brain | Coding | 3.17 | 2.83 | 2.87 | 1.23 | 0.2755 | 0.4846 | 3.65 | 3.54 | 3.3 | 1.27 | 0.0467 | 0.2 |
| TC0600010959.hg.1 | NHLRC1 | NHL repeat containing E3 ubiquitin protein ligase 1 | Coding | 2.98 | 2.53 | 2.68 | 1.23 | 0.1758 | 0.3582 | 3.04 | 3.02 | 2.87 | 1.13 | 0.2345 | 0.497 |
| TC0600013350.hg.1 | REPS1 | RALBP1 associated Eps domain containing 1 | Multiple_C | 10.09 | 9.53 | 9.79 | 1.23 | 0.221 | 0.4184 | 8.86 | 8.53 | 8.61 | 1.19 | 0.6103 | 0.8051 |
| TC0700009300.hg.1 | LOC10013088 | uncharacterized LOC100130880; novel transcript | Multiple_C | 3.78 | 3.36 | 3.48 | 1.23 | 0.584 | 0.7608 | 3.66 | 3.77 | 3.64 | 1.01 | 0.4869 | 0.7237 |
| TC0700009675.hg.1 | GIMAP8 | GTPase, IMAP family member 8 | Multiple_C | 3.21 | 2.74 | 2.91 | 1.23 | 0.0958 | 0.2335 | 4.32 | 4.36 | 4.12 | 1.15 | 0.9844 | 0.9935 |
| TC0700010412.hg.1 | SP8 | Sp8 transcription factor | Coding | 4.39 | 3.97 | 4.09 | 1.23 | 0.1656 | 0.3442 | 4.52 | 4.51 | 4.39 | 1.09 | 0.3046 | 0.5686 |
| TC0700012850.hg.1 | TAS2R38 | taste receptor, type 2, member 38 | Coding | 3.3 | 3.24 | 3 | 1.23 | 0.2959 | 0.5073 | 3.22 | 3.77 | 3.27 | 0.97 | 0.8673 | 0.9431 |

| | | | | | | | | | | | | | | | |
|---|---|---|---|---|---|---|---|---|---|---|---|---|---|---|---|
| TC0800006447.hg.1 | FBXO25 | F-box protein 25 | Multiple_C | 9.22 | 10.02 | 8.92 | 1.23 | 0.6455 | 0.8035 | 8.13 | 7.54 | 7.56 | 1.48 | 0.5113 | 0.7412 |
| TC0800007626.hg.1 | NPBWR1 | neuropeptides B/W receptor 1 | Coding | 3.09 | 3.08 | 2.79 | 1.23 | 0.1739 | 0.3559 | 3.33 | 3.18 | 3.11 | 1.16 | 0.0377 | 0.1769 |
| TC0800008448.hg.1 | ODF1 | outer dense fiber of sperm tails 1 | Coding | 3.37 | 2.91 | 3.07 | 1.23 | 0.3488 | 0.5618 | 3.48 | 3.34 | 3.22 | 1.20 | 0.3844 | 0.6433 |
| TC0800011872.hg.1 | SLA | Src-like-adaptor | Multiple_C | 4.59 | 4.2 | 4.29 | 1.23 | 0.5966 | 0.7701 | 4.81 | 4.77 | 4.73 | 1.06 | 0.997 | 0.9987 |
| TC0900008150.hg.1 | TMOD1 | tropomodulin 1 | Multiple_C | 5.62 | 5.13 | 5.32 | 1.23 | 0.1173 | 0.2704 | 5.72 | 5.56 | 5.24 | 1.39 | 0.4651 | 0.7068 |
| TC0900012065.hg.1 | ENTPD8 | ectonucleoside triphosphate diphosphohydrolase 8 | Multiple_C | 3.54 | 3.24 | 3.24 | 1.23 | 0.2402 | 0.4422 | 6.4 | 6.37 | 6.78 | 0.77 | 0.3476 | 0.6113 |
| TC0X00007267.hg.1 | MAGED1 | MAGE family member D1 | Multiple_C | 2.66 | 2.22 | 2.36 | 1.23 | 0.5937 | 0.7681 | 8.84 | 10.53 | 9.95 | 0.46 | 0.0033 | 0.0376 |
| TC0X00007382.hg.1 | RRAGB | Ras-related GTP binding B | Multiple_C | 6.4 | 6.1 | 6.1 | 1.23 | 0.0334 | 0.1068 | 6.93 | 7.22 | 6.64 | 1.22 | 0.2247 | 0.4855 |
| TC0X00007686.hg.1 | PBDC1 | polysaccharide biosynthesis domain containing 1 | Multiple_C | 4.54 | 4.02 | 4.24 | 1.23 | 0.28 | 0.4898 | 8.85 | 10.01 | 9.87 | 0.49 | 0.0002 | 0.0048 |
| TC0X00008796.hg.1 | ABCD1 | Transcript Identified by AceView, Entrez Gene ID(s) 215 | Coding | 4.23 | 3.88 | 3.93 | 1.23 | 0.0945 | 0.2311 | 5.03 | 4.97 | 4.18 | 1.80 | 0.0203 | 0.1216 |
| TC0X00009089.hg.1 | FAM9C | family with sequence similarity 9, member C | Multiple_C | 3.7 | 3.07 | 3.4 | 1.23 | 0.1054 | 0.2505 | 4.19 | 3.89 | 3.88 | 1.24 | 0.2883 | 0.5529 |
| TC0X00009632.hg.1 | PCSK1N | proprotein convertase subtilisin/kexin type 1 inhibitor | Multiple_C | 9.05 | 8.42 | 8.75 | 1.23 | 0.1213 | 0.2768 | 9.52 | 9.61 | 9.35 | 1.13 | 0.1675 | 0.4129 |
| TC0X00010098.hg.1 | KIAA2022 | KIAA2022 | Coding | 3.64 | 3.29 | 3.34 | 1.23 | 0.3932 | 0.607 | 3.9 | 3.69 | 3.74 | 1.12 | 0.9706 | 0.9879 |
| TC0Y00006909.hg.1 | SRY | sex determining region Y | Coding | 3.53 | 3.41 | 3.23 | 1.23 | 0.3719 | 0.5845 | 4.12 | 3.78 | 3.66 | 1.38 | 0.2527 | 0.5169 |
| TC1000007415.hg.1 | FXYD4 | FXYD domain containing ion transport regulator 4 | Multiple_C | 4.76 | 4.67 | 4.46 | 1.23 | 0.1757 | 0.3581 | 5.39 | 4.47 | 4.43 | 1.95 | 0.0005 | 0.0106 |
| TC1000007861.hg.1 | TET1 | tet methylcytosine dioxygenase 1 | Multiple_C | 3.54 | 3.13 | 3.24 | 1.23 | 0.6048 | 0.7764 | 8.66 | 8.53 | 8.58 | 1.06 | 0.677 | 0.8444 |
| TC1000009905.hg.1 | C1QL3 | complement component 1, q subcomponent-like 3 | Multiple_C | 3.59 | 3.4 | 3.29 | 1.23 | 0.2922 | 0.5035 | 4.31 | 4.38 | 4.23 | 1.06 | 0.6569 | 0.8324 |
| TC1000010022.hg.1 | PIP4K2A | phosphatidylinositol-5-phosphate 4-kinase, type II, alpha | Multiple_C | 10.96 | 10.62 | 10.66 | 1.23 | 0.0928 | 0.2281 | 11.61 | 11.17 | 12.1 | 0.71 | 0.0501 | 0.2085 |
| TC1000010642.hg.1 | A1CF | APOBEC1 complementation factor | Multiple_C | 3.47 | 3.12 | 3.17 | 1.23 | 0.2968 | 0.5084 | 4.29 | 5.74 | 5.22 | 0.52 | 0.0003 | 0.0075 |
| TC1000012498.hg.1 | GSTO2 | glutathione S-transferase omega 2 | Multiple_C | 11.63 | 12.66 | 11.33 | 1.23 | 0.3356 | 0.5496 | 8.9 | 8.87 | 9.24 | 0.79 | 0.7441 | 0.883 |
| TC1100006745.hg.1 | OR10A5 | olfactory receptor, family 10, subfamily A, member 5 | Coding | 3.04 | 2.84 | 2.74 | 1.23 | 0.4584 | 0.6646 | 3.26 | 3.24 | 3.42 | 0.90 | 0.1306 | 0.3579 |
| TC1100007257.hg.1 | CAT | catalase | Multiple_C | 12.99 | 13.13 | 12.69 | 1.23 | 0.2579 | 0.4643 | 8.16 | 8.33 | 8.57 | 0.75 | 0.7847 | 0.9035 |
| TC1100008041.hg.1 | CTSW | cathepsin W | Multiple_C | 4.65 | 4.39 | 4.35 | 1.23 | 0.1158 | 0.268 | 3.72 | 3.88 | 3.65 | 1.05 | 0.4471 | 0.6942 |
| TC1100008381.hg.1 | P2RY6 | pyrimidinergic receptor P2Y, G-protein coupled, 6 | Coding | 3.97 | 3.82 | 3.67 | 1.23 | 0.7674 | 0.8803 | 4.61 | 4.64 | 4.52 | 1.06 | 0.5043 | 0.7364 |
| TC1100009855.hg.1 | ART5 | ADP-ribosyltransferase 5 | Multiple_C | 3.69 | 3.32 | 3.39 | 1.23 | 0.1819 | 0.3666 | 3.69 | 3.54 | 3.54 | 1.11 | 0.2793 | 0.5454 |
| TC1100009929.hg.1 | OR51I1 | olfactory receptor, family 51, subfamily I, member 1 | Coding | 3.17 | 2.78 | 2.87 | 1.23 | 0.4773 | 0.6798 | 4.11 | 3.37 | 4.76 | 0.64 | 0.0094 | 0.0751 |
| TC1100010925.hg.1 | OR9I1 | olfactory receptor, family 9, subfamily I, member 1 | Coding | 3.56 | 3.16 | 3.26 | 1.23 | 0.5133 | 0.7087 | 4.15 | 3.97 | 3.66 | 1.40 | 0.073 | 0.259 |
| TC1100012208.hg.1 | CWF19L2 | CWF19-like 2, cell cycle control (S. pombe) | Multiple_C | 7.76 | 8.39 | 7.46 | 1.23 | 0.5063 | 0.7033 | 8.13 | 7.64 | 8.4 | 0.83 | 0.1562 | 0.3968 |
| TC1200009307.hg.1 | TMEM132B | transmembrane protein 132B | Multiple_C | 3.45 | 3.24 | 3.15 | 1.23 | 0.2355 | 0.4366 | 3.9 | 4.24 | 4.33 | 0.74 | 0.0411 | 0.1862 |
| TC1300010029.hg.1 | N4BP2L1 | NEDD4 binding protein 2-like 1 | Multiple_C | 5.47 | 6.1 | 5.17 | 1.23 | 0.4739 | 0.677 | 5.43 | 5.66 | 4.98 | 1.37 | 0.0079 | 0.0662 |
| TC1400007207.hg.1 | SAMD4A | sterile alpha motif domain containing 4A | Multiple_C | 9.71 | 9.12 | 9.41 | 1.23 | 0.2477 | 0.4514 | 8.09 | 7.78 | 8.04 | 1.04 | 0.2062 | 0.4628 |
| TC1400009766.hg.1 | IRF2BPL | interferon regulatory factor 2 binding protein-like | Multiple_C | 5.37 | 5.4 | 5.07 | 1.23 | 0.2342 | 0.435 | 6.99 | 6.68 | 6.23 | 1.69 | 0.1038 | 0.3157 |
| TC1400010615.hg.1 | MNAT1 | MNAT CDK-activating kinase assembly factor 1 | Multiple_C | 10.91 | 12.49 | 10.61 | 1.23 | 0.213 | 0.4079 | 11.07 | 10.12 | 10.29 | 1.72 | 0.0027 | 0.0332 |

| | | | | | | | | | | | | | | | |
|---|---|---|---|---|---|---|---|---|---|---|---|---|---|---|---|
| TC1400010704.hg.1 | CRIP1 | cysteine-rich protein 1 (intestinal) | Multiple_C | 6.69 | 6.49 | 6.39 | 1.23 | 0.1682 | 0.3479 | 4.75 | 4.13 | 3.88 | 1.83 | 0.0014 | 0.0215 |
| TC1400010772.hg.1 | DIO2 | deiodinase, iodothyronine, type II | Coding | 4.66 | 3.97 | 4.36 | 1.23 | 0.8566 | 0.9299 | 4.64 | 4.53 | 4.71 | 0.95 | 0.4533 | 0.6983 |
| TC1500006507.hg.1 | GOLGA6L22; C | golgin A6 family-like 22; golgin A6 family-like 1 | Coding | 4.8 | 4.43 | 4.5 | 1.23 | 0.136 | 0.3008 | 4.56 | 4.15 | 4.31 | 1.19 | 0.0646 | 0.2421 |
| TC1500009451.hg.1 | BCL2L10 | BCL2-like 10 (apoptosis facilitator) | Coding | 4.69 | 4.51 | 4.39 | 1.23 | 0.3156 | 0.5288 | 4.59 | 4.44 | 4.53 | 1.04 | 0.2022 | 0.4579 |
| TC1500010383.hg.1 | POLG | polymerase (DNA directed), gamma | Multiple_C | 8.18 | 8.15 | 7.88 | 1.23 | 0.2319 | 0.4323 | 7.23 | 7.49 | 7.61 | 0.77 | 0.0805 | 0.2734 |
| TC1500010730.hg.1 | HYPK | huntingtin interacting protein K | Multiple_C | 12.35 | 11.99 | 12.05 | 1.23 | 0.1758 | 0.3582 | 10.42 | 9.79 | 10.17 | 1.19 | 0.2456 | 0.51 |
| TC1600006469.hg.1 | CAPN15; MIR5 | calpain 15; microRNA 5587 | Multiple_C | 7.17 | 6.69 | 6.87 | 1.23 | 0.2012 | 0.3931 | 7.08 | 7.36 | 7.12 | 0.97 | 0.357 | 0.6202 |
| TC1600007931.hg.1 | LPCAT2 | lysophosphatidylcholine acyltransferase 2 | Multiple_C | 3.43 | 3.06 | 3.13 | 1.23 | 0.9043 | 0.954 | 12.08 | 10.48 | 10.78 | 2.46 | 0.0058 | 0.0544 |
| TC1600008126.hg.1 | CMTM2 | CKLF-like MARVEL transmembrane domain containing 2 | Multiple_C | 4.75 | 4.27 | 4.45 | 1.23 | 0.0506 | 0.1458 | 5.15 | 5.06 | 4.93 | 1.16 | 0.4708 | 0.7116 |
| TC1600009322.hg.1 | CARHSP1 | calcium regulated heat stable protein 1 | Multiple_C | 6.96 | 6.34 | 6.66 | 1.23 | 0.4596 | 0.6657 | 5.6 | 6.14 | 5.72 | 0.92 | 0.1784 | 0.4271 |
| TC1600010490.hg.1 | CNOT1; SNOR | CCR4-NOT transcription complex subunit 1; small nucleolar RNA | Multiple_C | 13.09 | 12.75 | 12.79 | 1.23 | 0.1156 | 0.2679 | 13.73 | 13.49 | 13.66 | 1.05 | 0.6499 | 0.829 |
| TC1600011414.hg.1 | EDC4 | enhancer of mRNA decapping 4 | Multiple_C | 7.49 | 7.54 | 7.19 | 1.23 | 0.6591 | 0.8131 | 9.41 | 10.25 | 9.54 | 0.91 | 0.1015 | 0.3117 |
| TC1700006574.hg.1 | OR1D4 | olfactory receptor, family 1, subfamily D, member 4 (gene/pseu | Multiple_C | 4.54 | 3.98 | 4.24 | 1.23 | 0.0957 | 0.2334 | 4.3 | 4.31 | 4.11 | 1.14 | 0.2474 | 0.512 |
| TC1700007852.hg.1 | GAST | gastrin | Multiple_C | 6 | 5.7 | 5.7 | 1.23 | 0.2891 | 0.4998 | 7.25 | 6.72 | 6.12 | 2.19 | 0.004 | 0.0433 |
| TC1700007983.hg.1 | ASB16 | ankyrin repeat and SOCS box containing 16 | Coding | 3.75 | 3.15 | 3.45 | 1.23 | 0.0427 | 0.1291 | 3.55 | 3.57 | 3.95 | 0.76 | 0.2602 | 0.5245 |
| TC1700008845.hg.1 | DNAI2 | dynein, axonemal, intermediate chain 2 | Multiple_C | 3.82 | 3.59 | 3.52 | 1.23 | 0.6028 | 0.7747 | 4.09 | 4.32 | 4.09 | 1.00 | 0.5939 | 0.795 |
| TC1700009881.hg.1 | NCOR1 | nuclear receptor corepressor 1 | Multiple_C | 11.65 | 11.32 | 11.35 | 1.23 | 0.6163 | 0.7849 | 10.61 | 10.40 | 10.73 | 0.92 | 0.3539 | 0.6174 |
| TC1700009992.hg.1 | FAM83G | family with sequence similarity 83, member G | Multiple_C | 8.04 | 7.02 | 7.74 | 1.23 | 0.5153 | 0.71 | 6.57 | 6.43 | 7.07 | 0.71 | 0.6929 | 0.8538 |
| TC1700010555.hg.1 | FBXO47 | F-box protein 47 | Coding | 3.53 | 3.56 | 3.23 | 1.23 | 0.4181 | 0.6297 | 3.26 | 3.08 | 3.03 | 1.17 | 0.0718 | 0.2565 |
| TC1700010576.hg.1 | NEUROD2 | neuronal differentiation 2 | Multiple_C | 3.63 | 3.23 | 3.33 | 1.23 | 0.122 | 0.2781 | 3.82 | 3.72 | 3.98 | 0.90 | 0.6767 | 0.8443 |
| TC1700011390.hg.1 | MARCHF10 | membrane associated ring finger 10 | Multiple_C | 3.59 | 3.29 | 3.29 | 1.23 | 0.4496 | 0.6574 | 4.38 | 4.11 | 4.04 | 1.27 | 0.4101 | 0.665 |
| TC1800007490.hg.1 | CDH20 | cadherin 20, type 2 | Multiple_C | 4.67 | 3.99 | 4.37 | 1.23 | 0.177 | 0.3596 | 4.48 | 4.45 | 4.21 | 1.21 | 0.2655 | 0.5301 |
| TC1800007986.hg.1 | LAMA1 | laminin, alpha 1 | Multiple_C | 4.07 | 3.4 | 3.77 | 1.23 | 0.6086 | 0.7793 | 4.2 | 4.10 | 4.02 | 1.13 | 0.4692 | 0.7103 |
| TC1900007314.hg.1 | OCEL1 | occludin/ELL domain containing 1 | Multiple_C | 4.64 | 4.34 | 4.34 | 1.23 | 0.3254 | 0.5392 | 3.89 | 3.64 | 3.3 | 1.51 | 0.0052 | 0.0503 |
| TC1900008415.hg.1 | CCDC9 | coiled-coil domain containing 9 | Multiple_C | 8.28 | 7.53 | 7.98 | 1.23 | 0.3431 | 0.5568 | 6.13 | 5.99 | 6.79 | 0.63 | 0.3309 | 0.595 |
| TC1900008633.hg.1 | CD33 | CD33 molecule | Multiple_C | 4.35 | 4.1 | 4.05 | 1.23 | 0.5124 | 0.7081 | 4.4 | 4.05 | 4.11 | 1.22 | 0.1763 | 0.4249 |
| TC1900008965.hg.1 | ZFP28 | ZFP28 zinc finger protein | Multiple_C | 4.59 | 3.94 | 4.29 | 1.23 | 0.1141 | 0.2652 | 4.3 | 4.69 | 4.58 | 0.82 | 0.4443 | 0.6923 |
| TC1900009639.hg.1 | AP1M2 | adaptor-related protein complex 1, mu 2 subunit | Multiple_C | 13.01 | 12.51 | 12.71 | 1.23 | 0.369 | 0.5819 | 4.46 | 4.06 | 4.13 | 1.26 | 0.1474 | 0.3836 |
| TC1900009740.hg.1 | DHPS | deoxyhypusine synthase | Multiple_C | 10.57 | 11.35 | 10.27 | 1.23 | 0.3676 | 0.5806 | 9.35 | 9.29 | 9.06 | 1.22 | 0.5335 | 0.7561 |
| TC1900009846.hg.1 | OR7A17 | olfactory receptor, family 7, subfamily A, member 17 | Multiple_C | 5.13 | 4.83 | 4.83 | 1.23 | 0.1796 | 0.3634 | 4.02 | 3.86 | 4.1 | 0.95 | 0.8609 | 0.9405 |
| TC1900009872.hg.1 | RASAL3 | RAS protein activator like 3 | Multiple_C | 7.02 | 6.57 | 6.72 | 1.23 | 0.7405 | 0.8656 | 6.46 | 6.58 | 6.43 | 1.02 | 0.5645 | 0.7745 |
| TC1900009959.hg.1 | ABHD8 | abhydrolase domain containing 8 | Multiple_C | 4.86 | 4.47 | 4.56 | 1.23 | 0.1546 | 0.3288 | 4.75 | 4.89 | 4.84 | 0.94 | 0.7618 | 0.8923 |
| TC1900010631.hg.1 | RINL | Ras and Rab interactor like | Multiple_C | 5.46 | 5.3 | 5.16 | 1.23 | 0.4572 | 0.6634 | 5.83 | 5.43 | 5.28 | 1.46 | 0.1194 | 0.3416 |

| | | | | | | | | | | | | | | | |
|---|---|---|---|---|---|---|---|---|---|---|---|---|---|---|---|
| TC1900010864.hg.1 | KCNN4 | potassium channel, calcium activated intermediate/small condu | Multiple_C | 11.15 | 11.31 | 10.85 | 1.23 | 0.3566 | 0.5699 | 5.67 | 5.88 | 5.43 | 1.18 | 0.538 | 0.759 |
| TC1900011255.hg.1 | SIGLEC10 | sialic acid binding Ig-like lectin 10 | Multiple_C | 5.83 | 5.1 | 5.53 | 1.23 | 0.8782 | 0.9408 | 4.17 | 4.09 | 4.27 | 0.93 | 0.8588 | 0.9397 |
| TC1900011334.hg.1 | ZNF677 | zinc finger protein 677 | Multiple_C | 4.06 | 3.6 | 3.76 | 1.23 | 0.4207 | 0.6319 | 4.28 | 3.90 | 4.25 | 1.02 | 0.1936 | 0.4472 |
| TC1900011379.hg.1 | NLRP12 | NLR family, pyrin domain containing 12 | Multiple_C | 3.46 | 2.81 | 3.16 | 1.23 | 0.3719 | 0.5846 | 3.99 | 3.99 | 3.91 | 1.06 | 0.995 | 0.9976 |
| TC1900011821.hg.1 | ZNF776 | zinc finger protein 776 | Multiple_C | 9.64 | 9.4 | 9.34 | 1.23 | 0.3852 | 0.5985 | 8.12 | 8.88 | 8.52 | 0.76 | 0.1969 | 0.4519 |
| TC1900011861.hg.1 | S1PR2 | sphingosine-1-phosphate receptor 2 | Multiple_C | 4.97 | 4.41 | 4.67 | 1.23 | 0.5665 | 0.7476 | 4.41 | 4.07 | 4.17 | 1.18 | 0.1005 | 0.3098 |
| TC1900011883.hg.1 | ZNF799 | zinc finger protein 799 | Multiple_C | 9.86 | 10.57 | 9.56 | 1.23 | 0.3463 | 0.5597 | 8.69 | 8.26 | 8.22 | 1.39 | 0.6467 | 0.8268 |
| TC1900011988.hg.1 | FAM83E | family with sequence similarity 83, member E | Coding | 5.64 | 5.42 | 5.34 | 1.23 | 0.1186 | 0.2724 | 4.91 | 5.04 | 4.92 | 0.99 | 0.8554 | 0.9384 |
| TC1900012023.hg.1 | ZNF836 | zinc finger protein 836 | Multiple_C | 7.87 | 8.22 | 7.57 | 1.23 | 0.2634 | 0.4706 | 5.94 | 5.96 | 6.1 | 0.90 | 0.8331 | 0.9258 |
| TC1900012049.hg.1 | TMEM86B | transmembrane protein 86B | Multiple_C | 4.58 | 4.18 | 4.28 | 1.23 | 0.2312 | 0.4316 | 4.45 | 4.30 | 4.27 | 1.13 | 0.5567 | 0.7706 |
| TC2000007923.hg.1 | NELFCD | negative elongation factor complex member C/D | Multiple_C | 11.34 | 11.04 | 11.04 | 1.23 | 0.2767 | 0.486 | 11.51 | 11.29 | 11.05 | 1.38 | 0.1364 | 0.367 |
| TC2000008210.hg.1 | AVP | arginine vasopressin | Multiple_C | 5.44 | 4.84 | 5.14 | 1.23 | 0.1076 | 0.2542 | 5.5 | 5.65 | 5.43 | 1.05 | 0.7631 | 0.8931 |
| TC2000008287.hg.1 | PROKR2 | prokineticin receptor 2 | Coding | 4.54 | 4.21 | 4.24 | 1.23 | 0.3368 | 0.5507 | 4.76 | 4.38 | 3.86 | 1.87 | 0.0191 | 0.117 |
| TC2000008960.hg.1 | EDEM2 | ER degradation enhancer, mannosidase alpha-like 2 | Multiple_C | 9.87 | 9.76 | 9.57 | 1.23 | 0.2885 | 0.4992 | 8.43 | 8.46 | 8.45 | 0.99 | 0.8651 | 0.9424 |
| TC2000009178.hg.1 | JPH2 | junctophilin 2 | Multiple_C | 5.06 | 4.52 | 4.76 | 1.23 | 0.5438 | 0.7304 | 4.61 | 4.61 | 4.48 | 1.09 | 0.3059 | 0.5699 |
| TC2000009916.hg.1 | TGIF2-C20orf2 | TGIF2-C20orf24 readthrough | Multiple_C | 13.62 | 13.18 | 13.32 | 1.23 | 0.1415 | 0.3091 | 10.75 | 11.04 | 11.22 | 0.72 | 0.1043 | 0.3166 |
| TC2200006654.hg.1 | ZNF74 | zinc finger protein 74 | Multiple_C | 4.02 | 3.63 | 3.72 | 1.23 | 0.1785 | 0.3618 | 4.37 | 4.33 | 4.24 | 1.09 | 0.4886 | 0.7247 |
| TC2200006935.hg.1 | ADRBK2 | adrenergic, beta, receptor kinase 2 | Multiple_C | 3.62 | 3.49 | 3.32 | 1.23 | 0.1343 | 0.298 | 6.34 | 4.49 | 4.04 | 4.92 | 6.93E-08 | 2.07E-05 |
| TC2200007109.hg.1 | DEPDC5 | DEP domain containing 5 | Multiple_C | 7.24 | 6.74 | 6.94 | 1.23 | 0.4178 | 0.6295 | 6.3 | 6.75 | 6.82 | 0.70 | 0.0703 | 0.2534 |
| TC2200007614.hg.1 | FAM118A | family with sequence similarity 118, member A | Multiple_C | 9.06 | 8.14 | 8.76 | 1.23 | 0.3492 | 0.5619 | 7.88 | 7.63 | 8.1 | 0.86 | 0.4432 | 0.6916 |
| TC2200007811.hg.1 | NCAPH2 | non-SMC condensin II complex subunit H2 | Multiple_C | 8.84 | 7.48 | 8.54 | 1.23 | 0.825 | 0.9121 | 7.69 | 8.88 | 9.16 | 0.36 | 0.012 | 0.0875 |
| TC2200008637.hg.1 | IL2RB | interleukin 2 receptor, beta | Multiple_C | 3.91 | 3.74 | 3.61 | 1.23 | 0.2782 | 0.4875 | 3.45 | 3.46 | 3.73 | 0.82 | 0.3287 | 0.5924 |
| TSUnmapped00000305.h | ADAMTS13 | ADAM metallopeptidase with thrombospondin type 1 motif 13 | Coding | 6.03 | 5.79 | 5.73 | 1.23 | 0.0713 | 0.188 | 6.22 | 6.06 | 6.02 | 1.15 | 0.1765 | 0.4252 |
| TSUnmapped00000393.h | ZNF546 | zinc finger protein 546 | Coding | 3.98 | 3.86 | 3.68 | 1.23 | 0.052 | 0.1486 | 4.52 | 4.93 | 4.35 | 1.13 | 0.515 | 0.7443 |
| TSUnmapped00000397.h | EIF3F | Eukaryotic translation initiation factor 3 subunit F [Source:UniP | Coding | 11.31 | 11.55 | 11.01 | 1.23 | 0.2656 | 0.4732 | 10.29 | 10.55 | 10.16 | 1.09 | 0.3881 | 0.6468 |
| TSUnmapped00000507.h | REXO4 | REX4 homolog, 3'-5' exonuclease [Source:HGNC Symbol;Acc:HG | Coding | 5.1 | 4.81 | 4.8 | 1.23 | 0.183 | 0.3683 | 4.49 | 4.65 | 4.59 | 0.93 | 0.6957 | 0.8555 |
| TSUnmapped00000661.h | INPP5D | inositol polyphosphate-5-phosphatase D | Coding | 3.12 | 3.19 | 2.82 | 1.23 | 0.3702 | 0.5828 | 7.21 | 7.54 | 7.91 | 0.62 | 0.0011 | 0.0182 |
| TC0100008321.hg.1 | GPX7 | glutathione peroxidase 7 | Multiple_C | 4.44 | 3.86 | 4.15 | 1.22 | 0.7032 | 0.8416 | 5.29 | 5.63 | 5.27 | 1.01 | 0.7159 | 0.8673 |
| TC0100009046.hg.1 | RPL5; SNORD2 | ribosomal protein L5; small nucleolar RNA, C/D box 21; small nu | Multiple_C | 18.41 | 18.42 | 18.12 | 1.22 | 0.1377 | 0.3032 | 16.27 | 15.84 | 15.8 | 1.39 | 0.085 | 0.2806 |
| TC0100009183.hg.1 | PALMD | palmdelphin | Multiple_C | 5.08 | 4.72 | 4.79 | 1.22 | 0.5361 | 0.725 | 5.17 | 4.99 | 4.81 | 1.28 | 0.0265 | 0.1423 |
| TC0100009394.hg.1 | CD53 | CD53 molecule | Multiple_C | 3.82 | 3.02 | 3.53 | 1.22 | 0.6315 | 0.7943 | 3.64 | 3.28 | 3.57 | 1.05 | 0.4777 | 0.7164 |
| TC0100009614.hg.1 | HSD3B1 | hydroxy-delta-5-steroid dehydrogenase, 3 beta- and steroid del | Multiple_C | 3.4 | 3.11 | 3.11 | 1.22 | 0.5937 | 0.7681 | 3.37 | 3.27 | 3.4 | 0.98 | 0.9874 | 0.9947 |
| TC0100010010.hg.1 | LCE1A | late cornified envelope 1A | Coding | 4.35 | 3.5 | 4.06 | 1.22 | 0.2972 | 0.5087 | 4.45 | 4.31 | 4.42 | 1.02 | 0.791 | 0.9067 |

| | | | | | | | | | | | | | | | |
|---|---|---|---|---|---|---|---|---|---|---|---|---|---|---|---|
| TC0100010332.hg.1 | NIT1 | nitrilase 1 | Multiple_C | 8.56 | 8.59 | 8.27 | 1.22 | 0.4894 | 0.6896 | 7.17 | 7.38 | 7.26 | 0.94 | 0.5482 | 0.7651 |
| TC0100010522.hg.1 | TIPRL | TOR signaling pathway regulator | Multiple_C | 11.18 | 11.53 | 10.89 | 1.22 | 0.3477 | 0.5608 | 9.79 | 9.68 | 9.66 | 1.09 | 0.3918 | 0.6502 |
| TC0100012588.hg.1 | MEGF6 | multiple EGF-like-domains 6 | Multiple_C | 4.72 | 4.44 | 4.43 | 1.22 | 0.2461 | 0.4496 | 4.63 | 4.30 | 4.02 | 1.53 | 0.2405 | 0.504 |
| TC0100012690.hg.1 | KLHL21 | kelch-like family member 21 | Multiple_C | 9.36 | 8.72 | 9.07 | 1.22 | 0.3381 | 0.552 | 7.47 | 7.48 | 8.13 | 0.63 | 0.0045 | 0.0459 |
| TC0100012935.hg.1 | PRAMEF10 | PRAME family member 10 | Coding | 3.63 | 3.64 | 3.34 | 1.22 | 0.0905 | 0.2239 | 3.76 | 3.78 | 3.99 | 0.85 | 0.337 | 0.6014 |
| TC0100013674.hg.1 | DLGAP3 | discs, large (Drosophila) homolog-associated protein 3 | Multiple_C | 4.61 | 4.16 | 4.32 | 1.22 | 0.2102 | 0.4041 | 3.89 | 4.14 | 4.28 | 0.76 | 0.1166 | 0.3375 |
| TC0100016218.hg.1 | RGS5 | regulator of G-protein signaling 5 | Multiple_C | 4.55 | 4.62 | 4.26 | 1.22 | 0.9438 | 0.974 | 4.18 | 4.13 | 3.87 | 1.24 | 0.291 | 0.5557 |
| TC0200010410.hg.1 | SPATS2L | spermatogenesis associated, serine-rich 2-like | Multiple_C | 13.23 | 12.42 | 12.94 | 1.22 | 0.2026 | 0.3949 | 12.02 | 11.98 | 12.88 | 0.55 | 0.003 | 0.0353 |
| TC0200010431.hg.1 | NIF3L1 | NIF3 NGG1 interacting factor 3-like 1 | Multiple_C | 8.94 | 9.3 | 8.65 | 1.22 | 0.5629 | 0.7448 | 7.56 | 8.04 | 8.07 | 0.70 | 0.0242 | 0.1346 |
| TC0200010527.hg.1 | ICOS | inducible T-cell co-stimulator | Coding | 3.73 | 3.52 | 3.44 | 1.22 | 0.6337 | 0.7959 | 4.2 | 3.66 | 3.84 | 1.28 | 0.0126 | 0.0901 |
| TC0200010982.hg.1 | DAW1 | dynein assembly factor with WDR repeat domains 1 | Multiple_C | 3.05 | 2.78 | 2.76 | 1.22 | 0.2287 | 0.4284 | 3.96 | 3.85 | 3.8 | 1.12 | 0.4747 | 0.7142 |
| TC0200014288.hg.1 | FAM168B | family with sequence similarity 168, member B | Multiple_C | 10.12 | 9.19 | 9.83 | 1.22 | 0.169 | 0.349 | 10.29 | 10.15 | 11.15 | 0.55 | 0.0002 | 0.005 |
| TC0300008103.hg.1 | RP11-325B23. | novel transcript; olfactory receptor, family 5, subfamily H, mem | Multiple_C | 2.9 | 2.52 | 2.61 | 1.22 | 0.2671 | 0.4751 | 3.18 | 3.43 | 3.47 | 0.82 | 0.3073 | 0.5715 |
| TC0300010561.hg.1 | SLC4A7 | solute carrier family 4, sodium bicarbonate cotransporter, mem | Multiple_C | 11.67 | 11.62 | 11.38 | 1.22 | 0.3326 | 0.5464 | 11.44 | 10.83 | 11.76 | 0.80 | 0.0372 | 0.1754 |
| TC0300012043.hg.1 | DRD3 | dopamine receptor D3 | Multiple_C | 3.69 | 3.33 | 3.4 | 1.22 | 0.1867 | 0.3737 | 3.58 | 3.73 | 3.81 | 0.85 | 0.2622 | 0.5265 |
| TC0300012784.hg.1 | COMMD2 | COMM domain containing 2 | Multiple_C | 12.15 | 12.88 | 11.86 | 1.22 | 0.2509 | 0.4552 | 10.43 | 10.77 | 10.95 | 0.70 | 0.136 | 0.3665 |
| TC0400006806.hg.1 | USP17L21 | ubiquitin specific peptidase 17-like family member 21 | Coding | 4.1 | 3.77 | 3.81 | 1.22 | 0.2959 | 0.5074 | 3.92 | 4.10 | 3.86 | 1.04 | 0.1658 | 0.4105 |
| TC0400008879.hg.1 | GAB1 | GRB2-associated binding protein 1 | Multiple_C | 7.65 | 6.96 | 7.36 | 1.22 | 0.9057 | 0.9548 | 6.67 | 6.00 | 5.23 | 2.71 | 0.0001 | 0.0045 |
| TC0400009735.hg.1 | NKX1-1 | NK1 homeobox 1 | Multiple_C | 6.87 | 6.64 | 6.58 | 1.22 | 0.486 | 0.6874 | 7.28 | 7.53 | 7.61 | 0.80 | 0.2606 | 0.5249 |
| TC0400010449.hg.1 | TLR10 | toll-like receptor 10 | Multiple_C | 3.68 | 3.2 | 3.39 | 1.22 | 0.3196 | 0.5334 | 3.95 | 4.18 | 3.55 | 1.32 | 0.1405 | 0.3726 |
| TC0400011136.hg.1 | GK2 | glycerol kinase 2 | Coding | 2.78 | 2.58 | 2.49 | 1.22 | 0.8846 | 0.9441 | 3.34 | 3.37 | 3.48 | 0.91 | 0.4615 | 0.7043 |
| TC0500008086.hg.1 | NR2F1 | nuclear receptor subfamily 2, group F, member 1 | Multiple_C | 9.65 | 10 | 9.36 | 1.22 | 0.4234 | 0.6346 | 6.62 | 8.69 | 8.91 | 0.20 | 3.02E-06 | 0.0003 |
| TC0500008852.hg.1 | SLC4A9 | solute carrier family 4, sodium bicarbonate cotransporter, mem | Multiple_C | 3.44 | 3.06 | 3.15 | 1.22 | 0.3486 | 0.5617 | 3.45 | 3.10 | 3.12 | 1.26 | 0.021 | 0.124 |
| TC0500011597.hg.1 | SLCO6A1 | solute carrier organic anion transporter family, member 6A1 | Multiple_C | 3.23 | 2.96 | 2.94 | 1.22 | 0.2496 | 0.4538 | 4.46 | 4.39 | 3.83 | 1.55 | 0.0101 | 0.0782 |
| TC0500012588.hg.1 | HAVCR1 | hepatitis A virus cellular receptor 1 | Multiple_C | 3.53 | 3.34 | 3.24 | 1.22 | 0.3471 | 0.5604 | 3.46 | 3.42 | 3.57 | 0.93 | 0.5617 | 0.7725 |
| TC0600007991.hg.1 | TREML4 | triggering receptor expressed on myeloid cells-like 4 | Coding | 3.84 | 3.65 | 3.55 | 1.22 | 0.0791 | 0.2032 | 3.77 | 3.62 | 3.77 | 1.00 | 0.8986 | 0.9576 |
| TC0600007996.hg.1 | NCR2 | natural cytotoxicity triggering receptor 2 | Coding | 3.02 | 2.51 | 2.73 | 1.22 | 0.5092 | 0.7053 | 3.63 | 3.36 | 3.33 | 1.23 | 0.1021 | 0.3128 |
| TC0600011376.hg.1 | PPP1R18 | protein phosphatase 1, regulatory subunit 18 | Multiple_C | 7.56 | 6.72 | 7.27 | 1.22 | 0.4282 | 0.6386 | 9.59 | 9.44 | 9.83 | 0.85 | 0.4629 | 0.7054 |
| TC0600011957.hg.1 | ADGRF1 | adhesion G protein-coupled receptor F1 | Multiple_C | 9.51 | 7.52 | 9.22 | 1.22 | 0.0476 | 0.1395 | 4.54 | 4.29 | 4.72 | 0.88 | 0.4085 | 0.664 |
| TC0600012529.hg.1 | RNGTT | RNA guanylyltransferase and 5-phosphatase | Multiple_C | 9.85 | 9.34 | 9.56 | 1.22 | 0.4575 | 0.6636 | 10.39 | 9.79 | 10.32 | 1.05 | 0.8041 | 0.9128 |
| TC0600012539.hg.1 | GABRR1 | gamma-aminobutyric acid (GABA) A receptor, rho 1 | Multiple_C | 3.52 | 3.3 | 3.23 | 1.22 | 0.3246 | 0.5387 | 3.76 | 3.94 | 3.96 | 0.87 | 0.7874 | 0.9051 |
| TC0700006619.hg.1 | SLC29A4 | solute carrier family 29 (equilibrative nucleoside transporter), m | Multiple_C | 4.28 | 3.85 | 3.99 | 1.22 | 0.1293 | 0.2899 | 3.52 | 3.35 | 3.36 | 1.12 | 0.1417 | 0.3745 |
| TC0700006652.hg.1 | ZNF316 | zinc finger protein 316 | Multiple_C | 8.24 | 7.84 | 7.95 | 1.22 | 0.4048 | 0.6177 | 8.53 | 8.55 | 8.57 | 0.97 | 0.1657 | 0.4105 |

| | | | | | | | | | | | | | | | |
|---|---|---|---|---|---|---|---|---|---|---|---|---|---|---|---|
| TC0700008261.hg.1 | RUNDC3B | RUN domain containing 3B | Multiple_C | 3.99 | 3.89 | 3.7 | 1.22 | 0.1093 | 0.2574 | 4.97 | 4.43 | 3.94 | 2.04 | 0.0032 | 0.0371 |
| TC0700009533.hg.1 | OR2A1 | olfactory receptor, family 2, subfamily A, member 1 | Coding | 4.36 | 3.98 | 4.07 | 1.22 | 0.3946 | 0.6082 | 5.11 | 5.02 | 4.57 | 1.45 | 0.0334 | 0.1642 |
| TC0700010924.hg.1 | NPC1L1 | NPC1-like 1 | Multiple_C | 3.49 | 2.89 | 3.2 | 1.22 | 0.1717 | 0.3529 | 4.29 | 4.35 | 4.46 | 0.89 | 0.1026 | 0.3136 |
| TC0700011318.hg.1 | ERV3-1; ZNF1 | endogenous retrovirus group 3, member 1; zinc finger protein 1 | Multiple_C | 7.58 | 7.73 | 7.29 | 1.22 | 0.0665 | 0.1788 | 6.16 | 6.44 | 6.44 | 0.82 | 0.289 | 0.5536 |
| TC0700012479.hg.1 | POT1 | protection of telomeres 1 | Multiple_C | 9.72 | 11.09 | 9.43 | 1.22 | 0.468 | 0.6721 | 9.47 | 8.71 | 9.38 | 1.06 | 0.7388 | 0.8798 |
| TC0700013254.hg.1 | PTPRN2 | protein tyrosine phosphatase, receptor type, N polypeptide 2 | Multiple_C | 8.82 | 9.21 | 8.53 | 1.22 | 0.6739 | 0.8225 | 6.77 | 7.82 | 8.07 | 0.41 | 0.0098 | 0.077 |
| TC0700013455.hg.1 | HYAL4 | hyaluronoglucosaminidase 4 | Coding | 3.74 | 3.12 | 3.45 | 1.22 | 0.1803 | 0.3644 | 3.48 | 3.42 | 3.28 | 1.15 | 0.1365 | 0.3671 |
| TC0800012427.hg.1 | STAU2 | staufen double-stranded RNA binding protein 2 | Multiple_C | 11.09 | 11.21 | 10.8 | 1.22 | 0.3988 | 0.6125 | 12.05 | 10.99 | 11.14 | 1.88 | 0.0778 | 0.2686 |
| TC0900009267.hg.1 | SSNA1 | Sjogren syndrome nuclear autoantigen 1 | Multiple_C | 10.03 | 9.51 | 9.74 | 1.22 | 0.2399 | 0.4419 | 7.87 | 8.42 | 8.53 | 0.63 | 0.0005 | 0.0105 |
| TC0900009895.hg.1 | C9orf24 | chromosome 9 open reading frame 24 | Multiple_C | 3.72 | 3.12 | 3.43 | 1.22 | 0.1903 | 0.3783 | 3.48 | 3.43 | 3.62 | 0.91 | 0.6253 | 0.8149 |
| TC0X00006687.hg.1 | MAGEB17 | MAGE family member B17 | Coding | 3.78 | 3.21 | 3.49 | 1.22 | 0.2588 | 0.4653 | 4.48 | 4.30 | 4.12 | 1.28 | 0.1111 | 0.3284 |
| TC0X00006816.hg.1 | PDK3 | pyruvate dehydrogenase kinase, isozyme 3 | Multiple_C | 8.43 | 8.08 | 8.14 | 1.22 | 0.5491 | 0.7343 | 6.77 | 7.48 | 7.09 | 0.80 | 0.4094 | 0.6646 |
| TC0X00007890.hg.1 | RPA4 | replication protein A4 | Coding | 3.97 | 3.86 | 3.68 | 1.22 | 0.1057 | 0.251 | 4.21 | 3.70 | 4.42 | 0.86 | 0.896 | 0.9561 |
| TC0X00008780.hg.1 | ZFP92 | ZFP92 zinc finger protein | Multiple_C | 4.96 | 4.38 | 4.67 | 1.22 | 0.3167 | 0.5301 | 5.57 | 5.72 | 5.48 | 1.06 | 0.466 | 0.7077 |
| TC0X00009684.hg.1 | SHROOM4 | shroom family member 4 | Multiple_C | 3.35 | 3.18 | 3.06 | 1.22 | 0.7542 | 0.8741 | 4.62 | 5.06 | 4.54 | 1.06 | 0.1451 | 0.3801 |
| TC0X00010399.hg.1 | BEX1 | brain expressed X-linked 1 | Multiple_C | 4.29 | 4.26 | 4 | 1.22 | 0.5012 | 0.6997 | 4.23 | 3.86 | 3.93 | 1.23 | 0.0627 | 0.2382 |
| TC0Y00006699.hg.1 | HSFY2; HSFY1 | heat shock transcription factor, Y-linked 2; heat shock transcript | Multiple_C | 3.74 | 3.48 | 3.45 | 1.22 | 0.2971 | 0.5086 | 3.74 | 3.49 | 3.19 | 1.46 | 0.1111 | 0.3285 |
| TC1000008591.hg.1 | GOLGA7B | golgin A7 family, member B | Multiple_C | 3.01 | 2.59 | 2.72 | 1.22 | 0.1794 | 0.3632 | 2.98 | 3.60 | 3.82 | 0.56 | 0.0002 | 0.0059 |
| TC1000008649.hg.1 | WNT8B | wingless-type MMTV integration site family, member 8B | Multiple_C | 4.61 | 4.46 | 4.32 | 1.22 | 0.4788 | 0.6809 | 4.54 | 4.03 | 4.06 | 1.39 | 0.0496 | 0.2073 |
| TC1000011635.hg.1 | TLX1NB | TLX1 neighbor | Multiple_C | 3.95 | 3.58 | 3.66 | 1.22 | 0.4554 | 0.6624 | 3.68 | 3.47 | 3.56 | 1.09 | 0.4083 | 0.664 |
| TC1000011720.hg.1 | CALHM3 | calcium homeostasis modulator 3 | Coding | 3.75 | 3.4 | 3.46 | 1.22 | 0.5635 | 0.7453 | 4.29 | 4.70 | 4.52 | 0.85 | 0.6559 | 0.8321 |
| TC1100006771.hg.1 | EIF3F | eukaryotic translation initiation factor 3, subunit F | Multiple_C | 13.6 | 14.28 | 13.31 | 1.22 | 0.2769 | 0.486 | 11.65 | 12.05 | 11.88 | 0.85 | 0.3371 | 0.6014 |
| TC1100007630.hg.1 | OR5T3 | olfactory receptor, family 5, subfamily T, member 3 | Coding | 3.1 | 2.72 | 2.81 | 1.22 | 0.4104 | 0.6227 | 3.45 | 3.25 | 3.13 | 1.25 | 0.0594 | 0.2301 |
| TC1100010091.hg.1 | MRVI1 | murine retrovirus integration site 1 homolog | Multiple_C | 5.73 | 5.17 | 5.44 | 1.22 | 0.0721 | 0.1894 | 5.28 | 5.37 | 5.08 | 1.15 | 0.1927 | 0.446 |
| TC1100011789.hg.1 | FAM181B | family with sequence similarity 181, member B | Coding | 4.65 | 4.16 | 4.36 | 1.22 | 0.2473 | 0.451 | 4.87 | 5.15 | 5 | 0.91 | 0.407 | 0.6629 |
| TC1100013004.hg.1 | CTNND1 | catenin (cadherin-associated protein), delta 1 | Multiple_C | 13.59 | 13.35 | 13.3 | 1.22 | 0.2674 | 0.4755 | 13.78 | 13.36 | 13.34 | 1.36 | 0.0275 | 0.1454 |
| TC1200006616.hg.1 | LTBR | lymphotoxin beta receptor (TNFR superfamily, member 3) | Multiple_C | 10.6 | 10.02 | 10.31 | 1.22 | 0.3188 | 0.5324 | 8.56 | 8.58 | 8.79 | 0.85 | 0.335 | 0.5992 |
| TC1200008615.hg.1 | MYBPC1 | myosin binding protein C, slow type | Multiple_C | 3.16 | 3.21 | 2.87 | 1.22 | 0.1703 | 0.351 | 3.56 | 3.76 | 3.09 | 1.39 | 0.0129 | 0.0914 |
| TC1200008845.hg.1 | RAD9B | RAD9 checkpoint clamp component B | Multiple_C | 5.68 | 6.17 | 5.39 | 1.22 | 0.7091 | 0.8456 | 4.73 | 5.05 | 4.62 | 1.08 | 0.718 | 0.8685 |
| TC1200009141.hg.1 | ACADS | acyl-CoA dehydrogenase, C-2 to C-3 short chain | Multiple_C | 5.64 | 5.8 | 5.35 | 1.22 | 0.2986 | 0.5103 | 3.39 | 3.74 | 3.47 | 0.95 | 0.4931 | 0.7276 |
| TC1200009397.hg.1 | FZD10 | frizzled class receptor 10 | Coding | 4.89 | 4.69 | 4.6 | 1.22 | 0.3581 | 0.5717 | 6.6 | 8.45 | 8.33 | 0.30 | 5.51E-07 | 9.62E-05 |
| TC1200009749.hg.1 | PIANP | PILR alpha associated neural protein | Coding | 3.92 | 3.42 | 3.63 | 1.22 | 0.553 | 0.737 | 4.06 | 4.12 | 4.16 | 0.93 | 0.8698 | 0.9439 |
| TC1200010018.hg.1 | SMCO3 | single-pass membrane protein with coiled-coil domains 3 | Coding | 3.68 | 3.41 | 3.39 | 1.22 | 0.302 | 0.5144 | 3.86 | 3.85 | 3.79 | 1.05 | 0.8206 | 0.9209 |

| | | | | | | | | | | | | | | |
|---|---|---|---|---|---|---|---|---|---|---|---|---|---|---|
| TC1200010299.hg.1 | H3F3C | H3 histone, family 3C | Coding | 10.13 | 9.7 | 9.84 | 1.22 | 0.8262 | 0.9127 | 8.35 | 8.64 | 8.65 | 0.81 | 0.186 | 0.4378 |
| TC1200010764.hg.1 | KRT73 | keratin 73, type II | Multiple_C | 3.23 | 2.93 | 2.94 | 1.22 | 0.4547 | 0.6621 | 3.41 | 3.43 | 3.25 | 1.12 | 0.8436 | 0.9317 |
| TC1200010766.hg.1 | KRT2 | keratin 2, type II | Multiple_C | 4.09 | 3.42 | 3.8 | 1.22 | 0.3295 | 0.5433 | 3.86 | 3.96 | 3.85 | 1.01 | 0.8971 | 0.9566 |
| TC1200011177.hg.1 | IFNG | interferon, gamma | Multiple_C | 3.38 | 3.38 | 3.09 | 1.22 | 0.8792 | 0.9413 | 3.74 | 3.28 | 3.48 | 1.20 | 0.2466 | 0.5111 |
| TC1200012694.hg.1 | CLLU1 | chronic lymphocytic leukemia up-regulated 1 | Multiple_C | 3.04 | 2.59 | 2.75 | 1.22 | 0.8868 | 0.9453 | 3.91 | 3.86 | 3.6 | 1.24 | 0.1019 | 0.3124 |
| TC1200012700.hg.1 | C12orf75 | chromosome 12 open reading frame 75 | Multiple_C | 13.29 | 13.79 | 13 | 1.22 | 0.2719 | 0.4805 | 10.29 | 9.35 | 9.65 | 1.56 | 0.1748 | 0.4228 |
| TC1300007861.hg.1 | PCCA | propionyl-CoA carboxylase alpha subunit | Multiple_C | 8.09 | 9.83 | 7.8 | 1.22 | 0.7441 | 0.8677 | 7.2 | 7.01 | 7.06 | 1.10 | 0.4263 | 0.6786 |
| TC1300008635.hg.1 | CSNK1A1L | casein kinase 1, alpha 1-like | Coding | 6.1 | 5.66 | 5.81 | 1.22 | 0.4478 | 0.6559 | 5.12 | 5.45 | 5.33 | 0.86 | 0.5508 | 0.7666 |
| TC1300009994.hg.1 | ERCC5 | excision repair cross-complementation group 5 | Multiple_C | 12.62 | 13.47 | 12.33 | 1.22 | 0.3179 | 0.5313 | 12.49 | 13.06 | 11.94 | 1.46 | 0.0028 | 0.0341 |
| TC1400007259.hg.1 | PELI2 | pellino E3 ubiquitin protein ligase family member 2 | Multiple_C | 9.42 | 9.27 | 9.13 | 1.22 | 0.3033 | 0.5161 | 3.91 | 4.39 | 3.51 | 1.32 | 0.0582 | 0.2275 |
| TC1400009912.hg.1 | GALC | galactosylceramidase | Multiple_C | 6.57 | 6.22 | 6.28 | 1.22 | 0.2724 | 0.4809 | 5.9 | 6.08 | 5.72 | 1.13 | 0.222 | 0.4823 |
| TC1400009978.hg.1 | GPR68 | G protein-coupled receptor 68 | Multiple_C | 3.85 | 3.49 | 3.56 | 1.22 | 0.1199 | 0.2745 | 3.49 | 3.61 | 3.54 | 0.97 | 0.7937 | 0.9082 |
| TC1400010643.hg.1 | SERPINA5 | serpin peptidase inhibitor, clade A (alpha-1 antiproteinase, antit | Multiple_C | 3.47 | 3.15 | 3.18 | 1.22 | 0.5103 | 0.7063 | 3.43 | 3.23 | 3.24 | 1.14 | 0.3352 | 0.5994 |
| TC1400010702.hg.1 | TEX22 | testis expressed 22 | Multiple_C | 4.27 | 3.97 | 3.98 | 1.22 | 0.3679 | 0.5807 | 4.15 | 4.11 | 4 | 1.11 | 0.5031 | 0.7349 |
| TC1400010742.hg.1 | RPS29 | ribosomal protein S29 | Multiple_C | 3.91 | 3.8 | 3.62 | 1.22 | 0.277 | 0.486 | 3.55 | 3.53 | 3.77 | 0.86 | 0.8188 | 0.9199 |
| TC1500007855.hg.1 | CYP1A2 | cytochrome P450, family 1, subfamily A, polypeptide 2 | Coding | 3.77 | 3.67 | 3.48 | 1.22 | 0.5742 | 0.7532 | 4.72 | 4.57 | 4.63 | 1.06 | 0.7208 | 0.8698 |
| TC1600008712.hg.1 | IRF8 | interferon regulatory factor 8 | Multiple_C | 7.93 | 9 | 7.64 | 1.22 | 0.971 | 0.9861 | 3.32 | 3.36 | 3.35 | 0.98 | 0.8592 | 0.9398 |
| TC1600009417.hg.1 | ZC3H7A | zinc finger CCCH-type containing 7A | Multiple_C | 10.63 | 10.11 | 10.34 | 1.22 | 0.1706 | 0.3514 | 10.3 | 10.31 | 10.65 | 0.78 | 0.1241 | 0.3479 |
| TC1600010869.hg.1 | BCAR1 | breast cancer anti-estrogen resistance 1 | Multiple_C | 9.91 | 7.38 | 9.62 | 1.22 | 0.4453 | 0.6538 | 10.99 | 11.43 | 12.1 | 0.46 | 0.0457 | 0.198 |
| TC1600011452.hg.1 | MC1R | melanocortin 1 receptor (alpha melanocyte stimulating hormon | Multiple_C | 4.74 | 4.8 | 4.45 | 1.22 | 0.9276 | 0.966 | 4.31 | 4.57 | 4.21 | 1.07 | 0.8409 | 0.9305 |
| TC1700006710.hg.1 | FBXO39 | F-box protein 39 | Coding | 4.25 | 3.88 | 3.96 | 1.22 | 0.471 | 0.6748 | 4.18 | 4.09 | 3.8 | 1.30 | 0.0705 | 0.2538 |
| TC1700007119.hg.1 | EVPLL | envoplakin-like | Multiple_C | 5.17 | 4.82 | 4.88 | 1.22 | 0.742 | 0.8666 | 4.25 | 4.75 | 4.55 | 0.81 | 0.284 | 0.5495 |
| TC1700008499.hg.1 | BCAS3 | breast carcinoma amplified sequence 3 | Multiple_C | 5.64 | 5.51 | 5.35 | 1.22 | 0.1544 | 0.3285 | 5.97 | 5.33 | 5.41 | 1.47 | 0.2071 | 0.4638 |
| TC1700010564.hg.1 | STAC2 | SH3 and cysteine rich domain 2 | Multiple_C | 4.53 | 4.15 | 4.24 | 1.22 | 0.3345 | 0.5484 | 4.44 | 4.30 | 3.9 | 1.45 | 0.0561 | 0.2227 |
| TC1700012091.hg.1 | RFNG | RFNG O-fucosylpeptide 3-beta-N-acetylglucosaminyltransferase | Multiple_C | 7.04 | 6.41 | 6.75 | 1.22 | 0.3142 | 0.5276 | 7.38 | 7.36 | 7.47 | 0.94 | 0.4822 | 0.7202 |
| TC1700012405.hg.1 | KRTAP4-8 | keratin associated protein 4-8 | Multiple_C | 5.43 | 4.97 | 5.14 | 1.22 | 0.2914 | 0.5025 | 5.52 | 5.53 | 5.38 | 1.10 | 0.5534 | 0.7684 |
| TC1800006786.hg.1 | RNMT | RNA (guanine-7-) methyltransferase | Multiple_C | 8.96 | 9.37 | 8.67 | 1.22 | 0.2162 | 0.412 | 10.51 | 10.30 | 10.11 | 1.32 | 0.1403 | 0.3723 |
| TC1800007963.hg.1 | EPB41L3 | erythrocyte membrane protein band 4.1-like 3 | Multiple_C | 3.29 | 3.06 | 3 | 1.22 | 0.3137 | 0.5271 | 3.97 | 3.90 | 3.44 | 1.44 | 0.0455 | 0.1973 |
| TC1800008456.hg.1 | ZSCAN30 | zinc finger and SCAN domain containing 30 | Multiple_C | 4.33 | 3.82 | 4.04 | 1.22 | 0.482 | 0.6836 | 4.12 | 4.30 | 3.91 | 1.16 | 0.201 | 0.4564 |
| TC1900006662.hg.1 | NMRK2 | nicotinamide riboside kinase 2 | Multiple_C | 4.21 | 4 | 3.92 | 1.22 | 0.1589 | 0.335 | 4.45 | 4.28 | 4.28 | 1.13 | 0.5927 | 0.7939 |
| TC1900007090.hg.1 | BEST2 | bestrophin 2 | Coding | 5.7 | 5.39 | 5.41 | 1.22 | 0.4125 | 0.6246 | 5.83 | 5.54 | 5.34 | 1.40 | 0.491 | 0.726 |
| TC1900007783.hg.1 | CHST8 | carbohydrate (N-acetylgalactosamine 4-0) sulfotransferase 8 | Multiple_C | 6.69 | 6.15 | 6.4 | 1.22 | 0.0977 | 0.2366 | 5.65 | 5.85 | 5.72 | 0.95 | 0.623 | 0.8136 |
| TC1900007829.hg.1 | GRAMD1A | GRAM domain containing 1A | Multiple_C | 5.28 | 4.84 | 4.99 | 1.22 | 0.2464 | 0.4499 | 8.01 | 7.95 | 7.44 | 1.48 | 0.1517 | 0.3902 |

| | | | | | | | | | | | | | | | |
|---|---|---|---|---|---|---|---|---|---|---|---|---|---|---|---|
| TC1900008384.hg.1 | CALM3 | calmodulin 3 (phosphorylase kinase, delta) | Multiple_C | 13.67 | 12.8 | 13.38 | 1.22 | 0.2592 | 0.4657 | 12.75 | 13.37 | 13.52 | 0.59 | 0.0009 | 0.0155 |
| TC1900008479.hg.1 | SPACA4 | sperm acrosome associated 4 | Coding | 4.84 | 4.3 | 4.55 | 1.22 | 0.1338 | 0.2973 | 4.9 | 4.74 | 4.61 | 1.22 | 0.0816 | 0.2753 |
| TC1900008967.hg.1 | ZNF470 | zinc finger protein 470 | Multiple_C | 7.88 | 7.21 | 7.59 | 1.22 | 0.2904 | 0.5013 | 5.55 | 5.29 | 5.79 | 0.85 | 0.432 | 0.6836 |
| TC1900009299.hg.1 | CACTIN | cactin, spliceosome C complex subunit | Multiple_C | 9.7 | 9.18 | 9.41 | 1.22 | 0.4749 | 0.6778 | 9.62 | 9.91 | 9.84 | 0.86 | 0.0689 | 0.2499 |
| TC1900010633.hg.1 | CCER2 | coiled-coil glutamate rich protein 2 | Coding | 10.93 | 10.56 | 10.64 | 1.22 | 0.0764 | 0.1977 | 10.77 | 10.98 | 10.57 | 1.15 | 0.5025 | 0.7346 |
| TC1900010956.hg.1 | RSPH6A | radial spoke head 6 homolog A (Chlamydomonas) | Coding | 5.06 | 4.56 | 4.77 | 1.22 | 0.293 | 0.5042 | 4.47 | 4.40 | 4.43 | 1.03 | 0.7148 | 0.8668 |
| TC1900011706.hg.1 | TDRD12 | tudor domain containing 12 | Coding | 3.74 | 3.45 | 3.45 | 1.22 | 0.2989 | 0.5106 | 4.07 | 4.09 | 3.93 | 1.10 | 0.7966 | 0.9092 |
| TC1900011830.hg.1 | ZNF446 | zinc finger protein 446 | Multiple_C | 4.43 | 4.11 | 4.14 | 1.22 | 0.1005 | 0.2418 | 4.1 | 4.52 | 4.38 | 0.82 | 0.2163 | 0.4754 |
| TC2000008909.hg.1 | EIF2S2 | eukaryotic translation initiation factor 2, subunit 2 beta, 38kDa | Multiple_C | 15.4 | 15.96 | 15.11 | 1.22 | 0.4768 | 0.6794 | 14.18 | 13.61 | 14.01 | 1.13 | 0.6661 | 0.8365 |
| TC2000009987.hg.1 | OVOL2 | ovo-like zinc finger 2 | Multiple_C | 9.09 | 9 | 8.8 | 1.22 | 0.6188 | 0.7867 | 5.31 | 4.71 | 4.87 | 1.36 | 0.2599 | 0.5242 |
| TC2000010026.hg.1 | TMEM189 | transmembrane protein 189 | Multiple_C | 11.91 | 10.34 | 11.62 | 1.22 | 0.0954 | 0.2328 | 10.44 | 10.28 | 11.25 | 0.57 | 0.003 | 0.0353 |
| TC2200009290.hg.1 | FAM19A5 | family with sequence similarity 19 (chemokine (C-C motif)-like), | Multiple_C | 4.8 | 4.45 | 4.51 | 1.22 | 0.3052 | 0.5182 | 4.63 | 4.59 | 4.5 | 1.09 | 0.1834 | 0.4342 |
| TSUnmapped00000034.h | TRAPPC4 | trafficking protein particle complex 4 | Coding | 4.12 | 3.69 | 3.83 | 1.22 | 0.341 | 0.5549 | 3.67 | 4.11 | 4.17 | 0.71 | 0.0379 | 0.1774 |
| TSUnmapped00000381.h | NDUFA10 | NADH dehydrogenase (ubiquinone) 1 alpha subcomplex, 10, 42l | Coding | 3.96 | 3.84 | 3.67 | 1.22 | 0.1666 | 0.3459 | 4.5 | 4.37 | 4.72 | 0.86 | 0.4951 | 0.729 |
| TSUnmapped00000815.h | RPS6KA1 | ribosomal protein S6 kinase, 90kDa, polypeptide 1 | Coding | 3.72 | 3.43 | 3.43 | 1.22 | 0.4224 | 0.6337 | 3.92 | 4.10 | 4.07 | 0.90 | 0.4048 | 0.6603 |
| TC0100006912.hg.1 | PRAMEF7; PR/ | PRAME family member 7; PRAME family member 8 | Coding | 3.99 | 3.71 | 3.71 | 1.21 | 0.2191 | 0.4161 | 3.64 | 3.62 | 3.6 | 1.03 | 0.8376 | 0.9285 |
| TC0100007675.hg.1 | FAM167B | family with sequence similarity 167, member B | Coding | 4.23 | 3.81 | 3.95 | 1.21 | 0.0416 | 0.1263 | 3.7 | 3.90 | 3.92 | 0.86 | 0.176 | 0.4244 |
| TC0100007705.hg.1 | AZIN2 | antizyme inhibitor 2 | Multiple_C | 5.15 | 5.69 | 4.87 | 1.21 | 0.2735 | 0.4821 | 4.96 | 4.97 | 4.48 | 1.39 | 0.05 | 0.2083 |
| TC0100007748.hg.1 | GJA4 | gap junction protein alpha 4 | Coding | 4.28 | 3.93 | 4 | 1.21 | 0.4233 | 0.6345 | 4.25 | 4.19 | 4.13 | 1.09 | 0.3677 | 0.6301 |
| TC0100008055.hg.1 | TIE1 | tyrosine kinase with immunoglobulin-like and EGF-like domains | Multiple_C | 4.85 | 4.43 | 4.57 | 1.21 | 0.4123 | 0.6244 | 5.53 | 5.44 | 5.73 | 0.87 | 0.22 | 0.4801 |
| TC0100008306.hg.1 | BTF3L4 | basic transcription factor 3-like 4 | Multiple_C | 12.65 | 13.17 | 12.37 | 1.21 | 0.1757 | 0.3581 | 11.81 | 11.45 | 11.46 | 1.27 | 0.0303 | 0.1545 |
| TC0100010625.hg.1 | FASLG | Fas ligand (TNF superfamily, member 6) | Coding | 4.1 | 4.14 | 3.82 | 1.21 | 0.1492 | 0.3208 | 4.54 | 4.77 | 4.71 | 0.89 | 0.4913 | 0.7263 |
| TC0100012334.hg.1 | OR2L8 | olfactory receptor, family 2, subfamily L, member 8 (gene/pseu | Multiple_C | 2.94 | 2.58 | 2.66 | 1.21 | 0.7072 | 0.8444 | 3.28 | 3.20 | 3.09 | 1.14 | 0.2766 | 0.5427 |
| TC0100013264.hg.1 | LACTBL1 | lactamase, beta-like 1 | Coding | 5.18 | 4.83 | 4.9 | 1.21 | 0.6387 | 0.7996 | 5.49 | 5.49 | 5.1 | 1.31 | 0.0713 | 0.2556 |
| TC0100013823.hg.1 | COL9A2 | collagen, type IX, alpha 2 | Multiple_C | 3.86 | 3.5 | 3.58 | 1.21 | 0.0446 | 0.1331 | 3.44 | 3.46 | 3.34 | 1.07 | 0.5444 | 0.7625 |
| TC0100015058.hg.1 | SASS6 | SAS-6 centriolar assembly protein | Multiple_C | 9.78 | 8.85 | 9.5 | 1.21 | 0.4897 | 0.6897 | 9.85 | 9.18 | 10.05 | 0.87 | 0.3676 | 0.63 |
| TC0100015982.hg.1 | SSR2 | signal sequence receptor, beta (translocon-associated protein b | Multiple_C | 16 | 16.1 | 15.72 | 1.21 | 0.1006 | 0.242 | 15.12 | 15.44 | 15.42 | 0.81 | 0.2352 | 0.4979 |
| TC0100016068.hg.1 | OR10K2 | olfactory receptor, family 10, subfamily K, member 2 | Coding | 4.55 | 4.27 | 4.27 | 1.21 | 0.9073 | 0.9556 | 4.5 | 4.33 | 4.56 | 0.96 | 0.8516 | 0.9358 |
| TC0100017358.hg.1 | SLC30A10 | solute carrier family 30, member 10 | Multiple_C | 3.39 | 3.03 | 3.11 | 1.21 | 0.2498 | 0.454 | 3.82 | 3.81 | 3.62 | 1.15 | 0.0931 | 0.2961 |
| TC0100018229.hg.1 | CYP4B1 | cytochrome P450, family 4, subfamily B, polypeptide 1 | Multiple_C | 3.98 | 3.64 | 3.7 | 1.21 | 0.4596 | 0.6656 | 4.47 | 4.21 | 4.2 | 1.21 | 0.461 | 0.7039 |
| TC0200006643.hg.1 | ASAP2 | ArfGAP with SH3 domain, ankyrin repeat and PH domain 2 | Multiple_C | 8.91 | 9.19 | 8.63 | 1.21 | 0.4413 | 0.65 | 7.74 | 7.74 | 7.35 | 1.31 | 0.1416 | 0.3742 |
| TC0200006707.hg.1 | KCNF1 | potassium channel, voltage gated modifier subfamily F, membe | Coding | 4.87 | 4.52 | 4.59 | 1.21 | 0.24 | 0.442 | 4.08 | 4.44 | 4.33 | 0.84 | 0.1687 | 0.4145 |
| TC0200010438.hg.1 | NDUFB3 | NADH dehydrogenase (ubiquinone) 1 beta subcomplex, 3, 12kD | Coding | 12.14 | 13.08 | 11.86 | 1.21 | 0.4432 | 0.652 | 8.19 | 8.77 | 8.02 | 1.13 | 0.0782 | 0.2692 |

| | | | | | | | | | | | | | | | |
|---|---|---|---|---|---|---|---|---|---|---|---|---|---|---|---|
| TC0200011287.hg.1 | ASB1 | ankyrin repeat and SOCS box containing 1 | Multiple_C | 7.05 | 6.35 | 6.77 | 1.21 | 0.0505 | 0.1457 | 7.41 | 7.02 | 7.81 | 0.76 | 0.0499 | 0.2083 |
| TC0200011402.hg.1 | FARP2 | FERM, ARH/RhoGEF and pleckstrin domain protein 2 | Multiple_C | 9.75 | 8.9 | 9.47 | 1.21 | 0.4527 | 0.6603 | 8.07 | 8.63 | 9.18 | 0.46 | 0.0001 | 0.0039 |
| TC0200011503.hg.1 | PXDN | peroxidasin | Multiple_C | 5.36 | 4.89 | 5.08 | 1.21 | 0.1385 | 0.3044 | 5.94 | 6.22 | 5.87 | 1.05 | 0.4985 | 0.7314 |
| TC0200012727.hg.1 | PUS10 | pseudouridylate synthase 10 | Multiple_C | 5.25 | 5.46 | 4.97 | 1.21 | 0.2551 | 0.4608 | 5.67 | 5.50 | 5.65 | 1.01 | 0.633 | 0.8193 |
| TC0200014273.hg.1 | TISP43; LOC64 | uncharacterized LOC150527; uncharacterized LOC646743; Trans | Multiple_C | 4.18 | 3.72 | 3.9 | 1.21 | 0.556 | 0.7392 | 4.11 | 3.89 | 3.89 | 1.16 | 0.3042 | 0.5679 |
| TC0200014688.hg.1 | ERMN | ermin | Coding | 3.38 | 3.03 | 3.1 | 1.21 | 0.9829 | 0.992 | 4.02 | 3.60 | 3.75 | 1.21 | 0.2663 | 0.5309 |
| TC0200015364.hg.1 | SATB2 | SATB homeobox 2 | Multiple_C | 10.92 | 10.3 | 10.64 | 1.21 | 0.4869 | 0.6881 | 6.4 | 6.67 | 7.06 | 0.63 | 0.0192 | 0.117 |
| TC0200015368.hg.1 | FTCDNL1 | formiminotransferase cyclodeaminase N-terminal like | Multiple_C | 5 | 4.79 | 4.72 | 1.21 | 0.0727 | 0.1904 | 4.63 | 4.60 | 4.71 | 0.95 | 0.7561 | 0.889 |
| TC0300006894.hg.1 | CMC1 | C-x(9)-C motif containing 1 | Multiple_C | 8.62 | 9.84 | 8.34 | 1.21 | 0.6183 | 0.7862 | 6.35 | 6.39 | 6.7 | 0.78 | 0.2296 | 0.4906 |
| TC0300006925.hg.1 | TGFBR2 | transforming growth factor beta receptor II | Multiple_C | 12.62 | 12.14 | 12.34 | 1.21 | 0.2092 | 0.4029 | 9.22 | 10.67 | 11.74 | 0.17 | 4.35E-12 | 1.42E-08 |
| TC0300008412.hg.1 | GAP43 | growth associated protein 43 | Coding | 4.39 | 3.66 | 4.11 | 1.21 | 0.7324 | 0.8603 | 6.26 | 5.97 | 6.21 | 1.04 | 0.4393 | 0.6896 |
| TC0300008539.hg.1 | SLC15A2 | solute carrier family 15 (oligopeptide transporter), member 2 | Multiple_C | 3.86 | 3.6 | 3.58 | 1.21 | 0.0472 | 0.1386 | 3.83 | 3.90 | 3.97 | 0.91 | 0.8499 | 0.9351 |
| TC0300008964.hg.1 | MRPS22 | mitochondrial ribosomal protein S22 | Multiple_C | 12.6 | 13.67 | 12.32 | 1.21 | 0.7192 | 0.852 | 10.77 | 10.95 | 10.64 | 1.09 | 0.2889 | 0.5536 |
| TC0300010709.hg.1 | DCLK3 | doublecortin-like kinase 3 | Multiple_C | 5.05 | 4.83 | 4.77 | 1.21 | 0.4011 | 0.6143 | 4.41 | 4.31 | 4.29 | 1.09 | 0.3214 | 0.5853 |
| TC0300011124.hg.1 | CISH | cytokine inducible SH2-containing protein | Multiple_C | 4.22 | 3.89 | 3.94 | 1.21 | 0.2866 | 0.4971 | 3.68 | 4.01 | 4.5 | 0.57 | 0.001 | 0.0168 |
| TC0300011362.hg.1 | FEZF2 | FEZ family zinc finger 2 | Coding | 4.11 | 3.57 | 3.83 | 1.21 | 0.685 | 0.8297 | 4.93 | 4.56 | 4.4 | 1.44 | 0.034 | 0.1659 |
| TC0300012206.hg.1 | ADCY5 | adenylate cyclase 5 | Multiple_C | 4.58 | 4.36 | 4.3 | 1.21 | 0.7169 | 0.8503 | 5.32 | 5.33 | 5.23 | 1.06 | 0.9859 | 0.9943 |
| TC0300012564.hg.1 | DZIP1L | DAZ interacting zinc finger protein 1-like | Multiple_C | 4.52 | 4.14 | 4.24 | 1.21 | 0.5616 | 0.7439 | 4.13 | 4.48 | 3.89 | 1.18 | 0.5158 | 0.7446 |
| TC0300013938.hg.1 | VGLL4 | vestigial-like family member 4 | Multiple_C | 9.48 | 8.9 | 9.2 | 1.21 | 0.1648 | 0.3432 | 11.41 | 11.42 | 11.15 | 1.20 | 0.0913 | 0.2924 |
| TC0400008106.hg.1 | HERC5 | HECT and RLD domain containing E3 ubiquitin protein ligase 5 | Multiple_C | 2.87 | 2.48 | 2.59 | 1.21 | 0.5672 | 0.7479 | 4.05 | 4.18 | 3.35 | 1.62 | 0.0577 | 0.2263 |
| TC0400010895.hg.1 | TMPRSS11D | transmembrane protease, serine 11D | Multiple_C | 3.32 | 3.46 | 3.04 | 1.21 | 0.7424 | 0.8667 | 4.47 | 4.30 | 4.25 | 1.16 | 0.2327 | 0.4947 |
| TC0400011056.hg.1 | NUP54 | nucleoporin 54kDa | Multiple_C | 11.61 | 11.84 | 11.33 | 1.21 | 0.3429 | 0.5568 | 9.87 | 9.39 | 10.01 | 0.91 | 0.1452 | 0.3802 |
| TC0400011298.hg.1 | NAP1L5 | nucleosome assembly protein 1-like 5 | Coding | 5.76 | 6.23 | 5.48 | 1.21 | 0.3775 | 0.5905 | 3.49 | 3.56 | 3.14 | 1.27 | 0.4249 | 0.6777 |
| TC0500008588.hg.1 | KIAA1024L | KIAA1024-like | Multiple_C | 2.87 | 2.75 | 2.59 | 1.21 | 0.4002 | 0.6137 | 3.02 | 3.04 | 3.25 | 0.85 | 0.5377 | 0.7589 |
| TC0500009058.hg.1 | AFAP1L1 | actin filament associated protein 1-like 1 | Multiple_C | 4.03 | 3.88 | 3.75 | 1.21 | 0.2121 | 0.4069 | 4 | 4.04 | 3.83 | 1.13 | 0.2772 | 0.5431 |
| TC0500010822.hg.1 | ACTBL2 | actin, beta-like 2 | Multiple_C | 3.78 | 3.7 | 3.5 | 1.21 | 0.3492 | 0.5619 | 3.51 | 3.79 | 3.58 | 0.95 | 0.565 | 0.7747 |
| TC0500012378.hg.1 | GPR151 | G protein-coupled receptor 151 | Coding | 3.87 | 3.47 | 3.59 | 1.21 | 0.4648 | 0.6696 | 3.81 | 3.77 | 3.59 | 1.16 | 0.3724 | 0.6336 |
| TC0500013058.hg.1 | C5orf60 | chromosome 5 open reading frame 60 | Multiple_C | 3.21 | 2.52 | 2.93 | 1.21 | 0.5165 | 0.7109 | 4.01 | 3.75 | 3.69 | 1.25 | 0.0719 | 0.2567 |
| TC0600007463.hg.1 | OR2H1 | olfactory receptor, family 2, subfamily H, member 1 | Multiple_C | 3.51 | 3.33 | 3.23 | 1.21 | 0.2299 | 0.4299 | 3.8 | 4.11 | 3.63 | 1.13 | 0.1093 | 0.3255 |
| TC0600008132.hg.1 | CDC5L | cell division cycle 5-like | Multiple_C | 11.96 | 12.09 | 11.68 | 1.21 | 0.1206 | 0.2756 | 11.72 | 11.69 | 11.61 | 1.08 | 0.5119 | 0.7416 |
| TC0600008527.hg.1 | DDX43 | DEAD (Asp-Glu-Ala-Asp) box polypeptide 43 | Multiple_C | 3.11 | 2.99 | 2.83 | 1.21 | 0.3092 | 0.5224 | 3.25 | 3.17 | 3.27 | 0.99 | 0.4923 | 0.727 |
| TC0600009800.hg.1 | GINM1 | glycoprotein integral membrane 1 | Multiple_C | 11 | 11.68 | 10.72 | 1.21 | 0.9357 | 0.97 | 10.28 | 10.23 | 10.47 | 0.88 | 0.4331 | 0.6841 |
| TC0600011491.hg.1 | HLA-DRB5 | major histocompatibility complex, class II, DR beta 5 | Multiple_C | 4.23 | 4.02 | 3.95 | 1.21 | 0.5096 | 0.7056 | 4.01 | 4.38 | 4.11 | 0.93 | 0.6112 | 0.8057 |

| | | | | | | | | | | | | | | | |
|---|---|---|---|---|---|---|---|---|---|---|---|---|---|---|---|
| TC0600012379.hg.1 | ELOVL4 | ELOVL fatty acid elongase 4 | Multiple_C | 2.53 | 2.29 | 2.25 | 1.21 | 0.9305 | 0.9672 | 3.1 | 3.04 | 2.92 | 1.13 | 0.0485 | 0.2047 |
| TC0600013272.hg.1 | BCLAF1 | BCL2-associated transcription factor 1 | Multiple_C | 12.41 | 11.82 | 12.13 | 1.21 | 0.2451 | 0.4486 | 12.54 | 13.10 | 13.08 | 0.69 | 0.0251 | 0.1376 |
| TC0600014217.hg.1 | SERPINB1 | serpin peptidase inhibitor, clade B (ovalbumin), member 1 | Multiple_C | 13.13 | 14.52 | 12.85 | 1.21 | 0.7438 | 0.8675 | 8.8 | 9.43 | 10.01 | 0.43 | 0.0504 | 0.209 |
| TC0600014271.hg.1 | NOTCH4 | notch 4 | Multiple_C | 6.58 | 6.14 | 6.3 | 1.21 | 0.3474 | 0.5605 | 6.01 | 6.52 | 6.42 | 0.75 | 0.3918 | 0.6502 |
| TC0600014370.hg.1 | RPS6KA2 | ribosomal protein S6 kinase, 90kDa, polypeptide 2 | Multiple_C | 4.95 | 5.03 | 4.67 | 1.21 | 0.3914 | 0.6052 | 4.95 | 4.92 | 4.9 | 1.04 | 0.2005 | 0.4558 |
| TC0700007984.hg.1 | VPS37D | vacuolar protein sorting 37 homolog D (S. cerevisiae) | Multiple_C | 6.75 | 6.53 | 6.47 | 1.21 | 0.4578 | 0.6639 | 5.6 | 5.69 | 5.63 | 0.98 | 0.6161 | 0.8096 |
| TC0700009134.hg.1 | CPA4 | carboxypeptidase A4 | Multiple_C | 3.82 | 3.39 | 3.54 | 1.21 | 0.1474 | 0.3183 | 4.76 | 4.65 | 4.42 | 1.27 | 0.2285 | 0.4894 |
| TC0700009513.hg.1 | OR2F2 | olfactory receptor, family 2, subfamily F, member 2 | Coding | 3.04 | 2.66 | 2.76 | 1.21 | 0.9947 | 0.9974 | 3.41 | 3.72 | 3.5 | 0.94 | 0.7004 | 0.858 |
| TC0700010141.hg.1 | MMD2 | monocyte to macrophage differentiation-associated 2 | Multiple_C | 2.93 | 2.45 | 2.65 | 1.21 | 0.1458 | 0.3159 | 3.44 | 3.40 | 3.16 | 1.21 | 0.083 | 0.2774 |
| TC0700010286.hg.1 | VWDE | von Willebrand factor D and EGF domains | Multiple_C | 3.97 | 3.73 | 3.69 | 1.21 | 0.3284 | 0.5422 | 7.83 | 7.40 | 7.08 | 1.68 | 0.0258 | 0.14 |
| TC0700011724.hg.1 | C7orf62 | chromosome 7 open reading frame 62 | Coding | 3.88 | 3.45 | 3.6 | 1.21 | 0.1283 | 0.2884 | 4.39 | 4.32 | 3.98 | 1.33 | 0.0178 | 0.1119 |
| TC0700012886.hg.1 | EPHA1 | EPH receptor A1 | Multiple_C | 7.37 | 7.04 | 7.09 | 1.21 | 0.3367 | 0.5507 | 4.03 | 3.82 | 3.99 | 1.03 | 0.6985 | 0.8571 |
| TC0700013371.hg.1 | ZNF138 | zinc finger protein 138 | Multiple_C | 9.64 | 9.65 | 9.36 | 1.21 | 0.4214 | 0.6325 | 7.93 | 7.59 | 8.28 | 0.78 | 0.3688 | 0.6308 |
| TC0800006439.hg.1 | ZNF596 | zinc finger protein 596 | Multiple_C | 6.49 | 6.7 | 6.21 | 1.21 | 0.2654 | 0.473 | 5.66 | 6.29 | 5.93 | 0.83 | 0.2909 | 0.5557 |
| TC0800009309.hg.1 | OR4F21 | olfactory receptor, family 4, subfamily F, member 21 | Coding | 4.22 | 3.92 | 3.94 | 1.21 | 0.6147 | 0.7842 | 3.93 | 3.81 | 3.71 | 1.16 | 0.3815 | 0.6408 |
| TC0800010379.hg.1 | EFCAB1 | EF-hand calcium binding domain 1 | Multiple_C | 4.54 | 4.28 | 4.26 | 1.21 | 0.1124 | 0.2625 | 3.88 | 4.00 | 3.79 | 1.06 | 0.6778 | 0.8449 |
| TC0800012416.hg.1 | PENK | proenkephalin | Multiple_C | 5.72 | 5.42 | 5.44 | 1.21 | 0.4722 | 0.6757 | 5.3 | 5.45 | 5.37 | 0.95 | 0.7064 | 0.8618 |
| TC0900007827.hg.1 | SPATA31E1 | SPATA31 subfamily E, member 1 | Coding | 3.87 | 3.7 | 3.59 | 1.21 | 0.0874 | 0.2182 | 4.08 | 4.13 | 4.04 | 1.03 | 0.8979 | 0.9571 |
| TC0900008817.hg.1 | TTC16 | tetratricopeptide repeat domain 16 | Multiple_C | 3.5 | 3.22 | 3.22 | 1.21 | 0.1179 | 0.2713 | 3.86 | 3.71 | 3.6 | 1.20 | 0.2381 | 0.5015 |
| TC0900011236.hg.1 | INIP | INTS3 and NABP interacting protein | Multiple_C | 9.73 | 9.78 | 9.45 | 1.21 | 0.1956 | 0.3856 | 10.38 | 9.99 | 10.97 | 0.66 | 0.0263 | 0.1417 |
| TC0900012185.hg.1 | CEL | carboxyl ester lipase | Multiple_C | 5.07 | 4.55 | 4.79 | 1.21 | 0.5321 | 0.722 | 5.32 | 5.15 | 5.06 | 1.20 | 0.3711 | 0.633 |
| TC0900012193.hg.1 | MAMDC4 | MAM domain containing 4 | Multiple_C | 4.73 | 4.23 | 4.45 | 1.21 | 0.612 | 0.7821 | 5.02 | 4.83 | 4.61 | 1.33 | 0.152 | 0.3906 |
| TC0X00007696.hg.1 | FGF16 | fibroblast growth factor 16 | Coding | 3.68 | 3.09 | 3.4 | 1.21 | 0.3264 | 0.5404 | 4.28 | 4.38 | 4.21 | 1.05 | 0.8184 | 0.9198 |
| TC0X00009271.hg.1 | ARX | aristaless related homeobox | Coding | 4.89 | 4.44 | 4.61 | 1.21 | 0.6022 | 0.7743 | 4.82 | 4.84 | 4.81 | 1.01 | 0.7801 | 0.9013 |
| TC0X00009836.hg.1 | SPIN3 | spindlin family, member 3 | Multiple_C | 5.85 | 5.46 | 5.57 | 1.21 | 0.3093 | 0.5224 | 7.29 | 7.72 | 7.83 | 0.69 | 0.0048 | 0.0479 |
| TC0Y00007321.hg.1 | ASMT | Homo sapiens acetylserotonin O-methyltransferase (ASMT), tra | Coding | 4.06 | 3.65 | 3.78 | 1.21 | 0.4967 | 0.6966 | 3.92 | 3.74 | 3.91 | 1.01 | 0.7018 | 0.8588 |
| TC1000007127.hg.1 | WAC | WW domain containing adaptor with coiled-coil | Multiple_C | 14.42 | 14 | 14.14 | 1.21 | 0.2835 | 0.4936 | 15.07 | 14.48 | 15.15 | 0.95 | 0.8231 | 0.9219 |
| TC1000008233.hg.1 | DYDC2 | DPY30 domain containing 2 | Multiple_C | 4.39 | 4.41 | 4.11 | 1.21 | 0.3364 | 0.5503 | 4.26 | 4.11 | 3.77 | 1.40 | 0.048 | 0.2033 |
| TC1100007889.hg.1 | SLC22A10 | solute carrier family 22, member 10 | Multiple_C | 4.08 | 3.9 | 3.8 | 1.21 | 0.778 | 0.8859 | 4.96 | 4.34 | 4.29 | 1.59 | 0.1208 | 0.3429 |
| TC1100008050.hg.1 | CST6 | cystatin E/M | Coding | 4.31 | 3.74 | 4.03 | 1.21 | 0.9407 | 0.9724 | 3.76 | 3.67 | 3.71 | 1.04 | 0.7209 | 0.8698 |
| TC1100008469.hg.1 | SERPINH1 | serpin peptidase inhibitor, clade H (heat shock protein 47), men | Multiple_C | 11.22 | 10.36 | 10.94 | 1.21 | 0.8576 | 0.9305 | 11.19 | 11.92 | 12.25 | 0.48 | 0.2082 | 0.4651 |
| TC1100009166.hg.1 | PAFAH1B2 | platelet-activating factor acetylhydrolase 1b, catalytic subunit 2 | Multiple_C | 14.01 | 14.23 | 13.73 | 1.21 | 0.398 | 0.6115 | 15 | 14.82 | 14.71 | 1.22 | 0.7524 | 0.8874 |
| TC1100009434.hg.1 | DDX25 | DEAD (Asp-Glu-Ala-Asp) box helicase 25 | Multiple_C | 5.01 | 4.87 | 4.73 | 1.21 | 0.1673 | 0.3468 | 4.7 | 4.80 | 4.65 | 1.04 | 0.6736 | 0.8419 |

| | | | | | | | | | | | | | | | |
|---|---|---|---|---|---|---|---|---|---|---|---|---|---|---|---|
| TC1100009906.hg.1 | OR52E2 | olfactory receptor, family 52, subfamily E, member 2 | Coding | 3.66 | 3.23 | 3.38 | 1.21 | 0.0751 | 0.195 | 3.95 | 3.85 | 3.61 | 1.27 | 0.0862 | 0.283 |
| TC1100010844.hg.1 | OR8J3 | olfactory receptor, family 8, subfamily J, member 3 | Coding | 3.17 | 3.07 | 2.89 | 1.21 | 0.394 | 0.6079 | 3.61 | 3.66 | 3.76 | 0.90 | 0.6061 | 0.8029 |
| TC1100011587.hg.1 | FAM168A | family with sequence similarity 168, member A | Multiple_C | 4.18 | 3.49 | 3.9 | 1.21 | 0.8183 | 0.9086 | 5.85 | 6.15 | 6.15 | 0.81 | 0.2415 | 0.5056 |
| TC1100011895.hg.1 | GRM5 | glutamate receptor, metabotropic 5 | Multiple_C | 2.92 | 2.63 | 2.64 | 1.21 | 0.3057 | 0.5188 | 3.33 | 2.90 | 3.13 | 1.15 | 0.2124 | 0.4702 |
| TC1100012666.hg.1 | OR8B2 | olfactory receptor, family 8, subfamily B, member 2 | Coding | 2.93 | 2.61 | 2.65 | 1.21 | 0.4133 | 0.6254 | 3.8 | 3.53 | 3.45 | 1.27 | 0.8699 | 0.944 |
| TC1200007536.hg.1 | CCDC65 | coiled-coil domain containing 65 | Multiple_C | 3.46 | 3.16 | 3.18 | 1.21 | 0.4461 | 0.6546 | 4.13 | 3.89 | 3.64 | 1.40 | 0.264 | 0.5288 |
| TC1200007737.hg.1 | HOXC11 | homeobox C11 | Multiple_C | 4.17 | 4.04 | 3.89 | 1.21 | 0.8388 | 0.9197 | 4.04 | 3.78 | 3.47 | 1.48 | 0.048 | 0.2033 |
| TC1200007789.hg.1 | OR6C75 | olfactory receptor, family 6, subfamily C, member 75 | Coding | 3.19 | 3.01 | 2.91 | 1.21 | 0.239 | 0.4409 | 3.69 | 3.74 | 3.81 | 0.92 | 0.7402 | 0.8807 |
| TC1200008133.hg.1 | LYZ | lysozyme | Multiple_C | 16.96 | 15.78 | 16.68 | 1.21 | 0.3598 | 0.5732 | 5.69 | 5.10 | 5.05 | 1.56 | 0.0026 | 0.032 |
| TC1200008299.hg.1 | MYF5 | myogenic factor 5 | Coding | 3.87 | 3.35 | 3.59 | 1.21 | 0.5593 | 0.742 | 4.04 | 4.13 | 3.93 | 1.08 | 0.7751 | 0.899 |
| TC1200009464.hg.1 | EP400NL | EP400 N-terminal like | Multiple_C | 6.08 | 6.09 | 5.8 | 1.21 | 0.0652 | 0.1762 | 5.38 | 5.68 | 5.51 | 0.91 | 0.5693 | 0.7779 |
| TC1200010699.hg.1 | POU6F1 | POU class 6 homeobox 1 | Multiple_C | 3.77 | 3.38 | 3.49 | 1.21 | 0.3214 | 0.535 | 4.27 | 4.66 | 4.69 | 0.75 | 0.037 | 0.1751 |
| TC1200010835.hg.1 | NFE2 | nuclear factor, erythroid 2 | Multiple_C | 3.08 | 2.67 | 2.8 | 1.21 | 0.1632 | 0.3413 | 3.6 | 4.62 | 4.06 | 0.73 | 0.0333 | 0.1641 |
| TC1200012739.hg.1 | PARP11 | poly(ADP-ribose) polymerase family member 11 | Multiple_C | 6.51 | 7.15 | 6.23 | 1.21 | 0.1333 | 0.2967 | 8.1 | 8.37 | 8.52 | 0.75 | 0.0424 | 0.1901 |
| TC1400006522.hg.1 | RNASE10 | ribonuclease, RNase A family, 10 (non-active) | Coding | 2.93 | 2.68 | 2.65 | 1.21 | 0.7012 | 0.8404 | 3.58 | 3.66 | 3.39 | 1.14 | 0.2697 | 0.5347 |
| TC1400008546.hg.1 | LINC01296; DI | long intergenic non-protein coding RNA 1296; double homeobo | Multiple_C | 5.38 | 5.18 | 5.1 | 1.21 | 0.4865 | 0.6878 | 9.02 | 9.57 | 9.38 | 0.78 | 0.9635 | 0.9846 |
| TC1400008764.hg.1 | GZMB | granzyme B | Coding | 6.47 | 5.99 | 6.19 | 1.21 | 0.1838 | 0.3694 | 6.95 | 7.29 | 6.91 | 1.03 | 0.6047 | 0.8023 |
| TC1400009100.hg.1 | NEMF | nuclear export mediator factor | Multiple_C | 9.13 | 9.47 | 8.85 | 1.21 | 0.6989 | 0.8388 | 7.16 | 7.55 | 7.88 | 0.61 | 0.1631 | 0.4067 |
| TC1400009299.hg.1 | C14orf105 | chromosome 14 open reading frame 105 | Multiple_C | 3.44 | 3.19 | 3.16 | 1.21 | 0.4206 | 0.6319 | 4 | 4.58 | 5.17 | 0.44 | 1.51E-06 | 0.0002 |
| TC1400010690.hg.1 | AMN | amnion associated transmembrane protein | Multiple_C | 8.95 | 8.69 | 8.67 | 1.21 | 0.5153 | 0.71 | 5.93 | 6.02 | 5.88 | 1.04 | 0.9593 | 0.9834 |
| TC1500007396.hg.1 | LDHAL6B | lactate dehydrogenase A-like 6B | Coding | 3.74 | 3.62 | 3.46 | 1.21 | 0.1775 | 0.3602 | 3.76 | 3.82 | 4.09 | 0.80 | 0.1801 | 0.4294 |
| TC1500008829.hg.1 | FAM189A1 | family with sequence similarity 189, member A1 | Multiple_C | 3.76 | 3.51 | 3.48 | 1.21 | 0.1632 | 0.3412 | 3.86 | 3.77 | 3.84 | 1.01 | 0.9759 | 0.9902 |
| TC1500009795.hg.1 | IGDCC3 | immunoglobulin superfamily, DCC subclass, member 3 | Multiple_C | 2.95 | 2.61 | 2.67 | 1.21 | 0.2726 | 0.4812 | 5.35 | 5.11 | 4.66 | 1.61 | 0.176 | 0.4244 |
| TC1500010134.hg.1 | CIB2 | calcium and integrin binding family member 2 | Multiple_C | 4.31 | 4.11 | 4.03 | 1.21 | 0.1572 | 0.3328 | 3.83 | 3.76 | 3.73 | 1.07 | 0.2473 | 0.512 |
| TC1500010871.hg.1 | ZNF280D | zinc finger protein 280D | Multiple_C | 10.68 | 11.27 | 10.4 | 1.21 | 0.1689 | 0.3488 | 9.81 | 8.99 | 9.43 | 1.30 | 0.1195 | 0.3416 |
| TC1600008568.hg.1 | ATMIN | ATM interactor | Multiple_C | 11.81 | 11.31 | 11.53 | 1.21 | 0.1107 | 0.2595 | 13.44 | 13.87 | 13.55 | 0.93 | 0.0613 | 0.235 |
| TC1600009198.hg.1 | NLRC3 | NLR family, CARD domain containing 3 | Multiple_C | 5 | 4.6 | 4.72 | 1.21 | 0.161 | 0.3378 | 5.37 | 5.18 | 5.45 | 0.95 | 0.6791 | 0.8455 |
| TC1600011221.hg.1 | MVD | mevalonate (diphospho) decarboxylase | Multiple_C | 11.28 | 12.18 | 11 | 1.21 | 0.1465 | 0.3168 | 6.65 | 6.57 | 6.51 | 1.10 | 0.6423 | 0.8246 |
| TC1600011320.hg.1 | WFIKKN1 | WAP, follistatin/kazal, immunoglobulin, kunitz and netrin domai | Coding | 6.01 | 5.63 | 5.73 | 1.21 | 0.0964 | 0.2345 | 5.93 | 6.31 | 6.46 | 0.69 | 0.0336 | 0.165 |
| TC1700006773.hg.1 | TMEM88 | transmembrane protein 88 | Coding | 4.76 | 4.34 | 4.48 | 1.21 | 0.1735 | 0.3554 | 5.13 | 5.37 | 5.28 | 0.90 | 0.4003 | 0.6572 |
| TC1700007883.hg.1 | STAT5A | signal transducer and activator of transcription 5A | Multiple_C | 9.22 | 9.11 | 8.94 | 1.21 | 0.7345 | 0.8613 | 7.83 | 8.04 | 8 | 0.89 | 0.6595 | 0.834 |
| TC1700008372.hg.1 | SCPEP1 | serine carboxypeptidase 1 | Multiple_C | 10.2 | 10.54 | 9.92 | 1.21 | 0.7413 | 0.8663 | 8.86 | 8.85 | 8.91 | 0.97 | 0.7857 | 0.9042 |
| TC1700008951.hg.1 | PRCD | progressive rod-cone degeneration | Multiple_C | 5.56 | 4.9 | 5.28 | 1.21 | 0.476 | 0.6787 | 4.88 | 4.67 | 4.78 | 1.07 | 0.9234 | 0.9685 |

| | | | | | | | | | | | | | | | |
|---|---|---|---|---|---|---|---|---|---|---|---|---|---|---|---|
| TC1700009032.hg.1 | TNRC6C | trinucleotide repeat containing 6C | Multiple_C | 4.81 | 4.51 | 4.53 | 1.21 | 0.3037 | 0.5166 | 6.63 | 6.92 | 6.98 | 0.78 | 0.2588 | 0.5231 |
| TC1700009743.hg.1 | MYH4 | myosin, heavy chain 4, skeletal muscle | Coding | 3.51 | 2.98 | 3.23 | 1.21 | 0.1871 | 0.3742 | 3.93 | 3.95 | 3.67 | 1.20 | 0.0735 | 0.2599 |
| TC1700010735.hg.1 | CCR10 | chemokine (C-C motif) receptor 10 | Coding | 4.16 | 4.1 | 3.88 | 1.21 | 0.2386 | 0.4403 | 3.91 | 4.14 | 3.83 | 1.06 | 0.9066 | 0.9614 |
| TC1700011257.hg.1 | HSF5 | heat shock transcription factor family member 5 | Coding | 3.16 | 2.94 | 2.88 | 1.21 | 0.1516 | 0.3246 | 2.9 | 3.25 | 3.02 | 0.92 | 0.6501 | 0.8291 |
| TC1800007340.hg.1 | DCC | DCC netrin 1 receptor | Multiple_C | 4.63 | 4.68 | 4.35 | 1.21 | 0.1617 | 0.3388 | 4.02 | 3.32 | 3.41 | 1.53 | 0.03 | 0.1535 |
| TC1800007911.hg.1 | MYOM1 | myomesin 1 | Multiple_C | 4.47 | 4.45 | 4.19 | 1.21 | 0.3027 | 0.5153 | 4.12 | 4.23 | 4.15 | 0.98 | 0.2403 | 0.5039 |
| TC1800009214.hg.1 | NDUFV2 | NADH dehydrogenase (ubiquinone) flavoprotein 2, 24kDa | Multiple_C | 10.72 | 11.18 | 10.44 | 1.21 | 0.8459 | 0.9243 | 9.35 | 10.28 | 9.38 | 0.98 | 0.8121 | 0.9166 |
| TC1800009281.hg.1 | IER3IP1 | immediate early response 3 interacting protein 1 | Multiple_C | 9.8 | 10.56 | 9.52 | 1.21 | 0.5662 | 0.7473 | 6.19 | 6.64 | 6.41 | 0.86 | 0.485 | 0.7222 |
| TC1900007294.hg.1 | NWD1 | NACHT and WD repeat domain containing 1 | Multiple_C | 2.65 | 2.66 | 2.37 | 1.21 | 0.2571 | 0.4633 | 3.19 | 2.86 | 2.8 | 1.31 | 0.0784 | 0.2697 |
| TC1900007300.hg.1 | F2RL3 | coagulation factor II (thrombin) receptor-like 3 | Multiple_C | 9.88 | 9.57 | 9.6 | 1.21 | 0.5406 | 0.7282 | 9.37 | 9.60 | 9.57 | 0.87 | 0.1436 | 0.3774 |
| TC1900011920.hg.1 | ZNF98 | zinc finger protein 98 | Multiple_C | 8.79 | 7.88 | 8.51 | 1.21 | 0.4118 | 0.624 | 7.4 | 7.03 | 7.62 | 0.86 | 0.376 | 0.6364 |
| TC1900011970.hg.1 | LYPD5 | LY6/PLAUR domain containing 5 | Multiple_C | 4.24 | 3.5 | 3.96 | 1.21 | 0.6106 | 0.7808 | 3.19 | 3.21 | 3.24 | 0.97 | 0.6985 | 0.8571 |
| TC2000007514.hg.1 | MMP9 | matrix metallopeptidase 9 | Multiple_C | 4.03 | 3.43 | 3.75 | 1.21 | 0.3038 | 0.5166 | 4.31 | 4.60 | 4.2 | 1.08 | 0.706 | 0.8617 |
| TC2000008146.hg.1 | RSPO4 | R-spondin 4 | Multiple_C | 6.46 | 5.86 | 6.18 | 1.21 | 0.2856 | 0.496 | 5.18 | 5.33 | 5.32 | 0.91 | 0.5469 | 0.7641 |
| TC2000008295.hg.1 | GPCPD1 | glycerophosphocholine phosphodiesterase 1 | Multiple_C | 8.44 | 8.66 | 8.16 | 1.21 | 0.1985 | 0.3895 | 6.02 | 5.89 | 6.47 | 0.73 | 0.3521 | 0.6154 |
| TC2000009961.hg.1 | SCRT2 | scratch family zinc finger 2 | Coding | 4.72 | 4.23 | 4.44 | 1.21 | 0.3144 | 0.5278 | 5.07 | 5.16 | 5.11 | 0.97 | 0.6827 | 0.8477 |
| TC2100007354.hg.1 | AIRE | autoimmune regulator | Multiple_C | 5.59 | 5.26 | 5.31 | 1.21 | 0.3399 | 0.5538 | 7.05 | 7.44 | 7.32 | 0.83 | 0.0412 | 0.1865 |
| TC2200007085.hg.1 | RNF185 | ring finger protein 185 | Multiple_C | 8.52 | 8.45 | 8.24 | 1.21 | 0.486 | 0.6874 | 8.16 | 8.81 | 8.83 | 0.63 | 0.0017 | 0.0237 |
| TC2200008427.hg.1 | OSM | oncostatin M | Coding | 4.94 | 4.53 | 4.66 | 1.21 | 0.4214 | 0.6325 | 5.07 | 5.09 | 5.11 | 0.97 | 0.8653 | 0.9424 |
| TC2200009028.hg.1 | CERK | ceramide kinase | Multiple_C | 7.63 | 7.56 | 7.35 | 1.21 | 0.2808 | 0.4908 | 10.77 | 10.62 | 10.52 | 1.19 | 0.6318 | 0.8187 |
| TSUnmapped00000171.h | TRAPPC4 | trafficking protein particle complex 4 | Coding | 6.32 | 6.2 | 6.04 | 1.21 | 0.2136 | 0.4089 | 5.4 | 5.03 | 5.56 | 0.90 | 0.1721 | 0.419 |
| TSUnmapped00000822.h | DUX3 | double homeobox 3 | Coding | 3.99 | 3.9 | 3.71 | 1.21 | 0.3701 | 0.5828 | 4.45 | 4.57 | 4.41 | 1.03 | 0.3013 | 0.5656 |
| TC0100010510.hg.1 | RCSD1 | RCSD domain containing 1 | Multiple_C | 4.22 | 3.83 | 3.95 | 1.21 | 0.5029 | 0.7006 | 5.01 | 5.56 | 5.22 | 0.86 | 0.0898 | 0.2899 |
| TC0100011520.hg.1 | PPP2R5A | protein phosphatase 2, regulatory subunit B, alpha | Multiple_C | 11.61 | 12.22 | 11.34 | 1.21 | 0.2916 | 0.5027 | 10.29 | 9.67 | 10.49 | 0.87 | 0.5028 | 0.7346 |
| TC0100011733.hg.1 | FBXO28 | F-box protein 28 | Multiple_C | 12.86 | 12.55 | 12.59 | 1.21 | 0.2533 | 0.4583 | 11.75 | 11.73 | 11.74 | 1.01 | 0.8844 | 0.9504 |
| TC0100012353.hg.1 | OR14C36 | olfactory receptor, family 14, subfamily C, member 36 | Coding | 3.54 | 3.26 | 3.27 | 1.21 | 0.4328 | 0.6428 | 4.2 | 4.46 | 4.22 | 0.99 | 0.789 | 0.9058 |
| TC0100013354.hg.1 | RHCE | Rh blood group, CcEe antigens | Multiple_C | 4.62 | 4.37 | 4.35 | 1.21 | 0.4506 | 0.6583 | 4.28 | 4.21 | 4.22 | 1.04 | 0.058 | 0.227 |
| TC0100013763.hg.1 | POU3F1 | POU class 3 homeobox 1 | Multiple_C | 2.49 | 2.14 | 2.22 | 1.21 | 0.4601 | 0.666 | 3.75 | 3.73 | 3.54 | 1.16 | 0.2553 | 0.5198 |
| TC0100014542.hg.1 | RPE65 | retinal pigment epithelium-specific protein 65kDa | Coding | 4.37 | 3.72 | 4.1 | 1.21 | 0.2421 | 0.4445 | 3.82 | 3.51 | 3.53 | 1.22 | 0.3241 | 0.5884 |
| TC0100015417.hg.1 | VTCN1 | V-set domain containing T cell activation inhibitor 1 | Multiple_C | 4.08 | 3.86 | 3.81 | 1.21 | 0.0574 | 0.1601 | 4.09 | 3.86 | 3.61 | 1.39 | 0.0748 | 0.2628 |
| TC0100018090.hg.1 | C1orf229 | chromosome 1 open reading frame 229 | Multiple_C | 4.33 | 4.13 | 4.06 | 1.21 | 0.4438 | 0.6523 | 4.08 | 4.11 | 3.93 | 1.11 | 0.7771 | 0.9001 |
| TC0100018201.hg.1 | MTFR1L | mitochondrial fission regulator 1-like | Multiple_C | 8.3 | 7.89 | 8.03 | 1.21 | 0.3251 | 0.539 | 9.16 | 9.26 | 9.24 | 0.95 | 0.4155 | 0.6697 |
| TC0100018257.hg.1 | GNAI3 | guanine nucleotide binding protein (G protein), alpha inhibiting | Multiple_C | 13.04 | 13.29 | 12.77 | 1.21 | 0.8346 | 0.9174 | 12.53 | 12.05 | 12.35 | 1.13 | 0.8708 | 0.9445 |

| | | | | | | | | | | | | | | | | |
|---|---|---|---|---|---|---|---|---|---|---|---|---|---|---|---|---|
| TC0100018280.hg.1 | FAM231D; LIN | family with sequence similarity 231, member D; long intergenic | Multiple_C | 10.06 | 10.47 | 9.79 | 1.21 | 0.4059 | 0.6186 | 10.95 | 11.01 | 10.93 | 1.01 | 0.8554 | 0.9384 |
| TC0100018323.hg.1 | IER5 | immediate early response 5 | Coding | 7.08 | 7.01 | 6.81 | 1.21 | 0.6177 | 0.7857 | 7.56 | 6.55 | 7.29 | 1.21 | 0.0263 | 0.1417 |
| TC0100018398.hg.1 | TNFRSF25 | tumor necrosis factor receptor superfamily, member 25 | Multiple_C | 7.5 | 7.37 | 7.23 | 1.21 | 0.2782 | 0.4875 | 7.61 | 7.31 | 7.11 | 1.41 | 0.0832 | 0.2777 |
| TC0200007399.hg.1 | PLEKHH2 | pleckstrin homology domain containing, family H (with MyTH4 c | Multiple_C | 4.44 | 4.19 | 4.17 | 1.21 | 0.347 | 0.5602 | 7.26 | 5.20 | 6.02 | 2.36 | 0.0171 | 0.1093 |
| TC0200010046.hg.1 | HOXD12 | homeobox D12 | Coding | 5.58 | 5.19 | 5.31 | 1.21 | 0.2253 | 0.4241 | 5.51 | 5.50 | 5.23 | 1.21 | 0.2487 | 0.5134 |
| TC0200011125.hg.1 | ATG16L1 | autophagy related 16-like 1 | Multiple_C | 6.83 | 6.68 | 6.56 | 1.21 | 0.0607 | 0.1671 | 6.48 | 5.86 | 6.14 | 1.27 | 0.0497 | 0.2076 |
| TC0200013516.hg.1 | TMEM127 | transmembrane protein 127 | Coding | 9.63 | 8.16 | 9.36 | 1.21 | 0.3093 | 0.5224 | 10.01 | 9.90 | 9.95 | 1.04 | 0.5299 | 0.7532 |
| TC0300007883.hg.1 | FRG2C | FSHD region gene 2 family, member C | Coding | 4.26 | 3.87 | 3.99 | 1.21 | 0.3424 | 0.5563 | 4.16 | 4.31 | 4.34 | 0.88 | 0.9128 | 0.9642 |
| TC0300008335.hg.1 | TMPRSS7 | transmembrane protease, serine 7 | Multiple_C | 3.02 | 2.7 | 2.75 | 1.21 | 0.3226 | 0.5363 | 3.4 | 3.23 | 3.07 | 1.26 | 0.0155 | 0.1026 |
| TC0300008991.hg.1 | SPSB4 | splA/ryanodine receptor domain and SOCS box containing 4 | Multiple_C | 3.98 | 3.61 | 3.71 | 1.21 | 0.8987 | 0.9518 | 4.93 | 4.97 | 5.1 | 0.89 | 0.3435 | 0.6072 |
| TC0300012392.hg.1 | EFCAB12 | EF-hand calcium binding domain 12 | Multiple_C | 3.12 | 2.62 | 2.85 | 1.21 | 0.3803 | 0.5932 | 3.2 | 2.97 | 3.27 | 0.95 | 0.7235 | 0.8711 |
| TC0300013707.hg.1 | WDR53 | WD repeat domain 53 | Coding | 8.92 | 8.39 | 8.65 | 1.21 | 0.344 | 0.5577 | 8.22 | 8.69 | 8.88 | 0.63 | 0.0264 | 0.1419 |
| TC0300013947.hg.1 | COLQ | collagen-like tail subunit (single strand of homotrimer) of asymr | Multiple_C | 3.84 | 3.73 | 3.57 | 1.21 | 0.6157 | 0.7848 | 3.52 | 3.73 | 3.62 | 0.93 | 0.5567 | 0.7706 |
| TC0400007534.hg.1 | GSX2 | GS homeobox 2 | Multiple_C | 5.37 | 4.61 | 5.1 | 1.21 | 0.1223 | 0.2786 | 6 | 6.21 | 6.14 | 0.91 | 0.8672 | 0.9431 |
| TC0400008725.hg.1 | PCDH10 | protocadherin 10 | Multiple_C | 3.09 | 2.82 | 2.82 | 1.21 | 0.891 | 0.9475 | 3.94 | 3.93 | 3.99 | 0.97 | 0.7953 | 0.9087 |
| TC0400009513.hg.1 | HELT | helt bHLH transcription factor | Multiple_C | 3.07 | 2.77 | 2.8 | 1.21 | 0.34 | 0.554 | 3.32 | 3.41 | 3.17 | 1.11 | 0.4442 | 0.6923 |
| TC0500007016.hg.1 | LSP1P3 | lymphocyte-specific protein 1 pseudogene 3 | Multiple_C | 5.18 | 4.66 | 4.91 | 1.21 | 0.2019 | 0.3941 | 7.23 | 7.00 | 7.42 | 0.88 | 0.1674 | 0.4128 |
| TC0500007185.hg.1 | GDNF-AS1 | GDNF antisense RNA 1 (head to head) | Multiple_C | 3.69 | 3.25 | 3.42 | 1.21 | 0.587 | 0.7633 | 3.4 | 3.11 | 3.44 | 0.97 | 0.9128 | 0.9642 |
| TC0500008175.hg.1 | RGMB | repulsive guidance molecule family member b | Multiple_C | 8.19 | 8.1 | 7.92 | 1.21 | 0.5955 | 0.7695 | 7.24 | 7.08 | 7.15 | 1.06 | 0.7166 | 0.8677 |
| TC0500008810.hg.1 | MATR3; SNOR | matrin 3; small nucleolar RNA, H/ACA box 74A; small nucleolar I | Multiple_C | 15.55 | 16.21 | 15.28 | 1.21 | 0.1248 | 0.2823 | 15.89 | 15.52 | 15.38 | 1.42 | 0.0102 | 0.0786 |
| TC0500009109.hg.1 | GPX3 | glutathione peroxidase 3 | Multiple_C | 3.43 | 3.03 | 3.16 | 1.21 | 0.5981 | 0.7711 | 4 | 3.80 | 3.99 | 1.01 | 0.2479 | 0.5124 |
| TC0500010489.hg.1 | CAPSL | calcyphosine-like | Coding | 3.06 | 2.69 | 2.79 | 1.21 | 0.5457 | 0.7313 | 3.43 | 3.80 | 3.66 | 0.85 | 0.8642 | 0.9422 |
| TC0500010523.hg.1 | GDNF | glial cell derived neurotrophic factor | Multiple_C | 6.04 | 5.63 | 5.77 | 1.21 | 0.1506 | 0.323 | 5.87 | 5.91 | 5.9 | 0.98 | 0.7436 | 0.8827 |
| TC0500012160.hg.1 | NME5 | NME/NM23 family member 5 | Multiple_C | 4.21 | 3.74 | 3.94 | 1.21 | 0.8385 | 0.9197 | 4.92 | 5.09 | 4.64 | 1.21 | 0.3654 | 0.6279 |
| TC0500012599.hg.1 | ADAM19 | ADAM metallopeptidase domain 19 | Multiple_C | 4.79 | 4.45 | 4.52 | 1.21 | 0.4332 | 0.6432 | 8.7 | 8.03 | 7.62 | 2.11 | 0.065 | 0.2427 |
| TC0500012956.hg.1 | HK3 | hexokinase 3 (white cell) | Multiple_C | 4.42 | 4.1 | 4.15 | 1.21 | 0.2736 | 0.4822 | 4.73 | 5.24 | 4.71 | 1.01 | 0.6631 | 0.8355 |
| TC0600007664.hg.1 | HLA-DQA2 | major histocompatibility complex, class II, DQ alpha 2 | Coding | 3.61 | 3.28 | 3.34 | 1.21 | 0.9583 | 0.9803 | 4.38 | 4.10 | 3.88 | 1.41 | 0.1353 | 0.3652 |
| TC0600008129.hg.1 | HSP90AB1 | heat shock protein 90kDa alpha (cytosolic), class B member 1 | Multiple_C | 16.32 | 16.2 | 16.05 | 1.21 | 0.2644 | 0.4718 | 17.13 | 17.04 | 17.37 | 0.85 | 0.4215 | 0.6745 |
| TC0600009871.hg.1 | ESR1 | estrogen receptor 1 | Multiple_C | 3.51 | 3.32 | 3.24 | 1.21 | 0.4206 | 0.6319 | 4.18 | 4.01 | 3.63 | 1.46 | 0.0955 | 0.3005 |
| TC0600011368.hg.1 | GNL1 | guanine nucleotide binding protein-like 1 | Multiple_C | 4.87 | 5.19 | 4.6 | 1.21 | 0.5545 | 0.738 | 5.27 | 5.50 | 5.27 | 1.00 | 0.9348 | 0.9725 |
| TC0600011489.hg.1 | BTNL2 | butyrophilin-like 2 | Multiple_C | 3.55 | 3.4 | 3.28 | 1.21 | 0.3728 | 0.5853 | 4.06 | 4.21 | 4.03 | 1.02 | 0.83 | 0.9246 |
| TC0600013951.hg.1 | FRMD1 | FERM domain containing 1 | Multiple_C | 4.86 | 4.55 | 4.59 | 1.21 | 0.402 | 0.6148 | 4.5 | 4.65 | 5.08 | 0.67 | 0.0511 | 0.211 |
| TC0600014193.hg.1 | SCAF8; TIAM2 | SR-related CTD-associated factor 8; T-cell lymphoma invasion ai | Multiple_C | 9.92 | 9.54 | 9.65 | 1.21 | 0.371 | 0.5837 | 10.21 | 10.63 | 10.62 | 0.75 | 0.4148 | 0.6693 |

| | | | | | | | | | | | | | | | |
|---|---|---|---|---|---|---|---|---|---|---|---|---|---|---|---|
| TC0600014196.hg.1 | SNX9 | sorting nexin 9 | Multiple_C | 12.16 | 12.24 | 11.89 | 1.21 | 0.4646 | 0.6695 | 11.26 | 11.53 | 11.61 | 0.78 | 0.717 | 0.8679 |
| TC0600014247.hg.1 | FAM65B | family with sequence similarity 65, member B | Multiple_C | 3.93 | 3.43 | 3.66 | 1.21 | 0.4977 | 0.6973 | 4.01 | 4.01 | 3.92 | 1.06 | 0.9547 | 0.9814 |
| TC0700008351.hg.1 | GNG11 | guanine nucleotide binding protein (G protein), gamma 11 | Multiple_C | 3.81 | 3.18 | 3.54 | 1.21 | 0.2149 | 0.4105 | 3.63 | 3.59 | 3.69 | 0.96 | 0.827 | 0.9237 |
| TC0700008560.hg.1 | GNB2 | guanine nucleotide binding protein (G protein), beta polypeptid | Multiple_C | 12.66 | 12.16 | 12.39 | 1.21 | 0.1334 | 0.2967 | 11.44 | 12.10 | 12.33 | 0.54 | 0.004 | 0.043 |
| TC0700009399.hg.1 | TAS2R4 | taste receptor, type 2, member 4 | Coding | 5.24 | 5.34 | 4.97 | 1.21 | 0.3328 | 0.5466 | 5.01 | 4.51 | 4.67 | 1.27 | 0.6511 | 0.8298 |
| TC0700009608.hg.1 | ZNF212 | zinc finger protein 212 | Coding | 7.72 | 7.26 | 7.45 | 1.21 | 0.337 | 0.5508 | 6.82 | 6.58 | 6.5 | 1.25 | 0.4306 | 0.6824 |
| TC0700010336.hg.1 | MEOX2 | mesenchyme homeobox 2 | Coding | 4.56 | 4.08 | 4.29 | 1.21 | 0.4013 | 0.6144 | 6.76 | 6.65 | 6.43 | 1.26 | 0.2183 | 0.4781 |
| TC0700011797.hg.1 | SAMD9L | sterile alpha motif domain containing 9-like | Multiple_C | 4.99 | 4.02 | 4.72 | 1.21 | 0.435 | 0.645 | 4.75 | 4.76 | 4.42 | 1.26 | 0.0673 | 0.2473 |
| TC0800007335.hg.1 | LETM2 | leucine zipper-EF-hand containing transmembrane protein 2 | Multiple_C | 5.62 | 5.11 | 5.35 | 1.21 | 0.5234 | 0.7161 | 7.1 | 6.69 | 7.33 | 0.85 | 0.3297 | 0.5939 |
| TC0800007809.hg.1 | YTHDF3 | YTH N(6)-methyladenosine RNA binding protein 3 | Multiple_C | 13.28 | 12.19 | 13.01 | 1.21 | 0.6836 | 0.8287 | 13.5 | 12.31 | 13.68 | 0.88 | 0.2349 | 0.4974 |
| TC0800010234.hg.1 | SFRP1 | secreted frizzled-related protein 1 | Multiple_C | 3.65 | 3.04 | 3.38 | 1.21 | 0.3566 | 0.5699 | 3.79 | 3.91 | 3.93 | 0.91 | 0.1271 | 0.3529 |
| TC0800011387.hg.1 | DPYS | dihydropyrimidinase | Multiple_C | 3.22 | 2.88 | 2.95 | 1.21 | 0.6078 | 0.7786 | 3.81 | 4.01 | 3.8 | 1.01 | 0.4656 | 0.7074 |
| TC0800012237.hg.1 | ZNF34 | zinc finger protein 34 | Multiple_C | 5.71 | 5.24 | 5.44 | 1.21 | 0.4099 | 0.6222 | 5.59 | 5.52 | 5.47 | 1.09 | 0.8738 | 0.9456 |
| TC0900009050.hg.1 | EEF1A1P5 | eukaryotic translation elongation factor 1 alpha 1 pseudogene 5 | Multiple_C | 18.39 | 18.18 | 18.12 | 1.21 | 0.0318 | 0.1028 | 18.81 | 18.82 | 19.03 | 0.86 | 0.4753 | 0.7146 |
| TC0900009084.hg.1 | ADAMTSL2 | ADAMTS like 2 | Coding | 3.09 | 2.55 | 2.82 | 1.21 | 0.4919 | 0.6919 | 4.84 | 5.16 | 4.78 | 1.04 | 0.4777 | 0.7164 |
| TC0900009832.hg.1 | TAF1L | TAF1 RNA polymerase II, TATA box binding protein (TBP)-associ | Coding | 4.52 | 4.93 | 4.25 | 1.21 | 0.6721 | 0.8212 | 4.47 | 4.15 | 4.32 | 1.11 | 0.7024 | 0.8591 |
| TC0900011064.hg.1 | OR13C4 | olfactory receptor, family 13, subfamily C, member 4 | Coding | 3.2 | 2.88 | 2.93 | 1.21 | 0.3482 | 0.5613 | 3.68 | 3.19 | 3.28 | 1.32 | 0.0383 | 0.178 |
| TC0900011432.hg.1 | RBM18 | RNA binding motif protein 18 | Multiple_C | 11.96 | 11.74 | 11.69 | 1.21 | 0.2995 | 0.5113 | 11.55 | 10.63 | 11.42 | 1.09 | 0.1177 | 0.3391 |
| TC0900011676.hg.1 | IER5L | immediate early response 5-like | Coding | 4.46 | 4.06 | 4.19 | 1.21 | 0.5116 | 0.7074 | 4.65 | 4.88 | 4.67 | 0.99 | 0.9624 | 0.9842 |
| TC0X00006775.hg.1 | PHEX | phosphate regulating endopeptidase homolog, X-linked | Multiple_C | 4.26 | 3.69 | 3.99 | 1.21 | 0.4276 | 0.6381 | 4.35 | 4.99 | 4.97 | 0.65 | 0.0496 | 0.2073 |
| TC0X00007733.hg.1 | FAM46D | family with sequence similarity 46, member D | Coding | 3.08 | 2.81 | 2.81 | 1.21 | 0.3817 | 0.5947 | 3.83 | 3.46 | 3.52 | 1.24 | 0.0452 | 0.1967 |
| TC0X00007949.hg.1 | ARMCX1 | armadillo repeat containing, X-linked 1 | Coding | 4.13 | 3.86 | 3.86 | 1.21 | 0.6939 | 0.8352 | 4.31 | 4.04 | 4.11 | 1.15 | 0.2334 | 0.4957 |
| TC0X00008120.hg.1 | RGAG1 | retrotransposon gag domain containing 1 | Coding | 3.5 | 3.11 | 3.23 | 1.21 | 0.1859 | 0.3724 | 3.3 | 3.44 | 3.73 | 0.74 | 0.1119 | 0.3294 |
| TC0X00008264.hg.1 | AKAP14 | A kinase (PRKA) anchor protein 14 | Multiple_C | 3.74 | 3.48 | 3.47 | 1.21 | 0.8184 | 0.9086 | 3.57 | 3.66 | 3.46 | 1.08 | 0.8663 | 0.9426 |
| TC0X00008824.hg.1 | TKTL1 | transketolase-like 1 | Multiple_C | 7.91 | 7.7 | 7.64 | 1.21 | 0.0771 | 0.199 | 7.59 | 7.67 | 7.68 | 0.94 | 0.2899 | 0.5547 |
| TC0X00009694.hg.1 | NUDT11 | nudix hydrolase 11 | Multiple_C | 3.98 | 3.74 | 3.71 | 1.21 | 0.1828 | 0.3679 | 4.08 | 3.98 | 3.85 | 1.17 | 0.1432 | 0.3765 |
| TC0X00010445.hg.1 | ESX1 | ESX homeobox 1 | Coding | 4.81 | 4.6 | 4.54 | 1.21 | 0.3785 | 0.5915 | 4.85 | 5.01 | 5.16 | 0.81 | 0.1576 | 0.3987 |
| TC0X00010804.hg.1 | ZNF280C | zinc finger protein 280C | Multiple_C | 8.9 | 8.35 | 8.63 | 1.21 | 0.1456 | 0.3156 | 9.5 | 9.43 | 10.3 | 0.57 | 5.45E-05 | 0.0024 |
| TC1000007545.hg.1 | RBP3 | retinol binding protein 3, interstitial | Multiple_C | 4.24 | 3.95 | 3.97 | 1.21 | 0.3451 | 0.5588 | 3.88 | 3.57 | 3.97 | 0.94 | 0.9171 | 0.9663 |
| TC1000012482.hg.1 | ENTPD1 | ectonucleoside triphosphate diphosphohydrolase 1 | Multiple_C | 4.81 | 4.59 | 4.54 | 1.21 | 0.0719 | 0.1891 | 4.83 | 4.51 | 4.55 | 1.21 | 0.1089 | 0.3247 |
| TC1000012510.hg.1 | ZNF511 | zinc finger protein 511 | Multiple_C | 12.15 | 11.97 | 11.88 | 1.21 | 0.2353 | 0.4364 | 9.96 | 9.99 | 9.47 | 1.40 | 0.0193 | 0.1174 |
| TC1100006759.hg.1 | OLFML1 | olfactomedin like 1 | Multiple_C | 3.36 | 3.01 | 3.09 | 1.21 | 0.4516 | 0.6592 | 3.75 | 3.93 | 4.02 | 0.83 | 0.7653 | 0.8944 |
| TC1100006773.hg.1 | TUB | tubby bipartite transcription factor | Multiple_C | 5.77 | 5.33 | 5.5 | 1.21 | 0.3932 | 0.607 | 4.75 | 4.44 | 4.57 | 1.13 | 0.5993 | 0.7989 |

| | | | | | | | | | | | | | | | |
|---|---|---|---|---|---|---|---|---|---|---|---|---|---|---|---|
| TC1100007608.hg.1 | OR5D13 | olfactory receptor, family 5, subfamily D, member 13 (gene/pse | Multiple_C | 3.3 | 3.07 | 3.03 | 1.21 | 0.0607 | 0.1671 | 3.8 | 3.33 | 3.41 | 1.31 | 0.0203 | 0.1215 |
| TC1100008181.hg.1 | LRP5 | LDL receptor related protein 5 | Multiple_C | 9.61 | 8.95 | 9.34 | 1.21 | 0.4096 | 0.6219 | 12.42 | 12.82 | 12.51 | 0.94 | 0.5547 | 0.7693 |
| TC1100011240.hg.1 | KCNK7 | potassium channel, two pore domain subfamily K, member 7 | Coding | 5.2 | 4.52 | 4.93 | 1.21 | 0.3552 | 0.5683 | 4.78 | 4.93 | 4.63 | 1.11 | 0.9603 | 0.9836 |
| TC1100011243.hg.1 | RELA | v-rel avian reticuloendotheliosis viral oncogene homolog A | Multiple_C | 9.97 | 8.29 | 9.7 | 1.21 | 0.2655 | 0.4732 | 8.78 | 8.63 | 8.92 | 0.91 | 0.6116 | 0.8059 |
| TC1100012123.hg.1 | MMP7 | matrix metallopeptidase 7 | Multiple_C | 4.48 | 4.03 | 4.21 | 1.21 | 0.1239 | 0.2808 | 4.39 | 3.91 | 4.32 | 1.05 | 0.5668 | 0.7761 |
| TC1100012130.hg.1 | MMP10 | matrix metallopeptidase 10 | Multiple_C | 4.25 | 4.12 | 3.98 | 1.21 | 0.0903 | 0.2235 | 3.9 | 3.53 | 3.51 | 1.31 | 0.3569 | 0.62 |
| TC1100012686.hg.1 | ESAM | endothelial cell adhesion molecule | Multiple_C | 6.8 | 6 | 6.53 | 1.21 | 0.2161 | 0.4118 | 4.65 | 4.33 | 4.51 | 1.10 | 0.6667 | 0.8369 |
| TC1100013141.hg.1 | OVCH2 | ovochymase 2 (gene/pseudogene) | Multiple_C | 3.34 | 3.32 | 3.07 | 1.21 | 0.9352 | 0.9697 | 3.38 | 3.35 | 3.25 | 1.09 | 0.7176 | 0.8682 |
| TC1200007061.hg.1 | CMAS | cytidine monophosphate N-acetylneuraminic acid synthetase | Multiple_C | 11.48 | 12.68 | 11.21 | 1.21 | 0.3829 | 0.596 | 10.81 | 10.52 | 10.19 | 1.54 | 0.0041 | 0.0435 |
| TC1200007775.hg.1 | MUCL1 | mucin-like 1 | Multiple_C | 3.17 | 2.8 | 2.9 | 1.21 | 0.2316 | 0.4321 | 3.72 | 3.34 | 3.28 | 1.36 | 0.018 | 0.1127 |
| TC1200007777.hg.1 | NEUROD4 | neuronal differentiation 4 | Coding | 3.91 | 3.45 | 3.64 | 1.21 | 0.2033 | 0.3954 | 3.75 | 3.85 | 4 | 0.84 | 0.2136 | 0.4717 |
| TC1200007880.hg.1 | INHBC | inhibin beta C | Coding | 4.99 | 4.74 | 4.72 | 1.21 | 0.4818 | 0.6834 | 4.92 | 4.83 | 4.94 | 0.99 | 0.8654 | 0.9424 |
| TC1200009239.hg.1 | KMT5A | lysine (K)-specific methyltransferase 5A | Multiple_C | 11.83 | 11.33 | 11.56 | 1.21 | 0.8309 | 0.9157 | 10.8 | 10.69 | 11.05 | 0.84 | 0.0403 | 0.184 |
| TC1200010653.hg.1 | CERS5 | ceramide synthase 5 | Multiple_C | 8.95 | 8.42 | 8.68 | 1.21 | 0.212 | 0.4066 | 8.37 | 9.12 | 9.67 | 0.41 | 7.63E-07 | 0.0001 |
| TC1200012206.hg.1 | KDM2B | lysine (K)-specific demethylase 2B | Multiple_C | 8.15 | 7.49 | 7.88 | 1.21 | 0.1887 | 0.3763 | 6.94 | 6.36 | 6.51 | 1.35 | 0.0105 | 0.0808 |
| TC1200012530.hg.1 | LRCOL1 | leucine rich colipase-like 1 | Multiple_C | 3.83 | 3.81 | 3.56 | 1.21 | 0.2358 | 0.437 | 3.58 | 3.70 | 3.77 | 0.88 | 0.353 | 0.6166 |
| TC1400007165.hg.1 | PTGER2 | prostaglandin E receptor 2 | Coding | 3.8 | 3.47 | 3.53 | 1.21 | 0.7103 | 0.8464 | 4.16 | 4.07 | 3.76 | 1.32 | 0.0616 | 0.2356 |
| TC1400007244.hg.1 | KTN1 | kinectin 1 (kinesin receptor) | Multiple_C | 9.91 | 11.18 | 9.64 | 1.21 | 0.3546 | 0.5677 | 8.34 | 7.81 | 7.52 | 1.77 | 0.0035 | 0.0397 |
| TC1400007566.hg.1 | SMOC1 | SPARC related modular calcium binding 1 | Multiple_C | 11.47 | 11.48 | 11.2 | 1.21 | 0.4128 | 0.6249 | 4.56 | 4.69 | 4.59 | 0.98 | 0.9469 | 0.9784 |
| TC1400007804.hg.1 | TSHR | thyroid stimulating hormone receptor | Multiple_C | 3.87 | 3.52 | 3.6 | 1.21 | 0.559 | 0.7417 | 3.58 | 3.46 | 3.35 | 1.17 | 0.0508 | 0.2101 |
| TC1400008762.hg.1 | CTSG | cathepsin G | Multiple_C | 4.13 | 3.76 | 3.86 | 1.21 | 0.6202 | 0.7875 | 3.9 | 4.28 | 4.22 | 0.80 | 0.0408 | 0.1856 |
| TC1500009393.hg.1 | USP50 | ubiquitin specific peptidase 50 | Multiple_C | 4.15 | 3.4 | 3.88 | 1.21 | 0.2799 | 0.4897 | 4.16 | 4.48 | 3.85 | 1.24 | 0.2624 | 0.5267 |
| TC1500010382.hg.1 | RLBP1 | retinaldehyde binding protein 1 | Multiple_C | 3.57 | 3.21 | 3.3 | 1.21 | 0.2476 | 0.4513 | 3.85 | 3.50 | 3.93 | 0.95 | 0.7173 | 0.8682 |
| TC1500010796.hg.1 | SYNM | synemin, intermediate filament protein | Multiple_C | 6.23 | 5.94 | 5.96 | 1.21 | 0.341 | 0.5549 | 5.77 | 5.76 | 5.49 | 1.21 | 0.3309 | 0.5949 |
| TC1600010409.hg.1 | BBS2 | Bardet-Biedl syndrome 2 | Multiple_C | 8.91 | 10.65 | 8.64 | 1.21 | 0.9167 | 0.9608 | 7.65 | 7.85 | 7.38 | 1.21 | 0.5053 | 0.7369 |
| TC1600011373.hg.1 | SLX1A-SULT1A | SLX1A-SULT1A3 readthrough (NMD candidate) | Multiple_C | 7.84 | 7.7 | 7.57 | 1.21 | 0.8877 | 0.9458 | 6.29 | 7.03 | 6.81 | 0.70 | 0.0846 | 0.2799 |
| TC1600011476.hg.1 | ZNF500 | zinc finger protein 500 | Multiple_C | 3.31 | 3.01 | 3.04 | 1.21 | 0.3403 | 0.5543 | 3.34 | 3.52 | 3.46 | 0.92 | 0.6501 | 0.8292 |
| TC1600011518.hg.1 | DOC2A | double C2-like domains, alpha | Multiple_C | 4.64 | 4.28 | 4.37 | 1.21 | 0.0737 | 0.1924 | 3.63 | 3.88 | 3.49 | 1.10 | 0.2081 | 0.465 |
| TC1600011554.hg.1 | LCAT | lecithin-cholesterol acyltransferase | Multiple_C | 4.91 | 4.65 | 4.64 | 1.21 | 0.3347 | 0.5486 | 6.3 | 6.42 | 6.7 | 0.76 | 0.044 | 0.1939 |
| TC1700006638.hg.1 | ARRB2 | arrestin, beta 2 | Multiple_C | 12.77 | 11.95 | 12.5 | 1.21 | 0.64 | 0.8006 | 12.82 | 12.69 | 12.73 | 1.06 | 0.8665 | 0.9427 |
| TC1700007620.hg.1 | CCL4L2; CCL4; | chemokine (C-C motif) ligand 4-like 2; chemokine (C-C motif) lig | Multiple_C | 3.27 | 3 | 3 | 1.21 | 0.2328 | 0.4335 | 3.35 | 3.19 | 3.16 | 1.14 | 0.51 | 0.7401 |
| TC1700008175.hg.1 | CALCOCO2 | calcium binding and coiled-coil domain 2 | Multiple_C | 12.07 | 13.17 | 11.8 | 1.21 | 0.5309 | 0.7211 | 9.76 | 9.37 | 9.87 | 0.93 | 0.9027 | 0.9596 |
| TC1700008489.hg.1 | C17orf64 | chromosome 17 open reading frame 64 | Multiple_C | 2.84 | 2.41 | 2.57 | 1.21 | 0.7088 | 0.8454 | 3.18 | 3.40 | 3.11 | 1.05 | 0.6674 | 0.8374 |

| | | | | | | | | | | | | | | | |
|---|---|---|---|---|---|---|---|---|---|---|---|---|---|---|---|
| TC1700009162.hg.1 | C17orf89 | chromosome 17 open reading frame 89 | Multiple_C | 10.58 | 10.31 | 10.31 | 1.21 | 0.7757 | 0.8844 | 10.05 | 10.75 | 10.41 | 0.78 | 0.6348 | 0.8208 |
| TC1700009597.hg.1 | SLC13A5 | solute carrier family 13 (sodium-dependent citrate transporter) | Multiple_C | 5.72 | 5.51 | 5.45 | 1.21 | 0.6379 | 0.7991 | 4.58 | 4.80 | 5.19 | 0.66 | 0.025 | 0.1373 |
| TC1700010714.hg.1 | KCNH4 | potassium channel, voltage gated eag related subfamily H, mem | Coding | 4.5 | 4.13 | 4.23 | 1.21 | 0.5437 | 0.7303 | 4.51 | 4.04 | 4.35 | 1.12 | 0.5118 | 0.7415 |
| TC1700012277.hg.1 | CDK5RAP3 | CDK5 regulatory subunit associated protein 3 | Multiple_C | 10.84 | 11.61 | 10.57 | 1.21 | 0.8275 | 0.9133 | 10.45 | 10.51 | 9.31 | 2.20 | 0.982 | 0.9926 |
| TC1700012361.hg.1 | CDRT1 | CMT1A duplicated region transcript 1 | Multiple_C | 5.28 | 4.8 | 5.01 | 1.21 | 0.6198 | 0.7874 | 4.06 | 3.78 | 3.57 | 1.40 | 0.1067 | 0.321 |
| TC1700012368.hg.1 | PLD6 | phospholipase D family, member 6 | Coding | 5.97 | 5.36 | 5.7 | 1.21 | 0.2031 | 0.3953 | 7.61 | 7.26 | 7.57 | 1.03 | 0.7887 | 0.9058 |
| TC1900006528.hg.1 | NDUFS7 | NADH dehydrogenase (ubiquinone) Fe-S protein 7, 20kDa (NAD | Multiple_C | 7.96 | 7.28 | 7.69 | 1.21 | 0.6136 | 0.7832 | 5.76 | 5.74 | 5.58 | 1.13 | 0.4874 | 0.7238 |
| TC1900006821.hg.1 | VAV1 | vav 1 guanine nucleotide exchange factor | Multiple_C | 6.8 | 6.66 | 6.53 | 1.21 | 0.4472 | 0.6554 | 6.02 | 5.96 | 5.94 | 1.06 | 0.3707 | 0.6328 |
| TC1900006890.hg.1 | RPS28 | ribosomal protein S28 | Multiple_C | 17.47 | 15.99 | 17.2 | 1.21 | 0.0486 | 0.1417 | 12.35 | 12.31 | 12.42 | 0.95 | 0.8412 | 0.9306 |
| TC1900006985.hg.1 | PDE4A | phosphodiesterase 4A, cAMP-specific | Multiple_C | 4.55 | 4.32 | 4.28 | 1.21 | 0.4642 | 0.6693 | 8.63 | 7.74 | 7.85 | 1.72 | 0.0064 | 0.0575 |
| TC1900008111.hg.1 | SPTBN4 | spectrin, beta, non-erythrocytic 4 | Multiple_C | 6.19 | 5.72 | 5.92 | 1.21 | 0.099 | 0.2392 | 6 | 6.26 | 6.05 | 0.97 | 0.7379 | 0.8792 |
| TC1900008910.hg.1 | ZNF628 | zinc finger protein 628 | Multiple_C | 4.75 | 4.32 | 4.48 | 1.21 | 0.4571 | 0.6633 | 5.36 | 5.77 | 5.49 | 0.91 | 0.4951 | 0.729 |
| TC1900009562.hg.1 | OR7G1 | olfactory receptor, family 7, subfamily G, member 1 | Coding | 3.67 | 3.75 | 3.4 | 1.21 | 0.1578 | 0.3337 | 3.66 | 3.48 | 3.53 | 1.09 | 0.385 | 0.644 |
| TC1900009564.hg.1 | OR7G3 | olfactory receptor, family 7, subfamily G, member 3 | Coding | 3.74 | 3.44 | 3.47 | 1.21 | 0.266 | 0.4737 | 3.67 | 3.63 | 3.73 | 0.96 | 0.979 | 0.9911 |
| TC1900010164.hg.1 | ZNF208 | zinc finger protein 208 | Multiple_C | 9.77 | 9.57 | 9.5 | 1.21 | 0.3176 | 0.5312 | 9.22 | 8.92 | 9.04 | 1.13 | 0.4086 | 0.664 |
| TC1900011327.hg.1 | ZNF160 | zinc finger protein 160 | Multiple_C | 8.73 | 8.17 | 8.46 | 1.21 | 0.1615 | 0.3385 | 7.55 | 7.52 | 7.75 | 0.87 | 0.4144 | 0.6689 |
| TC1900011524.hg.1 | ZNF667 | zinc finger protein 667 | Multiple_C | 4.91 | 4.43 | 4.64 | 1.21 | 0.2566 | 0.4629 | 4.69 | 4.74 | 4.33 | 1.28 | 0.1048 | 0.3176 |
| TC1900011723.hg.1 | ZNF793 | zinc finger protein 793 | Multiple_C | 4.31 | 4 | 4.04 | 1.21 | 0.3202 | 0.5337 | 4.4 | 4.76 | 4.37 | 1.02 | 0.8903 | 0.9532 |
| TC2100007254.hg.1 | UBASH3A | ubiquitin associated and SH3 domain containing A | Multiple_C | 4.65 | 4.46 | 4.38 | 1.21 | 0.4836 | 0.6851 | 4.85 | 4.76 | 4.63 | 1.16 | 0.6188 | 0.811 |
| TC2100007373.hg.1 | KRTAP10-4 | keratin associated protein 10-4 | Multiple_C | 2.97 | 2.62 | 2.7 | 1.21 | 0.8856 | 0.9448 | 3.5 | 3.23 | 3.19 | 1.24 | 0.1025 | 0.3136 |
| TC2100008536.hg.1 | PCBP3 | poly(rC) binding protein 3 | Multiple_C | 3.22 | 2.9 | 2.95 | 1.21 | 0.8327 | 0.9165 | 5.14 | 5.38 | 5.46 | 0.80 | 0.781 | 0.9017 |
| TC2200006517.hg.1 | CECR2 | cat eye syndrome chromosome region, candidate 2 | Multiple_C | 3.27 | 2.91 | 3 | 1.21 | 0.3128 | 0.5263 | 3.73 | 3.48 | 3.46 | 1.21 | 0.071 | 0.2549 |
| TC2200006688.hg.1 | LRRC74B | leucine rich repeat containing 74B | Multiple_C | 3.31 | 3.23 | 3.04 | 1.21 | 0.4342 | 0.6441 | 3.51 | 3.40 | 3.52 | 0.99 | 0.9844 | 0.9935 |
| TC2200007320.hg.1 | GALR3 | galanin receptor 3 | Coding | 4.84 | 4.47 | 4.57 | 1.21 | 0.5363 | 0.725 | 3.91 | 3.95 | 3.97 | 0.96 | 0.7554 | 0.8888 |
| TC2200008641.hg.1 | RAC2 | ras-related C3 botulinum toxin substrate 2 (rho family, small GT | Multiple_C | 6.57 | 6.08 | 6.3 | 1.21 | 0.4396 | 0.6486 | 5.09 | 5.00 | 5.21 | 0.92 | 0.5526 | 0.7681 |
| TC0100007564.hg.1 | RAB42 | RAB42, member RAS oncogene family | Multiple_C | 5.57 | 5.59 | 5.31 | 1.20 | 0.46 | 0.6659 | 6.46 | 5.66 | 5.22 | 2.36 | 3.78E-06 | 0.0004 |
| TC0100009866.hg.1 | FCGR1A | Fc fragment of IgG, high affinity Ia, receptor (CD64) | Multiple_C | 3.19 | 2.85 | 2.93 | 1.20 | 0.3884 | 0.6021 | 3.82 | 3.39 | 3.53 | 1.22 | 0.2297 | 0.4908 |
| TC0100010002.hg.1 | LCE4A | late cornified envelope 4A | Coding | 7.49 | 6.71 | 7.23 | 1.20 | 0.3319 | 0.5458 | 5.56 | 5.93 | 5.78 | 0.86 | 0.2407 | 0.5043 |
| TC0100011485.hg.1 | RCOR3 | REST corepressor 3 | Multiple_C | 6.71 | 5.76 | 6.45 | 1.20 | 0.5501 | 0.7351 | 6.7 | 6.46 | 6.64 | 1.04 | 0.5271 | 0.7512 |
| TC0100012219.hg.1 | C1orf100 | chromosome 1 open reading frame 100 | Multiple_C | 4.07 | 4.11 | 3.81 | 1.20 | 0.2742 | 0.4829 | 3.79 | 3.76 | 3.69 | 1.07 | 0.7998 | 0.9105 |
| TC0200008563.hg.1 | VWA3B | von Willebrand factor A domain containing 3B | Multiple_C | 4.77 | 4.44 | 4.51 | 1.20 | 0.5336 | 0.7233 | 3.65 | 3.67 | 3.74 | 0.94 | 0.5863 | 0.7902 |
| TC0200008614.hg.1 | NMS | neuromedin S | Coding | 4.24 | 3.77 | 3.98 | 1.20 | 0.4991 | 0.6985 | 3.95 | 3.85 | 3.89 | 1.04 | 0.3814 | 0.6408 |
| TC0200012383.hg.1 | HAAO | 3-hydroxyanthranilate 3,4-dioxygenase | Multiple_C | 4.02 | 3.76 | 3.76 | 1.20 | 0.8808 | 0.942 | 4.3 | 4.33 | 4.36 | 0.96 | 0.5856 | 0.7896 |

| | | | | | | | | | | | | | | | |
|---|---|---|---|---|---|---|---|---|---|---|---|---|---|---|---|
| TC0200012643.hg.1 | CCDC88A | coiled-coil domain containing 88A | Multiple_C | 10.8 | 10.97 | 10.54 | 1.20 | 0.6172 | 0.7854 | 11.04 | 11.65 | 10.92 | 1.09 | 0.4463 | 0.6935 |
| TC0200016706.hg.1 | MRPS5 | mitochondrial ribosomal protein S5 | Multiple_C | 9.96 | 9.86 | 9.7 | 1.20 | 0.6894 | 0.8328 | 7.76 | 7.41 | 7.86 | 0.93 | 0.6627 | 0.8353 |
| TC0300007063.hg.1 | VILL | villin-like | Multiple_C | 6.82 | 7.21 | 6.56 | 1.20 | 0.3976 | 0.6111 | 4.42 | 3.82 | 3.73 | 1.61 | 0.004 | 0.043 |
| TC0300008108.hg.1 | OR5K3 | olfactory receptor, family 5, subfamily K, member 3 | Coding | 3.1 | 2.84 | 2.84 | 1.20 | 0.3297 | 0.5435 | 3.69 | 3.77 | 3.64 | 1.04 | 0.2756 | 0.5417 |
| TC0300009012.hg.1 | GRK7 | G protein-coupled receptor kinase 7 | Multiple_C | 3.14 | 2.82 | 2.88 | 1.20 | 0.3925 | 0.6064 | 3.7 | 3.47 | 3.44 | 1.20 | 0.1957 | 0.4501 |
| TC0300010203.hg.1 | LHFPL4 | lipoma HMGIC fusion partner-like 4 | Multiple_C | 4.54 | 3.95 | 4.28 | 1.20 | 0.5791 | 0.7568 | 4.51 | 4.86 | 4.57 | 0.96 | 0.8223 | 0.9215 |
| TC0300010217.hg.1 | CIDEC | cell death-inducing DFFA-like effector c | Multiple_C | 4.78 | 4.69 | 4.52 | 1.20 | 0.5715 | 0.7512 | 3.8 | 4.37 | 3.66 | 1.10 | 0.9688 | 0.987 |
| TC0300012975.hg.1 | B3GALNT1 | beta-1,3-N-acetylgalactosaminyltransferase 1 (globoside blood | Multiple_C | 3.21 | 3.16 | 2.95 | 1.20 | 0.203 | 0.3953 | 4.84 | 4.39 | 4.92 | 0.95 | 0.217 | 0.4764 |
| TC0300013264.hg.1 | GNB4 | guanine nucleotide binding protein (G protein), beta polypeptid | Multiple_C | 4.36 | 3.93 | 4.1 | 1.20 | 0.2864 | 0.4968 | 4.12 | 4.07 | 3.85 | 1.21 | 0.302 | 0.5664 |
| TC0300013819.hg.1 | ZKSCAN7 | zinc finger with KRAB and SCAN domains 7 | Multiple_C | 5.03 | 4.76 | 4.77 | 1.20 | 0.2704 | 0.4788 | 4.47 | 5.07 | 4.94 | 0.72 | 0.1366 | 0.3673 |
| TC0400010251.hg.1 | DHX15 | DEAH (Asp-Glu-Ala-His) box helicase 15 | Multiple_C | 13.36 | 13.9 | 13.1 | 1.20 | 0.3075 | 0.5207 | 10.56 | 10.66 | 10.82 | 0.84 | 0.3353 | 0.5996 |
| TC0400011173.hg.1 | HNRNPDL | heterogeneous nuclear ribonucleoprotein D like | Multiple_C | 15.63 | 15.37 | 15.37 | 1.20 | 0.1141 | 0.2651 | 15.52 | 14.87 | 14.95 | 1.48 | 0.0571 | 0.225 |
| TC0400011422.hg.1 | DNAJB14 | DnaJ (Hsp40) homolog, subfamily B, member 14 | Multiple_C | 9.07 | 9.16 | 8.81 | 1.20 | 0.5418 | 0.7289 | 7.93 | 7.71 | 7.84 | 1.06 | 0.809 | 0.9155 |
| TC0400012754.hg.1 | ZNF141 | zinc finger protein 141 | Multiple_C | 5.77 | 5.72 | 5.51 | 1.20 | 0.3565 | 0.5698 | 8.28 | 8.05 | 8.25 | 1.02 | 0.6314 | 0.8185 |
| TC0500007590.hg.1 | FAM159B | family with sequence similarity 159, member B | Multiple_C | 3.56 | 3.44 | 3.3 | 1.20 | 0.2836 | 0.4937 | 4.66 | 4.55 | 3.72 | 1.92 | 0.0059 | 0.0545 |
| TC0500007895.hg.1 | CMYA5 | cardiomyopathy associated 5 | Multiple_C | 3.48 | 3.08 | 3.22 | 1.20 | 0.2092 | 0.4029 | 3.45 | 3.57 | 3.26 | 1.14 | 0.4167 | 0.6705 |
| TC0500013266.hg.1 | HMP19 | HMP19 protein; Neuron-specific protein family member 2 [Sou | Multiple_C | 3.1 | 3.1 | 2.84 | 1.20 | 0.8188 | 0.9089 | 3.52 | 3.40 | 3.01 | 1.42 | 0.0556 | 0.2215 |
| TC0600007084.hg.1 | RBM24 | RNA binding motif protein 24 | Multiple_C | 5.26 | 4.79 | 5 | 1.20 | 0.2386 | 0.4403 | 5.61 | 5.46 | 5.39 | 1.16 | 0.2054 | 0.4617 |
| TC0600008057.hg.1 | PTCRA | pre T-cell antigen receptor alpha | Coding | 4.17 | 3.87 | 3.91 | 1.20 | 0.0561 | 0.1574 | 3.94 | 3.92 | 3.95 | 0.99 | 0.8617 | 0.9408 |
| TC0600008346.hg.1 | KIAA1586 | KIAA1586 | Multiple_C | 9.99 | 10.06 | 9.73 | 1.20 | 0.4748 | 0.6778 | 11.31 | 10.74 | 11.27 | 1.03 | 0.9206 | 0.9672 |
| TC0600008870.hg.1 | FUT9 | fucosyltransferase 9 (alpha (1,3) fucosyltransferase) | Multiple_C | 2.72 | 2.41 | 2.46 | 1.20 | 0.2869 | 0.4974 | 3.71 | 3.68 | 3.41 | 1.23 | 0.092 | 0.2937 |
| TC0600009270.hg.1 | PLN | phospholamban | Coding | 3.41 | 3.38 | 3.15 | 1.20 | 0.6379 | 0.7991 | 3.92 | 3.51 | 3.6 | 1.25 | 0.1407 | 0.3728 |
| TC0600012050.hg.1 | GSTA2 | glutathione S-transferase alpha 2 | Coding | 3.7 | 3.82 | 3.44 | 1.20 | 0.5309 | 0.7211 | 4.31 | 4.14 | 3.97 | 1.27 | 0.053 | 0.2151 |
| TC0600012542.hg.1 | UBE2J1 | ubiquitin-conjugating enzyme E2, J1 | Multiple_C | 11.41 | 11.82 | 11.15 | 1.20 | 0.3505 | 0.5633 | 12.39 | 11.84 | 11.9 | 1.40 | 0.0033 | 0.0376 |
| TC0600012863.hg.1 | REV3L | REV3 like, DNA directed polymerase zeta catalytic subunit | Multiple_C | 8.48 | 8.57 | 8.22 | 1.20 | 0.946 | 0.9753 | 8.9 | 8.84 | 8.85 | 1.04 | 0.8063 | 0.9144 |
| TC0600013203.hg.1 | VNN1 | vanin 1 | Coding | 3.36 | 2.76 | 3.1 | 1.20 | 0.727 | 0.8563 | 3.56 | 3.42 | 3.43 | 1.09 | 0.2855 | 0.5505 |
| TC0600014255.hg.1 | UBD | ubiquitin D | Coding | 3.56 | 3.43 | 3.3 | 1.20 | 0.5107 | 0.7066 | 4.69 | 3.62 | 3.62 | 2.10 | 0.0062 | 0.0564 |
| TC0700007098.hg.1 | GARS | glycyl-tRNA synthetase | Multiple_C | 15.4 | 15.44 | 15.14 | 1.20 | 0.2514 | 0.4559 | 12.74 | 12.56 | 13.33 | 0.66 | 0.08 | 0.2727 |
| TC0700008563.hg.1 | EPO | erythropoietin | Multiple_C | 3.22 | 2.59 | 2.96 | 1.20 | 0.3266 | 0.5406 | 3.51 | 3.75 | 3.48 | 1.02 | 0.8892 | 0.9525 |
| TC0700009508.hg.1 | TCAF2 | TRPM8 channel-associated factor 2 | Multiple_C | 4.32 | 3.41 | 4.06 | 1.20 | 0.3462 | 0.5597 | 4.68 | 4.39 | 4.47 | 1.16 | 0.293 | 0.5579 |
| TC0700009725.hg.1 | GALNTL5 | polypeptide N-acetylgalactosaminyltransferase-like 5 | Multiple_C | 3.81 | 3.57 | 3.55 | 1.20 | 0.2857 | 0.4961 | 4.2 | 3.91 | 4.04 | 1.12 | 0.4674 | 0.7088 |
| TC0700011998.hg.1 | ZCWPW1 | zinc finger, CW type with PWWP domain 1 | Multiple_C | 4.22 | 3.97 | 3.96 | 1.20 | 0.8036 | 0.8997 | 3.94 | 4.20 | 4.15 | 0.86 | 0.7366 | 0.8786 |
| TC0700012037.hg.1 | NAT16 | N-acetyltransferase 16 (GCN5-related, putative) | Multiple_C | 5.78 | 5.5 | 5.52 | 1.20 | 0.2212 | 0.4185 | 5.78 | 6.18 | 5.9 | 0.92 | 0.9412 | 0.9757 |

| | | | | | | | | | | | | | | | |
|---|---|---|---|---|---|---|---|---|---|---|---|---|---|---|---|
| TC0700012242.hg.1 | C7orf66 | chromosome 7 open reading frame 66 | Multiple_C | 2.82 | 2.64 | 2.56 | 1.20 | 0.2333 | 0.434 | 3.65 | 3.47 | 3.22 | 1.35 | 0.0487 | 0.2051 |
| TC0700012728.hg.1 | PTN | pleiotrophin | Multiple_C | 6.35 | 5.72 | 6.09 | 1.20 | 0.4328 | 0.6428 | 5.77 | 5.66 | 5.72 | 1.04 | 0.6037 | 0.8019 |
| TC0800006623.hg.1 | DEFB103A | defensin, beta 103A | Coding | 5.44 | 4.96 | 5.18 | 1.20 | 0.1411 | 0.3087 | 5.18 | 5.23 | 5 | 1.13 | 0.4299 | 0.6817 |
| TC0800007196.hg.1 | SMIM18 | small integral membrane protein 18 | Coding | 4.13 | 4.09 | 3.87 | 1.20 | 0.3403 | 0.5543 | 4 | 4.04 | 3.46 | 1.45 | 0.0262 | 0.1416 |
| TC0800008124.hg.1 | CA3 | carbonic anhydrase III | Multiple_C | 4.51 | 4.54 | 4.25 | 1.20 | 0.5126 | 0.7083 | 4.25 | 3.80 | 3.83 | 1.34 | 0.2345 | 0.497 |
| TC0800009094.hg.1 | DENND3 | DENN/MADD domain containing 3 | Multiple_C | 5.91 | 5.84 | 5.65 | 1.20 | 0.0723 | 0.1897 | 5.99 | 5.89 | 5.77 | 1.16 | 0.1009 | 0.3106 |
| TC0900008490.hg.1 | C9orf43 | chromosome 9 open reading frame 43 | Multiple_C | 4.75 | 4.2 | 4.49 | 1.20 | 0.7982 | 0.8965 | 4.32 | 5.08 | 4.93 | 0.66 | 0.225 | 0.4858 |
| TC0900011260.hg.1 | ALAD | aminolevulinate dehydratase | Multiple_C | 4.99 | 4.61 | 4.73 | 1.20 | 0.1198 | 0.2745 | 3.82 | 3.94 | 3.81 | 1.01 | 0.8351 | 0.9272 |
| TC0X00007535.hg.1 | ARR3 | arrestin 3, retinal (X-arrestin) | Multiple_C | 4.47 | 4.17 | 4.21 | 1.20 | 0.3709 | 0.5836 | 4.3 | 4.39 | 4.79 | 0.71 | 0.591 | 0.7931 |
| TC0X00008223.hg.1 | WDR44 | WD repeat domain 44 | Multiple_C | 9.99 | 10.09 | 9.73 | 1.20 | 0.3085 | 0.5215 | 8.87 | 8.46 | 8.95 | 0.95 | 0.2879 | 0.5527 |
| TC0X00009250.hg.1 | ACOT9 | acyl-CoA thioesterase 9 | Multiple_C | 10.93 | 11.54 | 10.67 | 1.20 | 0.5507 | 0.7356 | 9.86 | 10.34 | 10.7 | 0.56 | 0.0224 | 0.1287 |
| TC0X00009541.hg.1 | SLC9A7 | solute carrier family 9, subfamily A (NHE7, cation proton antipo | Multiple_C | 8.33 | 8.4 | 8.07 | 1.20 | 0.6482 | 0.805 | 7.03 | 7.13 | 7.99 | 0.51 | 0.0621 | 0.2366 |
| TC0X00010989.hg.1 | LDOC1 | leucine zipper, down-regulated in cancer 1 | Multiple_C | 3.95 | 3.56 | 3.69 | 1.20 | 0.4617 | 0.667 | 3.91 | 3.83 | 4.12 | 0.86 | 0.2346 | 0.4971 |
| TC1000007068.hg.1 | GAD2 | glutamate decarboxylase 2 | Coding | 6.14 | 5.79 | 5.88 | 1.20 | 0.7665 | 0.8801 | 5.53 | 5.86 | 5.46 | 1.05 | 0.8935 | 0.9547 |
| TC1000007703.hg.1 | BICC1 | BicC family RNA binding protein 1 | Multiple_C | 2.94 | 2.68 | 2.68 | 1.20 | 0.6801 | 0.8261 | 8.68 | 10.15 | 9.94 | 0.42 | 0.0003 | 0.0068 |
| TC1000011003.hg.1 | PLA2G12B | phospholipase A2, group XIIB | Coding | 3.97 | 3.67 | 3.71 | 1.20 | 0.563 | 0.7449 | 4.69 | 5.08 | 4.35 | 1.27 | 0.1385 | 0.3695 |
| TC1000012169.hg.1 | ADAM12 | ADAM metallopeptidase domain 12 | Multiple_C | 3.27 | 2.81 | 3.01 | 1.20 | 0.1943 | 0.384 | 4.22 | 3.89 | 3.62 | 1.52 | 0.0174 | 0.1105 |
| TC1100007704.hg.1 | OR9Q2 | olfactory receptor, family 9, subfamily Q, member 2 | Coding | 3.72 | 3.52 | 3.46 | 1.20 | 0.1405 | 0.3077 | 3.88 | 3.68 | 3.46 | 1.34 | 0.1236 | 0.3471 |
| TC1100008665.hg.1 | CCDC81 | coiled-coil domain containing 81 | Multiple_C | 4.09 | 4.07 | 3.83 | 1.20 | 0.156 | 0.3309 | 4.55 | 4.56 | 4.41 | 1.10 | 0.4823 | 0.7202 |
| TC1100009240.hg.1 | C2CD2L | C2CD2-like | Multiple_C | 7.32 | 7.09 | 7.06 | 1.20 | 0.3482 | 0.5613 | 7.4 | 7.26 | 6.83 | 1.48 | 0.7268 | 0.8731 |
| TC1100009900.hg.1 | OR51G2 | olfactory receptor, family 51, subfamily G, member 2 | Coding | 3.1 | 3.15 | 2.84 | 1.20 | 0.3948 | 0.6084 | 2.85 | 3.25 | 3.06 | 0.86 | 0.1964 | 0.4511 |
| TC1100009931.hg.1 | UBQLN3 | ubiquilin 3 | Coding | 3.3 | 2.9 | 3.04 | 1.20 | 0.0428 | 0.1293 | 3.51 | 3.49 | 3.64 | 0.91 | 0.9347 | 0.9725 |
| TC1100010552.hg.1 | RAG2 | recombination activating gene 2 | Multiple_C | 2.93 | 2.68 | 2.67 | 1.20 | 0.6344 | 0.7965 | 4.84 | 4.27 | 3.74 | 2.14 | 0.0005 | 0.0105 |
| TC1100010654.hg.1 | SYT13 | synaptotagmin XIII | Coding | 3.53 | 3.29 | 3.27 | 1.20 | 0.4954 | 0.6952 | 3.69 | 3.82 | 3.71 | 0.99 | 0.9911 | 0.9963 |
| TC1100010741.hg.1 | CELF1 | CUGBP, Elav-like family member 1 | Multiple_C | 11.89 | 11.29 | 11.63 | 1.20 | 0.1613 | 0.3382 | 11.89 | 11.71 | 12.25 | 0.78 | 0.1354 | 0.3655 |
| TC1100011590.hg.1 | RAB6A | RAB6A, member RAS oncogene family | Multiple_C | 13.94 | 13.75 | 13.68 | 1.20 | 0.2252 | 0.424 | 14.24 | 13.56 | 13.94 | 1.23 | 0.2212 | 0.4813 |
| TC1100011754.hg.1 | TENM4 | teneurin transmembrane protein 4 | Multiple_C | 3.5 | 3.23 | 3.24 | 1.20 | 0.3093 | 0.5224 | 3.68 | 3.21 | 3.48 | 1.15 | 0.3285 | 0.5922 |
| TC1100012811.hg.1 | NFRKB | nuclear factor related to kappaB binding protein | Multiple_C | 8.8 | 8.01 | 8.54 | 1.20 | 0.616 | 0.7848 | 9.53 | 9.54 | 9.28 | 1.19 | 0.7602 | 0.8913 |
| TC1200008731.hg.1 | RFX4 | regulatory factor X, 4 (influences HLA class II expression) | Multiple_C | 4.71 | 4.54 | 4.45 | 1.20 | 0.6281 | 0.7924 | 5.18 | 5.56 | 5.24 | 0.96 | 0.2111 | 0.4687 |
| TC1200008789.hg.1 | DAO | D-amino-acid oxidase | Multiple_C | 3.82 | 3.55 | 3.56 | 1.20 | 0.6592 | 0.8131 | 3.64 | 3.35 | 3.53 | 1.08 | 0.2355 | 0.4982 |
| TC1200009432.hg.1 | LOC338797; R | uncharacterized LOC338797; novel transcript | Multiple_C | 3.71 | 3.5 | 3.45 | 1.20 | 0.3565 | 0.5698 | 4.28 | 4.16 | 4.09 | 1.14 | 0.1707 | 0.4171 |
| TC1200009876.hg.1 | CD69 | CD69 molecule | Multiple_C | 3.97 | 3.35 | 3.71 | 1.20 | 0.5461 | 0.7317 | 4.13 | 3.88 | 3.74 | 1.31 | 0.045 | 0.1963 |
| TC1200010626.hg.1 | C1QL4 | complement component 1, q subcomponent-like 4 | Coding | 6.14 | 5.64 | 5.88 | 1.20 | 0.3154 | 0.5286 | 6.08 | 5.83 | 5.8 | 1.21 | 0.3202 | 0.584 |

| | | | | | | | | | | | | | | | |
|---|---|---|---|---|---|---|---|---|---|---|---|---|---|---|---|
| TC1200010775.hg.1 | KRT3 | keratin 3, type II | Multiple_C | 3.29 | 3.08 | 3.03 | 1.20 | 0.1882 | 0.3756 | 3.54 | 3.44 | 3.43 | 1.08 | 0.5544 | 0.7691 |
| TC1200011453.hg.1 | C12orf50 | chromosome 12 open reading frame 50 | Multiple_C | 3.99 | 3.85 | 3.73 | 1.20 | 0.0633 | 0.1723 | 4.59 | 4.75 | 4.8 | 0.86 | 0.2897 | 0.5543 |
| TC1200011885.hg.1 | GLTP | glycolipid transfer protein | Coding | 9.87 | 9.11 | 9.61 | 1.20 | 0.2634 | 0.4706 | 6.83 | 7.28 | 7.25 | 0.75 | 0.0679 | 0.2481 |
| TC1200012792.hg.1 | KRT81 | keratin 81, type II | Multiple_C | 4.79 | 4.46 | 4.53 | 1.20 | 0.4511 | 0.6586 | 5.15 | 4.90 | 5.29 | 0.91 | 0.5367 | 0.7584 |
| TC1300009196.hg.1 | LINC00550 | long intergenic non-protein coding RNA 550 | Multiple_C | 3.42 | 3 | 3.16 | 1.20 | 0.9708 | 0.9861 | 3.72 | 3.68 | 3.39 | 1.26 | 0.2127 | 0.4706 |
| TC1300009522.hg.1 | UGGT2 | UDP-glucose glycoprotein glucosyltransferase 2 | Multiple_C | 10.82 | 11.56 | 10.56 | 1.20 | 0.413 | 0.6251 | 11.85 | 10.95 | 11.12 | 1.66 | 0.0028 | 0.0335 |
| TC1300009674.hg.1 | KDELC1 | KDEL (Lys-Asp-Glu-Leu) containing 1 | Multiple_C | 8.19 | 8.6 | 7.93 | 1.20 | 0.2576 | 0.4639 | 9.65 | 9.64 | 9.4 | 1.19 | 0.3501 | 0.614 |
| TC1300010011.hg.1 | LINC00452 | long intergenic non-protein coding RNA 452 | Multiple_C | 8.79 | 8.17 | 8.53 | 1.20 | 0.0583 | 0.1623 | 8.14 | 7.80 | 7.42 | 1.65 | 0.0637 | 0.2403 |
| TC1400010171.hg.1 | CCDC85C | coiled-coil domain containing 85C | Multiple_C | 7.87 | 7.31 | 7.61 | 1.20 | 0.8267 | 0.9129 | 6.77 | 7.10 | 7.14 | 0.77 | 0.0703 | 0.2534 |
| TC1400010593.hg.1 | FAM177A1 | family with sequence similarity 177, member A1 | Multiple_C | 10.87 | 11.09 | 10.61 | 1.20 | 0.2739 | 0.4826 | 9.8 | 9.70 | 9.62 | 1.13 | 0.3248 | 0.5885 |
| TC1500007231.hg.1 | GLDN | gliomedin | Multiple_C | 3.87 | 3.66 | 3.61 | 1.20 | 0.4581 | 0.6642 | 3.94 | 4.02 | 3.91 | 1.02 | 0.691 | 0.8526 |
| TC1500007972.hg.1 | CRABP1 | cellular retinoic acid binding protein 1 | Multiple_C | 4.31 | 4.17 | 4.05 | 1.20 | 0.6109 | 0.7811 | 4.29 | 3.68 | 4.25 | 1.03 | 0.1205 | 0.3425 |
| TC1500010769.hg.1 | GOLGA6C | golgin A6 family, member C | Coding | 4.51 | 4.14 | 4.25 | 1.20 | 0.0578 | 0.1612 | 5.34 | 4.62 | 4.62 | 1.65 | 0.0007 | 0.0126 |
| TC1500010846.hg.1 | GOLGA8K; ULI | golgin A8 family, member K; ULK4 pseudogene 1 | Multiple_C | 7.76 | 8.24 | 7.5 | 1.20 | 0.5496 | 0.7346 | 6.57 | 6.28 | 6.95 | 0.77 | 0.3197 | 0.5838 |
| TC1600007277.hg.1 | AQP8 | aquaporin 8 | Coding | 4.4 | 4.91 | 4.14 | 1.20 | 0.1643 | 0.3425 | 4.13 | 4.20 | 4.18 | 0.97 | 0.4732 | 0.7131 |
| TC1600007999.hg.1 | CX3CL1 | chemokine (C-X3-C motif) ligand 1 | Coding | 4.84 | 4.47 | 4.58 | 1.20 | 0.5119 | 0.7076 | 4.44 | 4.53 | 4.39 | 1.04 | 0.304 | 0.5679 |
| TC1600009246.hg.1 | ROGDI | rogdi homolog | Multiple_C | 7.55 | 6.87 | 7.29 | 1.20 | 0.3523 | 0.565 | 8.27 | 8.55 | 8.22 | 1.04 | 0.8434 | 0.9317 |
| TC1700006719.hg.1 | RNASEK; C17c | ribonuclease, RNase K; chromosome 17 open reading frame 49; | Multiple_C | 15.66 | 13.36 | 15.4 | 1.20 | 0.2374 | 0.4389 | 14.6 | 14.02 | 14.71 | 0.93 | 0.4453 | 0.6933 |
| TC1700007153.hg.1 | GRAPL | GRB2-related adaptor protein-like | Coding | 3.45 | 3.22 | 3.19 | 1.20 | 0.4855 | 0.6868 | 4.37 | 4.11 | 3.41 | 1.95 | 0.0049 | 0.0489 |
| TC1700007371.hg.1 | SLC13A2 | solute carrier family 13 (sodium-dependent dicarboxylate trans | Multiple_C | 3.89 | 3.49 | 3.63 | 1.20 | 0.3115 | 0.5248 | 4 | 3.95 | 4.04 | 0.97 | 0.7571 | 0.8898 |
| TC1700007561.hg.1 | CCL13 | chemokine (C-C motif) ligand 13 | Coding | 4.31 | 3.86 | 4.05 | 1.20 | 0.2539 | 0.4591 | 4.68 | 4.44 | 4.34 | 1.27 | 0.2298 | 0.4908 |
| TC1700008134.hg.1 | TBKBP1 | TBK1 binding protein 1 | Coding | 3.96 | 3.77 | 3.7 | 1.20 | 0.2974 | 0.5088 | 5.43 | 5.35 | 5.02 | 1.33 | 0.0688 | 0.2499 |
| TC1700008440.hg.1 | PPM1E | protein phosphatase, Mg2+/Mn2+ dependent, 1E | Multiple_C | 2.99 | 2.9 | 2.73 | 1.20 | 0.2528 | 0.4577 | 4.01 | 4.02 | 4.01 | 1.00 | 0.4686 | 0.7097 |
| TC1700009036.hg.1 | TMC8 | transmembrane channel like 8 | Multiple_C | 5.01 | 4.53 | 4.75 | 1.20 | 0.5265 | 0.7185 | 5.54 | 5.28 | 4.97 | 1.48 | 0.018 | 0.1127 |
| TC1700009248.hg.1 | TEX19 | testis expressed 19 | Coding | 4.64 | 4.85 | 4.38 | 1.20 | 0.5324 | 0.7221 | 4.91 | 3.30 | 3.93 | 1.97 | 0.0027 | 0.033 |
| TC1700009613.hg.1 | ASGR2 | asialoglycoprotein receptor 2 | Multiple_C | 4.39 | 3.98 | 4.13 | 1.20 | 0.3435 | 0.5571 | 4.4 | 4.35 | 3.9 | 1.41 | 0.015 | 0.1002 |
| TC1700009706.hg.1 | MFSD6L | major facilitator superfamily domain containing 6-like | Coding | 3.19 | 3.02 | 2.93 | 1.20 | 0.2071 | 0.4004 | 3.46 | 3.62 | 3.33 | 1.09 | 0.4031 | 0.6593 |
| TC1700009746.hg.1 | MYH3 | myosin, heavy chain 3, skeletal muscle, embryonic | Multiple_C | 3.66 | 3.42 | 3.4 | 1.20 | 0.5959 | 0.7698 | 3.38 | 3.49 | 3.14 | 1.18 | 0.7905 | 0.9065 |
| TC1700011262.hg.1 | C17orf47 | chromosome 17 open reading frame 47 | Coding | 4.51 | 4.03 | 4.25 | 1.20 | 0.3931 | 0.6069 | 5.05 | 5.06 | 4.66 | 1.31 | 0.0797 | 0.272 |
| TC1700012355.hg.1 | KRBA2 | KRAB-A domain containing 2 | Coding | 4.22 | 3.59 | 3.96 | 1.20 | 0.2225 | 0.4202 | 5.21 | 5.41 | 4.63 | 1.49 | 0.0038 | 0.0416 |
| TC1800009011.hg.1 | NETO1 | neuropilin (NRP) and tolloid (TLL)-like 1 | Multiple_C | 3.91 | 3.4 | 3.65 | 1.20 | 0.3897 | 0.6034 | 3.81 | 3.54 | 3.75 | 1.04 | 0.1255 | 0.3501 |
| TC1900007040.hg.1 | CNN1 | calponin 1, basic, smooth muscle | Multiple_C | 6.21 | 5.62 | 5.95 | 1.20 | 0.4467 | 0.6551 | 5.58 | 4.95 | 5.56 | 1.01 | 0.4361 | 0.6868 |
| TC1900007320.hg.1 | DDA1 | DET1 and DDB1 associated 1 | Multiple_C | 11.21 | 10.37 | 10.95 | 1.20 | 0.4421 | 0.6509 | 11.53 | 11.84 | 11.48 | 1.04 | 0.4458 | 0.6935 |

| | | | | | | | | | | | | | | | |
|---|---|---|---|---|---|---|---|---|---|---|---|---|---|---|---|
| TC1900007955.hg.1 | ZNF345 | zinc finger protein 345 | Multiple_C | 4.93 | 5.53 | 4.67 | 1.20 | 0.3835 | 0.5966 | 4.78 | 5.24 | 3.99 | 1.73 | 0.0137 | 0.0951 |
| TC1900009488.hg.1 | PCP2 | Purkinje cell protein 2 | Coding | 4.43 | 4.52 | 4.17 | 1.20 | 0.2761 | 0.4853 | 4.09 | 4.24 | 4.19 | 0.93 | 0.7957 | 0.9088 |
| TC1900009804.hg.1 | C19orf67 | chromosome 19 open reading frame 67 | Multiple_C | 5.48 | 5.19 | 5.22 | 1.20 | 0.1158 | 0.2681 | 4.37 | 4.33 | 4.36 | 1.01 | 0.599 | 0.7986 |
| TC1900011061.hg.1 | CARD8 | caspase recruitment domain family, member 8 | Multiple_C | 7.13 | 7.51 | 6.87 | 1.20 | 0.4619 | 0.667 | 7.1 | 7.59 | 7.33 | 0.85 | 0.1439 | 0.3778 |
| TC1900011318.hg.1 | ZNF888 | zinc finger protein 888 | Multiple_C | 9.06 | 9.2 | 8.8 | 1.20 | 0.3152 | 0.5285 | 7.37 | 8.01 | 7.65 | 0.82 | 0.2082 | 0.4651 |
| TC1900011481.hg.1 | SBK2 | SH3 domain binding kinase family, member 2 | Coding | 3.29 | 2.88 | 3.03 | 1.20 | 0.2448 | 0.4483 | 4.25 | 4.07 | 4.07 | 1.13 | 0.8255 | 0.923 |
| TC1900011901.hg.1 | SLC35E1 | solute carrier family 35, member E1 | Multiple_C | 10.84 | 9.67 | 10.58 | 1.20 | 0.2946 | 0.506 | 11.21 | 11.55 | 12.08 | 0.55 | 0.0004 | 0.0092 |
| TC2000006432.hg.1 | DEFB125 | defensin, beta 125 | Multiple_C | 3.34 | 3.25 | 3.08 | 1.20 | 0.2682 | 0.4763 | 3.84 | 3.43 | 3.55 | 1.22 | 0.2259 | 0.4863 |
| TC2000006694.hg.1 | SLX4IP | SLX4 interacting protein | Multiple_C | 7.3 | 6.66 | 7.04 | 1.20 | 0.2256 | 0.4244 | 10.34 | 11.04 | 10.34 | 1.00 | 0.8255 | 0.923 |
| TC2000009116.hg.1 | MAFB | v-maf avian musculoaponeurotic fibrosarcoma oncogene homo | Coding | 2.93 | 2.52 | 2.67 | 1.20 | 0.1278 | 0.2874 | 3.11 | 3.21 | 3.02 | 1.06 | 0.7686 | 0.8963 |
| TC2000009236.hg.1 | WFDC9 | WAP four-disulfide core domain 9 | Coding | 4.78 | 4.73 | 4.52 | 1.20 | 0.7726 | 0.8826 | 5.06 | 5.45 | 5.34 | 0.82 | 0.2337 | 0.4961 |
| TC2100006883.hg.1 | KRTAP20-3 | keratin associated protein 20-3 | Coding | 4.09 | 3.81 | 3.83 | 1.20 | 0.2291 | 0.4289 | 4.43 | 4.32 | 3.65 | 1.72 | 0.0012 | 0.0188 |
| TC2100008509.hg.1 | DSCR8 | Down syndrome critical region 8 | Multiple_C | 3.45 | 3.12 | 3.19 | 1.20 | 0.895 | 0.9498 | 3.84 | 4.11 | 4.04 | 0.87 | 0.363 | 0.6257 |
| TC2200006855.hg.1 | RGL4 | ral guanine nucleotide dissociation stimulator-like 4 | Multiple_C | 3.64 | 3.31 | 3.38 | 1.20 | 0.2207 | 0.4181 | 4.31 | 4.55 | 4.53 | 0.86 | 0.3265 | 0.5903 |
| TC2200007014.hg.1 | GAS2L1 | growth arrest-specific 2 like 1 | Multiple_C | 4.51 | 4.08 | 4.25 | 1.20 | 0.2024 | 0.3947 | 4.57 | 4.59 | 4.29 | 1.21 | 0.4528 | 0.6978 |
| TC2200007382.hg.1 | APOBEC3H | apolipoprotein B mRNA editing enzyme, catalytic polypeptide-li | Multiple_C | 3.51 | 3.19 | 3.25 | 1.20 | 0.1239 | 0.2808 | 4.56 | 4.25 | 4.21 | 1.27 | 0.5053 | 0.7369 |
| TC2200007490.hg.1 | MEI1 | meiotic double-stranded break formation protein 1 | Multiple_C | 2.99 | 2.73 | 2.73 | 1.20 | 0.3796 | 0.5927 | 3.55 | 3.53 | 3.41 | 1.10 | 0.1729 | 0.4202 |
| TC2200009218.hg.1 | MIR650; IGLV: | microRNA 650; immunoglobulin lambda variable 3-9 (gene/pseu | Multiple_C | 3.82 | 3.59 | 3.56 | 1.20 | 0.3468 | 0.56 | 3.29 | 3.23 | 3.18 | 1.08 | 0.289 | 0.5537 |
| TSUnmapped00000066.h | HMBS | hydroxymethylbilane synthase | Coding | 3.48 | 3.3 | 3.22 | 1.20 | 0.2283 | 0.428 | 3.54 | 3.79 | 3.61 | 0.95 | 0.6364 | 0.8215 |
| TSUnmapped00000117.h | ZNF660 | zinc finger protein 660 | Coding | 3.71 | 3.32 | 3.45 | 1.20 | 0.2431 | 0.4458 | 4.19 | 4.43 | 4.27 | 0.95 | 0.5979 | 0.7976 |
| TC0100006925.hg.1 | PRAMEF16; PI | PRAME family member 16; PRAME family member 17 | Coding | 3.44 | 3.05 | 3.19 | 1.19 | 0.9465 | 0.9754 | 3.42 | 3.38 | 3.35 | 1.05 | 0.7459 | 0.8834 |
| TC0100007017.hg.1 | C1orf64 | chromosome 1 open reading frame 64 | Coding | 4.35 | 3.95 | 4.1 | 1.19 | 0.5425 | 0.7293 | 5.52 | 5.24 | 4.95 | 1.48 | 0.0153 | 0.1016 |
| TC0100007739.hg.1 | C1orf94 | chromosome 1 open reading frame 94 | Multiple_C | 4.24 | 3.99 | 3.99 | 1.19 | 0.5067 | 0.7035 | 4.32 | 4.51 | 4.27 | 1.04 | 0.9704 | 0.9878 |
| TC0100011361.hg.1 | C1orf186 | chromosome 1 open reading frame 186 | Multiple_C | 3.64 | 3.53 | 3.39 | 1.19 | 0.2759 | 0.485 | 3.52 | 3.52 | 3.47 | 1.04 | 0.5451 | 0.7628 |
| TC0100011413.hg.1 | CR2 | complement component (3d/Epstein Barr virus) receptor 2 | Multiple_C | 3.88 | 3.21 | 3.63 | 1.19 | 0.5899 | 0.7653 | 3.72 | 3.51 | 3.62 | 1.07 | 0.5397 | 0.7594 |
| TC0100012535.hg.1 | HES5 | hes family bHLH transcription factor 5 | Coding | 4.19 | 4.01 | 3.94 | 1.19 | 0.3149 | 0.5283 | 4.17 | 4.41 | 4.51 | 0.79 | 0.6283 | 0.8166 |
| TC0100013800.hg.1 | HEYL | hes-related family bHLH transcription factor with YRPW motif-li | Coding | 4.19 | 3.82 | 3.94 | 1.19 | 0.7096 | 0.8459 | 4.52 | 4.46 | 4.58 | 0.96 | 0.6485 | 0.8283 |
| TC0100016828.hg.1 | F13B | coagulation factor XIII, B polypeptide | Multiple_C | 4.21 | 3.92 | 3.96 | 1.19 | 0.7159 | 0.8497 | 4.87 | 4.63 | 4 | 1.83 | 0.0032 | 0.0372 |
| TC0200007296.hg.1 | GALM | galactose mutarotase (aldose 1-epimerase) | Multiple_C | 8.55 | 8.24 | 8.3 | 1.19 | 0.5656 | 0.7469 | 7.23 | 7.08 | 7.31 | 0.95 | 0.8989 | 0.9577 |
| TC0200012134.hg.1 | C2orf71 | chromosome 2 open reading frame 71 | Multiple_C | 6.01 | 5.38 | 5.76 | 1.19 | 0.2373 | 0.4388 | 4.61 | 4.65 | 4.5 | 1.08 | 0.3375 | 0.6018 |
| TC0200013912.hg.1 | CKAP2L | cytoskeleton associated protein 2-like | Multiple_C | 9.56 | 9.18 | 9.31 | 1.19 | 0.3044 | 0.5172 | 9.06 | 9.65 | 9.35 | 0.82 | 0.1146 | 0.3341 |
| TC0200013922.hg.1 | IL36B | interleukin 36, beta | Coding | 3.66 | 3.26 | 3.41 | 1.19 | 0.6174 | 0.7854 | 3.68 | 3.41 | 3.27 | 1.33 | 0.031 | 0.1568 |
| TC0200016026.hg.1 | NMUR1 | neuromedin U receptor 1 | Coding | 4.19 | 3.79 | 3.94 | 1.19 | 0.7247 | 0.8551 | 4.21 | 4.11 | 4.15 | 1.04 | 0.5888 | 0.7918 |

| | | | | | | | | | | | | | | | |
|---|---|---|---|---|---|---|---|---|---|---|---|---|---|---|---|
| TC0200016569.hg.1 | HOXD4 | homeobox D4 | Coding | 4.31 | 4.37 | 4.06 | 1.19 | 0.3744 | 0.5872 | 3.93 | 3.73 | 3.67 | 1.20 | 0.6933 | 0.8541 |
| TC0200016690.hg.1 | DCTN1 | dynactin 1 | Multiple_C | 4.76 | 4.46 | 4.51 | 1.19 | 0.2113 | 0.4057 | 5.39 | 5.60 | 5.44 | 0.97 | 0.3454 | 0.6092 |
| TC0300006554.hg.1 | CPNE9 | copine family member IX | Multiple_C | 3.96 | 3.32 | 3.71 | 1.19 | 0.7337 | 0.8609 | 4.1 | 4.10 | 3.61 | 1.40 | 0.0683 | 0.2489 |
| TC0300008018.hg.1 | C3orf38 | chromosome 3 open reading frame 38 | Multiple_C | 9.04 | 9.63 | 8.79 | 1.19 | 0.6464 | 0.8042 | 7.23 | 6.74 | 7.48 | 0.84 | 0.9501 | 0.9795 |
| TC0300008242.hg.1 | ALCAM | activated leukocyte cell adhesion molecule | Multiple_C | 13.26 | 13.23 | 13.01 | 1.19 | 0.7836 | 0.8888 | 4.78 | 4.04 | 6.02 | 0.42 | 0.6915 | 0.8528 |
| TC0300008544.hg.1 | CASR | calcium-sensing receptor | Multiple_C | 5.6 | 5.28 | 5.35 | 1.19 | 0.0133 | 0.0522 | 5.32 | 5.39 | 5.35 | 0.98 | 0.6577 | 0.8328 |
| TC0300009916.hg.1 | HES1 | hes family bHLH transcription factor 1 | Multiple_C | 11.72 | 12.02 | 11.47 | 1.19 | 0.4187 | 0.6301 | 5.35 | 6.60 | 8.58 | 0.11 | 1.88E-12 | 8.06E-09 |
| TC0300011597.hg.1 | ZNF717 | zinc finger protein 717 | Multiple_C | 7.47 | 7.19 | 7.22 | 1.19 | 0.2798 | 0.4896 | 8.73 | 8.71 | 8.58 | 1.11 | 0.7349 | 0.8777 |
| TC0300013823.hg.1 | CLEC3B | C-type lectin domain family 3, member B | Coding | 3.71 | 3.36 | 3.46 | 1.19 | 0.1955 | 0.3856 | 3.69 | 3.65 | 3.7 | 0.99 | 0.9573 | 0.9823 |
| TC0400007785.hg.1 | ENAM | enamelin | Multiple_C | 3.34 | 2.83 | 3.09 | 1.19 | 0.9721 | 0.9864 | 3.46 | 3.36 | 3.53 | 0.95 | 0.4009 | 0.6575 |
| TC0400008391.hg.1 | EGF | epidermal growth factor | Multiple_C | 4.39 | 3.73 | 4.14 | 1.19 | 0.0756 | 0.196 | 4.73 | 4.85 | 4.04 | 1.61 | 0.0196 | 0.1186 |
| TC0400008504.hg.1 | MTRNR2L13 | MT-RNR2-like 13 | Coding | 3.77 | 3.35 | 3.52 | 1.19 | 0.4728 | 0.6761 | 3.68 | 3.57 | 3.78 | 0.93 | 0.463 | 0.7054 |
| TC0400009223.hg.1 | CPE | carboxypeptidase E | Coding | 3.77 | 3.44 | 3.52 | 1.19 | 0.5804 | 0.7579 | 6.81 | 5.96 | 6.41 | 1.32 | 0.3308 | 0.5949 |
| TC0400012437.hg.1 | SCRG1 | stimulator of chondrogenesis 1 | Coding | 2.9 | 2.73 | 2.65 | 1.19 | 0.1653 | 0.3437 | 3.38 | 3.13 | 2.91 | 1.39 | 0.0906 | 0.2913 |
| TC0400012836.hg.1 | ENPEP | glutamyl aminopeptidase (aminopeptidase A) | Multiple_C | 4.4 | 3.82 | 4.15 | 1.19 | 0.3249 | 0.5389 | 4.06 | 3.90 | 3.77 | 1.22 | 0.0334 | 0.1642 |
| TC0500007966.hg.1 | XRCC4 | X-ray repair complementing defective repair in Chinese hamste | Multiple_C | 9.74 | 11.81 | 9.49 | 1.19 | 0.2593 | 0.4657 | 9.53 | 9.64 | 9.05 | 1.39 | 0.0101 | 0.0785 |
| TC0600007287.hg.1 | HIST1H3E | histone cluster 1, H3e | Coding | 5.23 | 5.05 | 4.98 | 1.19 | 0.5411 | 0.7285 | 6.04 | 6.28 | 6.03 | 1.01 | 0.8673 | 0.9431 |
| TC0600009080.hg.1 | CEP57L1 | centrosomal protein 57kDa-like 1 | Multiple_C | 7.87 | 9.12 | 7.62 | 1.19 | 0.303 | 0.5157 | 7.16 | 6.74 | 7.49 | 0.80 | 0.2527 | 0.5169 |
| TC0600011875.hg.1 | LRRC73 | leucine rich repeat containing 73 | Coding | 3.85 | 3.56 | 3.6 | 1.19 | 0.8802 | 0.9419 | 4.26 | 4.04 | 3.72 | 1.45 | 0.0843 | 0.2795 |
| TC0600013033.hg.1 | TBC1D32 | TBC1 domain family, member 32 | Multiple_C | 6.89 | 8.04 | 6.64 | 1.19 | 0.3331 | 0.5468 | 6.79 | 6.46 | 6.01 | 1.72 | 0.0094 | 0.0749 |
| TC0600013878.hg.1 | PRR18 | proline rich 18 | Coding | 6.41 | 6.24 | 6.16 | 1.19 | 0.6704 | 0.8203 | 6.04 | 6.23 | 6.11 | 0.95 | 0.2729 | 0.5383 |
| TC0600014150.hg.1 | SMIM8 | small integral membrane protein 8 | Coding | 8.93 | 10.55 | 8.68 | 1.19 | 0.8007 | 0.8978 | 7.8 | 7.84 | 6.76 | 2.06 | 0.3361 | 0.6005 |
| TC0600014312.hg.1 | KHDC1L | KH homology domain containing 1-like | Multiple_C | 3.27 | 2.85 | 3.02 | 1.19 | 0.4998 | 0.699 | 3.21 | 2.95 | 2.77 | 1.36 | 0.2042 | 0.4603 |
| TC0700007905.hg.1 | AUTS2 | autism susceptibility candidate 2 | Multiple_C | 8.56 | 7.38 | 8.31 | 1.19 | 0.2368 | 0.4382 | 4.61 | 4.14 | 3.87 | 1.67 | 0.0006 | 0.0113 |
| TC0700010556.hg.1 | HOXA1 | homeobox A1 | Multiple_C | 3.98 | 3.51 | 3.73 | 1.19 | 0.4591 | 0.6652 | 6.77 | 5.70 | 5.3 | 2.77 | 0.0001 | 0.0044 |
| TC0700010760.hg.1 | ELMO1 | engulfment and cell motility 1 | Multiple_C | 4.34 | 3.95 | 4.09 | 1.19 | 0.3275 | 0.5413 | 5 | 4.87 | 4.53 | 1.39 | 0.0596 | 0.2307 |
| TC0700011477.hg.1 | TRIM74 | tripartite motif containing 74 | Coding | 5.69 | 5.74 | 5.44 | 1.19 | 0.3686 | 0.5815 | 5.6 | 5.34 | 4.86 | 1.67 | 0.0145 | 0.0984 |
| TC0700011502.hg.1 | ABHD11 | abhydrolase domain containing 11 | Multiple_C | 9.44 | 8.41 | 9.19 | 1.19 | 0.3708 | 0.5836 | 6.93 | 7.44 | 7.14 | 0.86 | 0.4101 | 0.665 |
| TC0700013500.hg.1 | ADAP1 | ArfGAP with dual PH domains 1 | Multiple_C | 11.42 | 11.37 | 11.17 | 1.19 | 0.791 | 0.893 | 8.91 | 8.60 | 9.03 | 0.92 | 0.831 | 0.9247 |
| TC0800006752.hg.1 | GATA4 | GATA binding protein 4 | Multiple_C | 3.11 | 2.62 | 2.86 | 1.19 | 0.3286 | 0.5425 | 3.25 | 3.18 | 3.06 | 1.14 | 0.0949 | 0.2994 |
| TC0800007057.hg.1 | DOCK5 | dedicator of cytokinesis 5 | Multiple_C | 10.79 | 10.34 | 10.54 | 1.19 | 0.6884 | 0.832 | 10.29 | 9.69 | 10.07 | 1.16 | 0.622 | 0.8128 |
| TC0800009483.hg.1 | PRR23D2 | proline rich 23 domain containing 2 | Coding | 3.9 | 3.28 | 3.65 | 1.19 | 0.2102 | 0.4041 | 3.66 | 3.50 | 3.48 | 1.13 | 0.1037 | 0.3157 |
| TC0800010880.hg.1 | IL7 | interleukin 7 | Multiple_C | 5.33 | 4.73 | 5.08 | 1.19 | 0.0816 | 0.2076 | 3.87 | 3.79 | 3.54 | 1.26 | 0.1972 | 0.452 |

| | | | | | | | | | | | | | | | |
|---|---|---|---|---|---|---|---|---|---|---|---|---|---|---|---|
| TC0800011002.hg.1 | REXO1L10P | REX1, RNA exonuclease 1 homolog-like 10, pseudogene | Multiple_C | 3.47 | 2.99 | 3.22 | 1.19 | 0.1584 | 0.3345 | 4.69 | 4.39 | 4.46 | 1.17 | 0.0472 | 0.2012 |
| TC0800012373.hg.1 | ZFP41 | ZFP41 zinc finger protein | Multiple_C | 4.99 | 4.68 | 4.74 | 1.19 | 0.297 | 0.5085 | 4.69 | 4.77 | 4.78 | 0.94 | 0.6814 | 0.8471 |
| TC0900006438.hg.1 | DOCK8 | dedicator of cytokinesis 8 | Multiple_C | 3.69 | 3.68 | 3.44 | 1.19 | 0.5704 | 0.7504 | 4.35 | 4.34 | 4.04 | 1.24 | 0.4935 | 0.728 |
| TC0900008250.hg.1 | ZNF189 | zinc finger protein 189 | Multiple_C | 8.61 | 9.52 | 8.36 | 1.19 | 0.2571 | 0.4633 | 7.34 | 8.01 | 7.83 | 0.71 | 0.0156 | 0.1028 |
| TC0900012285.hg.1 | CARD9 | caspase recruitment domain family, member 9 | Multiple_C | 4.4 | 4.21 | 4.15 | 1.19 | 0.3226 | 0.5363 | 5.23 | 5.43 | 5.15 | 1.06 | 0.4194 | 0.6729 |
| TC0X00006676.hg.1 | BMX | BMX non-receptor tyrosine kinase | Multiple_C | 3.08 | 2.56 | 2.83 | 1.19 | 0.3489 | 0.5618 | 3.65 | 3.56 | 3.32 | 1.26 | 0.2404 | 0.504 |
| TC0X00006801.hg.1 | CXorf58 | chromosome X open reading frame 58 | Coding | 3.38 | 3.05 | 3.13 | 1.19 | 0.3909 | 0.6046 | 3.39 | 3.57 | 3.4 | 0.99 | 0.7528 | 0.8876 |
| TC0X00007274.hg.1 | MAGED4B; M. | melanoma antigen family D4B; melanoma antigen family D4; sn | Multiple_C | 3.45 | 3.14 | 3.2 | 1.19 | 0.4501 | 0.6579 | 3.85 | 3.66 | 3.74 | 1.08 | 0.4442 | 0.6923 |
| TC0X00009980.hg.1 | CXorf65 | chromosome X open reading frame 65 | Multiple_C | 3.53 | 3.26 | 3.28 | 1.19 | 0.3395 | 0.5535 | 3.8 | 3.80 | 3.47 | 1.26 | 0.0422 | 0.1894 |
| TC0X00010050.hg.1 | PABPC1L2A | poly(A) binding protein, cytoplasmic 1-like 2A | Coding | 4.35 | 3.86 | 4.1 | 1.19 | 0.7792 | 0.8863 | 3.31 | 3.46 | 3.37 | 0.96 | 0.8678 | 0.9432 |
| TC0X00010806.hg.1 | GPR119 | G protein-coupled receptor 119 | Coding | 3.08 | 2.82 | 2.83 | 1.19 | 0.4076 | 0.6199 | 4.09 | 5.52 | 5.64 | 0.34 | 0.0006 | 0.0117 |
| TC0X00011336.hg.1 | IL9R | interleukin 9 receptor | Multiple_C | 5.98 | 5.51 | 5.73 | 1.19 | 0.288 | 0.4987 | 4.72 | 5.04 | 5.25 | 0.69 | 0.0713 | 0.2555 |
| TC0Y00006763.hg.1 | RBMY1F; RBN | RNA binding motif protein, Y-linked, family 1, member F; RNA bi | Multiple_C | 3.01 | 3.07 | 2.76 | 1.19 | 0.5488 | 0.734 | 3.47 | 3.50 | 3.29 | 1.13 | 0.0576 | 0.2261 |
| TC0Y00007332.hg.1 | IL9R | Homo sapiens interleukin 9 receptor (IL9R), transcript variant 1, | Multiple_C | 5.98 | 5.51 | 5.73 | 1.19 | 0.288 | 0.4987 | 4.72 | 5.04 | 5.25 | 0.69 | 0.0713 | 0.2555 |
| TC1000008045.hg.1 | SEC24C | SEC24 homolog C, COPII coat complex component | Multiple_C | 9.52 | 10.01 | 9.27 | 1.19 | 0.1266 | 0.2852 | 10.17 | 10.21 | 10.41 | 0.85 | 0.1505 | 0.3886 |
| TC1000008580.hg.1 | ANKRD2 | ankyrin repeat domain 2 (stretch responsive muscle) | Multiple_C | 5.34 | 4.8 | 5.09 | 1.19 | 0.5576 | 0.7403 | 5.03 | 5.21 | 5.05 | 0.99 | 0.679 | 0.8455 |
| TC1000008715.hg.1 | ELOVL3 | ELOVL fatty acid elongase 3 | Coding | 3.81 | 3.52 | 3.56 | 1.19 | 0.3138 | 0.5272 | 3.05 | 3.22 | 2.88 | 1.13 | 0.3672 | 0.6295 |
| TC1000010724.hg.1 | MRLN | myoregulin | Multiple_C | 3.46 | 3.26 | 3.21 | 1.19 | 0.8753 | 0.9394 | 3.93 | 3.75 | 3.68 | 1.19 | 0.9712 | 0.9882 |
| TC1000010887.hg.1 | NEUROG3 | neurogenin 3 | Coding | 4.99 | 4.5 | 4.74 | 1.19 | 0.6944 | 0.8355 | 5.16 | 5.31 | 4.95 | 1.16 | 0.6302 | 0.8177 |
| TC1000011567.hg.1 | CRTAC1 | cartilage acidic protein 1 | Multiple_C | 2.9 | 2.68 | 2.65 | 1.19 | 0.3251 | 0.539 | 3.57 | 3.37 | 3.25 | 1.25 | 0.0875 | 0.2855 |
| TC1000011774.hg.1 | SORCS1 | sortilin-related VPS10 domain containing receptor 1 | Multiple_C | 4.5 | 3.93 | 4.25 | 1.19 | 0.0685 | 0.1825 | 4.43 | 4.59 | 4.3 | 1.09 | 0.903 | 0.9598 |
| TC1000012530.hg.1 | EBLN1 | endogenous Bornavirus-like nucleoprotein 1 | Multiple_C | 4.47 | 4.43 | 4.22 | 1.19 | 0.5549 | 0.7384 | 4.35 | 3.93 | 3.85 | 1.41 | 0.0614 | 0.2352 |
| TC1100008704.hg.1 | TYR | tyrosinase | Multiple_C | 3.3 | 3 | 3.05 | 1.19 | 0.3641 | 0.5777 | 3.62 | 3.49 | 3.42 | 1.15 | 0.7983 | 0.9096 |
| TC1100009081.hg.1 | ANKK1 | ankyrin repeat and kinase domain containing 1 | Coding | 4.96 | 4.8 | 4.71 | 1.19 | 0.1497 | 0.3216 | 5.11 | 4.90 | 4.84 | 1.21 | 0.4415 | 0.6905 |
| TC1100011268.hg.1 | CATSPER1 | cation channel, sperm associated 1 | Multiple_C | 3.55 | 3.14 | 3.3 | 1.19 | 0.477 | 0.6796 | 3.93 | 4.00 | 3.9 | 1.02 | 0.4312 | 0.6826 |
| TC1100011651.hg.1 | KLHL35 | kelch-like family member 35 | Multiple_C | 3.91 | 3.69 | 3.66 | 1.19 | 0.097 | 0.2355 | 3.15 | 3.20 | 3.12 | 1.02 | 0.829 | 0.9243 |
| TC1100012615.hg.1 | CLMP | CXADR-like membrane protein | Multiple_C | 3.83 | 3.38 | 3.58 | 1.19 | 0.2571 | 0.4633 | 4.05 | 3.84 | 3.81 | 1.18 | 0.2563 | 0.5211 |
| TC1100012708.hg.1 | FEZ1 | fasciculation and elongation protein zeta 1 | Multiple_C | 3.55 | 3.47 | 3.3 | 1.19 | 0.3338 | 0.5476 | 4.39 | 4.22 | 3.92 | 1.39 | 0.1013 | 0.3114 |
| TC1100012791.hg.1 | C11orf45 | chromosome 11 open reading frame 45 | Multiple_C | 4.7 | 4.37 | 4.45 | 1.19 | 0.453 | 0.6605 | 4.13 | 4.12 | 4.06 | 1.05 | 0.8033 | 0.9122 |
| TC1100013069.hg.1 | FOLR3 | folate receptor 3 (gamma) | Multiple_C | 4.54 | 4.08 | 4.29 | 1.19 | 0.6278 | 0.7921 | 4.89 | 4.99 | 4.8 | 1.06 | 0.9049 | 0.9607 |
| TC1200008041.hg.1 | HMGA2 | high mobility group AT-hook 2 | Multiple_C | 5.76 | 5.9 | 5.51 | 1.19 | 0.1486 | 0.3199 | 5.81 | 5.53 | 5.41 | 1.32 | 0.636 | 0.8213 |
| TC1200009705.hg.1 | VWF | von Willebrand factor | Multiple_C | 5.94 | 5.47 | 5.69 | 1.19 | 0.2621 | 0.4692 | 6.79 | 6.47 | 5.94 | 1.80 | 0.0248 | 0.1366 |
| TC1200010840.hg.1 | GTSF1 | gametocyte specific factor 1 | Multiple_C | 3.42 | 3.2 | 3.17 | 1.19 | 0.5271 | 0.7187 | 3.6 | 3.50 | 3.69 | 0.94 | 0.9018 | 0.9592 |

| | | | | | | | | | | | | | | | | |
|---|---|---|---|---|---|---|---|---|---|---|---|---|---|---|---|---|
| TC1200010942.hg.1 | RDH16 | retinol dehydrogenase 16 (all-trans) | Multiple_C | 3.03 | 2.91 | 2.78 | 1.19 | 0.3128 | 0.5263 | 3.16 | 3.00 | 2.98 | 1.13 | 0.2883 | 0.5529 |
| TC1300007925.hg.1 | DAOA | D-amino acid oxidase activator | Coding | 4.09 | 3.72 | 3.84 | 1.19 | 0.8429 | 0.9227 | 4 | 4.06 | 3.75 | 1.19 | 0.0965 | 0.3026 |
| TC1400010574.hg.1 | BCL2L2-PABPN | BCL2L2-PABPN1 readthrough | Multiple_C | 8.94 | 8.65 | 8.69 | 1.19 | 0.591 | 0.766 | 8.24 | 8.36 | 8.27 | 0.98 | 0.908 | 0.962 |
| TC1500008343.hg.1 | UNC45A | unc-45 myosin chaperone A | Multiple_C | 9.29 | 9.21 | 9.04 | 1.19 | 0.6375 | 0.7989 | 8.19 | 8.25 | 7.96 | 1.17 | 0.7098 | 0.8638 |
| TC1500008772.hg.1 | GABRB3 | gamma-aminobutyric acid (GABA) A receptor, beta 3 | Multiple_C | 4.38 | 3.47 | 4.13 | 1.19 | 0.7217 | 0.8535 | 4.02 | 4.13 | 3.9 | 1.09 | 0.4314 | 0.6828 |
| TC1500009355.hg.1 | SHC4 | SHC (Src homology 2 domain containing) family, member 4 | Multiple_C | 3.43 | 3.43 | 3.18 | 1.19 | 0.4845 | 0.686 | 4.9 | 4.51 | 4.23 | 1.59 | 0.0086 | 0.0703 |
| TC1500009384.hg.1 | HDC | histidine decarboxylase | Multiple_C | 4.92 | 4.85 | 4.67 | 1.19 | 0.4066 | 0.6191 | 4.36 | 4.61 | 4.84 | 0.72 | 0.0319 | 0.1598 |
| TC1500010006.hg.1 | STRA6 | stimulated by retinoic acid 6 | Multiple_C | 3.65 | 3 | 3.4 | 1.19 | 0.816 | 0.9073 | 9.88 | 11.10 | 10.31 | 0.74 | 0.8066 | 0.9146 |
| TC1500010765.hg.1 | LMAN1L | lectin, mannose-binding, 1 like | Multiple_C | 3.11 | 2.88 | 2.86 | 1.19 | 0.1947 | 0.3845 | 3.02 | 3.19 | 3 | 1.01 | 0.8113 | 0.9166 |
| TC1600008168.hg.1 | RLTPR | RGD motif, leucine rich repeats, tropomodulin domain and proli | Multiple_C | 6.43 | 5.93 | 6.18 | 1.19 | 0.4425 | 0.6512 | 9.15 | 9.81 | 9.03 | 1.09 | 0.2503 | 0.5146 |
| TC1600009081.hg.1 | ZNF598 | zinc finger protein 598 | Multiple_C | 3.59 | 3.18 | 3.34 | 1.19 | 0.3095 | 0.5227 | 3.64 | 3.70 | 3.78 | 0.91 | 0.4037 | 0.6596 |
| TC1600009868.hg.1 | SULT1A2 | sulfotransferase family 1A member 2 | Multiple_C | 5.64 | 5.24 | 5.39 | 1.19 | 0.7248 | 0.8552 | 7.08 | 7.45 | 6.93 | 1.11 | 0.3584 | 0.6213 |
| TC1600011168.hg.1 | ZCCHC14 | zinc finger, CCHC domain containing 14 | Multiple_C | 8.53 | 7.57 | 8.28 | 1.19 | 0.9638 | 0.9824 | 8.93 | 8.93 | 9.1 | 0.89 | 0.8508 | 0.9357 |
| TC1600011474.hg.1 | ANKS3 | ankyrin repeat and sterile alpha motif domain containing 3 | Multiple_C | 6.27 | 6.33 | 6.02 | 1.19 | 0.629 | 0.793 | 6.84 | 7.21 | 7.21 | 0.77 | 0.0462 | 0.1987 |
| TC1700007784.hg.1 | RAPGEFL1 | Rap guanine nucleotide exchange factor like 1 | Multiple_C | 7.06 | 6.85 | 6.81 | 1.19 | 0.4695 | 0.6735 | 7.15 | 6.80 | 6.81 | 1.27 | 0.2357 | 0.4984 |
| TC1700008331.hg.1 | HLF | hepatic leukemia factor | Multiple_C | 3.25 | 2.81 | 3 | 1.19 | 0.2215 | 0.4189 | 3.7 | 3.46 | 3.43 | 1.21 | 0.8281 | 0.9241 |
| TC1700008923.hg.1 | UNK | unkempt family zinc finger | Multiple_C | 7.7 | 7.03 | 7.45 | 1.19 | 0.5788 | 0.7565 | 9.21 | 9.83 | 9.33 | 0.92 | 0.3706 | 0.6327 |
| TC1700009587.hg.1 | PITPNM3 | PITPNM family member 3 | Multiple_C | 5.05 | 4.69 | 4.8 | 1.19 | 0.2029 | 0.3952 | 3.75 | 3.66 | 3.81 | 0.96 | 0.6944 | 0.8546 |
| TC1700009637.hg.1 | PLSCR3; TMEM | phospholipid scramblase 3; transmembrane protein 256; TMEM | Multiple_C | 7.75 | 7.36 | 7.5 | 1.19 | 0.8638 | 0.9336 | 7.79 | 7.45 | 7.51 | 1.21 | 0.5946 | 0.7953 |
| TC1700010673.hg.1 | KRT35 | keratin 35, type I | Coding | 6.02 | 5.59 | 5.77 | 1.19 | 0.7541 | 0.8741 | 5.59 | 5.65 | 5.56 | 1.02 | 0.8686 | 0.9434 |
| TC1700012412.hg.1 | KRT33B | keratin 33B, type I | Coding | 3.53 | 2.97 | 3.28 | 1.19 | 0.5824 | 0.7598 | 3.71 | 3.84 | 3.5 | 1.16 | 0.2816 | 0.5474 |
| TC1800008549.hg.1 | RIT2 | Ras-like without CAAX 2 | Multiple_C | 3.87 | 3.65 | 3.62 | 1.19 | 0.2405 | 0.4426 | 3.46 | 3.39 | 3.24 | 1.16 | 0.6255 | 0.8149 |
| TC1800008595.hg.1 | LOXHD1 | lipoxygenase homology domains 1 | Multiple_C | 3.83 | 3.29 | 3.58 | 1.19 | 0.4158 | 0.6278 | 3.22 | 3.28 | 3.41 | 0.88 | 0.3587 | 0.6216 |
| TC1800008824.hg.1 | LMAN1 | lectin, mannose-binding, 1 | Multiple_C | 14.01 | 14.24 | 13.76 | 1.19 | 0.3475 | 0.5605 | 13.11 | 12.69 | 13.03 | 1.06 | 0.7175 | 0.8682 |
| TC1800009229.hg.1 | SMAD4 | SMAD family member 4 | Multiple_C | 10.35 | 10.12 | 10.1 | 1.19 | 0.2767 | 0.486 | 5.95 | 6.36 | 6.14 | 0.88 | 0.6751 | 0.843 |
| TC1900006852.hg.1 | RETN | resistin | Coding | 3.44 | 3.15 | 3.19 | 1.19 | 0.2004 | 0.3923 | 3.72 | 3.60 | 3.31 | 1.33 | 0.2247 | 0.4855 |
| TC1900006989.hg.1 | ATG4D | autophagy related 4D, cysteine peptidase | Multiple_C | 10.88 | 10.66 | 10.63 | 1.19 | 0.0848 | 0.2135 | 8.24 | 8.52 | 8.99 | 0.59 | 0.0187 | 0.1154 |
| TC1900007887.hg.1 | KIRREL2 | kin of IRRE like 2 (Drosophila) | Coding | 3.22 | 2.95 | 2.97 | 1.19 | 0.2816 | 0.4916 | 4.04 | 3.92 | 3.84 | 1.15 | 0.414 | 0.6684 |
| TC1900008351.hg.1 | FOXA3 | forkhead box A3 | Coding | 4.56 | 4.01 | 4.31 | 1.19 | 0.9136 | 0.9596 | 5.08 | 5.25 | 5.4 | 0.80 | 0.2581 | 0.5228 |
| TC1900008699.hg.1 | ZNF528 | zinc finger protein 528 | Multiple_C | 3.46 | 2.94 | 3.21 | 1.19 | 0.2571 | 0.4633 | 3.73 | 3.56 | 3.88 | 0.90 | 0.4512 | 0.6966 |
| TC1900008833.hg.1 | CNOT3 | CCR4-NOT transcription complex subunit 3 | Multiple_C | 8.84 | 8.2 | 8.59 | 1.19 | 0.5667 | 0.7477 | 8.48 | 8.70 | 8.74 | 0.84 | 0.2075 | 0.4642 |
| TC1900009084.hg.1 | SHC2 | SHC (Src homology 2 domain containing) transforming protein 2 | Multiple_C | 5.55 | 5.21 | 5.3 | 1.19 | 0.3394 | 0.5535 | 5.29 | 5.46 | 5.32 | 0.98 | 0.7461 | 0.8835 |
| TC1900009684.hg.1 | ACP5 | acid phosphatase 5, tartrate resistant | Multiple_C | 5.25 | 5.03 | 5 | 1.19 | 0.449 | 0.6569 | 4.24 | 4.25 | 4.28 | 0.97 | 0.7467 | 0.8838 |

| | | | | | | | | | | | | | | | |
|---|---|---|---|---|---|---|---|---|---|---|---|---|---|---|---|
| TC1900010370.hg.1 | SLC7A9 | solute carrier family 7 (amino acid transporter light chain, bo,+ s | Multiple_C | 3.26 | 2.89 | 3.01 | 1.19 | 0.3677 | 0.5806 | 3.78 | 3.90 | 3.69 | 1.06 | 0.4033 | 0.6594 |
| TC1900011475.hg.1 | ISOC2 | isochorismatase domain containing 2 | Multiple_C | 9.37 | 8.82 | 9.12 | 1.19 | 0.5277 | 0.7191 | 9.21 | 9.96 | 9.88 | 0.63 | 0.0319 | 0.1598 |
| TC1900011793.hg.1 | ZNF808; RPL39 | zinc finger protein 808; ribosomal protein L39 pseudogene 34 | Multiple_C | 11.18 | 11.19 | 10.93 | 1.19 | 0.3449 | 0.5585 | 8.82 | 8.79 | 8.72 | 1.07 | 0.6196 | 0.8117 |
| TC2000007973.hg.1 | CDH4 | cadherin 4, type 1, R-cadherin (retinal) | Multiple_C | 3.64 | 3.08 | 3.39 | 1.19 | 0.2603 | 0.4669 | 3.39 | 3.20 | 3.29 | 1.07 | 0.3184 | 0.5824 |
| TC2000009789.hg.1 | BHLHE23 | basic helix-loop-helix family, member e23 | Coding | 4.49 | 4.13 | 4.24 | 1.19 | 0.2411 | 0.4433 | 5.02 | 5.12 | 4.94 | 1.06 | 0.8512 | 0.9358 |
| TC2000009968.hg.1 | SIRPB2 | signal-regulatory protein beta 2 | Multiple_C | 3.77 | 3.2 | 3.52 | 1.19 | 0.711 | 0.847 | 3.59 | 3.95 | 3.96 | 0.77 | 0.1091 | 0.3252 |
| TC2100007888.hg.1 | KRTAP23-1 | keratin associated protein 23-1 | Coding | 4.29 | 3.81 | 4.04 | 1.19 | 0.2824 | 0.4926 | 3.79 | 3.69 | 3.84 | 0.97 | 0.5676 | 0.7765 |
| TC2100008128.hg.1 | DSCR4 | Down syndrome critical region 4 | Coding | 3.33 | 2.81 | 3.08 | 1.19 | 0.1092 | 0.2572 | 3.37 | 3.68 | 3.29 | 1.06 | 0.6113 | 0.8057 |
| TC2200006874.hg.1 | DDTL; DDT | D-dopachrome tautomerase-like; D-dopachrome tautomerase | Coding | 12.64 | 11.96 | 12.39 | 1.19 | 0.4064 | 0.6189 | 8.63 | 9.10 | 8.63 | 1.00 | 0.6836 | 0.8482 |
| TC2200007505.hg.1 | SEPT3 | septin 3 | Multiple_C | 4.06 | 3.64 | 3.81 | 1.19 | 0.1969 | 0.3876 | 6.95 | 6.17 | 5.7 | 2.38 | 0.0003 | 0.0079 |
| TC2200009170.hg.1 | ARSA | arylsulfatase A | Multiple_C | 4.02 | 3.7 | 3.77 | 1.19 | 0.1049 | 0.2495 | 4.6 | 4.70 | 4.4 | 1.15 | 0.7989 | 0.9101 |
| TSUnmapped00000119.h | HHAT | hedgehog acyltransferase | Coding | 4.95 | 4.66 | 4.7 | 1.19 | 0.7744 | 0.8837 | 5.05 | 4.74 | 4.32 | 1.66 | 0.0034 | 0.0383 |
| TSUnmapped00000124.h | ZNF546 | zinc finger protein 546 | Coding | 4.42 | 4.41 | 4.17 | 1.19 | 0.3603 | 0.5738 | 5 | 5.19 | 4.78 | 1.16 | 0.2765 | 0.5427 |
| TC0100007765.hg.1 | ZMYM4 | zinc finger, MYM-type 4 | Multiple_C | 9.54 | 9.22 | 9.3 | 1.18 | 0.9121 | 0.9584 | 9.31 | 9.45 | 9.71 | 0.76 | 0.2047 | 0.4609 |
| TC0100009403.hg.1 | CHI3L2 | chitinase 3-like 2 | Multiple_C | 3.71 | 3.58 | 3.47 | 1.18 | 0.625 | 0.7905 | 4.28 | 4.08 | 3.8 | 1.39 | 0.1831 | 0.434 |
| TC0100009937.hg.1 | C1orf56 | chromosome 1 open reading frame 56 | Multiple_C | 7.03 | 6.65 | 6.79 | 1.18 | 0.2199 | 0.417 | 5.47 | 6.03 | 5.76 | 0.82 | 0.251 | 0.5151 |
| TC0100010078.hg.1 | IL6R | interleukin 6 receptor | Multiple_C | 4.24 | 3.86 | 4 | 1.18 | 0.5927 | 0.7674 | 4.97 | 4.82 | 5.38 | 0.75 | 0.059 | 0.2289 |
| TC0100010497.hg.1 | DUSP27 | dual specificity phosphatase 27 (putative) | Multiple_C | 4.7 | 4.59 | 4.46 | 1.18 | 0.1729 | 0.3545 | 4.8 | 4.86 | 4.49 | 1.24 | 0.2024 | 0.4581 |
| TC0100013384.hg.1 | ZNF683 | zinc finger protein 683 | Coding | 3.66 | 3.72 | 3.42 | 1.18 | 0.6658 | 0.8173 | 3.71 | 3.80 | 3.61 | 1.07 | 0.1553 | 0.3955 |
| TC0100015275.hg.1 | ADORA3; TMI | adenosine A3 receptor; transmembrane and immunoglobulin do | Multiple_C | 3.47 | 3.31 | 3.23 | 1.18 | 0.2093 | 0.403 | 3.33 | 3.31 | 3.53 | 0.87 | 0.3686 | 0.6307 |
| TC0100015415.hg.1 | TRIM45 | tripartite motif containing 45 | Multiple_C | 4.05 | 3.81 | 3.81 | 1.18 | 0.3596 | 0.573 | 3.83 | 4.07 | 3.79 | 1.03 | 0.5026 | 0.7346 |
| TC0100015445.hg.1 | TBX15 | T-box 15 | Multiple_C | 3.12 | 3.2 | 2.88 | 1.18 | 0.2067 | 0.4 | 3.07 | 3.33 | 3.87 | 0.57 | 0.0109 | 0.0821 |
| TC0100017209.hg.1 | RD3 | retinal degeneration 3 | Multiple_C | 4.09 | 3.84 | 3.85 | 1.18 | 0.37 | 0.5827 | 4.13 | 3.88 | 3.87 | 1.20 | 0.9781 | 0.9908 |
| TC0100017435.hg.1 | HHIPL2 | HHIP-like 2 | Multiple_C | 3.5 | 3.19 | 3.26 | 1.18 | 0.7716 | 0.8821 | 3.61 | 4.02 | 3.67 | 0.96 | 0.7291 | 0.8742 |
| TC0100018529.hg.1 | SELE | selectin E | Multiple_C | 3.41 | 3.2 | 3.17 | 1.18 | 0.5736 | 0.7527 | 4 | 3.93 | 3.89 | 1.08 | 0.4277 | 0.6798 |
| TC0200007042.hg.1 | KCNK3 | potassium channel, two pore domain subfamily K, member 3 | Coding | 3.73 | 3.49 | 3.49 | 1.18 | 0.2964 | 0.5079 | 3.8 | 3.85 | 3.91 | 0.93 | 0.5244 | 0.7494 |
| TC0200007047.hg.1 | DPYSL5 | dihydropyrimidinase-like 5 | Multiple_C | 3.9 | 3.6 | 3.66 | 1.18 | 0.3262 | 0.5402 | 4.65 | 4.75 | 4.69 | 0.97 | 0.4167 | 0.6705 |
| TC0200007060.hg.1 | DNAJC5G | DnaJ (Hsp40) homolog, subfamily C, member 5 gamma | Multiple_C | 3.91 | 3.57 | 3.67 | 1.18 | 0.4907 | 0.6906 | 5.45 | 4.32 | 4.02 | 2.69 | 0.0005 | 0.011 |
| TC0200008793.hg.1 | SLC5A7 | solute carrier family 5 (sodium/choline cotransporter), member | Multiple_C | 3.61 | 3.33 | 3.37 | 1.18 | 0.2319 | 0.4323 | 3.86 | 4.12 | 3.91 | 0.97 | 0.9094 | 0.9628 |
| TC0200010784.hg.1 | RUFY4 | RUN and FYVE domain containing 4 | Multiple_C | 4.23 | 4.08 | 3.99 | 1.18 | 0.5023 | 0.7004 | 4.11 | 3.78 | 3.96 | 1.11 | 0.7945 | 0.9087 |
| TC0200011731.hg.1 | PDIA6 | protein disulfide isomerase family A, member 6 | Multiple_C | 14.01 | 14.22 | 13.77 | 1.18 | 0.1772 | 0.3598 | 12.08 | 11.68 | 12.43 | 0.78 | 0.018 | 0.1128 |
| TC0200012339.hg.1 | SLC8A1 | solute carrier family 8 (sodium/calcium exchanger), member 1 | Multiple_C | 3.24 | 3.21 | 3 | 1.18 | 0.44 | 0.6489 | 3.59 | 3.65 | 3.41 | 1.13 | 0.3139 | 0.578 |
| TC0200013286.hg.1 | SFTPB | surfactant protein B | Multiple_C | 4.67 | 4.19 | 4.43 | 1.18 | 0.474 | 0.6771 | 5.02 | 5.02 | 4.76 | 1.20 | 0.4644 | 0.7066 |

| | | | | | | | | | | | | | | | |
|---|---|---|---|---|---|---|---|---|---|---|---|---|---|---|---|
| TC0200015971.hg.1 | SLC16A14 | solute carrier family 16, member 14 | Coding | 5.72 | 5.56 | 5.48 | 1.18 | 0.4946 | 0.6945 | 6.91 | 6.40 | 6.91 | 1.00 | 0.3894 | 0.6481 |
| TC0200016202.hg.1 | RAB17 | RAB17, member RAS oncogene family | Multiple_C | 7.66 | 7.22 | 7.42 | 1.18 | 0.2347 | 0.4356 | 8.61 | 8.23 | 8.09 | 1.43 | 0.3398 | 0.6039 |
| TC0200016303.hg.1 | OR6B3 | olfactory receptor, family 6, subfamily B, member 3 | Coding | 3.28 | 2.88 | 3.04 | 1.18 | 0.8643 | 0.9337 | 3.57 | 3.81 | 3.69 | 0.92 | 0.842 | 0.9312 |
| TC0200016422.hg.1 | PPP1CB | protein phosphatase 1, catalytic subunit, beta isozyme | Multiple_C | 12.03 | 12.36 | 11.79 | 1.18 | 0.9682 | 0.9847 | 11.59 | 10.75 | 11.57 | 1.01 | 0.9343 | 0.9724 |
| TC0200016556.hg.1 | SLC4A10 | solute carrier family 4, sodium bicarbonate transporter, membe | Multiple_C | 3.72 | 3.07 | 3.48 | 1.18 | 0.241 | 0.4432 | 3.89 | 3.61 | 3.68 | 1.16 | 0.1266 | 0.352 |
| TC0300007256.hg.1 | CCR2 | chemokine (C-C motif) receptor 2 | Multiple_C | 4.66 | 4.19 | 4.42 | 1.18 | 0.5113 | 0.7072 | 4.43 | 4.87 | 4.53 | 0.93 | 0.917 | 0.9663 |
| TC0300007445.hg.1 | GRM2 | glutamate receptor, metabotropic 2 | Multiple_C | 3.58 | 3.47 | 3.34 | 1.18 | 0.594 | 0.7684 | 3.9 | 3.78 | 3.96 | 0.96 | 0.4135 | 0.668 |
| TC0300008094.hg.1 | OR5AC2 | olfactory receptor, family 5, subfamily AC, member 2 | Coding | 3.22 | 3.04 | 2.98 | 1.18 | 0.5709 | 0.7508 | 3.13 | 3.14 | 3.12 | 1.01 | 0.2305 | 0.4919 |
| TC0300008375.hg.1 | ZDHHC23 | zinc finger, DHHC-type containing 23 | Multiple_C | 7.38 | 6.87 | 7.14 | 1.18 | 0.3907 | 0.6043 | 8.28 | 7.12 | 7.07 | 2.31 | 2.14E-05 | 0.0012 |
| TC0300008705.hg.1 | ABTB1 | ankyrin repeat and BTB (POZ) domain containing 1 | Multiple_C | 6.39 | 6.63 | 6.15 | 1.18 | 0.3896 | 0.6034 | 5.47 | 5.25 | 4.6 | 1.83 | 0.0013 | 0.0198 |
| TC0300009688.hg.1 | HTR3E | 5-hydroxytryptamine (serotonin) receptor 3E, ionotropic | Coding | 3.87 | 3.88 | 3.63 | 1.18 | 0.346 | 0.5597 | 4.04 | 3.69 | 3.54 | 1.41 | 0.0094 | 0.0749 |
| TC0300009692.hg.1 | DVL3 | dishevelled segment polarity protein 3 | Multiple_C | 10.96 | 9.97 | 10.72 | 1.18 | 0.641 | 0.801 | 11.71 | 11.68 | 11.48 | 1.17 | 0.0586 | 0.2284 |
| TC0300009795.hg.1 | RTP1 | receptor (chemosensory) transporter protein 1 | Coding | 4.05 | 3.87 | 3.81 | 1.18 | 0.2787 | 0.4882 | 3.59 | 3.96 | 3.75 | 0.90 | 0.9704 | 0.9878 |
| TC0300012860.hg.1 | GPR149 | G protein-coupled receptor 149 | Coding | 3.18 | 3.01 | 2.94 | 1.18 | 0.3213 | 0.5349 | 4.04 | 3.71 | 3.9 | 1.10 | 0.2822 | 0.5477 |
| TC0300013820.hg.1 | ZNF660 | zinc finger protein 660 | Multiple_C | 3.22 | 2.83 | 2.98 | 1.18 | 0.2962 | 0.5077 | 3.68 | 3.45 | 3.43 | 1.19 | 0.0636 | 0.24 |
| TC0400006519.hg.1 | CRIPAK | cysteine-rich PAK1 inhibitor | Coding | 6.23 | 6.49 | 5.99 | 1.18 | 0.8654 | 0.9344 | 6.07 | 5.51 | 5.28 | 1.73 | 0.0688 | 0.2499 |
| TC0400008977.hg.1 | MAB21L2 | mab-21-like 2 (C. elegans) | Coding | 5.54 | 5.17 | 5.3 | 1.18 | 0.3376 | 0.5513 | 5.91 | 5.81 | 5.66 | 1.19 | 0.9904 | 0.9961 |
| TC0400009853.hg.1 | OTOP1 | otopetrin 1 | Coding | 3.28 | 3.03 | 3.04 | 1.18 | 0.6286 | 0.7928 | 3.3 | 3.29 | 3.25 | 1.04 | 0.3943 | 0.6524 |
| TC0400011535.hg.1 | DKK2 | dickkopf WNT signaling pathway inhibitor 2 | Multiple_C | 3.64 | 3.59 | 3.4 | 1.18 | 0.4863 | 0.6876 | 4.62 | 4.63 | 4.64 | 0.99 | 0.7192 | 0.8689 |
| TC0400011677.hg.1 | TRAM1L1 | translocation associated membrane protein 1-like 1 | Coding | 3.74 | 3.52 | 3.5 | 1.18 | 0.633 | 0.7953 | 3.67 | 3.66 | 3.65 | 1.01 | 0.9169 | 0.9662 |
| TC0400012940.hg.1 | SEC31A | SEC31 homolog A, COPII coat complex component | Multiple_C | 11.45 | 11.46 | 11.21 | 1.18 | 0.3678 | 0.5806 | 11.82 | 10.97 | 11.76 | 1.04 | 0.3127 | 0.5768 |
| TC0500009909.hg.1 | IRX2 | iroquois homeobox 2 | Coding | 4.16 | 3.95 | 3.92 | 1.18 | 0.2314 | 0.4318 | 3.9 | 4.01 | 3.79 | 1.08 | 0.5132 | 0.7428 |
| TC0500011435.hg.1 | LYSMD3 | LysM, putative peptidoglycan-binding, domain containing 3 | Multiple_C | 9.95 | 9.88 | 9.71 | 1.18 | 0.762 | 0.8778 | 10.5 | 9.56 | 10.46 | 1.03 | 0.6962 | 0.8558 |
| TC0500012463.hg.1 | CAMK2A | calcium/calmodulin-dependent protein kinase II alpha | Multiple_C | 6.02 | 5.5 | 5.78 | 1.18 | 0.0376 | 0.1171 | 6.47 | 6.23 | 6.07 | 1.32 | 0.1328 | 0.3617 |
| TC0500012791.hg.1 | KCNMB1 | potassium channel subfamily M regulatory beta subunit 1 | Coding | 4.91 | 4.54 | 4.67 | 1.18 | 0.4036 | 0.6165 | 4.82 | 4.86 | 4.77 | 1.04 | 0.901 | 0.9589 |
| TC0500013322.hg.1 | NAIP | NLR family, apoptosis inhibitory protein | Multiple_C | 6.33 | 6.89 | 6.09 | 1.18 | 0.198 | 0.3888 | 9.16 | 9.23 | 8.53 | 1.55 | 0.0277 | 0.146 |
| TC0600008247.hg.1 | IL17A | interleukin 17A | Coding | 6.05 | 5.84 | 5.81 | 1.18 | 0.9515 | 0.9777 | 4.85 | 4.97 | 5.01 | 0.90 | 0.7042 | 0.8603 |
| TC0600008310.hg.1 | TINAG | tubulointerstitial nephritis antigen | Multiple_C | 4.23 | 4.26 | 3.99 | 1.18 | 0.5537 | 0.7374 | 4.49 | 4.32 | 4.15 | 1.27 | 0.1621 | 0.4053 |
| TC0600011914.hg.1 | TCTE1 | t-complex-associated-testis-expressed 1 | Coding | 3.78 | 3.82 | 3.54 | 1.18 | 0.4077 | 0.62 | 3.49 | 3.69 | 3.7 | 0.86 | 0.4075 | 0.6634 |
| TC0600011938.hg.1 | CLIC5 | chloride intracellular channel 5 | Multiple_C | 4.53 | 4.29 | 4.29 | 1.18 | 0.361 | 0.5744 | 5.31 | 5.11 | 5.08 | 1.17 | 0.1824 | 0.433 |
| TC0600012009.hg.1 | DEFB114 | defensin, beta 114 | Multiple_C | 3.71 | 3.41 | 3.47 | 1.18 | 0.9894 | 0.9943 | 3.8 | 3.82 | 3.32 | 1.39 | 0.2114 | 0.469 |
| TC0600013740.hg.1 | RSPH3 | radial spoke 3 homolog (Chlamydomonas) | Multiple_C | 7.49 | 7.88 | 7.25 | 1.18 | 0.3466 | 0.5599 | 7.91 | 7.95 | 6.99 | 1.89 | 0.0133 | 0.0933 |
| TC0600013914.hg.1 | GPR31 | G protein-coupled receptor 31 | Multiple_C | 4.66 | 4.33 | 4.42 | 1.18 | 0.5182 | 0.7123 | 4.68 | 4.70 | 4.68 | 1.00 | 0.8519 | 0.9359 |

| | | | | | | | | | | | | | | | |
|---|---|---|---|---|---|---|---|---|---|---|---|---|---|---|---|
| TC0700006499.hg.1 | UNCX | UNC homeobox | Coding | 4.6 | 4.27 | 4.36 | 1.18 | 0.9853 | 0.9929 | 4.06 | 4.06 | 4.24 | 0.88 | 0.5429 | 0.7614 |
| TC0700007244.hg.1 | STARD3NL | STARD3 N-terminal like | Multiple_C | 10.97 | 11.91 | 10.73 | 1.18 | 0.7043 | 0.8423 | 8.98 | 9.10 | 9.44 | 0.73 | 0.1943 | 0.4482 |
| TC0700010437.hg.1 | RAPGEF5 | Rap guanine nucleotide exchange factor 5 | Multiple_C | 9.83 | 9.86 | 9.59 | 1.18 | 0.5191 | 0.7129 | 4.24 | 3.60 | 3.24 | 2.00 | 0.0035 | 0.0391 |
| TC0700011165.hg.1 | PHKG1 | phosphorylase kinase, gamma 1 (muscle) | Multiple_C | 3.47 | 3.34 | 3.23 | 1.18 | 0.4647 | 0.6695 | 3.52 | 3.51 | 3.49 | 1.02 | 0.6785 | 0.8452 |
| TC0700011706.hg.1 | ABCB1 | ATP binding cassette subfamily B member 1 | Multiple_C | 4.43 | 4.2 | 4.19 | 1.18 | 0.9576 | 0.9803 | 6.67 | 6.53 | 6.43 | 1.18 | 0.0762 | 0.2658 |
| TC0700011924.hg.1 | TMEM130 | transmembrane protein 130 | Multiple_C | 3.93 | 3.79 | 3.69 | 1.18 | 0.2058 | 0.3988 | 4.06 | 3.99 | 3.97 | 1.06 | 0.4369 | 0.6875 |
| TC0800006892.hg.1 | NAT2 | N-acetyltransferase 2 (arylamine N-acetyltransferase) | Coding | 3.34 | 3.25 | 3.1 | 1.18 | 0.1323 | 0.295 | 3.4 | 3.48 | 3.51 | 0.93 | 0.5923 | 0.7938 |
| TC0800008324.hg.1 | MATN2 | matrilin 2 | Multiple_C | 5.11 | 5.66 | 4.87 | 1.18 | 0.2799 | 0.4896 | 4.15 | 4.20 | 3.78 | 1.29 | 0.3518 | 0.6153 |
| TC0800008772.hg.1 | NDUFB9 | NADH dehydrogenase (ubiquinone) 1 beta subcomplex, 9, 22kD | Multiple_C | 14.61 | 13.81 | 14.37 | 1.18 | 0.305 | 0.518 | 11.52 | 11.35 | 11.03 | 1.40 | 0.015 | 0.1002 |
| TC0800010628.hg.1 | NKAIN3 | Jeck2013 ANTISENSE, coding, INTERNAL, intronic best transcript | Multiple_C | 6.25 | 5.97 | 6.01 | 1.18 | 0.3353 | 0.5494 | 5.92 | 6.13 | 6 | 0.95 | 0.5533 | 0.7684 |
| TC0800012093.hg.1 | CYP11B2 | cytochrome P450, family 11, subfamily B, polypeptide 2 | Coding | 4.26 | 3.97 | 4.02 | 1.18 | 0.4235 | 0.6347 | 4.75 | 4.72 | 4.37 | 1.30 | 0.5594 | 0.7716 |
| TC0900008295.hg.1 | OR13D1 | olfactory receptor, family 13, subfamily D, member 1 | Coding | 4.11 | 3.84 | 3.87 | 1.18 | 0.8376 | 0.9192 | 3.8 | 3.67 | 3.81 | 0.99 | 0.5049 | 0.7368 |
| TC0900009346.hg.1 | CBWD1 | COBW domain containing 1 | Multiple_C | 11.82 | 12.63 | 11.58 | 1.18 | 0.5353 | 0.7243 | 10.5 | 9.95 | 10.46 | 1.03 | 0.1686 | 0.4143 |
| TC0900009351.hg.1 | C9orf66 | chromosome 9 open reading frame 66 | Coding | 3.61 | 2.98 | 3.37 | 1.18 | 0.53 | 0.7204 | 3.6 | 4.10 | 3.78 | 0.88 | 0.4573 | 0.7014 |
| TC0900011473.hg.1 | DENND1A | DENN/MADD domain containing 1A | Multiple_C | 8.05 | 7.98 | 7.81 | 1.18 | 0.7342 | 0.8612 | 7.58 | 7.99 | 7.83 | 0.84 | 0.1979 | 0.453 |
| TC0X00007236.hg.1 | GAGE12C; GA | G antigen 12C; G antigen 12E; G antigen 12H | Coding | 3.53 | 3.09 | 3.29 | 1.18 | 0.4001 | 0.6136 | 3.74 | 3.58 | 3.55 | 1.14 | 0.4878 | 0.7242 |
| TC0X00008232.hg.1 | ZCCHC12 | zinc finger, CCHC domain containing 12 | Coding | 3.21 | 2.83 | 2.97 | 1.18 | 0.8803 | 0.9419 | 3.73 | 3.76 | 3.95 | 0.86 | 0.2277 | 0.4883 |
| TC0X00008365.hg.1 | PRR32 | proline rich 32 | Coding | 3.3 | 2.73 | 3.06 | 1.18 | 0.3944 | 0.608 | 3.7 | 3.47 | 3.48 | 1.16 | 0.265 | 0.5295 |
| TC0X00009767.hg.1 | HUWE1 | HECT, UBA and WWE domain containing 1, E3 ubiquitin protein | Multiple_C | 11.11 | 10.53 | 10.87 | 1.18 | 0.3054 | 0.5185 | 11.59 | 12.67 | 12.73 | 0.45 | 0.0085 | 0.0702 |
| TC0X00010408.hg.1 | TCEAL5 | transcription elongation factor A (SII)-like 5 | Coding | 5.56 | 4.93 | 5.32 | 1.18 | 0.2311 | 0.4315 | 5.23 | 5.41 | 5.07 | 1.12 | 0.3732 | 0.6341 |
| TC0X00011132.hg.1 | CSAG3 | CSAG family, member 3 | Multiple_C | 4.72 | 4.12 | 4.48 | 1.18 | 0.1048 | 0.2495 | 4.63 | 4.52 | 4.48 | 1.11 | 0.3398 | 0.6039 |
| TC0X00011237.hg.1 | RAB39B | RAB39B, member RAS oncogene family | Coding | 3.92 | 3.77 | 3.68 | 1.18 | 0.8233 | 0.9112 | 6.28 | 5.57 | 6.04 | 1.18 | 0.1456 | 0.3806 |
| TC1000006648.hg.1 | RBM17 | RNA binding motif protein 17 | Multiple_C | 11.26 | 10.99 | 11.02 | 1.18 | 0.6225 | 0.7889 | 11.67 | 11.26 | 11.59 | 1.06 | 0.4618 | 0.7045 |
| TC1000008502.hg.1 | TBC1D12 | TBC1 domain family, member 12 | Multiple_C | 7.44 | 8.3 | 7.2 | 1.18 | 0.5969 | 0.7702 | 7.05 | 6.50 | 6.39 | 1.58 | 0.0679 | 0.2481 |
| TC1000010132.hg.1 | MKX | mohawk homeobox | Coding | 3 | 2.56 | 2.76 | 1.18 | 0.3848 | 0.598 | 6.03 | 8.04 | 8.45 | 0.19 | 4.65E-12 | 1.42E-08 |
| TC1000010468.hg.1 | CXCL12 | chemokine (C-X-C motif) ligand 12 | Multiple_C | 5.99 | 5.79 | 5.75 | 1.18 | 0.306 | 0.5192 | 5.44 | 5.30 | 5.15 | 1.22 | 0.0704 | 0.2534 |
| TC1000011213.hg.1 | MAT1A | methionine adenosyltransferase I, alpha | Multiple_C | 2.9 | 2.66 | 2.66 | 1.18 | 0.5453 | 0.7312 | 3.26 | 3.14 | 3.1 | 1.12 | 0.1097 | 0.326 |
| TC1100006752.hg.1 | RBMXL2 | RNA binding motif protein, X-linked-like 2 | Coding | 3.79 | 3.88 | 3.55 | 1.18 | 0.349 | 0.5618 | 4.16 | 4.11 | 4.19 | 0.98 | 0.5133 | 0.7428 |
| TC1100007106.hg.1 | ANO3 | anoctamin 3 | Multiple_C | 4.13 | 3.7 | 3.89 | 1.18 | 0.6098 | 0.7801 | 5.07 | 4.63 | 4.52 | 1.46 | 0.0391 | 0.1803 |
| TC1100007736.hg.1 | OR4D10 | olfactory receptor, family 4, subfamily D, member 10 | Coding | 2.84 | 2.7 | 2.6 | 1.18 | 0.3943 | 0.6079 | 3.5 | 3.48 | 3.36 | 1.10 | 0.2861 | 0.5512 |
| TC1100009202.hg.1 | CD3G | CD3g molecule, gamma (CD3-TCR complex) | Multiple_C | 3.89 | 3.82 | 3.65 | 1.18 | 0.0768 | 0.1985 | 4.18 | 3.46 | 3.26 | 1.89 | 0.0005 | 0.0108 |
| TC1100009406.hg.1 | HEPN1 | hepatocellular carcinoma, down-regulated 1 | Coding | 4.54 | 4.31 | 4.3 | 1.18 | 0.6014 | 0.7736 | 3.56 | 3.70 | 3.67 | 0.93 | 0.7952 | 0.9087 |
| TC1100009904.hg.1 | OR51A2 | olfactory receptor, family 51, subfamily A, member 2 | Coding | 3.17 | 2.61 | 2.93 | 1.18 | 0.9567 | 0.9799 | 3.8 | 3.74 | 3.49 | 1.24 | 0.1244 | 0.3485 |

| | | | | | | | | | | | | | | | |
|---|---|---|---|---|---|---|---|---|---|---|---|---|---|---|---|
| TC1100010063.hg.1 | TMEM41B | transmembrane protein 41B | Multiple_C | 11.34 | 12.26 | 11.1 | 1.18 | 0.8007 | 0.8978 | 11.29 | 10.45 | 10.95 | 1.27 | 0.4681 | 0.7093 |
| TC1100010175.hg.1 | RRAS2 | related RAS viral (r-ras) oncogene homolog 2 | Multiple_C | 11.93 | 12.84 | 11.69 | 1.18 | 0.304 | 0.5169 | 10.55 | 10.00 | 10.74 | 0.88 | 0.2314 | 0.4928 |
| TC1100010456.hg.1 | PAX6 | paired box 6 | Multiple_C | 6.44 | 5.93 | 6.2 | 1.18 | 0.7839 | 0.8889 | 6.31 | 5.31 | 6 | 1.24 | 0.1045 | 0.317 |
| TC1100011270.hg.1 | GAL3ST3 | galactose-3-O-sulfotransferase 3 | Multiple_C | 4.54 | 4.24 | 4.3 | 1.18 | 0.457 | 0.6633 | 4.71 | 5.06 | 4.93 | 0.86 | 0.5064 | 0.7377 |
| TC1100012126.hg.1 | MMP8 | matrix metallopeptidase 8 | Coding | 4.01 | 3.71 | 3.77 | 1.18 | 0.1737 | 0.3556 | 3.42 | 3.37 | 3.47 | 0.97 | 0.4975 | 0.7308 |
| TC1100012906.hg.1 | IGSF9B | immunoglobulin superfamily, member 9B | Multiple_C | 7.43 | 6.9 | 7.19 | 1.18 | 0.7728 | 0.8827 | 6.91 | 6.29 | 6.71 | 1.15 | 0.7325 | 0.8763 |
| TC1200008659.hg.1 | STAB2 | stabilin 2 | Multiple_C | 4.19 | 3.93 | 3.95 | 1.18 | 0.81 | 0.904 | 4.05 | 4.14 | 4.29 | 0.85 | 0.249 | 0.5137 |
| TC1200011438.hg.1 | MGAT4C | MGAT4 family, member C | Multiple_C | 3.24 | 3.01 | 3 | 1.18 | 0.1046 | 0.249 | 3.74 | 3.55 | 3.49 | 1.19 | 0.3142 | 0.5781 |
| TC1200011752.hg.1 | GLT8D2 | glycosyltransferase 8 domain containing 2 | Multiple_C | 3.55 | 3.17 | 3.31 | 1.18 | 0.3763 | 0.5892 | 3.8 | 4.05 | 3.87 | 0.95 | 0.6492 | 0.8288 |
| TC1300006777.hg.1 | TEX26 | testis expressed 26 | Multiple_C | 2.78 | 2.6 | 2.54 | 1.18 | 0.253 | 0.4579 | 3.07 | 3.18 | 3.21 | 0.91 | 0.7065 | 0.8618 |
| TC1300007304.hg.1 | PRR20D; PRR2 | proline rich 20D; proline rich 20B | Coding | 2.91 | 2.66 | 2.67 | 1.18 | 0.3198 | 0.5334 | 3.47 | 3.28 | 3.26 | 1.16 | 0.5077 | 0.7384 |
| TC1300007305.hg.1 | PRR20D; PRR2 | proline rich 20D; proline rich 20B | Coding | 2.91 | 2.66 | 2.67 | 1.18 | 0.3198 | 0.5334 | 3.47 | 3.28 | 3.26 | 1.16 | 0.5077 | 0.7384 |
| TC1300007306.hg.1 | PRR20C; PRR2 | proline rich 20C; proline rich 20E | Coding | 2.91 | 2.66 | 2.67 | 1.18 | 0.3198 | 0.5334 | 3.47 | 3.28 | 3.26 | 1.16 | 0.5077 | 0.7384 |
| TC1300007843.hg.1 | TM9SF2 | transmembrane 9 superfamily member 2 | Multiple_C | 13.21 | 12.99 | 12.97 | 1.18 | 0.6072 | 0.7782 | 14.21 | 14.11 | 13.97 | 1.18 | 0.8901 | 0.9532 |
| TC1300009560.hg.1 | RNF113B | ring finger protein 113B | Coding | 3.5 | 3.05 | 3.26 | 1.18 | 0.3407 | 0.5546 | 4.66 | 4.55 | 4.16 | 1.41 | 0.0504 | 0.2091 |
| TC1300009961.hg.1 | PRR20B; PRR2 | proline rich 20B; proline rich 20E; proline rich 20A; proline rich 2 | Coding | 2.91 | 2.66 | 2.67 | 1.18 | 0.3198 | 0.5334 | 3.47 | 3.28 | 3.26 | 1.16 | 0.5077 | 0.7384 |
| TC1300009962.hg.1 | PRR20C; PRR2 | proline rich 20C; proline rich 20D | Coding | 2.91 | 2.66 | 2.67 | 1.18 | 0.3198 | 0.5334 | 3.47 | 3.28 | 3.26 | 1.16 | 0.5077 | 0.7384 |
| TC1400006739.hg.1 | NFATC4 | nuclear factor of activated T-cells, cytoplasmic, calcineurin-depe | Multiple_C | 5.18 | 4.98 | 4.94 | 1.18 | 0.1289 | 0.2891 | 4.66 | 4.76 | 4.78 | 0.92 | 0.8806 | 0.9487 |
| TC1400008209.hg.1 | SLC25A47 | solute carrier family 25, member 47 | Multiple_C | 7.3 | 7.28 | 7.06 | 1.18 | 0.2025 | 0.3947 | 6.52 | 6.60 | 6.36 | 1.12 | 0.581 | 0.7859 |
| TC1400010257.hg.1 | HSP90AA1 | heat shock protein 90kDa alpha (cytosolic), class A member 1 | Multiple_C | 16.02 | 15.8 | 15.78 | 1.18 | 0.6963 | 0.8369 | 15.67 | 15.02 | 15.81 | 0.91 | 0.3093 | 0.5733 |
| TC1500008620.hg.1 | GOLGA6L6 | golgin A6 family-like 6 | Coding | 2.89 | 2.63 | 2.65 | 1.18 | 0.3544 | 0.5674 | 3.05 | 3.24 | 3.3 | 0.84 | 0.3573 | 0.6202 |
| TC1500010292.hg.1 | SEC11A | SEC11 homolog A, signal peptidase complex subunit | Multiple_C | 15.78 | 17.29 | 15.54 | 1.18 | 0.1405 | 0.3077 | 13.36 | 12.85 | 13.03 | 1.26 | 0.4111 | 0.6657 |
| TC1500010739.hg.1 | SLC12A1 | solute carrier family 12 (sodium/potassium/chloride transporte | Multiple_C | 3.36 | 3.01 | 3.12 | 1.18 | 0.7379 | 0.8637 | 3.72 | 3.80 | 3.44 | 1.21 | 0.1125 | 0.3304 |
| TC1600006580.hg.1 | SYNGR3 | synaptogyrin 3 | Multiple_C | 5.92 | 5.42 | 5.68 | 1.18 | 0.2212 | 0.4185 | 6.16 | 5.99 | 5.74 | 1.34 | 0.0972 | 0.3038 |
| TC1600006926.hg.1 | SNN | stannin | Coding | 4.74 | 4.41 | 4.5 | 1.18 | 0.3934 | 0.6073 | 5.85 | 5.77 | 5.74 | 1.08 | 0.5398 | 0.7594 |
| TC1600008972.hg.1 | WDR24 | WD repeat domain 24 | Multiple_C | 5.33 | 4.88 | 5.09 | 1.18 | 0.4166 | 0.6284 | 5.44 | 5.78 | 5.87 | 0.74 | 0.3349 | 0.5992 |
| TC1600008973.hg.1 | FBXL16 | F-box and leucine-rich repeat protein 16 | Multiple_C | 4.16 | 3.78 | 3.92 | 1.18 | 0.3143 | 0.5277 | 5.62 | 6.18 | 5.97 | 0.78 | 0.5292 | 0.7527 |
| TC1600010012.hg.1 | PYDC1 | PYD (pyrin domain) containing 1 | Multiple_C | 3.61 | 2.89 | 3.37 | 1.18 | 0.3334 | 0.5472 | 3.68 | 3.68 | 3.75 | 0.95 | 0.7471 | 0.8841 |
| TC1700007326.hg.1 | TBC1D3P5 | TBC1 domain family, member 3 pseudogene 5 | Multiple_C | 4.03 | 3.79 | 3.79 | 1.18 | 0.4204 | 0.6318 | 4 | 4.34 | 4.16 | 0.90 | 0.4104 | 0.6652 |
| TC1700007381.hg.1 | SUPT6H | SPT6 homolog, histone chaperone | Multiple_C | 11.78 | 10.89 | 11.54 | 1.18 | 0.1721 | 0.3533 | 11.9 | 12.30 | 11.96 | 0.96 | 0.7158 | 0.8673 |
| TC1700008328.hg.1 | STXBP4 | syntaxin binding protein 4 | Multiple_C | 7.47 | 6.89 | 7.23 | 1.18 | 0.6962 | 0.8368 | 8.33 | 7.93 | 8.44 | 0.93 | 0.2345 | 0.497 |
| TC1700008378.hg.1 | MSI2 | musashi RNA binding protein 2 | Multiple_C | 10.69 | 11.05 | 10.45 | 1.18 | 0.4317 | 0.642 | 12.03 | 11.99 | 11.81 | 1.16 | 0.1906 | 0.4436 |
| TC1700009189.hg.1 | HGS | hepatocyte growth factor-regulated tyrosine kinase substrate | Multiple_C | 10.51 | 9.15 | 10.27 | 1.18 | 0.5058 | 0.703 | 11 | 11.60 | 11.59 | 0.66 | 0.0206 | 0.1228 |

| | | | | | | | | | | | | | | | |
|---|---|---|---|---|---|---|---|---|---|---|---|---|---|---|---|
| TC1700009669.hg.1 | ALOX12B | arachidonate 12-lipoxygenase, 12R type | Multiple_C | 4.58 | 4.02 | 4.34 | 1.18 | 0.1413 | 0.3089 | 4.5 | 4.60 | 4.73 | 0.85 | 0.4244 | 0.6772 |
| TC1700010426.hg.1 | SLFN13 | schlafen family member 13 | Multiple_C | 3.21 | 3.22 | 2.97 | 1.18 | 0.2946 | 0.5061 | 3.35 | 3.26 | 3.29 | 1.04 | 0.6566 | 0.8321 |
| TC1700010643.hg.1 | KRT40 | keratin 40, type I | Coding | 2.97 | 2.72 | 2.73 | 1.18 | 0.1133 | 0.2638 | 9.16 | 11.25 | 9.06 | 1.07 | 0.639 | 0.823 |
| TC1700012195.hg.1 | TBC1D26 | TBC1 domain family, member 26 | Multiple_C | 4.34 | 4.17 | 4.1 | 1.18 | 0.2168 | 0.4128 | 4.81 | 4.71 | 4.69 | 1.09 | 0.5637 | 0.774 |
| TC1700012257.hg.1 | KRTAP9-3 | keratin associated protein 9-3 | Coding | 3.94 | 3.49 | 3.7 | 1.18 | 0.6154 | 0.7848 | 4.11 | 3.72 | 3.82 | 1.22 | 0.4343 | 0.6852 |
| TC1700012268.hg.1 | CRHR1 | corticotropin releasing hormone receptor 1 | Multiple_C | 3.6 | 3.22 | 3.36 | 1.18 | 0.9861 | 0.9932 | 3.85 | 3.43 | 3.76 | 1.06 | 0.8666 | 0.9428 |
| TC1800006897.hg.1 | LAMA3 | laminin, alpha 3 | Multiple_C | 11.79 | 11.32 | 11.55 | 1.18 | 0.915 | 0.96 | 9.43 | 7.45 | 9.08 | 1.27 | 0.0543 | 0.2185 |
| TC1900006436.hg.1 | OR4F17 | olfactory receptor, family 4, subfamily F, member 17 | Multiple_C | 3.25 | 3.17 | 3.01 | 1.18 | 0.3586 | 0.5723 | 4.12 | 3.85 | 3.86 | 1.20 | 0.1378 | 0.3688 |
| TC1900006930.hg.1 | OR7E24 | olfactory receptor, family 7, subfamily E, member 24 | Coding | 3.18 | 3.1 | 2.94 | 1.18 | 0.6591 | 0.8131 | 3.63 | 3.56 | 3.31 | 1.25 | 0.0306 | 0.1556 |
| TC1900007176.hg.1 | PKN1 | protein kinase N1 | Multiple_C | 7.59 | 7.3 | 7.35 | 1.18 | 0.6192 | 0.787 | 7.15 | 7.42 | 7.3 | 0.90 | 0.3086 | 0.5727 |
| TC1900008050.hg.1 | IFNL2 | interferon, lambda 2 | Coding | 4.17 | 3.89 | 3.93 | 1.18 | 0.2993 | 0.5111 | 4.32 | 4.46 | 4.26 | 1.04 | 0.9323 | 0.9715 |
| TC1900009007.hg.1 | ZSCAN4 | zinc finger and SCAN domain containing 4 | Coding | 3.42 | 2.92 | 3.18 | 1.18 | 0.3086 | 0.5216 | 4.35 | 4.49 | 4 | 1.27 | 0.024 | 0.1339 |
| TC1900009371.hg.1 | ARRDC5 | arrestin domain containing 5 | Coding | 3.59 | 3.07 | 3.35 | 1.18 | 0.7489 | 0.8712 | 4.32 | 4.12 | 4.45 | 0.91 | 0.4395 | 0.6898 |
| TC1900011127.hg.1 | HRC | histidine rich calcium binding protein | Multiple_C | 5.37 | 5.3 | 5.13 | 1.18 | 0.0607 | 0.167 | 5.33 | 5.17 | 4.97 | 1.28 | 0.6364 | 0.8215 |
| TC2000006872.hg.1 | INSM1 | insulinoma-associated 1 | Coding | 4.34 | 3.99 | 4.1 | 1.18 | 0.4237 | 0.6348 | 4.53 | 4.24 | 4.08 | 1.37 | 0.0109 | 0.0821 |
| TC2000007329.hg.1 | ACTR5 | ARP5 actin-related protein 5 homolog (yeast) | Multiple_C | 8.66 | 9.36 | 8.42 | 1.18 | 0.4537 | 0.6612 | 7.19 | 7.46 | 8.18 | 0.50 | 0.0156 | 0.1029 |
| TC2000007519.hg.1 | CD40 | CD40 molecule, TNF receptor superfamily member 5 | Multiple_C | 4.33 | 4.05 | 4.09 | 1.18 | 0.217 | 0.4129 | 4.14 | 4.25 | 4.35 | 0.86 | 0.729 | 0.8742 |
| TC2000008436.hg.1 | TASP1 | taspase 1 | Multiple_C | 9.67 | 10.41 | 9.43 | 1.18 | 0.4122 | 0.6243 | 11.16 | 10.42 | 10.4 | 1.69 | 0.0045 | 0.0459 |
| TC2000009469.hg.1 | ZFP64 | ZFP64 zinc finger protein | Multiple_C | 10.33 | 9.63 | 10.09 | 1.18 | 0.781 | 0.8871 | 8.95 | 9.35 | 9.57 | 0.65 | 0.4012 | 0.6577 |
| TC2000009921.hg.1 | L3MBTL1 | l(3)mbt-like 1 (Drosophila) | Multiple_C | 3.26 | 2.68 | 3.02 | 1.18 | 0.5351 | 0.7243 | 3.44 | 3.53 | 3.58 | 0.91 | 0.805 | 0.9135 |
| TC2100006554.hg.1 | POTED | POTE ankyrin domain family, member D | Coding | 3.55 | 3.58 | 3.31 | 1.18 | 0.7542 | 0.8741 | 3.91 | 4.03 | 4.03 | 0.92 | 0.9555 | 0.9815 |
| TC2100007508.hg.1 | KCNE1 | potassium channel, voltage gated subfamily E regulatory beta su | Coding | 3.3 | 3.15 | 3.06 | 1.18 | 0.1447 | 0.3144 | 3.42 | 3.87 | 3.59 | 0.89 | 0.9109 | 0.9633 |
| TC2100008374.hg.1 | TSPEAR | thrombospondin-type laminin G domain and EAR repeats | Coding | 3.68 | 3.36 | 3.44 | 1.18 | 0.7552 | 0.8745 | 4.45 | 4.31 | 4.43 | 1.01 | 0.6819 | 0.8474 |
| TC2200007655.hg.1 | PPARA | peroxisome proliferator-activated receptor alpha | Multiple_C | 8.01 | 8.34 | 7.77 | 1.18 | 0.5188 | 0.7127 | 6.22 | 5.95 | 5.87 | 1.27 | 0.1233 | 0.3467 |
| TC2200008941.hg.1 | LDOC1L | leucine zipper, down-regulated in cancer 1-like | Coding | 5.22 | 4.98 | 4.98 | 1.18 | 0.2723 | 0.4808 | 6.3 | 7.05 | 7.16 | 0.55 | 0.0569 | 0.2245 |
| TCUn_GL000218v10000( | LOC389834; A | ankyrin repeat domain 57 pseudogene | Multiple_C | 3.7 | 3.41 | 3.46 | 1.18 | 0.6131 | 0.783 | 6.74 | 6.39 | 6.71 | 1.02 | 0.9661 | 0.9858 |
| TSUnmapped00000355.h | LEUTX | leucine twenty homeobox | Coding | 3.19 | 2.85 | 2.95 | 1.18 | 0.282 | 0.492 | 3.69 | 3.37 | 3.38 | 1.24 | 0.1706 | 0.4168 |
| TSUnmapped00000493.h | TRAPPC4 | trafficking protein particle complex 4 | Coding | 6.35 | 6.28 | 6.11 | 1.18 | 0.2336 | 0.4343 | 5.21 | 5.35 | 5.3 | 0.94 | 0.3685 | 0.6307 |
| TSUnmapped00000514.h | ZNF197 | zinc finger protein 197 | Coding | 5.2 | 5.15 | 4.96 | 1.18 | 0.5838 | 0.7608 | 4.71 | 4.69 | 4.75 | 0.97 | 0.3445 | 0.6081 |
| TC0100006822.hg.1 | PEX14 | peroxisomal biogenesis factor 14 | Multiple_C | 8.75 | 8.22 | 8.52 | 1.17 | 0.5243 | 0.7167 | 8.98 | 8.99 | 9.11 | 0.91 | 0.6532 | 0.8309 |
| TC0100006857.hg.1 | PTCHD2 | patched domain containing 2 | Multiple_C | 4.45 | 4.17 | 4.22 | 1.17 | 0.4607 | 0.6664 | 4.87 | 5.04 | 4.88 | 0.99 | 0.3649 | 0.6273 |
| TC0100007846.hg.1 | C1orf122 | chromosome 1 open reading frame 122 | Multiple_C | 7.16 | 7.05 | 6.93 | 1.17 | 0.4869 | 0.6881 | 4.62 | 5.01 | 4.57 | 1.04 | 0.6493 | 0.8288 |
| TC0100007991.hg.1 | FOXO6 | forkhead box O6 | Coding | 5.13 | 4.66 | 4.9 | 1.17 | 0.3363 | 0.5503 | 5.37 | 5.36 | 5.25 | 1.09 | 0.4835 | 0.7211 |

| | | | | | | | | | | | | | | | |
|---|---|---|---|---|---|---|---|---|---|---|---|---|---|---|---|
| TC0100009032.hg.1 | BTBD8 | BTB (POZ) domain containing 8 | Multiple_C | 6.58 | 7.91 | 6.35 | 1.17 | 0.3637 | 0.5772 | 6.31 | 6.13 | 6.01 | 1.23 | 0.2501 | 0.5146 |
| TC0100009881.hg.1 | BOLA1 | bolA family member 1 | Multiple_C | 7.82 | 7.34 | 7.59 | 1.17 | 0.1789 | 0.3624 | 7.14 | 7.12 | 6.89 | 1.19 | 0.2111 | 0.4687 |
| TC0100010713.hg.1 | PAPPA2 | pappalysin 2 | Multiple_C | 2.87 | 2.72 | 2.64 | 1.17 | 0.7081 | 0.8449 | 3.26 | 3.43 | 3.56 | 0.81 | 0.1958 | 0.4501 |
| TC0100011004.hg.1 | RGS21 | regulator of G-protein signaling 21 | Coding | 4.15 | 4.01 | 3.92 | 1.17 | 0.5465 | 0.7321 | 3.96 | 3.84 | 4.04 | 0.95 | 0.5338 | 0.7563 |
| TC0100012668.hg.1 | CHD5 | chromodomain helicase DNA binding protein 5 | Coding | 3.19 | 3.07 | 2.96 | 1.17 | 0.6896 | 0.8329 | 3.78 | 3.59 | 3.4 | 1.30 | 0.1133 | 0.332 |
| TC0100013194.hg.1 | EIF4G3 | eukaryotic translation initiation factor 4 gamma, 3 | Multiple_C | 11.78 | 10.78 | 11.55 | 1.17 | 0.8548 | 0.929 | 13.18 | 12.67 | 12.71 | 1.39 | 0.3011 | 0.5655 |
| TC0100013430.hg.1 | CD164L2 | CD164 sialomucin-like 2 | Multiple_C | 3.93 | 3.45 | 3.7 | 1.17 | 0.4036 | 0.6165 | 4.13 | 3.61 | 3.59 | 1.45 | 0.0351 | 0.1693 |
| TC0100013548.hg.1 | PUM1 | pumilio RNA binding family member 1 | Multiple_C | 12.21 | 11.43 | 11.98 | 1.17 | 0.9584 | 0.9803 | 10.88 | 10.88 | 11.08 | 0.87 | 0.78 | 0.9013 |
| TC0100014009.hg.1 | TESK2 | testis-specific kinase 2 | Multiple_C | 7.08 | 7.44 | 6.85 | 1.17 | 0.9407 | 0.9724 | 3.89 | 3.82 | 3.93 | 0.97 | 0.9648 | 0.985 |
| TC0100015750.hg.1 | HORMAD1 | HORMA domain containing 1 | Multiple_C | 3.4 | 2.99 | 3.17 | 1.17 | 0.9535 | 0.9784 | 3.83 | 3.87 | 3.56 | 1.21 | 0.3205 | 0.5844 |
| TC0100016052.hg.1 | FCRL1 | Fc receptor-like 1 | Multiple_C | 4.3 | 4.21 | 4.07 | 1.17 | 0.3924 | 0.6062 | 4.66 | 4.63 | 4.33 | 1.26 | 0.0363 | 0.173 |
| TC0100018230.hg.1 | CYP4B1 | cytochrome P450, family 4, subfamily B, polypeptide 1 | Multiple_C | 3.67 | 3.55 | 3.44 | 1.17 | 0.3273 | 0.5412 | 4.39 | 4.22 | 3.99 | 1.32 | 0.1743 | 0.422 |
| TC0100018294.hg.1 | LCE3C | late cornified envelope 3C | Coding | 3.07 | 3.13 | 2.84 | 1.17 | 0.8385 | 0.9197 | 3.55 | 3.40 | 3.4 | 1.11 | 0.6286 | 0.8168 |
| TC0100018308.hg.1 | FCGR2A | Fc fragment of IgG, low affinity IIa, receptor (CD32) | Multiple_C | 5.1 | 5.03 | 4.87 | 1.17 | 0.1683 | 0.3481 | 4.7 | 4.32 | 4.54 | 1.12 | 0.377 | 0.6374 |
| TC0200006540.hg.1 | DCDC2C | doublecortin domain containing 2C | Multiple_C | 4.08 | 3.65 | 3.85 | 1.17 | 0.1992 | 0.3904 | 4.41 | 4.56 | 4.17 | 1.18 | 0.1907 | 0.4438 |
| TC0200007054.hg.1 | ABHD1 | abhydrolase domain containing 1 | Multiple_C | 3.44 | 3.78 | 3.21 | 1.17 | 0.6844 | 0.8292 | 3.84 | 4.01 | 3.85 | 0.99 | 0.7559 | 0.8889 |
| TC0200010790.hg.1 | GPBAR1 | G protein-coupled bile acid receptor 1 | Coding | 3.35 | 2.88 | 3.12 | 1.17 | 0.5093 | 0.7054 | 3.78 | 3.82 | 3.62 | 1.12 | 0.2209 | 0.4812 |
| TC0200010794.hg.1 | CATIP | ciliogenesis associated TTC17 interacting protein | Multiple_C | 3.29 | 3.54 | 3.06 | 1.17 | 0.3759 | 0.5887 | 3.4 | 3.20 | 3.37 | 1.02 | 0.7044 | 0.8605 |
| TC0200014785.hg.1 | FIGN | fidgetin | Multiple_C | 3.09 | 3.07 | 2.86 | 1.17 | 0.2755 | 0.4846 | 3.42 | 3.08 | 3.18 | 1.18 | 0.3779 | 0.6379 |
| TC0200016073.hg.1 | KCNJ13 | potassium channel, inwardly rectifying subfamily J, member 13 | Multiple_C | 3.9 | 3.85 | 3.67 | 1.17 | 0.7663 | 0.88 | 4.13 | 4.05 | 4.12 | 1.01 | 0.9643 | 0.9848 |
| TC0300010566.hg.1 | EOMES | eomesodermin | Coding | 3.66 | 3.39 | 3.43 | 1.17 | 0.5253 | 0.7174 | 4.84 | 5.21 | 5.09 | 0.84 | 0.173 | 0.4202 |
| TC0300010581.hg.1 | AZI2 | 5-azacytidine induced 2 | Multiple_C | 8.64 | 9.31 | 8.41 | 1.17 | 0.8549 | 0.929 | 7.63 | 7.38 | 8.1 | 0.72 | 0.0462 | 0.1987 |
| TC0300013996.hg.1 | TMEM110-MU | TMEM110-MUSTN1 readthrough; transmembrane protein 110 | Multiple_C | 7.48 | 6.95 | 7.25 | 1.17 | 0.9612 | 0.9816 | 6.2 | 6.10 | 5.95 | 1.19 | 0.9849 | 0.9937 |
| TC0400006668.hg.1 | LINC01587 | long intergenic non-protein coding RNA 1587 | Multiple_C | 4.13 | 3.71 | 3.9 | 1.17 | 0.4257 | 0.6364 | 4.03 | 4.07 | 4.58 | 0.68 | 0.1141 | 0.3334 |
| TC0400007330.hg.1 | NSUN7 | NOP2/Sun domain family, member 7 | Multiple_C | 8.22 | 9.51 | 7.99 | 1.17 | 0.7778 | 0.8859 | 5.11 | 3.84 | 4.35 | 1.69 | 0.0242 | 0.1343 |
| TC0400008613.hg.1 | ADAD1 | adenosine deaminase domain containing 1 | Multiple_C | 2.98 | 2.83 | 2.75 | 1.17 | 0.4333 | 0.6433 | 3.33 | 3.34 | 3.39 | 0.96 | 0.2038 | 0.4595 |
| TC0400009741.hg.1 | FAM53A | family with sequence similarity 53, member A | Multiple_C | 4.93 | 4.53 | 4.7 | 1.17 | 0.1722 | 0.3535 | 5.34 | 5.29 | 5.13 | 1.16 | 0.0708 | 0.2544 |
| TC0400011580.hg.1 | CFI | complement factor I | Multiple_C | 4.77 | 4.44 | 4.54 | 1.17 | 0.4178 | 0.6295 | 4.83 | 4.80 | 4.03 | 1.74 | 0.1265 | 0.3518 |
| TC0400012350.hg.1 | SPOCK3 | sparc/osteonectin, cwcv and kazal-like domains proteoglycan (t | Coding | 4.25 | 3.88 | 4.02 | 1.17 | 0.2413 | 0.4436 | 3.89 | 3.73 | 3.75 | 1.10 | 0.2995 | 0.5639 |
| TC0500009411.hg.1 | RANBP17 | RAN binding protein 17 | Multiple_C | 7.22 | 8.08 | 6.99 | 1.17 | 0.616 | 0.7848 | 6.38 | 6.63 | 7.52 | 0.45 | 0.0005 | 0.0098 |
| TC0500009749.hg.1 | BTNL3 | butyrophilin-like 3 | Coding | 4.07 | 3.67 | 3.84 | 1.17 | 0.1898 | 0.3776 | 4.09 | 4.10 | 3.88 | 1.16 | 0.4285 | 0.6803 |
| TC0500010490.hg.1 | UGT3A1 | UDP glycosyltransferase 3 family, polypeptide A1 | Multiple_C | 4.22 | 4.24 | 3.99 | 1.17 | 0.4599 | 0.6659 | 4.21 | 4.45 | 3.98 | 1.17 | 0.6768 | 0.8443 |
| TC0500010498.hg.1 | RANBP3L | RAN binding protein 3-like | Coding | 3.86 | 3.57 | 3.63 | 1.17 | 0.3038 | 0.5166 | 3.78 | 3.49 | 3.72 | 1.04 | 0.8237 | 0.9222 |

| | | | | | | | | | | | | | | | |
|---|---|---|---|---|---|---|---|---|---|---|---|---|---|---|---|
| TC0500011408.hg.1 | TMEM161B | transmembrane protein 161B | Multiple_C | 10.12 | 11.44 | 9.89 | 1.17 | 0.3127 | 0.5262 | 10.61 | 9.71 | 10.07 | 1.45 | 0.0249 | 0.1372 |
| TC0500012107.hg.1 | CXCL14 | chemokine (C-X-C motif) ligand 14 | Multiple_C | 3.67 | 3.59 | 3.44 | 1.17 | 0.4577 | 0.6638 | 4.09 | 3.85 | 3.79 | 1.23 | 0.0227 | 0.1299 |
| TC0500013002.hg.1 | PROP1 | PROP paired-like homeobox 1 | Coding | 3.87 | 3.32 | 3.64 | 1.17 | 0.078 | 0.2009 | 3.77 | 3.55 | 3.33 | 1.36 | 0.0196 | 0.1185 |
| TC0500013371.hg.1 | DNAJC18 | DnaJ (Hsp40) homolog, subfamily C, member 18 | Multiple_C | 6.67 | 6.57 | 6.44 | 1.17 | 0.5282 | 0.7196 | 7.94 | 7.45 | 7.29 | 1.57 | 0.062 | 0.2366 |
| TC0600006873.hg.1 | BMP6 | bone morphogenetic protein 6 | Multiple_C | 4.3 | 4.21 | 4.07 | 1.17 | 0.634 | 0.7962 | 7.36 | 4.99 | 4.72 | 6.23 | 3.84E-06 | 0.0004 |
| TC0600011738.hg.1 | KCNK17 | potassium channel, two pore domain subfamily K, member 17 | Multiple_C | 6.38 | 6.07 | 6.15 | 1.17 | 0.141 | 0.3086 | 5.46 | 5.45 | 5.32 | 1.10 | 0.8845 | 0.9504 |
| TC0600014254.hg.1 | OR11A1 | olfactory receptor, family 11, subfamily A, member 1 | Multiple_C | 3.65 | 3.64 | 3.42 | 1.17 | 0.0945 | 0.2311 | 3.37 | 3.74 | 3.63 | 0.84 | 0.6272 | 0.8158 |
| TC0600014284.hg.1 | CPNE5 | copine V | Multiple_C | 3.8 | 3.38 | 3.57 | 1.17 | 0.8881 | 0.9459 | 4.18 | 4.19 | 4.25 | 0.95 | 0.7299 | 0.8746 |
| TC0700006567.hg.1 | TTYH3 | tweety family member 3 | Multiple_C | 7.29 | 6.45 | 7.06 | 1.17 | 0.0653 | 0.1765 | 10.93 | 12.15 | 11.89 | 0.51 | 0.0045 | 0.0459 |
| TC0700007475.hg.1 | C7orf57 | chromosome 7 open reading frame 57 | Multiple_C | 2.97 | 3.12 | 2.74 | 1.17 | 0.4694 | 0.6734 | 3.68 | 3.29 | 3.19 | 1.40 | 0.122 | 0.3447 |
| TC0700007499.hg.1 | VWC2 | von Willebrand factor C domain containing 2 | Coding | 3.3 | 3.04 | 3.07 | 1.17 | 0.2418 | 0.4441 | 3.26 | 3.13 | 3.24 | 1.01 | 0.4499 | 0.6961 |
| TC0700008274.hg.1 | ZNF804B | zinc finger protein 804B | Coding | 3.06 | 2.9 | 2.83 | 1.17 | 0.4436 | 0.6521 | 4.13 | 4.29 | 4.19 | 0.96 | 0.718 | 0.8685 |
| TC0700008527.hg.1 | LAMTOR4 | late endosomal/lysosomal adaptor, MAPK and MTOR activator | Multiple_C | 8.49 | 8.57 | 8.26 | 1.17 | 0.4507 | 0.6583 | 6.65 | 6.74 | 6.35 | 1.23 | 0.3199 | 0.5839 |
| TC0700009060.hg.1 | FAM71F1 | family with sequence similarity 71, member F1 | Multiple_C | 3.4 | 3.12 | 3.17 | 1.17 | 0.236 | 0.4373 | 3.82 | 3.67 | 3.52 | 1.23 | 0.0503 | 0.2089 |
| TC0700013405.hg.1 | BRI3 | brain protein I3 | Multiple_C | 8.49 | 8.21 | 8.26 | 1.17 | 0.2757 | 0.4849 | 7.26 | 7.21 | 7.27 | 0.99 | 0.4639 | 0.7061 |
| TC0800007866.hg.1 | MCMDC2 | minichromosome maintenance domain containing 2 | Multiple_C | 2.71 | 2.76 | 2.48 | 1.17 | 0.6187 | 0.7866 | 4.84 | 4.54 | 3.89 | 1.93 | 0.0068 | 0.0604 |
| TC0800009468.hg.1 | DEFB106B; DE | defensin, beta 106B; defensin, beta 106A | Coding | 5.68 | 5.41 | 5.45 | 1.17 | 0.9629 | 0.9823 | 5.22 | 4.94 | 5.21 | 1.01 | 0.3093 | 0.5733 |
| TC0800010648.hg.1 | CYP7B1 | cytochrome P450, family 7, subfamily B, polypeptide 1 | Multiple_C | 5.4 | 4.69 | 5.17 | 1.17 | 0.4095 | 0.6217 | 5.38 | 5.82 | 5.72 | 0.79 | 0.0965 | 0.3026 |
| TC0800011789.hg.1 | TMEM75 | transmembrane protein 75 | Multiple_C | 4.1 | 3.96 | 3.87 | 1.17 | 0.7418 | 0.8665 | 3.43 | 3.47 | 3.34 | 1.06 | 0.3711 | 0.633 |
| TC0800012308.hg.1 | ADHFE1; C8or | alcohol dehydrogenase, iron containing 1; chromosome 8 open | Multiple_C | 3.03 | 2.79 | 2.8 | 1.17 | 0.3531 | 0.5659 | 3.63 | 3.30 | 3.44 | 1.14 | 0.4542 | 0.699 |
| TC0900009345.hg.1 | FOXD4 | forkhead box D4 | Multiple_C | 3.72 | 3.16 | 3.49 | 1.17 | 0.8919 | 0.9481 | 3.55 | 3.62 | 3.52 | 1.02 | 0.4083 | 0.664 |
| TC0900009785.hg.1 | LINGO2 | leucine rich repeat and Ig domain containing 2 | Multiple_C | 3.14 | 2.53 | 2.91 | 1.17 | 0.6194 | 0.7872 | 2.95 | 3.24 | 3.4 | 0.73 | 0.0598 | 0.2313 |
| TC0900009902.hg.1 | ENHO | energy homeostasis associated | Multiple_C | 3.75 | 3.48 | 3.52 | 1.17 | 0.4013 | 0.6144 | 4.95 | 4.70 | 4.52 | 1.35 | 0.1136 | 0.3325 |
| TC0900009966.hg.1 | OR2S2 | olfactory receptor, family 2, subfamily S, member 2 (gene/pseu | Multiple_C | 4.08 | 3.87 | 3.85 | 1.17 | 0.3609 | 0.5743 | 3.84 | 4.00 | 3.91 | 0.95 | 0.8453 | 0.9325 |
| TC0X00006460.hg.1 | ASMT; AKAP1 | acetylserotonin O-methyltransferase; A kinase (PRKA) anchor p | Multiple_C | 3.78 | 3.44 | 3.55 | 1.17 | 0.6257 | 0.7911 | 3.83 | 3.97 | 4.15 | 0.80 | 0.1007 | 0.3102 |
| TC0X00007256.hg.1 | BMP15 | bone morphogenetic protein 15 | Coding | 3.38 | 3.07 | 3.15 | 1.17 | 0.3782 | 0.5912 | 3.24 | 3.32 | 3.47 | 0.85 | 0.3513 | 0.6151 |
| TC0X00007713.hg.1 | LPAR4 | lysophosphatidic acid receptor 4 | Multiple_C | 3.43 | 3.32 | 3.2 | 1.17 | 0.4721 | 0.6756 | 3.26 | 3.34 | 3.25 | 1.01 | 0.9723 | 0.9887 |
| TC0X00008401.hg.1 | RAB33A | RAB33A, member RAS oncogene family | Coding | 4.8 | 4.67 | 4.57 | 1.17 | 0.844 | 0.9231 | 4.56 | 4.72 | 4.39 | 1.13 | 0.1637 | 0.4076 |
| TC0X00008764.hg.1 | PNMA3 | paraneoplastic Ma antigen 3 | Multiple_C | 4.6 | 4.23 | 4.37 | 1.17 | 0.3454 | 0.5591 | 4.17 | 4.42 | 4.64 | 0.72 | 0.1783 | 0.427 |
| TC0X00008989.hg.1 | NLGN4X | neuroligin 4, X-linked | Multiple_C | 3.68 | 3.6 | 3.45 | 1.17 | 0.7034 | 0.8417 | 4.55 | 4.71 | 4.6 | 0.97 | 0.1967 | 0.4516 |
| TC0X00010387.hg.1 | TMSB15A | thymosin beta 15a | Multiple_C | 4.35 | 4.41 | 4.12 | 1.17 | 0.8023 | 0.8989 | 4.93 | 4.81 | 5.19 | 0.84 | 0.3436 | 0.6072 |
| TC0X00010933.hg.1 | ARHGEF6 | Rac/Cdc42 guanine nucleotide exchange factor 6 | Multiple_C | 4.45 | 3.93 | 4.22 | 1.17 | 0.0793 | 0.2037 | 4.73 | 4.58 | 4.26 | 1.39 | 0.015 | 0.1003 |
| TC0Y00007320.hg.1 | ASMT; SFRS17 | Homo sapiens acetylserotonin O-methyltransferase (ASMT), tra | Multiple_C | 3.78 | 3.44 | 3.55 | 1.17 | 0.6257 | 0.7911 | 3.83 | 3.97 | 4.15 | 0.80 | 0.1007 | 0.3102 |

| | | | | | | | | | | | | | | | |
|---|---|---|---|---|---|---|---|---|---|---|---|---|---|---|---|
| TC0Y00007342.hg.1 | RBMY1E; RBM | RNA binding motif protein, Y-linked, family 1, member E; RNA bi | Coding | 3.16 | 3.02 | 2.93 | 1.17 | 0.3461 | 0.5597 | 3.27 | 3.46 | 3.38 | 0.93 | 0.949 | 0.9793 |
| TC1000006616.hg.1 | UCN3 | urocortin 3 | Coding | 6.12 | 5.57 | 5.89 | 1.17 | 0.4185 | 0.63 | 5.66 | 5.96 | 5.97 | 0.81 | 0.1399 | 0.3716 |
| TC1000008212.hg.1 | NUTM2B | NUT family member 2B | Coding | 4.33 | 3.86 | 4.1 | 1.17 | 0.1118 | 0.2613 | 4.38 | 4.47 | 4.34 | 1.03 | 0.8116 | 0.9166 |
| TC1000008813.hg.1 | SORCS3 | sortilin-related VPS10 domain containing receptor 3 | Coding | 3.79 | 3.69 | 3.56 | 1.17 | 0.8558 | 0.9295 | 3.84 | 3.98 | 3.8 | 1.03 | 0.2768 | 0.5428 |
| TC1000011178.hg.1 | SFTPA2; SFTP/ | surfactant protein A2; surfactant protein A1 | Multiple_C | 5.49 | 5.01 | 5.26 | 1.17 | 0.2829 | 0.4929 | 5.22 | 5.23 | 5.18 | 1.03 | 0.5291 | 0.7526 |
| TC1000011681.hg.1 | C10orf95 | chromosome 10 open reading frame 95 | Multiple_C | 3.97 | 3.62 | 3.74 | 1.17 | 0.331 | 0.545 | 4.16 | 3.93 | 3.69 | 1.39 | 0.222 | 0.4823 |
| TC1000012586.hg.1 | SEC31B | SEC31 homolog B, COPII coat complex component | Multiple_C | 6.86 | 6.79 | 6.63 | 1.17 | 0.311 | 0.5245 | 5.74 | 5.21 | 5.39 | 1.27 | 0.1122 | 0.33 |
| TC1100006561.hg.1 | IGF2-AS | IGF2 antisense RNA | Multiple_C | 4.63 | 4.17 | 4.4 | 1.17 | 0.7387 | 0.8643 | 5.1 | 5.38 | 5.31 | 0.86 | 0.1575 | 0.3986 |
| TC1100007521.hg.1 | OR4A47 | olfactory receptor, family 4, subfamily A, member 47 | Coding | 3.29 | 2.92 | 3.06 | 1.17 | 0.7405 | 0.8656 | 3.42 | 3.51 | 3.42 | 1.00 | 0.4042 | 0.6599 |
| TC1100007614.hg.1 | OR5D16 | olfactory receptor, family 5, subfamily D, member 16 | Coding | 4.46 | 4.37 | 4.23 | 1.17 | 0.4913 | 0.6913 | 5.33 | 5.12 | 4.76 | 1.48 | 0.0095 | 0.0755 |
| TC1100007636.hg.1 | OR8J1 | olfactory receptor, family 8, subfamily J, member 1 | Coding | 4.02 | 3.56 | 3.79 | 1.17 | 0.5813 | 0.7588 | 4.19 | 4.07 | 3.62 | 1.48 | 0.0821 | 0.2762 |
| TC1100009678.hg.1 | HRAS | Harvey rat sarcoma viral oncogene homolog | Multiple_C | 6.46 | 6.17 | 6.23 | 1.17 | 0.0871 | 0.2176 | 8.41 | 8.79 | 8.47 | 0.96 | 0.8435 | 0.9317 |
| TC1100009883.hg.1 | C11orf40 | chromosome 11 open reading frame 40 | Coding | 4.05 | 3.57 | 3.82 | 1.17 | 0.2525 | 0.4573 | 3.78 | 3.64 | 3.52 | 1.20 | 0.0356 | 0.171 |
| TC1100010090.hg.1 | LYVE1 | lymphatic vessel endothelial hyaluronan receptor 1 | Multiple_C | 3.36 | 3.25 | 3.13 | 1.17 | 0.4299 | 0.6402 | 3.92 | 3.87 | 3.49 | 1.35 | 0.0611 | 0.2348 |
| TC1100010277.hg.1 | PTPN5 | protein tyrosine phosphatase, non-receptor type 5 (striatum-en | Multiple_C | 6.01 | 6.19 | 5.78 | 1.17 | 0.5028 | 0.7006 | 5.52 | 5.51 | 5.55 | 0.98 | 0.8358 | 0.9277 |
| TC1100011742.hg.1 | KCTD21 | potassium channel tetramerization domain containing 21 | Coding | 6.4 | 6.1 | 6.17 | 1.17 | 0.4977 | 0.6973 | 6.9 | 6.39 | 6.09 | 1.75 | 0.2212 | 0.4813 |
| TC1100012166.hg.1 | CASP5 | caspase 5 | Multiple_C | 3.68 | 3.31 | 3.45 | 1.17 | 0.2625 | 0.4696 | 4.27 | 4.07 | 4.18 | 1.06 | 0.8883 | 0.9524 |
| TC1100012852.hg.1 | LOC10361108 | uncharacterized LOC103611081; uncharacterized LOC10050743 | Multiple_C | 4.71 | 4.75 | 4.48 | 1.17 | 0.3411 | 0.5549 | 3.52 | 3.67 | 3.84 | 0.80 | 0.3324 | 0.5965 |
| TC1200009914.hg.1 | TAS2R7 | taste receptor, type 2, member 7 | Coding | 3.72 | 3.34 | 3.49 | 1.17 | 0.3271 | 0.541 | 3.57 | 3.80 | 3.9 | 0.80 | 0.0885 | 0.2878 |
| TC1200010741.hg.1 | C12orf80 | chromosome 12 open reading frame 80 | Multiple_C | 4.42 | 4.18 | 4.19 | 1.17 | 0.3384 | 0.5523 | 4.08 | 3.99 | 3.99 | 1.06 | 0.3063 | 0.5705 |
| TC1200011675.hg.1 | GOLGA2P5 | golgin A2 pseudogene 5 | Multiple_C | 5.85 | 5.89 | 5.62 | 1.17 | 0.8805 | 0.9419 | 4.84 | 5.06 | 4.95 | 0.93 | 0.9176 | 0.9663 |
| TC1300009597.hg.1 | GPR18 | G protein-coupled receptor 18 | Coding | 3.74 | 3.61 | 3.51 | 1.17 | 0.5239 | 0.7165 | 4.02 | 4.08 | 4.02 | 1.00 | 0.3257 | 0.5894 |
| TC1400006549.hg.1 | RNASE8 | ribonuclease, RNase A family, 8 | Coding | 2.74 | 2.58 | 2.51 | 1.17 | 0.4835 | 0.6851 | 3.81 | 3.78 | 3.61 | 1.15 | 0.5223 | 0.7487 |
| TC1400006652.hg.1 | ABHD4 | abhydrolase domain containing 4 | Multiple_C | 5.86 | 5.81 | 5.63 | 1.17 | 0.348 | 0.5611 | 4.98 | 4.88 | 5.02 | 0.97 | 0.6454 | 0.8258 |
| TC1400008427.hg.1 | KIF26A | kinesin family member 26A | Multiple_C | 4.18 | 3.55 | 3.95 | 1.17 | 0.311 | 0.5245 | 4.96 | 4.64 | 4.52 | 1.36 | 0.0618 | 0.2363 |
| TC1400008994.hg.1 | CLEC14A | C-type lectin domain family 14, member A | Coding | 4.4 | 4.01 | 4.17 | 1.17 | 0.1405 | 0.3077 | 5.15 | 5.32 | 5 | 1.11 | 0.408 | 0.6637 |
| TC1400010049.hg.1 | SERPINA12 | serpin peptidase inhibitor, clade A (alpha-1 antiproteinase, antit | Coding | 3.95 | 3.5 | 3.72 | 1.17 | 0.2386 | 0.4403 | 3.53 | 3.61 | 3.33 | 1.15 | 0.5751 | 0.7822 |
| TC1500010605.hg.1 | PGPEP1L | pyroglutamyl-peptidase I-like | Multiple_C | 5.68 | 5.59 | 5.45 | 1.17 | 0.1481 | 0.3191 | 5.36 | 5.23 | 5.36 | 1.00 | 0.9932 | 0.997 |
| TC1600006754.hg.1 | C16orf71 | chromosome 16 open reading frame 71 | Multiple_C | 3.42 | 2.97 | 3.19 | 1.17 | 0.9145 | 0.96 | 3.39 | 3.57 | 3.5 | 0.93 | 0.9706 | 0.9879 |
| TC1600007141.hg.1 | LOC81691; AC | exonuclease NEF-sp; Putative RNA exonuclease NEF-sp [Source | Multiple_C | 10.06 | 9.54 | 9.83 | 1.17 | 0.5611 | 0.7435 | 9.14 | 8.99 | 8.96 | 1.13 | 0.6623 | 0.8351 |
| TC1600008643.hg.1 | WFDC1 | WAP four-disulfide core domain 1 | Multiple_C | 4.11 | 3.49 | 3.88 | 1.17 | 0.22 | 0.4171 | 3.63 | 3.71 | 3.74 | 0.93 | 0.7614 | 0.8921 |
| TC1600009837.hg.1 | GTF3C1 | general transcription factor IIIC subunit 1 | Multiple_C | 8.25 | 7.69 | 8.02 | 1.17 | 0.5935 | 0.7679 | 9.42 | 9.23 | 9.14 | 1.21 | 0.4425 | 0.6912 |
| TC1600011316.hg.1 | ARHGDIG | Rho GDP dissociation inhibitor (GDI) gamma | Multiple_C | 4.33 | 4.81 | 4.1 | 1.17 | 0.7427 | 0.867 | 3.95 | 4.02 | 3.93 | 1.01 | 0.1037 | 0.3157 |

| | | | | | | | | | | | | | | | |
|---|---|---|---|---|---|---|---|---|---|---|---|---|---|---|---|
| TC1600011389.hg.1 | TP53TG3C | TP53 target 3C | Multiple_C | 3.76 | 3.52 | 3.53 | 1.17 | 0.4234 | 0.6346 | 3.96 | 3.71 | 4.12 | 0.90 | 0.8842 | 0.9504 |
| TC1600011415.hg.1 | NRN1L | neuritin 1-like | Multiple_C | 3.63 | 3.55 | 3.4 | 1.17 | 0.3846 | 0.5979 | 4.25 | 4.45 | 4.57 | 0.80 | 0.1907 | 0.4439 |
| TC1600011500.hg.1 | DCUN1D3 | DCN1, defective in cullin neddylation 1, domain containing 3 | Multiple_C | 9.42 | 9.43 | 9.19 | 1.17 | 0.5989 | 0.7714 | 8.39 | 8.18 | 8.69 | 0.81 | 0.2747 | 0.5405 |
| TC1700006653.hg.1 | GP1BA | glycoprotein Ib (platelet), alpha polypeptide | Coding | 5.59 | 4.89 | 5.36 | 1.17 | 0.1843 | 0.3699 | 5.08 | 5.15 | 5.11 | 0.98 | 0.8093 | 0.9156 |
| TC1700007408.hg.1 | PIPOX | pipecolic acid oxidase | Multiple_C | 4.18 | 4.17 | 3.95 | 1.17 | 0.8364 | 0.9186 | 4.15 | 3.80 | 3.97 | 1.13 | 0.5184 | 0.7463 |
| TC1700008303.hg.1 | C17orf112 | chromosome 17 open reading frame 112 | Multiple_C | 3.03 | 2.96 | 2.8 | 1.17 | 0.2425 | 0.4449 | 2.89 | 2.80 | 2.87 | 1.01 | 0.654 | 0.8315 |
| TC1700010004.hg.1 | LOC79999; LO | Homo sapiens uncharacterized LOC79999 (LOC79999), mRNA.; I | Multiple_C | 5.24 | 4.94 | 5.01 | 1.17 | 0.423 | 0.6342 | 4.78 | 4.84 | 4.89 | 0.93 | 0.5754 | 0.7822 |
| TC1700010551.hg.1 | RPL23; SNORA | ribosomal protein L23; small nucleolar RNA, H/ACA box 21 | Multiple_C | 18.15 | 18.82 | 17.92 | 1.17 | 0.0509 | 0.1464 | 14.62 | 14.48 | 14.04 | 1.49 | 0.0106 | 0.0809 |
| TC1700011249.hg.1 | MPO | myeloperoxidase | Multiple_C | 4.23 | 4.03 | 4 | 1.17 | 0.6127 | 0.7827 | 4.58 | 4.25 | 4.05 | 1.44 | 0.5217 | 0.7485 |
| TC1700011786.hg.1 | FOXJ1 | forkhead box J1 | Coding | 4.57 | 4.09 | 4.34 | 1.17 | 0.7705 | 0.8814 | 3.76 | 3.91 | 4.12 | 0.78 | 0.9089 | 0.9625 |
| TC1700012032.hg.1 | LINC00482 | long intergenic non-protein coding RNA 482 | Multiple_C | 4.46 | 4.07 | 4.23 | 1.17 | 0.342 | 0.5558 | 4.85 | 5.07 | 4.8 | 1.04 | 0.4989 | 0.7316 |
| TC1700012402.hg.1 | KRT222 | keratin 222, type II | Coding | 4.86 | 4.46 | 4.63 | 1.17 | 0.6407 | 0.8008 | 5.21 | 4.88 | 5.05 | 1.12 | 0.65 | 0.8291 |
| TC1800006447.hg.1 | CLUL1 | clusterin-like 1 (retinal) | Multiple_C | 4 | 3.96 | 3.77 | 1.17 | 0.2341 | 0.435 | 3.77 | 3.67 | 3.74 | 1.02 | 0.5789 | 0.7844 |
| TC1800008189.hg.1 | POTEC | POTE ankyrin domain family, member C | Multiple_C | 3.25 | 3.09 | 3.02 | 1.17 | 0.5575 | 0.7403 | 3.26 | 3.22 | 3.21 | 1.04 | 0.1529 | 0.3921 |
| TC1900007100.hg.1 | MAST1 | microtubule associated serine/threonine kinase 1 | Multiple_C | 5.14 | 4.56 | 4.91 | 1.17 | 0.2845 | 0.4948 | 5.42 | 5.16 | 5.12 | 1.23 | 0.1883 | 0.4404 |
| TC1900007679.hg.1 | PLEKHF1 | pleckstrin homology domain containing, family F (with FYVE dor | Coding | 5.23 | 5.07 | 5 | 1.17 | 0.5596 | 0.7423 | 5.83 | 5.84 | 5.58 | 1.19 | 0.3714 | 0.6331 |
| TC1900008141.hg.1 | CYP2S1 | cytochrome P450, family 2, subfamily S, polypeptide 1 | Multiple_C | 13.57 | 13.42 | 13.34 | 1.17 | 0.5484 | 0.7338 | 11.05 | 10.37 | 11.07 | 0.99 | 0.7822 | 0.9022 |
| TC1900008161.hg.1 | CEACAM3 | carcinoembryonic antigen-related cell adhesion molecule 3 | Multiple_C | 7.55 | 7.16 | 7.32 | 1.17 | 0.6307 | 0.7941 | 5.25 | 5.53 | 5.4 | 0.90 | 0.7887 | 0.9058 |
| TC1900008560.hg.1 | PTOV1; MIR47 | prostate tumor overexpressed 1; microRNA 4749 | Multiple_C | 9.45 | 9.05 | 9.22 | 1.17 | 0.8916 | 0.948 | 7.87 | 8.61 | 8.46 | 0.66 | 0.0551 | 0.2203 |
| TC1900009433.hg.1 | TUBB4A | tubulin, beta 4A class IVa | Coding | 10.59 | 9.5 | 10.36 | 1.17 | 0.5161 | 0.7107 | 8.61 | 8.89 | 9.16 | 0.68 | 0.0303 | 0.1545 |
| TC1900009455.hg.1 | MBD3L4 | methyl-CpG binding domain protein 3-like 4 | Coding | 3.44 | 3.19 | 3.21 | 1.17 | 0.8816 | 0.9425 | 3.62 | 3.73 | 3.65 | 0.98 | 0.7109 | 0.8644 |
| TC1900009888.hg.1 | OR10H1 | olfactory receptor, family 10, subfamily H, member 1 | Coding | 3.44 | 3.16 | 3.21 | 1.17 | 0.406 | 0.6186 | 4.03 | 4.15 | 3.86 | 1.13 | 0.3963 | 0.6542 |
| TC1900010466.hg.1 | LGI4 | leucine-rich repeat LGI family, member 4 | Multiple_C | 3.84 | 3.65 | 3.61 | 1.17 | 0.1229 | 0.2796 | 3.86 | 3.43 | 3.45 | 1.33 | 0.0944 | 0.2984 |
| TC1900011782.hg.1 | ADM5; CPT1C | adrenomedullin 5 (putative); carnitine palmitoyltransferase 1C | Multiple_C | 5.14 | 4.47 | 4.91 | 1.17 | 0.1595 | 0.3358 | 4.77 | 4.77 | 4.66 | 1.08 | 0.6206 | 0.8123 |
| TC1900012044.hg.1 | GP6 | glycoprotein VI (platelet) | Multiple_C | 5.02 | 4.46 | 4.79 | 1.17 | 0.5091 | 0.7052 | 5.42 | 5.52 | 5.32 | 1.07 | 0.8661 | 0.9425 |
| TC2000007091.hg.1 | MYLK2 | myosin light chain kinase 2 | Multiple_C | 3.44 | 2.97 | 3.21 | 1.17 | 0.321 | 0.5348 | 3.44 | 3.66 | 3.78 | 0.79 | 0.1017 | 0.3121 |
| TC2000007465.hg.1 | WISP2 | WNT1 inducible signaling pathway protein 2 | Multiple_C | 5.69 | 5.31 | 5.46 | 1.17 | 0.0657 | 0.1772 | 5.54 | 5.50 | 5.27 | 1.21 | 0.3891 | 0.6477 |
| TC2000008212.hg.1 | LZTS3; ProSAF | leucine zipper, putative tumor suppressor family member 3; Leu | Multiple_C | 4.25 | 4.02 | 4.02 | 1.17 | 0.3083 | 0.5214 | 5.56 | 4.98 | 4.31 | 2.38 | 0.0004 | 0.0095 |
| TC2000008856.hg.1 | C20orf203 | chromosome 20 open reading frame 203 | Multiple_C | 4.26 | 4.23 | 4.03 | 1.17 | 0.1958 | 0.3859 | 4.62 | 4.30 | 4.1 | 1.43 | 0.0363 | 0.173 |
| TC2000009823.hg.1 | STMN3 | stathmin-like 3 | Multiple_C | 5.82 | 5.62 | 5.59 | 1.17 | 0.3742 | 0.5868 | 6.6 | 7.00 | 6.6 | 1.00 | 0.5391 | 0.7594 |
| TC2000009944.hg.1 | RTFDC1 | replication termination factor 2 domain containing 1 | Multiple_C | 11.31 | 11.53 | 11.08 | 1.17 | 0.8783 | 0.9408 | 11.29 | 11.05 | 11.55 | 0.84 | 0.0993 | 0.308 |
| TC2100007910.hg.1 | KRTAP21-2 | keratin associated protein 21-2 | Coding | 2.97 | 2.77 | 2.74 | 1.17 | 0.1724 | 0.3536 | 4.31 | 3.75 | 3.64 | 1.59 | 0.0025 | 0.0316 |
| TC2200006432.hg.1 | BAGE5 | Synthetic construct Homo sapiens clone IMAGE:100062553, MC | Multiple_C | 5.97 | 5.58 | 5.74 | 1.17 | 0.4592 | 0.6653 | 5.04 | 5.48 | 5.66 | 0.65 | 0.1118 | 0.3294 |

| | | | | | | | | | | | | | | | |
|---|---|---|---|---|---|---|---|---|---|---|---|---|---|---|---|
| TC2200007132.hg.1 | RFPL3 | ret finger protein-like 3 | Coding | 3.91 | 3.54 | 3.68 | 1.17 | 0.3698 | 0.5825 | 4.35 | 4.24 | 3.98 | 1.29 | 0.4142 | 0.6687 |
| TC2200008620.hg.1 | IFT27 | intraflagellar transport 27 | Multiple_C | 5.47 | 5.91 | 5.24 | 1.17 | 0.441 | 0.6497 | 5.08 | 5.52 | 4.8 | 1.21 | 0.1788 | 0.4277 |
| TC2200009236.hg.1 | GGT1 | gamma-glutamyltransferase 1 | Multiple_C | 5.64 | 5.26 | 5.41 | 1.17 | 0.1172 | 0.2701 | 7.51 | 6.87 | 6.67 | 1.79 | 0.0041 | 0.0435 |
| TC2200009274.hg.1 | APOBEC3G | apolipoprotein B mRNA editing enzyme, catalytic polypeptide-li | Multiple_C | 3.48 | 3.26 | 3.25 | 1.17 | 0.3172 | 0.5306 | 9.46 | 5.30 | 4.84 | 24.59 | 1.40E-12 | 8.06E-09 |
| TSUnmapped00000049.h | PADI3 | peptidyl arginine deiminase, type III | Coding | 4.22 | 3.82 | 3.99 | 1.17 | 0.4506 | 0.6583 | 4.44 | 4.06 | 4.12 | 1.25 | 0.7015 | 0.8585 |
| TSUnmapped00000306.h | ZKSCAN7 | zinc finger with KRAB and SCAN domains 7 | Coding | 3.48 | 3.18 | 3.25 | 1.17 | 0.4435 | 0.6521 | 3.84 | 4.54 | 4.2 | 0.78 | 0.3327 | 0.5969 |
| TSUnmapped00000395.h | ATG16L1 | autophagy related 16-like 1 | Coding | 5.25 | 4.93 | 5.02 | 1.17 | 0.4532 | 0.6607 | 5.96 | 5.41 | 5.52 | 1.36 | 0.1941 | 0.448 |
| TSUnmapped00000587.h | PRAMEF11 | PRAME family member 11 | Coding | 3.22 | 2.67 | 2.99 | 1.17 | 0.3849 | 0.5981 | 3.52 | 3.36 | 3.28 | 1.18 | 0.3531 | 0.6166 |
| TSUnmapped00000592.h | FMN1 | formin 1 | Coding | 3.35 | 3.31 | 3.12 | 1.17 | 0.5771 | 0.7553 | 3.27 | 3.51 | 3.4 | 0.91 | 0.3457 | 0.6096 |
| TC0100007333.hg.1 | PITHD1 | PITH (C-terminal proteasome-interacting domain of thioredoxin | Multiple_C | 13.04 | 12.95 | 12.82 | 1.16 | 0.2789 | 0.4885 | 11.44 | 11.06 | 11.8 | 0.78 | 0.0306 | 0.1556 |
| TC0100008631.hg.1 | SGIP1 | SH3-domain GRB2-like (endophilin) interacting protein 1 | Multiple_C | 3.37 | 3.38 | 3.15 | 1.16 | 0.4061 | 0.6186 | 4.27 | 4.21 | 3.96 | 1.24 | 0.2607 | 0.525 |
| TC0100011066.hg.1 | CFHR3 | complement factor H-related 3 | Multiple_C | 3.87 | 3.57 | 3.65 | 1.16 | 0.1927 | 0.3816 | 3.97 | 3.85 | 3.65 | 1.25 | 0.1405 | 0.3725 |
| TC0100011458.hg.1 | TRAF3IP3 | TRAF3 interacting protein 3 | Multiple_C | 5.54 | 5.05 | 5.32 | 1.16 | 0.5414 | 0.7287 | 4.66 | 4.77 | 4.79 | 0.91 | 0.4748 | 0.7142 |
| TC0100014650.hg.1 | ZZZ3 | zinc finger, ZZ-type containing 3 | Multiple_C | 10.64 | 10.8 | 10.42 | 1.16 | 0.6416 | 0.8014 | 11.17 | 11.38 | 11.33 | 0.90 | 0.7108 | 0.8644 |
| TC0100015244.hg.1 | KCNA2 | potassium channel, voltage gated shaker related subfamily A, m | Multiple_C | 3.96 | 3.75 | 3.74 | 1.16 | 0.2117 | 0.4062 | 4.12 | 4.09 | 3.95 | 1.13 | 0.3521 | 0.6154 |
| TC0100018534.hg.1 | TNR | tenascin R | Multiple_C | 4.88 | 4.54 | 4.66 | 1.16 | 0.4044 | 0.6173 | 4.98 | 4.94 | 4.78 | 1.15 | 0.9275 | 0.9698 |
| TC0200007506.hg.1 | EPCAM | epithelial cell adhesion molecule | Multiple_C | 16.48 | 16.5 | 16.26 | 1.16 | 0.216 | 0.4118 | 14.8 | 14.96 | 14.81 | 0.99 | 0.6011 | 0.8002 |
| TC0200007618.hg.1 | EML6 | echinoderm microtubule associated protein like 6 | Multiple_C | 5.08 | 5.22 | 4.86 | 1.16 | 0.0622 | 0.1698 | 3.83 | 3.63 | 3.27 | 1.47 | 0.3664 | 0.6285 |
| TC0200007900.hg.1 | ARHGAP25 | Rho GTPase activating protein 25 | Multiple_C | 5.37 | 4.65 | 5.15 | 1.16 | 0.1649 | 0.3433 | 5.5 | 5.42 | 5.68 | 0.88 | 0.7609 | 0.8917 |
| TC0200007943.hg.1 | PCBP1 | poly(rC) binding protein 1 | Coding | 12.72 | 12.5 | 12.5 | 1.16 | 0.3429 | 0.5568 | 11.65 | 12.56 | 12.84 | 0.44 | 0.0002 | 0.0052 |
| TC0200008556.hg.1 | ZAP70 | zeta chain of T cell receptor associated protein kinase 70kDa | Multiple_C | 3.35 | 3.09 | 3.13 | 1.16 | 0.4216 | 0.6327 | 4 | 3.91 | 3.69 | 1.24 | 0.3317 | 0.5959 |
| TC0200009865.hg.1 | SCN2A | sodium channel, voltage gated, type II alpha subunit | Multiple_C | 3.41 | 3.38 | 3.19 | 1.16 | 0.1948 | 0.3846 | 3.33 | 3.63 | 3.57 | 0.85 | 0.657 | 0.8324 |
| TC0200010021.hg.1 | H3F3AP4; H3F | Homo sapiens H3 histone, family 3A, pseudogene 4 (H3F3AP4), | Multiple_C | 15.09 | 15.65 | 14.87 | 1.16 | 0.2814 | 0.4914 | 12.74 | 13.01 | 11.95 | 1.73 | 0.0061 | 0.0558 |
| TC0200011386.hg.1 | PPP1R7 | protein phosphatase 1, regulatory subunit 7 | Multiple_C | 10.88 | 10.64 | 10.66 | 1.16 | 0.8089 | 0.9035 | 10.76 | 10.20 | 10.76 | 1.00 | 0.1909 | 0.444 |
| TC0200012075.hg.1 | ZNF513 | zinc finger protein 513 | Multiple_C | 5.85 | 5.49 | 5.63 | 1.16 | 0.3964 | 0.61 | 6.83 | 6.44 | 6.34 | 1.40 | 0.9798 | 0.9914 |
| TC0200012129.hg.1 | TRMT61B | tRNA methyltransferase 61B | Multiple_C | 8.78 | 9.12 | 8.56 | 1.16 | 0.3065 | 0.5197 | 8.43 | 8.23 | 8.01 | 1.34 | 0.0285 | 0.1488 |
| TC0200012404.hg.1 | ZFP36L2 | ZFP36 ring finger protein-like 2 | Multiple_C | 12.96 | 12.35 | 12.74 | 1.16 | 0.7403 | 0.8655 | 11.34 | 11.96 | 12.14 | 0.57 | 0.0705 | 0.2536 |
| TC0200013357.hg.1 | KRCC1 | lysine-rich coiled-coil 1 | Multiple_C | 7.34 | 7.42 | 7.12 | 1.16 | 0.178 | 0.3609 | 6.78 | 6.63 | 6.67 | 1.08 | 0.8713 | 0.9446 |
| TC0200013925.hg.1 | PAX8 | paired box 8 | Multiple_C | 4.34 | 4.05 | 4.12 | 1.16 | 0.5367 | 0.7252 | 3.96 | 4.12 | 4.41 | 0.73 | 0.67 | 0.8387 |
| TC0200013943.hg.1 | SLC35F5 | solute carrier family 35, member F5 | Multiple_C | 9.93 | 10.51 | 9.71 | 1.16 | 0.3345 | 0.5484 | 10.43 | 10.24 | 10.67 | 0.85 | 0.3123 | 0.5765 |
| TC0200015790.hg.1 | CHPF | chondroitin polymerizing factor | Multiple_C | 8.19 | 7.92 | 7.97 | 1.16 | 0.549 | 0.7342 | 7.44 | 8.02 | 7.96 | 0.70 | 0.0276 | 0.1458 |
| TC0300008711.hg.1 | KBTBD12 | kelch repeat and BTB (POZ) domain containing 12 | Multiple_C | 5.12 | 4.78 | 4.9 | 1.16 | 0.7178 | 0.8509 | 4.93 | 4.95 | 4.86 | 1.05 | 0.6652 | 0.8363 |
| TC0300010315.hg.1 | IQSEC1 | IQ motif and Sec7 domain 1 | Multiple_C | 4.29 | 4.2 | 4.07 | 1.16 | 0.5854 | 0.7619 | 8.1 | 8.28 | 7.47 | 1.55 | 0.2522 | 0.5164 |

| | | | | | | | | | | | | | | | |
|---|---|---|---|---|---|---|---|---|---|---|---|---|---|---|---|
| TC0300011044.hg.1 | DALRD3 | DALR anticodon binding domain containing 3 | Multiple_C | 10.03 | 10.81 | 9.81 | 1.16 | 0.714 | 0.8491 | 7.68 | 7.56 | 7 | 1.60 | 0.0047 | 0.047 |
| TC0300011267.hg.1 | DNAH12 | dynein, axonemal, heavy chain 12 | Multiple_C | 3.6 | 3.61 | 3.38 | 1.16 | 0.652 | 0.8075 | 3.22 | 3.42 | 3.18 | 1.03 | 0.8946 | 0.9554 |
| TC0300011313.hg.1 | FAM3D | family with sequence similarity 3, member D | Coding | 4.66 | 4.28 | 4.44 | 1.16 | 0.2312 | 0.4315 | 3.71 | 3.82 | 3.69 | 1.01 | 0.2578 | 0.5227 |
| TC0300012483.hg.1 | C3orf36 | chromosome 3 open reading frame 36 | Coding | 5.11 | 4.99 | 4.89 | 1.16 | 0.5509 | 0.7358 | 5.23 | 5.23 | 4.5 | 1.66 | 0.0091 | 0.0732 |
| TC0300012804.hg.1 | ERICH6 | glutamate rich 6 | Multiple_C | 3.81 | 3.61 | 3.59 | 1.16 | 0.6203 | 0.7876 | 4.12 | 4.13 | 4.27 | 0.90 | 0.5737 | 0.7813 |
| TC0300013522.hg.1 | TMEM207 | transmembrane protein 207 | Coding | 4.55 | 4.51 | 4.33 | 1.16 | 0.525 | 0.7172 | 5.03 | 4.92 | 4.83 | 1.15 | 0.3787 | 0.6384 |
| TC0400007769.hg.1 | CSN1S1 | casein alpha s1 | Coding | 3.54 | 3.17 | 3.32 | 1.16 | 0.7557 | 0.8745 | 3.78 | 3.82 | 3.86 | 0.95 | 0.3823 | 0.6414 |
| TC0400009088.hg.1 | GUCY1B3 | guanylate cyclase 1, soluble, beta 3 | Multiple_C | 4.12 | 3.57 | 3.9 | 1.16 | 0.5275 | 0.7191 | 4.5 | 4.44 | 4.55 | 0.97 | 0.6242 | 0.8145 |
| TC0400012213.hg.1 | CTSO | cathepsin O | Coding | 4.65 | 4.32 | 4.43 | 1.16 | 0.1583 | 0.3343 | 3.58 | 3.50 | 3.52 | 1.04 | 0.585 | 0.789 |
| TC0500010930.hg.1 | TRIM23 | tripartite motif containing 23 | Multiple_C | 9.06 | 9.85 | 8.84 | 1.16 | 0.3676 | 0.5806 | 9.54 | 8.88 | 8.65 | 1.85 | 0.0059 | 0.0549 |
| TC0500012206.hg.1 | PROB1 | proline-rich basic protein 1 | Coding | 5.36 | 5.04 | 5.14 | 1.16 | 0.2827 | 0.4929 | 5.56 | 5.72 | 5.4 | 1.12 | 0.9538 | 0.9811 |
| TC0500012467.hg.1 | ARSI | arylsulfatase family, member I | Multiple_C | 4.51 | 3.9 | 4.29 | 1.16 | 0.5011 | 0.6996 | 5.54 | 5.07 | 4.61 | 1.91 | 0.0108 | 0.0818 |
| TC0500013350.hg.1 | RAPGEF6 | Rap guanine nucleotide exchange factor 6 | Multiple_C | 8.35 | 8.19 | 8.13 | 1.16 | 0.3972 | 0.611 | 8.75 | 8.16 | 8.5 | 1.19 | 0.2109 | 0.4685 |
| TC0500013353.hg.1 | ACSL6 | acyl-CoA synthetase long-chain family member 6 | Multiple_C | 4.5 | 4.16 | 4.28 | 1.16 | 0.4018 | 0.6147 | 3.93 | 3.92 | 3.96 | 0.98 | 0.3223 | 0.5861 |
| TC0600008303.hg.1 | LRRC1 | leucine rich repeat containing 1 | Multiple_C | 11.44 | 11.43 | 11.22 | 1.16 | 0.5024 | 0.7004 | 7.48 | 7.01 | 7.23 | 1.19 | 0.4489 | 0.6956 |
| TC0600009831.hg.1 | PPP1R14C | protein phosphatase 1, regulatory (inhibitor) subunit 14C | Coding | 3.18 | 2.87 | 2.96 | 1.16 | 0.6863 | 0.8308 | 3.35 | 3.49 | 3.22 | 1.09 | 0.2553 | 0.5199 |
| TC0600010210.hg.1 | UNC93A | unc-93 homolog A (C. elegans) | Multiple_C | 8.08 | 8.56 | 7.86 | 1.16 | 0.4935 | 0.6934 | 5.54 | 5.25 | 5.13 | 1.33 | 0.0453 | 0.1967 |
| TC0600011471.hg.1 | DXO | decapping exoribonuclease | Multiple_C | 4.9 | 4.56 | 4.68 | 1.16 | 0.5331 | 0.7228 | 6.12 | 6.37 | 6.37 | 0.84 | 0.8308 | 0.9247 |
| TC0600012413.hg.1 | IBTK | inhibitor of Bruton agammaglobulinemia tyrosine kinase | Multiple_C | 13.43 | 13.94 | 13.21 | 1.16 | 0.3592 | 0.5728 | 11.91 | 10.91 | 11.25 | 1.58 | 0.0054 | 0.0516 |
| TC0600012437.hg.1 | SNAP91 | synaptosome associated protein 91kDa | Multiple_C | 5.28 | 5.18 | 5.06 | 1.16 | 0.5375 | 0.7259 | 4.98 | 5.28 | 5.23 | 0.84 | 0.3878 | 0.6466 |
| TC0600014234.hg.1 | CAGE1 | cancer antigen 1 | Multiple_C | 3.51 | 3.45 | 3.29 | 1.16 | 0.4175 | 0.6292 | 3.97 | 3.44 | 3.48 | 1.40 | 0.0782 | 0.2692 |
| TC0700009479.hg.1 | OR6V1 | olfactory receptor, family 6, subfamily V, member 1 | Coding | 3.18 | 2.94 | 2.96 | 1.16 | 0.2062 | 0.3994 | 3.36 | 3.69 | 3.49 | 0.91 | 0.3193 | 0.5834 |
| TC0700009646.hg.1 | SSPO | SCO-spondin | Multiple_C | 5.04 | 4.98 | 4.82 | 1.16 | 0.2987 | 0.5103 | 4.88 | 4.67 | 4.33 | 1.46 | 0.0323 | 0.1609 |
| TC0700013060.hg.1 | TMUB1 | transmembrane and ubiquitin-like domain containing 1 | Multiple_C | 11.51 | 10.24 | 11.29 | 1.16 | 0.7652 | 0.8794 | 10.65 | 11.08 | 11.87 | 0.43 | 0.0008 | 0.0144 |
| TC0800006970.hg.1 | FAM160B2 | family with sequence similarity 160, member B2 | Multiple_C | 8.67 | 8.4 | 8.45 | 1.16 | 0.9555 | 0.9792 | 7.5 | 7.72 | 7.54 | 0.97 | 0.9264 | 0.9696 |
| TC0800011007.hg.1 | REXO1L4P | REX1, RNA exonuclease 1 homolog-like 4, pseudogene | Multiple_C | 3.79 | 3.46 | 3.57 | 1.16 | 0.4196 | 0.6309 | 5.15 | 5.00 | 4.86 | 1.22 | 0.13 | 0.3572 |
| TC0800011462.hg.1 | TMEM74 | transmembrane protein 74 | Multiple_C | 4.63 | 4.4 | 4.41 | 1.16 | 0.37 | 0.5827 | 4.35 | 4.27 | 4.32 | 1.02 | 0.873 | 0.9452 |
| TC0800012156.hg.1 | CCDC166 | coiled-coil domain containing 166 | Coding | 3.08 | 3.13 | 2.86 | 1.16 | 0.2182 | 0.4147 | 3.54 | 3.60 | 3.33 | 1.16 | 0.5173 | 0.7458 |
| TC0900007221.hg.1 | LOC10272423 | uncharacterized LOC102724238; uncharacterized LOC554249 | Multiple_C | 6.45 | 6.11 | 6.23 | 1.16 | 0.3483 | 0.5615 | 7.06 | 7.29 | 7.1 | 0.97 | 0.6349 | 0.8208 |
| TC0900008504.hg.1 | COL27A1 | collagen, type XXVII, alpha 1 | Multiple_C | 5.4 | 5.06 | 5.18 | 1.16 | 0.4363 | 0.6458 | 6.82 | 6.20 | 6.19 | 1.55 | 0.0168 | 0.1082 |
| TC0900009711.hg.1 | IFNE; MIR31H | interferon, epsilon; MIR31 host gene | Multiple_C | 5.04 | 4 | 4.82 | 1.16 | 0.1775 | 0.3603 | 5.08 | 4.30 | 4.37 | 1.64 | 0.029 | 0.1506 |
| TC0900010694.hg.1 | SHC3 | SHC (Src homology 2 domain containing) transforming protein 3 | Multiple_C | 3.52 | 3.15 | 3.3 | 1.16 | 0.4337 | 0.6438 | 4.94 | 5.08 | 5.05 | 0.93 | 0.6591 | 0.8338 |
| TC0900011259.hg.1 | HDHD3 | haloacid dehalogenase-like hydrolase domain containing 3 | Multiple_C | 9.62 | 9.06 | 9.4 | 1.16 | 0.4892 | 0.6895 | 5.14 | 5.89 | 5.6 | 0.73 | 0.0704 | 0.2534 |

| | | | | | | | | | | | | | | | |
|---|---|---|---|---|---|---|---|---|---|---|---|---|---|---|---|
| TC0900011431.hg.1 | LHX6 | LIM homeobox 6 | Multiple_C | 4.35 | 3.73 | 4.13 | 1.16 | 0.2295 | 0.4293 | 5.9 | 5.53 | 5.51 | 1.31 | 0.1834 | 0.4342 |
| TC0X00006593.hg.1 | CLCN4 | chloride channel, voltage-sensitive 4 | Coding | 7.55 | 7.3 | 7.33 | 1.16 | 0.8203 | 0.9099 | 6.89 | 7.02 | 7.53 | 0.64 | 0.0682 | 0.2487 |
| TC0X00006770.hg.1 | SMS | spermine synthase | Multiple_C | 14.4 | 14.41 | 14.18 | 1.16 | 0.4338 | 0.6438 | 13.43 | 13.55 | 14.08 | 0.64 | 0.0155 | 0.1025 |
| TC0X00006878.hg.1 | MAGEB1; MA( | MAGE family member B1; MAGE family member B4 | Coding | 4.53 | 3.77 | 4.31 | 1.16 | 0.0856 | 0.2148 | 4.37 | 4.03 | 3.99 | 1.30 | 0.081 | 0.2743 |
| TC0X00007251.hg.1 | CCNB3 | cyclin B3 | Multiple_C | 3.99 | 4.19 | 3.77 | 1.16 | 0.3892 | 0.6031 | 3.04 | 3.07 | 3.3 | 0.84 | 0.8729 | 0.9452 |
| TC0X00007820.hg.1 | TGIF2LX | TGFB-induced factor homeobox 2-like, X-linked | Coding | 2.95 | 2.55 | 2.73 | 1.16 | 0.5007 | 0.6994 | 3.55 | 3.44 | 3.28 | 1.21 | 0.1144 | 0.3337 |
| TC0X00011012.hg.1 | SLITRK4 | SLIT and NTRK-like family, member 4 | Multiple_C | 3.61 | 3.62 | 3.39 | 1.16 | 0.8921 | 0.9481 | 4.12 | 4.34 | 4.17 | 0.97 | 0.5763 | 0.7829 |
| TC0X00011191.hg.1 | TEX28 | testis expressed 28 | Multiple_C | 3.06 | 2.68 | 2.84 | 1.16 | 0.8425 | 0.9225 | 3.37 | 3.26 | 3.33 | 1.03 | 0.2332 | 0.4954 |
| TC0Y00006732.hg.1 | RPS4Y2 | ribosomal protein S4, Y-linked 2 | Coding | 3.31 | 2.99 | 3.09 | 1.16 | 0.1459 | 0.3161 | 3.45 | 3.58 | 3.34 | 1.08 | 0.25 | 0.5145 |
| TC1000007103.hg.1 | RAB18 | RAB18, member RAS oncogene family | Multiple_C | 12.11 | 13.03 | 11.89 | 1.16 | 0.1685 | 0.3485 | 11.53 | 10.74 | 11.16 | 1.29 | 0.2131 | 0.4713 |
| TC1000008896.hg.1 | SMC3 | structural maintenance of chromosomes 3 | Multiple_C | 14.06 | 13.4 | 13.84 | 1.16 | 0.9659 | 0.9836 | 13.44 | 12.89 | 13.37 | 1.05 | 0.3275 | 0.591 |
| TC1100006836.hg.1 | MRVI1-AS1 | MRVI1 antisense RNA 1 | Multiple_C | 5.15 | 4.75 | 4.93 | 1.16 | 0.0338 | 0.1078 | 5.01 | 5.19 | 5.17 | 0.90 | 0.5072 | 0.7382 |
| TC1100007675.hg.1 | RTN4RL2 | reticulon 4 receptor-like 2 | Coding | 4.87 | 4.44 | 4.65 | 1.16 | 0.1478 | 0.3187 | 4.08 | 4.28 | 4.06 | 1.01 | 0.8918 | 0.9539 |
| TC1100008013.hg.1 | TIGD3 | tigger transposable element derived 3 | Coding | 5.57 | 5.05 | 5.35 | 1.16 | 0.3431 | 0.5569 | 4.67 | 4.61 | 4.76 | 0.94 | 0.2985 | 0.5632 |
| TC1100008069.hg.1 | CNIH2 | cornichon family AMPA receptor auxiliary protein 2 | Multiple_C | 4.3 | 3.93 | 4.08 | 1.16 | 0.1742 | 0.3561 | 5.08 | 4.70 | 4.4 | 1.60 | 0.0778 | 0.2686 |
| TC1100009191.hg.1 | IL10RA | interleukin 10 receptor, alpha | Multiple_C | 3.36 | 3.1 | 3.14 | 1.16 | 0.9901 | 0.9947 | 3.37 | 3.41 | 3.52 | 0.90 | 0.8569 | 0.9392 |
| TC1100010316.hg.1 | DBX1 | developing brain homeobox 1 | Coding | 2.84 | 2.47 | 2.62 | 1.16 | 0.1952 | 0.3852 | 3.32 | 3.21 | 2.96 | 1.28 | 0.0954 | 0.3004 |
| TC1100010856.hg.1 | OR5M9 | olfactory receptor, family 5, subfamily M, member 9 | Coding | 3.13 | 2.64 | 2.91 | 1.16 | 0.315 | 0.5284 | 3.66 | 3.68 | 3.6 | 1.04 | 0.8686 | 0.9434 |
| TC1100012528.hg.1 | THY1 | Thy-1 cell surface antigen | Multiple_C | 3.71 | 3.38 | 3.49 | 1.16 | 0.6911 | 0.8335 | 5.04 | 4.59 | 3.81 | 2.35 | 0.0047 | 0.0475 |
| TC1100012647.hg.1 | OR6T1 | olfactory receptor, family 6, subfamily T, member 1 | Coding | 5.04 | 4.94 | 4.82 | 1.16 | 0.3356 | 0.5496 | 4.18 | 4.41 | 4.19 | 0.99 | 0.8061 | 0.9143 |
| TC1100012696.hg.1 | ROBO4 | roundabout guidance receptor 4 | Multiple_C | 3.96 | 3.58 | 3.74 | 1.16 | 0.7316 | 0.8598 | 3.9 | 4.19 | 4.07 | 0.89 | 0.5802 | 0.7853 |
| TC1100013170.hg.1 | EEF1G; MIR36 | eukaryotic translation elongation factor 1 gamma; microRNA 36 | Multiple_C | 18.04 | 17.87 | 17.82 | 1.16 | 0.0453 | 0.1345 | 18.11 | 18.09 | 18.2 | 0.94 | 0.8611 | 0.9406 |
| TC1200006676.hg.1 | ACSM4 | acyl-CoA synthetase medium-chain family member 4 | Multiple_C | 3.96 | 3.63 | 3.74 | 1.16 | 0.4237 | 0.6349 | 4.15 | 4.33 | 4.25 | 0.93 | 0.5925 | 0.7939 |
| TC1200006788.hg.1 | KLRD1 | killer cell lectin-like receptor subfamily D, member 1 | Multiple_C | 2.99 | 2.81 | 2.77 | 1.16 | 0.4261 | 0.6368 | 3.2 | 3.11 | 3 | 1.15 | 0.0881 | 0.2868 |
| TC1200007461.hg.1 | PCED1B | PC-esterase domain containing 1B | Multiple_C | 4.77 | 4.34 | 4.55 | 1.16 | 0.9175 | 0.9613 | 7.21 | 7.18 | 6.82 | 1.31 | 0.2964 | 0.561 |
| TC1200007731.hg.1 | HOXC13 | homeobox C13 | Multiple_C | 6.58 | 6.73 | 6.36 | 1.16 | 0.6152 | 0.7846 | 8.03 | 8.01 | 8.56 | 0.69 | 0.0354 | 0.1705 |
| TC1200008795.hg.1 | ACACB | acetyl-CoA carboxylase beta | Multiple_C | 6.79 | 6.44 | 6.57 | 1.16 | 0.1139 | 0.2648 | 5.87 | 5.96 | 5.81 | 1.04 | 0.1911 | 0.4441 |
| TC1200009959.hg.1 | LRP6 | LDL receptor related protein 6 | Multiple_C | 9.84 | 10.04 | 9.62 | 1.16 | 0.321 | 0.5348 | 10.33 | 10.29 | 10.18 | 1.11 | 0.7008 | 0.8581 |
| TC1200012068.hg.1 | C12orf49 | chromosome 12 open reading frame 49 | Multiple_C | 10.89 | 10.6 | 10.67 | 1.16 | 0.4783 | 0.6805 | 7.99 | 8.28 | 8.38 | 0.76 | 0.2092 | 0.4664 |
| TC1200012768.hg.1 | RERG | RAS-like, estrogen-regulated, growth inhibitor | Multiple_C | 6.03 | 5.2 | 5.81 | 1.16 | 0.1818 | 0.3664 | 5.46 | 5.53 | 5.46 | 1.00 | 0.1572 | 0.3982 |
| TC1300007074.hg.1 | SPERT | spermatid associated | Coding | 3.14 | 2.64 | 2.92 | 1.16 | 0.3114 | 0.5248 | 3.72 | 3.78 | 3.56 | 1.12 | 0.6003 | 0.7996 |
| TC1300007565.hg.1 | SLAIN1 | SLAIN motif family member 1 | Multiple_C | 8.19 | 8.41 | 7.97 | 1.16 | 0.5933 | 0.7678 | 8.37 | 7.20 | 7.21 | 2.23 | 0.0002 | 0.0063 |
| TC1300008381.hg.1 | AMER2 | APC membrane recruitment protein 2 | Multiple_C | 3.01 | 2.68 | 2.79 | 1.16 | 0.3152 | 0.5285 | 3.75 | 3.55 | 3.53 | 1.16 | 0.136 | 0.3665 |

| | | | | | | | | | | | | | | | |
|---|---|---|---|---|---|---|---|---|---|---|---|---|---|---|---|
| TC1300009288.hg.1 | KCTD12 | potassium channel tetramerization domain containing 12 | Multiple_C | 3.39 | 3.07 | 3.17 | 1.16 | 0.4483 | 0.6564 | 3.36 | 3.31 | 3.26 | 1.07 | 0.3991 | 0.6561 |
| TC1300009650.hg.1 | FGF14; FGF14 | fibroblast growth factor 14; FGF14 intronic transcript 1 | Multiple_C | 3.5 | 3.35 | 3.28 | 1.16 | 0.2885 | 0.4992 | 3.18 | 3.29 | 3.22 | 0.97 | 0.6443 | 0.8255 |
| TC1400006686.hg.1 | CMTM5 | CKLF-like MARVEL transmembrane domain containing 5 | Multiple_C | 3.78 | 3.33 | 3.56 | 1.16 | 0.9027 | 0.9531 | 3.8 | 3.71 | 3.4 | 1.32 | 0.442 | 0.6909 |
| TC1400009410.hg.1 | ZBTB25 | zinc finger and BTB domain containing 25 | Multiple_C | 8.13 | 7.8 | 7.91 | 1.16 | 0.6191 | 0.7869 | 8.3 | 8.10 | 8.09 | 1.16 | 0.8753 | 0.9465 |
| TC1400010333.hg.1 | PPP1R13B | protein phosphatase 1, regulatory subunit 13B | Multiple_C | 6.93 | 6.54 | 6.71 | 1.16 | 0.28 | 0.4897 | 5.87 | 6.27 | 6.62 | 0.59 | 0.0001 | 0.004 |
| TC1400010594.hg.1 | FAM177A1 | family with sequence similarity 177, member A1 | Multiple_C | 3.83 | 3.81 | 3.61 | 1.16 | 0.4424 | 0.6512 | 5.36 | 5.57 | 4.72 | 1.56 | 0.0145 | 0.0984 |
| TC1400010717.hg.1 | PPP1R3E | protein phosphatase 1, regulatory subunit 3E | Multiple_C | 4.92 | 4.34 | 4.7 | 1.16 | 0.2645 | 0.4719 | 6.22 | 6.63 | 6.31 | 0.94 | 0.2954 | 0.5604 |
| TC1500006522.hg.1 | GOLGA8DP | golgin A8 family, member D, pseudogene | Multiple_C | 5.15 | 4.89 | 4.93 | 1.16 | 0.051 | 0.1466 | 5.52 | 5.27 | 5.3 | 1.16 | 0.3402 | 0.604 |
| TC1500006811.hg.1 | CHRM5 | cholinergic receptor, muscarinic 5 | Coding | 3.73 | 3.17 | 3.51 | 1.16 | 0.6419 | 0.8014 | 3.86 | 3.76 | 3.73 | 1.09 | 0.3572 | 0.6202 |
| TC1500007532.hg.1 | FBXL22 | F-box and leucine-rich repeat protein 22 | Coding | 3.34 | 3.05 | 3.12 | 1.16 | 0.7199 | 0.8524 | 3.31 | 3.17 | 3.22 | 1.06 | 0.4106 | 0.6652 |
| TC1500008341.hg.1 | MAN2A2 | mannosidase, alpha, class 2A, member 2 | Multiple_C | 9.89 | 8.9 | 9.67 | 1.16 | 0.5145 | 0.7095 | 7.29 | 7.83 | 8.49 | 0.44 | 0.0003 | 0.0081 |
| TC16_KI270728v1_rando | TP53TG3B | TP53 target 3B | Multiple_C | 3.85 | 3.53 | 3.63 | 1.16 | 0.9969 | 0.9984 | 3.47 | 3.53 | 3.6 | 0.91 | 0.293 | 0.5578 |
| TC1600007511.hg.1 | ZNF646 | zinc finger protein 646 | Coding | 5.73 | 5.28 | 5.51 | 1.16 | 0.7545 | 0.8743 | 6.35 | 6.80 | 6.79 | 0.74 | 0.5715 | 0.7794 |
| TC1600007526.hg.1 | TRIM72 | tripartite motif containing 72, E3 ubiquitin protein ligase | Multiple_C | 3.13 | 2.8 | 2.91 | 1.16 | 0.3688 | 0.5817 | 3.15 | 3.17 | 3.18 | 0.98 | 0.8692 | 0.9436 |
| TC1600007621.hg.1 | TP53TG3B; TP | Homo sapiens TP53 target 3B (TP53TG3B), transcript variant 1, r | Multiple_C | 3.85 | 3.53 | 3.63 | 1.16 | 0.9969 | 0.9984 | 3.47 | 3.53 | 3.6 | 0.91 | 0.293 | 0.5578 |
| TC1600007985.hg.1 | CETP | cholesteryl ester transfer protein, plasma | Multiple_C | 3.15 | 2.82 | 2.93 | 1.16 | 0.4673 | 0.6717 | 3.71 | 3.62 | 3.34 | 1.29 | 0.3149 | 0.5788 |
| TC1600008504.hg.1 | CLEC3A | C-type lectin domain family 3, member A | Multiple_C | 3.47 | 3.17 | 3.25 | 1.16 | 0.3549 | 0.568 | 3.55 | 3.57 | 3.58 | 0.98 | 0.2934 | 0.5582 |
| TC1600008892.hg.1 | DPEP1 | dipeptidase 1 (renal) | Multiple_C | 7.56 | 7.22 | 7.34 | 1.16 | 0.1289 | 0.2891 | 9.07 | 10.55 | 8.67 | 1.32 | 0.258 | 0.5228 |
| TC1600009865.hg.1 | NUPR1 | nuclear protein 1, transcriptional regulator | Multiple_C | 8.9 | 10.35 | 8.68 | 1.16 | 0.4757 | 0.6784 | 7.32 | 7.34 | 7.21 | 1.08 | 0.4683 | 0.7093 |
| TC1600010244.hg.1 | ZNF423 | zinc finger protein 423 | Multiple_C | 3.92 | 4.3 | 3.7 | 1.16 | 0.6713 | 0.8209 | 4.7 | 4.84 | 4.64 | 1.04 | 0.9565 | 0.9819 |
| TC1600011390.hg.1 | TP53TG3B; TP | Homo sapiens TP53 target 3B (TP53TG3B), transcript variant 1, r | Multiple_C | 3.85 | 3.53 | 3.63 | 1.16 | 0.9969 | 0.9984 | 3.47 | 3.53 | 3.6 | 0.91 | 0.293 | 0.5578 |
| TC1700006535.hg.1 | HIC1 | hypermethylated in cancer 1 | Multiple_C | 6.72 | 6.52 | 6.5 | 1.16 | 0.863 | 0.9332 | 5.31 | 5.56 | 5.8 | 0.71 | 0.1786 | 0.4275 |
| TC1700006643.hg.1 | GLTPD2 | glycolipid transfer protein domain containing 2 | Coding | 5.38 | 5.14 | 5.16 | 1.16 | 0.4188 | 0.6301 | 5.22 | 5.57 | 5.57 | 0.78 | 0.7175 | 0.8682 |
| TC1700007931.hg.1 | IFI35 | interferon-induced protein 35 | Multiple_C | 8.42 | 8.21 | 8.2 | 1.16 | 0.457 | 0.6633 | 6 | 6.07 | 5.99 | 1.01 | 0.6236 | 0.814 |
| TC1700011033.hg.1 | PHOSPHO1 | phosphatase, orphan 1 | Coding | 4.41 | 4.17 | 4.19 | 1.16 | 0.5171 | 0.7113 | 5.3 | 5.19 | 5.2 | 1.07 | 0.662 | 0.835 |
| TC1700011653.hg.1 | SDK2 | sidekick cell adhesion molecule 2 | Multiple_C | 4.53 | 4.42 | 4.31 | 1.16 | 0.988 | 0.9936 | 4.32 | 4.20 | 4.01 | 1.24 | 0.1596 | 0.4016 |
| TC1700012084.hg.1 | NOTUM | notum pectinacetylesterase homolog (Drosophila) | Multiple_C | 3.87 | 3.78 | 3.65 | 1.16 | 0.4728 | 0.6761 | 12.83 | 13.70 | 12.68 | 1.11 | 0.8284 | 0.9241 |
| TC1700012193.hg.1 | NDEL1 | nudE neurodevelopment protein 1-like 1 | Multiple_C | 10.14 | 10.49 | 9.92 | 1.16 | 0.6797 | 0.8259 | 9.92 | 8.36 | 10.13 | 0.86 | 0.5508 | 0.7666 |
| TC1900007096.hg.1 | JUNB | jun B proto-oncogene | Multiple_C | 8.01 | 6.83 | 7.79 | 1.16 | 0.9663 | 0.9837 | 5.53 | 5.09 | 4.99 | 1.45 | 0.023 | 0.1306 |
| TC1900008600.hg.1 | GPR32 | G protein-coupled receptor 32 | Coding | 4.3 | 4.09 | 4.08 | 1.16 | 0.4881 | 0.6889 | 4.24 | 4.25 | 4.31 | 0.95 | 0.4546 | 0.6994 |
| TC1900009518.hg.1 | FBN3; mumar | Zhang2013 ALT_ACCEPTOR, ALT_DONOR, coding, INTERNAL, int | Multiple_C | 2.95 | 2.61 | 2.73 | 1.16 | 0.321 | 0.5348 | 3.66 | 3.45 | 3.27 | 1.31 | 0.1661 | 0.4109 |
| TC1900009822.hg.1 | PTGER1 | prostaglandin E receptor 1 | Coding | 5.54 | 5.35 | 5.32 | 1.16 | 0.4553 | 0.6624 | 6.12 | 6.41 | 6.4 | 0.82 | 0.2918 | 0.5564 |
| TC1900010510.hg.1 | THAP8 | THAP domain containing 8 | Multiple_C | 4.4 | 4.11 | 4.18 | 1.16 | 0.5488 | 0.734 | 3.76 | 3.68 | 3.83 | 0.95 | 0.5022 | 0.7344 |

| | | | | | | | | | | | | | | | |
|---|---|---|---|---|---|---|---|---|---|---|---|---|---|---|---|
| TC1900010604.hg.1 | RASGRP4 | RAS guanyl releasing protein 4 | Multiple_C | 3.53 | 3.14 | 3.31 | 1.16 | 0.6539 | 0.809 | 3.96 | 4.01 | 4.04 | 0.95 | 0.7868 | 0.9049 |
| TC1900010647.hg.1 | IFNL4 | interferon, lambda 4 (gene/pseudogene) | Multiple_C | 3.34 | 3.07 | 3.12 | 1.16 | 0.5965 | 0.7701 | 3.41 | 3.36 | 3.36 | 1.04 | 0.8316 | 0.9249 |
| TC1900011253.hg.1 | C19orf84 | chromosome 19 open reading frame 84 | Multiple_C | 9.09 | 8.83 | 8.87 | 1.16 | 0.5188 | 0.7127 | 9 | 9.30 | 8.86 | 1.10 | 0.8432 | 0.9317 |
| TC1900011336.hg.1 | VN1R4 | vomeronasal 1 receptor 4 | Coding | 3.22 | 3.33 | 3 | 1.16 | 0.4777 | 0.6801 | 3.19 | 3.27 | 3.04 | 1.11 | 0.694 | 0.8544 |
| TC1900011423.hg.1 | CDC42EP5 | CDC42 effector protein (Rho GTPase binding) 5 | Multiple_C | 5.2 | 4.88 | 4.98 | 1.16 | 0.9638 | 0.9824 | 4.42 | 4.40 | 4.26 | 1.12 | 0.4103 | 0.6652 |
| TC1900011648.hg.1 | CAPS | calcyphosine | Multiple_C | 4.58 | 4.41 | 4.36 | 1.16 | 0.4436 | 0.6521 | 4.61 | 4.95 | 4.91 | 0.81 | 0.1236 | 0.3471 |
| TC1900011655.hg.1 | PET100 | PET100 homolog | Multiple_C | 11.14 | 10.77 | 10.92 | 1.16 | 0.6796 | 0.8259 | 8.1 | 8.63 | 7.8 | 1.23 | 0.4097 | 0.6647 |
| TC1900011698.hg.1 | RPSAP58; RPS | ribosomal protein SA pseudogene 58; ribosomal protein SA | Multiple_C | 11.44 | 11.55 | 11.22 | 1.16 | 0.3507 | 0.5634 | 6.57 | 6.85 | 7.16 | 0.66 | 0.0271 | 0.1439 |
| TC1900011853.hg.1 | RFX2 | regulatory factor X, 2 (influences HLA class II expression) | Multiple_C | 4.43 | 4.06 | 4.21 | 1.16 | 0.4968 | 0.6967 | 4.58 | 4.51 | 4.38 | 1.15 | 0.797 | 0.9093 |
| TC1900011959.hg.1 | CEACAM8 | carcinoembryonic antigen-related cell adhesion molecule 8 | Coding | 3.86 | 3.4 | 3.64 | 1.16 | 0.2793 | 0.4889 | 4.3 | 4.42 | 4.25 | 1.04 | 0.8538 | 0.9371 |
| TC2000007105.hg.1 | TM9SF4 | transmembrane 9 superfamily protein member 4 | Multiple_C | 13.51 | 12.87 | 13.29 | 1.16 | 0.5526 | 0.7367 | 13.74 | 13.78 | 13.9 | 0.90 | 0.4704 | 0.7112 |
| TC2000009815.hg.1 | PTK6 | protein tyrosine kinase 6 | Multiple_C | 7.81 | 7.42 | 7.59 | 1.16 | 0.959 | 0.9806 | 7.27 | 6.41 | 6.11 | 2.23 | 0.0009 | 0.0149 |
| TC2100007336.hg.1 | TRAPPC10 | trafficking protein particle complex 10 | Multiple_C | 9.71 | 8.64 | 9.49 | 1.16 | 0.7092 | 0.8457 | 9.81 | 9.29 | 9.68 | 1.09 | 0.6447 | 0.8257 |
| TC2200006552.hg.1 | TMEM191B | transmembrane protein 191B | Multiple_C | 4.15 | 4.01 | 3.93 | 1.16 | 0.3611 | 0.5746 | 3.6 | 3.56 | 3.6 | 1.00 | 0.6055 | 0.8027 |
| TC2200006703.hg.1 | HIC2 | hypermethylated in cancer 2 | Multiple_C | 4.92 | 4.65 | 4.7 | 1.16 | 0.2608 | 0.4676 | 5.41 | 5.45 | 5.44 | 0.98 | 0.6257 | 0.815 |
| TC2200008654.hg.1 | MFNG | MFNG O-fucosylpeptide 3-beta-N-acetylglucosaminyltransferas | Multiple_C | 5.03 | 4.52 | 4.81 | 1.16 | 0.3035 | 0.5163 | 11.19 | 11.91 | 12.22 | 0.49 | 0.0019 | 0.026 |
| TC2200008680.hg.1 | BAIAP2L2 | BAI1-associated protein 2-like 2 | Multiple_C | 4.97 | 4.58 | 4.75 | 1.16 | 0.1176 | 0.271 | 4.92 | 5.69 | 5.38 | 0.73 | 0.0513 | 0.2116 |
| TC2200008907.hg.1 | SCUBE1 | signal peptide, CUB domain, EGF-like 1 | Multiple_C | 3.43 | 3.28 | 3.21 | 1.16 | 0.623 | 0.7892 | 3.61 | 3.51 | 3.41 | 1.15 | 0.4969 | 0.7304 |
| TSUnmapped00000113.h | PADI4 | peptidyl arginine deiminase, type IV | Coding | 3.74 | 3.12 | 3.52 | 1.16 | 0.3622 | 0.5757 | 4 | 3.98 | 3.96 | 1.03 | 0.8952 | 0.9556 |
| TSUnmapped00000369.h | ZNF780B | zinc finger protein 780B | Coding | 7.82 | 7.37 | 7.6 | 1.16 | 0.371 | 0.5837 | 7.04 | 6.82 | 6.96 | 1.06 | 0.9778 | 0.9908 |
| TSUnmapped00000469.h | INPP5D | inositol polyphosphate-5-phosphatase D | Coding | 3.22 | 3.08 | 3 | 1.16 | 0.2716 | 0.4802 | 3.45 | 3.59 | 3.28 | 1.13 | 0.6234 | 0.8139 |
| TSUnmapped00000594.h | GREM1 | gremlin 1, DAN family BMP antagonist [Source:HGNC Symbol;A | Coding | 4.17 | 3.66 | 3.95 | 1.16 | 0.395 | 0.6085 | 4.74 | 4.49 | 4.57 | 1.13 | 0.2587 | 0.523 |
| TC0100007445.hg.1 | CATSPER4 | cation channel, sperm associated 4 | Coding | 3.48 | 3.68 | 3.27 | 1.16 | 0.449 | 0.6569 | 4.08 | 3.79 | 3.58 | 1.41 | 0.0408 | 0.1855 |
| TC0100008653.hg.1 | IL12RB2 | interleukin 12 receptor, beta 2 | Multiple_C | 3.08 | 2.74 | 2.87 | 1.16 | 0.3009 | 0.5132 | 3.53 | 3.57 | 3.41 | 1.09 | 0.1605 | 0.403 |
| TC0100008779.hg.1 | ST6GALNAC3 | ST6 (alpha-N-acetyl-neuraminyl-2,3-beta-galactosyl-1,3)-N-acet | Multiple_C | 3.73 | 3.46 | 3.52 | 1.16 | 0.3731 | 0.5856 | 4.5 | 4.35 | 4.08 | 1.34 | 0.1101 | 0.3268 |
| TC0100012307.hg.1 | VN1R5 | vomeronasal 1 receptor 5 (gene/pseudogene) | Multiple_C | 3.35 | 3.26 | 3.14 | 1.16 | 0.658 | 0.8122 | 3.4 | 3.50 | 3.29 | 1.08 | 0.1796 | 0.4286 |
| TC0100012465.hg.1 | CPSF3L; MIR6 | cleavage and polyadenylation specific factor 3-like; microRNA 6 | Multiple_C | 11.11 | 11.24 | 10.9 | 1.16 | 0.4379 | 0.6473 | 10.89 | 11.49 | 11.07 | 0.88 | 0.6816 | 0.8472 |
| TC0100013057.hg.1 | FAM231A; FA | Homo sapiens family with sequence similarity 231, member A (F | Coding | 5.45 | 4.86 | 5.24 | 1.16 | 0.2349 | 0.4359 | 5.26 | 5.32 | 5.25 | 1.01 | 0.8289 | 0.9243 |
| TC0100013623.hg.1 | SYNC | syncoilin, intermediate filament protein | Multiple_C | 3.96 | 3.56 | 3.75 | 1.16 | 0.5113 | 0.7072 | 4.52 | 4.64 | 4.24 | 1.21 | 0.2876 | 0.5523 |
| TC0100013718.hg.1 | LSM10 | LSM10, U7 small nuclear RNA associated | Multiple_C | 8.64 | 8.49 | 8.43 | 1.16 | 0.5558 | 0.739 | 6.87 | 7.00 | 6.96 | 0.94 | 0.1139 | 0.3328 |
| TC0100014501.hg.1 | INSL5 | insulin-like 5 | Coding | 3.38 | 2.99 | 3.17 | 1.16 | 0.7592 | 0.8763 | 3.23 | 3.37 | 3.4 | 0.89 | 0.9417 | 0.9759 |
| TC0100014576.hg.1 | PTGER3 | prostaglandin E receptor 3 (subtype EP3) | Multiple_C | 3.79 | 3.42 | 3.58 | 1.16 | 0.3486 | 0.5617 | 3.83 | 3.54 | 3.43 | 1.32 | 0.037 | 0.1751 |
| TC0100014855.hg.1 | GBP4 | guanylate binding protein 4 | Multiple_C | 3.66 | 3.33 | 3.45 | 1.16 | 0.4882 | 0.689 | 3.83 | 4.01 | 3.88 | 0.97 | 0.8794 | 0.9483 |

| | | | | | | | | | | | | | | | |
|---|---|---|---|---|---|---|---|---|---|---|---|---|---|---|---|
| TC0100015093.hg.1 | OLFM3 | olfactomedin 3 | Multiple_C | 3.17 | 3.14 | 2.96 | 1.16 | 0.4241 | 0.635 | 4.07 | 4.00 | 3.59 | 1.39 | 0.0333 | 0.1641 |
| TC0100015717.hg.1 | OTUD7B | OTU deubiquitinase 7B | Multiple_C | 7.19 | 7.36 | 6.98 | 1.16 | 0.5087 | 0.705 | 7.03 | 6.95 | 7.25 | 0.86 | 0.5441 | 0.7624 |
| TC0100017064.hg.1 | NUAK2 | NUAK family, SNF1-like kinase, 2 | Multiple_C | 3.57 | 3.13 | 3.36 | 1.16 | 0.7625 | 0.8779 | 4.5 | 4.25 | 4.53 | 0.98 | 0.8713 | 0.9446 |
| TC0100018178.hg.1 | CELA2B | chymotrypsin-like elastase family, member 2B | Multiple_C | 2.95 | 2.62 | 2.74 | 1.16 | 0.7198 | 0.8523 | 3.27 | 3.23 | 3.03 | 1.18 | 0.309 | 0.5731 |
| TC0100018491.hg.1 | RORC | RAR-related orphan receptor C | Multiple_C | 3.83 | 3.36 | 3.62 | 1.16 | 0.7462 | 0.8695 | 4.41 | 4.17 | 4.25 | 1.12 | 0.2421 | 0.5059 |
| TC0200007102.hg.1 | PLB1 | phospholipase B1 | Multiple_C | 4.47 | 4.13 | 4.26 | 1.16 | 0.4339 | 0.6438 | 3.91 | 3.61 | 3.59 | 1.25 | 0.1118 | 0.3294 |
| TC0200008005.hg.1 | DYSF | dysferlin | Multiple_C | 2.95 | 2.91 | 2.74 | 1.16 | 0.3053 | 0.5184 | 3.08 | 3.15 | 3.37 | 0.82 | 0.5048 | 0.7368 |
| TC0200008462.hg.1 | PROM2 | prominin 2 | Multiple_C | 4 | 3.61 | 3.79 | 1.16 | 0.2279 | 0.4276 | 4.36 | 4.18 | 4.03 | 1.26 | 0.1546 | 0.3942 |
| TC0200008867.hg.1 | ACOXL | acyl-CoA oxidase-like | Multiple_C | 5.32 | 5.59 | 5.11 | 1.16 | 0.1814 | 0.3659 | 4.81 | 3.96 | 4.51 | 1.23 | 0.2939 | 0.5588 |
| TC0200011274.hg.1 | KLHL30 | kelch-like family member 30 | Coding | 3 | 2.78 | 2.79 | 1.16 | 0.9559 | 0.9794 | 3.58 | 3.47 | 3.65 | 0.95 | 0.4782 | 0.7168 |
| TC0200012550.hg.1 | FSHR | follicle stimulating hormone receptor | Multiple_C | 3.23 | 3.31 | 3.02 | 1.16 | 0.948 | 0.9761 | 3.54 | 3.38 | 3.45 | 1.06 | 0.8275 | 0.9237 |
| TC0200012617.hg.1 | TSPYL6 | TSPY-like 6 | Coding | 4.58 | 4.47 | 4.37 | 1.16 | 0.3109 | 0.5245 | 4.3 | 4.33 | 4.31 | 0.99 | 0.6499 | 0.829 |
| TC0200013192.hg.1 | LRRTM1 | leucine rich repeat transmembrane neuronal 1 | Coding | 3.06 | 2.78 | 2.85 | 1.16 | 0.5883 | 0.7642 | 3.01 | 3.08 | 3.23 | 0.86 | 0.5163 | 0.7449 |
| TC0200015307.hg.1 | DNAH7 | dynein, axonemal, heavy chain 7 | Multiple_C | 3.68 | 3.55 | 3.47 | 1.16 | 0.5686 | 0.7491 | 3.6 | 3.60 | 3.71 | 0.93 | 0.2816 | 0.5474 |
| TC0200015695.hg.1 | DIRC3 | disrupted in renal carcinoma 3 | Multiple_C | 4.35 | 3.95 | 4.14 | 1.16 | 0.2841 | 0.4944 | 4.96 | 4.85 | 4.37 | 1.51 | 0.0732 | 0.2593 |
| TC0200016065.hg.1 | ECEL1 | endothelin converting enzyme-like 1 | Multiple_C | 6.83 | 6.5 | 6.62 | 1.16 | 0.4308 | 0.6409 | 7.14 | 6.96 | 6.64 | 1.41 | 0.4814 | 0.7197 |
| TC0200016402.hg.1 | RSAD2 | radical S-adenosyl methionine domain containing 2 | Multiple_C | 4.26 | 3.52 | 4.05 | 1.16 | 0.1037 | 0.2475 | 4.48 | 4.51 | 4.3 | 1.13 | 0.4928 | 0.7275 |
| TC0200016455.hg.1 | C2orf74 | chromosome 2 open reading frame 74 | Multiple_C | 4.57 | 4.4 | 4.36 | 1.16 | 0.3991 | 0.6128 | 5.32 | 5.39 | 4.84 | 1.39 | 0.1846 | 0.4358 |
| TC0300012598.hg.1 | RBP1 | retinol binding protein 1, cellular | Multiple_C | 3.97 | 3.64 | 3.76 | 1.16 | 0.1269 | 0.2859 | 10.8 | 9.70 | 9.07 | 3.32 | 1.09E-08 | 5.43E-06 |
| TC0300013825.hg.1 | TDGF1 | teratocarcinoma-derived growth factor 1 | Multiple_C | 3.22 | 3.08 | 3.01 | 1.16 | 0.6 | 0.7723 | 2.82 | 2.79 | 2.81 | 1.01 | 0.357 | 0.6202 |
| TC0300013911.hg.1 | DNAJB11 | DnaJ (Hsp40) homolog, subfamily B, member 11 | Multiple_C | 12.89 | 13.64 | 12.68 | 1.16 | 0.3621 | 0.5755 | 11.75 | 11.38 | 11.68 | 1.05 | 0.455 | 0.6996 |
| TC0400006745.hg.1 | HTRA3 | HtrA serine peptidase 3 | Coding | 5.15 | 4.9 | 4.94 | 1.16 | 0.1158 | 0.2681 | 4.52 | 4.45 | 4.21 | 1.24 | 0.1567 | 0.3976 |
| TC0400009204.hg.1 | FAM218A | family with sequence similarity 218, member A | Coding | 3.41 | 3.11 | 3.2 | 1.16 | 0.1513 | 0.3241 | 3.26 | 3.32 | 3.15 | 1.08 | 0.481 | 0.7193 |
| TC0400010898.hg.1 | TMPRSS11F | transmembrane protease, serine 11F | Coding | 3.21 | 2.81 | 3 | 1.16 | 0.5291 | 0.72 | 3.55 | 3.56 | 3.46 | 1.06 | 0.2528 | 0.517 |
| TC0500006435.hg.1 | PLEKHG4B | pleckstrin homology domain containing, family G (with RhoGef ( | Multiple_C | 4.01 | 3.7 | 3.8 | 1.16 | 0.366 | 0.5794 | 7.93 | 8.11 | 7.82 | 1.08 | 0.0725 | 0.2582 |
| TC0500007120.hg.1 | TTC23L | tetratricopeptide repeat domain 23-like | Multiple_C | 3.44 | 3.36 | 3.23 | 1.16 | 0.2702 | 0.4785 | 4.28 | 4.25 | 3.8 | 1.39 | 0.0566 | 0.2239 |
| TC0500008589.hg.1 | CHSY3 | chondroitin sulfate synthase 3 | Multiple_C | 3.2 | 2.89 | 2.99 | 1.16 | 0.8878 | 0.9458 | 3.74 | 3.56 | 3.72 | 1.01 | 0.7731 | 0.898 |
| TC0500011347.hg.1 | HAPLN1 | hyaluronan and proteoglycan link protein 1 | Multiple_C | 3.53 | 2.84 | 3.32 | 1.16 | 0.8217 | 0.9104 | 3.31 | 3.30 | 3.26 | 1.04 | 0.549 | 0.7657 |
| TC0500012420.hg.1 | IL17B | interleukin 17B | Multiple_C | 4.62 | 4.49 | 4.41 | 1.16 | 0.3564 | 0.5698 | 4.79 | 4.61 | 4.52 | 1.21 | 0.0649 | 0.2427 |
| TC0500013220.hg.1 | FAM170A | family with sequence similarity 170, member A | Coding | 4.25 | 3.9 | 4.04 | 1.16 | 0.5891 | 0.7647 | 3.25 | 3.61 | 3.54 | 0.82 | 0.9714 | 0.9882 |
| TC0600007568.hg.1 | HCG27 | HLA complex group 27 (non-protein coding) | Multiple_C | 4.84 | 4.57 | 4.63 | 1.16 | 0.1596 | 0.336 | 4.64 | 4.63 | 4.5 | 1.10 | 0.1189 | 0.341 |
| TC0600008931.hg.1 | GRIK2 | glutamate receptor, ionotropic, kainate 2 | Multiple_C | 5.17 | 4.59 | 4.96 | 1.16 | 0.8849 | 0.9443 | 9.13 | 9.27 | 11.02 | 0.27 | 6.20E-07 | 0.0001 |
| TC0600009041.hg.1 | NR2E1 | nuclear receptor subfamily 2, group E, member 1 | Multiple_C | 3.6 | 3.08 | 3.39 | 1.16 | 0.7849 | 0.8894 | 3.56 | 3.91 | 3.82 | 0.84 | 0.3175 | 0.5815 |

| | | | | | | | | | | | | | | | |
|---|---|---|---|---|---|---|---|---|---|---|---|---|---|---|---|
| TC0600009243.hg.1 | RFX6 | regulatory factor X, 6 | Multiple_C | 2.96 | 2.81 | 2.75 | 1.16 | 0.443 | 0.6519 | 3.46 | 3.18 | 3.14 | 1.25 | 0.1252 | 0.3497 |
| TC0600011953.hg.1 | PLA2G7 | phospholipase A2, group VII (platelet-activating factor acetylhy | Multiple_C | 5.66 | 5.17 | 5.45 | 1.16 | 0.9002 | 0.9521 | 3.99 | 3.54 | 3.3 | 1.61 | 0.0075 | 0.064 |
| TC0600013193.hg.1 | STX7 | syntaxin 7 | Multiple_C | 9.17 | 9.61 | 8.96 | 1.16 | 0.8982 | 0.9515 | 8.84 | 8.88 | 8.17 | 1.59 | 0.0496 | 0.2073 |
| TC0600013719.hg.1 | DYNLT1 | dynein, light chain, Tctex-type 1 | Multiple_C | 11.64 | 11.65 | 11.43 | 1.16 | 0.7855 | 0.8899 | 9.48 | 9.72 | 9.23 | 1.19 | 0.3987 | 0.6559 |
| TC0700010941.hg.1 | MYO1G | myosin IG | Multiple_C | 3.42 | 3.11 | 3.21 | 1.16 | 0.5116 | 0.7074 | 4.44 | 4.28 | 4.29 | 1.11 | 0.6173 | 0.8099 |
| TC0700011167.hg.1 | NUPR2 | nuclear protein 2, transcriptional regulator | Multiple_C | 4.44 | 4.05 | 4.23 | 1.16 | 0.1727 | 0.3541 | 4.65 | 4.77 | 4.53 | 1.09 | 0.9807 | 0.9918 |
| TC0700013396.hg.1 | CLDN12 | claudin 12 | Multiple_C | 12.24 | 11.83 | 12.03 | 1.16 | 0.4 | 0.6135 | 9.76 | 8.98 | 10.16 | 0.76 | 0.0739 | 0.2609 |
| TC0800006620.hg.1 | DEFB106A | defensin, beta 106A | Coding | 4.58 | 4.17 | 4.37 | 1.16 | 0.9152 | 0.9601 | 4.29 | 4.17 | 4.26 | 1.02 | 0.1983 | 0.4533 |
| TC0800006983.hg.1 | PPP3CC | protein phosphatase 3, catalytic subunit, gamma isozyme | Multiple_C | 8.94 | 9.11 | 8.73 | 1.16 | 0.1595 | 0.3359 | 7.47 | 7.16 | 8.01 | 0.69 | 0.0036 | 0.0401 |
| TC0800007449.hg.1 | CHRNB3 | cholinergic receptor, nicotinic beta 3 | Multiple_C | 3.02 | 2.62 | 2.81 | 1.16 | 0.4859 | 0.6872 | 4.13 | 4.12 | 4.12 | 1.01 | 0.3116 | 0.5758 |
| TC0800007828.hg.1 | BHLHE22 | basic helix-loop-helix family, member e22 | Coding | 3.24 | 2.89 | 3.03 | 1.16 | 0.1543 | 0.3282 | 3.25 | 3.22 | 3.17 | 1.06 | 0.8759 | 0.9469 |
| TC0800009232.hg.1 | SPATC1 | spermatogenesis and centriole associated 1 | Coding | 3.31 | 3.18 | 3.1 | 1.16 | 0.2391 | 0.441 | 3.88 | 4.01 | 3.81 | 1.05 | 0.1575 | 0.3986 |
| TC0800012092.hg.1 | CYP11B1 | cytochrome P450, family 11, subfamily B, polypeptide 1 | Multiple_C | 6.06 | 5.6 | 5.85 | 1.16 | 0.8273 | 0.9133 | 6.66 | 6.64 | 6.47 | 1.14 | 0.6615 | 0.8347 |
| TC0900008314.hg.1 | FSD1L | fibronectin type III and SPRY domain containing 1-like | Multiple_C | 6.65 | 6.9 | 6.44 | 1.16 | 0.4459 | 0.6544 | 7.43 | 6.93 | 7.51 | 0.95 | 0.4428 | 0.6914 |
| TC0900009030.hg.1 | BARHL1 | BarH-like homeobox 1 | Coding | 3.31 | 3.14 | 3.1 | 1.16 | 0.5906 | 0.7658 | 3.76 | 3.72 | 3.84 | 0.95 | 0.9294 | 0.9702 |
| TC0900009078.hg.1 | CACFD1 | calcium channel flower domain containing 1 | Multiple_C | 5.21 | 5.02 | 5 | 1.16 | 0.7584 | 0.8758 | 4.81 | 4.92 | 4.82 | 0.99 | 0.7393 | 0.8802 |
| TC0900009746.hg.1 | IZUMO3 | IZUMO family member 3 | Multiple_C | 3.4 | 3.09 | 3.19 | 1.16 | 0.7929 | 0.8937 | 3.54 | 3.40 | 3.36 | 1.13 | 0.7706 | 0.8971 |
| TC0900010661.hg.1 | CDK20 | cyclin-dependent kinase 20 | Multiple_C | 3.57 | 3.3 | 3.36 | 1.16 | 0.8981 | 0.9514 | 5.28 | 4.87 | 5.11 | 1.13 | 0.069 | 0.2502 |
| TC0900011370.hg.1 | BRINP1 | bone morphogenetic protein/retinoic acid inducible neural-spec | Multiple_C | 3.95 | 3.61 | 3.74 | 1.16 | 0.9294 | 0.9665 | 3.86 | 4.02 | 3.89 | 0.98 | 0.7427 | 0.8822 |
| TC0X00006686.hg.1 | GRPR | gastrin-releasing peptide receptor | Coding | 3.29 | 2.62 | 3.08 | 1.16 | 0.7614 | 0.8775 | 4.62 | 4.91 | 5.02 | 0.76 | 0.2901 | 0.5549 |
| TC0X00007371.hg.1 | PAGE2B | P antigen family, member 2B | Multiple_C | 5.27 | 5.31 | 5.06 | 1.16 | 0.1262 | 0.2845 | 4.69 | 4.41 | 4.52 | 1.13 | 0.5165 | 0.745 |
| TC0X00007834.hg.1 | PCDH11X | protocadherin 11 X-linked | Multiple_C | 2.99 | 2.43 | 2.78 | 1.16 | 0.1882 | 0.3755 | 3.03 | 2.86 | 2.84 | 1.14 | 0.2117 | 0.4695 |
| TC0X00008002.hg.1 | NGFRAP1 | nerve growth factor receptor (TNFRSF16) associated protein 1 | Multiple_C | 5.23 | 4.59 | 5.02 | 1.16 | 0.8165 | 0.9075 | 9.91 | 10.25 | 9.86 | 1.04 | 0.7449 | 0.8832 |
| TC0X00008257.hg.1 | SOWAHD | sosondowah ankyrin repeat domain family member D | Coding | 3.43 | 3.01 | 3.22 | 1.16 | 0.4331 | 0.6431 | 3.09 | 3.34 | 3.18 | 0.94 | 0.6652 | 0.8363 |
| TC0X00008316.hg.1 | GRIA3 | glutamate receptor, ionotropic, AMPA 3 | Multiple_C | 3.73 | 3.47 | 3.52 | 1.16 | 0.1988 | 0.3897 | 4.32 | 4.28 | 4.37 | 0.97 | 0.7224 | 0.8706 |
| TC0X00008674.hg.1 | AFF2 | AF4/FMR2 family, member 2 | Multiple_C | 4.36 | 4.26 | 4.15 | 1.16 | 0.4875 | 0.6886 | 3.72 | 3.74 | 3.65 | 1.05 | 0.5757 | 0.7824 |
| TC0X00009208.hg.1 | CXorf23 | chromosome X open reading frame 23 | Multiple_C | 5.61 | 6.46 | 5.4 | 1.16 | 0.7193 | 0.8521 | 6.03 | 6.25 | 5.98 | 1.04 | 0.2858 | 0.5507 |
| TC0X00009803.hg.1 | MTRNR2L10 | MT-RNR2-like 10 | Coding | 4.34 | 4.37 | 4.13 | 1.16 | 0.7931 | 0.8938 | 4.09 | 3.94 | 4.12 | 0.98 | 0.8706 | 0.9444 |
| TC0X00010196.hg.1 | RPS6KA6 | ribosomal protein S6 kinase, 90kDa, polypeptide 6 | Multiple_C | 3.35 | 2.83 | 3.14 | 1.16 | 0.6681 | 0.8186 | 6.97 | 7.24 | 6.46 | 1.42 | 0.0804 | 0.2734 |
| TC0X00010759.hg.1 | DCAF12L1 | DDB1 and CUL4 associated factor 12-like 1 | Coding | 4.82 | 4.26 | 4.61 | 1.16 | 0.7276 | 0.8567 | 4.38 | 4.33 | 4.7 | 0.80 | 0.525 | 0.7498 |
| TC0X00011126.hg.1 | GABRA3 | gamma-aminobutyric acid (GABA) A receptor, alpha 3 | Multiple_C | 2.88 | 2.67 | 2.67 | 1.16 | 0.6156 | 0.7848 | 7.31 | 6.19 | 5.04 | 4.82 | 0.0009 | 0.0159 |
| TC0X00011411.hg.1 | PNCK | pregnancy up-regulated nonubiquitous CaM kinase | Multiple_C | 3.76 | 3.57 | 3.55 | 1.16 | 0.2342 | 0.435 | 3.86 | 3.73 | 4.02 | 0.90 | 0.2869 | 0.5521 |
| TC1000008380.hg.1 | LIPK | lipase, family member K | Coding | 3.34 | 3.01 | 3.13 | 1.16 | 0.573 | 0.7522 | 4.87 | 5.58 | 4.74 | 1.09 | 0.5054 | 0.737 |

| | | | | | | | | | | | | | | | |
|---|---|---|---|---|---|---|---|---|---|---|---|---|---|---|---|
| TC1000011168.hg.1 | ZCCHC24 | zinc finger, CCHC domain containing 24 | Coding | 4.2 | 4.49 | 3.99 | 1.16 | 0.3915 | 0.6053 | 6.31 | 5.94 | 5.87 | 1.36 | 0.0374 | 0.1763 |
| TC1000011203.hg.1 | ANXA11 | annexin A11 | Multiple_C | 14.91 | 14.27 | 14.7 | 1.16 | 0.1462 | 0.3164 | 12.62 | 12.60 | 12.72 | 0.93 | 0.1501 | 0.388 |
| TC1000012485.hg.1 | CC2D2B | coiled-coil and C2 domain containing 2B | Coding | 3.11 | 3.18 | 2.9 | 1.16 | 0.3724 | 0.585 | 3.57 | 3.49 | 3.01 | 1.47 | 0.0432 | 0.192 |
| TC1000012497.hg.1 | BORCS7-ASMT | BORCS7-ASMT readthrough (NMD candidate) | Multiple_C | 3.69 | 4.23 | 3.48 | 1.16 | 0.4194 | 0.6308 | 5.91 | 5.98 | 5.39 | 1.43 | 0.198 | 0.4531 |
| TC1100006707.hg.1 | OR52N2 | olfactory receptor, family 52, subfamily N, member 2 | Coding | 5.49 | 5.03 | 5.28 | 1.16 | 0.5984 | 0.7712 | 5.2 | 5.78 | 5.51 | 0.81 | 0.0733 | 0.2594 |
| TC1100006815.hg.1 | WEE1 | WEE1 G2 checkpoint kinase | Multiple_C | 11.48 | 11.17 | 11.27 | 1.16 | 0.6459 | 0.8038 | 10.78 | 10.24 | 11.34 | 0.68 | 0.1112 | 0.3287 |
| TC1100006986.hg.1 | MRGPRX3 | MAS-related GPR, member X3 | Coding | 2.95 | 2.73 | 2.74 | 1.16 | 0.5004 | 0.6992 | 3.23 | 3.54 | 3.18 | 1.04 | 0.8588 | 0.9397 |
| TC1100007625.hg.1 | OR8H2 | olfactory receptor, family 8, subfamily H, member 2 | Coding | 3.51 | 3.17 | 3.3 | 1.16 | 0.7491 | 0.8712 | 4.03 | 3.81 | 3.9 | 1.09 | 0.3144 | 0.5782 |
| TC1100008392.hg.1 | PLEKHB1 | pleckstrin homology domain containing, family B (evectins) mer | Multiple_C | 10.49 | 10.14 | 10.28 | 1.16 | 0.3224 | 0.5362 | 13.83 | 14.16 | 13.44 | 1.31 | 0.0099 | 0.0774 |
| TC1100011452.hg.1 | FGF3 | fibroblast growth factor 3 | Coding | 5.18 | 5.06 | 4.97 | 1.16 | 0.2969 | 0.5085 | 6.99 | 7.20 | 7.04 | 0.97 | 0.7233 | 0.871 |
| TC1100013054.hg.1 | RAD9A | RAD9 checkpoint clamp component A | Multiple_C | 8.51 | 7.35 | 8.3 | 1.16 | 0.2035 | 0.3958 | 9.24 | 9.33 | 9.74 | 0.71 | 0.1377 | 0.3687 |
| TC1200007380.hg.1 | PPHLN1 | periphilin 1 | Multiple_C | 13.12 | 13.49 | 12.91 | 1.16 | 0.4305 | 0.6407 | 12.93 | 12.89 | 12.66 | 1.21 | 0.0716 | 0.2561 |
| TC1200008126.hg.1 | CPSF6 | cleavage and polyadenylation specific factor 6 | Multiple_C | 11.04 | 10.66 | 10.83 | 1.16 | 0.1622 | 0.3396 | 11.04 | 11.59 | 11.53 | 0.71 | 0.0044 | 0.0457 |
| TC1200008517.hg.1 | METAP2 | methionyl aminopeptidase 2 | Multiple_C | 11.14 | 12.04 | 10.93 | 1.16 | 0.8309 | 0.9157 | 9.71 | 9.70 | 9.85 | 0.91 | 0.4819 | 0.7201 |
| TC1200008929.hg.1 | CFAP73 | cilia and flagella associated protein 73 | Multiple_C | 4.49 | 3.97 | 4.28 | 1.16 | 0.5065 | 0.7035 | 4.33 | 4.53 | 4.28 | 1.04 | 0.4873 | 0.7238 |
| TC1200009620.hg.1 | NRIP2 | nuclear receptor interacting protein 2 | Coding | 3.54 | 3.26 | 3.33 | 1.16 | 0.421 | 0.6321 | 3.2 | 3.06 | 3.14 | 1.04 | 0.8516 | 0.9358 |
| TC1200009834.hg.1 | MFAP5 | microfibrillar associated protein 5 | Multiple_C | 3.76 | 3.51 | 3.55 | 1.16 | 0.502 | 0.7002 | 3.21 | 3.35 | 3.12 | 1.06 | 0.3923 | 0.6506 |
| TC1200010022.hg.1 | MGP | matrix Gla protein | Multiple_C | 2.68 | 2.48 | 2.47 | 1.16 | 0.5484 | 0.7338 | 3.3 | 3.16 | 2.98 | 1.25 | 0.0447 | 0.1957 |
| TC1200010762.hg.1 | KRT74 | keratin 74, type II | Multiple_C | 3.92 | 3.45 | 3.71 | 1.16 | 0.7423 | 0.8667 | 3.9 | 3.79 | 3.73 | 1.13 | 0.6982 | 0.857 |
| TC1200010845.hg.1 | LACRT | lacritin | Multiple_C | 4.25 | 4.13 | 4.04 | 1.16 | 0.565 | 0.7465 | 3.97 | 4.26 | 4.29 | 0.80 | 0.1557 | 0.3961 |
| TC1200011699.hg.1 | ARL1 | ADP-ribosylation factor like GTPase 1 | Multiple_C | 11.86 | 13.28 | 11.65 | 1.16 | 0.7561 | 0.8745 | 9.5 | 8.98 | 9.09 | 1.33 | 0.1939 | 0.4477 |
| TC1200012258.hg.1 | PITPNM2 | phosphatidylinositol transfer protein, membrane-associated 2 | Multiple_C | 4.61 | 4.26 | 4.4 | 1.16 | 0.5034 | 0.701 | 4.1 | 4.38 | 4.66 | 0.68 | 0.0299 | 0.1535 |
| TC1300006601.hg.1 | SPATA13; C1C | spermatogenesis associated 13; C1q and tumor necrosis factor | Multiple_C | 6.59 | 6.29 | 6.38 | 1.16 | 0.8985 | 0.9517 | 8.95 | 9.83 | 8.77 | 1.13 | 0.1133 | 0.332 |
| TC1300007592.hg.1 | NDFIP2 | Nedd4 family interacting protein 2 | Multiple_C | 11.27 | 11.83 | 11.06 | 1.16 | 0.6379 | 0.7991 | 11.05 | 10.99 | 10.12 | 1.91 | 0.0486 | 0.2047 |
| TC1400007650.hg.1 | ACOT6 | acyl-CoA thioesterase 6 | Coding | 3.15 | 2.99 | 2.94 | 1.16 | 0.22 | 0.4171 | 3.39 | 3.64 | 3.48 | 0.94 | 0.6347 | 0.8208 |
| TC1400007944.hg.1 | KCNK13 | potassium channel, two pore domain subfamily K, member 13 | Coding | 3.35 | 2.96 | 3.14 | 1.16 | 0.3727 | 0.5853 | 3.86 | 3.93 | 3.86 | 1.00 | 0.9949 | 0.9976 |
| TC1400009776.hg.1 | NGB | neuroglobin | Coding | 4.43 | 4.05 | 4.22 | 1.16 | 0.4811 | 0.6828 | 4.78 | 4.41 | 4.25 | 1.44 | 0.0554 | 0.2211 |
| TC1500008271.hg.1 | WDR93 | WD repeat domain 93 | Multiple_C | 4.19 | 3.5 | 3.98 | 1.16 | 0.0472 | 0.1386 | 4.52 | 4.52 | 4.16 | 1.28 | 0.3641 | 0.6268 |
| TC1500008339.hg.1 | FES | FES proto-oncogene, tyrosine kinase | Multiple_C | 4.43 | 4.01 | 4.22 | 1.16 | 0.5944 | 0.7686 | 4.2 | 4.12 | 4.34 | 0.91 | 0.8683 | 0.9433 |
| TC1500010889.hg.1 | CELF6 | CUGBP, Elav-like family member 6 | Multiple_C | 4.26 | 4.02 | 4.05 | 1.16 | 0.3224 | 0.5363 | 4.17 | 4.39 | 4.33 | 0.90 | 0.5386 | 0.7592 |
| TC1600006940.hg.1 | TNFRSF17 | tumor necrosis factor receptor superfamily, member 17 | Multiple_C | 3.17 | 2.66 | 2.96 | 1.16 | 0.9005 | 0.9523 | 3.47 | 3.30 | 3.33 | 1.10 | 0.285 | 0.55 |
| TC1600008738.hg.1 | FOXC2 | forkhead box C2 | Coding | 3.93 | 3.4 | 3.72 | 1.16 | 0.5179 | 0.712 | 4.22 | 4.05 | 3.82 | 1.32 | 0.1022 | 0.3131 |
| TC1600009864.hg.1 | IL27 | interleukin 27 | Coding | 4.31 | 3.83 | 4.1 | 1.16 | 0.3661 | 0.5794 | 3.86 | 4.10 | 4.15 | 0.82 | 0.1193 | 0.3415 |

| | | | | | | | | | | | | | | | |
|---|---|---|---|---|---|---|---|---|---|---|---|---|---|---|---|
| TC1600010066.hg.1 | TP53TG3B; TP | TP53 target 3B; TP53 target 3; TP53 target 3C | Multiple_C | 3.9 | 4.22 | 3.69 | 1.16 | 0.7693 | 0.881 | 4.48 | 4.27 | 4.12 | 1.28 | 0.1624 | 0.4057 |
| TC1600010470.hg.1 | ZNF319 | zinc finger protein 319 | Coding | 5.65 | 5.3 | 5.44 | 1.16 | 0.5293 | 0.7201 | 7.72 | 7.71 | 7.75 | 0.98 | 0.7781 | 0.9008 |
| TC1600010868.hg.1 | CTRB2 | chymotrypsinogen B2 | Multiple_C | 3.38 | 3.19 | 3.17 | 1.16 | 0.5564 | 0.7393 | 3.41 | 3.77 | 3.82 | 0.75 | 0.0275 | 0.1451 |
| TC1700007790.hg.1 | CDC6 | cell division cycle 6 | Multiple_C | 11.65 | 9.17 | 11.44 | 1.16 | 0.9417 | 0.9729 | 11.29 | 11.99 | 12.19 | 0.54 | 0.0002 | 0.0061 |
| TC1700007997.hg.1 | GRN | granulin | Multiple_C | 12.81 | 11.43 | 12.6 | 1.16 | 0.6011 | 0.7734 | 11.88 | 12.65 | 11.72 | 1.12 | 0.9316 | 0.9711 |
| TC1700009535.hg.1 | CHRNE | cholinergic receptor, nicotinic epsilon | Multiple_C | 3.33 | 3.12 | 3.12 | 1.16 | 0.407 | 0.6194 | 4.56 | 3.79 | 3.45 | 2.16 | 3.68E-05 | 0.0018 |
| TC1700009948.hg.1 | SMCR5 | Smith-Magenis syndrome chromosome region, candidate 5 (nor | Multiple_C | 2.89 | 2.41 | 2.68 | 1.16 | 0.325 | 0.539 | 3.32 | 3.22 | 3.09 | 1.17 | 0.2288 | 0.4898 |
| TC1700010560.hg.1 | PLXDC1 | plexin domain containing 1 | Multiple_C | 4.34 | 4.15 | 4.13 | 1.16 | 0.2923 | 0.5035 | 4.56 | 4.53 | 4.39 | 1.13 | 0.4131 | 0.6677 |
| TC1700010638.hg.1 | KRT28 | keratin 28, type I | Coding | 3.46 | 3.2 | 3.25 | 1.16 | 0.4433 | 0.6521 | 3.69 | 3.96 | 3.74 | 0.97 | 0.9584 | 0.9831 |
| TC1700010828.hg.1 | FAM171A2 | family with sequence similarity 171, member A2 | Multiple_C | 5.71 | 5.21 | 5.5 | 1.16 | 0.6669 | 0.8177 | 5.55 | 5.42 | 5.24 | 1.24 | 0.087 | 0.2847 |
| TC1700011703.hg.1 | CD300LD | CD300 molecule-like family member d | Coding | 5.81 | 5.61 | 5.6 | 1.16 | 0.8108 | 0.9044 | 5.17 | 4.93 | 5.26 | 0.94 | 0.9362 | 0.9734 |
| TC1700011949.hg.1 | CBX8 | chromobox homolog 8 | Multiple_C | 7.03 | 6.81 | 6.82 | 1.16 | 0.2939 | 0.5052 | 7.48 | 7.44 | 7.48 | 1.00 | 0.7953 | 0.9087 |
| TC1700012383.hg.1 | OMG | oligodendrocyte myelin glycoprotein | Multiple_C | 3.36 | 3.35 | 3.15 | 1.16 | 0.3318 | 0.5458 | 3.77 | 3.41 | 3.08 | 1.61 | 0.0027 | 0.0331 |
| TC1700012387.hg.1 | SLFN12L | schlafen family member 12-like | Multiple_C | 3.41 | 3.3 | 3.2 | 1.16 | 0.4706 | 0.6746 | 3.84 | 3.50 | 3.28 | 1.47 | 0.0359 | 0.1717 |
| TC1800007354.hg.1 | POLI | polymerase (DNA directed) iota | Multiple_C | 5.19 | 6.46 | 4.98 | 1.16 | 0.9476 | 0.9759 | 6.74 | 6.83 | 6.25 | 1.40 | 0.0316 | 0.1589 |
| TC1800007863.hg.1 | ENOSF1 | enolase superfamily member 1 | Multiple_C | 8.84 | 9.01 | 8.63 | 1.16 | 0.6726 | 0.8214 | 7.24 | 7.97 | 7.19 | 1.04 | 0.7028 | 0.8594 |
| TC1800008628.hg.1 | ZBTB7C | zinc finger and BTB domain containing 7C | Multiple_C | 4.25 | 4.46 | 4.04 | 1.16 | 0.5632 | 0.7451 | 4.9 | 5.75 | 5.75 | 0.55 | 0.0271 | 0.1441 |
| TC1900006574.hg.1 | IZUMO4 | IZUMO family member 4 | Multiple_C | 5.82 | 5.73 | 5.61 | 1.16 | 0.4656 | 0.6703 | 5.76 | 5.93 | 5.65 | 1.08 | 0.7033 | 0.8597 |
| TC1900007063.hg.1 | ZNF788 | zinc finger family member 788 | Multiple_C | 3.64 | 3.1 | 3.43 | 1.16 | 0.558 | 0.7407 | 3.32 | 3.34 | 3.28 | 1.03 | 0.7518 | 0.8869 |
| TC1900008216.hg.1 | TEX101 | testis expressed 101 | Multiple_C | 5.19 | 4.79 | 4.98 | 1.16 | 0.381 | 0.5939 | 5.12 | 4.88 | 4.86 | 1.20 | 0.2269 | 0.4875 |
| TC1900008286.hg.1 | BCAM | basal cell adhesion molecule (Lutheran blood group) | Multiple_C | 5.52 | 5.29 | 5.31 | 1.16 | 0.1093 | 0.2573 | 6.55 | 5.95 | 5.29 | 2.39 | 2.00E-05 | 0.0012 |
| TC1900008668.hg.1 | FPR2 | formyl peptide receptor 2 | Coding | 4.18 | 3.74 | 3.97 | 1.16 | 0.2367 | 0.4381 | 4.49 | 4.53 | 4.03 | 1.38 | 0.0244 | 0.1352 |
| TC1900009266.hg.1 | ZNF77 | zinc finger protein 77 | Multiple_C | 6.63 | 6.38 | 6.42 | 1.16 | 0.4884 | 0.689 | 5.36 | 5.18 | 5.74 | 0.77 | 0.3646 | 0.6271 |
| TC1900009594.hg.1 | ZNF812P | zinc finger protein 812, pseudogene | Multiple_C | 4.51 | 4.56 | 4.3 | 1.16 | 0.5495 | 0.7346 | 4.42 | 4.44 | 4.47 | 0.97 | 0.8691 | 0.9436 |
| TC1900009651.hg.1 | YIPF2 | Yip1 domain family member 2 | Multiple_C | 6.66 | 6.38 | 6.45 | 1.16 | 0.5056 | 0.7029 | 6.1 | 5.69 | 6.04 | 1.04 | 0.7518 | 0.8869 |
| TC1900009791.hg.1 | C19orf57 | chromosome 19 open reading frame 57 | Multiple_C | 3.87 | 3.55 | 3.66 | 1.16 | 0.3753 | 0.588 | 4.9 | 5.09 | 4.62 | 1.21 | 0.5816 | 0.7862 |
| TC1900010644.hg.1 | SYCN | syncollin | Coding | 3.59 | 2.98 | 3.38 | 1.16 | 0.316 | 0.5292 | 3.45 | 3.68 | 3.74 | 0.82 | 0.2244 | 0.4851 |
| TC1900011093.hg.1 | MAMSTR | MEF2 activating motif and SAP domain containing transcription | Multiple_C | 3.96 | 3.59 | 3.75 | 1.16 | 0.1479 | 0.3187 | 3.5 | 3.59 | 3.62 | 0.92 | 0.579 | 0.7844 |
| TC1900011096.hg.1 | FUT1 | fucosyltransferase 1 (galactoside 2-alpha-L-fucosyltransferase, I | Multiple_C | 3.57 | 3.48 | 3.36 | 1.16 | 0.3945 | 0.6081 | 4.2 | 3.92 | 3.9 | 1.23 | 0.1347 | 0.3644 |
| TC1900011252.hg.1 | LIM2 | lens intrinsic membrane protein 2 | Coding | 2.86 | 2.57 | 2.65 | 1.16 | 0.3465 | 0.5599 | 3.35 | 3.40 | 3.47 | 0.92 | 0.6567 | 0.8322 |
| TC1900011716.hg.1 | LRFN3 | leucine rich repeat and fibronectin type III domain containing 3 | Coding | 5.77 | 5.35 | 5.56 | 1.16 | 0.3861 | 0.5994 | 4.13 | 4.03 | 3.94 | 1.14 | 0.3364 | 0.6008 |
| TC1900012012.hg.1 | KLK9 | kallikrein related peptidase 9 | Coding | 4.21 | 4.02 | 4 | 1.16 | 0.3115 | 0.5248 | 3.99 | 3.81 | 3.82 | 1.13 | 0.2416 | 0.5056 |
| TC2000007141.hg.1 | BPIFB3 | BPI fold containing family B, member 3 | Coding | 4.17 | 4.35 | 3.96 | 1.16 | 0.5647 | 0.7463 | 4.19 | 4.01 | 4.2 | 0.99 | 0.5521 | 0.7677 |

| | | | | | | | | | | | | | | | |
|---|---|---|---|---|---|---|---|---|---|---|---|---|---|---|---|
| TC2000009458.hg.1 | NFATC2 | nuclear factor of activated T-cells, cytoplasmic, calcineurin-depe | Multiple_C | 4.5 | 3.81 | 4.29 | 1.16 | 0.1524 | 0.3259 | 4.9 | 4.75 | 5.37 | 0.72 | 0.0389 | 0.1798 |
| TC2000009833.hg.1 | ZBTB46 | zinc finger and BTB domain containing 46 | Multiple_C | 7.33 | 6.85 | 7.12 | 1.16 | 0.5831 | 0.7603 | 7.82 | 7.85 | 7.38 | 1.36 | 0.1389 | 0.3701 |
| TC2100006674.hg.1 | CHODL | chondrolectin | Multiple_C | 4.88 | 4.99 | 4.67 | 1.16 | 0.501 | 0.6995 | 4.64 | 5.04 | 5.13 | 0.71 | 0.0458 | 0.1981 |
| TC2100007821.hg.1 | ADAMTS1 | ADAM metallopeptidase with thrombospondin type 1 motif 1 | Multiple_C | 3.47 | 3.49 | 3.26 | 1.16 | 0.8893 | 0.9467 | 3.14 | 3.18 | 3.32 | 0.88 | 0.4039 | 0.6596 |
| TC2100008549.hg.1 | KRTAP13-3 | keratin associated protein 13-3 | Coding | 3.12 | 2.82 | 2.91 | 1.16 | 0.3244 | 0.5384 | 3.14 | 3.27 | 3.57 | 0.74 | 0.0459 | 0.1982 |
| TC22_KI270734v1_rando | PRODH; LOC1( | Homo sapiens proline dehydrogenase (oxidase) 1 (PRODH), trar | Coding | 4.13 | 3.9 | 3.92 | 1.16 | 0.2019 | 0.3942 | 4.46 | 4.30 | 3.98 | 1.39 | 0.0032 | 0.0369 |
| TC2200007150.hg.1 | TIMP3 | TIMP metallopeptidase inhibitor 3 | Multiple_C | 4.55 | 4.01 | 4.34 | 1.16 | 0.4754 | 0.6783 | 6.42 | 5.46 | 5.65 | 1.71 | 0.0031 | 0.0365 |
| TC2200009272.hg.1 | APOBEC3D | apolipoprotein B mRNA editing enzyme, catalytic polypeptide-li | Coding | 3.25 | 2.96 | 3.04 | 1.16 | 0.8247 | 0.9119 | 7.46 | 5.80 | 6.1 | 2.57 | 7.41E-06 | 0.0006 |
| TSUnmapped00000472.h | RPS6KA1 | ribosomal protein S6 kinase, 90kDa, polypeptide 1 | Coding | 3.63 | 3.17 | 3.42 | 1.16 | 0.5223 | 0.7154 | 3.47 | 3.39 | 3.54 | 0.95 | 0.7271 | 0.8732 |
| TSUnmapped00000528.h | HNRNPCL1 | heterogeneous nuclear ribonucleoprotein C-like 1 | Coding | 3.72 | 3.75 | 3.51 | 1.16 | 0.8698 | 0.9367 | 3.92 | 3.93 | 4.09 | 0.89 | 0.434 | 0.6849 |
| TC0100006849.hg.1 | ANGPTL7 | angiopoietin like 7 | Multiple_C | 4.41 | 3.8 | 4.21 | 1.15 | 0.6739 | 0.8225 | 4.36 | 4.53 | 4.5 | 0.91 | 0.482 | 0.7201 |
| TC0100007288.hg.1 | EPHA8 | EPH receptor A8 | Multiple_C | 3.38 | 2.93 | 3.18 | 1.15 | 0.6245 | 0.7901 | 3.24 | 3.26 | 3.31 | 0.95 | 0.1376 | 0.3685 |
| TC0100007442.hg.1 | ZNF593 | zinc finger protein 593 | Multiple_C | 8.09 | 8.15 | 7.89 | 1.15 | 0.7691 | 0.881 | 6.21 | 6.60 | 6.32 | 0.93 | 0.291 | 0.5557 |
| TC0100007540.hg.1 | THEMIS2 | thymocyte selection associated family member 2 | Multiple_C | 3.56 | 3.74 | 3.36 | 1.15 | 0.4435 | 0.6521 | 3.42 | 3.51 | 3.47 | 0.97 | 0.8034 | 0.9123 |
| TC0100008266.hg.1 | C1orf185 | chromosome 1 open reading frame 185 | Multiple_C | 3.81 | 3.74 | 3.61 | 1.15 | 0.9461 | 0.9753 | 3.81 | 3.59 | 3.85 | 0.97 | 0.7452 | 0.8832 |
| TC0100010082.hg.1 | TDRD10 | tudor domain containing 10 | Multiple_C | 4.08 | 3.5 | 3.88 | 1.15 | 0.2579 | 0.4644 | 3.78 | 3.96 | 3.95 | 0.89 | 0.2913 | 0.556 |
| TC0100011267.hg.1 | ATP2B4 | ATPase, Ca++ transporting, plasma membrane 4 | Multiple_C | 3.77 | 3.43 | 3.57 | 1.15 | 0.4179 | 0.6295 | 3.46 | 3.62 | 4.02 | 0.68 | 0.3421 | 0.6059 |
| TC0100013024.hg.1 | FAM131C | family with sequence similarity 131, member C | Multiple_C | 4.72 | 4.62 | 4.52 | 1.15 | 0.5298 | 0.7204 | 4.44 | 4.20 | 3.99 | 1.37 | 0.2159 | 0.4749 |
| TC0100015352.hg.1 | NRAS | neuroblastoma RAS viral (v-ras) oncogene homolog | Multiple_C | 12.21 | 11.72 | 12.01 | 1.15 | 0.5546 | 0.7381 | 12.87 | 12.33 | 12.64 | 1.17 | 0.5802 | 0.7853 |
| TC0100015436.hg.1 | SPAG17 | sperm associated antigen 17 | Multiple_C | 3.01 | 2.78 | 2.81 | 1.15 | 0.5032 | 0.7008 | 4.09 | 3.93 | 3.31 | 1.72 | 0.0106 | 0.0811 |
| TC0100015471.hg.1 | ZNF697 | zinc finger protein 697 | Multiple_C | 6.12 | 6.18 | 5.92 | 1.15 | 0.1125 | 0.2625 | 7.21 | 6.65 | 7.1 | 1.08 | 0.5392 | 0.7594 |
| TC0100015828.hg.1 | RPTN | repetin | Coding | 3.61 | 3.23 | 3.41 | 1.15 | 0.3334 | 0.5472 | 4.05 | 3.93 | 3.78 | 1.21 | 0.4136 | 0.668 |
| TC0100015837.hg.1 | LCE3A | late cornified envelope 3A | Coding | 4.82 | 4.33 | 4.62 | 1.15 | 0.2157 | 0.4114 | 5.4 | 5.26 | 5.09 | 1.24 | 0.0887 | 0.2882 |
| TC0100015852.hg.1 | SPRR2F | small proline-rich protein 2F | Coding | 3.57 | 3.35 | 3.37 | 1.15 | 0.3197 | 0.5334 | 3.27 | 3.46 | 3.37 | 0.93 | 0.9291 | 0.9702 |
| TC0100016025.hg.1 | INSRR | insulin receptor-related receptor | Multiple_C | 4.1 | 3.82 | 3.9 | 1.15 | 0.2707 | 0.4792 | 3.8 | 3.85 | 3.76 | 1.03 | 0.6215 | 0.8128 |
| TC0100016930.hg.1 | LMOD1 | leiomodin 1 (smooth muscle) | Coding | 4.05 | 3.98 | 3.85 | 1.15 | 0.1379 | 0.3035 | 3.91 | 4.17 | 3.94 | 0.98 | 0.4282 | 0.6802 |
| TC0100017029.hg.1 | PIK3C2B | phosphatidylinositol-4-phosphate 3-kinase, catalytic subunit typ | Multiple_C | 7.28 | 7.08 | 7.08 | 1.15 | 0.8375 | 0.9192 | 5.82 | 6.62 | 6.84 | 0.49 | 0.0026 | 0.0322 |
| TC0100018142.hg.1 | SH3BP5L | SH3-binding domain protein 5-like | Multiple_C | 4.84 | 4.61 | 4.64 | 1.15 | 0.4745 | 0.6775 | 5.47 | 5.73 | 5.66 | 0.88 | 0.8209 | 0.9209 |
| TC0200010007.hg.1 | SP9 | Sp9 transcription factor | Coding | 3.06 | 2.7 | 2.86 | 1.15 | 0.3606 | 0.574 | 4.38 | 4.10 | 3.9 | 1.39 | 0.0495 | 0.2071 |
| TC0200010525.hg.1 | CTLA4 | cytotoxic T-lymphocyte-associated protein 4 | Multiple_C | 2.9 | 2.74 | 2.7 | 1.15 | 0.2505 | 0.4549 | 3.75 | 3.99 | 3.75 | 1.00 | 0.9454 | 0.9778 |
| TC0200010807.hg.1 | TTLL4 | tubulin tyrosine ligase-like family member 4 | Multiple_C | 8.96 | 7.88 | 8.76 | 1.15 | 0.4775 | 0.68 | 10.21 | 10.16 | 10.39 | 0.88 | 0.0826 | 0.2767 |
| TC0200010894.hg.1 | CCDC140 | coiled-coil domain containing 140 | Coding | 3.42 | 3.12 | 3.22 | 1.15 | 0.4935 | 0.6934 | 3.65 | 3.50 | 3.48 | 1.13 | 0.3508 | 0.6147 |
| TC0200011848.hg.1 | RAD51AP2 | RAD51 associated protein 2 | Coding | 3.46 | 3.9 | 3.26 | 1.15 | 0.1453 | 0.3153 | 3.64 | 4.01 | 3.64 | 1.00 | 0.9878 | 0.9949 |

| | | | | | | | | | | | | | | | |
|---|---|---|---|---|---|---|---|---|---|---|---|---|---|---|---|
| TC0200014362.hg.1 | NCKAP5 | NCK-associated protein 5 | Multiple_C | 3.88 | 3.48 | 3.68 | 1.15 | 0.2656 | 0.4732 | 4.47 | 4.57 | 3.96 | 1.42 | 0.0118 | 0.0864 |
| TC0200016713.hg.1 | ANKRD39 | ankyrin repeat domain 39 | Coding | 5.47 | 5.04 | 5.27 | 1.15 | 0.1343 | 0.298 | 5.72 | 6.01 | 5.93 | 0.86 | 0.6338 | 0.8202 |
| TC0300006596.hg.1 | SLC6A11 | solute carrier family 6 (neurotransmitter transporter), member | Multiple_C | 4.84 | 4.39 | 4.64 | 1.15 | 0.7839 | 0.8889 | 4.41 | 4.31 | 4.61 | 0.87 | 0.1356 | 0.3659 |
| TC0300007136.hg.1 | CTNNB1 | catenin (cadherin-associated protein), beta 1 | Multiple_C | 13.97 | 13.8 | 13.77 | 1.15 | 0.4799 | 0.682 | 14.51 | 15.08 | 14.6 | 0.94 | 0.2209 | 0.4812 |
| TC0300011386.hg.1 | PRICKLE2 | prickle homolog 2 | Multiple_C | 5.78 | 5.57 | 5.58 | 1.15 | 0.7405 | 0.8656 | 6.69 | 6.78 | 6.66 | 1.02 | 0.5314 | 0.7543 |
| TC0300011757.hg.1 | PROS1 | protein S (alpha) | Multiple_C | 7.9 | 8.96 | 7.7 | 1.15 | 0.9232 | 0.9642 | 6.24 | 6.32 | 6.05 | 1.14 | 0.7084 | 0.8629 |
| TC0300012935.hg.1 | SHOX2 | short stature homeobox 2 | Multiple_C | 4.91 | 4.94 | 4.71 | 1.15 | 0.592 | 0.7669 | 4.47 | 4.45 | 4.05 | 1.34 | 0.1086 | 0.3244 |
| TC0400006433.hg.1 | ZNF595 | zinc finger protein 595 | Multiple_C | 4.94 | 5.3 | 4.74 | 1.15 | 0.4071 | 0.6195 | 9.23 | 8.79 | 9.1 | 1.09 | 0.3871 | 0.646 |
| TC0400009784.hg.1 | TNIP2 | TNFAIP3 interacting protein 2 | Multiple_C | 8.26 | 8.31 | 8.06 | 1.15 | 0.4664 | 0.6709 | 8.43 | 8.24 | 8.35 | 1.06 | 0.6242 | 0.8145 |
| TC0400011727.hg.1 | PRDM5 | PR domain containing 5 | Multiple_C | 3.48 | 3.47 | 3.28 | 1.15 | 0.5718 | 0.7513 | 4.38 | 4.34 | 4.2 | 1.13 | 0.2195 | 0.4795 |
| TC0500006744.hg.1 | ROPN1L | rhophilin associated tail protein 1-like | Multiple_C | 3.73 | 3.36 | 3.53 | 1.15 | 0.9 | 0.9519 | 3.75 | 3.72 | 3.45 | 1.23 | 0.0801 | 0.2728 |
| TC0500007432.hg.1 | IL31RA | interleukin 31 receptor A | Multiple_C | 3.81 | 3.61 | 3.61 | 1.15 | 0.5067 | 0.7035 | 4.02 | 3.76 | 3.5 | 1.43 | 0.0176 | 0.1115 |
| TC0500011211.hg.1 | OTP | orthopedia homeobox | Multiple_C | 3.83 | 3.69 | 3.63 | 1.15 | 0.8814 | 0.9424 | 4.8 | 4.45 | 4.36 | 1.36 | 0.1075 | 0.3224 |
| TC0500011296.hg.1 | ACOT12 | acyl-CoA thioesterase 12 | Multiple_C | 3.31 | 3.25 | 3.11 | 1.15 | 0.2126 | 0.4074 | 3.67 | 3.39 | 3.33 | 1.27 | 0.7637 | 0.8932 |
| TC0500012044.hg.1 | FSTL4 | follistatin-like 4 | Multiple_C | 4.47 | 4.16 | 4.27 | 1.15 | 0.8432 | 0.9227 | 6.02 | 7.55 | 8.68 | 0.16 | 4.46E-05 | 0.0021 |
| TC0500012164.hg.1 | GFRA3 | GDNF family receptor alpha 3 | Multiple_C | 2.71 | 2.33 | 2.51 | 1.15 | 0.2566 | 0.4629 | 9.85 | 9.69 | 8.44 | 2.66 | 6.66E-06 | 0.0005 |
| TC0600006441.hg.1 | DUSP22 | dual specificity phosphatase 22 | Multiple_C | 7.61 | 7.9 | 7.41 | 1.15 | 0.4572 | 0.6634 | 8.18 | 8.60 | 8.75 | 0.67 | 0.0228 | 0.1301 |
| TC0600007588.hg.1 | MCCD1 | mitochondrial coiled-coil domain 1 | Coding | 4.64 | 4.73 | 4.44 | 1.15 | 0.8162 | 0.9073 | 3.57 | 3.49 | 3.41 | 1.12 | 0.9491 | 0.9793 |
| TC0600008324.hg.1 | GFRAL | GDNF family receptor alpha like | Coding | 3.36 | 3.22 | 3.16 | 1.15 | 0.4379 | 0.6473 | 3.66 | 3.72 | 3.66 | 1.00 | 0.6065 | 0.8032 |
| TC0600008505.hg.1 | RIMS1 | regulating synaptic membrane exocytosis 1 | Multiple_C | 4.1 | 4.05 | 3.9 | 1.15 | 0.3261 | 0.5402 | 5.21 | 4.44 | 4.21 | 2.00 | 0.0002 | 0.0049 |
| TC0600009248.hg.1 | VGLL2 | vestigial-like family member 2 | Coding | 3.21 | 2.93 | 3.01 | 1.15 | 0.6903 | 0.8331 | 3.25 | 3.49 | 3.45 | 0.87 | 0.3254 | 0.589 |
| TC0600010790.hg.1 | C6orf52 | chromosome 6 open reading frame 52 | Multiple_C | 3.97 | 3.88 | 3.77 | 1.15 | 0.4296 | 0.64 | 4.33 | 4.15 | 3.99 | 1.27 | 0.0502 | 0.2087 |
| TC0600010799.hg.1 | GCM2 | glial cells missing homolog 2 (Drosophila) | Coding | 4.97 | 4.22 | 4.77 | 1.15 | 0.13 | 0.291 | 4.93 | 4.80 | 4.97 | 0.97 | 0.8328 | 0.9257 |
| TC0600011454.hg.1 | VWA7 | von Willebrand factor A domain containing 7 | Multiple_C | 3.73 | 3.28 | 3.53 | 1.15 | 0.4238 | 0.6349 | 3.86 | 3.93 | 3.75 | 1.08 | 0.7442 | 0.883 |
| TC0600011770.hg.1 | TREML1 | triggering receptor expressed on myeloid cells-like 1 | Multiple_C | 4.73 | 4.18 | 4.53 | 1.15 | 0.2772 | 0.4864 | 4.57 | 4.38 | 4.48 | 1.06 | 0.6193 | 0.8114 |
| TC0600012042.hg.1 | TRAM2 | Transcript Identified by AceView, Entrez Gene ID(s) 9697 | Coding | 5.43 | 4.81 | 5.23 | 1.15 | 0.5193 | 0.713 | 4.67 | 4.87 | 4.81 | 0.91 | 0.3532 | 0.6167 |
| TC0600013330.hg.1 | PBOV1 | prostate and breast cancer overexpressed 1 | Coding | 3.11 | 3.38 | 2.91 | 1.15 | 0.4755 | 0.6784 | 3.58 | 4.20 | 3.53 | 1.04 | 0.5881 | 0.7916 |
| TC0700008104.hg.1 | LOC10013309 | uncharacterized LOC100133091; Salzman2013 ANNOTATED, IN | Multiple_C | 11.26 | 10.38 | 11.06 | 1.15 | 0.7642 | 0.8788 | 10.71 | 11.40 | 11.7 | 0.50 | 0.0206 | 0.1227 |
| TC0700008577.hg.1 | MUC17 | mucin 17, cell surface associated | Multiple_C | 3.96 | 3.74 | 3.76 | 1.15 | 0.5686 | 0.7491 | 4 | 3.94 | 3.81 | 1.14 | 0.0619 | 0.2363 |
| TC0700009518.hg.1 | OR2A5 | olfactory receptor, family 2, subfamily A, member 5 | Coding | 2.96 | 2.48 | 2.76 | 1.15 | 0.4589 | 0.6651 | 3.39 | 3.23 | 3.08 | 1.24 | 0.1911 | 0.4441 |
| TC0700010789.hg.1 | AMPH | amphiphysin | Multiple_C | 3.53 | 3.24 | 3.33 | 1.15 | 0.8008 | 0.8978 | 4.1 | 3.86 | 3.97 | 1.09 | 0.5601 | 0.7719 |
| TC0700010854.hg.1 | INHBA | inhibin beta A | Multiple_C | 5.35 | 5.2 | 5.15 | 1.15 | 0.1141 | 0.2652 | 5.78 | 6.03 | 5.65 | 1.09 | 0.5473 | 0.7644 |
| TC0700011489.hg.1 | TRIM50 | tripartite motif containing 50 | Multiple_C | 4.41 | 3.72 | 4.21 | 1.15 | 0.3709 | 0.5836 | 3.68 | 3.52 | 3.76 | 0.95 | 0.6292 | 0.8172 |

| | | | | | | | | | | | | | | | |
|---|---|---|---|---|---|---|---|---|---|---|---|---|---|---|---|
| TC0700012911.hg.1 | CTAGE8 | CTAGE family, member 8 | Coding | 7.52 | 7.25 | 7.32 | 1.15 | 0.6092 | 0.7796 | 7.89 | 8.36 | 7.7 | 1.14 | 0.8987 | 0.9576 |
| TC0800006837.hg.1 | TUSC3 | tumor suppressor candidate 3 | Multiple_C | 3.06 | 2.79 | 2.86 | 1.15 | 0.1854 | 0.3717 | 3.83 | 3.45 | 3.81 | 1.01 | 0.4517 | 0.697 |
| TC0800008140.hg.1 | ATP6V0D2 | ATPase, H+ transporting, lysosomal 38kDa, V0 subunit d2 | Coding | 4.57 | 4.37 | 4.37 | 1.15 | 0.4227 | 0.6339 | 4.09 | 4.28 | 4.32 | 0.85 | 0.6171 | 0.8099 |
| TC0800008321.hg.1 | LAPTM4B | lysosomal protein transmembrane 4 beta | Multiple_C | 8.01 | 8.01 | 7.81 | 1.15 | 0.5101 | 0.7061 | 10.67 | 10.85 | 10.81 | 0.91 | 0.8265 | 0.9234 |
| TC0800008955.hg.1 | WISP1 | WNT1 inducible signaling pathway protein 1 | Multiple_C | 4.01 | 3.79 | 3.81 | 1.15 | 0.4542 | 0.6617 | 4.67 | 4.61 | 4.72 | 0.97 | 0.8793 | 0.9483 |
| TC0800009244.hg.1 | MROH1 | maestro heat-like repeat family member 1 | Multiple_C | 7.49 | 7.15 | 7.29 | 1.15 | 0.7478 | 0.8706 | 6.69 | 6.71 | 6.27 | 1.34 | 0.505 | 0.7368 |
| TC0800009487.hg.1 | DEFB105A | defensin, beta 105A | Multiple_C | 3.83 | 4 | 3.63 | 1.15 | 0.3485 | 0.5616 | 3.75 | 3.93 | 3.89 | 0.91 | 0.4326 | 0.6839 |
| TC0900006714.hg.1 | CCDC171 | coiled-coil domain containing 171 | Multiple_C | 4.01 | 3.33 | 3.81 | 1.15 | 0.1956 | 0.3856 | 4.27 | 3.94 | 3.77 | 1.41 | 0.0087 | 0.071 |
| TC0900011835.hg.1 | OBP2B | odorant binding protein 2B | Multiple_C | 7.82 | 7.31 | 7.62 | 1.15 | 0.3724 | 0.585 | 6.82 | 6.84 | 6.71 | 1.08 | 0.9064 | 0.9614 |
| TC0X00006483.hg.1 | GYG2 | glycogenin 2 | Multiple_C | 5.06 | 4.64 | 4.86 | 1.15 | 0.3065 | 0.5196 | 3.98 | 3.33 | 3.49 | 1.40 | 0.0097 | 0.0765 |
| TC0X00007721.hg.1 | GPR174 | G protein-coupled receptor 174 | Coding | 3.24 | 3.02 | 3.04 | 1.15 | 0.3507 | 0.5634 | 3.36 | 3.69 | 3.51 | 0.90 | 0.5521 | 0.7677 |
| TC0X00008833.hg.1 | FAM50A | family with sequence similarity 50, member A | Multiple_C | 10.6 | 10.27 | 10.4 | 1.15 | 0.3412 | 0.5549 | 9.76 | 9.28 | 10.52 | 0.59 | 0.003 | 0.0353 |
| TC0X00009580.hg.1 | CFP | complement factor properdin | Multiple_C | 3.76 | 3.42 | 3.56 | 1.15 | 0.4536 | 0.6612 | 3.92 | 3.99 | 3.94 | 0.99 | 0.8708 | 0.9445 |
| TC0X00010531.hg.1 | LHFPL1 | lipoma HMGIC fusion partner-like 1 | Multiple_C | 3.11 | 3.01 | 2.91 | 1.15 | 0.4562 | 0.6629 | 3.79 | 3.48 | 3.44 | 1.27 | 0.1514 | 0.3895 |
| TC0X00011180.hg.1 | RENBP | renin binding protein | Multiple_C | 3.91 | 3.53 | 3.71 | 1.15 | 0.7939 | 0.8943 | 3.71 | 3.71 | 3.71 | 1.00 | 0.8885 | 0.9524 |
| TC1000008806.hg.1 | CFAP58 | cilia and flagella associated protein 58 | Coding | 4.18 | 4.2 | 3.98 | 1.15 | 0.6559 | 0.811 | 3.58 | 3.64 | 3.52 | 1.04 | 0.6909 | 0.8526 |
| TC1000010292.hg.1 | PARD3 | par-3 family cell polarity regulator | Multiple_C | 8.56 | 9.28 | 8.36 | 1.15 | 0.9036 | 0.9538 | 7.45 | 7.09 | 7.78 | 0.80 | 0.4814 | 0.7198 |
| TC1000012270.hg.1 | EBF3 | early B-cell factor 3 | Multiple_C | 3.89 | 3.66 | 3.69 | 1.15 | 0.2808 | 0.4908 | 4.53 | 4.64 | 4.42 | 1.08 | 0.7893 | 0.9059 |
| TC1000012309.hg.1 | TCERG1L | transcription elongation regulator 1-like | Multiple_C | 4.36 | 4.11 | 4.16 | 1.15 | 0.2531 | 0.458 | 4.42 | 4.73 | 4.89 | 0.72 | 0.4843 | 0.7219 |
| TC1100006473.hg.1 | LRRC56 | leucine rich repeat containing 56 | Multiple_C | 9.1 | 8.85 | 8.9 | 1.15 | 0.3271 | 0.541 | 6.62 | 6.96 | 6.85 | 0.85 | 0.605 | 0.8024 |
| TC1100007517.hg.1 | OR4X1 | olfactory receptor, family 4, subfamily X, member 1 (gene/pseu | Multiple_C | 3.4 | 3.13 | 3.2 | 1.15 | 0.4048 | 0.6177 | 4.24 | 4.71 | 4.54 | 0.81 | 0.0892 | 0.2891 |
| TC1100007610.hg.1 | OR5D14 | olfactory receptor, family 5, subfamily D, member 14 | Coding | 2.97 | 2.55 | 2.77 | 1.15 | 0.5243 | 0.7167 | 3.4 | 3.10 | 2.9 | 1.41 | 0.0633 | 0.2393 |
| TC1100007884.hg.1 | SLC3A2 | solute carrier family 3 (amino acid transporter heavy chain), me | Multiple_C | 15.3 | 14.83 | 15.1 | 1.15 | 0.1671 | 0.3467 | 14.53 | 13.38 | 14.16 | 1.29 | 0.0845 | 0.2798 |
| TC1100008400.hg.1 | DNAJB13 | DnaJ (Hsp40) homolog, subfamily B, member 13 | Multiple_C | 3.21 | 3.06 | 3.01 | 1.15 | 0.2017 | 0.3939 | 3.99 | 4.23 | 4.17 | 0.88 | 0.5232 | 0.7491 |
| TC1100008736.hg.1 | NAALAD2 | N-acetylated alpha-linked acidic dipeptidase 2 | Multiple_C | 3.42 | 3.26 | 3.22 | 1.15 | 0.8658 | 0.9346 | 3.66 | 3.16 | 3.45 | 1.16 | 0.2697 | 0.5347 |
| TC1100009225.hg.1 | CXCR5 | chemokine (C-X-C motif) receptor 5 | Coding | 5 | 4.73 | 4.8 | 1.15 | 0.4299 | 0.6401 | 5.69 | 5.49 | 5.35 | 1.27 | 0.0259 | 0.1403 |
| TC1100010948.hg.1 | GLYAT | glycine-N-acyltransferase | Multiple_C | 4.2 | 4.1 | 4 | 1.15 | 0.4679 | 0.6721 | 4.58 | 4.07 | 4.26 | 1.25 | 0.0791 | 0.2709 |
| TC1100011192.hg.1 | ATG2A | autophagy related 2A | Multiple_C | 8.98 | 7.97 | 8.78 | 1.15 | 0.4685 | 0.6724 | 8.88 | 8.76 | 8.06 | 1.77 | 0.2972 | 0.5618 |
| TC1100012197.hg.1 | GUCY1A2 | guanylate cyclase 1, soluble, alpha 2 | Multiple_C | 3.27 | 3.07 | 3.07 | 1.15 | 0.3593 | 0.5728 | 3.84 | 3.82 | 3.61 | 1.17 | 0.3243 | 0.5884 |
| TC1100012902.hg.1 | SPATA19 | spermatogenesis associated 19 | Coding | 3.68 | 3.67 | 3.48 | 1.15 | 0.7905 | 0.8929 | 3.34 | 3.31 | 3.16 | 1.13 | 0.6193 | 0.8114 |
| TC1200007043.hg.1 | SLCO1C1 | solute carrier organic anion transporter family, member 1C1 | Multiple_C | 3.63 | 3.77 | 3.43 | 1.15 | 0.0931 | 0.2286 | 3.7 | 3.81 | 3.62 | 1.06 | 0.9511 | 0.9797 |
| TC1200007236.hg.1 | METTL20 | methyltransferase like 20 | Multiple_C | 3.92 | 4.39 | 3.72 | 1.15 | 0.7779 | 0.8859 | 3.94 | 3.89 | 3.64 | 1.23 | 0.0997 | 0.3086 |
| TC1200007559.hg.1 | TROAP | trophinin associated protein | Multiple_C | 6.26 | 5.74 | 6.06 | 1.15 | 0.7041 | 0.8422 | 6.97 | 7.66 | 7.49 | 0.70 | 0.0395 | 0.1817 |

| | | | | | | | | | | | | | | | |
|---|---|---|---|---|---|---|---|---|---|---|---|---|---|---|---|
| TC1200007630.hg.1 | LETMD1 | LETM1 domain containing 1 | Multiple_C | 8.84 | 10.99 | 8.64 | 1.15 | 0.6642 | 0.8163 | 8.36 | 8.26 | 8.88 | 0.70 | 0.5703 | 0.7787 |
| TC1200007767.hg.1 | NCKAP1L | NCK-associated protein 1-like | Multiple_C | 4.16 | 4.13 | 3.96 | 1.15 | 0.7658 | 0.8797 | 3.89 | 3.72 | 3.55 | 1.27 | 0.025 | 0.1373 |
| TC1200010493.hg.1 | DBX2 | developing brain homeobox 2 | Coding | 5.18 | 4.85 | 4.98 | 1.15 | 0.4288 | 0.6391 | 5.26 | 4.99 | 4.95 | 1.24 | 0.2475 | 0.512 |
| TC1200012756.hg.1 | PRH1 | proline-rich protein HaeIII subfamily 1 | Multiple_C | 5.13 | 5 | 4.93 | 1.15 | 0.1939 | 0.3833 | 4.88 | 4.96 | 4.87 | 1.01 | 0.9405 | 0.9753 |
| TC1200012788.hg.1 | RAPGEF3 | Rap guanine nucleotide exchange factor 3 | Multiple_C | 4.33 | 3.89 | 4.13 | 1.15 | 0.5248 | 0.7171 | 4.28 | 4.51 | 4.21 | 1.05 | 0.6399 | 0.8234 |
| TC1300006861.hg.1 | NBEA | neurobeachin | Multiple_C | 3.69 | 3.43 | 3.49 | 1.15 | 0.4554 | 0.6624 | 5.48 | 5.58 | 4.88 | 1.52 | 0.0487 | 0.2051 |
| TC1300006996.hg.1 | FAM216B | family with sequence similarity 216, member B | Coding | 4.03 | 3.71 | 3.83 | 1.15 | 0.4493 | 0.6571 | 3.57 | 3.53 | 3.66 | 0.94 | 0.5352 | 0.7572 |
| TC1300010007.hg.1 | TMEM255B | transmembrane protein 255B | Multiple_C | 3.6 | 3.29 | 3.4 | 1.15 | 0.6278 | 0.7921 | 4.33 | 3.39 | 3.55 | 1.72 | 0.0061 | 0.056 |
| TC1400006540.hg.1 | RNASE2 | ribonuclease, RNase A family, 2 (liver, eosinophil-derived neuro | Coding | 4.34 | 3.91 | 4.14 | 1.15 | 0.4346 | 0.6445 | 3.9 | 4.29 | 4.03 | 0.91 | 0.7603 | 0.8914 |
| TC1400007307.hg.1 | ARID4A | AT rich interactive domain 4A (RBP1-like) | Multiple_C | 10.75 | 10.9 | 10.55 | 1.15 | 0.6919 | 0.834 | 9.74 | 9.42 | 9.2 | 1.45 | 0.1871 | 0.4389 |
| TC1400009770.hg.1 | ZDHHC22 | zinc finger, DHHC-type containing 22 | Multiple_C | 3.96 | 3.65 | 3.76 | 1.15 | 0.7633 | 0.8784 | 5.78 | 5.31 | 4.87 | 1.88 | 0.0002 | 0.0056 |
| TC1500006967.hg.1 | GCHFR | GTP cyclohydrolase I feedback regulator | Multiple_C | 7.7 | 7.25 | 7.5 | 1.15 | 0.3259 | 0.5399 | 7.9 | 8.36 | 7.62 | 1.21 | 0.2112 | 0.4687 |
| TC1500007985.hg.1 | LOC646938 | TBC1 domain family, member 2B pseudogene; Transcript Identi | Multiple_C | 5.8 | 5.57 | 5.6 | 1.15 | 0.3179 | 0.5313 | 7.1 | 7.00 | 6.71 | 1.31 | 0.6448 | 0.8257 |
| TC1500008052.hg.1 | MESDC1 | mesoderm development candidate 1 | Coding | 8.6 | 8.46 | 8.4 | 1.15 | 0.3601 | 0.5735 | 8.38 | 8.37 | 8.09 | 1.22 | 0.132 | 0.3605 |
| TC1500008727.hg.1 | NDN | necdin, MAGE family member | Coding | 3.71 | 3.39 | 3.51 | 1.15 | 0.2424 | 0.4448 | 3.5 | 3.41 | 3.14 | 1.28 | 0.1269 | 0.3525 |
| TC1500009001.hg.1 | GJD2 | gap junction protein delta 2 | Coding | 3.96 | 3.33 | 3.76 | 1.15 | 0.5882 | 0.7642 | 3.85 | 3.93 | 3.92 | 0.95 | 0.5198 | 0.7472 |
| TC1500009088.hg.1 | C15orf56 | chromosome 15 open reading frame 56 | Multiple_C | 3.52 | 3.3 | 3.32 | 1.15 | 0.2125 | 0.4074 | 3.74 | 3.85 | 3.51 | 1.17 | 0.2763 | 0.5424 |
| TC1500009182.hg.1 | VPS39 | vacuolar protein sorting 39 homolog (S. cerevisiae) | Multiple_C | 6.83 | 7.11 | 6.63 | 1.15 | 0.3896 | 0.6034 | 9.17 | 9.55 | 8.8 | 1.29 | 0.1283 | 0.3545 |
| TC1600007532.hg.1 | ITGAD | integrin alpha D | Multiple_C | 3.26 | 3.06 | 3.06 | 1.15 | 0.4049 | 0.6178 | 3.74 | 3.90 | 3.73 | 1.01 | 0.4486 | 0.6954 |
| TC1600008020.hg.1 | USB1 | U6 snRNA biogenesis 1 | Multiple_C | 8.71 | 7.94 | 8.51 | 1.15 | 0.861 | 0.9321 | 10.21 | 10.56 | 10.48 | 0.83 | 0.1315 | 0.3593 |
| TC1600009077.hg.1 | RPS2; SNORA( | ribosomal protein S2; small nucleolar RNA, H/ACA box 64; small | Multiple_C | 16.58 | 16.46 | 16.38 | 1.15 | 0.4294 | 0.6397 | 15.61 | 16.00 | 15.73 | 0.92 | 0.799 | 0.9101 |
| TC1600009642.hg.1 | GDE1 | glycerophosphodiester phosphodiesterase 1 | Multiple_C | 12.11 | 11.57 | 11.91 | 1.15 | 0.2895 | 0.5003 | 9.25 | 8.50 | 9.28 | 0.98 | 0.1297 | 0.3566 |
| TC1600011319.hg.1 | RAB40C | RAB40C, member RAS oncogene family | Multiple_C | 8.3 | 7.97 | 8.1 | 1.15 | 0.5829 | 0.7601 | 6.83 | 6.91 | 6.69 | 1.10 | 0.2646 | 0.5292 |
| TC1600011385.hg.1 | FBXL19 | F-box and leucine-rich repeat protein 19 | Multiple_C | 6.73 | 6.43 | 6.53 | 1.15 | 0.6333 | 0.7956 | 6.77 | 6.85 | 6.92 | 0.90 | 0.1921 | 0.4452 |
| TC1600011496.hg.1 | GPRC5B | G protein-coupled receptor, class C, group 5, member B | Multiple_C | 4.41 | 4.26 | 4.21 | 1.15 | 0.5943 | 0.7686 | 5.33 | 4.80 | 4.88 | 1.37 | 0.0859 | 0.2824 |
| TC1700010417.hg.1 | SLC35G3 | solute carrier family 35, member G3 | Coding | 3.94 | 3.87 | 3.74 | 1.15 | 0.7952 | 0.895 | 3.92 | 3.54 | 3.56 | 1.28 | 0.3178 | 0.5819 |
| TC1700010635.hg.1 | KRT24 | keratin 24, type I | Multiple_C | 4.06 | 3.74 | 3.86 | 1.15 | 0.7735 | 0.8832 | 4.46 | 4.71 | 4.33 | 1.09 | 0.9298 | 0.9703 |
| TC1700010692.hg.1 | KLHL11 | kelch-like family member 11 | Multiple_C | 7.61 | 7.28 | 7.41 | 1.15 | 0.7813 | 0.8872 | 7.33 | 7.16 | 7.37 | 0.97 | 0.8262 | 0.9234 |
| TC1700010993.hg.1 | HOXB5 | homeobox B5 | Multiple_C | 5.75 | 5.53 | 5.55 | 1.15 | 0.5339 | 0.7235 | 6.85 | 6.72 | 6.47 | 1.30 | 0.0134 | 0.0939 |
| TC1700011143.hg.1 | CA10 | carbonic anhydrase X | Multiple_C | 3.7 | 3.46 | 3.5 | 1.15 | 0.1486 | 0.3199 | 4.71 | 4.46 | 4.37 | 1.27 | 0.4859 | 0.723 |
| TC1700012025.hg.1 | CEP131 | centrosomal protein 131kDa | Multiple_C | 6.62 | 6.15 | 6.42 | 1.15 | 0.5411 | 0.7285 | 7.11 | 7.40 | 6.97 | 1.10 | 0.9385 | 0.9744 |
| TC1700012072.hg.1 | PPP1R27 | protein phosphatase 1, regulatory subunit 27 | Multiple_C | 4.61 | 4.38 | 4.41 | 1.15 | 0.4043 | 0.6173 | 3.83 | 3.62 | 3.85 | 0.99 | 0.9698 | 0.9875 |
| TC1700012073.hg.1 | P4HB | prolyl 4-hydroxylase, beta polypeptide | Multiple_C | 13.92 | 12.45 | 13.72 | 1.15 | 0.6208 | 0.7876 | 13.9 | 14.78 | 14.53 | 0.65 | 0.1683 | 0.414 |

| | | | | | | | | | | | | | | | |
|---|---|---|---|---|---|---|---|---|---|---|---|---|---|---|---|
| TC1700012265.hg.1 | MEIOC | meiosis specific with coiled-coil domain | Coding | 4.3 | 4.35 | 4.1 | 1.15 | 0.1651 | 0.3435 | 3.67 | 3.26 | 3.48 | 1.14 | 0.4197 | 0.6731 |
| TC1800006706.hg.1 | GNAL | guanine nucleotide binding protein (G protein), alpha activating | Multiple_C | 4.68 | 4.38 | 4.48 | 1.15 | 0.3688 | 0.5817 | 8.3 | 7.04 | 6.55 | 3.36 | 1.15E-06 | 0.0002 |
| TC1800007011.hg.1 | DSG4 | desmoglein 4 | Coding | 3.62 | 3.68 | 3.42 | 1.15 | 0.868 | 0.9356 | 3.59 | 3.99 | 3.73 | 0.91 | 0.8512 | 0.9358 |
| TC1800008472.hg.1 | RPRD1A | regulation of nuclear pre-mRNA domain containing 1A | Multiple_C | 10.6 | 10.82 | 10.4 | 1.15 | 0.6463 | 0.8042 | 10.35 | 9.93 | 9.49 | 1.82 | 0.0006 | 0.0123 |
| TC1900007267.hg.1 | AP1M1 | adaptor-related protein complex 1, mu 1 subunit | Multiple_C | 9.56 | 8.54 | 9.36 | 1.15 | 0.5272 | 0.7188 | 9.61 | 9.96 | 10.03 | 0.75 | 0.4562 | 0.7004 |
| TC1900007888.hg.1 | APLP1 | amyloid beta (A4) precursor-like protein 1 | Multiple_C | 5.47 | 4.97 | 5.27 | 1.15 | 0.8765 | 0.94 | 6.16 | 5.90 | 6.06 | 1.07 | 0.1098 | 0.3263 |
| TC1900010195.hg.1 | ZNF99 | zinc finger protein 99 | Multiple_C | 4.4 | 4.28 | 4.2 | 1.15 | 0.8659 | 0.9346 | 3.96 | 3.91 | 4.15 | 0.88 | 0.6813 | 0.847 |
| TC1900010789.hg.1 | POU2F2 | POU class 2 homeobox 2 | Multiple_C | 4.26 | 3.83 | 4.06 | 1.15 | 0.8087 | 0.9034 | 3.81 | 4.03 | 3.95 | 0.91 | 0.9156 | 0.9654 |
| TC1900010990.hg.1 | PNMAL2 | paraneoplastic Ma antigen family-like 2 | Multiple_C | 4.21 | 3.81 | 4.01 | 1.15 | 0.6967 | 0.8372 | 4.69 | 4.54 | 4.37 | 1.25 | 0.2652 | 0.5297 |
| TC1900011392.hg.1 | TFPT | TCF3 (E2A) fusion partner (in childhood Leukemia) | Coding | 7.93 | 7.53 | 7.73 | 1.15 | 0.1809 | 0.3652 | 7.66 | 7.86 | 7.78 | 0.92 | 0.2574 | 0.5224 |
| TC1900011464.hg.1 | TMEM150B | transmembrane protein 150B | Multiple_C | 3.53 | 3.26 | 3.33 | 1.15 | 0.8398 | 0.9204 | 3.45 | 3.72 | 3.3 | 1.11 | 0.5181 | 0.7463 |
| TC1900012043.hg.1 | LILRA4 | leukocyte immunoglobulin-like receptor, subfamily A (with TM c | Multiple_C | 2.72 | 2.48 | 2.52 | 1.15 | 0.4679 | 0.6721 | 3.5 | 3.27 | 3.09 | 1.33 | 0.1211 | 0.3435 |
| TC2000009275.hg.1 | OCSTAMP | osteoclast stimulatory transmembrane protein | Coding | 3.75 | 3.42 | 3.55 | 1.15 | 0.4358 | 0.6453 | 3.48 | 3.38 | 3.26 | 1.16 | 0.4841 | 0.7218 |
| TC2000009388.hg.1 | PTGIS | prostaglandin I2 (prostacyclin) synthase | Multiple_C | 4.13 | 3.69 | 3.93 | 1.15 | 0.4579 | 0.664 | 4.08 | 4.11 | 3.91 | 1.13 | 0.304 | 0.5679 |
| TC2000009618.hg.1 | ANKRD60 | ankyrin repeat domain 60 | Coding | 4.11 | 3.66 | 3.91 | 1.15 | 0.7956 | 0.8951 | 3.86 | 3.71 | 4.13 | 0.83 | 0.9236 | 0.9685 |
| TC2000009876.hg.1 | C20orf202 | chromosome 20 open reading frame 202 | Coding | 4 | 3.96 | 3.8 | 1.15 | 0.4679 | 0.6721 | 3.61 | 3.58 | 3.57 | 1.03 | 0.9088 | 0.9625 |
| TC2100007885.hg.1 | KRTAP26-1 | keratin associated protein 26-1 | Coding | 3.76 | 3.96 | 3.56 | 1.15 | 0.6898 | 0.8329 | 4.09 | 4.25 | 3.93 | 1.12 | 0.5399 | 0.7594 |
| TC2200007277.hg.1 | MPST | mercaptopyruvate sulfurtransferase | Multiple_C | 8.41 | 8.73 | 8.21 | 1.15 | 0.7321 | 0.8602 | 7.34 | 7.75 | 6.86 | 1.39 | 0.0716 | 0.2561 |
| TC2200007662.hg.1 | GTSE1 | G-2 and S-phase expressed 1 | Multiple_C | 6.99 | 6.59 | 6.79 | 1.15 | 0.5268 | 0.7187 | 6.86 | 7.74 | 7.46 | 0.66 | 0.0017 | 0.0243 |
| TC2200008238.hg.1 | TMEM211 | transmembrane protein 211 | Coding | 4.78 | 4.45 | 4.58 | 1.15 | 0.9378 | 0.9711 | 4.18 | 4.14 | 4.13 | 1.04 | 0.5909 | 0.7931 |
| TC2200008588.hg.1 | APOL3 | apolipoprotein L, 3 | Multiple_C | 4.1 | 3.57 | 3.9 | 1.15 | 0.6893 | 0.8328 | 3.65 | 3.93 | 3.89 | 0.85 | 0.2684 | 0.5331 |
| TSUnmapped00000018.h | ZNF35 | zinc finger protein 35 | Coding | 8.34 | 7.37 | 8.14 | 1.15 | 0.4395 | 0.6486 | 7.28 | 7.10 | 7.45 | 0.89 | 0.4069 | 0.6629 |
| TC0100006698.hg.1 | PHF13 | PHD finger protein 13 | Multiple_C | 8.78 | 8.57 | 8.59 | 1.14 | 0.6453 | 0.8034 | 7.33 | 7.14 | 7.18 | 1.11 | 0.6212 | 0.8128 |
| TC0100009897.hg.1 | CA14 | carbonic anhydrase XIV | Multiple_C | 3.41 | 2.79 | 3.22 | 1.14 | 0.4003 | 0.6138 | 3.59 | 3.81 | 3.35 | 1.18 | 0.5394 | 0.7594 |
| TC0100010148.hg.1 | LAMTOR2 | late endosomal/lysosomal adaptor, MAPK and MTOR activator | Multiple_C | 10.96 | 10.56 | 10.77 | 1.14 | 0.5484 | 0.7338 | 8.59 | 8.84 | 8.05 | 1.45 | 0.0991 | 0.3076 |
| TC0100011092.hg.1 | LHX9 | LIM homeobox 9 | Multiple_C | 4.45 | 4.03 | 4.26 | 1.14 | 0.9063 | 0.9551 | 4.56 | 4.46 | 4.16 | 1.32 | 0.5521 | 0.7677 |
| TC0100012441.hg.1 | RNF223 | ring finger protein 223 | Multiple_C | 7.82 | 7.53 | 7.63 | 1.14 | 0.1477 | 0.3185 | 7.72 | 7.55 | 7.32 | 1.32 | 0.5436 | 0.762 |
| TC0100012598.hg.1 | TP73-AS1 | TP73 antisense RNA 1 | Multiple_C | 4.36 | 4.17 | 4.17 | 1.14 | 0.7283 | 0.8572 | 3.66 | 3.37 | 3.4 | 1.20 | 0.3955 | 0.6534 |
| TC0100012930.hg.1 | PRAMEF11 | PRAME family member 11 | Coding | 3.75 | 3.72 | 3.56 | 1.14 | 0.3416 | 0.5554 | 3.7 | 3.49 | 3.32 | 1.30 | 0.2586 | 0.523 |
| TC0100014151.hg.1 | DMRTA2 | DMRT-like family A2 | Coding | 3.63 | 3.31 | 3.44 | 1.14 | 0.5036 | 0.7012 | 3.81 | 3.68 | 3.4 | 1.33 | 0.0703 | 0.2534 |
| TC0100016016.hg.1 | NES | nestin | Multiple_C | 3.03 | 2.58 | 2.84 | 1.14 | 0.5672 | 0.7479 | 8.97 | 9.45 | 9.34 | 0.77 | 0.1117 | 0.3293 |
| TC0100016027.hg.1 | ARHGEF11 | Rho guanine nucleotide exchange factor 11 | Multiple_C | 5.11 | 4.25 | 4.92 | 1.14 | 0.5781 | 0.7562 | 5.07 | 6.19 | 5.91 | 0.56 | 0.5161 | 0.7447 |
| TC0100016080.hg.1 | OR6N1 | olfactory receptor, family 6, subfamily N, member 1 | Coding | 4.53 | 4.34 | 4.34 | 1.14 | 0.4502 | 0.6579 | 4.2 | 4.29 | 4.18 | 1.01 | 0.6046 | 0.8023 |

| | | | | | | | | | | | | | | |
|---|---|---|---|---|---|---|---|---|---|---|---|---|---|---|
| TC0100018432.hg.1 | SFPQ | splicing factor proline/glutamine-rich | Multiple_C | 13.6 | 13.46 | 13.41 | 1.14 | 0.3446 | 0.5583 | 14.39 | 14.37 | 14.37 | 1.01 | 0.9045 | 0.9607 |
| TC0100018495.hg.1 | SHE | Src homology 2 domain containing E | Multiple_C | 4.35 | 4.33 | 4.16 | 1.14 | 0.4875 | 0.6886 | 3.96 | 3.75 | 3.65 | 1.24 | 0.1142 | 0.3335 |
| TC0200007825.hg.1 | CEP68 | centrosomal protein 68kDa | Multiple_C | 8.6 | 8.19 | 8.41 | 1.14 | 0.6039 | 0.7757 | 7.65 | 8.00 | 7.8 | 0.90 | 0.3556 | 0.6189 |
| TC0200008499.hg.1 | STARD7-AS1 | STARD7 antisense RNA 1 | Multiple_C | 4.27 | 3.84 | 4.08 | 1.14 | 0.235 | 0.436 | 4.19 | 4.59 | 4.59 | 0.76 | 0.1326 | 0.3614 |
| TC0200009424.hg.1 | ACMSD | aminocarboxymuconate semialdehyde decarboxylase | Multiple_C | 7.57 | 7.42 | 7.38 | 1.14 | 0.0891 | 0.2214 | 6.62 | 6.56 | 6.5 | 1.09 | 0.2792 | 0.5453 |
| TC0200009938.hg.1 | GORASP2 | golgi reassembly stacking protein 2 | Multiple_C | 11.84 | 11.46 | 11.65 | 1.14 | 0.42 | 0.6313 | 11.76 | 11.40 | 12.13 | 0.77 | 0.0209 | 0.1234 |
| TC0200014184.hg.1 | WDR33 | WD repeat domain 33 | Multiple_C | 12.75 | 12.65 | 12.56 | 1.14 | 0.5233 | 0.7161 | 12.74 | 12.67 | 12.91 | 0.89 | 0.2687 | 0.5335 |
| TC0300007220.hg.1 | ZNF502 | zinc finger protein 502 | Coding | 3.17 | 2.98 | 2.98 | 1.14 | 0.5231 | 0.716 | 3.65 | 3.29 | 3.48 | 1.13 | 0.0554 | 0.2211 |
| TC0300008142.hg.1 | TBC1D23 | TBC1 domain family, member 23 | Multiple_C | 11.61 | 12.28 | 11.42 | 1.14 | 0.363 | 0.5765 | 14.33 | 13.97 | 13.05 | 2.43 | 3.70E-06 | 0.0004 |
| TC0300008789.hg.1 | H1FOO | H1 histone family, member O, oocyte-specific | Coding | 3.52 | 3.33 | 3.33 | 1.14 | 0.2405 | 0.4425 | 4.19 | 4.10 | 3.99 | 1.15 | 0.5137 | 0.7432 |
| TC0300011171.hg.1 | SEMA3G | sema domain, immunoglobulin domain (Ig), short basic domain, | Multiple_C | 5.99 | 5.45 | 5.8 | 1.14 | 0.5408 | 0.7283 | 5.68 | 5.87 | 5.85 | 0.89 | 0.8929 | 0.9544 |
| TC0300011979.hg.1 | ZBED2 | zinc finger, BED-type containing 2 | Multiple_C | 4.02 | 3.79 | 3.83 | 1.14 | 0.2092 | 0.4029 | 4.03 | 4.42 | 4.31 | 0.82 | 0.1589 | 0.4004 |
| TC0400006517.hg.1 | UVSSA | UV stimulated scaffold protein A | Multiple_C | 5.75 | 5.45 | 5.56 | 1.14 | 0.9431 | 0.9736 | 6.03 | 6.31 | 6.15 | 0.92 | 0.793 | 0.9077 |
| TC0400006808.hg.1 | USP17L22 | ubiquitin specific peptidase 17-like family member 22 | Coding | 3.62 | 3.78 | 3.43 | 1.14 | 0.565 | 0.7465 | 4.02 | 4.05 | 3.82 | 1.15 | 0.1026 | 0.3137 |
| TC0400009068.hg.1 | LRAT | lecithin retinol acyltransferase (phosphatidylcholine--retinol O-a | Multiple_C | 4.52 | 3.93 | 4.33 | 1.14 | 0.5505 | 0.7354 | 4.05 | 4.00 | 3.89 | 1.12 | 0.4922 | 0.727 |
| TC0400012749.hg.1 | FRG2 | FSHD region gene 2 | Coding | 4.17 | 4.18 | 3.98 | 1.14 | 0.2698 | 0.4781 | 4.5 | 3.98 | 3.94 | 1.47 | 0.0766 | 0.2665 |
| TC0400012821.hg.1 | C4orf22 | chromosome 4 open reading frame 22 | Multiple_C | 3.87 | 3.66 | 3.68 | 1.14 | 0.3284 | 0.5422 | 3.72 | 3.81 | 3.89 | 0.89 | 0.251 | 0.5151 |
| TC0500007097.hg.1 | RXFP3 | relaxin/insulin-like family peptide receptor 3 | Coding | 4.05 | 3.85 | 3.86 | 1.14 | 0.5744 | 0.7535 | 4.54 | 4.29 | 4.19 | 1.27 | 0.0348 | 0.1684 |
| TC0500009100.hg.1 | SMIM3 | small integral membrane protein 3 | Multiple_C | 8.66 | 8.49 | 8.47 | 1.14 | 0.8352 | 0.9177 | 5.67 | 5.88 | 5.87 | 0.87 | 0.541 | 0.7601 |
| TC0500010511.hg.1 | C5orf42 | chromosome 5 open reading frame 42 | Multiple_C | 3.98 | 3.94 | 3.79 | 1.14 | 0.4869 | 0.6881 | 7.62 | 7.57 | 7.04 | 1.49 | 0.0348 | 0.1685 |
| TC0500011495.hg.1 | SPATA9 | spermatogenesis associated 9 | Multiple_C | 3.94 | 3.51 | 3.75 | 1.14 | 0.4484 | 0.6564 | 4.45 | 4.32 | 4.54 | 0.94 | 0.515 | 0.7442 |
| TC0500013399.hg.1 | PANK3 | pantothenate kinase 3 | Multiple_C | 11.75 | 12.66 | 11.56 | 1.14 | 0.9935 | 0.9967 | 9.86 | 9.23 | 10.74 | 0.54 | 0.0002 | 0.0058 |
| TC0600007006.hg.1 | RNF182 | ring finger protein 182 | Coding | 4.27 | 3.69 | 4.08 | 1.14 | 0.6349 | 0.7967 | 4.64 | 4.41 | 4.13 | 1.42 | 0.0813 | 0.275 |
| TC0600007092.hg.1 | FAM8A1 | family with sequence similarity 8, member A1 | Multiple_C | 9.91 | 10.35 | 9.72 | 1.14 | 0.1966 | 0.3871 | 10.69 | 10.29 | 10.58 | 1.08 | 0.2665 | 0.5309 |
| TC0600007302.hg.1 | BTN2A2 | butyrophilin, subfamily 2, member A2 | Multiple_C | 7.8 | 8.1 | 7.61 | 1.14 | 0.3472 | 0.5604 | 9.2 | 8.52 | 8.68 | 1.43 | 0.0143 | 0.0974 |
| TC0600007459.hg.1 | OR12D2 | olfactory receptor, family 12, subfamily D, member 2 (gene/pse | Multiple_C | 3.25 | 3.32 | 3.06 | 1.14 | 0.6906 | 0.8333 | 3.43 | 3.46 | 3.19 | 1.18 | 0.5061 | 0.7375 |
| TC0600007817.hg.1 | ARMC12 | armadillo repeat containing 12 | Coding | 6.59 | 6.99 | 6.4 | 1.14 | 0.4684 | 0.6724 | 4.91 | 5.13 | 5.11 | 0.87 | 0.3169 | 0.5809 |
| TC0600008955.hg.1 | LIN28B | lin-28 homolog B (C. elegans) | Multiple_C | 3.93 | 3.91 | 3.74 | 1.14 | 0.7418 | 0.8665 | 3.83 | 3.76 | 3.78 | 1.04 | 0.3965 | 0.6543 |
| TC0600009566.hg.1 | PEX7 | peroxisomal biogenesis factor 7 | Multiple_C | 7.23 | 8.31 | 7.04 | 1.14 | 0.5909 | 0.7659 | 6.08 | 7.24 | 6.23 | 0.90 | 0.472 | 0.7125 |
| TC0600010565.hg.1 | TUBB2A | tubulin, beta 2A class IIa | Multiple_C | 8.41 | 7.33 | 8.22 | 1.14 | 0.2643 | 0.4717 | 7.06 | 6.83 | 7.08 | 0.99 | 0.9968 | 0.9987 |
| TC0600011438.hg.1 | LTB | lymphotoxin beta (TNF superfamily, member 3) | Multiple_C | 6.76 | 6.45 | 6.57 | 1.14 | 0.4503 | 0.6581 | 6.52 | 6.51 | 6.76 | 0.85 | 0.927 | 0.9697 |
| TC0600011517.hg.1 | HLA-DPA1 | major histocompatibility complex, class II, DP alpha 1 | Multiple_C | 3.44 | 3.09 | 3.25 | 1.14 | 0.9764 | 0.9884 | 3.53 | 2.90 | 2.96 | 1.48 | 0.029 | 0.1507 |
| TC0600012175.hg.1 | KHDRBS2 | KH domain containing, RNA binding, signal transduction associa | Multiple_C | 3.54 | 3.36 | 3.35 | 1.14 | 0.3254 | 0.5393 | 3.61 | 3.52 | 3.59 | 1.01 | 0.133 | 0.362 |

| | | | | | | | | | | | | | | | |
|---|---|---|---|---|---|---|---|---|---|---|---|---|---|---|---|
| TC0700008974.hg.1 | SPAM1 | sperm adhesion molecule 1 (PH-20 hyaluronidase, zona pelluci | Coding | 2.97 | 2.86 | 2.78 | 1.14 | 0.6013 | 0.7736 | 3.29 | 3.47 | 3.48 | 0.88 | 0.2673 | 0.5318 |
| TC0700009643.hg.1 | KRBA1 | KRAB-A domain containing 1 | Multiple_C | 5.02 | 4.93 | 4.83 | 1.14 | 0.6103 | 0.7806 | 4.64 | 4.60 | 4.62 | 1.01 | 0.3161 | 0.5801 |
| TC0700011007.hg.1 | PKD1L1 | polycystic kidney disease 1 like 1 | Multiple_C | 3.37 | 3.28 | 3.18 | 1.14 | 0.6302 | 0.7938 | 3.87 | 3.95 | 3.81 | 1.04 | 0.9027 | 0.9596 |
| TC0700012731.hg.1 | DGKI | diacylglycerol kinase, iota | Multiple_C | 4.11 | 3.75 | 3.92 | 1.14 | 0.6501 | 0.8063 | 4.72 | 4.62 | 4.31 | 1.33 | 0.076 | 0.2653 |
| TC0700013397.hg.1 | CDK14 | cyclin-dependent kinase 14 | Multiple_C | 3.52 | 3.7 | 3.33 | 1.14 | 0.3508 | 0.5635 | 3.79 | 3.50 | 3.55 | 1.18 | 0.6251 | 0.8148 |
| TC0800007311.hg.1 | ADGRA2 | adhesion G protein-coupled receptor A2 | Multiple_C | 3.83 | 3.71 | 3.64 | 1.14 | 0.4561 | 0.6629 | 5.17 | 4.73 | 4.62 | 1.46 | 0.0139 | 0.0956 |
| TC0800007993.hg.1 | PI15 | peptidase inhibitor 15 | Multiple_C | 3.64 | 3.65 | 3.45 | 1.14 | 0.0499 | 0.1444 | 4.02 | 3.81 | 3.79 | 1.17 | 0.5459 | 0.7634 |
| TC0800008028.hg.1 | PKIA | protein kinase (cAMP-dependent, catalytic) inhibitor alpha | Multiple_C | 3.07 | 2.8 | 2.88 | 1.14 | 0.2839 | 0.4941 | 5.47 | 5.57 | 5.5 | 0.98 | 0.9738 | 0.9896 |
| TC0800008783.hg.1 | SQLE | squalene epoxidase | Multiple_C | 12.97 | 15.76 | 12.78 | 1.14 | 0.547 | 0.7326 | 8.11 | 7.49 | 8.95 | 0.56 | 0.0138 | 0.0955 |
| TC0800009717.hg.1 | FGF20 | fibroblast growth factor 20 | Coding | 3.51 | 3.32 | 3.32 | 1.14 | 0.5801 | 0.7576 | 4.46 | 5.07 | 4.32 | 1.10 | 0.6951 | 0.855 |
| TC0800012035.hg.1 | MROH5 | maestro heat-like repeat family member 5 | Multiple_C | 4.64 | 4.37 | 4.45 | 1.14 | 0.4291 | 0.6394 | 5.47 | 5.52 | 5.59 | 0.92 | 0.678 | 0.8449 |
| TC0900006554.hg.1 | INSL4 | insulin-like 4 (placenta) | Coding | 3.2 | 2.71 | 3.01 | 1.14 | 0.1574 | 0.3331 | 3.74 | 3.45 | 3.45 | 1.22 | 0.1387 | 0.3698 |
| TC0900009167.hg.1 | OBP2A | odorant binding protein 2A | Multiple_C | 5.07 | 5 | 4.88 | 1.14 | 0.2155 | 0.4111 | 5.65 | 5.49 | 5.27 | 1.30 | 0.1788 | 0.4276 |
| TC0900009700.hg.1 | IFNA14 | interferon, alpha 14 | Coding | 5.05 | 4.05 | 4.86 | 1.14 | 0.3995 | 0.6132 | 5.19 | 5.16 | 5.12 | 1.05 | 0.9385 | 0.9744 |
| TC0900010445.hg.1 | NMRK1 | nicotinamide riboside kinase 1 | Multiple_C | 9.12 | 11.2 | 8.93 | 1.14 | 0.57 | 0.7502 | 7.35 | 7.26 | 6.56 | 1.73 | 0.0079 | 0.0666 |
| TC0900011626.hg.1 | CIZ1 | CDKN1A interacting zinc finger protein 1 | Multiple_C | 12.19 | 11.44 | 12 | 1.14 | 0.4905 | 0.6905 | 11.6 | 12.47 | 12.48 | 0.54 | 0.0008 | 0.0139 |
| TC0X00008064.hg.1 | PIH1D3 | PIH1 domain containing 3 | Coding | 3.49 | 3.66 | 3.3 | 1.14 | 0.4727 | 0.6761 | 3.84 | 4.05 | 3.99 | 0.90 | 0.37 | 0.6322 |
| TC0X00008272.hg.1 | RHOXF2; RHO | Rhox homeobox family, member 2; Rhox homeobox family, me | Multiple_C | 3.52 | 3.39 | 3.33 | 1.14 | 0.2645 | 0.4719 | 3.76 | 3.76 | 3.58 | 1.13 | 0.45 | 0.6962 |
| TC0X00008822.hg.1 | OPN1MW2 | opsin 1 (cone pigments), medium-wave-sensitive 2 | Multiple_C | 4.83 | 4.83 | 4.64 | 1.14 | 0.7946 | 0.8946 | 4.27 | 4.01 | 3.97 | 1.23 | 0.3499 | 0.614 |
| TC0X00011289.hg.1 | GAGE2B; GAG | G antigen 2B; G antigen 2A; G antigen 2C | Coding | 3.94 | 3.5 | 3.75 | 1.14 | 0.4954 | 0.6952 | 3.8 | 3.93 | 3.79 | 1.01 | 0.9448 | 0.9776 |
| TC1000009012.hg.1 | ENO4 | enolase family member 4 | Multiple_C | 4.06 | 3.73 | 3.87 | 1.14 | 0.7171 | 0.8504 | 4.69 | 4.37 | 4.22 | 1.39 | 0.1234 | 0.3468 |
| TC1000009104.hg.1 | PLPP4 | phospholipid phosphatase 4 | Multiple_C | 3.74 | 3.46 | 3.55 | 1.14 | 0.4991 | 0.6985 | 4.41 | 4.35 | 4.1 | 1.24 | 0.0974 | 0.3043 |
| TC1000011266.hg.1 | LRIT2 | leucine-rich repeat, immunoglobulin-like and transmembrane d | Coding | 3.1 | 2.72 | 2.91 | 1.14 | 0.4522 | 0.6598 | 3.35 | 3.17 | 3.18 | 1.13 | 0.712 | 0.865 |
| TC1000011628.hg.1 | PDZD7 | PDZ domain containing 7 | Multiple_C | 5.22 | 5.24 | 5.03 | 1.14 | 0.4436 | 0.6521 | 5.84 | 5.58 | 5.52 | 1.25 | 0.4698 | 0.7107 |
| TC1100006662.hg.1 | OR51D1 | olfactory receptor, family 51, subfamily D, member 1 | Coding | 2.97 | 2.6 | 2.78 | 1.14 | 0.8782 | 0.9408 | 3.48 | 3.83 | 3.38 | 1.07 | 0.1286 | 0.3548 |
| TC1100006722.hg.1 | CCKBR | cholecystokinin B receptor | Multiple_C | 6.32 | 6.28 | 6.13 | 1.14 | 0.5786 | 0.7565 | 5.38 | 5.46 | 5.39 | 0.99 | 0.3914 | 0.65 |
| TC1100007772.hg.1 | MS4A12 | membrane-spanning 4-domains, subfamily A, member 12 | Multiple_C | 4.78 | 4.13 | 4.59 | 1.14 | 0.282 | 0.492 | 4.91 | 4.38 | 4.29 | 1.54 | 0.1903 | 0.4432 |
| TC1100008133.hg.1 | TBC1D10C | TBC1 domain family, member 10C | Multiple_C | 3.29 | 2.85 | 3.1 | 1.14 | 0.8968 | 0.9507 | 4.8 | 4.86 | 4.99 | 0.88 | 0.4187 | 0.6725 |
| TC1100009335.hg.1 | C11orf63 | chromosome 11 open reading frame 63 | Multiple_C | 3.55 | 3.35 | 3.36 | 1.14 | 0.5099 | 0.7059 | 5.87 | 6.35 | 6.09 | 0.86 | 0.3794 | 0.639 |
| TC1100009950.hg.1 | OR56A5 | olfactory receptor, family 56, subfamily A, member 5 | Coding | 3.36 | 2.87 | 3.17 | 1.14 | 0.9059 | 0.9548 | 3.38 | 3.56 | 3.39 | 0.99 | 0.3423 | 0.6061 |
| TC1100010391.hg.1 | MUC15 | mucin 15, cell surface associated | Coding | 3.66 | 3.78 | 3.47 | 1.14 | 0.6136 | 0.7832 | 3.84 | 3.73 | 3.82 | 1.01 | 0.7078 | 0.8624 |
| TC1100010393.hg.1 | SLC5A12 | solute carrier family 5 (sodium/monocarboxylate cotransporter | Multiple_C | 4.69 | 4.39 | 4.5 | 1.14 | 0.4816 | 0.6832 | 5.56 | 5.28 | 5.03 | 1.44 | 0.0803 | 0.2731 |
| TC1100010685.hg.1 | C11orf94 | chromosome 11 open reading frame 94 | Multiple_C | 3.32 | 2.94 | 3.13 | 1.14 | 0.4119 | 0.624 | 3.99 | 3.93 | 3.69 | 1.23 | 0.5168 | 0.7454 |

| | | | | | | | | | | | | | | | |
|---|---|---|---|---|---|---|---|---|---|---|---|---|---|---|---|
| TC1100012638.hg.1 | ZNF202 | zinc finger protein 202 | Multiple_C | 7.81 | 7.68 | 7.62 | 1.14 | 0.1105 | 0.2593 | 8.8 | 8.70 | 8.55 | 1.19 | 0.308 | 0.5721 |
| TC1200006570.hg.1 | KCNA1 | potassium channel, voltage gated shaker related subfamily A, m | Multiple_C | 3.21 | 3.03 | 3.02 | 1.14 | 0.3521 | 0.5648 | 4.65 | 4.67 | 4.29 | 1.28 | 0.2051 | 0.4614 |
| TC1200007832.hg.1 | NABP2 | nucleic acid binding protein 2 | Multiple_C | 10.06 | 9.43 | 9.87 | 1.14 | 0.7155 | 0.8497 | 10.03 | 11.15 | 11.36 | 0.40 | 0.0016 | 0.0231 |
| TC1200009198.hg.1 | LRRC43 | leucine rich repeat containing 43 | Multiple_C | 3.97 | 3.62 | 3.78 | 1.14 | 0.4721 | 0.6756 | 4.21 | 4.16 | 3.77 | 1.36 | 0.0135 | 0.094 |
| TC1200010415.hg.1 | SLC2A13 | solute carrier family 2 (facilitated glucose transporter), member | Multiple_C | 7.44 | 7.56 | 7.25 | 1.14 | 0.7154 | 0.8497 | 6.42 | 6.18 | 6.13 | 1.22 | 0.6335 | 0.8198 |
| TC1200010751.hg.1 | KRT85 | keratin 85, type II | Multiple_C | 4.1 | 3.8 | 3.91 | 1.14 | 0.3275 | 0.5413 | 4.43 | 4.03 | 4.17 | 1.20 | 0.3686 | 0.6307 |
| TC1200010768.hg.1 | KRT1 | keratin 1, type II | Multiple_C | 3.46 | 3.19 | 3.27 | 1.14 | 0.1787 | 0.3621 | 3.8 | 3.85 | 3.82 | 0.99 | 0.9849 | 0.9937 |
| TC1200010776.hg.1 | KRT4 | keratin 4, type II | Multiple_C | 4.43 | 4.14 | 4.24 | 1.14 | 0.2451 | 0.4486 | 3.38 | 3.53 | 3.43 | 0.97 | 0.9655 | 0.9854 |
| TC1200010793.hg.1 | CSAD | cysteine sulfinic acid decarboxylase | Multiple_C | 5.49 | 5.34 | 5.3 | 1.14 | 0.8275 | 0.9133 | 6.59 | 5.89 | 5.87 | 1.65 | 0.1584 | 0.4 |
| TC1200011054.hg.1 | PPM1H | protein phosphatase, Mg2+/Mn2+ dependent, 1H | Multiple_C | 5.55 | 6.09 | 5.36 | 1.14 | 0.6505 | 0.8065 | 6.95 | 6.69 | 6.42 | 1.44 | 0.0137 | 0.0948 |
| TC1200011588.hg.1 | USP44 | ubiquitin specific peptidase 44 | Multiple_C | 4.17 | 3.88 | 3.98 | 1.14 | 0.6272 | 0.792 | 3.28 | 3.48 | 3.29 | 0.99 | 0.5388 | 0.7593 |
| TC1200011882.hg.1 | TRPV4 | transient receptor potential cation channel, subfamily V, memb | Multiple_C | 3.79 | 3.27 | 3.6 | 1.14 | 0.6634 | 0.8156 | 6.53 | 4.79 | 4.61 | 3.78 | 4.51E-06 | 0.0004 |
| TC1200012754.hg.1 | KLRC1 | killer cell lectin-like receptor subfamily C, member 1 | Multiple_C | 3.3 | 3.15 | 3.11 | 1.14 | 0.4882 | 0.689 | 4.51 | 4.15 | 4.2 | 1.24 | 0.0865 | 0.2836 |
| TC1200012813.hg.1 | B4GALNT1 | beta-1,4-N-acetyl-galactosaminyl transferase 1 | Multiple_C | 3.67 | 3.37 | 3.48 | 1.14 | 0.3335 | 0.5473 | 4.67 | 4.82 | 4.9 | 0.85 | 0.7471 | 0.8841 |
| TC1200012853.hg.1 | NOS1 | nitric oxide synthase 1 (neuronal) | Multiple_C | 3.72 | 3.51 | 3.53 | 1.14 | 0.6635 | 0.8157 | 3.97 | 3.66 | 3.71 | 1.20 | 0.2361 | 0.4989 |
| TC1300007755.hg.1 | DNAJC3 | DnaJ (Hsp40) homolog, subfamily C, member 3 | Multiple_C | 11.32 | 11.54 | 11.13 | 1.14 | 0.811 | 0.9044 | 11.21 | 11.36 | 11.89 | 0.62 | 0.1654 | 0.4102 |
| TC1300008048.hg.1 | TEX29 | testis expressed 29 | Coding | 4.95 | 4.95 | 4.76 | 1.14 | 0.2341 | 0.435 | 4.42 | 4.29 | 4.22 | 1.15 | 0.6636 | 0.8355 |
| TC1300008834.hg.1 | SIAH3 | siah E3 ubiquitin protein ligase family member 3 | Coding | 3.57 | 3.42 | 3.38 | 1.14 | 0.4735 | 0.6767 | 4.24 | 3.96 | 3.74 | 1.41 | 0.1469 | 0.383 |
| TC1400009982.hg.1 | PPP4R3A | protein phosphatase 4, regulatory subunit 3A | Multiple_C | 11.45 | 11.03 | 11.26 | 1.14 | 0.5299 | 0.7204 | 10.83 | 10.30 | 11 | 0.89 | 0.8125 | 0.9166 |
| TC1400010270.hg.1 | ANKRD9 | ankyrin repeat domain 9 | Coding | 5.75 | 5.42 | 5.56 | 1.14 | 0.3789 | 0.5919 | 3.35 | 3.35 | 3.26 | 1.06 | 0.7774 | 0.9003 |
| TC1400010599.hg.1 | TTC6 | tetratricopeptide repeat domain 6 | Multiple_C | 3.14 | 3.15 | 2.95 | 1.14 | 0.5635 | 0.7453 | 3.61 | 3.63 | 3.44 | 1.13 | 0.246 | 0.5105 |
| TC1400010646.hg.1 | TCL1B; TCL6 | T-cell leukemia/lymphoma 1B; T-cell leukemia/lymphoma 6 (no | Multiple_C | 3.42 | 3.16 | 3.23 | 1.14 | 0.8763 | 0.9399 | 3.76 | 3.80 | 4 | 0.85 | 0.3154 | 0.5794 |
| TC1400010752.hg.1 | SLC35F4 | solute carrier family 35, member F4 | Multiple_C | 4.13 | 3.48 | 3.94 | 1.14 | 0.5041 | 0.7015 | 3.96 | 3.87 | 3.86 | 1.07 | 0.482 | 0.7201 |
| TC1500008304.hg.1 | GDPGP1 | GDP-D-glucose phosphorylase 1 | Multiple_C | 8.68 | 6.96 | 8.49 | 1.14 | 0.6439 | 0.8025 | 5.49 | 5.53 | 5.87 | 0.77 | 0.368 | 0.6303 |
| TC1500009928.hg.1 | THAP10 | THAP domain containing 10 | Coding | 7.15 | 6.84 | 6.96 | 1.14 | 0.5724 | 0.7518 | 6.58 | 6.83 | 6.81 | 0.85 | 0.416 | 0.6701 |
| TC1500009973.hg.1 | ADPGK | ADP-dependent glucokinase | Multiple_C | 8.21 | 8.16 | 8.02 | 1.14 | 0.3595 | 0.5729 | 6.36 | 6.25 | 6.39 | 0.98 | 0.8732 | 0.9453 |
| TC1600006530.hg.1 | TPSD1 | tryptase delta 1 | Multiple_C | 3.64 | 3.24 | 3.45 | 1.14 | 0.3775 | 0.5905 | 3.37 | 3.28 | 3.17 | 1.15 | 0.2733 | 0.5388 |
| TC1600006664.hg.1 | ZNF205 | zinc finger protein 205 | Multiple_C | 8.25 | 7.76 | 8.06 | 1.14 | 0.6764 | 0.8241 | 7.37 | 7.85 | 7.46 | 0.94 | 0.7731 | 0.898 |
| TC1600007431.hg.1 | ASPHD1 | aspartate beta-hydroxylase domain containing 1 | Multiple_C | 6.08 | 5.83 | 5.89 | 1.14 | 0.6285 | 0.7927 | 4.08 | 3.92 | 3.93 | 1.11 | 0.7606 | 0.8915 |
| TC1600009746.hg.1 | PALB2 | partner and localizer of BRCA2 | Multiple_C | 9.6 | 9.58 | 9.41 | 1.14 | 0.4538 | 0.6613 | 10.97 | 10.70 | 11.03 | 0.96 | 0.8637 | 0.9417 |
| TC1600011376.hg.1 | MAZ | MYC-associated zinc finger protein (purine-binding transcriptior | Multiple_C | 8.91 | 8.4 | 8.72 | 1.14 | 0.874 | 0.939 | 9.74 | 10.91 | 10.65 | 0.53 | 0.0001 | 0.004 |
| TC1700007361.hg.1 | TNFAIP1 | tumor necrosis factor, alpha-induced protein 1 (endothelial) | Multiple_C | 9.49 | 8.81 | 9.3 | 1.14 | 0.481 | 0.6827 | 8.66 | 8.34 | 8.34 | 1.25 | 0.1375 | 0.3685 |
| TC1700008878.hg.1 | CDR2L | cerebellar degeneration-related protein 2-like | Multiple_C | 5.3 | 4.74 | 5.11 | 1.14 | 0.4401 | 0.649 | 5.12 | 5.43 | 5.49 | 0.77 | 0.084 | 0.2789 |

| | | | | | | | | | | | | | | | |
|---|---|---|---|---|---|---|---|---|---|---|---|---|---|---|---|
| TC1700010647.hg.1 | KRTAP3-1 | keratin associated protein 3-1 | Multiple_C | 7.31 | 7.08 | 7.12 | 1.14 | 0.0757 | 0.1963 | 7.31 | 6.44 | 6.91 | 1.32 | 0.4145 | 0.669 |
| TC1700011687.hg.1 | BTBD17 | BTB (POZ) domain containing 17 | Coding | 3.1 | 2.93 | 2.91 | 1.14 | 0.5283 | 0.7196 | 3.28 | 3.46 | 3.35 | 0.95 | 0.8336 | 0.926 |
| TC1700012258.hg.1 | KRTAP9-8 | keratin associated protein 9-8 | Coding | 4.33 | 4.45 | 4.14 | 1.14 | 0.4226 | 0.6339 | 4.74 | 4.75 | 4.81 | 0.95 | 0.7259 | 0.8725 |
| TC1700012404.hg.1 | KRTAP1-1 | keratin associated protein 1-1 | Coding | 4.08 | 3.96 | 3.89 | 1.14 | 0.6452 | 0.8034 | 4.94 | 4.86 | 4.8 | 1.10 | 0.6111 | 0.8057 |
| TC1700012470.hg.1 | H3F3B | H3 histone, family 3B (H3.3B) | Multiple_C | 14.62 | 14.41 | 14.43 | 1.14 | 0.9191 | 0.9625 | 13.91 | 13.73 | 14.01 | 0.93 | 0.282 | 0.5476 |
| TC1800007955.hg.1 | C18orf42 | chromosome 18 open reading frame 42 | Coding | 4.63 | 4.32 | 4.44 | 1.14 | 0.2493 | 0.4532 | 4.02 | 3.90 | 3.71 | 1.24 | 0.4203 | 0.6735 |
| TC1900006866.hg.1 | SNAPC2 | small nuclear RNA activating complex polypeptide 2 | Multiple_C | 6.92 | 6.78 | 6.73 | 1.14 | 0.3307 | 0.5446 | 5.3 | 5.37 | 5.67 | 0.77 | 0.1601 | 0.4027 |
| TC1900007355.hg.1 | CCDC124 | coiled-coil domain containing 124 | Multiple_C | 10.77 | 9.81 | 10.58 | 1.14 | 0.6743 | 0.8228 | 9.88 | 10.19 | 10.33 | 0.73 | 0.1587 | 0.4002 |
| TC1900008912.hg.1 | SSC5D | scavenger receptor cysteine rich family, 5 domains | Multiple_C | 5.72 | 5.46 | 5.53 | 1.14 | 0.6172 | 0.7854 | 5.34 | 5.44 | 5.33 | 1.01 | 0.5216 | 0.7484 |
| TC1900009104.hg.1 | PRSS57 | protease, serine, 57 | Multiple_C | 5.46 | 4.9 | 5.27 | 1.14 | 0.8221 | 0.9105 | 5.81 | 5.90 | 5.96 | 0.90 | 0.4562 | 0.7004 |
| TC1900009155.hg.1 | GAMT | guanidinoacetate N-methyltransferase | Multiple_C | 3.91 | 3.7 | 3.72 | 1.14 | 0.4366 | 0.6461 | 7.48 | 7.62 | 7.05 | 1.35 | 0.0485 | 0.2047 |
| TC1900009297.hg.1 | TBXA2R | thromboxane A2 receptor | Multiple_C | 4.52 | 4.1 | 4.33 | 1.14 | 0.7709 | 0.8814 | 4.42 | 4.50 | 4.22 | 1.15 | 0.2682 | 0.5327 |
| TC1900010032.hg.1 | CERS1; GDF1 | ceramide synthase 1; growth differentiation factor 1 | Multiple_C | 3.19 | 2.88 | 3 | 1.14 | 0.5283 | 0.7196 | 5.54 | 5.74 | 5.68 | 0.91 | 0.5574 | 0.7711 |
| TC1900010286.hg.1 | UQCRFS1 | ubiquinol-cytochrome c reductase, Rieske iron-sulfur polypeptic | Coding | 12.48 | 13.2 | 12.29 | 1.14 | 0.5946 | 0.7689 | 9.12 | 9.70 | 9.45 | 0.80 | 0.3705 | 0.6327 |
| TC1900011101.hg.1 | PLEKHA4 | pleckstrin homology domain containing, family A (phosphoinosi | Multiple_C | 3.38 | 2.95 | 3.19 | 1.14 | 0.5716 | 0.7513 | 3.82 | 3.95 | 4.02 | 0.87 | 0.3011 | 0.5655 |
| TC1900011248.hg.1 | VSIG10L | V-set and immunoglobulin domain containing 10 like | Multiple_C | 4.02 | 3.57 | 3.83 | 1.14 | 0.6293 | 0.7931 | 3.55 | 3.53 | 3.54 | 1.01 | 0.8093 | 0.9156 |
| TC1900011414.hg.1 | LAIR1 | leukocyte-associated immunoglobulin-like receptor 1 | Multiple_C | 3.52 | 3.21 | 3.33 | 1.14 | 0.6672 | 0.818 | 3.95 | 4.09 | 3.93 | 1.01 | 0.2657 | 0.5304 |
| TC1900011961.hg.1 | PSG8 | pregnancy specific beta-1-glycoprotein 8 | Multiple_C | 3.74 | 3.41 | 3.55 | 1.14 | 0.6622 | 0.8146 | 3.52 | 3.41 | 3.69 | 0.89 | 0.48 | 0.7185 |
| TC1900012033.hg.1 | ZNF347 | zinc finger protein 347 | Multiple_C | 4.74 | 5.09 | 4.55 | 1.14 | 0.95 | 0.9768 | 3.7 | 3.72 | 3.76 | 0.96 | 0.5674 | 0.7764 |
| TC2000006802.hg.1 | BANF2 | barrier to autointegration factor 2 | Multiple_C | 2.96 | 3.01 | 2.77 | 1.14 | 0.78 | 0.8868 | 3.58 | 3.16 | 3.31 | 1.21 | 0.1788 | 0.4276 |
| TC2000009970.hg.1 | SIRPB1 | signal-regulatory protein beta 1 | Multiple_C | 4.07 | 4.02 | 3.88 | 1.14 | 0.2783 | 0.4877 | 3.74 | 3.78 | 4.05 | 0.81 | 0.174 | 0.4216 |
| TC2100006433.hg.1 | ICOSLG | inducible T-cell co-stimulator ligand | Coding | 4.75 | 4.56 | 4.56 | 1.14 | 0.4806 | 0.6825 | 4.14 | 4.00 | 4.51 | 0.77 | 0.1291 | 0.3557 |
| TC2100008382.hg.1 | KRTAP12-1 | keratin associated protein 12-1 | Coding | 6.51 | 6.01 | 6.32 | 1.14 | 0.9098 | 0.9569 | 5.52 | 5.80 | 6.06 | 0.69 | 0.0188 | 0.1156 |
| TC2100008467.hg.1 | C21orf58 | chromosome 21 open reading frame 58 | Multiple_C | 3.47 | 3.08 | 3.28 | 1.14 | 0.5589 | 0.7416 | 4.49 | 5.03 | 4.79 | 0.81 | 0.3458 | 0.6096 |
| TC2200009363.hg.1 | ODF3B | outer dense fiber of sperm tails 3B | Multiple_C | 4.39 | 4.04 | 4.2 | 1.14 | 0.5133 | 0.7087 | 4.83 | 4.72 | 4.84 | 0.99 | 0.5827 | 0.7873 |
| TSUnmapped00000153.h | AC208162.1 | | Coding | 3.96 | 3.66 | 3.77 | 1.14 | 0.2678 | 0.4758 | 4.01 | 4.33 | 4.16 | 0.90 | 0.8348 | 0.9269 |
| TSUnmapped00000573.h | PRAMEF25 | PRAME family member 25 | Coding | 4.45 | 4.25 | 4.26 | 1.14 | 0.475 | 0.6778 | 3.98 | 4.33 | 4.36 | 0.77 | 0.1104 | 0.3273 |
| TSUnmapped00000605.h | PRAMEF4 | PRAME family member 4 | Coding | 2.94 | 2.68 | 2.75 | 1.14 | 0.6156 | 0.7848 | 3.49 | 3.35 | 3.13 | 1.28 | 0.0547 | 0.2195 |
| TSUnmapped00000624.h | CYP2D6 | cytochrome P450, family 2, subfamily D, polypeptide 6 | Coding | 4.1 | 3.88 | 3.91 | 1.14 | 0.4737 | 0.6769 | 4.27 | 4.34 | 4.3 | 0.98 | 0.5325 | 0.7554 |
| TSUnmapped00000742.h | ZNF852 | zinc finger protein 852 | Coding | 6.4 | 6.61 | 6.21 | 1.14 | 0.6169 | 0.7853 | 5.82 | 5.74 | 6 | 0.88 | 0.0607 | 0.2337 |
| TSUnmapped00000793.h | PRAMEF6 | PRAME family member 6 [Source:HGNC Symbol;Acc:HGNC:305 | Coding | 5.21 | 4.75 | 5.02 | 1.14 | 0.2253 | 0.4241 | 5.05 | 5.08 | 5.14 | 0.94 | 0.8402 | 0.9302 |
| TC0100006620.hg.1 | TP73 | tumor protein p73 | Multiple_C | 4.51 | 3.84 | 4.33 | 1.13 | 0.8894 | 0.9467 | 3.68 | 3.89 | 3.72 | 0.97 | 0.8917 | 0.9539 |
| TC0100006926.hg.1 | PRAMEF20 | PRAME family member 20 | Coding | 2.82 | 2.7 | 2.64 | 1.13 | 0.268 | 0.4761 | 3.15 | 3.34 | 3.41 | 0.84 | 0.6154 | 0.809 |

| | | | | | | | | | | | | | | | |
|---|---|---|---|---|---|---|---|---|---|---|---|---|---|---|---|
| TC0100007108.hg.1 | PADI6 | peptidyl arginine deiminase, type VI | Multiple_C | 4.47 | 4.3 | 4.29 | 1.13 | 0.2018 | 0.394 | 4.5 | 4.10 | 4.46 | 1.03 | 0.9554 | 0.9814 |
| TC0100007147.hg.1 | PAX7 | paired box 7 | Multiple_C | 3.98 | 3.5 | 3.8 | 1.13 | 0.5462 | 0.7318 | 4.08 | 4.43 | 4.39 | 0.81 | 0.1849 | 0.4363 |
| TC0100007659.hg.1 | KHDRBS1 | KH domain containing, RNA binding, signal transduction associa | Multiple_C | 13.06 | 13.67 | 12.88 | 1.13 | 0.8319 | 0.9159 | 13.11 | 13.15 | 12.89 | 1.16 | 0.4789 | 0.7173 |
| TC0100008450.hg.1 | PRKAA2 | protein kinase, AMP-activated, alpha 2 catalytic subunit | Multiple_C | 3.02 | 2.63 | 2.84 | 1.13 | 0.4554 | 0.6624 | 9.3 | 8.85 | 9.42 | 0.92 | 0.5539 | 0.7688 |
| TC0100009181.hg.1 | PLPPR4 | phospholipid phosphatase related 4 | Multiple_C | 3.15 | 2.84 | 2.97 | 1.13 | 0.8013 | 0.8981 | 3.45 | 4.34 | 3.56 | 0.93 | 0.3169 | 0.5809 |
| TC0100009370.hg.1 | UBL4B | ubiquitin-like 4B | Coding | 3.25 | 3.03 | 3.07 | 1.13 | 0.3021 | 0.5145 | 3.57 | 3.61 | 3.64 | 0.95 | 0.5425 | 0.7614 |
| TC0100010121.hg.1 | HCN3 | hyperpolarization activated cyclic nucleotide gated potassium c | Multiple_C | 4.28 | 4.29 | 4.1 | 1.13 | 0.7996 | 0.8972 | 4.42 | 4.56 | 4.33 | 1.06 | 0.9647 | 0.985 |
| TC0100010227.hg.1 | CD1D | CD1d molecule | Coding | 8.09 | 7.71 | 7.91 | 1.13 | 0.5018 | 0.7002 | 8.24 | 8.06 | 7.9 | 1.27 | 0.0834 | 0.278 |
| TC0100012453.hg.1 | TNFRSF4 | tumor necrosis factor receptor superfamily, member 4 | Multiple_C | 3.37 | 2.99 | 3.19 | 1.13 | 0.545 | 0.7312 | 3.89 | 3.64 | 3.62 | 1.21 | 0.8834 | 0.95 |
| TC0100013647.hg.1 | TRIM62 | tripartite motif containing 62 | Multiple_C | 5.23 | 4.66 | 5.05 | 1.13 | 0.4233 | 0.6345 | 5.9 | 5.83 | 5.46 | 1.36 | 0.0509 | 0.2104 |
| TC0100013724.hg.1 | CSF3R | colony stimulating factor 3 receptor | Multiple_C | 3.69 | 3.32 | 3.51 | 1.13 | 0.1636 | 0.3419 | 4.33 | 3.98 | 3.83 | 1.41 | 0.0134 | 0.0938 |
| TC0100013872.hg.1 | EDN2 | endothelin 2 | Multiple_C | 2.72 | 2.01 | 2.54 | 1.13 | 0.1952 | 0.3852 | 3.49 | 3.06 | 2.87 | 1.54 | 0.0062 | 0.0566 |
| TC0100014245.hg.1 | YIPF1 | Yip1 domain family member 1 | Multiple_C | 7.07 | 6.73 | 6.89 | 1.13 | 0.2401 | 0.4422 | 6.7 | 6.76 | 7.41 | 0.61 | 0.0039 | 0.0419 |
| TC0100015350.hg.1 | AMPD1 | adenosine monophosphate deaminase 1 | Multiple_C | 3.2 | 2.83 | 3.02 | 1.13 | 0.8503 | 0.9265 | 3.86 | 3.78 | 3.31 | 1.46 | 0.1376 | 0.3685 |
| TC0100015896.hg.1 | NUP210L; MIF | nucleoporin 210kDa like; microRNA 5698 | Multiple_C | 3.12 | 2.91 | 2.94 | 1.13 | 0.6907 | 0.8333 | 4.09 | 4.11 | 3.91 | 1.13 | 0.3246 | 0.5885 |
| TC0200010586.hg.1 | CPO | carboxypeptidase O | Coding | 3.09 | 2.97 | 2.91 | 1.13 | 0.608 | 0.7787 | 3.1 | 3.44 | 3.35 | 0.84 | 0.6192 | 0.8113 |
| TC0200010972.hg.1 | MFF | mitochondrial fission factor | Multiple_C | 11.32 | 11.56 | 11.14 | 1.13 | 0.5333 | 0.723 | 8.75 | 8.68 | 8.66 | 1.06 | 0.661 | 0.8345 |
| TC0200011148.hg.1 | SPP2 | secreted phosphoprotein 2 | Multiple_C | 3.34 | 3.12 | 3.16 | 1.13 | 0.2699 | 0.4782 | 3.42 | 3.75 | 3.67 | 0.84 | 0.7108 | 0.8644 |
| TC0200011376.hg.1 | AGXT | alanine-glyoxylate aminotransferase | Multiple_C | 8.08 | 7.96 | 7.9 | 1.13 | 0.9716 | 0.9861 | 7.13 | 7.38 | 7.42 | 0.82 | 0.3022 | 0.5667 |
| TC0200011759.hg.1 | NTSR2 | neurotensin receptor 2 | Coding | 3.79 | 3.41 | 3.61 | 1.13 | 0.4973 | 0.6968 | 3.66 | 3.54 | 3.51 | 1.11 | 0.3154 | 0.5794 |
| TC0200012412.hg.1 | C1GALT1C1L | C1GALT1-specific chaperone 1 like | Multiple_C | 2.89 | 2.77 | 2.71 | 1.13 | 0.1683 | 0.3481 | 3.71 | 4.21 | 3.37 | 1.27 | 0.3092 | 0.5733 |
| TC0200013456.hg.1 | LOC442028; A | uncharacterized LOC442028; Transcript Identified by AceView, E | Multiple_C | 4.96 | 4.67 | 4.78 | 1.13 | 0.7717 | 0.8821 | 5.16 | 5.07 | 4.76 | 1.32 | 0.0673 | 0.2473 |
| TC0200013573.hg.1 | ACTR1B | ARP1 actin-related protein 1 homolog B, centractin beta (yeast) | Multiple_C | 10.1 | 9.95 | 9.92 | 1.13 | 0.9402 | 0.9723 | 9.71 | 10.24 | 9.74 | 0.98 | 0.4859 | 0.723 |
| TC0200015329.hg.1 | GTF3C3 | general transcription factor IIIC subunit 3 | Multiple_C | 10.2 | 12.11 | 10.02 | 1.13 | 0.6093 | 0.7797 | 10.88 | 10.36 | 10.69 | 1.14 | 0.1903 | 0.4432 |
| TC0200015514.hg.1 | GPR1 | G protein-coupled receptor 1 | Coding | 3.58 | 3.5 | 3.4 | 1.13 | 0.5612 | 0.7435 | 4.75 | 5.07 | 5.13 | 0.77 | 0.2386 | 0.5021 |
| TC0200015570.hg.1 | CRYGD | crystallin gamma D | Coding | 3.41 | 3.07 | 3.23 | 1.13 | 0.4762 | 0.6788 | 3.19 | 3.20 | 3.17 | 1.01 | 0.8585 | 0.9397 |
| TC0200015678.hg.1 | IGFBP5 | insulin like growth factor binding protein 5 | Multiple_C | 3.94 | 3.59 | 3.76 | 1.13 | 0.3662 | 0.5794 | 3.68 | 3.86 | 3.75 | 0.95 | 0.9213 | 0.9675 |
| TC0200015939.hg.1 | SPHKAP | SPHK1 interactor, AKAP domain containing | Multiple_C | 3.95 | 3.77 | 3.77 | 1.13 | 0.4338 | 0.6438 | 4.22 | 4.54 | 4.32 | 0.93 | 0.5746 | 0.7818 |
| TC0200016720.hg.1 | RNF149 | ring finger protein 149 | Multiple_C | 10.4 | 11.14 | 10.22 | 1.13 | 0.5324 | 0.7221 | 9.21 | 8.53 | 8.82 | 1.31 | 0.0211 | 0.124 |
| TC0300007294.hg.1 | PTPN23 | protein tyrosine phosphatase, non-receptor type 23 | Multiple_C | 8.43 | 8.31 | 8.25 | 1.13 | 0.9318 | 0.9679 | 8.68 | 8.72 | 8.56 | 1.09 | 0.2275 | 0.4882 |
| TC0300007409.hg.1 | SLC38A3 | solute carrier family 38, member 3 | Multiple_C | 4.4 | 4.23 | 4.22 | 1.13 | 0.5228 | 0.7158 | 7.34 | 7.69 | 7.24 | 1.07 | 0.6594 | 0.8339 |
| TC0300007673.hg.1 | SNTN | sentan, cilia apical structure protein | Coding | 3.66 | 3.53 | 3.48 | 1.13 | 0.449 | 0.6569 | 3.73 | 3.26 | 3.14 | 1.51 | 0.0186 | 0.1152 |
| TC0300008691.hg.1 | PLXNA1 | plexin A1 | Multiple_C | 8.61 | 7.9 | 8.43 | 1.13 | 0.7921 | 0.8935 | 9.53 | 9.52 | 9.36 | 1.13 | 0.36 | 0.6228 |

| | | | | | | | | | | | | | | | |
|---|---|---|---|---|---|---|---|---|---|---|---|---|---|---|---|
| TC0300010937.hg.1 | XCR1 | chemokine (C motif) receptor 1 | Multiple_C | 5.28 | 4.88 | 5.1 | 1.13 | 0.2323 | 0.433 | 5.57 | 5.83 | 5.61 | 0.97 | 0.5859 | 0.7898 |
| TC0300012140.hg.1 | GPR156 | G protein-coupled receptor 156 | Multiple_C | 3.28 | 3.25 | 3.1 | 1.13 | 0.2065 | 0.3998 | 4.48 | 4.34 | 4.32 | 1.12 | 0.3586 | 0.6216 |
| TC0300012275.hg.1 | KLF15 | Kruppel-like factor 15 | Multiple_C | 3.05 | 2.88 | 2.87 | 1.13 | 0.8058 | 0.9012 | 4.84 | 4.34 | 3.84 | 2.00 | 5.85E-05 | 0.0025 |
| TC0400007609.hg.1 | REST | RE1-silencing transcription factor | Multiple_C | 9.59 | 9.16 | 9.41 | 1.13 | 0.5775 | 0.7556 | 8.91 | 8.56 | 8.78 | 1.09 | 0.5565 | 0.7706 |
| TC0400007734.hg.1 | TMPRSS11E | transmembrane protease, serine 11E | Coding | 3.28 | 3.47 | 3.1 | 1.13 | 0.1963 | 0.3867 | 3.43 | 3.54 | 3.28 | 1.11 | 0.9411 | 0.9756 |
| TC0400008972.hg.1 | DCLK2 | doublecortin-like kinase 2 | Multiple_C | 3.61 | 3.29 | 3.43 | 1.13 | 0.2415 | 0.4438 | 3.92 | 3.67 | 3.58 | 1.27 | 0.3324 | 0.5965 |
| TC0400009958.hg.1 | ABLIM2 | actin binding LIM protein family, member 2 | Multiple_C | 4.57 | 4.45 | 4.39 | 1.13 | 0.4719 | 0.6755 | 5.55 | 5.34 | 5.24 | 1.24 | 0.052 | 0.2129 |
| TC0400011916.hg.1 | PCDH18 | protocadherin 18 | Multiple_C | 4.58 | 4.23 | 4.4 | 1.13 | 0.2591 | 0.4657 | 4.38 | 4.42 | 4.27 | 1.08 | 0.5399 | 0.7594 |
| TC0500006648.hg.1 | PAPD7 | PAP associated domain containing 7 | Multiple_C | 7.16 | 6.55 | 6.98 | 1.13 | 0.6667 | 0.8176 | 9.69 | 9.36 | 9.86 | 0.89 | 0.2276 | 0.4882 |
| TC0500008767.hg.1 | PKD2L2 | polycystic kidney disease 2-like 2 | Coding | 2.84 | 2.48 | 2.66 | 1.13 | 0.5357 | 0.7246 | 3.23 | 3.18 | 3.18 | 1.04 | 0.8041 | 0.9128 |
| TC0500009215.hg.1 | NIPAL4 | NIPA-like domain containing 4 | Multiple_C | 3.5 | 3.19 | 3.32 | 1.13 | 0.2801 | 0.4898 | 3.85 | 3.89 | 3.72 | 1.09 | 0.1583 | 0.3999 |
| TC0500009386.hg.1 | FOXI1 | forkhead box I1 | Coding | 3.87 | 3.34 | 3.69 | 1.13 | 0.2238 | 0.4221 | 4.38 | 4.49 | 4.26 | 1.09 | 0.9294 | 0.9702 |
| TC0500011759.hg.1 | ATG12 | autophagy related 12 | Multiple_C | 11.54 | 12.29 | 11.36 | 1.13 | 0.6471 | 0.8043 | 11.06 | 10.20 | 10.8 | 1.20 | 0.3227 | 0.5867 |
| TC0500012485.hg.1 | RBM22 | RNA binding motif protein 22 | Multiple_C | 12.56 | 12.13 | 12.38 | 1.13 | 0.1763 | 0.3588 | 12.17 | 11.84 | 12.51 | 0.79 | 0.1718 | 0.4184 |
| TC0500012506.hg.1 | SLC36A2 | solute carrier family 36 (proton/amino acid symporter), membe | Multiple_C | 3.56 | 3.42 | 3.38 | 1.13 | 0.4161 | 0.628 | 4.22 | 4.43 | 3.78 | 1.36 | 0.0428 | 0.1908 |
| TC0500012948.hg.1 | SNCB | synuclein beta | Multiple_C | 4.64 | 4.32 | 4.46 | 1.13 | 0.2479 | 0.4517 | 4.79 | 4.70 | 4.69 | 1.07 | 0.5945 | 0.7953 |
| TC0500013336.hg.1 | SSBP2 | single-stranded DNA binding protein 2 | Multiple_C | 9.85 | 9.2 | 9.67 | 1.13 | 0.9064 | 0.9551 | 10.42 | 9.83 | 9.83 | 1.51 | 0.0084 | 0.0698 |
| TC0600009455.hg.1 | ARG1 | arginase 1 | Multiple_C | 3.76 | 3.83 | 3.58 | 1.13 | 0.1053 | 0.2504 | 4.03 | 3.81 | 3.37 | 1.58 | 0.004 | 0.0433 |
| TC0600011065.hg.1 | KIAA0319 | KIAA0319 | Coding | 4.62 | 4.44 | 4.44 | 1.13 | 0.2488 | 0.4529 | 4.7 | 4.13 | 3.99 | 1.64 | 0.0008 | 0.0142 |
| TC0600011248.hg.1 | ZKSCAN4 | zinc finger with KRAB and SCAN domains 4 | Multiple_C | 3.05 | 2.77 | 2.87 | 1.13 | 0.6063 | 0.7774 | 4.35 | 4.36 | 4.19 | 1.12 | 0.4423 | 0.6911 |
| TC0600012011.hg.1 | DEFB110 | defensin, beta 110 | Coding | 3.28 | 3.1 | 3.1 | 1.13 | 0.64 | 0.8006 | 4.22 | 3.94 | 3.45 | 1.71 | 0.2028 | 0.4584 |
| TC0700008588.hg.1 | ZNHIT1 | zinc finger, HIT-type containing 1 | Multiple_C | 11.6 | 11.21 | 11.42 | 1.13 | 0.5046 | 0.702 | 7.9 | 8.27 | 8.58 | 0.62 | 0.0005 | 0.0104 |
| TC0700008617.hg.1 | SH2B2 | SH2B adaptor protein 2 | Multiple_C | 3.21 | 2.74 | 3.03 | 1.13 | 0.468 | 0.6721 | 3.18 | 3.59 | 3.45 | 0.83 | 0.9894 | 0.9956 |
| TC0700012883.hg.1 | FAM131B | family with sequence similarity 131, member B | Multiple_C | 3.7 | 3.43 | 3.52 | 1.13 | 0.4383 | 0.6477 | 3.76 | 3.89 | 3.8 | 0.97 | 0.3151 | 0.5789 |
| TC0800008338.hg.1 | KCNS2 | potassium voltage-gated channel, modifier subfamily S, membe | Coding | 2.73 | 2.36 | 2.55 | 1.13 | 0.4054 | 0.6182 | 3.43 | 3.35 | 3.26 | 1.13 | 0.1041 | 0.3162 |
| TC0800009626.hg.1 | DEFB134 | defensin, beta 134 | Coding | 4.01 | 3.77 | 3.83 | 1.13 | 0.5976 | 0.7708 | 4.02 | 4.00 | 4.12 | 0.93 | 0.3744 | 0.635 |
| TC0800010441.hg.1 | OPRK1 | opioid receptor, kappa 1 | Multiple_C | 3.14 | 2.85 | 2.96 | 1.13 | 0.4718 | 0.6753 | 3.21 | 3.39 | 3.54 | 0.80 | 0.8443 | 0.9318 |
| TC0800010685.hg.1 | MYBL1 | v-myb avian myeloblastosis viral oncogene homolog-like 1 | Multiple_C | 4.48 | 4.59 | 4.3 | 1.13 | 0.4288 | 0.6391 | 6.92 | 5.75 | 6.48 | 1.36 | 0.3398 | 0.6039 |
| TC0800010944.hg.1 | IMPA1 | inositol(myo)-1(or 4)-monophosphatase 1 | Multiple_C | 10.95 | 12.41 | 10.77 | 1.13 | 0.5693 | 0.7496 | 11.13 | 10.14 | 10.55 | 1.49 | 0.008 | 0.0674 |
| TC0800012097.hg.1 | LOC10013366 | Homo sapiens uncharacterized LOC100133669 (LOC100133669) | Multiple_C | 2.99 | 2.99 | 2.81 | 1.13 | 0.2732 | 0.4819 | 3.69 | 3.46 | 3.29 | 1.32 | 0.0831 | 0.2776 |
| TC0800012180.hg.1 | PARP10 | poly(ADP-ribose) polymerase family member 10 | Multiple_C | 5.62 | 5.26 | 5.44 | 1.13 | 0.8329 | 0.9165 | 5.29 | 5.49 | 5.74 | 0.73 | 0.0161 | 0.1054 |
| TC0900007092.hg.1 | CCDC107 | coiled-coil domain containing 107 | Coding | 5.29 | 4.74 | 5.11 | 1.13 | 0.4611 | 0.6667 | 4.06 | 4.63 | 4.88 | 0.57 | 0.3604 | 0.6232 |
| TC0900007318.hg.1 | SPATA31A7; S | SPATA31 subfamily A, member 7; SPATA31 subfamily A, membe | Multiple_C | 6.78 | 6.66 | 6.6 | 1.13 | 0.9453 | 0.975 | 6.77 | 6.82 | 6.93 | 0.90 | 0.9552 | 0.9814 |

| | | | | | | | | | | | | | | | |
|---|---|---|---|---|---|---|---|---|---|---|---|---|---|---|---|
| TC0900007819.hg.1 | CTSL3P | cathepsin L family member 3, pseudogene | Multiple_C | 3.78 | 3.93 | 3.6 | 1.13 | 0.6086 | 0.7793 | 3.22 | 3.11 | 3.1 | 1.09 | 0.274 | 0.5395 |
| TC0900009688.hg.1 | IFNB1 | interferon, beta 1, fibroblast | Coding | 3.76 | 3.39 | 3.58 | 1.13 | 0.6545 | 0.8095 | 4.2 | 4.19 | 3.97 | 1.17 | 0.0356 | 0.171 |
| TC0900009949.hg.1 | TPM2 | tropomyosin 2 (beta) | Multiple_C | 5.86 | 5.66 | 5.68 | 1.13 | 0.724 | 0.8548 | 10.87 | 10.90 | 10.82 | 1.04 | 0.3709 | 0.6329 |
| TC0900009958.hg.1 | FAM221B | family with sequence similarity 221, member B | Multiple_C | 3.14 | 2.72 | 2.96 | 1.13 | 0.6044 | 0.7761 | 4.02 | 4.06 | 4 | 1.01 | 0.4297 | 0.6817 |
| TC0900011277.hg.1 | KIF12 | kinesin family member 12 | Multiple_C | 3.62 | 3.56 | 3.44 | 1.13 | 0.6506 | 0.8066 | 3.95 | 3.86 | 3.85 | 1.07 | 0.9511 | 0.9797 |
| TC0X00006651.hg.1 | EGFL6 | EGF-like-domain, multiple 6 | Multiple_C | 4.3 | 4.24 | 4.12 | 1.13 | 0.6545 | 0.8095 | 4.44 | 4.06 | 3.99 | 1.37 | 0.0446 | 0.1954 |
| TC0X00007170.hg.1 | SPACA5B | sperm acrosome associated 5B | Coding | 4.42 | 4.67 | 4.24 | 1.13 | 0.5389 | 0.727 | 4.42 | 4.61 | 4.68 | 0.84 | 0.3454 | 0.6092 |
| TC0X00007209.hg.1 | PQBP1 | polyglutamine binding protein 1 | Multiple_C | 11.95 | 12.22 | 11.77 | 1.13 | 0.5559 | 0.7391 | 11.46 | 12.02 | 12.11 | 0.64 | 0.0015 | 0.0224 |
| TC0X00007301.hg.1 | XAGE5 | X antigen family, member 5 | Multiple_C | 3.46 | 3.19 | 3.28 | 1.13 | 0.7156 | 0.8497 | 3.52 | 3.84 | 3.32 | 1.15 | 0.0799 | 0.2724 |
| TC0X00008000.hg.1 | TCEAL7 | transcription elongation factor A (SII)-like 7 | Multiple_C | 4.6 | 4.52 | 4.42 | 1.13 | 0.2191 | 0.4161 | 5.02 | 4.85 | 4.78 | 1.18 | 0.2522 | 0.5164 |
| TC0X00008017.hg.1 | PLP1 | proteolipid protein 1 | Multiple_C | 4.34 | 4.27 | 4.16 | 1.13 | 0.2868 | 0.4973 | 4.93 | 4.96 | 5.2 | 0.83 | 0.6154 | 0.809 |
| TC0X00008207.hg.1 | AGTR2 | angiotensin II receptor, type 2 | Multiple_C | 3.88 | 3.32 | 3.7 | 1.13 | 0.6445 | 0.803 | 3.76 | 3.80 | 4.07 | 0.81 | 0.7703 | 0.8969 |
| TC0X00008820.hg.1 | OPN1LW | opsin 1 (cone pigments), long-wave-sensitive | Multiple_C | 4.85 | 4.97 | 4.67 | 1.13 | 0.8633 | 0.9334 | 4.74 | 4.56 | 4.5 | 1.18 | 0.4983 | 0.7314 |
| TC0X00008884.hg.1 | VAMP7 | vesicle associated membrane protein 7 | Multiple_C | 9.91 | 10.86 | 9.73 | 1.13 | 0.7245 | 0.8551 | 9.22 | 8.75 | 9.9 | 0.62 | 0.0018 | 0.0251 |
| TC0X00009179.hg.1 | RAI2 | retinoic acid induced 2 | Multiple_C | 3.94 | 3.6 | 3.76 | 1.13 | 0.6183 | 0.7862 | 3.2 | 3.14 | 3.24 | 0.97 | 0.2723 | 0.5378 |
| TC0X00009203.hg.1 | MAP3K15 | mitogen-activated protein kinase kinase kinase 15 | Multiple_C | 3.79 | 3.45 | 3.61 | 1.13 | 0.5517 | 0.7362 | 5.48 | 5.30 | 5.3 | 1.13 | 0.9502 | 0.9795 |
| TC0X00009718.hg.1 | XAGE1E; XAGI | X antigen family, member 1E; X antigen family, member 1B | Multiple_C | 3.75 | 3.36 | 3.57 | 1.13 | 0.2349 | 0.4359 | 3.64 | 3.58 | 3.62 | 1.01 | 0.3784 | 0.6381 |
| TC0X00009842.hg.1 | SPIN2A | spindlin family, member 2A | Coding | 4.49 | 4.45 | 4.31 | 1.13 | 0.5712 | 0.751 | 5.62 | 5.98 | 6.02 | 0.76 | 0.2092 | 0.4664 |
| TC0Y00006571.hg.1 | TSPY3 | testis specific protein, Y-linked 3 | Multiple_C | 3.64 | 3.41 | 3.46 | 1.13 | 0.8509 | 0.9269 | 3.83 | 3.65 | 3.78 | 1.04 | 0.2924 | 0.5572 |
| TC1000009106.hg.1 | LINC01561 | long intergenic non-protein coding RNA 1561 | Multiple_C | 3.87 | 3.72 | 3.69 | 1.13 | 0.4764 | 0.679 | 4.4 | 4.64 | 4.71 | 0.81 | 0.7683 | 0.8961 |
| TC1000009397.hg.1 | DPYSL4 | dihydropyrimidinase-like 4 | Multiple_C | 3.66 | 3.19 | 3.48 | 1.13 | 0.4843 | 0.6859 | 4.67 | 4.89 | 4.51 | 1.12 | 0.5645 | 0.7745 |
| TC1000010211.hg.1 | LYZL2 | lysozyme-like 2 | Coding | 3.12 | 2.84 | 2.94 | 1.13 | 0.3709 | 0.5836 | 3.42 | 3.25 | 3.19 | 1.17 | 0.2347 | 0.4972 |
| TC1000012198.hg.1 | C10orf90 | chromosome 10 open reading frame 90 | Multiple_C | 3.3 | 3.16 | 3.12 | 1.13 | 0.2546 | 0.4602 | 3.61 | 3.82 | 3.91 | 0.81 | 0.4756 | 0.7147 |
| TC1100006810.hg.1 | IPO7; SNORA2 | importin 7; small nucleolar RNA, H/ACA box 23 | Multiple_C | 11.87 | 11.8 | 11.69 | 1.13 | 0.3904 | 0.6041 | 15.02 | 14.63 | 14.58 | 1.36 | 0.0222 | 0.128 |
| TC1100007030.hg.1 | NAV2 | neuron navigator 2 | Multiple_C | 4.9 | 4.87 | 4.72 | 1.13 | 0.3169 | 0.5303 | 12.69 | 11.91 | 11.14 | 2.93 | 3.78E-05 | 0.0018 |
| TC1100007600.hg.1 | OR4P4 | olfactory receptor, family 4, subfamily P, member 4 | Coding | 3.43 | 3 | 3.25 | 1.13 | 0.4357 | 0.6452 | 3.74 | 3.51 | 3.42 | 1.25 | 0.1535 | 0.3928 |
| TC1100007700.hg.1 | OR9Q1 | olfactory receptor, family 9, subfamily Q, member 1 | Coding | 3.05 | 3.09 | 2.87 | 1.13 | 0.1137 | 0.2645 | 3.5 | 3.13 | 3.08 | 1.34 | 0.3124 | 0.5765 |
| TC1100007767.hg.1 | MS4A4A | membrane-spanning 4-domains, subfamily A, member 4A | Multiple_C | 3.02 | 3.07 | 2.84 | 1.13 | 0.4805 | 0.6825 | 3.22 | 3.24 | 3.16 | 1.04 | 0.4599 | 0.7031 |
| TC1100008328.hg.1 | RNF121 | ring finger protein 121 | Multiple_C | 8.94 | 8.97 | 8.76 | 1.13 | 0.4917 | 0.6918 | 9.72 | 9.81 | 9.79 | 0.95 | 0.4024 | 0.6586 |
| TC1100008978.hg.1 | RAB39A | RAB39A, member RAS oncogene family | Coding | 4.54 | 4.24 | 4.36 | 1.13 | 0.2431 | 0.4458 | 6.37 | 6.33 | 6.98 | 0.66 | 0.0185 | 0.1149 |
| TC1100009243.hg.1 | NLRX1 | NLR family member X1 | Multiple_C | 6.23 | 5.9 | 6.05 | 1.13 | 0.1975 | 0.3883 | 5.08 | 5.72 | 5.83 | 0.59 | 0.0015 | 0.0221 |
| TC1100009285.hg.1 | TMEM136 | transmembrane protein 136 | Multiple_C | 3.69 | 3.41 | 3.51 | 1.13 | 0.527 | 0.7187 | 8.36 | 7.74 | 7.53 | 1.78 | 0.0045 | 0.0459 |
| TC1100009384.hg.1 | OR8G1 | olfactory receptor, family 8, subfamily G, member 1 (gene/pseu | Multiple_C | 3.12 | 2.89 | 2.94 | 1.13 | 0.5086 | 0.705 | 4.03 | 4.42 | 3.8 | 1.17 | 0.4059 | 0.6614 |

| | | | | | | | | | | | | | | | |
|---|---|---|---|---|---|---|---|---|---|---|---|---|---|---|---|
| TC1100009947.hg.1 | OR52E6 | olfactory receptor, family 52, subfamily E, member 6 | Coding | 4.62 | 4.7 | 4.44 | 1.13 | 0.984 | 0.9924 | 4.17 | 4.31 | 4.29 | 0.92 | 0.7435 | 0.8827 |
| TC1100009951.hg.1 | OR52L1 | olfactory receptor, family 52, subfamily L, member 1 | Coding | 3.8 | 3.61 | 3.62 | 1.13 | 0.5369 | 0.7254 | 4.07 | 4.22 | 3.88 | 1.14 | 0.955 | 0.9814 |
| TC1100010732.hg.1 | MYBPC3 | myosin binding protein C, cardiac | Multiple_C | 5.07 | 4.37 | 4.89 | 1.13 | 0.4451 | 0.6536 | 5.19 | 4.96 | 4.76 | 1.35 | 0.3619 | 0.6246 |
| TC1100011090.hg.1 | EML3 | echinoderm microtubule associated protein like 3 | Multiple_C | 7.45 | 7.24 | 7.27 | 1.13 | 0.6331 | 0.7953 | 6.91 | 6.80 | 6.31 | 1.52 | 0.0518 | 0.2125 |
| TC1100011831.hg.1 | CCDC89 | coiled-coil domain containing 89 | Coding | 3.03 | 2.59 | 2.85 | 1.13 | 0.5377 | 0.726 | 3.05 | 3.19 | 2.84 | 1.16 | 0.1752 | 0.4232 |
| TC1100012165.hg.1 | CASP4 | caspase 4 | Multiple_C | 11.52 | 12.24 | 11.34 | 1.13 | 0.5308 | 0.7211 | 10.32 | 10.09 | 9.87 | 1.37 | 0.4116 | 0.6662 |
| TC1200009791.hg.1 | GDF3 | growth differentiation factor 3 | Coding | 3.69 | 3.12 | 3.51 | 1.13 | 0.362 | 0.5755 | 3.62 | 3.75 | 3.56 | 1.04 | 0.3018 | 0.5662 |
| TC1200010606.hg.1 | WNT10B | wingless-type MMTV integration site family, member 10B | Multiple_C | 5.16 | 4.56 | 4.98 | 1.13 | 0.6424 | 0.8016 | 5.13 | 5.10 | 5.09 | 1.03 | 0.5614 | 0.7723 |
| TC1200010769.hg.1 | KRT77 | keratin 77, type II | Multiple_C | 5.56 | 5.35 | 5.38 | 1.13 | 0.9352 | 0.9697 | 5.1 | 5.40 | 5.19 | 0.94 | 0.7743 | 0.8986 |
| TC1200012016.hg.1 | TBX3 | T-box 3 | Multiple_C | 10.55 | 9.19 | 10.37 | 1.13 | 0.4074 | 0.6198 | 7.23 | 7.44 | 7.43 | 0.87 | 0.8123 | 0.9166 |
| TC1300007312.hg.1 | PCDH17 | protocadherin 17 | Coding | 4.12 | 3.55 | 3.94 | 1.13 | 0.3827 | 0.5958 | 4.67 | 4.27 | 4.45 | 1.16 | 0.2799 | 0.5459 |
| TC1300008059.hg.1 | SOX1 | SRY box 1 | Coding | 3.31 | 3.21 | 3.13 | 1.13 | 0.692 | 0.834 | 3.75 | 3.84 | 3.54 | 1.16 | 0.1563 | 0.3969 |
| TC1300009330.hg.1 | RBM26 | RNA binding motif protein 26 | Multiple_C | 10.53 | 10.39 | 10.35 | 1.13 | 0.7472 | 0.8703 | 12.39 | 12.02 | 12.33 | 1.04 | 0.6655 | 0.8363 |
| TC1400006678.hg.1 | RNF212B | ring finger protein 212B | Multiple_C | 3.25 | 3.43 | 3.07 | 1.13 | 0.4977 | 0.6973 | 3.12 | 3.40 | 3.07 | 1.04 | 0.6675 | 0.8374 |
| TC1400006685.hg.1 | IL25 | interleukin 25 | Coding | 4.45 | 4.12 | 4.27 | 1.13 | 0.7119 | 0.8478 | 4.27 | 4.42 | 4.34 | 0.95 | 0.96 | 0.9835 |
| TC1400006821.hg.1 | G2E3 | G2/M-phase specific E3 ubiquitin protein ligase | Multiple_C | 7.83 | 8.52 | 7.65 | 1.13 | 0.5271 | 0.7187 | 6.4 | 5.13 | 6.92 | 0.70 | 0.074 | 0.261 |
| TC1400008642.hg.1 | OR10G2 | olfactory receptor, family 10, subfamily G, member 2 | Coding | 3.5 | 3.37 | 3.32 | 1.13 | 0.1594 | 0.3358 | 3.69 | 3.55 | 3.57 | 1.09 | 0.701 | 0.8583 |
| TC1400008763.hg.1 | GZMH | granzyme H | Coding | 4 | 4 | 3.82 | 1.13 | 0.6511 | 0.8068 | 3.47 | 3.68 | 3.53 | 0.96 | 0.2845 | 0.5496 |
| TC1400009698.hg.1 | LTBP2 | latent transforming growth factor beta binding protein 2 | Multiple_C | 4.31 | 4.25 | 4.13 | 1.13 | 0.3606 | 0.574 | 4.95 | 4.63 | 4.25 | 1.62 | 0.0116 | 0.0857 |
| TC1400010398.hg.1 | GPR132 | G protein-coupled receptor 132 | Multiple_C | 4.29 | 3.99 | 4.11 | 1.13 | 0.3748 | 0.5875 | 4.56 | 4.54 | 5.05 | 0.71 | 0.0721 | 0.2573 |
| TC1400010707.hg.1 | KIAA0125 | KIAA0125 | Multiple_C | 4.25 | 4.08 | 4.07 | 1.13 | 0.9546 | 0.9787 | 4.05 | 3.96 | 4.19 | 0.91 | 0.4762 | 0.7153 |
| TC1500009158.hg.1 | LTK | leukocyte receptor tyrosine kinase | Multiple_C | 3.83 | 3.54 | 3.65 | 1.13 | 0.6768 | 0.8243 | 4.12 | 3.84 | 3.91 | 1.16 | 0.6526 | 0.8305 |
| TC1500010256.hg.1 | BTBD1 | BTB (POZ) domain containing 1 | Multiple_C | 13.3 | 13.33 | 13.12 | 1.13 | 0.8818 | 0.9426 | 13.41 | 12.84 | 12.87 | 1.45 | 0.0229 | 0.1303 |
| TC1500010766.hg.1 | CPLX3 | complexin 3 | Coding | 4.71 | 4.41 | 4.53 | 1.13 | 0.1689 | 0.3489 | 6.11 | 5.83 | 5.49 | 1.54 | 0.0333 | 0.1639 |
| TC1600006550.hg.1 | TMEM204 | transmembrane protein 204 | Coding | 3.76 | 3.46 | 3.58 | 1.13 | 0.1636 | 0.3418 | 3.86 | 3.92 | 4.09 | 0.85 | 0.215 | 0.4736 |
| TC1600007014.hg.1 | NDE1; MIR48 | nudE neurodevelopment protein 1; microRNA 484 | Multiple_C | 10.49 | 9.55 | 10.31 | 1.13 | 0.5376 | 0.726 | 10.66 | 10.40 | 10.7 | 0.97 | 0.6775 | 0.8448 |
| TC1600007169.hg.1 | METTL9 | methyltransferase like 9 | Multiple_C | 12.23 | 13.14 | 12.05 | 1.13 | 0.5755 | 0.7542 | 12.37 | 11.27 | 11.28 | 2.13 | 0.0045 | 0.0463 |
| TC1600007819.hg.1 | NKD1 | naked cuticle homolog 1 (Drosophila) | Multiple_C | 3.47 | 3.03 | 3.29 | 1.13 | 0.5048 | 0.7021 | 16.33 | 16.66 | 15.84 | 1.40 | 0.0207 | 0.1231 |
| TC1600007829.hg.1 | CYLD | cylindromatosis (turban tumor syndrome) | Multiple_C | 6.09 | 5.92 | 5.91 | 1.13 | 0.814 | 0.9064 | 7.08 | 6.88 | 7.64 | 0.68 | 0.071 | 0.2549 |
| TC1600009545.hg.1 | ABCC6 | ATP binding cassette subfamily C member 6 | Multiple_C | 4.62 | 4.25 | 4.44 | 1.13 | 0.2634 | 0.4706 | 5.66 | 5.67 | 5.24 | 1.34 | 0.0239 | 0.1335 |
| TC1600009891.hg.1 | RABEP2 | rabaptin, RAB GTPase binding effector protein 2 | Multiple_C | 7.99 | 7.44 | 7.81 | 1.13 | 0.6214 | 0.788 | 7.72 | 7.62 | 7.43 | 1.22 | 0.2665 | 0.5309 |
| TC1600010561.hg.1 | CDH11 | cadherin 11, type 2, OB-cadherin (osteoblast) | Multiple_C | 4.29 | 4.1 | 4.11 | 1.13 | 0.4433 | 0.6521 | 4.55 | 4.32 | 4.01 | 1.45 | 0.0402 | 0.1837 |
| TC1600011321.hg.1 | FAM195A | family with sequence similarity 195, member A | Multiple_C | 5.38 | 5.32 | 5.2 | 1.13 | 0.1756 | 0.3581 | 5.21 | 5.29 | 5.3 | 0.94 | 0.6099 | 0.805 |

| | | | | | | | | | | | | | | | |
|---|---|---|---|---|---|---|---|---|---|---|---|---|---|---|---|
| TC1700006582.hg.1 | OR3A3 | olfactory receptor, family 3, subfamily A, member 3 | Coding | 5.23 | 4.71 | 5.05 | 1.13 | 0.2614 | 0.4683 | 4.7 | 4.77 | 4.71 | 0.99 | 0.8595 | 0.9399 |
| TC1700006763.hg.1 | ATP1B2 | ATPase, Na+/K+ transporting, beta 2 polypeptide | Multiple_C | 3.28 | 2.93 | 3.1 | 1.13 | 0.9792 | 0.9901 | 4.32 | 3.77 | 3.89 | 1.35 | 0.0089 | 0.0724 |
| TC1700006813.hg.1 | ODF4 | outer dense fiber of sperm tails 4 | Coding | 5.07 | 4.98 | 4.89 | 1.13 | 0.5076 | 0.7041 | 4.73 | 4.57 | 4.68 | 1.04 | 0.6212 | 0.8128 |
| TC1700007266.hg.1 | KCNJ12 | potassium channel, inwardly rectifying subfamily J, member 12 | Multiple_C | 5.79 | 5.43 | 5.61 | 1.13 | 0.5581 | 0.7407 | 5.31 | 5.91 | 5.87 | 0.68 | 0.5968 | 0.7969 |
| TC1700007775.hg.1 | CSF3 | colony stimulating factor 3 | Multiple_C | 3.5 | 3.15 | 3.32 | 1.13 | 0.3617 | 0.5752 | 3.27 | 3.34 | 3.36 | 0.94 | 0.6157 | 0.8091 |
| TC1700007993.hg.1 | RUNDC3A | RUN domain containing 3A | Multiple_C | 3.48 | 3.15 | 3.3 | 1.13 | 0.4304 | 0.6406 | 4.86 | 4.62 | 4.43 | 1.35 | 0.1197 | 0.3417 |
| TC1700009546.hg.1 | SLC52A1 | solute carrier family 52 (riboflavin transporter), member 1 | Multiple_C | 6.3 | 5.96 | 6.12 | 1.13 | 0.6591 | 0.8131 | 6.42 | 6.31 | 5.88 | 1.45 | 0.2229 | 0.4834 |
| TC1700010207.hg.1 | RAB34 | RAB34, member RAS oncogene family | Multiple_C | 4.87 | 4.64 | 4.69 | 1.13 | 0.8435 | 0.9229 | 4.46 | 4.62 | 4.54 | 0.95 | 0.6669 | 0.8371 |
| TC1700010550.hg.1 | C17orf98 | chromosome 17 open reading frame 98 | Coding | 3.46 | 3.02 | 3.28 | 1.13 | 0.4757 | 0.6784 | 3.44 | 3.58 | 3.69 | 0.84 | 0.967 | 0.9862 |
| TC1700010715.hg.1 | HCRT | hypocretin (orexin) neuropeptide precursor | Coding | 4.19 | 3.95 | 4.01 | 1.13 | 0.4407 | 0.6494 | 4.41 | 4.35 | 4.16 | 1.19 | 0.123 | 0.3462 |
| TC1700011305.hg.1 | HEATR6 | HEAT repeat containing 6 | Multiple_C | 7.9 | 7.65 | 7.72 | 1.13 | 0.3821 | 0.5952 | 8.77 | 8.72 | 8.93 | 0.90 | 0.5251 | 0.7498 |
| TC1700011575.hg.1 | ABCA9 | ATP binding cassette subfamily A member 9 | Multiple_C | 4.26 | 3.77 | 4.08 | 1.13 | 0.5872 | 0.7634 | 3.59 | 3.90 | 3.88 | 0.82 | 0.1152 | 0.3349 |
| TC1700012094.hg.1 | CCDC57 | coiled-coil domain containing 57 | Multiple_C | 5.07 | 5.07 | 4.89 | 1.13 | 0.5942 | 0.7686 | 6.18 | 6.35 | 5.92 | 1.20 | 0.1199 | 0.342 |
| TC1800007829.hg.1 | HSBP1L1 | heat shock factor binding protein 1-like 1 | Multiple_C | 10.68 | 10.38 | 10.5 | 1.13 | 0.3768 | 0.5899 | 7.6 | 7.10 | 6.76 | 1.79 | 0.0011 | 0.0184 |
| TC1800009034.hg.1 | FAM69C | family with sequence similarity 69, member C | Coding | 4.94 | 4.57 | 4.76 | 1.13 | 0.6166 | 0.7851 | 4.2 | 4.14 | 4.23 | 0.98 | 0.3462 | 0.61 |
| TC1900006466.hg.1 | CDC34 | cell division cycle 34 | Multiple_C | 5.7 | 5.57 | 5.52 | 1.13 | 0.9407 | 0.9724 | 4.99 | 4.86 | 5.02 | 0.98 | 0.9144 | 0.9649 |
| TC1900006834.hg.1 | MBD3L2 | methyl-CpG binding domain protein 3-like 2 | Coding | 5.12 | 4.82 | 4.94 | 1.13 | 0.2415 | 0.4438 | 4.96 | 4.95 | 5.03 | 0.95 | 0.9142 | 0.9648 |
| TC1900008276.hg.1 | CEACAM16 | carcinoembryonic antigen-related cell adhesion molecule 16 | Multiple_C | 3.64 | 3.43 | 3.46 | 1.13 | 0.3495 | 0.5622 | 3.83 | 3.79 | 3.84 | 0.99 | 0.2417 | 0.5056 |
| TC1900008763.hg.1 | DPRX | divergent-paired related homeobox | Coding | 3.73 | 3.48 | 3.55 | 1.13 | 0.3695 | 0.5823 | 3.44 | 3.40 | 3.48 | 0.97 | 0.98 | 0.9915 |
| TC1900008828.hg.1 | CACNG6 | calcium channel, voltage-dependent, gamma subunit 6 | Multiple_C | 4.72 | 4.45 | 4.54 | 1.13 | 0.1504 | 0.3226 | 5.08 | 5.05 | 4.75 | 1.26 | 0.2076 | 0.4642 |
| TC1900008906.hg.1 | TMEM190 | transmembrane protein 190 | Coding | 6.43 | 6.12 | 6.25 | 1.13 | 0.7246 | 0.8551 | 5.85 | 5.77 | 5.57 | 1.21 | 0.3407 | 0.6044 |
| TC1900008968.hg.1 | ZNF71 | zinc finger protein 71 | Multiple_C | 6.61 | 6.13 | 6.43 | 1.13 | 0.7105 | 0.8464 | 4.82 | 5.35 | 5.39 | 0.67 | 0.0094 | 0.075 |
| TC1900009269.hg.1 | TLE2 | transducin-like enhancer of split 2 | Multiple_C | 3.18 | 2.86 | 3 | 1.13 | 0.2956 | 0.507 | 3.1 | 3.02 | 3.05 | 1.04 | 0.2507 | 0.5149 |
| TC1900009755.hg.1 | KLF1 | Kruppel-like factor 1 (erythroid) | Multiple_C | 5.14 | 4.71 | 4.96 | 1.13 | 0.2398 | 0.4418 | 4.36 | 4.27 | 3.89 | 1.39 | 0.0936 | 0.297 |
| TC1900009896.hg.1 | CYP4F11 | cytochrome P450, family 4, subfamily F, polypeptide 11 | Multiple_C | 4.12 | 3.25 | 3.94 | 1.13 | 0.3821 | 0.5952 | 3.7 | 3.41 | 3.28 | 1.34 | 0.1059 | 0.3196 |
| TC1900010482.hg.1 | ATP4A | ATPase, H+/K+ exchanging, alpha polypeptide | Multiple_C | 6.19 | 5.83 | 6.01 | 1.13 | 0.1605 | 0.3371 | 6.24 | 6.23 | 5.96 | 1.21 | 0.0673 | 0.2473 |
| TC1900011067.hg.1 | TMEM143 | transmembrane protein 143 | Multiple_C | 5.74 | 4.85 | 5.56 | 1.13 | 0.4149 | 0.6271 | 4.64 | 4.73 | 4.68 | 0.97 | 0.4801 | 0.7186 |
| TC1900011196.hg.1 | NAPSA | napsin A aspartic peptidase | Multiple_C | 3.83 | 3.45 | 3.65 | 1.13 | 0.1953 | 0.3853 | 4.08 | 4.19 | 4.01 | 1.05 | 0.8581 | 0.9397 |
| TC1900011251.hg.1 | NKG7 | natural killer cell granule protein 7 | Coding | 7.02 | 6.82 | 6.84 | 1.13 | 0.134 | 0.2977 | 6.62 | 6.52 | 6.23 | 1.31 | 0.0331 | 0.1632 |
| TC1900011473.hg.1 | SHISA7 | shisa family member 7 | Coding | 3.77 | 3.77 | 3.59 | 1.13 | 0.3752 | 0.588 | 3.97 | 3.95 | 3.63 | 1.27 | 0.059 | 0.2291 |
| TC1900011882.hg.1 | ZNF442 | zinc finger protein 442 | Multiple_C | 6.46 | 6.26 | 6.28 | 1.13 | 0.5158 | 0.7105 | 5.59 | 6.01 | 5.92 | 0.80 | 0.1437 | 0.3775 |
| TC1900011946.hg.1 | FBXO27 | F-box protein 27 | Multiple_C | 3.83 | 3.27 | 3.65 | 1.13 | 0.9239 | 0.9644 | 9.16 | 8.54 | 9.2 | 0.97 | 0.196 | 0.4504 |
| TC2000006844.hg.1 | SCP2D1 | SCP2 sterol-binding domain containing 1 | Coding | 3.41 | 3.24 | 3.23 | 1.13 | 0.1023 | 0.245 | 3.86 | 3.59 | 3.22 | 1.56 | 0.0405 | 0.1846 |

| | | | | | | | | | | | | | | | |
|---|---|---|---|---|---|---|---|---|---|---|---|---|---|---|---|
| TC2000007929.hg.1 | EDN3 | endothelin 3 | Coding | 4.64 | 4.35 | 4.46 | 1.13 | 0.2797 | 0.4894 | 4.59 | 4.51 | 4.42 | 1.13 | 0.3642 | 0.6268 |
| TC2000009245.hg.1 | ACOT8 | acyl-CoA thioesterase 8 | Multiple_C | 8.51 | 7.96 | 8.33 | 1.13 | 0.4566 | 0.663 | 8.25 | 8.17 | 7.88 | 1.29 | 0.0224 | 0.1287 |
| TC2200006827.hg.1 | GNAZ | guanine nucleotide binding protein (G protein), alpha z polypep | Multiple_C | 2.53 | 2.05 | 2.35 | 1.13 | 0.8949 | 0.9497 | 7.86 | 8.51 | 7.67 | 1.14 | 0.4885 | 0.7247 |
| TC2200007506.hg.1 | WBP2NL | WBP2 N-terminal like | Multiple_C | 6.87 | 6.77 | 6.69 | 1.13 | 0.3899 | 0.6037 | 6.65 | 6.76 | 6.5 | 1.11 | 0.1422 | 0.3749 |
| TC2200007677.hg.1 | TBC1D22A | TBC1 domain family, member 22A | Multiple_C | 8.1 | 7.97 | 7.92 | 1.13 | 0.5525 | 0.7367 | 8.89 | 9.41 | 8.5 | 1.31 | 0.0547 | 0.2195 |
| TC2200007987.hg.1 | CLDN5 | claudin 5 | Coding | 5.14 | 5.24 | 4.96 | 1.13 | 0.7874 | 0.8913 | 4.46 | 4.57 | 4.5 | 0.97 | 0.3843 | 0.6433 |
| TSUnmapped00000048.h | ATG16L1 | autophagy related 16-like 1 | Coding | 4.74 | 4.46 | 4.56 | 1.13 | 0.3306 | 0.5445 | 4.55 | 4.54 | 4.61 | 0.96 | 0.9874 | 0.9947 |
| TSUnmapped00000115.h | NLRP10 | NLR family, pyrin domain containing 10 | Coding | 4.04 | 3.83 | 3.86 | 1.13 | 0.0577 | 0.161 | 4.12 | 4.19 | 4.19 | 0.95 | 0.6844 | 0.8486 |
| TSUnmapped00000135.h | INPP5D | inositol polyphosphate-5-phosphatase D | Coding | 3.25 | 3.28 | 3.07 | 1.13 | 0.5196 | 0.7132 | 9.03 | 9.18 | 9.42 | 0.76 | 0.0443 | 0.1948 |
| TSUnmapped00000292.h | TGM4 | transglutaminase 4 | Coding | 3.47 | 3.05 | 3.29 | 1.13 | 0.4154 | 0.6276 | 3.59 | 3.42 | 3.78 | 0.88 | 0.6543 | 0.8316 |
| TSUnmapped00000433.h | ATG16L1 | autophagy related 16-like 1 | Coding | 9.31 | 9.35 | 9.13 | 1.13 | 0.221 | 0.4184 | 8.09 | 7.55 | 7.91 | 1.13 | 0.5717 | 0.7796 |
| TC0100009109.hg.1 | SLC44A3 | solute carrier family 44, member 3 | Multiple_C | 8.14 | 7.92 | 7.97 | 1.13 | 0.9075 | 0.9557 | 6.65 | 8.25 | 7.71 | 0.48 | 0.0017 | 0.0234 |
| TC0100009452.hg.1 | FAM19A3 | family with sequence similarity 19 (chemokine (C-C motif)-like), | Coding | 8.87 | 8.41 | 8.7 | 1.13 | 0.4749 | 0.6778 | 7.42 | 7.78 | 7.58 | 0.90 | 0.26 | 0.5242 |
| TC0100009608.hg.1 | HAO2 | hydroxyacid oxidase 2 (long chain) | Multiple_C | 3.4 | 3.16 | 3.23 | 1.13 | 0.8556 | 0.9295 | 3.46 | 3.49 | 3.61 | 0.90 | 0.2485 | 0.5133 |
| TC0100010198.hg.1 | LRRC71 | leucine rich repeat containing 71 | Multiple_C | 3.86 | 3.93 | 3.69 | 1.13 | 0.7272 | 0.8565 | 4.41 | 4.17 | 3.86 | 1.46 | 0.1477 | 0.384 |
| TC0100010243.hg.1 | PYHIN1 | pyrin and HIN domain family, member 1 | Multiple_C | 4.29 | 3.86 | 4.12 | 1.13 | 0.1039 | 0.2478 | 3.45 | 3.59 | 3.63 | 0.88 | 0.3824 | 0.6414 |
| TC0100010532.hg.1 | XCL1 | chemokine (C motif) ligand 1 | Coding | 3.68 | 3.67 | 3.51 | 1.13 | 0.1884 | 0.3759 | 4.65 | 4.91 | 4.57 | 1.06 | 0.8005 | 0.9108 |
| TC0100010799.hg.1 | LHX4 | LIM homeobox 4 | Multiple_C | 5.01 | 4.73 | 4.84 | 1.13 | 0.4948 | 0.6947 | 6 | 5.67 | 5.77 | 1.17 | 0.2005 | 0.4558 |
| TC0100011325.hg.1 | TMCC2 | transmembrane and coiled-coil domain family 2 | Multiple_C | 3.85 | 3.98 | 3.68 | 1.13 | 0.2823 | 0.4924 | 4.32 | 4.49 | 4.8 | 0.72 | 0.146 | 0.3815 |
| TC0100011454.hg.1 | HSD11B1 | hydroxysteroid (11-beta) dehydrogenase 1 | Multiple_C | 3.71 | 3.35 | 3.54 | 1.13 | 0.6665 | 0.8176 | 4.49 | 4.27 | 4.33 | 1.12 | 0.6107 | 0.8054 |
| TC0100011472.hg.1 | HHAT | hedgehog acyltransferase | Multiple_C | 5.3 | 5.01 | 5.13 | 1.13 | 0.4959 | 0.6958 | 4.43 | 4.15 | 4.63 | 0.87 | 0.6934 | 0.8541 |
| TC0100012948.hg.1 | PRAMEF19 | PRAME family member 19 | Coding | 3.03 | 2.78 | 2.86 | 1.13 | 0.446 | 0.6545 | 3.63 | 3.73 | 3.25 | 1.30 | 0.1328 | 0.3617 |
| TC0100013169.hg.1 | PLA2G2C | phospholipase A2, group IIC | Multiple_C | 3.24 | 3.38 | 3.07 | 1.13 | 0.8842 | 0.9439 | 3.15 | 3.16 | 3.39 | 0.85 | 0.7453 | 0.8832 |
| TC0100015368.hg.1 | NGF | nerve growth factor (beta polypeptide) | Coding | 3.06 | 2.78 | 2.89 | 1.13 | 0.7885 | 0.8917 | 3.2 | 3.29 | 3.46 | 0.84 | 0.5041 | 0.7361 |
| TC0100015599.hg.1 | HFE2 | hemochromatosis type 2 (juvenile) | Coding | 3.12 | 2.98 | 2.95 | 1.13 | 0.7989 | 0.8967 | 3.19 | 3.03 | 3.07 | 1.09 | 0.5447 | 0.7627 |
| TC0100015950.hg.1 | FAM189B | family with sequence similarity 189, member B | Multiple_C | 6.54 | 6.04 | 6.37 | 1.13 | 0.7359 | 0.8621 | 6.67 | 6.73 | 7.19 | 0.70 | 0.0255 | 0.1392 |
| TC0100016339.hg.1 | DPT | dermatopontin | Coding | 3.77 | 3.28 | 3.6 | 1.13 | 0.4979 | 0.6975 | 4.4 | 4.50 | 4.23 | 1.13 | 0.147 | 0.3831 |
| TC0100018492.hg.1 | SPRR2A | small proline-rich protein 2A | Coding | 4.31 | 4.07 | 4.14 | 1.13 | 0.3658 | 0.5794 | 4.33 | 4.13 | 4.22 | 1.08 | 0.4183 | 0.6721 |
| TC0100018511.hg.1 | C1orf61 | chromosome 1 open reading frame 61 | Multiple_C | 4.3 | 3.98 | 4.13 | 1.13 | 0.3305 | 0.5445 | 5.41 | 5.60 | 5.08 | 1.26 | 0.1187 | 0.3408 |
| TC0200009464.hg.1 | THSD7B | thrombospondin type 1 domain containing 7B | Multiple_C | 4.99 | 4.95 | 4.82 | 1.13 | 0.6787 | 0.8255 | 5.2 | 5.21 | 5.03 | 1.13 | 0.5789 | 0.7844 |
| TC0200015529.hg.1 | MDH1B | malate dehydrogenase 1B | Multiple_C | 5.31 | 5.57 | 5.14 | 1.13 | 0.6089 | 0.7795 | 5.13 | 5.04 | 4.82 | 1.24 | 0.4606 | 0.7039 |
| TC0200015670.hg.1 | MARCHF4 | membrane associated ring finger 4 | Multiple_C | 3.11 | 2.82 | 2.94 | 1.13 | 0.418 | 0.6296 | 3.45 | 3.38 | 3.28 | 1.13 | 0.2937 | 0.5586 |
| TC0200015764.hg.1 | CNPPD1 | cyclin Pas1/PHO80 domain containing 1 | Multiple_C | 8.99 | 8.02 | 8.82 | 1.13 | 0.8311 | 0.9158 | 8.18 | 8.53 | 9.42 | 0.42 | 3.83E-05 | 0.0018 |

| | | | | | | | | | | | | | | | |
|---|---|---|---|---|---|---|---|---|---|---|---|---|---|---|---|
| TC0200016552.hg.1 | UPP2 | uridine phosphorylase 2 | Multiple_C | 2.73 | 2.63 | 2.56 | 1.13 | 0.1805 | 0.3647 | 2.99 | 2.98 | 3.02 | 0.98 | 0.4398 | 0.6898 |
| TC0300006565.hg.1 | IL17RC | interleukin 17 receptor C | Multiple_C | 8.58 | 8.64 | 8.41 | 1.13 | 0.5269 | 0.7187 | 7.26 | 7.19 | 6.79 | 1.39 | 0.9075 | 0.9617 |
| TC0300007438.hg.1 | RBM15B | RNA binding motif protein 15B | Coding | 9.97 | 9.56 | 9.8 | 1.13 | 0.3832 | 0.5963 | 10.22 | 10.52 | 9.83 | 1.31 | 0.7289 | 0.8742 |
| TC0300010762.hg.1 | SCN10A | sodium channel, voltage gated, type X alpha subunit | Coding | 3.15 | 2.76 | 2.98 | 1.13 | 0.8223 | 0.9107 | 3.91 | 3.99 | 3.77 | 1.10 | 0.313 | 0.5771 |
| TC0300010855.hg.1 | CYP8B1 | cytochrome P450, family 8, subfamily B, polypeptide 1 | Coding | 3.48 | 3.32 | 3.31 | 1.13 | 0.7087 | 0.8453 | 3.79 | 3.77 | 3.67 | 1.09 | 0.2497 | 0.5142 |
| TC0300010960.hg.1 | PRSS42 | protease, serine 42 | Coding | 4.17 | 4.04 | 4 | 1.13 | 0.1005 | 0.2418 | 4.53 | 4.40 | 4.33 | 1.15 | 0.3029 | 0.5671 |
| TC0300012282.hg.1 | UROC1 | urocanate hydratase 1 | Coding | 4.69 | 4.19 | 4.52 | 1.13 | 0.9266 | 0.9656 | 4.47 | 4.50 | 4.68 | 0.86 | 0.5309 | 0.7539 |
| TC0300012566.hg.1 | A4GNT | alpha-1,4-N-acetylglucosaminyltransferase | Coding | 3.4 | 3.35 | 3.23 | 1.13 | 0.194 | 0.3834 | 3.21 | 3.07 | 3.17 | 1.03 | 0.6006 | 0.7997 |
| TC0300012764.hg.1 | HLTF | helicase-like transcription factor | Multiple_C | 2.87 | 2.65 | 2.7 | 1.13 | 0.4636 | 0.6686 | 9.8 | 8.48 | 8.42 | 2.60 | 0.0001 | 0.0042 |
| TC0400007276.hg.1 | KLHL5 | kelch-like family member 5 | Multiple_C | 7.68 | 8.09 | 7.51 | 1.13 | 0.7351 | 0.8615 | 7.74 | 6.67 | 6.47 | 2.41 | 0.0359 | 0.1718 |
| TC0400007749.hg.1 | UGT2B7 | UDP glucuronosyltransferase 2 family, polypeptide B7 | Multiple_C | 3.33 | 3.2 | 3.16 | 1.13 | 0.5616 | 0.7439 | 3.59 | 4.14 | 3.57 | 1.01 | 0.9483 | 0.979 |
| TC0400009048.hg.1 | TLR2 | toll-like receptor 2 | Multiple_C | 2.85 | 2.53 | 2.68 | 1.13 | 0.8617 | 0.9324 | 3.76 | 3.82 | 3.96 | 0.87 | 0.5784 | 0.7843 |
| TC0400012011.hg.1 | FREM3 | FRAS1 related extracellular matrix 3 | Multiple_C | 3.46 | 3.41 | 3.29 | 1.13 | 0.4605 | 0.6663 | 3.53 | 3.91 | 3.7 | 0.89 | 0.4503 | 0.6962 |
| TC0500007376.hg.1 | FST | follistatin | Multiple_C | 4.48 | 4.24 | 4.31 | 1.13 | 0.6797 | 0.8259 | 4.17 | 3.72 | 3.99 | 1.13 | 0.4607 | 0.7039 |
| TC0500008872.hg.1 | PCDHB1 | protocadherin beta 1 | Coding | 3.22 | 2.99 | 3.05 | 1.13 | 0.4441 | 0.6525 | 3.34 | 3.45 | 3.36 | 0.99 | 0.7006 | 0.858 |
| TC0500009356.hg.1 | FBLL1 | fibrillarin-like 1 | Multiple_C | 4.78 | 4.34 | 4.61 | 1.13 | 0.2295 | 0.4293 | 4.31 | 4.51 | 4.5 | 0.88 | 0.378 | 0.638 |
| TC0500009490.hg.1 | BNIP1 | BCL2/adenovirus E1B 19kDa interacting protein 1 | Coding | 7.93 | 8.48 | 7.76 | 1.13 | 0.3184 | 0.532 | 7.03 | 6.93 | 7.7 | 0.63 | 0.0026 | 0.0325 |
| TC0500009671.hg.1 | ZFP2 | ZFP2 zinc finger protein | Coding | 4.6 | 5.34 | 4.43 | 1.13 | 0.1675 | 0.347 | 3.79 | 3.94 | 3.86 | 0.95 | 0.9126 | 0.9642 |
| TC0500009772.hg.1 | OR4F3 | olfactory receptor, family 4, subfamily F, member 3 | Coding | 4.72 | 4.49 | 4.55 | 1.13 | 0.8728 | 0.9383 | 3.96 | 4.47 | 3.94 | 1.01 | 0.136 | 0.3666 |
| TC0500010282.hg.1 | CDH12 | cadherin 12, type 2 (N-cadherin 2) | Multiple_C | 4.17 | 3.9 | 4 | 1.13 | 0.4934 | 0.6933 | 4.57 | 4.72 | 4.29 | 1.21 | 0.0468 | 0.2004 |
| TC0500012514.hg.1 | FAT2 | FAT atypical cadherin 2 | Multiple_C | 4.11 | 3.64 | 3.94 | 1.13 | 0.5673 | 0.7479 | 4.12 | 3.92 | 3.93 | 1.14 | 0.2636 | 0.5282 |
| TC0500012523.hg.1 | ATOX1 | antioxidant 1 copper chaperone | Multiple_C | 8.81 | 8.1 | 8.64 | 1.13 | 0.3395 | 0.5535 | 6.39 | 6.50 | 6.27 | 1.09 | 0.0762 | 0.2656 |
| TC0500012525.hg.1 | RP11-54C4.3; | uncharacterized protein LOC100652758 [Source:RefSeq peptid | Multiple_C | 5.68 | 5.82 | 5.51 | 1.13 | 0.4175 | 0.6292 | 5.04 | 4.95 | 4.74 | 1.23 | 0.5425 | 0.7614 |
| TC0500013258.hg.1 | ARHGEF37 | Rho guanine nucleotide exchange factor 37 | Multiple_C | 2.87 | 2.49 | 2.7 | 1.13 | 0.2931 | 0.5044 | 3.27 | 3.46 | 3.01 | 1.20 | 0.0533 | 0.2157 |
| TC0600007530.hg.1 | HLA-E | major histocompatibility complex, class I, E | Multiple_C | 7.86 | 8.47 | 7.69 | 1.13 | 0.7723 | 0.8825 | 6.51 | 6.19 | 5.53 | 1.97 | 0.004 | 0.0427 |
| TC0600007821.hg.1 | LHFPL5 | lipoma HMGIC fusion partner-like 5 | Multiple_C | 4.92 | 4.51 | 4.75 | 1.13 | 0.3942 | 0.6079 | 4.84 | 4.92 | 4.98 | 0.91 | 0.8486 | 0.9346 |
| TC0600008229.hg.1 | TFAP2D | transcription factor AP-2 delta (activating enhancer binding pro | Multiple_C | 4.24 | 4.01 | 4.07 | 1.13 | 0.3153 | 0.5285 | 3.51 | 3.52 | 3.51 | 1.00 | 0.5505 | 0.7664 |
| TC0600008897.hg.1 | POU3F2 | POU class 3 homeobox 2 | Coding | 2.81 | 2.64 | 2.64 | 1.13 | 0.5289 | 0.7199 | 3.68 | 4.22 | 3.48 | 1.15 | 0.3804 | 0.64 |
| TC0600014273.hg.1 | HLA-DRB1 | major histocompatibility complex, class II, DR beta 1 | Multiple_C | 3.44 | 3.15 | 3.27 | 1.13 | 0.4877 | 0.6888 | 3.86 | 3.74 | 3.48 | 1.30 | 0.0489 | 0.2057 |
| TC0700006690.hg.1 | NXPH1 | neurexophilin 1 | Multiple_C | 3.19 | 2.98 | 3.02 | 1.13 | 0.3516 | 0.5643 | 3.44 | 3.39 | 3.59 | 0.90 | 0.2613 | 0.5256 |
| TC0700007471.hg.1 | LINC00525 | long intergenic non-protein coding RNA 525 | Multiple_C | 4.53 | 4.18 | 4.36 | 1.13 | 0.6791 | 0.8257 | 5.1 | 5.43 | 4.86 | 1.18 | 0.4413 | 0.6905 |
| TC0700008149.hg.1 | PHTF2 | putative homeodomain transcription factor 2 | Multiple_C | 9.4 | 10.14 | 9.23 | 1.13 | 0.5704 | 0.7504 | 8.43 | 7.23 | 8.18 | 1.19 | 0.4192 | 0.6729 |
| TC0700008506.hg.1 | CYP3A43 | cytochrome P450, family 3, subfamily A, polypeptide 43 | Multiple_C | 3.51 | 4.03 | 3.34 | 1.13 | 0.2054 | 0.3983 | 3.59 | 3.50 | 3.44 | 1.11 | 0.7741 | 0.8985 |

| | | | | | | | | | | | | | | | |
|---|---|---|---|---|---|---|---|---|---|---|---|---|---|---|---|
| TC0700010398.hg.1 | TMEM196 | transmembrane protein 196 | Coding | 5.4 | 4.86 | 5.23 | 1.13 | 0.7868 | 0.8908 | 4.7 | 4.44 | 4.5 | 1.15 | 0.2557 | 0.5204 |
| TC0700011202.hg.1 | ZNF479 | zinc finger protein 479 | Coding | 4.12 | 3.98 | 3.95 | 1.13 | 0.8845 | 0.944 | 4.11 | 4.11 | 3.97 | 1.10 | 0.7187 | 0.8689 |
| TC0700011603.hg.1 | MAGI2 | membrane associated guanylate kinase, WW and PDZ domain c | Multiple_C | 3.45 | 2.97 | 3.28 | 1.13 | 0.9609 | 0.9814 | 3.61 | 3.49 | 3.85 | 0.85 | 0.2026 | 0.4582 |
| TC0700012996.hg.1 | ZNF746 | zinc finger protein 746 | Multiple_C | 7.91 | 7.52 | 7.74 | 1.13 | 0.9932 | 0.9965 | 8.53 | 9.20 | 9.31 | 0.58 | 0.0368 | 0.1746 |
| TC0800006606.hg.1 | PRR23D2; PRR | proline rich 23 domain containing 2; proline rich 23 domain con | Coding | 3.7 | 2.99 | 3.53 | 1.13 | 0.6529 | 0.808 | 3.57 | 3.60 | 3.7 | 0.91 | 0.4509 | 0.6963 |
| TC0800007042.hg.1 | ADAMDEC1 | ADAM-like, decysin 1 | Multiple_C | 3.92 | 3.63 | 3.75 | 1.13 | 0.0894 | 0.2218 | 3.76 | 3.89 | 3.58 | 1.13 | 0.3216 | 0.5855 |
| TC0800010746.hg.1 | PRDM14 | PR domain containing 14 | Multiple_C | 3.62 | 3.47 | 3.45 | 1.13 | 0.1525 | 0.326 | 4.05 | 4.23 | 4.05 | 1.00 | 0.9016 | 0.9591 |
| TC0800012323.hg.1 | CA13 | carbonic anhydrase XIII | Multiple_C | 8.91 | 8.44 | 8.74 | 1.13 | 0.5241 | 0.7165 | 4.24 | 4.28 | 3.95 | 1.22 | 0.3041 | 0.5679 |
| TC0900007854.hg.1 | C9orf47; S1PR | chromosome 9 open reading frame 47; sphingosine-1-phosphat | Multiple_C | 4.02 | 3.81 | 3.85 | 1.13 | 0.672 | 0.8212 | 4.02 | 4.27 | 3.95 | 1.05 | 0.8 | 0.9105 |
| TC0900009946.hg.1 | SIT1 | signaling threshold regulating transmembrane adaptor 1 | Multiple_C | 3.13 | 3.07 | 2.96 | 1.13 | 0.6613 | 0.8143 | 5.01 | 5.72 | 4.53 | 1.39 | 0.0879 | 0.2864 |
| TC0900009953.hg.1 | MSMP | microseminoprotein, prostate associated | Multiple_C | 4.41 | 4.08 | 4.24 | 1.13 | 0.2479 | 0.4517 | 4.18 | 4.50 | 4.51 | 0.80 | 0.073 | 0.259 |
| TC0900009956.hg.1 | SPAG8 | sperm associated antigen 8 | Multiple_C | 3.15 | 2.87 | 2.98 | 1.13 | 0.2229 | 0.4207 | 3.87 | 3.89 | 3.83 | 1.03 | 0.9294 | 0.9702 |
| TC0900011065.hg.1 | OR13C3 | olfactory receptor, family 13, subfamily C, member 3 | Coding | 3.07 | 2.93 | 2.9 | 1.13 | 0.8273 | 0.9133 | 3.48 | 3.28 | 3.43 | 1.04 | 0.1002 | 0.3094 |
| TC0900011163.hg.1 | PTPN3 | protein tyrosine phosphatase, non-receptor type 3 | Multiple_C | 10.25 | 9.88 | 10.08 | 1.13 | 0.8494 | 0.9261 | 9.37 | 8.78 | 9.21 | 1.12 | 0.0762 | 0.2656 |
| TC0900011442.hg.1 | OR1L8 | olfactory receptor, family 1, subfamily L, member 8 | Coding | 3.56 | 3.46 | 3.39 | 1.13 | 0.5987 | 0.7714 | 4.35 | 4.19 | 3.99 | 1.28 | 0.2343 | 0.4969 |
| TC0900011979.hg.1 | INPP5E | inositol polyphosphate-5-phosphatase E | Coding | 4.87 | 4.95 | 4.7 | 1.13 | 0.4571 | 0.6633 | 5.03 | 5.39 | 4.87 | 1.12 | 0.3976 | 0.6552 |
| TC0900012294.hg.1 | TPRN | taperin | Multiple_C | 5.73 | 6 | 5.56 | 1.13 | 0.8238 | 0.9114 | 4.16 | 4.81 | 4.59 | 0.74 | 0.1372 | 0.368 |
| TC0X00007368.hg.1 | TRO | trophinin | Multiple_C | 2.85 | 2.29 | 2.68 | 1.13 | 0.3749 | 0.5875 | 3.62 | 3.47 | 3.72 | 0.93 | 0.7975 | 0.9093 |
| TC0X00007790.hg.1 | DACH2 | dachshund family transcription factor 2 | Coding | 3.93 | 4.12 | 3.76 | 1.13 | 0.4839 | 0.6854 | 3.8 | 3.92 | 3.28 | 1.43 | 0.0357 | 0.1712 |
| TC0X00007951.hg.1 | ARMCX3 | armadillo repeat containing, X-linked 3 | Multiple_C | 3.58 | 3.17 | 3.41 | 1.13 | 0.6217 | 0.7884 | 7.64 | 7.41 | 8.25 | 0.66 | 0.8891 | 0.9525 |
| TC0X00008080.hg.1 | VSIG1 | V-set and immunoglobulin domain containing 1 | Multiple_C | 3.93 | 4.02 | 3.76 | 1.13 | 0.6619 | 0.8146 | 5.61 | 4.72 | 6.79 | 0.44 | 0.0002 | 0.0063 |
| TC0X00008754.hg.1 | CSAG3 | CSAG family, member 3 | Multiple_C | 6.23 | 5.81 | 6.06 | 1.13 | 0.3959 | 0.6094 | 5.83 | 5.87 | 5.88 | 0.97 | 0.4919 | 0.7268 |
| TC0X00010434.hg.1 | SLC25A53 | solute carrier family 25, member 53 | Multiple_C | 3.31 | 3.24 | 3.14 | 1.13 | 0.5371 | 0.7256 | 5 | 5.32 | 5.41 | 0.75 | 0.0764 | 0.2661 |
| TC0X00010448.hg.1 | TEX13A | testis expressed 13A | Coding | 4.58 | 4.16 | 4.41 | 1.13 | 0.2829 | 0.4929 | 4.72 | 4.54 | 4.7 | 1.01 | 0.6607 | 0.8345 |
| TC0X00011014.hg.1 | SPANXN2 | SPANX family, member N2 | Coding | 3.68 | 3.37 | 3.51 | 1.13 | 0.2937 | 0.505 | 3.62 | 3.85 | 3.45 | 1.13 | 0.2425 | 0.5061 |
| TC0X00011291.hg.1 | PAGE2 | P antigen family, member 2 (prostate associated) | Coding | 3.38 | 3.16 | 3.21 | 1.13 | 0.1922 | 0.3809 | 3.62 | 3.66 | 3.47 | 1.11 | 0.2942 | 0.5591 |
| TC0X00011319.hg.1 | TMSB15B | thymosin beta 15B | Coding | 3.23 | 3.17 | 3.06 | 1.13 | 0.8542 | 0.9288 | 4.37 | 4.75 | 4.63 | 0.84 | 0.1461 | 0.3815 |
| TC0Y00007117.hg.1 | CDY2B; CDY2A | chromodomain protein, Y-linked, 2B; chromodomain protein, Y- | Coding | 4.27 | 3.92 | 4.1 | 1.13 | 0.3728 | 0.5853 | 3.86 | 4.34 | 4.18 | 0.80 | 0.1692 | 0.4151 |
| TC1000009451.hg.1 | UTF1 | undifferentiated embryonic cell transcription factor 1 | Coding | 4.04 | 3.94 | 3.87 | 1.13 | 0.2805 | 0.4903 | 4.23 | 4.19 | 4.34 | 0.93 | 0.7656 | 0.8946 |
| TC1000010516.hg.1 | MSMB | microseminoprotein, beta- | Multiple_C | 3.32 | 3.11 | 3.15 | 1.13 | 0.5392 | 0.7272 | 3.73 | 3.48 | 3.19 | 1.45 | 0.0258 | 0.14 |
| TC1000010562.hg.1 | SYT15; ABC7-4 | Homo sapiens synaptotagmin XV (SYT15), transcript variant a, m | Coding | 4.75 | 4.52 | 4.58 | 1.13 | 0.7383 | 0.864 | 4.97 | 4.78 | 4.55 | 1.34 | 0.1355 | 0.3656 |
| TC1000011825.hg.1 | BBIP1 | BBSome interacting protein 1 | Multiple_C | 9.63 | 10.89 | 9.46 | 1.13 | 0.7987 | 0.8966 | 9.94 | 9.63 | 9.41 | 1.44 | 0.018 | 0.1127 |
| TC1100006717.hg.1 | OR56B4 | olfactory receptor, family 56, subfamily B, member 4 | Coding | 4.25 | 3.91 | 4.08 | 1.13 | 0.937 | 0.9708 | 4.12 | 4.04 | 4.21 | 0.94 | 0.4417 | 0.6907 |

| | | | | | | | | | | | | | | | |
|---|---|---|---|---|---|---|---|---|---|---|---|---|---|---|---|
| TC1100007996.hg.1 | ZFPL1 | zinc finger protein-like 1 | Multiple_C | 10.14 | 9.35 | 9.97 | 1.13 | 0.5718 | 0.7513 | 10.16 | 10.60 | 10.38 | 0.86 | 0.4038 | 0.6596 |
| TC1100009203.hg.1 | UBE4A | ubiquitination factor E4A | Multiple_C | 11.75 | 10.58 | 11.58 | 1.13 | 0.2241 | 0.4226 | 12.88 | 12.57 | 12.7 | 1.13 | 0.2782 | 0.5443 |
| TC1100010472.hg.1 | CCDC73 | coiled-coil domain containing 73 | Multiple_C | 4.83 | 4.79 | 4.66 | 1.13 | 0.2943 | 0.5058 | 4.74 | 4.50 | 4.72 | 1.01 | 0.3519 | 0.6153 |
| TC1100011288.hg.1 | MRPL11 | mitochondrial ribosomal protein L11 | Multiple_C | 10.93 | 11.08 | 10.76 | 1.13 | 0.7507 | 0.8719 | 9.97 | 10.61 | 10.09 | 0.92 | 0.114 | 0.333 |
| TC1100011294.hg.1 | CTSF | cathepsin F | Multiple_C | 3.24 | 3.07 | 3.07 | 1.13 | 0.6272 | 0.792 | 4.3 | 4.10 | 3.95 | 1.27 | 0.0778 | 0.2686 |
| TC1100013132.hg.1 | INS; INS-IGF2 | insulin; INS-IGF2 readthrough | Multiple_C | 3.65 | 3.46 | 3.48 | 1.13 | 0.4781 | 0.6804 | 3.74 | 3.55 | 3.56 | 1.13 | 0.1911 | 0.4441 |
| TC1100013221.hg.1 | CASP1 | caspase 1 | Multiple_C | 3.64 | 3.24 | 3.47 | 1.13 | 0.7063 | 0.8438 | 3.84 | 3.79 | 3.68 | 1.12 | 0.8725 | 0.9451 |
| TC1100013223.hg.1 | CARD17 | caspase recruitment domain family, member 17 | Coding | 3.91 | 3.65 | 3.74 | 1.13 | 0.3998 | 0.6133 | 4.06 | 4.11 | 3.99 | 1.05 | 0.8457 | 0.9325 |
| TC1200006475.hg.1 | LRTM2 | leucine-rich repeats and transmembrane domains 2 | Multiple_C | 5.82 | 5.41 | 5.65 | 1.13 | 0.2187 | 0.4155 | 4.76 | 4.39 | 4.23 | 1.44 | 0.0577 | 0.2263 |
| TC1200007523.hg.1 | OR8S1 | olfactory receptor, family 8, subfamily S, member 1 | Multiple_C | 3.06 | 2.93 | 2.89 | 1.13 | 0.4749 | 0.6778 | 3.74 | 3.44 | 3.41 | 1.26 | 0.1993 | 0.4546 |
| TC1200007690.hg.1 | TNS2 | tensin 2 | Multiple_C | 3.55 | 3.06 | 3.38 | 1.13 | 0.5324 | 0.7221 | 4.78 | 4.95 | 4.51 | 1.21 | 0.3261 | 0.5901 |
| TC1200009912.hg.1 | STYK1 | serine/threonine/tyrosine kinase 1 | Coding | 6.15 | 6.24 | 5.98 | 1.13 | 0.2413 | 0.4436 | 3.84 | 3.32 | 3.13 | 1.64 | 0.022 | 0.1274 |
| TC1200010062.hg.1 | RERGL | RERG/RAS-like | Multiple_C | 3.45 | 3.06 | 3.28 | 1.13 | 0.1597 | 0.3362 | 3.39 | 3.94 | 3.7 | 0.81 | 0.1118 | 0.3294 |
| TC1200010475.hg.1 | ADAMTS20 | ADAM metallopeptidase with thrombospondin type 1 motif 20 | Multiple_C | 3.32 | 3.43 | 3.15 | 1.13 | 0.4677 | 0.6721 | 4.58 | 4.74 | 4.5 | 1.06 | 0.2043 | 0.4603 |
| TC1200010846.hg.1 | DCD | dermcidin | Coding | 3.33 | 2.9 | 3.16 | 1.13 | 0.5803 | 0.7578 | 3.37 | 3.61 | 3.74 | 0.77 | 0.9037 | 0.9603 |
| TC1200012247.hg.1 | HCAR2 | hydroxycarboxylic acid receptor 2 | Coding | 3.69 | 3.36 | 3.52 | 1.13 | 0.8118 | 0.9049 | 4.16 | 4.27 | 4.24 | 0.95 | 0.247 | 0.5117 |
| TC1200012561.hg.1 | ZNF891 | zinc finger protein 891 | Multiple_C | 7 | 7.32 | 6.83 | 1.13 | 0.181 | 0.3654 | 4.77 | 4.70 | 4.6 | 1.13 | 0.2041 | 0.4601 |
| TC1200012698.hg.1 | CHPT1 | choline phosphotransferase 1 | Multiple_C | 12.14 | 12.91 | 11.97 | 1.13 | 0.7542 | 0.8741 | 7.76 | 7.87 | 8.19 | 0.74 | 0.0805 | 0.2734 |
| TC1200012877.hg.1 | ZNF605 | zinc finger protein 605 | Multiple_C | 7.79 | 8.3 | 7.62 | 1.13 | 0.6647 | 0.8167 | 5.85 | 5.62 | 5.63 | 1.16 | 0.4922 | 0.727 |
| TC1300008149.hg.1 | GRK1 | G protein-coupled receptor kinase 1 | Multiple_C | 4.13 | 3.98 | 3.96 | 1.13 | 0.1298 | 0.2908 | 4.2 | 4.21 | 4.09 | 1.08 | 0.8785 | 0.9479 |
| TC1300008602.hg.1 | MAB21L1 | mab-21-like 1 (C. elegans) | Multiple_C | 3.16 | 2.87 | 2.99 | 1.13 | 0.5343 | 0.7237 | 3.49 | 3.31 | 3.35 | 1.10 | 0.1766 | 0.4252 |
| TC1300009319.hg.1 | POU4F1 | POU class 4 homeobox 1 | Coding | 4.3 | 3.69 | 4.13 | 1.13 | 0.5745 | 0.7535 | 5.27 | 4.98 | 4.49 | 1.72 | 0.2091 | 0.4664 |
| TC1400010765.hg.1 | RDH11 | retinol dehydrogenase 11 (all-trans/9-cis/11-cis) | Multiple_C | 12.32 | 12.8 | 12.15 | 1.13 | 0.51 | 0.706 | 9.33 | 9.25 | 9.57 | 0.85 | 0.0833 | 0.2778 |
| TC1500006920.hg.1 | C15orf54 | chromosome 15 open reading frame 54 | Multiple_C | 4.19 | 3.84 | 4.02 | 1.13 | 0.1672 | 0.3467 | 4.42 | 4.43 | 4.31 | 1.08 | 0.6448 | 0.8257 |
| TC1500008154.hg.1 | ZSCAN2 | zinc finger and SCAN domain containing 2 | Multiple_C | 6.86 | 7.04 | 6.69 | 1.13 | 0.9652 | 0.9831 | 6.62 | 6.60 | 6.23 | 1.31 | 0.5821 | 0.7867 |
| TC1500008900.hg.1 | LOC283710; R | uncharacterized LOC283710; novel transcript; Transcript Identif | Multiple_C | 3.96 | 3.51 | 3.79 | 1.13 | 0.8037 | 0.8997 | 4.45 | 3.89 | 4.04 | 1.33 | 0.286 | 0.551 |
| TC1500009216.hg.1 | TGM7 | transglutaminase 7 | Multiple_C | 3.24 | 3.08 | 3.07 | 1.13 | 0.3698 | 0.5825 | 3.75 | 3.50 | 3.45 | 1.23 | 0.1814 | 0.4316 |
| TC1600007215.hg.1 | SCNN1G | sodium channel, non voltage gated 1 gamma subunit | Coding | 2.87 | 2.43 | 2.7 | 1.13 | 0.629 | 0.793 | 3.13 | 3.09 | 3.04 | 1.06 | 0.2094 | 0.4664 |
| TC1600007784.hg.1 | C16orf78 | chromosome 16 open reading frame 78 | Coding | 3.76 | 3.14 | 3.59 | 1.13 | 0.8343 | 0.9172 | 3.96 | 4.13 | 3.79 | 1.13 | 0.5981 | 0.7978 |
| TC1600008867.hg.1 | LOC400558; R | uncharacterized LOC400558; Transcript Identified by AceView, E | Multiple_C | 5.59 | 5.21 | 5.42 | 1.13 | 0.4583 | 0.6645 | 5 | 5.15 | 5.1 | 0.93 | 0.3625 | 0.6254 |
| TC1600008986.hg.1 | GNG13 | guanine nucleotide binding protein (G protein), gamma 13 | Coding | 4.51 | 4.37 | 4.34 | 1.13 | 0.5024 | 0.7004 | 4.85 | 4.73 | 4.58 | 1.21 | 0.4921 | 0.727 |
| TC1600009066.hg.1 | HAGH | hydroxyacylglutathione hydrolase | Multiple_C | 10.31 | 10.53 | 10.14 | 1.13 | 0.5075 | 0.7041 | 8.74 | 8.73 | 8.02 | 1.65 | 0.0078 | 0.0661 |
| TC1600011517.hg.1 | DOC2A | double C2-like domains, alpha | Multiple_C | 8.07 | 7.73 | 7.9 | 1.13 | 0.321 | 0.5348 | 4.86 | 4.54 | 4.77 | 1.06 | 0.3315 | 0.5955 |

| | | | | | | | | | | | | | | | |
|---|---|---|---|---|---|---|---|---|---|---|---|---|---|---|---|
| TC1700006768.hg.1 | EFNB3 | ephrin-B3 | Coding | 3.64 | 3.37 | 3.47 | 1.13 | 0.6648 | 0.8168 | 4.47 | 4.41 | 4.06 | 1.33 | 0.038 | 0.1775 |
| TC1700008282.hg.1 | NME1; NME1- | NME/NM23 nucleoside diphosphate kinase 1; NME1-NME2 rea | Multiple_C | 13.94 | 14.73 | 13.77 | 1.13 | 0.2242 | 0.4227 | 11.99 | 12.19 | 12.22 | 0.85 | 0.9493 | 0.9793 |
| TC1700010371.hg.1 | ASIC2 | acid sensing ion channel 2 | Multiple_C | 4.87 | 4.73 | 4.7 | 1.13 | 0.0422 | 0.1278 | 4.18 | 4.26 | 4.45 | 0.83 | 0.5275 | 0.7514 |
| TC1700010447.hg.1 | CCL5 | chemokine (C-C motif) ligand 5 | Coding | 4.05 | 3.55 | 3.88 | 1.13 | 0.06 | 0.1656 | 4.48 | 4.23 | 3.94 | 1.45 | 0.0275 | 0.1452 |
| TC1700010653.hg.1 | KRTAP2-3 | keratin associated protein 2-3 | Coding | 6.16 | 6.07 | 5.99 | 1.13 | 0.3159 | 0.5292 | 8.89 | 8.19 | 8.06 | 1.78 | 0.0654 | 0.2435 |
| TC1700011319.hg.1 | APPBP2 | amyloid beta precursor protein (cytoplasmic tail) binding protei | Multiple_C | 8.94 | 9.39 | 8.77 | 1.13 | 0.7304 | 0.859 | 8.87 | 8.39 | 8.42 | 1.37 | 0.0985 | 0.3065 |
| TC1700011723.hg.1 | USH1G | Usher syndrome 1G (autosomal recessive) | Coding | 3.38 | 3.22 | 3.21 | 1.13 | 0.559 | 0.7417 | 4.29 | 4.26 | 3.8 | 1.40 | 0.0061 | 0.0558 |
| TC1700012357.hg.1 | DHRS7C | dehydrogenase/reductase (SDR family) member 7C | Multiple_C | 3.83 | 3.68 | 3.66 | 1.13 | 0.523 | 0.7159 | 4 | 3.87 | 3.93 | 1.05 | 0.5973 | 0.7974 |
| TC1800006583.hg.1 | LRRC30 | leucine rich repeat containing 30 | Coding | 3.97 | 3.5 | 3.8 | 1.13 | 0.5356 | 0.7246 | 3.83 | 3.83 | 4 | 0.89 | 0.9517 | 0.9799 |
| TC1900006492.hg.1 | PRTN3 | proteinase 3 | Multiple_C | 4.08 | 3.8 | 3.91 | 1.13 | 0.3524 | 0.5652 | 4.91 | 4.90 | 4.8 | 1.08 | 0.3082 | 0.5723 |
| TC1900006757.hg.1 | ZNRF4 | zinc and ring finger 4 | Coding | 3.4 | 3.56 | 3.23 | 1.13 | 0.5137 | 0.7089 | 4.19 | 3.77 | 3.96 | 1.17 | 0.2767 | 0.5428 |
| TC1900006932.hg.1 | ZNF559-ZNF1 | ZNF559-ZNF177 readthrough; zinc finger protein 177; zinc finge | Multiple_C | 7.88 | 8.06 | 7.71 | 1.13 | 0.8195 | 0.9093 | 7.9 | 7.72 | 8.05 | 0.90 | 0.2257 | 0.4863 |
| TC1900007458.hg.1 | ZNF93 | zinc finger protein 93 | Multiple_C | 8.2 | 8.1 | 8.03 | 1.13 | 0.5795 | 0.7571 | 6.67 | 6.61 | 7.07 | 0.76 | 0.2722 | 0.5378 |
| TC1900007853.hg.1 | GPR42 | G protein-coupled receptor 42 (gene/pseudogene) | Coding | 3.67 | 3.61 | 3.5 | 1.13 | 0.4017 | 0.6147 | 3.87 | 4.13 | 3.97 | 0.93 | 0.6324 | 0.819 |
| TC1900007870.hg.1 | ETV2 | ets variant 2 | Coding | 4.65 | 4.32 | 4.48 | 1.13 | 0.5876 | 0.7639 | 4.58 | 4.87 | 4.66 | 0.95 | 0.6273 | 0.8158 |
| TC1900009022.hg.1 | ZNF135 | zinc finger protein 135 | Multiple_C | 3.54 | 3.28 | 3.37 | 1.13 | 0.2903 | 0.5012 | 3.71 | 4.12 | 4.02 | 0.81 | 0.4588 | 0.7024 |
| TC1900009287.hg.1 | SMIM24 | small integral membrane protein 24 | Multiple_C | 5.62 | 5.51 | 5.45 | 1.13 | 0.1446 | 0.3142 | 4.83 | 4.73 | 4.53 | 1.23 | 0.255 | 0.5194 |
| TC1900009311.hg.1 | ZFR2 | zinc finger RNA binding protein 2 | Multiple_C | 2.75 | 2.53 | 2.58 | 1.13 | 0.5065 | 0.7035 | 3.08 | 3.06 | 2.78 | 1.23 | 0.0909 | 0.2916 |
| TC1900009447.hg.1 | SH2D3A | SH2 domain containing 3A | Multiple_C | 7.32 | 7.07 | 7.15 | 1.13 | 0.9444 | 0.9745 | 5.83 | 5.67 | 5.5 | 1.26 | 0.1914 | 0.4444 |
| TC1900010158.hg.1 | ZNF100 | zinc finger protein 100 | Multiple_C | 11.58 | 10.97 | 11.41 | 1.13 | 0.8955 | 0.9501 | 11.35 | 11.44 | 11.74 | 0.76 | 0.2894 | 0.5542 |
| TC1900010782.hg.1 | ATP1A3 | ATPase, Na+/K+ transporting, alpha 3 polypeptide | Multiple_C | 8.52 | 8.3 | 8.35 | 1.13 | 0.6098 | 0.7801 | 6.68 | 6.27 | 6.38 | 1.23 | 0.0898 | 0.2897 |
| TC1900010971.hg.1 | IGFL3 | IGF like family member 3 | Multiple_C | 3.71 | 3.47 | 3.54 | 1.13 | 0.4963 | 0.6962 | 3.83 | 3.86 | 3.8 | 1.02 | 0.4343 | 0.6852 |
| TC1900011201.hg.1 | FAM71E1 | family with sequence similarity 71, member E1 | Multiple_C | 6.23 | 6.03 | 6.06 | 1.13 | 0.5828 | 0.7601 | 7.13 | 7.45 | 7.09 | 1.03 | 0.4279 | 0.6801 |
| TC1900011582.hg.1 | ZNF671 | zinc finger protein 671 | Multiple_C | 3.44 | 3.3 | 3.27 | 1.13 | 0.3622 | 0.5756 | 2.88 | 2.88 | 3.08 | 0.87 | 0.6164 | 0.8097 |
| TC1900011596.hg.1 | C19orf18 | chromosome 19 open reading frame 18 | Multiple_C | 3.63 | 3.61 | 3.46 | 1.13 | 0.6027 | 0.7747 | 4.13 | 3.63 | 3.6 | 1.44 | 0.1493 | 0.3869 |
| TC1900011763.hg.1 | APOC4-APOC | APOC4-APOC2 readthrough (NMD candidate); apolipoprotein C | Multiple_C | 6.06 | 5.82 | 5.89 | 1.13 | 0.146 | 0.3161 | 6.24 | 6.94 | 6.44 | 0.87 | 0.6273 | 0.8158 |
| TC1900011964.hg.1 | PSG7 | pregnancy specific beta-1-glycoprotein 7 (gene/pseudogene) | Multiple_C | 3.01 | 2.85 | 2.84 | 1.13 | 0.8837 | 0.9436 | 3.21 | 3.21 | 3.09 | 1.09 | 0.2369 | 0.5 |
| TC2000006852.hg.1 | SLC24A3 | solute carrier family 24 (sodium/potassium/calcium exchanger) | Coding | 3.59 | 3.88 | 3.42 | 1.13 | 0.7532 | 0.8738 | 3.92 | 3.98 | 3.75 | 1.13 | 0.3032 | 0.5675 |
| TC2000008055.hg.1 | ARFGAP1; MI | ADP-ribosylation factor GTPase activating protein 1; microRNA | Multiple_C | 12.71 | 12.13 | 12.54 | 1.13 | 0.4728 | 0.6761 | 14.39 | 14.32 | 14.4 | 0.99 | 0.5184 | 0.7463 |
| TC2000008267.hg.1 | RASSF2 | Ras association (RalGDS/AF-6) domain family member 2 | Multiple_C | 3.98 | 3.84 | 3.81 | 1.13 | 0.3854 | 0.5988 | 5.46 | 5.09 | 4.91 | 1.46 | 0.1586 | 0.4001 |
| TC2000009263.hg.1 | CDH22 | cadherin 22, type 2 | Multiple_C | 5.39 | 4.97 | 5.22 | 1.13 | 0.3226 | 0.5363 | 5.59 | 5.19 | 5.08 | 1.42 | 0.0533 | 0.2158 |
| TC2100008182.hg.1 | DSCAM | Down syndrome cell adhesion molecule | Multiple_C | 3.43 | 3.34 | 3.26 | 1.13 | 0.5431 | 0.7298 | 3.66 | 3.16 | 3.07 | 1.51 | 0.0782 | 0.2693 |
| TC2200007271.hg.1 | NCF4 | neutrophil cytosolic factor 4 | Multiple_C | 2.96 | 2.72 | 2.79 | 1.13 | 0.5907 | 0.7658 | 3.68 | 3.83 | 3.77 | 0.94 | 0.5439 | 0.7622 |

| | | | | | | | | | | | | | | | |
|---|---|---|---|---|---|---|---|---|---|---|---|---|---|---|---|
| TC2200007398.hg.1 | SYNGR1 | synaptogyrin 1 | Multiple_C | 5.67 | 5.39 | 5.5 | 1.13 | 0.8871 | 0.9454 | 6.41 | 5.93 | 5.65 | 1.69 | 0.0239 | 0.1335 |
| TC2200008589.hg.1 | APOL4 | apolipoprotein L, 4 | Multiple_C | 4.15 | 3.57 | 3.98 | 1.13 | 0.568 | 0.7485 | 4.18 | 3.69 | 3.85 | 1.26 | 0.6288 | 0.8169 |
| TC2200009219.hg.1 | IGLL5; MIR557 | immunoglobulin lambda-like polypeptide 5; microRNA 5571; im | Multiple_C | 3.92 | 3.82 | 3.75 | 1.13 | 0.4332 | 0.6432 | 4.19 | 4.09 | 4.25 | 0.96 | 0.3556 | 0.6189 |
| TSUnmapped00000435.h | RPS6KA1 | ribosomal protein S6 kinase, 90kDa, polypeptide 1 | Coding | 6.7 | 6.5 | 6.53 | 1.13 | 0.4599 | 0.6659 | 5.04 | 4.94 | 4.61 | 1.35 | 0.1088 | 0.3247 |
| TSUnmapped00000446.h | INPP5D | inositol polyphosphate-5-phosphatase D | Coding | 4.66 | 4.39 | 4.49 | 1.13 | 0.1022 | 0.2449 | 7.48 | 7.43 | 7.63 | 0.90 | 0.64 | 0.8234 |
| TSUnmapped00000458.h | HHAT | hedgehog acyltransferase | Coding | 3.5 | 3.41 | 3.33 | 1.13 | 0.3212 | 0.5349 | 4.33 | 4.21 | 4.05 | 1.21 | 0.2048 | 0.4611 |
| TC0100008152.hg.1 | UQCRH | ubiquinol-cytochrome c reductase hinge protein | Multiple_C | 13.78 | 14.6 | 13.62 | 1.12 | 0.6713 | 0.8209 | 8.87 | 8.88 | 8.69 | 1.13 | 0.0863 | 0.2833 |
| TC0100008787.hg.1 | ST6GALNAC5 | ST6 (alpha-N-acetyl-neuraminyl-2,3-beta-galactosyl-1,3)-N-acet | Multiple_C | 4.03 | 3.74 | 3.87 | 1.12 | 0.5346 | 0.7239 | 4.28 | 4.24 | 4.2 | 1.06 | 0.1567 | 0.3975 |
| TC0100009406.hg.1 | CHIA | chitinase, acidic | Multiple_C | 3.05 | 2.81 | 2.89 | 1.12 | 0.3877 | 0.6013 | 3.06 | 3.21 | 3.11 | 0.97 | 0.3706 | 0.6327 |
| TC0100010011.hg.1 | LCE6A | late cornified envelope 6A | Coding | 3.9 | 3.76 | 3.74 | 1.12 | 0.4229 | 0.6342 | 3.5 | 3.90 | 3.97 | 0.72 | 0.0676 | 0.2478 |
| TC0100010193.hg.1 | PRCC | papillary renal cell carcinoma (translocation-associated) | Multiple_C | 12.25 | 11.36 | 12.09 | 1.12 | 0.475 | 0.6778 | 11 | 11.29 | 11.78 | 0.58 | 0.0022 | 0.0286 |
| TC0100012341.hg.1 | OR2L5 | olfactory receptor, family 2, subfamily L, member 5 | Coding | 2.68 | 2.54 | 2.52 | 1.12 | 0.4628 | 0.668 | 3.28 | 2.98 | 2.91 | 1.29 | 0.1613 | 0.4041 |
| TC0100012864.hg.1 | C1orf127 | chromosome 1 open reading frame 127 | Multiple_C | 2.93 | 2.86 | 2.77 | 1.12 | 0.2318 | 0.4323 | 3.47 | 3.42 | 3.31 | 1.12 | 0.3164 | 0.5802 |
| TC0100013578.hg.1 | ADGRB2 | adhesion G protein-coupled receptor B2 | Multiple_C | 2.91 | 2.59 | 2.75 | 1.12 | 0.5416 | 0.7288 | 3.29 | 3.06 | 3.07 | 1.16 | 0.3036 | 0.5678 |
| TC0100015200.hg.1 | AMIGO1 | adhesion molecule with Ig-like domain 1 | Coding | 4.43 | 4.37 | 4.27 | 1.12 | 0.5397 | 0.7276 | 3.75 | 3.87 | 3.67 | 1.06 | 0.8731 | 0.9452 |
| TC0100017505.hg.1 | SDE2 | SDE2 telomere maintenance homolog (S. pombe) | Multiple_C | 9.97 | 10.01 | 9.81 | 1.12 | 0.4008 | 0.614 | 10.21 | 9.57 | 10.28 | 0.95 | 0.8471 | 0.9335 |
| TC0100017713.hg.1 | TTC13 | tetratricopeptide repeat domain 13 | Multiple_C | 10.21 | 10.36 | 10.05 | 1.12 | 0.9744 | 0.9874 | 8.69 | 8.68 | 8.7 | 0.99 | 0.9234 | 0.9685 |
| TC0100017926.hg.1 | GREM2 | gremlin 2, DAN family BMP antagonist | Coding | 4.56 | 4.14 | 4.4 | 1.12 | 0.788 | 0.8914 | 3.47 | 3.53 | 3.49 | 0.99 | 0.9134 | 0.9645 |
| TC0100018563.hg.1 | LEFTY1 | left-right determination factor 1 | Multiple_C | 6.27 | 6.05 | 6.11 | 1.12 | 0.4824 | 0.6838 | 6.22 | 6.32 | 6.1 | 1.09 | 0.6114 | 0.8058 |
| TC0200007070.hg.1 | GCKR | glucokinase (hexokinase 4) regulator | Multiple_C | 3.13 | 3.04 | 2.97 | 1.12 | 0.6357 | 0.7974 | 3.09 | 3.29 | 3.37 | 0.82 | 0.2692 | 0.5342 |
| TC0200007075.hg.1 | C2orf16 | chromosome 2 open reading frame 16 | Coding | 3.4 | 2.96 | 3.24 | 1.12 | 0.5104 | 0.7064 | 3.32 | 3.38 | 2.97 | 1.27 | 0.1035 | 0.3154 |
| TC0200007077.hg.1 | SLC4A1AP | solute carrier family 4 (anion exchanger), member 1, adaptor pr | Multiple_C | 10.73 | 10.61 | 10.57 | 1.12 | 0.4758 | 0.6784 | 11.04 | 10.56 | 10.92 | 1.09 | 0.602 | 0.8005 |
| TC0200010701.hg.1 | VWC2L | von Willebrand factor C domain containing protein 2-like | Multiple_C | 3.29 | 3.29 | 3.13 | 1.12 | 0.9305 | 0.9672 | 3.22 | 3.64 | 3.44 | 0.86 | 0.2968 | 0.5614 |
| TC0200011885.hg.1 | TTC32 | tetratricopeptide repeat domain 32 | Multiple_C | 7.06 | 9.24 | 6.9 | 1.12 | 0.6401 | 0.8006 | 6.11 | 6.52 | 5.01 | 2.14 | 0.0009 | 0.0155 |
| TC0200013075.hg.1 | TPRKB | TP53RK binding protein | Multiple_C | 8.32 | 9.24 | 8.16 | 1.12 | 0.8149 | 0.9069 | 6.21 | 7.00 | 6.96 | 0.59 | 0.0149 | 0.0999 |
| TC0300007167.hg.1 | KLHL40 | kelch-like family member 40 | Coding | 3.44 | 3.35 | 3.28 | 1.12 | 0.6401 | 0.8006 | 4.1 | 4.04 | 4.08 | 1.01 | 0.5183 | 0.7463 |
| TC0300008487.hg.1 | MAATS1 | MYCBP-associated, testis expressed 1 | Multiple_C | 3.11 | 2.8 | 2.95 | 1.12 | 0.7502 | 0.8717 | 3.72 | 3.64 | 3.85 | 0.91 | 0.8916 | 0.9539 |
| TC0300009136.hg.1 | TM4SF1-AS1 | TM4SF1 antisense RNA 1 | Multiple_C | 4.12 | 4.84 | 3.96 | 1.12 | 0.1965 | 0.387 | 4.11 | 4.46 | 4.2 | 0.94 | 0.8562 | 0.9388 |
| TC0300009497.hg.1 | TMEM212 | transmembrane protein 212 | Multiple_C | 4.07 | 3.61 | 3.91 | 1.12 | 0.4725 | 0.676 | 3.98 | 3.67 | 3.91 | 1.05 | 0.6401 | 0.8234 |
| TC0300010521.hg.1 | THRB | thyroid hormone receptor, beta | Multiple_C | 7.19 | 6.87 | 7.03 | 1.12 | 0.804 | 0.8999 | 4.35 | 3.74 | 3.68 | 1.59 | 0.0162 | 0.1058 |
| TC0300010841.hg.1 | LYZL4 | lysozyme-like 4 | Multiple_C | 4.14 | 4.09 | 3.98 | 1.12 | 0.9117 | 0.9583 | 4.04 | 3.71 | 3.5 | 1.45 | 0.0037 | 0.0409 |
| TC0300010933.hg.1 | FYCO1 | FYVE and coiled-coil domain containing 1 | Multiple_C | 8.72 | 8.41 | 8.56 | 1.12 | 0.4679 | 0.6721 | 8.5 | 7.75 | 7.9 | 1.52 | 0.0009 | 0.0156 |
| TC0300011081.hg.1 | UBA7; MIR519 | ubiquitin-like modifier activating enzyme 7; microRNA 5193 | Multiple_C | 4.03 | 3.86 | 3.87 | 1.12 | 0.7164 | 0.8501 | 3.48 | 3.46 | 3.49 | 0.99 | 0.8233 | 0.9219 |

| | | | | | | | | | | | | | | | |
|---|---|---|---|---|---|---|---|---|---|---|---|---|---|---|---|
| TC0300011145.hg.1 | IQCF1 | IQ motif containing F1 | Coding | 2.88 | 2.74 | 2.72 | 1.12 | 0.6039 | 0.7757 | 3.43 | 3.03 | 3.12 | 1.24 | 0.3833 | 0.6423 |
| TC0300012403.hg.1 | TMCC1 | transmembrane and coiled-coil domain family 1 | Multiple_C | 7.79 | 7.67 | 7.63 | 1.12 | 0.1556 | 0.3303 | 6.86 | 6.44 | 6.8 | 1.04 | 0.871 | 0.9445 |
| TC0300013846.hg.1 | GXYLT2 | glucoside xylosyltransferase 2 | Multiple_C | 8.68 | 8.27 | 8.52 | 1.12 | 0.6702 | 0.8201 | 7.34 | 8.25 | 7.61 | 0.83 | 0.8768 | 0.9471 |
| TC0300013918.hg.1 | SLC51A | solute carrier family 51, alpha subunit | Multiple_C | 4.38 | 3.88 | 4.22 | 1.12 | 0.3859 | 0.5993 | 4.87 | 4.75 | 5.06 | 0.88 | 0.5754 | 0.7822 |
| TC0300013990.hg.1 | TLR9 | toll-like receptor 9 | Coding | 3.14 | 2.9 | 2.98 | 1.12 | 0.51 | 0.706 | 3.51 | 3.26 | 3.23 | 1.21 | 0.096 | 0.3017 |
| TC0400006493.hg.1 | FGFRL1 | fibroblast growth factor receptor-like 1 | Multiple_C | 6.4 | 5.89 | 6.24 | 1.12 | 0.9203 | 0.9631 | 8.35 | 8.06 | 7.73 | 1.54 | 0.0016 | 0.0232 |
| TC0400007782.hg.1 | MUC7 | mucin 7, secreted | Multiple_C | 3.55 | 3.63 | 3.39 | 1.12 | 0.6205 | 0.7876 | 3.53 | 3.42 | 3.52 | 1.01 | 0.1683 | 0.414 |
| TC0400011411.hg.1 | ADH7 | alcohol dehydrogenase 7 (class IV), mu or sigma polypeptide | Multiple_C | 3.85 | 3.72 | 3.69 | 1.12 | 0.7648 | 0.8792 | 4.7 | 4.44 | 4.48 | 1.16 | 0.2163 | 0.4754 |
| TC0500006613.hg.1 | ADAMTS16 | ADAM metallopeptidase with thrombospondin type 1 motif 16 | Multiple_C | 3.62 | 3.33 | 3.46 | 1.12 | 0.5511 | 0.7359 | 3.18 | 3.00 | 3.41 | 0.85 | 0.2709 | 0.5363 |
| TC0500010910.hg.1 | HTR1A | 5-hydroxytryptamine (serotonin) receptor 1A, G protein-couple | Coding | 3.34 | 3.03 | 3.18 | 1.12 | 0.3523 | 0.565 | 3.65 | 3.87 | 3.96 | 0.81 | 0.1204 | 0.3425 |
| TC0500011150.hg.1 | GFM2 | G elongation factor, mitochondrial 2 | Multiple_C | 11.18 | 12.03 | 11.02 | 1.12 | 0.4412 | 0.6498 | 11.66 | 11.42 | 10.82 | 1.79 | 6.24E-05 | 0.0026 |
| TC0500013366.hg.1 | LECT2 | leukocyte cell-derived chemotaxin 2 | Multiple_C | 3.3 | 3.25 | 3.14 | 1.12 | 0.6483 | 0.805 | 3.16 | 3.27 | 3 | 1.12 | 0.1472 | 0.3834 |
| TC0600007552.hg.1 | DDR1; MIR46 | discoidin domain receptor tyrosine kinase 1; microRNA 4640 | Multiple_C | 9.59 | 9.27 | 9.43 | 1.12 | 0.7243 | 0.855 | 8.33 | 8.24 | 8.28 | 1.04 | 0.8294 | 0.9243 |
| TC0600009232.hg.1 | FAM26F | family with sequence similarity 26, member F | Coding | 3.65 | 3.28 | 3.49 | 1.12 | 0.6104 | 0.7807 | 3.5 | 3.77 | 3.64 | 0.91 | 0.5045 | 0.7366 |
| TC0600011121.hg.1 | HIST1H1A | histone cluster 1, H1a | Coding | 3.55 | 3.58 | 3.39 | 1.12 | 0.5416 | 0.7288 | 4.47 | 4.03 | 3.88 | 1.51 | 0.0043 | 0.0451 |
| TC0600011441.hg.1 | BAG6 | BCL2 associated athanogene 6 | Multiple_C | 12.08 | 11 | 11.92 | 1.12 | 0.5952 | 0.7693 | 13.17 | 13.43 | 13.28 | 0.93 | 0.7287 | 0.8742 |
| TC0600011564.hg.1 | MLN | motilin | Coding | 3.9 | 3.5 | 3.74 | 1.12 | 0.4725 | 0.676 | 4.17 | 4.08 | 3.88 | 1.22 | 0.1567 | 0.3975 |
| TC0600011740.hg.1 | KIF6 | kinesin family member 6 | Multiple_C | 3.78 | 3.37 | 3.62 | 1.12 | 0.409 | 0.6213 | 4.83 | 5.69 | 5.13 | 0.81 | 0.2829 | 0.5483 |
| TC0600013004.hg.1 | MCM9 | minichromosome maintenance 9 homologous recombination re | Multiple_C | 8.7 | 7.74 | 8.54 | 1.12 | 0.4693 | 0.6733 | 8.23 | 9.58 | 9.04 | 0.57 | 0.023 | 0.1307 |
| TC0600013165.hg.1 | EPB41L2 | erythrocyte membrane protein band 4.1-like 2 | Multiple_C | 13.19 | 13.19 | 13.03 | 1.12 | 0.9971 | 0.9986 | 11.61 | 12.41 | 11.56 | 1.04 | 0.3218 | 0.5857 |
| TC0700006906.hg.1 | KLHL7 | kelch-like family member 7 | Multiple_C | 8.83 | 10.25 | 8.67 | 1.12 | 0.4891 | 0.6894 | 8.58 | 8.55 | 8.51 | 1.05 | 0.2928 | 0.5578 |
| TC0700008519.hg.1 | ZSCAN21 | zinc finger and SCAN domain containing 21 | Multiple_C | 8.09 | 7.88 | 7.93 | 1.12 | 0.2663 | 0.4741 | 6.93 | 7.65 | 8 | 0.48 | 2.78E-06 | 0.0003 |
| TC0700009082.hg.1 | SMO | smoothened, frizzled class receptor | Multiple_C | 4.18 | 4.02 | 4.02 | 1.12 | 0.5933 | 0.7678 | 4.59 | 4.37 | 4.16 | 1.35 | 0.3479 | 0.6117 |
| TC0700009491.hg.1 | CLCN1 | chloride channel, voltage-sensitive 1 | Multiple_C | 5.21 | 5.09 | 5.05 | 1.12 | 0.7215 | 0.8535 | 5.2 | 5.38 | 5.3 | 0.93 | 0.4522 | 0.6975 |
| TC0700010749.hg.1 | KIAA0895 | KIAA0895 | Multiple_C | 8.57 | 8.76 | 8.41 | 1.12 | 0.5664 | 0.7475 | 8.06 | 7.95 | 7.98 | 1.06 | 0.3856 | 0.6447 |
| TC0700011658.hg.1 | SEMA3E | sema domain, immunoglobulin domain (Ig), short basic domain, | Coding | 4.01 | 4.15 | 3.85 | 1.12 | 0.6731 | 0.822 | 4.22 | 4.01 | 3.97 | 1.19 | 0.0161 | 0.1054 |
| TC0700011865.hg.1 | DLX5 | distal-less homeobox 5 | Multiple_C | 2.96 | 2.64 | 2.8 | 1.12 | 0.8429 | 0.9227 | 3.35 | 3.60 | 3.41 | 0.96 | 0.9406 | 0.9754 |
| TC0700013063.hg.1 | ASB10 | ankyrin repeat and SOCS box containing 10 | Coding | 5.35 | 5.03 | 5.19 | 1.12 | 0.9796 | 0.9902 | 5.97 | 5.86 | 5.84 | 1.09 | 0.1415 | 0.3742 |
| TC0700013586.hg.1 | C7orf76; SHFI | chromosome 7 open reading frame 76; split hand/foot malform | Multiple_C | 3.02 | 2.92 | 2.86 | 1.12 | 0.7878 | 0.8914 | 3.96 | 3.84 | 3.55 | 1.33 | 0.0316 | 0.1589 |
| TC0800008487.hg.1 | RIMS2 | regulating synaptic membrane exocytosis 2 | Multiple_C | 2.93 | 2.64 | 2.77 | 1.12 | 0.5671 | 0.7479 | 3.56 | 3.88 | 3.92 | 0.78 | 0.1018 | 0.3121 |
| TC0800008631.hg.1 | AARD | alanine and arginine rich domain containing protein | Multiple_C | 4.53 | 4.33 | 4.37 | 1.12 | 0.2137 | 0.4089 | 5.03 | 5.08 | 4.79 | 1.18 | 0.9908 | 0.9962 |
| TC0800009878.hg.1 | ENTPD4; LOXL | ectonucleoside triphosphate diphosphohydrolase 4; lysyl oxidas | Multiple_C | 9.56 | 10.08 | 9.4 | 1.12 | 0.5227 | 0.7156 | 9.32 | 8.38 | 8.81 | 1.42 | 0.1377 | 0.3687 |
| TC0800012087.hg.1 | LYNX1 | Ly6/neurotoxin 1 | Multiple_C | 4.43 | 4.29 | 4.27 | 1.12 | 0.3802 | 0.5931 | 5.39 | 5.72 | 5.35 | 1.03 | 0.7803 | 0.9013 |

| | | | | | | | | | | | | | | | |
|---|---|---|---|---|---|---|---|---|---|---|---|---|---|---|---|
| TC0800012470.hg.1 | TMEM71 | transmembrane protein 71 | Multiple_C | 3.76 | 3.54 | 3.6 | 1.12 | 0.3329 | 0.5467 | 3.81 | 3.53 | 3.74 | 1.05 | 0.8254 | 0.923 |
| TC0900009026.hg.1 | CFAP77 | cilia and flagella associated protein 77 | Coding | 3.96 | 3.65 | 3.8 | 1.12 | 0.9494 | 0.9764 | 4.57 | 4.63 | 4.32 | 1.19 | 0.2642 | 0.529 |
| TC0900009247.hg.1 | C8G | complement component 8, gamma polypeptide | Multiple_C | 3.18 | 2.73 | 3.02 | 1.12 | 0.2892 | 0.4998 | 3.47 | 3.49 | 3.31 | 1.12 | 0.3578 | 0.6206 |
| TC0900009846.hg.1 | B4GALT1 | UDP-Gal:betaGlcNAc beta 1,4- galactosyltransferase, polypeptic | Multiple_C | 7.75 | 7.34 | 7.59 | 1.12 | 0.9008 | 0.9524 | 5.98 | 6.45 | 6.52 | 0.69 | 0.0042 | 0.0443 |
| TC0900009905.hg.1 | CNTFR | ciliary neurotrophic factor receptor | Multiple_C | 3.33 | 3.04 | 3.17 | 1.12 | 0.2837 | 0.4938 | 3.28 | 3.06 | 3.29 | 0.99 | 0.53 | 0.7533 |
| TC0900010794.hg.1 | OGN | osteoglycin | Multiple_C | 3.81 | 3.82 | 3.65 | 1.12 | 0.3591 | 0.5727 | 3.62 | 3.82 | 3.76 | 0.91 | 0.9413 | 0.9757 |
| TC0900011510.hg.1 | RPL35 | ribosomal protein L35 | Multiple_C | 17.86 | 18.33 | 17.7 | 1.12 | 0.078 | 0.2009 | 15.41 | 15.33 | 15.04 | 1.29 | 0.022 | 0.1275 |
| TC0900012277.hg.1 | AK1 | adenylate kinase 1 | Coding | 7.81 | 6.84 | 7.65 | 1.12 | 0.4291 | 0.6394 | 6.19 | 6.63 | 6.76 | 0.67 | 0.0868 | 0.2841 |
| TC0X00006875.hg.1 | MAGEB2 | MAGE family member B2 | Coding | 4.93 | 4.69 | 4.77 | 1.12 | 0.5195 | 0.7132 | 4.32 | 4.36 | 5.26 | 0.52 | 0.0019 | 0.0256 |
| TC0X00007138.hg.1 | ZNF157 | zinc finger protein 157 | Coding | 3.27 | 3.16 | 3.11 | 1.12 | 0.357 | 0.5704 | 3.7 | 3.46 | 3.14 | 1.47 | 0.0172 | 0.1097 |
| TC0X00008178.hg.1 | HTR2C | 5-hydroxytryptamine (serotonin) receptor 2C, G protein-couple | Coding | 3.86 | 3.67 | 3.7 | 1.12 | 0.2694 | 0.4777 | 3.6 | 3.92 | 3.76 | 0.90 | 0.9454 | 0.9778 |
| TC0X00008351.hg.1 | LOC10012952 | testis expressed sequence 13-like | Multiple_C | 2.77 | 2.23 | 2.61 | 1.12 | 0.6855 | 0.8301 | 3.3 | 3.19 | 2.97 | 1.26 | 0.0824 | 0.2765 |
| TC0X00008415.hg.1 | ARHGAP36 | Rho GTPase activating protein 36 | Coding | 4.2 | 3.81 | 4.04 | 1.12 | 0.6027 | 0.7747 | 3.76 | 3.74 | 3.92 | 0.90 | 0.9227 | 0.9683 |
| TC0X00008631.hg.1 | UBE2NL | ubiquitin conjugating enzyme E2N-like (gene/pseudogene) | Multiple_C | 4.23 | 3.85 | 4.07 | 1.12 | 0.6208 | 0.7876 | 3.85 | 3.78 | 4.11 | 0.84 | 0.0712 | 0.2554 |
| TC0X00009737.hg.1 | FAM156A | family with sequence similarity 156, member A | Multiple_C | 7.89 | 7.77 | 7.73 | 1.12 | 0.5835 | 0.7606 | 7.46 | 8.67 | 8.66 | 0.44 | 0.0008 | 0.014 |
| TC0X00009786.hg.1 | ITIH6 | inter-alpha-trypsin inhibitor heavy chain family, member 6 | Multiple_C | 3.16 | 2.84 | 3 | 1.12 | 0.4254 | 0.6363 | 3.38 | 3.23 | 3.03 | 1.27 | 0.1233 | 0.3467 |
| TC0X00011409.hg.1 | MAGEA10 | MAGE family member A10 | Multiple_C | 3.26 | 3.12 | 3.1 | 1.12 | 0.3798 | 0.5928 | 3.35 | 2.97 | 3.21 | 1.10 | 0.5567 | 0.7706 |
| TC1000007875.hg.1 | DDX50 | DEAD (Asp-Glu-Ala-Asp) box polypeptide 50 | Multiple_C | 11.01 | 12.59 | 10.85 | 1.12 | 0.604 | 0.7758 | 11.2 | 11.17 | 11.17 | 1.02 | 0.5522 | 0.7678 |
| TC1000008406.hg.1 | KIF20B | kinesin family member 20B | Multiple_C | 10.37 | 9.84 | 10.21 | 1.12 | 0.9142 | 0.9598 | 11.69 | 11.21 | 12.18 | 0.71 | 0.1877 | 0.4397 |
| TC1000008476.hg.1 | CYP26C1 | cytochrome P450, family 26, subfamily C, polypeptide 1 | Coding | 2.79 | 2.68 | 2.63 | 1.12 | 0.2742 | 0.483 | 3.37 | 3.43 | 3.4 | 0.98 | 0.6067 | 0.8033 |
| TC1000008973.hg.1 | TDRD1 | tudor domain containing 1 | Coding | 3.46 | 3.28 | 3.3 | 1.12 | 0.1679 | 0.3475 | 4.18 | 3.79 | 4.04 | 1.10 | 0.7038 | 0.86 |
| TC1000012513.hg.1 | MTG1 | mitochondrial ribosome-associated GTPase 1 | Multiple_C | 7.44 | 7.46 | 7.28 | 1.12 | 0.3678 | 0.5806 | 7.91 | 7.95 | 7.77 | 1.10 | 0.5663 | 0.7759 |
| TC1100006713.hg.1 | OR56A3 | olfactory receptor, family 56, subfamily A, member 3 | Coding | 3.18 | 2.85 | 3.02 | 1.12 | 0.2326 | 0.4333 | 4 | 4.04 | 3.97 | 1.02 | 0.4773 | 0.7161 |
| TC1100007306.hg.1 | RAG1 | recombination activating gene 1 | Multiple_C | 4.05 | 4 | 3.89 | 1.12 | 0.9148 | 0.96 | 5.08 | 5.26 | 5.05 | 1.02 | 0.1905 | 0.4435 |
| TC1100007837.hg.1 | SCGB1D1 | secretoglobin, family 1D, member 1 | Coding | 4.9 | 4.74 | 4.74 | 1.12 | 0.4399 | 0.6489 | 5.14 | 4.78 | 5.3 | 0.90 | 0.546 | 0.7634 |
| TC1100009241.hg.1 | HINFP | histone H4 transcription factor | Multiple_C | 10.3 | 9.96 | 10.14 | 1.12 | 0.1574 | 0.3331 | 10.62 | 10.68 | 10.71 | 0.94 | 0.6579 | 0.8329 |
| TC1100010012.hg.1 | OR10A6 | olfactory receptor, family 10, subfamily A, member 6 (gene/pse | Multiple_C | 3.31 | 3.25 | 3.15 | 1.12 | 0.5313 | 0.7212 | 3.3 | 3.25 | 2.91 | 1.31 | 0.0874 | 0.2854 |
| TC1100012460.hg.1 | TMPRSS13 | transmembrane protease, serine 13 | Multiple_C | 2.91 | 2.96 | 2.75 | 1.12 | 0.8563 | 0.9298 | 3.46 | 3.60 | 3.65 | 0.88 | 0.1561 | 0.3966 |
| TC1200007786.hg.1 | OR6C1 | olfactory receptor, family 6, subfamily C, member 1 | Coding | 3.49 | 3.34 | 3.33 | 1.12 | 0.8908 | 0.9475 | 3.6 | 3.57 | 3.42 | 1.13 | 0.1826 | 0.4333 |
| TC1200007859.hg.1 | NAB2 | NGFI-A binding protein 2 (EGR1 binding protein 2) | Multiple_C | 6.45 | 6.22 | 6.29 | 1.12 | 0.5365 | 0.7251 | 6.55 | 6.78 | 6.7 | 0.90 | 0.1818 | 0.4319 |
| TC1200008582.hg.1 | FAM71C | family with sequence similarity 71, member C | Coding | 3.55 | 3.4 | 3.39 | 1.12 | 0.4263 | 0.6369 | 3.6 | 3.61 | 3.48 | 1.09 | 0.4492 | 0.6958 |
| TC1200008593.hg.1 | SLC17A8 | solute carrier family 17 (vesicular glutamate transporter), meml | Multiple_C | 3.15 | 3.24 | 2.99 | 1.12 | 0.6196 | 0.7872 | 3.7 | 3.44 | 3.39 | 1.24 | 0.188 | 0.4401 |
| TC1200009209.hg.1 | KNTC1 | kinetochore associated 1 | Multiple_C | 10.91 | 10.03 | 10.75 | 1.12 | 0.9861 | 0.9932 | 8.61 | 9.43 | 9.03 | 0.75 | 0.0655 | 0.2437 |

| | | | | | | | | | | | | | | | |
|---|---|---|---|---|---|---|---|---|---|---|---|---|---|---|---|
| TC1200011838.hg.1 | CMKLR1 | chemerin chemokine-like receptor 1 | Multiple_C | 3.57 | 3.89 | 3.41 | 1.12 | 0.8111 | 0.9044 | 3.92 | 4.26 | 3.95 | 0.98 | 0.3495 | 0.6137 |
| TC1200011931.hg.1 | FAM109A | family with sequence similarity 109, member A | Coding | 5.62 | 5.2 | 5.46 | 1.12 | 0.6143 | 0.7838 | 4.32 | 4.31 | 4.45 | 0.91 | 0.9991 | 0.9997 |
| TC1300008626.hg.1 | SMAD9 | SMAD family member 9 | Multiple_C | 5.18 | 4.69 | 5.02 | 1.12 | 0.1259 | 0.2842 | 5.55 | 5.22 | 4.75 | 1.74 | 0.001 | 0.0166 |
| TC1400008715.hg.1 | EFS | embryonal Fyn-associated substrate | Coding | 3.23 | 3.24 | 3.07 | 1.12 | 0.3675 | 0.5805 | 3.98 | 3.61 | 3.74 | 1.18 | 0.097 | 0.3034 |
| TC1400010648.hg.1 | BDKRB1 | bradykinin receptor B1 | Multiple_C | 4.42 | 3.99 | 4.26 | 1.12 | 0.9889 | 0.9942 | 4.65 | 4.54 | 4.21 | 1.36 | 0.0088 | 0.0719 |
| TC1500007551.hg.1 | TRIP4 | thyroid hormone receptor interactor 4 | Multiple_C | 8.06 | 8.56 | 7.9 | 1.12 | 0.5251 | 0.7172 | 8.41 | 8.23 | 8.05 | 1.28 | 0.3577 | 0.6205 |
| TC1500008350.hg.1 | SV2B | synaptic vesicle glycoprotein 2B | Multiple_C | 2.92 | 2.79 | 2.76 | 1.12 | 0.5151 | 0.7098 | 3.69 | 3.66 | 3.56 | 1.09 | 0.8993 | 0.958 |
| TC1500009335.hg.1 | MYEF2 | myelin expression factor 2 | Multiple_C | 3.3 | 2.97 | 3.14 | 1.12 | 0.7128 | 0.8481 | 10.95 | 9.90 | 9.76 | 2.28 | 0.0056 | 0.0532 |
| TC1500010688.hg.1 | OR4N4 | olfactory receptor, family 4, subfamily N, member 4 | Coding | 3.67 | 3.98 | 3.51 | 1.12 | 0.4019 | 0.6148 | 3.94 | 3.99 | 4.07 | 0.91 | 0.8853 | 0.9508 |
| TC1600006529.hg.1 | TPSAB1; TPSB | tryptase alpha/beta 1; tryptase beta 2 (gene/pseudogene) | Multiple_C | 4.54 | 4.16 | 4.38 | 1.12 | 0.432 | 0.642 | 4.49 | 4.67 | 4.51 | 0.99 | 0.4984 | 0.7314 |
| TC1600007335.hg.1 | SBK1 | SH3 domain binding kinase 1 | Multiple_C | 2.85 | 2.58 | 2.69 | 1.12 | 0.7534 | 0.8738 | 3.57 | 3.50 | 3.46 | 1.08 | 0.0677 | 0.2478 |
| TC1600008868.hg.1 | CDH15 | cadherin 15, type 1, M-cadherin (myotubule) | Multiple_C | 5.55 | 5.39 | 5.39 | 1.12 | 0.4617 | 0.667 | 5.08 | 5.02 | 4.94 | 1.10 | 0.6113 | 0.8057 |
| TC1700006746.hg.1 | C17orf74 | chromosome 17 open reading frame 74 | Coding | 3.51 | 3.55 | 3.35 | 1.12 | 0.1989 | 0.39 | 3.41 | 3.27 | 3.69 | 0.82 | 0.1102 | 0.327 |
| TC1700007170.hg.1 | MAPK7 | mitogen-activated protein kinase 7 | Multiple_C | 7.27 | 6.49 | 7.11 | 1.12 | 0.6474 | 0.8045 | 6.71 | 7.58 | 7.59 | 0.54 | 0.0194 | 0.118 |
| TC1700007474.hg.1 | RNF135 | ring finger protein 135 | Multiple_C | 6.09 | 6.05 | 5.93 | 1.12 | 0.7273 | 0.8565 | 4.71 | 4.60 | 4.27 | 1.36 | 0.0131 | 0.0925 |
| TC1700009179.hg.1 | FSCN2 | fascin actin-bundling protein 2, retinal | Multiple_C | 5.71 | 5.44 | 5.55 | 1.12 | 0.6038 | 0.7757 | 4.82 | 4.88 | 4.75 | 1.05 | 0.9356 | 0.973 |
| TC1700009627.hg.1 | YBX2 | Y box binding protein 2 | Multiple_C | 4.47 | 4.16 | 4.31 | 1.12 | 0.6208 | 0.7876 | 7.78 | 8.52 | 7.88 | 0.93 | 0.9895 | 0.9956 |
| TC1700009702.hg.1 | SPDYE4 | speedy/RINGO cell cycle regulator family member E4 | Coding | 3.19 | 3.08 | 3.03 | 1.12 | 0.5209 | 0.7144 | 3.75 | 3.83 | 3.53 | 1.16 | 0.3959 | 0.6539 |
| TC1700010800.hg.1 | MPP3 | membrane protein, palmitoylated 3 | Multiple_C | 5.04 | 4.82 | 4.88 | 1.12 | 0.8519 | 0.9273 | 4.9 | 5.05 | 4.93 | 0.98 | 0.5053 | 0.7369 |
| TC1700010962.hg.1 | SP6 | Sp6 transcription factor | Coding | 4.74 | 4.48 | 4.58 | 1.12 | 0.0852 | 0.2142 | 4.93 | 4.84 | 4.34 | 1.51 | 0.3582 | 0.6211 |
| TC1700011032.hg.1 | GNGT2 | guanine nucleotide binding protein (G protein), gamma transdu | Coding | 4.84 | 4.56 | 4.68 | 1.12 | 0.7193 | 0.8521 | 4.6 | 4.51 | 4.43 | 1.13 | 0.9477 | 0.979 |
| TC1700012309.hg.1 | METTL23 | methyltransferase like 23 | Multiple_C | 12.22 | 13.44 | 12.06 | 1.12 | 0.7833 | 0.8885 | 9.29 | 9.13 | 8.49 | 1.74 | 0.0009 | 0.0151 |
| TC1700012416.hg.1 | RAB5C | RAB5C, member RAS oncogene family | Multiple_C | 13.19 | 12.6 | 13.03 | 1.12 | 0.5478 | 0.7332 | 12.75 | 13.38 | 13.27 | 0.70 | 0.046 | 0.1984 |
| TC1800006446.hg.1 | CETN1 | centrin 1 | Coding | 4.37 | 3.97 | 4.21 | 1.12 | 0.5059 | 0.7031 | 4.27 | 4.63 | 4.24 | 1.02 | 0.3798 | 0.6395 |
| TC1900006480.hg.1 | PALM | paralemmin | Multiple_C | 5.31 | 4.91 | 5.15 | 1.12 | 0.452 | 0.6597 | 5.87 | 5.76 | 5.18 | 1.61 | 0.002 | 0.0267 |
| TC1900007021.hg.1 | TSPAN16 | tetraspanin 16 | Multiple_C | 4.07 | 3.83 | 3.91 | 1.12 | 0.4883 | 0.689 | 4.74 | 4.34 | 4.17 | 1.48 | 0.0126 | 0.0899 |
| TC1900007053.hg.1 | ZNF69 | zinc finger protein 69 | Coding | 7.16 | 7.25 | 7 | 1.12 | 0.6754 | 0.8234 | 5.37 | 5.66 | 5.66 | 0.82 | 0.9667 | 0.9861 |
| TC1900007570.hg.1 | ZNF492 | zinc finger protein 492 | Multiple_C | 6.44 | 6.46 | 6.28 | 1.12 | 0.6935 | 0.8351 | 6.42 | 6.49 | 6.39 | 1.02 | 0.9266 | 0.9697 |
| TC1900007675.hg.1 | VSTM2B | V-set and transmembrane domain containing 2B | Coding | 5.18 | 5.19 | 5.02 | 1.12 | 0.8751 | 0.9394 | 4.99 | 4.66 | 4.73 | 1.20 | 0.4462 | 0.6935 |
| TC1900008881.hg.1 | FCAR | Fc fragment of IgA receptor | Multiple_C | 2.89 | 2.7 | 2.73 | 1.12 | 0.8999 | 0.9519 | 4.02 | 3.76 | 3.72 | 1.23 | 0.0897 | 0.2896 |
| TC1900008999.hg.1 | ZNF549 | zinc finger protein 549 | Coding | 3.16 | 3.03 | 3 | 1.12 | 0.882 | 0.9427 | 3.38 | 3.27 | 3.31 | 1.05 | 0.9894 | 0.9956 |
| TC1900009361.hg.1 | DPP9 | dipeptidyl-peptidase 9 | Multiple_C | 10.87 | 9.81 | 10.71 | 1.12 | 0.6328 | 0.7951 | 10.95 | 11.52 | 11.54 | 0.66 | 0.0715 | 0.2559 |
| TC1900009454.hg.1 | MBD3L2; CTB- | Synthetic construct Homo sapiens clone IMAGE:100069019, MC | Multiple_C | 3.69 | 3.38 | 3.53 | 1.12 | 0.3491 | 0.5618 | 4.26 | 4.16 | 4.07 | 1.14 | 0.5839 | 0.788 |

| | | | | | | | | | | | | | | | |
|---|---|---|---|---|---|---|---|---|---|---|---|---|---|---|---|
| TC1900011029.hg.1 | MEIS3 | Meis homeobox 3 | Multiple_C | 3.54 | 3.38 | 3.38 | 1.12 | 0.1731 | 0.3548 | 5.23 | 4.77 | 4.35 | 1.84 | 0.0002 | 0.0054 |
| TC1900011077.hg.1 | LMTK3 | lemur tyrosine kinase 3 | Multiple_C | 3.97 | 3.73 | 3.81 | 1.12 | 0.5029 | 0.7006 | 3.74 | 3.49 | 3.76 | 0.99 | 0.8583 | 0.9397 |
| TC1900011772.hg.1 | CRX | cone-rod homeobox | Multiple_C | 4.62 | 4.31 | 4.46 | 1.12 | 0.4771 | 0.6796 | 5.18 | 5.51 | 5.35 | 0.89 | 0.6892 | 0.8515 |
| TC1900011785.hg.1 | NR1H2 | nuclear receptor subfamily 1, group H, member 2 | Multiple_C | 4.44 | 4.14 | 4.28 | 1.12 | 0.6663 | 0.8175 | 5.09 | 4.98 | 4.56 | 1.44 | 0.074 | 0.2611 |
| TC1900011850.hg.1 | FUT5 | fucosyltransferase 5 (alpha (1,3) fucosyltransferase) | Coding | 4.85 | 4.56 | 4.69 | 1.12 | 0.5887 | 0.7644 | 4.49 | 4.19 | 4.19 | 1.23 | 0.5301 | 0.7533 |
| TC1900012046.hg.1 | RDH13 | retinol dehydrogenase 13 (all-trans/9-cis) | Multiple_C | 8.35 | 7.59 | 8.19 | 1.12 | 0.3658 | 0.5794 | 7.64 | 7.90 | 8.21 | 0.67 | 0.0037 | 0.0411 |
| TC2000007224.hg.1 | SPAG4 | sperm associated antigen 4 | Multiple_C | 5.25 | 5.09 | 5.09 | 1.12 | 0.2307 | 0.4309 | 4.73 | 5.09 | 4.67 | 1.04 | 0.5489 | 0.7656 |
| TC2000007797.hg.1 | DOK5 | docking protein 5 | Multiple_C | 3 | 2.69 | 2.84 | 1.12 | 0.5504 | 0.7354 | 3.69 | 3.96 | 3.62 | 1.05 | 0.1872 | 0.439 |
| TC2000008107.hg.1 | TCEA2 | transcription elongation factor A (SII), 2 | Multiple_C | 4.57 | 4.47 | 4.41 | 1.12 | 0.8858 | 0.9449 | 6.71 | 6.50 | 5.87 | 1.79 | 0.0078 | 0.0661 |
| TC2000008870.hg.1 | SUN5 | Sad1 and UNC84 domain containing 5 | Multiple_C | 2.92 | 2.56 | 2.76 | 1.12 | 0.4148 | 0.6269 | 2.98 | 2.80 | 2.73 | 1.19 | 0.1399 | 0.3716 |
| TC2000009210.hg.1 | WFDC5 | WAP four-disulfide core domain 5 | Coding | 5.04 | 4.8 | 4.88 | 1.12 | 0.4332 | 0.6432 | 5.7 | 5.92 | 5.86 | 0.90 | 0.609 | 0.8043 |
| TC2000009448.hg.1 | KCNG1 | potassium channel, voltage gated modifier subfamily G, membe | Multiple_C | 5.07 | 4.64 | 4.91 | 1.12 | 0.1205 | 0.2754 | 4.33 | 4.01 | 4.01 | 1.25 | 0.2053 | 0.4617 |
| TC2100007668.hg.1 | C21orf91; C21 | chromosome 21 open reading frame 91; C21orf91 overlapping t | Multiple_C | 8.05 | 7.76 | 7.89 | 1.12 | 0.5149 | 0.7097 | 8.33 | 8.04 | 8.46 | 0.91 | 0.488 | 0.7244 |
| TC2200009153.hg.1 | DENND6B | DENN/MADD domain containing 6B | Multiple_C | 5.28 | 4.77 | 5.12 | 1.12 | 0.3492 | 0.5619 | 4.47 | 4.60 | 4.36 | 1.08 | 0.2229 | 0.4834 |
| TSUnmapped00000087.h | CACFD1 | calcium channel flower domain containing 1 | Coding | 5.34 | 5.08 | 5.18 | 1.12 | 0.1917 | 0.3802 | 5.4 | 5.25 | 5.22 | 1.13 | 0.5546 | 0.7692 |
| TSUnmapped00000181.h | HMBS | hydroxymethylbilane synthase | Coding | 5.51 | 5.12 | 5.35 | 1.12 | 0.2045 | 0.397 | 5.12 | 5.30 | 5.31 | 0.88 | 0.3395 | 0.6037 |
| TSUnmapped00000197.h | ATG16L1 | autophagy related 16-like 1 | Coding | 3.62 | 3.47 | 3.46 | 1.12 | 0.8339 | 0.9171 | 3.59 | 3.72 | 3.78 | 0.88 | 0.4873 | 0.7238 |
| TSUnmapped00000264.h | RPL7A | ribosomal protein L7a | Coding | 16.49 | 17 | 16.33 | 1.12 | 0.3938 | 0.6076 | 15.09 | 15.00 | 14.44 | 1.57 | 0.0118 | 0.0862 |
| TSUnmapped00000364.h | KAT6B | K(lysine) acetyltransferase 6B | Coding | 8.19 | 8.01 | 8.03 | 1.12 | 0.8495 | 0.9261 | 7.74 | 8.00 | 7.89 | 0.90 | 0.4749 | 0.7143 |
| TSUnmapped00000599.h | PRAMEF8 | PRAME family member 8 | Coding | 3.95 | 3.81 | 3.79 | 1.12 | 0.4894 | 0.6896 | 3.99 | 3.75 | 3.78 | 1.16 | 0.3219 | 0.5858 |
| TSUnmapped00000681.h | HMBS | hydroxymethylbilane synthase | Coding | 5.71 | 5.31 | 5.55 | 1.12 | 0.4374 | 0.6468 | 4.96 | 4.95 | 4.97 | 0.99 | 0.3153 | 0.5793 |
| TSUnmapped00000784.h | PADI1 | peptidyl arginine deiminase, type I | Coding | 3.4 | 3.38 | 3.24 | 1.12 | 0.502 | 0.7002 | 5.26 | 3.71 | 3.73 | 2.89 | 0.0003 | 0.0072 |
| TC0100007662.hg.1 | KPNA6 | karyopherin alpha 6 (importin alpha 7) | Multiple_C | 12.3 | 12.21 | 12.15 | 1.11 | 0.4484 | 0.6564 | 11.88 | 11.46 | 11.92 | 0.97 | 0.3496 | 0.6137 |
| TC0100010164.hg.1 | TMEM79 | transmembrane protein 79 | Multiple_C | 4.63 | 4.22 | 4.48 | 1.11 | 0.7076 | 0.8445 | 4.66 | 4.90 | 4.81 | 0.90 | 0.243 | 0.5067 |
| TC0100011014.hg.1 | RGS13 | regulator of G-protein signaling 13 | Multiple_C | 3.71 | 3.8 | 3.56 | 1.11 | 0.3831 | 0.5962 | 3.62 | 3.37 | 3.43 | 1.14 | 0.0852 | 0.2811 |
| TC0100012200.hg.1 | SDCCAG8 | serologically defined colon cancer antigen 8 | Multiple_C | 9.38 | 10.16 | 9.23 | 1.11 | 0.6954 | 0.8363 | 8.68 | 8.89 | 8.86 | 0.88 | 0.6161 | 0.8096 |
| TC0100012320.hg.1 | OR2G3 | olfactory receptor, family 2, subfamily G, member 3 | Coding | 4.02 | 3.69 | 3.87 | 1.11 | 0.7959 | 0.8952 | 4.83 | 4.72 | 4.61 | 1.16 | 0.2096 | 0.4666 |
| TC0100012332.hg.1 | OR2L13 | olfactory receptor, family 2, subfamily L, member 13 | Coding | 3.09 | 2.87 | 2.94 | 1.11 | 0.7298 | 0.8585 | 3.47 | 3.33 | 3.42 | 1.04 | 0.2364 | 0.4994 |
| TC0100012345.hg.1 | OR2L3 | olfactory receptor, family 2, subfamily L, member 3 | Coding | 3.65 | 3.44 | 3.5 | 1.11 | 0.7084 | 0.8451 | 3.12 | 3.11 | 3.33 | 0.86 | 0.7322 | 0.8763 |
| TC0100012503.hg.1 | CFAP74 | cilia and flagella associated protein 74 | Multiple_C | 4.04 | 3.79 | 3.89 | 1.11 | 0.4949 | 0.6948 | 3.86 | 3.95 | 4.31 | 0.73 | 0.1044 | 0.3167 |
| TC0100012938.hg.1 | HNRNPCL2 | heterogeneous nuclear ribonucleoprotein C-like 2 | Coding | 4.58 | 4.72 | 4.43 | 1.11 | 0.906 | 0.9549 | 4.19 | 4.21 | 4.11 | 1.06 | 0.8516 | 0.9358 |
| TC0100013049.hg.1 | NBPF1 | neuroblastoma breakpoint family, member 1 | Multiple_C | 11.33 | 10.31 | 11.18 | 1.11 | 0.9608 | 0.9814 | 10.05 | 10.13 | 10.39 | 0.79 | 0.2496 | 0.5141 |
| TC0100013128.hg.1 | AKR7L | aldo-keto reductase family 7-like (gene/pseudogene) | Multiple_C | 6.72 | 6.51 | 6.57 | 1.11 | 0.9715 | 0.9861 | 5.49 | 6.05 | 6 | 0.70 | 0.034 | 0.1659 |

| | | | | | | | | | | | | | | | |
|---|---|---|---|---|---|---|---|---|---|---|---|---|---|---|---|
| TC0100013896.hg.1 | CLDN19 | claudin 19 | Coding | 5.62 | 5.29 | 5.47 | 1.11 | 0.2955 | 0.5069 | 6.13 | 6.34 | 6.02 | 1.08 | 0.605 | 0.8024 |
| TC0100015180.hg.1 | WDR47 | WD repeat domain 47 | Multiple_C | 8.59 | 8.42 | 8.44 | 1.11 | 0.7147 | 0.8493 | 9.27 | 8.31 | 8.71 | 1.47 | 0.0273 | 0.1448 |
| TC0100015378.hg.1 | CASQ2 | calsequestrin 2 (cardiac muscle) | Multiple_C | 3.69 | 3.29 | 3.54 | 1.11 | 0.5652 | 0.7465 | 3.9 | 3.64 | 3.37 | 1.44 | 0.0037 | 0.0409 |
| TC0100015826.hg.1 | TCHH | trichohyalin | Multiple_C | 3.04 | 2.8 | 2.89 | 1.11 | 0.419 | 0.6302 | 2.97 | 3.05 | 3.12 | 0.90 | 0.8905 | 0.9533 |
| TC0100015836.hg.1 | LCE3D | late cornified envelope 3D | Multiple_C | 3.11 | 2.84 | 2.96 | 1.11 | 0.2066 | 0.4 | 3.94 | 4.04 | 3.68 | 1.20 | 0.0801 | 0.2728 |
| TC0100015862.hg.1 | S100A7 | S100 calcium binding protein A7 | Multiple_C | 4.03 | 4.22 | 3.88 | 1.11 | 0.3068 | 0.5198 | 3.35 | 3.48 | 3.21 | 1.10 | 0.6848 | 0.8489 |
| TC0100016045.hg.1 | FCRL4 | Fc receptor-like 4 | Multiple_C | 4.18 | 4.29 | 4.03 | 1.11 | 0.586 | 0.7624 | 3.77 | 4.02 | 3.67 | 1.07 | 0.6363 | 0.8215 |
| TC0100016156.hg.1 | ARHGAP30 | Rho GTPase activating protein 30 | Multiple_C | 3.86 | 3.43 | 3.71 | 1.11 | 0.7276 | 0.8567 | 3.81 | 4.04 | 3.43 | 1.30 | 0.0655 | 0.2437 |
| TC0100017043.hg.1 | LRRN2 | leucine rich repeat neuronal 2 | Multiple_C | 3.63 | 3.48 | 3.48 | 1.11 | 0.6214 | 0.788 | 3.57 | 3.68 | 3.68 | 0.93 | 0.5474 | 0.7644 |
| TC0100017952.hg.1 | MAP1LC3C | microtubule associated protein 1 light chain 3 gamma | Coding | 4.3 | 4.33 | 4.15 | 1.11 | 0.5607 | 0.7432 | 4.43 | 4.66 | 4.68 | 0.84 | 0.3448 | 0.6085 |
| TC0200009071.hg.1 | CFAP221 | cilia and flagella associated protein 221 | Multiple_C | 3.03 | 2.82 | 2.88 | 1.11 | 0.4482 | 0.6563 | 3.39 | 3.51 | 3.41 | 0.99 | 0.2118 | 0.4695 |
| TC0200009903.hg.1 | G6PC2 | glucose-6-phosphatase, catalytic, 2 | Multiple_C | 3.76 | 3.58 | 3.61 | 1.11 | 0.2192 | 0.4161 | 3.7 | 3.68 | 3.17 | 1.44 | 0.0382 | 0.178 |
| TC0200013700.hg.1 | MFSD9 | major facilitator superfamily domain containing 9 | Multiple_C | 8.2 | 8.17 | 8.05 | 1.11 | 0.2117 | 0.4061 | 7.44 | 6.89 | 7.25 | 1.14 | 0.4514 | 0.6967 |
| TC0200014017.hg.1 | EN1 | engrailed homeobox 1 | Multiple_C | 4.41 | 4.02 | 4.26 | 1.11 | 0.6614 | 0.8143 | 4.1 | 4.46 | 4.32 | 0.86 | 0.4938 | 0.7282 |
| TC0200015221.hg.1 | MSTN | myostatin | Coding | 3.09 | 3.14 | 2.94 | 1.11 | 0.2768 | 0.486 | 3.17 | 3.12 | 2.96 | 1.16 | 0.2259 | 0.4863 |
| TC0200015512.hg.1 | NDUFS1; GCSI | NADH dehydrogenase (ubiquinone) Fe-S protein 1, 75kDa (NAD | Multiple_C | 10.77 | 11.58 | 10.62 | 1.11 | 0.6845 | 0.8292 | 10.93 | 11.09 | 10.97 | 0.97 | 0.2826 | 0.548 |
| TC0200016433.hg.1 | TMEM178A | transmembrane protein 178A | Multiple_C | 3.58 | 3.82 | 3.43 | 1.11 | 0.6784 | 0.8254 | 5.1 | 4.25 | 3.67 | 2.69 | 6.28E-06 | 0.0005 |
| TC0200016774.hg.1 | ABCB6 | ATP binding cassette subfamily B member 6 (Langereis blood gr | Multiple_C | 5.84 | 5.65 | 5.69 | 1.11 | 0.617 | 0.7853 | 5.08 | 5.19 | 5.08 | 1.00 | 0.7003 | 0.858 |
| TC0300007225.hg.1 | TMEM42; MIF | transmembrane protein 42; microRNA 564 | Multiple_C | 5.36 | 6.42 | 5.21 | 1.11 | 0.4172 | 0.629 | 4.95 | 4.80 | 4.58 | 1.29 | 0.1188 | 0.341 |
| TC0300008962.hg.1 | FOXL2NB | FOXL2 neighbor | Multiple_C | 2.9 | 2.62 | 2.75 | 1.11 | 0.6286 | 0.7927 | 7.22 | 7.32 | 6.36 | 1.82 | 0.0025 | 0.0309 |
| TC0300009350.hg.1 | ARL14 | ADP-ribosylation factor like GTPase 14 | Coding | 3.8 | 3.63 | 3.65 | 1.11 | 0.5587 | 0.7414 | 3.52 | 3.44 | 3.51 | 1.01 | 0.7137 | 0.866 |
| TC0300009897.hg.1 | OPA1 | optic atrophy 1 (autosomal dominant) | Multiple_C | 12.58 | 13.13 | 12.43 | 1.11 | 0.9633 | 0.9824 | 12.7 | 12.17 | 12.41 | 1.22 | 0.9826 | 0.9927 |
| TC0300010372.hg.1 | GRIP2 | glutamate receptor interacting protein 2 | Multiple_C | 4.08 | 3.69 | 3.93 | 1.11 | 0.7096 | 0.8459 | 3.6 | 3.58 | 3.88 | 0.82 | 0.0863 | 0.2833 |
| TC0300010557.hg.1 | NEK10 | NIMA-related kinase 10 | Multiple_C | 3.14 | 2.76 | 2.99 | 1.11 | 0.366 | 0.5794 | 3.86 | 4.50 | 4.01 | 0.90 | 0.6074 | 0.8037 |
| TC0300012504.hg.1 | KY | kyphoscoliosis peptidase | Multiple_C | 4.72 | 4.43 | 4.57 | 1.11 | 0.9216 | 0.9636 | 4.55 | 4.36 | 4.59 | 0.97 | 0.4561 | 0.7004 |
| TC0300012676.hg.1 | PCOLCE2 | procollagen C-endopeptidase enhancer 2 | Multiple_C | 4.32 | 3.93 | 4.17 | 1.11 | 0.5255 | 0.7176 | 7.5 | 6.85 | 6.75 | 1.68 | 0.0075 | 0.0641 |
| TC0300012679.hg.1 | PAQR9 | progestin and adipoQ receptor family member IX | Coding | 3.34 | 2.55 | 3.19 | 1.11 | 0.4756 | 0.6784 | 3.13 | 3.59 | 3.36 | 0.85 | 0.4249 | 0.6777 |
| TC0300013428.hg.1 | TRA2B | transformer 2 beta homolog (Drosophila) | Multiple_C | 14.73 | 14.23 | 14.58 | 1.11 | 0.5746 | 0.7536 | 14.84 | 14.92 | 14.89 | 0.97 | 0.7022 | 0.8591 |
| TC0300013877.hg.1 | EPHB1 | EPH receptor B1 | Multiple_C | 4.11 | 3.89 | 3.96 | 1.11 | 0.6171 | 0.7854 | 3.98 | 4.30 | 4.19 | 0.86 | 0.1101 | 0.3268 |
| TC0300013878.hg.1 | NCK1 | NCK adaptor protein 1 | Multiple_C | 11.63 | 12.71 | 11.48 | 1.11 | 0.4848 | 0.6862 | 9.93 | 8.51 | 9.01 | 1.89 | 0.0313 | 0.158 |
| TC0400006812.hg.1 | USP17L24; US | ubiquitin specific peptidase 17-like family member 24; ubiquitin | Coding | 4.69 | 4.24 | 4.54 | 1.11 | 0.5756 | 0.7543 | 4.67 | 4.74 | 4.82 | 0.90 | 0.9821 | 0.9926 |
| TC0400006814.hg.1 | USP17L25; US | ubiquitin specific peptidase 17-like family member 25; ubiquitin | Coding | 4.69 | 4.24 | 4.54 | 1.11 | 0.5756 | 0.7543 | 4.67 | 4.74 | 4.82 | 0.90 | 0.9821 | 0.9926 |
| TC0400006816.hg.1 | USP17L26; US | ubiquitin specific peptidase 17-like family member 26; ubiquitin | Coding | 4.67 | 4.23 | 4.52 | 1.11 | 0.6058 | 0.7771 | 4.59 | 4.48 | 4.6 | 0.99 | 0.915 | 0.9652 |

| | | | | | | | | | | | | | | | |
|---|---|---|---|---|---|---|---|---|---|---|---|---|---|---|---|
| TC0400006820.hg.1 | USP17L27; US | ubiquitin specific peptidase 17-like family member 27; ubiquitin | Coding | 3.3 | 2.87 | 3.15 | 1.11 | 0.4115 | 0.6237 | 3.84 | 3.72 | 4.13 | 0.82 | 0.4983 | 0.7314 |
| TC0400006822.hg.1 | USP17L28; US | ubiquitin specific peptidase 17-like family member 28; ubiquitin | Coding | 3.3 | 2.87 | 3.15 | 1.11 | 0.4115 | 0.6237 | 3.84 | 3.72 | 4.13 | 0.82 | 0.4983 | 0.7314 |
| TC0400006824.hg.1 | USP17L29; US | ubiquitin specific peptidase 17-like family member 29; ubiquitin | Coding | 3.3 | 2.87 | 3.15 | 1.11 | 0.4115 | 0.6237 | 3.84 | 3.72 | 4.13 | 0.82 | 0.4983 | 0.7314 |
| TC0400006828.hg.1 | USP17L25; US | ubiquitin specific peptidase 17-like family member 25; ubiquitin | Coding | 3.3 | 2.87 | 3.15 | 1.11 | 0.4115 | 0.6237 | 3.84 | 3.72 | 4.13 | 0.82 | 0.4983 | 0.7314 |
| TC0400012018.hg.1 | GYPB | glycophorin B (MNS blood group) | Multiple_C | 2.93 | 2.86 | 2.78 | 1.11 | 0.6064 | 0.7775 | 2.83 | 3.02 | 3.16 | 0.80 | 0.8301 | 0.9246 |
| TC0400012060.hg.1 | TTC29 | tetratricopeptide repeat domain 29 | Multiple_C | 3.24 | 3 | 3.09 | 1.11 | 0.7638 | 0.8786 | 4.34 | 3.57 | 3.68 | 1.58 | 0.0088 | 0.0719 |
| TC0500008539.hg.1 | GRAMD3 | GRAM domain containing 3 | Multiple_C | 7.57 | 7.49 | 7.42 | 1.11 | 0.9314 | 0.9677 | 7.6 | 7.76 | 7.89 | 0.82 | 0.4101 | 0.665 |
| TC0500008620.hg.1 | CSF2 | colony stimulating factor 2 (granulocyte-macrophage) | Coding | 4.21 | 4.05 | 4.06 | 1.11 | 0.6366 | 0.7982 | 4.32 | 3.92 | 4.01 | 1.24 | 0.3008 | 0.5651 |
| TC0500008642.hg.1 | IL13 | interleukin 13 | Multiple_C | 4.59 | 4.57 | 4.44 | 1.11 | 0.2433 | 0.446 | 4.71 | 4.25 | 4.08 | 1.55 | 0.0823 | 0.2763 |
| TC0500009185.hg.1 | KIF4B | kinesin family member 4B | Coding | 4.56 | 4.21 | 4.41 | 1.11 | 0.8293 | 0.9145 | 3.93 | 4.48 | 4.41 | 0.72 | 0.3055 | 0.5695 |
| TC0500010148.hg.1 | DNAH5 | dynein, axonemal, heavy chain 5 | Multiple_C | 2.97 | 2.98 | 2.82 | 1.11 | 0.4801 | 0.6822 | 3.83 | 3.89 | 3.86 | 0.98 | 0.8687 | 0.9434 |
| TC0500012459.hg.1 | PDGFRB | platelet-derived growth factor receptor, beta polypeptide | Multiple_C | 4.09 | 3.88 | 3.94 | 1.11 | 0.6722 | 0.8212 | 4.85 | 5.16 | 4.92 | 0.95 | 0.7144 | 0.8666 |
| TC0500013155.hg.1 | BASP1 | brain abundant, membrane attached signal protein 1 | Coding | 4.61 | 4.31 | 4.46 | 1.11 | 0.7602 | 0.8766 | 4.31 | 4.31 | 4.52 | 0.86 | 0.3439 | 0.6075 |
| TC0600007771.hg.1 | UHRF1BP1 | UHRF1 binding protein 1 | Multiple_C | 7.44 | 7.04 | 7.29 | 1.11 | 0.2331 | 0.4339 | 7.95 | 7.77 | 8.29 | 0.79 | 0.0778 | 0.2686 |
| TC0600007986.hg.1 | APOBEC2 | apolipoprotein B mRNA editing enzyme, catalytic polypeptide-li | Coding | 3.48 | 3.26 | 3.33 | 1.11 | 0.5672 | 0.7479 | 4.15 | 4.07 | 3.78 | 1.29 | 0.2041 | 0.4601 |
| TC0600008323.hg.1 | HCRTR2 | hypocretin (orexin) receptor 2 | Coding | 3.11 | 2.85 | 2.96 | 1.11 | 0.4024 | 0.6152 | 3.72 | 3.65 | 3.58 | 1.10 | 0.2793 | 0.5453 |
| TC0600008674.hg.1 | RIPPLY2 | ripply transcriptional repressor 2 | Multiple_C | 5.18 | 4.8 | 5.03 | 1.11 | 0.1992 | 0.3903 | 5.21 | 4.97 | 4.84 | 1.29 | 0.5589 | 0.7714 |
| TC0600011777.hg.1 | TREM1 | triggering receptor expressed on myeloid cells 1 | Multiple_C | 5.09 | 4.99 | 4.94 | 1.11 | 0.5842 | 0.7611 | 4.32 | 4.24 | 4.21 | 1.08 | 0.2468 | 0.5115 |
| TC0600012952.hg.1 | FRK | fyn-related Src family tyrosine kinase | Coding | 10.13 | 10.21 | 9.98 | 1.11 | 0.9817 | 0.9914 | 8.41 | 8.89 | 9.01 | 0.66 | 0.0102 | 0.0787 |
| TC0700007434.hg.1 | IGFBP1 | insulin like growth factor binding protein 1 | Multiple_C | 3.88 | 4.11 | 3.73 | 1.11 | 0.7889 | 0.892 | 4.27 | 4.36 | 4 | 1.21 | 0.2309 | 0.4923 |
| TC0700008031.hg.1 | GTF2IRD2B | GTF2I repeat domain containing 2B | Multiple_C | 4.5 | 5.18 | 4.35 | 1.11 | 0.6966 | 0.837 | 4.06 | 3.89 | 4.06 | 1.00 | 0.8838 | 0.9503 |
| TC0700008328.hg.1 | RBM48 | RNA binding motif protein 48 | Coding | 8.31 | 8.77 | 8.16 | 1.11 | 0.5143 | 0.7093 | 8.85 | 7.99 | 8.72 | 1.09 | 0.5194 | 0.7469 |
| TC0700009770.hg.1 | DPP6 | dipeptidyl-peptidase 6 | Multiple_C | 3.16 | 2.94 | 3.01 | 1.11 | 0.6958 | 0.8366 | 3.79 | 3.67 | 3.74 | 1.04 | 0.1427 | 0.3758 |
| TC0800007132.hg.1 | PNOC | prepronociceptin | Multiple_C | 3.64 | 3.63 | 3.49 | 1.11 | 0.9084 | 0.956 | 4 | 4.26 | 4.14 | 0.91 | 0.3433 | 0.607 |
| TC0800007888.hg.1 | C8orf34 | chromosome 8 open reading frame 34 | Multiple_C | 5.11 | 5.15 | 4.96 | 1.11 | 0.2896 | 0.5005 | 4.7 | 4.71 | 4.87 | 0.89 | 0.8442 | 0.9318 |
| TC0800010144.hg.1 | ADRB3 | adrenoceptor beta 3 | Multiple_C | 2.93 | 2.6 | 2.78 | 1.11 | 0.49 | 0.69 | 2.98 | 3.02 | 2.87 | 1.08 | 0.6671 | 0.8372 |
| TC0800011690.hg.1 | KLHL38 | kelch-like family member 38 | Coding | 4.2 | 4.02 | 4.05 | 1.11 | 0.9773 | 0.9889 | 4.14 | 4.39 | 4.07 | 1.05 | 0.9203 | 0.9672 |
| TC0900009690.hg.1 | IFNW1 | interferon, omega 1 | Coding | 4.38 | 4.18 | 4.23 | 1.11 | 0.7574 | 0.8753 | 4.36 | 4.01 | 4.16 | 1.15 | 0.502 | 0.7343 |
| TC0900011199.hg.1 | OR2K2 | olfactory receptor, family 2, subfamily K, member 2 | Coding | 3.67 | 3.32 | 3.52 | 1.11 | 0.4729 | 0.6761 | 4.45 | 4.41 | 4.89 | 0.74 | 0.9774 | 0.9906 |
| TC0900011618.hg.1 | NAIF1 | nuclear apoptosis inducing factor 1 | Multiple_C | 5.26 | 5.36 | 5.11 | 1.11 | 0.5049 | 0.7023 | 5.56 | 5.73 | 5.41 | 1.11 | 0.6886 | 0.8511 |
| TC0900012245.hg.1 | ALDH1A1 | aldehyde dehydrogenase 1 family, member A1 | Multiple_C | 13.53 | 13.43 | 13.38 | 1.11 | 0.4634 | 0.6685 | 5.16 | 5.17 | 4.45 | 1.64 | 0.0046 | 0.0468 |
| TC0X00007230.hg.1 | GAGE2D; GAG | G antigen 2D; G antigen 13; G antigen 2E; G antigen 8; G antiger | Coding | 3.06 | 2.7 | 2.91 | 1.11 | 0.5476 | 0.7331 | 3.13 | 3.13 | 3.13 | 1.00 | 0.8729 | 0.9452 |
| TC0X00008509.hg.1 | ZNF449 | zinc finger protein 449 | Coding | 7.01 | 7.23 | 6.86 | 1.11 | 0.2986 | 0.5103 | 6.53 | 6.01 | 6.63 | 0.93 | 0.7291 | 0.8742 |

| | | | | | | | | | | | | | | | |
|---|---|---|---|---|---|---|---|---|---|---|---|---|---|---|---|
| TC0X00008829.hg.1 | RPL10; SNORA | ribosomal protein L10; small nucleolar RNA, H/ACA box 70 | Multiple_C | 18.51 | 18.43 | 18.36 | 1.11 | 0.1308 | 0.2926 | 15.28 | 15.55 | 16.35 | 0.48 | 0.0006 | 0.0115 |
| TC0X00009964.hg.1 | PDZD11 | PDZ domain containing 11 | Multiple_C | 11.1 | 10.65 | 10.95 | 1.11 | 0.9054 | 0.9547 | 8.8 | 9.55 | 9.58 | 0.58 | 0.0004 | 0.0096 |
| TC0X00011368.hg.1 | SSX2; SSX2B | synovial sarcoma, X breakpoint 2; synovial sarcoma, X breakpoi | Multiple_C | 4.4 | 4.17 | 4.25 | 1.11 | 0.4011 | 0.6143 | 4.56 | 4.76 | 4.58 | 0.99 | 0.9911 | 0.9963 |
| TC0X00011410.hg.1 | MAGEA10-MA | MAGEA10-MAGEA5 readthrough; novel transcript | Multiple_C | 4.58 | 4.42 | 4.43 | 1.11 | 0.4278 | 0.6383 | 4.48 | 4.63 | 4.57 | 0.94 | 0.4872 | 0.7237 |
| TC1000006861.hg.1 | MEIG1 | meiosis/spermiogenesis associated 1 | Multiple_C | 4.1 | 3.67 | 3.95 | 1.11 | 0.724 | 0.8548 | 3.99 | 4.04 | 3.84 | 1.11 | 0.2668 | 0.5312 |
| TC1000007936.hg.1 | SGPL1 | sphingosine-1-phosphate lyase 1 | Multiple_C | 9.38 | 9.64 | 9.23 | 1.11 | 0.6469 | 0.8043 | 9.84 | 9.55 | 10.28 | 0.74 | 0.0708 | 0.2546 |
| TC1000007986.hg.1 | ANAPC16 | anaphase promoting complex subunit 16 | Multiple_C | 12.24 | 13.47 | 12.09 | 1.11 | 0.9565 | 0.9798 | 9.57 | 9.51 | 9.81 | 0.85 | 0.5213 | 0.7482 |
| TC1000008270.hg.1 | C10orf99 | chromosome 10 open reading frame 99 | Multiple_C | 4.04 | 3.93 | 3.89 | 1.11 | 0.3618 | 0.5753 | 4.08 | 3.82 | 3.81 | 1.21 | 0.8942 | 0.9552 |
| TC1000009841.hg.1 | PHYH | phytanoyl-CoA 2-hydroxylase | Multiple_C | 10.26 | 10.41 | 10.11 | 1.11 | 0.7345 | 0.8613 | 6.94 | 7.22 | 7.18 | 0.85 | 0.1564 | 0.397 |
| TC1000010592.hg.1 | FAM170B | family with sequence similarity 170, member B | Coding | 3.16 | 2.95 | 3.01 | 1.11 | 0.3914 | 0.6052 | 3.49 | 3.40 | 3.35 | 1.10 | 0.4427 | 0.6914 |
| TC1000012416.hg.1 | FRG2B | FSHD region gene 2 family, member B | Coding | 3.87 | 3.83 | 3.72 | 1.11 | 0.5264 | 0.7184 | 4.32 | 4.05 | 4.01 | 1.24 | 0.2556 | 0.5202 |
| TC1100007017.hg.1 | TMEM86A | transmembrane protein 86A | Multiple_C | 4.85 | 4.8 | 4.7 | 1.11 | 0.096 | 0.2337 | 5.37 | 5.21 | 5.1 | 1.21 | 0.0985 | 0.3065 |
| TC1100007366.hg.1 | TTC17 | tetratricopeptide repeat domain 17 | Multiple_C | 11.15 | 11.6 | 11 | 1.11 | 0.886 | 0.945 | 11.17 | 10.27 | 10.53 | 1.56 | 0.0368 | 0.1747 |
| TC1100008933.hg.1 | DDI1 | DNA-damage inducible 1 homolog 1 | Coding | 3.35 | 3.13 | 3.2 | 1.11 | 0.5221 | 0.7152 | 3.85 | 4.03 | 3.83 | 1.01 | 0.9203 | 0.9672 |
| TC1100010033.hg.1 | TRIM66 | tripartite motif containing 66 | Multiple_C | 4.28 | 4.35 | 4.13 | 1.11 | 0.7743 | 0.8837 | 4.74 | 5.89 | 5.16 | 0.75 | 0.1871 | 0.4388 |
| TC1100010241.hg.1 | USH1C | Usher syndrome 1C | Multiple_C | 8.92 | 9.84 | 8.77 | 1.11 | 0.5561 | 0.7392 | 8.52 | 9.23 | 8.56 | 0.97 | 0.7255 | 0.8723 |
| TC1100010284.hg.1 | MRGPRX1 | MAS-related GPR, member X1 | Multiple_C | 2.76 | 2.8 | 2.61 | 1.11 | 0.2112 | 0.4056 | 3.3 | 3.20 | 3.18 | 1.09 | 0.8875 | 0.952 |
| TC1100011719.hg.1 | CLNS1A | chloride channel, nucleotide-sensitive, 1A | Multiple_C | 14.29 | 15.89 | 14.14 | 1.11 | 0.9374 | 0.9708 | 12.94 | 12.67 | 12.8 | 1.10 | 0.685 | 0.8489 |
| TC1200006643.hg.1 | PTMS | parathymosin | Multiple_C | 10.51 | 9.92 | 10.36 | 1.11 | 0.3419 | 0.5556 | 13.59 | 14.44 | 13.68 | 0.94 | 0.7533 | 0.8879 |
| TC1200007522.hg.1 | C12orf54 | chromosome 12 open reading frame 54 | Multiple_C | 3.51 | 3.18 | 3.36 | 1.11 | 0.5785 | 0.7565 | 3.58 | 3.93 | 3.9 | 0.80 | 0.3662 | 0.6283 |
| TC1200008606.hg.1 | UTP20 | UTP20 small subunit (SSU) processome component | Multiple_C | 9.79 | 9.56 | 9.64 | 1.11 | 0.9132 | 0.9593 | 8.9 | 8.61 | 9.61 | 0.61 | 0.0094 | 0.075 |
| TC1200010633.hg.1 | MCRS1 | microspherule protein 1 | Multiple_C | 9.45 | 8.99 | 9.3 | 1.11 | 0.4171 | 0.6289 | 8.49 | 8.86 | 9.14 | 0.64 | 0.0023 | 0.0293 |
| TC1200011099.hg.1 | GNS | glucosamine (N-acetyl)-6-sulfatase | Multiple_C | 13.02 | 12.35 | 12.87 | 1.11 | 0.3068 | 0.5198 | 11.84 | 11.80 | 12.17 | 0.80 | 0.0367 | 0.1745 |
| TC1200011690.hg.1 | SLC5A8 | solute carrier family 5 (sodium/monocarboxylate cotransporter | Coding | 3.8 | 3.6 | 3.65 | 1.11 | 0.5288 | 0.7199 | 3.09 | 3.04 | 3.28 | 0.88 | 0.7397 | 0.8805 |
| TC1200012612.hg.1 | MED21 | mediator complex subunit 21 | Multiple_C | 10.89 | 11.15 | 10.74 | 1.11 | 0.5034 | 0.701 | 11.82 | 11.69 | 12.09 | 0.83 | 0.7823 | 0.9022 |
| TC1200012752.hg.1 | KLRC3 | killer cell lectin-like receptor subfamily C, member 3 | Coding | 4.34 | 3.89 | 4.19 | 1.11 | 0.7675 | 0.8803 | 8.99 | 7.70 | 6.87 | 4.35 | 0.0001 | 0.0041 |
| TC1200012852.hg.1 | HRK | harakiri, BCL2 interacting protein | Multiple_C | 6.71 | 6.5 | 6.56 | 1.11 | 0.6 | 0.7723 | 5.09 | 4.93 | 4.8 | 1.22 | 0.1962 | 0.4508 |
| TC1400010044.hg.1 | SERPINA2 | serpin peptidase inhibitor, clade A (alpha-1 antiproteinase, anti | Multiple_C | 3.78 | 3.72 | 3.63 | 1.11 | 0.8945 | 0.9497 | 4.32 | 4.45 | 4.28 | 1.03 | 0.4662 | 0.7079 |
| TC1400010569.hg.1 | EDDM3A | epididymal protein 3A | Coding | 5.23 | 5.23 | 5.08 | 1.11 | 0.1663 | 0.3454 | 5.84 | 5.49 | 5.54 | 1.23 | 0.5723 | 0.78 |
| TC1500007009.hg.1 | MGA | MGA, MAX dimerization protein | Multiple_C | 9.29 | 9.51 | 9.14 | 1.11 | 0.6616 | 0.8144 | 9.5 | 9.24 | 9.75 | 0.84 | 0.0706 | 0.254 |
| TC1600006757.hg.1 | SMIM22 | small integral membrane protein 22 | Multiple_C | 7.04 | 6.81 | 6.89 | 1.11 | 0.5577 | 0.7404 | 5.66 | 5.39 | 5.07 | 1.51 | 0.0195 | 0.118 |
| TC1600007919.hg.1 | CTD-3032H12 | Transcript Identified by AceView, Entrez Gene ID(s) 10265; nove | Multiple_C | 3.41 | 3 | 3.26 | 1.11 | 0.5513 | 0.736 | 3.64 | 3.24 | 3.06 | 1.49 | 0.0083 | 0.0689 |
| TC1600008932.hg.1 | LUC7L | LUC7-like | Multiple_C | 9.33 | 9.86 | 9.18 | 1.11 | 0.817 | 0.9076 | 9.84 | 9.06 | 8.85 | 1.99 | 0.0263 | 0.1418 |

| | | | | | | | | | | | | | | | |
|---|---|---|---|---|---|---|---|---|---|---|---|---|---|---|---|
| TC1600009161.hg.1 | CLDN6 | claudin 6 | Coding | 3.7 | 3.35 | 3.55 | 1.11 | 0.2809 | 0.4909 | 3.59 | 3.47 | 3.41 | 1.13 | 0.1877 | 0.4397 |
| TC1600010262.hg.1 | BRD7 | bromodomain containing 7 | Multiple_C | 11.35 | 11.28 | 11.2 | 1.11 | 0.5399 | 0.7276 | 13.9 | 14.50 | 13.78 | 1.09 | 0.717 | 0.8679 |
| TC1700006810.hg.1 | ARHGEF15 | Rho guanine nucleotide exchange factor 15 | Multiple_C | 3.86 | 3.74 | 3.71 | 1.11 | 0.5551 | 0.7386 | 3.36 | 3.28 | 3.23 | 1.09 | 0.6328 | 0.8192 |
| TC1700008677.hg.1 | CACNG4 | calcium channel, voltage-dependent, gamma subunit 4 | Coding | 2.53 | 2.13 | 2.38 | 1.11 | 0.6896 | 0.8329 | 8.67 | 9.96 | 9.73 | 0.48 | 0.0005 | 0.0102 |
| TC1700009622.hg.1 | DVL2 | dishevelled segment polarity protein 2 | Multiple_C | 10.61 | 10.48 | 10.46 | 1.11 | 0.8949 | 0.9497 | 11.25 | 11.48 | 11.38 | 0.91 | 0.1012 | 0.311 |
| TC1700009895.hg.1 | LRRC75A | leucine rich repeat containing 75A | Multiple_C | 3.64 | 3.11 | 3.49 | 1.11 | 0.3234 | 0.5373 | 4.83 | 4.73 | 4.26 | 1.48 | 0.0022 | 0.0289 |
| TC1700010252.hg.1 | CORO6 | coronin 6 | Multiple_C | 3.44 | 3.48 | 3.29 | 1.11 | 0.3074 | 0.5206 | 5.17 | 4.77 | 4.07 | 2.14 | 0.0002 | 0.0061 |
| TC1700010393.hg.1 | CCL1 | chemokine (C-C motif) ligand 1 | Coding | 4.6 | 4.74 | 4.45 | 1.11 | 0.5122 | 0.708 | 4.14 | 3.92 | 3.9 | 1.18 | 0.9608 | 0.9837 |
| TC1700012177.hg.1 | WSCD1 | WSC domain containing 1 | Multiple_C | 5.35 | 5.51 | 5.2 | 1.11 | 0.7825 | 0.888 | 3.56 | 3.59 | 3.34 | 1.16 | 0.231 | 0.4923 |
| TC1800006720.hg.1 | ANKRD62 | ankyrin repeat domain 62 | Multiple_C | 4.17 | 4.07 | 4.02 | 1.11 | 0.4744 | 0.6774 | 4.51 | 3.98 | 4.24 | 1.21 | 0.0434 | 0.1926 |
| TC1800007968.hg.1 | TMEM200C | transmembrane protein 200C | Coding | 2.72 | 2.7 | 2.57 | 1.11 | 0.44 | 0.6489 | 2.83 | 2.85 | 2.7 | 1.09 | 0.6082 | 0.8041 |
| TC1800008070.hg.1 | PIEZO2 | piezo-type mechanosensitive ion channel component 2 | Multiple_C | 4.82 | 4.56 | 4.67 | 1.11 | 0.9941 | 0.9969 | 4.81 | 4.72 | 4.65 | 1.12 | 0.3814 | 0.6408 |
| TC1800008580.hg.1 | PSTPIP2 | proline-serine-threonine phosphatase interacting protein 2 | Multiple_C | 3.08 | 3.11 | 2.93 | 1.11 | 0.5725 | 0.7519 | 4.18 | 5.18 | 4.94 | 0.59 | 0.4465 | 0.6937 |
| TC1900007419.hg.1 | SLC25A42 | solute carrier family 25, member 42 | Multiple_C | 4.01 | 3.94 | 3.86 | 1.11 | 0.5366 | 0.7251 | 4.12 | 4.22 | 4.06 | 1.04 | 0.9636 | 0.9846 |
| TC1900007436.hg.1 | GATAD2A; MI | GATA zinc finger domain containing 2A; microRNA 640 | Multiple_C | 10.85 | 9.73 | 10.7 | 1.11 | 0.5084 | 0.7049 | 10.93 | 11.16 | 11.76 | 0.56 | 0.0709 | 0.2548 |
| TC1900008081.hg.1 | LEUTX | leucine twenty homeobox | Coding | 4.21 | 3.84 | 4.06 | 1.11 | 0.6189 | 0.7868 | 3.9 | 4.06 | 4.07 | 0.89 | 0.4251 | 0.6778 |
| TC1900008121.hg.1 | SNRPA | small nuclear ribonucleoprotein polypeptide A | Multiple_C | 13.3 | 12.67 | 13.15 | 1.11 | 0.2486 | 0.4527 | 13.12 | 13.36 | 13.33 | 0.86 | 0.07 | 0.2527 |
| TC1900008184.hg.1 | CIC | capicua transcriptional repressor | Multiple_C | 5.86 | 5.33 | 5.71 | 1.11 | 0.4367 | 0.6462 | 6.26 | 6.69 | 6.74 | 0.72 | 0.0947 | 0.2991 |
| TC1900008665.hg.1 | SPACA6 | sperm acrosome associated 6 | Multiple_C | 3.26 | 3.31 | 3.11 | 1.11 | 0.4224 | 0.6337 | 4.31 | 4.53 | 4.1 | 1.16 | 0.3726 | 0.6337 |
| TC1900008934.hg.1 | NLRP4 | NLR family, pyrin domain containing 4 | Multiple_C | 2.89 | 2.68 | 2.74 | 1.11 | 0.6811 | 0.8267 | 3.34 | 3.20 | 3.09 | 1.19 | 0.4347 | 0.6854 |
| TC1900009116.hg.1 | R3HDM4 | R3H domain containing 4 | Multiple_C | 8.12 | 7.51 | 7.97 | 1.11 | 0.8757 | 0.9395 | 7.16 | 7.02 | 6.55 | 1.53 | 0.1431 | 0.3764 |
| TC1900009188.hg.1 | REXO1; MIR19 | REX1, RNA exonuclease 1 homolog; microRNA 1909 | Multiple_C | 9.42 | 8.64 | 9.27 | 1.11 | 0.2862 | 0.4965 | 8.91 | 8.93 | 9.15 | 0.85 | 0.0445 | 0.1953 |
| TC1900009559.hg.1 | MUC16 | mucin 16, cell surface associated | Multiple_C | 3.78 | 3.52 | 3.63 | 1.11 | 0.3439 | 0.5575 | 3.25 | 3.32 | 3.29 | 0.97 | 0.7519 | 0.887 |
| TC1900009603.hg.1 | OLFM2 | olfactomedin 2 | Multiple_C | 3.58 | 3.76 | 3.43 | 1.11 | 0.6804 | 0.8263 | 4.06 | 3.67 | 3.68 | 1.30 | 0.1087 | 0.3246 |
| TC1900009867.hg.1 | AKAP8L | A kinase (PRKA) anchor protein 8-like | Multiple_C | 12.49 | 12.76 | 12.34 | 1.11 | 0.8557 | 0.9295 | 11.85 | 10.99 | 12 | 0.90 | 0.3716 | 0.6332 |
| TC1900009873.hg.1 | PGLYRP2 | peptidoglycan recognition protein 2 | Coding | 4.31 | 4.01 | 4.16 | 1.11 | 0.2567 | 0.4629 | 3.77 | 3.80 | 3.76 | 1.01 | 0.9251 | 0.9693 |
| TC1900009941.hg.1 | CPAMD8 | C3 and PZP-like, alpha-2-macroglobulin domain containing 8 | Multiple_C | 5.2 | 4.8 | 5.05 | 1.11 | 0.2369 | 0.4384 | 5.96 | 6.10 | 5.82 | 1.10 | 0.2643 | 0.529 |
| TC1900010931.hg.1 | EXOC3L2 | exocyst complex component 3-like 2 | Coding | 4.25 | 3.6 | 4.1 | 1.11 | 0.1671 | 0.3466 | 3.83 | 4.25 | 3.78 | 1.04 | 0.9571 | 0.9823 |
| TC1900011664.hg.1 | CCDC159 | coiled-coil domain containing 159 | Multiple_C | 2.77 | 2.52 | 2.62 | 1.11 | 0.5607 | 0.7432 | 3.04 | 3.23 | 2.8 | 1.18 | 0.4867 | 0.7237 |
| TC1900011837.hg.1 | UQCR11 | ubiquinol-cytochrome c reductase, complex III subunit XI | Multiple_C | 13.94 | 14.62 | 13.79 | 1.11 | 0.8387 | 0.9197 | 10.88 | 11.38 | 10.53 | 1.27 | 0.215 | 0.4736 |
| TC1900011869.hg.1 | RGL3 | ral guanine nucleotide dissociation stimulator-like 3 | Multiple_C | 3.75 | 3.41 | 3.6 | 1.11 | 0.7046 | 0.8425 | 6.73 | 6.27 | 5.72 | 2.01 | 0.0099 | 0.0774 |
| TC2000007005.hg.1 | CST7 | cystatin F (leukocystatin) | Coding | 4.18 | 4.14 | 4.03 | 1.11 | 0.1076 | 0.2542 | 14.06 | 12.39 | 12.79 | 2.41 | 0.0002 | 0.0047 |
| TC2000008614.hg.1 | NKX2-2 | NK2 homeobox 2 | Coding | 3.89 | 3.51 | 3.74 | 1.11 | 0.49 | 0.69 | 3.88 | 3.69 | 3.6 | 1.21 | 0.2837 | 0.5491 |

| | | | | | | | | | | | | | | | |
|---|---|---|---|---|---|---|---|---|---|---|---|---|---|---|---|
| TC2000009813.hg.1 | EEF1A2 | eukaryotic translation elongation factor 1 alpha 2 | Multiple_( | 5.43 | 5.28 | 5.28 | 1.11 | 0.8371 | 0.919 | 11.11 | 10.26 | 10.07 | 2.06 | 0.0012 | 0.0192 |
| TC2100008143.hg.1 | ERG | v-ets avian erythroblastosis virus E26 oncogene homolog | Multiple_( | 4.9 | 4.43 | 4.75 | 1.11 | 0.4411 | 0.6498 | 5.26 | 5.30 | 5.24 | 1.01 | 0.0938 | 0.2973 |
| TC2200007021.hg.1 | RFPL1 | ret finger protein-like 1 | Coding | 3.71 | 3.88 | 3.56 | 1.11 | 0.5633 | 0.7452 | 3.64 | 3.60 | 3.47 | 1.13 | 0.3706 | 0.6327 |
| TC2200007576.hg.1 | PARVG | parvin, gamma | Multiple_( | 3.97 | 3.65 | 3.82 | 1.11 | 0.4829 | 0.6844 | 3.76 | 3.92 | 3.61 | 1.11 | 0.2894 | 0.5542 |
| TC2200008302.hg.1 | TPST2 | tyrosylprotein sulfotransferase 2 | Multiple_( | 8.02 | 7.63 | 7.87 | 1.11 | 0.4216 | 0.6327 | 7.22 | 7.45 | 7.17 | 1.04 | 0.3646 | 0.6271 |
| TC2200008449.hg.1 | DUSP18 | dual specificity phosphatase 18 | Multiple_( | 3.9 | 3.55 | 3.75 | 1.11 | 0.7878 | 0.8914 | 5.04 | 4.90 | 4.28 | 1.69 | 0.0058 | 0.0543 |
| TC2200008576.hg.1 | RBFOX2 | RNA binding protein, fox-1 homolog (C. elegans) 2 | Multiple_( | 9.85 | 9.74 | 9.7 | 1.11 | 0.4657 | 0.6704 | 8.14 | 7.89 | 8.63 | 0.71 | 0.0303 | 0.1546 |
| TC2200009276.hg.1 | SGSM3 | small G protein signaling modulator 3 | Multiple_( | 6.89 | 7.53 | 6.74 | 1.11 | 0.5858 | 0.7622 | 7.16 | 6.93 | 6.88 | 1.21 | 0.0723 | 0.2577 |
| TC2200009357.hg.1 | PACSIN2 | protein kinase C and casein kinase substrate in neurons 2 | Multiple_( | 11.54 | 11.24 | 11.39 | 1.11 | 0.27 | 0.4783 | 11.77 | 11.08 | 11.28 | 1.40 | 0.0146 | 0.0986 |
| TSUnmapped00000657.h | PRAMEF25 | PRAME family member 25 | Coding | 3.57 | 3.44 | 3.42 | 1.11 | 0.5349 | 0.7241 | 4.14 | 4.28 | 4.21 | 0.95 | 0.5672 | 0.7763 |
| TSUnmapped00000724.h | ADAMTS13 | ADAM metallopeptidase with thrombospondin type 1 motif 13 | Coding | 2.81 | 2.5 | 2.66 | 1.11 | 0.8679 | 0.9356 | 3.28 | 3.56 | 3.25 | 1.02 | 0.9703 | 0.9878 |
| TSUnmapped00000795.h | PRAMEF25 | PRAME family member 25 | Coding | 3.57 | 3.44 | 3.42 | 1.11 | 0.5349 | 0.7241 | 4.14 | 4.28 | 4.21 | 0.95 | 0.5672 | 0.7763 |
| TC0100007922.hg.1 | PPIE | Transcript Identified by AceView, Entrez Gene ID(s) 10450 | Coding | 5.29 | 4.92 | 5.15 | 1.10 | 0.0702 | 0.1859 | 4.26 | 4.46 | 4.38 | 0.92 | 0.6421 | 0.8245 |
| TC0100008289.hg.1 | OSBPL9 | oxysterol binding protein-like 9 | Multiple_( | 12.24 | 11.96 | 12.1 | 1.10 | 0.3714 | 0.5841 | 11.89 | 11.60 | 12.01 | 0.92 | 0.1004 | 0.3098 |
| TC0100008402.hg.1 | MROH7; TTC4 | maestro heat-like repeat family member 7; tetratricopeptide re | Multiple_( | 4.41 | 4.39 | 4.27 | 1.10 | 0.0678 | 0.1813 | 3.83 | 3.74 | 3.64 | 1.14 | 0.4301 | 0.6819 |
| TC0100008454.hg.1 | C8A | complement component 8, alpha polypeptide | Coding | 2.82 | 2.5 | 2.68 | 1.10 | 0.6654 | 0.8171 | 3.31 | 3.34 | 3.13 | 1.13 | 0.6904 | 0.8524 |
| TC0100008924.hg.1 | CLCA4 | chloride channel accessory 4 | Multiple_( | 3.01 | 2.62 | 2.87 | 1.10 | 0.6579 | 0.8122 | 3.62 | 3.81 | 3.54 | 1.06 | 0.3412 | 0.6051 |
| TC0100010013.hg.1 | IVL | involucrin | Multiple_( | 4.25 | 3.94 | 4.11 | 1.10 | 0.2041 | 0.3966 | 3.69 | 3.79 | 3.76 | 0.95 | 0.5651 | 0.7747 |
| TC0100010341.hg.1 | FCER1G | Fc fragment of IgE, high affinity I, receptor for; gamma polypept | Multiple_( | 4.19 | 4.81 | 4.05 | 1.10 | 0.5277 | 0.7191 | 3.77 | 4.07 | 3.71 | 1.04 | 0.9552 | 0.9814 |
| TC0100010689.hg.1 | TNN | tenascin N | Coding | 2.9 | 2.76 | 2.76 | 1.10 | 0.9587 | 0.9805 | 3.4 | 3.23 | 3.26 | 1.10 | 0.5707 | 0.779 |
| TC0100012351.hg.1 | OR2M3 | olfactory receptor, family 2, subfamily M, member 3 | Coding | 3.38 | 3.18 | 3.24 | 1.10 | 0.6272 | 0.792 | 3.78 | 3.77 | 3.94 | 0.90 | 0.7575 | 0.8901 |
| TC0100012884.hg.1 | FBXO2 | F-box protein 2 | Multiple_( | 7.41 | 7.13 | 7.27 | 1.10 | 0.3534 | 0.5663 | 6.91 | 6.72 | 6.12 | 1.73 | 0.007 | 0.0609 |
| TC0100013159.hg.1 | RNF186 | ring finger protein 186 | Coding | 3.79 | 3.45 | 3.65 | 1.10 | 0.8661 | 0.9346 | 3.7 | 3.56 | 3.61 | 1.06 | 0.9376 | 0.9741 |
| TC0100013313.hg.1 | MYOM3 | myomesin 3 | Multiple_( | 3.03 | 2.62 | 2.89 | 1.10 | 0.3627 | 0.5762 | 3.66 | 4.05 | 3.51 | 1.11 | 0.4783 | 0.7169 |
| TC0100015287.hg.1 | FAM212B | family with sequence similarity 212, member B | Multiple_( | 4.18 | 3.88 | 4.04 | 1.10 | 0.5374 | 0.7259 | 4.61 | 4.44 | 4.1 | 1.42 | 0.8661 | 0.9425 |
| TC0100017878.hg.1 | ZP4 | zona pellucida glycoprotein 4 | Coding | 2.98 | 2.86 | 2.84 | 1.10 | 0.9535 | 0.9784 | 3.09 | 3.00 | 3.06 | 1.02 | 0.1623 | 0.4056 |
| TC0100018197.hg.1 | MDS2 | myelodysplastic syndrome 2 translocation associated | Multiple_( | 4.39 | 4.13 | 4.25 | 1.10 | 0.8741 | 0.939 | 4.8 | 4.95 | 4.46 | 1.27 | 0.0802 | 0.273 |
| TC0100018344.hg.1 | LYPLAL1 | lysophospholipase-like 1 | Multiple_( | 10.21 | 11.76 | 10.07 | 1.10 | 0.5879 | 0.7641 | 8.35 | 9.07 | 8.51 | 0.90 | 0.6386 | 0.823 |
| TC0200006714.hg.1 | C2orf50 | chromosome 2 open reading frame 50 | Coding | 5.24 | 5.13 | 5.1 | 1.10 | 0.6753 | 0.8233 | 6.24 | 6.09 | 6.08 | 1.12 | 0.0742 | 0.2615 |
| TC0200007895.hg.1 | PLEK | pleckstrin | Multiple_( | 3.67 | 3.32 | 3.53 | 1.10 | 0.6721 | 0.8212 | 3.72 | 3.55 | 3.75 | 0.98 | 0.4027 | 0.6589 |
| TC0200010796.hg.1 | CTDSP1 | CTD small phosphatase 1 | Multiple_( | 7.91 | 7.84 | 7.77 | 1.10 | 0.7782 | 0.8859 | 8.88 | 10.73 | 9.59 | 0.61 | 0.003 | 0.0357 |
| TC0200011379.hg.1 | SNED1 | sushi, nidogen and EGF-like domains 1 | Multiple_( | 4.34 | 4.22 | 4.2 | 1.10 | 0.1294 | 0.2899 | 4.83 | 4.61 | 4.41 | 1.34 | 0.5574 | 0.7711 |
| TC0200012959.hg.1 | NFU1 | NFU1 iron-sulfur cluster scaffold | Multiple_( | 11.07 | 11.55 | 10.93 | 1.10 | 0.6429 | 0.8019 | 8.7 | 9.54 | 9.01 | 0.81 | 0.4818 | 0.72 |

| | | | | | | | | | | | | | | | |
|---|---|---|---|---|---|---|---|---|---|---|---|---|---|---|---|
| TC0200013172.hg.1 | REG1B | regenerating islet-derived 1 beta | Multiple_C | 3.38 | 3.24 | 3.24 | 1.10 | 0.3155 | 0.5287 | 3.48 | 3.28 | 3.06 | 1.34 | 0.5054 | 0.737 |
| TC0200013323.hg.1 | CD8A | CD8a molecule | Multiple_C | 3.29 | 2.93 | 3.15 | 1.10 | 0.7973 | 0.896 | 3.59 | 3.61 | 3.45 | 1.10 | 0.3328 | 0.597 |
| TC0200014303.hg.1 | WTH3DI; LOC | RAB6C-like; Synthetic construct Homo sapiens clone IMAGE:10 | Multiple_C | 6.59 | 6.48 | 6.45 | 1.10 | 0.8998 | 0.9519 | 6.16 | 5.98 | 6.32 | 0.90 | 0.3587 | 0.6216 |
| TC0200016222.hg.1 | HES6 | hes family bHLH transcription factor 6 | Multiple_C | 6.33 | 5.87 | 6.19 | 1.10 | 0.9793 | 0.9901 | 7.34 | 8.07 | 7.86 | 0.70 | 0.0201 | 0.1211 |
| TC0200016412.hg.1 | MFSD2B | major facilitator superfamily domain containing 2B | Multiple_C | 3.88 | 3.78 | 3.74 | 1.10 | 0.3991 | 0.6129 | 5.13 | 4.85 | 4.4 | 1.66 | 0.0003 | 0.0072 |
| TC0200016424.hg.1 | LBH | limb bud and heart development | Multiple_C | 4.2 | 3.9 | 4.06 | 1.10 | 0.7232 | 0.8544 | 5.6 | 5.83 | 6.25 | 0.64 | 0.4989 | 0.7316 |
| TC0200016503.hg.1 | POU3F3 | POU class 3 homeobox 3 | Multiple_C | 5.09 | 4.7 | 4.95 | 1.10 | 0.5148 | 0.7097 | 5.47 | 5.52 | 5.36 | 1.08 | 0.4827 | 0.7204 |
| TC0200016587.hg.1 | PLCL1 | phospholipase C-like 1 | Multiple_C | 4.23 | 4.09 | 4.09 | 1.10 | 0.5866 | 0.763 | 4.51 | 4.27 | 4.56 | 0.97 | 0.142 | 0.3746 |
| TC0300009189.hg.1 | AADACL2 | arylacetamide deacetylase-like 2 | Coding | 3.54 | 3.42 | 3.4 | 1.10 | 0.4792 | 0.6814 | 3.49 | 3.72 | 3.53 | 0.97 | 0.9252 | 0.9693 |
| TC0300011566.hg.1 | CNTN3 | contactin 3 (plasmacytoma associated) | Multiple_C | 3.25 | 3.18 | 3.11 | 1.10 | 0.6274 | 0.792 | 4.32 | 3.75 | 3.81 | 1.42 | 0.1156 | 0.3355 |
| TC0300011852.hg.1 | IMPG2 | interphotoreceptor matrix proteoglycan 2 | Multiple_C | 3.17 | 3.15 | 3.03 | 1.10 | 0.3532 | 0.566 | 3.7 | 3.40 | 3.44 | 1.20 | 0.2978 | 0.5625 |
| TC0300012027.hg.1 | USF3 | upstream transcription factor family member 3 | Multiple_C | 8.94 | 9.39 | 8.8 | 1.10 | 0.6373 | 0.7988 | 8.22 | 9.23 | 8.76 | 0.69 | 0.3199 | 0.5839 |
| TC0300013704.hg.1 | SMCO1 | single-pass membrane protein with coiled-coil domains 1 | Coding | 2.74 | 2.48 | 2.6 | 1.10 | 0.9227 | 0.964 | 3.45 | 3.42 | 3.26 | 1.14 | 0.2201 | 0.4801 |
| TC0400008390.hg.1 | LRIT3 | leucine-rich repeat, immunoglobulin-like and transmembrane d | Coding | 4.43 | 4.01 | 4.29 | 1.10 | 0.2593 | 0.4657 | 3.54 | 3.37 | 3.6 | 0.96 | 0.375 | 0.6356 |
| TC0400010983.hg.1 | GC | group-specific component (vitamin D binding protein) | Multiple_C | 3.01 | 2.93 | 2.87 | 1.10 | 0.5452 | 0.7312 | 3.18 | 3.52 | 3.3 | 0.92 | 0.5842 | 0.7881 |
| TC0400011616.hg.1 | TIFA | TRAF-interacting protein with forkhead-associated domain | Coding | 9.02 | 9.68 | 8.88 | 1.10 | 0.7352 | 0.8616 | 6.51 | 6.19 | 6.78 | 0.83 | 0.407 | 0.6629 |
| TC0400012312.hg.1 | ANP32C | acidic nuclear phosphoprotein 32 family member C | Multiple_C | 5.97 | 5.97 | 5.83 | 1.10 | 0.5216 | 0.7149 | 5.51 | 5.74 | 5.86 | 0.78 | 0.0593 | 0.2298 |
| TC0400012462.hg.1 | GLRA3 | glycine receptor alpha 3 | Multiple_C | 2.87 | 2.31 | 2.73 | 1.10 | 0.9602 | 0.9811 | 3.16 | 3.01 | 3.09 | 1.05 | 0.9618 | 0.984 |
| TC0400012807.hg.1 | PRR27 | proline rich 27 | Coding | 3.6 | 3.32 | 3.46 | 1.10 | 0.8907 | 0.9474 | 3.7 | 3.49 | 3.57 | 1.09 | 0.5046 | 0.7366 |
| TC0500008479.hg.1 | SNX2 | sorting nexin 2 | Multiple_C | 12.86 | 13.12 | 12.72 | 1.10 | 0.7581 | 0.8756 | 11.66 | 10.10 | 10.92 | 1.67 | 0.0579 | 0.2269 |
| TC0500008774.hg.1 | WNT8A | wingless-type MMTV integration site family, member 8A | Multiple_C | 5.36 | 5.51 | 5.22 | 1.10 | 0.82 | 0.9096 | 5.48 | 4.92 | 4.93 | 1.46 | 0.0469 | 0.2004 |
| TC0500009409.hg.1 | GABRP | gamma-aminobutyric acid (GABA) A receptor, pi | Multiple_C | 4.92 | 4.48 | 4.78 | 1.10 | 0.8688 | 0.9361 | 5.27 | 5.14 | 5.25 | 1.01 | 0.8137 | 0.9172 |
| TC0500009432.hg.1 | SMIM23 | small integral membrane protein 23 | Multiple_C | 3.11 | 3.01 | 2.97 | 1.10 | 0.5218 | 0.7151 | 3.66 | 3.54 | 3.5 | 1.12 | 0.2341 | 0.4967 |
| TC0500009623.hg.1 | PRR7 | proline rich 7 (synaptic) | Coding | 6.06 | 5.69 | 5.92 | 1.10 | 0.7145 | 0.8493 | 5.97 | 6.07 | 6.08 | 0.93 | 0.7203 | 0.8697 |
| TC0500013212.hg.1 | SRP19; ZRSR1 | signal recognition particle 19kDa; zinc finger (CCCH type), RNA b | Multiple_C | 11.71 | 12.17 | 11.57 | 1.10 | 0.878 | 0.9407 | 11.4 | 10.73 | 11 | 1.32 | 0.0648 | 0.2426 |
| TC0500013244.hg.1 | PCDHB16 | protocadherin beta 16 | Coding | 5.54 | 4.71 | 5.4 | 1.10 | 0.9335 | 0.9687 | 5.11 | 5.57 | 5.27 | 0.90 | 0.5245 | 0.7495 |
| TC0500013292.hg.1 | MARCHF11 | membrane associated ring finger 11 | Coding | 5.2 | 5.36 | 5.06 | 1.10 | 0.6971 | 0.8376 | 4.4 | 4.75 | 4.65 | 0.84 | 0.4716 | 0.7122 |
| TC0500013430.hg.1 | GNB2L1; SNO | guanine nucleotide binding protein (G protein), beta polypeptid | Multiple_C | 17.63 | 17.43 | 17.49 | 1.10 | 0.3676 | 0.5806 | 16.81 | 16.75 | 16.88 | 0.95 | 0.7109 | 0.8644 |
| TC0600007169.hg.1 | HDGFL1 | hepatoma derived growth factor-like 1 | Coding | 4.69 | 4.39 | 4.55 | 1.10 | 0.4923 | 0.6924 | 4.24 | 4.45 | 4.17 | 1.05 | 0.571 | 0.779 |
| TC0600007756.hg.1 | PACSIN1 | protein kinase C and casein kinase substrate in neurons 1 | Multiple_C | 3.96 | 3.94 | 3.82 | 1.10 | 0.1876 | 0.3749 | 3.8 | 3.76 | 3.67 | 1.09 | 0.5745 | 0.7818 |
| TC0600008168.hg.1 | ANKRD66 | ankyrin repeat domain 66 | Multiple_C | 4.72 | 4.45 | 4.58 | 1.10 | 0.6659 | 0.8173 | 4.06 | 4.14 | 4.14 | 0.95 | 0.4756 | 0.7147 |
| TC0600009234.hg.1 | FAM26E | family with sequence similarity 26, member E | Coding | 4.03 | 3.75 | 3.89 | 1.10 | 0.701 | 0.8403 | 4.13 | 4.65 | 4.29 | 0.90 | 0.41 | 0.665 |
| TC0600010954.hg.1 | KIF13A | kinesin family member 13A | Multiple_C | 7.89 | 7.59 | 7.75 | 1.10 | 0.6782 | 0.8251 | 6.97 | 7.56 | 7.25 | 0.82 | 0.0369 | 0.1751 |

| | | | | | | | | | | | | | | | |
|---|---|---|---|---|---|---|---|---|---|---|---|---|---|---|---|
| TC0600011874.hg.1 | DLK2 | delta-like 2 homolog (Drosophila) | Multiple_C | 3.21 | 3.16 | 3.07 | 1.10 | 0.6998 | 0.8394 | 3.67 | 3.72 | 3.64 | 1.02 | 0.2814 | 0.5472 |
| TC0600014264.hg.1 | DDAH2 | dimethylarginine dimethylaminohydrolase 2 | Multiple_C | 8.9 | 9.13 | 8.76 | 1.10 | 0.1702 | 0.3509 | 9.3 | 9.55 | 8.48 | 1.77 | 0.0023 | 0.0292 |
| TC0700007336.hg.1 | HECW1 | HECT, C2 and WW domain containing E3 ubiquitin protein ligase | Multiple_C | 3.76 | 3.33 | 3.62 | 1.10 | 0.6845 | 0.8292 | 4.46 | 4.29 | 3.96 | 1.41 | 0.0569 | 0.2244 |
| TC0700011785.hg.1 | CDK6 | cyclin-dependent kinase 6 | Multiple_C | 9.47 | 8.48 | 9.33 | 1.10 | 0.7468 | 0.87 | 11.22 | 11.75 | 11.88 | 0.63 | 0.007 | 0.0609 |
| TC0700012370.hg.1 | ASZ1 | ankyrin repeat, SAM and basic leucine zipper domain containing | Multiple_C | 3.58 | 3.67 | 3.44 | 1.10 | 0.3459 | 0.5596 | 3.92 | 3.71 | 3.57 | 1.27 | 0.2538 | 0.518 |
| TC0700013329.hg.1 | AMZ1 | archaelysin family metallopeptidase 1 | Multiple_C | 5.4 | 5.27 | 5.26 | 1.10 | 0.608 | 0.7787 | 5.7 | 5.84 | 5.43 | 1.21 | 0.0325 | 0.1614 |
| TC0800008416.hg.1 | GRHL2 | grainyhead-like transcription factor 2 | Multiple_C | 10.91 | 11.05 | 10.77 | 1.10 | 0.7473 | 0.8704 | 5.2 | 5.00 | 5.17 | 1.02 | 0.7233 | 0.871 |
| TC0800009289.hg.1 | ZNF517 | zinc finger protein 517 | Multiple_C | 5.75 | 5.26 | 5.61 | 1.10 | 0.7684 | 0.8808 | 4.95 | 4.85 | 4.91 | 1.03 | 0.4118 | 0.6663 |
| TC0800011486.hg.1 | KCNV1 | potassium channel, voltage gated modifier subfamily V, membe | Multiple_C | 3.9 | 3.47 | 3.76 | 1.10 | 0.2611 | 0.4679 | 3.68 | 3.75 | 3.48 | 1.15 | 0.3104 | 0.5745 |
| TC0800012396.hg.1 | FBXO16 | F-box protein 16 | Multiple_C | 7.91 | 8 | 7.77 | 1.10 | 0.5704 | 0.7504 | 7.97 | 7.66 | 7.64 | 1.26 | 0.1693 | 0.4151 |
| TC0800012447.hg.1 | AZIN1 | antizyme inhibitor 1 | Multiple_C | 13.41 | 14.36 | 13.27 | 1.10 | 0.6624 | 0.8148 | 12.65 | 12.19 | 13.34 | 0.62 | 0.1536 | 0.3928 |
| TC0800012468.hg.1 | HHLA1 | HERV-H LTR-associating 1 | Multiple_C | 3.86 | 3.85 | 3.72 | 1.10 | 0.6805 | 0.8263 | 3.5 | 3.65 | 3.7 | 0.87 | 0.5397 | 0.7594 |
| TC0900008130.hg.1 | NUTM2G | NUT family member 2G | Coding | 4.06 | 3.97 | 3.92 | 1.10 | 0.418 | 0.6296 | 4.14 | 4.11 | 4.14 | 1.00 | 0.5114 | 0.7412 |
| TC0900008290.hg.1 | OR13C8 | olfactory receptor, family 13, subfamily C, member 8 | Coding | 2.99 | 3.1 | 2.85 | 1.10 | 0.3469 | 0.5602 | 3.55 | 3.35 | 3.16 | 1.31 | 0.0958 | 0.3013 |
| TC0900010200.hg.1 | LOC10272423 | Homo sapiens uncharacterized LOC102724238 (LOC102724238) | Multiple_C | 7.57 | 7.15 | 7.43 | 1.10 | 0.1058 | 0.251 | 8.22 | 8.88 | 8.3 | 0.95 | 0.4264 | 0.6786 |
| TC0900011992.hg.1 | AGPAT2 | 1-acylglycerol-3-phosphate O-acyltransferase 2 | Multiple_C | 9.22 | 8.98 | 9.08 | 1.10 | 0.318 | 0.5314 | 8.29 | 8.43 | 8.73 | 0.74 | 0.0129 | 0.0918 |
| TC0900012039.hg.1 | ANAPC2 | anaphase promoting complex subunit 2 | Multiple_C | 9.58 | 9.6 | 9.44 | 1.10 | 0.1971 | 0.3879 | 9.2 | 9.18 | 9.08 | 1.09 | 0.5328 | 0.7556 |
| TC0900012173.hg.1 | GARNL3 | GTPase activating Rap/RanGAP domain-like 3 | Multiple_C | 6.91 | 6.81 | 6.77 | 1.10 | 0.5298 | 0.7204 | 5.64 | 4.85 | 5.27 | 1.29 | 0.1996 | 0.455 |
| TC0X00006723.hg.1 | CDKL5 | cyclin-dependent kinase-like 5 | Multiple_C | 6.56 | 6.26 | 6.42 | 1.10 | 0.3642 | 0.5778 | 5.48 | 5.10 | 5.51 | 0.98 | 0.7175 | 0.8682 |
| TC0X00007381.hg.1 | FOXR2 | forkhead box R2 | Coding | 3.19 | 3.24 | 3.05 | 1.10 | 0.5964 | 0.7701 | 3.32 | 3.57 | 3.4 | 0.95 | 0.8574 | 0.9394 |
| TC0X00008512.hg.1 | SMIM10L2A | small integral membrane protein 10 like 2A | Multiple_C | 4.77 | 4.68 | 4.63 | 1.10 | 0.2969 | 0.5085 | 4.48 | 4.41 | 4.42 | 1.04 | 0.2242 | 0.4851 |
| TC0X00008752.hg.1 | MAGEA12; CS | MAGE family member A12; CSAG family, member 4 (pseudogen | Multiple_C | 3.95 | 3.73 | 3.81 | 1.10 | 0.7743 | 0.8837 | 4.06 | 3.91 | 3.9 | 1.12 | 0.7517 | 0.8869 |
| TC0X00009799.hg.1 | FAM104B | family with sequence similarity 104, member B | Multiple_C | 9.29 | 9 | 9.15 | 1.10 | 0.6359 | 0.7976 | 9.73 | 9.71 | 9.74 | 0.99 | 0.6282 | 0.8165 |
| TC0X00010369.hg.1 | ARMCX2 | armadillo repeat containing, X-linked 2 | Multiple_C | 4.31 | 4.02 | 4.17 | 1.10 | 0.527 | 0.7187 | 4.46 | 4.25 | 4.38 | 1.06 | 0.9807 | 0.9918 |
| TC0X00010385.hg.1 | NXF2B; TCP11 | nuclear RNA export factor 2B; t-complex 11 family, X-linked 2 | Multiple_C | 3.35 | 3.01 | 3.21 | 1.10 | 0.6894 | 0.8328 | 3.39 | 3.21 | 3.2 | 1.14 | 0.5017 | 0.7342 |
| TC0X00010516.hg.1 | DCX | doublecortin | Multiple_C | 3.3 | 3.3 | 3.16 | 1.10 | 0.5847 | 0.7614 | 3.86 | 3.72 | 3.9 | 0.97 | 0.9944 | 0.9975 |
| TC0X00010628.hg.1 | KIAA1210 | KIAA1210 | Coding | 3.74 | 3.25 | 3.6 | 1.10 | 0.1364 | 0.3011 | 3.5 | 3.54 | 3.64 | 0.91 | 0.4463 | 0.6935 |
| TC0X00010915.hg.1 | CT45A2 | cancer/testis antigen family 45, member A2 | Coding | 3.12 | 2.73 | 2.98 | 1.10 | 0.6828 | 0.828 | 3.56 | 3.55 | 3.59 | 0.98 | 0.299 | 0.5637 |
| TC0X00010917.hg.1 | CT45A8 | cancer/testis antigen family 45, member A8 | Coding | 3.14 | 2.85 | 3 | 1.10 | 0.6148 | 0.7842 | 3.57 | 3.61 | 3.61 | 0.97 | 0.3792 | 0.6388 |
| TC0X00010918.hg.1 | CT45A9 | cancer/testis antigen family 45, member A9 | Coding | 3.14 | 2.85 | 3 | 1.10 | 0.6148 | 0.7842 | 3.57 | 3.61 | 3.61 | 0.97 | 0.3792 | 0.6388 |
| TC0X00011365.hg.1 | SSX5 | synovial sarcoma, X breakpoint 5 | Coding | 3.39 | 2.99 | 3.25 | 1.10 | 0.6627 | 0.8151 | 3.79 | 3.64 | 3.57 | 1.16 | 0.3077 | 0.5717 |
| TC0Y00006569.hg.1 | TSPY3; TSPY4; | testis specific protein, Y-linked 3; testis specific protein, Y-linkec | Multiple_C | 3.28 | 3.04 | 3.14 | 1.10 | 0.7698 | 0.8811 | 3.7 | 3.50 | 3.63 | 1.05 | 0.6579 | 0.8329 |
| TC1000006880.hg.1 | PTER | phosphotriesterase related | Multiple_C | 10.88 | 11.31 | 10.74 | 1.10 | 0.8069 | 0.902 | 10.55 | 10.09 | 9.9 | 1.57 | 0.4411 | 0.6904 |

| | | | | | | | | | | | | | | | |
|---|---|---|---|---|---|---|---|---|---|---|---|---|---|---|---|
| TC1000007092.hg.1 | MASTL | microtubule associated serine/threonine kinase-like | Multiple_C | 8.82 | 8.45 | 8.68 | 1.10 | 0.9866 | 0.9933 | 6.5 | 6.95 | 7.58 | 0.47 | 0.0037 | 0.0412 |
| TC1000007277.hg.1 | GJD4 | gap junction protein delta 4 | Coding | 3.47 | 3.16 | 3.33 | 1.10 | 0.4144 | 0.6265 | 3.8 | 3.79 | 3.69 | 1.08 | 0.3578 | 0.6207 |
| TC1000008011.hg.1 | OIT3 | oncoprotein induced transcript 3 | Multiple_C | 4.07 | 3.86 | 3.93 | 1.10 | 0.6349 | 0.7967 | 4.35 | 4.25 | 4.7 | 0.78 | 0.3334 | 0.5976 |
| TC1000008383.hg.1 | LIPM | lipase, family member M | Coding | 3.32 | 3.28 | 3.18 | 1.10 | 0.7377 | 0.8636 | 3.22 | 3.38 | 3.34 | 0.92 | 0.9934 | 0.997 |
| TC1000008568.hg.1 | FRAT1 | frequently rearranged in advanced T-cell lymphomas 1 | Multiple_C | 5.95 | 5.47 | 5.81 | 1.10 | 0.8129 | 0.9057 | 4.93 | 5.10 | 4.76 | 1.13 | 0.8131 | 0.917 |
| TC1000008613.hg.1 | CNNM1 | cyclin and CBS domain divalent metal cation transport mediator | Multiple_C | 4.2 | 4.78 | 4.06 | 1.10 | 0.3668 | 0.5799 | 4.03 | 4.12 | 4.13 | 0.93 | 0.6746 | 0.8426 |
| TC1000008923.hg.1 | TECTB | tectorin beta | Multiple_C | 3.03 | 2.94 | 2.89 | 1.10 | 0.4658 | 0.6704 | 3.58 | 3.39 | 3.1 | 1.39 | 0.0817 | 0.2753 |
| TC1000010673.hg.1 | PCDH15 | protocadherin-related 15 | Multiple_C | 3.09 | 2.91 | 2.95 | 1.10 | 0.5024 | 0.7004 | 3.68 | 3.70 | 3.56 | 1.09 | 0.4019 | 0.6582 |
| TC1000010980.hg.1 | ASCC1 | activating signal cointegrator 1 complex subunit 1 | Multiple_C | 11.83 | 12.2 | 11.69 | 1.10 | 0.533 | 0.7227 | 11.79 | 11.75 | 11.33 | 1.38 | 0.4401 | 0.6898 |
| TC1000011599.hg.1 | CPN1 | carboxypeptidase N, polypeptide 1 | Coding | 4.18 | 3.88 | 4.04 | 1.10 | 0.126 | 0.2843 | 3.6 | 3.74 | 3.5 | 1.07 | 0.8267 | 0.9235 |
| TC1100006500.hg.1 | AP2A2 | adaptor-related protein complex 2, alpha 2 subunit | Multiple_C | 11.71 | 11.3 | 11.57 | 1.10 | 0.5624 | 0.7443 | 11.12 | 11.42 | 11.18 | 0.96 | 0.6517 | 0.8303 |
| TC1100006721.hg.1 | CNGA4 | cyclic nucleotide gated channel alpha 4 | Coding | 3.85 | 3.27 | 3.71 | 1.10 | 0.3797 | 0.5928 | 3.95 | 3.86 | 3.42 | 1.44 | 0.0436 | 0.1931 |
| TC1100006726.hg.1 | SMPD1 | sphingomyelin phosphodiesterase 1, acid lysosomal | Multiple_C | 4.29 | 4.01 | 4.15 | 1.10 | 0.332 | 0.5459 | 5.03 | 5.36 | 5.66 | 0.65 | 0.0046 | 0.0468 |
| TC1100007429.hg.1 | MAPK8IP1 | mitogen-activated protein kinase 8 interacting protein 1 | Multiple_C | 3.62 | 3.47 | 3.48 | 1.10 | 0.6061 | 0.7773 | 3.9 | 4.34 | 4.42 | 0.70 | 0.0446 | 0.1954 |
| TC1100007771.hg.1 | MS4A1 | membrane-spanning 4-domains, subfamily A, member 1 | Multiple_C | 3.18 | 2.94 | 3.04 | 1.10 | 0.6159 | 0.7848 | 3.62 | 3.38 | 3.71 | 0.94 | 0.8232 | 0.9219 |
| TC1100007778.hg.1 | MS4A18 | membrane-spanning 4-domains, subfamily A, member 18 | Multiple_C | 3.69 | 3.47 | 3.55 | 1.10 | 0.6763 | 0.8241 | 3.33 | 3.39 | 3.32 | 1.01 | 0.5965 | 0.7967 |
| TC1100008769.hg.1 | MTNR1B | melatonin receptor 1B | Multiple_C | 3.74 | 3.47 | 3.6 | 1.10 | 0.861 | 0.9321 | 4.17 | 4.29 | 4.15 | 1.01 | 0.6038 | 0.8019 |
| TC1100009244.hg.1 | PDZD3 | PDZ domain containing 3 | Multiple_C | 3.48 | 2.99 | 3.34 | 1.10 | 0.6454 | 0.8034 | 3.61 | 3.53 | 3.45 | 1.12 | 0.284 | 0.5495 |
| TC1100009370.hg.1 | OR10G8 | olfactory receptor, family 10, subfamily G, member 8 | Coding | 4.17 | 4.13 | 4.03 | 1.10 | 0.3583 | 0.572 | 4.44 | 4.55 | 4.54 | 0.93 | 0.8616 | 0.9408 |
| TC1100009880.hg.1 | TRIM21 | tripartite motif containing 21 | Coding | 6.23 | 6.52 | 6.09 | 1.10 | 0.6102 | 0.7806 | 4.24 | 4.45 | 4.8 | 0.68 | 0.0536 | 0.2168 |
| TC1100011622.hg.1 | CHRDL2 | chordin-like 2 | Multiple_C | 3.09 | 2.69 | 2.95 | 1.10 | 0.9825 | 0.9919 | 3.37 | 3.39 | 3.37 | 1.00 | 0.5429 | 0.7614 |
| TC1100012105.hg.1 | ANGPTL5 | angiopoietin like 5 | Coding | 3.92 | 3.9 | 3.78 | 1.10 | 0.3138 | 0.5273 | 4.59 | 4.43 | 4.24 | 1.27 | 0.1004 | 0.3098 |
| TC1100012786.hg.1 | KCNJ1 | potassium channel, inwardly rectifying subfamily J, member 1 | Multiple_C | 3.51 | 3.44 | 3.37 | 1.10 | 0.5848 | 0.7614 | 3.7 | 3.68 | 3.43 | 1.21 | 0.2394 | 0.5028 |
| TC1100013203.hg.1 | ALG1L9P | asparagine-linked glycosylation 1-like 9, pseudogene | Multiple_C | 5.63 | 5.34 | 5.49 | 1.10 | 0.7599 | 0.8763 | 5.36 | 5.43 | 5.25 | 1.08 | 0.8271 | 0.9237 |
| TC1200006816.hg.1 | PRH2 | proline-rich protein HaeIII subfamily 2 | Coding | 3.11 | 3.26 | 2.97 | 1.10 | 0.5007 | 0.6994 | 3.15 | 3.28 | 3.16 | 0.99 | 0.9641 | 0.9847 |
| TC1200008116.hg.1 | MDM2 | MDM2 proto-oncogene, E3 ubiquitin protein ligase | Multiple_C | 9.03 | 8.68 | 8.89 | 1.10 | 0.9466 | 0.9754 | 8.99 | 9.14 | 9.71 | 0.61 | 0.1053 | 0.3186 |
| TC1200011067.hg.1 | DPY19L2 | dpy-19-like 2 (C. elegans) | Multiple_C | 4.02 | 3.96 | 3.88 | 1.10 | 0.5747 | 0.7537 | 4.58 | 4.60 | 4.21 | 1.29 | 0.0853 | 0.2811 |
| TC1300007707.hg.1 | GPC5 | glypican 5 | Multiple_C | 3.82 | 3.71 | 3.68 | 1.10 | 0.7062 | 0.8438 | 3.52 | 3.32 | 3.45 | 1.05 | 0.776 | 0.8995 |
| TC1300008916.hg.1 | CAB39L | calcium binding protein 39-like | Multiple_C | 5.35 | 5.93 | 5.21 | 1.10 | 0.6036 | 0.7756 | 10.9 | 10.28 | 9.77 | 2.19 | 0.0193 | 0.1174 |
| TC1400007442.hg.1 | ZBTB1 | zinc finger and BTB domain containing 1 | Multiple_C | 9.48 | 10.02 | 9.34 | 1.10 | 0.6606 | 0.8138 | 9.87 | 9.71 | 9.42 | 1.37 | 0.0564 | 0.2234 |
| TC1400008597.hg.1 | RNASE12; RN/ | ribonuclease, RNase A family, 12 (non-active); ribonuclease, RN | Multiple_C | 3.8 | 3.51 | 3.66 | 1.10 | 0.6381 | 0.7992 | 4.42 | 3.80 | 3.95 | 1.39 | 0.0615 | 0.2355 |
| TC1400009992.hg.1 | FBLN5 | fibulin 5 | Multiple_C | 3.89 | 3.5 | 3.75 | 1.10 | 0.2507 | 0.4551 | 3.22 | 3.29 | 3.21 | 1.01 | 0.9976 | 0.9988 |
| TC1400010620.hg.1 | SNAPC1 | small nuclear RNA activating complex polypeptide 1 | Multiple_C | 8.64 | 10.58 | 8.5 | 1.10 | 0.6277 | 0.7921 | 9.03 | 8.76 | 9.2 | 0.89 | 0.7587 | 0.8905 |

| | | | | | | | | | | | | | | | |
|---|---|---|---|---|---|---|---|---|---|---|---|---|---|---|---|
| TC1500008003.hg.1 | TMED3 | transmembrane p24 trafficking protein 3 | Multiple_C | 10.07 | 9.8 | 9.93 | 1.10 | 0.4666 | 0.6711 | 4.1 | 4.20 | 3.93 | 1.13 | 0.1383 | 0.3692 |
| TC1600006453.hg.1 | PDIA2 | protein disulfide isomerase family A, member 2 | Multiple_C | 3.49 | 3.66 | 3.35 | 1.10 | 0.5138 | 0.7089 | 3.31 | 3.50 | 3.42 | 0.93 | 0.4899 | 0.7255 |
| TC1600007130.hg.1 | ACSM5 | acyl-CoA synthetase medium-chain family member 5 | Multiple_C | 3.27 | 3.19 | 3.13 | 1.10 | 0.7102 | 0.8463 | 3.99 | 3.79 | 3.92 | 1.05 | 0.1581 | 0.3996 |
| TC1600008889.hg.1 | CPNE7 | copine VII | Multiple_C | 6.36 | 6.37 | 6.22 | 1.10 | 0.386 | 0.5993 | 6.15 | 5.99 | 5.41 | 1.67 | 0.2658 | 0.5304 |
| TC1700007399.hg.1 | ERAL1 | Era-like 12S mitochondrial rRNA chaperone 1 | Multiple_C | 9.73 | 9.32 | 9.59 | 1.10 | 0.7148 | 0.8493 | 9.47 | 10.70 | 10.64 | 0.44 | 0.0105 | 0.0807 |
| TC1700008566.hg.1 | MAP3K3 | mitogen-activated protein kinase kinase kinase 3 | Multiple_C | 5.12 | 4.8 | 4.98 | 1.10 | 0.1841 | 0.3697 | 5.31 | 5.62 | 6.13 | 0.57 | 0.017 | 0.1091 |
| TC1700008915.hg.1 | SMIM6 | small integral membrane protein 6 | Multiple_C | 6.64 | 6.37 | 6.5 | 1.10 | 0.6854 | 0.83 | 7.35 | 7.59 | 7.33 | 1.01 | 0.4198 | 0.6731 |
| TC1700009600.hg.1 | TEKT1 | tektin 1 | Multiple_C | 4.06 | 4.1 | 3.92 | 1.10 | 0.5028 | 0.7006 | 4.52 | 4.28 | 4.06 | 1.38 | 0.0229 | 0.1303 |
| TC1700011074.hg.1 | DLX3 | distal-less homeobox 3 | Coding | 3.16 | 2.8 | 3.02 | 1.10 | 0.674 | 0.8226 | 5.96 | 5.98 | 5.75 | 1.16 | 0.5212 | 0.7482 |
| TC1700012367.hg.1 | TNFRSF13B | tumor necrosis factor receptor superfamily, member 13B | Multiple_C | 5.64 | 5.29 | 5.5 | 1.10 | 0.6881 | 0.8318 | 6.14 | 6.09 | 6 | 1.10 | 0.6016 | 0.8005 |
| TC1700012381.hg.1 | SGK494; FLJ25 | uncharacterized serine/threonine-protein kinase SgK494; Synth | Multiple_C | 6.07 | 6.18 | 5.93 | 1.10 | 0.9764 | 0.9885 | 5.91 | 6.30 | 5.77 | 1.10 | 0.3126 | 0.5768 |
| TC1700012386.hg.1 | RFFL | ring finger and FYVE-like domain containing E3 ubiquitin protein | Multiple_C | 8.64 | 8.18 | 8.5 | 1.10 | 0.6312 | 0.7943 | 6.45 | 7.14 | 7.22 | 0.59 | 0.0057 | 0.0534 |
| TC1800007052.hg.1 | ASXL3 | additional sex combs like transcriptional regulator 3 | Multiple_C | 3.85 | 3.56 | 3.71 | 1.10 | 0.6118 | 0.782 | 3.29 | 3.36 | 3.59 | 0.81 | 0.7124 | 0.8651 |
| TC1800008387.hg.1 | DSC1 | desmocollin 1 | Coding | 3.61 | 3.49 | 3.47 | 1.10 | 0.2628 | 0.4699 | 3.75 | 3.86 | 3.58 | 1.13 | 0.2491 | 0.5137 |
| TC1800008827.hg.1 | CCBE1 | collagen and calcium binding EGF domains 1 | Multiple_C | 3.77 | 3.54 | 3.63 | 1.10 | 0.8034 | 0.8996 | 3.55 | 3.59 | 3.43 | 1.09 | 0.417 | 0.6707 |
| TC1900006924.hg.1 | ZNF317 | zinc finger protein 317 | Multiple_C | 11.01 | 10.27 | 10.87 | 1.10 | 0.1781 | 0.361 | 9.74 | 9.40 | 10.02 | 0.82 | 0.3487 | 0.6127 |
| TC1900007344.hg.1 | B3GNT3 | UDP-GlcNAc:betaGal beta-1,3-N-acetylglucosaminyltransferase | Coding | 6.71 | 6.26 | 6.57 | 1.10 | 0.9751 | 0.9878 | 6.45 | 7.17 | 7.69 | 0.42 | 0.0019 | 0.0259 |
| TC1900008839.hg.1 | TSEN34 | TSEN34 tRNA splicing endonuclease subunit | Multiple_C | 5.66 | 5.57 | 5.52 | 1.10 | 0.4885 | 0.689 | 4.62 | 4.54 | 4.12 | 1.41 | 0.0836 | 0.2782 |
| TC1900009350.hg.1 | PLIN4 | perilipin 4 | Coding | 4.11 | 4 | 3.97 | 1.10 | 0.3148 | 0.5283 | 3.76 | 3.70 | 3.52 | 1.18 | 0.2796 | 0.5457 |
| TC1900009431.hg.1 | SLC25A23 | solute carrier family 25 (mitochondrial carrier; phosphate carrie | Multiple_C | 8.46 | 8.04 | 8.32 | 1.10 | 0.5437 | 0.7303 | 6.79 | 6.62 | 6.51 | 1.21 | 0.4492 | 0.6958 |
| TC1900010063.hg.1 | TSSK6 | testis-specific serine kinase 6 | Coding | 4.06 | 4.24 | 3.92 | 1.10 | 0.3935 | 0.6074 | 3.52 | 3.58 | 3.74 | 0.86 | 0.4056 | 0.6611 |
| TC1900010074.hg.1 | GMIP | GEM interacting protein | Multiple_C | 6.86 | 6.83 | 6.72 | 1.10 | 0.8565 | 0.9299 | 6.13 | 6.46 | 6.36 | 0.85 | 0.4227 | 0.6754 |
| TC1900010469.hg.1 | FAM187B | family with sequence similarity 187, member B | Coding | 3.02 | 2.67 | 2.88 | 1.10 | 0.5184 | 0.7125 | 3.16 | 3.39 | 3.47 | 0.81 | 0.1196 | 0.3417 |
| TC1900011114.hg.1 | LHB | luteinizing hormone beta polypeptide | Coding | 8.5 | 8.43 | 8.36 | 1.10 | 0.2076 | 0.4011 | 7.53 | 7.37 | 7.21 | 1.25 | 0.7345 | 0.8776 |
| TC1900011445.hg.1 | PPP1R12C | protein phosphatase 1, regulatory subunit 12C | Multiple_C | 7.45 | 7.29 | 7.31 | 1.10 | 0.4616 | 0.667 | 6.02 | 6.03 | 5.76 | 1.20 | 0.6779 | 0.8449 |
| TC1900011742.hg.1 | CEACAM6 | carcinoembryonic antigen-related cell adhesion molecule 6 (nor | Coding | 9.73 | 9.06 | 9.59 | 1.10 | 0.6928 | 0.8345 | 5.08 | 5.56 | 5.65 | 0.67 | 0.0247 | 0.1362 |
| TC1900011880.hg.1 | ZNF44 | zinc finger protein 44 | Multiple_C | 8.36 | 9.11 | 8.22 | 1.10 | 0.5109 | 0.7068 | 7.29 | 7.06 | 7.19 | 1.07 | 0.7791 | 0.9011 |
| TC1900011887.hg.1 | ZNF490 | zinc finger protein 490 | Multiple_C | 9.62 | 9.59 | 9.48 | 1.10 | 0.9469 | 0.9756 | 9.18 | 9.39 | 9.17 | 1.01 | 0.9994 | 0.9997 |
| TC1900011919.hg.1 | ZNF708 | zinc finger protein 708 | Multiple_C | 8.66 | 8.5 | 8.52 | 1.10 | 0.8633 | 0.9334 | 9.61 | 9.72 | 10.02 | 0.75 | 0.9728 | 0.989 |
| TC1900011993.hg.1 | CGB; CGB5 | chorionic gonadotropin, beta polypeptide; chorionic gonadotro | Multiple_C | 3.99 | 3.7 | 3.85 | 1.10 | 0.4846 | 0.686 | 4 | 3.94 | 3.96 | 1.03 | 0.2504 | 0.5148 |
| TC1900012031.hg.1 | ZNF816 | zinc finger protein 816 | Coding | 9.09 | 9.4 | 8.95 | 1.10 | 0.5764 | 0.7549 | 7.35 | 7.69 | 7.52 | 0.89 | 0.7663 | 0.8951 |
| TC2000007072.hg.1 | DEFB123 | defensin, beta 123 | Multiple_C | 4.35 | 4.21 | 4.21 | 1.10 | 0.7042 | 0.8422 | 5.16 | 5.28 | 5.4 | 0.85 | 0.9454 | 0.9778 |
| TC2000008686.hg.1 | CST5 | cystatin D | Coding | 4.6 | 4.07 | 4.46 | 1.10 | 0.5006 | 0.6994 | 5.16 | 5.18 | 4.83 | 1.26 | 0.0248 | 0.1366 |

| | | | | | | | | | | | | | | | | |
|---|---|---|---|---|---|---|---|---|---|---|---|---|---|---|---|---|
| TC2000009780.hg.1 | TCFL5 | transcription factor-like 5 (basic helix-loop-helix) | Multiple_C | 9.75 | 10.29 | 9.61 | 1.10 | 0.6826 | 0.8279 | 8.73 | 8.92 | 8.85 | 0.92 | 0.5007 | 0.7334 |
| TC2100006909.hg.1 | HUNK | hormonally up-regulated Neu-associated kinase | Multiple_C | 3.02 | 2.71 | 2.88 | 1.10 | 0.7417 | 0.8665 | 8.04 | 9.64 | 8.85 | 0.57 | 0.0008 | 0.0144 |
| TC2100007377.hg.1 | KRTAP10-10 | keratin associated protein 10-10 | Coding | 4.96 | 4.99 | 4.82 | 1.10 | 0.7229 | 0.8542 | 5.74 | 5.41 | 5.43 | 1.24 | 0.4135 | 0.668 |
| TC2100008081.hg.1 | CLDN14 | claudin 14 | Multiple_C | 4.89 | 4.65 | 4.75 | 1.10 | 0.1923 | 0.3811 | 4.61 | 4.70 | 4.96 | 0.78 | 0.7344 | 0.8776 |
| TC2200006570.hg.1 | DGCR6 | DiGeorge syndrome critical region gene 6 | Multiple_C | 7.54 | 6.99 | 7.4 | 1.10 | 0.5775 | 0.7557 | 7.5 | 7.36 | 6.79 | 1.64 | 0.0059 | 0.0545 |
| TC2200007043.hg.1 | HORMAD2 | HORMA domain containing 2 | Multiple_C | 3.4 | 3.32 | 3.26 | 1.10 | 0.4157 | 0.6277 | 3.7 | 3.89 | 3.84 | 0.91 | 0.9376 | 0.9741 |
| TC2200007483.hg.1 | CSDC2 | cold shock domain containing C2, RNA binding | Multiple_C | 4.59 | 4.47 | 4.45 | 1.10 | 0.4064 | 0.6189 | 5.7 | 5.73 | 5.41 | 1.22 | 0.319 | 0.583 |
| TC2200007787.hg.1 | MOV10L1 | Mov10 RISC complex RNA helicase like 1 | Multiple_C | 3.55 | 3.39 | 3.41 | 1.10 | 0.7074 | 0.8445 | 3.98 | 3.56 | 3.69 | 1.22 | 0.0621 | 0.2366 |
| TC2200007826.hg.1 | ACR | acrosin | Multiple_C | 2.79 | 2.75 | 2.65 | 1.10 | 0.5561 | 0.7392 | 3.37 | 3.26 | 3.27 | 1.07 | 0.5425 | 0.7614 |
| TC2200008234.hg.1 | PIWIL3 | piwi-like RNA-mediated gene silencing 3 | Multiple_C | 3.74 | 3.24 | 3.6 | 1.10 | 0.3461 | 0.5597 | 4.01 | 4.27 | 4.01 | 1.00 | 0.7091 | 0.8634 |
| TCUn_GL000219v10000( | LOC283788; A | FSHD region gene 1 pseudogene | Multiple_C | 7.41 | 7.6 | 7.27 | 1.10 | 0.4643 | 0.6693 | 6.52 | 6.31 | 6.28 | 1.18 | 0.4018 | 0.6582 |
| TSUnmapped00000204.h | OR10A6 | olfactory receptor, family 10, subfamily A, member 6 (gene/pse | Coding | 3.24 | 2.78 | 3.1 | 1.10 | 0.2031 | 0.3953 | 3.73 | 3.83 | 3.49 | 1.18 | 0.1194 | 0.3415 |
| TSUnmapped00000319.h | NDUFA6 | NADH dehydrogenase (ubiquinone) 1 alpha subcomplex, 6, 14kI | Coding | 5.6 | 5.4 | 5.46 | 1.10 | 0.4112 | 0.6234 | 4.48 | 4.44 | 4.52 | 0.97 | 0.8687 | 0.9434 |
| TSUnmapped00000626.h | FMN1 | formin 1 | Coding | 4.83 | 4.25 | 4.69 | 1.10 | 0.9465 | 0.9754 | 7.95 | 7.11 | 7.5 | 1.37 | 0.4033 | 0.6594 |
| TSUnmapped00000692.h | ZNF502 | zinc finger protein 502 | Coding | 3.4 | 3.19 | 3.26 | 1.10 | 0.8822 | 0.9428 | 3.75 | 3.54 | 4.05 | 0.81 | 0.3452 | 0.6091 |
| TSUnmapped00000714.h | MANSC1 | MANSC domain containing 1 | Coding | 4.76 | 4.23 | 4.62 | 1.10 | 0.7861 | 0.8902 | 3.59 | 3.51 | 3.64 | 0.97 | 0.9724 | 0.9887 |
| TSUnmapped00000720.h | PRAMEF10 | PRAME family member 10 | Coding | 3.42 | 3.42 | 3.28 | 1.10 | 0.2779 | 0.4872 | 3.17 | 3.16 | 3.02 | 1.11 | 0.5049 | 0.7368 |
| TC0100006931.hg.1 | PDPN | podoplanin | Multiple_C | 3.28 | 3.27 | 3.15 | 1.09 | 0.6666 | 0.8176 | 3.63 | 3.27 | 3.44 | 1.14 | 0.1842 | 0.4352 |
| TC0100008146.hg.1 | LURAP1 | leucine rich adaptor protein 1 | Multiple_C | 6.26 | 6.09 | 6.13 | 1.09 | 0.4702 | 0.6742 | 5.64 | 5.69 | 5.47 | 1.13 | 0.553 | 0.7684 |
| TC0100008334.hg.1 | ZYG11A | zyg-11 family member A, cell cycle regulator | Multiple_C | 2.98 | 3.15 | 2.85 | 1.09 | 0.6978 | 0.838 | 3.56 | 3.18 | 3.33 | 1.17 | 0.3634 | 0.6261 |
| TC0100008341.hg.1 | PODN | podocan | Multiple_C | 6.37 | 6.09 | 6.24 | 1.09 | 0.3316 | 0.5457 | 5.51 | 5.54 | 5.89 | 0.77 | 0.059 | 0.2289 |
| TC0100008496.hg.1 | FGGY | FGGY carbohydrate kinase domain containing | Multiple_C | 6.01 | 5.99 | 5.88 | 1.09 | 0.5456 | 0.7313 | 7.7 | 8.88 | 8.61 | 0.53 | 0.0082 | 0.0682 |
| TC0100012469.hg.1 | MXRA8 | matrix-remodelling associated 8 | Multiple_C | 3.02 | 2.77 | 2.89 | 1.09 | 0.6509 | 0.8067 | 3.73 | 3.59 | 3.65 | 1.06 | 0.9165 | 0.9659 |
| TC0100012942.hg.1 | PRAMEF18 | PRAME family member 18 | Coding | 3.32 | 3.33 | 3.19 | 1.09 | 0.7626 | 0.878 | 4.02 | 4.09 | 3.87 | 1.11 | 0.1705 | 0.4168 |
| TC0100014240.hg.1 | GLIS1 | GLIS family zinc finger 1 | Multiple_C | 3.87 | 3.41 | 3.74 | 1.09 | 0.4871 | 0.6883 | 3.83 | 3.72 | 3.62 | 1.16 | 0.3239 | 0.5882 |
| TC0100014852.hg.1 | GBP1 | guanylate binding protein 1, interferon-inducible | Multiple_C | 6.13 | 5.47 | 6 | 1.09 | 0.6831 | 0.8283 | 3.89 | 4.02 | 3.9 | 0.99 | 0.8661 | 0.9425 |
| TC0100016626.hg.1 | TEDDM1 | transmembrane epididymal protein 1 | Coding | 3.51 | 3.22 | 3.38 | 1.09 | 0.9687 | 0.9849 | 3.58 | 3.55 | 3.64 | 0.96 | 0.7276 | 0.8736 |
| TC0100016952.hg.1 | UBE2T | ubiquitin conjugating enzyme E2T | Multiple_C | 14.53 | 15.12 | 14.4 | 1.09 | 0.8555 | 0.9294 | 12.01 | 12.10 | 12 | 1.01 | 0.4697 | 0.7106 |
| TC0100017216.hg.1 | NEK2 | NIMA-related kinase 2 | Multiple_C | 11.36 | 12.66 | 11.23 | 1.09 | 0.3889 | 0.6028 | 10.44 | 10.60 | 10.37 | 1.05 | 0.7712 | 0.8972 |
| TC0100018353.hg.1 | SNAP47 | synaptosome associated protein 47kDa | Multiple_C | 7.9 | 7.41 | 7.77 | 1.09 | 0.596 | 0.7698 | 7.19 | 7.00 | 7.22 | 0.98 | 0.6664 | 0.8366 |
| TC0100018394.hg.1 | TTC34 | tetratricopeptide repeat domain 34 | Multiple_C | 8.09 | 7.6 | 7.96 | 1.09 | 0.5362 | 0.725 | 8.23 | 8.56 | 8.42 | 0.88 | 0.5918 | 0.7936 |
| TC0200008947.hg.1 | IL36A | interleukin 36, alpha | Coding | 3.28 | 3.03 | 3.15 | 1.09 | 0.4823 | 0.6838 | 3.66 | 3.70 | 3.42 | 1.18 | 0.4812 | 0.7196 |
| TC0200009095.hg.1 | INHBB | inhibin beta B | Coding | 3.34 | 3.13 | 3.21 | 1.09 | 0.5758 | 0.7544 | 11.3 | 11.74 | 12.08 | 0.58 | 0.0282 | 0.1477 |

| | | | | | | | | | | | | | | | |
|---|---|---|---|---|---|---|---|---|---|---|---|---|---|---|---|
| TC0200009616.hg.1 | KIF5C | kinesin family member 5C | Multiple_C | 3.36 | 2.96 | 3.23 | 1.09 | 0.6596 | 0.8133 | 3.82 | 3.65 | 3.72 | 1.07 | 0.5238 | 0.7491 |
| TC0200010741.hg.1 | SMARCAL1 | SWI/SNF related, matrix associated, actin dependent regulator c | Multiple_C | 7.68 | 7.22 | 7.55 | 1.09 | 0.7276 | 0.8567 | 7.5 | 7.68 | 7.12 | 1.30 | 0.1007 | 0.3103 |
| TC0200010795.hg.1 | SLC11A1 | solute carrier family 11 (proton-coupled divalent metal ion trans | Multiple_C | 3.78 | 3.61 | 3.65 | 1.09 | 0.9697 | 0.9853 | 4.22 | 4.40 | 4.34 | 0.92 | 0.6423 | 0.8245 |
| TC0200013061.hg.1 | PRADC1 | protease-associated domain containing 1 | Multiple_C | 8.61 | 8.09 | 8.48 | 1.09 | 0.612 | 0.7821 | 7.8 | 8.46 | 8.28 | 0.72 | 0.0176 | 0.1113 |
| TC0200013362.hg.1 | FABP1 | fatty acid binding protein 1, liver | Multiple_C | 3.32 | 3.21 | 3.19 | 1.09 | 0.8478 | 0.9256 | 3.6 | 3.69 | 3.28 | 1.25 | 0.12 | 0.3422 |
| TC0200014332.hg.1 | C2orf27B | chromosome 2 open reading frame 27B | Multiple_C | 3.13 | 2.94 | 3 | 1.09 | 0.7391 | 0.8646 | 3.2 | 3.29 | 3.24 | 0.97 | 0.3003 | 0.5646 |
| TC0200014821.hg.1 | SCN7A | sodium channel, voltage gated, type VII alpha subunit | Multiple_C | 2.88 | 2.76 | 2.75 | 1.09 | 0.6764 | 0.8241 | 3.48 | 3.23 | 3.21 | 1.21 | 0.0419 | 0.1884 |
| TC0200015214.hg.1 | OSGEPL1 | O-sialoglycoprotein endopeptidase-like 1 | Multiple_C | 7.15 | 9.94 | 7.02 | 1.09 | 0.8328 | 0.9165 | 6.98 | 6.23 | 6.87 | 1.08 | 0.8661 | 0.9425 |
| TC0200016752.hg.1 | TTN | titin | Multiple_C | 3.06 | 2.9 | 2.93 | 1.09 | 0.7592 | 0.8763 | 3.42 | 3.47 | 3.46 | 0.97 | 0.3144 | 0.5782 |
| TC0300006582.hg.1 | TATDN2; GHR | TatD DNase domain containing 2; ghrelin opposite strand/antise | Multiple_C | 5.18 | 4.59 | 5.05 | 1.09 | 0.6882 | 0.8318 | 6.42 | 6.54 | 5.92 | 1.41 | 0.0663 | 0.2456 |
| TC0300007033.hg.1 | MLH1 | mutL homolog 1 | Multiple_C | 3.47 | 3.15 | 3.34 | 1.09 | 0.5422 | 0.7291 | 9.26 | 10.16 | 10.39 | 0.46 | 5.59E-05 | 0.0025 |
| TC0300007257.hg.1 | CCR5 | chemokine (C-C motif) receptor 5 (gene/pseudogene) | Multiple_C | 3.43 | 3.33 | 3.3 | 1.09 | 0.5922 | 0.767 | 4.07 | 3.83 | 3.85 | 1.16 | 0.1414 | 0.3739 |
| TC0300007499.hg.1 | ITIH1 | inter-alpha-trypsin inhibitor heavy chain 1 | Multiple_C | 3.55 | 3.3 | 3.42 | 1.09 | 0.2968 | 0.5084 | 4.11 | 4.20 | 3.99 | 1.09 | 0.6689 | 0.8382 |
| TC0300009192.hg.1 | AADAC | arylacetamide deacetylase | Coding | 4.52 | 4.64 | 4.39 | 1.09 | 0.9638 | 0.9824 | 4.81 | 4.45 | 4.37 | 1.36 | 0.0109 | 0.0823 |
| TC0300009787.hg.1 | ADIPOQ | adiponectin, C1Q and collagen domain containing | Coding | 2.75 | 2.58 | 2.62 | 1.09 | 0.4729 | 0.6761 | 3.19 | 2.91 | 3.06 | 1.09 | 0.4651 | 0.7068 |
| TC0300010427.hg.1 | RFTN1 | raftlin, lipid raft linker 1 | Multiple_C | 3.48 | 3.21 | 3.35 | 1.09 | 0.5908 | 0.7659 | 3.64 | 3.96 | 3.83 | 0.88 | 0.5352 | 0.7572 |
| TC0300011247.hg.1 | WNT5A | wingless-type MMTV integration site family, member 5A | Multiple_C | 4.26 | 4.15 | 4.13 | 1.09 | 0.7143 | 0.8493 | 4.39 | 4.07 | 3.84 | 1.46 | 0.0166 | 0.1073 |
| TC0300012426.hg.1 | PIK3R4 | phosphoinositide-3-kinase, regulatory subunit 4 | Multiple_C | 8.72 | 8.86 | 8.59 | 1.09 | 0.6272 | 0.792 | 8.15 | 8.00 | 8.67 | 0.70 | 0.0479 | 0.2032 |
| TC0300013357.hg.1 | PARL | presenilin associated, rhomboid-like | Multiple_C | 11.04 | 11.78 | 10.91 | 1.09 | 0.6418 | 0.8014 | 10.14 | 10.31 | 10.17 | 0.98 | 0.3267 | 0.5903 |
| TC0300013964.hg.1 | PRSS50 | protease, serine 50 | Coding | 2.69 | 2.49 | 2.56 | 1.09 | 0.8945 | 0.9497 | 3.63 | 3.48 | 3.25 | 1.30 | 0.0274 | 0.1449 |
| TC0300013971.hg.1 | UCN2 | urocortin 2 | Coding | 3.02 | 2.7 | 2.89 | 1.09 | 0.8254 | 0.9123 | 4.66 | 4.72 | 4.35 | 1.24 | 0.022 | 0.1274 |
| TC0400008074.hg.1 | NUDT9 | nudix hydrolase 9 | Multiple_C | 9.34 | 9.56 | 9.21 | 1.09 | 0.4739 | 0.677 | 7.15 | 7.60 | 8.02 | 0.55 | 0.0122 | 0.0882 |
| TC0400009080.hg.1 | NPY2R | neuropeptide Y receptor Y2 | Coding | 4.94 | 4.92 | 4.81 | 1.09 | 0.7744 | 0.8837 | 4.62 | 4.56 | 4.41 | 1.16 | 0.5437 | 0.762 |
| TC0500007430.hg.1 | DDX4 | DEAD (Asp-Glu-Ala-Asp) box polypeptide 4 | Multiple_C | 3.21 | 2.95 | 3.08 | 1.09 | 0.6162 | 0.7849 | 3.24 | 3.42 | 3.35 | 0.93 | 0.8093 | 0.9156 |
| TC0500009183.hg.1 | MRPL22 | mitochondrial ribosomal protein L22 | Multiple_C | 10.13 | 10.66 | 10 | 1.09 | 0.6897 | 0.8329 | 7.62 | 7.96 | 7.51 | 1.08 | 0.2174 | 0.4768 |
| TC0500009801.hg.1 | EXOC3-AS1 | EXOC3 antisense RNA 1 | Multiple_C | 5.55 | 5.2 | 5.42 | 1.09 | 0.2077 | 0.4011 | 5.05 | 5.56 | 4.69 | 1.28 | 0.2566 | 0.5214 |
| TC0500012445.hg.1 | PDE6A | phosphodiesterase 6A, cGMP-specific, rod, alpha | Multiple_C | 3.54 | 3.49 | 3.41 | 1.09 | 0.6712 | 0.8209 | 3.47 | 3.65 | 3.22 | 1.19 | 0.7115 | 0.8647 |
| TC0500013241.hg.1 | PCDHB5 | protocadherin beta 5 | Coding | 2.9 | 2.56 | 2.77 | 1.09 | 0.4462 | 0.6546 | 3.22 | 3.35 | 3.83 | 0.66 | 0.0027 | 0.0334 |
| TC0600007087.hg.1 | CAP2 | CAP, adenylate cyclase-associated protein, 2 (yeast) | Multiple_C | 6.93 | 6.42 | 6.8 | 1.09 | 0.5638 | 0.7455 | 8 | 9.01 | 8.74 | 0.60 | 0.0162 | 0.106 |
| TC0600007608.hg.1 | C6orf25 | chromosome 6 open reading frame 25 | Multiple_C | 4.73 | 4.48 | 4.6 | 1.09 | 0.285 | 0.4953 | 4.71 | 4.87 | 4.83 | 0.92 | 0.711 | 0.8644 |
| TC0600009498.hg.1 | EYA4 | EYA transcriptional coactivator and phosphatase 4 | Multiple_C | 2.96 | 2.85 | 2.83 | 1.09 | 0.4715 | 0.6752 | 4.05 | 4.18 | 4.12 | 0.95 | 0.562 | 0.7727 |
| TC0600009795.hg.1 | SUMO4 | small ubiquitin-like modifier 4 | Multiple_C | 6.62 | 6.56 | 6.49 | 1.09 | 0.3699 | 0.5826 | 5.87 | 6.05 | 6.24 | 0.77 | 0.9218 | 0.9677 |
| TC0600010095.hg.1 | MAP3K4 | mitogen-activated protein kinase kinase kinase 4 | Multiple_C | 9.74 | 10.12 | 9.61 | 1.09 | 0.8311 | 0.9158 | 5.75 | 6.68 | 6.77 | 0.49 | 0.0382 | 0.1779 |

| | | | | | | | | | | | | | | | |
|---|---|---|---|---|---|---|---|---|---|---|---|---|---|---|---|
| TC0600012817.hg.1 | AK9 | adenylate kinase 9 | Multiple_C | 5.59 | 6 | 5.46 | 1.09 | 0.9193 | 0.9626 | 6.43 | 6.43 | 5.76 | 1.59 | 0.1784 | 0.4272 |
| TC0600013007.hg.1 | FAM184A | family with sequence similarity 184, member A | Multiple_C | 3.87 | 3.37 | 3.74 | 1.09 | 0.5519 | 0.7363 | 4.84 | 4.47 | 4.62 | 1.16 | 0.5987 | 0.7982 |
| TC0600014065.hg.1 | C6orf201 | chromosome 6 open reading frame 201 | Multiple_C | 2.97 | 2.76 | 2.84 | 1.09 | 0.5196 | 0.7132 | 4.02 | 3.77 | 3.72 | 1.23 | 0.3475 | 0.6113 |
| TC0700006890.hg.1 | IL6 | interleukin 6 | Multiple_C | 3.15 | 2.89 | 3.02 | 1.09 | 0.6679 | 0.8184 | 3.25 | 3.22 | 3.14 | 1.08 | 0.1195 | 0.3416 |
| TC0700006991.hg.1 | C7orf71 | chromosome 7 open reading frame 71 | Multiple_C | 3.05 | 2.84 | 2.92 | 1.09 | 0.6385 | 0.7995 | 3.64 | 3.75 | 3.84 | 0.87 | 0.2146 | 0.4732 |
| TC0700008555.hg.1 | FBXO24 | F-box protein 24 | Multiple_C | 4.7 | 4.69 | 4.57 | 1.09 | 0.7339 | 0.861 | 5.49 | 5.00 | 4.93 | 1.47 | 0.0184 | 0.1142 |
| TC0700008574.hg.1 | MUC3A | mucin 3A, cell surface associated | Multiple_C | 9.5 | 7.78 | 9.37 | 1.09 | 0.1734 | 0.3553 | 3.92 | 3.84 | 4 | 0.95 | 0.6172 | 0.8099 |
| TC0700008760.hg.1 | SLC26A4 | solute carrier family 26 (anion exchanger), member 4 | Multiple_C | 3.94 | 5.12 | 3.81 | 1.09 | 0.6241 | 0.7897 | 3.68 | 3.41 | 3.33 | 1.27 | 0.0309 | 0.1565 |
| TC0700008918.hg.1 | KCND2 | potassium channel, voltage gated Shal related subfamily D, mer | Multiple_C | 3.51 | 3.46 | 3.38 | 1.09 | 0.7652 | 0.8794 | 4.09 | 3.62 | 4 | 1.06 | 0.7116 | 0.8647 |
| TC0700010380.hg.1 | PRPS1L1 | phosphoribosyl pyrophosphate synthetase 1-like 1 | Coding | 3.17 | 3.1 | 3.04 | 1.09 | 0.7123 | 0.848 | 3.74 | 3.74 | 4.03 | 0.82 | 0.8189 | 0.9199 |
| TC0700010678.hg.1 | LSM5 | LSM5 homolog, U6 small nuclear RNA and mRNA degradation a | Multiple_C | 12.59 | 14.05 | 12.46 | 1.09 | 0.6775 | 0.8247 | 11.04 | 11.28 | 10.91 | 1.09 | 0.94 | 0.9753 |
| TC0700011803.hg.1 | CALCR | calcitonin receptor | Coding | 3.1 | 3.19 | 2.97 | 1.09 | 0.6191 | 0.7869 | 4 | 3.76 | 3.55 | 1.37 | 0.1076 | 0.3225 |
| TC0700012512.hg.1 | LRRC4 | leucine rich repeat containing 4 | Coding | 5.01 | 4.79 | 4.88 | 1.09 | 0.2766 | 0.4859 | 4.49 | 4.55 | 4.59 | 0.93 | 0.5139 | 0.7433 |
| TC0700013355.hg.1 | NME8 | NME/NM23 family member 8 | Multiple_C | 2.91 | 2.88 | 2.78 | 1.09 | 0.3417 | 0.5555 | 3.95 | 3.80 | 3.45 | 1.41 | 0.1195 | 0.3416 |
| TC0800008328.hg.1 | ERICH5 | glutamate rich 5 | Coding | 5.19 | 4.93 | 5.06 | 1.09 | 0.5701 | 0.7502 | 5.68 | 5.87 | 5.6 | 1.06 | 0.4655 | 0.7073 |
| TC0900007113.hg.1 | RECK | reversion-inducing-cysteine-rich protein with kazal motifs | Multiple_C | 4.8 | 4.91 | 4.67 | 1.09 | 0.4318 | 0.642 | 5.94 | 5.63 | 5.71 | 1.17 | 0.2171 | 0.4764 |
| TC0900007288.hg.1 | SPATA31A6 | SPATA31 subfamily A, member 6 | Multiple_C | 4.15 | 3.83 | 4.02 | 1.09 | 0.8224 | 0.9107 | 4.23 | 4.23 | 3.78 | 1.37 | 0.07 | 0.2527 |
| TC0900007517.hg.1 | C9orf135 | chromosome 9 open reading frame 135 | Multiple_C | 3.78 | 3.77 | 3.65 | 1.09 | 0.7811 | 0.8872 | 3.47 | 3.65 | 3.65 | 0.88 | 0.557 | 0.7707 |
| TC0900008930.hg.1 | PRRX2 | paired related homeobox 2 | Coding | 3.5 | 3.27 | 3.37 | 1.09 | 0.9441 | 0.9743 | 3.68 | 3.70 | 3.64 | 1.03 | 0.8197 | 0.9205 |
| TC0900009231.hg.1 | FAM69B | family with sequence similarity 69, member B | Multiple_C | 5.24 | 5.07 | 5.11 | 1.09 | 0.9806 | 0.9907 | 6.43 | 6.23 | 5.58 | 1.80 | 0.0081 | 0.0676 |
| TC0900009604.hg.1 | C9orf92 | chromosome 9 open reading frame 92 | Multiple_C | 3.26 | 3.63 | 3.13 | 1.09 | 0.7561 | 0.8745 | 3.63 | 3.37 | 3.56 | 1.05 | 0.3712 | 0.633 |
| TC0900011976.hg.1 | SNAPC4 | small nuclear RNA activating complex polypeptide 4 | Multiple_C | 8.81 | 8.18 | 8.68 | 1.09 | 0.881 | 0.9422 | 8.71 | 8.85 | 8.53 | 1.13 | 0.4268 | 0.6791 |
| TC0X00007034.hg.1 | NYX | nyctalopin | Multiple_C | 5.03 | 4.62 | 4.9 | 1.09 | 0.6756 | 0.8236 | 4.45 | 4.88 | 4.98 | 0.69 | 0.1688 | 0.4145 |
| TC0X00007557.hg.1 | MED12 | mediator complex subunit 12 | Multiple_C | 9.17 | 8.82 | 9.04 | 1.09 | 0.702 | 0.8409 | 10.03 | 11.16 | 10.69 | 0.63 | 0.0173 | 0.1103 |
| TC0X00007930.hg.1 | ARL13A | ADP-ribosylation factor like GTPase 13A | Multiple_C | 2.84 | 2.72 | 2.71 | 1.09 | 0.4882 | 0.689 | 3.38 | 3.34 | 3.72 | 0.79 | 0.0578 | 0.2266 |
| TC0X00008421.hg.1 | OR13H1 | olfactory receptor, family 13, subfamily H, member 1 | Coding | 3.33 | 3.14 | 3.2 | 1.09 | 0.6055 | 0.777 | 4.07 | 4.22 | 3.63 | 1.36 | 0.0312 | 0.1577 |
| TC0X00008611.hg.1 | MAGEC3 | MAGE family member C3 | Multiple_C | 3.07 | 2.91 | 2.94 | 1.09 | 0.9521 | 0.9779 | 3.56 | 3.57 | 3.58 | 0.99 | 0.538 | 0.759 |
| TC0X00008848.hg.1 | CTAG1B; CTA( | cancer/testis antigen 1B; cancer/testis antigen 1A | Coding | 5.52 | 5.44 | 5.39 | 1.09 | 0.5879 | 0.7641 | 5.08 | 4.86 | 4.92 | 1.12 | 0.6329 | 0.8192 |
| TC0X00009029.hg.1 | FAM9A | family with sequence similarity 9, member A | Coding | 3.01 | 2.84 | 2.88 | 1.09 | 0.0906 | 0.224 | 4.21 | 4.21 | 4.01 | 1.15 | 0.2591 | 0.5233 |
| TC0X00009867.hg.1 | SPIN4 | spindlin family, member 4 | Coding | 2.73 | 2.56 | 2.6 | 1.09 | 0.3077 | 0.5208 | 6.94 | 7.36 | 7.55 | 0.66 | 0.0136 | 0.0947 |
| TC0X00011135.hg.1 | MAGEA2 | MAGE family member A2 | Coding | 5.84 | 5.55 | 5.71 | 1.09 | 0.3104 | 0.5238 | 6.79 | 6.53 | 6.44 | 1.27 | 0.3833 | 0.6423 |
| TC0X00011144.hg.1 | PNMA5 | paraneoplastic Ma antigen family member 5 | Multiple_C | 4.49 | 4.33 | 4.36 | 1.09 | 0.8088 | 0.9034 | 3.37 | 3.23 | 3.34 | 1.02 | 0.8712 | 0.9445 |
| TC0X00011381.hg.1 | KCNE5; ACSL4 | potassium channel, voltage gated subfamily E regulatory beta su | Multiple_C | 4.38 | 3.79 | 4.25 | 1.09 | 0.6607 | 0.8138 | 5.12 | 5.34 | 5.01 | 1.08 | 0.9551 | 0.9814 |

| | | | | | | | | | | | | | | | |
|---|---|---|---|---|---|---|---|---|---|---|---|---|---|---|---|
| TC0Y00006744.hg.1 | RBMY1D; RBN | RNA binding motif protein, Y-linked, family 1, member D; RNA b | Coding | 3.1 | 3.34 | 2.97 | 1.09 | 0.6044 | 0.7761 | 4.18 | 4.20 | 3.6 | 1.49 | 0.0147 | 0.099 |
| TC1000006937.hg.1 | PLXDC2 | plexin domain containing 2 | Multiple_C | 3.67 | 3.29 | 3.54 | 1.09 | 0.879 | 0.9412 | 4.02 | 4.03 | 3.87 | 1.11 | 0.2544 | 0.5186 |
| TC1000007543.hg.1 | GDF10 | growth differentiation factor 10 | Coding | 4.64 | 4.27 | 4.51 | 1.09 | 0.9573 | 0.9801 | 4.87 | 4.67 | 4.76 | 1.08 | 0.7891 | 0.9058 |
| TC1000007590.hg.1 | SLC18A3 | solute carrier family 18 (vesicular acetylcholine transporter), me | Coding | 3.06 | 2.68 | 2.93 | 1.09 | 0.8857 | 0.9448 | 3.22 | 3.49 | 3.45 | 0.85 | 0.7691 | 0.8964 |
| TC1000007888.hg.1 | VPS26A | VPS26 retromer complex component A | Multiple_C | 13.11 | 13.31 | 12.98 | 1.09 | 0.6061 | 0.7773 | 13.15 | 12.80 | 13.22 | 0.95 | 0.3747 | 0.6355 |
| TC1000008335.hg.1 | NUTM2A | NUT family member 2A | Coding | 3.74 | 3.54 | 3.61 | 1.09 | 0.5306 | 0.7209 | 3.84 | 4.10 | 3.94 | 0.93 | 0.6059 | 0.8028 |
| TC1000009171.hg.1 | HMX2 | H6 family homeobox 2 | Coding | 5 | 4.92 | 4.87 | 1.09 | 0.4668 | 0.6713 | 4.65 | 4.60 | 4.54 | 1.08 | 0.7139 | 0.8662 |
| TC1000010531.hg.1 | NPY4R | neuropeptide Y receptor Y4 | Coding | 2.49 | 2.28 | 2.36 | 1.09 | 0.7762 | 0.8847 | 3.05 | 3.05 | 2.99 | 1.04 | 0.3458 | 0.6096 |
| TC1000011267.hg.1 | LRIT1 | leucine-rich repeat, immunoglobulin-like and transmembrane d | Multiple_C | 3.38 | 3.28 | 3.25 | 1.09 | 0.3322 | 0.546 | 3.99 | 3.74 | 3.53 | 1.38 | 0.0406 | 0.1847 |
| TC1000011563.hg.1 | SFRP5 | secreted frizzled-related protein 5 | Coding | 3.44 | 3.25 | 3.31 | 1.09 | 0.7034 | 0.8417 | 7.14 | 9.62 | 9.24 | 0.23 | 9.62E-07 | 0.0001 |
| TC1000011679.hg.1 | CUEDC2 | CUE domain containing 2 | Multiple_C | 8.79 | 8.85 | 8.66 | 1.09 | 0.9999 | 0.9999 | 7.36 | 7.79 | 7.76 | 0.76 | 0.0537 | 0.2169 |
| TC1000011948.hg.1 | PDZD8 | PDZ domain containing 8 | Multiple_C | 11.44 | 10.86 | 11.31 | 1.09 | 0.8358 | 0.9181 | 13.01 | 11.64 | 13.11 | 0.93 | 0.2702 | 0.5354 |
| TC1100006517.hg.1 | SYT8 | synaptotagmin VIII | Multiple_C | 4.5 | 4.3 | 4.37 | 1.09 | 0.9536 | 0.9784 | 4.96 | 5.01 | 4.72 | 1.18 | 0.6483 | 0.8281 |
| TC1100006973.hg.1 | OTOG | otogelin | Multiple_C | 3.74 | 3.38 | 3.61 | 1.09 | 0.4863 | 0.6876 | 3.57 | 3.42 | 3.61 | 0.97 | 0.787 | 0.9049 |
| TC1100007734.hg.1 | OR4D6 | olfactory receptor, family 4, subfamily D, member 6 | Coding | 3.05 | 3.18 | 2.92 | 1.09 | 0.4757 | 0.6784 | 3.22 | 3.39 | 3.35 | 0.91 | 0.5025 | 0.7346 |
| TC1100008779.hg.1 | CCDC67 | coiled-coil domain containing 67 | Multiple_C | 2.92 | 2.89 | 2.79 | 1.09 | 0.7293 | 0.8581 | 3.52 | 3.50 | 3.02 | 1.41 | 0.0187 | 0.1153 |
| TC1100009449.hg.1 | TIRAP | toll-interleukin 1 receptor (TIR) domain containing adaptor prot | Multiple_C | 5.85 | 5.55 | 5.72 | 1.09 | 0.566 | 0.7473 | 5.3 | 5.27 | 5.01 | 1.22 | 0.3572 | 0.6202 |
| TC1100009858.hg.1 | CHRNA10; NU | cholinergic receptor, nicotinic alpha 10; nucleoporin 98kDa | Multiple_C | 12.17 | 11.28 | 12.04 | 1.09 | 0.5455 | 0.7312 | 13.07 | 12.76 | 13.4 | 0.80 | 0.156 | 0.3966 |
| TC1100010661.hg.1 | CHST1 | carbohydrate (keratan sulfate Gal-6) sulfotransferase 1 | Multiple_C | 6.17 | 5.76 | 6.04 | 1.09 | 0.2595 | 0.4659 | 6.81 | 6.58 | 6.47 | 1.27 | 0.1495 | 0.3871 |
| TC1100010855.hg.1 | OR5R1 | olfactory receptor, family 5, subfamily R, member 1 (gene/pseu | Multiple_C | 3.88 | 3.59 | 3.75 | 1.09 | 0.5962 | 0.7699 | 3.41 | 3.90 | 3.75 | 0.79 | 0.4192 | 0.6729 |
| TC1100011549.hg.1 | PHOX2A | paired-like homeobox 2a | Multiple_C | 2.64 | 2.52 | 2.51 | 1.09 | 0.6793 | 0.8258 | 3.52 | 3.61 | 3.08 | 1.36 | 0.0469 | 0.2004 |
| TC1100012187.hg.1 | KBTBD3 | kelch repeat and BTB (POZ) domain containing 3 | Multiple_C | 5.18 | 5.87 | 5.05 | 1.09 | 0.7257 | 0.8557 | 4.24 | 4.55 | 3.95 | 1.22 | 0.4607 | 0.7039 |
| TC1100013109.hg.1 | GRAMD1B | GRAM domain containing 1B | Multiple_C | 3.79 | 3.66 | 3.66 | 1.09 | 0.8948 | 0.9497 | 9.31 | 10.65 | 11.33 | 0.25 | 7.56E-05 | 0.003 |
| TC1100013152.hg.1 | SAA2; SAA2-S. | serum amyloid A2; SAA2-SAA4 readthrough; serum amyloid A4, | Multiple_C | 3.98 | 3.4 | 3.85 | 1.09 | 0.2732 | 0.4819 | 4.01 | 3.76 | 3.87 | 1.10 | 0.5252 | 0.7498 |
| TC1100013229.hg.1 | FXYD6; FXYD6 | FXYD domain containing ion transport regulator 6; FXYD6-FXYD: | Multiple_C | 3.16 | 3.07 | 3.03 | 1.09 | 0.44 | 0.6489 | 5.27 | 5.17 | 4.65 | 1.54 | 0.018 | 0.1127 |
| TC1200007882.hg.1 | GLI1 | GLI family zinc finger 1 | Multiple_C | 5.06 | 4.46 | 4.93 | 1.09 | 0.6448 | 0.8032 | 5.33 | 5.21 | 4.78 | 1.46 | 0.014 | 0.0961 |
| TC1200008224.hg.1 | GLIPR1 | GLI pathogenesis-related 1 | Multiple_C | 4.16 | 3.96 | 4.03 | 1.09 | 0.8962 | 0.9504 | 3.74 | 3.70 | 3.76 | 0.99 | 0.2674 | 0.532 |
| TC1200009915.hg.1 | TAS2R8 | taste receptor, type 2, member 8 | Coding | 2.8 | 2.76 | 2.67 | 1.09 | 0.6503 | 0.8064 | 3.39 | 3.22 | 3.08 | 1.24 | 0.2169 | 0.4763 |
| TC1200010640.hg.1 | NCKAP5L | NCK-associated protein 5-like | Multiple_C | 4.06 | 3.93 | 3.93 | 1.09 | 0.6807 | 0.8265 | 5.55 | 6.15 | 5.84 | 0.82 | 0.081 | 0.2743 |
| TC1200010908.hg.1 | STAT2 | signal transducer and activator of transcription 2 | Multiple_C | 6.3 | 6.27 | 6.17 | 1.09 | 0.5065 | 0.7035 | 6.8 | 7.11 | 6.7 | 1.07 | 0.557 | 0.7707 |
| TC1400008816.hg.1 | PRKD1 | protein kinase D1 | Multiple_C | 3.22 | 3.15 | 3.09 | 1.09 | 0.453 | 0.6605 | 4.15 | 3.88 | 4.09 | 1.04 | 0.9587 | 0.9832 |
| TC1400009793.hg.1 | SNW1 | SNW domain containing 1 | Multiple_C | 10.81 | 11.15 | 10.68 | 1.09 | 0.9083 | 0.956 | 10.38 | 10.04 | 10.72 | 0.79 | 0.081 | 0.2743 |
| TC1400010377.hg.1 | AKT1 | v-akt murine thymoma viral oncogene homolog 1 | Multiple_C | 9.89 | 9.36 | 9.76 | 1.09 | 0.4844 | 0.6859 | 10.03 | 10.76 | 10.12 | 0.94 | 0.6242 | 0.8145 |

| | | | | | | | | | | | | | | | |
|---|---|---|---|---|---|---|---|---|---|---|---|---|---|---|---|
| TC1400010603.hg.1 | TTC6 | tetratricopeptide repeat domain 6 | Multiple_C | 3.85 | 3.74 | 3.72 | 1.09 | 0.4159 | 0.6279 | 4.25 | 3.96 | 4.16 | 1.06 | 0.5979 | 0.7976 |
| TC1400010647.hg.1 | BDKRB2 | bradykinin receptor B2 | Coding | 3.37 | 3.23 | 3.24 | 1.09 | 0.461 | 0.6666 | 4.03 | 3.72 | 3.51 | 1.43 | 0.0148 | 0.0993 |
| TC1500006495.hg.1 | OR4N4; OR4N | olfactory receptor, family 4, subfamily N, member 4; olfactory r | Multiple_C | 3.44 | 3.2 | 3.31 | 1.09 | 0.1247 | 0.2823 | 3.89 | 3.56 | 3.56 | 1.26 | 0.149 | 0.3862 |
| TC1500008276.hg.1 | MESP2 | mesoderm posterior bHLH transcription factor 2 | Multiple_C | 4.34 | 4.17 | 4.21 | 1.09 | 0.8778 | 0.9406 | 4.24 | 4.06 | 4.1 | 1.10 | 0.4764 | 0.7154 |
| TC1500010658.hg.1 | VIMP | VCP-interacting membrane selenoprotein | Multiple_C | 10.11 | 10.87 | 9.98 | 1.09 | 0.7249 | 0.8552 | 7.6 | 7.74 | 7.26 | 1.27 | 0.5805 | 0.7856 |
| TC1600007575.hg.1 | TP53TG3D | TP53 target 3D | Multiple_C | 2.93 | 3.03 | 2.8 | 1.09 | 0.502 | 0.7002 | 3.67 | 3.64 | 3.64 | 1.02 | 0.7223 | 0.8706 |
| TC1600008577.hg.1 | BCO1 | beta-carotene oxygenase 1 | Multiple_C | 5.79 | 6.09 | 5.66 | 1.09 | 0.958 | 0.9803 | 4.5 | 5.07 | 4.94 | 0.74 | 0.3758 | 0.6364 |
| TC1600009079.hg.1 | NOXO1 | NADPH oxidase organizer 1 | Multiple_C | 4.46 | 4.26 | 4.33 | 1.09 | 0.6152 | 0.7846 | 4.04 | 4.02 | 3.93 | 1.08 | 0.4473 | 0.6942 |
| TC1600010363.hg.1 | IRX3 | iroquois homeobox 3 | Multiple_C | 3.56 | 3.28 | 3.43 | 1.09 | 0.8309 | 0.9157 | 3.72 | 3.89 | 3.9 | 0.88 | 0.3299 | 0.5941 |
| TC1600010517.hg.1 | LOC729159; R | UPF0607 protein ENSP00000381418-like; Putative UPF0607 pro | Coding | 5.68 | 5.31 | 5.55 | 1.09 | 0.7142 | 0.8493 | 5.07 | 5.27 | 5.39 | 0.80 | 0.199 | 0.4542 |
| TC1600011567.hg.1 | CHST5 | carbohydrate (N-acetylglucosamine 6-O) sulfotransferase 5 | Coding | 4.68 | 5.06 | 4.55 | 1.09 | 0.2873 | 0.498 | 5.22 | 5.25 | 5.1 | 1.09 | 0.6786 | 0.8452 |
| TC1700006750.hg.1 | SLC35G6 | solute carrier family 35, member G6 | Coding | 4.26 | 4.45 | 4.13 | 1.09 | 0.6942 | 0.8354 | 4.05 | 4.16 | 4.14 | 0.94 | 0.5332 | 0.7558 |
| TC1700006781.hg.1 | CNTROB | centrobin, centrosomal BRCA2 interacting protein | Multiple_C | 8.09 | 7.91 | 7.96 | 1.09 | 0.8097 | 0.9039 | 7.05 | 7.45 | 7.55 | 0.71 | 0.4443 | 0.6923 |
| TC1700006839.hg.1 | NTN1 | netrin 1 | Multiple_C | 4.92 | 4.89 | 4.79 | 1.09 | 0.6718 | 0.8211 | 4.49 | 4.47 | 4.35 | 1.10 | 0.2709 | 0.5364 |
| TC1700007585.hg.1 | SLFN5 | schlafen family member 5 | Multiple_C | 6.24 | 7.27 | 6.11 | 1.09 | 0.9206 | 0.9633 | 4.38 | 4.13 | 4.49 | 0.93 | 0.4377 | 0.6883 |
| TC1700007617.hg.1 | CCL18 | chemokine (C-C motif) ligand 18 | Multiple_C | 3.9 | 3.78 | 3.77 | 1.09 | 0.6656 | 0.8172 | 4.73 | 4.58 | 4.4 | 1.26 | 0.1352 | 0.3652 |
| TC1700007730.hg.1 | LRRC37A11P | leucine rich repeat containing 37, member A11, pseudogene | Multiple_C | 3.33 | 3.1 | 3.2 | 1.09 | 0.8511 | 0.9269 | 3.6 | 3.71 | 3.65 | 0.97 | 0.2662 | 0.5307 |
| TC1700008350.hg.1 | NOG | noggin | Coding | 3.75 | 3.57 | 3.62 | 1.09 | 0.4664 | 0.6709 | 3.74 | 3.76 | 3.62 | 1.09 | 0.5272 | 0.7513 |
| TC1700008679.hg.1 | CACNG1 | calcium channel, voltage-dependent, gamma subunit 1 | Coding | 2.73 | 2.69 | 2.6 | 1.09 | 0.4781 | 0.6804 | 3.12 | 3.10 | 3.03 | 1.06 | 0.552 | 0.7677 |
| TC1700010684.hg.1 | HAP1 | huntingtin-associated protein 1 | Coding | 7.79 | 7.29 | 7.66 | 1.09 | 0.1123 | 0.2621 | 7.2 | 7.36 | 7.38 | 0.88 | 0.8971 | 0.9566 |
| TC1700011905.hg.1 | DNAH17 | dynein, axonemal, heavy chain 17 | Multiple_C | 3.82 | 3.69 | 3.69 | 1.09 | 0.6528 | 0.808 | 4.48 | 4.48 | 4.26 | 1.16 | 0.3149 | 0.5788 |
| TC1700012092.hg.1 | DUS1L | dihydrouridine synthase 1-like | Multiple_C | 8.2 | 8.01 | 8.07 | 1.09 | 0.6679 | 0.8184 | 9.55 | 9.78 | 9.63 | 0.95 | 0.3094 | 0.5734 |
| TC1800006827.hg.1 | GREB1L | growth regulation by estrogen in breast cancer-like | Multiple_C | 2.75 | 2.7 | 2.62 | 1.09 | 0.5316 | 0.7215 | 3.78 | 3.33 | 3.49 | 1.22 | 0.1331 | 0.362 |
| TC1800006915.hg.1 | HRH4 | histamine receptor H4 | Coding | 3.28 | 3.21 | 3.15 | 1.09 | 0.3567 | 0.5701 | 3.71 | 3.71 | 3.57 | 1.10 | 0.1194 | 0.3415 |
| TC1800007193.hg.1 | SLC14A2 | solute carrier family 14 (urea transporter), member 2 | Coding | 3.56 | 3.07 | 3.43 | 1.09 | 0.7114 | 0.8473 | 4.24 | 4.48 | 4.49 | 0.84 | 0.8538 | 0.9371 |
| TC1800007805.hg.1 | NFATC1 | nuclear factor of activated T-cells, cytoplasmic, calcineurin-depe | Multiple_C | 3.95 | 3.96 | 3.82 | 1.09 | 0.3129 | 0.5264 | 3.78 | 3.37 | 3.36 | 1.34 | 0.123 | 0.346 |
| TC1800009279.hg.1 | TCEB3CL; TCEI | transcription elongation factor B polypeptide 3C-like; transcripti | Coding | 5.82 | 5.61 | 5.69 | 1.09 | 0.2049 | 0.3976 | 6.33 | 6.47 | 6.55 | 0.86 | 0.6128 | 0.8066 |
| TC1900008920.hg.1 | ZNF524 | zinc finger protein 524 | Multiple_C | 4.4 | 4.46 | 4.27 | 1.09 | 0.1008 | 0.2423 | 3.79 | 4.28 | 4.07 | 0.82 | 0.4272 | 0.6792 |
| TC1900008988.hg.1 | ZNF805 | zinc finger protein 805 | Multiple_C | 6 | 6.45 | 5.87 | 1.09 | 0.5834 | 0.7604 | 5.56 | 4.88 | 5.24 | 1.25 | 0.3096 | 0.5735 |
| TC1900011172.hg.1 | VRK3 | vaccinia related kinase 3 | Multiple_C | 11.08 | 10.35 | 10.95 | 1.09 | 0.6763 | 0.8241 | 10.43 | 10.63 | 10.34 | 1.06 | 0.1235 | 0.347 |
| TC1900011704.hg.1 | ZNF536 | zinc finger protein 536 | Multiple_C | 2.83 | 2.51 | 2.7 | 1.09 | 0.534 | 0.7235 | 3.61 | 3.43 | 3.15 | 1.38 | 0.0582 | 0.2275 |
| TC1900011822.hg.1 | ZNF586 | zinc finger protein 586 | Multiple_C | 6.93 | 7.14 | 6.8 | 1.09 | 0.7328 | 0.8604 | 4.87 | 4.73 | 4.8 | 1.05 | 0.7966 | 0.9092 |
| TC1900011838.hg.1 | JSRP1 | junctional sarcoplasmic reticulum protein 1 | Multiple_C | 6.08 | 6.15 | 5.95 | 1.09 | 0.723 | 0.8543 | 5.41 | 5.34 | 5.14 | 1.21 | 0.0845 | 0.2798 |

| | | | | | | | | | | | | | | | |
|---|---|---|---|---|---|---|---|---|---|---|---|---|---|---|---|
| TC1900011884.hg.1 | ZNF443 | zinc finger protein 443 | Multiple_C | 9.15 | 9.93 | 9.02 | 1.09 | 0.6445 | 0.803 | 7.65 | 7.48 | 7.64 | 1.01 | 0.3967 | 0.6545 |
| TC1900011965.hg.1 | PSG2 | pregnancy specific beta-1-glycoprotein 2 | Multiple_C | 4.28 | 4.02 | 4.15 | 1.09 | 0.5414 | 0.7287 | 4.31 | 4.29 | 4.2 | 1.08 | 0.8514 | 0.9358 |
| TC2000007313.hg.1 | LBP | lipopolysaccharide binding protein | Coding | 3.4 | 3.12 | 3.27 | 1.09 | 0.5968 | 0.7702 | 3.37 | 2.90 | 2.96 | 1.33 | 0.0142 | 0.097 |
| TC2000008140.hg.1 | SLC52A3 | solute carrier family 52 (riboflavin transporter), member 3 | Multiple_C | 5.97 | 5.41 | 5.84 | 1.09 | 0.7198 | 0.8523 | 6.34 | 5.67 | 5.9 | 1.36 | 0.0447 | 0.1956 |
| TC2000009624.hg.1 | APCDD1L | adenomatosis polyposis coli down-regulated 1-like | Multiple_C | 4.14 | 3.78 | 4.01 | 1.09 | 0.6225 | 0.7889 | 3.77 | 3.77 | 3.75 | 1.01 | 0.9418 | 0.9759 |
| TC2100007599.hg.1 | SAMSN1 | SAM domain, SH3 domain and nuclear localization signals 1 | Multiple_C | 3.77 | 3.8 | 3.64 | 1.09 | 0.7159 | 0.8497 | 3.78 | 3.45 | 3.64 | 1.10 | 0.5002 | 0.7328 |
| TC2100007905.hg.1 | KRTAP6-2 | keratin associated protein 6-2 | Coding | 5.03 | 5.1 | 4.9 | 1.09 | 0.6801 | 0.8261 | 5.83 | 5.76 | 5.45 | 1.30 | 0.1641 | 0.4082 |
| TC2100007911.hg.1 | KRTAP21-1 | keratin associated protein 21-1 | Coding | 3.9 | 3.66 | 3.77 | 1.09 | 0.8007 | 0.8978 | 4.27 | 4.12 | 4.35 | 0.95 | 0.9374 | 0.9741 |
| TC2100008552.hg.1 | KRTAP19-6 | keratin associated protein 19-6 | Coding | 2.84 | 2.69 | 2.71 | 1.09 | 0.4561 | 0.6629 | 3.56 | 3.31 | 3.22 | 1.27 | 0.1578 | 0.3991 |
| TSUnmapped00000664.h | CACFD1 | calcium channel flower domain containing 1 | Coding | 3.95 | 3.84 | 3.82 | 1.09 | 0.6204 | 0.7876 | 4.15 | 4.14 | 4.13 | 1.01 | 0.4288 | 0.6806 |
| TC0100007175.hg.1 | HTR6 | 5-hydroxytryptamine (serotonin) receptor 6, G protein-coupled | Multiple_C | 3.12 | 2.78 | 3 | 1.09 | 0.4237 | 0.6348 | 3.55 | 3.58 | 3.47 | 1.06 | 0.2202 | 0.4803 |
| TC0100007556.hg.1 | PHACTR4 | phosphatase and actin regulator 4 | Multiple_C | 8.91 | 8.1 | 8.79 | 1.09 | 0.975 | 0.9877 | 9.9 | 9.97 | 9.86 | 1.03 | 0.2788 | 0.5451 |
| TC0100007573.hg.1 | OPRD1 | opioid receptor, delta 1 | Multiple_C | 4.01 | 3.73 | 3.89 | 1.09 | 0.618 | 0.7862 | 4.62 | 5.50 | 5.12 | 0.71 | 0.0403 | 0.184 |
| TC0100008975.hg.1 | GBP6 | guanylate binding protein family, member 6 | Coding | 3.27 | 3.12 | 3.15 | 1.09 | 0.624 | 0.7897 | 3.19 | 3.13 | 2.96 | 1.17 | 0.2545 | 0.5186 |
| TC0100010020.hg.1 | SPRR1B | small proline-rich protein 1B | Multiple_C | 3.19 | 3.1 | 3.07 | 1.09 | 0.6044 | 0.7761 | 2.97 | 3.06 | 3.1 | 0.91 | 0.8167 | 0.9187 |
| TC0100012093.hg.1 | EDARADD | EDAR-associated death domain | Multiple_C | 3.86 | 3.51 | 3.74 | 1.09 | 0.7394 | 0.8648 | 5.04 | 5.03 | 4.83 | 1.16 | 0.1488 | 0.3858 |
| TC0100012149.hg.1 | FMN2; ADH5P | formin 2; alcohol dehydrogenase 5 (class III), chi polypeptide, ps | Multiple_C | 3.44 | 3.51 | 3.32 | 1.09 | 0.6602 | 0.8137 | 3.79 | 3.92 | 3.45 | 1.27 | 0.081 | 0.2743 |
| TC0100015856.hg.1 | S100A12 | S100 calcium binding protein A12 | Coding | 3.3 | 3.29 | 3.18 | 1.09 | 0.741 | 0.866 | 3.55 | 3.76 | 3.68 | 0.91 | 0.7244 | 0.8717 |
| TC0100016067.hg.1 | OR10T2 | olfactory receptor, family 10, subfamily T, member 2 | Coding | 3.34 | 3.17 | 3.22 | 1.09 | 0.6559 | 0.811 | 3.26 | 3.46 | 3.4 | 0.91 | 0.7588 | 0.8905 |
| TC0100016091.hg.1 | OR10J3 | olfactory receptor, family 10, subfamily J, member 3 | Coding | 4.3 | 4.35 | 4.18 | 1.09 | 0.4206 | 0.6319 | 4.99 | 4.73 | 4.49 | 1.41 | 0.0432 | 0.1919 |
| TC0100016917.hg.1 | CSRP1 | cysteine and glycine-rich protein 1 | Multiple_C | 12.03 | 10.56 | 11.91 | 1.09 | 0.4451 | 0.6536 | 10.71 | 10.78 | 10.72 | 0.99 | 0.7559 | 0.8889 |
| TC0100018222.hg.1 | LOC10012992 | uncharacterized LOC100129924; Transcript Identified by AceVie | Multiple_C | 4.98 | 5.67 | 4.86 | 1.09 | 0.622 | 0.7886 | 4.77 | 4.31 | 4.67 | 1.07 | 0.366 | 0.6283 |
| TC0200007419.hg.1 | CAMKMT | calmodulin-lysine N-methyltransferase | Multiple_C | 9.25 | 9.99 | 9.13 | 1.09 | 0.5221 | 0.7152 | 7.98 | 7.05 | 7.95 | 1.02 | 0.961 | 0.9837 |
| TC0200007975.hg.1 | VAX2 | ventral anterior homeobox 2 | Coding | 3.8 | 3.68 | 3.68 | 1.09 | 0.4793 | 0.6815 | 3.47 | 3.89 | 3.85 | 0.77 | 0.6718 | 0.8404 |
| TC0200008090.hg.1 | HTRA2 | HtrA serine peptidase 2 | Multiple_C | 11.55 | 11.69 | 11.43 | 1.09 | 0.4256 | 0.6363 | 8.97 | 9.05 | 9.37 | 0.76 | 0.1191 | 0.3412 |
| TC0200009756.hg.1 | PKP4 | plakophilin 4 | Multiple_C | 10.63 | 10.4 | 10.51 | 1.09 | 0.8274 | 0.9133 | 10.88 | 11.26 | 11.88 | 0.50 | 0.0003 | 0.0074 |
| TC0200013096.hg.1 | MOB1A | MOB kinase activator 1A | Multiple_C | 13.3 | 13.41 | 13.18 | 1.09 | 0.5965 | 0.7701 | 13.5 | 13.10 | 12.99 | 1.42 | 0.6732 | 0.8415 |
| TC0200015350.hg.1 | RFTN2 | raftlin family member 2 | Multiple_C | 3.14 | 3.26 | 3.02 | 1.09 | 0.7519 | 0.8731 | 3.37 | 3.11 | 3.08 | 1.22 | 0.1462 | 0.3817 |
| TC0200015831.hg.1 | PAX3 | paired box 3 | Multiple_C | 4.03 | 4.09 | 3.91 | 1.09 | 0.634 | 0.7961 | 4.19 | 4.06 | 4.34 | 0.90 | 0.8926 | 0.9542 |
| TC0200015919.hg.1 | COL4A4 | collagen, type IV, alpha 4 | Multiple_C | 4.8 | 4.5 | 4.68 | 1.09 | 0.38 | 0.5929 | 4.79 | 4.65 | 4.52 | 1.21 | 0.6268 | 0.8156 |
| TC0200016530.hg.1 | PLEKHB2 | pleckstrin homology domain containing, family B (evectins) mer | Multiple_C | 12 | 11.5 | 11.88 | 1.09 | 0.6017 | 0.7738 | 10.79 | 10.10 | 10.51 | 1.21 | 0.1837 | 0.4347 |
| TC0200016666.hg.1 | LHCGR | luteinizing hormone/choriogonadotropin receptor | Multiple_C | 3.42 | 3.21 | 3.3 | 1.09 | 0.7457 | 0.8691 | 3.89 | 3.99 | 3.42 | 1.39 | 0.123 | 0.3462 |
| TC0300007392.hg.1 | FAM212A | family with sequence similarity 212, member A | Coding | 4.13 | 3.78 | 4.01 | 1.09 | 0.8399 | 0.9205 | 4.04 | 3.84 | 4 | 1.03 | 0.9417 | 0.9759 |

| | | | | | | | | | | | | | | | |
|---|---|---|---|---|---|---|---|---|---|---|---|---|---|---|---|
| TC0300007415.hg.1 | LSMEM2 | leucine-rich single-pass membrane protein 2 | Coding | 3.89 | 3.51 | 3.77 | 1.09 | 0.3433 | 0.557 | 4.35 | 4.32 | 4.23 | 1.09 | 0.3566 | 0.6198 |
| TC0300007437.hg.1 | MANF | mesencephalic astrocyte-derived neurotrophic factor | Multiple_C | 10.1 | 11.78 | 9.98 | 1.09 | 0.6241 | 0.7897 | 8.78 | 8.29 | 8.36 | 1.34 | 0.0158 | 0.104 |
| TC0300008686.hg.1 | CHCHD6 | coiled-coil-helix-coiled-coil-helix domain containing 6 | Multiple_C | 5.55 | 5.93 | 5.43 | 1.09 | 0.6256 | 0.7911 | 5.41 | 5.23 | 5.08 | 1.26 | 0.6372 | 0.822 |
| TC0300009328.hg.1 | IL12A | interleukin 12A | Multiple_C | 3.76 | 3.24 | 3.64 | 1.09 | 0.7812 | 0.8872 | 4.57 | 4.84 | 5.17 | 0.66 | 0.0614 | 0.2353 |
| TC0300011050.hg.1 | QARS; MIR689 | glutaminyl-tRNA synthetase; microRNA 6890 | Multiple_C | 12.07 | 12.4 | 11.95 | 1.09 | 0.6337 | 0.7959 | 10.15 | 10.33 | 10.14 | 1.01 | 0.1459 | 0.3812 |
| TC0300011309.hg.1 | ACOX2 | acyl-CoA oxidase 2, branched chain | Multiple_C | 4.1 | 3.95 | 3.98 | 1.09 | 0.3641 | 0.5778 | 4.19 | 4.45 | 4.45 | 0.84 | 0.8652 | 0.9424 |
| TC0300012009.hg.1 | C3orf17 | chromosome 3 open reading frame 17 | Multiple_C | 9.39 | 10.25 | 9.27 | 1.09 | 0.8929 | 0.9484 | 7.27 | 7.95 | 8.42 | 0.45 | 0.0095 | 0.0756 |
| TC0300012338.hg.1 | DNAJB8 | DnaJ (Hsp40) homolog, subfamily B, member 8 | Coding | 3.26 | 2.93 | 3.14 | 1.09 | 0.6093 | 0.7797 | 3.39 | 3.60 | 3.36 | 1.02 | 0.924 | 0.9688 |
| TC0300014065.hg.1 | SERP1 | stress-associated endoplasmic reticulum protein 1 | Coding | 11.48 | 12.17 | 11.36 | 1.09 | 0.4387 | 0.648 | 9.53 | 9.49 | 9.4 | 1.09 | 0.7462 | 0.8835 |
| TC0400007113.hg.1 | SMIM20 | small integral membrane protein 20 | Multiple_C | 12.15 | 14.8 | 12.03 | 1.09 | 0.5191 | 0.7129 | 9.04 | 8.75 | 8.5 | 1.45 | 0.4525 | 0.6977 |
| TC0400007456.hg.1 | ZAR1 | zygote arrest 1 | Coding | 4.37 | 4.25 | 4.25 | 1.09 | 0.911 | 0.9578 | 3.64 | 3.67 | 3.49 | 1.11 | 0.3791 | 0.6388 |
| TC0400008556.hg.1 | MYOZ2 | myozenin 2 | Coding | 3.77 | 3.7 | 3.65 | 1.09 | 0.5445 | 0.7308 | 3.8 | 3.61 | 3.84 | 0.97 | 0.5752 | 0.7822 |
| TC0400008616.hg.1 | BBS12 | Bardet-Biedl syndrome 12 | Coding | 5.75 | 6.42 | 5.63 | 1.09 | 0.8276 | 0.9133 | 4.85 | 4.89 | 5.28 | 0.74 | 0.0825 | 0.2766 |
| TC0400009644.hg.1 | DUX4L7 | double homeobox 4 like 7 | Multiple_C | 5.44 | 5.29 | 5.32 | 1.09 | 0.2657 | 0.4732 | 4.88 | 4.90 | 4.68 | 1.15 | 0.4647 | 0.7067 |
| TC0400009944.hg.1 | PSAPL1 | prosaposin-like 1 (gene/pseudogene) | Multiple_C | 3.53 | 3.56 | 3.41 | 1.09 | 0.6131 | 0.783 | 3.32 | 3.57 | 3.56 | 0.85 | 0.5912 | 0.7931 |
| TC0400010989.hg.1 | ADAMTS3 | ADAM metallopeptidase with thrombospondin type 1 motif 3 | Multiple_C | 3.35 | 2.95 | 3.23 | 1.09 | 0.3831 | 0.5962 | 3.22 | 3.37 | 3.56 | 0.79 | 0.1297 | 0.3566 |
| TC0400011756.hg.1 | IL21 | interleukin 21 | Coding | 4.31 | 3.94 | 4.19 | 1.09 | 0.308 | 0.5212 | 4.78 | 4.56 | 4.61 | 1.13 | 0.286 | 0.551 |
| TC0400012606.hg.1 | IRF2 | interferon regulatory factor 2 | Multiple_C | 8.12 | 8.17 | 8 | 1.09 | 0.8776 | 0.9404 | 8.36 | 7.27 | 7.38 | 1.97 | 0.0068 | 0.0601 |
| TC0500008454.hg.1 | PRR16 | proline rich 16 | Multiple_C | 2.96 | 2.72 | 2.84 | 1.09 | 0.7617 | 0.8777 | 3.38 | 3.40 | 3.21 | 1.13 | 0.3623 | 0.6252 |
| TC0500011712.hg.1 | EPB41L4A | erythrocyte membrane protein band 4.1 like 4A | Multiple_C | 4.58 | 4.1 | 4.46 | 1.09 | 0.6686 | 0.8188 | 4.8 | 3.84 | 3.88 | 1.89 | 0.0009 | 0.0149 |
| TC0500012592.hg.1 | FAM71B | family with sequence similarity 71, member B | Coding | 3.05 | 2.85 | 2.93 | 1.09 | 0.7759 | 0.8845 | 3.15 | 3.31 | 3.2 | 0.97 | 0.206 | 0.4624 |
| TC0500013374.hg.1 | APBB3; MIR68 | amyloid beta (A4) precursor protein-binding, family B, member | Multiple_C | 6.33 | 6.22 | 6.21 | 1.09 | 0.6787 | 0.8255 | 5.15 | 5.99 | 5.54 | 0.76 | 0.0434 | 0.1926 |
| TC0600007309.hg.1 | BTN1A1 | butyrophilin, subfamily 1, member A1 | Coding | 3.99 | 3.7 | 3.87 | 1.09 | 0.5761 | 0.7547 | 4.71 | 4.33 | 4.31 | 1.32 | 0.0227 | 0.1297 |
| TC0600008006.hg.1 | FOXP4; MIR46 | forkhead box P4; microRNA 4641 | Multiple_C | 7.21 | 6.41 | 7.09 | 1.09 | 0.3291 | 0.5428 | 8.46 | 8.88 | 9.1 | 0.64 | 0.0022 | 0.0284 |
| TC0600009107.hg.1 | GPR6 | G protein-coupled receptor 6 | Coding | 3.17 | 2.9 | 3.05 | 1.09 | 0.7841 | 0.889 | 3.38 | 3.29 | 3.61 | 0.85 | 0.9342 | 0.9724 |
| TC0600011524.hg.1 | COL11A2 | collagen, type XI, alpha 2 | Multiple_C | 4.43 | 4.39 | 4.31 | 1.09 | 0.5276 | 0.7191 | 4.84 | 3.94 | 3.61 | 2.35 | 0.0003 | 0.0078 |
| TC0600011971.hg.1 | PTCHD4 | patched domain containing 4 | Multiple_C | 2.51 | 2.33 | 2.39 | 1.09 | 0.6937 | 0.8352 | 3.03 | 3.34 | 3.04 | 0.99 | 0.1761 | 0.4245 |
| TC0600014097.hg.1 | MRPS18B | mitochondrial ribosomal protein S18B | Multiple_C | 11.59 | 11.78 | 11.47 | 1.09 | 0.8273 | 0.9133 | 11.53 | 11.28 | 11.66 | 0.91 | 0.0722 | 0.2574 |
| TC0700007191.hg.1 | EEPD1 | endonuclease/exonuclease/phosphatase family domain contair | Multiple_C | 10.48 | 10.81 | 10.36 | 1.09 | 0.7523 | 0.8733 | 10.79 | 11.03 | 11.22 | 0.74 | 0.1004 | 0.3098 |
| TC0700008525.hg.1 | MBLAC1 | metallo-beta-lactamase domain containing 1 | Coding | 4.01 | 4.08 | 3.89 | 1.09 | 0.7929 | 0.8937 | 3.15 | 3.41 | 3.39 | 0.85 | 0.2396 | 0.5031 |
| TC0700008557.hg.1 | MOSPD3 | motile sperm domain containing 3 | Multiple_C | 6 | 5.3 | 5.88 | 1.09 | 0.6948 | 0.8359 | 6.46 | 6.58 | 6.03 | 1.35 | 0.2826 | 0.548 |
| TC0700010671.hg.1 | PDE1C | phosphodiesterase 1C, calmodulin-dependent 70kDa | Multiple_C | 4.54 | 4.13 | 4.42 | 1.09 | 0.5147 | 0.7096 | 4.06 | 3.81 | 3.88 | 1.13 | 0.7245 | 0.8717 |
| TC0700011504.hg.1 | WBSCR27 | Williams Beuren syndrome chromosome region 27 | Multiple_C | 5.92 | 5.44 | 5.8 | 1.09 | 0.6805 | 0.8263 | 4.34 | 4.75 | 4.62 | 0.82 | 0.9562 | 0.9819 |

| | | | | | | | | | | | | | | | |
|---|---|---|---|---|---|---|---|---|---|---|---|---|---|---|---|
| TC0700013375.hg.1 | ZNF273 | zinc finger protein 273 | Multiple_C | 8.22 | 7.97 | 8.1 | 1.09 | 0.8659 | 0.9346 | 7.92 | 7.24 | 7.67 | 1.19 | 0.8836 | 0.9502 |
| TC0800007459.hg.1 | POMK | protein-O-mannose kinase | Multiple_C | 10.47 | 10.61 | 10.35 | 1.09 | 0.9959 | 0.998 | 10.66 | 10.26 | 10.87 | 0.86 | 0.3861 | 0.6451 |
| TC0800008131.hg.1 | REXO1L11P | REX1, RNA exonuclease 1 homolog-like 11, pseudogene | Multiple_C | 4.54 | 4.34 | 4.42 | 1.09 | 0.1182 | 0.2719 | 4.37 | 4.49 | 4.34 | 1.02 | 0.5224 | 0.7487 |
| TC0800009428.hg.1 | XKR5 | X-linked Kx blood group related 5 | Multiple_C | 5.26 | 4.98 | 5.14 | 1.09 | 0.9384 | 0.9713 | 4.63 | 4.55 | 4.58 | 1.04 | 0.4564 | 0.7004 |
| TC0800009826.hg.1 | NUDT18 | nudix hydrolase 18 | Multiple_C | 5.64 | 5.46 | 5.52 | 1.09 | 0.6719 | 0.8212 | 5.63 | 5.80 | 5.58 | 1.04 | 0.1302 | 0.3574 |
| TC0800011620.hg.1 | ENPP2 | ectonucleotide pyrophosphatase/phosphodiesterase 2 | Multiple_C | 3.71 | 4.11 | 3.59 | 1.09 | 0.638 | 0.7991 | 4.2 | 3.67 | 3.63 | 1.48 | 0.0378 | 0.1772 |
| TC0800012277.hg.1 | SFTPC | surfactant protein C | Multiple_C | 3.88 | 3.71 | 3.76 | 1.09 | 0.892 | 0.9481 | 3.81 | 3.80 | 4.03 | 0.86 | 0.7721 | 0.8976 |
| TC0900009168.hg.1 | PAEP | progestagen-associated endometrial protein | Multiple_C | 3.78 | 3.69 | 3.66 | 1.09 | 0.5537 | 0.7374 | 3.47 | 3.31 | 3.14 | 1.26 | 0.0432 | 0.1921 |
| TC0900009437.hg.1 | AK3 | adenylate kinase 3 | Multiple_C | 12.62 | 13.61 | 12.5 | 1.09 | 0.7991 | 0.8967 | 10.95 | 10.91 | 10.51 | 1.36 | 0.417 | 0.6707 |
| TC0900010865.hg.1 | FBP2 | fructose-1,6-bisphosphatase 2 | Coding | 3.44 | 3.32 | 3.32 | 1.09 | 0.8861 | 0.945 | 3.79 | 3.55 | 3.74 | 1.04 | 0.2099 | 0.467 |
| TC0900011302.hg.1 | TNFSF8 | tumor necrosis factor (ligand) superfamily, member 8 | Multiple_C | 3.61 | 3.54 | 3.49 | 1.09 | 0.3461 | 0.5597 | 3.57 | 3.61 | 3.63 | 0.96 | 0.6795 | 0.8459 |
| TC0900011969.hg.1 | LHX3 | LIM homeobox 3 | Coding | 3.11 | 2.98 | 2.99 | 1.09 | 0.6644 | 0.8164 | 3.2 | 3.27 | 3.16 | 1.03 | 0.7195 | 0.8692 |
| TC0900012187.hg.1 | KCNT1 | potassium channel, sodium activated subfamily T, member 1 | Multiple_C | 4.36 | 4.2 | 4.24 | 1.09 | 0.5444 | 0.7307 | 6.37 | 6.05 | 5.78 | 1.51 | 0.0678 | 0.2479 |
| TC0X00007568.hg.1 | TAF1 | TAF1 RNA polymerase II, TATA box binding protein (TBP)-associ | Multiple_C | 10.54 | 11.32 | 10.42 | 1.09 | 0.6924 | 0.8342 | 10.3 | 10.67 | 10.72 | 0.75 | 0.3117 | 0.5759 |
| TC0X00008332.hg.1 | STAG2 | stromal antigen 2 | Multiple_C | 13.03 | 14.06 | 12.91 | 1.09 | 0.4677 | 0.6721 | 13.27 | 13.69 | 14.02 | 0.59 | 0.0472 | 0.2012 |
| TC0X00009184.hg.1 | BEND2 | BEN domain containing 2 | Multiple_C | 3.24 | 3.24 | 3.12 | 1.09 | 0.5037 | 0.7012 | 3.97 | 3.93 | 3.98 | 0.99 | 0.4589 | 0.7024 |
| TC0X00009911.hg.1 | VSIG4 | V-set and immunoglobulin domain containing 4 | Coding | 3.59 | 3.49 | 3.47 | 1.09 | 0.5378 | 0.726 | 4.2 | 4.10 | 4.05 | 1.11 | 0.9678 | 0.9866 |
| TC0X00010919.hg.1 | CT45A10 | cancer/testis antigen family 45, member A10 | Coding | 3.13 | 3.01 | 3.01 | 1.09 | 0.7157 | 0.8497 | 3.36 | 3.44 | 3.58 | 0.86 | 0.9132 | 0.9645 |
| TC0X00011313.hg.1 | GPRASP2 | G protein-coupled receptor associated sorting protein 2 | Multiple_C | 3.89 | 3.6 | 3.77 | 1.09 | 0.6341 | 0.7962 | 4.73 | 5.39 | 6.37 | 0.32 | 6.52E-06 | 0.0005 |
| TC0Y00006440.hg.1 | SHOX | short stature homeobox | Multiple_C | 3.87 | 3.5 | 3.75 | 1.09 | 0.9173 | 0.9612 | 3.96 | 4.05 | 3.93 | 1.02 | 0.4041 | 0.6599 |
| TC1000007990.hg.1 | DDIT4 | DNA damage inducible transcript 4 | Multiple_C | 14.13 | 14.49 | 14.01 | 1.09 | 0.8153 | 0.9071 | 10.49 | 8.79 | 10.89 | 0.76 | 0.4344 | 0.6852 |
| TC1000009541.hg.1 | ADARB2 | adenosine deaminase, RNA-specific, B2 (inactive) | Multiple_C | 3.8 | 3.69 | 3.68 | 1.09 | 0.3962 | 0.6098 | 4.99 | 4.82 | 4.85 | 1.10 | 0.1161 | 0.3366 |
| TC1000009879.hg.1 | C10orf111 | chromosome 10 open reading frame 111 | Multiple_C | 4.15 | 3.82 | 4.03 | 1.09 | 0.8585 | 0.931 | 3.93 | 3.92 | 3.51 | 1.34 | 0.0314 | 0.1583 |
| TC1000011657.hg.1 | FGF8 | fibroblast growth factor 8 (androgen-induced) | Multiple_C | 7.46 | 6.99 | 7.34 | 1.09 | 0.2402 | 0.4422 | 5.93 | 6.24 | 6.12 | 0.88 | 0.4868 | 0.7237 |
| TC1000012433.hg.1 | SPAG6 | sperm associated antigen 6 | Multiple_C | 3.43 | 3.48 | 3.31 | 1.09 | 0.3753 | 0.588 | 3.66 | 3.63 | 3.55 | 1.08 | 0.3435 | 0.6071 |
| TC1100006746.hg.1 | OR10A2 | olfactory receptor, family 10, subfamily A, member 2 | Coding | 3.72 | 3.21 | 3.6 | 1.09 | 0.4761 | 0.6787 | 4.31 | 4.27 | 4.33 | 0.99 | 0.8056 | 0.9141 |
| TC1100007762.hg.1 | OOSP2 | oocyte secreted protein 2 | Multiple_C | 4.42 | 4.79 | 4.3 | 1.09 | 0.1892 | 0.377 | 4.62 | 4.53 | 4.52 | 1.07 | 0.4329 | 0.6841 |
| TC1100007795.hg.1 | PGA3; PGA4 | pepsinogen 3, group I (pepsinogen A); pepsinogen 4, group I (pe | Multiple_C | 4.71 | 4.44 | 4.59 | 1.09 | 0.8166 | 0.9075 | 4.82 | 4.49 | 4.65 | 1.13 | 0.5491 | 0.7657 |
| TC1100010495.hg.1 | LMO2 | LIM domain only 2 (rhombotin-like 1) | Multiple_C | 3.57 | 3.19 | 3.45 | 1.09 | 0.75 | 0.8716 | 5.61 | 5.16 | 4.63 | 1.97 | 0.0094 | 0.0749 |
| TC1100010623.hg.1 | ALX4 | ALX homeobox 4 | Coding | 2.54 | 2.57 | 2.42 | 1.09 | 0.665 | 0.8169 | 3.14 | 3.09 | 2.83 | 1.24 | 0.1151 | 0.3349 |
| TC1100010718.hg.1 | LRP4 | LDL receptor related protein 4 | Multiple_C | 4.14 | 3.66 | 4.02 | 1.09 | 0.6244 | 0.79 | 7.37 | 7.54 | 7.21 | 1.12 | 0.4894 | 0.7251 |
| TC1100010846.hg.1 | OR8K5 | olfactory receptor, family 8, subfamily K, member 5 | Coding | 3.44 | 2.81 | 3.32 | 1.09 | 0.3502 | 0.5631 | 4.33 | 4.00 | 3.72 | 1.53 | 0.0091 | 0.0735 |
| TC1100011558.hg.1 | PDE2A | phosphodiesterase 2A, cGMP-stimulated | Multiple_C | 3.41 | 3.51 | 3.29 | 1.09 | 0.9869 | 0.9934 | 3.66 | 3.64 | 3.39 | 1.21 | 0.5589 | 0.7714 |

| | | | | | | | | | | | | | | | |
|---|---|---|---|---|---|---|---|---|---|---|---|---|---|---|---|
| TC1100012834.hg.1 | ADAMTS8 | ADAM metallopeptidase with thrombospondin type 1 motif 8 | Multiple_C | 4.46 | 4.04 | 4.34 | 1.09 | 0.185 | 0.3711 | 4.93 | 4.66 | 4.68 | 1.19 | 0.8962 | 0.9562 |
| TC1100013025.hg.1 | TTC9C | tetratricopeptide repeat domain 9C | Multiple_C | 9.2 | 10.09 | 9.08 | 1.09 | 0.7948 | 0.8948 | 9.22 | 9.24 | 9.32 | 0.93 | 0.8626 | 0.9412 |
| TC1200006469.hg.1 | WNT5B | wingless-type MMTV integration site family, member 5B | Multiple_C | 5.67 | 5.07 | 5.55 | 1.09 | 0.0994 | 0.2398 | 5.15 | 5.34 | 5.24 | 0.94 | 0.8823 | 0.9495 |
| TC1200010690.hg.1 | CSRNP2 | cysteine-serine-rich nuclear protein 2 | Multiple_C | 6.08 | 5.75 | 5.96 | 1.09 | 0.7579 | 0.8755 | 6.59 | 6.70 | 6.42 | 1.13 | 0.1823 | 0.4329 |
| TC1200011495.hg.1 | LUM | lumican | Multiple_C | 4.7 | 4.73 | 4.58 | 1.09 | 0.7152 | 0.8496 | 7.82 | 8.27 | 9.31 | 0.36 | 5.78E-06 | 0.0005 |
| TC1200012543.hg.1 | ANKLE2 | ankyrin repeat and LEM domain containing 2 | Multiple_C | 11 | 10.26 | 10.88 | 1.09 | 0.7803 | 0.887 | 9.6 | 9.40 | 10.02 | 0.75 | 0.2851 | 0.5501 |
| TC1200012836.hg.1 | C12orf73 | chromosome 12 open reading frame 73 | Multiple_C | 9.83 | 10.95 | 9.71 | 1.09 | 0.7302 | 0.8588 | 6.83 | 7.77 | 7.82 | 0.50 | 0.0154 | 0.1021 |
| TC1300008417.hg.1 | GPR12 | G protein-coupled receptor 12 | Coding | 2.92 | 2.96 | 2.8 | 1.09 | 0.1835 | 0.3689 | 3.51 | 3.51 | 3.2 | 1.24 | 0.1587 | 0.4002 |
| TC1300009781.hg.1 | COL4A1 | collagen, type IV, alpha 1 | Multiple_C | 3.31 | 3.29 | 3.19 | 1.09 | 0.9091 | 0.9564 | 3.11 | 3.23 | 3.21 | 0.93 | 0.7166 | 0.8677 |
| TC1300009919.hg.1 | ATP4B | ATPase, H+/K+ exchanging, beta polypeptide | Coding | 4.2 | 3.89 | 4.08 | 1.09 | 0.7076 | 0.8445 | 4.04 | 4.17 | 4.14 | 0.93 | 0.7688 | 0.8964 |
| TC1300010043.hg.1 | PCDH20 | protocadherin 20 | Coding | 3.29 | 3.22 | 3.17 | 1.09 | 0.3552 | 0.5683 | 3.73 | 3.70 | 3.42 | 1.24 | 0.2421 | 0.5059 |
| TC1400009223.hg.1 | CNIH1 | cornichon family AMPA receptor auxiliary protein 1 | Multiple_C | 12.74 | 14.92 | 12.62 | 1.09 | 0.7655 | 0.8796 | 11.18 | 11.03 | 10.75 | 1.35 | 0.019 | 0.1166 |
| TC1400009381.hg.1 | KCNH5 | potassium channel, voltage gated eag related subfamily H, mem | Multiple_C | 3.88 | 3.87 | 3.76 | 1.09 | 0.4258 | 0.6365 | 3.4 | 3.25 | 3.36 | 1.03 | 0.9254 | 0.9693 |
| TC1400010100.hg.1 | ATG2B | autophagy related 2B | Multiple_C | 6.28 | 6.79 | 6.16 | 1.09 | 0.7621 | 0.8778 | 5.54 | 5.77 | 6.71 | 0.44 | 0.0629 | 0.2386 |
| TC1400010699.hg.1 | CEP170B | centrosomal protein 170B | Multiple_C | 7.8 | 7.29 | 7.68 | 1.09 | 0.3317 | 0.5457 | 6.87 | 7.16 | 7.39 | 0.70 | 0.0115 | 0.0852 |
| TC1500007788.hg.1 | TMEM202 | transmembrane protein 202 | Coding | 3.46 | 3.48 | 3.34 | 1.09 | 0.1611 | 0.338 | 4.27 | 4.06 | 3.93 | 1.27 | 0.0295 | 0.1522 |
| TC1500008370.hg.1 | ST8SIA2 | ST8 alpha-N-acetyl-neuraminide alpha-2,8-sialyltransferase 2 | Multiple_C | 3.24 | 2.6 | 3.12 | 1.09 | 0.8717 | 0.9377 | 4.44 | 4.49 | 4.5 | 0.96 | 0.723 | 0.8708 |
| TC1500010050.hg.1 | COX5A | cytochrome c oxidase subunit Va | Multiple_C | 15.02 | 14.77 | 14.9 | 1.09 | 0.6477 | 0.8047 | 11.58 | 11.12 | 11.27 | 1.24 | 0.727 | 0.8731 |
| TC1500010897.hg.1 | CHRNB4 | cholinergic receptor, nicotinic beta 4 | Coding | 4.59 | 4.34 | 4.47 | 1.09 | 0.2345 | 0.4354 | 4.23 | 4.47 | 4.47 | 0.85 | 0.1993 | 0.4546 |
| TC1600008019.hg.1 | TEPP | testis, prostate and placenta expressed | Multiple_C | 4.1 | 4.05 | 3.98 | 1.09 | 0.5733 | 0.7525 | 3.69 | 3.84 | 3.67 | 1.01 | 0.9545 | 0.9814 |
| TC1600008150.hg.1 | ELMO3 | engulfment and cell motility 3 | Multiple_C | 8.49 | 9.39 | 8.37 | 1.09 | 0.8557 | 0.9295 | 5.8 | 5.98 | 5.77 | 1.02 | 0.5624 | 0.773 |
| TC1600008858.hg.1 | LOC10012969 | uncharacterized LOC100129697; novel transcript, antisense to C | Multiple_C | 6.04 | 5.88 | 5.92 | 1.09 | 0.5303 | 0.7207 | 5.15 | 5.08 | 5.04 | 1.08 | 0.346 | 0.6098 |
| TC1600011407.hg.1 | CMTM1 | CKLF-like MARVEL transmembrane domain containing 1 | Coding | 4.72 | 3.97 | 4.6 | 1.09 | 0.7103 | 0.8464 | 7.22 | 7.34 | 7.01 | 1.16 | 0.2393 | 0.5028 |
| TC1700007372.hg.1 | FOXN1 | forkhead box N1 | Multiple_C | 5.63 | 5.43 | 5.51 | 1.09 | 0.7904 | 0.8929 | 5.58 | 5.51 | 5.39 | 1.14 | 0.4776 | 0.7164 |
| TC1700007603.hg.1 | RASL10B | RAS-like, family 10, member B | Multiple_C | 5.32 | 5.1 | 5.2 | 1.09 | 0.9339 | 0.9689 | 8.52 | 8.56 | 9.13 | 0.66 | 0.0355 | 0.1708 |
| TC1700008209.hg.1 | NGFR | nerve growth factor receptor | Multiple_C | 4.22 | 4.11 | 4.1 | 1.09 | 0.7478 | 0.8706 | 7.01 | 7.34 | 6.05 | 1.95 | 0.0002 | 0.0066 |
| TC1700008561.hg.1 | KCNH6 | potassium channel, voltage gated eag related subfamily H, mem | Coding | 3.29 | 3.05 | 3.17 | 1.09 | 0.3124 | 0.5258 | 3.83 | 4.36 | 3.98 | 0.90 | 0.4181 | 0.672 |
| TC1700009392.hg.1 | SCARF1 | scavenger receptor class F, member 1 | Multiple_C | 4.18 | 4.19 | 4.06 | 1.09 | 0.5833 | 0.7603 | 3.27 | 3.62 | 3.66 | 0.76 | 0.142 | 0.3747 |
| TC1700010659.hg.1 | KRTAP4-1 | keratin associated protein 4-1 | Coding | 3.27 | 2.99 | 3.15 | 1.09 | 0.7967 | 0.8956 | 4.16 | 3.70 | 3.92 | 1.18 | 0.0592 | 0.2294 |
| TC1700012206.hg.1 | LOC79999; LO | uncharacterized LOC79999; Homo sapiens uncharacterized prot | Multiple_C | 4.22 | 3.94 | 4.1 | 1.09 | 0.3314 | 0.5454 | 4.34 | 4.30 | 4.27 | 1.05 | 0.6176 | 0.8101 |
| TC1700012230.hg.1 | ZNF207; MIR6 | zinc finger protein 207; microRNA 632 | Multiple_C | 14.24 | 13.79 | 14.12 | 1.09 | 0.2839 | 0.4941 | 14.71 | 14.78 | 14.69 | 1.01 | 0.8441 | 0.9318 |
| TC1700012332.hg.1 | SPATA22 | spermatogenesis associated 22 | Multiple_C | 4.11 | 3.87 | 3.99 | 1.09 | 0.993 | 0.9964 | 3.53 | 3.75 | 3.76 | 0.85 | 0.1452 | 0.3802 |
| TC1700012397.hg.1 | KRT25 | keratin 25, type I | Coding | 4.51 | 4.49 | 4.39 | 1.09 | 0.2346 | 0.4354 | 3.89 | 3.81 | 3.69 | 1.15 | 0.3489 | 0.6129 |

| | | | | | | | | | | | | | | | |
|---|---|---|---|---|---|---|---|---|---|---|---|---|---|---|---|
| TC1800007543.hg.1 | SERPINB5 | serpin peptidase inhibitor, clade B (ovalbumin), member 5 | Multiple_C | 13.49 | 13.35 | 13.37 | 1.09 | 0.7878 | 0.8914 | 3.86 | 2.74 | 3.38 | 1.39 | 0.0569 | 0.2245 |
| TC1800008691.hg.1 | MRO | maestro | Multiple_C | 3.32 | 3.16 | 3.2 | 1.09 | 0.7287 | 0.8575 | 3.68 | 3.84 | 3.52 | 1.12 | 0.394 | 0.6521 |
| TC1800008943.hg.1 | CDH19 | cadherin 19, type 2 | Multiple_C | 3.22 | 3.17 | 3.1 | 1.09 | 0.7217 | 0.8535 | 3.12 | 3.27 | 3.2 | 0.95 | 0.6201 | 0.812 |
| TC1900006964.hg.1 | RDH8 | retinol dehydrogenase 8 (all-trans) | Multiple_C | 3.07 | 2.93 | 2.95 | 1.09 | 0.7961 | 0.8953 | 3.24 | 2.84 | 2.91 | 1.26 | 0.1374 | 0.3683 |
| TC1900007020.hg.1 | ANGPTL8 | angiopoietin like 8 | Multiple_C | 5.3 | 5.25 | 5.18 | 1.09 | 0.9629 | 0.9823 | 4.12 | 4.48 | 4.58 | 0.73 | 0.3389 | 0.603 |
| TC1900007208.hg.1 | SYDE1 | synapse defective 1, Rho GTPase, homolog 1 (C. elegans) | Multiple_C | 4.09 | 4.02 | 3.97 | 1.09 | 0.3067 | 0.5198 | 5.91 | 6.11 | 6.01 | 0.93 | 0.9535 | 0.981 |
| TC1900007905.hg.1 | OVOL3 | ovo-like zinc finger 3 | Multiple_C | 3.36 | 2.97 | 3.24 | 1.09 | 0.7974 | 0.896 | 3.24 | 3.27 | 3.11 | 1.09 | 0.7426 | 0.8821 |
| TC1900008558.hg.1 | MED25; MIR6 | mediator complex subunit 25; microRNA 6800 | Multiple_C | 5.88 | 5.43 | 5.76 | 1.09 | 0.3726 | 0.5853 | 4.6 | 5.06 | 5.1 | 0.71 | 0.0136 | 0.0945 |
| TC1900009176.hg.1 | TCF3 | transcription factor 3 | Multiple_C | 10.72 | 10.11 | 10.6 | 1.09 | 0.752 | 0.8731 | 11.42 | 11.70 | 11.59 | 0.89 | 0.2165 | 0.4756 |
| TC1900009989.hg.1 | INSL3 | insulin-like 3 (Leydig cell) | Coding | 4.77 | 4.53 | 4.65 | 1.09 | 0.2456 | 0.4492 | 4.4 | 4.53 | 4.67 | 0.83 | 0.4693 | 0.7104 |
| TC1900010013.hg.1 | LRRC25 | leucine rich repeat containing 25 | Coding | 3.72 | 3.67 | 3.6 | 1.09 | 0.6325 | 0.7949 | 3.89 | 3.71 | 3.58 | 1.24 | 0.6095 | 0.8046 |
| TC1900011194.hg.1 | KCNC3 | potassium channel, voltage gated Shaw related subfamily C, me | Multiple_C | 3.14 | 2.86 | 3.02 | 1.09 | 0.312 | 0.5254 | 3.49 | 3.43 | 3.24 | 1.19 | 0.068 | 0.2485 |
| TC1900011451.hg.1 | SYT5 | synaptotagmin V | Multiple_C | 3.54 | 3.49 | 3.42 | 1.09 | 0.2311 | 0.4314 | 3.73 | 3.56 | 3.46 | 1.21 | 0.3021 | 0.5665 |
| TC1900011495.hg.1 | NLRP11 | NLR family, pyrin domain containing 11 | Coding | 3.65 | 3.52 | 3.53 | 1.09 | 0.5038 | 0.7014 | 4.33 | 4.45 | 4.38 | 0.97 | 0.61 | 0.805 |
| TC1900011744.hg.1 | BCKDHA | branched chain keto acid dehydrogenase E1, alpha polypeptide | Multiple_C | 8.95 | 8.73 | 8.83 | 1.09 | 0.7504 | 0.8718 | 7.56 | 7.79 | 7.94 | 0.77 | 0.0232 | 0.1314 |
| TC1900011931.hg.1 | SYNE4 | spectrin repeat containing, nuclear envelope family member 4 | Multiple_C | 5.55 | 5.38 | 5.43 | 1.09 | 0.6997 | 0.8394 | 3.77 | 3.80 | 3.55 | 1.16 | 0.1144 | 0.3338 |
| TC2000006442.hg.1 | NRSN2 | neurensin 2 | Multiple_C | 3.36 | 3.3 | 3.24 | 1.09 | 0.8376 | 0.9192 | 8.82 | 9.18 | 8.82 | 1.00 | 0.5708 | 0.779 |
| TC2000007450.hg.1 | R3HDML | R3H domain containing-like | Coding | 5.75 | 5.63 | 5.63 | 1.09 | 0.1894 | 0.3771 | 6.75 | 6.41 | 6.36 | 1.31 | 0.1968 | 0.4516 |
| TC2000008016.hg.1 | MIR1-1HG; M | MIR1-1 host gene; microRNA 133a-2 | Multiple_C | 4.85 | 4.76 | 4.73 | 1.09 | 0.4756 | 0.6784 | 5.28 | 5.45 | 5.46 | 0.88 | 0.8001 | 0.9105 |
| TC2000009248.hg.1 | NEURL2 | neuralized E3 ubiquitin protein ligase 2 | Coding | 4.91 | 4.6 | 4.79 | 1.09 | 0.5873 | 0.7635 | 5.79 | 5.73 | 5.54 | 1.19 | 0.131 | 0.3587 |
| TC2000010010.hg.1 | BLCAP | bladder cancer associated protein | Multiple_C | 10.73 | 10.43 | 10.61 | 1.09 | 0.8003 | 0.8977 | 6.91 | 7.67 | 8.61 | 0.31 | 0.0041 | 0.0434 |
| TC2100007239.hg.1 | UMODL1 | uromodulin-like 1 | Multiple_C | 3.96 | 3.57 | 3.84 | 1.09 | 0.7561 | 0.8745 | 3.82 | 3.93 | 3.81 | 1.01 | 0.7068 | 0.8619 |
| TC2200006907.hg.1 | SGSM1 | small G protein signaling modulator 1 | Multiple_C | 3.34 | 3.31 | 3.22 | 1.09 | 0.1841 | 0.3698 | 3.75 | 3.71 | 3.82 | 0.95 | 0.7712 | 0.8972 |
| TC2200007214.hg.1 | APOL6 | apolipoprotein L, 6 | Multiple_C | 7.69 | 7.64 | 7.57 | 1.09 | 0.7986 | 0.8966 | 6.04 | 5.72 | 5.57 | 1.39 | 0.0212 | 0.1242 |
| TC2200008103.hg.1 | YPEL1 | yippee like 1 | Multiple_C | 2.95 | 2.78 | 2.83 | 1.09 | 0.3221 | 0.5359 | 3.81 | 4.34 | 3.67 | 1.10 | 0.0919 | 0.2937 |
| TC2200008112.hg.1 | TOP3B | topoisomerase (DNA) III beta | Multiple_C | 7.48 | 6.85 | 7.36 | 1.09 | 0.8351 | 0.9177 | 8.15 | 8.34 | 8.09 | 1.04 | 0.5263 | 0.7508 |
| TC2200008704.hg.1 | DMC1 | DNA meiotic recombinase 1 | Multiple_C | 3.65 | 3.54 | 3.53 | 1.09 | 0.6716 | 0.821 | 4.14 | 4.25 | 4.17 | 0.98 | 0.5382 | 0.759 |
| TC2200009324.hg.1 | DRICH1 | aspartate-rich 1 | Multiple_C | 3.94 | 3.57 | 3.82 | 1.09 | 0.6013 | 0.7735 | 3.92 | 3.95 | 4.17 | 0.84 | 0.8753 | 0.9465 |
| TC2200009361.hg.1 | MAPK12 | mitogen-activated protein kinase 12 | Multiple_C | 7.33 | 6.92 | 7.21 | 1.09 | 0.3765 | 0.5895 | 8.88 | 9.00 | 8.9 | 0.99 | 0.928 | 0.9698 |
| TSUnmapped00000229.h | ZKSCAN7 | zinc finger with KRAB and SCAN domains 7 | Coding | 4.03 | 3.65 | 3.91 | 1.09 | 0.6559 | 0.811 | 3.97 | 4.17 | 4.09 | 0.92 | 0.6771 | 0.8445 |
| TSUnmapped00000576.h | CACFD1 | calcium channel flower domain containing 1 | Coding | 4.81 | 4.59 | 4.69 | 1.09 | 0.5085 | 0.705 | 5 | 4.94 | 4.95 | 1.04 | 0.8244 | 0.9224 |
| TC0100006571.hg.1 | SKI; kerlabu | Memczak2013 ALT_ACCEPTOR, ALT_DONOR, coding, INTERNAL | Multiple_C | 4.17 | 3.94 | 4.06 | 1.08 | 0.5952 | 0.7693 | 3.68 | 3.63 | 3.61 | 1.05 | 0.6274 | 0.8158 |
| TC0100006650.hg.1 | AJAP1 | adherens junctions associated protein 1 | Multiple_C | 3.01 | 2.37 | 2.9 | 1.08 | 0.9186 | 0.9622 | 2.96 | 3.07 | 3.1 | 0.91 | 0.2323 | 0.4941 |

| | | | | | | | | | | | | | | | |
|---|---|---|---|---|---|---|---|---|---|---|---|---|---|---|---|
| TC0100007940.hg.1 | CAP1 | CAP, adenylate cyclase-associated protein 1 (yeast) | Multiple_C | 14.82 | 14.2 | 14.71 | 1.08 | 0.2016 | 0.3937 | 14.9 | 15.06 | 15.14 | 0.85 | 0.4659 | 0.7076 |
| TC0100008194.hg.1 | FOXD2 | forkhead box D2 | Coding | 3.24 | 3.1 | 3.13 | 1.08 | 0.6936 | 0.8351 | 3.62 | 3.66 | 3.39 | 1.17 | 0.5746 | 0.7818 |
| TC0100009715.hg.1 | PPIAL4F | peptidylprolyl isomerase A (cyclophilin A)-like 4F | Coding | 12.72 | 12.54 | 12.61 | 1.08 | 0.712 | 0.8478 | 9.77 | 9.96 | 10.31 | 0.69 | 0.1028 | 0.3141 |
| TC0100010331.hg.1 | KLHDC9 | kelch domain containing 9 | Multiple_C | 4.65 | 4.66 | 4.54 | 1.08 | 0.3663 | 0.5795 | 4.06 | 4.32 | 3.89 | 1.13 | 0.4022 | 0.6584 |
| TC0100011096.hg.1 | NEK7 | NIMA-related kinase 7 | Multiple_C | 11.61 | 11.7 | 11.5 | 1.08 | 0.7415 | 0.8665 | 9.62 | 9.07 | 9.61 | 1.01 | 0.8616 | 0.9408 |
| TC0100011270.hg.1 | LAX1 | lymphocyte transmembrane adaptor 1 | Multiple_C | 3.18 | 3.11 | 3.07 | 1.08 | 0.1248 | 0.2823 | 3.41 | 3.32 | 3.2 | 1.16 | 0.6592 | 0.8338 |
| TC0100011391.hg.1 | IL24 | interleukin 24 | Multiple_C | 3.06 | 3.05 | 2.95 | 1.08 | 0.6579 | 0.8122 | 3.53 | 3.69 | 3.54 | 0.99 | 0.5303 | 0.7534 |
| TC0100011453.hg.1 | G0S2 | G0/G1 switch 2 | Multiple_C | 4.22 | 4.15 | 4.11 | 1.08 | 0.8508 | 0.9269 | 6.11 | 6.34 | 6.11 | 1.00 | 0.6805 | 0.8463 |
| TC0100012350.hg.1 | OR2M2 | olfactory receptor, family 2, subfamily M, member 2 | Coding | 3.37 | 3.22 | 3.26 | 1.08 | 0.7953 | 0.895 | 4.38 | 4.67 | 4.24 | 1.10 | 0.3325 | 0.5967 |
| TC0100013161.hg.1 | PLA2G2E | phospholipase A2, group IIE | Coding | 5.35 | 4.75 | 5.24 | 1.08 | 0.6805 | 0.8263 | 5.51 | 5.80 | 5.69 | 0.88 | 0.6438 | 0.8254 |
| TC0100013429.hg.1 | FCN3 | ficolin (collagen/fibrinogen domain containing) 3 | Multiple_C | 4.06 | 3.77 | 3.95 | 1.08 | 0.529 | 0.72 | 4.29 | 4.36 | 4.49 | 0.87 | 0.3542 | 0.6177 |
| TC0100015379.hg.1 | NHLH2 | nescient helix-loop-helix 2 | Coding | 3.47 | 3.07 | 3.36 | 1.08 | 0.5899 | 0.7653 | 3.51 | 3.58 | 3.45 | 1.04 | 0.7996 | 0.9104 |
| TC0100016337.hg.1 | XCL2 | chemokine (C motif) ligand 2 | Coding | 3.34 | 3.49 | 3.23 | 1.08 | 0.7884 | 0.8916 | 5.26 | 5.21 | 4.56 | 1.62 | 0.0058 | 0.0543 |
| TC0100016919.hg.1 | CSRP1; marko | Memczak2013 ALT_ACCEPTOR, ALT_DONOR, coding, INTERNAL | Multiple_C | 4.5 | 4.57 | 4.39 | 1.08 | 0.7456 | 0.869 | 4.23 | 4.32 | 3.93 | 1.23 | 0.8151 | 0.9182 |
| TC0100017059.hg.1 | RBBP5 | retinoblastoma binding protein 5 | Multiple_C | 10.84 | 10.58 | 10.73 | 1.08 | 0.8705 | 0.9369 | 10.28 | 9.85 | 10.26 | 1.01 | 0.4346 | 0.6853 |
| TC0100018329.hg.1 | CFHR1 | complement factor H-related 1 | Multiple_C | 3.37 | 3.2 | 3.26 | 1.08 | 0.7076 | 0.8445 | 3.71 | 3.74 | 3.34 | 1.29 | 0.1074 | 0.3224 |
| TC0200006703.hg.1 | ATP6V1C2 | ATPase, H+ transporting, lysosomal 42kDa, V1 subunit C2 | Multiple_C | 5.13 | 4.8 | 5.02 | 1.08 | 0.6496 | 0.8059 | 4.72 | 4.34 | 4.16 | 1.47 | 0.0231 | 0.1308 |
| TC0200008669.hg.1 | IL1RL2 | interleukin 1 receptor-like 2 | Multiple_C | 3.84 | 3.59 | 3.73 | 1.08 | 0.7837 | 0.8889 | 4.45 | 4.17 | 3.92 | 1.44 | 0.1676 | 0.413 |
| TC0200009370.hg.1 | ZNF806 | zinc finger protein 806 | Multiple_C | 3.11 | 3.12 | 3 | 1.08 | 0.9445 | 0.9745 | 3.29 | 3.24 | 3.13 | 1.12 | 0.7429 | 0.8822 |
| TC0200010202.hg.1 | FSIP2 | fibrous sheath interacting protein 2 | Multiple_C | 4.01 | 3.67 | 3.9 | 1.08 | 0.9737 | 0.9874 | 3.94 | 4.10 | 3.72 | 1.16 | 0.5375 | 0.7589 |
| TC0200010658.hg.1 | CPS1 | carbamoyl-phosphate synthase 1 | Multiple_C | 4.09 | 4.31 | 3.98 | 1.08 | 0.3299 | 0.5437 | 4.45 | 4.50 | 4.55 | 0.93 | 0.5544 | 0.7691 |
| TC0200012159.hg.1 | CAPN13 | calpain 13 | Multiple_C | 2.96 | 2.98 | 2.85 | 1.08 | 0.613 | 0.7829 | 3.98 | 3.64 | 3.33 | 1.57 | 0.0018 | 0.0244 |
| TC0200013916.hg.1 | IL1B | interleukin 1 beta | Multiple_C | 3.31 | 2.91 | 3.2 | 1.08 | 0.6263 | 0.7915 | 3.37 | 3.40 | 3.39 | 0.99 | 0.3944 | 0.6525 |
| TC0200014382.hg.1 | TMEM163 | transmembrane protein 163 | Multiple_C | 3.96 | 3.61 | 3.85 | 1.08 | 0.8204 | 0.9099 | 8.41 | 7.57 | 7.51 | 1.87 | 0.0575 | 0.2261 |
| TC0200014620.hg.1 | CACNB4 | calcium channel, voltage-dependent, beta 4 subunit | Multiple_C | 3.51 | 3.04 | 3.4 | 1.08 | 0.2471 | 0.4507 | 4.19 | 3.81 | 3.87 | 1.25 | 0.059 | 0.2291 |
| TC0200014995.hg.1 | WIPF1 | WAS/WASL interacting protein family, member 1 | Multiple_C | 4.47 | 4.59 | 4.36 | 1.08 | 0.8709 | 0.9371 | 10.11 | 10.33 | 10.69 | 0.67 | 0.0442 | 0.1946 |
| TC0200016451.hg.1 | CCDC85A | coiled-coil domain containing 85A | Coding | 4.14 | 3.93 | 4.03 | 1.08 | 0.9337 | 0.9688 | 4.03 | 4.21 | 3.87 | 1.12 | 0.4562 | 0.7004 |
| TC0300007387.hg.1 | RNF123 | ring finger protein 123 | Multiple_C | 10.18 | 9.85 | 10.07 | 1.08 | 0.8141 | 0.9064 | 9.22 | 9.34 | 8.83 | 1.31 | 0.2347 | 0.4972 |
| TC0300007408.hg.1 | GNAT1 | guanine nucleotide binding protein (G protein), alpha transducir | Multiple_C | 3.81 | 3.38 | 3.7 | 1.08 | 0.8234 | 0.9112 | 4.88 | 5.16 | 4.94 | 0.96 | 0.1659 | 0.4107 |
| TC0300007479.hg.1 | PHF7 | PHD finger protein 7 | Multiple_C | 6.47 | 5.94 | 6.36 | 1.08 | 0.8299 | 0.915 | 4.93 | 5.00 | 4.4 | 1.44 | 0.2315 | 0.4928 |
| TC0300008467.hg.1 | ARHGAP31 | Rho GTPase activating protein 31 | Multiple_C | 3.25 | 3.3 | 3.14 | 1.08 | 0.3437 | 0.5574 | 3.51 | 3.40 | 3.59 | 0.95 | 0.6535 | 0.8312 |
| TC0300008824.hg.1 | ATP2C1 | ATPase, Ca++ transporting, type 2C, member 1 | Multiple_C | 12.03 | 12.2 | 11.92 | 1.08 | 0.9177 | 0.9614 | 11.26 | 10.38 | 10.58 | 1.60 | 0.0015 | 0.022 |
| TC0300010720.hg.1 | EPM2AIP1 | EPM2A (laforin) interacting protein 1 | Multiple_C | 2.68 | 2.62 | 2.57 | 1.08 | 0.7371 | 0.8632 | 5.64 | 5.95 | 5.41 | 1.17 | 0.1658 | 0.4105 |

| | | | | | | | | | | | | | | | |
|---|---|---|---|---|---|---|---|---|---|---|---|---|---|---|---|
| TC0300011058.hg.1 | CDHR4 | cadherin-related family member 4 | Multiple_C | 4.95 | 4.7 | 4.84 | 1.08 | 0.4629 | 0.6681 | 5.34 | 5.27 | 5.27 | 1.05 | 0.3728 | 0.6337 |
| TC0300012109.hg.1 | IGSF11 | immunoglobulin superfamily, member 11 | Multiple_C | 4.21 | 3.7 | 4.1 | 1.08 | 0.9086 | 0.9561 | 3.97 | 4.50 | 4.67 | 0.62 | 0.0046 | 0.047 |
| TC0300012212.hg.1 | MYLK | myosin light chain kinase | Multiple_C | 5.09 | 5.14 | 4.98 | 1.08 | 0.8217 | 0.9104 | 4.71 | 4.74 | 4.92 | 0.86 | 0.6264 | 0.8154 |
| TC0300012741.hg.1 | ZIC4 | Zic family member 4 | Multiple_C | 3.27 | 3.06 | 3.16 | 1.08 | 0.3905 | 0.6042 | 3.74 | 3.50 | 3.67 | 1.05 | 0.8851 | 0.9507 |
| TC0300013816.hg.1 | FAM198A | family with sequence similarity 198, member A | Multiple_C | 2.69 | 2.45 | 2.58 | 1.08 | 0.8996 | 0.9518 | 3 | 3.08 | 3.12 | 0.92 | 0.5101 | 0.7401 |
| TC0300014031.hg.1 | CCDC80; LINC | coiled-coil domain containing 80; long intergenic non-protein cc | Multiple_C | 3.84 | 3.57 | 3.73 | 1.08 | 0.9226 | 0.9639 | 4.31 | 4.12 | 4.15 | 1.12 | 0.5584 | 0.7714 |
| TC0300014092.hg.1 | TM4SF19-TCT | TM4SF19-TCTEX1D2 readthrough (NMD candidate) | Multiple_C | 5.17 | 4.94 | 5.06 | 1.08 | 0.6925 | 0.8342 | 6.37 | 6.37 | 6.85 | 0.72 | 0.0285 | 0.149 |
| TC0400006810.hg.1 | USP17L23 | ubiquitin specific peptidase 17-like family member 23 [Source:H | Coding | 3.65 | 3.64 | 3.54 | 1.08 | 0.4798 | 0.6819 | 3.9 | 3.96 | 3.88 | 1.01 | 0.5472 | 0.7643 |
| TC0400006936.hg.1 | C1QTNF7 | C1q and tumor necrosis factor related protein 7 | Multiple_C | 2.85 | 2.72 | 2.74 | 1.08 | 0.9191 | 0.9625 | 3.09 | 3.22 | 3.17 | 0.95 | 0.7163 | 0.8675 |
| TC0400008947.hg.1 | TMEM184C | transmembrane protein 184C | Multiple_C | 9.5 | 9.25 | 9.39 | 1.08 | 0.974 | 0.9874 | 11.21 | 10.88 | 11.61 | 0.76 | 0.7096 | 0.8637 |
| TC0400009064.hg.1 | FGB | fibrinogen beta chain | Multiple_C | 3.34 | 2.88 | 3.23 | 1.08 | 0.5043 | 0.7018 | 3.9 | 4.17 | 3.7 | 1.15 | 0.4909 | 0.726 |
| TC0400009708.hg.1 | GAK | cyclin G associated kinase | Multiple_C | 8.32 | 8.22 | 8.21 | 1.08 | 0.5983 | 0.7712 | 6.94 | 6.66 | 6.49 | 1.37 | 0.0564 | 0.2234 |
| TC0500006558.hg.1 | C5orf38 | chromosome 5 open reading frame 38 | Multiple_C | 4.15 | 3.95 | 4.04 | 1.08 | 0.946 | 0.9753 | 5.57 | 5.56 | 5.35 | 1.16 | 0.2733 | 0.5388 |
| TC0500007154.hg.1 | SLC1A3 | solute carrier family 1 (glial high affinity glutamate transporter), | Multiple_C | 6.91 | 6.72 | 6.8 | 1.08 | 0.3608 | 0.5742 | 4.72 | 5.24 | 5.31 | 0.66 | 0.8578 | 0.9397 |
| TC0500009032.hg.1 | SPINK14 | serine peptidase inhibitor, Kazal type 14 (putative) | Coding | 2.99 | 2.79 | 2.88 | 1.08 | 0.8767 | 0.9401 | 3.26 | 3.24 | 3.1 | 1.12 | 0.3593 | 0.622 |
| TC0500009400.hg.1 | KCNIP1 | Kv channel interacting protein 1 | Multiple_C | 3.65 | 3.25 | 3.54 | 1.08 | 0.6612 | 0.8142 | 3.49 | 3.66 | 3.74 | 0.84 | 0.8163 | 0.9185 |
| TC0500011513.hg.1 | PCSK1 | proprotein convertase subtilisin/kexin type 1 | Multiple_C | 3.42 | 3.22 | 3.31 | 1.08 | 0.9415 | 0.9729 | 3.57 | 3.60 | 3.45 | 1.09 | 0.9622 | 0.9842 |
| TC0500012646.hg.1 | IL12B | interleukin 12B | Coding | 2.8 | 2.46 | 2.69 | 1.08 | 0.7209 | 0.853 | 3.58 | 3.50 | 3.38 | 1.15 | 0.4131 | 0.6677 |
| TC0500012864.hg.1 | NKX2-5 | NK2 homeobox 5 | Coding | 4.47 | 4.31 | 4.36 | 1.08 | 0.7915 | 0.8932 | 5.82 | 6.17 | 6.34 | 0.70 | 0.0632 | 0.2393 |
| TC0500013237.hg.1 | HARS2 | histidyl-tRNA synthetase 2, mitochondrial | Multiple_C | 8.29 | 8.44 | 8.18 | 1.08 | 0.9239 | 0.9644 | 7.79 | 8.06 | 7.68 | 1.08 | 0.948 | 0.979 |
| TC0500013352.hg.1 | MEIKIN | meiotic kinetochore factor | Multiple_C | 3.84 | 3.51 | 3.73 | 1.08 | 0.4781 | 0.6804 | 4.3 | 4.45 | 4.62 | 0.80 | 0.4722 | 0.7127 |
| TC0500013389.hg.1 | MED7 | mediator complex subunit 7 | Multiple_C | 8.47 | 10.42 | 8.36 | 1.08 | 0.3581 | 0.5717 | 8.31 | 7.77 | 7.89 | 1.34 | 0.0028 | 0.0341 |
| TC0600006940.hg.1 | SMIM13 | small integral membrane protein 13 | Multiple_C | 7.18 | 6.88 | 7.07 | 1.08 | 0.9289 | 0.9663 | 8.81 | 8.11 | 8.31 | 1.41 | 0.0569 | 0.2244 |
| TC0600007449.hg.1 | OR2J2 | olfactory receptor, family 2, subfamily J, member 2 | Multiple_C | 3.3 | 3.01 | 3.19 | 1.08 | 0.6159 | 0.7848 | 3.68 | 3.73 | 3.86 | 0.88 | 0.973 | 0.9891 |
| TC0600008212.hg.1 | C6orf141 | chromosome 6 open reading frame 141 | Multiple_C | 7.43 | 7.28 | 7.32 | 1.08 | 0.6307 | 0.7941 | 4.85 | 4.89 | 4.99 | 0.91 | 0.4542 | 0.699 |
| TC0600008670.hg.1 | PRSS35 | protease, serine 35 | Coding | 3.59 | 3.68 | 3.48 | 1.08 | 0.7159 | 0.8497 | 3.85 | 3.91 | 3.46 | 1.31 | 0.0485 | 0.2047 |
| TC0600008764.hg.1 | ANKRD6 | ankyrin repeat domain 6 | Multiple_C | 3.73 | 3.84 | 3.62 | 1.08 | 0.932 | 0.9679 | 4.8 | 4.87 | 4.23 | 1.48 | 0.0031 | 0.0365 |
| TC0600009350.hg.1 | RNF217 | ring finger protein 217 | Multiple_C | 3.62 | 3.41 | 3.51 | 1.08 | 0.8699 | 0.9367 | 3.79 | 3.73 | 3.58 | 1.16 | 0.2806 | 0.5464 |
| TC0600011261.hg.1 | GPX6 | glutathione peroxidase 6 | Multiple_C | 5.36 | 4.76 | 5.25 | 1.08 | 0.5716 | 0.7513 | 4.53 | 4.41 | 4.27 | 1.20 | 0.2589 | 0.5233 |
| TC0600011465.hg.1 | EHMT2 | euchromatic histone-lysine N-methyltransferase 2 | Multiple_C | 8.51 | 8.14 | 8.4 | 1.08 | 0.6544 | 0.8095 | 10.19 | 10.79 | 10.84 | 0.64 | 0.0057 | 0.0535 |
| TC0700007416.hg.1 | RAMP3 | receptor (G protein-coupled) activity modifying protein 3 | Coding | 3.77 | 3.57 | 3.66 | 1.08 | 0.763 | 0.8782 | 3.62 | 3.56 | 3.64 | 0.99 | 0.5235 | 0.7491 |
| TC0700007975.hg.1 | FKBP6 | FK506 binding protein 6 | Multiple_C | 4.48 | 4.31 | 4.37 | 1.08 | 0.4472 | 0.6554 | 5 | 5.37 | 4.95 | 1.04 | 0.3245 | 0.5885 |
| TC0700010914.hg.1 | CAMK2B | calcium/calmodulin-dependent protein kinase II beta | Multiple_C | 3.42 | 3.18 | 3.31 | 1.08 | 0.7787 | 0.8862 | 3.74 | 3.52 | 3.17 | 1.48 | 0.1768 | 0.4255 |

| | | | | | | | | | | | | | | | |
|---|---|---|---|---|---|---|---|---|---|---|---|---|---|---|---|
| TC0700011796.hg.1 | SAMD9 | sterile alpha motif domain containing 9 | Coding | 6.49 | 4.74 | 6.38 | 1.08 | 0.945 | 0.9747 | 5.11 | 5.83 | 6.14 | 0.49 | 0.0206 | 0.1226 |
| TC0700012123.hg.1 | SLC26A5 | solute carrier family 26 (anion exchanger), member 5 | Multiple_C | 3.26 | 3.42 | 3.15 | 1.08 | 0.972 | 0.9864 | 4.58 | 4.11 | 3.59 | 1.99 | 0.0004 | 0.0089 |
| TC0700012126.hg.1 | RELN | reelin | Multiple_C | 3.26 | 2.88 | 3.15 | 1.08 | 0.6564 | 0.8112 | 3.4 | 3.26 | 3.5 | 0.93 | 0.6373 | 0.822 |
| TC0700012467.hg.1 | TMEM229A | transmembrane protein 229A | Coding | 3.6 | 3.76 | 3.49 | 1.08 | 0.6776 | 0.8247 | 3.9 | 4.37 | 4.26 | 0.78 | 0.267 | 0.5316 |
| TC0700012970.hg.1 | ZNF786 | zinc finger protein 786 | Multiple_C | 7.35 | 6.79 | 7.24 | 1.08 | 0.8517 | 0.9272 | 7.13 | 7.55 | 7.17 | 0.97 | 0.7402 | 0.8807 |
| TC0800007529.hg.1 | SPIDR | scaffolding protein involved in DNA repair | Multiple_C | 7.56 | 7.59 | 7.45 | 1.08 | 0.8062 | 0.9014 | 7.58 | 7.61 | 7.4 | 1.13 | 0.1493 | 0.3869 |
| TC0800010490.hg.1 | TMEM68 | transmembrane protein 68 | Multiple_C | 10.09 | 12.04 | 9.98 | 1.08 | 0.8786 | 0.9409 | 9.97 | 8.29 | 9.28 | 1.61 | 0.0725 | 0.2582 |
| TC0800011565.hg.1 | RAD21; MIR36 | RAD21 cohesin complex component; microRNA 3610 | Multiple_C | 13.14 | 13.38 | 13.03 | 1.08 | 0.8864 | 0.9452 | 12.83 | 12.81 | 12.89 | 0.96 | 0.9807 | 0.9918 |
| TC0800012362.hg.1 | KHDRBS3 | KH domain containing, RNA binding, signal transduction associa | Multiple_C | 10.49 | 11.1 | 10.38 | 1.08 | 0.5488 | 0.734 | 4.82 | 5.47 | 5.04 | 0.86 | 0.6906 | 0.8526 |
| TC0900008587.hg.1 | TLR4 | toll-like receptor 4 | Multiple_C | 4.32 | 3.98 | 4.21 | 1.08 | 0.647 | 0.8043 | 5.67 | 5.20 | 6.42 | 0.59 | 0.0627 | 0.2383 |
| TC0900008675.hg.1 | OR1L6 | olfactory receptor, family 1, subfamily L, member 6 | Coding | 3.73 | 3.51 | 3.62 | 1.08 | 0.8493 | 0.9261 | 4.21 | 4.59 | 4.74 | 0.69 | 0.584 | 0.788 |
| TC0900008763.hg.1 | PBX3 | pre-B-cell leukemia homeobox 3 | Multiple_C | 5.83 | 5.64 | 5.72 | 1.08 | 0.6426 | 0.8017 | 4.58 | 5.24 | 5.23 | 0.64 | 0.0293 | 0.1516 |
| TC0900008945.hg.1 | GPR107 | G protein-coupled receptor 107 | Multiple_C | 10.52 | 10.59 | 10.41 | 1.08 | 0.6361 | 0.7978 | 8.87 | 8.60 | 9.27 | 0.76 | 0.1043 | 0.3166 |
| TC0900009163.hg.1 | PPP1R26 | protein phosphatase 1, regulatory subunit 26 | Multiple_C | 6.25 | 6.4 | 6.14 | 1.08 | 0.4922 | 0.6923 | 6.65 | 6.36 | 6.03 | 1.54 | 0.0132 | 0.0928 |
| TC0900009606.hg.1 | BNC2 | basonuclin 2 | Multiple_C | 3.22 | 3.12 | 3.11 | 1.08 | 0.69 | 0.833 | 4.04 | 3.89 | 3.61 | 1.35 | 0.1122 | 0.33 |
| TC0900012156.hg.1 | PALM2 | paralemmin 2 | Multiple_C | 3.68 | 3.83 | 3.57 | 1.08 | 0.9402 | 0.9723 | 5.78 | 5.36 | 5.98 | 0.87 | 0.497 | 0.7305 |
| TC0900012199.hg.1 | GRIN1 | glutamate receptor, ionotropic, N-methyl D-aspartate 1 | Multiple_C | 5.31 | 4.88 | 5.2 | 1.08 | 0.9763 | 0.9884 | 4.24 | 3.96 | 4.12 | 1.09 | 0.321 | 0.585 |
| TC0X00007013.hg.1 | MPC1L | mitochondrial pyruvate carrier 1-like | Multiple_C | 2.67 | 2.48 | 2.56 | 1.08 | 0.9317 | 0.9678 | 3.37 | 3.42 | 3.4 | 0.98 | 0.5955 | 0.796 |
| TC0X00007112.hg.1 | CHST7 | carbohydrate (N-acetylglucosamine 6-O) sulfotransferase 7 | Coding | 3.82 | 3.59 | 3.71 | 1.08 | 0.9591 | 0.9806 | 4.92 | 4.88 | 4.83 | 1.06 | 0.775 | 0.8989 |
| TC0X00008526.hg.1 | FHL1 | four and a half LIM domains 1 | Multiple_C | 4.69 | 4.44 | 4.58 | 1.08 | 0.7256 | 0.8557 | 6.7 | 6.72 | 6.25 | 1.37 | 0.0294 | 0.1519 |
| TC0X00009648.hg.1 | TFE3 | transcription factor binding to IGHM enhancer 3 | Multiple_C | 7.42 | 7.03 | 7.31 | 1.08 | 0.705 | 0.8425 | 7.71 | 8.08 | 8.19 | 0.72 | 0.3888 | 0.6475 |
| TC0X00009672.hg.1 | PAGE1 | P antigen family, member 1 (prostate associated) | Coding | 2.73 | 2.87 | 2.62 | 1.08 | 0.3052 | 0.5182 | 3.23 | 2.95 | 3.1 | 1.09 | 0.3493 | 0.6135 |
| TC0X00010428.hg.1 | GLRA4 | glycine receptor alpha 4 | Multiple_C | 3.51 | 3.46 | 3.4 | 1.08 | 0.3997 | 0.6133 | 3.84 | 4.11 | 3.57 | 1.21 | 0.2991 | 0.5637 |
| TC0X00010712.hg.1 | THOC2 | THO complex 2 | Multiple_C | 10.22 | 10.43 | 10.11 | 1.08 | 0.7805 | 0.887 | 9.93 | 10.23 | 10.84 | 0.53 | 0.0088 | 0.0719 |
| TC0X00011136.hg.1 | MAGEA6 | MAGE family member A6 | Multiple_C | 5.71 | 5.37 | 5.6 | 1.08 | 0.6385 | 0.7995 | 5.07 | 5.07 | 4.94 | 1.09 | 0.5352 | 0.7572 |
| TC0X00011173.hg.1 | IDH3G | isocitrate dehydrogenase 3 (NAD+) gamma | Multiple_C | 13.7 | 12.19 | 13.59 | 1.08 | 0.7738 | 0.8834 | 11.96 | 12.55 | 13.43 | 0.36 | 0.0002 | 0.0059 |
| TC0Y00006525.hg.1 | TBL1Y | transducin (beta)-like 1, Y-linked | Multiple_C | 4.34 | 4.17 | 4.23 | 1.08 | 0.5929 | 0.7675 | 4.37 | 4.19 | 4.22 | 1.11 | 0.8885 | 0.9524 |
| TC0Y00006825.hg.1 | CDY1 | chromodomain protein, Y-linked, 1 | Coding | 3.53 | 3.72 | 3.42 | 1.08 | 0.4293 | 0.6395 | 3.57 | 3.73 | 3.39 | 1.13 | 0.4797 | 0.7181 |
| TC1000007603.hg.1 | FAM21A | family with sequence similarity 21, member A | Multiple_C | 11.11 | 10.23 | 11 | 1.08 | 0.3143 | 0.5277 | 12.7 | 12.62 | 12.83 | 0.91 | 0.4676 | 0.7088 |
| TC1000009006.hg.1 | PNLIPRP1 | pancreatic lipase-related protein 1 | Multiple_C | 3.49 | 3.78 | 3.38 | 1.08 | 0.9396 | 0.972 | 4.03 | 4.18 | 4.15 | 0.92 | 0.574 | 0.7814 |
| TC1000009535.hg.1 | IDI2 | isopentenyl-diphosphate delta isomerase 2 | Coding | 4.28 | 3.97 | 4.17 | 1.08 | 0.4046 | 0.6175 | 4.14 | 4.12 | 4.04 | 1.07 | 0.2016 | 0.4569 |
| TC1000010744.hg.1 | TMEM26 | transmembrane protein 26 | Multiple_C | 3.63 | 3.38 | 3.52 | 1.08 | 0.4114 | 0.6237 | 3.84 | 3.89 | 3.86 | 0.99 | 0.9713 | 0.9882 |
| TC1000012389.hg.1 | ADAM8 | ADAM metallopeptidase domain 8 | Multiple_C | 4.74 | 4.75 | 4.63 | 1.08 | 0.6253 | 0.7908 | 4.39 | 4.44 | 4.33 | 1.04 | 0.5614 | 0.7723 |

| | | | | | | | | | | | | | | | |
|---|---|---|---|---|---|---|---|---|---|---|---|---|---|---|---|
| TC1000012573.hg.1 | MMRN2 | multimerin 2 | Multiple_C | 3.45 | 3.23 | 3.34 | 1.08 | 0.6868 | 0.8311 | 3.73 | 3.68 | 3.45 | 1.21 | 0.0113 | 0.084 |
| TC1100006694.hg.1 | OR51Q1 | olfactory receptor, family 51, subfamily Q, member 1 (gene/pse | Coding | 2.6 | 2.51 | 2.49 | 1.08 | 0.6673 | 0.818 | 3.64 | 3.61 | 3.44 | 1.15 | 0.3732 | 0.6341 |
| TC1100006695.hg.1 | OR51I2 | olfactory receptor, family 51, subfamily I, member 2 | Coding | 3.13 | 2.86 | 3.02 | 1.08 | 0.8882 | 0.9459 | 3.72 | 3.46 | 3.11 | 1.53 | 0.0562 | 0.2228 |
| TC1100007554.hg.1 | OR4C13 | olfactory receptor, family 4, subfamily C, member 13 | Coding | 3.3 | 3.41 | 3.19 | 1.08 | 0.432 | 0.642 | 3.75 | 3.57 | 3.26 | 1.40 | 0.0716 | 0.2559 |
| TC1100007624.hg.1 | OR8I2 | olfactory receptor, family 8, subfamily I, member 2 | Coding | 4.11 | 3.79 | 4 | 1.08 | 0.7227 | 0.8542 | 4.21 | 4.32 | 4.47 | 0.84 | 0.9382 | 0.9743 |
| TC1100007897.hg.1 | LGALS12 | lectin, galactoside-binding, soluble, 12 | Coding | 2.8 | 2.76 | 2.69 | 1.08 | 0.5416 | 0.7288 | 3.04 | 3.11 | 3.1 | 0.96 | 0.8608 | 0.9405 |
| TC1100008015.hg.1 | FRMD8 | FERM domain containing 8 | Multiple_C | 3.55 | 3.25 | 3.44 | 1.08 | 0.5419 | 0.7289 | 3.37 | 3.74 | 3.71 | 0.79 | 0.2534 | 0.5176 |
| TC1100008727.hg.1 | TRIM64 | tripartite motif containing 64 | Coding | 3.36 | 3.22 | 3.25 | 1.08 | 0.6628 | 0.8152 | 3.8 | 3.55 | 3.62 | 1.13 | 0.2098 | 0.4668 |
| TC1100008821.hg.1 | KDM4E | lysine (K)-specific demethylase 4E | Coding | 3.72 | 3.61 | 3.61 | 1.08 | 0.7928 | 0.8937 | 3.72 | 3.58 | 3.52 | 1.15 | 0.1016 | 0.3118 |
| TC1100009374.hg.1 | VWA5A | von Willebrand factor A domain containing 5A | Multiple_C | 3.36 | 3.39 | 3.25 | 1.08 | 0.9477 | 0.976 | 6.01 | 6.08 | 5.33 | 1.60 | 0.0052 | 0.0502 |
| TC1100010261.hg.1 | HPS5 | Hermansky-Pudlak syndrome 5 | Multiple_C | 9.02 | 8.92 | 8.91 | 1.08 | 0.4998 | 0.699 | 8.66 | 8.23 | 8.5 | 1.12 | 0.5551 | 0.7697 |
| TC1100010778.hg.1 | OR4C5 | olfactory receptor, family 4, subfamily C, member 5 (gene/pseu | Coding | 3.23 | 3.28 | 3.12 | 1.08 | 0.6239 | 0.7897 | 3.34 | 3.28 | 3.37 | 0.98 | 0.7361 | 0.8784 |
| TC1100010792.hg.1 | FOLH1 | folate hydrolase (prostate-specific membrane antigen) 1 | Multiple_C | 3.82 | 3.86 | 3.71 | 1.08 | 0.8878 | 0.9458 | 4.23 | 3.81 | 3.87 | 1.28 | 0.118 | 0.3395 |
| TC1100011040.hg.1 | SYT7 | synaptotagmin VII | Multiple_C | 3.85 | 3.89 | 3.74 | 1.08 | 0.9975 | 0.9987 | 4.66 | 4.61 | 4.29 | 1.29 | 0.642 | 0.8245 |
| TC1100011141.hg.1 | RCOR2 | REST corepressor 2 | Multiple_C | 2.98 | 2.3 | 2.87 | 1.08 | 0.9013 | 0.9525 | 5.99 | 6.68 | 6.08 | 0.94 | 0.7457 | 0.8834 |
| TC1100012960.hg.1 | DNHD1 | dynein heavy chain domain 1 | Multiple_C | 3.43 | 3.45 | 3.32 | 1.08 | 0.237 | 0.4385 | 3.89 | 3.85 | 3.72 | 1.13 | 0.3132 | 0.5773 |
| TC1100013130.hg.1 | IGF2; INS-IGF2 | insulin-like growth factor 2; INS-IGF2 readthrough | Multiple_C | 4.26 | 3.76 | 4.15 | 1.08 | 0.8858 | 0.9449 | 5.11 | 4.54 | 4.43 | 1.60 | 0.0038 | 0.0417 |
| TC1100013135.hg.1 | OR51B5 | olfactory receptor, family 51, subfamily B, member 5 | Multiple_C | 3.91 | 3.6 | 3.8 | 1.08 | 0.9722 | 0.9865 | 11.21 | 7.79 | 9.68 | 2.89 | 0.0006 | 0.012 |
| TC1100013145.hg.1 | COPB1 | coatomer protein complex subunit beta 1 | Multiple_C | 11.2 | 12.23 | 11.09 | 1.08 | 0.6305 | 0.794 | 11.24 | 10.92 | 10.61 | 1.55 | 0.0944 | 0.2985 |
| TC1200007157.hg.1 | REP15 | RAB15 effector protein | Coding | 3.5 | 3.58 | 3.39 | 1.08 | 0.6427 | 0.8018 | 4.58 | 4.81 | 4.27 | 1.24 | 0.1827 | 0.4333 |
| TC1200007204.hg.1 | TSPAN11 | tetraspanin 11 | Multiple_C | 4.39 | 4.23 | 4.28 | 1.08 | 0.2023 | 0.3946 | 4.27 | 4.26 | 4.19 | 1.06 | 0.4631 | 0.7054 |
| TC1200007356.hg.1 | MUC19 | mucin 19, oligomeric | Multiple_C | 3.08 | 3.2 | 2.97 | 1.08 | 0.7162 | 0.85 | 3.54 | 3.37 | 3.02 | 1.43 | 0.0109 | 0.0821 |
| TC1200008683.hg.1 | CHST11 | carbohydrate (chondroitin 4) sulfotransferase 11 | Multiple_C | 5.84 | 5.33 | 5.73 | 1.08 | 0.6653 | 0.8171 | 14.14 | 13.20 | 12.1 | 4.11 | 1.67E-08 | 7.18E-06 |
| TC1200010703.hg.1 | BIN2 | bridging integrator 2 | Multiple_C | 2.97 | 2.96 | 2.86 | 1.08 | 0.927 | 0.9658 | 3.14 | 3.19 | 3.39 | 0.84 | 0.9823 | 0.9926 |
| TC1200010919.hg.1 | MIP | major intrinsic protein of lens fiber | Multiple_C | 3.89 | 4.15 | 3.78 | 1.08 | 0.8919 | 0.9481 | 4.06 | 3.91 | 3.82 | 1.18 | 0.2767 | 0.5428 |
| TC1200011083.hg.1 | C12orf56 | chromosome 12 open reading frame 56 | Multiple_C | 3.71 | 3.9 | 3.6 | 1.08 | 0.5365 | 0.7251 | 4.09 | 4.13 | 4.25 | 0.90 | 0.4994 | 0.732 |
| TC1200011991.hg.1 | IQCD | IQ motif containing D | Coding | 6.51 | 6.36 | 6.4 | 1.08 | 0.4878 | 0.6889 | 6.05 | 6.31 | 5.89 | 1.12 | 0.5837 | 0.788 |
| TC1200012159.hg.1 | MSI1 | musashi RNA binding protein 1 | Multiple_C | 3.76 | 3.55 | 3.65 | 1.08 | 0.329 | 0.5428 | 4.07 | 3.93 | 3.75 | 1.25 | 0.0879 | 0.2863 |
| TC1200012417.hg.1 | SLC15A4 | solute carrier family 15 (oligopeptide transporter), member 4 | Multiple_C | 10.43 | 10.6 | 10.32 | 1.08 | 0.5363 | 0.725 | 9.95 | 9.26 | 9.57 | 1.30 | 0.067 | 0.247 |
| TC1300009013.hg.1 | VPS36 | vacuolar protein sorting 36 homolog (S. cerevisiae) | Multiple_C | 8.3 | 8.63 | 8.19 | 1.08 | 0.5628 | 0.7447 | 9.9 | 9.74 | 9.42 | 1.39 | 0.0232 | 0.1314 |
| TC1400006998.hg.1 | LRFN5 | leucine rich repeat and fibronectin type III domain containing 5 | Multiple_C | 3.4 | 3.36 | 3.29 | 1.08 | 0.5747 | 0.7537 | 4.1 | 3.91 | 3.93 | 1.13 | 0.5375 | 0.7589 |
| TC1400007490.hg.1 | FAM71D | family with sequence similarity 71, member D | Multiple_C | 3.52 | 3.41 | 3.41 | 1.08 | 0.9526 | 0.9782 | 4.42 | 3.93 | 4.24 | 1.13 | 0.4942 | 0.7283 |
| TC1400008455.hg.1 | ZBTB42 | zinc finger and BTB domain containing 42 | Coding | 3.84 | 3.77 | 3.73 | 1.08 | 0.3249 | 0.5389 | 3.89 | 4.04 | 3.72 | 1.13 | 0.175 | 0.4229 |

| | | | | | | | | | | | | | | | |
|---|---|---|---|---|---|---|---|---|---|---|---|---|---|---|---|
| TC1400008752.hg.1 | CIDEB | cell death-inducing DFFA-like effector b | Multiple_C | 5.04 | 4.6 | 4.93 | 1.08 | 0.6272 | 0.792 | 5.8 | 6.15 | 6.05 | 0.84 | 0.2252 | 0.486 |
| TC1400009422.hg.1 | RAB15 | RAB15, member RAS oncogene family | Multiple_C | 8.73 | 8.67 | 8.62 | 1.08 | 0.4424 | 0.6512 | 11.5 | 11.38 | 10.54 | 1.95 | 6.79E-05 | 0.0028 |
| TC1400009694.hg.1 | SYNDIG1L | synapse differentiation inducing 1-like | Multiple_C | 7.9 | 7.52 | 7.79 | 1.08 | 0.3873 | 0.601 | 6.99 | 7.30 | 7.32 | 0.80 | 0.6337 | 0.8201 |
| TC1400009834.hg.1 | GTF2A1 | general transcription factor IIA 1 | Multiple_C | 10.66 | 10.37 | 10.55 | 1.08 | 0.8047 | 0.9003 | 12.98 | 12.60 | 12.6 | 1.30 | 0.6221 | 0.8128 |
| TC1500008236.hg.1 | ACAN | aggrecan | Multiple_C | 3.16 | 3.03 | 3.05 | 1.08 | 0.57 | 0.7502 | 3.25 | 3.13 | 3.26 | 0.99 | 0.5794 | 0.7849 |
| TC1500008267.hg.1 | TICRR | TOPBP1-interacting checkpoint and replication regulator | Multiple_C | 7.02 | 6.51 | 6.91 | 1.08 | 0.2487 | 0.4528 | 5.82 | 6.47 | 6.65 | 0.56 | 0.0008 | 0.0139 |
| TC1500009505.hg.1 | PRTG | protogenin | Multiple_C | 3.98 | 4.12 | 3.87 | 1.08 | 0.3705 | 0.5832 | 3.51 | 3.53 | 3.68 | 0.89 | 0.4503 | 0.6962 |
| TC1500010698.hg.1 | SNRPN; SNUR | small nuclear ribonucleoprotein polypeptide N; SNRPN upstrear | Multiple_C | 3.23 | 2.95 | 3.12 | 1.08 | 0.4384 | 0.6477 | 3.97 | 4.09 | 3.62 | 1.27 | 0.1129 | 0.331 |
| TC1600006448.hg.1 | HBQ1 | hemoglobin, theta 1 | Coding | 5.28 | 5.08 | 5.17 | 1.08 | 0.6422 | 0.8015 | 5.62 | 5.87 | 5.57 | 1.04 | 0.8516 | 0.9358 |
| TC1600006745.hg.1 | C16orf96 | chromosome 16 open reading frame 96 | Multiple_C | 3.48 | 3.7 | 3.37 | 1.08 | 0.5126 | 0.7083 | 3.75 | 3.88 | 3.63 | 1.09 | 0.3333 | 0.5975 |
| TC1600007988.hg.1 | CPNE2 | copine II | Multiple_C | 7.08 | 6.3 | 6.97 | 1.08 | 0.2226 | 0.4204 | 6.48 | 6.68 | 7 | 0.70 | 0.1413 | 0.3739 |
| TC1600009312.hg.1 | TMEM114 | transmembrane protein 114 | Multiple_C | 3.5 | 3.25 | 3.39 | 1.08 | 0.1985 | 0.3895 | 3.86 | 4.06 | 3.82 | 1.03 | 0.7484 | 0.885 |
| TC1600009846.hg.1 | GSG1L | GSG1-like | Coding | 3.05 | 2.8 | 2.94 | 1.08 | 0.604 | 0.7758 | 3.16 | 3.17 | 3.26 | 0.93 | 0.8874 | 0.9519 |
| TC1600011526.hg.1 | PRSS53 | protease, serine 53 | Multiple_C | 4.31 | 3.99 | 4.2 | 1.08 | 0.5262 | 0.7182 | 4.62 | 4.67 | 4.5 | 1.09 | 0.7473 | 0.8841 |
| TC1700006895.hg.1 | SHISA6 | shisa family member 6 | Multiple_C | 2.78 | 2.78 | 2.67 | 1.08 | 0.6637 | 0.8158 | 4.11 | 4.05 | 3.92 | 1.14 | 0.0811 | 0.2745 |
| TC1700008049.hg.1 | FMNL1 | formin like 1 | Multiple_C | 3.92 | 3.67 | 3.81 | 1.08 | 0.5061 | 0.7033 | 6.29 | 6.49 | 6.2 | 1.06 | 0.6808 | 0.8465 |
| TC1700008171.hg.1 | PRAC2 | prostate cancer susceptibility candidate 2 | Multiple_C | 4.3 | 4.07 | 4.19 | 1.08 | 0.3369 | 0.5508 | 5.29 | 5.27 | 4.78 | 1.42 | 0.1143 | 0.3336 |
| TC1700008513.hg.1 | C17orf82 | chromosome 17 open reading frame 82 | Multiple_C | 3.12 | 2.89 | 3.01 | 1.08 | 0.3477 | 0.5608 | 3.23 | 3.36 | 3.35 | 0.92 | 0.6366 | 0.8216 |
| TC1700009677.hg.1 | BORCS6 | BLOC-1 related complex subunit 6 | Multiple_C | 5.72 | 5.83 | 5.61 | 1.08 | 0.4239 | 0.6349 | 4.43 | 4.37 | 4.36 | 1.05 | 0.3254 | 0.589 |
| TC1700010206.hg.1 | PROCA1 | protein interacting with cyclin A1 | Multiple_C | 4.78 | 5 | 4.67 | 1.08 | 0.8833 | 0.9434 | 4.01 | 4.46 | 4.52 | 0.70 | 0.7686 | 0.8963 |
| TC1700011798.hg.1 | UBE2O | ubiquitin-conjugating enzyme E2O | Multiple_C | 8.41 | 8.12 | 8.3 | 1.08 | 0.5199 | 0.7134 | 9.72 | 9.59 | 9.48 | 1.18 | 0.6697 | 0.8385 |
| TC1700012271.hg.1 | GOSR2 | golgi SNAP receptor complex member 2 | Multiple_C | 7.12 | 7.1 | 7.01 | 1.08 | 0.4148 | 0.6269 | 8.35 | 8.87 | 9.05 | 0.62 | 0.0385 | 0.1787 |
| TC1800008259.hg.1 | CTAGE1 | cutaneous T-cell lymphoma-associated antigen 1 | Coding | 4.1 | 3.95 | 3.99 | 1.08 | 0.5983 | 0.7712 | 4.21 | 4.31 | 4.51 | 0.81 | 0.0405 | 0.1844 |
| TC1900006646.hg.1 | GIPC3 | GIPC PDZ domain containing family, member 3 | Multiple_C | 4.1 | 3.95 | 3.99 | 1.08 | 0.5221 | 0.7152 | 3.76 | 3.84 | 3.89 | 0.91 | 0.4797 | 0.7181 |
| TC1900007125.hg.1 | NACC1 | nucleus accumbens associated 1, BEN and BTB (POZ) domain cc | Multiple_C | 10.53 | 9.37 | 10.42 | 1.08 | 0.9747 | 0.9876 | 11.23 | 11.75 | 11.62 | 0.76 | 0.5401 | 0.7595 |
| TC1900007900.hg.1 | LOC10192757 | uncharacterized LOC101927572; Memczak2013 ANTISENSE, CD: | Multiple_C | 5.16 | 4.95 | 5.05 | 1.08 | 0.491 | 0.6909 | 4.7 | 5.13 | 5.1 | 0.76 | 0.1364 | 0.367 |
| TC1900008044.hg.1 | NCCRP1 | non-specific cytotoxic cell receptor protein 1 homolog (zebrafisl | Multiple_C | 4.19 | 3.85 | 4.08 | 1.08 | 0.6786 | 0.8255 | 4.64 | 4.72 | 4.62 | 1.01 | 0.3557 | 0.6189 |
| TC1900008075.hg.1 | LGALS16 | lectin, galactoside-binding, soluble, 16 | Coding | 3.67 | 3.73 | 3.56 | 1.08 | 0.5314 | 0.7212 | 3.83 | 3.62 | 3.82 | 1.01 | 0.3977 | 0.6552 |
| TC1900008826.hg.1 | CACNG7 | calcium channel, voltage-dependent, gamma subunit 7 | Multiple_C | 4.06 | 3.74 | 3.95 | 1.08 | 0.5409 | 0.7284 | 4.85 | 5.26 | 5.11 | 0.84 | 0.3903 | 0.6489 |
| TC1900009087.hg.1 | ODF3L2 | outer dense fiber of sperm tails 3-like 2 | Multiple_C | 4.41 | 4.14 | 4.3 | 1.08 | 0.3512 | 0.564 | 3.93 | 3.80 | 4.05 | 0.92 | 0.6561 | 0.8321 |
| TC1900009501.hg.1 | PRR36 | proline rich 36 | Multiple_C | 4.16 | 3.82 | 4.05 | 1.08 | 0.8077 | 0.9026 | 4.33 | 4.39 | 4.04 | 1.22 | 0.3018 | 0.5662 |
| TC1900011139.hg.1 | SLC17A7 | solute carrier family 17 (vesicular glutamate transporter), meml | Multiple_C | 4.61 | 4.43 | 4.5 | 1.08 | 0.2013 | 0.3933 | 4.2 | 3.92 | 4.17 | 1.02 | 0.6936 | 0.8542 |
| TC1900011741.hg.1 | CEACAM5 | carcinoembryonic antigen-related cell adhesion molecule 5 | Multiple_C | 13.81 | 13.01 | 13.7 | 1.08 | 0.901 | 0.9524 | 4.42 | 4.54 | 4.85 | 0.74 | 0.0459 | 0.1982 |

| | | | | | | | | | | | | | | | |
|---|---|---|---|---|---|---|---|---|---|---|---|---|---|---|---|
| TC1900011978.hg.1 | ZNF296 | zinc finger protein 296 | Coding | 4.66 | 4.19 | 4.55 | 1.08 | 0.7012 | 0.8404 | 4.88 | 5.33 | 5.4 | 0.70 | 0.4953 | 0.7291 |
| TC1900012036.hg.1 | LILRB3 | leukocyte immunoglobulin-like receptor, subfamily B (with TM a | Multiple_C | 3.33 | 3.06 | 3.22 | 1.08 | 0.4264 | 0.637 | 2.99 | 3.41 | 3.21 | 0.86 | 0.3528 | 0.6165 |
| TC2000009878.hg.1 | SNPH | syntaphilin | Coding | 2.78 | 2.51 | 2.67 | 1.08 | 0.7082 | 0.845 | 3.31 | 3.16 | 3.03 | 1.21 | 0.1465 | 0.3825 |
| TC2000009915.hg.1 | C20orf24 | chromosome 20 open reading frame 24 | Multiple_C | 13.87 | 13.55 | 13.76 | 1.08 | 0.6449 | 0.8032 | 11 | 11.26 | 11.37 | 0.77 | 0.1689 | 0.4145 |
| TC2000010017.hg.1 | EPPIN | epididymal peptidase inhibitor | Multiple_C | 3.47 | 3.4 | 3.36 | 1.08 | 0.9783 | 0.9896 | 3.89 | 3.80 | 3.81 | 1.06 | 0.5356 | 0.7576 |
| TC2100008355.hg.1 | ICOSLG | inducible T-cell co-stimulator ligand | Multiple_C | 5.36 | 5.15 | 5.25 | 1.08 | 0.249 | 0.453 | 4.89 | 4.57 | 4.86 | 1.02 | 0.2959 | 0.5607 |
| TC2200007544.hg.1 | BIK | BCL2-interacting killer (apoptosis-inducing) | Coding | 9.48 | 9.09 | 9.37 | 1.08 | 0.4204 | 0.6318 | 8.06 | 8.08 | 7.94 | 1.09 | 0.6585 | 0.8334 |
| TC2200008974.hg.1 | SMC1B | structural maintenance of chromosomes 1B | Coding | 4.03 | 3.71 | 3.92 | 1.08 | 0.4927 | 0.6927 | 4.8 | 4.94 | 5.01 | 0.86 | 0.2173 | 0.4766 |
| TC2200009142.hg.1 | MLC1 | megalencephalic leukoencephalopathy with subcortical cysts 1 | Multiple_C | 3.41 | 3.31 | 3.3 | 1.08 | 0.9806 | 0.9907 | 3.7 | 3.96 | 3.86 | 0.90 | 0.365 | 0.6274 |
| TSUnmapped00000278.h | ZNF501 | zinc finger protein 501 [Source:HGNC Symbol;Acc:HGNC:23717] | Coding | 3.77 | 3.76 | 3.66 | 1.08 | 0.5954 | 0.7694 | 3.75 | 3.45 | 3.73 | 1.01 | 0.5383 | 0.759 |
| TSUnmapped00000303.h | OBP2B | odorant binding protein 2B | Coding | 4.69 | 4.39 | 4.58 | 1.08 | 0.177 | 0.3596 | 4.92 | 4.87 | 4.78 | 1.10 | 0.4824 | 0.7202 |
| TSUnmapped00000775.h | PRAMEF25 | PRAME family member 25 | Coding | 4.21 | 3.92 | 4.1 | 1.08 | 0.6835 | 0.8287 | 4.5 | 4.72 | 4.46 | 1.03 | 0.9489 | 0.9793 |
| TC0100006497.hg.1 | SCNN1D | sodium channel, non voltage gated 1 delta subunit | Multiple_C | 3.1 | 2.91 | 3 | 1.07 | 0.6816 | 0.8271 | 4.53 | 4.59 | 4.36 | 1.13 | 0.3189 | 0.583 |
| TC0100006910.hg.1 | PRAMEF2 | PRAME family member 2 | Coding | 3.09 | 2.71 | 2.99 | 1.07 | 0.978 | 0.9894 | 3.85 | 3.82 | 3.75 | 1.07 | 0.5159 | 0.7446 |
| TC0100007412.hg.1 | MAN1C1 | mannosidase, alpha, class 1C, member 1 | Multiple_C | 2.78 | 2.42 | 2.68 | 1.07 | 0.4394 | 0.6485 | 3.64 | 3.46 | 3.44 | 1.15 | 0.1155 | 0.3355 |
| TC0100008106.hg.1 | BTBD19 | BTB (POZ) domain containing 19 | Multiple_C | 3.79 | 3.55 | 3.69 | 1.07 | 0.9135 | 0.9595 | 4 | 4.08 | 3.71 | 1.22 | 0.1211 | 0.3434 |
| TC0100009191.hg.1 | AGL | amylo-alpha-1, 6-glucosidase, 4-alpha-glucanotransferase | Multiple_C | 9.41 | 9.23 | 9.31 | 1.07 | 0.8165 | 0.9075 | 10.51 | 10.28 | 10.05 | 1.38 | 0.435 | 0.6857 |
| TC0100009907.hg.1 | TARS2; MIR68 | threonyl-tRNA synthetase 2, mitochondrial (putative); microRNA | Multiple_C | 10.25 | 10.57 | 10.15 | 1.07 | 0.9193 | 0.9626 | 9.18 | 9.45 | 9.3 | 0.92 | 0.6089 | 0.8043 |
| TC0100010003.hg.1 | C1orf68 | chromosome 1 open reading frame 68 | Multiple_C | 4.23 | 4.02 | 4.13 | 1.07 | 0.2636 | 0.4708 | 4.46 | 4.29 | 4.04 | 1.34 | 0.2444 | 0.5086 |
| TC0100010238.hg.1 | OR10Z1 | olfactory receptor, family 10, subfamily Z, member 1 | Coding | 3 | 2.63 | 2.9 | 1.07 | 0.6765 | 0.8242 | 3.32 | 3.34 | 3.32 | 1.00 | 0.9101 | 0.9628 |
| TC0100011463.hg.1 | SYT14 | synaptotagmin XIV | Multiple_C | 3.05 | 3.19 | 2.95 | 1.07 | 0.5724 | 0.7518 | 4.05 | 3.78 | 3.56 | 1.40 | 0.0383 | 0.178 |
| TC0100012895.hg.1 | MTHFR | methylenetetrahydrofolate reductase (NAD(P)H) | Multiple_C | 6.78 | 6.3 | 6.68 | 1.07 | 0.9569 | 0.98 | 5.38 | 5.28 | 5.6 | 0.86 | 0.6176 | 0.8101 |
| TC0100013373.hg.1 | SLC30A2 | solute carrier family 30 (zinc transporter), member 2 | Multiple_C | 3.95 | 3.79 | 3.85 | 1.07 | 0.3114 | 0.5248 | 4.88 | 4.61 | 4.46 | 1.34 | 0.3109 | 0.575 |
| TC0100015206.hg.1 | GNAT2 | guanine nucleotide binding protein (G protein), alpha transducin | Coding | 6.34 | 5.72 | 6.24 | 1.07 | 0.699 | 0.8389 | 6.29 | 6.58 | 6.05 | 1.18 | 0.9634 | 0.9845 |
| TC0100016051.hg.1 | FCRL2 | Fc receptor-like 2 | Multiple_C | 3.62 | 3.5 | 3.52 | 1.07 | 0.7599 | 0.8763 | 3.41 | 3.28 | 3.34 | 1.05 | 0.3969 | 0.6548 |
| TC0100016437.hg.1 | SLC9C2 | solute carrier family 9, member C2 (putative) | Multiple_C | 2.8 | 2.58 | 2.7 | 1.07 | 0.4138 | 0.6258 | 2.97 | 2.74 | 2.91 | 1.04 | 0.4331 | 0.6841 |
| TC0100017957.hg.1 | PLD5 | phospholipase D family, member 5 | Multiple_C | 3.45 | 3.47 | 3.35 | 1.07 | 0.7987 | 0.8966 | 4.73 | 4.63 | 4.4 | 1.26 | 0.1304 | 0.3577 |
| TC0100018288.hg.1 | PLEKHO1 | pleckstrin homology domain containing, family O member 1 | Multiple_C | 6.6 | 6.19 | 6.5 | 1.07 | 0.6746 | 0.8229 | 7.43 | 7.30 | 6.84 | 1.51 | 0.0125 | 0.0899 |
| TC0200008347.hg.1 | TEX37 | testis expressed 37 | Coding | 2.99 | 3.11 | 2.89 | 1.07 | 0.675 | 0.8231 | 4.02 | 3.79 | 3.59 | 1.35 | 0.0155 | 0.1025 |
| TC0200009312.hg.1 | POTEJ | POTE ankyrin domain family, member J | Multiple_C | 5.59 | 5.88 | 5.49 | 1.07 | 0.1616 | 0.3388 | 6.09 | 5.96 | 5.99 | 1.07 | 0.8773 | 0.9474 |
| TC0200012258.hg.1 | SULT6B1 | sulfotransferase family 6B member 1 | Multiple_C | 3.8 | 3.95 | 3.7 | 1.07 | 0.8003 | 0.8977 | 3.22 | 3.13 | 3.02 | 1.15 | 0.6287 | 0.8169 |
| TC0200012994.hg.1 | SNRPG | small nuclear ribonucleoprotein polypeptide G | Multiple_C | 12.78 | 13.92 | 12.68 | 1.07 | 0.6714 | 0.8209 | 11.67 | 12.36 | 11.56 | 1.08 | 0.5553 | 0.7697 |
| TC0200015575.hg.1 | C2orf80 | chromosome 2 open reading frame 80 | Coding | 3.2 | 2.9 | 3.1 | 1.07 | 0.8534 | 0.9284 | 4.15 | 4.16 | 4.03 | 1.09 | 0.6843 | 0.8486 |

| | | | | | | | | | | | | | | | |
|---|---|---|---|---|---|---|---|---|---|---|---|---|---|---|---|
| TC0300007025.hg.1 | STAC | SH3 and cysteine rich domain | Multiple_C | 4.57 | 4.33 | 4.47 | 1.07 | 0.2417 | 0.4441 | 4.03 | 4.33 | 4.49 | 0.73 | 0.0237 | 0.1329 |
| TC0300008173.hg.1 | PCNP | PEST proteolytic signal containing nuclear protein | Multiple_C | 13.56 | 14.03 | 13.46 | 1.07 | 0.9277 | 0.966 | 14.63 | 14.31 | 14.3 | 1.26 | 0.0709 | 0.2548 |
| TC0300008594.hg.1 | KALRN | kalirin, RhoGEF kinase | Multiple_C | 3.36 | 2.96 | 3.26 | 1.07 | 0.6291 | 0.793 | 3.81 | 3.79 | 3.71 | 1.07 | 0.5883 | 0.7917 |
| TC0300008948.hg.1 | ESYT3 | extended synaptotagmin-like protein 3 | Multiple_C | 2.73 | 2.64 | 2.63 | 1.07 | 0.9126 | 0.9588 | 4.46 | 4.06 | 3.97 | 1.40 | 0.0026 | 0.0323 |
| TC0300009468.hg.1 | PRKCI | protein kinase C, iota | Multiple_C | 13.35 | 14.64 | 13.25 | 1.07 | 0.6839 | 0.8288 | 13.08 | 12.30 | 12.4 | 1.60 | 0.0878 | 0.286 |
| TC0300009685.hg.1 | HTR3D | 5-hydroxytryptamine (serotonin) receptor 3D, ionotropic | Coding | 4.15 | 4.13 | 4.05 | 1.07 | 0.7612 | 0.8774 | 4.22 | 4.08 | 4.21 | 1.01 | 0.9796 | 0.9914 |
| TC0300009869.hg.1 | PYDC2 | pyrin domain containing 2 | Coding | 3.62 | 3.29 | 3.52 | 1.07 | 0.4136 | 0.6256 | 3.94 | 3.88 | 3.99 | 0.97 | 0.7396 | 0.8804 |
| TC0300011142.hg.1 | IQCF6 | IQ motif containing F6 | Coding | 3.45 | 3.33 | 3.35 | 1.07 | 0.7663 | 0.88 | 3.56 | 3.59 | 3.58 | 0.99 | 0.8218 | 0.9212 |
| TC0300013420.hg.1 | IGF2BP2 | insulin-like growth factor 2 mRNA binding protein 2 | Multiple_C | 12.39 | 12.95 | 12.29 | 1.07 | 0.3828 | 0.5959 | 12.62 | 11.24 | 11.64 | 1.97 | 0.0006 | 0.0117 |
| TC0300013470.hg.1 | RTP2 | receptor (chemosensory) transporter protein 2 | Coding | 4.12 | 3.99 | 4.02 | 1.07 | 0.4564 | 0.663 | 4.37 | 4.33 | 4.25 | 1.09 | 0.4431 | 0.6916 |
| TC0300013863.hg.1 | UPK1B | uroplakin 1B | Coding | 3.15 | 3.05 | 3.05 | 1.07 | 0.8032 | 0.8994 | 4.1 | 4.14 | 3.8 | 1.23 | 0.1085 | 0.3242 |
| TC0300013886.hg.1 | ZIC1 | Zic family member 1 | Coding | 3.33 | 3.21 | 3.23 | 1.07 | 0.5569 | 0.7397 | 3.3 | 3.33 | 3.27 | 1.02 | 0.3809 | 0.6405 |
| TC0400007466.hg.1 | CWH43 | cell wall biogenesis 43 C-terminal homolog | Multiple_C | 3.82 | 3.6 | 3.72 | 1.07 | 0.9574 | 0.9803 | 3.5 | 3.23 | 3.19 | 1.24 | 0.1441 | 0.3782 |
| TC0400008041.hg.1 | PTPN13 | protein tyrosine phosphatase, non-receptor type 13 (APO-1/CD | Multiple_C | 3.54 | 3.54 | 3.44 | 1.07 | 0.7099 | 0.8461 | 6.69 | 4.29 | 5.9 | 1.73 | 0.0004 | 0.0087 |
| TC0400011283.hg.1 | PPM1K | protein phosphatase, Mg2+/Mn2+ dependent, 1K | Multiple_C | 8.43 | 10.15 | 8.33 | 1.07 | 0.7188 | 0.8518 | 7.56 | 6.77 | 6.26 | 2.46 | 0.0004 | 0.0085 |
| TC0500009141.hg.1 | GRIA1 | glutamate receptor, ionotropic, AMPA 1 | Multiple_C | 4.52 | 4.72 | 4.42 | 1.07 | 0.5257 | 0.7178 | 5.31 | 5.43 | 5.26 | 1.04 | 0.7831 | 0.9026 |
| TC0500009181.hg.1 | CNOT8 | CCR4-NOT transcription complex subunit 8 | Multiple_C | 13.38 | 13.94 | 13.28 | 1.07 | 0.7841 | 0.889 | 11.59 | 10.98 | 11.11 | 1.39 | 0.0653 | 0.2433 |
| TC0500010553.hg.1 | FYB | FYN binding protein | Multiple_C | 3.76 | 4.05 | 3.66 | 1.07 | 0.2031 | 0.3953 | 3.22 | 3.27 | 3.39 | 0.89 | 0.7117 | 0.8648 |
| TC0500011579.hg.1 | ST8SIA4 | ST8 alpha-N-acetyl-neuraminide alpha-2,8-sialyltransferase 4 | Multiple_C | 3.56 | 2.99 | 3.46 | 1.07 | 0.7678 | 0.8804 | 3.56 | 3.57 | 3.76 | 0.87 | 0.2972 | 0.5618 |
| TC0500013301.hg.1 | DAB2 | Dab, mitogen-responsive phosphoprotein, homolog 2 (Drosoph | Multiple_C | 4.85 | 4.08 | 4.75 | 1.07 | 0.5192 | 0.7129 | 5.51 | 5.86 | 6.81 | 0.41 | 9.61E-05 | 0.0035 |
| TC0500013345.hg.1 | TMEM232 | transmembrane protein 232 | Multiple_C | 3.29 | 3.41 | 3.19 | 1.07 | 0.1261 | 0.2844 | 3.87 | 3.68 | 3.46 | 1.33 | 0.0228 | 0.13 |
| TC0500013376.hg.1 | DND1 | DND microRNA-mediated repression inhibitor 1 | Multiple_C | 5.44 | 5.29 | 5.34 | 1.07 | 0.7755 | 0.8844 | 5.82 | 5.91 | 5.5 | 1.25 | 0.5137 | 0.7431 |
| TC0600007591.hg.1 | NFKBIL1 | nuclear factor of kappa light polypeptide gene enhancer in B-ce | Multiple_C | 5.48 | 5.34 | 5.38 | 1.07 | 0.8806 | 0.9419 | 5.77 | 5.89 | 5.63 | 1.10 | 0.2941 | 0.559 |
| TC0600008075.hg.1 | TTBK1 | tau tubulin kinase 1 | Coding | 3.47 | 3.14 | 3.37 | 1.07 | 0.5754 | 0.7542 | 3.67 | 3.89 | 3.76 | 0.94 | 0.6251 | 0.8148 |
| TC0600008912.hg.1 | PRDM13 | PR domain containing 13 | Coding | 4.24 | 4.23 | 4.14 | 1.07 | 0.8523 | 0.9275 | 3.71 | 4.25 | 4.13 | 0.75 | 0.0923 | 0.2944 |
| TC0600009343.hg.1 | NKAIN2 | Na+/K+ transporting ATPase interacting 2 | Multiple_C | 4.79 | 5.01 | 4.69 | 1.07 | 0.8596 | 0.9315 | 4.58 | 4.23 | 4.38 | 1.15 | 0.2862 | 0.5512 |
| TC0600009393.hg.1 | RNF146 | ring finger protein 146 | Multiple_C | 10.65 | 10.99 | 10.55 | 1.07 | 0.9535 | 0.9784 | 10.26 | 10.31 | 9.9 | 1.28 | 0.1969 | 0.4519 |
| TC0600010241.hg.1 | KIF25 | kinesin family member 25 | Multiple_C | 6.01 | 5.89 | 5.91 | 1.07 | 0.905 | 0.9545 | 5.52 | 5.91 | 5.56 | 0.97 | 0.8647 | 0.9423 |
| TC0600010962.hg.1 | DEK | DEK proto-oncogene | Multiple_C | 14.78 | 14.82 | 14.68 | 1.07 | 0.9887 | 0.994 | 16.25 | 15.82 | 15.85 | 1.32 | 0.0367 | 0.1745 |
| TC0600011349.hg.1 | RNF39 | ring finger protein 39 | Coding | 4.32 | 3.86 | 4.22 | 1.07 | 0.6867 | 0.831 | 3.6 | 3.46 | 3.3 | 1.23 | 0.1585 | 0.4001 |
| TC0600011392.hg.1 | SFTA2 | surfactant associated 2 | Multiple_C | 3.83 | 3.55 | 3.73 | 1.07 | 0.3267 | 0.5406 | 4.75 | 4.33 | 4.31 | 1.36 | 0.0106 | 0.0811 |
| TC0600012514.hg.1 | AKIRIN2 | akirin 2 | Multiple_C | 8.75 | 9.24 | 8.65 | 1.07 | 0.7439 | 0.8676 | 7.1 | 7.64 | 8 | 0.54 | 0.0002 | 0.0059 |
| TC0600012521.hg.1 | CNR1 | cannabinoid receptor 1 (brain) | Multiple_C | 5.94 | 5.49 | 5.84 | 1.07 | 0.5291 | 0.72 | 6.38 | 6.50 | 6.55 | 0.89 | 0.193 | 0.4461 |

| | | | | | | | | | | | | | | | |
|---|---|---|---|---|---|---|---|---|---|---|---|---|---|---|---|
| TC0600012777.hg.1 | SNX3 | sorting nexin 3 | Multiple_C | 12.72 | 12.99 | 12.62 | 1.07 | 0.6512 | 0.8069 | 11.24 | 10.71 | 11.45 | 0.86 | 0.4624 | 0.7049 |
| TC0600013205.hg.1 | VNN2 | vanin 2 | Multiple_C | 2.85 | 2.79 | 2.75 | 1.07 | 0.624 | 0.7897 | 3.35 | 3.23 | 3.25 | 1.07 | 0.6164 | 0.8097 |
| TC0600013299.hg.1 | IL22RA2 | interleukin 22 receptor, alpha 2 | Coding | 3.38 | 3.44 | 3.28 | 1.07 | 0.933 | 0.9684 | 3.31 | 3.46 | 3.4 | 0.94 | 0.9685 | 0.9868 |
| TC0600014300.hg.1 | GCLC | glutamate-cysteine ligase, catalytic subunit | Multiple_C | 12.39 | 13.15 | 12.29 | 1.07 | 0.6443 | 0.8029 | 11.37 | 10.63 | 11.09 | 1.21 | 0.4985 | 0.7314 |
| TC0700007054.hg.1 | PRR15 | proline rich 15 | Coding | 9 | 8.81 | 8.9 | 1.07 | 0.8544 | 0.9289 | 9.02 | 9.43 | 9.31 | 0.82 | 0.2279 | 0.4886 |
| TC0700007426.hg.1 | ADCY1 | adenylate cyclase 1 (brain) | Multiple_C | 3.19 | 2.91 | 3.09 | 1.07 | 0.62 | 0.7874 | 3.92 | 3.88 | 3.67 | 1.19 | 0.3142 | 0.5781 |
| TC0700007552.hg.1 | POM121L12 | POM121 transmembrane nucleoporin-like 12 | Coding | 5.14 | 4.94 | 5.04 | 1.07 | 0.6347 | 0.7966 | 3.92 | 4.25 | 4.44 | 0.70 | 0.0067 | 0.0593 |
| TC0700008088.hg.1 | SRRM3 | serine/arginine repetitive matrix 3 | Multiple_C | 2.71 | 2.52 | 2.61 | 1.07 | 0.6749 | 0.8231 | 2.97 | 3.20 | 3.12 | 0.90 | 0.9332 | 0.972 |
| TC0700009222.hg.1 | LRGUK | leucine-rich repeats and guanylate kinase domain containing | Multiple_C | 4.58 | 4.01 | 4.48 | 1.07 | 0.197 | 0.3877 | 3.89 | 3.84 | 3.99 | 0.93 | 0.685 | 0.8489 |
| TC0700010281.hg.1 | THSD7A | thrombospondin type 1 domain containing 7A | Multiple_C | 3.95 | 4.14 | 3.85 | 1.07 | 0.9078 | 0.9557 | 3.39 | 3.77 | 3.83 | 0.74 | 0.432 | 0.6836 |
| TC0700012041.hg.1 | PLOD3 | procollagen-lysine, 2-oxoglutarate 5-dioxygenase 3 | Multiple_C | 9.07 | 8.91 | 8.97 | 1.07 | 0.7422 | 0.8666 | 7.98 | 8.41 | 8.73 | 0.59 | 0.0032 | 0.0369 |
| TC0700012780.hg.1 | HIPK2 | homeodomain interacting protein kinase 2 | Multiple_C | 10.45 | 9.67 | 10.35 | 1.07 | 0.4451 | 0.6536 | 13.27 | 13.88 | 13.04 | 1.17 | 0.3235 | 0.5878 |
| TC0700012917.hg.1 | TPK1 | thiamin pyrophosphokinase 1 | Multiple_C | 5.63 | 6.41 | 5.53 | 1.07 | 0.3927 | 0.6066 | 5.58 | 5.36 | 5.64 | 0.96 | 0.8515 | 0.9358 |
| TC0700013466.hg.1 | AKR1D1 | aldo-keto reductase family 1, member D1 | Multiple_C | 3.41 | 3.33 | 3.31 | 1.07 | 0.7268 | 0.8562 | 3.12 | 3.22 | 3.25 | 0.91 | 0.6383 | 0.8228 |
| TC0700013538.hg.1 | TRGJ1; TRGC2 | T cell receptor gamma joining 1; T cell receptor gamma constan | Multiple_C | 2.76 | 2.4 | 2.66 | 1.07 | 0.7219 | 0.8536 | 3.11 | 3.04 | 3.37 | 0.84 | 0.2014 | 0.4568 |
| TC0800008769.hg.1 | TRMT12 | tRNA methyltransferase 12 homolog (S. cerevisiae) | Multiple_C | 4.6 | 4.77 | 4.5 | 1.07 | 0.7168 | 0.8503 | 6.16 | 6.32 | 6.1 | 1.04 | 0.6216 | 0.8128 |
| TC0800009632.hg.1 | DEFB130 | defensin, beta 130 | Coding | 4.34 | 4.26 | 4.24 | 1.07 | 0.8657 | 0.9345 | 4.49 | 4.01 | 4.01 | 1.39 | 0.0907 | 0.2913 |
| TC0800009647.hg.1 | DEFB130 | defensin, beta 130 | Coding | 4.34 | 4.26 | 4.24 | 1.07 | 0.8657 | 0.9345 | 4.49 | 4.01 | 4.01 | 1.39 | 0.0907 | 0.2913 |
| TC0800009796.hg.1 | LZTS1 | leucine zipper, putative tumor suppressor 1 | Coding | 3.55 | 3.42 | 3.45 | 1.07 | 0.7657 | 0.8797 | 3.83 | 3.73 | 3.8 | 1.02 | 0.5702 | 0.7787 |
| TC0800010016.hg.1 | SARAF | store-operated calcium entry-associated regulatory factor | Multiple_C | 11.28 | 11.92 | 11.18 | 1.07 | 0.9827 | 0.992 | 9.29 | 9.25 | 9.47 | 0.88 | 0.3169 | 0.5809 |
| TC0800010918.hg.1 | ZNF704 | zinc finger protein 704 | Multiple_C | 3.52 | 3.16 | 3.42 | 1.07 | 0.9067 | 0.9553 | 8.41 | 8.85 | 7.58 | 1.78 | 0.0095 | 0.0755 |
| TC0800011137.hg.1 | RAD54B; FSBF | RAD54 homolog B (S. cerevisiae); fibrinogen silencer binding pr | Multiple_C | 9.99 | 10.3 | 9.89 | 1.07 | 0.9538 | 0.9784 | 9.64 | 9.59 | 9.86 | 0.86 | 0.5297 | 0.7532 |
| TC0900008145.hg.1 | CCDC180 | coiled-coil domain containing 180 | Multiple_C | 3.72 | 3.45 | 3.62 | 1.07 | 0.9199 | 0.9629 | 4.09 | 3.99 | 3.75 | 1.27 | 0.0663 | 0.2454 |
| TC0900008415.hg.1 | MUSK | muscle, skeletal, receptor tyrosine kinase | Coding | 4.38 | 4.23 | 4.28 | 1.07 | 0.9049 | 0.9545 | 4.4 | 4.26 | 4.2 | 1.15 | 0.4797 | 0.7181 |
| TC0900008851.hg.1 | DNM1 | dynamin 1 | Multiple_C | 7.59 | 7.68 | 7.49 | 1.07 | 0.6159 | 0.7848 | 11.78 | 11.59 | 11.58 | 1.15 | 0.3468 | 0.6104 |
| TC0900009424.hg.1 | GLIS3 | GLIS family zinc finger 3 | Multiple_C | 4.49 | 4.1 | 4.39 | 1.07 | 0.4599 | 0.6659 | 3.67 | 3.36 | 3.73 | 0.96 | 0.821 | 0.9209 |
| TC0X00006489.hg.1 | ARSF | arylsulfatase F | Coding | 4.21 | 3.82 | 4.11 | 1.07 | 0.2154 | 0.4111 | 4.14 | 4.36 | 4.21 | 0.95 | 0.8816 | 0.9494 |
| TC0X00007158.hg.1 | ZNF81 | zinc finger protein 81 | Multiple_C | 5.73 | 5.55 | 5.63 | 1.07 | 0.6978 | 0.838 | 5.69 | 6.48 | 6.9 | 0.43 | 0.012 | 0.0871 |
| TC0X00007559.hg.1 | GJB1 | gap junction protein beta 1 | Coding | 3.97 | 3.84 | 3.87 | 1.07 | 0.823 | 0.9111 | 12.35 | 13.10 | 12.47 | 0.92 | 0.9017 | 0.9591 |
| TC0X00008195.hg.1 | LUZP4 | leucine zipper protein 4 | Coding | 2.74 | 2.62 | 2.64 | 1.07 | 0.7373 | 0.8634 | 3.55 | 3.86 | 3.66 | 0.93 | 0.428 | 0.6801 |
| TC0X00010051.hg.1 | NAP1L6 | nucleosome assembly protein 1-like 6 | Multiple_C | 3.22 | 2.81 | 3.12 | 1.07 | 0.3808 | 0.5937 | 3.58 | 3.52 | 3.27 | 1.24 | 0.2063 | 0.4629 |
| TC0X00010926.hg.1 | MAP7D3 | MAP7 domain containing 3 | Multiple_C | 4.33 | 4.28 | 4.23 | 1.07 | 0.5402 | 0.7279 | 10.53 | 10.82 | 10.99 | 0.73 | 0.2971 | 0.5618 |
| TC0X00011174.hg.1 | PDZD4 | PDZ domain containing 4 | Multiple_C | 9.5 | 9.65 | 9.4 | 1.07 | 0.6314 | 0.7943 | 8.86 | 9.61 | 9.19 | 0.80 | 0.049 | 0.2058 |

| | | | | | | | | | | | | | | | |
|---|---|---|---|---|---|---|---|---|---|---|---|---|---|---|---|
| TC0X00011288.hg.1 | GAGE12I; GA( | G antigen 12I; G antigen 12F | Coding | 3.04 | 2.8 | 2.94 | 1.07 | 0.8234 | 0.9112 | 3.22 | 3.10 | 2.95 | 1.21 | 0.2254 | 0.4862 |
| TC1000006604.hg.1 | AKR1E2 | aldo-keto reductase family 1, member E2 | Multiple_( | 3.09 | 2.93 | 2.99 | 1.07 | 0.9316 | 0.9678 | 8.39 | 8.40 | 8.83 | 0.74 | 0.0448 | 0.1958 |
| TC1000006701.hg.1 | ITIH2 | inter-alpha-trypsin inhibitor heavy chain 2 | Multiple_( | 4.77 | 4.58 | 4.67 | 1.07 | 0.776 | 0.8846 | 4.38 | 4.42 | 4.68 | 0.81 | 0.2016 | 0.4571 |
| TC1000008582.hg.1 | C10orf62 | chromosome 10 open reading frame 62 | Coding | 3.37 | 3.18 | 3.27 | 1.07 | 0.4993 | 0.6986 | 3.7 | 3.89 | 3.66 | 1.03 | 0.4827 | 0.7204 |
| TC1000008619.hg.1 | NKX2-3 | NK2 homeobox 3 | Coding | 3.49 | 3.13 | 3.39 | 1.07 | 0.874 | 0.939 | 3.62 | 3.77 | 3.55 | 1.05 | 0.3758 | 0.6364 |
| TC1000009016.hg.1 | KCNK18 | potassium channel, two pore domain subfamily K, member 18 | Coding | 2.91 | 2.65 | 2.81 | 1.07 | 0.4925 | 0.6926 | 3.78 | 3.88 | 3.48 | 1.23 | 0.0729 | 0.2588 |
| TC1000009404.hg.1 | PWWP2B | PWWP domain containing 2B | Coding | 6.85 | 6.74 | 6.75 | 1.07 | 0.9485 | 0.9763 | 6.27 | 6.41 | 6.67 | 0.76 | 0.0442 | 0.1945 |
| TC1000010192.hg.1 | KIAA1462 | KIAA1462 | Multiple_( | 3.86 | 3.72 | 3.76 | 1.07 | 0.4739 | 0.677 | 5.06 | 4.46 | 4.77 | 1.22 | 0.0585 | 0.2282 |
| TC1000012403.hg.1 | SPRN | shadow of prion protein homolog (zebrafish) | Coding | 3.29 | 3.47 | 3.19 | 1.07 | 0.8285 | 0.9139 | 3.38 | 3.47 | 3.3 | 1.06 | 0.3037 | 0.5678 |
| TC1000012429.hg.1 | PRPF18 | pre-mRNA processing factor 18 | Multiple_( | 8.69 | 9.12 | 8.59 | 1.07 | 0.1642 | 0.3424 | 8.09 | 7.84 | 8.09 | 1.00 | 0.337 | 0.6014 |
| TC1100006663.hg.1 | OR51E1 | olfactory receptor, family 51, subfamily E, member 1 | Coding | 4.46 | 4.28 | 4.36 | 1.07 | 0.7935 | 0.8941 | 4.04 | 4.42 | 4.27 | 0.85 | 0.5715 | 0.7794 |
| TC1100008805.hg.1 | C11orf97 | chromosome 11 open reading frame 97 | Multiple_( | 3.88 | 4.05 | 3.78 | 1.07 | 0.6936 | 0.8351 | 3.12 | 3.38 | 3.31 | 0.88 | 0.7931 | 0.9077 |
| TC1100008969.hg.1 | ELMOD1 | ELMO/CED-12 domain containing 1 | Multiple_( | 3.72 | 3.24 | 3.62 | 1.07 | 0.4658 | 0.6704 | 5.68 | 4.21 | 4.04 | 3.12 | 1.30E-07 | 3.19E-05 |
| TC1100010818.hg.1 | OR4C46 | olfactory receptor, family 4, subfamily C, member 46 | Coding | 3.8 | 3.6 | 3.7 | 1.07 | 0.7976 | 0.8961 | 4.16 | 4.15 | 3.89 | 1.21 | 0.186 | 0.4378 |
| TC1100010883.hg.1 | APLNR | apelin receptor | Multiple_( | 2.86 | 2.73 | 2.76 | 1.07 | 0.7698 | 0.8811 | 3.3 | 3.31 | 3.24 | 1.04 | 0.3765 | 0.6369 |
| TC1100011188.hg.1 | CDC42BPG | CDC42 binding protein kinase gamma (DMPK-like) | Multiple_( | 4.58 | 4.33 | 4.48 | 1.07 | 0.5001 | 0.699 | 4.01 | 4.02 | 3.83 | 1.13 | 0.8336 | 0.926 |
| TC1100012029.hg.1 | FAM76B | family with sequence similarity 76, member B | Multiple_( | 9.8 | 9.97 | 9.7 | 1.07 | 0.5925 | 0.7672 | 10.85 | 10.74 | 10.57 | 1.21 | 0.0265 | 0.1423 |
| TC1100013065.hg.1 | KRTAP5-9 | keratin associated protein 5-9 | Coding | 3.78 | 3.4 | 3.68 | 1.07 | 0.7417 | 0.8665 | 3.98 | 4.17 | 4.05 | 0.95 | 0.596 | 0.7963 |
| TC1200006701.hg.1 | NECAP1 | NECAP endocytosis associated 1 | Multiple_( | 10.21 | 10.14 | 10.11 | 1.07 | 0.7917 | 0.8932 | 9.7 | 8.60 | 9.58 | 1.09 | 0.4396 | 0.6898 |
| TC1200009303.hg.1 | AACS | acetoacetyl-CoA synthetase | Multiple_( | 10.7 | 10.9 | 10.6 | 1.07 | 0.8345 | 0.9173 | 9.01 | 9.06 | 9.26 | 0.84 | 0.6657 | 0.8364 |
| TC1200010023.hg.1 | ERP27 | endoplasmic reticulum protein 27 | Multiple_( | 5.13 | 5.25 | 5.03 | 1.07 | 0.7173 | 0.8505 | 5.28 | 5.02 | 5.1 | 1.13 | 0.8716 | 0.9447 |
| TC1200011671.hg.1 | UHRF1BP1L | UHRF1 binding protein 1-like | Multiple_( | 10.29 | 10.45 | 10.19 | 1.07 | 0.3884 | 0.6021 | 8.94 | 9.03 | 9.53 | 0.66 | 0.0048 | 0.0479 |
| TC1400006533.hg.1 | EDDM3B | epididymal protein 3B | Coding | 3.51 | 3.64 | 3.41 | 1.07 | 0.2053 | 0.3983 | 3.6 | 3.54 | 3.6 | 1.00 | 0.4661 | 0.7078 |
| TC1400007285.hg.1 | NAA30 | N(alpha)-acetyltransferase 30, NatC catalytic subunit | Multiple_( | 10.77 | 11.04 | 10.67 | 1.07 | 0.7598 | 0.8763 | 8.93 | 9.03 | 9.7 | 0.59 | 0.0004 | 0.0084 |
| TC1400007712.hg.1 | BATF | basic leucine zipper transcription factor, ATF-like | Multiple_( | 4.79 | 4.47 | 4.69 | 1.07 | 0.3514 | 0.5642 | 8.23 | 8.71 | 8.95 | 0.61 | 0.0464 | 0.1993 |
| TC1400007774.hg.1 | NRXN3 | neurexin 3 | Multiple_( | 3.2 | 2.83 | 3.1 | 1.07 | 0.5188 | 0.7127 | 4.21 | 4.29 | 4.03 | 1.13 | 0.0908 | 0.2914 |
| TC1400008054.hg.1 | OTUB2 | OTU deubiquitinase, ubiquitin aldehyde binding 2 | Coding | 9.37 | 8.48 | 9.27 | 1.07 | 0.4121 | 0.6242 | 6.16 | 5.23 | 5.84 | 1.25 | 0.328 | 0.5915 |
| TC1400008688.hg.1 | C14orf93 | chromosome 14 open reading frame 93 | Multiple_( | 6.58 | 6.58 | 6.48 | 1.07 | 0.7687 | 0.8808 | 5.97 | 6.23 | 5.96 | 1.01 | 0.8498 | 0.9351 |
| TC1400010015.hg.1 | ITPK1 | inositol-tetrakisphosphate 1-kinase | Multiple_( | 12.1 | 12.15 | 12 | 1.07 | 0.6769 | 0.8244 | 11.37 | 10.81 | 11.1 | 1.21 | 0.0847 | 0.28 |
| TC1400010563.hg.1 | MIR5195; IGH | microRNA 5195; immunoglobulin heavy variable 5-78 (pseudog( | Multiple_( | 3.59 | 3.2 | 3.49 | 1.07 | 0.9637 | 0.9824 | 3.94 | 3.99 | 3.9 | 1.03 | 0.1603 | 0.4027 |
| TC1500007121.hg.1 | SPATA5L1 | spermatogenesis associated 5-like 1 | Multiple_( | 9.53 | 10.29 | 9.43 | 1.07 | 0.6564 | 0.8112 | 8.09 | 8.24 | 8.85 | 0.59 | 0.0646 | 0.2421 |
| TC1600006513.hg.1 | SOX8 | SRY box 8 | Multiple_( | 3.86 | 3.43 | 3.76 | 1.07 | 0.5756 | 0.7543 | 4.24 | 4.23 | 4.05 | 1.14 | 0.4716 | 0.7122 |
| TC1600008259.hg.1 | PDPR | pyruvate dehydrogenase phosphatase regulatory subunit | Multiple_( | 9.19 | 8.72 | 9.09 | 1.07 | 0.6342 | 0.7963 | 10.88 | 10.85 | 10.36 | 1.43 | 0.0323 | 0.1608 |

| | | | | | | | | | | | | | | | |
|---|---|---|---|---|---|---|---|---|---|---|---|---|---|---|---|
| TC1600008607.hg.1 | PLCG2 | phospholipase C, gamma 2 (phosphatidylinositol-specific) | Multiple_C | 4.07 | 3.85 | 3.97 | 1.07 | 0.5849 | 0.7614 | 5.34 | 4.94 | 5.17 | 1.13 | 0.148 | 0.3846 |
| TC1600010627.hg.1 | TPPP3 | tubulin polymerization-promoting protein family member 3 | Multiple_C | 4.57 | 4.4 | 4.47 | 1.07 | 0.6621 | 0.8146 | 7.16 | 5.75 | 4.84 | 4.99 | 1.31E-07 | 3.19E-05 |
| TC1600011384.hg.1 | PHKG2 | phosphorylase kinase, gamma 2 (testis) | Multiple_C | 8.47 | 7.43 | 8.37 | 1.07 | 0.9165 | 0.9608 | 6.26 | 6.80 | 7.04 | 0.58 | 0.0003 | 0.0083 |
| TC1700006741.hg.1 | KCTD11 | potassium channel tetramerization domain containing 11 | Multiple_C | 4.03 | 3.57 | 3.93 | 1.07 | 0.8918 | 0.9481 | 6.01 | 6.20 | 6.25 | 0.85 | 0.3006 | 0.565 |
| TC1700007605.hg.1 | C17orf50 | chromosome 17 open reading frame 50 | Coding | 6.61 | 6.43 | 6.51 | 1.07 | 0.6787 | 0.8255 | 6.56 | 6.71 | 6.36 | 1.15 | 0.5184 | 0.7463 |
| TC1700008152.hg.1 | SNX11 | sorting nexin 11 | Multiple_C | 9.28 | 9.73 | 9.18 | 1.07 | 0.5831 | 0.7603 | 8.15 | 8.14 | 8.36 | 0.86 | 0.4082 | 0.6639 |
| TC1700008719.hg.1 | KPNA2 | karyopherin alpha 2 (RAG cohort 1, importin alpha 1) | Multiple_C | 14.1 | 13.01 | 14 | 1.07 | 0.63 | 0.7936 | 15.17 | 15.41 | 15.46 | 0.82 | 0.1496 | 0.3872 |
| TC1700008945.hg.1 | SPHK1 | sphingosine kinase 1 | Multiple_C | 5.99 | 5.79 | 5.89 | 1.07 | 0.9966 | 0.9984 | 4.02 | 3.95 | 4.08 | 0.96 | 0.7709 | 0.8972 |
| TC1700008946.hg.1 | AANAT | aralkylamine N-acetyltransferase | Multiple_C | 3.95 | 3.78 | 3.85 | 1.07 | 0.8123 | 0.9052 | 4.4 | 4.42 | 4.02 | 1.30 | 0.2368 | 0.5 |
| TC1700009293.hg.1 | METRNL | meteorin, glial cell differentiation regulator-like | Multiple_C | 6.52 | 6.54 | 6.42 | 1.07 | 0.6421 | 0.8015 | 4.56 | 4.21 | 4.55 | 1.01 | 0.8644 | 0.9422 |
| TC1700009648.hg.1 | SOX15 | SRY box 15 | Multiple_C | 4.06 | 3.89 | 3.96 | 1.07 | 0.7404 | 0.8656 | 4.63 | 4.70 | 4.43 | 1.15 | 0.1778 | 0.4263 |
| TC1700011004.hg.1 | HOXB13 | homeobox B13 | Coding | 4.01 | 3.99 | 3.91 | 1.07 | 0.1855 | 0.3719 | 3.25 | 3.48 | 3.73 | 0.72 | 0.0381 | 0.1776 |
| TC1700012291.hg.1 | ACE | angiotensin I converting enzyme | Multiple_C | 4.77 | 5.28 | 4.67 | 1.07 | 0.4869 | 0.6881 | 3.92 | 3.91 | 3.65 | 1.21 | 0.0508 | 0.21 |
| TC1800007546.hg.1 | SERPINB12 | serpin peptidase inhibitor, clade B (ovalbumin), member 12 | Coding | 4.24 | 4.1 | 4.14 | 1.07 | 0.8667 | 0.9349 | 4.36 | 4.23 | 4.04 | 1.25 | 0.0832 | 0.2777 |
| TC1800007595.hg.1 | CCDC102B | coiled-coil domain containing 102B | Multiple_C | 4.21 | 4.46 | 4.11 | 1.07 | 0.3225 | 0.5363 | 4.88 | 4.55 | 4.48 | 1.32 | 0.1208 | 0.3429 |
| TC1800008750.hg.1 | TCF4 | transcription factor 4 | Multiple_C | 3.63 | 3.61 | 3.53 | 1.07 | 0.5163 | 0.7107 | 5.72 | 5.17 | 5.17 | 1.46 | 0.0557 | 0.2218 |
| TC1900006865.hg.1 | TGFBR3L | transforming growth factor beta receptor III like | Multiple_C | 4.7 | 4.74 | 4.6 | 1.07 | 0.2713 | 0.4799 | 5.06 | 5.02 | 4.95 | 1.08 | 0.6166 | 0.8098 |
| TC1900006978.hg.1 | ICAM4 | intercellular adhesion molecule 4 (Landsteiner-Wiener blood gr | Multiple_C | 4.68 | 4.56 | 4.58 | 1.07 | 0.8601 | 0.9318 | 4.21 | 4.44 | 4.39 | 0.88 | 0.4213 | 0.6745 |
| TC1900007115.hg.1 | NFIX | nuclear factor I/X (CCAAT-binding transcription factor) | Multiple_C | 10.97 | 10.37 | 10.87 | 1.07 | 0.2309 | 0.4311 | 11.81 | 12.47 | 12 | 0.88 | 0.3727 | 0.6337 |
| TC1900007447.hg.1 | ZNF101 | zinc finger protein 101 | Multiple_C | 7.99 | 8.23 | 7.89 | 1.07 | 0.761 | 0.8773 | 7.86 | 9.07 | 8.76 | 0.54 | 0.0016 | 0.0227 |
| TC1900008337.hg.1 | EML2-AS1 | EML2 antisense RNA 1 | Multiple_C | 8.72 | 8.59 | 8.62 | 1.07 | 0.2354 | 0.4366 | 8.55 | 8.75 | 8.7 | 0.90 | 0.7215 | 0.8701 |
| TC1900008489.hg.1 | FGF21 | fibroblast growth factor 21 | Coding | 3.76 | 3.74 | 3.66 | 1.07 | 0.7143 | 0.8493 | 4.34 | 4.27 | 4.25 | 1.06 | 0.837 | 0.9283 |
| TC1900008516.hg.1 | CGB5; CGB8 | chorionic gonadotropin, beta polypeptide 5; chorionic gonadotr | Coding | 4.81 | 4.55 | 4.71 | 1.07 | 0.8486 | 0.9257 | 4.66 | 4.65 | 4.57 | 1.06 | 0.1249 | 0.3493 |
| TC1900008594.hg.1 | C19orf81 | chromosome 19 open reading frame 81 | Multiple_C | 3.49 | 3.21 | 3.39 | 1.07 | 0.7059 | 0.8434 | 3.19 | 3.60 | 3.2 | 0.99 | 0.7705 | 0.897 |
| TC1900009308.hg.1 | RAX2 | retina and anterior neural fold homeobox 2 | Coding | 4.29 | 4.07 | 4.19 | 1.07 | 0.4995 | 0.6988 | 4.54 | 4.53 | 4.49 | 1.04 | 0.7072 | 0.8621 |
| TC1900009765.hg.1 | LYL1 | lymphoblastic leukemia associated hematopoiesis regulator 1 | Multiple_C | 4.33 | 4.24 | 4.23 | 1.07 | 0.7658 | 0.8797 | 3.79 | 3.77 | 3.72 | 1.05 | 0.686 | 0.8497 |
| TC1900010511.hg.1 | POLR2I | polymerase (RNA) II (DNA directed) polypeptide I, 14.5kDa | Multiple_C | 10.19 | 10.13 | 10.09 | 1.07 | 0.958 | 0.9803 | 6.57 | 6.45 | 6.38 | 1.14 | 0.2372 | 0.5004 |
| TC1900010651.hg.1 | GMFG | glia maturation factor, gamma | Multiple_C | 5.11 | 4.35 | 5.01 | 1.07 | 0.5736 | 0.7527 | 6.6 | 6.91 | 5.58 | 2.03 | 0.0137 | 0.0948 |
| TC1900010655.hg.1 | PAF1 | PAF1 homolog, Paf1/RNA polymerase II complex component | Multiple_C | 10.09 | 9.22 | 9.99 | 1.07 | 0.2672 | 0.4753 | 9.28 | 10.02 | 10.02 | 0.60 | 0.0032 | 0.0376 |
| TC1900011257.hg.1 | SIGLEC8 | sialic acid binding Ig-like lectin 8 | Multiple_C | 2.87 | 2.74 | 2.77 | 1.07 | 0.4905 | 0.6905 | 3.31 | 3.37 | 3.16 | 1.11 | 0.4337 | 0.6847 |
| TC1900011904.hg.1 | USHBP1 | Usher syndrome 1C binding protein 1 | Multiple_C | 6.41 | 6.43 | 6.31 | 1.07 | 0.6665 | 0.8176 | 5.4 | 5.25 | 5.32 | 1.06 | 0.4204 | 0.6736 |
| TC1900011960.hg.1 | PSG3 | pregnancy specific beta-1-glycoprotein 3 | Multiple_C | 4.15 | 4.2 | 4.05 | 1.07 | 0.5547 | 0.7381 | 3.52 | 3.68 | 3.86 | 0.79 | 0.194 | 0.448 |
| TC2000007149.hg.1 | BPIFA1 | BPI fold containing family A, member 1 | Coding | 3.32 | 3.14 | 3.22 | 1.07 | 0.6907 | 0.8333 | 3.26 | 3.22 | 3.18 | 1.06 | 0.6216 | 0.8128 |

| | | | | | | | | | | | | | | | |
|---|---|---|---|---|---|---|---|---|---|---|---|---|---|---|---|
| TC2000007687.hg.1 | LINC01270 | long intergenic non-protein coding RNA 1270 | Multiple_C | 3.86 | 3.5 | 3.76 | 1.07 | 0.7885 | 0.8917 | 5.36 | 4.93 | 5.01 | 1.27 | 0.1312 | 0.359 |
| TC2000008198.hg.1 | CPXM1 | carboxypeptidase X (M14 family), member 1 | Multiple_C | 4.02 | 3.77 | 3.92 | 1.07 | 0.5442 | 0.7306 | 4.9 | 4.94 | 4.67 | 1.17 | 0.066 | 0.2445 |
| TC2000010007.hg.1 | RBM12; CPNE | RNA binding motif protein 12; copine I | Multiple_C | 13.1 | 13.21 | 13 | 1.07 | 0.9147 | 0.96 | 12.29 | 13.00 | 12.57 | 0.82 | 0.0134 | 0.0937 |
| TC2100007467.hg.1 | DIP2A | disco-interacting protein 2 homolog A | Multiple_C | 8.61 | 8.62 | 8.51 | 1.07 | 0.7236 | 0.8546 | 7.65 | 7.33 | 7.46 | 1.14 | 0.7459 | 0.8834 |
| TC2100007506.hg.1 | C21orf140 | chromosome 21 open reading frame 140 | Multiple_C | 4.57 | 4.57 | 4.47 | 1.07 | 0.5068 | 0.7035 | 4.38 | 4.53 | 4.23 | 1.11 | 0.2875 | 0.5523 |
| TC2100008025.hg.1 | C21orf140 | chromosome 21 open reading frame 140 | Coding | 4.57 | 4.57 | 4.47 | 1.07 | 0.5068 | 0.7035 | 4.38 | 4.53 | 4.23 | 1.11 | 0.2875 | 0.5523 |
| TC2100008548.hg.1 | KRTAP13-2 | keratin associated protein 13-2 | Coding | 2.73 | 2.64 | 2.63 | 1.07 | 0.8822 | 0.9428 | 3.02 | 2.81 | 2.88 | 1.10 | 0.5152 | 0.7445 |
| TC2100008558.hg.1 | TCP10L | t-complex 10-like | Multiple_C | 4.18 | 4.31 | 4.08 | 1.07 | 0.9075 | 0.9557 | 5.38 | 4.42 | 4.71 | 1.59 | 0.0354 | 0.1705 |
| TC2100008569.hg.1 | TMPRSS2 | transmembrane protease, serine 2 | Multiple_C | 6.14 | 5.59 | 6.04 | 1.07 | 0.6478 | 0.8048 | 6.18 | 6.21 | 6.34 | 0.90 | 0.3612 | 0.6241 |
| TC2200006955.hg.1 | CRYBA4 | crystallin beta A4 | Multiple_C | 4.59 | 4.33 | 4.49 | 1.07 | 0.418 | 0.6296 | 4.52 | 4.69 | 4.39 | 1.09 | 0.5445 | 0.7625 |
| TC2200007776.hg.1 | ZBED4 | zinc finger, BED-type containing 4 | Coding | 9.99 | 9.85 | 9.89 | 1.07 | 0.7249 | 0.8552 | 9.54 | 9.74 | 9.97 | 0.74 | 0.0293 | 0.1516 |
| TC2200007979.hg.1 | C22orf39; HIR | chromosome 22 open reading frame 39; histone cell cycle regul | Multiple_C | 6.54 | 6.49 | 6.44 | 1.07 | 0.2867 | 0.4972 | 5.8 | 6.12 | 5.89 | 0.94 | 0.4017 | 0.6582 |
| TSUnmapped00000150.h | OBP2B | odorant binding protein 2B | Coding | 4.59 | 4.24 | 4.49 | 1.07 | 0.684 | 0.8289 | 4.11 | 4.18 | 4 | 1.08 | 0.7283 | 0.874 |
| TSUnmapped00000308.h | BCL2L14 | BCL2-like 14 (apoptosis facilitator) | Coding | 6.43 | 7.27 | 6.33 | 1.07 | 0.8103 | 0.9042 | 5.16 | 5.22 | 5.3 | 0.91 | 0.3988 | 0.656 |
| TSUnmapped00000392.h | FCGBP | Fc fragment of IgG binding protein | Coding | 4.04 | 3.83 | 3.94 | 1.07 | 0.1855 | 0.3719 | 4.62 | 4.12 | 3.61 | 2.01 | 0.002 | 0.0267 |
| TSUnmapped00000549.h | PRAMEF8 | PRAME family member 8 | Coding | 3.66 | 3.72 | 3.56 | 1.07 | 0.513 | 0.7086 | 3.71 | 3.49 | 3.6 | 1.08 | 0.8372 | 0.9283 |
| TSUnmapped00000765.h | HNRNPCL4 | heterogeneous nuclear ribonucleoprotein C-like 4 [Source:HGN | Coding | 4.87 | 5.06 | 4.77 | 1.07 | 0.5856 | 0.7621 | 4.41 | 4.33 | 4.43 | 0.99 | 0.5174 | 0.7458 |
| TC0100007063.hg.1 | FAM231C; FAI | family with sequence similarity 231, member C; family with sequ | Coding | 5.82 | 5.5 | 5.73 | 1.06 | 0.1812 | 0.3657 | 5.66 | 5.68 | 5.51 | 1.11 | 0.9841 | 0.9935 |
| TC0100007434.hg.1 | EXTL1 | exostosin-like glycosyltransferase 1 | Multiple_C | 2.77 | 2.49 | 2.68 | 1.06 | 0.8865 | 0.9453 | 3.48 | 3.58 | 3.36 | 1.09 | 0.4714 | 0.7122 |
| TC0100008073.hg.1 | KDM4A | lysine (K)-specific demethylase 4A | Multiple_C | 8.47 | 7.91 | 8.38 | 1.06 | 0.2626 | 0.4697 | 7.13 | 7.14 | 8.03 | 0.54 | 0.0095 | 0.0755 |
| TC0100008088.hg.1 | KLF17 | Kruppel-like factor 17 | Multiple_C | 4.09 | 3.76 | 4 | 1.06 | 0.4662 | 0.6708 | 4.33 | 3.98 | 3.9 | 1.35 | 0.153 | 0.3921 |
| TC0100010168.hg.1 | RHBG | Rh family, B glycoprotein (gene/pseudogene) | Multiple_C | 2.96 | 2.6 | 2.87 | 1.06 | 0.5524 | 0.7367 | 3.39 | 3.29 | 3.15 | 1.18 | 0.0676 | 0.2477 |
| TC0100010720.hg.1 | BRINP2 | bone morphogenetic protein/retinoic acid inducible neural-spec | Multiple_C | 4.04 | 3.78 | 3.95 | 1.06 | 0.6123 | 0.7823 | 3.47 | 3.45 | 3.42 | 1.04 | 0.7486 | 0.8852 |
| TC0100012002.hg.1 | KIAA1804; ML | mixed lineage kinase 4; Mitogen-activated protein kinase kinase | Multiple_C | 8.25 | 7.84 | 8.16 | 1.06 | 0.4551 | 0.6624 | 6.58 | 6.75 | 7.55 | 0.51 | 0.0012 | 0.0188 |
| TC0100012099.hg.1 | ACTN2 | actinin, alpha 2 | Multiple_C | 7.38 | 7.06 | 7.29 | 1.06 | 0.1025 | 0.2452 | 7.44 | 7.51 | 7.31 | 1.09 | 0.6523 | 0.8305 |
| TC0100014133.hg.1 | AGBL4 | ATP/GTP binding protein-like 4 | Multiple_C | 3.01 | 2.94 | 2.92 | 1.06 | 0.7169 | 0.8503 | 3.34 | 3.39 | 3.05 | 1.22 | 0.1776 | 0.4261 |
| TC0100014966.hg.1 | GCLM | glutamate-cysteine ligase, modifier subunit | Multiple_C | 8.76 | 8.81 | 8.67 | 1.06 | 0.7539 | 0.8741 | 6.98 | 6.30 | 7.68 | 0.62 | 0.0042 | 0.0444 |
| TC0100015193.hg.1 | MYBPHL | myosin binding protein H-like | Multiple_C | 4.72 | 4.49 | 4.63 | 1.06 | 0.8306 | 0.9156 | 4.53 | 4.43 | 3.95 | 1.49 | 0.0075 | 0.0645 |
| TC0100016049.hg.1 | FCRL3 | Fc receptor-like 3 | Multiple_C | 3.69 | 3.58 | 3.6 | 1.06 | 0.562 | 0.7441 | 4 | 4.05 | 3.99 | 1.01 | 0.4751 | 0.7145 |
| TC0100016189.hg.1 | FCGR3B | Fc fragment of IgG, low affinity IIIb, receptor (CD16b) | Multiple_C | 3.07 | 3.05 | 2.98 | 1.06 | 0.684 | 0.8288 | 3.08 | 2.64 | 3.08 | 1.00 | 0.7569 | 0.8897 |
| TC0100017060.hg.1 | DSTYK | dual serine/threonine and tyrosine protein kinase | Multiple_C | 6.89 | 7.39 | 6.8 | 1.06 | 0.3305 | 0.5445 | 5.31 | 5.30 | 5.84 | 0.69 | 0.1031 | 0.3145 |
| TC0100018438.hg.1 | MOB3C | MOB kinase activator 3C | Multiple_C | 5.57 | 5.43 | 5.48 | 1.06 | 0.9462 | 0.9753 | 5.99 | 5.66 | 5.56 | 1.35 | 0.0093 | 0.0747 |
| TC0100018553.hg.1 | PLEKHA6 | pleckstrin homology domain containing, family A member 6 | Multiple_C | 8.76 | 7.79 | 8.67 | 1.06 | 0.822 | 0.9104 | 6.87 | 8.18 | 9.19 | 0.20 | 5.61E-06 | 0.0005 |

| | | | | | | | | | | | | | | | |
|---|---|---|---|---|---|---|---|---|---|---|---|---|---|---|---|
| TC0200006476.hg.1 | SNTG2 | syntrophin, gamma 2 | Multiple_C | 5.6 | 5.75 | 5.51 | 1.06 | 0.139 | 0.3053 | 5.05 | 5.11 | 4.8 | 1.19 | 0.1899 | 0.4424 |
| TC0200006480.hg.1 | TPO | thyroid peroxidase | Multiple_C | 5.49 | 5.63 | 5.4 | 1.06 | 0.2224 | 0.4202 | 4.48 | 4.48 | 4.31 | 1.13 | 0.3836 | 0.6426 |
| TC0200008268.hg.1 | GNLY | granulysin | Multiple_C | 3.21 | 2.99 | 3.12 | 1.06 | 0.7385 | 0.8642 | 3.2 | 3.45 | 3.13 | 1.05 | 0.2295 | 0.4906 |
| TC0200010713.hg.1 | ATIC | 5-aminoimidazole-4-carboxamide ribonucleotide formyltransfe | Multiple_C | 12.24 | 12.33 | 12.15 | 1.06 | 0.9838 | 0.9924 | 11.73 | 11.50 | 11.74 | 0.99 | 0.5532 | 0.7684 |
| TC0200011146.hg.1 | MROH2A | maestro heat-like repeat family member 2A | Multiple_C | 3.49 | 3.65 | 3.4 | 1.06 | 0.4843 | 0.6859 | 3.7 | 4.00 | 3.93 | 0.85 | 0.5219 | 0.7485 |
| TC0200011509.hg.1 | MYT1L | myelin transcription factor 1-like | Multiple_C | 4.44 | 4.1 | 4.35 | 1.06 | 0.8715 | 0.9376 | 4.53 | 4.48 | 4.67 | 0.91 | 0.8084 | 0.9155 |
| TC0200012452.hg.1 | SIX2 | SIX homeobox 2 | Coding | 5.26 | 5.08 | 5.17 | 1.06 | 0.6 | 0.7723 | 4.93 | 4.98 | 4.97 | 0.97 | 0.5906 | 0.7931 |
| TC0200014345.hg.1 | ANKRD30BL | ankyrin repeat domain 30B-like | Multiple_C | 3.52 | 3.4 | 3.43 | 1.06 | 0.7683 | 0.8808 | 4.75 | 4.64 | 4.63 | 1.09 | 0.463 | 0.7054 |
| TC0200016659.hg.1 | ATP6V1E2 | ATPase, H+ transporting, lysosomal 31kDa, V1 subunit E2 | Multiple_C | 5.28 | 5.27 | 5.19 | 1.06 | 0.3848 | 0.5981 | 5.56 | 6.18 | 5.5 | 1.04 | 0.5532 | 0.7684 |
| TC0300006631.hg.1 | MKRN2 | makorin ring finger protein 2 | Multiple_C | 10.91 | 11.13 | 10.82 | 1.06 | 0.825 | 0.9121 | 9.85 | 9.56 | 9.8 | 1.04 | 0.8386 | 0.9291 |
| TC0300007106.hg.1 | MOBP | myelin-associated oligodendrocyte basic protein | Multiple_C | 5.68 | 5.54 | 5.59 | 1.06 | 0.363 | 0.5765 | 5.23 | 5.17 | 5.12 | 1.08 | 0.121 | 0.3432 |
| TC0300007273.hg.1 | PTH1R | parathyroid hormone 1 receptor | Multiple_C | 3.09 | 2.93 | 3 | 1.06 | 0.3429 | 0.5568 | 3.73 | 3.39 | 3.36 | 1.29 | 0.0731 | 0.2591 |
| TC0300007325.hg.1 | ZNF589 | zinc finger protein 589 | Multiple_C | 9.14 | 8.85 | 9.05 | 1.06 | 0.6994 | 0.8392 | 8.32 | 8.76 | 8.89 | 0.67 | 0.0139 | 0.0956 |
| TC0300009772.hg.1 | AHSG | alpha-2-HS-glycoprotein | Multiple_C | 2.99 | 2.83 | 2.9 | 1.06 | 0.859 | 0.9311 | 3.53 | 3.26 | 3.33 | 1.15 | 0.0822 | 0.2762 |
| TC0300011030.hg.1 | TMEM89 | transmembrane protein 89 | Coding | 5.85 | 5.74 | 5.76 | 1.06 | 0.3188 | 0.5324 | 6.31 | 6.09 | 6.01 | 1.23 | 0.1212 | 0.3438 |
| TC0300011951.hg.1 | MORC1 | MORC family CW-type zinc finger 1 | Coding | 3.48 | 3.34 | 3.39 | 1.06 | 0.4368 | 0.6463 | 3.35 | 3.28 | 3.21 | 1.10 | 0.4631 | 0.7054 |
| TC0300012346.hg.1 | RPN1 | ribophorin I | Multiple_C | 15.37 | 15.16 | 15.28 | 1.06 | 0.7033 | 0.8416 | 14.67 | 14.89 | 15.28 | 0.66 | 0.0013 | 0.0196 |
| TC0300013606.hg.1 | TMEM44 | transmembrane protein 44 | Multiple_C | 4.83 | 4.78 | 4.74 | 1.06 | 0.4069 | 0.6194 | 3.89 | 4.11 | 3.77 | 1.09 | 0.9009 | 0.9589 |
| TC0300013789.hg.1 | CAND2 | cullin-associated and neddylation-dissociated 2 (putative) | Multiple_C | 4.51 | 4.54 | 4.42 | 1.06 | 0.7522 | 0.8733 | 4.08 | 4.20 | 4.31 | 0.85 | 0.1786 | 0.4274 |
| TC0400007772.hg.1 | HTN1 | histatin 1 | Multiple_C | 3 | 2.84 | 2.91 | 1.06 | 0.7787 | 0.8862 | 4.02 | 3.89 | 4.03 | 0.99 | 0.2875 | 0.5523 |
| TC0400007913.hg.1 | SEPT11 | septin 11 | Multiple_C | 11.79 | 11.23 | 11.7 | 1.06 | 0.5904 | 0.7656 | 11.79 | 11.63 | 12.09 | 0.81 | 0.2633 | 0.5279 |
| TC0400007991.hg.1 | COPS4 | COP9 signalosome subunit 4 | Multiple_C | 10.82 | 12.07 | 10.73 | 1.06 | 0.6489 | 0.8054 | 8.26 | 8.65 | 9.12 | 0.55 | 0.0131 | 0.0924 |
| TC0400008483.hg.1 | UGT8 | UDP glycosyltransferase 8 | Multiple_C | 11.22 | 10.53 | 11.13 | 1.06 | 0.6987 | 0.8387 | 8.45 | 7.58 | 8.05 | 1.32 | 0.0675 | 0.2474 |
| TC0400008861.hg.1 | IL15 | interleukin 15 | Multiple_C | 3.99 | 4.12 | 3.9 | 1.06 | 0.7572 | 0.8753 | 4.71 | 4.42 | 3.88 | 1.78 | 0.0281 | 0.1476 |
| TC0400009592.hg.1 | TRIML1 | tripartite motif family like 1 | Multiple_C | 4.78 | 4.77 | 4.69 | 1.06 | 0.6433 | 0.802 | 4.79 | 4.48 | 4.4 | 1.31 | 0.1308 | 0.3584 |
| TC0400011052.hg.1 | CXCL9 | chemokine (C-X-C motif) ligand 9 | Coding | 3.5 | 3.1 | 3.41 | 1.06 | 0.413 | 0.625 | 3.84 | 3.68 | 3.45 | 1.31 | 0.0781 | 0.2692 |
| TC0400012193.hg.1 | MAP9 | microtubule-associated protein 9 | Multiple_C | 3.8 | 3.91 | 3.71 | 1.06 | 0.6427 | 0.8018 | 4.29 | 4.28 | 4.48 | 0.88 | 0.4042 | 0.6599 |
| TC0400012473.hg.1 | GPM6A | glycoprotein M6A | Multiple_C | 2.93 | 2.94 | 2.84 | 1.06 | 0.4991 | 0.6985 | 3.15 | 2.81 | 2.94 | 1.16 | 0.2435 | 0.5071 |
| TC0400012764.hg.1 | GPR78 | G protein-coupled receptor 78 | Multiple_C | 3.33 | 3.06 | 3.24 | 1.06 | 0.3463 | 0.5597 | 3.31 | 3.68 | 3.5 | 0.88 | 0.115 | 0.3348 |
| TC0400012948.hg.1 | EMCN | endomucin | Multiple_C | 4.52 | 4.53 | 4.43 | 1.06 | 0.5509 | 0.7358 | 4.06 | 4.16 | 4.37 | 0.81 | 0.0569 | 0.2245 |
| TC0500006437.hg.1 | LRRC14B | leucine rich repeat containing 14B | Coding | 2.81 | 2.77 | 2.72 | 1.06 | 0.6514 | 0.8071 | 3.37 | 3.46 | 3.38 | 0.99 | 0.5934 | 0.7945 |
| TC0500007491.hg.1 | GAPT | GRB2-binding adaptor protein, transmembrane | Multiple_C | 3.12 | 2.96 | 3.03 | 1.06 | 0.742 | 0.8666 | 3.77 | 3.68 | 3.22 | 1.46 | 0.1231 | 0.3463 |
| TC0500007591.hg.1 | CWC27 | CWC27 spliceosome-associated protein homolog | Multiple_C | 11.28 | 12.01 | 11.19 | 1.06 | 0.5544 | 0.738 | 11.8 | 11.19 | 10.87 | 1.91 | 0.0141 | 0.0965 |

| | | | | | | | | | | | | | | | |
|---|---|---|---|---|---|---|---|---|---|---|---|---|---|---|---|
| TC0500007681.hg.1 | OCLN | occludin | Multiple_C | 12.15 | 11.68 | 12.06 | 1.06 | 0.8218 | 0.9104 | 11.03 | 10.22 | 10.67 | 1.28 | 0.187 | 0.4388 |
| TC0500009046.hg.1 | ADRB2 | adrenoceptor beta 2, surface | Coding | 3.66 | 3.15 | 3.57 | 1.06 | 0.8703 | 0.9368 | 3.08 | 3.03 | 3.18 | 0.93 | 0.3705 | 0.6327 |
| TC0500010753.hg.1 | CDC20B | cell division cycle 20B | Coding | 3.38 | 3.34 | 3.29 | 1.06 | 0.3192 | 0.5329 | 4.32 | 4.20 | 4.3 | 1.01 | 0.3207 | 0.5846 |
| TC0500012760.hg.1 | SLIT3 | slit guidance ligand 3 | Multiple_C | 3.54 | 3.19 | 3.45 | 1.06 | 0.8637 | 0.9335 | 4.16 | 3.38 | 3.21 | 1.93 | 0.0046 | 0.0464 |
| TC0600009336.hg.1 | CLVS2 | clavesin 2 | Multiple_C | 2.86 | 2.55 | 2.77 | 1.06 | 0.6713 | 0.8209 | 3.47 | 3.15 | 3.37 | 1.07 | 0.577 | 0.7832 |
| TC0600012110.hg.1 | HMGCLL1 | 3-hydroxymethyl-3-methylglutaryl-CoA lyase-like 1 | Multiple_C | 3.71 | 3.74 | 3.62 | 1.06 | 0.7032 | 0.8416 | 4.18 | 3.51 | 3.87 | 1.24 | 0.2285 | 0.4894 |
| TC0600012188.hg.1 | LGSN | lengsin, lens protein with glutamine synthetase domain | Coding | 3.74 | 3.23 | 3.65 | 1.06 | 0.2702 | 0.4785 | 3.85 | 3.85 | 4.27 | 0.75 | 0.0675 | 0.2476 |
| TC0600012625.hg.1 | GPR63 | G protein-coupled receptor 63 | Coding | 5.89 | 5.32 | 5.8 | 1.06 | 0.806 | 0.9013 | 6.83 | 5.82 | 6.8 | 1.02 | 0.8862 | 0.9513 |
| TC0600014104.hg.1 | LY6G6D | lymphocyte antigen 6 complex, locus G6D | Multiple_C | 5.07 | 4.74 | 4.98 | 1.06 | 0.6885 | 0.8321 | 4.56 | 5.22 | 4.73 | 0.89 | 0.5258 | 0.7504 |
| TC0600014153.hg.1 | CFAP206 | cilia and flagella associated protein 206 | Multiple_C | 3.83 | 3.81 | 3.74 | 1.06 | 0.6775 | 0.8247 | 5.06 | 5.06 | 4.29 | 1.71 | 0.0049 | 0.0484 |
| TC0700008576.hg.1 | MUC12 | mucin 12, cell surface associated | Multiple_C | 4.66 | 4.42 | 4.57 | 1.06 | 0.4552 | 0.6624 | 4.15 | 4.35 | 4.33 | 0.88 | 0.6891 | 0.8515 |
| TC0700008707.hg.1 | RINT1 | RAD50 interactor 1 | Multiple_C | 10.04 | 10.32 | 9.95 | 1.06 | 0.7582 | 0.8757 | 9.87 | 9.23 | 10.93 | 0.48 | 0.0003 | 0.0068 |
| TC0700010945.hg.1 | NACAD | NAC alpha domain containing | Multiple_C | 4.36 | 3.87 | 4.27 | 1.06 | 0.2213 | 0.4187 | 4.85 | 4.54 | 4.41 | 1.36 | 0.1832 | 0.4342 |
| TC0700010989.hg.1 | TNS3 | tensin 3 | Multiple_C | 8.39 | 7.75 | 8.3 | 1.06 | 0.812 | 0.905 | 13.24 | 13.85 | 13.77 | 0.69 | 0.0015 | 0.0215 |
| TC0700011166.hg.1 | CHCHD2 | coiled-coil-helix-coiled-coil-helix domain containing 2 | Multiple_C | 14.95 | 14.34 | 14.86 | 1.06 | 0.8046 | 0.9003 | 12.86 | 13.55 | 13.02 | 0.90 | 0.8018 | 0.9116 |
| TC0700012163.hg.1 | EFCAB10 | EF-hand calcium binding domain 10 | Multiple_C | 5.12 | 4.58 | 5.03 | 1.06 | 0.3007 | 0.5129 | 4.31 | 4.26 | 4.72 | 0.75 | 0.2445 | 0.5089 |
| TC0700013439.hg.1 | BCAP29 | B-cell receptor-associated protein 29 | Multiple_C | 10.66 | 10.54 | 10.57 | 1.06 | 0.6811 | 0.8267 | 10.33 | 10.43 | 10.61 | 0.82 | 0.534 | 0.7563 |
| TC0700013508.hg.1 | RADIL | Ras association and DIL domains | Multiple_C | 3.22 | 3.18 | 3.13 | 1.06 | 0.4972 | 0.6968 | 3.19 | 3.09 | 3.07 | 1.09 | 0.5924 | 0.7938 |
| TC0800006769.hg.1 | ZNF705D | zinc finger protein 705D | Coding | 4.47 | 4.42 | 4.38 | 1.06 | 0.8277 | 0.9134 | 4.72 | 5.14 | 4.86 | 0.91 | 0.2479 | 0.5124 |
| TC0800007209.hg.1 | WRN | Werner syndrome, RecQ helicase-like | Multiple_C | 7.66 | 8.6 | 7.57 | 1.06 | 0.5316 | 0.7215 | 7.8 | 7.45 | 7.86 | 0.96 | 0.4624 | 0.7049 |
| TC0800007978.hg.1 | LY96 | lymphocyte antigen 96 | Coding | 4.54 | 4.9 | 4.45 | 1.06 | 0.5663 | 0.7475 | 3.81 | 3.40 | 4.11 | 0.81 | 0.3778 | 0.6378 |
| TC0800008299.hg.1 | SDC2 | syndecan 2 | Multiple_C | 3.55 | 3.9 | 3.46 | 1.06 | 0.7784 | 0.886 | 4.18 | 3.81 | 3.79 | 1.31 | 0.0183 | 0.1141 |
| TC0800011005.hg.1 | REXO1L2P | REX1, RNA exonuclease 1 homolog-like 2, pseudogene | Multiple_C | 3.75 | 3.49 | 3.66 | 1.06 | 0.5914 | 0.7662 | 5.16 | 4.96 | 4.83 | 1.26 | 0.092 | 0.2939 |
| TC0900008847.hg.1 | LCN2 | lipocalin 2 | Multiple_C | 5.92 | 5.6 | 5.83 | 1.06 | 0.5239 | 0.7165 | 4.58 | 4.55 | 4.44 | 1.10 | 0.6559 | 0.8321 |
| TC0900011145.hg.1 | ACTL7B | actin-like 7B | Coding | 5.55 | 5.31 | 5.46 | 1.06 | 0.1135 | 0.2643 | 5.15 | 5.16 | 5.13 | 1.01 | 0.5434 | 0.762 |
| TC0X00006590.hg.1 | WWC3 | WWC family member 3 | Multiple_C | 6.16 | 6.26 | 6.07 | 1.06 | 0.347 | 0.5602 | 5.09 | 5.70 | 5.32 | 0.85 | 0.5214 | 0.7482 |
| TC0X00007074.hg.1 | DUSP21 | dual specificity phosphatase 21 | Coding | 2.89 | 2.55 | 2.8 | 1.06 | 0.4391 | 0.6483 | 3.33 | 3.12 | 3.32 | 1.01 | 0.6346 | 0.8208 |
| TC0X00010426.hg.1 | MORF4L2 | mortality factor 4 like 2 | Multiple_C | 13.13 | 13.82 | 13.04 | 1.06 | 0.7355 | 0.8618 | 12.24 | 12.46 | 12.27 | 0.98 | 0.9544 | 0.9814 |
| TC0X00010481.hg.1 | TEX13B | testis expressed 13B | Coding | 3.63 | 3.43 | 3.54 | 1.06 | 0.7173 | 0.8505 | 3.41 | 3.62 | 3.49 | 0.95 | 0.5372 | 0.7587 |
| TC0X00011080.hg.1 | MAGEA9; MA | MAGE family member A9; MAGE family member A9B | Coding | 3.53 | 3.13 | 3.44 | 1.06 | 0.6913 | 0.8335 | 3.68 | 3.97 | 3.61 | 1.05 | 0.4989 | 0.7316 |
| TC0Y00007219.hg.1 | DAZ4; DAZ1 | deleted in azoospermia 4; deleted in azoospermia 1 | Multiple_C | 3.7 | 3.58 | 3.61 | 1.06 | 0.9495 | 0.9765 | 4.66 | 4.37 | 3.7 | 1.95 | 0.0021 | 0.0274 |
| TC1000009248.hg.1 | FANK1 | fibronectin type III and ankyrin repeat domains 1 | Multiple_C | 3.8 | 3.92 | 3.71 | 1.06 | 0.7305 | 0.859 | 4.66 | 4.17 | 3.55 | 2.16 | 0.0005 | 0.01 |
| TC1000011964.hg.1 | RAB11FIP2 | RAB11 family interacting protein 2 (class I) | Multiple_C | 8.45 | 8.31 | 8.36 | 1.06 | 0.9742 | 0.9874 | 10.54 | 10.16 | 10.1 | 1.36 | 0.1428 | 0.3759 |

| | | | | | | | | | | | | | | | |
|---|---|---|---|---|---|---|---|---|---|---|---|---|---|---|---|
| TC1000012483.hg.1 | C10orf131 | chromosome 10 open reading frame 131 | Coding | 3.34 | 2.94 | 3.25 | 1.06 | 0.721 | 0.853 | 4.26 | 4.59 | 4.27 | 0.99 | 0.8116 | 0.9166 |
| TC1100007634.hg.1 | OR8K1 | olfactory receptor, family 8, subfamily K, member 1 | Coding | 2.96 | 2.92 | 2.87 | 1.06 | 0.7 | 0.8395 | 3.71 | 3.31 | 3.31 | 1.32 | 0.0686 | 0.2494 |
| TC1100007650.hg.1 | OR9G1; OR9G | olfactory receptor, family 9, subfamily G, member 1; olfactory r | Coding | 5.44 | 5.28 | 5.35 | 1.06 | 0.3937 | 0.6076 | 6.07 | 5.57 | 5.43 | 1.56 | 0.0414 | 0.1872 |
| TC1100007965.hg.1 | SLC22A12 | solute carrier family 22 (organic anion/urate transporter), mem | Coding | 5.94 | 5.63 | 5.85 | 1.06 | 0.5633 | 0.7452 | 5.92 | 6.10 | 6 | 0.95 | 0.6695 | 0.8384 |
| TC1100008010.hg.1 | CDC42EP2 | CDC42 effector protein (Rho GTPase binding) 2 | Multiple_C | 8.21 | 7.63 | 8.12 | 1.06 | 0.3475 | 0.5605 | 7.01 | 6.91 | 7.04 | 0.98 | 0.5525 | 0.7681 |
| TC1100008323.hg.1 | LOC10012921 | beta-defensin 131-like | Multiple_C | 4.14 | 4.16 | 4.05 | 1.06 | 0.3979 | 0.6115 | 4.13 | 4.68 | 4.19 | 0.96 | 0.7715 | 0.8972 |
| TC1100008521.hg.1 | B3GNT6 | UDP-GlcNAc:betaGal beta-1,3-N-acetylglucosaminyltransferase | Coding | 3.95 | 3.81 | 3.86 | 1.06 | 0.8641 | 0.9337 | 4.26 | 4.21 | 4.05 | 1.16 | 0.3444 | 0.608 |
| TC1100008819.hg.1 | KDM4D | lysine (K)-specific demethylase 4D | Coding | 3.82 | 3.73 | 3.73 | 1.06 | 0.4318 | 0.642 | 3.1 | 3.27 | 3.17 | 0.95 | 0.6867 | 0.8499 |
| TC1100010350.hg.1 | CCDC179 | coiled-coil domain containing 179 | Multiple_C | 4.81 | 4.65 | 4.72 | 1.06 | 0.4252 | 0.6363 | 4.56 | 4.34 | 3.97 | 1.51 | 0.0106 | 0.0812 |
| TC1100010903.hg.1 | YPEL4 | yippee like 4 | Multiple_C | 3.79 | 3.22 | 3.7 | 1.06 | 0.7909 | 0.893 | 3.57 | 3.33 | 3.25 | 1.25 | 0.1526 | 0.3917 |
| TC1100010976.hg.1 | OSBP | oxysterol binding protein | Multiple_C | 10.47 | 10.85 | 10.38 | 1.06 | 0.5268 | 0.7187 | 10.92 | 10.92 | 10.95 | 0.98 | 0.8263 | 0.9234 |
| TC1100011197.hg.1 | BATF2 | basic leucine zipper transcription factor, ATF-like 2 | Multiple_C | 4.78 | 4.55 | 4.69 | 1.06 | 0.6906 | 0.8333 | 4.62 | 4.59 | 4.49 | 1.09 | 0.5333 | 0.756 |
| TC1100012285.hg.1 | COLCA1 | colorectal cancer associated 1 | Multiple_C | 3.77 | 3.63 | 3.68 | 1.06 | 0.7755 | 0.8844 | 3.63 | 3.82 | 3.44 | 1.14 | 0.2645 | 0.5291 |
| TC1100012478.hg.1 | CD3D | CD3d molecule, delta (CD3-TCR complex) | Multiple_C | 3.45 | 3 | 3.36 | 1.06 | 0.7902 | 0.8928 | 3.66 | 3.57 | 3.61 | 1.04 | 0.4845 | 0.722 |
| TC1100013084.hg.1 | AMOTL1 | angiomotin like 1 | Multiple_C | 3.66 | 3.8 | 3.57 | 1.06 | 0.3338 | 0.5475 | 3.8 | 3.93 | 3.83 | 0.98 | 0.6071 | 0.8036 |
| TC1100013131.hg.1 | IGF2 | insulin-like growth factor 2 | Multiple_C | 4.12 | 3.95 | 4.03 | 1.06 | 0.643 | 0.8019 | 5.53 | 4.72 | 4.55 | 1.97 | 0.0001 | 0.0037 |
| TC1200006555.hg.1 | CCND2 | cyclin D2 | Multiple_C | 12.97 | 12.6 | 12.88 | 1.06 | 0.9148 | 0.96 | 8.65 | 7.53 | 8.31 | 1.27 | 0.0634 | 0.2395 |
| TC1200006604.hg.1 | CD9 | CD9 molecule | Multiple_C | 16.51 | 16.84 | 16.42 | 1.06 | 0.5662 | 0.7473 | 13.02 | 13.11 | 13.26 | 0.85 | 0.6499 | 0.829 |
| TC1200006874.hg.1 | BORCS5 | BLOC-1 related complex subunit 5 | Multiple_C | 7.28 | 7.89 | 7.19 | 1.06 | 0.8847 | 0.9442 | 6.89 | 6.87 | 6.87 | 1.01 | 0.8649 | 0.9424 |
| TC1200007646.hg.1 | ACVRL1 | activin A receptor type IL | Multiple_C | 4.02 | 3.96 | 3.93 | 1.06 | 0.6052 | 0.7766 | 4.79 | 4.43 | 4.31 | 1.39 | 0.0205 | 0.1224 |
| TC1200007717.hg.1 | TARBP2 | TAR (HIV-1) RNA binding protein 2 | Multiple_C | 5.29 | 5.34 | 5.2 | 1.06 | 0.5233 | 0.7161 | 5.64 | 6.43 | 5.96 | 0.80 | 0.2198 | 0.4799 |
| TC1200007781.hg.1 | OR10A7 | olfactory receptor, family 10, subfamily A, member 7 | Coding | 3.56 | 3.15 | 3.47 | 1.06 | 0.2889 | 0.4996 | 3.32 | 3.14 | 3.16 | 1.12 | 0.1671 | 0.4124 |
| TC1200008669.hg.1 | TDG | thymine DNA glycosylase | Multiple_C | 9.26 | 8.52 | 9.17 | 1.06 | 0.7822 | 0.8879 | 7.87 | 8.02 | 7.77 | 1.07 | 0.632 | 0.8188 |
| TC1200008916.hg.1 | PTPN11 | protein tyrosine phosphatase, non-receptor type 11 | Multiple_C | 13.31 | 13.4 | 13.22 | 1.06 | 0.4193 | 0.6306 | 14.13 | 13.48 | 13.74 | 1.31 | 0.537 | 0.7585 |
| TC1200009903.hg.1 | KLRC4-KLRK1; | KLRC4-KLRK1 readthrough; killer cell lectin-like receptor subfam | Multiple_C | 5.06 | 4.99 | 4.97 | 1.06 | 0.7327 | 0.8604 | 5.14 | 4.53 | 4.72 | 1.34 | 0.0826 | 0.2768 |
| TC1200010510.hg.1 | SCAF11 | SR-related CTD-associated factor 11 | Multiple_C | 8.44 | 8.64 | 8.35 | 1.06 | 0.463 | 0.6681 | 8.46 | 8.86 | 9.21 | 0.59 | 0.0473 | 0.2017 |
| TC1200011423.hg.1 | TSPAN19 | tetraspanin 19 | Multiple_C | 3.34 | 3.39 | 3.25 | 1.06 | 0.6535 | 0.8086 | 3.65 | 3.42 | 3.46 | 1.14 | 0.8845 | 0.9505 |
| TC1200011557.hg.1 | CEP83 | centrosomal protein 83kDa | Multiple_C | 8.33 | 8.26 | 8.24 | 1.06 | 0.7596 | 0.8763 | 7.6 | 6.28 | 7.63 | 0.98 | 0.3299 | 0.5941 |
| TC1200012778.hg.1 | LMNTD1 | lamin tail domain containing 1 | Coding | 3.37 | 3.25 | 3.28 | 1.06 | 0.6467 | 0.8043 | 3.49 | 3.49 | 3.46 | 1.02 | 0.9172 | 0.9663 |
| TC1300006914.hg.1 | UFM1 | ubiquitin-fold modifier 1 | Multiple_C | 11.4 | 13.06 | 11.31 | 1.06 | 0.6731 | 0.822 | 11.21 | 10.53 | 9.91 | 2.46 | 0.0003 | 0.008 |
| TC1400006489.hg.1 | OR4K2 | olfactory receptor, family 4, subfamily K, member 2 | Coding | 3.01 | 3.1 | 2.92 | 1.06 | 0.5293 | 0.7201 | 3.38 | 3.16 | 3.11 | 1.21 | 0.0468 | 0.2004 |
| TC1400010605.hg.1 | CTAGE5 | CTAGE family, member 5 | Multiple_C | 8.44 | 9.78 | 8.35 | 1.06 | 0.2432 | 0.446 | 8.47 | 8.45 | 8.96 | 0.71 | 0.3341 | 0.5982 |
| TC1500007012.hg.1 | MAPKBP1 | mitogen-activated protein kinase binding protein 1 | Multiple_C | 6.85 | 6.5 | 6.76 | 1.06 | 0.2009 | 0.3929 | 6.86 | 6.82 | 6.96 | 0.93 | 0.6635 | 0.8355 |

| | | | | | | | | | | | | | | | |
|---|---|---|---|---|---|---|---|---|---|---|---|---|---|---|---|
| TC1500008978.hg.1 | EMC7 | ER membrane protein complex subunit 7 | Multiple_C | 12.89 | 13.21 | 12.8 | 1.06 | 0.8586 | 0.931 | 11.24 | 10.93 | 10.99 | 1.19 | 0.1015 | 0.3116 |
| TC1500009281.hg.1 | DUOX2 | dual oxidase 2 | Multiple_C | 3.24 | 2.91 | 3.15 | 1.06 | 0.8117 | 0.9048 | 3.67 | 3.67 | 3.4 | 1.21 | 0.0413 | 0.1868 |
| TC1500009282.hg.1 | DUOXA1 | dual oxidase maturation factor 1 | Multiple_C | 6.08 | 5.93 | 5.99 | 1.06 | 0.3841 | 0.5973 | 5.79 | 5.75 | 5.9 | 0.93 | 0.6637 | 0.8355 |
| TC1500010220.hg.1 | TMC3 | transmembrane channel like 3 | Multiple_C | 3.24 | 3.12 | 3.15 | 1.06 | 0.6482 | 0.805 | 3.52 | 3.53 | 3.55 | 0.98 | 0.7869 | 0.9049 |
| TC1500010732.hg.1 | TRIM69 | tripartite motif containing 69 | Multiple_C | 2.93 | 2.86 | 2.84 | 1.06 | 0.9899 | 0.9946 | 3.51 | 3.47 | 3.13 | 1.30 | 0.0794 | 0.2713 |
| TC1600006566.hg.1 | EME2 | essential meiotic structure-specific endonuclease subunit 2 | Multiple_C | 9.27 | 8.99 | 9.18 | 1.06 | 0.7397 | 0.865 | 7.41 | 7.65 | 7.6 | 0.88 | 0.7007 | 0.8581 |
| TC1600007927.hg.1 | IRX6 | iroquois homeobox 6 | Multiple_C | 3.71 | 3.57 | 3.62 | 1.06 | 0.4037 | 0.6166 | 3.3 | 3.34 | 3.3 | 1.00 | 0.9046 | 0.9607 |
| TC1600007997.hg.1 | CCL22 | chemokine (C-C motif) ligand 22 | Multiple_C | 3.16 | 3.01 | 3.07 | 1.06 | 0.8641 | 0.9337 | 3.42 | 3.39 | 3.5 | 0.95 | 0.7147 | 0.8667 |
| TC1600009722.hg.1 | USP31 | ubiquitin specific peptidase 31 | Multiple_C | 5.78 | 5.08 | 5.69 | 1.06 | 0.6621 | 0.8146 | 6.09 | 5.85 | 6.15 | 0.96 | 0.7639 | 0.8932 |
| TC1600011314.hg.1 | HBM | hemoglobin, mu | Coding | 3.5 | 3.24 | 3.41 | 1.06 | 0.9489 | 0.9763 | 4.81 | 4.53 | 4.4 | 1.33 | 0.0558 | 0.2219 |
| TC1600011330.hg.1 | ATP6V0C | ATPase, H+ transporting, lysosomal 16kDa, V0 subunit c | Multiple_C | 13.1 | 11.18 | 13.01 | 1.06 | 0.3062 | 0.5193 | 13.15 | 13.47 | 13.66 | 0.70 | 0.0365 | 0.1736 |
| TC1700006717.hg.1 | ALOX12 | arachidonate 12-lipoxygenase | Multiple_C | 3.61 | 3.35 | 3.52 | 1.06 | 0.851 | 0.9269 | 3.69 | 3.66 | 3.57 | 1.09 | 0.5841 | 0.788 |
| TC1700008452.hg.1 | GDPD1 | glycerophosphodiester phosphodiesterase domain containing 1 | Multiple_C | 7.45 | 9.41 | 7.36 | 1.06 | 0.8571 | 0.9301 | 7.97 | 6.92 | 6.28 | 3.23 | 4.93E-05 | 0.0022 |
| TC1700008514.hg.1 | TBX4 | T-box 4 | Multiple_C | 3.6 | 3.38 | 3.51 | 1.06 | 0.8432 | 0.9228 | 4.56 | 4.17 | 3.79 | 1.71 | 0.0511 | 0.211 |
| TC1700009236.hg.1 | SLC16A3; MIR | solute carrier family 16 (monocarboxylate transporter), membe | Multiple_C | 8.74 | 7.66 | 8.65 | 1.06 | 0.8841 | 0.9439 | 10.73 | 11.03 | 11.24 | 0.70 | 0.0897 | 0.2896 |
| TC1700009495.hg.1 | P2RX1 | purinergic receptor P2X, ligand gated ion channel, 1 | Multiple_C | 5.25 | 5.04 | 5.16 | 1.06 | 0.7201 | 0.8525 | 4.91 | 4.89 | 4.47 | 1.36 | 0.0614 | 0.2353 |
| TC1700010504.hg.1 | HNF1B | HNF1 homeobox B | Multiple_C | 3.62 | 3.38 | 3.53 | 1.06 | 0.5514 | 0.736 | 3.14 | 3.42 | 3.09 | 1.04 | 0.145 | 0.3797 |
| TC1700010790.hg.1 | ETV4 | ets variant 4 | Multiple_C | 8.64 | 8.2 | 8.55 | 1.06 | 0.9788 | 0.9899 | 9.11 | 9.38 | 9.17 | 0.96 | 0.9599 | 0.9835 |
| TC1700012056.hg.1 | PDE6G | phosphodiesterase 6G, cGMP-specific, rod, gamma | Multiple_C | 5.6 | 5.68 | 5.51 | 1.06 | 0.6156 | 0.7848 | 4.67 | 4.90 | 4.76 | 0.94 | 0.519 | 0.7466 |
| TC1700012057.hg.1 | OXLD1 | oxidoreductase-like domain containing 1 | Multiple_C | 5.48 | 5.65 | 5.39 | 1.06 | 0.8218 | 0.9104 | 6.61 | 6.55 | 5.61 | 2.00 | 0.0004 | 0.0085 |
| TC1700012450.hg.1 | GH1 | growth hormone 1 | Multiple_C | 3.9 | 3.71 | 3.81 | 1.06 | 0.3321 | 0.5459 | 4.34 | 4.45 | 4.46 | 0.92 | 0.9461 | 0.9781 |
| TC1800009252.hg.1 | GALR1 | galanin receptor 1 | Multiple_C | 3.71 | 3.38 | 3.62 | 1.06 | 0.4545 | 0.662 | 3.8 | 3.96 | 3.72 | 1.06 | 0.9821 | 0.9926 |
| TC1900006620.hg.1 | GNA15 | guanine nucleotide binding protein (G protein), alpha 15 (Gq cla | Multiple_C | 6.43 | 6.12 | 6.34 | 1.06 | 0.6749 | 0.8231 | 5.44 | 5.68 | 5.63 | 0.88 | 0.4147 | 0.6692 |
| TC1900006684.hg.1 | SHD | Src homology 2 domain containing transforming protein D | Multiple_C | 4.47 | 4.17 | 4.38 | 1.06 | 0.6019 | 0.7739 | 4.48 | 4.58 | 4.5 | 0.99 | 0.3587 | 0.6216 |
| TC1900007849.hg.1 | MAG | myelin associated glycoprotein | Multiple_C | 5.22 | 5.01 | 5.13 | 1.06 | 0.7225 | 0.854 | 4.75 | 4.96 | 4.89 | 0.91 | 0.5225 | 0.7487 |
| TC1900007947.hg.1 | ZNF382 | zinc finger protein 382 | Multiple_C | 4.24 | 3.84 | 4.15 | 1.06 | 0.94 | 0.9722 | 4.24 | 4.01 | 4.47 | 0.85 | 0.2587 | 0.523 |
| TC1900008476.hg.1 | SULT2B1 | sulfotransferase family 2B member 1 | Multiple_C | 5.45 | 5.36 | 5.36 | 1.06 | 0.8212 | 0.9103 | 4.73 | 4.77 | 4.62 | 1.08 | 0.6492 | 0.8288 |
| TC1900009393.hg.1 | SAFB2 | scaffold attachment factor B2 | Multiple_C | 10.36 | 10.19 | 10.27 | 1.06 | 0.9677 | 0.9844 | 9.93 | 9.90 | 10.32 | 0.76 | 0.0894 | 0.2892 |
| TC1900009729.hg.1 | ZNF563 | zinc finger protein 563 | Multiple_C | 5.96 | 5.99 | 5.87 | 1.06 | 0.428 | 0.6384 | 5.79 | 5.90 | 5.4 | 1.31 | 0.1677 | 0.4131 |
| TC1900009917.hg.1 | EPS15L1 | epidermal growth factor receptor pathway substrate 15-like 1 | Multiple_C | 6.68 | 5.54 | 6.59 | 1.06 | 0.6074 | 0.7783 | 5.83 | 5.20 | 6.07 | 0.85 | 0.253 | 0.5172 |
| TC1900010612.hg.1 | MAP4K1 | mitogen-activated protein kinase kinase kinase kinase 1 | Multiple_C | 2.56 | 2.58 | 2.47 | 1.06 | 0.6697 | 0.8197 | 3.26 | 3.25 | 3.06 | 1.15 | 0.252 | 0.5163 |
| TC1900011094.hg.1 | RASIP1 | Ras interacting protein 1 | Multiple_C | 3.9 | 4.17 | 3.81 | 1.06 | 0.6315 | 0.7943 | 4.88 | 4.80 | 4.33 | 1.46 | 0.0162 | 0.1058 |
| TC1900011223.hg.1 | KLK1 | kallikrein 1 | Multiple_C | 3.64 | 3.52 | 3.55 | 1.06 | 0.7742 | 0.8837 | 5.71 | 5.29 | 4.89 | 1.77 | 0.0068 | 0.06 |

| | | | | | | | | | | | | | | | |
|---|---|---|---|---|---|---|---|---|---|---|---|---|---|---|---|
| TC1900011933.hg.1 | ZNF585A | zinc finger protein 585A | Multiple_C | 11.08 | 11.75 | 10.99 | 1.06 | 0.8241 | 0.9115 | 6.89 | 7.11 | 7.53 | 0.64 | 0.0264 | 0.1419 |
| TC2200007523.hg.1 | SERHL | serine hydrolase-like (pseudogene) | Multiple_C | 5.01 | 4.96 | 4.92 | 1.06 | 0.7479 | 0.8706 | 6.54 | 6.72 | 7.28 | 0.60 | 0.0107 | 0.0816 |
| TC2200007953.hg.1 | PRODH | proline dehydrogenase (oxidase) 1 | Multiple_C | 6.86 | 6.73 | 6.77 | 1.06 | 0.9798 | 0.9903 | 7.27 | 6.73 | 6.58 | 1.61 | 0.0031 | 0.0362 |
| TC2200008679.hg.1 | SLC16A8 | solute carrier family 16 (monocarboxylate transporter), membe | Multiple_C | 2.95 | 2.83 | 2.86 | 1.06 | 0.6466 | 0.8043 | 3.79 | 3.89 | 3.8 | 0.99 | 0.908 | 0.962 |
| TC2200008908.hg.1 | SCUBE1 | signal peptide, CUB domain, EGF-like 1 | Multiple_C | 6.08 | 6.05 | 5.99 | 1.06 | 0.6167 | 0.7851 | 5.58 | 5.83 | 5.61 | 0.98 | 0.7276 | 0.8736 |
| TSUnmapped00000232.h | ZKSCAN7 | zinc finger with KRAB and SCAN domains 7 | Coding | 3.83 | 3.75 | 3.74 | 1.06 | 0.5676 | 0.7482 | 3.68 | 3.94 | 3.82 | 0.91 | 0.9062 | 0.9613 |
| TSUnmapped00000271.h | OR10A3 | olfactory receptor, family 10, subfamily A, member 3 | Coding | 3.17 | 2.71 | 3.08 | 1.06 | 0.5291 | 0.72 | 3.44 | 3.63 | 3.45 | 0.99 | 0.7965 | 0.9092 |
| TSUnmapped00000321.h | RPS25 | ribosomal protein S25 | Coding | 11.72 | 12.14 | 11.63 | 1.06 | 0.5705 | 0.7504 | 9.86 | 10.06 | 9.8 | 1.04 | 0.6745 | 0.8425 |
| TSUnmapped00000704.h | FMN1 | formin 1 | Coding | 3.92 | 4.16 | 3.83 | 1.06 | 0.5797 | 0.7573 | 4.21 | 3.47 | 4.25 | 0.97 | 0.7109 | 0.8644 |
| TSUnmapped00000820.h | CFHR2 | complement factor H-related 2 | Coding | 4.66 | 4.55 | 4.57 | 1.06 | 0.5723 | 0.7518 | 4.54 | 4.60 | 4.3 | 1.18 | 0.1982 | 0.4532 |
| TC0100008054.hg.1 | TMEM125 | transmembrane protein 125 | Multiple_C | 5.82 | 5.58 | 5.74 | 1.06 | 0.9877 | 0.9935 | 4.18 | 4.32 | 4.31 | 0.91 | 0.8241 | 0.9222 |
| TC0100008816.hg.1 | IFI44 | interferon-induced protein 44 | Multiple_C | 5.31 | 3.99 | 5.23 | 1.06 | 0.7128 | 0.8481 | 3.92 | 3.65 | 3.74 | 1.13 | 0.5594 | 0.7716 |
| TC0100008922.hg.1 | CLCA1 | chloride channel accessory 1 | Coding | 3.66 | 3.27 | 3.58 | 1.06 | 0.9966 | 0.9984 | 4.16 | 4.27 | 4.15 | 1.01 | 0.7336 | 0.8771 |
| TC0100009553.hg.1 | C1orf137 | chromosome 1 open reading frame 137 | Multiple_C | 3.63 | 3.57 | 3.55 | 1.06 | 0.7254 | 0.8555 | 3.7 | 3.29 | 3.33 | 1.29 | 0.0593 | 0.2298 |
| TC0100010043.hg.1 | S100A1 | S100 calcium binding protein A1 | Multiple_C | 5.47 | 5.32 | 5.39 | 1.06 | 0.2431 | 0.4458 | 5.56 | 5.71 | 5.51 | 1.04 | 0.9625 | 0.9842 |
| TC0100010310.hg.1 | SLAMF7 | SLAM family member 7 | Multiple_C | 3.83 | 3.6 | 3.75 | 1.06 | 0.8441 | 0.9232 | 3.92 | 4.02 | 3.76 | 1.12 | 0.2074 | 0.4641 |
| TC0100011414.hg.1 | CR1 | complement component (3b/4b) receptor 1 (Knops blood grour | Multiple_C | 3.28 | 2.87 | 3.2 | 1.06 | 0.7578 | 0.8754 | 3.39 | 3.02 | 3.14 | 1.19 | 0.0285 | 0.1489 |
| TC0100012355.hg.1 | OR2T6 | olfactory receptor, family 2, subfamily T, member 6 | Coding | 5.33 | 4.95 | 5.25 | 1.06 | 0.8279 | 0.9135 | 4.46 | 4.21 | 4.17 | 1.22 | 0.1339 | 0.3634 |
| TC0100012674.hg.1 | GPR153 | G protein-coupled receptor 153 | Multiple_C | 5.72 | 5.45 | 5.64 | 1.06 | 0.1214 | 0.277 | 5.94 | 6.19 | 5.84 | 1.07 | 0.9759 | 0.9902 |
| TC0100012947.hg.1 | PRAMEF14 | PRAME family member 14 | Multiple_C | 2.88 | 2.7 | 2.8 | 1.06 | 0.6838 | 0.8288 | 3.35 | 3.18 | 3 | 1.27 | 0.1303 | 0.3574 |
| TC0100013837.hg.1 | RIMS3 | regulating synaptic membrane exocytosis 3 | Multiple_C | 3.65 | 3.36 | 3.57 | 1.06 | 0.3375 | 0.5513 | 4.12 | 4.13 | 4.11 | 1.01 | 0.8862 | 0.9513 |
| TC0100014048.hg.1 | POMGNT1 | protein O-linked mannose N-acetylglucosaminyltransferase 1 (b | Multiple_C | 8.4 | 8.5 | 8.32 | 1.06 | 0.6669 | 0.8177 | 8.17 | 8.17 | 8.31 | 0.91 | 0.0509 | 0.2102 |
| TC0100014608.hg.1 | LRRC53 | leucine rich repeat containing 53 | Coding | 3.39 | 3.09 | 3.31 | 1.06 | 0.9343 | 0.9692 | 3.92 | 3.98 | 3.81 | 1.08 | 0.8444 | 0.9318 |
| TC0100015481.hg.1 | ADAM30 | ADAM metallopeptidase domain 30 | Coding | 2.8 | 2.57 | 2.72 | 1.06 | 0.5885 | 0.7642 | 3.33 | 3.28 | 3.07 | 1.20 | 0.1204 | 0.3425 |
| TC0100015551.hg.1 | FAM72D | Transcript Identified by AceView, Entrez Gene ID(s) 728833 | Multiple_C | 5.34 | 5.18 | 5.26 | 1.06 | 0.6062 | 0.7773 | 5.01 | 5.15 | 4.89 | 1.09 | 0.0987 | 0.3067 |
| TC0100015714.hg.1 | SV2A | synaptic vesicle glycoprotein 2A | Coding | 5.74 | 5.81 | 5.66 | 1.06 | 0.2339 | 0.4347 | 6.36 | 7.06 | 7.51 | 0.45 | 0.0001 | 0.0044 |
| TC0100015902.hg.1 | C1orf43 | chromosome 1 open reading frame 43 | Multiple_C | 13.83 | 13.84 | 13.75 | 1.06 | 0.6057 | 0.7771 | 12.02 | 12.03 | 12.63 | 0.66 | 0.0135 | 0.094 |
| TC0100016318.hg.1 | MPC2 | mitochondrial pyruvate carrier 2 | Multiple_C | 11.57 | 12.42 | 11.49 | 1.06 | 0.8388 | 0.9197 | 8.71 | 9.28 | 8.85 | 0.91 | 0.0711 | 0.2551 |
| TC0100016356.hg.1 | F5 | coagulation factor V (proaccelerin, labile factor) | Multiple_C | 6.05 | 5.67 | 5.97 | 1.06 | 0.4646 | 0.6695 | 5.65 | 6.92 | 6.66 | 0.50 | 0.0004 | 0.0084 |
| TC0100017663.hg.1 | ACTA1 | actin, alpha 1, skeletal muscle | Coding | 10.37 | 9.75 | 10.29 | 1.06 | 0.8478 | 0.9256 | 8.17 | 8.94 | 8.93 | 0.59 | 0.0053 | 0.0511 |
| TC0100018310.hg.1 | FCGR2B | Fc fragment of IgG, low affinity IIb, receptor (CD32) | Multiple_C | 3.42 | 3.96 | 3.34 | 1.06 | 0.7739 | 0.8835 | 3.54 | 3.61 | 3.32 | 1.16 | 0.105 | 0.3179 |
| TC0200006665.hg.1 | TAF1B | TATA box binding protein (TBP)-associated factor, RNA polymer | Multiple_C | 6.86 | 8.06 | 6.78 | 1.06 | 0.8157 | 0.9072 | 8.21 | 7.66 | 8.2 | 1.01 | 0.9375 | 0.9741 |
| TC0200010264.hg.1 | MFSD6 | major facilitator superfamily domain containing 6 | Multiple_C | 11.48 | 11.45 | 11.4 | 1.06 | 0.7118 | 0.8476 | 11.62 | 11.36 | 10.72 | 1.87 | 0.0219 | 0.1271 |

| | | | | | | | | | | | | | | | |
|---|---|---|---|---|---|---|---|---|---|---|---|---|---|---|---|
| TC0200010971.hg.1 | COL4A3 | collagen, type IV, alpha 3 (Goodpasture antigen) | Multiple_C | 3.81 | 3.8 | 3.73 | 1.06 | 0.216 | 0.4118 | 4.01 | 3.70 | 3.58 | 1.35 | 0.151 | 0.389 |
| TC0200013578.hg.1 | TMEM131 | transmembrane protein 131 | Multiple_C | 9.58 | 8.7 | 9.5 | 1.06 | 0.8046 | 0.9003 | 9.9 | 9.71 | 10.29 | 0.76 | 0.0948 | 0.2992 |
| TC0200015216.hg.1 | ORMDL1 | ORMDL sphingolipid biosynthesis regulator 1 | Multiple_C | 10.23 | 11.17 | 10.15 | 1.06 | 0.9946 | 0.9973 | 9.11 | 8.61 | 9.18 | 0.95 | 0.3208 | 0.5847 |
| TC0300007435.hg.1 | DOCK3 | dedicator of cytokinesis 3 | Multiple_C | 3.34 | 3.26 | 3.26 | 1.06 | 0.5712 | 0.751 | 3.93 | 4.05 | 4.06 | 0.91 | 0.9729 | 0.989 |
| TC0300008755.hg.1 | GP9 | glycoprotein IX (platelet) | Coding | 3.12 | 3.25 | 3.04 | 1.06 | 0.1516 | 0.3246 | 3.07 | 3.17 | 2.92 | 1.11 | 0.4344 | 0.6852 |
| TC0300011203.hg.1 | DCP1A | decapping mRNA 1A | Multiple_C | 8.31 | 7.78 | 8.23 | 1.06 | 0.5942 | 0.7686 | 6.44 | 6.49 | 7.03 | 0.66 | 0.0024 | 0.0306 |
| TC0300011802.hg.1 | GABRR3 | gamma-aminobutyric acid (GABA) A receptor, rho 3 (gene/pseu | Multiple_C | 3.21 | 3.18 | 3.13 | 1.06 | 0.8813 | 0.9424 | 3.66 | 4.25 | 3.5 | 1.12 | 0.138 | 0.3689 |
| TC0300013966.hg.1 | PRSS45 | protease, serine 45 | Coding | 5.39 | 4.88 | 5.31 | 1.06 | 0.5412 | 0.7286 | 5.22 | 5.25 | 5.54 | 0.80 | 0.9185 | 0.9666 |
| TC0400006552.hg.1 | C4orf48 | chromosome 4 open reading frame 48 | Multiple_C | 4.5 | 4.47 | 4.42 | 1.06 | 0.985 | 0.9928 | 5.36 | 5.31 | 5.12 | 1.18 | 0.9756 | 0.9902 |
| TC0400006990.hg.1 | MED28 | mediator complex subunit 28 | Multiple_C | 10.76 | 11.49 | 10.68 | 1.06 | 0.7478 | 0.8706 | 10.49 | 10.47 | 10.33 | 1.12 | 0.1771 | 0.4259 |
| TC0400011012.hg.1 | PF4 | platelet factor 4 | Coding | 5.21 | 4.79 | 5.13 | 1.06 | 0.3278 | 0.5417 | 4.79 | 5.10 | 5.09 | 0.81 | 0.2301 | 0.4913 |
| TC0400011054.hg.1 | CXCL11 | chemokine (C-X-C motif) ligand 11 | Coding | 3.6 | 3.99 | 3.52 | 1.06 | 0.698 | 0.8381 | 3.57 | 3.83 | 3.51 | 1.04 | 0.6778 | 0.8449 |
| TC0400011409.hg.1 | ADH1C | alcohol dehydrogenase 1C (class I), gamma polypeptide | Multiple_C | 3.46 | 3.12 | 3.38 | 1.06 | 0.5384 | 0.7266 | 3.77 | 3.68 | 3.83 | 0.96 | 0.3143 | 0.5782 |
| TC0400012796.hg.1 | PDGFRA | platelet-derived growth factor receptor, alpha polypeptide | Multiple_C | 4.2 | 3.97 | 4.12 | 1.06 | 0.8767 | 0.9401 | 4.29 | 4.30 | 4.21 | 1.06 | 0.4865 | 0.7236 |
| TC0500010308.hg.1 | CDH10 | cadherin 10, type 2 (T2-cadherin) | Multiple_C | 3.51 | 3.5 | 3.43 | 1.06 | 0.5793 | 0.757 | 2.99 | 2.95 | 3.03 | 0.97 | 0.6392 | 0.8231 |
| TC0500012947.hg.1 | GPRIN1 | G protein regulated inducer of neurite outgrowth 1 | Multiple_C | 5.98 | 5.58 | 5.9 | 1.06 | 0.8427 | 0.9225 | 5.69 | 6.51 | 6.56 | 0.55 | 4.66E-05 | 0.0021 |
| TC0500013234.hg.1 | FAM53C | family with sequence similarity 53, member C | Multiple_C | 3.59 | 3.22 | 3.51 | 1.06 | 0.8253 | 0.9122 | 3.52 | 3.65 | 3.53 | 0.99 | 0.2354 | 0.4981 |
| TC0600007820.hg.1 | CLPSL1 | colipase-like 1 | Coding | 3.22 | 3.06 | 3.14 | 1.06 | 0.9472 | 0.9758 | 2.87 | 2.98 | 3.02 | 0.90 | 0.7626 | 0.8928 |
| TC0600012257.hg.1 | B3GAT2 | beta-1,3-glucuronyltransferase 2 | Coding | 3.5 | 3.51 | 3.42 | 1.06 | 0.7552 | 0.8745 | 3.85 | 3.86 | 4.14 | 0.82 | 0.3596 | 0.6224 |
| TC0700007585.hg.1 | VSTM2A; VSTI | V-set and transmembrane domain containing 2A; VSTM2A over | Multiple_C | 4.56 | 4.08 | 4.48 | 1.06 | 0.8432 | 0.9227 | 4.41 | 4.73 | 4.75 | 0.79 | 0.1764 | 0.425 |
| TC0700009132.hg.1 | CPA2 | carboxypeptidase A2 (pancreatic) | Multiple_C | 2.96 | 2.47 | 2.88 | 1.06 | 0.9541 | 0.9785 | 3.83 | 3.68 | 3.61 | 1.16 | 0.2359 | 0.4985 |
| TC0700009140.hg.1 | CPA1 | carboxypeptidase A1 (pancreatic) | Multiple_C | 4.47 | 3.94 | 4.39 | 1.06 | 0.9284 | 0.9663 | 3.7 | 3.63 | 3.77 | 0.95 | 0.6765 | 0.8442 |
| TC0700010198.hg.1 | FAM220A | family with sequence similarity 220, member A | Multiple_C | 6.46 | 6.63 | 6.38 | 1.06 | 0.4318 | 0.642 | 6.63 | 6.86 | 6.89 | 0.84 | 0.6788 | 0.8453 |
| TC0700010751.hg.1 | AOAH | acyloxyacyl hydrolase (neutrophil) | Multiple_C | 3.4 | 3.39 | 3.32 | 1.06 | 0.7552 | 0.8745 | 3.25 | 3.57 | 3.69 | 0.74 | 0.0992 | 0.3077 |
| TC0700011955.hg.1 | CYP3A4 | cytochrome P450, family 3, subfamily A, polypeptide 4 | Multiple_C | 3.11 | 3.01 | 3.03 | 1.06 | 0.5516 | 0.7361 | 4.31 | 3.61 | 3.32 | 1.99 | 0.0036 | 0.0399 |
| TC0700012038.hg.1 | MOGAT3 | monoacylglycerol O-acyltransferase 3 | Multiple_C | 3.6 | 3.35 | 3.52 | 1.06 | 0.6048 | 0.7764 | 4.49 | 4.73 | 4.44 | 1.04 | 0.6803 | 0.8462 |
| TC0700013366.hg.1 | ZNF735 | zinc finger protein 735 | Coding | 5.13 | 4.89 | 5.05 | 1.06 | 0.5929 | 0.7675 | 4.69 | 4.65 | 4.98 | 0.82 | 0.2735 | 0.5389 |
| TC0700013423.hg.1 | ZNF655 | zinc finger protein 655 | Multiple_C | 4.03 | 3.9 | 3.95 | 1.06 | 0.9711 | 0.9861 | 4.15 | 4.11 | 3.86 | 1.22 | 0.0934 | 0.2968 |
| TC0800007995.hg.1 | CRISPLD1 | cysteine-rich secretory protein LCCL domain containing 1 | Multiple_C | 3.83 | 3.55 | 3.75 | 1.06 | 0.5784 | 0.7565 | 4.34 | 4.15 | 4.01 | 1.26 | 0.4252 | 0.6779 |
| TC0800010084.hg.1 | DUSP26 | dual specificity phosphatase 26 (putative) | Coding | 4.52 | 4.11 | 4.44 | 1.06 | 0.904 | 0.9538 | 4.56 | 4.74 | 4.59 | 0.98 | 0.995 | 0.9976 |
| TC0800011006.hg.1 | REXO1L2P; RE | Homo sapiens REX1, RNA exonuclease 1 homolog-like 2, pseudo | Multiple_C | 3.73 | 3.46 | 3.65 | 1.06 | 0.5575 | 0.7403 | 5.18 | 5.09 | 4.81 | 1.29 | 0.0905 | 0.2913 |
| TC0800011075.hg.1 | TMEM55A | transmembrane protein 55A | Multiple_C | 7.52 | 8.09 | 7.44 | 1.06 | 0.9744 | 0.9874 | 5.99 | 5.39 | 5.83 | 1.12 | 0.7433 | 0.8825 |
| TC0800011951.hg.1 | FAM135B | family with sequence similarity 135, member B | Multiple_C | 3.33 | 3.44 | 3.25 | 1.06 | 0.5288 | 0.7199 | 4.12 | 4.30 | 3.6 | 1.43 | 0.027 | 0.1437 |

| | | | | | | | | | | | | | | | |
|---|---|---|---|---|---|---|---|---|---|---|---|---|---|---|---|
| TC0800012086.hg.1 | LYPD2 | LY6/PLAUR domain containing 2 | Coding | 4.2 | 3.91 | 4.12 | 1.06 | 0.7151 | 0.8495 | 4.65 | 4.57 | 4.43 | 1.16 | 0.2562 | 0.5211 |
| TC0900007165.hg.1 | TRMT10B | tRNA methyltransferase 10B | Multiple_C | 6.63 | 6.69 | 6.55 | 1.06 | 0.2843 | 0.4945 | 6.07 | 6.61 | 6.73 | 0.63 | 0.0035 | 0.0391 |
| TC0900009155.hg.1 | C9orf62 | chromosome 9 open reading frame 62 | Multiple_C | 4.27 | 4.24 | 4.19 | 1.06 | 0.5379 | 0.7261 | 3.45 | 3.52 | 3.64 | 0.88 | 0.9549 | 0.9814 |
| TC0900011246.hg.1 | ZNF883 | zinc finger protein 883 | Multiple_C | 3.88 | 3.94 | 3.8 | 1.06 | 0.7786 | 0.8862 | 4.94 | 4.31 | 4.9 | 1.03 | 0.7384 | 0.8794 |
| TC0900011382.hg.1 | FBXW2 | F-box and WD repeat domain containing 2 | Multiple_C | 12.6 | 12.32 | 12.52 | 1.06 | 0.9466 | 0.9754 | 12.05 | 11.89 | 12.46 | 0.75 | 0.1337 | 0.3631 |
| TC0900012272.hg.1 | RC3H2 | ring finger and CCCH-type domains 2 | Multiple_C | 10.8 | 10.54 | 10.72 | 1.06 | 0.5353 | 0.7243 | 11.08 | 10.23 | 10.79 | 1.22 | 0.3592 | 0.622 |
| TC0X00007053.hg.1 | MAOA | monoamine oxidase A | Multiple_C | 7.71 | 8.19 | 7.63 | 1.06 | 0.3638 | 0.5774 | 5.22 | 4.31 | 4.48 | 1.67 | 0.0009 | 0.0152 |
| TC0X00007767.hg.1 | POU3F4 | POU class 3 homeobox 4 | Multiple_C | 4.37 | 4.23 | 4.29 | 1.06 | 0.1928 | 0.3818 | 4.62 | 4.98 | 4.66 | 0.97 | 0.9533 | 0.981 |
| TC0X00008468.hg.1 | CCDC160 | coiled-coil domain containing 160 | Multiple_C | 3.96 | 4.01 | 3.88 | 1.06 | 0.1311 | 0.2931 | 3.57 | 3.39 | 3.41 | 1.12 | 0.7256 | 0.8724 |
| TC0X00009018.hg.1 | PNPLA4 | patatin-like phospholipase domain containing 4 | Coding | 6.28 | 6.52 | 6.2 | 1.06 | 0.7904 | 0.8929 | 6.42 | 7.24 | 6.98 | 0.68 | 0.1054 | 0.3186 |
| TC0X00009707.hg.1 | MAGED4; MA | melanoma antigen family D4; melanoma antigen family D4B; sn | Multiple_C | 4.26 | 4.24 | 4.18 | 1.06 | 0.7808 | 0.8871 | 3.75 | 3.75 | 3.58 | 1.13 | 0.2429 | 0.5066 |
| TC0X00010460.hg.1 | RIPPLY1 | ripply transcriptional repressor 1 | Coding | 3.06 | 3.14 | 2.98 | 1.06 | 0.5878 | 0.7641 | 3.55 | 3.55 | 3.5 | 1.04 | 0.6169 | 0.8098 |
| TC0X00010847.hg.1 | TFDP3 | transcription factor Dp family, member 3 | Coding | 4.9 | 4.61 | 4.82 | 1.06 | 0.6671 | 0.818 | 4.34 | 4.49 | 4.22 | 1.09 | 0.5582 | 0.7714 |
| TC1000012489.hg.1 | HOGA1 | 4-hydroxy-2-oxoglutarate aldolase 1 | Multiple_C | 5.07 | 5.07 | 4.99 | 1.06 | 0.7559 | 0.8745 | 4.64 | 4.63 | 4.73 | 0.94 | 0.7713 | 0.8972 |
| TC1100007386.hg.1 | ACCSL | 1-aminocyclopropane-1-carboxylate synthase (inactive)-like | Multiple_C | 2.92 | 2.88 | 2.84 | 1.06 | 0.4909 | 0.6908 | 3.3 | 3.17 | 3.32 | 0.99 | 0.2073 | 0.4641 |
| TC1100007585.hg.1 | OR4A15 | olfactory receptor, family 4, subfamily A, member 15 | Coding | 3.34 | 3 | 3.26 | 1.06 | 0.7629 | 0.8782 | 3.73 | 3.94 | 3.86 | 0.91 | 0.4901 | 0.7257 |
| TC1100008004.hg.1 | SPDYC | speedy/RINGO cell cycle regulator family member C | Multiple_C | 3.66 | 3.51 | 3.58 | 1.06 | 0.2948 | 0.5062 | 4.22 | 4.51 | 3.99 | 1.17 | 0.4085 | 0.664 |
| TC1100008101.hg.1 | RCE1; C11orf8 | Ras converting CAAX endopeptidase 1; chromosome 11 open re | Multiple_C | 6.11 | 5.9 | 6.03 | 1.06 | 0.7207 | 0.8529 | 6.12 | 6.01 | 6.28 | 0.90 | 0.1101 | 0.3268 |
| TC1100009287.hg.1 | ARHGEF12 | Rho guanine nucleotide exchange factor (GEF) 12 | Multiple_C | 11.45 | 11.49 | 11.37 | 1.06 | 0.5473 | 0.7328 | 13.48 | 13.30 | 12.9 | 1.49 | 0.0907 | 0.2914 |
| TC1100009426.hg.1 | PATE1 | prostate and testis expressed 1 | Multiple_C | 4.99 | 4.95 | 4.91 | 1.06 | 0.7968 | 0.8957 | 4.59 | 4.82 | 5.04 | 0.73 | 0.0826 | 0.2768 |
| TC1100009495.hg.1 | KCNJ5 | potassium channel, inwardly rectifying subfamily J, member 5 | Multiple_C | 4.03 | 3.53 | 3.95 | 1.06 | 0.3924 | 0.6062 | 3.69 | 3.40 | 3.51 | 1.13 | 0.3786 | 0.6383 |
| TC1100010157.hg.1 | PTH | parathyroid hormone | Coding | 4.61 | 4.54 | 4.53 | 1.06 | 0.7643 | 0.8788 | 4.56 | 4.59 | 4.11 | 1.37 | 0.2377 | 0.5012 |
| TC1100010946.hg.1 | LPXN | leupaxin | Multiple_C | 6.26 | 6.14 | 6.18 | 1.06 | 0.6942 | 0.8354 | 5.77 | 5.95 | 6.74 | 0.51 | 0.0167 | 0.1079 |
| TC1100012100.hg.1 | TRPC6 | transient receptor potential cation channel, subfamily C, memb | Multiple_C | 3.68 | 3.79 | 3.6 | 1.06 | 0.5688 | 0.7492 | 3.58 | 3.54 | 3.94 | 0.78 | 0.2423 | 0.506 |
| TC1200006782.hg.1 | CLEC9A | C-type lectin domain family 9, member A | Multiple_C | 4.44 | 4.1 | 4.36 | 1.06 | 0.093 | 0.2286 | 4.38 | 4.40 | 4.23 | 1.11 | 0.7906 | 0.9065 |
| TC1200007792.hg.1 | OR6C65 | olfactory receptor, family 6, subfamily C, member 65 | Coding | 3.56 | 3.39 | 3.48 | 1.06 | 0.3374 | 0.5512 | 3.92 | 3.97 | 3.86 | 1.04 | 0.6662 | 0.8365 |
| TC1200008184.hg.1 | TBC1D15 | TBC1 domain family, member 15 | Multiple_C | 10.9 | 12.33 | 10.82 | 1.06 | 0.8409 | 0.9214 | 8.55 | 7.99 | 8.32 | 1.17 | 0.7112 | 0.8645 |
| TC1200008275.hg.1 | SYT1 | synaptotagmin I | Multiple_C | 6.46 | 6.98 | 6.38 | 1.06 | 0.3325 | 0.5464 | 13.52 | 13.34 | 13.94 | 0.75 | 0.0608 | 0.234 |
| TC1200008860.hg.1 | CUX2 | cut-like homeobox 2 | Multiple_C | 3.8 | 3.42 | 3.72 | 1.06 | 0.8542 | 0.9288 | 3.91 | 3.44 | 3.68 | 1.17 | 0.3966 | 0.6544 |
| TC1200009096.hg.1 | CCDC60 | coiled-coil domain containing 60 | Multiple_C | 3.27 | 2.95 | 3.19 | 1.06 | 0.2818 | 0.4918 | 3.54 | 3.41 | 3.44 | 1.07 | 0.5441 | 0.7624 |
| TC1200011301.hg.1 | KRR1 | KRR1, small subunit (SSU) processome component, homolog (ye | Multiple_C | 12.67 | 13.2 | 12.59 | 1.06 | 0.9079 | 0.9557 | 11.99 | 11.48 | 11.76 | 1.17 | 0.255 | 0.5195 |
| TC1200011996.hg.1 | LHX5 | LIM homeobox 5 | Multiple_C | 2.89 | 2.63 | 2.81 | 1.06 | 0.7628 | 0.8782 | 3.34 | 3.32 | 3.09 | 1.19 | 0.4477 | 0.6948 |
| TC1200012641.hg.1 | RDH5 | retinol dehydrogenase 5 (11-cis/9-cis) | Multiple_C | 5.2 | 4.54 | 5.12 | 1.06 | 0.7564 | 0.8746 | 5.21 | 5.52 | 5.74 | 0.69 | 0.0476 | 0.2025 |

| | | | | | | | | | | | | | | | |
|---|---|---|---|---|---|---|---|---|---|---|---|---|---|---|---|
| TC1200012643.hg.1 | ERBB3 | erb-b2 receptor tyrosine kinase 3 | Multiple_C | 12.13 | 12.28 | 12.05 | 1.06 | 0.7541 | 0.8741 | 11.49 | 12.57 | 11.16 | 1.26 | 0.4495 | 0.6959 |
| TC1300006699.hg.1 | ATP5EP2 | ATP synthase, H+ transporting, mitochondrial F1 complex, epsilo | Multiple_C | 11.78 | 12.77 | 11.7 | 1.06 | 0.3434 | 0.5571 | 8 | 7.98 | 7.77 | 1.17 | 0.8802 | 0.9486 |
| TC1300006994.hg.1 | TNFSF11 | tumor necrosis factor (ligand) superfamily, member 11 | Multiple_C | 3.59 | 3.47 | 3.51 | 1.06 | 0.4097 | 0.622 | 3.5 | 4.00 | 4.07 | 0.67 | 0.014 | 0.0959 |
| TC1300009922.hg.1 | LINC00552 | long intergenic non-protein coding RNA 552 | Multiple_C | 4.36 | 3.97 | 4.28 | 1.06 | 0.4538 | 0.6613 | 5.28 | 5.47 | 5.34 | 0.96 | 0.4362 | 0.6868 |
| TC1300010031.hg.1 | CCDC169-SOH | CCDC169-SOHLH2 readthrough | Coding | 3.08 | 3 | 3 | 1.06 | 0.6468 | 0.8043 | 3.69 | 3.63 | 3.72 | 0.98 | 0.4346 | 0.6853 |
| TC1300010033.hg.1 | CCDC169 | coiled-coil domain containing 169 | Multiple_C | 3.66 | 3.96 | 3.58 | 1.06 | 0.3366 | 0.5504 | 3.84 | 3.85 | 3.76 | 1.06 | 0.7778 | 0.9005 |
| TC1400006501.hg.1 | OR4K17 | olfactory receptor, family 4, subfamily K, member 17 | Coding | 2.78 | 2.61 | 2.7 | 1.06 | 0.7752 | 0.8843 | 3.26 | 3.09 | 3.24 | 1.01 | 0.4246 | 0.6774 |
| TC1400006718.hg.1 | PSME1 | proteasome activator subunit 1 | Multiple_C | 11.9 | 11.23 | 11.82 | 1.06 | 0.4287 | 0.6391 | 7.79 | 7.67 | 7.35 | 1.36 | 0.0423 | 0.1899 |
| TC1400007555.hg.1 | PLEKHD1 | pleckstrin homology domain containing, family D (with coiled-cc | Multiple_C | 3.53 | 3.34 | 3.45 | 1.06 | 0.7933 | 0.894 | 3.68 | 3.50 | 3.43 | 1.19 | 0.0339 | 0.1658 |
| TC1400008599.hg.1 | OR6S1 | olfactory receptor, family 6, subfamily S, member 1 | Coding | 2.96 | 2.84 | 2.88 | 1.06 | 0.8086 | 0.9033 | 3.39 | 3.17 | 3.33 | 1.04 | 0.2655 | 0.5301 |
| TC1400009402.hg.1 | ESR2 | estrogen receptor 2 (ER beta) | Multiple_C | 3.05 | 2.79 | 2.97 | 1.06 | 0.6989 | 0.8388 | 3.07 | 2.97 | 3.17 | 0.93 | 0.9536 | 0.981 |
| TC1500007583.hg.1 | SLC51B | solute carrier family 51, beta subunit | Coding | 3.08 | 3.26 | 3 | 1.06 | 0.6766 | 0.8242 | 4 | 3.88 | 3.5 | 1.41 | 0.0129 | 0.0918 |
| TC1500009214.hg.1 | TGM5 | transglutaminase 5 | Multiple_C | 3.81 | 3.63 | 3.73 | 1.06 | 0.4545 | 0.662 | 4.05 | 4.34 | 4.37 | 0.80 | 0.4168 | 0.6706 |
| TC1500009921.hg.1 | UACA | uveal autoantigen with coiled-coil domains and ankyrin repeats | Multiple_C | 11.88 | 12.95 | 11.8 | 1.06 | 0.882 | 0.9427 | 10.16 | 8.94 | 9.29 | 1.83 | 0.2956 | 0.5604 |
| TC1500010738.hg.1 | CTXN2 | cortexin 2 | Coding | 3.08 | 2.94 | 3 | 1.06 | 0.8891 | 0.9466 | 3.64 | 3.44 | 3.38 | 1.20 | 0.1172 | 0.3385 |
| TC1500010836.hg.1 | GOLGA6L22 | golgin A6 family-like 22 | Coding | 3 | 3 | 2.92 | 1.06 | 0.4709 | 0.6748 | 3.36 | 3.27 | 3.3 | 1.04 | 0.4941 | 0.7282 |
| TC1500010886.hg.1 | CALML4 | calmodulin-like 4 | Multiple_C | 11.89 | 13.44 | 11.81 | 1.06 | 0.6315 | 0.7943 | 8.57 | 8.50 | 8.57 | 1.00 | 0.9377 | 0.9741 |
| TC1500010901.hg.1 | ST20 | suppressor of tumorigenicity 20 | Multiple_C | 10.3 | 9.88 | 10.22 | 1.06 | 0.4172 | 0.629 | 7.52 | 7.77 | 7.96 | 0.74 | 0.0391 | 0.1803 |
| TC1600006686.hg.1 | OR2C1 | olfactory receptor, family 2, subfamily C, member 1 | Coding | 3.4 | 3.48 | 3.32 | 1.06 | 0.7679 | 0.8805 | 3.62 | 3.44 | 3.38 | 1.18 | 0.2241 | 0.4851 |
| TC1600007424.hg.1 | ZG16 | zymogen granule protein 16 | Multiple_C | 3.84 | 3.35 | 3.76 | 1.06 | 0.7432 | 0.8672 | 3.2 | 3.44 | 3.47 | 0.83 | 0.2659 | 0.5305 |
| TC1600010532.hg.1 | CDH8 | cadherin 8, type 2 | Multiple_C | 3.6 | 3.21 | 3.52 | 1.06 | 0.8261 | 0.9127 | 3.94 | 4.03 | 4.21 | 0.83 | 0.5267 | 0.7511 |
| TC1600011238.hg.1 | CBFA2T3 | core-binding factor, runt domain, alpha subunit 2; translocated | Multiple_C | 5.36 | 5.14 | 5.28 | 1.06 | 0.6383 | 0.7993 | 4.06 | 4.10 | 4 | 1.04 | 0.2293 | 0.4905 |
| TC1700006432.hg.1 | SCGB1C2 | secretoglobin, family 1C, member 2 | Coding | 8.19 | 7.96 | 8.11 | 1.06 | 0.1728 | 0.3544 | 7.57 | 7.79 | 7.96 | 0.76 | 0.0295 | 0.1522 |
| TC1700006523.hg.1 | SERPINF2 | serpin peptidase inhibitor, clade F (alpha-2 antiplasmin, pigmen | Multiple_C | 5.57 | 5.29 | 5.49 | 1.06 | 0.8952 | 0.9499 | 4.9 | 5.16 | 5.18 | 0.82 | 0.1587 | 0.4002 |
| TC1700007454.hg.1 | GOSR1 | golgi SNAP receptor complex member 1 | Multiple_C | 12.92 | 12.46 | 12.84 | 1.06 | 0.7701 | 0.8811 | 13.37 | 13.73 | 13.58 | 0.86 | 0.6752 | 0.8431 |
| TC1700008313.hg.1 | KIF2B | kinesin family member 2B | Coding | 3.44 | 3.39 | 3.36 | 1.06 | 0.9515 | 0.9777 | 4.05 | 4.34 | 4.13 | 0.95 | 0.5488 | 0.7655 |
| TC1700010674.hg.1 | KRT36 | keratin 36, type I | Coding | 3.86 | 3.48 | 3.78 | 1.06 | 0.6694 | 0.8195 | 3.63 | 3.61 | 3.55 | 1.06 | 0.9337 | 0.972 |
| TC1700012263.hg.1 | DHX8 | DEAH (Asp-Glu-Ala-His) box polypeptide 8 | Multiple_C | 12.78 | 12.34 | 12.7 | 1.06 | 0.663 | 0.8152 | 12.15 | 11.91 | 12.24 | 0.94 | 0.5409 | 0.7601 |
| TC1700012449.hg.1 | CSHL1 | chorionic somatomammotropin hormone-like 1 | Multiple_C | 4.83 | 4.43 | 4.75 | 1.06 | 0.6275 | 0.792 | 5.21 | 5.07 | 5.08 | 1.09 | 0.8928 | 0.9543 |
| TC1700012471.hg.1 | MIR4738; H3F | microRNA 4738; H3 histone, family 3B (H3.3B) | Multiple_C | 3.15 | 2.95 | 3.07 | 1.06 | 0.3735 | 0.586 | 3.95 | 4.15 | 3.97 | 0.99 | 0.5428 | 0.7614 |
| TC1900006493.hg.1 | ELANE | elastase, neutrophil expressed | Coding | 3.43 | 3.35 | 3.35 | 1.06 | 0.7658 | 0.8797 | 3.62 | 3.73 | 3.68 | 0.96 | 0.2162 | 0.4753 |
| TC1900007184.hg.1 | CLEC17A | C-type lectin domain family 17, member A | Multiple_C | 5.28 | 5.01 | 5.2 | 1.06 | 0.9073 | 0.9556 | 5.34 | 5.16 | 5.27 | 1.05 | 0.2962 | 0.561 |
| TC1900007233.hg.1 | CYP4F22 | cytochrome P450, family 4, subfamily F, polypeptide 22 | Coding | 2.86 | 2.87 | 2.78 | 1.06 | 0.5461 | 0.7317 | 3.32 | 3.01 | 2.99 | 1.26 | 0.0847 | 0.2801 |

| | | | | | | | | | | | | | | | |
|---|---|---|---|---|---|---|---|---|---|---|---|---|---|---|---|
| TC1900007405.hg.1 | CRTC1 | CREB regulated transcription coactivator 1 | Multiple_C | 7.49 | 7.16 | 7.41 | 1.06 | 0.8942 | 0.9495 | 8.14 | 8.33 | 8.13 | 1.01 | 0.9887 | 0.9952 |
| TC1900008062.hg.1 | SELV | selenoprotein V; selenoprotein V [Source:EntrezGene;Acc:3483 | Multiple_C | 3.14 | 2.83 | 3.06 | 1.06 | 0.4473 | 0.6554 | 3.21 | 3.35 | 3.52 | 0.81 | 0.9855 | 0.994 |
| TC1900008072.hg.1 | LGALS13 | lectin, galactoside-binding, soluble, 13 | Multiple_C | 2.79 | 2.69 | 2.71 | 1.06 | 0.3317 | 0.5457 | 3.06 | 3.08 | 2.99 | 1.05 | 0.97 | 0.9877 |
| TC1900008537.hg.1 | GFY | golgi-associated, olfactory signaling regulator | Coding | 3.14 | 3.08 | 3.06 | 1.06 | 0.8805 | 0.9419 | 3.88 | 3.95 | 3.61 | 1.21 | 0.4078 | 0.6637 |
| TC1900008879.hg.1 | KIR2DS5; KIR3 | killer cell immunoglobulin-like receptor, two domains, short cyt | Multiple_C | 3.18 | 2.9 | 3.1 | 1.06 | 0.3343 | 0.5481 | 4.05 | 3.74 | 3.64 | 1.33 | 0.1617 | 0.4048 |
| TC1900008963.hg.1 | ZNF471 | zinc finger protein 471 | Multiple_C | 3.4 | 3.43 | 3.32 | 1.06 | 0.8746 | 0.9391 | 3.78 | 3.85 | 3.9 | 0.92 | 0.5472 | 0.7643 |
| TC1900010676.hg.1 | FCGBP | Fc fragment of IgG binding protein | Multiple_C | 3.86 | 3.74 | 3.78 | 1.06 | 0.403 | 0.6158 | 3.73 | 3.54 | 3.78 | 0.97 | 0.3417 | 0.6053 |
| TC1900011440.hg.1 | NLRP7 | NLR family, pyrin domain containing 7 | Coding | 2.81 | 2.68 | 2.73 | 1.06 | 0.6207 | 0.7876 | 3.58 | 3.01 | 3.2 | 1.30 | 0.0507 | 0.2098 |
| TC1900011482.hg.1 | SBK3 | SH3 domain binding kinase family, member 3 | Multiple_C | 4.91 | 4.71 | 4.83 | 1.06 | 0.6143 | 0.7838 | 4.13 | 4.19 | 4.28 | 0.90 | 0.8782 | 0.9479 |
| TC1900011967.hg.1 | PSG9 | pregnancy specific beta-1-glycoprotein 9 | Coding | 3.92 | 3.25 | 3.84 | 1.06 | 0.3411 | 0.5549 | 4.05 | 3.44 | 3.71 | 1.27 | 0.1001 | 0.3094 |
| TC2000006956.hg.1 | NXT1 | nuclear transport factor 2-like export factor 1 | Multiple_C | 7.51 | 7.01 | 7.43 | 1.06 | 0.8246 | 0.9117 | 6.73 | 7.01 | 6.94 | 0.86 | 0.3244 | 0.5884 |
| TC2000007466.hg.1 | KCNK15 | potassium channel, two pore domain subfamily K, member 15 | Multiple_C | 4.07 | 3.82 | 3.99 | 1.06 | 0.8875 | 0.9456 | 3.64 | 3.59 | 3.57 | 1.05 | 0.3052 | 0.5691 |
| TC2000008682.hg.1 | CST1 | cystatin SN | Coding | 2.53 | 2.53 | 2.45 | 1.06 | 0.7681 | 0.8806 | 3.32 | 3.24 | 3.03 | 1.22 | 0.025 | 0.1373 |
| TC2000009026.hg.1 | RBL1 | retinoblastoma-like 1 | Multiple_C | 10.75 | 9.43 | 10.67 | 1.06 | 0.9855 | 0.993 | 10.4 | 10.51 | 10.8 | 0.76 | 0.1267 | 0.3522 |
| TC2000009250.hg.1 | PLTP | phospholipid transfer protein | Multiple_C | 4.86 | 4.8 | 4.78 | 1.06 | 0.6575 | 0.812 | 8.36 | 7.26 | 6.91 | 2.73 | 1.22E-05 | 0.0008 |
| TC2100007204.hg.1 | FAM3B | family with sequence similarity 3, member B | Multiple_C | 4.24 | 4.48 | 4.16 | 1.06 | 0.9581 | 0.9803 | 6.25 | 6.34 | 5.53 | 1.65 | 0.0084 | 0.0693 |
| TC2100007916.hg.1 | KRTAP7-1 | keratin associated protein 7-1 (gene/pseudogene) | Multiple_C | 4.59 | 4.47 | 4.51 | 1.06 | 0.7348 | 0.8613 | 4.56 | 4.58 | 4.34 | 1.16 | 0.3674 | 0.6296 |
| TC2200006894.hg.1 | UPB1 | ureidopropionase, beta | Multiple_C | 2.8 | 2.68 | 2.72 | 1.06 | 0.9241 | 0.9644 | 3.07 | 2.87 | 2.94 | 1.09 | 0.4356 | 0.6863 |
| TC2200007327.hg.1 | POLR2F; MIR4 | polymerase (RNA) II (DNA directed) polypeptide F; microRNA 45 | Multiple_C | 11.73 | 11.59 | 11.65 | 1.06 | 0.8771 | 0.9403 | 11.29 | 11.67 | 11.24 | 1.04 | 0.9482 | 0.979 |
| TC2200008613.hg.1 | CACNG2 | calcium channel, voltage-dependent, gamma subunit 2 | Multiple_C | 2.75 | 2.71 | 2.67 | 1.06 | 0.6923 | 0.8342 | 2.87 | 2.87 | 2.95 | 0.95 | 0.4092 | 0.6646 |
| TC2200008759.hg.1 | ENTHD1 | ENTH domain containing 1 | Multiple_C | 3.12 | 3.4 | 3.04 | 1.06 | 0.6407 | 0.8008 | 3.62 | 3.30 | 3.35 | 1.21 | 0.3002 | 0.5646 |
| TSUnmapped00000240.h | TMEM42 | transmembrane protein 42 | Coding | 6.48 | 8.52 | 6.4 | 1.06 | 0.6656 | 0.8172 | 5.42 | 5.35 | 5.22 | 1.15 | 0.295 | 0.5599 |
| TSUnmapped00000571.h | GREM1 | gremlin 1, DAN family BMP antagonist [Source:HGNC Symbol;A | Coding | 4.03 | 3.46 | 3.95 | 1.06 | 0.4521 | 0.6597 | 5.02 | 4.83 | 4.87 | 1.11 | 0.2191 | 0.479 |
| TSUnmapped00000732.h | SLC37A4 | solute carrier family 37 (glucose-6-phosphate transporter), men | Coding | 8.89 | 9.01 | 8.81 | 1.06 | 0.9436 | 0.974 | 8.84 | 9.71 | 9.46 | 0.65 | 0.0073 | 0.0631 |
| TSUnmapped00000754.h | FMN1 | formin 1 | Coding | 4.95 | 4.5 | 4.87 | 1.06 | 0.4671 | 0.6716 | 6.44 | 5.62 | 6.12 | 1.25 | 0.477 | 0.716 |
| TSUnmapped00000821.h | DUX5 | double homeobox 5 | Coding | 3.25 | 3.4 | 3.17 | 1.06 | 0.573 | 0.7522 | 3.46 | 3.70 | 3.64 | 0.88 | 0.8524 | 0.9361 |
| TC0100007020.hg.1 | CLCNKB | chloride channel, voltage-sensitive Kb | Coding | 3.42 | 3.31 | 3.35 | 1.05 | 0.3639 | 0.5775 | 3.96 | 3.47 | 3.51 | 1.37 | 0.2995 | 0.5639 |
| TC0100007670.hg.1 | IQCC | IQ motif containing C | Multiple_C | 6.69 | 5.87 | 6.62 | 1.05 | 0.6106 | 0.7808 | 6.76 | 6.86 | 6.94 | 0.88 | 0.6246 | 0.8146 |
| TC0100008216.hg.1 | SLC5A9 | solute carrier family 5 (sodium/sugar cotransporter), member 9 | Multiple_C | 3.16 | 3.04 | 3.09 | 1.05 | 0.9236 | 0.9644 | 3.22 | 3.34 | 3.31 | 0.94 | 0.4804 | 0.7189 |
| TC0100010047.hg.1 | NPR1 | natriuretic peptide receptor 1 | Multiple_C | 4.18 | 3.83 | 4.11 | 1.05 | 0.8451 | 0.9238 | 5.02 | 4.56 | 4.7 | 1.25 | 0.5667 | 0.7761 |
| TC0100010230.hg.1 | CD1C | CD1c molecule | Multiple_C | 4.06 | 3.87 | 3.99 | 1.05 | 0.4187 | 0.6301 | 3.82 | 3.99 | 4.2 | 0.77 | 0.5121 | 0.7417 |
| TC0100010565.hg.1 | GORAB | golgin, RAB6-interacting | Multiple_C | 7.82 | 8.71 | 7.75 | 1.05 | 0.9726 | 0.9867 | 6.87 | 6.74 | 6.71 | 1.12 | 0.6382 | 0.8228 |
| TC0100010577.hg.1 | MROH9 | maestro heat-like repeat family member 9 | Coding | 2.93 | 2.88 | 2.86 | 1.05 | 0.4492 | 0.657 | 3.37 | 3.31 | 3.31 | 1.04 | 0.976 | 0.9902 |

| | | | | | | | | | | | | | | | |
|---|---|---|---|---|---|---|---|---|---|---|---|---|---|---|---|
| TC0100011263.hg.1 | OPTC | opticin | Coding | 3.77 | 3.3 | 3.7 | 1.05 | 0.7156 | 0.8497 | 3.61 | 3.77 | 3.69 | 0.95 | 0.9385 | 0.9744 |
| TC0100012319.hg.1 | OR2G2 | olfactory receptor, family 2, subfamily G, member 2 | Coding | 3.11 | 3.07 | 3.04 | 1.05 | 0.7984 | 0.8966 | 3.47 | 3.69 | 3.82 | 0.78 | 0.074 | 0.261 |
| TC0100012358.hg.1 | OR2T2 | olfactory receptor, family 2, subfamily T, member 2 | Coding | 5.68 | 5.62 | 5.61 | 1.05 | 0.6093 | 0.7797 | 5.83 | 5.84 | 5.5 | 1.26 | 0.1569 | 0.3977 |
| TC0100013036.hg.1 | RSG1 | REM2 and RAB-like small GTPase 1 | Multiple_C | 4.16 | 4.27 | 4.09 | 1.05 | 0.7559 | 0.8745 | 3.83 | 3.87 | 3.73 | 1.07 | 0.3987 | 0.6559 |
| TC0100016402.hg.1 | MYOC | myocilin, trabecular meshwork inducible glucocorticoid respons | Coding | 3 | 2.98 | 2.93 | 1.05 | 0.875 | 0.9393 | 3.24 | 3.63 | 3.18 | 1.04 | 0.7793 | 0.9011 |
| TC0100017374.hg.1 | RAB3GAP2; AI | RAB3 GTPase activating protein subunit 2 (non-catalytic); aurora | Multiple_C | 9.86 | 9.91 | 9.79 | 1.05 | 0.9755 | 0.988 | 11.38 | 10.95 | 11.04 | 1.27 | 0.2541 | 0.5182 |
| TC0200006569.hg.1 | SOX11 | SRY box 11 | Multiple_C | 3.39 | 3.76 | 3.32 | 1.05 | 0.6953 | 0.8363 | 3.62 | 3.68 | 3.68 | 0.96 | 0.5082 | 0.7386 |
| TC0200006583.hg.1 | RNF144A | ring finger protein 144A | Multiple_C | 4.71 | 4.34 | 4.64 | 1.05 | 0.8086 | 0.9033 | 3.87 | 3.99 | 3.74 | 1.09 | 0.2128 | 0.4706 |
| TC0200006936.hg.1 | KLHL29 | kelch-like family member 29 | Multiple_C | 4.2 | 4.4 | 4.13 | 1.05 | 0.9581 | 0.9803 | 5.18 | 4.61 | 4.83 | 1.27 | 0.0365 | 0.1738 |
| TC0200007775.hg.1 | MDH1 | malate dehydrogenase 1 | Multiple_C | 11.01 | 12.27 | 10.94 | 1.05 | 0.7622 | 0.8778 | 8.47 | 8.81 | 8.38 | 1.06 | 0.0761 | 0.2656 |
| TC0200008728.hg.1 | GPR45 | G protein-coupled receptor 45 | Coding | 3.44 | 3.51 | 3.37 | 1.05 | 0.1516 | 0.3246 | 3.89 | 3.60 | 3.51 | 1.30 | 0.0728 | 0.2586 |
| TC0200011100.hg.1 | ALPI | alkaline phosphatase, intestinal | Multiple_C | 3.28 | 3.37 | 3.21 | 1.05 | 0.5451 | 0.7312 | 3.9 | 3.73 | 3.65 | 1.19 | 0.3818 | 0.641 |
| TC0200011361.hg.1 | DUSP28 | dual specificity phosphatase 28 | Multiple_C | 3.42 | 3.61 | 3.35 | 1.05 | 0.5421 | 0.7291 | 3.56 | 3.66 | 3.27 | 1.22 | 0.2928 | 0.5578 |
| TC0200012459.hg.1 | SRBD1 | S1 RNA binding domain 1 | Multiple_C | 8.61 | 9.06 | 8.54 | 1.05 | 0.9545 | 0.9787 | 8.36 | 8.92 | 8.55 | 0.88 | 0.2378 | 0.5013 |
| TC0200012804.hg.1 | VPS54 | vacuolar protein sorting 54 homolog (S. cerevisiae) | Multiple_C | 7.32 | 9.12 | 7.25 | 1.05 | 0.7581 | 0.8756 | 6.36 | 6.55 | 6.51 | 0.90 | 0.4886 | 0.7247 |
| TC0200013489.hg.1 | TRIM43B | tripartite motif containing 43B | Multiple_C | 3.46 | 3.77 | 3.39 | 1.05 | 0.8955 | 0.9501 | 3.9 | 3.55 | 3.56 | 1.27 | 0.0862 | 0.2831 |
| TC0200014192.hg.1 | SAP130 | Sin3A associated protein 130kDa | Multiple_C | 9.83 | 7.96 | 9.76 | 1.05 | 0.6776 | 0.8247 | 10.83 | 10.76 | 11.44 | 0.66 | 0.0586 | 0.2284 |
| TC0200014468.hg.1 | LRP1B | LDL receptor related protein 1B | Multiple_C | 3.67 | 3.59 | 3.6 | 1.05 | 0.8895 | 0.9467 | 4.3 | 4.24 | 4.11 | 1.14 | 0.4095 | 0.6647 |
| TC0200015401.hg.1 | ORC2 | origin recognition complex subunit 2 | Multiple_C | 10.06 | 10.05 | 9.99 | 1.05 | 0.4642 | 0.6693 | 10.68 | 10.44 | 10.85 | 0.89 | 0.5589 | 0.7714 |
| TC0200016000.hg.1 | GPR55 | G protein-coupled receptor 55 | Multiple_C | 3.38 | 3.26 | 3.31 | 1.05 | 0.7341 | 0.8612 | 4.09 | 4.14 | 3.8 | 1.22 | 0.1652 | 0.4099 |
| TC0200016555.hg.1 | TBR1 | T-box, brain, 1 | Multiple_C | 3.53 | 3.23 | 3.46 | 1.05 | 0.3919 | 0.6057 | 3.57 | 3.42 | 3.35 | 1.16 | 0.1583 | 0.3999 |
| TC0200016684.hg.1 | TIA1 | TIA1 cytotoxic granule-associated RNA binding protein | Multiple_C | 9.33 | 10.36 | 9.26 | 1.05 | 0.6682 | 0.8187 | 9.96 | 9.71 | 9.62 | 1.27 | 0.4929 | 0.7276 |
| TC0300007251.hg.1 | CXCR6 | chemokine (C-X-C motif) receptor 6 | Multiple_C | 3.16 | 3.22 | 3.09 | 1.05 | 0.3343 | 0.5481 | 3.71 | 3.25 | 3.14 | 1.48 | 0.1077 | 0.3227 |
| TC0300008106.hg.1 | OR5H2 | olfactory receptor, family 5, subfamily H, member 2 | Coding | 3.59 | 3.52 | 3.52 | 1.05 | 0.6882 | 0.8318 | 3.3 | 3.38 | 3.22 | 1.06 | 0.8608 | 0.9405 |
| TC0300008804.hg.1 | TRH | thyrotropin-releasing hormone | Coding | 4.81 | 4.74 | 4.74 | 1.05 | 0.2511 | 0.4555 | 4.27 | 4.43 | 4.64 | 0.77 | 0.1156 | 0.3355 |
| TC0300012765.hg.1 | HLTF | Transcript Identified by AceView, Entrez Gene ID(s) 6596 | Coding | 4.45 | 4.19 | 4.38 | 1.05 | 0.6422 | 0.8015 | 5.38 | 5.03 | 5.05 | 1.26 | 0.322 | 0.5858 |
| TC0300013597.hg.1 | CPN2 | carboxypeptidase N, polypeptide 2 | Coding | 3.3 | 3.44 | 3.23 | 1.05 | 0.7256 | 0.8556 | 3.53 | 3.78 | 3.4 | 1.09 | 0.5249 | 0.7497 |
| TC0300013984.hg.1 | HYAL2 | hyaluronoglucosaminidase 2 | Multiple_C | 4.71 | 4.43 | 4.64 | 1.05 | 0.8215 | 0.9104 | 5.11 | 5.13 | 5.09 | 1.01 | 0.8768 | 0.9471 |
| TC0300014049.hg.1 | ACAD11 | acyl-CoA dehydrogenase family, member 11 | Multiple_C | 7.16 | 9.56 | 7.09 | 1.05 | 0.9923 | 0.9961 | 6.78 | 5.75 | 5.8 | 1.97 | 0.0006 | 0.0114 |
| TC0300014086.hg.1 | ATP13A4 | ATPase type 13A4 | Multiple_C | 3.31 | 3.41 | 3.24 | 1.05 | 0.4323 | 0.6423 | 3.42 | 3.46 | 3.51 | 0.94 | 0.5618 | 0.7725 |
| TC0400007839.hg.1 | PF4V1 | platelet factor 4 variant 1 | Coding | 5.18 | 5.08 | 5.11 | 1.05 | 0.9875 | 0.9935 | 5.11 | 4.97 | 4.92 | 1.14 | 0.5289 | 0.7525 |
| TC0400008803.hg.1 | RAB33B | RAB33B, member RAS oncogene family | Multiple_C | 9.84 | 10.4 | 9.77 | 1.05 | 0.7734 | 0.8832 | 7.61 | 7.21 | 7.01 | 1.52 | 0.1812 | 0.4313 |
| TC0400009642.hg.1 | DUX4L8 | double homeobox 4 like 8 | Multiple_C | 4.41 | 4.32 | 4.34 | 1.05 | 0.4808 | 0.6826 | 3.98 | 4.23 | 4.37 | 0.76 | 0.0268 | 0.143 |

| | | | | | | | | | | | | | | | |
|---|---|---|---|---|---|---|---|---|---|---|---|---|---|---|---|
| TC0400009712.hg.1 | DGKQ | diacylglycerol kinase theta | Multiple_C | 5.17 | 5.42 | 5.1 | 1.05 | 0.6938 | 0.8352 | 4.86 | 5.26 | 5.09 | 0.85 | 0.1554 | 0.3956 |
| TC0400010205.hg.1 | KCNIP4 | Kv channel interacting protein 4 | Multiple_C | 3.08 | 2.74 | 3.01 | 1.05 | 0.8315 | 0.9158 | 4.16 | 4.40 | 4.41 | 0.84 | 0.4327 | 0.6839 |
| TC0400010553.hg.1 | BEND4 | BEN domain containing 4 | Multiple_C | 2.99 | 3.13 | 2.92 | 1.05 | 0.6637 | 0.8158 | 3.31 | 3.32 | 3.31 | 1.00 | 0.6564 | 0.8321 |
| TC0400010944.hg.1 | CSN2 | casein beta | Coding | 3.12 | 2.94 | 3.05 | 1.05 | 0.9277 | 0.966 | 3.31 | 3.28 | 3.2 | 1.08 | 0.7227 | 0.8707 |
| TC0500007933.hg.1 | CKMT2 | creatine kinase, mitochondrial 2 (sarcomeric) | Multiple_C | 3.63 | 3.46 | 3.56 | 1.05 | 0.7484 | 0.8708 | 4.26 | 4.38 | 4.07 | 1.14 | 0.1532 | 0.3922 |
| TC0500009250.hg.1 | UBLCP1 | ubiquitin-like domain containing CTD phosphatase 1 | Multiple_C | 12.21 | 11.53 | 12.14 | 1.05 | 0.8253 | 0.9122 | 11.01 | 10.35 | 10.69 | 1.25 | 0.3114 | 0.5756 |
| TC0500010662.hg.1 | HCN1 | hyperpolarization activated cyclic nucleotide gated potassium c | Coding | 3.35 | 2.95 | 3.28 | 1.05 | 0.6511 | 0.8068 | 4.07 | 3.42 | 3.69 | 1.30 | 0.2549 | 0.5194 |
| TC0500011160.hg.1 | ANKRD31 | ankyrin repeat domain 31 | Multiple_C | 4.92 | 4.64 | 4.85 | 1.05 | 0.1852 | 0.3715 | 3.84 | 4.23 | 3.88 | 0.97 | 0.9363 | 0.9735 |
| TC0500011881.hg.1 | CEP120 | centrosomal protein 120kDa | Multiple_C | 8.93 | 8.73 | 8.86 | 1.05 | 0.8232 | 0.9112 | 8.98 | 8.63 | 8.6 | 1.30 | 0.0671 | 0.247 |
| TC0500013309.hg.1 | ESM1 | endothelial cell-specific molecule 1 | Coding | 5.06 | 4.6 | 4.99 | 1.05 | 0.5221 | 0.7152 | 5.06 | 5.13 | 5.55 | 0.71 | 0.0714 | 0.2558 |
| TC0600007313.hg.1 | ABT1 | activator of basal transcription 1 | Multiple_C | 6.21 | 6.39 | 6.14 | 1.05 | 0.4693 | 0.6733 | 6.65 | 7.22 | 7.04 | 0.76 | 0.1075 | 0.3224 |
| TC0600007395.hg.1 | ZNF165 | zinc finger protein 165 | Coding | 6.13 | 7.45 | 6.06 | 1.05 | 0.6649 | 0.8169 | 6.04 | 5.06 | 6.21 | 0.89 | 0.9501 | 0.9795 |
| TC0600007657.hg.1 | HLA-DQA1 | major histocompatibility complex, class II, DQ alpha 1 | Multiple_C | 4.25 | 4.29 | 4.18 | 1.05 | 0.5852 | 0.7617 | 4.47 | 4.28 | 4.43 | 1.03 | 0.2955 | 0.5604 |
| TC0600010609.hg.1 | PXDC1 | PX domain containing 1 | Multiple_C | 2.89 | 2.77 | 2.82 | 1.05 | 0.7926 | 0.8936 | 5.39 | 5.16 | 5.55 | 0.90 | 0.2846 | 0.5496 |
| TC0600010797.hg.1 | MAK | male germ cell-associated kinase | Coding | 4.43 | 4.23 | 4.36 | 1.05 | 0.8607 | 0.9321 | 5.08 | 4.40 | 4.11 | 1.96 | 0.0146 | 0.0984 |
| TC0600011840.hg.1 | PRPH2 | peripherin 2 (retinal degeneration, slow) | Multiple_C | 3.44 | 3.29 | 3.37 | 1.05 | 0.4189 | 0.6301 | 4.37 | 4.24 | 4.16 | 1.16 | 0.1912 | 0.4442 |
| TC0600013204.hg.1 | VNN3 | vanin 3 | Multiple_C | 3.11 | 3.21 | 3.04 | 1.05 | 0.2936 | 0.505 | 3.39 | 3.73 | 3.5 | 0.93 | 0.8151 | 0.9182 |
| TC0600013228.hg.1 | SLC2A12 | solute carrier family 2 (facilitated glucose transporter), member | Multiple_C | 6.28 | 6.75 | 6.21 | 1.05 | 0.8155 | 0.9071 | 6.73 | 6.63 | 5.68 | 2.07 | 0.0002 | 0.0061 |
| TC0700006603.hg.1 | FOXK1 | forkhead box K1 | Multiple_C | 9.42 | 8.78 | 9.35 | 1.05 | 0.7291 | 0.8579 | 9.12 | 9.28 | 9.71 | 0.66 | 0.0951 | 0.2999 |
| TC0700006984.hg.1 | SNX10 | sorting nexin 10 | Multiple_C | 8.84 | 9 | 8.77 | 1.05 | 0.4768 | 0.6794 | 7.77 | 7.40 | 7.64 | 1.09 | 0.4227 | 0.6754 |
| TC0700008000.hg.1 | LAT2 | linker for activation of T-cells family member 2 | Multiple_C | 6.12 | 6.47 | 6.05 | 1.05 | 0.9087 | 0.9562 | 8.31 | 8.14 | 8.29 | 1.01 | 0.6145 | 0.8082 |
| TC0700009130.hg.1 | SSMEM1 | serine-rich single-pass membrane protein 1 | Coding | 3.19 | 2.58 | 3.12 | 1.05 | 0.4666 | 0.6711 | 4.15 | 4.12 | 4.29 | 0.91 | 0.6574 | 0.8327 |
| TC0700009139.hg.1 | CPA5 | carboxypeptidase A5 | Multiple_C | 3.62 | 3.37 | 3.55 | 1.05 | 0.9249 | 0.9648 | 4.17 | 4.24 | 4.06 | 1.08 | 0.6913 | 0.8527 |
| TC0700011010.hg.1 | SUN3 | Sad1 and UNC84 domain containing 3 | Multiple_C | 4.34 | 4.07 | 4.27 | 1.05 | 0.2714 | 0.4799 | 4.04 | 4.29 | 4.03 | 1.01 | 0.795 | 0.9087 |
| TC0700012085.hg.1 | ALKBH4 | alkB homolog 4, lysine demthylase | Multiple_C | 6.31 | 6.08 | 6.24 | 1.05 | 0.9394 | 0.972 | 5.29 | 5.79 | 5.86 | 0.67 | 0.1154 | 0.3352 |
| TC0700013043.hg.1 | GIMAP6 | GTPase, IMAP family member 6 | Multiple_C | 4.87 | 4.47 | 4.8 | 1.05 | 0.5088 | 0.705 | 4.58 | 4.67 | 4.3 | 1.21 | 0.3026 | 0.5669 |
| TC0700013367.hg.1 | ZNF679 | zinc finger protein 679 | Coding | 4.61 | 4.42 | 4.54 | 1.05 | 0.7944 | 0.8945 | 5.07 | 4.70 | 5.23 | 0.90 | 0.8753 | 0.9465 |
| TC0800006654.hg.1 | CLDN23 | claudin 23 | Multiple_C | 5.09 | 4.83 | 5.02 | 1.05 | 0.6195 | 0.7872 | 4.4 | 4.65 | 4.44 | 0.97 | 0.7638 | 0.8932 |
| TC0800007043.hg.1 | ADAM7 | ADAM metallopeptidase domain 7 | Coding | 3.24 | 3.44 | 3.17 | 1.05 | 0.7824 | 0.888 | 3.52 | 3.78 | 3.8 | 0.82 | 0.197 | 0.4519 |
| TC0800007899.hg.1 | SULF1 | sulfatase 1 | Multiple_C | 4.81 | 4.9 | 4.74 | 1.05 | 0.5364 | 0.7251 | 4.87 | 4.55 | 4.72 | 1.11 | 0.3979 | 0.6554 |
| TC0800009154.hg.1 | PSCA | prostate stem cell antigen | Multiple_C | 5.02 | 4.64 | 4.95 | 1.05 | 0.5284 | 0.7196 | 4.55 | 4.64 | 4.56 | 0.99 | 0.9939 | 0.9974 |
| TC0800009265.hg.1 | GPT | glutamic-pyruvate transaminase (alanine aminotransferase) | Multiple_C | 4.27 | 4.26 | 4.2 | 1.05 | 0.8563 | 0.9298 | 3.55 | 3.57 | 3.73 | 0.88 | 0.5395 | 0.7594 |
| TC0800009967.hg.1 | NUGGC | nuclear GTPase, germinal center associated | Coding | 3.24 | 3.14 | 3.17 | 1.05 | 0.811 | 0.9044 | 3.57 | 4.03 | 3.7 | 0.91 | 0.3661 | 0.6283 |

| | | | | | | | | | | | | | | | |
|---|---|---|---|---|---|---|---|---|---|---|---|---|---|---|---|
| TC0800010045.hg.1 | TEX15 | testis expressed 15 | Multiple_C | 3.01 | 3.08 | 2.94 | 1.05 | 0.8935 | 0.949 | 5.21 | 4.53 | 3.76 | 2.73 | 0.0013 | 0.0194 |
| TC0900009696.hg.1 | IFNA7 | interferon, alpha 7 | Coding | 3.17 | 3.15 | 3.1 | 1.05 | 0.7969 | 0.8958 | 3.64 | 3.78 | 3.67 | 0.98 | 0.7947 | 0.9087 |
| TC0900010271.hg.1 | FOXD4L5 | forkhead box D4-like 5 | Coding | 4.8 | 4.42 | 4.73 | 1.05 | 0.731 | 0.8594 | 4.25 | 4.10 | 3.9 | 1.27 | 0.0629 | 0.2387 |
| TC0900011872.hg.1 | BRD3 | bromodomain containing 3 | Multiple_C | 9.16 | 8.48 | 9.09 | 1.05 | 0.3954 | 0.6089 | 9.09 | 9.67 | 9.3 | 0.86 | 0.1842 | 0.4353 |
| TC0900012151.hg.1 | ANKRD19P | ankyrin repeat domain 19, pseudogene | Multiple_C | 5.58 | 5.88 | 5.51 | 1.05 | 0.806 | 0.9013 | 5.63 | 5.80 | 5.72 | 0.94 | 0.8649 | 0.9424 |
| TC0X00006791.hg.1 | PTCHD1 | patched domain containing 1 | Coding | 2.87 | 2.93 | 2.8 | 1.05 | 0.938 | 0.9712 | 3.34 | 3.46 | 3.4 | 0.96 | 0.7896 | 0.9059 |
| TC0X00006935.hg.1 | MAGEB16 | MAGE family member B16 | Coding | 3.98 | 3.76 | 3.91 | 1.05 | 0.8958 | 0.9502 | 4.44 | 4.52 | 4.62 | 0.88 | 0.7068 | 0.8619 |
| TC0X00007176.hg.1 | SSX1 | synovial sarcoma, X breakpoint 1 | Multiple_C | 3.66 | 3.77 | 3.59 | 1.05 | 0.5528 | 0.7368 | 4.34 | 4.33 | 4.4 | 0.96 | 0.9493 | 0.9793 |
| TC0X00007190.hg.1 | PORCN | porcupine homolog (Drosophila) | Multiple_C | 9.73 | 9.22 | 9.66 | 1.05 | 0.8905 | 0.9474 | 9.18 | 8.19 | 9.68 | 0.71 | 0.9506 | 0.9797 |
| TC0X00007240.hg.1 | CLCN5 | chloride channel, voltage-sensitive 5 | Multiple_C | 7.27 | 7.27 | 7.2 | 1.05 | 0.8751 | 0.9394 | 5.92 | 6.33 | 6.58 | 0.63 | 0.0068 | 0.0598 |
| TC0X00007704.hg.1 | COX7B | cytochrome c oxidase subunit VIIb | Multiple_C | 15.47 | 16.89 | 15.4 | 1.05 | 0.5274 | 0.719 | 10.68 | 11.55 | 11.48 | 0.57 | 0.0463 | 0.1989 |
| TC0X00008014.hg.1 | TMEM31 | transmembrane protein 31 | Coding | 4.93 | 4.62 | 4.86 | 1.05 | 0.4463 | 0.6547 | 6.01 | 5.79 | 5.69 | 1.25 | 0.2135 | 0.4717 |
| TC0X00008147.hg.1 | ZCCHC16 | zinc finger, CCHC domain containing 16 | Coding | 2.66 | 2.37 | 2.59 | 1.05 | 0.7594 | 0.8763 | 3.13 | 3.38 | 2.99 | 1.10 | 0.2008 | 0.4562 |
| TC0X00008387.hg.1 | XPNPEP2 | X-prolyl aminopeptidase (aminopeptidase P) 2, membrane-bou | Coding | 3.04 | 2.78 | 2.97 | 1.05 | 0.4763 | 0.679 | 3.24 | 3.53 | 3.22 | 1.01 | 0.8571 | 0.9393 |
| TC0X00008655.hg.1 | CXorf51A; CX | chromosome X open reading frame 51A; chromosome X open r | Multiple_C | 4.55 | 4.23 | 4.48 | 1.05 | 0.4278 | 0.6383 | 4.78 | 5.22 | 4.91 | 0.91 | 0.5522 | 0.7677 |
| TC0X00009795.hg.1 | ALAS2 | 5-aminolevulinate synthase 2 | Multiple_C | 3.63 | 3.4 | 3.56 | 1.05 | 0.8501 | 0.9264 | 4.29 | 4.17 | 3.82 | 1.39 | 0.1031 | 0.3145 |
| TC0X00010914.hg.1 | CT45A7; CT45 | cancer/testis antigen family 45, member A7; cancer/testis antig | Coding | 3.37 | 3.33 | 3.3 | 1.05 | 0.7279 | 0.8569 | 3.52 | 3.41 | 3.62 | 0.93 | 0.8443 | 0.9318 |
| TC0X00010943.hg.1 | GPR101 | G protein-coupled receptor 101 | Coding | 3.98 | 4.01 | 3.91 | 1.05 | 0.7737 | 0.8833 | 4.54 | 4.15 | 3.83 | 1.64 | 0.0075 | 0.0644 |
| TC0X00011219.hg.1 | CTAG2 | cancer/testis antigen 2 | Multiple_C | 3.67 | 3.58 | 3.6 | 1.05 | 0.7729 | 0.8828 | 3.86 | 3.93 | 4.05 | 0.88 | 0.3316 | 0.5957 |
| TC0Y00006858.hg.1 | VAMP7 | Homo sapiens vesicle-associated membrane protein 7 (VAMP7) | Multiple_C | 10.33 | 11.27 | 10.26 | 1.05 | 0.7734 | 0.8832 | 9.57 | 9.14 | 10.16 | 0.66 | 0.0034 | 0.0385 |
| TC0Y00007343.hg.1 | RBMY1J; RBM | RNA binding motif protein, Y-linked, family 1, member J; RNA bi | Multiple_C | 3.02 | 3.16 | 2.95 | 1.05 | 0.3734 | 0.586 | 3.03 | 3.12 | 3.02 | 1.01 | 0.3858 | 0.6449 |
| TC1000007574.hg.1 | WDFY4 | WDFY family member 4 | Multiple_C | 5.32 | 5.38 | 5.25 | 1.05 | 0.7793 | 0.8863 | 4.57 | 4.55 | 4.67 | 0.93 | 0.2909 | 0.5557 |
| TC1000010602.hg.1 | ERCC6; ERCC6 | excision repair cross-complementation group 6; ERCC6-PGBD3 | Multiple_C | 9.14 | 9.27 | 9.07 | 1.05 | 0.87 | 0.9368 | 9.1 | 9.01 | 8.87 | 1.17 | 0.8552 | 0.9382 |
| TC1000010661.hg.1 | MBL2 | mannose-binding lectin (protein C) 2, soluble | Multiple_C | 4.1 | 3.97 | 4.03 | 1.05 | 0.8797 | 0.9417 | 3.54 | 3.49 | 3.54 | 1.00 | 0.6208 | 0.8125 |
| TC1000011072.hg.1 | DUSP13 | dual specificity phosphatase 13 | Multiple_C | 3.07 | 2.85 | 3 | 1.05 | 0.5322 | 0.722 | 3.56 | 3.68 | 3.68 | 0.92 | 0.4827 | 0.7204 |
| TC1000012481.hg.1 | CYP2C19 | cytochrome P450, family 2, subfamily C, polypeptide 19 | Coding | 4.74 | 4.36 | 4.67 | 1.05 | 0.437 | 0.6464 | 4.32 | 4.47 | 4.57 | 0.84 | 0.1287 | 0.355 |
| TC1100006976.hg.1 | MYOD1 | myogenic differentiation 1 | Coding | 2.84 | 2.79 | 2.77 | 1.05 | 0.7988 | 0.8967 | 3.2 | 3.42 | 3.32 | 0.92 | 0.7928 | 0.9077 |
| TC1100007026.hg.1 | ZDHHC13 | zinc finger, DHHC-type containing 13 | Multiple_C | 8.34 | 9.06 | 8.27 | 1.05 | 0.7977 | 0.8961 | 8.81 | 8.80 | 8.09 | 1.65 | 0.001 | 0.0166 |
| TC1100007110.hg.1 | FIBIN | fin bud initiation factor homolog (zebrafish) | Coding | 5.04 | 4.91 | 4.97 | 1.05 | 0.8009 | 0.8978 | 4.92 | 5.08 | 4.83 | 1.06 | 0.6437 | 0.8253 |
| TC1100008435.hg.1 | RNF169 | ring finger protein 169 | Multiple_C | 9.64 | 9.94 | 9.57 | 1.05 | 0.7751 | 0.8842 | 9.68 | 9.34 | 9.77 | 0.94 | 0.2026 | 0.4582 |
| TC1100009110.hg.1 | NNMT | nicotinamide N-methyltransferase | Multiple_C | 3.85 | 3.69 | 3.78 | 1.05 | 0.7665 | 0.8801 | 3.85 | 3.90 | 3.99 | 0.91 | 0.5909 | 0.7931 |
| TC1100009932.hg.1 | UBQLNL | ubiquilin-like | Coding | 3.02 | 2.87 | 2.95 | 1.05 | 0.7419 | 0.8665 | 3.02 | 3.15 | 3.1 | 0.95 | 0.7582 | 0.8903 |
| TC1200007660.hg.1 | KRT86 | keratin 86, type II | Multiple_C | 5.98 | 5.73 | 5.91 | 1.05 | 0.3902 | 0.6039 | 5.63 | 5.59 | 5.63 | 1.00 | 0.8748 | 0.9462 |

| | | | | | | | | | | | | | | | |
|---|---|---|---|---|---|---|---|---|---|---|---|---|---|---|---|
| TC1200009740.hg.1 | IFFO1 | intermediate filament family orphan 1 | Multiple_C | 5.66 | 5.33 | 5.59 | 1.05 | 0.596 | 0.7699 | 5.9 | 6.13 | 5.82 | 1.06 | 0.8786 | 0.948 |
| TC1200010016.hg.1 | WBP11 | WW domain binding protein 11 | Multiple_C | 12.87 | 12.13 | 12.8 | 1.05 | 0.5642 | 0.7458 | 13.98 | 13.91 | 14.12 | 0.91 | 0.5564 | 0.7706 |
| TC1200010592.hg.1 | ADCY6; MIR47 | adenylate cyclase 6; microRNA 4701 | Multiple_C | 7.87 | 7.59 | 7.8 | 1.05 | 0.8263 | 0.9128 | 7.98 | 8.62 | 8.42 | 0.74 | 0.0101 | 0.0785 |
| TC1200010799.hg.1 | SP7 | Sp7 transcription factor | Coding | 2.9 | 3.06 | 2.83 | 1.05 | 0.7398 | 0.8651 | 3.29 | 2.96 | 3.09 | 1.15 | 0.2825 | 0.548 |
| TC1200011535.hg.1 | UBE2N | ubiquitin conjugating enzyme E2N | Multiple_C | 12.9 | 12.72 | 12.83 | 1.05 | 0.5538 | 0.7374 | 12 | 12.50 | 12.34 | 0.79 | 0.1409 | 0.3733 |
| TC1200011597.hg.1 | CCDC38 | coiled-coil domain containing 38 | Multiple_C | 4.86 | 4.99 | 4.79 | 1.05 | 0.7924 | 0.8935 | 4.71 | 4.77 | 4.83 | 0.92 | 0.6257 | 0.815 |
| TC1300007232.hg.1 | CCDC70 | coiled-coil domain containing 70 | Coding | 3.82 | 3.61 | 3.75 | 1.05 | 0.7337 | 0.8609 | 4.14 | 4.29 | 3.91 | 1.17 | 0.3051 | 0.5691 |
| TC1300007706.hg.1 | MIR17HG; MI | miR-17-92 cluster host gene; microRNA 17; microRNA 18a; micr | Multiple_C | 5.69 | 7.06 | 5.62 | 1.05 | 0.6347 | 0.7966 | 6.92 | 7.04 | 6.85 | 1.05 | 0.902 | 0.9592 |
| TC1300008963.hg.1 | DLEU7 | deleted in lymphocytic leukemia, 7 | Multiple_C | 4.29 | 4 | 4.22 | 1.05 | 0.9417 | 0.9729 | 4.73 | 4.81 | 5.07 | 0.79 | 0.8369 | 0.9282 |
| TC1300009309.hg.1 | EDNRB | endothelin receptor type B | Multiple_C | 6.48 | 6.27 | 6.41 | 1.05 | 0.6518 | 0.8073 | 6.81 | 6.65 | 6.43 | 1.30 | 0.0618 | 0.2362 |
| TC1400006544.hg.1 | SLC39A2 | solute carrier family 39 (zinc transporter), member 2 | Multiple_C | 4.07 | 4.09 | 4 | 1.05 | 0.5557 | 0.739 | 3.7 | 3.53 | 3.88 | 0.88 | 0.5772 | 0.7834 |
| TC1400006791.hg.1 | FOXG1 | forkhead box G1 | Coding | 4.17 | 3.93 | 4.1 | 1.05 | 0.8533 | 0.9283 | 6.64 | 6.88 | 6.87 | 0.85 | 0.439 | 0.6893 |
| TC1400007666.hg.1 | LIN52 | lin-52 DREAM MuvB core complex component | Multiple_C | 9.95 | 10.02 | 9.88 | 1.05 | 0.7092 | 0.8457 | 9.83 | 9.84 | 9.86 | 0.98 | 0.9975 | 0.9988 |
| TC1400008182.hg.1 | CYP46A1 | cytochrome P450, family 46, subfamily A, polypeptide 1 | Multiple_C | 4.34 | 4.48 | 4.27 | 1.05 | 0.6925 | 0.8342 | 3.98 | 4.20 | 4.24 | 0.84 | 0.7405 | 0.8807 |
| TC1400008625.hg.1 | HNRNPC | heterogeneous nuclear ribonucleoprotein C (C1/C2) | Multiple_C | 16.3 | 16.86 | 16.23 | 1.05 | 0.2682 | 0.4763 | 17.34 | 17.57 | 17.48 | 0.91 | 0.2432 | 0.5068 |
| TC1400008716.hg.1 | MYH6 | myosin, heavy chain 6, cardiac muscle, alpha | Multiple_C | 4.33 | 4.31 | 4.26 | 1.05 | 0.4571 | 0.6633 | 4.17 | 4.34 | 4.07 | 1.07 | 0.4895 | 0.7252 |
| TC1400009957.hg.1 | NRDE2 | NRDE-2, necessary for RNA interference, domain containing | Multiple_C | 5.31 | 5.37 | 5.24 | 1.05 | 0.5885 | 0.7642 | 4.21 | 4.78 | 5.06 | 0.55 | 0.0194 | 0.1179 |
| TC1400010631.hg.1 | IFT43 | intraflagellar transport 43 | Multiple_C | 5.43 | 5.72 | 5.36 | 1.05 | 0.861 | 0.9321 | 4.36 | 4.46 | 4.09 | 1.21 | 0.2652 | 0.5297 |
| TC1400010758.hg.1 | CCDC175 | coiled-coil domain containing 175 | Multiple_C | 3.97 | 3.98 | 3.9 | 1.05 | 0.805 | 0.9006 | 3.89 | 3.56 | 3.91 | 0.99 | 0.7453 | 0.8832 |
| TC1500007735.hg.1 | LRRC49 | leucine rich repeat containing 49 | Multiple_C | 7.6 | 8.85 | 7.53 | 1.05 | 0.8317 | 0.9159 | 7.1 | 6.28 | 6.92 | 1.13 | 0.3693 | 0.6314 |
| TC1500008053.hg.1 | CFAP161 | cilia and flagella associated protein 161 | Multiple_C | 2.77 | 2.59 | 2.7 | 1.05 | 0.9242 | 0.9645 | 3.58 | 3.82 | 3.9 | 0.80 | 0.3618 | 0.6246 |
| TC1500009123.hg.1 | DNAJC17 | DnaJ (Hsp40) homolog, subfamily C, member 17 | Multiple_C | 6.99 | 7.17 | 6.92 | 1.05 | 0.8849 | 0.9443 | 6.15 | 6.97 | 6.74 | 0.66 | 0.0134 | 0.0937 |
| TC1500009776.hg.1 | RASL12 | RAS-like, family 12 | Coding | 4.06 | 3.91 | 3.99 | 1.05 | 0.4724 | 0.6759 | 4.22 | 4.37 | 4.53 | 0.81 | 0.0347 | 0.1684 |
| TC1500009865.hg.1 | ANP32A | acidic nuclear phosphoprotein 32 family member A | Multiple_C | 14.83 | 14.66 | 14.76 | 1.05 | 0.7023 | 0.841 | 15.78 | 15.97 | 15.83 | 0.97 | 0.657 | 0.8324 |
| TC1600008661.hg.1 | CRISPLD2 | cysteine-rich secretory protein LCCL domain containing 2 | Multiple_C | 5.7 | 5.44 | 5.63 | 1.05 | 0.7477 | 0.8705 | 5.65 | 5.40 | 5.65 | 1.00 | 0.7338 | 0.8772 |
| TC1600008832.hg.1 | IL17C | interleukin 17C | Multiple_C | 4.64 | 4.34 | 4.57 | 1.05 | 0.7956 | 0.8951 | 5.23 | 5.22 | 5.13 | 1.07 | 0.7297 | 0.8745 |
| TC1600009156.hg.1 | PKMYT1 | Transcript Identified by AceView, Entrez Gene ID(s) 9088 | Multiple_C | 3.99 | 3.57 | 3.92 | 1.05 | 0.4559 | 0.6628 | 4.43 | 4.32 | 4.14 | 1.22 | 0.2414 | 0.5055 |
| TC1600010239.hg.1 | CBLN1 | cerebellin 1 precursor | Coding | 2.58 | 2.6 | 2.51 | 1.05 | 0.5667 | 0.7477 | 3.22 | 3.21 | 3.42 | 0.87 | 0.7601 | 0.8913 |
| TC1600010914.hg.1 | ADAMTS18 | ADAM metallopeptidase with thrombospondin type 1 motif 18 | Multiple_C | 3.01 | 2.67 | 2.94 | 1.05 | 0.7943 | 0.8945 | 3.19 | 4.21 | 3.95 | 0.59 | 0.0066 | 0.0588 |
| TC1600011257.hg.1 | SLC22A31 | solute carrier family 22, member 31 | Multiple_C | 3.13 | 3.08 | 3.06 | 1.05 | 0.705 | 0.8425 | 4.11 | 3.96 | 3.72 | 1.31 | 0.0767 | 0.2667 |
| TC1600011402.hg.1 | POLR2C | polymerase (RNA) II (DNA directed) polypeptide C, 33kDa | Multiple_C | 12.68 | 12.04 | 12.61 | 1.05 | 0.8696 | 0.9366 | 12.49 | 12.72 | 12.66 | 0.89 | 0.0731 | 0.2591 |
| TC1700007100.hg.1 | MYO15A | myosin XVA | Multiple_C | 3.69 | 3.67 | 3.62 | 1.05 | 0.9838 | 0.9924 | 3.71 | 3.42 | 3.26 | 1.37 | 0.0067 | 0.0592 |
| TC1700007864.hg.1 | TTC25 | tetratricopeptide repeat domain 25 | Multiple_C | 4.27 | 4.26 | 4.2 | 1.05 | 0.6961 | 0.8367 | 4.49 | 4.61 | 4.05 | 1.36 | 0.5203 | 0.7477 |

| | | | | | | | | | | | | | | | |
|---|---|---|---|---|---|---|---|---|---|---|---|---|---|---|---|
| TC1700009049.hg.1 | PGS1 | phosphatidylglycerophosphate synthase 1 | Multiple_C | 6.85 | 6.54 | 6.78 | 1.05 | 0.8274 | 0.9133 | 7.4 | 7.19 | 7.22 | 1.13 | 0.7114 | 0.8646 |
| TC1700009750.hg.1 | TMEM220 | transmembrane protein 220 | Multiple_C | 3.45 | 3.57 | 3.38 | 1.05 | 0.8617 | 0.9324 | 3.87 | 3.72 | 3.75 | 1.09 | 0.6315 | 0.8185 |
| TC1700009781.hg.1 | ZNF18 | zinc finger protein 18 | Multiple_C | 4.6 | 5.31 | 4.53 | 1.05 | 0.8563 | 0.9298 | 5.98 | 6.43 | 5.63 | 1.27 | 0.4713 | 0.7121 |
| TC1700010459.hg.1 | CCL3 | chemokine (C-C motif) ligand 3 | Multiple_C | 3.65 | 3.59 | 3.58 | 1.05 | 0.8594 | 0.9314 | 4.14 | 4.11 | 4.35 | 0.86 | 0.4548 | 0.6996 |
| TC1700010644.hg.1 | KRTAP3-3 | keratin associated protein 3-3 | Coding | 4.01 | 3.94 | 3.94 | 1.05 | 0.4919 | 0.692 | 6.78 | 6.88 | 5.55 | 2.35 | 9.72E-07 | 0.0001 |
| TC1700012033.hg.1 | TMEM105 | transmembrane protein 105 | Multiple_C | 4.15 | 4.11 | 4.08 | 1.05 | 0.3612 | 0.5747 | 4.56 | 4.41 | 4.19 | 1.29 | 0.2138 | 0.4721 |
| TC1700012220.hg.1 | TBC1D29 | TBC1 domain family, member 29 | Multiple_C | 4.41 | 4.64 | 4.34 | 1.05 | 0.9057 | 0.9548 | 4.49 | 4.66 | 4.34 | 1.11 | 0.2515 | 0.5156 |
| TC1800006655.hg.1 | TXNDC2 | thioredoxin domain containing 2 (spermatozoa) | Multiple_C | 5.47 | 5.7 | 5.4 | 1.05 | 0.5493 | 0.7343 | 5.16 | 5.05 | 5.03 | 1.09 | 0.878 | 0.9478 |
| TC1800008399.hg.1 | B4GALT6 | UDP-Gal:betaGlcNAc beta 1,4- galactosyltransferase, polypeptic | Multiple_C | 6.51 | 6.54 | 6.44 | 1.05 | 0.9834 | 0.9922 | 5.53 | 6.17 | 5.85 | 0.80 | 0.0206 | 0.1227 |
| TC1900006468.hg.1 | GZMM | granzyme M | Coding | 4 | 3.84 | 3.93 | 1.05 | 0.6829 | 0.8281 | 3.6 | 3.62 | 3.6 | 1.00 | 0.5105 | 0.7403 |
| TC1900006475.hg.1 | FGF22 | fibroblast growth factor 22 | Multiple_C | 3.76 | 3.59 | 3.69 | 1.05 | 0.8855 | 0.9448 | 3.89 | 4.01 | 3.98 | 0.94 | 0.8334 | 0.9259 |
| TC1900008528.hg.1 | TRPM4 | transient receptor potential cation channel, subfamily M, meml | Multiple_C | 6.23 | 6.25 | 6.16 | 1.05 | 0.6322 | 0.7947 | 5.47 | 5.56 | 5.14 | 1.26 | 0.0294 | 0.1519 |
| TC1900008543.hg.1 | FCGRT | Fc fragment of IgG, receptor, transporter, alpha | Multiple_C | 4.65 | 4.69 | 4.58 | 1.05 | 0.5678 | 0.7484 | 4.73 | 4.67 | 4.37 | 1.28 | 0.084 | 0.2789 |
| TC1900009031.hg.1 | ZNF274 | zinc finger protein 274 | Multiple_C | 4.81 | 4.59 | 4.74 | 1.05 | 0.4552 | 0.6624 | 4.95 | 5.28 | 4.97 | 0.99 | 0.897 | 0.9566 |
| TC1900010894.hg.1 | ZNF180 | zinc finger protein 180 | Multiple_C | 4.44 | 4.45 | 4.37 | 1.05 | 0.496 | 0.6959 | 4.57 | 4.81 | 4.73 | 0.90 | 0.6438 | 0.8254 |
| TC1900011259.hg.1 | SIGLEC12 | sialic acid binding Ig-like lectin 12 (gene/pseudogene) | Multiple_C | 3.35 | 3.09 | 3.28 | 1.05 | 0.7809 | 0.8871 | 3.61 | 3.93 | 3.68 | 0.95 | 0.4683 | 0.7093 |
| TC1900011907.hg.1 | PDE4C | phosphodiesterase 4C, cAMP-specific | Multiple_C | 3.55 | 3.77 | 3.48 | 1.05 | 0.9759 | 0.9882 | 3.72 | 3.99 | 3.76 | 0.97 | 0.7111 | 0.8644 |
| TC2000006434.hg.1 | DEFB126 | defensin, beta 126 | Multiple_C | 3.06 | 3.02 | 2.99 | 1.05 | 0.7672 | 0.8802 | 3.55 | 3.28 | 3.11 | 1.36 | 0.1945 | 0.4485 |
| TC2000006894.hg.1 | KIZ | kizuna centrosomal protein | Multiple_C | 5.58 | 6.24 | 5.51 | 1.05 | 0.8208 | 0.9102 | 5.9 | 6.53 | 6.2 | 0.81 | 0.2468 | 0.5114 |
| TC2000007269.hg.1 | TLDC2 | TBC/LysM-associated domain containing 2 | Coding | 4.62 | 4.63 | 4.55 | 1.05 | 0.7578 | 0.8754 | 6.34 | 6.89 | 6.24 | 1.07 | 0.6526 | 0.8305 |
| TC2000007707.hg.1 | BCAS4 | breast carcinoma amplified sequence 4 | Multiple_C | 6.2 | 6.16 | 6.13 | 1.05 | 0.7503 | 0.8718 | 5.62 | 5.59 | 5.59 | 1.02 | 0.871 | 0.9445 |
| TC2000007887.hg.1 | C20orf85 | chromosome 20 open reading frame 85 | Coding | 3.53 | 3.37 | 3.46 | 1.05 | 0.8501 | 0.9264 | 4.12 | 4.00 | 3.67 | 1.37 | 0.1108 | 0.3281 |
| TC2000008650.hg.1 | THBD | thrombomodulin | Coding | 3.35 | 3.13 | 3.28 | 1.05 | 0.9902 | 0.9948 | 3.39 | 3.44 | 3.46 | 0.95 | 0.7638 | 0.8932 |
| TC2000009748.hg.1 | CABLES2 | Cdk5 and Abl enzyme substrate 2 | Multiple_C | 6.79 | 5.54 | 6.72 | 1.05 | 0.4476 | 0.6557 | 5.51 | 5.82 | 5.88 | 0.77 | 0.1009 | 0.3106 |
| TC2100006784.hg.1 | JAM2 | junctional adhesion molecule 2 | Multiple_C | 3.29 | 3.45 | 3.22 | 1.05 | 0.4007 | 0.614 | 3.49 | 3.42 | 3.6 | 0.93 | 0.694 | 0.8544 |
| TC2100006880.hg.1 | KRTAP20-1 | keratin associated protein 20-1 | Coding | 4.56 | 4.42 | 4.49 | 1.05 | 0.3062 | 0.5193 | 4.27 | 4.08 | 4.35 | 0.95 | 0.3451 | 0.6089 |
| TC2100007534.hg.1 | TEKT4P2 | tektin 4 pseudogene 2 | Multiple_C | 3.34 | 2.74 | 3.27 | 1.05 | 0.7669 | 0.8801 | 5.03 | 4.94 | 4.94 | 1.06 | 0.7519 | 0.887 |
| TC2200007051.hg.1 | CCDC157 | coiled-coil domain containing 157 | Multiple_C | 3.6 | 3.47 | 3.53 | 1.05 | 0.901 | 0.9524 | 4.22 | 4.44 | 4.67 | 0.73 | 0.5324 | 0.7552 |
| TC2200007278.hg.1 | KCTD17 | potassium channel tetramerization domain containing 17 | Multiple_C | 5.58 | 5.24 | 5.51 | 1.05 | 0.9606 | 0.9813 | 6.05 | 6.42 | 6.28 | 0.85 | 0.2536 | 0.5178 |
| TC2200008110.hg.1 | PPM1F | protein phosphatase, Mg2+/Mn2+ dependent, 1F | Multiple_C | 4.86 | 4.85 | 4.79 | 1.05 | 0.6461 | 0.804 | 7.12 | 7.35 | 7.46 | 0.79 | 0.4868 | 0.7237 |
| TSUnmapped00000006.h | RPS6KA1 | ribosomal protein S6 kinase, 90kDa, polypeptide 1 | Coding | 6.24 | 5.99 | 6.17 | 1.05 | 0.4859 | 0.6873 | 4.8 | 4.79 | 5 | 0.87 | 0.5965 | 0.7967 |
| TSUnmapped00000187.h | ZNF197 | zinc finger protein 197 | Coding | 3.67 | 3.61 | 3.6 | 1.05 | 0.9213 | 0.9636 | 4.07 | 4.16 | 4.07 | 1.00 | 0.7945 | 0.9087 |
| TSUnmapped00000622.h | GREM1 | gremlin 1, DAN family BMP antagonist [Source:HGNC Symbol;Ac | Coding | 3.18 | 3.16 | 3.11 | 1.05 | 0.7689 | 0.881 | 3.67 | 3.55 | 3.44 | 1.17 | 0.138 | 0.3689 |

| | | | | | | | | | | | | | | | |
|---|---|---|---|---|---|---|---|---|---|---|---|---|---|---|---|
| TC0100007103.hg.1 | PADI3 | peptidyl arginine deiminase, type III | Coding | 3.9 | 3.76 | 3.84 | 1.04 | 0.8861 | 0.945 | 4 | 4.08 | 4.23 | 0.85 | 0.3423 | 0.6061 |
| TC0100007144.hg.1 | KLHDC7A | kelch domain containing 7A | Multiple_C | 2.64 | 2.67 | 2.58 | 1.04 | 0.497 | 0.6968 | 4.11 | 3.51 | 3.55 | 1.47 | 0.0087 | 0.0709 |
| TC0100007510.hg.1 | SYTL1 | synaptotagmin like 1 | Multiple_C | 4.63 | 4.73 | 4.57 | 1.04 | 0.8727 | 0.9382 | 4.54 | 4.17 | 4.02 | 1.43 | 0.0372 | 0.1754 |
| TC0100009038.hg.1 | C1orf146 | chromosome 1 open reading frame 146 | Coding | 2.93 | 2.86 | 2.87 | 1.04 | 0.4817 | 0.6833 | 3.09 | 3.19 | 3.02 | 1.05 | 0.3075 | 0.5716 |
| TC0100012348.hg.1 | OR2M5 | olfactory receptor, family 2, subfamily M, member 5 | Coding | 3.39 | 3.34 | 3.33 | 1.04 | 0.5111 | 0.7069 | 2.9 | 3.25 | 3.21 | 0.81 | 0.2916 | 0.5562 |
| TC0100012818.hg.1 | CTNNBIP1 | catenin, beta interacting protein 1 | Multiple_C | 6.9 | 6.99 | 6.84 | 1.04 | 0.9681 | 0.9846 | 7.01 | 7.52 | 7.2 | 0.88 | 0.0635 | 0.2397 |
| TC0100012946.hg.1 | PRAMEF8 | PRAME family member 8 | Coding | 2.91 | 2.61 | 2.85 | 1.04 | 0.5077 | 0.7043 | 3.54 | 3.46 | 3.24 | 1.23 | 0.277 | 0.5429 |
| TC0100013303.hg.1 | HMGCL | 3-hydroxymethyl-3-methylglutaryl-CoA lyase | Multiple_C | 10.38 | 11.53 | 10.32 | 1.04 | 0.9216 | 0.9636 | 6.76 | 7.05 | 6.1 | 1.58 | 0.1198 | 0.3418 |
| TC0100013339.hg.1 | RUNX3 | runt-related transcription factor 3 | Multiple_C | 3.55 | 3.16 | 3.49 | 1.04 | 0.9822 | 0.9918 | 8.99 | 8.82 | 9.74 | 0.59 | 0.0053 | 0.0509 |
| TC0100015102.hg.1 | COL11A1 | collagen, type XI, alpha 1 | Multiple_C | 3.57 | 3.47 | 3.51 | 1.04 | 0.7882 | 0.8915 | 3.85 | 3.77 | 3.86 | 0.99 | 0.8612 | 0.9406 |
| TC0100016145.hg.1 | CD244 | CD244 molecule, natural killer cell receptor 2B4 | Multiple_C | 3.12 | 2.86 | 3.06 | 1.04 | 0.9302 | 0.9671 | 3.32 | 3.38 | 3.14 | 1.13 | 0.3216 | 0.5855 |
| TC0100016639.hg.1 | SHCBP1L | SHC SH2-domain binding protein 1-like | Multiple_C | 3.22 | 3.23 | 3.16 | 1.04 | 0.4911 | 0.691 | 3.37 | 3.40 | 3.32 | 1.04 | 0.4054 | 0.6608 |
| TC0200006715.hg.1 | PQLC3 | PQ loop repeat containing 3 | Multiple_C | 7.06 | 6.95 | 7 | 1.04 | 0.8313 | 0.9158 | 5.14 | 5.52 | 5.48 | 0.79 | 0.0262 | 0.1414 |
| TC0200014414.hg.1 | CXCR4 | chemokine (C-X-C motif) receptor 4 | Multiple_C | 3.89 | 3.54 | 3.83 | 1.04 | 0.609 | 0.7796 | 6.79 | 6.64 | 6.32 | 1.39 | 0.1207 | 0.3429 |
| TC0200015314.hg.1 | STK17B | serine/threonine kinase 17b | Multiple_C | 11.71 | 12.29 | 11.65 | 1.04 | 0.9225 | 0.9639 | 7.43 | 7.64 | 7.87 | 0.74 | 0.2284 | 0.4893 |
| TC0300007065.hg.1 | DLEC1 | deleted in lung and esophageal cancer 1 | Multiple_C | 4.02 | 3.96 | 3.96 | 1.04 | 0.8071 | 0.9022 | 3.64 | 3.69 | 3.63 | 1.01 | 0.9786 | 0.991 |
| TC0300008110.hg.1 | OR5K1 | olfactory receptor, family 5, subfamily K, member 1 | Multiple_C | 4.21 | 4.29 | 4.15 | 1.04 | 0.3804 | 0.5933 | 4.32 | 4.02 | 3.6 | 1.65 | 0.0057 | 0.0534 |
| TC0300008826.hg.1 | NEK11 | NIMA-related kinase 11 | Multiple_C | 5.33 | 5.2 | 5.27 | 1.04 | 0.8899 | 0.947 | 4.47 | 4.43 | 4.34 | 1.09 | 0.5515 | 0.7672 |
| TC0300013068.hg.1 | MECOM | MDS1 and EVI1 complex locus | Multiple_C | 11.23 | 11.59 | 11.17 | 1.04 | 0.6776 | 0.8247 | 3.87 | 4.16 | 3.9 | 0.98 | 0.8306 | 0.9247 |
| TC0300013959.hg.1 | CCDC13 | coiled-coil domain containing 13 | Multiple_C | 7.15 | 6.96 | 7.09 | 1.04 | 0.7554 | 0.8745 | 6.81 | 6.73 | 6.69 | 1.09 | 0.0778 | 0.2686 |
| TC0400007779.hg.1 | CABS1 | calcium-binding protein, spermatid-specific 1 | Coding | 2.87 | 2.79 | 2.81 | 1.04 | 0.8347 | 0.9175 | 3.14 | 3.27 | 3.34 | 0.87 | 0.5078 | 0.7384 |
| TC0400008362.hg.1 | OSTC | oligosaccharyltransferase complex subunit (non-catalytic) | Multiple_C | 11.26 | 13.87 | 11.2 | 1.04 | 0.8282 | 0.9137 | 8.01 | 8.28 | 8.02 | 0.99 | 0.6322 | 0.819 |
| TC0400009588.hg.1 | ZFP42 | ZFP42 zinc finger protein | Multiple_C | 3.04 | 3.03 | 2.98 | 1.04 | 0.5611 | 0.7435 | 3.21 | 2.99 | 2.97 | 1.18 | 0.3463 | 0.6101 |
| TC0400010465.hg.1 | UGDH | UDP-glucose 6-dehydrogenase | Multiple_C | 13.47 | 14.13 | 13.41 | 1.04 | 0.5903 | 0.7656 | 11.42 | 11.36 | 12.02 | 0.66 | 0.0012 | 0.0191 |
| TC0400010940.hg.1 | SULT1B1 | sulfotransferase family 1B member 1 | Coding | 3.28 | 3.16 | 3.22 | 1.04 | 0.6865 | 0.8309 | 3.55 | 3.54 | 3.31 | 1.18 | 0.0523 | 0.2137 |
| TC0400011473.hg.1 | SLC9B1 | solute carrier family 9, subfamily B (NHA1, cation proton antipo | Multiple_C | 3.62 | 3.58 | 3.56 | 1.04 | 0.6692 | 0.8193 | 4.12 | 4.17 | 3.68 | 1.36 | 0.0211 | 0.1242 |
| TC0400011974.hg.1 | MGAT4D | MGAT4 family, member D | Multiple_C | 4.59 | 4.37 | 4.53 | 1.04 | 0.3911 | 0.6048 | 4.39 | 4.40 | 4.5 | 0.93 | 0.6709 | 0.8395 |
| TC0500007972.hg.1 | VCAN | versican | Multiple_C | 4.49 | 4.4 | 4.43 | 1.04 | 0.3837 | 0.5967 | 4.28 | 4.08 | 3.98 | 1.23 | 0.1645 | 0.409 |
| TC0500009192.hg.1 | SGCD | sarcoglycan delta | Multiple_C | 3.19 | 3 | 3.13 | 1.04 | 0.7847 | 0.8894 | 3.77 | 3.88 | 3.97 | 0.87 | 0.299 | 0.5637 |
| TC0500009861.hg.1 | SLC6A3 | solute carrier family 6 (neurotransmitter transporter), member | Multiple_C | 4.07 | 3.72 | 4.01 | 1.04 | 0.5934 | 0.7679 | 3.87 | 3.88 | 3.76 | 1.08 | 0.5174 | 0.7458 |
| TC0500010760.hg.1 | DHX29 | DEAH (Asp-Glu-Ala-His) box polypeptide 29 | Multiple_C | 10.98 | 11.37 | 10.92 | 1.04 | 0.7961 | 0.8953 | 10.34 | 10.65 | 10.8 | 0.73 | 0.0476 | 0.2025 |
| TC0500012826.hg.1 | SH3PXD2B | SH3 and PX domains 2B | Multiple_C | 7.36 | 6.53 | 7.3 | 1.04 | 0.7445 | 0.8679 | 6.63 | 6.67 | 6.09 | 1.45 | 0.0101 | 0.0783 |
| TC0500013130.hg.1 | TRIM52 | tripartite motif containing 52 | Multiple_C | 7.48 | 7.34 | 7.42 | 1.04 | 0.6206 | 0.7876 | 6.75 | 6.67 | 6.24 | 1.42 | 0.0336 | 0.165 |

| | | | | | | | | | | | | | | | |
|---|---|---|---|---|---|---|---|---|---|---|---|---|---|---|---|
| TC0600007959.hg.1 | DAAM2 | dishevelled associated activator of morphogenesis 2 | Multiple_C | 3.32 | 3.13 | 3.26 | 1.04 | 0.9165 | 0.9608 | 3.33 | 3.29 | 3.45 | 0.92 | 0.2995 | 0.5639 |
| TC0600008062.hg.1 | GNMT | glycine N-methyltransferase | Coding | 3.81 | 3.47 | 3.75 | 1.04 | 0.8015 | 0.8983 | 4.09 | 4.08 | 4.04 | 1.04 | 0.1285 | 0.3548 |
| TC0600010078.hg.1 | SLC22A3 | solute carrier family 22 (organic cation transporter), member 3 | Multiple_C | 8.03 | 8.34 | 7.97 | 1.04 | 0.8451 | 0.9238 | 4.87 | 7.92 | 8.64 | 0.07 | 1.20E-10 | 2.57E-07 |
| TC0600010709.hg.1 | F13A1 | coagulation factor XIII, A1 polypeptide | Multiple_C | 3.52 | 3.46 | 3.46 | 1.04 | 0.9272 | 0.966 | 2.92 | 3.34 | 3.47 | 0.68 | 0.0112 | 0.0835 |
| TC0600011116.hg.1 | SLC17A3; HIST | solute carrier family 17 (organic anion transporter), member 3; | Multiple_C | 4.1 | 4.02 | 4.04 | 1.04 | 0.8863 | 0.9451 | 4.46 | 4.70 | 4.19 | 1.21 | 0.1957 | 0.4501 |
| TC0600011634.hg.1 | TULP1 | tubby like protein 1 | Multiple_C | 3 | 3.05 | 2.94 | 1.04 | 0.9176 | 0.9614 | 3.44 | 3.50 | 3.36 | 1.06 | 0.5009 | 0.7335 |
| TC0600011753.hg.1 | LRFN2 | leucine rich repeat and fibronectin type III domain containing 2 | Coding | 3.14 | 2.98 | 3.08 | 1.04 | 0.6975 | 0.8377 | 3.82 | 3.97 | 3.81 | 1.01 | 0.9694 | 0.9875 |
| TC0600013251.hg.1 | HBS1L | HBS1-like translational GTPase | Multiple_C | 11.92 | 12.7 | 11.86 | 1.04 | 0.9542 | 0.9785 | 12 | 11.70 | 11.98 | 1.01 | 0.7961 | 0.909 |
| TC0600013962.hg.1 | DACT2 | dishevelled-binding antagonist of beta-catenin 2 | Multiple_C | 5.75 | 5.77 | 5.69 | 1.04 | 0.4204 | 0.6318 | 6.41 | 6.46 | 6.24 | 1.13 | 0.5795 | 0.7849 |
| TC0600014086.hg.1 | ZNF391 | zinc finger protein 391 | Multiple_C | 5.67 | 6.26 | 5.61 | 1.04 | 0.599 | 0.7715 | 4.64 | 4.44 | 4.45 | 1.14 | 0.0525 | 0.214 |
| TC0700007472.hg.1 | C7orf69 | chromosome 7 open reading frame 69 | Multiple_C | 3.15 | 3.03 | 3.09 | 1.04 | 0.5201 | 0.7136 | 3.53 | 3.27 | 3.37 | 1.12 | 0.7402 | 0.8807 |
| TC0700008252.hg.1 | CROT | carnitine O-octanoyltransferase | Multiple_C | 4.16 | 3.97 | 4.1 | 1.04 | 0.8383 | 0.9197 | 5.35 | 5.44 | 6.94 | 0.33 | 0.039 | 0.18 |
| TC0700008659.hg.1 | NFE4 | nuclear factor, erythroid 4 | Multiple_C | 3.16 | 3.12 | 3.1 | 1.04 | 0.6463 | 0.8042 | 3.47 | 3.36 | 3.28 | 1.14 | 0.8515 | 0.9358 |
| TC0700008753.hg.1 | GPR22 | G protein-coupled receptor 22 | Multiple_C | 3.29 | 3.44 | 3.23 | 1.04 | 0.757 | 0.8752 | 3.49 | 3.27 | 2.88 | 1.53 | 0.0302 | 0.1545 |
| TC0700010058.hg.1 | GRIFIN | galectin-related inter-fiber protein | Multiple_C | 5.52 | 5.19 | 5.46 | 1.04 | 0.7915 | 0.8932 | 4.55 | 4.72 | 4.5 | 1.04 | 0.6404 | 0.8236 |
| TC0700010644.hg.1 | CRHR2 | corticotropin releasing hormone receptor 2 | Multiple_C | 3.63 | 3.46 | 3.57 | 1.04 | 0.7287 | 0.8575 | 4.04 | 3.45 | 3.49 | 1.46 | 0.0254 | 0.1388 |
| TC0700012416.hg.1 | TSPAN12 | tetraspanin 12 | Multiple_C | 9.49 | 10.71 | 9.43 | 1.04 | 0.6477 | 0.8047 | 7.54 | 7.34 | 7.82 | 0.82 | 0.1917 | 0.4446 |
| TC0700012708.hg.1 | SLC13A4 | solute carrier family 13 (sodium/sulfate symporter), member 4 | Multiple_C | 5.64 | 5.56 | 5.58 | 1.04 | 0.6467 | 0.8043 | 6.02 | 5.95 | 6.02 | 1.00 | 0.8009 | 0.9111 |
| TC0700013182.hg.1 | CNPY1 | canopy FGF signaling regulator 1 | Multiple_C | 3.59 | 3.6 | 3.53 | 1.04 | 0.7472 | 0.8703 | 9.62 | 8.83 | 8.66 | 1.95 | 0.0036 | 0.0403 |
| TC0700013313.hg.1 | VIPR2 | vasoactive intestinal peptide receptor 2 | Multiple_C | 2.63 | 2.62 | 2.57 | 1.04 | 0.7548 | 0.8745 | 3.31 | 3.32 | 3.27 | 1.03 | 0.686 | 0.8497 |
| TC0700013616.hg.1 | GCC1 | GRIP and coiled-coil domain containing 1 | Multiple_C | 6.54 | 7.07 | 6.48 | 1.04 | 0.8843 | 0.9439 | 5.95 | 6.08 | 6.28 | 0.80 | 0.4296 | 0.6816 |
| TC0800006860.hg.1 | MICU3 | mitochondrial calcium uptake family, member 3 | Multiple_C | 3.53 | 3.41 | 3.47 | 1.04 | 0.8448 | 0.9236 | 4.15 | 3.93 | 3.62 | 1.44 | 0.0612 | 0.2348 |
| TC0800007269.hg.1 | UNC5D | unc-5 netrin receptor D | Multiple_C | 3.25 | 3.28 | 3.19 | 1.04 | 0.7875 | 0.8913 | 3.97 | 3.72 | 3.89 | 1.06 | 0.9403 | 0.9753 |
| TC0800008474.hg.1 | BAALC | brain and acute leukemia, cytoplasmic | Multiple_C | 3.4 | 3.12 | 3.34 | 1.04 | 0.9693 | 0.9851 | 3.83 | 3.64 | 3.47 | 1.28 | 0.2231 | 0.4837 |
| TC0800008540.hg.1 | TRHR | thyrotropin-releasing hormone receptor | Coding | 2.81 | 2.64 | 2.75 | 1.04 | 0.7595 | 0.8763 | 3.39 | 3.58 | 3.54 | 0.90 | 0.382 | 0.6412 |
| TC0800009178.hg.1 | C8orf31 | chromosome 8 open reading frame 31 | Multiple_C | 2.89 | 2.88 | 2.83 | 1.04 | 0.5171 | 0.7113 | 3.5 | 3.52 | 3.28 | 1.16 | 0.2387 | 0.5021 |
| TC0800009241.hg.1 | WDR97 | WD repeat domain 97 | Multiple_C | 3.05 | 2.83 | 2.99 | 1.04 | 0.579 | 0.7567 | 3.33 | 3.34 | 3.3 | 1.02 | 0.3194 | 0.5834 |
| TC0800009444.hg.1 | DEFA5 | defensin, alpha 5, Paneth cell-specific | Coding | 3.39 | 3.38 | 3.33 | 1.04 | 0.9288 | 0.9663 | 3.41 | 3.28 | 3.44 | 0.98 | 0.876 | 0.9469 |
| TC0800010224.hg.1 | ZMAT4 | zinc finger, matrin-type 4 | Multiple_C | 2.72 | 2.7 | 2.66 | 1.04 | 0.5264 | 0.7184 | 3.25 | 3.36 | 3.08 | 1.13 | 0.173 | 0.4202 |
| TC0800010802.hg.1 | C8orf89 | chromosome 8 open reading frame 89 | Multiple_C | 3.89 | 4.29 | 3.83 | 1.04 | 0.8371 | 0.919 | 4.61 | 4.45 | 4.16 | 1.37 | 0.1896 | 0.4422 |
| TC0800012185.hg.1 | OPLAH | 5-oxoprolinase (ATP-hydrolysing) | Multiple_C | 6.04 | 6.37 | 5.98 | 1.04 | 0.6881 | 0.8318 | 5.15 | 5.24 | 5.08 | 1.05 | 0.8644 | 0.9422 |
| TC0900007047.hg.1 | DNAI1 | dynein, axonemal, intermediate chain 1 | Multiple_C | 4.04 | 3.86 | 3.98 | 1.04 | 0.1614 | 0.3384 | 4.62 | 4.37 | 4.24 | 1.30 | 0.2579 | 0.5228 |
| TC0900008425.hg.1 | ZNF483 | zinc finger protein 483 | Multiple_C | 6.04 | 6.53 | 5.98 | 1.04 | 0.6471 | 0.8043 | 6.16 | 5.91 | 6.05 | 1.08 | 0.3265 | 0.5903 |

| | | | | | | | | | | | | | | | |
|---|---|---|---|---|---|---|---|---|---|---|---|---|---|---|---|
| TC0900008708.hg.1 | LHX2 | LIM homeobox 2 | Coding | 3.58 | 3.34 | 3.52 | 1.04 | 0.9038 | 0.9538 | 3.62 | 3.57 | 3.46 | 1.12 | 0.5941 | 0.7951 |
| TC0900011067.hg.1 | OR13C2 | olfactory receptor, family 13, subfamily C, member 2 | Coding | 3.29 | 3.42 | 3.23 | 1.04 | 0.5185 | 0.7126 | 3.69 | 3.96 | 3.47 | 1.16 | 0.2987 | 0.5633 |
| TC0900011445.hg.1 | PDCL | phosducin like | Coding | 10.66 | 10.87 | 10.6 | 1.04 | 0.7975 | 0.8961 | 9.62 | 9.59 | 9.46 | 1.12 | 0.8919 | 0.9539 |
| TC0900012220.hg.1 | CDKN2B | cyclin-dependent kinase inhibitor 2B (p15, inhibits CDK4) | Multiple_C | 6.77 | 6.51 | 6.71 | 1.04 | 0.8199 | 0.9096 | 8.21 | 8.13 | 7.66 | 1.46 | 0.0171 | 0.1095 |
| TC0X00007390.hg.1 | KLF8 | Kruppel-like factor 8 | Multiple_C | 3.26 | 3.23 | 3.2 | 1.04 | 0.4734 | 0.6766 | 3.86 | 3.68 | 3.73 | 1.09 | 0.2798 | 0.5459 |
| TC0X00008586.hg.1 | LOC389895 | chromosome 16 open reading frame 72-like | Multiple_C | 4.92 | 4.94 | 4.86 | 1.04 | 0.9992 | 0.9997 | 6 | 5.96 | 5.83 | 1.13 | 0.1474 | 0.3836 |
| TC0X00008612.hg.1 | MAGEC1 | MAGE family member C1 | Coding | 4.22 | 4.09 | 4.16 | 1.04 | 0.3701 | 0.5828 | 4.18 | 4.03 | 3.9 | 1.21 | 0.3752 | 0.6358 |
| TC0X00008694.hg.1 | HSFX1 | heat shock transcription factor family, X-linked 1 | Coding | 3.85 | 3.84 | 3.79 | 1.04 | 0.9214 | 0.9636 | 4.59 | 4.38 | 4.27 | 1.25 | 0.1418 | 0.3745 |
| TC0X00009191.hg.1 | RS1 | retinoschisin 1 | Multiple_C | 3.28 | 3.24 | 3.22 | 1.04 | 0.4665 | 0.671 | 3.14 | 3.12 | 3.31 | 0.89 | 0.541 | 0.7601 |
| TC0X00009498.hg.1 | FUNDC1 | FUN14 domain containing 1 | Multiple_C | 9.09 | 10.75 | 9.03 | 1.04 | 0.767 | 0.8801 | 8.35 | 8.60 | 8.69 | 0.79 | 0.0196 | 0.1188 |
| TC0X00009661.hg.1 | SYP | synaptophysin | Multiple_C | 4.75 | 4.65 | 4.69 | 1.04 | 0.908 | 0.9558 | 5.59 | 5.95 | 5.85 | 0.84 | 0.2049 | 0.4612 |
| TC0X00009917.hg.1 | EDA2R | ectodysplasin A2 receptor | Multiple_C | 3.36 | 3.35 | 3.3 | 1.04 | 0.3992 | 0.6129 | 3.37 | 3.56 | 3.5 | 0.91 | 0.3657 | 0.628 |
| TC0X00010130.hg.1 | MAGT1 | magnesium transporter 1 | Multiple_C | 13.56 | 13.82 | 13.5 | 1.04 | 0.8148 | 0.9069 | 13.64 | 14.49 | 14.92 | 0.41 | 0.0009 | 0.0156 |
| TC0X00010131.hg.1 | PGAM4 | phosphoglycerate mutase family member 4 | Coding | 4.94 | 4.19 | 4.88 | 1.04 | 0.3176 | 0.5311 | 4.74 | 5.48 | 5.43 | 0.62 | 0.0106 | 0.0809 |
| TC0X00010440.hg.1 | H2BFWT | H2B histone family, member W, testis-specific | Coding | 2.48 | 2.11 | 2.42 | 1.04 | 0.7686 | 0.8808 | 3.09 | 3.01 | 2.88 | 1.16 | 0.2866 | 0.5517 |
| TC1000007275.hg.1 | CCNY | cyclin Y | Multiple_C | 9.91 | 10.17 | 9.85 | 1.04 | 0.9649 | 0.983 | 10.18 | 10.50 | 10.37 | 0.88 | 0.8228 | 0.9219 |
| TC1000008658.hg.1 | PAX2 | paired box 2 | Multiple_C | 3.12 | 2.88 | 3.06 | 1.04 | 0.531 | 0.7212 | 3.67 | 3.24 | 3.47 | 1.15 | 0.4893 | 0.7251 |
| TC1000008913.hg.1 | ADRA2A | adrenoceptor alpha 2A | Coding | 3.06 | 2.77 | 3 | 1.04 | 0.8478 | 0.9256 | 2.98 | 3.13 | 2.96 | 1.01 | 0.8393 | 0.9294 |
| TC1000009450.hg.1 | KNDC1 | Transcript Identified by AceView, Entrez Gene ID(s) 85442 | Coding | 4.28 | 3.86 | 4.22 | 1.04 | 0.3684 | 0.5812 | 4.32 | 4.77 | 5.24 | 0.53 | 0.0185 | 0.1149 |
| TC1000011490.hg.1 | SORBS1 | Transcript Identified by AceView, Entrez Gene ID(s) 10580 | Coding | 2.93 | 2.7 | 2.87 | 1.04 | 0.7524 | 0.8733 | 3.32 | 3.38 | 3.23 | 1.06 | 0.2245 | 0.4852 |
| TC1000011940.hg.1 | VAX1 | ventral anterior homeobox 1 | Coding | 5.49 | 5.34 | 5.43 | 1.04 | 0.8342 | 0.9172 | 5.21 | 5.49 | 5.46 | 0.84 | 0.1357 | 0.3662 |
| TC1100006525.hg.1 | KRTAP5-5 | keratin associated protein 5-5 | Coding | 4.97 | 4.46 | 4.91 | 1.04 | 0.9497 | 0.9767 | 4.37 | 4.17 | 4.49 | 0.92 | 0.6346 | 0.8208 |
| TC1100006541.hg.1 | LSP1; MIR784 | lymphocyte-specific protein 1; microRNA 7847 | Multiple_C | 9.68 | 9.34 | 9.62 | 1.04 | 0.2752 | 0.4842 | 8.83 | 8.85 | 8.57 | 1.20 | 0.3717 | 0.6332 |
| TC1100007167.hg.1 | FSHB | follicle stimulating hormone, beta polypeptide | Coding | 3.64 | 3.35 | 3.58 | 1.04 | 0.5012 | 0.6997 | 4.53 | 4.59 | 4.69 | 0.90 | 0.5913 | 0.7933 |
| TC1100007285.hg.1 | FJX1 | four jointed box 1 | Multiple_C | 6.27 | 6.15 | 6.21 | 1.04 | 0.3579 | 0.5714 | 7.87 | 8.15 | 7.82 | 1.04 | 0.9362 | 0.9734 |
| TC1100007620.hg.1 | OR5AS1 | olfactory receptor, family 5, subfamily AS, member 1 | Coding | 2.7 | 2.67 | 2.64 | 1.04 | 0.8318 | 0.9159 | 3.27 | 2.96 | 3.27 | 1.00 | 0.3643 | 0.6269 |
| TC1100007847.hg.1 | SCGB1A1 | secretoglobin, family 1A, member 1 (uteroglobin) | Coding | 4.02 | 3.66 | 3.96 | 1.04 | 0.7717 | 0.8821 | 3.53 | 3.61 | 3.71 | 0.88 | 0.823 | 0.9219 |
| TC1100008102.hg.1 | LRFN4 | leucine rich repeat and fibronectin type III domain containing 4 | Multiple_C | 6.99 | 6.89 | 6.93 | 1.04 | 0.9756 | 0.988 | 6.29 | 6.37 | 6.42 | 0.91 | 0.1793 | 0.4283 |
| TC1100008289.hg.1 | SHANK2-AS3 | SHANK2 antisense RNA 3 | Multiple_C | 5.4 | 5.49 | 5.34 | 1.04 | 0.2758 | 0.4849 | 4.9 | 4.91 | 4.84 | 1.04 | 0.8305 | 0.9247 |
| TC1100008532.hg.1 | AQP11 | aquaporin 11 | Multiple_C | 5.3 | 5.5 | 5.24 | 1.04 | 0.797 | 0.8958 | 4.25 | 4.63 | 4.48 | 0.85 | 0.1272 | 0.3531 |
| TC1100008899.hg.1 | CEP126 | centrosomal protein 126kDa | Coding | 4.86 | 5.25 | 4.8 | 1.04 | 0.9522 | 0.9779 | 6.03 | 5.83 | 5.33 | 1.62 | 0.0029 | 0.0343 |
| TC1100009960.hg.1 | OR52B2 | olfactory receptor, family 52, subfamily B, member 2 | Coding | 3.4 | 3.19 | 3.34 | 1.04 | 0.9278 | 0.966 | 3.99 | 3.63 | 3.64 | 1.27 | 0.1848 | 0.436 |
| TC1100010288.hg.1 | MRGPRX2 | MAS-related GPR, member X2 | Coding | 3.62 | 3.4 | 3.56 | 1.04 | 0.9536 | 0.9784 | 4.34 | 4.20 | 3.87 | 1.39 | 0.0114 | 0.0845 |

| | | | | | | | | | | | | | | | |
|---|---|---|---|---|---|---|---|---|---|---|---|---|---|---|---|
| TC1100010440.hg.1 | MPPED2 | metallophosphoesterase domain containing 2 | Multiple_C | 3.86 | 3.63 | 3.8 | 1.04 | 0.9423 | 0.9732 | 3.83 | 3.55 | 3.61 | 1.16 | 0.2471 | 0.5117 |
| TC1100010833.hg.1 | OR5I1 | olfactory receptor, family 5, subfamily I, member 1 | Coding | 2.8 | 3.18 | 2.74 | 1.04 | 0.4297 | 0.64 | 3.85 | 3.86 | 3.4 | 1.37 | 0.0801 | 0.2728 |
| TC1100010932.hg.1 | OR10W1 | olfactory receptor, family 10, subfamily W, member 1 | Coding | 4.27 | 4.22 | 4.21 | 1.04 | 0.8077 | 0.9026 | 3.73 | 4.03 | 3.64 | 1.06 | 0.496 | 0.7298 |
| TC1100011058.hg.1 | FTH1 | ferritin, heavy polypeptide 1 | Multiple_C | 17.76 | 17.4 | 17.7 | 1.04 | 0.1709 | 0.3518 | 17.34 | 16.94 | 17.38 | 0.97 | 0.7741 | 0.8985 |
| TC1100011083.hg.1 | AHNAK | AHNAK nucleoprotein | Multiple_C | 8.01 | 8.13 | 7.95 | 1.04 | 0.8767 | 0.9401 | 6.93 | 6.81 | 6.42 | 1.42 | 0.3342 | 0.5982 |
| TC1100011113.hg.1 | SLC22A8 | solute carrier family 22 (organic anion transporter), member 8 | Multiple_C | 3.57 | 3.23 | 3.51 | 1.04 | 0.657 | 0.8117 | 4.4 | 4.16 | 3.59 | 1.75 | 0.0013 | 0.0198 |
| TC1100011291.hg.1 | ZDHHC24 | zinc finger, DHHC-type containing 24 | Multiple_C | 5.21 | 4.95 | 5.15 | 1.04 | 0.257 | 0.4633 | 5.42 | 5.42 | 5.05 | 1.29 | 0.1705 | 0.4168 |
| TC1100011339.hg.1 | CABP2 | calcium binding protein 2 | Coding | 5.61 | 5.62 | 5.55 | 1.04 | 0.2461 | 0.4497 | 5.39 | 5.79 | 5.54 | 0.90 | 0.4645 | 0.7066 |
| TC1100011446.hg.1 | FGF19 | fibroblast growth factor 19 | Coding | 4.41 | 4.35 | 4.35 | 1.04 | 0.1936 | 0.3829 | 13.43 | 11.20 | 11.29 | 4.41 | 2.02E-07 | 4.55E-05 |
| TC1100012698.hg.1 | HEPACAM | hepatic and glial cell adhesion molecule | Multiple_C | 2.84 | 2.79 | 2.78 | 1.04 | 0.3586 | 0.5723 | 3.15 | 3.03 | 2.95 | 1.15 | 0.2886 | 0.5532 |
| TC1100013080.hg.1 | FOLH1B | folate hydrolase 1B | Multiple_C | 3.67 | 3.79 | 3.61 | 1.04 | 0.811 | 0.9044 | 3.56 | 3.68 | 3.63 | 0.95 | 0.3871 | 0.646 |
| TC1200007418.hg.1 | ANO6 | anoctamin 6 | Multiple_C | 11.32 | 10.75 | 11.26 | 1.04 | 0.6654 | 0.8171 | 12.2 | 11.19 | 12.06 | 1.10 | 0.2012 | 0.4566 |
| TC1200007714.hg.1 | AMHR2 | anti-Mullerian hormone receptor, type II | Multiple_C | 2.78 | 2.69 | 2.72 | 1.04 | 0.5422 | 0.7291 | 3.08 | 2.87 | 3.11 | 0.98 | 0.8274 | 0.9237 |
| TC1200007796.hg.1 | OR6C68 | olfactory receptor, family 6, subfamily C, member 68 | Coding | 3.92 | 4.31 | 3.86 | 1.04 | 0.9771 | 0.9889 | 4.19 | 4.07 | 3.84 | 1.27 | 0.1508 | 0.3887 |
| TC1200009452.hg.1 | MMP17 | matrix metallopeptidase 17 (membrane-inserted) | Multiple_C | 4.43 | 4.41 | 4.37 | 1.04 | 0.4866 | 0.6879 | 5.95 | 5.97 | 5.88 | 1.05 | 0.9914 | 0.9963 |
| TC1200009847.hg.1 | A2M | alpha-2-macroglobulin | Multiple_C | 3.59 | 3.75 | 3.53 | 1.04 | 0.5676 | 0.7482 | 9.21 | 5.40 | 4.14 | 33.59 | 2.03E-11 | 4.83E-08 |
| TC1200010572.hg.1 | OR10AD1 | olfactory receptor, family 10, subfamily AD, member 1 | Coding | 2.87 | 3.22 | 2.81 | 1.04 | 0.7869 | 0.8909 | 3.71 | 3.47 | 3.65 | 1.04 | 0.7016 | 0.8586 |
| TC1200010865.hg.1 | ITGA7 | integrin alpha 7 | Multiple_C | 4.35 | 4.33 | 4.29 | 1.04 | 0.721 | 0.853 | 5.81 | 5.70 | 5.68 | 1.09 | 0.6819 | 0.8474 |
| TC1200011051.hg.1 | LINC01465 | long intergenic non-protein coding RNA 1465 | Multiple_C | 2.98 | 3.01 | 2.92 | 1.04 | 0.7977 | 0.8961 | 3.05 | 3.39 | 2.96 | 1.06 | 0.2587 | 0.523 |
| TC1200012747.hg.1 | RBP5 | retinol binding protein 5, cellular | Multiple_C | 4.95 | 4.54 | 4.89 | 1.04 | 0.8033 | 0.8995 | 5.32 | 5.31 | 5.29 | 1.02 | 0.5782 | 0.7843 |
| TC1400006510.hg.1 | OR11H4 | olfactory receptor, family 11, subfamily H, member 4 | Coding | 3.08 | 2.88 | 3.02 | 1.04 | 0.8776 | 0.9404 | 3.11 | 3.19 | 3.11 | 1.00 | 0.1606 | 0.4032 |
| TC1400008669.hg.1 | OR6J1 | olfactory receptor, family 6, subfamily J, member 1 (gene/pseuc | Coding | 3.95 | 3.92 | 3.89 | 1.04 | 0.7692 | 0.881 | 3.6 | 3.51 | 3.5 | 1.07 | 0.4427 | 0.6913 |
| TC1400008760.hg.1 | SDR39U1 | short chain dehydrogenase/reductase family 39U, member 1 | Multiple_C | 9.48 | 9.66 | 9.42 | 1.04 | 0.9066 | 0.9552 | 8.28 | 8.50 | 8.42 | 0.91 | 0.522 | 0.7487 |
| TC1400009703.hg.1 | RPS6KL1 | ribosomal protein S6 kinase-like 1 | Multiple_C | 4.62 | 4.59 | 4.56 | 1.04 | 0.6777 | 0.8247 | 4.06 | 4.14 | 3.95 | 1.08 | 0.8215 | 0.9211 |
| TC1400010361.hg.1 | TMEM179 | transmembrane protein 179 | Coding | 4.01 | 3.72 | 3.95 | 1.04 | 0.7673 | 0.8802 | 4.05 | 3.72 | 3.81 | 1.18 | 0.1645 | 0.409 |
| TC1500006959.hg.1 | CHST14 | carbohydrate (N-acetylgalactosamine 4-0) sulfotransferase 14 | Coding | 5.84 | 5.83 | 5.78 | 1.04 | 0.7833 | 0.8885 | 6.97 | 7.58 | 7.47 | 0.71 | 0.014 | 0.0959 |
| TC1500007109.hg.1 | DUOXA2 | dual oxidase maturation factor 2 | Multiple_C | 2.69 | 2.47 | 2.63 | 1.04 | 0.3376 | 0.5513 | 3.76 | 3.71 | 3.5 | 1.20 | 0.1665 | 0.4116 |
| TC1500008670.hg.1 | POTEB | POTE ankyrin domain family, member B | Multiple_C | 3.29 | 3.38 | 3.23 | 1.04 | 0.8364 | 0.9186 | 4.63 | 4.45 | 4.02 | 1.53 | 0.0024 | 0.0305 |
| TC1500009720.hg.1 | HERC1 | HECT and RLD domain containing E3 ubiquitin protein ligase fan | Multiple_C | 7.76 | 7.78 | 7.7 | 1.04 | 0.4303 | 0.6406 | 7.43 | 7.10 | 7.47 | 0.97 | 0.182 | 0.4323 |
| TC1500009796.hg.1 | IGDCC4 | immunoglobulin superfamily, DCC subclass, member 4 | Multiple_C | 3.91 | 4 | 3.85 | 1.04 | 0.9004 | 0.9522 | 3.57 | 3.37 | 3.2 | 1.29 | 0.0121 | 0.0881 |
| TC1500009861.hg.1 | ITGA11 | integrin alpha 11 | Multiple_C | 3.7 | 3.36 | 3.64 | 1.04 | 0.55 | 0.735 | 3.97 | 4.19 | 4.18 | 0.86 | 0.9664 | 0.9859 |
| TC1500010591.hg.1 | FAM169B | family with sequence similarity 169, member B | Coding | 4.33 | 4.29 | 4.27 | 1.04 | 0.6412 | 0.8012 | 4.39 | 4.34 | 4.22 | 1.13 | 0.2811 | 0.5469 |
| TC1500010877.hg.1 | PARP16 | poly(ADP-ribose) polymerase family member 16 | Multiple_C | 5.65 | 6.36 | 5.59 | 1.04 | 0.8885 | 0.9462 | 5.79 | 6.51 | 6.39 | 0.66 | 0.0137 | 0.0949 |

| | | | | | | | | | | | | | | | |
|---|---|---|---|---|---|---|---|---|---|---|---|---|---|---|---|
| TC1500010911.hg.1 | CPEB1 | cytoplasmic polyadenylation element binding protein 1 | Multiple_C | 4.24 | 3.97 | 4.18 | 1.04 | 0.3444 | 0.5581 | 4.5 | 4.21 | 4.14 | 1.28 | 0.0837 | 0.2785 |
| TC1600007345.hg.1 | APOBR | apolipoprotein B receptor | Coding | 4.06 | 3.49 | 4 | 1.04 | 0.985 | 0.9928 | 3.17 | 3.39 | 3.31 | 0.91 | 0.3819 | 0.641 |
| TC1600007436.hg.1 | TAOK2 | TAO kinase 2 | Multiple_C | 8.26 | 7.92 | 8.2 | 1.04 | 0.8283 | 0.9138 | 7.89 | 8.90 | 8.77 | 0.54 | 0.002 | 0.0269 |
| TC1600008330.hg.1 | HPR | haptoglobin-related protein | Multiple_C | 3.23 | 3.44 | 3.17 | 1.04 | 0.8338 | 0.9171 | 4.13 | 3.54 | 3.49 | 1.56 | 0.0131 | 0.0924 |
| TC1600008840.hg.1 | LOC339059; R | uncharacterized LOC339059; novel transcript, antisense to FAM | Multiple_C | 3.64 | 3.31 | 3.58 | 1.04 | 0.6954 | 0.8363 | 3.37 | 3.56 | 3.49 | 0.92 | 0.9409 | 0.9756 |
| TC1600009047.hg.1 | PTX4 | pentraxin 4, long | Coding | 3.49 | 3.15 | 3.43 | 1.04 | 0.6869 | 0.8311 | 3.53 | 3.40 | 3.52 | 1.01 | 0.6364 | 0.8215 |
| TC1600010603.hg.1 | RRAD | Ras-related associated with diabetes | Multiple_C | 2.77 | 2.77 | 2.71 | 1.04 | 0.8491 | 0.926 | 3.87 | 3.69 | 3.65 | 1.16 | 0.2206 | 0.4807 |
| TC1600010778.hg.1 | HYDIN | HYDIN, axonemal central pair apparatus protein | Multiple_C | 2.66 | 2.58 | 2.6 | 1.04 | 0.8242 | 0.9116 | 3.57 | 3.13 | 2.98 | 1.51 | 0.0414 | 0.1871 |
| TC1600011050.hg.1 | KCNG4 | potassium channel, voltage gated modifier subfamily G, membe | Coding | 2.91 | 3 | 2.85 | 1.04 | 0.8174 | 0.9079 | 3.98 | 3.54 | 3.09 | 1.85 | 0.0003 | 0.007 |
| TC1700007634.hg.1 | ZNHIT3 | zinc finger, HIT-type containing 3 | Multiple_C | 9.91 | 12.13 | 9.85 | 1.04 | 0.8746 | 0.9391 | 6.15 | 6.81 | 6.8 | 0.64 | 0.0532 | 0.2155 |
| TC1700007754.hg.1 | PNMT | phenylethanolamine N-methyltransferase | Multiple_C | 4.98 | 4.69 | 4.92 | 1.04 | 0.7576 | 0.8753 | 5.39 | 5.94 | 5.28 | 1.08 | 0.2433 | 0.5069 |
| TC1700009173.hg.1 | BAHCC1 | BAH domain and coiled-coil containing 1 | Multiple_C | 6.32 | 6.18 | 6.26 | 1.04 | 0.9896 | 0.9944 | 6.29 | 6.39 | 6.43 | 0.91 | 0.5887 | 0.7918 |
| TC1700009472.hg.1 | OR1G1 | olfactory receptor, family 1, subfamily G, member 1 | Coding | 2.94 | 2.81 | 2.88 | 1.04 | 0.7434 | 0.8674 | 3.38 | 3.29 | 3.42 | 0.97 | 0.8854 | 0.9508 |
| TC1700010195.hg.1 | SLC46A1 | solute carrier family 46 (folate transporter), member 1 | Multiple_C | 4.77 | 4.35 | 4.71 | 1.04 | 0.928 | 0.9661 | 5.81 | 5.85 | 6.14 | 0.80 | 0.0197 | 0.1191 |
| TC1700010587.hg.1 | IKZF3 | IKAROS family zinc finger 3 | Multiple_C | 4.4 | 3.97 | 4.34 | 1.04 | 0.7881 | 0.8915 | 4.89 | 4.14 | 4.36 | 1.44 | 0.2227 | 0.4833 |
| TC1700010658.hg.1 | KRTAP4-2 | keratin associated protein 4-2 | Coding | 4.4 | 4.26 | 4.34 | 1.04 | 0.8251 | 0.9121 | 4.23 | 4.03 | 3.93 | 1.23 | 0.062 | 0.2366 |
| TC1700012378.hg.1 | SEBOX | SEBOX homeobox | Coding | 2.6 | 2.58 | 2.54 | 1.04 | 0.7806 | 0.887 | 3.12 | 2.87 | 2.98 | 1.10 | 0.0887 | 0.2882 |
| TC1800007392.hg.1 | BOD1L2 | biorientation of chromosomes in cell division 1-like 2 | Coding | 3.39 | 3.39 | 3.33 | 1.04 | 0.524 | 0.7165 | 3.46 | 3.13 | 3.26 | 1.15 | 0.2767 | 0.5428 |
| TC1800008431.hg.1 | CCDC178 | coiled-coil domain containing 178 | Multiple_C | 3.23 | 3.39 | 3.17 | 1.04 | 0.3399 | 0.5538 | 3.29 | 3.82 | 3.42 | 0.91 | 0.663 | 0.8355 |
| TC1900007085.hg.1 | ZNF791 | zinc finger protein 791 | Multiple_C | 9.17 | 9.71 | 9.11 | 1.04 | 0.5311 | 0.7212 | 9.12 | 8.72 | 8.31 | 1.75 | 0.0013 | 0.0204 |
| TC1900007688.hg.1 | CCNE1 | cyclin E1 | Multiple_C | 8.6 | 7.73 | 8.54 | 1.04 | 0.6957 | 0.8366 | 6.04 | 5.89 | 6.46 | 0.75 | 0.1294 | 0.3562 |
| TC1900007859.hg.1 | FFAR2 | free fatty acid receptor 2 | Multiple_C | 3.56 | 3.74 | 3.5 | 1.04 | 0.7562 | 0.8745 | 4.38 | 4.08 | 3.73 | 1.57 | 0.0062 | 0.0564 |
| TC1900008058.hg.1 | PLEKHG2 | pleckstrin homology domain containing, family G (with RhoGef ( | Multiple_C | 4.51 | 4.79 | 4.45 | 1.04 | 0.7991 | 0.8967 | 4.15 | 4.35 | 3.86 | 1.22 | 0.2033 | 0.4588 |
| TC1900008825.hg.1 | PRKCG | protein kinase C, gamma | Coding | 3.71 | 3.7 | 3.65 | 1.04 | 0.6794 | 0.8258 | 3.8 | 3.62 | 3.41 | 1.31 | 0.0535 | 0.2162 |
| TC1900009164.hg.1 | PCSK4 | proprotein convertase subtilisin/kexin type 4 | Multiple_C | 5.74 | 5.64 | 5.68 | 1.04 | 0.4137 | 0.6258 | 6.08 | 5.98 | 5.86 | 1.16 | 0.771 | 0.8972 |
| TC1900009226.hg.1 | LINGO3 | leucine rich repeat and Ig domain containing 3 | Coding | 4.31 | 4.56 | 4.25 | 1.04 | 0.7692 | 0.881 | 4.74 | 4.69 | 4.68 | 1.04 | 0.7544 | 0.8884 |
| TC1900009396.hg.1 | LONP1 | lon peptidase 1, mitochondrial | Multiple_C | 9.42 | 10.07 | 9.36 | 1.04 | 0.8115 | 0.9048 | 9.1 | 9.25 | 9.19 | 0.94 | 0.3212 | 0.5852 |
| TC1900009492.hg.1 | CLEC4G | C-type lectin domain family 4, member G | Multiple_C | 5.2 | 4.89 | 5.14 | 1.04 | 0.9309 | 0.9675 | 5.3 | 5.38 | 5.28 | 1.01 | 0.9472 | 0.9786 |
| TC1900009517.hg.1 | FBN3 | fibrillin 3 | Multiple_C | 3.8 | 3.68 | 3.74 | 1.04 | 0.9513 | 0.9776 | 4.11 | 4.15 | 4.3 | 0.88 | 0.8847 | 0.9505 |
| TC1900009608.hg.1 | EIF3G | eukaryotic translation initiation factor 3, subunit G | Multiple_C | 13.14 | 13.76 | 13.08 | 1.04 | 0.8737 | 0.9389 | 12.11 | 12.78 | 12.14 | 0.98 | 0.1942 | 0.4481 |
| TC1900009832.hg.1 | ADGRE3 | adhesion G protein-coupled receptor E3 | Coding | 4 | 3.7 | 3.94 | 1.04 | 0.5801 | 0.7577 | 3.66 | 3.48 | 3.68 | 0.99 | 0.9175 | 0.9663 |
| TC1900009893.hg.1 | CYP4F2 | cytochrome P450, family 4, subfamily F, polypeptide 2 | Multiple_C | 4.61 | 4.29 | 4.55 | 1.04 | 0.6456 | 0.8035 | 4.96 | 4.81 | 4.78 | 1.13 | 0.4694 | 0.7105 |
| TC1900009991.hg.1 | JAK3 | Janus kinase 3 | Multiple_C | 7.41 | 7.25 | 7.35 | 1.04 | 0.6913 | 0.8335 | 6.44 | 6.37 | 6.51 | 0.95 | 0.5127 | 0.7424 |

| | | | | | | | | | | | | | | | |
|---|---|---|---|---|---|---|---|---|---|---|---|---|---|---|---|
| TC1900010009.hg.1 | JUND | jun D proto-oncogene | Coding | 8.96 | 8.45 | 8.9 | 1.04 | 0.8866 | 0.9453 | 8.07 | 8.17 | 8.08 | 0.99 | 0.996 | 0.9982 |
| TC1900010386.hg.1 | CEBPA | CCAAT/enhancer binding protein (C/EBP), alpha | Coding | 3.49 | 3.41 | 3.43 | 1.04 | 0.7686 | 0.8808 | 3.49 | 3.95 | 3.61 | 0.92 | 0.9316 | 0.9711 |
| TC1900010765.hg.1 | CEACAM7 | carcinoembryonic antigen-related cell adhesion molecule 7 | Multiple_C | 3.08 | 3.3 | 3.02 | 1.04 | 0.7889 | 0.8921 | 3 | 2.91 | 3.23 | 0.85 | 0.9883 | 0.995 |
| TC1900011055.hg.1 | BSPH1 | binder of sperm protein homolog 1 | Coding | 2.72 | 2.85 | 2.66 | 1.04 | 0.6255 | 0.791 | 3.52 | 3.73 | 3.38 | 1.10 | 0.1766 | 0.4253 |
| TC1900011270.hg.1 | HAS1 | hyaluronan synthase 1 | Multiple_C | 3.13 | 2.97 | 3.07 | 1.04 | 0.9883 | 0.9937 | 4.21 | 3.70 | 3.86 | 1.27 | 0.0238 | 0.1333 |
| TC1900011652.hg.1 | ZNF358 | zinc finger protein 358 | Multiple_C | 3.47 | 3.4 | 3.41 | 1.04 | 0.6433 | 0.802 | 6.05 | 7.32 | 7.31 | 0.42 | 0.0048 | 0.0477 |
| TC1900011892.hg.1 | RTBDN | retbindin | Coding | 3.68 | 3.44 | 3.62 | 1.04 | 0.8079 | 0.9027 | 3.09 | 3.17 | 3.29 | 0.87 | 0.2575 | 0.5225 |
| TC1900011900.hg.1 | CHERP | calcium homeostasis endoplasmic reticulum protein | Multiple_C | 9.66 | 9.48 | 9.6 | 1.04 | 0.7323 | 0.8603 | 8.82 | 9.60 | 9.58 | 0.59 | 0.0013 | 0.0202 |
| TC2000006601.hg.1 | CDS2 | CDP-diacylglycerol synthase 2 | Multiple_C | 8.66 | 8.39 | 8.6 | 1.04 | 0.8191 | 0.9091 | 10.5 | 10.89 | 10.35 | 1.11 | 0.425 | 0.6777 |
| TC2000007633.hg.1 | DDX27 | DEAD (Asp-Glu-Ala-Asp) box polypeptide 27 | Multiple_C | 9.83 | 10.33 | 9.77 | 1.04 | 0.965 | 0.9831 | 10.49 | 10.63 | 10.82 | 0.80 | 0.2464 | 0.5109 |
| TC2000007905.hg.1 | LOC79160; RP | uncharacterized LOC79160; novel transcript | Multiple_C | 3.75 | 3.58 | 3.69 | 1.04 | 0.7236 | 0.8547 | 4.42 | 3.99 | 4.2 | 1.16 | 0.3615 | 0.6242 |
| TC2000008027.hg.1 | NTSR1 | neurotensin receptor 1 (high affinity) | Multiple_C | 4.62 | 4.4 | 4.56 | 1.04 | 0.5188 | 0.7127 | 11.73 | 10.82 | 11.29 | 1.36 | 0.0378 | 0.1771 |
| TC2000008824.hg.1 | FOXS1 | forkhead box S1 | Multiple_C | 3.58 | 3.15 | 3.52 | 1.04 | 0.7553 | 0.8745 | 3.88 | 3.84 | 4.01 | 0.91 | 0.9354 | 0.9728 |
| TC2000009014.hg.1 | NDRG3 | NDRG family member 3 | Multiple_C | 12.42 | 11.83 | 12.36 | 1.04 | 0.8573 | 0.9303 | 12.51 | 11.78 | 12.3 | 1.16 | 0.4964 | 0.7301 |
| TC2100007176.hg.1 | PCP4 | Purkinje cell protein 4 | Multiple_C | 6.11 | 6.09 | 6.05 | 1.04 | 0.9548 | 0.9788 | 5.13 | 4.67 | 4.43 | 1.62 | 0.0674 | 0.2474 |
| TC2100007362.hg.1 | LRRC3 | leucine rich repeat containing 3 | Coding | 4.84 | 4.84 | 4.78 | 1.04 | 0.6402 | 0.8006 | 4.55 | 4.44 | 4.49 | 1.04 | 0.8924 | 0.954 |
| TC2100008252.hg.1 | TMPRSS3 | transmembrane protease, serine 3 | Multiple_C | 4.82 | 5.34 | 4.76 | 1.04 | 0.5342 | 0.7237 | 5.16 | 4.80 | 5.3 | 0.91 | 0.8023 | 0.9117 |
| TC2200006630.hg.1 | TANGO2 | transport and golgi organization 2 homolog | Multiple_C | 7.79 | 7.04 | 7.73 | 1.04 | 0.9436 | 0.974 | 8.39 | 8.73 | 9.08 | 0.62 | 0.1216 | 0.3442 |
| TC2200007494.hg.1 | CCDC134 | coiled-coil domain containing 134 | Multiple_C | 8.57 | 8.93 | 8.51 | 1.04 | 0.9262 | 0.9655 | 7.96 | 8.20 | 8.74 | 0.58 | 0.0003 | 0.0081 |
| TC2200008367.hg.1 | CHEK2 | checkpoint kinase 2 | Multiple_C | 8.1 | 9 | 8.04 | 1.04 | 0.2262 | 0.4251 | 5.64 | 5.52 | 6.02 | 0.77 | 0.0787 | 0.2703 |
| TC2200008635.hg.1 | TMPRSS6 | transmembrane protease, serine 6 | Multiple_C | 3.07 | 3 | 3.01 | 1.04 | 0.8139 | 0.9063 | 3.59 | 3.73 | 3.52 | 1.05 | 0.0662 | 0.2454 |
| TC2200009269.hg.1 | APOBEC3A | apolipoprotein B mRNA editing enzyme, catalytic polypeptide-li | Multiple_C | 4.09 | 3.7 | 4.03 | 1.04 | 0.9011 | 0.9524 | 3.84 | 3.85 | 3.75 | 1.06 | 0.4393 | 0.6896 |
| TSUnmapped00000099.h | OR5P2 | olfactory receptor, family 5, subfamily P, member 2 | Coding | 2.52 | 2.6 | 2.46 | 1.04 | 0.7157 | 0.8497 | 2.94 | 2.89 | 3.17 | 0.85 | 0.9708 | 0.9879 |
| TSUnmapped00000317.h | HHAT | hedgehog acyltransferase | Coding | 4.51 | 4.45 | 4.45 | 1.04 | 0.9825 | 0.9919 | 4.31 | 4.15 | 4.25 | 1.04 | 0.8773 | 0.9474 |
| TSUnmapped00000794.h | PRAMEF9 | PRAME family member 9 | Coding | 3.49 | 3.64 | 3.43 | 1.04 | 0.8151 | 0.907 | 3.98 | 4.07 | 4.21 | 0.85 | 0.6001 | 0.7995 |
| TC0100006479.hg.1 | KLHL17 | kelch-like family member 17 | Multiple_C | 6.06 | 5.92 | 6.01 | 1.04 | 0.9295 | 0.9665 | 5.81 | 5.97 | 5.76 | 1.04 | 0.2521 | 0.5163 |
| TC0100007570.hg.1 | YTHDF2 | YTH N(6)-methyladenosine RNA binding protein 2 | Multiple_C | 12.32 | 11.48 | 12.27 | 1.04 | 0.5493 | 0.7343 | 10.98 | 11.08 | 11.31 | 0.80 | 0.0182 | 0.1136 |
| TC0100007778.hg.1 | TFAP2E | transcription factor AP-2 epsilon (activating enhancer binding pr | Coding | 3.75 | 3.38 | 3.7 | 1.04 | 0.5114 | 0.7073 | 4.67 | 4.51 | 4.44 | 1.17 | 0.0427 | 0.1905 |
| TC0100008163.hg.1 | DMBX1 | diencephalon/mesencephalon homeobox 1 | Coding | 4.57 | 4.44 | 4.52 | 1.04 | 0.1484 | 0.3195 | 4 | 4.15 | 3.95 | 1.04 | 0.8605 | 0.9404 |
| TC0100008797.hg.1 | AK5 | adenylate kinase 5 | Multiple_C | 4.13 | 4.13 | 4.08 | 1.04 | 0.5833 | 0.7603 | 3.37 | 3.46 | 3.7 | 0.80 | 0.4456 | 0.6935 |
| TC0100009364.hg.1 | CSF1 | colony stimulating factor 1 (macrophage) | Multiple_C | 4.06 | 3.8 | 4.01 | 1.04 | 0.5736 | 0.7527 | 3.82 | 3.27 | 3.71 | 1.08 | 0.5451 | 0.7628 |
| TC0100011777.hg.1 | H3F3A; H3F3A | H3 histone, family 3A; H3 histone, family 3A, pseudogene 4 | Multiple_C | 15.73 | 16.27 | 15.68 | 1.04 | 0.5763 | 0.7549 | 13.09 | 13.59 | 12.79 | 1.23 | 0.094 | 0.2979 |
| TC0100012055.hg.1 | GGPS1 | geranylgeranyl diphosphate synthase 1 | Multiple_C | 9.59 | 11.43 | 9.54 | 1.04 | 0.6366 | 0.7981 | 8.72 | 8.00 | 8.35 | 1.29 | 0.8714 | 0.9446 |

| | | | | | | | | | | | | | | | |
|---|---|---|---|---|---|---|---|---|---|---|---|---|---|---|---|
| TC0100013314.hg.1 | IL22RA1 | interleukin 22 receptor, alpha 1 | Multiple_C | 9.5 | 8.52 | 9.45 | 1.04 | 0.7265 | 0.8561 | 4.16 | 4.80 | 4.5 | 0.79 | 0.1523 | 0.391 |
| TC0100015288.hg.1 | KCND3 | potassium channel, voltage gated Shal related subfamily D, mer | Multiple_C | 3.03 | 2.8 | 2.98 | 1.04 | 0.7793 | 0.8863 | 3.8 | 3.57 | 3.52 | 1.21 | 0.2768 | 0.5428 |
| TC0100016138.hg.1 | SLAMF1 | signaling lymphocytic activation molecule family member 1 | Multiple_C | 2.82 | 2.61 | 2.77 | 1.04 | 0.7194 | 0.8521 | 3.47 | 3.59 | 3.04 | 1.35 | 0.0631 | 0.2391 |
| TC0100017107.hg.1 | IL10 | interleukin 10 | Multiple_C | 3.28 | 2.92 | 3.23 | 1.04 | 0.6346 | 0.7966 | 4.48 | 4.10 | 4.17 | 1.24 | 0.083 | 0.2774 |
| TC0100018158.hg.1 | MMP23B; MM | matrix metallopeptidase 23B; matrix metallopeptidase 23A (pse | Multiple_C | 3.23 | 3.12 | 3.18 | 1.04 | 0.8588 | 0.9311 | 3.34 | 3.36 | 3.16 | 1.13 | 0.8733 | 0.9453 |
| TC0100018408.hg.1 | PRAMEF27 | PRAME family member 27 | Coding | 4.15 | 3.97 | 4.1 | 1.04 | 0.2643 | 0.4717 | 4.61 | 4.88 | 4.69 | 0.95 | 0.545 | 0.7628 |
| TC0200006440.hg.1 | ACP1 | acid phosphatase 1, soluble | Multiple_C | 13.48 | 14.44 | 13.43 | 1.04 | 0.7191 | 0.852 | 12.77 | 13.01 | 12.52 | 1.19 | 0.9894 | 0.9956 |
| TC0200008167.hg.1 | REG3G | regenerating islet-derived 3 gamma | Multiple_C | 4.41 | 4.43 | 4.36 | 1.04 | 0.3878 | 0.6014 | 4.1 | 4.03 | 4.17 | 0.95 | 0.8229 | 0.9219 |
| TC0200008447.hg.1 | TEKT4 | tektin 4 | Multiple_C | 4.53 | 4.11 | 4.48 | 1.04 | 0.5226 | 0.7156 | 4.58 | 4.59 | 4.56 | 1.01 | 0.758 | 0.8903 |
| TC0200008501.hg.1 | ITPRIPL1 | inositol 1,4,5-trisphosphate receptor interacting protein-like 1 | Coding | 2.97 | 2.8 | 2.92 | 1.04 | 0.9894 | 0.9943 | 10.17 | 9.77 | 10.1 | 1.05 | 0.5839 | 0.788 |
| TC0200010823.hg.1 | CDK5R2 | cyclin-dependent kinase 5, regulatory subunit 2 (p39) | Coding | 5.51 | 5.49 | 5.46 | 1.04 | 0.8329 | 0.9165 | 5.6 | 5.63 | 5.42 | 1.13 | 0.1812 | 0.4313 |
| TC0200013008.hg.1 | CD207 | CD207 molecule, langerin | Coding | 4.35 | 4.23 | 4.3 | 1.04 | 0.6975 | 0.8377 | 5.35 | 5.36 | 5.17 | 1.13 | 0.4848 | 0.7222 |
| TC0200014584.hg.1 | MMADHC | methylmalonic aciduria (cobalamin deficiency) cblD type, with h | Multiple_C | 12.51 | 13.44 | 12.46 | 1.04 | 0.1635 | 0.3417 | 10.74 | 10.04 | 10.15 | 1.51 | 0.0069 | 0.0606 |
| TC0200016688.hg.1 | RAB11FIP5 | RAB11 family interacting protein 5 (class I) | Multiple_C | 5.02 | 4.98 | 4.97 | 1.04 | 0.4407 | 0.6494 | 5.18 | 4.92 | 4.7 | 1.39 | 0.0067 | 0.0592 |
| TC0300007500.hg.1 | ITIH3 | inter-alpha-trypsin inhibitor heavy chain 3 | Multiple_C | 3.53 | 3.42 | 3.48 | 1.04 | 0.8384 | 0.9197 | 3.09 | 3.00 | 3.04 | 1.04 | 0.7906 | 0.9065 |
| TC0300008114.hg.1 | GPR15 | G protein-coupled receptor 15 | Coding | 3.73 | 3.45 | 3.68 | 1.04 | 0.8809 | 0.9422 | 3.86 | 3.88 | 4.21 | 0.78 | 0.4042 | 0.6599 |
| TC0300008979.hg.1 | CLSTN2 | calsyntenin 2 | Multiple_C | 3.34 | 3.6 | 3.29 | 1.04 | 0.7935 | 0.8941 | 4.16 | 3.86 | 4.01 | 1.11 | 0.097 | 0.3034 |
| TC0300011266.hg.1 | ASB14 | ankyrin repeat and SOCS box containing 14 | Multiple_C | 3.19 | 2.54 | 3.14 | 1.04 | 0.3469 | 0.5602 | 4.24 | 4.38 | 4.13 | 1.08 | 0.473 | 0.713 |
| TC0300012449.hg.1 | CPNE4 | copine IV | Multiple_C | 3.28 | 3.45 | 3.23 | 1.04 | 0.5146 | 0.7095 | 3.48 | 3.34 | 3.57 | 0.94 | 0.6982 | 0.857 |
| TC0300013019.hg.1 | SI | sucrase-isomaltase (alpha-glucosidase) | Multiple_C | 3.17 | 3.24 | 3.12 | 1.04 | 0.7 | 0.8395 | 3.68 | 3.77 | 3.89 | 0.86 | 0.428 | 0.6801 |
| TC0300013162.hg.1 | SPATA16 | spermatogenesis associated 16 | Coding | 2.55 | 2.53 | 2.5 | 1.04 | 0.8306 | 0.9156 | 3.59 | 3.71 | 3.34 | 1.19 | 0.1283 | 0.3545 |
| TC0300013930.hg.1 | SSUH2 | ssu-2 homolog (C. elegans) | Multiple_C | 3.41 | 3.18 | 3.36 | 1.04 | 0.5141 | 0.7091 | 3.45 | 3.48 | 3.22 | 1.17 | 0.0392 | 0.1804 |
| TC0400009086.hg.1 | GUCY1A3 | guanylate cyclase 1, soluble, alpha 3 | Multiple_C | 3.05 | 3.14 | 3 | 1.04 | 0.7774 | 0.8856 | 3.65 | 3.15 | 3.31 | 1.27 | 0.1477 | 0.384 |
| TC0400009892.hg.1 | CRMP1 | collapsin response mediator protein 1 | Multiple_C | 4.93 | 4.65 | 4.88 | 1.04 | 0.9494 | 0.9764 | 6.26 | 7.73 | 8.23 | 0.26 | 1.34E-08 | 6.27E-06 |
| TC0400010260.hg.1 | SEPSECS | Sep (O-phosphoserine) tRNA:Sec (selenocysteine) tRNA synthas | Multiple_C | 7.65 | 9.04 | 7.6 | 1.04 | 0.8229 | 0.9111 | 7.09 | 7.50 | 7.06 | 1.02 | 0.4113 | 0.666 |
| TC0400011015.hg.1 | CXCL3 | chemokine (C-X-C motif) ligand 3 | Multiple_C | 7.53 | 7.41 | 7.48 | 1.04 | 0.7378 | 0.8637 | 5.95 | 5.16 | 5.47 | 1.39 | 0.0185 | 0.1149 |
| TC0400012696.hg.1 | TRIML2 | tripartite motif family like 2 | Multiple_C | 3.55 | 3.32 | 3.5 | 1.04 | 0.9143 | 0.9599 | 4.1 | 3.63 | 3.24 | 1.82 | 0.0091 | 0.0735 |
| TC0500006950.hg.1 | PRDM9 | PR domain containing 9 | Coding | 3.05 | 3.5 | 3 | 1.04 | 0.8465 | 0.9247 | 3.25 | 3.29 | 3.3 | 0.97 | 0.9561 | 0.9818 |
| TC0500007912.hg.1 | ZFYVE16 | zinc finger, FYVE domain containing 16 | Multiple_C | 8.7 | 8.66 | 8.65 | 1.04 | 0.8645 | 0.9338 | 9.49 | 9.42 | 8.85 | 1.56 | 0.0023 | 0.0296 |
| TC0500008307.hg.1 | TSLP | thymic stromal lymphopoietin | Multiple_C | 3.03 | 3.15 | 2.98 | 1.04 | 0.2164 | 0.4122 | 3.53 | 3.16 | 3.17 | 1.28 | 0.3553 | 0.6186 |
| TC0500013118.hg.1 | OR2V1 | olfactory receptor, family 2, subfamily V, member 1 | Coding | 2.55 | 2.5 | 2.5 | 1.04 | 0.6062 | 0.7773 | 3.05 | 2.88 | 2.86 | 1.14 | 0.1776 | 0.4261 |
| TC0600006653.hg.1 | RIPK1 | receptor (TNFRSF)-interacting serine-threonine kinase 1 | Multiple_C | 7.63 | 7.36 | 7.58 | 1.04 | 0.8885 | 0.9462 | 7.8 | 8.50 | 8.08 | 0.82 | 0.0236 | 0.1328 |
| TC0600006793.hg.1 | CDYL | chromodomain protein, Y-like | Multiple_C | 8.3 | 8.54 | 8.25 | 1.04 | 0.6953 | 0.8363 | 8.6 | 8.08 | 8.2 | 1.32 | 0.2833 | 0.5487 |

| | | | | | | | | | | | | | | | |
|---|---|---|---|---|---|---|---|---|---|---|---|---|---|---|---|
| TC0600007555.hg.1 | DPCR1 | diffuse panbronchiolitis critical region 1 | Coding | 3.67 | 3.62 | 3.62 | 1.04 | 0.8601 | 0.9318 | 4.51 | 4.36 | 4.25 | 1.20 | 0.1988 | 0.4541 |
| TC0600008167.hg.1 | TDRD6 | tudor domain containing 6 | Coding | 4.98 | 5.1 | 4.93 | 1.04 | 0.6574 | 0.8118 | 4.6 | 4.42 | 4.45 | 1.11 | 0.3484 | 0.6123 |
| TC0600008509.hg.1 | KCNQ5 | potassium channel, voltage gated KQT-like subfamily Q, membe | Multiple_C | 3.51 | 3.61 | 3.46 | 1.04 | 0.5455 | 0.7312 | 4.3 | 4.27 | 4.96 | 0.63 | 0.0231 | 0.131 |
| TC0600010071.hg.1 | SLC22A1 | solute carrier family 22 (organic cation transporter), member 1 | Multiple_C | 5.69 | 5.82 | 5.64 | 1.04 | 0.4702 | 0.6742 | 5.32 | 5.02 | 4.99 | 1.26 | 0.222 | 0.4823 |
| TC0600014374.hg.1 | PHF10 | PHD finger protein 10 | Multiple_C | 11.05 | 11.31 | 11 | 1.04 | 0.9227 | 0.964 | 10.08 | 10.72 | 10.22 | 0.91 | 0.5499 | 0.7662 |
| TC0700007065.hg.1 | PLEKHA8 | pleckstrin homology domain containing, family A (phosphoinosi | Multiple_C | 8.65 | 8.73 | 8.6 | 1.04 | 0.935 | 0.9697 | 8.22 | 8.14 | 8.69 | 0.72 | 0.0387 | 0.1789 |
| TC0700007105.hg.1 | ADCYAP1R1 | adenylate cyclase activating polypeptide 1 (pituitary) receptor t | Multiple_C | 3.22 | 3.56 | 3.17 | 1.04 | 0.9432 | 0.9736 | 3.58 | 3.54 | 3.49 | 1.06 | 0.5233 | 0.7491 |
| TC0700009499.hg.1 | TAS2R60 | taste receptor, type 2, member 60 | Coding | 2.98 | 2.87 | 2.93 | 1.04 | 0.7616 | 0.8777 | 3.99 | 4.07 | 3.89 | 1.07 | 0.3554 | 0.6187 |
| TC0700009514.hg.1 | OR2F1 | olfactory receptor, family 2, subfamily F, member 1 (gene/pseu | Multiple_C | 3.7 | 3.68 | 3.65 | 1.04 | 0.8635 | 0.9335 | 4.09 | 4.20 | 4.38 | 0.82 | 0.1481 | 0.3847 |
| TC0700009816.hg.1 | EN2 | engrailed homeobox 2 | Coding | 2.68 | 2.67 | 2.63 | 1.04 | 0.6907 | 0.8333 | 5.9 | 6.38 | 6.57 | 0.63 | 0.1378 | 0.3688 |
| TC0800008044.hg.1 | STMN2 | stathmin 2 | Coding | 4.06 | 4.32 | 4.01 | 1.04 | 0.5312 | 0.7212 | 4.61 | 4.34 | 4.54 | 1.05 | 0.5506 | 0.7665 |
| TC0800008758.hg.1 | FAM91A1 | family with sequence similarity 91, member A1 | Multiple_C | 10.25 | 10.51 | 10.2 | 1.04 | 0.3729 | 0.5854 | 8.65 | 7.86 | 9.09 | 0.74 | 0.0202 | 0.1214 |
| TC0800012213.hg.1 | VPS28 | vacuolar protein sorting 28 homolog (S. cerevisiae) | Multiple_C | 9.72 | 10.5 | 9.67 | 1.04 | 0.8926 | 0.9483 | 7.26 | 7.04 | 6.51 | 1.68 | 0.0214 | 0.1252 |
| TC0800012393.hg.1 | ADRA1A | adrenoceptor alpha 1A | Multiple_C | 3.94 | 3.7 | 3.89 | 1.04 | 0.5867 | 0.763 | 4.54 | 4.51 | 4.31 | 1.17 | 0.7472 | 0.8841 |
| TC0900007452.hg.1 | ZNF658 | zinc finger protein 658 | Coding | 4.59 | 5.85 | 4.54 | 1.04 | 0.8614 | 0.9323 | 3.85 | 4.00 | 3.79 | 1.04 | 0.1215 | 0.3442 |
| TC0900007457.hg.1 | CNTNAP3P2; ( | contactin associated protein-like 3 pseudogene 2; contactin ass | Multiple_C | 3.42 | 3.09 | 3.37 | 1.04 | 0.9531 | 0.9784 | 4.03 | 5.08 | 4.71 | 0.62 | 0.1835 | 0.4344 |
| TC0900009453.hg.1 | INSL6 | insulin-like 6 | Multiple_C | 3.15 | 3.08 | 3.1 | 1.04 | 0.7122 | 0.8479 | 4.14 | 4.06 | 3.96 | 1.13 | 0.4919 | 0.7268 |
| TC0900009581.hg.1 | FREM1 | FRAS1 related extracellular matrix 1 | Multiple_C | 3.13 | 2.86 | 3.08 | 1.04 | 0.3391 | 0.5531 | 4.24 | 3.95 | 3.65 | 1.51 | 0.024 | 0.1338 |
| TC0900009702.hg.1 | IFNA5 | interferon, alpha 5 | Coding | 3.53 | 3.75 | 3.48 | 1.04 | 0.7418 | 0.8665 | 4.21 | 4.17 | 3.93 | 1.21 | 0.4166 | 0.6705 |
| TC0900010637.hg.1 | GAS1 | growth arrest-specific 1 | Coding | 3.82 | 3.56 | 3.77 | 1.04 | 0.7171 | 0.8504 | 3.95 | 4.36 | 4.3 | 0.78 | 0.4177 | 0.6715 |
| TC0900010825.hg.1 | NINJ1 | ninjurin 1 | Multiple_C | 6.68 | 6.19 | 6.63 | 1.04 | 0.8539 | 0.9287 | 7.14 | 6.98 | 7.39 | 0.84 | 0.1908 | 0.4439 |
| TC0900011857.hg.1 | FAM163B | family with sequence similarity 163, member B | Coding | 5.03 | 4.93 | 4.98 | 1.04 | 0.6201 | 0.7874 | 5.32 | 5.35 | 5.08 | 1.18 | 0.1516 | 0.3899 |
| TC0900012139.hg.1 | PGM5 | phosphoglucomutase 5 | Multiple_C | 3.22 | 2.99 | 3.17 | 1.04 | 0.7781 | 0.8859 | 3.91 | 5.29 | 4.01 | 0.93 | 0.4526 | 0.6977 |
| TC0X00006488.hg.1 | ARSH | arylsulfatase family, member H | Multiple_C | 3.39 | 3.41 | 3.34 | 1.04 | 0.8218 | 0.9104 | 4.32 | 3.86 | 3.58 | 1.67 | 0.0195 | 0.1182 |
| TC0X00006970.hg.1 | OTC | ornithine carbamoyltransferase | Multiple_C | 3.13 | 3.06 | 3.08 | 1.04 | 0.9201 | 0.963 | 4.06 | 4.23 | 3.84 | 1.16 | 0.0568 | 0.2242 |
| TC0X00007918.hg.1 | TNMD | tenomodulin | Multiple_C | 3.55 | 3.71 | 3.5 | 1.04 | 0.8562 | 0.9298 | 3.91 | 3.46 | 3.55 | 1.28 | 0.1607 | 0.4032 |
| TC0X00008276.hg.1 | ATP1B4 | ATPase, Na+/K+ transporting, beta 4 polypeptide | Coding | 4.04 | 4.12 | 3.99 | 1.04 | 0.8361 | 0.9183 | 3.55 | 3.45 | 3.42 | 1.09 | 0.3321 | 0.5962 |
| TC0X00009302.hg.1 | DCAF8L1 | DDB1 and CUL4 associated factor 8-like 1 | Coding | 3.93 | 3.94 | 3.88 | 1.04 | 0.8929 | 0.9484 | 4.02 | 3.97 | 3.87 | 1.11 | 0.4849 | 0.7222 |
| TC0X00010893.hg.1 | CT55 | cancer/testis antigen 55 | Multiple_C | 2.86 | 2.84 | 2.81 | 1.04 | 0.6703 | 0.8202 | 3.27 | 3.77 | 3.94 | 0.63 | 0.0693 | 0.2512 |
| TC0Y00006574.hg.1 | TSPY10 | testis specific protein, Y-linked 10 | Multiple_C | 3.35 | 3.21 | 3.3 | 1.04 | 0.9265 | 0.9656 | 3.7 | 3.51 | 3.57 | 1.09 | 0.7 | 0.8578 |
| TC0Y00006767.hg.1 | PRY2; PRY | PTPN13-like, Y-linked 2; PTPN13-like, Y-linked | Coding | 3.79 | 3.7 | 3.74 | 1.04 | 0.835 | 0.9177 | 3.91 | 4.00 | 3.92 | 0.99 | 0.5156 | 0.7446 |
| TC0Y00006861.hg.1 | GTPBP6 | Homo sapiens GTP binding protein 6 (putative) (GTPBP6), mRNA | Multiple_C | 5.45 | 5.35 | 5.4 | 1.04 | 0.7701 | 0.8811 | 5.98 | 6.73 | 6.61 | 0.65 | 0.0013 | 0.0198 |
| TC1000007010.hg.1 | MSRB2 | methionine sulfoxide reductase B2 | Multiple_C | 8.03 | 9.7 | 7.98 | 1.04 | 0.6082 | 0.7789 | 4.8 | 5.61 | 4.7 | 1.07 | 0.3627 | 0.6256 |

| | | | | | | | | | | | | | | | |
|---|---|---|---|---|---|---|---|---|---|---|---|---|---|---|---|
| TC1000009057.hg.1 | NANOS1 | nanos homolog 1 (Drosophila) | Coding | 4.69 | 4.37 | 4.64 | 1.04 | 0.865 | 0.9341 | 6.48 | 6.98 | 6.86 | 0.77 | 0.7337 | 0.8771 |
| TC1000009997.hg.1 | CASC10 | cancer susceptibility candidate 10 | Multiple_C | 6.34 | 6.16 | 6.29 | 1.04 | 0.884 | 0.9438 | 5.24 | 5.30 | 5.12 | 1.09 | 0.2418 | 0.5056 |
| TC1000009999.hg.1 | SKIDA1 | SKI/DACH domain containing 1 | Multiple_C | 4.04 | 3.69 | 3.99 | 1.04 | 0.6528 | 0.808 | 4.06 | 3.86 | 4.08 | 0.99 | 0.8251 | 0.9228 |
| TC1000010548.hg.1 | ZNF488 | zinc finger protein 488 | Coding | 3.86 | 3.64 | 3.81 | 1.04 | 0.5526 | 0.7367 | 3.98 | 4.13 | 4.15 | 0.89 | 0.2493 | 0.5138 |
| TC1000011190.hg.1 | SFTPD | surfactant protein D | Coding | 5.49 | 5.4 | 5.44 | 1.04 | 0.1205 | 0.2756 | 6.03 | 6.14 | 5.84 | 1.14 | 0.3105 | 0.5746 |
| TC1000012570.hg.1 | NDST2 | N-deacetylase/N-sulfotransferase (heparan glucosaminyl) 2 | Multiple_C | 6.86 | 7.07 | 6.81 | 1.04 | 0.6269 | 0.792 | 7.41 | 7.65 | 7.78 | 0.77 | 0.1331 | 0.3621 |
| TC1100007626.hg.1 | OR8H3 | olfactory receptor, family 8, subfamily H, member 3 | Coding | 2.67 | 2.4 | 2.62 | 1.04 | 0.5599 | 0.7425 | 3.57 | 3.34 | 3.25 | 1.25 | 0.112 | 0.3296 |
| TC1100007701.hg.1 | OR6Q1 | olfactory receptor, family 6, subfamily Q, member 1 (gene/pseu | Multiple_C | 3.98 | 3.7 | 3.93 | 1.04 | 0.2686 | 0.4768 | 3.59 | 3.67 | 3.69 | 0.93 | 0.3766 | 0.637 |
| TC1100008524.hg.1 | OMP | olfactory marker protein | Coding | 5.1 | 4.9 | 5.05 | 1.04 | 0.5529 | 0.7368 | 4.88 | 5.01 | 4.91 | 0.98 | 0.8434 | 0.9317 |
| TC1100008525.hg.1 | MYO7A | myosin VIIA | Multiple_C | 4.02 | 3.85 | 3.97 | 1.04 | 0.3011 | 0.5134 | 4.67 | 4.81 | 4.39 | 1.21 | 0.3582 | 0.6211 |
| TC1100008653.hg.1 | CCDC83 | coiled-coil domain containing 83 | Multiple_C | 3.16 | 3.07 | 3.11 | 1.04 | 0.5378 | 0.726 | 3.71 | 3.54 | 3.45 | 1.20 | 0.0613 | 0.235 |
| TC1100011629.hg.1 | XRRA1 | X-ray radiation resistance associated 1 | Multiple_C | 7.48 | 7.42 | 7.43 | 1.04 | 0.7237 | 0.8547 | 9.16 | 9.73 | 9.47 | 0.81 | 0.0847 | 0.28 |
| TC1200008595.hg.1 | NR1H4 | nuclear receptor subfamily 1, group H, member 4 | Coding | 2.9 | 3.07 | 2.85 | 1.04 | 0.5541 | 0.7377 | 2.96 | 3.18 | 3.08 | 0.92 | 0.8511 | 0.9358 |
| TC1200009076.hg.1 | SUDS3 | SDS3 homolog, SIN3A corepressor complex component | Multiple_C | 8.85 | 9.8 | 8.8 | 1.04 | 0.998 | 0.999 | 8.22 | 7.80 | 8.17 | 1.04 | 0.6637 | 0.8355 |
| TC1200009746.hg.1 | ACRBP | acrosin binding protein | Multiple_C | 2.73 | 2.49 | 2.68 | 1.04 | 0.7697 | 0.8811 | 3.39 | 3.22 | 3.22 | 1.13 | 0.4261 | 0.6785 |
| TC1200009747.hg.1 | ING4 | inhibitor of growth family member 4 | Multiple_C | 7.72 | 9.21 | 7.67 | 1.04 | 0.6618 | 0.8146 | 8.67 | 9.05 | 7.72 | 1.93 | 0.0011 | 0.0177 |
| TC1200009753.hg.1 | MLF2 | myeloid leukemia factor 2 | Multiple_C | 14.09 | 13.49 | 14.04 | 1.04 | 0.5612 | 0.7435 | 14.56 | 14.39 | 14.69 | 0.91 | 0.3941 | 0.6521 |
| TC1200009891.hg.1 | CLEC7A | C-type lectin domain family 7, member A | Multiple_C | 3.73 | 3.33 | 3.68 | 1.04 | 0.8704 | 0.9369 | 3.83 | 3.70 | 3.75 | 1.06 | 0.4728 | 0.7128 |
| TC1200011598.hg.1 | HAL | histidine ammonia-lyase | Multiple_C | 4.78 | 4.55 | 4.73 | 1.04 | 0.235 | 0.4361 | 5.53 | 5.43 | 5.06 | 1.39 | 0.3357 | 0.5999 |
| TC1200011846.hg.1 | CORO1C | coronin, actin binding protein, 1C | Multiple_C | 13.73 | 12.84 | 13.68 | 1.04 | 0.9873 | 0.9935 | 14.76 | 14.66 | 14.84 | 0.95 | 0.4087 | 0.664 |
| TC1200012748.hg.1 | CD163L1 | CD163 molecule-like 1 | Multiple_C | 3.86 | 3.69 | 3.81 | 1.04 | 0.7733 | 0.8832 | 6.71 | 6.29 | 8.36 | 0.32 | 0.0063 | 0.057 |
| TC1300006989.hg.1 | AKAP11 | A kinase (PRKA) anchor protein 11 | Multiple_C | 9.71 | 10.02 | 9.66 | 1.04 | 0.7698 | 0.8811 | 9.91 | 9.48 | 8.58 | 2.51 | 0.0016 | 0.0227 |
| TC1300008614.hg.1 | SPG20 | spastic paraplegia 20 (Troyer syndrome) | Multiple_C | 3.88 | 3.84 | 3.83 | 1.04 | 0.6277 | 0.7921 | 4.04 | 3.96 | 3.92 | 1.09 | 0.3023 | 0.5667 |
| TC1300009492.hg.1 | TGDS | TDP-glucose 4,6-dehydratase | Multiple_C | 11.27 | 12.45 | 11.22 | 1.04 | 0.6989 | 0.8388 | 11.22 | 11.18 | 11.09 | 1.09 | 0.9765 | 0.9904 |
| TC1400007155.hg.1 | C14orf166 | chromosome 14 open reading frame 166 | Multiple_C | 14.46 | 15.63 | 14.41 | 1.04 | 0.7638 | 0.8786 | 12.06 | 11.71 | 11.96 | 1.07 | 0.9495 | 0.9793 |
| TC1400008214.hg.1 | WDR25 | WD repeat domain 25 | Multiple_C | 5.32 | 5.19 | 5.27 | 1.04 | 0.8312 | 0.9158 | 5.2 | 5.24 | 5.27 | 0.95 | 0.732 | 0.8761 |
| TC1400008780.hg.1 | NOVA1 | neuro-oncological ventral antigen 1 | Multiple_C | 3.58 | 3.28 | 3.53 | 1.04 | 0.7831 | 0.8884 | 5.89 | 6.92 | 5.39 | 1.41 | 0.027 | 0.1435 |
| TC1500006748.hg.1 | FAN1 | FANCD2/FANCI-associated nuclease 1 | Multiple_C | 7.88 | 8.46 | 7.83 | 1.04 | 0.7045 | 0.8424 | 8.06 | 7.90 | 8.12 | 0.96 | 0.0873 | 0.2851 |
| TC1500007946.hg.1 | LINGO1-AS1 | LINGO1 antisense RNA 1 | Multiple_C | 2.73 | 2.64 | 2.68 | 1.04 | 0.7754 | 0.8844 | 3.46 | 3.37 | 3.48 | 0.99 | 0.451 | 0.6963 |
| TC1500009002.hg.1 | ACTC1 | actin, alpha, cardiac muscle 1 | Multiple_C | 5.45 | 5.29 | 5.4 | 1.04 | 0.3667 | 0.5799 | 5.26 | 5.51 | 5.52 | 0.84 | 0.173 | 0.4202 |
| TC1500009127.hg.1 | PPP1R14D | protein phosphatase 1, regulatory (inhibitor) subunit 14D | Coding | 3.68 | 3.64 | 3.63 | 1.04 | 0.3347 | 0.5486 | 4.71 | 4.57 | 4.09 | 1.54 | 0.0229 | 0.1303 |
| TC1500009170.hg.1 | SPTBN5 | spectrin, beta, non-erythrocytic 5 | Multiple_C | 5.83 | 5.76 | 5.78 | 1.04 | 0.4968 | 0.6967 | 5.99 | 5.81 | 5.69 | 1.23 | 0.21 | 0.467 |
| TC1600006492.hg.1 | METRN | meteorin, glial cell differentiation regulator | Multiple_C | 2.53 | 2.51 | 2.48 | 1.04 | 0.7653 | 0.8795 | 3.76 | 3.87 | 3.63 | 1.09 | 0.6636 | 0.8355 |

| | | | | | | | | | | | | | | | |
|---|---|---|---|---|---|---|---|---|---|---|---|---|---|---|---|
| TC1600006656.hg.1 | MMP25 | matrix metallopeptidase 25 | Multiple_C | 3.99 | 3.84 | 3.94 | 1.04 | 0.743 | 0.8672 | 4.59 | 4.66 | 4.38 | 1.16 | 0.3386 | 0.6028 |
| TC1600007307.hg.1 | C16orf82 | chromosome 16 open reading frame 82 | Multiple_C | 3.66 | 3.48 | 3.61 | 1.04 | 0.7227 | 0.8542 | 3.84 | 3.94 | 3.89 | 0.97 | 0.6739 | 0.842 |
| TC1600010811.hg.1 | PMFBP1 | polyamine modulated factor 1 binding protein 1 | Multiple_C | 2.89 | 2.83 | 2.84 | 1.04 | 0.9694 | 0.9852 | 3.94 | 3.14 | 3.54 | 1.32 | 0.0128 | 0.0913 |
| TC1700006767.hg.1 | WRAP53 | WD repeat containing, antisense to TP53 | Multiple_C | 5.85 | 5.35 | 5.8 | 1.04 | 0.1975 | 0.3883 | 5.25 | 5.84 | 5.86 | 0.66 | 0.0176 | 0.1112 |
| TC1700007108.hg.1 | MIEF2 | mitochondrial elongation factor 2 | Multiple_C | 4.38 | 4.23 | 4.33 | 1.04 | 0.6844 | 0.8292 | 4.42 | 4.69 | 4.71 | 0.82 | 0.1206 | 0.3428 |
| TC1700007565.hg.1 | TMEM132E | transmembrane protein 132E | Multiple_C | 4.26 | 4.06 | 4.21 | 1.04 | 0.8386 | 0.9197 | 6.12 | 6.08 | 6.1 | 1.01 | 0.2831 | 0.5486 |
| TC1700008768.hg.1 | KCNJ16 | potassium channel, inwardly rectifying subfamily J, member 16 | Multiple_C | 3.37 | 3.39 | 3.32 | 1.04 | 0.7354 | 0.8617 | 3.65 | 3.75 | 3.68 | 0.98 | 0.7832 | 0.9026 |
| TC1700009194.hg.1 | GCGR | glucagon receptor | Multiple_C | 2.91 | 2.84 | 2.86 | 1.04 | 0.6366 | 0.7981 | 3.33 | 3.60 | 3.54 | 0.86 | 0.9818 | 0.9925 |
| TC1700011722.hg.1 | FADS6 | fatty acid desaturase 6 | Multiple_C | 2.91 | 2.65 | 2.86 | 1.04 | 0.6203 | 0.7875 | 3.83 | 4.15 | 3.75 | 1.06 | 0.8574 | 0.9394 |
| TC1700012054.hg.1 | NPLOC4 | NPL4 homolog, ubiquitin recognition factor | Multiple_C | 9.98 | 9.3 | 9.93 | 1.04 | 0.591 | 0.7659 | 10.49 | 10.10 | 10.54 | 0.97 | 0.3606 | 0.6234 |
| TC1800006461.hg.1 | ADCYAP1 | adenylate cyclase activating polypeptide 1 (pituitary) | Multiple_C | 3.43 | 3.45 | 3.38 | 1.04 | 0.8558 | 0.9295 | 3.75 | 3.64 | 3.73 | 1.01 | 0.8488 | 0.9347 |
| TC1800007685.hg.1 | ZNF407 | zinc finger protein 407 | Multiple_C | 7.11 | 7.1 | 7.06 | 1.04 | 0.5782 | 0.7563 | 7.6 | 8.07 | 7.71 | 0.93 | 0.5631 | 0.7736 |
| TC1800007696.hg.1 | TSHZ1 | teashirt zinc finger homeobox 1 | Multiple_C | 3.55 | 3.26 | 3.5 | 1.04 | 0.6406 | 0.8008 | 3.92 | 4.05 | 3.96 | 0.97 | 0.7086 | 0.863 |
| TC1800008166.hg.1 | MC2R | melanocortin 2 receptor (adrenocorticotropic hormone) | Multiple_C | 4.61 | 4.54 | 4.56 | 1.04 | 0.7153 | 0.8497 | 4.25 | 4.38 | 4.17 | 1.06 | 0.5829 | 0.7874 |
| TC1800008609.hg.1 | TCEB3C | transcription elongation factor B polypeptide 3C (elongin A3) | Coding | 4.96 | 4.7 | 4.91 | 1.04 | 0.8545 | 0.9289 | 6.05 | 6.08 | 6.29 | 0.85 | 0.6697 | 0.8385 |
| TC1900006892.hg.1 | ANGPTL4 | angiopoietin like 4 | Multiple_C | 3.42 | 3.2 | 3.37 | 1.04 | 0.8472 | 0.9253 | 3.06 | 3.55 | 3.3 | 0.85 | 0.2261 | 0.4865 |
| TC1900007142.hg.1 | ZSWIM4 | zinc finger, SWIM-type containing 4 | Multiple_C | 4.86 | 4.59 | 4.81 | 1.04 | 0.4231 | 0.6343 | 4.93 | 5.04 | 4.72 | 1.16 | 0.5245 | 0.7495 |
| TC1900007247.hg.1 | OR10H4 | olfactory receptor, family 10, subfamily H, member 4 | Coding | 3.06 | 2.94 | 3.01 | 1.04 | 0.8421 | 0.9221 | 3.75 | 3.85 | 3.59 | 1.12 | 0.5443 | 0.7625 |
| TC1900007950.hg.1 | ZNF567 | zinc finger protein 567 | Multiple_C | 10.16 | 9.9 | 10.11 | 1.04 | 0.8627 | 0.9331 | 7.88 | 7.07 | 7.58 | 1.23 | 0.1821 | 0.4325 |
| TC1900008214.hg.1 | CD177 | CD177 molecule | Coding | 3.76 | 3.66 | 3.71 | 1.04 | 0.4945 | 0.6945 | 4.83 | 4.76 | 4.62 | 1.16 | 0.2834 | 0.5488 |
| TC1900009405.hg.1 | FUT3 | fucosyltransferase 3 (galactoside 3(4)-L-fucosyltransferase, Lew | Multiple_C | 3.57 | 3.53 | 3.52 | 1.04 | 0.7757 | 0.8844 | 3.8 | 3.65 | 3.73 | 1.05 | 0.6265 | 0.8154 |
| TC1900010798.hg.1 | PAFAH1B3 | platelet-activating factor acetylhydrolase 1b, catalytic subunit 3 | Multiple_C | 11.59 | 10.92 | 11.54 | 1.04 | 0.8192 | 0.9091 | 10.45 | 10.81 | 10.68 | 0.85 | 0.1556 | 0.396 |
| TC1900010836.hg.1 | PSG4 | pregnancy specific beta-1-glycoprotein 4 | Multiple_C | 5.29 | 4.99 | 5.24 | 1.04 | 0.9236 | 0.9644 | 4.96 | 4.81 | 4.88 | 1.06 | 0.5727 | 0.7804 |
| TC1900010988.hg.1 | PNMAL1 | paraneoplastic Ma antigen family-like 1 | Coding | 2.95 | 2.91 | 2.9 | 1.04 | 0.8241 | 0.9115 | 3.41 | 3.50 | 3.64 | 0.85 | 0.4997 | 0.7323 |
| TC1900010994.hg.1 | PTGIR | prostaglandin I2 (prostacyclin) receptor (IP) | Multiple_C | 6.48 | 6.03 | 6.43 | 1.04 | 0.5572 | 0.74 | 6.2 | 6.30 | 6.04 | 1.12 | 0.6721 | 0.8407 |
| TC1900011902.hg.1 | SMIM7; MED2 | small integral membrane protein 7; mediator complex subunit 2 | Multiple_C | 13.9 | 14.02 | 13.85 | 1.04 | 0.4047 | 0.6176 | 14.43 | 14.63 | 14.38 | 1.04 | 0.6283 | 0.8166 |
| TC1900011976.hg.1 | ZNF285 | zinc finger protein 285 | Multiple_C | 2.84 | 2.68 | 2.79 | 1.04 | 0.8604 | 0.9319 | 2.94 | 3.26 | 2.89 | 1.04 | 0.7954 | 0.9087 |
| TC2000008227.hg.1 | ADAM33 | ADAM metallopeptidase domain 33 | Multiple_C | 4.55 | 4.58 | 4.5 | 1.04 | 0.8195 | 0.9093 | 4.65 | 4.76 | 4.51 | 1.10 | 0.7008 | 0.8581 |
| TC2100007066.hg.1 | RIPPLY3 | ripply transcriptional repressor 3 | Multiple_C | 3.71 | 3.71 | 3.66 | 1.04 | 0.6724 | 0.8212 | 3.86 | 3.84 | 3.78 | 1.06 | 0.9974 | 0.9988 |
| TC2100007491.hg.1 | CBS | cystathionine-beta-synthase | Multiple_C | 4.06 | 3.8 | 4.01 | 1.04 | 0.7562 | 0.8745 | 8.62 | 7.92 | 7.25 | 2.58 | 0.0068 | 0.0601 |
| TC2200006678.hg.1 | SNAP29 | synaptosome associated protein 29kDa | Multiple_C | 10.28 | 9.15 | 10.23 | 1.04 | 0.3484 | 0.5615 | 10.44 | 10.56 | 10.22 | 1.16 | 0.3918 | 0.6502 |
| TC2200006862.hg.1 | SMARCB1 | SWI/SNF related, matrix associated, actin dependent regulator ( | Multiple_C | 11.01 | 10.7 | 10.96 | 1.04 | 0.8247 | 0.9119 | 11.53 | 12.32 | 11.98 | 0.73 | 0.0041 | 0.0434 |
| TSUnmapped00000140.h | RPS6KA1 | ribosomal protein S6 kinase, 90kDa, polypeptide 1 | Coding | 9.58 | 9.21 | 9.53 | 1.04 | 0.5449 | 0.7311 | 7.16 | 7.43 | 7.27 | 0.93 | 0.1414 | 0.3739 |

| | | | | | | | | | | | | | | | |
|---|---|---|---|---|---|---|---|---|---|---|---|---|---|---|---|
| TSUnmapped00000330.h | ZNF502 | zinc finger protein 502 | Coding | 2.86 | 2.83 | 2.81 | 1.04 | 0.7596 | 0.8763 | 3.42 | 3.25 | 3.67 | 0.84 | 0.9065 | 0.9614 |
| TSUnmapped00000478.h | ZNF502 | zinc finger protein 502 | Coding | 3.07 | 3.15 | 3.02 | 1.04 | 0.9953 | 0.9977 | 3.91 | 3.35 | 4.1 | 0.88 | 0.9421 | 0.9761 |
| TSUnmapped00000521.h | OBP2B | odorant binding protein 2B | Coding | 4.15 | 3.97 | 4.1 | 1.04 | 0.4066 | 0.6191 | 4 | 4.01 | 3.96 | 1.03 | 0.6104 | 0.8052 |
| TSUnmapped00000569.h | ZDHHC3 | zinc finger, DHHC-type containing 3 | Coding | 9.58 | 9.06 | 9.53 | 1.04 | 0.6307 | 0.7941 | 6.92 | 6.95 | 7.33 | 0.75 | 0.727 | 0.8731 |
| TSUnmapped00000757.h | SLC25A26 | solute carrier family 25 (S-adenosylmethionine carrier), membe | Coding | 4.24 | 3.9 | 4.19 | 1.04 | 0.2318 | 0.4323 | 4.71 | 4.37 | 4.57 | 1.10 | 0.4251 | 0.6778 |
| TC0100007646.hg.1 | HCRTR1 | hypocretin (orexin) receptor 1 | Multiple_C | 4.31 | 3.84 | 4.27 | 1.03 | 0.8539 | 0.9287 | 4.47 | 4.13 | 4.1 | 1.29 | 0.3142 | 0.5781 |
| TC0100009355.hg.1 | GPR61 | G protein-coupled receptor 61 | Multiple_C | 3.78 | 3.61 | 3.74 | 1.03 | 0.2865 | 0.497 | 4.09 | 4.10 | 3.88 | 1.16 | 0.4677 | 0.7089 |
| TC0100010007.hg.1 | LCE1F | late cornified envelope 1F | Coding | 3.6 | 3.37 | 3.56 | 1.03 | 0.4306 | 0.6408 | 3.93 | 3.92 | 3.75 | 1.13 | 0.344 | 0.6076 |
| TC0100010301.hg.1 | VANGL2 | VANGL planar cell polarity protein 2 | Multiple_C | 4.44 | 4.18 | 4.4 | 1.03 | 0.5661 | 0.7473 | 4.74 | 4.51 | 4.52 | 1.16 | 0.1401 | 0.3718 |
| TC0100010379.hg.1 | UHMK1 | U2AF homology motif (UHM) kinase 1 | Multiple_C | 11.95 | 11.76 | 11.91 | 1.03 | 0.9309 | 0.9675 | 11.23 | 10.40 | 11.49 | 0.84 | 0.097 | 0.3034 |
| TC0100012354.hg.1 | OR2T4 | olfactory receptor, family 2, subfamily T, member 4 | Coding | 2.82 | 2.73 | 2.78 | 1.03 | 0.6622 | 0.8147 | 3.27 | 3.19 | 3.33 | 0.96 | 0.7248 | 0.872 |
| TC0100012940.hg.1 | HNRNPCL4 | heterogeneous nuclear ribonucleoprotein C-like 4 | Coding | 5.36 | 5.37 | 5.32 | 1.03 | 0.437 | 0.6464 | 4.9 | 5.27 | 5.43 | 0.69 | 0.0381 | 0.1776 |
| TC0100014927.hg.1 | EVI5 | ecotropic viral integration site 5 | Multiple_C | 8.52 | 9.28 | 8.48 | 1.03 | 0.8993 | 0.9518 | 8.65 | 7.70 | 8.86 | 0.86 | 0.1777 | 0.4263 |
| TC0100015342.hg.1 | SYT6 | synaptotagmin VI | Multiple_C | 7.98 | 7.39 | 7.94 | 1.03 | 0.5115 | 0.7073 | 7.69 | 8.05 | 8.35 | 0.63 | 0.0179 | 0.1126 |
| TC0100015636.hg.1 | GJA5 | gap junction protein alpha 5 | Coding | 3.96 | 3.99 | 3.92 | 1.03 | 0.9706 | 0.986 | 4.16 | 4.02 | 4.07 | 1.06 | 0.2648 | 0.5294 |
| TC0100016960.hg.1 | SYT2 | synaptotagmin II | Multiple_C | 5.84 | 5.74 | 5.8 | 1.03 | 0.9658 | 0.9836 | 5.6 | 5.59 | 5.58 | 1.01 | 0.5566 | 0.7706 |
| TC0100017020.hg.1 | REN | renin | Multiple_C | 3.12 | 2.85 | 3.08 | 1.03 | 0.8521 | 0.9273 | 3.85 | 3.58 | 3.35 | 1.41 | 0.115 | 0.3348 |
| TC0100018112.hg.1 | OR11L1 | olfactory receptor, family 11, subfamily L, member 1 | Coding | 3.74 | 3.48 | 3.7 | 1.03 | 0.923 | 0.9641 | 4.25 | 4.42 | 3.97 | 1.21 | 0.4583 | 0.7021 |
| TC0100018171.hg.1 | PRAMEF25; PI | PRAME family member 25; PRAME family member 26 | Coding | 4.98 | 4.71 | 4.94 | 1.03 | 0.851 | 0.9269 | 4.58 | 4.87 | 4.72 | 0.91 | 0.9528 | 0.9808 |
| TC0100018321.hg.1 | TEX35 | testis expressed 35 | Coding | 5.51 | 5.9 | 5.47 | 1.03 | 0.2471 | 0.4507 | 5.14 | 5.05 | 4.71 | 1.35 | 0.3128 | 0.577 |
| TC0200008950.hg.1 | IL1F10 | interleukin 1 family, member 10 (theta) | Multiple_C | 3.55 | 3.5 | 3.51 | 1.03 | 0.7825 | 0.888 | 3.95 | 3.79 | 3.6 | 1.27 | 0.0325 | 0.1614 |
| TC0200008974.hg.1 | FOXD4L1 | forkhead box D4-like 1 | Coding | 5 | 4.77 | 4.96 | 1.03 | 0.748 | 0.8706 | 3.85 | 3.91 | 3.84 | 1.01 | 0.6402 | 0.8234 |
| TC0200009927.hg.1 | MYO3B | myosin IIIB | Multiple_C | 3.13 | 2.77 | 3.09 | 1.03 | 0.7915 | 0.8932 | 3.7 | 3.84 | 3.69 | 1.01 | 0.2146 | 0.4732 |
| TC0200010119.hg.1 | PLEKHA3 | pleckstrin homology domain containing, family A (phosphoinosi | Multiple_C | 10.36 | 10.98 | 10.32 | 1.03 | 0.9351 | 0.9697 | 10.2 | 9.20 | 10.32 | 0.92 | 0.1976 | 0.4526 |
| TC0200010284.hg.1 | MYO1B | myosin IB | Multiple_C | 8.52 | 8.05 | 8.48 | 1.03 | 0.9898 | 0.9946 | 10.41 | 10.41 | 11.11 | 0.62 | 0.0002 | 0.0061 |
| TC0200010834.hg.1 | FAM134A | family with sequence similarity 134, member A | Multiple_C | 7.92 | 7.44 | 7.88 | 1.03 | 0.886 | 0.945 | 7.83 | 8.34 | 7.75 | 1.06 | 0.1893 | 0.4419 |
| TC0200011753.hg.1 | E2F6 | E2F transcription factor 6 | Multiple_C | 7.9 | 8.04 | 7.86 | 1.03 | 0.9113 | 0.958 | 6.96 | 7.18 | 7.07 | 0.93 | 0.1848 | 0.4361 |
| TC0200012063.hg.1 | PRR30 | proline rich 30 | Coding | 3.39 | 3.17 | 3.35 | 1.03 | 0.4303 | 0.6406 | 3.62 | 3.37 | 3.81 | 0.88 | 0.2358 | 0.4985 |
| TC0200013129.hg.1 | TACR1 | tachykinin receptor 1 | Multiple_C | 3.05 | 3.15 | 3.01 | 1.03 | 0.6917 | 0.8338 | 3.79 | 4.00 | 3.73 | 1.04 | 0.4359 | 0.6865 |
| TC0200014512.hg.1 | ZEB2 | zinc finger E-box binding homeobox 2 | Multiple_C | 4.09 | 3.91 | 4.05 | 1.03 | 0.8409 | 0.9213 | 4.82 | 4.62 | 4.3 | 1.43 | 0.0143 | 0.0973 |
| TC0300007677.hg.1 | C3orf49 | chromosome 3 open reading frame 49 | Multiple_C | 3.62 | 3.32 | 3.58 | 1.03 | 0.4884 | 0.689 | 4.01 | 4.20 | 4.2 | 0.88 | 0.8158 | 0.9183 |
| TC0300009168.hg.1 | SELT; SELT.1 | selenoprotein T; SELT protein; Selenoprotein T; cDNA FLJ90525 | Multiple_C | 14.47 | 14.85 | 14.43 | 1.03 | 0.9772 | 0.9889 | 13.95 | 13.70 | 14.04 | 0.94 | 0.4829 | 0.7205 |
| TC0300013598.hg.1 | LRRC15 | leucine rich repeat containing 15 | Coding | 3.72 | 3.71 | 3.68 | 1.03 | 0.7597 | 0.8763 | 4.19 | 4.54 | 4.3 | 0.93 | 0.9906 | 0.9962 |

| | | | | | | | | | | | | | | | |
|---|---|---|---|---|---|---|---|---|---|---|---|---|---|---|---|
| TC0300014069.hg.1 | CLRN1 | clarin 1 | Multiple_C | 3.74 | 3.3 | 3.7 | 1.03 | 0.7057 | 0.8432 | 4.61 | 4.88 | 4.38 | 1.17 | 0.0761 | 0.2656 |
| TC0300014081.hg.1 | TMEM41A | transmembrane protein 41A | Multiple_C | 13.11 | 12.04 | 13.07 | 1.03 | 0.4114 | 0.6237 | 12.16 | 11.44 | 11.75 | 1.33 | 0.2589 | 0.5232 |
| TC0400008020.hg.1 | CDS1 | CDP-diacylglycerol synthase 1 | Multiple_C | 10.12 | 10.41 | 10.08 | 1.03 | 0.9255 | 0.9651 | 4.46 | 3.47 | 4.06 | 1.32 | 0.0079 | 0.0664 |
| TC0400009318.hg.1 | GALNTL6 | polypeptide N-acetylgalactosaminyltransferase-like 6 | Multiple_C | 2.84 | 2.78 | 2.8 | 1.03 | 0.8584 | 0.9309 | 3.04 | 3.10 | 3.07 | 0.98 | 0.7182 | 0.8686 |
| TC0400009879.hg.1 | CYTL1 | cytokine like 1 | Coding | 3.55 | 3.5 | 3.51 | 1.03 | 0.9482 | 0.9761 | 5.65 | 6.67 | 6.08 | 0.74 | 0.0258 | 0.1401 |
| TC0400011414.hg.1 | TRMT10A | tRNA methyltransferase 10A | Multiple_C | 7.74 | 9.02 | 7.7 | 1.03 | 0.9018 | 0.9527 | 5.64 | 6.54 | 6.49 | 0.55 | 0.0016 | 0.0225 |
| TC0400012309.hg.1 | TKTL2 | transketolase-like 2 | Coding | 3.66 | 3.62 | 3.62 | 1.03 | 0.8816 | 0.9425 | 4.23 | 4.06 | 3.71 | 1.43 | 0.2853 | 0.5503 |
| TC0500007876.hg.1 | BHMT | betaine--homocysteine S-methyltransferase | Multiple_C | 4.16 | 4.26 | 4.12 | 1.03 | 0.8363 | 0.9185 | 4.74 | 4.66 | 4.53 | 1.16 | 0.459 | 0.7024 |
| TC0500008470.hg.1 | SNCAIP | synuclein alpha interacting protein | Multiple_C | 3.81 | 3.6 | 3.77 | 1.03 | 0.7313 | 0.8597 | 3.65 | 3.22 | 3.65 | 1.00 | 0.9392 | 0.9749 |
| TC0500012394.hg.1 | C5orf46 | chromosome 5 open reading frame 46 | Multiple_C | 3.75 | 3.55 | 3.71 | 1.03 | 0.842 | 0.9221 | 3.36 | 3.34 | 3.42 | 0.96 | 0.7974 | 0.9093 |
| TC0500013059.hg.1 | CBY3 | chibby homolog 3 (Drosophila) | Coding | 3.11 | 3.03 | 3.07 | 1.03 | 0.9994 | 0.9997 | 3.37 | 3.22 | 3.26 | 1.08 | 0.2474 | 0.512 |
| TC0600007461.hg.1 | OR10C1 | olfactory receptor, family 10, subfamily C, member 1 (gene/pse | Multiple_C | 4.27 | 4.52 | 4.23 | 1.03 | 0.9427 | 0.9734 | 4.81 | 4.62 | 4 | 1.75 | 0.0052 | 0.0507 |
| TC0600008343.hg.1 | BEND6 | BEN domain containing 6 | Multiple_C | 3.15 | 3.09 | 3.11 | 1.03 | 0.6805 | 0.8263 | 3.74 | 3.84 | 3.83 | 0.94 | 0.7406 | 0.8807 |
| TC0600009476.hg.1 | TAAR9 | trace amine associated receptor 9 (gene/pseudogene) | Coding | 4.4 | 4.35 | 4.36 | 1.03 | 0.9879 | 0.9936 | 4.73 | 4.29 | 4.55 | 1.13 | 0.686 | 0.8497 |
| TC0600010825.hg.1 | ADTRP | androgen-dependent TFPI-regulating protein | Multiple_C | 3.93 | 3.9 | 3.89 | 1.03 | 0.7178 | 0.8508 | 3.66 | 3.81 | 3.46 | 1.15 | 0.8598 | 0.9401 |
| TC0600011843.hg.1 | TBCC | tubulin folding cofactor C | Coding | 8.52 | 8.85 | 8.48 | 1.03 | 0.7134 | 0.8486 | 6.38 | 7.22 | 7.15 | 0.59 | 0.0028 | 0.0342 |
| TC0600012006.hg.1 | CRISP1 | cysteine-rich secretory protein 1 | Coding | 3.76 | 3.62 | 3.72 | 1.03 | 0.9092 | 0.9564 | 3.57 | 3.59 | 3.53 | 1.03 | 0.4022 | 0.6584 |
| TC0600012763.hg.1 | SCML4 | sex comb on midleg-like 4 (Drosophila) | Multiple_C | 3.15 | 3.07 | 3.11 | 1.03 | 0.7787 | 0.8862 | 3.61 | 3.61 | 3.57 | 1.03 | 0.1364 | 0.367 |
| TC0600014263.hg.1 | LY6G6E | lymphocyte antigen 6 complex, locus G6E (pseudogene) | Multiple_C | 5.28 | 5.12 | 5.24 | 1.03 | 0.4973 | 0.6968 | 5.28 | 5.52 | 5.11 | 1.13 | 0.7578 | 0.8901 |
| TC0700006632.hg.1 | OCM | oncomodulin | Coding | 4.59 | 4.37 | 4.55 | 1.03 | 0.6264 | 0.7915 | 3.28 | 3.01 | 3.29 | 0.99 | 0.2982 | 0.563 |
| TC0700007755.hg.1 | ZNF727 | zinc finger protein 727 | Coding | 4.53 | 4.53 | 4.49 | 1.03 | 0.8085 | 0.9033 | 3.35 | 3.47 | 3.64 | 0.82 | 0.0707 | 0.2543 |
| TC0700008141.hg.1 | PTPN12 | protein tyrosine phosphatase, non-receptor type 12 | Multiple_C | 11.49 | 12.15 | 11.45 | 1.03 | 0.8101 | 0.9041 | 10.71 | 10.06 | 10.55 | 1.12 | 0.9964 | 0.9983 |
| TC0700010139.hg.1 | PAPOLB | poly(A) polymerase beta | Coding | 2.97 | 2.59 | 2.93 | 1.03 | 0.408 | 0.6202 | 3.73 | 3.46 | 3.43 | 1.23 | 0.5939 | 0.7949 |
| TC0700011655.hg.1 | PCLO | piccolo presynaptic cytomatrix protein | Multiple_C | 3.93 | 4.09 | 3.89 | 1.03 | 0.3084 | 0.5214 | 4.38 | 4.75 | 4.81 | 0.74 | 0.4127 | 0.6674 |
| TC0700011799.hg.1 | HEPACAM2 | HEPACAM family member 2 | Multiple_C | 3.63 | 3.51 | 3.59 | 1.03 | 0.5634 | 0.7452 | 4.73 | 4.58 | 4.17 | 1.47 | 0.0438 | 0.1934 |
| TC0800008183.hg.1 | OSGIN2 | oxidative stress induced growth inhibitor family member 2 | Multiple_C | 10.56 | 11.01 | 10.52 | 1.03 | 0.9213 | 0.9636 | 9.37 | 7.84 | 8.94 | 1.35 | 0.0208 | 0.1231 |
| TC0800008531.hg.1 | EMC2 | ER membrane protein complex subunit 2 | Multiple_C | 12.05 | 14.19 | 12.01 | 1.03 | 0.9685 | 0.9848 | 10.43 | 9.33 | 9.22 | 2.31 | 0.0485 | 0.2047 |
| TC0800009745.hg.1 | FGL1 | fibrinogen like 1 | Multiple_C | 3.34 | 3.2 | 3.3 | 1.03 | 0.8585 | 0.931 | 4.56 | 4.42 | 4.17 | 1.31 | 0.0728 | 0.2586 |
| TC0800012240.hg.1 | RPL8; MIR685 | ribosomal protein L8; microRNA 6850 | Multiple_C | 18.36 | 18.29 | 18.32 | 1.03 | 0.1668 | 0.3462 | 15.3 | 15.42 | 15.3 | 1.00 | 0.8846 | 0.9505 |
| TC0900009922.hg.1 | FAM205C | family with sequence similarity 205, member C | Multiple_C | 4.66 | 4.78 | 4.62 | 1.03 | 0.6167 | 0.7851 | 3.9 | 3.43 | 4 | 0.93 | 0.6742 | 0.8422 |
| TC0900010396.hg.1 | C9orf57 | chromosome 9 open reading frame 57 | Coding | 2.67 | 2.55 | 2.63 | 1.03 | 0.869 | 0.9361 | 3.92 | 3.59 | 3.22 | 1.62 | 0.0066 | 0.0586 |
| TC0900011247.hg.1 | ZFP37 | ZFP37 zinc finger protein | Coding | 6.79 | 7.61 | 6.75 | 1.03 | 0.9553 | 0.979 | 6.45 | 6.54 | 6.13 | 1.25 | 0.1705 | 0.4168 |
| TC0X00007239.hg.1 | USP27X | ubiquitin specific peptidase 27, X-linked | Coding | 5.65 | 6.05 | 5.61 | 1.03 | 0.4255 | 0.6363 | 5.14 | 5.08 | 4.47 | 1.59 | 0.0136 | 0.0946 |

| | | | | | | | | | | | | | | | |
|---|---|---|---|---|---|---|---|---|---|---|---|---|---|---|---|
| TC0X00007768.hg.1 | CYLC1 | cylicin, basic protein of sperm head cytoskeleton 1 | Coding | 3.7 | 3.5 | 3.66 | 1.03 | 0.8716 | 0.9376 | 3.68 | 3.85 | 3.74 | 0.96 | 0.8183 | 0.9197 |
| TC0X00007970.hg.1 | NXF2B; NXF2 | nuclear RNA export factor 2B; nuclear RNA export factor 2 | Coding | 3.03 | 2.83 | 2.99 | 1.03 | 0.904 | 0.9538 | 3.13 | 3.07 | 2.89 | 1.18 | 0.1101 | 0.3268 |
| TC0X00008518.hg.1 | SAGE1 | sarcoma antigen 1 | Multiple_C | 3.04 | 2.64 | 3 | 1.03 | 0.6897 | 0.8329 | 3.72 | 3.63 | 3.78 | 0.96 | 0.4179 | 0.6717 |
| TC0X00008645.hg.1 | SLITRK2 | SLIT and NTRK-like family, member 2 | Coding | 2.82 | 2.72 | 2.78 | 1.03 | 0.5131 | 0.7086 | 2.91 | 2.89 | 2.96 | 0.97 | 0.2559 | 0.5207 |
| TC0X00008759.hg.1 | NSDHL | NAD(P) dependent steroid dehydrogenase-like | Multiple_C | 8.94 | 9.42 | 8.9 | 1.03 | 0.8121 | 0.9052 | 7.25 | 7.73 | 8.98 | 0.30 | 2.14E-07 | 4.78E-05 |
| TC0X00009375.hg.1 | FTH1P18 | ferritin, heavy polypeptide 1 pseudogene 18 | Multiple_C | 3.78 | 3.59 | 3.74 | 1.03 | 0.6122 | 0.7822 | 4.12 | 3.74 | 3.66 | 1.38 | 0.0461 | 0.1987 |
| TC0X00009882.hg.1 | MTMR8 | myotubularin related protein 8 | Multiple_C | 3.44 | 3.26 | 3.4 | 1.03 | 0.9261 | 0.9655 | 5.05 | 4.37 | 4.4 | 1.57 | 0.0229 | 0.1304 |
| TC0X00009952.hg.1 | AWAT2 | acyl-CoA wax alcohol acyltransferase 2 | Multiple_C | 4.15 | 4.22 | 4.11 | 1.03 | 0.9395 | 0.972 | 3.66 | 3.74 | 3.47 | 1.14 | 0.3069 | 0.5711 |
| TC0X00010023.hg.1 | RGAG4 | retrotransposon gag domain containing 4 | Coding | 3.11 | 3.17 | 3.07 | 1.03 | 0.9969 | 0.9984 | 3.61 | 3.86 | 3.42 | 1.14 | 0.3021 | 0.5665 |
| TC0X00010452.hg.1 | SERPINA7 | serpin peptidase inhibitor, clade A (alpha-1 antiproteinase, antit | Multiple_C | 2.96 | 2.63 | 2.92 | 1.03 | 0.4562 | 0.6629 | 3.8 | 3.65 | 3.69 | 1.08 | 0.5347 | 0.7568 |
| TC0X00011302.hg.1 | MSN | moesin | Multiple_C | 5.69 | 5.72 | 5.65 | 1.03 | 0.5211 | 0.7145 | 12.68 | 13.59 | 14.32 | 0.32 | 1.45E-05 | 0.0009 |
| TC0X00011378.hg.1 | RAB40A | RAB40A, member RAS oncogene family | Coding | 3.7 | 3.58 | 3.66 | 1.03 | 0.7677 | 0.8803 | 3.87 | 3.74 | 3.67 | 1.15 | 0.3278 | 0.5912 |
| TC1000007959.hg.1 | CDH23 | cadherin-related 23 | Multiple_C | 4.77 | 4.71 | 4.73 | 1.03 | 0.6104 | 0.7806 | 5.01 | 5.01 | 5.45 | 0.74 | 0.2126 | 0.4705 |
| TC1000009286.hg.1 | NPS | neuropeptide S | Coding | 3.59 | 3.79 | 3.55 | 1.03 | 0.8844 | 0.944 | 4.07 | 4.06 | 3.97 | 1.07 | 0.6335 | 0.8199 |
| TC1000009452.hg.1 | VENTX | VENT homeobox | Coding | 4.28 | 3.96 | 4.24 | 1.03 | 0.6896 | 0.8329 | 4.79 | 4.88 | 4.66 | 1.09 | 0.7946 | 0.9087 |
| TC1000010609.hg.1 | OGDHL | oxoglutarate dehydrogenase-like | Multiple_C | 4.84 | 4.47 | 4.8 | 1.03 | 0.8902 | 0.9472 | 5.37 | 5.56 | 5.14 | 1.17 | 0.4413 | 0.6905 |
| TC1000010818.hg.1 | CTNNA3 | catenin (cadherin-associated protein), alpha 3 | Multiple_C | 3 | 2.79 | 2.96 | 1.03 | 0.8268 | 0.9129 | 3.6 | 3.61 | 3.45 | 1.11 | 0.2823 | 0.5478 |
| TC1000011606.hg.1 | BLOC1S2 | biogenesis of lysosomal organelles complex-1, subunit 2 | Multiple_C | 10.95 | 12.11 | 10.91 | 1.03 | 0.81 | 0.904 | 9.68 | 9.44 | 9.67 | 1.01 | 0.8066 | 0.9146 |
| TC1000011932.hg.1 | C10orf82 | chromosome 10 open reading frame 82 | Multiple_C | 3.95 | 3.75 | 3.91 | 1.03 | 0.9749 | 0.9877 | 4.86 | 5.12 | 5.18 | 0.80 | 0.7837 | 0.9026 |
| TC1000012529.hg.1 | NSUN6 | NOP2/Sun domain family, member 6 | Multiple_C | 5.17 | 6.17 | 5.13 | 1.03 | 0.5167 | 0.7109 | 5.28 | 4.68 | 4.39 | 1.85 | 0.0471 | 0.201 |
| TC1000012585.hg.1 | CHUK | conserved helix-loop-helix ubiquitous kinase | Multiple_C | 7.78 | 8.37 | 7.74 | 1.03 | 0.4786 | 0.6808 | 7.71 | 6.99 | 7.11 | 1.52 | 0.0264 | 0.142 |
| TC1100006508.hg.1 | MUC5AC | mucin 5AC, oligomeric mucus/gel-forming | Multiple_C | 4.78 | 4.26 | 4.74 | 1.03 | 0.5057 | 0.7029 | 3.3 | 3.26 | 3.29 | 1.01 | 0.7603 | 0.8914 |
| TC1100006696.hg.1 | OR52D1 | olfactory receptor, family 52, subfamily D, member 1 | Coding | 4.32 | 4.32 | 4.28 | 1.03 | 0.4715 | 0.6752 | 4.62 | 3.94 | 4.98 | 0.78 | 0.0524 | 0.2137 |
| TC1100007519.hg.1 | OR4C3 | olfactory receptor, family 4, subfamily C, member 3 | Coding | 3.95 | 3.77 | 3.91 | 1.03 | 0.8168 | 0.9075 | 3.71 | 3.84 | 3.83 | 0.92 | 0.9611 | 0.9837 |
| TC1100007573.hg.1 | OR4A5 | olfactory receptor, family 4, subfamily A, member 5 | Coding | 3.19 | 3.24 | 3.15 | 1.03 | 0.7387 | 0.8643 | 3.37 | 3.50 | 3.27 | 1.07 | 0.4807 | 0.7191 |
| TC1100008109.hg.1 | C11orf86 | chromosome 11 open reading frame 86 | Coding | 3.9 | 3.58 | 3.86 | 1.03 | 0.7735 | 0.8832 | 4.13 | 4.27 | 4.28 | 0.90 | 0.7793 | 0.9011 |
| TC1100009334.hg.1 | CRTAM | cytotoxic and regulatory T-cell molecule | Multiple_C | 3.16 | 2.98 | 3.12 | 1.03 | 0.9415 | 0.9729 | 3.44 | 3.98 | 3.74 | 0.81 | 0.6024 | 0.8008 |
| TC1100009943.hg.1 | OR52N5 | olfactory receptor, family 52, subfamily N, member 5 | Coding | 3.17 | 3.51 | 3.13 | 1.03 | 0.8642 | 0.9337 | 3.42 | 3.59 | 3.47 | 0.97 | 0.8135 | 0.9171 |
| TC1100010639.hg.1 | TP53I11 | tumor protein p53 inducible protein 11 | Multiple_C | 9.68 | 9.01 | 9.64 | 1.03 | 0.7049 | 0.8425 | 5.62 | 5.58 | 5.24 | 1.30 | 0.2752 | 0.5414 |
| TC1100013039.hg.1 | ARL2-SNX15 | ARL2-SNX15 readthrough (NMD candidate) | Multiple_C | 9.58 | 9.3 | 9.54 | 1.03 | 0.472 | 0.6755 | 7.2 | 7.52 | 7.39 | 0.88 | 0.1117 | 0.3293 |
| TC1100013150.hg.1 | TPH1 | tryptophan hydroxylase 1 | Multiple_C | 3.31 | 2.53 | 3.27 | 1.03 | 0.9541 | 0.9785 | 3.54 | 3.70 | 3.46 | 1.06 | 0.8013 | 0.9112 |
| TC1100013222.hg.1 | CARD16 | caspase recruitment domain family, member 16 | Multiple_C | 3.99 | 3.84 | 3.95 | 1.03 | 0.2601 | 0.4666 | 3.81 | 4.01 | 4.09 | 0.82 | 0.3264 | 0.5903 |
| TC1200009420.hg.1 | ADGRD1 | adhesion G protein-coupled receptor D1 | Multiple_C | 3.23 | 3.32 | 3.19 | 1.03 | 0.3406 | 0.5546 | 4.31 | 4.10 | 4.19 | 1.09 | 0.2735 | 0.5389 |

| | | | | | | | | | | | | | | | |
|---|---|---|---|---|---|---|---|---|---|---|---|---|---|---|---|
| TC1200009981.hg.1 | HEBP1 | heme binding protein 1 | Multiple_C | 12.1 | 13.18 | 12.06 | 1.03 | 0.8476 | 0.9255 | 7.68 | 7.29 | 6.86 | 1.77 | 0.0056 | 0.0531 |
| TC1200010614.hg.1 | DHH | desert hedgehog | Multiple_C | 4.09 | 3.73 | 4.05 | 1.03 | 0.9431 | 0.9736 | 4.39 | 4.41 | 4.21 | 1.13 | 0.3647 | 0.6273 |
| TC1200010713.hg.1 | FIGNL2 | fidgetin-like 2 | Multiple_C | 2.94 | 2.89 | 2.9 | 1.03 | 0.6379 | 0.7991 | 3.49 | 3.29 | 3.31 | 1.13 | 0.2557 | 0.5204 |
| TC1200010976.hg.1 | AGAP2 | ArfGAP with GTPase domain, ankyrin repeat and PH domain 2 | Multiple_C | 3.45 | 3.31 | 3.41 | 1.03 | 0.6118 | 0.7819 | 4.59 | 4.61 | 4.42 | 1.13 | 0.3437 | 0.6072 |
| TC1200012767.hg.1 | PRB2 | proline-rich protein BstNI subfamily 2 | Multiple_C | 3.94 | 3.76 | 3.9 | 1.03 | 0.7522 | 0.8733 | 4.42 | 4.65 | 4.7 | 0.82 | 0.2507 | 0.5149 |
| TC1300007561.hg.1 | SCEL | sciellin | Multiple_C | 3.93 | 4.23 | 3.89 | 1.03 | 0.1741 | 0.3561 | 6.28 | 4.88 | 4.61 | 3.18 | 7.05E-08 | 2.07E-05 |
| TC1300008023.hg.1 | CARKD | carbohydrate kinase domain containing | Multiple_C | 7.4 | 7.46 | 7.36 | 1.03 | 0.3409 | 0.5549 | 9.86 | 9.85 | 9.59 | 1.21 | 0.337 | 0.6014 |
| TC1400007098.hg.1 | ARF6 | ADP-ribosylation factor 6 | Multiple_C | 13.42 | 13.47 | 13.38 | 1.03 | 0.565 | 0.7465 | 11.1 | 11.46 | 12.19 | 0.47 | 0.0011 | 0.0173 |
| TC1400008058.hg.1 | PPP4R4 | protein phosphatase 4, regulatory subunit 4 | Multiple_C | 3.57 | 3.29 | 3.53 | 1.03 | 0.596 | 0.7698 | 4.24 | 3.84 | 3.65 | 1.51 | 0.0076 | 0.0648 |
| TC1400009076.hg.1 | MDGA2 | MAM domain containing glycosylphosphatidylinositol anchor 2 | Multiple_C | 3.94 | 3.68 | 3.9 | 1.03 | 0.759 | 0.8761 | 3.83 | 3.48 | 3.73 | 1.07 | 0.3179 | 0.582 |
| TC1400010637.hg.1 | C14orf178 | chromosome 14 open reading frame 178 | Coding | 2.92 | 3 | 2.88 | 1.03 | 0.6593 | 0.8132 | 3.92 | 3.66 | 3.72 | 1.15 | 0.3549 | 0.6181 |
| TC1500006890.hg.1 | TMCO5A | transmembrane and coiled-coil domains 5A | Multiple_C | 3.76 | 3.83 | 3.72 | 1.03 | 0.8894 | 0.9467 | 3.77 | 3.76 | 3.57 | 1.15 | 0.444 | 0.6922 |
| TC1500008114.hg.1 | TM6SF1 | transmembrane 6 superfamily member 1 | Multiple_C | 4.65 | 4.7 | 4.61 | 1.03 | 0.6583 | 0.8124 | 5.46 | 5.71 | 5.29 | 1.13 | 0.175 | 0.4229 |
| TC1500008172.hg.1 | SLC28A1 | solute carrier family 28 (concentrative nucleoside transporter), | Multiple_C | 2.94 | 2.77 | 2.9 | 1.03 | 0.9415 | 0.9729 | 3.37 | 3.39 | 3.16 | 1.16 | 0.1252 | 0.3497 |
| TC1500009466.hg.1 | ONECUT1 | one cut homeobox 1 | Multiple_C | 4.31 | 3.92 | 4.27 | 1.03 | 0.7812 | 0.8872 | 4.62 | 4.58 | 4.06 | 1.47 | 0.0069 | 0.0608 |
| TC1500010404.hg.1 | MESP1 | mesoderm posterior bHLH transcription factor 1 | Multiple_C | 4.08 | 4.11 | 4.04 | 1.03 | 0.9077 | 0.9557 | 4.08 | 4.25 | 4.14 | 0.96 | 0.6789 | 0.8453 |
| TC1600008134.hg.1 | CA7 | carbonic anhydrase VII | Coding | 3.21 | 3.16 | 3.17 | 1.03 | 0.8949 | 0.9497 | 3.77 | 3.46 | 3.51 | 1.20 | 0.1101 | 0.3268 |
| TC1600009651.hg.1 | GPR139 | G protein-coupled receptor 139 | Coding | 2.72 | 2.49 | 2.68 | 1.03 | 0.7836 | 0.8888 | 3.63 | 3.69 | 3.68 | 0.97 | 0.7966 | 0.9092 |
| TC1600010179.hg.1 | DNAJA2 | DnaJ (Hsp40) homolog, subfamily A, member 2 | Multiple_C | 13.15 | 14.2 | 13.11 | 1.03 | 0.8803 | 0.9419 | 12.69 | 12.28 | 12.79 | 0.93 | 0.1625 | 0.4058 |
| TC1600010861.hg.1 | FA2H | fatty acid 2-hydroxylase | Multiple_C | 9.74 | 8.97 | 9.7 | 1.03 | 0.7143 | 0.8493 | 4.16 | 3.53 | 4.36 | 0.87 | 0.6746 | 0.8426 |
| TC1700006720.hg.1 | BCL6B | B-cell CLL/lymphoma 6, member B | Multiple_C | 3.46 | 3.36 | 3.42 | 1.03 | 0.9677 | 0.9844 | 3.17 | 3.11 | 2.94 | 1.17 | 0.386 | 0.645 |
| TC1700006909.hg.1 | MYOCD | myocardin | Multiple_C | 3.59 | 3.43 | 3.55 | 1.03 | 0.774 | 0.8835 | 4.02 | 4.10 | 3.79 | 1.17 | 0.1612 | 0.4041 |
| TC1700008376.hg.1 | AKAP1 | A kinase (PRKA) anchor protein 1 | Multiple_C | 11.37 | 10.84 | 11.33 | 1.03 | 0.7202 | 0.8526 | 10.58 | 10.72 | 10.7 | 0.92 | 0.7832 | 0.9026 |
| TC1700009081.hg.1 | ENGASE | endo-beta-N-acetylglucosaminidase | Multiple_C | 6.23 | 6.61 | 6.19 | 1.03 | 0.9248 | 0.9648 | 6.68 | 6.60 | 6.6 | 1.06 | 0.3636 | 0.6264 |
| TC1700009215.hg.1 | ASPSCR1 | alveolar soft part sarcoma chromosome region, candidate 1 | Multiple_C | 4.74 | 4.48 | 4.7 | 1.03 | 0.852 | 0.9273 | 10.25 | 11.03 | 9.44 | 1.75 | 0.0042 | 0.0442 |
| TC1700011121.hg.1 | TOB1 | transducer of ERBB2, 1 | Multiple_C | 12.73 | 12.45 | 12.69 | 1.03 | 0.9873 | 0.9935 | 12.48 | 11.62 | 11.83 | 1.57 | 0.2012 | 0.4565 |
| TC1700011442.hg.1 | PECAM1 | platelet/endothelial cell adhesion molecule 1 | Multiple_C | 3.48 | 3.32 | 3.44 | 1.03 | 0.5899 | 0.7653 | 3.78 | 4.05 | 3.81 | 0.98 | 0.8404 | 0.9303 |
| TC1700011566.hg.1 | FAM20A | family with sequence similarity 20, member A | Multiple_C | 3.49 | 3.5 | 3.45 | 1.03 | 0.8564 | 0.9298 | 3.97 | 4.21 | 3.57 | 1.32 | 0.1114 | 0.329 |
| TC1800007014.hg.1 | DSG2 | desmoglein 2 | Multiple_C | 13.25 | 13.77 | 13.21 | 1.03 | 0.9709 | 0.9861 | 10.98 | 11.29 | 10.53 | 1.37 | 0.4021 | 0.6584 |
| TC1900006470.hg.1 | BSG | basigin (Ok blood group) | Multiple_C | 15.73 | 15.05 | 15.69 | 1.03 | 0.5083 | 0.7048 | 15.73 | 16.17 | 15.92 | 0.88 | 0.9088 | 0.9625 |
| TC1900006508.hg.1 | ABCA7 | ATP binding cassette subfamily A member 7 | Multiple_C | 7.21 | 6.76 | 7.17 | 1.03 | 0.7746 | 0.8839 | 7.64 | 6.81 | 6.6 | 2.06 | 0.0004 | 0.0087 |
| TC1900007819.hg.1 | ZNF302 | zinc finger protein 302 | Multiple_C | 8.59 | 9.87 | 8.55 | 1.03 | 0.5405 | 0.7282 | 8.52 | 9.99 | 8.66 | 0.91 | 0.4617 | 0.7044 |
| TC1900008503.hg.1 | DHDH | dihydrodiol dehydrogenase (dimeric) | Multiple_C | 3.46 | 3.66 | 3.42 | 1.03 | 0.1718 | 0.3531 | 3.58 | 3.61 | 3.64 | 0.96 | 0.6357 | 0.8212 |

| | | | | | | | | | | | | | | |
|---|---|---|---|---|---|---|---|---|---|---|---|---|---|---|
| TC1900008522.hg.1 | SNRNP70 | small nuclear ribonucleoprotein, U1 70kDa subunit | Multiple_C | 12.95 | 13.41 | 12.91 | 1.03 | 0.7131 | 0.8483 | 13.23 | 13.57 | 12.96 | 1.21 | 0.9086 | 0.9624 |
| TC1900008535.hg.1 | DKKL1 | dickkopf-like 1 | Coding | 4.72 | 4.58 | 4.68 | 1.03 | 0.6908 | 0.8333 | 4.24 | 4.23 | 4.15 | 1.06 | 0.63 | 0.8175 |
| TC1900010037.hg.1 | SUGP2 | SURP and G-patch domain containing 2 | Multiple_C | 7.94 | 7.85 | 7.9 | 1.03 | 0.69 | 0.833 | 9.75 | 9.45 | 9.42 | 1.26 | 0.0734 | 0.2596 |
| TC1900010582.hg.1 | WDR87 | WD repeat domain 87 | Multiple_C | 3.42 | 3.34 | 3.38 | 1.03 | 0.5807 | 0.7581 | 4.08 | 3.98 | 4.05 | 1.02 | 0.4695 | 0.7106 |
| TC1900011034.hg.1 | ZNF541 | zinc finger protein 541 | Multiple_C | 3.88 | 3.78 | 3.84 | 1.03 | 0.8436 | 0.9229 | 4.05 | 3.88 | 3.72 | 1.26 | 0.1064 | 0.3203 |
| TC1900011331.hg.1 | ZNF665 | zinc finger protein 665 | Multiple_C | 5.61 | 5.39 | 5.57 | 1.03 | 0.8094 | 0.9038 | 3.58 | 3.74 | 3.52 | 1.04 | 0.2413 | 0.5054 |
| TC1900011389.hg.1 | VSTM1 | V-set and transmembrane domain containing 1 | Multiple_C | 3.02 | 2.81 | 2.98 | 1.03 | 0.9779 | 0.9893 | 3.51 | 3.11 | 3.24 | 1.21 | 0.0249 | 0.137 |
| TC1900011406.hg.1 | LILRB5 | leukocyte immunoglobulin-like receptor, subfamily B (with TM a | Multiple_C | 2.84 | 2.74 | 2.8 | 1.03 | 0.8007 | 0.8978 | 3.24 | 3.30 | 3.38 | 0.91 | 0.6079 | 0.804 |
| TC1900011731.hg.1 | LGALS17A | Charcot-Leyden crystal protein pseudogene | Multiple_C | 5.3 | 5.28 | 5.26 | 1.03 | 0.7937 | 0.8942 | 4.98 | 4.87 | 5.06 | 0.95 | 0.6416 | 0.8242 |
| TC2000007481.hg.1 | SEMG1 | semenogelin I | Multiple_C | 3.47 | 3.44 | 3.43 | 1.03 | 0.6744 | 0.8228 | 4.51 | 3.95 | 3.95 | 1.47 | 0.0489 | 0.2055 |
| TC2000007830.hg.1 | TFAP2C | transcription factor AP-2 gamma (activating enhancer binding p | Coding | 5.65 | 5.59 | 5.61 | 1.03 | 0.6404 | 0.8007 | 3.78 | 3.00 | 3.26 | 1.43 | 0.0098 | 0.0766 |
| TC2000008175.hg.1 | PDYN | prodynorphin | Coding | 3.93 | 4.26 | 3.89 | 1.03 | 0.9612 | 0.9816 | 3.97 | 3.79 | 3.87 | 1.07 | 0.6921 | 0.853 |
| TC2000009877.hg.1 | RAD21L1 | RAD21 cohesin complex component like 1 | Multiple_C | 3.26 | 2.81 | 3.22 | 1.03 | 0.8436 | 0.9229 | 4.12 | 3.97 | 3.55 | 1.48 | 0.0313 | 0.1579 |
| TC2100007816.hg.1 | CYYR1 | cysteine/tyrosine-rich 1 | Coding | 3.13 | 2.6 | 3.09 | 1.03 | 0.9905 | 0.995 | 3.83 | 3.78 | 3.79 | 1.03 | 0.9262 | 0.9696 |
| TC2200008033.hg.1 | DGCR6L | DiGeorge syndrome critical region gene 6-like | Coding | 5.8 | 5.47 | 5.76 | 1.03 | 0.6087 | 0.7793 | 5.62 | 5.84 | 5.38 | 1.18 | 0.4714 | 0.7122 |
| TC2200009323.hg.1 | RIMBP3C | RIMS binding protein 3C | Coding | 4.33 | 3.95 | 4.29 | 1.03 | 0.7325 | 0.8603 | 4.15 | 4.22 | 4.18 | 0.98 | 0.1705 | 0.4168 |
| TSUnmapped00000275.h | NDUFA6 | NADH dehydrogenase (ubiquinone) 1 alpha subcomplex, 6, 14kI | Coding | 7.7 | 8.23 | 7.66 | 1.03 | 0.9455 | 0.9751 | 5.27 | 5.53 | 5.34 | 0.95 | 0.6996 | 0.8576 |
| TSUnmapped00000284.h | LRP6 | LDL receptor related protein 6 | Coding | 9.22 | 10.59 | 9.18 | 1.03 | 0.8124 | 0.9053 | 10.27 | 9.77 | 9.65 | 1.54 | 0.2352 | 0.4979 |
| TSUnmapped00000761.h | PRAMEF7 | PRAME family member 7 | Coding | 2.98 | 2.94 | 2.94 | 1.03 | 0.8706 | 0.9369 | 3.58 | 3.48 | 3.54 | 1.03 | 0.9589 | 0.9833 |
| TC0100006602.hg.1 | ACTRT2 | actin-related protein T2 | Coding | 4.57 | 4.43 | 4.54 | 1.02 | 0.8993 | 0.9518 | 4.58 | 4.68 | 4.97 | 0.76 | 0.9512 | 0.9797 |
| TC0100006696.hg.1 | ZBTB48 | zinc finger and BTB domain containing 48 | Multiple_C | 4 | 4.11 | 3.97 | 1.02 | 0.9486 | 0.9763 | 3.87 | 3.83 | 3.81 | 1.04 | 0.7287 | 0.8742 |
| TC0100006880.hg.1 | TNFRSF8 | tumor necrosis factor receptor superfamily, member 8 | Multiple_C | 5.05 | 4.84 | 5.02 | 1.02 | 0.8401 | 0.9206 | 5.55 | 5.56 | 5.51 | 1.03 | 0.8867 | 0.9516 |
| TC0100009965.hg.1 | SNX27 | sorting nexin family member 27 | Multiple_C | 10.71 | 10.78 | 10.68 | 1.02 | 0.9151 | 0.96 | 11.06 | 9.76 | 10.69 | 1.29 | 0.4154 | 0.6697 |
| TC0100010592.hg.1 | PRRC2C | proline-rich coiled-coil 2C | Multiple_C | 12.55 | 11.77 | 12.52 | 1.02 | 0.9011 | 0.9524 | 12.53 | 11.83 | 12.49 | 1.03 | 0.6991 | 0.8573 |
| TC0100011945.hg.1 | CAPN9 | calpain 9 | Multiple_C | 3.72 | 3.55 | 3.69 | 1.02 | 0.8772 | 0.9403 | 4 | 4.44 | 4.34 | 0.79 | 0.931 | 0.9709 |
| TC0100011993.hg.1 | MAP10 | microtubule-associated protein 10 | Coding | 3.01 | 2.91 | 2.98 | 1.02 | 0.955 | 0.9789 | 3.79 | 3.72 | 3.51 | 1.21 | 0.1732 | 0.4205 |
| TC0100013608.hg.1 | MARCKSL1 | MARCKS-like 1 | Multiple_C | 10.81 | 9.84 | 10.78 | 1.02 | 0.4751 | 0.678 | 7.02 | 7.93 | 7.74 | 0.61 | 0.0344 | 0.167 |
| TC0100013881.hg.1 | GUCA2A | guanylate cyclase activator 2A (guanylin) | Coding | 4.63 | 4.38 | 4.6 | 1.02 | 0.5 | 0.699 | 4.25 | 4.31 | 4.28 | 0.98 | 0.8672 | 0.9431 |
| TC0100015925.hg.1 | KCNN3 | potassium channel, calcium activated intermediate/small condu | Multiple_C | 3.76 | 3.55 | 3.73 | 1.02 | 0.8371 | 0.919 | 4.23 | 4.34 | 4.3 | 0.95 | 0.8431 | 0.9317 |
| TC0100017307.hg.1 | USH2A | Usher syndrome 2A (autosomal recessive, mild) | Multiple_C | 3.46 | 3.08 | 3.43 | 1.02 | 0.71 | 0.8461 | 3.78 | 3.82 | 3.58 | 1.15 | 0.5759 | 0.7826 |
| TC0100017365.hg.1 | EPRS | glutamyl-prolyl-tRNA synthetase | Multiple_C | 12.76 | 13.55 | 12.73 | 1.02 | 0.703 | 0.8415 | 10.9 | 10.60 | 10.9 | 1.00 | 0.971 | 0.9881 |
| TC0100017467.hg.1 | NVL | nuclear VCP-like | Multiple_C | 10.81 | 11.53 | 10.78 | 1.02 | 0.6141 | 0.7836 | 11.75 | 11.47 | 11.8 | 0.97 | 0.9678 | 0.9866 |
| TC0100018161.hg.1 | PLCH2 | phospholipase C, eta 2 | Multiple_C | 5.39 | 5.39 | 5.36 | 1.02 | 0.9625 | 0.9821 | 6.66 | 6.57 | 6.64 | 1.01 | 0.7804 | 0.9013 |

| | | | | | | | | | | | | | | | |
|---|---|---|---|---|---|---|---|---|---|---|---|---|---|---|---|
| TC0100018416.hg.1 | TAS1R2 | taste receptor, type 1, member 2 | Coding | 3.76 | 3.51 | 3.73 | 1.02 | 0.804 | 0.8999 | 3.66 | 3.60 | 3.66 | 1.00 | 0.8614 | 0.9407 |
| TC0100018461.hg.1 | AKNAD1 | AKNA domain containing 1 | Multiple_C | 3.55 | 3.59 | 3.52 | 1.02 | 0.6138 | 0.7834 | 3.57 | 3.46 | 3.55 | 1.01 | 0.8315 | 0.9249 |
| TC0100018514.hg.1 | VSIG8 | V-set and immunoglobulin domain containing 8 | Coding | 3.41 | 3.23 | 3.38 | 1.02 | 0.8767 | 0.9401 | 4.09 | 3.79 | 3.75 | 1.27 | 0.1789 | 0.4278 |
| TC0200008334.hg.1 | SMYD1 | SET and MYND domain containing 1 | Multiple_C | 3.52 | 3.52 | 3.49 | 1.02 | 0.544 | 0.7305 | 4.24 | 4.62 | 3.82 | 1.34 | 0.0306 | 0.1556 |
| TC0200009270.hg.1 | RAB6C | RAB6C, member RAS oncogene family | Coding | 8.76 | 8.87 | 8.73 | 1.02 | 0.4816 | 0.6832 | 6.32 | 6.41 | 6.65 | 0.80 | 0.195 | 0.4492 |
| TC0200010726.hg.1 | TMEM169 | transmembrane protein 169 | Coding | 5.46 | 5.24 | 5.43 | 1.02 | 0.8154 | 0.9071 | 8.12 | 8.16 | 7.8 | 1.25 | 0.1061 | 0.3198 |
| TC0200011070.hg.1 | C2orf57 | chromosome 2 open reading frame 57 | Coding | 6.04 | 5.94 | 6.01 | 1.02 | 0.5441 | 0.7305 | 5.64 | 5.80 | 5.75 | 0.93 | 0.9847 | 0.9937 |
| TC0200012030.hg.1 | HADHA | hydroxyacyl-CoA dehydrogenase/3-ketoacyl-CoA thiolase/enoy | Multiple_C | 13.32 | 13.81 | 13.29 | 1.02 | 0.9046 | 0.9543 | 11.29 | 11.57 | 11.75 | 0.73 | 0.2316 | 0.4928 |
| TC0200015012.hg.1 | ATP5G3 | ATP synthase, H+ transporting, mitochondrial Fo complex subur | Multiple_C | 13.19 | 13.72 | 13.16 | 1.02 | 0.621 | 0.7877 | 10.77 | 10.76 | 10.61 | 1.12 | 0.3189 | 0.583 |
| TC0200015431.hg.1 | TMEM237 | transmembrane protein 237 | Multiple_C | 7.68 | 7.57 | 7.65 | 1.02 | 0.2355 | 0.4366 | 8.87 | 9.64 | 9.54 | 0.63 | 0.0131 | 0.0923 |
| TC0200015776.hg.1 | RESP18 | regulated endocrine-specific protein 18 | Multiple_C | 3.94 | 3.84 | 3.91 | 1.02 | 0.6251 | 0.7906 | 4.41 | 4.15 | 3.77 | 1.56 | 0.0689 | 0.2499 |
| TC0200016345.hg.1 | PASK | PAS domain containing serine/threonine kinase | Multiple_C | 5.2 | 5.26 | 5.17 | 1.02 | 0.7909 | 0.893 | 4.95 | 5.02 | 4.8 | 1.11 | 0.4044 | 0.66 |
| TC0200016507.hg.1 | LIMS3L; LIMS3 | LIM and senescent cell antigen-like domains 3-like; LIM and sen | Multiple_C | 4.22 | 4.08 | 4.19 | 1.02 | 0.9585 | 0.9803 | 4.52 | 4.38 | 4.52 | 1.00 | 0.9534 | 0.981 |
| TC0200016559.hg.1 | BBS5 | Bardet-Biedl syndrome 5 | Multiple_C | 3.14 | 2.85 | 3.11 | 1.02 | 0.9508 | 0.9773 | 5.34 | 5.40 | 5.49 | 0.90 | 0.8313 | 0.9249 |
| TC0200016562.hg.1 | PHOSPHO2 | phosphatase, orphan 2 | Multiple_C | 5.08 | 6.05 | 5.05 | 1.02 | 0.8577 | 0.9305 | 5.35 | 5.98 | 5.22 | 1.09 | 0.2402 | 0.5039 |
| TC0200016570.hg.1 | HOXD3 | homeobox D3 | Coding | 4.28 | 4.03 | 4.25 | 1.02 | 0.9425 | 0.9733 | 3.81 | 3.64 | 3.47 | 1.27 | 0.2239 | 0.4847 |
| TC0300008111.hg.1 | OR5K2 | olfactory receptor, family 5, subfamily K, member 2 | Coding | 3.31 | 3.15 | 3.28 | 1.02 | 0.8995 | 0.9518 | 3.85 | 3.67 | 3.75 | 1.07 | 0.1494 | 0.3869 |
| TC0300009010.hg.1 | RNF7 | ring finger protein 7 | Multiple_C | 12.85 | 14.13 | 12.82 | 1.02 | 0.7953 | 0.895 | 9.52 | 9.80 | 9.51 | 1.01 | 0.7202 | 0.8697 |
| TC0300009334.hg.1 | C3orf80 | chromosome 3 open reading frame 80 | Multiple_C | 4.33 | 4.15 | 4.3 | 1.02 | 0.5431 | 0.7298 | 4.19 | 3.93 | 3.82 | 1.29 | 0.0171 | 0.1094 |
| TC0300009704.hg.1 | FAM131A | family with sequence similarity 131, member A | Multiple_C | 3.49 | 3.47 | 3.46 | 1.02 | 0.964 | 0.9824 | 3.88 | 4.07 | 3.95 | 0.95 | 0.9191 | 0.9668 |
| TC0300010390.hg.1 | RBSN | rabenosyn, RAB effector | Multiple_C | 9.52 | 9.24 | 9.49 | 1.02 | 0.9993 | 0.9997 | 9.14 | 8.81 | 9.66 | 0.70 | 0.0436 | 0.1931 |
| TC0300011311.hg.1 | FAM107A | family with sequence similarity 107, member A | Multiple_C | 4.02 | 3.72 | 3.99 | 1.02 | 0.4357 | 0.6452 | 4.39 | 4.38 | 4.46 | 0.95 | 0.8151 | 0.9182 |
| TC0300012047.hg.1 | ZNF80 | zinc finger protein 80 | Coding | 3.63 | 3.55 | 3.6 | 1.02 | 0.6076 | 0.7784 | 4.07 | 3.66 | 3.95 | 1.09 | 0.6059 | 0.8028 |
| TC0300012585.hg.1 | FOXL2 | forkhead box L2 | Coding | 2.8 | 2.43 | 2.77 | 1.02 | 0.966 | 0.9836 | 4.15 | 4.78 | 4.37 | 0.86 | 0.1564 | 0.397 |
| TC0300013920.hg.1 | NRROS | negative regulator of reactive oxygen species | Multiple_C | 3.3 | 3.44 | 3.27 | 1.02 | 0.9342 | 0.9691 | 3.62 | 3.60 | 3.62 | 1.00 | 0.592 | 0.7938 |
| TC0300013934.hg.1 | EMC3 | ER membrane protein complex subunit 3 | Multiple_C | 10.14 | 10.2 | 10.11 | 1.02 | 0.647 | 0.8043 | 8.78 | 9.02 | 9.2 | 0.75 | 0.0243 | 0.1346 |
| TC0400006818.hg.1 | USP17L28; US | ubiquitin specific peptidase 17-like family member 28; ubiquitin | Coding | 3.91 | 3.57 | 3.88 | 1.02 | 0.9908 | 0.9951 | 4.56 | 4.23 | 4.47 | 1.06 | 0.4208 | 0.6739 |
| TC0400007744.hg.1 | UGT2B10 | UDP glucuronosyltransferase 2 family, polypeptide B10 | Coding | 3.17 | 3.15 | 3.14 | 1.02 | 0.98 | 0.9903 | 3.3 | 3.06 | 3.07 | 1.17 | 0.0723 | 0.2577 |
| TC0400008662.hg.1 | SLC25A31 | solute carrier family 25 (mitochondrial carrier; adenine nucleoti | Coding | 3.85 | 3.93 | 3.82 | 1.02 | 0.7128 | 0.8481 | 4.08 | 4.31 | 3.95 | 1.09 | 0.9913 | 0.9963 |
| TC0400011563.hg.1 | ETNPPL | ethanolamine-phosphate phospho-lyase | Multiple_C | 2.81 | 2.76 | 2.78 | 1.02 | 0.802 | 0.8987 | 3.71 | 3.53 | 3.41 | 1.23 | 0.184 | 0.4351 |
| TC0400011932.hg.1 | ELF2 | E74-like factor 2 (ets domain transcription factor) | Multiple_C | 8.84 | 9.02 | 8.81 | 1.02 | 0.8151 | 0.907 | 8.45 | 8.79 | 8.32 | 1.09 | 0.5115 | 0.7413 |
| TC0400012182.hg.1 | FGG | fibrinogen gamma chain | Multiple_C | 3.46 | 3.75 | 3.43 | 1.02 | 0.9064 | 0.9551 | 3.51 | 3.37 | 3.23 | 1.21 | 0.0959 | 0.3014 |
| TC0400012873.hg.1 | FAM149A | family with sequence similarity 149, member A | Multiple_C | 6.64 | 6.66 | 6.61 | 1.02 | 0.9995 | 0.9998 | 4.95 | 4.77 | 5.11 | 0.90 | 0.5605 | 0.7721 |

| | | | | | | | | | | | | | | | |
|---|---|---|---|---|---|---|---|---|---|---|---|---|---|---|---|
| TC0500007413.hg.1 | GZMA | granzyme A | Multiple_C | 3.1 | 3.08 | 3.07 | 1.02 | 0.8392 | 0.9201 | 3.37 | 3.58 | 3.49 | 0.92 | 0.8746 | 0.946 |
| TC0500009559.hg.1 | SFXN1 | sideroflexin 1 | Multiple_C | 12.35 | 12.25 | 12.32 | 1.02 | 0.7931 | 0.8938 | 12.38 | 12.14 | 12.89 | 0.70 | 0.0641 | 0.2409 |
| TC0500011100.hg.1 | ZNF366 | zinc finger protein 366 | Multiple_C | 3.47 | 3.43 | 3.44 | 1.02 | 0.6571 | 0.8117 | 3.48 | 3.45 | 3.33 | 1.11 | 0.3096 | 0.5735 |
| TC0500012388.hg.1 | DPYSL3 | dihydropyrimidinase-like 3 | Multiple_C | 2.37 | 2.42 | 2.34 | 1.02 | 0.9838 | 0.9924 | 9.76 | 10.30 | 9.06 | 1.62 | 0.3515 | 0.6152 |
| TC0500013280.hg.1 | ZDHHC11B | zinc finger, DHHC-type containing 11B | Multiple_C | 4.26 | 4.25 | 4.23 | 1.02 | 0.5165 | 0.7108 | 5.48 | 4.42 | 4.36 | 2.17 | 1.06E-05 | 0.0008 |
| TC0600007410.hg.1 | ZSCAN9 | zinc finger and SCAN domain containing 9 | Multiple_C | 5.94 | 6.08 | 5.91 | 1.02 | 0.8257 | 0.9124 | 6.11 | 6.54 | 6.58 | 0.72 | 0.0741 | 0.2611 |
| TC0600007562.hg.1 | PSORS1C1 | psoriasis susceptibility 1 candidate 1 | Multiple_C | 3.25 | 3.29 | 3.22 | 1.02 | 0.4615 | 0.6669 | 3.64 | 3.80 | 3.81 | 0.89 | 0.7691 | 0.8964 |
| TC0600007833.hg.1 | PNPLA1 | patatin-like phospholipase domain containing 1 | Coding | 3.19 | 3.1 | 3.16 | 1.02 | 0.5924 | 0.7672 | 3.64 | 3.41 | 3.7 | 0.96 | 0.715 | 0.8669 |
| TC0600010647.hg.1 | ECI2 | enoyl-CoA delta isomerase 2 | Multiple_C | 10.98 | 11.42 | 10.95 | 1.02 | 0.7316 | 0.8599 | 11.35 | 11.41 | 11.43 | 0.95 | 0.9591 | 0.9834 |
| TC0600012731.hg.1 | ATG5 | autophagy related 5 | Multiple_C | 10.19 | 10.93 | 10.16 | 1.02 | 0.9214 | 0.9636 | 8.89 | 8.54 | 8.74 | 1.11 | 0.2065 | 0.4631 |
| TC0700006583.hg.1 | SDK1 | sidekick cell adhesion molecule 1 | Multiple_C | 2.87 | 2.74 | 2.84 | 1.02 | 0.7558 | 0.8745 | 3.12 | 3.02 | 3.1 | 1.01 | 0.2223 | 0.4826 |
| TC0700007294.hg.1 | SUGCT | succinyl-CoA:glutarate-CoA transferase | Multiple_C | 6.07 | 5.92 | 6.04 | 1.02 | 0.346 | 0.5597 | 4.96 | 4.41 | 4.53 | 1.35 | 0.0628 | 0.2384 |
| TC0700009696.hg.1 | CHPF2; MIR67 | chondroitin polymerizing factor 2; microRNA 671 | Multiple_C | 7.21 | 7.28 | 7.18 | 1.02 | 0.4239 | 0.6349 | 8.42 | 8.81 | 8.73 | 0.81 | 0.1188 | 0.3409 |
| TC0700010887.hg.1 | COA1 | cytochrome c oxidase assembly factor 1 homolog | Multiple_C | 13.34 | 14.63 | 13.31 | 1.02 | 0.907 | 0.9555 | 12.46 | 12.54 | 12.53 | 0.95 | 0.8291 | 0.9243 |
| TC0700011497.hg.1 | MLXIPL | MLX interacting protein-like | Multiple_C | 5.1 | 4.99 | 5.07 | 1.02 | 0.6876 | 0.8315 | 7.01 | 7.06 | 7.27 | 0.84 | 0.2564 | 0.5212 |
| TC0700012228.hg.1 | LAMB4 | laminin, beta 4 | Multiple_C | 4.48 | 4.35 | 4.45 | 1.02 | 0.4374 | 0.6468 | 4.37 | 4.34 | 4.3 | 1.05 | 0.8557 | 0.9385 |
| TC0700013291.hg.1 | ESYT2 | extended synaptotagmin-like protein 2 | Multiple_C | 11.36 | 11.53 | 11.33 | 1.02 | 0.5914 | 0.7662 | 10.51 | 10.01 | 10.39 | 1.09 | 0.6646 | 0.8359 |
| TC0700013554.hg.1 | ZPBP | zona pellucida binding protein | Coding | 3 | 2.97 | 2.97 | 1.02 | 0.8775 | 0.9404 | 3.64 | 3.17 | 3.08 | 1.47 | 0.028 | 0.147 |
| TC0800009247.hg.1 | HSF1 | heat shock transcription factor 1 | Multiple_C | 10.03 | 9.29 | 10 | 1.02 | 0.9147 | 0.96 | 9.24 | 9.69 | 9.9 | 0.63 | 0.0018 | 0.0253 |
| TC0800011000.hg.1 | REXO1L1P; RE | REX1, RNA exonuclease 1 homolog-like 1, pseudogene; REX1, RI | Multiple_C | 3.5 | 3.19 | 3.47 | 1.02 | 0.8074 | 0.9024 | 4.23 | 4.14 | 4.06 | 1.13 | 0.5286 | 0.7524 |
| TC0800011561.hg.1 | EIF3H | eukaryotic translation initiation factor 3, subunit H | Multiple_C | 16.01 | 16.5 | 15.98 | 1.02 | 0.8643 | 0.9337 | 14.75 | 14.30 | 14.47 | 1.21 | 0.1034 | 0.3151 |
| TC0800011972.hg.1 | KCNK9 | potassium channel, two pore domain subfamily K, member 9 | Multiple_C | 4.27 | 4.11 | 4.24 | 1.02 | 0.9449 | 0.9747 | 4.46 | 4.15 | 4.06 | 1.32 | 0.0379 | 0.1775 |
| TC0900008022.hg.1 | ZNF169 | zinc finger protein 169 | Multiple_C | 4.52 | 4.58 | 4.49 | 1.02 | 0.8482 | 0.9257 | 4.56 | 4.37 | 3.96 | 1.52 | 0.0145 | 0.0981 |
| TC0900008816.hg.1 | CFAP157 | cilia and flagella associated protein 157 | Multiple_C | 4.65 | 4.87 | 4.62 | 1.02 | 0.5445 | 0.7308 | 5.05 | 4.55 | 4.53 | 1.43 | 0.0746 | 0.2625 |
| TC0900009461.hg.1 | PLGRKT | plasminogen receptor, C-terminal lysine transmembrane protei | Multiple_C | 7.95 | 9.59 | 7.92 | 1.02 | 0.7719 | 0.8822 | 6.04 | 5.81 | 5.54 | 1.41 | 0.1291 | 0.3556 |
| TC0900010301.hg.1 | SPATA31A3 | SPATA31 subfamily A, member 3 | Multiple_C | 6.19 | 5.99 | 6.16 | 1.02 | 0.9616 | 0.9819 | 6.05 | 6.00 | 6.22 | 0.89 | 0.911 | 0.9634 |
| TC0900011611.hg.1 | PIP5KL1 | phosphatidylinositol-4-phosphate 5-kinase-like 1 | Multiple_C | 3.75 | 3.8 | 3.72 | 1.02 | 0.6464 | 0.8042 | 3.6 | 3.79 | 3.41 | 1.14 | 0.2713 | 0.5367 |
| TC0900011837.hg.1 | ABO | ABO blood group (transferase A, alpha 1-3-N-acetylgalactosami | Multiple_C | 3.9 | 3.67 | 3.87 | 1.02 | 0.402 | 0.6148 | 3.82 | 3.82 | 3.87 | 0.97 | 0.9867 | 0.9943 |
| TC0X00007191.hg.1 | EBP | emopamil binding protein (sterol isomerase) | Multiple_C | 14.46 | 14.55 | 14.43 | 1.02 | 0.7605 | 0.8769 | 11.56 | 12.49 | 13.01 | 0.37 | 1.15E-05 | 0.0008 |
| TC0X00009129.hg.1 | TMEM27 | transmembrane protein 27 | Coding | 3.47 | 4.31 | 3.44 | 1.02 | 0.7336 | 0.8609 | 4.09 | 4.75 | 4.2 | 0.93 | 0.6862 | 0.8497 |
| TC0X00009199.hg.1 | ADGRG2 | adhesion G protein-coupled receptor G2 | Multiple_C | 2.98 | 2.7 | 2.95 | 1.02 | 0.785 | 0.8894 | 3.55 | 3.63 | 3.56 | 0.99 | 0.4 | 0.657 |
| TC0X00009268.hg.1 | PCYT1B | phosphate cytidylyltransferase 1, choline, beta | Multiple_C | 2.81 | 2.61 | 2.78 | 1.02 | 0.8225 | 0.9108 | 7.24 | 6.81 | 5.96 | 2.43 | 0.0007 | 0.0131 |
| TC0X00009878.hg.1 | ASB12 | ankyrin repeat and SOCS box containing 12 | Coding | 2.8 | 3.03 | 2.77 | 1.02 | 0.6474 | 0.8045 | 3.76 | 3.52 | 3.71 | 1.04 | 0.6645 | 0.8358 |

| | | | | | | | | | | | | | | | |
|---|---|---|---|---|---|---|---|---|---|---|---|---|---|---|---|
| TC0X00010198.hg.1 | HDX | highly divergent homeobox | Multiple_C | 4.9 | 4.73 | 4.87 | 1.02 | 0.9248 | 0.9648 | 7.9 | 7.52 | 7.66 | 1.18 | 0.5046 | 0.7366 |
| TC0X00010442.hg.1 | TMSB15B; RP: | Homo sapiens thymosin beta 15B (TMSB15B), mRNA.; Thymosi | Coding | 2.75 | 2.72 | 2.72 | 1.02 | 0.9358 | 0.97 | 3.7 | 3.83 | 3.96 | 0.84 | 0.4752 | 0.7146 |
| TC0X00010962.hg.1 | MCF2 | MCF.2 cell line derived transforming sequence | Multiple_C | 3.27 | 2.98 | 3.24 | 1.02 | 0.6961 | 0.8367 | 3.66 | 3.48 | 3.62 | 1.03 | 0.5153 | 0.7446 |
| TC0X00011367.hg.1 | SSX7 | synovial sarcoma, X breakpoint 7 | Coding | 3.94 | 4.19 | 3.91 | 1.02 | 0.6963 | 0.8369 | 4.25 | 4.53 | 4.47 | 0.86 | 0.248 | 0.5126 |
| TC0X00011399.hg.1 | FAM127B | family with sequence similarity 127, member B | Multiple_C | 3.33 | 3.25 | 3.3 | 1.02 | 0.4055 | 0.6183 | 3.62 | 3.58 | 3.58 | 1.03 | 0.9923 | 0.9968 |
| TC0Y00007327.hg.1 | TSPY10 | testis specific protein, Y-linked 10 | Coding | 2.86 | 2.61 | 2.83 | 1.02 | 0.6701 | 0.8201 | 3.57 | 3.38 | 3.43 | 1.10 | 0.2476 | 0.5121 |
| TC1000008663.hg.1 | SEMA4G | sema domain, immunoglobulin domain (Ig), transmembrane do | Multiple_C | 6.61 | 6.9 | 6.58 | 1.02 | 0.9094 | 0.9566 | 5.8 | 6.28 | 5.94 | 0.91 | 0.6351 | 0.8208 |
| TC1000009170.hg.1 | HMX3 | H6 family homeobox 3 | Coding | 3.48 | 3.3 | 3.45 | 1.02 | 0.5398 | 0.7276 | 3.61 | 4.04 | 4.25 | 0.64 | 0.019 | 0.1166 |
| TC1000011376.hg.1 | CH25H | cholesterol 25-hydroxylase | Coding | 3.06 | 2.99 | 3.03 | 1.02 | 0.3661 | 0.5794 | 3.13 | 3.12 | 3.11 | 1.01 | 0.857 | 0.9392 |
| TC1000012156.hg.1 | TEX36 | testis expressed 36 | Coding | 3.58 | 3.53 | 3.55 | 1.02 | 0.7942 | 0.8945 | 3.9 | 3.69 | 4.07 | 0.89 | 0.333 | 0.5972 |
| TC1000012590.hg.1 | PSD | pleckstrin and Sec7 domain containing | Multiple_C | 4.81 | 4.67 | 4.78 | 1.02 | 0.7861 | 0.8902 | 3.74 | 3.74 | 3.73 | 1.01 | 0.5951 | 0.7959 |
| TC1100007425.hg.1 | DKFZp779M0( | uncharacterized DKFZp779M0652; Transcript Identified by AceV | Multiple_C | 3.94 | 3.88 | 3.91 | 1.02 | 0.909 | 0.9564 | 4.01 | 3.98 | 3.83 | 1.13 | 0.4583 | 0.7021 |
| TC1100007906.hg.1 | RTN3 | reticulon 3 | Multiple_C | 13.16 | 13.52 | 13.13 | 1.02 | 0.9862 | 0.9932 | 13.62 | 13.19 | 13.14 | 1.39 | 0.079 | 0.2709 |
| TC1100008724.hg.1 | TRIM49D1; TR | tripartite motif containing 49D1; tripartite motif containing 49D | Coding | 4.14 | 3.88 | 4.11 | 1.02 | 0.2032 | 0.3954 | 3.92 | 3.70 | 3.93 | 0.99 | 0.9603 | 0.9836 |
| TC1100009896.hg.1 | OR51S1 | olfactory receptor, family 51, subfamily S, member 1 | Coding | 4.37 | 4.66 | 4.34 | 1.02 | 0.7116 | 0.8475 | 4.5 | 4.41 | 4.06 | 1.36 | 0.0994 | 0.3082 |
| TC1100010026.hg.1 | LMO1 | LIM domain only 1 (rhombotin 1) | Multiple_C | 5.39 | 5.39 | 5.36 | 1.02 | 0.7555 | 0.8745 | 5.56 | 5.55 | 5.6 | 0.97 | 0.8463 | 0.933 |
| TC1100010089.hg.1 | RNF141 | ring finger protein 141 | Multiple_C | 10.74 | 11.75 | 10.71 | 1.02 | 0.7882 | 0.8915 | 10.32 | 10.34 | 9.83 | 1.40 | 0.0721 | 0.2572 |
| TC1100011008.hg.1 | PTGDR2 | prostaglandin D2 receptor 2 | Multiple_C | 5.17 | 4.72 | 5.14 | 1.02 | 0.8715 | 0.9376 | 6.28 | 6.35 | 6.85 | 0.67 | 0.0762 | 0.2656 |
| TC1100012958.hg.1 | OR56B1 | olfactory receptor, family 56, subfamily B, member 1 | Coding | 2.78 | 2.68 | 2.75 | 1.02 | 0.6605 | 0.8138 | 3.1 | 2.96 | 3.18 | 0.95 | 0.6834 | 0.8482 |
| TC1100013027.hg.1 | COX8A | cytochrome c oxidase subunit VIIIA (ubiquitous) | Multiple_C | 16.48 | 13.95 | 16.45 | 1.02 | 0.3631 | 0.5765 | 11.9 | 12.37 | 12.01 | 0.93 | 0.8773 | 0.9474 |
| TC1200008346.hg.1 | ALX1 | ALX homeobox 1 | Coding | 2.6 | 2.53 | 2.57 | 1.02 | 0.686 | 0.8305 | 3.14 | 3.16 | 3.19 | 0.97 | 0.4414 | 0.6905 |
| TC1200010577.hg.1 | ZNF641 | zinc finger protein 641 | Multiple_C | 4.98 | 4.96 | 4.95 | 1.02 | 0.2718 | 0.4803 | 5.16 | 5.46 | 5.18 | 0.99 | 0.2529 | 0.5171 |
| TC1200010667.hg.1 | FAM186A | family with sequence similarity 186, member A | Coding | 2.98 | 2.77 | 2.95 | 1.02 | 0.5369 | 0.7254 | 3.57 | 3.57 | 3.86 | 0.82 | 0.3124 | 0.5765 |
| TC1200011246.hg.1 | PTPRR | protein tyrosine phosphatase, receptor type, R | Multiple_C | 8.95 | 10.82 | 8.92 | 1.02 | 0.8351 | 0.9177 | 4.05 | 3.68 | 3.63 | 1.34 | 0.375 | 0.6356 |
| TC1200011920.hg.1 | MYL2 | myosin light chain 2 | Multiple_C | 3 | 3.04 | 2.97 | 1.02 | 0.8589 | 0.9311 | 3.67 | 3.44 | 3.2 | 1.39 | 0.0118 | 0.0864 |
| TC1200012587.hg.1 | GPR162 | G protein-coupled receptor 162 | Multiple_C | 3.77 | 4 | 3.74 | 1.02 | 0.4608 | 0.6664 | 3.97 | 4.29 | 4.18 | 0.86 | 0.934 | 0.9723 |
| TC1200012645.hg.1 | ZC3H10 | zinc finger CCCH-type containing 10 | Multiple_C | 5.88 | 5.65 | 5.85 | 1.02 | 0.8678 | 0.9356 | 7.02 | 6.93 | 6.66 | 1.28 | 0.1854 | 0.437 |
| TC1200012714.hg.1 | HSPB8 | heat shock 22kDa protein 8 | Multiple_C | 3.66 | 3.53 | 3.63 | 1.02 | 0.8159 | 0.9072 | 4.82 | 4.86 | 4.71 | 1.08 | 0.8712 | 0.9445 |
| TC1200012806.hg.1 | MYO1A | myosin IA | Multiple_C | 3.73 | 3.81 | 3.7 | 1.02 | 0.5704 | 0.7504 | 3.79 | 3.98 | 3.9 | 0.93 | 0.5418 | 0.7608 |
| TC1300006594.hg.1 | TNFRSF19 | tumor necrosis factor receptor superfamily, member 19 | Multiple_C | 3.66 | 3.51 | 3.63 | 1.02 | 0.6337 | 0.7959 | 6.85 | 7.58 | 6.77 | 1.06 | 0.964 | 0.9847 |
| TC1300007187.hg.1 | TRIM13; KCNI | tripartite motif containing 13; potassium channel regulator | Multiple_C | 9.4 | 10.63 | 9.37 | 1.02 | 0.7652 | 0.8794 | 9.09 | 9.23 | 8.97 | 1.09 | 0.2957 | 0.5605 |
| TC1300009514.hg.1 | DZIP1 | DAZ interacting zinc finger protein 1 | Multiple_C | 3.11 | 3.07 | 3.08 | 1.02 | 0.3574 | 0.5709 | 3.59 | 3.78 | 3.54 | 1.04 | 0.6545 | 0.8317 |
| TC1400006534.hg.1 | RNASE6 | ribonuclease, RNase A family, k6 | Coding | 3.63 | 3.52 | 3.6 | 1.02 | 0.8636 | 0.9335 | 3.49 | 3.33 | 3.34 | 1.11 | 0.0917 | 0.2933 |

| | | | | | | | | | | | | | | | |
|---|---|---|---|---|---|---|---|---|---|---|---|---|---|---|---|
| TC1500009143.hg.1 | EXD1 | exonuclease 3-5 domain containing 1 | Multiple_C | 3.12 | 3.18 | 3.09 | 1.02 | 0.6911 | 0.8335 | 3.57 | 3.51 | 3.26 | 1.24 | 0.1404 | 0.3725 |
| TC1500009760.hg.1 | PIF1 | PIF1 5-to-3 DNA helicase | Multiple_C | 7.23 | 7.21 | 7.2 | 1.02 | 0.8314 | 0.9158 | 7.18 | 7.46 | 7.34 | 0.90 | 0.5489 | 0.7655 |
| TC1500010689.hg.1 | HERC2P2 | hect domain and RLD 2 pseudogene 2 | Multiple_C | 6.49 | 6.43 | 6.46 | 1.02 | 0.8305 | 0.9155 | 6.85 | 6.67 | 6.72 | 1.09 | 0.9207 | 0.9672 |
| TC1600007739.hg.1 | PHKB | phosphorylase kinase, beta | Multiple_C | 10.26 | 10.83 | 10.23 | 1.02 | 0.7498 | 0.8715 | 9.86 | 10.61 | 10.75 | 0.54 | 0.0093 | 0.0744 |
| TC1600007827.hg.1 | NOD2 | nucleotide-binding oligomerization domain containing 2 | Multiple_C | 4.53 | 4.54 | 4.5 | 1.02 | 0.3842 | 0.5975 | 5.05 | 4.89 | 4.66 | 1.31 | 0.0493 | 0.2067 |
| TC1600009953.hg.1 | GDPD3 | glycerophosphodiester phosphodiesterase domain containing 3 | Multiple_C | 3.54 | 3.3 | 3.51 | 1.02 | 0.5943 | 0.7686 | 3.48 | 3.22 | 3.1 | 1.30 | 0.4489 | 0.6956 |
| TC1600011356.hg.1 | MPV17L | MPV17 mitochondrial membrane protein-like | Multiple_C | 3.97 | 3.83 | 3.94 | 1.02 | 0.4983 | 0.6979 | 3.75 | 3.90 | 3.58 | 1.13 | 0.3802 | 0.6398 |
| TC1600011570.hg.1 | CMC2 | C-x(9)-C motif containing 2 | Multiple_C | 12.99 | 14.73 | 12.96 | 1.02 | 0.8195 | 0.9093 | 10.6 | 10.05 | 10.27 | 1.26 | 0.0504 | 0.209 |
| TC1700006445.hg.1 | LOC10050638 | uncharacterized LOC100506388; Transcript Identified by AceVie | Multiple_C | 4.48 | 4.33 | 4.45 | 1.02 | 0.9618 | 0.9819 | 3.71 | 4.01 | 3.91 | 0.87 | 0.951 | 0.9797 |
| TC1700006642.hg.1 | TM4SF5 | transmembrane 4 L six family member 5 | Multiple_C | 4.98 | 4.86 | 4.95 | 1.02 | 0.6923 | 0.8342 | 4.23 | 4.35 | 4.26 | 0.98 | 0.7304 | 0.875 |
| TC1700006665.hg.1 | ZFP3 | ZFP3 zinc finger protein | Coding | 4.96 | 5.04 | 4.93 | 1.02 | 0.8564 | 0.9298 | 4.78 | 4.95 | 4.59 | 1.14 | 0.2991 | 0.5637 |
| TC1700007137.hg.1 | FBXW10 | F-box and WD repeat domain containing 10 | Multiple_C | 3.98 | 3.71 | 3.95 | 1.02 | 0.9592 | 0.9806 | 3.35 | 3.23 | 3.01 | 1.27 | 0.0829 | 0.2772 |
| TC1700008151.hg.1 | NFE2L1 | nuclear factor, erythroid 2-like 1 | Multiple_C | 12.42 | 12.26 | 12.39 | 1.02 | 0.9322 | 0.968 | 12.11 | 11.50 | 12.2 | 0.94 | 0.1281 | 0.3542 |
| TC1700008191.hg.1 | B4GALNT2 | beta-1,4-N-acetyl-galactosaminyl transferase 2 | Multiple_C | 2.9 | 2.66 | 2.87 | 1.02 | 0.7721 | 0.8823 | 3.82 | 3.94 | 3.64 | 1.13 | 0.0903 | 0.2907 |
| TC1700009742.hg.1 | MYH8 | myosin, heavy chain 8, skeletal muscle, perinatal | Coding | 3.68 | 3.78 | 3.65 | 1.02 | 0.5355 | 0.7245 | 3.55 | 3.49 | 3.48 | 1.05 | 0.3979 | 0.6554 |
| TC1700010637.hg.1 | KRT26 | keratin 26, type I | Coding | 4.03 | 4.14 | 4 | 1.02 | 0.4674 | 0.6717 | 3.99 | 3.76 | 3.61 | 1.30 | 0.1584 | 0.4 |
| TC1700011101.hg.1 | CHAD | chondroadherin | Coding | 3.7 | 3.76 | 3.67 | 1.02 | 0.5536 | 0.7374 | 3.65 | 3.84 | 4.1 | 0.73 | 0.02 | 0.1205 |
| TC1700012431.hg.1 | HOXB2 | homeobox B2 | Multiple_C | 4.52 | 4.21 | 4.49 | 1.02 | 0.8315 | 0.9158 | 3.79 | 3.80 | 3.92 | 0.91 | 0.8359 | 0.9277 |
| TC1800006882.hg.1 | CABLES1 | Cdk5 and Abl enzyme substrate 1 | Multiple_C | 11.1 | 11.04 | 11.07 | 1.02 | 0.9252 | 0.965 | 9.18 | 10.91 | 11.01 | 0.28 | 7.63E-08 | 2.14E-05 |
| TC1800007682.hg.1 | CNDP1 | carnosine dipeptidase 1 (metallopeptidase M20 family) | Multiple_C | 3.75 | 3.47 | 3.72 | 1.02 | 0.6816 | 0.8271 | 3.85 | 3.55 | 3.7 | 1.11 | 0.5458 | 0.7634 |
| TC1800008317.hg.1 | ZNF521 | zinc finger protein 521 | Multiple_C | 7.98 | 8.63 | 7.95 | 1.02 | 0.3482 | 0.5613 | 3.92 | 3.73 | 3.68 | 1.18 | 0.1386 | 0.3698 |
| TC1900007113.hg.1 | DAND5 | DAN domain family member 5, BMP antagonist | Coding | 3.29 | 3.24 | 3.26 | 1.02 | 0.5415 | 0.7288 | 3.56 | 3.59 | 3.62 | 0.96 | 0.6374 | 0.822 |
| TC1900007265.hg.1 | FAM32A | family with sequence similarity 32, member A | Coding | 10.63 | 10.82 | 10.6 | 1.02 | 0.949 | 0.9763 | 9.98 | 10.79 | 10.53 | 0.68 | 0.2229 | 0.4834 |
| TC1900008146.hg.1 | CCDC97 | coiled-coil domain containing 97 | Coding | 8.4 | 7.91 | 8.37 | 1.02 | 0.5055 | 0.7029 | 8.9 | 9.49 | 9.38 | 0.72 | 0.0955 | 0.3006 |
| TC1900008163.hg.1 | DMRTC2 | DMRT-like family C2 | Multiple_C | 4.71 | 4.52 | 4.68 | 1.02 | 0.9027 | 0.9531 | 5.34 | 5.46 | 5.48 | 0.91 | 0.7904 | 0.9065 |
| TC1900008259.hg.1 | ZNF233 | zinc finger protein 233 | Multiple_C | 5.55 | 5.59 | 5.52 | 1.02 | 0.3798 | 0.5928 | 4.85 | 4.43 | 4.47 | 1.30 | 0.1891 | 0.4417 |
| TC1900008649.hg.1 | CEACAM18 | carcinoembryonic antigen-related cell adhesion molecule 18 | Coding | 4.1 | 3.85 | 4.07 | 1.02 | 0.7813 | 0.8872 | 3.82 | 3.61 | 3.59 | 1.17 | 0.2991 | 0.5637 |
| TC1900010984.hg.1 | CCDC8 | coiled-coil domain containing 8 | Coding | 3.39 | 3.18 | 3.36 | 1.02 | 0.8179 | 0.9083 | 3.89 | 3.79 | 3.58 | 1.24 | 0.1101 | 0.3268 |
| TC1900011138.hg.1 | PTH2 | parathyroid hormone 2 | Multiple_C | 5.68 | 5.49 | 5.65 | 1.02 | 0.4448 | 0.6534 | 5.18 | 5.18 | 5.09 | 1.06 | 0.8069 | 0.9148 |
| TC1900011228.hg.1 | KLK5 | kallikrein related peptidase 5 | Multiple_C | 4.3 | 4.88 | 4.27 | 1.02 | 0.5273 | 0.7189 | 4.38 | 4.17 | 3.96 | 1.34 | 0.2433 | 0.5069 |
| TC1900011800.hg.1 | LILRB4 | leukocyte immunoglobulin-like receptor, subfamily B (with TM a | Multiple_C | 3.67 | 3.25 | 3.64 | 1.02 | 0.6319 | 0.7945 | 3.83 | 3.81 | 3.85 | 0.99 | 0.8459 | 0.9327 |
| TC1900012040.hg.1 | LILRA5 | leukocyte immunoglobulin-like receptor, subfamily A (with TM c | Multiple_C | 5.69 | 5.93 | 5.66 | 1.02 | 0.9531 | 0.9784 | 5.55 | 5.75 | 5.53 | 1.01 | 0.7206 | 0.8698 |
| TC2000006961.hg.1 | CSTL1 | cystatin-like 1 | Multiple_C | 3.13 | 3.12 | 3.1 | 1.02 | 0.7574 | 0.8753 | 3.32 | 3.21 | 3.1 | 1.16 | 0.4071 | 0.6629 |

| | | | | | | | | | | | | | | | |
|---|---|---|---|---|---|---|---|---|---|---|---|---|---|---|---|
| TC2000007819.hg.1 | CSTF1 | cleavage stimulation factor, 3 pre-RNA, subunit 1 | Multiple_C | 11.49 | 11.91 | 11.46 | 1.02 | 0.5105 | 0.7064 | 11.28 | 11.52 | 11.78 | 0.71 | 0.1255 | 0.3501 |
| TC2000008546.hg.1 | C20orf78 | chromosome 20 open reading frame 78 | Multiple_C | 3.97 | 4.28 | 3.94 | 1.02 | 0.9396 | 0.972 | 3.91 | 3.80 | 3.75 | 1.12 | 0.1535 | 0.3928 |
| TC2000009169.hg.1 | GTSF1L | gametocyte specific factor 1-like | Coding | 5.4 | 5.11 | 5.37 | 1.02 | 0.355 | 0.5681 | 5.42 | 5.60 | 5.58 | 0.90 | 0.5368 | 0.7584 |
| TC2000009228.hg.1 | SPINT3 | serine peptidase inhibitor, Kunitz type, 3 | Coding | 2.67 | 2.65 | 2.64 | 1.02 | 0.7047 | 0.8425 | 3.09 | 3.04 | 3.14 | 0.97 | 0.515 | 0.7442 |
| TC2000009954.hg.1 | ZGPAT; LIME1 | zinc finger, CCCH-type with G-patch domain; Lck interacting trar | Multiple_C | 6.71 | 6.53 | 6.68 | 1.02 | 0.899 | 0.9518 | 7.84 | 7.80 | 7.6 | 1.18 | 0.7971 | 0.9093 |
| TC2100008477.hg.1 | S100B | S100 calcium binding protein B | Coding | 4.4 | 3.96 | 4.37 | 1.02 | 0.97 | 0.9856 | 4.11 | 4.24 | 3.97 | 1.10 | 0.3775 | 0.6376 |
| TC2200006793.hg.1 | GGTLC2 | gamma-glutamyltransferase light chain 2 | Multiple_C | 6.75 | 6.55 | 6.72 | 1.02 | 0.94 | 0.9722 | 6.89 | 6.63 | 6.38 | 1.42 | 0.1126 | 0.3305 |
| TC2200008000.hg.1 | TXNRD2 | Transcript Identified by AceView, Entrez Gene ID(s) 10587 | Coding | 5.4 | 5.54 | 5.37 | 1.02 | 0.2509 | 0.4552 | 5.55 | 5.39 | 4.98 | 1.48 | 0.0403 | 0.184 |
| TC2200008639.hg.1 | SSTR3 | somatostatin receptor 3 | Coding | 7.58 | 7.38 | 7.55 | 1.02 | 0.2307 | 0.4309 | 7.61 | 7.65 | 7.63 | 0.99 | 0.8722 | 0.945 |
| TC2200009289.hg.1 | TRMU | tRNA 5-methylaminomethyl-2-thiouridylate methyltransferase | Multiple_C | 6.81 | 6.79 | 6.78 | 1.02 | 0.3727 | 0.5853 | 7.62 | 7.81 | 7.76 | 0.91 | 0.581 | 0.7859 |
| TSUnmapped00000016.h | PRAMEF7 | PRAME family member 7 | Coding | 3.47 | 3.21 | 3.44 | 1.02 | 0.5605 | 0.743 | 3.42 | 3.29 | 3.21 | 1.16 | 0.2848 | 0.5498 |
| TSUnmapped00000241.h | ZNF502 | zinc finger protein 502 | Coding | 3.42 | 3.66 | 3.39 | 1.02 | 0.8673 | 0.9353 | 4.93 | 4.37 | 4.29 | 1.56 | 0.0159 | 0.1048 |
| TSUnmapped00000270.h | TRAPPC4 | trafficking protein particle complex 4 | Coding | 11.77 | 12.09 | 11.74 | 1.02 | 0.4277 | 0.6383 | 9.26 | 9.05 | 9.05 | 1.16 | 0.4744 | 0.714 |
| TSUnmapped00000641.h | PRAMEF2 | PRAME family member 2 | Coding | 3.01 | 2.63 | 2.98 | 1.02 | 0.7073 | 0.8444 | 3.88 | 3.70 | 3.78 | 1.07 | 0.268 | 0.5326 |
| TC0100006514.hg.1 | TMEM88B | transmembrane protein 88B | Multiple_C | 3.37 | 3.37 | 3.35 | 1.01 | 0.565 | 0.7465 | 3.46 | 3.44 | 3.45 | 1.01 | 0.2948 | 0.5598 |
| TC0100006683.hg.1 | HES3 | hes family bHLH transcription factor 3 | Coding | 4.15 | 3.87 | 4.13 | 1.01 | 0.7278 | 0.8568 | 3.96 | 3.94 | 4.4 | 0.74 | 0.0317 | 0.1594 |
| TC0100006903.hg.1 | AADACL3 | arylacetamide deacetylase-like 3 | Multiple_C | 4.1 | 3.76 | 4.08 | 1.01 | 0.5476 | 0.7331 | 5.11 | 4.80 | 5.33 | 0.86 | 0.579 | 0.7844 |
| TC0100007440.hg.1 | FAM110D | family with sequence similarity 110, member D | Coding | 2.96 | 3 | 2.94 | 1.01 | 0.4985 | 0.6979 | 3.93 | 3.34 | 3.57 | 1.28 | 0.0287 | 0.1493 |
| TC0100008182.hg.1 | CYP4X1 | cytochrome P450, family 4, subfamily X, polypeptide 1 | Multiple_C | 3.24 | 3.15 | 3.22 | 1.01 | 0.6804 | 0.8263 | 3.97 | 3.64 | 3.54 | 1.35 | 0.2819 | 0.5475 |
| TC0100008591.hg.1 | PGM1 | phosphoglucomutase 1 | Multiple_C | 9.29 | 9.73 | 9.27 | 1.01 | 0.9405 | 0.9724 | 7.24 | 8.03 | 7.26 | 0.99 | 0.3467 | 0.6103 |
| TC0100010012.hg.1 | SMCP | sperm mitochondria-associated cysteine-rich protein | Coding | 3.68 | 3.92 | 3.66 | 1.01 | 0.495 | 0.6948 | 3.48 | 3.58 | 3.59 | 0.93 | 0.789 | 0.9058 |
| TC0100010584.hg.1 | FMO1 | flavin containing monooxygenase 1 | Multiple_C | 2.97 | 3.08 | 2.95 | 1.01 | 0.478 | 0.6804 | 3.59 | 3.57 | 3.18 | 1.33 | 0.0714 | 0.2557 |
| TC0100010784.hg.1 | TDRD5 | tudor domain containing 5 | Coding | 3.26 | 3.42 | 3.24 | 1.01 | 0.8064 | 0.9017 | 3.22 | 3.23 | 3.07 | 1.11 | 0.2029 | 0.4586 |
| TC0100011262.hg.1 | PRELP | proline/arginine-rich end leucine-rich repeat protein | Coding | 4.2 | 4.32 | 4.18 | 1.01 | 0.4315 | 0.6417 | 3.92 | 3.70 | 3.86 | 1.04 | 0.9155 | 0.9654 |
| TC0100011746.hg.1 | CNIH4 | cornichon family AMPA receptor auxiliary protein 4 | Multiple_C | 12.83 | 13.89 | 12.81 | 1.01 | 0.8959 | 0.9502 | 11.16 | 10.96 | 10.91 | 1.19 | 0.6739 | 0.842 |
| TC0100011957.hg.1 | TRIM67 | tripartite motif containing 67 | Coding | 3.47 | 3.07 | 3.45 | 1.01 | 0.8354 | 0.9179 | 4.35 | 4.53 | 4.35 | 1.00 | 0.872 | 0.9449 |
| TC0100014543.hg.1 | DEPDC1 | DEP domain containing 1 | Multiple_C | 9.83 | 10.78 | 9.81 | 1.01 | 0.7792 | 0.8863 | 9.23 | 9.32 | 9.33 | 0.93 | 0.4001 | 0.657 |
| TC0100014904.hg.1 | HFM1 | HFM1, ATP-dependent DNA helicase homolog | Multiple_C | 4.29 | 4.37 | 4.27 | 1.01 | 0.6429 | 0.8019 | 4.4 | 4.60 | 4.14 | 1.20 | 0.4335 | 0.6846 |
| TC0100015364.hg.1 | TSPAN2 | tetraspanin 2 | Multiple_C | 4.25 | 4.49 | 4.23 | 1.01 | 0.6614 | 0.8143 | 4.13 | 3.55 | 3.92 | 1.16 | 0.1768 | 0.4255 |
| TC0100015868.hg.1 | S100A2 | S100 calcium binding protein A2 | Multiple_C | 9.28 | 9.53 | 9.26 | 1.01 | 0.8538 | 0.9287 | 7.98 | 7.53 | 7.4 | 1.49 | 0.0375 | 0.1763 |
| TC0100016099.hg.1 | CRP | C-reactive protein, pentraxin-related | Multiple_C | 2.87 | 2.68 | 2.85 | 1.01 | 0.9799 | 0.9903 | 3.5 | 3.63 | 3.27 | 1.17 | 0.1875 | 0.4392 |
| TC0200009189.hg.1 | GYPC | glycophorin C (Gerbich blood group) | Multiple_C | 3.91 | 3.87 | 3.89 | 1.01 | 0.6582 | 0.8124 | 3.92 | 3.87 | 3.8 | 1.09 | 0.4513 | 0.6966 |
| TC0200011257.hg.1 | PRLH | prolactin releasing hormone | Coding | 3.96 | 4.02 | 3.94 | 1.01 | 0.9213 | 0.9636 | 3.98 | 4.11 | 4.04 | 0.96 | 0.8917 | 0.9539 |

| | | | | | | | | | | | | | | | |
|---|---|---|---|---|---|---|---|---|---|---|---|---|---|---|---|
| TC0200012731.hg.1 | LOC339803; A | uncharacterized LOC339803; Transcript Identified by AceView, E | Multiple_C | 3.86 | 3.87 | 3.84 | 1.01 | 0.9635 | 0.9824 | 3.95 | 4.26 | 4.47 | 0.70 | 0.1741 | 0.4216 |
| TC0200013284.hg.1 | C2orf68 | chromosome 2 open reading frame 68 | Multiple_C | 6.16 | 6.03 | 6.14 | 1.01 | 0.7909 | 0.893 | 6.61 | 7.20 | 7.06 | 0.73 | 0.0965 | 0.3024 |
| TC0200014226.hg.1 | LOC151121; A | uncharacterized LOC151121; novel transcript | Multiple_C | 3.4 | 3.27 | 3.38 | 1.01 | 0.8475 | 0.9255 | 3.48 | 3.47 | 3.37 | 1.08 | 0.7758 | 0.8993 |
| TC0200014771.hg.1 | FAP | fibroblast activation protein alpha | Multiple_C | 2.76 | 2.57 | 2.74 | 1.01 | 0.5537 | 0.7374 | 4.06 | 4.00 | 3.88 | 1.13 | 0.4591 | 0.7024 |
| TC0200014860.hg.1 | CCDC173 | coiled-coil domain containing 173 | Coding | 4.42 | 4.34 | 4.4 | 1.01 | 0.189 | 0.3767 | 4.22 | 4.21 | 3.8 | 1.34 | 0.2585 | 0.523 |
| TC0200015603.hg.1 | ACADL | acyl-CoA dehydrogenase, long chain | Multiple_C | 5.34 | 5.4 | 5.32 | 1.01 | 0.9644 | 0.9827 | 5.2 | 5.23 | 4.95 | 1.19 | 0.1128 | 0.3308 |
| TC0200015757.hg.1 | CFAP65 | cilia and flagella associated protein 65 | Multiple_C | 2.79 | 2.99 | 2.77 | 1.01 | 0.8435 | 0.9229 | 3.44 | 3.16 | 3.21 | 1.17 | 0.4106 | 0.6652 |
| TC0200016347.hg.1 | HDLBP | high density lipoprotein binding protein | Multiple_C | 14.93 | 14.32 | 14.91 | 1.01 | 0.9937 | 0.9968 | 14.97 | 14.88 | 15.44 | 0.72 | 0.2431 | 0.5067 |
| TC0300007051.hg.1 | ITGA9 | integrin alpha 9 | Multiple_C | 3.47 | 3.59 | 3.45 | 1.01 | 0.8833 | 0.9434 | 4.03 | 4.08 | 4.13 | 0.93 | 0.6222 | 0.8128 |
| TC0300007566.hg.1 | SPATA12 | spermatogenesis associated 12 | Coding | 4.16 | 4.39 | 4.14 | 1.01 | 0.6881 | 0.8318 | 5.96 | 5.77 | 5.29 | 1.59 | 0.0124 | 0.0894 |
| TC0300008303.hg.1 | LINC01205 | long intergenic non-protein coding RNA 1205 | Multiple_C | 4.24 | 4.02 | 4.22 | 1.01 | 0.3835 | 0.5966 | 4.31 | 4.48 | 4.45 | 0.91 | 0.6562 | 0.8321 |
| TC0300008385.hg.1 | TIGIT | T-cell immunoreceptor with Ig and ITIM domains | Multiple_C | 4.63 | 5.08 | 4.61 | 1.01 | 0.6616 | 0.8144 | 5.32 | 5.12 | 4.85 | 1.39 | 0.3044 | 0.5682 |
| TC0300008550.hg.1 | CSTA | cystatin A (stefin A) | Multiple_C | 4.47 | 4.51 | 4.45 | 1.01 | 0.8969 | 0.9508 | 4.82 | 4.32 | 4.48 | 1.27 | 0.1464 | 0.3821 |
| TC0300009774.hg.1 | HRG | histidine-rich glycoprotein | Multiple_C | 4.63 | 4.21 | 4.61 | 1.01 | 0.5295 | 0.7203 | 3.85 | 3.97 | 3.87 | 0.99 | 0.7979 | 0.9094 |
| TC0300010431.hg.1 | DAZL | deleted in azoospermia-like | Multiple_C | 3.82 | 3.92 | 3.8 | 1.01 | 0.6733 | 0.822 | 3.86 | 4.26 | 4.33 | 0.72 | 0.32 | 0.584 |
| TC0300010775.hg.1 | CX3CR1 | chemokine (C-X3-C motif) receptor 1 | Coding | 3.79 | 4 | 3.77 | 1.01 | 0.8199 | 0.9096 | 4.49 | 4.30 | 4.2 | 1.22 | 0.1736 | 0.421 |
| TC0300010943.hg.1 | CCR1 | chemokine (C-C motif) receptor 1 | Coding | 3.38 | 3.48 | 3.36 | 1.01 | 0.9738 | 0.9874 | 3.88 | 4.02 | 4.04 | 0.90 | 0.9052 | 0.9607 |
| TC0300013815.hg.1 | KRBOX1 | KRAB box domain containing 1 | Multiple_C | 3.62 | 3.05 | 3.6 | 1.01 | 0.7799 | 0.8868 | 3.82 | 3.58 | 3.66 | 1.12 | 0.3734 | 0.6341 |
| TC0300013915.hg.1 | TPRG1 | tumor protein p63 regulated 1 | Multiple_C | 4.13 | 3.73 | 4.11 | 1.01 | 0.9039 | 0.9538 | 4.74 | 4.38 | 4.37 | 1.29 | 0.4867 | 0.7237 |
| TC0300013954.hg.1 | ZNF385D | zinc finger protein 385D | Multiple_C | 3.03 | 2.75 | 3.01 | 1.01 | 0.3266 | 0.5406 | 3.75 | 3.81 | 3.69 | 1.04 | 0.5983 | 0.7979 |
| TC0400007597.hg.1 | THEGL | theg spermatid protein-like | Multiple_C | 4.26 | 4.33 | 4.24 | 1.01 | 0.7522 | 0.8733 | 4.15 | 3.92 | 3.88 | 1.21 | 0.1177 | 0.3391 |
| TC0400010785.hg.1 | IGFBP7 | insulin like growth factor binding protein 7 | Multiple_C | 3.98 | 4.07 | 3.96 | 1.01 | 0.5677 | 0.7482 | 4.22 | 3.74 | 3.81 | 1.33 | 0.1741 | 0.4217 |
| TC0400011407.hg.1 | ADH1A | alcohol dehydrogenase 1A (class I), alpha polypeptide | Multiple_C | 2.89 | 2.92 | 2.87 | 1.01 | 0.8994 | 0.9518 | 3.42 | 3.85 | 3.64 | 0.86 | 0.9379 | 0.9742 |
| TC0400011618.hg.1 | NEUROG2 | neurogenin 2 | Coding | 3.7 | 3.44 | 3.68 | 1.01 | 0.8992 | 0.9518 | 4.18 | 4.15 | 4.15 | 1.02 | 0.5607 | 0.7721 |
| TC0400012027.hg.1 | ANAPC10 | anaphase promoting complex subunit 10 | Multiple_C | 10.52 | 12.28 | 10.5 | 1.01 | 0.9567 | 0.9799 | 8.55 | 8.42 | 8.85 | 0.81 | 0.2582 | 0.5228 |
| TC0500009224.hg.1 | LSM11 | LSM11, U7 small nuclear RNA associated | Coding | 6.39 | 7 | 6.37 | 1.01 | 0.5359 | 0.7249 | 5.04 | 5.44 | 6.04 | 0.50 | 0.0008 | 0.0146 |
| TC0500009304.hg.1 | GABRG2 | gamma-aminobutyric acid (GABA) A receptor, gamma 2 | Coding | 2.76 | 2.73 | 2.74 | 1.01 | 0.9244 | 0.9645 | 4.75 | 4.99 | 4.57 | 1.13 | 0.5555 | 0.7698 |
| TC0500009378.hg.1 | DOCK2 | dedicator of cytokinesis 2 | Multiple_C | 2.77 | 2.53 | 2.75 | 1.01 | 0.9838 | 0.9924 | 3.54 | 3.09 | 3.16 | 1.30 | 0.211 | 0.4687 |
| TC0500010813.hg.1 | MIER3 | mesoderm induction early response 1, family member 3 | Multiple_C | 9.93 | 10.5 | 9.91 | 1.01 | 0.9154 | 0.9602 | 9.87 | 9.35 | 9.61 | 1.20 | 0.2726 | 0.538 |
| TC0500012059.hg.1 | VDAC1 | voltage-dependent anion channel 1 | Multiple_C | 14.2 | 14.19 | 14.18 | 1.01 | 0.4494 | 0.6573 | 13.98 | 13.40 | 13.83 | 1.11 | 0.8155 | 0.9182 |
| TC0500012248.hg.1 | NDUFA2 | NADH dehydrogenase (ubiquinone) 1 alpha subcomplex, 2, 8kD | Multiple_C | 12.17 | 12.57 | 12.15 | 1.01 | 0.9623 | 0.982 | 7.82 | 8.22 | 7.75 | 1.05 | 0.9312 | 0.971 |
| TC0500012526.hg.1 | GLRA1 | glycine receptor alpha 1 | Multiple_C | 2.44 | 2.44 | 2.42 | 1.01 | 0.9527 | 0.9782 | 2.98 | 3.07 | 3.23 | 0.84 | 0.7132 | 0.8657 |
| TC0500012605.hg.1 | SOX30 | SRY box 30 | Coding | 4.24 | 3.42 | 4.22 | 1.01 | 0.1325 | 0.2953 | 4.95 | 4.52 | 4.13 | 1.77 | 0.0234 | 0.1322 |

| | | | | | | | | | | | | | | | |
|---|---|---|---|---|---|---|---|---|---|---|---|---|---|---|---|
| TC0500013089.hg.1 | GFPT2 | glutamine-fructose-6-phosphate transaminase 2 | Multiple_ | 3.63 | 3.43 | 3.61 | 1.01 | 0.8755 | 0.9395 | 3.47 | 3.89 | 3.83 | 0.78 | 0.1861 | 0.4379 |
| TC0500013392.hg.1 | CCNJL | cyclin J-like | Multiple_ | 7.63 | 6.62 | 7.61 | 1.01 | 0.7299 | 0.8586 | 7.77 | 7.84 | 8.51 | 0.60 | 0.0049 | 0.0486 |
| TC0500013414.hg.1 | THOC3 | THO complex 3 | Multiple_ | 13.46 | 13.3 | 13.44 | 1.01 | 0.6826 | 0.8279 | 11.03 | 11.18 | 11.6 | 0.67 | 0.0115 | 0.0854 |
| TC0600008099.hg.1 | POLH | polymerase (DNA directed), eta | Multiple_ | 8.24 | 8.02 | 8.22 | 1.01 | 0.7215 | 0.8535 | 7.72 | 7.73 | 7.92 | 0.87 | 0.2681 | 0.5326 |
| TC0600009310.hg.1 | GJA1 | gap junction protein alpha 1 | Multiple_ | 3.9 | 3.94 | 3.88 | 1.01 | 0.6509 | 0.8067 | 4.51 | 4.02 | 3.81 | 1.62 | 0.0237 | 0.1328 |
| TC0600009667.hg.1 | VTA1 | vesicle (multivesicular body) trafficking 1 | Multiple_ | 12.18 | 13 | 12.16 | 1.01 | 0.5085 | 0.705 | 12.18 | 11.95 | 11.9 | 1.21 | 0.1782 | 0.427 |
| TC0600010111.hg.1 | PACRG | PARK2 co-regulated | Multiple_ | 3.73 | 4.07 | 3.71 | 1.01 | 0.5753 | 0.7542 | 3.69 | 3.66 | 3.55 | 1.10 | 0.7972 | 0.9093 |
| TC0600011237.hg.1 | OR2B2 | olfactory receptor, family 2, subfamily B, member 2 | Multiple_ | 3.52 | 3.53 | 3.5 | 1.01 | 0.7108 | 0.8468 | 3.44 | 3.25 | 3.12 | 1.25 | 0.1235 | 0.3471 |
| TC0600013190.hg.1 | MOXD1 | monooxygenase, DBH-like 1 | Multiple_ | 3.2 | 2.89 | 3.18 | 1.01 | 0.8563 | 0.9298 | 3.72 | 3.70 | 3.31 | 1.33 | 0.0193 | 0.1174 |
| TC0600013743.hg.1 | TAGAP | T-cell activation RhoGTPase activating protein | Coding | 4.88 | 4.71 | 4.86 | 1.01 | 0.7097 | 0.846 | 5.92 | 5.87 | 5.69 | 1.17 | 0.3636 | 0.6264 |
| TC0700006931.hg.1 | FAM221A | family with sequence similarity 221, member A | Multiple_ | 6.92 | 8.37 | 6.9 | 1.01 | 0.4855 | 0.6869 | 6 | 5.77 | 5.12 | 1.84 | 0.0046 | 0.0468 |
| TC0700007501.hg.1 | C7orf72 | chromosome 7 open reading frame 72 | Coding | 2.65 | 2.29 | 2.63 | 1.01 | 0.7707 | 0.8814 | 3.6 | 3.47 | 3.45 | 1.11 | 0.1657 | 0.4105 |
| TC0700008094.hg.1 | ZP3 | zona pellucida glycoprotein 3 (sperm receptor) | Multiple_ | 8.06 | 7.86 | 8.04 | 1.01 | 0.8483 | 0.9257 | 6.65 | 6.21 | 6.47 | 1.13 | 0.283 | 0.5485 |
| TC0700010376.hg.1 | SNX13 | sorting nexin 13 | Multiple_ | 11.78 | 12.16 | 11.76 | 1.01 | 0.9859 | 0.9932 | 10.33 | 9.44 | 10.28 | 1.04 | 0.4216 | 0.6745 |
| TC0700011003.hg.1 | HUS1 | HUS1 checkpoint clamp component | Multiple_ | 9.82 | 9.5 | 9.8 | 1.01 | 0.9517 | 0.9777 | 12.41 | 11.44 | 12.81 | 0.76 | 0.0334 | 0.1641 |
| TC0700011458.hg.1 | CALN1 | calneuron 1 | Multiple_ | 2.79 | 2.84 | 2.77 | 1.01 | 0.9619 | 0.9819 | 3.3 | 3.12 | 3.01 | 1.22 | 0.1507 | 0.3887 |
| TC0700012443.hg.1 | FEZF1 | FEZ family zinc finger 1 | Coding | 4.92 | 4.49 | 4.9 | 1.01 | 0.6773 | 0.8247 | 3.2 | 3.33 | 3.55 | 0.78 | 0.2069 | 0.4637 |
| TC0800006625.hg.1 | ZNF705B | zinc finger protein 705B | Coding | 4.81 | 4.94 | 4.79 | 1.01 | 0.6174 | 0.7854 | 4.24 | 4.13 | 4.64 | 0.76 | 0.0763 | 0.2659 |
| TC0800008940.hg.1 | PHF20L1 | PHD finger protein 20-like 1 | Multiple_ | 9 | 9.01 | 8.98 | 1.01 | 0.6688 | 0.8189 | 8.92 | 8.71 | 8.89 | 1.02 | 0.512 | 0.7417 |
| TC0800009143.hg.1 | ADGRB1 | adhesion G protein-coupled receptor B1 | Multiple_ | 5.48 | 5.56 | 5.46 | 1.01 | 0.5299 | 0.7204 | 5.7 | 5.67 | 5.44 | 1.20 | 0.371 | 0.633 |
| TC0800009463.hg.1 | DEFB4B | defensin, beta 4B | Coding | 3.1 | 3.21 | 3.08 | 1.01 | 0.8123 | 0.9052 | 3.34 | 3.35 | 3.2 | 1.10 | 0.5203 | 0.7477 |
| TC0800009486.hg.1 | DEFB107A; DE | defensin, beta 107A; defensin, beta 107B | Coding | 4.28 | 4.14 | 4.26 | 1.01 | 0.8629 | 0.9332 | 3.82 | 3.83 | 3.71 | 1.08 | 0.455 | 0.6996 |
| TC0800009958.hg.1 | CHRNA2 | cholinergic receptor, nicotinic alpha 2 | Multiple_ | 3.59 | 3.53 | 3.57 | 1.01 | 0.6685 | 0.8188 | 3.27 | 3.35 | 3.84 | 0.67 | 0.0301 | 0.1539 |
| TC0800011107.hg.1 | C8orf87 | chromosome 8 open reading frame 87 | Multiple_ | 3.52 | 3.4 | 3.5 | 1.01 | 0.8341 | 0.9172 | 3.41 | 3.33 | 3.28 | 1.09 | 0.3916 | 0.6501 |
| TC0800012142.hg.1 | MROH6 | maestro heat-like repeat family member 6 | Multiple_ | 3.6 | 3.53 | 3.58 | 1.01 | 0.9874 | 0.9935 | 3.58 | 3.82 | 3.69 | 0.93 | 0.5441 | 0.7624 |
| TC0900006481.hg.1 | VLDLR | very low density lipoprotein receptor | Multiple_ | 2.95 | 2.93 | 2.93 | 1.01 | 0.394 | 0.6078 | 6.86 | 6.16 | 6.55 | 1.24 | 0.6603 | 0.8342 |
| TC0900008694.hg.1 | CRB2 | crumbs family member 2 | Multiple_ | 3.15 | 3 | 3.13 | 1.01 | 0.6637 | 0.8158 | 3.86 | 3.73 | 3.53 | 1.26 | 0.1176 | 0.3391 |
| TC0900008805.hg.1 | LRSAM1 | leucine rich repeat and sterile alpha motif containing 1 | Multiple_ | 7.01 | 7 | 6.99 | 1.01 | 0.8483 | 0.9257 | 6.32 | 6.23 | 6.09 | 1.17 | 0.1501 | 0.388 |
| TC0900010833.hg.1 | C9orf129 | chromosome 9 open reading frame 129 | Multiple_ | 4.27 | 3.95 | 4.25 | 1.01 | 0.9516 | 0.9777 | 5.32 | 5.65 | 5.85 | 0.69 | 0.1727 | 0.42 |
| TC0X00006671.hg.1 | MOSPD2 | motile sperm domain containing 2 | Multiple_ | 9.17 | 9.96 | 9.15 | 1.01 | 0.7594 | 0.8763 | 8.43 | 7.69 | 8.38 | 1.04 | 0.4019 | 0.6582 |
| TC0X00007122.hg.1 | RGN | regucalcin | Multiple_ | 5.24 | 5.15 | 5.22 | 1.01 | 0.323 | 0.5368 | 5.04 | 5.02 | 4.74 | 1.23 | 0.3505 | 0.6145 |
| TC0X00007919.hg.1 | SRPX2 | sushi-repeat containing protein, X-linked 2 | Multiple_ | 3.26 | 3.15 | 3.24 | 1.01 | 0.9994 | 0.9997 | 4.26 | 4.28 | 4.37 | 0.93 | 0.5672 | 0.7763 |
| TC0X00009493.hg.1 | EFHC2 | EF-hand domain (C-terminal) containing 2 | Multiple_ | 2.79 | 3.09 | 2.77 | 1.01 | 0.7672 | 0.8802 | 3.21 | 3.38 | 3.5 | 0.82 | 0.6052 | 0.8026 |

| | | | | | | | | | | | | | | | |
|---|---|---|---|---|---|---|---|---|---|---|---|---|---|---|---|
| TC0X00009680.hg.1 | DGKK | diacylglycerol kinase, kappa | Multiple_C | 2.75 | 2.52 | 2.73 | 1.01 | 0.9715 | 0.9861 | 3.17 | 3.36 | 3.18 | 0.99 | 0.711 | 0.8644 |
| TC0X00009776.hg.1 | FAM120C | family with sequence similarity 120C | Multiple_C | 4.77 | 4.47 | 4.75 | 1.01 | 0.7306 | 0.8591 | 5.27 | 5.55 | 5.46 | 0.88 | 0.1237 | 0.3473 |
| TC0X00009778.hg.1 | WNK3 | WNK lysine deficient protein kinase 3 | Multiple_C | 3.51 | 3.4 | 3.49 | 1.01 | 0.9545 | 0.9787 | 3.29 | 3.79 | 3.29 | 1.00 | 0.7711 | 0.8972 |
| TC0X00011290.hg.1 | GAGE1 | G antigen 1 | Multiple_C | 2.93 | 3.02 | 2.91 | 1.01 | 0.9319 | 0.9679 | 3.17 | 3.17 | 3.24 | 0.95 | 0.9773 | 0.9906 |
| TC0Y00006512.hg.1 | TSPY2 | testis specific protein, Y-linked 2 | Multiple_C | 3.66 | 3.48 | 3.64 | 1.01 | 0.711 | 0.847 | 4.36 | 4.25 | 4.14 | 1.16 | 0.4231 | 0.6758 |
| TC0Y00006837.hg.1 | PRYP4 | PTPN13-like, Y-linked pseudogene 4 | Multiple_C | 4.63 | 4.53 | 4.61 | 1.01 | 0.9633 | 0.9824 | 4.55 | 4.46 | 4.51 | 1.03 | 0.5783 | 0.7843 |
| TC0Y00007338.hg.1 | HSFY2 | heat shock transcription factor, Y-linked 2 | Multiple_C | 3.58 | 3.45 | 3.56 | 1.01 | 0.0835 | 0.211 | 4 | 3.66 | 3.94 | 1.04 | 0.5955 | 0.796 |
| TC1000007530.hg.1 | FRMPD2B; FR | FERM and PDZ domain containing 2B, pseudogene; FERM and P | Multiple_C | 4.15 | 4.39 | 4.13 | 1.01 | 0.3801 | 0.593 | 4.19 | 4.29 | 4.27 | 0.95 | 0.9837 | 0.9934 |
| TC1000009145.hg.1 | BTBD16 | BTB (POZ) domain containing 16 | Multiple_C | 5.28 | 5.84 | 5.26 | 1.01 | 0.9394 | 0.972 | 5.2 | 5.70 | 5.54 | 0.79 | 0.2423 | 0.506 |
| TC1000009830.hg.1 | CCDC3 | coiled-coil domain containing 3 | Multiple_C | 3.77 | 3.33 | 3.75 | 1.01 | 0.8699 | 0.9367 | 3.81 | 4.05 | 3.9 | 0.94 | 0.726 | 0.8726 |
| TC1000011933.hg.1 | HSPA12A | heat shock 70kDa protein 12A | Multiple_C | 3.24 | 3.12 | 3.22 | 1.01 | 0.964 | 0.9824 | 7.24 | 8.07 | 7.52 | 0.82 | 0.7397 | 0.8805 |
| TC1100007602.hg.1 | OR4C6 | olfactory receptor, family 4, subfamily C, member 6 | Coding | 3.01 | 3.02 | 2.99 | 1.01 | 0.5641 | 0.7458 | 4.5 | 4.15 | 4.28 | 1.16 | 0.4084 | 0.664 |
| TC1100007670.hg.1 | P2RX3 | purinergic receptor P2X, ligand gated ion channel, 3 | Multiple_C | 4.38 | 4.36 | 4.36 | 1.01 | 0.966 | 0.9836 | 3.89 | 3.82 | 3.98 | 0.94 | 0.5396 | 0.7594 |
| TC1100009098.hg.1 | HTR3A | 5-hydroxytryptamine (serotonin) receptor 3A, ionotropic | Multiple_C | 3.49 | 3.56 | 3.47 | 1.01 | 0.6505 | 0.8066 | 3.47 | 3.43 | 3.48 | 0.99 | 0.9942 | 0.9975 |
| TC1100009884.hg.1 | TRIM68 | tripartite motif containing 68 | Multiple_C | 5.67 | 5.39 | 5.65 | 1.01 | 0.3971 | 0.6109 | 6.88 | 7.12 | 7.01 | 0.91 | 0.1163 | 0.3369 |
| TC1100009893.hg.1 | OR51F1 | olfactory receptor, family 51, subfamily F, member 1 (gene/pseu | Multiple_C | 2.92 | 2.76 | 2.9 | 1.01 | 0.8136 | 0.9061 | 3.31 | 3.21 | 3.35 | 0.97 | 0.9082 | 0.9622 |
| TC1100011116.hg.1 | SLC22A24 | solute carrier family 22, member 24 | Coding | 2.98 | 3.11 | 2.96 | 1.01 | 0.6264 | 0.7915 | 3.06 | 3.12 | 3.3 | 0.85 | 0.5176 | 0.7459 |
| TC1100011182.hg.1 | PYGM | phosphorylase, glycogen, muscle | Multiple_C | 3.68 | 3.63 | 3.66 | 1.01 | 0.4565 | 0.663 | 3.56 | 4.13 | 3.59 | 0.98 | 0.7812 | 0.9017 |
| TC1100011684.hg.1 | PRKRIR | protein-kinase, interferon-inducible double stranded RNA depe | Multiple_C | 9.87 | 10.32 | 9.85 | 1.01 | 0.9638 | 0.9824 | 11.42 | 11.44 | 11.76 | 0.79 | 0.5269 | 0.7512 |
| TC1100012094.hg.1 | PGR | progesterone receptor | Multiple_C | 2.98 | 2.86 | 2.96 | 1.01 | 0.916 | 0.9606 | 3.47 | 3.56 | 3.82 | 0.78 | 0.0677 | 0.2479 |
| TC1100012469.hg.1 | SCN4B | sodium channel, voltage gated, type IV beta subunit | Multiple_C | 5.1 | 5.09 | 5.08 | 1.01 | 0.0804 | 0.2057 | 4.96 | 4.86 | 4.66 | 1.23 | 0.3655 | 0.6279 |
| TC1200007893.hg.1 | SLC26A10 | solute carrier family 26, member 10 | Multiple_C | 2.82 | 2.48 | 2.8 | 1.01 | 0.9373 | 0.9708 | 4 | 3.78 | 3.8 | 1.15 | 0.4994 | 0.7321 |
| TC1200010753.hg.1 | KRT82 | keratin 82, type II | Coding | 4.01 | 3.95 | 3.99 | 1.01 | 0.5769 | 0.7553 | 3.59 | 3.66 | 3.4 | 1.14 | 0.4472 | 0.6942 |
| TC1200010850.hg.1 | TESPA1 | thymocyte expressed, positive selection associated 1 | Multiple_C | 3.06 | 2.68 | 3.04 | 1.01 | 0.3689 | 0.5819 | 3.19 | 3.52 | 3.26 | 0.95 | 0.4469 | 0.694 |
| TC1200011179.hg.1 | IL22 | interleukin 22 | Multiple_C | 3.23 | 3.11 | 3.21 | 1.01 | 0.8157 | 0.9072 | 4.24 | 4.06 | 3.81 | 1.35 | 0.1242 | 0.3482 |
| TC1200011310.hg.1 | PHLDA1 | pleckstrin homology-like domain, family A, member 1 | Multiple_C | 15.07 | 14.88 | 15.05 | 1.01 | 0.4951 | 0.6949 | 15.68 | 15.42 | 15.49 | 1.14 | 0.3928 | 0.6511 |
| TC1200011474.hg.1 | ATP2B1 | ATPase, Ca++ transporting, plasma membrane 1 | Multiple_C | 12.03 | 11.89 | 12.01 | 1.01 | 0.6172 | 0.7854 | 7.45 | 6.80 | 7.62 | 0.89 | 0.0788 | 0.2704 |
| TC1200011506.hg.1 | BTG1; LINC01 | B-cell translocation gene 1, anti-proliferative; long intergenic nc | Multiple_C | 6.03 | 6.32 | 6.01 | 1.01 | 0.8485 | 0.9257 | 8.05 | 6.93 | 6.43 | 3.07 | 1.19E-06 | 0.0002 |
| TC1200011844.hg.1 | TMEM119 | transmembrane protein 119 | Multiple_C | 6.54 | 6.19 | 6.52 | 1.01 | 0.9274 | 0.966 | 6.67 | 6.78 | 6.73 | 0.96 | 0.8345 | 0.9268 |
| TC1200012215.hg.1 | HPD | 4-hydroxyphenylpyruvate dioxygenase | Multiple_C | 3.07 | 2.76 | 3.05 | 1.01 | 0.8538 | 0.9287 | 3.23 | 3.38 | 3.31 | 0.95 | 0.9624 | 0.9842 |
| TC1200012578.hg.1 | KCNA6; GALN | potassium channel, voltage gated shaker related subfamily A, m | Multiple_C | 5.28 | 5.36 | 5.26 | 1.01 | 0.7702 | 0.8812 | 5.32 | 5.28 | 5.21 | 1.08 | 0.2731 | 0.5386 |
| TC1300009583.hg.1 | DOCK9 | dedicator of cytokinesis 9 | Multiple_C | 8.65 | 8.65 | 8.63 | 1.01 | 0.8758 | 0.9395 | 9.73 | 9.00 | 8.66 | 2.10 | 9.18E-05 | 0.0034 |
| TC1300010037.hg.1 | KIAA0226L | KIAA0226-like | Multiple_C | 2.65 | 2.5 | 2.63 | 1.01 | 0.5721 | 0.7516 | 3.33 | 3.77 | 3.22 | 1.08 | 0.3777 | 0.6378 |

| | | | | | | | | | | | | | | | |
|---|---|---|---|---|---|---|---|---|---|---|---|---|---|---|---|
| TC1400008761.hg.1 | CMA1 | chymase 1, mast cell | Coding | 3.5 | 3.56 | 3.48 | 1.01 | 0.8337 | 0.9171 | 4.03 | 3.96 | 3.84 | 1.14 | 0.413 | 0.6677 |
| TC1400009131.hg.1 | MAP4K5 | mitogen-activated protein kinase kinase kinase kinase 5 | Multiple_C | 11.29 | 10.8 | 11.27 | 1.01 | 0.9249 | 0.9648 | 13.13 | 12.20 | 13.21 | 0.95 | 0.7817 | 0.9021 |
| TC1400010261.hg.1 | MOK | MOK protein kinase | Multiple_C | 5.99 | 5.74 | 5.97 | 1.01 | 0.3593 | 0.5728 | 5.32 | 5.55 | 5.43 | 0.93 | 0.8432 | 0.9317 |
| TC1400010734.hg.1 | NKX2-1 | NK2 homeobox 1 | Coding | 4.42 | 4.19 | 4.4 | 1.01 | 0.5284 | 0.7196 | 5.09 | 4.76 | 4.49 | 1.52 | 0.0602 | 0.2322 |
| TC1500007644.hg.1 | C15orf61 | chromosome 15 open reading frame 61 | Multiple_C | 5.68 | 6.91 | 5.66 | 1.01 | 0.906 | 0.9549 | 4.73 | 4.66 | 4.78 | 0.97 | 0.1917 | 0.4446 |
| TC1500008038.hg.1 | ARNT2; MIR5! | aryl-hydrocarbon receptor nuclear translocator 2; microRNA 55 | Multiple_C | 5.05 | 4.89 | 5.03 | 1.01 | 0.3237 | 0.5377 | 9.33 | 10.12 | 8.87 | 1.38 | 0.0404 | 0.1842 |
| TC1500008703.hg.1 | CYFIP1 | cytoplasmic FMR1 interacting protein 1 | Multiple_C | 11.31 | 11.66 | 11.29 | 1.01 | 0.9877 | 0.9935 | 9.98 | 9.69 | 9.85 | 1.09 | 0.5339 | 0.7563 |
| TC1500010888.hg.1 | PARP6 | poly(ADP-ribose) polymerase family member 6 | Multiple_C | 4.38 | 4.3 | 4.36 | 1.01 | 0.703 | 0.8415 | 8.31 | 8.83 | 8.28 | 1.02 | 0.4238 | 0.6765 |
| TC1600006483.hg.1 | MSLN | mesothelin | Multiple_C | 3.48 | 3.64 | 3.46 | 1.01 | 0.9987 | 0.9994 | 6.37 | 6.24 | 6.31 | 1.04 | 0.1955 | 0.4498 |
| TC1600006600.hg.1 | E4F1 | E4F transcription factor 1 | Multiple_C | 9.15 | 9.23 | 9.13 | 1.01 | 0.9803 | 0.9905 | 8.2 | 8.21 | 7.97 | 1.17 | 0.6676 | 0.8375 |
| TC1600007113.hg.1 | C16orf62 | chromosome 16 open reading frame 62 | Multiple_C | 4.04 | 3.93 | 4.02 | 1.01 | 0.398 | 0.6115 | 5.19 | 5.64 | 5.72 | 0.69 | 0.0378 | 0.1771 |
| TC1600009656.hg.1 | PDILT | protein disulfide isomerase-like, testis expressed | Coding | 5.97 | 5.76 | 5.95 | 1.01 | 0.601 | 0.7734 | 5.79 | 5.63 | 5.33 | 1.38 | 0.146 | 0.3814 |
| TC1600010276.hg.1 | SNX20 | sorting nexin 20 | Multiple_C | 3.1 | 3.15 | 3.08 | 1.01 | 0.9976 | 0.9989 | 3.32 | 3.29 | 3.04 | 1.21 | 0.0452 | 0.1966 |
| TC1700007145.hg.1 | SLC5A10 | solute carrier family 5 (sodium/sugar cotransporter), member 1 | Multiple_C | 4.7 | 4.65 | 4.68 | 1.01 | 0.9286 | 0.9663 | 5.17 | 5.10 | 5.12 | 1.04 | 0.8729 | 0.9452 |
| TC1700007557.hg.1 | CCL2 | chemokine (C-C motif) ligand 2 | Multiple_C | 4.12 | 4.06 | 4.1 | 1.01 | 0.2918 | 0.503 | 4.32 | 3.78 | 4.11 | 1.16 | 0.1935 | 0.4471 |
| TC1700007626.hg.1 | CCL4L2; CCL4L | chemokine (C-C motif) ligand 4-like 2; chemokine (C-C motif) lig | Multiple_C | 3.96 | 3.97 | 3.94 | 1.01 | 0.8682 | 0.9357 | 3.93 | 3.83 | 3.85 | 1.06 | 0.7314 | 0.8755 |
| TC1700008123.hg.1 | MYL4 | myosin light chain 4 | Multiple_C | 4.2 | 4.15 | 4.18 | 1.01 | 0.7723 | 0.8825 | 4 | 3.96 | 3.97 | 1.02 | 0.4436 | 0.6918 |
| TC1700008862.hg.1 | RAB37 | RAB37, member RAS oncogene family | Multiple_C | 3.58 | 3.58 | 3.56 | 1.01 | 0.7601 | 0.8765 | 4.71 | 4.48 | 3.81 | 1.87 | 0.0822 | 0.2762 |
| TC1700009122.hg.1 | SLC26A11 | solute carrier family 26 (anion exchanger), member 11 | Multiple_C | 5.24 | 5.23 | 5.22 | 1.01 | 0.372 | 0.5846 | 5.09 | 5.16 | 5.35 | 0.84 | 0.558 | 0.7714 |
| TC1700009409.hg.1 | RTN4RL1 | reticulon 4 receptor-like 1 | Coding | 4.58 | 4.23 | 4.56 | 1.01 | 0.4944 | 0.6943 | 4.5 | 4.25 | 4.26 | 1.18 | 0.1047 | 0.3174 |
| TC1700010223.hg.1 | SEZ6 | seizure related 6 homolog (mouse) | Multiple_C | 5.09 | 4.82 | 5.07 | 1.01 | 0.6181 | 0.7862 | 4.59 | 4.70 | 5.15 | 0.68 | 0.0047 | 0.047 |
| TC1700012178.hg.1 | WSCD1 | WSC domain containing 1 | Multiple_C | 3.09 | 3.1 | 3.07 | 1.01 | 0.6289 | 0.7929 | 3.9 | 3.75 | 3.71 | 1.14 | 0.3067 | 0.571 |
| TC1700012351.hg.1 | KCNAB3 | potassium channel, voltage gated subfamily A regulatory beta s | Multiple_C | 3.95 | 3.71 | 3.93 | 1.01 | 0.8813 | 0.9424 | 4.5 | 5.17 | 4.6 | 0.93 | 0.7264 | 0.8729 |
| TC1700012410.hg.1 | KRTAP4-3 | keratin associated protein 4-3 | Coding | 3.62 | 3.5 | 3.6 | 1.01 | 0.9023 | 0.953 | 4.28 | 3.84 | 3.64 | 1.56 | 0.0116 | 0.0854 |
| TC1800007096.hg.1 | FHOD3 | formin homology 2 domain containing 3 | Multiple_C | 3.6 | 3.24 | 3.58 | 1.01 | 0.3713 | 0.5841 | 5.94 | 5.68 | 6.02 | 0.95 | 0.6976 | 0.8567 |
| TC1900007087.hg.1 | WDR83 | WD repeat domain 83 | Multiple_C | 8.41 | 8.21 | 8.39 | 1.01 | 0.6395 | 0.8003 | 6.68 | 7.05 | 6.78 | 0.93 | 0.7076 | 0.8624 |
| TC1900007456.hg.1 | ZNF253 | zinc finger protein 253 | Multiple_C | 11.59 | 11.26 | 11.57 | 1.01 | 0.8057 | 0.9012 | 9.75 | 10.23 | 9.81 | 0.96 | 0.737 | 0.8788 |
| TC1900009310.hg.1 | MATK | megakaryocyte-associated tyrosine kinase | Multiple_C | 3.28 | 3.13 | 3.26 | 1.01 | 0.6908 | 0.8333 | 4.08 | 4.08 | 4.17 | 0.94 | 0.1709 | 0.4173 |
| TC1900009320.hg.1 | ZBTB7A | zinc finger and BTB domain containing 7A | Multiple_C | 10.04 | 9.38 | 10.02 | 1.01 | 0.8911 | 0.9476 | 8.68 | 8.80 | 9.3 | 0.65 | 0.0376 | 0.1768 |
| TC1900009891.hg.1 | LOC10272427 | uncharacterized LOC102724279; Transcript Identified by AceVie | Multiple_C | 4.44 | 4.48 | 4.42 | 1.01 | 0.9841 | 0.9925 | 5 | 5.26 | 5.18 | 0.88 | 0.3592 | 0.622 |
| TC1900011494.hg.1 | NLRP9 | NLR family, pyrin domain containing 9 | Coding | 4.5 | 3.96 | 4.48 | 1.01 | 0.784 | 0.8889 | 4.96 | 4.93 | 4.83 | 1.09 | 0.6297 | 0.8175 |
| TC1900011519.hg.1 | ZNF582 | zinc finger protein 582 | Coding | 4.07 | 4.35 | 4.05 | 1.01 | 0.3705 | 0.5832 | 3.8 | 3.64 | 3.65 | 1.11 | 0.5052 | 0.7369 |
| TC1900011549.hg.1 | DUXA | double homeobox A | Multiple_C | 2.87 | 2.8 | 2.85 | 1.01 | 0.9816 | 0.9913 | 3.39 | 3.81 | 3.6 | 0.86 | 0.6292 | 0.8172 |

| | | | | | | | | | | | | | | | |
|---|---|---|---|---|---|---|---|---|---|---|---|---|---|---|---|
| TC1900011697.hg.1 | ZNF730 | zinc finger protein 730 | Multiple_C | 5.61 | 5.53 | 5.59 | 1.01 | 0.5858 | 0.7621 | 5.87 | 5.74 | 5.81 | 1.04 | 0.7167 | 0.8677 |
| TC1900011969.hg.1 | IRGQ | immunity-related GTPase family, Q | Multiple_C | 5.34 | 5.49 | 5.32 | 1.01 | 0.815 | 0.907 | 4.99 | 5.31 | 5.28 | 0.82 | 0.0628 | 0.2385 |
| TC2000007516.hg.1 | SLC12A5 | solute carrier family 12 (potassium/chloride transporter), memb | Multiple_C | 5.72 | 5.56 | 5.7 | 1.01 | 0.7163 | 0.85 | 5.47 | 5.98 | 6.13 | 0.63 | 0.0181 | 0.113 |
| TC2000009200.hg.1 | RIMS4 | regulating synaptic membrane exocytosis 4 | Coding | 4.93 | 4.83 | 4.91 | 1.01 | 0.9618 | 0.9819 | 4.54 | 4.67 | 4.58 | 0.97 | 0.5948 | 0.7954 |
| TC2000009317.hg.1 | SULF2 | sulfatase 2 | Multiple_C | 13.14 | 12.68 | 13.12 | 1.01 | 0.7034 | 0.8417 | 10.21 | 8.85 | 9.73 | 1.39 | 0.0294 | 0.1518 |
| TC2000009433.hg.1 | FAM65C | family with sequence similarity 65, member C | Multiple_C | 4.95 | 4.8 | 4.93 | 1.01 | 0.5306 | 0.7209 | 4.5 | 4.58 | 4.73 | 0.85 | 0.8088 | 0.9155 |
| TC2000009597.hg.1 | CTCFL | CCCTC-binding factor (zinc finger protein)-like | Multiple_C | 3.58 | 3.6 | 3.56 | 1.01 | 0.8756 | 0.9395 | 3.69 | 3.69 | 4.02 | 0.80 | 0.1002 | 0.3094 |
| TC2000009782.hg.1 | DIDO1 | death inducer-obliterator 1 | Multiple_C | 10.72 | 9.84 | 10.7 | 1.01 | 0.9792 | 0.9901 | 11.76 | 11.72 | 11.64 | 1.09 | 0.9392 | 0.9749 |
| TC2000009913.hg.1 | CNBD2 | cyclic nucleotide binding domain containing 2 | Multiple_C | 2.75 | 2.74 | 2.73 | 1.01 | 0.5984 | 0.7712 | 3.5 | 3.95 | 3.81 | 0.81 | 0.8814 | 0.9492 |
| TC2100007883.hg.1 | KRTAP24-1 | keratin associated protein 24-1 | Coding | 3.62 | 3.83 | 3.6 | 1.01 | 0.7787 | 0.8862 | 3.69 | 3.41 | 3.64 | 1.04 | 0.2206 | 0.4807 |
| TC2100008550.hg.1 | KRTAP19-1 | keratin associated protein 19-1 | Coding | 5.43 | 5.11 | 5.41 | 1.01 | 0.7327 | 0.8604 | 5.34 | 5.39 | 5.24 | 1.07 | 0.6428 | 0.8248 |
| TC2200008346.hg.1 | MN1 | meningioma (disrupted in balanced translocation) 1 | Multiple_C | 3.49 | 3.26 | 3.47 | 1.01 | 0.7093 | 0.8457 | 3.68 | 4.03 | 3.81 | 0.91 | 0.5755 | 0.7823 |
| TC2200008956.hg.1 | PHF21B | PHD finger protein 21B | Multiple_C | 3.32 | 3.09 | 3.3 | 1.01 | 0.9951 | 0.9976 | 3.6 | 3.69 | 3.65 | 0.97 | 0.9661 | 0.9858 |
| TC2200009021.hg.1 | CELSR1 | cadherin, EGF LAG seven-pass G-type receptor 1 | Multiple_C | 5.24 | 5.21 | 5.22 | 1.01 | 0.8171 | 0.9076 | 4.75 | 5.26 | 5.13 | 0.77 | 0.1276 | 0.3536 |
| TC2200009164.hg.1 | SYCE3 | synaptonemal complex central element protein 3 | Coding | 3.9 | 4.01 | 3.88 | 1.01 | 0.3519 | 0.5647 | 3.85 | 4.04 | 4.19 | 0.79 | 0.357 | 0.6202 |
| TSUnmapped00000281.h | HYOU1 | hypoxia up-regulated 1 | Coding | 6.9 | 7.14 | 6.88 | 1.01 | 0.8287 | 0.9142 | 6.51 | 6.61 | 6.75 | 0.85 | 0.9409 | 0.9756 |
| TSUnmapped00000324.h | CYP2D6 | cytochrome P450, family 2, subfamily D, polypeptide 6 | Coding | 5.48 | 5.46 | 5.46 | 1.01 | 0.9659 | 0.9836 | 5.53 | 6.20 | 6.05 | 0.70 | 0.1412 | 0.3738 |
| TC0100008882.hg.1 | DNASE2B | deoxyribonuclease II beta | Coding | 3.5 | 3.6 | 3.49 | 1.01 | 0.8411 | 0.9214 | 4.82 | 4.77 | 4.69 | 1.09 | 0.7886 | 0.9058 |
| TC0100009449.hg.1 | MOV10 | Mov10 RISC complex RNA helicase | Multiple_C | 7.53 | 7.24 | 7.52 | 1.01 | 0.9565 | 0.9798 | 7.24 | 7.79 | 7.19 | 1.04 | 0.6661 | 0.8365 |
| TC0100010144.hg.1 | RXFP4 | relaxin/insulin-like family peptide receptor 4 | Coding | 4.22 | 4.14 | 4.21 | 1.01 | 0.9591 | 0.9806 | 4.82 | 4.74 | 4.84 | 0.99 | 0.3744 | 0.635 |
| TC0100010185.hg.1 | APOA1BP | apolipoprotein A-I binding protein | Multiple_C | 12.55 | 11.99 | 12.54 | 1.01 | 0.7574 | 0.8753 | 10.73 | 11.19 | 10.99 | 0.84 | 0.206 | 0.4624 |
| TC0100010672.hg.1 | RABGAP1L | RAB GTPase activating protein 1-like | Multiple_C | 9.02 | 10.16 | 9.01 | 1.01 | 0.6849 | 0.8296 | 7.44 | 6.89 | 6.92 | 1.43 | 0.0906 | 0.2913 |
| TC0100012317.hg.1 | OR2W5 | olfactory receptor, family 2, subfamily W, member 5 (gene/pseu | Multiple_C | 3.65 | 3.7 | 3.64 | 1.01 | 0.5155 | 0.7102 | 3.68 | 3.61 | 3.67 | 1.01 | 0.9084 | 0.9623 |
| TC0100013458.hg.1 | EYA3 | EYA transcriptional coactivator and phosphatase 3 | Multiple_C | 7.03 | 7.26 | 7.02 | 1.01 | 0.7706 | 0.8814 | 7.82 | 7.63 | 8.18 | 0.78 | 0.1632 | 0.4068 |
| TC0100014766.hg.1 | LPAR3 | lysophosphatidic acid receptor 3 | Multiple_C | 4.05 | 3.97 | 4.04 | 1.01 | 0.9748 | 0.9877 | 4.96 | 4.49 | 4.39 | 1.48 | 0.0101 | 0.0782 |
| TC0100015597.hg.1 | ANKRD34A | ankyrin repeat domain 34A | Coding | 3.37 | 3.11 | 3.36 | 1.01 | 0.9106 | 0.9575 | 4.15 | 4.26 | 4.07 | 1.06 | 0.5234 | 0.7491 |
| TC0100016071.hg.1 | OR6Y1 | olfactory receptor, family 6, subfamily Y, member 1 | Coding | 3.5 | 3.36 | 3.49 | 1.01 | 0.821 | 0.9102 | 3.68 | 3.72 | 3.59 | 1.06 | 0.7072 | 0.8621 |
| TC0100016618.hg.1 | ZNF648 | zinc finger protein 648 | Coding | 4.39 | 4.27 | 4.38 | 1.01 | 0.916 | 0.9606 | 4.92 | 4.54 | 4.84 | 1.06 | 0.5848 | 0.7888 |
| TC0200006537.hg.1 | COLEC11 | collectin subfamily member 11 | Multiple_C | 4.94 | 4.65 | 4.93 | 1.01 | 0.535 | 0.7241 | 5.31 | 5.26 | 5.05 | 1.20 | 0.191 | 0.444 |
| TC0200007028.hg.1 | GAREM2 | GRB2 associated regulator of MAPK1 2 | Multiple_C | 3.5 | 3.36 | 3.49 | 1.01 | 0.8566 | 0.9299 | 3.99 | 3.73 | 3.48 | 1.42 | 0.0252 | 0.1379 |
| TC0200008811.hg.1 | SH3RF3 | SH3 domain containing ring finger 3 | Multiple_C | 4.99 | 4.93 | 4.98 | 1.01 | 0.6501 | 0.8063 | 5.63 | 5.30 | 5.3 | 1.26 | 0.1018 | 0.3121 |
| TC0200009341.hg.1 | TUBA3D | tubulin, alpha 3d | Multiple_C | 3.04 | 3.06 | 3.03 | 1.01 | 0.9213 | 0.9636 | 3.49 | 3.33 | 3.38 | 1.08 | 0.9038 | 0.9603 |
| TC0200014845.hg.1 | SPC25 | SPC25, NDC80 kinetochore complex component | Multiple_C | 8.26 | 7.55 | 8.25 | 1.01 | 0.7148 | 0.8493 | 7.44 | 8.32 | 7.79 | 0.78 | 0.1174 | 0.3388 |

| | | | | | | | | | | | | | | | |
|---|---|---|---|---|---|---|---|---|---|---|---|---|---|---|---|
| TC0200015793.hg.1 | OBSL1 | obscurin-like 1 | Multiple_C | 3.22 | 3.07 | 3.21 | 1.01 | 0.2463 | 0.4498 | 7.38 | 8.68 | 7.14 | 1.18 | 0.2532 | 0.5175 |
| TC0200016220.hg.1 | ILKAP | ILK associated serine/threonine phosphatase | Multiple_C | 7.22 | 6.94 | 7.21 | 1.01 | 0.819 | 0.909 | 6.84 | 6.75 | 7.12 | 0.82 | 0.219 | 0.4789 |
| TC0200016582.hg.1 | NABP1 | nucleic acid binding protein 1 | Multiple_C | 7.56 | 7.29 | 7.55 | 1.01 | 0.8484 | 0.9257 | 8.91 | 8.12 | 8.77 | 1.10 | 0.7889 | 0.9058 |
| TC0200016647.hg.1 | DPY30 | dpy-30 histone methyltransferase complex regulatory subunit | Multiple_C | 14.19 | 15.65 | 14.18 | 1.01 | 0.8432 | 0.9227 | 11.6 | 10.87 | 10.95 | 1.57 | 0.1939 | 0.4476 |
| TC0200016712.hg.1 | ANKRD23 | ankyrin repeat domain 23 | Multiple_C | 4.08 | 4.03 | 4.07 | 1.01 | 0.9068 | 0.9553 | 4.34 | 4.64 | 4.65 | 0.81 | 0.9634 | 0.9845 |
| TC0200016714.hg.1 | FAM178B | family with sequence similarity 178, member B | Multiple_C | 3.28 | 3 | 3.27 | 1.01 | 0.9872 | 0.9935 | 4.59 | 4.83 | 4.37 | 1.16 | 0.1936 | 0.4472 |
| TC0300006552.hg.1 | MTMR14 | myotubularin related protein 14 | Multiple_C | 9.37 | 9.6 | 9.36 | 1.01 | 0.7011 | 0.8403 | 8.84 | 8.93 | 9.43 | 0.66 | 0.0041 | 0.0435 |
| TC0300006658.hg.1 | FBLN2 | fibulin 2 | Multiple_C | 3.13 | 2.91 | 3.12 | 1.01 | 0.9902 | 0.9948 | 3.52 | 3.49 | 3.28 | 1.18 | 0.1405 | 0.3726 |
| TC0300008105.hg.1 | OR5H6 | olfactory receptor, family 5, subfamily H, member 6 (gene/pseu | Multiple_C | 3.18 | 3.17 | 3.17 | 1.01 | 0.7504 | 0.8718 | 3.99 | 4.32 | 3.86 | 1.09 | 0.9962 | 0.9982 |
| TC0300008559.hg.1 | PARP15 | poly(ADP-ribose) polymerase family member 15 | Multiple_C | 3.58 | 3.95 | 3.57 | 1.01 | 0.551 | 0.7358 | 3.62 | 3.73 | 3.77 | 0.90 | 0.6776 | 0.8448 |
| TC0300009459.hg.1 | GPR160 | G protein-coupled receptor 160 | Multiple_C | 11.74 | 13.02 | 11.73 | 1.01 | 0.991 | 0.9953 | 6.45 | 5.34 | 5.72 | 1.66 | 0.0242 | 0.1346 |
| TC0300011048.hg.1 | QRICH1 | glutamine-rich 1 | Multiple_C | 12.06 | 11.66 | 12.05 | 1.01 | 0.5203 | 0.7137 | 11.18 | 11.98 | 11.53 | 0.78 | 0.1125 | 0.3304 |
| TC0300011628.hg.1 | ROBO1 | roundabout guidance receptor 1 | Multiple_C | 7.86 | 7.78 | 7.85 | 1.01 | 0.7314 | 0.8598 | 13.76 | 13.74 | 13.64 | 1.09 | 0.5663 | 0.7759 |
| TC0300012345.hg.1 | LINC01565 | long intergenic non-protein coding RNA 1565 | Multiple_C | 4.32 | 3.98 | 4.31 | 1.01 | 0.7647 | 0.8791 | 4.67 | 4.37 | 4.68 | 0.99 | 0.669 | 0.8382 |
| TC0300013344.hg.1 | KLHL6 | kelch-like family member 6 | Multiple_C | 3.06 | 2.88 | 3.05 | 1.01 | 0.973 | 0.9869 | 3.6 | 3.44 | 3.39 | 1.16 | 0.0482 | 0.2039 |
| TC0400010083.hg.1 | NKX3-2 | NK3 homeobox 2 | Coding | 3.78 | 3.89 | 3.77 | 1.01 | 0.3799 | 0.5928 | 4.99 | 5.05 | 5.04 | 0.97 | 0.472 | 0.7125 |
| TC0400012926.hg.1 | SULT1E1 | sulfotransferase family 1E member 1 | Multiple_C | 3.42 | 3.69 | 3.41 | 1.01 | 0.1769 | 0.3596 | 3.78 | 3.71 | 3.7 | 1.06 | 0.1783 | 0.427 |
| TC0500008814.hg.1 | PAIP2 | poly(A) binding protein interacting protein 2 | Multiple_C | 14.46 | 15.94 | 14.45 | 1.01 | 0.7533 | 0.8738 | 13.58 | 13.17 | 12.65 | 1.91 | 0.0005 | 0.0104 |
| TC0500009033.hg.1 | SPINK6 | serine peptidase inhibitor, Kazal type 6 | Coding | 3.56 | 3.58 | 3.55 | 1.01 | 0.9184 | 0.9619 | 3.6 | 3.56 | 3.61 | 0.99 | 0.5772 | 0.7834 |
| TC0500009221.hg.1 | C5orf52 | chromosome 5 open reading frame 52 | Coding | 3 | 2.88 | 2.99 | 1.01 | 0.7978 | 0.8961 | 3.61 | 3.48 | 3.44 | 1.13 | 0.346 | 0.6098 |
| TC0500009301.hg.1 | GABRA6 | gamma-aminobutyric acid (GABA) A receptor, alpha 6 | Multiple_C | 2.73 | 2.52 | 2.72 | 1.01 | 0.9556 | 0.9792 | 3.58 | 3.51 | 3.32 | 1.20 | 0.4259 | 0.6783 |
| TC0500009886.hg.1 | IRX4 | iroquois homeobox 4 | Multiple_C | 2.93 | 2.65 | 2.92 | 1.01 | 0.9611 | 0.9816 | 3.29 | 3.46 | 3.26 | 1.02 | 0.6897 | 0.852 |
| TC0500010491.hg.1 | UGT3A2 | UDP glycosyltransferase 3 family, polypeptide A2 | Multiple_C | 4.45 | 4.47 | 4.44 | 1.01 | 0.5884 | 0.7642 | 4.73 | 4.32 | 4.34 | 1.31 | 0.2065 | 0.4631 |
| TC0500012105.hg.1 | NEUROG1 | neurogenin 1 | Coding | 2.97 | 2.9 | 2.96 | 1.01 | 0.851 | 0.9269 | 3.33 | 3.22 | 3.02 | 1.24 | 0.0869 | 0.2843 |
| TC0500012162.hg.1 | BRD8 | bromodomain containing 8 | Multiple_C | 11.14 | 11.45 | 11.13 | 1.01 | 0.9336 | 0.9687 | 11.89 | 12.20 | 11.59 | 1.23 | 0.0586 | 0.2284 |
| TC0500012354.hg.1 | YIPF5 | Yip1 domain family member 5 | Multiple_C | 11.33 | 11.97 | 11.32 | 1.01 | 0.7782 | 0.8859 | 10.52 | 10.25 | 10.59 | 0.95 | 0.3107 | 0.5747 |
| TC0500012818.hg.1 | STK10 | serine/threonine kinase 10 | Multiple_C | 9.23 | 8.48 | 9.22 | 1.01 | 0.7686 | 0.8808 | 9.54 | 9.69 | 10.15 | 0.66 | 0.0385 | 0.1787 |
| TC0500013310.hg.1 | LOC10246708 | uncharacterized LOC102467081; novel transcript, antisense to C | Multiple_C | 2.96 | 3.04 | 2.95 | 1.01 | 0.4617 | 0.667 | 3.31 | 3.32 | 3.33 | 0.99 | 0.5408 | 0.7601 |
| TC0600007477.hg.1 | MOG | myelin oligodendrocyte glycoprotein | Multiple_C | 3.6 | 3.49 | 3.59 | 1.01 | 0.9316 | 0.9678 | 3.54 | 3.46 | 3.52 | 1.01 | 0.9219 | 0.9677 |
| TC0600009235.hg.1 | FAM26D | family with sequence similarity 26, member D | Coding | 2.72 | 2.69 | 2.71 | 1.01 | 0.6718 | 0.8211 | 2.92 | 3.01 | 3.02 | 0.93 | 0.5946 | 0.7953 |
| TC0600009606.hg.1 | ARFGEF3 | ARFGEF family member 3 | Multiple_C | 8.61 | 8.56 | 8.6 | 1.01 | 0.9729 | 0.9869 | 6.39 | 5.98 | 5.91 | 1.39 | 0.0806 | 0.2736 |
| TC0600009910.hg.1 | OPRM1 | opioid receptor, mu 1 | Multiple_C | 3.27 | 3.32 | 3.26 | 1.01 | 0.5845 | 0.7612 | 3.19 | 3.33 | 3.36 | 0.89 | 0.563 | 0.7735 |
| TC0600010343.hg.1 | TBP | TATA box binding protein | Multiple_C | 10.7 | 10.14 | 10.69 | 1.01 | 0.9661 | 0.9836 | 9.55 | 10.13 | 10.12 | 0.67 | 0.0857 | 0.2821 |

| | | | | | | | | | | | | | | | |
|---|---|---|---|---|---|---|---|---|---|---|---|---|---|---|---|
| TC0600011354.hg.1 | TRIM10 | tripartite motif containing 10 | Coding | 5.16 | 5.1 | 5.15 | 1.01 | 0.6052 | 0.7766 | 5.66 | 5.35 | 5.29 | 1.29 | 0.1502 | 0.3881 |
| TC0600011487.hg.1 | C6orf10 | chromosome 6 open reading frame 10 | Coding | 3.84 | 3.96 | 3.83 | 1.01 | 0.9517 | 0.9777 | 3.18 | 3.47 | 3.56 | 0.77 | 0.187 | 0.4388 |
| TC0600011561.hg.1 | IP6K3 | inositol hexakisphosphate kinase 3 | Multiple_C | 3.53 | 3.25 | 3.52 | 1.01 | 0.8702 | 0.9368 | 3.93 | 4.15 | 3.81 | 1.09 | 0.5504 | 0.7664 |
| TC0600011657.hg.1 | C6orf222 | chromosome 6 open reading frame 222 | Coding | 4.52 | 4.61 | 4.51 | 1.01 | 0.7699 | 0.8811 | 4.18 | 4.31 | 4.34 | 0.90 | 0.7834 | 0.9026 |
| TC0600012713.hg.1 | PREP | prolyl endopeptidase | Multiple_C | 12.35 | 13.19 | 12.34 | 1.01 | 0.9431 | 0.9736 | 10.47 | 10.61 | 10.82 | 0.78 | 0.2975 | 0.5622 |
| TC0600012833.hg.1 | SLC22A16 | solute carrier family 22 (organic cation/carnitine transporter), m | Multiple_C | 3.43 | 3.66 | 3.42 | 1.01 | 0.3939 | 0.6078 | 4.01 | 4.16 | 4.23 | 0.86 | 0.3039 | 0.5679 |
| TC0600013536.hg.1 | RAET1E | retinoic acid early transcript 1E | Multiple_C | 4.11 | 4.41 | 4.1 | 1.01 | 0.4971 | 0.6968 | 4.26 | 4.41 | 4.53 | 0.83 | 0.2074 | 0.4641 |
| TC0600014246.hg.1 | C6orf229 | chromosome 6 open reading frame 229 | Multiple_C | 3.09 | 2.99 | 3.08 | 1.01 | 0.3314 | 0.5455 | 3.28 | 3.35 | 3.42 | 0.91 | 0.3987 | 0.6559 |
| TC0700009079.hg.1 | TSPAN33 | tetraspanin 33 | Multiple_C | 4.84 | 4.31 | 4.83 | 1.01 | 0.762 | 0.8778 | 6.95 | 6.53 | 6.56 | 1.31 | 0.1274 | 0.3534 |
| TC0700009676.hg.1 | GIMAP7 | GTPase, IMAP family member 7 | Coding | 5 | 4.79 | 4.99 | 1.01 | 0.8912 | 0.9476 | 5.01 | 5.14 | 5.1 | 0.94 | 0.6261 | 0.8152 |
| TC0700009677.hg.1 | GIMAP4 | GTPase, IMAP family member 4 | Multiple_C | 3.29 | 3.24 | 3.28 | 1.01 | 0.6058 | 0.7771 | 3.3 | 3.38 | 3.27 | 1.02 | 0.9787 | 0.991 |
| TC0700009682.hg.1 | AOC1 | amine oxidase, copper containing 1 | Multiple_C | 3.64 | 3.21 | 3.63 | 1.01 | 0.9316 | 0.9678 | 5.45 | 5.43 | 5.29 | 1.12 | 0.3037 | 0.5678 |
| TC0700012010.hg.1 | TFR2 | transferrin receptor 2 | Multiple_C | 6.62 | 6.37 | 6.61 | 1.01 | 0.9649 | 0.983 | 5.98 | 5.97 | 5.55 | 1.35 | 0.0894 | 0.2893 |
| TC0700012054.hg.1 | MYL10 | myosin light chain 10 | Multiple_C | 2.83 | 2.62 | 2.82 | 1.01 | 0.393 | 0.6068 | 3.19 | 3.14 | 3.22 | 0.98 | 0.7497 | 0.8859 |
| TC0700012453.hg.1 | SLC13A1 | solute carrier family 13 (sodium/sulfate symporter), member 1 | Coding | 3.75 | 3.95 | 3.74 | 1.01 | 0.7004 | 0.8397 | 3.91 | 4.01 | 3.89 | 1.01 | 0.6162 | 0.8096 |
| TC0700012869.hg.1 | TRPV5 | transient receptor potential cation channel, subfamily V, memb | Coding | 3.05 | 3.08 | 3.04 | 1.01 | 0.9408 | 0.9724 | 3.09 | 3.16 | 3.21 | 0.92 | 0.8811 | 0.949 |
| TC0700013368.hg.1 | ZNF736 | zinc finger protein 736 | Multiple_C | 9.55 | 9.62 | 9.54 | 1.01 | 0.7667 | 0.8801 | 7.49 | 7.73 | 8.18 | 0.62 | 0.0045 | 0.0462 |
| TC0800007848.hg.1 | TRIM55 | tripartite motif containing 55 | Multiple_C | 4.5 | 4.41 | 4.49 | 1.01 | 0.3492 | 0.5619 | 4.1 | 4.16 | 3.86 | 1.18 | 0.4603 | 0.7035 |
| TC0800008235.hg.1 | FAM92A1 | family with sequence similarity 92, member A1 | Multiple_C | 3.68 | 3.22 | 3.67 | 1.01 | 0.6277 | 0.7921 | 9 | 8.58 | 8.43 | 1.48 | 0.0028 | 0.0337 |
| TC0800012247.hg.1 | ZNF16 | zinc finger protein 16 | Multiple_C | 6.8 | 6.71 | 6.79 | 1.01 | 0.3951 | 0.6086 | 5.57 | 5.85 | 5.75 | 0.88 | 0.2812 | 0.5469 |
| TC0900009212.hg.1 | C9orf163 | chromosome 9 open reading frame 163 | Multiple_C | 4.51 | 4.43 | 4.5 | 1.01 | 0.6246 | 0.7902 | 4.83 | 4.91 | 4.87 | 0.97 | 0.7948 | 0.9087 |
| TC0900009557.hg.1 | MPDZ | multiple PDZ domain protein | Multiple_C | 3.66 | 3.65 | 3.65 | 1.01 | 0.4399 | 0.6489 | 4.2 | 4.41 | 3.78 | 1.34 | 0.0443 | 0.1948 |
| TC0900009660.hg.1 | SLC24A2 | solute carrier family 24 (sodium/potassium/calcium exchanger), | Multiple_C | 3.85 | 3.71 | 3.84 | 1.01 | 0.871 | 0.9372 | 3.37 | 3.50 | 3.41 | 0.97 | 0.9792 | 0.9912 |
| TC0900011182.hg.1 | SVEP1 | sushi, von Willebrand factor type A, EGF and pentraxin domain ( | Multiple_C | 4.42 | 4.32 | 4.41 | 1.01 | 0.9756 | 0.988 | 4.19 | 4.23 | 4.32 | 0.91 | 0.755 | 0.8886 |
| TC0X00006964.hg.1 | HYPM | huntingtin interacting protein M | Coding | 3.8 | 3.56 | 3.79 | 1.01 | 0.3846 | 0.5979 | 4.35 | 4.04 | 4.01 | 1.27 | 0.2147 | 0.4733 |
| TC0X00007206.hg.1 | ERAS | ES cell expressed Ras | Coding | 5.51 | 5.41 | 5.5 | 1.01 | 0.9096 | 0.9567 | 4.71 | 4.95 | 4.96 | 0.84 | 0.9499 | 0.9795 |
| TC0X00007416.hg.1 | FAAH2 | fatty acid amide hydrolase 2 | Multiple_C | 4.33 | 4.78 | 4.32 | 1.01 | 0.9967 | 0.9984 | 4.74 | 4.48 | 4.13 | 1.53 | 0.0114 | 0.0848 |
| TC0X00007480.hg.1 | HEPH | hephaestin | Multiple_C | 4.38 | 4.47 | 4.37 | 1.01 | 0.8461 | 0.9244 | 4.25 | 4.34 | 4.09 | 1.12 | 0.6802 | 0.8462 |
| TC0X00007531.hg.1 | DGAT2L6 | diacylglycerol O-acyltransferase 2-like 6 | Coding | 3.09 | 3.04 | 3.08 | 1.01 | 0.7708 | 0.8814 | 3.13 | 3.19 | 3.42 | 0.82 | 0.2162 | 0.4753 |
| TC0X00007999.hg.1 | BEX4 | brain expressed X-linked 4 | Multiple_C | 3.69 | 3.65 | 3.68 | 1.01 | 0.5556 | 0.7389 | 3.77 | 4.04 | 3.72 | 1.04 | 0.3365 | 0.6009 |
| TC0X00008671.hg.1 | FMR1NB | fragile X mental retardation 1 neighbor | Coding | 2.89 | 3.37 | 2.88 | 1.01 | 0.6605 | 0.8138 | 3.19 | 3.38 | 3.22 | 0.98 | 0.6827 | 0.8477 |
| TC0X00009321.hg.1 | NR0B1 | nuclear receptor subfamily 0, group B, member 1 | Coding | 2.72 | 2.77 | 2.71 | 1.01 | 0.9281 | 0.9662 | 3.66 | 3.52 | 3.56 | 1.07 | 0.5875 | 0.7912 |
| TC0X00011354.hg.1 | FIGF | c-fos induced growth factor (vascular endothelial growth factor | Multiple_C | 3.85 | 3.91 | 3.84 | 1.01 | 0.6281 | 0.7924 | 4.23 | 3.69 | 4.28 | 0.97 | 0.5098 | 0.7399 |

| | | | | | | | | | | | | | | | |
|---|---|---|---|---|---|---|---|---|---|---|---|---|---|---|---|
| TC0X00011397.hg.1 | FAM127C | family with sequence similarity 127, member C | Coding | 4.35 | 4.04 | 4.34 | 1.01 | 0.5245 | 0.7168 | 4.56 | 4.74 | 4.63 | 0.95 | 0.6018 | 0.8005 |
| TC1000007748.hg.1 | CDK1 | cyclin-dependent kinase 1 | Multiple_C | 13.07 | 13.99 | 13.06 | 1.01 | 0.782 | 0.8878 | 11.73 | 12.24 | 12.16 | 0.74 | 0.0612 | 0.2348 |
| TC1000009802.hg.1 | UPF2 | UPF2 regulator of nonsense transcripts homolog (yeast) | Multiple_C | 9.16 | 9.49 | 9.15 | 1.01 | 0.889 | 0.9466 | 9 | 8.77 | 9.12 | 0.92 | 0.3952 | 0.6532 |
| TC1000011026.hg.1 | USP54 | ubiquitin specific peptidase 54 | Multiple_C | 8.18 | 7.93 | 8.17 | 1.01 | 0.8388 | 0.9198 | 8.56 | 8.67 | 8.52 | 1.03 | 0.8343 | 0.9266 |
| TC1000011363.hg.1 | RNLS | renalase, FAD-dependent amine oxidase | Multiple_C | 3.14 | 2.67 | 3.13 | 1.01 | 0.7048 | 0.8425 | 7.2 | 6.74 | 7 | 1.15 | 0.492 | 0.7269 |
| TC1000011839.hg.1 | GPAM | glycerol-3-phosphate acyltransferase, mitochondrial | Multiple_C | 6.77 | 6.87 | 6.76 | 1.01 | 0.5981 | 0.7711 | 6.98 | 7.21 | 7.76 | 0.58 | 0.001 | 0.0165 |
| TC1100006988.hg.1 | MRGPRX4 | MAS-related GPR, member X4 | Multiple_C | 4.13 | 3.94 | 4.12 | 1.01 | 0.6745 | 0.8228 | 4.37 | 4.63 | 4.73 | 0.78 | 0.4448 | 0.6927 |
| TC1100007063.hg.1 | ANO5 | anoctamin 5 | Multiple_C | 3.57 | 3.7 | 3.56 | 1.01 | 0.7424 | 0.8667 | 3.64 | 3.53 | 3.51 | 1.09 | 0.3755 | 0.636 |
| TC1100007612.hg.1 | OR5D18 | olfactory receptor, family 5, subfamily D, member 18 | Coding | 2.79 | 2.9 | 2.78 | 1.01 | 0.7573 | 0.8753 | 3.03 | 3.11 | 3.2 | 0.89 | 0.4845 | 0.7221 |
| TC1100007818.hg.1 | DAGLA | diacylglycerol lipase, alpha | Multiple_C | 6.41 | 5.66 | 6.4 | 1.01 | 0.9869 | 0.9934 | 7.18 | 7.27 | 7.82 | 0.64 | 0.0007 | 0.0127 |
| TC1100007892.hg.1 | SLC22A9 | solute carrier family 22 (organic anion transporter), member 9 | Coding | 4.25 | 3.96 | 4.24 | 1.01 | 0.4785 | 0.6807 | 4 | 3.94 | 3.66 | 1.27 | 0.2083 | 0.4652 |
| TC1100007938.hg.1 | FERMT3 | fermitin family member 3 | Multiple_C | 3.94 | 3.78 | 3.93 | 1.01 | 0.9076 | 0.9557 | 3.24 | 3.53 | 3.44 | 0.87 | 0.9403 | 0.9753 |
| TC1100008553.hg.1 | KCTD21-AS1 | KCTD21 antisense RNA 1 | Multiple_C | 5.8 | 5.76 | 5.79 | 1.01 | 0.9493 | 0.9764 | 5.05 | 5.84 | 5.36 | 0.81 | 0.189 | 0.4415 |
| TC1100009042.hg.1 | HSPB2; C11or | heat shock 27kDa protein 2; chromosome 11 open reading fram | Multiple_C | 7.98 | 7.64 | 7.97 | 1.01 | 0.582 | 0.7594 | 7.89 | 8.81 | 8.39 | 0.71 | 0.1184 | 0.3401 |
| TC1100009418.hg.1 | LOC403312; A | putative uncharacterized protein MGC39545; novel transcript, a | Multiple_C | 3.32 | 3.34 | 3.31 | 1.01 | 0.4998 | 0.699 | 3.61 | 3.42 | 3.6 | 1.01 | 0.8998 | 0.9583 |
| TC1100009617.hg.1 | GLB1L3 | galactosidase beta 1 like 3 | Multiple_C | 3.31 | 3.57 | 3.3 | 1.01 | 0.3848 | 0.5981 | 2.98 | 2.79 | 3.07 | 0.94 | 0.6169 | 0.8098 |
| TC1100010863.hg.1 | OR5M11 | olfactory receptor, family 5, subfamily M, member 11 | Coding | 4.34 | 4.71 | 4.33 | 1.01 | 0.8828 | 0.9431 | 3.9 | 3.94 | 3.8 | 1.07 | 0.1888 | 0.4413 |
| TC1100011190.hg.1 | EHD1 | EH domain containing 1 | Multiple_C | 9.07 | 8.72 | 9.06 | 1.01 | 0.5568 | 0.7397 | 9.45 | 8.79 | 8.77 | 1.60 | 0.01 | 0.0778 |
| TC1100011250.hg.1 | AP5B1 | adaptor-related protein complex 5, beta 1 subunit | Coding | 4.66 | 4.57 | 4.65 | 1.01 | 0.6836 | 0.8287 | 6.09 | 6.21 | 5.94 | 1.11 | 0.7376 | 0.8791 |
| TC1200008895.hg.1 | MAPKAPK5; A | mitogen-activated protein kinase-activated protein kinase 5; AD | Multiple_C | 14 | 14.89 | 13.99 | 1.01 | 0.9724 | 0.9866 | 12.39 | 13.00 | 12.55 | 0.90 | 0.4185 | 0.6723 |
| TC1200009941.hg.1 | PRB3 | proline-rich protein BstNI subfamily 3 | Multiple_C | 3.9 | 3.83 | 3.89 | 1.01 | 0.9163 | 0.9607 | 3.87 | 3.72 | 3.95 | 0.95 | 0.6876 | 0.8503 |
| TC1200010251.hg.1 | OVCH1 | ovochymase 1 | Multiple_C | 3.91 | 3.71 | 3.9 | 1.01 | 0.3765 | 0.5895 | 3.87 | 3.70 | 3.63 | 1.18 | 0.22 | 0.4801 |
| TC1200012667.hg.1 | TPH2 | tryptophan hydroxylase 2 | Multiple_C | 2.88 | 2.98 | 2.87 | 1.01 | 0.9763 | 0.9884 | 3.47 | 3.35 | 3.64 | 0.89 | 0.5881 | 0.7916 |
| TC1300008084.hg.1 | ATP11A | ATPase, class VI, type 11A | Multiple_C | 6.48 | 6.05 | 6.47 | 1.01 | 0.5253 | 0.7174 | 9.92 | 9.18 | 8.31 | 3.05 | 1.36E-05 | 0.0009 |
| TC1300008264.hg.1 | N6AMT2 | N-6 adenine-specific DNA methyltransferase 2 (putative) | Multiple_C | 6.89 | 7.02 | 6.88 | 1.01 | 0.8266 | 0.9129 | 7.08 | 6.66 | 5.82 | 2.39 | 0.0174 | 0.1105 |
| TC1400006506.hg.1 | OR11G2 | olfactory receptor, family 11, subfamily G, member 2 | Coding | 2.78 | 2.67 | 2.77 | 1.01 | 0.5652 | 0.7465 | 3.89 | 4.05 | 3.92 | 0.98 | 0.3687 | 0.6308 |
| TC1400009315.hg.1 | TIMM9 | translocase of inner mitochondrial membrane 9 homolog (yeast | Multiple_C | 9.84 | 12.16 | 9.83 | 1.01 | 0.943 | 0.9736 | 8.5 | 7.48 | 7.87 | 1.55 | 0.077 | 0.2674 |
| TC1400009786.hg.1 | ISM2 | isthmin 2 | Multiple_C | 3.58 | 3.52 | 3.57 | 1.01 | 0.5668 | 0.7477 | 4.09 | 3.98 | 3.95 | 1.10 | 0.7538 | 0.8881 |
| TC15_KI270727v1_randc | AC171558.1 | Olfactory receptor 4N4 [Source:UniProtKB/Swiss-Prot;Acc:Q8N | Coding | 2.58 | 3.13 | 2.57 | 1.01 | 0.6722 | 0.8212 | 2.99 | 2.93 | 3.07 | 0.95 | 0.6692 | 0.8382 |
| TC1500006925.hg.1 | THBS1 | thrombospondin 1 | Multiple_C | 4.41 | 4.26 | 4.4 | 1.01 | 0.9852 | 0.9929 | 9.1 | 8.47 | 9.37 | 0.83 | 0.4052 | 0.6606 |
| TC1500008127.hg.1 | ADAMTSL3 | ADAMTS like 3 | Multiple_C | 2.58 | 2.51 | 2.57 | 1.01 | 0.9166 | 0.9608 | 3.24 | 3.05 | 3.18 | 1.04 | 0.1008 | 0.3105 |
| TC1500009781.hg.1 | UBAP1L | ubiquitin associated protein 1 like | Multiple_C | 3.31 | 3.26 | 3.3 | 1.01 | 0.9358 | 0.97 | 3.6 | 3.62 | 3.42 | 1.13 | 0.6739 | 0.842 |
| TC1600006462.hg.1 | RAB11FIP3 | RAB11 family interacting protein 3 (class II) | Multiple_C | 5.71 | 5.4 | 5.7 | 1.01 | 0.8259 | 0.9126 | 6.13 | 6.30 | 6.07 | 1.04 | 0.2296 | 0.4906 |

| | | | | | | | | | | | | | | | |
|---|---|---|---|---|---|---|---|---|---|---|---|---|---|---|---|
| TC1600008000.hg.1 | CCL17 | chemokine (C-C motif) ligand 17 | Coding | 5.22 | 5.21 | 5.21 | 1.01 | 0.5557 | 0.7389 | 5.61 | 5.67 | 5.52 | 1.06 | 0.7927 | 0.9077 |
| TC1600009675.hg.1 | ZP2 | zona pellucida glycoprotein 2 (sperm receptor) | Multiple_C | 3.04 | 3.06 | 3.03 | 1.01 | 0.9486 | 0.9763 | 3.7 | 3.60 | 3.43 | 1.21 | 0.2807 | 0.5465 |
| TC1600010003.hg.1 | ZNF668 | zinc finger protein 668 | Multiple_C | 4.32 | 3.94 | 4.31 | 1.01 | 0.9303 | 0.9672 | 4.47 | 4.61 | 4.48 | 0.99 | 0.8244 | 0.9224 |
| TC1600010122.hg.1 | LINC00273 | long intergenic non-protein coding RNA 273 | Multiple_C | 4.87 | 4.73 | 4.86 | 1.01 | 0.5091 | 0.7052 | 5.58 | 5.76 | 5.53 | 1.04 | 0.4427 | 0.6913 |
| TC1600010341.hg.1 | AKTIP | AKT interacting protein | Multiple_C | 9.17 | 9.26 | 9.16 | 1.01 | 0.9457 | 0.9752 | 7.44 | 7.37 | 7.89 | 0.73 | 0.0815 | 0.2751 |
| TC1600010789.hg.1 | TAT | tyrosine aminotransferase | Multiple_C | 3.66 | 3.31 | 3.65 | 1.01 | 0.7 | 0.8395 | 3.7 | 3.87 | 3.53 | 1.13 | 0.7292 | 0.8742 |
| TC1600010866.hg.1 | LDHD | lactate dehydrogenase D | Multiple_C | 6.15 | 6.17 | 6.14 | 1.01 | 0.8959 | 0.9502 | 5.13 | 5.19 | 4.94 | 1.14 | 0.3248 | 0.5885 |
| TC1600011497.hg.1 | UMOD | uromodulin | Multiple_C | 4.34 | 4.36 | 4.33 | 1.01 | 0.6511 | 0.8068 | 4.89 | 4.69 | 4.72 | 1.13 | 0.4442 | 0.6923 |
| TC1700006630.hg.1 | SMTNL2 | smoothelin-like 2 | Multiple_C | 4.72 | 4.74 | 4.71 | 1.01 | 0.879 | 0.9412 | 4.59 | 4.64 | 4.37 | 1.16 | 0.1341 | 0.3636 |
| TC1700006770.hg.1 | DNAH2 | dynein, axonemal, heavy chain 2 | Multiple_C | 3.62 | 3.45 | 3.61 | 1.01 | 0.9263 | 0.9655 | 3.51 | 3.44 | 3.29 | 1.16 | 0.4573 | 0.7014 |
| TC1700007127.hg.1 | LGALS9C; LGA | lectin, galactoside-binding, soluble, 9C; lectin, galactoside-bindi | Multiple_C | 5.85 | 4.63 | 5.84 | 1.01 | 0.1918 | 0.3804 | 5.02 | 4.97 | 4.91 | 1.08 | 0.8357 | 0.9277 |
| TC1700007780.hg.1 | CASC3; MIR68 | cancer susceptibility candidate 3; microRNA 6866 | Multiple_C | 11.69 | 10.94 | 11.68 | 1.01 | 0.8745 | 0.9391 | 11.37 | 10.38 | 11.8 | 0.74 | 0.0328 | 0.1623 |
| TC1700010019.hg.1 | MFAP4 | microfibrillar associated protein 4 | Multiple_C | 2.55 | 2.64 | 2.54 | 1.01 | 0.7981 | 0.8964 | 3.03 | 3.11 | 3.19 | 0.90 | 0.3939 | 0.6521 |
| TC1700010397.hg.1 | C17orf102 | chromosome 17 open reading frame 102 | Multiple_C | 3.11 | 2.99 | 3.1 | 1.01 | 0.6454 | 0.8034 | 3.24 | 3.29 | 3.07 | 1.13 | 0.0726 | 0.2582 |
| TC1700010440.hg.1 | MMP28 | matrix metallopeptidase 28 | Multiple_C | 3.65 | 4.39 | 3.64 | 1.01 | 0.9925 | 0.9962 | 3.22 | 3.13 | 3.1 | 1.09 | 0.6384 | 0.8228 |
| TC1700011439.hg.1 | TEX2 | testis expressed 2 | Multiple_C | 8.08 | 7.9 | 8.07 | 1.01 | 0.9996 | 0.9998 | 9.86 | 9.78 | 9.78 | 1.06 | 0.4849 | 0.7222 |
| TC1700011984.hg.1 | NPTX1 | neuronal pentraxin I | Multiple_C | 4.85 | 4.86 | 4.84 | 1.01 | 0.4995 | 0.6988 | 5 | 4.94 | 4.99 | 1.01 | 0.9785 | 0.991 |
| TC1700012158.hg.1 | B3GNTL1 | UDP-GlcNAc:betaGal beta-1,3-N-acetylglucosaminyltransferase | Multiple_C | 4.1 | 4.03 | 4.09 | 1.01 | 0.8494 | 0.9261 | 4.37 | 4.64 | 5.06 | 0.62 | 0.9437 | 0.9771 |
| TC1700012205.hg.1 | PRPSAP2 | phosphoribosyl pyrophosphate synthetase-associated protein 2 | Multiple_C | 10.91 | 11.56 | 10.9 | 1.01 | 0.5074 | 0.7041 | 10.6 | 10.55 | 10.5 | 1.07 | 0.4745 | 0.7141 |
| TC1700012426.hg.1 | WNT3 | wingless-type MMTV integration site family, member 3 | Multiple_C | 4.4 | 4.37 | 4.39 | 1.01 | 0.9967 | 0.9984 | 4.87 | 4.79 | 4.88 | 0.99 | 0.2478 | 0.5123 |
| TC1800006571.hg.1 | ARHGAP28 | Rho GTPase activating protein 28 | Multiple_C | 3.05 | 3.06 | 3.04 | 1.01 | 0.3239 | 0.5378 | 4.41 | 4.40 | 4.46 | 0.97 | 0.8921 | 0.9539 |
| TC1900006534.hg.1 | APC2 | adenomatosis polyposis coli 2 | Multiple_C | 6.42 | 6.23 | 6.41 | 1.01 | 0.7496 | 0.8715 | 6.52 | 6.39 | 6.07 | 1.37 | 0.0448 | 0.1958 |
| TC1900006682.hg.1 | EBI3 | Epstein-Barr virus induced 3 | Multiple_C | 2.82 | 2.67 | 2.81 | 1.01 | 0.8413 | 0.9216 | 2.97 | 2.95 | 2.77 | 1.15 | 0.2178 | 0.4772 |
| TC1900007205.hg.1 | CASP14 | caspase 14 | Multiple_C | 2.71 | 2.74 | 2.7 | 1.01 | 0.4311 | 0.6413 | 3.28 | 3.42 | 3.17 | 1.08 | 0.547 | 0.7641 |
| TC1900007678.hg.1 | POP4 | POP4 homolog, ribonuclease P/MRP subunit | Multiple_C | 9.49 | 9.35 | 9.48 | 1.01 | 0.5898 | 0.7653 | 9.54 | 10.41 | 9.62 | 0.95 | 0.8606 | 0.9404 |
| TC1900007876.hg.1 | ZBTB32 | zinc finger and BTB domain containing 32 | Multiple_C | 5.96 | 6.06 | 5.95 | 1.01 | 0.1335 | 0.2968 | 6.05 | 6.04 | 5.75 | 1.23 | 0.5637 | 0.774 |
| TC1900008297.hg.1 | CLPTM1 | cleft lip and palate associated transmembrane protein 1 | Multiple_C | 9.4 | 7.87 | 9.39 | 1.01 | 0.5433 | 0.73 | 8.94 | 8.92 | 9.64 | 0.62 | 0.0061 | 0.0557 |
| TC1900010025.hg.1 | CRLF1 | cytokine receptor-like factor 1 | Multiple_C | 3.52 | 3.39 | 3.51 | 1.01 | 0.893 | 0.9485 | 3.76 | 3.32 | 3.56 | 1.15 | 0.2031 | 0.4587 |
| TC1900011057.hg.1 | PLA2G4C | phospholipase A2, group IVC (cytosolic, calcium-independent) | Multiple_C | 3.47 | 3.55 | 3.46 | 1.01 | 0.8899 | 0.947 | 3.44 | 3.80 | 3.51 | 0.95 | 0.7167 | 0.8677 |
| TC1900011391.hg.1 | OSCAR | osteoclast associated, immunoglobulin-like receptor | Multiple_C | 5.45 | 5.05 | 5.44 | 1.01 | 0.7622 | 0.8778 | 4.41 | 4.71 | 4.67 | 0.84 | 0.235 | 0.4976 |
| TC1900011705.hg.1 | TDRD12 | tudor domain containing 12 | Multiple_C | 2.76 | 2.69 | 2.75 | 1.01 | 0.6705 | 0.8204 | 3.91 | 3.87 | 3.82 | 1.06 | 0.6478 | 0.8278 |
| TC1900011770.hg.1 | C5AR1 | complement component 5a receptor 1 | Multiple_C | 3.18 | 3.32 | 3.17 | 1.01 | 0.761 | 0.8773 | 3.86 | 3.97 | 3.86 | 1.00 | 0.6329 | 0.8192 |
| TC1900011812.hg.1 | ZNF547; TRAP | zinc finger protein 547; trafficking protein particle complex 2B; | Multiple_C | 6.79 | 7.44 | 6.78 | 1.01 | 0.7016 | 0.8405 | 4.77 | 5.07 | 4.77 | 1.00 | 0.567 | 0.7763 |

| | | | | | | | | | | | | | | | |
|---|---|---|---|---|---|---|---|---|---|---|---|---|---|---|---|
| TC2000006756.hg.1 | MACROD2; R | MACRO domain containing 2; ribosomal protein S3 pseudogene | Multiple_C | 2.99 | 3 | 2.98 | 1.01 | 0.9597 | 0.9808 | 3.35 | 3.65 | 3.23 | 1.09 | 0.0802 | 0.2729 |
| TC2000006913.hg.1 | PAX1 | paired box 1 | Multiple_C | 4.05 | 3.96 | 4.04 | 1.01 | 0.6551 | 0.8102 | 4.11 | 4.17 | 3.95 | 1.12 | 0.6032 | 0.8015 |
| TC2000009211.hg.1 | WFDC12 | WAP four-disulfide core domain 12 | Coding | 3.79 | 3.68 | 3.78 | 1.01 | 0.9138 | 0.9597 | 4.02 | 4.21 | 3.91 | 1.08 | 0.749 | 0.8855 |
| TC2000009242.hg.1 | WFDC3 | WAP four-disulfide core domain 3 | Multiple_C | 5.34 | 5.59 | 5.33 | 1.01 | 0.1747 | 0.3566 | 7.11 | 6.21 | 6.37 | 1.67 | 0.0299 | 0.1533 |
| TC2000009602.hg.1 | ZBP1 | Z-DNA binding protein 1 | Multiple_C | 3.2 | 2.96 | 3.19 | 1.01 | 0.4681 | 0.6722 | 3.48 | 3.30 | 3.17 | 1.24 | 0.1138 | 0.3327 |
| TC2200007010.hg.1 | EMID1 | EMI domain containing 1 | Multiple_C | 3.22 | 3.33 | 3.21 | 1.01 | 0.9487 | 0.9763 | 4.04 | 4.35 | 4.15 | 0.93 | 0.7052 | 0.8611 |
| TC2200007023.hg.1 | NEFH | neurofilament, heavy polypeptide | Coding | 4.09 | 4.12 | 4.08 | 1.01 | 0.6287 | 0.7928 | 4.34 | 4.55 | 4.54 | 0.87 | 0.5026 | 0.7346 |
| TC2200008717.hg.1 | NPTXR | neuronal pentraxin receptor | Multiple_C | 4.01 | 3.83 | 4 | 1.01 | 0.5307 | 0.721 | 4.85 | 4.84 | 4.88 | 0.98 | 0.7464 | 0.8836 |
| TC2200008914.hg.1 | EFCAB6 | EF-hand calcium binding domain 6 | Multiple_C | 3.3 | 3.32 | 3.29 | 1.01 | 0.8678 | 0.9356 | 4.13 | 3.88 | 3.83 | 1.23 | 0.1549 | 0.3948 |
| TC2200009282.hg.1 | SAMM50 | SAMM50 sorting and assembly machinery component | Multiple_C | 12.39 | 12.75 | 12.38 | 1.01 | 0.5003 | 0.6992 | 8.92 | 9.10 | 8.82 | 1.07 | 0.6835 | 0.8482 |
| TSUnmapped00000173.h | CACFD1 | calcium channel flower domain containing 1 | Coding | 5.21 | 5.07 | 5.2 | 1.01 | 0.9736 | 0.9874 | 5.82 | 5.70 | 5.51 | 1.24 | 0.142 | 0.3746 |
| TSUnmapped00000508.h | HHAT | hedgehog acyltransferase | Coding | 4.43 | 4.32 | 4.42 | 1.01 | 0.8608 | 0.9321 | 5.06 | 5.16 | 4.75 | 1.24 | 0.96 | 0.9835 |
| TSUnmapped00000552.h | FBL | fibrillarin | Coding | 4.8 | 4.87 | 4.79 | 1.01 | 0.9892 | 0.9943 | 4.33 | 4.52 | 4.69 | 0.78 | 0.0881 | 0.2867 |
| TSUnmapped00000770.h | DGKD | diacylglycerol kinase, delta 130kDa | Coding | 4.88 | 5.28 | 4.87 | 1.01 | 0.8705 | 0.9369 | 4.92 | 5.03 | 4.81 | 1.08 | 0.2452 | 0.5094 |
| TC0100006907.hg.1 | PRAMEF12 | PRAME family member 12 | Coding | 4.96 | 5.01 | 4.96 | 1.00 | 0.6317 | 0.7944 | 4.26 | 4.28 | 4.35 | 0.94 | 0.4188 | 0.6725 |
| TC0100007105.hg.1 | PADI4 | peptidyl arginine deiminase, type IV | Multiple_C | 3.47 | 3.25 | 3.47 | 1.00 | 0.6567 | 0.8114 | 3.69 | 3.60 | 3.6 | 1.06 | 0.6807 | 0.8465 |
| TC0100007481.hg.1 | PIGV | phosphatidylinositol glycan anchor biosynthesis class V | Multiple_C | 6.53 | 7.29 | 6.53 | 1.00 | 0.9474 | 0.9758 | 4.16 | 4.78 | 4.76 | 0.66 | 0.0283 | 0.1482 |
| TC0100008079.hg.1 | IPO13 | importin 13 | Multiple_C | 6.53 | 6.24 | 6.53 | 1.00 | 0.7774 | 0.8856 | 7.1 | 7.89 | 8.12 | 0.49 | 0.005 | 0.0489 |
| TC0100009799.hg.1 | GJA8 | gap junction protein alpha 8 | Coding | 2.95 | 2.67 | 2.95 | 1.00 | 0.9908 | 0.9951 | 3.98 | 3.53 | 3.12 | 1.82 | 0.0194 | 0.1179 |
| TC0100010365.hg.1 | FCRLA | Fc receptor-like A | Multiple_C | 6.7 | 6.64 | 6.7 | 1.00 | 0.984 | 0.9924 | 6.19 | 5.93 | 5.86 | 1.26 | 0.134 | 0.3636 |
| TC0100011811.hg.1 | PSEN2 | presenilin 2 | Multiple_C | 7.53 | 7.74 | 7.53 | 1.00 | 0.6724 | 0.8212 | 7.31 | 7.47 | 7.34 | 0.98 | 0.4885 | 0.7247 |
| TC0100013374.hg.1 | TRIM63 | tripartite motif containing 63, E3 ubiquitin protein ligase | Multiple_C | 4.24 | 3.96 | 4.24 | 1.00 | 0.386 | 0.5993 | 4.72 | 4.72 | 4.73 | 0.99 | 0.8601 | 0.9402 |
| TC0100013533.hg.1 | MATN1 | matrilin 1, cartilage matrix protein | Multiple_C | 3.49 | 3.28 | 3.49 | 1.00 | 0.6877 | 0.8316 | 3.87 | 3.83 | 3.8 | 1.05 | 0.1746 | 0.4227 |
| TC0100016173.hg.1 | CFAP126 | cilia and flagella associated protein 126 | Coding | 3.22 | 3.03 | 3.22 | 1.00 | 0.6752 | 0.8233 | 3.77 | 3.62 | 3.77 | 1.00 | 0.9502 | 0.9795 |
| TC0100018452.hg.1 | GBP7 | guanylate binding protein 7 | Coding | 3.49 | 3.58 | 3.49 | 1.00 | 0.8314 | 0.9158 | 3.85 | 3.75 | 3.68 | 1.13 | 0.132 | 0.3605 |
| TC0200007243.hg.1 | VIT | vitrin | Coding | 3.05 | 2.66 | 3.05 | 1.00 | 0.9378 | 0.9711 | 2.9 | 2.99 | 2.96 | 0.96 | 0.2721 | 0.5377 |
| TC0200007388.hg.1 | LINC01126 | long intergenic non-protein coding RNA 1126 | Multiple_C | 3.69 | 3.76 | 3.69 | 1.00 | 0.8765 | 0.94 | 3.23 | 3.57 | 3.59 | 0.78 | 0.0455 | 0.1973 |
| TC0200008080.hg.1 | WDR54 | WD repeat domain 54 | Multiple_C | 7.85 | 7.63 | 7.85 | 1.00 | 0.9651 | 0.9831 | 6.51 | 7.16 | 6.36 | 1.11 | 0.4537 | 0.6986 |
| TC0200008285.hg.1 | MRPL35 | mitochondrial ribosomal protein L35 | Multiple_C | 11.99 | 12.19 | 11.99 | 1.00 | 0.7719 | 0.8822 | 9.6 | 9.89 | 9.98 | 0.77 | 0.0214 | 0.1253 |
| TC0200009959.hg.1 | DYNC1I2 | dynein, cytoplasmic 1, intermediate chain 2 | Multiple_C | 10.53 | 11.67 | 10.53 | 1.00 | 0.692 | 0.834 | 11.55 | 11.30 | 11.48 | 1.05 | 0.2373 | 0.5006 |
| TC0200015754.hg.1 | FEV | FEV (ETS oncogene family) | Multiple_C | 3.09 | 3.2 | 3.09 | 1.00 | 0.7016 | 0.8405 | 3.14 | 3.06 | 2.85 | 1.22 | 0.157 | 0.3979 |
| TC0200016047.hg.1 | NPPC | natriuretic peptide C | Coding | 4.1 | 3.93 | 4.1 | 1.00 | 0.9417 | 0.9729 | 3.61 | 3.56 | 3.49 | 1.09 | 0.4976 | 0.7309 |
| TC0200016560.hg.1 | KLHL41 | kelch-like family member 41 | Multiple_C | 2.73 | 2.68 | 2.73 | 1.00 | 0.7921 | 0.8935 | 3.68 | 3.59 | 3.46 | 1.16 | 0.3311 | 0.5951 |

| | | | | | | | | | | | | | | | |
|---|---|---|---|---|---|---|---|---|---|---|---|---|---|---|---|
| TC0200016637.hg.1 | SMC6 | structural maintenance of chromosomes 6 | Multiple_C | 10.68 | 11.43 | 10.68 | 1.00 | 0.6418 | 0.8014 | 10.96 | 9.59 | 10.44 | 1.43 | 0.8856 | 0.9509 |
| TC0300009139.hg.1 | TM4SF4 | transmembrane 4 L six family member 4 | Multiple_C | 5.49 | 5.63 | 5.49 | 1.00 | 0.3908 | 0.6045 | 5.18 | 5.71 | 5.34 | 0.90 | 0.9241 | 0.9688 |
| TC0300009474.hg.1 | CLDN11 | claudin 11 | Multiple_C | 3.3 | 3.2 | 3.3 | 1.00 | 0.9398 | 0.9722 | 13.46 | 12.41 | 13.33 | 1.09 | 0.2097 | 0.4666 |
| TC0300011251.hg.1 | ERC2 | ELKS/RAB6-interacting/CAST family member 2 | Multiple_C | 4.74 | 4.49 | 4.74 | 1.00 | 0.7543 | 0.8742 | 4.91 | 4.95 | 5.23 | 0.80 | 0.3868 | 0.6457 |
| TC0300011955.hg.1 | DPPA2 | developmental pluripotency associated 2 | Coding | 2.75 | 2.91 | 2.75 | 1.00 | 0.7443 | 0.8678 | 3.73 | 3.71 | 3.37 | 1.28 | 0.0832 | 0.2777 |
| TC0400007280.hg.1 | WDR19 | WD repeat domain 19 | Multiple_C | 8.29 | 9.93 | 8.29 | 1.00 | 0.8803 | 0.9419 | 10.19 | 9.31 | 8.62 | 2.97 | 1.04E-05 | 0.0007 |
| TC0400007315.hg.1 | CHRNA9 | cholinergic receptor, nicotinic alpha 9 | Multiple_C | 3.02 | 3.43 | 3.02 | 1.00 | 0.47 | 0.6739 | 3.42 | 3.63 | 3.5 | 0.95 | 0.4973 | 0.7307 |
| TC0400009234.hg.1 | TLL1 | tolloid like 1 | Multiple_C | 3.48 | 3.81 | 3.48 | 1.00 | 0.4662 | 0.6708 | 3.78 | 3.18 | 3.18 | 1.52 | 0.0609 | 0.2344 |
| TC0500008122.hg.1 | GPR150 | G protein-coupled receptor 150 | Coding | 3.3 | 3.03 | 3.3 | 1.00 | 0.7479 | 0.8706 | 3.18 | 3.18 | 3.14 | 1.03 | 0.4908 | 0.726 |
| TC0500009023.hg.1 | JAKMIP2-AS1 | JAKMIP2 antisense RNA 1 | Multiple_C | 2.72 | 2.98 | 2.72 | 1.00 | 0.9618 | 0.9819 | 9.35 | 11.01 | 9.33 | 1.01 | 0.9987 | 0.9997 |
| TC0500011875.hg.1 | PPIC | peptidylprolyl isomerase C (cyclophilin C) | Multiple_C | 10.66 | 10.67 | 10.66 | 1.00 | 0.6587 | 0.8127 | 6.21 | 5.17 | 5.04 | 2.25 | 0.0002 | 0.0062 |
| TC0500012504.hg.1 | SLC36A3 | solute carrier family 36, member 3 | Multiple_C | 3.5 | 3.62 | 3.5 | 1.00 | 0.6067 | 0.7777 | 3.03 | 3.02 | 3.15 | 0.92 | 0.967 | 0.9862 |
| TC0500013140.hg.1 | AHRR; PDCD6 | aryl-hydrocarbon receptor repressor; programmed cell death 6 | Multiple_C | 6.99 | 6.61 | 6.99 | 1.00 | 0.4469 | 0.6552 | 10.04 | 10.17 | 10.34 | 0.81 | 0.0829 | 0.2773 |
| TC0500013224.hg.1 | SLC27A6 | solute carrier family 27 (fatty acid transporter), member 6 | Coding | 3.47 | 3.3 | 3.47 | 1.00 | 0.4534 | 0.661 | 4.05 | 3.99 | 4.25 | 0.87 | 0.1011 | 0.3108 |
| TC0500013421.hg.1 | GRM6 | glutamate receptor, metabotropic 6 | Multiple_C | 2.73 | 2.78 | 2.73 | 1.00 | 0.6642 | 0.8163 | 3.4 | 3.79 | 3.23 | 1.13 | 0.3265 | 0.5903 |
| TC0600007143.hg.1 | CDKAL1 | CDK5 regulatory subunit associated protein 1-like 1 | Multiple_C | 7.04 | 7.01 | 7.04 | 1.00 | 0.9636 | 0.9824 | 11.27 | 10.42 | 11.35 | 0.95 | 0.3083 | 0.5724 |
| TC0600007597.hg.1 | LST1 | leukocyte specific transcript 1 | Multiple_C | 4.96 | 5.04 | 4.96 | 1.00 | 0.9747 | 0.9876 | 4.4 | 4.38 | 4.29 | 1.08 | 0.5496 | 0.766 |
| TC0600009477.hg.1 | TAAR8 | trace amine associated receptor 8 | Coding | 3.17 | 3.15 | 3.17 | 1.00 | 0.6305 | 0.794 | 3.83 | 3.81 | 3.93 | 0.93 | 0.4767 | 0.7156 |
| TC0600011192.hg.1 | POM121L2 | POM121 transmembrane nucleoporin-like 2 | Coding | 3.02 | 3.1 | 3.02 | 1.00 | 0.865 | 0.9341 | 3.28 | 3.23 | 3.29 | 0.99 | 0.6911 | 0.8526 |
| TC0600012512.hg.1 | RARS2 | arginyl-tRNA synthetase 2, mitochondrial | Multiple_C | 11.31 | 12.63 | 11.31 | 1.00 | 0.8536 | 0.9286 | 10.53 | 10.10 | 10.11 | 1.34 | 0.5603 | 0.772 |
| TC0700006813.hg.1 | HDAC9 | histone deacetylase 9 | Multiple_C | 7.53 | 7.13 | 7.53 | 1.00 | 0.9584 | 0.9803 | 6.83 | 6.86 | 7.35 | 0.70 | 0.0192 | 0.1171 |
| TC0700006852.hg.1 | ABCB5 | ATP binding cassette subfamily B member 5 | Multiple_C | 4.24 | 4.03 | 4.24 | 1.00 | 0.4489 | 0.6569 | 4.52 | 4.30 | 4.32 | 1.15 | 0.2298 | 0.4908 |
| TC0700009122.hg.1 | KLHDC10 | kelch domain containing 10 | Multiple_C | 9.08 | 8.93 | 9.08 | 1.00 | 0.7069 | 0.8442 | 8.49 | 8.44 | 8.76 | 0.83 | 0.1886 | 0.441 |
| TC0700011560.hg.1 | CCL26 | chemokine (C-C motif) ligand 26 | Coding | 3.84 | 3.51 | 3.84 | 1.00 | 0.9004 | 0.9522 | 3.93 | 3.60 | 3.86 | 1.05 | 0.4085 | 0.664 |
| TC0700012907.hg.1 | OR2A42; OR2A | olfactory receptor, family 2, subfamily A, member 42; olfactory | Coding | 4.24 | 4.22 | 4.24 | 1.00 | 0.6864 | 0.8308 | 4.15 | 4.24 | 4.37 | 0.86 | 0.3145 | 0.5783 |
| TC0700013421.hg.1 | ZNF789 | zinc finger protein 789 | Multiple_C | 6.65 | 6.53 | 6.65 | 1.00 | 0.4625 | 0.6678 | 7.53 | 8.02 | 7.57 | 0.97 | 0.6575 | 0.8327 |
| TC0700013424.hg.1 | GS1-259H13.2 | transmembrane protein 225-like; Jeck2013 ALT_ACCEPTOR, ALT | Multiple_C | 3.64 | 3.44 | 3.64 | 1.00 | 0.6609 | 0.814 | 3.61 | 3.63 | 3.73 | 0.92 | 0.563 | 0.7735 |
| TC0700013583.hg.1 | MTERF1 | mitochondrial transcription termination factor 1 | Multiple_C | 3.67 | 3.52 | 3.67 | 1.00 | 0.9915 | 0.9957 | 8.02 | 8.26 | 8.3 | 0.82 | 0.715 | 0.8668 |
| TC0800008679.hg.1 | COL14A1 | collagen, type XIV, alpha 1 | Multiple_C | 2.94 | 2.48 | 2.94 | 1.00 | 0.9244 | 0.9645 | 3.78 | 3.98 | 3.63 | 1.11 | 0.2464 | 0.5109 |
| TC0800009523.hg.1 | MFHAS1 | malignant fibrous histiocytoma amplified sequence 1 | Multiple_C | 9.64 | 10.5 | 9.64 | 1.00 | 0.5523 | 0.7367 | 8.75 | 9.02 | 9.62 | 0.55 | 0.0004 | 0.0094 |
| TC0800010696.hg.1 | TCF24 | transcription factor 24 | Multiple_C | 2.73 | 2.67 | 2.73 | 1.00 | 0.9918 | 0.9958 | 4.04 | 3.92 | 3.5 | 1.45 | 0.0633 | 0.2394 |
| TC0800011015.hg.1 | PSKH2 | protein serine kinase H2 | Multiple_C | 4.19 | 3.92 | 4.19 | 1.00 | 0.6008 | 0.7732 | 4.63 | 4.59 | 4.57 | 1.04 | 0.7709 | 0.8972 |
| TC0800011037.hg.1 | DCAF4L2 | DDB1 and CUL4 associated factor 4-like 2 | Coding | 2.7 | 2.82 | 2.7 | 1.00 | 0.8023 | 0.8989 | 3.32 | 3.31 | 3.08 | 1.18 | 0.7381 | 0.8792 |

| | | | | | | | | | | | | | | | |
|---|---|---|---|---|---|---|---|---|---|---|---|---|---|---|---|
| TC0900007816.hg.1 | CTSL | cathepsin L | Multiple_C | 3.6 | 3.64 | 3.6 | 1.00 | 0.6454 | 0.8034 | 6.94 | 6.85 | 7.06 | 0.92 | 0.5898 | 0.7925 |
| TC0900007844.hg.1 | SPIN1 | spindlin 1 | Multiple_C | 10.98 | 10.52 | 10.98 | 1.00 | 0.7782 | 0.8859 | 12.1 | 11.07 | 11.56 | 1.45 | 0.0755 | 0.2641 |
| TC0900008289.hg.1 | OR13F1 | olfactory receptor, family 13, subfamily F, member 1 | Coding | 3.11 | 3.32 | 3.11 | 1.00 | 0.662 | 0.8146 | 3.49 | 3.46 | 3.1 | 1.31 | 0.0436 | 0.1931 |
| TC0900008666.hg.1 | OR1J4 | olfactory receptor, family 1, subfamily J, member 4 | Coding | 3.51 | 3.59 | 3.51 | 1.00 | 0.8871 | 0.9454 | 3.8 | 3.81 | 3.64 | 1.12 | 0.553 | 0.7684 |
| TC0900009942.hg.1 | FAM166B | family with sequence similarity 166, member B | Multiple_C | 3.66 | 3.5 | 3.66 | 1.00 | 0.1672 | 0.3467 | 3.94 | 4.04 | 3.83 | 1.08 | 0.5582 | 0.7714 |
| TC0900010322.hg.1 | TMEM252 | transmembrane protein 252 | Coding | 3.12 | 3.05 | 3.12 | 1.00 | 0.873 | 0.9384 | 3.35 | 3.43 | 3.24 | 1.08 | 0.312 | 0.5762 |
| TC0X00006669.hg.1 | GLRA2 | glycine receptor alpha 2 | Coding | 3.73 | 3.58 | 3.73 | 1.00 | 0.5921 | 0.767 | 3.34 | 3.45 | 3.56 | 0.86 | 0.584 | 0.788 |
| TC0X00007263.hg.1 | CXorf67 | chromosome X open reading frame 67 | Coding | 3.12 | 3 | 3.12 | 1.00 | 0.723 | 0.8543 | 3.19 | 3.28 | 3.51 | 0.80 | 0.0711 | 0.2552 |
| TC0X00007805.hg.1 | CPXCR1 | CPX chromosome region, candidate 1 | Coding | 3.52 | 3.49 | 3.52 | 1.00 | 0.7584 | 0.8758 | 3.81 | 3.43 | 3.57 | 1.18 | 0.3613 | 0.6242 |
| TC0X00009328.hg.1 | CXorf21 | chromosome X open reading frame 21 | Coding | 2.94 | 3.01 | 2.94 | 1.00 | 0.7585 | 0.8758 | 4.4 | 3.84 | 3.86 | 1.45 | 0.0235 | 0.1324 |
| TC0X00009340.hg.1 | FTHL17 | ferritin, heavy polypeptide-like 17 | Coding | 5.28 | 5.27 | 5.28 | 1.00 | 0.9026 | 0.9531 | 5.22 | 5.44 | 5.34 | 0.92 | 0.6637 | 0.8355 |
| TC0X00010770.hg.1 | ACTRT1 | actin-related protein T1 | Coding | 3.53 | 3.8 | 3.53 | 1.00 | 0.6345 | 0.7965 | 3.93 | 3.62 | 3.6 | 1.26 | 0.1162 | 0.3368 |
| TC0X00011311.hg.1 | ARMCX5-GPR/ | ARMCX5-GPRASP2 readthrough | Multiple_C | 5.59 | 5.84 | 5.59 | 1.00 | 0.4473 | 0.6554 | 5.54 | 6.09 | 6.73 | 0.44 | 0.0006 | 0.0114 |
| TC0Y00007341.hg.1 | RBMY1E; RBM | RNA binding motif protein, Y-linked, family 1, member E; RNA bi | Coding | 2.65 | 2.81 | 2.65 | 1.00 | 0.9823 | 0.9918 | 3.31 | 3.45 | 3.31 | 1.00 | 0.6908 | 0.8526 |
| TC1000008239.hg.1 | SH2D4B | SH2 domain containing 4B | Multiple_C | 2.94 | 2.92 | 2.94 | 1.00 | 0.969 | 0.985 | 3.59 | 3.67 | 3.7 | 0.93 | 0.738 | 0.8792 |
| TC1000009396.hg.1 | JAKMIP3 | Janus kinase and microtubule interacting protein 3 | Multiple_C | 5.29 | 5.23 | 5.29 | 1.00 | 0.691 | 0.8335 | 7.62 | 7.17 | 6.85 | 1.71 | 0.0232 | 0.1311 |
| TC1000010914.hg.1 | PPA1 | pyrophosphatase (inorganic) 1 | Multiple_C | 13.52 | 14.36 | 13.52 | 1.00 | 0.9774 | 0.9889 | 11.64 | 11.80 | 12.28 | 0.64 | 0.0123 | 0.0887 |
| TC1000010921.hg.1 | NODAL | nodal growth differentiation factor | Coding | 3.97 | 3.46 | 3.97 | 1.00 | 0.8671 | 0.9352 | 3.69 | 3.73 | 3.82 | 0.91 | 0.3464 | 0.6101 |
| TC1000011029.hg.1 | SYNPO2L | synaptopodin 2-like | Coding | 4.13 | 4.29 | 4.13 | 1.00 | 0.5366 | 0.7252 | 3.61 | 3.76 | 3.49 | 1.09 | 0.3018 | 0.5661 |
| TC1000012122.hg.1 | NKX1-2 | NK1 homeobox 2 | Multiple_C | 3.48 | 3.42 | 3.48 | 1.00 | 0.6219 | 0.7885 | 3.33 | 3.50 | 3.68 | 0.78 | 0.0919 | 0.2937 |
| TC1100006906.hg.1 | FAR1 | fatty acyl CoA reductase 1 | Multiple_C | 12.81 | 12.65 | 12.81 | 1.00 | 0.8876 | 0.9457 | 13.46 | 12.48 | 13.26 | 1.15 | 0.3278 | 0.5912 |
| TC1100007628.hg.1 | OR5J2 | olfactory receptor, family 5, subfamily J, member 2 | Multiple_C | 3.15 | 3.05 | 3.15 | 1.00 | 0.9672 | 0.9842 | 3.86 | 3.67 | 3.77 | 1.06 | 0.566 | 0.7756 |
| TC1100007770.hg.1 | MS4A5 | membrane-spanning 4-domains, subfamily A, member 5 | Multiple_C | 3.89 | 3.46 | 3.89 | 1.00 | 0.7551 | 0.8745 | 4.86 | 4.83 | 4.53 | 1.26 | 0.08 | 0.2728 |
| TC1100007812.hg.1 | LRRC10B | leucine rich repeat containing 10B | Coding | 3.35 | 3.09 | 3.35 | 1.00 | 0.795 | 0.8948 | 4.08 | 3.84 | 3.85 | 1.17 | 0.0217 | 0.1267 |
| TC1100008454.hg.1 | TPBGL | trophoblast glycoprotein-like | Multiple_C | 2.42 | 2.21 | 2.42 | 1.00 | 0.9609 | 0.9814 | 3.12 | 3.05 | 2.72 | 1.32 | 0.0631 | 0.2391 |
| TC1100008733.hg.1 | UBTFL1 | upstream binding transcription factor, RNA polymerase I-like 1 | Coding | 2.98 | 2.54 | 2.98 | 1.00 | 0.7145 | 0.8493 | 3.55 | 3.54 | 3.09 | 1.38 | 0.0447 | 0.1956 |
| TC1100009799.hg.1 | TRPM5 | transient receptor potential cation channel, subfamily M, memb | Coding | 3.35 | 3.11 | 3.35 | 1.00 | 0.9892 | 0.9943 | 3.57 | 3.44 | 3.44 | 1.09 | 0.4832 | 0.7207 |
| TC1100010008.hg.1 | OR5P2 | olfactory receptor, family 5, subfamily P, member 2 | Coding | 2.62 | 2.54 | 2.62 | 1.00 | 0.9078 | 0.9557 | 3.3 | 3.44 | 3.26 | 1.03 | 0.4982 | 0.7314 |
| TC1100010054.hg.1 | NRIP3 | nuclear receptor interacting protein 3 | Multiple_C | 3.07 | 2.89 | 3.07 | 1.00 | 0.8291 | 0.9144 | 4.22 | 3.07 | 4.08 | 1.10 | 0.1314 | 0.3592 |
| TC1100010876.hg.1 | OR5G5P; OR5 | olfactory receptor, family 5, subfamily G, member 5 pseudogen | Multiple_C | 3.01 | 2.88 | 3.01 | 1.00 | 0.7048 | 0.8425 | 3.72 | 3.46 | 3.39 | 1.26 | 0.1411 | 0.3736 |
| TC1100011265.hg.1 | EIF1AD | eukaryotic translation initiation factor 1A domain containing | Multiple_C | 7.87 | 7.22 | 7.87 | 1.00 | 0.9287 | 0.9663 | 8.64 | 8.51 | 9.52 | 0.54 | 0.003 | 0.0354 |
| TC1100011346.hg.1 | NUDT8 | nudix hydrolase 8 | Multiple_C | 7.37 | 7.29 | 7.37 | 1.00 | 0.7725 | 0.8825 | 6 | 6.21 | 6.15 | 0.90 | 0.4984 | 0.7314 |
| TC1100011677.hg.1 | WNT11 | wingless-type MMTV integration site family, member 11 | Multiple_C | 5.42 | 5.86 | 5.42 | 1.00 | 0.2233 | 0.4214 | 6.99 | 5.34 | 4.64 | 5.10 | 5.22E-08 | 1.72E-05 |

| | | | | | | | | | | | | | | | |
|---|---|---|---|---|---|---|---|---|---|---|---|---|---|---|---|
| TC1100011901.hg.1 | NOX4 | NADPH oxidase 4 | Multiple_C | 3.05 | 3.22 | 3.05 | 1.00 | 0.9094 | 0.9566 | 3.32 | 3.50 | 3.23 | 1.06 | 0.5835 | 0.7878 |
| TC1100012137.hg.1 | MMP13 | matrix metallopeptidase 13 | Coding | 3.17 | 2.98 | 3.17 | 1.00 | 0.8397 | 0.9204 | 3.7 | 4.12 | 3.72 | 0.99 | 0.7953 | 0.9087 |
| TC1100012853.hg.1 | SNX19 | sorting nexin 19 | Multiple_C | 6.85 | 6.54 | 6.85 | 1.00 | 0.5384 | 0.7266 | 7.96 | 7.90 | 7.6 | 1.28 | 0.1868 | 0.4387 |
| TC1100013185.hg.1 | FAU | Finkel-Biskis-Reilly murine sarcoma virus (FBR-MuSV) ubiquitou | Multiple_C | 14.59 | 14.9 | 14.59 | 1.00 | 0.7621 | 0.8778 | 11.3 | 11.44 | 10.61 | 1.61 | 0.0022 | 0.0289 |
| TC1100013193.hg.1 | PTPRCAP | protein tyrosine phosphatase, receptor type, C-associated prote | Multiple_C | 4.04 | 4.06 | 4.04 | 1.00 | 0.5557 | 0.7389 | 4.28 | 4.45 | 4.35 | 0.95 | 0.3784 | 0.6381 |
[truncated: 1,537,975 more chars]
