# Supplementary material for: Loss of cadherin 17 downregulates LGR5 expression, stem cell properties and drug resistance in metastatic colorectal cancer cells
Source: Cell Death Dis. 2025 Jul 1;16(1):475. doi: 10.1038/s41419-025-07811-w (PMC12217925; doi:10.1038/s41419-025-07811-w)
Supplement: Supplementary file 3 — Original WB data [file 41419_2025_7811_MOESM3_ESM.pdf]

Figure 1A

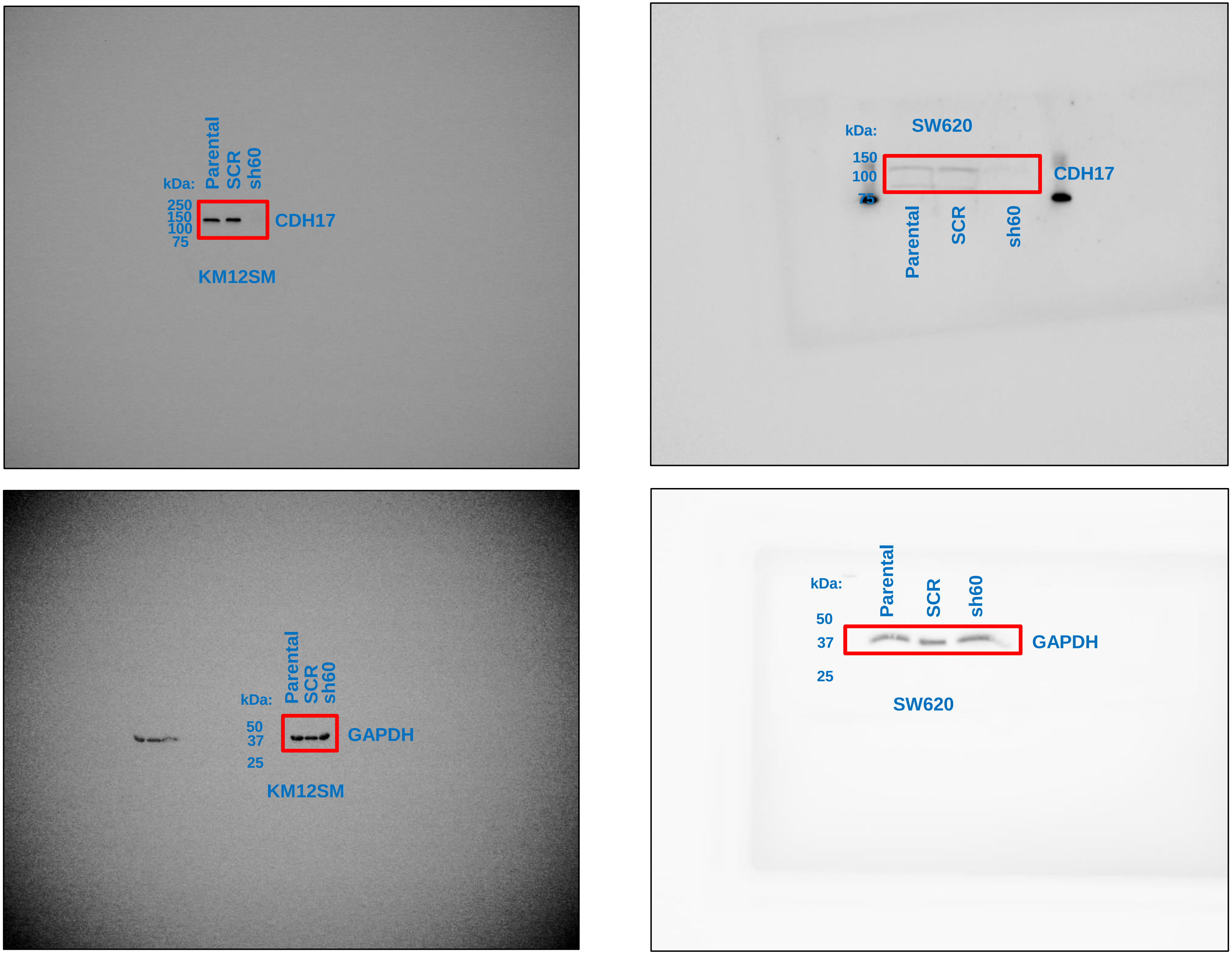

kDa:
Parental
SCR
sh60
250
150
100
75
CDH17
KM12SM
kDa:
SW620
150
100
75
CDH17
Parental
SCR
sh60
kDa:
Parental
SCR
sh60
50
37
25
GAPDH
KM12SM
kDa:
Parental
SCR
sh60
50
37
GAPDH
25
SW620

Figure 2C

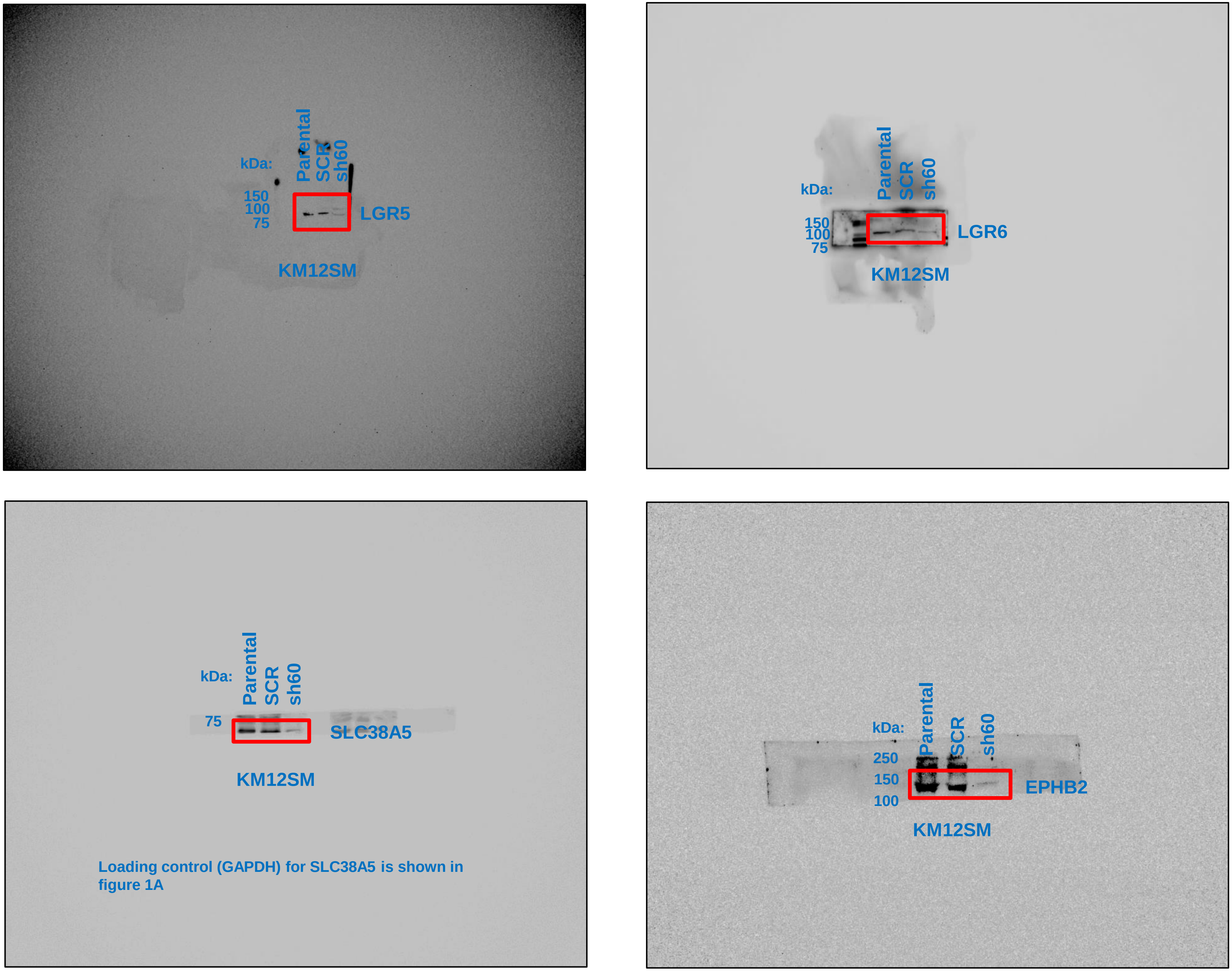

kDa:
Parental
SCR
sh60
150
100
75
LGR5
KM12SM
kDa:
Parental
SCR
sh60
150
100
75
LGR6
KM12SM
kDa:
Parental
SCR
sh60
75
SLC38A5
KM12SM
Loading control (GAPDH) for SLC38A5 is shown in figure 1A
kDa:
Parental
SCR
sh60
250
150
100
EPHB2
KM12SM

Figure 2C

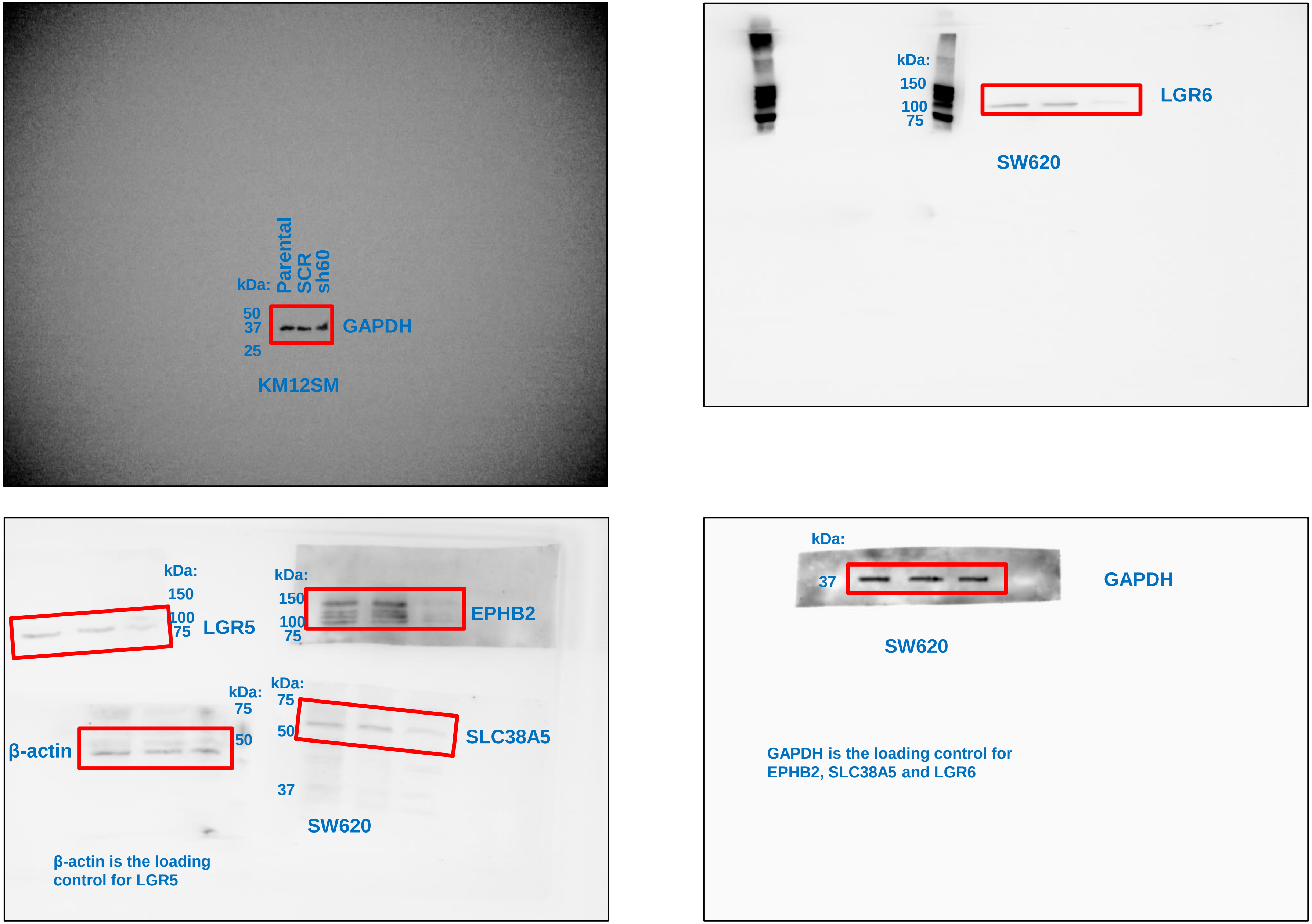
kDa:
Parental
SCR
sh60
50
37
25
GAPDH
KM12SM
kDa:
150
100
75
LGR6
SW620
kDa:
150
100
75
LGR5
kDa:
150
100
75
EPHB2
kDa:
75
50
β-actin
kDa:
75
50
37
SLC38A5
SW620
β-actin is the loading control for LGR5
kDa:
37
GAPDH
SW620
GAPDH is the loading control for EPHB2, SLC38A5 and LGR6

Figure 3D

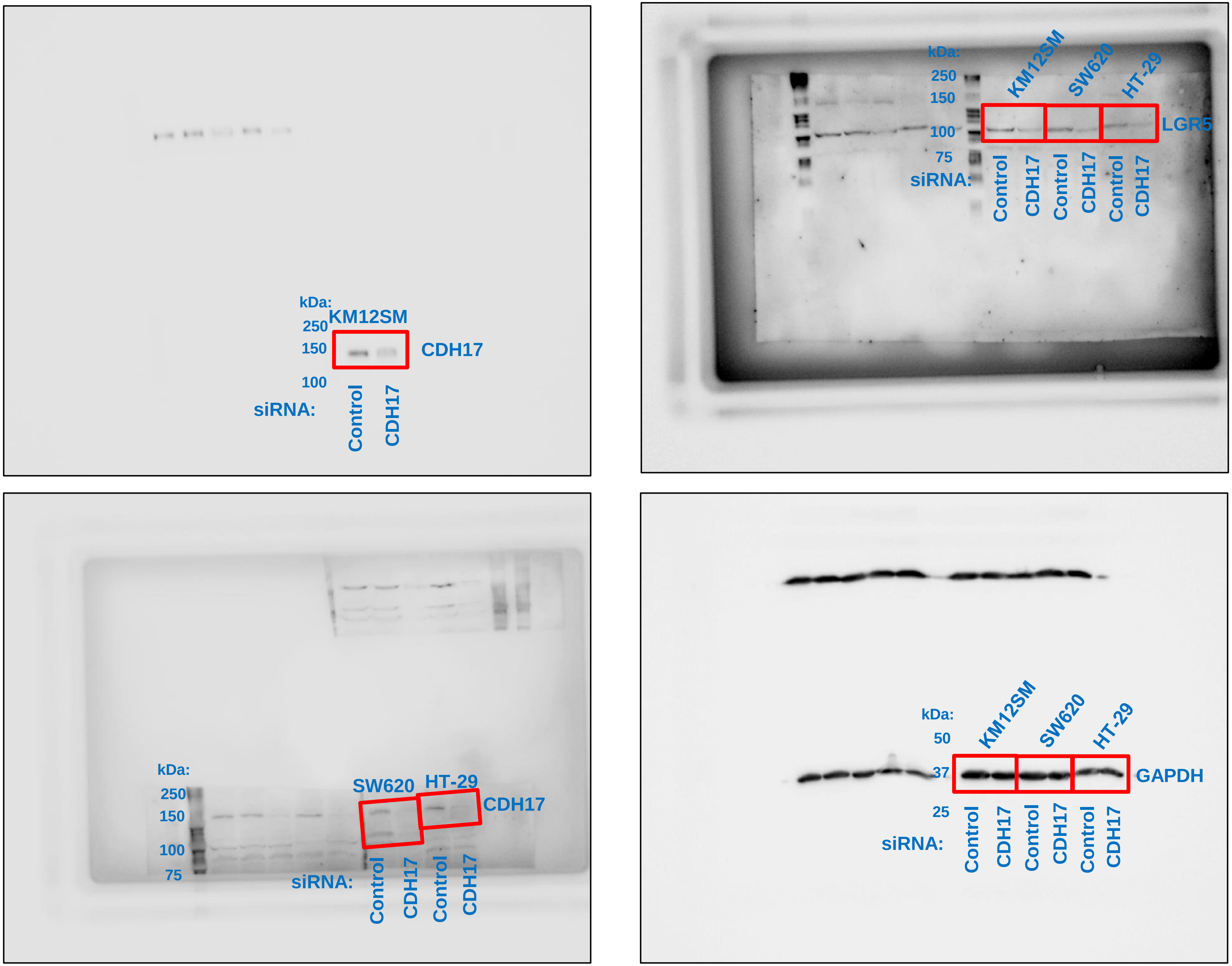

kDa:
250
150
100
KM12SM
CDH17
siRNA:
Control
CDH17
kDa:
250
150
100
75
KM12SM
SW620
HT-29
LGR5
siRNA:
Control
CDH17
Control
CDH17
Control
CDH17
kDa:
250
150
100
75
SW620
HT-29
CDH17
siRNA:
Control
CDH17
Control
CDH17
kDa:
50
37
25
KM12SM
SW620
HT-29
GAPDH
siRNA:
Control
CDH17
Control
CDH17
Control
CDH17

Figure 3E

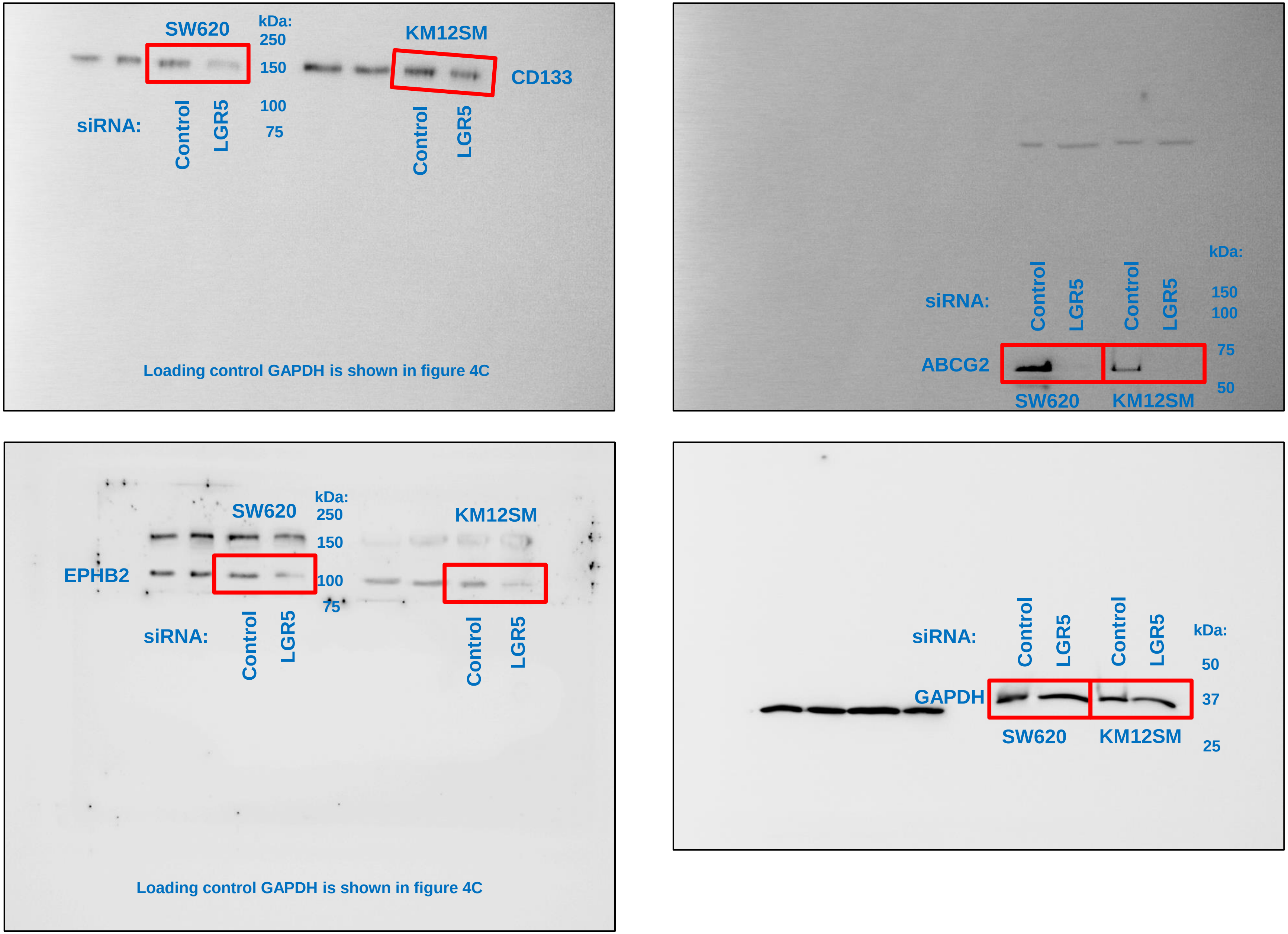
SW620
kDa:
250
150
100
75
KM12SM
CD133
siRNA:
Control
LGR5
Control
LGR5
Loading control GAPDH is shown in figure 4C
siRNA:
Control
LGR5
Control
LGR5
kDa:
150
100
75
50
ABCG2
SW620
KM12SM
SW620
kDa:
250
150
100
75
KM12SM
EPHB2
siRNA:
Control
LGR5
Control
LGR5
Loading control GAPDH is shown in figure 4C
siRNA:
Control
LGR5
Control
LGR5
kDa:
50
37
25
GAPDH
SW620
KM12SM

Figure 3F

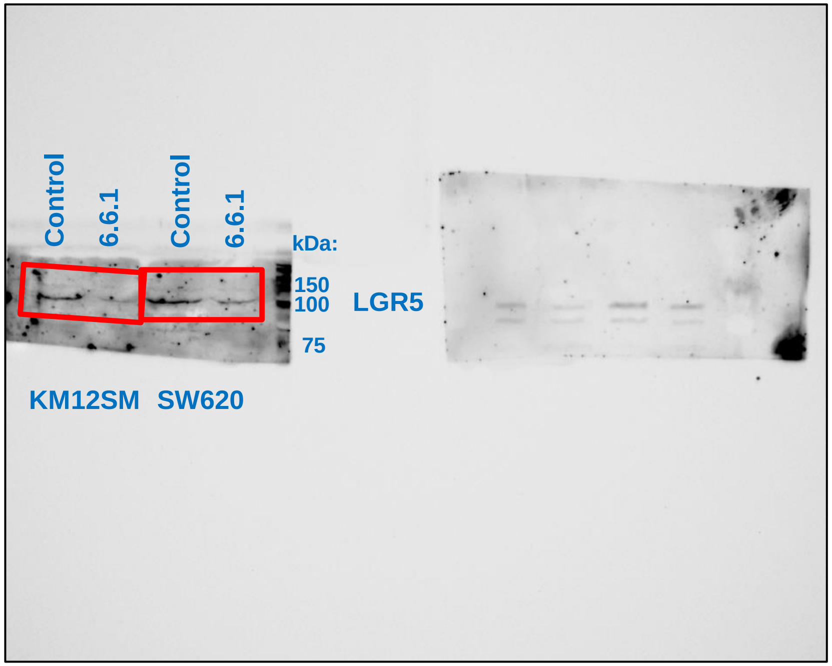

Control
6.6.1
Control
6.6.1
kDa:
150
100
75
LGR5
KM12SM
SW620

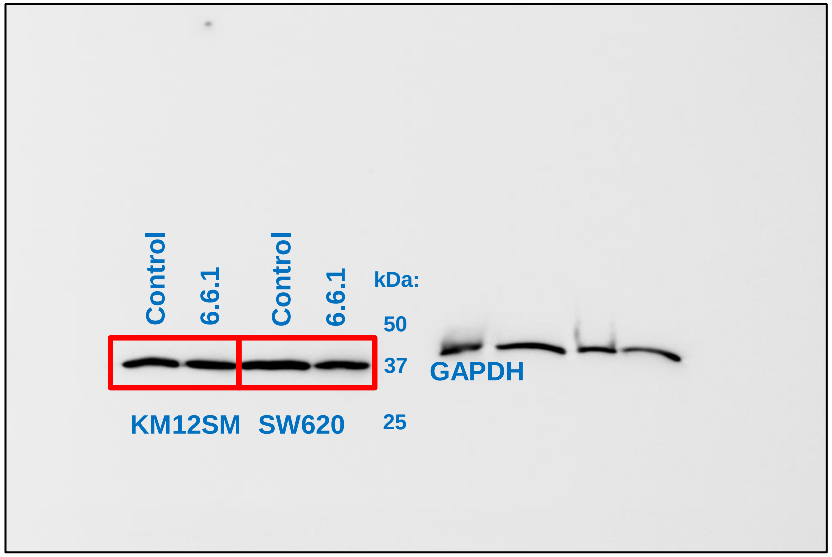

Control
6.6.1
Control
6.6.1
kDa:
50
37
25
GAPDH
KM12SM
SW620

Figure 3H

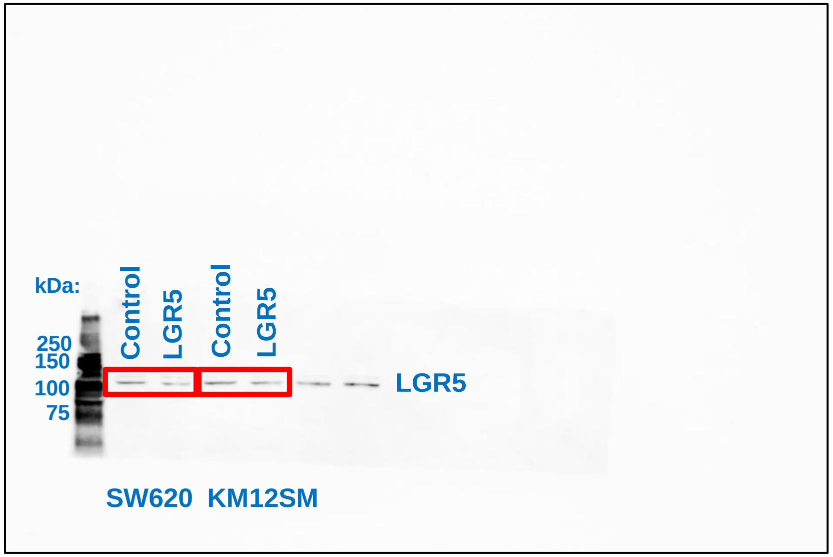
kDa:
250
150
100
75
Control
LGR5
Control
LGR5
LGR5
SW620 KM12SM

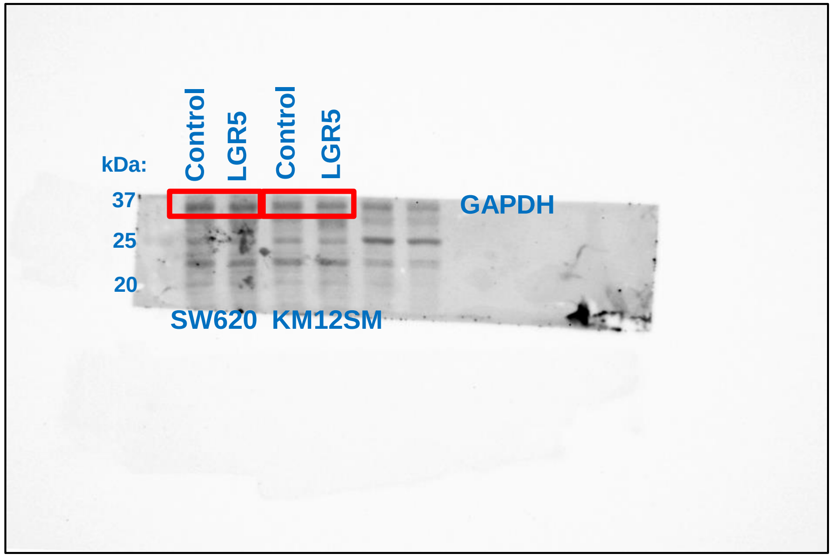
kDa:
37
25
20
Control
LGR5
Control
LGR5
GAPDH
SW620 KM12SM

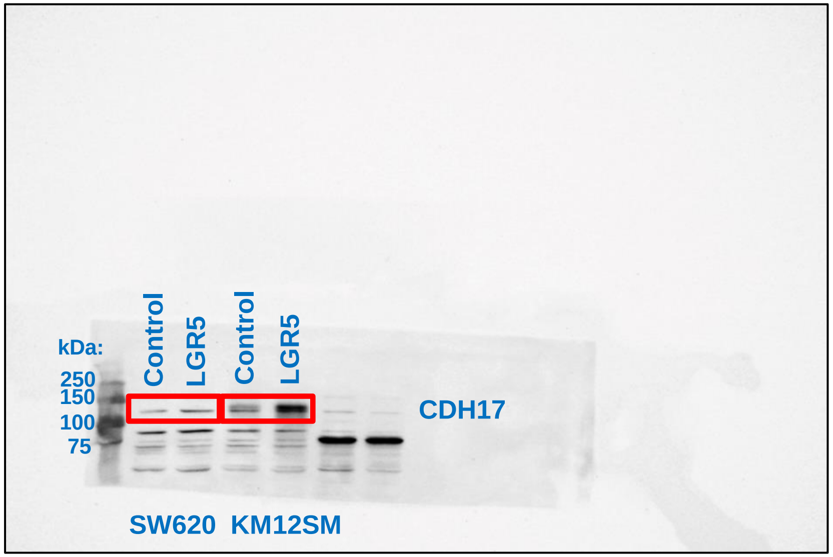
kDa:
250
150
100
75
Control
LGR5
Control
LGR5
CDH17
SW620 KM12SM

Figure 4C

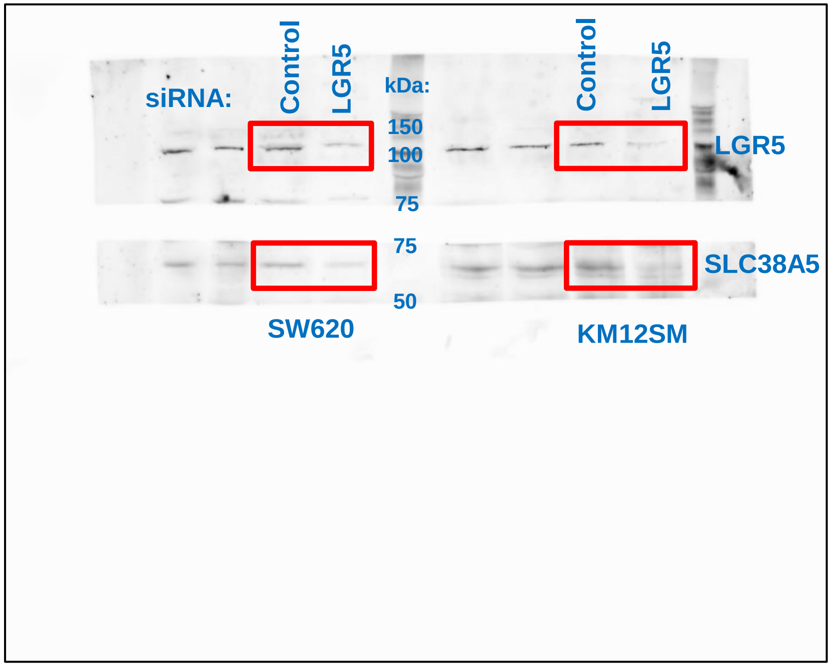
siRNA:
Control
LGR5
kDa:
150
100
75
75
50
Control
LGR5
LGR5
SLC38A5
SW620
KM12SM

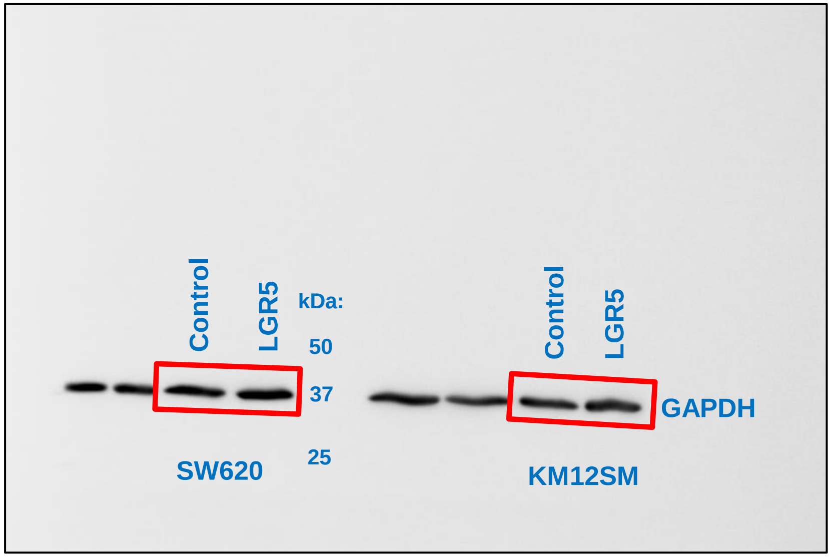
Control
LGR5
kDa:
50
37
25
Control
LGR5
GAPDH
SW620
KM12SM

Figure 4E

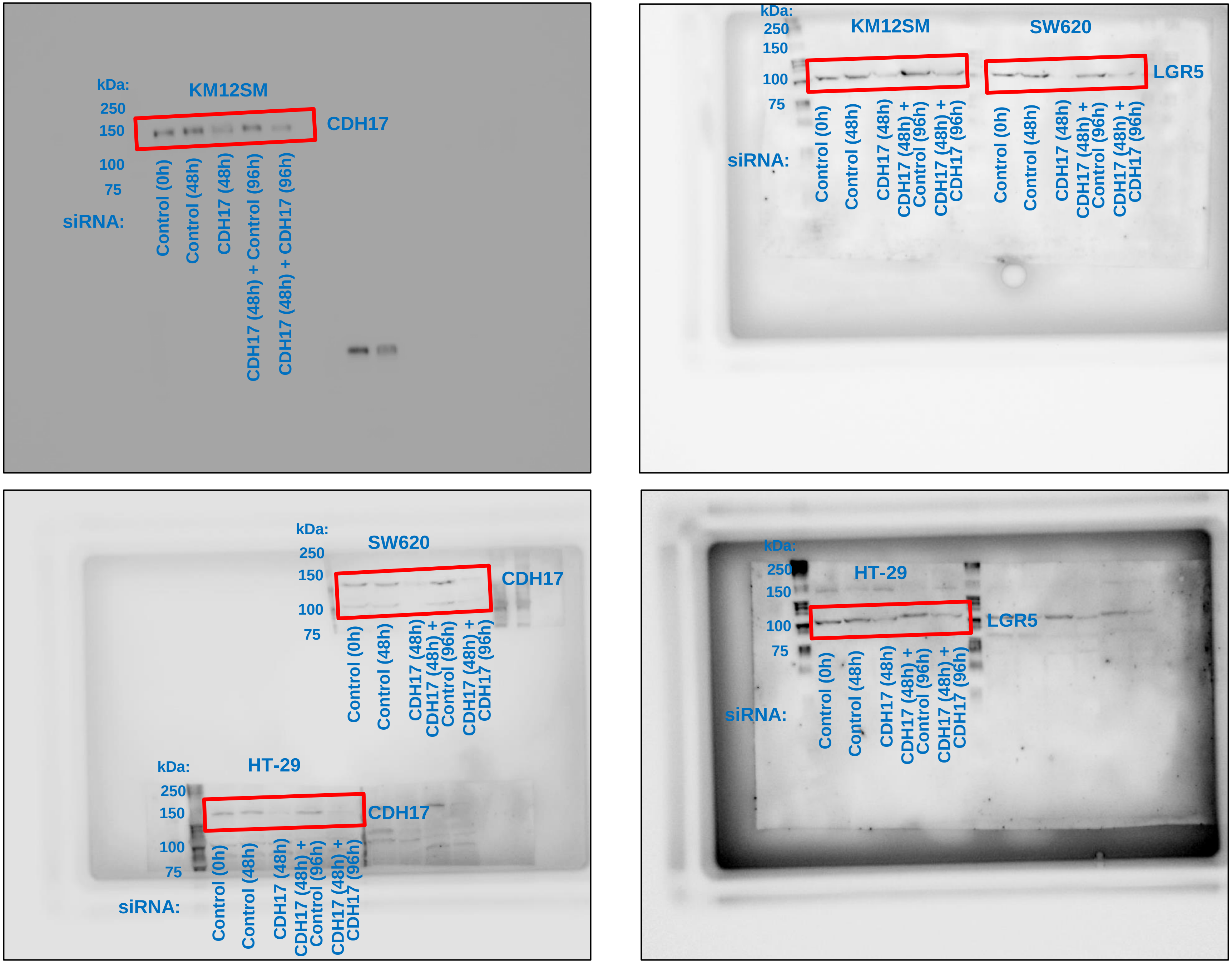
kDa:
KM12SM
250
150
100
75
CDH17
siRNA:
Control (0h)
Control (48h)
CDH17 (48h)
CDH17 (48h) + Control (96h)
CDH17 (48h) + CDH17 (96h)
kDa:
KM12SM
SW620
250
150
100
75
LGR5
siRNA:
Control (0h)
Control (48h)
CDH17 (48h)
CDH17 (48h) + Control (96h)
CDH17 (48h) + CDH17 (96h)
kDa:
SW620
250
150
100
75
CDH17
Control (0h)
Control (48h)
CDH17 (48h)
CDH17 (48h) + Control (96h)
CDH17 (48h) + CDH17 (96h)
kDa:
HT-29
250
150
100
75
CDH17
siRNA:
Control (0h)
Control (48h)
CDH17 (48h)
CDH17 (48h) + Control (96h)
CDH17 (48h) + CDH17 (96h)
kDa:
HT-29
250
150
100
75
LGR5
siRNA:
Control (0h)
Control (48h)
CDH17 (48h)
CDH17 (48h) + Control (96h)
CDH17 (48h) + CDH17 (96h)

Figure 4E

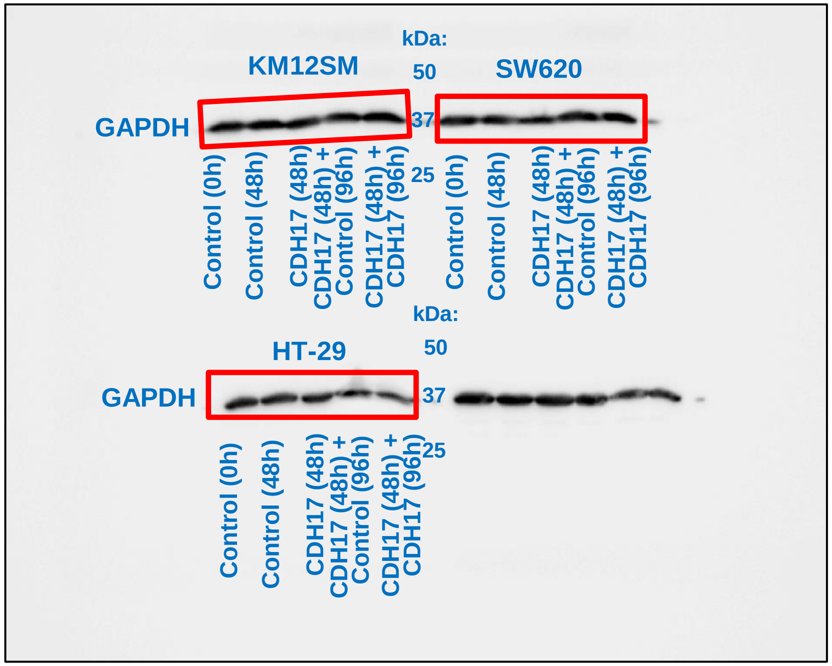

kDa:
KM12SM
50
SW620
GAPDH
37
25
Control (0h)
Control (48h)
CDH17 (48h)
CDH17 (48h) +
Control (96h)
CDH17 (48h) +
CDH17 (96h)
Control (0h)
Control (48h)
CDH17 (48h)
CDH17 (48h) +
Control (96h)
CDH17 (48h) +
CDH17 (96h)
kDa:
50
HT-29
GAPDH
37
25
Control (0h)
Control (48h)
CDH17 (48h)
CDH17 (48h) +
Control (96h)
CDH17 (48h) +
CDH17 (96h)

Figure 6C

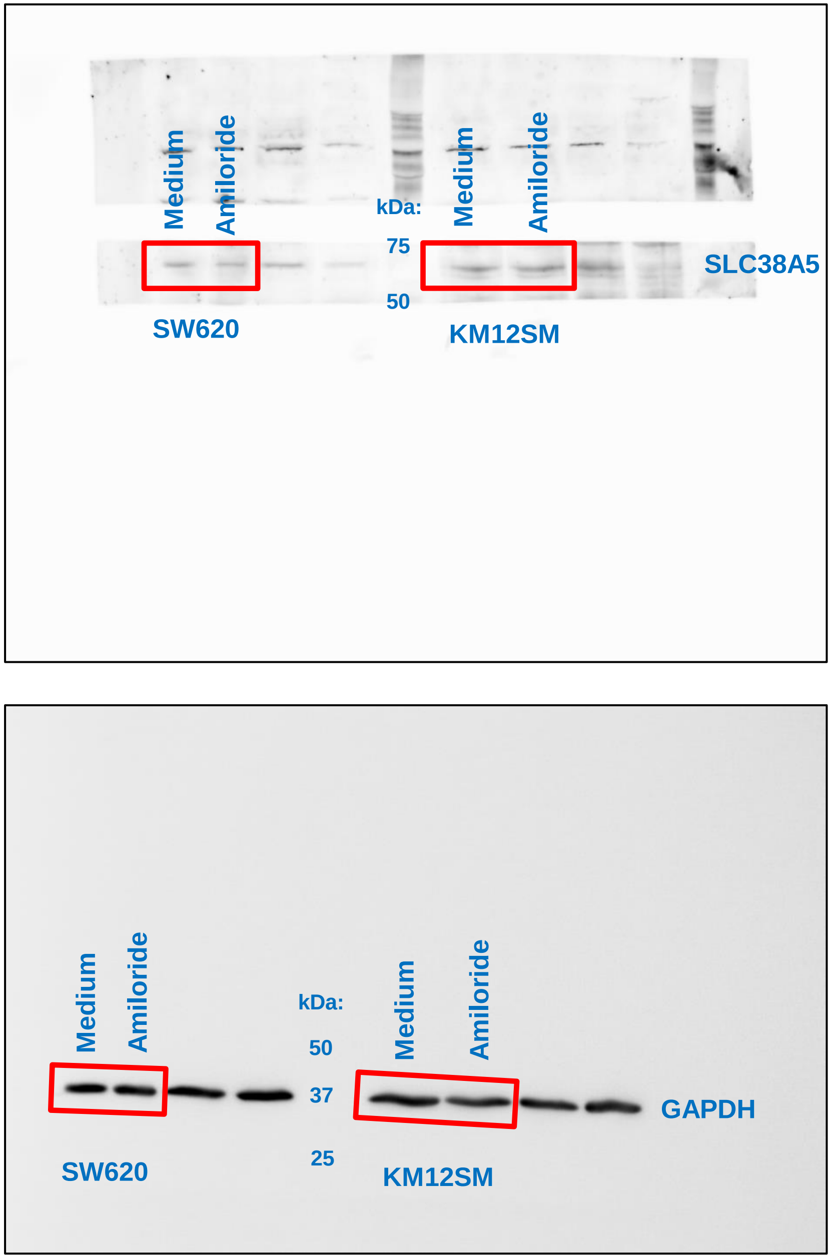
Medium
Amiloride
kDa:
75
50
Medium
Amiloride
SLC38A5
SW620
KM12SM
Medium
Amiloride
kDa:
50
37
25
Medium
Amiloride
GAPDH
SW620
KM12SM

Figure S3A

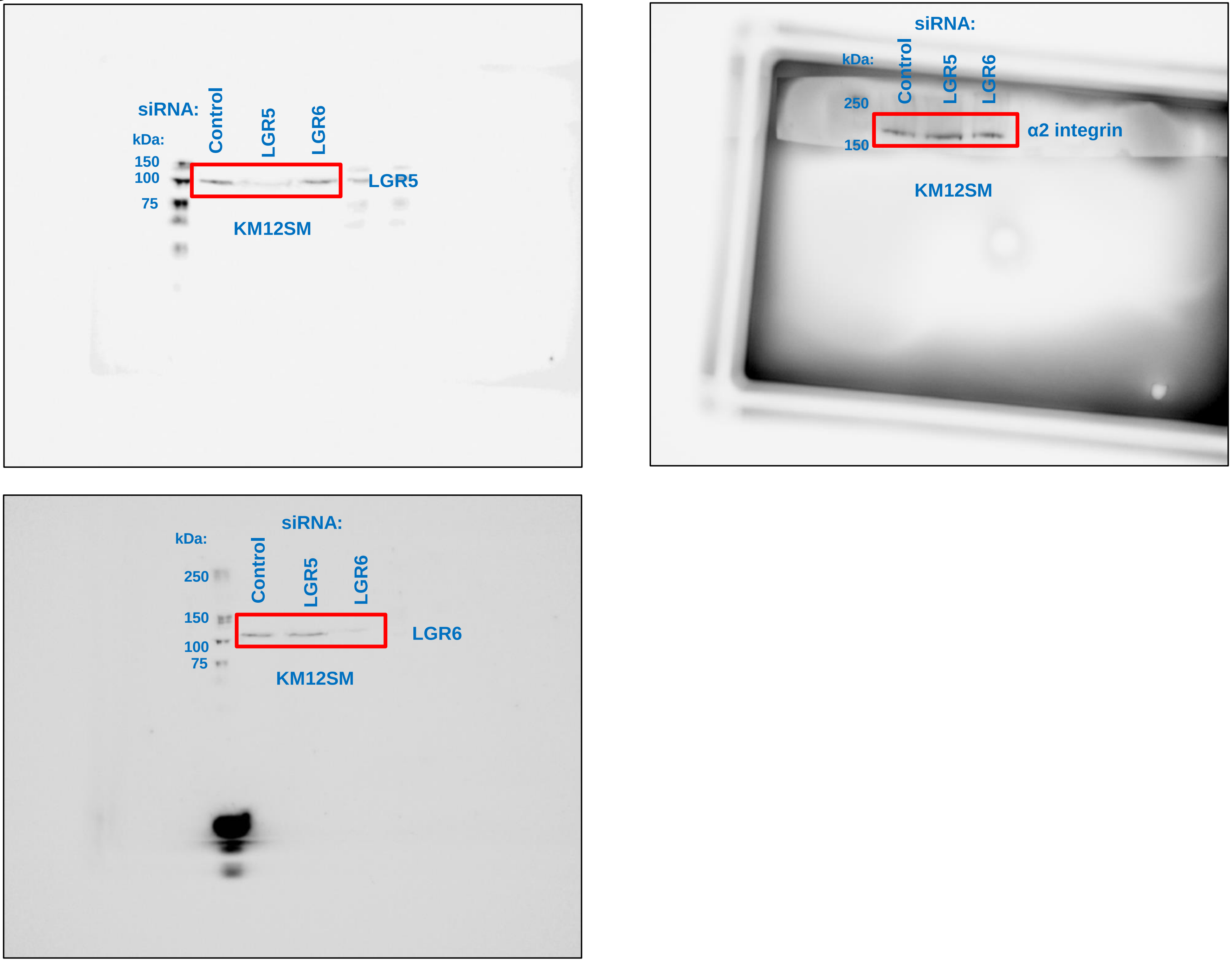
siRNA:
kDa:
150
100
75
Control
LGR5
LGR6
LGR5
KM12SM
siRNA:
kDa:
250
150
Control
LGR5
LGR6
α2 integrin
KM12SM
siRNA:
kDa:
250
150
100
75
Control
LGR5
LGR6
LGR6
KM12SM

Figure S3A

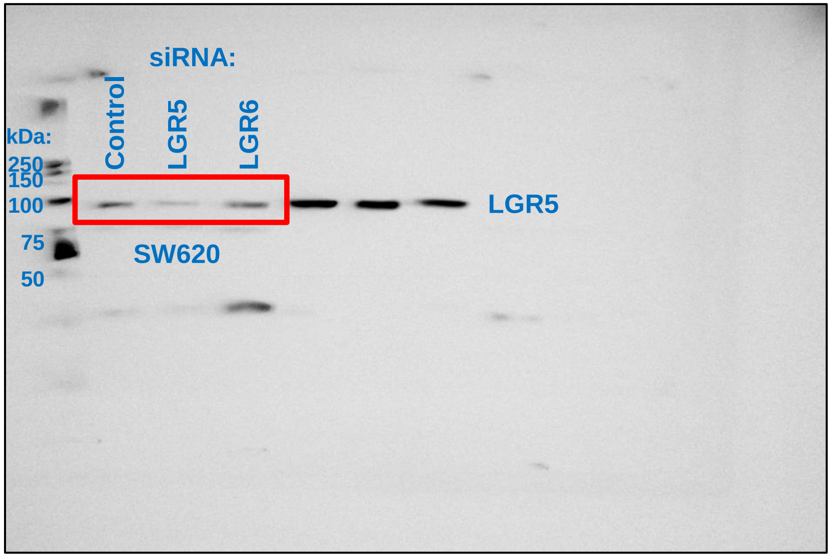

siRNA:
Control
LGR5
LGR6
kDa:
250
150
100
75
50
LGR5
SW620

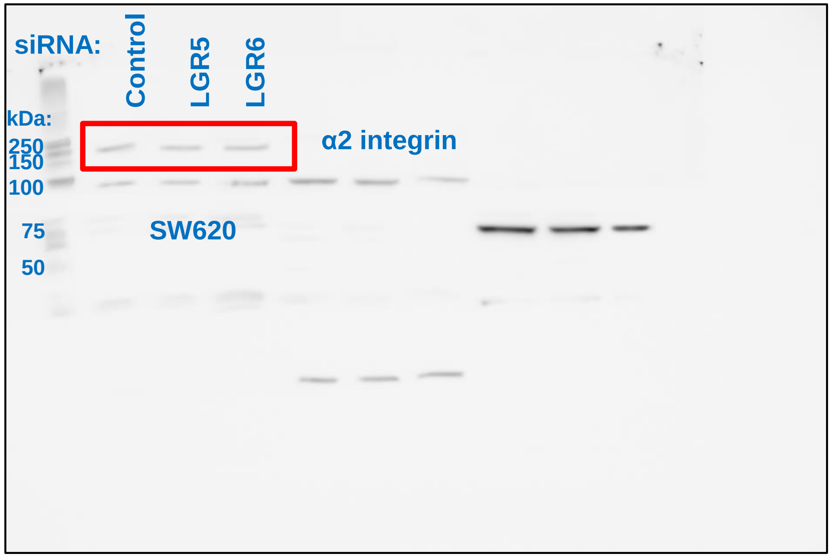

siRNA:
Control
LGR5
LGR6
kDa:
250
150
100
75
50
α2 integrin
SW620

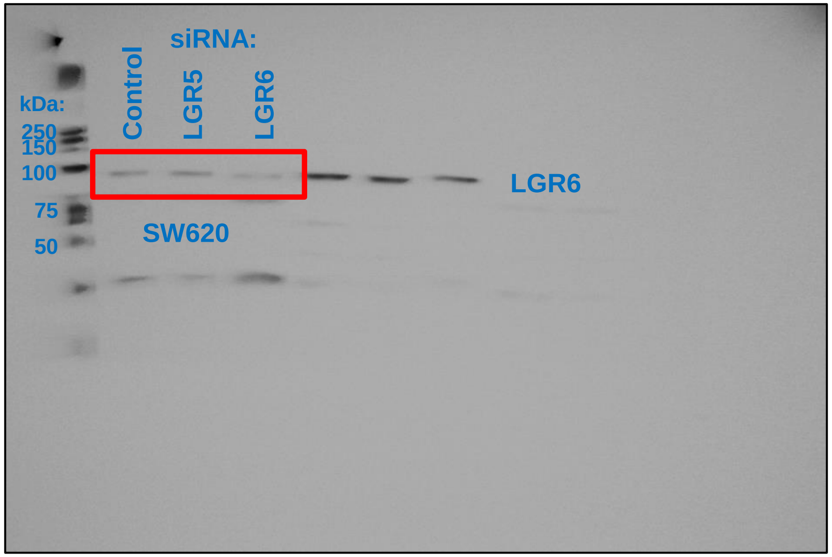

siRNA:
Control
LGR5
LGR6
kDa:
250
150
100
75
50
LGR6
SW620

Figure S4A

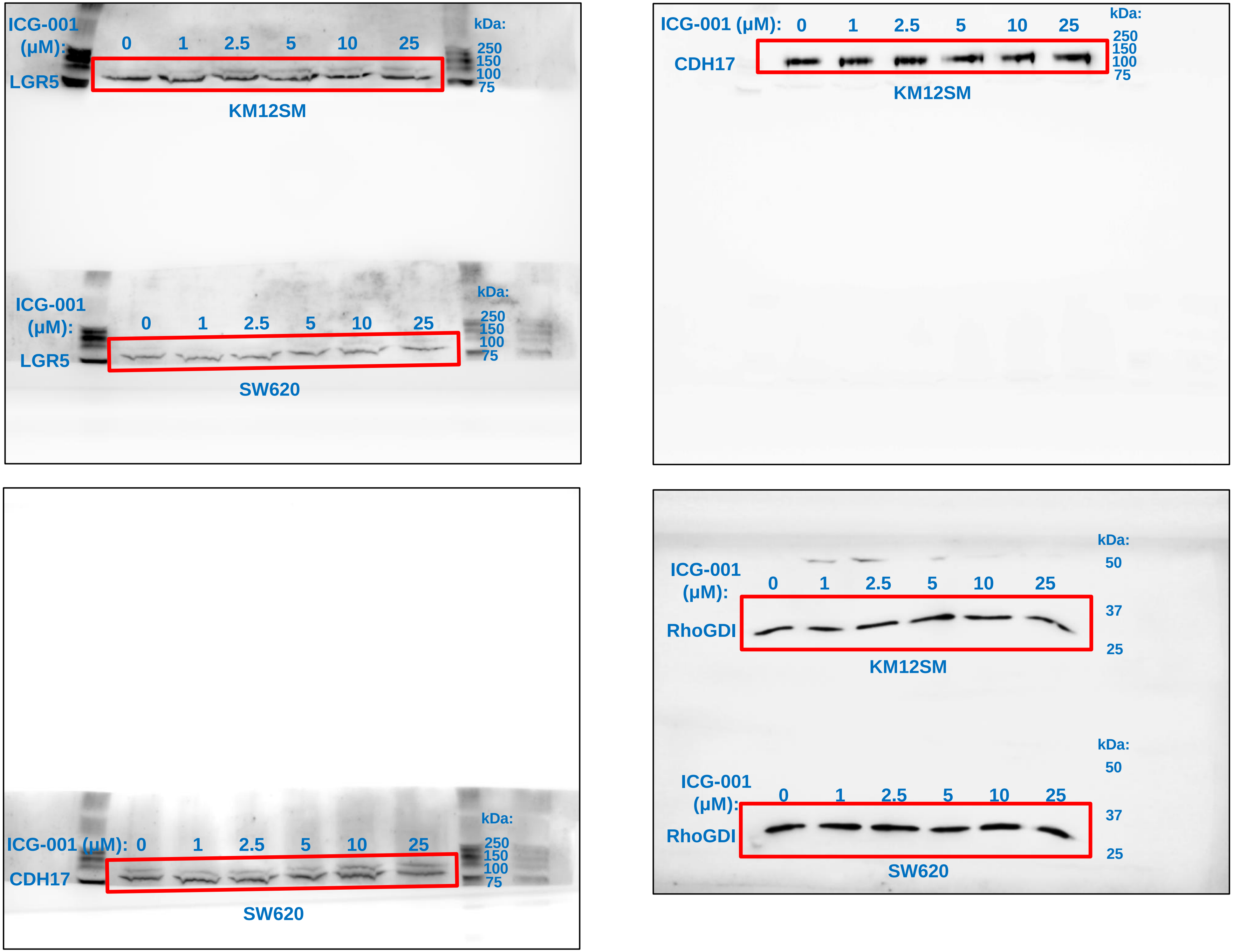
ICG-001 (µM):
0
1
2.5
5
10
25
kDa:
250
150
100
75
LGR5
KM12SM
ICG-001 (µM): 0 1 2.5 5 10 25
CDH17
KM12SM
ICG-001 (µM):
0 1 2.5 5 10 25
kDa:
250
150
100
75
LGR5
SW620
ICG-001 (µM): 0 1 2.5 5 10 25
kDa:
50
37
25
RhoGDI
KM12SM
ICG-001 (µM): 0 1 2.5 5 10 25
kDa:
250
150
100
75
CDH17
SW620
ICG-001 (µM): 0 1 2.5 5 10 25
kDa:
50
37
25
RhoGDI
SW620

Figure S4B

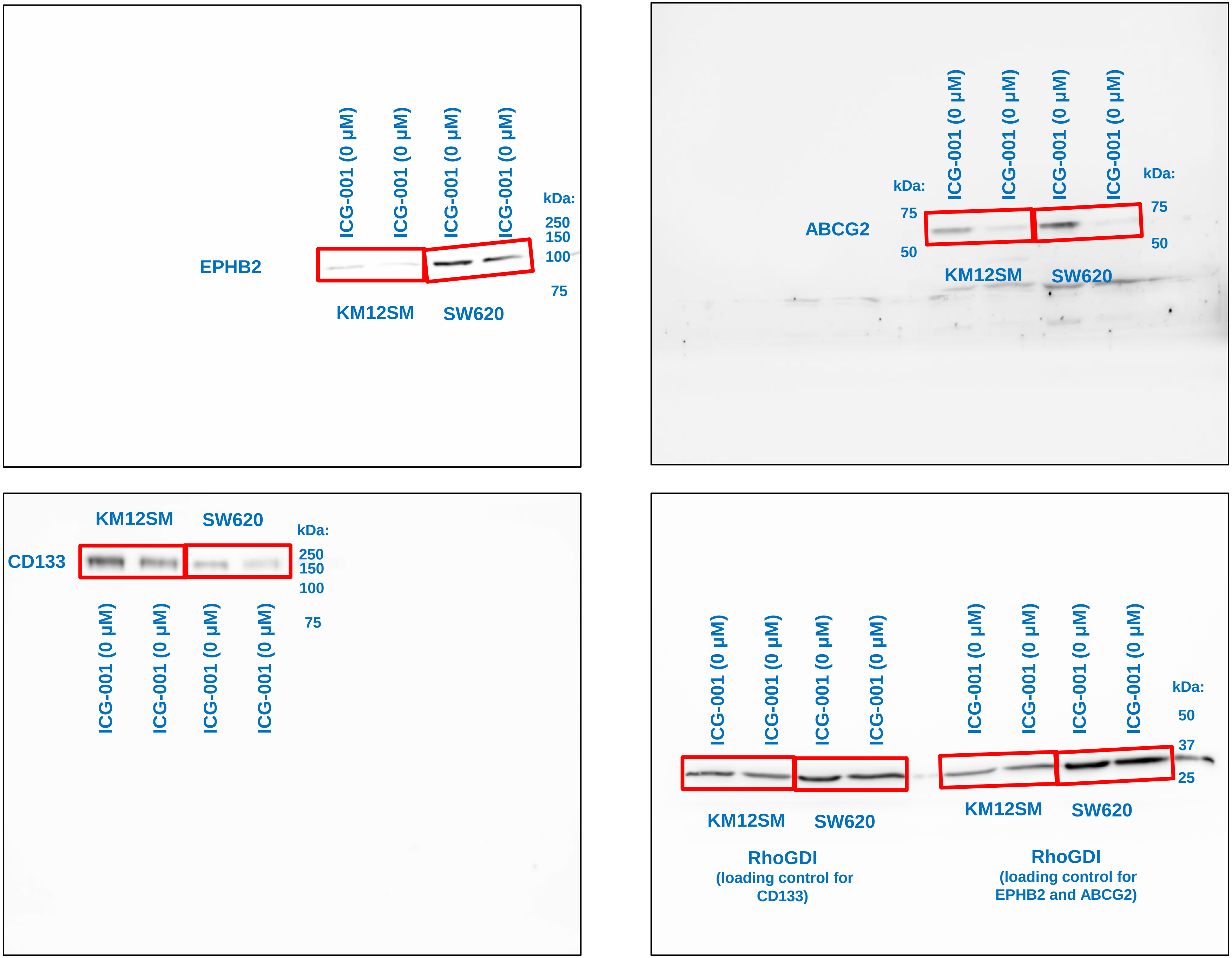
ICG-001 (0 µM)
ICG-001 (0 µM)
ICG-001 (0 µM)
ICG-001 (0 µM)
kDa:
250
150
100
75
EPHB2
KM12SM
SW620
ICG-001 (0 µM)
ICG-001 (0 µM)
ICG-001 (0 µM)
ICG-001 (0 µM)
kDa:
75
50
kDa:
75
50
ABCG2
KM12SM
SW620
KM12SM
SW620
kDa:
CD133
250
150
100
75
ICG-001 (0 µM)
ICG-001 (0 µM)
ICG-001 (0 µM)
ICG-001 (0 µM)
ICG-001 (0 µM)
ICG-001 (0 µM)
ICG-001 (0 µM)
ICG-001 (0 µM)
ICG-001 (0 µM)
ICG-001 (0 µM)
ICG-001 (0 µM)
ICG-001 (0 µM)
kDa:
50
37
25
KM12SM
SW620
KM12SM
SW620
RhoGDI
(loading control for
CD133)
RhoGDI
(loading control for
EPHB2 and ABCG2)

Figure S6A

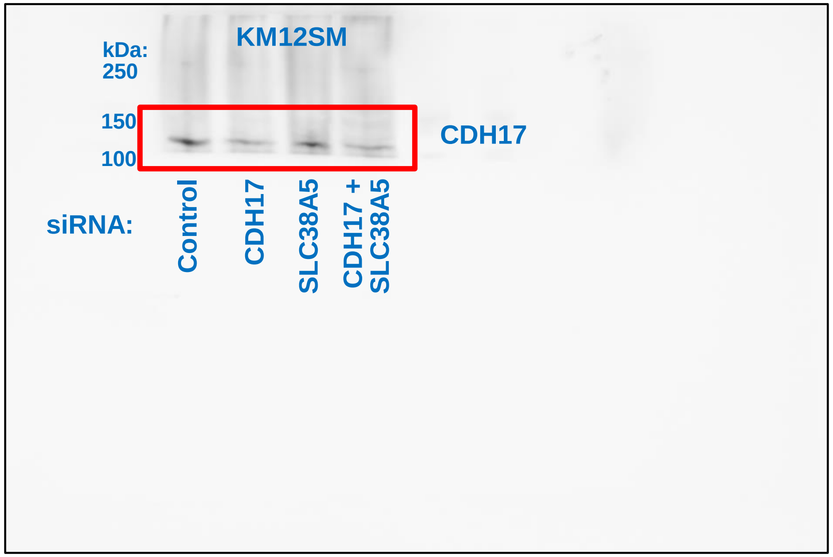
KM12SM
kDa:
250
150
100
CDH17
siRNA:
Control
CDH17
SLC38A5
CDH17 + SLC38A5

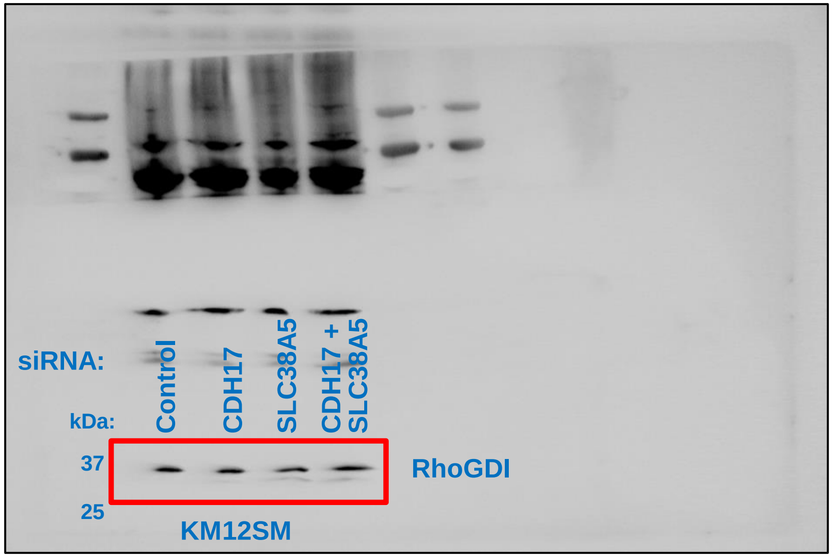
siRNA:
Control
CDH17
SLC38A5
CDH17 + SLC38A5
kDa:
37
25
RhoGDI
KM12SM

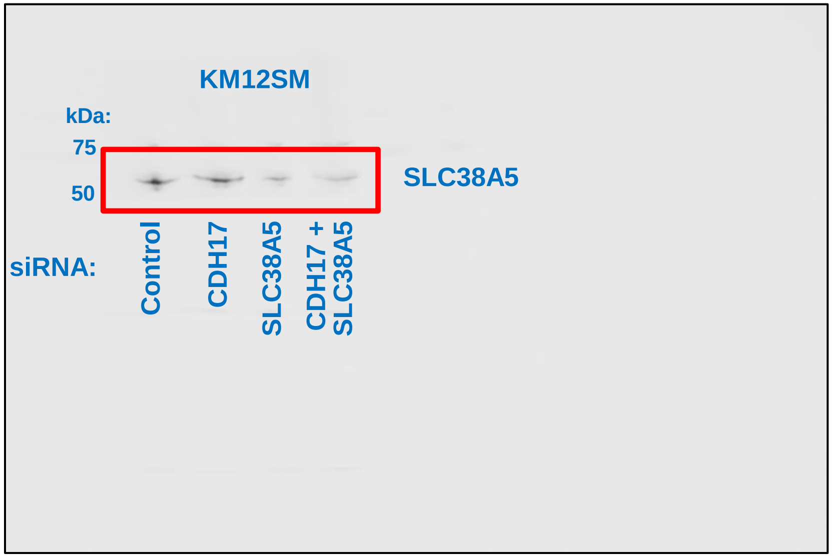
KM12SM
kDa:
75
50
SLC38A5
siRNA:
Control
CDH17
SLC38A5
CDH17 + SLC38A5

Figure S6A

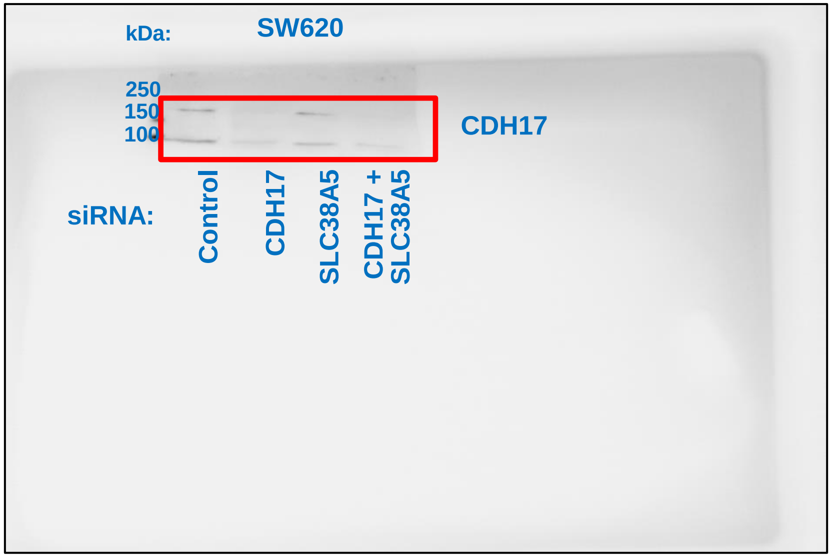
kDa:
SW620
250
150
100
CDH17
siRNA:
Control
CDH17
SLC38A5
CDH17 +
SLC38A5

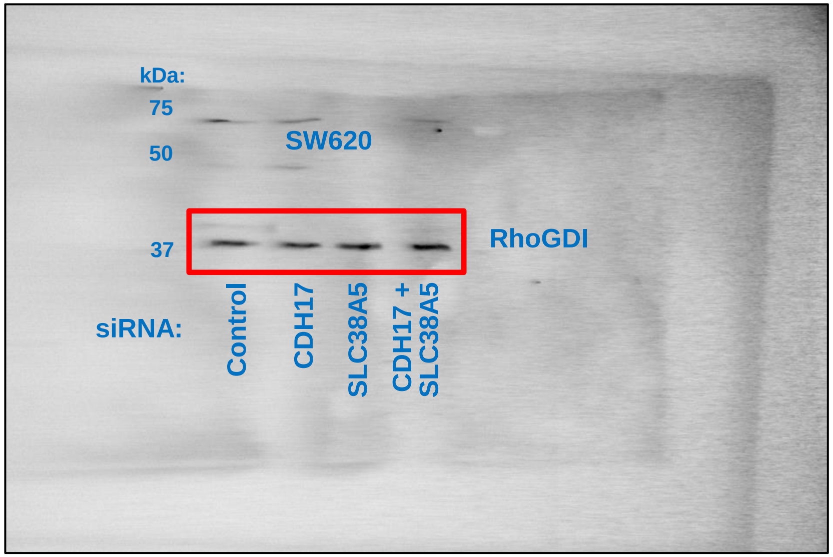
kDa:
75
SW620
50
37
RhoGDI
siRNA:
Control
CDH17
SLC38A5
CDH17 +
SLC38A5

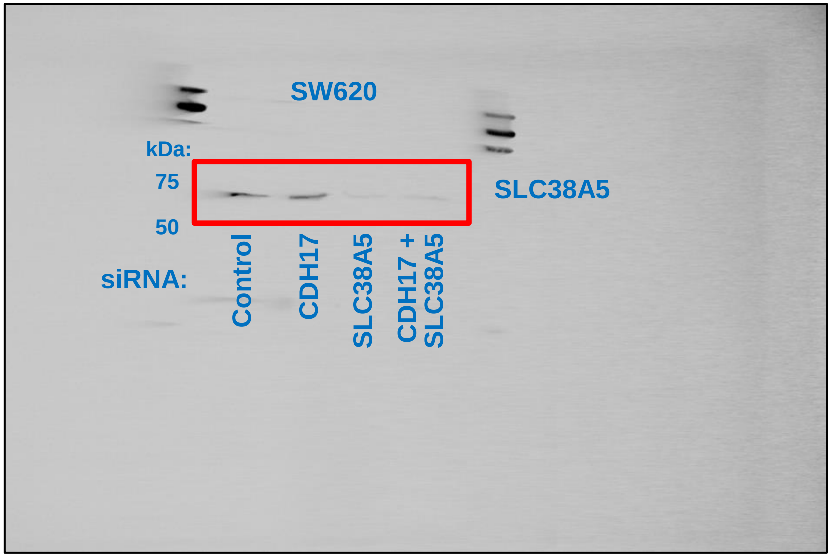
SW620
kDa:
75
50
SLC38A5
siRNA:
Control
CDH17
SLC38A5
CDH17 +
SLC38A5

Figure S6B

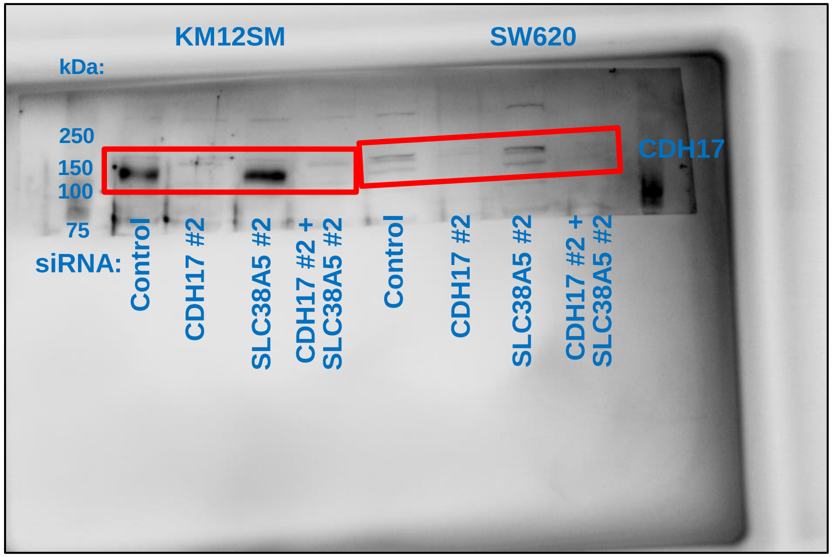
KM12SM
SW620
kDa:
250
150
100
75
CDH17
siRNA:
Control
CDH17 #2
SLC38A5 #2
CDH17 #2 + SLC38A5 #2
Control
CDH17 #2
SLC38A5 #2
CDH17 #2 + SLC38A5 #2

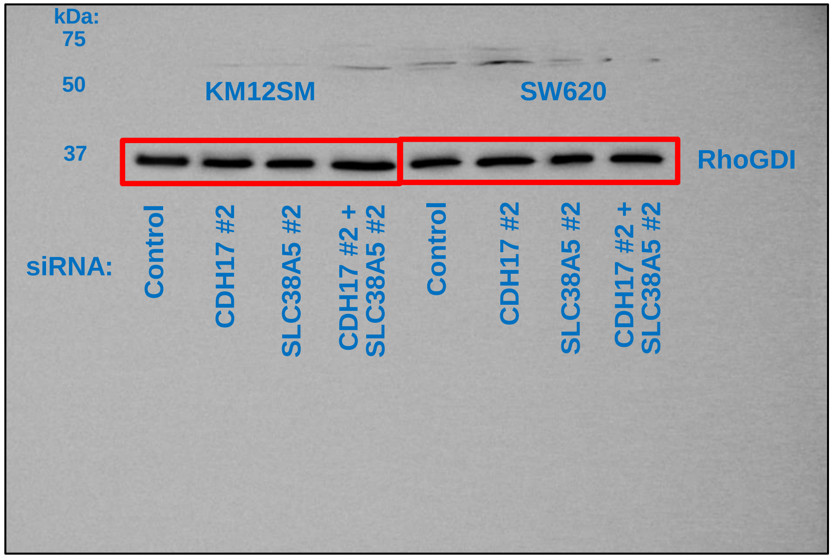
kDa:
75
50
KM12SM
SW620
37
RhoGDI
siRNA:
Control
CDH17 #2
SLC38A5 #2
CDH17 #2 + SLC38A5 #2
Control
CDH17 #2
SLC38A5 #2
CDH17 #2 + SLC38A5 #2

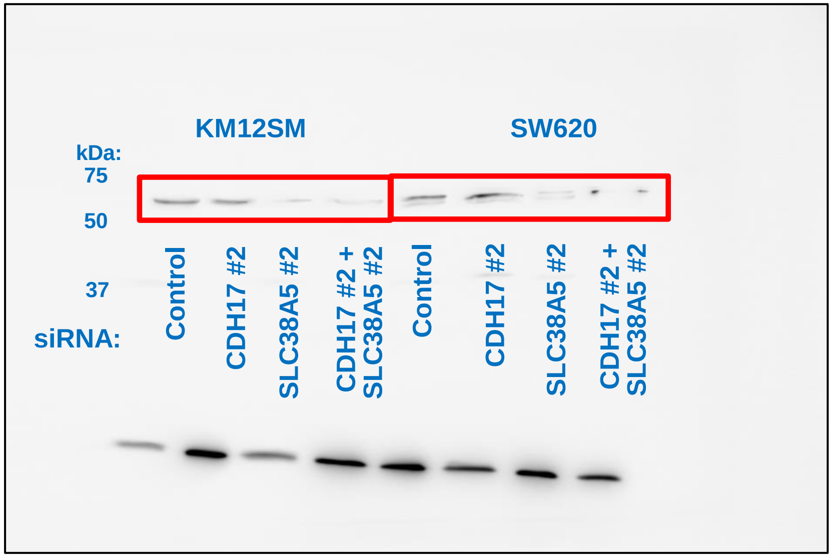
KM12SM
SW620
kDa:
75
50
37
siRNA:
Control
CDH17 #2
SLC38A5 #2
CDH17 #2 + SLC38A5 #2
Control
CDH17 #2
SLC38A5 #2
CDH17 #2 + SLC38A5 #2
